# Global, regional, and national incidence, prevalence, and years lived with disability for 354 diseases and injuries for 195 countries and territories, 1990–2017: a systematic analysis for the Global Burden of Disease Study 2017

**DOI:** 10.1016/S0140-6736(18)32279-7

**Published:** 2018-11-10

**Authors:** GBD 2017 Disease and Injury Incidence and Prevalence Collaborators, Spencer L James, Degu Abate, Kalkidan Hassen Abate, Solomon M Abay, Cristiana Abbafati, Nooshin Abbasi, Hedayat Abbastabar, Foad Abd-Allah, Jemal Abdela, Ahmed Abdelalim, Ibrahim Abdollahpour, Rizwan Suliankatchi Abdulkader, Zegeye Abebe, Semaw F Abera, Olifan Zewdie Abil, Haftom Niguse Abraha, Laith Jamal Abu-Raddad, Niveen M E Abu-Rmeileh, Manfred Mario Kokou Accrombessi, Dilaram Acharya, Pawan Acharya, Ilana N Ackerman, Abdu A Adamu, Oladimeji M Adebayo, Victor Adekanmbi, Olatunji O Adetokunboh, Mina G Adib, Jose C Adsuar, Kossivi Agbelenko Afanvi, Mohsen Afarideh, Ashkan Afshin, Gina Agarwal, Kareha M Agesa, Rakesh Aggarwal, Sargis Aghasi Aghayan, Sutapa Agrawal, Alireza Ahmadi, Mehdi Ahmadi, Hamid Ahmadieh, Muktar Beshir Ahmed, Amani Nidhal Aichour, Ibtihel Aichour, Miloud Taki Eddine Aichour, Tomi Akinyemiju, Nadia Akseer, Ziyad Al-Aly, Ayman Al-Eyadhy, Hesham M Al-Mekhlafi, Rajaa M Al-Raddadi, Fares Alahdab, Khurshid Alam, Tahiya Alam, Alaa Alashi, Seyed Moayed Alavian, Kefyalew Addis Alene, Mehran Alijanzadeh, Reza Alizadeh-Navaei, Syed Mohamed Aljunid, Ala'a Alkerwi, François Alla, Peter Allebeck, Mohamed M L Alouani, Khalid Altirkawi, Nelson Alvis-Guzman, Azmeraw T Amare, Leopold N Aminde, Walid Ammar, Yaw Ampem Amoako, Nahla Hamed Anber, Catalina Liliana Andrei, Sofia Androudi, Megbaru Debalkie Animut, Mina Anjomshoa, Mustafa Geleto Ansha, Carl Abelardo T Antonio, Palwasha Anwari, Jalal Arabloo, Antonio Arauz, Olatunde Aremu, Filippo Ariani, Bahroom Armoon, Johan Ärnlöv, Amit Arora, Al Artaman, Krishna K Aryal, Hamid Asayesh, Rana Jawad Asghar, Zerihun Ataro, Sachin R Atre, Marcel Ausloos, Leticia Avila-Burgos, Euripide F G A Avokpaho, Ashish Awasthi, Beatriz Paulina Ayala Quintanilla, Rakesh Ayer, Peter S Azzopardi, Arefeh Babazadeh, Hamid Badali, Alaa Badawi, Ayele Geleto Bali, Katherine E Ballesteros, Shoshana H Ballew, Maciej Banach, Joseph Adel Mattar Banoub, Amrit Banstola, Aleksandra Barac, Miguel A Barboza, Suzanne Lyn Barker-Collo, Till Winfried Bärnighausen, Lope H Barrero, Bernhard T Baune, Shahrzad Bazargan-Hejazi, Neeraj Bedi, Ettore Beghi, Masoud Behzadifar, Meysam Behzadifar, Yannick Béjot, Abate Bekele Belachew, Yihalem Abebe Belay, Michelle L Bell, Aminu K Bello, Isabela M Bensenor, Eduardo Bernabe, Robert S Bernstein, Mircea Beuran, Tina Beyranvand, Neeraj Bhala, Suraj Bhattarai, Soumyadeep Bhaumik, Zulfiqar A Bhutta, Belete Biadgo, Ali Bijani, Boris Bikbov, Ver Bilano, Nigus Bililign, Muhammad Shahdaat Bin Sayeed, Donal Bisanzio, Brigette F Blacker, Fiona M Blyth, Ibrahim R Bou-Orm, Soufiane Boufous, Rupert Bourne, Oliver J Brady, Michael Brainin, Luisa C Brant, Alexandra Brazinova, Nicholas J K Breitborde, Hermann Brenner, Paul Svitil Briant, Andrew M Briggs, Andrey Nikolaevich Briko, Gabrielle Britton, Traolach Brugha, Rachelle Buchbinder, Reinhard Busse, Zahid A Butt, Lucero Cahuana-Hurtado, Jorge Cano, Rosario Cárdenas, Juan J Carrero, Austin Carter, Félix Carvalho, Carlos A Castañeda-Orjuela, Jacqueline Castillo Rivas, Franz Castro, Ferrán Catalá-López, Kelly M Cercy, Ester Cerin, Yazan Chaiah, Alex R Chang, Hsing-Yi Chang, Jung-Chen Chang, Fiona J Charlson, Aparajita Chattopadhyay, Vijay Kumar Chattu, Pankaj Chaturvedi, Peggy Pei-Chia Chiang, Ken Lee Chin, Abdulaal Chitheer, Jee-Young J Choi, Rajiv Chowdhury, Hanne Christensen, Devasahayam J Christopher, Flavia M Cicuttini, Liliana G Ciobanu, Massimo Cirillo, Rafael M Claro, Daniel Collado-Mateo, Cyrus Cooper, Josef Coresh, Paolo Angelo Cortesi, Monica Cortinovis, Megan Costa, Ewerton Cousin, Michael H Criqui, Elizabeth A Cromwell, Marita Cross, John A Crump, Abel Fekadu Dadi, Lalit Dandona, Rakhi Dandona, Paul I Dargan, Ahmad Daryani, Rajat Das Gupta, José Das Neves, Tamirat Tesfaye Dasa, Gail Davey, Adrian C Davis, Dragos Virgil Davitoiu, Barbora De Courten, Fernando Pio De La Hoz, Diego De Leo, Jan-Walter De Neve, Meaza Girma Degefa, Louisa Degenhardt, Selina Deiparine, Robert P Dellavalle, Gebre Teklemariam Demoz, Kebede Deribe, Nikolaos Dervenis, Don C Des Jarlais, Getenet Ayalew Dessie, Subhojit Dey, Samath Dhamminda Dharmaratne, Mesfin Tadese Dinberu, M Ashworth Dirac, Shirin Djalalinia, Linh Doan, Klara Dokova, David Teye Doku, E Ray Dorsey, Kerrie E Doyle, Tim Robert Driscoll, Manisha Dubey, Eleonora Dubljanin, Eyasu Ejeta Duken, Bruce B Duncan, Andre R Duraes, Hedyeh Ebrahimi, Soheil Ebrahimpour, Michelle Marie Echko, David Edvardsson, Andem Effiong, Joshua R Ehrlich, Charbel El Bcheraoui, Maysaa El Sayed Zaki, Ziad El-Khatib, Hajer Elkout, Iqbal R F Elyazar, Ahmadali Enayati, Aman Yesuf Endries, Benjamin Er, Holly E Erskine, Babak Eshrati, Sharareh Eskandarieh, Alireza Esteghamati, Sadaf Esteghamati, Hamed Fakhim, Vahid Fallah Omrani, Mahbobeh Faramarzi, Mohammad Fareed, Farzaneh Farhadi, Talha A Farid, Carla Sofia E sá Farinha, Andrea Farioli, Andre Faro, Maryam S Farvid, Farshad Farzadfar, Valery L Feigin, Netsanet Fentahun, Seyed-Mohammad Fereshtehnejad, Eduarda Fernandes, Joao C Fernandes, Alize J Ferrari, Garumma Tolu Feyissa, Irina Filip, Florian Fischer, Christina Fitzmaurice, Nataliya A Foigt, Kyle J Foreman, Jack Fox, Tahvi D Frank, Takeshi Fukumoto, Nancy Fullman, Thomas Fürst, João M Furtado, Neal D Futran, Seana Gall, Morsaleh Ganji, Fortune Gbetoho Gankpe, Alberto L Garcia-Basteiro, William M Gardner, Abadi Kahsu Gebre, Amanuel Tesfay Gebremedhin, Teklu Gebrehiwo Gebremichael, Tilayie Feto Gelano, Johanna M Geleijnse, Ricard Genova-Maleras, Yilma Chisha Dea Geramo, Peter W Gething, Kebede Embaye Gezae, Keyghobad Ghadiri, Khalil Ghasemi Falavarjani, Maryam Ghasemi-Kasman, Mamata Ghimire, Rakesh Ghosh, Aloke Gopal Ghoshal, Simona Giampaoli, Paramjit Singh Gill, Tiffany K Gill, Ibrahim Abdelmageed Ginawi, Giorgia Giussani, Elena V Gnedovskaya, Ellen M Goldberg, Srinivas Goli, Hector Gómez-Dantés, Philimon N Gona, Sameer Vali Gopalani, Taren M Gorman, Alessandra C Goulart, Bárbara Niegia Garcia Goulart, Ayman Grada, Morgan E Grams, Giuseppe Grosso, Harish Chander Gugnani, Yuming Guo, Prakash C Gupta, Rahul Gupta, Rajeev Gupta, Tanush Gupta, Bishal Gyawali, Juanita A Haagsma, Vladimir Hachinski, Nima Hafezi-Nejad, Hassan Haghparast Bidgoli, Tekleberhan B Hagos, Gessessew Bugssa Hailu, Arvin Haj-Mirzaian, Arya Haj-Mirzaian, Randah R Hamadeh, Samer Hamidi, Alexis J Handal, Graeme J Hankey, Yuantao Hao, Hilda L Harb, Sivadasanpillai Harikrishnan, Josep Maria Haro, Mehedi Hasan, Hadi Hassankhani, Hamid Yimam Hassen, Rasmus Havmoeller, Caitlin N Hawley, Roderick J Hay, Simon I Hay, Akbar Hedayatizadeh-Omran, Behzad Heibati, Delia Hendrie, Andualem Henok, Claudiu Herteliu, Sousan Heydarpour, Desalegn Tsegaw Hibstu, Huong Thanh Hoang, Hans W Hoek, Howard J Hoffman, Michael K Hole, Enayatollah Homaie Rad, Praveen Hoogar, H Dean Hosgood, Seyed Mostafa Hosseini, Mehdi Hosseinzadeh, Mihaela Hostiuc, Sorin Hostiuc, Peter J Hotez, Damian G Hoy, Mohamed Hsairi, Aung Soe Htet, Guoqing Hu, John J Huang, Chantal K Huynh, Kim Moesgaard Iburg, Chad Thomas Ikeda, Bogdan Ileanu, Olayinka Stephen Ilesanmi, Usman Iqbal, Seyed Sina Naghibi Irvani, Caleb Mackay Salpeter Irvine, Sheikh Mohammed Shariful Islam, Farhad Islami, Kathryn H Jacobsen, Leila Jahangiry, Nader Jahanmehr, Sudhir Kumar Jain, Mihajlo Jakovljevic, Mehdi Javanbakht, Achala Upendra Jayatilleke, Panniyammakal Jeemon, Ravi Prakash Jha, Vivekanand Jha, John S Ji, Catherine O Johnson, Jost B Jonas, Jacek Jerzy Jozwiak, Suresh Banayya Jungari, Mikk Jürisson, Zubair Kabir, Rajendra Kadel, Amaha Kahsay, Rizwan Kalani, Tanuj Kanchan, Manoochehr Karami, Behzad Karami Matin, André Karch, Corine Karema, Narges Karimi, Seyed M Karimi, Amir Kasaeian, Dessalegn H Kassa, Getachew Mullu Kassa, Tesfaye Dessale Kassa, Nicholas J Kassebaum, Srinivasa Vittal Katikireddi, Norito Kawakami, Ali Kazemi Karyani, Masoud Masoud Keighobadi, Peter Njenga Keiyoro, Laura Kemmer, Grant Rodgers Kemp, Andre Pascal Kengne, Andre Keren, Yousef Saleh Khader, Behzad Khafaei, Morteza Abdullatif Khafaie, Alireza Khajavi, Ibrahim A Khalil, Ejaz Ahmad Khan, Muhammad Shahzeb Khan, Muhammad Ali Khan, Young-Ho Khang, Mohammad Khazaei, Abdullah T Khoja, Ardeshir Khosravi, Mohammad Hossein Khosravi, Aliasghar A Kiadaliri, Daniel N Kiirithio, Cho-Il Kim, Daniel Kim, Pauline Kim, Young-Eun Kim, Yun Jin Kim, Ruth W Kimokoti, Yohannes Kinfu, Adnan Kisa, Katarzyna Kissimova-Skarbek, Mika Kivimäki, Ann Kristin Skrindo Knudsen, Jonathan M Kocarnik, Sonali Kochhar, Yoshihiro Kokubo, Tufa Kolola, Jacek A Kopec, Soewarta Kosen, Georgios A Kotsakis, Parvaiz A Koul, Ai Koyanagi, Michael A Kravchenko, Kewal Krishan, Kristopher J Krohn, Barthelemy Kuate Defo, Burcu Kucuk Bicer, G Anil Kumar, Manasi Kumar, Hmwe Hmwe Kyu, Deepesh P Lad, Sheetal D Lad, Alessandra Lafranconi, Ratilal Lalloo, Tea Lallukka, Faris Hasan Lami, Van C Lansingh, Arman Latifi, Kathryn Mei-Ming Lau, Jeffrey V Lazarus, Janet L Leasher, Jorge R Ledesma, Paul H Lee, James Leigh, Janni Leung, Miriam Levi, Sonia Lewycka, Shanshan Li, Yichong Li, Yu Liao, Misgan Legesse Liben, Lee-Ling Lim, Stephen S Lim, Shiwei Liu, Rakesh Lodha, Katharine J Looker, Alan D Lopez, Stefan Lorkowski, Paulo A Lotufo, Nicola Low, Rafael Lozano, Tim C D Lucas, Lydia R Lucchesi, Raimundas Lunevicius, Ronan A Lyons, Stefan Ma, Erlyn Rachelle King Macarayan, Mark T Mackay, Fabiana Madotto, Hassan Magdy Abd El Razek, Muhammed Magdy Abd El Razek, Dhaval P Maghavani, Narayan Bahadur Mahotra, Hue Thi Mai, Marek Majdan, Reza Majdzadeh, Azeem Majeed, Reza Malekzadeh, Deborah Carvalho Malta, Abdullah A Mamun, Ana-Laura Manda, Helena Manguerra, Treh Manhertz, Mohammad Ali Mansournia, Lorenzo Giovanni Mantovani, Chabila Christopher Mapoma, Joemer C Maravilla, Wagner Marcenes, Ashley Marks, Francisco Rogerlândio Martins-Melo, Ira Martopullo, Winfried März, Melvin B Marzan, Tivani Phosa Mashamba-Thompson, Benjamin Ballard Massenburg, Manu Raj Mathur, Kunihiro Matsushita, Pallab K Maulik, Mohsen Mazidi, Colm McAlinden, John J McGrath, Martin McKee, Man Mohan Mehndiratta, Ravi Mehrotra, Kala M Mehta, Varshil Mehta, Fabiola Mejia-Rodriguez, Tesfa Mekonen, Addisu Melese, Mulugeta Melku, Michele Meltzer, Peter T N Memiah, Ziad A Memish, Walter Mendoza, Desalegn Tadese Mengistu, Getnet Mengistu, George A Mensah, Seid Tiku Mereta, Atte Meretoja, Tuomo J Meretoja, Tomislav Mestrovic, Naser Mohammad Gholi Mezerji, Bartosz Miazgowski, Tomasz Miazgowski, Anoushka I Millear, Ted R Miller, Benjamin Miltz, G K Mini, Mojde Mirarefin, Erkin M Mirrakhimov, Awoke Temesgen Misganaw, Philip B Mitchell, Habtamu Mitiku, Babak Moazen, Bahram Mohajer, Karzan Abdulmuhsin Mohammad, Noushin Mohammadifard, Mousa Mohammadnia-Afrouzi, Mohammed A Mohammed, Shafiu Mohammed, Farnam Mohebi, Modhurima Moitra, Ali H Mokdad, Mariam Molokhia, Lorenzo Monasta, Yoshan Moodley, Mahmood Moosazadeh, Ghobad Moradi, Maziar Moradi-Lakeh, Mehdi Moradinazar, Paula Moraga, Lidia Morawska, Ilais Moreno Velásquez, Joana Morgado-Da-Costa, Shane Douglas Morrison, Marilita M Moschos, Seyyed Meysam Mousavi, Kalayu Brhane Mruts, Achenef Asmamaw Muche, Kindie Fentahun Muchie, Ulrich Otto Mueller, Oumer Sada Muhammed, Satinath Mukhopadhyay, Kate Muller, John Everett Mumford, Manoj Murhekar, Jonah Musa, Kamarul Imran Musa, Ghulam Mustafa, Ashraf F Nabhan, Chie Nagata, Mohsen Naghavi, Aliya Naheed, Azin Nahvijou, Gurudatta Naik, Nitish Naik, Farid Najafi, Luigi Naldi, Hae Sung Nam, Vinay Nangia, Jobert Richie Nansseu, Bruno Ramos Nascimento, Gopalakrishnan Natarajan, Nahid Neamati, Ionut Negoi, Ruxandra Irina Negoi, Subas Neupane, Charles Richard James Newton, Josephine W Ngunjiri, Anh Quynh Nguyen, Ha Thu Nguyen, Huong Lan Thi Nguyen, Huong Thanh Nguyen, Long Hoang Nguyen, Minh Nguyen, Nam Ba Nguyen, Son Hoang Nguyen, Emma Nichols, Dina Nur Anggraini Ningrum, Molly R Nixon, Nomonde Nolutshungu, Shuhei Nomura, Ole F Norheim, Mehdi Noroozi, Bo Norrving, Jean Jacques Noubiap, Hamid Reza Nouri, Malihe Nourollahpour Shiadeh, Mohammad Reza Nowroozi, Elaine O Nsoesie, Peter S Nyasulu, Christopher M Odell, Richard Ofori-Asenso, Felix Akpojene Ogbo, In-Hwan Oh, Olanrewaju Oladimeji, Andrew T Olagunju, Tinuke O Olagunju, Pedro R Olivares, Helen Elizabeth Olsen, Bolajoko Olubukunola Olusanya, Kanyin L Ong, Sok King Ong, Eyal Oren, Alberto Ortiz, Erika Ota, Stanislav S Otstavnov, Simon Øverland, Mayowa Ojo Owolabi, Mahesh P A, Rosana Pacella, Amir H Pakpour, Adrian Pana, Songhomitra Panda-Jonas, Andrea Parisi, Eun-Kee Park, Charles D H Parry, Shanti Patel, Sanghamitra Pati, Snehal T Patil, Ajay Patle, George C Patton, Vishnupriya Rao Paturi, Katherine R Paulson, Neil Pearce, David M Pereira, Norberto Perico, Konrad Pesudovs, Hai Quang Pham, Michael R Phillips, David M Pigott, Julian David Pillay, Michael A Piradov, Meghdad Pirsaheb, Farhad Pishgar, Oleguer Plana-Ripoll, Dietrich Plass, Suzanne Polinder, Svetlana Popova, Maarten J Postma, Akram Pourshams, Hossein Poustchi, Dorairaj Prabhakaran, Swayam Prakash, V Prakash, Caroline A Purcell, Manorama B Purwar, Mostafa Qorbani, D Alex Quistberg, Amir Radfar, Anwar Rafay, Alireza Rafiei, Fakher Rahim, Kazem Rahimi, Afarin Rahimi-Movaghar, Vafa Rahimi-Movaghar, Mahfuzar Rahman, Mohammad Hifz ur Rahman, Muhammad Aziz Rahman, Sajjad Ur Rahman, Rajesh Kumar Rai, Fatemeh Rajati, Usha Ram, Prabhat Ranjan, Anna Ranta, Puja C Rao, David Laith Rawaf, Salman Rawaf, K Srinath Reddy, Robert C Reiner, Nickolas Reinig, Marissa Bettay Reitsma, Giuseppe Remuzzi, Andre M N Renzaho, Serge Resnikoff, Satar Rezaei, Mohammad Sadegh Rezai, Antonio Luiz P Ribeiro, Stephen R Robinson, Leonardo Roever, Luca Ronfani, Gholamreza Roshandel, Ali Rostami, Gregory A Roth, Ambuj Roy, Enrico Rubagotti, Perminder S Sachdev, Nafis Sadat, Basema Saddik, Ehsan Sadeghi, Sahar Saeedi Moghaddam, Hosein Safari, Yahya Safari, Roya Safari-Faramani, Mahdi Safdarian, Sare Safi, Saeid Safiri, Rajesh Sagar, Amirhossein Sahebkar, Mohammad Ali Sahraian, Haniye Sadat Sajadi, Nasir Salam, Joseph S Salama, Payman Salamati, Komal Saleem, Zikria Saleem, Yahya Salimi, Joshua A Salomon, Sundeep Santosh Salvi, Inbal Salz, Abdallah M Samy, Juan Sanabria, Yingying Sang, Damian Francesco Santomauro, Itamar S Santos, João Vasco Santos, Milena M Santric Milicevic, Bruno Piassi Sao Jose, Mayank Sardana, Abdur Razzaque Sarker, Nizal Sarrafzadegan, Benn Sartorius, Shahabeddin Sarvi, Brijesh Sathian, Maheswar Satpathy, Arundhati R Sawant, Monika Sawhney, Sonia Saxena, Mete Saylan, Elke Schaeffner, Maria Inês Schmidt, Ione J C Schneider, Ben Schöttker, David C Schwebel, Falk Schwendicke, James G Scott, Mario Sekerija, Sadaf G Sepanlou, Edson Serván-Mori, Seyedmojtaba Seyedmousavi, Hosein Shabaninejad, Azadeh Shafieesabet, Mehdi Shahbazi, Amira A Shaheen, Masood Ali Shaikh, Mehran Shams-Beyranvand, Mohammadbagher Shamsi, Morteza Shamsizadeh, Heidar Sharafi, Kiomars Sharafi, Mehdi Sharif, Mahdi Sharif-Alhoseini, Meenakshi Sharma, Rajesh Sharma, Jun She, Aziz Sheikh, Peilin Shi, Kenji Shibuya, Mika Shigematsu, Rahman Shiri, Reza Shirkoohi, Kawkab Shishani, Ivy Shiue, Farhad Shokraneh, Haitham Shoman, Mark G Shrime, Si Si, Soraya Siabani, Tariq J Siddiqi, Inga Dora Sigfusdottir, Rannveig Sigurvinsdottir, João Pedro Silva, Dayane Gabriele Alves Silveira, Narayana Sarma Venkata Singam, Jasvinder A Singh, Narinder Pal Singh, Virendra Singh, Dhirendra Narain Sinha, Eirini Skiadaresi, Erica Leigh N Slepak, Karen Sliwa, David L Smith, Mari Smith, Adauto Martins Soares Filho, Badr Hasan Sobaih, Soheila Sobhani, Eugène Sobngwi, Samir S Soneji, Moslem Soofi, Masoud Soosaraei, Reed J D Sorensen, Joan B Soriano, Ireneous N Soyiri, Luciano A Sposato, Chandrashekhar T Sreeramareddy, Vinay Srinivasan, Jeffrey D Stanaway, Dan J Stein, Caitlyn Steiner, Timothy J Steiner, Mark A Stokes, Lars Jacob Stovner, Michelle L Subart, Agus Sudaryanto, Mu'awiyyah Babale Sufiyan, Bruno F Sunguya, Patrick John Sur, Ipsita Sutradhar, Bryan L Sykes, Dillon O Sylte, Rafael Tabarés-Seisdedos, Santosh Kumar Tadakamadla, Birkneh Tilahun Tadesse, Nikhil Tandon, Segen Gebremeskel Tassew, Mohammad Tavakkoli, Nuno Taveira, Hugh R Taylor, Arash Tehrani-Banihashemi, Tigist Gashaw Tekalign, Shishay Wahdey Tekelemedhin, Merhawi Gebremedhin Tekle, Habtamu Temesgen, Mohamad-Hani Temsah, Omar Temsah, Abdullah Sulieman Terkawi, Mebrahtu Teweldemedhin, Kavumpurathu Raman Thankappan, Nihal Thomas, Binyam Tilahun, Quyen G To, Marcello Tonelli, Roman Topor-Madry, Fotis Topouzis, Anna E Torre, Miguel Tortajada-Girbés, Mathilde Touvier, Marcos Roberto Tovani-Palone, Jeffrey A Towbin, Bach Xuan Tran, Khanh Bao Tran, Christopher E Troeger, Thomas Clement Truelsen, Miltiadis K Tsilimbaris, Derrick Tsoi, Lorainne Tudor Car, E Murat Tuzcu, Kingsley N Ukwaja, Irfan Ullah, Eduardo A Undurraga, Jurgen Unutzer, Rachel L Updike, Muhammad Shariq Usman, Olalekan A Uthman, Muthiah Vaduganathan, Afsane Vaezi, Pascual R Valdez, Santosh Varughese, Tommi Juhani Vasankari, Narayanaswamy Venketasubramanian, Santos Villafaina, Francesco S Violante, Sergey Konstantinovitch Vladimirov, Vasily Vlassov, Stein Emil Vollset, Kia Vosoughi, Isidora S Vujcic, Fasil Shiferaw Wagnew, Yasir Waheed, Stephen G Waller, Yafeng Wang, Yuan-Pang Wang, Elisabete Weiderpass, Robert G Weintraub, Daniel J Weiss, Fitsum Weldegebreal, Kidu Gidey Weldegwergs, Andrea Werdecker, T Eoin West, Harvey A Whiteford, Justyna Widecka, Tissa Wijeratne, Lauren B Wilner, Shadrach Wilson, Andrea Sylvia Winkler, Alison B Wiyeh, Charles Shey Wiysonge, Charles D A Wolfe, Anthony D Woolf, Shouling Wu, Yun-Chun Wu, Grant M A Wyper, Denis Xavier, Gelin Xu, Simon Yadgir, Ali Yadollahpour, Seyed Hossein Yahyazadeh Jabbari, Tomohide Yamada, Lijing L Yan, Yuichiro Yano, Mehdi Yaseri, Yasin Jemal Yasin, Alex Yeshaneh, Ebrahim M Yimer, Paul Yip, Engida Yisma, Naohiro Yonemoto, Seok-Jun Yoon, Marcel Yotebieng, Mustafa Z Younis, Mahmoud Yousefifard, Chuanhua Yu, Vesna Zadnik, Zoubida Zaidi, Sojib Bin Zaman, Mohammad Zamani, Zohreh Zare, Ayalew Jejaw Zeleke, Zerihun Menlkalew Zenebe, Kai Zhang, Zheng Zhao, Maigeng Zhou, Sanjay Zodpey, Inbar Zucker, Theo Vos, Christopher J L Murray

## Abstract

**Background:**

The Global Burden of Diseases, Injuries, and Risk Factors Study 2017 (GBD 2017) includes a comprehensive assessment of incidence, prevalence, and years lived with disability (YLDs) for 354 causes in 195 countries and territories from 1990 to 2017. Previous GBD studies have shown how the decline of mortality rates from 1990 to 2016 has led to an increase in life expectancy, an ageing global population, and an expansion of the non-fatal burden of disease and injury. These studies have also shown how a substantial portion of the world's population experiences non-fatal health loss with considerable heterogeneity among different causes, locations, ages, and sexes. Ongoing objectives of the GBD study include increasing the level of estimation detail, improving analytical strategies, and increasing the amount of high-quality data.

**Methods:**

We estimated incidence and prevalence for 354 diseases and injuries and 3484 sequelae. We used an updated and extensive body of literature studies, survey data, surveillance data, inpatient admission records, outpatient visit records, and health insurance claims, and additionally used results from cause of death models to inform estimates using a total of 68 781 data sources. Newly available clinical data from India, Iran, Japan, Jordan, Nepal, China, Brazil, Norway, and Italy were incorporated, as well as updated claims data from the USA and new claims data from Taiwan (province of China) and Singapore. We used DisMod-MR 2.1, a Bayesian meta-regression tool, as the main method of estimation, ensuring consistency between rates of incidence, prevalence, remission, and cause of death for each condition. YLDs were estimated as the product of a prevalence estimate and a disability weight for health states of each mutually exclusive sequela, adjusted for comorbidity. We updated the Socio-demographic Index (SDI), a summary development indicator of income per capita, years of schooling, and total fertility rate. Additionally, we calculated differences between male and female YLDs to identify divergent trends across sexes. GBD 2017 complies with the Guidelines for Accurate and Transparent Health Estimates Reporting.

**Findings:**

Globally, for females, the causes with the greatest age-standardised prevalence were oral disorders, headache disorders, and haemoglobinopathies and haemolytic anaemias in both 1990 and 2017. For males, the causes with the greatest age-standardised prevalence were oral disorders, headache disorders, and tuberculosis including latent tuberculosis infection in both 1990 and 2017. In terms of YLDs, low back pain, headache disorders, and dietary iron deficiency were the leading Level 3 causes of YLD counts in 1990, whereas low back pain, headache disorders, and depressive disorders were the leading causes in 2017 for both sexes combined. All-cause age-standardised YLD rates decreased by 3·9% (95% uncertainty interval [UI] 3·1–4·6) from 1990 to 2017; however, the all-age YLD rate increased by 7·2% (6·0–8·4) while the total sum of global YLDs increased from 562 million (421–723) to 853 million (642–1100). The increases for males and females were similar, with increases in all-age YLD rates of 7·9% (6·6–9·2) for males and 6·5% (5·4–7·7) for females. We found significant differences between males and females in terms of age-standardised prevalence estimates for multiple causes. The causes with the greatest relative differences between sexes in 2017 included substance use disorders (3018 cases [95% UI 2782–3252] per 100 000 in males *vs* s1400 [1279–1524] per 100 000 in females), transport injuries (3322 [3082–3583] *vs* 2336 [2154–2535]), and self-harm and interpersonal violence (3265 [2943–3630] *vs* 5643 [5057–6302]).

**Interpretation:**

Global all-cause age-standardised YLD rates have improved only slightly over a period spanning nearly three decades. However, the magnitude of the non-fatal disease burden has expanded globally, with increasing numbers of people who have a wide spectrum of conditions. A subset of conditions has remained globally pervasive since 1990, whereas other conditions have displayed more dynamic trends, with different ages, sexes, and geographies across the globe experiencing varying burdens and trends of health loss. This study emphasises how global improvements in premature mortality for select conditions have led to older populations with complex and potentially expensive diseases, yet also highlights global achievements in certain domains of disease and injury.

**Funding:**

Bill & Melinda Gates Foundation.

## Introduction

Measuring non-fatal health loss is one of the most complex endeavours in population health research. The evolution of modern health-care systems has led to an increasing number of diseases and injuries being diagnosed and treated in individual patients, and developments such as antihypertensive and statin medications, percutaneous coronary intervention, and antiretroviral therapies have led to averted deaths and longer lives. In parallel with the increasing complexity of clinical medicine in the past century, measuring non-fatal health loss has necessitated continuous refinement as diagnostic classification systems expand, new diseases emerge, and metrics of disability improve. Across the global landscape, increased non-fatal health loss paradoxically reflects both success in terms of diminishing rates of premature death but also failure in terms of maintaining health care for diseased and injured individuals. It is increasingly evident that differential access to care, economic inequality, and imbalanced risk factor profiles can and do challenge the ability of health systems to achieve equitable health outcomes in the face of complex and resource-draining diseases and injuries. Addressing such lapses in health equity can pose a burden to under-resourced health care systems and economies.

Global progress in improving the burden of non-fatal health outcomes has been limited, in part by a predominant focus on mortality rates as a common metric of tracking global health progress.^1^, ^2^, ^3^ In the latter part of the 20th century, the global community focused on communicable diseases such as tuberculosis, HIV, malaria, and other conditions that cause premature mortality. In the past decade, it has become evident that measuring non-fatal health loss is important for tracking progress as the disease burden, in terms of disability-adjusted life-years (DALYs), evolves toward being dominated by years lived with disability (YLDs). Transitions in ageing populations and reduced mortality in many areas of the world have created dynamic temporal patterns, particularly within the past decade, and measuring such time patterns is important because advents such as developing a cure for hepatitis C, discovering new therapies for cancer, and improving treatments for HIV can rapidly transform the burden in populations with access to these developments, and as conditions such as diabetes and non-alcoholic fatty liver disease become increasingly prevalent in lower-income countries.^4^

**Research in context**

**Evidence before this study**

The Global Burden of Diseases, Injuries, and Risk Factors (GBD) study is a comprehensive study of health loss designed to capture complex patterns of disease and injury burden; for non-fatal health outcomes, these are measured in terms of incidence, prevalence, and years lived with disability (YLDs). Previous versions of the study have increased the estimation detail for conditions, locations, ages, and years. This study is a reassessment of the incidence, prevalence, and YLDs of diseases and injuries from 1990 to 2017 and updates results from previous GBD studies. There are no alternative measurements of non-fatal health loss that include the level of detail provided in the GBD study.

**Added value of this study**

This study adds new knowledge on non-fatal burden globally and improves upon previous iterations of the GBD study in the following ways. We expanded our database of non-fatal health outcomes by adding 2842 collaborator-provided data sources and incorporating new clinical data representing an additional 149 million admissions and 3·7 billion outpatient visits for use in GBD modelling. This resulted in a total of 68 781 sources being used in the estimation process for GBD 2017. We improved estimation methods including updating the calculation of the Socio-demographic Index (SDI), adding the ability to report the statistical differences in non-fatal health outcomes for males and females, using internally consistent GBD estimates of population and fertility, and adopting several cause-specific modelling improvements. Cause-specific improvements included the following; for diarrhoea, we added additional literature informing aetiological attribution; for HIV/AIDS, we updated absolute neutrophil count bias adjustments, antiretroviral therapy coverage data, and sex-specific survey estimates. For hepatitis, we added case fatality rates and hepatitis B vaccine coverage to viral hepatitis incidence models. For maternal, neonatal, and child health causes, we added in-facility delivery rates to the inpatient admission per-capita estimates to more accurately measure the denominator for incident cases and expanded the age range affected by protein-energy malnutrition. For cancer, we applied mortality-incidence ratios directly to cause-specific mortality rates to estimate incidence, and then calculated prevalence on the basis of incidence and survival estimates. For mental and substance abuse disorders, we adopted new covariates for opioid use and updated autism spectrum disorder designations to be consistent with the most recent Diagnostic and Statistical Manual of Mental Disorders. We also added 19 new causes to our cause hierarchy, including type 1 and type 2 diabetes, chronic kidney disease due to type 1 diabetes, and chronic kidney disease due to type 2 diabetes; cirrhosis due to non-alcoholic steatohepatitis (NASH); liver cancer due to NASH; invasive non-typhoidal salmonella; myelodysplastic, myeloproliferative, and other haemopoietic neoplasms; subarachnoid haemorrhage; non-rheumatic valvular heart disease including calcific aortic and degenerative mitral subtypes; aggregates of vision disorders and hearing loss; poisoning by carbon monoxide; poisoning by other means; and estimates for natures of injury (eg, fractures).

**Implications of all the available evidence**

Global non-fatal burden is continuing to increase despite minor improvements in age-standardised rates. Three causes (low back pain, headache disorders, and depressive disorders) have prevailed as leading causes of non-fatal health loss for nearly three decades, while diabetes has emerged as the fourth leading cause of disability globally. The increase in YLDs reflects an ageing global population commensurate with declines in premature mortality across the development spectrum. Globally, patterns of non-fatal health loss vary dynamically by sex, age, location, SDI, and cause. The increasing burden of non-fatal diseases, injuries, and impairments could pose considerable challenges to health systems and economies not equipped to care for complex and expensive conditions.

Estimates reported in recent iterations of the Global Burden of Diseases, Injuries, and Risk Factors Study (GBD) have also illustrated differential health outcomes in males and females in certain locations and conditions. This topic has received attention in terms of mortality rates for sex-specific conditions such as maternal causes,^5^, ^6^, ^7^, ^8^, ^9^ gynaecological and breast malignancies,^10^, ^11^, ^12^, ^13^ and long-term complications of obstructed labour, such as obstetric fistula.^14^, ^15^, ^16^, ^17^, ^18^ GBD 2016 also highlighted how global, age-standardised, all-cause YLD rates are approximately 10% higher in females than males, emphasising how there may be sex-specific characteristics of the non-fatal burden that have not been explored in detail, particularly with respect to the differences in sex-specific health outcomes.^2^ It is increasingly of interest to measure differences in male and female non-fatal health loss.

This year's GBD study represents the continued effort of quantifying non-fatal health outcomes in terms of incidence, prevalence, and YLDs for a list of 354 GBD causes for the years 1990–2017. Because the study is remeasured and published on an annual basis, new estimates are provided not only for new estimation years but also for all previous estimation years and supersede any previous results. This year's study on non-fatal burden incorporates improvements in study design, estimation strategy, and data availability, and focuses on areas of non-fatal burden that are emerging as topical issues in measuring and improving health outcomes. We also explore the patterns of non-fatal health loss over time and estimate the statistical differences in non-fatal health loss for males and females.

## Methods

### Overview

The GBD study provides a standardised approach for estimating incidence, prevalence, and YLDs by cause, age, sex, year, and location. The study aims to use all accessible information on disease occurrence, natural history, and severity that passes a set of inclusion criteria. Our objective is to maximise the comparability of data, despite different collection methods or case definitions; to find a consistent set of estimates between data on prevalence, incidence, and causes of death; and to predict estimates for locations and causes with sparse or absent data by borrowing information from other locations and covariates.

The study conducts annual updates to incorporate new causes and data (including published literature, surveillance data, survey data, hospital and clinical data, and other types of data) and to improve demographic and statistical methods. In this study, we apply different methods to utilise available data and to measure specific epidemiological patterns of each cause of non-fatal burden. Our standard approach uses the Bayesian meta-regression tool DisMod-MR 2.1. Subsequently, we use data for severity and the occurrence of particular consequences of diseases, or sequelae, to establish the proportion of prevalent cases experiencing each sequela. There are several classes of alternative approaches for estimating non-fatal health outcomes, including for injuries, cancers, HIV/AIDS, other infectious diseases, and neonatal disorders. Presented below is a high-level description of our study methods; the supplementary methods (appendix 1 section 4) provide further detail on inputs, analytical processes, and outputs and methods specific to each cause in GBD 2017.

Analyses were completed using Python version 2.7, Stata version 13.1, or R version 3.3. Statistical code used for GBD estimation is publicly available online. All rates are expressed as age-standardised based on the GBD reference population^19^ unless otherwise specified. This study complies with the Guidelines for Accurate and Transparent Health Estimates Reporting (GATHER)^20^ recommendations (appendix 1).

### Geographical units, time periods, and demographics

GBD 2017 is based on a geographical hierarchy that includes 195 countries and territories grouped into 21 regions and seven GBD super-regions (appendix 1). Each year, GBD includes subnational analyses for a few new countries and continues to provide subnational estimates for countries that were added in previous cycles. Subnational estimation in GBD 2017 includes five new countries (Ethiopia, Iran, New Zealand, Norway, Russia) and countries previously estimated at subnational levels (GBD 2013: China, Mexico, and the UK [regional level]; GBD 2015: Brazil, India, Japan, Kenya, South Africa, Sweden, and the USA; GBD 2016: Indonesia and the UK [local government authority level]). All analyses are at the first level of administrative organisation within each country except for New Zealand (by Māori ethnicity), Sweden (by Stockholm and non-Stockholm), and the UK (by local government authorities). All subnational estimates for these countries were incorporated into model development and evaluation as part of GBD 2017. To meet data use requirements, in this publication we present all subnational estimates excluding those pending publication (Brazil, India, Japan, Kenya, Mexico, Sweden, the UK, and the USA); given space constraints these results are presented in appendix tables and figures instead of in the main text (appendix 2). Subnational estimates for countries with populations larger than 200 million people (measured using our most recent year of published estimates) that have not yet been published elsewhere are presented wherever estimates are illustrated with maps but are not included in data tables. Cause-specific results for non-fatal estimates for GBD 2017 cover the years 1990–2017. A subset of areas in this analysis focuses on 1990, 2007, and 2017 to show changes over time to better inform policy assessments.

GBD 2017 is the first time that estimation of fertility and population has been done within the GBD framework. Previously, the GBD study used external sources^21^, ^22^ for fertility and population estimates, which affect estimates throughout the GBD study, particularly estimates expressed in terms of population rates. The purpose of using internally derived demographic estimates is to ensure internal consistency across all GBD estimates. That is, mortality rates and fertility rates have to match population rate change such that there should be no births, deaths, or migrations that are not accounted for in our population estimates.

### GBD cause list

In GBD 2017, we further refined the existing cause list, and added 19 new causes, increasing the number of estimated causes in GBD to 359 with 282 causes of death estimated and 354 causes of non-fatal health loss estimated. In the GBD study, causes and their sequelae are organised into hierarchical levels. Level 1 contains three broad cause groups: communicable, maternal, neonatal, and nutritional diseases (CMNN); non-communicable diseases (NCDs); and injuries. For non-fatal health estimates, there are 22 Level 2 causes, 167 Level 3 causes, and 288 Level 4 causes. We also report estimates for 3484 sequelae, nine impairments, and seven nature of injury aggregates.

### New for GBD 2017

In GBD 2017, we report on 381 Level 5 sequelae. We have opted to include aggregate sequelae for GBD 2017 to foster more nuanced interpretations of groups of health outcomes that are relevant to policy makers and clinical users of the GBD. In addition, this reporting list allows for more detailed evaluation of aetiologies and outcomes from GBD causes.

For the first time in the GBD study, we present the burden of injuries in terms of nature of injury as well as external cause of injury. Previously, we reported the incidence, prevalence, and YLDs of injuries expressed only in terms of what caused the injury—eg, those caused by falls. However, the burden that results from falls is experienced in terms of the bodily harm that the fall itself causes—eg, spinal injury or skeletal fracture. We have grouped the 47 nature of injury sequelae into seven combined categories that represent 1410 sequelae. The supplementary methods (appendix 1) includes the full GBD 2017 non-fatal reporting hierarchy from Level 1 to Level 6.

### Data sources

The process for non-fatal estimation begins with the compilation of data sources from a diverse set of possible sources, which include 21 possible Global Health Data Exchange (GHDx) data types ranging from scientific literature to survey data to epidemiological surveillance data. Our collaborator network provided 2842 data sources for GBD 2017. We analysed 21 100 sources of epidemiological surveillance data (country-years of disease reporting) for GBD 2017 and 4734 sources of disease registry data. For non-fatal estimation, we did systematic data and literature searches for 82 non-fatal causes and one impairment, which were updated to Feb 11, 2018. Search terms used for cause-specific systematic reviews, inclusion and exclusion criteria, preferred and alternative case definitions, and study methods detailed by cause are available in the supplementary methods (appendix 1 section 4). This search process contributed to the use of 15 449 scientific literature sources and 3126 survey sources used in non-fatal estimation, reflecting our updated counting criteria for GBD 2017. Household survey data archived in the GHDx were systematically screened together with sources suggested by country-level experts, surveys located in multinational survey data catalogues, and Ministry of Health and Central Statistical Office websites. Primary data sources containing disease prevalence, incidence, mortality risk, duration, remission, or severity were then combined in the estimation process. The supplementary methods section provides further details on gold standard data sources, adjustments, correction factors, and standardisations employed when incorporating these different types of non-fatal data (appendix 1 section 4).

In addition to data sources based on primary literature, surveys, and surveillance, the GBD study has used an increasing number of hospital discharge records, outpatient visit records, and health insurance claims to inform various steps of the non-fatal modelling process. This year, we received hospital discharge records for an additional 30 country-years, specifically discharge records from India (3 country-years), Iran (10), Japan (6), Jordan (1), Nepal (1), Brazil (2), China (1), and Italy (6); inpatient and outpatient claims from Taiwan (province of China); additional years of inpatient and outpatient claims from the USA; and inpatient claims from Singapore, representing an additional 148 842 107 hospital admissions globally and bringing the total number of admissions that inform GBD estimation to more than 2·6 billion. Additionally, we received 10 years of outpatient visit records from Norway, representing a total of 153 351 282 outpatient visits over a 10-year period. Overall, the study now uses hospital data from 335 country-years, outpatient visit data from 45 country-years, and health insurance claims data from 33 country-years between the USA, Taiwan (province of China), and Singapore. These data inform multiple cause models in various ways, mainly by providing incidence and prevalence estimates adjusted for readmission, non-primary diagnosis, outpatient utilisation, or a combination of the above, but also by estimating parameters such as case fatality rates, remission rates, procedure rates, and distribution of disease subtypes. The supplementary methods provide a more detailed description of how the clinical data adjustments are calculated and how admission and outpatient visit data are processed and utilised (appendix 1 section 2).

In the supplementary methods (appendix 1), we show the geographical coverage of non-fatal data, both incidence and prevalence, for GBD 2017. In addition, we illustrate the non-fatal data density and availability for GBD 2017 from 1990 to 2017 by GBD region and year for each of the three Level 1 GBD cause groups. The GHDx provides the metadata for all sources used for non-fatal estimation.

### Non-fatal disease models

For GBD 2017, we modelled non-fatal disease burden using DisMod-MR 2.1, a meta-analysis tool that uses a compartmental model structure with a series of differential equations that synthesise sparse and heterogeneous epidemiological data for non-fatal disease and injury outcomes. Estimation occurred at the five levels of the GBD location hierarchy—global, super-regional, regional, national, and subnational—with results of each higher level providing guidance for the analysis at the lower geographical level. Important modelling strategy changes from GBD 2016 to GBD 2017 for specific causes, as well as further details on these causes and their respective models, can be found in the supplementary methods (appendix 1 section 4).

Custom models were created if DisMod-MR 2.1 did not capture the complexity of the disease or if incidence and prevalence needed to be calculated from other data, or both. Further details of these custom models can be found in the cause-specific methods sections of the supplementary methods (appendix 1 section 4).

Prevalence was estimated for nine impairments, defined as sequelae of multiple causes for which better data were available to estimate the overall occurrence than for each underlying cause: anaemia, intellectual disability, epilepsy, hearing loss, vision loss, heart failure, infertility, pelvic inflammatory disease, and Guillain-Barré syndrome. Different methodological approaches were used for each impairment estimation process; these details are described in the supplementary methods (appendix 1 section 4).

### Severity distributions and disability weights

Severity splits apply a set of proportions that represent the distribution of cases of a given non-fatal cause by its underlying severities. Severity splits are typically categorised as asymptomatic, mild, moderate, and severe. This distinction is important for conditions such as asthma that have a broad spectrum of symptomatic severities. Severity splits for most conditions use the Medical Expenditure Panel Survey (MEPS) data or literature sources identified through systematic reviews. Further detail on the severity splits for each cause, including changes from GBD 2016, are available in the cause-specific modelling write-ups in the supplementary methods (appendix 1 section 4).

Disability weight estimation is described in more detail elsewhere in the literature,^23^ but in summary, these represent the severity of health loss associated with a single given health state. The supplementary methods (appendix 1) provide a complete listing of the lay descriptions of all 234 health states used in the estimation of non-fatal results for GBD 2017.

### Comorbidity

A combined disability weight is required to account for individuals with more than one condition. To calculate a combined disability weight, the health loss associated with two disability weights are multiplied together and then a weighted average of each constituent disability weight is calculated. The adjusted disability weight is proportional to the magnitude of the original disability weight. A simulation of 40 000 distinct individuals is done that calculates the distribution of comorbid conditions on the basis of the expected distribution of each condition's sequelae in the population. Then, the resulting distributions of comorbidity-adjusted disability weights are used to calculate YLDs. This process did not change from GBD 2016.

### YLD computation

YLDs were estimated as the product of prevalence estimate and a disability weight for health states of each mutually exclusive sequela, adjusted for comorbidity as described above. The GBD cause hierarchy also includes 35 residual disease categories to capture YLDs from conditions that lack specific estimation models.

### Uncertainty analysis

We apply the same technique for propagating uncertainty as used elsewhere in the GBD study design.^19^, ^24^, ^25^ The distribution of every step in the computation process is stored in 1000 draws that are used for every other step in the process. The distributions are determined from the sampling error of data inputs, the uncertainty of the model coefficients, and the uncertainty of severity distributions and disability weights. Final estimates are computed using the mean estimate across 1000 draws, and the 95% uncertainty intervals (UIs) are determined on the basis of the 25th and 975th ranked values across all 1000 draws.

### The Socio-demographic Index

The Socio-demographic Index (SDI) is a summary measure that estimates a location's position on a spectrum of development.^26^ The SDI was originally constructed for GBD 2015 using the Human Development Index (HDI) methodology, wherein a 0–1 index value was determined for each of the original three covariate inputs (total fertility rate in women aged 15–49 years, educational attainment over the age of 15 years, and lag-distributed income per capita) using the observed minima and maxima over the estimation period to set the scales. In response to feedback from collaborators, we have refined the indicator with each GBD cycle. For GBD 2017, we replaced the total fertility rate with the total fertility rate in women under the age of 25 years. The GBD 2017 Population and Fertility^24^ analysis of age-specific fertility rates revealed that through the process of development, many countries exhibited a decline in age-specific fertility rates over the age of 30 years and then increased, creating a U-shaped pattern; however, age-specific fertility rates in ages 10–14 years, 15–19 years, 20–24 years, and total fertility under 25 years did not exhibit this pattern. Total fertility under 25 years remains highly correlated with mortality measures including under-5 mortality rates (Pearson's correlation coefficient *r*=0·873), and results from this revised method for computing SDI and results from GBD 2016 are also correlated (*r*=0·992).^24^ We computed the composite SDI as the geometric mean of the three indices for each location-year. The cutoff values used to determine quintiles for analysis were then computed using country-level estimates of SDI for 2017, excluding countries with populations of less than 1 million. These quintiles are used to categorise and present GBD 2017 results on the basis of sociodemographic status. The SDI values ranged from a low of 0·191 in Niger to a high of 0·918 in Denmark in 2017. Additional details on and results from the SDI calculation are available in the supplementary methods (appendix 1 section 2).

### Role of the funding source

The funder of the study had no role in study design, data collection, data analysis, data interpretation, or writing the report. All authors had full access to the data in the study and had final responsibility for the decision to submit for publication.

## Results

### Global prevalence, incidence, and YLDs

Non-fatal estimates by cause for 354 causes and nine impairments for the years 1990, 2007, and 2017 are available by age and sex through the online results tool. Results and findings mentioned in the discussion can also be viewed interactively through an online data visualisation tool.

**Figure 1:**
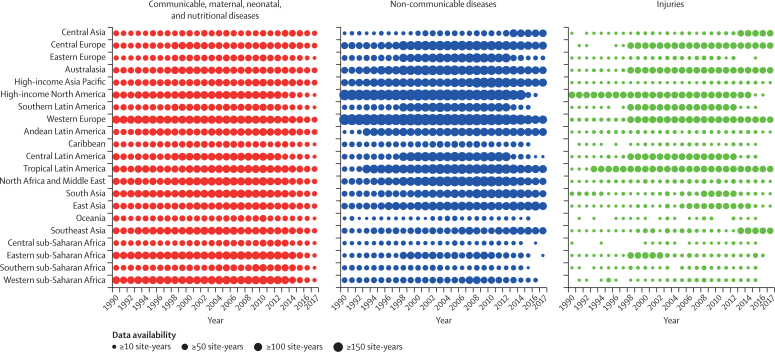
Non-fatal data availability in terms of site-years by GBD region and year for Level 1 causes of burden, 1990–2017 This figure represents non-fatal data from 1990 to 2017, showing the number of site-years for each location-year combination for each Level 1 cause of burden by GBD region.

Figure 1 shows the data density in terms of site-years by GBD region, cause group, and year. The figure shows how data density generally improves over time and how certain regions, particularly higher income regions, are more data dense than others. Additionally, the figure highlights how injuries data are generally less available than for the other two cause groups.

Table 1 reports cause-specific global estimates of prevalence, incidence, and YLDs for causes at Levels 1–5 of the GBD hierarchy for 2017, as well as the percentage change in YLDs and age-standardised YLD rates between 1990, 2007, and 2017. Unless otherwise specified, all rates reported in this analysis are age standardised.

**Table 1:** Global prevalence, incidence, and YLDs for 2017; percentage change of YLD counts; and percentage change of age-standardised YLD rates for 1990–2007 and 2007–17 for both sexes combined for all Level 5 causes, nature of injury aggregates, and nine impairments

				**Prevalence (thousands) 2017 counts**	**Incidence (thousands) 2017 counts**	**YLDs (thousands)**				
						2017 counts	Percentage change in counts, 1990–2007	Percentage change in counts, 2007–17	Percentage change in age-standardised rates, 1990–2007	Percentage change in age-standardised rates, 2007–17
**All causes**				**7 369 526·2 (7 344 769·0 to 7 392 430·8)**	**38 480 253·2 (36 469 390·1 to 40 567 963·0)**	**853 042·6 (642 084·6 to 1 097 347·2)**	29·8% (28·8 to 30·8)*	17·0% (16·4 to 17·6)*	−3·0% (−3·5 to −2·5)*	−0·9% (−1·4 to −0·4)*
**Communicable, maternal, neonatal, and nutritional diseases**				**4 767 056·2 (4 646 620·9 to 4 904 464·9)**	**27 145 980·3 (25 247 991·1 to 29 151 315·9)**	**117 573·7 (86 670·4 to 154 424·2)**	10·6% (7·4 to 14·8)*	2·6% (0·5 to 5·5)*	−7·8% (−10·4 to −4·5)*	−7·6% (−9·6 to −5·0)*
	**HIV/AIDS and sexually transmitted infections**			**1 238 129·2 (1 129 539·6 to 1 359 466·0)**	**769 111·2 (694 471·1 to 850 896·0)**	**5369·7 (3783·6 to 7272·2)**	204·0% (136·7 to 302·7)*	**−6·0% (−20·6 to 8·3)**	130·4% (79·8 to 202·8)*	−17·6% (−30·5 to −4·7)*
	HIV/AIDS			36 822·2 (34 794·9 to 39 199·8)	1942·1 (1632·1 to 2287·5)†	3949·0 (2746·5 to 5419·1)	372·9% (299·6 to 489·2)*	−11·5% (−26·2 to 5·4)	257·8% (204·6 to 343·0)*	−22·6% (−35·7 to −7·8)*
		HIV/AIDS and drug-susceptible tuberculosis co-infection		1049·5 (956·6 to 1149·6)	1321·6 (1203·1 to 1454·8)	404·7 (272·3 to 546·5)	329·9% (316·0 to 345·1)*	−19·6% (−21·8 to −17·3)*	229·1% (219·0 to 240·1)*	−29·2% (−31·1 to −27·2)*
		HIV/AIDS and multidrug-resistant tuberculosis without extensive drug resistance co-infection		37·6 (25·2 to 54·5)	52·0 (37·5 to 71·2)	15·3 (9·0 to 24·4)	3509·4% (1734·6 to 6384·8)*	−23·4% (−47·9 to 12·8)	2591·5% (1256·0 to 4788·1)*	−32·9% (−54·5 to −1·1)*
		HIV/AIDS and extensively drug-resistant tuberculosis co-infection		1·4 (0·9 to 2·3)	1·7 (1·2 to 2·3)	0·6 (0·3 to 1·0)	..	37·1% (−12·3 to 116·5)	..	19·5% (−23·8 to 88·6)
		HIV/AIDS resulting in other diseases		35 733·7 (33 669·3 to 38 076·0)	1942·1 (1632·1 to 2287·5)	3528·5 (2439·7 to 4941·4)	376·9% (292·0 to 510·8)*	−10·4% (−26·9 to 9·3)	260·2% (199·6 to 357·9)*	−21·7% (−36·3 to −4·5)*
			HIV/AIDS not on antiretroviral treatment without tuberculosis	14 763·0 (13 278·6 to 16 643·9)	1942·1 (1632·1 to 2287·5)	1911·1 (1240·9 to 2928·7)	343·8% (257·7 to 481·8)*	−47·8% (−52·9 to −42·9)*	235·0% (172·5 to 332·7)*	−53·9% (−58·4 to −49·5)*
			HIV/AIDS on antiretroviral treatment without tuberculosis	20 970·7 (19 876·1 to 22 058·6)	..	1617·4 (1079·5 to 2267·1)	2 265 649·4% (1 200 289·1 to 5 966 377·3)*	491·3% (420·4 to 581·8)*	1 847 617·4% (1 009 483·5 to 4 566 992·3)*	404·7% (343·7 to 482·1)*
	Sexually transmitted infections excluding HIV			1 216 425·2 (1 107 618·8 to 1 337 882·9)	767 169·1 (692 748·8 to 849 178·3)	1420·7 (764·5 to 2552·2)	33·8% (32·1 to 35·3)*	13·4% (12·1 to 14·8)*	0·8% (−0·2 to 1·5)	0·8% (−0·3 to 1·8)
		Syphilis		36 388·6 (31 030·7 to 42 960·2)	10 263·8 (8574·2 to 11 991·1)	72·9 (50·9 to 98·3)	28·7% (25·1 to 33·0)*	18·5% (15·7 to 21·5)*	−10·8% (−13·8 to −7·4)*	−3·5% (−5·8 to −1·2)*
			Early syphilis	36 018·0 (30 662·1 to 42 602·0)	10 263·8 (8574·2 to 11 991·1)	8·6 (2·6 to 21·9)	36·6% (32·5 to 40·8)*	13·7% (10·1 to 17·1)*	5·0% (2·2 to 7·8)*	2·2% (−1·0 to 5·3)
			Tertiary syphilis	370·6 (319·8 to 420·3)	..	64·3 (43·9 to 88·2)	27·7% (23·9 to 32·2)*	19·2% (16·1 to 22·5)*	−12·5% (−15·3 to −9·4)*	−4·2% (−6·5 to −1·8)*
		Chlamydial infection		109 822·0 (93 827·4 to 128 829·4)	297 131·3 (247 050·0 to 358 150·1)	314·6 (179·4 to 565·4)	30·9% (28·5 to 33·7)*	10·1% (7·7 to 12·7)*	0·3% (−1·0 to 2·0)	−0·7% (−2·7 to 1·6)
			Chlamydia episode	104 561·0 (88 447·0 to 123 536·5)	297 131·3 (247 050·0 to 358 150·1)	175·0 (68·4 to 379·5)	29·0% (26·4 to 31·4)*	9·0% (7·7 to 10·3)*	−1·0% (−1·7 to −0·3)*	−1·7% (−2·4 to −1·0)*
			Chlamydial infection complications	5261·1 (4960·3 to 5607·8)	..	139·6 (92·2 to 195·6)	33·5% (28·8 to 38·4)*	11·6% (6·5 to 16·6)*	2·1% (−1·0 to 5·2)	0·7% (−3·8 to 5·2)
		Gonococcal infection		47 269·2 (36 099·9 to 61 106·1)	137 221·5 (105 854·1 to 173 538·4)	190·3 (102·2 to 356·6)	27·0% (24·1 to 30·5)*	10·2% (6·8 to 14·4)*	1·3% (−0·5 to 3·3)	1·9% (−1·3 to 5·6)
			Gonococcal infection complications	1705·4 (1596·2 to 1824·5)	..	68·9 (45·3 to 97·9)	31·9% (26·9 to 36·9)*	16·9% (9·7 to 24·5)*	2·6% (−1·1 to 6·4)	6·6% (0·1 to 13·5)*
			Gonorrhoea episode	45 563·8 (34 373·4 to 59 361·5)	137 221·5 (105 854·1 to 173 538·4)	121·3 (46·5 to 271·5)	24·7% (22·1 to 28·0)*	6·7% (2·9 to 9·9)*	0·7% (−0·9 to 2·6)	−0·5% (−4·2 to 2·7)
		Trichomoniasis		142 114·5 (118 989·2 to 170 489·8)	244 855·9 (208 226·8 to 289 024·3)	242·8 (97·6 to 523·8)	37·7% (35·4 to 40·1)*	16·0% (14·2 to 17·7)*	2·9% (1·9 to 3·8)*	2·2% (1·1 to 3·2)*
		Genital herpes		955 894·8 (847 327·5 to 1 087 446·6)	77 696·7 (68 687·0 to 87 707·7)	247·4 (79·8 to 593·7)	41·7% (39·0 to 43·3)*	19·8% (18·1 to 21·0)*	1·9% (1·1 to 2·8)*	1·5% (0·9 to 2·3)*
		Other sexually transmitted infections		11 860·5 (11 121·7 to 12 735·7)	..	352·7 (214·9 to 598·5)	33·9% (31·9 to 36·2)*	11·2% (9·6 to 13·1)*	1·8% (0·7 to 3·1)*	0·8% (−0·6 to 2·3)
			Other sexually transmitted diseases residual	..	..	193·3 (105·6 to 361·3)	27·2% (25·3 to 29·5)*	7·8% (5·7 to 10·4)*	−0·0% (−1·2 to 1·4)	−0·6% (−2·6 to 1·7)
			Other sexually transmitted diseases	11 860·5 (11 121·7 to 12 735·7)	..	352·7 (214·9 to 598·5)	33·9% (31·9 to 36·2)*	11·2% (9·6 to 13·1)*	1·8% (0·7 to 3·1)*	0·8% (−0·6 to 2·3)
	**Respiratory infections and tuberculosis**			**2 187 290·0 (1 979 143·1 to 2 449 760·7)**	**17 942 622·2 (16 102 037·4 to 20 038 445·4)**	**11 670·3 (7845·9 to 16 749·7)**	16·2% (14·9 to 17·6)*	9·8% (8·9 to 10·7)*	−6·9% (−8·3 to −5·7)*	−2·6% (−3·6 to −1·7)*
	Tuberculosis			1 929 208·6 (1 710 952·7 to 2 199 199·9)	8965·8 (8191·8 to 9820·8)	3120·4 (2133·6 to 4230·6)	16·3% (14·6 to 18·0)*	9·4% (7·8 to 11·2)*	−13·9% (−15·1 to −12·7)*	−7·6% (−8·8 to −6·5)*
		Latent tuberculosis infection		1 918 892·1 (1 701 127·1 to 2 187 433·5)	..	..	..	..	..	..
		Drug-susceptible tuberculosis		9828·6 (8860·7 to 10 773·9)	8508·6 (7808·6 to 9371·0)	2969·7 (2011·4 to 4077·3)	11·4% (9·4 to 13·5)*	9·6% (5·2 to 12·7)*	−17·5% (−18·9 to −16·1)*	−7·5% (−11·1 to −5·1)*
		Multidrug-resistant tuberculosis without extensive drug resistance		464·1 (229·1 to 863·3)	432·8 (254·6 to 726·9)	142·8 (66·6 to 281·1)	589·4% (189·9 to 1708·7)*	4·8% (−45·2 to 76·4)	399·5% (110·5 to 1218·1)*	−11·8% (−53·5 to 48·1)
		Extensively drug-resistant tuberculosis		23·7 (13·9 to 44·1)	24·5 (17·7 to 35·0)	7·9 (4·1 to 15·1)	..	44·8% (−11·7 to 157·8)	..	20·9% (−26·3 to 115·5)
	Lower respiratory infections			10 638·1 (9729·1 to 11 559·4)	471 825·5 (429 571·3 to 516 976·9)	648·9 (432·6 to 927·7)	3·2% (0·3 to 6·3)*	15·8% (11·8 to 20·1)*	−11·1% (−13·0 to −9·3)*	4·4% (0·5 to 8·4)*
		Guillain-Barré syndrome due to lower respiratory infections		12·3 (6·9 to 19·9)	..	3·6 (1·7 to 6·6)	29·2% (25·3 to 33·5)*	17·9% (15·5 to 20·6)*	2·7% (1·1 to 4·3)*	3·1% (1·9 to 4·2)*
		Lower respiratory infection episode		10 625·8 (9719·1 to 11 547·2)	471 825·5 (429 571·3 to 516 976·9)	645·3 (429·9 to 925·0)	3·1% (0·1 to 6·1)*	15·8% (11·8 to 20·1)*	−11·2% (−13·1 to −9·3)*	4·5% (0·5 to 8·4)*
	Upper respiratory infections			236 084·8 (211 064·1 to 264 360·3)	17 144 182·9 (15 334 493·4 to 19 211 715·4)	5866·0 (3422·5 to 9336·4)	19·6% (17·3 to 21·9)*	11·5% (10·3 to 12·8)*	−2·5% (−3·3 to −1·8)*	0·2% (−0·6 to 1·1)
		Guillain-Barré syndrome due to upper respiratory infections		33·4 (24·4 to 44·7)	..	9·9 (5·8 to 15·5)	29·2% (25·3 to 33·4)*	17·9% (15·5 to 20·6)*	2·7% (1·1 to 4·3)*	3·1% (1·9 to 4·2)*
		Upper respiratory infection episode		236 051·4 (211 015·2 to 264 325·0)	17 144 182·9 (15 334 493·4 to 19 211 715·4)	5856·2 (3414·4 to 9325·9)	19·6% (17·3 to 21·8)*	11·4% (10·3 to 12·8)*	−2·5% (−3·3 to −1·8)*	0·2% (−0·6 to 1·1)
	Otitis media			101 690·4 (92 570·7 to 111 633·5)	317 648·0 (254 458·5 to 397 736·6)	2034·8 (1230·7 to 3227·8)	11·6% (8·9 to 14·5)*	4·3% (1·8 to 7·0)*	−5·8% (−8·0 to −3·7)*	−4·6% (−6·9 to −2·2)*
		Acute otitis media		18 153·8 (14 592·6 to 22 589·9)	317 625·1 (254 441·2 to 397 715·6)	238·4 (117·1 to 437·1)	10·7% (8·2 to 13·6)*	7·5% (5·2 to 9·7)*	4·6% (2·5 to 7·1)*	0·2% (−2·1 to 2·2)
		Chronic otitis media		83 536·6 (75 211·7 to 92 279·1)	22·8 (0·8 to 81·8)	1796·4 (1107·7 to 2821·4)	11·7% (8·7 to 14·9)*	3·9% (1·0 to 7·0)*	−7·1% (−9·4 to −4·6)*	−5·2% (−7·8 to −2·5)*
	**Enteric infections**			**93 304·4 (86 780·5 to 99 732·5)**	**6 307 792·4 (5 822 111·3 to 6 830 241·4)**	**10 583·7 (7283·3 to 14 516·1)**	16·4% (13·6 to 19·4)*	23·6% (20·6 to 26·9)*	−2·5% (−4·2 to −0·7)*	9·7% (6·8 to 12·7)*
	Diarrhoeal diseases			93 472·8 (86 857·2 to 99 961·1)	6 292 936·7 (5 808 374·7 to 6 816 675·4)	10 465·1 (7203·1 to 14 386·3)	17·6% (14·8 to 20·7)*	24·5% (21·5 to 27·9)*	−1·6% (−3·4 to 0·3)	10·4% (7·6 to 13·4)*
		Guillain-Barré syndrome due to diarrhoeal diseases		11·4 (7·8 to 15·7)	..	3·4 (2·0 to 5·4)	29·2% (25·3 to 33·5)*	17·9% (15·5 to 20·6)*	2·7% (1·1 to 4·3)*	3·1% (2·0 to 4·2)*
		Diarrhoeal disease episode		93 461·4 (86 846·3 to 99 951·5)	6 292 936·7 (5 808 374·7 to 6 816 675·4)	10 461·7 (7201·2 to 14 382·7)	17·6% (14·7 to 20·7)*	24·5% (21·5 to 27·9)*	−1·6% (−3·4 to 0·3)	10·4% (7·6 to 13·4)*
	Typhoid and paratyphoid			387·5 (312·6 to 467·9)	14 321·1 (12 540·3 to 16 337·4)	114·9 (77·7 to 164·2)	−26·3% (−32·0 to −20·7)*	−24·9% (−30·9 to −18·4)*	−35·2% (−39·8 to −30·4)*	−30·4% (−36·1 to −24·2)*
		Typhoid fever		691·5 (582·2 to 808·9)	10 924·3 (9343·0 to 12 597·1)	105·5 (70·3 to 151·0)	−26·4% (−32·5 to −20·4)*	−25·6% (−32·0 to −18·5)*	−35·2% (−40·2 to −29·8)*	−31·1% (−37·2 to −24·4)*
			Typhoid fever complications	144·0 (120·1 to 173·7)	1880·1 (1605·0 to 2191·0)	45·9 (29·9 to 66·3)	−26·0% (−35·3 to −15·4)*	−25·7% (−35·6 to −14·3)*	−34·9% (−43·0 to −25·5)*	−31·1% (−40·6 to −20·4)*
			Typhoid fever episode	547·5 (457·7 to 640·9)	9044·1 (7759·9 to 10 439·8)	59·6 (39·3 to 87·0)	−26·7% (−33·8 to −18·5)*	−25·6% (−33·2 to −16·9)*	−35·4% (−41·6 to −28·5)*	−31·0% (−38·4 to −22·7)*
		Paratyphoid fever		149·0 (117·0 to 185·1)	3396·9 (2666·5 to 4184·1)	9·4 (5·9 to 13·9)	−25·8% (−32·5 to −18·1)*	−15·8% (−23·8 to −7·1)*	−35·6% (−41·2 to −29·2)*	−22·0% (−29·5 to −13·6)*
			Intestinal perforation due to paratyphoid	6·7 (5·2 to 8·4)	173·9 (135·4 to 215·9)	0·8 (0·5 to 1·1)	−26·4% (−34·5 to −17·6)*	−16·0% (−23·8 to −6·2)*	−36·0% (−42·9 to −28·8)*	−22·1% (−29·7 to −12·6)*
			Paratyphoid fever episode	142·3 (111·5 to 176·8)	3222·9 (2537·7 to 3976·5)	8·6 (5·4 to 12·8)	−25·8% (−32·7 to −17·7)*	−15·8% (−24·2 to −6·5)*	−35·6% (−41·4 to −28·7)*	−21·9% (−29·8 to −13·2)*
	Invasive non-typhoidal salmonella			20·5 (14·5 to 28·6)	534·6 (409·0 to 705·0)	2·7 (1·6 to 4·3)	97·4% (71·1 to 127·3)*	−20·9% (−30·1 to −9·7)*	75·1% (52·6 to 101·3)*	−26·9% (−36·1 to −16·0)*
	Other intestinal infectious diseases			..	..	1·0 (0·6 to 1·4)	−40·9% (−47·2 to −33·9)*	−41·4% (−47·4 to −34·8)*	−45·3% (−50·8 to −39·2)*	−47·0% (−52·6 to −40·9)*
	**Neglected tropical diseases and malaria**			**1 278 896·5 (1 223 506·1 to 1 343 059·2)**	**357 652·1 (301 519·2 to 431 965·1)**	**13 622·9 (9498·3 to 18 673·3)**	**2·4% (−1·9 to 7·7)**	−10·3% (−15·0 to −5·6)*	−19·2% (−22·5 to −15·4)*	−20·6% (−24·6 to −16·5)*
	Malaria			136 085·1 (126 471·7 to 145 009·3)	208 768·2 (170 214·0 to 257 506·0)	1468·0 (1034·0 to 2020·6)	14·1% (4·7 to 24·4)*	−22·6% (−28·0 to −15·7)*	0·3% (−8·0 to 9·1)	−28·4% (−33·4 to −22·0)*
		Malaria complications		794·8 (723·8 to 875·7)	..	328·3 (255·8 to 405·9)	43·2% (38·1 to 49·0)*	26·8% (22·2 to 31·8)*	17·3% (13·1 to 21·9)*	17·4% (13·1 to 22·1)*
		Malaria episode		12 152·1 (7883·1 to 17 229·6)	208 768·2 (170 214·0 to 257 506·0)	423·2 (217·3 to 710·8)	10·0% (−2·3 to 20·4)	−22·1% (−29·0 to −13·9)*	−0·4% (−11·4 to 9·4)	−27·5% (−34·2 to −19·6)*
		Malaria parasitaemia		123 138·2 (112 779·9 to 133 815·6)	..	716·6 (471·7 to 1056·9)	10·9% (0·3 to 23·5)*	−34·5% (−40·4 to −27·0)*	−2·5% (−11·9 to 8·4)	−39·3% (−44·8 to −32·4)*
	Chagas disease			6197·0 (5248·5 to 7243·9)	162·5 (139·0 to 189·0)	57·3 (38·3 to 82·5)	10·1% (6·9 to 13·2)*	2·0% (−1·4 to 5·7)	−24·0% (−26·1 to −21·8)*	−17·8% (−20·6 to −15·0)*
		Acute Chagas disease		0·9 (0·4 to 1·5)	162·5 (139·0 to 189·0)	0·0 (0·0 to 0·1)	−16·2% (−22·1 to −12·7)*	−11·8% (−14·4 to −8·9)*	−32·1% (−36·3 to −29·6)*	−20·8% (−23·1 to −18·5)*
		Asymptomatic Chagas disease		5274·6 (4437·8 to 6166·6)	..	..	..	..	..	..
		Symptomatic chronic Chagas infection		921·4 (731·1 to 1128·3)	..	57·2 (38·3 to 82·4)	10·1% (7·0 to 13·3)*	2·0% (−1·4 to 5·7)	−24·0% (−26·1 to −21·8)*	−17·8% (−20·6 to −15·0)*
	Leishmaniasis			4130·2 (3515·7 to 4966·8)	669·1 (506·6 to 874·3)	264·4 (172·4 to 389·6)	7·6% (−8·7 to 29·4)	30·1% (20·2 to 42·3)*	−16·2% (−28·2 to 1·1)	14·1% (4·6 to 25·9)*
		Visceral leishmaniasis		10·6 (8·2 to 16·5)	42·4 (32·9 to 66·1)	0·8 (0·5 to 1·3)	−96·0% (−97·1 to −94·0)*	−72·4% (−79·4 to −58·3)*	−96·4% (−97·4 to −94·7)*	−74·5% (−81·0 to −61·3)*
		Cutaneous and mucocutaneous leishmaniasis		4166·6 (3560·7 to 4992·8)	626·6 (460·0 to 834·2)	263·6 (171·9 to 388·8)	65·8% (35·6 to 126·1)*	31·5% (21·2 to 44·2)*	21·9% (0·0 to 66·3)*	15·3% (5·2 to 27·4)*
	African trypanosomiasis			4·9 (1·3 to 19·8)	3·3 (2·0 to 8·1)	1·3 (0·3 to 5·3)	−60·9% (−69·0 to −47·0)*	−79·1% (−94·4 to −11·2)*	−68·9% (−75·4 to −58·4)*	−81·2% (−94·9 to −20·5)*
		Trypanosomiasis Gambiense		4·8 (1·3 to 19·6)	3·1 (1·8 to 8·0)	1·3 (0·3 to 5·3)	−60·3% (−69·1 to −44·4)*	−78·4% (−94·6 to −7·0)*	−68·5% (−75·6 to −56·3)*	−80·5% (−95·1 to −16·4)*
		Trypanosomiasis Rhodesiense		0·1 (0·0 to 0·3)	0·2 (0·1 to 0·6)	0·0 (0·0 to 0·1)	−67·7% (−81·9 to −46·6)*	−91·7% (−97·3 to −73·1)*	−74·2% (−85·6 to −57·6)*	−92·5% (−97·6 to −75·4)*
	Schistosomiasis			142 788·5 (131 656·9 to 155 480·2)	..	1089·1 (535·8 to 2082·0)	48·4% (44·0 to 51·5)*	−20·7% (−22·2 to −19·2)*	10·6% (7·3 to 13·0)*	−30·0% (−31·5 to −28·6)*
		Mild schistosomiasis		114 409·2 (106 010·4 to 124 045·9)	..	642·0 (259·2 to 1341·1)	53·2% (51·5 to 54·7)*	−21·8% (−23·3 to −20·4)*	13·1% (11·5 to 14·6)*	−31·5% (−32·9 to −30·1)*
		Anaemia due to schistosomiasis		7618·2 (6901·9 to 8321·6)	..	180·6 (119·1 to 268·6)	33·0% (24·1 to 42·6)*	−30·4% (−35·1 to −25·9)*	−1·3% (−7·6 to 5·6)	−38·6% (−42·8 to −34·6)*
		Schistosomiasis complications		20 785·2 (18 564·0 to 23 286·9)	..	266·5 (146·7 to 472·7)	50·9% (47·6 to 54·5)*	−9·0% (−11·9 to −4·3)*	15·7% (12·7 to 18·5)*	−17·9% (−20·4 to −14·4)*
	Cysticercosis			5417·9 (4662·0 to 6190·3)	..	1568·5 (1015·3 to 2181·0)	13·5% (8·2 to 18·7)*	8·5% (3·7 to 12·8)*	−17·7% (−21·3 to −14·2)*	−9·1% (−13·1 to −5·6)*
	Cystic echinococcosis			589·5 (373·9 to 926·5)	139·6 (90·2 to 213·9)	48·3 (25·4 to 85·0)	33·5% (26·3 to 41·9)*	18·9% (12·4 to 25·5)*	−1·0% (−7·6 to 4·8)	3·4% (−1·1 to 6·9)
	Lymphatic filariasis			64 623·4 (59 178·2 to 70 866·1)	..	1364·0 (752·0 to 2157·6)	25·5% (4·6 to 37·4)*	−37·0% (−48·7 to −26·4)*	−6·4% (−22·0 to 2·3)	−44·8% (−54·7 to −35·5)*
		Prevalence of detectable microfilaria due to lymphatic filariasis		52 285·4 (48 689·8 to 55 843·9)	..	..	..	..	..	..
		Lymphatic filariasis complications		12 338·1 (8403·3 to 17 434·1)	..	1364·0 (752·0 to 2157·6)	25·5% (4·6 to 37·4)*	−37·0% (−48·7 to −26·4)*	−6·4% (−22·0 to 2·3)	−44·8% (−54·7 to −35·5)*
	Onchocerciasis			20 938·1 (12 882·3 to 37 227·7)	..	1342·9 (639·1 to 2371·9)	−10·6% (−15·5 to −4·3)*	3·9% (−15·1 to 19·9)	−32·4% (−36·4 to −27·3)*	−8·0% (−25·8 to 6·7)
		Asymptomatic onchocerciasis		5131·9 (35·8 to 18 859·4)	..	..	..	..	..	..
		Skin disease due to onchocerciasis		14 654·2 (10 690·5 to 19 713·6)	..	1246·9 (552·7 to 2254·6)	−8·6% (−14·3 to −0·6)*	3·7% (−18·4 to 20·3)	−30·0% (−34·7 to −23·6)*	−7·5% (−27·7 to 7·9)
		Vision loss due to onchocerciasis		1152·1 (829·0 to 1703·6)	..	96·1 (60·6 to 141·5)	−31·6% (−36·5 to −26·5)*	7·0% (−4·8 to 21·4)	−52·9% (−56·4 to −49·3)*	−15·0% (−24·3 to −3·8)*
	Trachoma			3818·9 (2842·6 to 5135·2)	..	302·9 (201·7 to 425·1)	−12·8% (−18·2 to −6·4)*	−5·5% (−13·1 to 2·0)	−41·8% (−45·6 to −37·6)*	−28·2% (−33·8 to −22·5)*
	Dengue			6267·4 (3416·1 to 10 611·9)	104 771·9 (63 759·0 to 158 870·0)	1019·8 (447·3 to 1909·6)	178·9% (68·9 to 8404·5)*	61·1% (41·3 to 148·0)*	128·0% (38·1 to 6804·6)*	45·2% (27·4 to 123·4)*
		Post-dengue chronic fatigue syndrome		4418·2 (2064·8 to 8078·2)	..	911·1 (380·7 to 1726·5)	179·0% (69·0 to 8459·3)*	61·1% (41·1 to 149·3)*	128·2% (38·2 to 6850·0)*	45·2% (27·3 to 124·6)*
		Dengue episode		1849·2 (1117·6 to 2774·7)	104 771·9 (63 759·0 to 158 870·0)	108·7 (56·0 to 189·7)	177·7% (68·1 to 8513·3)*	61·1% (42·2 to 140·9)*	126·7% (37·4 to 6942·1)*	45·0% (28·0 to 116·7)*
	Yellow fever			2·6 (0·8 to 7·1)	97·4 (28·0 to 251·7)	0·1 (0·0 to 0·2)	−53·3% (−57·7 to −47·9)*	−15·8% (−25·4 to −4·4)*	−61·1% (−64·7 to −56·7)*	−22·4% (−31·4 to −11·4)*
		Asymptomatic yellow fever		1·5 (0·4 to 4·2)	54·4 (14·0 to 152·9)	..	..	..	..	..
		Yellow fever episode		1·2 (0·3 to 3·0)	43·0 (12·5 to 115·1)	0·1 (0·0 to 0·2)	−53·3% (−57·7 to −47·9)*	−15·8% (−25·4 to −4·4)*	−61·1% (−64·7 to −56·7)*	−22·4% (−31·4 to −11·4)*
	Rabies			0·5 (0·4 to 0·6)	13·4 (10·9 to 16·2)	0·1 (0·0 to 0·1)	−46·8% (−57·6 to −36·0)*	−35·4% (−45·7 to −23·8)*	−56·9% (−66·1 to −48·1)*	−43·3% (−52·3 to −32·9)*
	Intestinal nematode infections			894 917·5 (836 669·5 to 961 911·6)	..	1661·4 (960·3 to 2708·6)	−35·8% (−41·3 to −30·1)*	−30·1% (−33·9 to −26·1)*	−47·3% (−51·8 to −42·6)*	−36·3% (−39·8 to −32·7)*
		Ascariasis		447 009·0 (394 765·2 to 508 585·1)	..	603·8 (325·2 to 1037·6)	−38·3% (−47·2 to −28·7)*	−34·2% (−41·2 to −26·8)*	−48·3% (−55·9 to −40·2)*	−39·9% (−46·2 to −33·1)*
			Asymptomatic ascariasis	414 347·5 (365 611·9 to 472 277·1)	..	..	..	..	..	..
			Ascariasis complications	32 661·5 (28 939·0 to 36 737·1)	..	603·8 (325·2 to 1037·6)	−38·3% (−47·2 to −28·7)*	−34·2% (−41·2 to −26·8)*	−48·3% (−55·9 to −40·2)*	−39·9% (−46·2 to −33·1)*
		Trichuriasis		289 617·7 (254 640·5 to 330 724·5)	..	212·7 (120·0 to 353·7)	−43·0% (−50·2 to −35·7)*	−23·1% (−29·3 to −15·8)*	−53·4% (−59·4 to −47·5)*	−29·3% (−35·0 to −22·5)*
			Asymptomatic trichuriasis	278 887·2 (244 650·6 to 318 878·2)	..	..	..	..	..	..
			Trichuriasis complications	10 730·6 (9782·7 to 11 693·0)	..	212·7 (120·0 to 353·7)	−43·0% (−50·2 to −35·7)*	−23·1% (−29·3 to −15·8)*	−53·4% (−59·4 to −47·5)*	−29·3% (−35·0 to −22·5)*
		Hookworm disease		229 217·1 (212 538·1 to 246 731·6)	..	845·0 (510·0 to 1340·3)	−31·6% (−39·5 to −23·8)*	−28·5% (−34·0 to −22·7)*	−44·8% (−51·3 to −38·5)*	−35·2% (−40·2 to −30·0)*
			Asymptomatic hookworm disease	190 730·4 (176 950·0 to 205 624·5)	..	..	..	..	..	..
			Anaemia due to hookworm disease	9536·1 (8764·4 to 10 362·8)	..	245·9 (164·0 to 360·4)	−41·2% (−49·1 to −32·8)*	−35·4% (−41·8 to −28·5)*	−51·7% (−58·1 to −44·7)*	−41·3% (−47·1 to −34·9)*
			Hookworm disease complications	28 950·6 (26 952·9 to 31 087·4)	..	599·1 (334·2 to 993·7)	−25·8% (−33·7 to −17·7)*	−25·2% (−30·2 to −19·5)*	−40·8% (−47·2 to −34·4)*	−32·2% (−36·8 to −27·1)*
		Food-borne trematodiases		82 532·4 (74 596·1 to 91 774·9)	40 746·0 (35 650·0 to 46 019·1)	1870·7 (1070·9 to 3149·7)	9·4% (−9·4 to 31·8)	8·5% (4·8 to 12·0)*	−16·6% (−30·0 to −0·8)*	−6·2% (−9·1 to −3·5)*
			Asymptomatic food-borne trematodiases	65 832·6 (56 442·3 to 75 378·7)	30 998·0 (23 711·7 to 37 759·6)	..	..	..	..	..
			Food-borne trematodiases complications	16 699·7 (11 172·6 to 25 636·1)	9748·1 (5025·4 to 16 377·4)	1870·7 (1070·9 to 3149·7)	9·4% (−9·4 to 31·8)	8·5% (4·8 to 12·0)*	−16·6% (−30·0 to −0·8)*	−6·2% (−9·1 to −3·5)*
		Leprosy		518·5 (487·7 to 552·5)	48·5 (45·8 to 51·4)	31·5 (21·5 to 44·6)	35·0% (31·7 to 38·2)*	−1·3% (−3·7 to 1·1)	−5·5% (−7·8 to −3·4)*	−20·4% (−22·4 to −18·5)*
		Ebola virus disease		..	..	..	..	−96·8% (−97·5 to −94·7)*	..	−97·1% (−97·8 to −95·3)*
			Ebola cases	..	..	..	..	−97·8% (−97·9 to −97·7)*	..	−98·1% (−98·1 to −98·0)*
			Post-Ebola chronic fatigue syndrome	..	..	..	..	−96·7% (−97·5 to −94·6)*	..	−97·1% (−97·7 to −95·2)*
		Zika virus disease		37·6 (28·2 to 52·0)	2232·2 (1659·6 to 3097·6)	1·2 (0·8 to 1·8)	..	..	..	..
			Zika virus complications	0·9 (0·7 to 1·5)	0·6 (0·4 to 1·2)	0·5 (0·3 to 0·8)	..	..	..	..
			Zika virus episode	36·7 (27·3 to 50·9)	2231·6 (1659·1 to 3097·0)	0·7 (0·4 to 1·1)	..	..	..	..
		Guinea worm disease		..	..	..	−99·6% (−99·6 to −99·6)*	−99·5% (−99·6 to −99·3)*	−99·7% (−99·7 to −99·7)*	−99·5% (−99·7 to −99·3)*
			Moderate pain and limited mobility due to guinea worm	..	..	..	−99·6% (−99·6 to −99·6)*	−99·5% (−99·6 to −99·3)*	−99·7% (−99·7 to −99·7)*	−99·5% (−99·7 to −99·4)*
			Guinea worm disease complications	..	..	..	−99·6% (−99·7 to −99·6)*	−99·5% (−99·6 to −99·2)*	−99·7% (−99·7 to −99·7)*	−99·5% (−99·7 to −99·3)*
		Other neglected tropical diseases		52 797·1 (51 667·9 to 54 034·5)	..	1531·2 (1027·0 to 2201·6)	2·2% (−1·2 to 5·5)	−5·7% (−9·7 to −1·5)*	−10·9% (−13·7 to −8·0)*	−13·3% (−17·1 to −9·4)*
			Acute infection due to other neglected tropical diseases	..	..	13·3 (6·9 to 23·0)	164·3% (61·7 to 303·2)*	107·6% (83·3 to 199·8)*	135·1% (44·5 to 257·7)*	86·9% (64·7 to 169·5)*
			Anaemia due to other neglected tropical diseases	52 797·1 (51 667·9 to 54 034·5)	..	1517·9 (1018·7 to 2185·7)	1·9% (−1·4 to 5·3)	−6·2% (−10·2 to −2·0)*	−11·1% (−14·0 to −8·3)*	−13·7% (−17·6 to −9·8)*
	**Other infectious diseases**			**101 451·5 (97 425·1 to 105 559·6)**	**478 720·6 (450 498·3 to 511 601·6)**	**4056·6 (2835·5 to 5535·8)**	5·0% (2·1 to 7·5)*	**−0·5% (−2·9 to 1·6)**	−13·3% (−15·3 to −11·5)*	−10·6% (−12·7 to −8·9)*
	Meningitis			10 572·9 (8836·7 to 12 552·2)	5045·4 (4435·1 to 5877·8)	933·9 (653·0 to 1255·1)	10·6% (8·4 to 13·2)*	−3·2% (−5·9 to −0·3)*	−10·3% (−12·2 to −8·1)*	−12·4% (−14·7 to −9·7)*
		Pneumococcal meningitis		3557·0 (2932·0 to 4337·6)	444·9 (357·8 to 552·1)	325·0 (219·2 to 440·0)	19·9% (16·6 to 23·3)*	−24·8% (−27·4 to −22·1)*	−3·7% (−6·3 to −1·0)*	−32·1% (−34·6 to −29·6)*
			Acute pneumococcal meningitis	19·9 (15·8 to 25·0)	444·9 (357·8 to 552·1)	2·6 (1·6 to 3·9)	9·7% (3·6 to 15·9)*	−28·4% (−34·2 to −22·4)*	−0·9% (−6·0 to 4·7)	−34·1% (−39·8 to −28·4)*
			Pneumococcal meningitis complications	3537·1 (2915·9 to 4314·0)	..	322·4 (217·6 to 436·2)	20·0% (16·7 to 23·4)*	−24·7% (−27·4 to −22·1)*	−3·7% (−6·4 to −1·0)*	−32·1% (−34·5 to −29·5)*
		*H influenzae* type B meningitis		924·2 (668·2 to 1229·3)	262·3 (195·1 to 351·1)	84·3 (57·6 to 115·4)	−2·7% (−5·7 to 0·5)	−48·1% (−50·4 to −45·8)*	−20·3% (−22·8 to −17·6)*	−52·5% (−54·6 to −50·3)*
			Acute *H influenzae* type B meningitis	11·3 (8·4 to 15·2)	262·3 (195·1 to 351·1)	1·5 (0·9 to 2·4)	−9·6% (−14·9 to −4·3)*	−48·4% (−54·2 to −42·2)*	−16·3% (−21·4 to −11·4)*	−51·7% (−57·2 to −45·6)*
			*H influenzae* type B meningitis complications	912·9 (657·9 to 1216·9)	..	82·8 (56·6 to 113·3)	−2·6% (−5·6 to 0·7)	−48·1% (−50·4 to −45·8)*	−20·4% (−23·0 to −17·6)*	−52·5% (−54·6 to −50·2)*
		Meningococcal infection		1076·7 (764·8 to 1424·7)	402·5 (312·5 to 517·6)	99·0 (67·5 to 135·4)	12·4% (9·4 to 15·4)*	−3·2% (−6·4 to −0·1)*	−9·8% (−12·3 to −7·3)*	−12·9% (−15·9 to −10·0)*
			Acute meningococcal meningitis	18·0 (13·9 to 23·1)	402·5 (312·5 to 517·6)	2·4 (1·4 to 3·7)	5·8% (−1·3 to 13·5)	−4·1% (−12·3 to 4·5)	−3·6% (−9·8 to 3·0)	−12·0% (−19·8 to −3·9)*
			Meningococcal meningitis complications	1058·6 (749·6 to 1404·6)	..	96·6 (65·7 to 132·1)	12·6% (9·6 to 15·6)*	−3·2% (−6·3 to −0·1)*	−10·0% (−12·5 to −7·4)*	−12·9% (−15·9 to −10·1)*
		Other meningitis		5015·1 (3735·5 to 6370·4)	3935·7 (3466·6 to 4569·8)	425·7 (292·3 to 570·1)	5·6% (2·9 to 8·6)*	58·6% (53·4 to 64·1)*	−13·3% (−15·7 to −10·7)*	43·4% (38·7 to 48·4)*
			Other acute bacterial meningitis	64·0 (54·6 to 76·8)	1519·8 (1296·4 to 1836·4)	8·5 (5·4 to 12·6)	−1·8% (−6·8 to 4·1)	54·1% (41·2 to 68·0)*	−8·3% (−13·1 to −3·2)*	42·9% (30·7 to 56·3)*
			Acute viral meningitis	109·0 (95·5 to 125·5)	2416·0 (2142·8 to 2745·8)	14·5 (9·3 to 20·8)	6·8% (3·0 to 10·6)*	5·8% (0·5 to 11·4)*	−9·2% (−12·4 to −5·9)*	−3·9% (−9·0 to 1·6)
			Other bacterial meningitis complications	4842·0 (3574·3 to 6179·3)	..	402·7 (275·3 to 539·5)	5·7% (2·8 to 9·0)*	61·6% (56·3 to 67·5)*	−13·6% (−16·2 to −10·8)*	46·1% (41·1 to 51·4)*
	Encephalitis			6724·9 (3731·2 to 10 760·4)	2220·5 (2189·1 to 2255·2)	524·1 (365·5 to 691·3)	9·0% (6·6 to 11·2)*	6·7% (4·6 to 8·9)*	−15·8% (−17·5 to −14·1)*	−6·2% (−8·1 to −4·3)*
		Acute encephalitis		116·9 (115·1 to 118·8)	2220·5 (2189·1 to 2255·2)	15·5 (10·4 to 22·2)	14·1% (13·3 to 14·8)*	13·9% (13·4 to 14·3)*	−5·2% (−5·8 to −4·7)*	1·2% (0·8 to 1·6)*
		Encephalitis complications		6608·0 (3613·0 to 10 644·0)	..	508·6 (355·4 to 672·4)	8·8% (6·4 to 11·1)*	6·5% (4·3 to 8·8)*	−16·1% (−17·8 to −14·3)*	−6·5% (−8·4 to −4·5)*
	Diphtheria			1·1 (0·7 to 1·7)	14·4 (9·7 to 22·4)	0·1 (0·0 to 0·1)	−76·4% (−81·7 to −69·1)*	−32·3% (−55·3 to 7·2)	−77·6% (−82·6 to −70·7)*	−36·7% (−58·7 to 1·1)
	Whooping cough			1974·5 (1525·2 to 2490·2)	14 413·5 (11 134·0 to 18 178·7)	98·1 (58·2 to 154·8)	−26·1% (−27·9 to −24·0)*	−8·2% (−10·3 to −6·3)*	−26·5% (−28·4 to −24·5)*	−12·9% (−14·8 to −11·0)*
	Tetanus			59·6 (56·7 to 62·6)	79·2 (53·4 to 105·3)	1·7 (1·1 to 2·5)	−59·8% (−66·6 to −51·9)*	−28·6% (−39·7 to −17·5)*	−64·3% (−70·0 to −57·5)*	−36·2% (−46·2 to −26·1)*
		Severe tetanus		4·4 (3·0 to 5·9)	79·2 (53·4 to 105·3)	0·6 (0·3 to 0·9)	−73·0% (−77·3 to −66·9)*	−57·0% (−67·1 to −42·6)*	−75·4% (−79·2 to −70·1)*	−61·3% (−70·2 to −48·0)*
		Neonatal tetanus complications		55·2 (52·9 to 57·6)	..	1·1 (0·6 to 1·8)	12·2% (9·5 to 14·1)*	8·6% (6·6 to 10·3)*	−7·3% (−9·0 to −6·1)*	−2·0% (−3·7 to −0·5)*
	Measles			572·3 (203·7 to 1267·9)	20 888·3 (7433·5 to 46 276·7)	51·4 (17·4 to 118·2)	−44·0% (−47·2 to −41·0)*	−46·7% (−51·4 to −41·5)*	−44·6% (−47·8 to −41·6)*	−49·7% (−54·1 to −44·8)*
	Varicella and herpes zoster			6836·5 (6151·0 to 7510·6)	95 660·6 (91 657·3 to 99 992·6)	311·4 (187·5 to 471·2)	38·1% (34·9 to 41·5)*	21·7% (19·1 to 24·3)*	1·8% (0·0 to 3·7)*	1·0% (−0·6 to 2·5)
		Chickenpox		1236·7 (1200·1 to 1273·0)	64 530·2 (62 619·2 to 66 422·9)	7·1 (2·8 to 15·0)	1·4% (−1·1 to 3·8)	5·8% (3·3 to 8·1)*	−0·9% (−3·1 to 1·4)	0·1% (−2·4 to 2·3)
		Herpes zoster		5599·7 (4913·3 to 6277·7)	31 130·4 (27 271·7 to 35 058·2)	304·3 (183·4 to 461·1)	39·5% (36·3 to 42·8)*	22·1% (19·5 to 24·8)*	1·9% (0·1 to 3·8)*	1·0% (−0·6 to 2·6)
	Acute hepatitis			31 960·4 (29 698·0 to 34 406·8)	340 398·7 (319 758·5 to 362 492·1)	511·8 (334·5 to 739·0)	32·1% (21·7 to 42·8)*	7·5% (−0·8 to 17·2)	4·9% (−3·5 to 13·5)	−3·8% (−11·0 to 4·7)
		Acute hepatitis A		13 087·1 (12 396·2 to 13 831·2)	170 132·3 (161 150·2 to 179 805·9)	211·2 (134·0 to 308·2)	24·2% (15·6 to 33·9)*	6·8% (0·2 to 13·5)*	5·6% (−1·7 to 13·6)	−0·4% (−6·9 to 6·3)
		Acute hepatitis B		16 793·7 (14 752·6 to 19 222·1)	145 731·0 (128 012·1 to 166 802·5)	263·5 (169·6 to 398·1)	42·1% (21·9 to 65·7)*	8·4% (−6·2 to 26·7)	5·5% (−9·4 to 22·7)	−6·7% (−18·9 to 8·6)
		Acute hepatitis C		587·4 (532·0 to 649·7)	5091·1 (4610·8 to 5631·1)	8·2 (4·0 to 15·8)	5·7% (0·7 to 10·9)*	3·1% (−1·3 to 7·5)	−8·6% (−12·4 to −4·9)*	−7·1% (−11·4 to −3·0)*
		Acute hepatitis E		1492·1 (1330·2 to 1674·3)	19 444·3 (17 332·9 to 21 836·3)	28·9 (17·9 to 43·5)	20·6% (10·1 to 32·1)*	6·5% (−1·8 to 15·7)	−0·4% (−8·6 to 8·9)	−1·9% (−9·6 to 6·2)
	Other unspecified infectious diseases			53 643·7 (52 688·5 to 54 671·1)	..	1624·1 (1084·1 to 2337·5)	−0·4% (−3·3 to 2·5)	−3·5% (−6·7 to −0·2)*	−16·0% (−18·3 to −13·8)*	−12·1% (−15·1 to −9·0)*
		Guillain-Barré syndrome due to other infectious diseases		7·4 (5·0 to 10·7)	..	2·2 (1·2 to 3·6)	29·2% (25·3 to 33·5)*	18·0% (15·5 to 20·6)*	2·7% (1·1 to 4·3)*	3·1% (2·0 to 4·2)*
		Other infectious diseases		..	..	187·2 (114·4 to 291·1)	12·3% (8·1 to 15·9)*	18·0% (15·7 to 20·2)*	−11·2% (−14·3 to −8·6)*	6·0% (4·1 to 7·8)*
		Anaemia due to other infectious diseases		53 636·3 (52 678·9 to 54 662·5)	..	1434·7 (967·8 to 2073·7)	−1·6% (−4·7 to 1·4)	−5·7% (−9·1 to −2·2)*	−16·5% (−19·0 to −14·1)*	−13·9% (−17·2 to −10·6)*
	**Maternal and neonatal disorders**			**158 835·8 (140 427·7 to 179 076·8)**	**101 962·6 (94 726·5 to 109 284·9)**	**29 894·3 (22 429·9 to 38 381·6)**	49·5% (44·8 to 54·4)*	22·6% (16·2 to 29·9)*	24·5% (20·8 to 28·3)*	11·3% (5·5 to 18·0)*
	Maternal disorders			8532·0 (7424·5 to 9856·9)	79 812·2 (72 665·3 to 87 135·8)	805·6 (570·1 to 1084·3)	0·7% (−8·3 to 10·5)	−3·3% (−12·0 to 7·1)	−21·9% (−28·6 to −14·6)*	−12·5% (−20·6 to −3·3)*
		Maternal haemorrhage		1659·9 (1538·9 to 1777·6)	6988·4 (5611·4 to 8568·4)	60·9 (40·9 to 87·1)	−16·6% (−28·5 to −3·5)*	−4·5% (−18·5 to 11·5)	−32·4% (−42·0 to −21·7)*	−12·6% (−25·1 to 2·2)
			Maternal haemorrhage complications	1506·9 (1412·6 to 1601·5)	..	35·5 (23·6 to 51·8)	−20·9% (−28·3 to −13·0)*	−6·7% (−14·7 to 1·1)	−35·8% (−41·7 to −29·6)*	−14·6% (−21·7 to −7·5)*
			Maternal haemorrhage episode	153·0 (100·5 to 227·6)	6988·4 (5611·4 to 8568·4)	25·5 (15·0 to 40·0)	−9·3% (−35·3 to 30·5)	−1·2% (−32·3 to 41·8)	−26·6% (−47·7 to 5·4)	−9·5% (−37·8 to 30·0)
		Maternal sepsis and other pregnancy-related infections		2989·5 (2502·7 to 3623·3)	12 060·9 (9668·8 to 14 890·9)	57·0 (29·3 to 101·3)	−1·7% (−39·7 to 68·6)	5·9% (−34·4 to 65·1)	−21·4% (−51·3 to 33·9)	−3·0% (−40·4 to 51·3)
			Infertility due to puerperal sepsis	2073·0 (1839·6 to 2335·1)	113·0 (100·6 to 127·1)	10·4 (3·9 to 21·8)	39·6% (36·9 to 42·7)*	21·8% (19·2 to 24·3)*	−0·3% (−2·2 to 1·7)	6·6% (4·3 to 8·8)*
			Maternal sepsis and other maternal infection episode	916·5 (511·3 to 1514·1)	11 947·9 (9553·3 to 14 781·0)	46·6 (22·8 to 85·5)	−6·9% (−46·6 to 75·8)	2·9% (−42·8 to 75·5)	−24·5% (−56·7 to 42·2)	−4·8% (−47·1 to 61·2)
		Maternal hypertensive disorders		2849·8 (1890·6 to 3972·4)	15 830·2 (13 530·0 to 18 779·5)	143·1 (80·7 to 231·8)	−6·3% (−35·5 to 40·3)	−0·9% (−34·3 to 41·0)	−24·7% (−48·6 to 12·5)	−9·1% (−39·6 to 29·3)
			Maternal hypertensive disorder complications	102·0 (97·7 to 107·0)	1600·2 (1535·3 to 1675·1)	6·7 (4·1 to 10·2)	38·4% (31·0 to 46·7)*	17·9% (10·7 to 24·7)*	9·7% (3·8 to 16·4)*	7·3% (0·7 to 13·4)*
			Maternal hypertensive disorder episode	2747·7 (1789·8 to 3869·6)	14 230·0 (11 902·3 to 17 192·4)	136·4 (75·7 to 220·6)	−7·5% (−37·4 to 40·2)	−1·7% (−36·0 to 41·7)	−25·7% (−49·9 to 12·4)	−9·8% (−41·1 to 30·4)
		Maternal obstructed labour and uterine rupture		1232·6 (1048·2 to 1449·1)	7915·2 (6032·2 to 10 169·3)	397·6 (260·5 to 551·0)	−0·9% (−6·0 to 5·2)	−10·7% (−15·8 to −5·0)*	−24·9% (−28·6 to −20·6)*	−20·0% (−24·5 to −14·9)*
			Obstructed labour, acute event	105·1 (64·7 to 163·8)	7694·5 (5811·6 to 9959·4)	33·0 (16·8 to 55·9)	−9·9% (−42·7 to 51·8)	0·8% (−40·1 to 61·4)	−26·4% (−53·6 to 23·6)	−7·0% (−44·2 to 48·6)
			Maternal obstructed labour complications	1127·5 (939·1 to 1338·2)	220·7 (179·2 to 267·7)	364·6 (238·5 to 506·1)	−0·1% (−4·2 to 4·3)	−11·6% (−15·7 to −7·2)*	−24·8% (−27·7 to −21·6)*	−21·1% (−24·7 to −17·3)*
		Maternal abortive outcome		164·2 (109·5 to 234·1)	20 052·1 (16 262·0 to 25 281·8)	18·1 (10·2 to 29·7)	2·5% (−32·1 to 54·5)	2·4% (−34·5 to 51·8)	−18·0% (−45·6 to 24·0)	−5·6% (−39·4 to 39·1)
		Ectopic pregnancy		138·7 (90·1 to 199·9)	16 965·4 (13 647·7 to 20 936·5)	15·1 (8·3 to 24·0)	35·5% (−10·4 to 115·0)	14·6% (−26·3 to 72·1)	6·5% (−29·1 to 68·3)	5·1% (−32·4 to 58·5)
		Other maternal disorders		..	..	113·8 (80·5 to 154·3)	45·4% (32·0 to 60·0)*	20·1% (8·9 to 33·5)*	12·9% (3·0 to 23·8)*	9·0% (−1·0 to 21·2)
	Neonatal disorders			150 455·9 (131 734·1 to 170 851·4)	22 150·4 (20 975·1 to 23 700·8)	29 088·7 (21 771·2 to 37 499·1)	52·1% (47·2 to 57·4)*	23·5% (16·8 to 31·2)*	27·0% (22·9 to 31·3)*	12·1% (6·2 to 19·2)*
		Neonatal preterm birth		92 585·6 (81 049·6 to 106 597·3)	17 416·9 (17 182·7 to 17 646·4)	13 156·9 (9307·3 to 17 833·1)	46·0% (39·6 to 52·0)*	26·1% (10·6 to 41·4)*	22·2% (17·0 to 27·1)*	14·8% (0·7 to 28·6)*
			Preterm birth complications	67 508·3 (56 539·6 to 81 115·5)	..	12 537·7 (8824·7 to 17 161·8)	48·4% (41·1 to 55·5)*	27·3% (10·6 to 43·7)*	24·7% (18·7 to 30·8)*	16·0% (0·8 to 30·8)*
			Retinopathy of prematurity	11 361·8 (8979·6 to 13 871·7)	..	619·2 (415·3 to 861·4)	14·4% (10·0 to 18·7)*	6·1% (2·4 to 10·3)*	−8·6% (−12·3 to −5·0)*	−5·9% (−9·5 to −2·0)*
			Uncomplicated preterm birth	13 715·6 (12 775·0 to 14 383·3)	17 416·9 (17 182·7 to 17 646·4)	..	..	..	..	..
		Neonatal encephalopathy due to birth asphyxia and trauma		53 823·7 (39 817·7 to 70 951·7)	2753·4 (1840·4 to 4017·0)	9703·5 (5872·1 to 15 081·3)	69·8% (57·8 to 84·9)*	23·0% (19·3 to 27·6)*	39·5% (30·0 to 51·5)*	11·1% (7·7 to 14·9)*
		Neonatal sepsis and other neonatal infections		14 268·5 (8908·0 to 21 573·1)	1326·7 (792·7 to 2284·9)	4171·9 (2230·5 to 7009·0)	44·7% (39·1 to 50·6)*	19·5% (15·9 to 22·5)*	17·5% (13·0 to 22·1)*	7·5% (4·3 to 10·1)*
			Neonatal sepsis and other neonatal infection complications	13 944·6 (8561·4 to 21 248·8)	996·6 (472·4 to 1951·3)	4171·9 (2230·5 to 7009·0)	44·7% (39·1 to 50·6)*	19·5% (15·9 to 22·5)*	17·5% (13·0 to 22·1)*	7·5% (4·3 to 10·1)*
			Neonatal sepsis and other neonatal infection episode	323·9 (254·4 to 405·5)	330·1 (259·0 to 413·2)	..	..	..	..	..
		Haemolytic disease and other neonatal jaundice		2355·4 (2032·4 to 2684·1)	653·3 (269·5 to 1211·6)	877·7 (679·2 to 1090·3)	37·1% (33·2 to 40·9)*	20·2% (16·6 to 23·3)*	14·8% (11·4 to 17·9)*	9·3% (6·0 to 12·2)*
		Other neonatal disorders		..	..	1178·7 (893·2 to 1519·2)	35·3% (31·1 to 39·6)*	16·0% (9·2 to 23·8)*	35·6% (31·4 to 39·9)*	9·7% (3·3 to 17·2)*
	**Nutritional deficiencies**			**1 862 030·8 (1 806 258·9 to 1 921 493·5)**	**1 186 745·8 (1 089 728·9 to 1 283 530·0)**	**42 376·2 (28 774·0 to 61 009·9)**	−8·3% (−10·4 to −6·2)*	−7·8% (−10·6 to −4·9)*	−20·9% (−22·7 to −19·3)*	−16·1% (−18·5 to −13·4)*
	Protein-energy malnutrition			96 454·6 (93 495·9 to 99 611·1)	79 726·7 (76 183·7 to 83 392·8)	1798·0 (1167·7 to 2545·0)	−19·5% (−23·4 to −15·5)*	−3·4% (−6·3 to −0·7)*	−22·0% (−26·0 to −18·1)*	−10·1% (−12·8 to −7·6)*
	Iodine deficiency			116 021·9 (104 300·5 to 128 878·3)	3027·9 (2734·2 to 3363·7)	2057·7 (1247·3 to 3255·6)	−33·6% (−38·2 to −27·8)*	−6·9% (−10·1 to −4·3)*	−46·4% (−50·2 to −41·9)*	−17·4% (−20·1 to −14·9)*
		Visible goitre without symptoms		113 393·1 (101 868·1 to 126 335·9)	3027·9 (2734·2 to 3363·7)	1208·8 (590·5 to 2248·6)	−41·9% (−45·1 to −38·3)*	−6·1% (−8·2 to −4·4)*	−53·3% (−55·6 to −50·6)*	−17·4% (−19·2 to −15·9)*
		Visible goitre with complications		2628·8 (1500·7 to 3684·3)	..	848·9 (445·5 to 1286·3)	−17·0% (−22·5 to −11·1)*	−8·1% (−15·8 to −3·1)*	−32·7% (−37·2 to −27·6)*	−17·3% (−24·2 to −12·8)*
	Vitamin A deficiency			818 420·2 (748 849·9 to 892 688·1)	1 103 991·2 (1 007 169·1 to 1 199 987·2)	8313·0 (5398·6 to 12 150·6)	−19·7% (−22·9 to −16·7)*	−17·3% (−20·8 to −13·6)*	−23·0% (−26·0 to −20·2)*	−22·3% (−25·7 to −18·8)*
		Asymptomatic vitamin A deficiency		600 048·0 (547 539·5 to 655 650·3)	1 103 991·2 (1 007 169·1 to 1 199 987·2)	..	..	..	..	..
		Vitamin A deficiency complications		5403·4 (4329·4 to 6754·9)	..	304·0 (195·6 to 442·0)	23·8% (19·4 to 28·3)*	13·4% (9·9 to 17·1)*	8·8% (5·3 to 12·1)*	3·7% (0·6 to 7·1)*
		Vitamin A deficiency with anaemia		212 971·7 (189 376·4 to 240 436·1)	..	8009·0 (5196·4 to 11 716·1)	−20·5% (−23·7 to −17·4)*	−18·1% (−21·8 to −14·3)*	−23·6% (−26·7 to −20·7)*	−23·0% (−26·5 to −19·5)*
	Dietary iron deficiency			1 136 043·5 (1 117 204·2 to 1 156 043·7)	..	30 013·9 (20 323·3 to 43 628·0)	−0·2% (−2·8 to 2·2)	−5·2% (−8·3 to −1·9)*	−17·4% (−19·4 to −15·5)*	−14·3% (−17·2 to −11·4)*
	Other nutritional deficiencies			..	..	193·6 (125·3 to 272·6)	−13·7% (−20·8 to −7·2)*	2·2% (−2·6 to 6·2)	−25·8% (−32·2 to −19·9)*	−9·6% (−13·9 to −6·2)*
**Non-communicable diseases**				**7 011 916·8 (6 965 421·4 to 7 057 358·0)**	**10 813 562·6 (10 375 088·4 to 11 286 148·0)**	**678 294·4 (510 467·3 to 875 605·3)**	35·0% (34·4 to 35·7)*	19·3% (18·8 to 19·9)*	−1·3% (−1·7 to −0·9)*	**0·1% (−0·3 to 0·5)**
	**Neoplasms**			**100 482·9 (98 189·8 to 102 850·5)**	**24 361·6 (21 911·3 to 27 310·3)**	**7775·2 (5747·9 to 10 028·9)**	59·3% (55·6 to 63·3)*	40·6% (38·3 to 43·2)*	8·5% (6·0 to 11·2)*	9·7% (7·6 to 12·0)*
	Lip and oral cavity cancer			1631·9 (1570·3 to 1691·6)	389·8 (374·5 to 404·4)	163·3 (120·3 to 212·3)	49·6% (44·6 to 54·6)*	39·8% (34·0 to 45·1)*	1·1% (−2·3 to 4·4)	9·2% (4·7 to 13·4)*
		Diagnosis and primary therapy phase of mouth cancer		95·7 (92·1 to 99·1)	389·8 (374·5 to 404·4)	26·6 (18·0 to 36·6)	50·8% (44·8 to 57·1)*	40·1% (34·2 to 46·3)*	2·2% (−1·8 to 6·4)	9·8% (5·2 to 14·5)*
		Controlled phase of mouth cancer		1364·1 (1312·8 to 1414·1)	..	62·4 (40·8 to 89·4)	51·8% (46·5 to 56·9)*	41·0% (35·3 to 46·7)*	3·6% (0·0 to 6·8)*	11·2% (6·7 to 15·6)*
		Metastatic phase of mouth cancer		149·4 (143·4 to 154·9)	..	62·1 (42·6 to 81·2)	47·5% (41·0 to 54·6)*	38·5% (31·9 to 45·2)*	−1·0% (−5·3 to 3·6)	7·4% (2·3 to 12·5)*
		Terminal phase of mouth cancer		22·7 (21·8 to 23·6)	..	12·2 (8·5 to 15·7)	47·4% (42·5 to 51·7)*	39·5% (33·9 to 44·5)*	−1·4% (−4·4 to 1·4)	7·8% (3·4 to 11·6)*
	Nasopharynx cancer			508·7 (480·9 to 539·0)	109·8 (104·4 to 115·6)	52·3 (37·8 to 69·6)	6·6% (−1·2 to 14·3)	20·3% (13·7 to 28·1)*	−24·7% (−29·8 to −19·7)*	−1·6% (−7·0 to 4·5)
		Diagnosis and primary therapy phase of nasopharynx cancer		29·1 (27·5 to 30·9)	109·8 (104·4 to 115·6)	8·3 (5·6 to 11·7)	7·7% (−0·9 to 17·5)	20·3% (12·0 to 29·9)*	−23·4% (−29·4 to −16·9)*	−0·7% (−7·4 to 6·9)
		Controlled phase of nasopharynx cancer		427·7 (403·8 to 453·3)	..	20·3 (13·1 to 29·9)	6·7% (−1·8 to 15·3)	19·7% (12·1 to 28·1)*	−23·7% (−29·5 to −18·0)*	−0·7% (−6·9 to 6·0)
		Metastatic phase of nasopharynx cancer		46·2 (43·9 to 48·7)	..	20·6 (14·1 to 27·7)	6·5% (−1·3 to 14·9)	20·9% (13·3 to 29·5)*	−25·6% (−30·6 to −19·9)*	−2·6% (−8·6 to 4·2)
		Terminal phase of nasopharynx cancer		5·7 (5·4 to 5·9)	..	3·1 (2·1 to 4·0)	3·5% (−3·1 to 9·8)	20·7% (15·4 to 27·1)*	−27·8% (−32·2 to −23·8)*	−3·5% (−7·6 to 1·6)
	Other pharynx cancer			440·8 (399·7 to 462·4)	179·3 (160·3 to 188·6)	59·2 (42·3 to 77·6)	61·7% (54·3 to 70·4)*	44·3% (35·0 to 51·2)*	10·1% (5·1 to 15·8)*	11·7% (4·4 to 16·9)*
		Diagnosis and primary therapy phase of other pharynx cancer		24·6 (22·3 to 25·7)	179·3 (160·3 to 188·6)	7·0 (4·7 to 9·8)	69·9% (62·7 to 77·5)*	46·7% (38·2 to 53·0)*	15·7% (10·8 to 20·8)*	13·5% (7·0 to 18·4)*
		Controlled phase of other pharynx cancer		334·1 (304·3 to 350·1)	..	15·5 (10·0 to 22·9)	72·0% (63·3 to 81·8)*	46·5% (37·3 to 54·0)*	17·5% (11·7 to 24·1)*	13·8% (6·6 to 19·6)*
		Metastatic phase of other pharynx cancer		69·6 (62·2 to 73·2)	..	29·9 (20·4 to 39·7)	56·4% (48·1 to 66·2)*	42·7% (32·2 to 51·5)*	6·4% (0·6 to 12·8)*	10·3% (2·3 to 16·9)*
		Terminal phase of other pharynx cancer		12·6 (11·2 to 13·3)	..	6·8 (4·7 to 8·7)	56·7% (49·4 to 64·7)*	44·2% (34·9 to 51·0)*	6·6% (1·5 to 11·8)*	11·3% (4·1 to 16·6)*
	Oesophageal cancer			806·3 (782·1 to 829·8)	472·5 (459·5 to 485·3)	130·3 (94·5 to 166·4)	34·6% (29·1 to 40·2)*	19·0% (14·5 to 23·9)*	−9·1% (−12·7 to −5·3)*	−9·1% (−12·6 to −5·4)*
		Diagnosis and primary therapy phase of oesophageal cancer		67·0 (65·0 to 69·0)	472·5 (459·5 to 485·3)	18·4 (12·4 to 25·4)	45·2% (37·3 to 53·9)*	23·9% (17·5 to 31·3)*	−1·3% (−6·7 to 4·5)	−5·0% (−9·9 to 0·8)
		Controlled phase of oesophageal cancer		535·4 (518·8 to 552·0)	..	24·6 (16·0 to 35·8)	52·6% (45·9 to 58·7)*	26·1% (20·9 to 31·5)*	4·7% (0·1 to 8·8)*	−2·8% (−6·8 to 1·2)
		Metastatic phase of oesophageal cancer		167·5 (162·9 to 171·9)	..	68·2 (47·0 to 89·1)	28·6% (22·5 to 35·2)*	16·1% (10·5 to 21·8)*	−13·3% (−17·4 to −8·9)*	−11·6% (−15·8 to −7·3)*
		Terminal phase of oesophageal cancer		36·4 (35·4 to 37·4)	..	19·0 (13·4 to 24·3)	29·0% (21·9 to 36·6)*	16·7% (10·7 to 23·1)*	−13·1% (−17·9 to −8·2)*	−11·2% (−15·7 to −6·3)*
	Stomach cancer			2823·2 (2740·2 to 2914·9)	1220·7 (1189·0 to 1254·6)	348·8 (255·9 to 448·5)	17·6% (14·4 to 20·9)*	32·7% (28·1 to 37·7)*	−20·8% (−23·0 to −18·6)*	1·2% (−2·3 to 5·0)
		Diagnosis and primary therapy phase of stomach cancer		268·1 (260·2 to 276·6)	1220·7 (1189·0 to 1254·6)	70·1 (47·9 to 95·2)	22·1% (17·7 to 26·4)*	39·7% (34·3 to 45·6)*	−17·8% (−20·7 to −15·0)*	6·6% (2·5 to 11·1)*
		Controlled phase of stomach cancer		2122·2 (2057·1 to 2193·0)	..	96·9 (62·7 to 140·7)	31·4% (28·3 to 34·8)*	54·0% (48·8 to 59·4)*	−11·0% (−13·1 to −8·8)*	18·1% (14·2 to 22·2)*
		Metastatic phase of stomach cancer		344·3 (335·7 to 353·8)	..	137·3 (95·8 to 178·7)	11·2% (7·5 to 15·1)*	21·4% (16·9 to 26·2)*	−25·3% (−27·7 to −22·7)*	−7·4% (−10·8 to −3·9)*
		Terminal phase of stomach cancer		88·7 (86·5 to 91·2)	..	44·6 (31·3 to 56·9)	11·8% (7·7 to 15·9)*	21·1% (16·3 to 26·8)*	−24·8% (−27·6 to −22·0)*	−7·7% (−11·4 to −3·5)*
	Colon and rectum cancer			9352·3 (9143·0 to 9558·6)	1833·5 (1791·9 to 1873·5)	877·6 (650·2 to 1133·2)	70·2% (65·4 to 74·2)*	39·8% (35·9 to 43·6)*	12·3% (9·3 to 15·1)*	6·0% (3·1 to 9·0)*
		Diagnosis and primary therapy phase of colon and rectum cancers		401·1 (391·8 to 410·2)	1833·5 (1791·9 to 1873·5)	103·1 (70·4 to 139·0)	78·5% (72·3 to 84·4)*	43·5% (38·3 to 48·8)*	18·7% (14·7 to 22·5)*	9·4% (5·4 to 13·4)*
		Controlled phase of colon and rectum cancers		7946·7 (7764·5 to 8129·2)	..	453·5 (304·5 to 637·7)	83·2% (79·5 to 86·7)*	42·8% (38·9 to 47·0)*	22·9% (20·6 to 25·1)*	9·2% (6·3 to 12·3)*
		Metastatic phase of colon and rectum cancers		653·8 (639·1 to 667·7)	..	253·1 (176·5 to 327·7)	54·5% (49·5 to 59·3)*	35·5% (31·2 to 40·2)*	0·6% (−2·4 to 3·5)	1·8% (−1·4 to 5·2)
		Terminal phase of colon and rectum cancers		92·9 (90·8 to 94·9)	..	45·9 (32·6 to 57·9)	48·8% (42·5 to 54·6)*	33·5% (28·3 to 38·2)*	−2·9% (−6·8 to 0·7)	0·2% (−3·7 to 3·8)
		Stoma from colon and rectum cancers, beyond 10 years		257·8 (249·3 to 266·6)	..	22·1 (15·3 to 30·0)	52·3% (47·4 to 57·0)*	28·3% (25·1 to 31·6)*	−3·5% (−6·5 to −0·6)*	−5·2% (−7·6 to −2·9)*
	Liver cancer			803·4 (753·1 to 856·7)	953·1 (916·5 to 997·0)	229·5 (163·9 to 301·7)	52·2% (44·8 to 60·5)*	39·8% (34·2 to 47·3)*	4·4% (−0·6 to 9·9)	8·1% (3·8 to 13·7)*
		Liver cancer due to hepatitis B		661·2 (617·3 to 713·0)	404·0 (378·3 to 434·1)	98·1 (69·9 to 129·6)	42·1% (34·2 to 52·6)*	38·1% (30·2 to 48·7)*	−1·5% (−7·0 to 5·6)	8·6% (2·5 to 17·0)*
			Diagnosis and primary therapy phase of liver cancer due to hepatitis B	97·9 (91·6 to 105·1)	404·0 (378·3 to 434·1)	26·6 (18·0 to 37·2)	40·5% (29·7 to 53·4)*	35·2% (24·8 to 48·0)*	−2·5% (−9·9 to 6·5)	6·4% (−1·6 to 16·5)
			Controlled phase of liver cancer due to hepatitis B	453·6 (422·8 to 490·7)	..	21·3 (13·7 to 31·5)	61·5% (50·8 to 75·1)*	70·3% (58·3 to 84·7)*	12·0% (4·8 to 21·1)*	35·0% (25·4 to 46·4)*
			Metastatic phase of liver cancer due to hepatitis B	78·5 (73·5 to 84·4)	..	33·5 (22·9 to 44·7)	37·4% (27·3 to 50·4)*	29·1% (19·7 to 40·9)*	−4·7% (−11·8 to 4·0)	1·5% (−6·0 to 10·7)
			Terminal phase of liver cancer due to hepatitis B	31·3 (29·3 to 33·6)	..	16·7 (11·5 to 21·7)	37·9% (31·2 to 47·0)*	29·3% (22·3 to 39·6)*	−4·4% (−9·0 to 1·7)	1·5% (−4·0 to 9·5)
		Liver cancer due to hepatitis C		366·5 (341·7 to 391·6)	257·9 (241·3 to 274·5)	60·6 (43·0 to 79·7)	62·2% (54·8 to 68·9)*	38·8% (34·3 to 44·5)*	9·7% (4·6 to 14·2)*	4·9% (1·5 to 9·2)*
			Diagnosis and primary therapy phase of liver cancer due to hepatitis C	60·8 (56·9 to 64·7)	257·9 (241·3 to 274·5)	16·8 (11·3 to 23·4)	61·1% (53·2 to 68·2)*	37·3% (31·9 to 43·9)*	8·8% (3·5 to 13·5)*	3·7% (−0·3 to 8·7)
			Controlled phase of liver cancer due to hepatitis C	234·4 (217·9 to 250·9)	..	10·9 (6·9 to 15·9)	82·3% (72·8 to 92·3)*	56·4% (48·6 to 65·8)*	25·9% (19·4 to 32·6)*	19·6% (13·6 to 26·7)*
			Metastatic phase of liver cancer due to hepatitis C	51·0 (47·7 to 54·3)	..	22·1 (14·9 to 29·5)	57·6% (49·8 to 64·5)*	34·4% (29·1 to 40·1)*	6·2% (1·0 to 10·8)*	1·4% (−2·6 to 5·7)
			Terminal phase of liver cancer due to hepatitis C	20·3 (19·0 to 21·6)	..	10·9 (7·6 to 14·1)	58·4% (51·0 to 64·7)*	34·7% (30·9 to 39·7)*	6·7% (1·8 to 10·7)*	1·5% (−1·4 to 5·2)
		Liver cancer due to alcohol use		206·3 (180·7 to 239·9)	143·9 (127·2 to 165·0)	34·7 (24·8 to 47·1)	62·6% (52·2 to 70·8)*	39·4% (33·7 to 46·1)*	9·6% (2·7 to 15·0)*	7·2% (2·9 to 12·1)*
			Diagnosis and primary therapy phase of liver cancer due to alcohol use	34·0 (30·0 to 39·1)	143·9 (127·2 to 165·0)	9·7 (6·4 to 13·9)	61·1% (50·9 to 69·6)*	38·5% (32·0 to 45·2)*	8·5% (1·7 to 14·1)*	6·4% (1·7 to 11·4)*
			Controlled phase of liver cancer due to alcohol use	132·6 (115·1 to 155·3)	..	6·2 (3·8 to 9·2)	88·0% (74·5 to 101·3)*	55·3% (45·5 to 65·6)*	28·8% (19·5 to 37·8)*	20·5% (13·1 to 28·5)*
			Metastatic phase of liver cancer due to alcohol use	28·4 (25·2 to 32·6)	..	12·7 (8·6 to 17·4)	57·1% (47·4 to 65·0)*	35·4% (29·9 to 41·7)*	5·7% (−0·7 to 10·8)	3·9% (−0·2 to 8·5)
			Terminal phase of liver cancer due to alcohol use	11·3 (10·0 to 13·0)	..	6·1 (4·2 to 8·1)	57·4% (47·4 to 65·1)*	35·3% (30·3 to 41·2)*	5·9% (−0·8 to 10·8)	3·8% (0·2 to 8·2)*
		Liver cancer due to NASH		97·4 (86·8 to 108·0)	72·2 (64·6 to 79·9)	17·5 (12·3 to 23·1)	74·4% (63·9 to 83·0)*	52·5% (47·5 to 58·9)*	18·2% (11·2 to 23·6)*	16·1% (12·4 to 20·9)*
			Diagnosis and primary therapy phase of liver cancer due to NASH	16·9 (15·1 to 18·6)	72·2 (64·6 to 79·9)	4·8 (3·2 to 6·7)	73·3% (62·6 to 81·8)*	51·4% (46·5 to 57·5)*	17·3% (10·3 to 22·7)*	15·2% (11·6 to 19·8)*
			Controlled phase of liver cancer due to NASH	60·5 (53·5 to 67·4)	..	2·9 (1·8 to 4·4)	93·7% (82·0 to 103·9)*	75·6% (66·7 to 85·7)*	34·2% (26·1 to 40·9)*	35·6% (28·9 to 43·7)*
			Metastatic phase of liver cancer due to NASH	14·3 (12·8 to 15·9)	..	6·5 (4·4 to 8·7)	70·7% (60·0 to 79·0)*	47·3% (42·7 to 53·3)*	15·3% (8·4 to 20·6)*	11·9% (8·4 to 16·3)*
			Terminal phase of liver cancer due to NASH	5·7 (5·1 to 6·3)	..	3·2 (2·2 to 4·2)	70·7% (60·0 to 79·0)*	47·3% (42·6 to 53·3)*	15·3% (8·3 to 20·6)*	11·9% (8·4 to 16·3)*
		Liver cancer due to other causes		114·8 (102·2 to 128·7)	75·1 (67·5 to 83·3)	18·6 (13·2 to 25·1)	43·9% (36·4 to 52·0)*	42·3% (36·1 to 50·4)*	−0·3% (−5·4 to 5·0)	11·0% (6·5 to 17·2)*
			Diagnosis and primary therapy phase of liver cancer due to other causes	18·0 (16·1 to 20·0)	75·1 (67·5 to 83·3)	5·2 (3·5 to 7·3)	42·7% (35·2 to 50·5)*	40·6% (34·9 to 47·9)*	−1·1% (−6·0 to 4·0)	9·7% (5·4 to 15·3)*
			Controlled phase of liver cancer due to other causes	76·3 (67·5 to 85·8)	..	3·7 (2·3 to 5·5)	61·3% (51·7 to 72·7)*	68·1% (56·9 to 81·0)*	12·3% (5·8 to 20·1)*	32·7% (23·7 to 42·5)*
			Metastatic phase of liver cancer due to other causes	14·7 (13·2 to 16·3)	..	6·6 (4·5 to 9·0)	40·0% (32·7 to 47·7)*	35·3% (30·0 to 42·2)*	−3·1% (−7·8 to 2·0)	5·4% (1·4 to 10·6)*
			Terminal phase of liver cancer due to other causes	5·8 (5·3 to 6·5)	..	3·2 (2·2 to 4·1)	40·0% (32·7 to 47·7)*	35·3% (30·0 to 42·2)*	−3·1% (−7·8 to 2·0)	5·4% (1·4 to 10·6)*
	Gallbladder and biliary tract cancer			235·9 (209·6 to 252·6)	210·9 (186·1 to 225·4)	49·1 (34·4 to 64·3)	33·4% (23·2 to 38·9)*	34·6% (28·1 to 41·4)*	−12·1% (−18·8 to −8·5)*	1·4% (−3·7 to 6·8)
		Diagnosis and primary therapy phase of gallbladder and biliary tract cancer		33·7 (29·9 to 35·9)	210·9 (186·1 to 225·4)	9·3 (6·2 to 12·9)	34·1% (24·3 to 39·7)*	35·5% (28·5 to 42·6)*	−11·7% (−18·1 to −7·9)*	2·1% (−3·3 to 7·8)
		Controlled phase of gallbladder and biliary tract cancer		126·7 (113·2 to 136·4)	..	5·8 (3·7 to 8·5)	36·9% (28·6 to 44·3)*	53·1% (42·0 to 64·3)*	−8·1% (−13·8 to −3·1)*	17·5% (8·9 to 26·4)*
		Metastatic phase of gallbladder and biliary tract cancer		58·6 (51·7 to 62·7)	..	25·0 (16·8 to 33·4)	32·9% (21·7 to 39·8)*	31·7% (25·3 to 38·3)*	−12·6% (−20·0 to −8·0)*	−1·0% (−6·1 to 4·3)
		Terminal phase of gallbladder and biliary tract cancer		16·9 (14·9 to 18·1)	..	8·9 (6·2 to 11·6)	32·3% (21·9 to 37·2)*	31·4% (25·4 to 37·6)*	−13·3% (−20·0 to −9·8)*	−1·4% (−6·0 to 3·6)
	Pancreatic cancer			380·6 (372·7 to 388·4)	447·7 (438·6 to 456·3)	91·9 (64·7 to 119·3)	65·8% (62·2 to 69·6)*	38·5% (34·8 to 42·2)*	9·8% (7·3 to 12·4)*	4·7% (1·8 to 7·6)*
		Diagnosis and primary therapy phase of pancreatic cancer		99·0 (97·0 to 101·0)	447·7 (438·6 to 456·3)	26·5 (18·1 to 36·2)	65·4% (60·1 to 70·8)*	38·7% (33·2 to 43·5)*	9·6% (6·0 to 13·2)*	4·8% (0·6 to 8·4)*
		Controlled phase of pancreatic cancer		151·7 (148·4 to 155·0)	..	7·1 (4·6 to 10·3)	102·1% (96·0 to 109·5)*	35·1% (30·2 to 40·4)*	37·5% (33·3 to 42·6)*	5·1% (1·4 to 9·2)*
		Metastatic phase of pancreatic cancer		93·2 (91·3 to 95·0)	..	38·9 (26·7 to 51·3)	62·5% (57·6 to 67·8)*	39·1% (34·2 to 44·0)*	7·7% (4·3 to 11·2)*	4·8% (1·0 to 8·6)*
		Terminal phase of pancreatic cancer		36·7 (35·9 to 37·4)	..	19·4 (13·6 to 24·8)	62·1% (59·3 to 65·0)*	38·6% (34·9 to 41·9)*	6·6% (4·6 to 8·6)*	4·4% (1·6 to 7·0)*
	Larynx cancer			1094·9 (1074·7 to 1118·4)	210·6 (206·4 to 215·5)	109·4 (79·7 to 142·5)	25·1% (21·7 to 28·5)*	34·2% (30·6 to 37·9)*	−14·9% (−17·2 to −12·6)*	2·8% (0·0 to 5·6)*
		Diagnosis and primary therapy phase of larynx cancer		65·3 (64·1 to 66·8)	210·6 (206·4 to 215·5)	18·1 (12·3 to 24·9)	27·6% (22·0 to 33·4)*	38·1% (32·8 to 43·5)*	−13·1% (−17·0 to −9·1)*	5·8% (1·7 to 9·9)*
		Controlled phase of larynx cancer		882·3 (864·9 to 902·3)	..	48·7 (32·0 to 70·1)	28·2% (24·5 to 31·8)*	36·5% (32·5 to 40·5)*	−12·3% (−14·7 to −9·8)*	4·9% (1·9 to 8·0)*
		Metastatic phase of larynx cancer		79·7 (78·1 to 81·6)	..	33·8 (23·2 to 44·7)	19·9% (14·9 to 24·9)*	30·8% (25·3 to 36·1)*	−18·8% (−22·2 to −15·5)*	−0·1% (−4·3 to 4·0)
		Terminal phase of larynx cancer		11·3 (11·0 to 11·6)	..	6·1 (4·2 to 7·8)	16·3% (13·9 to 19·0)*	28·2% (25·1 to 31·3)*	−21·3% (−22·9 to −19·6)*	−2·2% (−4·5 to 0·2)
		Laryngectomy from larynx cancer, beyond 10 years		56·3 (54·4 to 58·2)	..	2·7 (1·7 to 4·1)	48·5% (43·6 to 53·7)*	25·6% (21·0 to 30·3)*	−0·8% (−4·3 to 2·7)	−4·5% (−8·0 to −0·9)*
	Tracheal, bronchus, and lung cancer			3343·1 (3267·9 to 3422·5)	2163·1 (2117·0 to 2212·9)	537·1 (393·6 to 677·2)	51·6% (47·5 to 55·5)*	41·0% (36·8 to 45·4)*	2·9% (0·1 to 5·5)*	7·2% (3·9 to 10·4)*
		Diagnosis and primary therapy phase of tracheal, bronchus, and lung cancer		219·5 (214·6 to 224·8)	2163·1 (2117·0 to 2212·9)	57·0 (39·1 to 77·4)	64·3% (58·4 to 69·9)*	51·1% (45·1 to 57·5)*	11·4% (7·3 to 15·2)*	14·9% (10·3 to 19·7)*
		Controlled phase of tracheal, bronchus, and lung cancer		2188·1 (2137·3 to 2241·4)	..	99·3 (64·4 to 144·3)	87·5% (83·3 to 91·7)*	65·8% (61·2 to 70·5)*	28·0% (25·1 to 30·7)*	26·4% (22·9 to 30·0)*
		Metastatic phase of tracheal, bronchus, and lung cancer		765·7 (749·5 to 783·3)	..	299·0 (208·3 to 387·8)	44·3% (40·1 to 48·2)*	34·5% (30·1 to 38·7)*	−2·0% (−4·8 to 0·5)	2·2% (−1·1 to 5·3)
		Terminal phase of tracheal, bronchus, and lung cancer		169·8 (166·2 to 173·7)	..	81·8 (58·1 to 103·2)	44·3% (38·9 to 49·6)*	34·0% (28·8 to 39·6)*	−2·2% (−5·7 to 1·3)	1·8% (−2·1 to 6·0)
	Malignant skin melanoma			2324·4 (1794·8 to 2796·2)	308·7 (237·6 to 365·9)	140·9 (90·8 to 201·6)	104·3% (82·1 to 111·6)*	32·3% (27·0 to 36·9)*	41·3% (24·4 to 46·9)*	4·9% (0·9 to 8·6)*
		Diagnosis and primary therapy phase of malignant skin melanoma		64·1 (49·0 to 76·8)	308·7 (237·6 to 365·9)	17·5 (11·0 to 25·2)	110·3% (89·4 to 118·3)*	32·4% (26·1 to 38·8)*	46·4% (30·5 to 52·6)*	5·4% (0·5 to 10·6)*
		Controlled phase of malignant skin melanoma		2208·7 (1709·0 to 2662·4)	..	101·2 (59·9 to 154·2)	113·8% (93·9 to 121·4)*	32·9% (27·7 to 37·9)*	50·2% (34·9 to 55·4)*	6·4% (2·3 to 10·7)*
		Metastatic phase of malignant skin melanoma		44·3 (32·7 to 49·6)	..	18·4 (11·3 to 25·1)	68·4% (40·0 to 80·0)*	29·5% (22·6 to 35·5)*	12·5% (−7·3 to 20·9)	−1·6% (−6·9 to 3·2)
		Terminal phase of malignant skin melanoma		7·3 (5·4 to 8·2)	..	3·9 (2·4 to 5·2)	64·2% (37·2 to 73·7)*	28·7% (21·6 to 33·9)*	9·0% (−9·7 to 15·6)	−2·6% (−7·8 to 1·3)
	Non-melanoma skin cancer			2537·1 (1666·4 to 3696·8)	7663·6 (5251·1 to 10 570·3)	90·2 (49·5 to 149·0)	211·2% (142·3 to 305·0)*	32·7% (25·3 to 40·0)*	96·8% (46·7 to 162·7)*	−2·0% (−7·7 to 3·9)
		Non-melanoma skin cancer (squamous-cell carcinoma)		2158·9 (1294·8 to 3255·8)	1778·8 (1068·8 to 2620·9)	87·7 (46·9 to 146·3)	221·7% (148·5 to 334·5)*	32·8% (25·0 to 40·0)*	102·5% (47·3 to 180·3)*	−2·0% (−7·8 to 4·0)
		Non-melanoma skin cancer (basal-cell carcinoma)		596·8 (325·9 to 947·0)	5884·8 (3702·9 to 8742·9)	2·5 (0·9 to 5·2)	44·5% (20·3 to 76·5)*	30·8% (23·1 to 37·0)*	−3·8% (−21·1 to 18·1)	−1·0% (−6·4 to 3·2)
	Breast cancer			16 697·3 (16 178·9 to 17 171·7)	1960·7 (1891·4 to 2023·2)	1307·9 (932·0 to 1769·3)	67·7% (60·6 to 72·7)*	33·8% (29·0 to 37·7)*	11·7% (7·1 to 15·0)*	4·5% (0·7 to 7·5)*
		Diagnosis and primary therapy phase of breast cancer		416·5 (401·4 to 430·2)	1960·7 (1891·4 to 2023·2)	107·8 (75·1 to 146·7)	71·1% (61·6 to 79·5)*	36·1% (29·4 to 42·2)*	15·1% (9·0 to 20·4)*	7·3% (2·2 to 11·9)*
		Controlled phase of breast cancer		13 532·2 (13 027·8 to 13 977·4)	..	843·4 (538·9 to 1218·3)	74·5% (66·8 to 79·7)*	34·4% (29·3 to 38·7)*	17·7% (12·9 to 21·0)*	6·0% (2·1 to 9·3)*
		Metastatic phase of breast cancer		676·3 (654·5 to 697·0)	..	263·8 (183·5 to 341·1)	57·5% (49·2 to 64·5)*	33·4% (28·0 to 38·2)*	2·8% (−2·1 to 7·0)	2·3% (−1·8 to 5·8)
		Terminal phase of breast cancer		52·0 (50·4 to 54·0)	..	26·9 (18·8 to 34·2)	49·6% (40·4 to 57·4)*	31·4% (25·7 to 36·4)*	−2·1% (−7·7 to 2·8)	0·5% (−3·9 to 4·4)
		Mastectomy from breast cancer, beyond 10 years		2020·3 (1957·2 to 2080·1)	..	66·0 (38·5 to 102·5)	38·5% (35·8 to 41·3)*	25·8% (23·9 to 27·7)*	−9·5% (−11·2 to −7·8)*	−6·2% (−7·5 to −4·7)*
	Cervical cancer			3657·9 (3364·6 to 3803·1)	601·2 (554·5 to 625·4)	288·1 (205·6 to 381·5)	24·2% (12·3 to 32·3)*	18·9% (12·6 to 23·2)*	−14·2% (−22·3 to −8·7)*	−1·7% (−6·8 to 1·8)
		Diagnosis and primary therapy phase of cervical cancer		187·6 (172·7 to 195·1)	601·2 (554·5 to 625·4)	51·4 (34·5 to 70·5)	26·3% (13·6 to 35·9)*	19·2% (12·2 to 25·5)*	−12·2% (−21·1 to −6·0)*	−0·8% (−6·7 to 4·2)
		Controlled phase of cervical cancer		3275·4 (3011·5 to 3405·2)	..	151·8 (96·7 to 222·6)	25·7% (13·5 to 34·1)*	19·7% (13·5 to 24·1)*	−12·4% (−20·8 to −6·8)*	0·1% (−5·2 to 3·7)
		Metastatic phase of cervical cancer		171·3 (158·6 to 178·3)	..	72·2 (48·9 to 95·3)	20·9% (10·1 to 29·0)*	17·4% (10·9 to 22·9)*	−17·6% (−24·7 to −12·4)*	−5·1% (−10·3 to −0·6)*
		Terminal phase of cervical cancer		23·6 (21·9 to 24·6)	..	12·8 (8·8 to 16·4)	17·5% (7·5 to 24·9)*	17·4% (11·7 to 21·6)*	−20·4% (−27·0 to −15·7)*	−6·4% (−10·9 to −3·0)*
	Uterine cancer			3084·6 (3005·7 to 3171·3)	406·8 (396·7 to 418·0)	210·9 (151·5 to 279·7)	65·8% (60·6 to 71·6)*	36·5% (32·7 to 41·1)*	11·0% (7·5 to 14·9)*	4·9% (1·9 to 8·2)*
		Diagnosis and primary therapy phase of uterine cancer		141·8 (138·2 to 145·8)	406·8 (396·7 to 418·0)	37·5 (25·9 to 51·1)	69·1% (60·6 to 78·2)*	37·4% (30·9 to 44·1)*	13·5% (7·8 to 19·7)*	5·9% (1·0 to 10·9)*
		Controlled phase of uterine cancer		2838·0 (2765·2 to 2918·0)	..	129·3 (84·2 to 188·0)	72·5% (67·5 to 78·1)*	38·3% (34·6 to 43·0)*	16·6% (13·3 to 20·2)*	6·9% (4·1 to 10·5)*
		Metastatic phase of uterine cancer		94·9 (92·6 to 97·4)	..	38·8 (26·7 to 50·7)	48·4% (40·9 to 56·2)*	31·1% (25·7 to 37·3)*	−2·3% (−7·2 to 2·9)	−1·0% (−5·2 to 3·8)
		Terminal phase of uterine cancer		9·9 (9·7 to 10·2)	..	5·3 (3·7 to 6·8)	42·2% (38·7 to 46·5)*	29·0% (25·9 to 33·0)*	−6·6% (−8·8 to −3·9)*	−3·1% (−5·4 to −0·1)*
	Ovarian cancer			1353·0 (1313·7 to 1401·1)	286·1 (278·1 to 295·3)	176·1 (127·9 to 224·2)	48·9% (42·2 to 56·6)*	27·7% (22·9 to 32·9)*	1·6% (−2·6 to 6·5)	2·2% (−1·6 to 6·4)
		Diagnosis and primary therapy phase of ovarian cancer		48·1 (46·7 to 49·8)	286·1 (278·1 to 295·3)	13·8 (9·3 to 19·2)	51·7% (45·6 to 58·8)*	28·4% (23·9 to 33·0)*	4·4% (0·5 to 8·9)*	3·7% (0·1 to 7·4)*
		Controlled phase of ovarian cancer		1027·2 (997·4 to 1064·3)	..	47·3 (30·3 to 69·3)	52·7% (45·2 to 60·7)*	28·6% (23·2 to 33·9)*	5·8% (0·9 to 11·0)*	4·5% (0·2 to 8·9)*
		Metastatic phase of ovarian cancer		261·4 (253·9 to 270·1)	..	106·2 (72·2 to 139·2)	47·4% (40·1 to 55·7)*	27·2% (21·3 to 33·5)*	0·1% (−4·7 to 5·5)	1·2% (−3·5 to 6·3)
		Terminal phase of ovarian cancer		16·3 (15·8 to 16·8)	..	8·8 (6·1 to 11·2)	42·6% (37·6 to 48·5)*	29·0% (25·2 to 32·9)*	−4·6% (−7·6 to −1·0)*	−0·1% (−2·9 to 3·1)
	Prostate cancer			9901·9 (8810·8 to 12 762·0)	1334·3 (1170·9 to 1697·9)	843·2 (614·4 to 1146·4)	97·5% (87·3 to 125·6)*	41·0% (36·3 to 50·9)*	27·7% (20·5 to 46·5)*	5·6% (2·1 to 13·2)*
		Diagnosis and primary therapy phase of prostate cancer		342·9 (303·1 to 446·1)	1334·3 (1170·9 to 1697·9)	86·1 (57·9 to 121·6)	115·8% (103·5 to 149·5)*	44·0% (37·3 to 56·3)*	42·5% (34·4 to 65·2)*	8·6% (3·6 to 17·9)*
		Controlled phase of prostate cancer		8443·6 (7481·1 to 11 011·9)	..	476·0 (309·6 to 698·6)	123·7% (113·6 to 156·9)*	43·3% (38·4 to 54·7)*	49·5% (42·7 to 72·3)*	8·3% (4·5 to 17·1)*
		Metastatic phase of prostate cancer		653·8 (568·1 to 817·9)	..	244·6 (168·2 to 321·2)	63·3% (56·2 to 86·0)*	36·4% (31·6 to 44·7)*	2·9% (−1·5 to 17·2)	1·1% (−2·4 to 7·5)
		Terminal phase of prostate cancer		39·7 (34·2 to 48·1)	..	18·5 (13·0 to 23·8)	57·9% (47·9 to 75·1)*	35·1% (28·7 to 43·8)*	−0·7% (−6·9 to 10·3)	−0·5% (−5·1 to 6·2)
		Impotence and incontinence after 10-year survival from prostate cancer		421·9 (411·4 to 432·6)	..	18·1 (12·6 to 25·0)	61·8% (57·7 to 66·2)*	35·0% (31·2 to 38·9)*	0·5% (−1·9 to 3·1)	−1·6% (−4·2 to 1·1)
	Testicular cancer			578·0 (556·4 to 603·6)	71·3 (68·8 to 74·4)	36·2 (24·8 to 49·6)	51·2% (44·3 to 58·7)*	23·8% (18·3 to 29·5)*	14·7% (9·4 to 20·4)*	9·0% (4·1 to 14·1)*
		Diagnosis and primary therapy phase of testicular cancer		18·9 (18·2 to 19·7)	71·3 (68·8 to 74·4)	5·4 (3·6 to 7·5)	55·1% (48·5 to 62·3)*	24·9% (19·2 to 30·8)*	18·2% (13·3 to 23·5)*	10·4% (5·4 to 15·6)*
		Controlled phase of testicular cancer		549·1 (528·5 to 573·5)	..	26·1 (16·8 to 38·7)	55·3% (47·5 to 64·2)*	25·0% (19·0 to 31·4)*	18·7% (12·7 to 25·4)*	10·7% (5·2 to 16·2)*
		Metastatic phase of testicular cancer		9·0 (8·7 to 9·4)	..	4·1 (2·8 to 5·4)	31·0% (26·1 to 35·8)*	16·9% (13·1 to 21·2)*	−3·8% (−7·2 to −0·3)*	−0·3% (−3·5 to 3·4)
		Terminal phase of testicular cancer		1·0 (1·0 to 1·1)	..	0·6 (0·4 to 0·7)	22·7% (17·7 to 27·7)*	9·9% (6·2 to 14·3)*	−8·8% (−12·4 to −5·2)*	−5·7% (−8·8 to −1·8)*
	Kidney cancer			2184·1 (2081·8 to 2249·0)	393·0 (371·2 to 404·6)	141·0 (101·2 to 190·1)	50·2% (44·0 to 58·6)*	24·1% (18·8 to 28·9)*	7·2% (3·5 to 12·3)*	−2·2% (−6·3 to 1·6)
		Diagnosis and primary therapy phase of kidney cancer		..	393·0 (371·2 to 404·6)	..	..	..	..	..
		Controlled phase of kidney cancer		2080·7 (1985·3 to 2143·1)	..	95·6 (62·2 to 139·5)	47·6% (39·9 to 57·7)*	22·6% (16·8 to 27·7)*	8·2% (3·8 to 14·7)*	−2·0% (−6·7 to 2·0)
		Metastatic phase of kidney cancer		86·7 (81·4 to 89·3)	..	36·4 (24·6 to 48·3)	55·9% (49·4 to 63·7)*	27·2% (21·8 to 33·1)*	5·1% (0·6 to 10·3)*	−2·6% (−6·7 to 1·9)
		Terminal phase of kidney cancer		16·7 (15·7 to 17·2)	..	9·0 (6·3 to 11·6)	57·3% (52·5 to 62·9)*	27·0% (23·3 to 31·5)*	5·8% (2·7 to 9·3)*	−3·0% (−5·8 to 0·4)
	Bladder cancer			2632·4 (2566·9 to 2717·2)	473·8 (462·2 to 491·8)	247·0 (179·4 to 322·8)	47·3% (43·4 to 53·6)*	33·0% (29·9 to 36·2)*	−1·9% (−4·6 to 2·2)	0·9% (−1·4 to 3·3)
		Diagnosis and primary therapy phase of bladder cancer		141·8 (138·1 to 146·6)	473·8 (462·2 to 491·8)	37·4 (25·4 to 50·8)	47·4% (41·0 to 56·0)*	34·2% (29·3 to 39·3)*	−1·3% (−5·4 to 4·2)	2·2% (−1·5 to 6·0)
		Controlled phase of bladder cancer		2269·1 (2209·3 to 2343·1)	..	139·9 (95·0 to 195·4)	50·9% (46·7 to 58·3)*	34·1% (30·8 to 37·6)*	2·0% (−0·8 to 6·6)	2·7% (0·1 to 5·3)*
		Metastatic phase of bladder cancer		121·5 (118·5 to 126·6)	..	48·2 (33·4 to 63·1)	37·7% (31·9 to 44·9)*	29·9% (25·5 to 34·6)*	−10·3% (−14·0 to −5·9)*	−3·1% (−6·4 to 0·3)
		Terminal phase of bladder cancer		22·6 (22·0 to 23·5)	..	11·6 (8·1 to 14·8)	38·0% (32·5 to 45·2)*	29·5% (25·2 to 34·1)*	−10·4% (−14·0 to −5·8)*	−3·5% (−6·8 to 0·0)
		Incontinence from bladder cancer, beyond 10 years		77·5 (74·8 to 80·5)	..	10·0 (6·8 to 14·1)	63·5% (57·7 to 69·4)*	32·3% (27·9 to 37·3)*	5·2% (1·6 to 9·2)*	−0·8% (−4·1 to 2·9)
	Brain and nervous system cancer			1705·7 (1471·0 to 1894·8)	405·2 (351·0 to 442·6)	166·9 (117·5 to 223·0)	56·9% (34·3 to 85·1)*	49·9% (41·3 to 58·9)*	19·1% (2·2 to 37·8)*	24·8% (17·7 to 32·8)*
		Diagnosis and primary therapy phase of brain and nervous system cancers		100·0 (86·4 to 110·7)	405·2 (351·0 to 442·6)	28·6 (19·0 to 40·4)	76·5% (51·1 to 107·8)*	61·2% (51·3 to 71·1)*	34·8% (15·7 to 55·6)*	35·4% (27·5 to 44·1)*
		Controlled phase of brain and nervous system cancers		1453·1 (1251·8 to 1625·0)	..	68·5 (43·3 to 102·1)	72·1% (45·5 to 109·1)*	61·9% (50·1 to 74·9)*	35·8% (16·1 to 61·4)*	38·0% (28·0 to 49·2)*
		Metastatic phase of brain and nervous system cancers		130·1 (112·7 to 139·8)	..	57·7 (38·4 to 78·2)	41·8% (22·1 to 62·1)*	36·5% (30·0 to 42·0)*	4·9% (−9·4 to 17·6)	10·9% (5·6 to 15·2)*
		Terminal phase of brain and nervous system cancers		22·6 (19·6 to 24·3)	..	12·2 (8·4 to 15·9)	38·7% (18·9 to 59·0)*	34·3% (27·8 to 39·3)*	3·0% (−10·8 to 15·6)	8·8% (3·7 to 12·9)*
	Thyroid cancer			2144·9 (2059·5 to 2287·8)	255·5 (245·7 to 272·5)	132·0 (91·7 to 180·8)	95·1% (87·1 to 104·5)*	37·7% (32·4 to 44·0)*	33·4% (27·9 to 39·2)*	11·4% (7·1 to 16·5)*
		Diagnosis and primary therapy phase of thyroid cancer		57·8 (55·5 to 61·6)	255·5 (245·7 to 272·5)	16·6 (11·1 to 23·1)	99·5% (91·6 to 109·7)*	38·5% (32·3 to 45·3)*	37·5% (32·6 to 43·9)*	12·7% (7·6 to 18·1)*
		Controlled phase of thyroid cancer		2040·7 (1958·4 to 2176·9)	..	94·5 (61·6 to 138·2)	101·9% (93·0 to 113·0)*	38·1% (32·4 to 45·2)*	40·1% (34·3 to 46·8)*	12·9% (8·2 to 18·7)*
		Metastatic phase of thyroid cancer		42·9 (41·6 to 45·4)	..	19·0 (12·9 to 25·3)	67·5% (60·9 to 73·2)*	35·7% (31·4 to 40·1)*	9·9% (5·2 to 13·3)*	4·3% (1·1 to 7·7)*
		Terminal phase of thyroid cancer		3·5 (3·4 to 3·8)	..	1·9 (1·3 to 2·5)	63·9% (57·0 to 70·0)*	34·7% (30·4 to 39·1)*	8·6% (4·2 to 12·1)*	3·9% (0·6 to 7·3)*
	Mesothelioma			78·6 (76·1 to 81·1)	34·6 (33·5 to 35·7)	14·9 (10·6 to 19·7)	33·7% (17·7 to 56·9)*	23·9% (16·2 to 30·9)*	−10·0% (−20·4 to 5·2)	−4·6% (−10·3 to 0·7)
		Diagnosis and primary therapy phase of mesothelioma		9·1 (8·8 to 9·4)	34·6 (33·5 to 35·7)	2·6 (1·7 to 3·6)	35·5% (20·0 to 58·2)*	24·6% (17·0 to 31·2)*	−8·7% (−19·0 to 6·2)	−4·1% (−9·8 to 1·0)
		Controlled phase of mesothelioma		46·8 (45·3 to 48·3)	..	2·2 (1·4 to 3·2)	34·9% (18·0 to 58·3)*	23·6% (14·8 to 32·4)*	−8·6% (−19·8 to 7·0)	−4·3% (−11·0 to 2·3)
		Metastatic phase of mesothelioma		20·0 (19·3 to 20·6)	..	8·7 (5·9 to 11·6)	33·3% (17·0 to 56·9)*	23·7% (15·3 to 32·1)*	−10·4% (−20·9 to 4·9)	−4·8% (−11·1 to 1·4)
		Terminal phase of mesothelioma		2·8 (2·7 to 2·9)	..	1·5 (1·0 to 1·9)	31·6% (16·2 to 53·5)*	24·1% (17·1 to 30·2)*	−11·7% (−21·7 to 2·5)	−4·6% (−9·8 to −0·2)*
	Hodgkin lymphoma			657·1 (568·9 to 780·8)	101·1 (88·0 to 118·7)	50·5 (35·1 to 69·3)	31·2% (18·9 to 59·3)*	19·1% (13·8 to 23·7)*	−1·0% (−10·3 to 19·8)	2·5% (−2·2 to 6·5)
		Diagnosis and primary therapy phase of Hodgkin lymphoma		21·7 (18·8 to 25·9)	101·1 (88·0 to 118·7)	6·2 (4·1 to 9·0)	44·2% (30·3 to 77·5)*	22·8% (17·3 to 27·3)*	10·8% (0·9 to 35·5)*	6·6% (2·0 to 10·6)*
		Controlled phase of Hodgkin lymphoma		601·2 (520·5 to 716·2)	..	28·6 (17·5 to 43·0)	43·8% (29·2 to 77·6)*	22·4% (16·1 to 27·8)*	11·1% (0·4 to 36·7)*	6·7% (1·4 to 11·3)*
		Metastatic phase of Hodgkin lymphoma		31·0 (26·7 to 36·1)	..	14·0 (9·1 to 19·0)	13·5% (4·6 to 33·3)*	13·3% (8·3 to 17·3)*	−16·9% (−23·0 to −3·0)*	−5·0% (−9·1 to −1·6)*
		Terminal phase of Hodgkin lymphoma		3·2 (2·7 to 3·7)	..	1·7 (1·1 to 2·2)	−2·7% (−10·9 to 13·0)	4·6% (0·8 to 8·2)*	−28·0% (−33·5 to −17·1)*	−12·1% (−15·3 to −9·1)*
	Non-Hodgkin lymphoma			2371·9 (2325·0 to 2418·1)	488·0 (478·9 to 496·9)	193·1 (140·2 to 254·2)	77·8% (70·5 to 84·3)*	41·4% (37·7 to 45·0)*	26·1% (21·4 to 30·3)*	11·3% (8·2 to 14·2)*
		Diagnosis and primary therapy phase of non-Hodgkin lymphoma		87·8 (86·0 to 89·4)	488·0 (478·9 to 496·9)	24·2 (16·4 to 33·1)	90·1% (82·5 to 97·4)*	45·3% (40·5 to 49·9)*	35·2% (30·7 to 39·7)*	14·7% (10·8 to 18·3)*
		Controlled phase of non-Hodgkin lymphoma		2119·9 (2077·3 to 2161·3)	..	97·3 (62·9 to 140·5)	91·9% (83·9 to 99·1)*	45·4% (41·4 to 49·4)*	38·6% (33·9 to 42·8)*	15·6% (12·3 to 18·9)*
		Metastatic phase of non-Hodgkin lymphoma		140·1 (137·6 to 142·6)	..	58·8 (40·5 to 77·5)	60·4% (53·7 to 66·4)*	35·4% (31·3 to 39·6)*	12·0% (7·9 to 15·8)*	5·1% (1·9 to 8·4)*
		Terminal phase of non-Hodgkin lymphoma		24·1 (23·7 to 24·6)	..	12·9 (9·0 to 16·5)	57·3% (51·5 to 62·0)*	34·0% (30·5 to 37·2)*	9·4% (6·2 to 11·9)*	3·8% (1·2 to 6·2)*
	Multiple myeloma			449·3 (414·6 to 520·9)	152·7 (140·6 to 172·7)	90·8 (64·2 to 118·4)	93·4% (85·2 to 102·7)*	44·3% (38·6 to 50·3)*	29·7% (24·3 to 35·4)*	10·3% (5·9 to 14·7)*
		Diagnosis and primary therapy phase of multiple myeloma		28·1 (26·0 to 32·7)	152·7 (140·6 to 172·7)	8·0 (5·3 to 11·2)	116·1% (110·1 to 130·1)*	48·6% (43·2 to 54·8)*	47·0% (43·2 to 55·9)*	14·4% (10·2 to 19·0)*
		Controlled phase of multiple myeloma		252·3 (233·7 to 294·6)	..	11·7 (7·5 to 17·3)	118·2% (107·1 to 135·2)*	48·9% (42·0 to 56·3)*	48·8% (41·5 to 60·1)*	15·0% (9·7 to 20·7)*
		Metastatic phase of multiple myeloma		157·9 (145·3 to 181·5)	..	65·1 (44·2 to 85·7)	90·5% (80·8 to 100·4)*	43·8% (37·3 to 50·4)*	27·7% (21·3 to 34·0)*	9·8% (4·8 to 14·8)*
		Terminal phase of multiple myeloma		10·9 (10·1 to 12·3)	..	5·9 (4·1 to 7·6)	63·8% (53·4 to 68·1)*	35·8% (31·5 to 39·8)*	8·5% (0·7 to 11·3)*	2·4% (−0·9 to 5·5)
	Leukaemia			2432·4 (2190·3 to 2591·6)	518·5 (472·2 to 548·0)	263·3 (191·2 to 342·0)	27·8% (13·5 to 38·0)*	25·1% (19·7 to 29·5)*	−3·6% (−12·7 to 2·1)	2·3% (−2·3 to 6·5)
		Acute lymphoid leukaemia		521·1 (433·1 to 579·5)	64·2 (56·4 to 70·4)	43·0 (30·6 to 58·6)	29·9% (3·6 to 46·7)*	31·6% (11·9 to 47·7)*	11·1% (−9·4 to 24·0)	18·6% (0·3 to 33·7)*
			Diagnosis and primary therapy phase of acute lymphoid leukaemia	32·0 (28·1 to 35·1)	64·2 (56·4 to 70·4)	9·2 (6·1 to 13·0)	18·6% (−6·0 to 33·6)	11·8% (−3·0 to 21·9)	0·6% (−18·0 to 11·8)	−1·0% (−14·5 to 8·0)
			Controlled phase of acute lymphoid leukaemia	465·2 (383·8 to 521·0)	..	22·6 (14·0 to 34·1)	39·6% (10·2 to 59·8)*	48·4% (20·2 to 69·2)*	24·4% (−0·3 to 41·3)	36·8% (10·2 to 56·6)*
			Metastatic phase of acute lymphoid leukaemia	20·1 (17·3 to 21·7)	..	9·1 (6·0 to 12·4)	26·3% (3·9 to 42·1)*	21·6% (10·0 to 31·5)*	2·4% (−13·6 to 13·8)	5·8% (−4·5 to 14·3)
			Terminal phase of acute lymphoid leukaemia	3·7 (3·2 to 4·0)	..	2·0 (1·4 to 2·6)	25·4% (2·9 to 41·5)*	20·8% (9·0 to 30·9)*	2·0% (−14·1 to 13·7)	5·2% (−5·2 to 13·9)
		Chronic lymphoid leukaemia		567·7 (534·1 to 600·4)	90·6 (85·6 to 95·6)	74·6 (54·8 to 96·5)	72·3% (65·5 to 80·3)*	42·7% (36·5 to 49·1)*	12·5% (7·8 to 17·7)*	7·3% (2·5 to 12·3)*
			Diagnosis and primary therapy phase of chronic lymphoid leukaemia	30·4 (28·6 to 32·1)	90·6 (85·6 to 95·6)	8·6 (5·8 to 11·9)	78·0% (71·6 to 84·8)*	45·4% (39·0 to 52·0)*	18·1% (13·6 to 22·7)*	10·6% (5·5 to 15·7)*
			Controlled phase of chronic lymphoid leukaemia	424·3 (399·1 to 449·4)	..	19·5 (12·7 to 28·2)	83·4% (75·4 to 91·3)*	46·6% (39·4 to 54·0)*	23·1% (18·0 to 28·5)*	12·2% (6·9 to 17·9)*
			Metastatic phase of chronic lymphoid leukaemia	108·9 (103·0 to 114·8)	..	44·4 (31·2 to 58·5)	67·8% (59·1 to 77·9)*	40·8% (33·9 to 48·3)*	8·5% (2·8 to 14·8)*	5·1% (0·0 to 10·7)*
			Terminal phase of chronic lymphoid leukaemia	4·0 (3·8 to 4·2)	..	2·2 (1·5 to 2·8)	58·4% (52·4 to 64·5)*	35·9% (30·4 to 41·8)*	1·6% (−2·4 to 5·5)	0·5% (−3·6 to 5·0)
		Acute myeloid leukaemia		150·9 (137·0 to 160·0)	119·6 (108·4 to 125·9)	28·8 (20·2 to 37·7)	52·6% (24·2 to 72·6)*	22·9% (12·9 to 30·0)*	13·5% (−4·6 to 25·4)	1·0% (−7·1 to 6·7)
			Diagnosis and primary therapy phase of acute myeloid leukaemia	..	119·6 (108·4 to 125·9)	..	..	..	..	..
			Controlled phase of acute myeloid leukaemia	99·2 (90·5 to 105·8)	..	4·8 (3·0 to 7·2)	63·3% (25·1 to 91·8)*	18·7% (7·4 to 27·0)*	26·4% (1·9 to 44·0)*	0·0% (−9·5 to 7·1)
			Metastatic phase of acute myeloid leukaemia	42·5 (38·5 to 44·7)	..	19·0 (12·5 to 25·5)	50·4% (23·7 to 69·1)*	23·8% (14·0 to 30·8)*	11·1% (−6·0 to 22·1)	1·1% (−6·4 to 6·8)
			Terminal phase of acute myeloid leukaemia	9·2 (8·4 to 9·7)	..	5·0 (3·4 to 6·5)	51·0% (24·2 to 69·8)*	24·0% (14·2 to 30·8)*	11·6% (−5·6 to 22·6)	1·2% (−6·2 to 6·8)
		Chronic myeloid leukaemia		95·8 (88·3 to 103·2)	34·2 (31·5 to 36·7)	11·7 (8·4 to 15·4)	6·5% (1·3 to 11·2)*	6·6% (3·0 to 10·0)*	−28·1% (−31·5 to −24·8)*	−15·0% (−17·9 to −12·1)*
			Diagnosis and primary therapy phase of chronic myeloid leukaemia	6·9 (6·4 to 7·4)	34·2 (31·5 to 36·7)	2·0 (1·3 to 2·8)	13·6% (8·5 to 18·5)*	7·5% (3·9 to 11·0)*	−23·4% (−26·6 to −20·3)*	−13·7% (−16·6 to −10·8)*
			Controlled phase of chronic myeloid leukaemia	76·1 (70·1 to 82·0)	..	3·7 (2·4 to 5·6)	17·9% (12·1 to 23·7)*	7·8% (3·9 to 11·5)*	−19·3% (−23·2 to −15·2)*	−12·6% (−15·8 to −9·5)*
			Metastatic phase of chronic myeloid leukaemia	10·6 (9·7 to 11·3)	..	4·8 (3·2 to 6·4)	−1·3% (−6·1 to 2·9)	5·5% (2·0 to 9·1)*	−33·5% (−36·6 to −30·5)*	−16·7% (−19·7 to −13·8)*
			Terminal phase of chronic myeloid leukaemia	2·3 (2·1 to 2·5)	..	1·2 (0·9 to 1·6)	−1·7% (−6·3 to 2·6)	5·5% (1·9 to 9·1)*	−33·9% (−36·7 to −31·1)*	−16·8% (−19·6 to −13·9)*
		Other leukaemia		1096·8 (930·7 to 1204·2)	209·9 (180·8 to 227·5)	105·3 (74·7 to 137·1)	9·1% (−6·7 to 25·2)	15·4% (8·5 to 22·4)*	−14·8% (−25·1 to −5·3)*	−4·0% (−9·7 to 1·9)
			Diagnosis and primary therapy phase of other leukaemia	..	209·9 (180·8 to 227·5)	..	..	..	..	..
			Controlled phase of other leukaemia	957·9 (808·7 to 1058·8)	..	45·4 (28·5 to 67·6)	7·6% (−13·7 to 33·7)	13·1% (1·7 to 25·4)*	−8·8% (−24·9 to 9·7)	−1·1% (−11·7 to 10·0)
			Metastatic phase of other leukaemia	130·2 (114·4 to 139·3)	..	55·1 (37·6 to 72·8)	10·8% (−0·2 to 20·4)	17·4% (13·1 to 23·7)*	−18·7% (−25·8 to −13·5)*	−6·0% (−9·4 to −1·3)*
			Terminal phase of other leukaemia	8·8 (7·7 to 9·4)	..	4·7 (3·3 to 6·1)	6·4% (−4·0 to 17·1)	15·2% (11·1 to 20·7)*	−20·7% (−27·2 to −15·4)*	−7·6% (−10·9 to −3·4)*
	Other malignant cancers			9176·2 (8080·3 to 10 439·8)	715·5 (656·3 to 740·0)	678·1 (481·1 to 910·4)	99·7% (84·8 to 118·8)*	149·2% (122·1 to 179·0)*	46·4% (35·6 to 60·0)*	107·7% (84·2 to 133·3)*
		Diagnosis and primary therapy phase of other malignant neoplasms		205·1 (181·3 to 231·1)	715·5 (656·3 to 740·0)	56·6 (38·0 to 80·5)	126·8% (107·3 to 150·6)*	177·7% (146·5 to 210·3)*	67·9% (55·4 to 83·7)*	133·3% (107·0 to 161·9)*
		Controlled phase of other malignant neoplasms		8451·7 (7437·1 to 9683·5)	..	397·1 (252·9 to 592·8)	140·6% (114·5 to 174·5)*	204·9% (166·8 to 245·8)*	86·8% (68·6 to 109·9)*	161·4% (127·6 to 198·8)*
		Metastatic phase of other malignant neoplasms		460·4 (413·4 to 501·3)	..	188·6 (128·3 to 248·2)	67·0% (58·2 to 80·1)*	86·3% (71·8 to 101·9)*	18·3% (12·2 to 27·4)*	49·1% (37·2 to 61·1)*
		Terminal phase of other malignant neoplasms		68·0 (61·1 to 73·8)	..	35·8 (24·8 to 46·0)	62·1% (54·1 to 73·7)*	77·1% (65·3 to 90·1)*	14·2% (8·8 to 22·0)*	40·7% (31·0 to 51·3)*
	Other neoplasms			25 223·0 (24 682·1 to 25 725·3)	11 159·5 (10 928·2 to 11 416·2)	55·1 (35·2 to 80·9)	47·4% (42·6 to 52·3)*	34·5% (31·6 to 37·3)*	4·7% (1·8 to 7·7)*	6·0% (4·0 to 7·9)*
		Myelodysplastic, myeloproliferative, and other haemopoietic neoplasms		1212·2 (1098·1 to 1332·1)	134·3 (118·4 to 148·9)	55·1 (35·2 to 80·9)	47·4% (42·6 to 52·3)*	34·5% (31·6 to 37·3)*	4·7% (1·8 to 7·7)*	6·0% (4·0 to 7·9)*
		Benign and in-situ intestinal neoplasms		3966·3 (3840·7 to 4110·3)	1010·9 (980·1 to 1049·4)	..	..	..	..	..
		Benign and in-situ cervical and uterine neoplasms		1180·0 (1138·1 to 1223·0)	299·4 (287·6 to 312·8)	..	..	..	..	..
		Other benign and in-situ neoplasms		19 417·1 (18 926·5 to 19 882·6)	9715·0 (9488·7 to 9966·6)	..	..	..	..	..
	**Cardiovascular diseases**			**485 620·9 (468 031·7 to 504 964·4)**	**72 721·2 (70 388·1 to 75 264·1)**	**35 697·3 (26 428·2 to 45 510·3)**	39·8% (38·8 to 40·8)*	34·3% (32·0 to 37·0)*	−5·5% (−6·2 to −4·8)*	4·5% (2·9 to 6·4)*
	Rheumatic heart disease			39 345·4 (37 960·8 to 40 828·7)	1311·3 (1269·4 to 1354·9)	1901·0 (1232·8 to 2766·0)	40·9% (40·0 to 41·8)*	16·0% (15·3 to 16·7)*	7·3% (6·7 to 7·9)*	2·4% (1·9 to 3·1)*
		Rheumatic heart disease without heart failure		38 145·4 (36 705·9 to 39 639·2)	1311·3 (1269·4 to 1354·9)	1797·9 (1160·7 to 2619·0)	40·0% (39·2 to 40·9)*	15·4% (14·8 to 16·1)*	7·3% (6·7 to 7·9)*	2·6% (2·0 to 3·2)*
		Heart failure due to rheumatic heart disease		1199·9 (1006·1 to 1403·8)	..	103·1 (69·7 to 145·1)	58·9% (54·7 to 62·6)*	27·2% (24·4 to 30·2)*	7·9% (5·3 to 10·3)*	−0·1% (−2·2 to 1·9)
	Ischaemic heart disease			126 451·5 (118 587·5 to 134 706·5)	10 636·5 (9573·2 to 11 794·5)	5291·9 (3657·3 to 7238·9)	35·8% (34·5 to 37·0)*	23·5% (22·9 to 24·2)*	−10·9% (−11·9 to −10·0)*	−5·2% (−5·7 to −4·7)*
		Asymptomatic ischaemic heart disease following myocardial infarction		37 908·6 (31 907·4 to 44 225·6)	..	..	..	..	..	..
		Angina due to ischaemic heart disease		70 969·3 (66 224·1 to 75 830·1)	..	3850·4 (2615·6 to 5381·2)	35·1% (34·3 to 35·9)*	22·9% (22·2 to 23·5)*	−10·0% (−10·4 to −9·5)*	−4·6% (−5·0 to −4·2)*
		Heart failure due to ischaemic heart disease		17 007·2 (14 512·0 to 19 849·5)	..	1388·9 (951·7 to 1947·2)	37·2% (33·2 to 41·7)*	25·9% (24·3 to 27·5)*	−13·5% (−16·2 to −10·7)*	−6·4% (−7·6 to −5·1)*
		Myocardial infarction due to ischaemic heart disease		566·4 (505·5 to 634·4)	10 636·5 (9573·2 to 11 794·5)	52·7 (36·6 to 72·9)	48·9% (46·1 to 51·7)*	13·6% (10·5 to 16·6)*	−4·2% (−5·9 to −2·5)*	−14·6% (−17·0 to −12·3)*
	Stroke			104 178·7 (98 454·0 to 110 125·0)	11 931·1 (11 118·4 to 12 825·8)‡	18 695·4 (13 574·3 to 23 686·9)	40·0% (38·4 to 41·4)*	43·6% (39·6 to 47·8)*	−4·7% (−5·8 to −3·7)*	11·2% (8·1 to 14·3)*
		Ischaemic stroke		82 417·3 (76 967·8 to 88 516·9)	7737·5 (6951·1 to 8677·6)‡	14 304·4 (10 297·5 to 18 176·0)	42·8% (40·6 to 44·9)*	54·3% (49·1 to 59·4)*	−4·1% (−5·5 to −2·7)*	18·1% (14·1 to 22·0)*
			Acute ischaemic stroke	587·5 (529·1 to 655·7)	7737·5 (6951·1 to 8677·6)‡	144·3 (100·1 to 188·1)	40·1% (37·4 to 42·9)*	43·0% (37·0 to 49·4)*	−6·9% (−8·5 to −5·2)*	8·8% (4·3 to 13·6)*
			Chronic ischaemic stroke	81 829·7 (76 384·9 to 87 926·6)	..	14 160·1 (10 204·3 to 17 970·9)	42·8% (40·6 to 45·0)*	54·4% (49·2 to 59·6)*	−4·1% (−5·4 to −2·7)*	18·2% (14·1 to 22·2)*
		Intracerebral haemorrhage		17 914·9 (16 190·6 to 19 666·6)	3128·6 (2874·6 to 3418·0)‡	2946·9 (2121·6 to 3794·1)	32·1% (29·9 to 34·3)*	13·9% (8·0 to 20·3)*	−6·8% (−8·2 to −5·4)*	−9·5% (−14·2 to −4·8)*
			Acute intracerebral haemorrhage	168·7 (154·2 to 184·6)	3128·6 (2874·6 to 3418·0)‡	44·3 (31·0 to 57·8)	39·4% (36·8 to 42·4)*	−1·9% (−6·8 to 3·2)	−5·5% (−7·3 to −3·7)*	−24·4% (−28·1 to −20·6)*
			Chronic intracerebral haemorrhage	17 746·2 (16 029·3 to 19 493·5)	..	2902·6 (2090·3 to 3738·5)	32·0% (29·8 to 34·1)*	14·2% (8·1 to 20·6)*	−6·8% (−8·3 to −5·4)*	−9·3% (−14·0 to −4·4)*
		Subarachnoid haemorrhage		9321·9 (8523·7 to 10 242·4)	1064·9 (953·3 to 1182·6)‡	1444·2 (1031·6 to 1845·4)	36·8% (34·7 to 38·9)*	25·0% (21·3 to 28·6)*	−5·7% (−7·1 to −4·3)*	−1·6% (−4·4 to 1·2)
			Acute subarachnoid haemorrhage	80·5 (71·7 to 89·5)	1064·9 (953·3 to 1182·6)‡	21·1 (14·5 to 28·0)	40·2% (31·1 to 43·9)*	25·2% (19·8 to 27·2)*	−4·6% (−10·2 to −2·5)*	−1·6% (−5·5 to −0·2)*
			Chronic subarachnoid haemorrhage	9241·4 (8444·1 to 10 171·4)	..	1423·1 (1016·6 to 1817·9)	36·7% (34·6 to 38·8)*	25·0% (21·2 to 28·6)*	−5·7% (−7·1 to −4·3)*	−1·6% (−4·4 to 1·2)
	Hypertensive heart disease			17 067·7 (14 426·9 to 19 874·3)	..	1408·0 (966·6 to 1982·4)	63·8% (60·5 to 67·2)*	34·9% (33·2 to 36·6)*	6·0% (3·8 to 8·2)*	1·9% (0·7 to 2·9)*
	Non-rheumatic valvular heart disease			29 729·1 (28 505·4 to 31 022·5)	30 691·2 (29 436·4 to 32 002·2)	360·8 (207·1 to 559·6)	54·9% (49·0 to 61·0)*	35·8% (30·9 to 40·7)*	−4·5% (−7·7 to −1·7)*	−1·0% (−4·3 to 2·1)
		Non-rheumatic calcific aortic valve disease		12 570·2 (11 411·8 to 13 841·1)	12 570·2 (11 411·8 to 13 841·1)	168·0 (96·6 to 259·9)	64·8% (56·5 to 73·5)*	38·9% (30·9 to 46·2)*	−0·2% (−5·1 to 4·3)	0·2% (−5·9 to 5·3)
			Heart failure due to calcific aortic valve disease	1386·0 (896·6 to 2029·9)	..	111·0 (63·7 to 173·8)	42·3% (30·1 to 56·6)*	30·6% (13·2 to 47·6)*	−14·1% (−21·4 to −6·0)*	−6·0% (−19·0 to 6·2)
			Non-rheumatic calcific aortic valve disease	11 184·2 (9987·3 to 12 425·7)	12 570·2 (11 411·8 to 13 841·1)	56·9 (30·4 to 93·2)	163·9% (124·2 to 209·0)*	58·7% (36·3 to 84·3)*	62·6% (37·1 to 93·2)*	15·0% (−1·6 to 34·3)
		Non-rheumatic degenerative mitral valve disease		18 121·0 (17 682·3 to 18 586·4)	18 121·0 (17 682·3 to 18 586·4)	190·3 (109·2 to 295·3)	47·4% (41·5 to 52·7)*	33·2% (28·1 to 38·0)*	−8·1% (−11·5 to −5·4)*	−2·0% (−5·6 to 1·2)
			Heart failure due to degenerative mitral valve disease	1723·6 (1111·4 to 2494·0)	..	139·9 (81·5 to 217·1)	33·3% (25·0 to 43·1)*	26·7% (15·5 to 38·6)*	−17·1% (−22·2 to −11·4)*	−6·8% (−15·0 to 2·0)
			Non-rheumatic degenerative mitral valve disease	16 397·4 (15 574·9 to 17 179·2)	18 121·0 (17 682·3 to 18 586·4)	50·5 (26·7 to 84·0)	129·8% (95·6 to 171·4)*	55·2% (33·1 to 78·0)*	44·9% (22·1 to 72·2)*	14·0% (−2·5 to 30·9)
		Other non-rheumatic valve diseases		26·9 (22·1 to 32·2)	..	2·5 (1·6 to 3·6)	63·1% (58·9 to 67·2)*	37·7% (34·1 to 41·1)*	7·4% (5·1 to 9·4)*	6·1% (4·1 to 8·1)*
			Mild heart failure due to other non-rheumatic valve disease	5·0 (3·5 to 6·8)	..	0·2 (0·1 to 0·3)	63·6% (59·2 to 68·1)*	38·0% (34·5 to 41·4)*	7·7% (5·4 to 9·9)*	6·2% (4·2 to 8·1)*
			Moderate heart failure due to other non-rheumatic valve disease	3·3 (2·2 to 4·4)	..	0·2 (0·1 to 0·4)	63·6% (59·0 to 68·0)*	38·0% (34·5 to 41·6)*	7·7% (5·3 to 10·0)*	6·2% (4·0 to 8·2)*
			Severe heart failure due to other non-rheumatic valve disease	8·8 (6·9 to 10·9)	..	1·5 (1·0 to 2·3)	63·0% (58·7 to 67·4)*	37·7% (33·6 to 41·6)*	7·3% (4·8 to 9·8)*	6·1% (3·7 to 8·8)*
			Controlled, medically managed heart failure due to other non-rheumatic valve disease	9·9 (8·0 to 12·1)	..	0·5 (0·3 to 0·7)	63·2% (58·8 to 67·6)*	37·6% (33·5 to 41·6)*	7·4% (5·0 to 10·0)*	6·1% (3·3 to 8·7)*
	Cardiomyopathy and myocarditis			5429·9 (4694·4 to 6257·1)	3071·0 (2745·0 to 3451·2)	623·8 (424·7 to 859·3)	27·4% (24·5 to 30·9)*	21·2% (19·2 to 23·0)*	−12·6% (−14·9 to −10·3)*	−3·9% (−5·0 to −2·9)*
		Myocarditis		1804·6 (1635·3 to 1983·8)	3071·0 (2745·0 to 3451·2)	131·4 (90·1 to 183·0)	26·6% (23·9 to 29·6)*	15·9% (13·9 to 18·0)*	−5·4% (−6·8 to −4·1)*	−1·9% (−3·0 to −0·9)*
			Acute myocarditis	740·8 (656·3 to 830·8)	3071·0 (2745·0 to 3451·2)	35·7 (22·5 to 52·6)	33·3% (30·5 to 36·2)*	19·1% (16·7 to 21·6)*	−2·4% (−4·0 to −0·6)*	−1·3% (−2·9 to 0·2)
			Heart failure due to myocarditis	1063·7 (914·1 to 1215·2)	..	95·7 (63·9 to 133·5)	24·4% (21·0 to 28·2)*	14·8% (12·3 to 17·3)*	−6·5% (−8·1 to −4·9)*	−2·1% (−3·4 to −0·8)*
		Alcoholic cardiomyopathy		1621·8 (1370·5 to 1902·4)	..	139·1 (95·1 to 196·1)	20·7% (16·8 to 25·1)*	15·5% (12·9 to 18·1)*	−17·9% (−20·5 to −15·2)*	−9·5% (−11·2 to −7·7)*
		Other cardiomyopathy		4212·7 (3634·6 to 4869·9)	..	353·3 (237·9 to 493·9)	30·8% (27·2 to 34·9)*	25·7% (23·4 to 27·9)*	−12·8% (−15·6 to −9·9)*	−2·5% (−3·9 to −1·0)*
	Atrial fibrillation and flutter			37 574·2 (32 548·8 to 42 588·4)	3046·0 (2605·8 to 3507·2)	2921·5 (1992·6 to 4034·2)	49·0% (46·7 to 51·7)*	31·4% (30·3 to 32·5)*	−3·7% (−5·1 to −1·9)*	−1·2% (−1·9 to −0·6)*
		Asymptomatic atrial fibrillation and flutter		22 545·7 (19 530·0 to 25 680·2)	3046·0 (2605·8 to 3507·2)	..	..	..	..	..
		Symptomatic atrial fibrillation and flutter		15 028·5 (13 016·1 to 17 142·8)	..	2921·5 (1992·6 to 4034·2)	49·0% (46·7 to 51·7)*	31·4% (30·3 to 32·5)*	−3·7% (−5·1 to −1·9)*	−1·2% (−1·9 to −0·6)*
	Peripheral vascular disease			118 123·6 (102 706·9 to 134 350·4)	10 811·7 (9282·1 to 12 503·4)	515·6 (237·6 to 938·4)	40·0% (37·6 to 42·8)*	27·8% (26·6 to 29·3)*	−8·5% (−9·6 to −7·0)*	−4·7% (−5·5 to −3·6)*
		Asymptomatic peripheral arterial disease		78 631·6 (66 970·1 to 91 233·4)	10 811·7 (9282·1 to 12 503·4)	..	..	..	..	..
		Symptomatic claudication due to peripheral arterial disease		39 492·0 (31 074·7 to 50 227·2)	..	515·6 (237·6 to 938·4)	40·0% (37·6 to 42·8)*	27·8% (26·6 to 29·3)*	−8·5% (−9·6 to −7·0)*	−4·7% (−5·5 to −3·6)*
	Endocarditis			654·1 (567·5 to 743·8)	1222·5 (1097·6 to 1334·0)	53·8 (36·6 to 75·3)	31·1% (27·3 to 35·6)*	24·6% (22·1 to 27·3)*	−10·7% (−14·3 to −7·2)*	−2·5% (−4·4 to −0·8)*
		Acute endocarditis		87·9 (78·3 to 96·3)	1222·5 (1097·6 to 1334·0)	4·9 (3·2 to 7·3)	34·3% (31·7 to 36·9)*	26·2% (23·2 to 29·2)*	2·1% (1·4 to 2·9)*	4·3% (2·3 to 6·4)*
		Heart failure due to endocarditis		566·2 (480·9 to 655·8)	..	48·9 (33·3 to 68·7)	30·8% (26·5 to 35·7)*	24·5% (21·8 to 27·4)*	−11·7% (−15·5 to −8·1)*	−3·2% (−5·1 to −1·3)*
	Other cardiovascular and circulatory diseases			75 599·8 (64 068·9 to 88 170·3)	..	3925·4 (2650·1 to 5544·1)	32·5% (30·0 to 35·5)*	24·4% (22·5 to 26·2)*	−9·1% (−10·9 to −6·9)*	−0·5% (−1·4 to 0·4)
		Heart failure due to other cardiovascular diseases		1562·0 (1279·4 to 1882·1)	..	129·6 (86·6 to 185·3)	32·6% (28·4 to 37·1)*	30·0% (27·5 to 32·6)*	−15·0% (−18·0 to −12·0)*	−2·0% (−3·8 to −0·2)*
		Other cardiovascular and circulatory disease episodes		74 037·8 (62 741·1 to 86 283·3)	..	3795·9 (2564·1 to 5373·7)	32·4% (30·0 to 35·5)*	24·2% (22·3 to 26·0)*	−8·8% (−10·7 to −6·7)*	−0·4% (−1·3 to 0·5)
	**Chronic respiratory diseases**			**544 899·2 (506 937·5 to 584 858·4)**	**62 161·4 (55 134·8 to 69 320·7)**	**44 311·8 (36 751·6 to 51 407·1)**	21·9% (19·7 to 24·3)*	22·8% (19·0 to 26·5)*	−12·5% (−13·7 to −11·3)*	**−0·4% (−3·3 to 2·5)**
	Chronic obstructive pulmonary disease			299 398·2 (269 025·2 to 330 073·8)	18 475·7 (16 736·0 to 20 255·6)	30 611·5 (26 034·9 to 34 813·0)	24·9% (22·7 to 27·2)*	23·8% (18·6 to 28·6)*	−14·1% (−15·5 to −12·6)*	−2·8% (−6·9 to 0·9)
		Chronic obstructive pulmonary disease with heart failure		14 890·7 (12 183·6 to 17 715·3)	..	5673·6 (4163·8 to 7049·6)	66·5% (61·8 to 71·3)*	36·1% (32·1 to 39·1)*	5·4% (2·6 to 8·1)*	1·3% (−1·1 to 3·4)
		Chronic obstructive pulmonary disease without heart failure		284 507·4 (254 368·4 to 315 515·1)	18 475·7 (16 736·0 to 20 255·6)	24 937·9 (21 040·1 to 28 738·5)	18·9% (16·3 to 21·5)*	21·3% (15·4 to 26·8)*	−17·6% (−19·3 to −15·8)*	−3·7% (−8·4 to 0·6)
	Pneumoconiosis			527·5 (470·0 to 593·2)	60·1 (53·1 to 67·0)	80·5 (54·5 to 111·5)	41·3% (36·5 to 46·3)*	27·0% (22·8 to 31·2)*	−3·8% (−6·9 to −0·7)*	−2·0% (−5·2 to 1·1)
		Silicosis		162·4 (127·5 to 202·6)	23·7 (19·1 to 29·0)	24·9 (16·0 to 36·0)	45·4% (36·4 to 54·7)*	29·1% (21·3 to 37·1)*	−0·0% (−5·9 to 6·1)	0·2% (−5·8 to 6·4)
			Heart failure due to silicosis	11·0 (9·1 to 13·1)	..	5·0 (3·4 to 6·9)	64·3% (58·4 to 70·9)*	31·0% (26·8 to 35·3)*	8·7% (5·1 to 13·1)*	−1·6% (−4·8 to 1·9)
			Silicosis without heart failure	151·4 (117·1 to 190·0)	23·7 (19·1 to 29·0)	19·8 (12·2 to 29·4)	41·3% (30·6 to 52·9)*	28·6% (19·0 to 38·5)*	−2·1% (−9·3 to 5·9)	0·6% (−6·9 to 8·3)
		Asbestosis		92·1 (73·1 to 118·3)	9·4 (7·7 to 11·6)	14·4 (9·2 to 21·6)	42·6% (32·3 to 53·9)*	35·0% (29·6 to 41·3)*	−2·1% (−9·1 to 5·2)	4·4% (0·6 to 8·6)*
			Asbestosis without heart failure	86·6 (67·7 to 112·7)	9·4 (7·7 to 11·6)	11·9 (7·4 to 18·2)	38·3% (27·2 to 50·6)*	36·6% (30·1 to 44·7)*	−3·9% (−11·9 to 4·7)	7·1% (2·4 to 12·5)*
			Heart failure due to asbestosis	5·6 (4·7 to 6·5)	..	2·5 (1·7 to 3·5)	65·2% (54·7 to 77·6)*	27·6% (23·8 to 31·8)*	5·5% (−1·3 to 13·3)	−6·0% (−8·9 to −3·0)*
		Coal worker pneumoconiosis		147·9 (117·5 to 190·1)	15·1 (12·0 to 19·8)	21·9 (14·2 to 31·8)	39·1% (28·8 to 50·1)*	23·1% (13·8 to 32·3)*	−6·0% (−12·4 to 1·1)	−5·6% (−12·7 to 1·2)
			Coal worker pneumoconiosis without heart failure	140·9 (110·7 to 182·6)	15·1 (12·0 to 19·8)	18·7 (12·0 to 27·8)	36·5% (24·8 to 48·9)*	22·4% (11·6 to 33·0)*	−7·3% (−14·7 to 0·9)	−6·0% (−14·1 to 1·7)
			Heart failure due to coal worker pneumoconiosis	7·0 (5·9 to 8·2)	..	3·2 (2·2 to 4·3)	57·8% (51·8 to 64·5)*	27·5% (23·0 to 32·1)*	2·7% (−1·6 to 7·1)	−3·1% (−6·6 to 0·4)
		Other pneumoconiosis		125·1 (105·0 to 149·3)	11·9 (9·9 to 14·3)	19·3 (12·6 to 28·5)	38·2% (31·9 to 45·3)*	23·3% (18·5 to 27·9)*	−6·9% (−10·9 to −2·7)*	−5·0% (−8·4 to −1·6)*
			Heart failure due to other pneumoconiosis	4·1 (3·4 to 4·7)	..	1·9 (1·3 to 2·6)	80·7% (75·2 to 86·3)*	27·8% (23·5 to 31·9)*	15·2% (12·3 to 18·2)*	−4·5% (−7·5 to −1·9)*
			Other pneumoconiosis without heart failure	121·0 (100·9 to 145·2)	11·9 (9·9 to 14·3)	17·5 (11·2 to 26·0)	34·9% (28·3 to 42·1)*	22·8% (17·7 to 27·8)*	−8·8% (−12·9 to −4·5)*	−5·0% (−8·8 to −1·2)*
	Asthma			272 677·5 (242 295·9 to 304 699·6)	43 123·4 (36 191·5 to 50 226·4)	10 622·9 (7057·0 to 15 056·4)	8·0% (5·2 to 10·9)*	19·3% (15·3 to 23·1)*	−13·5% (−14·9 to −11·9)*	4·9% (1·0 to 8·4)*
		Asymptomatic asthma		99 070·1 (84 654·0 to 114 607·7)	15 631·4 (12 827·3 to 18 539·7)	..	..	..	..	..
		Symptomatic asthma		173 607·4 (151 655·5 to 198 619·2)	27 492·0 (22 746·9 to 32 507·6)	10 622·9 (7057·0 to 15 056·4)	8·0% (5·2 to 10·9)*	19·3% (15·3 to 23·1)*	−13·5% (−14·9 to −11·9)*	4·9% (1·0 to 8·4)*
	Interstitial lung disease and pulmonary sarcoidosis			6234·2 (5661·0 to 6848·2)	502·2 (458·8 to 550·0)	648·2 (440·3 to 923·2)	44·3% (41·7 to 47·3)*	29·1% (26·5 to 31·8)*	−2·3% (−3·9 to −0·5)*	0·1% (−1·8 to 2·0)
		Heart failure due to interstitial lung disease and pulmonary sarcoidosis		416·8 (322·3 to 505·6)	..	169·8 (114·4 to 236·1)	60·8% (56·2 to 65·6)*	31·4% (28·6 to 34·2)*	5·5% (2·8 to 8·4)*	−1·2% (−3·3 to 0·9)
		Interstitial lung disease and pulmonary sarcoidosis without heart failure		5817·3 (5242·6 to 6431·9)	502·2 (458·8 to 550·0)	478·5 (320·7 to 690·8)	39·3% (36·3 to 42·8)*	28·2% (25·3 to 31·5)*	−4·9% (−6·9 to −2·7)*	0·5% (−1·7 to 2·8)
	Other chronic respiratory diseases			..	..	2348·7 (1930·8 to 2739·6)	62·5% (58·1 to 67·0)*	23·8% (19·2 to 28·5)*	21·4% (17·8 to 25·1)*	9·2% (5·0 to 13·3)*
	**Digestive diseases**			**2 049 831·2 (1 983 314·3 to 2 122 941·6)**	**465 978·6 (429 600·4 to 500 015·1)**	**19 939·7 (13 858·2 to 27 973·1)**	31·1% (29·3 to 32·7)*	20·5% (19·5 to 21·6)*	−3·4% (−4·1 to −2·7)*	1·3% (0·6 to 2·0)*
	Cirrhosis and other chronic liver diseases			1 500 585·1 (1 448 741·6 to 1 556 007·5)	5154·9 (4935·4 to 5366·5)	1745·6 (1221·5 to 2387·3)	50·7% (49·4 to 52·1)*	34·8% (33·0 to 36·3)*	8·4% (7·6 to 9·3)*	10·4% (9·0 to 11·6)*
		Cirrhosis and other chronic liver diseases due to hepatitis B		431 116·3 (395 729·0 to 468 718·9)	1531·5 (1419·0 to 1643·9)	488·4 (337·9 to 665·7)	53·7% (50·9 to 56·5)*	33·2% (30·7 to 35·6)*	8·7% (6·9 to 10·5)*	9·0% (7·1 to 10·9)*
			Cirrhosis and other chronic liver diseases due to hepatitis B, decompensated	2974·2 (2810·2 to 3124·4)	539·5 (509·2 to 570·8)	488·4 (337·9 to 665·7)	53·7% (50·9 to 56·5)*	33·2% (30·7 to 35·6)*	8·7% (6·9 to 10·5)*	9·0% (7·1 to 10·9)*
			Chronic hepatitis B without cirrhosis	391 475·2 (356 323·1 to 429 032·9)	..	..	..	..	..	..
			Cirrhosis and other chronic liver diseases due to hepatitis B, compensated	36 666·9 (34 057·4 to 39 502·9)	992·1 (890·6 to 1095·8)	..	..	..	..	..
		Cirrhosis and other chronic liver diseases due to hepatitis C		134 493·9 (118 558·2 to 153 823·8)	1178·2 (1086·0 to 1272·2)	431·3 (300·3 to 596·4)	56·1% (53·5 to 58·4)*	36·5% (33·9 to 38·9)*	8·4% (6·7 to 9·9)*	10·2% (8·2 to 11·9)*
			Cirrhosis and other chronic liver diseases due to hepatitis C, decompensated	2641·5 (2487·0 to 2806·1)	467·3 (438·1 to 496·2)	431·3 (300·3 to 596·4)	56·1% (53·5 to 58·4)*	36·5% (33·9 to 38·9)*	8·4% (6·7 to 9·9)*	10·2% (8·2 to 11·9)*
			Chronic hepatitis C without cirrhosis	104 133·4 (88 178·6 to 123 899·7)	..	..	..	..	..	..
			Cirrhosis and other chronic liver diseases due to hepatitis C, compensated	27 719·0 (25 515·4 to 29 987·7)	710·9 (628·8 to 800·8)	..	..	..	..	..
		Cirrhosis and other chronic liver diseases due to alcohol use		26 041·9 (24 252·8 to 28 011·2)	903·7 (839·9 to 973·5)	400·1 (280·3 to 551·5)	58·1% (55·4 to 61·0)*	37·7% (35·1 to 40·5)*	8·1% (6·3 to 9·9)*	9·8% (7·8 to 11·9)*
			Cirrhosis and other chronic liver diseases due to alcohol, decompensated	2457·4 (2313·9 to 2610·3)	433·1 (407·6 to 458·5)	400·1 (280·3 to 551·5)	58·1% (55·4 to 61·0)*	37·7% (35·1 to 40·5)*	8·1% (6·3 to 9·9)*	9·8% (7·8 to 11·9)*
			Cirrhosis and other chronic liver diseases due to alcohol, compensated	23 584·5 (21 876·0 to 25 478·8)	470·6 (417·4 to 525·3)	..	..	..	..	..
		Cirrhosis due to NASH		892 322·8 (858 624·9 to 927 954·4)	367·8 (334·5 to 403·7)	148·6 (102·8 to 205·0)	83·2% (78·8 to 87·5)*	54·6% (51·0 to 58·3)*	25·6% (22·8 to 28·5)*	23·5% (20·7 to 26·4)*
			Non-alcoholic fatty liver disease/NASH	882 058·3 (847 914·0 to 917 435·7)	..	..	..	..	..	..
			Cirrhosis and other chronic liver diseases due to NASH	10 264·5 (9358·1 to 11 216·7)	367·8 (334·5 to 403·7)	148·6 (102·8 to 205·0)	83·2% (78·8 to 87·5)*	54·6% (51·0 to 58·3)*	25·6% (22·8 to 28·5)*	23·5% (20·7 to 26·4)*
		Cirrhosis and other chronic liver diseases due to other causes		16 616·0 (15 165·6 to 17 954·6)	1173·7 (1084·6 to 1260·7)	277·1 (192·1 to 386·4)	24·0% (20·6 to 27·3)*	22·7% (20·0 to 25·7)*	2·6% (−0·0 to 5·0)	8·1% (5·8 to 10·6)*
			Cirrhosis and other chronic liver diseases due to other cause, decompensated	1653·0 (1550·5 to 1755·9)	248·4 (231·4 to 265·9)	277·1 (192·1 to 386·4)	24·0% (20·6 to 27·3)*	22·7% (20·0 to 25·7)*	2·6% (−0·0 to 5·0)	8·1% (5·8 to 10·6)*
			Cirrhosis and other chronic liver diseases due to other cause, compensated	14 963·1 (13 571·8 to 16 245·0)	925·4 (840·5 to 1008·8)	..	..	..	..	..
	Upper digestive system diseases			807 369·3 (728 919·0 to 888 668·9)	384 665·9 (348 550·3 to 419 766·4)	13 042·1 (8272·3 to 19 986·6)	32·0% (29·9 to 33·8)*	19·3% (18·1 to 20·6)*	−3·6% (−4·5 to −2·7)*	0·6% (−0·2 to 1·4)
		Peptic ulcer disease		17 161·2 (15 032·4 to 19 357·3)	6719·1 (6078·7 to 7434·1)	832·8 (555·4 to 1184·1)	22·2% (20·1 to 24·6)*	18·9% (16·5 to 21·1)*	−14·2% (−15·6 to −12·6)*	−1·6% (−3·5 to −0·1)*
			Acute peptic ulcer disease	7·8 (6·8 to 8·9)	..	2·5 (1·7 to 3·6)	32·0% (25·8 to 39·1)*	12·6% (10·9 to 14·4)*	−10·3% (−14·4 to −5·4)*	−12·2% (−13·1 to −11·3)*
			Chronic peptic ulcer disease	17 153·4 (15 023·5 to 19 349·4)	6719·1 (6078·7 to 7434·1)	830·2 (553·1 to 1180·9)	22·2% (20·1 to 24·5)*	18·9% (16·5 to 21·2)*	−14·2% (−15·6 to −12·6)*	−1·6% (−3·5 to −0·0)*
		Gastritis and duodenitis		140 449·0 (120 336·3 to 161 832·9)	96 720·4 (85 853·2 to 108 445·4)	6196·0 (3976·7 to 8960·7)	27·7% (25·0 to 30·3)*	18·2% (16·1 to 20·4)*	−4·5% (−5·8 to −3·3)*	0·3% (−1·2 to 1·7)
			Acute gastritis and duodenitis	40·1 (34·5 to 46·3)	..	13·1 (8·6 to 18·7)	27·8% (25·2 to 30·5)*	15·4% (13·9 to 16·9)*	−10·2% (−11·4 to −8·9)*	−6·1% (−7·0 to −5·3)*
			Chronic gastritis and duodenitis	140 408·8 (120 292·9 to 161 787·1)	96 720·4 (85 853·2 to 108 445·4)	6182·8 (3967·9 to 8944·1)	27·7% (25·0 to 30·3)*	18·2% (16·1 to 20·4)*	−4·5% (−5·8 to −3·3)*	0·3% (−1·2 to 1·7)
	Gastro-oesophageal reflux disease			709 264·3 (625 708·7 to 794 604·2)	281 226·3 (246 633·4 to 315 761·3)	6013·4 (3215·9 to 10 192·9)	38·5% (36·3 to 40·4)*	20·7% (19·1 to 22·3)*	−0·7% (−1·5 to −0·0)*	1·3% (0·6 to 2·0)*
	Appendicitis			722·7 (673·2 to 775·6)	19 016·1 (17 716·4 to 20 344·0)	223·6 (149·1 to 306·5)	27·7% (23·9 to 31·9)*	10·3% (6·6 to 14·0)*	2·3% (−0·7 to 5·5)	0·2% (−3·1 to 3·7)
	Paralytic ileus and intestinal obstruction			140·2 (128·1 to 152·3)	3855·4 (3520·1 to 4187·5)	44·2 (30·0 to 60·4)	36·0% (32·4 to 39·3)*	25·5% (22·8 to 28·4)*	2·9% (1·2 to 4·5)*	4·2% (2·1 to 6·4)*
	Inguinal, femoral, and abdominal hernia			26 490·8 (24 196·8 to 28 760·4)	41 182·8 (36 372·8 to 46 265·0)	2567·0 (1713·5 to 3550·5)	21·2% (17·9 to 24·8)*	14·5% (12·3 to 17·0)*	−7·1% (−8·9 to −5·3)*	−2·9% (−4·4 to −1·2)*
	Inflammatory bowel disease			6848·9 (6421·4 to 7304·4)	4048·8 (3776·1 to 4377·4)	1019·4 (705·4 to 1381·8)	48·5% (44·4 to 52·7)*	21·7% (18·0 to 25·1)*	4·7% (1·9 to 7·6)*	−0·2% (−3·1 to 2·3)
		Ulcerative colitis		4701·0 (4318·4 to 5113·6)	2690·9 (2440·5 to 2994·3)	684·0 (468·6 to 935·0)	54·8% (50·4 to 59·5)*	29·5% (24·9 to 33·9)*	7·7% (4·8 to 10·9)*	4·6% (1·2 to 7·8)*
		Crohn's disease		2147·9 (2007·7 to 2301·8)	1357·8 (1240·2 to 1484·4)	335·4 (227·1 to 460·4)	38·8% (34·0 to 43·8)*	8·6% (5·0 to 11·8)*	−0·1% (−3·5 to 3·4)	−8·7% (−11·5 to −6·2)*
	Vascular intestinal disorders			89·8 (80·0 to 100·8)	619·8 (555·7 to 686·7)	27·1 (18·5 to 36·7)	56·5% (50·4 to 63·2)*	25·2% (20·2 to 30·8)*	12·4% (8·9 to 16·2)*	−1·3% (−5·1 to 2·8)
	Gallbladder and biliary diseases			30 575·9 (27 503·2 to 34 125·0)	5790·7 (5195·0 to 6386·5)	26·4 (18·0 to 37·5)	29·3% (26·7 to 32·1)*	27·5% (24·1 to 31·3)*	−9·6% (−11·0 to −8·1)*	3·9% (1·7 to 6·3)*
	Pancreatitis			6115·8 (5533·9 to 6704·1)	1644·2 (1525·6 to 1769·5)	364·4 (186·3 to 612·8)	52·0% (48·7 to 54·8)*	26·6% (23·3 to 29·7)*	6·5% (4·1 to 8·3)*	2·5% (0·0 to 4·9)*
		Acute pancreatitis		118·6 (107·5 to 130·0)	1300·9 (1181·4 to 1426·0)	47·7 (34·2 to 63·9)	33·9% (30·5 to 37·3)*	17·5% (15·7 to 19·4)*	−6·6% (−8·6 to −4·2)*	−4·4% (−5·6 to −3·2)*
		Chronic pancreatitis		5997·3 (5412·4 to 6583·8)	343·3 (311·5 to 375·0)	316·7 (145·2 to 556·1)	55·5% (52·7 to 58·8)*	28·1% (24·5 to 31·8)*	9·0% (7·2 to 11·1)*	3·7% (0·9 to 6·5)*
	Other digestive diseases			..	..	879·9 (605·6 to 1191·4)	0·9% (−2·8 to 5·0)	30·6% (28·9 to 32·1)*	−19·4% (−21·5 to −17·3)*	9·5% (8·2 to 10·6)*
	**Neurological disorders**			**3 121 435·3 (2 951 124·5 to 3 316 268·0)**	**1 006 294·5 (907 590·7 to 1 098 468·9)**	**73 161·8 (50 721·9 to 100 409·9)**	35·1% (31·9 to 38·1)*	17·8% (15·8 to 20·2)*	**0·5% (−1·6 to 2·4)**	**1·4% (−0·3 to 3·4)**
	Alzheimer's disease and other dementias			44 988·8 (39 716·2 to 50 377·8)	7300·6 (6515·7 to 8133·4)	6570·4 (4678·1 to 8588·5)	62·7% (60·6 to 64·9)*	36·9% (35·2 to 38·8)*	−2·3% (−3·3 to −1·2)*	−1·7% (−2·3 to −1·1)*
	Parkinson's disease			8525·4 (7037·3 to 10 185·6)	1025·9 (854·1 to 1229·9)	1219·0 (823·7 to 1662·2)	69·2% (66·5 to 72·6)*	34·3% (32·5 to 36·2)*	8·9% (7·1 to 11·0)*	1·0% (−0·1 to 2·1)
	Epilepsy			27 288·3 (21 576·0 to 33 443·8)	2470·8 (1905·5 to 3062·9)	8561·9 (5380·6 to 12 551·5)	24·1% (8·0 to 42·9)*	15·9% (3·5 to 31·2)*	0·1% (−12·3 to 14·9)	3·4% (−7·8 to 16·8)
	Multiple sclerosis			1761·1 (1598·2 to 1947·9)	54·9 (50·1 to 60·8)	456·6 (327·7 to 595·0)	41·6% (38·2 to 45·2)*	18·3% (16·1 to 20·5)*	−0·9% (−3·2 to 1·4)	−2·4% (−4·2 to −0·4)*
	Motor neuron disease			237·1 (211·2 to 264·1)	67·3 (60·7 to 74·3)	50·4 (35·7 to 67·5)	39·6% (36·3 to 43·2)*	25·9% (23·1 to 28·9)*	4·6% (3·5 to 5·9)*	4·5% (3·0 to 6·1)*
	Headache disorders			3 072 812·1 (2 904 886·2 to 3 266 065·0)	995 374·9 (896 864·3 to 1 087 335·4)	54 341·8 (35 570·5 to 76 780·4)	34·0% (33·0 to 35·1)*	15·4% (14·6 to 16·2)*	0·4% (−0·2 to 1·0)	1·0% (0·5 to 1·5)*
		Migraine		1 331 364·6 (1 237 219·6 to 1 433 357·2)	112 933·5 (102 829·9 to 122 899·8)	47 245·4 (29 986·7 to 68 669·3)	34·2% (33·1 to 35·4)*	15·3% (14·5 to 16·2)*	0·6% (−0·0 to 1·2)	1·1% (0·6 to 1·7)*
			Medication overuse headache due to migraine	83 755·8 (70 723·8 to 97 287·8)	..	9166·1 (5759·6 to 13 329·2)	35·5% (33·6 to 37·3)*	17·5% (16·2 to 18·7)*	−1·4% (−2·5 to −0·5)*	0·1% (−0·3 to 0·6)
			Migraine	1 247 608·8 (1 154 817·3 to 1 347 980·9)	112 933·5 (102 829·9 to 122 899·8)	38 079·3 (23 576·3 to 57 421·4)	33·9% (32·6 to 35·3)*	14·8% (13·8 to 15·8)*	1·1% (0·4 to 1·9)*	1·3% (0·7 to 2·0)*
		Tension-type headache		2 331 334·7 (2 110 373·3 to 2 575 461·4)	882 441·4 (783 241·2 to 975 064·3)	7096·4 (4044·6 to 11 213·5)	32·7% (31·0 to 34·6)*	15·6% (14·5 to 16·9)*	−0·9% (−1·7 to −0·3)*	0·3% (−0·1 to 0·6)
			Medication overuse headache due to tension-type headache	31 134·3 (21 220·1 to 41 990·3)	..	3382·8 (1753·7 to 5875·5)	35·5% (33·5 to 37·5)*	17·5% (16·0 to 18·8)*	−1·4% (−2·5 to −0·4)*	0·1% (−0·6 to 0·8)
			Tension-type headache	2 300 200·4 (2 079 425·9 to 2 547 509·0)	882 441·4 (783 241·2 to 975 064·3)	3713·6 (1525·8 to 6887·1)	30·3% (28·5 to 32·4)*	14·0% (12·9 to 15·3)*	−0·5% (−1·4 to 0·4)	0·4% (0·0 to 0·7)*
	Other neurological disorders			38·9 (25·6 to 53·2)	..	1961·8 (1276·3 to 2823·8)	35·7% (19·8 to 54·5)*	31·9% (18·8 to 47·1)*	11·8% (−0·5 to 26·1)	19·5% (7·2 to 33·4)*
		Other neurological disorders		..	..	1950·3 (1267·2 to 2813·5)	35·8% (19·7 to 54·7)*	32·0% (18·8 to 47·3)*	11·9% (−0·5 to 26·4)	19·6% (7·2 to 33·6)*
		Guillain-Barré syndrome due to other neurological disorders		38·9 (25·6 to 53·2)	..	11·5 (6·4 to 18·1)	29·7% (25·8 to 34·1)*	18·2% (15·8 to 20·8)*	2·7% (1·0 to 4·3)*	3·1% (2·0 to 4·3)*
	**Mental disorders**			**970 812·4 (923 455·4 to 1 020 930·6)**	**336 996·3 (315 596·6 to 362 049·6)**	**122 746·3 (91 620·8 to 157 883·6)**	31·6% (30·5 to 32·7)*	13·5% (12·9 to 14·1)*	−0·6% (−1·2 to −0·0)*	−1·1% (−1·5 to −0·7)*
	Schizophrenia			19 776·9 (17 578·3 to 22 210·7)	1130·5 (1004·6 to 1281·9)	12 657·9 (9481·9 to 15 563·7)	38·6% (37·1 to 40·2)*	17·2% (16·1 to 18·3)*	−0·0% (−0·8 to 0·7)	−0·3% (−1·1 to 0·4)
	Depressive disorders			264 455·6 (246 380·1 to 286 312·0)	258 164·5 (238 280·7 to 281 665·5)	43 099·9 (30 536·4 to 58 895·6)	33·4% (31·0 to 35·8)*	14·3% (13·1 to 15·6)*	−1·9% (−3·2 to −0·4)*	−2·6% (−3·5 to −1·8)*
		Major depressive disorder		163 044·1 (149 530·9 to 178 929·1)	241 893·3 (222 032·7 to 265 574·7)	32 846·7 (23 081·1 to 44 291·2)	32·1% (29·2 to 35·0)*	12·6% (11·3 to 14·0)*	−2·4% (−4·0 to −0·6)*	−3·6% (−4·6 to −2·7)*
		Dysthymia		106 904·4 (93 445·9 to 122 812·3)	16 271·1 (14 236·4 to 18 420·1)	10 253·2 (6878·0 to 14 982·1)	38·3% (35·5 to 41·0)*	20·4% (18·2 to 22·6)*	−0·3% (−1·8 to 1·3)	0·8% (−0·8 to 2·3)
	Bipolar disorder			45 549·4 (39 864·5 to 52 852·8)	4525·2 (3944·7 to 5237·5)	9293·8 (5868·2 to 13 748·5)	34·0% (32·6 to 35·4)*	15·2% (14·0 to 16·6)*	0·4% (−0·3 to 1·1)	1·2% (0·6 to 1·8)*
	Anxiety disorders			284 360·1 (265 607·5 to 304 531·7)	42 340·0 (39 597·5 to 45 199·5)	27 121·4 (19 248·3 to 36 106·3)	32·3% (30·6 to 34·0)*	12·8% (11·7 to 14·0)*	0·4% (−0·5 to 1·3)	−1·2% (−2·0 to −0·4)*
	Eating disorders			15 801·7 (12 596·2 to 19 488·6)	9589·7 (6950·1 to 13 126·9)	3351·9 (2149·3 to 4871·6)	37·7% (35·4 to 40·0)*	18·9% (17·3 to 20·5)*	7·9% (6·8 to 8·9)*	9·4% (8·2 to 10·5)*
		Anorexia nervosa		3360·3 (2533·8 to 4321·5)	1027·8 (777·5 to 1329·1)	715·8 (440·9 to 1066·2)	30·9% (28·0 to 33·9)*	13·5% (11·0 to 15·7)*	5·4% (3·3 to 7·2)*	6·2% (4·1 to 8·1)*
		Bulimia nervosa		12 509·7 (9480·4 to 15 976·5)	8561·9 (5957·9 to 12 142·7)	2636·2 (1660·3 to 3937·1)	39·7% (36·9 to 42·7)*	20·5% (18·6 to 22·2)*	8·6% (7·5 to 9·8)*	10·3% (9·0 to 11·6)*
	Autism spectrum disorders			31 179·7 (28 000·9 to 34 413·8)	670·7 (602·2 to 739·1)	4731·3 (3238·8 to 6518·6)	22·8% (22·1 to 23·5)*	11·4% (10·8 to 12·1)*	−0·7% (−1·2 to −0·2)*	−0·3% (−0·8 to 0·3)
	Attention-deficit/hyperactivity disorder			73 317·5 (62 267·0 to 85 889·6)	3302·7 (2683·9 to 4031·4)	888·8 (543·2 to 1411·8)	20·1% (17·5 to 22·6)*	6·9% (5·2 to 8·5)*	−0·2% (−2·1 to 1·7)	−0·6% (−2·1 to 0·8)
	Conduct disorder			53 228·4 (41 976·6 to 65 750·3)	17 273·1 (13 809·1 to 20 989·8)	6445·2 (3869·6 to 10 299·8)	16·1% (14·4 to 17·8)*	4·8% (3·1 to 6·5)*	1·5% (0·1 to 2·9)*	2·4% (0·9 to 3·8)*
	Idiopathic developmental intellectual disability			100 572·2 (58 404·5 to 143 878·6)	..	4046·4 (1940·2 to 6859·5)	13·0% (9·1 to 15·9)*	0·0% (−2·8 to 1·5)	−5·8% (−9·0 to −3·1)*	−8·7% (−11·1 to −7·5)*
	Other mental disorders			149 478·6 (130 882·3 to 170 462·4)	..	11 109·6 (7355·5 to 15 324·6)	37·7% (36·9 to 38·6)*	17·6% (17·0 to 18·3)*	0·0% (−0·5 to 0·4)	0·0% (−0·4 to 0·5)
	**Substance use disorders**			**175 588·8 (161 747·6 to 189 304·3)**	**60 099·6 (53 685·6 to 67 048·7)**	**31 052·9 (22 217·1 to 40 499·7)**	34·3% (32·2 to 36·2)*	16·7% (14·4 to 19·2)*	2·2% (1·1 to 3·4)*	2·9% (0·8 to 5·1)*
	Alcohol use disorders			107 420·2 (95 908·0 to 119 660·2)	52 406·2 (45 976·6 to 59 250·1)	10 712·5 (7368·0 to 14 760·1)	35·9% (33·8 to 38·3)*	10·1% (8·5 to 11·7)*	0·9% (−0·1 to 1·9)	−4·3% (−5·4 to −3·1)*
		Alcohol dependence		107 118·2 (95 586·5 to 119 383·0)	52 397·0 (45 967·6 to 59 241·9)	10 701·5 (7361·4 to 14 747·1)	36·0% (33·8 to 38·4)*	10·1% (8·5 to 11·7)*	0·9% (−0·1 to 2·0)	−4·3% (−5·4 to −3·1)*
		Fetal alcohol syndrome		302·0 (240·9 to 370·5)	9·1 (7·5 to 11·1)	11·1 (6·6 to 17·0)	2·5% (−3·2 to 7·2)	−4·7% (−10·1 to −0·2)*	−17·6% (−22·1 to −13·9)*	−14·7% (−19·4 to −10·6)*
	Drug use disorders			71 244·4 (63 963·8 to 79 771·6)	7693·4 (6880·2 to 8628·5)	20 340·3 (14 276·7 to 26 665·5)	33·3% (30·3 to 36·3)*	20·4% (17·4 to 23·6)*	3·1% (1·3 to 4·8)*	7·1% (4·3 to 9·9)*
		Opioid use disorders		40 484·6 (34 271·4 to 47 941·6)	4085·2 (3463·6 to 4794·2)	16 844·7 (11 549·8 to 22 534·5)	38·4% (35·4 to 41·9)*	23·7% (20·2 to 27·2)*	6·2% (4·5 to 8·2)*	9·3% (6·0 to 12·5)*
		Cocaine use disorders		5017·2 (4521·0 to 5610·4)	260·3 (227·9 to 301·6)	680·0 (446·1 to 974·8)	24·7% (21·2 to 28·6)*	12·6% (11·0 to 14·4)*	−4·7% (−6·9 to −2·1)*	0·3% (−1·2 to 2·0)
		Amphetamine use disorders		7382·6 (5376·5 to 9821·8)	887·2 (666·2 to 1170·2)	977·5 (560·1 to 1537·4)	5·8% (1·9 to 10·9)*	2·7% (−0·4 to 5·7)	−13·8% (−16·6 to −10·4)*	−4·0% (−6·8 to −1·5)*
		Cannabis use disorders		17 857·3 (14 597·9 to 21 953·6)	2460·7 (2061·0 to 3022·0)	517·7 (329·1 to 765·6)	23·8% (19·7 to 28·5)*	4·4% (2·2 to 6·6)*	−2·2% (−4·7 to 0·6)	−3·7% (−5·7 to −1·7)*
		Other drug use disorders		2237·7 (1984·3 to 2509·6)	..	1320·5 (888·2 to 1854·3)	16·8% (12·0 to 22·4)*	8·2% (5·2 to 11·2)*	−8·3% (−11·3 to −4·9)*	−1·9% (−4·3 to 0·6)
	**Diabetes and kidney diseases**			**1 011 116·6 (962 767·9 to 1 065 061·5)**	**43 444·6 (40 700·3 to 46 375·6)**	**45 884·4 (32 018·9 to 62 235·3)**	68·4% (66·5 to 70·4)*	29·1% (24·8 to 34·0)*	15·4% (14·2 to 16·7)*	**3·2% (−0·4 to 6·9)**
	Diabetes mellitus			475 995·8 (436 590·5 to 522 782·8)	22 935·6 (21 082·9 to 25 040·9)	38 575·4 (26 083·6 to 53 398·2)	75·9% (73·5 to 78·3)*	30·1% (25·0 to 35·8)*	20·3% (18·8 to 21·9)*	3·9% (−0·1 to 8·3)
		Type 1 diabetes mellitus		13 019·0 (11 698·8 to 14 614·6)	400·3 (362·3 to 441·7)	964·3 (659·9 to 1356·0)	40·6% (38·2 to 43·1)*	15·1% (13·8 to 16·7)*	3·5% (2·0 to 4·9)*	−1·2% (−2·3 to −0·1)*
			Uncomplicated type 1 diabetes mellitus	8762·6 (7743·9 to 9877·9)	400·3 (362·3 to 441·7)	412·4 (256·1 to 601·4)	34·7% (32·1 to 37·3)*	11·9% (10·1 to 13·6)*	3·5% (1·8 to 5·1)*	−1·2% (−2·7 to 0·1)
			Vision loss due to type 1 diabetes mellitus retinopathy	82·5 (65·3 to 102·7)	..	6·9 (4·5 to 10·1)	61·4% (56·5 to 66·9)*	4·0% (−0·9 to 9·0)	10·4% (7·4 to 14·1)*	−15·1% (−19·2 to −11·1)*
			Diabetic neuropathy due to type 1 diabetes mellitus	4173·9 (3612·2 to 4837·5)	..	545·0 (365·7 to 762·7)	45·4% (42·2 to 48·6)*	17·9% (15·2 to 20·7)*	3·3% (1·4 to 5·3)*	−1·0% (−3·3 to 1·2)
		Type 2 diabetes mellitus		462 976·9 (423 591·9 to 509 485·3)	22 535·3 (20 693·8 to 24 626·7)	37 611·1 (25 451·4 to 52 049·5)	77·2% (74·7 to 79·8)*	30·5% (25·3 to 36·4)*	20·9% (19·3 to 22·5)*	4·0% (−0·1 to 8·6)
			Uncomplicated type 2 diabetes mellitus	263 762·9 (232 967·8 to 297 029·0)	22 535·3 (20 693·8 to 24 626·7)	12 152·7 (7658·0 to 17 914·1)	74·0% (70·9 to 77·3)*	28·5% (22·8 to 34·5)*	20·6% (18·5 to 22·8)*	4·0% (−0·7 to 8·9)
			Vision loss due to type 2 diabetes mellitus retinopathy	4509·0 (3835·7 to 5282·0)	..	362·5 (252·6 to 500·2)	97·1% (90·5 to 104·5)*	21·1% (18·2 to 23·6)*	31·3% (27·0 to 36·3)*	−5·7% (−7·7 to −3·8)*
			Diabetic neuropathy due to type 2 diabetes mellitus	194 704·9 (169 971·1 to 224 314·8)	..	25 095·9 (16 801·1 to 34 866·6)	78·5% (75·0 to 82·2)*	31·6% (25·7 to 38·1)*	20·9% (18·6 to 23·3)*	4·2% (−0·4 to 9·2)
	Chronic kidney disease			697 509·5 (649 209·4 to 752 050·7)	19 735·6 (17 726·6 to 21 982·7)	7306·3 (5434·9 to 9214·6)	38·8% (35·2 to 42·7)*	24·4% (19·7 to 29·5)*	−4·1% (−6·9 to −1·3)*	−0·2% (−3·8 to 3·8)
		Chronic kidney disease due to type 1 diabetes mellitus		3246·5 (2916·0 to 3615·5)	118·3 (100·5 to 141·5)	264·7 (187·5 to 353·0)	58·2% (52·8 to 63·9)*	21·7% (17·4 to 26·3)*	10·3% (6·8 to 14·0)*	0·3% (−3·0 to 3·7)
			Albuminuria with preserved GFR due to type 1 diabetes mellitus	1406·8 (1223·8 to 1605·5)	..	..	..	..	..	..
			End-stage chronic kidney disease due to type 1 diabetes mellitus	412·3 (326·6 to 512·2)	..	168·0 (110·5 to 233·7)	67·5% (60·5 to 75·9)*	22·1% (16·5 to 27·4)*	13·8% (8·8 to 19·2)*	−0·2% (−4·4 to 3·9)
			Stage III chronic kidney disease due to type 1 diabetes mellitus	1186·3 (1002·9 to 1413·2)	118·3 (100·5 to 141·5)	22·8 (15·1 to 33·1)	12·2% (5·2 to 23·4)*	14·8% (5·8 to 24·0)*	−13·6% (−18·6 to −5·9)*	0·4% (−7·6 to 8·5)
			Stage IV chronic kidney disease due to type 1 diabetes mellitus	149·4 (118·7 to 187·7)	..	20·5 (12·8 to 29·7)	54·8% (47·9 to 62·3)*	25·6% (16·0 to 34·9)*	7·4% (2·8 to 12·3)*	1·6% (−6·2 to 9·2)
			Stage V chronic kidney disease due to type 1 diabetes mellitus	91·7 (74·9 to 111·7)	..	53·5 (36·6 to 73·1)	61·3% (54·0 to 69·3)*	22·1% (15·2 to 29·4)*	14·7% (10·1 to 19·9)*	1·5% (−4·2 to 7·5)
		Chronic kidney disease due to type 2 diabetes mellitus		125 811·6 (116 308·7 to 135 941·1)	2352·5 (2063·9 to 2680·9)	1450·3 (1080·6 to 1875·1)	49·9% (41·4 to 55·6)*	29·6% (23·0 to 35·6)*	0·7% (−3·5 to 4·3)	−0·5% (−5·2 to 4·0)
			Albuminuria with preserved GFR due to type 2 diabetes mellitus	85 416·5 (76 825·4 to 94 433·7)	..	..	..	..	..	..
			End-stage chronic kidney disease due to type 2 diabetes mellitus	1046·2 (889·3 to 1236·3)	..	432·3 (296·9 to 564·4)	94·0% (85·7 to 104·3)*	32·2% (27·7 to 36·7)*	31·7% (26·2 to 38·3)*	0·8% (−2·5 to 4·0)
			Stage III chronic kidney disease due to type 2 diabetes mellitus	35 357·3 (31 403·1 to 39 849·1)	2352·5 (2063·9 to 2680·9)	156·1 (102·1 to 234·4)	10·9% (5·6 to 17·2)*	19·0% (10·9 to 27·0)*	−26·2% (−29·6 to −22·1)*	−8·1% (−14·2 to −1·9)*
			Stage IV chronic kidney disease due to type 2 diabetes mellitus	2906·1 (2314·3 to 3621·8)	..	308·5 (198·6 to 443·0)	37·1% (31·0 to 41·1)*	30·5% (21·2 to 40·3)*	−9·4% (−11·7 to −7·4)*	−0·8% (−7·6 to 6·7)
			Stage V chronic kidney disease due to type 2 diabetes mellitus	1085·5 (915·0 to 1281·7)	..	553·5 (383·2 to 745·7)	47·7% (30·4 to 55·4)*	30·4% (19·5 to 39·2)*	0·2% (−7·0 to 4·8)	1·2% (−6·6 to 7·8)
		Chronic kidney disease due to hypertension		23 605·4 (21 734·7 to 25 994·1)	911·2 (810·0 to 1033·5)	1390·7 (1036·0 to 1772·1)	47·7% (44·0 to 52·1)*	28·0% (23·0 to 33·1)*	0·8% (−1·7 to 3·7)	1·4% (−2·4 to 5·4)
			Albuminuria with preserved GFR due to hypertension	4310·3 (3891·2 to 4793·2)	..	..	..	..	..	..
			End-stage chronic kidney disease due to hypertension	825·3 (700·2 to 968·4)	..	341·2 (232·8 to 450·8)	78·0% (70·5 to 86·4)*	33·5% (28·9 to 37·6)*	20·2% (15·4 to 25·5)*	2·6% (−0·7 to 5·4)
			Stage III chronic kidney disease due to hypertension	14 545·5 (13 086·5 to 16 401·2)	911·2 (810·0 to 1033·5)	123·0 (81·8 to 181·1)	16·0% (10·7 to 22·4)*	16·6% (9·1 to 23·6)*	−22·9% (−26·2 to −18·7)*	−9·3% (−14·9 to −3·8)*
			Stage IV chronic kidney disease due to hypertension	2743·0 (2268·8 to 3328·2)	..	299·7 (200·7 to 418·4)	39·4% (36·2 to 42·9)*	29·0% (20·5 to 37·3)*	−7·1% (−8·7 to −5·1)*	0·4% (−5·9 to 6·7)
			Stage V chronic kidney disease due to hypertension	1181·3 (1017·6 to 1370·2)	..	626·7 (432·1 to 829·9)	47·3% (43·9 to 52·4)*	27·2% (20·9 to 33·9)*	3·3% (1·3 to 6·5)*	3·6% (−1·2 to 9·0)
		Chronic kidney disease due to glomerulonephritis		28 809·4 (26 470·2 to 31 575·8)	1509·5 (1345·4 to 1699·8)	1047·9 (753·5 to 1372·2)	38·1% (34·5 to 43·1)*	20·4% (15·5 to 25·6)*	0·8% (−1·4 to 3·8)	2·2% (−1·8 to 6·8)
			Albuminuria with preserved GFR due to glomerulonephritis	5008·3 (4410·3 to 5616·9)	..	..	..	..	..	..
			End-stage chronic kidney disease due to glomerulonephritis	691·9 (594·2 to 812·8)	..	270·4 (188·0 to 360·3)	57·3% (52·0 to 63·4)*	22·7% (18·4 to 27·2)*	10·2% (6·6 to 14·3)*	−0·4% (−3·6 to 2·8)
			Stage III chronic kidney disease due to glomerulonephritis	20 751·9 (18 673·1 to 23 071·0)	1509·5 (1345·4 to 1699·8)	85·8 (55·6 to 125·3)	7·4% (1·1 to 17·8)*	8·3% (0·1 to 16·9)*	−17·2% (−21·6 to −10·0)*	−4·7% (−12·1 to 2·5)
			Stage IV chronic kidney disease due to glomerulonephritis	1435·8 (1169·9 to 1762·1)	..	172·4 (110·5 to 246·3)	30·8% (27·3 to 35·6)*	21·3% (13·0 to 29·7)*	−6·1% (−8·3 to −3·2)*	2·2% (−5·4 to 9·3)
			Stage V chronic kidney disease due to glomerulonephritis	921·6 (765·1 to 1104·5)	..	519·3 (351·4 to 713·8)	39·4% (35·4 to 44·9)*	21·2% (14·8 to 28·3)*	2·8% (0·7 to 6·2)*	4·9% (−0·7 to 10·7)
		Chronic kidney disease due to other and unspecified causes		516 036·7 (480 273·8 to 555 659·4)	14 844·1 (13 340·7 to 16 532·6)	3152·6 (2327·5 to 4005·4)	30·2% (27·0 to 34·6)*	22·2% (17·2 to 27·6)*	−10·3% (−13·0 to −7·3)*	−1·5% (−5·5 to 2·8)
			Albuminuria with preserved GFR due to other and unspecified causes	285 109·9 (256 788·7 to 314 368·4)	..	..	..	..	..	..
			End-stage chronic kidney disease due to other and unspecified causes	955·4 (822·5 to 1111·7)	..	380·7 (257·9 to 504·2)	59·9% (54·4 to 66·0)*	23·9% (19·3 to 28·3)*	13·4% (9·5 to 17·3)*	0·2% (−3·1 to 3·2)
			Stage III chronic kidney disease due to other and unspecified causes	222 956·2 (200 778·0 to 247 902·5)	14 844·1 (13 340·7 to 16 532·6)	1105·1 (727·3 to 1640·7)	11·4% (7·2 to 15·7)*	15·6% (9·4 to 22·0)*	−24·8% (−27·6 to −22·1)*	−8·4% (−13·4 to −3·2)*
			Stage IV chronic kidney disease due to other and unspecified causes	4825·1 (4024·2 to 5754·4)	..	520·5 (346·0 to 725·0)	37·3% (34·0 to 40·9)*	27·9% (20·0 to 36·0)*	−7·8% (−9·3 to −5·9)*	0·3% (−5·5 to 6·5)
			Stage V chronic kidney disease due to other and unspecified causes	2190·0 (1894·3 to 2551·5)	..	1146·3 (788·6 to 1517·9)	43·4% (39·4 to 49·2)*	25·9% (19·9 to 32·2)*	2·9% (0·8 to 6·3)*	4·5% (−0·4 to 9·7)
	Acute glomerulonephritis			52·4 (45·5 to 60·7)	773·4 (672·8 to 877·3)	2·6 (1·6 to 4·0)	−1·3% (−13·9 to 2·3)	1·0% (−2·1 to 3·5)	−24·3% (−34·2 to −21·4)*	−12·6% (−15·3 to −10·1)*
	**Skin and subcutaneous diseases**			**1 974 238·4 (1 916 671·8 to 2 034 645·7)**	**4 185 971·3 (3 971 760·5 to 4 391 218·2)**	**41 621·9 (27 371·7 to 61 859·5)**	24·0% (22·9 to 25·3)*	13·0% (12·5 to 13·6)*	0·9% (0·4 to 1·4)*	0·6% (0·1 to 1·1)*
	Dermatitis			291 689·4 (276 520·7 to 308 059·0)	274 034·1 (246 120·0 to 302 497·5)	11 128·1 (6484·1 to 17 733·4)	19·3% (18·2 to 20·7)*	12·1% (11·3 to 12·8)*	−0·1% (−0·7 to 0·5)	1·1% (0·4 to 1·8)*
		Atopic dermatitis		205 517·2 (193 701·2 to 218 582·4)	27 134·4 (25 282·9 to 29 055·0)	9003·4 (4887·0 to 14 981·0)	17·0% (16·1 to 17·9)*	11·6% (10·8 to 12·5)*	0·4% (−0·4 to 1·1)	1·7% (0·9 to 2·6)*
		Contact dermatitis		79 666·7 (70 425·7 to 89 554·9)	221 252·8 (193 775·3 to 249 726·6)	1989·2 (1304·4 to 2950·5)	31·0% (28·8 to 33·2)*	14·4% (12·6 to 16·2)*	−1·6% (−2·3 to −1·0)*	−1·1% (−1·7 to −0·5)*
		Seborrhoeic dermatitis		10 035·9 (9450·2 to 10 668·4)	25 646·9 (23 981·6 to 27 315·9)	135·6 (78·1 to 215·0)	20·8% (18·5 to 23·3)*	8·4% (6·6 to 10·3)*	−7·7% (−9·0 to −6·4)*	−7·1% (−8·3 to −5·8)*
	Psoriasis			64 609·6 (62 454·3 to 66 767·5)	7846·6 (7564·9 to 8162·8)	5569·5 (3956·1 to 7354·3)	43·1% (42·0 to 44·2)*	21·1% (20·3 to 21·9)*	5·1% (4·3 to 5·8)*	2·3% (1·6 to 2·9)*
	Bacterial skin diseases			11 397·9 (11 061·4 to 11 741·7)	266 779·7 (260 229·3 to 273 665·3)	177·9 (112·3 to 274·1)	29·2% (27·2 to 31·0)*	15·5% (14·0 to 17·1)*	2·0% (−0·5 to 4·4)	0·8% (−1·1 to 2·9)
		Cellulitis		2071·5 (1952·2 to 2186·2)	42 958·6 (40 535·7 to 45 172·9)	118·2 (78·7 to 167·8)	26·7% (24·9 to 28·6)*	12·9% (11·7 to 14·2)*	−2·6% (−3·8 to −1·3)*	−2·9% (−3·8 to −2·0)*
		Pyoderma		10 557·2 (10 245·5 to 10 853·2)	223 821·2 (217 649·0 to 230 259·5)	59·6 (23·9 to 123·5)	35·0% (33·7 to 36·4)*	20·9% (19·8 to 22·2)*	13·0% (11·9 to 14·2)*	8·8% (7·8 to 9·9)*
			Impetigo	4620·7 (4358·2 to 4870·5)	84 007·5 (79 485·3 to 88 529·9)	26·4 (10·4 to 55·8)	35·7% (33·6 to 37·8)*	20·7% (18·7 to 22·7)*	24·1% (22·2 to 26·0)*	13·4% (11·5 to 15·2)*
			Abscess and other bacterial skin diseases	5936·4 (5770·4 to 6098·7)	139 813·6 (135 314·1 to 143 965·8)	33·2 (13·4 to 69·5)	34·5% (32·8 to 36·3)*	21·1% (19·7 to 22·6)*	5·5% (4·2 to 6·8)*	5·1% (3·8 to 6·4)*
	Scabies			175 406·7 (154 517·9 to 198 404·1)	527 476·5 (462 050·9 to 598 087·9)	4528·7 (2506·4 to 7414·6)	16·1% (13·8 to 18·5)*	6·6% (5·3 to 8·0)*	−4·3% (−5·0 to −3·6)*	−3·1% (−3·6 to −2·6)*
	Fungal skin diseases			743 458·4 (681 568·4 to 808 149·7)	2 126 927·9 (1 917 361·6 to 2 317 274·7)	4154·5 (1651·4 to 8633·2)	21·5% (19·0 to 24·0)*	10·9% (9·2 to 12·5)*	−3·1% (−3·8 to −2·4)*	−4·4% (−5·4 to −3·4)*
		Tinea capitis		160 239·3 (133 390·6 to 194 439·3)	303 016·6 (245 340·4 to 369 760·2)	916·5 (357·3 to 1965·4)	−5·8% (−7·3 to −4·3)*	−13·0% (−14·5 to −11·3)*	−13·8% (−15·0 to −12·7)*	−19·0% (−20·4 to −17·3)*
		Other fungal skin diseases		583 219·1 (526 500·8 to 645 363·9)	1 823 911·3 (1 638 297·9 to 2 009 788·9)	3238·0 (1305·4 to 6694·4)	37·1% (35·2 to 39·3)*	20·2% (19·0 to 21·6)*	1·8% (1·5 to 2·1)*	1·3% (1·1 to 1·5)*
	Viral skin diseases			130 639·2 (125 604·0 to 136 047·9)	116 329·8 (111 012·4 to 121 710·1)	4033·0 (2595·4 to 5995·6)	10·4% (9·8 to 11·1)*	6·4% (6·0 to 6·9)*	−2·8% (−3·2 to −2·3)*	−1·8% (−2·2 to −1·4)*
		Viral warts		54 309·6 (52 104·2 to 56 403·2)	30 140·5 (29 078·5 to 31 211·0)	1662·2 (1066·0 to 2439·8)	13·7% (12·8 to 14·6)*	5·6% (4·8 to 6·3)*	−6·8% (−7·3 to −6·3)*	−4·8% (−5·4 to −4·3)*
		Molluscum contagiosum		76 329·6 (71 596·6 to 81 134·1)	86 189·3 (80 984·6 to 91 427·2)	2370·7 (1511·7 to 3525·9)	8·2% (7·5 to 8·9)*	7·1% (6·5 to 7·6)*	0·2% (−0·3 to 0·8)	0·3% (−0·3 to 0·8)
	Acne vulgaris			119 082·3 (107 127·9 to 133 651·4)	60 118·8 (53 260·2 to 68 180·7)	2547·6 (1518·8 to 4056·6)	46·1% (44·6 to 47·6)*	16·2% (15·2 to 17·2)*	19·8% (18·6 to 20·9)*	11·4% (10·3 to 12·5)*
	Alopecia areata			15 981·0 (15 477·3 to 16 515·9)	28 185·2 (27 302·2 to 29 126·3)	523·1 (334·9 to 774·8)	28·8% (27·6 to 30·1)*	12·7% (11·8 to 13·8)*	−2·3% (−3·1 to −1·4)*	−1·5% (−2·3 to −0·6)*
	Pruritus			71 224·3 (63 948·2 to 80 034·2)	55 643·1 (49 208·3 to 62 689·4)	755·6 (356·1 to 1433·2)	36·4% (34·1 to 38·8)*	18·9% (17·5 to 20·4)*	2·9% (2·5 to 3·4)*	1·6% (1·2 to 2·0)*
	Urticaria			83 610·0 (73 335·4 to 95 162·9)	147 198·5 (129 941·2 to 166 345·6)	5014·8 (3321·0 to 7046·4)	19·3% (17·4 to 21·5)*	10·8% (9·8 to 11·9)*	0·7% (0·2 to 1·2)*	0·3% (−0·2 to 0·8)
	Decubitus ulcer			1143·7 (1022·6 to 1288·5)	4199·3 (3752·4 to 4741·3)	181·2 (125·8 to 244·3)	45·2% (42·7 to 47·7)*	28·9% (26·3 to 31·9)*	−3·3% (−4·9 to −1·8)*	−0·4% (−2·2 to 1·5)
	Other skin and subcutaneous diseases			550 810·3 (538 490·7 to 563 961·3)	571 231·9 (558 726·3 to 584 649·5)	3008·1 (1446·7 to 5557·4)	45·1% (44·6 to 45·6)*	25·6% (25·2 to 26·0)*	6·6% (6·4 to 6·8)*	4·3% (4·1 to 4·5)*
	**Sense organ diseases**			**2 035 737·0 (1 994 115·8 to 2 079 908·6)**	**..**	**66 576·1 (44 700·8 to 95 675·1)**	42·0% (41·1 to 42·8)*	24·1% (23·4 to 24·8)*	−1·0% (−1·6 to −0·5)*	−0·9% (−1·5 to −0·3)*
	Blindness and vision impairment			1 193 642·8 (1 154 777·0 to 1 237 118·0)	..	29 895·2 (20 277·7 to 44 036·0)	39·3% (37·8 to 40·5)*	23·1% (22·0 to 24·0)*	−2·7% (−3·5 to −1·9)*	−1·8% (−2·7 to −0·9)*
		Glaucoma		5993·5 (5157·5 to 6984·8)	..	686·1 (462·9 to 948·8)	41·5% (39·1 to 44·2)*	27·9% (26·4 to 29·4)*	−6·0% (−7·4 to −4·2)*	−4·7% (−5·8 to −3·6)*
		Cataract		107 987·7 (95 775·3 to 122 319·3)	..	8005·8 (5579·2 to 10 820·9)	49·4% (46·9 to 52·0)*	29·6% (27·4 to 31·7)*	−2·8% (−4·2 to −1·2)*	−2·0% (−3·6 to −0·5)*
		Age-related macular degeneration		6758·8 (5612·6 to 8183·0)	..	530·7 (363·6 to 728·6)	59·3% (54·5 to 63·9)*	30·7% (28·6 to 32·9)*	2·8% (−0·7 to 6·1)	−3·7% (−5·3 to −2·1)*
		Refraction disorders		185 392·8 (165 673·9 to 208 575·2)	..	7984·3 (5353·0 to 11 487·0)	24·6% (21·7 to 27·7)*	15·3% (13·6 to 17·0)*	−6·1% (−7·8 to −4·3)*	−3·1% (−4·2 to −1·9)*
		Near vision loss		969 669·5 (932 613·8 to 1 009 067·7)	..	9802·9 (4669·6 to 17 922·5)	46·4% (45·6 to 47·1)*	24·6% (24·0 to 25·2)*	1·2% (0·8 to 1·7)*	0·5% (0·1 to 0·9)*
		Other vision loss		34 607·5 (30 956·5 to 38 397·4)	..	2885·3 (2013·7 to 3884·0)	36·7% (34·6 to 38·8)*	21·0% (19·4 to 22·6)*	−4·6% (−5·8 to −3·3)*	−3·7% (−4·8 to −2·6)*
	Age-related and other hearing loss			1 317 431·7 (1 276 962·6 to 1 356 229·1)	..	34 229·6 (23 176·8 to 48 684·1)	44·4% (43·0 to 45·7)*	24·9% (23·9 to 26·0)*	0·4% (−0·4 to 1·1)	−0·2% (−1·0 to 0·5)
	Other sense organ diseases			103 750·5 (100 613·2 to 107 050·4)	..	2451·3 (1519·6 to 3573·5)	42·1% (41·1 to 43·1)*	25·0% (24·2 to 25·8)*	1·8% (1·3 to 2·3)*	0·9% (0·4 to 1·4)*
		Chronic other sense organ diseases		87 788·5 (84 748·6 to 91 039·5)	..	2232·4 (1381·5 to 3246·2)	44·3% (43·4 to 45·3)*	26·1% (25·4 to 26·9)*	1·8% (1·3 to 2·3)*	0·9% (0·4 to 1·4)*
		Acute other sense organ diseases		15 962·1 (15 501·3 to 16 458·6)	..	218·9 (133·2 to 332·7)	24·3% (22·6 to 25·9)*	14·6% (13·2 to 16·2)*	1·7% (0·5 to 2·9)*	1·0% (−0·3 to 2·4)
	**Musculoskeletal disorders**			**1 312 131·3 (1 248 058·7 to 1 383 422·6)**	**334 744·9 (309 934·0 to 363 175·8)**	**135 881·3 (99 022·6 to 179 645·0)**	38·4% (36·4 to 40·2)*	19·9% (18·8 to 21·2)*	−2·3% (−3·3 to −1·2)*	−1·1% (−1·7 to −0·5)*
	Rheumatoid arthritis			19 965·1 (17 990·5 to 21 955·7)	1204·6 (1071·1 to 1331·7)	2626·0 (1783·4 to 3529·9)	45·8% (44·1 to 47·5)*	33·5% (30·6 to 36·4)*	0·9% (−0·1 to 1·9)	6·7% (4·7 to 8·8)*
	Osteoarthritis			303 096·5 (273 279·8 to 338 632·0)	14 933·5 (13 386·3 to 16 739·6)	9604·0 (4808·6 to 19 139·0)	63·1% (61·5 to 64·9)*	31·4% (30·7 to 32·1)*	8·5% (7·5 to 9·6)*	1·0% (0·5 to 1·6)*
		Osteoarthritis of the hip		40 010·0 (37 473·8 to 42 725·7)	2045·1 (1913·7 to 2195·9)	1265·4 (648·2 to 2609·3)	59·1% (57·9 to 60·4)*	35·3% (34·5 to 36·2)*	5·7% (4·9 to 6·4)*	3·8% (3·2 to 4·4)*
		Osteoarthritis of the knee		263 086·5 (232 661·9 to 298 640·0)	12 888·4 (11 375·0 to 14 656·0)	8338·6 (4152·3 to 16 431·9)	63·7% (61·8 to 65·8)*	30·8% (30·1 to 31·6)*	9·0% (7·7 to 10·3)*	0·6% (0·1 to 1·2)*
	Low back pain			576 980·9 (518 940·4 to 637 177·9)	245 858·9 (221 816·5 to 272 419·6)	64 946·7 (46 512·3 to 87 417·1)	30·0% (27·9 to 31·9)*	17·5% (16·2 to 19·0)*	−7·2% (−8·4 to −6·0)*	−2·1% (−2·6 to −1·6)*
		Low back pain with leg pain		187 103·9 (167 946·8 to 209 695·8)	78 640·7 (69 782·0 to 87 714·4)	26 843·2 (19 173·1 to 36 078·7)	31·2% (29·0 to 33·4)*	19·0% (17·6 to 20·6)*	−7·8% (−8·9 to −6·5)*	−2·2% (−2·7 to −1·6)*
		Low back pain without leg pain		389 877·0 (350 624·1 to 429 901·0)	167 218·2 (150 726·4 to 185 159·5)	38 103·5 (27 216·0 to 51 524·8)	29·1% (27·1 to 31·1)*	16·5% (15·1 to 18·0)*	−6·9% (−8·1 to −5·7)*	−2·0% (−2·5 to −1·6)*
	Neck pain			288 718·6 (254 715·3 to 323 483·0)	65 310·3 (57 678·2 to 73 917·2)	28 631·1 (19 951·8 to 40 202·3)	44·4% (41·9 to 47·0)*	21·4% (19·5 to 23·6)*	−0·0% (−1·2 to 1·2)	−0·7% (−1·6 to 0·5)
	Gout			41 217·7 (36 699·8 to 46 101·3)	7437·7 (6573·8 to 8457·4)	1285·0 (867·4 to 1768·9)	54·7% (52·5 to 56·9)*	30·9% (29·2 to 32·6)*	4·4% (3·1 to 5·8)*	2·7% (1·6 to 3·7)*
	Other musculoskeletal disorders			336 467·8 (285 415·0 to 383 783·9)	..	28 788·6 (19 498·0 to 39 995·7)	46·1% (43·6 to 48·6)*	19·0% (15·5 to 22·4)*	4·3% (3·3 to 5·4)*	−0·8% (−3·3 to 1·4)
	**Other non-communicable diseases**			**4 916 184·8 (4 793 498·0 to 5 046 526·7)**	**4 209 631·0 (3 838 652·6 to 4 611 876·0)**	**53 645·9 (36 899·7 to 74 479·3)**	25·7% (24·2 to 27·5)*	12·9% (11·8 to 14·2)*	−5·3% (−6·0 to −4·6)*	−3·2% (−3·9 to −2·4)*
	Congenital anomalies			100 366·3 (95 153·7 to 106 524·9)	5445·3 (5088·8 to 5844·4)	12 056·9 (8552·1 to 16 062·8)	23·1% (22·0 to 24·2)*	12·7% (11·7 to 13·7)*	−0·1% (−0·9 to 0·8)	0·8% (−0·2 to 1·6)
		Neural tube defects		2874·4 (2566·3 to 3205·2)	163·9 (145·4 to 185·1)	854·7 (600·1 to 1133·9)	25·1% (22·7 to 27·5)*	17·0% (13·8 to 20·3)*	2·1% (0·2 to 4·1)*	5·1% (2·2 to 8·0)*
			Severe motor and cognitive impairment due to anencephaly	0·1 (0·1 to 0·1)	12·8 (10·2 to 16·3)	0·0 (0·0 to 0·1)	−5·3% (−7·2 to −3·3)*	−6·5% (−8·6 to −4·5)*	−4·5% (−6·4 to −2·4)*	−8·9% (−11·0 to −7·0)*
			Encephalocoele due to neural tube defects	300·6 (246·9 to 354·9)	28·8 (22·3 to 37·4)	51·8 (34·6 to 72·3)	17·0% (14·3 to 20·0)*	12·8% (9·8 to 15·6)*	−6·3% (−8·1 to −4·4)*	−0·7% (−3·4 to 1·9)
			Spina bifida due to neural tube defects	2573·8 (2296·6 to 2874·9)	122·3 (106·0 to 141·4)	802·9 (557·1 to 1069·3)	25·7% (23·1 to 28·3)*	17·3% (14·1 to 20·6)*	2·8% (0·7 to 4·8)*	5·5% (2·6 to 8·5)*
		Congenital heart anomalies		11 998·3 (10 958·7 to 13 123·9)	2481·7 (2203·2 to 2775·4)	589·5 (287·2 to 973·4)	12·2% (9·7 to 13·9)*	7·6% (5·7 to 9·1)*	1·9% (0·4 to 3·2)*	0·2% (−1·5 to 1·5)
			Critical malformations of great vessels, congenital valvular heart disease, and patent ductus arteriosus	2075·2 (1788·4 to 2407·9)	519·5 (406·2 to 650·6)	124·7 (62·7 to 201·6)	10·0% (7·1 to 12·6)*	6·9% (4·3 to 9·1)*	0·9% (−1·5 to 2·9)	−0·1% (−2·3 to 2·1)
			Other congenital heart anomalies	2540·8 (2333·1 to 2769·7)	398·3 (353·7 to 445·6)	142·9 (62·3 to 243·7)	14·2% (11·1 to 16·6)*	8·9% (6·5 to 11·1)*	1·5% (−0·6 to 3·5)	0·4% (−1·8 to 2·4)
			Severe congenital heart anomalies excluding single ventricle heart defects	2899·7 (2567·0 to 3266·8)	565·8 (453·1 to 699·6)	170·9 (86·1 to 282·1)	20·6% (17·0 to 23·4)*	9·2% (6·5 to 11·6)*	6·5% (4·1 to 8·7)*	0·7% (−1·7 to 3·0)
			Single ventricle and single ventricle pathway congenital heart anomalies	344·0 (285·0 to 413·7)	263·5 (186·1 to 356·8)	21·1 (11·3 to 33·7)	13·0% (9·0 to 17·2)*	7·2% (3·2 to 11·1)*	5·5% (1·8 to 9·4)*	0·8% (−3·1 to 4·6)
			Ventricular septal defect and atrial septal defect	4138·6 (3401·3 to 4864·5)	734·5 (588·6 to 908·7)	129·8 (62·0 to 225·5)	2·9% (−0·0 to 5·7)	5·1% (2·5 to 7·5)*	−2·4% (−4·7 to −0·0)*	−0·7% (−3·3 to 1·6)
		Orofacial clefts		10 816·4 (9945·7 to 11 654·1)	195·5 (152·0 to 249·3)	320·8 (211·9 to 468·2)	8·7% (3·4 to 14·7)*	13·5% (7·5 to 20·0)*	−11·2% (−15·5 to −6·4)*	1·5% (−4·0 to 7·3)
		Down syndrome		3535·7 (3092·1 to 4015·5)	110·5 (95·2 to 130·6)	326·4 (222·7 to 452·6)	22·5% (21·3 to 23·8)*	11·8% (9·1 to 14·7)*	−1·5% (−2·6 to −0·5)*	−0·2% (−2·6 to 2·4)
		Turner syndrome		572·3 (502·0 to 652·8)	19·6 (17·0 to 22·9)	10·1 (5·0 to 16·5)	21·6% (19·5 to 24·1)*	9·1% (7·3 to 10·9)*	1·1% (−0·6 to 2·7)	0·9% (−0·7 to 2·5)
		Klinefelter syndrome		866·8 (747·4 to 994·2)	28·3 (24·2 to 32·8)	5·2 (2·4 to 9·7)	28·6% (24·5 to 33·0)*	7·4% (4·3 to 10·7)*	4·4% (1·4 to 7·6)*	−1·7% (−4·5 to 1·1)
		Other chromosomal abnormalities		6379·1 (5052·5 to 7827·4)	301·3 (236·2 to 371·0)	593·1 (389·9 to 833·1)	24·2% (22·1 to 26·3)*	19·6% (16·9 to 22·4)*	2·3% (0·7 to 4·1)*	7·1% (4·7 to 9·5)*
		Congenital musculoskeletal and limb anomalies		31 506·6 (28 137·2 to 36 022·4)	1537·0 (1327·4 to 1777·0)	4622·5 (3030·7 to 6345·8)	24·8% (23·9 to 25·8)*	13·4% (12·5 to 14·2)*	−0·6% (−1·4 to 0·1)	0·4% (−0·4 to 1·1)
			Polydactyly and syndactyly	432·6 (342·4 to 538·9)	218·7 (158·9 to 295·2)	4·7 (2·1 to 9·1)	0·3% (−3·9 to 5·2)	7·1% (3·1 to 10·9)*	−4·4% (−8·1 to −0·2)*	0·9% (−3·2 to 4·7)
			Congenital limb deficiency	14 811·3 (13 154·2 to 17 016·1)	389·3 (343·2 to 449·2)	2217·3 (1452·1 to 3057·6)	22·4% (21·2 to 23·6)*	11·8% (10·8 to 12·9)*	−0·1% (−1·1 to 0·8)	0·5% (−0·4 to 1·5)
			Other congenital musculoskeletal anomalies	16 262·8 (14 519·3 to 18 611·7)	929·0 (801·7 to 1074·0)	2400·4 (1574·9 to 3296·1)	27·3% (26·1 to 28·5)*	14·8% (13·8 to 15·8)*	−1·1% (−2·1 to −0·2)*	0·2% (−0·7 to 1·0)
		Urogenital congenital anomalies		5725·5 (4984·9 to 6504·8)	249·7 (216·9 to 285·3)	141·0 (82·3 to 233·3)	13·0% (11·5 to 14·6)*	4·2% (2·2 to 6·3)*	−4·7% (−5·8 to −3·6)*	−4·1% (−6·0 to −2·3)*
		Digestive congenital anomalies		17 662·5 (15 899·6 to 19 748·8)	357·9 (323·3 to 398·8)	794·3 (528·2 to 1134·1)	16·3% (15·0 to 17·5)*	8·7% (4·3 to 13·2)*	−8·5% (−9·5 to −7·5)*	−4·3% (−8·3 to −0·4)*
			Congenital diaphragmatic hernia	315·6 (276·8 to 357·7)	15·5 (12·9 to 17·9)	6·2 (3·6 to 10·1)	13·9% (12·9 to 14·9)*	6·5% (5·6 to 7·3)*	−1·3% (−2·0 to −0·5)*	−1·8% (−2·6 to −1·0)*
			Congenital atresia or stenosis of the digestive tract	111·9 (83·4 to 142·7)	137·3 (96·5 to 174·1)	8·9 (5·4 to 13·7)	−5·9% (−8·5 to −3·4)*	6·1% (3·2 to 9·1)*	−7·8% (−10·3 to −5·4)*	0·7% (−2·1 to 3·6)
			Congenital malformations of the abdominal wall after treatment	1258·9 (1109·6 to 1431·5)	84·7 (71·3 to 99·3)	54·0 (34·5 to 79·4)	15·2% (14·3 to 16·2)*	7·4% (6·6 to 8·2)*	0·1% (−0·6 to 0·8)	−0·8% (−1·6 to −0·1)*
			Other congenital malformations of the digestive tract	15 976·1 (14 219·3 to 18 092·5)	120·3 (88·9 to 169·7)	725·2 (480·5 to 1035·6)	16·7% (15·2 to 18·2)*	8·8% (4·0 to 13·7)*	−9·2% (−10·3 to −8·1)*	−4·7% (−9·0 to −0·4)*
		Other congenital anomalies		25 911·9 (22 431·7 to 30 006·9)	..	3799·4 (2595·5 to 5226·3)	25·9% (23·0 to 28·7)*	12·1% (9·6 to 14·5)*	2·9% (0·5 to 5·3)*	0·8% (−1·4 to 3·0)
			Other congenital birth defects	..	..	446·4 (309·1 to 601·8)	34·8% (32·6 to 37·3)*	19·3% (17·3 to 22·0)*	19·0% (17·6 to 20·5)*	9·8% (8·0 to 11·9)*
			Hearing loss due to other congenital anomalies	25 911·9 (22 431·7 to 30 006·9)	..	3353·0 (2227·2 to 4697·0)	24·8% (21·6 to 28·0)*	11·2% (8·4 to 13·9)*	1·1% (−1·4 to 3·6)	−0·4% (−2·8 to 2·1)
	Urinary diseases and male infertility			110 267·4 (99 363·2 to 120 483·9)	366 721·5 (337 838·9 to 396 838·4)	3135·6 (2053·6 to 4529·0)	33·7% (31·9 to 35·6)*	26·9% (25·5 to 28·4)*	−7·6% (−9·0 to −6·2)*	−0·8% (−1·7 to 0·1)
		Urinary tract infections		5250·9 (4741·8 to 5801·2)	274 626·5 (247 558·6 to 303 427·3)	173·0 (106·4 to 256·5)	29·8% (27·1 to 32·4)*	16·6% (14·0 to 19·3)*	0·3% (−1·2 to 1·9)	1·9% (0·1 to 3·7)*
		Urolithiasis		3147·5 (2808·1 to 3483·6)	83 173·6 (74 603·1 to 91 918·7)	230·9 (159·6 to 317·0)	31·2% (28·7 to 34·1)*	18·6% (16·2 to 20·9)*	−7·5% (−9·3 to −5·7)*	−2·0% (−3·5 to −0·3)*
		Benign prostatic hyperplasia		74 541·9 (65 358·9 to 84 081·8)	8921·5 (7859·8 to 10 046·2)	2427·3 (1562·8 to 3460·0)	36·7% (34·1 to 39·5)*	32·0% (30·7 to 33·3)*	−8·0% (−9·5 to −6·2)*	−0·2% (−1·1 to 0·8)
		Male infertility		30 432·7 (24 686·0 to 36 812·7)	..	180·9 (74·8 to 373·9)	33·1% (29·7 to 36·8)*	17·0% (13·7 to 20·1)*	1·3% (−0·5 to 3·3)	6·9% (4·1 to 9·7)*
		Other urinary diseases		..	..	123·5 (84·7 to 170·6)	8·4% (6·5 to 10·3)*	−7·1% (−8·6 to −5·7)*	−18·0% (−19·3 to −16·7)*	−20·3% (−21·5 to −19·2)*
	Gynaecological diseases			815 073·8 (787 646·5 to 843 595·7)	195 631·4 (189 141·1 to 202 275·1)	11 619·6 (7966·6 to 16 279·1)	33·9% (31·3 to 36·5)*	10·2% (9·0 to 11·6)*	−1·9% (−3·4 to −0·5)*	−2·4% (−3·3 to −1·4)*
		Uterine fibroids		159 444·4 (139 576·9 to 182 588·5)	8445·5 (7062·0 to 10 077·7)	1473·4 (866·6 to 2386·3)	44·3% (39·5 to 48·4)*	8·1% (3·9 to 11·7)*	−2·5% (−5·7 to 0·4)	−8·0% (−11·5 to −5·1)*
		Polycystic ovarian syndrome		52 159·9 (41 652·7 to 66 540·8)	2129·0 (1727·3 to 2716·3)	459·4 (203·7 to 886·2)	33·2% (31·8 to 34·6)*	13·1% (12·1 to 14·1)*	0·6% (−0·3 to 1·4)	1·8% (1·0 to 2·7)*
		Female infertility		61 238·4 (43 321·2 to 82 668·2)	..	342·6 (129·5 to 723·4)	32·1% (26·8 to 37·5)*	27·5% (22·2 to 32·7)*	−0·0% (−2·6 to 2·7)	16·5% (12·0 to 20·8)*
		Endometriosis		44 656·0 (37 289·1 to 52 852·4)	6291·6 (5083·7 to 7555·2)	4121·5 (2752·3 to 5940·5)	31·2% (26·0 to 37·5)*	9·2% (7·9 to 10·5)*	−3·1% (−6·3 to 0·5)	−3·0% (−3·9 to −2·0)*
		Genital prolapse		108 559·4 (96 272·0 to 121 882·8)	12 319·0 (10 947·3 to 13 860·3)	337·3 (161·9 to 634·2)	26·0% (25·0 to 27·2)*	19·9% (18·5 to 21·3)*	−13·7% (−14·4 to −12·9)*	−3·6% (−4·4 to −2·8)*
		Premenstrual syndrome		472 189·7 (451 411·0 to 493 266·2)	127 157·4 (121 707·4 to 132 919·3)	3930·0 (2533·7 to 5900·3)	32·3% (30·9 to 33·7)*	8·8% (7·2 to 10·1)*	−0·6% (−1·4 to 0·3)	−2·2% (−3·6 to −1·0)*
		Other gynaecological diseases		50 421·8 (48 042·0 to 52 824·5)	39 288·9 (37 076·3 to 41 538·6)	955·4 (643·6 to 1384·7)	42·2% (36·5 to 47·7)*	14·5% (11·6 to 17·4)*	3·0% (−0·6 to 6·7)	1·3% (−1·5 to 3·9)
			Other gynaecological diseases	45 859·4 (43 492·6 to 48 226·9)	39 288·9 (37 076·3 to 41 538·6)	855·8 (576·7 to 1238·9)	41·5% (35·6 to 47·2)*	15·5% (12·2 to 18·6)*	2·5% (−1·2 to 6·4)	1·9% (−1·1 to 5·0)
			Other gynaecological diseases with anaemia	4562·4 (4423·1 to 4703·7)	..	99·6 (66·3 to 146·8)	47·3% (39·9 to 55·6)*	6·6% (1·1 to 11·8)*	6·4% (0·9 to 12·5)*	−4·2% (−8·9 to 0·6)
	Haemoglobinopathies and haemolytic anaemias			1 922 603·3 (1 878 676·9 to 1 967 411·4)	42 237·7 (40 961·6 to 43 660·7)	5313·7 (3608·1 to 7683·0)	1·1% (−2·0 to 4·1)	−5·3% (−8·4 to −2·3)*	−17·8% (−20·2 to −15·5)*	−15·2% (−17·9 to −12·5)*
		Thalassaemias		411·4 (384·1 to 441·7)	106·3 (99·7 to 113·5)	17·2 (11·4 to 25·3)	−38·9% (−42·9 to −34·6)*	−20·0% (−25·4 to −15·4)*	−41·0% (−44·8 to −36·6)*	−24·4% (−29·6 to −20·0)*
		Thalassaemias trait		298 566·6 (287 549·2 to 309 863·6)	4868·2 (4697·5 to 5051·2)	1908·8 (1282·8 to 2781·8)	−8·2% (−12·8 to −3·6)*	−12·1% (−16·1 to −7·7)*	−25·7% (−29·1 to −22·1)*	−21·6% (−25·1 to −18·0)*
		Sickle cell disorders		3131·7 (2845·5 to 3434·9)	613·1 (514·6 to 732·0)	252·7 (173·0 to 354·4)	42·0% (34·4 to 50·4)*	15·1% (8·0 to 21·2)*	28·0% (21·0 to 35·3)*	8·4% (1·8 to 14·2)*
		Sickle cell trait		460 730·8 (416 642·3 to 511 272·8)	10 822·4 (9887·9 to 11 861·3)	1203·4 (812·3 to 1731·4)	20·5% (16·4 to 24·3)*	−1·3% (−5·1 to 2·9)	1·2% (−1·9 to 4·2)	−10·3% (−13·9 to −6·4)*
		G6PD deficiency		359 180·8 (344 591·4 to 374 393·5)	7844·9 (7507·8 to 8199·3)	24·7 (17·1 to 34·4)	13·8% (8·1 to 21·1)*	2·3% (−4·6 to 8·7)	−6·9% (−11·5 to −1·1)*	−8·8% (−15·2 to −3·1)*
		G6PD trait		940 308·4 (924 830·5 to 955 818·2)	17 982·7 (17 673·9 to 18 299·5)	0·4 (0·3 to 0·6)	28·3% (22·4 to 34·0)*	3·0% (−1·7 to 7·8)	1·3% (−3·2 to 5·7)	−8·1% (−12·2 to −3·9)*
		Other haemoglobinopathies and haemolytic anaemias		79 442·9 (77 864·8 to 81 099·0)	..	1906·4 (1289·0 to 2768·5)	−0·4% (−3·3 to 2·5)	−2·5% (−5·8 to 1·0)	−20·8% (−23·0 to −18·7)*	−13·8% (−16·8 to −10·6)*
	Endocrine, metabolic, blood, and immune disorders			109 922·2 (106 937·4 to 113 100·4)	..	3215·7 (2232·5 to 4501·4)	6·1% (4·1 to 8·2)*	3·2% (1·0 to 5·4)*	−16·0% (−17·4 to −14·5)*	−9·5% (−11·4 to −7·4)*
		Anaemia due to endocrine, metabolic, blood, and immune disorders		81 387·3 (80 146·9 to 82 729·4)	..	2177·1 (1481·4 to 3130·1)	0·0% (−2·5 to 2·6)	−4·9% (−8·1 to −1·5)*	−19·4% (−21·4 to −17·4)*	−14·3% (−17·2 to −11·3)*
		Endocrine, metabolic, blood, and immune disorders		28 144·8 (25 572·5 to 30 946·4)	..	1005·7 (684·2 to 1398·9)	27·2% (24·6 to 30·0)*	25·4% (22·2 to 28·7)*	−5·4% (−6·6 to −4·0)*	3·3% (1·0 to 5·5)*
		Heart failure due to endocrine, metabolic, blood, and immune disorders		390·1 (322·2 to 465·9)	..	32·9 (21·8 to 46·4)	34·0% (27·5 to 40·8)*	28·1% (24·7 to 31·5)*	−13·9% (−18·2 to −9·7)*	−4·6% (−6·9 to −2·1)*
	Oral disorders			3 466 894·0 (3 271 733·1 to 3 676 485·6)	3 599 595·0 (3 233 032·9 to 3 992 824·0)	18 304·4 (10 992·8 to 28 338·1)	38·4% (36·9 to 39·8)*	21·4% (20·5 to 22·3)*	−2·7% (−3·8 to −1·8)*	−1·3% (−2·0 to −0·6)*
		Caries of deciduous teeth		531 801·7 (443 844·0 to 622 463·7)	1 057 534·3 (756 100·5 to 1 400 908·8)	138·9 (59·4 to 278·1)	−4·5% (−6·2 to −2·9)*	4·9% (3·1 to 6·3)*	−7·0% (−8·6 to −5·6)*	−2·1% (−3·7 to −0·8)*
		Caries of permanent teeth		2 301 999·2 (2 104 931·2 to 2 525 509·0)	2 452 124·9 (2 233 639·1 to 2 665 441·2)	1618·9 (697·9 to 3087·7)	20·6% (18·9 to 22·5)*	9·4% (8·6 to 10·3)*	−8·2% (−9·3 to −7·0)*	−4·0% (−4·8 to −3·3)*
		Periodontal disease		796 122·9 (670 981·0 to 930 283·0)	71 483·7 (61 593·0 to 81 227·2)	5185·6 (2039·3 to 10 653·7)	50·2% (48·8 to 51·6)*	26·6% (25·1 to 27·9)*	3·2% (2·6 to 3·7)*	2·8% (2·3 to 3·2)*
		Edentulism and severe tooth loss		267 457·5 (235 214·4 to 300 011·3)	18 452·1 (16 192·9 to 20 997·1)	7345·9 (4894·2 to 10 408·2)	40·8% (39·4 to 42·1)*	24·6% (23·7 to 25·5)*	−6·3% (−7·1 to −5·5)*	−4·1% (−4·7 to −3·4)*
		Other oral disorders		139 086·3 (133 117·0 to 145 643·0)	..	4015·1 (2507·7 to 5900·3)	32·7% (31·9 to 33·6)*	15·7% (15·0 to 16·3)*	0·0% (−0·3 to 0·4)	0·2% (−0·1 to 0·5)
**Injuries**				**1 507 481·4 (1 439 758·0 to 1 587 209·4)**	**520 710·3 (493 430·2 to 547 988·6)**	**57 174·5 (42 073·9 to 75 427·0)**	23·7% (22·1 to 25·3)*	23·4% (22·0 to 24·8)*	−10·4% (−11·2 to −9·5)*	2·9% (2·1 to 3·7)*
	**Transport injuries**			**226 305·6 (209 529·5 to 244 291·1)**	**63 920·6 (56 848·5 to 71 592·2)**	**13 394·4 (9586·9 to 17 861·0)**	32·9% (30·5 to 35·3)*	21·6% (20·4 to 22·9)*	−4·0% (−5·4 to −2·7)*	1·2% (0·4 to 2·0)*
	Road injuries			174 209·6 (162 042·0 to 187 472·1)	54 192·3 (47 381·6 to 61 645·9)	10 159·7 (7272·0 to 13 618·8)	42·8% (41·0 to 44·8)*	21·7% (20·5 to 22·9)*	1·7% (0·6 to 2·8)*	0·5% (−0·3 to 1·3)
		Pedestrian road injuries		46 000·9 (39 776·2 to 54 538·8)	11 038·9 (9097·7 to 13 168·8)	2710·1 (1912·5 to 3722·5)	35·6% (32·3 to 38·5)*	18·1% (16·2 to 19·8)*	−3·1% (−4·8 to −1·5)*	−2·4% (−3·6 to −1·3)*
		Cyclist road injuries		30 602·6 (25 887·6 to 35 843·6)	11 912·9 (9669·7 to 14 669·8)	1701·1 (1203·1 to 2311·2)	49·1% (45·4 to 52·5)*	27·8% (25·6 to 29·8)*	6·6% (4·5 to 8·4)*	5·1% (3·9 to 6·4)*
		Motorcyclist road injuries		43 911·6 (37 802·2 to 50 950·2)	10 099·8 (8264·3 to 12 257·1)	2563·7 (1759·1 to 3510·3)	61·6% (58·6 to 64·8)*	22·5% (20·7 to 24·4)*	15·6% (14·0 to 17·3)*	2·0% (0·6 to 3·4)*
		Motor vehicle road injuries		42 004·6 (36 541·9 to 49 398·3)	16 209·2 (13 485·6 to 19 383·3)	2568·1 (1842·3 to 3439·8)	30·8% (29·3 to 32·4)*	16·3% (14·8 to 17·7)*	−7·7% (−8·5 to −6·9)*	−4·5% (−5·6 to −3·5)*
		Other road injuries		11 689·9 (9728·7 to 14 348·1)	4931·4 (3777·3 to 6241·2)	616·6 (438·2 to 853·2)	52·6% (49·6 to 55·3)*	45·4% (42·4 to 47·9)*	9·0% (7·4 to 10·5)*	20·3% (18·6 to 21·8)*
	Other transport injuries			52 096·1 (43 127·5 to 61 355·1)	9728·3 (8120·6 to 11 627·0)	3234·7 (2315·7 to 4345·0)	9·0% (6·9 to 11·4)*	21·4% (19·8 to 23·3)*	−18·5% (−19·8 to −17·2)*	3·5% (2·0 to 5·0)*
	**Unintentional injuries**			**935 298·2 (876 022·5 to 1 008 077·1)**	**415 410·3 (390 092·6 to 441 943·0)**	**36 509·7 (26 384·7 to 49 052·5)**	19·8% (18·0 to 21·6)*	26·5% (25·2 to 27·7)*	−13·8% (−14·7 to −12·8)*	4·5% (3·7 to 5·3)*
	Falls			411 712·0 (366 391·0 to 465 355·0)	171 691·2 (152 472·7 to 194 061·9)	19 252·7 (13 725·4 to 26 140·4)	23·1% (21·3 to 24·9)*	27·7% (26·5 to 29·0)*	−12·7% (−13·6 to −11·8)*	3·9% (3·0 to 4·6)*
	Drowning			2207·3 (1940·6 to 2531·3)	357·5 (311·4 to 411·9)	131·6 (94·3 to 175·8)	−11·2% (−14·0 to −8·2)*	14·2% (10·4 to 17·9)*	−33·1% (−34·9 to −31·2)*	−3·8% (−6·6 to −1·3)*
	Fire, heat, and hot substances			99 746·8 (85 298·5 to 115 988·1)	8991·5 (7481·2 to 10 740·9)	3177·0 (2210·4 to 4396·7)	0·8% (−4·4 to 6·2)	17·8% (13·9 to 21·7)*	−25·3% (−28·5 to −22·0)*	1·1% (−1·6 to 3·7)
	Poisonings			4556·0 (3808·4 to 5352·3)	4079·6 (3287·6 to 4973·7)	467·4 (308·0 to 663·8)	8·6% (7·2 to 10·0)*	29·9% (28·3 to 31·8)*	−18·1% (−19·0 to −17·1)*	12·2% (10·8 to 13·9)*
		Poisoning by carbon monoxide		854·2 (717·2 to 1034·9)	1043·5 (770·9 to 1392·2)	75·5 (50·4 to 109·9)	16·0% (14·1 to 18·0)*	33·4% (30·2 to 36·5)*	−13·1% (−14·4 to −11·9)*	14·7% (12·0 to 17·5)*
		Poisoning by other means		3701·8 (2951·6 to 4490·9)	3036·0 (2379·5 to 3782·1)	391·9 (252·7 to 576·0)	7·3% (5·8 to 8·8)*	29·3% (27·2 to 31·4)*	−19·0% (−19·9 to −18·0)*	11·8% (9·9 to 13·7)*
	Exposure to mechanical forces			194 479·9 (169 045·7 to 225 589·9)	72 503·4 (62 042·8 to 84 320·6)	4959·6 (3445·4 to 6978·5)	16·5% (14·3 to 18·6)*	29·0% (27·6 to 30·5)*	−16·0% (−17·2 to −14·9)*	7·4% (6·5 to 8·4)*
		Unintentional firearm injuries		8469·2 (6941·4 to 9838·8)	1911·8 (1390·6 to 2544·6)	320·1 (224·6 to 436·7)	13·7% (12·2 to 15·2)*	28·0% (26·5 to 29·5)*	−16·5% (−17·4 to −15·7)*	8·6% (7·7 to 9·6)*
		Other exposure to mechanical forces		186 010·7 (159 749·5 to 216 401·4)	70 591·6 (60 734·7 to 82 371·3)	4639·5 (3204·1 to 6560·6)	16·7% (14·4 to 18·9)*	29·1% (27·7 to 30·6)*	−16·0% (−17·2 to −14·8)*	7·3% (6·3 to 8·4)*
	Adverse effects of medical treatment			2673·1 (2059·9 to 3308·5)	34 975·0 (29 997·9 to 40 308·2)	356·5 (223·9 to 533·8)	60·5% (55·4 to 65·6)*	48·0% (41·2 to 54·5)*	18·5% (16·0 to 21·0)*	19·6% (15·3 to 24·0)*
	Animal contact			38 403·6 (34 169·0 to 43 179·8)	43 844·6 (37 631·9 to 51 149·2)	1075·6 (732·1 to 1484·0)	11·3% (9·7 to 12·9)*	17·9% (16·1 to 19·6)*	−17·0% (−17·9 to −16·2)*	1·1% (−0·2 to 2·3)
		Venomous animal contact		15 812·1 (13 211·3 to 18 557·7)	21 097·9 (17 255·4 to 25 592·6)	717·2 (487·1 to 1004·2)	14·7% (12·7 to 17·1)*	18·2% (16·2 to 20·3)*	−13·6% (−14·8 to −12·2)*	2·7% (1·1 to 4·3)*
		Non-venomous animal contact		22 591·5 (19 045·6 to 26 729·8)	22 746·6 (18 607·8 to 27 654·5)	358·4 (224·3 to 555·6)	5·0% (2·4 to 7·6)*	17·2% (14·8 to 19·1)*	−22·9% (−24·3 to −21·8)*	−1·8% (−3·5 to −0·5)*
	Foreign body			19 350·9 (16 787·2 to 22 108·8)	23 101·7 (19 925·9 to 26 454·1)	949·3 (683·3 to 1285·0)	16·3% (13·6 to 19·1)*	21·5% (20·3 to 22·9)*	−13·0% (−15·0 to −11·0)*	3·4% (2·4 to 4·5)*
		Pulmonary aspiration and foreign body in airway		3008·1 (2442·0 to 3662·4)	1346·3 (1115·6 to 1607·1)	150·4 (103·1 to 204·4)	12·2% (6·2 to 19·3)*	23·8% (20·7 to 26·9)*	−14·1% (−17·9 to −9·5)*	5·8% (3·6 to 8·2)*
		Foreign body in eyes		3190·4 (1644·6 to 4857·3)	17 672·8 (14 633·0 to 20 947·8)	201·6 (104·6 to 334·5)	20·3% (17·7 to 23·1)*	20·3% (18·8 to 22·1)*	−9·4% (−12·1 to −7·4)*	2·7% (1·3 to 3·6)*
		Foreign body in other body part		13 152·5 (10 991·9 to 15 298·2)	4082·5 (3342·2 to 5009·6)	597·3 (427·3 to 783·4)	16·0% (13·3 to 19·2)*	21·4% (20·0 to 22·9)*	−13·9% (−15·6 to −11·9)*	3·1% (1·8 to 4·3)*
	Environmental heat and cold exposure			34 896·7 (29 946·9 to 40 886·4)	8769·0 (7357·0 to 10 370·5)	1509·5 (1072·5 to 2023·8)	13·6% (11·0 to 16·4)*	24·5% (22·3 to 26·6)*	−16·7% (−18·2 to −15·1)*	4·9% (3·4 to 6·4)*
	Exposure to forces of nature			15 757·0 (11 656·5 to 20 853·3)	689·2 (587·4 to 817·5)	725·5 (524·9 to 975·9)	220·3% (200·7 to 238·8)*	22·6% (19·0 to 26·1)*	156·4% (140·2 to 171·7)*	7·8% (5·0 to 10·4)*
	Other unintentional injuries			115 519·2 (98 844·3 to 134 764·7)	46 407·6 (39 513·5 to 53 786·8)	3904·9 (2715·6 to 5485·4)	20·4% (18·4 to 22·2)*	28·4% (27·0 to 29·7)*	−13·9% (−14·9 to −13·0)*	5·7% (4·9 to 6·6)*
	**Self-harm and interpersonal violence**			**351 859·6 (316 749·7 to 390 321·7)**	**41 379·4 (37 344·5 to 45 834·4)**	**7270·4 (5637·2 to 9008·3)**	27·2% (24·7 to 30·0)*	13·0% (10·8 to 15·0)*	−3·5% (−5·4 to −1·3)*	−2·0% (−3·7 to −0·5)*
	Self-harm			8502·0 (7072·4 to 10 011·6)	3939·8 (3338·1 to 4603·3)	435·3 (306·0 to 574·8)	18·5% (15·2 to 21·9)*	6·3% (4·8 to 7·9)*	−16·0% (−18·2 to −13·9)*	−12·1% (−13·1 to −11·1)*
		Self-harm by firearm		114·4 (100·5 to 130·9)	41·6 (26·1 to 63·2)	4·8 (3·4 to 6·5)	13·5% (11·6 to 15·7)*	7·6% (5·3 to 9·9)*	−21·3% (−22·7 to −19·9)*	−12·9% (−14·7 to −11·1)*
		Self-harm by other specified means		8387·6 (6963·0 to 9894·9)	3898·2 (3299·6 to 4570·6)	430·5 (301·7 to 568·8)	18·6% (15·2 to 22·0)*	6·3% (4·8 to 7·9)*	−16·0% (−18·1 to −13·9)*	−12·1% (−13·1 to −11·1)*
	Interpersonal violence			297 781·5 (268 378·9 to 330 722·3)	22 919·4 (19 569·3 to 26 696·3)	4561·8 (3522·0 to 5756·8)	24·0% (21·7 to 26·3)*	14·6% (13·1 to 15·9)*	−7·4% (−8·7 to −5·9)*	−0·8% (−1·6 to −0·0)*
		Assault by firearm		2599·6 (2158·5 to 3151·0)	551·9 (421·7 to 725·6)	116·9 (83·8 to 159·3)	41·0% (38·7 to 43·5)*	20·2% (18·3 to 22·0)*	1·1% (−0·3 to 2·4)	0·5% (−0·9 to 1·9)
		Assault by sharp object		14 754·5 (12 033·6 to 20 359·8)	4233·0 (3265·4 to 5366·7)	475·1 (333·8 to 659·8)	21·0% (18·2 to 23·7)*	13·8% (12·1 to 15·7)*	−12·0% (−13·5 to −10·5)*	−3·4% (−4·9 to −1·9)*
		Sexual violence		238 200·3 (209 368·6 to 270 335·5)	..	2142·0 (1447·1 to 3106·7)	26·1% (22·5 to 30·1)*	12·4% (10·2 to 14·3)*	−1·9% (−3·5 to −0·1)*	0·6% (−0·6 to 1·6)
		Assault by other means		42 227·1 (35 479·3 to 50 750·5)	18 134·5 (15 426·3 to 21 180·0)	1827·8 (1295·0 to 2446·7)	21·3% (18·4 to 24·5)*	17·1% (15·7 to 18·6)*	−12·4% (−14·1 to −10·6)*	−1·9% (−3·0 to −0·7)*
	Conflict and terrorism			41 912·3 (28 964·3 to 59 365·8)	12 492·6 (10 797·4 to 15 087·4)	2134·0 (1438·2 to 3191·0)	35·1% (30·0 to 40·8)*	10·1% (4·9 to 15·5)*	8·2% (4·0 to 12·7)*	−3·2% (−7·6 to 1·4)
	Executions and police conflict			3680·5 (2459·9 to 5304·1)	2027·6 (1683·1 to 2486·1)	139·3 (99·1 to 197·0)	59·4% (45·9 to 74·0)*	32·9% (27·9 to 51·1)*	22·3% (11·9 to 33·5)*	14·6% (9·9 to 31·1)*
	**Nature of injury aggregates**									
	Amputations			371 422·5 (343 523·2 to 407 748·4)	9847·8 (8414·6 to 11 519·6)	6409·0 (4283·7 to 9318·6)	17·4% (12·9 to 21·9)*	19·6% (15·7 to 23·2)*	−15·5% (−18·6 to −12·2)*	−0·4% (−3·5 to 2·4)
	Burns			208 679·9 (192 327·8 to 227 204·0)	14 307·6 (12 437·8 to 16 359·2)	6720·1 (4841·1 to 9083·9)	5·8% (0·9 to 11·2)*	14·3% (10·1 to 18·6)*	−21·0% (−24·2 to −17·8)*	−1·5% (−4·3 to 1·2)
	Fractures			360 136·5 (338 070·9 to 385 693·0)	139 646·3 (128 305·0 to 152 504·8)	19 793·8 (13 702·1 to 27 655·9)	30·3% (29·1 to 31·5)*	27·3% (26·1 to 28·3)*	−8·6% (−9·3 to −7·8)*	3·0% (2·1 to 3·7)*
	Head injuries			46 873·2 (44 984·0 to 48 892·5)	21 652·5 (19 206·0 to 24 416·0)	6898·0 (4883·9 to 9277·6)	35·6% (34·5 to 36·8)*	27·9% (27·1 to 28·7)*	−2·5% (−3·2 to −1·8)*	6·2% (5·6 to 6·9)*
	Spinal injuries			22 489·5 (20 671·6 to 25 115·6)	775·8 (643·3 to 957·7)	6633·4 (4708·0 to 8639·5)	26·1% (23·4 to 28·9)*	25·0% (22·9 to 26·6)*	−6·6% (−8·7 to −4·1)*	6·3% (5·0 to 7·3)*
	Minor injuries			198 921·3 (187 143·0 to 213 281·5)	181 177·5 (167 026·3 to 195 833·2)	1752·5 (831·1 to 3319·5)	25·5% (24·1 to 27·1)*	27·5% (26·8 to 28·4)*	−10·2% (−11·1 to −9·2)*	5·1% (4·6 to 5·8)*
	Other injuries			71 339·1 (67 479·1 to 75 358·0)	153 302·9 (143 603·2 to 162 955·6)	6825·7 (4830·6 to 9168·5)	20·4% (18·7 to 22·4)*	23·1% (21·0 to 24·8)*	−10·7% (−11·8 to −9·4)*	4·1% (2·6 to 5·4)*
	**Impairments**									
	Anaemia			1 950 345·6 (1 909 153·0 to 1 990 086·8)	..	58 197·1 (39 535·4 to 83 046·0)	−0·9% (−3·3 to 1·5)	−6·7% (−9·5 to −3·8)*	−17·1% (−18·9 to −15·2)*	−16·2% (−18·8 to −13·5)*
	Epilepsy			63 783·6 (55 496·5 to 73 199·5)	..	25 957·3 (20 369·4 to 32 900·4)	34·1% (25·9 to 42·1)*	16·3% (10·6 to 22·1)*	8·8% (1·9 to 15·2)*	4·5% (−0·7 to 9·8)
	Guillain-Barré syndrome			103·7 (82·7 to 127·2)	..	30·7 (19·3 to 44·4)	29·4% (25·5 to 33·8)*	18·3% (15·9 to 21·0)*	2·7% (1·0 to 4·3)*	3·4% (2·2 to 4·5)*
	Hearing loss			1 428 450·1 (1 388 276·9 to 1 465 389·9)	..	39 454·3 (27 100·6 to 55 310·2)	40·0% (38·7 to 41·0)*	22·4% (21·4 to 23·3)*	0·0% (−0·7 to 0·6)	−0·5% (−1·2 to 0·1)
	Heart failure			64 343·9 (57 187·0 to 71 648·3)	..	9906·9 (7261·9 to 12 443·6)	55·4% (51·7 to 59·1)*	32·6% (30·3 to 34·6)*	−0·4% (−2·6 to 1·9)	−0·5% (−2·0 to 0·7)
	Infertility			123 084·9 (99 543·3 to 150 556·1)	..	956·9 (487·4 to 1791·9)	31·7% (28·0 to 34·8)*	17·3% (13·6 to 20·5)*	−0·7% (−3·0 to 1·0)	6·8% (3·5 to 9·8)*
	Developmental intellectual disability			188 585·1 (145 641·8 to 230 402·8)	..	25 301·2 (19 706·5 to 31 400·2)	30·2% (25·9 to 35·0)*	12·9% (10·0 to 16·1)*	7·1% (3·7 to 11·0)*	2·1% (−0·5 to 5·1)
	Pelvic inflammatory disease			1064·1 (906·7 to 1249·0)	..	141·0 (94·8 to 197·2)	42·3% (39·0 to 45·9)*	16·0% (13·6 to 18·7)*	4·5% (2·5 to 6·7)*	2·5% (0·4 to 4·7)*
	Vision loss			1 339 970·3 (1 291 077·0 to 1 393 811·0)	..	34 125·4 (23 206·6 to 48 924·6)	38·3% (36·9 to 39·6)*	22·2% (21·2 to 23·3)*	−2·4% (−3·2 to −1·6)*	−1·5% (−2·3 to −0·7)*

Data in parentheses are 95% uncertainty intervals. We did not calculate incidence for the nine impairments and certain neglected tropical diseases. Blank cells mean that no estimate is available or that the estimate has a magnitude less than 50. G6PD=glucose-6-phosphate dehydrogenase. GFR=glomerular filtration rate. *H influenzae=Haemophilus influenzae*. NASH=non-alcoholic steatohepatitis. YLDs=years lived with disability.

* Percentage changes that are statistically significant.

† Incidence of HIV/AIDS represents new infections of HIV only and does not include new infections of tuberculosis in HIV positive cases.

‡ Incidence estimates for stroke represent first-ever stroke only.

### Prevalence

For all ages and both sexes combined, globally, in 2017, the three most common causes at Level 3 of the GBD cause hierarchy in terms of all-age prevalent cases were oral disorders (3·47 billion, 95% UI 3·27–3·68), headache disorders (3·07 billion, 2·90–3·27), and tuberculosis including latent tuberculosis infection (1·93 billion, 1·71–2·20; table 1).

Global age-standardised prevalence rankings remained unchanged for the top two Level 3 causes in the GBD hierarchy from 1990 to 2017, with oral disorders and headache disorders remaining the two most common causes. Tuberculosis including latent tuberculosis infection was the third leading cause in 1990 and became the fourth leading cause in 2017, whereas haemoglobinopathies were the fourth leading cause in 1990 and became the third leading cause in 2017. Between 1990 and 2017, the age-standardised prevalence decreased for oral disorders by 5·5% (95% UI 4·9 to 6·0) but increased for headache disorders by 0·3% (–0·2 to 0·9) and for haemoglobinopathies by 4·7% (4·3 to 5·1).

### Incidence

Globally, in 2017, for all ages and both sexes combined, the three leading Level 3 causes in terms of incident cases were upper respiratory infections (17·1 billion, 95% UI 15·3 to 19·2), diarrhoeal diseases (6·29 billion, 5·81 to 6·82), and oral disorders (3·60 billion, 3·23 to 3·99; table 1). These case rankings remained unchanged for the top three causes between 1990 and 2017 despite a decrease in age-standardised incidence rates of upper respiratory infections of 2·6% (95% UI 2·0 to 3·1), from 232 815 new cases (95% UI 207 461 to 260 397) to 226 802 new cases (201 716 to 253 367) per 100 000, and in age-standardised incident rates of oral disorders of 0·3% (–1·1 to 0·6), from 48 423 new cases (43 233 to 53 971) to 48 276 new cases (43 109 to 53 919) per 100 000, and an increase in the number of new cases per 100 000 of diarrhoeal diseases of 11·7% (8·8 to 14·6), from 75 087 new cases (69 475 to 81 367) to 83 846 new cases (77 402 to 90 965) per 100 000.

### YLDs

The global number of YLDs increased from 562 million (95% UI 421–723) to 853 million (642–1097) between 1990 and 2017, representing a 51·8% (50·2–53·5) increase and a 7·2% (6·0–8·4) increase in the all-age YLD rate, while age-standardised YLD rates decreased from 11 310 YLDs (8485–14 506) to 10 871 YLDs (8171–13 980) per 100 000, representing a 3·9% (3·1–4·6) decrease. CMNN causes accounted for 13·8% (12·5–15·1) of total YLDs in 2017, while NCDs accounted for 79·5% (77·8–81·1) and injuries for 6·7% (6·2–7·3), with a total of 118 million (86·7–154) YLDs for CMNN causes, 678 million (510–876) for NCDs, and 57·2 million (42·1–75·4) for injuries. The number of YLDs from CMNN causes increased from 1990 to 2017 by 13·6% (9·15–19·2), and the YLD rates from CMNN causes decreased by 14·8% (10·7–18·0) from 1846 YLDs (1343–2472) to 1573 YLDs (1159–2067) per 100 000 during the same period. The number of YLDs from NCD causes increased between 1990 and 2017 by 61·1% (60·0–62·4), and the YLD rate from these causes decreased by 1·2% (0·66–1·8) from 8684 YLDs (6540–11 223) to 8579 YLDs (6454–11 084) per 100 000. The number of YLDs from injuries increased between 1990 and 2017 by 52·7% (49·3–56·4), and the YLD rate from injuries decreased by 7·8% (6·27–9·28) from 779 (577–1023) YLDs to 719 YLDs (529–948) per 100 000. In 2017, the YLD rate for all causes ranged from 9120 YLDs (6877–11 622) per 100 000 in Columbia to 14 824 YLDs (11 080–19 203) per 100 000 in Yemen.

Globally, in 1990, for all ages and both sexes, the leading Level 3 causes of YLDs were low back pain (42·5 million YLDs, 95% UI 30·2 to 57·2), headache disorders (35·1 million, 22·8 to 49·7), and dietary iron deficiency (31·7 million, 21·6 to 45·5). Between 1990 and 2007, the number of all-age YLDs attributed to low back pain increased by 30·0% (27·9 to 31·9) and those attributed to headache disorders increased by 34·0% (33·0 to 35·1), while the number of all-age YLDs for dietary iron deficiency decreased by 0·2% (–2·8 to 2·2). Between 1990 and 2007, the number of all-age YLDs attributed to depressive disorders increased by 33·4% (31·0 to 35·8), becoming the third leading cause of all-age YLDs in 2007, and shifting dietary iron deficiency to fourth; the rankings for low back pain and headache disorders did not change from 1990 to 2007. From 2007 to 2017, we observed further increases in the number of all-age YLDs attributable to the leading three causes: low back pain (17·5%, 95% UI 16·2–19·0), headache disorders (15·4%, 14·6–16·2), and depressive disorders (14·3%, 13·1–15·6).

**Figure 2:**
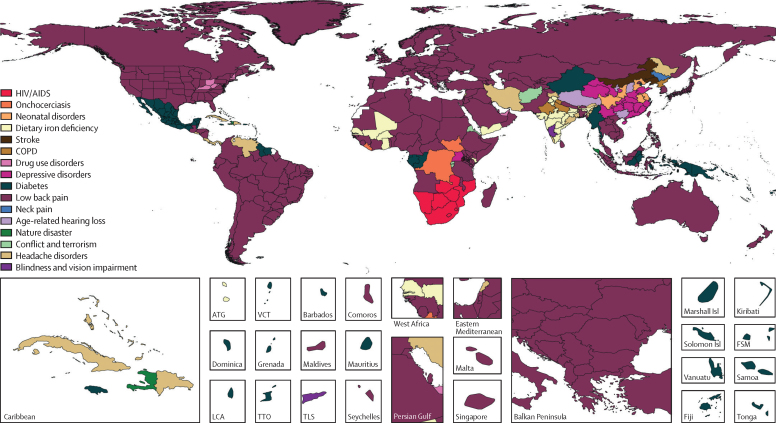
Leading Level 3 causes of age-standardised YLD rates by location for both sexes combined, 2017 ATG=Antigua and Barbuda. COPD=chronic obstructive pulmonary disease. FSM=Federated States of Micronesia. Isl=Islands. LCA=Saint Lucia. TLS=Timor-Leste. TTO=Trinidad and Tobago. VCT=Saint Vincent and the Grenadines. YLD=years lived with disability.

Figure 2 illustrates the leading Level 3 causes of YLD rates by GBD country and select subnational locations in 2017 for both sexes combined. The geographical variation in the leading Level 3 causes of YLD rates across countries is shown: low back pain was the leading cause in 126 of the 195 countries and territories whereas diabetes was the leading cause of YLD rates in Mexico, Equatorial Guinea, Congo (Brazzaville), Myanmar, Mauritius, and Gabon, as well as parts of the Caribbean and most of Oceania. Dietary iron deficiency was the leading cause of YLD rates in Yemen, India, Antigua and Barbuda, and in parts of western sub-Saharan Africa. Conflict and terrorism was the leading cause of YLDs in Afghanistan, Eritrea, Rwanda, and Burundi.

The most common Level 3 causes of YLD rates by country and subnational locations by sex are shown in appendix 2. In both females and males, low back pain was the leading Level 3 cause by country. In females, the second most commonly leading Level 3 cause was headache disorders, which was the leading cause in 57 countries, followed by diabetes, which was the leading cause in 20 countries. In males, the second most commonly leading Level 3 cause was diabetes, highest in 39 countries, followed by drug use disorders and conflict and terror, each leading in five countries.

### Temporal YLD trends in causes and impairments by geographical regions and SDI quintiles

**Figure 3:**
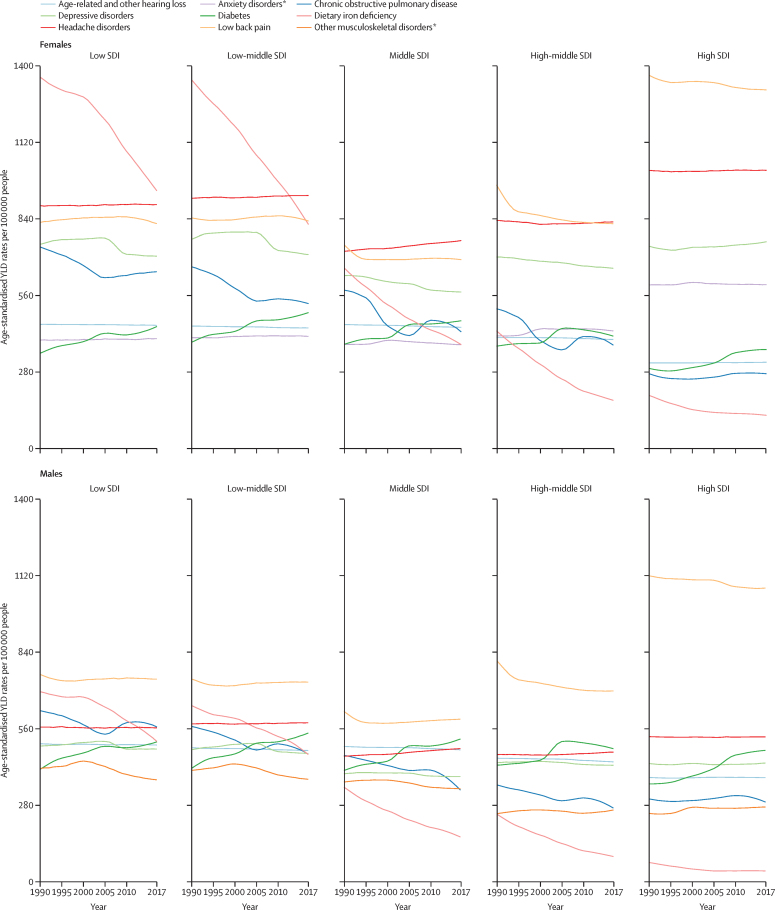
Trends of age-standardised YLD rates per 100 000 for the top eight Level 3 causes of non-fatal burden in 2017 for each sex by SDI quintile, 1990–2017 Mean estimates are shown. SDI=Socio-demographic Index. YLDs=years lived with disability. *One legend is shown for females and males. Anxiety disorders are a top cause of non-fatal burden for females and other musculoskeletal disorders are a top cause of non-fatal burden for males.

Figure 3 shows rankings and trends of the top causes of YLDs by SDI quintile and by sex. SDI-based patterns varied by sex: the top cause of YLD rates between 1990 and 2017 in the lowest SDI quintile was dietary iron deficiency for females and low back pain for males, whereas in the highest SDI quintile it was low back pain for both females and males. In the lowest SDI quintile, for both sexes combined, the top three causes of YLD rates were low back pain, dietary iron deficiency, and headache disorders, while in the highest SDI quintile the top three causes were low back pain, headache disorders, and depressive disorders (appendix 2). The supplementary results show these results for the leading six impairments estimated in GBD: anaemia, heart failure, vision loss, hearing loss, epilepsy, and infertility (appendix 2). The immense burden of anaemia is shown, particularly for females in low-SDI regions. The differences in the burden of impairments by sex are also provided (appendix 2).

**Figure 4:**
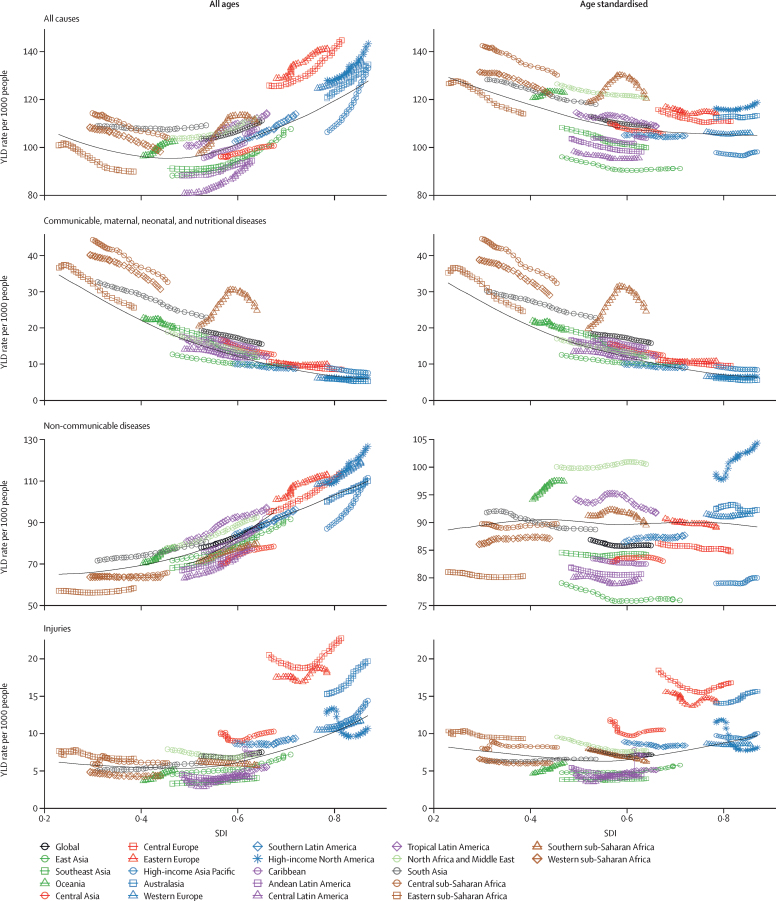
Co-evolution of all-age and age-standardised YLD rates with SDI globally and for GBD regions for Level 1 causes for both sexes combined, 1990–2017 Coloured lines are global and region values for YLDs. Each point in a line represents 1 year, starting from 1990 and ending in 2017. In all regions, SDI has increased over time so progress in SDI is associated with points further to the right and later years for a given region, with a downwards gradient indicating a reduction in YLD rate. The black lines indicate the trajectories for each geography expected on the basis of SDI alone. SDI=Socio-demographic Index. YLDs=years lived with disability.

Figure 4 shows the all-age and age-standardised YLD rates by SDI and GBD region between 1990 and 2017 for males and females combined for all causes and then separately for Level 1 causes. In general, many regions experienced decreases in all-cause age-standardised YLD rates as SDI increased (figure 4). However, there were important exceptions to this finding. First, some regions did not follow this trend consistently. Southern sub-Saharan Africa showed an increase in YLD rates for more than a decade before beginning a more precipitous decline coinciding with the apex of the HIV epidemic, whereas regions with a higher baseline SDI have generally experienced minimal changes or increases in age-standardised YLD rates over the past decade despite advances in SDI. We found that trends also varied over time depending on cause. As SDI increased, age-standardised and all-age YLDs improved for CMNN causes in most regions, but this relation was less reliable for NCDs. The NCD pattern also differed markedly between all-age and age-standardised rates for NCDs, with all-age rates increasing as SDI improved with relatively little change over time observed in age-standardised rates. For injuries, some regions initially experienced a declining burden as SDI increased, followed by an increasing burden as SDI continued to increase over time, as seen in Central Europe, for example.

### Age-specific and sex-specific patterns in prevalence and YLDs

Table 2 shows global age-standardised prevalence for females and males for all Level 2 GBD causes as well as the relative difference between the sexes in 1990 and 2017. In both 1990 and 2017, the cause with the greatest relative difference between sexes was substance use disorders, which had a relative difference of 1·09 (95% UI 1·03–1·15) in 1990, which increased to 1·16 (1·09–1·23) in 2017. In 1990, 302 million (226–388) YLDs were in females and 260 million (195–336) were in males. The all-age YLD rate for females was 11 273·6 (8455·6–14 492·5) per 100 000 and the all-age YLD for males was 9571·4 (7160·9–12 356·1) per 100 000 in 1990. Among the global sum of YLDs, 53·6% (53·3–53·9) or 457 million (344–587) YLDs were in females and 46·4% (46·1–46·7) or 396 million (297–510) YLDs were in males. In the subset of causes that occur in both males and females, there were 761 million (565–987) total YLDs, with 53·0% (52·8–53·2) or 444 million (334–571) YLDs in females and 47·0% (46·8–47·2) or 395 million (296–510) YLDs in males. Between 1990 and 2017, all-age YLD rates increased for females and males, by 6·5% (5·4–7·7) to 12 007·7 (9036·7–15352·0) YLDs per 100 000 females and by 7·9% (6·6–9·2) to 10 328·1 (7744·1–13 306·9) YLDs per 100 000 males.

**Table 2:** Global age-standardised prevalence rates per 100 000 for males and females for Level 2 GBD causes with the relative difference between the sexes for 1990 and 2017

	**Prevalence in thousands, 1990**			**Prevalence in thousands, 2017**			**Percentage change, 1990–2017**	
	Males	Females	Sex difference	Males	Females	Sex difference	Males	Females
Substance use disorders	3008 (2773 to 3237)	1439 (1319 to 1557)	1·09 (1·03 to 1·15)	3018 (2782 to 3252)	1400 (1279 to 1524)	1·16 (1·09 to 1·23)	0·3% (−1·2 to 2·0)	−2·7% (−4·1 to −1·2)
Unintentional injuries	14 678 (13 770 to 15 758)	10 230 (9589 to 10 981)	0·43 (0·40 to 0·47)	13 773 (12 888 to 14 884)	9747 (9148 to 10 484)	0·41 (0·38 to 0·45)	−6·2% (−6·8 to −5·4)	−4·7% (−5·6 to −3·8)
Self-harm and interpersonal violence	3193 (2893 to 3529)	5637 (5058 to 6278)	0·43 (0·39 to 0·48)	3265 (2943 to 3630)	5643 (5057 to 6302)	0·42 (0·37 to 0·47)	2·3% (−0·1 to 4·9)	0·1% (−1·5 to 1·7)
HIV/AIDS and sexually transmitted infections	11 433 (10 355 to 12 703)	18 320 (16 789 to 19 998)	0·38 (0·36 to 0·39)	11 902 (10 826 to 13 196)	18 936 (17 366 to 20 631)	0·37 (0·36 to 0·39)	4·1% (3·3 to 4·9)	3·4% (2·6 to 4·1)
Transport injuries	3150 (2924 to 3384)	2367 (2189 to 2576)	0·33 (0·30 to 0·37)	3322 (3082 to 3582)	2336 (2154 to 2535)	0·42 (0·39 to 0·47)	5·4% (4·1 to 7·2)	−1·3% (−2·6 to 0·0)
Neoplasms	917 (871 to 938)	1191 (1167 to 1219)	0·23 (0·21 to 0·27)	1188 (1146 to 1244)	1355 (1318 to 1391)	0·12 (0·07 to 0·16)	29·6% (26·4 to 34·9)	13·8% (9·4 to 17·7)
Other non-communicable diseases	57 424 (55 351 to 59 629)	73 370 (72 026 to 74 850)	0·22 (0·20 to 0·23)	55 565 (53 542 to 57 701)	72 470 (71 183 to 73 891)	0·23 (0·22 to 0·25)	−3·2% (−3·7 to −2·8)	−1·2% (−1·5 to −1·0)
Maternal and neonatal disorders	1435 (1264 to 1629)	1820 (1643 to 2018)	0·21 (0·18 to 0·25)	1953 (1712 to 2208)	2288 (2027 to 2586)	0·15 (0·11 to 0·18)	36·1% (29·3 to 43·0)	25·7% (18·9 to 32·2)
Digestive diseases	26 590 (25 678 to 27 503)	22 435 (21 585 to 23 377)	0·19 (0·17 to 0·20)	28 165 (27 283 to 29 064)	23 417 (22 581 to 24 342)	0·20 (0·19 to 0·21)	5·9% (5·1 to 6·8)	4·4% (3·7 to 5·1)
Neurological disorders	36 664 (34 401 to 39 294)	42 703 (40 642 to 45 164)	0·14 (0·13 to 0·16)	36 952 (34 695 to 39 469)	42 718 (40 688 to 45 034)	0·14 (0·12 to 0·15)	0·8% (−0·1 to 1·6)	0·0% (−0·6 to 0·7)
Musculoskeletal disorders	15 247 (14 414 to 16 131)	17 770 (16 882 to 18 766)	0·14 (0·13 to 0·15)	14 918 (14 143 to 15 761)	17 581 (16 745 to 18 503)	0·15 (0·14 to 0·16)	−2·2% (−3·0 to −1·4)	−1·1% (−2·0 to −0·2)
Nutritional deficiencies	28 066 (27 246 to 28 935)	31 354 (30 605 to 32 153)	0·11 (0·09 to 0·12)	23 473 (22 648 to 24 309)	26 059 (25 332 to 26 864)	0·10 (0·08 to 0·11)	−16·4% (−18·0 to −14·6)	−16·9% (−18·0 to −15·8)
Diabetes and kidney diseases	10 920 (10 428 to 11 489)	12 120 (11 558 to 12 743)	0·10 (0·09 to 0·11)	12 036 (11 453 to 12 688)	13 044 (12 444 to 13 733)	0·08 (0·07 to 0·09)	10·2% (8·1 to 12·5)	7·6% (5·6 to 9·6)
Enteric infections	1115 (1043 to 1189)	1216 (1141 to 1292)	0·08 (0·07 to 0·09)	1180 (1096 to 1261)	1313 (1221 to 1405)	0·10 (0·09 to 0·11)	5·8% (2·8 to 9·1)	8·0% (5·0 to 11·3)
Respiratory infections and tuberculosis	33 220 (29 889 to 37 160)	31 051 (27 950 to 34 713)	0·07 (0·06 to 0·08)	29 021 (26 238 to 32 454)	27 301 (24 756 to 30 428)	0·06 (0·06 to 0·07)	−12·6% (−14·2 to −11·0)	−12·1% (−13·5 to −10·7)
Mental disorders	12 264 (11 533 to 13 003)	13 237 (12 584 to 13 938)	0·07 (0·05 to 0·10)	12 010 (11 338 to 12 694)	12 834 (12 214 to 13 494)	0·06 (0·04 to 0·09)	−2·1% (−2·7 to −1·4)	−3·0% (−3·6 to −2·5)
Cardiovascular diseases	6496 (6261 to 6744)	6149 (5904 to 6414)	0·06 (0·05 to 0·07)	6253 (6031 to 6482)	5939 (5716 to 6177)	0·05 (0·04 to 0·06)	−3·8% (−4·4 to −3·1)	−3·4% (−4·1 to −2·7)
Chronic respiratory diseases	7926 (7441 to 8420)	8411 (7890 to 8935)	0·06 (0·05 to 0·07)	6731 (6248 to 7268)	7267 (6742 to 7825)	0·07 (0·06 to 0·09)	−15·1% (−17·7 to −12·5)	−13·6% (−16·0 to −11·3)
Skin and subcutaneous diseases	25 139 (24 328 to 26 021)	26 565 (25 776 to 27 407)	0·05 (0·05 to 0·06)	25 221 (24 454 to 26 042)	26 592 (25 832 to 27 377)	0·05 (0·05 to 0·06)	0·3% (−0·4 to 1·0)	0·1% (−0·5 to 0·6)
Other infectious diseases	1574 (1497 to 1661)	1633 (1563 to 1709)	0·04 (0·01 to 0·07)	1336 (1273 to 1404)	1381 (1330 to 1437)	0·03 (0·00 to 0·07)	−15·1% (−18·8 to −11·3)	−15·4% (−18·1 to −12·7)
Neglected tropical diseases and malaria	29 748 (28 431 to 31 138)	28 954 (27 613 to 30 320)	0·03 (0·02 to 0·03)	17 001 (16 259 to 17 864)	16 834 (16 104 to 17 681)	0·01 (0·00 to 0·02)	−42·8% (−46·3 to −38·7)	−41·9% (−45·5 to −37·6)
Sense organ diseases	25 031 (24 477 to 25 581)	25 473 (24 949 to 26 007)	0·02 (0·01 to 0·02)	24 868 (24 325 to 25 411)	25 892 (25 375 to 26 438)	0·04 (0·03 to 0·04)	−0·7% (−0·8 to −0·5)	1·6% (1·4 to 1·9)

Data in parentheses are 95% uncertainty intervals. The relative difference for each of the 22 Level 2 causes between sexes were calculated using the values for females as the denominator. The causes are ranked by the value of the sex difference in 1990. All changes are significant.

**Figure 5:**
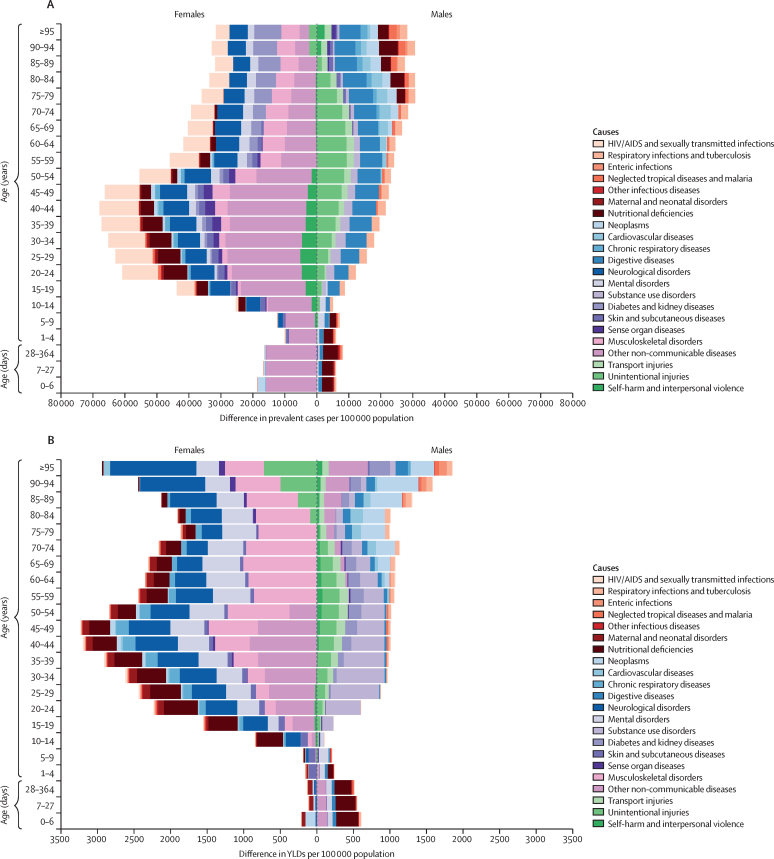
Sex differences in global prevalence and YLD rates per 100 000 for 22 Level 2 causes by age, 2017 The figure represents the difference in prevalence (A) and YLD rates (B) between males and females as well as the cause composition of those differences for each GBD age group for the Level 2 causes of non-fatal burden. GBD=Global Burden of Diseases, Injuries, and Risk Factors Study. YLDs=years lived with disability.

Figure 5A illustrates the differences in global prevalence for females and males for 2017 by age group for the 22 Level 2 GBD causes of non-fatal health loss, calculated as the female-specific estimate subtracted from the male-specific estimate such that causes on the left side of the chart are more prevalent in females while causes on the right side of the chart are more prevalent in males in a given age group. Females experience overall higher prevalence in every age group and the highest age-specific global prevalence differences for female-predominant causes occur between the ages of 20 years and 49 years. From birth to 4 years of age, females have higher prevalence of other NCDs, which continues through all age groups, and of neoplasms than males, whereas neurological disorders are female predominant in all ages, becoming notably evident from age 5–9 years and continuing throughout all subsequent age groups. Similarly, HIV/AIDS and sexually transmitted infections are female predominant in all ages, a trend that becomes evident from age 10–14 years. Although nutritional deficiencies are more prevalent in males in the earliest age groups up to age 9 years, they are more prevalent in females in subsequent age groups up to age 74 years, at which point they once again become more prevalent in males up to the oldest age group. Females also have higher prevalence of self-harm and interpersonal violence starting from age 1–4 years and continuing until age 84 years, after which they become more prevalent in males. Digestive diseases are more prevalent in males in all age groups, whereas unintentional injuries emerge as a male-predominant cause from 5 years of age and remain as such until age 89 years, after which they become female predominant. Transport injuries are male predominant in all age groups starting from age 1–4 years. Respiratory infections and tuberculosis are male predominant in all ages. Cardiovascular diseases are male predominant starting from age 50–54 years, whereas chronic respiratory diseases emerge as a male-predominant cause from age 65–69 years.

Figure 5B shows the sex differences in the global burden of YLDs for 2017 by age group for the 22 Level 2 GBD causes of non-fatal health loss. Before age 10–14 years, males have greater YLD rates than females, driven largely by higher rates of nutritional deficiencies. From age 10–14 years and older, females have greater overall YLD rates in every age group. Under the age of 1 year, females experience higher YLD rates due to neoplasms and maternal and neonatal disorders, whereas male infants experience higher YLD rates due to nutritional deficiencies and other NCDs. Starting at age 10 years, females experience a higher YLD rate due to other NCDs (until age 50–54 years), musculoskeletal disorders, mental disorders, neurological disorders, and chronic respiratory diseases. Males experience higher YLD rates due to unintentional injuries, transport injuries, and substance use disorders for most of life, although females older than 80 years of age experience a higher rate of unintentional injuries than do males. For self-harm and interpersonal violence, females experience higher rates than males until age 30–34 years, at which point males experience higher YLD rates. Males also experience higher YLD rates from neoplasms, cardiovascular diseases, and other NCDs starting in older age groups (≥60 years), and from digestive diseases from age 25–29 years.

**Figure 6:**
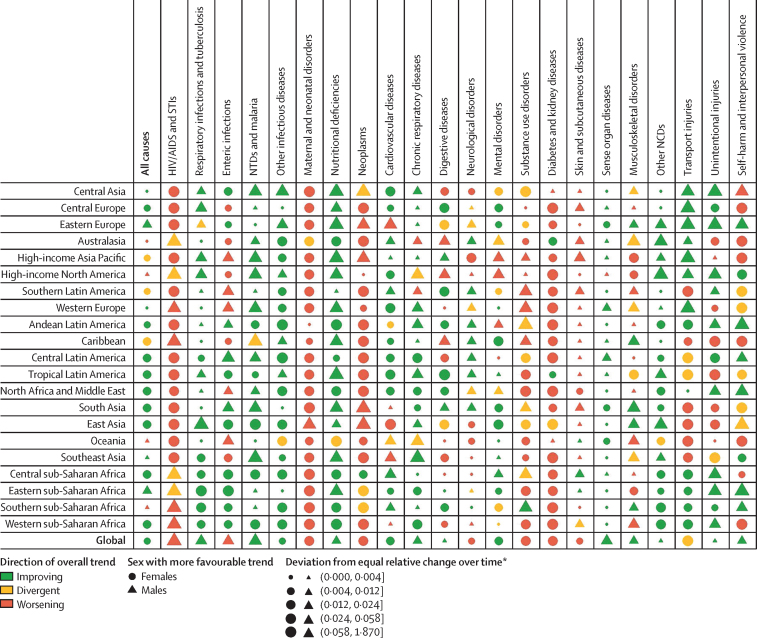
Absolute difference in non-fatal trend equality for males and females in terms of age-standardised YLD rates, 1990–2017 This figure shows whether females or males experienced more favourable trends between 1990 and 2017 in terms of age-standardised YLDs by GBD region and Level 2 cause. Circles indicate females experienced more favourable trends and triangles indicate males experienced more favourable trends, where more favourable refers to either decreasing more or increasing less. Green indicates that the overall trend is improving (ie, decreasing age-standardised YLDs) and red indicates that the overall trend is worsening (ie, increasing age-standardised YLDs). Yellow indicates cause-regions where one sex is increasing and the other sex is decreasing. For example, in Andean Latin America for substance use, the large yellow triangle means that males have experienced decreasing age-standardised YLDs whereas the trend for females is the opposite (ie, increasing age-standardised YLDs). Different sizes refer to greater deviations from equal trends between 1990 and 2017. For example, for chronic respiratory conditions, males have experienced more favourable trends in both Andean Latin America and the Caribbean, with both regions having decreasing trends over time for both sexes, but the Caribbean is closer to having equal trends for males and females between 1990 and 2017. GBD=Global Burden of Diseases, Injuries, and Risk Factors Study. STIs=sexually transmitted infections. NCDs=non-communicable diseases. NTDs=neglected tropical diseases. YLDs=years lived with disability. *Round brackets indicate excluded endpoints whereas square brackets indicate included endpoints.

Figure 6 shows the extent to which males and females in region–cause combinations have diverged in terms of achieving equal change over time between 1990 and 2017. Each region–cause combination shows which sex has performed better over time either by decreasing more or by increasing less in terms of age-standardised YLD rates. Among the 462 region–cause combinations (excluding any “Global” or “All causes” combinations), females had more favourable outcomes over time for 260 combinations (56·3%), and males had more favourable outcomes for 202 combinations (43·7%). The Z score, that is, the number of SDs from equality, was 0·433 for the mean deviation of female-favourable causes and 0·313 for the mean deviation of male-favourable causes.

**Figure 7:**
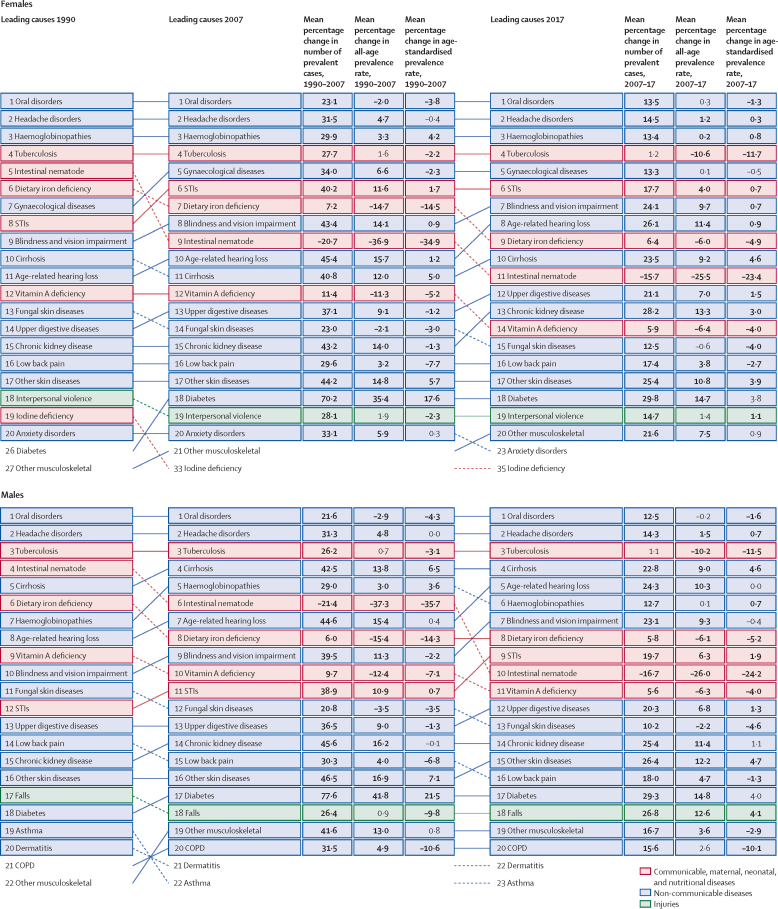
Leading 20 Level 3 causes of global prevalence for 1990, 2007, and 2017, with percentage change in number of cases and all-age and age-standardised rates for each sex Causes are connected by lines between time periods; solid lines are increases and dashed lines are decreases. For the time periods 1990–2007 and 2007–17, three measures of change are shown: percentage change in the number of cases, percentage change in the all-age prevalence rate, and percentage change in the age-standardised prevalence rate. Communicable, maternal, neonatal, and nutritional diseases are shown in red; non-communicable causes in blue; and injuries in green. Statistically significant changes are shown in bold. COPD=chronic obstructive pulmonary disease. STIs=sexually transmitted infections.

Figure 7 shows the leading 20 causes of prevalence and YLDs for females and males separately for 1990, 2007, and 2017, with percentage change. In 1990, the most common causes for females were oral disorders, headache disorders, and haemoglobinopathies and haemolytic anaemias, whereas for males, the most common causes were oral disorders, headache disorders, and tuberculosis including latent tuberculosis. The number of prevalent cases in the top three leading causes for females increased by 23·1% (95% UI 21·8 to 24·5) for oral disorders, 31·5% (30·2 to 32·8) for headache disorders, and 29·9% (29·6 to 30·1) for haemoglobinopathies and haemolytic anaemias between 1990 and 2007. Between 2007 and 2017, the number of cases increased further by 13·5% (12·9 to 14·1), 14·5% (13·8 to 15·3), and 13·4% (13·2 to 13·6), respectively (figure 7A). Between 1990 and 2007, for males, the number of prevalent cases of oral disorders increased by 21·6% (20·1 to 23·1), the number of headache disorders increased by 31·3% (29·9 to 32·8), and the number of prevalent cases of tuberculosis including latent tuberculosis infection increased by 26·2% (21·7 to 30·3). Between 2007 and 2017, the number of prevalent cases for these three causes increased by 12·5% (11·9 to 13·1), 14·3% (13·6 to 15·2), and 1·1% (–0·67 to 3·01), respectively (figure 7B). The age-standardised prevalence of the top cause for females, oral disorders, decreased by 3·8% (3·3 to 4·3) between 1990 and 2007, and decreased by 1·3% (1·0 to 1·7) between 2007 and 2017. For the second and third top causes for females, the age-standardised prevalence decreased by 0·4% (–1·0 to 0·3) for headache disorders and increased by 4·2% (4·0 to 4·4) for haemoglobinopathies and haemolytic anaemias between 1990 and 2007 and increased by 0·3% (0·1 to 0·6) and 0·8% (0·6 to 1·0), respectively, between 2007 and 2017. In 2017, oral disorders, headache disorders, and haemoglobinopathies and haemolytic anaemias remained the top three leading Level 3 causes of global age-standardised prevalence for females. Between 1990 and 2007, the age-standardised prevalence for the top three leading causes for males decreased by 4·3% (3·8 to 4·9) for oral disorders, increased by less than 0·1% (–0·85 to 0·85) for headache disorders, and decreased by 3·1% (0·8 to 5·4) for tuberculosis including latent tuberculosis infection. Between 2007 and 2017, the age-standardised prevalence for males for oral disorders and tuberculosis further decreased by 1·6% (1·2 to 2·0) and 11·5% (10·1 to 12·8), respectively, whereas the age-standardised prevalence for headache disorders increased by 0·7% (0·4 to 0·9). In 2017, oral disorders, headache disorders, and tuberculosis including latent tuberculosis remained the three Level 3 causes with greatest global age-standardised prevalence for males.

**Figure 8:**
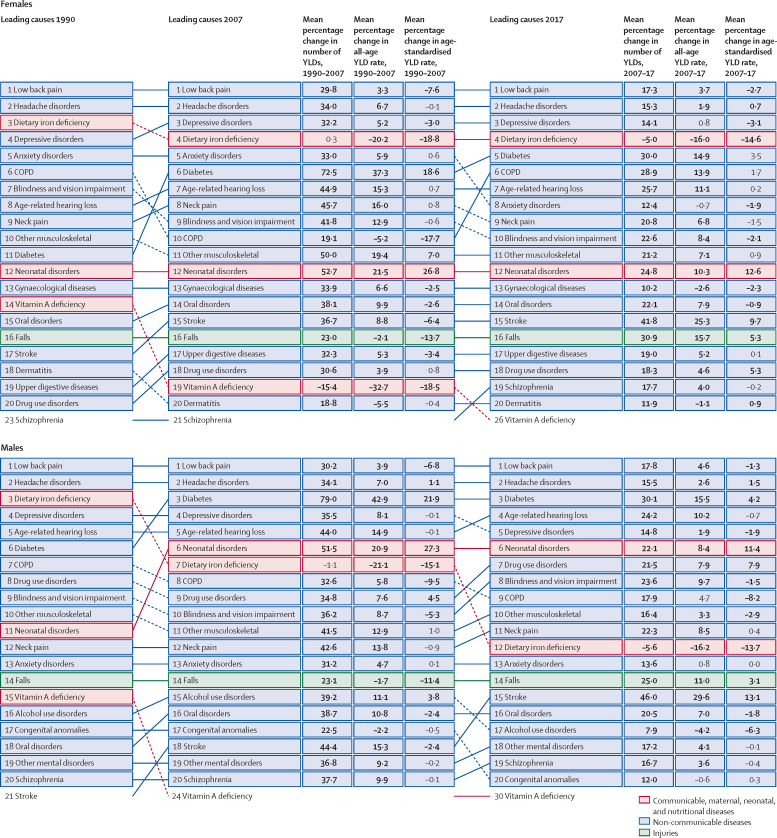
Leading 20 Level 3 causes of global YLDs for 1990, 2007, and 2017, with percentage change in number of YLDs and all age and age-standardised rates for each sex Causes are connected by lines between time periods; solid lines are increases and dashed lines are decreases. For the time periods 1990–2007 and 2007–17, three measures of change are shown: percentage change in the number of YLDs, percentage change in the all-age YLD rate, and percentage change in the age-standardised YLD rate. Communicable, maternal, neonatal, and nutritional diseases are shown in red; non-communicable causes in blue; and injuries in green. Statistically significant changes are shown in bold. COPD=chronic obstructive pulmonary disease. YLDs=years lived with disability.

In terms of numbers of YLDs, in 1990, the leading causes for both females and males were low back pain, headache disorders, and dietary iron deficiency (figure 8). For both males and females, the top two leading causes of global YLDs remained consistent between both time periods, during which the total number of YLDs for each of these causes increased. The third top cause in 2017 was depressive disorders for females and diabetes for males. From 1990 to 2007, for females, the number of YLDs attributable to low back pain increased by 29·8% (95% UI 27·7–31·8) and headache disorders increased by 34·0% (32·8–35·3). From 1990 to 2007, for females, the number of YLDs attributable to depressive disorders increased by 32·2% (29·8–34·4), causing depressive disorders to become the third leading cause in 2007. The number of YLDs for the three leading causes for females continued to increase from 2007 to 2017, by 17·3% (15·8–18·8) for low back pain, 15·3% (14·4–16·2) for headache disorders, and 14·1% (12·8–15·5) for depressive disorders. From 1990 to 2007, for males, the number of YLDs attributable to the top two leading causes increased by 30·2% (28·1–32·2) for low back pain and 34·1% (32·7–35·4) for headache disorders. From 1990 to 2007, for males, the number of YLDs attributable to diabetes increased by 79·0% (75·8–82·2), causing diabetes to become the third leading cause. The number of YLDs for the three leading causes continued to climb from 2007 to 2017, increasing by 17·8% (16·5–19·3) for low back pain, 15·5% (14·5–16·5) for headache disorders, and 30·1% (24·8–36·1) for diabetes.

Between 1990 and 2007, age-standardised YLD rates for females decreased for the three leading causes: low back pain (by 7·6%, 95% UI 6·2 to 8·9), headache disorders (by 0·1%, −0·80 to 0·69), and dietary iron deficiency (by 18·8%, 17·0 to 20·7). Between 1990 and 2007, the YLD rate for depressive disorders decreased by 3·0% (1·6 to 4·5) and became the third leading cause of YLDs in females in 2007 due to the simultaneous large decrease in dietary iron deficiency (figure 8A). Between 1990 and 2017, YLD rates for females decreased for low back pain (by 10·1%, 95% UI 8·8 to 11·3), depressive disorders (by 6·0%, 5·0 to 7·1), and dietary iron deficiency (by 30·7%, 27·4 to 33·7), while headache disorders increased by 0·6% (–0·19 to 1·42). Between 1990 and 2007, the YLD rates for males decreased for low back pain (6·8%, 5·6 to 7·8) and dietary iron deficiency (15·1%, 10·9 to 18·9), and increased for headache disorders (1·1%, 0·15 to 1·9). Between 1990 and 2007, the YLD rate for diabetes increased by 21·9% (20·0 to 24·0) and became the third leading cause of YLDs in males in 2007. From 2007 to 2017, the YLD rate for headache disorders increased by 1·5% (0·8 to 2·3) and that for diabetes increased by 4·2% (0·0 to 8·7), whereas the YLD rate for low back pain decreased by 1·3% (0·7 to 1·9). Between 1990 and 2017, the YLD rate for low back pain decreased by 8·0% (6·**9** to 9·1) and that for dietary iron deficiency decreased by 26·7% (20·6 to 32·5), whereas the rates for headache disorders and diabetes increased by 2·6% (1·6–3·6) and 27·0% (22·2–32·4), respectively.

## Discussion

### Main findings

While age-standardised all-cause global YLD rates decreased by less than 4% over the nearly three-decade period from 1990 to 2017, the number of total YLDs has increased by more than 50% during this time. This pattern is concerning given the lack of substantial improvement in age-standardised rates over time as well as the increased magnitude of total health loss. These patterns are probably related to population growth and ageing as well as increasing numbers of YLDs from conditions such as type 2 diabetes and opioid use disorders, which were less common in 1990. YLD increases, even when age-standardised rates are slightly improving, might pose a burden to economies and health-care systems that have not expanded proportionally to population growth or in populations where economic improvements have not been equitably distributed.

Globally, over the 28-year period studied, three chronic NCDs (low back pain, headache disorders, and depressive disorders) have prevailed as three of the top four leading causes of YLDs, collectively causing 162 million (95% UI 118–216) YLDs in 2017 and representing nearly one in five YLDs globally. This substantial portion of global YLDs might be amenable to treatment and care access, because headache disorders and depressive disorders can be treated with low-cost therapeutics. The persistence of depressive disorders and low back pain is also concerning given the former's relation with self-harm and the latter's relation with potential loss of functional status in the work force.

### Emerging trends

For the first time in the GBD study, we estimated the burdens of type 1 and type 2 diabetes separately. Our estimates illustrate the emergence of diabetes as a leading cause of disability globally, ranking as the fourth leading cause of age-standardised YLDs in 2017. This increased burden was observed across all levels of development. Age-standardised YLDs increased for both females and males across all SDI quintiles between 1990 and 2017 (figure 3). Diabetes poses complicated care challenges even in areas with reliable access to medical services. Preventive measures such as prediabetes screening, lifestyle modification, and treatment with low-cost medications such as metformin could avert worsening incidence rates, but as prevalence increases, health-care systems will also need to provide access to services such as wound care, surgical resources, medications including insulin, care for diabetic retinopathy, and increased focus on heightened cardiovascular disease risk.

This is also the first year of GBD estimation for non-alcoholic fatty liver disease (NAFLD) and cirrhosis and liver cancer caused by non-alcoholic steatohepatitis (NASH). For cirrhosis and liver cancer caused by NASH, we estimated 892 million (95% UI 859–928) global cases for cirrhosis due to NASH and 97 400 cases (86 800–108 000) for liver cancer due to NASH, and an age-standardised prevalence of 11 061 cases (10 651–11 493) per 100 000 for cirrhosis due to NASH and 1·20 cases (1·07–1·33) per 100 000 for liver cancer due to NASH in 2017 (appendix 2), identifying NASH as an increasingly important cause of liver disease. We observed that these cases were distributed across all GBD regions, although more commonly in North Africa and the Middle East and in Oceania in terms of age-standardised rates. This expansion of NAFLD and liver disease due to NASH reflects the worsening burden of metabolic conditions globally. Given the complexity of chronic liver disease and the difficulty of effectively treating obesity, efforts to prevent obesity and to develop therapeutics for NAFLD will be increasingly important.

After initial steep reductions in HIV incidence between 1998 and 2005, the global rate of decline in new HIV infections continues, although it has slowed in recent years and global prevalence has increased slightly since 2010, a trend which is probably driven by rapid expansion of antiretroviral therapy in high-prevalence countries and extended life expectancy of people living with HIV. These longer lifespans call for increased resources for treatment and continued prevention and treatment interventions to maintain declines in incidence.^27^ For tuberculosis, it is a notable success that drug-susceptible tuberculosis has declined in terms of age-standardised incidence since 1990. The burden of malaria has also declined since the mid-2000s in terms of age-standardised incidence rates. High age-standardised incidence rates of acute viral hepatitis have persisted, however, with age-standardised incidence rates of hepatitis A and B combined exceeding malaria incidence in 2017 despite availability of vaccines. The age-standardised incidence rate of acute hepatitis did not significantly change from 1990 to 2007, from 2007 to 2017, or from 1990 to 2017, suggesting that more proactive global initiatives are important, particularly with respect to vaccine coverage^28^ and cost-effective access to hepatitis C curative treatments.^29^, ^30^, ^31^

Incidence rates of lower respiratory infections (LRIs) declined among children younger than 5 years by 32·4% (95% UI 27·2–37·5) since 1990 and caused 83·0 million (66·4–101) episodes in children younger than 5 years in 2017, while the age-standardised incidence rate decreased by 5·8% (1·5–10·0) between 1990 and 2017 (appendix 1). Among all ages, pneumococcal pneumonia has prevailed as the leading aetiological subtype of LRI, accounting for more than five times more YLDs than the second leading cause (influenza). Diarrhoeal episodes have persisted as the second most common incident cause of health loss after upper respiratory infection among all ages, with 6·29 billion (5·81–6·82) total episodes in 2017, with rotavirus prevailing as the leading aetiology. The age-standardised incidence of diarrhoea varied nine-fold across GBD regions, with the lowest rates in Australasia and the highest rates in Oceania (appendix 2). The burdens of pneumococcal pneumonia and rotavirus diarrhoea are notable given the availability of vaccines for these pathogens, highlighting the need for continued expansion of vaccine coverage and delivery systems.

While the burden of musculoskeletal disorders, vision loss, and hearing loss in older ages has been a consistent theme in past GBD studies due to their substantial contribution to non-fatal health loss, the burden of medically complex, high-resource conditions has also become increasingly concerning. Cardiovascular disease, chronic respiratory conditions, and neurological disorders sum to form more than 15% of age-standardised YLDs in every GBD region except two (southern Latin America and eastern sub-Saharan Africa; appendix 2). GBD risk factor estimates show that the drivers for atherosclerotic cardiovascular diseases—high systolic blood pressure,^32^, ^33^, ^34^ high cholesterol, poor diet,^35^ high fasting plasma glucose,^35^ and low physical activity^36^—prevail in many geographies, indicating that this burden can be expected to persist and possibly expand as populations age. While the efforts of primary prevention approaches such as statin therapy and smoking cessation are evident in the gradual declines in age-standardised prevalence in high-income countries over the past 28 years, the prevalence of cardiovascular disease in these populations still remains high, which might also reflect improved survival from acute events due to improved response systems and interventions such as percutaneous coronary intervention, coronary artery bypass grafting, and rapid stroke treatment guidelines,^37^, ^38^, ^39^, ^40^ as well as secondary prevention with statins, antihypertensives, and smoking cessation.^41^, ^42^, ^43^, ^44^, ^45^, ^46^ As developing economies increasingly experience cardiovascular disease burden, investing in health system infrastructure to ensure access to these interventions as well as to primary prevention therapeutics could help to mitigate disability and improve survival into older ages.^40^, ^47^, ^48^ Chronic respiratory conditions also inflict considerable health loss in older people, contributing to more than 5% of YLDs starting in the 50–54 year age group, globally, who were exposed to risk factors such as smoking and indoor air pollution and similarly pose challenges to health systems with ageing populations, given the importance of oxygen therapy,^49^, ^50^, ^51^, ^52^ access to medical care,^53^, ^54^, ^55^ and the risk of respiratory infections among these patient populations.^56^, ^57^ Neurological conditions continue to show large numbers of prevalent cases and YLDs, with consistent increases in recent years. We anticipate that this burden will also expand as populations age, creating challenges for health-care systems given the lack of modifiable risk factors for some neurological conditions and effective treatments for conditions such as Alzheimer's disease. Given the extensive care requirements these conditions—in isolation or as comorbidities—demand from both health systems and social and familial supports, their morbidity can reflect not only diminished health for an individual and population but also lost capital in an economy and among caretakers.^58^, ^59^, ^60^, ^61^, ^62^, ^63^

Opioid use disorders including opioid dependence have become an era-defining epidemic in the USA with more than 4 million prevalent cases in 2017. Outside the USA, opioid dependence was estimated to cause more than 1% of age-standardised YLDs in 135 countries in 2017, whereas in 12 out of 21 GBD regions, more than 5% of YLDs in the 15–49 year age group were caused by substance use disorders overall (appendix 2). Cumulatively, these results show pervasive health loss from substance use, which might also cause lost human capital given the concentration of the burden in working ages. The burden of mental disorders is present in both sexes and across all age groups after emerging in childhood with idiopathic intellectual disability and autism spectrum disorders and continuing into older ages with depressive disorders, anxiety disorders, and schizophrenia. Mental disorders have consistently formed more than 14% of age-standardised YLDs for nearly three decades, and have greater than 10% prevalence in all 21 GBD regions. These findings substantiate a global need for increased mental health and substance abuse treatment resources, improved judiciousness of opioid prescribing patterns particularly for chronic pain, and expanded harm-reduction strategies such as opioid substitution therapy and needle programmes.

### Cross-cutting themes

Among the 22 causes in the Level 2 GBD cause hierarchy, 14 causes had a relative difference between males and females greater than or equal to 10% in terms of prevalent cases in 2017, while 12 causes had a relative difference greater than 10% in terms of age-standardised prevalence. Notably, females had significantly higher prevalence of HIV/AIDS and sexually transmitted infections (driven primarily by higher burden from sexually transmitted infections) and self-harm and interpersonal violence, among other causes, whereas males had significantly higher prevalence of substance use disorders and transport injuries, among other causes. Figure 6 illustrates how divergent trends were nearly evenly distributed between males and females, despite the overall higher numbers of YLDs experienced by females. As indicated by the higher Z score in female-favourable causes, the cause-regions where females did better over time tended to have greater improvements in age-standardised YLD rates than did the cause-regions where males did better over time. There were important exceptions, however: for example, with HIV/AIDS and sexually transmitted infections in central sub-Saharan Africa, the age-standardised YLD rates for females increased by 30·0% (95% UI 6·8–68·1), while rates for males decreased by 43·0% (23·2–58·5) between 1990 and 2017. This particular trend reveals a striking lapse in health equity given the extensive knowledge of treatment and prevention methods for HIV/AIDS and many sexually transmitted infections.

These observations imply that health systems might have achieved improvements in non-fatal burden that have not been shared across sexes. The underlying reasons for these imbalances are likely to be complex. For causes that are inherently more common in males or females, improvements in one sex over the other might reflect these causes differing in susceptibility to prevention, intervention, and treatment. For example, autoimmune conditions such as rheumatoid arthritis can affect both males and females but are ordinarily more common in females.^64^, ^65^ However, such a cause still leads to increased disability that might also have ramifications not captured in a health study, such as income loss due either to disability at young ages or to the cost of modern immunotherapies. Similarly, females of childbearing age experience risk of pregnancy-related and maternal conditions, risks that could be pronounced in lower-resource settings without sufficient access to modern obstetric facilities and follow-up care.^66^, ^67^ Other causes have firmly established causal risk factors such as smoking and chronic obstructive pulmonary disease (COPD), where the higher COPD rates in males are probably due to historically higher rates of smoking compared with females. Additionally, some causes may be susceptible to systematic misdiagnosis due to conventional clinical heuristics based on flawed or non-existent evidence. For example, in some areas, females presenting with acute coronary syndrome are more commonly misdiagnosed,^68^ whereas other causes, such as autism spectrum disorders, might be systematically underdiagnosed in females compared with males.^69^, ^70^, ^71^ These biases in sex-specific misdiagnosis emphasise the need for continued refinement in sex-specific burden estimation.

While YLD rates are highest in older ages, globally, YLD counts are heavily concentrated in working-age males and females (ie, from 20–54 years), a pattern which is particularly evident among causes such as mental disorders, neurological disorders, and musculoskeletal disorders, which sum to more than 45% of all YLDs in these age groups. This finding is notable for two main reasons. First, these age groups have a considerable number of years to live that would otherwise be in full health, emphasising how conditions at these ages, even if having lower disability weights, can still contribute substantially to the non-fatal burden. Second, a disabling condition that occurs during this period of life could represent lost human capital. This loss will be increasingly important to characterise with human capital emerging as an important area of focus at the World Bank.^72^, ^73^

In GBD 2017, anaemia is classified as an impairment, with aetiologies ranging from rare genetic haemoglobinopathies to iron deficiency. Because these aetiologies alone could cause a relatively small proportion of all anaemia cases, the magnitude of anaemia's burden would be obscured by the granularity of these aetiologies if it was not also estimated in aggregate. In 2017, the sum of YLDs across all causes of anaemia was 58·2 million (95% UI 39·5–83·0), with a global age-standardised rate of 783·9 YLDs (531·3–1117·8) per 100 000 for both sexes, with sex-specific rates of 1012·8 YLDs (689·4–1436·5) per 100 000 females, and 555·9 YLDs (370·4–801·8) per 100 000 males. Globally, in terms of YLD rates in 2017, anaemia is the leading impairment for females, males, and both sexes combined. If anaemia were treated as a cause, it would rank as the fourth leading Level 2 cause and as the first leading Level 3 cause in terms of age-standardised YLD rates globally for females in 2017. The third leading impairment for males, females, and both sexes combined was blindness and vision impairment (1·34 billion [1·29–1·39] cases in 2017), which is notable given the economic consequences of vision loss,^74^, ^75^ the relative treatability of many vision loss conditions, and the increased risk of injury that stems from visual impairment.^76^, ^77^ Summed together, the impairments included in the GBD cause hierarchy comprise a total of more than 5 billion prevalent cases worldwide in 2017, making it evident that health-care systems and policy makers should evaluate non-fatal burden not only for diseases and injuries but also for impairments and their aetiologies.

### Important changes in GBD 2017 compared with GBD 2016

*Overview*

Overall, the most important systemic change to this year's study is the estimation of population and fertility in the GBD framework. In addition, we made changes to the GBD cause hierarchy (for example adding an aggregate Level 3 headache disorders cause), which limits certain cause-specific comparisons with past GBD cycles; this is discussed in more detail in the Limitations section below. Other notable cause-specific considerations, comparisons, and limitations that are new as of GBD 2017 are as follows. More details for each cause can be found in the supplementary methods (appendix 1 section 4).

#### Cause-specific considerations, comparisons, and limitations

In GBD 2017, we added the Malnutrition and Enteric Disease (MAL-ED) study to our existing use of the Global Enteric Multicenter Study (GEMS)^78^, ^79^, ^80^ to measure diarrhoeal aetiologies, which affected our aetiological distributions with increased norovirus and rotavirus attributions. Our overall diarrhoea incidence estimates were not affected. The Walker and colleagues' study^81^ estimated 1·7 billion episodes of diarrhoea among children younger than 5 years in 2010, a value that is greater than the GBD 2017 estimate in the same year (1·16 billion, 95% UI 1·00–1·34). For pneumonia, a study by Rudan and colleagues^82^ estimated a global pneumonia incidence of 0·22 episodes (IQR 0·11–0·51) per child-year among children younger than 5 years in 2010, accounting for 122·6 million episodes, a value that is greater than the GBD 2017 estimate of 94·5 million (79·5–112). These two studies are part of the Maternal and Child Epidemiology Estimation (MCEE). The MCEE methodology for diarrhoea and pneumonia incidence differs from GBD in that MCEE uses only community-based studies. By contrast, the GBD uses a greater volume of data including Demographic and Health Surveys, Multiple Indicator Cluster Surveys, and clinical data records. We also used diarrhoea-specific and LRI-specific summary exposure values as covariates in our models, which include location-specific and year-specific estimates of risk factors associated with diarrhoea or LRI. We observed that these covariates had significant relationships with diarrhoea and LRI incidence rates and therefore strengthened our estimates, particularly in data-sparse areas. Further modelling details are described in the supplementary methods (appendix 1 section 4).

For HIV/AIDS, we used new methods to adjust for representativeness bias in ANC surveillance data,^83^ which led to our models predicting a lower peak and flatter trend of the epidemic in southern sub-Saharan Africa compared with GBD 2016. We updated our inputs of antiretroviral therapy coverage distribution informed by data from the International Epidemiology Databases to Evaluate AIDS and CD4+ progression parameters for children.^84^, ^85^ We also improved our sex-specific modelling strategy in Spectrum by sex-splitting incidence based on a model fit to the sex ratio of prevalence observed in countries with representative surveys. Our estimated global prevalence results are similar to UNAIDS 2017 values, with some variation at the national level. Estimates in recent years are expected to be less stable due to improvements in HIV treatment coverage and prevention. Further modelling details are described in the supplementary methods (appendix 1 section 4).

The hepatitis estimation process has changed to improve internal consistency between mortality and non-fatal estimates for GBD 2017. First, we included case-fatality rates from clinical data in our mortality models to improve the distribution of hepatitis deaths by virus. Second, we developed a time series of hepatitis B virus vaccine coverage in infants to use as a covariate. Third, the aggregate cause-specific mortality rate of acute hepatitis, cirrhosis, and liver cancer due to hepatitis B and hepatitis C was combined with virus-specific seroprevalence data to ensure internal consistency among incidence, prevalence, remission, and excess mortality rates. Fourth, the prevalence of chronic hepatitis B and C are now captured in the cirrhosis and other chronic liver diseases cause group rather than in the prevalence estimates for acute hepatitis B and acute hepatitis C. Overall, this caused prevalent cases of hepatitis B and C to be shifted from the acute hepatitis B and C causes to the cirrhosis and other chronic liver diseases cause. Specifically, in terms of global age-standardised prevalence rates for both sexes in 2016, acute hepatitis B decreased from 6295 cases (95% UI 5349–7436) per 100 000 in GBD 2016 to 219 cases (193–248) per 100 000 in GBD 2017, while its corresponding chronic cause, cirrhosis and other chronic liver diseases due to hepatitis B, increased from 170 cases (159–184) to 5625 cases (5159–6118) per 100 000. Similarly, acute hepatitis C decreased from 2152 cases (1927–2382) per 100 000 in GBD 2016 to 8·11 cases (7·37–8·98) per 100 000, while its corresponding chronic cause, cirrhosis and other chronic liver diseases due to hepatitis C, increased from 149 cases (136–164) to 1723 cases (1517–1969) per 100 000 (appendix 2).

#### Maternal, neonatal, and child health

Several changes were adopted for GBD 2017. Adding clinical and claims records from outside the USA to our models affected congenital birth defects, haemoglobinopathies, and maternal disorders. Updating our inpatient admission per-capita estimates to include in-facility deliveries as a newborn admission affected neonatal disorders and congenital birth defects. Adopting in-facility delivery estimates for processing clinical data for maternal disorders led to lower estimates of pregnancy complications, especially in high-utilisation settings. We refined our method for estimating age-specific preterm birth and its complications to be consistent with GBD risk factor analysis, which resulted in preterm birth prevalence being higher than in previous GBD estimates. We enforced internal consistency among our estimates such that, for example, the sum of all specific types of congenital heart disease must equal the total number of cases of congenital heart disease. These methodological changes have lowered global estimates for many of the specific causes of birth defects, enforced the internal consistency of mortality and non-fatal estimates, narrowed the unexplained geographical variation in disease incidence, and strengthened the empirical relationship with known environmental, nutritional, and behavioural determinants of these conditions. For nutritional deficiencies, we extended our analysis of protein-energy malnutrition past age 5 years, incorporated cause-specific mortality rates, and included the prevalent cases of moderate wasting without oedema. Although the net result might seem to be an increase in the number of protein-energy malnutrition cases, it instead reflects a more comprehensive assessment of acute malnutrition than was estimated previously.

#### Diabetes

Diabetes has been estimated in previous GBD studies, but this is the first year that type 1 and type 2 diabetes were estimated and reported separately. The estimation strategy for diabetes is provided in more detail in the supplementary methods (appendix 1 section 4), but in summary, we measured overall diabetes and type 1 diabetes. We subtracted estimates of type 1 from overall diabetes to produce estimates for type 2 diabetes. One of the main limitations to measuring type 2 diabetes is that surveys of diabetes in adults do not make a distinction between cases of type 1 and type 2 diabetes.

#### NASH and NAFLD

In GBD 2017, for the first time we estimated NASH as a cause of liver cancer and cirrhosis and NAFLD as an asymptomatic health state. Our global age-standardised prevalence estimate of NAFLD and NASH that leads to cirrhosis or liver cancer was 10 935 cases (95% UI 10 522–11 365) per 100 000 in 2017, which was lower than a study by Younossi and colleagues,^86^ which estimated a global prevalence of 24%. This difference is due to how we adjust for alcohol use in the population, which helps to distinguish these conditions from alcohol-driven liver disease. Our study shows a similar geographical pattern to the Younossi study, with higher rates in North America and the Middle East, corresponding to higher rates of obesity.

#### Cardiovascular diseases

We split haemorrhagic stroke into subarachnoid haemorrhage and intracerebral haemorrhage as subtypes of stroke and added non-rheumatic valve disease as an additional cause and aetiology of heart failure. The overarching limitation in cardiovascular estimation is that low-income and middle-income locations are less likely to have diagnostic tests such as treadmill tests, ankle-brachial index measurement, and other modern diagnostics. This also limits the identification of preclinical atherosclerotic disease for individuals who have not had clinical events but who might have ischaemic changes with provocative cardiac testing. For stroke, there were few sources of incidence data in many low-income and middle-income countries, and many clinical records used the ICD code for “acute, unspecified stroke”, which does not differentiate between ischaemic stroke, intracerebral haemorrhage, and subarachnoid haemorrhage. Future incorporation of more clinical record data might help to address these limitations by providing more diagnostic detail for cause subtypes.

#### Chronic respiratory diseases

We added several data sources that had lower estimates than the International Study of Asthma and Allergies in Childhood studies, which were the main source of asthma prevalence previously. Additionally, the adjustment factor for studies reporting on physician-diagnosed asthma without an additional question on wheezing changed between GBD 2016 and GBD 2017 after new data sources were added.

#### Neurological conditions

For dementia, we added covariates for whether studies had common features present in their diagnostic protocol, such as a review of clinical records or a diagnosis by a clinician to try to correct for some of the heterogeneity between studies, because only a very small fraction of studies used the same methods. We do not have reliable covariates for prevalence or incidence of dementia and other neurological disorders, although in future studies, estimates of neurological disorders will benefit from the increased use of clinical and claims records.

#### Cancer

For GBD 2017, we added the new cause “myelodysplastic, myeloproliferative, and other haemopoietic neoplasms”, which were previously estimated as part of the “other neoplasms” group. In addition, we added new causes for three categories of benign and in-situ neoplasms: intestinal, cervical and uterine, and other.

#### Mental and substance use disorders

We estimated burden for a combined group of autism spectrum disorders consistent with the Diagnostic and Statistical Manual of Mental Disorders 5 designations in GBD 2017 as opposed to separately estimating autism and Asperger's syndrome and other autism disorders.^87^ Modelling improvements were made for opioid use disorders by incorporating new country-level covariates, including a measure of opioid analgesic consumption by country and the prevalence of injection drug use. Enhancements in the fatal modelling of opioid use disorders also contributed to these improved estimates. Improvements were also made in terms of data additions, particularly for cocaine and amphetamine dependence, where new data showed greater subnational variation in Mexico and Brazil.

#### Injuries

For GBD 2017, improvements were made to the computational framework required for injuries non-fatal measurement. These updates included incorporating GBD 2017 age groups and updating the differential equation solver for converting incidence to prevalence. Poisoning was divided into the two subcauses of poisoning by carbon monoxide and poisoning by other agents. Our estimate of 172 million (95% UI 152–194) falls per year resulting in any short-term or long-term disability was higher than those of WHO, which estimated 37 million falls^88^ that required medical attention per year—a difference that might be explained by GBD's inclusion of a wide spectrum of injury severities including minor injuries that result from falls. The WHO and GBD estimates on road injuries were more similar, with WHO estimating 20–50 million people experiencing non-fatal road injuries per year^89^ and GBD 2017 estimating 54·2 million (47·4–61·6) road injuries in 2017. For fires, WHO and GBD 2017 had similar estimates when comparisons were available—for example, in the USA, WHO reported 410 000 burn injuries^90^ in 2008 and GBD 2017 reported 360 000 (313 000–380 000) fire, heat, and hot substance injuries.

### Limitations

As is evidenced in figure 1 and the supplementary methods, there is considerable heterogeneity in terms of data density for each Level 1 location-cause combination in the GBD study. Data availability does not consistently correlate with burden—that is, high-burden causes and locations can have relatively sparse data for non-fatal outcomes. This will also be increasingly problematic as more countries start to experience greater burden from NCDs.

Currently, we inform most of the severity distributions that affect YLD calculation using the Medical Expenditure Panel Survey from the USA. This poses a limitation as the severities experienced in this population probably do not reflect global severity distributions or reflect that severity distributions are likely to vary over time, location, age, and by treatment access. As such, YLDs estimated might not reflect improvements in disability over time as SDI increases. Even for the limited diseases such as COPD, epilepsy, and stroke for which data from epidemiological studies allow us to differentiate severity by age, time, and location, the underlying data sources are still extremely limited in coverage by age, time, and location. Relying on published data on severity is unlikely to provide improved estimates in the near future. We also plan to access linked data between administrative records with diagnostic information and health surveys using health status measures to quantify severity of disease and how this changes over time and by age and sex.

Comorbidity adjustment in GBD 2017 assumes independent distributions of comorbid conditions. This is a limitation because comorbidity distributions are known to vary by cause, age, sex, location, and time. For example, diabetes and cardiovascular disease are more likely to be comorbid than asthma and gynaecological disorders but currently our comorbidity adjustment does not capture these correlations.

Clinical data records have known selection bias for subsets of the population that have access to health care. Some GBD causes rely heavily on clinical data given the lack of other sources, with efforts made to correct for this bias by using representative studies as a reference group. In recent years, the GBD study has used USA claims records to inform hospital data adjustments and various other elements of GBD estimation. Relying on data from a privately insured cohort in a high-income country poses limitations to the generalisability of these adjustments. This year, we also used tabulated claims records from Taiwan (province of China) for the first time in the GBD study, and in future publications we plan to continue incorporating claims data from additional countries to address this limitation. Acquiring further health insurance claims data will not only inform estimates in terms of incidence and prevalence measures but will also help to develop more accurate parameters that affect GBD estimates in other modelling steps.

With regard to age-time patterns, in DisMod-MR 2.1, we start by estimating a global model that uses all the available data and covariates with hierarchical random effects for geographies to estimate a prior distribution that is passed down the geographical cascade. Time trends in our estimates result from where the covariates are changing over time, when year is included as a covariate or when data in different time periods lead to variation in the posterior estimates. This framework, however, does not capture true cohort effects. For example, we do not directly take into consideration prevalence in 30-year-olds in 1990 when estimating prevalence in 50-year-olds in 2010. This is a limitation for some diseases with known cohort effects such as the high incidence of hepatitis C in the USA in younger adults in the 1970s. We are continuing to develop a version of DisMod that explicitly incorporates a full age-time-period model in a Bayesian framework that will help to address this issue.

While our cause list currently specifies 354 diseases and injuries, there is great interest in incorporating a greater level of detail. Going forward, we intend to become more granular in our cause list, but every new disease requires a substantial amount of new reviews and model building. In consultation with our Scientific Council, we will need to balance our available resources with this growing list of priorities for new additions.

Similar to the cause reporting list, the hierarchy of the cause list and the method by which conditions are classified and grouped is a complex component of the GBD framework that has undergone nearly continuous improvement and refinement in years past. Grouping GBD causes into broad cause groups is a necessary part of the study's estimation and reporting process, but it can also cause limitations and challenges in the absence of broad consensus on objective frameworks for organising cause hierarchies. Other studies that measure burden of disease exemplify how disease groupings can affect a study's implications and interpretation. A recent study^91^ cited medical errors as the third leading cause of death in the USA but grouped all heart disease and all cancer as the first and second leading causes, respectively. A different aggregation or level of detail in terms of the disease grouping in such a study could change these rankings, which become important in the realm of health policy and resource allocation as well as popular and political perception of what causes death and disability in a population. In the GBD study, we have strived to retain as much detail as is methodologically possible given available data, and additionally we have incorporated input from the GBD collaborator network in terms of refining our cause hierarchy to represent a heterogeneous mix of medical specialties, research expertise, and location-specific knowledge. In addition, our framework allows us to revise the cause hierarchy for specific specialties such as the burden of neurological disorders.^92^

### Future directions

The methods and data that inform YLD estimation in the GBD study will continue to evolve. Several specific improvements have been suggested by the GBD collaborator network and the global research community, and the following future directions are planned to be incorporated in the coming cycles.

Because the GBD cycle continues to be published on an annual basis, it will be important to incorporate updated data sources for older estimation years but also to add sources for the most recent estimation year. This necessitates data inclusion immediately after the end of the calendar year in order to be incorporated for the following year's estimation. This is challenging because some surveys, surveillance systems, and literature studies have a lag period between data collection and publication. One solution to this challenge would be adding, updating, and incorporating clinical data records from inpatient and outpatient visits from hospitals and clinics from more locations. Clinical data provide a level of detail, demographic information, and diagnostic accuracy that enhances the GBD study's ability to produce accurate estimates in the more recent estimation years. Relying more heavily on clinical data will require improved methods for adjusting for known representativeness biases. In the future, we plan to link medical records to representative survey results, which is possible with certain claims databases such as the Medicare Current Beneficiary Survey in the USA.^93^

In the next iteration of the GBD study, we plan to incorporate improved age-time estimation using a new tool, DisMod-AT, that simultaneously quantifies the relationships over time, by age, and by age cohorts. DisMod-AT will address the age-time measurement limitation described above.

We plan to continue updating and refining our cause and location hierarchies including the causes described above. This is the first year that estimates for a non-geographical subnational grouping (ie, Māori and non-Māori populations in New Zealand) were modelled, and in future GBD studies it would be compelling to similarly estimate disease burden in groups within a location not strictly determined by geographies—for example, race groups within the USA or refugee populations in host countries.

### Conclusion

The global burden of non-fatal health loss in 2017 represents a complex culmination of nearly three decades of evolving disease patterns and risk factor profiles, improving health care, and dynamic populations. This study provides comprehensive updates from prior GBD studies and identifies topical themes that can be used to guide future research and discussion in the arena of non-fatal health loss. Continuing a trend from throughout the history of the GBD study, we show how YLDs continue to increase as countries improve along the development spectrum and emphasise how economies should anticipate difficulties in caring for older populations suffering from complex conditions such as diabetes, respiratory and cardiovascular diseases, and neurological conditions. Additionally, we report heterogeneity in outcomes experienced across different sexes, geographies, and income levels, and identify regions and causes where sexes have had divergent health trends over time. These findings should help to focus prevention and treatment efforts on groups and areas that have experienced inequitable health outcomes. Continuing the theme of health equity, we highlight the extensive amount of health loss experienced globally for conditions that are treatable or preventable such as viral hepatitis, emphasising the profound impact that health-care systems can produce with increasing investment in and access to health-care resources. Finally, we emphasise the burden of non-fatal health loss in younger and middle ages, where it is speculated that non-fatal health loss could ultimately lead to loss in human capital.

Correspondence to: Prof Christopher J L Murray, Institute for Health Metrics Evaluation, University of Washington, Seattle, WA 98121, USA cjlm@uw.edu

## Data sharing
