# Department of Error

**DOI:** 10.1016/S0140-6736(19)31047-5

**Published:** 2019-06-22

*GBD 2017 Disease and Injury Incidence and Prevalence Collaborators. Global, regional, and national incidence, prevalence, and years lived with disability for 354 diseases and injuries for 195 countries and territories, 1990–2017: a systematic analysis for the Global Burden of Disease Study 2017.* Lancet *2018; **392:** 1789–858—*In this Global Health Metrics paper, Nicholas L S Roberts and W Cliff Mountjoy-Venning have been added to the collaborators list; affiliation details have been amended for Mina Anjomshoa, Joseph Adel Mattar Banoub, and Yasin Jemal Yasin; and the declaration of interests statement has been amended for Boris Bikbov. These corrections have been made to the online version as of June 20, 2019.

