# Supplementary material for: Global, regional, and national incidence, prevalence, and years lived with disability for 354 diseases and injuries for 195 countries and territories, 1990–2017: a systematic analysis for the Global Burden of Disease Study 2017
Source: Lancet. 2018 Nov 10;392(10159):1789–858. doi: 10.1016/S0140-6736(18)32279-7 (PMC6227754; doi:10.1016/S0140-6736(18)32279-7)
Supplement: Supplementary appendix 1 [file mmc1.pdf]

THE LANCET

Supplementary appendix 1

This appendix formed part of the original submission and has been peer reviewed. We post it as supplied by the authors.

Supplement to: GBD 2017 Disease and Injury Incidence and Prevalence Collaborators. Global, regional, and national incidence, prevalence, and years lived with disability for 354 diseases and injuries for 195 countries and territories, 1990–2017: a systematic analysis for the Global Burden of Disease Study 2017. *Lancet* 2018; **392:** 1789–858.

Appendix to “Global, regional, and national incidence, prevalence, and years lived with disability for 354 diseases and injuries for 195 countries, 1990–2017: a systematic analysis for the Global Burden of Disease Study 2017”

This appendix provides further methodological detail, supplemental figures, and more detailed results for “Global, regional, and national incidence, prevalence, and years lived with disability for 354 diseases and injuries for 195 countries, 1990–2017: a systematic analysis for the Global Burden of Disease Study 2017.” This appendix is organized into sections that follow the structure of the main paper.

Preamble

This appendix provides further methodological detail for "Global, regional, and national incidence, prevalence, and years lived with disability for 354 diseases and injuries for 195 countries, 1990–2017: a systematic analysis for the Global Burden of Disease Study 2017." This study complies with the Guidelines for Accurate and Transparent Health Estimates Reporting (GATHER) recommendations. It includes detailed tables and information on data in an effort to maximize transparency in our estimation processes and provide a comprehensive description of analytical steps. We intend this appendix to be a living document, to be updated with each iteration of the Global Burden of Disease Study.

Table of Contents

Appendix to "Global, regional, and national incidence, prevalence, and years lived with disability for 354 diseases and injuries for 195 countries, 1990–2017: a systematic analysis for the Global Burden of Disease Study 2017" 1

Preamble 2

Table of Contents 3

Authors' Contributions i

List of Appendix Tables and Figures........ 5

Appendix Figure 1a: Overview analytical flowchart for DisMod-MR 2.1 modelling strategies and injuries, GBD 2017 819

Appendix Figure 1b: Analytical flowchart for modelling strategies other than DisMod-MR 2.1 and injuries for selected nonfatal cause groups 820

Appendix Figure 2: DisMod-MR 2.1 analytical cascade 821

Appendix Figure 3: Number of most detailed GBD causes with any incidence or prevalence data by location, 1980-2017 822

Appendix Table 1: GBD 2017 location hierarchy with levels 823

Appendix Table 2: GATHER checklist of information that should be included in reports of global health estimates, with description of compliance and location of information for GBD 2017 837

Appendix Table 3: GBD 2017 cause and sequela hierarchy 840

Appendix Table 4: List of International Classification of Diseases (ICD) codes mapped to the GBD 2017 non-fatal cause list 1014

Appendix Table 5: GBD 2017 sequelae, health states, health state lay descriptions, and disability weights 1022

Appendix Table 6: GBD 2017 methods of estimating years lived with disability (YLDs) for 35 residual categories 1099

Appendix Table 7: List of GBD 2017 non-fatal causes with prevalence at birth 1102

Appendix Table 8: Socio-demographic Index values for all estimated GBD locations, 1990–2017 1103

Appendix Table 9: Socio-demographic Index groupings by location, based on 2016 values 1115

Section 1. GBD overview 7
1.1 Geographic units of the analysis 7
1.2 Time period of the analysis 7
1.3 Statement of GATHER compliance 7
1.4 List of abbreviations 7
1.5 GBD results overview 10
1.6 Data input sources overview 10
1.7 Level 5 sequelae 11
1.8 Funding sources 11
Section 2. Nonfatal outcome estimation 11
2.1 Data sources, identification, and extraction 12
2.1.1 Systematic reviews 12
2.1.2 Survey data preparation 12
2.1.3 Disease registries 12
22.1.4 Estimation of hospital envelope 13
2.1.5 Claims, inpatient hospital, and outpatient data 17
2.1.6 Case notifications 21
2.2 Data adjustment 21
2.3 DisMod-MR 2.1 estimation 22
2.4 Impairment and underlying cause estimation 25
2.4.1 Impairment squeeze 26
2.5 Severity distribution 27
2.6 Disability weights 29
2.7 Comorbidity correction (COMO) 32
2.8 YLD computation, uncertainty, and residual YLDs 34
2.9 Birth prevalence 34
2.10 Socio-demographic Index (SDI) analysis and epidemiological transition 35
Section 3. GBD cause list 38
Section 4. Nonfatal cause-specific estimation process 39
Section 5. References 1133

Authors' Contributions

Managing the estimation process

Ashkan Afshin, Tahiya Alam, Brigette Blacker, Elizabeth Cromwell, Lalit Dandona, Rakhi Dandona, Louisa Degenhardt, Samath Dharmaratne, Charbel El Bcheraoui, Valery L Feigin, Christina Fitzmaurice, Nancy Fullman, Caitlin Hawley, Simon Hay, Spencer James, Nicholas Kassebaum, Ibrahim Khalil, Hmwe Kyu, Stephen Lim, Alan Lopez, Rafael Lozano, Ashley Marks, Awoke Misganaw, Ali Mokdad, Christopher Murray, Mohsen Naghavi, Elaine Nsoesie, Helen Olsen, David Pigott, Puja Rao, Robert Reiner, Joseph Salama, David Smith, Mari Smith, Jeffrey Stanaway, Caitlyn Steiner, Stein Emil Vollset, Theo Vos, and Harvey Whiteford

Writing the first draft of the manuscript

Jack Fox, Spencer L James, Kristopher J Krohn, Kathryn Lau, Benjamin Miltz, Christopher J L Murray, Helen E Olsen, and Theo Vos.

Providing data or critical feedback on data sources

Aliasghar A Kiadaliri, Kalkidan Hassen Abate, Solomon Mequanente Abay, Cristiana Abbafati, Zegeye Abebe, Semaw Ferede Abera, Laith Abu-Raddad, Niveen Abu-Rmeileh, Manfred Accrombessi, Abdu Adamu, Oladimeji Adebayo, Olatunji Adetokunboh, Mina Adib, José C. Adsuar, Mohsen Afarideh, Ashkan Afshin, Rakesh Aggarwal, Sargis Aghayan, Mehdi Ahmadi, Muktar Ahmed, Miloud Taki Eddine Aichour, Tomi Akinyemiju, Nadia Akseer, Fares Alahdab, Khurshid Alam, Kefyalew Addis Alene, Syed Aljunid, François Alla, Peter Allebeck, Dayane Gabriele Alves Silveira, Nelson Alvis-Guzman, Azmeraw T. Amare, Walid Ammar, Yaw Ampem Amoako, Catalina Liliana Andrei, Mustafa Geleto Ansha, Carl Abelardo Antonio, Olatunde Aremu, Bahram Armoon , Johan Ärnlöv, Al Artaman, Krishna Kumar Aryal, Hamid Asayesh, Rana Asghar, Marcel Ausloos, Ashish Awasthi, Beatriz Paulina Ayala Quintanilla, Rakesh Ayer, Peter Azzopardi, Nam Ba Nguyen, Arefeh Babazadeh, Alaa Badawi, Shoshana Ballew, Maciej Banach, Joseph Banoub, Amrit Banstola, Aleksandra Barac, Miguel A. Barboza, Till Bärnighausen, Lope Barrero, Bernhard Baune, Neeraj Bedi, Masoud Behzadifar, Meysam Behzadifar, Yihalem Abebe Belay, Aminu Bello, Isabela Bensenor, Eduardo Bernabe, Robert Bernstein, Mircea Beuran, Tina Beyranvand, Neeraj Bhala, Suraj Bhattarai, Belete Biadgo, Boris Bikbov, Ver Bilano, Nigus Bililign, Muhammad Shahdaat Bin Sayeed, Ibrahim Bou-Orm, Rupert Bourne, Luisa Brant, Andrey Briko, Gabrielle Britton, Traolach Brugha, Lucero Cahuana-Hurtado, Jorge Cano, Juan J Carrero, Deborah Carvalho Malta, Carlos Castañeda-Orjuela, Jacqueline Castillo Rivas, Franz Castro, Ferrán Catalá-López, Yazan Chaiah, Alex Chang, Hsing-Yi Chang, Jung-Chen Chang, Fiona Charlson, Aparajita Chattopadhyay, Pankaj Chaturvedi, Peggy Chiang, Abdulaal Chitheer, Rajiv Chowdhury, Devasahayam Christopher, Massimo Cirillo, Rafael Claro, Daniel Collado-Mateo, Josef Coresh, Paolo Angelo Cortesi, Monica Cortinovis, Michael Criqui , Marita Cross, John Crump, Lalit Dandona, Rakhi Dandona, Ahmad Daryani, José Das Neves, Tamirat Tesfaye Dasa, Adrian Davis, Barbora De Courten, Fernando De La Hoz, Louisa Degenhardt, Kebede Deribe, Nikolaos Dervenis, Getenet Dessie, Samath Dharmaratne, M.Ashworth Dirac, Klara Dokova, David Teye Doku, Kerrie Doyle, Tim Driscoll, Manisha Dubey, Eyasu Ejeta Duken, Andre Duraes, Hedyeh Ebrahimi, Soheil Ebrahimpour, Andem Effiong, Joshua Ehrlich, Maysaa El El Sayed Zaki, Ziad El-Khatib, Iqbal Elyazar, Benjamin Er, Holly Erskine, Babak Eshrati, Sharareh Eskandarieh, Alireza Esteghamati, Sadaf Esteghamati, Mohammad Fareed, Carla Farinha, Andre Faro, Farshad Farzadfar, Valery Feigin, Netsanet Fentahun, Seyed-Mohammad Fereshtehnejad, Joao Fernandes, Irina Filip, Nataliya Foigt, Takeshi Fukumoto, Thomas Fürst, Neal Futran, Seana Gall, Morsaleh Ganji, Gbetoho Fortune Gankpe, Tigist Gashaw, Abadi Kahsu Gebre, Amanuel Tesfay Gebremedhin, Merhawi Gebremedhin, Teklu

Gebremichael, Tilayie Feto Gelano, Johanna Geleijnse, Ayele Geleto, Ricard Genova-Maleras, Khalil Ghasemi Falavarjani, Mamata Ghimire, Simona Giampaoli, Kidu Gidey, Tiffany Gill, Ibrahim Ginawi, Srinivas Goli, Hector Gomez-Dantes, Philimon Gona, Sameer Gopalani, Bárbara Goulart, Ayman Grada, Morgan Grams, Giuseppe Grosso, Prakash Gupta, Rahul Gupta, Rajat Das Gupta, Rajeev Gupta, Tanush Gupta, Juanita Haagsma, Nima Hafezi-Nejad, Tekleberhan Beyene Hagos, Arvin Haj-Mirzaian, Arya Haj-Mirzaian, Yuantao Hao, Hilda Harb, Sivadasanpillai Harikrishnan, Josep Maria Haro, Hadi Hassankhani, Hamid Y. Hassen, Rasmus Havmoeller, Roderick Hay, Delia Hendrie, Andualem Henok, Claudiu Herteliu, Mohammad Hifz Ur Rahman, Huong Hoang, Long Hoang Nguyen, Son Hoang Nguyen, Hans Hoek, Howard J. Hoffman, Michael Hole, Enayatollah Homaie Rad, Praveen Hoogar, H Dean Hosgood, Mehdi Hosseinzadeh, Sorin Hostiuc, Damian Hoy, Mohamed Hsairi, Aung Soe Htet, Guoqing Hu, Kim Moesgaard Iburg, Bogdan Ileanu, Farhad Islami, Kathryn H. Jacobsen, Nader Jahanmehr, Mihajlo Jakovljevic, Spencer James, Mehdi Javanbakht, Achala Jayatilleke, Panniyammakal Jeemon, Vivekanand Jha, Catherine Johnson, Jost B. Jonas, Jacek Jozwiak, Mikk Jürisson, Zubair Kabir, Rajendra Kadel, Rizwan Kalani, Tanuj Kanchan, André Karch, Corine Karema, Narges Karimi, Seyed M. Karimi, Amir Kasaeian, Getachew Mullu Kassa, Nicholas J Kassebaum, Srinivasa Vittal Katikireddi, Norito Kawakami, Peter Keiyoro, Andre Keren, Yousef Khader, Morteza Abdullatif Khafaie, Ibrahim Khalil, Muhammad Shahzeb Khan, Young-Ho Khang, Abdullah T. Khoja, Ardeshir Khosravi, Mohammad Hossein Khosravi, Daniel Kiirithio, Cho-Il Kim, Daniel Kim, Young-Eun Kim, Yun Jin Kim, Adnan Kisa, Ann Kristin Knudsen, Soewarta Kosen, Parvaiz Koul, Ai Koyanagi, Michael Kravchenko, Kewal Krishan, Kristopher Krohn, Barthelemy Kuate Defo, Burcu Kucuyk Bicer, Manasi Kumar, Deepesh P. Lad, Sheetal D. Lad, Alessandra Lafranconi, Huong Lan Nguyen, Van Lansingh, Janet Leasher, Misgan Legesse , James Leigh, Janni Leung, Yichong Li, Yu Liao, Lee-Ling Lim, Shiwei Liu, Alan Lopez, Stefan Lorkowski, Paulo Lotufo, Nicola Low, Ronan Lyons, Stefan Ma, Erlyn Rachelle Macarayan, Dhaval Maghavani, P A Mahesh, Marek Majdan, Reza Majdzadeh, Azeem Majeed, Reza Malekzadeh, Mohammad Ali Mansournia, Lorenzo Mantovani, Joemer Maravilla, Wagner Marcenes, Francisco Rogerlândio Martins-Melo, Winfried März, Melvin Marzan, Benjamin Massenburg, Manu Mathur, Kunihiro Matsushita, Mohsen Mazidi, John Mcgrath, Fabiola Mejia-Rodriguez, Addisu Melese, Mulugeta Melku, Peter Memiah, Ziad Memish, Walter Mendoza, Getnet Mengistu, Zerihun Menlkalew, Atte Meretoja, Tuomo Meretoja, Tomislav Mestrovic, Tomasz Miazgowski, Ted R Miller, Erkin Mirrakhimov, Babak Moazen, Noushin Mohammadifard, Shafiu Mohammed, Farnam Mohebi, Ali Mokdad, Lorenzo Monasta, Yoshan Moodley, Ghobad Moradi, Maziar Moradi-Lakeh, Mehdi Moradinazar, Lidia Morawska, Ilais Moreno, Joana Morgado-Da-Costa, Marilita Moschos, Seyyed Meysam Mousavi, Ulrich Mueller, Manoj Murhekar, Kamarul Imran Musa, Ghulam Mustafa, Ashraf Nabhan, Mohsen Naghavi, Seyed Sina Naghibi Irvani, Azin Nahvijou, Nitish Naik, Farid Najafi, Luigi Naldi, Hae-Sung Nam, Bruno Nascimento, Ionut Negoi, Ruxandra Irina Negoi, Charles Newton, Josephine Ngunjiri, Anh Nguyen, Ha Nguyen, Huong Nguyen, Shuhei Nomura, Mehdi Noroozi, Bo Norrving, Jean Jacques Noubiap, Hamid Reza Nouri, Malihe Nourollahpour, Felix Ogbo, In-Hwan Oh, Olanrewaju Oladimeji, Tinuke O. Olagunju, Bolajoko Olusanya, Kanyin Ong, Sok King Ong, Eyal Oren, Alberto Ortiz, Stanislav Otstavnov, Simon Øverland, Mayowa Owolabi, Adrian Pana, Songhomitra Panda-Jonas, Andrea Parisi, Shanti Patel, Sanghamitra Pati, Snehal Patil, Ajay Patle, Neil Pearce, David Pereira, Norberto Perico, Linh Phuong Doan, David Pigott, Meghdad Pirsaheb, Farhad Pishgar, Dietrich Plass, Suzanne Polinder, Hossein Poustchi, Swayam Prakash, Mostafa Qorbani, Hai Quang Pham, D. Alex Quistberg, Amir Radfar, Anwar Rafay, Alireza Rafiei, Fakher Rahim, Afarin Rahimi-Movaghar, Vafa Rahimi-Movaghar, Sajjad Rahman, Rajesh Kumar Rai, Usha Ram, Prabhat Ranjan, Paturi Rao, David Laith Rawaf, Salman Rawaf, Kolli Srinath Reddy, Nickolas Reinig, Giuseppe Remuzzi, Andre Renzaho, Serge Resnikoff,

Mohammad Sadegh Rezai, Antonio Luiz Ribeiro, Leonardo Roever, Luca Ronfani, Gholamreza Roshandel, Ambuj Roy, Enrico Rubagotti, Perminder Sachdev, Ehsan Sadeghi, Hosein Safari, Yahya Safari, Saeid Safiri, Mohammad Ali Sahraian, Nasir Salam, Joseph Salama, Payman Salamati, Komal Saleem , Yahya Salimi, Joshua A Salomon, Abdallah M. Samy, Juan Sanabria, Yingying Sang, Milena Santric Milicevic, Bruno Sao Jose, Mayank Sardana, Nizal Sarrafzadegan, Maheswar Satpathy, Monika Sawhney, Sonia Saxena, Mete Saylan, Elke Schaffner, David C Schwebel, Falk Schwendicke, James Scott, Mario Šekerija, Sadaf Sepanlou, Edson Serván-Mori, Seyedmojtaba Seyedmousavi, Amira Shaheen, Masood Ali Shaikh, Mehran Shams-Beyranvand, Kiomars Sharafi, Mehdi Sharif, Sheikh Mohammed Shariful Islam, Meenakshi Sharma, Aziz Sheikh, Peilin Shi, Kawkab Shishani, Ivy Shiue, Farhad Shokraneh, Si Si, Soraya Siabani, Tariq Jamal Siddiqi, Jasvinder Singh, Narinder Pal Singh, Virendra Singh, Dhirendra Narain Sinha, Adauto Martins Soares Filho, Soheila Sobhani, Eugene Sobngwi, Reed Sorensen, Luciano Sposato, Chandrashekhar T Sreeramareddy, Timothy Steiner, Mark Stokes, Agus Sudaryanto, Muawiyyah Babale Sufiyan, Rizwan Suliankatchi Abdulkader, Bryan L. Sykes, Dillon Sylte, Rafael Tabarés-Seisdedos, Mesfin Tadese, Nuno Taveira, Hugh Taylor, Gebre Teklemariam Demoz, Awoke Temesgen, Mohamad-Hani Temsah, Omar Temsah, Mebrahtu Teweldemedhin, Hue Thi Mai, Nihal Thomas, Marcello Tonelli, Miguel Tortajada-Girbés, Mathilde Touvier, Marcos Roberto Tovani-Palone, Bach Tran, Khanh Bao Tran, Thomas Truelsen, Derrick Tsoi, Kingsley Nnanna Ukwaja, Irfan Ullah, Eduardo Undurraga, Muhammad Shariq Usman, Olalekan A Uthman, Muthiah Vaduganathan, Afsane Vaezi, Pascual Valdez, Tommi Vasankari, Narayanaswamy Venketasubramanian, Santos Villafaina, Francesco S Violante, Sergey Vladimirov, Vasiliy Vlassov, Stein Emil Vollset, Theo Vos, Fasil Wagnew, Shishay Wahdey, Yasir Waheed, Yuan-Pang Wang, Elisabete Weiderpass, Robert Weintraub, Inbal Weiss Salz, Harvey Whiteford, Justyna Widecka, Tissa Wijeratne, Charles Shey Wiysonge, Charles Wolfe, Shouling Wu, Yun-Chun Wu, Grant Wyper, Denis Xavier , Gelin Xu, Ali Yadollahpour, Hossein Yahyazadeh, Lijing Yan, Mehdi Yaseri, Yasin Jemal Yasin, Alex Yeshaneh, Ebrahim M. Yimer, Naohiro Yonemoto, Seok-Jun Yoon, Marcel Yotebieng, Mustafa Younis, Chuanhua Yu, Vesna Zadnik, Zoubida Zaidi, Sojib Bin Zaman, Mohammad Zamani, Zohreh Zare, and Sanjay Zodpey.

Developing methods or computational machinery

Cristiana Abbafati, Kareha Agesa, Mehran Alijanzadeh, Donal Bisanzio, Kelly Cercy, Cyrus Cooper , Josef Coresh, Ahmad Daryani, Maysaa El El Sayed Zaki, Farshad Farzadfar, Christina Fitzmaurice, Kyle Foreman, Tahvi Frank, Ellen Goldberg, Taren Gorman, Morgan Grams, Mehdi Hosseinzadeh, Chantal Huynh, Bogdan Ileanu, Spencer James, Catherine Johnson, Nicholas J Kassebaum, Adnan Kisa, Jonathan Kocarnik, Kathryn Lau, Misgan Legesse , James Leigh, Tim Lucas, Treh Manhertz, Kunihiro Matsushita, Zerihun Menlkalew, Anoushka Millear, Benjamin Miltz, Muhammed Mmagdy Abd El Razek, Mohsen Naghavi, Kanyin Ong, Charles Parry, David Pigott, Robert Reiner, Gregory Roth, Joshua A Salomon, Abdallah M. Samy, Yingying Sang, Damian Santomauro, Shahabeddin Sarvi, Maheswar Satpathy, David C Schwebel, Mehdi Sharif, David Smith, Masoud Soosaraei, Vinay Srinivasan, Jeffrey Stanaway, Patrick Sur, Dillon Sylte, Christopher Troeger, Derrick Tsoi, Rachel L. Updike, Theo Vos, Daniel Weiss, Shadrach Wilson, Simon Yadgir, and Lijing Yan.

Applying analytical methods to produce estimates

Kareha Agesa, Sutapa Agrawal, Muktar Ahmed, Mohamed Lamine Alouani, Azmeraw T. Amare, Olatunde Aremu, Amit Arora, Katherine Ballesteros, Donal Bisanzio, Kelly Cercy, Fiona Charlson, Elizabeth Cromwell, Ahmad Daryani, Getenet Dessie, M.Ashworth Dirac, Michelle Echko, Aman Endries, Holly Erskine, Alize Ferrari, Christina Fitzmaurice, Tahvi Frank, William Gardner, Segen Gebremeskel, Ayele

Geleto, Rakesh Ghosh, Ellen Goldberg, Hamid Y. Hassen, Mehdi Hosseinzadeh, Chad Ikeda, Bogdan Ileanu, Spencer James, Catherine Johnson, Manoochehr Karami, Nicholas J Kassebaum, Laura Kemmer, Yun Jin Kim, Adnan Kisa, Jonathan Kocarnik, Hmwe Kyu, Kathryn Lau, Jorge Ledesma, Misgan Legesse , James Leigh, Janni Leung, Tim Lucas, Lydia Lucchesi, Helena Manguerra, Treh Manhertz, Ira Martopullo, Mohsen Mazidi, Anoushka Millear, Benjamin Miltz, Shafiu Mohammed, Ali Mokdad, Maziar Moradi-Lakeh, Mohsen Naghavi, Emma Nichols, Bolajoko Olusanya, Kanyin Ong, Katherine Paulson, David Pigott, Caroline Purcell, Fakher Rahim, Marissa Reitsma, Gregory Roth, Nafis Sadat, Abdallah M. Samy, Yingying Sang, Damian Santomauro, Maheswar Satpathy, Seyedmojtaba Seyedmousavi, Mehdi Sharif, Soraya Siabani, Virendra Singh, Karen Sliwa, Reed Sorensen, Chandrashekhar T Sreeramareddy, Vinay Srinivasan, Jeffrey Stanaway, Lars Jacob Stovner, Patrick Sur, Bryan L. Sykes, Dillon Sylte, Christopher Troeger, Derrick Tsoi, Rachel L. Updike, Theo Vos, Daniel Weiss, Inbal Weiss Salz, Tissa Wijeratne, Lauren Wilner, Shadrach Wilson, Yun-Chun Wu, Simon Yadgir, Naohiro Yonemoto, and Mustafa Younis.

Providing critical feedback on methods or results

Aliasghar A Kiadaliri, Degu Abate, Kalkidan Hassen Abate, Solomon Mequanente Abay, Cristiana Abbafati, Nooshin Abbasi, Hedayat Abbastabar, Ibrahim Abdollahpour, Zegeye Abebe, Semaw Ferede Abera, Haftom Abraha, Niveen Abu-Rmeileh, Pawan Acharya, Ilana Ackerman, Abdu Adamu, Oladimeji Adebayo, Victor Adekanmbi, Olatunji Adetokunboh, Mina Adib, José C. Adsuar, Kossivi Afanvi, Mohsen Afarideh, Gina Agarwal, Sargis Aghayan, Sutapa Agrawal, Alireza Ahmadi, Mehdi Ahmadi, Hamid Ahmadieh, Muktar Ahmed, Amani Nidhal Aichour, Ibtihel Aichour, Miloud Taki Eddine Aichour, Tomi Akinyemiju, Nadia Akseer, Fares Alahdab, Ziyad Al-Aly, Khurshid Alam, Tahiya Alam, Seyed Moayed Alavian, Kefyalew Addis Alene, Ayman Al-Eyadhy, Mehran Alijanzadeh, Reza Alizadeh-Navaei, Syed Aljunid, Ala'a Alkerwi, Peter Allebeck, Hesham Al-Mekhlafi, Rajaa Al-Raddadi, Khalid Altirkawi, Dayane Gabriele Alves Silveira, Nelson Alvis-Guzman, Azmeraw T. Amare, Leopold N. Aminde, Walid Ammar, Yaw Ampem Amoako, Catalina Liliana Andrei, Sofia Androudi, Mina Anjomshoa, Mustafa Geleto Ansha, Carl Abelardo Antonio, Palwasha Anwari, Olatunde Aremu, Filippo Ariani, Bahram Armoon , Johan Ärnlöv, Amit Arora, Al Artaman, Krishna Kumar Aryal, Hamid Asayesh, Zerihun Ataro, Sachin Atre, Marcel Ausloos, Leticia Avila-Burgos, Euripide Avokpaho, Ashish Awasthi, Beatriz Paulina Ayala Quintanilla, Peter Azzopardi, Nam Ba Nguyen, Arefeh Babazadeh, Hamid Badali, Alaa Badawi, Shoshana Ballew, Joseph Banoub, Amrit Banstola, Aleksandra Barac, Miguel A. Barboza, Suzanne Barker-Collo, Till Bärnighausen, Lope Barrero, Bernhard Baune, Shahrzad Bazargan-Hejazi, Neeraj Bedi, Ettore Beghi, Yannick Béjot, Abate Bekele, Yihalem Abebe Belay, Michelle Bell, Aminu Bello, Isabela Bensenor, Eduardo Bernabe, Robert Bernstein, Mircea Beuran, Tina Beyranvand, Neeraj Bhala, Suraj Bhattarai, Soumyadeep Bhaumik, Belete Biadgo, Ali Bijani, Boris Bikbov, Nigus Bililign, Muhammad Shahdaat Bin Sayeed, Donal Bisanzio, Brigette Blacker, Fiona Blyth, Soufiane Boufous, Oliver Brady, Luisa Brant, Alexandra Brazinova, Nicholas Breitborde, Hermann Brenner, Andrew Briggs, Andrey Briko, Gabrielle Britton, Traolach Brugha, Rachelle Buchbinder, Reinhard Busse, Zahid Butt, Alessandra C Goulart, Lucero Cahuana-Hurtado, Jorge Cano, Rosario Cárdenas, Juan J Carrero, Deborah Carvalho Malta, Carlos Castañeda-Orjuela, Jacqueline Castillo Rivas, Franz Castro, Ferrán Catalá-López, Ester Cerin, Yazan Chaiah, Alex Chang, Jung-Chen Chang, Aparajita Chattopadhyay, Vijay Kumar Chattu, Peggy Chiang, Ken Chin, Yilma Chisha, Jee-Young Choi, Hanne Christensen, Devasahayam Christopher, Flavia Cicuttini, Liliana G Ciobanu, Massimo Cirillo, Rafael Claro, Daniel Collado-Mateo, Josef Coresh, Paolo Angelo Cortesi, Monica Cortinovis, Megan Costa, Ewerton Cousin, Michael Criqui , John Crump, Abel Dadi, Lalit Dandona, Rakhi Dandona, Paul Dargan, Ahmad Daryani, José Das Neves, Tamirat Tesfaye Dasa, Gail Davey, Adrian Davis, Barbora De Courten, Diego De Leo, Jan-Walter De Neve, Megbaru Debalkie, Louisa

Degenhardt, Robert Dellavalle, Kebede Deribe, Nikolaos Dervenis, Don Des Jarlais, Getenet Dessie, Subhojit Dey, Samath Dharmaratne, M.Ashworth Dirac, Shiri Djalalinia, Klara Dokova, David Teye Doku, E. Ray Dorsey, Kerrie Doyle, Tim Driscoll, Manisha Dubey, Eleonora Dubljanin, Eyasu Ejeta Duken, Bruce Duncan, Andre Duraes, Hedyeh Ebrahimi, Soheil Ebrahimpour, Michelle Echko, David Edvardsson, Andem Effiong, Joshua Ehrlich, Ziad El-Khatib, Iqbal Elyazar, Ahmadali Enayati, Aman Endries, Benjamin Er, Holly Erskine, Babak Eshrati, Sharareh Eskandarieh, Alireza Esteghamati, Sadaf Esteghamati, Hamed Fakhim, Mahbobeh Faramarzi, Mohammad Fareed, Farzaneh Farhadi, Talha Farid, Carla Farinha, Andrea Farioli, Andre Faro, Farshad Farzadfar, Valery Feigin, Netsanet Fentahun, Seyed-Mohammad Fereshtehnejad, Joao Fernandes, Garumma Tolu Feyissa, Irina Filip, Florian Fischer, Christina Fitzmaurice, Nataliya Foigt, Takeshi Fukumoto, Nancy Fullman, Thomas Fürst, Joao M Furtado, Neal Futran, Morsaleh Ganji, Gbetoho Fortune Gankpe, Alberto L. Garcia-Basteiro, Tigist Gashaw, Abadi Kahsu Gebre, Amanuel Tesfay Gebremedhin, Merhawi Gebremedhin, Segen Gebremeskel, Teklu Gebremichael, Tilayie Feto Gelano, Johanna Geleijnse, Ayele Geleto, Ricard Genova-Maleras, Kebede Embaye Gezae, Maryam Ghasemi-Kasman, Mamata Ghimire, Rakesh Ghosh, Kidu Gidey, Paramjit Gill, Tiffany Gill, Meaza Girma, Giorgia Giussani, Elena Gnedovskaya, Srinivas Goli, Philimon Gona, Aloke Gopal, Sameer Gopalani, Taren Gorman, Bárbara Goulart, Ayman Grada, Morgan Grams, Giuseppe Grosso, Harish Gugnani, Yuming Guo, Rahul Gupta, Rajat Das Gupta, Rajeev Gupta, Tanush Gupta, Bishal Gyawali, Juanita Haagsma, Vladimir Hachinski, Nima Hafezi-Nejad, Hassan Haghparast-Bidgoli, Tekleberhan Beyene Hagos, Dessalegn Haile, Gessessew Bugssa Hailu, Arvin Haj-Mirzaian, Arya Haj-Mirzaian, Randah Hamadeh, Samer Hamidi, Alexis Jeannine Handal, Graeme Hankey, Yuantao Hao, Hilda Harb, Sivadasanpillai Harikrishnan, Josep Maria Haro, Mehedi Hasan, Hadi Hassankhani, Hamid Y. Hassen, Rasmus Havmoeller, Caitlin Hawley, Roderick Hay, Akbar Hedayatizadeh-Omran, Behzad Heibati, Delia Hendrie, Claudiu Herteliu, Sousan Heydarpour, Desalegn Hibstu, Long Hoang Nguyen, Son Hoang Nguyen, Michael Hole, Enayatollah Homaie Rad, Praveen Hoogar, H Dean Hosgood, Mostafa Hossini, Mihaela Hostiuc, Sorin Hostiuc, Damian Hoy, Guoqing Hu, John Huang, Bogdan Ileanu, Olayinka Ilesanmi, Usman Iqbal, Farhad Islami, Kathryn H. Jacobsen, Sudhir Kumar Jain, Mihajlo Jakovljevic, Spencer James, Mehdi Javanbakht, Achala Jayatilleke, Panniyammakal Jeemon, Ayalew Jejaw Zeleke, Ravi Prakash Jha, Vivekanand Jha, Catherine Johnson, Jost B. Jonas, Jacek Jozwiak, Suresh Jungari, Mikk Jürisson, Zubair Kabir, Rajendra Kadel, Rizwan Kalani, Tanuj Kanchan, Manoochehr Karami, Behzad Karami Matin, André Karch, Seyed M. Karimi, Amir Kasaeian, Getachew Mullu Kassa, Tesfaye Kassa, Nicholas J Kassebaum, Srinivasa Vittal Katikireddi, Ali Kazemi Karyani, Peter Keiyoro, Yousef Khader, Morteza Abdullatif Khafaie, Ibrahim Khalil, Ejaz Khan, Muhammad Shahzeb Khan, Young-Ho Khang, Abdullah T. Khoja, Ardeshir Khosravi, Mohammad Hossein Khosravi, Daniel Kiirithio, Daniel Kim, Young-Eun Kim, Yun Jin Kim, Ruth Kimokoti, Adnan Kisa, Katarzyna Kissimova-Skarbek, Mika Kivimaki, Ann Kristin Knudsen, Jonathan Kocarnik, Sonali Kochhar, Yoshihiro Kokubo, Tufa Kolola, Jacek Kopec, Soewarta Kosen, Georgios Kotsakis, Parvaiz Koul, Ai Koyanagi, Kewal Krishan, Kristopher Krohn, Barthelemy Kuate Defo, Manasi Kumar, Hmwe Kyu, Deepesh P. Lad, Sheetal D. Lad, Alessandra Lafranconi, Ratilal Lalloo, Tea Lallukka, Faris Lami, Huong Lan Nguyen, Arman Latifi, Jeffrey Lazarus, Janet Leasher, Paul Lee, Misgan Legesse , James Leigh, Janni Leung, Miriam Levi, Sonia Lewycka, Shanshan Li, Yu Liao, Lee-Ling Lim, Shiwei Liu, Rakesh Lodha, Katharine Looker, Alan Lopez, Stefan Lorkowski, Paulo Lotufo, Nicola Low, Raimundas Lunevicius, Ronan Lyons, Kala M. Mehta, Stefan Ma, Erlyn Rachelle Macarayan, Mark Mackay, F Madotto, Hassan Magdy Abd El Razek, P A Mahesh, Marek Majdan, Reza Majdzadeh, Azeem Majeed, Reza Malekzadeh, Abdullah Mamun, Mohammad Ali Mansournia, Lorenzo Mantovani, Chabila C. Mapoma, Joemer Maravilla, Wagner Marcenes, Francisco

Rogerlândio Martins-Melo, Melvin Marzan, Tivani Mashamba-Thompson, Benjamin Massenburg, Manu Mathur, Kunihiro Matsushita, Pallab K Maulik, Mohsen Mazidi, Colm Mcalinden, John Mcgrath, Martin Mckee, Ravi Mehrotra, Varshil Mehta, Tesfa Mekonen, Addisu Melese, Mulugeta Melku, Michele Meltzer, Peter Memiah, Ziad Memish, Walter Mendoza, Desalegn Tadese Mengistu, Getnet Mengistu, Zerihun Menlkalew, George Mensah, Seid Tiku Mereta, Atte Meretoja, Tuomo Meretoja, Tomislav Mestrovic, Tomasz Miazgowski, Ted R Miller, Gk Mini, Mojde Mirarefin, Erkin Mirrakhimov, Philip B Mitchell, Habtamu Mitiku , Muhammed Mmagdy Abd El Razek, Babak Moazen, Bahram Mohajer, Mohammed Mohammed, Shafiu Mohammed, Farnam Mohebi, Ali Mokdad, Mariam Molokhia, Lorenzo Monasta, Mahmood Moosazadeh, Ghobad Moradi, Maziar Moradi-Lakeh, Mehdi Moradinazar, Paula Moraga, Lidia Morawska, Joana Morgado-Da-Costa, Shane Morrison, Marilita Moschos, Seyyed Meysam Mousavi, Kalayu Brhane Mruts, Kindie Fentahun Muchie, Ulrich Mueller, Satinath Mukhopadhyay, Jonah Musa, Kamarul Imran Musa, Ghulam Mustafa, Ashraf Nabhan, Chie Nagata, Mohsen Naghavi, Seyed Sina Naghibi Irvani, Aliya Naheed, Azin Nahvijou, Nitish Naik, Farid Najafi, Vinay Nangia, Jobert Richie Nansseu, Bruno Nascimento, Ionut Negoi, Ruxandra Irina Negoi, Subas Neupane, Charles Newton, Josephine Ngunjiri, Emma Nichols, Nomonde Nolutshungu, Ole F. Norheim, Mehdi Noroozi, Bo Norrving, Jean Jacques Noubiap, Hamid Reza Nouri, Malihe Nourollahpour, Mohammad Reza Nowroozi, Dina Nur Anggraini Ningrum, Peter Nyasulu, Richard Ofori-Asenso, Felix Ogbo, In-Hwan Oh, Olanrewaju Oladimeji, Tinuke O. Olagunju, Pedro Olivares, Bolajoko Olusanya, Kanyin Ong, Eyal Oren, Alberto Ortiz, Erika Ota, Stanislav Otstavnov, Simon Øverland, Mayowa Owolabi, Rosana Pacella, Amir Pakpour, Adrian Pana, Songhomitra Panda-Jonas, Eun-Kee Park, Shanti Patel, Sanghamitra Pati, Snehal Patil, George Patton, Katherine Paulson, Neil Pearce, David Pereira, Norberto Perico, Linh Phuong Doan, David Pigott, Julian Pillay, Michael Piradov, Meghdad Pirsaheb, Farhad Pishgar, Oleguer Plana-Ripoll, Dietrich Plass, Suzanne Polinder, Svetlana Popova, Maarten Postma, Akram Pourshams, Hossein Poustchi, Dorairaj Prabhakaran, Swayam Prakash, V Prakash, Manorama Purwar, Mostafa Qorbani, Hai Quang Pham, D. Alex Quistberg, Amir Radfar, Anwar Rafay, Alireza Rafiei, Fakher Rahim, Kazem Rahimi, Afarin Rahimi-Movaghar, Vafa Rahimi-Movaghar, Mahfuzar Rahman, Muhammad Aziz Rahman, Sajjad Rahman, Rajesh Kumar Rai, Fatemeh Rajati, Usha Ram, Prabhat Ranjan, Annemarei Ranta, David Laith Rawaf, Salman Rawaf, Kolli Srinath Reddy, Giuseppe Remuzzi, Andre Renzaho, Serge Resnikoff, Satar Rezaei, Mohammad Sadegh Rezai, Antonio Luiz Ribeiro, Stephen Robinson, Leonardo Roever, Luca Ronfani, Gholamreza Roshandel, Gregory Roth, Ambuj Roy, Enrico Rubagotti, Perminder Sachdev, Basema Saddik, Hosein Safari, Yahya Safari, Roya Safari-Faramani, Mahdi Safdarian, Sare Safi, Saeid Safiri, Rajesh Sagar, Amirhossein Sahebkar, Mohammad Ali Sahraian, Haniye Sadat Sajadi, Nasir Salam, Komal Saleem , Yahya Salimi, Joshua A Salomon, Sundeep Salvi, Abdallah M. Samy, Juan Sanabria, Yingying Sang, Itamar Santos, João Vasco Santos, Milena Santric Milicevic, Bruno Sao Jose, Mayank Sardana, Abdur Razzaque Sarker, Benn Sartorius, Shahabeddin Sarvi, Brijesh Sathian, Maheswar Satpathy, Monika Sawhney, Sonia Saxena, Mete Saylan, Elke Schaffner, Maria Inês Schmidt, Ione Schneider, Ben Schöttker, David C Schwebel, Falk Schwendicke, James Scott, Sadaf Sepanlou, Edson Serván-Mori, Seyedmojtaba Seyedmousavi, Hosein Shabaninejad, Azadeh Shafieesabet, Amira Shaheen, Masood Ali Shaikh, Mehran Shams-Beyranvand, Mohammadbagher Shamsi, Heidar Sharafi, Kiomars Sharafi, Mehdi Sharif, Mahdi Sharif-Alhoseini, Sheikh Mohammed Shariful Islam, Rajesh Sharma, Jun She, Aziz Sheikh, Mika Shigematsu, Rahman Shiri, Ivy Shiue, Haitham Shoman, Mark Shrime, Si Si, Soraya Siabani, Tariq Jamal Siddiqi, Inga Dora Sigfusdottir, Rannveig Sigurvinsdottir, João Pedro Silva, Narayana Sarma Singam, Jasvinder Singh, Virendra Singh, Dhirendra Narain Sinha, Eirini Skiadaresi, Badr Sobaih, Soheila Sobhani, Eugene Sobngwi, Samir Soneji, Moslem Soofi, Reed Sorensen, Joan B Soriano, Ireneous Soyiri, Luciano

Sposato, Chandrashekhar T Sreeramareddy, Dan J Stein, Timothy Steiner, Mark Stokes, Muawiyyah Babale Sufiyan, Rizwan Suliankatchi Abdulkader, Bruno Sunguya, Patrick Sur, Ipsita Sutradhar, Bryan L. Sykes, Dillon Sylte, Rafael Tabarés-Seisdedos, Santosh Tadakamadla, Mesfin Tadese, Birkneh Tilahun Tadesse, Nikhil Tandon, Nuno Taveira, Hugh Taylor, Arash Tehrani-Banihashemi, Gebre Teklemariam Demoz, Awoke Temesgen, Habtamu Temesgen, Mohamad-Hani Temsah, Omar Temsah, Abdullah Terkawi, Mebrahtu Teweldemedhin, Kavumpurathu Thankappan, Hue Thi Mai, Nihal Thomas, Binyam Tilahun, Quyen G To, Marcello Tonelli, Roman Topor-Madry, Fotis Topouzis, Miguel Tortajada-Girbés, Mathilde Touvier, Marcos Roberto Tovani-Palone, Bach Tran, Khanh Bao Tran, Christopher Troeger, Thomas Truelsen, Miltiadis Tsilimbaris, Derrick Tsoi, Lorainne Tudor Car, E Murat Tuzcu, Kingsley Nnanna Ukwaja, Irfan Ullah, Eduardo Undurraga, Jurgen Unutzer, Muhammad Shariq Usman, Olalekan A Uthman, Muthiah Vaduganathan, Afsane Vaezi, Pascual Valdez, Santosh Varughese, Tommi Vasankari, Narayanaswamy Venketasubramanian, Santos Villafaina, Francesco S Violante, Sergey Vladimirov, Vasiliy Vlassov, Stein Emil Vollset, Theo Vos, Kia Vosoughi, Isidora Vujcic, Fasil Wagnew, Shishay Wahdey, Yasir Waheed, Stephen Waller, Yafeng Wang, Yuan-Pang Wang, Elisabete Weiderpass, Robert Weintraub, Inbal Weiss Salz, Fitsum Weldegebreal, T. Eoin West, Harvey Whiteford, Tissa Wijeratne, Andrea Winkler, Alison. B Wiyeh, Charles Shey Wiysonge, Charles Wolfe, Anthony Woolf, Grant Wyper, Denis Xavier , Gelin Xu, Ali Yadollahpour, Tomohide Yamada, Lijing Yan, Yuichiro Yano, Mehdi Yaseri, Yasin Jemal Yasin, Alex Yeshaneh, Ebrahim M. Yimer, Engida Yisma, Naohiro Yonemoto, Seok-Jun Yoon, Marcel Yotebieng, Mustafa Younis, Mahmoud Yousefifard, Chuanhua Yu, Vesna Zadnik, Zoubida Zaidi, Sojib Bin Zaman, Mohammad Zamani, Zohreh Zare, Olifan Zewdie, Kai Zhang, Zheng Zhao, and Inbar Zucker.

Drafting the work or revising is critically for important intellectual content

Nooshin Abbasi, Olatunji Adetokunboh, Mohsen Afarideh, Muktar Ahmed, Tomi Akinyemiju, Fares Alahdab, Khurshid Alam, Syed Aljunid, Mohamed Lamine Alouani, Yaw Ampem Amoako, Amit Arora, Peter Azzopardi, Nam Ba Nguyen, Hamid Badali, Lope Barrero, Shahrzad Bazargan-Hejazi, Yihalem Abebe Belay, Neeraj Bhala, Oliver Brady, Andrew Briggs, Alessandra C Goulart, Carlos Castañeda-Orjuela, Franz Castro, Fiona Charlson, Devasahayam Christopher, Flavia Cicuttini, John Crump, Ahmad Daryani, Gail Davey, Dragos Davitoiu, Jan-Walter De Neve, Louisa Degenhardt, Selina Deiparine, Getenet Dessie, Samath Dharmaratne, M.Ashworth Dirac, David Teye Doku, David Edvardsson, Maysaa El El Sayed Zaki, Holly Erskine, Sadaf Esteghamati, Vahid Fallah Omrani, Andre Faro, Seyed-Mohammad Fereshtehnejad, Joao Fernandes, Alize Ferrari, Nataliya Foigt, Takeshi Fukumoto, Segen Gebremeskel, Maryam Ghasemi-Kasman, Rakesh Ghosh, Ibrahim Ginawi, Rajat Das Gupta, Alexis Jeannine Handal, Hadi Hassankhani, Rasmus Havmoeller, Long Hoang Nguyen, Son Hoang Nguyen, Enayatollah Homaie Rad, Farhad Islami, Spencer James, Ravi Prakash Jha, Mikk Jürisson, Manoochehr Karami, Nicholas J Kassebaum, Grant Rodgers Kemp, Morteza Abdullatif Khafaie, Behzad Khafaie , Alireza Khajavi, Yun Jin Kim, Jonathan Kocarnik, Kristopher Krohn, Huong Lan Nguyen, Kathryn Lau, Alan Lopez, Hassan Magdy Abd El Razek, Ana Laura Manda, Treh Manhertz, Joemer Maravilla, Francisco Rogerlândio Martins-Melo, Melvin Marzan, Benjamin Massenburg, Colm Mcalinden, Tuomo Meretoja, Muhammed Mmagdy Abd El Razek, Bahram Mohajer, Karzan Mohammad, Mehdi Moradinazar, Ghulam Mustafa, Nahid Neamati, Olanrewaju Oladimeji, Tinuke O. Olagunju, Helen E. Olsen, Bolajoko Olusanya, Kanyin Ong, Simon Øverland, Konrad Pesudovs, Micahel R Phillips, Linh Phuong Doan, Hai Quang Pham, Anwar Rafay, Alireza Rafiei, Vafa Rahimi-Movaghar, Muhammad Aziz Rahman, Fatemeh Rajati, Leonardo Roever, Juan Sanabria, Damian Santomauro, Milena Santric Milicevic, Bruno Sao Jose, Abdur Razzaque Sarker, Shahabeddin Sarvi, Maheswar Satpathy, Monika Sawhney, Seyedmojtaba Seyedmousavi, Mehdi Sharif, Mika Shigematsu, Inga Dora Sigfusdottir, Rannveig Sigurvinsdottir, Virendra Singh, Dhirendra Narain

Sinha, Eirini Skiadaresi, Karen Sliwa, Masoud Soosaraei, Nuno Taveira, Mebrahtu Teweldemedhin, Hue Thi Mai, Nihal Thomas, Marcos Roberto Tovani-Palone, Bach Tran, Thomas Truelsen, Olalekan A Uthman, Theo Vos, Yuan-Pang Wang, and Robert Weintraub.

Extracting, cleaning, or cataloging data; designing or coding figures and tables

Hedayat Abbastabar, Olatunji Adetokunboh, Kareha Agesa, Rakesh Aggarwal, Khurshid Alam, Mohamed Lamine Alouani, Boris Bikbov, Donal Bisanzio, Rupert Bourne, Paul Briant, Ferrán Catalá-López, Devasahayam Christopher, Monica Cortinovis, Marita Cross, Ahmad Daryani, Selina Deiparine, Getenet Dessie, M.Ashworth Dirac, Aman Endries, Benjamin Er, Holly Erskine, Hamed Fakhim, Andre Faro, Alize Ferrari, Jack Fox, Takeshi Fukumoto, Gbetoho Fortune Gankpe, William Gardner, Segen Gebremeskel, Johanna Geleijnse, Ayele Geleto, Rakesh Ghosh, Ellen Goldberg, Taren Gorman, Yuming Guo, Tanush Gupta, Mehedi Hasan, Roderick Hay, Behzad Heibati, Chad Ikeda, Bogdan Ileanu, Caleb Irvine, Spencer James, Catherine Johnson, Manoochehr Karami, André Karch, Tesfaye Kassa, Nicholas J Kassebaum, Laura Kemmer, Grant Rodgers Kemp, Andre Keren, Yun Jin Kim, Jonathan Kocarnik, Kristopher Krohn, Barthelemy Kuate Defo, Kathryn Lau, Misgan Legesse , Janni Leung, Shanshan Li, Shiwei Liu, Lydia Lucchesi, Stefan Ma, Reza Malekzadeh, Helena Manguerra, Treh Manhertz, Mohammad Ali Mansournia, Mohsen Mazidi, Anoushka Millear, Benjamin Miltz, Modhurima Moitra, Maziar Moradi-Lakeh, Bruno Nascimento, Minh Nguyen, Emma Nichols, Kanyin Ong, Andrea Parisi, Katherine Paulson, Norberto Perico, David Pigott, Hossein Poustchi, Caroline Purcell, Nickolas Reinig, Giuseppe Remuzzi, Enrico Rubagotti, Hosein Safari, Saeid Safiri, Abdallah M. Samy, Damian Santomauro, Shahabeddin Sarvi, Maheswar Satpathy, Monika Sawhney, Sadaf Sepanlou, Mehdi Sharif, Farhad Shokraneh, Virendra Singh, Erica Leigh Slepak, Masoud Soosaraei, Jeffrey Stanaway, Lars Jacob Stovner, Michelle Subart, Patrick Sur, Dillon Sylte, Mohammad Tavakkoli, Awoke Temesgen, Anna Torre, Derrick Tsoi, Irfan Ullah, Rachel L. Updike, Theo Vos, Daniel Weiss, Inbal Weiss Salz, Tissa Wijeratne, Shadrach Wilson, Simon Yadgir, Ali Yadollahpour, Hossein Yahyazadeh, Mahmoud Yousefifard, and Zheng Zhao.

Managing the overall research enterprise

Ashkan Afshin, Tahiya Alam, Peter Allebeck, Brigette Blacker, Elizabeth Cromwell, Lalit Dandona, Rakhi Dandona, Adrian Davis, Louisa Degenhardt, Samath Dharmaratne, Charbel El Bcheraoui, Valery Feigin, Christina Fitzmaurice, Kyle Foreman, Nancy Fullman, Thomas Fürst, Caitlin Hawley, Simon Hay, Spencer James, Nicholas Kassebaum, Ibrahim Khalil, Kristopher Krohn, Hmwe Kyu, Stephen Lim, Alan Lopez, Rafael Lozano, Ashley Marks, George Mensah, Awoke Misganaw, Ali Mokdad, Kate Muller, Christopher Murray, Mohsen Naghavi, Molly Nixon, Elaine Nsoesie, Helen Olsen, David Pigott, Puja Rao, Robert Reiner, Joseph Salama, Joshua A Salomon, Benn Sartorius, David Smith, Mari Smith, Jeff Stanaway, Caitlyn Steiner, Roman Topor-Madry, Stein Emil Vollset, Theo Vos, and Harvey Whiteford.

Did not provide contribution information

Jemal Abdela, Dilaram Acharya, Nahla Anber, Jalal Arabloo, Michael Brainin, Felix Carvalho, Hajer Elkout, Maryam Farvid, Eduarda Fernandes, John Ji, Masoud Keighobadi, Mohammad Khazaei, Narayan Mahotra, Man Mohan Mehndiratta, Mousa Mohammadnia-Afrouzi, Gopalakrishnan Natarajan, Puja Rao, Sahar Saeedi Moghaddam, Zikria Saleem, Mehdi Shahbazi, Reza Shirkoohi, Jeffrey Towbin, and Paul Yip.

List of Appendix Tables and Figures

Appendix Figure 1a: Overview analytical flowchart for DisMod-MR 2.1 modelling strategies and injuries, GBD 2017

Appendix Figure 1b: Analytical flowchart for modelling strategies other than DisMod-MR 2.1 and injuries for selected nonfatal cause groups

Appendix Figure 2: DisMod-MR 2.1 analytical cascade

Appendix Figure 3: Number of most detailed GBD causes with any incidence or prevalence data by location, 1980-2017

Appendix Table 1: GBD 2017 location hierarchy with levels

Appendix Table 2: GATHER checklist of information that should be included in reports of global health estimates, with description of compliance and location of information for GBD 2017

Appendix Table 3: GBD 2017 cause and sequela hierarchy

Appendix Table 4: List of International Classification of Diseases (ICD) codes mapped to the GBD 2017 non-fatal cause list

Appendix Table 5: GBD 2017 sequelae, health states, health state lay descriptions, and disability weights

Appendix Table 6: GBD 2017 methods of estimating years lived with disability (YLDs) for 35 residual categories

Appendix Table 7: List of GBD 2017 non-fatal causes with prevalence at birth

Appendix Table 8: Socio-demographic Index values for all estimated GBD locations, 1990–2017

Appendix Table 9: Socio-demographic Index groupings by location, based on 2016 values

Section 1. GBD overview

1.1 Geographic units of the analysis

The locations included in GBD 2017 have been arranged into a set of hierarchical categories composed of seven super-regions and a further nested set of 21 regions containing 195 countries and territories (Appendix Table 1). Subnational estimation in GBD 2017 includes Brazil, China, India, Indonesia, Japan, Kenya, Mexico, South Africa, Sweden, the United Kingdom, and the United States, and new subnational assessments at the administrative one level for Ethiopia, Iran, Norway, and Russia and by Maori ethnicity for New Zealand. For this publication, we present subnational estimates in figures only for all subnational countries with the exception of the new assessments which will be reported in separate publications. Select subnational estimates are also included in supplementary results appendix. Combined, there are a total of 390 locations at the first subnational unit level. Included in subnational Level 1 locations are countries that have been subdivided into the first subnational level, such as states or provinces, for the GBD analysis; subnational Level 2 only applies to India, England, and Russia. For this paper we present data at the national and territory level.

1.2 Time period of the analysis

A complete set of cause-specific incidence, prevalence, and YLD numbers and rates were computed for the years 1990, 1995, 2000, 2005, 2010, and 2017. All GBD 2017 results and online data visualisations are available at http://vizhub.healthdata.org/gbd-compare[1] with access to results for all GBD metrics.

1.3 Statement of GATHER compliance

This study complies with the Guidelines for Accurate and Transparent Health Estimates Reporting (GATHER) recommendations. We have documented the steps involved in our analytical procedures and detailed the data sources used in compliance with the GATHER. For additional GATHER reporting, please refer to Appendix Table 1.

The GATHER recommendations may be found here: http://gather-statement.org/

1.4 List of abbreviations

ADHD: attention-deficit/hyperactivity disorder
AG: acute glomerulonephritis
AN: anorexia nervosa
ANC: antenatal care
ART: antiretroviral therapy
ASD: autism spectrum disorder
AU: acute urolithiasis
BCG: bacille Calmette-Guerin vaccine
BFGS: Broyden-Fletcher-Goldfarb-Shanno optimization
BMI: body mass index
BN: bulimia nervosa
BPH: benign prostatic hypertrophy
BRFSS: Behavioral Risk Factor Surveillance Survey
BTL: basic tabulation list

CD: conduct disorder
CD4: CD4+ T lymphocyte; an indicator of immune function
CDC: United States Centers for Disease Control and Prevention
CDR: Clinical Dementia Rating scale
CE: cystic echinococcosis
CIBA: cohort incidence bias adjustment
CKD: chronic kidney disease
CL: cutaneous leishmaniasis
CoD: cause of death
CODEm: cause of death ensemble modelling
COMO: comorbidity correction
COPD: chronic obstructive pulmonary disease
CSMR: cause-specific mortality rate
CVD: cardiovascular disease
CZS: congenital Zika syndrome
DALYs: disability-adjusted life-years
DHS: Demographic and Health Survey
DM: diabetes mellitus
DSM: Diagnostic and Statistical Manual of Mental Disorders
DSP: Disease Surveillance Points
DST: drug sensitivity testing
DW: Disability weights
EHB: extreme hyperbilirunemia
EMBID: endrocrine, metabolic, blood, and immune disorders
EMR: excess mortality rate
EPEC: enteropathogenic *Escherichia coli*
EPP: Estimation and Projection Package
ETEC: enterotoxigenic *Escherichia coli*
FAS: fetal alcohol syndrome
FBT: foodborne trematodiases
GAHI: Global Atlas of Helminth Infections
GATHER: Guidelines for Accurate and Transparent Health Estimates Reporting
GBS: Guillain-Barré syndrome
GBD: Global Burden of Diseases, Injuries, and Risk Factors Study
GEMS: Global Enteric Multicenter Study
GHDx: Global Health Data Exchange
HAQ: Healthcare Access and Quality
HAT: human African trypanosomiasis
Hib: *Haemophilus influenzae* type B
ICD: International Classification of Disease
IDA: iron-deficiency anaemia
IHD: ischaemic heart disease
ILD: interstitial lung disease
IMIS: incremental mixture importance sampling
IPUMS: Integrated Public Use Microdata Series
IPV: intimate partner violence
IRS: indoor residual spraying
INI: intestinal nematode infection

iNTS: invasive non-typhoidal salmonella
ITN: insecticide-treated bed net
LBP: low back pain
LDI: lag distributed income
LF: lymphatic filariasis
LFTU: loss-to-follow-up
LN-LDI: natural log of lag-distributed income
LRI: lower respiratory infection
LTBI: latent tuberculosis infection
MAD: median absolute deviation
MAP: Malaria Atlas Project
MDA: mass drug administration
MDD: major depressive disorder
MDR-TB: multidrug-resistant tuberculosis
MEPS: Medical Expenditure Panel Surveys
MICS: Multiple Indicator Cluster Surveys
MI: myocardial infarction
MIRU-VNTR: mycobacterial interspersed repetitive units-variable number of tandem repeats
MND: motor neuron disease
MS: multiple sclerosis
MSK: musculoskeletal disorders
MSM: men who have sex with men
MTBC: *mycobacterium tuberculosis* complex
NAFLD: non-alcoholic fatty liver disease
NCC: neurocysticercosis
NCD: non-communicable disease
NE: neonatal encephalopathy
NESARC: National Epidemiological Survey on Alcohol and Related Conditions
NIDR: Notifiable Infectious Disease Reporting
NMSC: non-melanoma skin cancer
NP: neck pain
NSMHWB: Australian National Survey of Mental Health and Wellbeing of Adults
NTD: neglected tropical disease
OA: osteoarthritis
OECD: Organisation for Economic Co-operation and Development
PAF: population attributable fraction
PAHO: Pan American Health Organization
PCOS: polycystic ovarian syndrome
PCR: polymerase chain reaction
PCV3: three-dose pneumococcal conjugate vaccine
PEM: protein energy malnutrition
PfPR: malaria prevalence
PID: pelvis inflammatory disease
PLWH: people living with HIV
PM2.5: particulate matter <2.5μm in diameter
PMS: premenstrual syndrome
PPD: purified protein derivative
qPRC: quantitative polymerase chain reaction

RA: rheumatoid arthritis
RCT: randomised controlled trial
RHD: rheumatic heart disease
RHS: Reproductive Health Surveys
RMSE: root mean square error
RSV: respiratory syncytial virus
SD: standard deviation
SDG: Sustainable Development Goal
SDI: Socio-demographic Index
SEER: Surveillance, Epidemiology, and End Results Program
SEV: summary exposure value
SRS: Sample Registration System
ST-GPR: spatiotemporal Gaussian process regression
STH: soil-transmitted helminthiasis
STIs: sexually transmitted infections
TB: tuberculosis
TMREL: theoretical minimum-risk exposure level
TST: tuberculin skin test
TTH: tension-type headache
UN: United Nations
UNAIDS: Joint United Nations Programme on HIV/AIDS
UNICEF: United Nations Children's Fund
URI: upper respiratory infection
UTI: urinary tract infection
VA: verbal autopsy
VL: visceral leishmaniasis
WFS: World Fertility Surveys
WHO: World Health Organization
PCT: preventive chemotherapy and transmission control
XDR-TB: extensively drug-resistant tuberculosis
YLDs: years lived with disability
YLLs: years of life lost

1.5 GBD results overview

Results from the Global Burden of Disease Study (GBD) are now measured in terabytes. Results are available in an interactive data downloading tool on the Global Health Data exchange (GHDx). Data and underlying code used for this analysis will be made publicly available pending manuscript acceptance.

The core summary results include deaths, years of life lost (YLLs), years lived with disability (YLDs), and disability-adjusted life-years (DALYs). The GHDx includes data for causes, risks, cause-risk attribution, aetiologies, and impairments.

1.6 Data input sources overview

GBD 2017 incorporated a large number and wide variety of input sources to estimate mortality, population, fertility, causes of death and illness, and risk factors for 195 countries and territories from 1990–2017. These input sources are accessible through an interactive citation tool available in the GHDx.

Users can retrieve citations for a specific GBD component, cause or risk, and geography by choosing from the available selection boxes. They can then view and access GHDx records for input sources and export a CSV file that includes the GHDx metadata, citations, and information about where the data were used in GBD. Additional metadata for each input source are available through the citation tool, as required by the GATHER statement.

The citation tool will be made publicly available pending manuscript acceptance.

1.7 Level 5 sequelae

It is difficult to meaningfully report on the large number of sequelae that are estimated for some of the diseases and injuries in GBD. Moreover, most interest is not in this level of detail. For this reason, we have grouped sequelae into meaningful aggregates, making decisions disease by disease. For instance, for infectious diseases we grouped sequelae for the acute infectious disease episode and the long-term sequelae into two categories. Another example is for injuries for which we estimate 47 different sequelae but have grouped these into cohesive categories such as all fractures, all traumatic brain injury and all burns. These aggregates of sequelae fit into level 5 of the GBD hierarchy of causes and sequelae between the most detailed causes at level 4 and the most detailed sequela at level 6. We reported on level 5 sequelae aggregates for GBD 2015 but were unable to do so in GBD 2016. We have reviewed all decision made in GBD 2015 and made some alterations based on feedback from collaborators and dealing with new causes and sequelae.

1.8 Funding sources

Research reported in this publication was supported by the Bill & Melinda Gates Foundation, the National Institute on Aging of the National Institutes of Health (award P30AG047845), and the National Institute of Mental Health of the National Institutes of Health (award R01MH110163). The content is solely the responsibility of the authors and does not necessarily represent the official views of the Bill & Melinda Gates Foundation or the National Institutes of Health.

Section 2. Nonfatal outcome estimation

The GBD 2017 nonfatal estimation process is visually represented in Appendix Figures 1a and 1b illustrating the steps necessary to estimate incidence, prevalence, and YLDs for disease and injury sequelae in GBD 2017. Appendix Figure 1a outlines the general process of nonfatal outcome estimation from data inputs to finalization of YLD burden results; steps 3b and 3c of that process identifies alternative modelling approaches employed for certain causes and injuries. Alternative approaches are illustrated in greater detail in Appendix Figure 1b. Conceptually, the estimation effort is divided into eight major components: (1) compiling data sources through data identification and extraction; (2) data adjustment; (3) estimation of prevalence and incidence by cause and sequelae using DisMod-MR 2.1 or alternative modelling strategies for selected cause groups; (4) estimation by impairment; (5) severity distributions; (6) incorporation of disability weights; (7) comorbidity adjustment; and (8) the estimation of YLDs by sequelae and causes. Appendix Section 4 contains additional detail specific to each disease,

impairment, and injury, and their sequelae. Nonfatal modelling strategies vary significantly between causes.

2.1 Data sources, identification, and extraction

2.1.1 Systematic reviews

For GBD 2016, updated systematic reviews were conducted for 116 causes and 4 impairments. For GBD 2017, we conducted literature reviews for 82 non-fatal causes and one impairment through February 2018. For other disease sequelae, only a small fraction of the existing data appears in the published literature and other sources predominate such as survey data, disease registers, notification data or hospital inpatient data. As was done in GBD 2016, data were systematically screened from household surveys archived in the Global Health Data Exchange (http://ghdx.healthdata.org/), including Demographic and Health Surveys, Multiple Indicator Cluster Surveys, Living Standards Measurement Surveys, and Reproductive Health Surveys. Other national health surveys were identified based on survey series that had yielded usable data for past rounds of GBD, sources suggested to us by in-country collaborators, and surveys identified in major multinational survey data catalogs, such as the International Household Survey Network and the World Health Organization (WHO) Central Data Catalog, as well as through country Ministry of Health and Central Statistical Office websites. Case notifications reported to the WHO were updated through 2017. Citations for all data sources used for nonfatal estimation in GBD 2017 are provided in searchable form through a web tool (http://ghdx.healthdata.org/). A description of the search terms employed for cause-specific systematic reviews are detailed by cause in Appendix Section 4.

2.1.2 Survey data preparation

For GBD 2017, survey data for which we have access to the unit record data constitute a substantial part of the underlying data used in the estimation process. During extraction, we concentrate on demographic variables (such as location, sex, age), survey design variables (such as sampling strategy and sampling weights), and the variables used to define the population estimate (such as prevalence or a proportion) and a measure of uncertainty (standard error, confidence interval or sample size and number of cases).

2.1.3 Disease registries

For GBD 2017 nonfatal estimation, disease registries were an important source for a select number of conditions such as cancers, end-stage renal disease, and congenital disorders.

Registry data is particularly key in the estimation of neoplasms given the increasing attention to non-communicable diseases, particularly cancers, in low and middle-income areas of the world. The GHDx source tool (http://ghdx.healthdata.org/data-type/disease-registry)[2] provides a comprehensive list of registry data used in GBD estimation processes.

22.1.4 Estimation of hospital envelope

Figure. Overview process of estimation of hospital envelope. This process utilizes administrative data, reported tabulations, and survey microdata to estimate the rates of inpatient admissions per capita for every location and demographic group in the GBD hierarchy.

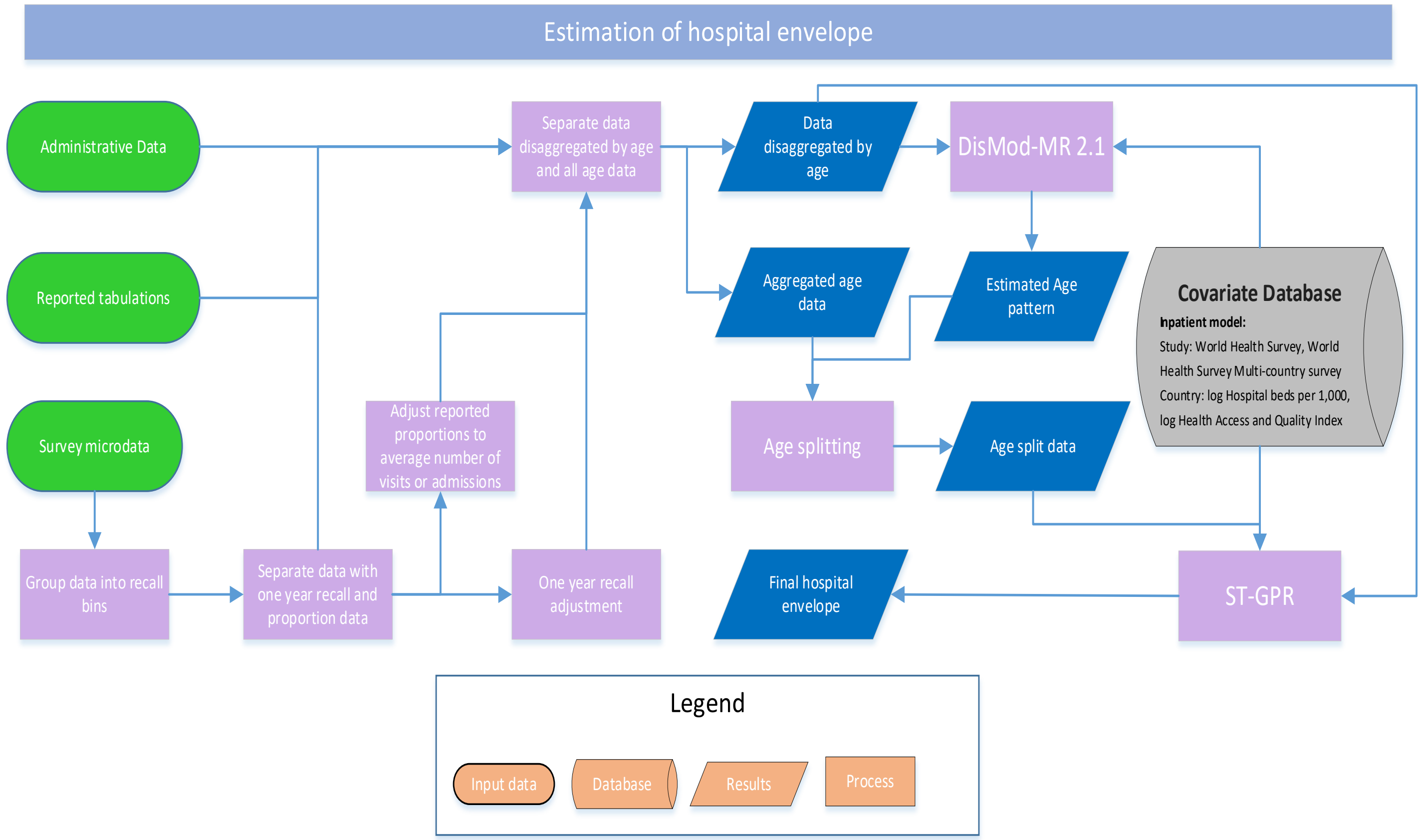

Input data and methodological summary

Case definition

We defined a hospital admission as the overnight admission into a formal health care facility but excluded admissions to long-term care (>120 days), nursing care facilities, and traditional or spiritual healers.

Input data

We searched the Global Health Data Exchange (GHDx) for population surveys, administrative records, and censuses from January 1990 to September 2017. We applied five secondary data filters: “discharge”, “health facility”, “nationally representative”, “household”, or “outpatient.” We also applied ten keyword filters: “healthcare access”, “health care costs”, “healthcare economics”, “healthcare expenditure”, “healthcare services”, “healthcare use”, “outpatient facilities”, “patient counts”, “hospitals”, or “length of stay”. We applied no language restrictions to our search and required all returned records to either contain microdata or tabulated reports. We searched the returned records’ metadata looking for measures of inpatient care. For inclusion, we required all measures to be nationally or subnationally

representative. Additionally, we consulted with experts and GBD collaborators to gather data sources that were not within the GHDx.

To estimate inpatient admission rates for newborns, we input estimates of the in-facility delivery (IFD) rates for every subnational and national location at 5-year intervals starting at 1990 and including the most recent 2017 estimate. IFD was estimated using an ST-GPR model based off of population-representative surveys and administrative data. In total, we accepted data sources from 8,947 location-years (1,413 from administrative records and 7,534 from population surveys).

Modelling strategy

Data adjustment

We classified each of the accepted data sources into four data types: (a) proportion of survey respondents who were admitted into the hospital in the past 30 days; (b) proportion of survey respondents who were admitted to the hospital in the past year; (c) average number of admissions (utilization rate) reported by survey respondents in the past year; and (d) average number of visits reported by annual administrative records. We assigned measures reported by annual administrative records as our reference group as these data types were free from recall bias and most closely matched our case definition. In data sources where microdata was available, we extracted and binned the data based on sex and age groups of under-1, 1-4, 4-9, 10-14, through till 95+ years of age.

We crosswalked each of the three non-reference (survey) data types to the reference (administrative record) data type through the use of penalized spline regressions to account for non-systematic differences between the data types. For each non-reference data type and each sex, we looked for overlap between the non-reference data type and reference data type on the basis of location, year, age group, and sex. With the overlapping data, we calculated the ratio of the point estimate from the reference data type, μ_{ref}, to the non-reference data type, μ_s. We fit these ratios with a penalized spline regression shown below

$$\ln\left(\frac{\mu_{ref,i}}{\mu_{s,i}}\right) = h(age_i) + \varepsilon_i \ (1)$$

Where i denotes a given matched observation, $h(age_i)$ represents a basis function which estimated a cross-validated penalized spline over the population weighted mean age of the age group, and ε represents the residual. In the below figures, for each non-reference data type, we plot the ratio of μ_{ref} and μ_s across age and by sex and the predictions from the penalized spline regressions.

Figure. Global age-sex specific crosswalks to equate each non-reference data type top the reference data type. For each non-reference data type and each sex, we plot the ratio of reference data points to non-reference data points, which were matched based on location, age group, year, and sex. Using a penalized spline regression, we estimated the crosswalk between each non-reference data type and the reference type. We plot the crosswalk and the associated prediction error below.

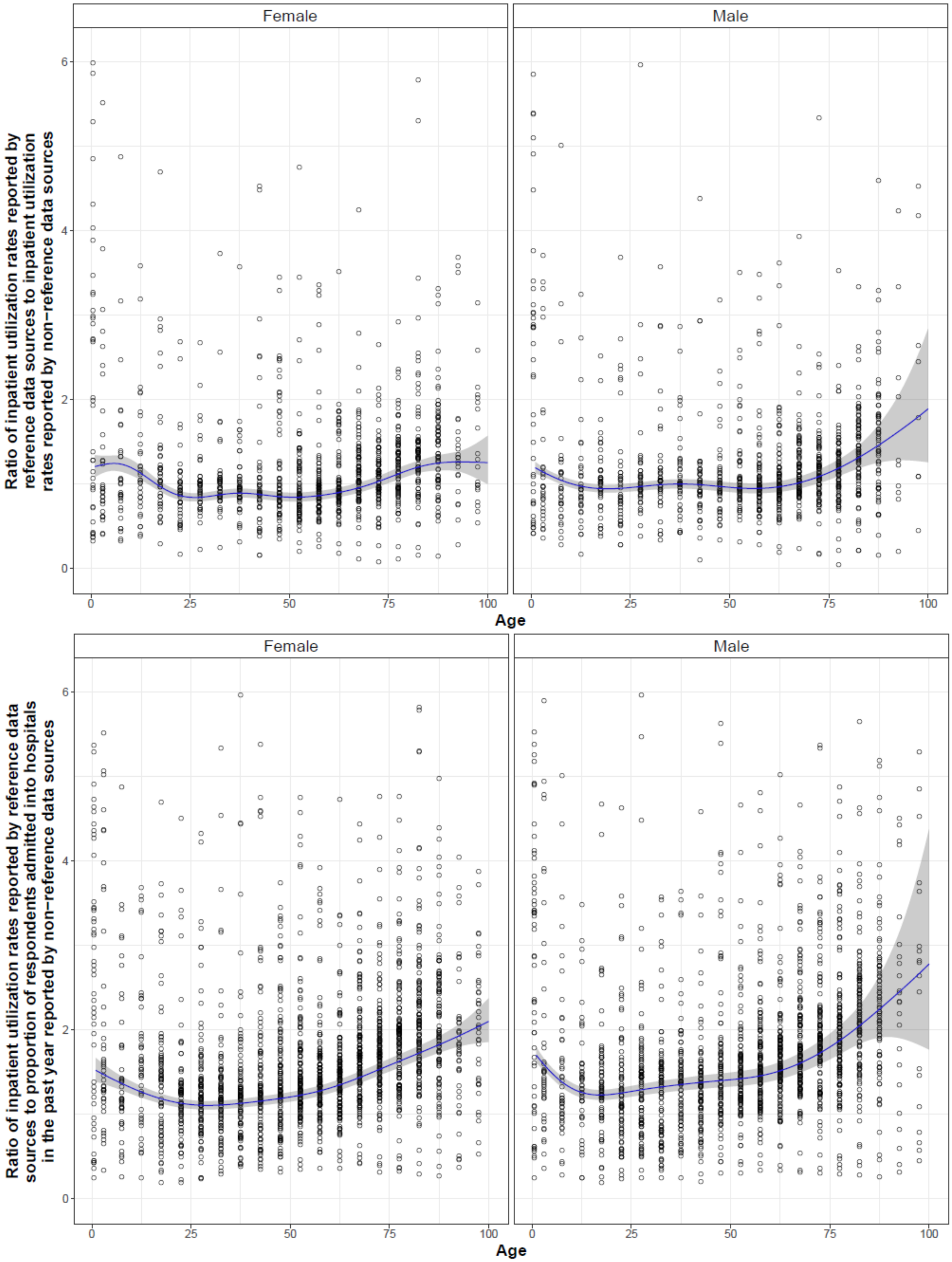
Female
Male
Ratio of inpatient utilization rates reported by reference data sources to inpatient utilization rates reported by non−reference data sources
Age
Female
Male
Ratio of inpatient utilization rates reported by reference data sources to proportion of respondents admitted into hospitals in the past year reported by non−reference data sources
Age

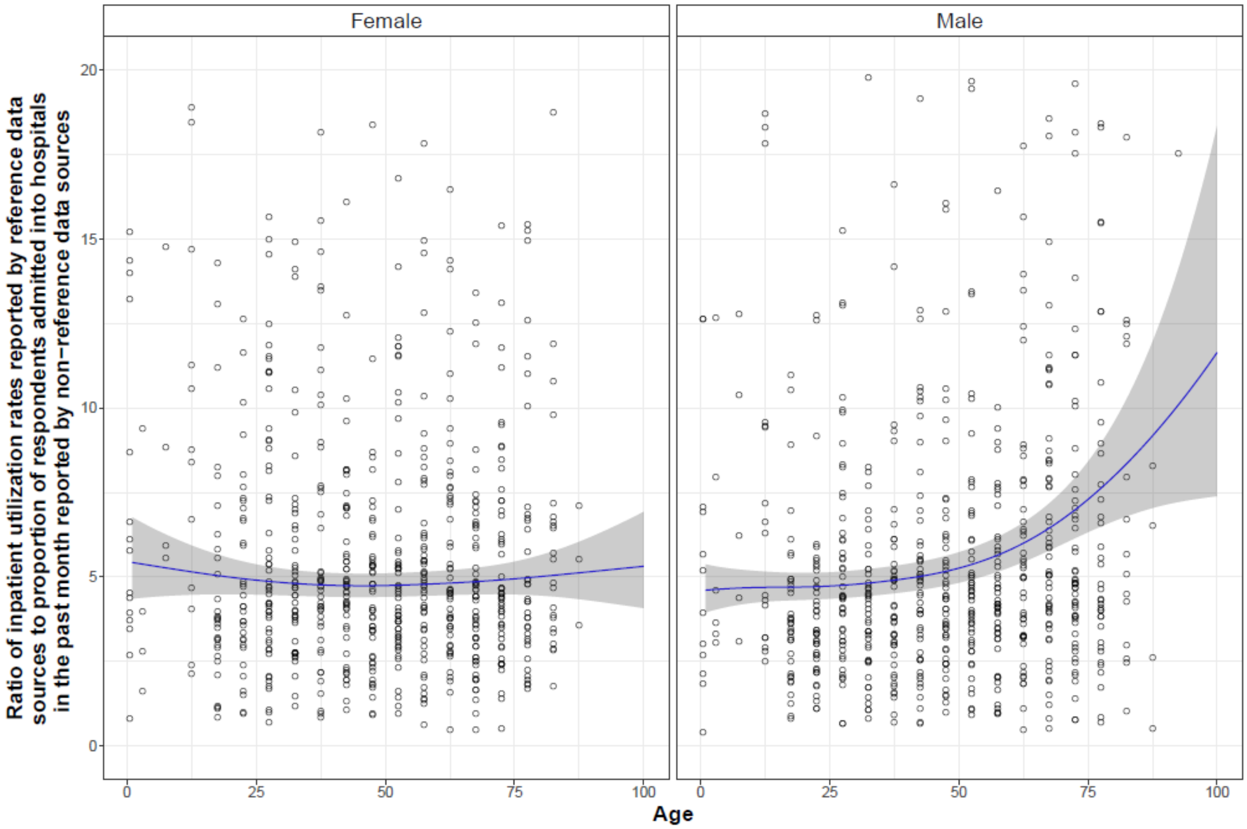

To crosswalk non-reference data types to reference data types, we multiplied non-reference data types by the exponentiated predictions from respective penalized spline regressions. Uncertainty from the adjustments were accounted for using the following equation

$$se_a = \sqrt{se_m^2 \cdot se_s^2 + se_m^2 \cdot \mu_s^2 + se_s^2 \cdot \mu_m^2} \ (2)$$

Where $se_a, se_m,$ and se_s are the standard errors of the adjusted non-reference data point, the exponentiated crosswalk prediction, and the non-reference data point, respectively. μ_s and μ_m are the means of the non-reference data point and the exponentiated crosswalk predictions from the penalized spline regressions.

Age-sex splitting

Prior to modelling, we ran a DisMod-MR 2.1 model with data that was disaggregated by age to estimate countries' age-pattern and then applied the estimated age-pattern to split aggregated all age data into the age groups that are necessary 5-year age groups that are encouraged by ST-GPR. This procedure was operationalized by calculating a constant, k, which was the ratio of the aggregated all age data point, $\mu_{all\ age}$, to the all age estimated utilization rate from the DisMod-MR 2.1 model, $\widehat{\mu_d}$

$$k = \frac{\mu_{all\ age}}{\widehat{\mu_d}} \ (3)$$

The constant, k, was then multiplied by age specific utilization rates from the DisMod-MR 2.1 model. The uncertainty from the data and the age-pattern were propagated following equation 2. The split data were then incorporated into the final DisMod-MR 2.1 model.

ST-GPR modelling

The input data was modelled using space-time Gaussian process regression (ST-GPR) to allow for smoothing over age, time, and location in locations that were missing complete datasets. To further help explain variation in geographies with little to no data, we used the covariates of natural log of hospital beds per 1,000 and the healthcare access and quality index for every location. Hospital beds per 1,000 was estimated using ST-GPR on data sourced from the World Bank. Coefficients for the covariates are presented in the below table.

Table. Estimated coefficients of the hospital envelope model.

Covariate	Sex	Coefficient	Exponentiated coefficient
log Hospital beds per 1,000	Male	0.40<br>(0.36 — 0.43)	1.49<br>(1.44 — 1.54)
log Hospital beds per 1,000	Female	0.42<br>(0.39 — 0.46)	1.53<br>(1.47 — 1.58)
Healthcare Access and Quality Index	Male	0.013<br>(0.011 — 0.013)	1.012<br>(1.011 — 1.013)
Healthcare Access and Quality Index	Female	0.014<br>(0.013 — 0.016)	1.014<br>(1.013 – 1.015)

2.1.5 Claims, inpatient hospital, and outpatient data

For GBD 2017, claims (linkage) data, inpatient hospital, and outpatient data played a key role in the nonfatal estimation process of many GBD causes.

Claims data

For GBD 2017, we accessed aggregate data derived from claims information in a database of US private health insurance and public insurance schemes of Medicaid and Medicare, for the years 2000, 2010, 2011, 2012, 2013, and 2014. The population covered in each year was 3.3 million in 2000, 41.1 million in 2010, 44.4 million in 2011, 41.5 million in 2012, 42.2 in 2013, and 36.4 in 2014. For each of these individuals, information on every health service encounter was collected and all episodes of care were linked to individuals by unique identifiers. Outpatient claims could have up to 4 diagnoses (15 for inpatient claims). We mapped all ICD-9 diagnoses to GBD causes (see Appendix table 4). GBD conditions were categorized as "prevalent" or "incident" depending on cause duration and based on the specification of the research team responsible for the cause. In a given year, for each individual in the claims data, a prevalent case was defined as any mention in any diagnostic field associated with any claim, including inpatient and outpatient encounters. An incident case was defined the same way but assumed that claims within a condition-specific duration were the same case. In this way, an individual could have multiple incident cases in a given year, while avoiding double-counting cases with multiple claims from a single illness episode.

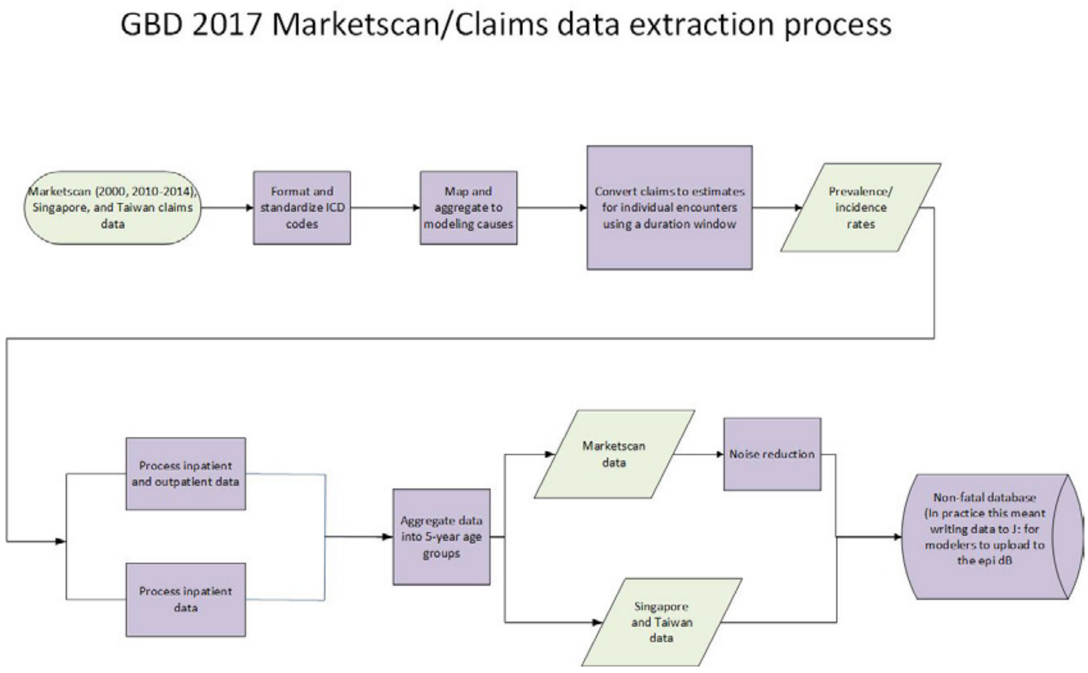

Inpatient hospital admissions

Inpatient hospital data were extracted from 335 country-years. ICD coding was standardized across sources, and versions of ICD.

For GBD 2015, one limitation of our use of hospital data in non-fatal disease estimation was the challenge of accessing accurate information on coverage populations for any given data source. Section 2.1.4 of the appendix describes the modelling strategy for the hospital envelope, an estimate of hospital utilization. In GBD 2016, we used the hospital utilization envelope in place of information on coverage population. We calculate age- and sex-specific cause fractions in each inpatient hospital data source and multiply these by the hospital utilization envelope to produce incidence or prevalence rates. In GBD 2017, we used the same approach except the hospital envelope was measured in space-time Gaussian process regression to accommodate admission data reflecting newborns in a birthing facility.

Using the Marketscan claims data described above, we generated three scalars that were applied to the inpatient hospital data on a cause by cause basis. The scalars account for bias in inpatient hospital data from sources which were aggregated by ICD code and by primary diagnosis only. First, we corrected to account for multiple admissions for an individual. Second, we adjusted for non-primary diagnoses. Third, we corrected to account for inpatient and outpatient care. Combined with the uncorrected version (no scalar applied), this resulted in four stages of incidence and prevalence estimates from inpatient hospital data: 1) (un-corrected) inpatient admissions by episode, primary diagnosis, 2) inpatient admissions by individual, primary diagnosis only, 3) inpatient hospital admissions, accounting for all diagnoses, 4) an estimate of inpatient admissions and outpatient visits by individual, accounting for all diagnoses. These

data were reviewed in conjunction with data from all other sources for each model that utilizes hospital data to determine which stage of data was most appropriate as an input to non-fatal disease estimation.

We created alternative estimates for the readmission and comorbidity correction factors by incorporating four other sources of inpatient discharge records that included unique patient identifiers linked to individuals and multiple ICD-coded diagnoses. These sources were the Healthcare Cost and Utilization Project State Inpatient Databases, the New Zealand National Minimum Dataset, the Philippine Health Insurance Corporation Claims, and Taiwanese national claims data. With the scalars from all five sources, we created a mixed-effects linear model to predict the correction factor at the national level for every inpatient hospital data source, as an alternative to using the ratio derived only from Marketscan. Annual outpatient visits per capita was used as a country-level covariate in the readmission scalar models, and inpatient admissions per capita was used as a country-level covariate in both the readmission and comorbidity scalar models.

An alternative estimate for the total number of inpatient and outpatient cases of each cause was also created incorporating the Taiwanese claims dataset with Marketscan. With the scalars from both sources, we created a mixed-effects linear model to predict the ratio to the super-region level. The model used the super-region level covariate of annual outpatient visits per capita, which was estimated using DisMod-MR 2.1. The equations we used for each of the three scalars can be found below:

a) Correction to account for multiple admissions which gives us inpatient admissions by individual, primary diagnosis only
    a. $inpatient^{1^\circ}_{admin} * \left(\frac{MS\ inpatient^{1^\circ}_{indiv}}{MS\ inpatient^{1^\circ}_{admin}}\right) = inpatient^{1^\circ}_{indiv}$
b) Correction to adjust for non-primary diagnoses which gives us inpatient admissions by individual, all diagnoses
    a. $inpatient^{1^\circ}_{admin} * \left(\frac{MS\ inpatient^{all}_{indiv}}{MS\ inpatient^{1^\circ}_{admin}}\right) = inpatient^{all}_{indiv}$
c) Correction to account for inpatient and outpatient care which gives us inpatient admissions and outpatient visits by individual, all diagnoses
    a. $inpatient^{1^\circ}_{admin} * \left(\frac{MS\ inpatient^{all}_{indiv} \cup MS\ outpatient^{all}_{indiv}}{MS\ inpatient^{1^\circ}_{admin}}\right) = inpatient|outpatient^{all}_{indiv}$

Maternal causes used separate cause-fractions, and a different scalar calculated from a maternal hospital admissions rate instead of the hospital envelope, using the equation below:

$$\left(\frac{events}{\#\ of\ total\ hospital\ visits}\right) * \left(\frac{hospital\ visits}{live\ births}\right) * \left(\frac{births}{population}\right)$$

Congenital and neonatal causes and injuries used a separate correction factor as well.

In cases where the third scalar, accounting for inpatient and outpatient care, was greater than 50, we determined it to be unstable and did not apply this scalar to the hospital data. Two exceptions to this rule

were for congenital and neonatal causes, where infants hospitalized with these conditions often have comorbid states that make it very likely that the given code would not be listed as primary.

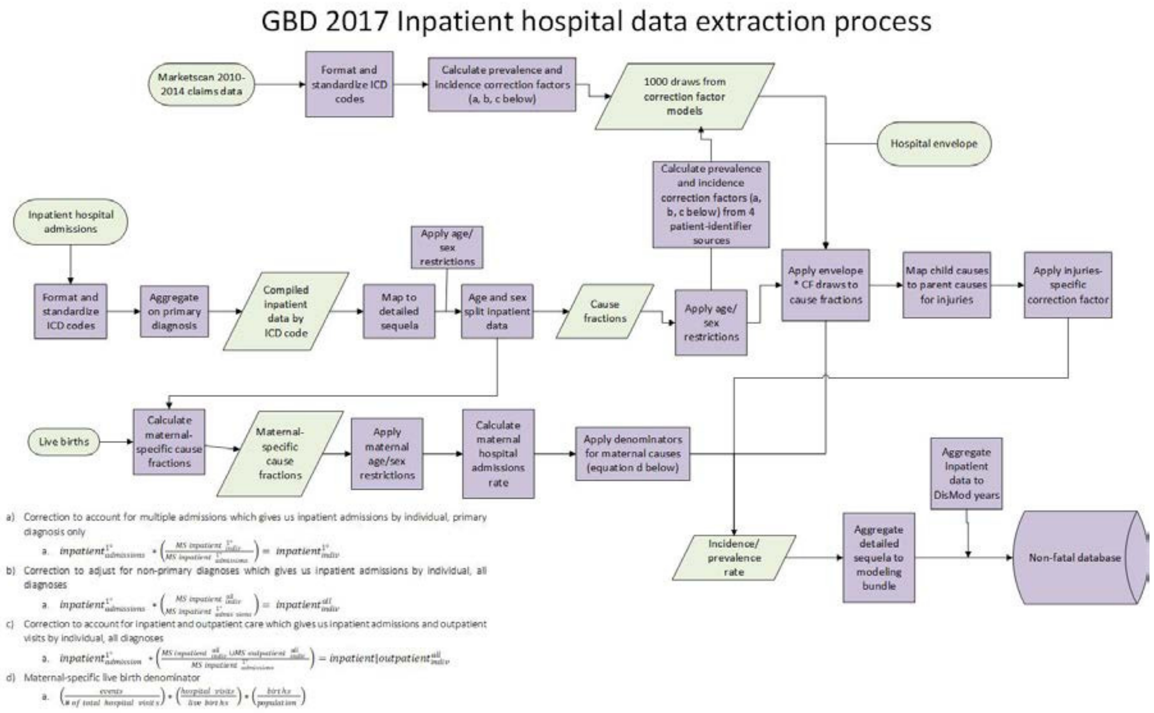

Outpatient

Outpatient encounter data were available from the USA, Sweden and Norway for 45 location-years. No changes were made in the processing of outpatient data from GBD 2016, aside from updates to the ICD mappings to GBD cause.

Similar to the inpatient hospital data, a scalar was calculated using Marketscan claims data to adjust for multiple visits per individual within one year (for prevalent conditions), and within a cause-specific duration (for incident causes).

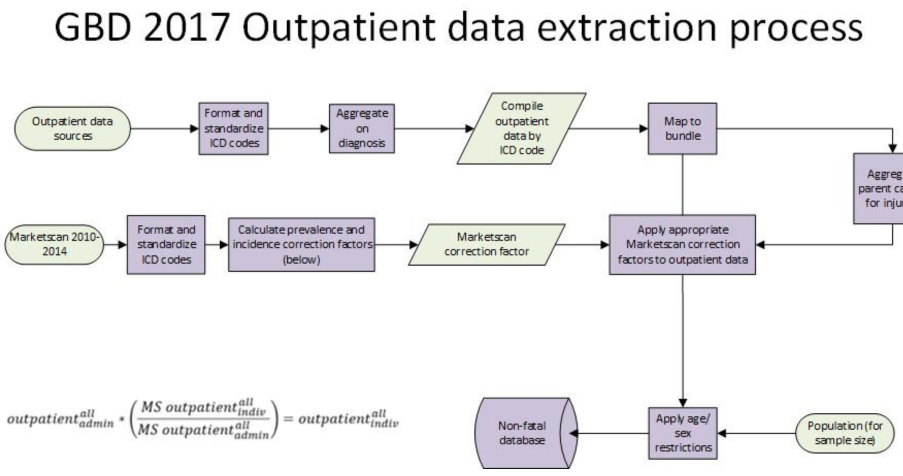

2.1.6 Case notifications

Case notifications, active screening, intervention coverage studies, and surveillance contributed to estimates of infectious diseases. If available, we extracted data from survey and administrative microdata; otherwise, data were extracted from published literature and reports. For many infectious diseases and neglected tropical diseases (NTDs), we make use of cases notified by countries to the World Health Organization (WHO) and other global monitoring entities. The causes for which we use WHO case notification data include tuberculosis (TB), measles, yellow fever, rabies, dengue, cholera, whooping cough, human African trypanosomiasis (HAT), meningitis, all sexually transmitted infections (STIs), and other infectious and NTDs, such as Ebola.

2.2 Data adjustment

In addition to the corrections applied to claims and hospital data, a number of other adjustments were applied to extracted nonfatal sources in order to make the data more consistent and suitable for modelling. In this second step of nonfatal estimation, commonly applied adjustments included age-sex splitting, bias correction, adjustments for underreporting of notification data, and computing expected values of excess mortality. Age-sex splitting was commonly applied to literature data reported by age or sex but not by age and sex. For GBD 2017, we split all data reported in age groups with a width greater than 20 years, using age patterns from available survey microdata or regional patterns derived from an initial run of main modelling tool, DisMod-MR 2.1. We relied on the meta-regression component of DisMod-MR 2.1 for most of the bias correction of data for variations in study attributes such as case definitions and measurement method. DisMod-MR 2.1 calculates a single adjustment that is applied regardless of age, sex, or location. If enough data were available to differentiate these adjustments by age, sex, or location, or if detailed survey data were available to make more precise adjustments between different thresholds on a biochemical measure, we applied bias corrections to the data before entry into DisMod-MR 2.1. For instance, we crosswalked between 12 different case definitions with different thresholds of fasting plasma glucose or glycated hemoglobin levels for diabetes mellitus based on available survey data with individual records of the actual measurements. In another example, we corrected data on COPD from surveys applying different thresholds on spirometry measurements using studies that had reported on prevalence of COPD for the reference and alternative thresholds. As this relationship varied with age, age-specific correction factors were derived. The correction of notification data for underreporting relied on studies that had examined the gap between true incidence and notified cases.

In GBD 2017, we estimated expected values of excess mortality from prevalence or incidence and cause-specific mortality rate (CSMR) data for every cause for which deaths were estimated with the exception of a few causes with very low mortality rates such as uterine fibroids. We matched every prevalence data point (or incidence data for short-duration conditions) with the CSMR value corresponding to the age range, sex, year, and location of the data point. We restricted this to data points reporting age-groups spanning 20 years or less. The ratio of CSMR to prevalence (or incidence times a short duration) is conceptually equivalent to an excess mortality rate. To reflect a gradient in excess mortality, we added in

21

all relevant models the log of lag distributed income (LDI) or the Healthcare Access and Quality (HAQ) index as a covariate, with a strong prior that as LDI or HAQ Index increases, excess mortality declines.

2.3 DisMod-MR 2.1 estimation

a. Estimation of sequelae and causes

The most extensively used estimation method is the Bayesian meta-regression method DisMod-MR 2.1. For some causes such as HIV/AIDS or hepatitis B and C, disease-specific natural history models have been used where the underlying three state model in DisMod-MR 2.1 (susceptible, cases, dead) is insufficient to capture the complexity of a disease process. For some diseases with a range of sequelae differentiated by severity, such as chronic obstructive pulmonary disease (COPD) or diabetes mellitus, DisMod-MR 2.1 is used to meta-analyze the data on overall prevalence with separate DisMod-MR 2.1 models of the proportions of cases with different severity levels or sequelae. Likewise, DisMod-MR 2.1 is used to meta-analyze data on the proportions of liver cancer and cirrhosis due to underlying etiologies such as hepatitis B, hepatitis C, and alcohol use.

b. DisMod-MR 2.1 description

Until GBD 2010, nonfatal estimates in burden of disease assessments were based on a single data source on prevalence, incidence, remission or a mortality risk selected by the researcher as most relevant to a particular location and time. For GBD 2010, we set a more ambitious goal: to evaluate all available information on a disease that passes a minimum quality standard. That required a different analytical tool that would be able to pool disparate information presented in varying age groupings and from data sources using different methods. The DisMod-MR 1.0 tool used in GBD 2010 evaluated and pooled all available data, adjusted data for systematic bias associated with methods that varied from the reference and produced estimates by world regions with uncertainty intervals using Bayesian statistical methods. For GBD 2013, the improved DisMod-MR 2.0 had increased computational speed allowing computations that were consistent between all disease parameters at the country rather than region level. The hundred-fold increase in speed of DisMod-MR 2.0 was partly due to a more efficient rewrite of the code in C++ but also by changing to a model specification using log rates rather than a negative binomial model used in DisMod-MR 1.0. In cross-validation tests, the log rates specification worked as well or better than the negative binomial specification.[3] For GBD 2015, we rewrote the 'wrapper' code that organizes the flow of data and settings at each level of the analytical cascade. The sequence of estimation occurs at five levels: global, super-region, region, country and, where applicable, subnational location. The super-region priors are generated at the global level with mixed-effects, nonlinear regression using all available data; the super-region fit, in turn, informs the region fit, and so on down the cascade. The wrapper gives analysts the choice to branch the cascade in terms of time and sex at different levels depending on data density. The default used in most models is to branch by sex after the global fit but to retain all years of data until the lowest level in the cascade. Appendix Figure 2 summarizes the DisMod-MR process.

In updating the 'wrapper,' we consolidated the code base into a single language, Python, to make the code more transparent and efficient and to better deal with subnational estimation. The computational

engine is limited to three levels of random effects; we differentiate estimates at the super-region, region and country level. In GBD 2013, the subnational units of China, the UK and Mexico were treated as 'countries' such that a random effect was estimated for every location with contributing data. However, the lack of a hierarchy between country and subnational units meant that the fit to country data contributed as much to the estimation of a subnational unit as the fits for all other countries in the region. We found inconsistency between the country fit and the aggregation of subnational estimates when the country's epidemiology varied from the average of the region. Adding an additional level of random effects required a prohibitively comprehensive rewrite of the underlying DisMod-MR engine. Instead, we added a fifth layer to the cascade, with subnational estimation informed by the country fit and country covariates, plus an adjustment based on the average of the residuals between the subnational location's available data and its prior. This mimicked the impact of a random effect on estimates between subnationals.

For GBD 2016, the computational engine (DisMod-MR 2.1) remained substantively unchanged from GBD 2015. We updated the age prediction sets to include age groups 80-84, 85-89, 90-94, and 95+, to comply with changes across all functional areas of the GBD. We also expanded the set of locations where subnational units are modelled; the set in 2016 included: Brazil, China, England, India, Indonesia, Japan, Kenya, Mexico, Saudi Arabia, South Africa, Sweden, and the United States.

In GBD 2017, we continued to use DisMod-MR 2.1, as there were no substantial changes. Updates to computation include extending the terminal prediction year to 2017 and additional subnational units in Ethiopia, Iran, New Zealand, Norway, and Russia. Saudi Arabia was also modelled only at the national level in 2017.

Appendix Figure 2 - GBD 2017 DisMod-MR 2.1 analytical cascade

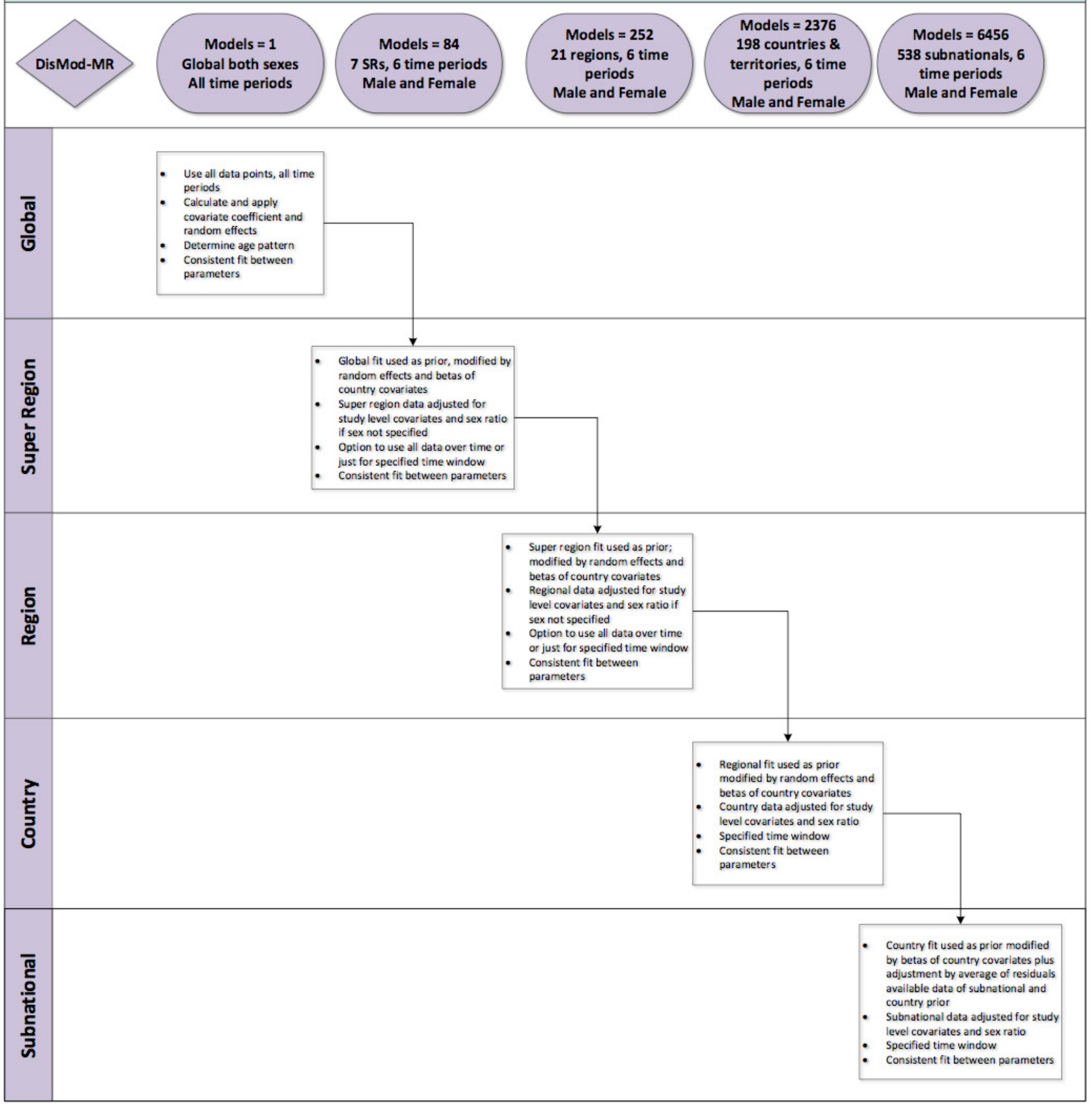

c. DisMod-MR 2.1 likelihood estimation

Analysts have the choice of using a Gaussian, log-Gaussian, Laplace or Log-Laplace likelihood function in DisMod-MR 2.1. The default log-Gaussian equation for the data likelihood is:

$$-log\left[p\left(y_j|\Phi\right)\right] = \log\left(\sqrt{2\pi}\right) + \log\left(\delta_j + s_j\right) + \frac{1}{2}\left(\frac{\log\left(a_j + \eta_j\right) - \log\left(m_j + \eta_j\right)}{\delta_j + s_j}\right)^2$$

where, y_j is a 'measurement value' (i.e., data point); Φ denotes all model random variables; η_j is the offset value, eta, for a particular 'integrand' (prevalence, incidence, remission, excess mortality rate, with-condition mortality rate, cause-specific mortality rate, relative risk or standardized mortality ratio) and a_j is the adjusted measurement for data point j, defined by:

$$a_j = e^{(-u_j - c_j)} y_j$$

where u_j is the total 'area effect' (i.e., the sum of the random effects at three levels of the cascade: super-region, region and country) and c_j is the total covariate effect (i.e., the mean combined fixed effects for sex, study covariates, and country level covariates), defined by:

$$c_j = \sum_{k=0}^{K[I(j)]-1} \beta_{I(j),k} \hat{X}_{k,j}$$

with standard deviation

$$s_j = \sum_{l=0}^{L[I(j)]-1} \zeta_{I(j),l} \hat{Z}_{k,j}$$

where k denotes the mean value of each data point in relation to a covariate (also called x-covariate); I(j) denotes a data point for a particular integrand, j; $\beta_{I(j),k}$ is the multiplier of the kth x-covariate for the ith integrand; $\hat{X}_{k,j}$ is the covariate value corresponding to the data point j for covariate k; l denotes the standard deviation of each data point in relation to a covariate (also called z-covariate); $\zeta_{I(j),k}$ is the multiplier of the lth z-covariate for the ith integrand; and δ_j is the standard deviation for adjusted measurement j, defined by:

$$\delta_j = log\left[y_j + e^{(-u_j - c_j)}\eta_j + c_j\right] - log\left[y_j + e^{(-u_j - c_j)}\eta_j\right]$$

Where m_j denotes the model for the jth measurement, not counting effects or measurement noise and defined by:

$$m_j = \frac{1}{B(j) - A(j)} \int_{A(j)}^{B(j)} I_j(\mathrm{a})\, \mathrm{da}$$

where A(j) is the lower bound of the age range for a data point; B(j) is the upper bound of the age range for a data point; and I_j denotes the function of age corresponding to the integrand for data point j.

2.4 Impairment and underlying cause estimation

For GBD 2017, as in GBD 2016, we estimated the country-age-sex-year prevalence of nine impairments – step 4 of Appendix Figure 1a. Impairments in GBD are conditions or specific domains of functional health loss which are spread across many GBD causes as sequelae and for which there are better data to estimate the occurrence of the overall impairment than for each sequela based on the underlying cause. These impairments included: anaemia, epilepsy, hearing loss, heart failure, intellectual disability,

infertility, vision loss, Guillain-Barré syndrome, and pelvic inflammatory disease. Overall impairment prevalence was estimated using DisMod-MR 2.1. We constrained cause-specific estimates of impairments, as in the 19 causes of blindness, to sum to the total prevalence estimated for that impairment. Anaemia, epilepsy, hearing loss, heart failure, and intellectual disability were estimated at different levels of severity. Estimates were made separately for primary infertility (those unable to conceive), secondary infertility (those having trouble conceiving again), and whether the impairment affected men and/or women. In the case of epilepsy, we determined the proportions with idiopathic and secondary epilepsy as well as for the proportions with severe and less severe epilepsy using mixed effects regressions. The sparse data for the proportion of seizure-free, treated epilepsy were pooled in a random effects meta-analysis. DisMod-MR 2.1 models produced country-, age-, sex-, and year-specific severity levels of hearing loss and vision loss. Due to limited information on the severity levels of intellectual disability, we assumed a similar distribution of severity globally, based on random effects meta-analysis of IQ-specific data for the overall impairment. This was supplemented by cause-specific severity distributions for chromosomal causes and iodine deficiency; the severity of intellectual disability included in the long-term sequelae of causes including neonatal disorders, meningitis, encephalitis, neonatal tetanus, and malaria was estimated in combined health states of multiple impairments such as motor impairment, blindness, and/or seizures.[14] We changed the name of the intellectual disability impairment to specify that estimates reflect cases arising during the developmental period which we have defined as ages below 20. The severity of heart failure was derived from our Medical Expenditure Panel Surveys (MEPS) analysis and therefore was not specific for country, year, age, or sex.

A detailed description of the methods of each impairment can be found at the end of Section 4 of this appendix.

2.4.1 Impairment squeeze

For impairments like epilepsy, intellectual disability, and blindness, mentioned above in Step 4, we often have better information regarding the total prevalence of the impairment rather than the prevalence of said impairment due to its various causes. For example, we have more data and a better idea of the total number of blind individuals (which we refer to herein as the blindness "envelope") in the world than we do the number of individuals who are blind due to a specific cause like retinopathy of prematurity or cataract. We achieve this consistency by either "squeezing" or inflating the individual sequela prevalence values so that their sums fit into each appropriate envelope. Blindness, epilepsy and/or intellectual disability appear in various combinations with motor impairment levels as sequelae for a number of neonatal disorders and infectious diseases like malaria and neonatal tetanus ("Moderate motor impairment with blindness and epilepsy due to neonatal tetanus," for example). This present an extra challenge as any squeeze or inflation of one of the impairments making up a sequela will affect the others. We set some rules on how to do these adjustments sequentially. First, when the envelope of an impairment is smaller than the sum of all contributing causes, we redistribute the ‘excess’ prevalent cases of combined impairment sequelae onto the sequelae that only have motor impairment (at mild, moderate or severe level) within the same cause grouping. Second, we apply the adjustments in a

particular order such that we always fit at least one of the envelopes exactly where the other one or two envelopes may be exceeded by some amount. We first enforce a fit to the epilepsy impairment envelope, then intellectual disability and, lastly, blindness. Thus, the epilepsy envelope will always match exactly, whereas the intellectual disability and blindness envelopes may occasionally be exceeded on a draw-by-draw basis.

2.5 Severity distribution

In GBD 2017, we report on 381 sequelae at Level 5 of the hierarchy (Appendix Table 3). We generally followed the same approach for estimating the distribution of severity as in GBD 2016. For Zika, we included sequelae for those with symptomatic acute infection, a small proportion with Guillain-Barré syndrome and the number of neonates with congenital Zika as reported to Pan American Health Organization (PAHO). For sexual violence, we estimated concurrent physical injuries and the more immediate psychological outcomes following sexual violence. For the added causes that were split from broader cause categories, the differentiation between drug-sensitive and drug-resistant tuberculosis, the creation of other leukemia, alcoholic and other cardiomyopathy, and self-harm by firearm or other means each follow the same pattern of assigning sequelae as for their parent causes. In cases in which severity was related to a particular impairment, such as mild, moderate, and severe heart failure due to ischemic heart disease, the analysis was driven by impairment estimation methods. Severity levels for conditions such as chronic kidney disease and COPD were modelled using DisMod-MR 2.1, while we performed meta-analyses to estimate the allocation of severity for causes such as rheumatoid arthritis, dementia, and multiple sclerosis.

For many causes we had inadequate data on severity from surveys or the epidemiological literature. For those diseases, we made use of three population surveys: the US Medical Expenditure Panel Survey (MEPS) 2000–2014, the [US] National Epidemiological Survey on Alcohol and Related Conditions (NESARC) 2000–2001 and 2004–2005, and the Australian National Survey of Mental Health and Wellbeing of Adults (NSMHWB) 1997.[20–22] Each dataset contained individual-level measurements of functional health status using the SF-12 Health Survey as well as diagnostic information on the conditions affecting each individual.

In order to use the data collected using SF-12 for measuring the distribution of severity, the individual SF-12 summary scores were mapped to an equivalent disability weight. A convenience sample of respondents was asked to complete SF-12 for the hypothetical individual living in a health state described using a selection of 60 of the 235 health states with their lay descriptions from the GBD disability weights (DW) surveys, reflecting the full range of severity. Each of these health states has a measured disability weight associated with it on a zero to one scale. In total, we collected 1,980 usable responses. To deal with heterogeneity in responses, we excluded from the statistical analysis responses that were more than two median absolute deviations from the median for each health state. After correcting for outliers, the rank order correlation between SF-12 scores for the hypothetical individuals in each health state characterized by the lay description with the measured disability weight was -0.815. The health states served as random effect groups, such that the composite score would be equal to the intercept plus the

random effect estimated for that health state, or

$$DW_i = \alpha + U_{health\ state}.$$

The final relationship between SF-12 score and disability weight is shown below:

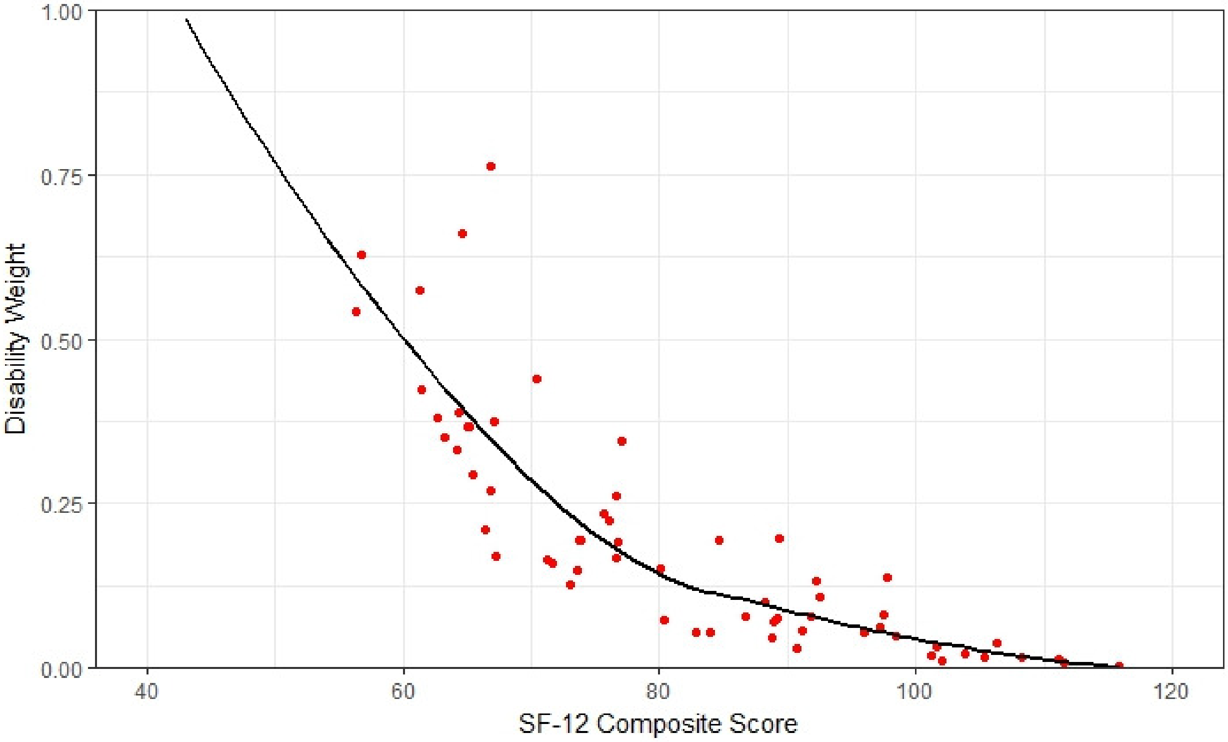

To generate a smooth mapping from SF-12 combined scores to the GBD disability weight space, we used LOESS regression on the random effects for each health state. Because disability weights are defined in the range from 0 to 1, we truncated the function at a combined SF-12 score of 116.36 (any combined score above this level was set to 0) and truncated the function at 42.7 so that any combined score less than that value was set to 1. All SF-12 survey data were thus transformed into disability weight space.

The second stage of the analysis was to build models predicting the transformed SF-12 scores as a function of the number of conditions suffered by each individual. First, variable selection was performed using LASSO regression to penalize the regression coefficients of highly correlated conditions. The tuning parameter, λ, controls the strength of the least-squares penalty. When λ=0, LASSO regression returns the same results as ordinary least-squares regression. Higher values of λ impose a stronger penalty and constrain a greater number of model parameters to 0. A 10-fold cross-validation was used to find the value of the λ that minimized the mean cross-validated error. This process resulted in a λ value of 0.0013 and eliminated 10 conditions from the analysis. Transformed SF-12 scores into the disability weight scale for the remaining 190 conditions were then modelled for each measure *m* of each individual *i* over *n* total conditions in the survey, as follows:

$$logit(DW)_{im} = \beta_0 + \beta_1 Condition1_{im} + \cdots + \beta_n Condition\ n_{im}$$

This equation effectively assumes that comorbid conditions act to change SF-12 scores in a multiplicative fashion rather than an additive fashion.

To estimate the comorbidity-corrected effect of each condition (i.e., in isolation) on total disability, we compared the predicted disability weight without the condition of interest ("counterfactual DW") with the predicted disability weight including the condition of interest. Following the multiplicative comorbidity equation, the joint effect can be written:

$$Condition\ specificDW = 1 - \frac{1 - predictedDW_m}{1 - counterfactualDW_m}$$

The mean of this condition-specific effect over all observations is the population marginal effect of a condition.

Using the model above, we estimate a counterfactual disability weight – the total individual disability weight excluding the effect of the condition of interest. We compared the observed distribution of functional health status with this counterfactual distribution to determine the marginal effect of the condition of interest. In other words, we estimate the health state for each individual and for each condition as the cumulative individual weight minus the effects of all comorbid conditions.

$$Health\ state\ DW = 1 - \frac{1 - individual\ cumulativeDW_m}{1 - counterfactualDW_m}$$

The estimation strategy for health state-specific severity distributions where there are multiple severity categories involved binning individuals' weights into severity cutoffs (e.g., mild, moderate, and severe) for which disability weights were derived. These bins were defined using results from the GBD Disability Weights Studies[23] for conditions which had multiple health states defined. Cutoffs were taken as the midpoints between levels of health state and cases distributed into severity bins accordingly. Cases were considered asymptomatic if the counterfactual weight was equal to or exceeded the individual cumulative weight.

2.6 Disability weights

To compute YLDs for a particular health outcome in a given population, the number of people living with that outcome is multiplied by a disability weight that represents the magnitude of health loss associated with the outcome. Disability weights are measured on a scale from 0 to 1, with 0 implying a state that is equivalent to full health and 1 a state equivalent to death.

Disability weights used in GBD studies prior to GBD 2010 have been criticized for the method used (person trade-off), the small elite panel of international public health experts who determined the weights and the lack of consistency over time as the GBD cause list expanded and additional disability

weights from a study in the Netherlands[24] were added or others derived by ad-hoc methods.

GBD 2010 disability weights measurement study

For GBD 2010, a primary data collection effort focused on measuring health loss rather than welfare loss using a standardized approach of simple comparison questions directed to the general public across diverse communities.

Multicountry household surveys were conducted between Oct 28, 2009 and June 23, 2010 in five countries (Bangladesh, Indonesia, Peru, Tanzania, and the US) selected to provide diversity across culture, language and socioeconomic status.

Personal face-to-face computer-assisted interviews were conducted for all household surveys with the exception of the survey in the US which was conducted as computer-assisted telephone interviews. Households were randomly selected using a multistage stratified sampling design where the probability of selection was proportional to the population size. In all cases, samples were designed to be representative for a given geographical area with national representation in the case of the US.

For every contacted household, an adult respondent aged 18 years or older was randomly selected by the survey program using the Kish approach. For face to face interviews, up to three visits were made to selected households to establish contact. When a respondent was identified, up to three return visits were made in order to do the survey at a time when the respondent was available. For the US telephone surveys, repeat calls were made up to seven times.

A web-based survey was posted at a dedicated URL between July 26, 2010 and May 16, 2011. The survey was initially available in English with subsequent availability in Spanish and Mandarin. Recruitment of respondents occurred through several channels, such as news items and editorials in scientific journals, announcements at scientific meetings, postings on websites of institutions participating in the GBD, social networking and communication mobilization channels as well as direct contact with individuals and groups with known global health interests by tapping into the professional networks of the study investigators and their colleagues. Participants in the web-based survey were required to be aged 18 or older. Household surveys obtained oral informed consent from all participants; written informed consent was obtained from participants in the web survey. Ethical review board approval was obtained from each household survey site and at the University of Washington, Seattle, WA.

Standardized survey instruments were developed to obtain comparative assessments of the full array of disease and injury sequelae, parsimoniously captured in 220 unique health states. Lay descriptions of health states formed the basis for all comparisons. These descriptions used simple, non-clinical vocabulary that emphasized the major functional consequences and symptoms associated with each health state. Development of these descriptions involved an iterative process of detailed consultation with experts participating in the GBD 2010 study with the goal of both capturing the most relevant details of each health state while avoiding ambiguity and ensuring consistency. Where possible, health states were grounded in standard clinical classifications systems, for example, the Canadian Cardiovascular Society grading scale was referenced for descriptions of stages of angina,[25] while the New York Heart

Association functional classification was referenced for severity of heart failure.[26] Pilot testing indicated that the lay descriptions in face-to-face interviews should not exceed 30 words.

A paired comparison question formed the basis of all surveys. The questions in the survey were framed with the following statement, "A person's health may limit how well parts of his body or mind work. As a result, some people are not able to do all of the things in life that others may do, and some people are more severely limited than others. I am going to ask you a series of questions about different health problems. In each question, I will describe two different people…" Descriptions of two hypothetical people, each with a particular health state, were presented to respondents who were then asked which person they regarded as the healthier. Health pairs in all surveys were selected by a randomizing computer algorithm. In the five household surveys, paired comparisons were presented for a subset of 108 health states pertaining to chronic conditions. The framing of chronic and acute conditions is different as they were presented as causing life-long or temporary health loss. We chose to only field health states that could be framed as lasting a lifetime in the household surveys as we hypothesized that presenting differently framed comparisons would be difficult to convey in face-to-face interviews. In the web survey we considered this more feasible as respondents could read and refer to the framing of the question for each pair-wise comparison. All 220 health states were thus evaluated in the web survey.

In addition, the web survey included questions relating to population health and health programs specifically – such as "Imagine two different health programs. The first program prevented 1,000 people from getting an illness that causes rapid death. The second program prevented 2,000 people from getting an illness that is not fatal but causes lifelong health problems resulting in moderate to severe disability. Which program would you say produced the greater overall health benefits?" This information was used to anchor the results from the pair-wise comparisons on the 0–1 disability weight scale.

GBD 2013 European disability weights measurement study

The GBD 2010 disability weights were critically dependent on the ways that outcomes were described to survey respondents. Descriptions for health states were designed to balance validity and parsimony and this necessarily meant that some details of different health states had to be omitted. As lay descriptions were developed collaboratively through individual expert groups organized around a particular set of health issues – some amount of variability in language and detail inevitably occurred. Criticisms and suggestions for improvement came from a number of commentators on the GBD 2010 disability weights measurement study.[27–29]

The GBD 2013 Study expanded the list of disease and injury causes and sequelae which were mapped to 235 unique health states. Additional data for the European Disability Weights Measurement Study were collected between September 23, 2013 and November 11, 2013 in Hungary, Italy, the Netherlands and Sweden. The initiation of these surveys was connected to a project sponsored by the European Centre for Disease Prevention and Control (the Burden of Communicable Diseases in Europe project).[30] The four selected countries were chosen to be representative of the four regions of Europe (east, south, middle, and north) in terms of age, sex and education of the respondents. Respondents were recruited from standing internet panels in each country on the basis of quota sampling with reference to age, sex and education in such a way as to maintain population representativeness of these characteristics. Eligible participants were aged 18–65 and were preselected in the case of the Netherlands, where age, sex and

education of respondents were already known, or in the case of the other three countries, invited to participate via a web-link and then selected on the basis of their individual characteristics.

The protocol for the European disability weights measurement study followed the protocol that was developed and implemented in the GBD 2010 disability weights measurement study. Lay descriptions for some health states that lacked mention of an important symptom or for which consistency of wording across different levels of severity had been noted were reworded. The European disability weights measurement study included 255 health states, of which 183 were used in the analyses of GBD 2013. Those 183 consisted of 135 of the 220 health states that were included in the European disability weights measurement study with unmodified lay descriptions; 30 from GBD 2010 for which alternative lay descriptions were included. Disability weights were estimated for additional sequelae that were incorporated into GBD 2013 but had not been included in GBD 2010.

Finding high correlation in resulting disability weight values between the country surveys and the web survey, we analyzed the results of all surveys together. We ran probit regression analyses on the answers to the pair-wise comparison questions, with dummies for each health state with a value of 1 for the first state in a pair, –1 for the second of a pair being chosen, and 0 for all states other than the pair being considered. This method formalizes the intuition that if two health states in a pair produce similar health loss, the answers are likely to be evenly split; a pair of health states with very different health loss, will get many more responses favoring one over the other. The statistical methods infer the distances between values attached to different health states based on the frequencies of responses to the paired comparisons. A second analytic step is needed to anchor the resulting estimates onto the 0–1 disability weights scale. We anchored results from the probit regression analysis onto the 0–1 scale using population health equivalence data from the GBD 2010 web survey using a linear regression of the probit coefficients from the analysis of paired comparisons on the logit-transformed disability weight estimates derived from interval regression of the population health equivalence responses. Using numerical integration, we then estimated mean values for disability weights on the natural 0–1 scale. Uncertainty was estimated by bootstrapping with 1000 samples.

A complete listing of the lay descriptions and values for the 234 health states (including combined health states) used in GBD 2017 is provided in Appendix Table 5.

2.7 Comorbidity correction (COMO)

The final stage in the estimation of YLDs is a micro-simulation, which adjusts for comorbidity. We refer to this micro-simulation process as "COMO." For GBD 2017, we estimated the co-occurrence of different diseases by simulating 40,000 individuals in each location-age-sex-year combination as exposed to the independent probability of having any of the sequelae included in GBD 2017 based on disease prevalence. We tested the contribution of dependent and independent comorbidity in the US MEPS data, and found that independent comorbidity was the dominant factor even though there are well-known examples of dependent comorbidity, i.e., clustering of conditions such as diabetes and stroke or anxiety and alcohol use disorders. Age was the main predictor of comorbidity such that age-specific microsimulations accommodated most of the required comorbidity correction.[31]

The two components necessary for the computation of YLDs, prevalence of each disease sequelae and disability weights, are the two inputs into COMO. The prevalence values are primarily produced using DisMod-MR 2.1. The disability weights have been described above.

The micro-simulation, as performed for each age-sex-location-year, can best be represented as a four-step process. First, simulants are exposed to independent probabilities of having each sequela, where the probability is equal to the prevalence estimate. For each simulant, the probability of having a disease sequela is equal to the estimated prevalence from that draw from the uncertainty distribution. Each simulant is determined to have or not have the disease sequelae based on a draw from a binomial distribution. From this simulation, simulants end up having from none to multiple disease sequelae. Second, the disability weight for each simulant is estimated based on the disease sequelae that they have acquired. The formula for the cumulative disability weight for a simulant is one minus the multiplicative sum of one minus each disability weight present:

$$Simulant\ DW_l = 1 - \prod_{k=i}^{j}(1 - DW_k)$$

where the DW_k is the disability weight for the kth disease sequela that the simulant *l* has acquired. Once the simulant disability weight is computed, the disability weight attributable to each sequela for the simulant is calculated using the following formula:

$$ADW_{lk} = \frac{DW_k}{\sum_{k=i}^{k=j} DW_k} * Simulant\ DW_l$$

where ADW_{lk} is the attributable DW for disease sequela *k* in simulant *l*; DW_k is the disability weight for disease sequela *k*, and simulant DW_l is the disability weight for simulant *l* from the combination of all sequelae that they have acquired. This formula apportions the overall simulant disability weight to each condition in proportion to the disability weight of each condition in isolation.

Finally, YLDs per capita in an age-sex-country-year are computed by taking the sum of the attributable disability weights for a disease sequela across simulants.

$$YLD\ Rate_k = \frac{\sum_{l=1}^{n} ADW_{lk}}{n}$$

The actual number of YLDs from disease sequela *k* in an age-sex-location-year is then computed as the YLD rate *k* times the appropriate age-sex- location-year population.

By repeating the simulation process for each age-sex-country-year 1,000 times, the uncertainty in the prevalence of each disease sequela and the disability weight is propagated into the final comorbidity corrected YLD results. We selected 40,000 simulants for each age-sex- location-year group on the basis of simulation testing, which has shown that results are stable for YLDs at this number of simulants even in the younger age groups when prevalence is relatively low. Mean results for YLDs which reflect 40 million simulants (40,000 simulants multiplied by 1,000 iterations to capture uncertainty) are very stable in each

age-sex-location-year. For any given location-year-age-sex group, sequelae with prevalence of less than one in 20,000 were excluded from the microsimulation.

2.8 YLD computation, uncertainty, and residual YLDs

For GBD 2017, we computed YLDs by sequela as prevalence multiplied by the disability weight for the health state associated with that sequela. The uncertainty ranges reported around YLDs incorporates uncertainty in prevalence and uncertainty in the disability weight. To do this, we take the 1,000 samples of comorbidity-corrected YLDs and 1,000 samples of the disability weight to generate 1,000 samples of the YLD distribution. We assume no correlation in the uncertainty in prevalence and disability weights. The 95% uncertainty interval is reported as the 25th and 975th values of the distribution. Uncertainty intervals for YLDs at different points in time (1990, 1995, 2000, 2005, 2010, and 2016) for a given disease or sequela are correlated because of the shared uncertainty in the disability weight. For this reason, changes in YLDs over time can be significant even if the uncertainty intervals of the two estimates of YLDs largely overlap as significance is determined by the uncertainty around the prevalence estimates.

Residual YLDs

Despite expanding our list of causes and sequelae in successive GBD iterations, many diseases remain for which we do not explicitly estimate disease prevalence and YLDs. Less common diseases and their sequelae were included in 35 residual categories (Appendix Table 6). For 22 of these residual categories, epidemiological data on incidence or prevalence were available and so these were modelled accordingly. For 13 residual categories, epidemiological data on incidence and prevalence were not available but sufficient cause of death data allowed for cause of death estimates. For these residual categories, we estimated YLDs by multiplying the residual YLL estimates by the ratio of YLDs to YLL from the estimates level 3 causes in the same disease category that were explicitly modelled. This scaling was undertaken for each country-sex-year. This approach made the simplifying assumption that the residual diseases caused disability proportionate to the ratio of disability to mortality in explicitly modelled diseases. We did not include causes with large disability but no or little mortality in estimating these ratios. For example, we estimated the YLDs from other neurological disorders from the YLD to YLL ratios for dementia, multiple sclerosis, and Parkinson's disease, but did not include the YLDs from headaches and epilepsy in the ratio.

2.9 Birth prevalence

A number of conditions are present at birth and quantifying them is important to fully describing the epidemiology of diseases within populations (Appendix Table 7). These include many conditions included in the GBD cause group of neonatal disorders, infections that are transmitted from mother to child either transplacentally or during birth, and congenital birth defects arising either *de novo* or as the result of maternal exposures. While included in the underlying models informing from previous GBD iterations, we have developed a system for reporting them for the first time in GBD 2017.

Mathematically (i.e., in the models), conditions present at birth are equivalent to "birth prevalence."

However, we report these as “incidence” in recognition of how GBD defines incidence as a new case of a disease or injury entering the population. To process these results for publication in GBD, we used a three step process. First, the number of cases at birth was calculated as birth prevalence rate multiplied by number of live births for each location, sex, and year. Second, the number of cases present at birth were summed with incident cases during the early neonatal period (calculated as 0-6 days incidence rate times 0-6 days population), and early neonatal incidence rate was recalculated by re-dividing by 0-6 days population. Third, incidence rates for aggregate age groups were re-calculated using the revised incidence figures for the early neonatal period.

Causes included in reporting are all of those where birth prevalence has been estimated in GBD 2017 as part of existing modelling processes (Appendix Table 7). While extensive, this list should not be considered to be exhaustive of all of the conditions that can be present at birth. Future efforts in GBD in this arena will focus on identifying and comprehensively including all conditions present at birth, including revision of model frameworks as necessary. These efforts will also be facilitated by continuing improvements in the resolution of epidemiologic estimates of disease burden during pregnancy. These efforts are also expected to themselves facilitate subsequent analyses derived from GBD that evaluate how maternal interventions, including pregnancy surveillance, can influence patterns of neonatal, infant, and child health.

2.10 Socio-demographic Index (SDI) analysis and epidemiological transition

The Socio-demographic Index (SDI) is a composite indicator of development status strongly correlated with health outcomes. In short, it is the geometric mean of 0 to 1 indices of total fertility rate under the age of 25 (TFU25), mean education for those aged 15 and older (EDU15+), and lag distributed income (LDI) per capita.

a. Development of revised SDI indicator

SDI was originally constructed for GBD 2015 using the Human Development Index (HDI) methodology, wherein a 0 to 1 index value was determined for each of the original three covariate inputs (total fertility rate in ages 15 to 49, EDU15+, and LDI per capita) using the observed minima and maxima over the estimation period to set the scales.

In response to feedback from collaborators and the evolution of the GBD, we have refined the indicator with each GBD cycle. For GBD 2017, in conjunction with our expanded estimation of age-specific fertility, we chose to replace the total fertility rate as one of the three component indices with the total fertility rate under 25 (TFU25). The TFU25 provides a better measure of women’s status in society, as it focuses on ages where childbearing disrupts the pursuit of education and entrance into the workforce.

During GBD 2016 we moved from using relative index scales to absolute scales to enhance the stability of SDI’s interpretation over time, as we noticed that the measure was highly sensitive to the addition of subnational units that tended to stretch the empirical minima and maxima. We selected the minima and maxima of the scales by examining the relationships each of the inputs had with life expectancy at birth and under-5 mortality and identifying points of limiting returns at both high and low values, if they occurred prior to theoretical limits (e.g., a TFU25 of 0).

Thus, an index score of 0 represents the minimum level of each covariate input past which selected health

outcomes can get no worse, while an index score of 1 represents the maximum level of each covariate input past which selected health outcomes cease to improve. As a composite, a location with an SDI of 0 would have a theoretical minimum level of development relevant to these health outcomes, while a location with an SDI of 1 would have a theoretical maximum level of development relevant to these health outcomes.

We summarize the final scales for GBD 2017 in the table below.

Input	Lower Bound	Upper Bound
TFU25	0	3
LDI per capita	250 USD (5.52 log USD)[b]	60,000 USD (11.00 log USD)
EDU15+	0 years	17 years

[b] The minimum for the LDI scale was originally set at the theoretical limit of 0 USD, as we did not observe an asymptotic relationship between log(LDI) and E_0 or 5q0 at lower values of log(LDI). Empirically, however, we also did not observe an LDI below 350 USD (5.86 log USD) for the estimation period 1970-2016. In log-space, this meant that approximately half of our scale was not being utilized, compressing the observed variation in LDI and diminishing its meaningful contribution to SDI. Accordingly, we set the lower limit on LDI to 250 USD (5.52 log USD) to ensure we were fully utilizing the range of the scale to capture its variation across space and time, as is the case with the other two inputs.

Using scales described above, we computed the index scores underlying SDI as follows:

$$I_{Cly} = \frac{(C_{ly} - C_{low})}{(C_{high} - C_{low})}$$

Where I_{cly} – the index for covariate *C*, location *l*, and year *y* – is equal to the difference between the value of that covariate in that location-year and the lower bound of the covariate divided by the difference between the upper and lower bounds for that covariate. If the values of input covariates fell outside the upper or lower bounds (e.g. LDI per capita greater than 60,000 USD), they were mapped to the respective upper or lower bounds. We also note that the index value for TFU25 was computed as $1 - I_{TFU25ly}$, as lower TFU25s correspond to higher levels of development, and thus higher index scores. For GBD 2017 we expanded the computation of SDI to 890 national and subnational locations spanning the time period 1950-2017.

The composite Socio-Demographic Index is the geometric mean of these three indices for a given location-year. The cutoff values used to determine quintiles for analysis were then computed using country-level estimates of SDI for the year 2017, excluding countries with populations less than 1 million.

Example calculation

Below we present the calculation of SDI for Mexico in the year 2010:

$$TFU25 = 1.09;\ Mean\ educ\ yrs\ pc = 8.23;\ lnLDI = 9.60$$

$$I_{TFU25} = 1 - \frac{1.09 - 0}{3 - 0} = .637$$

$$I_{Educ} = \frac{8.23 - 0}{17 - 0} = .484$$

$$I_{lnLDI} = \frac{9.60 - 5.52}{11.00 - 5.52} = .744$$

$$SDI = \sqrt[3]{I_{TFU25} * I_{Educ} * I_{lnLDI}} = \sqrt[3]{.637 * .484 * .744} = .611$$

$$I_{lnLDI} = \frac{9.58 - 5.52}{11.00 - 5.52} = .741$$

$$SDI = \sqrt[3]{I_{TFR} * I_{Educ} * I_{lnLDI}} = \sqrt[3]{.855 * .543 * .741} = .701$$

SDI location groupings and SDI values are displayed in Appendix Tables 8 and 9.

Section 3. GBD cause list

The GBD cause and sequelae list is organized hierarchically (see Appendix Table 3) to accommodate different purposes and needs of various users.

The first two levels aggregate causes into general grouping. At Level 1 there are three cause groups: communicable, maternal, neonatal, and nutritional diseases (Group 1 diseases); non-communicable diseases (Group 2); and injuries (Group 3). These Level 1 aggregates are subdivided at Level 2 of the hierarchy into 21 cause groupings (e.g., neonatal disorders, neurological disorders, and transport injuries). The disaggregation into Levels 3 and 4 contains the finest level of detail for causes captured in GBD 2017. The greatest detail available for some causes, such as anxiety disorders or rheumatoid arthritis, is at Level 3 of the hierarchy while other specific causes are at Level 4 of the hierarchy with an aggregate category at Level 3 (for example, depressive disorders at Level 3 which encompasses major depressive disorders and dysthymia at Level 4). Sequelae of diseases and injuries are organized at Levels 5 and 6 of the hierarchy. In GBD, sequelae are defined as distinct, mutually exclusive, categories of health consequences that can be directly attributed to a cause. For example, both neuropathy and blindness due to diabetic retinopathy are sequelae of diabetes; stroke and ischaemic heart disease are not, as these consequences cannot be categorically ascribed to diabetes in an individual despite good evidence for increased risk of these outcomes. The finest detail for all sequelae estimated in GBD is at Level 6 and is aggregated into summary sequelae categories (Level 5) for causes with large numbers of sequelae. Examples include the grouping of the infectious disease episodes and long-term sequelae of meningitis, and the grouping of 47 injury sequelae into seven summary categories (for example all fractures, spinal cord lesions, and head injuries). Sequelae in GBD are mutually exclusive and collectively exhaustive and thus our YLD estimates at each level of the hierarchy sum to the total of the level above. Prevalence and incidence aggregation is estimated at the level of individuals who may have more than one sequela or disease, and therefore are not additive.

The GBD cause list continues to evolve to reflect the policy relevance, public health, and medical care importance of the causes of major losses of health. The cause and sequelae list expanded following input from the Scientific Council and GBD collaborator network. The cause list has expanded from 327 causes with nonfatal estimates in GBD 2016 to 354 causes in GBD 2017. As in GBD 2016, we made no estimates for YLDs for just five causes, either because no disability is possible, as is the case with sudden infant death syndrome; because disability may occur rarely but at levels too low for accurate estimation given the data, as for aortic aneurysm; or because the disability is captured by the complicating causes that led to that cause of death, as for indirect maternal deaths, late maternal deaths, and maternal deaths aggravated by HIV/AIDS. Appendix Table 4 provides a list of International Classification of Diseases version 9 (ICD-9) and International Classification of Diseases version 10 (ICD-10) codes for all GBD causes and the nature of injury categories.

Section 4. Nonfatal cause-specific estimation process

- HIV/AIDS
- Sexually transmitted diseases excluding HIV
- Tuberculosis
- Lower respiratory infections
- Upper respiratory infections
- Otitis media
- Diarrheal diseases
- Typhoid and paratyphoid
- Invasive Non-typhoidal Salmonella (iNTS)
- Other intestinal infectious diseases
- Malaria
- Chagas disease
- Visceral leishmaniasis
- Cutaneous and mucocutaneous leishmaniasis
- African trypanosomiasis
- Schistosomiasis
- Cysticercosis
- Cystic echinococcosis
- Lymphatic filariasis
- Onchocerciasis
- Dengue
- Yellow fever
- Rabies
- Ascariasis
- Trichuriasis
- Hookworm disease
- Food-borne trematodiases
- Leprosy
- Ebola virus disease
- Zika virus disease
- Guinea worm disease
- Other neglected tropical diseases
- Meningitis
- Encephalitis
- Diphtheria
- Whooping cough
- Tetanus
- Measles
- Varicella and herpes zoster
- Acute hepatitis
- Other unspecified infectious diseases
- Maternal disorders

- Maternal obstructed labor and uterine rupture
- Neonatal preterm birth
- Neonatal encephalopathy due to birth asphyxia and trauma
- Neonatal sepsis and other neonatal infections
- Hemolytic disease and other neonatal jaundice
- Other neonatal disorders
- Protein-energy malnutrition
- Iodine deficiency
- Vitamin A deficiency
- Other nutritional deficiencies
- Cancer
- Rheumatic heart disease
- Ischemic heart disease
- Ischaemic Stroke, Intracerebral Haemorrhage, and Subarachnoid Haemorrhage
- Non-rheumatic valvular heart disease
- Myocarditis
- Atrial fibrillation and flutter
- Peripheral artery disease
- Endocarditis
- Other cardiovascular and circulatory diseases
- Chronic obstructive pulmonary disease
- Pneumoconiosis
- Asthma
- Interstitial lung disease and pulmonary sarcoidosis
- Other chronic respiratory diseases
- Cirrhosis and other chronic liver diseases
- Non-alcoholic fatty liver disease/Non-alcoholic steatohepatitis (NFLD/NASH)
- Peptic ulcer disease
- Gastritis and duodenitis
- Gastroesophageal reflux disease
- Appendicitis
- Paralytic ileus and intestinal obstruction
- Inguinal, femoral, and abdominal hernia
- Inflammatory bowel disease
- Vascular intestinal disorders
- Gallbladder and biliary diseases
- Pancreatitis
- Other digestive diseases
- Alzheimer’s disease and other dementias
- Parkinson’s disease
- Multiple sclerosis
- Motor neuron disease
- Migraine

- Tension-type headache
- Other neurological disorders
- Schizophrenia
- Major depressive disorder
- Dysthymia
- Bipolar disorder
- Anxiety disorders
- Anorexia nervosa
- Bulimia nervosa
- Autistic spectrum disorders
- Attention-deficit/hyperactivity disorder
- Conduct disorder
- Other mental disorders
- Alcohol use disorders
- Fetal alcohol syndrome
- Opioid use disorders
- Cocaine use disorders
- Amphetamine use disorders
- Cannabis use disorders
- Other drug use disorders
- Diabetes mellitus
- Chronic kidney disease
- Acute glomerulonephritis
- Dermatitis
- Psoriasis
- Cellulitis
- Pyoderma
- Scabies
- Fungal skin diseases
- Viral skin diseases
- Acne vulgaris
- Alopecia areata
- Pruritus
- Urticaria
- Decubitus ulcer
- Other skin and subcutaneous diseases
- Other sense organ diseases
- Rheumatoid arthritis
- Osteoarthritis
- Low back pain
- Neck pain
- Gout
- Other musculoskeletal disorders
- Congenital anomalies
- Urinary tract infections

- Urolithiasis
- Benign prostatic hyperplasia
- Other urinary diseases
- Uterine fibroids
- Polycystic ovarian syndrome
- Endometriosis
- Genital prolapse
- Premenstrual syndrome
- Other gynecological conditions
- Hemoglobinopathies and hemolytic anemias
- Endocrine, metabolic, blood, and immune disorders
- Oral disorders
- Injuries
- Sexual violence
- Anemia
- Epilepsy
- GBS
- Hearing loss
- Heart failure
- Infertility
- Development intellectual disability
- Pelvic inflammatory disease
- Blindness and vision impairment

Section 5. References

1 GBD Compare | IHME Viz Hub. http://vizhub.healthdata.org/gbd-compare (accessed June 24, 2016)

2 Disease registry | GHDx. http://ghdx.healthdata.org/data-type/disease-registry (accessed June 24, 2016).

3 An Integrative Metaregression Framework for Descriptive Epidemiology (Publications on Global Health, Institute for Health Metrics and Evaluation): Abraham D. Flaxman, Theo Vos, Christopher J.L. Murray, Patricia Kiyono: 9780295991849: Amazon.com: Books. https://www.amazon.com/Integrative-Metaregression-Descriptive-Epidemiology-Publications/dp/0295991844 (accessed June 24, 2016).

4 A Haagsma J. Posttraumatic Stress Disorder Following Injury: Trajectories and Impact on Health-Related Quality of Life. *J Depress Anxiety* 2013. DOI:10.4172/2167-1044.S4-002.

5 Polinder S, van Beeck EF, Essink-Bot ML, *et al.* Functional outcome at 2.5, 5, 9, and 24 months after injury in the Netherlands. *J Trauma* 2007; 62: 133–41.

6 Sullivan PW, Ghushchyan V. Mapping the EQ-5D Index from the SF-12. *Med Decis Mak Int J Soc Med Decis Mak* 2006; 26: 401–9.

7 Burstein R, Fleming T, Haagsma J, Salomon JA, Vos T, Murray CJ. Estimating distributions of health state severity for the global burden of disease study. *Popul Health Metr* 2015; 13: 31.

8 Global, regional, and national age-sex specific all-cause and cause-specific mortality for 240 causes of death, 1990-ReadReadyRExp2013: a systematic analysis for the Global Burden of Disease Study 2013. *Lancet* 2015; 385: 117–71.

9 Haagsma JA, Graetz N, Bolliger I, *et al.* The global burden of injury: incidence, mortality, disability-adjusted life years and time trends from the Global Burden of Disease study 2013. *Inj Prev* 2015; : injuryprev-2015-041616.

10 Pickelsimer EE, Selassie AW, Gu JK, Langlois JA. A population-based outcomes study of persons hospitalized with traumatic brain injury: operations of the South Carolina Traumatic Brain Injury Follow-up Registry. *J Head Trauma Rehabil* 2006; 21: 491–504.

11 Mackenzie EJ, Rivara FP, Jurkovich GJ, *et al.* The National Study on Costs and Outcomes of Trauma. *J Trauma* 2007; 63: S54-67-86.

12 van Loey NE, van Beeck EF, Faber BW, van de Schoot R, Bremer M. Health-Related Quality of Life After Burns: A Prospective Multicentre Cohort Study With 18 Months Follow-Up. *J Trauma* 2011; published online Oct 24. DOI:10.1097/TA.0b013e3182199072.

13 Medical Expenditure Panel Survey Home. https://meps.ahrq.gov/mepsweb/ (accessed June 25, 2016).

14 Burstein R, Fleming T, Haagsma J, Salomon JA, Vos T, Murray CJ. Estimating distributions of health state severity for the global burden of disease study. *Popul Health Metr* 2015; 13: 31.

15 Clark DV, Kibuuka H, Millard M, *et al.* Long-term sequelae after Ebola virus disease in Bundibugyo, Uganda: a retrospective cohort study. *Lancet Infect Dis* 2015; 15: 905–12.

16 Qureshi AI, Chughtai M, Loua TO, *et al.* Study of Ebola Virus Disease Survivors in Guinea. *Clin Infect Dis* 2015; 61: 1035–42.

17 Rowe AK, Bertolli J, Khan AS, *et al.* Clinical, Virologic, and Immunologic Follow-Up of Convalescent Ebola

Hemorrhagic Fever Patients and Their Household Contacts, Kikwit, Democratic Republic of the Congo. *J Infect Dis* 1999; 179: S28–35.

18 Bwaka MA, Bonnet M-J, Calain P, *et al.* Ebola Hemorrhagic Fever in Kikwit, Democratic Republic of the Congo: Clinical Observations in 103 Patients. *J Infect Dis* 1999; 179: S1–7.

19 PRO-ACT - HOME. https://nctu.partners.org/ProACT/ (accessed May 26, 2016).

20 Medical Expenditure Panel Survey Home. https://meps.ahrq.gov/mepsweb/ (accessed June 25, 2016).

21 NIAAA Publications. http://pubs.niaaa.nih.gov/publications/arh29-2/74-78.htm (accessed June 25, 2016).

22 Statistics c=AU; o=Commonwealth of A ou=Australian B of. Main Features - Main Features. 1998; published online March 12. http://www.abs.gov.au/ausstats/abs@.nsf/ProductsbyReleaseDate/D5A0AC778746378FCA2574EA00122887?OpenDocument (accessed June 25, 2016).

23 Salomon JA, Haagsma JA, Davis A, *et al.* Disability weights for the Global Burden of Disease 2013 study. *Lancet Glob Health* 2015; 3: e712–23.

24 Stouthard M, Essink-Bot ML, Bonsel G, Barendregt J, Kramers P. Disability weights for diseases in the Netherlands. *Eramus Univ Rotterdam* 1997.

25 Campeau L. The Canadian Cardiovascular Society grading of angina pectoris revisited 30 years later. *Can J Cardiol* 2002; 18: 371–9.

26 Nomenclature and criteria for diagnosis of diseases of the heart and great vessels / the Criteria Committee of the New York Heart Association. - Version details. Trove. http://trove.nla.gov.au/version/13288061 (accessed June 25, 2016).

27 Nord E. Disability weights in the Global Burden of Disease 2010: Unclear meaning and overstatement of international agreement. *Health Policy* 2013; 111: 99–104.

28 Taylor HR, Jonas JB, Keeffe J, *et al.* Disability weights for vision disorders in Global Burden of Disease study. *The Lancet* 2013; 381: 23.

29 Voigt K, King NB. Disability weights in the global burden of disease 2010 study: two steps forward, one step back? *Bull World Health Organ* 2014; 92: 226–8.

30 Kretzschmar M, Mangen M-JJ, Pinheiro P, *et al.* New methodology for estimating the burden of infectious diseases in Europe. *PLoS Med* 2012; 9: e1001205.

31 Vos T, Flaxman AD, Naghavi M, *et al.* Years lived with disability (YLDs) for 1160 sequelae of 289 diseases and injuries 1990–2010: a systematic analysis for the Global Burden of Disease Study 2010. *The Lancet* 2012; 380: 2163–96.

32 Gupta PD. A general method of decomposing a difference between two rates into several components. *Demography* 1978; 15: 99–112.

HIV/AIDS

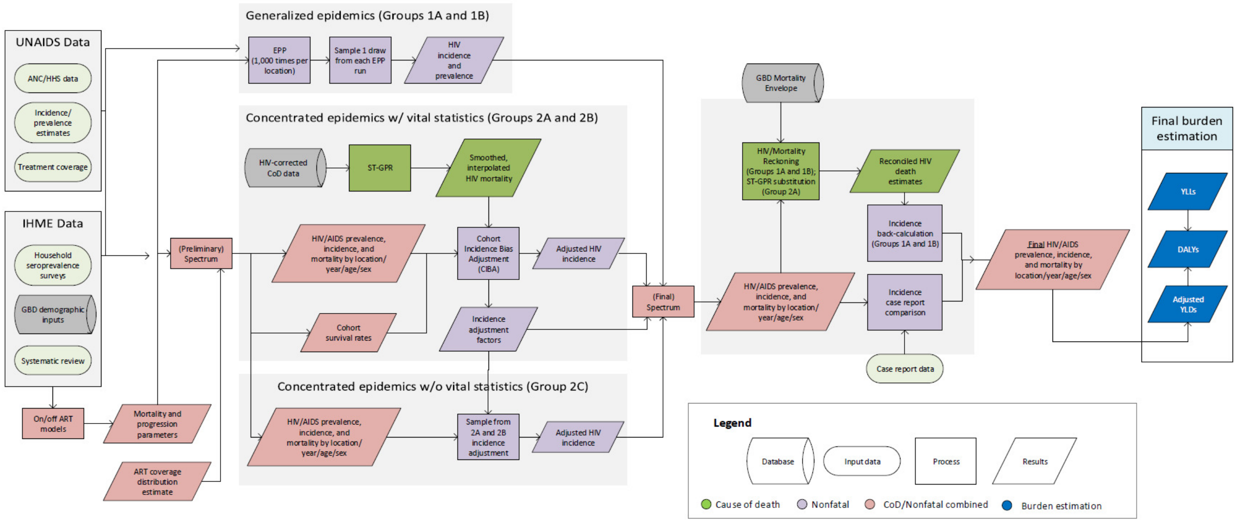

Case definition

Infection with the human immunodeficiency virus (HIV) causes influenza-like symptoms during the acute period following infection and can lead to acquired immunodeficiency syndrome (AIDS) if untreated. HIV attacks the immune system of its host, leaving infected individuals more susceptible to opportunistic infections like tuberculosis. Although there are two different subtypes of HIV, HIV-1 and HIV-2, no distinction is made in our estimation process or presentation of results. For HIV, ICD 10 codes are B20-B24, C46-C469, D84.9; ICD 9 codes are 042-044, 112-118 (after 1980), 130 (after 1980), 136.3-136.8 (after 1980), 176.0-176.9 (after 1980), 279 (after 1980); and ICD9 BTL codes are B184-B185.

Input data

Household seroprevalence surveys
Geographically representative HIV seroprevalence survey results were used as inputs to the model for countries with generalised HIV epidemics where available.

GBD demographic inputs
Location-specific population, fertility, and HIV-free survival rates from GBD 2017 and migration data from UNAIDS were used as inputs in modelling all locations.

UNAIDS data
The files compiled by UNAIDS for their HIV/AIDS estimation process were our main source of data for producing estimates of HIV burden. These files are typically country-specific and contain both demographic data (population, fertility, migration, and HIV-free survival rates) and HIV-specific information. In all cases except migration, we substituted in our own, internally consistent demographic estimates. The HIV-specific information includes what is needed to run both the Spectrum and Estimation and Projection Package (EPP) models. Spectrum requires the following input data: AIDS mortality among people living with HIV with and without ART, CD4 progression among people living with HIV not on-ART, ART coverage among adults and children, Cotrimoxazole coverage among children,

coverage of breastfeeding among women living with HIV, prevention of mother-to-child transmission coverage, and CD4 thresholds for treatment eligibility. EPP uses many of the same assumptions as Spectrum but fits a simpler model to HIV prevalence data from surveillance sites and representative surveys. Antenatal care, incidence, prevalence, and treatment coverage data from UNAIDS were used in modelling for all locations. We extracted all of these data from the proprietary format used by UNAIDS.

For GBD 2017, we received updated national-level files for 97 countries and updated subnational-level files for eight countries. For many of the GBD locations not covered by these files, we had UNAIDS files from an earlier year of estimation, which we used again. After combining, we were left with a set of 35 countries for which we have never received a UNAIDS file, many of them countries with small populations and/or low HIV prevalence. In those places, we generated regional averages of all needed inputs. This enabled us to run Spectrum for every GBD location.

In several cases, we have modified the structure or data in the UNAIDS files. In South Africa, Russia, Iran, New Zealand, Great Britain, Kenya, Japan, Indonesia, Mexico, United States, Norway, Brazil, Ukraine, and China, which we estimate at the subnational level, we split the national-level UNAIDS file into subnational datasets using assumptions from GBD 2017 demographics and GBD 2016 HIV prevalence. We also estimate at the subnational level in Ethiopia, Kenya, and India, but have subnational-level UNAIDS files for these locations; however, in Kenya and India, we must split larger subnationals to more granular locations. The subnational locations in Ethiopia, Kenya, India, and South Africa are fit as separate subpopulations in EPP, so we extracted the prevalence data for the individual subnationals. In Benin, Cote d'Ivoire, Haiti, Moldova, Mozambique, Nigeria, Togo, Zambia, and Zimbabwe, the country files that we received from UNAIDS contained only subnational data without national-level aggregates. In these cases, we aggregated the UNAIDS files and ran EPP and Spectrum at the national level.

Vital registration data

We used all available sources of vital registration and sample registration data from the GBD Causes of Death database after garbage code redistribution and HIV/AIDS mis-coding correction, except in Group 1A countries as described below.[1, 2] There are two different cause of death data sources for HIV/AIDS in China: the Disease Surveillance Point (DSP) system and the Notifiable Infectious Disease Reporting (NIDR) system. Both systems are administered by the Chinese Center for Disease Control and Prevention, but the reported number of deaths due to HIV is significantly lower in DSP. Therefore, we have used the provincial-level ratio of deaths due to HIV/AIDS from NIDR to those from DSP, choosing the larger ratio between years 2013 and 2014, and scaled the reported deaths in the DSP system, which is in turn used in the spatiotemporal Gaussian process regression (ST-GPR).

On-ART literature data

Data were identified by using search terms "HIV," "mortality," and "antiretroviral therapy" in PubMed searches across the literature. To be included, studies must include only HIV-positive people who receive antiretroviral therapy (ART) but who were ART-naïve prior to the study. In addition, studies must report either a duration-specific mortality proportion or a hazard ratio across age or sex, and must not include children.

For duration-specific survival data, studies must report uncertainty on mortality estimates or provide stratum-specific sample sizes and must include duration-specific data to allow for calculation of 0-6, 7-12, or 13-24 month conditional mortality. In addition, studies must either report separate mortality and loss-to-follow-up (LTFU) curves, be corrected for LTFU using vital registration data or double sampling, or be conducted in a high-income setting. Finally, studies must report the percent of participants who are male and the median age of participants.

Hazard ratio data for ages or sexes can only be used if the hazard ratios are controlled for other variables of interest (age, sex, and CD4 category). In GBD 2013, we identified 102 papers for extraction. For GBD 2015, we included 13 additional studies informing the duration-specific mortality estimation process and 26 studies informing the age and sex hazard ratio estimation process (some studies were used and counted in both). We also added one study to our LTFU analysis. For GBD 2016, we included 12 additional studies informing the duration-specific mortality estimation process and 11 studies informing the age and sex hazard ratio estimation process (some studies were used and counted in both). For GBD 2017, we included 17 additional studies informing the duration-specific mortality estimation process and 13 studies informing the age and sex hazard ratio estimation process (some studies were used and counted in both). We also included two new studies in our LTFU analysis.

Off-ART literature data

In GBD 2013, to characterise uncertainty in the progression and death rates, we systematically reviewed the literature on mortality without ART. We searched terms related to pre-ART or ART-naive survival since seroconversion.[3] After screening, we identified 13 cohort studies that included the cohorts used by UNAIDS from which we extracted survival at each one-year point after infection. Screening for additional, recently published studies in GBD 2015, GBD 2016, and GBD 2017 identified no new cohort studies for inclusion in this analysis.

Severity splits and disability weights

The basis of the GBD disability weight survey assessments are lay descriptions of sequelae highlighting major functional consequences and symptoms. The lay descriptions and disability weights for HIV/AIDS severity levels are shown below.

**Severity level**	**Lay description**	**DW (95% CI)**
Symptomatic HIV	has weight loss, fatigue, and frequent infections.	0.274 (0.184–0.377)
AIDS with antiretroviral treatment	has occasional fevers and infections. The person takes daily medication that sometimes causes diarrhoea.	0.078 (0.052–0.111)
AIDS without antiretroviral treatment	has severe weight loss, weakness, fatigue, cough and fever, and frequent infections, skin rashes, and diarrhoea.	0.582 (0.406–0.743)

The proportion of people living with HIV/AIDS who are being treated with antiretroviral therapy is an output of Spectrum, the compartmental model used to make consistent incidence, prevalence, and mortality estimates described below.

Modelling strategy

In GBD 2017, our general modelling strategy for estimating HIV incidence, prevalence, and mortality is very similar to the strategy used in GBD 2016. We continue to use the Spectrum program rewritten in

Python for GBD 2013 to facilitate faster and more flexible execution necessary for our more intensive computational needs. We made several changes to the modelling strategy in Spectrum comparing to the Spectrum software used by UNAIDS. We also, again, ran EPP using an open-source computer program in R written by Jeffrey Eaton.[4] We ran EPP for all Group 1 countries in order to produce incidence and prevalence estimates that were consistent with the demographic and epidemiological assumptions used in GBD 2017.

On-ART
First, we corrected reported probabilities of death for loss to follow-up using an update of the approach developed by Verguet and colleagues.[5] Verguet and colleagues used tracing and follow-up studies to empirically estimate the relationship between death in LTFU and the rate of LTFU.
To create estimates of age-specific hazard ratios, we synthesised hazard ratio data in five broad age groups: 15-25, 25-35, 35-45, 45-55, 55-100, and modelled the data using DisMod-MR 2.1.

To create estimates of sex-specific hazard ratios, we use the *metan* function in Stata to create estimates of relative risks separately by region, using female age groups as the reference group.
The age and sex hazard ratios were applied to the study-level mortality rates, accounting for the distribution of ages and sexes in the mortality data. We then subtracted HIV-free mortality from the model life table process to calculate study-level age-sex HIV-specific mortality.

We used DisMod-MR 2.1 to synthesise the age-sex-split study-level data into estimates of conditional probability of death over initial CD4 count.[3] We modelled the data separately by duration, age, sex and region and added a fixed effect on whether the study was conducted prior to 2002. Finally, we replaced our on-ART mortality rates with those estimated off treatment if they were higher.

Off-ART
Following UNAIDS assumptions, no-ART mortality is modelled as shown in the figure below.[3]

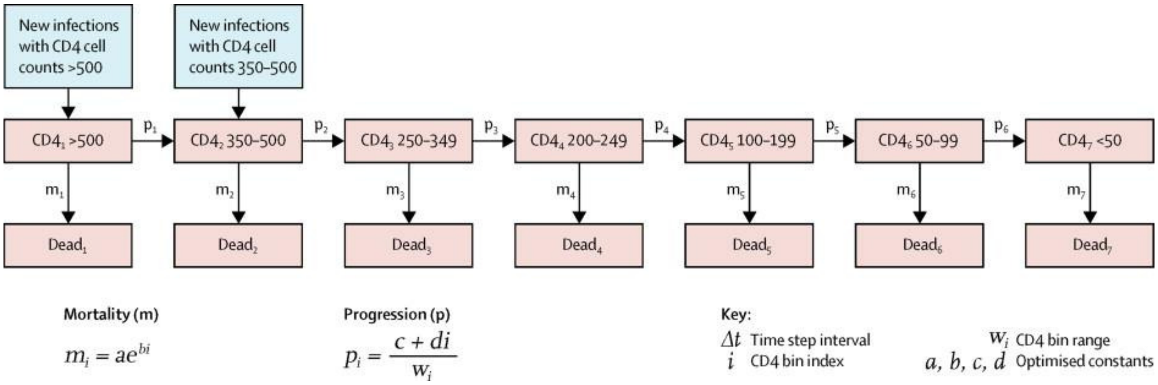

The death and progression rates between CD4 categories vary by age according to four age groups: 15–24 years, 25–34 years, 35–44 years, and 45 years or older. We modelled the logit of the conditional probability of death between years in these studies using the following formula:

$$logit\ (m_{ijk}) = \beta_0 + \sum_{i=1}^{4} \beta_{1i}\, a_i + \sum_{j=1}^{12} \beta_{2j} t_j + u_k + \varepsilon_{ijk}$$

In the formula, m is conditional probability of death from year t_j to t_{j+1}, a_i is an indicator variable for age group at seroconversion (15–24 years, 25–34 years, 35–44 years, and 45 years or older), t_j is an indicator variable of year since seroconversion, and u_K is a study-level random effect.

By sampling the variance-covariance matrix of the regression coefficients and the study-level random effect, we generated 1,000 survival curves for each age group that capture the systematic variation in survival across the available studies. For each of the 1,000 survival curves, we used a framework modelled after the UNAIDS optimisation framework in which we find a set of progression and death rates that minimises the sum of the squared errors for the fit to the survival curve.[8, 9]

Changes for GBD 2017

In GBD 2017, we chose to estimate mortality for each region in its own DisMod model, whereas previous GBD iterations estimated all regions together with fixed effects. This change was driven by new results from the IeDEA cohort collaboration which provided enough data to estimate mortality trends by CD4 in each region separately.[6] We also added a year covariate to our LTFU model reflecting evidence from a large meta-analysis by Zurcher and colleagues, which showed that mortality among the LTFU has declined in recent years.[7] Finally, we replaced our estimated on-ART mortality rates by rates off ART, accounting for progression to lower CD4 categories, if the on-ART rates were higher. This was done to ensure individuals would not experience higher mortality when they entered treatment in Spectrum.

Burden estimation overview

UNAIDS uses two key analytical components in their epidemiological estimation. EPP is used to estimate incidence and prevalence trajectories that are consistent with prevalence surveys and other prevalence measurements such as antenatal clinic sero-surveillance. Spectrum is a compartmental HIV progression model used to generate age-specific incidence, prevalence, and death rates from the EPP incidence and prevalence curves and assumptions about intervention scale-up and local variation in epidemiology.

Due to the substantial differences in the quality and types of data available across different countries, we used three different methodologies to produce year-, age-, and sex-specific estimates of HIV incidence, prevalence, and mortality.

Spectrum

For GBD 2013, we created an exact replica of Spectrum in Python. This enabled us to run thousands of iterations of the model at once on our computing cluster and allowed for more flexible input data structures. Additionally, in order to generate estimates with more realistic ranges of uncertainty than those in UNAIDS 2012, we adjusted all input data by uniformly sampled factors between 0.9 and 1.1. These changes, along with our new estimation of with- and without-ART mortality and CD4 progression parameters, persist into GBD 2017.

Changes for GBD 2017

For GBD 2017, we implemented a new approach to address selection bias resulting from temporal and geographic variation in ANC reporting, which has the potential to skew unadjusted estimates, especially early in the epidemic when there are no nationally representative prevalence surveys to anchor estimates.[14] To address this issue, the specification of the likelihood of observed ANC clinic data within EPP includes random intercepts for each clinic. While this approach largely accounts for differences in level between clinics, it does not impact the estimated shape of the epidemic. In order to leverage available information from nearby geographies, we developed a model for data imputation which establishes an epidemic peak from a first-stage model fit to ANC clinic data from a location and its nearest neighbours. The model included random effects for country, clinic, and time. The year of the largest random effect was used as t_{max} the location of a single knot in an imputation model which predicted the

logit of prevalence in each year for a clinic as a linear spline. We can write this method mathematically in the following way:

$$logit(\rho_i(t)) = \beta_0 + S(t) + \beta_3 X_i + \epsilon$$

$$S(t) = \beta_1 S_1(t) + \beta_2 S_2(t)$$

$$S_1(t) = \begin{cases} t_{max} - t & t \leq t_{max} \\ 0 & t > t_{max} \end{cases}$$

$$S_2(t) = \begin{cases} t - t_{max} & t \geq t_{max} \\ 0 & t < t_{max} \end{cases}$$

$\rho_i(t)$ is prevalence among ANC attending pregnant women in clinic *i*, with location-level intercept β_0, linear spline *S(t)* with a knot at t_{max}, and site-specific fixed effects X_i.

One thousand draws of imputed clinic prevalence, accounting for covariance between predictors, were generated for clinic-years where at least one clinic had an observation in a given year. These draws were used for each of the 1,000 EPP runs we ran for each location.

Additionally, for GBD 2017 we improved our sex-specific modelling strategy in Spectrum by sex-splitting incidence based on a model fit to the sex ratio of prevalence observed in countries with representative surveys. We also updated the Spectrum pediatric module to reflect changes made by UNAIDS.[10] Our child module was revised to include CD4 progression and CD4-specific mortality rates taken from a model fit to survival data from IeDEA. We also updated child initiation of ART to include data on-ART distribution from IeDEA.

ART coverage distribution

In UNAIDS' implementation of Spectrum, initiation of ART is constrained by eligibility criteria and distributed across CD4 count groups according to both the expected number of deaths and the number of people in each untreated CD4 count group – groups with a large proportion of PLHIV and high numbers of expected deaths initiated the most individuals into treatment. Three surveys were available at the time of publication that contained questions which can help inform the CD4 count distribution of ART coverage, Uganda AIS 2011 and Kenya AIS 2007 and 2012. Both of these surveys conducted CD4 count measurements and include a question regarding the amount of time that an individual receiving ART had been enrolled in treatment. Survey data provide cross-sectional CD4 count information; however, the Spectrum modelling framework tracks individuals by categorical CD4 count at the initiation of treatment. In order to crosswalk the cross-sectional survey data into estimates of CD4 count at treatment initiation, we built a model using relevant cohort data which tracked changes in CD4 count after initiation of treatment to translate an individual's current CD4 count and duration on treatment into CD4 count at initiation of treatment. The functional form for changes in CD4 count as a function of duration on treatment was a natural spline on duration with knots at 3, 12, 24 and 36 months, and an interaction between initial CD4 count and duration.

After crosswalking, we predicted the probability of being on treatment as a function of individual income (measured through an asset-based index), stratified by CD4 count, age, and sex. The results of this prediction were translated into country-specific age-sex-year-CD4 count probabilities of coverage using a conversion factor between individual income and LDI. We used stochastic frontier analysis to constrain the maximum possible coverage for a given degree of income and CD4 count. Predicted probabilities of coverage were input to Spectrum to inform the distribution, and not the overall level, of ART treatment by CD4 count. Spectrum converted counts of expected individuals on treatment in each

CD4 count group and scaled the distribution across CD4 count groups to match the input data on the number of people on ART coming from UNAIDS country files. In cases where the predicted number of individuals initiating treatment exceeds the total number of untreated individuals in a CD4 count group, we reallocate treatment evenly to other CD4 count groups.

Countries with seroprevalence surveys and antenatal clinic data (Groups 1A and 1B)
We identified 50 countries – as well as subnational locations in India, Kenya, Ethiopia, and South Africa – with at least 0.5% adult HIV prevalence and at least one geographically representative HIV seroprevalence survey or available antenatal care clinic (ANC) data. In order to ensure that our estimates of incidence and prevalence in these places were consistent with our estimates of HIV progression, we used a version of EPP written in R and C++ by Jeffrey Eaton to create new fits to the available prevalence data. The version of EPP used in GBD 2017 was updated in 2017 by Jeffrey Eaton. In this new version, an ANC prevalence adjustment was included and incorporated with the 2016 lookup database for the relative risk between pregnant women and the whole adult population and an additional parameter to estimate ANC variance inflation was included as well.

For adjusting ANC data to align with the national 15 to 49 both sexes population, we extracted data on HIV prevalence among pregnant women who gave birth within the last year and attended an ANC clinic from available DHS surveys. A simple model with regional random effects was run to generate location-specific prior distributions for the ANC bias adjustment where surveys were available, and regional priors for locations without a survey. The adjustment using a time-series of relative risk between pregnant women and the adult population was removed, and the ANC bias parameter was changed to account for all of the biases observed between these two populations.

In the new version of EPP, in addition to the equilibrium prior assumption of the force of infection in projection, a random walk approach is available as an alternative method. For locations with two or more prevalence surveys and a declining trend between the mean of the most recent two surveys, the random walk approach was chosen to project the force of infection. We assumed the change of the log scaled force of infection was following a normal distribution with mean equal to the median of the change of the modelled force of infection among the years having ART implemented or prevalence data, and the SD was equal to the default setting as the mean SD of the change of the modelled force of infections among the years having prevalence data. The projection year was chosen from the most recent year between the year with the lowest model force of infection and the year of the second latest survey data.

In the new EPP code, an optimisation step was added into IMIS function to speed up the parameter sampling step based on Raftery and Bao.[10] Two optimisation methods have been introduced. The main algorithm is Broyden–Fletcher–Goldfarb–Shanno (BFGS) optimisation. If BFGS fails, Nelder-Mead optimum is used instead. In our 2016 EPP model, by substituting in our own assumptions about HIV progression rates and on/off ART mortality, we were able to ensure that the implied relationship between incidence and mortality/prevalence in EPP is similar to that in Spectrum.

To incorporate uncertainty in our mortality and progression parameters, we run EPP with separate draws of each of these parameters. This process produced 1,000 sets of EPP output for each of the locations that make up the 48 countries in the group. Every set of EPP outputs contains 500 consistent draws of HIV incidence and prevalence in adults aged 15-49.

For every location in the group, we sampled one of the 500 incidence/prevalence draws from each of the sets of EPP results. By sampling one draw from each set, we ensured that the distribution of

progression parameters dictating the relationship between incidence and prevalence was exactly the same as the distribution of the sorted parameters generated in the previous step. At the end of this process, for every location in the set of 48 countries, we were left with 1,000 linked draws of adult incidence and prevalence and the exact mortality and progression parameters that generated those draws. We then ran these results, along with the previously described demographic and HIV-specific inputs, through Spectrum to produce location-, year-, age-, and sex-specific estimates of HIV incidence, prevalence, and mortality.

The HIV/mortality reckoning process is intended as a method of reconciling separate estimates of HIV mortality (and its resulting effect on estimates of HIV-free and all-cause mortality) in Group 1 countries by averaging estimates of HIV mortality from the model life table process and EPP-Spectrum. Additional details on the reckoning can be found in the GBD 2017 mortality manuscript.[11]

Since Spectrum produces HIV incidence, prevalence, and deaths that are consistent with one another over time, the reckoning process results in death numbers that are no longer consistent with the incidence and prevalence produced in Spectrum. In order to recreate this consistency, we recalculated incidence for all Group 1 locations using reckoned deaths and prevalence produced by Spectrum. The updated incidence is calculated by aggregating counts of new infections, HIV deaths from Spectrum, and HIV deaths after reckoning at the year-sex level. The difference between reckoned HIV deaths and HIV deaths from Spectrum is added to Spectrum incidence, and we calculate the ratio between updated incidence and Spectrum incidence. Age-specific counts of new infections are then scaled by their corresponding sex-year ratios.

Countries with vital registration data (Group 2A and 2B)
Vital registration is one of the highest-quality sources of data on HIV burden in many countries, so generating estimates that are consistent with these data with necessary adjustment to account for any potential underreporting is critical. We identified 108 countries – as well as 574 subnational locations from Brazil, China, Japan, Indonesia, India, Mexico, Sweden, the United Kingdom, Ukraine, Russia, New Zealand, Iran, Norway and the United States – with usable points of vital registration data, verbal autopsy (VA) data, or sample registration system (SRS) data. In India, Vietnam, and Indonesia, we used SRS and VA data, respectively, as input mortality for CIBA. For India we extracted the resulting age-sex distribution of incidence but scaled the level to match the adult incidence rate estimated from EPP for each state.

We imputed missing years of data to generate a complete time series for HIV from the estimated start year of the epidemic using ST-GPR. We analysed mortality trends using ST-GPR starting in 1981, the year that HIV was first identified in the United States.[12] For ST-GPR, we adjusted the lambda (time weight) and GPR scale according to the completeness of vital registration data, with 4- and 5-star quality VR using parameters designed to follow the data more closely. We produced separate splines by country/age group, up to the peak year of death rate. We then ran a linear regression with fixed effects on region, age, and sex. Following this, we ran space-time residual smoothing, in which time, age, and space weights are used to inform smoothing of the residuals between data points and the linear regression estimate. From this process, we generated space-time estimates with the applied weights, along with the median absolute deviation (MAD) of the space-time estimates from the data. The MAD was calculated at various levels of the geographic hierarchy (eg, subnational and national), and was added into the data variance term. The data variance and space-time estimates were then analysed using Gaussian process regression to return a final estimate of mortality along with uncertainty.

Although Spectrum produces HIV mortality estimates that are within the realm of possibility in most countries using the incidence curves provided in the UNAIDS country files, it is a deterministic model that has not yet been integrated into an optimisable framework. Therefore, in order to “fit” it to vital registration data, we need to adjust input incidence.

To improve the fit of this process, in GBD 2015, we restructured Spectrum to track cohorts by year of HIV infection. With this version of Spectrum we can output, among many other metrics, HIV deaths by year, age, sex, and infection cohort. This enables us to adjust incidence to fit to death much more precisely and without making any rigid assumptions about the time from HIV infection to HIV death.

We have incorporated these improvements into a cohort incidence bias adjustment (CIBA) process. First, we ran Spectrum normally to produce 1,000 draws of incidence, prevalence, and mortality. Then, by year, age, and sex, we took the ratio of VR deaths to Spectrum deaths to quantify the amount of bias in Spectrum. Using draw-level duration data from the new version of Spectrum, for every year-, age-, and sex-specific infection cohort, we calculated the share of all HIV deaths observed over the course of the projection period in that cohort that would occur in each year after the year of infection. For example, projecting from 1970 through 2016, we identified the cohort of men infected in 1992 at the age of 16, calculated the total number of HIV deaths in that cohort in all subsequent years through the end of 2016, and divided the annual number of deaths by that total. This showed us the distribution of deaths among that cohort over the projection period. In the most extreme case (infections in 2015), we could only produce one point of that distribution (2016), so that single value is exactly 1·0; 100% of the deaths observed in that cohort occurred in 2016.

We then used these distributions of death to weigh the ratio of VR deaths to Spectrum deaths, meaning that ratios in the years where we expect the largest share of deaths were weighed most heavily. We then multiplied the initial size of that cohort from the normal run of Spectrum by the sum of the combined ratios to get a new estimate of new cases in that year/age/sex combination.

We can write this method mathematically in the following way:

$$r_t = \frac{VR_t}{D_t}$$

$$\rho_t^{t-i} = \frac{d_t^{t-i}}{\sum_{k=t-i+1}^{n} d_k^{t-i}}$$

$$\alpha^{t-i} = \sum_{k=t-i+1}^{n} r_k * \rho_k^{t-i}$$

$$n_{\text{adjusted}}^{t-i} = \alpha^{t-i} * n^{t-i}$$

VR_t is the number of HIV/AIDS deaths in year t from ST-GPR, and D_t is the number of HIV/AIDS deaths from the first run of Spectrum. In the second equation, d_t^{t-i} is the number of HIV/AIDS deaths among members of infection cohort $t - i$ in year t, with $i \geq 1$, from the new, duration-tracking version of Spectrum, and n is final year of the projection. Therefore, ρ_t^{t-i} is the share of observed deaths in cohort $t - i$ that we expect to occur in year t. It follows that α^{t-i} is the weighted adjustment ratio described above, which we multiply by the estimated initial size of infection cohort $t - i$ as calculated in the first-stage Spectrum run to get the adjusted number of new cases, $n_{\text{adjusted}}^{t-i}$. This process is run separately for every sex, single-age, and draw.

CIBA allows ratios in each year after a given infection year to influence the final adjustment to incidence. The size of that influence is determined by the relative importance of that year in the cohort-year's distribution of deaths over time. The result is a new set of 1,000 draws of incidence and a set of 1,000 ratios of post-adjustment incidence to pre-adjustment incidence. We perform this adjustment using mean durations from the new version of Spectrum in order to try to shift the mean of the regular distribution of deaths.

Finally, to produce location-, year-, age-, and sex-specific estimates of HIV incidence, prevalence, and mortality, we ran the new estimates of incidence and all previously input data through Spectrum.

Countries without survey data and vital registration data (Group 2C)
The remaining 30 countries – as well as Macao Special Administrative Region of China – had neither geographically representative seroprevalence surveys nor reliable vital registration systems. To produce estimates of HIV burden in these countries, we assumed that Spectrum is similarly biased as in other Group 2 countries within the same super-region. This involved running Spectrum, adjusting incidence using 1,000 adjustment ratios randomly sampled from CIBA results from the same super-region, and rerunning Spectrum using the new draws of adjusted incidence. As above, the estimates of incidence, prevalence, and mortality were incorporated into the rest of the machinery via the reckoning process.

HIV/AIDS resulting in other diseases

There are two Level 4 causes under the HIV/AIDS Level 3 cause in the GBD 2015 cause hierarchy. The modeling process for HIV/AIDS-tuberculosis is detailed in a separate part of this appendix. We computed deaths for HIV resulting in other diseases by subtracting HIV/AIDS-tuberculosis deaths from all HIV deaths at the 1,000-draw level.

Source Counts

HIV on/off treatment	**Other**
**Site-years (total)**	17,045
**Number of countries with data**	148
**Number of GBD regions with data (out of 21 regions)**	21
**Number of GBD super-regions with data (out of 7 super-regions)**	7

HIV Prevalence	**Other**
**Site-years (total)**	2,037
**Number of countries with data**	51
**Number of GBD regions with data (out of 21 regions)**	9
**Number of GBD super-regions with data (out of 7 super-regions)**	5

HIV Incidence case reports	**Other**
**Site-years (total)**	666
**Number of countries with data**	54
**Number of GBD regions with data (out of 21 regions)**	7
**Number of GBD super-regions with data (out of 7 super-regions)**	3

References

1. Global, regional, and national age–sex specific all-cause and cause-specific mortality for 240 causes of death, 1990–2013: a systematic analysis for the Global Burden of Disease Study 2013. *The Lancet* 2015; **385**: 117–71.

2. Birnbaum JK, Murray CJ, Lozano R. Exposing misclassified HIV/AIDS deaths in South Africa. *Bull World Health Organ* 2011; **89**: 278–85.

3. Murray CJL, Ortblad KF, Guinovart C, *et al.* Global, regional, and national incidence and mortality for HIV, tuberculosis, and malaria during 1990–2013: a systematic analysis for the Global Burden of Disease Study 2013. *The Lancet* 2014; **384**: 1005–70.

4. jeffeaton/epp. GitHub. https://github.com/jeffeaton/epp (accessed April 21, 2016).

5. Verguet S, Lim SS, Murray CJL, Gakidou E, Salomon JA. Incorporating Loss to Follow-up in Estimates of Survival Among HIV-Infected Individuals in Sub-Saharan Africa Enrolled in Antiretroviral Therapy Programs. *J Infect Dis* 2013; **207**: 72–9.

6. Anderegg N, Johnson LF, Zaniewski E, *et al*. All-cause mortality in HIV-positive adults starting combination antiretroviral therapy: correcting for loss to follow-up. *AIDS* 2017; **31 Suppl 1:** S31-40.

7. Zürcher K, Mooser A, Anderegg N, *et* al. Outcomes of HIV-positive patients lost to follow-up in African treatment programmes. *Trop Med Int Health* 2017; **22**: 375-387.

8. Ghys PD, Zaba B, Prins M. Survival and mortality of people infected with HIV in low and middle income countries: results from the extended ALPHA network. *AIDS Lond Engl* 2007; **21 Suppl 6**: S1–4.

9. Hallett TB, Zaba B, Todd J, *et al.*, ALPHA Network. Estimating incidence from prevalence in generalised HIV epidemics: methods and validation. *PLoS Med* 2008; **5**: e80.

10. Raftery AE, Bao L. Estimating and Projecting Trends in HIV/AIDS Generalized Epidemics Using Incremental Mixture Importance Sampling. *Biometrics* 2010; **66**: 1162–73.

11. Wang H, Murray CJ, Carter A, He F. Global, regional, and national under-5 mortality, adult mortality, age-specific mortality, and life expectancy, 1970-2016: a systematic analysis for the Global Burden of Disease Study 2016. *The Lancet* 2017; **390**: 1151-1210.

12. CDC. Pneumocystis Pneumonia --- Los Angeles. MMWR Wkly. 1981; published online June 5. http://www.cdc.gov/mmwr/preview/mmwrhtml/june_5.htm (accessed April 21, 2016).

13. CDC. Pneumocystis Pneumonia --- Los Angeles. MMWR Wkly. 1981; published online June 5. http://www.cdc.gov/mmwr/preview/mmwrhtml/june_5.htm (accessed April 21, 2016).

14. Ng M, Gakidou E, Murray CJL, Lim S. A comparison of missing data procedures for addressing selection bias in HIV sentinel surveillance data. *Population Health Metrics* 2013; **11:** 12.

Sexually transmitted infections (STIs), excluding HIV:

Chlamydia, gonorrhoea, trichomonas, genital herpes due to HSV-2, syphilis (early infection and adult tertiary syphilis), and other STIs

Flowchart

Sexually-transmitted infections excluding HIV:
Chlamydia, Gonorrhea, Trichomonas, Genital herpes due to HSV-2, Syphilis (early and adult tertiary)

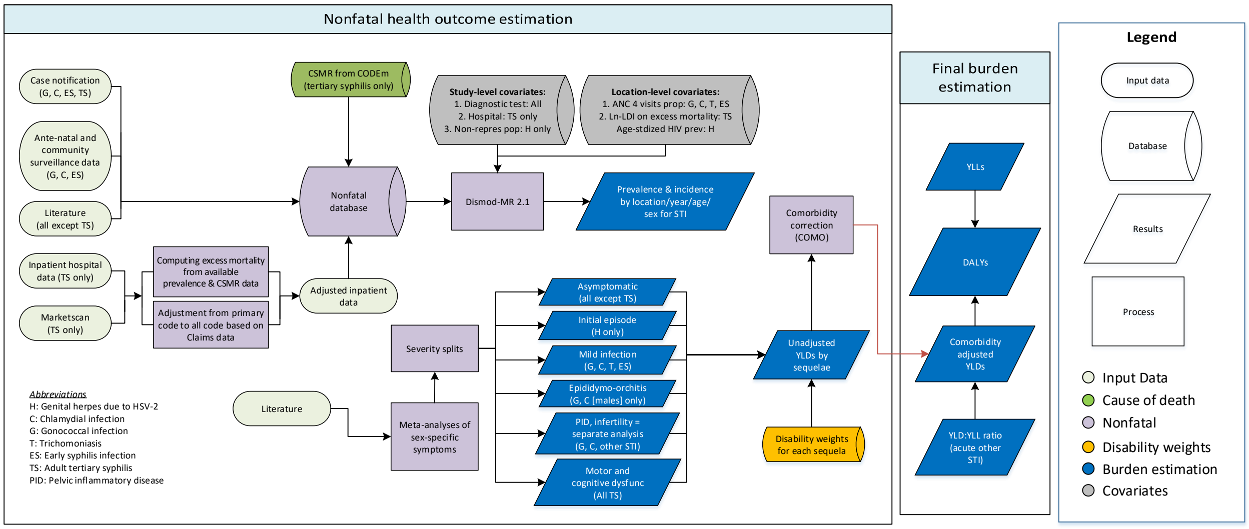

Input data and methodological summary

Case definition

For GBD 2017, we estimated the prevalence and incidence of genital and reproductive tract infection with several common sexually transmitted infections, including *Chlamydia trachomatis*, *Neisseria gonorrhoea*, *Trichomonas vaginalis*, *Treponema pallidum* (syphilis), and genital herpes associated with seroprevalent HSV-2. Separate estimates were completed for early syphilis and adult tertiary syphilis. ICD-9 and ICD-10 codes associated with each cause in the GBD 2017 cause-specific mortality analyses are listed below. Of note, mortality was assumed to be zero in trichomoniasis and genital herpes infection, and YLDs due to congenital syphilis were not estimated.

Table 1. International classification of diseases codes for sexually transmitted infections in GBD 2015 cause of death analysis

Condition	ICD10 code	ICD9 code
Sexually transmitted infections (STI) excl HIV	A50-A60, A63-A64, I98.0, K67.0-K67.2, M73.0-M73.1	090-099,131,614
Syphilis	A50-A53, I98.0, K67.0-K67.2, M73.0-M73.1	090-097
Congenital syphilis	A50	090
Early syphilis	A51	091
Adult tertiary syphilis	A52, I98.0	093-095

Chlamydial infection	A55-A56,K67.0	099.41,099.5
Gonococcal infection	A54,K67.1	098
Trichomoniasis	A59,K67.0	131
Genital herpes	A60	054.1
Other STI	A57-A58, A63-A64, M73.0	099 (except 099.41, 099.5)

Input data

Model inputs

Systematic literature reviews were completed on April 17, 2015, for chlamydia, gonorrhoea, trichomonas, genital herpes, and early syphilis. Three related search strings were used as many studies report on multiple infections. With the exception of the early syphilis literature review, which was completed in GBD 2015, these were the same search strings and strategies as were employed in GBD 2013.

462 initial hits; 54 sources selected for full text review and data extraction: (((chlamydia[Title/Abstract] OR chlamydia tracomatis[Title/Abstract] OR trachoma[Title/Abstract]) AND prevalence[Title/Abstract]) AND ('2013'[Date - Publication] : '2015'[Date - Publication])) /// ((gonorrhea[Title/Abstract] OR Neisseria[Title/Abstract] OR gonococcal[Title/Abstract]) AND prevalence[Title/Abstract]) AND ("2013"[PDAT] : "2015"[PDAT]) /// ((trichomonal[Title/Abstract] OR trichomonas[Title/Abstract]) AND prevalence[Title/Abstract]) AND ('2013'[PDAT] : '2015'[PDAT])

1265 initial hits; 178 sources selected for full text review and data extraction: ("syphilis"[MeSH] OR "Treponema pallidum"[MeSH]) NOT "Yaws"[MeSH] AND "prevalence"[MeSH] AND "1990"[PDAT] : "2015"[PDAT] AND "humans"[MeSH] **///** ("syphilis"[MeSH] OR "Treponema pallidum"[Mesh]) NOT “Yaws”[MeSH] AND ("incidence"[MeSH]) AND ("1990"[PDAT] : "2015"[PDAT]) AND "humans"[MeSH])

13 initial hits; 1 selected for full text review and data extraction: herpes"[Title/Abstract] OR "Herpesvirus 2, Human"[Mesh]) AND ("Prevalence"[Title/Abstract] OR "Incidence"[Title/Abstract] AND ("2015"[PDAT] : "2015"[PDAT])

The genital herpes dataset was supplemented by those sources contained in recent published estimates completed by Looker and colleagues. For all other STIs, most notably early syphilis, we supplemented our datasets with manual search of national ministry of health websites, antenatal clinic surveillance reports, and case-notification data from locations where centralised reporting is mandatory.

To be included, a study must report on laboratory-confirmed diagnosis of an STI. For each STI, the reference category for diagnostic modality was a DNA-based test (eg, PCR or other nucleic acid amplification test) or a blood-serology test (for syphilis: treponemal, non-treponemal) and data using any other diagnostic test were quantitatively crosswalked to the reference category using binary study-level covariates in DisMod-MR 2.1. For genital herpes, any sources that did not use nucleic acid amplification were excluded. Given the potential variability in immunological reasons for false positives in each of the other STI categories, we also did not perform any adjustment for published sensitivity and specificity of tests, as these results are often drawn from particular populations that may not be representative of the global experience for each infection.

For all STI, including genital herpes, sources were excluded if the sample population was drawn exclusively from a high-risk group (eg, HIV-positive, men who have sex with men [MSM], or sex workers).

In GBD 2016, all surveillance data were considered prevalence when modelling chlamydia and gonorrhoea, and all incidence data were converted to prevalence when modelling early syphilis by dividing by the percent symptomatic (~5%). However, this decision was reversed for GBD 2017. Due to difficulty in reconciling differences between prevalence and incidence sources, likely due to underreporting in surveillance data, incidence data were ignored for all STIs.

To better reflect disease duration differences by sex and location for each STI excluding genital herpes, consistent duration ranges were calculated using a sum of the duration of untreated and treated disease, weighted by the percent of individuals that are symptomatic and the probability of receiving treatment if symptomatic with the formula below. These estimates were calculated from published numbers in GBD

$$\boldsymbol{Duration} = (\%\,Symptomatic)(Prob_{Rx})(Duration_{Rx}) + (1 - \%\,Symptomatic)(Duration_{not\ Rx}) + (\%\,Symptomatic)(1 - Prob_{Rx})\,(Duration_{not\ Rx})$$

2000 and WHO 2005, which were largely expert-driven. The probability of treatment if symptomatic was scaled using the Healthcare Access and Quality (HAQ) index to compute this probability for each location and year. For syphilis, durations per stage (primary, secondary, latent, and tertiary) were calculated individually and summed along with the average seroreversion by stage, weighing by the proportion of cases remaining at each stage and including the time it would take to serorevert after adequate treatment.

Data were outliered or excluded if we found them unreasonable when compared to regional, super-regional, and global rates.

Composition of final datasets are shown below for each of the different STI models.

Gonococcal infection

	**Incidence**	**Prevalence**
**Site-years (total)**	2,993	150
**Number of countries with data**	46	61
**Number of GBD regions with data (out of 21 regions)**	9	19
**Number of GBD super-regions with data (out of 7 super-regions)**	4	7

Chlamydial infection

	**Incidence**	**Prevalence**
**Site-years (total)**	2,154	291
**Number of countries with data**	14	91
**Number of GBD regions with data (out of 21 regions)**	6	20
**Number of GBD super-regions with data (out of 7 super-regions)**	3	7

Early syphilis

	**Incidence**	**Prevalence**
**Site-years (total)**	2,812	830
**Number of countries with data**	57	137

**Number of GBD regions with data (out of 21 regions)**	14	20
**Number of GBD super-regions with data (out of 7 super-regions)**	6	7

Adult tertiary syphilis

	**Incidence**	**Prevalence**
**Site-years (total)**	271	641
**Number of countries with data**	2	35
**Number of GBD regions with data (out of 21 regions)**	2	15
**Number of GBD super-regions with data (out of 7 super-regions)**	2	6

Trichomoniasis infection

	**Incidence**	**Prevalence**
**Site-years (total)**	2	162
**Number of countries with data**	1	55
**Number of GBD regions with data (out of 21 regions)**	1	19
**Number of GBD super-regions with data (out of 7 super-regions)**	1	7

Genital herpes

	**Incidence**	**Prevalence**
**Site-years (total)**	48	312
**Number of countries with data**	17	76
**Number of GBD regions with data (out of 21 regions)**	7	20
**Number of GBD super-regions with data (out of 7 super-regions)**	4	7

Modelling strategy

We estimated the nonfatal burden of STI in three parts. For the first part we estimated the prevalence, incidence, remission, and case fatality from acute infection associated with gonococcus, chlamydia, trichomoniasis, genital herpes (from herpes simplex virus 2), early syphilis, and adult tertiary syphilis using the data above and DisMod-MR 2.1. Not all cases of STI are symptomatic, so we used literature review to guide splitting prevalent cases into asymptomatic and symptomatic health states. Specific modelling considerations in DisMod-MR 2.1 for each STI are described below.

The second part, which is estimation of prevalence, incidence, remission and case fatality from pelvic inflammatory disease (PID) and PID-induced primary and secondary infertility, is described in a separate section on those conditions. Briefly, for PID we used ICD-9 and ICD-10 coded discharge datasets, Marketscan claims database, and systematic literature review to develop a dataset that was subsequently modelled for all geographies using DisMod-MR 2.1. Processing steps for discharge and claims data are described separately. PID was then split into underlying ideologies (chlamydia, gonorrhoea, and other) using results of supplemental literature review and DisMod-MR 2.1 models of the proportion due to each

etiology. PID-induced primary and secondary infertility assuming only a fixed subset of incident PID cases develop infertility and that there is no remission in these cases.

The third and final part involved finding the ratio of YLD to YLL ratio for all STI (excluding other STI) and then applying that same ratio to other STI YLLs.

Gonococcal infection

Prevalence data from literature were the primary inputs, reversing the decision to interpret notifications as prevalence in GBD 2016. Incidence data from case notification systems and surveillance were ignored in the final model as they were magnitudes below prevalence studies. However, a model using only incidence data was used to inform an age-split on the prevalence data as the notifications had more detailed age-specific data available.

Incidence was restricted to occur only between ages 10 and 69. EMR was set to have a maximum value of 0.0001. Remission rates were calculated for each location-year by combining percent of individuals that show symptoms, the proportion that receive treatment if symptomatic, and the disease duration if untreated/treated as described previously. Study covariates included crosswalks for data where case diagnosis was based on culture or other non-nucleic acid amplification tests (PCR is the gold-standard diagnostic method). Female-to-male ratio of prevalence and incidence was restricted to not exceed 2.01:1, an approach that was used to account for relatively sparse data from males. Coverage proportion of four visits to antenatal care clinics (ANC4) was the only country covariate and was assigned to prevalence.

We assigned the proportion of persons who developed symptoms of infection and/or epididymo-orchitis. Both were unchanged from GBD 2010 and are the same as used in WHO analyses. Males were assigned a fixed proportion of each of the following health states. Epididymo-orchitis differed for geographies with long time series of high-quality vital registration data ("data-rich") compared to geographies with poor data ("all others"). This split was performed for developed versus developing countries in GBD 2013 and GBD 2010. Data-rich proportion was 0.03 (0.015–0.045) and all others was 0.0975 (0.0483–0.143). To adjust prevalence and YLDs from epididymo-orchitis due to gonococcal infection for the increased disability weight but much shorter duration, epididymo-orchitis incidence was taken from the split and modelled separately in DisMod with a duration of three weeks. A proportion of those not assigned epididymo-orchitis, 0.5875 (0.5288–0.6463), were assigned a health state of mild, acute infectious disease. The remainder were assumed to be asymptomatic. Females were estimated to have a proportion with mild, acute infectious disease of 0.34 (0.306–0.374) and the remainder asymptomatic.

Gonococcal infection

Study-level covariate	Parameter	Geography level	beta	Exponentiated beta
Sex	prevalence	Global	-0.043 (-0.31 to 0.21)	0.96 (0.74–1.24)
Culture-positive	prevalence	Global	-0.03 (-0.097 to -0.0012)	0.97 (0.91–1.00)
Diagnosis other	prevalence	Global	-0.16 (-0.29 to -0.012)	0.85 (0.75–0.99)

Country-level covariate	Parameter	beta	Exponentiated beta

Antenatal Care (4 visits) Coverage (proportion)	prevalence	-0.054 (-0.098 to -0.0032)	0.95 (0.91–1.00)

Chlamydial infection

Chlamydia estimation methods were the same as used for gonococcal infection described above, with a few exceptions. No age-split was performed as the age pattern without an age-split was reasonable. The female-to-male sex ratio of prevalence and incidence was restricted to not exceed 1.35:1. The proportion of antenatal care coverage (four visits) was applied as a country-level covariate on prevalence.

The approach to estimating sequelae was the same as for gonorrhoea, including the adjustment for shorter epididymo-orchitis duration, although the proportions in each state were different. Data-rich proportion was 0.02 (0.01–0.03) and all others was 0.0625 (0.0325–0.0975). A proportion of the remainder, 0.505 (0.4545–0.5555), were assigned a health state of mild, acute infectious disease. The remainder were assumed to be asymptomatic. Females were estimated to have proportion with mild, acute infectious disease of 0.17 (0.153–0.187) and the remainder asymptomatic. Again, despite a higher number of cases among females, the YLD per case among males was higher.

Chlamydial infection

Study-level covariate	Parameter	Geography level	beta	Exponentiated beta
Sex	prevalence	Global	0.17 (-0.29 to 0.0097)	0.85 (0.75–1.01)
Diagnosis other	prevalence	Global	0.38 (0.14–0.59)	1.46 (1.15–1.81)
Culture-positive	prevalence	Global	-0.072 (-0.23 to -0.0012)	0.93 (0.80–1.00)

Country-level covariate	Parameter	beta	Exponentiated beta
Antenatal Care (4 visits) Coverage (proportion)	prevalence	-0.047 (-0.097 to -0.0024)	0.95 (0.91–1.00)

Trichomoniasis

Trichomoniasis estimation methods were similar to those used for gonococcal and chlamydia infection. However, the female-to-male sex ratio was unbounded, and excess mortality rate was assumed to be zero. Like the other STIs, surveillance and case-notification data for trichomonas was considered to be unreliable and not used. Accordingly, no age-split using incidence data was used. The reference definition for diagnostic was PCR, and wet mount and culture positive were crosswalked against the reference definition.

Only a small proportion of males were assumed to be symptomatic, 0.067 (0.063 – 0.073), with trichomoniasis and assigned a health state of mild, acute infectious disease. For females, 0.34 (0.306–0.374) were assigned a health state of mild, acute infectious disease.

Trichomonas infection

Study-level covariate	Parameter	Geography level	beta	Exponentiated beta

Sex	prevalence	Global	-1.62 (-1.98 to -0.85)	0.20 (0.14–0.43)
Diagnostic wet mount	prevalence	Global	-0.43 (-0.65 to -0.18)	0.65 (0.52–0.83)
Culture-positive	prevalence	Global	-0.22 (-0.49 to 0.065)	0.80 (0.61–1.07)

Country-level covariate	Parameter	beta	Exponentiated beta
Antenatal care (4 visits) coverage (proportion)	prevalence	-0.053 (-0.098 to -0.0031)	0.95 (0.91–1.00)

Genital herpes due to HSV-2

Genital herpes estimation assumed mortality is zero and remission is a small value (0–0.02) to account for a subset of herpes-infected patients becoming test-negative over time due to seroreversion. Incidence estimates were based on mathematical integration of age-specific prevalence data using DisMod-MR 2.1. Incidence was restricted to occur between ages 10 and 79. After careful analysis, the modelling strategy for genital herpes has evolved to exclude high-risk populations including MSM, HIV-positive, prisoners, sex workers, drug users, and STI clinic samples. However, blood donor samples were included and crosswalked to the general population assuming the prevalence is lower than that of the general population. Data on pregnant populations were also crosswalked, without a priori knowledge of direction. Crosswalks were performed at the regional level to allow for variation in the quantitative relationship. A single country covariate, age-standardised HIV prevalence, was used to guide estimates in geographies with sparse data in recognition of the strong relationship between HSV-2 and HIV transmission.

Many with initial genital herpes infection have a painful rash that, while not as severe as that accompanying zoster reactivation, is more severe than that associated with recurrent genital herpes episodes. A systematic literature review revealed a few studies that informed our estimation that 0.175 (0.10–0.25) of initial herpes cases have symptoms of moderate, acute infectious disease lasting 3 (2–4) weeks and 0.189 have prevalent persons have 6 (5–7) recurrent episodes per year each lasting 2 (1–3) weeks.[5–7]

Genital herpes

Study-level covariate	Parameter	Geography level	beta	Exponentiated beta
Sex	prevalence	Global	-0.49 (-0.58 to -0.4)	0.61 (0.56–0.67)
Study population blood donors	Prevalence	Region	-0.37 (-0.61 to -0.14)	0.69 (0.54–0.87)
Study population pregnant	Prevalence	Region	-0.099 (-0.24 to 0.047)	0.91 (0.79–1.05)

Country-level covariate	Parameter	beta	Exponentiated beta
HIV age-standardised prevalence	Prevalence	0.88 (0.56–1.00)	2.41 (1.75–2.71)

Early syphilis

Prevalence data from literature and ANC clinic reports were the primary inputs. Similar to other STIs, incidence data from case notification systems and surveillance were excluded due to difficulty reconciling prevalence data and the low incidence data reported by surveillance sources. In order to discourage DisMod from calculating implausible sex differences in regions with sparse male data, stub male data were created in Argentina and the Solomon Islands by copying female data in those locations. The age range was restricted to be from 10 to 64 years. Excess mortality was assumed to be 0. Remission values for each location and year were calculated as described above. Blood donor samples consistently had prevalence values that were lower than that of the general population, and were crosswalked accordingly. Data from studies using only treponemal tests, or only non-treponemal tests, were additionally crosswalked to the reference definition of both treponemal and non-treponemal testing. HIV age-standardised prevalence was applied as a country-level covariate on prevalence.

Our review of literature on proportion of early syphilis cases with symptoms led to assignment of a 0.043 (0.014–0.073) of prevalent cases to manifest primary and secondary symptoms. The remainder were considered asymptomatic.

Early syphilis infection

Study-level covariate	Parameter	Geography level	beta	Exponentiated beta
Study population blood donors	prevalence	Country	-0.41 (-0.41 to -0.41)	0.66 (0.66–0.66)
Non-treponemal only diagnosis	prevalence	Country	0.83 (0.83–0.83)	2.29 (2.29–2.29)
Treponemal only diagnosis	prevalence	Country	0.68 (0.53–0.82)	1.97 (1.70–2.28)

Country-level covariate	Parameter	beta	Exponentiated beta
HIV age-standardised prevalence	prevalence	0.021 (0.00033–0.093)	1.02 (1.00–1.10)

Adult tertiary syphilis

Prevalence data were the primary input. Incidence was assumed to not occur until age 15. Excess mortality rate was capped at 0.1, which equates to minimum duration of five years, and no remission was assumed. Cause-specific mortality rate (CSMR) results from GBD 2017 mortality and causes of death analysis were included in the model, which was also used to back-calculate excess mortality rate (EMR) data to match each input prevalence datum.[12] CSMR priors were not passed on through the cascade so as to restrict the utility of these data to back-calculating prevalence at the country level. The reference source was hospital data, which were adjusted for the likelihood of tertiary syphilis to appear as an inpatient or outpatient case. A study-level covariate on MarketScan 2000 data was used to crosswalk older data to the reference source of more recent hospital data. Natural log of lag-distributed income (LN-LDI) was used as a country-level covariate on EMR.

There are eight sequelae, including asymptomatic adult tertiary syphilis.

Adult tertiary syphilis

Study-level covariate	Parameter	Geography level	beta	Exponentiated beta
Sex	prevalence	Global	0.51 (0.48–0.55)	1.67 (1.61–1.73)
All MarketScan, year 2000	prevalence	Global	0.16 (0.10–0.23)	1.18 (1.11–1.26)

Country-level covariate	Parameter	beta	Exponentiated beta
LDI (I$ per capita)	excess mortality rate	-0.19 (-0.22 to -0.16)	0.83 (0.80–0.85)

<u>Other sexually transmitted infections</u>

To calculate YLDs due to acute infection with other STI, we calculated the YLD to YLL ratio for all STI (excluding other STI) and then applied that same ratio to other STI YLLs. YLDs were also estimated to other STI as a result of the proportion of PID and PID-induced infertility that was not due to gonorrhoea or chlamydia.

<u>Uncertainty and model selection</u>

For all STI estimates, uncertainty bounds include uncertainty due to input data, including CSMR from GBD 2017 mortality and causes of death analysis, crosswalks from non-reference definitions, uncertainty in numerical solutions (posteriors) of each DisMod-MR 2.1 model, and proportion of all infections with each type of symptom.

In consultation with GBD researchers and collaborators, final models were selected on a combination of qualitative and quantitative goodness of fit to input data, plausibility of geographic and temporal trends, consistency of age pattern, and, when available, comparison with other published studies on STI epidemiology. Directionality, magnitude, and plausibility of study-level and country-level covariates were also considered in the process of model development. Of note, due to the nature of statistical modelling, final results do not always cover the values reported in input data.

Tuberculosis

Flowchart

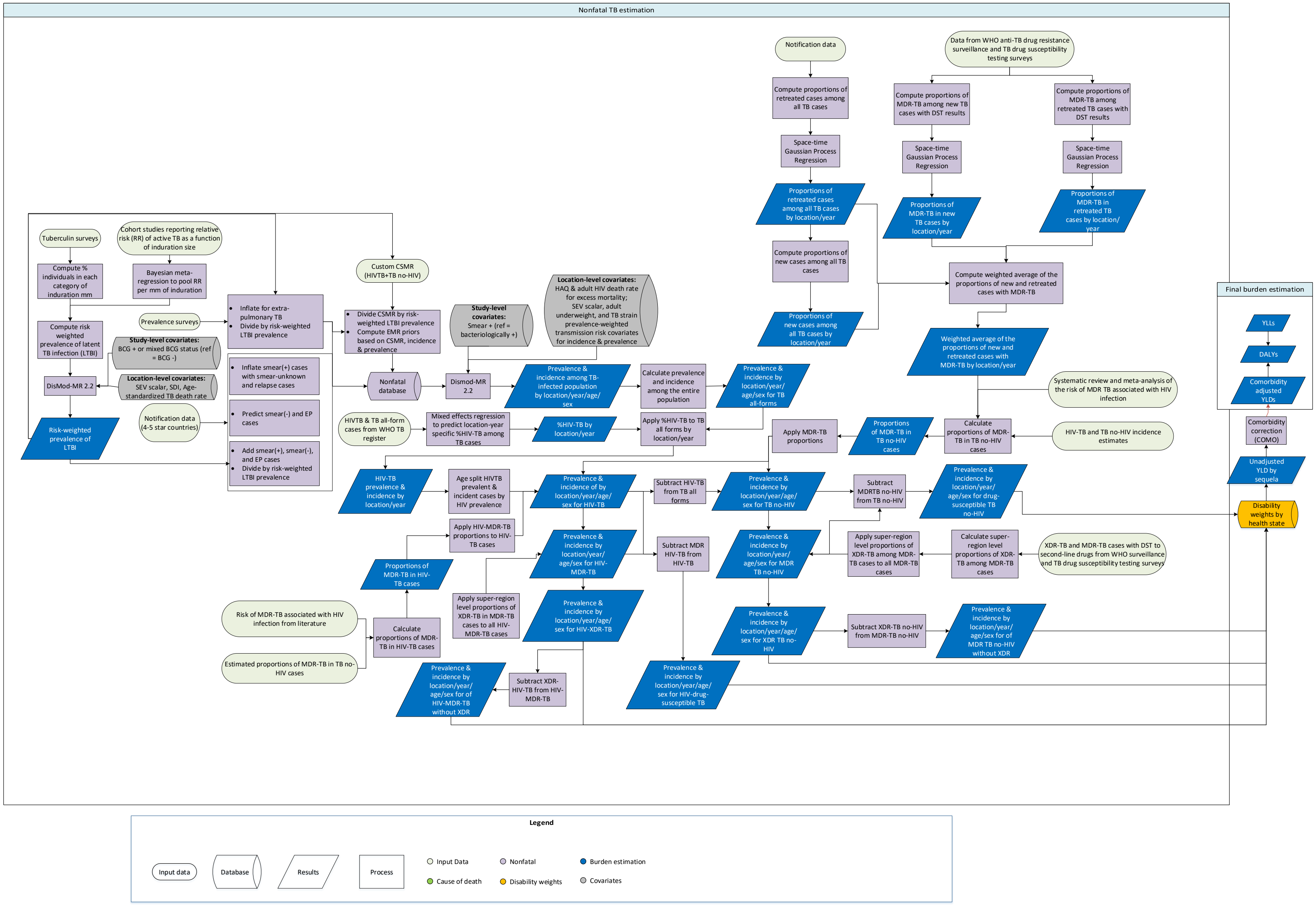

Tuberculosis

Case Definition

Tuberculosis (TB) is an infectious disease caused by *Mycobacterium tuberculosis*. The case definition includes all forms of TB, including pulmonary TB and extrapulmonary TB, which are bacteriologically confirmed or clinically diagnosed. For TB, the ICD 10 codes are A10-A19.9, B90-B90.9, K67.3, K93.0, M49.0, P37.0, and ICD 9 codes are 010-019.9, 137-137.9, 138.0, 138.9, 139.9, 320.4, 730.4-730.6. For HIV-TB, the ICD 10 code is B20.0.

Latent TB infection is defined as an infection with *Mycobacterium tuberculosis*, without any symptoms or signs of active TB disease.

We separately estimated the incidence and prevalence of multidrug-resistant tuberculosis and extensively drug-resistant tuberculosis by HIV status. The case definitions are shown below.

(1) Multidrug-resistant TB without extensive drug resistance: a form of TB (among HIV-negative individuals) that is resistant to the two most effective first-line anti-tuberculosis drugs (isoniazid and rifampicin), but is not resistant to any fluoroquinolone and any second-line injectable drugs (amikacin, kanamycin, or capreomycin).

(2) Extensively drug-resistant TB: a form of TB (among HIV-negative individuals) that is resistant to isoniazid and rifampicin, plus any fluoroquinolone and any second-line injectable drugs.

(3) Drug-susceptible TB: TB (among HIV-negative individuals) that is susceptible to isoniazid and rifampicin.

(4) HIV/AIDS - Multidrug-resistant TB without extensive drug resistance: a form of TB (among HIV-positive individuals) that is resistant to the two most effective first-line anti-tuberculosis drugs (isoniazid and rifampicin), but is not resistant to any fluoroquinolone and any second-line injectable drugs (amikacin, kanamycin, or capreomycin).

(5) HIV/AIDS - Extensively drug-resistant TB: a form of TB (among HIV-positive individuals) that is resistant to isoniazid and rifampicin, plus any fluoroquinolone and any second-line injectable drugs.

(6) HIV/AIDS - Drug-susceptible TB: TB (among HIV-positive individuals) that is susceptible to isoniazid and rifampicin.

Input data

Model inputs

Input data for TB include annual case notifications, data from prevalence surveys, and estimated cause-specific mortality rates (CSMR) of TB among HIV-positive and HIV-negative individuals. From these inputs, we calculated “priors” (expected values) on excess mortality to give more guidance to the model. Input data for latent TB infection (LTBI) include: (1) population-based tuberculin surveys, and (2) cohort studies examining the risk of developing active TB disease as a function of induration size. An updated systematic review was done for GBD 2017. The search terms, number of studies identified, and number of studies included are shown in the table below.

Outcome	Search Terms	Total number of studies identified	Number of studies included
Tuberculosis	Pubmed: ("tuberculosis"[MeSH] OR tuberculosis[Title/Abstract]) OR TB[Title/Abstract] OR Mycobacterium tuberculosis[Title/Abstract] AND prevalence[Title/Abstract] AND ("2016/08/01"[PDAT] : "2017/09/15[PDAT]) NOT (animals[MESH] NOT humans[MESH])	732	29
LTBI (tuberculin surveys)	Pubmed: ("tuberculin survey"[tiab] OR (("risk"[MeSH Terms] OR "risk"[tiab] OR "risk of"[tiab]) AND ("tuberculosis"[MeSH Terms] OR "tuberculosis"[tiab] OR "tuberculous"[tiab]) AND ("infection"[MeSH Terms] OR "infection"[tiab])) OR (("risk"[MeSH Terms] OR "risk"[tiab] OR "risk of"[tiab]) AND TB[tiab] AND ("infection"[MeSH Terms] OR "infection"[tiab])) OR "latent tuberculosis infection"[tiab] OR "latent TB infection"[tiab] OR "latent tuberculosis"[MESH]) AND ("survey"[tiab] OR "surveys"[tiab]) NOT (animals[MESH] NOT humans[MESH]) ("2016/08/01"[PDAT] : "2017/09/07"[PDAT])<br><br>Google Scholar: ("tuberculin survey" OR "risk of tuberculous infection" OR "risk of tuberculosis infection" OR "risk of TB infection" OR "latent tuberculosis infection" OR "latent TB infection") AND "survey". (01-01-2016 to 09-08-2017).	54;<br>1326	3;<br>5
LTBI (cohort studies)	Pubmed: ("tuberculin"[tiab] OR ("tuberculin"[tiab] AND "positive"[tiab]) OR "Mantoux"[tiab] OR ("Mantoux"[tiab] AND "positive"[tiab]) OR "induration"[tiab]) AND (active[tiab] AND ("tuberculosis"[MeSH] OR "tuberculosis"[tiab])) AND ("risk"[MeSH] OR "risk"[tiab]) AND ("prospective"[tiab] OR "follow up"[tiab] OR "longitudinal"[tiab]) NOT (animals[MESH] NOT humans[MESH]) ("2016/08/01"[PDAT] : "2017/09/21"[PDAT])	20	0

Input data for multidrug-resistant TB (MDR-TB) and extensively drug-resistant TB (XDR-TB) include: (i) the number of MDR-TB cases, XDR-TB cases, new and retreated TB cases with a drug sensitivity testing (DST) result for isoniazid and rifampicin, and MDR-TB cases with DST for second-line drugs from routine surveillance and surveys reported to the World Health Organization, and (ii) the risk of MDR-TB associated with HIV infection from the literature.[1]

Modelling Strategy

Overview

Our TB modelling strategy has not changed substantially from GBD 2016, but we made a refinement in the MI ratio regression approach: we used the Healthcare Access and Quality (HAQ) index instead of using SDI in the MI ratio regression (as HAQ is a better health-related index than SDI for TB). First, we estimated risk-weighted prevalence of LTBI by location, year, age, and sex using data from population-based tuberculin surveys and cohort studies reporting the risk of developing active TB disease as a function of induration size. Next, we divided the inputs on prevalence (from surveys in low- and middle-income countries), incidence (notification data from countries with a four- or five-star rating, and estimated incidence for countries with a less than four-star rating), and cause-specific mortality rate (CSMR) by the risk-weighted LTBI prevalence in order to model TB among those at risk in each country. We first ran a mixed effects regression (with region random effects) using MI ratios (logit transformed) from locations with a 4- or 5-star rating on causes of death with HAQ index as a covariate anchoring the lower end of the HAQ index scale with a data point from the Bangalore study[2] reporting that 49.2% of 126 untreated new pulmonary TB cases were dead at the end of the five-year follow up period, to predict age-sex specific MI ratios for all locations and years. We then estimated age-sex-specific incidence using the predicted MI ratios and CSMR estimates. We used DisMod-MR 2.1, the GBD Bayesian meta-regression tool to generate consistent trends in all parameters. We then multiplied the DisMod-MR 2.1 outputs by the risk-weighted prevalence of LTBI to get population-level estimates of incidence and prevalence. Because the outputs from DisMod-MR 2.1 are for all forms of TB, we split them into MDR-TB and XDR-TB by HIV status. To do so, we estimated the proportions of TB cases with MDR-TB for all locations and years, using data from notifications and survey data. We then estimated the proportions of MDR-TB among HIV-negative individuals and MDR-TB among HIV-positive individuals based on the risk of MDR-TB associated with HIV infection from a meta-analysis[1]. To split MDR-TB into MDR-TB with and without extensive drug resistance, we pooled the limited notification and survey data on the proportion of MDR-TB cases with extensive drug resistance by super-region, and applied these proportions to MDR-TB cases among HIV-negative and HIV-positive individuals, respectively.

Modelling risk-weighted latent TB infection prevalence

Input data for modelling risk-weighted LTBI prevalence were from two sources: (i) population-based tuberculin skin test (TST) surveys, and (ii) cohort studies examining the risk of developing active TB disease as a function of induration size. First, we extracted the prevalence of tuberculin skin testing results by induration size using the most detailed induration categories reported by studies. Second, from cohort studies reporting on the relative risk of developing active TB disease as a function of induration size, we pooled the risk of developing active TB by induration size in millimeters using the DisMod Ode computational engine. Third, we multiplied the LTBI prevalence by induration in millimeters ranging from 0-20+ with the relative risk of developing active TB at each induration size, and summed them up to derive risk-weighted LTBI prevalence for each age group.

Available evidence[3] suggests that people with very advanced HIV infection (CD4 counts <200 cells/mm^3) may have a false-negative TST (0mm induration) due to profound immune suppression, but still have very high risk for TB. For those who are HIV-positive, but with higher CD4 counts, the risk for active TB

increases with greater induration size as in HIV-negative individuals (ie, the shape of the tuberculin response curve is similar to that for the general population). To take into account the false-negative TST response in HIV cases with profound immune suppression, we first computed the proportion of HIV-positive individuals with CD4 counts <200 cells/mm for the 0 mm induration group using our HIV prevalence estimates for that particular category. We then multiplied that proportion by the relative risk of developing active TB disease in the 0 mm induration group compared with the 20+ mm induration group among HIV-positive individuals. The relative risk was computed using data from a prospective, multicenter cohort study of HIV-positive people in the United States.[3]

Using the risk-weighted LTBI prevalence (adjusting for a false-negative TST among people with advanced HIV infection) as input data, we ran a DisMod-MR 2.1 model with the HAQ index covariate to help inform variation over year and geography, with priors that at higher HAQ index values, LTBI prevalence decreases. We included two study covariates (BCG-positive, and mixed BCG status) where the reference category is BCG-negative. We found no statistically significant difference between studies using different dosages of tuberculin purified protein derivative (PPD). We therefore did not include different PPD dosages as study covariates but added more uncertainty to data points from studies that used dosages larger or smaller than the standard dose of 5 tuberculin units per test dose of 0.1 ml, by entering them as z-covariates in DisMod.

Modelling TB incidence

Incidence inputs were from two different sources: (1) incidence from notification data for countries with a four- or five-star rating on their cause of death data[4] as a proxy for the quality of health-related administrative data systems, and (2) estimated incidence for countries with a less than four-star rating. We used the age- and sex-specific notifications (all new and relapse cases combined) in our analysis. Prior to 2013, notification data were available by case type (new pulmonary smear-positive, new pulmonary smear-negative, and new extra-pulmonary) and there were missing age data, especially for younger age groups in some countries. We imputed the missing age groups for the three forms of TB notifications. Smear-positive age-specific notifications were inflated with the proportion smear-unknown and relapsed cases only reported at the country-year level. Some countries reported only pulmonary smear-positive cases for selected years. Missing smear-negative and extrapulmonary cases were predicted from the adjusted smear-positive cases using a seemingly unrelated regression. All three types of notifications were added together to represent TB-all-form incidence for countries with a four- or five-star rating.

To generate incidence estimates for locations with a less than four-star rating, we ran a mixed effects regression (with region random effects), using MI ratios (logit transformed) from locations with a 4- or 5-star rating on causes of death as input data with HAQ index as a covariate anchoring the lower end of the HAQ index scale with a data point from a cohort study in the 1960s[2] reporting that 49.2% of 126 untreated new pulmonary TB cases were dead at the end of the five-year follow-up period, in order to predict age-sex-specific MI ratios for all locations and years. We then used the MI ratios and cause-specific mortality estimates to compute the incidence input for DisMod-MR 2.1 for locations with a less than four-star rating. In locations where estimated MI ratios were greater than notification-based MI ratios, we used the latter to compute the incidence input. Notification-based MI ratios were computed using notification data and estimated CSMR for 2010. For other years, we assumed a similar proportional difference between predicted MI ratios and notifications-based MI ratios as in 2010 and adjusted the

predicted MI ratios accordingly, which were then used to compute the incidence input. We computed the age-sex-specific incidence of TB among the latent TB-infected population, using TB incidence as the numerator and our estimated risk-weighted latent TB infection prevalence as the denominator.

Modelling TB prevalence

Data from prevalence surveys reporting on pulmonary smear-positive TB and bacteriologically positive TB were included. Because incidence data are for all forms of TB, we adjusted prevalence surveys to account for extrapulmonary cases. We ran a spatiotemporal Gaussian process regression to predict location-year-age-sex-specific proportions of extrapulmonary TB among all TB cases using data on the three forms of TB from the incidence data above. We then computed the extrapulmonary inflation factor as *1+(proportion of extrapulmonary TB /(1- proportion of extrapulmonary TB))*, and applied it to data from prevalence surveys. We then computed the prevalence of TB among the TB-infected population, using TB prevalence as the numerator and our estimated risk-weighted LTBI prevalence as the denominator. We included a study covariate indicating whether it was bacteriologically positive TB (reference category) or smear-positive TB. We found no systematic bias between studies that used both symptoms and chest X-ray as screening methods and studies that used only one of the methods. We therefore did not adjust them for systematic bias but added more uncertainty to data points from studies that used only one of the screening methods (by using it as a z-covariate in DisMod). We also added more uncertainty to data points from subnational surveys. We included two location-level covariates, namely, age-standardised adult underweight prevalence and log-transformed age-standardised Summary Exposure Variable (SEV) scalar for TB (a summary variable of the exposure levels of TB risk factors weighted by relative risk) to help inform variation of TB prevalence over year and geography.

Modelling TB excess mortality

We matched each prevalence data point and TB CSMR (TB and HIV-TB combined) by location, year, age, and sex to calculate excess mortality rate (EMR) as *EMR=CSMR/prevalence*. We also matched each incidence data point and TB CSMR by location, year, age, and sex to calculate EMR for countries with a four- or five-star rating on their cause of death data. To reflect a gradient in EMR, we added the HAQ index and adult HIV death rates as country-level covariates.

DisMod-MR 2.1

For each location, we included the following as input in the DisMod model: case notifications for locations with a four- or five-star rating, predicted MI-ratio-based incidence for locations with a less than four-star rating, prevalence survey data where available, excess mortality estimates, and CSMR (TB and HIV-TB combined) by age and sex.

The output from the DisMod model was for all forms of TB in TB-infected populations, including both HIV-negative and HIV-positive individuals. We computed the incidence and prevalence of TB among the entire population, by multiplying the prevalence of LTBI with the DisMod model estimates.

Betas and exponentiated values from the DisMod model are shown in the table below.

Covariate	Parameter	Beta (95% CI)	Exponentiated beta (95% CI)
Smear positive TB	Prevalence	-0.75	0.47 (0.47–0.47)
Sex (male)	Prevalence	0.17	1.18 (1.10–1.27)
Sex (male)	Incidence	0.32	1.38 (1.38–1.38)
Age-standardised proportion adult underweight	Prevalence	2.65	14.16 (8.81–19.47)
Age-standardised SEV scalar (log-transformed)	Prevalence	0.76	2.13 (2.12–2.17)
HAQI (log-transformed)	Excess mortality	-1.50	0.22 (0.21–0.23)
Adult HIV death rate	Excess mortality	0.64	1.89 (1.03–6.23)

HIV-TB incidence and prevalence

To distinguish HIV-TB from all forms of TB, we first estimated the proportions of HIV-TB cases among all TB cases using data on the number of TB cases recorded as HIV-positive and the number of TB cases with an HIV test result recorded in the WHO TB notifications register. We ran a mixed effects regression using the adult HIV death rate as a covariate to predict location-year-specific HIV-TB proportions, which were then applied to TB incident and prevalent cases from DisMod, to generate HIV-TB incident and prevalent cases by location and year. These cases were then age-sex split based on the age-sex pattern of estimated HIV prevalence by location-year to generate location-year-age-sex-specific HIV-TB incident and prevalent cases.

Multidrug-resistant TB, extensively drug-resistant TB, and drug-susceptible TB

We ran spatiotemporal Gaussian process regressions to predict the proportions of new TB cases with MDR-TB, proportions of retreated TB cases with MDR-TB, and proportions of retreated cases among all TB cases for all locations and years. We calculated the proportions of new TB cases among all TB cases as *1- estimated proportions of retreated cases*. Next, we computed the weighted average of the proportions of new and retreated cases with MDR-TB at the 1000 draw level. We then used the weighted average proportions of MDR-TB, along with the HIV-TB and TB no-HIV incidence estimates, and the relative risk of MDR-TB associated with HIV infection from the literature[1] to compute the proportions of MDR-TB cases among HIV-negative TB cases $\left(PnoHIV_{c,y,a,s}\right)$ by location, year, age, and sex using the following formula:

$$PnoHIV_{c,y,a,s} = \frac{MDR_{c,y}}{\left(1 + \left(RR\frac{HIVTB_{c,y,a,s}}{TBnoHIV_{c,y,a,s}}\right)\right) TBnoHIV_{c,y,a,s}}$$

where $MDR_{c,y}$ is the number of all MDR-TB cases among HIV-positive and HIV-negative individuals by location and year, *RR* is the relative risk of MDR-TB associated with HIV infection, $HIVTB_{c,y,a,s}$ is the

number of HIV-TB incident cases by location, year, age, and sex, and $TBnoHIV_{c,y,a,s}$ is the number of TB no-HIV incident cases by location, year, age, and sex.

We then applied the predicted proportions of MDR-TB cases among HIV-negative TB cases to our predicted HIV-negative TB incident and prevalent cases to generate MDR-TB incident and prevalent cases by location, year, age, and sex. Next, we subtracted MDR-TB cases from all HIV-negative TB cases to generate drug-susceptible TB cases by location, year, age, and sex. To distinguish XDR-TB from MDR-TB, we aggregated the XDR-TB cases and MDR-TB cases (with drug sensitivity testing for second-line drugs) up to the super-region level and calculated the super-region-level proportions of XDR-TB among MDR-TB cases, which were then applied to MDR-TB cases in corresponding countries within the super-regions to produce XDR-TB cases by location, year, age, and sex. We linearly extrapolated XDR-TB prevalence and incidence back assuming the rates were zero in 1992, one year before 1993 when XDR-TB was first recorded in USA surveillance data.[5] Finally, we subtracted XDR-TB cases from MDR-TB cases to generate MDR-TB (without XDR) cases by location, year, age, and sex.

HIV/AIDS - Multidrug-resistant TB, HIV/AIDS - extensively drug-resistant TB, and HIV/AIDS - drug-susceptible TB

To split HIV-TB into HIV-MDR-TB and HIV-drug-susceptible-TB, we first calculated the proportions of HIV-MDR-TB among all HIV-TB cases ($PHIV_{c,y,a,s}$) for each location, year, age, and sex using the following formula:

$$PHIV_{c,y,a,s} = PnoHIV_{c,y,a,s}RR$$

where $PnoHIV_{c,y,a,s}$ is the proportions of MDR-TB among all HIV-negative TB cases for each location, year, age, and sex and RR is the relative risk of MDR-TB associated with HIV infection. We then applied the predicted proportions of MDR-TB cases among HIV-TB cases to our estimated HIV-TB incident and prevalent cases to generate HIV-MDR-TB incident and prevalent cases by location, year, age, and sex. Next, we subtracted HIV-MDR-TB cases from all HIV-TB cases to generate HIV-drug-susceptible-TB cases by location, year, age, and sex. To separate out HIV-XDR-TB from HIV-MDR-TB, we applied the super-region level proportions of XDR-TB among MDR-TB cases, to HIV-MDR-TB cases in corresponding countries within the super-regions to produce HIV-XDR-TB cases by location, year, age, and sex. We linearly extrapolated HIV-XDR-TB prevalence and incidence back assuming the rates were zero in 1992, one year before 1993 when XDR-TB was first recorded in USA surveillance data.[5] Finally, we subtracted HIV-XDR-TB cases from HIV-MDR-TB cases to generate HIV-MDR-TB (without extensive drug resistance) cases by location, year, age, and sex.

New MDR-TB and XDR-TB cases among retreated cases by HIV status

Because we split TB incidence (new and relapse cases combined) by drug-resistance type, the above estimation did not capture new MDR-TB and XDR-TB cases arising from retreated TB cases other than relapse cases. We therefore separately estimated new MDR-TB and XDR-TB cases arising from retreated TB cases and added them to the incident cases estimated above. To do so, we first ran a spatiotemporal Gaussian process regression using notification data and HAQ index as a covariate to predict the proportion of retreated cases (excluding relapse cases) among all TB patients for all locations and years.

Next, we computed retreated cases as *(retreated proportion*estimated incident cases)/(1-retreated proportion)*. We then computed the total number of TB cases by summing estimated incident cases and retreated cases. Similar to our estimation for MDR-TB and XDR-TB among TB incident cases by HIV status, we estimated MDR-TB and XDR-TB cases among all TB cases (incident cases and retreated cases combined) by HIV status. Finally, the number of retreated cases with MDR-TB was computed by subtracting MDR-TB among TB incident cases from MDR-TB among all TB cases (incident cases and retreated cases combined), separately for HIV-positive and HIV-negative individuals. Similarly, the number of retreated cases with XDR-TB was computed by subtracting XDR-TB among TB incident cases from XDR-TB among all TB cases, separately for HIV-positive and HIV-negative individuals. All computations were done at the 1000-draw level.

Disability weights

The lay descriptions and disability weights for severity levels derived from the GBD disability weights study are shown below.

Health state name	Lay description	Disability Weights (95% CI)
Tuberculosis, not HIV infected	has a persistent cough and fever, is short of breath, feels weak, and has lost a lot of weight	0.333 (0.224–0.454)
Tuberculosis, HIV infected	has a persistent cough and fever, shortness of breath, night sweats, weakness and fatigue and severe weight loss	0.408 (0.274–0.549)

For drug-susceptible TB, MDR-TB without extensive drug resistance, and XDR-TB, we used the same disability weight [0.333 (0.224–0.454)] as in non-HIV-infected TB. For HIV-drug-susceptible-TB, HIV-MDR-TB without extensive drug resistance, and HIV-XDR-TB, we used the same disability weight [0.408 (0.274–0.549))] as in HIV-infected TB.

Source Counts

Allforms tuberculosis	**Incidence**	**Prevalence**
**Site-years (total)**	1,751	166
**Number of countries with data**	76	28
**Number of GBD regions with data (out of 21 regions)**	15	10
**Number of GBD super-regions with data (out of 7 super-regions)**	5	7

Latent tuberculosis	**Prevalence**
**Site-years (total)**	228
**Number of countries with data**	48
**Number of GBD regions with data (out of 21 regions)**	15
**Number of GBD super-regions with data (out of 7 super-regions)**	7

Proportion of HIV-TB among all TB cases	Other
**Site-years (total)**	1,533
**Number of countries with data**	167
**Number of GBD regions with data (out of 21 regions)**	21
**Number of GBD super-regions with data (out of 7 super-regions)**	7

MDR-TB and MDR-HIV-TB proportions	Proportion
**Site-years (total)**	853
**Number of countries with data**	139
**Number of GBD regions with data (out of 21 regions)**	21
**Number of GBD super-regions with data (out of 7 super-regions)**	7

XDR-TB and XDR-HIV-TB proportions	Other
**Site-years (total)**	85
**Number of countries with data**	84
**Number of GBD regions with data (out of 21 regions)**	19
**Number of GBD super-regions with data (out of 7 super-regions)**	7

Risk of MDR-TB associated with HIV infection	Other
**Site-years (total)**	1
**Number of countries with data**	0
**Number of GBD regions with data (out of 21 regions)**	0
**Number of GBD super-regions with data (out of 7 super-regions)**	0

Latent tuberculosis relative risk	Other
**Site-years (total)**	27
**Number of countries with data**	21
**Number of GBD regions with data (out of 21 regions)**	11
**Number of GBD super-regions with data (out of 7 super-regions)**	6

References

1. Mesfin YM, Hailemariam D, Biadglign S, Kibret KT. Association between HIV/AIDS and multi-drug resistance tuberculosis: a systematic review and meta-analysis. *PLoS One*. 2014 Jan 8;9(1):e82235.
2. Institute NT. Tuberculosis in a rural population of South India: a five-year epidemiological study. *Bulletin of the World Health Organization*. 1974;51(5):473.
3. Markowitz N, Hansen NI, Hopewell PC, Glassroth J, Kvale PA, Mangura BT, Wilcosky TC, Wallace JM, Rosen MJ, Reichman LB. Incidence of tuberculosis in the United States among HIV-infected persons. *Annals of internal medicine*. 1997 Jan 15;126(2):123-32.
4. GBD 2016 Mortality and Causes of Death Collaborators. Global, regional, and national age-sex specific mortality for 264 causes of death, 1980–2016: a systematic analysis for the Global Burden of Disease Study 2016. *The Lancet* (under review)
5. Centers for Disease Control and Prevention (CDC). Extensively Drug-Resistant Tuberculosis --- United States, 1993—2006. *MMWR*. 2007; 56(11);250-253.

Lower respiratory infections (LRI)

Flowchart

Lower respiratory infections

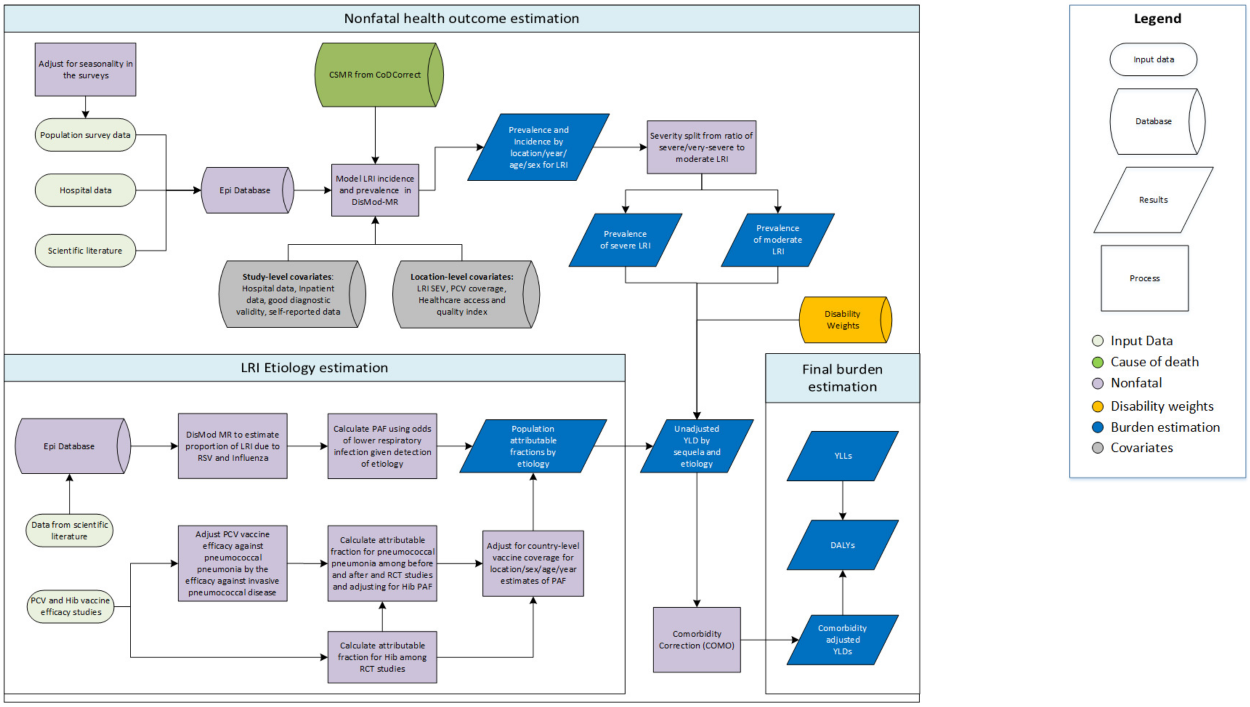

Case definition

We used clinician-diagnosed pneumonia or bronchiolitis as our case definition for lower respiratory infections (LRI). We included ICD9 codes 073.0-073.6, 079.82, 466-469, 480-489, 513.0, and 770.0 and ICD10 codes A48.1, J09-J22, J85.1, P23-P23.9, and U04. LRI aetiologies are modelled separately from overall LRI incidence and prevalence. The aetiologies include influenza, respiratory syncytial virus, *Streptococcus pneumoniae,* and *Haemophilus influenzae* type b and are episodes of LRI where the aetiology is the causal pathogen in the infection.

Input data

Model inputs

Input data included all data used in GBD 2016. We used two primary types of input data for lower respiratory infections. The first is lower respiratory infection incidence and prevalence data. These data come from a systematic literature review, hospital inpatient and outpatient data, claims data from the US, and population-representative surveys (**Table 1**). The second type of data is on the aetiologies of LRI. Influenza and respiratory syncytial virus (RSV) population attributable fractions were informed by a systematic literature review of the proportion of LRI cases that are positive for each pathogen. *Haemophilus influenzae* type B (Hib) and *Streptococcus pneumoniae* (pneumococcal pneumonia) are informed by a systematic review of vaccine efficacy and effectiveness studies.

To estimate the non-fatal burden of lower respiratory infections (LRI), we conducted a systematic review of scientific literature for LRI incidence and prevalence (Search String: ('lower respiratory' [title/abstract] OR pneumonia[title/abstract] AND ('2016/01/01'[PDat] : '2017/5/31'[PDat])AND Humans[MeSH Terms] NOT(autoimmune[title/abstract] OR COPD [title/abstract] OR 'cystic fibrosis'[title/abstract]). Our inclusion criteria were studies that were published between January 2016 and May 2017, had a sample size of at least 100, were at least one year in duration, and included lower respiratory infections, pneumonia, or bronchiolitis in the case definition. During our literature review we identified 595 studies, of which 75 met our inclusion criteria and were extracted. We excluded studies that described pandemic H1N1 influenza solely and studies that used influenza-like illness as the case definition. We assigned an age range based on the prevalence-weighted mean age of LRI in the appropriate year/sex/location if the ages of the study participants were not reported.

We also used self-reported prevalence of LRI symptoms from population-representative surveys, such as the Demographic and Health Survey and the Multiple Indicator Cluster Survey. When possible, we extracted survey data by one-year age group and by sex. We converted these data from two-week period prevalence to point prevalence. The equation for this adjustment is

$$1)\quad Point\ Prevalence = \frac{Period\ Prevalence * Duration}{(Recall\ Period + Duration - 1)}$$

We accepted four survey definitions for the prevalence of symptoms of LRI: 1) Cough with difficulty breathing with the symptoms in the chest with a fever was our gold standard but we also accepted 2) Cough with difficulty breathing with the symptoms in the chest *without* fever, 3) Cough with difficulty breathing with fever, and 4) Cough with difficulty breathing *without* fever. To make these definitions comparable, we identified the surveys that met the best case definition (definition 1). Within these surveys, we calculated the ratio of the prevalence of the best case definition to the prevalence of the alternate definitions. This ratio was regressed using age in years (factor), year of the survey, and GBD region (random intercept). The predicted values were used to adjust the prevalence for all the surveys that reported alternate case definitions.

Survey data were adjusted for seasonality. An inclusion criterion for scientific literature is a study duration longer than one year to avoid bias in the seasonal timing of LRI. Surveys are frequently conducted over several months. To account for seasonal variation in LRI symptom prevalence, we fit a generalised additive model with a forced periodicity for each GBD region. The model is mixed-effects with random effects on each country. The model accounts for the year of the survey and the case definition used. The percent difference between the monthly model fit LRI prevalence and the mean fitted LRI prevalence is a scalar to adjust survey data by month and geography.

In addition to survey data, hospital inpatient, outpatient data, and US claims data were included in the LRI modelling. To make the data more consistent in the modelling process, we converted all incidence data to prevalence. We found the ratio of the prevalence of LRI in hospitalisation records to the prevalence of LRI in our case definition (clinician-diagnosed pneumonia or bronchiolitis) for locations that contained data on both these prevalence values. We then regressed this ratio using age in years (factor) and GBD region (random intercept) to predict the adjustment factor for hospitalisation data to make them compatible with the reference case definition for our modelling.

Table 1: Data Inputs for LRI morbidity modelling by parameter

	**Prevalence**
**Site-years (total)**	3,114
**Number of countries with data**	159
**Number of GBD regions with data (out of 21 regions)**	21
**Number of GBD super-regions with data (out of 7 super-regions)**	7

Figure 1. The number of geography-years of data used in the LRI non-fatal modelling by GBD geography is shown

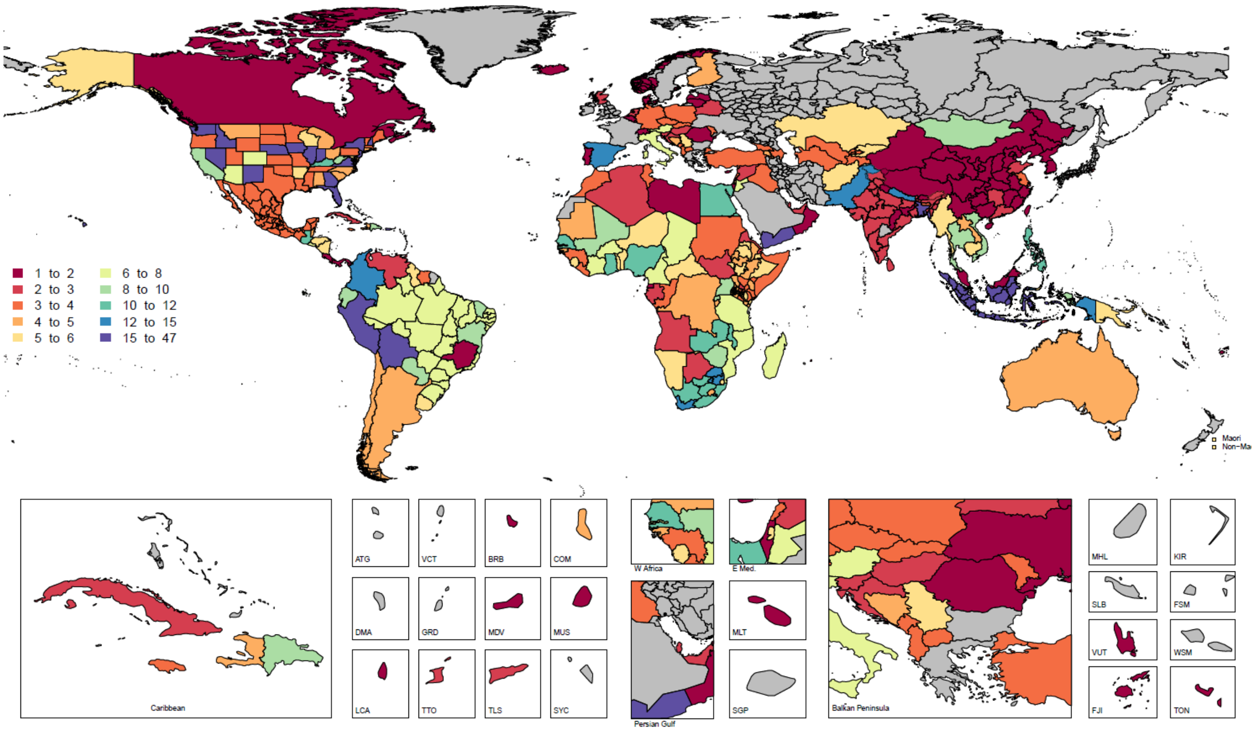

We performed a systematic review of the duration of symptoms of LRI. We sought consistency with our case definition of LRI and defined our duration as the time between the onset of symptoms to the resolution of increased work of breathing. Although crucial, there were very limited data on spatial, temporal, or age-specific duration, which may vary based on severity, aetiology, and treatment. We identified 485 titles from PubMed and extracted six studies which were used in a meta-analysis (mean duration 7.79 days, 6.2–9.64 days).

We also conducted a systematic literature review of studies on the Hib vaccine and PCV effectiveness studies against X-ray-confirmed pneumonia and against pneumococcal and Hib disease until May 2017. For PCV studies, we extracted, if available, the distribution of pneumococcal pneumonia serotypes and the serotypes included in the PCV used in the study. No new studies were identified for GBD 2017. We excluded observational and case-control studies due to implausibly high vaccine efficacy estimates. Hib trial data were exclusively from children <5 years so we did not include the effect of Hib on ages over 5

years of age. PCV trial data are also frequently limited to younger populations. To understand the contribution of pneumococcal pneumonia in older populations, we also included PCV efficacy studies that used before-after approaches.

Severity splits

The distribution of moderate (85%) and severe (15%) lower respiratory infections is determined by a meta-analysis of the ratio of severe to all LRI from studies that report the incidence of moderate and severe lower respiratory infections.

We used the health states of acute infectious disease episode, moderate and severe, with the lay descriptions and disability weight values shown in table below:

Table 2: Severity Splits

Severity level	Lay description	DW (95% CI)
Moderate	Has a fever and aches and feels weak which causes some difficulty with daily activities.	0.051 (0.032–0.074)
Severe	Has a high fever and pain and feels very weak, which causes great difficulty with daily activities.	0.133 (0.088–0.19)

Modelling strategy

The non-fatal lower respiratory infection burden is modelled in model-MR, a Bayesian meta-regression modelling framework. DisMod-MR produces estimates of the incidence, prevalence, and remission of LRI for each age, sex, geographic location, and year. We defined the time to recovery as an average of 10 days (5-15 days), which corresponds with a remission 36.5. The models are informed by study-level covariates and by country-level covariates.

Input data are adjusted to our standard case definition. Data are adjusted, as described previously, and Marketscan data are adjusted within DisMod to be compatible with the standard case definition (**Table 3**).

Table 3. Model covariates

Study covariate	Type	Parameter	Exponentiated beta
Self-reported	Study-level	Prevalence	3.58 (3.36–3.73)
Marketscan	Study-level	Prevalence	2.39 (2.29–2.45)
LRI SEV	Country-level	Prevalence	1.00 (1.00–1.01)
Socio-demographic Index	Country-level	Prevalence	0.47 (0.42–0.52)
Healthcare access and quality index	Country-level	Excess mortality	0.99 (0.99–0.99)

Aetiologies

We estimated LRI aetiologies separately from overall LRI mortality using two distinct counterfactual modelling strategies to estimate population attributable fractions (PAFs), described in detail below. The PAF represents the relative reduction in LRI mortality if there was no exposure to a given aetiology. As LRIs can be caused by multiple pathogens and the pathogens may co-infect, PAFs can overlap and add up to more than 100%. Separate strategies were used for viral – influenza and respiratory syncytial virus (RSV) – and bacterial – *Streptococcus pneumoniae* and *Haemophilus influenzae* type B – aetiologies. We did not attribute aetiologies to neonatal pneumonia deaths due to a dearth of reliable data in this age group. We calculated uncertainty of our PAF estimates from 1,000 draws of each parameter using normal distributions in log space.

Influenza and RSV. We calculated the population attributable fraction (PAF) from the proportion of severe LRI cases positive for influenza and RSV. We assumed that hospitalised LRI cases are a proxy of severe cases. We used the following formula to estimate PAF:[2]

$$PAF = Proportion * (1-1/OR)$$

Where Proportion is the proportion of LRI cases that test positive for influenza or RSV and OR is the odds ratio of LRI given the presence of the pathogen. We used an odds ratio of 5.1 (3.19–8.14) for influenza and 9.79 (4.98–19.27) for RSV from a published meta-analysis.[3]

We modelled the proportion data using the meta-regression tool DisMod-MR to estimate the proportion of LRI cases that are positive for influenza and RSV, separately, by location/year/age/sex. We accounted for study-level covariates in our models such as PCR as the diagnostic technique, studies that investigated RSV or influenza exclusively, and studies from inpatient populations.

Pneumococcal pneumonia and Hib. For *Streptococcus pneumoniae* (pneumococcal pneumonia) and *Haemophilus influenzae* type B (Hib), we calculated the population attributable fraction using a vaccine probe design.[4,5] The ratio of vaccine effectiveness against nonspecific pneumonia to pathogen-specific disease represents the fraction of pneumonia cases attributable to each pathogen.

To estimate the PAF for Hib and pneumococcal pneumonia, we calculated the ratio of vaccine effectiveness against nonspecific pneumonia to pathogen-specific pneumonia (Equations 1 and 3). We estimated a study-level estimate of PAF from a meta-analysis of these ratios. To estimate the PAF for Hib, we only used randomised controlled trials because of implausibly high values of vaccine efficacy in case-control studies. To estimate the PAF for pneumococcal pneumonia, we included RCTs and before and after vaccine introduction longitudinal studies.

We adjusted the study-level PAF estimate by vaccine coverage and expected vaccine performance to estimate country- and year-specific PAF values. For pneumococcal pneumonia, we adjusted the PAF by the final Hib PAF estimate and by vaccine serotype coverage. Finally, we used an age distribution of PAF modelled in DisMod to determine the PAF by age. Because of an absence of data describing vaccine efficacy against Hib in children older than two years, we did not attribute Hib to episodes of LRI in ages five years and older.

We used a vaccine probe design to estimate the PAF for pneumococcal pneumonia and (Hib) by first calculating the ratio of vaccine effectiveness against nonspecific pneumonia to pathogen-specific pneumonia at the study level (Equations 1 and 2).[4–6] We then adjusted this estimate by vaccine coverage and expected vaccine performance to estimate country- and year-specific PAF values (Equations 3 and 4).

1) $HibPAF_{Base} = \frac{VE_{Pneumonia}}{VE_{Hib}}$

2) $PneumoPAF_{Base} = \frac{VE_{Pneumonia} *(1-PAF_{Hib}*VE_{Hib\ Optimal})}{VE_{Streptococcus}*Cov_{Serotype}}$

3) $PAF_{Hib} = PAF_{Base} * \frac{(1-Cov_{Hib}*VE_{Hib\ Optimal})}{(1-PAF_{Base}*Cov_{Hib}*VE_{Hib\ Optimal})}$

4) $PAF_{Pneumo} = \frac{PAF_{Base}*(1-Cov_{PCV}*VE_{PCV\ Optimal})}{(1-PAF_{Hib}*Cov_{Hib}*VE_{Hib\ Optimal})*\left(1-\frac{PAF_{Base}Cov_{PCV}*VE_{PCV\ Optimal}}{(1-PAF_{Hib}*Cov_{Hib}*VE_{Hib\ Optimal})}\right)}$

Where $VE_{Pneumonia}$ is the vaccine efficacy against nonspecific pneumonia, VE_{Hib} is the vaccine efficacy against invasive Hib disease, $VE_{Streptococcus}$ is the vaccine efficacy against serotype-specific pneumococcal pneumonia, $Cov_{serotype}$ is the serotype-specific vaccine coverage for PCV,[7] $VE_{Hib\ Optimal}$ is the Hib effectiveness in the community (0.8)[8], PAF_{Hib} is the final PAF for Hib, Cov_{PCV} is the PCV coverage, Cov_{Hib} is the Hib coverage by country, and $VE_{PCV\ Optimal}$ is the vaccine effectiveness in the community (0.8).[9]

For Hib, we assumed that the vaccine efficacy against invasive Hib disease is the same against Hib pneumonia. For pneumococcal pneumonia, a recent study in adults[10] found that the vaccine efficacy against invasive pneumococcal disease may be significantly higher than against pneumococcal pneumonia. We used this ratio to adjust estimates of vaccine efficacy against invasive pneumococcal disease from other studies. However, recognising that the study is unique in that it uses a urine antigen test among adults, we added uncertainty around our adjustment using a wide uniform distribution (median 0.65, 0.3–1.0).

The major modelling change from GBD 2016 to GBD 2017 was the introduction of the survey and hospital data adjustment process outside of DisMod. DisMod is able to reconcile differences between a reference and non-reference binary covariate. However, this adjustment occurs just once globally and for all ages. We determined that there was significant variation in age and geography in these data adjustments and introduced a new mixed-effects regression to predict the ratio of the non-standard to the standard case definitions.

References

1 World Health Organization: Department of Child and Adolescent Health and Development. Handbook Integrated Management of Childhood Illness. 2005.

2 Miettinen OS. Proportion of disease caused or prevented by a given exposure, trait or intervention. *Am J Epidemiol* 1974; **99**: 325–32.

3 Shi T, McLean K, Campbell H, Nair H. Aetiological role of common respiratory viruses in acute lower respiratory infections in children under five years: A systematic review and meta-analysis. *J Glob Health* 2015; **5**: 10408.

4 Feikin DR, Scott JAG, Gessner BD. Use of vaccines as probes to define disease burden. *Lancet Lond Engl* 2014; **383**: 1762–70.

5 O'Brien KL, Wolfson LJ, Watt JP, *et al.* Burden of disease caused by Streptococcus pneumoniae in children younger than 5 years: global estimates. *Lancet Lond Engl* 2009; **374**: 893–902.

6 Watt JP, Wolfson LJ, O'Brien KL, *et al.* Burden of disease caused by Haemophilus influenzae type b in children younger than 5 years: global estimates. *Lancet Lond Engl* 2009; **374**: 903–11.

7 Johnson HL, Deloria-Knoll M, Levine OS, *et al.* Systematic evaluation of serotypes causing invasive pneumococcal disease among children under five: the pneumococcal global serotype project. *PLoS Med* 2010; **7**. DOI:10.1371/journal.pmed.1000348.

8 Swingler G, Fransman D, Hussey G. Conjugate vaccines for preventing Haemophilus influenzae type B infections. *Cochrane Database Syst Rev* 2007; : CD001729.

9 Lucero MG, Dulalia VE, Nillos LT, *et al.* Pneumococcal conjugate vaccines for preventing vaccine-type invasive pneumococcal disease and X-ray defined pneumonia in children less than two years of age. *Cochrane Database Syst Rev* 2009; : CD004977.

10 Bonten MJM, Huijts SM, Bolkenbaas M, *et al.* Polysaccharide conjugate vaccine against pneumococcal pneumonia in adults. *N Engl J Med* 2015; **372**: 1114–25.

Upper respiratory infections

Flowchart

Upper respiratory infections (URI)

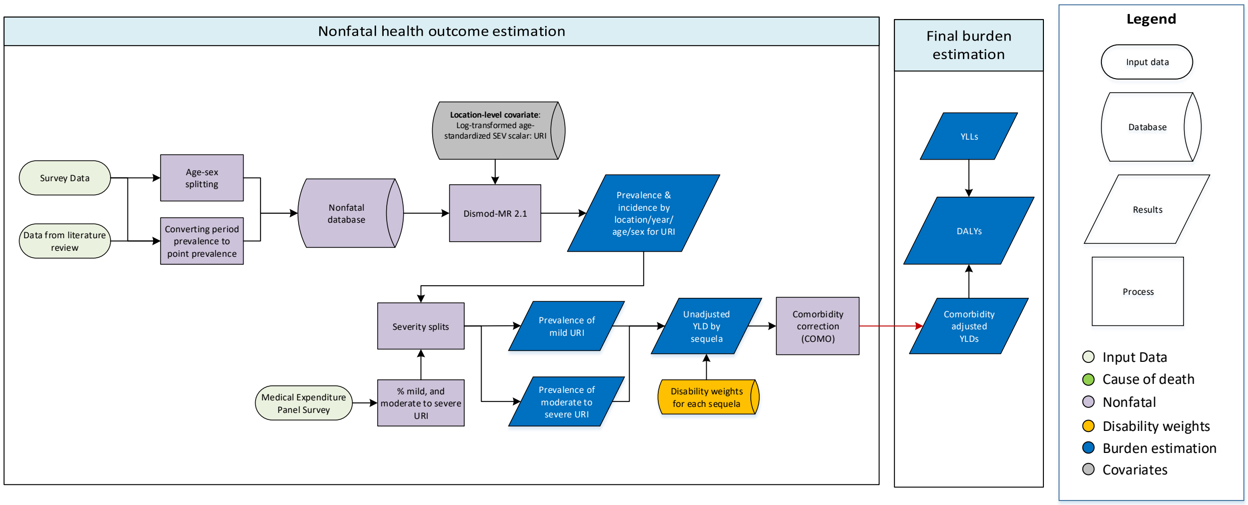

Case Definition

Upper respiratory infections (URI) include acute nasopharyngitis, sinusitis, pharyngitis, tonsillitis, laryngitis/tracheitis and epiglottitis. For URI, ICD 10 codes are J00-J02, J02.8-J03, J03.8-J06.9, J36, J36.0, and ICD 9 codes are 460-465.9, 475-475.9, 476.9.

Input data

Model Inputs

For GBD 2013, a systematic review of the prevalence of URI was conducted. The PubMed search string was: ((upper respiratory infection[Title/Abstract] or rhinitis[Title/Abstract] or rhinosinusitis[Title/Abstract] or sinusitis[Title/Abstract] or nasopharyngitis[Title/Abstract] or rhinopharyngitis[Title/Abstract] or common cold[Title/Abstract] or pharyngitis[Title/Abstract] or tonsillitis[Title/Abstract] or epiglottitis[Title/Abstract] or supraglottitis[Title/Abstract] or laryngitis[Title/Abstract] or laryngotracheitis[Title/Abstract] or tracheitis[Title/Abstract]) AND (prevalence[Title/Abstract] OR incidence[Title/Abstract]) NOT (allergies or allergy or allergic rhinitis or asthma) AND ("2009"[Date – Publication] : "2013"[Date – Publication]))

For GBD 2017, another systematic review of URI was conducted. The PubMed search string was: ((upper respiratory infection[Title/Abstract] or rhinitis[Title/Abstract] or rhinitis[MeSH] or rhinosinusitis[Title/Abstract] or sinusitis[Title/Abstract] or sinusitis[MeSH] or nasopharyngitis[Title/Abstract] or rhinopharyngitis[Title/Abstract] or common cold[Title/Abstract] or common cold[MeSH] or pharyngitis[Title/Abstract] or pharyngitis[MeSH] or tonsillitis[Title/Abstract] or epiglottitis[Title/Abstract] or supraglottitis[Title/Abstract] or supraglottitis[MeSH] or laryngitis[Title/Abstract] or laryngitis[MeSH] or laryngotracheitis[Title/Abstract] or tracheitis[Title/Abstract] or tracheitis[MeSH]) AND (prevalence[Title/Abstract] OR incidence[Title/Abstract] OR remission[Title/Abstract] OR duration[Title/Abstract]) NOT (allergies or allergy or allergic rhinitis or asthma) AND (2013/09/01[PDAT] : 2017[PDAT]))

The exclusion criteria for both systematic reviews were:

1. Studies that were not population-based, eg, hospital or clinic-based studies
2. Studies that did not provide primary data on epidemiological parameters, eg, a commentary piece
3. Studies with a sample size of less than 150
4. Reviews

Ultimately, no articles from the GBD 2017 systematic review were selected for incorporation into the URI model. However, three data sources provided by collaborators were added to the model for GBD 2017. Given the low yield of the most recent systematic review, we will prioritise adding data from national surveys as opposed to journal articles in future rounds, given that we expect comprehensive, national surveys to be more likely to estimate the burden of URI.

The table below shows the number of site-years of data included in GBD 2017, as well as the number of countries, regions, and super-regions represented.

	**Incidence**	**Prevalence**
**Site-years (total)**	3	211
**Number of countries with data**	1	73
**Number of GBD regions with data (out of 21 regions)**	1	18
**Number of GBD super-regions with data (out of 7 super-regions)**	1	7

In addition, data from nationally representative surveys including United States National Health Interview Surveys and Demographic and Health Surveys were included.

Severity Splits

The table below shows the severity distributions based on the data from Medical Expenditure Panel Surveys where we categorised "acute nasopharyngitis or acute uri multi sites/nos" as mild URI and "acute sinusitis, acute pharyngitis, acute tonsillitis, and acute laryngitis/tracheitis and epiglottitis" as moderate URI.

**Mild URI Proportion**	**Moderate URI Proportion**
.56 (.43 - .68)	.44 (.32 - .57)

The lay descriptions and disability weights for severity levels derived from the GBD disability weights study are shown below.

Severity level	Lay description		DW (95% CI)
Mild upper respiratory infections	has a low fever and mild discomfort , but no difficulty with daily activities.		0.006 (0.002–0.012)
Moderate/severe upper respiratory infections	has a fever and aches, and feels weak, which causes		0.051 (0.032–0.074)

	some difficulty with daily activities.		

Modelling Strategy

URI was modeled using a standard DisMod MR 2.1 model. We used secondhand smoke as the only location-level covariate in the final model. We tested other location-level covariates as well, such as the log-transformed age-standardised URI SEV scalar, indoor air pollution (all cooking fuels), and outdoor air pollution ($PM_{2.5}$).

Betas and exponentiated values are shown in the table below:

Covariate	Parameter	beta	Exponentiated beta
Secondhand smoke	Prevalence	0.11	1.12 (0.80–1.48)
Sex	Prevalence	-0.027	0.97 (0.95–0.99)

Otitis media

Flowchart

Otitis media (OM)

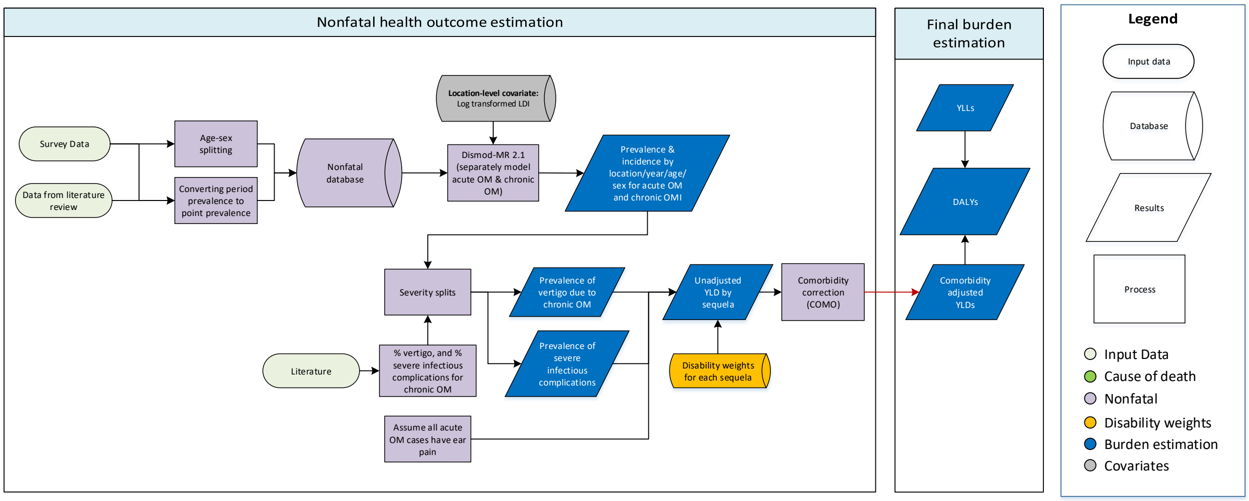

Case Definition

Otitis media is an infection of the middle ear space. We included acute otitis media, chronic otitis media, and hearing loss due to chronic otitis media in the GBD non-fatal outcome modelling. Hearing loss due to chronic otitis media estimation is included in the hearing loss report provided separately. The ICD 10 codes are H65-H75.83, and ICD 9 codes are 381-384.9.

Input data

Model Inputs

A systematic review of the prevalence of otitis media was conducted for GBD 2013. The PubMed search terms were: (((otitis media[Title/Abstract] AND (incidence[Title/Abstract] OR prevalence[Title/Abstract])) AND ("2009"[Date – Publication] : "2013"[Date – Publication])).

The exclusion criteria were:

1. Studies that were not population-based, eg, hospital or clinic-based studies
2. Studies that did not provide primary data on epidemiological parameters, eg, commentaries
3. Studies with a sample size of less than 150
4. Reviews
5. Case series

Updates to systematic reviews are performed on an ongoing schedule across all GBD causes, and an update for otitis media will be performed in the next one to two iterations.

The tables below show the number of site-years of data included in GBD 2017, as well as the number of countries, regions, and super-regions represented.

Acute otitis media		
	**Incidence**	**Prevalence**
**Site-years (total)**	339	8
**Number of countries with data**	9	7
**Number of GBD regions with data (out of 21 regions)**	5	5
**Number of GBD super-regions with data (out of 7 super-regions)**	3	4

Chronic otitis media			
	**Incidence**	**Prevalence**	**Remission**
**Site-years (total)**	1	30	5
**Number of countries with data**	1	15	4
**Number of GBD regions with data (out of 21 regions)**	1	9	4
**Number of GBD super-regions with data (out of 7 super-regions)**	1	5	3

In addition, data from the United States Medical Expenditure Panel Surveys and Australia National Health Surveys were included. The addition of US claims data in the acute otitis model is one of the main changes for GBD 2017.

Severity splits

We assume that all acute otitis media cases would experience ear pain. The severity distributions for chronic otitis media based on the study by Lin and colleagues (2009) were as follows: (i) vertigo (2.9%, 95% CI: 2.4–3.6%), and (ii) severe infectious complications (0.05%, 95% CI: 0.01–0.2%). We assumed that all chronic otitis media cases experience either mild or moderate hearing loss. The lay descriptions and disability weights for severity levels derived from the GBD disability weights study are shown below.

Severity level	Lay description	DW (95% CI)
Ear pain	has an earache that causes some difficulty with daily activities	0.013 (0.007–0.024)
Vertigo due to chronic otitis media*		
Severe infectious complications due to chronic otitis media	has an earache that causes some difficulty with daily activities	0.013 (0.007–0.024)

* See the hearing loss report for the lay descriptions and disability weights for different severity levels.

Modelling Strategy

We modelled acute and chronic otitis media as separate non-fatal health outcomes using DisMod-MR 2.1. Log-transformed LDI covariate was used as a location-level covariate to model chronic otitis media.

Betas and exponentiated values are shown in the table below:

Acute otitis media DisMod model

Covariate	Parameter	Beta (95% CI)	Exponentiated beta (95% CI)
Sex	Prevalence	0.39	1.47 (0.91–2.28)
Sex	Incidence	-0.21	0.81 (0.80–0.82)

Chronic otitis media DisMod model

Covariate	Parameter	Beta (95% CI)	Exponentiated beta (95% CI)
Log LDI	Prevalence	-0.39	0.68 (0.61–0.78)
Sex	Prevalence	0.048	1.05 (0.75–1.45)

Reference

Lin, Y. S., Lin, L. C., Lee, F. P., & Lee, K. J. (2009). The prevalence of chronic otitis media and its complication rates in teenagers and adult patients. *Otolaryngology-Head and Neck Surgery*, *140*(2), 165-170.

Diarrhoeal diseases

Flowchart

Diarrheal diseases

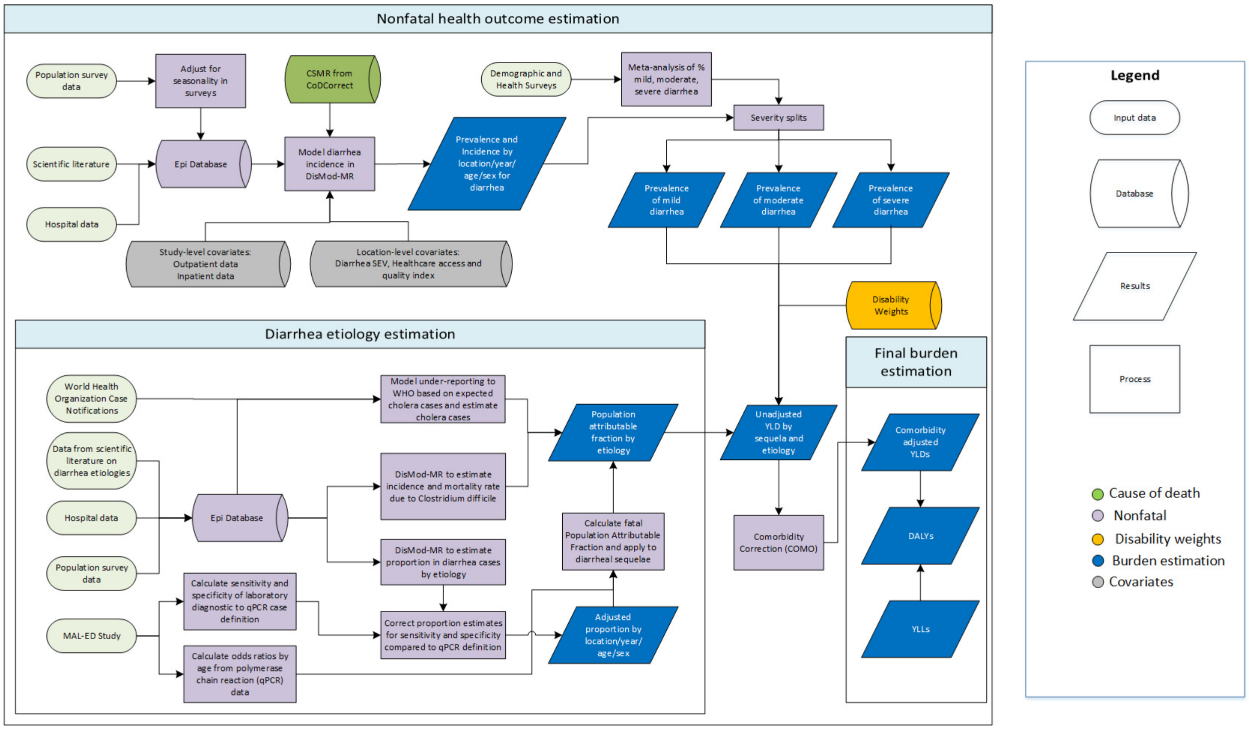

Case definition

We defined diarrhoeal disease episodes as three or more loose stools in a 24-hour period. In the diarrhoea models, self-reported prevalence is the reference category for all data adjustments. Hospital input data use ICD9 codes 001-009.9 and ICD10 codes A00-A09. We also split diarrhoea episodes into three severity levels: mild, moderate, and severe.

Input data

Model inputs

We used two main types of data in the diarrhoea non-fatal burden estimation and the attribution of diarrhoeal aetiologies. Moreover, we included all data sources used in GBD 2016 and conducted new reviews of scientific literature, surveys, and hospitalisation data. We presented a summary of the data sources in **Table 1**.

The first type of data is the incidence and prevalence of diarrhoea in community and hospital settings. Hospital data and healthcare utilisation data were identified using the ICD9 codes 001-009.9 and ICD10 codes A00-A09. To be consistent with the survey data, we transformed the hospital and health care data from incidence to prevalence. The second type of data are from population-representative surveys, such as the Demographic and Health Surveys and the Multiple Indicator Cluster Surveys. We converted the

prevalence of maternal-reported two-week period from surveys to point prevalence in one-year age groups using this equation:

$$Point\ Prevalence = Period\ Prevalence * \frac{Duration}{(Recall\ Period + Duration - 1)}$$

Where the mean duration was the duration in days, an average of 4.3 (4.2–4.4).[1]

Survey data were adjusted for seasonality. An inclusion criterion for scientific literature is a study duration longer than one year to avoid bias in the seasonal timing of diarrhoea. Surveys are frequently conducted over several months. To account for seasonal variation in diarrhoea prevalence, we fit a mixed-effects generalised additive model for each GBD region with a forced periodicity and a random intercept by country. The percent difference between the monthly model fit diarrhoea prevalence and the mean fitted diarrhoeal prevalence is a scalar to adjust survey data by month and geography.

Table 1: Data Inputs for diarrhoea morbidity modelling by parameter.

	Prevalence
**Site-years (total)**	3,247
**Number of countries with data**	169
**Number of GBD regions with data (out of 21 regions)**	21
**Number of GBD super-regions with data (out of 7 super-regions)**	7

Figure 1. The number of geography-years used in diarrhoea non-fatal modelling for each country is shown

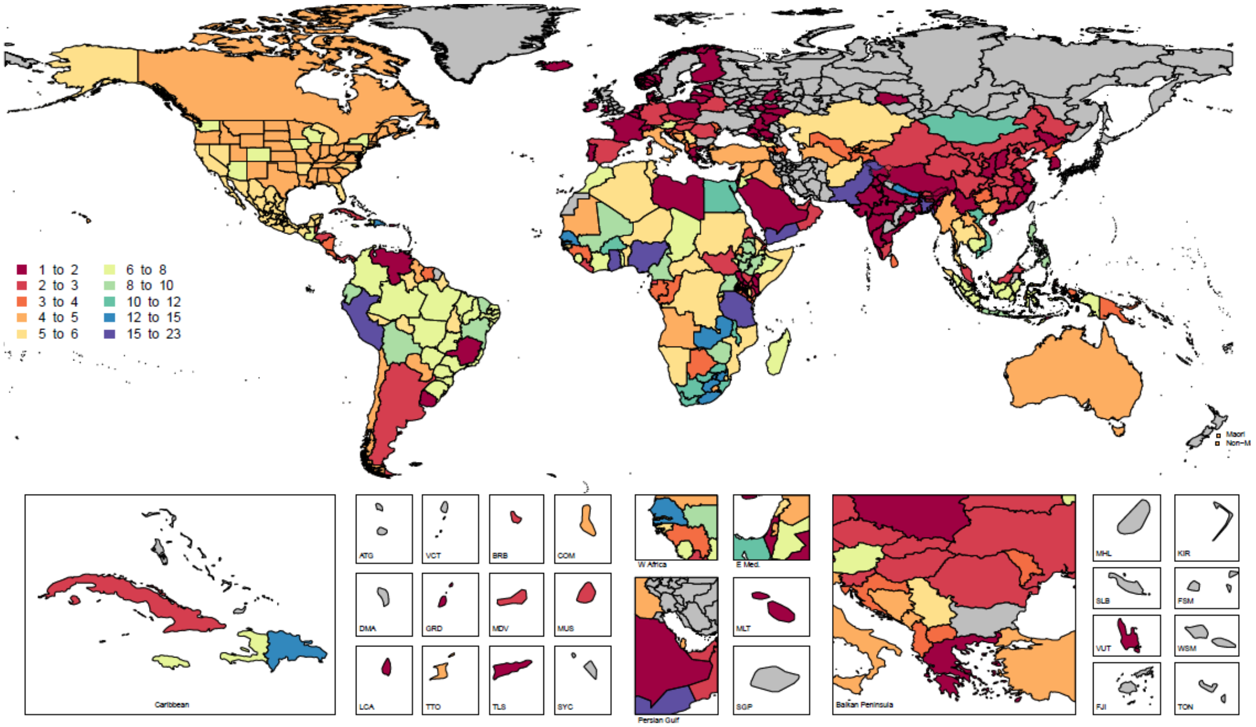

Aetiologies

The second type of data describes diarrhoea aetiologies. We extracted data on all aetiologies except *C. difficile* from scientific literature that reported the proportion of diarrhoea cases that tested positive for each pathogen. We completed a systematic literature review covering the years January 2016 to May 2017 for diarrhoea prevalence, incidence, and all diarrhoea aetiologies. Inclusion criteria included diarrhoea as the case definition, studies with a sample size of at least 100, and studies with at least one year of follow-up. We excluded studies that reported on diarrhoeal outbreaks exclusively and those that used acute gastroenteritis with or without diarrhoea.

We pulled all articles using a PubMed search term that combined non-specific and aetiology-specific diarrhoea on May 25, 2017 (Search string: (diarrhoea[title/abstract] OR diarrhoea[title/abstract]) AND (2016/05/01:2017/12/31[PDat]) AND Humans[MeSH Terms] AND (incidence[title/abstract] OR prevalence[title/abstract] OR epidemiology[title/abstract] OR salmonella[title/abstract] OR aeromonad*[title/abstract] OR shigell*[title/abstract] OR enteropathogenic[title/abstract] OR enterotoxigenic[title/abstract] OR campylobacter[title/abstract] OR amoebiasis[title/abstract] OR entamoeb*[title/abstract] OR cryptosporid*[title/abstract] OR rotavirus[title/abstract] OR norovirus[title/abstract] OR adenovirus[title/abstract] OR etiology[title/abstract]) NOT (appendicitis[title/abstract] OR esophag*[title/abstract] OR surger*[title/abstract] OR gastritis[title/abstract] OR liver[title/abstract] OR case report[title] OR case-report[title] OR therapy[title] OR treatment[title] Crohn[title/abstract] OR “inflammatory bowel”[title/abstract] OR irritable[title/abstract] OR travel*[title] OR Outbreak[title] OR Review[ptyp] OR vomiting[title/abstract)).

We identified 225 studies, of which 51 met our inclusion criteria. We extracted data points for location, sex, year, and age. For the data that describe proportion of episodes positive for a given pathogen, we assigned an age range based on the prevalence-weighted mean age of diarrhoea in the appropriate year/sex/location if the age of the study participants was not reported.

We used the Global Enteric Multicenter Study (GEMS), a seven-site, case-control study of moderate-to-severe diarrhoea in children under 5 years,[2] and the MAL-ED study,[3] a multi-site birth cohort, to calculate odds ratios for the diarrhoeal pathogens. We analysed raw data for a systematic reanalysis, representative of the distribution of cases and controls by age and site that were tested for the presence of pathogen using quantitative polymerase chain reaction (qPCR).[4]

Severity split inputs

Diarrhoeal diseases have three severity levels: mild, moderate, and severe (Table 2). The proportion of diarrhoea cases that are assigned to each comes from a systematic review of diarrhoea severity.[1] Mild cases are the proportion of diarrhoea cases that did not seek medical care (64.8%); moderate cases are the proportion that sought medical care but did not have severe dehydration or bloody stool (28.9%); and severe cases are the proportion that sought medical care with severe dehydration or bloody stool (6.9%). These proportions are based on the frequency of dehydration and bloody stool among community-based studies reported in the systematic review.

Table 2. Severity splits, details on the severity levels for diarrhoea in GBD 2017 and the associated disability weight (DW) with that severity.

Severity level	Lay description	DW (95% CI)
Mild	Has diarrhoea defined as 3 or more loose stools in a 24-hour period with no dehydration	0.074 (0.049–0.104)
Moderate	Has diarrhoea defined as 3 or more loose stools in a 24-hour period with painful cramps and feeling thirsty and any dehydration	0.188 (0.125–0.264)
Severe	Has diarrhoea defined as 3 or more loose stools in a 24-hour period with painful cramps and is very thirsty or feels nauseated or tired and/or severely dehydrated	0.247 (0.164–0.348)

Modelling strategy

The non-fatal diarrhoeal disease burden is modelled in DisMod-MR, a Bayesian meta-regression modelling framework. DisMod-MR produces estimates of the incidence, prevalence, and remission of diarrhoea for each age, sex, geographic location, and year. We defined remission, or the time to recovery, as five days average. Diarrhoeal disease episodes are characterised as three or more loose stools in a 24-hour period. The reference category for our input data is community-based diarrhoea episodes such as data from population-representative surveys or community cohorts. Input data that are from a different population, such as hospital outpatient or inpatient groups, are adjusted by study-level covariates so that they are consistent with the reference category. In contrast to GBD 2016, the adjustment for inpatient populations occurred outside of DisMod using a mixed-effects regression model with a random intercept by GBD super-region to determine the relative prevalence in hospitalised to non-hospitalised diarrhoea.

Country-level covariates also inform the model. These include the proportion of the population that have access to improved sanitation, access to improved water sources, health system access, income per capita, and the SEV for diarrhoea (**Table 3**).

Table 3. Covariates. Summary of covariates used in the diarrhoea DisMod-MR meta-regression model

Covariate	Type	Parameter	Exponentiated beta (95% Uncertainty Interval)
Hospital data	Study-level		0.11 (0.094–0.12)
Marketscan data	Study-level		1.20 (1.17–1.23)
Diarrhoea SEV	Country-level	Prevalence	1.04 (1.01–1.08)
Rotavirus vaccine coverage	Country-level	Prevalence	1.00 (1.00–1.00)

Healthcare access and quality index	Country-level	Excess mortality	0.95 (0.95–0.95)

We estimated diarrhoeal disease aetiologies independently from overall diarrhoea mortality using a counterfactual strategy for enteric adenovirus, *Aeromonas*, *Entamoeba histolytica*, *Campylobacter*, *Cryptosporidium*, typical enteropathogenic *Escherichia coli* (t-EPEC), enterotoxigenic *Escherichia coli* (ETEC), norovirus, non-typhoidal salmonella, rotavirus, and *Shigella*. *Vibrio cholerae* and *Clostridium difficile* were modelled separately.

Diarrhoeal aetiologies are attributed to diarrhoeal deaths using a counterfactual approach. We calculated a population attributable fraction (PAF) from the proportion of severe diarrhoea cases that are positive for each aetiology. The PAF represents the relative reduction in diarrhoea mortality if there was no exposure to a given aetiology. As diarrhoea can be caused by multiple pathogens and the pathogens may co-infect, PAFs can overlap and add up to more than 100%. We used the following formula to estimate PAF:[5]

$$PAF = Proportion * (1 - \frac{1}{OR})$$

Where *Proportion* is the proportion of diarrhoea cases positive for an aetiology and *OR* is the odds ratio of diarrhoea given the presence of the pathogen.

We dichotomized the continuous qPCR test result using the value of the cycle threshold (Ct) that most accurately discriminated between cases and controls. The Ct values range from 0 to 35 cycles representing the relative concentration of the target gene in the stool sample. A low value indicates a higher concentration of the pathogen while a value of 35 indicates the absence of the target in the sample. We used the lower Ct value when we had multiple Ct values for the cutpoint. The case definition for each pathogen is a Ct value that is below the established cutoff point.

We used a generalised linear mixed effects logistic regression model to calculate the odds ratio for under 1 year and 1-2 years old for each of our pathogens from the MAL-ED study. The MAL-ED study was used exclusively because the samples tested from that study are from community-based samples, which we determined were more representative of non-fatal diarrhoea than the GEMS samples, which tested only moderate-to-severe diarrhoea. The odds ratio for 1-2 years was applied to all GBD age groups over 5 years. There were three pathogen-age odds ratios that were not statistically significant: Aeromonas and Amoebiasis in under 1 year and Campylobacter in 1-2 years. If the odds ratio was not statistically significant, we transformed the odds ratios only for those aetiologies in log-space such that exponentiated values could not be below 1. The transformation was:

Odds ratio = exp(log(or) – 1)) + 1

We modelled the proportion data using the meta-regression tool DisMod-MR to estimate the proportion of positive diarrhoea cases for each separate aetiology by location/year/age/sex and to adjust for the covariates.

We used the estimated sensitivity and specificity of the laboratory diagnostic technique used in a pooled dataset of the GEMS and MAL-ED data compared to the qPCR case definition to adjust our proportion before we computed the PAF:[6]

$$Proportion_{True} = \frac{(Proportion_{Observed} + Specificity - 1)}{(Sensitivity + Specificity - 1)}$$

We used this correction to account for the fact that the proportions we used are based on a new test that is not consistent with the laboratory-based case definition (qPCR versus conventional laboratory testing for pathogens).[7] Because differences in the type of PCR used in the original (non-reference qPCR diagnostic) between GEMS and MALED in detecting norovirus, we combined the sensitivity and specificity results for norovirus such that 50% of the draws were coming from GEMS test results exclusively and 50% of the draws were coming from MALED test results exclusively. Additionally, because the original laboratory diagnostic technique used for *Campylobacter* in MALED was one not commonly used, we only used GEMS to determine the sensitivity and specificity of bacterial culture compared to qPCR in detecting *Campylobacter.*[8]

Our literature review extracted the proportion of any enteropathogenic *Escherichia coli* (EPEC) without differentiating between typical (tEPEC) and atypical (aEPEC). In order to be consistent with the odds ratios that we obtained, we adjusted our proportion estimates of any EPEC to typical EPEC only. This adjustment was informed by a subset of our literature review that reported both atypical and typical EPEC. We estimated a ratio by super-region of tEPEC to any EPEC and adjusted our proportion estimates accordingly. We found that the majority of EPEC diarrhoea cases were positive for atypical EPEC, consistent with other published work.[9]

For *Vibrio cholerae* (cholera), we used the literature review to estimate expected number of cholera cases for each country-year using the incidence of diarrhoea, estimated using DisMod-MR, and the proportion of diarrhoea cases that are positive for cholera. We assigned cholera PAF using odds ratios from the qPCR results to estimate a number of cholera-attributable cases. We compared this expected number of cholera cases to the number reported to the World Health Organization at the country-year level.[10] We modelled the underreporting fraction to correct the cholera case notification data for all countries using health system access and the diarrhoea SEV scalar to predict total cholera cases. We used the age-specific proportion of positive cholera samples in DisMod and our incidence estimates to predict the number of cholera cases for each age/sex/year/location. Finally, we modelled the case fatality ratio of cholera using DisMod-MR and to estimate the number of cholera deaths.

For *C. difficile*, we modelled incidence and mortality in DisMod-MR for each age, sex, year, location. DisMod-MR is a Bayesian meta-regression tool that uses spatiotemporal information as priors to estimate prevalence, incidence, remission, and mortality for *C. difficile* infection. DisMod-MR uses a compartmental model to relate prevalence, incidence, remission, and mortality. We set remission in our model to 1 month.

There are no major modelling updates from GBD 2016.

References

1 Lamberti LM, Fischer Walker CL, Black RE. Systematic review of diarrhea duration and severity in children and adults in low- and middle-income countries. *BMC Public Health* 2012; **12**: 276.

2 Kotloff KL, Nataro JP, Blackwelder WC, *et al.* Burden and aetiology of diarrhoeal disease in infants and young children in developing countries (the Global Enteric Multicenter Study, GEMS): a prospective, case-control study. *Lancet Lond Engl* 2013; **382**: 209–22.

3 Platts-Mills J, Liu J, Rogawski E. Aetiology, burden and clinical characteristics of diarrhoea in children in low-resource settings using quantitative molecular diagnostics: results from the MAL-ED cohort study. *Lancet Glob Health* 2018; : Accepted.

4 Liu J, Gratz J, Amour C, *et al.* A laboratory-developed TaqMan Array Card for simultaneous detection of 19 enteropathogens. *J Clin Microbiol* 2013; **51**: 472–80.

5 Miettinen OS. Proportion of disease caused or prevented by a given exposure, trait or intervention. *Am J Epidemiol* 1974; **99**: 325–32.

6 Reiczigel J, Földi J, Ozsvári L. Exact confidence limits for prevalence of a disease with an imperfect diagnostic test. *Epidemiol Infect* 2010; **138**: 1674–8.

7 Platts-Mills JA, Operario DJ, Houpt ER. Molecular diagnosis of diarrhea: current status and future potential. *Curr Infect Dis Rep* 2012; **14**: 41–6.

8 Platts-Mills JA, Liu J, Gratz J, *et al.* Detection of Campylobacter in stool and determination of significance by culture, enzyme immunoassay, and PCR in developing countries. *J Clin Microbiol* 2014; **52**: 1074–80.

9 Ochoa TJ, Barletta F, Contreras C, Mercado E. New insights into the epidemiology of enteropathogenic Escherichia coli infection. *Trans R Soc Trop Med Hyg* 2008; **102**: 852–6.

10 World Health Organization. Global Health Observatory data repository: Cholera. 2016. http://apps.who.int/gho/data/node.main.174?lang=en (accessed Aug 25, 2016).

Typhoid and paratyphoid fevers

Flowchart

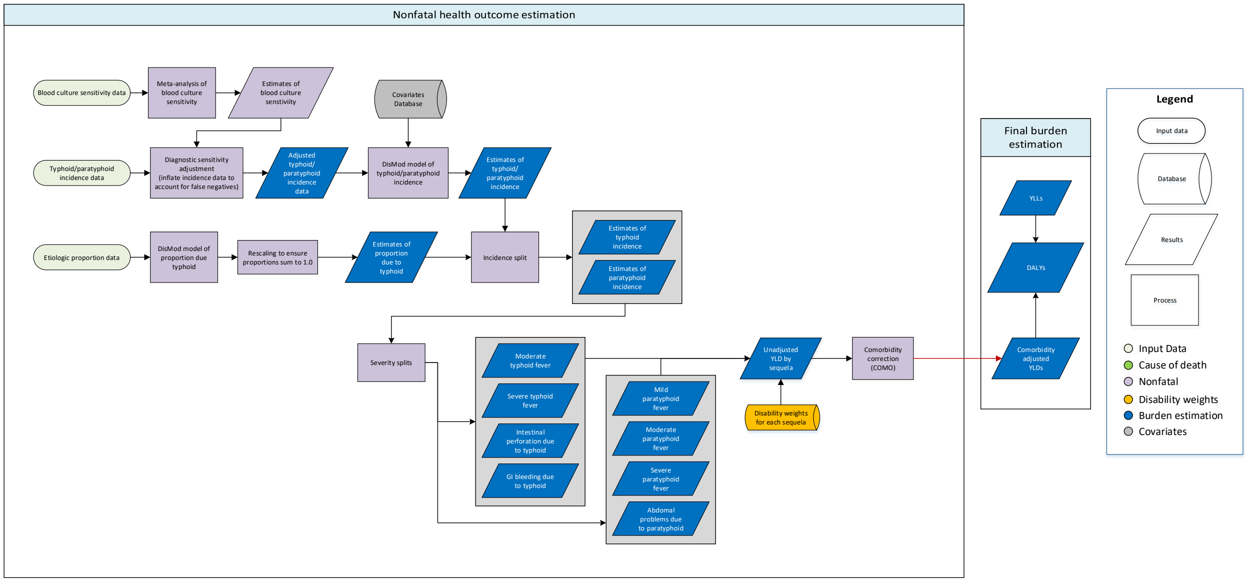

Case definition

Typhoid and paratyphoid are acute bacterial infections that most commonly cause febrile illness and gastrointestinal symptoms. Severe cases are associated with intestinal bleeding and perforation, altered mental state and, in some cases, death. We define a confirmed case as one for which there has been a positive blood culture test for either *Salmonella enterica typhi* or *paratyphi*. Diagnostic criteria do not typically accompany national surveillance reports; however, with blood culture being the standard diagnostic, we treat reported cases as confirmed. Given the poor sensitivity of blood culture, however, we estimated case definition as simply febrile illness resulting from an infection with *Salmonella enterica typhi* or *paratyphi*. This is effectively a counterfactual definition in which we attempt to estimate the number of true infections regardless of test result. These causes include all ICD-10 codes under the heading A01 (Typhoid and paratyphoid fevers).

Input data

Model inputs

Our incidence dataset included a combination of data from prospective cohort studies and national surveillance systems. Similarly, data on proportions due to typhoid and paratyphoid included a combination of prospective cohort studies and national surveillance systems.

	**Incidence**	**Proportion**
**Site-years (total)**	453	380
**Number of countries with data**	24	23
**Number of GBD regions with data (out of 21 regions)**	14	11
**Number of GBD super-regions with data (out of 7 super-regions)**	7	6

Updates to systematic reviews are performed on an ongoing schedule across all GBD causes; an update for typhoid and paratyphoid fevers will be performed in the next one to two iterations. While no systematic update was conducted, we did incorporate new data that were provided by collaborators.

Severity splits
For GBD 2017, we derived severity splits based on a published review of enteric fever outcomes from (Azmatullah A, Qamar FN, Thaver D, et al. 2005).

Paratyphoid is split into four sequelae: mild (28.5% [15.6–44.2]), moderate (52.25% [27.2–77.7]), severe (14.25% [8.2–21.8]), and abdominal pain and distention (5.0% [2.8–7.6]):

Sequela	Description	Disability weight
Mild	Has a low fever and mild discomfort, but no difficulty with daily activities.	0.006 (0.002–0.012)
Moderate	Has a fever and aches, and feels weak, which causes some difficulty with daily activities.	0.051 (0.032–0.074)
Severe	Has a high fever and pain, and feels very weak, which causes great difficulty with daily activities.	0.133 (0.088–0.19)
Abdominal pain & distention due to paratyphoid	Has pain in the belly and feels nauseated. The person has difficulties with daily activities.	0.114 (0.078–0.159)

Similarly, typhoid is split into four sequelae: moderate (35.0% [26.0–44.3]), severe (47.75% [38.0–57.4]), severe abdominal pain and distention (17.0% [10.0–25.7]), and intestinal bleeding (0.25% [0–2.0]):

Sequela	Description	Disability weight
Moderate	Has a fever and aches, and feels weak, which causes some difficulty with daily activities.	0.051 (0.032–0.074)
Severe	Has a high fever and pain, and feels very weak, which causes great difficulty with daily activities.	0.133 (0.088–0.19)
Gastrointestinal bleeding	Vomits blood and feels nauseated.	0.325 (0.209–0.462)
Abdominal pain and distention (includes intestinal perforation)	Has severe pain in the belly and feels nauseated. The person is anxious and unable to carry out daily activities.	0.324 (0.22–0.442)

Modelling strategy

We first model total incidence of typhoid and paratyphoid combined. Second, we model the proportion of this total due to typhoid and the proportion due to paratyphoid. Finally, we split the case estimates into sequelae representing different major symptoms and levels of severity.

Total incidence was modelled using DisMod-MR, using the summary exposure values (SEV) for unsafe water, and the proportion of the population living in the Indian Ocean monsoon belt as covariates. We performed a crosswalk using a study-level covariate indicating sources that were based on passive versus active surveillance, with active surveillance as the reference. This adjusts for incomplete case capture by passive surveillance. Incidence data were inflated to account for poor diagnostic sensitivity, based on an internal meta-analysis of the sensitivity of blood culture, the most common diagnostic used for typhoid. Similarly, we used two DisMod models to estimate aetiologic proportions: one for the proportion of total incidence due to typhoid, and one for the proportion due to paratyphoid.

Typhoid cases are split between four sequelae: moderate typhoid fever, severe typhoid fever, severe typhoid fever with intestinal bleeding, and typhoid fever with abdominal complications. Paratyphoid cases are split between four sequelae: mild paratyphoid fever, moderate paratyphoid fever, severe paratyphoid fever, and paratyphoid fever with abdominal complications.

Changes from GBD 2016 to GBD 2017

We made no major changes in our methods between GBD 2016 and 2017.

Invasive non-typhoidal salmonella (iNTS)

Flowchart

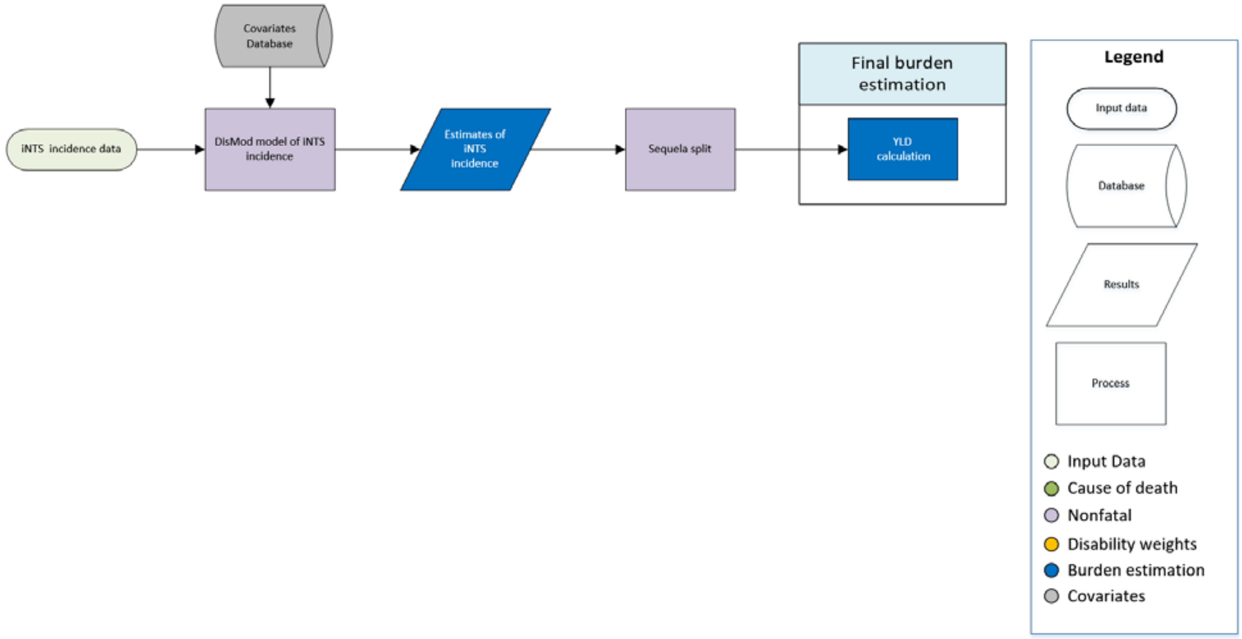

Case definition

Non-typhoidal salmonella infections are typically associated with diarrhoea. When these bacteria invade a typically sterile site like blood, they produce invasive non-typhoidal salmonella (iNTS) disease. Whereas non-typhoidal salmonella infections typically produce diarrhoeal illness, iNTS is typically febrile and can manifest in diverse symptoms that vary with severity and the exact site of the infection. Blood culture is the standard diagnostic for iNTS, and has good sensitivity and specificity. We thus define a case of iNTS as any blood-culture-confirmed non-typhoidal salmonella infection.

Input data

Model inputs

We conducted a systematic review for studies of iNTS incidence, including sources that provided iNTS incidence rates derived from either active surveillance or, more commonly, hospital- or clinic-based surveillance with adjustments for health care utilisation. Studies of special populations (eg, people living with HIV/AIDS) were excluded. In total, we found 34 sources meeting our inclusion criteria.

	Incidence
**Site-years (total)**	135
**Number of countries with data**	25
**Number of GBD regions with data (out of 21 regions)**	11
**Number of GBD super-regions with data (out of 7 super-regions)**	6

Severity splits

Given the typical severity of iNTS and the breadth of potential symptoms and manifestations, we assign all cases to the severe acute infectious disease episode health state, with a disability weight of 0.133 (0.088–0.19)

Sequela	Description	Disability weight
Severe acute infectious disease episode	Has a high fever and pain, and feels very weak, which causes great difficulty with daily activities.	0.133 (0.088–0.19)

Modelling strategy

We modelled incidence using DisMod-MR, using HIV mortality rate, malaria incidence adjusted for antimalarial coverage and drug effectiveness, and the summary exposure values (SEV) for sanitation as country-level covariates. We use no study-level covariates in the model. We estimated prevalence as the product of incidence times duration. We assumed a mean duration of 14 days (95% CI: 7–21).

Changes from GBD 2016 to GBD 2017

iNTS is a new cause for GBD 2017.

Other intestinal infectious diseases

In addition to the intestinal infectious diseases described above, there are many diverse types of intestinal infectious diseases. Because these intestinal infectious diseases are diverse in their underlying causes and risk factors as well as in their associated health outcomes, modelling them together in a DisMod-MR model would not produce reliable estimates of prevalence or excess mortality. Instead, we calculated the YLDs caused by intestinal infectious diseases directly using a YLD/YLL ratio.

We calculated the ratio of YLDs to YLLs across the specified intestinal infectious diseases for which nonfatal outcomes were modelled, using YLL estimates from the GBD 2017 cause of death (CoD) analysis. We then multiplied this YLD/YLL ratio by the YLL estimates for other intestinal infectious diseases from the GBD 2017 CoD analysis, providing us with an estimate of the YLDs associated with other intestinal infectious diseases.

Malaria

Flowchart

Malaria non-fatal outcomes (parasite rate and case incidence) in Sub-Saharan Africa

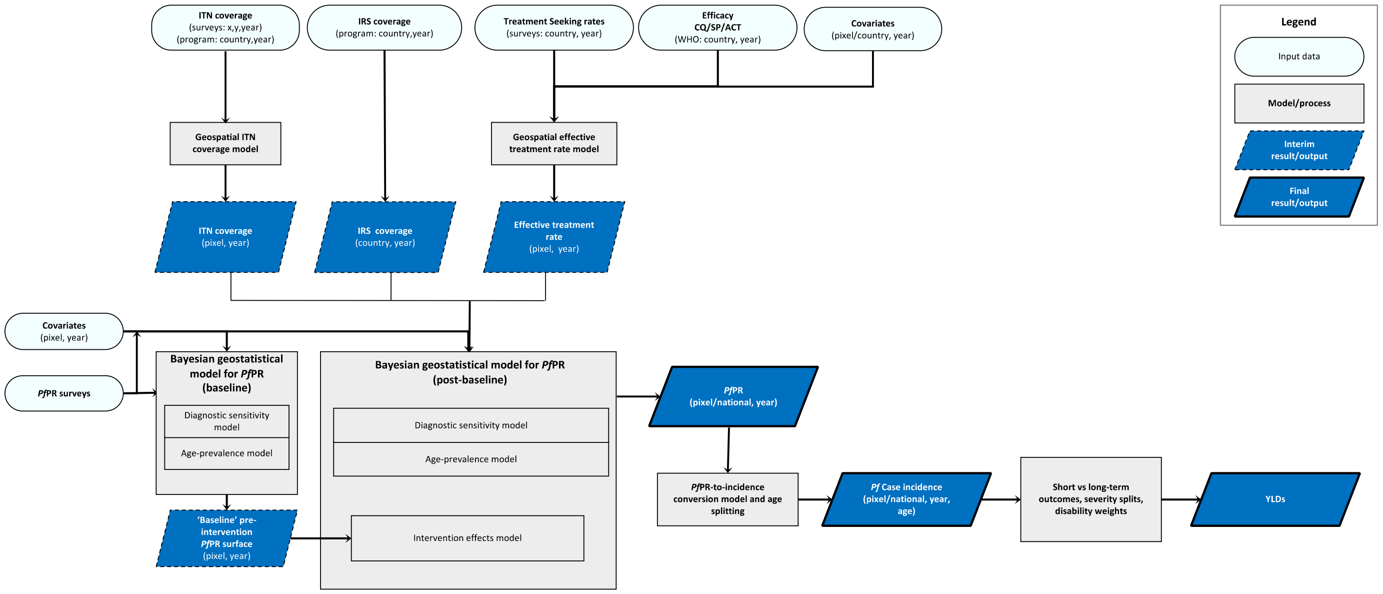

Malaria non-fatal outcomes (parasite rate and case incidence) outside Sub-Saharan Africa

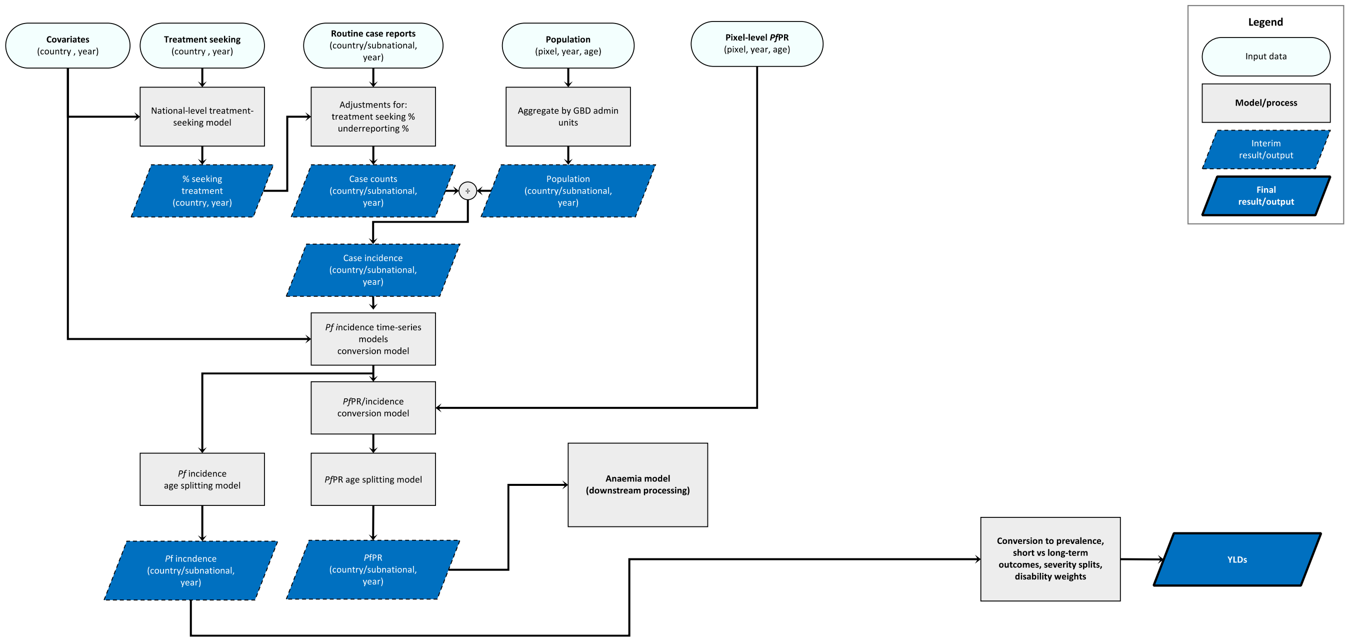

Case definition

Malaria is an acute parasitic mosquito-borne disease. An individual with uncomplicated malaria experiences one to two weeks of persistent fever, chills/shivering, sweating, joint pains, and headache. The individual will likely be lethargic and feverish, causing loss of daily function during the attack. Individuals with an untreated *P. falciparum* infection may develop severe malaria, which includes the symptoms of uncomplicated malaria but may also involve swelling, difficulty breathing, unconsciousness, and death. Microscopy is considered the gold-standard diagnostic approach for the purposes of GBD. The relevant ICD-10 codes are B50-B54.

Data input

Primary data inputs were:

(i) Routine malaria case reports from national routine surveillance systems. These were obtained at the national level from the WHO World Malaria Report and at the subnational administrative level, wherever possible, via an exhaustive search of published and grey literature sources along with online data portals hosted by national ministries of health. Each retained record consisted of an annual count of malaria cases along with a distinction between confirmed and unconfirmed diagnoses, and differentiation by malaria parasite species.

(ii) Cross-sectional, geolocated, and community-representative observations of infection prevalence for *Plasmodium falciparum* (referred to hereafter as *P. falciparum* parasite rate, *Pf*PR).

These malaria epidemiological metrics were augmented in the modelling by:

(iii) Malaria Atlas Project (MAP) modelled estimates of malaria control intervention population coverage (ITNs, IRS, and effective treatment with an antimalarial drug) resolved to 5 km x 5 km pixel-year level (for sub-Saharan Africa) and country-year level (outside sub-Saharan Africa).

(iv) A large suite of environmental, sociodemographic, and economic covariates resolved to 5 km x 5 km pixel-year level (for sub-Saharan Africa) and country-year level (outside sub-Saharan Africa).

Modelling strategy

The suitability, availability, and quality of *Pf*PR and routine case reporting data, as well as detailed intervention coverage information, differ markedly inside versus outside sub-Saharan Africa. As such, we developed separate modelling strategies for countries inside sub-Saharan Africa versus those outside. The exceptions were Algeria, Egypt, Morocco, Comoros, Mauritius, Cape Verde, Sao Tome and Principe, Rwanda, Botswana, Namibia, Eritrea, Djibouti, and South Africa. Despite being part of Africa, these countries exhibit epidemiological trends and have data availability/quality more akin to non-African settings.

*Pf*PR and case incidence modelling: Africa

Modelling was conducted in the following steps:

(i) The large assembly of geolocated *Pf*PR surveys maintained by MAP was used in a Bayesian spatiotemporal geostatistical model to predict *Pf*PR for every pixel-year in sub-Saharan Africa, representing an update to earlier work (Bhatt et al Nature, Gething et al NEJM). The model took into account (i) *Pf*PR survey participant age ranges and diagnostic type; (ii) coverage of ITNs, IRS, and effective antimalarial drug coverage, and how these metrics changed through time at each date and prediction location; (iii) environmental conditions at each date and prediction location (including density of vegetation, temperature, humidity, rainfall, elevation, and proximity to populated areas). The outcome was a predicted space-time "cube" of *Pf*PR, standardized to the 2-10 age range, for each year 1980–2017.

(ii) The *Pf*PR cube was then converted into an equivalent cube of the predicted incidence rate of clinical malaria. This conversion was achieved using an established model (Cameron et al Nature Communications) and provided estimates stratified first into three broad age bins (0-5; 5-15; <15) and then into the final 23 GBD 2017 age bins.

*Pf*PR and case incidence modelling: Outside Africa

Malaria endemic countries outside Africa tend to have less *Pf*PR data than those inside, in part because prevalence is generally lower. Thus, *Pf*PR becomes an inefficient way to measure malaria risk. Routine surveillance systems outside Africa are generally stronger, meaning that reports of malaria cases from health systems are more reliable and provide some insight into the total malaria burden in the community. Modelling outside Africa was carried out in the following steps:

(i) National and subnational case reports were first subject to adjustments to identify and minimise bias. Bias in reported case numbers arises from various sources. First, a fraction of cases in the community will fail to seek treatment or will attend a private or informal health care provider who will not provide a record of that case to the routine surveillance system. We adjusted for these factors by modelling the fraction of cases seeking care from different provider categories based on data from nationally representative cross-sectional household surveys (primarily from the Demographic and Health Survey (DHS) program and the Multiple Indicator Cluster Survey program). Another factor for which we must adjust is cases reaching formal clinics may not be subject to a confirmatory diagnostic test. We adjusted for this by assuming the fraction of unconfirmed cases that were truly malaria would equal the fraction of positives among all those tested. A final factor we adjust for is incomplete data as many routine surveillance systems fail to capture all case reports, with facilities/regions missing from the national totals in a given year. We adjusted for this based on reporting completeness statistics published nationally by WHO.

(ii) These adjusted routine case reports were georeferenced using digitised administrative boundary data using a spatial database of such boundaries collated and maintained by MAP.

(iii) Each case report was converted to an estimate of clinical incidence rate by dividing it by the estimated population in each unit, with the latter quantity derived by combing high-resolution gridded population data and the aforementioned administrative boundaries.

(iv) Bayesian time-series models were then applied to the case reports for each country to impute incidence rates for years with missing data. The results from this analysis, in conjunction with the adjusted case reports, constitute the incidence values delivered for GBD 2017.

(v) The incidence rate for each country-year was then converted to an inferred *Pf*PR value using the same model described earlier (Cameron et al). This allowed us to utilise these polygon-

level surveillance data and the *Pf*PR point-level data (where present) within the same modelling framework.

(vi) The combined *Pf*PR survey point data and (pseudo) *Pf*PR administrative unit data were then used in a Bayesian spatiotemporal geostatistical model to predict *Pf*PR at pixel-year level across all countries. As for the Africa model, *Pf*PR was standardised by age and diagnostic type and informed by a wide suite of covariates. An additional mechanism was developed to allow polygon (ie, administrative unit) and point (ie, survey) data to be used jointly to infer the predicted space-time surfaces.

(vii) The predicted *Pf*PR cube was then adjusted to ensure that, after conversion to pixel-level incidence, the incidence counts per country-year would precisely match the incidence results from step (iv). The summarised *Pf*PR values (ie, population-weighted and tallied for each country-year) from the adjusted *Pf*PR cube constitute the *Pf*PR values delivered for GBD 2017.

Total malaria cases by country, year, sex

The pixel-level predictions of clinical incidence rate (both inside and outside Africa) were combined with high-resolution gridded population data to estimate total cases per pixel-year. These were then aggregated to GBD national/subnational geographies. Inside sub-Saharan Africa, for countries endemic for *P. vivax* and *P. falciparum*, we calculated the number of cases due to *P. vivax* by applying the fraction of *P. vivax* and *P. falciparum* obtained from WHO and a literature review. Outside sub-Saharan Africa we followed the identical procedure for *P. vivax* and *P. falciparum.* Final age-splitting was accomplished using age-versus-incidence rate relationships gleaned from the paper by Cameron and colleagues (2014).

Determining YLDs for malaria

As in GBD 2016, we use a two-step process for determining malaria severity. For acute cases, severity splits for mild, moderate, and severe malaria were produced by analysis of MEPS data. These sequelae and their associated disability weights are presented below.

Table 1. Severity level, lay description, and DW

Severity level	Lay description	DW (95% CI)
Mild	Has a low fever and mild discomfort but no difficulty with daily activities.	0.006 (0.002–0.012)
Moderate	Has a fever and aches and feels weak, which causes some difficulty with daily activities.	0.051 (0.032–0.074)
Severe	Has a high fever and pain and feels very weak, which causes great difficulty with daily activities.	0.133 (0.088–0.19)

To determine long-term neurological burden due to malaria, we use the work by Roca-Felter and colleagues (2008) that examined the number of uncomplicated cases that led to longer-term impairment. Analytically, this means multiplying incidence estimates (described in the section above for persons under 20 by 0.00029 (0.000077–0.00057). This adjusted case estimate is then combined with excess mortality rates derived from all-cause mortality and standardised mortality ratios for neonatal encephalopathy (NE) in a DisMod model to produce prevalence estimates of long-term sequelae for all estimation years. Implicit in this process is an assumption that the disability and trend of impairment due to severe malaria follow NE. The subsequent severity splitting follows NE as well.

To determine the burden of acute (short-term) malaria, the incidence estimation results are combined and converted to prevalence by matching each draw with a draw of duration of clinical illness. Consistent with GBD 2016, we use a uniform distribution between 14 and 28 days for duration.

References

Bhatt S, Weiss DJ, Cameron E, et al. The effect of malaria control on Plasmodium falciparum in Africa between 2000 and 2015. *Nature* 2015; 526: 207–11.

Cameron, E., K.E. Battle, S. Bhatt, D.J. Weiss, D. Bisanzio, B. Mappin, U. Dalrymple, S.I. Hay, D.L. Smith, J.T. Griffin, E.A. Wenger, P.A. Eckhoff, T.A. Smith, M.A. Penny, and P.W. Gething. 2015. Defining the relationship between infection prevalence and clinical incidence of Plasmodium falciparum malaria. *Nature Communications* 6:8170.

Gething, P.W., D.C. Casey, D.J. Weiss, D. Bisanzio, S. Bhatt, E. Cameron, K.E. Battle, U. Dalrymple, J. Rozier, P.C. Rao, M.J. Kutz, R.M. Barber, C. Huynh, K.A. Shackelford, M.M. Coates, G. Nguyen, M.S. Fraser, R. Kulikoff, H. Wang, M. Naghavi, D.L. Smith, C.J.L. Murray, S.I. Hay, and S.S. Lim. 2016. "Mapping Plasmodium falciparum Mortality in Africa between 1990 and 2015." New England Journal of Medicine 375 (25):2435-2445.

Chagas disease

Flowchart

Chagas disease

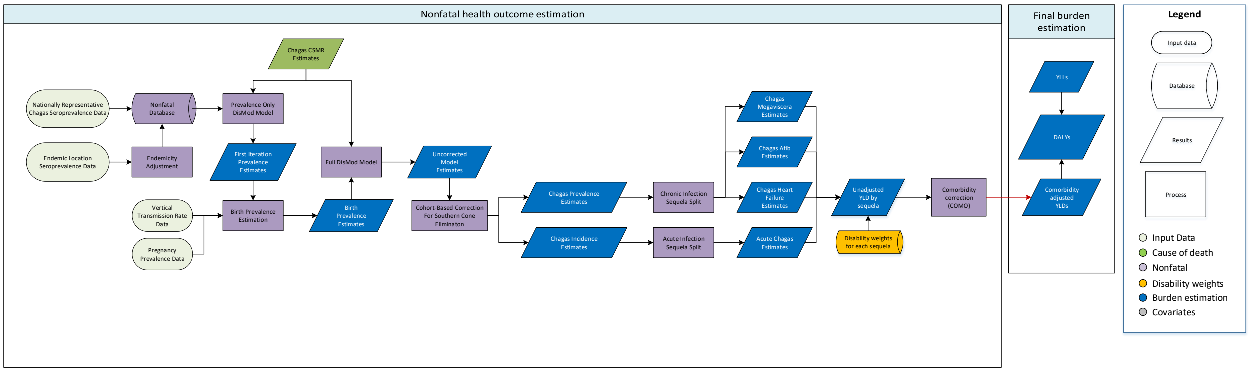

Case definition

Chagas disease is defined by infection with the protozoa *Trypanosoma cruzi,* which is transmitted by *Triatominae* insect vectors (most common), blood transfusion, organ transplant, and congenital transmission. It includes an acute phase corresponding with the time of infection, and is typically asymptomatic. Chronic infection may be latent (ie, asymptomatic), or result in cardiovascular or digestive sequelae. It includes all ICD-10 codes under the heading B57 (Chagas disease), with codes B57.0-B75.1 corresponding to the acute phase, B57.2 corresponding to chronic cardiovascular sequelae, and B57.3 corresponding to chronic digestive sequelae.

Input data

Model inputs

For GBD 2017 estimation, we used seroprevalence data to model Chagas. The table below illustrates the geographic distribution of model input data for the estimation process.

Table 1. Data Coverage

	Prevalence
Site-years (total)	78
Number of countries with data	13
Number of GBD regions with data (out of 21 regions)	4
Number of GBD super-regions with data (out of 7 super-regions)	2

We also use CSMR estimates in the modelling process, which will be addressed in further detail below.

Modelling strategy

We modelled Chagas disease using a full DisMod-MR 2.1 Bayesian meta-regression model incorporating seroprevalence data, as above, and CSMR estimates. We assume no remission. We eliminate all new infections, except those via vertical transmission, in Chile and Uruguay for years after the interruption of vector-based transmission (Abad-Franch F, Diotaiuti L, Gurgel-Gonçalves R, Gürtler RE. Certifying the interruption of Chagas disease transmission by native vectors: cui bono? Mem Inst Oswaldo Cruz 2013;108:251–4.; Coura JR. Chagas disease: control, elimination and eradication. Is it possible? Mem Inst Oswaldo Cruz 2013;108:962–7.). For non-endemic countries, we estimate the prevalence of imported chronic infections based on migration. For each non-endemic country, we estimate the total number of people infected with Chagas as the sum of the number of immigrants from each endemic country multiplied by the corresponding prevalence of Chagas in that endemic country.

We estimate five sequelae: symptomatic acute infection from incidence; and megaviscera, heart failure, atrial fibrillation, and chronic asymptomatic infection from prevalence. We assume that 5% of acute infections will be symptomatic (Teixeira AR, Nitz N, Guimaro MC, Gomes C, Santos-Buch CA. Chagas disease. Postgrad Med J 2006;82:788–98.). The proportion of chronic infections resulting in a given sequela varies by sex and age: the prevalence of megaviscera among those infected with Chagas ranges from 0% in children to nearly 10% among older adults (Coura JR, Naranjo MA, Willcox HP. Chagas' disease in the Brazilian Amazon: II. A serological survey. Rev Inst Med Trop São Paulo 1995; 37:103–7.); the prevalence of atrial fibrillation attributable to Chagas ranges from 0% among children to approximately 10% in men over 80 years of age (Ribeiro AL, Marcolino MS, Prineas RJ, Lima-Costa MF. Electrocardiographic abnormalities in elderly Chagas disease patients: 10-year follow-up of the Bambuí Cohort Study of Aging. J Am Heart Assoc 2014;3:e000632.); and the prevalence of heart failure attributable to Chagas among those who are infected ranges from 0% among young children, to a maximum of 23% among men over 80 years of age (Sabino EC, Ribeiro AL, Salemi VM, et al., for the National Heart, Lung, and Blood Institute Retrovirus Epidemiology Donor Study-II (REDS-II), International Component. Ten-year incidence of Chagas cardiomyopathy among asymptomatic Trypanosoma cruzi-seropositive former blood donors. Circulation 2013;127:1105–15.).

Severity splits and disability weights

The table below illustrates the sequelae, lay descriptions, and DWs for Chagas disease.

Table 2. Sequelae, lay description and DWs

Sequelae	Description	Disability Weight
Atrial fibrillation and flutter due to Chagas disease	Has periods of rapid and irregular heartbeats and occasional fainting.	0.224 (0.151–0.312)

Mild heart failure due to Chagas disease	Is short of breath and easily tires with moderate physical activity, such as walking uphill or more than a quarter-mile on level ground. The person feels comfortable at rest or during activities requiring less effort.	0.041 (0.026–0.062)
Moderate heart failure due to Chagas disease	Is short of breath and easily tires with minimal physical activity, such as walking only a short distance. The person feels comfortable at rest but avoids moderate activity.	0.072 (0.047–0.103)
Severe heart failure due to Chagas disease	Is short of breath and feels tired when at rest. The person avoids any physical activity, for fear of worsening the breathing problems.	0.179 (0.122–0.251)
Mild chronic digestive disease due to Chagas disease	Has some pain in the belly that causes nausea but does not interfere with daily activities.	0.011 (0.005–0.021)
Moderate chronic digestive disease due to Chagas disease	Has pain in the belly and feels nauseated. The person has difficulties with daily activities.	0.114 (0.078–0.159)
Acute Chagas disease	Has a fever and aches, and feels weak, which causes some difficulty with daily activities.	0.051 (0.032–0.074)
Asymptomatic Chagas disease	Latent Chagas infection (ie, chronic infection with no apparent symptoms)	NA

Changes from GBD 2016 to GBD 2017

We have made no substantive changes in the modelling strategy for endemic countries from GBD 2016 to GBD 2017.

Visceral leishmaniasis

Visceral leishmaniasis – GBD2017

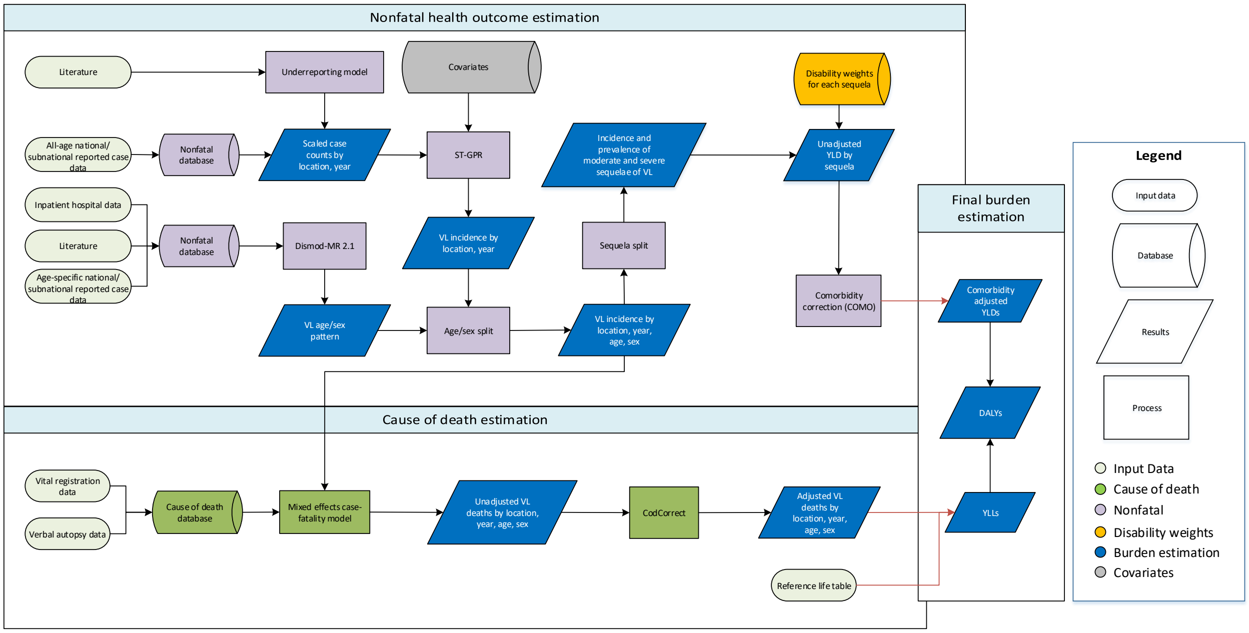

Visceral leishmaniasis (VL) is the most serious manifestation of disease caused by the *Leishmania* parasite, transmitted through the bite of phlebotomine sandflies. Those infected typically present with fever, weight loss, anaemia, leukopenia, thrombocytopenia, and enlargement of the spleen and liver. If left untreated, it can be fatal. Transmission varies by geographic region, with a variety of reservoir hosts implicated, and different vector species associated, maintaining both zoonotic and anthroponotic transmission cycles. The ICD9 code related to visceral leishmaniasis is 085.0, and the ICD10 code is B55.0.

Description of general methodology

The fatal estimation process for visceral leishmaniasis is built from incident case notification data representative of the GBD geographic location, which is adjusted for underreporting. The upscaled all-age, both-sex case counts are modelled using spatiotemporal Gaussian process regression (ST-GPR) in order to impute for missing location-year combinations as well as to account for further biases and inaccuracies in reporting. Datasets that disaggregate VL cases by age and sex are modelled using DisMod to produce a global age-sex split which is applied to the all-age, both-sex envelope estimates resulting from ST-GPR. The mean incidence estimates are compared with estimated death counts to generate a case-fatality rate model that is subsequently used to estimate deaths for each age, sex, location, year.

Input Data – Case Notification time series

Current estimation for the all-age, both-sex incidence envelope is based upon location-representative information rather than site-specific epidemiological measures due to the absence of global foci maps allowing for upscaling of geographically precise information. The primary data resource therefore is the case notification time-series reported by National Control Programs and Ministries of Health to the World Health Organization. This is supplemented by systematic literature review (last updated for GBD 2015) to identify alternate sources of data for years missing information. For countries with subnational

estimates, in-country collaborators have compiled information for respective programs, or identified key resources. Notifications from 1,151 location-years were available.

Input Data – Underreporting assessments

It is recognised that case notification series record only a subset of the true cases present. A review was undertaken to identify articles that compared reported cases with alternate measures to estimate the degree of underreporting. The following search strings were used: 'leish* AND under*'; 'active passive leish*'. Inclusion criteria were broad to maximise spatiotemporal coverage in potential estimates – any report that compared reported statistics with some notion of "truth" (whether capture-recapture, active surveillance, etc.) were extracted. Values for both cutaneous and visceral leishmaniasis were included. For GBD 2017, 12 articles were included, summarised in Table 1.

Citation	GBD location	Time period	Pathogen	Method synopsis	Proportion of "true" cases reported
Copeland *et al.*, 1990 "Comparison of active and passive case detection of cutaneous leishmaniasis in Guatemala" (Copeland, Arana, and Navin 1990)	Guatemala	1990	CL	Comparison of Ministry of Health data with cross-sectional population-based survey to inform estimated number of cases	64/2574
Yadon *et al.* 2001 "Assessment of Leishmaniasis notification system in Santiago del Estero, Argentina, 1990-1993" (Yadón et al. 2001)	Argentina	1990–1993	CL	Capture-recapture methods were used to evaluate four reporting sources.	94/210
Sesma *et al.* 1997 "Leishmaniasis in Navarra: a review of activities" (Sesma and Barricarte 1997)	Spain	1990–1997	CL, VL	Comparison of active searching within the region with reporting via Epidemiological Surveillance System	8/21
Maia-Elkhoury *et al.* 2007 "Analysis of visceral leishmaniasis reports by the capture-recapture method" (Maia-Elkhoury et al. 2007)	Brazil	2002–2003	VL	Comparison of three notification systems for completeness	5896/10691
Singh *et al.* 2006 "Serious underreporting of visceral leishmaniasis through passive case reporting in Bihar, India" (S. P. Singh et al. 2006)	Bihar, India	2003	VL	Comparison of actively detected cases (identified via household surveys) and governmental health system records. Estimate is among study population	8/65
Singh *et al.* 2006 "Serious underreporting of visceral leishmaniasis through passive case reporting in Bihar, India" (S. P. Singh et al. 2006)	Bihar, India	2003	VL	Comparison of actively detected cases (identified via household surveys) and governmental health system records.	109/876
Gkolfinopoulou *et al.* 2013 "Epidemiology of human leishmaniasis in Greece, 1981-2011" (Gkolfinopoulou et al. 2013)	Greece	2004–2009	VL	Comparing number of cases identified at national reference laboratory with mandatory notification system.	260/361

Singh *et al.* 2010 “Estimation of under-reporting of Visceral Leishmaniasis cases in Bihar India” (V. P. Singh et al. 2010)	Bihar, India	2006	VL	Comparison of actual reported number of cases with estimates age-sex stratified incidence proportions for a cohort of 31,324 persons	34/177
Hirve *et al.* 2010 “Effectiveness and feasibility of active and passive case detection in the Visceral Leishmaniasis Elimination Initiative in India, Bangladesh, and Nepal” (Hirve et al. 2010)	Bihar, India<br>Nepal<br>Bangladesh	2008	VL	Comparing active case detection evaluations (conducting via house-to-house screening) with passive case detection systems	111/130<br>119/127<br>18/25<br>20/32
Faraj *et al.* 2016 “Effectiveness and cost of insecticide-treated bed nets and indoor residual spraying for the control of cutaneous leishmaniasis: A cluster-randomized control trial in Morocco” (Faraj et al. 2016)	Morocco	2008–2013	CL	Comparison of incidence of new CL cases by both active and passive case detection	409/670
Das *et al.* 2014 “Active and passive case detection strategies for the control of leishmaniasis in Bangladesh” (Das et al. 2014)	Bangladesh	2010–2011	VL	Comparing two districts’ estimates [identified in the paper as being directly comparable] of cases, one via active case detection, the other via passive case detection. Active case detection was via community education and outreach workers targeting households	756/1087
Rahman *et al.* 2015 “Performance of Kala-azar surveillance in Gaffargaon subdistrict of Mymensingh, Bangladesh” (Rahman et al. 2015)	Bangladesh	2010–2011	VL	Comparison of cases reported to the local health complex versus active search for kala-azar cases	29/58
Eid *et al.* 2017 “Assessment of a Leishmaniasis reporting system in tropical Bolivia using the capture-recapture method” (Eid et al. 2017)	Bolivia	2013–2014	CL	Active surveillance during medical campaigns were compared to registered cases reported by the National Program of Leishmaniasis Control	23/86.4

Table 1: Metadata for underreporting scalars used in GBD 2017. For each record, a citation, GBD location of relevance, year, pathogen, brief summary of methods, and output values used in modelling are listed.

Input data – age/sex-split data

Where possible, information disaggregating location-level statistics by age and sex were extracted.

	Incidence
Site-years (total)	1519
Number of countries with data	70
Number of GBD regions with data (out of 21 regions)*	14
Number of GBD super-regions with data (out of 7 super-regions)	7

Table 2: Summary statistics for data used to inform age/sex split modelling. *It should be noted that not all GBD regions have leishmaniasis-endemic countries within them.

Method – geographic restrictions

There are strong climatic and biogeographic constraints on the geographic distribution of VL resulting in a focal rather than cosmopolitan global distribution. As a result, it is necessary to identify locations burdened by the disease through space and time as distinct from countries were VL is absent. Tags were assigned to each location-year based upon the outcome of a search of IHME databases, as well as location-specific searches of PubMed. Each location-year is tagged as follows:

- Present – where a specific citation of either an autochthonous laboratory-confirmed case (ie, a case with PCR, serological, or parasitological diagnosis), reported case (ie, a case noted as VL, but with no supporting diagnostic), or supporting evidence (ie, confirmed infection in animal reservoirs or sandfly vectors)
- Protocol Present – for a given location-year, where no specific citation is used, but is present for another year in the same location, it is assumed that VL is present given that eradication of the pathogen has not been achieved
- Absent – where PubMed location-specific searches returned zero relevant results, in locations scoring -25 or lower as evaluated by Pigott *et al.* (2014) [the threshold for "absence" in that study (Pigott et al. 2014)], locations were tagged as Absent
- Protocol Absent – as with Absent, locations with zero relevant PubMed results, but with greater than -25 as evaluated by Pigott *et al.* (2014), were tagged as Protocol Absent (Pigott et al. 2014)

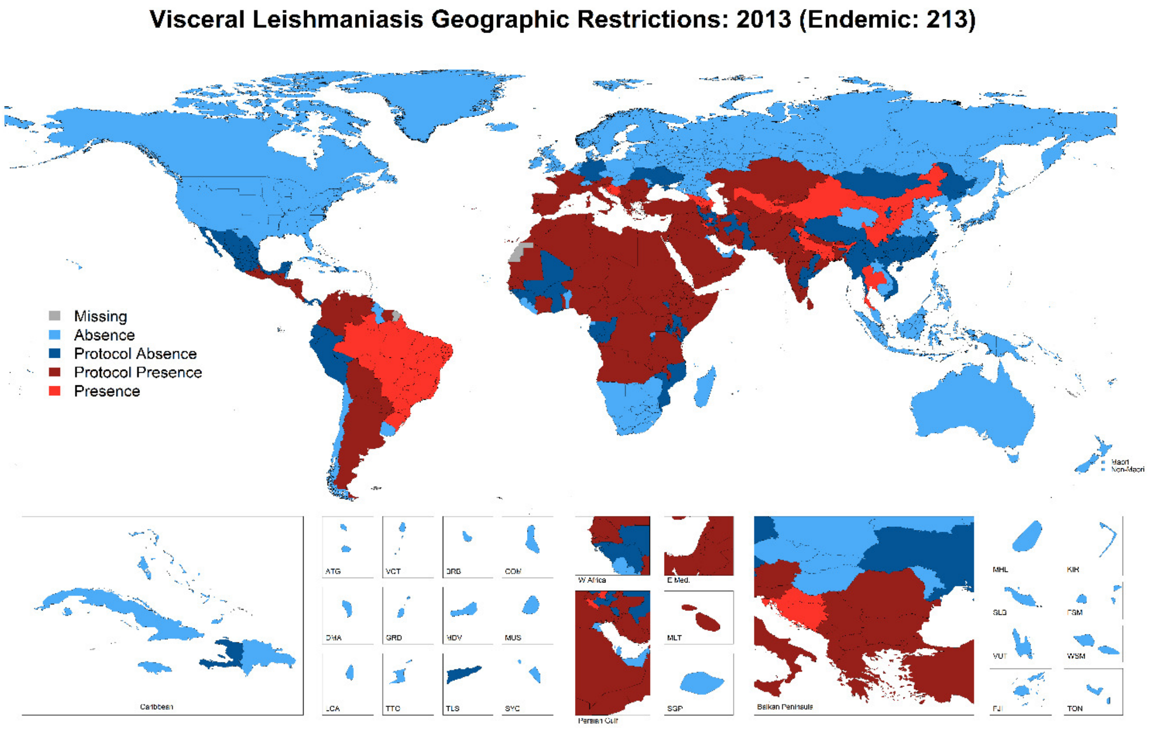

Figure 1: Visceral Leishmaniasis geographic restrictions for the year 2013. GBD locations tagged as present are coloured in red, yellow represents protocol presence, dark blue represents protocol absence, and absence is represented by light blue. Locations missing tags are presented in grey.

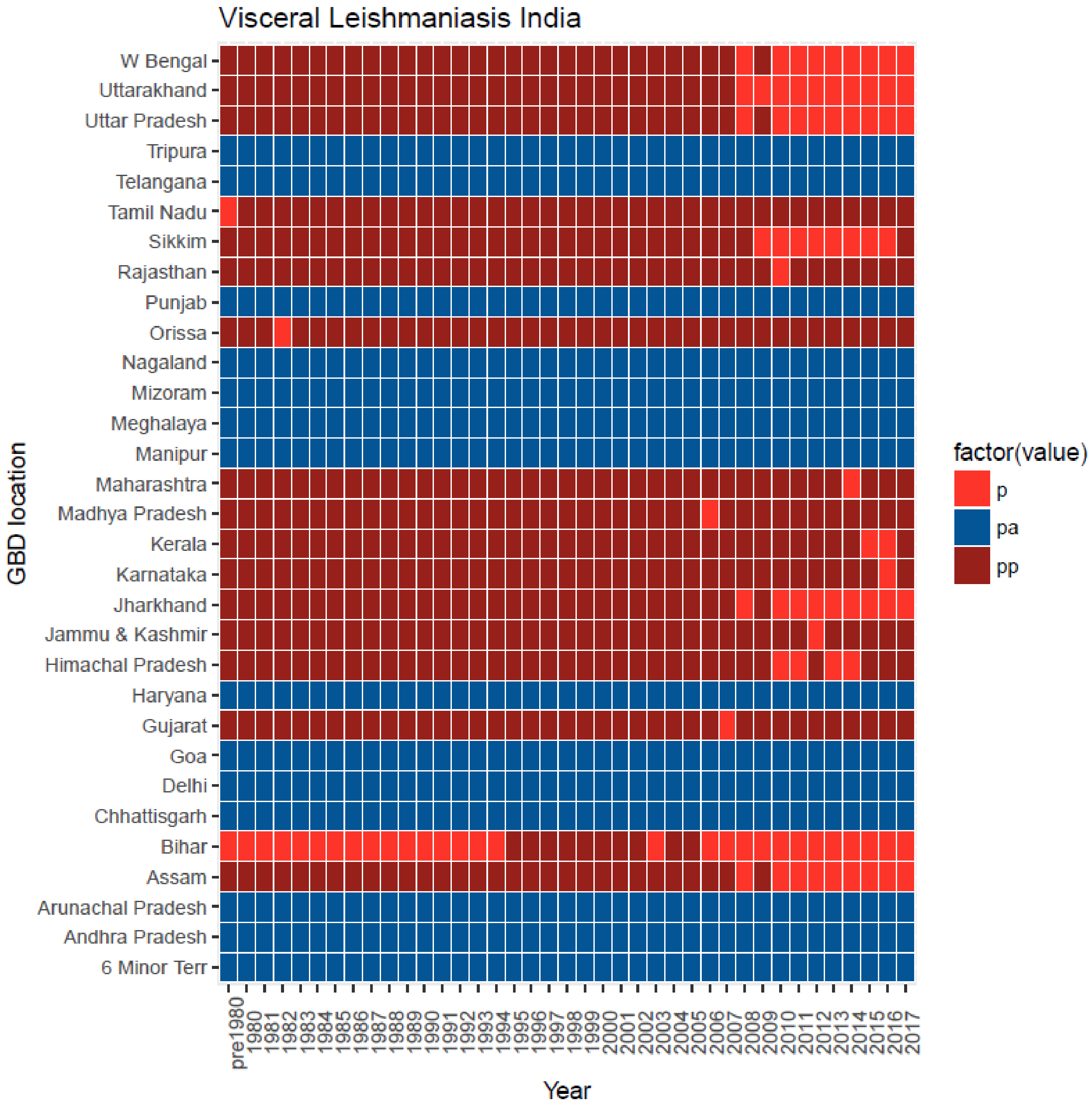

Figure 2: Visceral Leishmaniasis geographic restrictions for Indian subnationals. Locations tagged as present are coloured in red, yellow represents protocol presence, and dark blue represents protocol absence.

Full time series of maps and tables, with relevant GHDx NIDs, are available upon request from gbdsec@uw.edu.

Method – underreporting modelling and scaled case counts

Underreporting scalars were modelled as a generalised linear model estimating the proportion of true cases captured by reporting systems: a value of 1 therefore represents all actual cases of leishmaniasis being reported through notification systems. The specific models is as follows:

$$\frac{reported\ cases}{\text{"true" cases}} = Pathogen + Year + Sociodemographic\ Index$$

To account for potential biases inherently present based upon differing survey methods or location-specific confounders, 1,000 models were run, with each model randomly dropping all data from a specific location, and then one additional data point from the remaining dataset. Similarly, for estimates that spanned multiple years, for each model one of the years within the range of possible years was randomly assigned.

To generate scaled case counts, for each of the 1,000 models a random number was generated, using a normal distribution with mean being that of the mean estimated scalar bounded by the upper and lower confidence interval. With these 1,000 scalars, 1,000 scaled case counts were calculated and summarised for modelling within ST-GPR.

Method – ST-GPR

Using existing IHME tools, the summarised values were modelled using ST-GPR to produce a complete time series of estimates for each location-year tagged “Present” or “Protocol Present”. In short, ST-GPR attempts to model non-linear trends utilising a Gaussian process to fit a trend, rather than a definitive functional form. The following model specifications were used:

$$Incidence = \ Healthcare\ Access\ and\ Quality\ Index + Sociodemographic\ Index + (1|level\ 1) + (1|level\ 2) + (1|level\ 3)$$

where levels 1, 2, and 3, referring to GBD location hierarchies, treated as random effects. The following hyperparameters were used: st-lambda = 0.4, st-omega = 1, st-zeta = 0.01, gpr-scale = 10.

Method – DisMod

DisMod was used to generate an age-sex curve to disaggregate all-age, both-sex incidence data. DisMod is an integrated meta-regression framework that allows for multiple datasets to be integrated into a singular analysis regardless of age-binning, sources, and geographies. As a consequence, a variety of differently aggregated information can be evaluated to generate a consensus output. From this model, the global fit was used.

Method – YLD estimation (incorporating duration and disability weighting) / COMO

Following standard GBD estimation protocols, incidence estimates were used to calculate disease prevalence (by multiplication with duration), disaggregated by disease sequelae. In total, two health states are assigned to visceral leishmaniasis, “moderate visceral leishmaniasis” and “severe visceral leishmaniasis” [Table 3]. Duration values were taken from Murray et *al.* (2005).

Sequela	Health state lay description	Disability weight	Duration
Moderate visceral leishmaniasis	Infectious disease, acute episode, moderate “has a fever and aches, and feels weak, which causes some difficulty in daily activities”	0.051 (0.032–0.074)	2.5 months

Severe visceral leishmaniasis	Infectious disease, acute episode, severe<br>"has a high fever and pain, and feels very weak, which causes great difficulty with daily activities"	0.133 (0.088–0.19)	15 days

Table 3: Sequelae and associated metadata. For the sequelae used in GBD 2017, the lay descriptor health state, disability weight, and duration are listed.

Central processing is used to generate the final estimates, including co-morbidity simulations.

Changes from GBD 2016

A number of changes to the methodology were implemented for GBD 2017:

Geographic restrictions – to improve transparency and tractability of geographic restrictions, maps of restricted locations and years are available, with clear designation of data (or assumptions) used to inform a GBD location-year's status. As a result of updating, the status of some GBD locations has changed in the light of new evidence (eg, Angola). While we explore how best to host this information, it is currently available upon request to gbdsec@uw.edu.

All-age, both-sex incidence envelope – new data were acquired and an ST-GPR methodology implemented consistently across the globe. Relevant covariates were updated from GBD 2016. One important change was the removal of the "High endemicity" covariate, which constrained predictions, particularly in low SDI countries in Africa, since its construction and subsequent use in models are not independent of each other.

Age-sex breakdown – age-sex curves were taken from a DisMod model using an updated dataset of age-sex specific information

Underreporting model – considerable changes were undertaken from GBD 2016 for underreporting. Rather than using a single scalar, taken from expert opinion (Alvar et al. 2012), applied across the entire time series, a model was developed, parameterised by real data, allowing for spatiotemporal variation in estimates. These variable scalars were then applied to their relevant location-year case count values.

Results specific to visceral leishmaniasis model

The aim here is to provide insights in some of the sub-models that are involved in the VL estimation process that are not published as part of the GBD capstones or readily available via the supplemental materials. For further questions, please direct toward gbdsec@uw.edu.

<u>Underreporting</u>

Coefficients
Pathogen: 0.6371 (-0.0456 to 1.5868) (where pathogen order is CL, VL)
Year: 0.1350 (0.0714–0.2058)
SDI: 4.6230 (2.0290–9.3287)

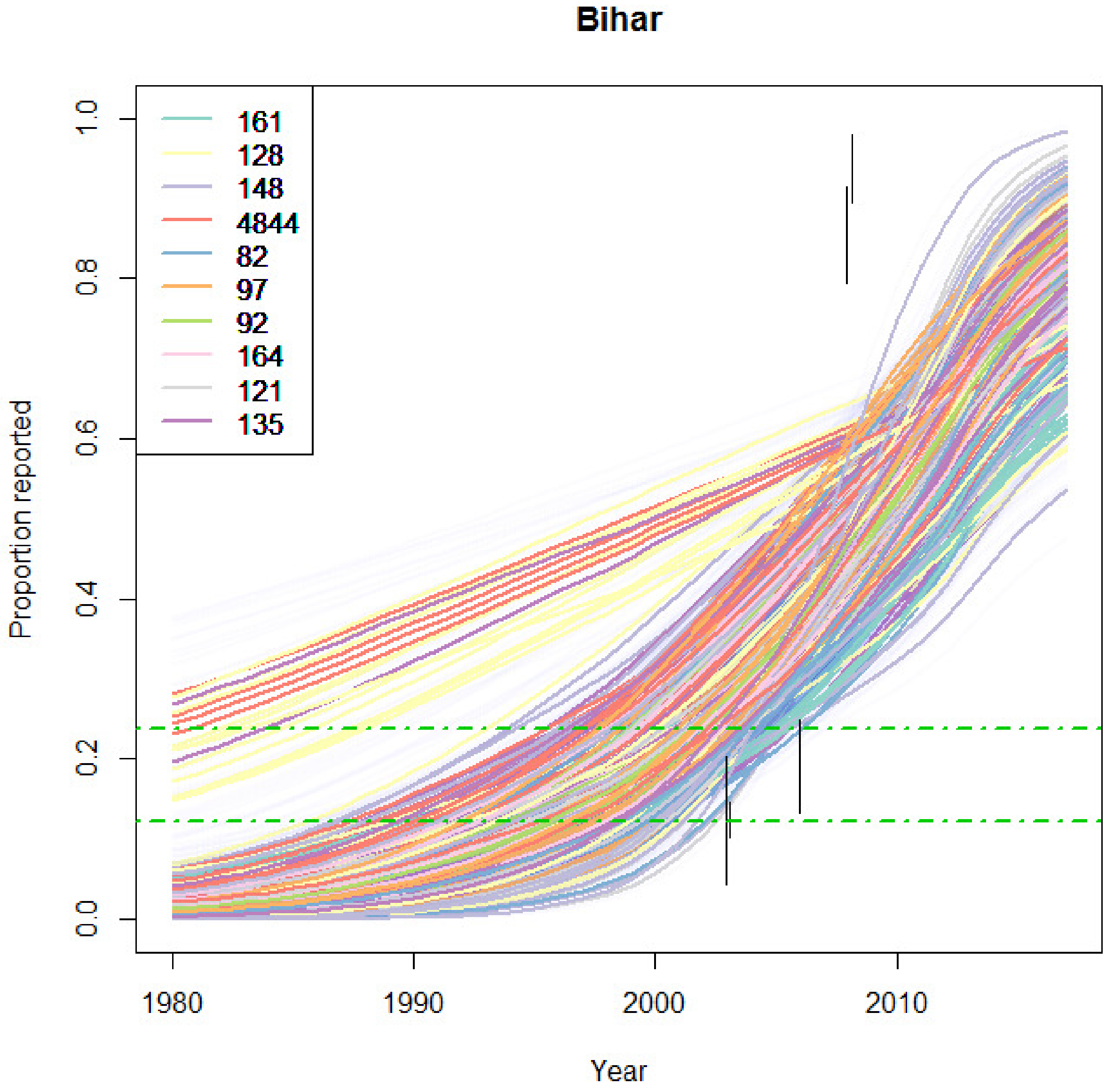

Figure 3: Example of VL underreporting model for Bihar, India. Plot showing each of the 1,000 iterations of the underreporting model run, coloured by the location that has been held out [colours coded by their GBD location id: 161 = Bangladesh, 128 = Guatemala, 148 = Morocco, 4844 = Bihar, India, 82 = Greece, 97 = Argentina, 92 = Spain, 164 = Nepal, 121 = Bolivia, 135 = Brazil]. The black vertical lines represent data points (with standard errors) for Bihar as listed in Table 1, and the green dashed line is the upper and lower bound of the underreporting factor recorded by Alvar *et al.* (2012), which was applied across all time in GBD 2016.

Age- and sex-specific trends in incidence rate

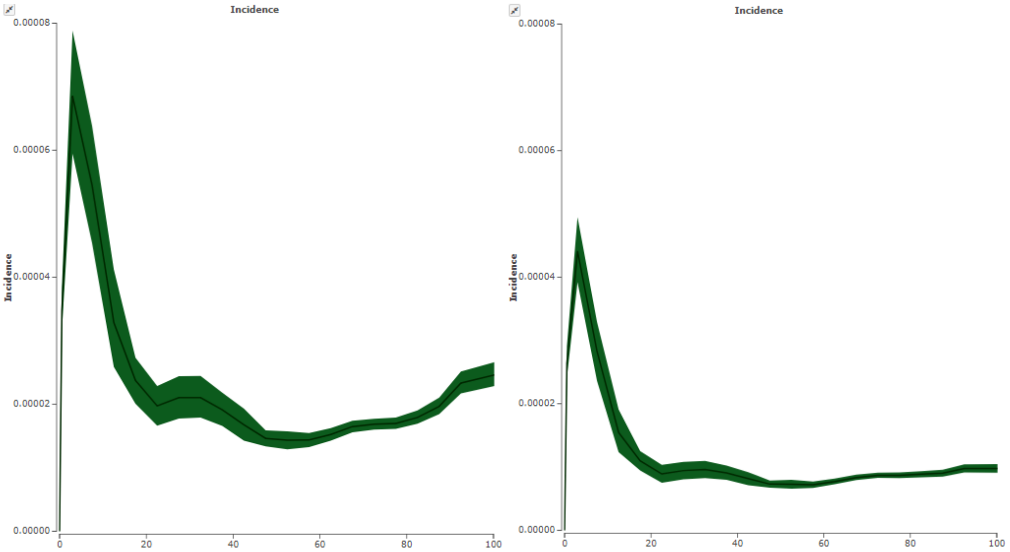

Figure 4: Global age-specific incidence estimates for males (left) and females (right) for the year 2010. Incidence is on the Y-axis (rate per total population), and age in years on the X-axis. Screenshot from EpiViz.

Figure 4 shows the age-specific variation in incidence rates, differentiated by sex. When considered as a global aggregate, we see that reported male incident rates are approximately double those of females, with highest rates observed in younger age groupings. In adults, levels are comparatively flat, but there is an uptick in older age groups.

<u>ST-GPR</u>

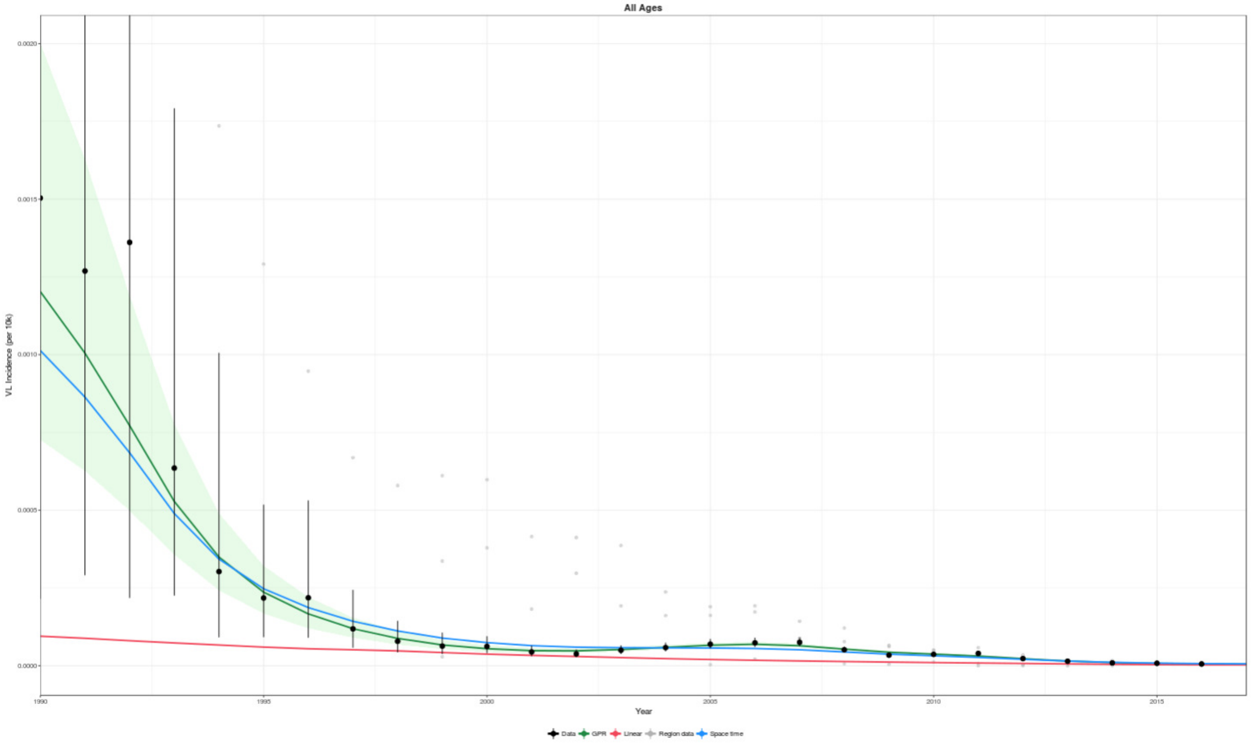

Figure 5: ST-GPR estimates for India (all-age, both sex) for years 1990–2017. Black dots represent input data points (post processing for underreporting) with the black lines indicating variance. The green line

represents the mean GPR estimated value, with uncertainty shown by the green polygon. The blue line indicates the space-time component of the ST-GPR; the red indicates the linear regression component derived off of global data. Transparent black dots represent data from other locations in the GBD region CFR.

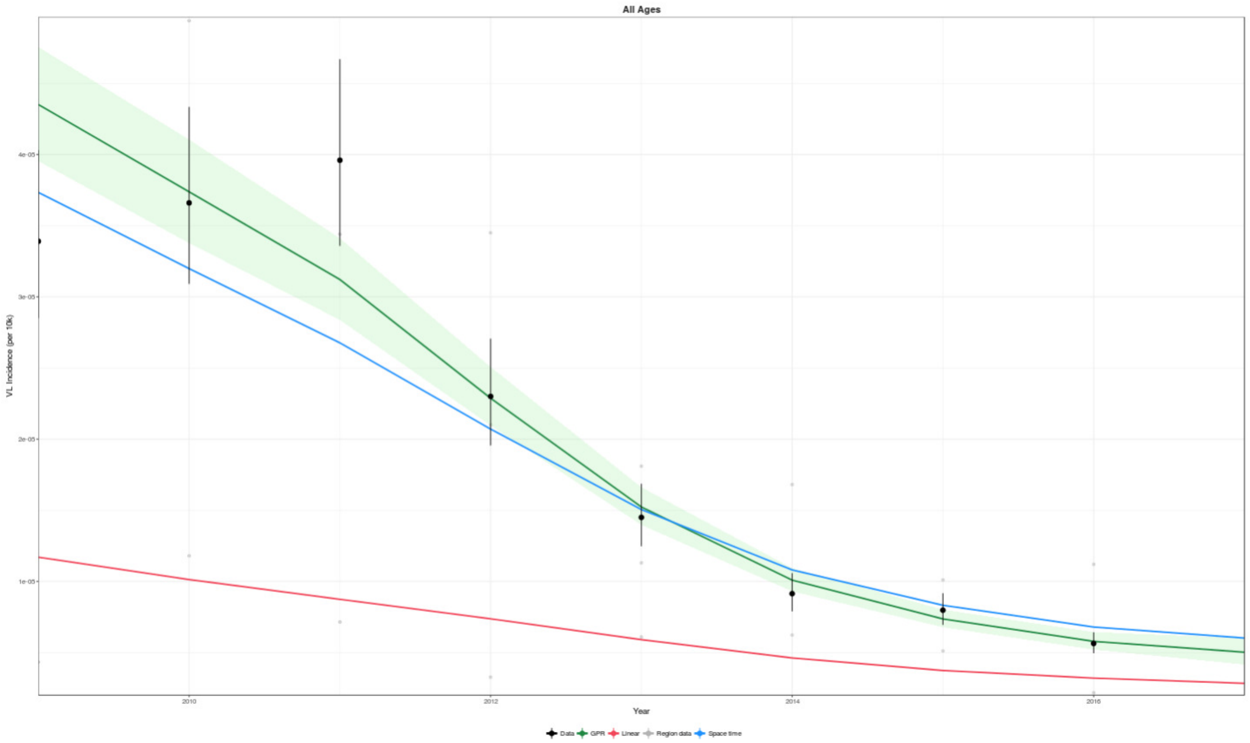

Figure 6: ST-GPR estimates for India (all-age, both sex) for years 2009–2017. Colouration and symbols are as stated in caption for Figure 5.

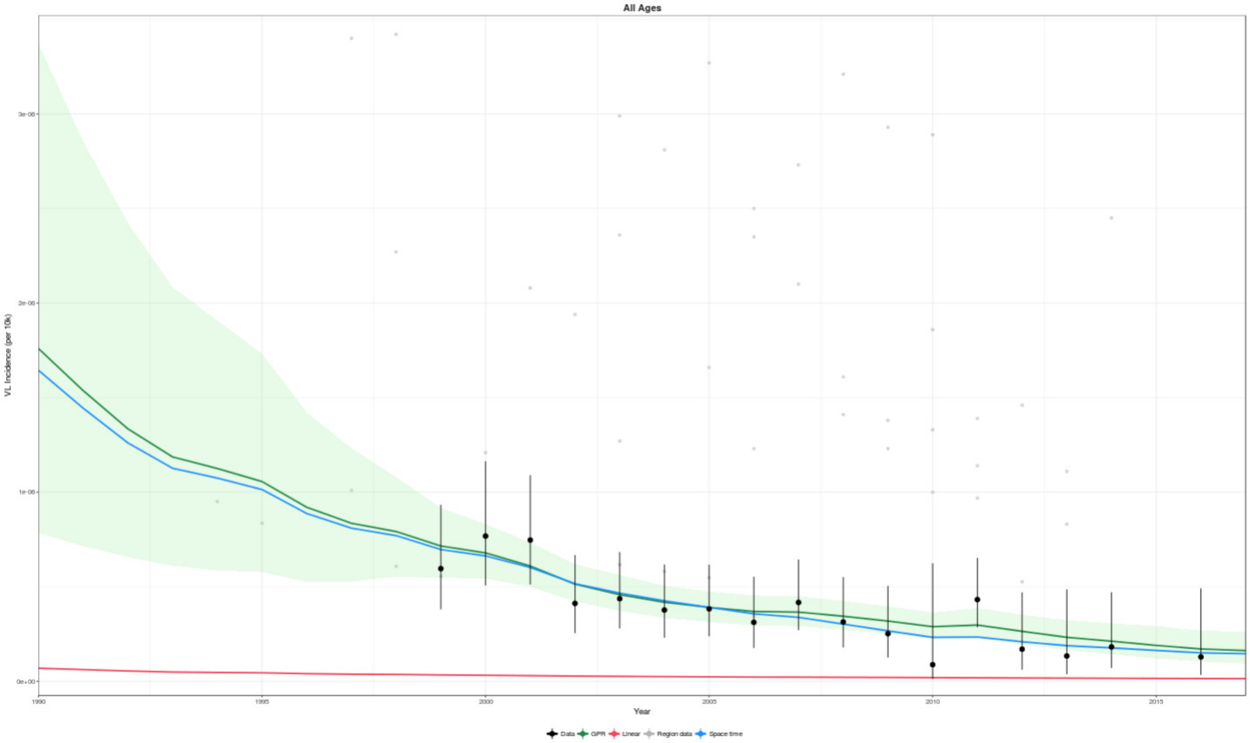

Figure 7: ST-GPR estimates for France (all-age, both sex) for years 1990–2017. Colouration and symbols are as stated in caption for Figure 5.

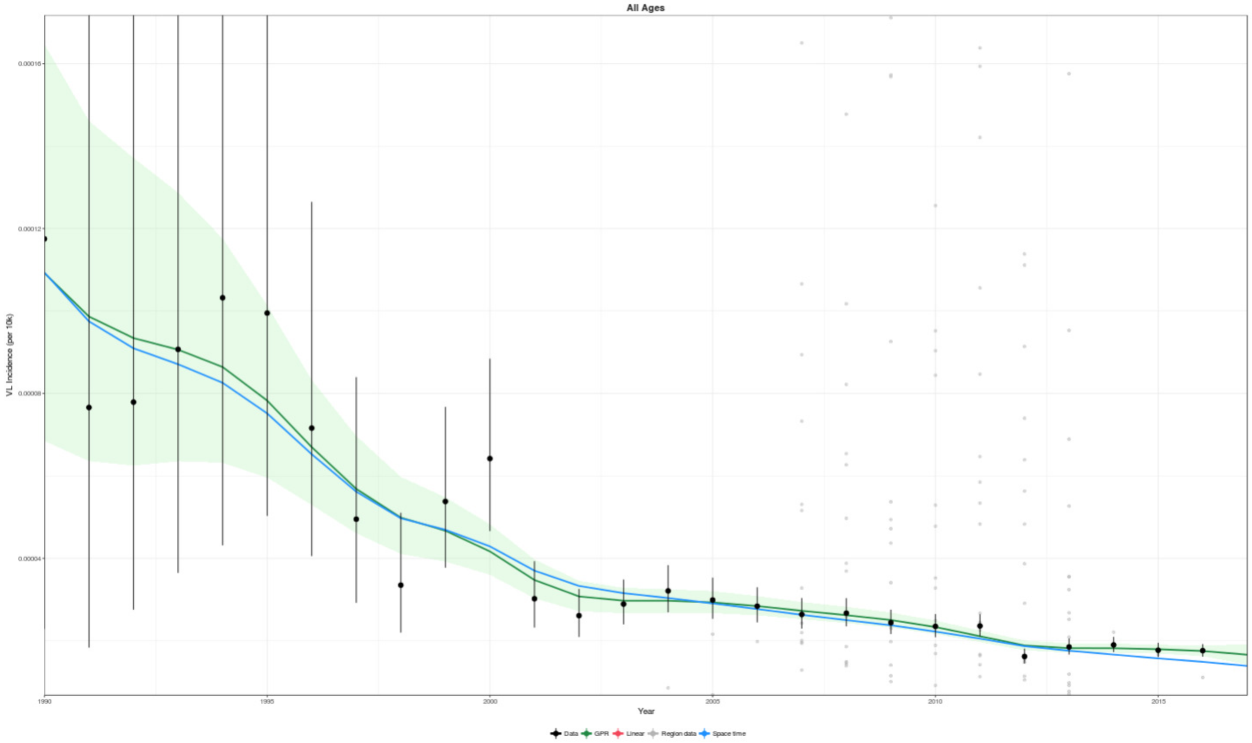

Figure 8: ST-GPR estimates for Brazil (all-age, both sex) for years 1990–2017. Colouration and symbols are as stated in caption for Figure 5.

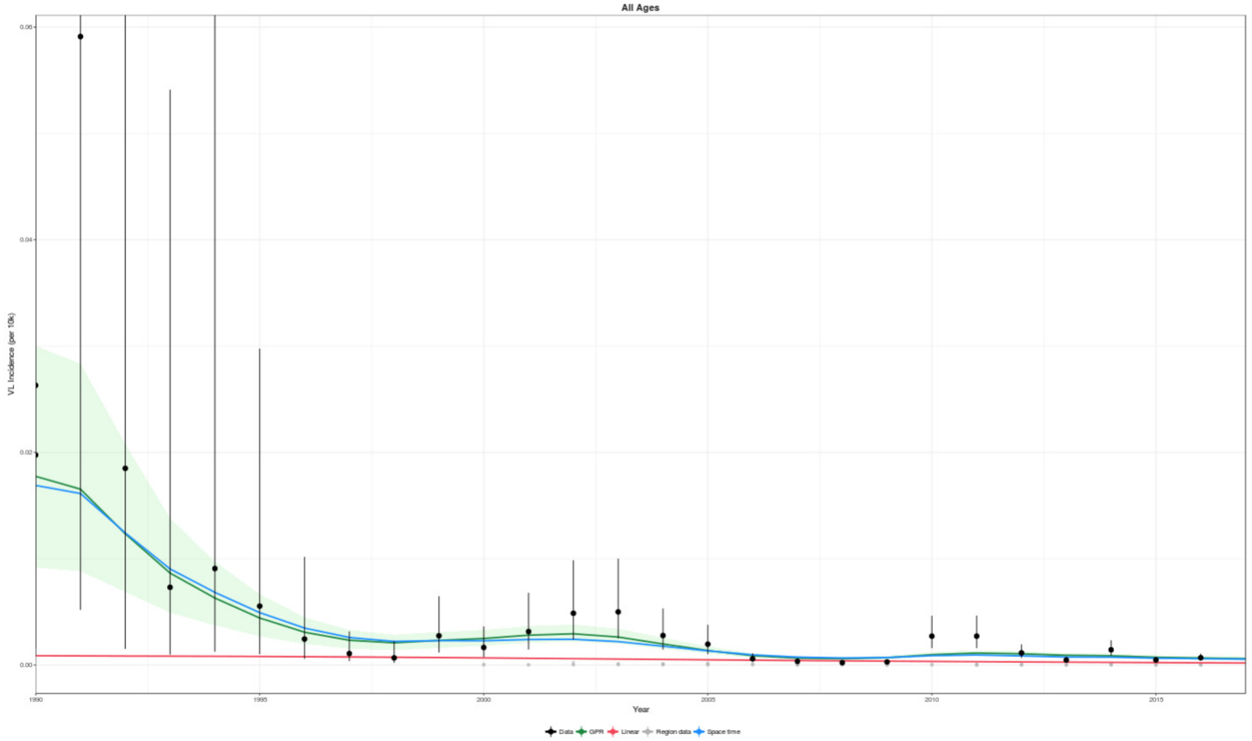

Figure 9: ST-GPR estimates for South Sudan (all-age, both sex) for years 1990–2017. Colouration and symbols are as stated in caption for Figure 5.

Limitations

As with any modelling process, a number of limitations are known, which will be the focus of additional effort in upcoming GBD cycles and engagement with collaborators. Given the focus on location-representative estimates, the existing model is based upon national case counts. This excludes a large resource of published literature and grey literature focused on site-specific surveillance or surveys. While some pathogens have integrated subnational approaches as a building block for national estimates (eg, schistosomiasis), this is yet to be implemented for visceral leishmaniasis. Regardless of contribution to the global incidence model, these data can be used to inform age-sex splits, as well as a variety of other key parameters, particularly duration parameters, which are currently lacking uncertainty and support from a full literature review.

The removal of the "high endemicity" dataset in the ST-GPR framework led to some sub-Saharan African nations having considerably higher burden estimates than prior cycles. For many nations, this is reflective of the sporadic reporting of cases in these countries (eg, in Angola and the Democratic Republic of Congo), and a consensus on pathogen presence is highly uncertain. It was important to remove this covariate, however, as it was a prior imposed on the model, a model designed to evaluate this status that leveraged the same data that were in the model itself. This high degree of dependence we aimed to eliminate. In the next cycle of GBD, there is a need to identify an independent resource to aid in quantifying the population-at-risk, which the high endemicity layer was designed to approximate, as well as engaging with self-identified pathogen-specific and country-specific collaborators to re-evaluate the presence/protocol presence status assigned to these nations.

Similarly, existing death data are limited in geographic distribution (due to primarily coming from countries with robust vital registration systems), and could lack in external validity when extrapolated to other nations. While region-level random effects help account for some of this (for instance, mitigating some of the higher case fatality rates in immunocompromised individuals in the high-income region in GBD), this doesn't eliminate all possible confounding, and furthermore, does not negate the fact that most of the high-incidence countries do not report a full time series of deaths. Further cycles should explicitly consider the reported case fatality rates in the literature, many of which come from those VR data-poor regions.

Age-sex patterns are highly reflective of the countries from which data are obtained. Importantly, there is a large skew in information coming from Brazil. This information has potential biases due to the nature of the data inputs (notification and hospital data) and the corresponding age-sex variation in health-seeking behaviours. Yet again, consulting some of the detailed household surveys that do exist will increase geographic coverage of these estimates, and provide an important independent comparator to determine whether these disparities are genuine, or an artefact of the reporting systems consulted in this current model.

References

Alvar, Jorge, Iván D Vélez, Caryn Bern, Mercé Herrero, Philippe Desjeux, Jorge Cano, Jean Jannin, and Margriet den Boer. 2012. "Leishmaniasis Worldwide and Global Estimates of Its Incidence." *PLoS One* 7 (5): e35671.

Copeland, H W, B A Arana, and T R Navin. 1990. "Comparison of Active and Passive Case Detection of Cutaneous Leishmaniasis in Guatemala." *Am. J. Trop. Med. Hyg.* 43 (3): 257–259.

Das, A K, A D Harries, S G Hinderaker, R Zachariah, B Ahmed, G N Shah, M A Khogali, G I Das, E M Ahmed, and K Ritmeijer. 2014. "Active and Passive Case Detection Strategies for the Control of Leishmaniasis in Bangladesh." *Public Health Action* 4 (1): 15–21.

Eid, Daniel, Miguel Guzman-Rivero, Ernesto Rojas, Isabel Goicolea, Anna-Karin Hurtig, Daniel Illanes, and Miguel San Sebastian. 2017. “Assessment of a Leishmaniasis Reporting System in Tropical Bolivia Using the Capture-Recapture Method,” October, tpmd170308.

Faraj, Chafika, Joshua Yukich, El Bachir Adlaoui, Rachid Wahabi, Abraham Peter Mnzava, Mustapha Kaddaf, Abderrahmane Laamrani El Idrissi, Btissam Ameur, and Immo Kleinschmidt. 2016. “Effectiveness and Cost of Insecticide-Treated Bed Nets and Indoor Residual Spraying for the Control of Cutaneous Leishmaniasis: A Cluster-Randomized Control Trial in Morocco.” *Am. J. Trop. Med. Hyg.* 94 (3): 679–685.

Gkolfinopoulou, K, N Bitsolas, S Patrinos, L Veneti, A Marka, G Dougas, D Pervanidou, et al. 2013. “Epidemiology of Human Leishmaniasis in Greece, 1981-2011.” *Euro Surveill.* 18 (29): 20532.

Hirve, S, S P Singh, N Kumar, M R Banjara, P Das, S Sundar, S Rijal, et al. 2010. “Effectiveness and Feasibility of Active and Passive Case Detection in the Visceral Leishmaniasis Elimination Initiative in India, Bangladesh, and Nepal.” *Am. J. Trop. Med. Hyg.* 83 (3): 507–511.

Maia-Elkhoury, Ana Nilce Silveira, Eduardo Hage Carmo, Marcia Leite Sousa-Gomes, and Eduardo Mota. 2007. “[Analysis of visceral leishmaniasis reports by the capture-recapture method].” *Rev. Saude Publica* 41 (6): 931–937.

Pigott, David M, Samir Bhatt, Nick Golding, Kirsten A Duda, Katherine E Battle, Oliver J Brady, Jane P Messina, et al. 2014. “Global Distribution Maps of the Leishmaniases.” *Elife* 3 (January): e02851.

Rahman, Kazi Mizanur, Indira V M Samarawickrema, David Harley, Anna Olsen, Colin D Butler, Shariful Amin Sumon, Subrata Kumar Biswas, Stephen P Luby, and Adrian C Sleigh. 2015. “Performance of Kala-Azar Surveillance in Gaffargaon Subdistrict of Mymensingh, Bangladesh.” Edited by Carlos Franco-Paredes. *PLoS Negl. Trop. Dis.* 9 (4): e0003531.

Sesma, B, and A Barricarte. 1997. “[Leishmaniasis in Navarra: review of activities].” *An. Sist. Sanit. Navar.* 20 (2): 209–216.

Singh, S P, D C S Reddy, M Rai, and S Sundar. 2006. “Serious Underreporting of Visceral Leishmaniasis through Passive Case Reporting in Bihar, India.” *Trop. Med. Int. Health* 11 (6): 899–905.

Singh, V P, A Ranjan, R K Topno, R B Verma, N A Siddique, V N Ravidas, N Kumar, K Pandey, and P Das. 2010. “Estimation of Under-Reporting of Visceral Leishmaniasis Cases in Bihar, India.” *Am. J. Trop. Med. Hyg.* 82 (1): 9–11.

Yadón, Z E, M A Quigley, C R Davies, L C Rodrigues, and E L Segura. 2001. “Assessment of Leishmaniasis Notification System in Santiago Del Estero, Argentina, 1990-1993.” *Am. J. Trop. Med. Hyg.* 65 (1): 27–30.

Cutaneous leishmaniasis

Cutaneous & Mucocutaneous Leishmaniasis

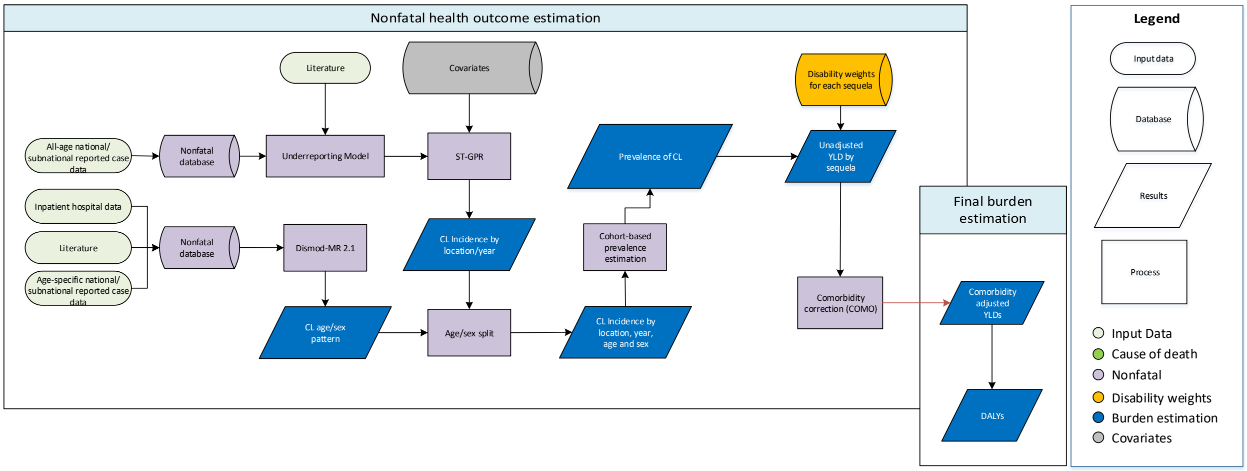

Description of general methodology

The non-fatal estimation process for cutaneous leishmaniasis is built from incident case notification data representative of the GBD geographic location, which are adjusted for underreporting. The upscaled all-age, both sex, case counts are modelled using spatiotemporal Gaussian process regression (ST-GPR) in order to impute for missing location-year combinations as well as to account for further biases and inaccuracies in reporting. Datasets that disaggregate CL cases by age and sex are modelled using DisMod to produce a global age-sex split which is applied to the all-age, both-sex envelope estimates resulting from ST-GPR. These incidence estimates are used to derive prevalence measures, as well as compute the resulting years lived with disability values.

Input Data – Case Notification time series

Current estimation for the all-age, both-sex, incidence envelope is based upon location-representative information rather than site-specific epidemiological measures due to the absence of global foci maps allowing for upscaling of geographically precise information. The primary data resource therefore is the case notification time-series reported by National Control Programs and Ministries of Health to the World Health Organization. This is supplemented by systematic literature review (last updated for GBD 2015) to identify alternate sources of data for years missing information. For countries with subnational estimates, in-country collaborators have compiled information for respective programs, or identified key resources, again supplemented by literature reviews. Where possible, information disaggregating location-level statistics by age and sex were extracted

	Incidence
Site-years (total)	848
Number of countries with data	56
Number of GBD regions with data (out of 21 regions)	13
Number of GBD super-regions with data (out of 7 super-regions)	7

Table 1: Summary statistics for data used in cutaneous leishmaniasis estimation.

Method – Geographic restrictions

There are strong climatic and biogeographic constraints on the geographic distribution of CL resulting in a focal, rather than cosmopolitan global distribution. As a result, it is necessary to identify locations burdened by the disease through space and time as distinct from countries where CL is absent. Tags were assigned to each location-year based upon the outcome of a search of IHME databases, as well as location-specific searches of PubMed. Each location-year is tagged as follows:

- Present – where a specific citation of either an autochthonous laboratory-confirmed case (ie, a case with PCR, serological, or parasitological diagnosis), reported case (ie, a case noted as CL, but with no supporting diagnostic), or supporting evidence (ie, confirmed infection in animal reservoirs or sandfly vectors)
- Protocol Present – for a given location-year, where no specific citation is used, but is present for another year in the same location, it is assumed that CL is present given that eradication of the pathogen has not been achieved
- Absent – where PubMed location-specific searches returned zero relevant results, in locations scoring -25 or lower as evaluated by Pigott and colleagues (2014) [the threshold for "absence" in that study], locations were tagged as Absent
- Protocol Absent – as with Absent, locations with zero relevant PubMed results, but with greater than -25 as evaluated by Pigott and colleagues (2014), were tagged as Protocol Absent

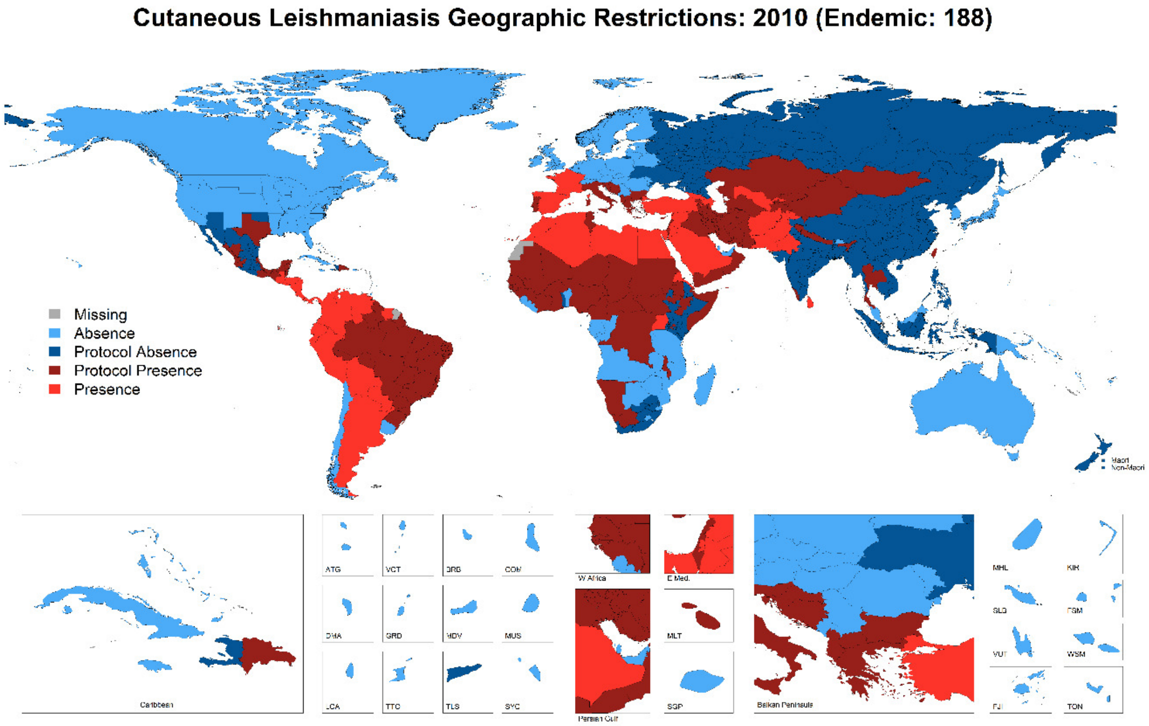

Figure 1: Cutaneous Leishmaniasis geographic restrictions for the year 2010. GBD locations tagged as present are coloured in red, dark red represents protocol presence, dark blue represents protocol absence, and absence is represented by light blue. Locations missing tags are presented in grey.

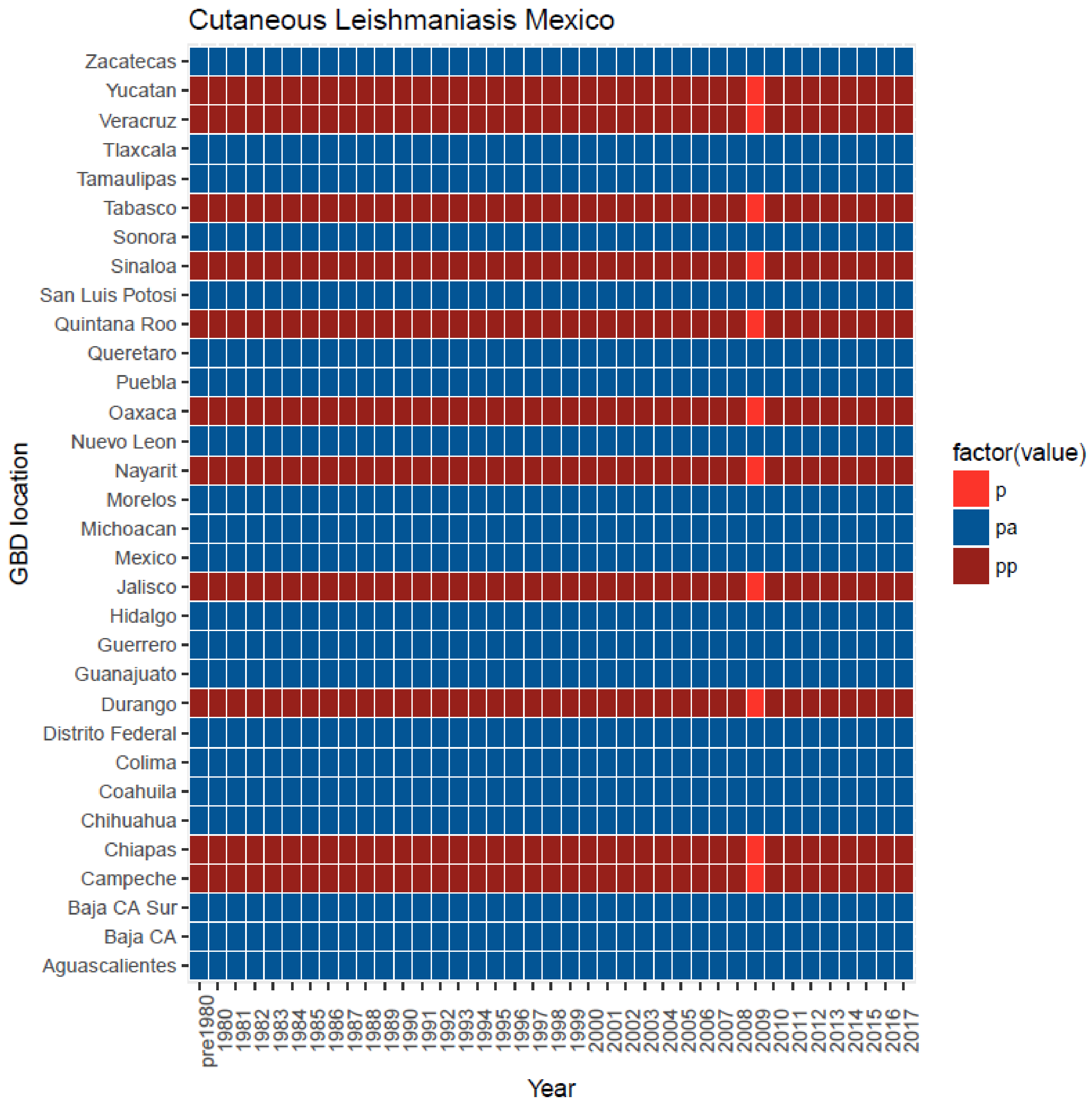

Figure 2: Cutaneous Leishmaniasis geographic restrictions for Mexican subnationals. Locations tagged as present are coloured in red, dark red represents protocol presence, and dark blue represents protocol absence.

Full time series of maps and tables, with relevant GHDx NIDs are available upon request from gbdsec@uw.edu.

Method – ST-GPR

Using existing IHME tools, the summarised values were modelled using ST-GPR to produce a complete time series of estimates for each location-year tagged “Present” or “Protocol Present”. In short, ST-GPR attempts to model non-linear trends utilizing a Gaussian process to fit a trend, rather than a definitive

functional form. Case count data were translated into estimates of true case counts by using underreporting scalars as identified by Alvar et al. (2012).

Method – DisMod

DisMod was used to generate an age-sex curve to disaggregate all-age, both-sex, incidence data. DisMod is an integrated meta-regression framework that allows for multiple datasets to be integrated into a singular analysis regardless of age-binning, sources, and geographies. As a consequence, a variety of differently aggregated information can be evaluated to generate a consensus output. From this model, the global fit was used.

Method – YLD estimation (incorporating duration and disability weighting) / COMO

Following standard GBD estimation protocols, incidence estimates were used to calculate disease prevalence (by multiplication with duration), disaggregated by disease sequelae. One health state is assigned to Cutaneous Leishmaniasis, [Table 2]. Duration value of initial acute infection was set to six months (Reithinger et al. 2007). Prevalence of long-term sequelae was based upon the proportion of cases that would result in facial scarring. The average proportion of sores that occurred on the face was calculated based upon a sample-weighted average of the proportion from four studies conducted in North Africa/Middle East. This proportion was 0.476. Of these people, only those who did not have appropriate access to health care were assigned long-term sequelae, estimated via the Healthcare Access and Quality Index. CL incidence, multiplied by proportion of people with facial sores, times the proportion of people without adequate health care access in each location-year, was used to obtain incidence of people with long-term sequelae, with cohorts streamed through time.

Sequela	Health state lay description	Disability weight	Duration
Cutaneous and mucocutaneous leishmaniasis	"has a slight, visible physical deformity that others notice, which causes some worry and discomfort"	0.011 (0.005–0.021)	6 months (46.7% * HAQ Index) Lifelong

Table 2: Sequelae and associated metadata. For the sequelae used in GBD 2017, the lay descriptor health state, disability weight, and duration are listed.

Central processing is used to generate the final estimates, including co-morbidity simulations.

Changes from GBD 2016

There were no substantive changes from the GBD 2016 methodology

Limitations

As with any modelling process, a number of limitations are known, which will be the focus of additional effort in upcoming GBD cycles and engagement with collaborators. Given the focus on location-representative estimates, the existing model is focused on national case counts. This excludes a large resource of published literature and grey literature focused on site-specific surveillance or surveys. While some pathogens have integrated subnational approaches as a building block for national estimates (eg, schistosomiasis) this has yet to be implemented for cutaneous leishmaniasis. Regardless of contribution

to the global incidence model, these data can be used to inform age-sex splits, as well as a variety of other key parameters, particularly duration parameters, which are currently lacking uncertainty.

References

Alvar et al. (2012) Leishmaniasis Worldwide and Global Estimates of Its Incidence. PLoS One 7(5):e35671

Pigott et al. (2014) Global Distribution Maps of the Leishmaniases. eLife 3:e02851

Reithinger et al. (2007) Cutaneous Leishmaniasis. Lancet Infect Dis 7(9):581-96

Human African Trypanosomiasis (HAT)

Flowchart

African trypanosomiasis

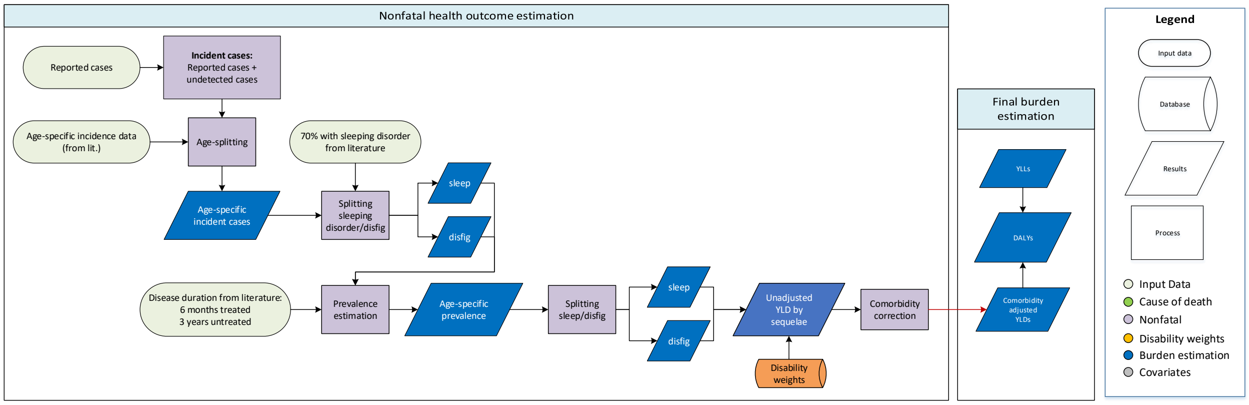

Input Data & Methodological Summary

Case Definition

Human African trypanosomiasis (HAT), also known as sleeping sickness, is a vector-borne disease which is transmitted by the bite of the tsetse fly. It is caused by the parasite *Trypanosoma brucei* with two subspecies, namely *T.b. rhodesience* (makes up less than 5% of total HAT cases) and *T.b. gambiense*. Cases are diagnosed through laboratory methods which rest on finding the parasite in body fluid or tissue by microscopy. In highly endemic or epidemic areas where the likelihood of false positives in serological tests is deemed lower, a seropositive individual is considered affected even in the absence of parasitological confirmation. The ICD-10 codes for HAT are B56.0, B56.1 and B56.9.

Input data

Model inputs

Data sources for GBD 2017:

1) Annual case totals 1980–2016: National-level annual case totals from 1990–2016 were obtained from the publicly available data via WHO, available here: http://apps.who.int/gho/data/node.main.A1635?lang=en

Table of case data counts

	Incidence
**Site-years (total)**	611
**Number of countries with data**	25
**Number of GBD regions with data (out of 21 regions)**	4
**Number of GBD super-regions with data (out of 7 super-regions)**	1

Subnational data:

Kenya: Kenyan subnational estimates are attributed to Busia County. Identification of subnational locations for Kenyan case data were obtained via studies published in the peer-reviewed literature[1] and review of maps published from via the WHO HAT Atlas[2]: http://www.who.int/entity/trypanosomiasis_african/country/Kenya_whole_0014.jpg?ua=1.

2) Age/sex data: Data on the age and sex distribution of HAT cases were extracted from the peer-reviewed literature via a systematic review of sources identified in PubMed using the following search string:

((African trypanosomiasis[Title/Abstract] AND (incidence[Title/Abstract] OR burden[Title/Abstract] OR prevalence[Title/Abstract] OR community[Title/Abstract])) AND (“1990”[Date – Publication] : “2017”[Date – Publication]))

This yielded 219 studies, of which only three met the inclusion criteria and were extracted[3-5]. The inclusion criteria were:

1. Studies representative of the national population
2. Population-based studies
3. Studies with primary data on incidence
4. Studies of human African trypanosomiasis (excluded studies on animal African trypanosomiasis)

Table data counts for age/sex-specific prevalence and incidence

	Incidence	Prevalence
**Site-years (total)**	2	1
**Number of countries with data**	2	1
**Number of GBD regions with data (out of 21 regions)**	2	1
**Number of GBD super-regions with data (out of 7 super-regions)**	1	1

3) Population at risk estimates 1980–2015: population at risk estimates from GBD 2010 ArcGIS analysis using geocoded case notifications for 2000 to 2009[2] and population Count Grid estimates from Gridded Population of the World.

Table of data counts for population at risk estimates

	Other
**Site-years (total)**	751
**Number of countries with data**	24
**Number of GBD regions with data (out of 21 regions)**	4
**Number of GBD super-regions with data (out of 7 super-regions)**	1

4) Screening coverage: Data on active versus passive screening coverage were obtained from a Weekly Epidemiological Report[6] identifying the population screened from 1997 to 2004 at the national level.

Table of data counts for screening coverage data

	Other
**Site-years (total)**	109
**Number of countries with data**	29
**Number of GBD regions with data (out of 21 regions)**	4
**Number of GBD super-regions with data (out of 7 super-regions)**	1

5) Geographic restrictions: Data file of all GBD locations, defining location as either endemic or non-endemic for HAT. Estimates are not produced for non-endemic countries, nor are they generated for countries with a history of HAT transmission but no data reported by WHO from 1990 to 2016.

Modelling strategy

Geographic restrictions

For countries historically considered endemic for HAT, but which have no reported case data or estimate of the population at risk, estimates are not produced. These countries include Botswana, Ethiopia, Guinea-Bissau, and Rwanda.

Among countries where population at-risk data are available, if no cases were reported to WHO, we assume the incidence of HAT is zero for those years and generate model estimates accordingly.

Modelling steps
Non-fatal estimates for HAT were generated as follows:

1. The incidence of reported HAT cases among the population at-risk was calculated as the total number of reported cases divided by the population at-risk estimates generated by the GBD working group for the period 1980–2015. Population at-risk estimates for 2016–2017 were generated by assuming an annual 2% rate of population growth.

2. To estimate the number of cases that were likely undetected by country and year, a multi-level mixed-effects linear regression of log-transformed incidence rate (ratio of reported HAT cases to population at risk) on log-transformed screening coverage (ratio of number screened for HAT to population at risk), with country random effects, was performed. Gaps were then filled using interpolation between years and extrapolation from 2016 to 2017 for reported cases. This model generates a beta-coefficient which is used to estimate the case detection rate (see step 4).

   For country-years in which no screening coverage data were reported:
   - Among countries with data reported, 1997–2004, the proportion of the at-risk population screened from 1997 was used retrospectively for the period 1980–1996 and the screening coverage from 2004 was carried forward from 2005–2017.
   - For countries with no screening data reported, the mean screening coverage for the region was used to impute a value over time.

3. Assuming the same proportion in treated (reported) and untreated (undetected) cases, the incidence estimates were then split into the two sequelae, skin disfigurement and sleeping disorder. This was done by generating 1,000 draws of the splitting proportion for the sequelae (70%–74% with sleeping disorder) based on a study that reported presence of symptoms at admission of patients in treatment centers[7]. Draws were generated from a beta distribution with alpha parameter = 1884 and beta parameter = 649.

4. To compute prevalence of HAT, 1,000 draws of total duration of symptoms in untreated cases were generated from a normal distribution with mean = [ln(3) – 0.5 * sigma^2], and standard deviation = sigma, where sigma = [ln(4.39)-ln(1.92))/(invnormal(0.975)*2)]: these parameters were based on a study of *T.b. gambiense*[7] which estimated an average duration of three years to untreated cases. An estimated duration of six months was applied to cases that received treatment, based on findings from a paper about *T.b. rhodesiense* in Uganda[8].

5. Prevalence was then estimated from the incident cases before applying age pattern. Prevalence of treated and untreated cases were summed up, assuming that untreated cases have been prevalent up to their death for a certain duration[9]. For untreated cases, it was assumed that half the duration is spent with sleeping disorder (severe motor and cognitive impairment) and disfigurement[7]. Treated (ie, reported) cases are assumed to have been prevalent for 0.5 years, and for the fraction of treated cases that present with sleeping disorder, it was assumed that this is present for half the total duration and that the rest of the duration is spent suffering from disfiguring skin disease. Among reported cases assumed to be detected prior to stage 2 infection, we do not attribute any of the duration of morbidity to sleeping disorder.

6. Finally, an age-pattern was applied to the prevalence estimates using the incidence studies from Sudan[5], DRC[3], and Uganda[4]. The age-pattern in GBD 2017 employed a cubic spline to account for the higher risk of infection among working-age adults.

Severity splits/sequelae

The basis of the GBD disability weight (DW) survey assessments are lay descriptions of sequelae highlighting major functional consequences and symptoms. The lay descriptions and disability weights for HAT sequelae due to HAT are shown below.

Sequela	Lay description	DW (95% CI)
Skin disfigurement, level 1	has a slight, visible physical deformity that is sometimes sore or itchy. Others notice the deformity, which causes some worry and discomfort.	0.027 (0.015–0.042)
Motor plus cognitive impairments, severe	cannot move around without help, and cannot lift or hold objects, get dressed or sit upright. The person also has very low intelligence, speaks few words, and needs constant supervision and help with all daily activities	0.542 (0.37–0.702)

References

1. Rutto JJ, Osano O, Thuranira EG, Kurgat RK, Odenyo VA. Socio-economic and cultural determinants of human african trypanosomiasis at the Kenya - Uganda transboundary. *PLoS Negl Trop Dis* 2013; **7**(4): e2186.

2. Simarro PP, Cecchi G, Paone M, et al. The Atlas of human African trypanosomiasis: a contribution to global mapping of neglected tropical diseases. *Int J Health Geogr* 2010; **9**: 57.

3. Lutumba P, Makieya E, Shaw A, Meheus F, Boelaert M. Human African trypanosomiasis in a rural community, Democratic Republic of Congo. *Emerg Infect Dis* 2007; **13**(2): 248-54.

4. Fevre EM, Odiit M, Coleman PG, Woolhouse ME, Welburn SC. Estimating the burden of rhodesiense sleeping sickness during an outbreak in Serere, eastern Uganda. *BMC Public Health* 2008; **8**: 96.

5. Moore A, Richer M, Enrile M, Losio E, Roberts J, Levy D. Resurgence of sleeping sickness in Tambura County, Sudan. *Am J Trop Med Hyg* 1999; **61**(2): 315-8.

6. World Health Organization. Human African trypanosomiasis (sleeping sickness): epidemiological update. *Weekly epidemiological record* 2006; **February 24**(8): 69-80.

7. Blum J, Schmid C, Burri C. Clinical aspects of 2541 patients with second stage human African trypanosomiasis. *Acta Trop* 2006; **97**(1): 55-64.

8. Odiit M, Kansiime F, Enyaru JC. Duration of symptoms and case fatality of sleeping sickness caused by Trypanosoma brucei rhodesiense in Tororo, Uganda. *East Afr Med J* 1997; **74**(12): 792-5.

9. Checchi F, Filipe JA, Haydon DT, Chandramohan D, Chappuis F. Estimates of the duration of the early and late stage of gambiense sleeping sickness. *BMC Infect Dis* 2008; **8**: 16.

Schistosomiasis

Flowchart

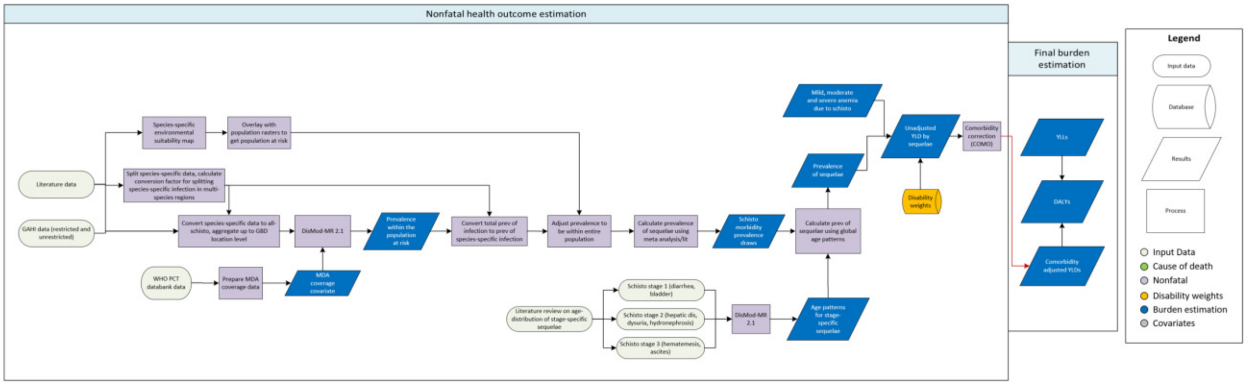

Case definition

Schistosomiasis, also known as bilharzia or "snail fever," is a helminth disease caused by infection with five species of the parasite *Schistosoma,* namely, *S mansoni, S japonicum, S haematobium, S mekongi, and S intercalatuma*. It is considered a neglected tropical disease (NTD). The first three species cause the most infection and the last two rarely cause disease. Diagnosis is made by microscopic exam of stool or urine for parasite eggs. For less advanced infections, serologic techniques are used. The ICD-10 codes for schistosomiasis are B65-B65.9.

Input data

Model inputs

To model non-fatal outcomes due to schistosomiasis, we conducted a systematic literature review, extracting prevalence data from 1980 to 2016 for the five species of schistosomiasis listed above. The search string used in the systematic review is (schistosom*[Title/Abstract] OR bilharzia*[Title/Abstract] OR "snail fever"[Title/Abstract]) AND ("1990"[Date - Publication] : "3000"[Date - Publication]) AND (epidemiolog* OR inciden* OR prevalen* OR seroprevalen*) NOT (animals[mesh] NOT humans[mesh]). Additionally, we used data compiled by the Global Atlas of Helminth Infections (GAHI), which includes grey literature and unpublished data.

Mass drug administration data

Mass drug administration data were extracted from the WHO PCT Databank [1].

Severity splits/sequelae

The table below shows the list of clinical sequelae (including mild, moderate, and severe anaemia) due to schistosomiasis, their lay descriptions, and the associated disease stages and disability weights. Using literature [1], a list of eight possible clinical sequelae and anaemia sequelae were defined (mild infection, mild diarrhoea, haematemesis (vomiting blood), hepatomegaly, ascites (buildup of fluid in the

peritoneal cavity), dysuria (painful urination), bladder pathology, hydronephrosis (swelling of kidney due to buildup of urine in the kidney), mild anaemia, moderate anaemia, and severe anaemia).

Table 2. Clinical sequela, lay descriptions, disease stages, and DWs

Clinical sequela	Lay description	Disease stage	Disability weights (DWs)
Mild infection	has a low fever and mild discomfort , but no difficulty with daily activities	1	0.006 (0.002–0.012)
Mild diarrhoea		1	0.056
Hepatomegaly	has some pain in the belly that causes nausea but does not interfere with daily activities	2	0.011 (0.005–0.021)
Dysuria	has some pain in the belly that causes nausea but does not interfere with daily activities	2	0.011 (0.005–0.021)
Hydronephrosis	has some pain in the belly that causes nausea but does not interfere with daily activities	2	0.011 (0.005–0.021)
Haematemesis	vomits blood and feels nauseated	3	0.325 (0.209–0.463)
Ascites	has pain in the belly and feels nauseated. The person has difficulties with daily activities	3	0.114 (0.078–0.159)
Bladder pathology	has some pain in the belly that causes nausea but does not interfere with daily activities	3	0.011 (0.005–0.021)
Mild anaemia	feels slightly tired and weak at times, but this does not interfere with normal daily activities	NA	0.004 (0.001–0.008)
Moderate anaemia	feels moderate fatigue, weakness, and shortness of breath after exercise, making daily activities more difficult	NA	0.052 (0.034–0.076)
Severe anaemia	feels very weak, tired, and short of breath, and has problems with activities that require physical effort or deep concentration	NA	0.149 (0.101–0.210)

Modelling strategy

The morbidity model for schistosomiasis involved a multi-step process. First, we ran a single-parameter prevalence model in DisMod-MR 2.1 using the prevalence data extracted in the systematic review and from the GAHI database. We make the assumption that all of our data are measured within a population at risk – therefore, the estimates from the DisMod model represent prevalence estimates among the population at risk for schistosomiasis. Additionally, we included the MDA treatment data from WHO as a country-level covariate in the DisMod model. Second, we ran three separate ecological niche maps for the three major species of schistosomiasis (*S mansoni, S haematobium,* and *S japonicum*) using a boosted regression tree and all geolocated data that were extracted from both the literature review and the GAHI database. The output was 1,000 maps (representing 1,000 draws) for each of the three species representing the suitability for schistosomiasis to exist in each 5x5 km square. Then, we extracted population at risk by optimising the area under the curve for each of the 1,000 maps for each of the three species, overlaid the three species maps over one another, and extracted 1,000 draws of proportion of the population at risk for schistosomiasis at the GBD location level.

The BRT was overestimating in Brazil and China. In Brazil, we masked out urban areas from the population at risk rasters, and in China we used year-specific masks based off of published literature on county-specific elimination of schistosomiasis, allowing the geographic restrictions to be implemented at a more detailed level where information is available (5).

We then scaled the prevalence estimates to the population at risk estimates from the ecological niche map to get age/sex/location/year all-schistosomiasis prevalence envelopes. 4) We ran a generalised linear model to get species-specific proportional prevalence on data from literature that reported both *S. haematobium* and *S. mansoni* infection, and 5) literature-informed parameters (a, b, c) for translating infection (x) to morbidity (y): $y = (a + bx^c)/(1 + bx^c) – a$ [2-4]. We used the species-specific conversion factors calculated in step (4) to split the all-schistosomiasis envelope into species-specific schistosomiasis. We then used the parameters determined in step (5) to translate infection into morbidity to get age/sex/year/location-specific prevalence of sequelae. The burden of anaemia due to schistosomiasis was estimated (see anaemia documentation for details).

Model evaluation was done by separately assessing the fit of the single-parameter DisMod models and checking the final estimates produced after age-sex splits. Plots of time trends of prevalence across locations and age were used to evaluate the results. In addition, maps of the global distribution of total schistosomiasis prevalence and prevalence of sequelae due to schistosomiasis were also assessed across time.

Changes from GBD 2016 to GBD 2017

The boosted-regression tree environmental suitability maps were further developed in 2017 and were selected based off of improved area under the curve (AUC) statistics. The urban mask for Brazil was also newly implemented this year.

References

1. World Health Organization (WHO). WHO PCT Databank - Schistosomiasis. Geneva, Switzerland: World Health Organization (WHO).
2. van der Werf MJ, de Vlas SJ, Brooker S, et al. Quantification of clinical morbidity associated with schistosome infection in sub-Saharan Africa. Acta Trop. 2003;86(2-3):125-39
3. van der Werf MJ, de Vlas SJ, Looman CW, Nagelkerke NJ, Habbema JD, Engels D. Associating community prevalence of Schistosoma mansoni infection with prevalence of signs and symptoms. Acta Trop. 2002;82(2):127-37
4. van der Werf MJ, de Vlas SJ. Diagnosis of urinary schistosomiasis: A novel approach to compare bladder pathology measured by ultrasound and three methods for hematuria detection. Am. J. Trop. Med. Hyg. 2004;82:98-106
5. Zhou, Xiao-Nong & Bergquist, Robert & Leonardo, Lydia & Olveda, Remigio. (2018). Schistosomiasis: The Disease and its Control.

Cysticercosis

Flowchart

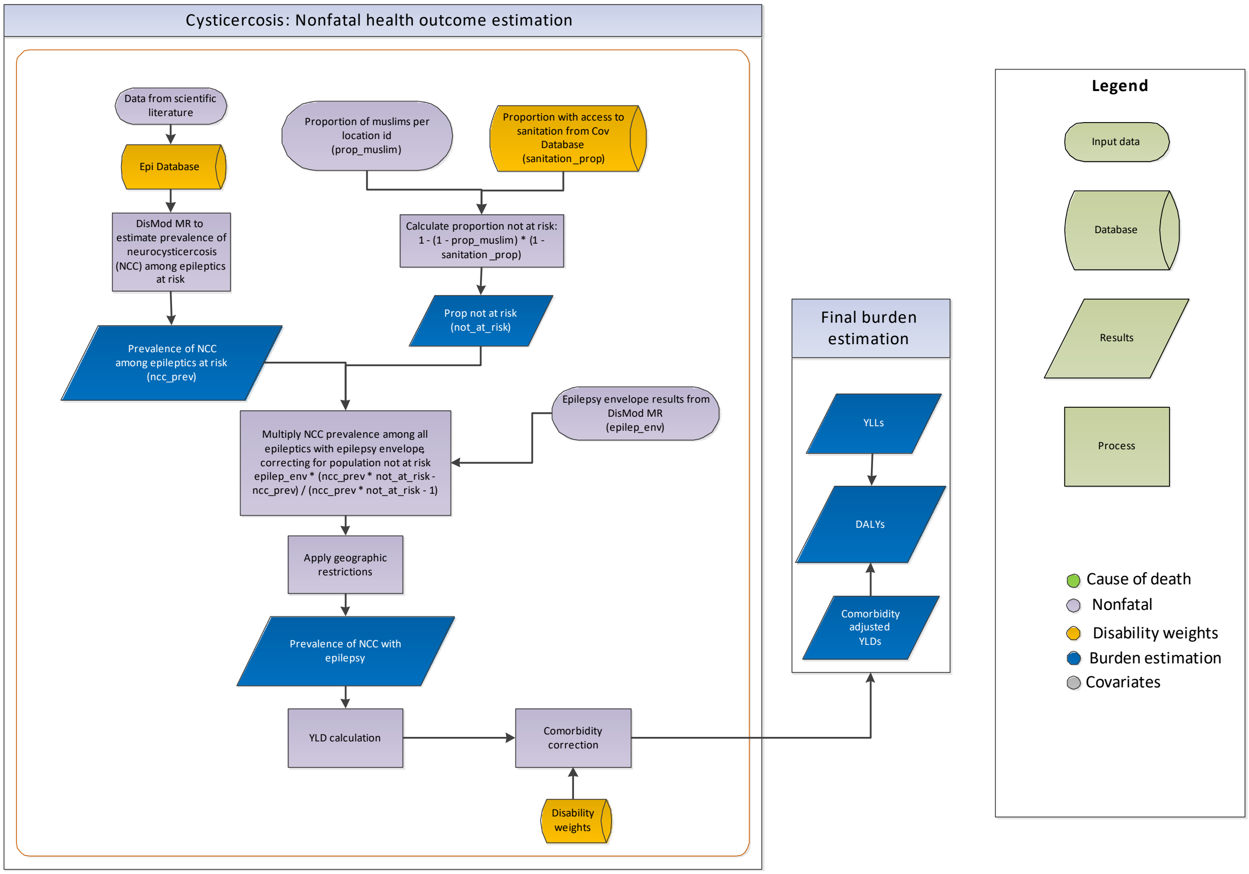

Input Data & Methodological Summary

Case Definition

Cysticercosis, or neurocysticercosis (NCC), is a parasitic disease caused by the pig tapeworm *Taenia solium.* It is transmitted via ingestion of eggs or gravid proglottids shed by a human or non-human host with an intestinal infection of the same helminth known as Taeniasis. In rare cases, auto-infection is also possible among people with intestinal infections. Diagnosis is made by magnetic resonance imaging (MRI) or computerized tomography (CT) brain scans to identify cysts. The ICD-10 codes for cysticercosis are B69-B69.9.

Input data

Systematic literature review

The nonfatal estimation for cysticercosis focused on estimating prevalence of NCC among epileptics at risk as well as the prevalence of NCC with epilepsy. A systematic review of literature was conducted in PubMed for GBD 2015 using the following search string:

("cysticercosis"[Title/Abstract] OR "neurocysticercosis"[Title/Abstract] OR "cysticerciasis"[Title/Abstract] OR "Taenia solium"[Title/Abstract]) AND (“1990”[Date – Publication] : “2015”[Date – Publication]) AND (epidemiology OR prevalence)).

This yielded 1,038 studies, of which 166 were included during the title/abstract screening. Following the full-text screening, 17 studies were included and extracted – studies were excluded because of one or more of the following reasons:

1. study not in epileptics
2. study not population-based
3. study does not have primary data on prevalence of NCC among epileptics at risk
4. study not in humans (some studies were on cysticercosis in pigs)
5. study on comorbidities with NCC (other than epilepsy)
6. study on sub-population, eg, patients with neurological disorders
7. review study

The table below displays the number of site-years by geography:

Table 1. Site-years for GBD 2017

	Prevalence
Site-years (total)	31
Number of countries with data	14
Number of GBD regions with data (out of 21 regions)	8
Number of GBD super-regions with data (out of 7 super-regions)	4

A study-level covariate was also created in GBD 2015 to indicate the type of diagnosis for each study, ie, definitive or probable. Of the 77 rows of country-year-age-sex data, there were 15 rows with definitive diagnosis and 62 rows with probable diagnosis.

Covariates

Data were ascertained from the PEW Research Center [1] on the proportion of the population that is Muslim and incorporated as a continuous covariate with a range between 0 and 1.

Epilepsy envelope

The modelling process incorporates 1,000 draws of epilepsy envelope prevalence from the GBD 2017 epilepsy DisMod-MR model – details on this modelling process can be found elsewhere.

Geographic restrictions

We conducted a literature review to determine the geographic extent of the disease and classify locations based on whether the disease is absent or present in each year. Locations that were geographically restricted in any given year did not have estimates made for them. Of note, we did not attempt a complete systematic review, since a single high-quality source could offer sufficient evidence of presence. Evidence of absence or presence was not available for every location for each year, and so

assumptions were made for missing years by taking into consideration the epidemiological characteristics of the disease.

If evidence indicated disease presence for two non-consecutive years, we assumed presence for all years between the two. If evidence indicated disease absence for two non-consecutive years, we assumed absence for all years between the two. If evidence indicated a change in status (ie, from absent to present, or present to absent) between two non-consecutive years, then we conducted targeted searches to ascertain the relevant year of introduction or elimination for that location. In the cases where presence or absence information was missing for the start or end years of our study interval (1990–2017) without evidence of any introduction or elimination events within the interval, we applied the status of the first and last presence/absence observations, respectively, to all years between the interval bound and the observation year. For cysticercosis, we performed targeted searches to classify location-years in PubMed and Google Scholar. In our searches, we compiled 21 peer-reviewed articles, meta-analyses, and WHO reports.

Modelling strategy

DisMod-MR was used to model the prevalence of NCC among epileptics at risk. In the model, pigs raised in extensive agricultural systems per capita, SDI, and religion (binary, >50% Muslim) were used as country-level covariates. In addition, the prevalence of "definitive diagnosis" was transformed to that of "probable and definitive diagnosis" so as to not underestimate overall prevalence.

Table 2. DisMod model covariates

Covariate	Type	Parameter	Exponentiated beta
Sex	Study-level	Prevalence	0.76 (0.31–1.73)
Definitive diagnosis	Study-level	Prevalence	0.56 (0.37–0.87)
Religion (binary, > 50% Muslim)	Country-level	Prevalence	0.48 (0.17–0.98)
Socio-demographic Index	Country-level	Prevalence	0.35 (0.14–0.95)
Pigs raised in extensive agricultural systems per capita	Country-level	Prevalence	2.31 (1.02–6.79)

After running DisMod, we adjusted the fraction of people with epilepsy attributable to cysticercosis in endemic countries for the population at risk based on the proportion of the population without access to sanitation and the proportion of the population that is Muslim. The following is the computation for estimating NCC prevalence among epileptics at risk:

$$Prevalence_{NCC\ prevalence} = Prevalence_{epilepsy} * \frac{NM - N}{NM - 1}$$

Where prevalence = prevalence of all-cause epilepsy in total population, N = proportion of NCC among epileptics at risk (non-Muslims without access to sanitation), and M = proportion of population not at risk of contracting NCC. It was assumed that the prevalence of epilepsy due to causes other than NCC is the same regardless of whether a population is at risk or not. It was also assumed that Muslims and non-

Muslims have equal access to sanitation. Geographic restrictions were applied to set prevalence to zero in non-endemic locations.

Model evaluation was done by separately assessing the fit of the DisMod-MR model and checking the estimates produced after estimating prevalence of NCC with epilepsy. Plots of time trends of prevalence across locations and age were used to evaluate the results. In addition, maps of the global distribution of prevalence of NCC among epileptics at risk and prevalence of NCC with epilepsy were also assessed across time.

Several changes were made compared to the GBD 2016 modelling strategy. First, we made slight changes to model parameters in DisMod-MR to improve model fit. Second, we incorporated two new covariates (ie, pigs raised in extensive agricultural systems per capita, SDI) to better inform the model. Lastly, we updated geographic restrictions and updated proportion of population with Muslim data by imputing subnational locations with national proportions due to a lack of data at the subnational level.

References:

1. "Table: Muslim Population by Country Pew Research Center, Washington, D.C." (July 7, 2017). http://www.pewforum.org/2011/01/27/table-muslim-population-by-country/

Cystic Echinococcosis

Flowchart

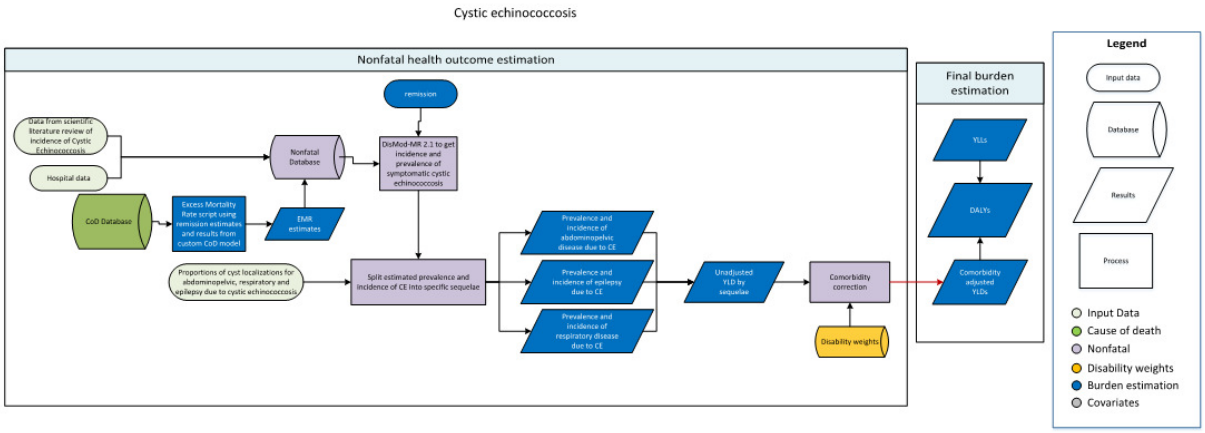

Input Data & Methodological Summary

Case definition

Cystic echinococcosis is a parasitic disease caused by infection with the *Echinococcus granulosis* tapeworm. It is a natural parasite of canines, with sheep being the most common intermediate host in the two-stage lifecycle, but can be spread to humans through ingestion of soil, water, or food contaminated with the fecal matter of an infected dog containing infective eggs. Diagnosis is made by clinical findings, imaging, serology, and tissue pathology. The ICD-9 and ICD-10 codes for echinococcosis are 122.0-122.9 and B67-B67.9, respectively.

Input data

Systematic Literature Review

The non-fatal estimation for cystic echinococcosis (CE) focused on estimating incidence and prevalence of CE and its sequelae. A systematic review of literature was conducted in PubMed for GBD 2015 using the following search string:

> ("echinococcosis"[Title/Abstract] OR "hydatid disease"[Title/Abstract] OR "hydatidosis"[Title/Abstract] OR "echinococcal disease"[Title/Abstract] OR "Echinococcus granulosus infection"[Title/Abstract]) AND (“1990”[Date – Publication] : “2015”[Date – Publication]) AND (epidemiology OR incidence OR prevalence).

This yielded 1,619 studies of which 279 were included during the title/abstract screening. Following the full-text screening, 77 studies (32 incidence, 43 prevalence, and 2 both) were included and extracted – studies were excluded because of one or more of the following reasons:

1. study not population-based
2. study does not have primary data on prevalence and/or incidence
3. study not in humans
4. study on sub-populations

5. review study

Since we were interested in modelling symptomatic CE cases, we only used data on incidence of patients diagnosed by imaging techniques (mainly ultrasonography). Therefore, we excluded prevalence data, which were mostly from serological studies. Data from these extracted studies were combined with data from studies extracted during GBD 2013.

Hospital data

Hospital data prepared by the GBD team were used as additional input into our models. These data were adjusted to account for multiple hospital episodes of a single case and non-primary diagnoses. The table below displays the site-years by geography for both the systematic review and hospital data combined.

Table 1a. Site-years from systematic review and hospital data for GBD 2017

	**Incidence**
Site-years (total)	1,338
Number of countries with data	49
Number of GBD regions with data (out of 21 regions)	17
Number of GBD super-regions with data (out of 7 super-regions)	7

Geographic restrictions

We conducted a literature review to determine the geographic extent of the disease and classify locations based on whether the disease is absent or present in each year. Locations that were geographically restricted in any given year did not have estimates made. Of note, we did not attempt a complete systematic review, since a single high-quality source could offer sufficient evidence of presence. Evidence of absence or presence was not available for every location for each year, and so assumptions were made for missing years by taking into consideration the epidemiological characteristics of the disease.

If evidence indicated disease presence for two non-consecutive years, we assumed presence for all years between the two. If evidence indicated disease absence for two non-consecutive years, we assumed absence for all years between the two. If evidence indicated a change in status (ie, from absent to present, or present to absent) between two non-consecutive years, then we conducted targeted searches to ascertain the relevant year of introduction or elimination for that location. In the cases where presence or absence information was missing for the start or end years of our study interval (1990–2017) without evidence of any introduction or elimination events within the interval, we applied the status of the first and last presence/absence observations respectively to all years between the interval bound and the observation year. For cystic echinococcosis, we performed targeted searches to classify location-years in PubMed and Google Scholar. Geographic restrictions were populated by reviewing sources referenced by Deplazes and colleagues along with ad hoc searches in PubMed for evidence of active transmission of cystic echinococcosis in respective countries [1].

Sequelae due to cystic echinococcosis

The table below shows the sequelae due to echinococcosis and their associated disability weights.

Table 2. Sequelae, lay descriptions, and disability weights (DWs)

Sequela	Lay description	DW (95% CI)
Chronic respiratory disease	"has cough and shortness of breath after heavy physical activity, but is able to walk long distances and climb stairs."	0.019 (0.011–0.033)
Abdominal problems	"has pain in the belly and feels nauseated. The person has difficulties with daily activities."	0.114 (0.078–0.159)
Epilepsy	(Combined DW)	NA

Modelling strategy

The morbidity model for cystic echinococcosis involved a multi-step process. First, DisMod-MR was used to model incidence and prevalence of symptomatic cystic echinococcosis using incidence data from systematic reviews in GBD 2013 and 2015 and hospital data, excess mortality rate estimates, and an assumed remission of 0.15–0.25 per case per year (duration 2–6.7 years, average 5 years). Estimates of excess mortality rate were obtained by pulling death estimates from our CoD model. The following steps were followed to estimate excess mortality rate: 1) create custom age groups for CE deaths with uncertainty; 2) calculate CSMR as CSMR=deaths/population at the 1,000 draw level – calculate mean CSMR, uncertainty interval, and standard error; and 3) calculate EMR as EMR=CSMR/(prevalence), where prevalence = (incidence*5) – standard error of EMR was calculated taking into consideration the standard errors of both prevalence and CSMR. Geographic restrictions were applied to set incidence and prevalence to zero in location-years where the disease was not endemic. These computations provided 655 site-years of EMR data.

Table 3. DisMod model covariates

Covariate	Type	Parameter	Exponentiated beta
Sex	Study-level	Incidence	0.66 (0.63–0.70)
Urbanicity	Country-level	Incidence	1.00 (0.98–1.00)
Echinococcosis endemicity	Country-level	Incidence	6.03 (5.75–6.37)
Proportion of population involved in agricultural activities	Country-level	Incidence	1.00 (1.00–1.00)
Sex	Study-level	Excess mortality rate	1.63 (1.56–1.70)

After producing all-case prevalence draws, 1,000 draws of proportions for abdominal, respiratory, and epileptic symptoms among echinococcosis cases adding up to 1 were generated. Uncertainty in the splitting proportions was captured by drawing them from a Dirichlet distribution, informed by published data on cysts localization [2]. On average, the proportions of abdominal, respiratory, and epileptic

symptoms due to echinococcosis were 0.5, 0.47, and 0.03, respectively. These proportions were used to split the prevalence and incidence from DisMod into the three sequelae.

Model evaluation was done by separately assessing the fit of the DisMod MR model and checking the estimates produced after estimating incidence and prevalence of sequelae due to cystic echinococcosis. Plots of time trends of incidence and prevalence across locations and age were used to evaluate the results. In addition, maps of the global distribution of incidence and prevalence were assessed across time.

References

1. Deplazes P, Rinaldi L, Alvarez Rojas CA, Torgerson PR, Harandi MF, Romig T, Antolova D, Schrurer JM, Lahmar S, Cringoli G, Magambo J, Thompson RC, Jenkins EJ. Global Distribution of Alveolar and Cystic Echinococcosis. Advanced Parasitology. 2017. 95: 315-493.
2. Raether W, Hänel H. Epidemiology, clinical manifestations and diagnosis of zoonotic cestode infections: an update. Parasitology Research. 2003. 91:412-438.

Lymphatic Filariasis

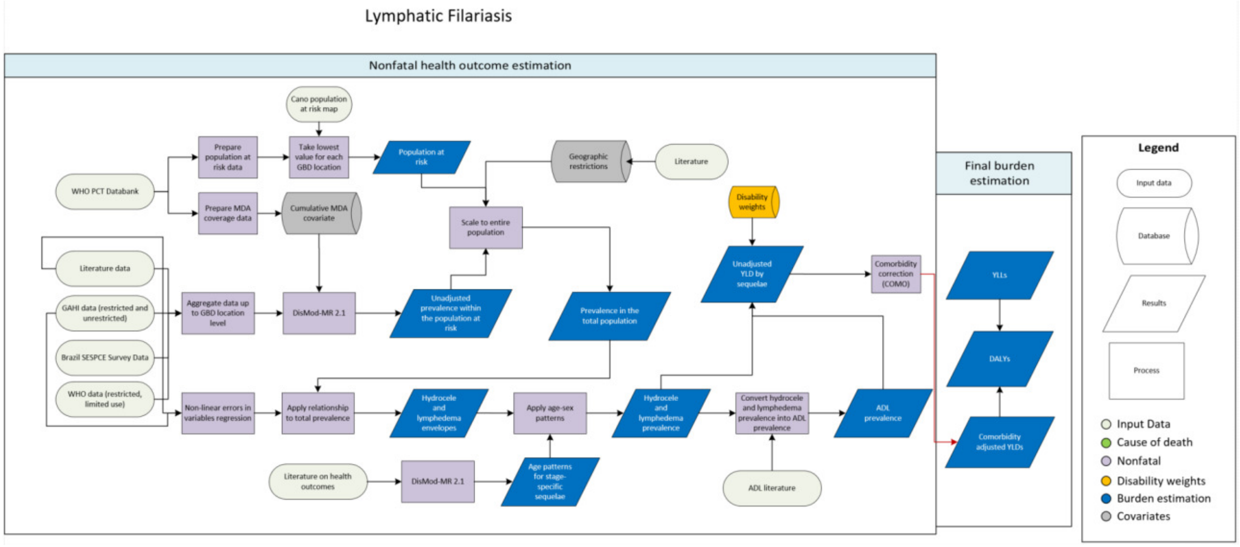

Input Data and Methodological Summary

Case Definition

Lymphatic filariasis (LF) is a neglected tropical disease in which threadlike nematodes invade the lymphatic system. The worms responsible – *Wuchereria bancrofti*, *Brugia malayi,* and *Brugia timori* – are spread from human to human via mosquitoes. The most prominent clinical manifestations of LF are lymphoedema (a swelling of the legs, also known in its more extreme manifestation as elephantiasis) and hydrocele (a collection of fluid in the sac around the testicles).

Input data

A systematic review of literature for GBD 2016 in the PubMed database was done on October 14, 2016, for prevalence and incidence data using the search (Lymphatic filariasis AND prevalence) OR (Lymphatic filariasis AND (prevalence OR incidence OR "mass drug administration" OR MDA OR coverage)) OR (Lymphedema, hydrocele) OR (Transmission Assessment Survey (TAS)) OR (Lymphatic filariasis AND mapping).

Population at risk and MDA coverage data come from the WHO PCT Databank [1].

Modelling strategy

Data on prevalence of microfilaria is modelled using Dismod-MR 2.1. Due to the focal nature of lymphatic filariasis, we make the assumption that data collected are from endemic locations unless specified in literature or survey methods. If the data are nationally representative, we adjust the data points by multiplying by the inverse of the proportion of the population at risk. Due to the fact that data are collected in endemic locations or we adjust them so that they are within the population at risk,

we then scaled the DisMod-MR 2.1 estimates according to at-risk population in order to attain nationally representative values. We developed a new MDA location-level covariate that is used in the DisMod model based off WHO PCT Databank data, informing prevalence estimates.

For lymphoedema and hydrocele, we incorporate survey data from the Global LF Atlas in a non-linear error-in-variables regression that determines the prevalence of lymphoedema and hydrocele as functions of microfilaria prevalence, which is then applied to the total microfilaria DisMod model in order to attain an envelope of cases by location-year. Separately, all available prevalence data for these conditions are modelled in DisMod in order to determine an age-sex pattern.

In the estimation of lymphoedema and hydrocele prevalence, we perform the same population at-risk correction that is done on microfilaria prevalence. For hydrocele prevalence after treatment, we take the value before MDA rollout in 2000 and reduce that by the same treatment efficacy function described for microfilaria prevalence, using dosage-reduction data specific to hydrocele along with the location-year-specific MDA coverage. For lymphoedema, we assume no new cases appear among treated individuals. As such, we reduce lymphoedema prevalence in post-treatment years in accordance with MDA coverage.

Sequela	Data points	Regions	Countries	Subnational units
Prevalence of detectable microfilaria	1,552	10	40	28
Lymphoedema due to lymphatic filariasis	511	10	25	15
Hydrocele due to lymphatic filariasis	265	8	22	12

Changes from GBD 2016 to GBD 2017

We conducted a new literature review, and utilised data from recent years and the MDA covariate to predict the time trend rather than last year's non-linear regression to estimate the reduction of microfilaria as a function of treatments per person. Additionally, we used age-specific data extracted from communities that were pre-MDA and post-MDA to develop age-trends specific to MDA status. We then split out all-age data according to MDA status to provide more granular data to the unadjusted prevalence model.

Onchocerciasis

Flowchart

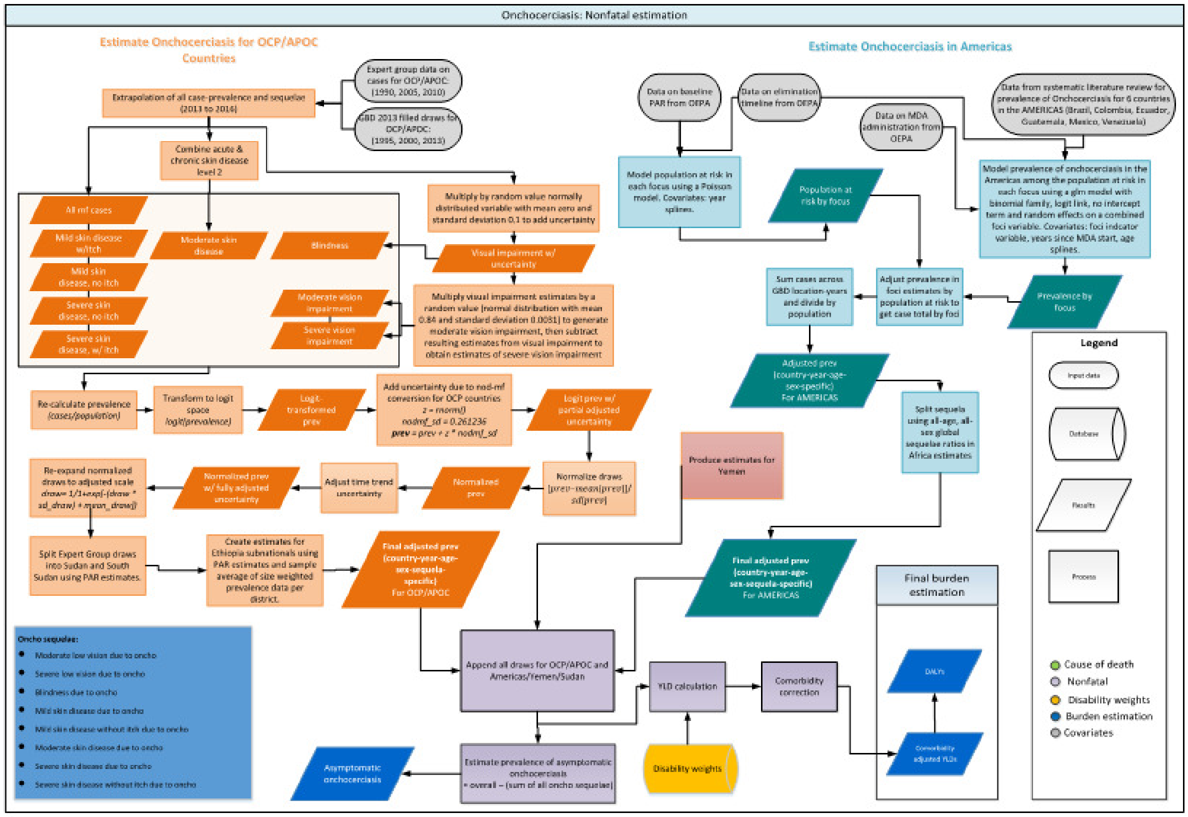

Input data & methodological summary

Case definition

Onchocerciasis, also known as river blindness, is a parasitic disease caused by *Onchocerca volvulus*. It is transmitted via the bite of one of several species of *Similium* blackflies that have historically bred in fast-moving freshwater rivers and tributaries throughout sub-Saharan Africa, Central America, and South America. Diagnosis can be made by skin snip biopsy to identify larvae, surgical removal of nodules and exam for adult worms, slit lamp exam of anterior part of the eye where larvae or lesions caused by them are visible, and antibody tests (mostly useful to visitors to areas with parasites). The ICD-10 code for onchocerciasis is B73.

Input data

Model inputs

Prevalence data prepared by the GBD 2010 expert group (EG) was used for modelling the nonfatal outcomes resulting from onchocerciasis in Africa. This included 1,000 draws of infection and morbidity (visual impairment, blindness, and skin conditions) cases with confidence intervals categorised by

country, age, and sex for years 1990, 1995, 2000, 2005, and 2010. Details about the materials and methods used by the EG to generate these draws can be found elsewhere [1-5]. These data represented all African countries included in the African Programme for Onchocerciasis Control (APOC) and the Onchocerciasis Control Programme (OCP) for which initial Rapid Epidemiological Mapping of Onchocerciasis (REMO) assessments demonstrated a need for Community-Directed Treatment with Ivermectin (CDTI) (defined as having a prevalence of skin nodules greater than 20%). Four countries – Rwanda, Mozambique, Kenya, and Gabon – were designated as hypo-endemic countries after initial REMO assessments and not included due to sparsity of cases and paucity of data. Estimates for Sudan from GBD 2010 were reassigned to South Sudan in GBD 2013 after its independence in 2011 since REMO assessments indicated that the vast majority of cases occurred in that area of the former Sudan. The tables below show the countries included in each program and the number of corresponding GBD locations they represent.

	**APOC Countries**	**OCP Countries**
Countries included	Angola, Burundi, Cameroon, Central African Republic, Chad, Congo, Democratic Republic of Congo, Ethiopia, Equatorial Guinea, Liberia, Malawi, Nigeria, South Sudan, Tanzania, and Uganda	Benin, Burkina Faso, Côte d'Ivoire, Ghana, Guinea Bissau, Guinea, Mali, Niger, Senegal, Sierra Leone, and Togo
Hypo-endemic countries not included	Rwanda, Mozambique, Kenya, Gabon, Sudan	
GBD countries & subnationals provided by EG	15	11
GBD world regions	3	1

Prevalence data for modelling non-fatal outcomes resulting from onchocerciasis in the Americas was extracted via a systematic literature review. Web of Science, Scopus, and PubMed were searched with the following search strings:

**Database**	**Search string**	**Yield**
PubMed	(oncho*[Title/Abstract] OR "river blindness"[Title/Abstract] OR "O. volvulus"[Title/Abstract] OR "robles disease"[Title/Abstract] OR "blinding filariasis"[Title/Abstract] OR "coast erysipelas"[Title/Abstract] OR “sowda” [Title/Abstract] OR “nodding syndrome”[Title/Abstract]) AND (“1980”[Date – Publication] : “2016”[Date – Publication]) AND (epidemiology[Title/Abstract] OR prevalence[Title/Abstract] OR incidence[Title/Abstract] OR surveillance[Title/Abstract] OR”MDA”[Title/Abstract] OR “Mass Drug Administration”[Title/Abstract] OR “Community-directed treatment with ivermectin”[Title/Abstract] OR “CDTI”[Title/Abstract] OR “mass treatment”[Title/Abstract] OR “multiple ivermectin treatments”[Title/Abstract] OR “monthly doses of ivermectin”[Title/Abstract] OR “large scale treatment”[Title/Abstract] OR REMO[Title/Abstract] OR “Rapid epidemiological mapping of onchocerciasis”[Title/Abstract] OR APOC[Title/Abstract] OR “African Programme for Onchocerciasis Control”[Title/Abstract] OR OCP[Title/Abstract] OR “Onchocerciasis Control Programme”[Title/Abstract]) NOT(Animals[MeSH] NOT Humans[MeSH])	986
Web of Science	TS=(oncho* OR "river blindness" OR "O. volvulus" OR "robles disease" OR "blinding filariasis" OR "coast erysipelas" OR sowda OR “nodding syndrome”) AND TS=(epidemiology	1,144

	OR prevalence OR incidence OR surveillance OR MDA OR "Mass Drug Administration" OR "Community-directed treatment with ivermectin" OR CDTI OR "mass treatment" OR "multiple ivermectin treatments" OR "monthly doses of ivermectin" OR "large scale treatment" OR REMO OR "Rapid epidemiological mapping of onchocerciasis" OR APOC OR "African Programme for Onchocerciasis Control" OR OCP OR "Onchocerciasis Control Programme") NOT TS=((Animals NOT Humans))	
SCOPUS	(TITLE-ABS-KEY(oncho* OR "river blindness" OR "O. volvulus" OR "robles disease" OR "blinding filariasis" OR "coast erysipelas")) AND TITLE-ABS-KEY(epidemiology OR prevalence OR incidence OR surveillance OR MDA OR "Mass Drug Administration" OR "Community-directed treatment with ivermectin" OR CDTI OR "mass treatment" OR "multiple ivermectin treatments" OR "monthly doses of ivermectin" OR "large scale treatment" OR REMO OR "Rapid epidemiological mapping of onchocerciasis" OR APOC OR "African Programme for Onchocerciasis Control" OR OCP OR "Onchocerciasis Control Programme") AND NOT KEY(Animals NOT Humans) AND PUBYEAR > 1979	2,000

This yielded 4,130 results in total, which was reduced to 2,502 after removing duplicates. The title and abstracts were screened for inclusion or exclusion with the following criteria:

Exclusion criteria:

- Pre-1980
- Non-original source
- Non-representative population
  - Vulnerable populations (eg, slum-dwellers, prisoners, orphans, high-risk jobs, etc.)
  - Hospital-based samples (including saved stool samples)
  - Non-native peoples (eg, migrants, expats, nomads, etc.)
  - Immunosuppression/illness (eg, HIV, TB, CA, RA, asthma, malaria, handicap, etc.)
- Non-human population
- Does not meet case definition
- Case-control study

Sixty-one articles were identified for full text screening and extraction from the historically endemic American countries: Guatemala, Brazil, Ecuador, Venezuela, Mexico, and Colombia.

Severity splits/sequelae

The table below shows the list of common clinical manifestations of onchocerciasis and the sequelae to which they have been mapped along with the lay description and the associated disability weight (DW) of each sequela.

**Clinical manifestation**	**Sequela name**	**Lay description**	**DW**
Uveitis; Punctate keratitis; Optic neuritis; Torpid Iritis; Onchochorioretinitis	Moderate vision impairment	"has vision problems that make it difficult to recognize faces or objects across a room"	0.031 (0.019–0.049)
Sclerosing keratitis; Optic neuropathy; Optic atrophy;	Severe vision impairment	"has severe vision loss, which causes difficulty in daily activities, some emotional impact (for example worry), and some	0.184 (0.125–0.258)

Choroidoretinopathy; Cataracts		difficulty going outside the home without assistance”	
Blindness	Blindness	“is completely blind, which causes great difficulty in some daily activities, worry and anxiety, and great difficulty going outside the home without assistance”	0.187 (0.124–0.260)
Acute papular onchodermatitis; Onchocercomata (subcutaneous nodules)	Mild skin disease	“has a slight, visible physical deformity that is sometimes sore or itchy. Others notice the deformity, which causes some worry and discomfort”	0.027 (0.015–0.042)
Chronic papular onchodermatitis; Lichenified onchodermatitis (“sowda”); Lymphadenopathy	Mild skin disease without itch	“has a slight, visible physical deformity that others notice, which causes some worry and discomfort”	0.011 (0.005–0.021)
Skin atrophy; Depigmentation (“leopard skin”)	Moderate skin disease	“has a visible physical deformity that is sore and itchy. Other people stare and comment, which causes the person to worry. The person has trouble sleeping and concentrating”	0.188 (0.124–0.267)
Hanging groin; Lymphoedema	Severe skin disease without itch	“has an obvious physical deformity that makes others uncomfortable, which causes the person to avoid social contact, feel worried, sleep poorly, and think about suicide”	0.405 (0.275–0.546)
	Asymptomatic onchocerciasis	NA	NA

Modelling strategy

The nonfatal modelling for onchocerciasis included six major steps. In the first step, GBD 2010 prevalence was exponentially extrapolated to obtain GBD 2017 estimates. Acute skin disease level 2 and chronic skin disease level 2 were summed to create the moderate skin disease sequela. Uncertainty was quantified and provided by the EG for all estimates except those of visual impairment and blindness. In these cases, for each of the OCP draws the number of cases were multiplied by a random value (the exponent of a normally distributed variable with mean zero and standard deviation 0.1) in order to add uncertainty. Within each draw, the same randomly drawn value was applied to all country-year-age-sex estimates. Visual impairment was then split into moderate and severe vision impairment by first multiplying the visual impairment estimates by a random value (from a normal distribution with mean 0.84 and standard deviation 0.0031) to generate moderate vision impairment, and then subtracting the resulting estimates from visual impairment to obtain estimates of severe vision impairment. Prevalence of sequelae was calculated by dividing the cases by the population.

The second step in modelling morbidity due to onchocerciasis was the adjustment of uncertainty in the conversion of nodule prevalence to microfilaria (mf) prevalence and in the effects of mass drug

administration (MDA). To adjust for uncertainty in translation of nodule prevalence to mf prevalence, the final OCP draws from the first step were logit transformed and uncertainty was added from a random value drawn from a normal distribution to the transformed estimates. The resulting estimates were then normalised and scaled using estimates published elsewhere [1]. To adjust for uncertainty due to MDA, the year when MDA with ivermectin started was set according to the table below.

Country	MDA start year
Angola, Burundi, South Sudan	2005
Congo, Ethiopia, DRC	2001
Cameroon, Central African Republic, Equatorial Guinea, Liberia, Nigeria, Uganda	1999
Chad, Niger, Tanzania	1998
Malawi	1997
All others	1990

The uncertainty in the time trend was then multiplied by the normalised prevalence estimates and the final prevalence was obtained by re-expanding the scaled normalised draws and adjusting the scale back from logit scale.

Third, since EG draws were provided before the independence of South Sudan in 2011, Sudan estimates from the EG were partitioned between Sudan and South Sudan. Population at risk (PAR) estimates pre- and post-Abu Hamed foci elimination in 2015 in Sudan were used to proportionally split cases between the two countries [2]. REMO maps showing definite needs for community-directed treatment with ivermectin (CTDI) were digitised and overlaid with population per pixel rasters to produce estimates of PAR pre-Abu Hamed elimination. Post-Abu Hamed elimination in 2015, REMO maps were edited to remove the foci as a definite CDTI areas and estimates were reproduced.

In the fourth step, prevalence in the Ethiopia subnationals was estimated separately and appended to the Africa model. Subnational draws were split proportionally based on sample size weighted prevalence from prevalence data, using population at risk estimates derived from digitising a map of onchocerciasis endemic districts in 2015 from Meribo and colleagues to convert into case space [3]. A proportion of cases falling into each subnational was then used to split national case numbers provided by EG draws into each subnational.

In the fifth step, prevalence of onchocerciasis in Yemen was modelled separately and combined with the Africa model. Due to limited data, this was done utilising one data point from the Ministry of Health published in 1991 only accounting for population change [22]. Furthermore, the global age-sex trend was imposed to produce age-sex-specific estimates. The clinical manifestation of Yemeni onchocerciasis is different from other regions, notably the atypical and most severe cutaneous manifestation known as sowda [23]. Therefore, all cases of onchocerciasis are being mapped to mild skin disease due to onchocerciasis without itch.

In the sixth step, prevalence of onchocerciasis in the Americas was modelled separately and combined with the Africa and Yemen models. For the GBD estimation period, onchocerciasis is known to have occurred in six countries of Central and Southern America: Mexico, Guatemala, Colombia, Ecuador, Brazil and Venezuela. The epidemiology of onchocerciasis is very different in these countries than in Africa because it has only occurred in relatively small, well defined foci. These foci have been mapped

and thoroughly monitored since the early 1990s with the formation of the Onchocerciasis Elimination Program of the Americas (OEPA) and all of the prevalence surveys conducted are only representative of these areas. Additionally, certain foci are geographically continuous across national boundaries. Therefore, we modelled onchocerciasis in these countries at the focus level among the population at risk in each focus instead of at the national level.

Population at risk for each focus was modelled using data from OEPA on baseline population at risk [6] and data from OEPA and peer-reviewed studies on dates of elimination in each focus [6-19]. This was done with a Poisson model using year splines as a covariate, and 1,000 draws of the population at risk were drawn from the predicted mean and standard error. The prevalence of disease among the population at risk was subsequently modelled using a generalised linear model with a binomial family, logit link, no intercept term, and random effects on a combined-foci variable created by grouping foci by geographic contiguity and nearness when data were sparse. Covariates included an indicator term on the foci, the number of years since MDA began, and splines on age. One thousand draws of prevalence were calculated from 1,000 draws of beta values from the variance-covariance matrix and adjusted by the estimated population at risk in each focus-year to determine the number of cases. The cases were then summed by GBD geography and year and divided by national population to find the national prevalence. While the model predicted case values very close to zero in the countries where elimination has occurred, these were overwritten to zero values for all years after certified elimination. The ratio of global all-age, all-sex prevalence of each sequela to the all-cases prevalence from the Africa estimates was applied to all-cases prevalence from the Americas to calculate prevalence of each sequelae.

Lastly, to estimate the prevalence of asymptomatic onchocerciasis, the prevalence of morbidity (vision loss, blindness and skin conditions) was subtracted from the overall onchocerciasis prevalence. Moderate vision impairment, severe vision impairment, and blindness estimates were each multiplied by a factor of 8/33 before subtraction to account for cases that have concurring symptoms.

References

1. Zouré HG, Noma M, Tekle AH, Amazigo UV, Diggle PJ, Giorgi E, Remme JH. The geographic distribution of onchocerciasis in the 20 participating countries of the African Programme for Onchocerciasis Control: (2) pre-control endemicity levels and estimated number infected. Parasites & Vectors. 2014. 7-326.
2. Zarroung IM, Hashim K, ElMubark WA, Shumo ZA, Salih KA, ElNojomi NA, Awad HA, Aziz N, Katabarwa M, Hassan HK, Unnasch TR, Machenzie CD, Richards F, Higazi TB. The First Confirmed Elimination of an Onchocerciasis Focus in Africa: Abu Hamed, Sudan. The American Journal of Tropical Medicine and Hygeine. 2016. 95(5):1037-1040.
3. Meribo K, Kebede B, Mekasha Feleke S, Mengistu B, Mulugeta A, Sileshi M, Samuel A, Deribe K, Tadesse Z. Review of Ethiopian Onchocerciasis Elimination Program. Ethiopian Medical Journa. 2017. 55(Suppl 1): 55-63.
4. Coffeng L, Stolk W, Hoerauf A, Habbema D, Bakker R, Hopkins A, de Vlas S. Elimination of African onchocerciasis: modeling the impact of increasing the frequency of ivermectin mass treatment. PLoS One. 2014. 9(12):e115886.
5. Coffeng LE, Stolk WA, Zouré HG, Veerman JL, Agblewonu KB, Murdoch ME, Noma M, Fobi G, Richardus JH, Bundy DA, Habbema D, de Vlas SJ, Amazigo UV. African Programme For

Onchocerciasis Control 1995-2015: model-estimated health impact and cost. PLoS Negl Trop Dis. 2013; 7(1): e2032.
6. Murdoch ME, Asuzu MC, Hagan M, Makunde WH, Ngoumou P, Ogbuagu KF, Okello D, Ozoh G, Remme J. Onchocerciasis: the clinical and epidemiological burden of skin disease in Africa. Ann Trop Med Parasitol. 2002; 96(3): 283-296.
7. Brieger WR, Awedoba AK, Eneanya CI, Hagan M, Ogbuagu KF, Okello DO, Ososanya OO, Ovuga EB, Noma M, Kale OO, Burnham GM, Remme JH. The effects of ivermectin on onchocercal skin disease and severe itching: results of a multicentre trial. Trop Med Int Health. 1998; 3(12): 951-61.
8. México. http://www.oepa.net/Mexico.htm (accessed July 7, 2017).
9. Guatemala. http://www.oepa.net/guatemala.html (accessed July 7, 2017).
10. Venezuela. http://www.oepa.net/venezuela.html (accessed July 7, 2017).
11. Colombia. http://www.oepa.net/colombia.html (accessed July 7, 2017).
12. Ecuador. http://www.oepa.net/ecuador.html (accessed July 7, 2017).
13. Rodríguez-Pérez MA, Unnasch TR, Domínguez-Vázquez A, *et al.* Lack of Active Onchocerca volvulus Transmission in the Northern Chiapas Focus of Mexico. *The American Journal of Tropical Medicine and Hygiene* 2010; **83**: 15–20.
14. Rodríguez-Pérez MA, Domínguez-Vázquez A, Unnasch TR, *et al.* Interruption of Transmission of Onchocerca volvulus in the Southern Chiapas Focus, México. *PLOS Neglected Tropical Diseases* 2013; **7**: e2133.
15. Rodríguez-Pérez MA, Unnasch TR, Domínguez-Vázquez A, *et al.* Interruption of Transmission of Onchocerca volvulus in the Oaxaca Focus, Mexico. *The American Journal of Tropical Medicine and Hygiene* 2010; **83**: 21–7.
16. Cruz-Ortiz N, Gonzalez RJ, Lindblade KA, *et al.* Elimination of Onchocerca volvulus Transmission in the Huehuetenango Focus of Guatemala. Journal of Parasitology Research. 2012. https://www.hindawi.com/journals/jpr/2012/638429/abs/ (accessed July 7, 2017).
17. Jr FR, Rizzo N, Espinoza CED, *et al.* One Hundred Years After Its Discovery in Guatemala by Rodolfo Robles, Onchocerca volvulus Transmission Has Been Eliminated from the Central Endemic Zone. *The American Journal of Tropical Medicine and Hygiene* 2015; **93**: 1295–304.
18. Gonzalez RJ, Cruz-Ortiz N, Rizzo N, *et al.* Successful interruption of transmission of Onchocerca volvulus in the Escuintla-Guatemala focus, Guatemala. *PLoS Negl Trop Dis* 2009; **3**: e404.
19. Lindblade KA, Arana B, Zea-Flores G, *et al.* Elimination of Onchocercia volvulus transmission in the Santa Rosa focus of Guatemala. *Am J Trop Med Hyg* 2007; **77**: 334–41.
20. Convit J, Schuler H, Borges R, *et al.* Interruption of Onchocerca volvulus transmission in Northern Venezuela. *Parasites & Vectors* 2013; **6**: 289.
21. WHO | WHO declares Ecuador free of onchocerciasis (river blindness). WHO. http://www.who.int/neglected_diseases/ecuador_free_from_onchocerciasis/en/ (accessed July 7, 2017).
22. Onchocerciasis and its control: report of a WHO Expert Committee on Onchocerciasis Control. WHO. http://apps.who.int/iris/bitstream/handle/10665/37346/WHO_TRS_852.pdf;jsessionid=023018C4198968F3E918A2EA8334432C?sequence=1
23. Al-Kubati A, Mackenzie CD, Boakye D, Al-Qubati Y, Al-Samie A, Awad IE, Thylefors B, Hopkins A. Onchocerciasis in Yemen: moving forward towards an elimination program. *International Health*. March 2018; 10(1): i89–i96.

Dengue

Flowchart

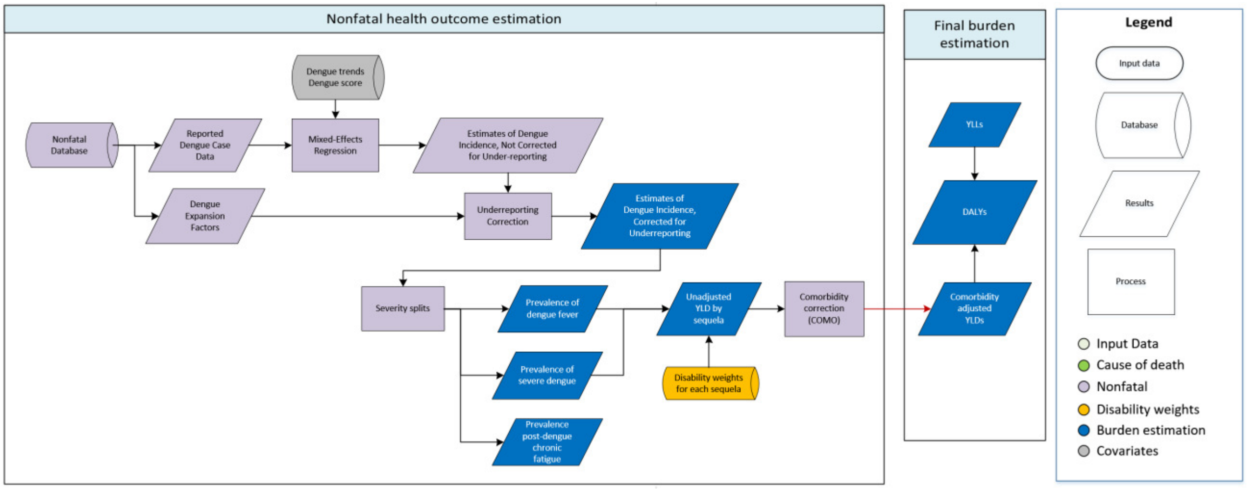

Case definition

Dengue is mosquito-borne viral infection that causes febrile illness and, in severe cases, jaundice, haemorrhage, and death. It includes all ICD-10 codes under the heading A90 (Dengue fever [classical dengue]) and A91 (Dengue haemorrhagic fever).

Input data

Model inputs

For GBD 2016, we modelled dengue incidence based on officially reported cases. The table below illustrates the geographic distribution of data points used in our analysis.

Table 1. Geographies

Level	Incidence
Data points	2,920
Studies	70
Locations	201
Regions	15

While no systematic update was conducted, we did incorporate new expansion factor data that were provided by collaborators and have updated to the latest available case reports for GBD 2017.

Modelling strategy

The methods used to model dengue incidence remain unchanged from GBD 2016, and are an improved variant of the methods used for GBD 2013 that were described by Stanaway and colleagues. Briefly, we

derive two dengue-specific covariates: first a variable to define the expected spatial distribution of the disease based on principal components analysis of dengue CSMR estimates and dengue transmission probability and, second, a variable to define the country-specific trends, based on a mixed-effects model of reported cases. We then estimate a mixed-effects negative binomial model with number of reported cases as the dependent variable, fixed effects on the aforementioned spatial and temporal covariates, and random effects on location. These random effects are assumed to correspond to deviations in reporting completeness and, calibrating against published expansion factor data (ie, estimates of the degree of underreporting), they are inflated to adjust for underreporting estimates. The resulting incidence estimates are split into moderate (94.5%) and severe (5.5%) sequelae, based on the proportion of reported cases that were severe. We assume that 8.4% of symptomatic infections will produce post-acute chronic fatigue lasting an average of six months (Teixeira L de AS, Lopes JSM, Martins AG da C, Campos FAB, Miranzi S de SC, Nascentes GAN. Persistence of dengue symptoms in patients in Uberaba, Minas Gerais State, Brazil. *Cad Saúde Pública* 2010; **26**: 624–30.).

Severity splits and disability weights

Table 2. Sequelae, lay descriptions, and DWs

Sequela	Lay description	Disability Weight (DW)
Moderate	Has a fever and aches, and feels weak, which causes some difficulty with daily activities.	0.051 (0.032–0.074)
Severe	Has a high fever and pain, and feels very weak, which causes great difficulty with daily activities.	0.133 (0.088–0.19)
Asymptomatic	Infection with no apparent illness.	NA

Changes from GBD 2016 to GBD 2017

We have made no substantive changes in the modelling strategy from GBD 2016 to GBD 2017.

References

1. Stanaway JD, Shepard DS, Undurraga EA, Halasa YA, Coffeng LE, Brady OJ, et al. The global burden of dengue: an analysis from the Global Burden of Disease Study 2013. *The Lancet Infectious Diseases* [Internet]. 2016 Feb [cited 2016 May 23].

2. Bhatt S, Gething PW, Brady OJ, Messina JP, Farlow AW, Moyes CL, et al. The global distribution and burden of dengue. *Nature*. 2013 Apr 25;496(7446):504–7.

Yellow Fever

Flowchart

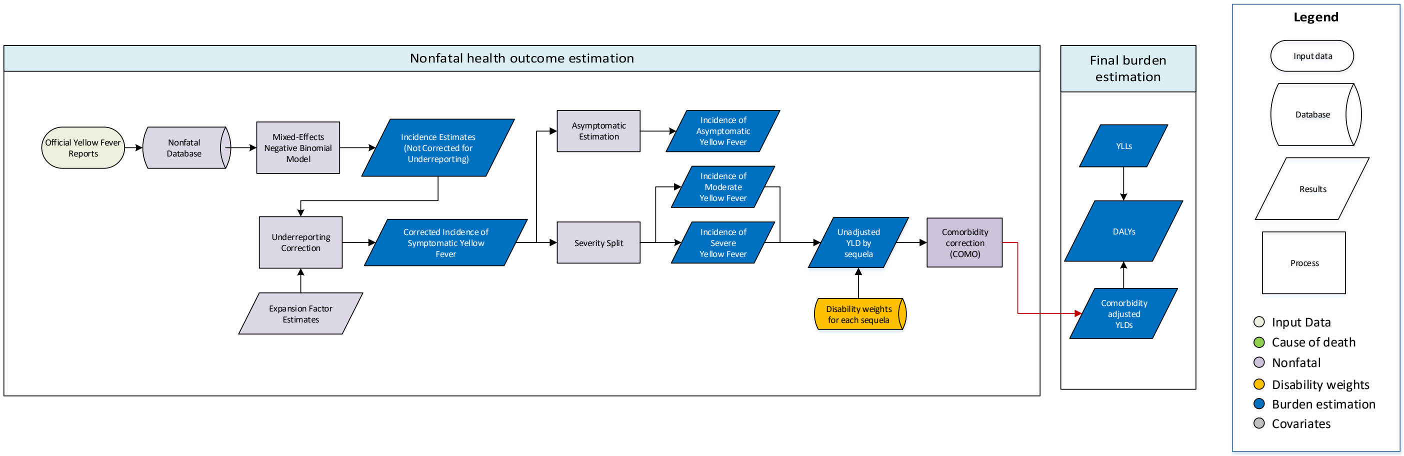

Case definition

Yellow fever is mosquito-borne viral infection that causes febrile illness and, in severe cases, jaundice, haemorrhage, and death. It is considered a neglected tropical disease (NTD). It includes all ICD-10 codes under the heading A95 (yellow fever).

Input data

Model inputs

Case data for the yellow fever estimate process comes from official case reports filed with the World Health Organization. The table below shows the distribution of said data geographically for the GBD 2017 estimation process.

Table 1. Data spread

Level	Incidence
Data points	909
Studies	19
Locations	47
Regions	9

We have updated to the latest available case reports for GBD 2017.

Severity splits

Yellow fever is split into three levels of severity: moderate (33% [13–52]), severe (12% [5–26]), and asymptomatic (55% [37–74]). The table below illustrates this breakdown.

Table 2. Sequela, description, and disability weight (DW)

Sequela	Description	Disability weight (DW)
Moderate	Has a fever and aches, and feels weak, which causes some difficulty with daily activities.	0.051 (0.032–0.074)
Severe	Has a high fever and pain, and feels very weak, which causes great difficulty with daily activities.	0.133 (0.088–0.19)
Asymptomatic	Infection with no apparent illness.	NA

Modelling strategy

We modelled reported cases of yellow fever using a mixed-effects negative binomial model, with fixed effects for year and random effects for super-region, region, and country. We assume that yellow fever cases are underreported, and that this underreporting mirrors that for dengue (a disease for which we have better data on underreporting). With that, we estimate symptomatic cases as the product of our base case estimates and dengue expansion factors (ie, the factor by which you must multiply reported cases to derive true cases). Based on published estimates from Johansson and colleagues (2014), we assume that 27% of symptomatic cases will be severe.

Changes from GBD 2016 to GBD 2017

We have made no substantive changes in the modelling strategy for endemic countries from GBD 2016 to GBD 2017.

Rabies

Flowchart

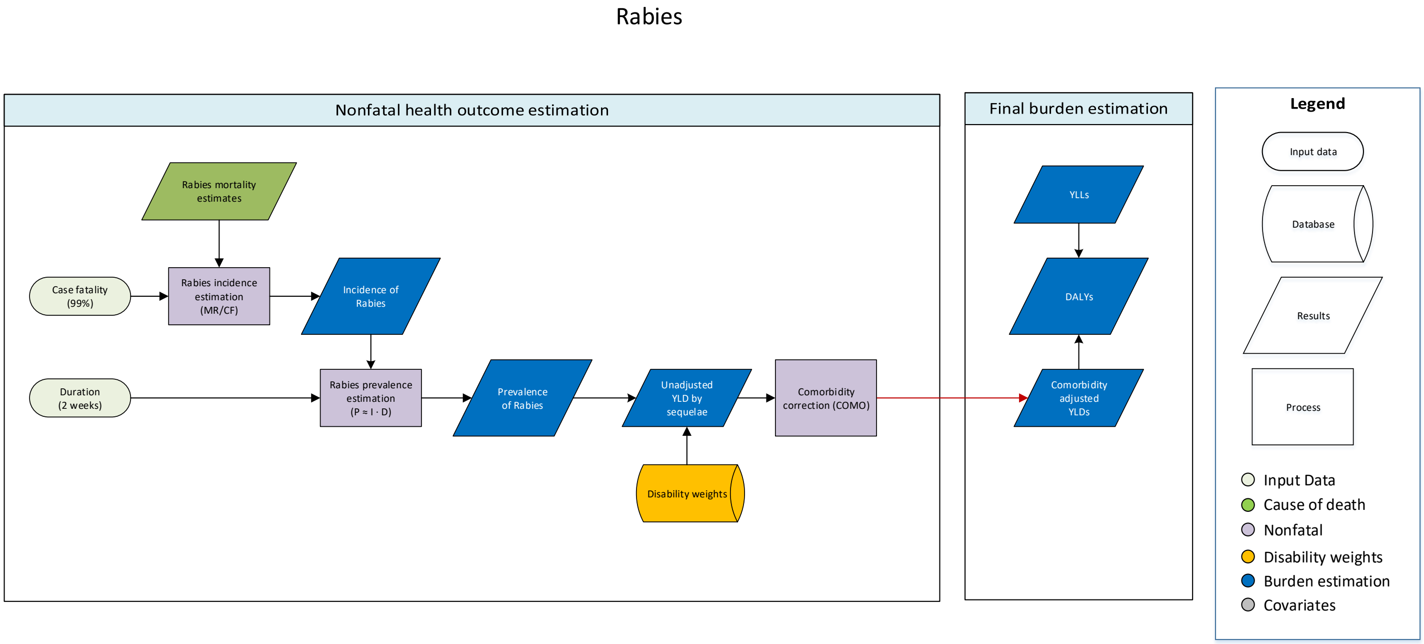

Input data and methodological summary

Case definition

Rabies is a fatal viral infection transmitted by animal bites. Without prophylactic vaccination the disease is almost universally fatal. The disease has a long incubation period (1-3 months), and early intervention with prophylactic vaccination is nearly 100% effective in preventing symptomatic disease. It is considered a neglected tropical disease (NTD). We model symptomatic infections, not including those infections in which intervention prevented the onset of symptomatic disease, corresponding to the ICD10 code A82.

Input data

Model inputs

As we derive our estimate of cases from our estimate of deaths, no incidence data are used in the model. For GBD 2017, we modelled rabies mortality using all available data in the cause of death database. Data points were outliered if they reported an improbable number of rabies deaths (eg, zero rabies deaths in a hyperendemic country) or if their inclusion in the model yielded distorted trends. In some cases, multiple data sources for the same location differed dramatically both in their quality and reported rabies mortality (eg, a verbal autopsy and vital registration source). In these cases, the lower-quality data source was outliered.

Modelling strategy

We derive estimates of the number of symptomatic rabies infections (ie, those not averted through prophylactic vaccination) based on rabies mortality estimates, assuming 99% case fatality. All cases are assumed to be severe.

We modelled rabies mortality using a two-model hybrid approach 1) a global CODEm model of all locations, using all data in the CoD database; and 2) a CODEm model restricted to data-rich countries.

Sequela description and DW

There is only one sequela and associated disability weight for rabies, which is severe. The lay description is included in the table below.

Table 2. Sequela, description, and DW

Sequela	Description	Disability Weight (95% CI)
Severe	Has a high fever and pain, and feels very weak, which causes great difficulty with daily activities.	0.133 (0.088–0.19)

Changes from GBD 2016 to GBD 2017

We have made no substantive changes in the modelling strategy for rabies from GBD 2016.

Ascariasis

Flowchart

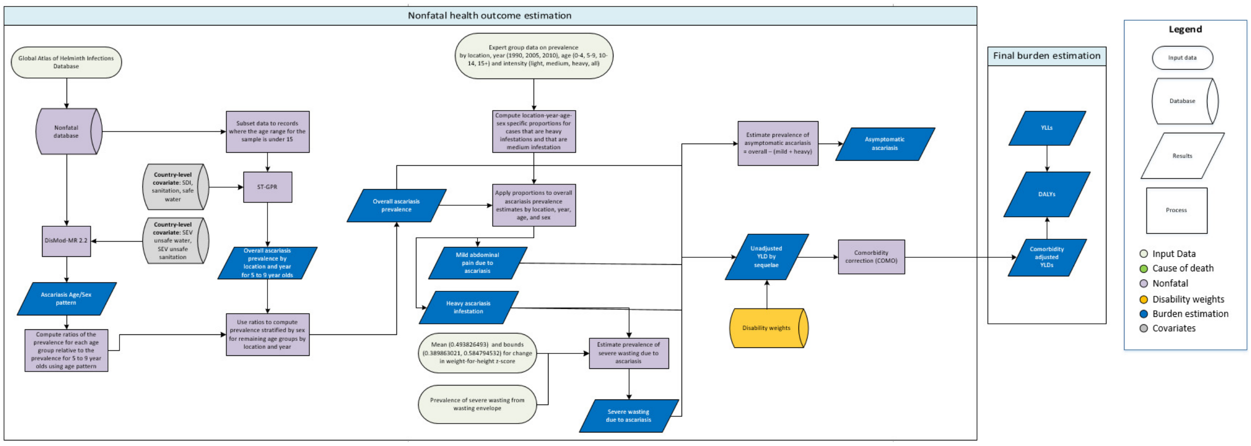

Input data and methodological summary

Case definition

Ascariasis is a helminthic disease caused by the parasitic roundworm *Ascaris lumbricoides*. It is one of the three intestinal nematode infections (INI), or soil-transmitted helminthiasis (STH), that are modelled in GBD. Diagnosis is made by examination of stool by microscope or PCR, with or without concentration procedures. The ICD-10 codes for ascariasis are B77-B77.9.

Input data

Global Atlas of Helminth Infections Data

Input data for this model were primarily compiled from the Global Atlas of Helminth Infections (GAHI) database. The GAHI database collates an exhaustive catalog of surveys and studies conducted by scientists that attempt to estimate the burden of STH [1]. Each record in the database contained metadata (ie, location, year, age range, sex) of each study sample and the prevalence of ascariasis in that sample. We excluded data points where the age range of the sample was unknown and retained only those surveys where Kato-Katz diagnostics were used. The table below displays the number of site-years by geography:

Table 1a. Site-years for ascariasis from the Global Atlas of Helminth Infections data used in GBD 2017

	Prevalence
Site-years (total)	353
Number of countries with data	62
Number of GBD regions with data (out of 21 regions)	13
Number of GBD super-regions with data (out of 7 super-regions)	6

Expert Group Data

Since GBD 2010, we have used prevalence data prepared by the GBD expert group (EG) containing mean prevalence with confidence intervals, stratified by location, year (1990, 2005, 2010), age group (0-4, 5-9, 10-14, 15+ years) and intensity of infection (light, medium, heavy, all). In order to move toward updating inputs and methods, we altered our use of these data. For some stages of our processes, we retain information from previous GBD cycles and the expert group, detailed below.

Geographic restrictions

We conducted a literature review to determine the geographic extent of the disease and classify locations based on whether the disease is absent or present in each year. Locations that were geographically restricted in any given year did not have estimates made for them. Of note, we did not attempt a complete systematic review, since a single high-quality source could offer sufficient evidence of presence. Evidence of absence or presence was not available for every location for each year, and so assumptions were made for missing years by taking into consideration the epidemiological characteristics of the disease.

If evidence indicated disease presence for two non-consecutive years, we assumed presence for all years between the two. If evidence indicated disease absence for two non-consecutive years, we assumed absence for all years between the two. If evidence indicated a change in status (ie, from absent to present, or present to absent) between two non-consecutive years, than we conducted targeted searches to ascertain the relevant year of introduction or elimination for that location. In the cases where presence or absence information was missing for the start or end years of our study interval (1990–2017) without evidence of any introduction or elimination events within the interval, we applied the status of the first and last presence/absence observations, respectively, to all years between the interval bound and the observation year. Our search was done in conjunction with the title/abstract screening portion of a systematic literature review for prevalence data. The search strings and yield can be viewed in the table below for each of the databases queried.

Table 1b. Geographic restriction search strings

Database	Search String	Yield
PubMed	(Ascariasis[Title/Abstract] OR Ascaris[Title/Abstract] OR "A. lumbricoides"[Title/Abstract] OR Ascaris[MeSH] OR Trichuris[Title/Abstract] OR Trichuriasis[Title/Abstract] OR "Whip Worm"[Title/Abstract] OR "T. trichura"[Title/Abstract] OR Trichuris[MeSH] OR Hookworm[Title/Abstract] OR "A. duodenale"[Title/Abstract] OR "Ancylostoma duodenale"[Title/Abstract] OR ancylostomiasis[Title/Abstract] OR "N. americanus"[Title/Abstract] OR "Necator americanus"[Title/Abstract] OR necatoriasis[Title/Abstract] OR Ancylostoma [MeSH] OR Necator[MeSH]) AND (prevalence[Title/Abstract] OR incidence[Title/Abstract] OR epidemiology[Title/Abstract] OR surveillance[Title/Abstract]) NOT(Animals[MeSH] NOT Humans[MeSH])	2,376
Web of Science	(Ascariasis OR Ascaris OR A. lumbricoides OR Trichuris OR Trichuriasis OR Whip Worm OR T. trichura OR Hookworm OR A. duodenale OR Ancylostoma duodenale OR anclyostomiasis OR N. americanus OR Necator americanus OR necatoriasis) AND TOPIC:(prevalence OR incidence OR epidemiology OR surveillance) NOTTOPIC: ((Animals NOT Humans))<br>Timespan: 1980-2016. Indexes: SCI-EXPANDED, SSCI, A&HCI, ESCI.	2,266

SCOPUS	TITLE-ABS_KEY (ascariasis OR ascaris OR a. lumbricoides OR trichuris OR trichuriasis OR whip worm OR t. trichura OR hookworm OR a. duodenale OR ancylostoma duodenale OR anclyostomiasis OR n. americanus OR necator americanus OR necatoriasis) AND PUBYEAR>1979	29

These papers were used to classify location-years for all locations and years present in the literature. We only utilised papers that are explicitly concerned with ascariasis. Additionally, systematic literature reviews, meta-analyses, national health statistics publications, and collaborator input were used to classify location-years not present in the literature review wherever possible.

Health states/sequelae

The table below shows the list of sequelae due to ascariasis and the associated disability weights (DW). Prevalence of medium infection and heavy infection were mapped to *mild abdominopelvic problems* and *heavy infestation of ascariasiss,* respectively. Light infection or asymptomatic was not attributed any disability. To inform the wasting model, 1,000 draws of severe wasting prevalence among children under 5 years were ascertained from GBD 2017 estimates – the methods used to generate estimates of wasting prevalence are detailed elsewhere (part of risk factors documentation) [2].

Table 2. Sequelae, lay descriptions, and disability weights (DWs)

Sequela	Lay description	DW
Mild abdominopelvic problems	"has some pain in the belly that causes nausea but does not interfere with daily activities"	0.011 (0.005–0.021)
Heavy infestation	"has cramping pain and a bloated feeling in the belly"	0.027 (0.015–0.043)
Severe wasting	"is extremely skinny and has no energy"	0.128 (0.082–0.183)
Asymptomatic ascariasis	N/A	N/A

Modelling strategy

DisMod-MR 2.2

In the estimation of overall morbidity due to ascariasis, we implemented a three-stage modelling framework. The first stage of the modelling process was using DisMod-MR to generate a global age-sex curve to disaggregate all-age, both-sex prevalence data. DisMod is an integrated meta-regression framework that allows for multiple datasets to be used within a singular analysis regardless of age-binning, sources, and geographies. As a result, a variety of differently aggregated information can be evaluated to generate a consensus output. Our final model contained all processed GAHI data as input and was informed by two country-level covariates (ie, all risk factors SEV for unsafe water, and all risk factors SEV for unsafe sanitation). From this model, the global fits were used.

Table 3a. DisMod model covariates

Covariate	Type	Parameter	Exponentiated beta
SEV unsafe water	Country-level	Proportion	4.41 (4.22–4.48)

SEV unsafe sanitation	Country-level	Proportion	4.45 (4.35–4.48)

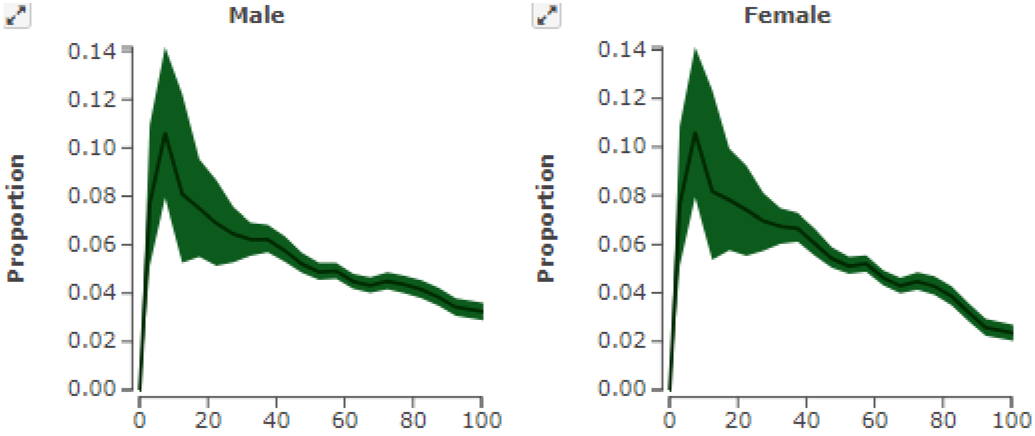

Figure 1: Global age-specific prevalence estimates for males (left) and females (right) for the year 2017. Proportion (prevalence) is on the Y-axis, and age in years on the X-axis. Screenshot from EpiViz tool.

Figure 1 shows the age-specific variation in prevalence rates, differentiated by sex. When considered as a global aggregate, we see that reported male and female prevalence are very similar. This is mostly a function of data used for modelling mainly being reported for both sexes. The highest prevalence rates are among adolescents and then decline among adults.

ST-GPR

After obtaining a global age-sex pattern from DisMod, we utilise a spatiotemporal Gaussian process regression (ST-GPR) to generate a complete time series of estimates for each location where there are no geographic restrictions. ST-GPR attempts to model non-linear trends utilising a Gaussian process to fit a trend. The following model specifications were used:

$$Prevalence = Proportion\ Sanitation + Proportion\ Safe\ Water + Sociodemographic\ Index + (1|level\ 2) + (1|level\ 3)$$

Where Levels 2 and 3 refer to GBD location hierarchies, or random effects for region and location. Notably, the covariates for the model were sanitation or proportion of population with access to improved toilet types, and safe water or proportion of population with access to improved water sources. Improved toilet types and improved water sources are defined by the Joint Monitoring Program. The following hyperparameters were used: st-lambda = 0.25, st-omega =2, st-zeta = 0.01, gpr-scale = 15. We selected these hyperparameters as they provided more weight to country-level data rather than region-level data when estimating the prevalence for a given location-year. In other words, these hyperparameters ensure that the Gaussian process regressions follow country-specific data rather than region-specific data when estimating a time series for a location.

It is important to note that we did not use all processed GAHI data for the ST-GPR model. We opted to run a child-only model because the bulk of our data is among adolescents and there is more granular age information that we can leverage during modelling processes. More specifically, any data points that had

age bins between 0 and 15 years were assigned to the 5 to 9 age group. We selected all data with age bins between 0 and 15 because they fall within the peak in prevalence across all age groups; this is where a majority of data are, and this provides sufficient statistical power for our model. This left us with 210 site-years of global input data for ST-GPR.

Table 3b. ST-GPR model covariates

Covariate	Exponentiated beta	Standard error
Socio-demographic Index	-9.99	2.09
Safe water	-2.56	1.06
Sanitation	3.95	0.79

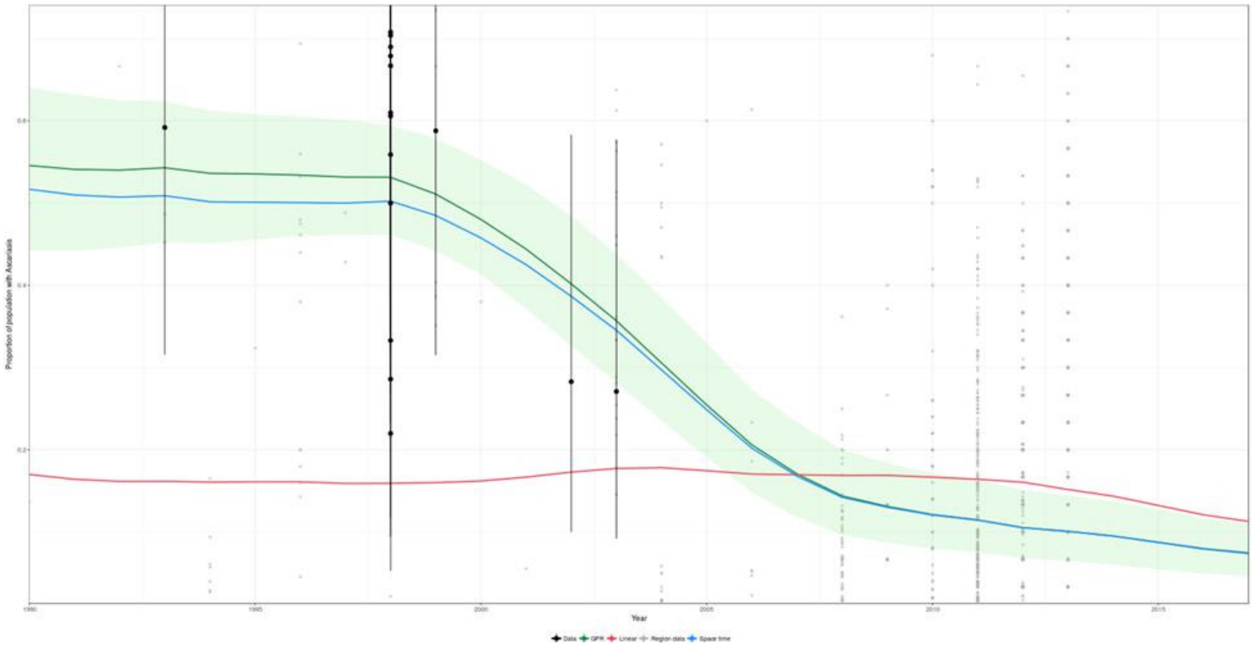

Figure 2: ST-GPR estimates for Cameroon (0- to 15-year-olds, both sex) for years 1990–2017. Black dots represent input data points, with the black lines indicating variance. The green line represents the mean GPR estimated values, with uncertainty shown by the green polygon. The blue line indicates the space-time component of the ST-GPR; the red line indicates the linear regression component derived from global data. Transparent black dots represent data from other locations in the GBD region (Western sub-Saharan Africa).

Figures 2 displays the time trends as computed by ST-GPR. For the most part, locations looked similar to Cameroon, where we see consistent declines in prevalence throughout time.

Imputations

The final stage of the overall prevalence modelling process is to impute the remaining age groups by borrowing information from the ST-GPR time series for 5- to 9-year-olds and the DisMod global age-sex pattern. First, we assign each age group a ratio of how much larger or smaller the prevalence is compared

to the prevalence for 5- to 9-year-olds using the DisMod global age-sex pattern. More specifically, the following is the computation for each age group:

$$Ratio = \frac{prevalence_{[age\ start]to\ [age\ end]}}{prevalence_{5\ to\ 9}}$$

We opted not to use the age-sex curves by location or region, because DisMod performed better at disaggregating our heterogeneous data at the global level. With a ratio for every age group by sex, we multiplied the ratio by the ST-GPR location-year estimates to impute estimates for the remaining age groups.

Health states/sequelae

Following computations of location-year-age-sex-specific prevalence of ascariasis, we leverage information from the 2010 EG data to conduct sequelae splits. The 2010 EG data provided estimates for heavy infestation, mild abdominopelvic problems, and asymptomatic ascariasis by location and for 1990, 2005, and 2010. These three values add up to all cases of ascariasis. Thus, for heavy infestation and mild abdominopelvic problems, we computed the proportion of cases that belong to our sequelae of interest over all cases of ascariasis. More specifically, the following is the computation by heavy infestation and mild abdominopelvic problems:

$$Proportion_{sequelae} = \frac{prevalence_{sequelae}}{prevalence_{all\ cases}}$$

This calculation was done for every location, year, and age group available. Because the EG data only had four age groups (0-4, 5-9, 10-14, 15+ years), we applied the 15+ age group proportion for all remaining age groups. In addition, for 1995 and 2000 we applied the 1990 proportions, and for 2017 we applied the 2010 proportions. Using these location-year-age-specific proportions, we multiplied the total ascariasis estimates to compute heavy infestation and mild abdominopelvic prevalence. To estimate the prevalence of asymptomatic ascariasis, prevalence of mild and heavy infestation was subtracted from the overall ascariasis prevalence.

The final step in the modelling process was to estimate the prevalence of severe wasting due to ascariasis in age groups 28–364 days and 1–4 years. This was done separately using 1,000 draws of prevalence of heavy infestation due to ascariasis and the wasting envelope prevalence. The initial step in determining prevalence of severe wasting due to ascariasis was generating 1,000 draws of change in weight-for-height z-score per heavy prevalent case from a random normal distribution with mean = 0.493826493 and standard deviation = 0.04972834 (calculated from upper and lower bounds of the mean estimate). The mean, upper, and lower bounds were based on a published article [2]. The prevalence of severe wasting due to ascariasis was then obtained as a function of change in weight-for-height z-score. The following are the computations:

$$Prevalence_{wasting\ due\ to\ ascariasis} = wasting - \Phi(\Phi^{-1}(\text{wasting}) - \text{z score} * \text{heavy infestation})$$

Where Φ is the standard normal cumulative distribution function and Φ^{-1} is the inverse standard normal cumulative distribution function.

Changes from GBD 2016

Significant changes have been made compared to prior GBD cycles in an effort to build a database of ascariasis data points and to continuously update methodologies. The following are changes since prior versions of GBD:

Data – In prior years we used estimates from the 2010 EG data. Here, we transition to utilising a comprehensive database of ascariasis data points so we may annually update our inputs to reflect up-to-date data.

Age-sex pattern – Given a substantial amount of heterogeneity in our input data, age-sex curves were generated from a DisMod model.

Gaussian process regressions – To obtain location-year prevalence estimates, we implemented ST-GPR methodology consistently across the globe.

Limitations

As we attempt to improve the modelling processes for ascariasis, we recognise that there are several limitations. A substantial limitation is with regard to our data. While the GAHI database represents a comprehensive synthesis of ascariasis data points, numerous data points were excluded due to our specific case definition. We opted to only include studies where Kato-Katz was used to identify infected individuals, forcing us to drop a large proportion of the GAHI database. Inclusion of these studies may provide substantially more information with regard to our age patterns and time trends. Upcoming GBD cycles will explore methods for combining data with idiosyncratic diagnostic tools.

A secondary limitation to our data is that several included studies are not considered to be nationally representative, and therefore at a location level, the data are highly heterogeneous (Figure 3). Numerous studies within the database were conducted in districts or townships, and in some cases the studies were done in known areas where prevalence is high. Upcoming GBD cycles will continue to rigorously vet these data points and update the database with literature searches. In addition, exploring the use of methodologies, such as model-based geostatistics, that implicitly model the spatial heterogeneity with this focal condition, are likely necessary.

Furthermore, we made a large assumption that the global age-sex distributions were applicable to all locations. While we believe that prevalence should peak among adolescents and slowly decline afterward, there is likely variation across regions and locations. Given that our data are either among children or all-age, it is very difficult to build an age trend at granular location levels. Thus, we allowed DisMod to disaggregate our heterogeneous data in an effort to provide sensible age-sex curves.

We believe that more work needs to be done to improve our sequelae split methods. Since the EG data do not provide all estimation years and age groups, several assumptions had to be made. Thus, we will explore conducting literature searches to provide novel data points for sequelae estimations. Lastly, we ought to explore covariate effects in our ST-GPR model. Surprisingly, the sanitation covariate had a positive relationship with ascariasis prevalence, suggesting that our covariates may need to be reevaluated for future GBD cycles.

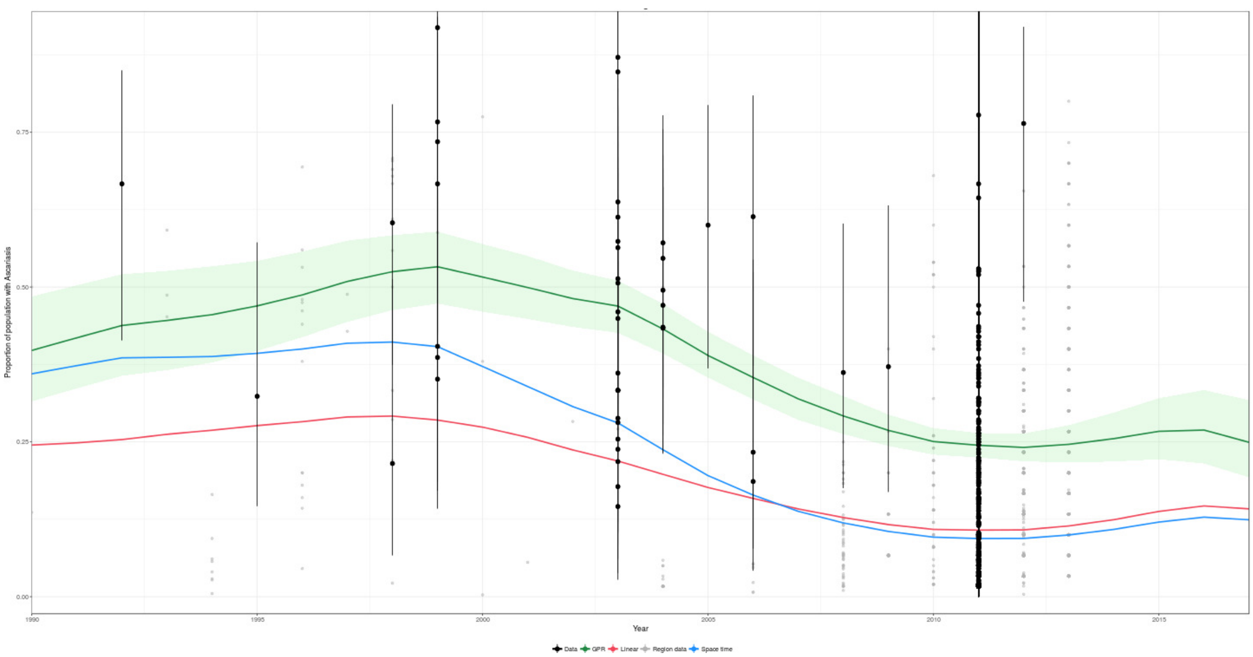

Figure 3: ST-GPR estimates for Nigeria (0 to 15 year olds, both sex) for years 1990–2017. Coloration and symbols are as stated in caption for Figure 2.

Figure 3 show the time trend for Nigeria as computed by ST-GPR. For some locations, we estimate this fluctuating time trend which is a function of the heterogeneity in our input data. As explained above, some of these data points are representative of townships in Nigeria, causing a great deal of heterogeneity throughout time.

References

1. London School of Hygiene and Tropical Medicine. Global Atlas of Helminth Infections – Soil Transmitted Helminths. London, United Kingdom: London School of Hygiene and Tropical Medicine.
2. Hall A, Hewitt G, Tuffrey V, de Silva N. A review and meta-analysis of the impact of intestinal worms on child growth and nutrition. *Maternal and Child Nutrition*. 2008. 4. 118-236.

Trichuriasis

Flowchart

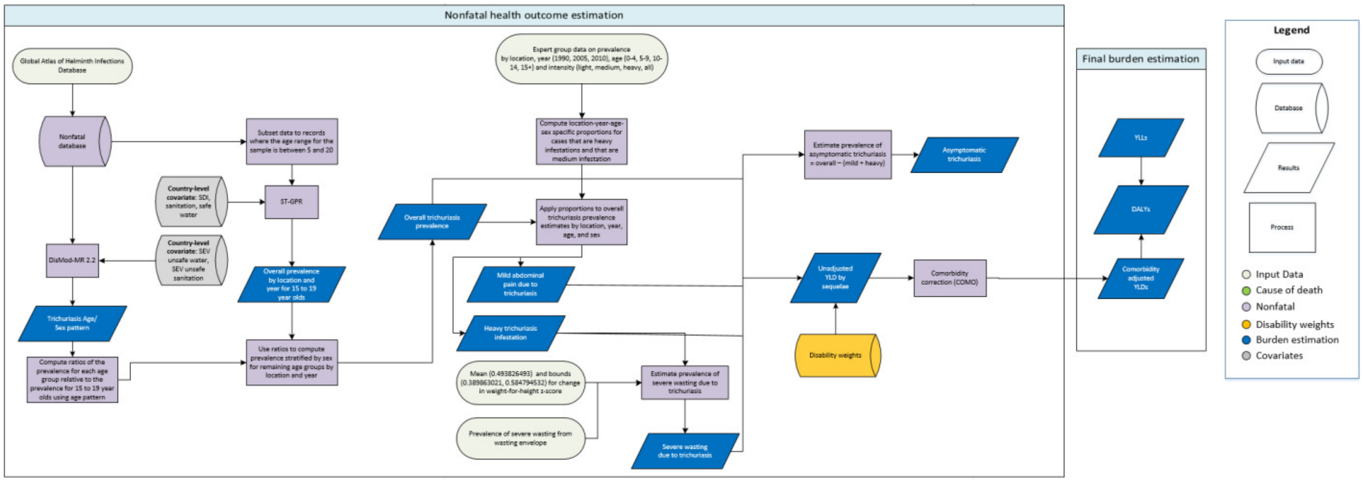

Input data and methodological summary

Case definition

Trichuriasis is a helminth diseases caused by the parasitic whipworm *Trichuris trichiura*. It is one of the three intestinal nematode infections (INI), or soil-transmitted helminthiasis (STH), that we model in GBD. Diagnosis is made by examination of stool by microscope or PCR, with or without concentration procedures. The ICD-10 code for trichuriasis is B79.

Input data

Global Atlas of Helminth Infections Data

Input data for this model were primarily compiled from the Global Atlas of Helminth Infections (GAHI) database. The GAHI database collates an exhaustive catalog of surveys and studies conducted by scientists that attempt to estimate the burden of STH [1]. Each record in the database contained metadata (ie, location, year, age range, sex) of each study sample and the prevalence of trichuriasis in that sample. We excluded data points where the age range of the sample was unknown and retained only those surveys where Kato-Katz diagnostics were used. The table below displays the number of site-years by geography:

Table 1a. Site-years for trichuriasis from the Global Atlas of Helminth Infections data used in GBD 2017

	Prevalence
Site-years (total)	315
Number of countries with data	60
Number of GBD regions with data (out of 21 regions)	13
Number of GBD super-regions with data (out of 7 super-regions)	6

Expert group data

Since GBD 2010, we have used prevalence data prepared by the GBD expert group (EG) containing mean prevalence with confidence intervals, stratified by location, year (1990, 2005, 2010), age group (0-4, 5-9, 10-14, 15+ years) and intensity of infection (light, medium, heavy, all). In order to move toward updating inputs and methods, we altered our use of this data. For some stages of our processes, we retain information from previous GBD cycles and the expert group, detailed below.

Geographic restrictions

We conducted a literature review to determine the geographic extent of the disease and classify locations based on whether the disease is absent or present in each year. Locations that were geographically restricted in any given year did not have estimates made for them. Of note, we did not attempt a complete systematic review, since a single high-quality source could offer sufficient evidence of presence. Evidence of absence or presence was not available for every location for each year, and so assumptions were made for missing years by taking into consideration the epidemiological characteristics of the disease.

If evidence indicated disease presence for two non-consecutive years, we assumed presence for all years between the two. If evidence indicated disease absence for two non-consecutive years, we assumed absence for all years between the two. If evidence indicated a change in status (ie, from absent to present, or present to absent) between two non-consecutive years, then we conducted targeted searches to ascertain the relevant year of introduction or elimination for that location. In the cases where presence or absence information was missing for the start or end years of our study interval (1990–2017) without evidence of any introduction or elimination events within the interval, we applied the status of the first and last presence/absence observations, respectively, to all years between the interval bound and the observation year. Our search was done in conjunction with the title/abstract screening portion of a systematic literature review for prevalence data. The search strings and yield can be viewed in the table below for each of the databases queried.

Table 1b. Geographic restriction search strings

Database	Search String	Yield
PubMed	(Ascariasis[Title/Abstract] OR Ascaris[Title/Abstract] OR "A. lumbricoides"[Title/Abstract] OR Ascaris[MeSH] OR Trichuris[Title/Abstract] OR Trichuriasis[Title/Abstract] OR "Whip Worm"[Title/Abstract] OR "T. trichura"[Title/Abstract] OR Trichuris[MeSH] OR Hookworm[Title/Abstract] OR "A. duodenale"[Title/Abstract] OR "Ancylostoma duodenale”[Title/Abstract] OR ancylostomiasis[Title/Abstract] OR "N. americanus"[Title/Abstract] OR “Necator americanus”[Title/Abstract] OR necatoriasis[Title/Abstract] OR Ancylostoma [MeSH] OR Necator[MeSH]) AND (prevalence[Title/Abstract] OR incidence[Title/Abstract] OR epidemiology[Title/Abstract] OR surveillance[Title/Abstract]) NOT(Animals[MeSH] NOT Humans[MeSH])	2,376
Web of Science	(Ascariasis OR Ascaris OR A. lumbricoides OR Trichuris OR Trichuriasis OR Whip Worm OR T. trichura OR Hookworm OR A. duodenale OR Ancylostoma duodenale OR anclyostomiasis OR N. americanus OR Necator americanus OR necatoriasis) AND TOPIC:(prevalence OR incidence OR epidemiology OR surveillance) NOTTOPIC: ((Animals NOT Humans))<br>Timespan: 1980-2016. Indexes: SCI-EXPANDED, SSCI, A&HCI, ESCI.	2,266

SCOPUS	TITLE-ABS_KEY (ascariasis OR ascaris OR a. lumbricoides OR trichuris OR trichuriasis OR whip worm OR t. trichura OR hookworm OR a. duodenale OR ancylostoma duodenale OR anclyostomiasis OR n. americanus OR necator americanus OR necatoriasis) AND PUBYEAR>1979	29

These papers were used to classify location-years for all locations and years present in the literature. We only utilised papers that are explicitly concerned with trichuriasis. Additionally, systematic literature reviews, meta-analyses, national health statistics publications, and collaborator input were used to classify location-years not present in the literature review wherever possible.

Health states/sequelae

The table below shows the list of sequelae due to trichuriasis and the associated disability weights (DW). Prevalence of medium infection and heavy infection were mapped to *mild abdominopelvic problems* and *heavy infestation of trichuriasis,* respectively. Light infection was not attributed any disability. To inform the wasting model, 1,000 draws of severe wasting prevalence among children under 5 years were ascertained from GBD 2017 estimates – the methods used to generate estimates of wasting prevalence are detailed elsewhere (part of risk factors documentation) [2].

Table 2. Sequelae, lay description, and disability weights (DWs)

Sequela	Lay description	DW (95% CI)
Mild abdominopelvic problems	"has some pain in the belly that causes nausea but does not interfere with daily activities"	0.011 (0.005–0.021)
Heavy infestation	"has cramping pain and a bloated feeling in the belly"	0.027 (0.015–0.044)
Severe wasting	"is extremely skinny and has no energy"	0.128 (0.082–0.183)
Asymptomatic trichuriasis	N/A	N/A

Modelling strategy

DisMod-MR 2.2

In the estimation of overall morbidity due to trichuriasis, we implemented a three-stage modelling framework. The first stage of the modelling process was using DisMod-MR to generate a global age-sex curve to disaggregate all-age, both-sex prevalence data. DisMod is an integrated meta-regression framework that allows for multiple datasets to be used within a singular analysis regardless of age-binning, sources, and geographies. As a result, a variety of differently aggregated information can be evaluated to generate a consensus output. Our final model contained all processed GAHI data as input and was informed by two country-level covariates (ie, all risk factors SEV for unsafe water, and all risk factors SEV for unsafe sanitation). From this model, the global fits were used.

Table 3a. DisMod model covariates

Covariate	Type	Parameter	Exponentiated beta
SEV unsafe water	Country-level	Proportion	4.40 (4.17–4.48)
SEV unsafe sanitation	Country-level	Proportion	4.44 (4.36–4.48)

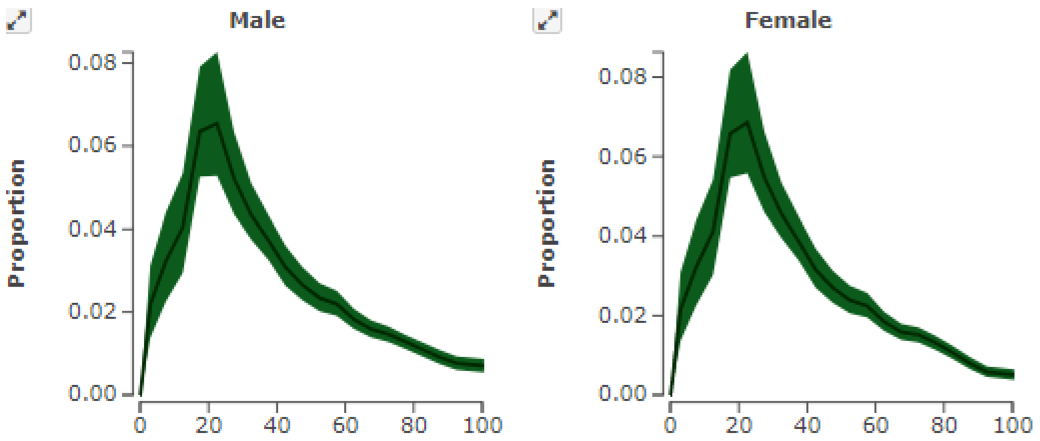

Figure 1: Global age-specific prevalence estimates for males (left) and females (right) for the year 2017. Proportion (prevalence) is on the Y-axis, and age in years on the X-axis. Screenshot from EpiViz tool.

Figure 1 shows the age-specific variation in prevalence rates, differentiated by sex. When considered as a global aggregate, we see that reported male and female prevalence are very similar. This is mostly a function of data used for modelling mainly being reported for both sexes. The highest prevalence rates are among young adults and then decline among adults.

ST-GPR

After obtaining a global age-sex pattern from DisMod, we utilise a spatiotemporal Gaussian process regression (ST-GPR) to generate a complete time series of estimates for each location where there are no geographic restrictions. ST-GPR attempts to model non-linear trends utilising a Gaussian process to fit a trend. The following model specifications were used:

$$Prevalence = Proportion\ Sanitation + Proportion\ Safe\ Water + Sociodemographic\ Index + (1|level\ 2) + (1|level\ 3)$$

Where Levels 2 and 3 refer to GBD location hierarchies, or random effects for region and location. Notably, the covariates for the model were sanitation or proportion of population with access to improved toilet types, and safe water or proportion of population with access to improved water sources. Improved toilet types and improved water sources are defined by the Joint Monitoring Programme. The following hyperparameters were used: st-lambda = 0.25, st-omega =2, st-zeta = 0.01, gpr-scale = 15. We selected these hyperparameters as they provided more weight to country-level data rather than region-level data when estimating the prevalence for a given location-year. In other words, these hyperparameters ensure that the Gaussian process regressions follow country-specific data rather than region-specific data when estimating a time series for a location.

It is important to note that we did not use all processed GAHI data for the ST-GPR model. We opted to run an adolescent-only model because the bulk of our data are among children and there is more granular age information that we can leverage during modelling processes. More specifically, any data points that had age bins between 5 and 20 years were assigned to the 15 to 19 age group. We selected all data with age bins between 5 and 20 because it falls right below the peak in prevalence across all age

groups, this is where a majority of data are, and it provides sufficient statistical power for our model. This left us with 188 site-years of global input data for ST-GPR.

Table 3b. ST-GPR model covariates

Covariate	Exponentiated beta	Standard error
Socio-demographic Index	-2.89	1.77
Safe water	0.20	0.87
Sanitation	0.20	0.76

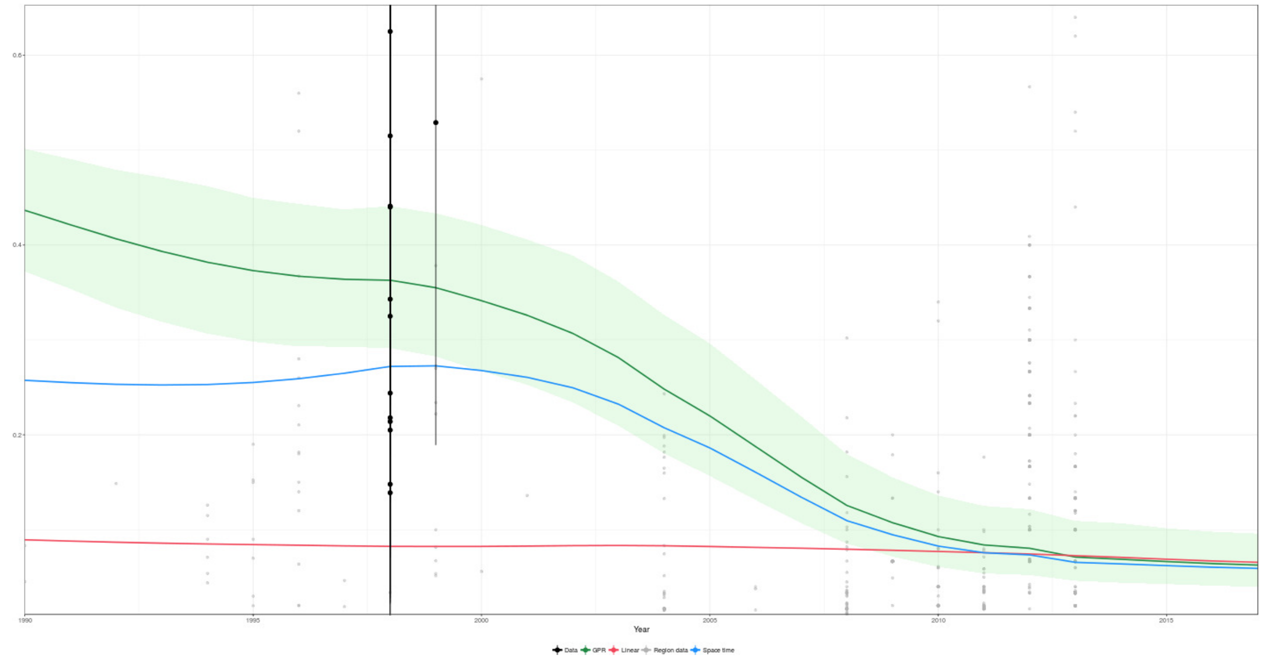

Figure 2: ST-GPR estimates for Cameroon (5- to 20-year-olds, both sexes) for years 1990–2017. Black dots represent input data points, with the black lines indicating variance. The green line represents the mean GPR estimated values, with uncertainty shown by the green polygon. The blue line indicates the space-time component of the ST-GPR; the red line indicates the linear regression component derived from global data. Transparent black dots represent data from other locations in the GBD region (Western sub-Saharan Africa).

Figures 2 displays the time trends as computed by ST-GPR. For the most part, locations looked similar to Cameroon, where we see consistent declines in prevalence throughout time.

Imputation

The final stage of the overall prevalence modelling process is to impute the remaining age groups by borrowing information from the ST-GPR time series for 15- to 19-year-olds and the DisMod global age-sex pattern. First, we assign each age group a ratio of how much larger or smaller the prevalence is compared to the prevalence for 15- to 19-year-olds using the DisMod global age-sex pattern. More specifically, the following is the computation for each age group:

$$Ratio = \frac{prevalence_{[age\ start] to\ [age\ end]}}{prevalence_{15\ to\ 19}}$$

We opted not to use the age-sex curves by location or region, because DisMod performed better at disaggregating our heterogeneous data at the global level. With a ratio for every age group by sex, we multiplied the ratio by the ST-GPR location-year estimates to impute estimates for the remaining age groups.

Health states/sequelae

Following computations of location-year-age-sex-specific prevalence of trichuriasis, we leverage information from the 2010 EG data to conduct sequelae splits. The 2010 EG data provided estimates for heavy infestation, mild abdominopelvic problems, and asymptomatic trichuriasis by location and for 1990, 2005, and 2010. These three values add up to all cases of trichuriasis. Thus, for heavy infestation and mild abdominopelvic problems, we computed the proportion of cases that belong to our sequelae of interest over all cases of trichuriasis. More specifically, the following is the computation by heavy infestation and mild abdominopelvic problems:

$$Proportion_{sequelae} = \frac{prevalence_{sequelae}}{prevalence_{all\ cases}}$$

This calculation was done for every location, year, and age group available. Because the EG data only had four age groups (0-4, 5-9, 10-14, 15+ years), we applied the 15+ age group proportion for all remaining age groups. In addition, for 1995 and 2000 we applied the 1990 proportions, and for 2017 we applied the 2010 proportions. Using these location-year-age-specific proportions, we multiplied the total trichuriasis estimates to compute heavy infestation and mild abdominopelvic prevalence. To estimate the prevalence of asymptomatic trichuriasis, prevalence of mild and heavy infestation was subtracted from the overall trichuriasis prevalence.

The final step in the modelling process was to estimate the prevalence of severe wasting due to trichuriasis in age groups 28–364 days and 1–4 years. This was done separately using 1,000 draws of prevalence of heavy infestation due to trichuriasis and the wasting envelope prevalence. The initial step in determining prevalence of severe wasting due to trichuriasis was generating 1,000 draws of change in weight-for-height z-score per heavy prevalent case from a random normal distribution with mean = 0.493826493 and standard deviation = 0.04972834 (calculated from upper and lower bounds of the mean estimate). The mean, upper, and lower bounds were based on a published article [2]. The prevalence of severe wasting due to trichuriasis was then obtained as a function of change in weight-for-height z-score. The following are the computations:

$$Prevalence_{wasting\ due\ to\ trichuriasis} = wasting - \Phi(\Phi^{-1}(\text{wasting}) - \text{z score} * \text{heavy infestation})$$

Where Φ is the standard normal cumulative distribution function and Φ^{-1} is the inverse standard normal cumulative distribution function.

Changes from GBD 2016

Significant changes have been made compared to prior GBD cycles in an effort to build a database of trichuriasis data points and to continuously update methodologies. The following changes have been made since prior versions of GBD:

Data – In prior years we used estimates from the 2010 EG data. Here, we transition to utilising a comprehensive database of trichuriasis data points so we may annually update our inputs to reflect up-to-date data.

Age-sex pattern – Given a substantial amount of heterogeneity in our input data, age-sex curves were generated from a DisMod model.

Gaussian process regressions – To obtain location-year prevalence estimates, we implemented ST-GPR methodology consistently across the globe.

Limitations

As we attempt to improve the modelling processes for trichuriasis, we recognise that there are several limitations. A substantial limitation is with regard to our data. While the GAHI database represents a comprehensive synthesis of trichuriasis data points, numerous data points were excluded due to our specific case definition. We opted to only include studies where Kato-Katz was used to identify infected individuals, forcing us to drop a large proportion of the GAHI database. Inclusion of these studies may provide substantially more information with regard to our age patterns and time trends. Upcoming GBD cycles will explore methods for combining data with idiosyncratic diagnostic tools.

A secondary limitation to our data is that several included studies are not considered to be nationally representative, and therefore at a location level, the data are highly heterogeneous (Figure 3). Numerous studies within the database were conducted in districts or townships, and in some cases the studies were done in known areas where prevalence is high. Upcoming GBD cycles will continue to rigorously vet these data points and update the database with literature searches. In addition, exploring the use of methodologies, such as model-based geostatistics, that implicitly model the spatial heterogeneity with this focal condition, are likely necessary.

Furthermore, we made a large assumption that the global age-sex distributions were applicable to all locations. While we believe that prevalence should peak among young adults and slowly decline afterward, there is likely variation across regions and locations. Given that our data are either among adolescents or all-age, it is very difficult to build an age trend at granular location levels. Thus, we allowed DisMod to disaggregate our heterogeneous data in an effort to provide sensible age-sex curves.

We believe that more work needs to be done to improve our sequelae split methods. Since the EG data do not provide all estimation years and age groups, several assumptions had to be made. Thus, we will explore conducting literature searches to provide novel data points for sequelae estimations. Lastly, we ought to explore covariate effects in our ST-GPR model. While safe water and sanitation were not considered to be statistically significant in the model, they had positive relationships with trichuriasis prevalence, suggesting that our covariates may need to be reevaluated for future GBD cycles.

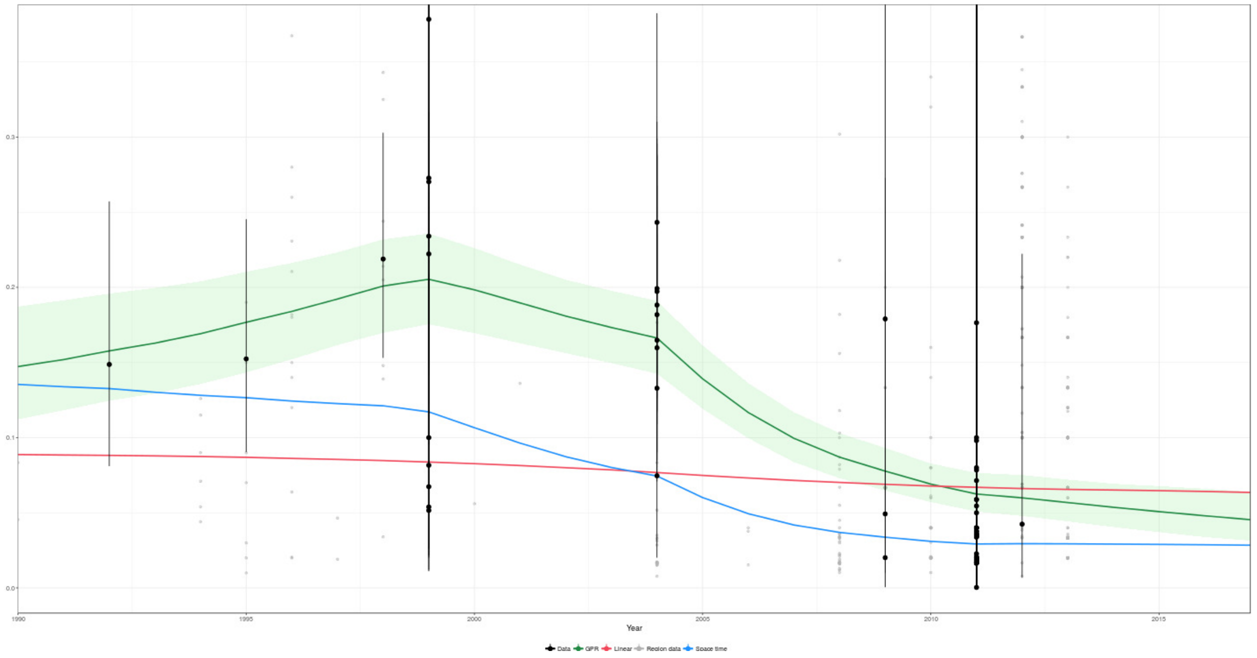

Figure 3: ST-GPR estimates for Nigeria (5- to 20-year-olds, both sexes) for years 1990–2017. Coloration and symbols are as stated in caption for Figure 2.

Figure 3 shows the time trend for Nigeria as computed by ST-GPR. For some locations, we estimate this fluctuating time trend which is a function of the heterogeneity in our input data. As explained above, some of these data points are representative of townships in Nigeria causing a great deal of heterogeneity throughout time.

References

1. London School of Hygiene and Tropical Medicine. Global Atlas of Helminth Infections – Soil Transmitted Helminths. London, United Kingdom: London School of Hygiene and Tropical Medicine.
2. Hall A, Hewitt G, Tuffrey V, de Silva N. A review and meta-analysis of the impact of intestinal worms on child growth and nutrition. *Maternal and Child Nutrition*. 2008. 4. 118-236.

Hookworm Disease

Flowchart

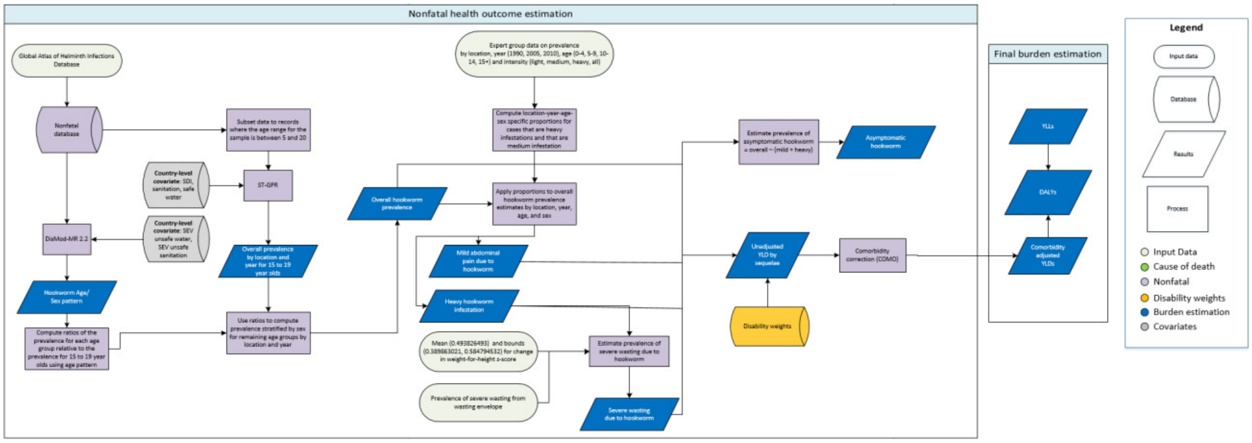

Input data and methodological summary

Case Definition

Hookworm disease is a helminthic disease caused by intestinal parasites in the roundworm group, *Ancylostoma duodenale* and *Necator americanus*. It is one of the three intestinal nematode infections (INI), or soil-transmitted helminthiasis (STH), that we model in GBD. Diagnosis is made by examination of stool by microscope or PCR, with or without concentration procedures. The ICD-10 codes for hookworm disease are B76-B76.9.

Input data

Global Atlas of Helminth Infections Data

Input data for this model were primarily compiled from the Global Atlas of Helminth Infections (GAHI) database. The GAHI database collates an exhaustive catalog of surveys and studies conducted by scientists that attempt to estimate the burden of STH [1]. Each record in the database contained metadata (ie, location, year, age range, sex) of each study sample and the prevalence of hookworm in that sample. We excluded data points where the age range of the sample was unknown and retained only those surveys where Kato-Katz diagnostics were used. The table below displays the number of site-years by geography:

Table 1a. Site-years for hookworm from the Global Atlas of Helminth Infections data used in GBD 2017

	Prevalence
Site-years (total)	312
Number of countries with data	60
Number of GBD regions with data (out of 21 regions)	10
Number of GBD super-regions with data (out of 7 super-regions)	5

Expert Group Data

Since GBD 2010, we have used prevalence data prepared by the GBD expert group (EG) containing mean prevalence with confidence intervals, stratified by location, year (1990, 2005, 2010), age group (0-4, 5-9, 10-14, 15+ years) and intensity of infection (light, medium, heavy, all). In order to move toward updating inputs and methods, we altered our use of these data. For some stages of our processes, we retain information from previous GBD cycles and the expert group, detailed below.

Geographic Restrictions

We conducted a literature review to determine the geographic extent of the disease and classify locations based on whether the disease is absent or present in each year. Locations that were geographically restricted in any given year did not have estimates made for them. Of note, we did not attempt a complete systematic review, since a single high-quality source could offer sufficient evidence of presence. Evidence of absence or presence was not available for every location for each year, and so assumptions were made for missing years by taking into consideration the epidemiological characteristics of the disease.

If evidence indicated disease presence for two non-consecutive years, we assumed presence for all years between the two. If evidence indicated disease absence for two non-consecutive years, we assumed absence for all years between the two. If evidence indicated a change in status (ie, from absent to present, or present to absent) between two non-consecutive years, then we conducted targeted searches to ascertain the relevant year of introduction or elimination for that location. In the cases where presence or absence information was missing for the start or end years of our study interval (1990–2017) without evidence of any introduction or elimination events within the interval, we applied the status of the first and last presence/absence observations, respectively, to all years between the interval bound and the observation year. Our search was done in conjunction with the title/abstract screening portion of a systematic literature review for prevalence data. The search strings and yield can be viewed in the table below for each of the databases queried.

Table 1b. Geographic Restriction Search Strings

Database	Search String	Yield
PubMed	(Ascariasis[Title/Abstract] OR Ascaris[Title/Abstract] OR "A. lumbricoides"[Title/Abstract] OR Ascaris[MeSH] OR Trichuris[Title/Abstract] OR Trichuriasis[Title/Abstract] OR "Whip Worm"[Title/Abstract] OR "T. trichura"[Title/Abstract] OR Trichuris[MeSH] OR Hookworm[Title/Abstract] OR "A. duodenale"[Title/Abstract] OR "Ancylostoma duodenale"[Title/Abstract] OR ancylostomiasis[Title/Abstract] OR "N. americanus"[Title/Abstract] OR "Necator americanus"[Title/Abstract] OR necatoriasis[Title/Abstract] OR Ancylostoma [MeSH] OR Necator[MeSH]) AND (prevalence[Title/Abstract] OR incidence[Title/Abstract] OR epidemiology[Title/Abstract] OR surveillance[Title/Abstract]) NOT(Animals[MeSH] NOT Humans[MeSH])	2,376
Web of Science	(Ascariasis OR Ascaris OR A. lumbricoides OR Trichuris OR Trichuriasis OR Whip Worm OR T. trichura OR Hookworm OR A. duodenale OR Ancylostoma duodenale OR anclyostomiasis OR N. americanus OR Necator americanus OR necatoriasis) AND TOPIC:(prevalence OR incidence OR epidemiology OR surveillance) NOTTOPIC: ((Animals NOT Humans))<br>Timespan: 1980-2016. Indexes: SCI-EXPANDED, SSCI, A&HCI, ESCI.	2,266

SCOPUS	TITLE-ABS_KEY (ascariasis OR ascaris OR a. lumbricoides OR trichuris OR trichuriasis OR whip worm OR t. trichura OR hookworm OR a. duodenale OR ancylostoma duodenale OR anclyostomiasis OR n. americanus OR necator americanus OR necatoriasis) AND PUBYEAR>1979	29

These papers were used to classify location-years for all locations and years present in the literature. We only utilised papers that are explicitly concerned with hookworm. Additionally, systematic literature reviews, meta-analyses, national health statistics publications and collaborator input were used to classify location-years not present in the literature review wherever possible.

Health states/sequelae

The table below shows the list of sequelae due to hookworm and the associated disability weights (DW). Prevalence of medium infection and heavy infection were mapped to *mild abdominopelvic problems* and *heavy infestation of hookworm,* respectively. Light infection was not attributed any disability. To inform the wasting model, 1,000 draws of severe wasting prevalence among children under 5 years were ascertained from GBD 2017 estimates – the methods used to generate estimates of wasting prevalence are detailed elsewhere (part of risk factors documentation) [2].

Table 2. Sequelae, lay descriptions, and disability weights (DWs)

Sequela	Lay description	DW
Mild abdominopelvic problems	"has some pain in the belly that causes nausea but does not interfere with daily activities"	0.011 (0.005–0.021)
Heavy infestation	"has cramping pain and a bloated feeling in the belly"	0.027 (0.015–0.044)
Severe wasting	"is extremely skinny and has no energy"	0.128 (0.082–0.183)
Asymptomatic hookworm disease	NA	NA
Mild anaemia	"feels slightly tired and weak at times, but this does not interfere with normal daily activities"	0.004 (0.001–0.008)
Moderate anaemia	"feels moderate fatigue, weakness, and shortness of breath after exercise, making daily activities more difficult"	0.052 (0.034–0.076)
Severe anaemia	"feels very weak, tired and short of breath, and has problems with activities that require physical effort or deep concentration"	0.149 (0.101–0.210)

Modelling strategy

DisMod-MR 2.2

In the estimation of overall morbidity due to hookworm, we implemented a three-stage modelling framework. The first stage of the modelling process was using DisMod-MR to generate a global age-sex curve to disaggregate all-age, both-sex prevalence data. DisMod is an integrated meta-regression framework that allows for multiple datasets to be used within a singular analysis regardless of age-binning, sources, and geographies. As a result, a variety of differently aggregated information can be

evaluated to generate a consensus output. Our final model contained all processed GAHI data as input and was informed by two country-level covariates (ie, all risk factors SEV for unsafe water, and all risk factors SEV for unsafe sanitation). From this model, the global fits were used.

Table 3a. DisMod model covariates

Covariate	Type	Parameter	Exponentiated beta
SEV unsafe water	Country-level	Proportion	4.38 (4.14–4.48)
SEV unsafe sanitation	Country-level	Proportion	4.44 (4.25–4.48)

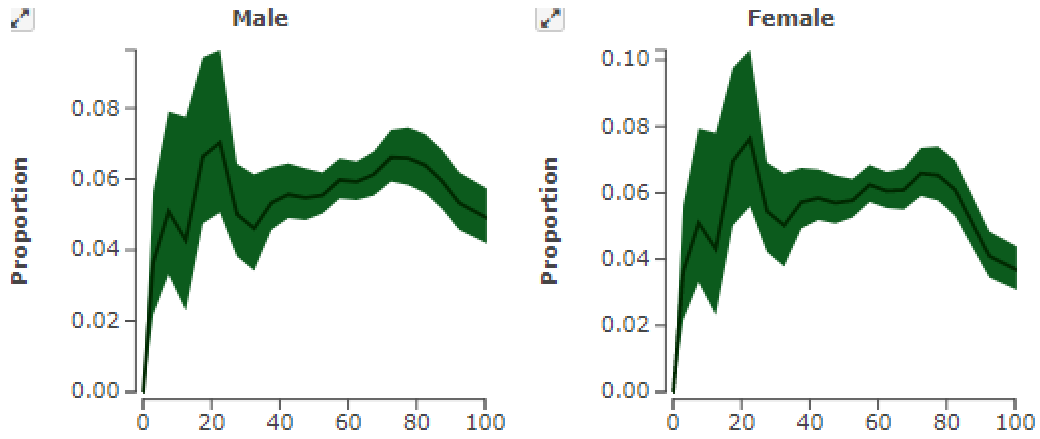

Figure 1: Global age-specific prevalence estimates for males (left) and females (right) for the year 2017. Proportion (prevalence) is on the Y-axis, and age in years on the X-axis. Screenshot from EpiViz tool.

Figure 1 shows the age-specific variation in prevalence rates, differentiated by sex. When considered as a global aggregate, we see that reported male and female prevalence are very similar. This is mostly a function of data used for modelling mainly being reported for both sexes. Prevalence peaks among young adults, followed by a decline and then stabilising during adulthood. These age-sex curves are similar to what has been reported in the literature [3, 4].

ST-GPR

After obtaining a global age-sex pattern from DisMod, we utilise a spatiotemporal Gaussian process regression (ST-GPR) to generate a complete time series of estimates for each location where there are no geographic restrictions. ST-GPR attempts to model non-linear trends utilising a Gaussian process to fit a trend. The following model specifications were used:

$$Prevalence = \ Proportion\ Sanitation + Proportion\ Safe\ Water + Sociodemographic\ Index + (1|level\ 2) + (1|level\ 3)$$

Where levels 2 and 3 refer to GBD location hierarchies, or random effects for region and location. Notably, the covariates for the model were sanitation or proportion of population with access to improved toilet types, and safe water or proportion of population with access to improved water sources. Improved toilet types and improved water sources are defined by the Joint Monitoring Programme. The following hyperparameters were used: st-lambda = 0.25, st-omega =2, st-zeta = 0.01, gpr-scale = 15. We

selected these hyperparameters as they provided more weight to country-level data rather than region-level data when estimating the prevalence for a given location-year. In other words, these hyperparameters ensure that the Gaussian process regressions follow country-specific data rather than region-specific data when estimating a time series for a location.

It is important to note that we did not use all processed GAHI data for the ST-GPR model. We opted to run an adolescent-only model because the bulk of our data are among children and there is more granular age information that we can leverage during modelling processes. More specifically, any data points that had age bins between 5 and 20 years were assigned to the 15 to 19 age group. We selected all data with age bins between 5 and 20 because this falls right below the peak in prevalence across all age groups, this is where a majority of data are, and it provides sufficient statistical power for our model. This left us with 199 site-years of global input data for ST-GPR.

Table 3b. ST-GPR model covariates

Covariate	Exponentiated beta	Standard error
Socio-demographic Index	-0.12	1.69
Safe water	-2.89	0.66
Sanitation	-2.40	0.82

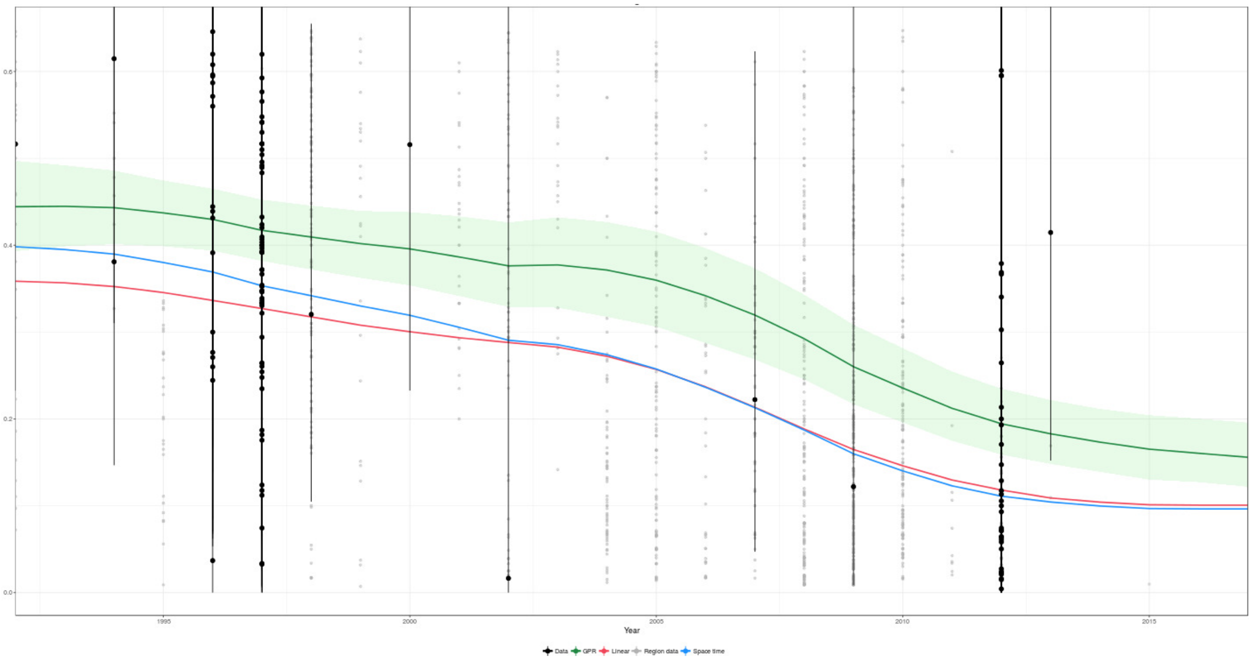

Figure 2: ST-GPR estimates for Tanzania (5- to 20-year-olds, both sexes) for years 1990–2017. Black dots represent input data points, with the black lines indicating variance. The green line represents the mean GPR estimated values, with uncertainty shown by the green polygon. The blue line indicates the space-time component of the ST-GPR; the red line indicates the linear regression component derived from global data. Transparent black dots represent data from other locations in the GBD region (Western sub-Saharan Africa).

Figures 2 displays the time trends as computed by ST-GPR. For the most part, locations looked similar to Tanzania, where we see steady declines in prevalence throughout time.

Imputation

The final stage of the overall prevalence modelling process is to impute the remaining age groups by borrowing information from the ST-GPR time series for 15- to 19-year-olds and the DisMod global age-sex pattern. First, we assign each age group a ratio of how much larger or smaller the prevalence is compared to the prevalence for 15- to 19-year-olds using the DisMod global age-sex pattern. More specifically, the following is the computation for each age group:

$$Ratio = \frac{prevalence_{[age\ start] to\ [age\ end]}}{prevalence_{15\ to\ 19}}$$

We opted not to use the age-sex curves by location or region, because DisMod performed better at disaggregating our heterogeneous data at the global level. With a ratio for every age group by sex, we multiplied the ratio by the ST-GPR location-year estimates to impute estimates for the remaining age groups.

Health states/sequelae

Following computations of location-year-age-sex-specific prevalence of hookworm, we leverage information from the 2010 EG data to conduct sequelae splits. The 2010 EG data provided estimates for heavy infestation, mild abdominopelvic problems, and asymptomatic hookworm by location and for 1990, 2005, and 2010. These three values add up to all cases of hookworm. Thus, for heavy infestation and mild abdominopelvic problems, we computed the proportion of cases that belong to our sequelae of interest over all cases of hookworm. More specifically, the following is the computation by heavy infestation and mild abdominopelvic problems:

$$Proportion_{sequelae} = \frac{prevalence_{sequelae}}{prevalence_{all\ cases}}$$

This calculation was done for every location, year, and age group available. Because the EG data only had four age groups (0-4, 5-9, 10-14, 15+ years), we applied the 15+ age group proportion for all remaining age groups. In addition, for 1995 and 2000 we applied the 1990 proportions, and for 2017 we applied the 2010 proportions. Using these location-year-age specific proportions, we multiplied the total hookworm estimates to compute heavy infestation and mild abdominopelvic prevalence. To estimate the prevalence of asymptomatic hookworm, prevalence of mild and heavy infestation was subtracted from the overall hookworm prevalence.

The final step in the modelling process was to estimate the prevalence of severe wasting due to hookworm in age groups 28–364 days and 1–4 years. This was done separately using 1,000 draws of prevalence of heavy infestation due to hookworm and the wasting envelope prevalence. The initial step in determining prevalence of severe wasting due to hookworm was generating 1,000 draws of change in weight-for-height z-score per heavy prevalent case from a random normal distribution with mean = 0.493826493 and standard deviation = 0.04972834 (calculated from upper and lower bounds of the mean estimate). The mean, upper, and lower bounds were based on a published article [2]. The prevalence of severe wasting due to hookworm was then obtained as a function of change in weight-for-height z-score. The following are the computations:

$$Prevalence_{wasting\ due\ to\ hookworm} = wasting - \Phi(\Phi^{-1}(\text{wasting}) - \text{z score} * \text{heavy infestation})$$

Where Φ is the standard normal cumulative distribution function and Φ^{-1} is the inverse standard normal cumulative distribution function. Finally, the burden of anaemia due to hookworm disease was estimated separately (see anaemia documentation for details).

Changes from GBD 2016

Significant changes have been made compared to prior GBD cycles in an effort to build a database of hookworm data points and to continuously update methodologies. The following changes have been made since prior versions of GBD:

Data – In prior years we used estimates from the 2010 EG data. Here, we transition to utilising a comprehensive database of hookworm data points so we may annually update our inputs to reflect up-to-date data.

Age-sex pattern – Given a substantial amount of heterogeneity in our input data, age-sex curves were generated from a DisMod model.

Gaussian process regression – To obtain location-year prevalence estimates, we implemented ST-GPR methodology consistently across the globe.

Limitations

As we attempt to improve the modelling processes for hookworm, we recognise that there are several limitations. A substantial limitation is with regard to our data. While the GAHI database represents a comprehensive synthesis of hookworm data points, numerous data points were excluded due to our specific case definition. We opted to only include studies where Kato-Katz was used to identify infected individuals, forcing us to drop a large proportion of the GAHI database. Inclusion of these studies may provide substantially more information with regard to our age patterns and time trends. Upcoming GBD cycles will explore methods for combining data with idiosyncratic diagnostic tools.

A secondary limitation to our data is that several included studies are not considered to be nationally representative, and therefore, at a location level, the data are highly heterogeneous (Figure 3). Numerous studies within the database were conducted in districts or townships, and in some cases the studies were done in known areas where prevalence is high. Upcoming GBD cycles will continue to rigorously vet these data points and update the database with literature searches. In addition, exploring the use of methodologies, such as model-based geostatistics, that implicitly model the spatial heterogeneity with this focal condition, are likely necessary.

Furthermore, we made a large assumption that the global age-sex distributions were applicable to all locations. While we believe that prevalence should peak among young adults and slowly decline afterward, there is likely variation across regions and locations. Given that our data are either among adolescents or all-age, it is very difficult to build an age trend at granular location levels. Thus, we allowed DisMod to disaggregate our heterogeneous data in an effort to provide sensible age-sex curves.

Lastly, we believe that more work needs to be done to improve our sequelae split methods. Since the EG data do not provide all estimation years and age groups, several assumptions had to be made. Thus, we will explore conducting literature searches to provide novel data points for sequelae estimations.

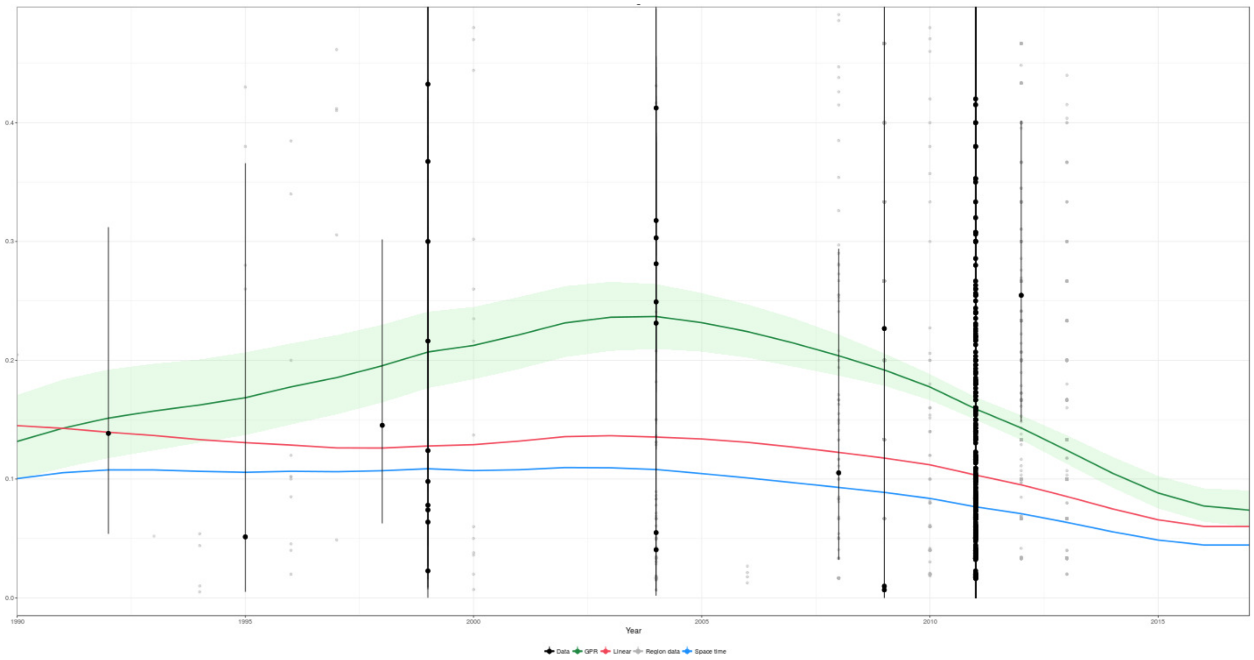

Figure 3: ST-GPR estimates for Nigeria (5- to 20-year-olds, both sexes) for years 1990–2017. Colouration and symbols are as stated in caption for Figure 2.

Figure 3 show the time trend for Nigeria as computed by ST-GPR. For some locations, we estimate this fluctuating time trend, which is a function of the heterogeneity in our input data. As explained above, some of these data points are representative of townships in Nigeria causing a great deal of heterogeneity throughout time.

References

1. London School of Hygiene and Tropical Medicine. Global Atlas of Helminth Infections – Soil Transmitted Helminths. London, United Kingdom: London School of Hygiene and Tropical Medicine.
2. Hall A, Hewitt G, Tuffrey V, de Silva N. A review and meta-analysis of the impact of intestinal worms on child growth and nutrition. *Maternal and Child Nutrition*. 2008. 4. 118-236.
3. Riess H, Clowes P, Kroidl, Kowuor D, Nsojo A, Mangu C, Schule S, Mansmann U, Geldmacher C, Mhina S, Maboko L, Hoelscher M, Saathoff E. Hookworm Infection and Environmental Factors in Mbeya Region, Tanzania: A Cross-Sectional, Population-Based Study. *PLoS Neglected Tropical Diseases*. 2013. 7. e2408.
4. Pullan R, Kabatereine N, Quinnell R, Brooker S. Spatial and Genetic Epidemiology of Hookworm in a Rural Community in Uganda. *PLoS Neglected Tropical Diseases*. 2010. 4. e713.

Foodborne Trematodiases

Clonorchiasis

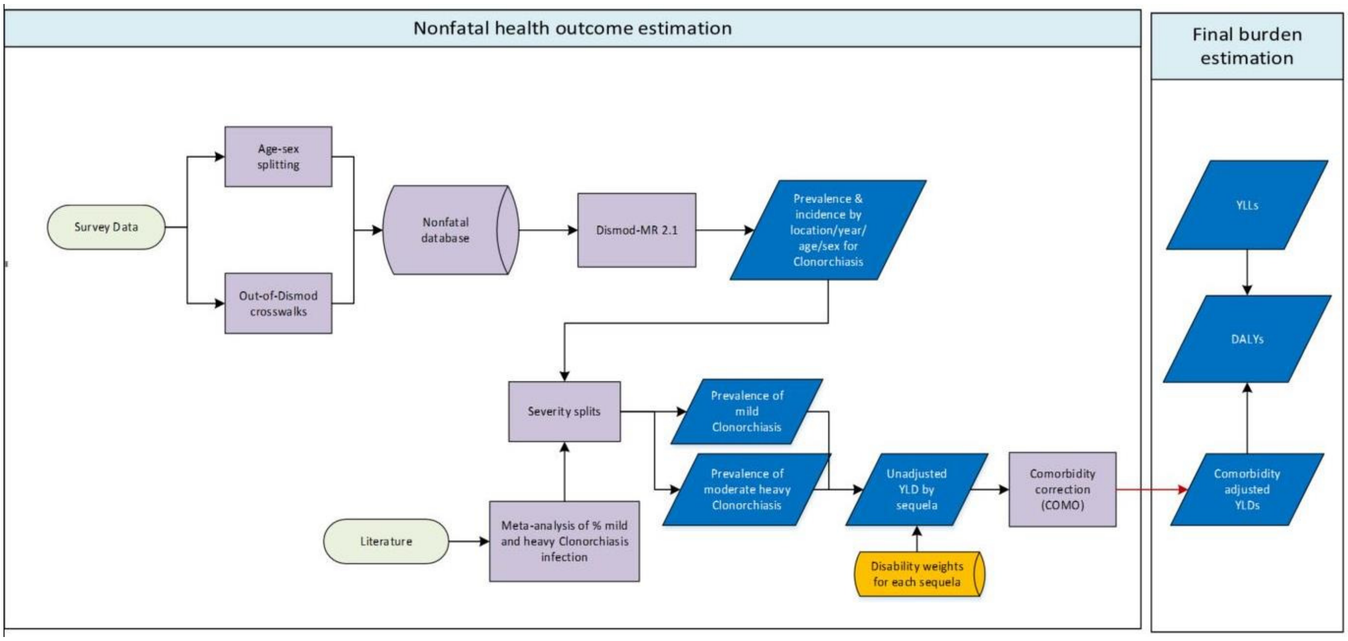

Fascioliasis

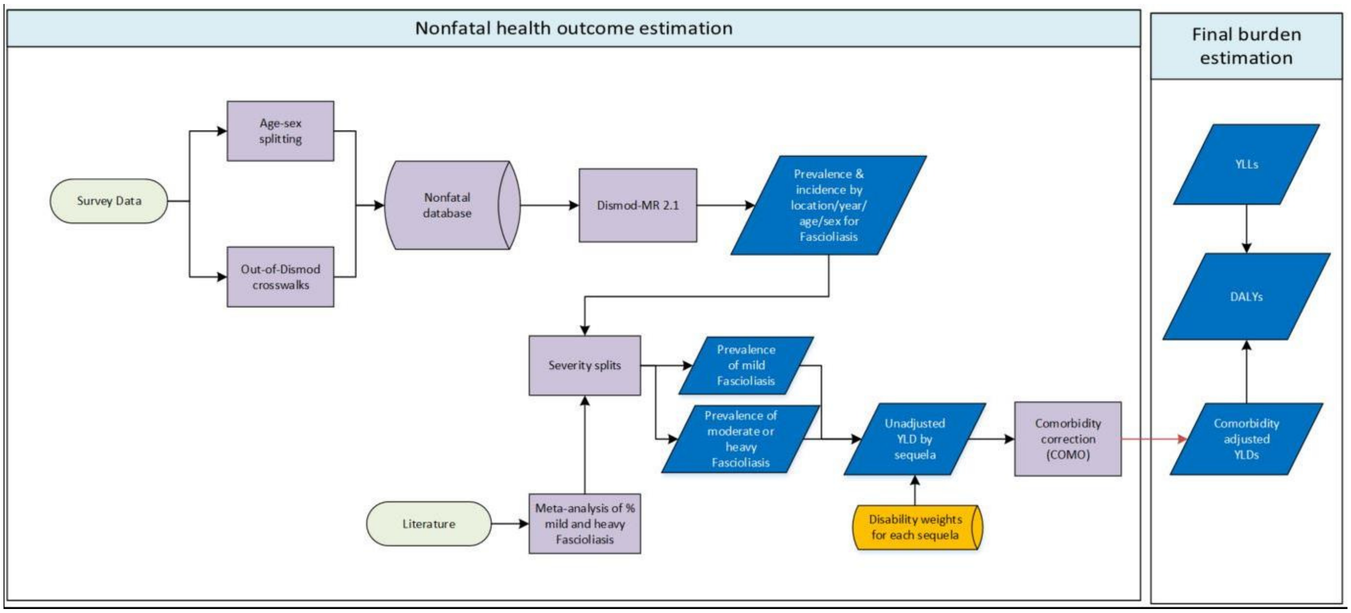

Intestinal Fluke

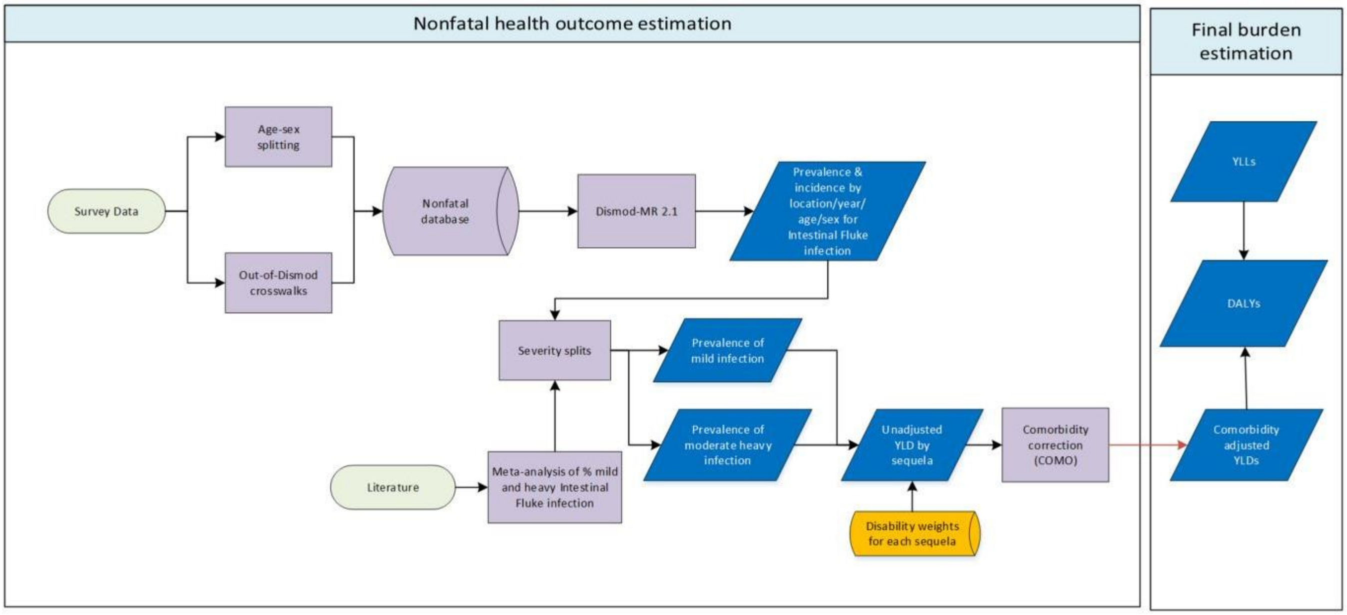

Opisthorchiasis

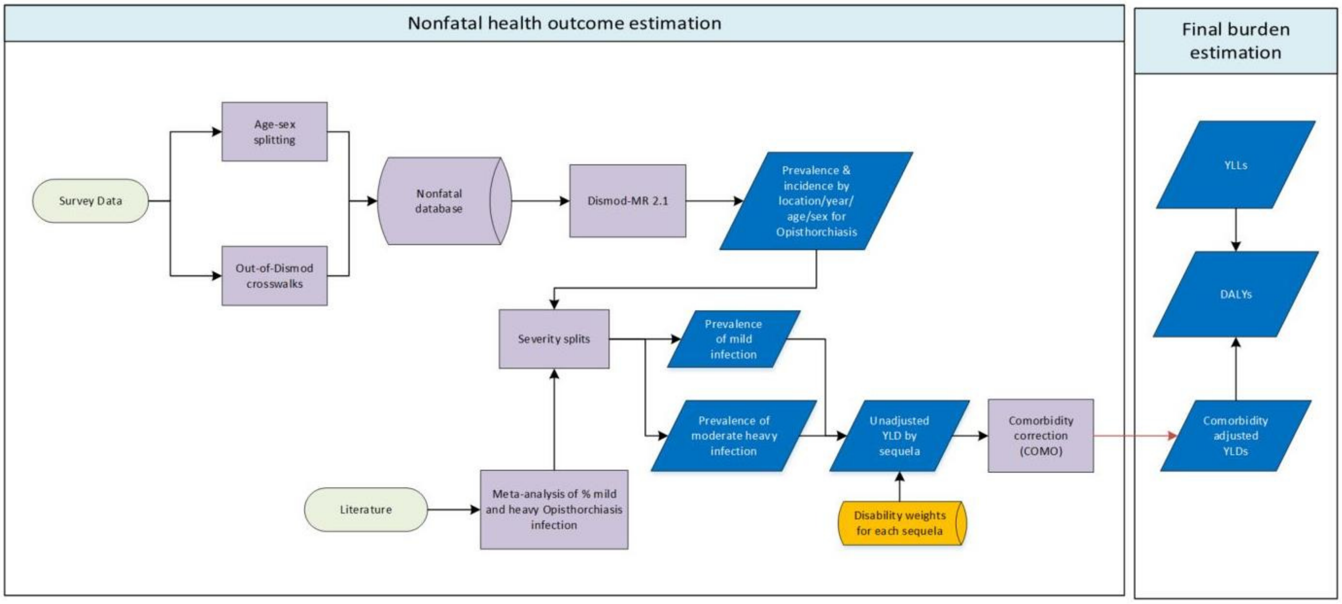

Paragonimiasis

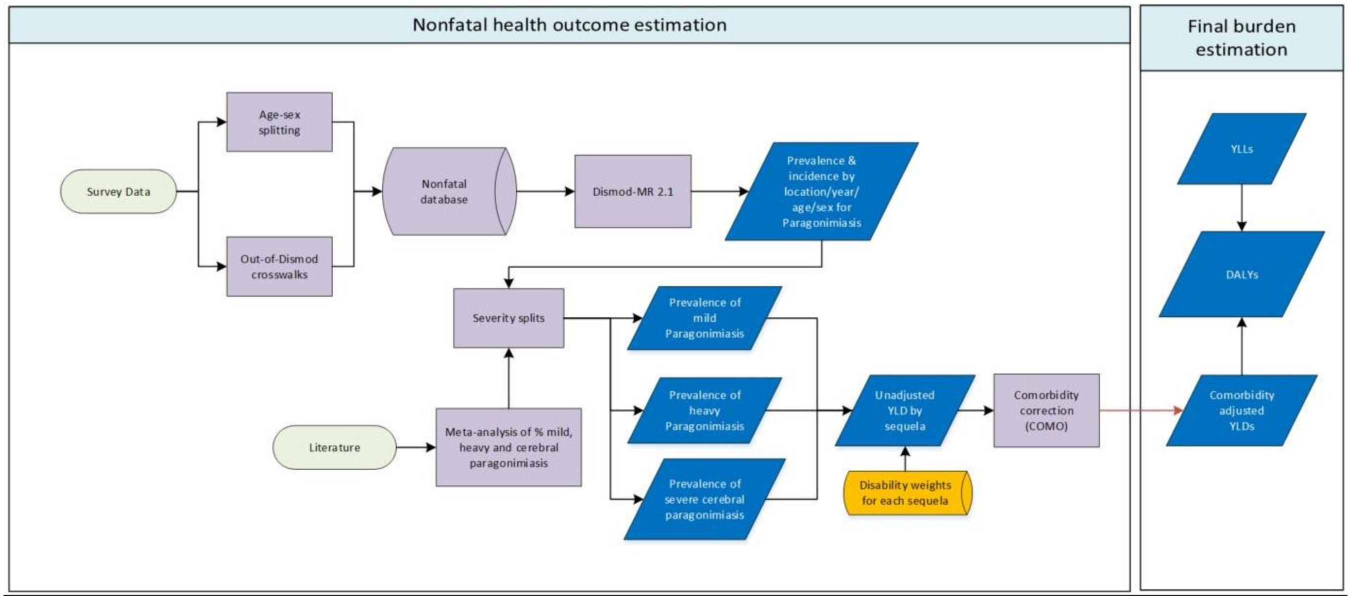

Input Data & Methodological Summary

Case definition

Human foodborne trematodiases (FBT) is defined as the infection with parasitic worms of the class trematoda, which are also known as flukes. Trematodes are transmitted via contaminated food, and infection is highly related to food habits. Definitive hosts, including humans, become infected when ingesting viable metacercariae by consuming contaminated aquatic products (eg, watercress). In the ICD-10, FBT are listed under code B66 [1].

FBT is subdivided into six types of FBT (see Table 1):

- Clonorchiasis
- Fascioliasis
- Intestinal fluke
- Opisthorchiasis
- Paragonimiasis (normal and cerebral infections)

Table 1. Subtypes of FBT

	Species of FBT	Also known as:	Carcinogen
1	Chlonorchiasis	(Chinese) Liver fluke	Associated with choliangiocarcinoma
2	Opisthorchiasis<br><br>(*O viverrini* & *O felineus*)	Liver fluke	Associated with choliangiocarcinoma<br><br>(*O viverrini*)
3	Fascioliasis	Liver fluke	No available evidence

4	Intenstinal fluke	Liver fluke	No available evidence
5	Paragonimiasis	Lung fluke	

Thresholds for heavy infection and duration by species of FBT

The majority of people infected with FBTs are asymptomatic. When symptoms do occur, they are often non-specific. Among the clinical symptomatic group, severity is associated with worm burden, typically measured by fecal egg counts, and the duration of infection. The thresholds for heavy infection and duration by species of FBT are shown in Table 2. The clinical presentation of FBT depends on the target organs (liver, lung, or intestines). Clonorchiasis and opisthorchiasis patients may suffer from loss of appetite, fullness, indigestion, diarrhoea, pain in the right upper quadrant, lassitude, weight loss, ascites, and oedema.[2, 3] Cholangitis, obstructive jaundice, intra-abdominal mass, cholecystitis, and gallbladder or intrahepatic stones may occur as complications.[3, 4]

Table 2. Thresholds for heavy infection and duration by species of FBT

	Species of FBT	Case thresholds for heavy infection	Duration
1	Chlonorchiasis	10,000 eggs per g of feces	lifelong
2	Opisthorchiasis	10,000 eggs per g of feces	lifelong
3	Fascioliasis	1,000 eggs per g of faces	lifelong
4	Intenstinal fluke	1,000 eggs per g of faces	lifelong
5	Paragonimiasis	100 eggs per 5 ml sputum	lifelong
6	Cerebral paragonimiasis	Any infection of the brain with flukes and/or eggs of *Paragonimus* spp.	lifelong

Input data

Model inputs

For GBD 2010, the data came from the expert group and is the result of their analysis. The expert group analysis used the results of a systematic literature review performed by Furst and colleagues as a starting point for the analysis.[5] Furst and colleagues searched PubMed, WHOLIS, FAOBIB, Embase, CAB Abstracts, Literatura Latino Americana e do Caribe em Ciências de Saùde (LILACS), ISI Web of Science, BIOSIS preview, Science Direct, African Journals OnLine (AJOL), and the System for Information on Grey Literature in Europe (SIGLE), period Jan 1, 1980, to Dec 31, 2008. The initial number of studies identified through the literature review was ~34,000 references. The literature review included extracted data from 181 studies. For GBD 2013 and GBD 2015, the search strategy was replicated to capture epidemiological studies published between 2008 and 2015.

Input data for the assessment of the total national number of infected people

Only studies that used countrywide surveys to estimate the national prevalence rates were included (or for China, province-wide surveys). Reason for choosing only national studies is that FBT shows a highly focal spatial distribution and local cross-sectional surveys would profoundly under- or overestimate true national prevalences. We decided not to model national and subnational together and get a coefficient on subnational, because there is not a one-fits-all relationship across the world. Infection is highly related to food habits, and there are highly varying differences between national and subnational prevalence rates. The final GBD 2016 dataset contained 29 prevalence studies from 17 countries. We used raw data from the selected studies as input for DisMod.

Prevalence of intestinal fluke infection

Intestinal fluke is different from the other types of FBT, because there are several pathogens that fall under intestinal fluke infection. It can be caused by pathogens, such as *Metagonimus* spp., *Echinostoma* spp., and *Neodiplostomatidae*.[6] When assessing the prevalence of intestinal fluke infection, we added the identified prevalence for each parasite species in order to obtain the overall prevalence of intestinal fluke infections. This approach may lead to a certain overestimation of the true prevalence, because people may be co-infected with more than one intestinal fluke species. There is no sufficient evidence about the proportion of co-infections, but the resulting overestimation of the true prevalence may be more than offset by the assumptions made in our previous modelling approach and the many challenges in generating the underlying epidemiological parameters (eg, diagnostic inaccuracy in the detection of infections with the more than 50 intestinal fluke species). Also of note: the transmission source of intestinal fluke infections are species-specific and therefore vary. For instance, *Fasciolopsis buski* is usually transmitted by eating raw water plants with the infective parasite stage attached to the water plants, whereas *Neodiplostomatidae* are transmitted by eating undercooked and infested frogs, snakes, and tadpoles. Because of these different transmission pathways, the rate of co-infection might in fact be smaller than expected.

Input data to differentiate between asymptomatic and heavy infections

We estimated the proportion of heavily infected among all infected in all available national and regional cross-sectional surveys. It is expected that heavy infection increases with age and there are data available on heavy infection by age group. We therefore decided to include age-dependent rates of heavy infection for clonorchiasis, opisthorchiasis, and intenstinal fluke infection. For (cerebral) paragonimiasis and fascioliasis there were not sufficient age-dependent data on high intensity FBT infection.

Total data inputs – Chlonorchiasis

	**Prevalence**
**Site-years (total)**	121
**Number of countries with data**	4
**Number of GBD regions with data (out of 21 regions)**	4
**Number of GBD super-regions with data (out of 7 super-regions)**	3

Total data inputs – Fascioliasis

	**Prevalence**
**Site-years (total)**	65
**Number of countries with data**	8
**Number of GBD regions with data (out of 21 regions)**	4
**Number of GBD super-regions with data (out of 7 super-regions)**	4

Total data inputs – Intestinal flukes

	Prevalence
**Site-years (total)**	101
**Number of countries with data**	7
**Number of GBD regions with data (out of 21 regions)**	5
**Number of GBD super-regions with data (out of 7 super-regions)**	4

Total data inputs – Opisthorchiasis

	Prevalence
**Site-years (total)**	10
**Number of countries with data**	5
**Number of GBD regions with data (out of 21 regions)**	3
**Number of GBD super-regions with data (out of 7 super-regions)**	2

Total data inputs – Paragonimiasis

	Prevalence
**Site-years (total)**	74
**Number of countries with data**	5
**Number of GBD regions with data (out of 21 regions)**	4
**Number of GBD super-regions with data (out of 7 super-regions)**	3

Total data inputs – Cerebral paragonimiasis

	Prevalence
**Site-years (total)**	4
**Number of countries with data**	2
**Number of GBD regions with data (out of 21 regions)**	2
**Number of GBD super-regions with data (out of 7 super-regions)**	2

Modelling strategy

We used a three-step process for the disease modelling of FBT. In the first step we used DisMod-MR 2.0 to estimate the prevalence of FBT by age, sex, year, and country. In the second we differentiated between asymptomatic and heavy infections. MetaXL (a meta-analysis add-in for Microsoft Excel) was used to estimate the proportion of heavily infected among all infected by age group for clonorchiasis, opisthorchiasis, and intenstinal fluke infection (see Table 3 and 4). These proportions were used to estimate the prevalence of heavy FBT infection.

The third step consisted of deselecting countries that have no autochtonous case reports of FBT (input 34,000 references from literature review).

Table 3. Percentage of high-intensity infection by age group and type of FBT (based on eight FBT prevalence studies)

Age category	Clonorchiasis			Opisthorchiasis			Intestinal fluke infection		
	Mean	Low	High	Mean	Low	High	Mean	Low	High
0-9	30%	17%	44%	10%	0%	29%	8%	3%	14%
10-19	15%	0%	43%	15%	0%	69%	11%	8%	14%
20-29	18%	10%	29%	16%	0%	52%	18%	15%	21%
30-39	17%	5%	34%	21%	0%	56%	22%	17%	28%
40-49	22%	13%	32%	28%	1%	68%	22%	13%	32%
50-59	18%	0%	49%	29%	0%	75%	17%	9%	28%
60+	32%	18%	47%	25%	0%	64%	15%	8%	23%

Table 4. Percentage of high-intensity infection by type of FBT (based on four FBT prevalence studies)

Type of FBT	Mean	Low	High
Paragonimiasis	23%	0%	59%
Fascioliasis	19%	3%	41%

Cerebral paragonimiasis

It was assumed that 0.8% of paragonimiasis cases have cerebral involvement. This proportion was used to estimate the prevalence of cerebral paragonimiasis. This proportion is based on one study. The data are from Oh SJ. The rate of cerebral involvement in paragonimiasis: an epidemiologic study. *Jpn J Parasitol* 1969;18:211-14. The study was performed in Paju, South Korea. This is an area with 6,738 inhabitants, and according to the survey, it was estimated that 29.6% of all individuals would react to intradermal test (= an immunological reaction indicating previous or current contact with the parasite). 25% of all "positive reactors" may have eggs in their sputum (= active infection with the parasite currently present in the human host). If these rates are applied to the community as a whole, the number of patients with active paragonimiasis would be at least 498 (=6,738*0.296*0.250). Furthermore, four cases of cerebral paragonimiasis were found in this community. Therefore, four out of 498 individuals with active paragonimus infection suffered from cerebral infection (=0.80%; 95% confidence interval 0.019%–1.587%).

Severity splits and disability weights

For GBD 2016, FBT was not split into health states with different severities. The table below shows the GBD 2016 disability weights that were used to calculate the burden of FBT in YLDs.

Table 5. Disability weights that were used to calculate FBT YLDs

Sequelae	Severity description	Health state name	Disability weight
Asymptomatic clonorchiasis	Clonorchiasis, currently without symptoms	N/A	0.000 (0.000–0.000)

Heavy clonorchiasis	Abdominal pain and nausea reported as moderate	Abdominopelvic problem, moderate	0.114 (0.078–0.159)
Asymptomatic opisthorchiasis	Opisthorchiasis, currently without symptoms	N/A	0.000 (0.000–0.000)
Heavy opisthorchiasis	Abdominal pain and nausea reported as moderate	Abdominopelvic problem, moderate	0.114 (0.078–0.159)
Asymptomatic fascioliasis	Fascioliasis, currently without symptoms	N/A	0.000 (0.000–0.000)
Heavy fascioliasis	Abdominal pain and nausea reported as moderate	Abdominopelvic problem, moderate	0.114 (0.078–0.159)
Asymptomatic intestinal fluke infection	Intestinal fluke infection, currently without symptoms	N/A	0.000 (0.000–0.000)
Heavy intestinal fluke infection	Abdominal pain and nausea reported as moderate	Abdominopelvic problem, moderate	0.114 (0.078–0.159)
Asymptomatic paragonimiasis	Paragonimiasis, currently without symptoms	N/A	0.000 (0.000–0.000)
Heavy paragonimiasis	Cough, fever, and weight loss	Tuberculosis, not HIV-infected	0.333 (0.224–0.454)
Cerebral paragonimiasis	Epilepsy due to cerebral paragonimiasis	Epilepsy, less severe (seizures < once per month)	0.263 (0.173–0.367)
		Epilepsy, severe (seizures >= once per month	0.552 (0.375–0.710)

Note. N/A: not applicable

Changes from GBD 2016 to GBD 2017

We conducted an abbreviated literature search based on citations included in published reviews (5) and recommendations by Dr. Thomas Furst. We completed extractions for all but five records during the allotted timeline. Sources were unable to be extracted due to language barriers and lack of detailed citation information.

References

1. WHO. *International Statistical Classification of Diseases and Related Health Problems. 10th Revision. Version for 2007.* 2007 [cited 2009 October 14, 2009]; Available from: http://apps.who.int/classifications/apps/icd/icd10online/.
2. Rim, H.J., *Clonorchiasis: an update.* J Helminthol, 2005. **79**(3): p. 269-81.
3. Pungpak, S., et al., *Clinical features in severe opisthorchiasis viverrini.* Southeast Asian J Trop Med Public Health, 1985. **16**(3): p. 405-9.
4. Rim, H.J., *The current pathobiology and chemotherapy of clonorchiasis.* Korean J Parasitol, 1986. **24**(Suppl.): p. 1-141.

5. Furst, T., J. Keiser, and J. Utzinger, *Global burden of human food-borne trematodiasis: a systematic review and meta-analysis.* Lancet Infect Dis, 2012. **12**(3): p. 210-21.
6. Furst, T., et al., *Manifestation, diagnosis, and management of foodborne trematodiasis.* BMJ, 2012. **344**: p. e4093.

Leprosy

Leprosy

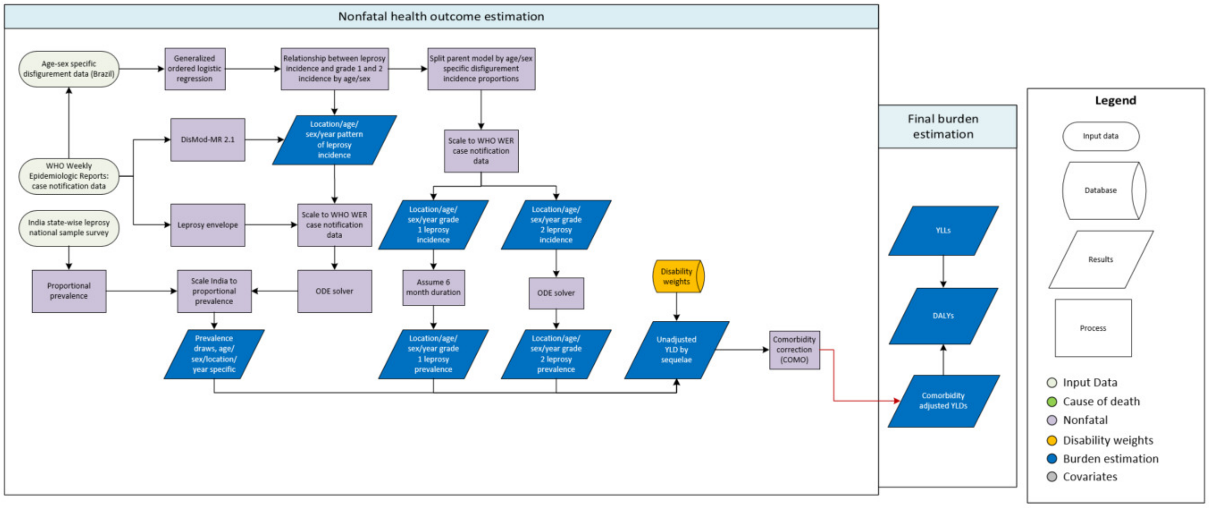

Input Data and Methodological Summary

Case definition

Leprosy is a chronic bacterial infection caused by *Mycobacterium leprae*, primarily affecting the nervous system, skin, respiratory tract, and eyes. Transmission is facilitated through contact with fluid from the nose and mouth of an infected individual. The ICD-10 code for leprosy is A30.9.

Input data

To model non-fatal outcomes due to leprosy, WHO Weekly Epidemiological Record (WER) case notification data were used from 1987 to 2016 to capture incident cases of leprosy. Stage-specific incidence data for grade 1 and grade 2 leprosy that are used to define age-sex patterns came from Brazil case notification data.

Table of data counts for leprosy incidence data

	**Incidence**
**Site-years (total)**	1,374
**Number of countries with data**	147
**Number of GBD regions with data (out of 21 regions)**	21
**Number of GBD super-regions with data (out of 7 super-regions)**	7

Table of data counts for leprosy grade inputs – grade 1

	Prevalence
**Site-years (total)**	12
**Number of countries with data**	1
**Number of GBD regions with data (out of 21 regions)**	1
**Number of GBD super-regions with data (out of 7 super-regions)**	1

Table of data counts for leprosy grade inputs – grade 2

	Prevalence
**Site-years (total)**	710
**Number of countries with data**	121
**Number of GBD regions with data (out of 21 regions)**	17
**Number of GBD super-regions with data (out of 7 super-regions)**	6

Modelling strategy

We used a multi-step process for the disease modelling of leprosy. In the first step, we ran a single-parameter model using DisMod-MR 2.1 to estimate the leprosy incidence age pattern by age, sex, year, and country. Then, we scaled the incidence outputs to the WHO WER cases, and used the ordinary differential equations (ODE) solver to calculate prevalence from the scaled DisMod-MR 2.0 incidence outputs.

Severity data were prepared by running a generalised ordered logistic regression using Brazil case notification data to get the relationship between leprosy incidence and grade 1 and grade 2 incidence by age and sex. We then used this relationship to split the parent DisMod-MR 2.1 model, and again scaled to WHO WER severity-specific cases. For disfigurement grade 1, we apply a duration of six months to get prevalence estimates. For disfigurement grade 2, we again use the ODE solver to get prevalence estimates.

Model evaluation was done by separately assessing the fit of the parent DisMod model and checking the final estimates produced after age-sex splits. Plots of time trends of prevalence across locations and age were used to evaluate the results. In addition, maps of the global distribution of leprosy prevalence and prevalence of sequelae due to leprosy were also assessed across time.

Changes from GBD 2016 to GBD 2017

We extracted WHO WER data from 2013 to 2016 to update prevalence and incidence estimates.

Ebola virus disease

Flowchart

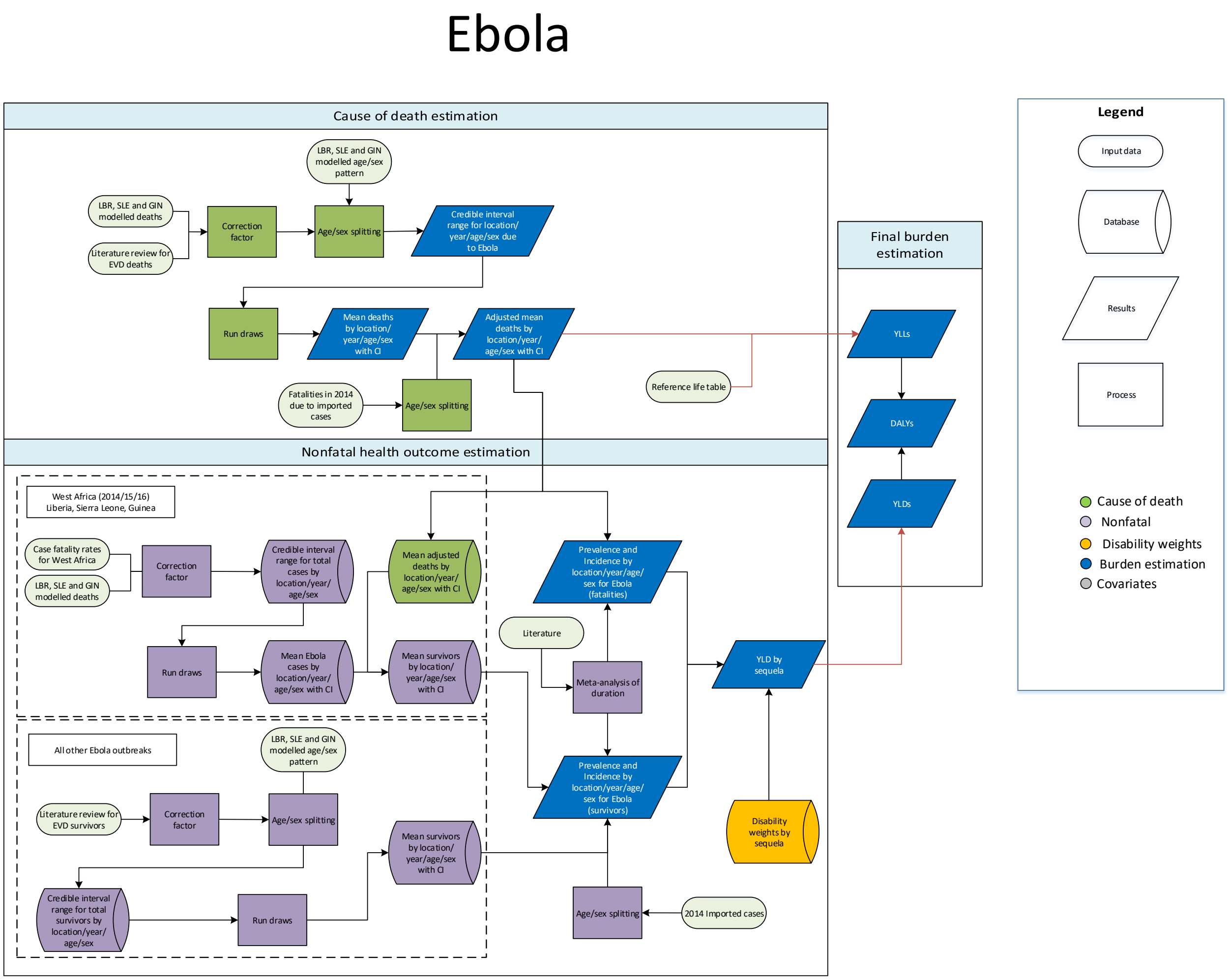

Input data and methodological summary

Background and case definition

Ebola virus is a relatively rare viral pathogen linked with high case fatality rates in both humans and non-human primates. The disease is zoonotic, and while bats have been implicated as reservoirs, definitive host species are yet to be identified. Once a human becomes infected after viral transmission from animal sources either directly or indirectly, secondary human-to-human transmission is possible, primarily through exchange of infectious bodily fluids and secretions. Clinical cases typically present initially as a febrile illness, similar to a number of different pathogens, which can be subsequently followed by haemorrhagic complications and death. Historically there have been a number of outbreaks, usually no more than a few hundred cases, typically constrained to one country, focused in Central Africa. The West African outbreak, however, which started in Guinea in 2013, claimed more lives than all previous

outbreaks combined, and spread across the region seeding additional outbreaks. There is an ICD code for Ebola, A98.4, but no data used in the modelling reference that coding (ie, all the data are from literature extractions). Data for Ebola virus disease were only included if the case was identified as either "probable" or "confirmed" as per WHO definitions [http://www.who.int/csr/resources/publications/ebola/ebola-case-definition-contact-en.pdf]. A confirmed case is any suspected or probable case with a positive laboratory result through either detection of virus RNA via reverse transcriptase-polymerase chain reaction, or by detection of IgM antibodies directed against Ebola. A probable case is any suspected case evaluated by a clinician or any deceased suspected case with an epidemiological link to a confirmed case.

Input data

Model inputs

Two distinct sequelae were assigned to Ebola virus disease (EVD) to be incorporated into the YLD estimation process: (i) sequela associated with the initial symptomatic phase of the infection (associated with all cases of Ebola virus disease) and (ii) sequela characterising the long-term post-EVD consequences of infection. As such, data were required both to ascertain the number of deaths as well as those surviving from each outbreak.

Data on fatal cases were inherited from the GBD 2017 mortality estimation process and were converted into incidence of cases of Ebola (with fatal outcomes) by cross-referencing locational annualised population estimates.

In order to calculate the numbers of survivors from each outbreak, two data sources were referenced, one based upon modelled estimates of the main three countries in the West African Ebola outbreak (namely Sierra Leone, Liberia, and Guinea), supplemented by WHO Situation Reports covering the clusters of 2016 cases and literature references covering all other subsequent outbreaks.

Researchers from Imperial College London (UK), as part of the WHO Ebola response team, provided modelled estimates for the number of fatalities that result from a given number of reported cases (provided by line lists from WHO). This method was used in a variety of papers to generate baseline estimates of case fatality rates and other key epidemiological measures while correcting for the lag period between initially reporting a case and the final outcome of that case (whether it be death or survival). The full data cleaning and methodology are reported elsewhere.[1,2] Age-sex patterns derived from this analysis were applied to total envelope estimates as reported by WHO and CDC. Raw number of survivors were estimated by subtracting total deaths as reported by WHO and CDC from total cases.

For all other outbreaks, numbers of survivors were directly evaluated based upon numbers published in a previous review[3,4] and consulting original documents describing these outbreaks. This initial review was also updated to include the outbreak that occurred in the Democratic Republic of the Congo in 2014,[5] and cases in 2016 and 2017. This resulted in datasets describing each outbreak with variable degrees of detail: some fully describing the age and sex breakdown of all survivors [eg, Rosello et al.[6]] and others simply providing the final total. Only confirmed or probable cases were included as per the case definition. Outbreaks that spanned multiple years, in the absence of sufficient data providing an accurate breakdown, were apportioned between the years by evenly assigning a uniform number of survivors to

each month of the outbreak's duration. An additional search was conducted to identify imported cases from the West African outbreak during 2014 and 2015.

Table 1. Sequelae and disability weights (DWs) associated with Ebola

Sequelae	Description	Disability weight
Infectious disease, acute episode, severe	Has a high fever and pain and feels very weak, which causes great difficulty with daily activities	0.133 (0.088–0.19)
Infectious disease, post-acute consequences (fatigue, emotional lability, insomnia)	Is always tired and easily upset. The person feels pain all over the body and is depressed	0.219 (0.148–0.308)

It was not possible to create bespoke disability weights for the more specific sequelae often associated with Ebola virus disease (eg, haemorrhaging or ocular complications in survivors), so existing disability weights were co-opted. General high fevers and weakness characterise the majority of presenting cases[7] with long-term complications generally related to weakness and arthralgia.[8]

Modelling strategy

Data on cases (both survivors and fatalities) resulting from imported cases from 2014 and 2015 were used as specific count data as it was assumed to be an accurate representation of the cases and outbreaks in these countries, all of which were on high alert for importation of cases.[9,10]

The other input data were processed prior to inclusion in GBD to account for any potential underreporting of deaths. A meta-analysis of existing underreporting studies from the literature was performed, using a random effects model with a DerSimonian-Laird estimator. A variety of sources were included, capturing a number of different estimation processes, all identified by literature review. The figure below shows the different effect sizes of the different studies, as well as the resulting GBD 2016 (used in GBD 2017) correction factor, with the GBD 2015 correction factor for reference. The correction factor ranged from 1.5147 to 2.5720 with a mean of 2.0433.

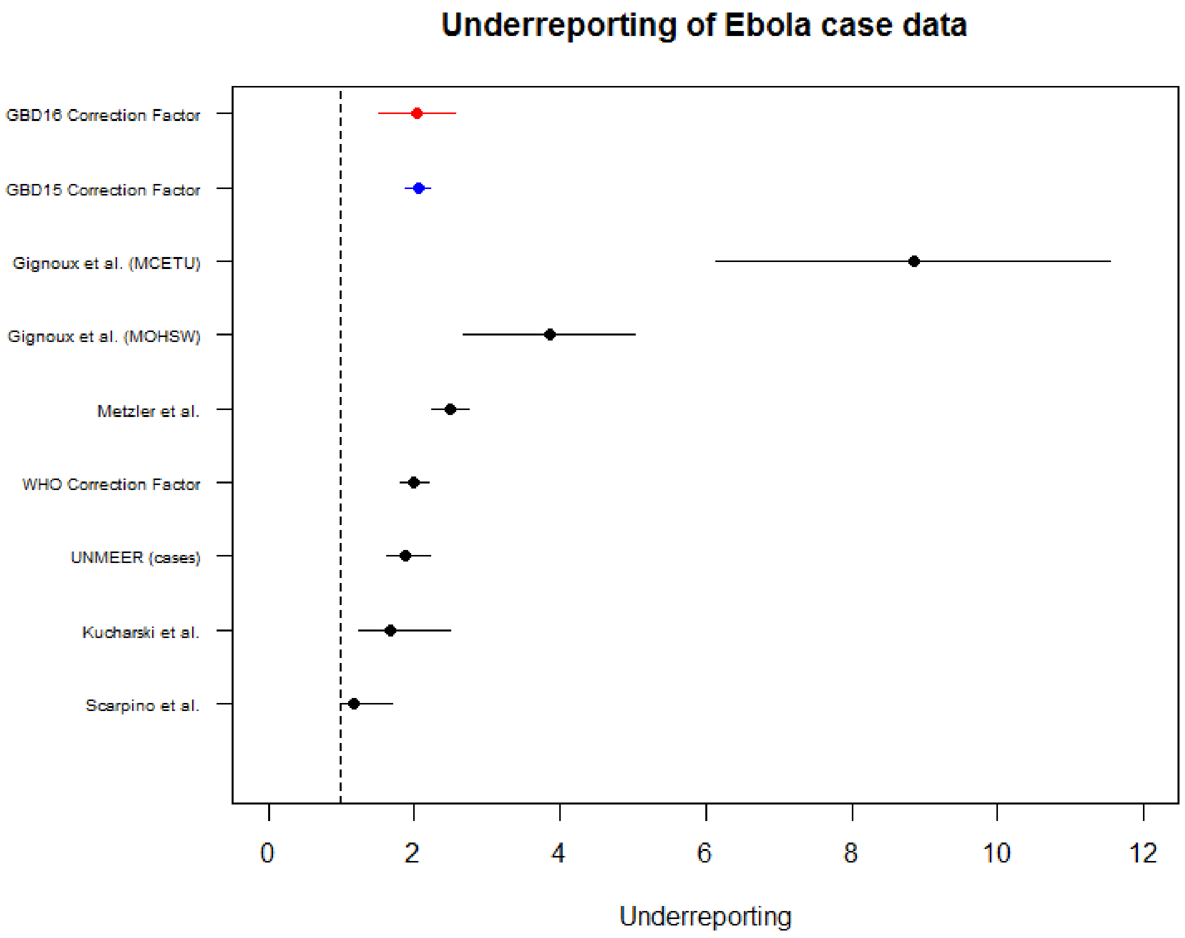

In order to capture this potential variation, all input data were multiplied by the lower and upper limit of this estimated correction factor; these numbers then provided the lower and upper bounds from which draw values were taken. For outbreaks where no data were supplied for age and/or sex, the pattern observed in the West African outbreak (for which there were the most comprehensive data) was used to apportion these total values.

One thousand draws were taken from a normal distribution fitted between these lower and upper bound values, which generated mean estimates stratified by age, sex, location, and year along with credible intervals for these numbers. For the West African outbreak, this generated total case numbers, from which the estimated number of deaths was subtracted in order to provide an estimate for the total number of survivors. For all other outbreaks, this data processing directly estimated the total number of survivors from each outbreak. These count data were converted into prevalence estimates by cross-referencing estimates of population size.

In order to estimate the duration of the sequelae categories, previous modelled assessments of the West African outbreak were consulted.[1,2] The duration of initial infection for patients was calculated as the total time period between onset of symptoms to death or to discharge from hospital (8.2 days [7.9–8.4] and 15.1 [14.6–15.6], respectively). These time periods were assumed to be appropriate for

characterising all other outbreaks. This time period was then assigned a disability weight corresponding to "infectious disease, acute episode, severe."

For long-term sequelae estimation, the proportion of survivors still suffering post-acute consequences was modelled using an exponential function with proportions of survivors still reporting poor health states (derived from a number of survivor studies[8,12–21]) reported over different time periods. The average duration of post-Ebola sequelae was then calculated as 0.9042 years (0.3673–1.4268).

The final combination of YLDs associated with prevalent initial onset of disease and prevalent post-EVD consequences was then calculated to provide an overall YLD estimate stratified by age, sex, location, and year. Estimates were provided for the years 1990, 1995, 2000, 2005, 2010, 2015, and 2016 as per non-fatal GBD estimation protocols.

Potential limitations

Data on Ebola outbreaks prior to 2014 are sparse, and as a result many values derived from the West African outbreak were assumed to be valid for historical outbreaks as well. This may mask significant differences that exist between these outbreaks, some of which were caused by different species of Ebola virus. In order to minimize this problem, we chose to implement a data-driven approach – for those outbreaks where sufficiently detailed historical data could be obtained, this was used in preference to any assumed age/sex breakdown.

Haemorrhagic manifestations are currently not considered as an explicit health state for disability weighting, and as a result, the current classification (of infectious disease, acute episode, severe) may be an underestimate. In contrast, the post-Ebola disease sequelae disability weighting may overestimate this burden, particularly when applied over a long period of time. In both instances, however, these disability weightings represent the most relevant linkages in the absence of bespoke values being generated.

Due to so few historical survivors of Ebola virus disease, only a handful of studies have tracked the long-term sequelae among cohorts of survivors beyond a two-year period. Given the large number of survivors from the West African outbreak, it is likely that future parameterization of this component will become much better data-driven. The current log-linear regression model extends for a period of 20 years and therefore could prove to be an overestimate of duration. In addition, ocular manifestations are not currently considered within the sequelae envelope – future iterations will consider health states identified by ongoing cohort analyses of Ebola survivors. Comments from collaborators in previous cycles have highlighted ocular conditions for inclusion; however, definitive evidence of a linkage with Ebola remains inconclusive. A study (conducted in West Africa) comparing Ebola survivors with background prevalence rates of many of the symptoms reported in survivors (eg, uveitis), suggested no difference in rates of these ophthalmic complications[22]. Understanding which of the many observed clinical outcomes in patients are caused by the virus, as opposed to incidentally co-morbid, is a necessary prerequisite for inclusion in the GBD.

References

1 Agua-Agum J, Ariyarajah A, Aylward B, *et al.* West African Ebola Epidemic after One Year — Slowing but Not Yet under Control. *N Engl J Med* 2015; **372**: 584–7.

2 Ebola Virus Disease in West Africa - The First 9 Months of the Epidemic and Forward Projections. *N*

Engl J Med 2014; **371**: 1481–95.

3 Pigott DM, Golding N, Mylne A, *et al.* Mapping the zoonotic niche of Ebola virus disease in Africa. *Elife* 2014; **3**: e04395.

4 Mylne A, Brady OJ, Huang Z, *et al.* A comprehensive database of the geographic spread of past human Ebola outbreaks. *Sci Data* 2014; **1**: 140042.

5 Maganga GD, Kapetshi J, Berthet N, *et al.* Ebola virus disease in the Democratic Republic of Congo. *N Engl J Med* 2014; **371**: 2083–91.

6 Rosello A, Mossoko M, Flasche S, *et al.* Ebola virus disease in the Democratic Republic of the Congo, 1976-2014. *Elife* 2015; **4**. DOI:10.7554/eLife.09015.

7 Schieffelin JS, Shaffer JG, Goba A, *et al.* Clinical Illness and Outcomes in Patients with Ebola in Sierra Leone. *N Engl J Med* 2014; **371**: 2092–100.

8 Tiffany A, Vetter P, Mattia J, *et al.* Ebola Virus Disease Complications as Experienced by Survivors in Sierra Leone. *Clin Infect Dis* 2016; **62**: 1360–6.

9 Fasina FO, Shittu A, Lazarus D, *et al.* Transmission dynamics and control of Ebola virus disease outbreak in Nigeria, July to September 2014. *Euro Surveill* 2014; **19**: 20920.

10 Althaus CL, Low N, Musa EO, Shuaib F, Gsteiger S. Ebola virus disease outbreak in Nigeria: Transmission dynamics and rapid control. *Epidemics* 2015; **11**: 80–4.

11 UNMEER. Sierra Leone: Ebola emergency Weekly Situation Report No. 7. 2014 https://www.humanitarianresponse.info/system/files/documents/files/UNMEER_NERC_SitRep_07Dec.pdf.

12 Clark D V, Kibuuka H, Millard M, *et al.* Long-term sequelae after Ebola virus disease in Bundibugyo, Uganda: a retrospective cohort study. *Lancet Infect Dis* 2015; **15**: 905–12.

13 Qureshi AI, Chughtai M, Loua TO, *et al.* Study of Ebola Virus Disease Survivors in Guinea. *Clin Infect Dis* 2015; **61**: 1035–42.

14 Rowe AK, Bertolli J, Khan AS, *et al.* Clinical, virologic, and immunologic follow-up of convalescent Ebola hemorrhagic fever patients and their household contacts, Kikwit, Democratic Republic of the Congo. Commission de Lutte contre les Epidémies à Kikwit. *J Infect Dis* 1999; **179 Suppl**: S28-35.

15 Bwaka MA, Bonnet MJ, Calain P, *et al.* Ebola hemorrhagic fever in Kikwit, Democratic Republic of the Congo: clinical observations in 103 patients. *J Infect Dis* 1999; **179 Suppl**: S1-7.

16 Mohammed H, Vandy AO, Stretch R, *et al.* Sequelae and Other Conditions in Ebola Virus Disease Survivors, Sierra Leone, 2015. *Emerg Infect Dis* 2017; **23**: 66–73.

17 Nanyonga M, Saidu J, Ramsay A, Shindo N, Bausch DG. Sequelae of Ebola Virus Disease, Kenema District, Sierra Leone. *Clin Infect Dis* 2016; **62**: 125–6.

18 Mattia JG, Vandy MJ, Chang JC, *et al.* Early clinical sequelae of Ebola virus disease in Sierra Leone: a cross-sectional study. *Lancet Infect Dis* 2016; **16**: 331–8.

19 Epstein L, Wong KK, Kallen AJ, Uyeki TM. Post-Ebola Signs and Symptoms in U.S. Survivors. *N Engl J Med* 2015; **373**: 2484–6.

20 Etard J-F, Sow MS, Leroy S, *et al.* Multidisciplinary assessment of post-Ebola sequelae in Guinea (Postebogui): an observational cohort study. *Lancet Infect Dis* 2017. DOI:10.1016/S1473-3099(16)30516-3.

21 Scott JT, Sesay FR, Massaquoi TA, Idriss BR, Sahr F, Semple MG. Post-Ebola Syndrome, Sierra Leone. *Emerg Infect Dis* 2016; **22**: 641–6.

22 Steptoe, PJ, Scott JT, Baxter, JM, *et al.* Novel retinal lesion in Ebola survivors, Sierra Leone, 2016. *Emerg Infect Dis* 2017; **23**: 1102-9

Zika

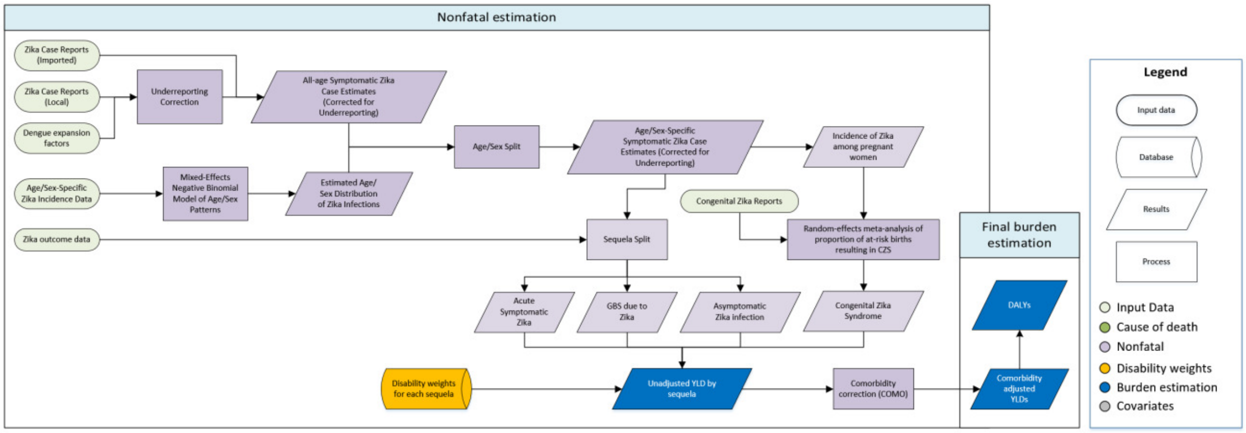

Input data

Data on cases of acute Zika and Congenital Zika Syndrome (CZS) come from official reports, primarily from the Pan American Health Organization (PAHO).

Modelling strategy

We estimate the all-age incidence of symptomatic Zika as the product of reported Zika cases and country-specific expansion factors that adjust for underreporting. Those expansion factors are derived from our dengue model, and the methods used for their estimation are detailed in the dengue model documentation and by Stanaway and colleagues.(1) A subset of incidence data were age/sex-specific, and we used a mixed-effects negative binomial model with cubic splines on age and interaction terms with sex to estimate the age/sex distribution of cases. We then split total incidence based on the age/sex-distribution model to estimate the incidence of symptomatic Zika by location, year, age, and sex.

We conducted a meta-analysis of three studies(2–4) to estimate the proportion of all Zika infections that are symptomatic. We estimate that 41% of Zika infections are symptomatic (14–68%), with 59% being asymptomatic. We then estimated incidence of asymptomatic infections as

$$I_{asymp} = \frac{I_{symp}}{Pr_{symp}} - I_{symp}$$

Where I_{asymp} is the incidence of asymptomatic infections, I_{symp} is the incidence of symptomatic Zika, and Pr_{symp} is the proportion of infections that are symptomatic (ie, 41%).

We assume that the incidence of Zika among pregnant women equals the incidence of Zika among all women, within a given location, year, and age group. We then estimate the number of pregnant women infected with Zika as the product of incidence of Zika and the number of pregnant women in every location, year, and age group. Finally, we used an intercept only, mixed-effects Poisson regression model, with random effects on location, the number of at-risk births as the exposure term, and the

number of reported CZS cases as the outcome to estimate proportion of at-risk births (ie, those in which the mother was infected with Zika during pregnancy) resulting in CZS.

Changes from GBD 2016 to GBD 2017

We have made no substantive changes in the modelling strategy from GBD 2016 to GBD 2017.

References

1. Stanaway JD, Shepard DS, Undurraga EA, Halasa YA, Coffeng LE, Brady OJ, et al. The global burden of dengue: an analysis from the Global Burden of Disease Study 2013. Lancet Infect Dis [Internet]. 2016 Feb [cited 2016 May 23]; Available from: http://linkinghub.elsevier.com/retrieve/pii/S1473309916000268

2. Gallian P, Cabié A, Richard P, Paturel L, Charrel RN, Pastorino B, et al. Zika virus in asymptomatic blood donors in Martinique. Blood. 2017 Jan 12;129(2):263–6.

3. Duffy MR, Chen T-H, Hancock WT, Powers AM, Kool JL, Lanciotti RS, et al. Zika virus outbreak on Yap Island, Federated States of Micronesia. N Engl J Med. 2009 Jun 11;360(24):2536–43.

4. Aubry M, Teissier A, Huart M, Merceron S, Vanhomwegen J, Roche C, et al. Zika Virus Seroprevalence, French Polynesia, 2014–2015. Emerg Infect Dis. 2017 Apr;23(4):669–72.

Dracunculiasis (Guinea worm)

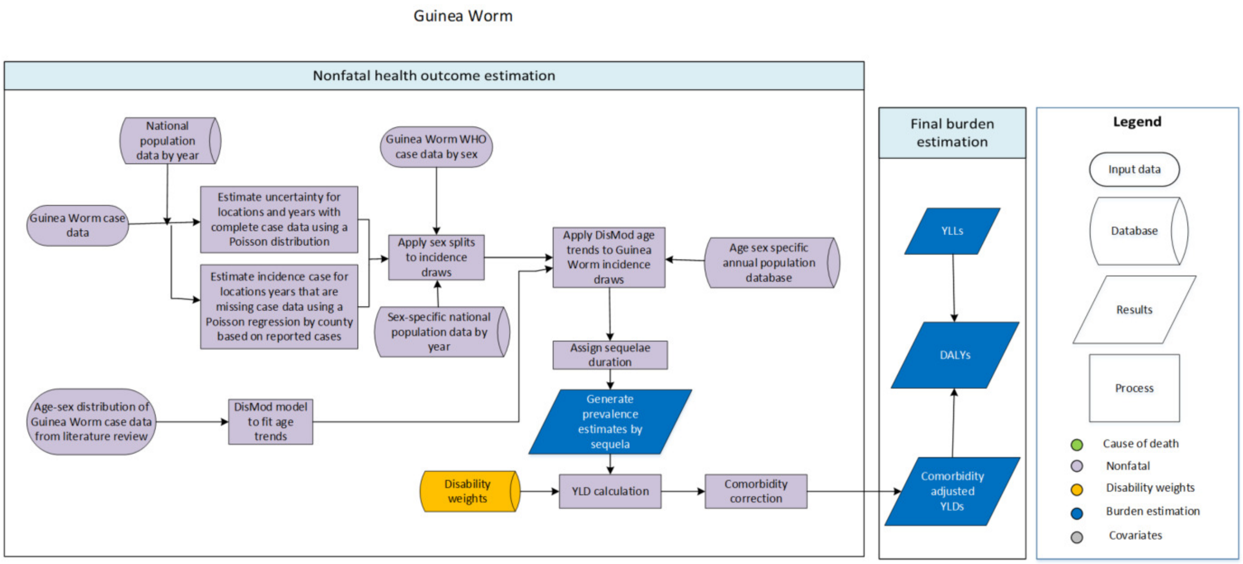

Background

Guinea-worm disease is caused by the parasitic worm *Dracunculus medinensis*. The transmission cycle begins when Guinea worm larvae are released in stagnant water (e.g., ponds, lakes, open wells) where they are ingested by freshwater copepods (small crustaceans sometimes called water fleas) of the genus *Cyclops* [1]. When a person consumes water containing *Cyclops*, the copepods are dissolved by gastric acids and intestinal enzymes and the larvae are released. Larvae then migrate through the intestinal wall and travel to the connective tissues. The larvae mature and mate 60–90 days after infection; shortly thereafter, the male dies and the pregnant female worm continues to move through the victim's connective tissues. Approximately 10–14 months post-infection, the adult worm creates a painful burning blister on the skin that develops and enlarges over several days, usually from the feet or lower limbs. Blister formation may be preceded by a slight fever, itchy rash, nausea, vomiting, and diarrhoea. To relieve the pain associated with the worm's emergence, infected persons immerse the infected part of their body in local stagnant water sources, such as ponds. Upon entering the water, the female worm will expel her larvae and the cycle can begin again [1-4].

The global campaign to eradicate Guinea worm began in 1980, when the US Centers for Disease Control and Prevention (CDC) suggested that Guinea worm eradication would be an ideal indicator of the success of the International Drinking Water Supply and Sanitation Decade of 1981–1990; in 1981, Guinea worm eradication was adopted as a sub-goal of this United Nations advocacy effort [1, 5]. In 1986, the World Health Assembly adopted a resolution to eliminate Guinea worm disease, and since then, the Carter Center has led a coalition that includes ministries of health of endemic countries, CDC, the World Health Organization (WHO), the United Nations Children's Fund (UNICEF), thousands of village volunteers, and supervisory staff supported by numerous donors [5].

To break the cycle of transmission, ministries of health in endemic countries implement a suite of interventions: case detection and containment, provision of safe water sources, distribution of filter cloths and pipe filters, water source treatment with Abate® (a larvacide), and health education.

By design, the Guinea worm eradication programmatic infrastructure covers the entire at-risk population in endemic countries. Since case containment[6] is a key intervention designed to not only interrupt transmission but also monitor progress toward eradication, incident cases of Guinea worm disease are nationally representative. To implement case containment as an intervention, all cases of Guinea worm disease are identified. Containment is defined as detection within 24 hours of the worm's emergence; the patient did not contaminate any water source; the patient received proper wound care and health education on not entering any water source; a supervisor verified the case as dracunculiasis within seven days; and Abate® is used if there is any uncertainty about contamination of water sources or known contamination of water sources [7]. Case reporting occurs at the village level on a monthly basis; case data are then aggregated within the national Guinea Worm Eradication Program and reported to WHO. In settings where annual case reports are low (suggesting no transmission) or transmission has been interrupted, cash rewards are promoted to enhance surveillance activities.

Input Data & Methodological Summary

Case Definition

A Guinea worm case is defined as an individual with Guinea worm disease. A person is counted as a case only once in a calendar year, ie, when the first Guinea worm emerged from that person, although an individual may have more than one worm emerge at a time and/or more than one worm emerge during the year. These cases are confirmed through the Guinea worm eradication program infrastructure by clinical exam and verification by local supervisors. All specimens from case-patients are sent to the CDC for laboratory evaluation and confirmation [7].

Input data

Model inputs

Geographic restrictions

Only the following countries were identified as guinea-worm endemic as of 1990[8]: Benin, Burkina Faso, Cameroon, Central African Republic, Chad, Cote d'Ivoire, Ethiopia, Ghana, India, Kenya, Mali, Mauritania, Niger, Nigeria, Pakistan, Senegal, Sudan, South Sudan, Togo, Uganda, and Yemen[8]. Any country not reporting Guinea worm as of 1990 is not included in the GBD model.

Geographic restrictions by year were also implemented to account for the period post-transmission to reflect the accomplishments of the Guinea worm eradication campaign. Geographic restriction for countries that were endemic in 1990 was defined based on data reported post-interruption of transmission. In the GBD analysis, Guinea worm disease was no longer modelled for the year that followed the last reported case (imported or indigenous) provided that the subsequent years through 2017 also had no case reports. To ensure that cases were attributed to burden in the country in which the case was detected, both indigenous and imported cases were included. For example, Kenya reported its last (imported) case in 2005, and as no other cases were reported through 2017, incidence from 2006

onward is zero. For Chad, a country that had years during which no cases were reported, the model covers the entire period 1990–2017.

Data sources

1) Case data by geography, by year
2) Literature review of age/sex distribution
3) Literature review for sequelae (type, duration, and proportion)

Case data: Annual case data were reported by WHO in the Weekly Epidemiological Record for the period 1990–2017. For years or geographies for which WER reports were not published, the following sources were also used to extract case counts:

1) CDC's MMWR reports
2) 1990–1999 total country reports from Hopkins *et al*[8]
3) India subnational estimates: India MOH report (1984–1999)
4) The Carter Center's Guinea worm wrap-up: disaggregation of case totals for Sudan and South Sudan pre-2011 (independence) to ensure case totals from 1990–2010 are consistent with current national boundaries; 2016 provisional case data.

The number of cases annually was compared to official total numbers published in WER 2016 to ensure accuracy of data entry.

Table of incident case data counts

	**Incidence**
**Site-years (total)**	816
**Number of countries with data**	21
**Number of GBD regions with data (out of 21 regions)**	5
**Number of GBD super-regions with data (out of 7 super-regions)**	3

Subnational data

India: Subnational data for India were obtained from the Ministry of Health for the period 1984–1999; cases were reported by year and state: http://www.ncdc.gov.in/index2.asp?slid=329&sublinkid=216.

Kenya: Subnational data from Kenya were requested from the MOH but not obtained. To split cases by subnational unit, the Carter Center Guinea Worm Wrap-Up was reviewed to identify districts with endemic villages. A national survey conducted 1993/1994 found cases in Turkana and West Pokot counties, but case totals were not reported by county. Indigenous transmission was interrupted in 1995, with imported cases reported until 2005. WER reports from 1999 to 2006 document that all imported cases from 1998 to 2005 occurred in Turkana County. All cases in Kenya are currently analysed in GBD as occurring in Turkana County as we are unable to disaggregate the data.

Accounting for possible under-reporting

Once national eradication programs were initiated, national case searches were conducted to improve the accuracy of national case estimates. These searches were designed to enumerate prevalent Guinea worm disease cases and identify endemic villages to direct intervention and surveillance activities. For the majority of years included in the GBD analysis, the total number of Guinea worm cases reported is equivalent to a national census, as all cases are identified and reported. Nevertheless, not all endemic countries were able to initiate full national surveillance as of 1990.

The model does not account for the possibility that cases occurred in communities that were not included in routine surveillance or did not achieve 100% reporting coverage over time. However, any cases that may have been undetected would likely not have been a significant increase over annual totals given the comprehensive nature of Guinea worm disease surveillance activities. Nevertheless, there are years for which the annual case data is inconsistent with preceding/following annual case totals and could not be accounted for in our model. For example, Niger reported 500 cases in 1992, despite reporting 32,829 cases in 1991 and 25,346 cases in 1993. In those instances, the following data points were identified as outliers and excluded from analysis as follows:

Table 1. List of reported case data outliered in the analysis to account for possible under-reporting

Country	Year	Reported Cases
Central African Republic	1996	9
Central African Republic	1997	5
Ethiopia	1992	303
Kenya (Turkana County)	1990	6
Uganda	1990	4,704
Uganda*	1992	126,369
Benin	1991	4,006
Benin	1992	4,315
Chad	1992	156
Cote d'Ivoire	1990	1,360
Mali	1990	884
Mauritania	1992	1,557
Niger	1992	500
Senegal	1990	38
Togo	1990	3,042
Togo	1991	5,118
South Sudan*	1996	116,844
Sudan	1994	132

*For these two data points, we do not dispute that over 100,000 cases of Guinea worm likely occurred. However, given the amount of missing data in the early time series for these two countries, inclusion of these resulted in implausibly high case predictions (over 1 million cases in Uganda in 1990 and over 1.5 million for South Sudan from 1990 to 1995).

Age/sex distribution

Generally, the risk of Guinea worm infection varies according to sex- or age-specific differences in access to safe drinking water. A study in Ethiopia found women were more likely to experience Guinea worm disease than men; in India, men experienced greater risk of infection [1]. Exposure to unsafe water sources varies largely on mobility patterns and type of water sources: communities in which infected water is carried in for consumption are more likely to see more Guinea worm disease in children and older adults [9]. Once interventions to control the spread of Guinea worm infection are implemented, the age and sex distribution likely changes to reflect variation in coverage and uptake of eradication interventions, such as larvacide of water sources and case-containment rates; age/sex case data are currently not available.

Table of age-specific prevalence data inputs

	**Prevalence**
**Site-years (total)**	7
**Number of countries with data**	4
**Number of GBD regions with data (out of 21 regions)**	3
**Number of GBD super-regions with data (out of 7 super-regions)**	2

The evidence base available to describe risk of infection by age is as follows:

1) Studies from Nigeria:
    a. Adeyeba *et al* [10]: Guinea worm disease not common among children <1 year of age; increase in risk by age
    b. Kale *et al* [11]: More boys ages 5-9 years than girls were infected (11.9% v. 6.8%); Women ages 20-29 years had higher prevalence of infection than men (13.4% v. 4.7%); Overall, the prevalence in both men and women was highest in ages 10-14 years and 30 years or older.
    c. Greenwood *et al* [12]: The mean age of male cases was 25.8 years (95% CI: 23.9, 27.7) and 26.9 years for females (95% CI: 23.7, 30.1).
2) Other countries:
    a. Sudan [13]: No significant age trend among lower-endemicity villages; higher-endemicity villages (n=4) had higher prevalence in children and older adults. This study attributes the difference in age trends to community-level water source.
    b. Ghana [14]: The trend in age of first infection reported was similar for males and females, with more females experiencing first infection between 15 and 19 years and males between 20 and 24 years of age. The proportion of men with Guinea worm disease was much higher than among women 25-54 years of age. Adults >15 years of age were more likely to be infected than children.

The evidence base available to describe the risk of infection by gender is as follows:

1) Studies from Nigeria:
    a. Adeyeba *et al* [10]: No difference among males and females.
    b. Kale *et al* [11]: No overall gender difference comparing total males infected to total females infected, although gender differences for certain age groups (see notes above).
    c. Greenwood *et al* [12]: Two-thirds of cases reported among 47 villages from 1971 to 1974 were male.

WHO Weekly Epidemiological Record (WER) age reports: Age and sex data were reported by country for 2009 onward; these data capture the age distribution for Chad, Ethiopia, Ghana, Mali, and South Sudan. We excluded these data as the age/sex distribution is only described for children <15 years or adults, which does not permit fitting an age trend across multiple categories.

WER sex-specific data: Sex-specific differences in the burden of Guinea worm disease could reflect differing levels of access to eradication program interventions, in addition to risk factors associated with local transmission dynamics. Since the data reported from 2009 to 2015 are the only available nationally representative data, we used the overall sex difference to generate sex-specific incidence and prevalence, with females experiencing a slightly higher risk (53%) compared to males (47%):

Table 2. WHO Weekly Epidemiological Record total worm burden by gender, by year

Year	Female	Male	Total	% Fem	% Male
**2009**	1699	1490	3189	53%	47%
**2010**	976	821	1797	54%	46%
**2011**	524	534	1058	50%	50%
**2012**	273	269	542	50%	50%
**2013**	79	69	148	53%	47%
**2014**	63	63	126	50%	50%
**2015**	9	13	22	41%	59%
**Total**	3623	3259	6882	53%	47%

There is limited evidence to suggest that risk varies jointly by sex and age; however, evidence for this modification also suggests that such age- and sex-specific risks may vary by endemic community within a given geography (in some settings, women at higher risk, in others men, but not for all age strata). Without additional data sources in which cases are disaggregated by age and sex, this joint relationship is not modelled.

To model age-specific variation, we used data from seven studies with age-specific case data to generate an age-trend in a DisMod model. We further assumed no Guinea worm disease occurred in infants less than 1 year of age.

Severity splits/sequelae

Sequelae associated with Guinea worm relate to the wound at the site of the worm's emergence, which can include abscesses and chronic ulcerations. Joint and tissue damage can occur, as well as secondary

infection in connective tissues [15]. During the worm's emergence, which takes approximately one month to exit the body, the ulcer is painful and itchy [1]. The wound is subject to secondary infection and scarring. Possible long-term consequences of Guinea worm infection include arthritis or other permanent damage to connective tissues; however, data on this are limited. In the Greenwood study, 41.7% of all cases experienced infection at the site of emergence, and the annual proportion of cases with definite arthritis ranged from 1.6% to 7.3% of all cases.

While an individual experiences Guinea worm disease, they are generally unable to work and have limited mobility at the time prior and during emergence and in the subsequent period in which they are healing. Although most worms emerge in the feet and lower legs, there are reports of worms exiting at other sites [15], which could cause other disability not accounted for here. A study in Nigeria found that 98% of worms emerged in the lower limbs[16]. The Greenwood study also observed that 88.4% emerged in the lower limbs. Therefore, for the purposes of estimating the burden of Guinea worm disease in GBD, all disability associated with Guinea worm disease is attributed to lower limb conditions, pain, and lack of mobility. Due to limited data, we cannot account for differential disability based on number of worms emerging at the same time.

The following evidence base was reviewed to determine the proportion of cases attributed to each sequela, as well as duration of sequelae.

Duration of disability and type of disability:

Studies from Nigeria:

1) Adeyeba *et al* [10]: 93.4% incapacitated for an average of 26 days.
2) Smith *et al* [17]: Average disability duration 12.7 weeks; 58% unable to leave the home for a mean duration of 4.2 weeks; duration of disability greater among those older than 50 years compared to those younger than 50 years.
3) Okoye *et al* [16]: 21% of cases were totally incapacitated due to their infection (not permanently disabled).
4) Kate *et al* [11]: A survey of 17 villages from 1971 to 1975 found that duration of disability was approximately 100 days.
5) Greenwood *et al* [12]: Weekly visits to 47 villages from 1971 to 1974 reported mean duration of illness ranging from 4.2 weeks to 7.2 weeks. 17.4% of cases had an active infection which persisted for 10 weeks or more.

Other countries:

6) Benin [18]: From two villages in highly endemic areas, estimated 39-59 days of disability experienced after worm emergence.
7) Ghana [19]: 28.2% experienced pain 12-18 months post-emergence; 5% unable to carry out at least one daily activity, 0.5% permanently impaired (ligament damage to thumb).
8) Ghana [14]: Complete disability experienced among males with Guinea worm disease lasted approximately 5 weeks among those untreated. Among cases provided supportive care (wound management), the duration of disability was 2.5 weeks.

For all cases, we assume each experiences pain and disfigurement (level 2), and musculoskeletal problems, lower limb (moderate) for a period of one month, followed by two months of pain and

disfigurement (mild). We then assume that 30% of all cases will then experience disfigurement level 1 with itch/pain for an additional nine months (approximately a year of disability) to account for longer-term disability associated with recovery.

Table 3. Sequela associated with Guinea worm disease in the Global Burden of Disease study

Sequela	Lay description	DW (95% CI)
Disfigurement, level 2, with itch/pain	Has a visible physical deformity that is sore and itchy. Other people stare and comment, which causes the person to worry. The person has trouble sleeping and concentrating.	0.188 (0.125–0.267)
Disfigurement, level 1, with itch/pain	Has a slight, visible physical deformity that is sometimes sore or itchy. Others notice the deformity, which causes some worry and discomfort.	0.027 (0.015–0.042)
Musculoskeletal problems, lower limbs, moderate	Has moderate pain in the leg, which makes the person limp, and causes some difficulty walking, standing, lifting and carrying heavy things, getting up and down and sleeping.	0.079 (0.054–0.11)

Modelling strategy

Total incidence

The incidence of Guinea worm disease is modelled in GBD using two approaches: for years and locations for which case data were reported, 1,000 draws of incidence were estimated using a beta distribution of cases and total population minus cases. For years and locations for which case data were missing (largely the early 1990s) a Poisson regression of all case data was implemented per country, using the total population as the offset. The predicted incidence and standard error were used to generate a random distribution of 1,000 incidence draws. Incidence is multiplied by duration of sequelae to calculate prevalence. Country-level incidence predictions are shown in the following figures.

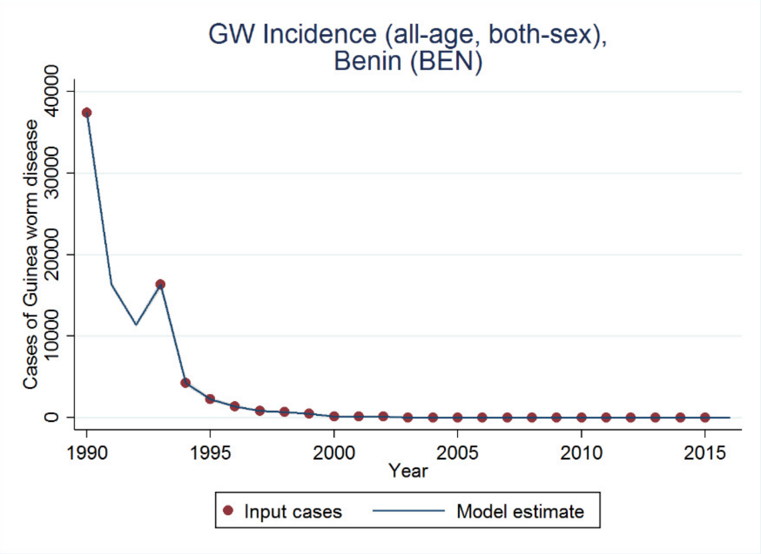
GW Incidence (all-age, both-sex),
Benin (BEN)
Cases of Guinea worm disease
0
10000
20000
30000
40000
1990
1995
2000
2005
2010
2015
Year
Input cases
Model estimate

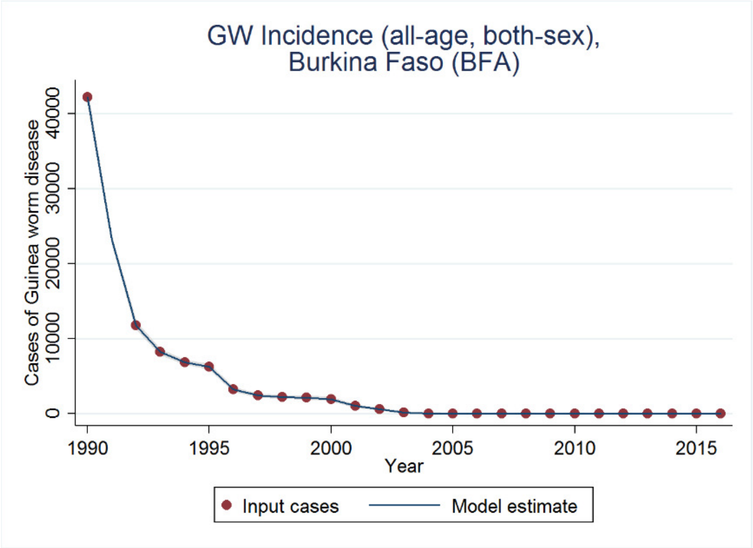
GW Incidence (all-age, both-sex),
Burkina Faso (BFA)
Cases of Guinea worm disease
0
10000
20000
30000
40000
1990
1995
2000
2005
2010
2015
Year
Input cases
Model estimate

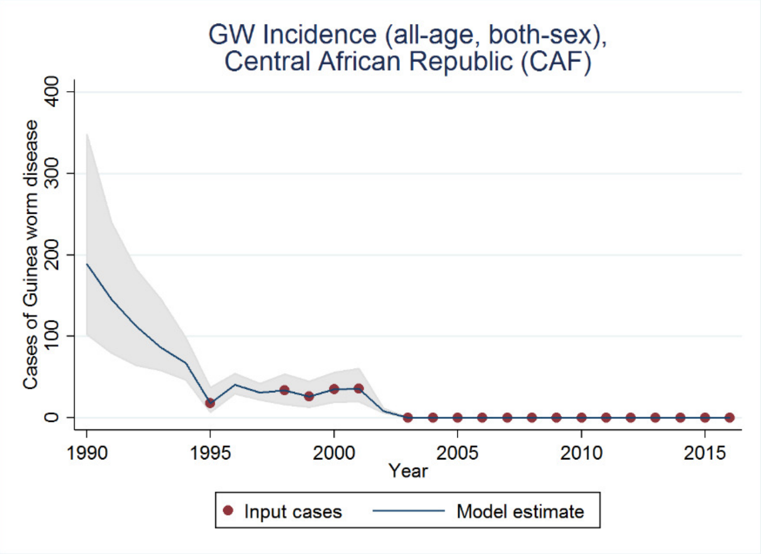
GW Incidence (all-age, both-sex),
Central African Republic (CAF)
Cases of Guinea worm disease
0
100
200
300
400
1990
1995
2000
2005
2010
2015
Year
Input cases
Model estimate

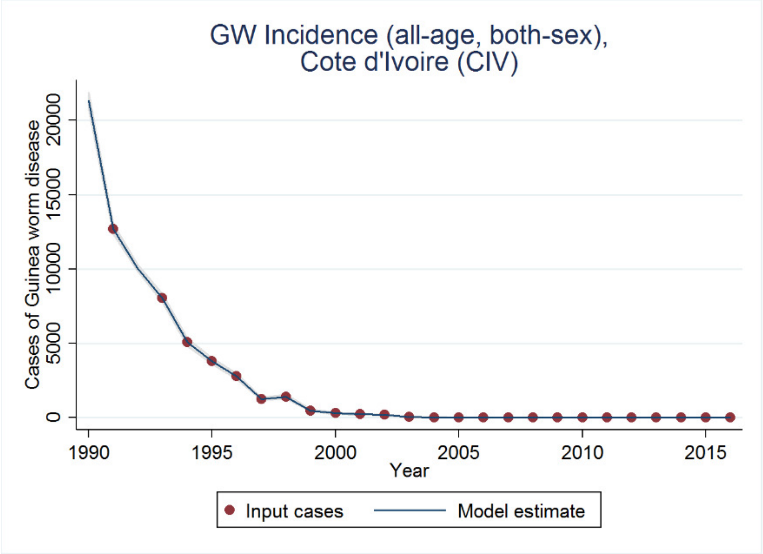
GW Incidence (all-age, both-sex),
Cote d'Ivoire (CIV)
Cases of Guinea worm disease
0
5000
10000
15000
20000
1990
1995
2000
2005
2010
2015
Year
Input cases
Model estimate

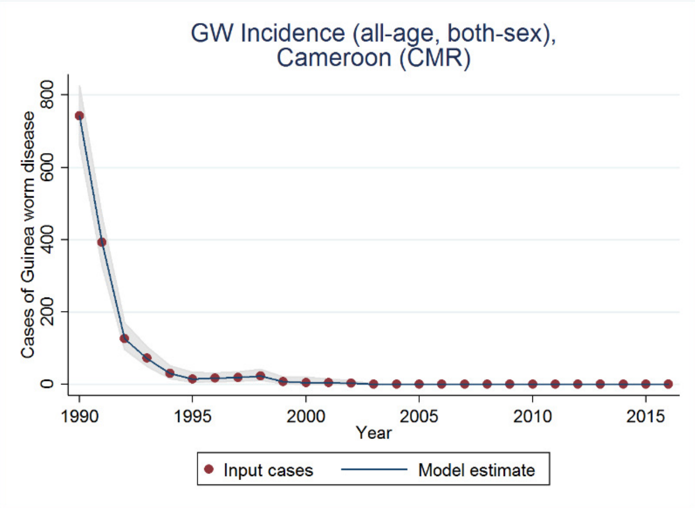

GW Incidence (all-age, both-sex),
Cameroon (CMR)
Cases of Guinea worm disease
0
200
400
600
800
1990
1995
2000
2005
2010
2015
Year
Input cases
Model estimate

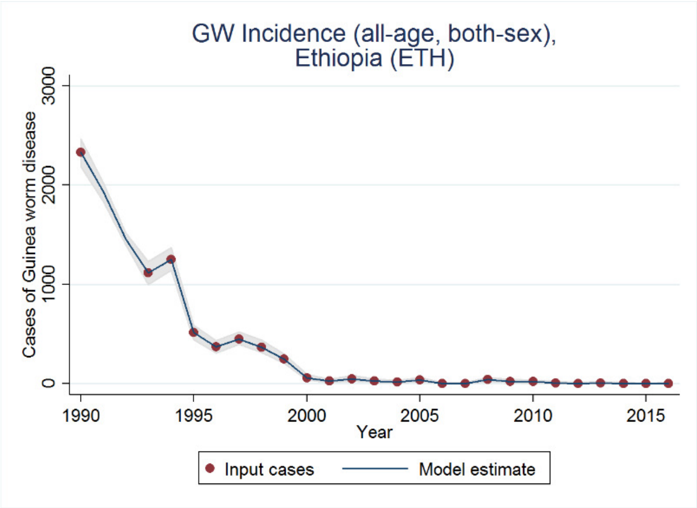

GW Incidence (all-age, both-sex),
Ethiopia (ETH)
Cases of Guinea worm disease
0
1000
2000
3000
1990
1995
2000
2005
2010
2015
Year
Input cases
Model estimate

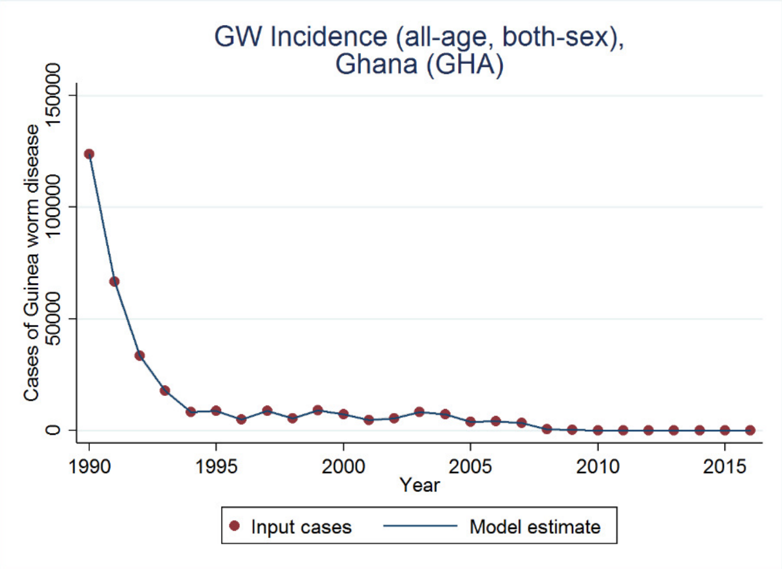

GW Incidence (all-age, both-sex),
Ghana (GHA)
Cases of Guinea worm disease
0
50000
100000
150000
1990
1995
2000
2005
2010
2015
Year
Input cases
Model estimate

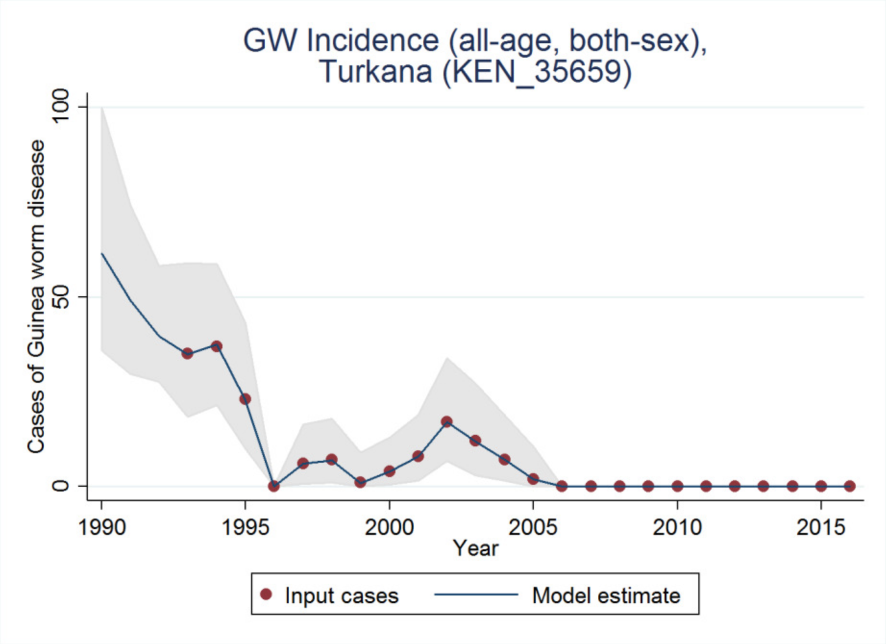

GW Incidence (all-age, both-sex),
Turkana (KEN_35659)
Cases of Guinea worm disease
0
50
100
1990
1995
2000
2005
2010
2015
Year
Input cases
Model estimate

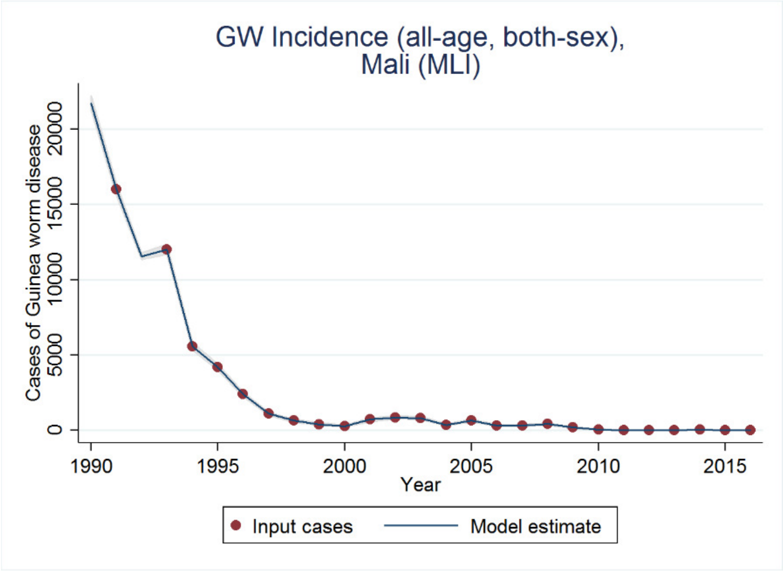

GW Incidence (all-age, both-sex), Mali (MLI)
Cases of Guinea worm disease
0
5000
10000
15000
20000
1990
1995
2000
2005
2010
2015
Year
Input cases
Model estimate

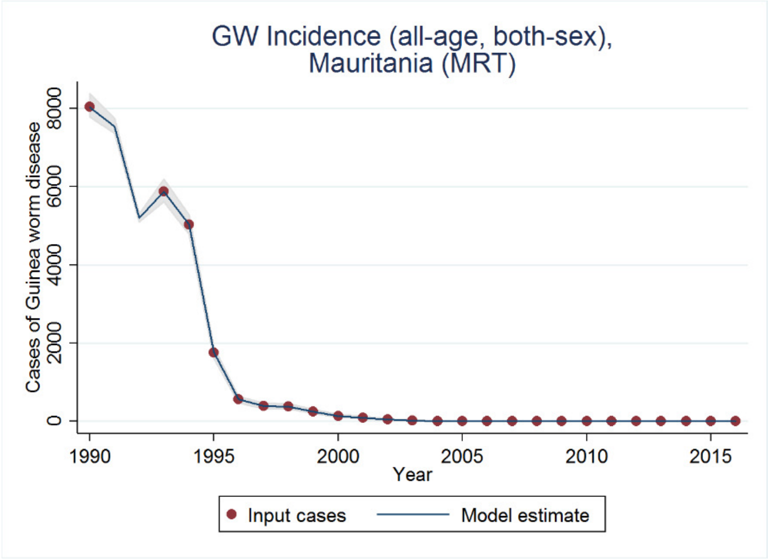

GW Incidence (all-age, both-sex), Mauritania (MRT)
Cases of Guinea worm disease
0
2000
4000
6000
8000
1990
1995
2000
2005
2010
2015
Year
Input cases
Model estimate

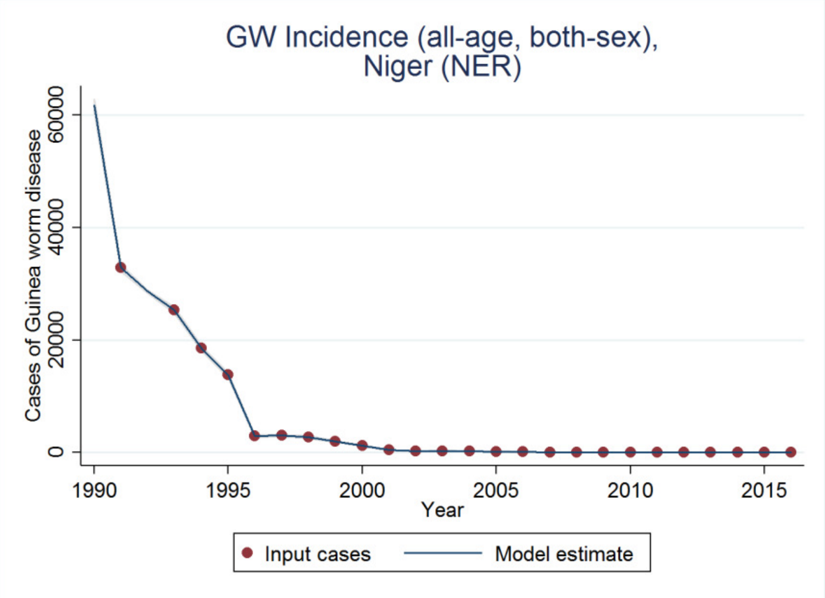
GW Incidence (all-age, both-sex),
Niger (NER)
Cases of Guinea worm disease
0
20000
40000
60000
1990
1995
2000
2005
2010
2015
Year
Input cases
Model estimate

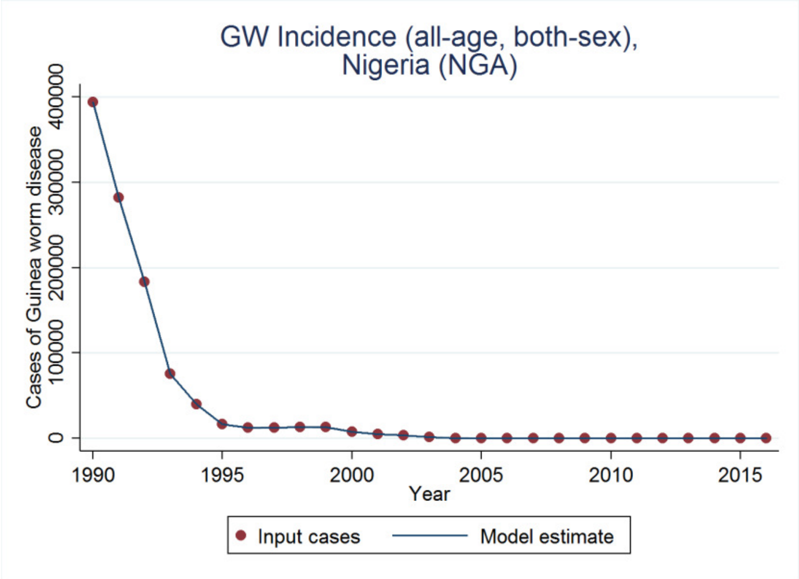
GW Incidence (all-age, both-sex),
Nigeria (NGA)
Cases of Guinea worm disease
0
100000
200000
300000
400000
1990
1995
2000
2005
2010
2015
Year
Input cases
Model estimate

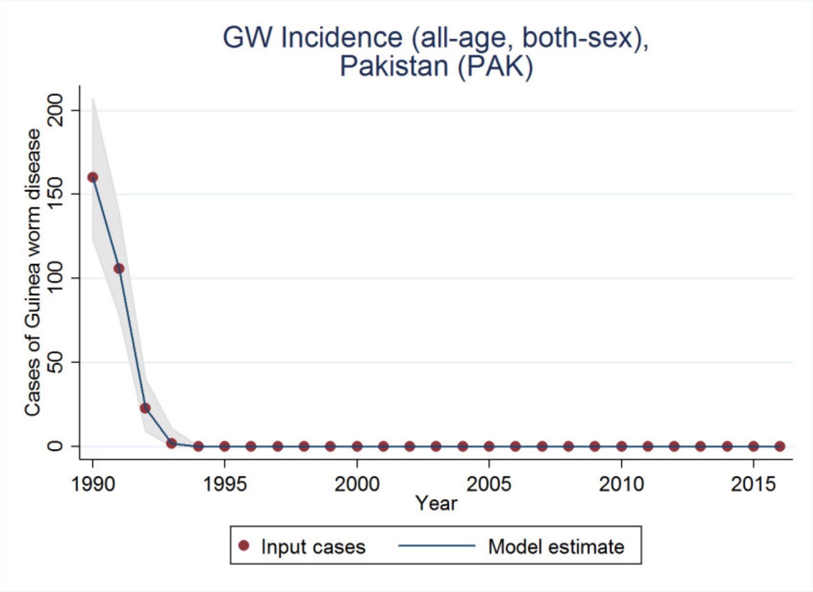
GW Incidence (all-age, both-sex),
Pakistan (PAK)
Cases of Guinea worm disease
0
50
100
150
200
1990
1995
2000
2005
2010
2015
Year
Input cases
Model estimate

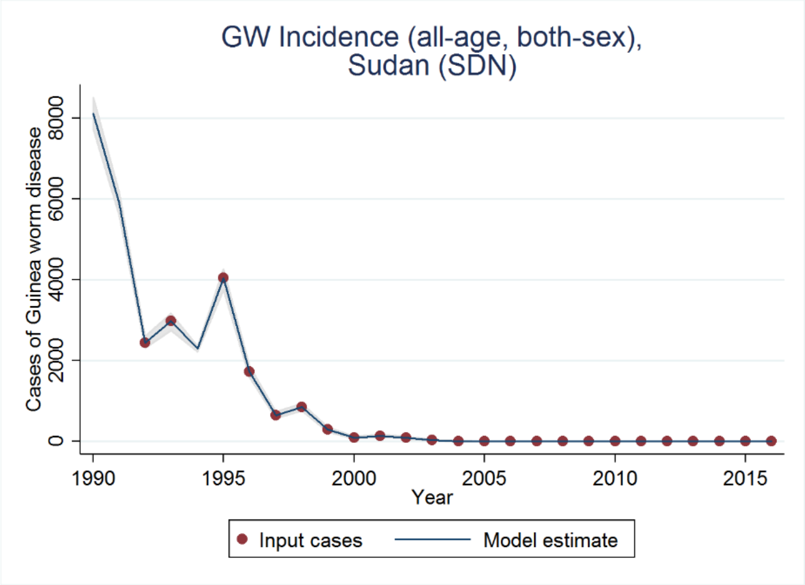
GW Incidence (all-age, both-sex),
Sudan (SDN)
Cases of Guinea worm disease
0
2000
4000
6000
8000
1990
1995
2000
2005
2010
2015
Year
Input cases
Model estimate

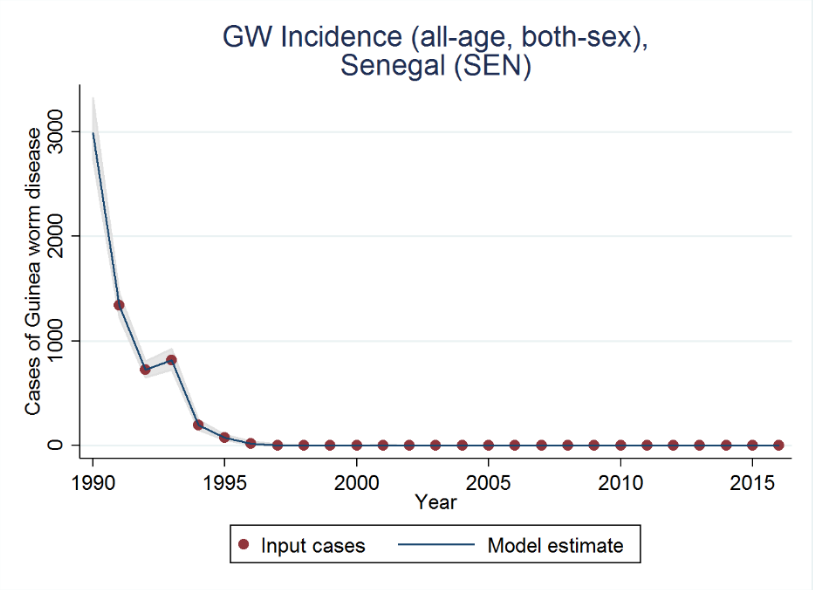
GW Incidence (all-age, both-sex),
Senegal (SEN)
Cases of Guinea worm disease
0
1000
2000
3000
1990
1995
2000
2005
2010
2015
Year
Input cases
Model estimate

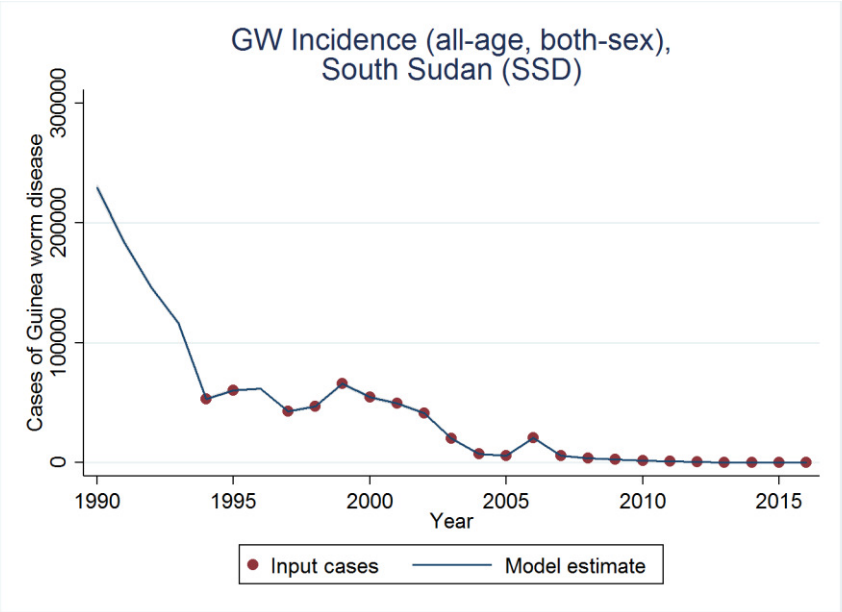
GW Incidence (all-age, both-sex),
South Sudan (SSD)
Cases of Guinea worm disease
0
100000
200000
300000
1990
1995
2000
2005
2010
2015
Year
Input cases
Model estimate

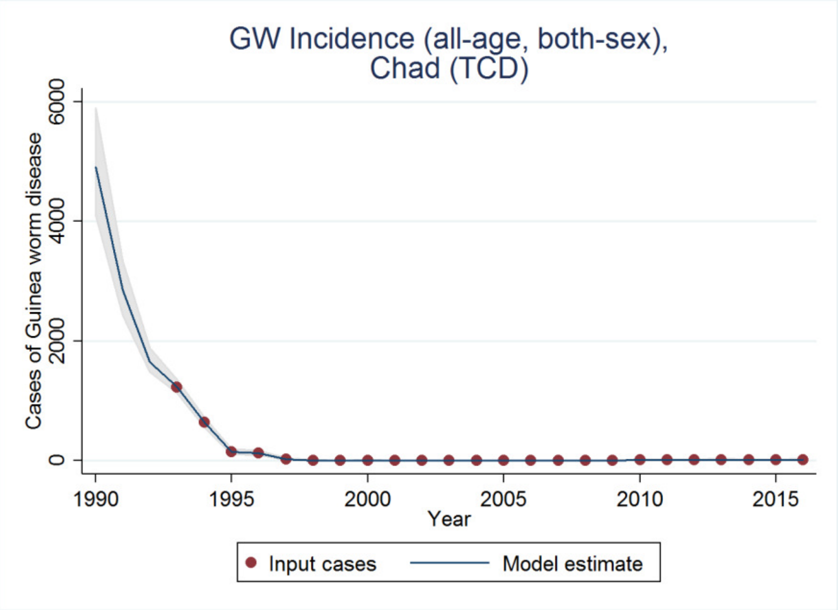
GW Incidence (all-age, both-sex),
Chad (TCD)
Cases of Guinea worm disease
0
2000
4000
6000
1990
1995
2000
2005
2010
2015
Year
Input cases
Model estimate

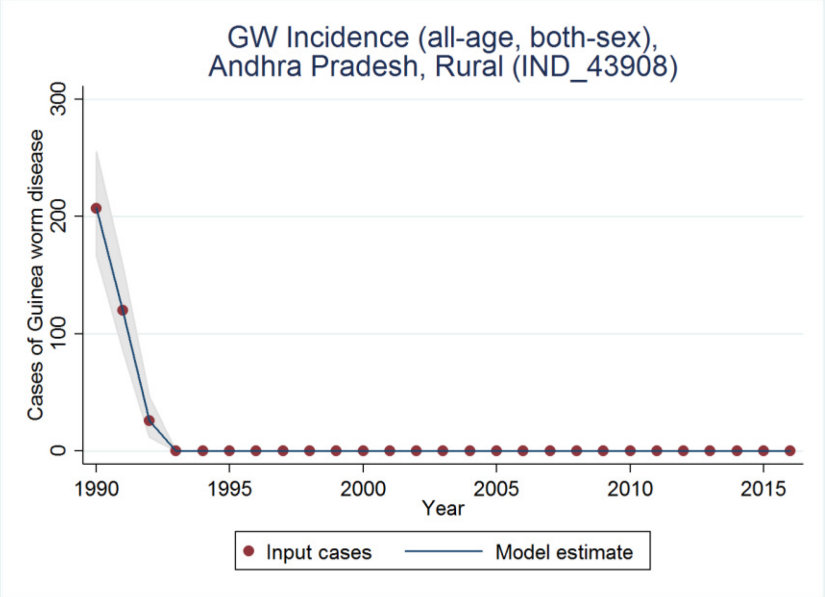
GW Incidence (all-age, both-sex),
Andhra Pradesh, Rural (IND_43908)
Cases of Guinea worm disease
0
100
200
300
1990
1995
2000
2005
2010
2015
Year
Input cases
Model estimate

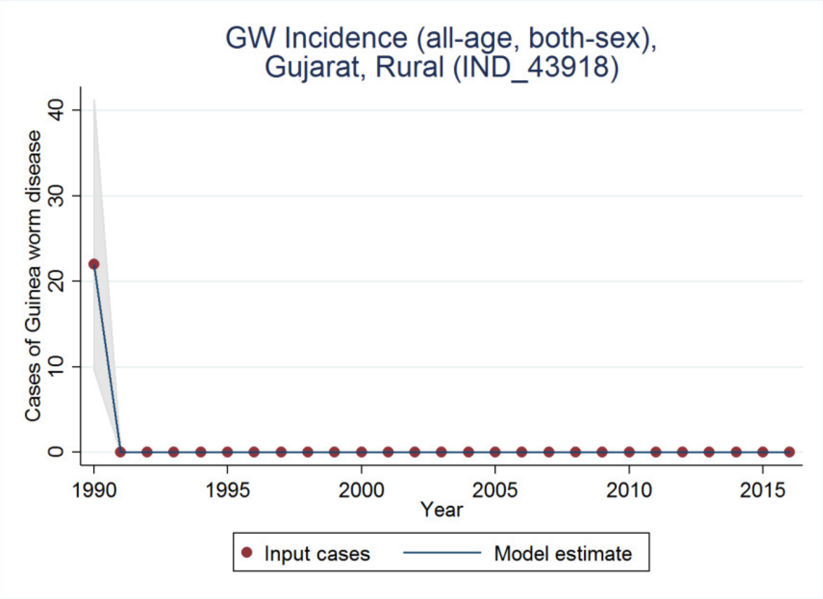
GW Incidence (all-age, both-sex),
Gujarat, Rural (IND_43918)
Cases of Guinea worm disease
0
10
20
30
40
1990
1995
2000
2005
2010
2015
Year
Input cases
Model estimate

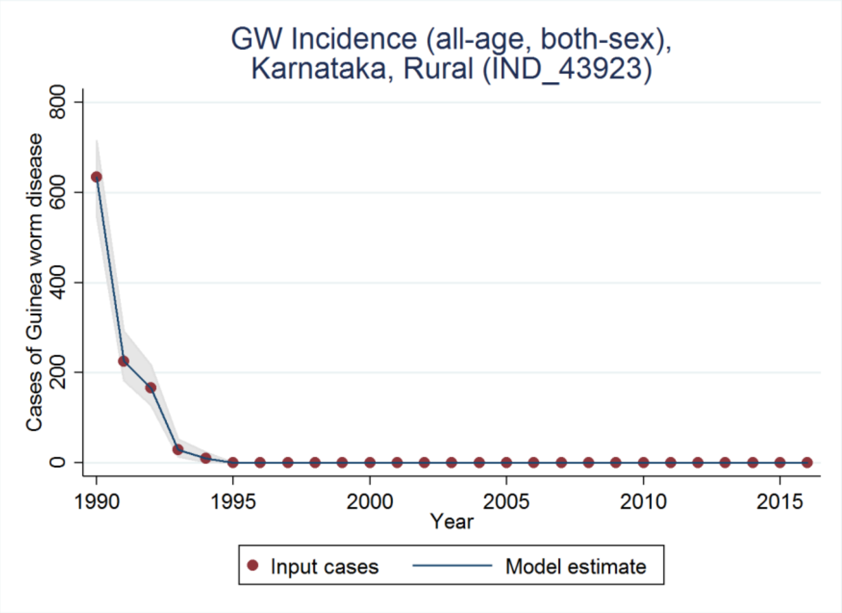
GW Incidence (all-age, both-sex),
Karnataka, Rural (IND_43923)
Cases of Guinea worm disease
0
200
400
600
800
1990
1995
2000
2005
2010
2015
Year
Input cases
Model estimate

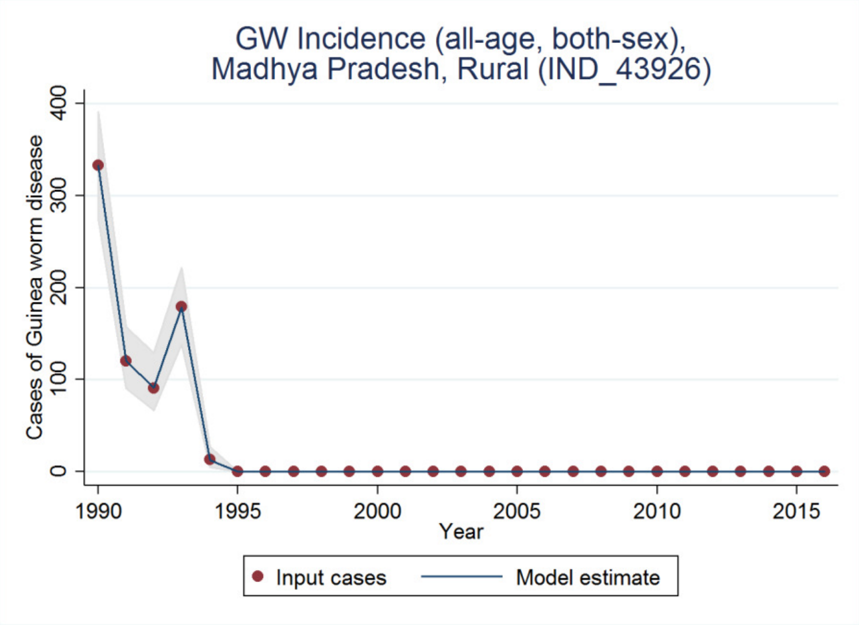
GW Incidence (all-age, both-sex),
Madhya Pradesh, Rural (IND_43926)
Cases of Guinea worm disease
0
100
200
300
400
1990
1995
2000
2005
2010
2015
Year
Input cases
Model estimate

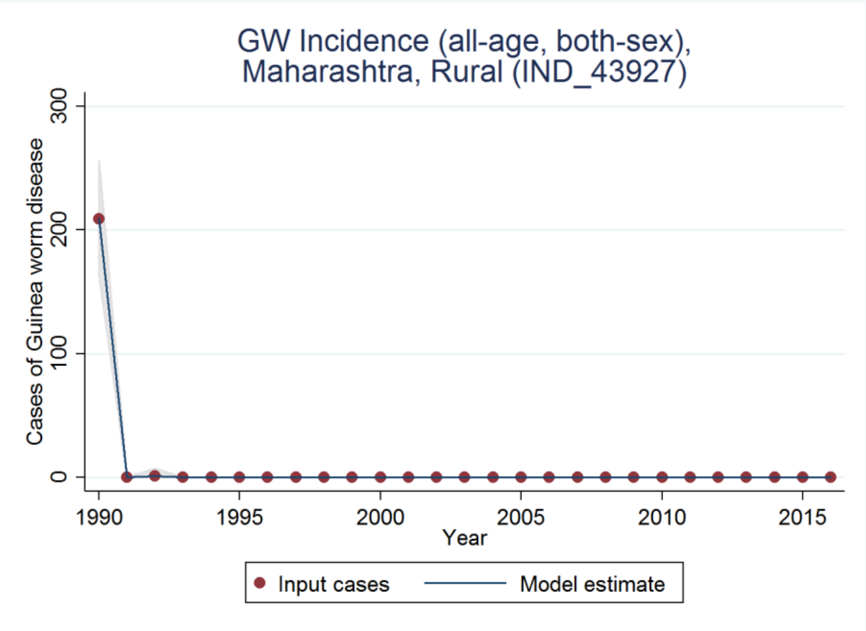
GW Incidence (all-age, both-sex),
Maharashtra, Rural (IND_43927)
Cases of Guinea worm disease
0
100
200
300
1990
1995
2000
2005
2010
2015
Year
Input cases
Model estimate

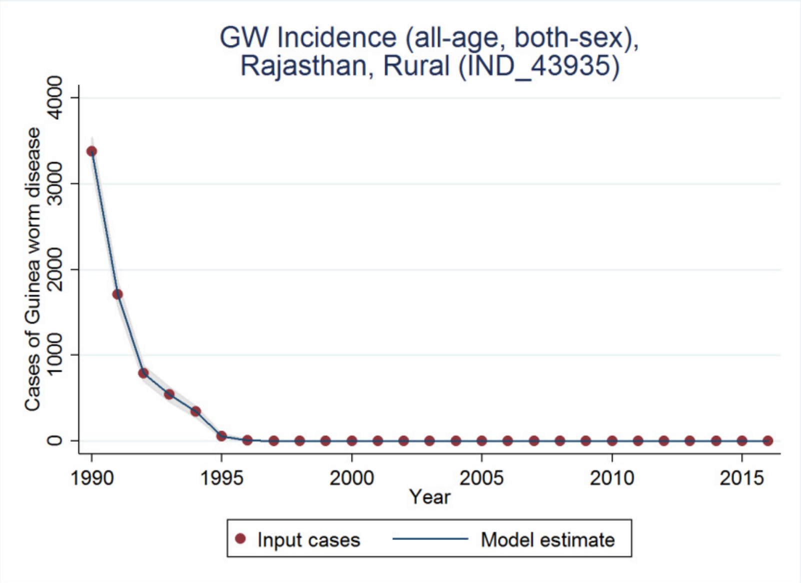
GW Incidence (all-age, both-sex),
Rajasthan, Rural (IND_43935)
Cases of Guinea worm disease
0
1000
2000
3000
4000
1990
1995
2000
2005
2010
2015
Year
Input cases
Model estimate

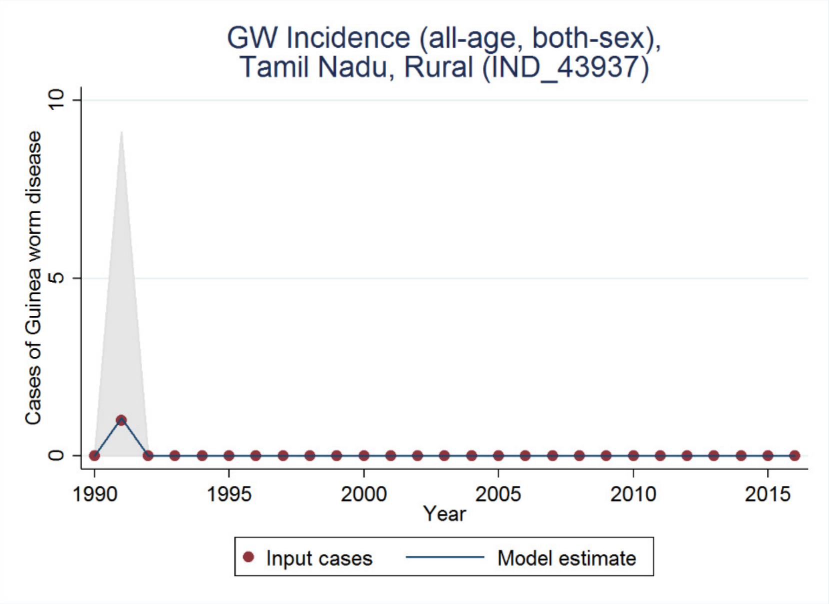
GW Incidence (all-age, both-sex),
Tamil Nadu, Rural (IND_43937)
Cases of Guinea worm disease
0
5
10
1990
1995
2000
2005
2010
2015
Year
Input cases
Model estimate

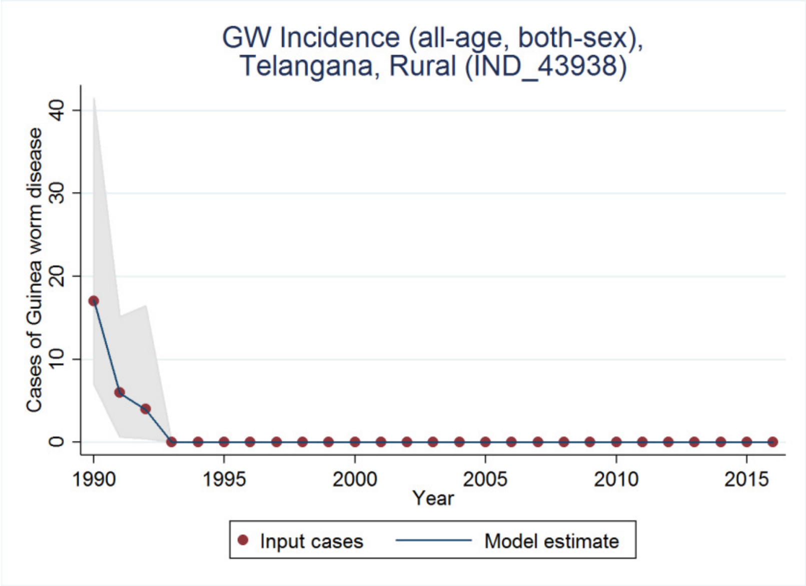
GW Incidence (all-age, both-sex),
Telangana, Rural (IND_43938)
Cases of Guinea worm disease
0
10
20
30
40
1990
1995
2000
2005
2010
2015
Year
Input cases
Model estimate

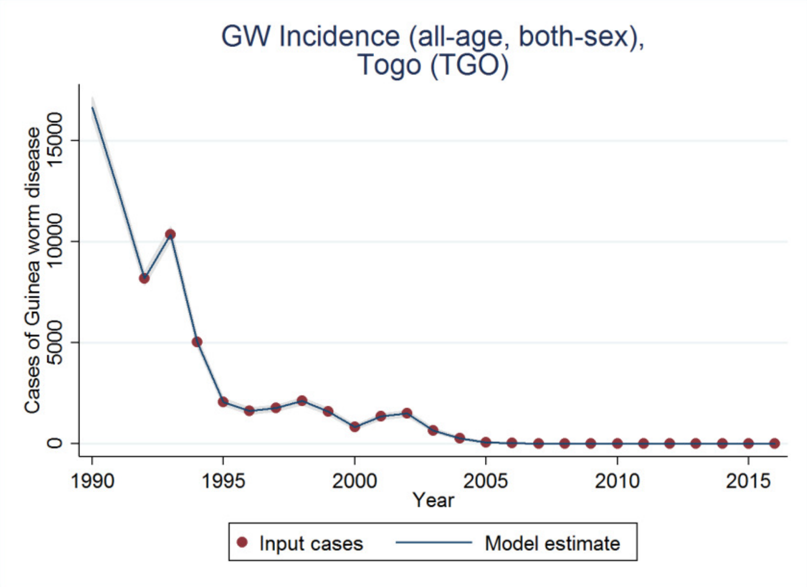
GW Incidence (all-age, both-sex),
Togo (TGO)
Cases of Guinea worm disease
0
5000
10000
15000
1990
1995
2000
2005
2010
2015
Year
Input cases
Model estimate

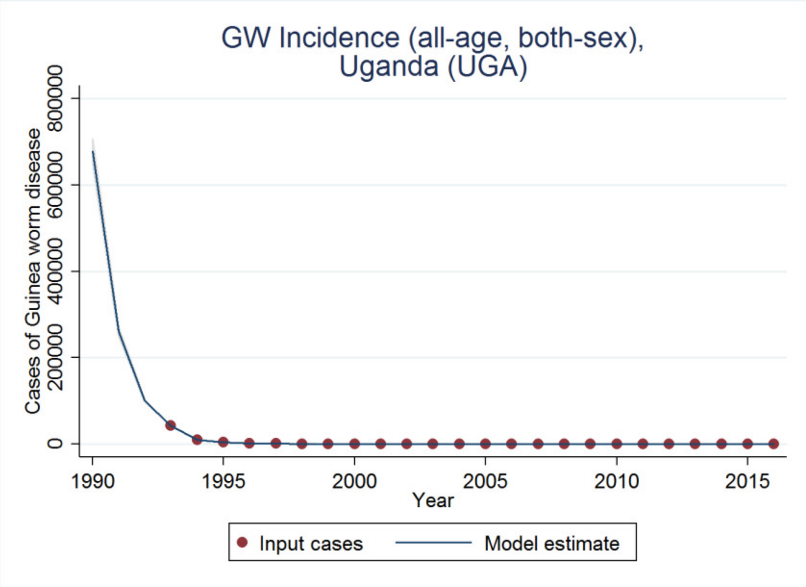
GW Incidence (all-age, both-sex),
Uganda (UGA)
Cases of Guinea worm disease
0
200000
400000
600000
800000
1990
1995
2000
2005
2010
2015
Year
Input cases
Model estimate

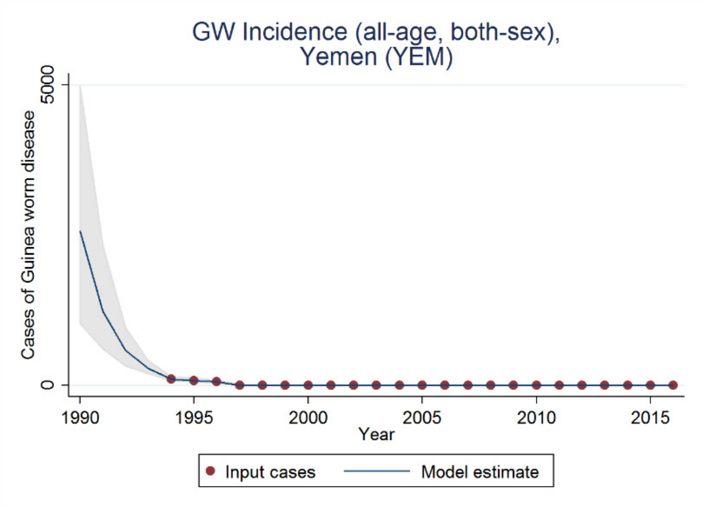
GW Incidence (all-age, both-sex),
Yemen (YEM)
Cases of Guinea worm disease
0
5000
1990
1995
2000
2005
2010
2015
Year
Input cases
Model estimate

Figure 1. Overall comparison of model versus reported cases (excluding outliers)

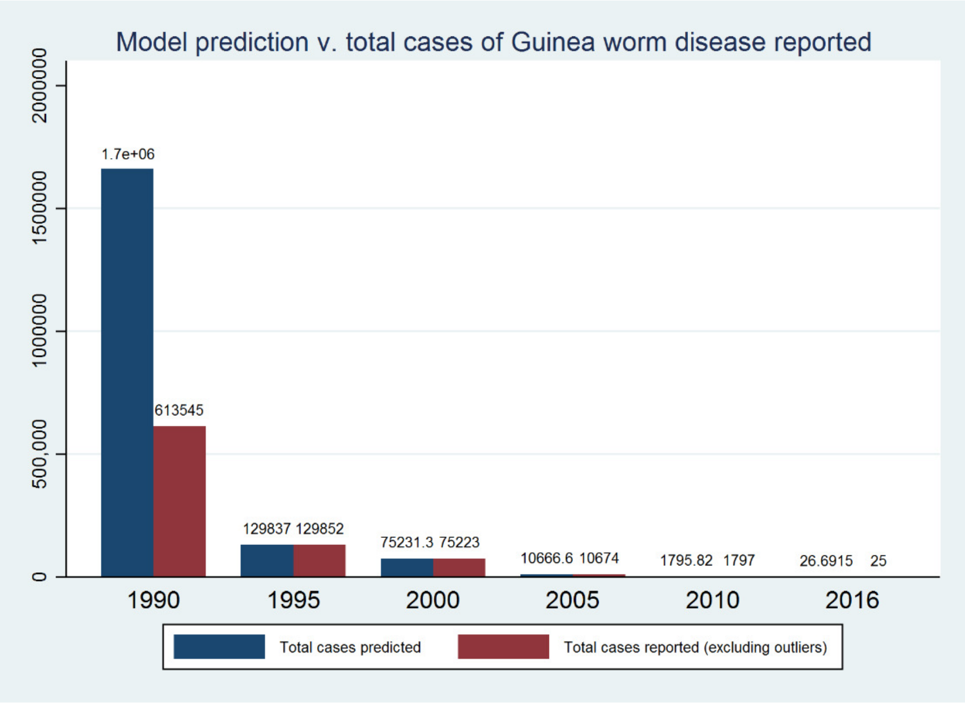

Sex-specific incidence

To account for the proportion of cases in females compared to males (53% to 47%), the incidence draws were multiplied by the sex proportion and the total population (to estimate number of cases by sex), then divided by the sex-specific total population for that year to calculate sex-specific incidence.

Age-specific incidence

In order to generate age-specific incidence, a literature search was conducted to identify national and subnational data sources in which age-specific prevalence was reported. The only nationally representative data available were WER reports from 2009 onward; however, age was only reported as less than 15 years of age or older than 15 years of age. In order to generate a trend over the life course, eight subnational data sources were identified. The prevalence of Guinea worm disease was extracted by age category reported in the original paper. An age trend was then fit using DisMod 2.0, with the following model settings:

Age mesh points: 0 0.01 5 10 15 20 25 30 35 40 45 50 55 60 65 70 75 80 85 90 1000

Drill year: 2000; Drill location: Global; no birth prevalence; 30 year time window

The age data were used to generate one single-age trend that we assumed applied to all geographies and all estimation periods from 1990 to 2017.

Figure 2. Age-specific prevalence model generated by DisMod

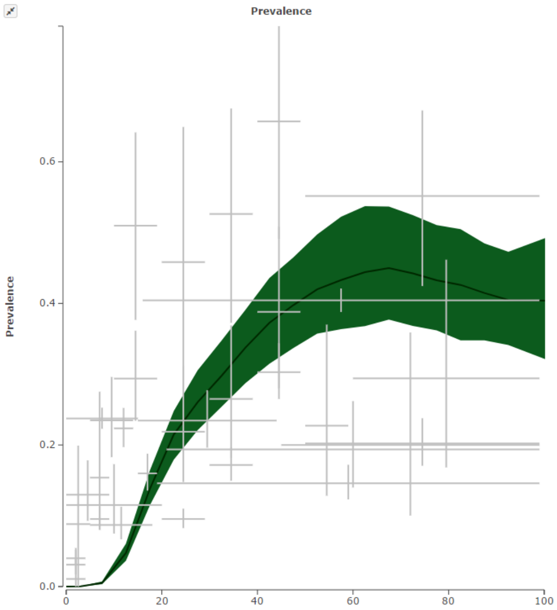

To apply this age prevalence curve to the sex-split incidence draws, 1,000 draws of output were downloaded from DisMod and applied to the incidence data as follows:

j indexes the age strata

i indexes the draw (1 to 1,000)

sex cases draw is the total number of cases for the sex stratum (all ages)

$$age\ cases_j = DisMod\ Draw_{i,j} * age\ population_j$$

$$age\ incidence\ draw_i = \frac{age\ cases_j \left(\frac{sex\ cases\ draw_i}{total\ cases}\right)}{age\ population_j}$$

Under the assumption that Guinea worm disease occurs approximately one year post-infection, incidence among children aged less than 1 year was set to zero.

Sequelae splits

Prevalence of the sequelae listed in Table 3 was calculated by multiplying the age- and sex-specific incidence draw by the duration of the health state (in years).

1) Guinea worm pain associated with worm emergence (Level 2): all cases, 1 month
2) Guinea worm pain associated with worm emergence (Level 1): all cases, 2 months plus 30% of cases for an additional 9 months
3) Lower limb musculoskeletal problems: all cases, 1 month

References

1. Cairncross S, Muller R, Zagaria N. Dracunculiasis (Guinea worm disease) and the eradication initiative. Clin Microbiol Rev. 2002;15(2):223-46.
2. Biswas G, Sankara DP, Agua-Agum J, Maiga A. Dracunculiasis (guinea worm disease): eradication without a drug or a vaccine. Philos Trans R Soc Lond B Biol Sci. 2013;368(1623):20120146.
3. Ruiz-Tiben E, Hopkins DR. Dracunculiasis (Guinea worm disease) eradication. Adv Parasitol. 2006;61:275-309.
4. Greenaway C. Dracunculiasis (guinea worm disease). CMAJ. 2004;170(4):495-500.
5. Hopkins DR, Ruiz-Tiben E, Downs P, Withers PC, Jr., Roy S. Dracunculiasis eradication: neglected no longer. Am J Trop Med Hyg. 2008;79(4):474-9.
6. Kappus KD, Hopkins DR, Ruiz-Tiben E, Imtiaz R, Andersen J, Azam M, et al. A strategy to speed the eradication of dracunculiasis. World Health Forum. 1991;12(2):220-5.
7. Prevention CfDCa. Guinea worm wrap-up Atlanta, GA: WHO Collborating center for Research, Training and Eradication of Dracunculiasis, CDC; 2015.
8. Hopkins DR, Ruiz-Tiben E, Diallo N, Withers PC, Jr., Maguire JH. Dracunculiasis eradication: and now, Sudan. Am J Trop Med Hyg. 2002;67(4):415-22.
9. Watts SJ, Brieger WR, Yacoob M. Guinea worm: an in-depth study of what happens to mothers, families and communities. Soc Sci Med. 1989;29(9):1043-9.
10. Adeyeba OA, Kale OO. Epidemiology of dracunculiasis and its socio-economic impact in a village in south-west Nigeria. West Afr J Med. 1991;10(3-4):208-15.
11. Kale OO. The clinico-epidemiological profile of guinea worm in the Ibadan district of Nigeria. Am J Trop Med Hyg. 1977;26(2):208-14.
12. Greenwood B, Greenwood A, Bradley A. Guinea worm infection in northern Nigeria: reflections on a disease approaching eradication. Trop Med Int Health. 2017.
13. Tayeh A, Cairncross S. The impact of dracunculiasis on the nutritional status of children in South Kordofan, Sudan. Ann Trop Paediatr. 1996;16(3):221-6.
14. Belcher DW, Wurapa FK, Ward WB, Lourie IM. Guinea worm in southern Ghana: its epidemiology and impact on agricultural productivity. Am J Trop Med Hyg. 1975;24(2):243-9.
15. Muller R. Guinea worm disease: epidemiology, control, and treatment. Bull World Health Organ. 1979;57(5):683-9.
16. Okoye SN, Onwuliri CO, Anosike JC. A survey of predilection sites and degree of disability associated with guineaworm (Dracunculus medinensis). Int J Parasitol. 1995;25(9):1127-9.
17. Smith GS, Blum D, Huttly SR, Okeke N, Kirkwood BR, Feachem RG. Disability from dracunculiasis: effect on mobility. Ann Trop Med Parasitol. 1989;83(2):151-8.
18. Chippaux JP, Banzou A, Agbede K. [Social and economic impact of dracunculosis: a longitudinal study carried out in 2 villages in Benin]. Bull World Health Organ. 1992;70(1):73-8.
19. Hours M, Cairncross S. Long-term disability due to guinea worm disease. Trans R Soc Trop Med Hyg. 1994;88(5):559-60.

Other neglected tropical diseases

In addition to the neglected tropical diseases described above, there are many diverse types of neglected tropical diseases, which are encompassed by the following ICD 10 codes: A68-A68.9, A69.2-A69.5, A75-A75.9, A77-A79.9, A92-A94.0, A96-A96.9, A98-A98.8, B58, B59-B60.8, B68-B68.9, B70-B72.0, B74.3-B75, B78-B78.9, B80-B81.8, B83-B83.8, P37.1.

Because these neglected tropical diseases are diverse in their underlying causes and risk factors as well as in their associated health outcomes, modelling them together in a DisMod-MR model would not produce reliable estimates of prevalence or excess mortality. Instead, we calculated the YLDs caused by neglected tropical diseases directly using a YLD/YLL ratio.

We calculated the ratio of YLDs to YLLs across the specified neglected tropical diseases for which non-fatal outcomes were modelled, using YLL estimates from the GBD 2017 cause of death (CoD) analysis. We then multiplied this YLD/YLL ratio by the YLL estimates for other neglected tropical diseases from the GBD 2017 CoD analysis, providing us with an estimate of the YLDs associated with other neglected tropical diseases.

Meningitis

Flowchart

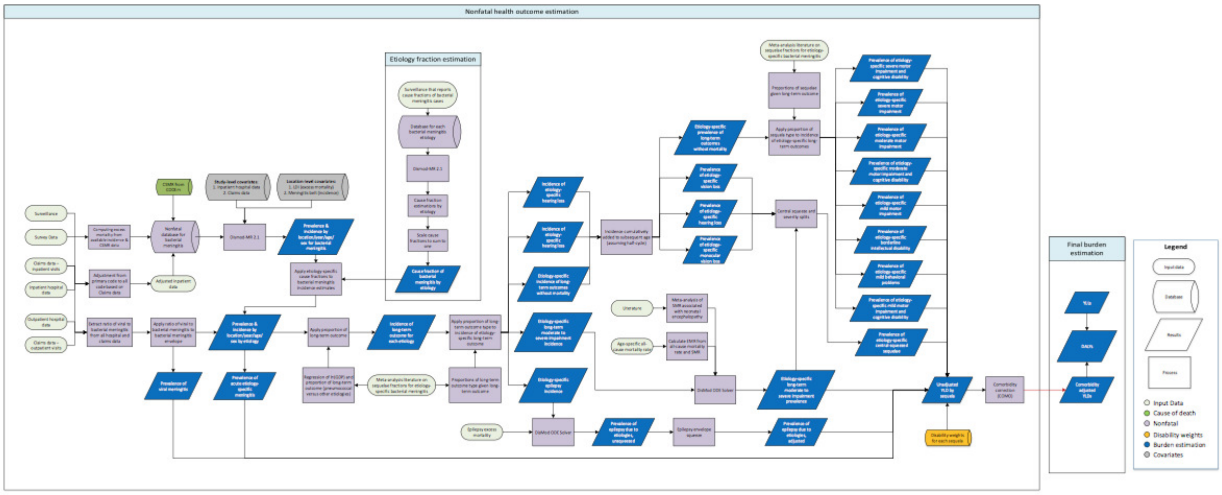

Case definition

Meningitis is a disease caused by inflammation of the meninges, the protective membrane surrounding the brain and spinal cord, and is typically caused by an infection in the cerebrospinal fluid. Symptoms include headache, fever, stiff neck, and sometimes seizures. Included in the GBD modelling were cases meeting ICD-10 diagnostic criteria for meningitis due to bacteria, viruses, or other causes (A39-A39.9, A87-A87.9, D86.81, G00.0-G00.8, G03-G03.8, Z20.811, and Z22.31). In GBD 2017, meningitis encompasses viral meningitis and four bacterial aetiologies: pneumococcal, *Haemophilus influenzae* type B (HiB), meningococcal, and other.

Input data

Model inputs

In the GBD 2010 study, a systematic review of literature was conducted to capture studies of incidence and excess mortality rate for all bacterial meningitis cases. For each of the four aetiologies, literature included excess mortality rate, incidence, proportion, remission, and standardised mortality ratio. The inclusion criteria stipulated that: (1) the publication year must be between 1980 and 2010; (2) "caseness" was based on diagnoses by antigen test, blood test, cerebrospinal fluid test, polymerase chain reaction test, or latex agglutination test; (3) sufficient information must be provided on study method and sample characteristics to assess the quality of the study; and (4) study samples must be representative of the general population. No limitation was set on the language of publication. For GBD 2013, the search strategy was replicated to capture epidemiological studies published between 2010 and 2013. The search strategy was repeated in 2015 only to capture excess mortality – updates to systematic reviews are performed on an ongoing schedule across all GBD causes. An update for meningitis literature is scheduled for GBD 2018.

Additional sources we included in the acute bacterial meningitis model were inpatient-only hospital data and USA claims data from 2000, 2010, and 2012, primary diagnosis and inpatient only. Sequelae and severity splits were informed by a meta-analysis, Edmond and colleagues (1), while an internal meta-analysis informed mortality estimates for long-term moderate to severe impairments.

Data were outliered or excluded if we found them unreasonable when compared to regional, super-regional, and global rates.

The tables below show the total number of specific site-years of data included in GBD 2017, as well as the number of countries and GBD world regions represented for the bacterial meningitis model and each aetiology model that informs an aetiology split.

Table 1a. Acute bacterial meningitis

	**Excess mortality rate**	**Incidence**
**Site-years (total)**	74	1535
**Number of countries with data**	42	75
**Number of GBD regions with data (out of 21 regions)**	14	21
**Number of GBD super-regions with data (out of 7 super-regions)**	6	7

Table 1b. Pneumococcal meningitis proportion

	**Proportion**
**Site-years (total)**	92
**Number of countries with data**	44
**Number of GBD regions with data (out of 21 regions)**	18
**Number of GBD super-regions with data (out of 7 super-regions)**	7

Table 1c. Meningococcal meningitis proportion

	**Proportion**
**Site-years (total)**	86
**Number of countries with data**	41
**Number of GBD regions with data (out of 21 regions)**	17
**Number of GBD super-regions with data (out of 7 super-regions)**	7

Table 1d. *H influenzae* type B meningitis proportion

	**Proportion**
**Site-years (total)**	93
**Number of countries with data**	44
**Number of GBD regions with data (out of 21 regions)**	18
**Number of GBD super-regions with data (out of 7 super-regions)**	7

Table 1e. Other bacterial meningitis proportion

	Proportion
**Site-years (total)**	85
**Number of countries with data**	39
**Number of GBD regions with data (out of 21 regions)**	16
**Number of GBD super-regions with data (out of 7 super-regions)**	7

Modelling strategy

Non-fatal outcomes were modelled using a combination of custom models, DisMod-MR 2.1, and in GBD 2017, we added the use of an ordinary differential equations solver (ODE) for more timely and accurate estimates. First, the overall incidence and prevalence of bacterial meningitis were modelled to estimate the short-term morbidity due to acute infection. This DisMod model had a set duration (1/remission) of four weeks with a range ±2 weeks. We also imposed caps on excess mortality for neonates and elders based on the highest excess mortality estimates from GBD 2013. Hospital data were flagged with a covariate for inpatient hospital data, as were USA claims data with year-specific covariates to be crosswalked to the reference data, which we extracted from literature. We used the function in DisMod-MR 2.1 to pull in cause-specific mortality rate (CSMR) data from our CODEm and CODcorrect analyses and match with prevalence data points for the same geography. We calculated excess mortality rate to estimate priors by dividing CSMR by prevalence, calculated from remission and incidence. To help inform trends where we lack data, we applied a country-level covariate for proportion of the population at the subnational and country levels that lives within the meningitis belt in sub-Saharan Africa (2). In GBD 2017 we added country-level covariates for coverage of Hib3 vaccine and the MenAfriVac vaccine initiative to the parent meningitis model. We also outliered incidence input data points with zero cases that were pulling down final estimates. Betas and exponentiated values (which can be interpreted as an odd ratio) are shown in the tables below for study-level covariates and country-level covariates.

Table 2. Study covariates

Study covariate	Parameter	Beta	Exponentiated beta
Literature	Incidence	-0.83 (-0.93 to -0.74)	0.44 (0.39–0.48)
Surveillance	Incidence	-1.89 (-2 to -1.7)	0.15 (0.14–0.18)
Claims data – 2000	Incidence	-0.096 (-0.15 to -0.038)	0.91 (0.86–0.96)
Claims data – other	Incidence	0.13 (0.083–0.17)	1.14 (1.09–1.19)

Table 3. Country-level covariates

Country-level covariate	Parameter	Beta	Exponentiated beta
Hib3 vaccine coverage	Incidence	-0.61 (-0.66 to -0.57)	0.54 (0.52–0.57)
Meningitis belt	Incidence	1.93 (1.76–2.00)	6.88 (5.84–7.37)
MenAfriVac initiative	Incidence	-1.01 (-2 to -0.019)	0.36 (0.14–0.98)

LDI	Excess mortality	-0.00051 (-0.0013 to -0.000058)	1.00 (1.00–1.00)
Healthcare Access and Quality index	Excess mortality	-0.027 (-0.028 to -0.027)	0.97 (0.97–0.97)

Incidence and prevalence of bacterial meningitis were split into four aetiologies (pneumococcal, meningococcal, *H influenzae* type B, and other bacterial meningitis) using four proportion models run in DisMod-MR 2.1. Results from these models were squeezed to sum to 1 at the draw level for each location, year, age, and sex. We applied a Hib3 vaccine coverage covariate to the *H. influenzae* type B proportion model, the proportion of the population living in the meningitis belt covariate and the proportion of the population living in areas covered by the MenAfriVac initiative (meningitis meningococcal type A) to the meningococcal meningitis proportion model, and a PCV3 coverage covariate to the pneumococcal meningitis model. Betas and exponentiated values are shown in the table below.

Table 4. Beta and exponentiated beta values

Country-level covariate	Etiology	Parameter	beta	Exponentiated beta
Hib3 vaccine coverage	Hib	Incidence	-1.73 (-1.99 to -1.26)	0.18 (0.14–0.28)
Meningitis belt (proportion of population)	Meningococcal	Incidence	0.93 (0.065–1.82)	2.53 (1.07–6.17)
MenAfriVac coverage	Meningococcal	Incidence	-0.49 (-1.31 to 0.32)	0.61 (0.27–1.37)
PCV3 vaccine coverage	Pneumococcal	Incidence	-1.03 (-1.96 to -0.061)	0.36 (0.14–0.94)

Data for viral meningitis were only available from hospitals or USA claims data, and not from population studies, so incidence and prevalence of viral meningitis were extrapolated from bacterial meningitis incidence by applying age- and sex-specific ratios between bacterial and viral cases from a combination of hospital data and USA claims data. In addition to short-term sequelae as a result of acute bacterial and viral meningitis, we also modelled the long-term outcomes from bacterial meningitis infection. In GBD 2017, we moved to produce both prevalence and incidence estimates of the viral meningitis outcome.

Sequelae splits

We first split the long-term sequelae among survivors of acute infection. We calculated the acute-phase survivors by applying the excess mortality (calculated by the acute meningitis DisMod model) to the incidence of each aetiology (excess mortality was converted to case fatality rate by $e^{(\text{-excess mortality x 1/(excess mortality + remission))}}$. The survivors were then subject for long-term sequelae by applying the post-discharge proportions of health consequences calculated by a meta-analysis by Edmond and colleagues (1). We calculated the ratio of acute meningitis survivors that experience major long-term impairments for all aetiologies, and the ratio of minor impairments to major impairments for pneumococcal meningitis versus all other aetiologies (because pneumococcal meningitis showed significantly higher risk of

morbidity than other aetiologies). This ratio was based off a regression of log-transformed GDP and ratio values from Edmonds and colleagues – this was different from last year, which used GNI. The regression is shown below:

$$y = -0.33590 \ln(GDP) + 1.15230$$

We used these two ratios to calculate the proportions of survivors who contract a long-term minor impairment and those who contract a long-term major impairment. The proportion with major impairments were further split (again using pooled proportions from Edmond and colleagues) into specific major impairments, which were grouped into vision loss, hearing loss, moderate-to-severe cognitive impairments, and epilepsy.

The calculated incidence of long-term sequelae was then converted to prevalence by two different approaches. For the sequelae not associated with excess mortality, which were vision loss, hearing loss, intellectual disability, motor impairment, and behavioural problems, the incidence of each age was cumulatively added up to the subsequent age (assuming half-cycle) to construct prevalence at each age. If the sequela is associated with excess mortality (epilepsy and moderate-to-severe cognitive impairments), the calculated incidence was used as in input to the ODE solver together with the corresponding mortality parameters (excess mortality data from the epilepsy envelope DisMod model, and standardised mortality ratio data from a neonatal encephalopathy meta-analysis, converted to excess mortality using all-cause mortality estimates) to estimate the prevalence. Vision loss, hearing loss, and epilepsy estimates were squeezed and severity split centrally.

Disability weights

The basis of the GBD disability weight survey assessments are lay descriptions of sequelae highlighting major functional consequences and symptoms. The lay descriptions and disability weights for sequelae associated with each aetiology are shown below.

Table 5. Severity splits, lay descriptions, DWs

Severity split	Lay description	DW (95% CI)
Mild behaviour problems	This person is hyperactive and has difficulty concentrating, remembering things, and completing tasks.	0.045 (0.028–0.066)
Mild hearing loss	This person has great difficulty hearing and understanding another person talking in a noisy place (for example, on an urban street).	0.01 (0.004–0.019)
Mild hearing loss with ringing	This person is unable to hear and understand another person talking, even in a quiet place, is unable to take part in a phone conversation. Difficulties with communicating and relating to others cause emotional impact at times (for example worry or depression).	0.021 (0.012–0.036)
Moderate hearing loss	This person has great difficulty hearing and understanding another person talking in a noisy place	0.027 (0.015–0.042)

	(for example, on an urban street), and sometimes has annoying ringing in the ears.	
Moderate hearing loss with ringing	This person is unable to hear and understand another person talking, even in a quiet place, is unable to take part in a phone conversation. Difficulties with communicating and relating to others often cause worry, depression, or loneliness.	0.074 (0.048–0.107)
Moderately severe hearing loss	Custom DW from hearing loss impairment envelope	
Severe hearing loss	This person is unable to hear and understand another person talking, even in a quiet place, and unable to take part in a phone conversation. Difficulties with communicating and relating to others cause emotional impact at times (for example worry or depression).	0.158 (0.105–0.227)
Profound hearing loss	This person is unable to hear and understand another person talking, even in a quiet place, is unable to take part in a phone conversation, and has great difficulty hearing anything in any other situation. Difficulties with communicating and relating to others often cause worry, depression, or loneliness.	0.204 (0.134–0.288)
Complete hearing loss	This person cannot hear at all in any situation, including even the loudest sounds, and cannot communicate verbally or use a phone. Difficulties with communicating and relating to others often cause worry, depression, or loneliness.	0.215 (0.144–0.307)
Severe hearing loss with ringing	This person is unable to hear and understand another person talking, even in a quiet place, is unable to take part in a phone conversation, and has annoying ringing in the ears for more than 5 minutes at a time, almost every day. Difficulties with communicating and relating to others cause emotional impact at times (for example worry or depression).	0.261 (0.175–0.36)
Profound hearing loss with ringing	This person is unable to hear and understand another person, even in a quiet place, is unable to take part in a phone conversation, has great difficulty hearing anything in any other situation, and has annoying ringing in the ears for more than 5 minutes at a time, several times a day. Difficulties with communicating and relating to others often cause worry, depression, or loneliness,	0.277 (0.182–0.387)
Complete hearing loss with ringing	This person cannot hear at all in any situation, including even the loudest sounds, and cannot communicate verbally or use a phone, and has very annoying ringing in the ears for more than half of the	0.316 (0.212–0.435)

	day. Difficulties with communicating and relating to others often cause worry, depression, or loneliness.	
Moderate motor impairment	This person has some difficulty in moving around, and difficulty in lifting and holding objects, dressing and sitting upright, but is able to walk without help.	0.061 (0.04–0.089)
Moderate motor plus cognitive impairments	This person has some difficulty in moving around, holding objects, dressing and sitting upright, but can walk without help. This person has low intelligence and is slow in learning to speak and to do simple tasks.	0.203 (0.134–0.29)
Long-term mild motor impairment	This person has some difficulty in moving around but is able to walk without help.	0.01 (0.005–0.02)
Borderline intellectual disability	This person is slow in learning at school. As an adult, the person has some difficulty doing complex or unfamiliar tasks but otherwise functions independently.	0.011 (0.005–0.02)
Severe motor impairment	This person is unable to move around without help, and is not able to lift or hold objects, get dressed or sit upright.	0.402 (0.268–0.545)
Epilepsy	(combined DW)	NA
Blindness	Is completely blind, which causes great difficulty in some daily activities, worry and anxiety, and great difficulty going outside the home without assistance.	0.187 (0.124–0.26)
Severe acute episode of infectious disease	This person has a high fever and pain, and feels very weak, which causes great difficulty with daily activities.	0.133 (0.088–0.19)
Mild intellectual disability	This person has low intelligence and is slow in learning at school. As an adult, the person can live independently, but often needs help to raise children and can only work at simple supervised jobs.	0.043 (0.026–0.065)
Monocular distance vision loss	This person is blind in one eye and has difficulty judging distances.	0.017 (0.009–0.029)
Mild motor plus cognitive impairments	This person has some difficulty in moving around but is able to walk without help. The person is slow in learning at school. As an adult, the person has some difficulty doing complex or unfamiliar tasks but otherwise functions independently.	0.031 (0.018–0.05)
Severe motor plus cognitive impairments	This person cannot move around without help, and cannot lift or hold objects, get dressed or sit upright. The person also has very low intelligence, speaks few words, and needs constant supervision and help with all daily activities.	0.542 (0.37–0.702)

References

(1) Edmond, K. *et al.* Global and regional risk of disabling sequelae from bacterial meningitis: a systematic review and meta-analysis. *The Lancet Infectious Diseases* **10,** 317–328 (2010).

(2) Centers for Disease Control (CDC). CDC health information for international travel 2016: the yellow book. New York City, United States: Oxford University Press, USA, 2016.

Encephalitis

Flowchart

Encephalitis

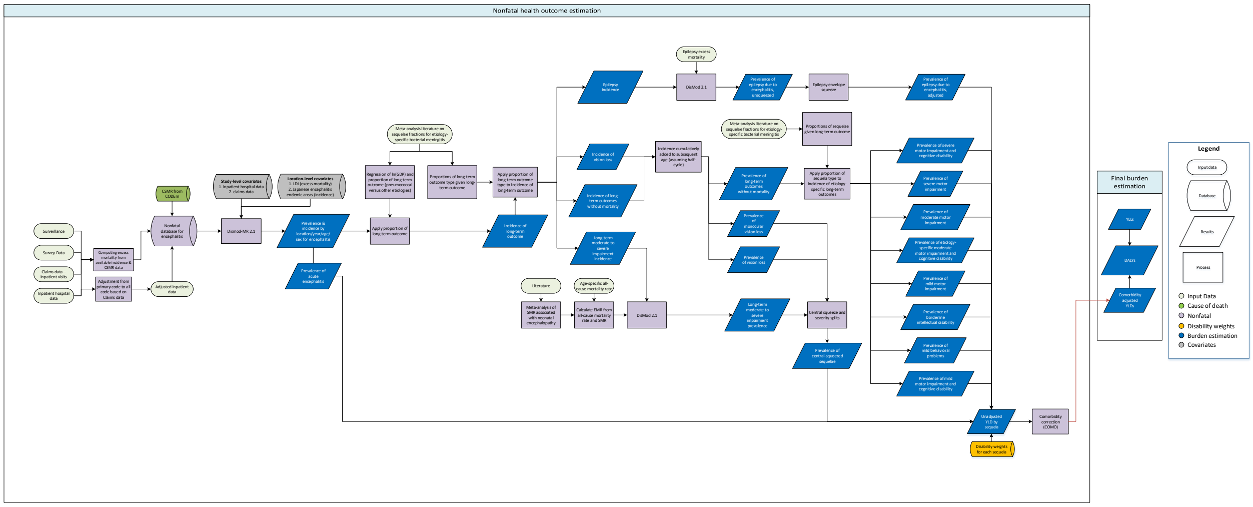

Case definition

Encephalitis is a disease caused by an acute inflammation of the brain. Symptoms of encephalitis can include flu-like symptoms like headache, fever, drowsiness, and fatigue, and at times, seizures, hallucinations, or stroke. Included in the GBD modelling were cases meeting ICD-10 diagnostic criteria for encephalitis (A83-A86.4, B94.1, F07.1, G04-G05.8).

Input data

Model inputs

In the GBD 2015 study, a systematic review of literature was conducted to capture studies of incidence, excess mortality rate, remission, and standardised mortality ratio for encephalitis. These data sources included hospital data and literature. The inclusion criteria stipulated that: (1) the publication year must be between 1980 and 2013; (2) sufficient information must be provided on study method and sample characteristics to assess the quality of the study; and (3) study samples must be representative of the general population. No limitation was set on the language of publication. We did not perform an updated literature review for GBD 2017.

Additional sources we included in the acute bacterial meningitis model were inpatient hospital data and USA claims data from 2000, 2010, and 2012, primary diagnosis and inpatient only. Sequelae and severity splits were informed by a meta-analysis, Edmond and colleagues (1), while an internal meta-analysis informed mortality estimates for long-term moderate-to-severe impairments.

Data were outliered or excluded if we found they differed significantly when compared to regional, super-regional, and global rates.

The tables below show the number of measure specific site-years included in GBD 2017, as well as the number of countries and GBD world regions represented for encephalitis.

Table 1. Acute encephalitis

	Incidence
**Site-years (total)**	1446
**Number of countries with data**	51
**Number of GBD regions with data (out of 21 regions)**	16
**Number of GBD super-regions with data (out of 7 super-regions)**	7

Modelling strategy

Non-fatal outcomes were modelled using a combination of custom models and DisMod-MR 2.1. First, the overall incidence and prevalence of encephalitis were modelled to estimate the short-term morbidity due to acute infection. This DisMod model had a set duration (1/remission) of three weeks. We also imposed caps on excess mortality for ages 10–50. USA claims data were grouped into year-specific covariates based on quality, and were crosswalked to the reference data, which we extracted from literature and inpatient hospital data. We used the function in DisMod-MR 2.1 to pull in cause-specific mortality rate (CSMR) data from our CODEm and CODcorrect analyses and match with incidence data points for the same geography. We calculated excess mortality rate to estimate priors by dividing CSMR by prevalence, calculated from remission and incidence. To help inform trends where we lack data, we applied a binary country-level covariate at the subnational and country level that indicates if the location is in a Japanese Encephalitis endemic area (2). We also applied a lag-distributed income covariate to excess mortality. Betas and exponentiated values (which can be interpreted as an odds ratio) are shown in the tables below for study-level covariates and country-level covariates. In GBD 2017 we updated the Japanese Encephalitis covariate to include areas of Russia. We outliered incidence input data points with zero cases that were dragging down final estimates. We also improved our time efficiency and estimation accuracy by using an ordinary differential equations solver (ODE solver) in place of traditional DisMod-MR.

Table 2. Study covariates

Study covariate	Parameter	beta	Exponentiated beta
Claims data – 2000	Incidence	-0.22 (-0.27 to -0.18)	0.81 (0.76–0.83)
Claims data - other	Incidence	0.15 (0.097–0.19)	1.16 (1.10–1.20)
Literature	Incidence	-0.55 (-0.62 to -0.48)	0.58 (0.54–0.62)

Table 3. Country-level covariates

Country-level covariate	Parameter	beta	Exponentiated beta
Japanese Encephalitis endemic area	Incidence	0.052 (0.0041–0.097)	1.05 (1.00–1.10)
LDI (log transformed)	Excess mortality	-0.3 (-0.31 to -0.29)	0.74 (0.73–0.75)

DTP3 Coverage	Incidence	-1.56 (-1.68 to -1.45)	0.21 (0.19–0.23)

In addition to short-term sequelae as a result of acute encephalitis, we also modelled the long-term outcomes from encephalitis.

Sequelae splits

We first split the long-term sequelae among survivors of acute infection. We calculated the acute phase survivors by applying the excess mortality (calculated by the acute meningitis DisMod model) to the incidence of each aetiology (excess mortality was converted to case fatality rate by $e^{(\text{-excess mortality x 1/(excess mortality + remission))}}$. The survivors were then subject to long-term sequelae by applying the post-discharge proportions of health consequences calculated by a meta-analysis by Edmond and colleagues (2). We calculated the ratio of acute encephalitis survivors that result in a major long-term impairment, and the ratio of minor impairments to major impairments, based off a regression of log-transformed GDP and ratio values from Edmonds and colleagues. This regression was done differently from last year, which previously used GNI. The regression is shown below:

$$y = -0.33590 \ln(GDP) + 1.15230$$

We assumed a similar pattern of health outcomes for encephalitis infection survivors as with other bacterial meningitis survivors (except hearing loss, as we could not find evidence of hearing loss as a consequence of encephalitis infection). We used these two ratios to calculate the proportions of survivors who contract a long-term minor impairment and those who contract a long-term major impairment. The proportion with major impairments were further split (again using pooled proportions from Edmond and colleagues) into specific major impairments, which were grouped into vision loss, moderate to severe cognitive impairments, and epilepsy.

The calculated incidence of long-term sequelae was then converted to prevalence by two different approaches. For the sequelae not associated with excess mortality, which were vision loss, intellectual disability, motor impairment, and behavioural problems, the incidence of each age was cumulatively added up to the subsequent age (assuming half-cycle) to construct prevalence at each age. If the sequela is associated with excess mortality (epilepsy and moderate-to-severe cognitive impairments), the calculated incidence was used as an input the ODE solver, together with the corresponding mortality parameters (excess mortality data from the epilepsy envelope DisMod model, and standardised mortality ratio data from a neonatal encephalopathy meta-analysis, converted to excess mortality using all-cause mortality estimates) to estimate the prevalence. Vision loss and epilepsy estimates were squeezed and severity split centrally.

Disability weights

The basis of the GBD disability weight survey assessments is lay descriptions of sequelae highlighting major functional consequences and symptoms. The lay descriptions and disability weights for sequelae associated with encephalitis are shown below.

Table 4. Severity splits, lay descriptions, and DWs

Severity split	Lay description	DW (95% CI)

Mild behaviour problems	This person is hyperactive and has difficulty concentrating, remembering things, and completing tasks.	0.045 (0.028–0.066)
Moderate motor impairment	This person has some difficulty in moving around, and difficulty in lifting and holding objects, dressing and sitting upright, but is able to walk without help.	0.061 (0.04–0.089)
Moderate motor plus cognitive impairments	This person has some difficulty in moving around, holding objects, dressing and sitting upright, but can walk without help. This person has low intelligence and is slow in learning to speak and to do simple tasks.	0.203 (0.134–0.29)
Long-term mild motor impairment	This person has some difficulty in moving around but is able to walk without help.	0.01 (0.005–0.02)
Borderline intellectual disability	This person is slow in learning at school. As an adult, the person has some difficulty doing complex or unfamiliar tasks but otherwise functions independently.	0.011 (0.005–0.02)
Severe motor impairment	This person is unable to move around without help, and is not able to lift or hold objects, get dressed, or sit upright.	0.402 (0.268–0.545)
Epilepsy	(combined DW)	NA
Blindness	Is completely blind, which causes great difficulty in some daily activities, worry and anxiety, and great difficulty going outside the home without assistance.	0.187 (0.124–0.26)
Acute encephalitis	This person has a high fever and pain, and feels very weak, which causes great difficulty with daily activities.	0.133 (0.088–0.19)
Mild intellectual disability	This person has low intelligence and is slow in learning at school. As an adult, the person can live independently but often needs help to raise children and can only work at simple supervised jobs.	0.043 (0.026–0.065)
Monocular distance vision loss	This person is blind in one eye and has difficulty judging distances.	0.017 (0.009–0.029)
Mild motor plus cognitive impairments	This person has some difficulty in moving around but is able to walk without help. The person is slow in learning at school. As an adult, the person has some difficulty doing complex or unfamiliar tasks but otherwise functions independently.	0.031 (0.018–0.05)
Severe motor plus cognitive impairments	This person cannot move around without help, and cannot lift or hold objects, get dressed or sit upright. The person also has very low intelligence, speaks few words, and needs constant supervision and help with all daily activities.	0.542 (0.37–0.702)

References

(1) Edmond, K. *et al.* Global and regional risk of disabling sequelae from bacterial meningitis: a systematic review and meta-analysis. *The Lancet Infectious Diseases* **10,** 317–328 (2010).

(2) Centers for Disease Control (CDC). CDC health information for international travel 2016: the yellow book. New York City, United States: Oxford University Press, USA, 2016.

Diphtheria

Model flowchart

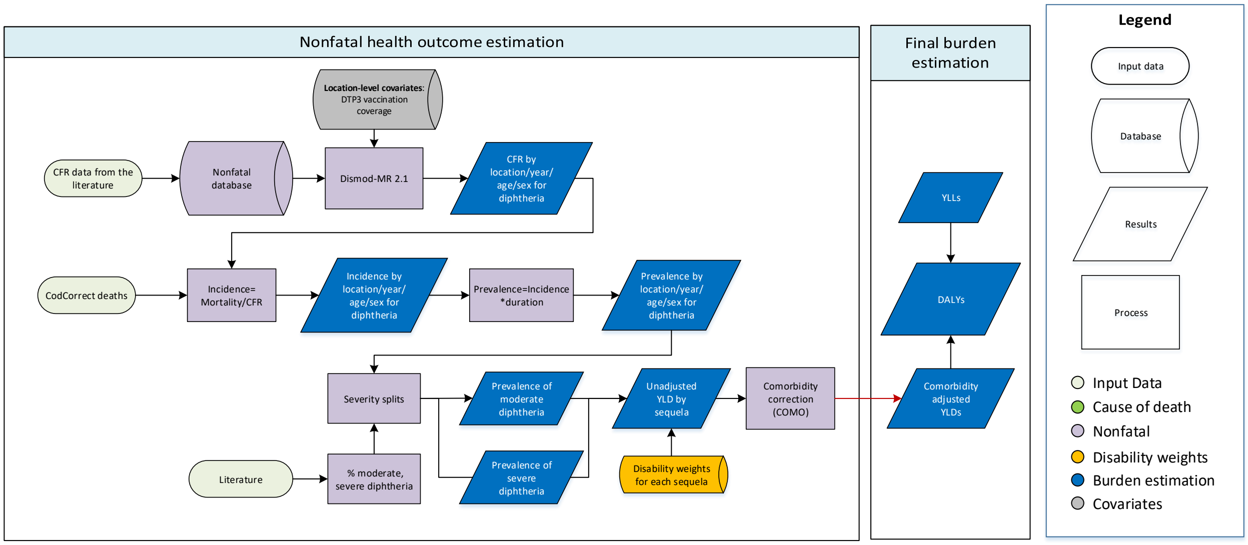

Case definition

Diphtheria is a bacterial infection caused by *Corynebacterium diphtheriae*. For diphtheria, ICD 10 codes are A36-A36.9, Z22.2, Z23.6, and ICD9 codes are 032-032.9, V02.4, V03.5, and V74.3.

Input data

Model inputs

Input data for non-fatal diphtheria outcomes include case fatality ratio data from a systematic review of the literature and diphtheria mortality estimates from the GBD cause of death analysis (for diphtheria, a negative binomial regression model).

A systematic review of diphtheria case fatality was conducted for GBD 2016 to add to the literature used in previous iterations of the GBD. The PubMed search terms were: ((diphtheria[Title/Abstract] AND case fatality[Title/Abstract])) AND ("2013"[Date - Publication]: "2016"[Date - Publication]). We did not update this review for GBD 2017.

Severity split & disability weights

We draw primarily on the literature, as well as patterns in data observed in South Asia and Central Asia, to assign the following severity distributions for diphtheria: cases are proportionally split into 70% (95% CI:66.5–73.5%) moderate outcomes and 30% (95% CI: 26.5–33.5%) severe outcomes. The lay descriptions and disability weights for diphtheria severity levels derived from the GBD disability weights study are shown in Table 1.

Table 1. Severity splits, lay descriptions, and disability weights

Severity level	Lay description	DW (95% CI)
Moderate diphtheria	Has a fever and aches, and feels weak, which causes some difficulty with daily activities.	0.051 (0.032–0.074)
Severe diphtheria	Has a high fever and pain, and feels very weak, which causes great difficulty with daily activities.	0.133 (0.088–0.19)

Modelling strategy

We used DisMod-MR 2.1 as a meta-regression tool to pool the case fatality data and generate location-, year-, age-, and sex-specific case fatality ratio estimates. We used the Healthcare Access and Quality (HAQ) index as a location-level covariate. Diphtheria mortality was modelled using a negative binomial regression and data from the cause of death database with the DTP3 coverage covariate and age dummy variables (see cause of death capstone paper).

Incidence was calculated as mortality rate divided by case fatality ratio. Prevalence was calculated as the product of estimates for incidence and case duration (a mean of 27.5 days, based on a meta-analysis of duration data from the literature).

Table 2 shows model covariate coefficients and exponentiated values (from the DisMod case-fatality model), which can be interpreted as odds ratios.

Table 2. Model coefficient values (raw and exponentiated)

Covariate	Parameter	Coefficient (95% CI)	Exponentiated coefficient (95% CI)
Healthcare Access and Quality (HAQ) index	Case fatality ratio	0.50 (0.026, 1.31)	1.64 (1.03, 3.70)
Sex	Case fatality ratio	0.098 (-0.76, 0.88)	1.10 (0.47, 2.41)

Counts of unique input data sources for the diphtheria case fatality ratio model are shown in Table 3.

Table 3. Input source counts

	**Case fatality ratio**
**Site-years (total)**	28
**Number of countries with data**	12
**Number of GBD regions with data (out of 21 regions)**	6
**Number of GBD super-regions with data (out of 7 super-regions)**	5

No other significant changes were made to the modelling strategy from previous iterations of GBD.

Pertussis (whooping cough)

Flowchart

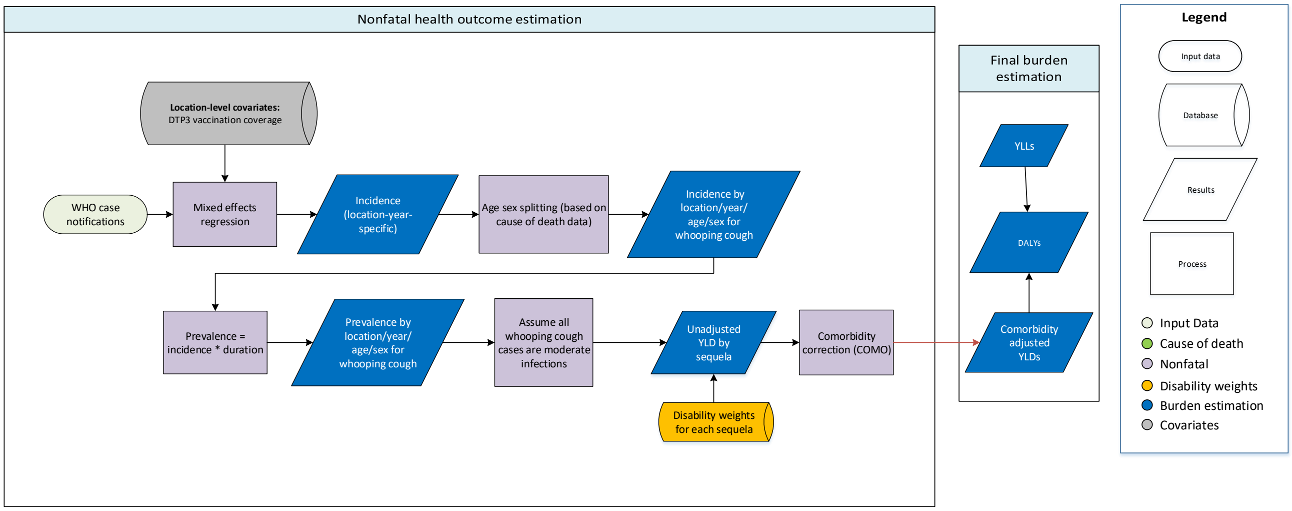

Case definition

Pertussis (whooping cough), is a contagious respiratory disease caused by the bacterium *Bordetella pertussis*. For pertussis, ICD 10 codes are A37-A37.91, Z23.7, and ICD 9 codes are 033-033.9, 484.3, V03.6.

Input data

Model inputs

For our whooping cough non-fatal estimation process, the primary source of input data was pertussis case notifications from the World Health Organization (WHO) through the Joint Reporting Form (JRF). For GBD 2017, we included case notifications through December 31, 2017 (released in June of 2018). Historical case notifications and vaccination coverage for the United Kingdom back to 1940 were included to better inform the natural history model.

Severity splits

We assumed all pertussis cases were moderate episodes of acute infectious disease because of associated symptoms and MEPS data. The lay description and disability weight derived from the GBD Disability Weights study are shown in Table 1.

Table 1. Severity splits, lay descriptions, and disability weights

Severity level	Lay description	DW (95% CI)
Moderate	Has a fever and aches and feels weak, which causes some difficulty with daily activities.	0.051 (0.032–0.074)

Modelling strategy

We modelled log-transformed incidence with a mixed-effects linear regression of case notifications on diphtheria-tetanus-pertussis third-dose (DTP3) vaccination coverage. The random effect by country allowed us to account for variation in registration completeness. We used the following functional form:

$$Y_{ij} = \beta_0 + \beta_1 \, DTP3_{ij} + u_j + e_{ij},$$

where Y_{ij} is the log-transformed incidence rate (in cases per 100,000 persons using WHO case notifications and GBD populations), β_0 is the fixed effect intercept, β_1 is the fixed effects slope on the log-transformed proportion of unvaccinated individuals, u_j is the country random effect, e_{ij} is the residual, i is the year, and j is the location.

Counts of unique input data sources that inform the pertussis incidence rate model are shown in Table 2.

Table 2. Input source counts

	**Incidence**
**Site-years (total)**	5,599
**Number of countries with data**	191
**Number of GBD regions with data (out of 21 regions)**	21
**Number of GBD super-regions with data (out of 7 super-regions)**	7

The results of this model were then used to predict incidence as a function of vaccine coverage. To correct for underreporting in case notifications, we used a value of the random effect that matched the highest random effect in a high income region – Switzerland (which has a pertussis monitoring system that captures a high percentage of cases) – to get an implied attack rate assumed to be the same for all unvaccinated populations. Uncertainty was estimated by taking 1,000 iterations of the predictions based on the variance-covariance matrix.

Prevalence was calculated by multiplying incidence rate by the average case duration, which was assumed to be 50 days.

Tetanus

Flowchart

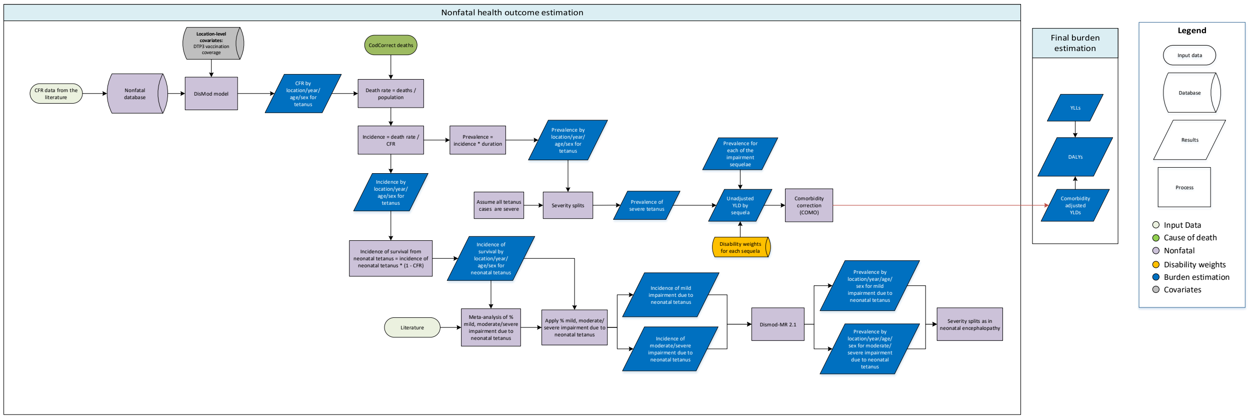

Case definition

Tetanus is a serious bacterial disease caused by the bacterium *Clostridium tetani.* For tetanus, the ICD 10 codes are A33-A35.0, Z23.5, and ICD 9 codes are 037-037.9,771.3,V03.7.

Input data

Model inputs

Input data for the estimation of tetanus included case fatality rate data extracted from a systematic review of the literature and GBD estimates of tetanus cause-specific mortality calculated through the GBD cause of death estimation process (here, estimates were produced with the GBD ensemble modelling framework CODEm).

A systematic review was conducted for GBD 2016 to supplement previous data extractions. The PubMed search terms were: (tetanus[Title/Abstract]) AND (case fatality[Title/Abstract]) AND ("2013"[Date - Publication]: "2016"[Date - Publication]). An update to this systematic review was not conducted for GBD 2017.

Severity splits and disability weights

We assume that all tetanus cases are severe episodes of acute infectious diseases. The lay descriptions and disability weights for tetanus derived from the GBD disability weights study are shown in Table 1.

Table 1. Severity splits, lay descriptions, and disability weights (DW)

Severity level	Lay description	DW (95% CI)
Severe	Has a high fever and pain, and feels very weak, which causes great difficulty with daily activities.	0.133 (0.088–0.19)

Regarding the severity level of impairment due to neonatal tetanus, we assume the same distribution as observed in neonatal encephalopathy.

Modelling strategy

We used DisMod-MR 2.0 as a meta-regression tool to pool the case fatality ratio data and generate location-, year-, age-, and sex-specific case fatality estimates. We used the Healthcare Access and Quality (HAQ) index as a location-level covariate. Mortality was modelled using the standard CODEm tool on neonatal tetanus (children under age 1) and non-neonatal tetanus (ages 1-80) separately for males and females. Table 2 shows model coefficients and exponentiated values for the covariates used in the estimation process for the DisMod case-fatality model, which can be interpreted as odds ratios.

Table 2. Model coefficient values (raw and exponentiated)

Covariate	Parameter	Beta (95% CI)	Exponentiated beta (95% CI)
Healthcare Access and Quality (HAQ) index	Case fatality ratio	0.16 (-0.0048 to 0.50)	1.17 (1.00–1.64)
Sex	Case fatality ratio	-0.067 (-0.33 to 0.19)	0. 39 (0.72–1.20)

Counts of unique input data sources for the tetanus case fatality ratio model are shown in Table 3.

Table 3. Input source counts

	**Case fatality ratio**
**Site-years (total)**	76
**Number of countries with data**	17
**Number of GBD regions with data (out of 21 regions)**	10
**Number of GBD super-regions with data (out of 7 super-regions)**	7

Incidence was then calculated as:

$$incidence = \frac{mortality\ rate}{case\ fatality\ rate}.$$

Prevalence was computed from the product of estimated incidence rates and case duration derived from a literature review. All computation was performed in draw space to propagate uncertainty.

To estimate mild and moderate impairment due to neonatal tetanus, we first computed the incidence of survival from neonatal tetanus as:

$$incidence\ of\ survival = incidence * (1 - CFR).$$

We then conducted a meta-analysis of published studies to estimate the proportion of mild impairment due to neonatal tetanus and moderate-to-severe impairment due to neonatal tetanus. We applied these proportions to the estimated incidence of survival to generate incidence rates of impairments due to neonatal tetanus (mild and moderate-to-severe), which were used as input data in DisMod 2.0. We ran two separate DisMod models (one for the mild severity and another for moderate-to-severe cases) on these outputs.

Measles

Flowchart

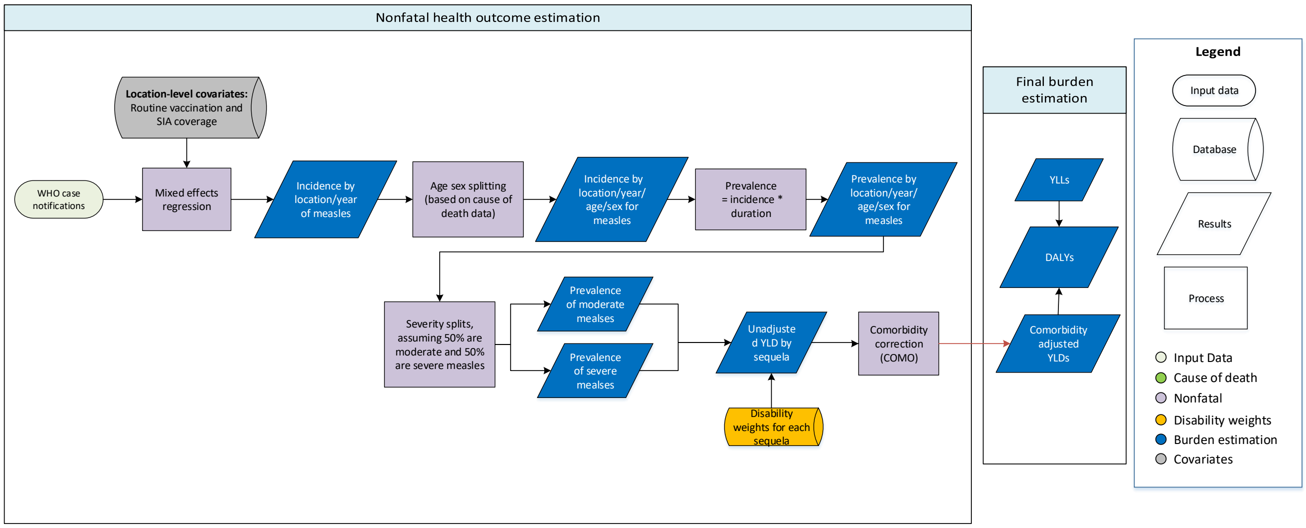

Case definition

Measles is a contagious infection caused by the measles virus. Symptoms include cough, runny nose, fever, conjunctivitis, and red, blotchy skin. For measles, ICD 10 codes are B05-B05.9, Z24.4, and ICD 9 codes are 055-055.9, 484.0, V04.2, V73.2.

Input data

Model inputs

The primary source of input data was measles case notifications from the World Health Organization (WHO) through the Joint Reporting Form (JRF). For GBD 2017, we included case notifications through December 31, 2017 (released in June of 2018). We supplemented these WHO input data with subnational case notifications for the United States and Japan, where available, from national ministries of health.

Severity splits

We assume 50% of measles cases were acute episodes of moderate infectious disease and 50% were acute episodes of severe infectious disease. The lay descriptions and disability weights for measles severity levels derived from the GBD disability weights study are shown in Table 1.

Table 1. Severity splits, lay descriptions, and disability weights

Severity level	Lay description	DW (95% CI)
Moderate	Has a fever and aches, and feels weak, which causes some difficulty with daily activities.	0.051 (0.032–0.074)
Severe	Has a high fever and pain, and feels very weak, which causes great difficulty with daily activities.	0.133 (0.088–0.19)

Modelling strategy

We modelled measles incidence in count space with a mixed-effects linear regression of case notifications on routine measles vaccination rates (first- and second-dose measles-containing vaccines) and coverage of supplementary immunisation activities (SIAs). More precisely, log-transformed incidence rates were regressed on the log of the proportion unvaccinated with first- and second-dose measles-containing vaccine, and additional SIA coverage lagged by one, two, three, four, and five years, with super-region, region, and country-level random effects:

$$Y_{ij} = \beta_0 + \beta_1\, MCV1_{ij} + \beta_2\, MCV2_{ij} + \beta_{a3}\, SIA_{aij} + u_j + e_{ij},$$

where Y_{ij} is the natural log of measles incidence rate per 100,000 people, β_0 is the fixed-effect intercept, β_1 is the fixed-effects slope on 1st dose measles vaccine coverage, β_2 is the fixed-effects slope on 2nd dose measles vaccine coverage, β_{a3} is the fixed-effects slope on supplementary measles immunisation campaign coverage (administered doses over the target population of all under-15s) lagged by a=1-5 years, u_j is the location-level random effects, e_{ij} is the residual, i is the year, and j is the location.

Counts of unique input data sources that inform the measles incidence rate model are shown in Table 2.

Table 2. Input source counts

	**Incidence**
**Site-years (total)**	3,984
**Number of countries with data**	186
**Number of GBD regions with data (out of 21 regions)**	21
**Number of GBD super-regions with data (out of 7 super-regions)**	7

The results of this mixed effects regression model were then used to predict location- and year-specific incidence rate as a function of routine vaccine coverage and SIAs. To correct for underreporting in case notifications, we added the effect of a 95% attack rate, assumed to be the same across all unvaccinated populations. We validated the attack rate assumption on high-income regions and Latin America, presumed to have complete case notifications, and found that it appropriately predicted measles morbidity in these regions and is thus a good assumption for a global model. Uncertainty was estimated by taking 1,000 iterations of the predictions based on the variance-covariance matrix. For locations in three super-regions – high-income, Central Europe/Eastern Europe/Central Asia, and Latin America and Caribbean – we used reported measles cases from WHO and country sources as a direct measure of incident measles cases.

Prevalence was calculated by multiplying incidence by an average case duration, which was assumed to be 10 days.

Varicella (chickenpox) and herpes zoster

Flowchart

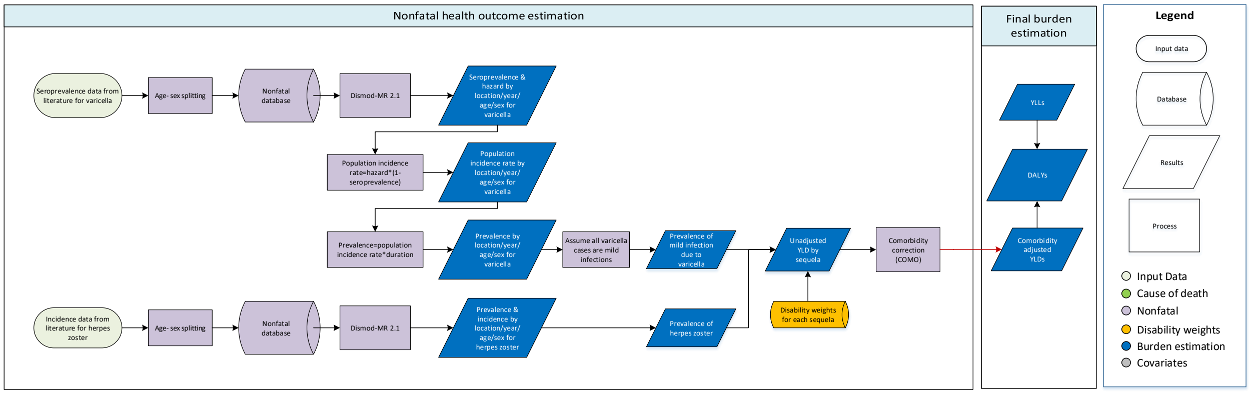

Case definition

Varicella (also known as chicken pox) is an acute infectious disease caused by *Varicella zoster* virus. *Herpes zoster* (also known as shingles), is caused by the reactivation of the same virus that causes varicella. For varicella and herpes zoster, the ICD 10 codes are B01-B02.9, P35.8, Z20.820, and ICD 9 codes are 052-053.9, V01.71, V01.79, V05.4.

Input data

Model inputs

Input data for varicella were from published seroprevalence studies, and those for herpes zoster were from published incidence studies.

Systematic reviews of the literature were conducted for both varicella and herpes zoster and updated for GBD 2016. The PubMed search query for varicella was as follows: (varicella[Title/Abstract] AND seroprevalence[Title/Abstract]) AND (incidence[Title/Abstract] OR prevalence[Title/Abstract]) NOT (herpes zoster[Title/Abstract] OR shingles[Title/Abstract]) AND ("2013"[Date - Publication] : "2016"[Date - Publication]). The PubMed search query for herpes zoster was as follows: ((herpes zoster[Title/Abstract] OR shingles[Title/Abstract]) AND (incidence[Title/Abstract)) NOT (varicella[Title/Abstract] OR chicken pox[Title/Abstract]) AND ("2013"[Date - Publication] : "2016"[Date - Publication]).

We excluded studies that were: (1) not population-based, eg, hospital or clinic-based studies; (2) did not provide primary data on epidemiological parameters, eg, commentaries; (3) review articles; (4) case series; or (5) self-reported cases.

These systematic reviews were not updated for GBD 2017.

Severity splits & disability weights

We assume all varicella cases are mild episodes of acute infectious disease. Herpes zoster was a sequela studied in the GBD disability weight study. The lay descriptions and corresponding disability weights are presented in Table 1.

Table 1. Severity level, lay description, and disability weights (DW)

Severity level	Lay description	DW (95% CI)
Mild acute infectious disease	Has a low fever and mild discomfort but no difficulty with daily activities.	0.006 (0.002–0.012)
Herpes zoster	Has a blistering skin rash that causes pain, with some burning and itching.	0.058 (0.035–0.09)

Modelling strategy

The modelling strategies for varicella and herpes zoster are outlined below.

Data for varicella seroprevalence by country were first run through the GBD non-fatal estimation framework DisMod-MR 2.1. First, we modelled varicella seroprevalence data as prevalence in DisMod, after specifying zero remission and no excess mortality. Next, we calculate population incidence rate as:

$$incidence\ rate\ =\ hazard\ *(1-prevalence)$$

from the DisMod incidence rate estimates (ie, hazards).

Then, we calculate prevalence as:

$$prevalence = incidence\ rate * duration$$

assuming a mean case duration of seven days.

Herpes zoster morbidity was modelled using a standard DisMod model. We assume no excess mortality associated with herpes zoster.

Raw and exponentiated coefficients values on country- and study-level covariates are shown in Table 2. Exponentiated values can be interpreted as odds ratios.

Table 2

a. Varicella DisMod model coefficients

Covariate	Parameter	Beta (95% CI)	Exponentiated beta (95% CI)
Sex	Seroprevalence	-0.091 (-0.69 to 0.47)	0.91 (0.50–1.60)
Healthcare Access and Quality (HAQ) index	Seroprevalence	-0.50 (-1.00 to 0)	0.61 (0.37–1.00)

b. Herpes zoster DisMod model coefficients

Covariate	Parameter	Beta (95% CI)	Exponentiated beta (95% CI)
Sex	Incidence	-0.084 (-0.23 to -0.056)	0.92 (0.30–1.06)

Counts of unique input data sources are shown in Table 3 for both varicella (prevalence) and herpes zoster (incidence).

Table 3. Input source counts

	Varicella prevalence	Herpes zoster incidence
**Site-years (total)**	67	79
**Number of countries with data**	34	20
**Number of GBD regions with data (out of 21 regions)**	15	6
**Number of GBD super-regions with data (out of 7 super-regions)**	7	3

Acute Hepatitis: A, B, C, and E

Acute hepatitis A

Flowchart

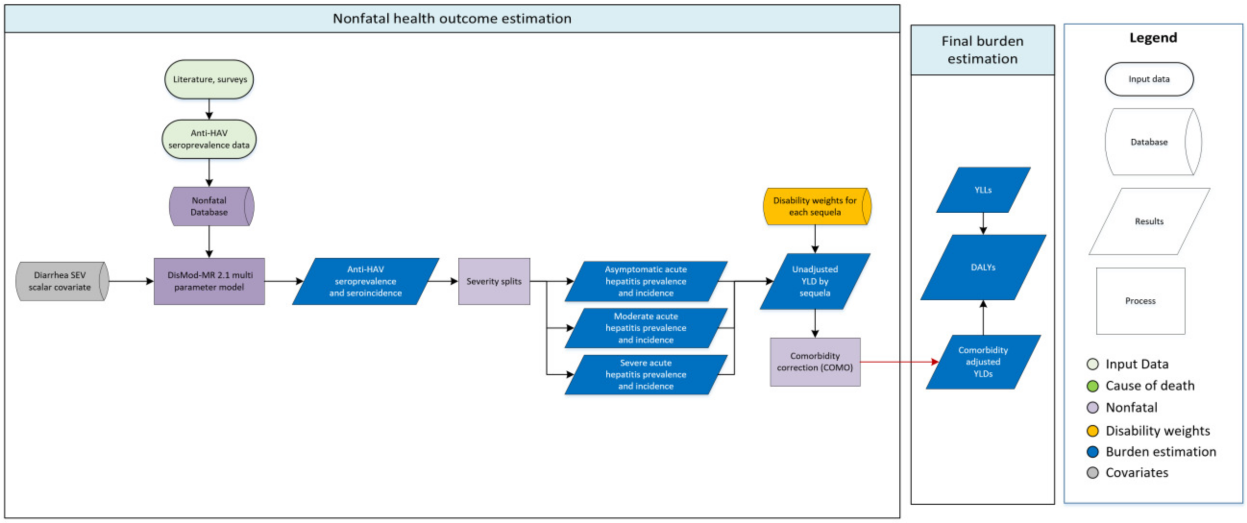

Case definition

We define acute hepatitis A as an infection with the hepatitis A virus resulting in anti-HAV IgG seroconversion, regardless of symptoms. It includes all ICD-10 codes under the heading B15 (Acute hepatitis A).

Input data

Model inputs

Our case definition is infection with hepatitis A, irrespective of symptomology. We use anti-hepatitis A virus (HAV) seroprevalence data from population-based studies and surveys to inform these estimates. We present a summary of the data sources in Table 1 below. Updates to systematic reviews are performed on an ongoing schedule across all GBD causes; an update for hepatitis A will be performed in the next one to two iterations. The last systematic review was performed as part of GBD 2013.

Table 1: Data inputs for acute hepatitis A morbidity modelling by parameter

	Prevalence
Site-years (total)	562
Number of countries/subnational locations with data	119
Number of GBD regions with data (out of 21 regions)	21
Number of GBD super-regions with data (out of 7 super-regions)	7

Severity splits & disability weights

The table below illustrates the sequelae associated with acute hepatitis A, as well as the lay descriptions and associated disability weights.

Sequela	Description	Disability Weight
Moderate	Has a fever and aches, and feels weak, which causes some difficulty with daily activities.	0.051 (0.032–0.074)
Severe	Has a high fever and pain, and feels very weak, which causes great difficulty with daily activities.	0.133 (0.088–0.19)
Asymptomatic	Infection with no apparent illness of	NA

We calculate acute symptomatic infections by multiplying incidence of acute infection by the probability of acute symptomatic infection. The probability of symptomatic infection comes from Armstrong and Bell and is shown in the figure below (where probability of symptomatic infection is represented as "probability of jaundice") [1]. The probability increases with age from ~1% in the first year of life to ~85% in adulthood. The probability function is:

$$Prob\ (symptomatic) = 0.852 * (1 - \ e^{-0.01244\ *\ age^{1.903}})$$

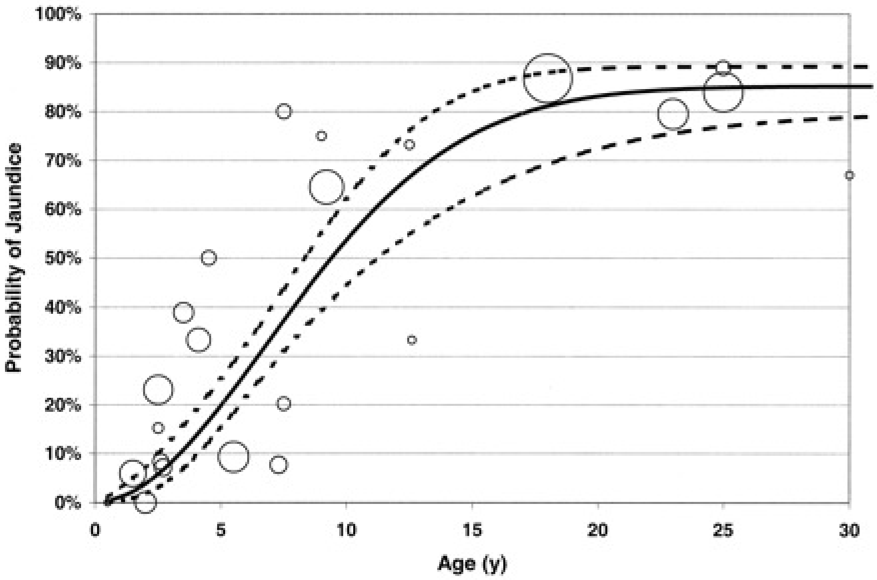

The remainder of acute infections are assumed to be asymptomatic.

We then base severity splits for moderate and severe on expert opinion that the probability of severe infection follows a beta distribution with mean 0.6% (table 2 reports percentiles of this distribution.) We assume the rest of symptomatic infections are moderate.

Table 2. Percentiles of the probability distribution of severe acute hepatitis A

0 percentile	25 percentile	50 percentile	75 percentile	100 percentile
0.0024	0.0054	0.006	0.007	0.01

Modelling strategy

We model the seroprevalence of anti-hepatitis A virus IgG using a DisMod-MR 2.1 model of anti-HAV seroprevalence. Remission and excess mortality value priors of zero were used, and an incidence value prior range between 0 and 0.5 was used. Given its reasonably stable force of infection among susceptible people across age groups, we derive incidence from the prevalence estimates using the following formula:

$$incid = \frac{-\ln(1 - prev)}{\text{age}_{\text{mid}}} * (1 - prev)$$

Changes from GBD 2016 to GBD 2017

This approach is a modification from GBD 2016, where instead of utilising DisMod-MR 2.1, we estimated anti-HAV seroprevalence via a catalytic binomial generalised linear model with a complementary log-log link, and an offset term for log-age. That previous model used a predictive covariate derived from principal components analysis of lag-distributed income (LDI) and the proportion of the population with access to improved water.

Acute hepatitis B and C

Flowchart

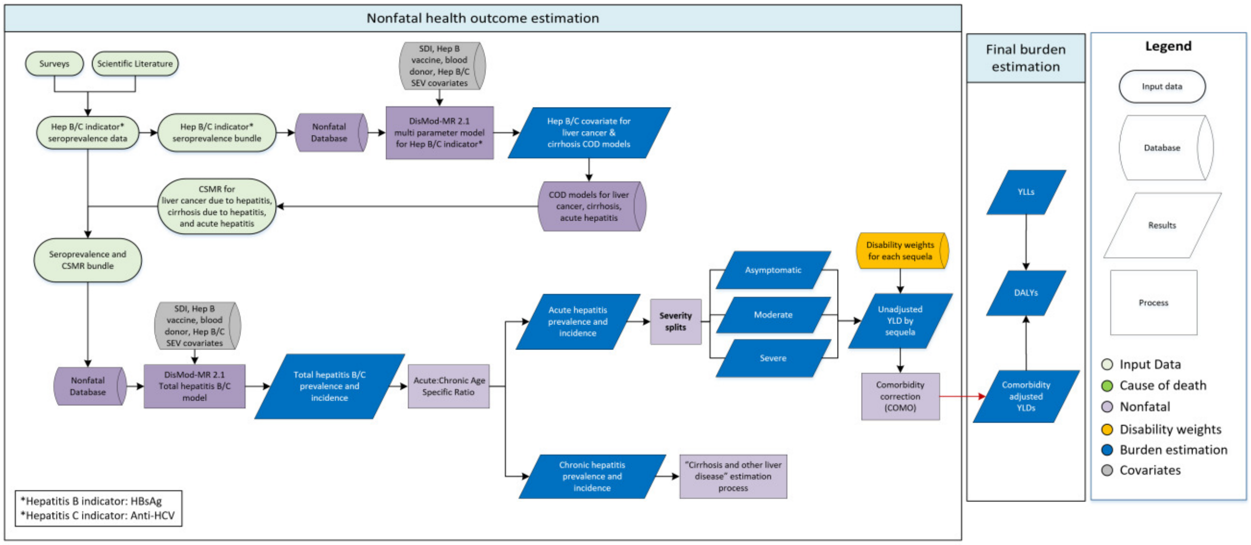

Acute hepatitis B

Case definition

We define acute hepatitis B as the period corresponding to initial infection with the hepatitis B virus, regardless of symptoms. It includes all ICD-10 codes under the heading B16 (Acute hepatitis B).

Input data

Model inputs

We use hepatitis B surface antigen (HBsAg) seroprevalence data from population-based studies and surveys. We present a summary of the seroprevalence data sources in Table 3 below. The last systematic review was performed as part of GBD 2013. Updates to systematic reviews are performed on an ongoing schedule across all GBD causes; an update for hepatitis B will be performed in the next one to two iterations.

Table 3: Data Inputs for acute hepatitis B morbidity modelling by parameter.

	Prevalence
Site-years (total)	420
Number of countries/subnational locations with data	74
Number of GBD regions with data (out of 21 regions)	19
Number of GBD super-regions with data (out of 7 super-regions)	7

We also use cause-specific mortality rate (CSMR) data, which is obtained through the hepatitis mortality modelling process. The generation and incorporation of the CSMR data is described further in the modelling strategy section below.

Modelling strategy

We model the incidence of chronic HBsAg carriage using a full DisMod-MR 2.1 model of HBsAg seroprevalence. We then convert incidence of chronic carriage to total incidence of hepatitis B infection by dividing age-specific estimates of the incidence of chronic carriage by age-specific estimates of the probability of infection resulting in carriage based on Edmunds and colleagues [2]:

$$P(carrier \mid age \leq 6\ months) = 0.885$$

$$P(carrier \mid 6\ months \leq age < 25\ years) = e^{-0.645 \times age^{0.455}}$$

$$P(carrier \mid age \geq 25\ years) = e^{-0.645 \times 25^{0.455}} = 0.061$$

Starting this round, we introduced a second modelling step after the initial estimation of prevalence and incidence. The initial prevalence and incidence estimates are used as covariates for hepatitis B mortality due to acute hepatitis, cirrhosis, and liver cancer, as detailed in the causes of death paper. Following completion of CoDCorrect, we summed the cause-specific mortality rate (CSMR) due to all three and combined the aggregated data with the same dataset of HBsAg seroprevalence. This step ensured internal consistency between all hepatitis prevalence and acute hepatitis incidence estimates. We convert the incidence of chronic HBsAg carriage to incidence of acute hepatitis B through the same calculation described above.

We then split symptomatic cases into moderate (73%) and severe (27%) severities based on data from McMahon and colleagues [3].

Sequela	Description	Disability Weight
Moderate	Has a fever and aches, and feels weak, which causes some difficulty with daily activities.	0.051 (0.032–0.074)
Severe	Has a high fever and pain, and feels very weak, which causes great difficulty with daily activities.	0.133 (0.088–0.19)
Asymptomatic	Infection with no apparent illness.	NA

A limitation of this model is that it does not account for lifetime immunity after initial HBsAg infection, which may cause incidence estimates to be inflated. In future iterations of GBD, we plan to update the model so that individuals can only be considered infected once.

Changes from GBD 2016 to GBD 2017

We have introduced a second round of modelling incidence and prevalence that combines the seroprevalence data with cause-specific mortality rate data from the hepatitis mortality estimation process. A new covariate for hepatitis B childhood vaccination was also added to the DisMod-MR 2.1 model.

Acute hepatitis C

Case definition

We define acute hepatitis C as the period corresponding to initial infection with the hepatitis C virus, resulting in anti-HCV IgG seroconversion, regardless of symptoms. It includes all ICD-10 codes under the heading B17.1 (Acute hepatitis C).

Input data

Model inputs

To estimate morbidity for hepatitis C, we use anti-HCV seroprevalence data from population-based studies and surveys to estimate incidence and prevalence of hepatitis C infection. The last systematic review was performed as part of GBD 2013. Updates to systematic reviews are performed on an ongoing schedule across all GBD causes; an update for hepatitis C will be performed in the next one to two iterations. We present a summary of the data sources in Table 4 below.

Table 4: Data Inputs for acute hepatitis C morbidity modelling by parameter.

	Prevalence
Site-years (total)	333

**Number of countries/subnational locations with data**	75
**Number of GBD regions with data (out of 21 regions)**	20
**Number of GBD super-regions with data (out of 7 super-regions)**	7

We also use cause-specific mortality rate (CSMR) data, which is obtained through the hepatitis mortality modelling process. The generation and incorporation of the CSMR data is described further in the modelling strategy section below.

Modelling strategy

We model the incidence and prevalence of hepatitis C infection using a full DisMod-MR 2.1 model of anti-HCV seroprevalence data. Starting this round, we introduce a new modelling step after the initial estimation of prevalence and incidence. The initial prevalence and incidence estimates are used to estimate hepatitis C mortality, as detailed in the causes of death paper. We then run a second DisMod-MR 2.1 model using anti-HCV seroprevalence data, with the addition of cause-specific mortality rate data derived from the mortality estimates for acute hepatitis C, liver cancer due to hepatitis C, and cirrhosis due to hepatitis C.

We estimate chronic infections from total incident infections by multiplying by the probability an incident infection will be chronic. We estimate this probability using cases reported in Guadagnino and colleagues 1997, sampling from a beta distribution (table 6) [4].

Table 5. Percentiles of the probability distribution of chronic hepatitis C

0 percentile	25 percentile	50 percentile	75 percentile	100 percentile
0.65	0.73	0.75	0.76	0.83

Of the remaining acute infections, we divide incident infections into asymptomatic (75%), moderate (24%), and severe (1%) states based on expert opinion.

Sequela	Description	Disability Weight
Moderate	Has a fever and aches, and feels weak, which causes some difficulty with daily activities.	0.051 (0.032–0.074)
Severe	Has a high fever and pain, and feels very weak, which causes great difficulty with daily activities.	0.133 (0.088–0.19)
Asymptomatic	Infection with no apparent illness.	NA

Changes from GBD 2016 to GBD 2017

We have introduced a second round of modelling incidence and prevalence that combines the seroprevalence data with cause-specific mortality rate data from the hepatitis mortality estimation process.

Acute hepatitis E

Flowchart

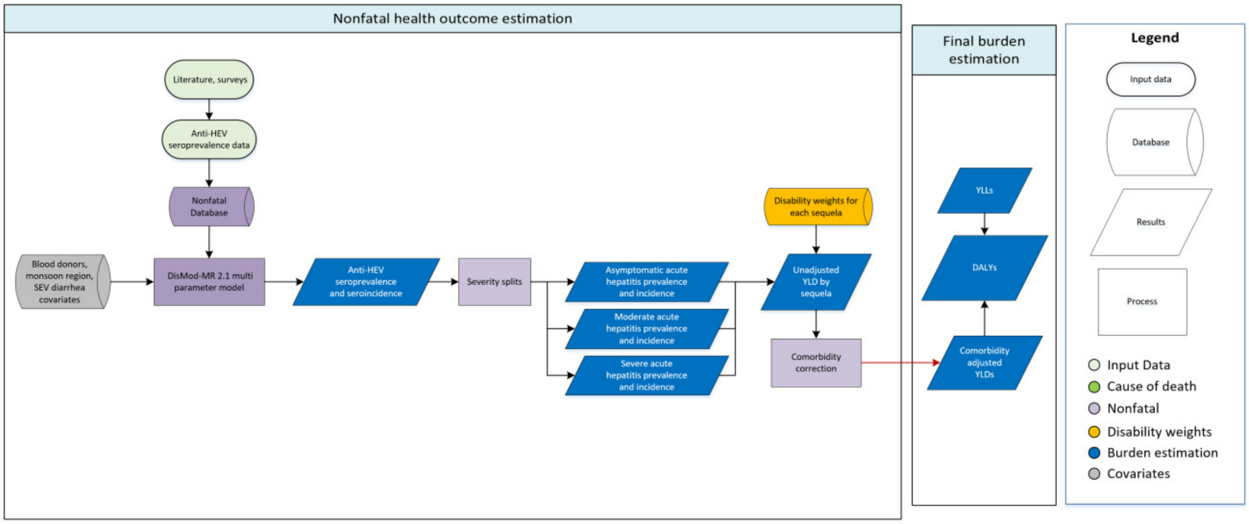

Case definition

We define acute hepatitis E as an infection with the hepatitis E virus resulting in anti-HEV IgG seroconversion, regardless of symptoms. It includes all ICD-10 codes under the heading B17.2 (Acute hepatitis E).

Input data

Model inputs

We use anti-HEV seroprevalence data from population-based studies and surveys to estimate incidence of infection. The last systematic review was performed as part of GBD 2013. Updates to systematic reviews are performed on an ongoing schedule across all GBD causes; an update for hepatitis E will be performed in the next one to two iterations. We present a summary of the data sources in Table 5 below.

Table 5: Data Inputs for acute hepatitis E morbidity modelling by parameter.

	Prevalence
Site-years (total)	91
Number of countries/subnational locations with data	43
Number of GBD regions with data (out of 21 regions)	19
Number of GBD super-regions with data (out of 7 super-regions)	7

Modelling Strategy

We model the incidence of hepatitis E using a full DisMod-MR 2.1 model of anti-HEV seroprevalence, assuming no remission. Based on information published by Rein and colleagues [5], we assume that the probability of symptomatic infection increases with age from ~1% in the first year of life to ~60% in adulthood.

The table below illustrates the sequelae associated with acute hepatitis E, along with their descriptions and disability weights.

Sequela	Description	Disability Weight
Moderate	Has a fever and aches, and feels weak, which causes some difficulty with daily activities.	0.051 (0.032–0.074)
Severe	Has a high fever and pain, and feels very weak, which causes great difficulty with daily activities.	0.133 (0.088–0.19)
Asymptomatic	Infection with no apparent illness.	NA

Changes from GBD 2016 to GBD 2017

We have made no substantive changes in the modelling strategy from GBD 2016.

References

1. Armstrong GL, Bell BP. Hepatitis A Virus Infections in the United States: Model-Based Estimates and Implications for Childhood Immunization. Pediatrics. 2002 May 1;109(5):839–45.
2. Edmunds WJ, Medley GF, Nokes DJ, Hall AJ, Whittle HC. The influence of age on the development of the hepatitis B carrier state. Proc Biol Sci. 1993 Aug 23;253(1337):197–201.
3. McMahon BJ, Alward WL, Hall DB, Heyward WL, Bender TR, Francis DP, et al. Acute hepatitis B virus infection: relation of age to the clinical expression of disease and subsequent development of the carrier state. J Infect Dis. 1985 Apr;151(4):599–603).
4. Guadagnino, Vincenzo, et al. "Prevalence, risk factors, and genotype distribution of hepatitis C virus infection in the general population: a community-based survey in southern Italy." Hepatology 26.4 (1997): 1006-1011.
5. Rein DB, Stevens GA, Theaker J, Wittenborn JS, Wiersma ST. The global burden of hepatitis E virus genotypes 1 and 2 in 2005. Hepatology. 2012 Apr 1;55(4):988–97.

Other unspecified infectious diseases

Flowchart

Anemia Impairment Estimation

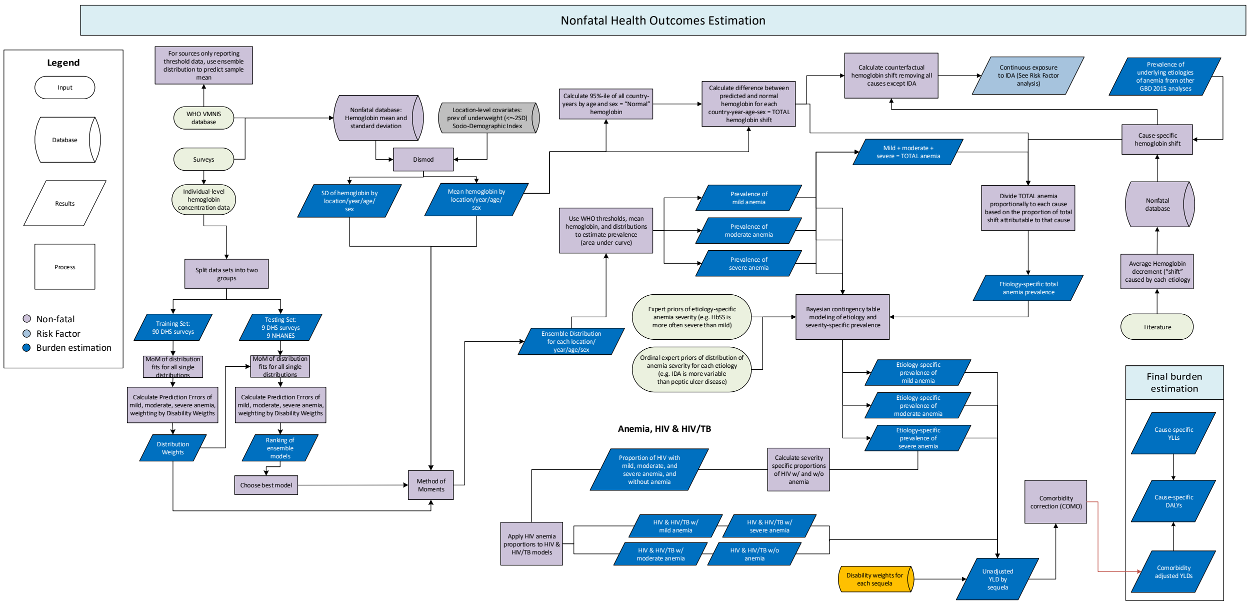

Input data and methodological summary

For GBD 2017, we estimate other unspecified infectious diseases using the residual anaemia impairment envelope based on a fixed proportion of redistribution. The resulting models of mild anaemia due to other infectious diseases, moderate anaemia due to other infectious diseases, and severe anaemia due to other infectious diseases go into our central computation to generate YLDs based on our prevalence values.

Causes for which allocation of the residual anaemia envelope was based on fixed proportion redistribution methods*:
Iron-deficiency anaemia (IDA)
Other infectious diseases
Other neglected tropical diseases
Other endocrine, nutrition, blood, and immune disorders
Other haemoglobinopathies and haemolytic anaemias

* A minimum of 10% of all anaemia was assigned to residual categories based on analysis of NHANES-III data from the United States

References

1. Kassebaum NJ. The Global Burden of Anemia. *Hematology/Oncology Clinics* 2016; **30**: 247–308.

2. Kassebaum NJ, Jasrasaria R, Naghavi M, *et al.* A systematic analysis of global anemia burden from 1990 to 2010. *Blood* 2014; **123**: 615–24.

Maternal disorders

1) Abortion and miscarriage; 2) Ectopic pregnancy; 3) Obstructed labour and uterine rupture; 4) Maternal haemorrhage; 5) Maternal sepsis and other maternal infections; 6) Maternal hypertensive disorders; 7) Other maternal disorders. Obstetric fistula is captured separately.

Flowchart

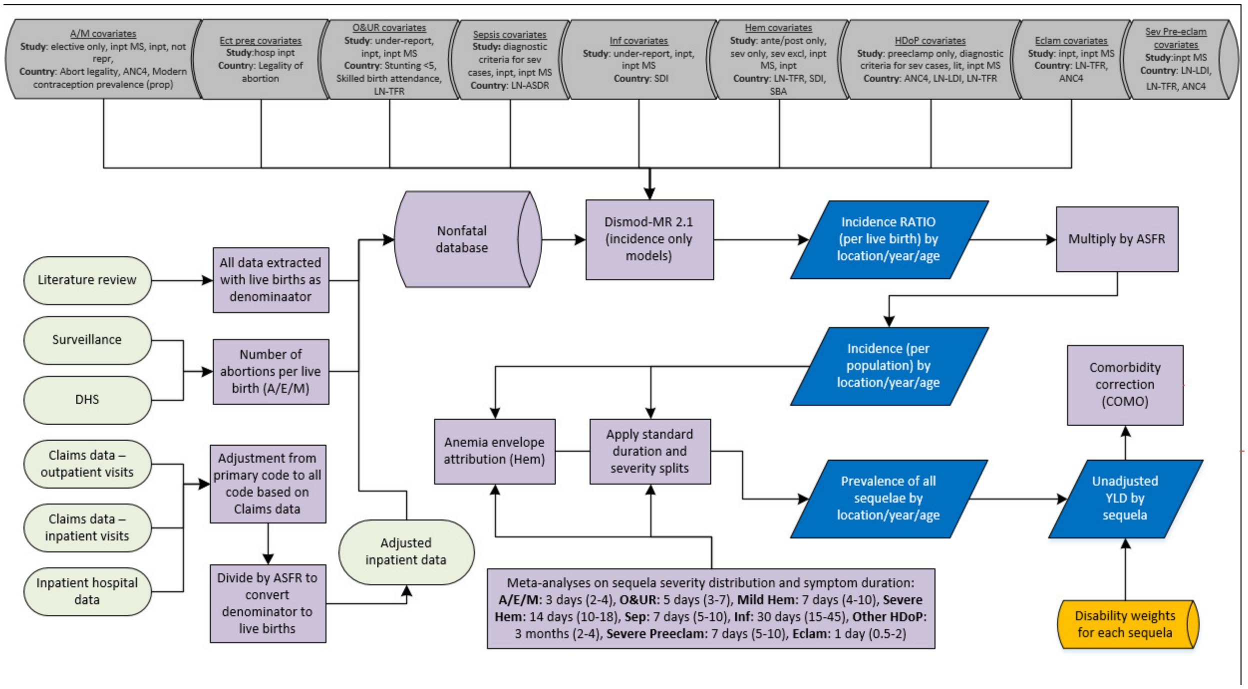

Input data and methodological summary

Case definition

Maternal disorders are those complications occurring during pregnancy, childbirth, and the postpartum period. Nine different statistical models were completed for GBD 2017. These included 1) abortion and miscarriage, 2) obstructed labour and uterine rupture, 3) maternal haemorrhage (including placental disorders), 4) maternal sepsis, 5) other maternal infections, 6) hypertensive disorders of pregnancy, 7) eclampsia, 8) severe pre-eclampsia, 9) ectopic pregnancy. Other direct maternal disorders were estimated using a YLD-to-YLL ratio approach used for multiple different GBD causes. Late maternal death and indirect maternal disorders, including HIV-related maternal death, were not assigned any disability as the disability associated with them was assumed to be included in estimates for the underlying conditions. The table below lists the ICD-9 and ICD-10 codes for each of the statistical models.

Table 1. ICD codes for maternal disorders

International classification of diseases codes for maternal disorders in GBD 2017 non-fatal analysis		
**Model**	**ICD10 code**	**ICD9 code**
Abortion, miscarriage	O01-O08, O36.4	631, 634-639
Ectopic pregnancy	O00	633-633.91
Maternal haemorrhage	O20, O43.2, O44-O46, O62.2, O67, O72	640-641, 661.0, 666
Eclampsia	O15	642.6
Hypertensive disorders of pregnancy	O11-O16	642.3, 642.4, 642.5, 642.6, 642.7, 642.9
Severe pre-eclampsia	O14.1-O14.13	642.5-642.54
Obstructed labour and uterine rupture	O64-O66, O71, O83	659-660, 662, 665, 669.5, 669.6
Other maternal infections	O23, O41, O75.2-3, O86, O91	646.5, 646.6, 659.2, 672.0, 674.1, 674.2, 674.3, 675
Maternal sepsis	O85	659.3, 670

Input data

Systematic literature reviews were completed for GBD 2010, GBD 2013, GBD 2015, GBD 2016, and GBD 2017. These were updated on July 20, 2017, using the combined search string below. In addition, we searched ministry of health websites for pregnancy complication data and used Confidential Enquiry and other sources used in our maternal mortality analyses when they presented data on pregnancy complications. We also performed snowball searches for abortion reporting and surveillance data systems, finding multiple such systems throughout high-income countries and several geographies in Central and Eastern Europe. Inpatient and outpatient data were used, as were claims data from Taiwan and Singapore as well as MarketScan in the United States. These data were extracted and processed as described in the section on hospital data, including use of primary-to-any inpatient ratio to correct for under-reporting of pregnancy complications in hospital datasets that rely only on primary discharge codes. All data were extracted in standard fashion, and were uploaded and stored on a centralised SQL database. The final dataset contents for each of the models is shown below as well.

Maternal disorders search string:

(((((("Postpartum Hemorrhage" OR "Uterine Hemorrhage") OR (maternal[Title/Abstract] OR pregnant[Title/Abstract] OR pregnancy[Title/Abstract] OR mothers) AND (haemorrhag*[Title/Abstract] OR hemorrhag*[Title/Abstract]) NOT "case report"[All fields]) OR (("induced abortion" OR

"Therapeutic abortion" OR "legal Abortion" OR "medical abortion" OR "miscarriage" OR "Abortion, Induced"[Mesh] OR "Abortion, Therapeutic"[Mesh] OR "Abortion, Legal"[Mesh] OR "ectopic Pregnancy") NOT ("case report"[Title/Abstract] OR "birth defect"[Title/Abstract] OR congenital[Title/Abstract])) OR ("obstructed labour" OR "obstructed labor" OR "labour dystocia" OR "labor dystocia" OR dystocia OR "cephalopelvic disproportion" OR "cephalo-pelvic disproportion") OR (("obstetric fistula" OR "vesicovaginal fistula") OR "rectovaginal fistula") OR (("Puerperal Infection"[Mesh] OR "Puerperal Infection" OR ((maternal[Title/Abstract] OR pregnant[Title/Abstract] OR pregnancy[Title/Abstract]) AND (Sepsis OR infection[Title/Abstract]))) NOT "case report") OR ((Pre-Eclampsia[Title/Abstract] OR preeclampsia[Title/Abstract] OR Eclampsia[Title/Abstract] OR Pre-Eclampsia[Mesh] OR Eclampsia[Mesh] OR "Hypertension, Pregnancy-Induced"[Mesh] OR "pregnancy induced hypertension"[Title/Abstract] OR "gestational hypertension"[Title/Abstract] OR "Hypertensive disorders of pregnancy"[Title/Abstract]) NOT ("case report" OR "kidney don*"[Title/Abstract] OR polymorphism*[Title/Abstract] OR endotheli*[Title/Abstract]))) AND (2016/08/01[PDat] : 2017/12/31[PDat]) NOT (animals[MeSH] NOT humans[MeSH]))) OR (((((("maternal mortality"[Title/Abstract] OR "maternal death"[Title/Abstract] OR "MM"[Title/Abstract] OR "confidential enquiry"[Title/Abstract] OR ((obstetric[Title/Abstract] OR pregnancy[Title/Abstract]) AND (etiology[Title/Abstract] OR cause[Title/Abstract] or pattern[Title/Abstract]) AND (death[Title/Abstract] OR mortality[Title/Abstract]))) NOT (fetal[Title/Abstract] OR newborns[Title/Abstract] OR newborn[Title/Abstract] OR neonatal[Title/Abstract] OR “case report”[Title/Abstract] OR “case study”[Title/Abstract] OR pathogenesis[Title/Abstract] OR thromboprophylaxis[Title/Abstract])) NOT (animals[MeSH] NOT humans[MeSH]) OR (((“maternal mortality”[Title/Abstract] OR “maternal death*”[Title/Abstract] OR “MMR”[Title/Abstract]) AND (“Afghanistan”[Title/Abstract] OR “Albania”[Title/Abstract] OR “Algeria”[Title/Abstract] OR “Andorra”[Title/Abstract] OR “Angola”[Title/Abstract] OR “Antigua and Barbuda”[Title/Abstract] OR “Argentina”[Title/Abstract] OR “Armenia”[Title/Abstract] OR “Azerbaijan”[Title/Abstract] OR “Bahrain”[Title/Abstract] OR “Bangladesh”[Title/Abstract] OR “Barbados”[Title/Abstract] OR “Belarus”[Title/Abstract] OR “Belize”[Title/Abstract] OR “Benin”[Title/Abstract] OR “Bhutan”[Title/Abstract] OR “Bolivia”[Title/Abstract] OR “Bosnia and Herzegovina”[Title/Abstract] OR “Botswana”[Title/Abstract] OR “Brazil”[Title/Abstract] OR “Brunei”[Title/Abstract] OR “Bulgaria”[Title/Abstract] OR “Burkina Faso”[Title/Abstract] OR “Burundi”[Title/Abstract] OR “Cambodia”[Title/Abstract] OR “Cameroon”[Title/Abstract] OR “Cape Verde”[Title/Abstract] OR “Central African Republic”[Title/Abstract] OR “Chad”[Title/Abstract] OR “China”[Title/Abstract] OR “Colombia”[Title/Abstract] OR “Comoros”[Title/Abstract] OR “Congo”[Title/Abstract] OR “Costa Rica”[Title/Abstract] OR “Croatia”[Title/Abstract] OR “Cuba”[Title/Abstract] OR “Cyprus”[Title/Abstract] OR “Côte d’Ivoire”[Title/Abstract] OR “Democratic Republic of the Congo”[Title/Abstract] OR “Djibouti”[Title/Abstract] OR “Dominica”[Title/Abstract] OR “Dominican Republic”[Title/Abstract] OR “Ecuador”[Title/Abstract] OR “Egypt”[Title/Abstract] OR “El Salvador”[Title/Abstract] OR “Equatorial Guinea”[Title/Abstract] OR “Eritrea”[Title/Abstract] OR “Ethiopia”[Title/Abstract] OR “Federated States of Micronesia”[Title/Abstract] OR “Fiji”[Title/Abstract] OR “Gabon”[Title/Abstract] OR “Georgia”[Title/Abstract] OR “Ghana”[Title/Abstract] OR “Grenada”[Title/Abstract] OR “Guatemala”[Title/Abstract] OR “Guinea”[Title/Abstract] OR “Guinea-Bissau”[Title/Abstract] OR “Guyana”[Title/Abstract] OR “Haiti”[Title/Abstract] OR “Honduras”[Title/Abstract] OR “India”[Title/Abstract] OR “Indonesia”[Title/Abstract] OR “Iran”[Title/Abstract] OR “Iraq”[Title/Abstract] OR “Jamaica”[Title/Abstract] OR “Jordan”[Title/Abstract] OR “Kazakhstan”[Title/Abstract] OR “Kenya”[Title/Abstract] OR “Kiribati”[Title/Abstract] OR

“Kuwait”[Title/Abstract] OR “Kyrgyzstan”[Title/Abstract] OR “Laos”[Title/Abstract] OR “Latvia”[Title/Abstract] OR “Lebanon”[Title/Abstract] OR “Lesotho”[Title/Abstract] OR “Liberia”[Title/Abstract] OR “Libya”[Title/Abstract] OR “Lithuania”[Title/Abstract] OR “Macedonia”[Title/Abstract] OR “Madagascar”[Title/Abstract] OR “Malawi”[Title/Abstract] OR “Malaysia”[Title/Abstract] OR “Maldives”[Title/Abstract] OR “Mali”[Title/Abstract] OR “Malta”[Title/Abstract] OR “Marshall Islands”[Title/Abstract] OR “Mauritania”[Title/Abstract] OR “Mauritius”[Title/Abstract] OR “Moldova”[Title/Abstract] OR “Mongolia”[Title/Abstract] OR “Montenegro”[Title/Abstract] OR “Morocco”[Title/Abstract] OR “Mozambique”[Title/Abstract] OR “Myanmar”[Title/Abstract] OR “Namibia”[Title/Abstract] OR “Nepal”[Title/Abstract] OR “Nicaragua”[Title/Abstract] OR “Niger”[Title/Abstract] OR “Nigeria”[Title/Abstract] OR “North Korea”[Title/Abstract] OR “Oman”[Title/Abstract] OR “Pakistan”[Title/Abstract] OR “Palestine”[Title/Abstract] OR “Panama”[Title/Abstract] OR “Papua New Guinea”[Title/Abstract] OR “Paraguay”[Title/Abstract] OR “Peru”[Title/Abstract] OR “Philippines”[Title/Abstract] OR “Qatar”[Title/Abstract] OR “Romania”[Title/Abstract] OR “Russia”[Title/Abstract] OR “Rwanda”[Title/Abstract] OR “Saint Lucia”[Title/Abstract] OR “Saint Vincent and the Grenadines”[Title/Abstract] OR “Samoa”[Title/Abstract] OR “Saudi Arabia”[Title/Abstract] OR “Senegal”[Title/Abstract] OR “Serbia”[Title/Abstract] OR “Seychelles”[Title/Abstract] OR “Sierra Leone”[Title/Abstract] OR “Singapore”[Title/Abstract] OR “Solomon Islands”[Title/Abstract] OR “Somalia”[Title/Abstract] OR “South Africa”[Title/Abstract] OR "South Sudan"[Title/Abstract] OR “Sri Lanka”[Title/Abstract] OR “Sudan”[Title/Abstract] OR “Suriname”[Title/Abstract] OR “Swaziland”[Title/Abstract] OR “Syria”[Title/Abstract] OR “São Tomé and Príncipe”[Title/Abstract] OR “Taiwan”[Title/Abstract] OR “Tajikistan”[Title/Abstract] OR “Tanzania”[Title/Abstract] OR “Thailand”[Title/Abstract] OR “The Bahamas”[Title/Abstract] OR “The Gambia”[Title/Abstract] OR “Timor-Leste”[Title/Abstract] OR “Togo”[Title/Abstract] OR “Tonga”[Title/Abstract] OR “Trinidad and Tobago”[Title/Abstract] OR “Tunisia”[Title/Abstract] OR “Turkmenistan”[Title/Abstract] OR “Uganda”[Title/Abstract] OR “Ukraine”[Title/Abstract] OR “United Arab Emirates”[Title/Abstract] OR “Uruguay”[Title/Abstract] OR “Uzbekistan”[Title/Abstract] OR “Vanuatu”[Title/Abstract] OR “Venezuela”[Title/Abstract] OR “Vietnam”[Title/Abstract] OR “Yemen”[Title/Abstract] OR “Zambia”[Title/Abstract] OR “Zimbabwe”[Title/Abstract]) NOT (animals[MeSH] NOT humans[MeSH]) NOT (“demographic and health survey*”[Title/Abstract] OR DHS[Title/Abstract] OR “reproductive health survey*”[Title/Abstract] OR RHS[Title/Abstract]))) AND (2016/08/15[PDat] : 2017/12/31[PDat]))))) OR ((HIV[Title/Abstract] OR "Acquired Immunodeficiency Syndrome"[Title/Abstract] OR AIDS[Title/Abstract]) AND (“pregnant”[Title/Abstract] OR “pregnancy”[Title/Abstract] OR “postpartum”[Title/Abstract] OR "post partum"[Title/Abstract]) AND (“mortality”[Title/Abstract] OR “death”[Title/Abstract]) NOT "case report" NOT (animals[MeSH] NOT humans[MeSH]) AND (2016/08/15[PDat] : 2017/12/31[PDat]))

Maternal Disorders

	Incidence
Studies	22
Countries/subnationals	21/3

GBD world regions	10

The table below shows the number of site-years by maternal cause:

Maternal haemorrhage

	Case fatality rate	Incidence
**Site-years (total)**	18	763
**Number of countries with data**	12	62
**Number of GBD regions with data (out of 21 regions)**	8	19
**Number of GBD super-regions with data (out of 7 super-regions)**	6	7

Hypertensive disorders of pregnancy

	Case fatality rate	Incidence
**Site-years (total)**	14	1,416
**Number of countries with data**	11	71
**Number of GBD regions with data (out of 21 regions)**	7	19
**Number of GBD super-regions with data (out of 7 super-regions)**	5	7

Eclampsia

	Case fatality rate	Incidence
**Site-years (total)**	56	787
**Number of countries with data**	24	70
**Number of GBD regions with data (out of 21 regions)**	12	19
**Number of GBD super-regions with data (out of 7 super-regions)**	7	7

Severe pre-eclampsia

	Case fatality rate	Incidence
**Site-years (total)**	7	656
**Number of countries with data**	7	30
**Number of GBD regions with data (out of 21 regions)**	6	14
**Number of GBD super-regions with data (out of 7 super-regions)**	5	6

Obstructed labour

	Incidence

**Site-years (total)**	1,301
**Number of countries with data**	42
**Number of GBD regions with data (out of 21 regions)**	15
**Number of GBD super-regions with data (out of 7 super-regions)**	7

Abortion and miscarriage

	**Incidence**
**Site-years (total)**	2,477
**Number of countries with data**	51
**Number of GBD regions with data (out of 21 regions)**	16
**Number of GBD super-regions with data (out of 7 super-regions)**	7

Ectopic pregnancy

	**Case fatality rate**	**Incidence**
**Site-years (total)**	1	573
**Number of countries with data**	1	40
**Number of GBD regions with data (out of 21 regions)**	1	14
**Number of GBD super-regions with data (out of 7 super-regions)**	1	7

Puerperal sepsis

	**Case fatality rate**	**Incidence**
**Site-years (total)**	14	1,310
**Number of countries with data**	13	57
**Number of GBD regions with data (out of 21 regions)**	10	18
**Number of GBD super-regions with data (out of 7 super-regions)**	7	7

Other maternal infections

	**Incidence**
**Site-years (total)**	710
**Number of countries with data**	42
**Number of GBD regions with data (out of 21 regions)**	15
**Number of GBD super-regions with data (out of 7 super-regions)**	7

Modelling strategy

We estimated the incidence ratio of each category of pregnancy complications using DisMod-MR 2.1, with the exception of other maternal disorders, which we estimated using a YLD-to-YLL ratio approach used in multiple causes across GBD 2017. The reason is that most literature and surveillance data are expressed in terms of number of events per livebirth rather than per population. Hospital and claims data, which were centrally processed for all GBD 2017 causes to have population as the denominator, were transformed to have livebirths as the denominator by dividing by age-specific fertility rate (ASFR; live births per population).

We used the datasets described above to estimate incidence ratio for each age-sex-location-year in the GBD 2017 location hierarchy using DisMod-MR 2.1. A number of study-level covariates were used to crosswalk from non-standard sub-populations or case definitions. For most conditions, MarketScan claims data or literature data were considered to be the closest approximation of the true incidence of complications, and so were identified as the reference category. A series of country covariates were then chosen to help drive the magnitude of estimates in areas of sparse or absent data. As fertility rate is partially related to the number of cases of a given complication, the natural log of total fertility rate (TFR) from our demographics analysis was used in most of the models. No specific age or slope priors were used. All models were run with a time window of five years. The quantitative results of study-level and country-level covariates for each condition are shown below.

Abortion and miscarriage

**Study-level covariate**	**Parameter**	**Geography level**	**beta**	**Exponentiated beta**
Not representative	Incidence	Global	-1 (-1.97 to 0)	0.37 (0.14–1.00)
Hospital inpatient	Incidence	Global	-4.3e-04 (-5.9e-04 to -1.2e-04)	1.00 (1.00–1.00)
DHS survey data	Incidence	Global	-8.7e-04 (-0.0023 to -1.4e-04)	1.00 (1.00–1.00)

**Country-level covariate**	**Parameter**	**Geography level**	**beta**	**Exponentiated beta**
Legality of abortion	Incidence	Global	0.020 (0.019 to 0.021)	1.02 (1.02–1.02)
Antenatal care (4 visits) coverage (proportion)	Incidence	Global	-2.3e-05 (-2.8e-05 to -4.6e-06)	1.00 (1.00–1.00)
Contraception (modern) prevalence (proportion)	Incidence	Global	-0.1 (-0.1 to -0.1)	0.90 (0.90–0.90)

Ectopic pregnancy

Country-level covariate	Parameter	Geography level	beta	Exponentiated beta
Legality of abortion	Incidence	Global	-0.0086 (-0.0096 to -0.0075)	0.99 (0.99–0.99)
Pelvic inflammatory disease age-standardised prevalence	Incidence	Global	0.60 (0.050 to 0.99)	1.82 (1.05–2.68)
Ectopic pregnancy lnASDR	Incidence	Global	0.0013 (0.000041 to 0.0043)	1.00 (1.00–1.00)

Maternal haemorrhage

**Study-level covariate**	**Parameter**	**Geography level**	**beta**	**Exponentiated beta**
Postpartum haemorrhage only	Incidence	Global	-0.0092 (-0.034 to -0.00015)	0.99 (0.97–1.00)
Antepartum haemorrhage only	Incidence	Global	-0.34 (-0.77 to -0.032)	0.71 (0.46–0.97)
Severe haemorrhage only	Incidence	Global	-0.19 (-0.4 to -0.018)	0.82 (0.67–0.98)
Severe haemorrhage excluded	Incidence	Global	-0.075 (-0.26 to -0.0027)	0.93 (0.77–1.00)

**Country-level covariate**	**Parameter**	**Geography level**	**beta**	**Exponentiated beta**
Skilled birth attendance (proportion)	Incidence	Global	-0.0039 (-0.016 to -0.00037)	1.00 (0.98–1.00)
Socio-demographic Index	Incidence	Global	-0.1 (-0.11 to -0.1)	0.90 (0.90–0.90)
Total fertility rate	Incidence	Global	0.0027 (0.00012 to 0.0099)	1.00 (1.00–1.01)

Hypertensive disorders of pregnancy

**Study-level covariate**	**Parameter**	**Geography level**	**beta**	**Exponentiated beta**
Hospital inpatient	Incidence	Global	-0.3 (-0.3 to -0.3)	0.74 (0.74–0.74)
Literature	Incidence	Global	-0.23 (-0.24 to -0.22)	0.80 (0.79–0.80)
Diagnostic criteria for severe cases	Incidence	Global	-2 (-4 to 0)	0.14 (0.018–1.00)
Includes only pre-eclampsia data (no other	Incidence	Global	-0.69 (-0.71 to -0.67)	0.50 (0.49–0.51)

gestational hypertension)				
Chronic hypertension combined with hypertensive disorders of pregnancy	Incidence	Global	1.01 (0.97–1.05)	2.75 (2.64–2.86)

**Country-level covariate**	**Parameter**	**Geography level**	**beta**	**Exponentiated beta**
Antenatal vare (4 visits) voverage (proportion)	Incidence	Global	-5.1e-06 (-5.7e-06 to -3.5e-06)	1.00 (1.00–1.00)
Systolic blood pressure (mmHg)	Incidence	Global	-0.0098 (-0.013 to -0.0063)	0.99 (0.99–0.99)
Total fertility rate	Incidence	Global	-1.7e-04 (-1.7e-04 to -1.7e-04)	1.00 (1.00–1.00)

Eclampsia

**Study-level covariate**	**Parameter**	**Geography level**	**beta**	**Exponentiated beta**
Hospital inpatient	Incidence	Global	4.6e-04 (9.4e-05 to 0.0011)	1.00 (1.00–1.00)
MarketScan	Incidence	Global	-0.37 (-0.39 to -0.36)	0.69 (0.68–0.70)
Inpatient-only MarketScan, year 2000	Incidence	Global	-1.54 (-1.59 to -1.49)	0.21 (0.20–0.23)

**Country-level covariate**	**Parameter**	**Geography level**	**beta**	**Exponentiated beta**
Antenatal care (4 visits) coverage (proportion)	Incidence	Global	-1.76 (-3.7 to -1.11)	0.17 (0.025–0.33)
Total fertility rate	Incidence	Global	-0.24 (-0.33 to -0.21)	0.79 (0.72–0.81)

Severe pre-eclampsia

**Study-level covariate**	**Parameter**	**Geography level**	**beta**	**Exponentiated beta**
Includes only pre-eclampsia data (no other gestational hypertension)	Incidence	Global	-0.34 (-0.52 to -0.21)	0.71 (0.59–0.81)
MarketScan	Incidence	Global	0.67 (0.55–0.83)	1.94 (1.73–2.28)

**Country-level covariate**	**Parameter**	**Geography level**	**beta**	**Exponentiated beta**
Antenatal Care (4 visits) Coverage (proportion)	Incidence	Global	-0.014 (-0.055 to -2.1-04)	1.00 (0.99–1.00)

Total fertility rate	Incidence	Global	-0.058 (-0.16 to -0.0021)	0.94 (0.85–1.00)
LDI (I$ per capita)	Incidence	Global	-0.052 (-0.1 to -0.013)	0.95 (0.90–0.99)

Obstructed labour and uterine rupture

**Study-level covariate**	**Parameter**	**Geography level**	**beta**	**Exponentiated beta**
Risk of bias high	Incidence	Global	1.50 (1.50–1.50)	4.48 (4.48–4.48)
MarketScan	Incidence	Global	0.95 (0.92–0.98)	2.59 (2.51–2.67)
Marketscan, year 2000	Incidence	Global	1.99 (1.95–2.00)	7.29 (7.04–7.39)

**Country-level covariate**	**Parameter**	**Geography level**	**beta**	**Exponentiated beta**
Stunting (proportion <2SD height for age, <5 years)	Incidence	Global	0.0014 (0.00023–0.0064)	1.00 (1.00–1.01)
Skilled birth attendance (proportion)	Incidence	Global	-9.6e-04 (-0.0042 to -1.4e-04)	1.00 (1.00–1.00)

Maternal sepsis

**Study-level covariate**	**Parameter**	**Geography level**	**beta**	**Exponentiated beta**
Hospital inpatient	Incidence	Global	0.098 (0.0066–0.22)	1.10 (1.01–1.24)
Diagnostic criteria for severe cases	Incidence	Global	-1.32 (-1.67 to -1)	0.27 (0.19–0.37)
MarketScan, year 2000	Incidence	Global	-0.0033 (-0.42 to 0.44)	1.00 (0.66–1.55)
MarketScan	Incidence	Global	-0.75 (-0.86 to -0.65)	0.47 (0.42–0.52)

**Country-level covariate**	**Parameter**	**Geography level**	**beta**	**Exponentiated beta**
Diabetes age-Standardised prevalence (proportion)	Incidence	Global	0.14 (0.0031–0.51)	1.15 (1.00–1.66)
Maternal sepsis and other maternal infections LN-ASDR	Incidence	Global	0.12 (0.11–0.14)	1.13 (1.11–1.15)

Other maternal infections:

Study-level covariate	Parameter	Geography level	beta	Exponentiated beta
Hospital inpatient	Incidence	Global	-0.25 (-0.3 to -0.2)	0.78 (0.74–0.82)
Under-reported	Incidence	Global	-0.45 (-1.02 to -0.024)	0.64 (0.36–0.98)

Country-level covariate	Parameter	Geography level	beta	Exponentiated beta
Socio-demographic Index	Incidence	Global	-0.0028 (-0.008 to -8.5e-05)	1.00 (0.99–1.00)

Severity splits

After completion of DisMod-MR 2.1 models, all age-specific ratios were then converted to population rates by multiplying by ASFR. Maternal haemorrhage was split between moderate (500 to <1000 ml blood loss) and severe (>1000 ml blood loss) on the basis of a meta-analysis of 19 studies[1]. Data on the average duration of acute symptoms were not available so, after consultation with clinician collaborators, we assigned a duration of seven days (+/-3) for moderate haemorrhage and 14 days (+/- 4) for severe haemorrhage. The total maternal haemorrhage incidence served as input to the causal attribution process of the overall anaemia envelope, which is described separately. This is a change from GBD 2013, when only the prevalence of maternal haemorrhage was considered in anaemia causal attribution, which was inappropriate because the disability from haemorrhage-induced anaemia is longer-lasting than the acute haemorrhagic event, a situation that was not previously reflected. Acute disability was calculated assuming incident cases of abortion and miscarriage persist for an average of three days (+/-1). The same was calculated for ectopic pregnancy. Obstructed labour was assigned a duration of five days (+/-2). Again, these determinations were based on clinical expert determination as we could not identify any data to inform this.

Hypertensive disorders of pregnancy (HDoP) and maternal sepsis and other maternal infections were estimated in two models each. HDoP YLD estimates included severe pre-eclampsia and other HDoP which were derived from two models of total HDoP and severe pre-eclampsia. The duration of severe pre-eclampsia was assigned to be 7 days (+/-2) and other HDoP was assigned a duration of three months (2-4). Eclampsia was a separate model, assigned a duration of one day (+/-1). A large number of those with severe pre-eclampsia go on to have long-term sequelae of the condition[2], as do those with eclampsia[3,4], both of which were included in the estimates of HDoP and made up the bulk of the YLDs for those conditions. Maternal sepsis was assigned a duration of five days (+/-2) and, based on the same data identified in our review of pelvic inflammatory disease (PID; described separately), a proportion of incident cases were estimated to continue on to have secondary infertility due to maternal sepsis. Other maternal infections were assigned a wide potential duration of 15 to 45 days (mean 30).

Uncertainty and model selection

For all maternal disorders, uncertainty bounds include uncertainty due to input data, crosswalks from non-reference definitions, uncertainty in numerical solutions (posteriors) of each DisMod-MR 2.1 model, duration of symptoms, and proportion of all persons with each type of symptom.

In consultation with GBD researchers and collaborators, final models were selected on a combination of qualitative and quantitative goodness of fit to input data, plausibility of geographic and temporal trends, consistency of age pattern, and, when available, comparison with other published studies on the epidemiology of pregnancy complications. Directionality, magnitude, and plausibility of study-level and country-level covariates were also considered in the process of model development. Of note, due to the nature of statistical modelling, final results do not always cover the values reported in input data.

Maternal disorders (Obstetric fistula)

Flowchart

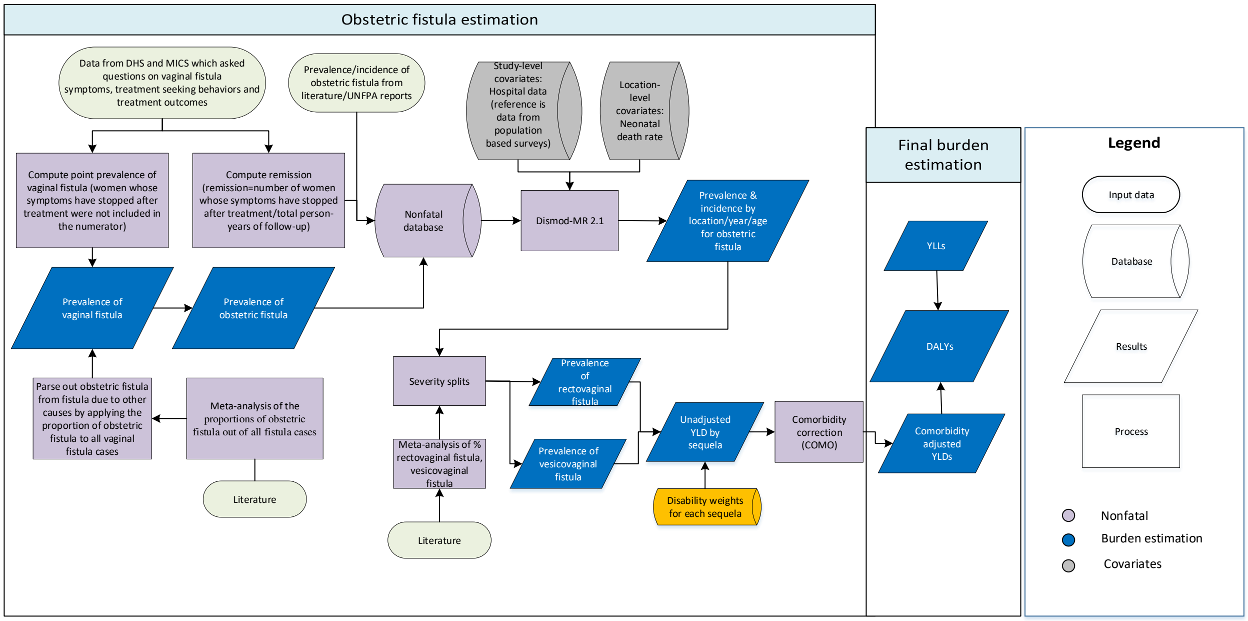

Case Definition

This is estimated as a component of maternal obstructed labour. Obstetric fistula is a severe long-term complication of prolonged obstructed labour in which a fistula (hole) develops between the birth canal and the bladder and/or rectum.

Input data

Model Inputs

A systematic review was conducted for GBD 2015. The PubMed search terms were: (('obstetric fistula'[All Fields] OR 'vesicovaginal fistula'[All Fields]) OR 'rectovaginal fistula'[All Fields]) AND ('2013'[PDAT] : '2015'[PDAT]) AND 'humans'[MeSH Terms].

The exclusion criteria were:

1. Studies that did not provide primary data on epidemiological parameters, eg, commentaries
2. Case series
3. Reviews

The table below shows the number of literature studies included in GBD 2017, as well as the number of countries or subnational units and GBD world regions represented.

	Prevalence	Incidence	Mortality risk

Studies	8	3	-
Countries/subnationals	8	3	-
GBD world regions	3	1	-

In addition to using data from published studies, we also included data from UNFPA reports and nationally representative Demographic and Health Surveys and Multiple Indicator Cluster Surveys.

The data used in these models are summarised in the table below:

	Incidence	Prevalence	Remission
**Site-years (total)**	4	32	10
**Number of countries with data**	4	26	10
**Number of GBD regions with data (out of 21 regions)**	1	5	3
**Number of GBD super-regions with data (out of 7 super-regions)**	1	3	1

Severity Splits

The following severity distributions were assigned based on a meta-analysis of published studies[1-4] and Pakistan Demographic and Health survey (2006–2007): vesicovaginal fistula (90.8%, 95% CI: 85.0 to 95.4%); rectovaginal fistula (9.2%, 95% CI: 4.6 to 15.0%). The lay descriptions and disability weights for severity levels derived from the GBD disability weights study are shown below.

Severity level	Lay description	DW (95% CI)
vesicovaginal fistula	has an abnormal opening between the bladder and the vagina, which makes her unable to control urinating. The woman is anxious and depressed.	0.342 (0.227–0.478)
rectovaginal fistula	has an abnormal opening between her vagina and rectum causing flatulence and feces to escape through the vagina. The person gets infections in her vagina, and has pain when urinating.	0.501 (0.339–0.657)

Modelling Strategy

For GBD 2017, obstetric fistula was modelled using DisMod-MR 2.1. We used neonatal mortality rate as a country-level covariate. We also included a study-level covariate indicating whether it was a hospital-based or community-based study. We assume obstetric fistula is restricted to sub-Saharan Africa, South Asia, Yemen, Afghanistan, and Sudan. Remission was calculated, using the cure data from 11 Demographic and Health surveys, by dividing the number of cured obstetric fistula cases by total person-years of follow-up of all cases (cured, uncured, and untreated). The person-year of follow-up for uncured or untreated fistula cases was calculated as the time interval (in years) between the last birth and the date of interview. For cured cases, we assumed that the person-year of follow up was half the time interval (in years) between the last birth and the date of interview.

Betas and exponentiated values (which can be interpreted as an odds ratio) are shown in the table below:

Covariate	Parameter	Beta (95% CI)	Exponentiated beta (95% CI)
Neonatal mortality rate	Prevalence	1.95 (1.78–2.00)	7.00 (5.92–7.38)
Neonatal mortality rate	Incidence	0.39 (0.018–1.08)	1.48 (1.02–2.95)
Neonatal mortality rate	Remission	-0.78 (-0.99 to -0.38)	0.46 (0.37–0.68)
Hospital data	Prevalence	-1.75 (-1.99 to -1.29)	0.17 (0.14–0.28)
Hospital data	Incidence	-0.79 (-1.93 to 0.32)	0.46 (0.14–1.38)

References

1. Danso KA, Martey J, Wall LL, Elkins TE. The epidemiology of genitourinary fistulae in Kumasi, Ghana, 1977–1992. International Urogynecology Journal. 1996;7(3):117-120.

2. Wall LL, Karshima JA, Kirschner C, Arrowsmith SD. The obstetric vesicovaginal fistula: characteristics of 899 patients from Jos, Nigeria. Am. J. Obstet. Gynecol. 2004;190(4):1011-1016.

3. Das R, Sengupta S. Vesico-vaginal fistula of obstetric origin. J. Obstet. Gynaecol. India. 1969;19:383-389.

4. Mahfouz NP. Urinary and Faecal Fistulae. BJOG. 1938;45(3):405-424.

Neonatal preterm birth complications

Flowchart

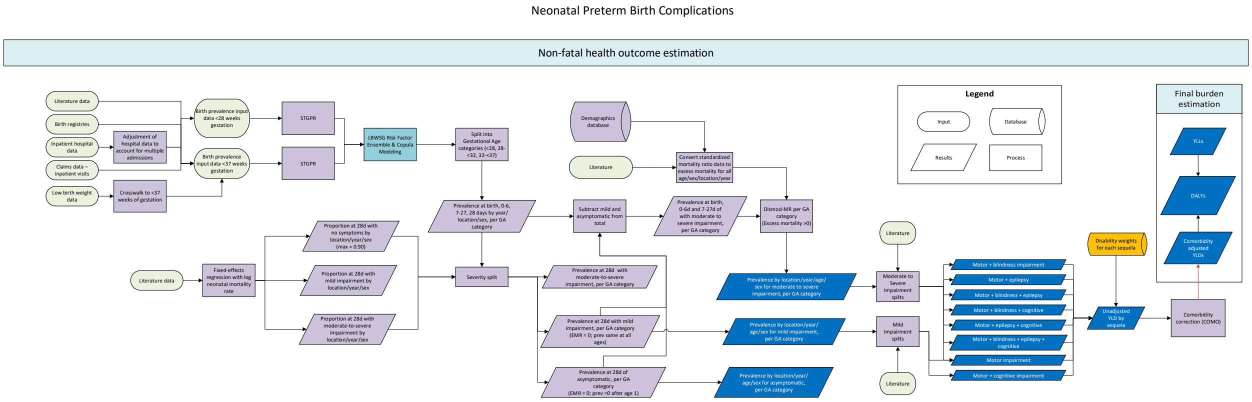

Input data and methodological summary

Case definition

Preterm birth is defined as live birth before 37 completed weeks of gestation. In our analysis, we further break down this cause into three sub-categories of preterm birth, based on gestational age: extremely preterm (<28 weeks), very preterm (28 to <32 weeks), and moderate to late preterm (32 to <37 weeks). These categories are based on the World Health Organization (WHO) definition of preterm birth.

Modelling Strategy

Modeling for neonatal preterm birth is in five parts:

Process 1: Estimation of birth prevalence distributions of extremely preterm birth (<28 weeks) and preterm birth (<37 weeks)

Process 2: Ageing birth prevalence distribution through early and late neonatal periods to 28 days and aggregating into preterm categories (<28 weeks, 28-<32 weeks, and 32-<37 weeks)

Process 3: Estimation of asymptomatic, mild, and moderate-severe impairment proportions at 28 days

Process 4: Splitting Neonatal Preterm Birth Prevalence Envelopes into asymptomatic, mild, and moderate-severe prevalence across all ages

Process 5: Splitting mild and moderate-severe impairment prevalence into sequelae

Process 1: Estimation of birth prevalence distributions of extremely preterm birth (<28 weeks) and preterm birth (<37 weeks)

Spatiotemporal Gaussian process regression was used to estimate a birth prevalence envelope of preterm (<37 weeks) birth and a birth prevalence envelope of extremely preterm (<28 weeks) birth for all locations, years, and sexes estimated in GBD.

Input data: preterm birth (<28 and <37 weeks)

Preterm birth (<28 and <37 weeks) prevalence data were sourced from literature, registry, survey, and clinical informatics data.

Clinical informatics prevalence data

Only inpatient data were included from clinical informatics datasets, because we believed it would be more representative of the true prevalence of preterm birth than outpatient data: preterm birth in the countries from which hospital data were available are almost sure to be admitted to the hospital, whereas outpatient data are more likely to capture repeated visits by the same child as they grow. Hospital data were adjusted to account for multiple admissions before transformation into incidence and upload into the DisMod model.

Claims data were not included in either preterm model, as they were was implausibly low, and high-quality registry data were available for all locations where claims data were available (United States, Taiwan, and Singapore).

Literature prevalence data

An initial systematic review was completed for GBD 2010, and for GBD 2013 and GBD 2016, a review was conducted on literature published since the previous addition. A second systematic review was done in GBD 2017 to capture any data not yet included in the previous searches. The PubMed database was searched using the following search string: ((("Infant, Premature"[Mesh] OR ("infant"[All Fields] AND "premature"[All Fields]) OR "premature infant"[All Fields] OR ("preterm"[All Fields] AND "infant"[All Fields]) OR "preterm infant"[All Fields] OR ("infant, newborn"[MeSH Terms] OR ("infant"[All Fields] AND "newborn"[All Fields]) OR "newborn infant"[All Fields] OR ("newborn"[All Fields] AND "infant"[All Fields])) AND (premature[All Fields] OR preterm[All Fields]) OR "premature birth"[MeSH Terms] OR ("premature"[All Fields] AND "birth"[All Fields]) OR "premature birth"[All Fields] OR ("preterm"[All Fields] AND "birth"[All Fields]) OR "preterm birth"[All Fields]) ((("Infant, Premature"[Mesh] OR ("infant"[All Fields] AND "premature"[All Fields]) OR "premature infant"[All Fields] OR ("preterm"[All Fields] AND "infant"[All Fields]) OR "preterm infant"[All Fields] OR ("infant, newborn"[MeSH Terms] OR ("infant"[All Fields] AND "newborn"[All Fields]) OR "newborn infant"[All Fields] OR ("newborn"[All Fields] AND "infant"[All Fields])) AND (premature[All Fields] OR preterm[All Fields]) OR "premature birth"[MeSH Terms] OR ("premature"[All Fields] AND "birth"[All Fields]) OR "premature birth"[All Fields] OR ("preterm"[All Fields] AND "birth"[All Fields]) OR "preterm birth"[All Fields]) AND ("2012"[PDAT] : "3000"[PDAT]) AND "humans"[MeSH Terms].

The exclusion criteria were:

1. Studies that did not provide primary data on epidemiological parameters, eg, a commentary piece
2. Non-representative studies (eg, only high-risk pregnancies)
3. Reviews

Low birth weight data

Low birth weight (<2500 grams) data was extracted from literature, vital registration systems, and surveys. DHS survey data were observed to have high missingness; to correct for the missingness, birth weight was imputed using the Amelia package in R. Birth weight was predicted using standard Amelia imputation methods from the following variables also in the DHS surveys: urbanicity, sex, birthweight recorded on card, birth order, maternal education, paternal education, child age, child weight, child height, mother's age at birth, mother's weight, shared toilet facility, and household water treated.

The preterm birth (<37 weeks) ST-GPR model was informed by low birth weight (<2500 grams) data. Low birth weight data are more readily available than preterm birth data, especially in low- and middle-income countries. In data sources where both preterm birth (<37 weeks) and low birth weight data were available for the same location, year, and sex, preterm birth was regressed on low birth weight. The regression was then used to predict the preterm birth estimate for all low birth weight data that were used in the low birth weight ST-GPR model (outliers excluded, post-imputation for missing birth weight). Figure 1 shows the preterm birth mean prevalence estimated from the low birth weight mean.

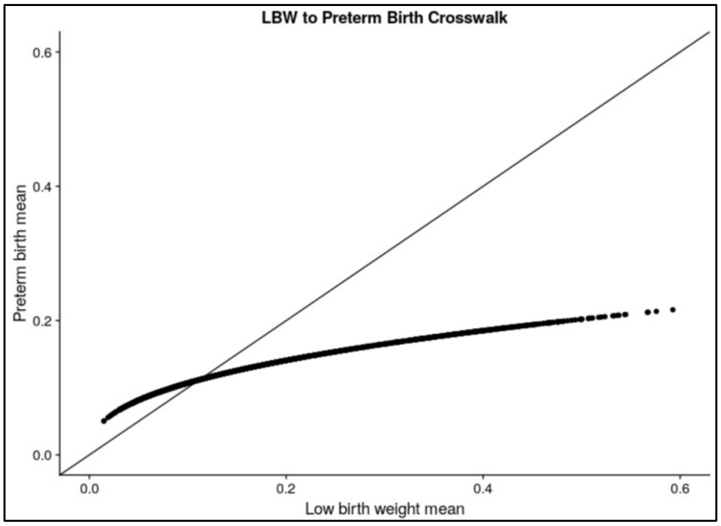

Table 1 shows the number of site-years of data by type included in GBD 2017, as well as the number of countries, GBD regions, and GBD super-regions represented.

Table 1. Geographic representation for preterm models

	Preterm <28 wks	Preterm <37 wks	LBW <2500g
Site-years (total)	1,872	2,420	2,980
Number of countries with data	45	75	154
Number of GBD regions with data (out of 21 regions)	14	21	21
Number of GBD super-regions with data (out of 7 super-regions)	6	7	7

Modelling steps

ST-GPR models were used to estimate categorical estimates of preterm birth (<37 weeks) and extreme preterm birth (<28 weeks) for every sex/location/year estimated in GBD. Mean gestational age for every sex/location/year is also estimated from the categorical prevalence estimates. Ordinary least squares was used to model mean gestational age for all location-year-sexes by regressing mean gestational age on prevalence of <37 weeks' gestation per location-year. All data in Table 2 were used to fit the model.

Table 2: Summary of data inputs

Location	Years of data	Total births*	Format of data
BRA	2016	2,854,380	Microdata
ECU	2003-2015	2,473,039	Microdata
ESP	1990-2014	8,537,220	Microdata
JPN	1995-2015	23,644,506	Tabulations
MEX	2008-2012	10,256,117	Microdata
NOR	1990-2014	1,489,210	Microdata
NZL	1990-2016	1,600,501	Microdata
SGP	1993-2015	972,775	Tabulations
TWN	1998-2002	1,331,760	Tabulations

URY	1996-2014	698,622	Microdata
USA	1990-2014	81,929,879	Microdata

** Pooled across all year and sexes, excluding data missing year of birth, gestational age, or birth weight*

Ensemble model methods standard to GBD are used to model the distribution at birth of gestational age. Gestational age ensemble distribution models used the prevalence of <37 weeks' gestation, the prevalence of <28 weeks' gestation, and mean gestational age per each location-year-sex as inputs into the model.

Global ensemble weights for gestational age were derived by using a 3 million sample of all available microdata in Table 2 to select the ensemble weights. Of the exponential, gamma, inverse gamma, Weibull, log normal, and normal distributions, the three distribution families that received the highest weights were the Weibull (87%), normal (4%), and inverse gamma (4%) distributions.

The gestational birth prevalence distributions were then paired with low birth weight prevalence distributions using copulae methodology to create joint distributions of gestational age and birth weight. These were then split into 500g and 2wk joint "bins" (eg, 4000–4500g and 34–36wks of gestation). More information on this methodology can be found in the Low Birth Weight & Short Gestation (LBWSG) risk factor appendix of the GBD 2017 Risk Factor capstone.

Process 2: Aging birth prevalence distribution through early and late neonatal periods to 28 days and aggregating into <28 wk, 28-<32 wk, and 32-<37 wk preterm categories

Early neonatal prevalence and late neonatal prevalence were estimated using life table approaches for each 500g & 2-week bin. Using the all-cause early neonatal mortality rate for each location-year-sex, births per location-year-sex-bin, and the relative risks for each location-year-sex-bin in the early neonatal period (estimated in a separate process of the LBWSG methods), the all-cause early neonatal mortality rate was calculated for each location-year-sex-bin. The early neonatal mortality rate per bin was used to calculate the number of survivors at seven days and prevalence in the early neonatal period. Using the same process, the all-cause late neonatal mortality rate for each location-year-sex was paired with the number of survivors at seven days and late neonatal relative risks per bin to calculate late neonatal prevalence and survivors at 28 days.

The 500g and 2-week bins at birth, early neonatal, late neonatal, and 28 days were re-aggregated into three preterm categories: <28 weeks of gestation, 28-<32 weeks of gestation, and 32-<37 weeks of gestation. These estimates formed the neonatal preterm prevalence envelope estimates for each gestational age category.

Process 3: Estimation of asymptomatic, mild, and moderate-severe impairment proportions at 28 days

Using mild impairment proportion and moderate-to-severe impairment proportion data, we ran a single mixed-effects regression model, with a dummy variable on each gestational age and proportion type, to generate country-year-sex-specific estimates of both parameters for each gestational age. The remainder of 1 – (mild proportion + moderate-severe proportion) was assigned to asymptomatic proportion, by gestational age.

Figure 1: Impairment proportions by gestational age and severity type

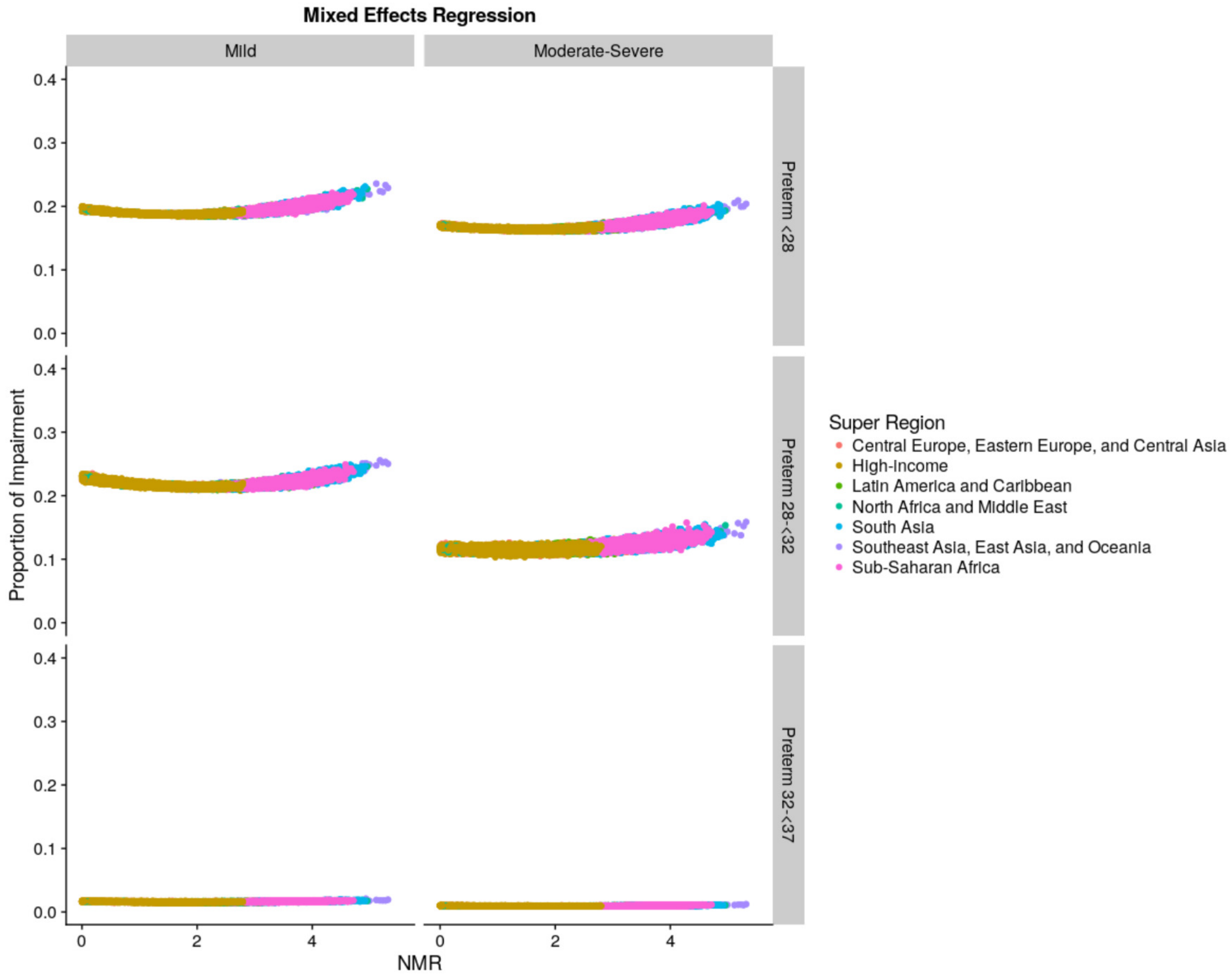

Process 4: Splitting neonatal preterm birth prevalence envelopes into asymptomatic, mild, and moderate-severe prevalence across all ages

Asymptomatic, mild, and moderate-severe impairment proportions at 28 days were applied to prevalence at 28 days. Asymptomatic prevalence was assumed to be the same at early neonatal, late neonatal, and post-neonatal period as at 28 days. Asymptomatic prevalence was set to 0 after the post-neonatal period, as no burden is assumed after 1 year. Mild prevalence was assumed to be the same at all GBD age groups as at 28 days, reflecting the assumption that there is no excess mortality among those born with mild impairment due to neonatal preterm birth.

For moderate/severe impairment, moderate-severe prevalence calculated at birth, early neonatal, and late neonatal periods were combined with excess mortality estimates derived from the standard mortality ratios (SMR) of cerebral palsy and used as inputs into a second DisMod-MR model. SMR was converted to EMR by multiplying age-specific mortality by age-specific standardised mortality ratios - 1. For this model, remission and incidence were also set to zero. For mild impairment, we assumed no excess mortality and no remission, and as such simply applied the birth prevalence of mild impairment to every age group.

The sum of asymptomatic and mild impairment in the early and late neonatal periods was subtracted from the neonatal preterm birth envelope estimates for each gestational age in the early and late neonatal periods,

respectively, in order to estimate moderate-severe impairment. This reflects the assumption that all deaths in the early and late neonatal period were among those with moderate-severe impairment, and all newborns born with asymptomatic or mild impairment due to preterm birth did not experience excess mortality.

Process 5: Splitting mild and moderate-severe impairment prevalence into sequelae

Mild impairment and moderate-severe impairment due to neonatal preterm birth are split into the following sequelae. To determine the proportion of people within each of these severity levels, one study informed moderate-to-severe impairment splits, and mild impairments cases were divided equally into both categories.[1]

Table 3: Health states by severity (identical across gestational ages)

Health State	Mild	Moderate	Severe
Motor only	X	X	X
Motor + Cognitive	X		
Motor + Epilepsy		X	X
Motor + Blindness		X	X
Motor + Blindness + Epilepsy		X	X
Motor + Blindness + Cognitive		X	X
Motor + Epilepsy + Cognitive		X	X
Motor + Blindness + Epilepsy + Cognitive		X	X

**For disability weights and combined disability weights, please refer to the disability weights table in the appendix.

Retinopathy of prematurity

The proportion of infants born with prematurity and surviving to the end of the neonatal period who go onto develop retinopathy of prematurity is applied to prevalence of preterm birth at 28 days. Proportional splits were estimated by regressing proportion of ROP among preterm infants on log NMR from 55 studies in 19 countries. The prevalence of infants with ROP is then split into five vision sequelae of varying severity: asymptomatic, mild, moderate, severe, and complete vision loss (blindness).

[1] Badawi et al Developmental Medicine & Child Neurology, 2005, 47:293-8

Neonatal encephalopathy due to birth asphyxia and birth trauma

Flowchart

Neonatal Encephalopathy due to birth asphyxia and birth trauma

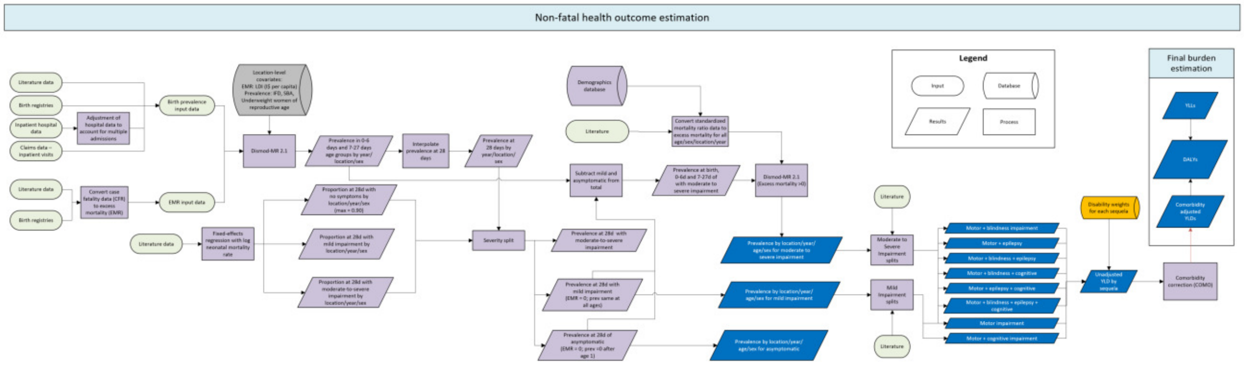

Input data and methodological summary

Case definition

Neonatal encephalopathy (NE) due to birth asphyxia and birth trauma, also called hypoxic-ischaemic encephalopathy, is defined as injury to the brain in the first few moments or days of life in an infant born at term. NE has multiple aetiologies and is defined more by its symptoms – abnormal neurological function, including reduced level of consciousness, seizures, depression of tone and reflexes, or difficulty maintaining respiration – than its origin. NE can occur when an infant is deprived of oxygen during delivery or sustains physical trauma to the head, among other causes.

Modelling Strategy

Modelling for NE is in four parts:

Process 1: Estimation of NE prevalence envelope at birth, neonatal, and 28 days

Process 2: Estimation of asymptomatic, mild, and moderate-severe impairment proportions at 28 days

Process 3: Splitting NE prevalence envelope into asymptomatic, mild, and moderate-severe prevalence across all ages

Process 4: Splitting mild and moderate-severe impairment prevalence into sequelae

Process 1: Estimation of NE prevalence envelope at birth, neonatal, and 28 days

DisMod-MR is used to estimate an envelope of neonatal encephalopathy prevalence at birth, early neonatal, and late neonatal periods for all locations, years, and sexes estimated in GBD. Two types of input data inform the DisMod model: prevalence data and case fatality ratio (CFR) data.

Input data: prevalence

Prevalence data were sourced from literature and clinical informatics data.

Clinical informatics prevalence data

Clinical informatics data (hospital and claims) formed the bulk of the input data for the NE envelope model. Only inpatient data were included from these datasets, because we believed it would be more representative of the true prevalence of neonatal encephalopathy than outpatient data: infants with neonatal encephalopathy in the countries from which hospital data were available are almost sure to be admitted to the hospital, whereas outpatient data are more likely to capture repeated visits by the same child as they grow.

After noticing instability in hospital data across locations when using the ICD codes mapped to neonatal encephalopathy in GBD 2016, we chose this year to use only hospital data coded to P21 (birth asphyxia), P24 (neonatal aspiration syndromes), and P91 (hypoxic ischaemic encephalopathy, unspecified), and their more detailed sub-codes. These codes are believed to be most representative of neonatal encephalopathy in the hospital datasets used in GBD; subsetting to these ICD codes led to stable data that matched available literature data. Hospital data were then adjusted to account for multiple admissions before upload into the DisMod model.

Literature prevalence data

A systematic review for NE was completed for GBD 2010. For GBD 2013 and GBD 2015, a review was conducted on literature published since the previous edition. The PubMed database was searched using the following search string**:** ((("infant"[Title/Abstract] OR "newborn"[Title/Abstract] OR "newborn infant"[Title/Abstract]) AND ("encephalopathy"[Title/Abstract] OR "neonatal encephalopathy"[Title/Abstract] OR "perinatal asphyxia"[Title/Abstract] OR "asphyxia neonatorum"[Title/Abstract] OR "newborn encephalopathy"[Title/Abstract] OR "hypoxic ischaemic encephalopathy"[Title/Abstract] OR ("birth trauma"[Title/Abstract] AND "birth asphyxia"[Title/Abstract]))) AND ("2012"[PDAT] : "3000"[PDAT]) AND "humans"[MeSH Terms])

The exclusion criteria were:

1. Studies that did not provide primary data on epidemiological parameters, eg, a commentary piece
2. Non-representative studies (eg, only high-risk pregnancies)
3. Reviews

No additional literature searches were done for GBD 2016 and 2017.

<u>Input data: case fatality ratio</u>

Prior to input into DisMod, CFR data are transformed into excess mortality rate (EMR) using the formula $EMR = -\frac{\ln(1-CFR)}{\frac{days\ of\ observation\ period}{365}}$, which is analogous to the transformation of cumulative incidence to incidence rate. The denominator in this equation is the number of days in the observation period for the data point – for example, data that followed newborns with neonatal encephalopathy for one year would have a denominator of 1.

Table 1 shows the number of site-years of data by type included in GBD 2017, as well as the number of countries, GBD regions, and GBD super-regions represented.

Table 1. Geographic representation for NE envelope model

	Case fatality rate	Prevalence
Site-years (total)	36	1,329
Number of countries with data	24	51
Number of GBD regions with data (out of 21 regions)	12	20
Number of GBD super-regions with data (out of 7 super-regions)	6	7

Modelling Steps

A DisMod model estimated prevalence at birth, and early and late neonatal age groups. Remission and incidence are both set to zero, as no one can develop encephalopathy after birth, and no one can cease to have been born with encephalopathy after the fact. Three country-level covariates informed prevalence estimated: in-facility delivery, skilled birth attendance, and underweight women of reproductive age. Excess mortality was informed by the country-level covariate lag-distributed income. A study-level covariate was set on all claims data.

After estimating birth prevalence and early neonatal and late neonatal age groups, prevalence at 28 days was estimated by linearly extrapolating early neonatal and late neonatal prevalence. Prevalence at 28 days is not an age group that is reported in GBD, but it is required for modelling since the proportional severity splits from literature, which determine asymptomatic, mild, and moderate-severe prevalence, are based on prevalence at 28 days.

Process 2: Estimation of asymptomatic, mild, and moderate-severe impairment proportions at 28 days

Using mild impairment proportion and moderate-to-severe impairment proportion data, we ran mixed-effect hierarchical regressions with log neonatal mortality rate as a predictor and a dummy variable on proportion severity (mild or moderate-severe) to generate country-year-sex-specific estimates of both parameters. The remainder of 1 – (mild proportion + moderate-severe proportion) was assigned to asymptomatic proportion.

As mild impairment and moderate-severe impairment were calculated separately, it was possible that they could sum to a value greater than one. To address this, we checked the sum of the two values in any of the 1,000 iterations of the uncertainty analysis, and if greater than 0.9, proportionately rescaled both estimates to sum to 0.9 in any of the 1,000 iterations of the uncertainty analysis (we picked 0.9 rather than 1 to allow at least some probability of a child having no impairment).

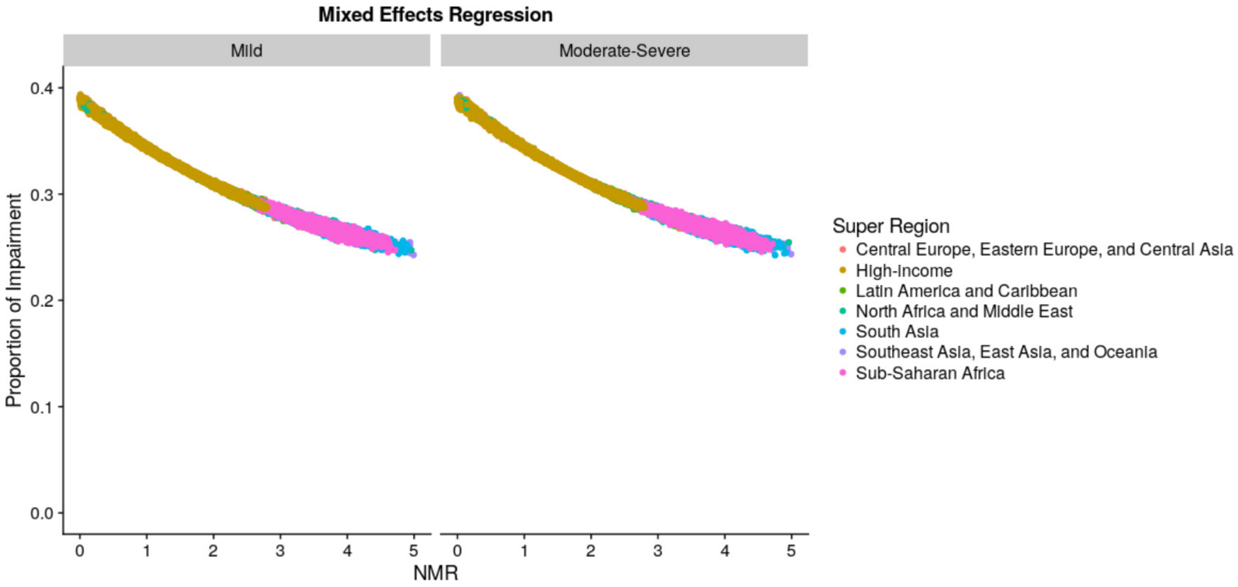

Process 3: Splitting NE prevalence envelope into asymptomatic, mild, and moderate-severe prevalence across all ages

Asymptomatic, mild, and moderate-severe impairment proportions at 28 days were applied to prevalence at 28 days. Asymptomatic prevalence was assumed to be the same at early neonatal, late neonatal, and post-neonatal period as at 28 days. Asymptomatic prevalence was set to 0 after the post-neonatal period, as no burden is

assumed after 1 year. Mild prevalence was assumed to be the same at all GBD age groups as at 28 days, reflecting the assumption that there is no excess mortality among those born with mild neonatal encephalopathy.

For moderate/severe impairment, moderate-severe prevalence calculated at birth, early neonatal, and late neonatal periods were combined with excess mortality estimates derived from the standard mortality ratios (SMR) of cerebral palsy and used as inputs into a second DisMod-MR model. SMR was converted to EMR by multiplying age-specific mortality by age-specific standardised mortality ratios - 1. For this model, remission and incidence were also set to zero. For mild impairment, we assumed no excess mortality and no remission, and as such simply applied the birth prevalence of mild impairment to every age group.

The sum of asymptomatic and mild impairment in the early and late neonatal periods was subtracted from the NE envelope estimates (Process 1) in the early and late neonatal periods in order to estimate moderate-severe impairment. This reflects the assumption that all deaths in the early and late neonatal period were among those with moderate-severe impairment, and all newborns born with asymptomatic or mild NE did not experience excess mortality.

Process 4: Splitting mild and moderate-severe impairment prevalence into sequelae

Mild impairment and moderate-severe impairment due to NE are split into the following sequelae. To determine the proportion of people within each of these severity levels, one study informed moderate-to-severe impairment splits, and mild impairments cases were divided equally into both categories.[1]

Health State	Mild	Moderate	Severe
Motor only	X	X	X
Motor + Cognitive	X		
Motor + Epilepsy		X	X
Motor + Blindness		X	X
Motor + Blindness + Epilepsy		X	X
Motor + Blindness + Cognitive		X	X
Motor + Epilepsy + Cognitive		X	X
Motor + Blindness + Epilepsy + Cognitive		X	X

**For disability weights and combined disability weights, please refer to the disability weights table in the appendix.

[1] Badawi et al Developmental Medicine & Child Neurology, 2005, 47:293-8

Neonatal sepsis and other neonatal infections

Flowchart

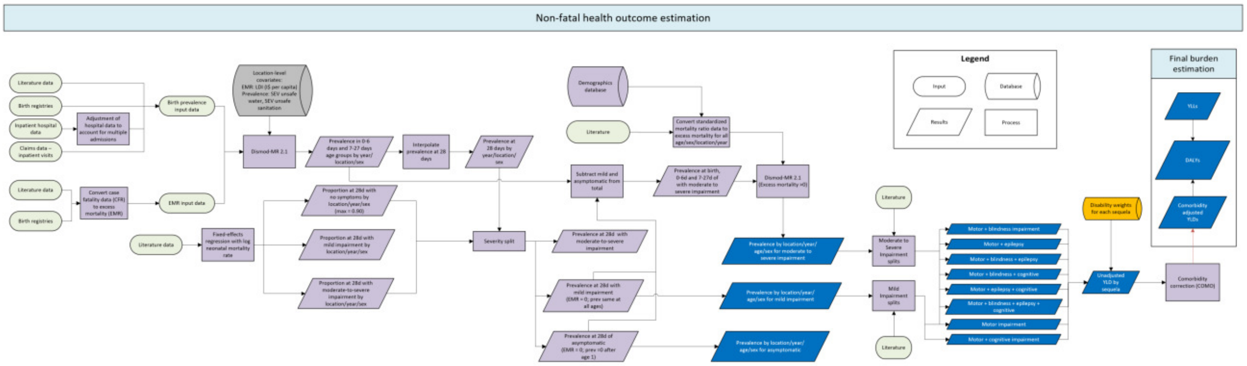

Input data and methodological summary

Case definition

Neonatal sepsis and other neonatal infections are infections during the neonatal period that advance to a systemic bloodstream infection, the underlying cause of which can be meningitis, gastroenteritis, or other aetiologies. Neonatal pneumonia, however, is not included – it is captured in our modelling of pneumonia as a separate entity.

Modelling Strategy

Modelling for NS is in four parts:

Process 1: Estimation of NS prevalence envelope at early neonatal, late neonatal, and 28 days

Process 2: Estimation of asymptomatic, mild, and moderate-severe impairment proportions at 28 days

Process 3: Splitting NS prevalence envelope into asymptomatic, mild, and moderate-severe prevalence across all ages

Process 4: Splitting mild and moderate-severe impairment prevalence into sequelae

Process 1: Estimation of NS prevalence envelope at birth, neonatal, and 28 days

DisMod-MR is used to estimate an envelope of neonatal sepsis prevalence at birth, early neonatal, and late neonatal periods for all locations, years, and sexes estimated in GBD. Two types of input data inform the DisMod model: incidence data and case fatality ratio (CFR) data.

<u>**Input data: incidence**</u>

Prevalence data were sourced from literature and clinical informatics data. All prevalence data were then converted to incidence data before input into the model.

Clinical informatics prevalence data

Clinical informatics data (hospital and claims) formed the bulk of the input data for the NS envelope model. Only inpatient data were included from these datasets, because we believed they would be more representative of the true prevalence of neonatal sepsis than outpatient data: infants with neonatal sepsis in the countries from which hospital data were available are almost sure to be admitted to the hospital, whereas outpatient data are more likely to capture repeated visits by the same child as they grow. Hospital data were adjusted to account for multiple admissions before transformation into incidence and upload into the DisMod model.

Literature prevalence data

A systematic review was completed for GBD 2010, and for GBD 2013 and GBD 2015, a review was conducted on literature published since the previous edition. The PubMed database was searched using the following search string: (("infant"[Title/Abstract] OR "newborn"[Title/Abstract] OR "newborn infant"[Title/Abstract]) AND ("neonatal sepsis"[All Fields] OR "neonatal septicaemia"[All Fields] OR "neonatal meningitis"[All Fields] OR "early sepsis"[All Fields] OR "early septicaemia"[All Fields] OR "tetanus"[All Fields] OR "meningitis"[All Fields] OR "sepsis"[All Fields])) AND ("2012"[PDAT] : "3000"[PDAT]) AND "humans"[MeSH Terms]

The exclusion criteria were:

1. Studies that did not provide primary data on epidemiological parameters, eg, a commentary piece
2. Non-representative studies (eg, only high-risk pregnancies)
3. Reviews

No additional literature searches were done for GBD 2016 and 2017.

<u>Input data: case fatality ratio</u>

Prior to input into DisMod, CFR data are transformed into excess mortality rate (EMR) using the formula $EMR = -\frac{\ln(1-CFR)}{\frac{days\ of\ observation\ period}{365}}$, which is analogous to the transformation of cumulative incidence to incidence rate. The denominator in this equation is the number of days in the observation period for the data point – for example, data that followed newborns with neonatal sepsis for one year would have a denominator of 1.

Table 1 shows the number of site-years of data by type included in GBD 2017, as well as the number of countries, GBD regions, and GBD super-regions represented.

Table 1. Geographic representation for NS envelope model

	Case fatality rate	Prevalence
Site-years (total)	18	1,305
Number of countries with data	15	37
Number of GBD regions with data (out of 21 regions)	10	14
Number of GBD super-regions with data (out of 7 super-regions)	6	5

Modelling Steps

A DisMod model estimated prevalence in early, late, and post-neonatal age groups. Unlike other neonatal cause models using similar modelling strategies (preterm birth and encephalopathy), no birth prevalence is estimated for NS. Remission was set to 26, as a cause of neonatal sepsis is assumed to last two weeks. Incidence is set to 0 after 27 days, as by definition, neonatal sepsis must occur within the neonatal period (0-27 days). Two country-level

covariates informed prevalence estimated: SEV unsafe water and SEV unsafe sanitation. Excess mortality was informed by the country-level covariate lag-distributed income. A study-level covariate was set on all claims and literature data.

After estimating early, late, and post-neonatal age groups, prevalence at 28 days was estimated by linearly interpolating early late and post-neonatal prevalence. Prevalence at 28 days is not an age group that is reported in GBD, but it is required for modelling since the proportional severity splits from literature, which determine asymptomatic, mild, and moderate-severe prevalence, are based on prevalence at 28 days. The post-neonatal age group estimated in this model is dropped and not used in further modelling steps; only the early neonatal, late neonatal, and 28-day prevalence estimates are retained for the envelope.

Process 2: Estimation of asymptomatic, mild, and moderate-severe impairment proportions at 28 days

Using mild impairment proportion and moderate-to-severe impairment proportion data, we ran meta-analysis to generate country-year-sex-specific estimates of both parameters. The remainder of 1 – (mild proportion + moderate-severe proportion) was assigned to asymptomatic proportion.

Mild Impairment Proportion: 10.2% (7.2% – 12.9%)

Figure 1: Mild impairment regression, based on three studies

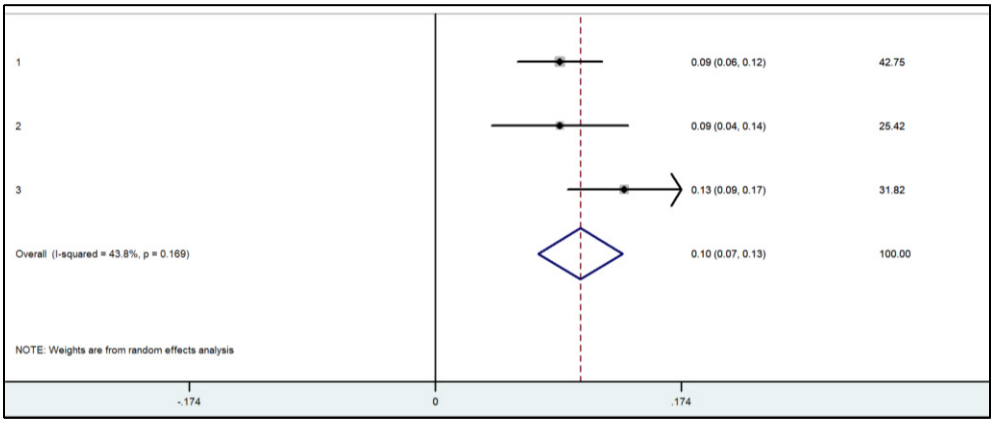

Moderate-severe impairment proportion: 4.3% (2.5% – 6.0%)

Figure 2: Mild impairment regression, based on two studies

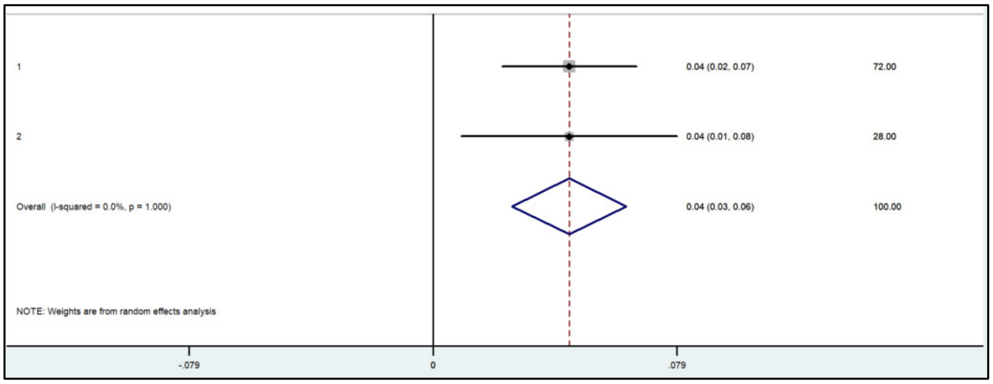

Process 3: Splitting NS prevalence envelope into asymptomatic, mild, and moderate-severe prevalence across all ages

Asymptomatic, mild, and moderate-severe impairment proportions at 28 days were applied to prevalence at 28 days. Asymptomatic prevalence was assumed to be the same at early neonatal, late neonatal, and post-neonatal periods as at 28 days. Asymptomatic prevalence was set to 0 after the post-neonatal period, as no burden is assumed after 1 year. Mild prevalence was assumed to be the same at all GBD age groups as at 28 days, reflecting the assumption that there is no excess mortality among those born with mild neonatal sepsis.

For moderate/severe impairment, moderate-severe prevalence calculated at birth and in the early neonatal and late neonatal periods were combined with excess mortality estimates derived from the standard mortality ratios (SMR) of cerebral palsy and used as inputs into a second DisMod-MR model. SMR was converted to EMR by multiplying age-specific mortality by age-specific standardised mortality ratios - 1. For this model, remission and incidence were also set to zero. For mild impairment, we assumed no excess mortality and no remission, and as such simply applied the birth prevalence of mild impairment to every age group.

The sum of asymptomatic and mild impairment in the early and late neonatal periods were subtracted from the NS envelope estimates (Process 1) in the early and late neonatal periods, respectively, in order to estimate moderate-severe impairment. This reflects the assumption that all deaths in the early and late neonatal period were among those with moderate-severe impairment, and all newborns born with asymptomatic or mild NS did not experience excess mortality.

Process 4: Splitting mild and moderate-severe impairment prevalence into sequelae

Mild impairment and moderate-severe impairment due to NS are split into the following sequelae. To determine the proportion of people within each of these severity levels, one study informed moderate-to-severe impairment splits, and mild impairments cases were divided equally into both categories.[1]

Table 1: Health states by severity

**Health State**	**Mild**	**Moderate**	**Severe**
Motor only	X	X	X
Motor + Cognitive	X		
Motor + Epilepsy		X	X
Motor + Blindness		X	X
Motor + Blindness + Epilepsy		X	X
Motor + Blindness + Cognitive		X	X
Motor + Epilepsy + Cognitive		X	X
Motor + Blindness + Epilepsy + Cognitive		X	X

**For disability weights and combined disability weights, please refer to the disability weights table in the appendix.

[1] Badawi et al Developmental Medicine & Child Neurology, 2005, 47:293-8

Haemolytic disease and other neonatal jaundice

Flowchart

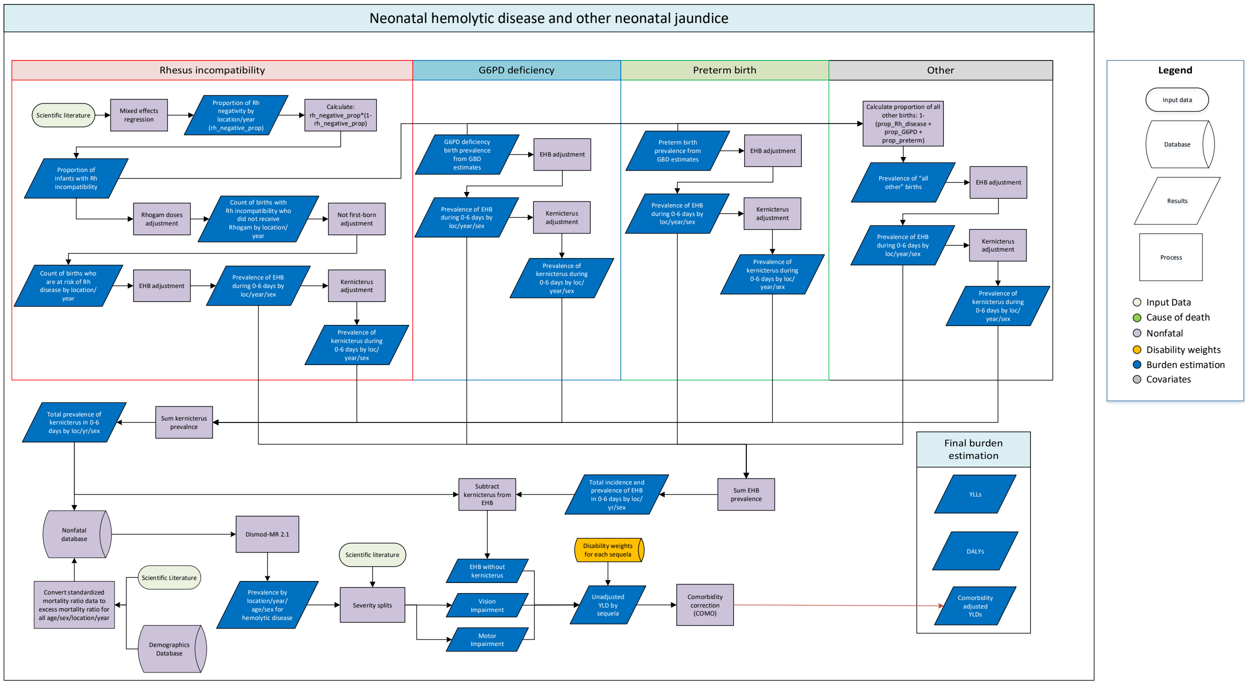

Case definition

Haemolytic disease of the newborn and other neonatal jaundice refers to several aetiologies by which an infant develops extreme hyperbilirubinemia (EHB) and can then go on to develop kernicterus. The aetiologies that we model for GBD are EHB from Rhesus (Rh) disease, preterm birth, glucose-6-phosphate dehydrogenase deficiency (G6PD), and other causes.

Input data

Model inputs

A systematic review was completed for GBD 2010, and for GBD 2013 and GBD 2015, a review was conducted on literature published since the previous edition. The PubMed database was searched using the following search string: (("infant"[Title/Abstract] OR "newborn"[Title/Abstract] OR "newborn infant"[Title/Abstract]) AND ("haemolytic"[All Fields] OR "hyperbilirubinemia"[All Fields] OR "jaundice"[All Fields] OR "glucose-6-phosphate dehydrogenase deficiency"[All Fields] OR "G6PD deficiency"[All Fields] OR "hyperbilirubinemia"[All Fields] OR "EHB"[All Fields] OR "phototherapy"[All Fields] OR "ABO incompatibility"[All Fields] OR "RH incompatibility"[All Fields] OR "rh blood group system"[All Fields] OR "Rhesus"[All Fields] OR "Rhesus disease"[All Fields] OR "erythroblastosis fetalis"[All Fields] OR "kernicterus"[All Fields])) AND ("2012"[PDAT] : "3000"[PDAT]) Sort by: PublicationDate

The exclusion criteria were:

1. Studies that did not provide primary data on epidemiological parameters, eg, a commentary piece
2. Non-representative studies (eg, only high-risk pregnancies)
3. Reviews

For haemolytic disease, much of the input data comes from other GBD models, which are described in detail in the “modelling strategy” section. However, the modelling process for EHB from Rh disease involves literature data for several parameters, the breadth of which are described in the tables below:

Table 1a. Rh negativity

	Prevalence
Site-years (total)	53
Number of countries with data	45
Number of GBD regions with data (out of 21 regions)	13
Number of GBD super-regions with data (out of 7 super-regions)	7

Table 1b. Rhogam (Rh0 immune globulin) doses distributed to countries in 2010

	Proportion
Site-years (total)	138
Number of countries with data	138
Number of GBD regions with data (out of 21 regions)	19
Number of GBD super-regions with data (out of 7 super-regions)	7

Table 1c. Children who are not first-born

	Proportion
Site-years (total)	1033
Number of countries with data	81
Number of GBD regions with data (out of 21 regions)	14
Number of GBD super-regions with data (out of 7 super-regions)	6

**Please note that US claims data and hospital data were not included in the haemolytic disease modelling process because they are not coded separately by aetiology. They could not be slotted into the existing modelling framework.

Modelling strategy

For each of the four aetiologies (Rh disease, G6PD deficiency, preterm birth, and other causes) we estimated the prevalence of extreme hyperbilirubinemia (EHB or bilirubin>25 or exchange transfusion). Then, we multiplied this prevalence by an estimated proportion of EHB cases who go on to develop kernicterus. We used development of kernicterus as our criterion for incidence of long-term moderate/severe impairment.

In GBD 2016, all impairment due to haemolytic disease and other neonatal jaundice was captured in kernicterus. In GBD 2017, disability due to EHB without kernicterus was added.

Rh Disease

We began with data on the prevalence of Rh negativity in the population (Table 1a), the number of Rhogam (Rh_0 immune globulin) doses distributed to countries in 2010 (Table 1b), and the proportion of children who are not firstborn (Table 1c). Mixed effect regressions were run on Rh negativity and birth order greater than one to generate estimates of these values for every country-year. We made the assumptions that the proportion of Rhogam doses to Rh-incompatible children stayed constant over time, and that countries with NMR<5 had complete Rhogam coverage. This yields the following equation for the prevalence of EHB in countries with NMR>5:

EHB Prevalence = Rh negative prevalence * (1 – Rh negative prevalence) * (2010 Rhogam doses / 2010 Rh incompatible babies) * (not-firstborn prevalence) * 0.15

The 0.15 multiplier represents results of previous calculations showing the proportion of women developing Rh isoimmunisation with a risk for anti-Rh antibodies complicating subsequent pregnancies.

Finally, to generate estimates of kernicterus prevalence, we multiplied the prevalence of EHB by 0.0072 (0.0038, 0.112) – the proportion of children with EHB who develop kernicterus.[1,2,3]

G6PD, preterm, and other

The other three pathways of kernicterus modelling simply involve multiplication by scalars. For each of these, given a complete set of prevalence estimates from other GBD models, we multiplied by a scalar representing the proportion of children who are expected to develop EHB (see the table below for the values of these scalars). We then adjusted that estimate upward by a factor of 2.45 (1.44, 4.16) for countries in which the NMR is greater than 15, a value utilised in previous publications to reflect heightened risk in those countries where access to phototherapy for prevention of EHB is not standard.[4,5]

---

[1] Walker WC. Haemolytic Disease of the Newborn. In: Gairdner D, Hull D, eds. Recent Advances in Paediatrics, 4th ed. London, United Kingdom: Churchill, 1971.
[2] Vaughan VC. Kernicterus in erythroblastosis fetalis. J Pediatr. 1946.
[3] Mollison PL, Cutbush M. Exchange transfusion in haemolytic disease of the newborn. Lancet. 1948; 252(6527): 522-7.
[4] Mah MP, Clark SL, Akhigbe E, Englebright J, Frye DK, Meyers JA, Perlin JB, Rodriguez M, Shepard A. Reduction of severe hyperbilirubinemia after institution of predischarge bilirubin screening. Pediatrics. 2010; 125(5): e1143-8.
[5] Brown AK, Kim MH, Wu PY, Bryla DA. Efficacy of phototherapy in prevention and management of neonatal hyperbilirubinemia. Pediatrics. 1985; 75(2 Pt 2): 393-400.

Finally, these EHB prevalence values were multiplied by a set of NMR-dependent scalars representing the proportion of EHB cases that go on to develop kernicterus. See table below, for those values.

Table 2. EHB proportion and CIs

Cause	Source of birth prevalence estimates	EHB proportion	95% CI
G6PD	Congenital DisMod model, GBD 2017.	0.0013	(0.00085, 0.002)
Preterm birth complications	Custom modelling for preterm conditions, discussed elsewhere in this document (summed over all gestational ages).	0.00045	(0.00029, 0.0007)
Other	Global births – (Rh disease birth prevalence + preterm complications birth prevalence + G6PD birth prevalence)	0.00038	(0.00033, 0.00163)

Table 3: NMR-dependent kernicterus proportions applied to G6PD, preterm, and other EHB estimates.

NMR	Kernicterus proportion	95% CI
<5	0.23	(0.099, 0.361)
5-15	0.35	(0.12, 0.58)
>=15	0.438	(0.255, 0.621)

Final kernicterus prevalence

We estimated preterm kernicterus in order to arrive at a proper value for the number of "other" children at risk of kernicterus, but we do not actually include our preterm estimates in our measures of kernicterus since we assume that its disability is captured in our preterm models. Thus, we generate our final birth prevalence of kernicterus by:

Kernicterus birth prevalence = (kernicterus prevalence from Rh disease) + (kernicterus prevalence from G6PD) + (kernicterus prevalence from other disorders).

These estimates, along with estimates of excess mortality associated with kernicterus, are then used as inputs into DisMod to generate estimates of moderate to severe impairment starting at age 7 days.

Prevalence of moderate-to-severe impairment was then split into health states. One study informed the moderate-to-severe impairment splits.[6] Disability due to extreme hyperbilirubinemia due to haemolytic disease and other neonatal jaundice was added as a sequela in GBD 2017 to capture disability due to EHB

[6] Badawi et al. Developmental Medicine & Child Neurology, 2005, 47:293-8

without kernicterus in the neonatal period. This was calculated by subtracting kernicterus prevalence from total EHB prevalence in the neonatal period. The basis of the GBD disability weight (DW) survey assessments are lay descriptions of sequelae highlighting major functional consequences and symptoms.

Table 4: Health states by severity

Health State	Moderate	Severe
Motor only	X	X
Motor + Cognitive		
Motor + Epilepsy	X	X
Motor + Blindness	X	X
Motor + Blindness + Epilepsy	X	X
Motor + Blindness + Cognitive	X	X
Motor + Epilepsy + Cognitive	X	X
Motor + Blindness + Epilepsy + Cognitive	X	X
Extreme hyperbilirubinemia due to hemolytic disease and other neonatal jaundice, without kernicterus	-	-

**For disability weights and combined disability weights, please refer to the disability weights table in the appendix.

Other neonatal disorders

In addition to the neonatal disorders described above, there are many diverse types of neonatal disorders with a range of severities and associated sequelae. Because these other neonatal disorders are diverse in their underlying causes and risk factors as well as in their associated health outcomes, modelling them together in a DisMod-MR model would not produce reliable estimates of prevalence or excess mortality. Instead, we calculated the YLDs caused by other neonatal disorders directly using a YLD/YLL ratio.

We calculated the ratio of YLDs to YLLs across the specified neonatal disorders for which non-fatal outcomes were modelled, using YLL estimates from the GBD 2017 cause of death (CoD) analysis. We then multiplied this YLD/YLL ratio by the YLL estimates for other chronic respiratory diseases from the GBD 2017 CoD analysis, providing us with an estimate of the YLDs associated with other neonatal disorders.

Protein-energy malnutrition

Flowchart

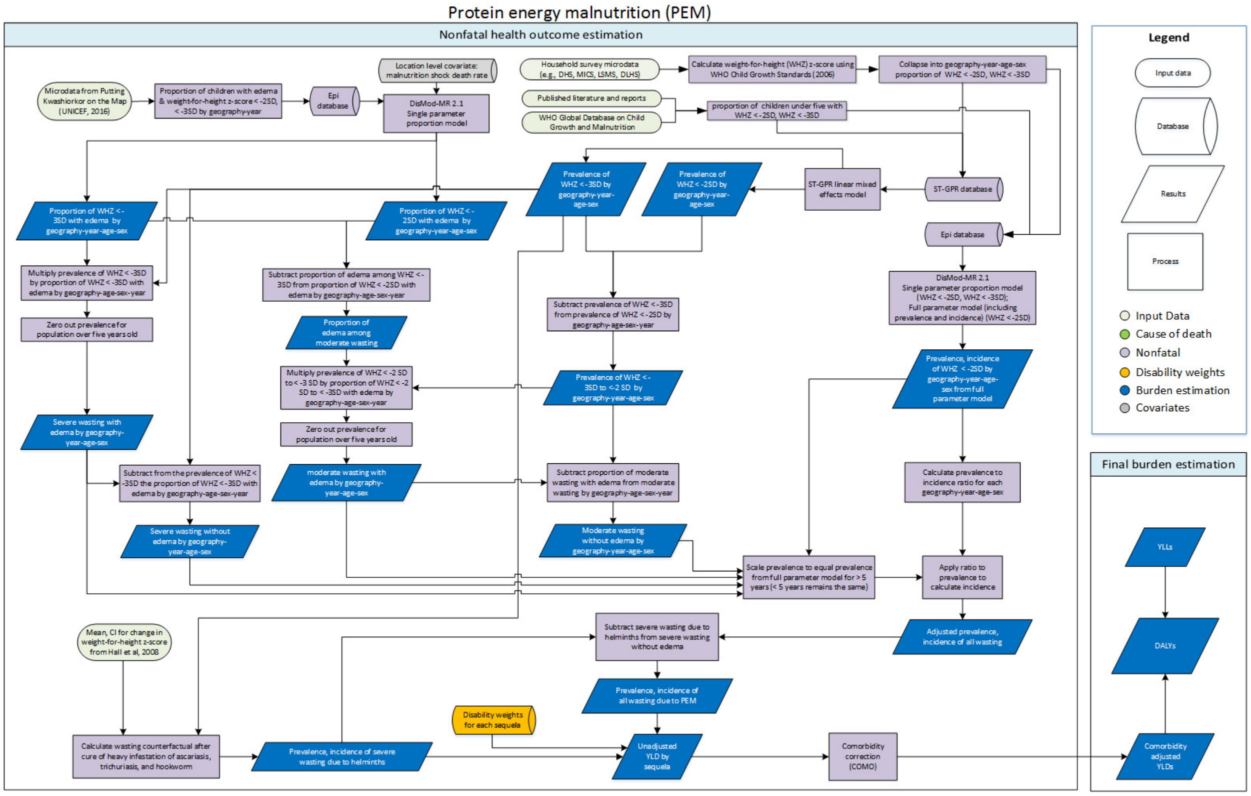

Input data and methodological summary

Case Definition

For protein-energy malnutrition (PEM), ICD 10 codes are E40-E46.9, E64.0, and ICD 9 codes are 260-263.9. Our assessment of non-fatal PEM includes the quantification of non-fatal health loss associated with moderate and severe acute malnutrition, commonly referred to as "wasting," and was defined in terms of weight-for-height Z-scores (WHZ) on the WHO 2006 growth standard for children. We quantified non-fatal PEM burden in four mutually exclusive and collectively exhaustive categories, reflecting distinct gradations of disability that can occur: moderate wasting ***without oedema*** (WHZ < -2SD to < -3 SD), moderate wasting ***with oedema*** (WHZ < -2SD to < -3 SD), severe wasting ***without oedema*** (WHZ < -3SD), and severe wasting ***with oedema*** (WHZ < -3SD). The aggregate of categories that include "oedema" can be considered equivalent to the disease state commonly referred to as "kwashiorkor" and severe wasting can likewise be considered equivalent to "marasmus."

This classification reflects a moderate shift from GBD 2015, when moderate wasting without oedema was not included in our non-fatal estimates, and by definition is associated with higher prevalence estimates than previously published by GBD. The other GBD 2015 categories – kwashiorkor, marasmus, and severe wasting – have unchanged case definitions, but have been renamed for clarity and consistency. This revised GBD 2016 case definition more closely aligns with other and allows for better application to the international nutrition community's programming and estimates related to non-fatal PEM. This change has been continued in GBD 2017.

Input data

The input data for this model come in three primary streams. First, we used individual-level and tabulated child anthropometry data from health surveys, literature, and national reports, and centralised them to inform the prevalence of WHZ decrement in each category corresponding to our case definitions. Second, to inform the proportion of children under 5 years who have signs of organ failure manifested as oedema (ie, kwashiorkor), we used a compiled dataset of surveys conducted using Standardized Monitoring and Assessment of Relief and Transitions (SMART) methods. Third, we used the cause-specific mortality rate (CSMR) results of our causes-of-death GBD analyses to inform spatiotemporal and age patterns in PEM mortality, which helped also inform patterns of PEM morbidity. These data sources and modelling process are described in other GBD publications on cause-specific mortality. All data were extracted with the most detailed standard demographic identifiers available, including age, sex, country, year, and subnational location if available.

Table 1a: Dataset contents for total wasting (moderate + severe, with and without oedema)

	Prevalence
Site-years (total)	2960
Number of countries with data	157
Number of GBD regions with data (out of 21 regions)	21
Number of GBD super-regions with data (out of 7 super-regions)	7

Table 1b: Covariate effects for total wasting (moderate + severe, with and without oedema)

Measure	Covariate	Covariate type	Beta Value	Exponentiated
Prevalence	Sex	Study-level (x-cov)	0.062 (0.052–0.072)	1.06 (1.05–1.07)
Prevalence	Sanitation (prop access)	Country-level	-0.059 (-0.066 to -0.051)	0.94 (0.94–0.95)
Prevalence	Socio-demographic Index	Country-level	-0.087 (-0.2 to -0.0026)	0.92 (0.82–1.00)
Prevalence	Malnutrition Shock log-trans mort rt	Country-level	0.00048 (0.000015–0.0015)	1.00 (1.00–1.00)
Excess mortality rate	Sex	Study-level (x-cov)	-0.3 (-0.34 to -0.27)	0.74 (0.71–0.77)
Excess mortality rate	Healthcare Access and Quality index	Country-level	-0.049 (-0.052 to -0.045)	0.95 (0.95–0.96)
Cause-specific mortality rate	Sex	Study-level (x-cov)	0.18 (0.17–0.19)	1.20 (1.18–1.21)

Table 2a: Dataset contents for proportion of oedema among total wasting

	Proportion
Site-years (total)	240
Number of countries with data	45
Number of GBD regions with data (out of 21 regions)	12
Number of GBD super-regions with data (out of 7 super-regions)	6

Table 2b: Covariate effects for proportion of oedema among total wasting

Measure	Covariate	Covariate type	Beta value	Exponentiated
Proportion	Sex	Study-level (x-cov)	0.064 (-0.02 to 0.098)	1.07 (0.98–1.10)
Proportion	energy unadjusted(kcal)	Country-level	-1 (-1 to -1)	0.37 (0.37–0.37)
Proportion	Malnutrition shock log-transformed mortality rate	Country-level	0.53 (0.49–0.57)	1.70 (1.63–1.76)

Table 3a: Dataset contents for proportion of oedema among severe wasting

	Proportion
Site-years (total)	240
Number of countries with data	45
Number of GBD regions with data (out of 21 regions)	12
Number of GBD super-regions with data (out of 7 super-regions)	6

Table 3b: Covariate effects for proportion of oedema among severe wasting

Measure	Covariate	Covariate type	Beta Value	Exponentiated
Proportion	Sex	Study-level (x-cov)	2.00 (1.99–2.00)	7.36 (7.29–7.39)
Proportion	energy unadjusted(kcal)	Country-level	-1 (-1 to -1)	0.37 (0.37–0.37)
Proportion	Malnutrition shock log-transformed mortality rate	Country-level	0.99 (0.97–1.00)	2.70 (2.64–2.72)

Modelling Strategy

We used five parallel models to inform our estimates, the first two of which were completed in ST-GPR and the second three of which were completed in DisMod-MR 2.1: 1) Prevalence of WHZ <-2 in children under 5 years, 2) Prevalence of WHZ <-3 in children under 5 years, 3) Proportion of those with WHZ <-2 who have oedema in under 5 years, 4) Proportion of those with WHZ <-3 who have oedema in under 5 years, and 5) Prevalence, incidence, and excess mortality of WHZ <-2 in all ages. As a final step, we subtracted a number of cases of PEM where the underlying aetiology is severe worm infestation.

The results of the first four models were used for children under 5 years. Arithmetic transformations were performed to ensure that the final results fit into the mutually exclusive, collectively exhaustive categories described in the first section above. We assumed zero prevalence of oedema in people over 5

years old. The results of the final model were used for all age groups 5 years and older and the proportion of moderate versus severe wasting in each of those age groups was derived from the first set of models.

Using available information from scientific publications, which suggest the mean duration of illness is nine months, and conversations with collaborators and nutrition experts, we applied what we consider a plausible set of remission rate bounds of 0.25–1.25 (# of remitted cases of PEM per person-year of illness) to the final of the five models. These bounds allowed DisMod-MR 2.1 to mathematically derive an internally consistent solution for incidence, prevalence, remission, excess mortality, and cause-specific mortality using all available data. This could only be done for the aggregate PEM definition (prevalence of WHZ <-2) to ensure that the case definition for prevalence matched that of the mortality results. The incidence-to-prevalence ratio derived from the final model was applied equally across all the categories of non-fatal PEM. Future work in systematically evaluating longitudinal datasets on nutrition and growth failure will allow us to improve the empirical basis for PEM incidence estimates, including improved resolution for the component categories.

We applied disability weights from the GBD disability weight survey to the prevalence of the above sequelae according to their corresponding health state and severity level. The sequelae, along with their lay descriptions and disability weights for health states derived from the GBD disability weights study, are shown below. We assumed that those with moderate wasting, but no oedema, did not have any direct disability due to this condition.

Table 4. Sequelae, severity, lay description, and DWs

Sequela	Health state name	Lay description	DW (95% CI)
Moderate wasting without oedema	Asymptomatic	--	--
Moderate wasting with oedema	Kwashiorkor	Is very tired and irritable and has diarrhoea.	0.051 (0.031–0.079)
Severe wasting without oedema	Severe wasting	Is extremely skinny and has no energy.	0.128 (0.082–0.183)
Severe wasting with oedema	Kwashiorkor + severe wasting	Is very tired and irritable and has diarrhoea.	0.051 (0.031–0.079)
		Is extremely skinny and has no energy.	0.128 (0.082–0.183)

Because both worms and PEM can cause wasting, we needed to divide out the wasting envelope to attribute wasting to both PEM and worms. We determined the amount of wasting attributable to worms by referencing Hall and colleagues 2008[1] to determine the mean and confidence interval estimates of the z-score shift. We then calculated the counterfactual wasting prevalence given no worms, according to the z-score shift. From this, we calculated the fraction of wasting that is attributable to worms and assigned the remainder of wasting to PEM. We assumed no oedema due to worms and the same prevalence-to-incidence ratio as in each of the other models.

Following the assignment of disability weights to the various sequelae, the resulting years lived with disability (YLDs) go through the comorbidity simulator, which accounts for any comorbidity and corrects

[1] Hall A, Hewitt G, Tuffrey V, de Silva N. A review and meta-analysis of the impact of intestinal worms on child growth and nutrition. Maternal and Child Nutrition. 2008. 4. 118-236.

accordingly. The final outputs are comorbidity-adjusted YLDs, which are combined with years of life lost (YLLs) for final disability-adjusted life-years (DALYs).

Iodine deficiency

Flowchart

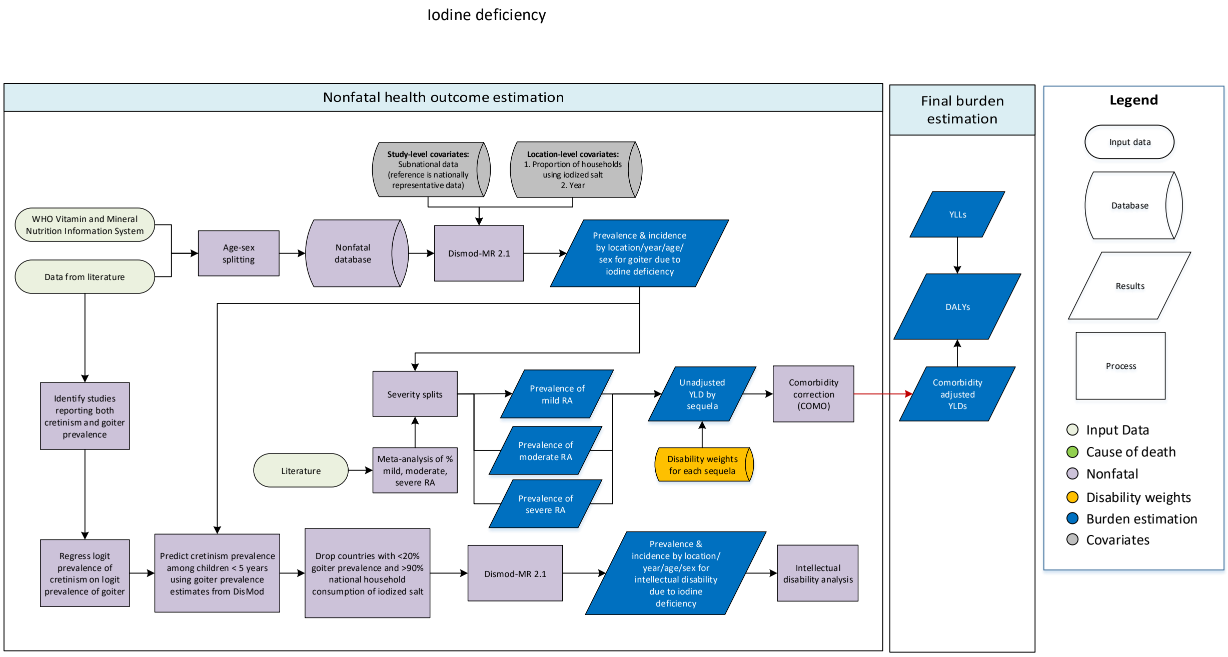

Input data and methodological summary

Case definition

Our assessment of the non-fatal burden of iodine deficiency includes estimates of only the subset of iodine deficiency associated with visible goiter (grade 2) and its associated sequelae, including thyroid dysfunction, heart failure, and intellectual disability (historically referred to as "cretinism"). It does not include estimates of sub-clinical iodine deficiency or non-visible goiter (grade 1) induced by iodine deficiency. Expanding to include all forms of subclinical iodine deficiency is a goal of future iterations of GBD analyses. The corresponding ICD-10 codes from the causes of death analysis for iodine deficiency are E00-E02. Further details of mortality modelling can be found in the GBD 2017 cause-specific mortality publication.

Input data

For GBD 2017, data from the WHO Vitamin and Mineral Nutrition Information System and published studies were used. This extraction and an accompanying systematic review were last conducted for GBD 2013. The PubMed search terms were: ((iodine deficiency[Title/Abstract] AND prevalence[Title/Abstract]) AND ("2009"[Date – Publication] : "2013"[Date – Publication]))

The exclusion criteria were:

1. Studies that were not population-based, eg, hospital or clinic-based studies
2. Studies that did not provide primary data on epidemiological parameters, eg, commentaries
3. Review articles
4. Case series

5. Self-reported cases

Updates to systematic reviews are performed on an ongoing schedule across all GBD causes; an update for iodine deficiency will be performed in the next one to two iterations. The table below shows the number of literature studies included in GBD 2016, as well as the number of countries or subnational units and GBD world regions represented.

Table 1. Dataset contents for goiter due to iodine deficiency

	Prevalence
Site-years (total)	294
Number of countries with data	75
Number of GBD regions with data (out of 21 regions)	17
Number of GBD super-regions with data (out of 7 super-regions)	7

Table 2. Dataset contents for intellectual disability due to iodine deficiency

	Other (RR or SMR)
Site-years (total)	6
Number of countries with data	5
Number of GBD regions with data (out of 21 regions)	4
Number of GBD super-regions with data (out of 7 super-regions)	2

Modelling strategy

For GBD 2017, we modelled the prevalence of grade 2 goiter in DisMod-MR 2.1. We chose to model grade 2 goiter over grade 1 goiter because of the greater reliability and consistency of the clinical diagnosis of grade 2 goiter worldwide. We used a study-level covariate to indicate national and subnational observations, where nationally representative studies were set as the reference category. We used household iodised salt consumption proportion as a country-level covariate.

We estimated the prevalence of intellectual disability due to iodine deficiency (cretinism) by regressing data points from studies reporting both cretinism and goiter prevalence in the same population. To do so, we first transformed cretinism prevalence and goiter prevalence into logit space, regressed the logit prevalence of cretinism on the logit prevalence of goiter, and predicted for all locations using the goiter estimates from the DisMod-MR 2.1 model above. We dropped locations with total goiter prevalence less than 20% and locations with household iodised salt consumption greater than 90%. We kept observations in children younger than 5 years and use these data as incidence input in a second DisMod-MR 2.1 to generate location-year-age-sex-specific estimates. This was combined with relative risk (RR) and standardised mortality ratio (SMR) data identified in the literature review described above. We assumed zero remission as once intellectual disability develops as a result of iodine deficiency, the disease is a lifelong condition, and zero incidence after age 5. We repeated the dropout criteria of total goiter prevalence and iodised salt consumption on the DisMod-MR 2.1 output. The severity split for intellectual disability due to iodine deficiency is presented separately in the section for intellectual disability.

Heart failure attributable to iodine deficiency was modelled separately, and the methods for this outcome are presented separately in the section for heart failure and its aetiologies.

Betas and exponentiated values (which can be interpreted as an odds ratio) for the grade 2 goiter DisMod-MR 2.1 model are shown in the table below.

Table 3. Beta and exponentiated beta values for goiter due to iodine deficiency

Covariate	Parameter	beta	Exponentiated beta
Proportions of households using iodised salts	Prevalence	1.03 (-0.81 to 1.95)	2.80 (0.44–7.00)
Subnational	Prevalence	0.043 (0.0037–0.16)	1.04 (1.00–1.17)
Sex	Prevalence	-0.56 (-0.77 to -0.36)	0.57 (0.46–0.70)
Health system access 2 (unitless)	Prevalence	-0.43 (-0.51 to -0.25)	0.65 (0.60–0.78)

Table 4. Beta and exponentiated beta values for intellectual disability due to iodine deficiency

Covariate	Parameter	beta	Exponentiated beta
Proportions of households using iodized salts	Incidence	-0.6 (-0.72 to -0.46)	0.55 (0.49–0.63)
Sex	Incidence	-0.42 (-0.45 to -0.4)	0.66 (0.64–0.67)
Sex	Relative risk	0.10 (-0.6 to 0.91)	1.11 (0.55–2.48)

Severity splits & disability weights

Our approach to apportioning cases of grade 2 goiter was modified in GBD 2016 to improve internal consistency of YLD estimates. The primary change was that in GBD 2015, the types of symptoms (heart failure, thyroid dysfunction, intellectual disability) were considered to be mutually exclusive, but recognising they are not, we constructed independent joint probabilities for each. Initial severity proportions have not changed since GBD 2010: visible goiter without symptoms of thyroid dysfunction (proportion=0.915, 95% confidence interval (CI): 0.904–0.926); goiter with symptoms of thyroid dysfunction (proportion=0.085, 95% confidence interval (CI): 0.084–0.086). The regression results for intellectual disability (ID) were applied to the total estimates of iodine deficiency. All of those with ID were assumed to have symptoms of thyroid dysfunction. Heart failure due to iodine deficiency, which was estimated separately, was assumed to only occur in the subset of persons with profound ID. These changes continued into GBD 2017.

The lay descriptions and disability weights for severity levels derived from the GBD disability weights study are shown below.

Table 5. Severity, health state, lay description, and DWs for sequelae specific to iodine deficiency

Sequela	Health state name	Lay description	Disability weight
Visible goiter without symptoms	Disfigurement, level 1	has a slight, visible physical deformity that others notice, which causes some worry and discomfort.	0.011 (0.005–0.021)
Visible goiter with symptoms without intellectual disability or heart failure	Iodine-deficiency goiter	has a large mass in the front of the neck. The person sometimes has weakness and fatigue, constipation and weight gain.	0.199 (0.133–0.276)
Visible goiter with ***severe*** *intellectual disability due to iodine deficiency*	Intellectual disability / mental retardation, severe	has very low intelligence and cannot speak more than a few words, needs constant supervision and help with most daily activities, and can do only the simplest tasks.	0.326 (0.233–0.438)*

	Iodine-deficiency goiter	(see above)	
Visible goiter with ***profound*** *intellectual disability due to iodine deficiency*	Intellectual disability / mental retardation, profound	has very low intelligence, has almost no language, and does not understand even the most basic requests or instructions. The person requires constant supervision and help for all activities.	0.358 (0.252–0.475)*
	Iodine-deficiency goiter	(see above)	
Visible goiter with profound intellectual disability and ***mild*** *heart failure due to iodine deficiency*	Intellectual disability / mental retardation, profound	(see above)	0.384 (0.276–0.502)*
	Iodine-deficiency goiter	(see above)	
	Heart failure, mild	is short of breath and easily tires with moderate physical activity, such as walking uphill or more than a quarter-mile on level ground. The person feels comfortable at rest or during activities requiring less effort.	
Visible goiter with profound intellectual disability and ***moderate*** *heart failure due to iodine deficiency*	Intellectual disability / mental retardation, profound	(see above)	0.403 (0.293–0.524)*
	Iodine-deficiency goiter	(see above)	
	Heart failure, moderate	is short of breath and easily tires with minimal physical activity, such as walking only a short distance. The person feels comfortable at rest but avoids moderate activity.	
Visible goiter with profound intellectual disability with ***severe*** *heart failure due to iodine deficiency*	Intellectual disability / mental retardation, profound	(see above)	0.471 (0.344–0.602)*
	Iodine-deficiency goiter	(see above)	
	Heart failure, severe	is short of breath and feels tired when at rest. The person avoids any physical activity, for fear of worsening the breathing problems.	

No other significant changes were made to the modelling strategy for GBD 2017.

Vitamin A deficiency

Flowchart

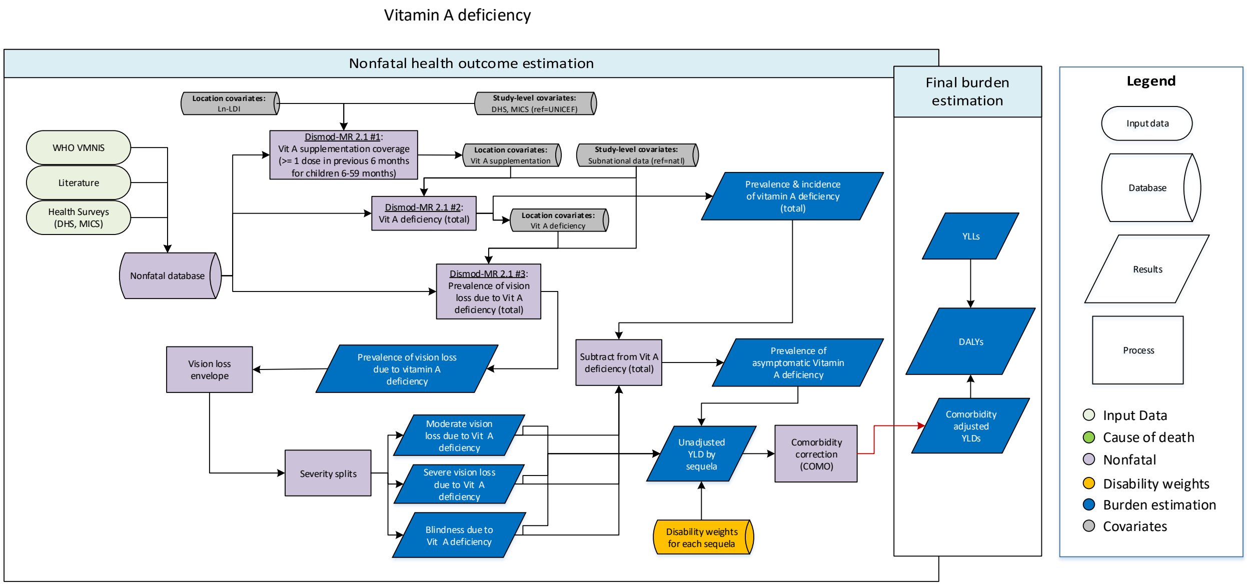

Input data and methodological summary

Case definition

For GBD 2017, the case assessment of vitamin A deficiency involves the quantification of total vitamin A deficiency (serum retinol < 0.7 µmol/L) as well as blindness and vision loss due to vitamin A deficiency, which are associated with corneal ulcerations and corneal scars. ICD 10 codes are E50-E50.9, E64.1, and ICD 9 codes are 264-264.9.

To ensure we were using as much information as possible, and therefore maximise the data basis of our estimates, we modelled vitamin A deficiency sequentially. The first step was to estimate the coverage of vitamin A supplementation. Although the typical metric on which supplementation is tracked is 2+ doses of vitamin A in the previous 12 months for children under 5 years, most existing health surveys do not routinely provide sufficient information to calculate it. Our case definition for the supplementation model was therefore the proportion of children 6-59 months of age who received at least one dose of vitamin A in the previous six months. Supplementation estimates were then used as a location-level covariate to guide incidence and prevalence models of overall vitamin A deficiency, which was subsequently used as a location-level covariate to guide prevalence estimates of vision loss due to vitamin A deficiency. The difference between total vitamin A deficiency and vision loss due to vitamin A deficiency is considered asymptomatic. Total vitamin A deficiency is separately considered as a risk factor in the GBD 2016 comparative risk assessment analysis.

Input data

For GBD 2017, we used data from the WHO Vitamin and Mineral Nutrition Information System, health surveys such as DHS and MICS, and studies identified through literature review. A systematic review was last conducted for GBD 2013. The PubMed search terms were: ((vitamin A deficiency[Title/Abstract] AND

prevalence[Title/Abstract]) AND ("2009"[Date – Publication] : "2013"[Date – Publication])). The table below shows the number of data points included in the final datasets. Exclusion criteria were:

1. Studies that were not population-based, eg, hospital or clinic-based studies
2. Studies that did not provide primary data on epidemiological parameters, eg, commentaries
3. Review articles
4. Case series
5. Self-reported cases

Table 1. Geographic representation of datasets used for three stages of Vitamin A deficiency non-fatal burden estimation (number of data points per geography)

Geography	Supplementation (proportion)	Deficiency (prevalence)	Vision loss (relative risk)	Vision loss (prevalence)
Global	900	1540	1	81
East Asia	12	27		
Southeast Asia	102	212		21
Oceania	24	54		
Central Asia	51	66		
Central Europe	2	13		
Eastern Europe		3		
Australasia		1		
Western Europe		38		
Southern Latin America		16		
High-income North America		33		
Caribbean	17	34		1
Andean Latin America	25	70		
Central Latin America	33	212		1
Tropical Latin America	1	52		2
North Africa and Middle East	49	148		18
South Asia	61	96		21
Central sub-Saharan Africa	60	8		1
Eastern sub-Saharan Africa	182	220		10
Southern sub-Saharan Africa	49	57		1
Western sub-Saharan Africa	232	180		5

Modelling strategy

All vitamin A deficiency estimates were made using DisMod-MR 2.1. As described above, we first estimated vitamin A supplementation coverage. Although all data were from ages 6-59 months, we assumed no difference in age pattern of supplementation coverage and used the natural log of lag-distributed income per capita (LN-LDI) as a location-level covariate to inform estimates where data were absent. DHS and MICS data was crosswalked to the reference data source, which came from UNICEF (http://data.worldbank.org/indicator/SN.ITK.VITA.ZS).

Table 2a: Dataset contents for vitamin A supplementation model

	Prevalence
Site-years (total)	845
Number of countries with data	94
Number of GBD regions with data (out of 21 regions)	15
Number of GBD super-regions with data (out of 7 super-regions)	6

Table 2b: Covariate effects for vitamin A supplementation model

Measure	Covariate	Type	Value	Exponentiated
Prevalence	MICS	Study-level	-0.6 (-0.76 to -0.45)	0.55 (0.47–0.64)
Prevalence	DHS	Study-level	-0.09 (-0.2 to 0.025)	0.91 (0.82–1.03)
Prevalence	LDI (I$ per capita)	Country-level	0.013 (0.00033–0.042)	1.01 (1.00–1.04)

Second, we estimated the age- and sex-specific prevalence of vitamin A deficiency (serum retinol < 0.7 µmol/L). WHO VMNIS was the primary data source for this model and was supplemented with data from DHS and other health surveys where testing was performed. We assumed the following in our model: no excess mortality, birth prevalence is possible, and that incidence and remission are both decreasing after age 5. Incidence estimates were therefore derived from the changing age pattern of prevalence with some allowance for remission. Data from subnational locations were crosswalked to the reference data sources of nationally representative data. Location-level covariates were used for vitamin A supplementation coverage from the above model as well as Socio-demographic Index (SDI) numbers and grams per capita of vegetables available in foods.

Table 3a: Dataset contents for vitamin A deficiency model

	Prevalence
Site-years (total)	295
Number of countries with data	96
Number of GBD regions with data (out of 21 regions)	20
Number of GBD super-regions with data (out of 7 super-regions)	7

Table 3b: Covariate effects for vitamin A deficiency model

Measure	Covariate	Type	Value	Exponentiated
Prevalence	Sex	Study-level	-0.0091 (-0.088 to 0.065)	0.99 (0.92–1.07)
Prevalence	Subnational	Study-level	-0.28 (-0.44 to -0.1)	0.76 (0.64–0.90)
Prevalence	Vit A suppl. coverage	Country-level	-0.028 (-0.1 to -0.00071)	0.97 (0.90–1.00)
Prevalence	SDI	Country-level	-2.98 (-3 to -2.92)	0.051 (0.050–0.054)
Prevalence	Veget unadj (g/pop)	Country-level	-1.36 (-1.53 to -1.12)	0.26 (0.22–0.33)

Third, for models of prevalence of blindness and vision loss due to vitamin A deficiency, this was run as a single-parameter meta-regression on prevalence, so incidence estimates were not generated. Data from subnational locations were crosswalked to the reference data sources of nationally representative data. Vitamin A deficiency prevalence was used as a location-level covariate. Two covariates that were used in GBD 2015 were removed in the GBD 2016 models, and this was carried over into GBD 2017. These were 1) proportion of children who are underweight, and 2) year. The first was removed to eliminate a modelling circularity where, because of timing of processes, the only available results for this covariate at the time of modelling vitamin A deficiency were from the previous round of GBD estimation. The second

was removed because it functionally assumes a fixed time trend in the prevalence of vision loss, which may not be the case everywhere.

Table 4a: Dataset contents for vision loss due to vitamin A deficiency model

	Prevalence
**Site-years (total)**	44
**Number of countries with data**	27
**Number of GBD regions with data (out of 21 regions)**	10
**Number of GBD super-regions with data (out of 7 super-regions)**	5

Table 4b: Covariate effects for vision loss due to vitamin A deficiency model

Measure	Covariate	Type	Value	Exponentiated
Prevalence	Sex	Study-level	-0.044 (-1.4 to 1.32)	0.96 (0.25–3.73)
Prevalence	Subnational	Study-level	0.24 (0.015–0.48)	1.27 (1.02–1.62)
Prevalence	Vit A def prev (age-stdised)	Country-level	1.11 (0.069–1.97)	3.05 (1.07–7.14)

Severity splits and disability weights

Our GBD 2017 results include explicit estimates of total vitamin A deficiency, although those without vision loss are assumed to be asymptomatic. Description of how our estimates of total vision loss described above are parsed into moderate vision loss, severe vision loss, and blindness can be found in the modelling description for the "vision loss impairment". Sequelae and corresponding disability weights for each of the health states associated with vitamin A deficiency are shown in the table below.

Table 5. Severity, lay description, and disability weight (DW)

Sequela	Health state name	Lay description	Disability weight
Moderate vision impairment loss due to vitamin A deficiency	Distance vision, moderate impairment	has vision problems that make it difficult to recognise faces or objects across a room.	0.031 (0.019–0.049)
Severe vision impairment loss due to vitamin A deficiency	Distance vision, severe impairment	has severe vision loss, which causes difficulty in daily activities, some emotional impact (for example worry), and some difficulty going outside the home without assistance.	0.184 (0.125–0.258)
Blindness due to vitamin A deficiency	Distance vision blindness	is completely blind, which causes great difficulty in some daily activities, worry and anxiety, and great difficulty going outside the home without assistance.	0.187 (0.124–0.26)
Asymptomatic vitamin A deficiency	Asymptomatic	--	--

No other changes were made for GBD 2017.

Other nutritional deficiencies

Other nutritional deficiencies encompass a wide variety of causes of morbidity, ranging from vitamin deficiencies to other nutritional anaemias. GBD 2017 treats these causes as a single category, given their relatively limited burden, diversity in underlying causes and risk factors, and data availability. Instead of modelling them in a traditional modelling format, we calculate the YLDs associated with other nutritional deficiencies using a YLD/YLL ratio.

The first input for this non-fatal portion of other nutritional deficiencies burden is the YLL estimates from the GBD 2017 causes of death (CoD) analysis. The causes and their associated ICD-10 codes that constitute other nutritional deficiencies for CoD are listed below. Additionally, CoD includes specific models for protein-energy malnutrition, another nutritional cause of morbidity and mortality; as protein-energy malnutrition has a specific non-fatal model that results in YLDs, we can calculate the YLD/YLL ratio for protein-energy malnutrition. We multiply the YLL estimates for other nutritional deficiencies from CoD by the YLD/YLL ratio for PEM, providing us with an estimate of the YLDs associated with other nutritional deficiencies.

GBD cause	ICD-10 code
Other nutritional deficiencies	D51-D52.0 (vitamin B12 deficiency anaemia and folate deficiency anaemia)
Other nutritional deficiencies	D52.8-D53.9 (other nutritional anaemias)
Other nutritional deficiencies	D64.3 (other sideroblastic anaemias)
Other nutritional deficiencies	E51-E61.9 (thiamine, niacin, other B group vitamins, ascorbic acid, vitamin D, other vitamin, dietary calcium, dietary selenium, dietary zinc, and other nutrient element deficiencies)
Other nutritional deficiencies	E63-E64.0 (other nutritional deficiencies and sequelae of protein-calorie malnutrition)
Other nutritional deficiencies	E64.2-E64.9 (sequelae of vitamin C deficiency, rickets, other nutritional deficiencies, and unspecified nutritional deficiencies)
Other nutritional deficiencies	M12.1-M12.19 (Kaschin-Beck disease)

Cancer

Neoplasms

The general framework for the GBD 2017 cancer estimation applies to all malignant neoplasms (aka cancers) except for non-melanoma skin cancer (basal cell carcinoma and squamous cell carcinoma) and myelodysplastic, myeloproliferative, and other haemopoietic neoplasms;

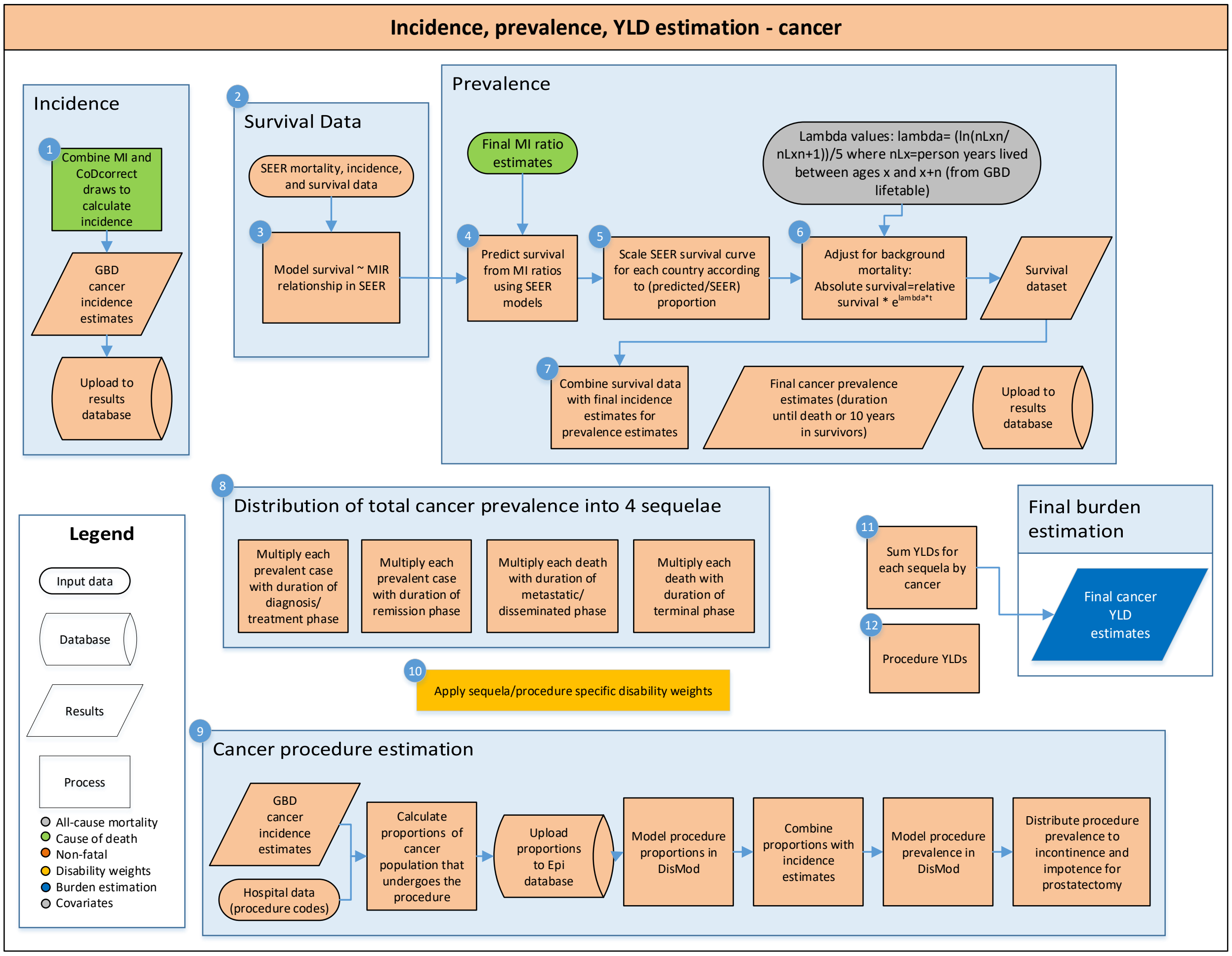

Input data and methodological appendix

Case definition

For GBD 2017, incidence, prevalence, and disability are estimated for all cancers and benign neoplasms as defined in ICD-10 (C00-D49). The associated ICD codes for neoplasms estimated for GBD 2017 are listed in Appendix Table 4. Prevalence for all cancers is estimated for a maximum of ten years after incidence, as in GBD 2013, GBD 2015, and GBD 2016. Prevalence extending beyond the ten-year period is only estimated for permanent sequelae resulting from procedures.

To estimate disability for each cancer, total prevalence is split into four sequelae: 1. diagnosis and primary therapy; 2. controlled phase; 3. metastatic phase; and 4. terminal phase. The diagnosis and primary therapy phase is defined as the time from the onset of symptoms to the end of treatment. The controlled phase is defined as the time between finishing primary treatment and the earliest of either: cure (defined as recurrence- and progression-free survival after ten years); death from another cause; or progression to the metastatic phase. The metastatic phase is defined as the time period of intensive treatment for metastatic disease, as determined for each cancer by SEER (Surveillance, Epidemiology, and End Results Program) averages (Table 3). The terminal phase is defined as the one-month period prior to death. Each of these four sequelae has a separate disability weight, which are the same across cancers (**Error! Reference source not found.**). Because of long-term disability associated with treatment-related procedures, additional disability beyond these four sequelae is estimated for five cancers: breast cancer (disability due to mastectomy), larynx cancer (disability due to laryngectomy), colon and rectum cancer (disability due to stoma), bladder cancer (disability due to incontinence from cystectomy), and prostate cancer (disability due to incontinence and impotence from prostatectomy).

Input data

Cancer incidence is directly estimated from cancer mortality using mortality to incidence ratios (MIRs). Data sources for cancer mortality are described in detail elsewhere.[1] To estimate the proportion of cancer patients undergoing procedures we used SEER data form 1983 to 2008[2] and Mexico Hospital Data from 2001 to 2009[3]. Data sources used to adjust procedure sequelae will be listed below.

Modelling strategy

Estimation of cancer mortality and MIR estimation has been described in the GBD 2017 Mortality and Causes of Death capstone papers. The final GBD cancer mortality estimates are transformed to incidence estimates by using separately estimated MIRs. To summarize the MIR estimation process, incidence and mortality data from cancer registries were matched by cancer, age, sex, year, and location to generate MI ratios. We used a fixed effect logistic regression model to estimate MIRs, with covariates for sex, categorical age, and the Healthcare Access and Quality index (HAQ index)[4] (which replaced the Socio-demographic Index [SDI] used in this model in GBD 2016):

$$\text{logit}\left(MI\ ratio_{c,a,s,t}\right) = \alpha + \beta_1 HAQI_{c,t} + \sum_{a}^{A} \beta_2 I_a + \beta_3 I_s + \epsilon_{c,a,s,t}$$

c: country, a: age group, t: time (years); s: sex
HAQI: Healthcare Access and Quality index
I: indicator variable
$\epsilon_{c,a,s,t}$: error term

Data points were outliered manually if they clearly influenced the model in an unrealistic way. For example, a data point was marked as an outlier if it created a single-year, single-age-group spike in model predictions that was inconsistent with the trend suggested by surrounding data points. Results from the final linear model were used as input for space-time smoothing and a Gaussian process regression (ST-GPR). The ST-GPR process has been updated for GBD 2017.

Final MIR estimates at the 1,000-draw level were combined with final mortality estimates (also at the 1,000-draw level) to generate incidence estimates. It was assumed that uncertainty in the MIR is independent of uncertainty in the estimated mortality.

After transforming the final GBD cancer mortality estimates to incidence estimates (step 1 in the general cancer flowchart), incidence was combined with the relative yearly survival estimates up to ten years (step 7 in the flowchart). For GBD 2017 we updated our methods to more directly utilise MIRs to generate these yearly cancer relative survival estimates. Previous reports suggest that the value of (1 – MIR) may serve as a proxy for five-year relative survival, with the exact correlation varying slightly by cancer type.[5] We used SEER*Stat[6] to obtain mortality, incidence, and relative survival statistics from the nine SEER registries[7] reporting from 1980–2014 (step 2), by cancer type, sex, five-year blocks (ie, 1980–1984, 1985–1989, etc.), and five-year age groups (except combining 80+). For each cancer, we modelled five-year relative survival with the SEER MIRs using Poisson regression, weighted by the number of incident cases (step 3). To reduce variability due to small samples, we only included MIRs based on at least 25 incident cases (except for the rarer cancers mesothelioma, nasopharyngeal cancer, and acute myeloid leukaemia, where MIRs based on at least ten cases were included). These models were then applied to the GBD MIR estimates to predict an estimated five-year survival for each age/sex/year/location (step 4). To prevent unrealistic values, predicted survival values were winsorised to be between 100% survival and the worst-case survival scenario from SurvCan and USA 1950 survival data[8,9] utilised in previous GBD cycles. To obtain yearly survival estimates up to ten years, we compared these five-year relative survival estimates to the SEER sex-specific all-ages relative five-year survival data from 2004 (the latest year with ten-year survival available).[10] The proportion of the predicted GBD five-year survival estimate to the SEER five-year survival statistic was used as a scalar to generate yearly survival estimates from the GBD survival predictions under the proportional hazard assumption (step 5).

To transform relative to absolute survival (adjusting for background mortality), GBD 2017 life tables were used (step 6 and 7 in the flowchart) to calculate lambda values: lambda= (ln(nLxn/nLxn+1))/5, where nLx=person-years lived between ages x and x+n (from GBD lifetable). Absolute survival was then calculated using an exponential survival function (absolute survival = relative survival * $e^{lambda*t}$).

For the purposes of calculating disability due to cancer, survivors beyond ten years were considered cured. For this group, the survivor population prevalence was divided into two sequelae (1. diagnosis and primary therapy; 2. controlled phase). For the population that did not survive beyond ten years, the yearly prevalence was divided into the four sequelae by assigning the fixed durations for each of the diagnosis and primary therapy phase, metastatic phase, and terminal phase, and assigning the remaining prevalence to the controlled phase (step 9 in the flowchart). Duration of these four sequelae remained the same as for GBD 2013, GBD 2015, and GBD 2016.[11] Table 3 lists the duration of each, along with the sources used to determine their length.

Table 3. Duration of four prevalence sequelae by cancer					
	Diagnosis/ Treatment (months)	Remission	Disseminated/metastatic (months)	Note	Terminal (months)
Oesophageal cancer	5[12]	Calculated based on remainder of time after attributing other sequelae.	4.6[10]	SEER Summary Stage 1997 (Distant site/node involved) 1995–2000	1 month
Stomach cancer	5.2[12]	Calculated based on remainder of time after attributing other sequelae.	3.88[10]	SEER Summary Stage 1997 (Distant site/node involved) 1995–2000	1 month
Liver cancer	4	Calculated based on remainder of time after attributing other sequelae.	2.51[10]	SEER Summary Stage 1997 (Distant site/node involved) 1995–2000	1 month
Larynx cancer	5.3[12]	Calculated based on remainder of time after attributing other sequelae.	8.84[10]	SEER Stage IVc	1 month
Lung cancer	3.3[13]	Calculated based on remainder of time after attributing other sequelae.	4.51[10]	SEER Summary Stage 1997 (Distant site/node involved) 1995–2000	1 month
Breast cancer	3[13]	Calculated based on remainder of time after attributing other sequelae.	17.7[10]	SEER Summary Stage 1997 (Distant site/node involved) 1995–2000	1 month
Cervical cancer	4.8[12]	Calculated based on remainder of time after attributing other sequelae.	9.21[10]	SEER Summary Stage 1997 (Distant site/node involved) 1995–2000	1 month
Uterine cancer	4.6[12]	Calculated based on remainder of time after attributing other sequelae.	11.6[10]	SEER Summary Stage 1997 (Distant site/node involved) 1995–2000	1 month
Prostate cancer	4[13]	Calculated based on remainder of time after attributing other sequelae.	30.35[10]	SEER Summary Stage 1997 (Distant site/node involved) 1995–2000	1 month
Colorectal cancer	4[13]	Calculated based on remainder of time after attributing other sequelae.	9.69[10]	SEER Summary Stage 1997 (Distant site/node involved) 1995–2000	1 month
Oral cancer	5.3[12]	Calculated based on remainder of time after attributing other sequelae.	9.33[10]	SEER Stage IVc	1 month
Nasopharyngeal cancer	5.3[12]	Calculated based on remainder of time after attributing other sequelae.	13.19[10]	SEER Stage IVc	1 month
Cancer of other part of pharynx	5.3[12]	Calculated based on remainder of time after attributing other sequelae.	7.91[10]	SEER Stage IVc	1 month
Gallbladder cancer	4	Calculated based on remainder of time after attributing other sequelae.	3.47[10]	SEER Summary Stage 1997 (Distant site/node involved) 1995–2000	1 month
Pancreas cancer	4.1[12]	Calculated based on remainder of time after attributing other sequelae.	2.54[10]	SEER Summary Stage 1997 (Distant site/node involved) 1995–2000	1 month

Melanoma	2.9[14]	7.18[10]	SEER Summary Stage 1997 (Distant site/node involved) 1995–2000
Ovarian cancer	3.2[13]	25.6[10]	SEER Summary Stage 1997 (Distant site/node involved) 1995–2000
Testicular cancer	3.7[12]	19.47[10]	SEER Stage III
Kidney cancer	5.3[12]	5.38[10]	SEER Summary Stage 1997 (Distant site/node involved) 1995–2000
Bladder cancer	5.1[12]	5.8[10]	SEER Summary Stage 1997 (Distant site/node involved) 1995–2000
Brain cancer	5	6.93[10]	SEER Median age standardised survival, all patients, all years
Thyroid cancer	3	19.39[10]	SEER Stage IVc
Mesothelioma	4	7.75[10]	SEER Summary Stage 1997 (Distant site/node involved) 1995–2000
Hodgkin's lymphoma	3.7[13]	26[15]	
Non Hodgkin lymphoma	3.7[13]	7.7[15]	
Multiple myeloma	7[12]	36.82[10]	SEER Median age-standardised survival, all patients, all years
Leukaemia[12]	5	43.67[10]	SEER Median age-standardised survival, all patients, all years
ALL	12	7.02[10]	SEER Median age-standardised survival, all patients, all years
AML	6	4.6[10]	SEER Median age-standardised survival, all patients, all years
CLL	6	48[16]	SEER Median age-standardised survival, all patients, all years
CML	6	4.6[10]	SEER Median age-standardised survival for AML (patients with CML die in blast crisis, which is treated like AML) all patients, all years

Leukaemia other	6		48[16]	SEER Median age-standardised survival, all patients, all years	
Other	4.4 (mean of other cancer durations)		15.81[10]	SEER Median age-standardised survival, all patients, all years	

For cancer-specific procedure sequelae, hospital data were used to estimate the number of cancer patients undergoing mastectomy, laryngectomy, stoma, prostatectomy, and cystectomy (step 10 in the flowchart). These proportions remained the same as in GBD 2013, GBD 2015, and GBD 2016.[11] Proportions were generated by dividing the rate of procedures generated from the diagnostic codes in the hospital dataset and the coverage population by the GBD age- and sex-specific disease incidence rates for that country. Diagnostic codes used are listed in table 4:

**Table 4. Procedure codes used to estimate cancer procedure proportions**		
Procedure	Cancer	Procedure code (ICD-9_CM)
Mastectomy	Breast cancer	854, 8541, 8542, 8543, 8544, 8545<br>8546, 8547, 8548
Laryngectomy	Larynx cancer	301, 303, 304, 3029
Stoma	Colon and rectum cancer	461, 4610, 4611, 4613, 4862
Cystectomy	Bladder cancer	5771, 5779
Prostatectomy	Prostate	603, 604, 605, 606, 6062

To estimate procedure-related disability for each of these five cancers, the procedure proportions (proportion of each cancer population that undergo these procedures) from hospital data were used as input for a proportion model in DisMod-MR 2.1 to estimate the proportions for all locations, by age, year, and by sex.

Since colostomy or ileostomy procedures are done for reasons other than cancer, a literature review was conducted to determine the proportion of ostomies due to colorectal cancer. The "all-cause" colostomy proportions were multiplied by 0.58, based on the results of the literature review that an average of 58% of ostomies are done for colorectal cancer.[17–19]

The final procedure proportions were applied to the incidence cases of the respective cancers and multiplied with the proportion of the incidence population surviving for ten years to determine the incident cases of the cancer population that underwent procedures and that survived beyond ten years. These incident cases were used again as an input for DisMod-MR 2.1, with a remission specification of zero and an excess mortality rate prior of 0 to 0.1, as well as with increasing the age of the population and the year by ten years to reflect prevalence after that population has survived ten years. This approach was updated compared to GBD 2016, where we did not include an age or time shift. The results from this model are incidence and lifetime prevalent cases of persons with these cancer-related sequelae who have survived beyond ten years.

Since disability associated with prostatectomy comes from impotence and incontinence, and not from the prostatectomy itself, 18% of the prostatectomy prevalence was assumed to have incontinence and 55%

was assumed to have impotence, based on a literature review done for GBD 2013.[20–27] Cases were assigned disability for either impotence or incontinence, but no cases were assigned disability from both.

We assumed that for the population surviving up to ten years, only the prevalence population being in remission experiences additional disability due to procedures (eg, women suffering from metastatic breast cancer do not experience additional disability due to a mastectomy during this phase). To estimate the prevalence of the cancer population in remission during the first ten years after diagnosis with and without procedure-related disability, we multiplied the prevalence of the population in the remission phase with the proportion of the population undergoing a procedure. This step allowed us to estimate disability during the remission phase for both the population experiencing disability due to the remission phase alone, as well as the population experiencing disability from the remission phase and the additional procedure-related disability.

Lastly, the procedure sequelae prevalence and general sequelae prevalence were multiplied with their respective disability weights (Table 5) to obtain the number of YLDs (steps 11 and 12 in the flowchart). The sum of these YLDs is the final YLD estimate associated with each cancer.

Table 5. Lay description and disability weights

Health state	Lay description	Estimate	Uncertainty interval	
Cancer, diagnosis and primary therapy (cancer_diagnosis)	This person has pain, nausea, fatigue, weight loss and high anxiety.	0.288	0.193	0.399
Cancer, controlled phase (generic_medication)	This person has a chronic disease that requires medication every day and causes some worry but minimal interference with daily activities.	0.049	0.031	0.072
Cancer, metastatic (cancer_metastatic)	This person has severe pain, extreme fatigue, weight loss and high anxiety.	0.451	0.307	0.600
Terminal phase, with medication (cancer_terminal_treat)	This person has lost a lot of weight and regularly uses strong medication to avoid constant pain. The person has no appetite, feels nauseous, and needs to spend most of the day in bed.	0.540	0.377	0.687
Mastectomy (cancer_mastectomy)	This person had one of her breasts removed and sometimes has pain or swelling in the arms.	0.036	0.020	0.057
Stoma (cancer_stoma)	This person has a pouch attached to an opening in the belly to collect and empty stools.	0.095	0.063	0.131

Laryngectomy (speech_problems)	This person has difficulty speaking, and others find it difficult to understand.	0.051	0.032	0.078
Urinary incontinence (incontinence)	This person cannot control urinating.	0.139	0.094	0.198
Impotence (impotence)	This person has difficulty in obtaining or maintaining an erection.	0.017	0.009	0.030

There are no other significant changes to the GBD 2017 neoplasm modelling process compared to GBD 2016.

Non-melanoma skin cancer (squamous and basal cell carcinoma)

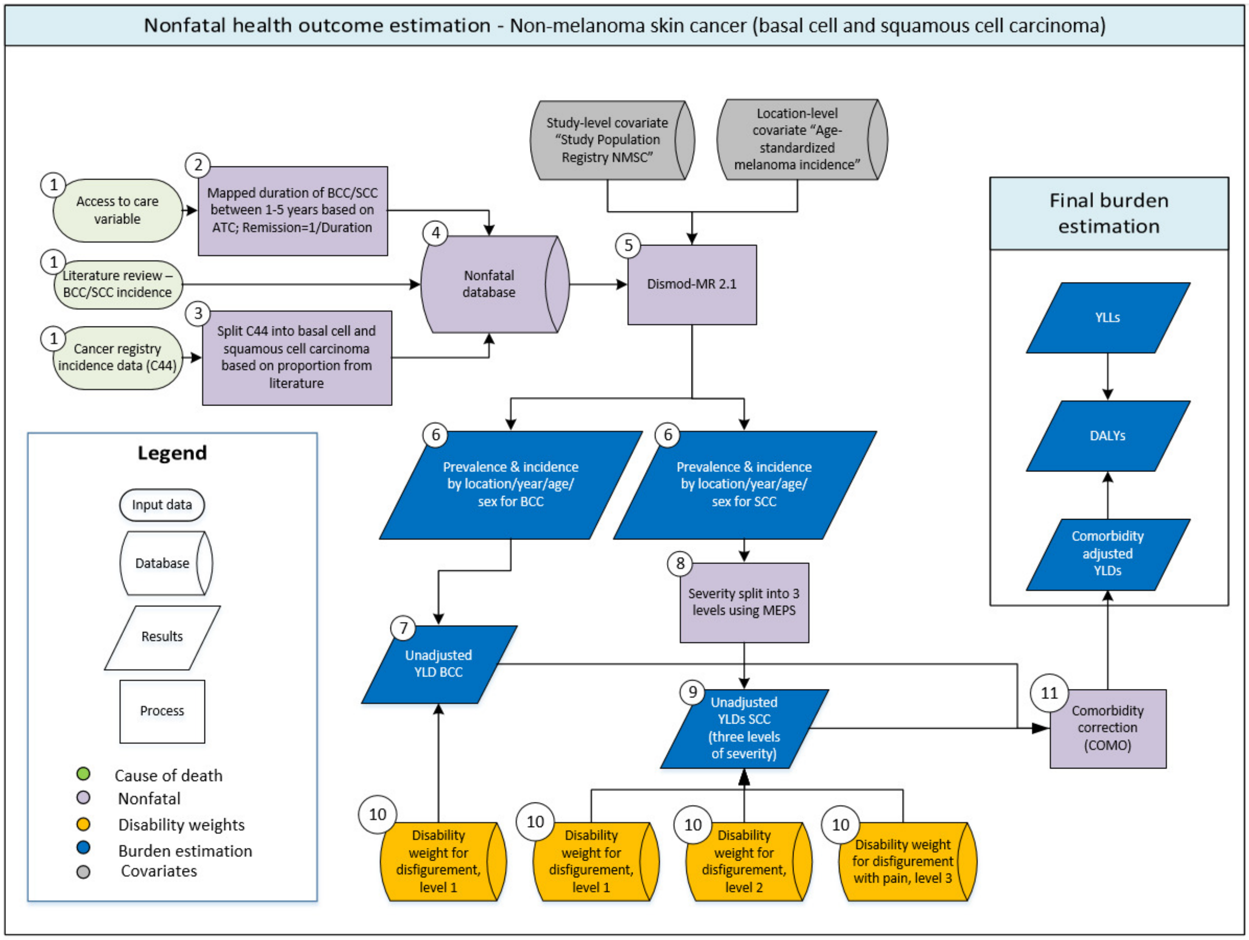

Case definition

Non-melanoma skin cancer (NMSC) is defined as basal cell carcinoma and squamous cell carcinoma. NMSC does not include other types of skin cancer (eg, melanoma, Merkel cell carcinoma).

Input data

We estimated squamous cell and basal cell skin cancer incidence by using cancer registry as well as primary literature, and MarketScan data for incidence. Only cancer registries that were listed in CI5 VIII as registering squamous cell carcinoma or basal cell carcinoma, respectively, were included in the analysis.

Modelling strategy

For cancer registry data reported at the three-digit level (ie, C44: Other and unspecified malignant neoplasm of skin), proportions from Karagas and colleagues were used to split C44 into squamous cell carcinoma and basal cell carcinoma.[28] The only new data we added compared to GBD 2016 were MarketScan data. DisMod-MR 2.1 was used to model incidence and prevalence. Prevalence was calculated as a function of two extreme scenarios (duration 1 versus 5 years). Country, age, sex and year-specific duration was estimated using a country-age-sex-year specific relative access-to-care-score.

The access to care score was based on the melanoma mortality to incidence ratio:

$$Access\ to\ care = 1 - \frac{Age\ standardized\ MIR_{cys} - Age\ standardized\ MIR_{min}}{Age\ standardized\ MIR_{max} - Age\ standardized\ MIR_{min}}$$

c=country; y=year; s=sex; Age-standardized MI $ratio_{min}$=lowest MIR for all countries and years; Age standardized MIR_{max}=highest MIR for all countries and years

Remission was calculated as the inverse of the duration estimates and used as additional input for DisMod-MR 2.1.

To reflect differing degrees of disability due to squamous cell carcinoma we used three levels of severity that were derived from MEPS (Medical Expenditure Panel Survey). Prevalence was multiplied by distinct disability weights (Table 6) to generate YLDs.

Table 6. Lay description and disability weights

Cause	Health state		Estimate with uncertainty interval
Cutaneous squamous cell carcinoma, mild	Disfigurement, level 1	has a slight, visible physical deformity that others notice, which causes some worry and discomfort.	0.011 (0.005–0.021)
Cutaneous squamous cell carcinoma, moderate	Disfigurement, level 2	has a visible physical deformity that causes others to stare and comment. As a result, the person is worried and has trouble sleeping and concentrating.	0.067 (0.044–0.096)

Cutaneous squamous cell carcinoma, severe	Disfigurement, level 3, with itch/pain	has an obvious physical deformity that is very painful and itchy. The physical deformity makes others uncomfortable, which causes the person to avoid social contact, feel worried, sleep poorly, and think about suicide.	0.576 (0.401–0.731)
Disfigurement due to basal cell carcinoma	Disfigurement, level 1	has a slight, visible physical deformity that others notice, which causes some worry and discomfort.	0.011 (0.005–0.021)

There are no other significant changes to the GBD 2017 NMSC modelling process compared to GBD 2016.

Non-melanoma skin cancer, squamous cell carcinoma

	Incidence
Site-years (total)	1448
Number of countries with data	88
Number of GBD regions with data (out of 21 regions)	20
Number of GBD super-regions with data (out of 7 super-regions)	7

Non-melanoma skin cancer, basal cell carcinoma

	Prevalence
Site-years (total)	1608
Number of countries with data	89
Number of GBD regions with data (out of 21 regions)	20
Number of GBD super-regions with data (out of 7 super-regions)	7

Myelodysplastic, myeloproliferative, and other haematological neoplasms

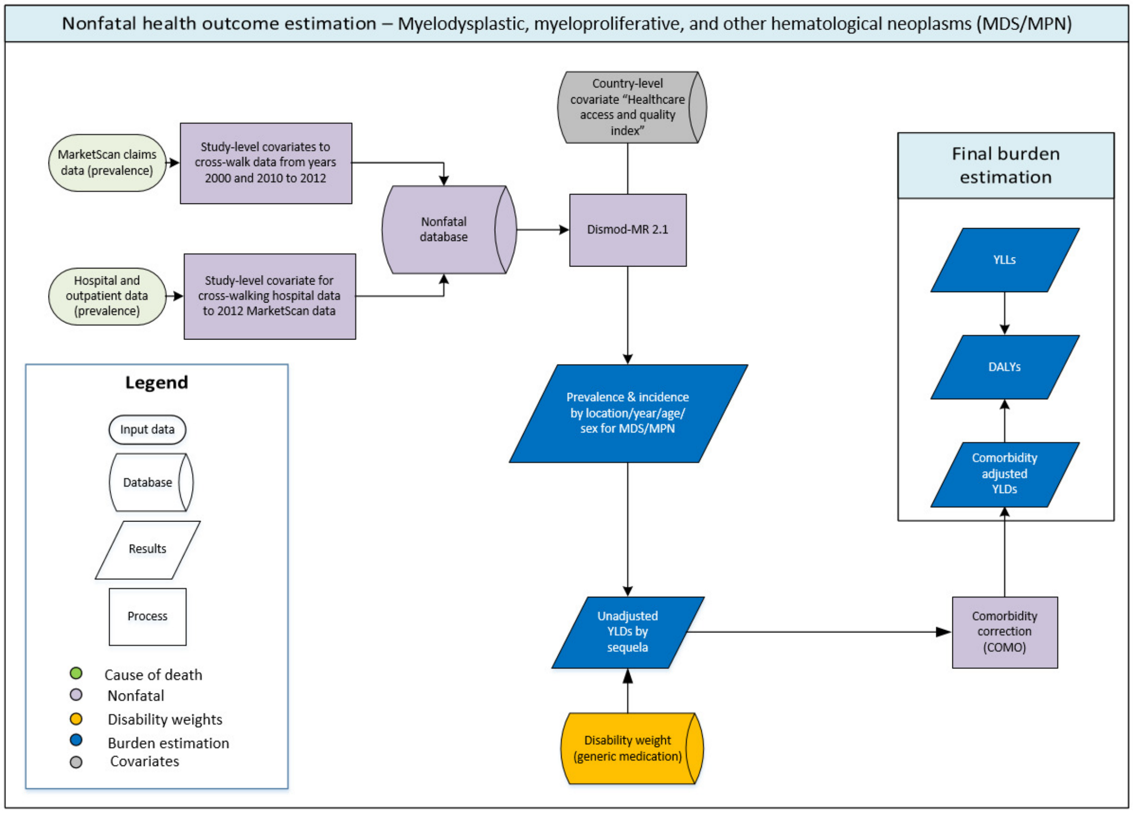

Case definition

For GBD 2017 we newly estimated the myelodysplastic, myeloproliferative, and other haematological neoplasms (MDS/MPN). While these neoplasms comprise a wide variety of diseases and outcomes, we have modelled them together as a single group for GBD 2017.

Input data

We estimated MDS/MPN deaths using vital registration data (as outlined above). We did not use cancer registry data for these neoplasms, as it has only been reported within cancer registries since 2001 and is recognised to be underreported.[29] We estimated MDS/MPN prevalence using MarketScan claims data from the United States in the years 2000, 2010, and 2012, as well as hospital and outpatient data from other health systems worldwide.

Modelling strategy

We modelled deaths for all locations and years, by age and by sex, using CODEm. As MDS/MPN can be a precursor to leukaemia, our MDS/MPN CODEm model used the same covariates as the CODEm model for acute myeloid leukaemia.

We modelled the prevalence of these diseases for all locations, by age, year, and by sex using a prevalence model in DisMod-MR 2.1. Each of the MarketScan 2000, 2010, and hospital data sources were crosswalked to the 2012 MarketScan data. For DisMod model specifications, cause-specific mortality

rates came from the CODEm model, remission was specified to be zero, and the excess mortality rate was set to be inversely related to the Healthcare Access and Quality index covariate.

While this broad category of haematological neoplasms is heterogeneous in its components' severity or propensity for transformation to leukaemia, modelling these components separately was not feasible for 2017. This is an admitted limitation, and an area of desired future improvement as data availability improves. For GBD 2017, the generic medication disability weight was assigned for all MDS/MPN cases (see Table 5).

Myelodysplastic, myeloproliferative, and other haemopoietic neoplasms

	Prevalence
Site-years (total)	1381
Number of countries with data	41
Number of GBD regions with data (out of 21 regions)	16
Number of GBD super-regions with data (out of 7 super-regions)	7

Benign and in situ intestinal neoplasms; Benign and in situ cervical and uterine neoplasms; Other benign and in situ neoplasms

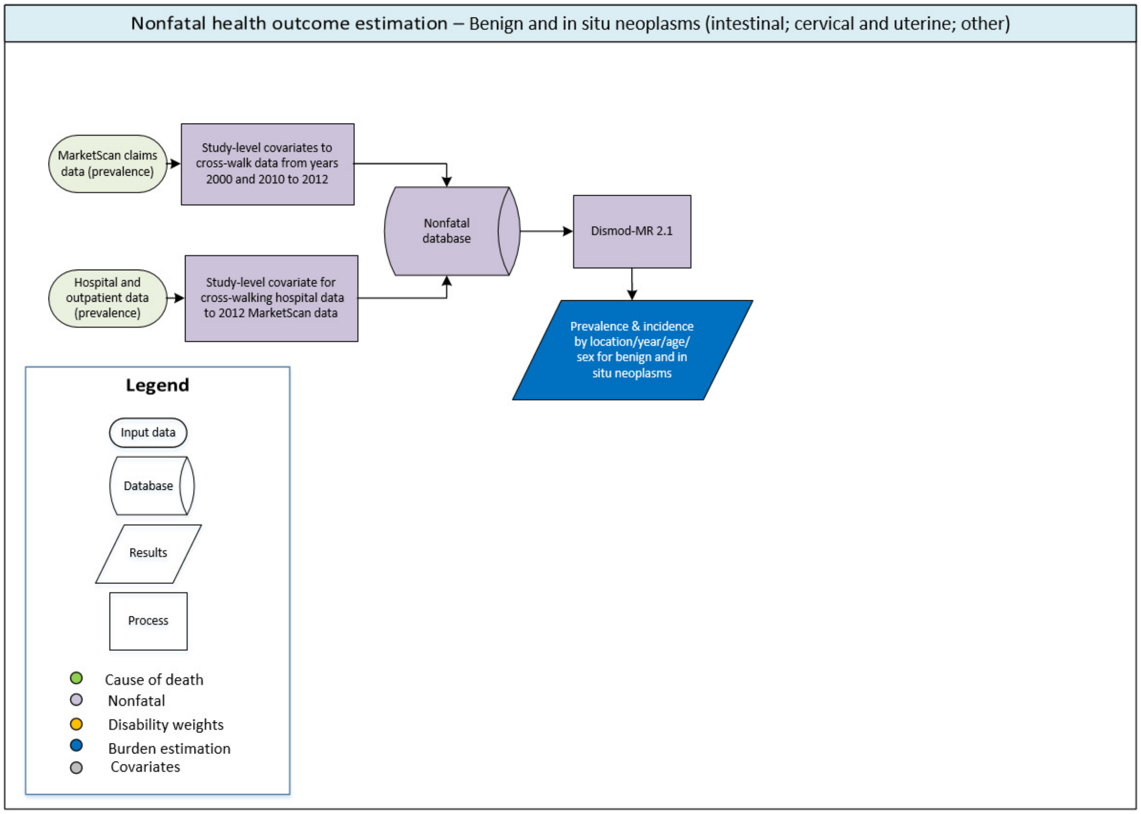

Case definition

For GBD 2017 we newly estimated three categories of benign and in situ neoplasms: intestinal neoplasms; cervical and uterine neoplasms; and other benign and in situ neoplasms. Benign and in situ intestinal neoplasms were defined as any non-invasive intestinal growth. Benign and in situ cervical and uterine neoplasms were defined as any non-invasive cervical and uterine growth, except for uterine fibroids. Other benign and in situ neoplasms were defined as any non-invasive neoplasms not covered by other causes.

Input data

To estimate the prevalence of each of these categories for all locations, by age, year, and sex, the prevalence of these neoplasms from hospital data was used as input for a prevalence model in DisMod-MR 2.1. These inputs included MarketScan claims data from the United States in the years 2000, 2010, and 2012, as well as hospital and outpatient data from other health systems worldwide. Each of these data sources was crosswalked to the 2012 MarketScan data.

Modelling strategy

In the DisMod model, excess mortality rate was specified to be zero, and remission was allowed to vary from 0 to 1. For benign and in situ cervical and uterine neoplasms, in the DisMod model, excess mortality rate was specified to be zero, and remission was allowed to vary from 0 to 0.75. For other benign and in situ neoplasms, in the DisMod model, excess mortality rate was specified to be zero, and remission was allowed to vary from 0 to 1.

All three of these benign and in situ neoplasms are by definition benign and localised. As such, no deaths or disability were attributed to their occurrence in GBD 2017.

Benign and in situ intestinal neoplasms

	Prevalence
Site-years (total)	1414
Number of countries with data	44
Number of GBD regions with data (out of 21 regions)	16
Number of GBD super-regions with data (out of 7 super-regions)	7

Benign and in situ uterine neoplasms

	Prevalence
Site-years (total)	1411
Number of countries with data	42
Number of GBD regions with data (out of 21 regions)	16
Number of GBD super-regions with data (out of 7 super-regions)	7

Other benign and in situ neoplasms

	Prevalence
Site-years (total)	1415
Number of countries with data	45
Number of GBD regions with data (out of 21 regions)	16
Number of GBD super-regions with data (out of 7 super-regions)	7

References

1 GBD 2017 Causes of Death Collaborators. Global, regional, and national age-sex specific mortality for 264 causes of death, 1980-2017: a systematic analysis for the Global Burden of Disease Study 2017. *Lancet Lond Engl*; **submitted**.

2 National Cancer Institute (United States). United States SEER Cancer Data 1973-2010. Bethesda, United States: National Cancer Institute (United States). .

3 Ministry of Health (Mexico). Mexico Ministry of Health Hospital Discharges 2000-2012. Mexico City, México: Ministry of Health (Mexico). .

4 Barber RM, Fullman N, Sorensen RJD, *et al.* Healthcare Access and Quality Index based on mortality from causes amenable to personal health care in 195 countries and territories, 1990–2015: a novel analysis from the Global Burden of Disease Study 2015. *The Lancet* 2017; **390**: 231–66.

5 Asadzadeh Vostakolaei F, Karim-Kos HE, Janssen-Heijnen MLG, Visser O, Verbeek ALM, Kiemeney LALM. The validity of the mortality to incidence ratio as a proxy for site-specific cancer survival. *Eur J Public Health* 2011; **21**: 573–7.

6 SEER*Stat Software. 2014 http://seer.cancer.gov/seerstat/.

7 Surveillance, Epidemiology, and End Results (SEER) Program (www.seer.cancer.gov) SEER*Stat Database: Incidence - SEER 18 Regs Research Data + Hurricane Katrina Impacted Louisiana Cases, Nov 2012 Sub (1973-2010 varying) - Linked To County Attributes - Total U.S., 1969-2011 Counties, National Cancer Institute, DCCPS, Surveillance Research Program, Surveillance Systems Branch, released April 2013, based on the November 2012 submission. .

8 Sankaranarayanan R, Swaminathan R, Lucas E. Cancer survival in Africa, Asia, the Caribbean and Central America (SurvCan). .

9 National Center for Health Statistics, Centers for Disease Control and, Prevention. US Mortality Files. 61-Year Trends in U.S. Cancer Death Rates. http://seer.cancer.gov/archive/csr/1975_2010/results_merged/topic_historical_mort_trends.pdf.

10 SEER Cancer Statistics Review 1975-2011. http://seer.cancer.gov/csr/1975_2011/results_merged/topic_survival_by_year_dx.pdf.

11 Fitzmaurice C, Dicker D, Pain A, *et al.* The Global Burden of Cancer 2013. *JAMA Oncol* 2015; published online May 28. DOI:10.1001/jamaoncol.2015.0735.

12 Neal RD, Din NU, Hamilton W, *et al.* Comparison of cancer diagnostic intervals before and after implementation of NICE guidelines: analysis of data from the UK General Practice Research Database. *Br J Cancer* 2014; **110**: 584–92.

13 Allgar VL, Neal RD. Delays in the diagnosis of six cancers: analysis of data from the National Survey of NHS Patients: Cancer. *Br J Cancer* 2005; **92**: 1959–70.

14 Neal RD, Cannings-John R, Hood K, *et al.* Excision of malignant melanomas in North Wales: effect of location and surgeon on time to diagnosis and quality of excision. *Fam Pract* 2008; **25**: 221–7.

15 Kewalramani T, Nimer SD, Zelenetz AD, *et al.* Progressive disease following autologous transplantation in patients with chemosensitive relapsed or primary refractory Hodgkin's disease or aggressive non-Hodgkin's lymphoma. *Bone Marrow Transplant* 2003; **32**: 673–9.

16 Esteban D, Tovar N, Jiménez R, *et al.* Patients with relapsed/refractory chronic lymphocytic leukaemia may benefit from inclusion in clinical trials irrespective of the therapy received: a case-control retrospective analsysis. *Blood Cancer J* 2015; **5**: e356.

17 Canova C, Giorato E, Roveron G, Turrini P, Zanotti R. Validation of a stoma-specific quality of life questionnaire in a sample of patients with colostomy or ileostomy. *Colorectal Dis Off J Assoc Coloproctology G B Irel* 2013; **15**: e692-698.

18 Caricato M, Ausania F, Ripetti V, Bartolozzi F, Campoli G, Coppola R. Retrospective analysis of long-term defunctioning stoma complications after colorectal surgery. *Colorectal Dis Off J Assoc Coloproctology G B Irel* 2007; **9**: 559–61.

19 Erwin-Toth P, Thompson SJ, Davis JS. Factors impacting the quality of life of people with an ostomy in North America: results from the Dialogue Study. *J Wound Ostomy Cont Nurs Off Publ Wound Ostomy Cont Nurses Soc WOCN* 2012; **39**: 417–22; quiz 423–4.

20 Catalona WJ, Carvalhal GF, Mager DE, Smith DS. Potency, continence and complication rates in 1,870 consecutive radical retropubic prostatectomies. *J Urol* 1999; **162**: 433–8.

21 Donnellan SM, Duncan HJ, MacGregor RJ, Russell JM. Prospective assessment of incontinence after radical retropubic prostatectomy: objective and subjective analysis. *Urology* 1997; **49**: 225–30.

22 Eastham JA, Kattan MW, Rogers E, *et al.* Risk factors for urinary incontinence after radical prostatectomy. *J Urol* 1996; **156**: 1707–13.

23 Kundu SD, Roehl KA, Eggener SE, Antenor JAV, Han M, Catalona WJ. Potency, continence and complications in 3,477 consecutive radical retropubic prostatectomies. *J Urol* 2004; **172**: 2227–31.

24 Potosky AL, Davis WW, Hoffman RM, *et al.* Five-Year Outcomes After Prostatectomy or Radiotherapy for Prostate Cancer: The Prostate Cancer Outcomes Study. *JNCI J Natl Cancer Inst* 2004; **96**: 1358–67.

25 Sacco E, Prayer-Galetti T, Pinto F, *et al.* Urinary incontinence after radical prostatectomy: incidence by definition, risk factors and temporal trend in a large series with a long-term follow-up. *BJU Int* 2006; **97**: 1234–41.

26 Stanford JL, Feng Z, Hamilton AS, *et al.* Urinary and sexual function after radical prostatectomy for clinically localized prostate cancer: the Prostate Cancer Outcomes Study. *JAMA* 2000; **283**: 354–60.

27 Walsh PC, Marschke P, Ricker D, Burnett AL. Patient-reported urinary continence and sexual function after anatomic radical prostatectomy. *Urology* 2000; **55**: 58–61.

28 Karagas MR, Greenberg ER, Spencer SK, Stukel TA, Mott LA. Increase in incidence rates of basal cell and squamous cell skin cancer in New Hampshire, USA. New Hampshire Skin Cancer Study Group. *Int J Cancer J Int Cancer* 1999; **81**: 555–9.

29 Cogle CR, Craig BM, Rollison DE, List AF. Incidence of the myelodysplastic syndromes using a novel claims-based algorithm: high number of uncaptured cases by cancer registries. *Blood* 2011; **117**: 7121–5.

Rheumatic Heart Disease

Flowchart

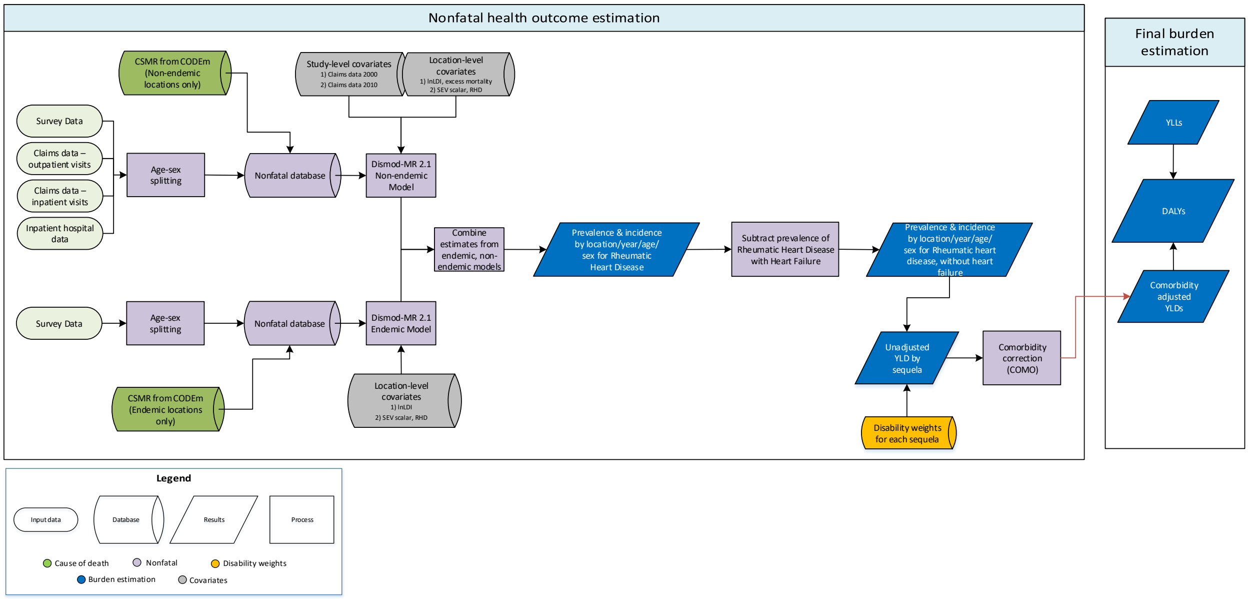

Input data and methodological appendix

Case definition

Rheumatic heart disease (RHD) was defined as a clinical diagnosis by a physician with or without confirmation using echocardiography. This case definition for echocardiographic confirmation of RHD follows the World Heart Federation criteria for echocardiographic diagnosis of rheumatic heart disease (1).

Criterion	Definition
1. Echocardiography	Prevalent rheumatic heart disease based on echocardiographic assessment and clinical confirmation
2. Clinical diagnosis	Prevalent rheumatic heart disease based on physician diagnosis

ICD codes for data included from hospital records can be found in Methods Appendix Table 4.

Input data

Model inputs

We did not perform a systematic review for GBD 2017. A systematic review was performed for GBD 2013 and updated for GBD 2015. The GBD 2015 search information encompassed the following:

- Search terms: ('rheumatic heart disease' AND epidemiology[MeSH Subheading]) OR ('acute rheumatic fever' AND epidemiology[MeSH Subheading]) OR ('rheumatic fever' AND

epidemiology[MeSH Subheading]) OR (RHD AND epidemiology[MeSH Subheading]) OR ('valvular heart disease' AND epidemiology[MeSH Subheading]) OR (((streptococcus OR streptococci) AND heart) AND epidemiology[MeSH Subheading]) OR (heart AND valve AND disease AND epidemiology[MeSH Subheading]) OR ('mitral valve stenosis' AND epidemiology[MeSH Subheading]) OR (('rheumatic heart disease' OR 'rheumatic fever') AND prevalence) OR (('rheumatic heart disease' OR 'rheumatic fever') AND incidence) OR (('rheumatic heart disease' OR 'rheumatic fever') AND ('standardized mortality ratio' OR SMR)) OR ('rheumatic heart disease' OR 'rheumatic fever' AND 'case fatality')
- Dates included in search: 1/1/2013 – 3/16/2015
- Number of initial hits: 2,045
- Number of sources included: 17

These differed from the GBD 2013 search terms:
- (hasabstract[text] AND Humans[Mesh] AND middle age[MeSH])) OR 21) AND ((rheumatic heart disease/epidemiology[Mesh] OR rheumatic heart disease/mortality[Mesh]) AND (prevalence[Title/Abstract] OR incidence[Title/Abstract]) AND ("2010"[Date - Publication] : "3000"[Date - Publication]) AND (hasabstract[text] AND Humans[Mesh] AND middle age[MeSH]))

The number of data sources used in the endemic and non-endemic DisMod models are reported by integrand in the tables below. The endemic and non-endemic DisMod models are described below.

Endemic model data counts

	Prevalence	Incidence
Site-years (total)	70	0
Number of countries with data	28	0
Number of GBD regions with data (out of 21 regions)	9	0
Number of GBD super-regions with data (out of 7 super-regions)	5	0

Non-endemic model data counts

	Prevalence	Incidence
Site-years (total)	443	12
Number of countries with data	34	2
Number of GBD regions with data (out of 21 regions)	11	2
Number of GBD super-regions with data (out of 7 super-regions)	5	1

We did not include any non-literature-based data types other than the hospital and claims data described elsewhere. Hospital and claims data were available only for the non-endemic country model. An individual was only counted as a case in the claims data if RHD was the primary diagnosis. We used

uncorrected inpatient hospital data as the correction factors generated using US claims data were exceptionally high (~100X) and could not be validated using data sources from other geographies. As patterns of disease and access to care vary widely, using uncorrected hospital data avoids inappropriately scaling inpatient data in other countries based on the very high use of RHD codes in the US in all-position diagnosis and outpatient claims data. We excluded all outpatient data, as they were implausibly low when compared with inpatient data from the same locations and claims data.

For the non-endemic country model, we included study-level covariates for inpatient hospital data and claims data from 2000 and 2010 to adjust these data points, using as reference the data obtained from literature and claims data from 2012.

Severity splits and disability weights

Severity level	Lay description	DW (95% CI)
Rheumatic heart disease, not including heart failure	Has a chronic disease that requires medication every day and causes some worry but minimal interference with daily activities.	0.049 (0.031–0.072)

Modelling strategy

For GBD 2017 estimation, we ran two models using DisMod-MR – one for non-endemic countries and one for endemic countries. For GBD 2016, we identified locations as endemic if the estimated death rate due to RHD was greater than 0.15 per 100,000 in the 5 to 9 age group, or if that location had an SDI less than 0.6. For GBD 2017, we identified locations as endemic if the estimated death rate due to RHD was greater than 0.15 per 100,000 in the 10 to 14 age group, or if that location had an SDI less than 0.6. This change in age group was made based on feedback from RHD expert reviewers due to concerns that the death rate in 5 to 9 age group would not capture endemicity in locations where RHD is common only in later age groups. Each location estimated as part of GBD 2017 is listed below as either “Endemic” or “Non-endemic”.

Remission

In GBD 2016, we assumed that there was no remission from RHD. For GBD 2017, we estimated remission in both the endemic and non-endemic DisMod models. This decision was based on two studies[2,3] that observed remission among confirmed RHD cases. We used the equation below to convert reported proportion of remitted individuals in each study to a remission rate, defined as the number of remitted cases divided by the total person-years of disease:

$$remission\,rate = \frac{\log(1 - proportion\,remitted)}{years\;of\;followup}$$

Where *proportion remitted* is the reported proportion of all individuals with RHD at baseline who ended up remitting, and *years of followup* is the mean follow-up time in the study. The relevant values for the two papers and the calculated remission rates are listed in the table below.

Study	Remitted proportion	Mean follow-up time	Calculated remission rate
Beaton et al[2]	0.3	2.4 years	0.14 cases per person-year

Engelman et al[3]	0.1	7.5 years	0.014 cases per person-year

In order to acknowledge the uncertainty in these calculated remission rates and to allow DisMod flexibility in estimating remission, we input 0.15 as the upper bound for remission the remission prior and 0.01 as the lower bound for remission the remission prior. Because the two studies used to estimate remission were done only in children, we applied these remission priors to only those younger than age 20, and setting a remission prior of zero for adults older than age 20.

DisMod models

Non-endemic model: We included hospital data, claims data, and limited literature data on prevalence. We also included CSMR from our mortality estimates of RHD for non-endemic locations only. A prior of no remission was set, and excess mortality was capped at 0.1 for all ages. We included study-level covariates for claims data from 2000 and 2010, crosswalking them to data from the literature and claims data in 2012. We also included the log-transformed age-standardised SEV scalar for RHD and the natural log of lagged distributed income (lnLDI, I$ per capita) as country-level covariates for prevalence and excess mortality, respectively.

Endemic model: We included prevalence data from surveys published in the literature. As with the high-income model, we included CSMR from our mortality estimates of RHD for endemic locations only. A prior of no remission was set for all ages, and excess mortality was capped at 0.07, the highest observed mean excess mortality rate data point observed in this model. We also set priors of 0 on incidence for ages 0 to 1 and 50 to 100 to account for patterns of incidence in endemic countries. We used lnLDI as a fixed-effect country-level covariate on prevalence and excess mortality, enforcing an inverse relationship for both. The log-transformed, age-standardised SEV scalar was also used as a fixed-effect country-level covariate on prevalence.

We combined estimates from the endemic and non-endemic models, selecting estimates for the locations identified as non-endemic from the non-endemic model and estimates for the locations identified as endemic from the endemic model. Estimates of heart failure due to RHD were then subtracted from the estimates for RHD, giving the overall prevalence of RHD without heart failure. A description of the modelling strategy for heart failure due to RHD can be found in the heart failure appendix. We evaluated models based on comparing estimates with input data as well as estimates from previous rounds of GBD.

The table below shows the country covariates, parameters, betas, and exponentiated betas:

Covariate	Parameter	beta	Exponentiated beta
Endemic model			
Log-transformed age-standardised SEV scalar: RHD	Prevalence	1.00 (0.76–1.24)	2.71 (2.14–3.45)
LDI (I$ per capita)	Excess mortality rate	-0.26 (-0.48 to -0.11)	0.76 (0.62–0.90)
Non-endemic model			
Inpatient MarketScan, year 2000	Study-level	1.12 (0.95 to 1.30)	3.07 (2.59–3.69)
Inpatient MarketScan, year 2010	Study-level	-0.001 (-0.11 to 0.1)	1 (0.9–1.11)

Log-transformed age-standardised SEV scalar: RHD	Prevalence	0.82 (0.75–0.99)	2.27 (2.12–2.70)
LDI (I$ per capita)	Excess mortality rate	-0.54 (-0.57 to -0.5)	0.58 (0.57–0.61)

Endemic locations: Aceh, Acre, Addis Ababa, Afar, Afghanistan, Alagoas, Albania, Alborz, Algeria, Amapá, Amazonas, American Samoa, Amhara, Andean Latin America, Andhra Pradesh, Andhra Pradesh, Rural, Andhra Pradesh, Urban, Angola, Anhui, Antigua and Barbuda, Ardebil, Argentina, Armenia, Arunachal Pradesh, Arunachal Pradesh, Rural, Assam, Assam, Rural, Assam, Urban, Azerbaijan, Bahia, Bangladesh, Barbados, Baringo, Belize, Bengkulu, Benin, Benishangul-Gumuz, Bhutan, Bihar, Bihar, Rural, Bihar, Urban, Bolivia, Bomet, Botswana, Brazil, Bungoma, Burkina Faso, Burundi, Busia, Cambodia, Cameroon, Cape Verde, Caribbean, Ceará, Central African Republic, Central Asia, Central Europe, Eastern Europe, and Central Asia, Central Kalimantan, Central Sub-Saharan Africa, Chad, Chahar Mahaal and Bakhtiari, Chhattisgarh, Chhattisgarh, Rural, Chhattisgarh, Urban, Chiapas, China, Chongqing, Comoros, Congo, Costa Rica, Cote d'Ivoire, Cuba, Delhi, Delhi, Rural, Delhi, Urban, Democratic Republic of the Congo, Dire Dawa, Distrito Federal, Djibouti, Dominica, Dominican Republic, East Asia, East Azarbayejan, East Nusa Tenggara, Eastern Cape, Eastern Sub-Saharan Africa, Ecuador, Egypt, El Salvador, Elgeyo-Marakwet, Embu, Equatorial Guinea, Eritrea, Espírito Santo, Ethiopia, Fars, Federated States of Micronesia, Fiji, Free State, Gabon, Gambella, Gansu, Garissa, Gauteng, Georgia, Ghana, Gilan, Global, Goa, Goa, Rural, Goa, Urban, Goiás, Golestan, Gorontalo, Grenada, Guam, Guangxi, Guatemala, Guerrero, Guinea, Guinea-Bissau, Guizhou, Gujarat, Gujarat, Rural, Gujarat, Urban, Guyana, Hainan, Haiti, Hamadan, Harari, Haryana, Haryana, Rural, Haryana, Urban, Hebei, Heilongjiang, Henan, Hidalgo, Himachal Pradesh, Himachal Pradesh, Rural, Himachal Pradesh, Urban, HomaBay, Honduras, Hormozgan, Hubei, Hunan, Ilam, India, Inner Mongolia, Iran, Iraq, Isfahan, Isiolo, Jamaica, Jammu and Kashmir, Jammu and Kashmir, Rural, Jammu and Kashmir, Urban, Jharkhand, Jharkhand, Rural, Jharkhand, Urban, Jiangxi, Jilin, Kajiado, Kakamega, Karnataka, Karnataka, Rural, Karnataka, Urban, Kenya, Kerala, Kerala, Rural, Kerala, Urban, Kericho, Kerman, Kermanshah, Khorasan-e-Razavi, Khuzestan, Kiambu, Kilifi, Kiribati, Kirinyaga, Kisii, Kisumu, Kitui, Kohgiluyeh and Boyer-Ahmad, Kurdistan, Kwale, KwaZulu-Natal, Kyrgyzstan, Laikipia, Lamu, Laos, Latin America and Caribbean, Lesotho, Liaoning, Liberia, Libya, Limpopo, Lorestan, Machakos, Madagascar, Madhya Pradesh, Madhya Pradesh, Rural, Madhya Pradesh, Urban, Maharashtra, Maharashtra, Rural, Maharashtra, Urban, Makueni, Malawi, Malaysia, Maldives, Mali, Maluku, Mandera, Manipur, Manipur, Rural, Manipur, Urban, Maranhão, Markazi, Marsabit, Marshall Islands, Mato Grosso, Mato Grosso do Sul, Mauritania, Mauritius, Mazandaran, Meghalaya, Meghalaya, Rural, Meghalaya, Urban, Meru, Mexico City, Michoacán de Ocampo, Migori, Minas Gerais, Mizoram, Rural, Mombasa, Mongolia, Morocco, Mozambique, Mpumalanga, Murang'a, Myanmar, Nagaland, Nagaland, Rural, Nairobi, Nakuru, Namibia, Nandi, Narok, Nepal, Nicaragua, Niger, Nigeria, Ningxia, North Africa and Middle East, North Africa and Middle East, North Khorasan, North Korea, North Maluku, North-West, Northern Cape, Northern Mariana Islands, Nyamira, Nyandarua, Nyeri, Oaxaca, Oceania, Odisha, Odisha, Rural, Odisha, Urban, Oromia, Pakistan, Palestine, Panama, Papua, Papua New Guinea, Pará, Paraguay, Paraíba, Paraná, Pernambuco, Peru, Philippines, Piaui, Puebla, Punjab, Punjab, Rural, Punjab, Urban, Qazvin, Qinghai, Rajasthan, Rajasthan, Rural, Rajasthan, Urban, Republic of Tuva, Riau Islands, Rio de Janeiro, Rio Grande do Norte, Rio Grande do Sul, Rondônia, Roraima, Rwanda, Saint Lucia, Saint Vincent and the Grenadines, Samburu, Samoa, Santa Catarina, São Paulo, Sao Tome and Principe, Semnan,

Senegal, Sergipe, Seychelles, Shaanxi, Shandong, Shanxi, Siaya, Sichuan, Sierra Leone, Sikkim, Sikkim, Rural, Sikkim, Urban, Sistan and Baluchistan, Solomon Islands, Somali, Somalia, South Africa, South Asia, South Asia, South Kalimantan, South Khorasan, South Sudan, Southeast Asia, Southeast Asia, East Asia, and Oceania, Southeast Sulawesi, Southern Nations, Nationalities, and Peoples, Southern Sub-Saharan Africa, Sub-Saharan Africa, Sudan, Suriname, Swaziland, Syria, TaitaTaveta, Tajikistan, Tamil Nadu, Tamil Nadu, Rural, Tamil Nadu, Urban, TanaRiver, Tanzania, Tehran, Telangana, Telangana, Rural, Telangana, Urban, Thailand, TharakaNithi, The Bahamas, The Gambia, Tianjin, Tibet, Tigray, Timor-Leste, Tocantins, Togo, Tonga, TransNzoia, Trinidad and Tobago, Tripura, Tripura, Rural, Tripura, Urban, Tropical Latin America, Turkana, Turkmenistan, Tyumen oblast without autonomous areas, UasinGishu, Uganda, Union Territories other than Delhi, Union Territories other than Delhi, Rural, Union Territories other than Delhi, Urban, United Arab Emirates, Uttar Pradesh, Uttar Pradesh, Rural, Uttar Pradesh, Urban, Uttarakhand, Uttarakhand, Rural, Uttarakhand, Urban, Uzbekistan, Vanuatu, Veracruz de Ignacio de la Llave, Vihiga, Wajir, West Azarbayejan, West Bengal, West Bengal, Rural, West Bengal, Urban, West Kalimantan, West Nusa Tenggara, West Papua, West Sulawesi, West Sumatra, Western Cape, Western Sub-Saharan Africa, WestPokot, Xinjiang, Yemen, Yunnan, Zambia, Zanjan, Zimbabwe

Non-endemic locations: Aguascalientes, Aichi, Akershus, Akita, Alabama, Alaska, Altai kray, Amur oblast, Andorra, Aomori, Arizona, Arkansas, Arkhangelsk oblast without Nenets autonomous district, Arunachal Pradesh, Urban, Astrakhan oblast, Aust-Agder, Australasia, Australia, Austria, Bahrain, Baja California, Baja California Sur, Bali, Bangka-Belitung Islands, Banten, Barking and Dagenham, Barnet, Barnsley, Bath and North East Somerset, Bedford, Beijing, Belarus, Belgium, Belgorod oblast, Bermuda, Bexley, Birmingham, Blackburn with Darwen, Blackpool, Bolton, Bosnia and Herzegovina, Bournemouth, Bracknell Forest, Bradford, Brent, Brighton and Hove, Bristol, City of, Bromley, Brunei, Bryansk oblast, Buckinghamshire, Bulgaria, Bury, Bushehr, Buskerud, Calderdale, California, Cambridgeshire, Camden, Campeche, Canada, Central Bedfordshire, Central Europe, Central Java, Central Latin America, Central Sulawesi, Chechen Republic, Chelyabinsk oblast, Cheshire East, Cheshire West and Chester, Chiba, Chihuahua, Chile, Chukchi autonomous area, Chuvash Republic, Coahuila, Colima, Colombia, Colorado, Connecticut, Cornwall, County Durham, Coventry, Croatia, Croydon, Cumbria, Cyprus, Czech Republic, Darlington, Delaware, Denmark, Derby, Derbyshire, Devon, District of Columbia, Doncaster, Dorset, Dudley, Durango, Ealing, East Java, East Kalimantan, East Midlands, East of England, East Riding of Yorkshire, East Sussex, Eastern Europe, Ehime, Enfield, England, Essex, Estonia, Finland, Finnmark, Florida, France, Fujian, Fukui, Fukuoka, Fukushima, Gateshead, Georgia, Germany, Gifu, Gloucestershire, Greater London, Greece, Greenland, Greenwich, Guanajuato, Guangdong, Gunma, Hackney, Halton, Hammersmith and Fulham, Hampshire, Haringey, Harrow, Hartlepool, Havering, Hawaii, Hedmark, Herefordshire, County of, Hertfordshire, High-income, High-income Asia Pacific, High-income North America, Hillingdon, Hiroshima, Hokkaidō, Hong Kong Special Administrative Region of China, Hordaland, Hounslow, Hungary, Hyōgo, Ibaraki, Iceland, Idaho, Illinois, Indiana, Indonesia, Iowa, Ireland, Irkutsk oblast, Ishikawa, Isle of Wight, Islington, Israel, Italy, Ivanovo oblast, Iwate, Jakarta, Jalisco, Jambi, Japan, Jewish autonomous oblast, Jiangsu, Jordan, Kabardian-Balkar Republic, Kagawa, Kagoshima, Kaliningrad oblast, Kaluga oblast, Kamchatka kray, Kanagawa, Kansas, Karachaev-Chercassian Republic, Kazakhstan, Kemerovo oblast, Kensington and Chelsea, Kent, Kentucky, Khabarovsk kray, Khanty-Mansi autonomous area, Kingston upon Hull, City of, Kingston upon Thames, Kirklees, Kirov oblast, Knowsley, Kōchi, Komi Republic, Kostroma oblast, Krasnodar kray, Krasnoyarsk kray, Kumamoto, Kurgan oblast, Kursk oblast, Kuwait, Kyōto, Lambeth, Lampung, Lancashire, Latvia, Lebanon, Leeds, Leicester, Leicestershire, Leningrad oblast, Lewisham,

Lincolnshire, Lipetzk oblast, Lithuania, Liverpool, Louisiana, Luton, Luxembourg, Macao Special Administrative Region of China, Macedonia, Magadan oblast, Maine, Malta, Manchester, Maryland, Massachusetts, Medway, Merton, Mexico, México, Michigan, Middlesbrough, Mie, Milton Keynes, Minnesota, Mississippi, Missouri, Miyagi, Miyazaki, Mizoram, Mizoram, Urban, Moldova, Montana, Montenegro, Møre og Romsdal, Morelos, Moscow City, Moscow oblast, Murmansk oblast, Nagaland, Urban, Nagano, Nagasaki, Nara, Nayarit, Nebraska, Nenets autonomous district, Netherlands, Nevada, New Hampshire, New Jersey, New Mexico, New York, New Zealand, New Zealand Maori population, New Zealand non-Maori population, Newcastle upon Tyne, Newham, Niigata, Nizhny Novgorod oblast, Nordland, Norfolk, North Carolina, North Dakota, North East England, North East Lincolnshire, North Kalimantan, North Lincolnshire, North Somerset, North Sulawesi, North Sumatra, North Tyneside, North West England, North Yorkshire, Northamptonshire, Northern Ireland, Northumberland, Norway, Nottingham, Nottinghamshire, Novgorod oblast, Novosibirsk oblast, Nuevo León, Ohio, Ōita, Okayama, Okinawa, Oklahoma, Oldham, Oman, Omsk oblast, Oppland, Oregon, Orenburg oblast, Oryol oblast, Ōsaka, Oslo, Østfold, Oxfordshire, Pennsylvania, Penza oblast, Perm kray, Peterborough, Plymouth, Poland, Poole, Portsmouth, Portugal, Primorsky kray, Pskov oblast, Puerto Rico, Qatar, Qom, Querétaro, Quintana Roo, Reading, Redbridge, Redcar and Cleveland, Republic of Adygeya, Republic of Altai, Republic of Bashkortostan, Republic of Buryatia, Republic of Crimea, Republic of Dagestan, Republic of Ingushetia, Republic of Kalmykia, Republic of Karelia, Republic of Khakasia, Republic of Mariy El, Republic of Mordovia, Republic of North Ossetia-Alania, Republic of Sakha (Yakutia), Republic of Tatarstan, Rhode Island, Riau, Richmond upon Thames, Rochdale, Rogaland, Romania, Rostov oblast, Rotherham, Russian Federation, Rutland, Ryazan oblast, Saga, Saitama, Sakhalin oblast, Salford, Samara oblast, San Luis Potosí, Sandwell, Sankt-Petersburg, Saratov oblast, Saudi Arabia, Scotland, Sefton, Serbia, Sevastopol, Shanghai, Sheffield, Shiga, Shimane, Shizuoka, Shropshire, Sinaloa, Singapore, Slough, Slovakia, Slovenia, Smolensk oblast, Sogn og Fjordane, Solihull, Somerset, Sonora, South Carolina, South Dakota, South East England, South Gloucestershire, South Korea, South Sulawesi, South Sumatra, South Tyneside, South West England, Southampton, Southend-on-Sea, Southern Latin America, Southwark, Spain, Sri Lanka, St Helens, Staffordshire, Stavropol kray, Stockholm, Stockport, Stockton-on-Tees, Stoke-on-Trent, Suffolk, Sunderland, Surrey, Sutton, Sverdlovsk oblast, Sweden, Sweden except Stockholm, Swindon, Switzerland, Tabasco, Taiwan, Tamaulipas, Tambov oblast, Tameside, Telemark, Telford and Wrekin, Tennessee, Texas, Thurrock, Tlaxcala, Tochigi, Tokushima, Tōkyō, Tomsk oblast, Torbay, Tottori, Tower Hamlets, Toyama, Trafford, Troms, Trøndelag, Tula oblast, Tunisia, Turkey, Tver oblast, Udmurt Republic, Ukraine, Ukraine (without Crimea & Sevastopol), Ulyanovsk oblast, United Kingdom, United States, Uruguay, Utah, Venezuela, Vermont, Vest-Agder, Vestfold, Vietnam, Virgin Islands, U.S., Virginia, Vladimir oblast, Volgograd oblast, Vologda oblast, Voronezh oblast, Wakayama, Wakefield, Wales, Walsall, Waltham Forest, Wandsworth, Warrington, Warwickshire, Washington, West Berkshire, West Java, West Midlands, West Sussex, West Virginia, Western Europe, Westminster, Wigan, Wiltshire, Windsor and Maidenhead, Wirral, Wisconsin, Wokingham, Wolverhampton, Worcestershire, Wyoming, Yamagata, Yamaguchi, Yamalo-Nenets autonomous area, Yamanashi, Yaroslavl oblast, Yazd, Yogyakarta, York, Yorkshire and the Humber, Yucatán, Zabaikalsk kray, Zacatecas, Zhejiang

1. Reményi, B. et al. Nat. Rev. Cardiol. 9, 297–309 (2012); published online 28 February 2012
2. Beaton A, Aliku T, Dewyer A, et al. Latent Rheumatic Heart Disease: Identifying the Children at Highest Risk of Unfavorable Outcome. Circulation. 2017;136(23):2233-2244.

3. Engelman D, Wheaton GR, Mataika RL, et al. Screening-detected rheumatic heart disease can progress to severe disease. Heart Asia. 2016;8(2):67-73.

Ischaemic heart disease

Flowchart

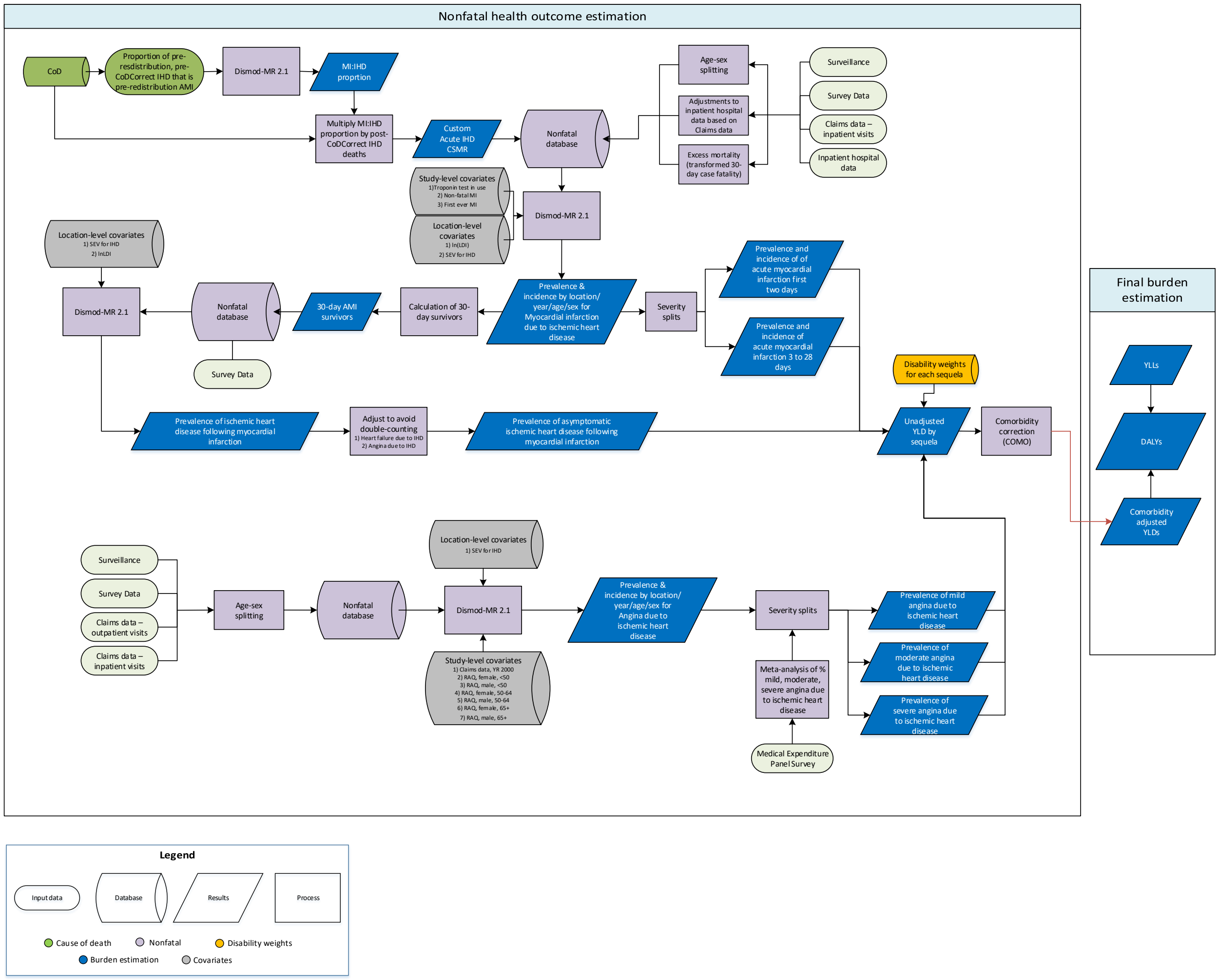

Input data and methodological summary

Case definition

Case definitions:

1) Acute myocardial infarction (MI): Definite and possible MI according to the third universal definition of myocardial infarction:
    a. When there is clinical evidence of myocardial necrosis in a clinical setting consistent with myocardial ischaemia or
    b. Detection of a rise and/or fall of cardiac biomarker values and with at least one of the following: i) symptoms of ischaemia, ii) new or presumed new ST-segment-T wave changes or new left bundle branch block, iii) development of pathological Q waves in the ECG, iv) imaging evidence of new loss of viable myocardium or new regional wall motion abnormality, or v) identification of an intracoronary thrombus by angiography or autopsy.
    c. Sudden (abrupt) unexplained cardiac death, involving cardiac arrest or no evidence of a non-coronary cause of death
    d. Prevalent MI is considered to last from the onset of the event to 28 days after the event and is divided into an acute phase (0–2 days) and subacute (3–28 days).

2) Chronic IHD
    a. Angina; clinically diagnosed stable exertional angina pectoris or definite angina pectoris according to the Rose Angina Questionnaire, physician diagnosis, or taking nitrate medication for the relief of chest pain.
    b. Asymptomatic ischaemic heart disease following myocardial infarction; survival to 28 days following incident MI. The GBD study does not use estimates based on ECG evidence for prior MI, due to its limited specificity and sensitivity (1).

ICD codes used for inclusion of hospital and claims data for MI and angina can be found in Methods Appendix Table 4.

Input data

Model inputs

Myocardial infarction

A systematic review for myocardial infarction was not performed for GBD 2017. Updates to systematic reviews are performed on an ongoing schedule across all GBD causes; an update for myocardial infarction will be performed in the next one to two iterations.

A systematic review was done for myocardial infarction for GBD 2015. The search strings used were extensive; a full list will be provided on request.

The dates of the search were 1/1/2009 – 2/3/2015. 38,522 studies were returned; 194 were extracted (this number includes extractions that were done for STEMI/NSTEMI models and revascularisation models that are not currently part of the MI modelling process but may be in the future).

A systematic review for myocardial infarction was also done for GBD 2013. The extensive search terms for that review will be provided on request.

The table below shows the number of site-years of data included in the estimation process and the geographic distribution of the data.

	Case fatality rate	Excess mortality rate	Incidence
**Site-years (total)**	70	117	520
**Number of countries with data**	3	18	41
**Number of GBD regions with data (out of 21 regions)**	3	8	11
**Number of GBD super-regions with data (out of 7 super-regions)**	2	4	5

Apart from inpatient hospital and inpatient claims data, we did not include any data from sources other than the literature for myocardial infarction. We excluded data with broad age ranges where it was impossible to obtain more granular data, as these data caused the known age pattern for increased risk of myocardial infarction to be masked in the estimates generated from DisMod.

We included a study-level covariate to correct for the change in diagnostic criteria to include troponin measurements within DisMod. This adjustment was applied to data collected before 2000. We also included a study-level covariate to adjust data points within DisMod that captured only first-ever MI, using studies where all events were included as the reference. We also adjusted estimates within DisMod from studies that only included non-fatal cases using study-level covariates with sources that included fatal and non-fatal cases as reference.

Asymptomatic ischaemic heart disease following myocardial infarction

As the main input for this model are 28-day survivors calculated from the excess mortality estimates for the myocardial infarction model, a systematic review was not performed for GBD 2017. We included data for excess mortality and standardised mortality ratio to inform the estimates of survival after myocardial infarction.

The table below shows the number of site-years of data included in the estimation process and the geographic distribution of the data.

	Excess mortality rate	Standardised mortality ratio
**Site-years (total)**	32	1
**Number of countries with data**	12	1
**Number of GBD regions with data (out of 21 regions)**	4	1
**Number of GBD super-regions with data (out of 7 super-regions)**	7	1

Angina

A systematic review was not performed for GBD 2017. Updates to systematic reviews are performed on an ongoing schedule across all GBD causes; an update for angina will be performed in the next one to two iterations.

A systematic review for angina was last performed for GBD 2013. The search terms for that are: (Angina Pectoris/epidemiology[Mesh] OR Angina Pectoris/mortality[Mesh]) AND (prevalence[Title/Abstract] OR incidence[Title/Abstract]) AND ("2010"[Date - Publication] : "3000"[Date - Publication])

The table below shows the number of site-years of data included in the estimation process and the geographic distribution of the data.

	Excess mortality rate	Mortality risk	Prevalence
Site-years (total)	7	5	252
Number of countries with data	0	5	61
Number of GBD regions with data (out of 21 regions)	0	4	18
Number of GBD super-regions with data (out of 7 super-regions)	7	4	7

We included survey data (including NHANES and World Health Study questionnaires) which included the RAQ items. Prevalence of angina was calculated using the standard algorithm to determine whether the RAQ was positive or negative.

We excluded data with broad age ranges where it was impossible to obtain more granular data, as these data caused the known age pattern for increased risk of angina to be masked in the estimates generated from DisMod.

We included sex- and age-group-specific covariates to adjust prevalence data points obtained from the RAQ using the claims data as the reference since the RAQ has been shown to be neither sensitive nor specific.

We also included US claims data, but did not include inpatient hospital data from any locations. Stable angina (unstable angina is modeled as part of MI) is expected to be rare in inpatient but common in outpatient data as it is a condition usually managed on an outpatient basis, except for specific surgical interventions. This discrepancy leads to implausible correction factors based on inpatient/outpatient information from claims data (~150X); thus adjusted data cannot be used. Including uncorrected data in the model is likely to lead to incorrect estimates as hospitalisation and procedure rates are likely to vary between geographies based on access to and patterns of care. All outpatient data were excluded as they were implausibly low for all locations when compared with literature and claims data.

Severity split inputs

Acute myocardial infarction was split into two severity levels by length of time since the event – days 1 and 2 versus days 3 through 28. Disability weights were established for these two severities using the standard approach for GBD 2017.

Asymptomatic ischaemic heart disease following myocardial infarction was all assigned to the asymptomatic severity level. No disability weight is assigned to this level.

Angina was split into asymptomatic, mild, moderate, and severe groups using information from MEPS. Disability weights were established for these severities using the standard approach for GBD 2017.

Acute myocardial infarction

Severity level	Lay description	DW (95% CI)
Acute myocardial infarction, days 1-2	Has severe chest pain that becomes worse with any physical activity. The person feels nauseated, short of breath, and very anxious.	0.432 (0.288–0.579)
Acute myocardial infarction, days 3-28	Gets short of breath after heavy physical activity, and tires easily, but has no problems when at rest. The person has to take medication every day and has some anxiety.	0.074 (0.049–0.105)

Asymptomatic ischaemic heart disease following myocardial infarction

Severity level	Lay description	DW (95% CI)
Asymptomatic ischaemic heart disease		N/A

Angina pectoris

Severity level	Lay description	DW (95% CI)
Asymptomatic angina		N/A
Mild angina	Has chest pain that occurs with strenuous physical activity, such as running or lifting heavy objects. After a brief rest, the pain goes away.	0.033 (0.02–0.052)
Moderate angina	Has chest pain that occurs with moderate physical activity, such as walking uphill or more than half a kilometer (around a quarter-mile) on level ground. After a brief rest, the pain goes away.	0.08 (0.052–0.113)
Severe angina	Has chest pain that occurs with minimal physical activity, such as walking only a short distance. After a brief rest, the pain goes away. The person avoids most physical activities because of the pain.	0.167 (0.11–0.24)

Modelling strategy

Myocardial infarction

- We first calculated custom cause-specific mortality estimates using cause of death data prior to garbage code redistribution, generating age-sex-country-specific proportions of IHD deaths that were due to MI (acute IHD) versus those due to other causes of IHD (chronic IHD). Estimates of this proportion for all locations were then generated using a DisMod proportion-only model. Due to a high degree of variability in pre-redistribution coding practices by location, we used the global age-, sex-, and year-specific proportions of acute deaths in subsequent calculations. The global proportions

were multiplied by post-CoDCorrect (final GBD estimates) IHD deaths to generate CSMR estimates for MI, even though GBD reports only deaths for all IHD taken together. These data, along with incidence and excess mortality data, informed a DisMod model to estimate the prevalence and incidence of myocardial infarction due to ischaemic heart disease.

- These estimates were split into prevalence and incidence estimates for days 1-2 and days 3-28 post-event. Disability weights were assigned to each of these two groupings.
- We set a value prior of one month for remission (11/13) from the MI health state. We also set a value prior for the maximum excess mortality rate of 10 for all ages. We included the Healthcare Access and Quality (HAQ) Index as a fixed-effect country-level covariate on excess mortality, forcing an inverse relationship.

Covariate	Parameter	Beta	Exponentiated beta
Diagnostic blood sample (troponin)	Incidence	-0.44 (-0.45 to -0.44)	0.64 (0.64 to 0.64)
First ever MI	Incidence	-0.65 (-0.66 to -0.65)	0.52 (0.52 to 0.52)
Non-fatal MI	Incidence	-0.40 (-0.41 to -0.40)	0.67 (0.67 to 0.67)
Healthcare Access and Quality (HAQ) Index	Excess mortality rate	-0.1 (-0.1 to -0.1)	0.90 (0.90 to 0.90)
Log-transformed age-standardised SEV scalar: IHD	Incidence	0.75 (0.75 to 0.76)	2.12 (2.12 to 2.14)

<u>Asymptomatic ischaemic heart disease</u>

- Excess mortality estimates from the myocardial infarction model were used to generate data of the incidence of surviving 28 days post-event.
- We used these data, along with the estimates of CSMR due to chronic IHD (the other part of the proportion described in step 1) and excess mortality data in a DisMod model to estimate the prevalence of persons with IHD following myocardial infarction. This estimate included subjects with angina and heart failure; a proportion of this prevalence was removed in order to avoid double-counting based on evidence from the literature (2). The result of this step generates estimates of asymptomatic ischaemic heart disease following myocardial infarction.
- We set a value prior of 0 for remission for all ages.
- We also included the log-transformed, age-standardised SEV scalar for IHD as a fixed effect, country-level covariate on prevalence and LDI (I$ per capita) as a fixed-effect country-level covariate on excess mortality, forcing an inverse relationship for LDI.

Covariate	Parameter	Beta	Exponentiated beta
LDI (I$ per capita)	Excess mortality rate	-0.4 (-0.5 to -0.25)	0.67 (0.61 to 0.78)
Log-transformed age-standardised SEV scalar: IHD	Incidence	1.00 (0.75 to 1.25)	2.72 (2.12 to 3.49)

<u>Angina</u>

- We used prevalence data from the literature and USA claims databases, along with data on mortality risk to estimate the prevalence and incidence of angina for all locations.
- The proportion of mild, moderate, and severe angina was determined by the standard approach for severity splitting for GBD 2017.
- We included a value prior of 0 for remission for all ages. We also included a value prior of 1 for excess mortality for all ages.

- We included age- and sex-specific study-level covariates to adjust data points based on RAQ, using data points from the claims database as the reference.
- We also included the log-transformed, age-standardised SEV scalar for IHD as a fixed effect, country-level covariate on prevalence and LDI (I$ per capita) as a fixed effect, country-level covariate on excess mortality, forcing an inverse relationship LDI.

Covariate	Parameter	Beta	Exponentiated beta
RAQ, female, less than 50	Prevalence	2.34 (2.30 to 2.42)	10.34 (9.98 to 11.20)
RAQ, male, less than 50	Prevalence	0.94 (0.93 to 0.95)	2.57 (2.52 to 2.59)
RAQ, female, 50 to 64	Prevalence	1.46 (1.40 to 1.50)	4.33 (4.04 to 4.47)
RAQ, male, 50 to 64	Prevalence	0.95 (0.86 to 1.00)	2.58 (2.36 to 2.71)
RAQ, female, 65 plus	Prevalence	0.29 (0.27 to 0.30)	1.34 (1.32 to 1.35)
RAQ, male, 65 plus	Prevalence	0.27 (0.20 to 0.30)	1.31 (1.22 to 1.35)
Log-transformed age-standardised SEV scalar: IHD	Prevalence	1.18 (1.10 to 1.24)	3.25 (3.00 to 3.47)
LDI (I$ per capita)	Excess mortality rate	-0.55 (-1 to -0.1)	0.58 (0.37 to 0.90)

There have been no substantive changes in the modelling strategy for myocardial infarction, asymptomatic ischaemic heart disease following myocardial infarction, and angina from GBD 2016.

Ischaemic Stroke, Intracerebral Haemorrhage, and Subarachnoid Haemorrhage

Flowchart

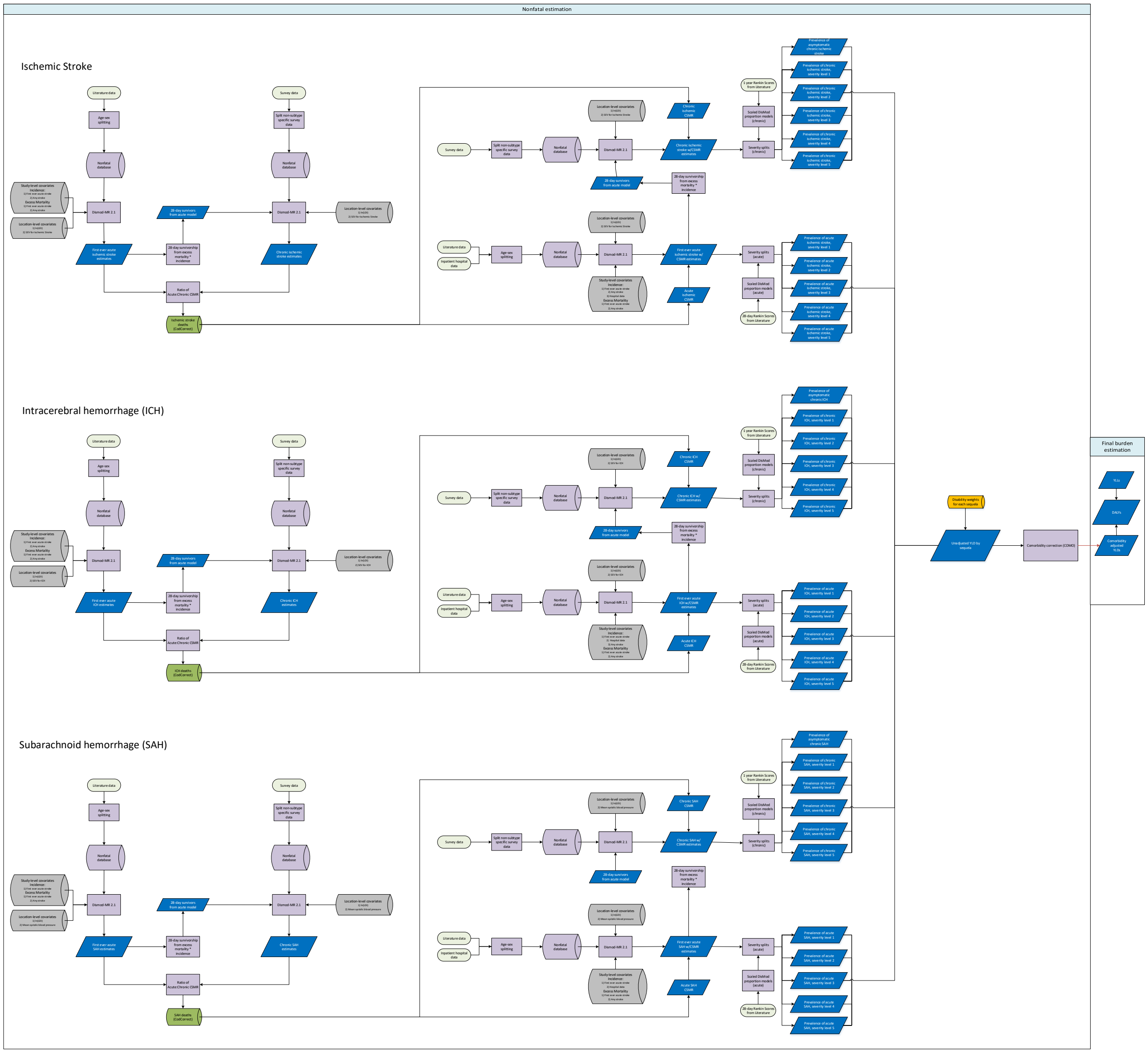

Input data and methodological summary

Case definition

Stroke was defined according to WHO criteria – rapidly developing clinical signs of focal (at times global) disturbance of cerebral function lasting more than 24 hours or leading to death with no apparent cause other than that of vascular origin (1). Data on transient ischaemic attack (TIA) were not included.

Acute stroke: Stroke cases are considered acute from the day of incidence of a first-ever stroke through day 28 following the event.

Chronic stroke: Stroke cases are considered chronic beginning 28 days following the occurrence of an event. Chronic stroke includes the sequelae of an acute stroke AND all recurrent stroke events. GBD 2015 adopts this broader definition of chronic stroke than was used in prior iterations in order to model acute strokes using only first-ever incident events.

Ischaemic stroke: an episode of neurological dysfunction caused by focal cerebral, spinal, or retinal infarction

Intracerebral haemorrhage: a focal collection of blood within the brain parenchyma or ventricular system that is not caused by trauma

Subarachnoid haemorrhage: bleeding into the subarachnoid space (the space between the arachnoid membrane and the pia mater of the brain or spinal cord)

ICD codes used for inclusion of hospital and claims data can be found in Appendix Table 4.

Input data

Model inputs

A systematic review was performed for GBD 2017. Search terms, dates of search, and databases queried follow:

1) Ischaemic stroke
    a. Google scholar: ("ischemic stroke" OR “cerebral infarction” OR “ischaemic stroke”) AND (incidence OR prevalence OR mortality OR epidemiology). Reviewed first 1000 hits, sorted by relevance
    b. Global Index Medicus search: (tw:("ischemic stroke") OR tw:(“cerebral infarction” OR tw:(“ischaemic stroke”)) AND (tw:(incidence) OR tw:(prevalence) OR tw:(mortality) OR tw:(epidemiology)) AND NOT (tw:(rats) OR tw:(mice) OR tw:(dogs) OR tw:(apes) OR tw:(monkeys)). Dates of search: 01Jan2010 – 31Aug2017
2) Intracerebral haemorrhage
    a. Google scholar: ("hemorrhagic stroke" OR “intracerebral hemorrhage” OR “haemorrhagic stroke” OR “intracerebral haemorrhage”) AND (incidence OR prevalence OR mortality OR epidemiology). Reviewed first 1000 hits, sorted by relevance
    b. GIM search: (tw:("intracerebral hemorrhage") OR tw:(“intracerebral haemorrhage”) OR tw:(“hemorrhagic stroke”) OR tw:(“haemorrhagic stroke”)) AND (tw:(incidence) OR tw:(prevalence) OR tw:(mortality) OR tw:(epidemiology)) AND NOT (tw:(rats) OR tw:(mice) OR tw:(dogs) OR tw:(apes) OR tw:(monkeys)). Dates of search: 01Jan2010 – 31Aug2017
3) Subarachnoid haemorrhage

a. Google scholar search: ("subarachnoid hemorrhage" OR “subarachnoid haemorrhage”) AND (incidence OR prevalence OR mortality OR epidemiology). Reviewed first 1000 hits, sorted by relevance.
b. GIM search: (tw:("subarachnoid hemorrhage") OR tw:(“subarachnoid haemorrhage”)) AND (tw:(incidence) OR tw:(prevalence) OR tw:(mortality) OR tw:(epidemiology)) AND NOT (tw:(rats) OR tw:(mice) OR tw:(dogs) OR tw:(apes) OR tw:(monkeys)). Dates of search: 01Jan2010 – 31Aug2017

The tables below show the number of site-years and types of data included in the estimation process.

Acute ischaemic stroke

	**Case fatality rate**	**Excess mortality rate**	**Incidence**
**Site-years (total)**	158	158	428
**Number of countries with data**	47	47	62
**Number of GBD regions with data (out of 21 regions)**	17	17	17
**Number of GBD super-regions with data (out of 7 super-regions)**	7	7	7

Acute intracerebral haemorrhage

	**Case fatality rate**	**Excess mortality rate**	**Incidence**
**Site-years (total)**	148	148	417
**Number of countries with data**	41	41	61
**Number of GBD regions with data (out of 21 regions)**	16	16	17
**Number of GBD super-regions with data (out of 7 super-regions)**	7	7	7

Acute subarachnoid haemorrhage

	**Case fatality rate**	**Excess mortality rate**	**Incidence**
**Site-years (total)**	39	106	342
**Number of countries with data**	17	28	47
**Number of GBD regions with data (out of 21 regions)**	11	13	14
**Number of GBD super-regions with data (out of 7 super-regions)**	5	6	6

Chronic ischaemic stroke

	Prevalence
**Site-years (total)**	605
**Number of countries with data**	24
**Number of GBD regions with data (out of 21 regions)**	15
**Number of GBD super-regions with data (out of 7 super-regions)**	6

Chronic intracerebral haemorrhage

	Prevalence
**Site-years (total)**	605
**Number of countries with data**	24
**Number of GBD regions with data (out of 21 regions)**	15
**Number of GBD super-regions with data (out of 7 super-regions)**	6

Chronic subarachnoid haemorrhage

	Prevalence
**Site-years (total)**	605
**Number of countries with data**	24
**Number of GBD regions with data (out of 21 regions)**	15
**Number of GBD super-regions with data (out of 7 super-regions)**	6

We included inpatient hospital data, adjusted for readmission and primary to any diagnosis using correction factors estimated from US claims data. We excluded data for locations where the data points were implausibly low (Vietnam, Philippines, India). In addition, we included unpublished stroke registry data for acute ischaemic stroke, acute intracerebral haemorrhage, and acute subarachnoid haemorrhage. We also included survey data for chronic stroke. These surveys were identified based on expert opinion and review of major survey series focused on world health that included questions regarding self-reported history of stroke.

As with many models in GBD, the diversity of data sources available means that we needed to adjust available data to our preferred or reference case definition (2). For the first-ever acute stroke models we used DisMod to estimate the statistical association between measurements taken using different case definitions and then used these estimates to adjust the non-referent data points. We included study-level covariates to adjust data points for first and recurrent strokes combined, using data for first strokes only as reference. We also included study-level covariates to adjust data points that reported all stroke subtypes combined, using as reference studies with subtype-specific information.

Severity split inputs

The table below illustrates the severity level, lay description, and disability weights for GBD 2017. In previous iterations of GBD, severity splits for stroke were based on the standard approach described elsewhere (3). For GBD 2016, we undertook a review to identify epidemiologic literature which reported the degree of disability at 28 days (for acute stroke) or one year (for chronic stroke) using the modified Rankin scale (mRS) and the Mini-Mental State Examination (MMSE) or the Montreal Cognitive

Assessment (MoCA). The mRS assesses functional capabilities, while the MMSE and MoCA tests provide evaluations of cognitive functioning. We then mapped these measures to the existing GBD categories as indicated below. This approach allowed us to include location-specific information and can be updated as more data on functional or cognitive status become available.

Acute stroke severity splits

Severity level	Lay description	Modified Rankin score	Cognitive status	DW (95% CI)
Stroke, mild	Has some difficulty in moving around and some weakness in one hand, but is able to walk without help.	1	N/A	0.019 (0.01–0.032)
Stroke, moderate	Has some difficulty in moving around, and in using the hands for lifting and holding things, dressing, and grooming.	2, 3	MoCA>=24 or MMSE>=26	0.07 (0.046–0.099)
Stroke, moderate plus cognition problems	Has some difficulty in moving around, in using the hands for lifting and holding things, dressing and grooming, and in speaking. The person is often forgetful and confused.	2, 3	MoCA<24 or MMSE<26	0.316 (0.206–0.437)
Stroke, severe	Is confined to bed or a wheelchair, has difficulty speaking, and depends on others for feeding, toileting, and dressing.	4, 5	MoCA>=24 or MMSE>=26	0.552 (0.377–0.707)
Stroke, severe plus cognition problems	Is confined to bed or a wheelchair, depends on others for feeding, toileting, and dressing, and has difficulty speaking, thinking clearly, and remembering things.		MoCA<24 or MMSE<26	0.588 (0.411–0.744)

Chronic stroke severity splits

Severity level	Lay description	Modified Rankin score	Cognitive status	DW (95% CI)
Stroke, asymptomatic		0	N/A	N/A
Stroke, long-term consequences, mild	Has some difficulty in moving around and some weakness in one hand, but is able to walk without help.	1	N/A	0.019 (0.01–0.032)
Stroke, long-term consequences, moderate	Has some difficulty in moving around, and in using the hands for lifting and holding things, dressing, and grooming.	2, 3	MoCA>=24 or MMSE>=26	0.07 (0.046–0.099)
Stroke, long-term consequences,	Has some difficulty in moving around, in using the hands for	2, 3	MoCA<24 or MMSE<26	0.316 (0.206–0.437)

moderate plus cognition problems	lifting and holding things, dressing and grooming, and in speaking. The person is often forgetful and confused.			
Stroke, long-term consequences, severe	Is confined to bed or a wheelchair, has difficulty speaking, and depends on others for feeding, toileting, and dressing.	4, 5	MoCA>=24 or MMSE>=26	0.552 (0.377–0.707)
Stroke, long-term consequences, severe plus cognition problems	Is confined to bed or a wheelchair, depends on others for feeding, toileting, and dressing, and has difficulty speaking, thinking clearly, and remembering things.	4, 5	MoCA<24 or MMSE<26	0.588 (0.411–0.744)

The table below shows the number of site-years and types of data included in the estimation process.

	**Acute proportion**	**Chronic proportion**
**Site-years (total)**	9	16
**Number of countries with data**	6	13
**Number of GBD regions with data (out of 21 regions)**	6	7
**Number of GBD super-regions with data (out of 7 super-regions)**	4	5

We used DisMod-MR 2.1, a Bayesian meta-regression tool, to model the six severity levels, with an independent proportion model for each. Reports which grouped mRS scores differently than our mapping (eg, 0-2) were adjusted in DisMod by estimating the association between these alternate groupings and our preferred mappings. These statistical associations were used to adjust data points to the referent category as necessary. The six models were scaled such that the sum of the proportions for all levels equaled 1.

Modelling strategy

Three general approaches were employed for all of the components of the stroke modelling process, detailed in the table below.

- Data points were adjusted from nonstandard to standard case definitions using estimates from statistical models generated by DisMod for the acute models. Coefficients for these crosswalks can be found in the tables for fixed effects located below.
- The GBD summary exposure values (SEV), which are the relative risk-weighted prevalence of exposure, were included as covariates for the ischaemic stroke or intracerebral haemorrhage models as appropriate, and a covariate for country income was used as a country-level covariate for both models (4). As subarachnoid haemorrhage is new in GBD 2017, the SEV for this cause has not been generated. Instead, we included the covariate for mean systolic blood pressure as a country-level covariate for incidence in the acute models or prevalence in the chronic models, along with a covariate for country income for excess mortality. Coefficients for these covariates can be found in the tables for fixed effects located below.

- Two versions of each stroke model were run, referred to as step 1 and step 2 models. First, we ran the step 1 DisMod-MR models for acute and chronic subtype-specific stroke using only incidence, prevalence, and case fatality data as inputs. We then used the ratio of acute:chronic cause-specific mortality estimated by these models to divide GBD stroke deaths into acute and chronic stroke deaths, using the global average for the proportion of acute:chronic stroke mortality. The acute and chronic models were then run (step 2) using the same incidence, prevalence, and case fatality data as well as the custom cause-specific mortality rates as input data.

Step 1

- We generated estimates for first-ever acute ischaemic stroke, first-ever acute intracerebral haemorrhage, and first-ever acute subarachnoid haemorrhage using DisMod-MR 2.1 with data collected on stroke incidence and excess mortality. We set value priors of 11 to 13 on remission for all ages to establish a one-month duration for these acute sequelae.
- We then calculated the rate of surviving until 28 days after an acute event for all three subtypes using the modelled estimates of excess mortality and incidence.
- These survivor data were used in the chronic subtype-specific models as incidence inputs.
- We then ran the chronic stroke models, using the survivor incidence data and excess mortality data. Non-subtype-specific prevalence data were split into ischaemic stroke, intracerebral haemorrhage, and subarachnoid haemorrhage components using the ratio of 28-day survivors from the first-stage acute models.
- We set a value prior of 0 on remission for all ages.
- Implausible or extreme outliers in input data were dropped from these estimation results.
- From these six models, we generated the proportions of deaths due to acute ischaemic stroke, chronic ischaemic stroke, acute intracerebral haemorrhage, chronic intracerebral haemorrhage, acute subarachnoid haemorrhage, and chronic subarachnoid haemorrhage, and split the post-CoDCorrect subtype-specific stroke deaths generated from the GBD mortality estimates into these six parts, by multiplying the location-, sex-, age- and year-specific CSMR results by the global proportions estimated from the DisMod models. Thus, the mortality rates due to acute ischaemic stroke, chronic ischaemic stroke, acute intracerebral haemorrhage, chronic intracerebral haemorrhage, acute subarachnoid haemorrhage, and chronic subarachnoid haemorrhage are driven by all available data on incidence, prevalence, and excess mortality data for stroke. These CSMR estimates were then uploaded into the non-fatal database and used as inputs for models in Step 2.

Step 2

- We re-ran the first-ever acute subtype-specific models with CSMR as derived from CoDCorrect and epidemiological data as described above. Twenty-eight-day survivorship was recalculated from these models and uploaded into the chronic subtype-specific with CSMR models. These chronic models also use CSMR as derived from CoDCorrect and epidemiological data as described above.

Models were evaluated based on expert opinion, comparison with previous iterations, and model fit.

Changes in the modelling of stroke for GBD 2017

For GBD 2017, we separated haemorrhagic stroke into intracerebral haemorrhage and subarachnoid haemorrhage, generating estimates for each subtype separately. The same two-step approach was used for all three subtypes. Other than this change, there have been no substantive changes to the modeling process.

1) Hatano S. Experience from a multicentre stroke register: a preliminary report. Bull WHO 54, 541-553. 1976.
2) GBD 2015 Disease and Injury Incidence and Prevalence Collaborators. Global, regional, and national incidence, prevalence, and years lived with disability for 310 diseases and injuries, 1990-2015: a systematic analysis for the Global Burden of Disease Study 2015. Lancet. 2016 Oct 8;388(10053):1545-1602. doi: 10.1016/S0140-6736(16)31678-6.
3) Burstein et al. Estimating distributions of health state severity for the global burden of disease study. Population Health Metrics (2015) 13:31
4) GBD 2015 Risk Factors Collaborators. Global, regional, and national comparative risk assessment of 79 behavioural, environmental and occupational, and metabolic risks or clusters of risks, 1990-2015: a systematic analysis for the Global Burden of Disease Study 2015. Lancet. 2016 Oct 8;388(10053):1659-1724. doi: 10.1016/S0140-6736(16)31679-8.

The tables below indicate the covariates used by cause in the estimation process, as well as the beta and exponentiated beta values.

Step 1:

Cause	Variable name	Measure	beta	Exponentiated beta
Chronic ischaemic stroke	Log-transformed SEV scalar: Isch Stroke	Prevalence	0.75 (0.75 to 0.75)	2.12 (2.12 to 2.12)
Chronic ischaemic stroke	LDI (I$ per capita)	Excess mortality rate	-0.11 (-0.13 to -0.1)	0.90 (0.88 to 0.90)
Chronic intracerebral haemorrhage	Log-transformed SEV scalar: Hem Stroke	Prevalence	0.75 (0.75 to 0.76)	2.12 (2.12 to 2.14)
Chronic intracerebral haemorrhage	LDI (I$ per capita)	Excess mortality rate	-0.11 (-0.15 to -0.1)	0.89 (0.86 to 0.90)
Chronic subarachnoid haemorrhage	LDI (I$ per capita)	Excess mortality rate	-0.13 (-0.21 to -0.1)	0.88 (0.81 to 0.90)
First ever acute intracerebral haemorrhage	Hospital data	Incidence	-0.25 (-0.26 to -0.25)	0.78 (0.77 to 0.78)
First ever acute intracerebral haemorrhage	Any stroke	Incidence	1.61 (1.60 to 1.61)	4.99 (4.96 to 5.00)
First ever acute intracerebral haemorrhage	First-ever acute stroke, ischaemic or haemorrhagic	Incidence	0.53 (0.52 to 0.54)	1.69 (1.68 to 1.71)
First ever acute intracerebral haemorrhage	Log-transformed age-standardised SEV scalar: haemorrhagic stroke	Incidence	0.77 (0.75 to 0.80)	2.15 (2.12 to 2.22)
First ever acute intracerebral haemorrhage	Any stroke	Excess mortality rate	0.0094 (0.000077 to 0.033)	1.01 (1.00 to 1.03)
First ever acute intracerebral haemorrhage	First-ever acute stroke, ischaemic or haemorrhagic	Excess mortality rate	0.19 (0.066 to 0.33)	1.21 (1.07 to 1.39)
First ever acute intracerebral haemorrhage	LDI (I$ per capita)	Excess mortality rate	-0.11 (-0.14 to -0.1)	0.89 (0.87 to 0.90)

First ever acute subarachnoid haemorrhage	Hospital data	Incidence	0.52 (0.52 to 0.53)	1.69 (1.68 to 1.71)
First ever acute subarachnoid haemorrhage	Systolic blood pressure (mmHg)	Incidence	0.062 (0.050 to 0.073)	1.06 (1.05 to 1.08)
First ever acute subarachnoid haemorrhage	First ever acute stroke, ischaemic or haemorrhagic	Incidence	0.53 (0.52 to 0.54)	1.70 (1.68 to 1.71)
First ever acute subarachnoid haemorrhage	Aneurysmal subarachnoid haemorrhage only	Incidence	-0.12 (-0.16 to -0.1)	0.89 (0.86 to 0.90)
First ever acute subarachnoid haemorrhage	LDI (I$ per capita)	Excess mortality rate	-0.46 (-0.5 to -0.37)	0.63 (0.61 to 0.69)
First ever acute subarachnoid haemorrhage	First ever acute stroke, ischaemic or haemorrhagic	Excess mortality rate	0.36 (0.21 to 0.49)	1.43 (1.24 to 1.63)
First ever acute ischaemic stroke	Hospital data	Incidence	0.36 (0.36 to 0.37)	1.44 (1.43 to 1.45)
First ever acute ischaemic stroke	Any stroke	Incidence	0.33 (0.31 to 0.33)	1.38 (1.37 to 1.39)
First ever acute ischaemic stroke	First ever acute stroke, ischaemic or haemorrhagic	Incidence	0.37 (0.36 to 0.38)	1.44 (1.43 to 1.46)
First ever acute ischaemic stroke	Log-transformed age-standardised SEV scalar: ischaemic stroke	Incidence	0.86 (0.76 to 1.01)	2.36 (2.13 to 2.75)
First ever acute ischaemic stroke	LDI (I$ per capita)	Excess mortality rate	-0.11 (-0.13 to -0.1)	0.90 (0.88 to 0.90)
First ever acute ischaemic stroke	Any stroke	Excess mortality rate	0.85 (0.74 to 0.96)	2.35 (2.09 to 2.62)
First ever acute ischaemic stroke	First ever acute stroke, ischaemic or haemorrhagic	Excess mortality rate	0.29 (0.17 to 0.40)	1.33 (1.18 to 1.50)

Step 2:

Cause	Variable name	Measure	beta	Exponentiated beta
Chronic ischaemic stroke with CSMR	Log-transformed SEV scalar: Ischaemic stroke	Prevalence	1.13 (1.04 to 1.23)	3.11 (2.83 to 3.42)
Chronic ischaemic stroke with CSMR	LDI (I$ per capita)	Excess mortality rate	-0.5 (-0.5 to -0.5)	0.61 (0.61 to 0.61)
Chronic intracerebral haemorrhage with CSMR	Log-transformed SEV scalar: Haemorrhagic stroke	Prevalence	0.76 (0.75 to 0.79)	2.14 (2.12 to 2.21)
Chronic intracerebral haemorrhage with CSMR	LDI (I$ per capita)	Excess mortality rate	-0.5 (-0.5 to -0.48)	0.61 (0.61 to 0.62)
First-ever acute intracerebral haemorrhage with CSMR	Hospital data	Incidence	-0.27 (-0.32 to -0.22)	0.76 (0.72 to 0.80)

First-ever acute intracerebral haemorrhage with CSMR	Hospital Inpatient	Incidence	0.5 (0.49 to 0.50)	1.65 (1.64 to 1.65)
First-ever acute intracerebral haemorrhage with CSMR	Any stroke	Incidence	1.60 (1.59 to 1.61)	4.98 (4.83 to 5.00)
First-ever acute intracerebral haemorrhage with CSMR	First-ever acute stroke, ischaemic or haemorrhagic	Incidence	0.52 (0.52 to 0.53)	1.69 (1.68 — 1.71)
First-ever acute intracerebral haemorrhage with CSMR	Log-transformed SEV scalar: Hem stroke	Incidence	0.76 (0.75 to 0.80)	2.14 (2.12 to 2.22)
First-ever acute intracerebral haemorrhage with CSMR	Any stroke	Excess mortality rate	-0.65 (-0.76 to -0.53)	0.52 (0.47 to 0.59)
First-ever acute intracerebral haemorrhage with CSMR	First-ever acute stroke, ischaemic or haemorrhagic	Excess mortality rate	-0.092 (-0.11 to -0.0052)	0.91 (0.83 to 0.99)
First-ever acute intracerebral haemorrhage with CSMR	LDI (I$ per capita)	Excess mortality rate	-0.11 (-0.14 to -0.42)	0.90 (0.87 to 0.90)
First-ever acute subarachnoid haemorrhage with CSMR	First-ever acute stroke, ischaemic or haemorrhagic	Incidence	0.53 (0.52 to 0.54)	1.69 (1.68 to 1.70)
First-ever acute subarachnoid haemorrhage with CSMR	Any stroke	Excess mortality rate	0.0050 (-1.00 to 1.00)	1.01 (0.37 to 2.72)
First-ever acute subarachnoid haemorrhage with CSMR	First-ever acute stroke, ischaemic or haemorrhagic	Excess mortality rate	1.00 (0.99 to 1.00)	2.71 (2.68 to 2.72)
First-ever acute subarachnoid haemorrhage with CSMR	LDI (I$ per capita)	Excess mortality rate	-0.3 (-0.5 to -0.1)	0.74 (0.61 to 0.90)
First-ever acute subarachnoid haemorrhage with CSMR	Hospital data	Incidence	-0.95 (-1.03 to -0.88)	0.39 (0.36 to 0.42)
First-ever acute subarachnoid haemorrhage with CSMR	Hospital Inpatient	Incidence	0.20 (0.20 to 0.20)	1.22 (1.22 to 1.22)
First-ever acute ischaemic stroke with CSMR	First-ever acute stroke, ischaemic or haemorrhagic	Incidence	0.37 (0.36 to 0.38)	1.44 (1.43 to 1.46)
First-ever acute ischaemic stroke with CSMR	Log-transformed age-standardised SEV scalar: Ischaemic stroke	Incidence	0.79 (0.75 to 0.84)	2.20 (2.12 to 2.33)
First-ever acute ischaemic stroke with CSMR	Hospital data	Incidence	-0.26 (-0.3 to -0.21)	0.77 (0.74 to 0.81)
First-ever acute ischaemic stroke with CSMR	Hospital Inpatient	Incidence	0.30 (0.30 to 0.30)	1.35 (1.34 to 1.35)

First-ever acute ischaemic stroke with CSMR	Any stroke	Incidence	0.30 (0.29 to 0.32)	1.35 (1.34 to 1.38)
First-ever acute ischaemic stroke with CSMR	Any stroke	Excess mortality rate	0.98 (0.94 to 1.00)	2.68 (2.56 to 2.72)
First-ever acute ischaemic stroke with CSMR	First-ever acute stroke, ischaemic or haemorrhagic	Excess mortality rate	0.94 (0.86 to 0.99)	2.55 (2.35 to 2.70)
First-ever acute ischaemic stroke with CSMR	LDI (I$ per capita)	Excess mortality rate	-0.5 (-0.5 to -0.5)	0.61 (0.61 to 0.61)

Non-rheumatic valvular heart diseases

Flowchart

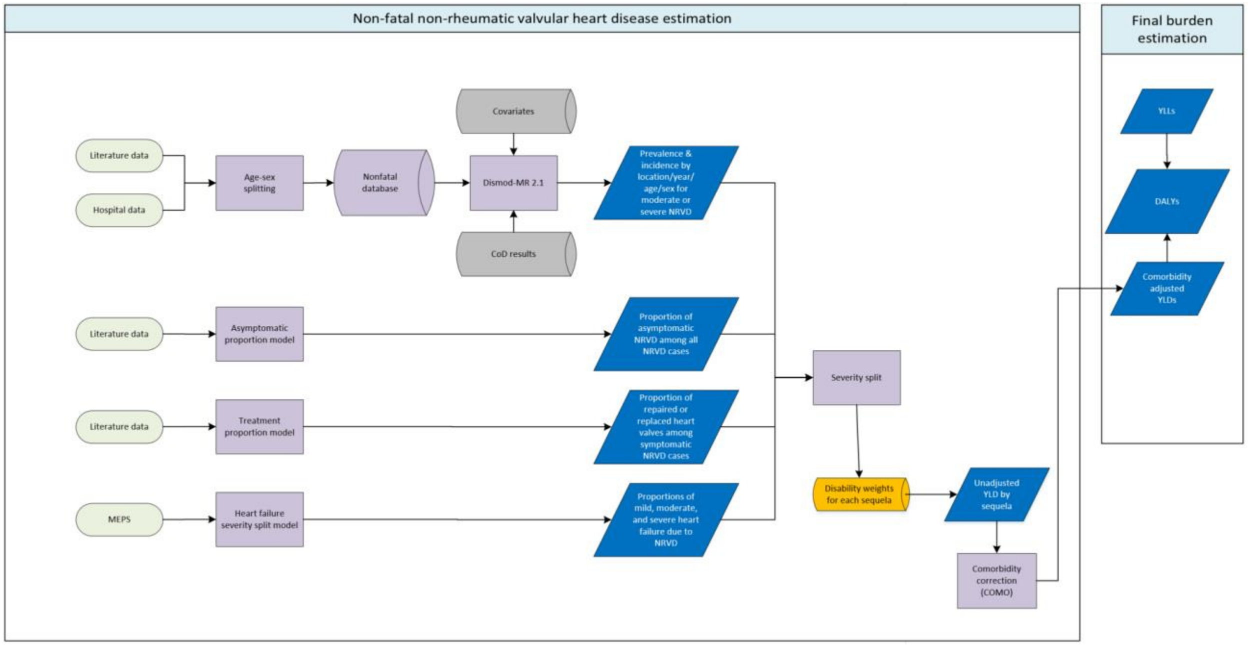

Case definitions

Calcific aortic valve disease

Calcific aortic valve disease was defined as clinical diagnosis of aortic valve stenosis or regurgitation due to progressive calcification of the aortic valve or annulus leading to hemodynamically moderate or severe aortic stenosis or regurgitation. Cases were determined by echocardiography. Calcific aortic valve disease in the GBD did not include aortic valve disease with an aetiology that was congenital, rheumatic, or infectious. Disease due to these aetiologies are modelled in other causes in the GBD. Information on unicuspid or bicuspid valves was generally not available and is often unknown in advanced calcific disease. Therefore, we included cases of unicuspid or bicuspid valves in our case definition if they developed clinically significant aortic stenosis. The criteria for aortic stenosis follow the American Heart Association/American College of Cardiology definition of haemodynamically moderate or severe aortic stenosis and are listed in Table 1. The criteria for aortic regurgitation follow the American Heart Association/American College of Cardiology definition of haemodynamically moderate or severe aortic regurgitation and are listed in Table 2. Mild haemodynamic aortic stenosis or regurgitation was not included in our case definition because mildly abnormal haemodynamic parameters are difficult to differentiate from non-pathological stenosis and/or regurgitation, and are generally not reported in population-based studies.

Table 1

**AHA/ACC definitions of aortic stenosis**
Maximum jet velocity ≥ 3 m/s

Mean pressure gradient ≥ 20 mmHg

Table 2

**AHA/ACC definitions of aortic regurgitation**
Central jet mitral regurgitation ≥ 25% of the left ventricular outflow tract
Vena contracta ≥ 0.3 cm
Regurgitant volume ≥ 30 mL/beat
Regurgitant fraction ≥ 30%
Angiography grade ≥ 2+

Degenerative mitral valve disease

Degenerative mitral valve disease was defined as myxomatous degeneration of the mitral valve leading to regurgitation or prolapse. Cases were determined by echocardiography by a physician. Degenerative mitral valve disease did not include mitral valve disease with an aetiology that was congenital, rheumatic, infectious, traumatic, carcinoid, or functional (ie, secondary to left ventricular remodeling due to heart failure from another cause). Mitral valve stenosis was always considered to have a rheumatic aetiology and therefore was not included in the definition of degenerative mitral valve disease. Degenerative mitral valve disease was restricted to persons at or above the age of 15 in order to exclude congenital mitral valve disorders. This age restriction is consistent with other progressive cardiovascular diseases modelled in the GBD. The criteria for mitral regurgitation follow the American Heart Association/American College of Cardiology definition of haemodynamically progressive or severe mitral regurgitation and are listed in Table 3. Mild haemodynamic mitral regurgitation was not included in our case definition because mild mitral valve disease cannot be differentiated from nonpathological regurgitation and is generally not reported in population-based studies..

Table 3

**AHA/ACC definitions of mitral regurgitation**
Central jet mitral regurgitation > 20% of the left atrium
Vena contracta ≥ 0.7 cm
Regurgitant volume ≥ 60 mL/beat
Regurgitant fraction ≥ 50%
Effective regurgitant orifice ≥ 0.4 cm^2
Angiography grade ≥ 2+

Other non-rheumatic valve disease

Other non-rheumatic valve disease is a residual category that captures non-rheumatic, non-congenital valve disorders of the tricuspid and pulmonary valves. This includes tricuspid regurgitation, tricuspid stenosis, pulmonary regurgitation, and pulmonary stenosis. Other non-rheumatic valve disease did not

include tricuspid or pulmonary valve disease with an setiology that was congenital, rheumatic, infectious, traumatic, carcinoid, or functional (ie, secondary to heart failure due to another cause).

Input data

Data on the prevalence, incidence, treatment, hsemodynamic severity, and asymptomatic status were collected from PubMed using the following search strings on 8/21/2017:

Calcific aortic valve disease

("aortic stenosis"[Title/Abstract] OR "aortic regurgitation"[Title/Abstract]) NOT ("Transcatheter Aortic Valve Replacement"[MeSH] OR "Transcatheter aortic valve implantation"[KEYWORD]) AND (epidemiology[MeSH Major Topic] OR epidemiology[Subheading] OR epidemiology[MeSH Terms] OR prevalence[Title/Abstract] OR mortality[Title/Abstract]) NOT (animals[MeSH] NOT humans[MeSH]) AND ("1980/1/01"[PDAT] : "2017/12/31"[PDAT]) NOT Comment[ptyp] NOT Case Reports[ptyp]

Degenerative mitral valve disease

("mitral stenosis"[Title/Abstract] OR "mitral regurgitation"[Title/Abstract]) AND ("epidemiology"[MeSH Major Topic] OR "epidemiology"[Subheading] OR "epidemiology"[MeSH Terms] OR prevalence[Title/Abstract] OR mortality[Title/Abstract]) NOT (animals[MeSH] NOT humans[MeSH]) AND ("1980/1/01"[PDAT] : "2017/12/31"[PDAT]) NOT Comment[ptyp] NOT Case Reports[ptyp]

We excluded literature that was not representative, included rheumatic, endocarditic, or congenital heart disease in its case definition, or included haemodynamically mild valve disease in its case definition. We did not run a literature review for "other non-rheumatic valve diseases" because we did not directly model non-fatal burden due to this cause.

Data on the prevalence of non-rheumatic valvular heart diseases was also obtained from inpatient hospital data. These data were adjusted for multiple visits and non-primary diagnoses. An adjustment to capture cases that were not seen inpatient was also done during the modelling process and is described below. Hospital data were excluded below age 30 or if the age-series for a given hospital data source was implausible. Prevalence data from both inpatient and outpatient hospital claims were used in the United States.

Modelling strategy

For other non-rheumatic valve diseases, we estimated nonfatal burden using the cause of death heart failure approach. This method is used for most cardiovascular diseases that cause heart failure and is described above.

In order to estimate non-fatal burden for calcific aortic valve disease and degenerative mitral valve disease, we first determined the sequelae and corresponding health states that result from these conditions. This information, along with the disability weights applied to each health state, are displayed in Table 4.

Table 4: Sequelae, health state lay descriptions, and disability weights

Sequela	Health state name	Health state lay description	Disability weight
Asymptomatic non-rheumatic valve disease	Asymptomatic	--	0
Non-rheumatic valve disease after treatment	Generic uncomplicated disease: worry and daily medication	Has a chronic disease that requires medication every day and causes some worry but minimal interference with daily activities.	0.049 (0.031–0.072)
Mild heart failure due to non-rheumatic valve disease	Heart failure, mild	Is short of breath and easily tires with moderate physical activity, such as walking uphill or more than a quarter-mile on level ground. The person feels comfortable at rest or during activities requiring less effort.	0.041 (0.026–0.062)
Moderate heart failure due to non-rheumatic valve disease	Heart failure, moderate	Is short of breath and easily tires with minimal physical activity, such as walking only a short distance. The person feels comfortable at rest but avoids moderate activity.	0.072 (0.047–0.103)
Severe heart failure due to non-rheumatic valve disease	Heart failure, severe	Is short of breath and feels tired when at rest. The person avoids any physical activity, for fear of worsening the breathing problems.	0.179 (0.122–0.251)

To model the burden due to each of the sequela above, we first modelled the overall prevalences of haemodynamically moderate and severe calcific aortic valve disease and degenerative mitral valve disease. We then estimated the proportion of those with prevalent disease who were haemodynamically moderate, assuming that this would approximate the proportion who were asymptomatic. We then estimated the proportion of those with symptomatic disease (ie, those with haemodynamically severe disease) who were treated. The remaining proportion – those with untreated symptomatic disease – was split into three proportions: those with mild, moderate, and severe heart failure. All proportions were calculated and converted to population prevalences at the draw level, thus propagating uncertainty from each step through to all subsequent steps. Population prevalences for each severity are necessary in order to calculate the burden for each severity. Figure 1 visualises this framework. Each of these modelling steps is outlined in greater detail below.

Figure 1: Modelling framework for calcific aortic valve disease and degenerative mitral valve disease

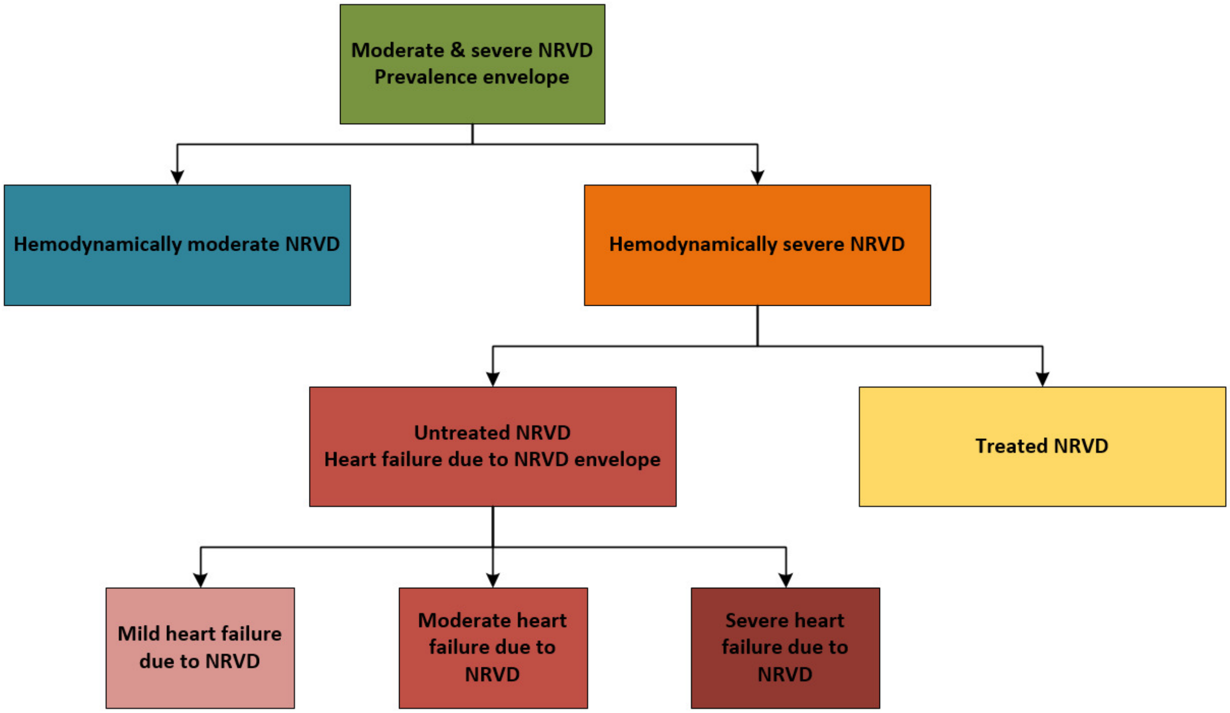

Prevalence envelope

We separately modelled the overall prevalences of calcific aortic valve disease and degenerative mitral valve disease in DisMod-MR 2.1. We used cause-specific mortality rates from the fatal modelling process as inputs. These two models estimate the prevalences of these two valve diseases for each age, sex, location, and year. The number of site-years of data points used are reported in Table 5 for CAVD and Table 6 for DMVD.

Table 5: Number of data points used for the CAVD prevalence envelope DisMod model

	Prevalence	Case fatality rate	Other
Site-years (total)	1096	1	5
Number of countries with data	34	1	4
Number of GBD regions with data (out of 21 regions)	9	1	3
Number of GBD super-regions with data (out of 7 super-regions)	4	1	1

Table 6: Number of data points used for the DMVD prevalence envelope DisMod model

	Prevalence	Case fatality rate	Other
Site-years (total)	1096	2	4
Number of countries with data	29	2	3
Number of GBD regions with data (out of 21 regions)	9	2	3
Number of GBD super-regions with data (out of 7 super-regions)	4	1	1

As mentioned above, we needed to adjust inpatient hospital data in order to account for prevalent cases that were never seen as inpatients. Additionally, we decided to adjust for USA claims data from the year 2000, which was determined to have a characteristically different sample than other years of USA claims data. We included an indicator variable for hospital data claims data in 2000 as study-level covariates in the DisMod models to account for this. However, the compartmental nature of the model prevented convergence. We therefore estimated the appropriate coefficients for these two study-level covariates outside of DisMod, and then input these coefficients as hard priors in the DisMod models. The out-of-DisMod regression used to estimate these coefficients was:

$$logit(y_{sr,r}) = \beta_1 * female + midage + \beta_2 * hospital + \beta_3 * claims_{2000} + \beta_4 * claims + \eta_{sr} + \gamma_r$$

Where $y_{sr,r}$ is the estimated prevalence of calcific aortic or degenerative mitral valve disease for super-region sr and region r, $midage$ is the midpoint of the age range of a data point, $hospital$ is an indicator variable for hospital data, $claims_{2000}$ is an indicator variable for USA claims data in the year 2000, $claims$ is an indicator variable for all claims data, η_{sr} is a super-region specific random effect, and γ_r is a region-specific random effect. The random effects allow pooling of geographically similar data to estimate β_2 and β_3. These two coefficients were passed into DisMod, and their values are reported in tables 7 and 8 for calcific aortic valve disease and degenerative mitral valve disease, respectively. All other covariates used are also included in these tables. The integrand that used the covariate as well as the coefficients are also reported. For calcific aortic valve disease, we initially ran models that included mean systolic blood pressure, mean total cholesterol, and mean fasting plasma glucose as country-level covariates based on biological plausibility. However, these covariates were not significant and were therefore removed from the model.

Table 7: Covariates and resulting coefficients for calcific aortic valve disease

Level	Covariate	Integrand	Coefficients	Exponentiated coefficients
Study-level	US claims data, year 2000	Prevalence	-0.56 (-0.57 to -0.56)	0.57 (0.56 to 0.57)
	All claims data	Prevalence	-0.68 (-0.69 to -0.68)	0.50 (0.50 to 0.51)
	Hospital data	Prevalence	-2.15 (-2.15 to -2.15)	0.12 (0.12 to 0.12)
Country-level	Mean BMI	Prevalence	0.088 (0.068 to 0.12)	1.09 (1.07 to 1.13)
	Smoking Prevalence	Prevalence	0.006 (0.0001 to 0.030)	1.01 (1.00 to 1.03)
	HAQ index	Excess mortality rate	-0.019 (-0.025 to -0.017)	0.98 (0.98 to 0.98)

Table 7: Covariates and resulting coefficients for degenerative mitral valve disease

Level	Covariate	Integrand	Coefficients	Exponentiated coefficients
Study-level	Claims data, 2000	Prevalence	-0.42 (-0.45 to -0.39)	0.66 (0.64 to 0.68)
	All claims data	Prevalence	-0.57 (-0.69 to -0.52)	.56 (0.50 to 0.59)
	Hospital data	Prevalence	-2.11 (-2.13 to -2.10)	0.12 (0.12 to 0.12)

Country-level	HAQ index	Excess mortality rate	-1.11<br>(-1.39 to –0.77)	0.33<br>(0.25 to 0.46)

Haemodynamically moderate proportion

We estimated the proportion of individuals with haemodynamically moderate or severe valve disease who were haemodynamically moderate. As mentioned above, we assumed that individuals with haemodynamically moderate disease were asymptomatic. There were a total of five data sources that reported the proportion of individuals who were haemodynamically moderate. Because of the sparsity of data, we modelled the haemodynamically moderate proportion together for both calcific aortic valve disease and degenerative mitral valve disease. We modelled a proportion with uncertainty that varied by age with the following regression:

$$logit(y) = \beta_0 + \beta_1 age + \gamma$$

Where y is the proportion of haemodynamically moderate disease, *age* is the midpoint age for each data point, and γ is a random effect for each data source. The regression coefficients are reported in Table 9.

Table 9: Moderate NRVD regression coefficients

Covariate	Coefficients	Transformed coefficients
Intercept (β_0)	6.6<br>(4.9 to 8.4)	0.998<br>(0.992 to 0.999)
Age (β_1)	-0.07<br>(-.093 to -.047)	0.932<br>(0.911 to 0.954)

The prevalence of those with haemodynamically moderate valve disease and the prevalence of those with haemodynamically severe disease were calculated using the prevalence envelope and the proportion of those with haemodynamically moderate disease for each five-year age group, sex, location, and year.

Treated proportion

We estimated the proportion of individuals who had haemodynamically severe disease who had been treated. Treatment was defined as valve replacement or repair. We assumed that treatment was not performed on any individuals with only haemodynamically moderate disease. The number of data points are reported in Table 10.

Table 10: Data on treated NRVD

Input data	Number of data points

**Unique sources**	**23**
**Geography-years**	**35**

These data were all from relatively high-income geographies, yet it is important that we capture the difference in treatment between high- and low-income locations. Because of this challenge, we ran a regression using the Healthcare Access and Quality (HAQ) index predicting the level of treatment and set a prior that the proportion of individuals with a valve replacement or repair was zero where HAQ index was equal to zero. This assumption allowed us to estimate an increasing relationship between HAQ index and proportion treated, where the estimated proportion treated was based on data where HAQ index was high. We used the regression equation:

$$logit(y) = \alpha + \beta_1 * haqi + \beta_2 * age + \beta_3 * severity$$

where y is the proportion of individuals with haemodynamically severe disease who had a valve replacement or repair, $haqi$ is the Healthcare Access and Quality index, age is the midpoint of the age range for a given data point, and $severity$ is an indicator variable to adjust for data points where the denominator of the proportion treated included both haemodynamically moderate and haemodynamically severe individuals. The prevalence of those with treated valve disease and the prevalence of those with untreated haemodynamically severe disease were calculated using the prevalence of haemodynamically severe disease and the proportion of those with treated valve disease. The results of this regression are reported in Table 11 and plotted for three ages in Figure 2.

Table 11: Treated NRVD regression coefficients

Covariate	Coefficients	Transformed coefficients
Intercept (β_0)	-4.69<br>(-5.90 to -3.43)	0.009<br>(0.003 to 0.032)
HAQI (β_1)	0.080<br>(0.070 to 0.089)	1.083<br>(1.073 to 1.093)
Age (β_2)	-0.029<br>(-0.04 to -0.015)	0.971<br>(0.957 to 0.985)
Severity (β_3)	-0.947<br>(-1.40 to -0.54)	0.377<br>(0.246 to 0.578)

Figure 2: Results of treatment model for three ages

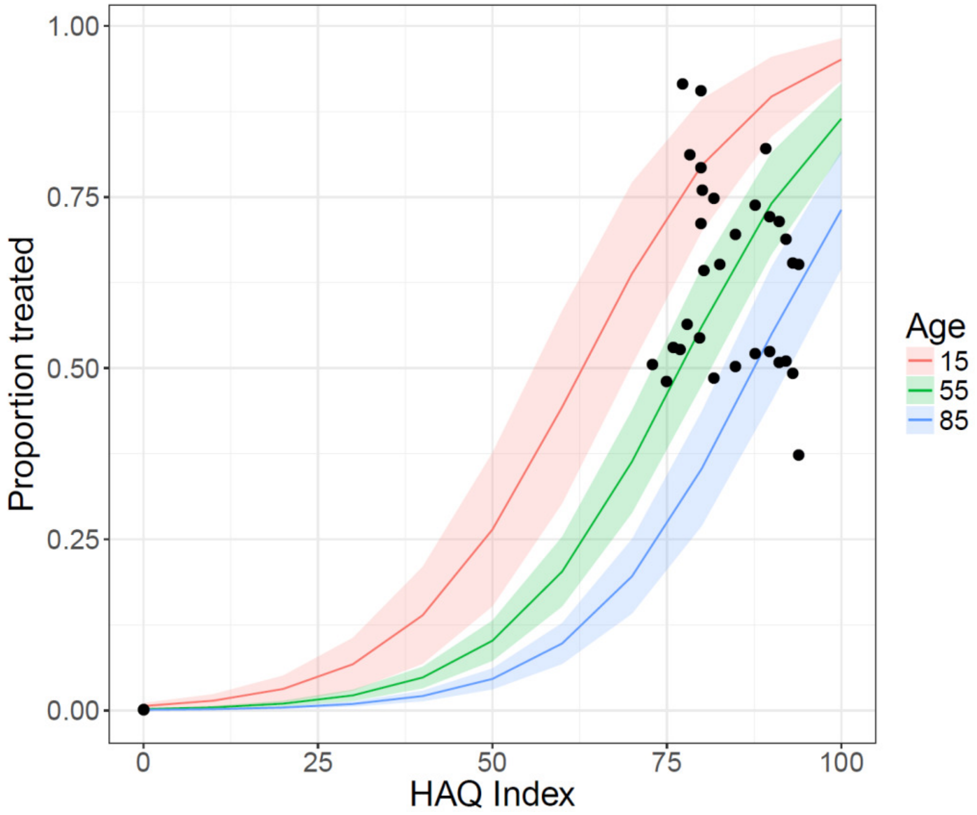

Final burden estimation

The proportions of mild, moderate, and severe heart failure due to valve disease were estimated using the heart failure analysis described above. The prevalences of these diseases were estimated using the prevalence of haemodynamically severe disease and the corresponding proportion for each severity of heart failure. Burden due to each severity of valve disease was estimated by multiplying the prevalence of each severity by the corresponding disability weight.

Acute Myocarditis

Flowchart

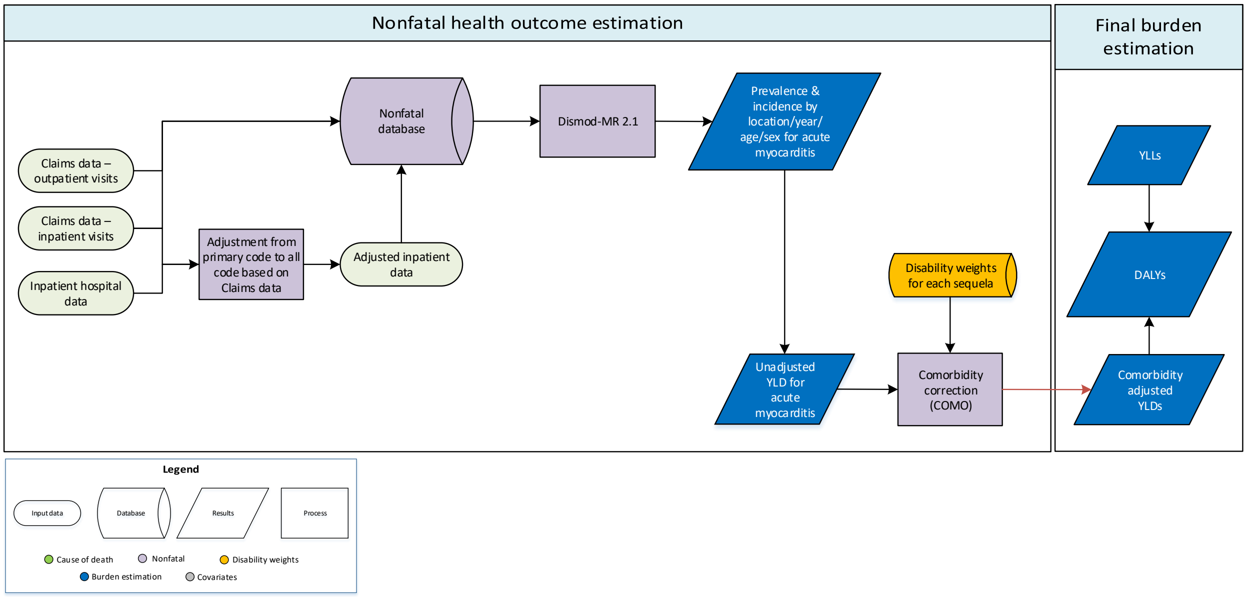

Input data and methodological summary

Case definition

Myocarditis refers to a heterogenous group of diseases with variable clinical and pathological features. Acute myocarditis was defined for GBD as the acute and time-limited symptoms of myocarditis separate from its chronic heart failure-related sequelae. Heart failure due to myocarditis is estimated separately in GBD (see methods for heart failure). Symptoms of acute myocarditis are nonspecific and include a flu-like or gastrointestinal syndrome, followed by anginal-type chest pain, arrhythmias, syncope, or heart failure.

A list of the ICD codes included can be found in Methods Appendix Table 4.

Input data

Model inputs

The preferred data sources for acute myocarditis were hospital admission data and other health facility data identifying cases of acute myocarditis.

A systematic review was performed for GBD 2013 and updated for GBD 2015. A systematic review was not performed for GBD 2017.

The GBD 2015 search terms included: (cardiomyopathy AND epidemiology[MeSH Subheading]) OR (myocarditis AND epidemiology[MeSH Subheading]) OR (cardiomyopathy AND (incidence OR prevalence OR “case fatality”)) OR (myocarditis AND (incidence OR prevalence OR “case fatality”))

- Dates included in search: 1/1/2013 – 3/16/2015
- Number of initial hits: 3,598
- Number of sources included: 0

The GBD 2013 search terms included: (hasabstract[text] AND Humans[Mesh] AND middle age[MeSH])) OR 21) AND ((cardiomyopathy/epidemiology[Mesh] OR cardiomyopathy/mortality[Mesh]) AND (prevalence[Title/Abstract] OR incidence[Title/Abstract]) AND ("2010"[Date - Publication] : "3000"[Date - Publication]) AND (hasabstract[text] AND Humans[Mesh] AND middle age[MeSH]))

The table below shows the number of site-years of data included in the estimation process.

	**Incidence**
**Site-years (total)**	482
**Number of countries with data**	32
**Number of GBD regions with data (out of 21 regions)**	12
**Number of GBD super-regions with data (out of 7 super-regions)**	6

We did not include any non-literature-based data, apart from the hospital and claims data described elsewhere. We used inpatient hospital data adjusted for readmission, primary to any diagnosis, and inpatient to outpatient utilisation based on correction factors generated using USA claims data. We excluded all outpatient data, as they were implausibly low when compared with inpatient data from the same locations and with claims data. Inpatient hospital data points that were more than two-fold higher or 0.5-fold lower than the median absolute deviation value for high-income North America, Central Europe, and Western Europe for that age-sex group were excluded.

Severity splits and disability weights

Severity level	Lay description	DW (95% CI)
Acute myocarditis	Has a fever and aches, and feels weak, which causes some difficulty with daily activities.	0.051 (0.032–0.074)

Modelling strategy

For GBD 2017, we estimated myocarditis using a DisMod-MR 2.1 Bayesian meta-regression model, setting a minimum of 3 and maximum of 5 as value priors on remission to establish an average duration of three months. We set a value prior of 0 for all ages on excess mortality. Country-level covariates used included the cardiomyopathy and myocarditis summary exposure variable (SEV) on incidence and the Healthcare Access and Quality index (HAQ Index) on excess mortality.

The table below gives the parameters, betas, and exponentiated betas for study-level and country-level covariates used in the model

Study covariate	Parameter	beta	Exponentiated beta
Healthcare Access and Quality index	Excess mortality rate	-0.55 (-0.99 to -0.1)	0.58 (0.37 to 0.90)
Log-transformed age-standardised SEV scalar: CMP	Incidence	0.76 (0.75 to 0.77)	2.13 (2.12 to 2.16)

No substantive changes were made to the modelling approach for GBD 2017.

Atrial Fibrillation and Flutter

Flowchart

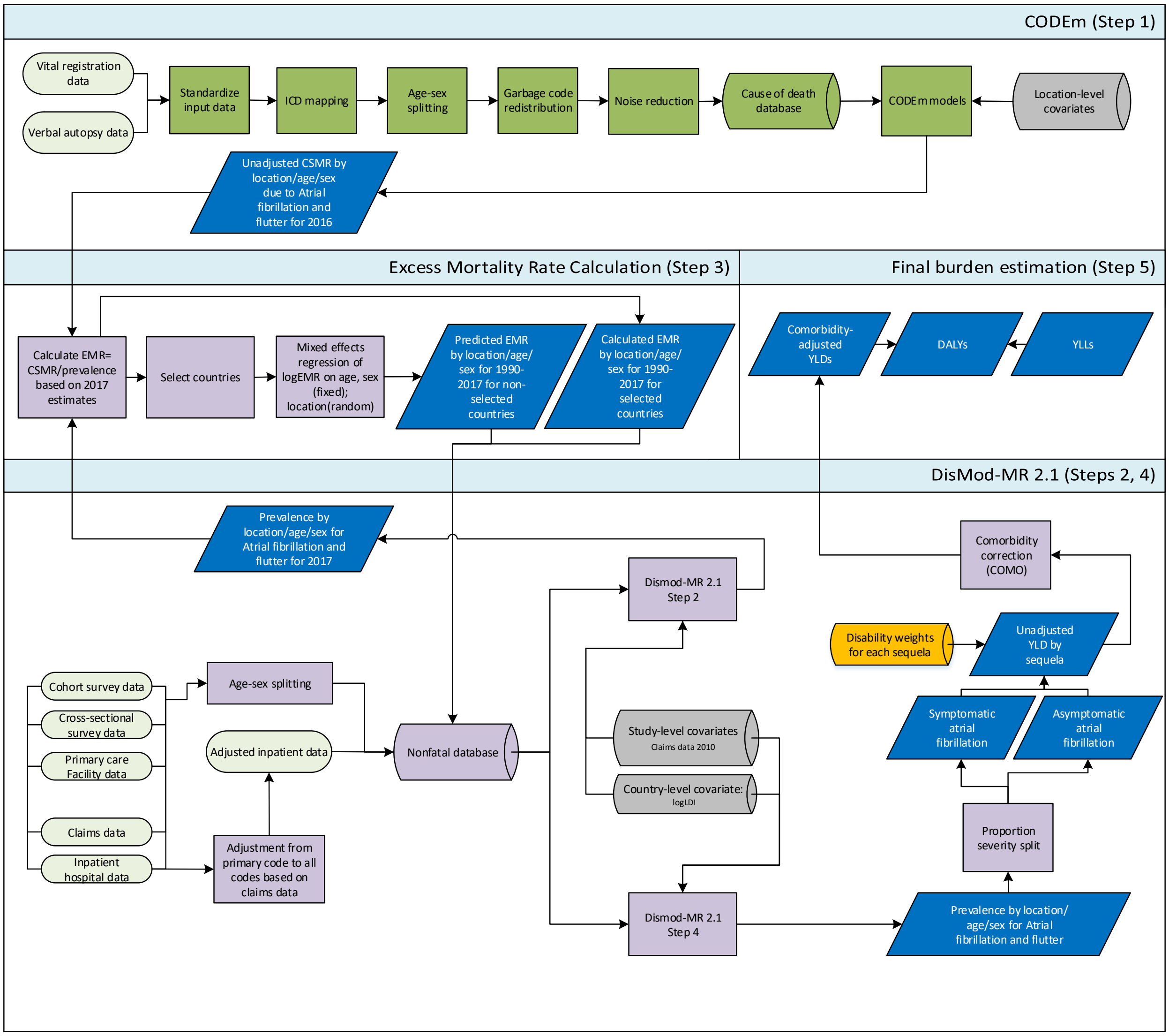

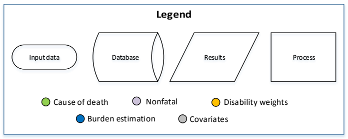

Input data and methodological summary

Case definition

Atrial fibrillation is a supraventricular arrhythmia due to disorganised depolarisation of the atrium. Atrial flutter is a macro-reentrant supraventricular arrhythmia, usually involving the cavo-tricuspid isthmus. Diagnosis requires an ECG demonstrating: 1) irregularly irregular RR intervals (in the absence of complete

AV block); 2) no distinct P waves on the surface ECG, and; 3) an atrial cycle length (when visible) that is usually variable and less than 200 milliseconds.

ICD codes used for inclusion of hospital and claims data can be found in Methods Appendix Table 4.

Input data

Model inputs

We did not perform a systematic review for GBD 2017. A systematic review was performed for GBD 2015 with the following search terms: (“atrial fibrillation” AND epidemiology[MeSH Subheading]) OR (“atrial flutter” AND epidemiology[MeSH Subheading]) OR (“atrial fibrillation” AND (prevalence OR incidence OR “case fatality”)) OR (“atrial flutter” AND (prevalence OR incidence OR “case fatality”)) OR (“heart atrium fibrillation” AND epidemiology[MeSH Subheading]) OR (“heart atrium fibrillation” AND (prevalence OR incidence OR “case fatality”))

The dates of the search were 1/1/2013 – 3/15/2016. There were 5,630 studies returned and, of those, 27 were extracted.

A systematic review was also performed for GBD 2013, with the search terms: (hasabstract[text] AND Humans[Mesh] AND middle age[MeSH])) OR 21) AND ((atrial fibrillation/epidemiology[Mesh] OR atrial fibrillation/mortality[Mesh]) AND (prevalence[Title/Abstract] OR incidence[Title/Abstract]) AND ("2010"[Date - Publication] : "3000"[Date - Publication]) AND (hasabstract[text] AND Humans[Mesh] AND middle age[MeSH]))

The table below shows the number of site-years of data and data types included in the estimation process.

	**Incidence**	**Mortality risk**	**Prevalence**
**Site-years (total)**	19	6	1425
**Number of countries with data**	8	5	40
**Number of GBD regions with data (out of 21 regions)**	3	5	12
**Number of GBD super-regions with data (out of 7 super-regions)**	2	3	6

Apart from hospital and claims data points on prevalence, no non-literature-based data were included. We included hospital data corrected for readmission, primary to any diagnosis, and inpatient to outpatient utilisation ratios using adjustment factors calculated from US claims data. We excluded hospital data in certain geographies (eg, Philippines, China, India) where the data were implausibly low. We also excluded all outpatient administrative data as the values for all locations were implausibly low. We included study-level covariates to adjust the claims data from 2010 within DisMod, using as reference literature data and the claims data from 2012.

Severity splits & disability weights

Atrial fibrillation is split into symptomatic and asymptomatic based on standard GBD proportion information. The table below includes lay descriptions and disability weights for the severity levels of atrial fibrillation:

Severity level	Lay description	DW (95% CI)
Asymptomatic	No symptoms	N/A
Symptomatic	Has periods of rapid and irregular heartbeats and occasional fainting	0.224 (0.151–0.312)

Modelling strategy

In order to address changes in coding practices for atrial fibrillation that resulted in an implausible trend of increasing death-certificate-based mortality rates, we used a prevalence-based modelling approach that combined DisMod-MR and CODEm models to generate estimates for atrial fibrillation and flutter. This approach, first used in GBD 2015, allowed us to more generate more accurate estimates, using observed prevalence and incidence rates along with modelled excess mortality rates generated from prevalence and cause-specific mortality estimates.

The modelling steps are illustrated in the above flowchart. Effect sizes for covariates included in both the DisMod-MR 2.1 and CODEm models can be found in the table below.

- In Step 1, we estimated deaths for atrial fibrillation using a standard CODEm approach.

- In Step 2, we estimated prevalence rates in DisMod-MR 2.1 using data from published reports of cross-sectional and cohort surveys, as well as primary care facility data. We also used claims data covering inpatient and outpatient visits for the United States along with inpatient hospital data from 163 locations in 15 countries. For GBD 2017, inpatient hospital data were adjusted using age- and sex-specific information from claims data for: 1) readmission within one year; 2) primary diagnosis code to secondary codes; and, 3) the ratio of inpatient to outpatient visits. We set priors of no remission and capped excess mortality at 0.4 for all ages. We included the Healthcare Access and Quality (HAQ) index as a country-level, fixed-effect covariate on excess mortality and the log-transformed, age-standardised SEV scalar for atrial fibrillation and flutter as a country-level, fixed-effect covariate on prevalence.

- In Step 3, we calculated the excess mortality rate (EMR) for 2017 (defined as the cause-specific mortality rate [CSMR] estimated from CODEm divided by the prevalence rate from DisMod-MR 2.1). We then selected 17 countries based on four conditions: 1) ranking of 4 or 5 stars on the system for assessing the quality of VR data; 2) prevalence data available from the literature were included in the DisMod-MR 2.1 estimation; 3) prevalence rate ≥ 0.005; and, 4) CSMR ≥ 0.00002. Using information from these countries as input data, we ran a linear mixed-effects regression of logEMR on sex, age, and location. Sex and age were treated as fixed effects for the regression, while location was considered a random effect. We then predicted age- and sex-specific EMR using the results of this regression for all non-selected countries. Countries included in the regression were assigned their directly calculated values. These EMR data points were assigned to the time period 1990–2017 and uploaded into the non-fatal database in order to be used in modelling.

- In Step 4, we re-ran DisMod-MR using the input data described in Step 2 along with the EMR estimated in Step 3. We included log-transformed lagged distributed income (LDI) as a fixed-effect, country-level covariate on excess mortality and the log-transformed, age-standardised SEV scalar for atrial fibrillation and flutter as a fixed-effect, country-level covariate on prevalence. We also included study-level covariates to crosswalk the USA claims data from 2010 to the reference data, which included literature data and claims data from 2012. We included a value prior of 0 for remission for all ages and set a value prior of 0 for excess mortality for ages 0-30.

The prevalence from the DisMod-MR model in Step 4 was used as the finalised output for upload to COMO and further processing into YLDs and DALYs.

Models were evaluated based on expert opinion, comparison with results from previous rounds of GBD, and model fit.

The tables below include the study covariates, parameters, betas, and exponentiated betas.

DisMod covariates – Step 2

Covariate	Parameter	Beta	Exponentiated beta
All MarketScan, year 2010	Prevalence	-0.077 (-0.099 to -0.051)	0.93 (0.91 to 0.95)
Log-transformed age-standardised SEV scalar: A Fib	Prevalence	0.75 (0.75 to 0.75)	2.12 (2.12 to 2.12)
Healthcare Access and Quality Index	Excess mortality rate	-0.11 (-0.13 to -0.088)	0.90 (0.88 to 0.92)

DisMod Covariates – Step 4

Covariate	Parameter	Beta	Exponentiated beta
All MarketScan, year 2010	Prevalence	0.017 (-0.013 to 0.040)	1.02 (0.99 to 1.04)
Log-transformed age-standardised SEV scalar: A Fib	Prevalence	0.75 (0.75 to 0.75)	2.12 (2.12 to 2.12)
LDI (I$ per capita)	Excess mortality rate	-0.1 (-0.1 to -0.1)	0.90 (0.90 to 0.90)

No substantive changes were made to the modelling strategy for GBD 2017.

Peripheral arterial disease

Flowchart

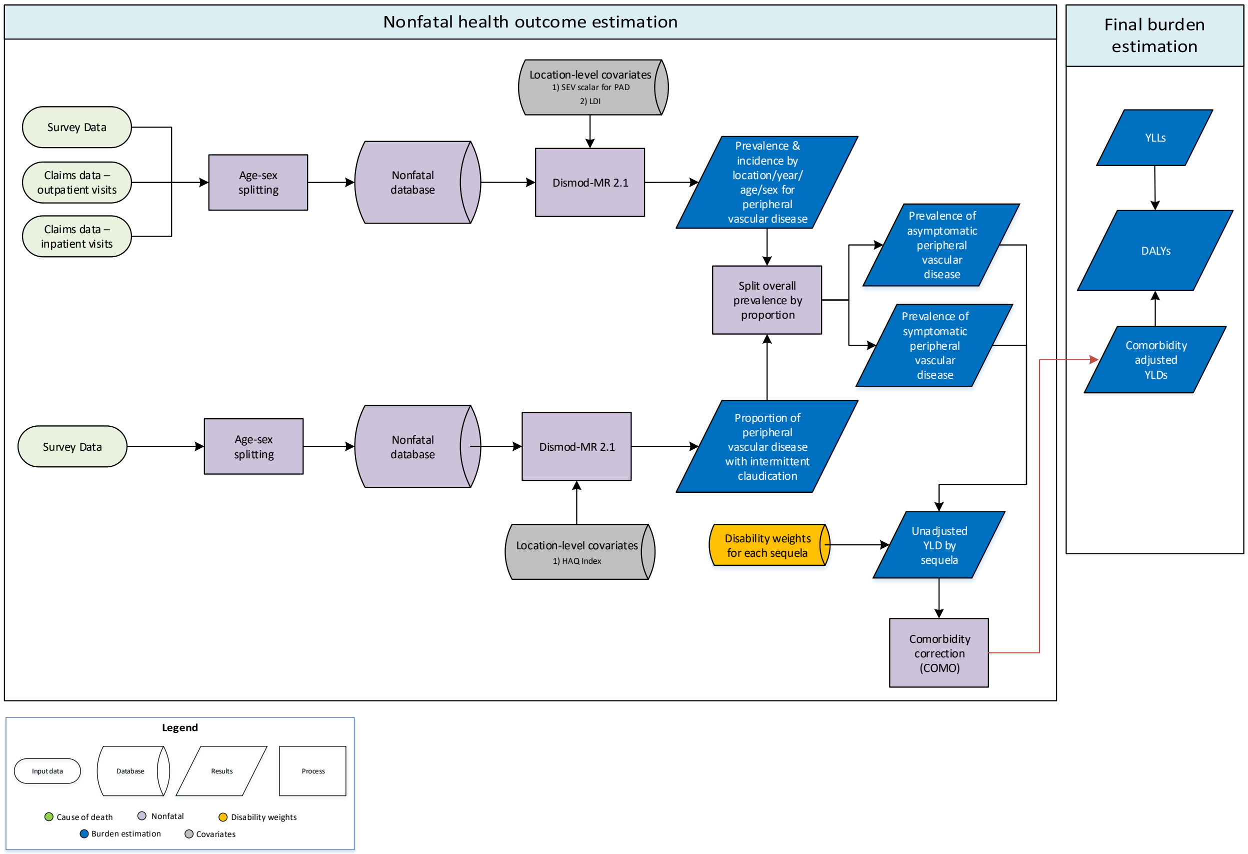

Input data and methodological appendix

Case definition

For GBD 2017, peripheral arterial disease was defined as having an ankle-brachial index (ABI) <0.9. Intermittent claudication was defined clinically.

Specific ICD codes for claims data included can be found in Methods Appendix Table 4.

Input data

Model inputs

We did not perform a systematic review for GBD 2017.

A systematic review was performed for peripheral arterial disease and intermittent claudication for GBD 2015. The search terms were: ('peripheral vascular disease'[TIAB] AND 'epidemiology'[Subheading]) OR ('peripheral arterial disease'[TIAB] AND 'epidemiology'[Subheading]) OR ('peripheral artery disease'[TIAB] AND 'epidemiology'[Subheading]) OR ('intermittent claudication'[TIAB] AND 'epidemiology'[Subheading]) OR ('ankle-brachial index'[TIAB] AND 'epidemiology'[Subheading]) OR ('ankle brachial index'[TIAB] AND

'epidemiology'[Subheading]) OR ('peripheral artery occlusive disease'[TIAB] AND 'epidemiology'[Subheading]) OR ('peripheral obliterative arteriopathy'[TIAB] AND 'epidemiology'[Subheading]) OR ('peripheral vascular disease'[TIAB] AND 'prevalence'[MeSH Terms]) OR ('peripheral vascular disease'[TIAB] AND 'incidence'[MeSH Terms]) OR ('peripheral vascular disease'[TIAB] AND 'case fatality'[All Fields]) OR ('symptomatic claudication'[TIAB] AND (proportion[All Fields] OR percent[All Fields]))

The search was conducted from 1/1/2013 to 3/16/2015. 1,658 results were returned, of which six were extracted.

A systematic review was also performed for peripheral arterial disease and intermittent claudication for GBD 2013. Search terms can be provided upon request.

The table below shows the number of literature studies included in GBD 2017, as well as the number of countries or subnational units and GBD world regions represented.

Peripheral arterial disease

	**Case fatality rate**	**Excess mortality rate**	**Incidence**	**Mortality risk**	**Prevalence**
**Site-years (total)**	3	1	3	19	443
**Number of countries with data**	3	1	3	11	15
**Number of GBD regions with data (out of 21 regions)**	3	1	2	7	8
**Number of GBD super-regions with data (out of 7 super-regions)**	2	1	1	5	5

Proportion with intermittent claudication

	**Proportion**
**Site-years (total)**	9
**Number of countries with data**	4
**Number of GBD regions with data (out of 21 regions)**	3
**Number of GBD super-regions with data (out of 7 super-regions)**	2

Apart from the claims data from the United States and primary care data from Norway, we did not include any non-literature-based data types. We did not use inpatient hospital data, as peripheral arterial disease is expected to be rare in inpatient data but common in outpatient data as it is a condition usually managed on an outpatient basis, except for specific surgical interventions. This discrepancy leads to implausible correction factors based on inpatient/outpatient information from claims data (~150X); thus adjusted data cannot be used. Including uncorrected data in the model is likely to lead to incorrect estimates as hospitalisation and procedure rates are likely to vary between geographies based on access to and patterns of care.

Outpatient data were not included as the rates were implausibly low for all locations.

Severity splits and disability weights

We used the proportion of intermittent claudication to split the overall prevalence of peripheral arterial disease into symptomatic and asymptomatic peripheral vascular disease. The table below illustrates these values:

Severity level	Lay description	DW (95% CI)
Asymptomatic	No symptoms	No DW assigned
Symptomatic	Has cramping pains in the legs after walking a medium distance. The pain goes away after a short rest.	0.014 (0.007–0.025)

Modelling strategy

For GBD 2017, we used DisMod MR 2.1 to model the overall prevalence of peripheral arterial disease using prevalence data from literature studies and claims data.

We corrected US claims data outside of DisMod, using as reference literature reports of prevalence. Using as a model the adjustment factors developed to translate tobacco consumption prevalence to tobacco consumption frequency, we matched administrative claims data to population-based literature data based on age group, sex, and super-region (1). For the adjustment factor, we developed the following generalised additive model on matched data

$$\ln\left(\mu_{ref,i}\right) = \beta_0 + \beta_1 \ln\left(\mu_{b,i}\right) + s(age_i) + \varepsilon_i$$

Where i represents a given matched observation, age signifies the data point's age group, $s(x)$ represents a penalised spline where the smoothing parameter is chosen through cross-validation and μ_{ref} and μ_b denote the mean of the data point from literature and the mean of the claims data point, respectively. Predictions from the model were then taken as the adjusted data points. The standard error of each corrected data point was adjusted to account for the uncertainty due to the correction.

We included a study-level covariate in DisMod to adjust primary care data from Norway. We also included the log-transformed, age-standardised SEV scalar for PAD and log-transformed LDI as fixed-effect, country-level covariates. We set value priors of 0 for incidence from ages 0 to 30. We also set a value prior of 0 for remission for all ages. Finally, we set a value prior of a maximum value of 0.25 on excess mortality for all ages.

The table below illustrates the study covariates, parameters, beta, and exponentiated beta values for the overall peripheral vascular disease model.

Covariate	Parameter	Beta	Exponentiated beta
Log-transformed age-standardised SEV scalar: PVD	Prevalence	1.20 (1.10 to 1.25)	3.34 (2.99 to 3.49)
LDI (I$ per capita)	Excess mortality rate	-0.3 (-0.5 to -0.1)	0.74 (0.61 to 0.90)
ICPC data (Norway)	Prevalence	-2 (-2 to -1.99)	0.14 (0.14 to 0.14)

We used DisMod MR-2.1 to model the proportion of peripheral vascular disease with intermittent claudication. We set a value prior of 0 for proportion for ages 0 to 40.

Covariate	Parameter	Beta	Exponentiated beta
Healthcare Access and Quality index	Proportion	-0.0072 (-0.023 to -0.00025)	0.99 (0.98–1.00)

To obtain final estimates for the sequelae of interest, we multiplied the prevalence model by the proportion model at the draw level to generate the prevalence of symptomatic and asymptomatic peripheral vascular disease.

Models were evaluated based on expert review, comparisons with estimates from prior rounds of GBD, and assessing model fit.

There have been no substantive changes from GBD 2016 in terms of modelling strategy for peripheral arterial disease.

1. Reitsma, Marissa B., et al. “Smoking prevalence and attributable disease burden in 195 countries and territories, 1990–2015: a systematic analysis from the Global Burden of Disease Study 2015.” The Lancet.

Acute endocarditis

Flowchart

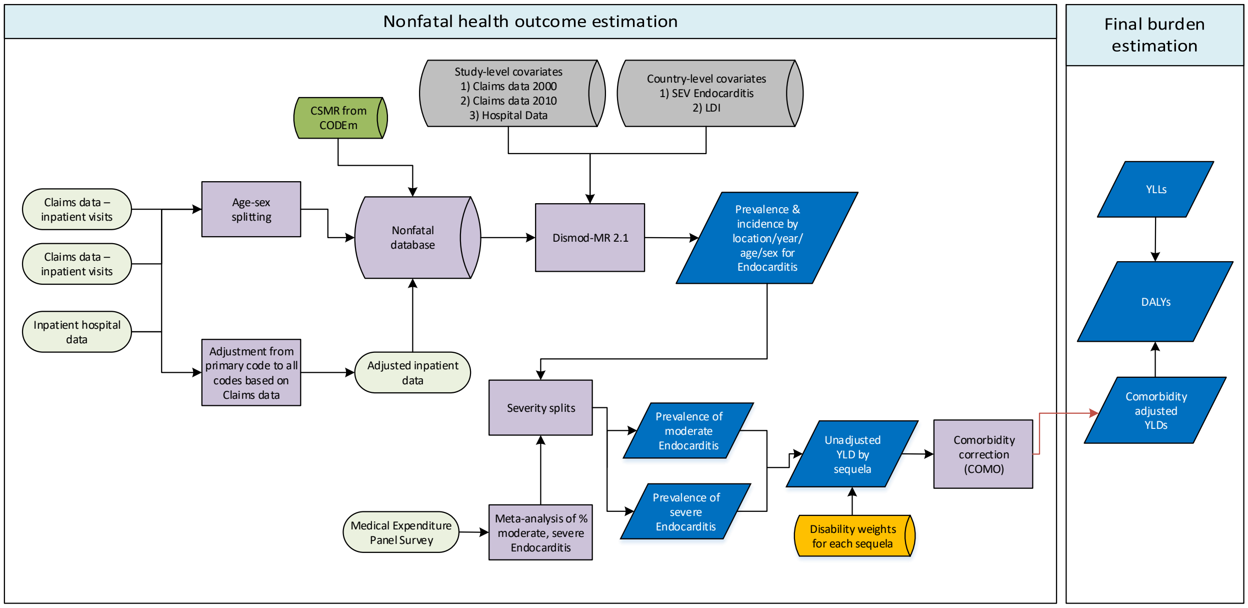

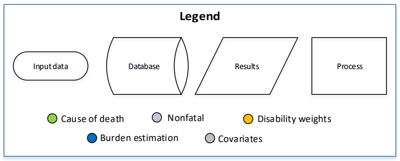

Input data and methodological appendix

Case definition

Our case definition for acute endocarditis was a clinical diagnosis of infective endocarditis. The ICD codes included can be found in Methods Appendix Table 4.

Input data

Model inputs

A systematic review was performed for GBD 2013 and updated for GBD 2015. We did not perform a systematic review for GBD 2017. The following search terms were used: ((‘endocarditis’[MeSH Terms] OR ‘endocarditis’[All Fields]) AND ‘epidemiology’[Subheading]) OR ((‘endocarditis’[MeSH Terms] OR ‘endocarditis’[All Fields]) AND ((‘epidemiology’[Subheading] OR ‘epidemiology’[All Fields] OR ‘incidence’[All Fields] OR ‘incidence’[MeSH Terms]) OR (‘epidemiology’[Subheading] OR ‘epidemiology’[All Fields] OR ‘prevalence’[All Fields] OR ‘prevalence’[MeSH Terms]) OR ‘case fatality’[All Fields])) OR ((‘endocardium’[MeSH Terms] OR ‘endocardium’[All Fields]) AND inflammation[TIAB] AND ‘epidemiology’[Subheading]) OR ((‘endocardium’[MeSH Terms] OR ‘endocardium’[All Fields]) AND inflammation[TIAB] AND ((‘epidemiology’[Subheading] OR ‘epidemiology’[All Fields] OR ‘incidence’[All

Fields] OR 'incidence'[MeSH Terms]) OR ('epidemiology'[Subheading] OR 'epidemiology'[All Fields] OR 'prevalence'[All Fields] OR 'prevalence'[MeSH Terms]) OR 'case fatality'[All Fields]))

- Dates included in search: 1/1/2013 – 3/16/2015
- Number of initial hits: 1,246
- Number of sources included: 6

The table below shows the number of site-years of data included in GBD 2017, as well as the number of countries or subnational units and GBD world regions represented.

	Case fatality rate	Excess mortality rate	Incidence
**Site-years (total)**	7	1	620
**Number of countries with data**	5	1	37
**Number of GBD regions with data (out of 21 regions)**	3	1	12
**Number of GBD super-regions with data (out of 7 super-regions)**	2	1	5

We did not include any non-literature-based data types, apart from the hospital and claims data described elsewhere. We excluded all outpatient data, as they were implausibly low when compared with inpatient data from the same locations and claims data. We used hospital data corrected for readmission and primary to any diagnosis based on the correction factors generated using USA claims data. We excluded any inpatient hospital data points which were more than two-fold higher or 0.5-fold lower than the median absolute deviation value for high-income North America, Central Europe, and Western Europe for that age-sex group.

Severity split inputs

We used the standard GBD approach, which utilises MEPS data to split overall estimates of endocarditis into moderate and severe categories. The table below includes the severity level, lay descriptions, and DWs associated with acute endocarditis.

Severity level	Lay description	DW (95% CI)
Moderate	Has a fever and aches, and feels weak, which causes some difficulty with daily activities.	0.051 (0.032–0.074)
Severe	Has a high fever and pain, and feels very weak, which causes great difficulty with daily activities.	0.133 (0.088–0.19)

Modelling strategy

For GBD 2017, we estimated endocarditis using a DisMod-MR-2.1 Bayesian meta-regression model, setting a minimum of 11 and maximum of 13 as value priors on remission to establish an average duration of one month. Country-level covariates used included the endocarditis summary exposure variable (SEV) on incidence and lag distributed income (LDI) per capita (I$) on excess mortality.

We evaluated models by comparing model fits with the data and with results from previous GBD estimation cycles.

The table below gives the parameters, betas, and exponentiated betas for study-level and country-level covariates used in the model

Covariate	Parameter	Beta	Exponentiated beta
LDI (I$per capita)	Excess mortality rate	-0.1 (-0.1 to -0.1)	0.90 (0.90 to 0.90)
Log-transformed age-standardised SEV scalar: endocarditis	Incidence	0.91 (0.76 to 1.20)	2.50 (2.13 to 3.31)

No significant changes were made to the modelling strategy from GBD 2016.

Other cardiovascular disease

Flowchart

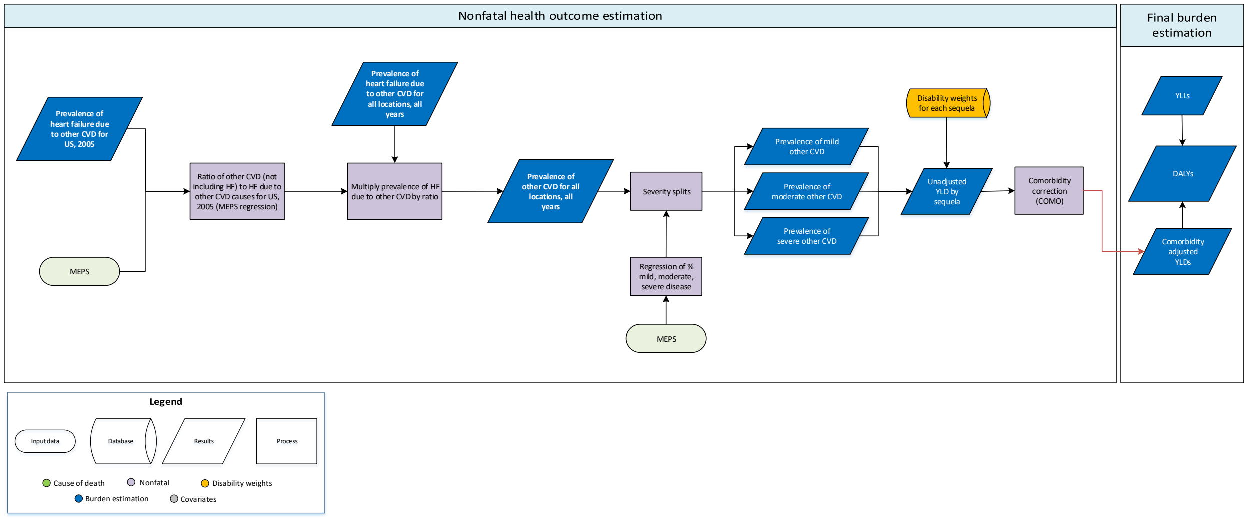

Case definition

Other cardiovascular disease is a residual category resulting from the GBD approach of estimating the total burden of all causes. Prevalence estimates are produced in order to provide YLDs consistent with the estimated YLLs from the death modelling process and to enable the calculation of DALYs.

Conditions included in this cause, based on ICD codes used for both fatal and non-fatal modelling, are Other diseases of pulmonary vessels; Acute pericarditis; Other diseases of pericardium; Pericarditis in diseases classified elsewhere; Paroxysmal tachycardia; Cardiac septal defect, acquired; Rupture of chordae tendineae, not elsewhere classified; Rupture of papillary muscle, not elsewhere classified; Intracardiac thrombosis, not elsewhere classified; Cerebral amyloid angiopathy; Other aneurysm; Other disorders of arteries and arterioles; Diseases of capillaries; Disorders of arteries, arterioles, and capillaries in diseases classified elsewhere; Phlebitis and thrombophlebitis; Portal vein thrombosis; Other venous embolism and thrombosis; Varicose veins of lower extremities; Varicose veins of other sites; Other disorders of veins; Nonspecific lymphadenitis; Other non-infective disorders of lymphatic vessels and lymph nodes; Other disorders of circulatory system in diseases classified elsewhere.

Input data

As this is a residual category, we used data from the Medical Expenditure Panel Survey and modelled estimates from heart failure due to other cardiovascular disease to estimate prevalence of other cardiovascular disease.

Severity split inputs

The table below includes lay descriptions and disability weights for the severity levels of other cardiovascular disease for GBD 2017.

Severity level	Lay description	DW (95% CI)
Asymptomatic		N/A
Mild	Is short of breath and easily tires with moderate physical activity, such as walking uphill or more than a quarter-mile on level ground. The person feels comfortable at rest or during activities requiring less effort.	0.041 (0.026–0.062)
Moderate	Is short of breath and easily tires with minimal physical activity, such as walking only a short distance. The person feels comfortable at rest but avoids moderate activity.	0.072 (0.047–0.103)
Severe	Is short of breath and feels tired when at rest. The person avoids any physical activity, for fear of worsening the breathing problems.	0.179 (0.122–0.251)

Modelling strategy

To obtain prevalence estimates of other cardiovascular disease, we used MEPS data combined with prevalence estimates of heart failure due to other CVD for the USA in 2005 to estimate the ratio of the prevalence of heart failure due to other CVD causes to the prevalence of other CVD causes. We then applied this ratio to the age-, sex-, and year-specific prevalence estimates for heart failure due to other CVD causes for all locations to generate prevalence estimates of other cardiovascular disease.

Non-rheumatic valve disease, including mitral and aortic valve disease, previously included as part of the other cardiovascular disease aggregate, was modelled as a separate cause for GBD 2017; a write-up of the modelling strategy can be found in this appendix. No other significant changes were made to the modelling strategy for GBD 2017.

Chronic obstructive pulmonary disease (COPD)

Flowchart

Chronic Obstructive Pulmonary Disease (COPD)

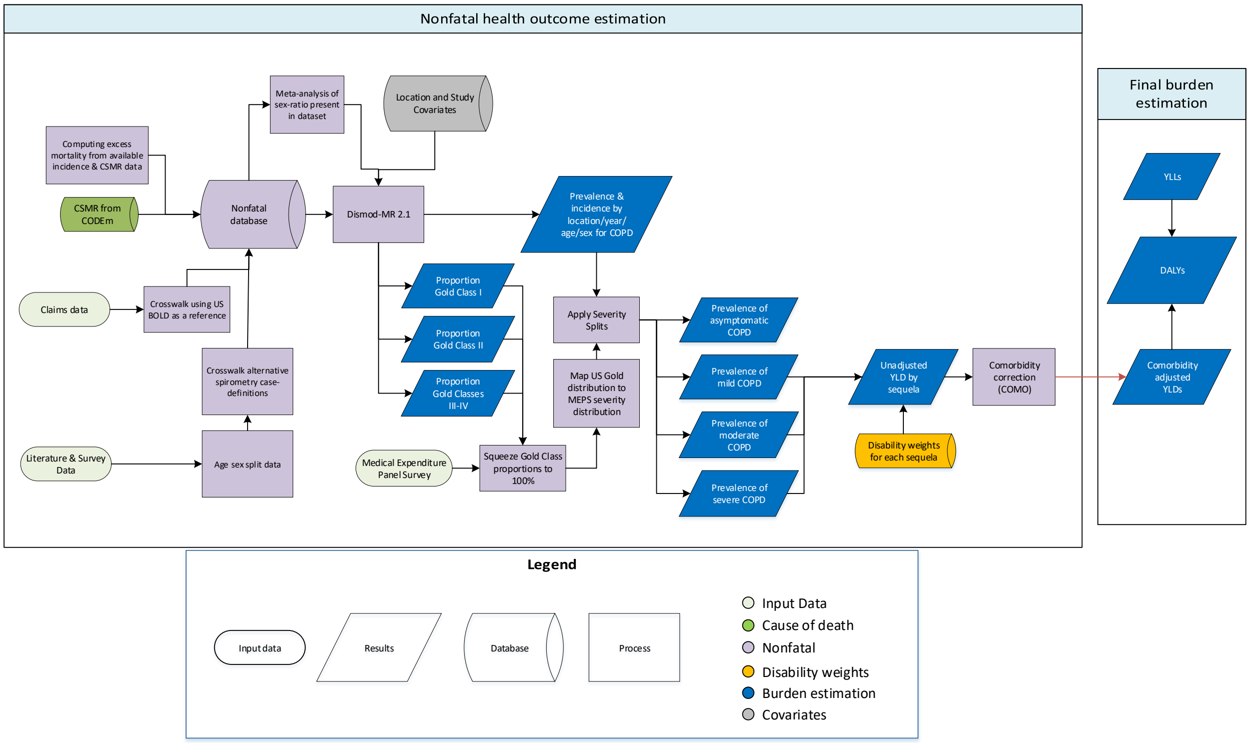

Input data and methodological summary

Case definition

COPD is defined as in the Global Initiative for Chronic Obstructive Lung Disease (GOLD) classification: a measurement of <0.7 FEV$_1$/FVC (one second of forceful exhalation/total forced expiration) on spirometry after bronchodilation. It should be noted that this is the same reference definition as was used for GBD 2015 and GBD 2016, but it is different from GBD 2013, where the "Lower Limit of Normal (LLN)," ie, relative to an age- and sex-specific norm for the FEV$_1$/FVC ratio, was the reference. We made this decision because the severity grading of COPD follows the GOLD Class definition rather than the LLN concept. The definitions of the severity classes in the GOLD classification are provided below.

GOLD CLASS	FEV$_1$ Score
I: Mild	>=80% of normal
II: Moderate	50-79% of normal
IV: Severe	<50% of normal

ICD-10 codes associated with COPD include J41, J42, J43, J44, and J47. The corresponding ICD-9 codes are 491-492, and 496. J40 & 490 (Bronchitis, not specified as acute or chronic) and J47 & 494 (Bronchiectasis) were mapped to COPD for GBD 2016 but excluded for GBD 2017 based on expert feedback.

Input data

No systematic review of the literature was completed for GBD 2017; however, for GBD 2016, we updated the systematic review from previous iterations. The full search term was:

(chronic obstructive pulmonary disease[Title/Abstract] AND (prevalence[Title/Abstract] or incidence [Title/Abstract] or mortality [Title/Abstract] or death [Title/Abstract]) AND "Cross-Sectional Studies"[MeSH Terms]) Filters: Publication date from 04/01/2015 to 11/01/2016; Humans

For GBD 2017, we reviewed the papers listed in the following meta-analysis of COPD prevalence estimates:

Adeloye D, Chua S, Lee C, Basquill C, Papana A, Theodoratou E, Nair H, Gasevic D, Sridhar D, Campbell H, Chan KY. Global and regional estimates of COPD prevalence: Systematic review and meta–analysis. Journal of global health. 2015 Dec;5(2).

In addition to scientific literature, we included survey data with spirometry measurements, such as the National Health and Nutrition Examination Study series in the United States. The Study of Aging and Global Health (SAGE) series, the Korean NHANES, the English Longitudinal Study of Aging (ELSA), and the Turkey Chronic Diseases and Risk Factors Study 2011 were all added for GBD 2017.

Data using alternative case-definitions of COPD prevalence (ie, LLN or FEV1/FVC<0.7 pre-bronchodilator) were crosswalked to the reference case-definition with age-specific ratios derived from studies reporting prevalence using both the alternative and reference case-definitions.

Furthermore, claims data for the United States were included. Additional information on the claims data collection and pre-corrections are provided elsewhere. Briefly, we determined USA national and state-level estimates of COPD prevalence from a database of individual-level ICD-coded health service encounters. Persons with any inpatient claim or at least two outpatient claims associated with COPD were marked as a prevalent case for that year.

For GBD 2016, a correction was made for COPD USA claims data. Under the assumption that NHANES estimates are more accurate than claims data estimates because they use spirometry measurements, we derived an age-specific crosswalk to adjust USA claims data according to the ratio between NHANES and the national-level USA claims estimates. However, for GBD 2017 we decided the age-pattern apparent in NHANES is unreliable and perhaps implausibly high in individuals under 30 years old, due to the fact that NHANES spirometry measurements are taken without the use of a bronchodilator. Instead, we derived an age-specific crosswalk using a comparison of BOLD study results from Kentucky to claims data from Kentucky. Claims data are valuable for the subnational variation they can provide; however, the challenge of correcting the systematic bias present in claims data relative to spirometry-based prevalence data has no clear or singular resolution.

The volume of claims data is sufficiently large to have a ripple effect throughout the model. One way this effect manifests is in the sex-ratio. The GBD 2016 NHANES-based crosswalk was both age and sex-specific. The GBD 2017 BOLD-based crosswalk, on the other hand, is not sex-specific, and this decision

was made because BOLD estimates in Kentucky are greater in females than in males, whereas USA NHANES and claims data suggest greater prevalence in females. As a result of using a non-sex-specific crosswalk, the sex-ratio present in the claims data is preserved by the crosswalk. This ratio, while in the direction we expect (larger prevalence in males), is smaller in magnitude than the ratio from NHANES, and therefore smaller than the ratio present in our adjusted data for GBD 2016. This modelling decision had the effect of increasing prevalence in females in the US, and this, combined with new UK data that are higher in females than the GBD 2016 models, resulted in higher modelled prevalence for females in many other GBD regions as well. A table describing the density and distribution of the available data informing the COPD estimation process is provided below.

	Prevalence	Incidence	Proportion by GOLD class
Site-years (total)	504	5	39
Number of countries with data	53	5	31
Number of GBD regions with data (out of 21 regions)	16	3	15
Number of GBD super-regions with data (out of 7 super-regions)	7	2	7

Modelling strategy

As described above, the estimation of COPD burden occurs in three main steps. The first is the estimation of prevalence and incidence using a DisMod-MR 2.1 model. The second is the separate estimation of the proportions by three GOLD class groupings in DisMod-MR 2.1. The third is the combination of these two processes to derive prevalence by severity.

Step 1: Main COPD model

Prior settings include remission of 0 and an incidence ceiling of 0.0002 before age 20. The latter was necessary to avoid a kick-up of estimates in childhood at an age range with few or no primary data.

Similar to other causes, we included estimates of cause-specific mortality rate (CSMR) and derived estimates of excess mortality rate (EMR) by dividing every prevalence data point by the CSMR value for the corresponding location, age, sex, and year. We did not estimate EMR for data points with an age range greater than 20 years.

To assist estimation, each model includes a series of country-level covariates that describe spatiotemporal patterns. For example, we use the COPD standardised exposure variables (SEV), which aggregates multiple risk factors into a single variable. We also use the log of LDI and the Healthcare Access and Quality (HAQ) index on EMR to capture country-level variation of EMR, assuming a negative coefficient (ie, lower mortality with rising GDP and HAQ). For this GBD cycle, the proportion of elevation over 1500m was also added as a country-level covariate on prevalence and EMR based on its significance in the COPD cause of death models.

For GBD 2017, with the new adjustment strategy for claims data, it appeared that DisMod was calculating a sex-coefficient that placed too much weight on the sex-ratio from the claims. The claims ratio is smaller than the ratio from the remainder of the dataset, so this had an undesirable effect. In response, we performed a random-effects meta-analysis of the male:female ratio in our dataset and fixed the sex-coefficient in the DisMod prevalence model accordingly.

Step 2: GOLD class models

The GOLD class models use data from surveys that specified prevalence by GOLD class after expressing the values as a proportion of all COPD cases. For GBD 2016 we used fixed effects from the SEV scalar and the log of lag-distributed income (LDI) per capita to assist estimation. For GBD 2017, we dropped these covariates because they did not produce significant coefficients. We also restricted random effects to +/- 0.5 to control implausible geographical variation.

Table of model coefficients for COPD

Model	Variable name	Measure	Beta	Exponentiated
COPD	Elevation over 1500m (proportion)	excess mortality rate	0.21 (0.12–0.31)	1.23 (1.12–1.36)
COPD	LDI (I$ per capita)	excess mortality rate	-0.5 (-0.5 to -0.5)	0.61 (0.60–0.61)
COPD	Log age-standardised SEV scalar: COPD	prevalence	0.90 (0.90–0.90)	2.46 2.46–2.46)

Severity

The three GOLD class groupings reflect a grading based on a physiological measurement rather than a direct measurement of disease severity. In order to map the epidemiological findings by GOLD class into the three COPD health states for which we have disability weights (DW), we used the 2001–2011 Medical Expenditure Panel Survey (MEPS) data from the United States. Specifically, we convert the GOLD class designations estimated for the USA in 2005 (the midpoint of MEPS years of analyses) into GBD classifications of asymptomatic, mild, moderate, and severe COPD.

The table below shows the three health states of COPD and the corresponding lay descriptions and disability weights. The graph shows the average proportion by GOLD class (after scaling to 100%) across all ages for USA in 2005. We also show the proportion of MEPS respondents reporting any health service contact in the past year for COPD with a DW value attributable to COPD of 0, mild range (0 to midpoint between DWs for mild and moderate), moderate range (midpoint of DW values mild and moderate to midpoint of DW values for moderate and severe) and severe range (midpoint between DW values moderate and severe or higher). The DW value for COPD was derived from a regression with indicator variables for all health states reported by MEPS respondents and their reported overall level of disability derived from a conversion of 12-Item Short Form Surveys (SF-12) answers to GBD DW values. This analysis gave the severity distribution for each GBD cause reported in MEPS after correcting for any comorbid causes individual respondents reported during a year.

Health state	Lay description	DW (95% CI)

Mild COPD	This person has cough and shortness of breath after heavy physical activity, but is able to walk long distances and climb stairs.	0.019 (0.011–0.033)
Moderate COPD	This person has cough, wheezing, and shortness of breath, even after light physical activity. The person feels tired and can walk only short distances or climb only a few stairs.	0.225 (0.153–0.31)
Severe COPD	This person has cough, wheezing, and shortness of breath all the time. The person has great difficulty walking even short distances or climbing any stairs, feels tired when at rest, and is anxious.	0.408 (0.273–0.556)

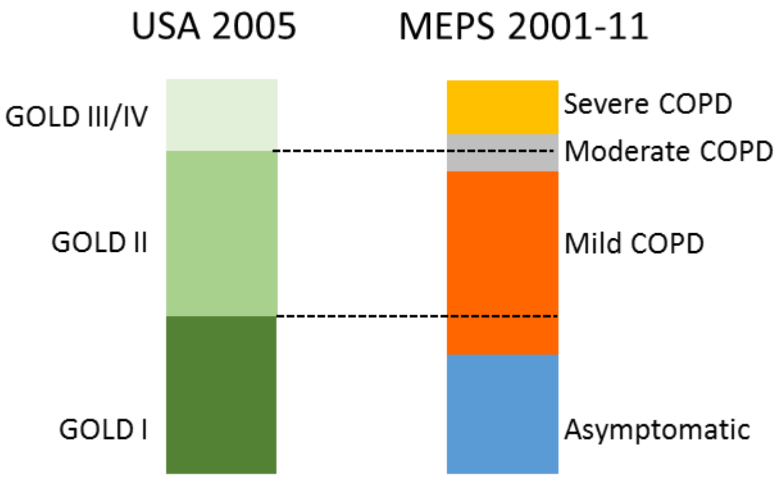

The algorithm to translate GOLD class to COPD DW categories first assigns GOLD III&IV to severe COPD and what remains to moderate. Next, GOLD class I is assigned to the asymptomatic category first and what remains goes to mild COPD. This algorithm is repeated for each age and sex category and for all 1,000 draws from the DisMod models of GOLD classes and the MEPS analyses. We end up with proportions of each of the GOLD class categories that map onto GBD COPD health states with uncertainty bounds determined by the 25th and 975th values of the 1,000 draws. These values are then applied to the estimates of the proportion of cases by GOLD class category, after scaling to 100%, by location, year, age, and sex. This assumes that the relationship between GOLD class and GBD COPD health states in the United States applies everywhere.

Pneumoconiosis

Coal Worker's Pneumoconiosis, Asbestosis, Silicosis, and Other Pneumoconiosis

Flowchart

Pneumoconiosis:
Coal Worker's, Asbestosis, Silicosis, Other

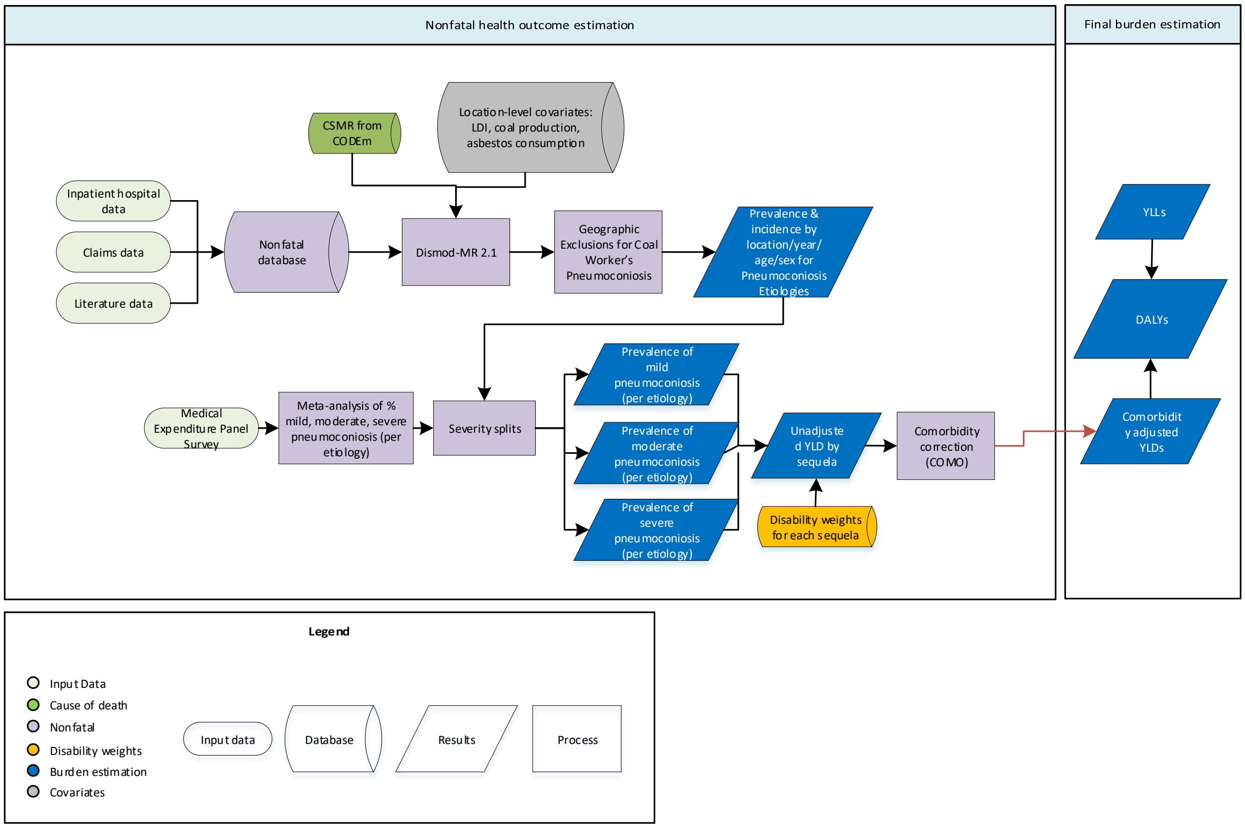

Input data and methodological appendix

Case definition

Pneumoconiosis is a chronic lung disease typified by lung scarring and other interstitial damage caused by exposure to dust and other containments – usually through occupational exposure. For GBD, we model pneumoconiosis by exposure type: coal, asbestos, silica, and other.

Input data

Data used to make estimates of pneumoconiosis are predominantly from three main sources. The first is literature data from systematic reviews, usually from smaller-scale studies of prevalence. One challenge with literature data is that most studies are conducted in high-risk populations that are not representative of the general population. No systematic review of the literature was conducted for GBD

2017. The second source of data is inpatient hospital reports, and the third is claims data for the United States and Taiwan. For all aetiologies, we use a sex-specific correction factor of the hospital inpatient data where numbers are adjusted upward by the ratio of primary diagnosis to secondary diagnosis present in the claims data. Greater detail on the preparation of the inpatient and claims data is provided elsewhere.

The table below includes details regarding input data counts. All data are for prevalence. Data which have been marked as outliers are not included in these counts.

	Asbestosis	Coal worker's Pneumoconiosis	Silicosis	Other Pneumoconiosis
Site-years (total)	945	769	744	934
Number of countries with data	32	29	33	38
Number of GBD regions with data (out of 21 regions)	12	13	13	15
Number of GBD super-regions with data (out of 7 super-regions)	5	6	6	7

Severity split inputs

Data to inform estimates of the severity gradient due to pneumoconiosis etiologies are derived from previous analyses of the Medical Expenditure Panel Survey (MEPS). The disability weights are also shared.

Severity level	Lay description	DW (95% CI)
Mild	Has cough and shortness of breath after heavy physical activity, but is able to walk long distances and climb stairs.	0.019 (0.011–0.033)
Moderate	Has cough, wheezing, and shortness of breath, even after light physical activity. The person feels tired and can walk only short distances or climb only a few stairs.	0.225 (0.153–0.312)
Severe	Has cough, wheezing, and shortness of breath all the time. The person has great difficulty walking even short distances or climbing any stairs, feels tired when at rest, and is anxious.	0.408 (0.273–0.556)

Modelling strategy

Estimates for the pneumoconiosis aetiologies are produced using a standard DisMod-MR 2.1 approach.

For all aetiologies, we use prior settings of zero remission. Additionally, we assume no incidence and prevalence before the age of 10.

To assist estimation, each model includes a series of country-level covariates that describe spatiotemporal patterns. The standardised exposure variable (SEV) covariates, which were used for GBD

2016, were removed because the associated risk-outcome pairs for the new calculation resulted in undefined SEV values. However, we added the SEV scalar for mesothelioma in the asbestosis model, as asbestosis and mesothelioma have a common risk factor in asbestos exposure. The gold production covariate, which was used for the GBD 2016 silicosis model, was removed because DisMod was assigning it implausible coefficient values. Subnational updates were made to coal production and asbestos consumption to account for new subnational locations for GBD 2017.

Cause	Measure	Variable name	Beta	Exponentiated
Asbestosis	Prevalence	Asbestos consumption (per capita)	0.47 (0.015–1.70)	1.60 (1.02–5.47)
Asbestosis	Prevalence	Log-transformed age-standardised SEV scalar: Mesothelioma	0.029 (0.000016–0.32)	1.03 (1.00–1.38)
Coal worker's	Prevalence	Coal production (per capita)	0.0017 (-0.00025 to 0.0045)	1.00 (1.00–1.00)

Prevalence and incidence of coal worker's pneumoconiosis were set to zero in locations without a history of coal mining given the causal and necessary relationship between respective occupational exposure and disease. For GBD 2016 these locations were values with zero coal production for 30 years in the GBD coal production covariate, but for GBD 2017 we cross-referenced these locations with vital registration data to ensure that we are not setting prevalence and incidence to zero for any locations where vital registration codes greater than zero deaths due to coal worker's pneumoconiosis.

Asthma

Flowchart

Asthma

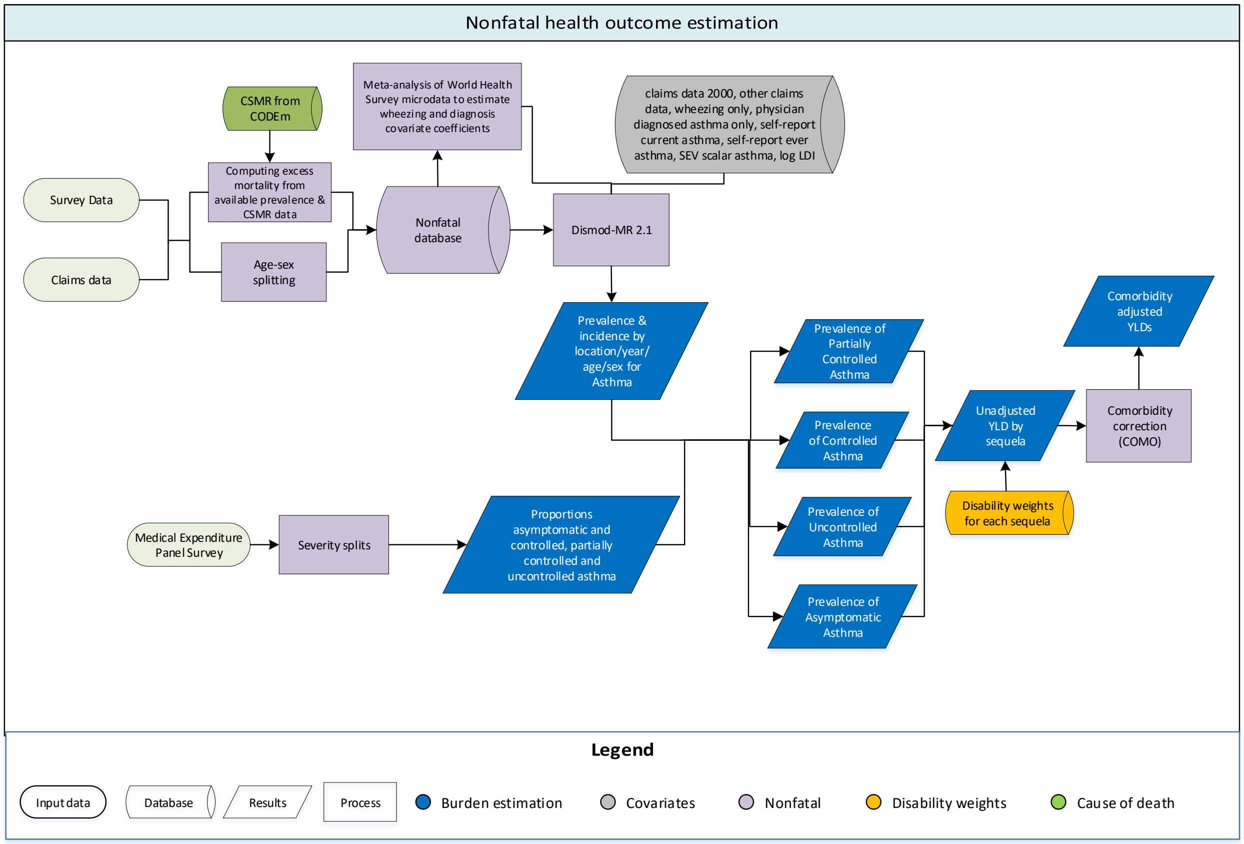

Case definition

Asthma is a chronic lung disease marked by spasms in the bronchi usually resulting from an allergic reaction or hypersensitivity and causing difficulty in breathing. We define asthma as a doctor's diagnosis and wheezing in the past year. The relevant ICD-10 codes are J45 and J46. ICD-9 code is 493.

Input data

No systematic review of the literature was completed for this GBD cycle. However, for GBD 2016, we did a full systematic review of the literature on asthma. We used the following search string in PubMed and filtered by studies of humans published between January 2012 and November 2016.

(Asthma[Title/Abstract] AND prevalence[Title/Abstract] AND "Cross-Sectional Studies"[MeSH Terms])

Survey data added for GBD 2016 include the Survey of Health, Ageing and Retirement in Europe (SHARE), the Russian Ural Eye and Medical Study, the South Africa National Income Dynamics Study, the South

Africa General Household Survey 2009, and the WHO Study on Global Ageing and Adult Health series (SAGE), among others.

Surveys carried out as part of the International Study of Asthma and Allergies in Childhood (ISAAC) collaboration are the most important source of prevalence data in children.

The following table provides a description of the data density and distribution by geography and epidemiological measure (including the claims data discussed below).

	Prevalence	Incidence	Remission	Other
Site-years (total)	1389	10	32	9
Number of countries with data	136	5	15	6
Number of GBD regions with data (out of 21 regions)	21	1	7	3
Number of GBD super-regions with data (out of 7 super-regions)	7	1	5	3

In addition to literature and survey data, we use claims data from the United States. Information on the source and preparation of these data are provided in detail elsewhere.

Modelling strategy

We use DisMod-MR 2.1 as the main modelling tool for asthma. Prior settings include a maximum remission of 0.3 (reflecting the upper bound of the highest observed data) and no incidence between the ages of 0 and 0.5 year, as a diagnosis cannot be made in young infants.

Data points from the ISAAC studies were reported for both sexes combined. We sex-split before modelling using the ratios derived from the 2012 US claims data.

Data that describe wheezing in the past year but do not report presence/absence of an accompanying diagnosis are crosswalked to the reference category using a study-level covariate in DisMod. As the table below shows, studies that only report wheezing are systematically higher than reference data points and are adjusted down – dividing by the exponentiated coefficient. Data that describe prevalence of lifetime diagnosis of asthma but not accompanying wheezing in the past year are also crosswalked to the reference category using a study-level covariate. For GBD 2016, we allowed DisMod to estimate these coefficients. For GBD 2017 we performed an analysis of World Health Survey microdata to estimate the coefficients and used these values as priors in the DisMod model.

To account for country-level differences in excess mortality as a function of available medical care we use log lag-distributed income (LDI) as a covariate and assume a negative coefficient. The effect size is shown below.

For GBD 2016, claims data for 2000 and 2010 were adjusted via study covariates to account for systematically lower estimates relative to the 2012 claims data. Implicit in this adjustment is the assumption that variation between years of claims data is a function of data-collection inconsistencies. However, an analysis for GBD 2017 showed that even the 2012 claims data were systematically lower than asthma survey data. To account for this, we estimated a MarketScan 2000 coefficient and a separate MarketScan coefficient for the remaining years of data, by comparing the national values in these datasets to national asthma estimates from the USA National Health and Nutrition Examination Survey and National Health Interview Surveys.

Similar to other causes, we include estimates of cause-specific mortality rate (CSMR) and excess mortality rate (EMR) derived as a matched value for each prevalence data point dividing CSMR by prevalence. We restrict these EMR calculations to data points of 20-year age span or less.

To assist estimation, the model includes a series of country-level covariates that describe spatiotemporal patterns. Specifically, we use log LDI and the asthma standardised exposure variable (SEV), a scalar that combines exposure of all GBD risks that influence asthma. A full covariate list, including the study-level covariates described above, are presented in the following table with their associated effects:

Variable name	Measure	Beta	Exponentiated
Wheezing only	prevalence	1.05<br>(1.05–1.05)	2.85<br>(2.85–2.85)
Physician-diagnosed asthma only	prevalence	0.60<br>(0.60–0.60)	1.82<br>(1.82–1.82)
Self-reported currently have asthma	prevalence	0.22<br>(0.16–0.28)	1.24<br>(1.17–1.32)
Self-reported ever having asthma	prevalence	0.24<br>(0.20–0.28)	1.28<br>(1.23–1.32)
Claims data 2000	prevalence	-1.25<br>(-1.25 to -1.25)	0.29<br>(0.29–0.29)
Claims data post-2000	prevalence	-0.79<br>(-0.79 to -0.79)	0.45<br>(0.45–0.45)
Log SEV scalar: asthma	prevalence	0.75<br>(0.75–0.76)	2.13<br>(2.12–2.14)
Log LDI (I$ per capita)	excess mortality rate	-0.5<br>(-0.5 to -0.5)	0.61<br>(0.61–0.61)

Severity split inputs

Lay descriptions and disability weights for the asthma health states are shown in the table below. The distribution between the three health states is derived from an analysis of the USA Medical Expenditure Panel Surveys (MEPS). The methods are described in full in a separate section of this appendix. Briefly, MEPS is an ongoing survey of health service encounters with as its main objective to collect data on health expenditure. Panels are recruited every year and followed up for a period of two years. Diagnostic information provided by respondents on the reasons for any health care contact are coded into three-digit ICD-9 codes by professional coders.

Twice over the two-year follow-up period, respondents are asked to fill in 12-Item Short Form Surveys (SF-12). From convenience samples asking respondents to fill in SF-12 for 60 of the GBD health states, IHME has created a mapping from SF-12 scores to GBD disability weights (DW). We perform a regression with indicator variables for all GBD causes that we can identify from the ICD codes in MEPS to derive for each individual with a diagnosis the amount of disability that can be attributed to that condition after controlling for any comorbid conditions. Anyone with a diagnosis of asthma in whom the disability assigned to asthma is negative or zero we assume is asymptomatic (at the time of asking SF-12 question relating to their health status in the past four weeks). Non-zero values we bin into the three health states assuming a split between these at the midpoint between DW values. The table below gives the proportions in MEPS in each of the health states and an asymptomatic state.

Severity level	Lay description	DW (95% CI)	Severity distribution
Asymptomatic			36.2% (35.0–37.3%)
Controlled	This person has wheezing and cough once a month, which does not cause difficulty with daily activities.	0.015 (0.007–0.026)	19.9% (13.6–27.8%)
Partially controlled	This person has wheezing and cough once a week, which causes some difficulty with daily activities.	0.036 (0.022–0.055)	20.6% (15.1–25.8%)
Uncontrolled	This person has wheezing, cough, and shortness of breath more than twice a week, which causes difficulty with daily activities and sometimes wakes the person at night.	0.133 (0.086–0.192)	23.3% (18.7–30.3%)

Interstitial lung disease and pulmonary sarcoidosis (ILD)

Flowchart

Interstitial Lung Disease and Pulmonary Sarcoidosis (ILD)

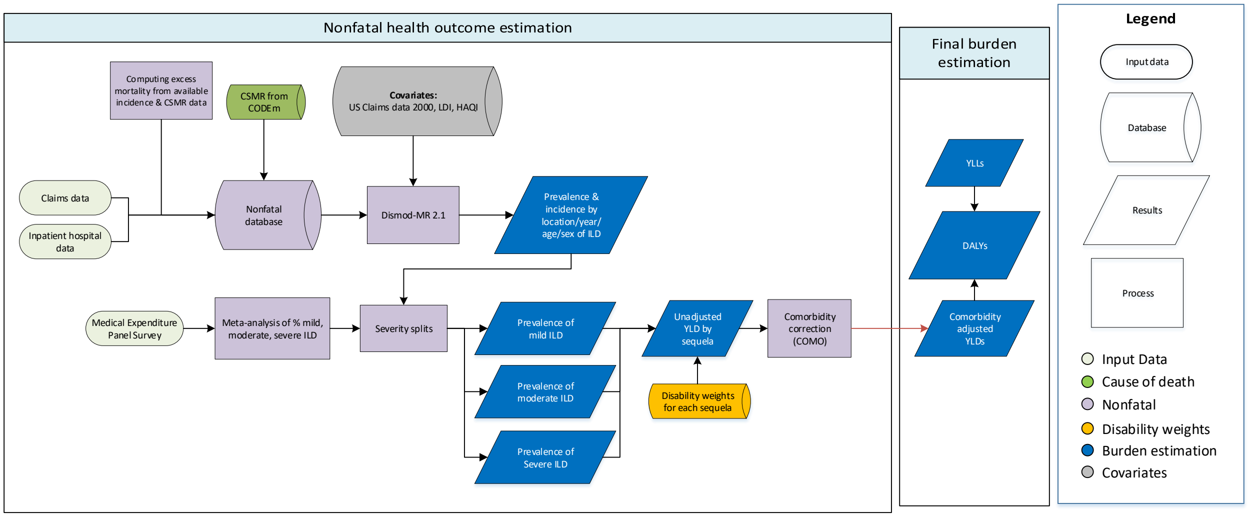

Case definition

Interstitial lung diseases and pulmonary sarcoidosis are a collection of chronic respiratory diseases that impair lung function and oxygen uptake through scarring and/or inflammation. The relevant ICD codes are D86 and J84. For interstitial lung disease, we use the American Thoracic Society as the gold standard definition.

Input data

Model Inputs

No systematic review of the literature was conducted for ILD for this iteration of the Global Burden of Disease. These reviews are done on a rotating basis and updates will be made for a future iteration.

Data used to make estimates of ILD are predominantly from three main sources. The first is literature data from previous systematic reviews – usually from smaller-scale studies of prevalence or incidence. The second main data type is claims data for the United States. The source and preparation of these data is described elsewhere. The third main data type is adjusted hospital inpatient records. Because these records only report primary diagnosis, we a priori adjust the numbers by a sex-specific factor based on the observed ratio between USA claims data and USA inpatient hospital data.

The following table provides a picture of the number of available studies along with their distribution globally and by epidemiological profile. In short, the ILD data landscape is rather sparse. The available data are largely skewed toward high-income countries like the United States or the member countries of

the European Union. The relatively high number of subnational units with data is largely a function of claims data in the United States and hospital data from Mexico and Brazil.

	Prevalence	Incidence	Other
Site-years (total)	1380	54	2
Number of countries with data	39	16	2
Number of GBD regions with data (out of 21 regions)	15	7	2
Number of GBD super-regions with data (out of 7 super-regions)	7	4	2

Severity splits

Data to inform estimates of the severity gradient due to ILD are derived from previously analyses of the Medical Expenditure Panel Survey (MEPS). The table below illustrates the lay descriptions and disability weights associated with different levels of severity of interstitial lung disease.

Severity level	Lay description	DW (95% CI)
Mild	Has cough and shortness of breath after heavy physical activity, but is able to walk long distances and climb stairs.	0.019 (0.011–0.033)
Moderate	Has cough, wheezing, and shortness of breath, even after light physical activity. The person feels tired and can walk only short distances or climb only a few stairs.	0.225 (0.153–0.312)
Severe	Has cough, wheezing, and shortness of breath all the time. The person has great difficulty walking even short distances or climbing any stairs, feels tired when at rest, and is anxious.	0.408 (0.273–0.556)

Modelling strategy

Estimates for ILD are produced using a standard DisMod-MR 2.1 approach. We use prior settings of zero remission and we constrain the super-region random effects to -0.5 to 0.5 to ensure model stability.

As described above, we use an a priori adjustment of hospital inpatient data.

Similar to other causes, we include estimates of cause-specific mortality rate (CSMR) and Excess Mortality Rate (EMR). The source and estimation of these rates are discussed elsewhere.

Variable name	Measure	Beta	Exponentiated
All MarketScan, year 2000	prevalence	-0.25 (-0.27 to -0.23)	0.78 (0.76–0.79)

LDI (I$ per capita)	excess mortality rate	-0.2<br>(-0.2 to -0.2)	0.82<br>(0.82–0.82)
Healthcare Access and Quality index	excess mortality rate	0.012<br>(0.012–0.013)	1.01<br>(1.01–1.01)

A study-level covariate was used for MarketScan 2000 data to adjust for systematically low values. To account for country-level differences in excess mortality (perhaps as a function of available medical care) we use ln(lag distributed income) and Healthcare Access and Quality (HAQ) index as proxy measures. The effect sizes are shown above.

Other chronic respiratory diseases

In addition to the chronic respiratory diseases described above, there are many diverse types of chronic respiratory diseases with a range of severities and associated sequelae. Because these chronic respiratory diseases are diverse in their underlying causes and risk factors as well as in their associated health outcomes, modelling them together in a DisMod-MR model would not produce reliable estimates of prevalence or excess mortality. Instead, we calculated the YLDs caused by other chronic respiratory diseases directly using a YLD/YLL ratio.

We calculated the ratio of YLDs to YLLs across the specified chronic respiratory diseases for which non-fatal outcomes were modelled, using YLL estimates from the GBD 2017 cause of death (CoD) analysis. We then multiplied this YLD/YLL ratio by the YLL estimates for other chronic respiratory diseases from the GBD 2017 CoD analysis, providing us with an estimate of the YLDs associated with other chronic respiratory diseases.

Cirrhosis

Flowchart

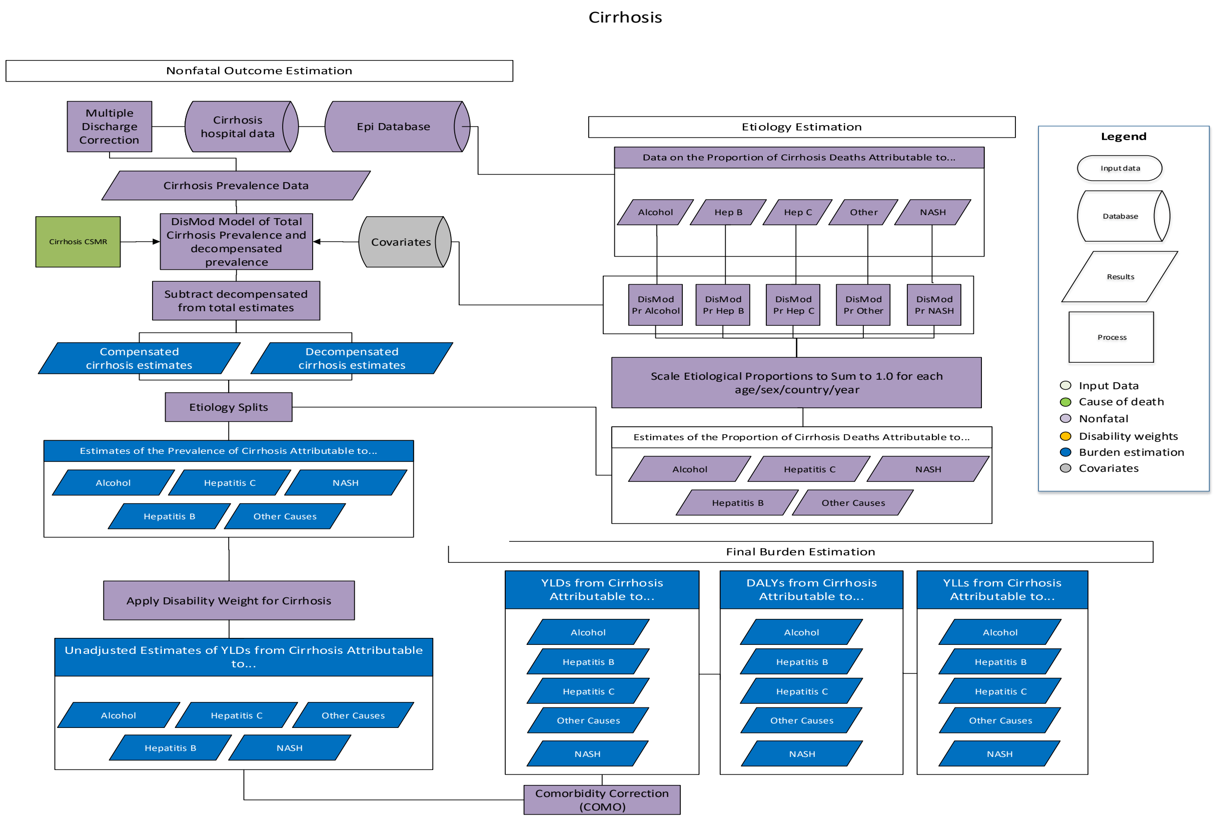

Input data and methodological summary

Case definition

Cirrhosis is a chronic liver disease most often caused by alcohol use or chronic infection with hepatitis B or C. Early disease is typically asymptomatic as the liver's resilience compensates for cirrhotic damage. Decompensated cirrhosis occurs when the disease progresses beyond the capacity of the liver to compensate for the damage, and is marked by profound symptoms, health loss and, often, death. We model decompensated cirrhosis, defined by cirrhosis (or a closely related diagnosis code) as the primary diagnosis in hospital data. This includes ICD1-0 codes K70-K77, I85, P78.81.

Input data

Model inputs

We modelled total cirrhosis prevalence and decompensated cirrhosis based on hospital data and claims data, using different definitions (any diagnoses of cirrhosis and primary diagnoses as cirrhosis, respectively) for each model. The table below indicates the number of site-years used for both the total and decompensated models based on measure and location type.

Total cirrhosis data input

	Incidence	Prevalence
**Site-years (total)**	1	1406
**Number of countries with data**	1	41
**Number of GBD regions with data (out of 21 regions)**	1	16
**Number of GBD super-regions with data (out of 7 super-regions)**	1	7

Decompensated cirrhosis data input

	Incidence	Prevalence
**Site-years (total)**	1	1432
**Number of countries with data**	1	42
**Number of GBD regions with data (out of 21 regions)**	1	16
**Number of GBD super-regions with data (out of 7 super-regions)**	1	7

A systematic review of the literature was conducted to capture studies of the proportion of cirrhosis attributable to alcohol, hepatitis B, hepatitis C, and other causes. In GBD 2017, we added cirrhosis due to non-alcoholic steatohepatitis as a fifth aetiology. We re-extracted all literature within our databases on cirrhosis aetiology proportions for cirrhosis due to NASH. We assigned cases of cryptogenic cirrhosis to the NASH etiology. We searched the peer-reviewed literature via PubMed and solicited sources from GBD collaborators.

The inclusion criteria stipulated that: 1) the publication year must be from 1980 onward; 2) the sample had to be a representative sample of those with decompensated cirrhosis (eg, studies of patients with both HCC and hepatitis were excluded); 3) sufficient information must be provided on study method and sample characteristics to assess the quality of the study; 4) hepatitis B and C were confirmed via HBsAg, in the case of hepatitis B, and anti-HCV IgG, in the case of hepatitis C. The table below summarises the number of site-years of data for each cirrhosis aetiology, and also includes the total number of countries and GBD regions represented.

Cirrhosis aetiology proportion input

	**HepB**	**HepC**	**Alcohol**	**Other**	**NASH**
**Site-years (total)**	76	80	55	34	24

**Number of countries with data**	32	35	23	18	14
**Number of GBD regions with data (out of 21 regions)**	17	17	13	11	10
**Number of GBD super-regions with data (out of 7 super-regions)**	7	7	6	6	5

Modelling strategy

We modelled cirrhosis prevalence using hospital data and CSMR estimates, assuming no remission. To estimate the prevalence of cirrhosis due to alcohol, cirrhosis due to hepatitis B, cirrhosis due to hepatitis C, cirrhosis due to other causes, and cirrhosis due to NASH, we developed aetiological proportion models using DisMod-MR 2.1, and used the results of these models to split the parent total cirrhosis and decompensated cirrhosis prevalence estimates.

Given the similar aetiologies for liver cancer and cirrhosis we integrated the aetiology models for these two causes. We have more data for liver cancer aetiologies than we do for cirrhosis. Therefore, we first developed five single-parameter DisMod models, each to estimate the proportion of liver cancer due to a given cause (ie, alcohol, hepatitis B, hepatitis C, other, and NASH). Estimates from these liver cancer models were then used as covariates in the five corresponding cirrhosis aetiology models. Proportions from the five aetiology models were then rescaled to sum to one at the draw level, and used to split the parent cirrhosis estimates. Listed below are covariate tables for each model: total cirrhosis, decompensated cirrhosis, and the five aetiology proportions, used in the published model for GBD 2017.

Total cirrhosis covariate table

Study covariate	Type	Parameter	Beta	Exponentiated beta
Claims data – 2000	Study level	Prevalence	-1.08 (-1.12 to -1.05)	0.34 (0.33–0.35)
Claims data – Other	Study level	Prevalence	-0.25 (-0.25 to -0.25)	0.78 (0.78–0.78)
Alcohol (LPC)	Country level	Prevalence	0.0055 (0.0021–0.0085)	1.01 (1.00–1.01)
Obesity prevalence	Country level	Prevalence	0.022 (0.0010–0.066)	1.02 (1.00–1.07)
Hep B Seroprevalence	Country level	Prevalence	1.97 (1.88–2.00)	7.18 (6.55–7.38)
Hep C Seroprevalence	Country level	Prevalence	0.12 (0.0047–0.44)	1.13 (1.00–1.56)
Healthcare Access and Quality index	Country level	Excess mortality	-0.03 (-0.042 to -0.027)	0.97 (0.96–0.97)

Decompensated cirrhosis covariate table

Study covariate	Type	Parameter	Beta	Exponentiated beta
Claims data – 2000	Study level	Prevalence	-0.88 (-0.92 to -0.83)	0.42 (0.40–0.43)
Hospital Inpatient Data	Study level	Prevalence	-1.53 (-1.57 to -1.39)	0.22 (0.21–0.25)
Alcohol (LPC)	Country level	Prevalence	0.0074 (0.0044–0.010)	1.01 (1.00–1.01)
Obesity prevalence	Country level	Prevalence	0.63 (0.41–1.06)	1.87 (1.51–2.89)
Hep B Seroprevalence	Country level	Prevalence	1.97 (1.93–2.00)	7.20 (6.89–7.39)
Hep C Seroprevalence	Country level	Prevalence	0.073 (0.0033–0.25)	1.08 (1.00–1.28)
Healthcare access and quality index	Country level	Excess mortality	-0.04 (-0.041 to -0.04)	0.96 (0.96–0.96)

Proportion of cirrhosis due to hepatitis B covariate table

Study covariate	Type	Parameter	Beta	Exponentiated beta
Hep B Seroprevalence	Country level	Proportion	0.53 (0.033–0.97)	1.70 (1.03–2.65)
Proportion of liver cancer due to Hep B	Country level	Proportion	0.62 (0.059–0.98)	1.87 (1.06–2.68)
Hep B 3-dose coverage (10-year lag)	Country level	Proportion	-0.18 (-0.58 to -0.007)	0.84 (0.56–0.99)

Proportion of cirrhosis due to hepatitis C covariate table

Study covariate	Type	Parameter	Beta	Exponentiated beta
Hep C Seroprevalence	Country level	Proportion	0.50 (0.022–0.98)	1.65 (1.02–2.66)
Proportion of liver cancer due to Hep C	Country level	Proportion	0.57 (0.039–0.98)	1.76 (1.04–2.67)

Proportion of cirrhosis due to alcohol covariate table

Study covariate	Type	Parameter	Beta	Exponentiated beta
Alcohol (LPC)	Country level	Proportion	0.022 (0.00080–0.067)	1.02 (1.00–1.07)

Proportion of liver cancer due to alcohol	Country level	Proportion	0.49 (0.022–0.97)	1.63 (1.02–2.64)
Alcohol abstainer proportion, age-standardised	Country-level	Proportion	-0.44 (-0.95 to -0.019)	0.64 (0.39–0.98)

Proportion of cirrhosis due to NASH covariate table

Study covariate	Type	Parameter	Beta	Exponentiated beta
BMI (mean)	Country level	Proportion	0.074 (0.0018–0.25)	1.08 (1.00–1.29)
Obesity (prevalence)	Country level	Proportion	0.81 (0.030–1.92)	2.25 (1.03–6.79)
NAFLD prevalence	Country level	Proportion	0.93 (0.032–1.94)	2.53 (1.03–6.92)

Proportion of cirrhosis due to other causes covariate table

Study covariate	Type	Parameter	Beta	Exponentiated beta
Proportion of liver cancer due to other causes	Country level	Proportion	0.50 (0.026–0.97)	1.65 (1.03–2.64)

Disability weight

Decompensated cirrhosis of each aetiology has one disability weight listed below.

Sequelae	Lay description	DW (95% CI)
Decompensated cirrhosis of the liver	Has a swollen belly and swollen legs. The person feels weakness, fatigue and loss of appetite.	0.178 (0.122–0.25)

Non-alcoholic fatty liver disease

Flowchart

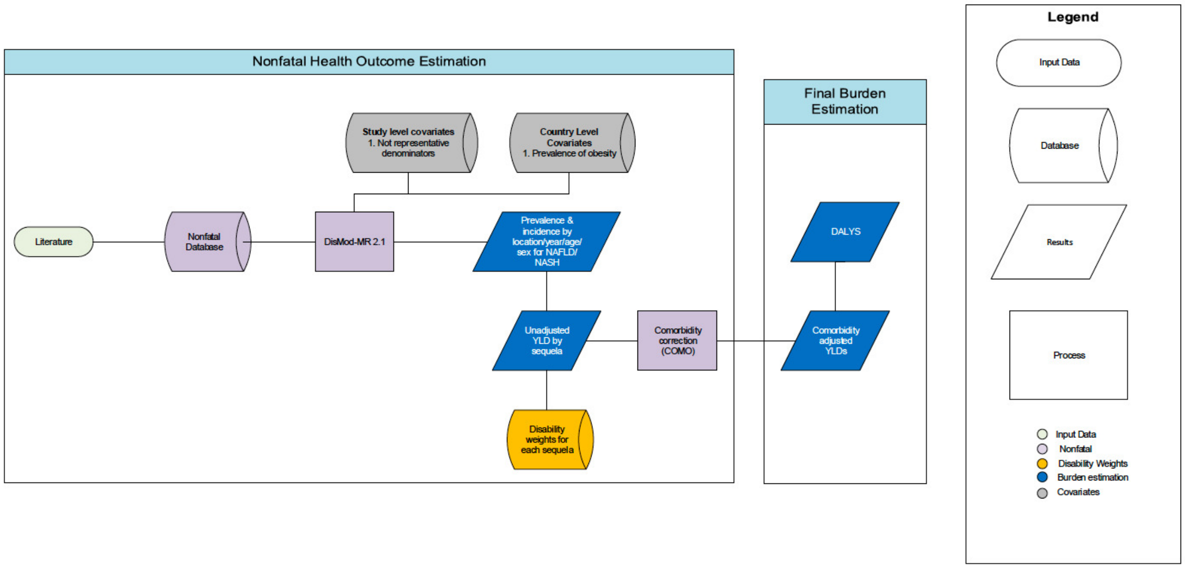

Case definition

Non-alcoholic fatty liver disease (NAFLD) is an umbrella term for a range of liver conditions affecting people who drink little to no alcohol. The main characteristic of non-alcoholic fatty liver disease is an excess of fat stored in liver cells. ICD 10 codes include K76.0

Input data

Model inputs

NAFLD is a new cause added to the hierarchy of digestive diseases in GBD 2017. We performed a systematic literature review using PubMed capturing all causes of NAFLD prevalence from 1990 to 2016. The following inclusion criteria were used:

(1) Sample size greater than 100
(2) Representative of locale or area and sufficient methods
(3) Does not exclude comorbidities
(4) NAFLD diagnosed by ultrasound (USS) or other imaging diagnostic

The majority of NAFLD cases are asymptomatic. Therefore, we excluded any hospital and claims data which severely underestimated NAFLD prevalence. Data were outliered or excluded if we found they differed significantly when compared to regional, super-regional, and global rates. We made ultrasonography our gold standard per expert opinion of our GI collaborators. We excluded any studies using serum diagnostics or fatty liver indexes and scores to diagnose NAFLD. Clinical data from studies for

patients with GI distress, in specialty outpatient clinics, and with comorbid selection processes were excluded. The caveat to this was that we accepted city/region-specific studies for large Asian studies pooling data from general checkups, where USS is a part of the checkup regiment (eg, South Korea, Japan, and some parts of China). There was no significant difference between these studies with population-based random samples.

The tables below show the number of measure specific site-years included in GBD 2017, as well as the number of countries and GBD world regions represented for the NAFLD.

Table 1. NAFLD prevalence inputs

	Incidence	Prevalence
**Site-years (total)**	2	53
**Number of countries with data**	2	18
**Number of GBD regions with data (out of 21 regions)**	2	9
**Number of GBD super-regions with data (out of 7 super-regions)**	2	5

Modelling strategy

We modelled prevalence and incidence of NAFLD using DisMod-MR 2.1. Our prior inputs include zero excess mortality for all ages and zero incidence from age 0-5. By disease definition, many epidemiological studies excluded persons who drink excessive quantities of alcohol or have other chronic liver disease and hepatitis. We first ran models adjusting different definitions of alcohol exclusion toward the most frequently used definition: 70 grams per week for men and 140 grams per week for women. The effect of these adjustments was insignificant and dropped in the final model. We applied a specific age, sex, year and location proportion of <70g/140g alcohol intake to generate results applicable to the general population. Our final estimates are alcohol-adjusted but have not been adjusted for those excluded due to chronic liver disease and hepatitis status. A full list of covariates and coefficients used in the NAFLD model are listed below.

Table 2. Country-level covariates

Country-level covariate	Parameter	beta	Exponentiated beta
BMI (mean)	Prevalence	0.25 (0.13–0.36)	1.28 (1.14–1.43)

Disability weights

There is no associated sequelae or disability weight with NAFLD. Cases of NAFLD are assigned asymptomatic with zero disability weight.

Peptic Ulcer Disease

Flowchart

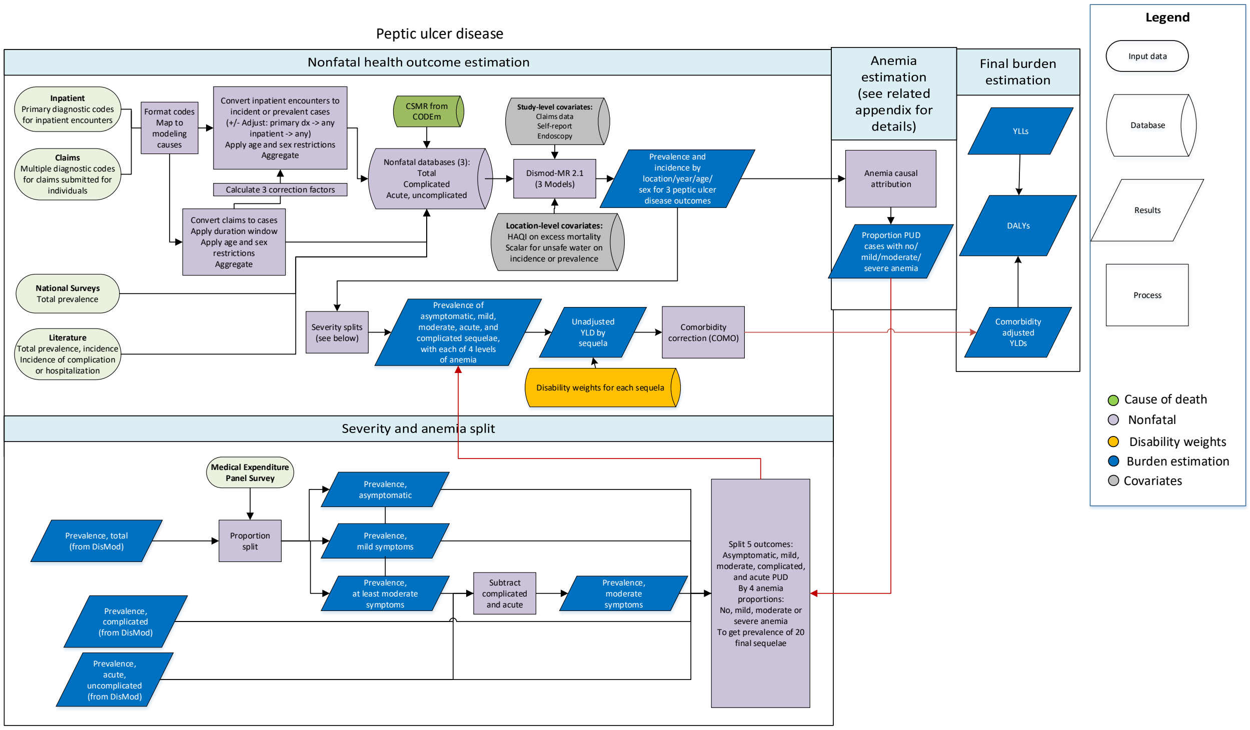

Case definition

Peptic ulcer disease is a digestive disorder defined by defects in the lining of the stomach (gastric ulcers) or the duodenum (duodenal ulcers) that extend through the muscularis mucosa. Gold-standard diagnosis is by endoscopy, although in some cases contrast imaging is sufficient to establish the diagnosis. Peptic ulcers can develop marked abdominal pain acutely or can have a more insidious onset and develop into a chronic disease with asymptomatic and symptomatic periods. Symptomatic periods of chronic peptic ulcer disease are characterised by abdominal pain, bloating, nausea, early satiety, belching, and fatty food intolerance. Regardless of the duration of the disease, acute, life-threatening complications of bleeding, perforation, or gastric outlet obstruction can develop. Chronic gastric ulcer disease predisposes to gastric cancer.

ICD10 codes used to identify cases of peptic ulcer disease are K25, K26, K27, K28, and K31. ICD10 codes for complicated peptic ulcer disease are K25.0-2, K25.4-6, K26.0-2, K26.4-6, K27.0-2, K27.4-6, K28.0-2, and K28.4-6. ICD10 codes for acute peptic ulcer disease without complication are K25.3, K26.3, K27.3 and K28.3. Equivalent ICD9 codes were used where appropriate.

Input data

Model inputs

In GBD 2016, two systematic searches of peer-reviewed literature were conducted using PubMed. The first looked for prevalence and incidence of total peptic ulcer disease and the second looked for studies of the incidence of peptic ulcer disease cases with bleeding, perforation, or hospital admission. The results of these literature searches were included in two databases – one for total peptic ulcer disease and one for symptomatic peptic ulcer disease – along with data from national surveys and facilities and claims data. The details of these databases can be found in the appendix to GBD 2016.

In GBD 2017, three separate databases were developed to model total peptic ulcer disease, peptic ulcer disease with complication (such as haemorrhage or perforation), and peptic ulcer disease, acute, without complication (but with sufficient severity and diagnostic uncertainty to require hospitalisation).

The total peptic ulcer disease dataset included peer-reviewed literature and national surveys from the GBD 2016 total peptic ulcer disease database, international inpatient facility data, and claims data from the United States and Taiwan. In claims data, multiple encounters are linked to individuals and include multiple diagnostic codes per encounter. An individual was extracted from claims data as a prevalent case if they had any peptic ulcer disease code as any diagnosis in one or more inpatient encounters or two or more outpatient encounters. Inpatient encounters were extracted from facilities data if an appropriate code appeared as a discharge diagnosis. Correction factors from claims data were then applied to the inpatient encounters to estimate the number of cases represented by the encounters, adjusting for the fact that some facilities only provide the primary discharge diagnosis, and estimating the number of outpatient cases represented by each inpatient case. The included national surveys reported doctor-confirmed, self-reported diagnoses of peptic ulcer disease. Literature studies included studies that ascertained cases from administrative data using diagnostic codes, from self-report, and from study-specific evaluations including history, physical, and endoscopic exam.

The peptic ulcer disease with complication dataset included the results of the GBD 2016 literature review of peptic ulcer disease with bleeding, perforation, or hospitalisation. It also included data from insurance claims for inpatient encounters and hospital discharge data. Claims were extracted as incident cases, linking multiple encounters for an individual and assuming multiple encounters within a 60-day window represented a single episode. Discharges with an appropriate ICD code as any diagnosis were extracted as encounters and adjusted using a correction factor from claims data to estimate the number of incident cases, and another to account for some sources only providing primary diagnoses.

The peptic ulcer disease, uncomplicated, acute dataset included data from insurance claims for inpatient encounters and hospital discharge data. Claims were extracted as incident cases, linking multiple encounters for an individual and assuming multiple encounters within a 60-day window represented a single episode. Discharges with an appropriate ICD code as any diagnosis were extracted as encounters and adjusted using a correction factor from claims data to estimate the number of incident cases, and another to account for some sources only providing primary diagnoses.

Data from outpatient encounters from facilities in the United States and Sweden were considered for inclusion in the total peptic ulcer disease database, but these data violated established regional trends and age distributions and were excluded. Hospital discharge data were highly heterogeneous; for each source-location-year-sex combination, age-standardised mean was calculated, and the series was excluded if this was 0 or was greater than three times the median absolute deviation above or below the median. Data were further considered for exclusion if relatively high values in young age groups led to

overestimation of subnational pseudo-random effects and poor model fit, or if we found them unreasonable when compared to regional, super-regional, and global rates, but no data for these models met these criteria for exclusion.

The tables below show the number of studies included in GBD 2017, as well as the number of countries and GBD world regions represented.

Total peptic ulcer disease

	Prevalence	Incidence
Site-years (total)	1427	1
Number of countries with data	48	1
Number of GBD regions with data (out of 21 regions)	16	1
Number of GBD super-regions with data (out of 7 super-regions)	7	1

Based on facilities and claims data refresh 8

Peptic ulcer disease, with complication

	Incidence
Site-years (total)	756
Number of countries with data	26
Number of GBD regions with data (out of 21 regions)	13
Number of GBD super-regions with data (out of 7 super-regions)	6

Based on facilities and claims data refresh 8

Peptic ulcer disease, uncomplicated, acute

	Incidence
Site-years (total)	1304
Number of countries with data	40
Number of GBD regions with data (out of 21 regions)	16
Number of GBD super-regions with data (out of 7 super-regions)	7

Based on facilities and claims refresh 8

Severity split & disability weight

The basis of the GBD disability weight survey assessments are lay descriptions of sequelae highlighting major functional consequences and symptoms. The lay descriptions and disability weights for peptic ulcer disease were determined as follows.

Peptic ulcer disease, with complication, and peptic ulcer disease, uncomplicated, acute, were assigned the following lay descriptions and disability weights.

Severity level	Lay description	DW (95% CI)
Peptic ulcer disease, with complication	This person vomits blood and feels nauseous.	0.325 (0.209–0.462)
Peptic ulcer disease, uncomplicated, acute	This person has severe pain in the belly and feels nauseous. The person is anxious and unable to carry out daily activities.	0.324 (0.220–0.442)

Prevalence draws from the total peptic ulcer disease model were divided into asymptomatic, mild, and moderate using proportions derived from the Medical Expenditure Panel Survey (MEPS). It must be noted that the MEPS analysis uses quality-of-life data from individuals who had a health care encounter for peptic ulcer disease within the preceding 12 months, and were interviewed about their quality of life in the preceding four weeks, so the asymptomatic proportion represents those with diagnosed disease who were asymptomatic in a given period of time, not those always asymptomatic who may have peptic ulcer disease on endoscopy if examined for study or screening purposes. After dividing the total prevalence draws by these three proportions, the complicated and uncomplicated, acute prevalence draws were subtracted from the moderate draws. The asymptomatic, mild, and remaining moderate prevalent cases were then assigned the following lay descriptions and disability weights.

Severity level	Lay description	DW (95% CI)
Diagnosed peptic ulcer disease, not in a symptomatic episode	--	0
Mild peptic ulcer disease episode	This person has some pain in the belly that causes nausea but does not interfere with daily activities.	0.011 (0.005–0.021)
Moderate peptic ulcer disease episode	This person has pain in the belly and feels nauseous. The person has difficulties with daily activities.	0.114 (0.080–0.159)

*The numerous sequelae generated from exclusive combinations of anaemia and peptic ulcer disease each contain custom disability weights. More information can be found in the appendix detailing disability weights.

Methods for causal attribution of anaemia due to peptic ulcer can be found elsewhere in the appendix detailing strategies for impairments. Each final combination of sequela of anaemia and peptic ulcer disease underwent exclusivity adjustments to prevent double counting.

Modelling strategy

Total peptic ulcer disease, symptomatic and asymptomatic

The DisMod model for total peptic ulcer disease used prevalence data as described above. Reference data were from hospital discharges and those literature studies that used diagnostic codes for case ascertainment. United States claims data, Taiwanese claims data, self-reported data (from literature and surveys), and data from literature studies that used endoscopy were all marked with study-level covariates. The model employed the function in DisMod-MR 2.1 to pull in cause-specific mortality rate (CSMR) data from CODEm and CoDCorrect analyses and match with prevalence data points for the same geography. The prior value of remission was bounded from 0.1 to 0.5 (a duration of two to ten years), the prior value of excess mortality rate was bounded 0 to 0.1, and the prior value of incidence was bounded to 0 to 0 for those aged 0 to 5 years of age. A location-level covariate for a Healthcare Access and Quality index was applied to excess mortality, and a scaled exposure variable for access to safe water was applied as a location-level covariate on prevalence. Betas and exponentiated values (which can be interpreted as an odds ratio) are shown in the tables below for study-level covariates and location-level covariates.

**Covariate type**	**Covariate**	**Parameter**	**beta**	**Exponentiated beta**
Study-level covariate	Diagnosis by endoscopy	Prevalence	3.75	42.6

			(3.38 to 4.11)	(29.4 to 61.1)
Study-level covariate	Self-reported diagnosis	Prevalence	1.52<br>(1.45 to 1.59)	4.57<br>(4.25 to 4.92)
Study-level covariate	USA claims data	Prevalence	-0.66<br>(-0.69 to -0.62)	0.52<br>(0.50 to 0.54)
Study-level covariate	TWN claims data	Prevalence	4.91<br>(4.68 to 5.00)	136<br>(107 to 148)
Location covariate	Scaled exposure variable for unsafe water	Prevalence	2.98<br>(2.94 to 3.00)	19.7<br>(19.0 to 20.1)
Location covariate	Healthcare Access and Quality index	Excess mortality rate	-0.028<br>(-0.028 to -0.028)	0.97<br>(0.97 to 0.97)

Based on model # 334847

Complicated peptic ulcer disease

The DisMod model for complicated peptic ulcer disease included incidence data as described above. The prior value of incidence was set to 0 from birth to age 5 years, the prior value of excess mortality rate was bounded to 0.1 to 10, and the prior value of remission was bounded to 6 to 13 cases of remission per person-year (disease duration 4 to 8.7 weeks). A location-level covariate for a Healthcare Access and Quality index was applied to excess mortality ratio, and a location-level covariate for the log-transformed age-standardised death rate due to peptic ulcer disease was applied to incidence, but neither of these were found to be predictive.

Covariate type	Covariate	Parameter	beta	Exponentiated beta
Location covariate	Natural log of age-standardised death rate	Incidence	0.00060<br>(0.000058 to 0.0029)	1.00<br>(1.00 to 1.00)
Location covariate	Healthcare Access and Quality index	Excess mortality rate	0.022<br>(-1.88 to 1.95)	1.02<br>(0.15 to 6.99)

Based on model # 338777

Acute peptic ulcer disease, without complication

The DisMod model for acute, uncomplicated peptic ulcer disease included incidence data as described above. Incidence was forced to 0 through age 5 years, the range of prior values on excess mortality rate was bounded to 0 to 0.1, and the range of prior values on remission was bounded to 16.5 to 17.5 cases per person-year (duration of approximately three weeks). Location-level covariates were applied for Healthcare Access and Quality index (on excess mortality rate), log-transformed age-standardised death rate due to peptic ulcer disease (on incidence), and unsafe water (on incidence).

Covariate type	Covariate	Parameter	beta	Exponentiated beta
Location covariate	Healthcare Access and Quality index	Excess mortality rate	-0.49<br>(-1 to 0)	0.61<br>(0.37 to 1.00)

Location covariate	Scaled exposure variable for unsafe water	Incidence	-5<br>(-5 to -5)	0.0067<br>(0.0067 to 0.0068)
Location covariate	Natural log of age-standardised death rate	Incidence	0.000091<br>(0.000012 to 0.00013)	1.00<br>(1.00 to 1.00)

Based on model # 339515

Summary of changes

Compared to GBD 2016, GBD 2017 differs in data inputs and strategy. The peptic ulcer disease database includes more location-years of hospital discharge data and more years of United States claims data, and adds the new source of Taiwanese claims data. Claims data were extracted with a more stringent case definition, in that two outpatient encounters, rather than one, were required to meet the definition of a prevalent case for a given year in claims data; this decreased the prevalent cases in claims data and the magnitude of the correction factor applied to hospital discharge data to estimate outpatient cases. The GBD 2017 model also differed by using hospital discharge data as the reference case.

Additionally, in GBD 2016, two DisMod models were done, one for total peptic ulcer disease and one for peptic ulcer disease, symptomatic episodes, and draws of the two models were subtracted to determine the asymptomatic portion. For the GBD 2016 symptomatic model, encounters in claims and hospital data were extracted as incident symptomatic episodes lasting 4-6 weeks. In GBD 2017, total, complicated, and acute, uncomplicated disease requiring hospitalisation were explicitly modelled in DisMod. Draws from the total model were divided into asymptomatic, mild, and at least moderate disease using proportions and disability weights derived from MEPS; the use of MEPS to derive disability weights is derived in a separate appendix, but in brief, MEPS used a general population sample to identify individuals who had health care encounters of any kind in the preceding 12 months with peptic ulcer disease codes. These individuals responded to a validated quality-of-life instrument with reference to the four preceding weeks, and disability weights were derived from these responses.

Gastritis and duodenitis

Flowchart

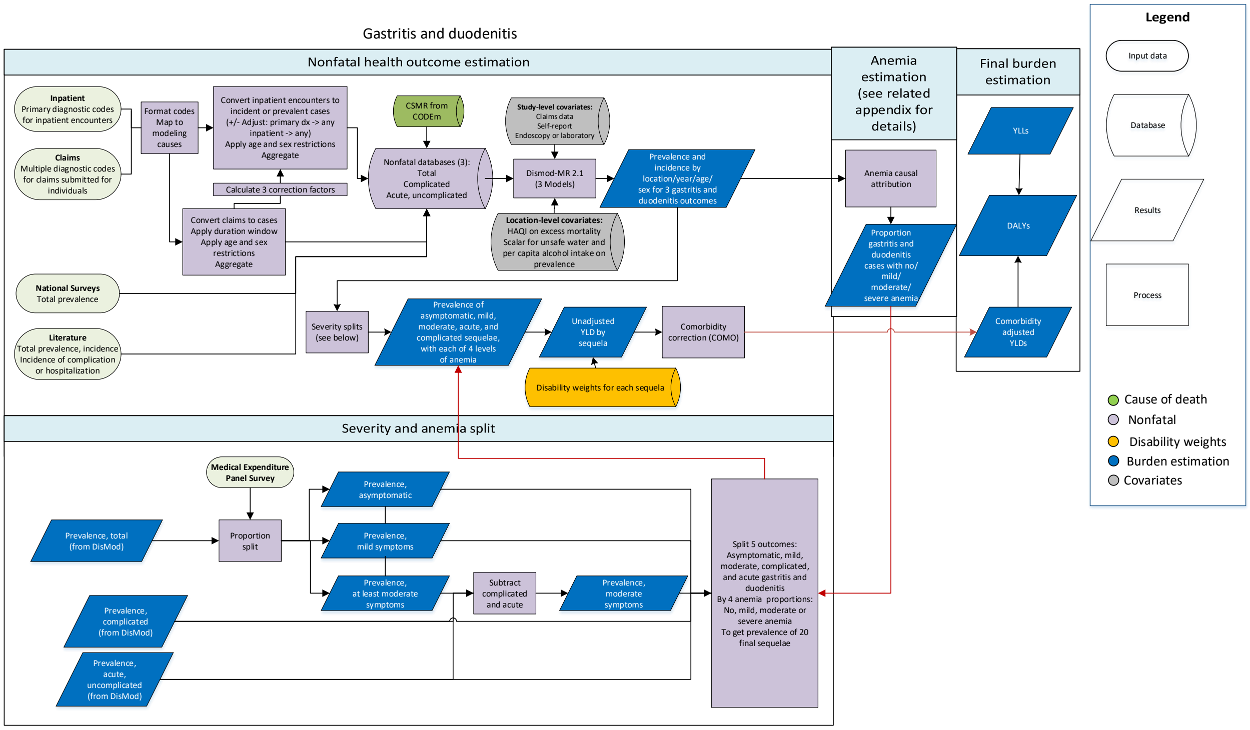

Case definition

Gastritis and duodenitis refer to inflammation of the lining of the stomach and duodenum, respectively, often with damage to epithelial cells lining the gut that is visible via endoscope. Gold standard diagnosis is by biopsy, although a number of biochemical and microbiological tests have good predictive value. This inflammation can acutely produce severe symptoms, or have a subtle onset and evolve into a chronic illness characterised by asymptomatic periods and periods of abdominal pain, bloating, nausea, early satiety, and belching. Complications such as haemorrhage may develop. Chronic gastritis predisposes to gastric cancer.

The ICD10 code for gastritis and duodenitis is K29. ICD10 codes for complicated gastritis and duodenitis are K29.01, K29.21, K29.31, K29.41, K29.51, K29.61, K29.71, K29.81, K29.91. ICD10 codes for acute gastritis are K29.0, K 29.00, K29.1, K29.2, and K29.20. Equivalent ICD9 codes were used where appropriate.

Input data

Model inputs

In GBD 2017, three separate databases were developed to model total gastritis and duodenitis, gastritis and duodenitis with complication (such as haemorrhage), and gastritis and duodenitis, acute, without complication (but with sufficient severity and diagnostic uncertainty to require hospitalisation).

The total gastritis and duodenitis dataset included peer-reviewed literature from a systematic review performed for GBD 2016, international inpatient facility data, and claims data from the United States and Taiwan. In claims data, multiple encounters are linked to individuals and include multiple diagnostic codes per encounter. An individual was extracted from claims data as a prevalent case if they had any gastritis or duodenitis code as any diagnosis in one or more inpatient encounters or two or more outpatient encounters. Inpatient encounters were extracted from facilities data if an appropriate code appeared as a discharge diagnosis. Correction factors from claims data were then applied to the inpatient encounters to estimate the number of cases represented by the encounters, adjusting for the fact that some facilities only provide the primary discharge diagnosis, and estimating the number of outpatient cases represented by each inpatient case. Literature studies included studies that ascertained cases from administrative data using diagnostic codes, from self-report, and from study-specific evaluations including some combination of history, physical, serum tests (for pepsinogen, gastrin, or *Helicobacter pylori* antibodies), and endoscopic exam.

The gastritis and duodenitis, with complication dataset included insurance claims for inpatient encounters and hospital discharges. Claims were extracted as incident cases, linking multiple encounters for an individual and assuming multiple encounters within a 60-day window represented a single episode. Discharges with an appropriate ICD code as a primary diagnosis were extracted as encounters and adjusted using a correction factor from claims data to estimate the number of incident cases.

The gastritis and duodenitis, uncomplicated, acute dataset included insurance claims for inpatient encounters and hospital discharges. Claims were extracted as incident cases, linking multiple encounters for an individual and assuming multiple encounters within a 60-day window represented a single episode. Discharges with an appropriate ICD code as a primary diagnosis were extracted as encounters and adjusted using a correction factor from claims data to estimate the number of incident cases.

Data from outpatient encounters from facilities in the United States and Sweden were considered for inclusion in the total gastritis and duodenitis database, but these data violated established regional trends and age distributions and were excluded. Hospital discharge data were highly heterogeneous; for each source-location-year-sex combination, age-standardised mean was calculated, and the series was excluded if this was zero or was greater than three times the median absolute deviation above or below the median. Further data were excluded if relatively high values in young age groups led to overestimation of subnational pseudo-random effects and poor model fit, or if we found them unreasonable when compared to regional, super-regional, and global rates.

The tables below show the number of studies included in GBD 2017, as well as the number of countries and GBD world regions represented.

Total gastritis and duodenitis

	Prevalence
Site-years (total)	1402
Number of countries with data	47
Number of GBD regions with data (out of 21 regions)	17
Number of GBD super-regions with data (out of 7 super-regions)	7

Based on facilities and claims data refresh 8

Gastritis and duodenitis, with complication

	Incidence
Site-years (total)	285
Number of countries with data	2
Number of GBD regions with data (out of 21 regions)	2
Number of GBD super-regions with data (out of 7 super-regions)	1

Based on facilities and claims data refresh 8

Gastritis and duodenitis, uncomplicated, acute

	Incidence
Site-years (total)	730
Number of countries with data	21
Number of GBD regions with data (out of 21 regions)	13
Number of GBD super-regions with data (out of 7 super-regions)	6

Based on facilities and claims data refresh 8

Severity split & disability weight

The basis of the GBD disability weight survey assessments are lay descriptions of sequelae highlighting major functional consequences and symptoms. The lay descriptions and disability weights for gastritis and duodenitis were determined as follows.

Gastritis and duodenitis, with complication, and gastritis and duodenitis, uncomplicated, acute, were assigned the following lay descriptions and disability weights.

Severity level	Lay description	DW (95% CI)
Gastritis and duodenitis, with complication	This person vomits blood and feels nauseous.	0.325 (0.209–0.462)
Gastritis and duodenitis, acute	This person has severe pain in the belly and feels nauseous. The person is anxious and unable to carry out daily activities.	0.324 (0.220–0.442)

Prevalence draws from the total gastritis and duodenitis model were divided into asymptomatic, mild, and moderate using proportions derived from the Medical Expenditure Panel Survey (MEPS). It must be noted that the MEPS analysis uses quality-of-life data from individuals who had a health care encounter for gastritis and duodenitis within the preceding 12 months and were interviewed about their quality of life in the preceding four weeks, so the asymptomatic proportion represents those with diagnosed disease who were asymptomatic in a given period of time, not those always asymptomatic who may have gastritis and duodenitis on lab tests or endoscopy if examined for study or screening purposes. After dividing the total prevalence draws by these three proportions, the complicated and uncomplicated acute prevalence draws were subtracted from the moderate draws.

The asymptomatic, mild, and remaining moderate prevalent cases were then assigned the following lay descriptions and disability weights.

Severity level	Lay description	DW (95% CI)
Diagnosed gastritis and duodenitis, not in a symptomatic episode	--	0
Mild gastritis and duodenitis episode	This person has some pain in the belly that causes nausea but does not interfere with daily activities.	0.011 (0.005–0.021)
Moderate gastritis and duodenitis episode	This person has pain in the belly and feels nauseous. The person has difficulties with daily activities.	0.114 (0.080–0.159)

*The numerous sequelae generated from exclusive combinations of anaemia and gastritis and duodenitis each contain custom disability weights. More information can be found in the appendix detailing disability weights.

Methods for causal attribution of anaemia due to gastritis and duodenitis can be found elsewhere in the appendix detailing strategies for impairments. Each final combination of sequela of anaemia and gastritis and duodenitis underwent exclusivity adjustments to prevent double counting.

Modelling strategy

The DisMod model for total gastritis and duodenitis used prevalence data as described above, and the function in DisMod-MR 2.1 to pull in cause-specific mortality rate (CSMR) data from CODEm and CoDCorrect analyses and match with prevalence data points for the same geography. Reference data for prevalence were adjusted hospital discharge data and literature studies that ascertained cases using diagnostic codes. Claims data, literature that ascertained cases by self-report, and literature that ascertained cases by laboratory exams or endoscopy were marked with study-level covariates. Prior value of remission was bounded from 0 to 1 (a minimum duration of one year), and prior value of excess mortality rate was bounded from 0 to 0.1. Location-level covariates for alcohol consumption and access to safe water were applied to prevalence, which we forced positive with a lower bound of 0 on the priors. Additionally, a Healthcare Access and Quality index was applied as a location-level covariate on excess mortality rate.

Betas and exponentiated values (which can be interpreted as odds ratios) are shown in the table below for study-level and location-level covariates.

**Covariate type**	**Covariate**	**Parameter**	**beta**	**Exponentiated beta**
Study-level covariate	Diagnosis by endoscopy, biopsy, and/or serology	Prevalence	2.00 (1.84 to 2.16)	7.40 (6.33 to 8.67)
Study-level covariate	Self-reported	Prevalence	-1.06 (-2.13 to -0.083)	0.35 (0.12 to 0.92)
Study-level covariate	USA claims	Prevalence	-1.01 (-1.04 to -0.97)	0.36 (0.35 to 0.38)
Study-level covariate	TWN claims	Prevalence	-2.17 (-4.77 to -0.31)	0.11 (0.0085 to 0.73)
Location covariate	Alcohol	Prevalence	0.0014	1.00

	(litres per capita)		(0.000057 to 0.0036)	(1.00 to 1.00)
Location covariate	Scaled exposure variable for unsafe water access	Prevalence	4.97 (4.92 to 5.00)	144 (137 to 148)
Location covariate	Healthcare Access and Quality index	Excess mortality rate	-0.033 (-0.033 to -0.032)	0.97 (0.97 to 0.97)

Based on model # 334739

Complicated gastritis and duodenitis

The DisMod model for complicated gastritis and duodenitis included incidence data as described above. The prior value of incidence was bounded to 0 to 0.3, the prior value of excess mortality rate was bounded to 0.1 to 10, and the prior value of remission was bounded to 6 to 13 cases of remission per person-year (disease duration 4 to 8.7 weeks). A location-level covariate for a Healthcare Access and Quality index was applied to excess mortality ratio, and location-level covariates for the log-transformed age-standardised death rate due to gastritis and duodenitis, per capita alcohol consumption, and unsafe water access were applied to incidence.

Betas and exponentiated values (which can be interpreted as odds ratios) are shown in the table below for study-level and location-level covariates.

Covariate type	Covariate	Parameter	beta	Exponentiated beta
Location covariate	Natural log of age-standardised death rate	Incidence	0.000088 (0.000013 to 0.00041)	1.00 (1.00 to 1.00)
Location covariate	Alcohol (litres per capita)	Incidence	0.0016 (0.000056 to 0.0055)	1.00 (1.00 to 1.00)
Location covariate	Scaled exposure variable for unsafe water access	Incidence	2.97 (2.91 to 3.00)	19.5 (18.3 to 20.1)
Location covariate	Healthcare Access and Quality index	Excess mortality rate	-0.5 (-1 to 0)	0.61 (0.37 to 1.00)

Based on model # 346799

Acute gastritis and duodenitis, without complication

The DisMod model for acute, uncomplicated gastritis and duodenitis included incidence data as described above. The reference case was adjusted hospital discharge data and Taiwanese claims data, and a study-level covariate was applied to United States claims data. Incidence was forced to 0 through age 5 years, the range of prior values on excess mortality rate was bounded to 0 to 0.1, and the range of prior values on remission was bounded to 6 to 13 cases per person-year. Location-level covariates were applied for log-transformed, lag-distributed income (on excess mortality rate), log-transformed age-standardised

death rate due to gastritis and duodenitis (on incidence), and for per capita alcohol consumption (on incidence).

Betas and exponentiated values (which can be interpreted as odds ratios) are shown in the tables below for study-level and location-level covariates.

Covariate type	Covariate	Parameter	beta	Exponentiated beta
Location covariate	Log-transformed lag-distributed income	Excess mortality rate	-0.51 (-1 to -0.0017)	0.60 (0.37 to 1.00)
Location covariate	Alcohol (litres per capita)	Incidence	0.055 (0.048 to 0.061)	1.06 (1.05 to 1.06)
Location covariate	Natural log of age-standardised death rate	Incidence	0.0012 (0.0000099 to 0.0039)	1.00 (1.00 to 1.00)

Based on model # 330032

Summary of changes

Compared to GBD 2016, GBD 2017 differs in data inputs and strategy. The total gastritis and duodenitis database includes more location-years of hospital discharge data and more years of United States claims data, and adds the new source of Taiwanese claims data. Claims data were extracted with a more stringent case definition, in that two outpatient encounters, rather than one, were required to meet the definition of a prevalent case for a given year in claims data; this decreased the total prevalent cases in claims data. Hospital discharge data preparation was improved; in GBD 2016, hospital encounters with gastritis and duodenitis ICDs as any diagnosis were adjusted to estimate individuals from encounters and adjusted to reflect that some facilities only provide primary diagnosis codes. However, there was no adjustment made to estimate outpatient cases, so this data input only captured hospitalised cases. In GBD 2017, the correction factors used to estimate outpatient cases from hospital discharges were improved as described in the related appendix, and this new correction factor was applied to hospital data for total gastritis and duodenitis; this increased the total prevalence in hospital data. The GBD 2017 model also differed by using hospital discharge data as the reference case.

Additionally, in GBD 2016, two DisMod models were done, one for total gastritis and duodenitis and one for gastritis and duodenitis, symptomatic episodes, and draws of the two models were subtracted to determine the asymptomatic portion. For the GBD 2016 symptomatic model, encounters in claims and hospital data were extracted as incident symptomatic episodes lasting 4-6 weeks, and hospital data were not adjusted to estimate outpatient episodes. In GBD 2017, total, complicated, and acute, uncomplicated disease requiring hospitalisation were explicitly modelled in DisMod. Draws from the total model were divided into asymptomatic, mild, and at least moderate disease using proportions and disability weights derived from MEPS; the use of MEPS to derive disability weights is derived in a separate appendix, but in brief, MEPS used a general population sample to identify individuals who had health care encounters of any kind in the preceding 12 months with gastritis or duodenitis codes. These individuals responded to a validated quality-of-life instrument with reference to the four preceding weeks, and disability weights were derived from these responses.

Gastroesophageal reflux disease

Flowchart

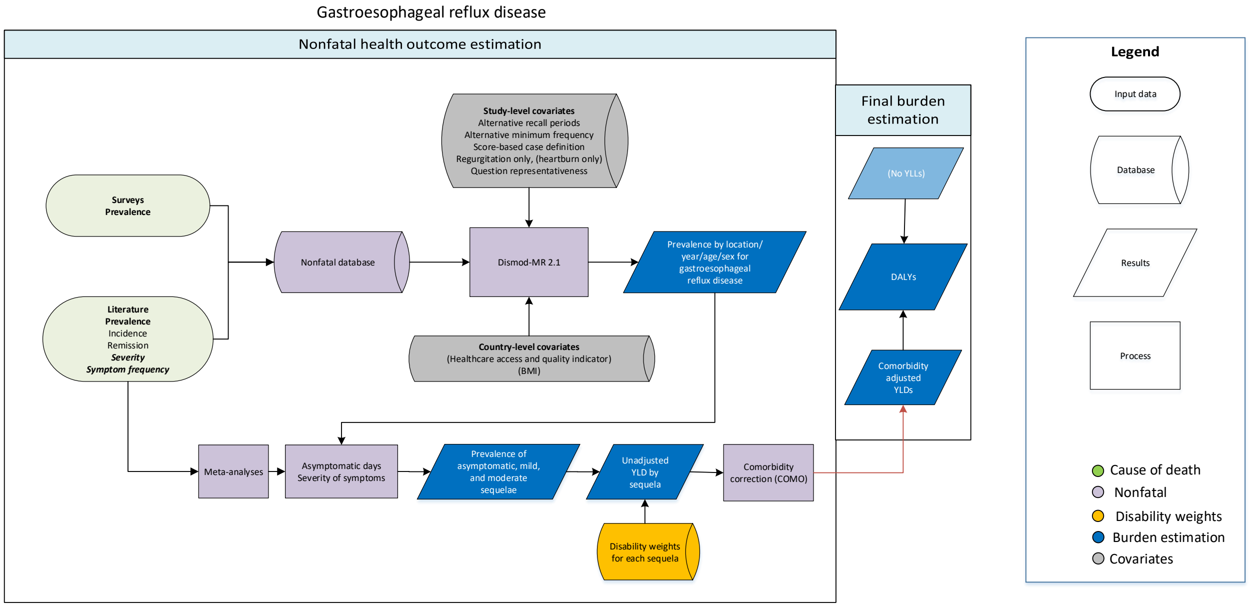

Case definition

Gastroesophageal reflux disease (GERD) is a digestive disorder that develops when the reflux of stomach contents causes troublesome symptoms, complications, or both. The cardinal symptoms of typical GERD are heartburn (a burning feeling behind the breastbone) and regurgitation (the unpleasant sensation of material moving upward from the stomach toward the mouth). In GBD 2017 individuals with heartburn, regurgitation, or both, at least once weekly over a six- to 12-month recall period were regarded as having GERD.

Individuals who experience oesophageal complications (ulceration, metaplasia, etc.) without symptoms, whose sole symptom of gastroesophageal reflux is chest pain without typical reflux symptoms, or who experience reflux primarily as a trigger or exacerbating factor in respiratory or head and neck diseases (chronic cough, dental erosion, etc.) are not included. This strategy avoids double-counting disability already attributed to other underlying diseases modelled in GBD. Likewise, we regarded newborn reflux as a separate disease, which is modelled elsewhere and excluded from this analysis.

Input data

Model inputs

GERD was not modelled in rounds of GBD prior to 2017. A systematic review of peer-reviewed literature was conducted via PubMed to capture studies of GERD prevalence, incidence, and remission. The search terms were:

("gastroesophageal reflux disease"[MeSH Terms]
OR "heartburn"[MeSH Terms]
OR "GERD"[Title/Abstract]

OR "gastroesophageal reflux disease"[Title/Abstract]
OR "heartburn"[Title/Abstract]
OR "GORD"[Title/Abstract]
OR "GER"[Title/Abstract]
OR "acid reflux"[Title/Abstract]
OR "barett's esophagus"[Title/Abstract]
OR "reflux disease"[Title/Abstract]
OR "hiatal hernia"[Title/Abstract]
OR **("achalasia"[Title/Abstract] AND "cardia"[Title/Abstract]**)
OR (
("gastroesophageal"[Title/Abstract] OR "gastro oesophageal"[Title/Abstract] OR "esophagus"[Title/Abstract] OR "esophageal"[Title/Abstract])
AND ***("hernia"[Title/Abstract] OR "ulcer"[Title/Abstract] OR "obstruction"[Title/Abstract] OR "perforation"[Title/Abstract] OR "laceration"[Title/Abstract] OR "dyskinesia"[Title/Abstract] OR "diverticulum"[Title/Abstract])***
)

AND ("prevalence"[Title/Abstract] OR "incidence"[Title/Abstract)
AND ("1990/01/01"[PDAT] : "2017/07/26"[PDAT])
NOT(animals[MeSH] NOT humans[MeSH])

)

Studies were included if cases were identified from a general population defined only by location and years of observation, or the source population was arguably representative of such a population, and results included one or more estimates of prevalence, incidence, or remission of GERD. Due to diverse case definitions encountered, studies that defined cases based on symptom-based questionnaires, self-reported diagnosis, diagnostic codes in facilities or claims data, or using one of several diagnostic tests (pH manometry, oesophagoduodenoscopy, trial of empiric therapy) were all included, but studies had to include sufficient information on case definition and diagnostics to identify those needing adjustment to our standard case-definition. Studies that were conducted in a clinically or demographically defined subpopulation or were otherwise non-representative, or where source population was not adequately described, were excluded. Studies that did not provide sufficient uncertainty information were excluded (percent prevalence reported without sample size, standard deviation, or confidence interval). All studies of incidence and remission provided inadequate information on person-time of observation and loss to follow-up, and so they were excluded. Some studies could not be extracted due to lack of translation. Modelling studies and meta-analyses were not included as input data.

The USA National Health Interview Surveys in 2007 and 2012 asked participants about the occurrence of typical reflux symptoms, and these data were included in our analysis.

Due to the indolent course of illness and widespread availability of effective over-the-counter medications to treat typical reflux symptoms, clinical data from outpatient or inpatient facilities and claims data were considered poorly representative of GERD and were not used in this analysis.

Data were marked as outliers and excluded if relatively high values in young age groups led to overestimation of subnational pseudo-random effects and poor model fit, or if we found them unreasonable when compared to regional, super-regional, and global rates.

Gastroesophageal reflux disease

	Prevalence
Site-years (total)	140
Number of countries with data	39
Number of GBD regions with data (out of 21 regions)	11
Number of GBD super-regions with data (out of 7 super-regions)	6

Severity split & disability weight

Throughout the literature, the severity of GERD is often divided into three or four categories, using definitions such as those in the table below. We reviewed the studies in the literature review above (population-based studies of prevalence) and, if provided, extracted counts of cases of each severity as reported. These cases were then mapped to one of two GBD 2017 GERD severities (also shown in the table below). These categories were mapped to GBD health states, which are associated with disability weights. The basis of the GBD disability weight survey assessments are lay descriptions of sequelae highlighting major functional consequences and symptoms, also shown below. The proportion of cases in each of the GBD 2017 GERD severities was calculated for the pooled total cases, along with standard deviations based on a simple proportion model.

Literature severity levels	GBD severity level	Lay description
Mild: can be ignored	Mild/moderate	Often has a burning sensation in the back of the chest after eating
Moderate: cannot be ignored but does not affect lifestyle	Mild/moderate	Often has a burning sensation in the back of the chest after eating
Severe: affects lifestyle	Severe (abdom_mod)	Has pain in the belly* and feels nauseous. Has difficulty with daily activities.
Very severe: has marked effect on lifestyle	Severe (abdom_mod)	Has pain in the belly* and feels nauseous. Has difficulty with daily activities.

*We acknowledge that gastroesophageal reflux symptoms are felt in the chest, not the belly, but opine that a health state that incorporates other gastrointestinal symptoms and indicates interference with daily activities, such as difficulty eating and sleeping, better represents more severe gastroesophageal reflux disease than a health state that describes only post-prandial heartburn.

Many studies in the literature also report the frequency of GERD symptoms as the proportions of cases in each of a set of mutually exclusive and collectively exhaustive frequency categories. Examples include: 1-6 days/week and daily; 1 day/week, 2-6 days/week and daily; 1-3 days/week, 4-6 days/week and daily; etc. For each study, for each frequency category, 1,000 proportion draws were generated using a beta distribution with case counts in and out of the frequency category as shape parameters. These proportion draws were combined with the assumption that the number of days symptomatic within a category are uniformly distributed to produce draws of the mean number of days/week symptomatic across all cases in a study. Means and standard deviations of these draws were combined in a meta-analysis, and final mean and standard deviation were divided by 7 to estimate the proportion of cases symptomatic on a given day, with uncertainty.

Severity and frequency distributions were determined only for cases that already met minimum severity and frequency criteria to meet our reference case definition, as given above. Severity and frequency categories were combined to generate four categories, as shown below.

GBD severity-frequency category	DW (95% CI)
Mild/moderate GERD, asymptomatic days	None
Mild/moderate GERD, symptomatic days	0.027 (0.015–0.046)
Severe GERD, asymptomatic days	None
Severe GERD, symptomatic days	0.114 (0.080–0.159)

Modelling strategy

A full compartmental model of GERD epidemiology was developed using DisMod-MR 2.1. Only prevalence data were included. Excess mortality was assumed a priori to be 0, and remission prior was set to 0.2 to 0.5 cases per person-year. Incidence was forced to 0 from birth to age 5 years, and after this age prior was set to 0 to 0.2 cases per person-year. We included study-level covariates for alternative recall periods (unspecified, 3 months, or 1 month or less), for alternative minimum symptom frequencies (no minimum frequency, frequency once per month or more), for use of a score-based case-definition that synthesised severity, number, and frequency of symptoms, for use of a case-definition based on a single cardinal reflux symptom (regurgitation only), for studies where the sample's representativeness was considered questionable, and for data extracted from a report from a national survey, rather than a peer-reviewed publication. We considered study-level covariates for six-month versus 12-month recall, but these were not found to be predictive. We also considered country-level covariates for mean body-mass index and for Healthcare Access and Quality index, but these were also not predictive after accounting for study design variation, so were removed from the model. Betas and exponentiated values (which can be interpreted as odds ratios) are shown in the tables below for study-level covariates and country-level covariates.

**Covariate type**	**Covariate**	**Parameter**	**Beta**	**Exp(beta)**
Study-level	Recall unspecified	Prevalence	0.087<br>(-0.13 to 0.28)	1.09<br>(0.88 to 1.32)
Study-level	Recall <= 1 month	Prevalence	-0.65<br>(-0.96 to -0.35)	0.52<br>(0.38 to 0.70)
Study-level	Recall 3 months	Prevalence	-0.3<br>(-0.55 to -0.066)	0.74<br>(0.58 to 0.94)
Study-level	No minimum symptom frequency	Prevalence	0.57<br>(0.32 to 0.83)	1.76<br>(1.37 to 2.30)
Study-level	Once monthly or more frequent symptoms	Prevalence	0.22<br>(-0.12 to 0.57)	1.24<br>(0.89 to 1.78)
Study-level	Diagnostic score	Prevalence	0.66<br>(0.41 to 0.91)	1.94<br>(1.51 to 2.48)
Study-level	Only asked about regurgitation	Prevalence	-0.62<br>(-0.93 to -0.31)	0.54<br>(0.39 to 0.73)
Study-level	Representativeness questionable	Prevalence	0.19<br>(-0.014 to 0.39)	1.21<br>(0.99 to 1.48)
Study-level	Survey data	Pravalence	-0.39<br>(-0.79 to -0.036)	0.67<br>(0.045 to 0.97)

Based on model # 331784

Appendicitis

Flowchart

Appendicitis

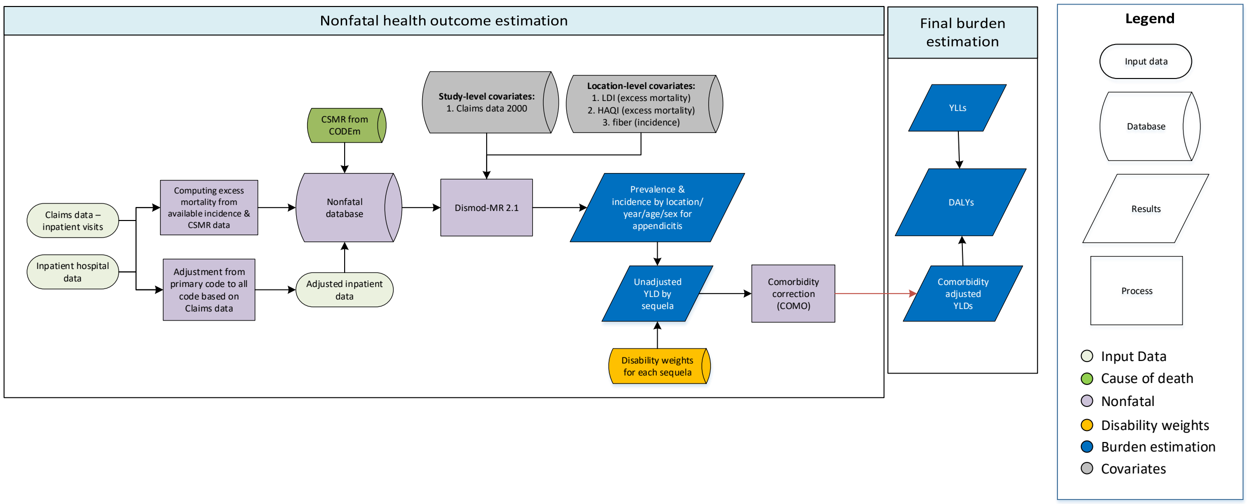

Case definition

Appendicitis is an inflammation of the appendix that causes nausea, vomiting, and sharp pain in the right lower abdomen. Appendicitis requires surgery, or septic shock may set in and the patient will be at risk for severe complications, including sepsis and death. ICD-10 codes included are K35-K35.3, K35.8, K35.80, K35.89, K35.9, K36, K36.0, K37, K37.0, K37.9, and K38.3.

Input data

Model inputs

For GBD 2017, the data used for appendicitis are hospital inpatient data, USA claims data for 2000 and 2010 through 2014 by USA state, and Taiwan claims data for 2016. Descriptions of search strategies for hospital and claims data are included elsewhere in the appendix. The agreed-upon approach for appendicitis was to rely primarily on these data sources and not conduct a formal literature review. Data were outliered or excluded if we found them unreasonable when compared to regional, super-regional, and global rates.

Table 1. Site-years of data by different measures and geographical coverage

	Incidence
Site-years (total)	1408
Number of countries with data	43
Number of GBD regions with data (out of 21 regions)	16
Number of GBD super-regions with data (out of 7 super-regions)	7

Severity splits

The basis of the GBD disability weight survey assessments are lay descriptions of sequelae highlighting major functional consequences and symptoms.

Table 2. Severity level and lay description.

Severity split	Lay description	DW (95% CI)
Appendicitis, severe	This person has severe pain in the belly and feels nauseated. The person is anxious and unable to carry out daily activities.	0.324 (0.219–0.442)

Modelling strategy

DisMod-MR 2.1, a Bayesian meta-regression tool, was used to estimate appendicitis prevalence by age, sex, year, and geography (subnational [select countries], country, region, super-region). Prior settings in the DisMod model included bounding remission from 25 to 27 (a duration of about two weeks) for all age groups and capping excess mortality at 0.31. We used study-level covariates to adjust incidence derived from USA claims data for 2000 toward the level of other incidence data points, which were more representative of the general population.

We used the function in DisMod-MR 2.1 to pull in cause-specific mortality rate (CSMR) data from our CODEm and CODcorrect analyses and match with prevalence data points for the same geography. We calculated excess mortality rate to estimate priors by dividing CSMR by prevalence. We also applied a fibre (g per day) country-level covariate to incidence, forcing a positive relationship with a lower bound of 0. A lag-distributed income (LDI) covariate was applied to excess mortality, log-transformed and forced negative with an upper bound of 0 and a lower bound of -1. Similarly, a Healthcare Access and Quality index (HAQI) covariate was also forced negative (-2, 0) on excess mortality.

Betas and exponentiated values (which can be interpreted as an odds ratio) are shown in the tables below for study-level covariates and country-level covariates:

Table 3. Study-level beta and exponentiated values

Covariate	Parameter	beta	Exponentiated beta
Inpatient-only MarketScan, year 2000	Incidence	0.057 (0.019–0.096)	1.06 (1.02–1.10)

Table 4. Location-level beta and exponentiated values

Covariate	Parameter	beta	Exponentiated beta
Fibre adjusted (g)	Incidence	-0.033 (-0.036 to -0.029)	0.97 (0.96–0.97)
LDI (I$ per capita)	Excess mortality rate	-0.00072 (-0.0021 to -0.000075)	1.00 (1.00–1.00)
Healthcare Access and Quality index	Excess mortality rate	-0.0045 (-0.0052 to -0.0037)	1.00 (0.99–1.00)

Paralytic ileus and intestinal obstruction

Flowchart

Paralytic ileus and intestinal obstruction (ileus)

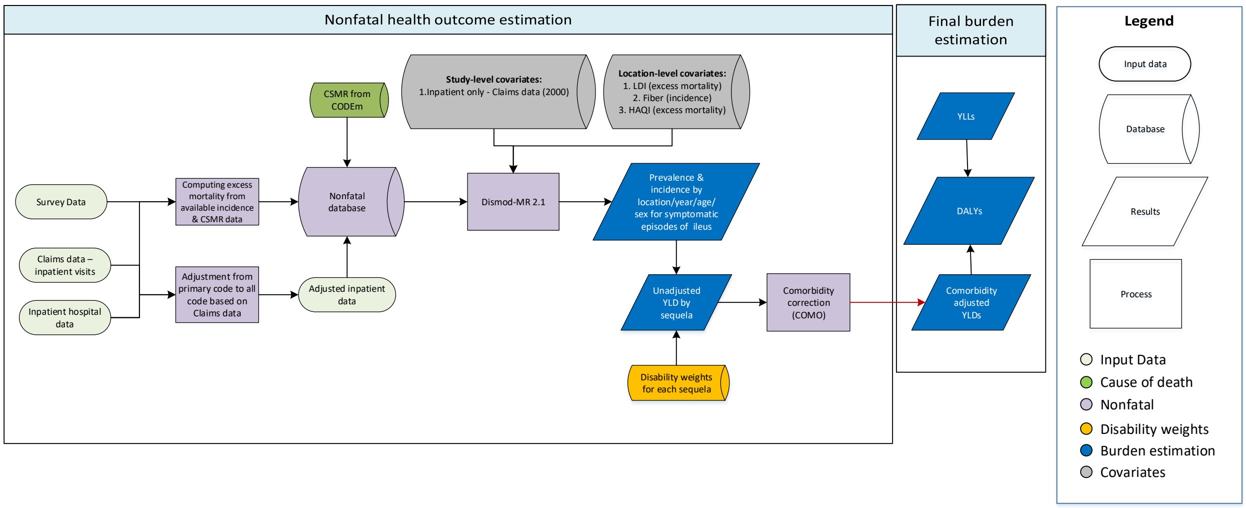

Case definition

Paralytic ileus and intestinal obstruction is a lack of digestive propulsion caused by failed peristalsis, typically requiring surgery. ICD code for paralytic ileus and intestinal obstruction is K56.

Input data

Model inputs

For GBD 2017, the data used for paralytic ileus and intestinal obstruction were hospital inpatient data, USA claims data for 2000 and 2010 through 2014 by USA state, and Taiwan claims data for 2016 primary diagnoses only. Descriptions of search strategies for hospital and claims data are included elsewhere in the appendix. The agreed-upon approach for paralytic ileus and intestinal obstruction was to only use these data sources, and not conduct a literature review. Data were outliered or excluded if we found them unreasonable when compared to regional, super-regional, and global rates.

Table 1. Site-years of data by different measures and geographical coverage

	Incidence
Site-years (total)	1437
Number of countries with data	45
Number of GBD regions with data (out of 21 regions)	16
Number of GBD super-regions with data (out of 7 super-regions	7

Severity splits

The basis of the GBD disability weight survey assessments is lay descriptions of sequelae highlighting major functional consequences and symptoms.

Table 2. Severity level and lay description.

Severity level	Lay description	DW (95% CI)
Paralytic ileus and intestinal obstruction, severe	This person has severe pain in the belly and feels nauseated. The person is anxious and unable to carry out daily activities.	0.324 (0.219–0.442)

Modelling strategy

DisMod-MR 2.1, a Bayesian meta-regression tool, was used to estimate paralytic ileus and intestinal obstruction prevalence by age, sex, year, and geography (subnational [select countries], country, region, super-region).

Paralytic ileus and intestinal obstruction was modelled with remission set between 25 and 26 for all age groups, implying a duration of approximately two weeks. We also set a prior for the maximum incidence of 0.002 for ages 0 to 5. The reference data were hospital inpatient data and USA claims (2010–2014) and Taiwan claims (2016). Study-level covariates were used to adjust incidence derived from USA claims data for 2010 toward these reference data points.

We used the function in DisMod-MR 2.1 to pull in cause-specific mortality rate (CSMR) data from our CODEm and CODcorrect analyses and match with incidence data points for the same geography. We calculated the excess mortality rate to estimate priors by dividing CSMR by incidence/remission. We also applied location-level covariates: fibre consumption to incidence with an upper bound of 0 and a lower bound of -2, a lag-distributed income covariate to excess mortality, log-transformed and forced negative with an upper bound of 0 and a lower bound of -1, and a Healthcare Access and Quality (HAQ) index covariate with an upper bound of 0 and lower bound of -2. We restricted location random effects to (-0.5, 0.5) in various GBD regions and super-regions (Central Europe, Eastern Europe, Central Asia, South Asia, Latin America and the Caribbean, North Africa and Middle East, high-Income, Southeast Asia, East Asia, Oceania and sub-Saharan Africa).

Betas and exponentiated values (which can be interpreted as an odds ratio) are shown in the tables below for study-level covariates and country-level covariates:

Table 3. Study-level beta and exponentiated values

Covariate	Parameter	beta	Exponentiated beta
Inpatient-only MarketScan, year 2000	Incidence	-0.2 (-0.23 to -0.18)	0.82 (0.79–0.84)

Table 4. Location-level beta and exponentiated values

Covariate	Parameter	beta	Exponentiated beta
Fibre adjusted (g)	Incidence	-0.075 (-0.12 to -0.055)	0.93 (0.89–0.95)
LDI (I$ per capita)	Excess mortality rate	-0.24 (-0.27 to -0.21)	0.79 (0.76–0.81)
Healthcare Access and Quality index	Excess mortality rate	-0.032 (-0.034 to -0.031)	0.97 (0.97–0.97)

Inguinal, femoral, and abdominal hernias

Flowchart

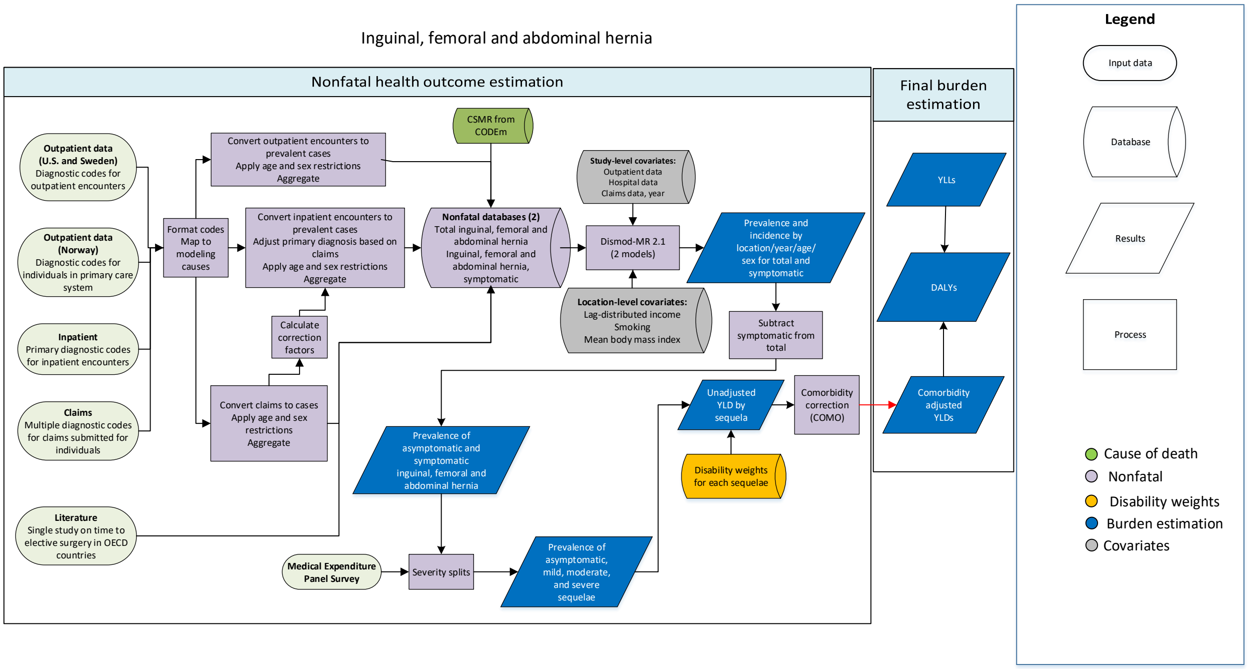

Case definition

Hernia refers to when an internal organ protrudes through an opening in the tissue that holds it in place. Inguinal, femoral, and abdominal hernia comprises the disorders in which portions of the digestive tract protrude through defects in the walls of the abdominal cavity. These occasionally lead to life-threatening acute complications, but more commonly are asymptomatic or cause chronic or intermittent pain. Symptomatic hernias are surgically repaired. ICD10 codes are K40, K41, K42, K44, K45, and K46 and all their 4-digit and 5-digit constituents. The ICD9 codes are 550, 551, 552, 553 and their constituents, with the exceptions of 551.1-3, 552.1-3, and 553.1-3.

Input data

Model inputs

For GBD 2017, two databases were developed for inguinal, femoral, and abdominal hernia: total and symptomatic.

Data for total hernia included outpatient data from the USA, Sweden, and Norway; claims for inpatient and outpatient encounters submitted to commercial insurance in the USA in 2000, 2010, and 2012; and international hospital discharge data. Outpatient data from Norway and claims data from the USA are provided for individuals and link multiple encounters. Individuals from claims datasets were extracted as prevalent cases if they had one or more inpatient encounters or two or more outpatient encounters. Outpatient data from the USA and Sweden and all inpatient data are provided at the encounter level; encounters with a relevant ICD code as any diagnosis were adjusted using claims data to estimate

prevalent cases. Remission estimates were derived from a single, large study of mean wait times for elective surgical repair in OECD countries performed by Siciliani and colleagues. Survey and literature data were otherwise not included.

To model symptomatic hernia, data from international hospital discharges and claims data for inpatient encounters submitted to commercial insurance in the USA in 2000, 2010, and 2012 were used. Inpatient claims were extracted as prevalent cases if one or more encounter had a hernia ICD code as any diagnosis. Hospital discharges were extracted if hernia was the primary diagnostic code, and they were adjusted using claims data to estimate the number of unique cases represented by the discharges. Remission was again estimated based on elective surgery wait times as described above.

Data were considered for exclusion as outliers if they were found to be unreasonable when compared to regional, super-regional, and global rates, but none were found to meet these criteria and all data were included.

The tables below show the number of studies or sources included in GBD 2017, as well as the number of countries and GBD world regions represented.

Total inguinal, femoral, and abdominal hernias

	Prevalence	Incidence	Remission
Site-years (total)	1499	1	570
Number of countries with data	45	1	33
Number of GBD regions with data (out of 21 regions)	16	1	9
Number of GBD super-regions with data (out of 7 super-regions)	7	1	4

Based on facilities and claims data refresh 6

Inguinal, femoral, and abdominal hernias, symptomatic episodes

	Prevalence	Remission
Site-years (total)	1162	1122
Number of countries with data	42	33
Number of GBD regions with data (out of 21 regions)	15	9
Number of GBD super-regions with data (out of 7 super-regions)	7	4

Based on facilities and claims data refresh 3

Severity split and disability weights

The basis of the GBD disability weight survey assessments are lay descriptions of sequelae highlighting major functional consequences and symptoms. Prevalent cases of symptomatic hernia were divided according to severity distributions derived from data from the Medical Expenditure Panel Survey (MEPS) to assign them to mild, moderate, and severe sequelae. Asymptomatic cases were assigned no disability. The lay descriptions and disability weights for inguinal, abdominal, and femoral hernias are shown below.

Severity split	Lay description	DW (95% CI)
Asymptomatic inguinal, abdominal, and femoral hernia	--	0

Mild inguinal, abdominal, and femoral hernia, symptomatic episodes	This person has some pain in the belly that causes nausea but does not interfere with daily activities.	0.011 (0.005–0.021)
Moderate inguinal, abdominal, and femoral hernia, symptomatic episodes	This person has pain in the belly and feels nauseous. The person has difficulties with daily activities.	0.114 (0.080–0.159)
Severe inguinal, abdominal, and femoral hernia, symptomatic episodes	This person has severe pain in the belly and feels nauseated. The person is anxious and unable to carry out daily activities.	0.324 (0.219–0.442)

Modelling strategy

Two separate, full, compartmental models for inguinal, femoral, and abdominal hernia – total and symptomatic – were developed, using the databases described above as inputs into DisMod-MR 2.1. For the total model, reference data were hospital discharge data, and claims and outpatient data were marked with study-level covariates; DisMod was allowed to solve for the coefficients for these covariates with broad range of prior values (-2 to 2). For the symptomatic model, reference data were claims from the United States in 2012, and hospital discharge data and claims from 2000 and 2010 were marked with study-level covariates; the prior values on the coefficients for these covariates were restricted to a range of negative values near the coefficient from GBD 2016, and these coefficients were determined by a regression analysis that used only data from locations with multiple sources. DisMod-MR 2.1 was set to pull in cause-specific mortality rate (CSMR) data from our CODEm and CoDcorrect analyses and match with geography.

For total hernia, the model assumed some hernias were prevalent at birth, with incidence of new hernia set to 0 for the first year of life. The prior value on remission was bounded from 0 to 5 (a minimum duration of about ten weeks). The incidence prior on symptomatic hernia was bounded to 0 to 0.02.

A lag-distributed income covariate was applied to remission and excess mortality in both total and symptomatic models. Additional covariates on prevalence were included in the total model: lag-distributed income, smoking prevalence, and mean body-mass index. Betas and exponentiated values (which can be interpreted as an odds ratio) are shown in the tables below for study-level covariates and location-level covariates.

<u>Total inguinal, femoral, and abdominal hernias</u>

**Covariate type**	**Covariate**	**Parameter**	**beta**	**Exponentiated beta**
Study-level covariate	Claims data - 2000	Prevalence	0.35 (0.30 to 0.40)	1.42 (1.36 to 1.48)
Study-level covariate	Claims data - 2010	Prevalence	0.67 (0.63 to 0.72)	1.96 (1.88 to 2.05)
Study-level covariate	Claims data - 2012	Prevalence	0.72 (0.68 to 0.76)	2.05 (1.97 to 2.14)

Study-level covariate	Outpatient data	Prevalence	-1.99 (-2 to -1.96)	0.14 (0.14 to 0.14)
Country covariate	LDI (I$ per capita)	Incidence	-0.0013 (-1.94 to 1.99)	1.00 (0.14 to 7.31)
Country covariate	LDI (I$ per capita)	Excess mortality rate	-0.00052 (-0.0016 to -0.00003)	1.00 (1.00 to 1.00)
Country covariate	LDI (I$ per capita)	Remission	1.03 (0.89 to 1.14)	2.81 (2.43 to 3.14)
Country covariate	Smoking prevalence	Prevalence	0.40 (0.17 to 0.64)	1.48 (1.19 to 1.91)
Country covariate	Mean body-mass index	Prevalence	-0.00017 (-0.00067 to -0.000024)	1.00 (1.00 to 1.00)

Based on model #277430

Inguinal, femoral, and abdominal hernias, symptomatic episodes

**Covariate type**	**Covariate**	**Parameter**	**beta**	**Exponentiated beta**
Study-level covariate	Hospital data	Prevalence	-1.23 (-1.27 to -1.2)	0.29 (0.28 to 0.30)
Study-level covariate	Claims data - 2000	Prevalence	-0.64 (-0.68 to -0.6)	0.53 (0.50 to 0.55)
Country covariate	LDI (I$ per capita)	Excess mortality rate	-0.00049 (-0.0016 to -0.00002)	1.00 (1.00 to 1.00)
Country covariate	LDI (I$ per capita)	Remission	0.90 (0.64 to 0.97)	2.45 (1.89 to 2.65)

Based on model #211640

We calculated prevalence of asymptomatic hernias by subtracting the estimated prevalence of symptomatic hernias from our estimated prevalence of total hernias.

Summary of changes

Compared to GBD 2016, GBD 2017 differs by including more location-years of hospital discharge data, including outpatient data from United States, Sweden, and Norway in estimation of total prevalence, choosing hospital discharge data as the reference case for total prevalence, and excluding fewer data as outliers in both total and symptomatic models. In GBD 2016, all NID-sex-location-year series with age-standardised mean prevalence more than 1.75x higher or less than 0.25x lower than the mean among USA states were marked as outliers, and this exclusion criterion was not employed this year.

Inflammatory bowel disease

Flowchart

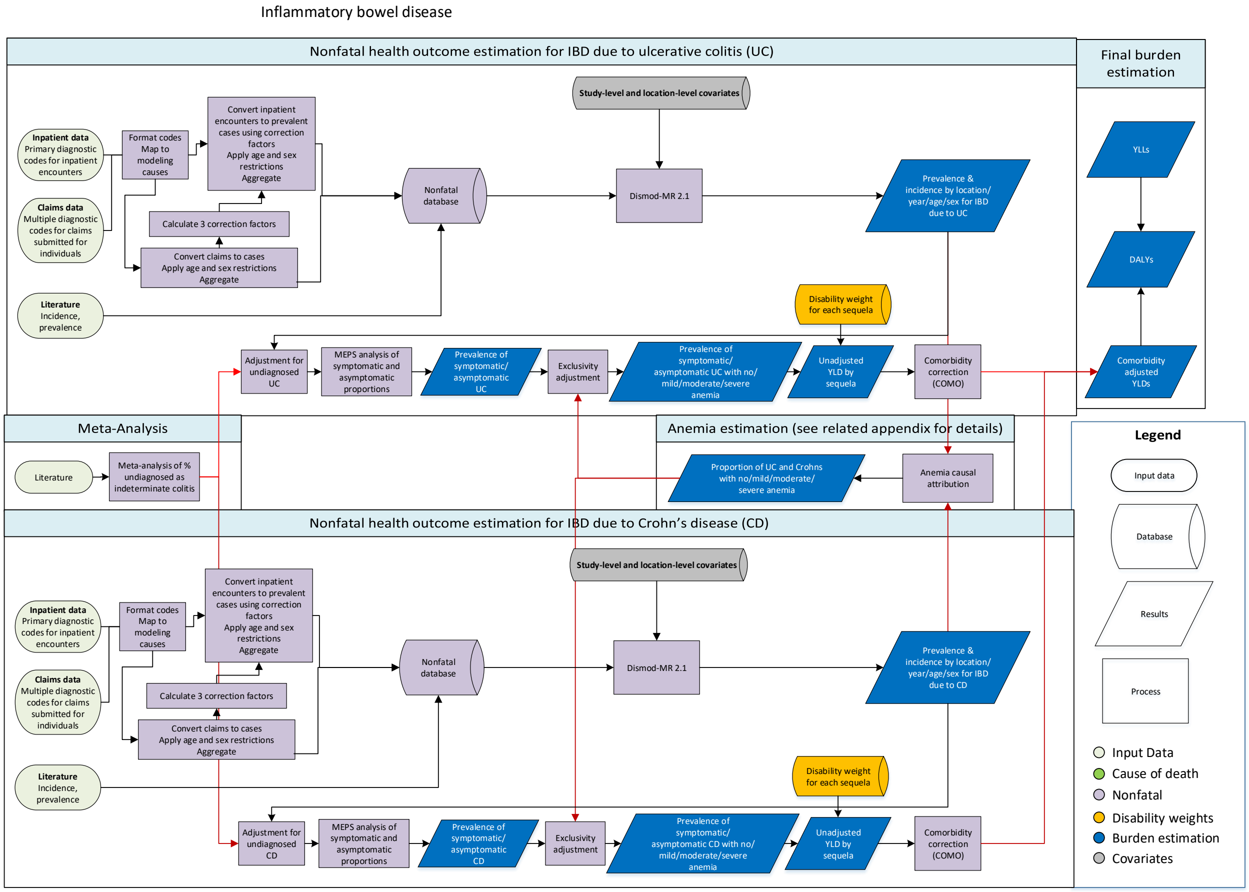

Case definition

Inflammatory bowel disease comprises digestive disorders resulting from inflammation of the colon and gastrointestinal tract, predominantly Crohn's disease (inflammation of the small and large intestine) and ulcerative colitis (inflammation of the colon and rectum). These disorders are diagnosed by endoscopy, imaging studies, or biopsy, in a patient with appropriate clinical signs and symptoms. In some cases of inflammatory bowel disease, neither Crohn's disease nor ulcerative colitis can be definitively diagnosed, and a diagnosis of indeterminate colitis is applied, indefinitely, or until definitive features of Crohn's or ulcerative colitis declare themselves. ICD codes are K50 for Crohn's disease, K51 for ulcerative colitis, and K52 for indeterminate colitis.

Input data

Model inputs

GBD 2017, like GBD 2016, utilised two separate databases for inflammatory bowel disease, one each for ulcerative colitis and Crohn's disease. Both include data from literature, hospital discharges, and claims data from the United States from 2000, 2010 and 2012. Claims data link multiple inpatient and outpatient

claims to a single individual; prevalent cases were extracted if an individual had at least one inpatient or outpatient encounter with an appropriate ICD code as any diagnosis, and correction factors were derived to apply to other data sources. Data from hospital discharges were adjusted using correction factors from claims, converting encounters to estimates of cases, correcting for some facilities providing only primary diagnostic codes, and estimating outpatient cases from inpatient cases. Literature data came from a systematic review conducted for GBD 2016. In brief, a single systematic review of literature was conducted to capture studies of prevalence and incidence for all inflammatory bowel diseases. Studies were excluded if they were not representative of the national population, or if they had insufficient study and sampling methods. Reviews were excluded from the search results.

Data were marked as outliers or excluded if we found them unreasonable when compared to regional, super-regional, and global rates.

The tables below show the number of studies or sources included in GBD 2017, as well as the number of countries and GBD world regions represented.

Non-infective inflammatory bowel disease due to ulcerative colitis

	Prevalence	Incidence	Case fatality
Site-years (total)	1241	282	3
Number of countries with data	51	52	2
Number of GBD regions with data (out of 21 regions)	17	13	1
Number of GBD super-regions with data (out of 7 super-regions)	7	6	1

From facilities and claims data refresh 3

Non-infective inflammatory bowel disease due to Crohn's disease

	Prevalence	Incidence	Case fatality	Excess mortality
Site-years (total)	1183	270	1	1
Number of countries with data	50	49	1	1
Number of GBD regions with data (out of 21 regions)	17	12	1	1
Number of GBD super-regions with data (out of 7 super-regions)	7	5	1	1

From facilities and claims data refresh 3

Disability weights

The basis of the GBD disability weight survey assessments are lay descriptions of sequelae highlighting major functional consequences and symptoms. For GBD 2017, we used the Medical Expenditure Panel Survey to find the proportion of ulcerative colitis and Crohn's disease asymptomatic versus symptomatic during a given four-week period. The lay descriptions and disability weights for sequelae associated with inflammatory bowel disease are shown below.

Severity split	Lay description	DW (95% CI)
Crohn's disease, currently asymptomatic	--	0

Crohn's disease, symptomatic	This person has cramping abdominal pain, has diarrhoea several times a day, and feels very tired for two months every year. When the person does not have symptoms, there is anxiety about them returning.	0.231 (0.156–0.32)
Ulcerative colitis, currently asymptomatic	--	0
Ulcerative colitis, symptomatic	This person has cramping abdominal pain, has diarrhoea several times a day, and feels very tired for two months every year. When the person does not have symptoms, there is anxiety about them returning.	0.231 (0.156–0.32)

*The numerous sequelae generated from exclusive combinations of anaemia and inflammatory bowel disease each contain custom disability weights. More information can be found in the appendix detailing disability weights

Modelling strategy

The modelling strategy for all inflammatory bowel disease encompasses separate DisMod models for ulcerative colitis and Crohn's disease, which are then adjusted to account for inflammatory bowel disease due to indeterminate colitis.

Non-infective inflammatory bowel disease due to ulcerative colitis, pre-adjustment (for indeterminant colitis)

The DisMod model for ulcerative colitis used prevalence and incidence data as described above. Reference data were claims from the United States from 2012, and study-level covariates were used to mark data from literature, hospital discharges, United States claims in 2000 and 2010, and the Medical Expenditure Panel Survey. Prior value on remission was set to 0 for all age groups, and an incidence prior value was set to 0 only for ages 0 to 1. Location-level covariates were log-transformed lag-distributed income and a Healthcare Access and Quality index (both on excess mortality) and absolute value of average latitude and log-transformed age-standardised death rate due to IBD (on prevalence). Betas and exponentiated values (which can be interpreted as an odds ratio) are shown in the tables below for study-level covariates and location-level covariates.

Covariate type	Covariate	Parameter	beta	Exponentiated beta
Study-level covariate	Hospital inpatient	Prevalence	0.40 (0.40 to 0.40)	1.49 (1.49 to 1.49)
Study-level covariate	Medical Expenditure Panel Survey	Prevalence	-0.0092 (-0.044 to -0.00013)	0.99 (0.96 to 1.00)
Study-level covariate	Literature	Prevalence	-0.0043 (-0.013 to -0.000047)	1.00 (0.99 to 1.00)
Study-level covariate	USA claims data - 2000	Prevalence	-0.012 (-0.04 to -0.00056)	0.99 (0.96 to 1.00)
Study-level covariate	USA claims data - 2010	Prevalence	-0.0012 (-0.0035 to -0.0000015)	1.00 (1.00 to 1.00)

Location covariate	Absolute value of average latitude	Prevalence	0.049 (0.048 to 0.050)	1.05 (1.05 to 1.05)
Location covariate	Natural log of age-standardised death rate due to IBD	Prevalence	0.24 (0.21 to 0.26)	1.27 (1.23 to 1.30)
Location covariate	LDI (I$ per capita)	Excess mortality rate	-0.5 (-0.99 to -0.017)	0.61 (0.37 to 0.98)
Location covariate	Healthcare Access and Quality index	Excess mortality rate	-0.5 (-1 to -0.029)	0.60 (0.37 to 0.97)

Based on model: 213476

<u>Non-infective inflammatory bowel disease due to Crohn disease, pre-adjustment (for indeterminant colitis)</u>

The DisMod model for Crohn's disease used incidence and prevalence data as described above. Reference data were hospital discharge data and United States claims data from 2012. Study-level covariates were used to mark data from literature, the Medical Expenditure Panel Survey, and United States claims data from 2000 and 2010. Prior value on remission was set to 0 for all age groups and on incidence prior value was set to 0 only for ages 0 to 2. Location-level covariates were log-transformed lag-distributed income and a Healthcare Access and Quality index (both on excess mortality) and log-transformed age-standardised death rate due to IBD (on prevalence).

**Covariate type**	**Covariate**	**Parameter**	**beta**	**Exponentiated beta**
Study-level covariate	Medical Expenditure Panel Survey	Prevalence	-0.0072 (-0.028 to -0.00016)	0.99 (0.97 to 1.00)
Study-level covariate	Literature	Prevalence	-0.0035 (-0.01 to -0.000079)	1.00 (0.99-1.00)
Study-level covariate	USA claims data – 2000	Prevalence	-0.00073 (-0.0026 to -0.00012)	1.00 (1.00 to 1.00)
Study-level covariate	USA claims data - 2010	Prevalence	-0.000067 (-0.000067 to -0.000067)	1.00 (1.00 to 1.00)
Study-level covariate	Natural log of age-standardised death rate due to IBD	Prevalence	0.99 (0.96 to 1.02)	2.69 (2.62 to 2.76)
Location covariate	LDI (I$ per capita)	Excess mortality rate	-0.88 (-1 to -0.59)	0.42 (0.37 to 0.56)
Location covariate	Healthcare Access and Quality index	Excess mortality rate	-0.63 (-0.99 to -0.052)	0.53 (0.37 to 0.95)

Based on model: 213515

In GBD 2015, a meta-analysis was conducted to determine the ratio of inflammatory bowel disease cases that are categorised as indeterminate colitis to cases that are characterised as either Crohn's disease or ulcerative colitis. The result of this meta-analysis was a mean ratio of 0.059 (0.047–0.071). The prevalence results of the DisMod models for ulcerative colitis and Crohn's disease were therefore

adjusted by multiplying by a normal distribution of draws from 1.059 (1.047–1.071) to account for all inflammatory bowel disease cases.

Summary of changes

Compared to GBD 2016, GBD 2017 added additional location-years of hospital discharge data.

Vascular intestinal disorders

Flowchart

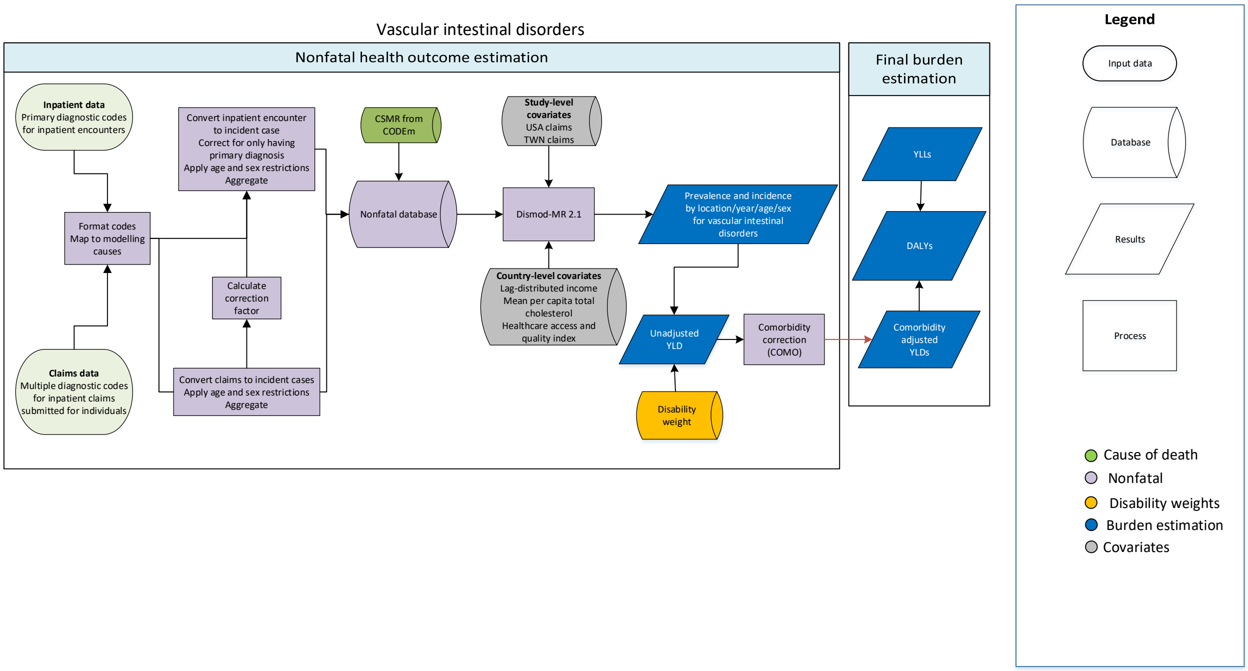

Case definition

Vascular intestinal disorders comprise ischaemic disorders and vascular malformations (ie, angiodysplasias). Ischaemia occurs when there is decreased blood supply to the gastrointestinal tract, causing injury to the bowel, and vascular malformations occur when blood vessels in the bowel grow inappropriately, predisposing to bleeding. Vascular intestinal disorders typically require surgical treatment. The ICD10 code for vascular intestinal disorders is K55; ischaemia and angiodysplasia are only distinguished at the level of 4-digit and 5-digit codes. Equivalent codes for ICD9 are 569.84, 569.85 and 569.86 (for angiodysplasia), and 557 and its 4- and 5-digit constituents (for ischaemia).

Input data

Model inputs

For GBD 2017, the data used for vascular intestinal disorders are hospital inpatient data and claims data from the United States and Taiwan. Literature and survey data, and data from outpatient facilities, are not included in this dataset. Inpatient claims are linked at the individual level and were extracted as incident cases, assuming multiple claims within a duration window relate to the same episode. Hospital discharges were extracted as encounters if any discharge diagnosis was an appropriate ICD code; these data were then adjusted using ratios from claims data to estimate the number of individuals represented and to account for some facilities only recording primary diagnoses.

Hospital discharge data were highly heterogeneous; for each source-location-year-sex combination, age-standardised mean was calculated, and the series was excluded if this was zero or was greater than three

times the median absolute deviation above or below the median. Data were further considered for exclusion if relatively high values in young age groups led to overestimation of subnational pseudo-random effects and poor model fit, or if we found them unreasonable when compared to regional, super-regional, and global rates, but no data for these models met these criteria for exclusion.

The table below shows the number of data sources included in GBD 2017, as well as the number of countries or subnational units and GBD world regions represented.

	Incidence
Site-years (total)	1374
Number of countries with data	41
Number of GBD regions with data (out of 21 regions)	15
Number of GBD super-regions with data (out of 7 super-regions)	7

Based on facilities and claims data refresh 8

Severity split and disability weights

The basis of the GBD disability weight survey assessments are lay descriptions of sequelae highlighting major functional consequences and symptoms. The lay description and disability weights for vascular intestinal disorders are shown below. All cases are assumed to be severe.

Severity split	Lay description	DW (95% CI)
Vascular intestinal disorders, severe	This person has severe pain in the belly and feels nauseated. The person is anxious and unable to carry out daily activities.	0.324 (0.219–0.442)

Modelling strategy

The DisMod model for vascular intestinal disorders used incidence data as described above. The reference data were adjusted hospital discharges, and claims from the United States and Taiwan were marked with study-level covariates. DisMod-MR 2.1 was set to pull in cause-specific mortality rate (CSMR) data from CODEm and CoDcorrect analyses and match with incidence data points for the same geography.

Prior settings included bounding remission from 2 to 12 (a duration from about four weeks to half a year) for all age groups and capping excess mortality at 10. A lag-distributed income covariate (log transformed) and a mean total cholesterol covariate were applied to incidence. A Healthcare Access and Quality index covariate was applied to excess mortality rate.

Betas and exponentiated values (which can be interpreted as an odds ratio) are shown in the tables below for study-level covariates and location-level covariates.

**Covariate type**	**Covariate**	**Parameter**	**Beta**	**Exponentiated beta**
Study-level covariate	USA claims	Incidence	-0.075 (-0.12 to -0.036)	0.93 (0.88 to 0.97)
Study-level covariate	TWN claims	Incidence	2.93 (2.74 to 3.00)	18.7 (15.5 to 20.0)
Location covariate	Cholesterol (total, mean per capita)	Incidence	0.0041 (-0.066 to 0.062)	1.00 (0.94 to 1.06)

Location covariate	LDI (I$ per capita)	Incidence	0.14 (0.12 to 0.16)	1.15 (1.12 to 1.17)
Location covariate	Healthcare Access and Quality index	Excess mortality rate	-0.042 (-0.043 to -0.041)	0.96 (0.96 to 0.96)

Based on model version: 326390

Summary of changes

Compared to GBD 2016, GBD 2017 modelling of vascular intestinal disorders differed primarily by adding additional location-years of hospital discharge data, additional years of United States claims data and Taiwan claims data, and by making adjusted hospital discharge data the reference case.

Gallbladder and biliary diseases

Flowchart

Gallbladder and biliary diseases

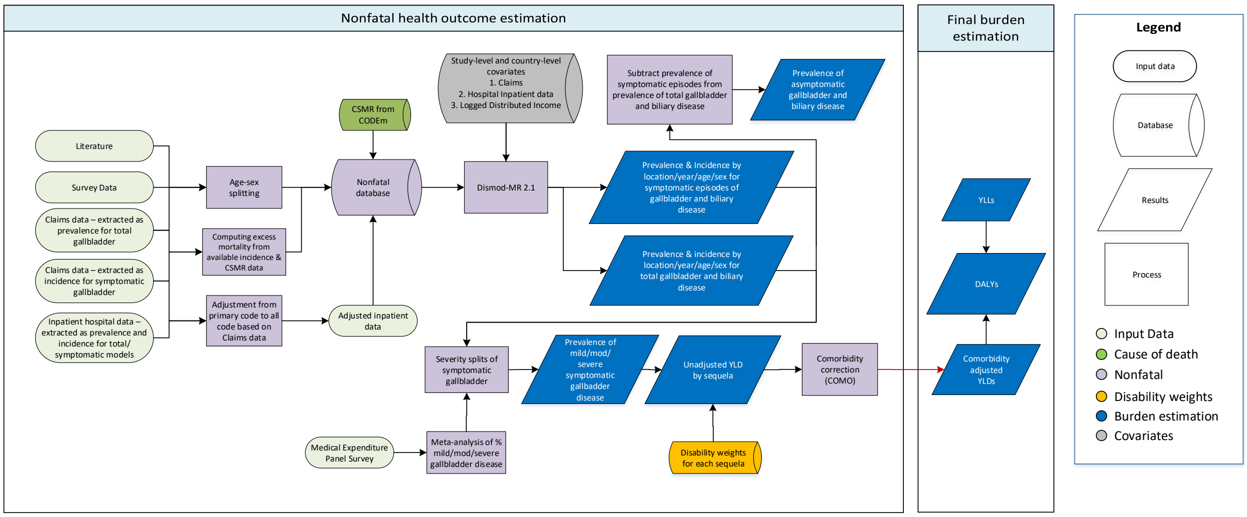

Input data and methodological summary

Case definition

Gallbladder and biliary diseases are digestive disorders including gallstones, cholecystitis, cholangitis, and other diseases of the gallbladder and biliary tract. Cholecystitis is an inflammation of the gallbladder, and cholangitis is an infection or inflammation of the bile duct, the result of a bacterial infection – both are often the result of gallstones. Gallbladder and biliary diseases, especially the presence of gallstones, can often be asymptomatic with periodic symptomatic episodes of severe abdominal pain, nausea, vomiting, and at times fever. ICD codes included are K80, K81, K82, and K83.

Input data

Model inputs

In GBD 2016, chronic gallbladder and biliary diseases was renamed to total gallbladder and biliary diseases (total gallbladder disease). Data inputs are separate for the total gallbladder disease and symptomatic episode models. A systematic review of literature was conducted to capture studies of prevalence and incidence of gallbladder and biliary diseases. For GBD 2016, we performed a systematic literature search using PubMed. Studies not representative of the national population (ie, *H. pylori* cohorts, patients presenting with pain), studies without sufficient information on study and sampling methods, and reviews were excluded. For GBD 2017, in addition to previous literature data, hospital inpatient, USA claims data from 2000 and 2010 through 2014 at the USA state level, and Taiwan claims data for 2016 were included, extracted as prevalence for the total gallbladder model, and extracted an

incident case for the symptomatic gallbladder model. Data were outliered or excluded if we found them unreasonable when compared to regional, super-regional, and global rates.

The tables below show the number of studies included in GBD 2017, as well as the number of countries or subnational units and GBD world regions represented.

Table 1. Site-years of data by different measures and geographical coverage

Cause		Prevalence	Incidence
Total gallbladder and biliary diseases	Site-years (total)	1447	13
	Number of countries with data	51	2
	Number of GBD regions with data (out of 21 regions)	16	2
	Number of GBD super-regions with data (out of 7 super-regions	7	2
Symptomatic gallbladder and biliary diseases	Site-years (total)	1	1413
	Number of countries with data	1	44
	Number of GBD regions with data (out of 21 regions)	1	16
	Number of GBD super-regions with data (out of 7 super-regions)	1	7

Disability weights

The basis of the GBD disability weight survey assessments are lay descriptions of sequelae highlighting major functional consequences and symptoms. In line with GBD 2016, we included the mild and severe sequela and applied the disability weights to prevalent cases in the symptomatic gallbladder model. The lay descriptions and disability weights for gallbladder and biliary disease are shown below:

Table 2. Severity level and lay descriptions

Severity split	Lay description	DW (95% CI)
Mild gallbladder and biliary disease, symptomatic episodes	This person has some pain in the belly that causes nausea but does not interfere with daily activities.	0.011 (0.005–0.021)
Moderate gallbladder and biliary disease, symptomatic episodes	This person has pain in the belly and feels nauseous. The person has difficulties with daily activities.	0.114 (0.080–0.159)
Severe gallbladder and biliary disease, symptomatic episodes	This person has severe pain in the belly and feels nauseated. The person is anxious and unable to carry out daily activities.	0.324 (0.219–0.442)

Modelling strategy

The DisMod model for total gallbladder and biliary diseases included bounding remission from 0 to 1 (a minimum duration of one year). Reference data were from literature, USA claims data from 2010 to 2014, and Taiwan claims data from 2016, and we marked inpatient hospital and USA claims data years 2000 with separate year-specific study-level covariates. We used the function in DisMod-MR 2.1 to pull in

cause-specific mortality rate (CSMR) data from our CODEm and CODcorrect analyses and match with prevalence data points for the same geography. We calculated excess mortality rate to estimate priors by dividing CSMR by prevalence. We also applied a lag-distributed income covariate to excess mortality, log transformed and forced negative with an upper bound of 0 and a lower bound of -1. Betas and exponentiated values (which can be interpreted as an odds ratio) are shown in the tables below for study-level covariates and country-level covariates.

Table 3. Beta and exponentiated values for total gallbladder

**Covariate type**	**Covariate**	**Parameter**	**beta**	**Exponentiated beta**
Study-level covariate	Hospital inpatient	Prevalence	0.55 (0.53–0.57)	1.74 (1.70–1.77)
Study-level covariate	Claims data - 2000	Prevalence	-0.25 (-0.29 to -0.22)	0.78 (0.75–0.81)
Country covariate	LDI (I$ per capita)	Excess mortality rate	-0.8 (-0.81 to -0.79)	0.45 (0.44–0.45)

The symptomatic episodes of gallbladder and biliary diseases DisMod model bounded remission from 9 to 26 (a duration of about two to six weeks). The reference data were US claims data from 2010 to 2014 and Taiwan claims data from 2016, and we marked other USA claims years, inpatient data, and literature with specific study-level covariates. We used the function in DisMod-MR 2.1 to pull in cause-specific mortality rate (CSMR) data from our CODEm and CODcorrect analyses and match with incidence data points for the same geography. We calculated excess mortality rate to estimate priors by dividing CSMR by incidence/remission. We also applied a lag-distributed income covariate to excess mortality, log transformed and forced negative with an upper bound of 0 and a lower bound of -1. Betas and exponentiated values (which can be interpreted as an odds ratio) are shown in the tables below for study-level covariates and country-level covariates.

Table 4. Beta and exponentiated values for symptomatic gallbladder

**Covariate type**	**Covariate**	**Parameter**	**beta**	**Exponentiated beta**
Study-level covariate	Hospital inpatient	Incidence	0.50 (0.50–0.50)	1.65 (1.65–1.66)
Study-level covariate	Literature	Incidence	0.84 (0.60–1.08)	2.31 (1.82–2.96)
Study-level covariate	Inpatient-only claims data – 2000	Incidence	-0.033 (-0.062 to -0.0021)	0.97 (0.94–1.00)
Country covariate	LDI (I$ per capita)	Excess mortality rate	-0.82 (-0.83 to -0.81)	0.44 (0.44–0.45)

To calculate prevalence of asymptomatic gallbladder and biliary diseases, we took the estimated prevalence of symptomatic episodes and subtracted it from our estimated prevalence of total gallbladder and biliary diseases.

Pancreatitis

Flowcharts for Chronic pancreatitis and Pancreatitis, acute episodes

Pancreatitis

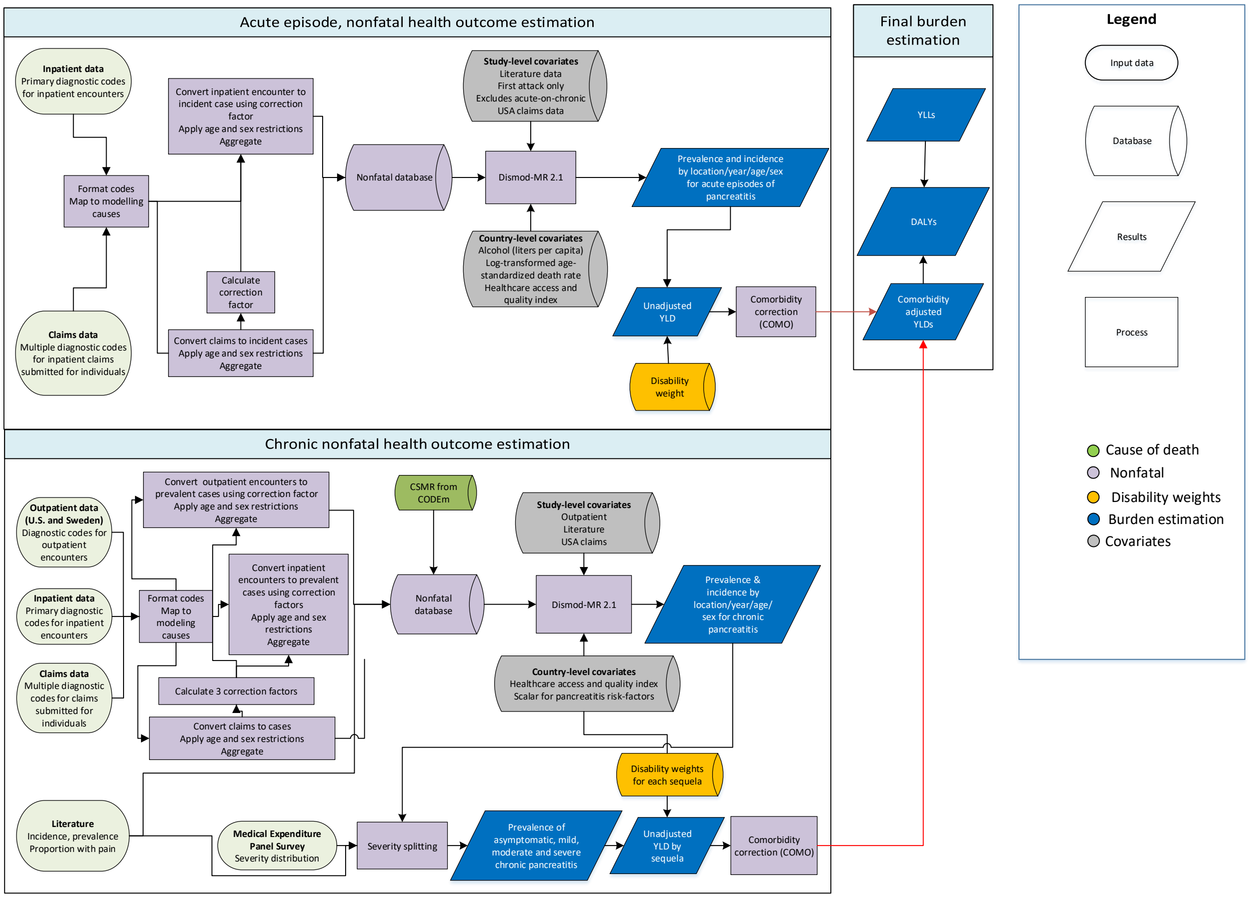

Input data and methodological summary

Case definition

Pancreatitis is the inflammation of the pancreas. Acute pancreatitis involves active inflammation and injury to the pancreas, resulting in severe upper abdominal pain and nausea, inappropriate release of pancreatic contents, and a systemic inflammatory response with fever, low blood pressure, and, in some cases, failure of one or more organs. Chronic pancreatitis involves permanent damage to the pancreas from longstanding or recurrent inflammation; this produces chronic or episodic abdominal pain and nausea and ultimately failure of the pancreas to produce and release digestive enzymes and hormones, leading to chronic diarrhoea, poor absorption of nutrients from food, and diabetes. Individuals with chronic pancreatitis can have superimposed episodes of acute pancreatitis. In prior rounds of GBD, we modelled acute and chronic pancreatitis together, but in GBD 2017 we developed separate models for these two diseases.

ICD10 codes are K85 for acute and K86 for chronic pancreatitis. ICD9 code 577.0 corresponds to acute pancreatitis, and 577 and the remainder of its four-digit and five-digit constituents refer to chronic or unspecified pancreatitis.

Input data

Model inputs

Two databases were developed for pancreatitis in GBD 2017. Scientific literature previously used to model acute and chronic pancreatitis together was divided. For GBD 2016, a systematic review of the prevalence and incidence of pancreatitis throughout the world was conducted. The exclusion criteria were studies clearly not representative of the national population (ie, alcoholics or smokers), self-reported data, and reviews. Studies were added to the acute database if they measured the incidence of acute pancreatitis as defined by appropriate ICD codes, or by a combination of clinical, biochemical, and radiographic criteria. Likewise, studies were added to the chronic database if they employed appropriate ICD codes or appropriate clinical, biochemical, and radiographic criteria. Some studies reported incidence of acute and chronic disease separately and data were extracted to both databases, but those few studies that reported only a single measure for both disorders were dropped.

The acute database included scientific literature and hospital discharge data (from numerous countries) and data from insurance claims for inpatient encounters (in the United States and Taiwan). Claims data are linked to individuals, and when multiple claims for the same individual were submitted outside of a duration window, they were regarded as separate episodes. Hospital discharges were included only if the primary discharge diagnosis was a code for acute pancreatitis, and incident cases were estimated from number of discharges using a correction factor from claims data. Scientific studies included studies that measured incidence of first episode of acute pancreatitis only, and studies that measured incidence of all acute pancreatitis, including recurrent episodes. Scientific studies also included both those that excluded and those that included individuals with underlying chronic pancreatitis. Study-level covariates were used to adjust for these differences in design (details below).

The chronic database included scientific literature and hospital discharges from numerous countries, outpatient facility data from the United States and Sweden, and insurance claims data for both inpatient and outpatient encounters in the United States and Taiwan. Claims data are linked at the individual level; individuals were counted as prevalent cases if they had at least two outpatient or one inpatient encounter with a chronic pancreatitis ICD code as any diagnosis. Claims data were used to develop correction factors to apply to hospital discharge data. Outpatient encounters from facilities were extracted if any encounter diagnosis was for chronic pancreatitis, and they were adjusted to estimate prevalent cases using correction factors from claims data. Likewise, hospital discharges were extracted if any discharge diagnosis was an ICD code for chronic pancreatitis, and encounters were adjusted to estimate the number of prevalent cases, to account for some facilities only providing primary diagnostic codes, and to estimate the number of outpatient cases from the number of inpatient cases, all using correction factors from claims data.

Hospital discharge data were highly heterogeneous; for each source-location-year-sex combination, age-standardised mean was calculated, and the series was excluded if this was zero or was greater than three times the median absolute deviation above or below the median. Further data were excluded if relatively high values in young age groups led to overestimation of subnational pseudo-random effects and poor

model fit, or if we found them unreasonable when compared to regional, super-regional, and global rates.

The table below shows the number of studies or sources included in GBD 2017, as well as the number of countries and GBD world regions represented.

Acute pancreatitis episodes

	Prevalence	Incidence
Site-years (total)	16	1362
Number of countries with data	5	42
Number of GBD regions with data (out of 21 regions)	4	16
Number of GBD super-regions with data (out of 7 super-regions)	2	7

Based on facilities and claims data refresh 8

Chronic pancreatitis

	Prevalence	Incidence
Site-years (total)	1401	14
Number of countries with data	35	6
Number of GBD regions with data (out of 21 regions)	13	4
Number of GBD super-regions with data (out of 7 super-regions)	7	2

Based on facilities and claims data refresh 8

Severity split and disability weights

The basis of the GBD disability weight survey assessments are lay descriptions of sequelae highlighting major functional consequences and symptoms. The lay descriptions and disability weights for pancreatitis are shown below. All prevalent cases from the pancreatitis, acute episodes model were assigned a single, combined disability weight for severe abdominal pain and severe infectious disease symptoms. Prevalent cases from the chronic pancreatitis disease model were divided into symptomatic and asymptomatic groups using proportions found in a review of published studies of the natural history of chronic pancreatitis. The symptomatic group was divided into mild, moderate, and severe groups using proportions from the Medical Expenditure Panel Survey (MEPS).

Severity split	Lay description	DW (95% CI)
Acute pancreatitis episodes	This person has severe pain in the belly and feels nauseated. The person has high fevers, pain and feels very weak. This causes great difficulty with daily activities.	*Combined DW:<br>0.324 (0.220–0.442)<br>0.133 (0.088–0.190)
Asymptomatic chronic pancreatitis	--	0
Mild chronic pancreatitis	This person has some pain in the belly that causes nausea but does not interfere with daily activities.	0.011 (0.005–0.021)
Moderate chronic pancreatitis	This person has pain in the belly and feels nauseous. The person has difficulties with daily activities.	0.114 (0.080–0.159)

Severe chronic pancreatitis	This person has severe pain in the belly and feels nauseated. The person is anxious and unable to carry out daily activities.	0.324 (0.219–0.442)

*Acute pancreatitis episodes have a custom disability weight combining abdominal pain and infectious disease. More information can be found in the appendix detailing disability weights.

Modelling strategy

Acute pancreatitis episodes

The DisMod model for acute pancreatitis episodes used incidence and prevalence data as described above. The reference data for incidence were adjusted hospital discharge data and Taiwan claims data. Literature data and United States claims data were marked with study-level covariates; additional study-level covariates were used to indicate studies from the literature that only measured first attack of pancreatitis or those that excluded acute pancreatitis cases with pre-existing chronic pancreatitis. The prior value of remission was bounded from 8 to 9 (a duration from about six weeks) for all ages. Location-level covariates included per capita alcohol consumption and the log-normalised age-standardised death rate due to pancreatitis (both on incidence) and a Healthcare Access and Quality index (on excess mortality rate).

Betas and exponentiated values (which can be interpreted as an odds ratio) are shown in the tables below for study-level covariates and location-level covariates.

Acute pancreatitis

**Covariate type**	**Covariate**	**Parameter**	**beta**	**Exponentiated beta**
Study-level covariate	Literature	Incidence	-0.096 (-0.32 to 0.17)	0.91 (0.72 to 1.18)
Study-level covariate	First attack only	Incidence	0.075 (-0.33 to 0.51)	1.08 (0.72 to 1.66)
Study-level covariate	Excludes acute-on-chronic	Incidence	1.73 (1.25 to 2.00)	5.65 (3.49 to 7.36)
Study-level covariate	USA claims data	Incidence	0.23 (0.21 to 0.25)	1.26 (1.24 to 1.29)
Location covariate	Alcohol (litres per capita)	Incidence	0.00015 (0.0000090 to 0.00051)	1.00 (1.00 to 1.00)
Location covariate	Natural log of age-standardised death rate due to pancreatitis	Incidence	0.39 (0.36 to 0.42)	1.47 (1.44 to 1.52)
Location covariate	Healthcare Access and Quality index	Excess mortality rate	-0.0069 (-2 to 2)	0.99 (0.14 to 7.39)

Based on model #349700

Chronic pancreatitis

The DisMod model for chronic pancreatitis used incidence and prevalence data as described above. The reference data for prevalence were adjusted hospital discharge data and Taiwan claims data, and other sources – outpatient data from the United States and Sweden, literature, and United States claims data – were marked with study-level covariates. DisMod-MR 2.1 was set to pull in cause-specific mortality rate (CSMR) data from CODEm and CODcorrect analyses and match with incidence and prevalence data points for the same geography. The value prior on remission was set to 0. Location-level covariates included a log-transformed age-standardised SEV scalar covariate for pancreatitis on prevalence, and a Healthcare Access and Quality index on excess mortality rate.

Betas and exponentiated values (which can be interpreted as an odds ratio) are shown in the tables below for study-level covariates and location-level covariates.

Chronic pancreatitis

**Covariate type**	**Covariate**	**Parameter**	**beta**	**Exponentiated beta**
Study-level covariate	Outpatient	Prevalence	-3.41 (-3.47 to -3.35)	0.033 (0.031 to 0.035)
Study-level covariate	Literature	Prevalence	-1.7 (-1.98 to -1.32)	0.18 (0.14 to 0.27)
Study-level covariate	USA claims	Prevalence	-0.65 (-0.68 to -0.61)	0.52 (0.51 to 0.54)
Location covariate	Log-transformed age-standardised scaled exposure variable for pancreatitis risk factors	Prevalence	-0.11 (-0.15 to -0.076)	0.89 (0.86 to 0.93)
Location covariate	Healthcare Access and Quality index	Excess mortality rate	-0.038 (-0.039 to -0.038)	0.96 (0.96 to 0.96)

Based on model #341345

Summary of changes

Compared to GBD 2016, GBD 2017 pancreatitis models differ primarily by separately modelling acute pancreatitis episodes and chronic pancreatitis, which by definition, does not remit. In GBD 2016, data inputs that ascertained acute pancreatitis cases, chronic pancreatitis cases, or both were all included in a single model with prior value on remission bounded to 8 to 9, thereby assuming all cases remit or die. Lesser additional changes include adding Taiwan claims data, more location-years of hospital discharge data, outpatient facilities data, and more years of USA claims data. The reference case was also changed to hospital discharge data in GBD 2017, in contrast to using claims data as reference case in GBD 2016.

Other digestive diseases

In addition to the digestive diseases described above, there are many diverse types of digestive diseases with a range of severities and associated sequelae. Because these digestive diseases are diverse in their underlying causes and risk factors as well as in their associated health outcomes, modelling them together in a DisMod-MR model would not produce reliable estimates of prevalence or excess mortality. Instead, we calculated the YLDs caused by digestive diseases directly using a YLD/YLL ratio.

We calculated the ratio of YLDs to YLLs across the specified digestive diseases for which non-fatal outcomes were modelled, using YLL estimates from the GBD 2017 cause of death (CoD) analysis. We then multiplied this YLD/YLL ratio by the YLL estimates for other digestive diseases from the GBD 2017 CoD analysis, providing us with an estimate of the YLDs associated with other digestive diseases.

Alzheimer's disease and other dementias

Flowchart

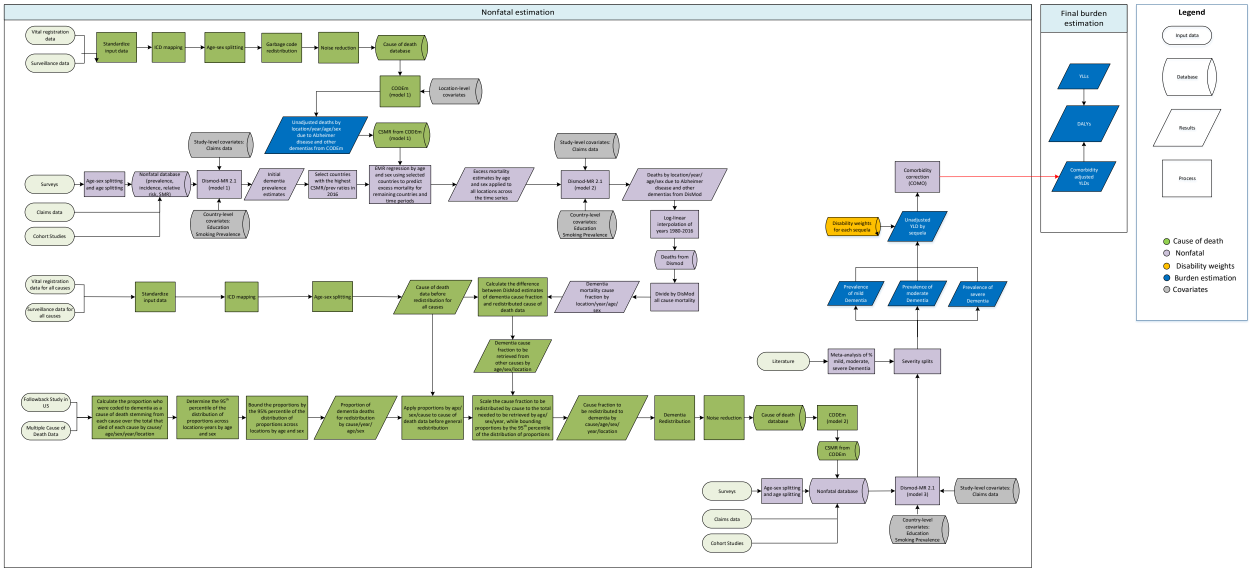

Input data and methodological summary

Case definition

Dementia is a progressive, degenerative, and chronic neurological disorder typified by memory impairment and other neurological dysfunctions. For the purposes of GBD 2017, we use the Diagnostic and Statistical Manual of Mental Disorders III, IV or V, or ICD case definitions as the reference. A wide array of diagnostic and screening instruments exists, including Clinical Dementia Rating scale (CDR), Mini Mental State Examination (MMSE), and the Geriatric Mental State (GMS). For severity rating purposes we use the CDR as the reference. The relevant ICD-10 codes for dementia are F00, F01, F02, F03, G30, and G31. The ICD-9 codes are 290, 291.2, 291.8, 294 and 331.

Unlike most causes in the Global Burden of Disease project, dementia mortality and morbidity estimates are modelled jointly. This is because of marked discrepancies between prevalence data and cause of death data. Specifically, prevalence data suggest little to no variation over time (eg, 1990–2017), whereas age-standardised mortality rates in vital registrations in high-income countries have increased multiple times over this same period. Additionally, prevalence variation between countries is much smaller than the variation in death rates assigned to dementia in vital registration. We attribute these discrepancies to changing coding practices rather than epidemiological change.

Because of this joint procedure, descriptions of the mortality estimation process are included where relevant.

Input data

Model inputs

To inform our estimates of burden due to dementia, we use mortality data from vital registration systems, as well as prevalence data from surveys and administrative data such as claims sources.

An updated systematic review was conducted covering September 2016 to August 2017, and search terms[1] were set to capture studies for all dementia, including its subtypes. The search yielded 970 initial hits, and 38 were marked for extraction. Inclusion criteria comprised studies that reported prevalence, incidence, remission rate, excess mortality rate, relative risk of mortality, standardised mortality ratio, or with-condition mortality rate. Studies with no clearly defined sample were excluded. A flow chart documenting this review is displayed below.

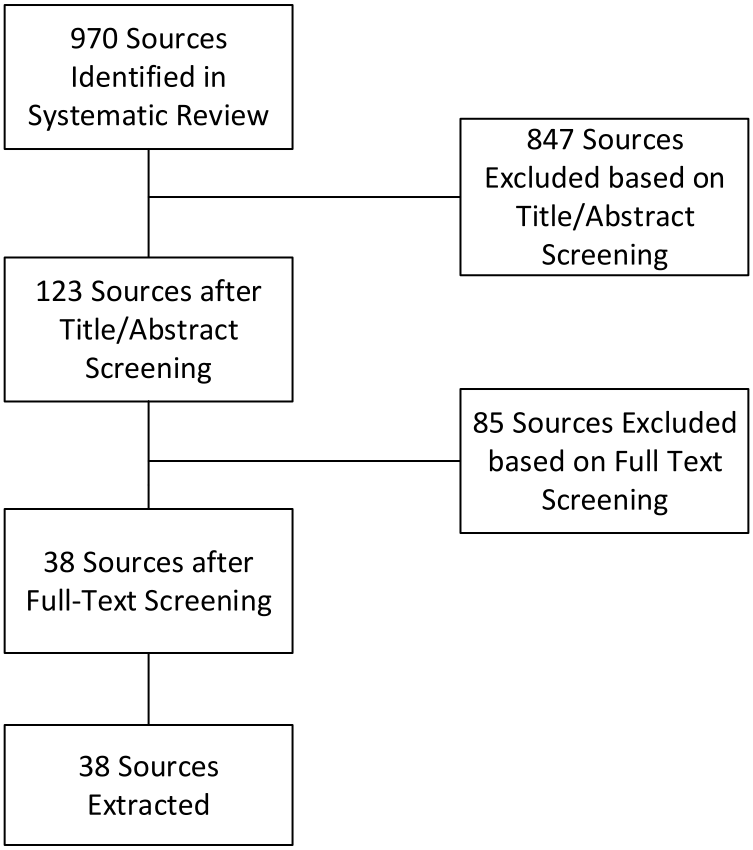

[1] ((dementia[Title/Abstract]) AND (incidence[Title/Abstract] OR prevalence[Title/Abstract] OR epidemiology[Title/Abstract])) AND ("2016/09/01"[Date - Publication] : "2017/08/29"[Date - Publication])

Additionally, a table describing the density and distribution of the epidemiological data available for GBD 2017 is presented below:

	Prevalence	Incidence	Mortality Risk
**Site-years (total)**	477	115	23
**Number of countries with data**	45	24	17
**Number of GBD regions with data (out of 21 regions)**	17	10	10
**Number of GBD super-regions with data (out of 7 super-regions)**	7	4	6

Studies with age and sex detail separately were split into age- and sex-specific data points. We also split data points where the age range was greater than 20 years using the age pattern from the United States, where we had the most detail by age.

This year we also added two additional years of claims data from the USA, and excluded claims data from 2000, which were systematically lower than other years. We also changed the algorithm for deriving prevalence from the claims data. Previously, an individual with any inpatient or outpatient visit in a given year would be counted as a previous case. However, this would pick up visits where a doctor may have used a potential or probable diagnosis without confirmation. Therefore, the new algorithm will only count a prevalent case where an individual has one inpatient visit, two outpatient visits, or one outpatient and one inpatient visit. This change to the algorithm resulted in a decrease in prevalence estimates for the United States.

Severity splits

In GBD 2013 (and still used in GBD 2017), we extracted data from studies reporting on mild, moderate, and severe dementia. As the data indicate an age pattern with greater proportions with more severe disease in the very old, we restricted our analyses to studies reporting on severity <70, 70-79, and 80+ ages. Most of these studies reported severity based on the CDR scale: CDR=1 as mild, CDR=2 as moderate, and CDR=3 as severe dementia. Other studies report staging of dementia according to MMSE; the Functional capacity scale; the Cambridge Mental Disorders of the Elderly Examination (CAMDEX); the scale of Hughes, and the Geriatric Mental State (GMS). We excluded all studies that used the DSM III criteria, as we found that these sources reported systematically higher severities. We used a random effects meta-analysis to pool the data by severity level.

We multiplied estimations of prevalence (country-year-sex-age-specific) by the fractions of mild, moderate, and severe dementia and estimated 95% uncertainty intervals at the 1,000-draw level.

Severity level	Lay description	DW (95% CI)	Severity distribution
Mild	The person has some trouble remembering recent events and finds it hard to concentrate and make decisions and plans.	0.069 (0.046–0.099)	<70: 79% (71–85%)<br>70-79: 77% (70–82%)<br>80+: 68% (59–76%)
Moderate	The person has memory problems and confusion, feels disoriented, at times hears voices that are not real, and needs help with some daily activities.	0.377 (0.252–0.508)	<70: 15% (10–21%)<br>70-79: 16% (12–22%)<br>80+: 23% (19–28%)

Severe	The person has complete memory loss, no longer recognises close family members, and requires help with all daily activities.	0.449 (0.304–0.595)	<70: 6% (3–11%)<br>70-79: 7% (4–12%)<br>80+: 9% (6–13%)

Modelling strategy

As mentioned above, the estimation of morbidity due to dementia occurs in conjunction with the mortality estimation.

First, we ran a CODEm model for dementia and extracted the mortality rates by age, sex, and geography for 2017.

Second, we ran a DisMod-MR 2.1 model with all data on incidence, prevalence, and mortality risk (RR, SMR, or with-condition mortality rates) and a setting of zero remission and extracted 2017 prevalence by age, sex, and geography. To account for potential systematic differences between medical claims and survey data, we added a covariate to crosswalk USA claims data and also added a covariate for European GP data. Additionally, to account for systematic differences in different methods of case ascertainment between different studies, this year we added study-level covariates on whether or not studies used a doctor-given diagnosis, whether or not they used clinical records to ascertain a diagnosis, and whether or not a study used an algorithmic diagnosis.

Third, we selected countries where the sum of cause-specific mortality rate to prevalence ratio for males and females exceeded 2.5 (excluding small island nations, those without vital registration, and countries without data on prevalence). This resulted in choosing Finland, Scotland, Sweden, the United States, England, the Netherlands, and Canada. The choice to pick fewer countries for this regression compared to GBD 2015, which used 30 countries in the EMR regression, was motivated by a desire to reduce the spread in EMR values, as countries used in the regression retain their original EMR values. We decided to exclude Finland from the regression, as it is a high outlier compared to the other countries included in the regression. However, we still allowed Finland to retain its original EMR values.

Fourth, we used a linear effects regression with dummies on age group and sex to predict excess mortality (ie, the ratio of cause-specific mortality rate and prevalence) by age and sex, the results of which are found in the tables below.

Table: Fixed effect coefficients of EMR regression. Outcome: ln(EMR)

Independent variables	Coef	Std. error	P value	95% Confidence interval	
Male	0.004	0.035	0.910	-0.064	0.072
Age 40-59	-2.897	0.074	0.000	-3.042	-2.752
Age 60-64	-2.267	0.074	0.000	-2.412	-2.122
Age 65-69	-2.103	0.074	0.000	-2.248	-1.958
Age 70-74	-1.845	0.074	0.000	-1.99	-1.7
Age 75- 80	-1.524	0.074	0.000	-1.669	-1.379
Age 80-84	-1.217	0.074	0.000	-1.362	-1.072
Age 85-89	-0.865	0.074	0.000	-1.01	-0.72
Age 90-94	-0.406	0.074	0.000	-0.551	-0.261

Constant	-1.748	0.055	0.000	-1.856	-1.64

Table: Predicted EMR values by age and sex (95% CI)

	Male	Female
Age 40-59	0.01 (0.009 - 0.011)	0.01 (0.009 - 0.011)
Age 60-64	0.018 (0.016 - 0.02)	0.018 (0.016 - 0.02)
Age 65-69	0.021 (0.019 - 0.024)	0.021 (0.019 - 0.024)
Age 70-74	0.028 (0.025 - 0.031)	0.028 (0.025 - 0.031)
Age 75- 80	0.038 (0.034 - 0.042)	0.038 (0.034 - 0.042)
Age 80-84	0.052 (0.046 - 0.057)	0.052 (0.047 - 0.057)
Age 85-89	0.074 (0.067 - 0.082)	0.074 (0.066 - 0.082)
Age 90-94	0.116 (0.105 - 0.13)	0.116 (0.104 - 0.129)
Age 95+	0.175 (0.157 - 0.196)	0.175 (0.157 - 0.194)

Fifth, these estimates were added to a second DisMod-MR 2.1 model as pertaining to the full 1990–2017 estimation period. For the countries included in the regression where the age-standardised EMR calculated from the country-specific data was higher than the age-standardised EMR calculated in the regression, we retained their age- and sex-specific ratios and entered those also as pertaining to the full 1990–2017 estimation period. Thus, the model reflects the cause-specific mortality rate if all countries over time would have had the average propensity to code to dementia as an underlying cause of death similar to the selected countries in 2017.

In this model, we assumed zero remission as well as zero excess mortality and incidence until age 40. We also did not allow random effects in the model in order to reduce spurious inflation of regional differences due to differences in measurement and measurement error. Because of lack of consistency between prevalence and incidence data, we excluded incidence data from the final model. In a few locations we found good consistency between prevalence and incidence, and these were locations where incidence and prevalence were collected as part of the same study. In other locations (Beijing, Australia, Italy, Canada, various states in the USA, Mexico, and Nigeria) we noted that DisMod-MR 2.1 was pushing the fit above the available prevalence data and below incidence – "averaging the difference." In all cases the incidence and prevalence data were collected by different studies. We decided to drop the incidence estimates, as measuring incidence of dementia when symptoms are still mild is more prone to measurement bias than measuring prevalence when the diagnosis has become more obvious over time.

The table below provides additional information on the country covariates and study-level covariates used in this model, as well as beta and exponentiated beta values.

Variable	Measure	Beta	Exponentiated beta value (CI)
Mean years of education, age-standardised	prevalence	-0.083	0.92 (0.92 to 0.92)

Smoking prevalence, age-standardised, both sexes	prevalence	0.34	1.40 (1.33 to 1.46)
USA claims data	prevalence	-0.38	0.68 (0.63 to 0.71)
European GP data	prevalence	-0.93	0.39 (0.36 to 0.42)
Diagnosed using cutoff score algorithm	prevalence	0.19	1.20 (1.10 to 1.31)
Diagnosed using only clinical records	prevalence	-0.11	0.90 (0.81 to 0.99)

We did a meta-analysis of the effect of education (in years) on dementia to set bounds on the covariate value. A literature search yielded six sources reporting specifically the effect of a single year of education. The meta-analysis estimated a relative risk of 0.92 for each additional year of education, which was used to set bounds on the country-level covariate in DisMod. The forest plot is shown below.

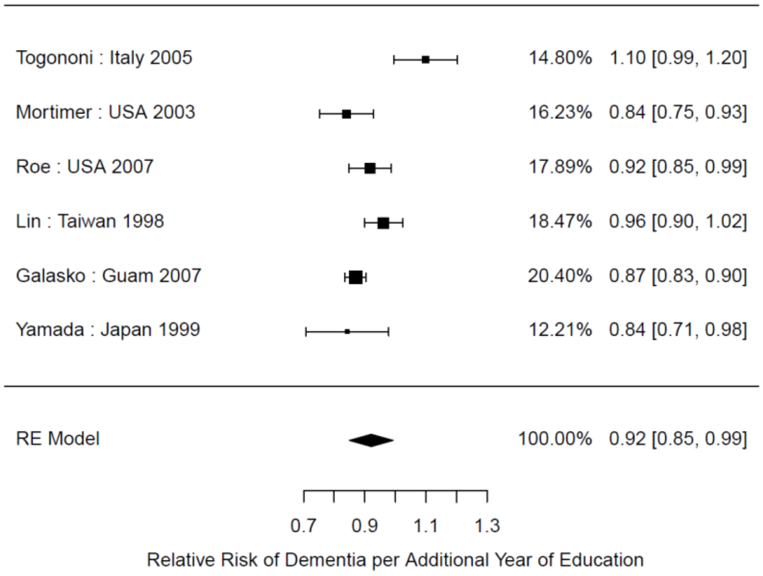

As described above, we used a crosswalk to standardise the claims data relative to existing literature data. This year we also added study-level covariates for studies that did not complete an in-person doctor diagnosis, that diagnosed dementia using a cutoff score algorithm, or that diagnosed dementia using only clinical records. We tested all covariates, and where a covariate was significant we retained it in the final model. The covariate on no in-person doctor diagnosis was not significant so was added as a z-cov, which increases the uncertainty. Age-standardised education was used as a proxy for general brain health/use that may be protective of dementia – specifically Alzheimer's disease. Smoking prevalence (age-

standardised, both sexes) was also used as a covariate to guide estimates, as the literature has shown a positive relationship between smoking and dementia.

We then use this model to set the level of dementia cause-specific mortality to be retrieved through the dementia redistribution process, which uses multiple cause of death data to determine from which causes the excess mortality should be retrieved. The COD data are then adjusted to reflect the cause-specific mortality rates predicted from the second DisMod-MR 2.1 model. Additional details on this process can be found in the COD capstone appendix.

We then run a second CODEm model after adjusting the data through the redistribution and noise-reduction processes and pull the cause-specific mortality results from this model into a final DisMod model, with the same settings as the models previous, but with the exclusion of the estimated excess mortality data. The table below provides additional information on the country covariates and study-level covariates used in this model, as well as beta and exponentiated beta values.

Variable	Measure	Beta	Exponentiated beta value (CI)
Mean years of education, age-standardised	prevalence	-0.083	0.92 (0.92 to 0.92)
Smoking prevalence, age-standardised, both sexes	prevalence	0.29	1.34 (1.27 to 1.41)
USA claims data	prevalence	-0.42	0.65 (0.62 to 0.69)
European GP data	prevalence	-0.95	0.38 (0.36 to 0.41)
Diagnosed using cutoff score algorithm	prevalence	0.15	1.17 (1.07 to 1.26)
Diagnosed using only clinical records	prevalence	-0.14	0.87 (0.78 to 0.96)

Parkinson's Disease

Flowchart

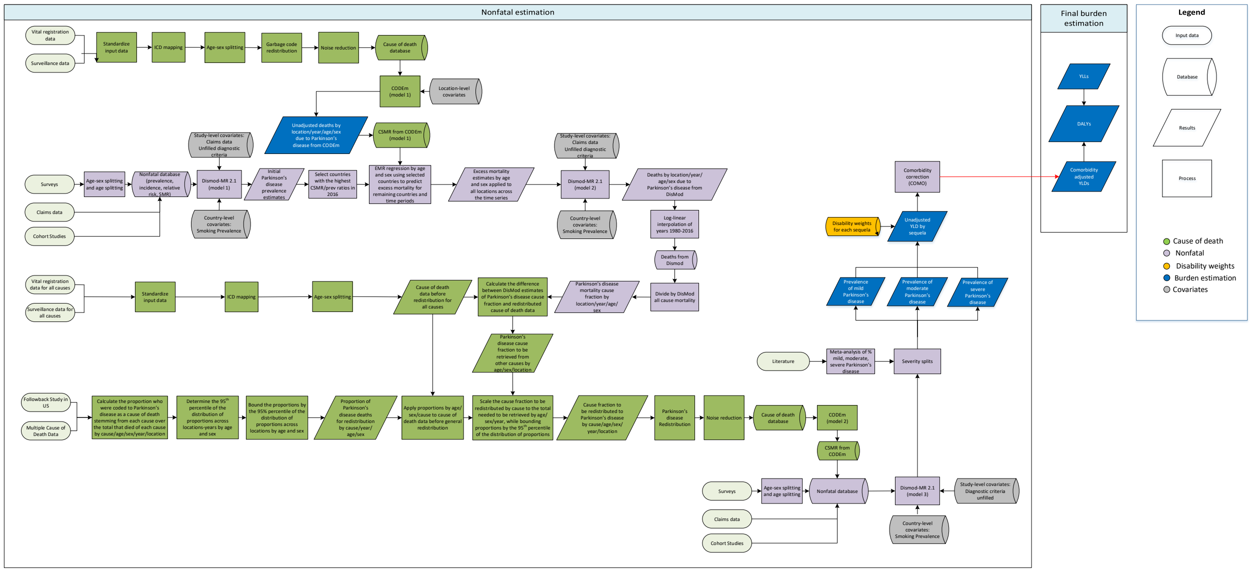

Case definition

Parkinson's disease is a chronic, degenerative, and progressive neurological condition typified by the loss of motor mobility and control – most notably tremors. The corresponding ICD-10 codes are G20, G21, and G22. Our case definition for GBD is the presence of at least two of the four primary symptoms: (1) tremors/trembling, (2) bradykinesia, (3) stiffness of limbs and torso, and (4) posture instability.

Unlike most causes in the Global Burden of Disease project, Parkinson's disease mortality and morbidity estimates are modelled jointly. This is because of marked discrepancies between prevalence data and cause of death data. Specifically, prevalence data suggest little to no variation over time (eg, 1990–2017) whereas age-standardised mortality rates in vital registrations in high-income countries have increased multiple times over this same period. Additionally, prevalence variation between countries is much smaller than the variation in death rates assigned to Parkinson's disease in vital registration. We attribute these discrepancies to changing coding practices rather than epidemiological change.

Because of this joint procedure, descriptions of the mortality estimation process are included where relevant.

Input data

Model inputs

To inform our estimates of burden due to Parkinson's disease, we use mortality data from vital registration systems, as well as prevalence data from surveys and administrative data such as claims sources.

An updated systematic review was conducted from September 2015 to August 2017, and the search terms were set to capture studies for Parkinson's disease.[1] This search term resulted in 660 initial hits with 20 sources marked for extraction. Studies with no clearly defined sample or that drew from specific clinic/patient organizations were excluded. A flowchart documenting this review is displayed below.

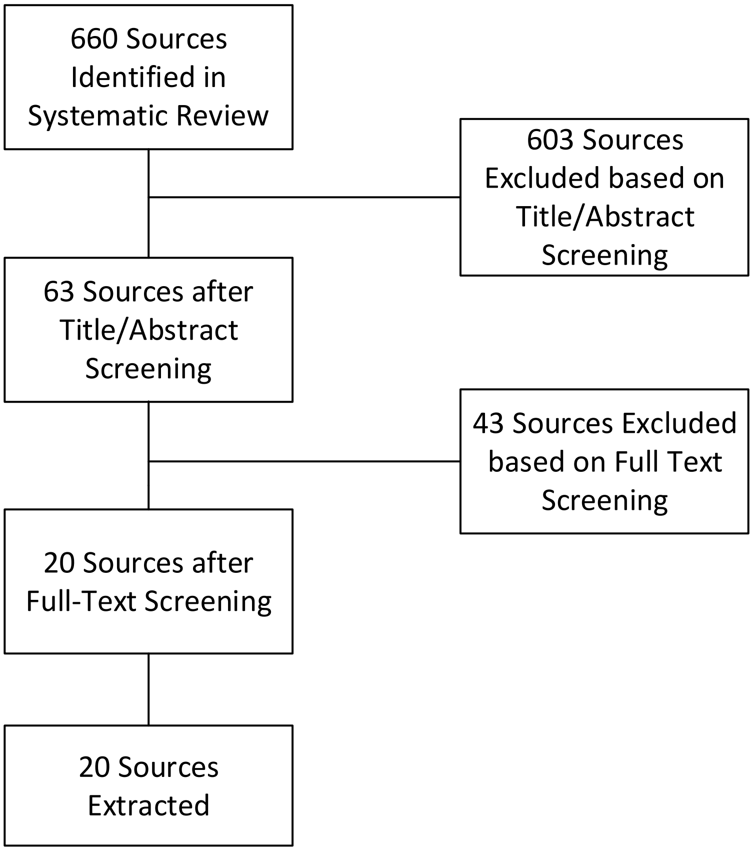

The following table provides a description of the density and distribution of literature data informing the Parkinson's estimates:

	Prevalence	Incidence	Mortality Risk
Site-years (total)	394	67	15
Number of countries with data	36	22	9
Number of GBD regions with data (out of 21 regions)	14	9	4
Number of GBD super-regions with data (out of 7 super-regions)	7	4	3

Beyond the exclusion of studies using non-representative populations, there are no substantial adjustment or outliering criteria for the Parksinson's model. Certain studies have been outliered on a

[1] (Parkinson disease[Title/Abstract] OR Parkinson's disease[Title/Abstract]) AND (epidemiology[Title/Abstract] OR prevalence[Title/Abstract] OR incidence[Title/Abstract]) AND ("2015/09/31"[PDAT] : "2017/08/23"[PDAT])

case-by-case basis due to subsequent review and exclusion due to inappropriateness of the study design, or case ascertainment that conflict with existing gold-standard data – where possible.

Studies with age and sex detail separately were split into age- and sex-specific data points. We also split data points where the age range was greater than 20 years using the age pattern from the United States, where we had the most detail by age.

This year we also added two additional years of claims data from the USA, and excluded claims data from 2000, which were systematically lower than other years. We also changed the algorithm for deriving prevalence from the claims data. Previously, an individual with any inpatient or outpatient visit in a given year would be counted as a previous case. However, this would pick up visits where a doctor may have used a potential or probable diagnosis without confirmation. Therefore, the new algorithm will only count a prevalent case where an individual has one inpatient visit, two outpatient visits, or one outpatient and one inpatient visit. This change to the algorithm resulted in a decrease in prevalence estimates for the United States.

Severity splits

As in GBD 2013, we use Hoehn and Yahr stages to determine severity. However, for GBD 2017, the cutpoints were updated in order to more accurately correspond with the lay descriptions of severities. Specifically, a Hoehn and Yahr stage 4 now corresponds to a designation of severe, where before it was classified as moderate.

Severity	Stage
Mild	≤2.0
Moderate	2.5-3.5
Severe	≥4

The following figures show the results of the meta-analysis on Hoehn and Yahr stage

Figure 1. Percentage of mild cases of Parkinson's disease in population-based studies

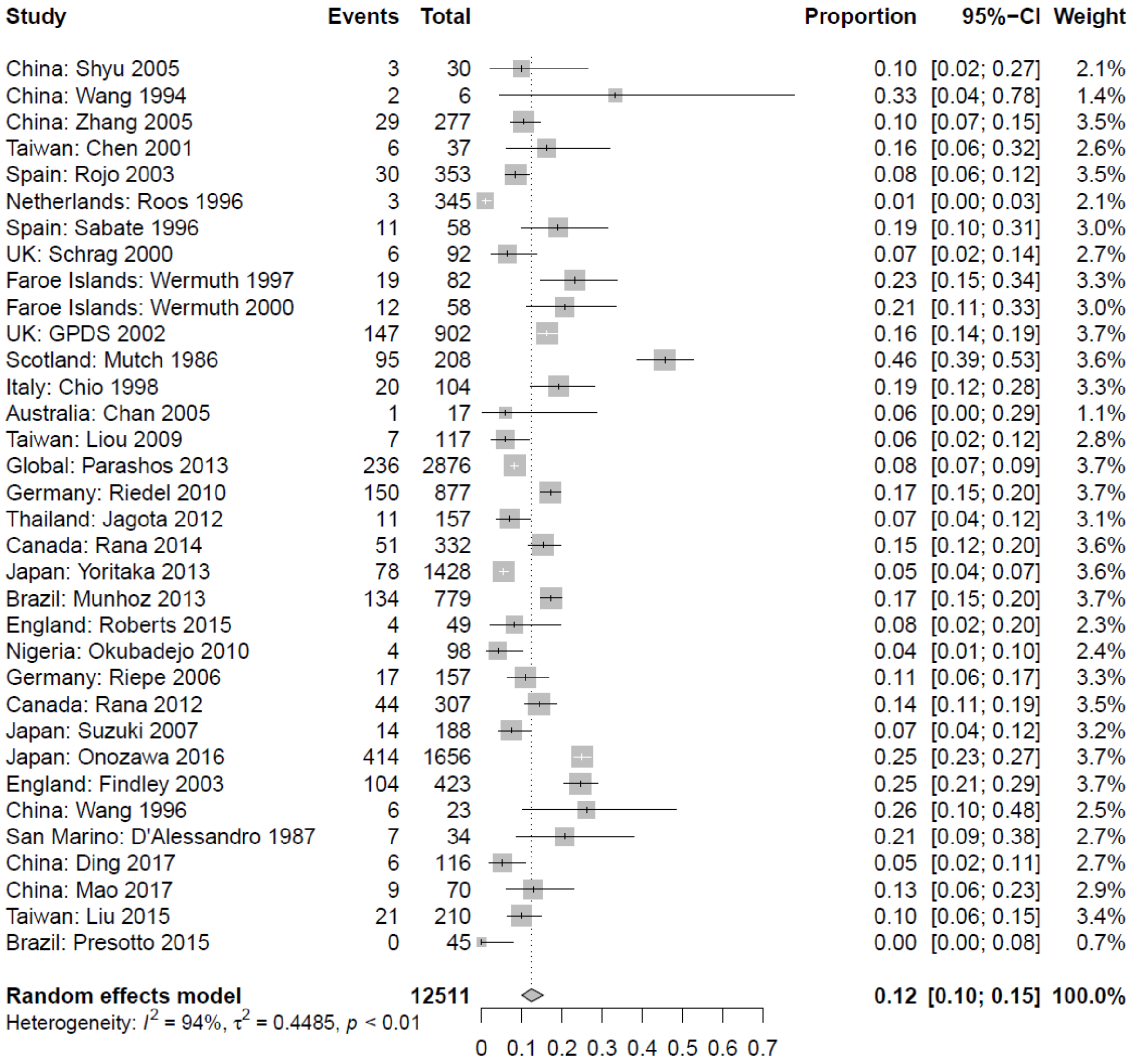

Figure 2. Percentage of moderate cases of Parkinson's disease in population-based studies

Study	Events	Total	Proportion	95%−CI	Weight
China: Shyu 2005	15	30	0.50	[0.31; 0.69]	2.4%
China: Wang 1994	2	6	0.33	[0.04; 0.78]	0.9%
China: Zhang 2005	51	277	0.18	[0.14; 0.23]	3.3%
Taiwan: Chen 2001	12	37	0.32	[0.18; 0.50]	2.5%
Spain: Rojo 2003	144	353	0.41	[0.36; 0.46]	3.5%
Netherlands: Roos 1996	94	345	0.27	[0.23; 0.32]	3.4%
Spain: Sabate 1996	12	58	0.21	[0.11; 0.33]	2.6%
UK: Schrag 2000	45	92	0.49	[0.38; 0.60]	3.1%
Faroe Islands: Wermuth 1997	16	82	0.20	[0.12; 0.30]	2.8%
Faroe Islands: Wermuth 2000	3	58	0.05	[0.01; 0.14]	1.6%
UK: GPDS 2002	444	902	0.49	[0.46; 0.53]	3.6%
Scotland: Mutch 1986	52	208	0.25	[0.19; 0.31]	3.3%
Italy: Chio 1998	15	104	0.14	[0.08; 0.23]	2.8%
Australia: Chan 2005	5	17	0.29	[0.10; 0.56]	1.8%
Taiwan: Liou 2009	18	117	0.15	[0.09; 0.23]	2.9%
Global: Parashos 2013	929	2876	0.32	[0.31; 0.34]	3.6%
Germany: Riedel 2010	339	877	0.39	[0.35; 0.42]	3.6%
Thailand: Jagota 2012	84	157	0.54	[0.45; 0.61]	3.3%
Canada: Rana 2014	121	332	0.36	[0.31; 0.42]	3.5%
Japan: Yoritaka 2013	414	1428	0.29	[0.27; 0.31]	3.6%
Brazil: Munhoz 2013	379	779	0.49	[0.45; 0.52]	3.6%
England: Roberts 2015	23	49	0.47	[0.33; 0.62]	2.8%
Nigeria: Okubadejo 2010	29	98	0.30	[0.21; 0.40]	3.1%
Germany: Riepe 2006	55	157	0.35	[0.28; 0.43]	3.3%
Canada: Rana 2012	105	307	0.34	[0.29; 0.40]	3.4%
Japan: Suzuki 2007	89	188	0.47	[0.40; 0.55]	3.4%
Japan: Onozawa 2016	959	1656	0.58	[0.55; 0.60]	3.6%
England: Findley 2003	120	423	0.28	[0.24; 0.33]	3.5%
China: Wang 1996	3	23	0.13	[0.03; 0.34]	1.5%
San Marino: D'Alessandro 1987	4	34	0.12	[0.03; 0.27]	1.8%
China: Ding 2017	57	116	0.49	[0.40; 0.59]	3.2%
China: Mao 2017	23	70	0.33	[0.22; 0.45]	2.9%
Taiwan: Liu 2015	44	210	0.21	[0.16; 0.27]	3.3%
Brazil: Presotto 2015	21	45	0.47	[0.32; 0.62]	2.7%
**Random effects model**		**12511**	**0.33**	**[0.29; 0.37]**	**100.0%**

Heterogeneity: $I^2 = 95\%$, $\tau^2 = 0.2645$, $p < 0.01$

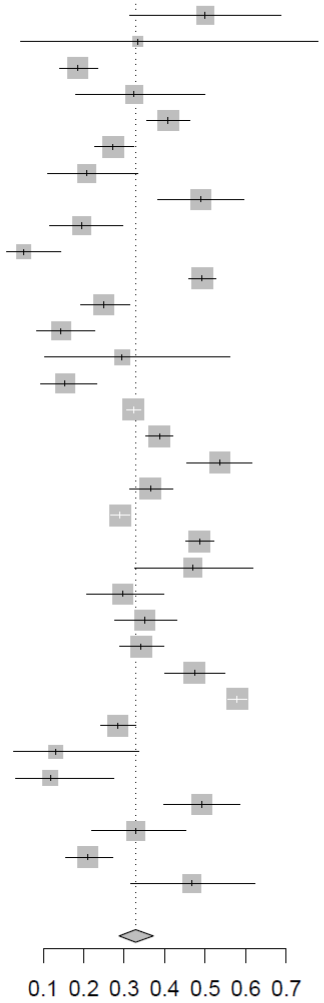

Figure 3. Percentage of severe cases of Parkinson's disease in population-based studies

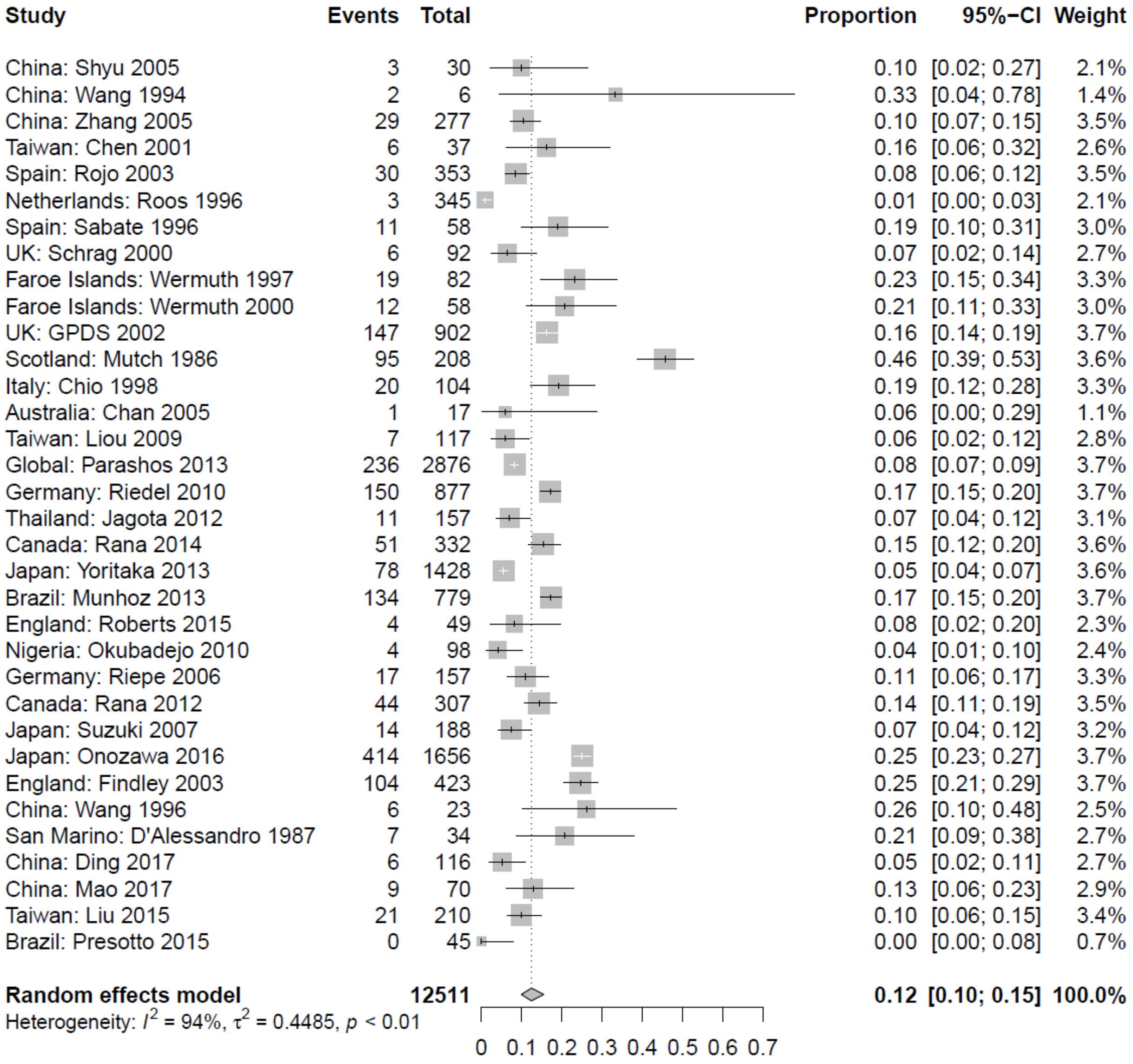

Severity estimates were generated by multiplying estimates of prevalence (country-year-sex-age-specific) by the fractions of mild, moderate, and severe PD, and 95% confidence intervals were estimated by taking 1,000 draws.

The following table provides the lay description and disability weights associated with Parkinson's disease.

Severity level	Lay description	DW (95% CI)
Mild	Has mild tremors and moves a little slowly, but is able to walk and do daily activities without assistance.	0.01 (0.005–0.019)
Moderate	Has moderate tremors and moves slowly, which causes some difficulty in walking and daily activities. The person has some trouble swallowing, talking, sleeping, and remembering things.	0.267 (0.181–0.372)
Severe	Has severe tremors and moves very slowly, which causes great difficulty in walking and daily activities. The person falls easily and has a lot of difficulty talking, swallowing, sleeping, and remembering things.	0.575 (0.396–0.73)

Modelling strategy

First, we ran a CODEm model for Parkinson's disease and extracted the mortality rates by age, sex, and geography for 2017.

Second, we ran a DisMod-MR 2.1 model with all data on incidence, prevalence, and mortality risk (relative risk, standardised mortality ratio, or with-condition mortality rates) and a setting of zero remission, and extracted 2017 prevalence by age, sex, and geography. To account for potential systematic differences between claims and survey data, we crosswalked for each year of claims data.

Third, we selected the countries (France, England, the United States, the Netherlands, Finland, Scotland, and Wales) with the highest cause-specific mortality rate (from step 1) to prevalence (from step two) ratio, which also had data on prevalence, prevalence rates above 0.0005, and a population greater than 1 million.

Fourth, we used a linear effects regression with dummies on age group and sex to predict excess mortality (ie, the ratio of cause-specific mortality rate and prevalence) by age and sex, the results of which are found in the tables below.

**Table: Fixed-effect coefficients of EMR regression. Outcome: ln(EMR)**					
Independent variables	Coef	Std. error	P value	95% Confidence interval	
Male	0.288	0.036	0.000	0.218	0.358
Age 40-59	-3.25	0.076	0.000	-3.399	-3.101
Age 60-64	-2.557	0.076	0.000	-2.706	-2.407
Age 65-69	-2.021	0.076	0.000	-2.17	-1.871
Age 70-74	-1.42	0.076	0.000	-1.57	-1.271

Age 75- 80	-0.898	0.076	0.000	-1.047	-0.749
Age 80-84	-0.502	0.076	0.000	-0.651	-0.352
Age 85-89	-0.248	0.076	0.001	-0.397	-0.099
Age 90-94	-0.047	0.076	0.537	-0.196	0.102
Constant	-2.357	0.057	0.000	-2.469	-2.246

Table: Predicted EMR values by age and sex (95% CI)		
	Male	Female
Age 40-59	0.005 (0.004 - 0.005)	0.004 (0.003 - 0.004)
Age 60-64	0.01 (0.009 - 0.011)	0.007 (0.007 - 0.008)
Age 65-69	0.017 (0.015 - 0.019)	0.013 (0.011 - 0.014)
Age 70-74	0.031 (0.027 - 0.034)	0.023 (0.02 - 0.025)
Age 75- 80	0.051 (0.046 - 0.057)	0.039 (0.035 - 0.043)
Age 80-84	0.076 (0.068 - 0.085)	0.058 (0.052 - 0.064)
Age 85-89	0.099 (0.089 - 0.111)	0.074 (0.066 - 0.083)
Age 90-94	0.12 (0.108 - 0.135)	0.09 (0.081 - 0.1)
Age 95+	0.126 (0.113 - 0.142)	0.095 (0.085 - 0.106)

Fifth, these estimates were added to a second DisMod-MR 2.1 model as pertaining to the full 1990–2017 estimation period. We allowed the countries included in the regression to retain their original EMR values when the age-standardised EMR for a country was higher than the age-standardised EMR prediction generated from the regression. These countries retained their age- and sex-specific ratios and entered those also as pertaining to the full 1990–2017 estimation period. Thus, the model reflects the cause-specific mortality rate if all countries over time would have had the average propensity to code to dementia as an underlying cause of death similar to the selected countries in 2017.

In this model, we assumed zero remission among all ages, with no incidence or excess mortality for ages zero to 20 years old. We ignored data on incidence, relative risk, standardised mortality ratio, and with-condition mortality, as these were shown to be inconsistent with prevalence estimates. This year, we decided not to allow random effects in the model in order to prevent spurious inflation of regional differences due to differences in measurement and measurement error.

We made one study-level crosswalk: diagnostic criteria. Studies that do not use the gold-standard case definition of presence of at least two of the four main symptoms are crosswalked to meet this gold standard definition. The table below shows the effect of this crosswalk, which results in a downward adjustment of non-standard data points. We have an additional study-level covariate for case ascertainment, or studies that ascertain cases on clinical record review rather than using live diagnostic processes. However, this adjustment was not significant for GBD 2017 and was therefore changed to a z-cov, which effects the uncertainty of the estimates.

In previous rounds of GBD, we used Socio-demographic Index as a country covariate proxy to capture possible social and cultural risk factors or modifiers of Parkinson's prevalence. However, the potential effect of SDI is likely confounded by study quality and case ascertainment, so we decided to remove that covariate. Due to the removal of the covariate on SDI, our estimates for Latin America and sub-Saharan Africa have significantly increased. We also included a covariate for smoking prevalence, as studies have found an inverse relationship between smoking and Parkinson's disease.

The following table provides an overview of the study-level and country covariates used in the second Parkinson's disease DisMod MR-2.1 model.

Covariate	Measure	Beta	Exponentiated
Smoking prevalence	prevalence	-0.66	0.52 (0.43 to 0.62)
(Un)Filled diagnostic criteria	prevalence	0.16	1.18 (1.11 to 1.24)

We then use this model to set the level of dementia cause-specific mortality to be retrieved through the dementia redistribution process, which uses multiple cause of death data to determine from which causes the excess mortality should be retrieved. The COD data are then adjusted to reflect the cause-specific mortality rates predicted from the second DisMod-MR 2.1 model. Additional details on this process can be found in the COD capstone appendix.

We then run a second CODEm model after adjusting the data through the redistribution and noise-reduction processes, and pull the cause-specific mortality results from this model into a final DisMod model, with the same settings as the models previous, but with the exclusion of the estimated excess mortality data.

The following table provides an overview of the study-level and country covariates used in the third Parkinson's disease DisMod MR-2.1 model.

Covariate	Measure	Beta	Exponentiated
Smoking prevalence	prevalence	-0.62	0.54 (0.44 to 0.63)
(Un)Filled diagnostic criteria	prevalence	0.15	1.16 (1.09 to 1.23)

Multiple sclerosis (MS)

Flowchart

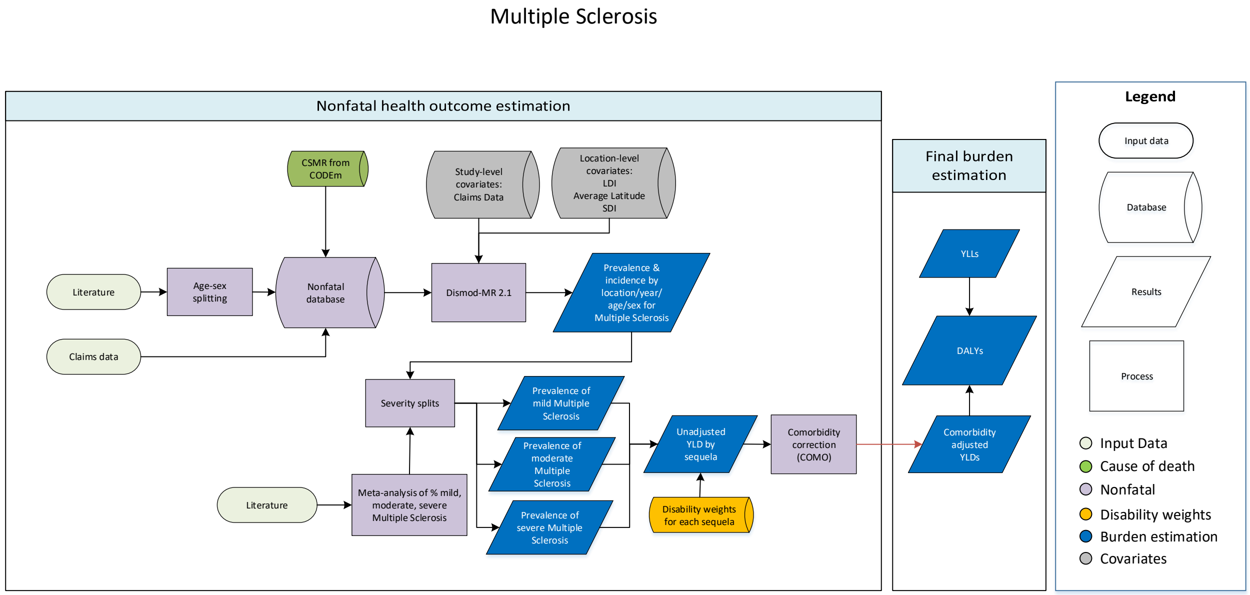

Input data and methodological summary

Case definition

Multiple sclerosis is a chronic, degenerative, and progressive neurological condition typified by the damaging of the myelin sheaths. For GBD, the McDonald's criteria for diagnosis are considered the gold standard, but other definitions such as Poser Committee's criteria and self-report of a doctor's diagnosis are also included. The ICD-10 code for MS is G35.

Input data

An updated systematic review was done using the search string ((("multiple sclerosis"[Title/Abstract]) AND ((epidemiology[Title/Abstract] OR prevalence[Title/Abstract] OR incidence[Title/Abstract]))) AND ("2015/06/15"[Date - Publication] : "2017/09/13"[Date - Publication])) from 5/15/2015-9/13/17 yielded 733 hits with 28 sources marked for extraction. A flowchart documenting this review is displayed below.

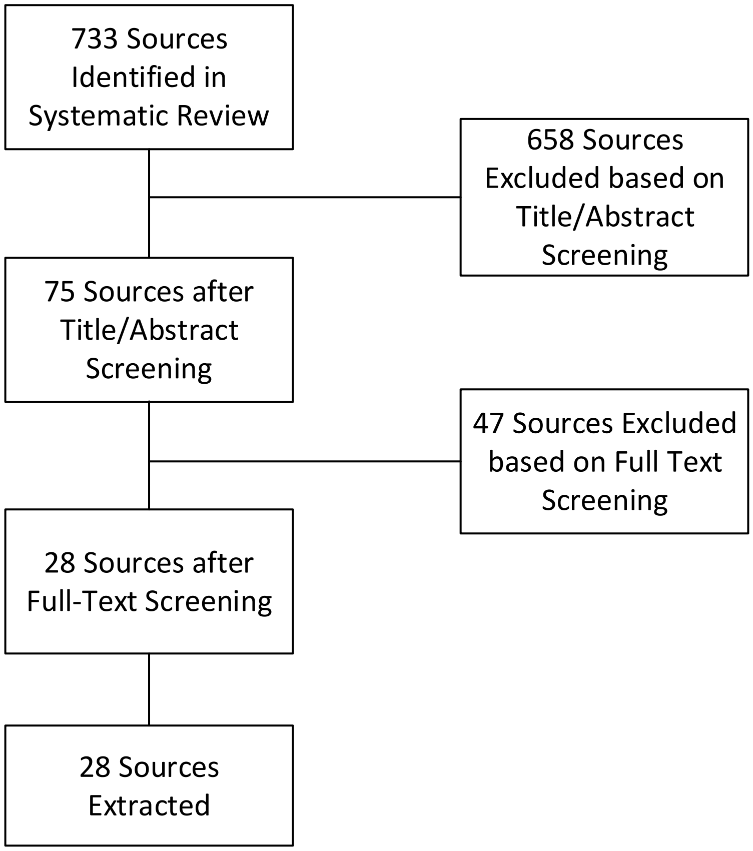

The data underpinning estimates of burden due to MS are generally of two types. The first are representative, population-based surveys. This includes retrospective case/hospital report analysis, nationally representative health studies, and the like. Studies with no clearly defined sample or that draw from specific clinic/patient organisations were excluded during the systematic review phase.

The second type are claims data. This year we added three years of claims data from the USA and one year of claims data from Taiwain. We also changed the algorithm for deriving prevalence from the claims data. Previously, an individual with any inpatient or outpatient visit in a given year would be counted as a previous case. However, this would pick up visits where a doctor may have used a potential or probable diagnosis without confirmation. Therefore, the new algorithm will only count a prevalent case where an individual has one inpatient visit, two outpatient visits, or one outpatient and one inpatient visit. This change to the algorithm resulted in a decrease in prevalence estimates for the United States.

The following table provides a description of the density and distribution of literature data informing the MS estimates:

	Prevalence	Incidence	Excess mortality rate	Age-standardised mortality risk
Site-years (total)	489	267	6	8
Number of countries with data	38	28	5	6
Number of GBD regions with data (out of 21 regions)	10	8	2	2
Number of GBD super-regions with data (out of 7 super-regions)	5	4	1	1

Beyond the exclusion of studies using non-representative populations, there are no substantial adjustment or outliering criteria for the MS model. Certain studies have been outliered on a case-by-case basis due to: (1) subsequent review and exclusion due to inappropriate of the study design, and overly broad age and sex groups that conflict with existing gold standard age-sex specific data – where possible.

Studies with age and sex detail separately were split into age- and sex-specific data points. We also split data points where the age range was greater than 20 years using the age pattern from the United States, where we had the most detail by age.

Severity splits

As in GBD 2013, we used Kurtzke's Expanded Disability Status Scale (EDSS) to determine severity splits for MS. The EDSS scores corresponding to each severity are as follows:

Asymptomatic: EDSS = 0
Mild: 0 < EDSS ≤ 3.5
Moderate: 3.5 < EDSS ≤ 6.5
Severe: 6.5 < EDSS ≤ 9.5

The table below illustrates severity levels, lay descriptions, and DWs.

Severity level	Lay description	DW (95% CI)
Asymptomatic	-	0 (0-0)
Mild	Has mild loss of feeling in one hand, is a little unsteady while walking, has slight loss of vision in one eye, and often needs to urinate urgently.	0.183 (0.124–0.253)
Moderate	Needs help walking, has difficulty with writing and arm coordination, has loss of vision in one eye and cannot control urinating.	0.463 (0.313–0.613)
Severe	Has slurred speech and difficulty swallowing. The person has weak arms and hands, very limited and stiff leg movement, has loss of vision in both eyes and cannot control urinating.	0.719 (0.534–0.858)

Because not all sources had information on the number of cases with EDSS stage 0, instead reporting on a mild category, we implemented a two-step meta-analysis strategy. First, we subsetted the studies to those that reported on the number of cases with EDSS stage 0, and did meta-analyses on the proportion of asymptomatic and mild cases. Then, we conducted meta-analyses on the full dataset to get the proportion mild, moderate, and severe, and we squeezed the asymptomatic and mild categories from the previous meta-analyses into the mild category established by the meta-analysis on the full dataset.

The following figures provide the result of the first meta-analysis on the asymptomatic and mild categories.

Figure 1. Asymptomatic cases of MS

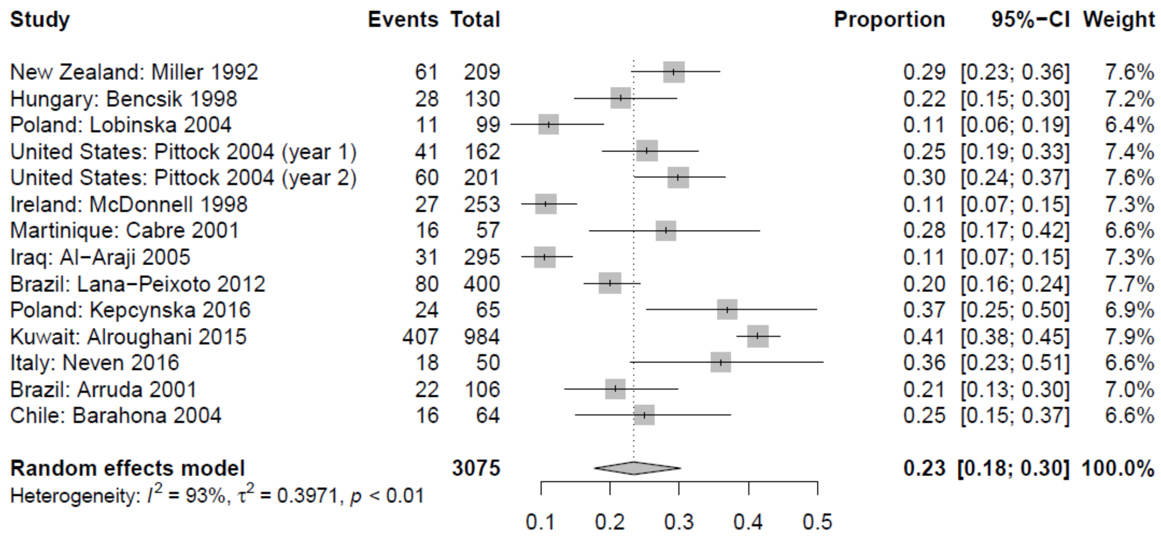

Figure 2. Mild cases of MS

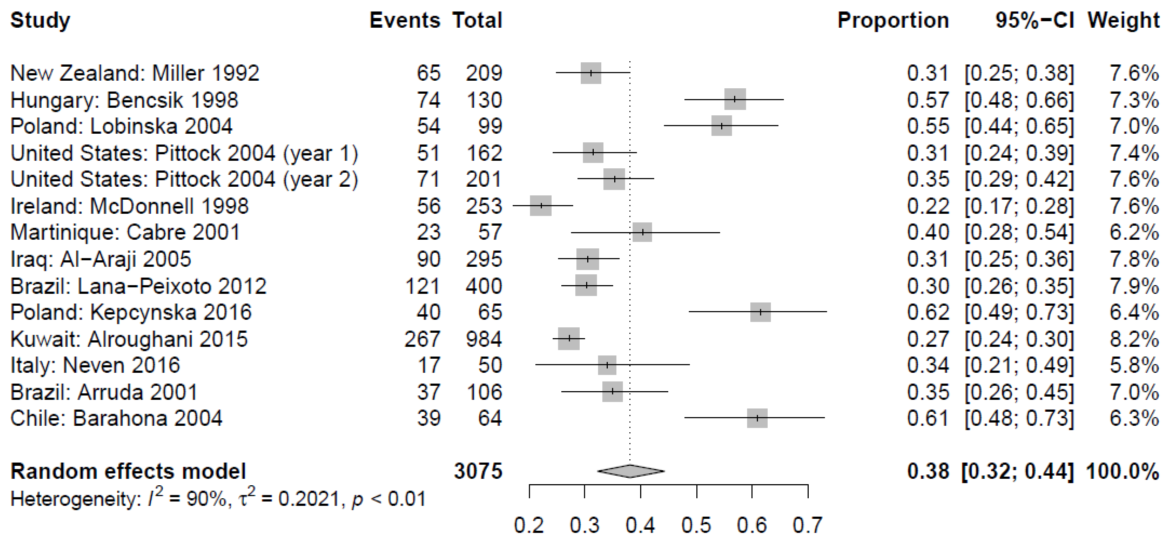

The following figures provide the result of the second meta-analysis on the mild, moderate, and severe categories.

Figure 3. Mild cases of MS (including both asymptomatic and mild categories)

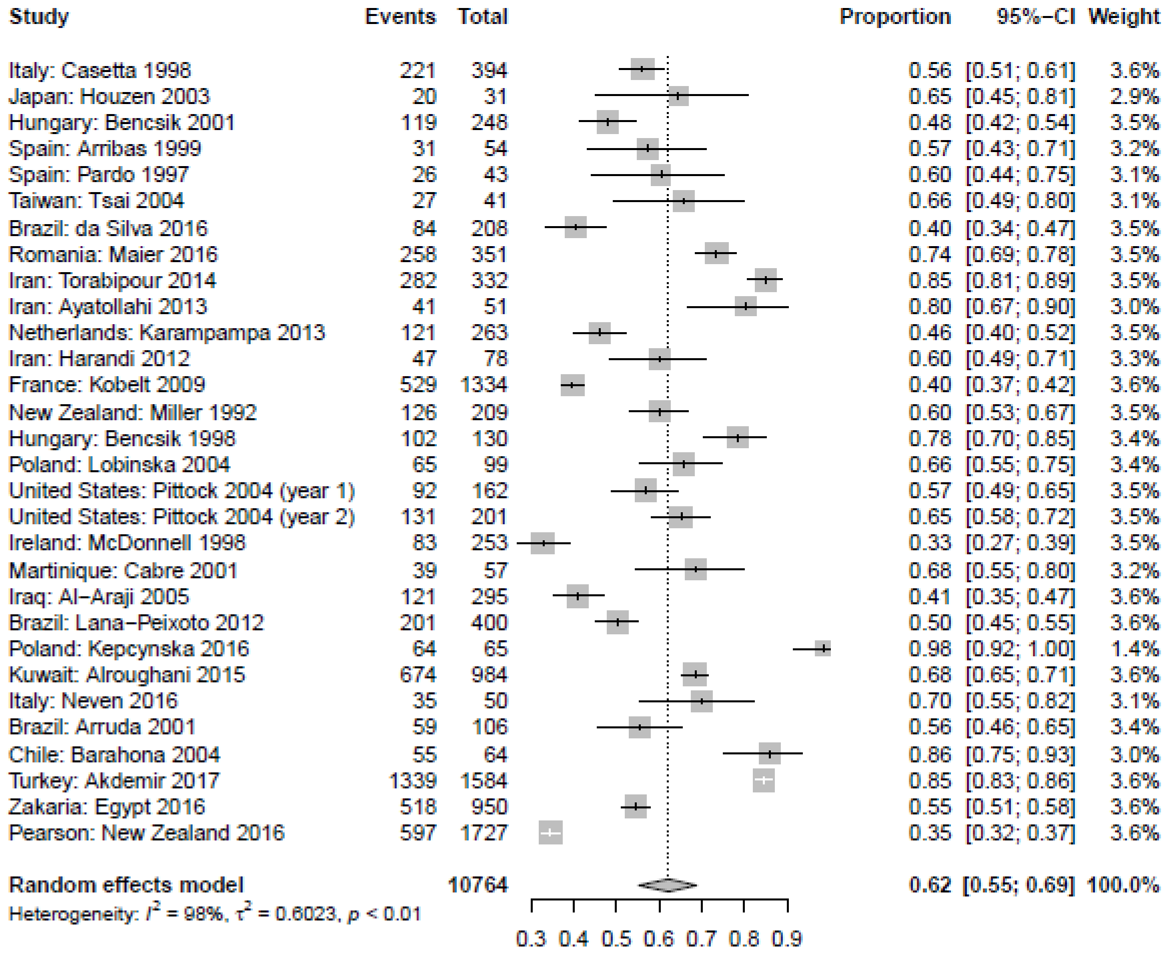

Figure 4. Moderate cases of MS

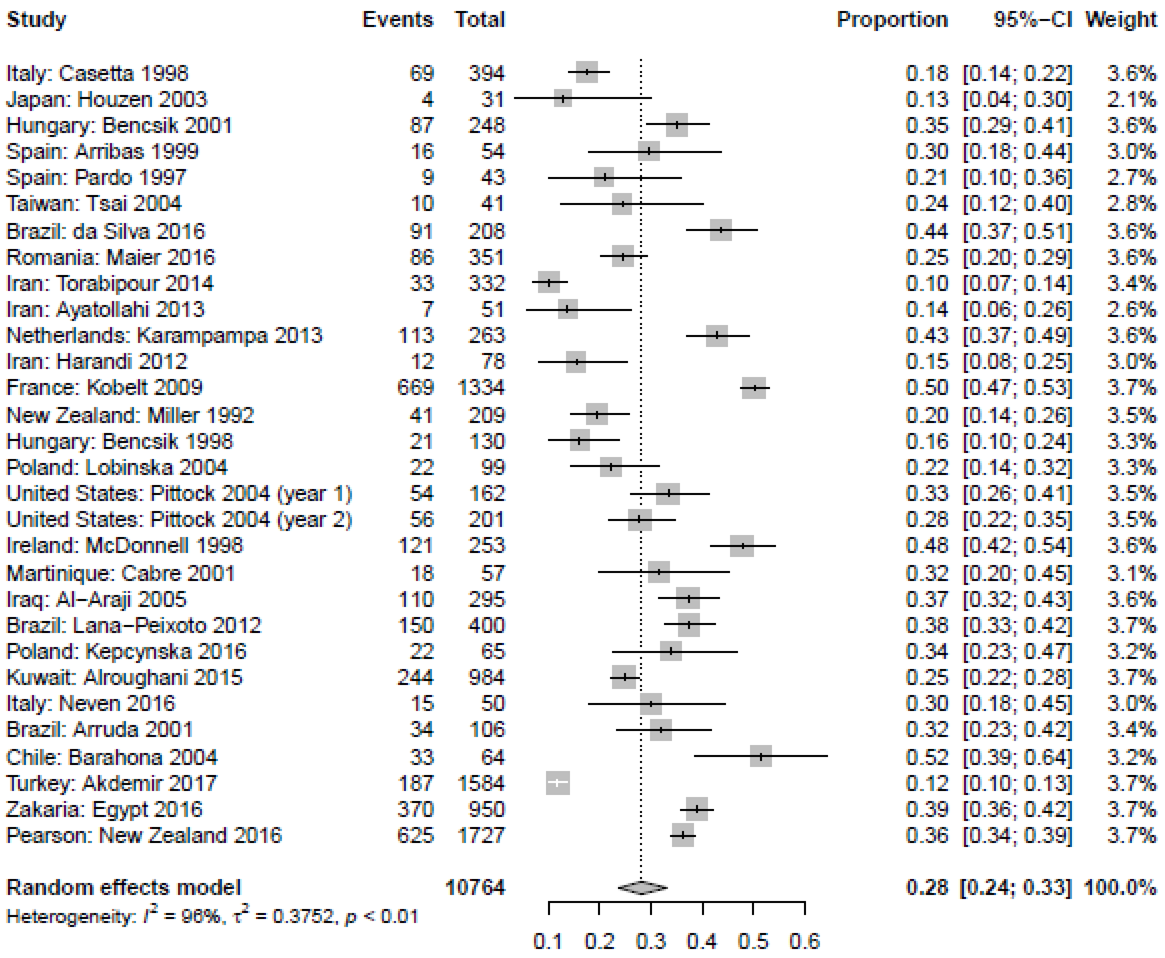

Figure 5. Severe cases of MS

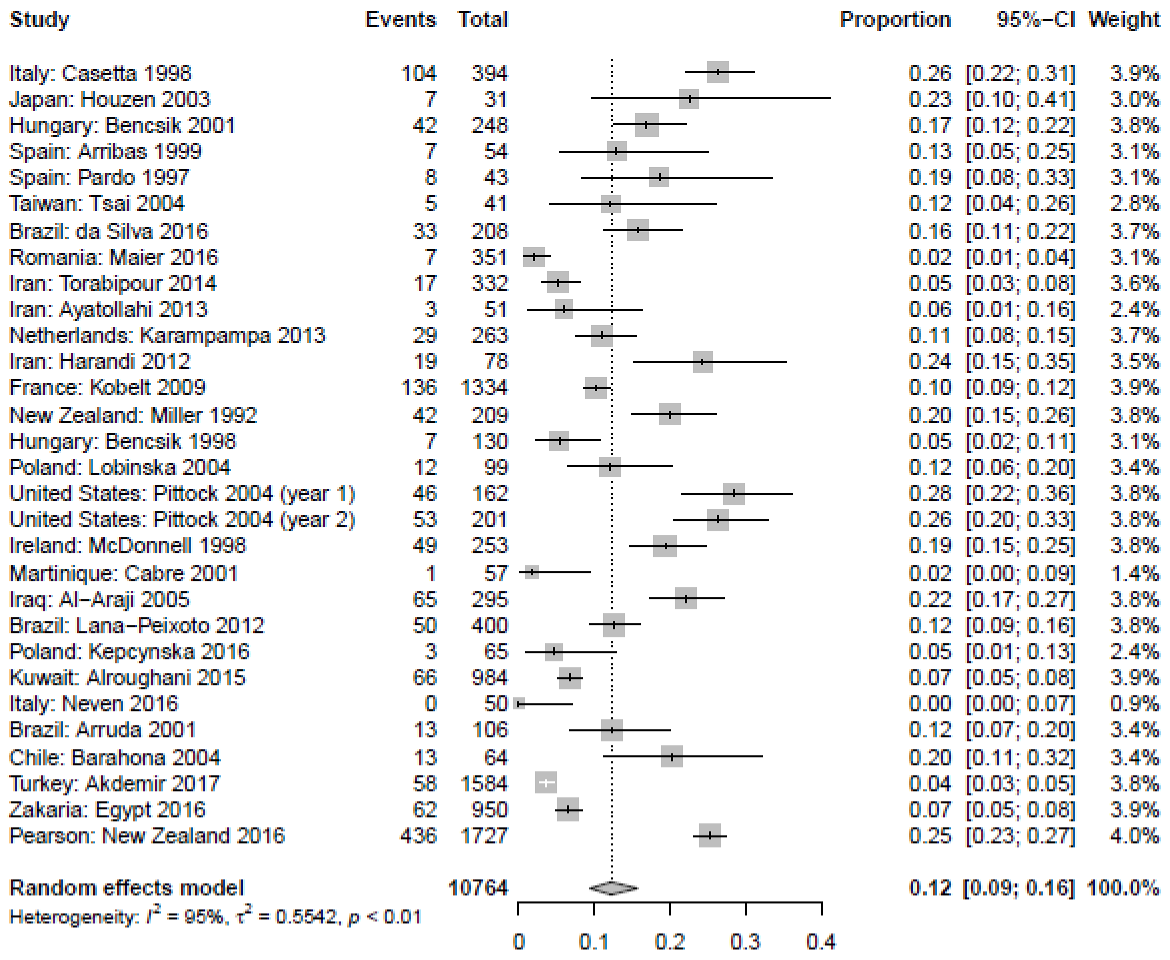

Modelling strategy

We use DisMod 2.1 as the main analytical tool for the MS estimation process. Prior settings include zero remission for all ages, and no incidence or excess mortality for persons under 4 years old. We also constrain the super-region random effects for prevalence and incidence to -0.5 and 0.5 to account for spurious inflation of regional differences.

USA claims data for 2000 are adjusted via a study covariate to account for systematically low estimates relative to other years of data. Implicit in this adjustment is the assumption that variation between years of claims data is a function of data-collection inconsistencies and noise.

Similar to other cases we use GBD estimates of cause-specific mortality rate (CSMR) and excess mortality rate (EMR) in this model.

To assist the estimation process, we use several country-level covariates. These effects plus those of the study covariates are presented below.

Covariate	Measure	Beta	Exponentiated	Parameter type

Absolute value of average latitude	prevalence	0.065	1.07 (1.03 to 1.24)	Country-level
Absolute value of average latitude	incidence	0.029	1.03 (1.02 to 1.04)	Country-level
All MarketScan, year 2000	prevalence	-0.34	0.71 (0.64 to 1.00)	x-cov
Healthcare Access and Quality index	excess mortality rate	-0.084	0.92 (0.66 to 0.97)	Country-level

As described in the literature, extreme latitude is associated with higher prevalence and incidence of MS. While the pathway that affects MS is not fully understood, our results suggest a sizable relationship. Our operationalisation of latitude is created by a population-weighted average of latitude by country and taking the absolute value. The underlying population distribution rasters are part of the Gridded Population of the World dataset.

Although there are no known cures for MS, we expect disease management to differ globally – largely as a function of available resources. To capture this, we use the Healthcare Access and Quality index covariate to capture this relationship in the estimation of excess mortality.

Motor neuron diseases

Flowchart

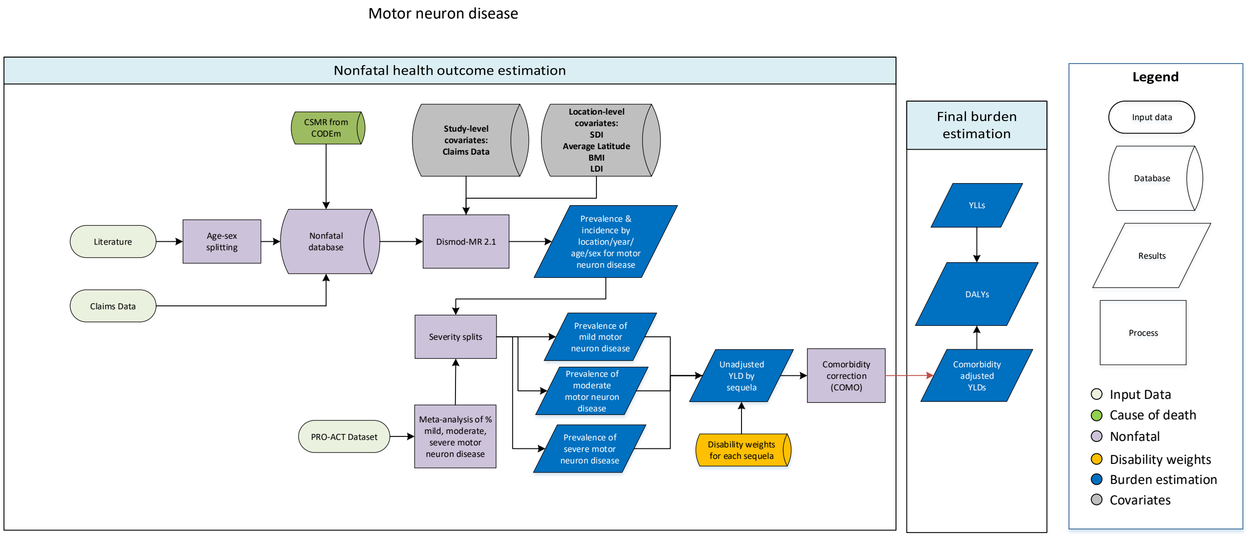

Case definition

Motor neuron diseases (MND) are a set of chronic, degenerative, and progressive neurological conditions typified by the destruction of motor neurons and the subsequent deterioration of voluntary muscle activity. The most common MND is amyotrophic lateral sclerosis. The ICD-10 code corresponding to motor neuron diseases is G12. Our gold standard diagnostic criteria are the El Escorial Criteria, with other similar criteria (eg, the original set from World Federation of Neurology) if necessary.

Input data

A full systematic review was conducted for GBD 2015. The following search string guided our search, which resulted in 3,146 hits with 58 sources meeting extraction criteria: (1) the study is a representative population-based study, (2) reports on prevalence, incidence, remission, excess mortality, relative risk of mortality, standardised mortality ratio, or with-condition mortality rate. Studies with no clearly defined sample were excluded.

(('motor neuron disease'[MeSH Terms] OR ('motor'[All Fields] AND 'neuron'[All Fields] AND 'disease'[All Fields]) OR 'motor neuron disease'[All Fields] OR ('motor'[All Fields] AND 'neuron'[All Fields] AND 'diseases'[All Fields]) OR 'motor neuron diseases'[All Fields]) OR ('amyotrophic lateral sclerosis'[MeSH Terms] OR ('amyotrophic'[All Fields] AND 'lateral'[All Fields] AND 'sclerosis'[All Fields]) OR 'amyotrophic lateral sclerosis'[All Fields]) OR ALS[All Fields] OR ('motor neuron disease'[MeSH Terms] OR ('motor'[All Fields] AND 'neuron'[All Fields] AND 'disease'[All Fields]) OR 'motor neuron disease'[All Fields] OR ('primary'[All Fields] AND 'lateral'[All Fields] AND 'sclerosis'[All Fields]) OR 'primary lateral sclerosis'[All Fields]) OR ('Politics Life Sci'[Journal] OR 'pls'[All Fields]) OR ('muscular atrophy, spinal'[MeSH Terms] OR ('muscular'[All Fields] AND 'atrophy'[All Fields] AND 'spinal'[All Fields]) OR 'spinal muscular atrophy'[All Fields] OR ('progressive'[All Fields] AND 'muscular'[All Fields] AND 'atrophy'[All Fields]) OR 'progressive muscular atrophy'[All Fields]) OR PBP[All Fields] OR ('pseudobulbar palsy'[MeSH Terms] OR ('pseudobulbar'[All Fields] AND 'palsy'[All Fields]) OR 'pseudobulbar palsy'[All Fields])) AND

(('epidemiology'[Subheading] OR 'epidemiology'[All Fields] OR 'epidemiology'[MeSH Terms]) OR population-based[All Fields])

The following table provides an overview of the density and distribution of the data used for GBD 2017.

	Prevalence	Incidence	Excess mortality rate	Other
Site-years (total)	315	101	2	1
Number of countries with data	7	17	2	1
Number of GBD regions with data (out of 21 regions)	6	7	1	1
Number of GBD super-regions with data (out of 7 super-regions)	5	4	1	1

Beyond the literature data, we also make use of claims data from the United States. This year we also added three additional years of claims data from the USA, so we now make use of claims data from 2000 and 2010–2014. We also changed the algorithm for deriving prevalence from the claims data. Previously, an individual with any inpatient or outpatient visit in a given year would be counted as a previous case. However, this would pick up visits where a doctor may have used a potential or probable diagnosis without confirmation. Therefore, the new algorithm will only count a prevalent case where an individual has one inpatient visit, two outpatient visits, or one outpatient and one inpatient visit. This change to the algorithm resulted in a decrease in prevalence estimates for the United States. We also added one year of claims data for Taiwan.

Except for excluding studies using non-representative populations, there are no substantial adjustments or outliering criteria for the MND model. Certain studies have been outliered on a case-by-case basis due to subsequent review and exclusion due to inappropriateness of the study design and case definition.

Severity splits

To calculate severity and disability due to MND we analysed a dataset from Pooled Resource Open-access ALS Clinical Trials (PRO-ACT). This dataset contains the largest ALS clinical trials dataset, with a total of 8,635 ALS patient records from multiple completed clinical trials. Among these, we conducted the final analysis with n=4838 (56%) of the patients with complete ALS Function Rating Score (ALSFRS) with average follow-up time of 184 days (min: -22, max: 648), in which 2,999 (62%) received experimental (medication) treatments and 1,301 (27%) received placebo (in these trials, the medications tested were found to be no better than placebo with respect to their effects on ALS progressions).

The ALSFRS is an instrument for evaluating the functional status of patients with amyotrophic lateral sclerosis. It can be used to monitor functional changes in a patient over time. It measures (1) speech, (2) salivation, (3) swallowing, (4) handwriting, (5) cutting food and handling utensils (with or without gastrostomy), (6) dressing and hygiene, (7) turning in bed and adjusting bed clothes, (8) walking, (9) climbing stairs, and (10) breathing. Each task is rated on a 5-point scale from 0 = can't do, to 4 = normal ability. Individual item scores are summed to produce a reported total score of between 0 and 40 (worst to best). ALSFRS has been revised to ALSFRS-R, which includes 12 questions (ALSFRS Q10 changes to (10)

Dyspnea, (11) Orthopnea, and (12) Respiratory insufficiency), with individual item scores summed to a score between 0 and 48.

In order to eliminate any bias from the treatment effects on the ALSFRS, only the first observation at the time of trial is selected. If the first observation is missing at the time of trial (or prior), the next non-missing observation is selected to be included in the final analysis.

We subsequently mapped ALSFRS scores into GBD severities, and sequelae into different combinations of speech problems, chronic obstructive pulmonary disease, and motor impairment using the following logic:

Motor impairment

The ALSFRS assess motor function of the legs through questions on walking (Q8) and stair climbing (Q9).

Combined score	Severity level
8	None
5-7	Mild
2-4	Moderate
0-1	Severe

The ALSFRS also assesses motor impairment through questions on handwriting (Q4), cutting food and handling utensils (Q5), and dressing and hygiene (Q6).

Combined score	Severity level
12	None
9-11	Mild
3-8	Moderate
0-2	Severe

After determining case severity on these two separate metrics, we aggregate by taking the most severe ranking (eg, severe + mild = a severe case).

Respiratory problems:

Question 10 of the ALSFRS describes breathing difficulty as a function of MND.

ALSFRS score	Description	Severity level
4	Normal	None
3	Shortness of breath with minimal exertion	Mild
2	Shortness of breath at rest	Moderate
0-1	Intermittent ventilator assistance required/ventilator-dependent	Severe

Speech problems

Speech impairment due to MND is derived from ALSFRS question 1, which describes speech impediments. A score of 4 on this question denotes no impairment, while all other values suggest some impairment.

Creating sequelae

After determining the severity status of each case for the three symptom umbrellas, we subsequently estimated the relative proportion of each combination of symptom class and their respective severities. Those without any symptoms (eg, no severity) were categorised as having worry about the diagnosis for disability estimation. The following table displays the various sequelae and their associated proportions.

Sequela	Proportion (Mean)	Proportion (Lower)	Proportion (Upper)
Mild motor impairment, mild respiratory problems and speech problems due to motor neuron disease	0.01779	0.01658	0.01909
Mild motor impairment, moderate respiratory problems and speech problems due to motor neuron disease	0.00270	0.00225	0.00324
Mild motor impairment, severe respiratory problems and speech problems due to motor neuron disease	0.00082	0.00059	0.00113
Mild motor impairment, and speech problems due to motor neuron disease	0.02052	0.01922	0.02190
Moderate motor impairment, mild respiratory problems and speech problems due to motor neuron disease	0.03377	0.03210	0.03552
Moderate motor impairment, moderate respiratory problems and speech problems due to motor neuron disease	0.00715	0.00640	0.00799
Moderate motor impairment, severe respiratory problems and speech problems due to motor neuron disease	0.00286	0.00240	0.00342
Moderate motor impairment, and speech problems due to motor neuron disease	0.03041	0.02883	0.03208
Severe motor impairment, mild respiratory problems and speech problems due to motor neuron disease	0.05242	0.05035	0.05457
Severe motor impairment, moderate respiratory problems and speech problems due to motor neuron disease	0.02247	0.02111	0.02392
Severe motor impairment, severe respiratory problems and speech problems due to motor neuron disease	0.01365	0.01259	0.01479
Severe motor impairment and speech problems due to motor neuron disease	0.04765	0.04567	0.04970
Mild respiratory problems and speech problems due to motor neuron disease	0.01157	0.01060	0.01263
Moderate respiratory problems and speech problems due to motor neuron disease	0.00142	0.00111	0.00182
Severe respiratory problems and speech problems due to motor neuron disease	0.00023	0.00013	0.00043
Speech problems due to motor neuron disease	0.02457	0.02315	0.02608
Mild motor impairment and mild respiratory problems due to motor neuron disease	0.02245	0.02109	0.02389
Mild motor impairment and moderate respiratory problems due to motor neuron disease	0.00275	0.00230	0.00329

Mild motor impairment and severe respiratory problems due to motor neuron disease	0.00068	0.00047	0.00097
Mild motor impairment due to motor neuron disease	0.10388	0.10103	0.10681
Moderate motor impairment and mild respiratory problems due to motor neuron disease	0.06744	0.06511	0.06985
Moderate motor impairment and moderate respiratory problems due to motor neuron disease	0.01302	0.01199	0.01413
Moderate motor impairment and severe respiratory problems due to motor neuron disease	0.00412	0.00356	0.00477
Moderate motor impairment due to motor neuron disease	0.20136	0.19760	0.20518
Severe motor impairment and mild respiratory problems due to motor neuron disease	0.06902	0.06666	0.07146
Severe motor impairment and moderate respiratory problems due to motor neuron disease	0.02000	0.01872	0.02137
Severe motor impairment and severe respiratory problems due to motor neuron disease	0.01062	0.00969	0.01163
Severe motor impairment due to motor neuron disease	0.15037	0.14702	0.15378
Mild respiratory problems due to motor neuron disease	0.00643	0.00571	0.00723
Moderate respiratory problems due to motor neuron disease	0.00044	0.00028	0.00069
Severe respiratory problems due to motor neuron disease	0.00005	0.00001	0.00017
Asymptomatic, but worry about diagnosis due to motor neuron disease	0.03738	0.03562	0.03921

To determine disability due to these sequelae, we use the standard multiplicative aggregation formula as described in the main text. The following table provides description and disability weight assigned to the sequelae as appropriate.

Symptom group	Severity level	Lay description	DW (95%)
Respiratory problems	Asymptomatic		
Respiratory problems	Mild	Has cough and shortness of breath after heavy physical activity, but is able to walk long distances and climb stairs.	0.019 (0.011–0.033)
Respiratory problems	Moderate	Has cough, wheezing, and shortness of breath, even after light physical activity. The person feels tired and can walk only short distances or climb only a few stairs.	0.225 (0.153–0.31)
Respiratory problems	Severe	Has cough, wheezing, and shortness of breath all the time. The person has great difficulty walking even short distances or climbing any stairs, feels tired when at rest, and is anxious.	0.408 (0.273–0.556)
Motor impairment	Asymptomatic		

Motor impairment	Mild	Has some difficulty in moving around but is able to walk without help.	0.01 (0.005–0.019)
Motor impairment	Moderate	Has some difficulty in moving around and difficulty in lifting and holding objects, dressing, and sitting upright, but is able to walk without help.	0.061 (0.04–0.089)
Motor impairment	Severe	Is unable to move around without help, and is not able to lift or hold objects, get dressed, or sit upright.	0.402 (0.268–0.545)
Speech problems	No		
Speech problems	Yes	Has difficulty speaking, and others find it difficult to understand.	0.051 (0.032–0.078)
Asymptomatic, but worry	Yes	Has a disease diagnosis that causes some worry but minimal interference with daily activities.	0.012 (0.006–0.023)

Modelling strategy

We use DisMod 2.1 as the main analytical tool for MND estimation. Prior settings are limited to 0 remission at all ages. We also constrain the super-region random effects for prevalence and incidence to -0.5 and 0.5 to account for spurious inflation of regional differences.

Claims data for 2000 are adjusted via a study covariate to account for systematically low estimates relative to other available claims data. Implicit in this adjustment is the assumption that variation between years of claims data is a function of data-collection inconsistencies and noise.

Similar to other cases we use GBD estimates of cause-specific mortality rate (CSMR) and excess mortality rate (EMR) in this model. The source and estimation of these rates are discussed elsewhere.

To assist the estimation process we use several country-level covariates.

Covariate	Measure	Beta	Exponentiated
Absolute value of average latitude	Prevalence	0.004	1.00 (1.00 to 1.01)
LDI (I$ per capita)	Excess mortality rate	-0.50	0.61 (0.61 to 0.61)
All MarketScan, year 2000	Prevalence	-0.038	0.96 (0.94 to 0.98)

Although there are no known cures for MND, we expect disease management to differ globally – largely as a function of available resources. To capture this, we use the natural log of lagged distributed income per capita as a proxy to capture this relationship in the estimation of excess mortality.

As described in the literature, extreme latitude may be associated with higher prevalence and incidence of motor neuron disease. While the pathway that affects motor neuron disease is not fully understood,

our results suggest a relationship. Our operationalisation of latitude is created by a population-weighted average of latitude by country and taking the absolute value. The underlying population distribution rasters are part of the Gridded Population of the World dataset.

Headaches

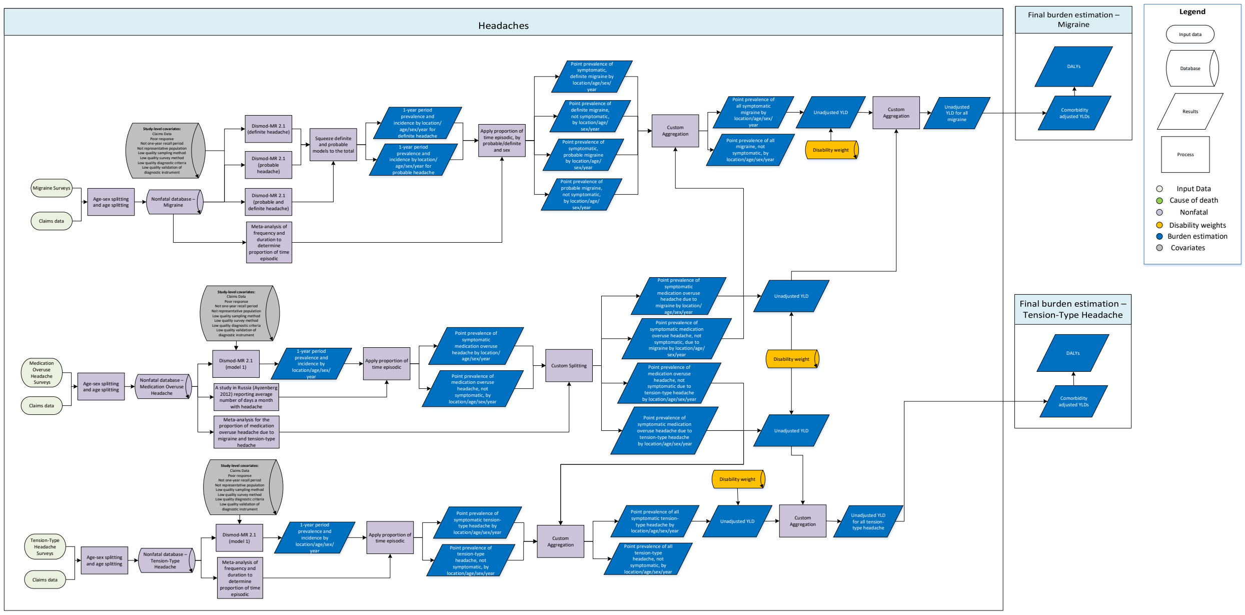

Case Definitions

Migraine

Migraine is a disabling primary headache disorder, typically characterised by recurrent moderate or severe unilateral pulsatile headaches. The ICD-10 code for migraine is G43 and ICD-9 code is 346. The two major types are migraine without aura and migraine with aura (transient neurological symptoms). In GBD, we do not distinguish between migraine with and without aura as most epidemiological studies report on overall migraine only. The reference diagnostic criteria for migraine are from the International Classification of Headache Disorders (ICHD)-3, which describe five criteria:

At least five attacks fulfilling criteria 2-5

1. Headache attacks lasting 4-72 hr (untreated or unsuccessfully treated)
2. Headache has at least two of the following four characteristics:
    a. Unilateral location
    b. Pulsating quality
    c. Moderate or severe pain intensity
    d. Aggravation by or causing avoidance of routine physical activity
3. During headache at least one of the following:
    a. Nausea and/or vomiting
    b. Photophobia and phonophobia
4. Not better accounted for by another ICHD-3 diagnosis

Definite migraine is headache that satisfies all the criteria outlined above, while probable migraine satisfies all criteria except one. Studies that have looked at the reasons for cases with probable headache not fulfilling criteria definite diagnosis have suggested that most often it is the duration criterion that is

left unfilled.[1,2,3,4,5] In previous rounds of GBD we have not distinguished between probable and definite migraine, but for GBD 2017 we have both accounted for the varying case definitions used by different sources and have also accounted for the difference in average duration of episodes through our calculation of the proportion of time symptomatic ("time symptomatic") by headache type (probable/definite).

Tension-type headache

Tension-type headache (TTH) is characterised by a dull, non-pulsatile, diffuse, band-like (or vice-like) pain of mild to moderate intensity in the head or neck. The ICD-10 code for TTH is G44.2 and the ICD-9 code is 339.1. The reference diagnostic criteria for tension-type headache are from the ICHD-3, which describe five criteria:

1. At least 10 attacks fulfilling criteria 2-5
2. Lasing from 30 minutes to 7 days
3. At least two of the following four characteristics:
    a. Bilateral location
    b. Pressing or tightening (non-pulsating) quality
    c. Mild or moderate intensity
    d. Not aggravated by routine physical activity such as walking or climbing stairs
4. Both of the following:
    a. No nausea or vomiting
    b. No more than one of photophobia or phonophobia
5. Not better accounted for by another ICHD-3 diagnosis

Definite tension-type headache is headache that satisfies all criteria outlined above, while probable tension-type headache satisfies all criteria except one. In previous rounds of GBD we have not distinguished between probable and definite tension-type headache, but for GBD 2017 we have accounted for varying case definitions used by different sources. There was not sufficient evidence pointing to a difference in duration between probable and definite tension-type headache, so we applied a single value for the proportion of time symptomatic for tension-type headache.

Medication overuse headache

Both migraine and tension-type headache can give rise to medication overuse headache (MOH), with the following International Classification of Headache Disorders (ICHD-3) diagnostic criteria:

1. Headache occurring on ≥15 days/month in a patient with a pre-existing headache disorder

[1] Kim B-K, Chung YK, Kim J-M, Lee K-S, Chu MK. Prevalence, clinical characteristics and disability of migraine and probable migraine: A nationwide population-based survey in Korea. *Cephalalgia* 2013; **33**: 1106–16.

[2] Lantéri-Minet M, Valade D, Géraud G, Chautard M, Lucas C. Migraine and probable migraine – results of FRAMIG 3, a French nationwide survey carried out according to the 2004 IHS classification. *Cephalalgia*; **25**: 1146–58.

[3] Pfaffenrath V, Fendrich K, Vennemann M, *et al.* Regional variations in the prevalence of migraine and tension-type headache applying the new IHS criteria: the German DMKG Headache Study. *Cephalalgia*; **29**: 48–57.

[4] Rasmussen BK, Jensen R, Olesen J. A Population-Based Analysis of the Diagnostic Criteria of the International Headache Society. *Cephalalgia* 1991; **11**: 129–34.

[5] Fendrich K, Vennemann M, Pfaffenrath V, *et al.* Headache Prevalence Among Adolescents — The German DMKG Headache Study. *Cephalalgia* 2007; **27**: 347–54.

2. Regular overuse for >3 months of one or more drugs that can be taken for acute and/or symptomatic treatment of headache
3. Not better accounted for by another ICHD-3 diagnosis.

ICHD-3 explicitly states that, when a person fulfils criteria for both migraine and MOH, both diagnoses should be given. However, our GBD headache collaborators, Steiner and Stovner, say that in survey practice, a screening question on chronic headache is used first, followed by questions to determine if medication overuse is present. This means the diagnoses of migraine and MOH become mutually exclusive (obviating any potential problem of double-counting).

Input Data

Migraine

We conducted an update to the systematic review of migraine for GBD 2017, which covered September 2015 to September 2017. The search string for this review was ((((("migraine disorders"[MeSH Terms] OR migraine[All Fields]) AND ((prevalence[Title/Abstract] OR incidence[Title/Abstract] OR remission[Title/Abstract] OR epidemiology[Title/Abstract])) AND ("2015/09/01"[Date - Publication] : "2017/09/19"[Date - Publication])))))). The search yielded 490 hits, with 29 ultimately marked for extraction. A flowchart documenting this review is displayed below.

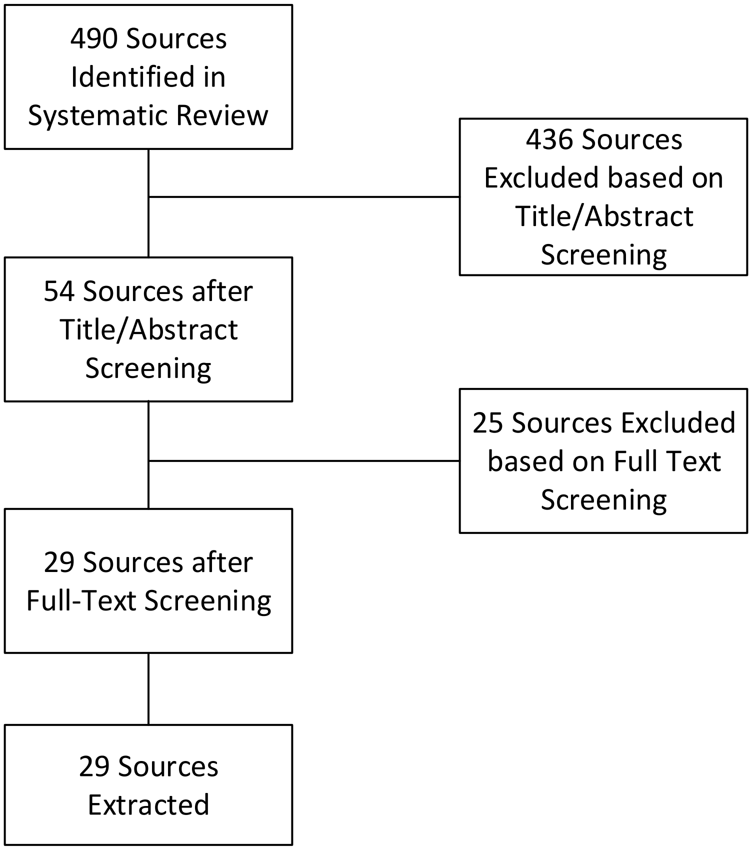

Inclusion criteria of the systematic reviews were:

- Representative, population-based surveys
- Reporting of prevalence of migraine headache

In past GBD rounds, we have utilised medical claims data from the US. However, for this round of GBD we decided to exclude these data as the adjustment needed make the claims data comparable to population representative surveys was unstable.

The table below illustrates the geographic distribution of migraine data.

	Prevalence
**Site-years (total)**	124
**Number of countries with data**	48
**Number of GBD regions with data (out of 21 regions)**	16
**Number of GBD super-regions with data (out of 7 super-regions)**	7

For this round of GBD, we also went back to each data source to document whether or not the study reported on definite or probable headache and re-extracted extra information where it was available. If a study reported a more detailed age or sex pattern for one type of headache and not the other, we applied the age/sex pattern from the type with more detailed data to the type with less detailed data. Additionally, if there were data points where the age range spanned greater than 20 years, we age split the data using the DisMod age pattern from the super-region containing that data point.

Tension-type headache

We conducted an update to the systematic review of TTH for GBD 2017, which covered January 2015 to September 2017. The search string for this review was ((((("headache"[MeSH Terms]) OR ("headache"[Title/Abstract] AND "tension"[Title/Abstract])) AND ("epidemiology"[Title/Abstract] OR "prevalence"[Title/Abstract] OR "incidence"[Title/Abstract] OR "remission"[Title/Abstract])) AND ("2015/04/01"[Date - Publication] : "2017/09/25"[Date - Publication]))). The search yielded 299 initial hits and 19 studies were ultimately extracted. A flow chart documenting this review is displayed below.

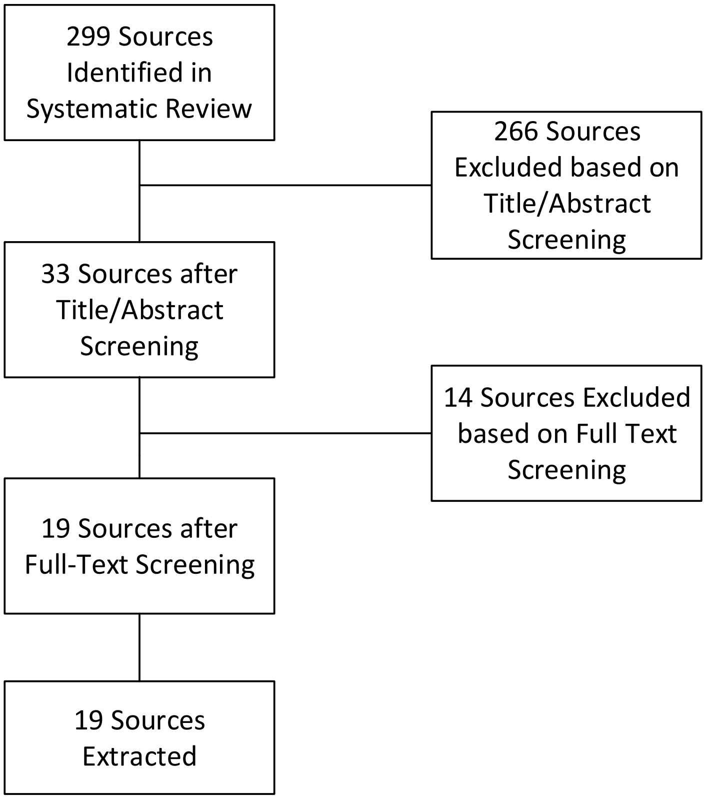

Inclusion criteria of the systematic reviews were:

- Representative, population-based surveys
- Reporting of prevalence of TTH headache

In past GBD rounds, we utilised medical claims data from the US. However, for this round of GBD we decided to exclude these data, as the adjustment needed make the claims data comparable to population representative surveys was unstable.

The table below illustrates the geographic distribution of TTH data.

	Prevalence
Site-years (total)	80
Number of countries with data	36
Number of GBD regions with data (out of 21 regions)	15
Number of GBD super-regions with data (out of 7 super-regions)	7

For this round of GBD, we also went back to each data source to document whether or not the study reported on definite or probable headache and re-extracted extra information where it was available. If a study reported a more detailed age or sex pattern for one type of headache and not the other, we applied the age/sex pattern from the type with more detailed data to the type with less detailed data. Additionally, if there were data points where the age range spanned greater than 20 years, we age split the data using the DisMod age pattern from the super-region containing that data point.

Medication overuse headache

We conducted an update to the systematic review of MOH for GBD 2017, which covered January 2014 to September 2017. The search string for this review was (("headache"[MeSH Terms] OR "headache"[Title/Abstract]) AND ("pharmaceutical preparations"[MeSH Terms] OR "pharmaceutical preparations"[Title/Abstract] OR "medication"[Title/Abstract]) AND ("epidemiology"[Title/Abstract] OR "prevalence"[Title/Abstract] OR "incidence"[Title/Abstract] OR "remission"[Title/Abstract]) AND ("2014/06/01"[PDAT] : "2017/09/25"[PDAT])). The search yielded 202 initial hits, and nine studies were ultimately extracted. A flow chart documenting this review is displayed below.

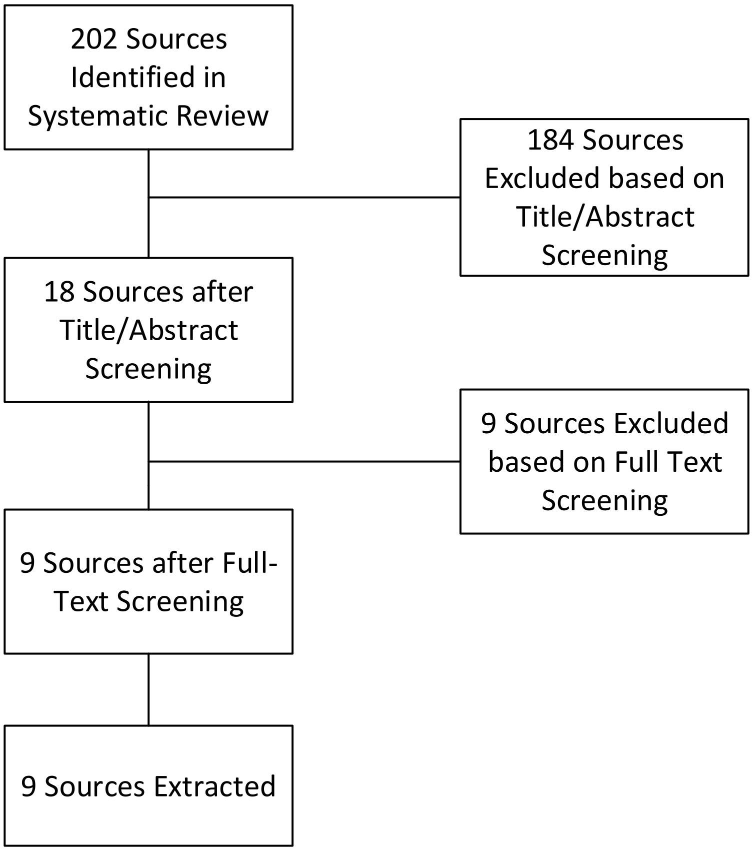

Inclusion criteria of the systematic reviews were:

- Representative, population-based surveys
- Reporting of prevalence of MOH headache

The table below shows the geographic distribution of MOH data.

	Prevalence	Remission
Site-years (total)	41	9
Number of countries with data	25	6
Number of GBD regions with data (out of 21 regions)	9	2
Number of GBD super-regions with data (out of 7 super-regions)	7	2

Study-level covariates

We used a list of binary covariates which are a modified version of quality indicators of epidemiological studies on headache (Steiner TJ, Stovner LJ et al [2013]. Improving quality in population surveys of headache prevalence, burden, and cost: key methodological considerations. J Headache Pain, 14: 87) and shown in the table below.

Study covariate	Notation	
	**Less desirable (1)**	**Reference (zero)**
**Other than one-year recall period**	Point prevalence	One-year prevalence
**Not representative**	Selected population	General population or community-based sample from whole country OR general population or community-based sample from defined region within a country, or school-based (for children)
**Low-quality sampling method**	Not stated OR no (or failed) attempt to secure representativeness	Total defined population, or random sample corrected for population demographics OR random sample uncorrected for population demographics
**Poor response**	Not stated, or <70%	70–100%
**Low-quality survey method and type of interviewer**	Not stated OR self-administered (unsupervised) questionnaire OR telephone or face-to-face interview by untrained or unspecified interviewer(s)	Face-to-face interview with headache expert or trained interviewer
**Low-quality validation of diagnostic instrument**	Instrument not specified or not validated OR validated, but sensitivity and/or specificity <70% OR validated only in screen-positive sub-sample, or in clinic or unspecified sample, but sensitivity and specificity ≥70%	Validated in target population or similar, and sensitivity and specificity ≥70%, or all diagnoses made in face-to-face or telephone interviews by headache expert
**Low-quality diagnostic criteria**	Not stated OR stated, other than ICHD OR ICHD (or reasonable modification)	ICHD (or reasonable modification)

We also added covariates for whether or not the estimates from the study were based on lifetime recall of headaches and whether or not the study was conducted in a school setting. For migraine and tension-type headache, we additionally included a covariate to mark studies where the type of headache (probable/definite) was not explicitly mentioned in the study but was determined based on the diagnostic criteria stated.

All study covariates were initially evaluated as x-covs (which means that data points were adjusted to the reference value if a systematic bias is detected); those that did not have a significant coefficient were

entered as z-covs (which means that a multiplier was applied to the standard error of such data points to indicate they were less certain values because they did not meet the reference criteria for study quality).

Modelling Strategy

Migraine

For GBD 2017, we ran separate DisMod models for definite migraine, probable migraine, and the total migraine category. After running the separate models, we then scaled the results of probable and definite headache to the total headache envelope to ensure consistency.

Because some data sources, especially earlier data from before ICHD became the standard (the initial criteria were published in 1988), largely report on definite migraine, we also adjusted studies that reported only on definite migraine to the total migraine category in order to better inform that model. All data that reported on both definite and total migraine were used in regression models by sex in order to derive an age- and sex-specific adjustment. The adjustment is shown in the graphs below.

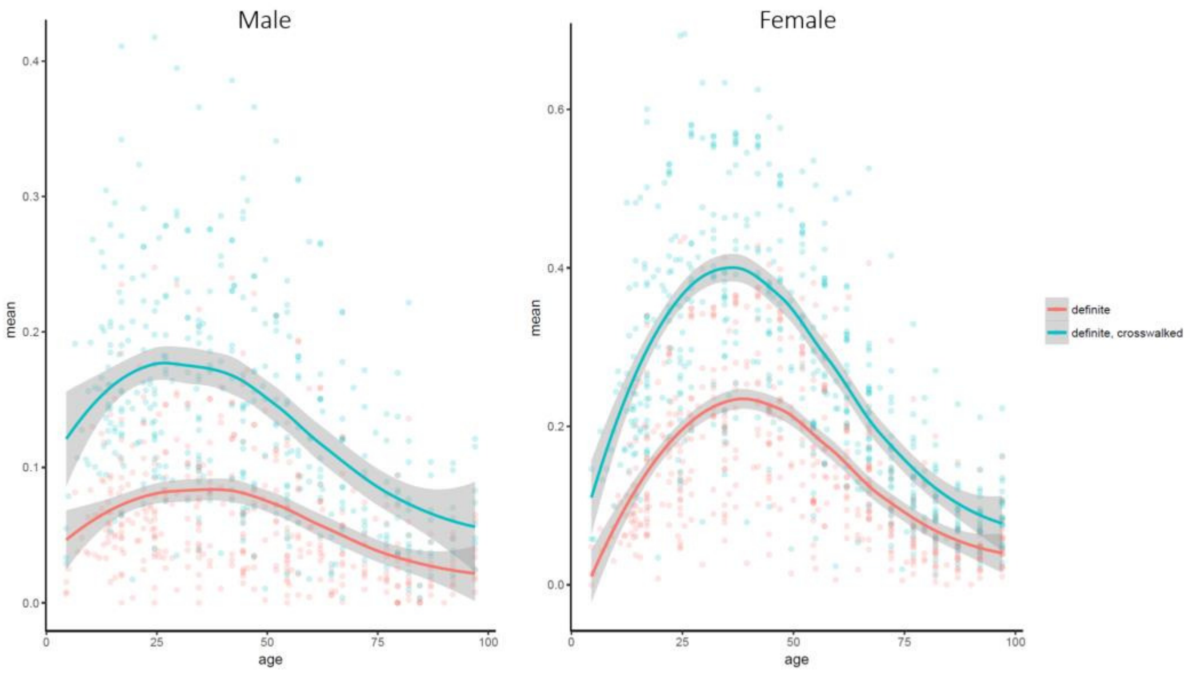

In all models, we decided to exclude the covariate on SDI, because the coefficient was not significant and was unstable. For all models we also assumed no excess mortality due to migraine, and no incidence from 0 to age 5 based on expert advice. We also set remission to be between 0 and 0.1.

The tables below showsthe fixed-effect values of the x-covs which are in log space (since DisMod uses an offset lognormal model) as well as the exponentiated values, which for an x-cov can be interpreted as an odds ratio.

Definite migraine

Study covariate	Parameter	beta	Exponentiated beta
Study school population	Prevalence	0.71	2.03 (1.99 to 2.12)

Low-quality sampling method	Prevalence	0.27	1.31 (1.19 to 1.44)
Recall lifetime	Prevalence	0.021	1.02 (1.00 to 1.07)

Probable migraine

Study covariate	Parameter	beta	Exponentiated beta
Study school population	Prevalence	1.01	2.74 (2.04 to 4.20)
Low-quality sampling method	Prevalence	1.02	2.78 (2.42 to 3.16)
Recall lifetime	Prevalence	0.93	2.53 (2.08 to 3.07)

Total migraine

Study covariate	Parameter	beta	Exponentiated beta
Study school population	Prevalence	0.70	2.01 (1.99 to 2.07)
Recall lifetime	Prevalence	0.01	1.11 (1.00 to 1.22)

Tension-type headache

As we do not have different estimates of time symptomatic by headache type for TTH, we only ran one DisMod model for total tension-type headache. However, we adjusted the data for definite tension-type headache to reflect the level of total headache to better inform the model. The adjustment was determined through sex-specific regression models informed by studies that reported on both total headache and definite headache. The result of that adjustment is shown below.

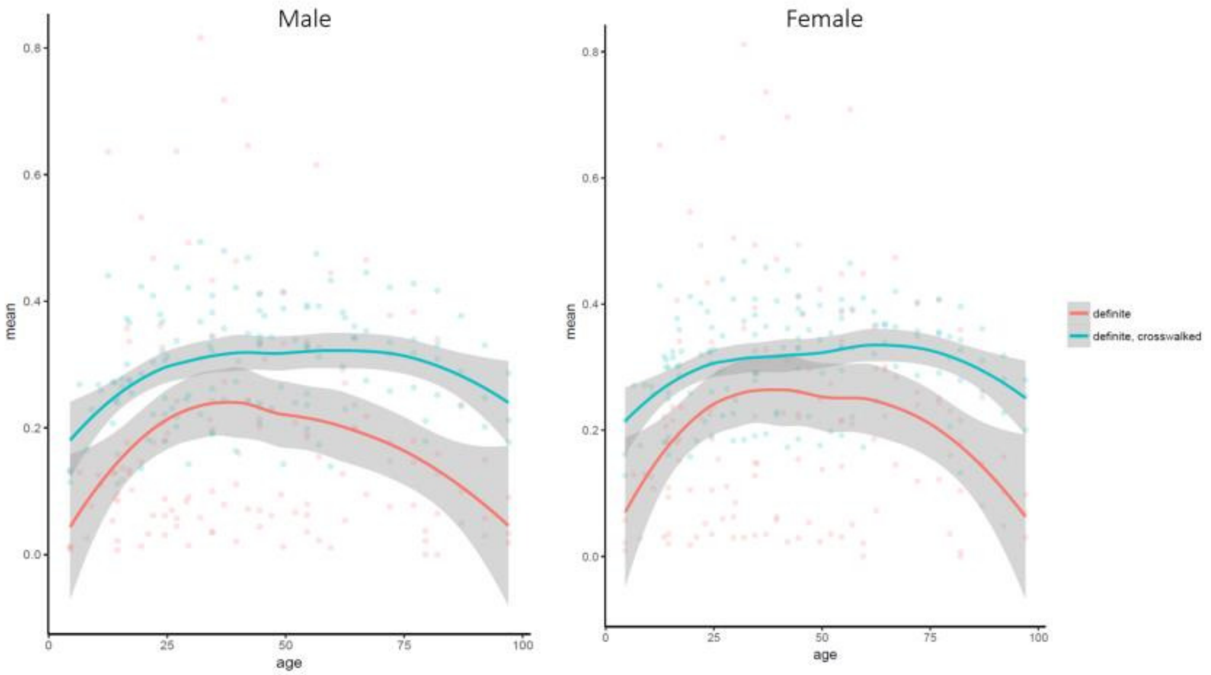

Prior settings in the DisMod model included setting incidence to 0 before age 5, based on expert advice. We also assumed no excess mortality due to TTH. In the absence of any data on remission, we set bounds

between 0 and 0.5, ie, ensuring an average duration of at least two years. We decided to exclude the covariate on SDI, because the coefficient was not significant and was unstable.

The table below shows the fixed-effect values of the x-covs which are in log space (since DisMod uses an offset lognormal model) as well as the exponentiated values, which for an x-cov can be interpreted as an odds ratio.

Study covariate	Parameter	beta	Exponentiated beta
Study school population	Prevalence	0.034	1.03 (1.00 to 1.11)
Other than 1-year period recall	Prevalence	-0.32	0.73 (0.62 to 0.85)

Medication overuse headache

MOH was initially modelled separately in DisMod, then included as a sequela of TTH in the proportion estimated as due to TTH. Prior settings in the DisMod model included setting incidence to 0 before age 5 based on expert advice. We also assume no excess mortality due to MOH. Based on seven literature sources on remission (listed in references below), we set bounds of the remission to be between 0 and 0.4. SDI was removed as a country-level covariate because the coefficient was not significant and was unstable.

The table below shows the fixed-effect values of the x-covs which are in log space (since DisMod uses an offset lognormal model) as well as the exponentiated values, which for an x-cov can be interpreted as an odds ratio.

Study covariate	Parameter	beta	Exponentiated beta
Low-quality survey method	Prevalence	-0.73	0.48 (0.42 to 0.54)

Calculation of time symptomatic

In previous iterations of GBD, estimates of the proportion of time over a year spent with headache ("time symptomatic") used meta-analyses of studies reporting on both frequency and duration. An estimate of time symptomatic was derived by study, and these results were meta-analysed to obtain a final estimate. However, for GBD 2017 we decided to separately meta-analyse estimates of duration and frequency, which allowed for the inclusion of additional studies that reported on either one value or the other.

Migraine

To determine the proportion of time over a year spent with migraine headache ("time symptomatic"), we meta-analysed data on the frequency and duration of definite headache and total headache combined. There were not enough data available to obtain reliable estimates on the frequency and duration of probable headache from the literature. A meta-analysis of 20 studies on the frequency of definite migraine yielded a frequency of 39.68 attacks per year and a meta-analysis of 12 studies on the duration of definite migraine yielded a duration of 20.83 hours, which indicates a proportion of time symptomatic of 0.094. A meta-analysis of 11 studies on the frequency of the total migraine category yielded a frequency of 43.8 attacks per year and a meta-analysis on nine studies on the duration of the total migraine category yielded a duration of 14.87 hours, which indicates a proportion of time symptomatic of

0.074. As the proportion of time symptomatic for the total migraine category is the weighted average of time symptomatic for definite and probable migraine, weighted by the prevalence of each headache type, the proportion of time symptomatic for probable migraine can be calculated as shown below.

$$Time\ Symp_{Probable} = \frac{Time\ Symp_{Total} - Prevalence_{Definite} * Time\ Symp_{Definite}}{Prevalence_{Probable}}$$

Using this equation, we calculated the proportion of time spent symptomatic for probable migraine is 0.049.

Tension-type headache

As there were not enough data to determine whether or not the proportion of time symptomatic varied by headache type, we calculated one value for the proportion of time symptomatic and applied this proportion to the entire category of tension-type headache. A meta-analysis of seven studies on the frequency of the total tension-type headache category yielded an estimate of 37.83 attacks per year and a meta-analysis on six studies on the duration of the total tension-type headache category yielded an estimate of a duration of 12.93 hours. Using these estimates, we calculated the proportion of time symptomatic as 0.046.

Medication overuse headache

While previously we had used one study reporting on the number of days with headache per month to determine the proportion of time spent over a year with medication overuse headache, this year we decided to employ the same strategy used for migraine and tension-type headache, wherein an estimate is derived by meta-analysing frequency and duration data. To determine the proportion of time over a year spent with medication overuse headache, we meta-analysed the two available studies on frequency and used the one available study on duration. The result of the meta-analysis on frequency gave an estimate of 250.83 attacks per year, and the available source on duration estimated an average duration of 18.59 hours. From this data we estimated that the proportion of time symptomatic for medication overuse headache was 0.532.

Medication overuse headache split

As medication overuse headache can develop from migraine or tension-type headache, we split medication overuse into sequelae of both primary headache disorders. Based on a meta-analysis of three sources, 73.2% (63.7–81.0) of medication-overuse headache is assigned to medication overuse headache due to tension-type headache. The forest plot is shown below.

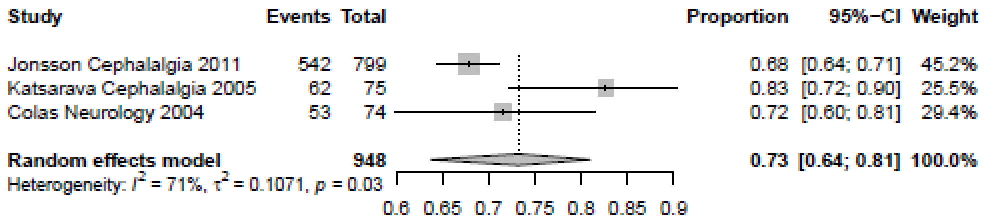

Other neurological disorders

In addition to the neurological disorders described above, there are many diverse types of neurological disorders with a range of severities and associated sequelae. Because these neurological disorders are diverse in their underlying causes and risk factors as well as in their associated health outcomes, modelling them together in a DisMod-MR model would not produce reliable estimates of prevalence or excess mortality. Instead, we calculated the YLDs caused by neurological disorders directly using a YLD/YLL ratio.

We calculated the ratio of YLDs to YLLs across the specified neurological disorders for which non-fatal outcomes were modelled, using YLL estimates from the GBD 2017 cause of death (CoD) analysis. We then multiplied this YLD/YLL ratio by the YLL estimates for other neurological disorders from the GBD 2017 CoD analysis, providing us with an estimate of the YLDs associated with other neurological disorders.

Schizophrenia

Flowchart

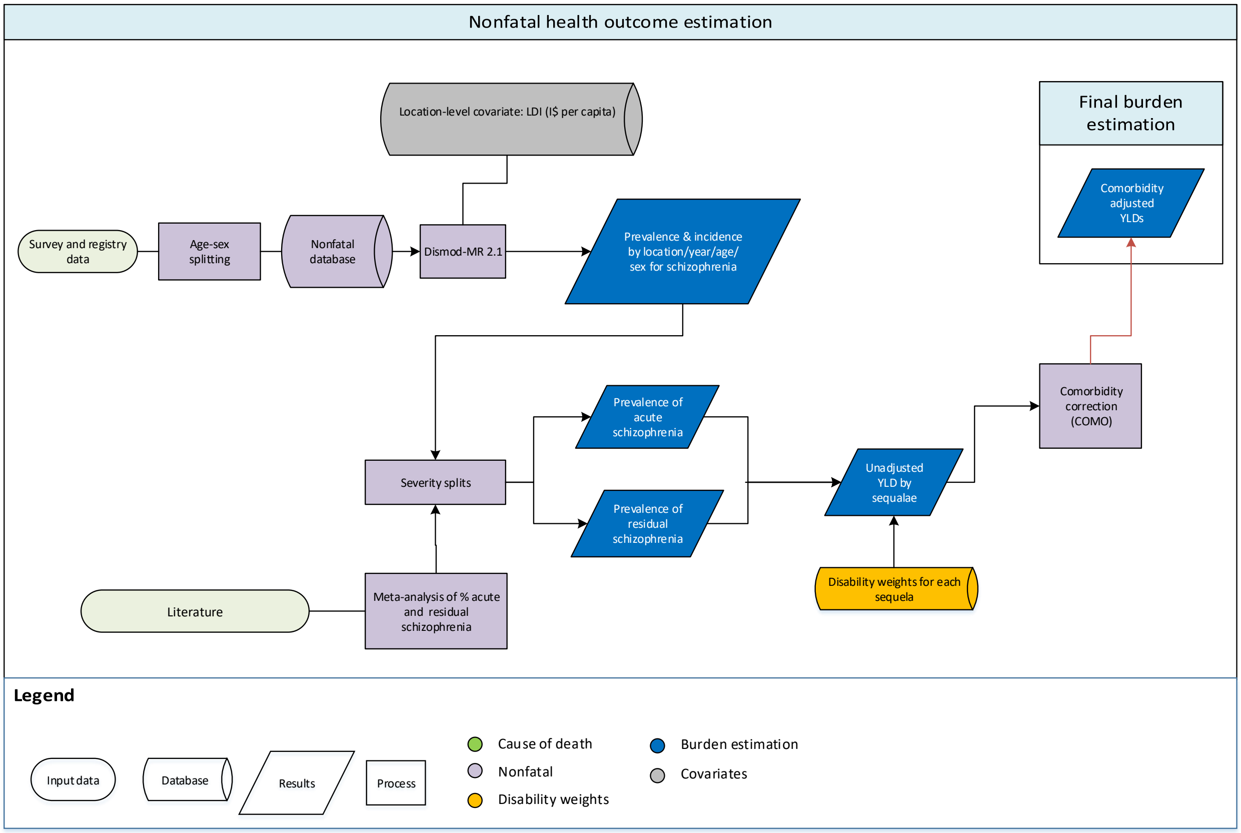

Case Definition

Schizophrenia is a chronic psychotic disorder which involves the experience of positive symptoms (eg, delusions, hallucinations, thought disorder) and negative symptoms (eg, flat affect, loss of interest, and emotional withdrawal). Included in the GBD disease modelling were cases meeting the Diagnostic and Statistical Manual of Mental Disorders (DSM-IV-TR) or the International Classification of Diseases (ICD-10) diagnostic criteria for schizophrenia (DSM: 295.10-295.30, 295.60, 295.90; ICD: F20).[1,2] Diagnostic criteria are:

A. Two (or more) of the following, each present for a significant portion of time during a one-month period (or less if successfully treated):

1. Delusions
2. Hallucinations
3. Disorganised speech
4. Grossly disorganised or catatonic behavior
5. Negative symptoms

B. Social/occupational dysfunction

C. Continuous signs of the disturbance persist for at least 6 months.

D. Exclusions must be met for schizoaffective and mood disorders, substance and general medical conditions, and a relationship to a pervasive development disorder.

Input data

Model inputs

For GBD 2010, a systematic review of the literature was conducted to capture studies of prevalence, incidence, duration, and excess mortality associated with schizophrenia. In summary, the search was conducted in three stages involving electronic searches of the peer-reviewed literature (via PsycInfo, Embase and PubMed), the grey literature, and expert consultation. The inclusion criteria stipulated that (1) the publication year must be from 1980 onward; (2) "caseness" must be based on clinical threshold as established by the DSM or ICD; (3) sufficient information must be provided on study method and sample characteristics to assess the quality of the study; and (4) study samples must be representative of the general population (ie, inpatient or pharmacological treatment samples, case studies, veterans, or refugee samples were excluded). No limitation was set on the language of publication.

The agreed-upon approach for mental and substance use disorders was to conduct electronic database searches on a rolling basis. All three stages of GBD 2010's literature review were repeated for GBD 2015 and GBD 2017. Additionally, two targeted systematic reviews were conducted to further supplement the schizophrenia dataset. The first review captured studies reporting on the epidemiology of schizophrenia within Maori versus non-Maori populations (as opposed to New Zealand more broadly), given the inclusion of these two sub-groups in GBD 2017. The second review searched for studies on the epidemiology of schizophrenia in China using primarily the China National Knowledge Infrastructure database. The focus was to search for studies published in Chinese journals that would not typically be captured in mainstream databases such as PsycInfo, Embase and PubMed.

The table below shows the number of site-years of data represented in the GBD 2017 model, as well as the number of countries and GBD world regions represented.

	Prevalence	Incidence	Remission	Other
Site-years (total)	120	57	31	62
Number of countries with data	24	17	13	27
Number of GBD regions with data (out of 21 regions)	10	8	8	14
Number of GBD super-regions with data (out of 7 super-regions)	5	6	5	8

Age and sex splitting

In GBD 2017, reported estimates of prevalence were split by age and sex where possible. First, if studies reported prevalence for broad age groups by sex (eg, prevalence in 15- to 65-year-old males and females separately), and also by specific age groups but for both sexes combined (eg, prevalence in 15- to 30-year-olds, then in 31- to 65-year-olds, for males and females combined); age-specific estimates were split by sex using the reported sex ratio and bounds of uncertainty. Second, where studies reported estimates across age groups spanning 20 years or more, these were split into five-year age groups using the prevalence age pattern estimated by DisMod-MR.

Severity splits

The basis of the GBD disability weight survey assessments are lay descriptions of sequelae highlighting major functional consequences and symptoms. The lay descriptions and disability weights for schizophrenia severity levels are shown below.

Severity level	Lay description	DW (95% CI)
acute state	Hears and sees things that are not real and is afraid, confused, and sometimes violent. The person has great difficulty with communication and daily activities, and sometimes wants to harm or kill himself (or herself).	0.778 (0.606–0.9)
residual state	Hears and sees things that are not real and has trouble communicating. The person can be forgetful, has difficulty with daily activities, and thinks about hurting himself (or herself).	0.588 (0.411–0.754)

Severity splits used in GBD 2017 were consistent with those used in GBD 2016 for schizophrenia. Information on the distribution of acute and residual states of schizophrenia was obtained from a separate systematic review of the literature.[4] Meta-XL (a Microsoft Excel add-in for meta-analysis) was used to pool estimates across all studies to calculate the overall proportion of schizophrenia cases in each health state – acute 63% (29%–91%) and residual state 37% (9%–71%).

Modelling strategy

The GBD 2017 epidemiological modelling strategy for schizophrenia made use of DisMod-MR 2.1 to estimate prevalence by age, sex, year, and country. Data across all epidemiological parameters were included in the modelling process. We assumed no incidence before age 10 and after age 80. This minimum age of onset was corroborated with expert feedback and existing literature on schizophrenia. Remission was also restricted to a maximum of 0.04 as guided by data available in the dataset.

Study-level covariates can be used in DisMod-MR 2.1 to accommodate for between-study variability in the raw prevalence data. In GBD 2017, tested covariates failed to demonstrate significance, resulting in a model without the inclusion of any covariates.

A location-level covariate, LDI, was also included. This covariate represents a moving average of gross domestic product (GDP) over time. LDI was also applied to excess mortality data with a negative relationship assumed. The table below illustrates the covariate, parameter, beta and exponentiated beta values for the model.

Location-level covariate	Parameter	beta	Exponentiated beta
LDI	Excess mortality rate	-0.55 (-1 to -0.1)	0.58 (0.37–0.90)

Citations

1. American Psychiatric Association. Diagnostic and Statistical Manual of Mental Disorders (DSM-IV-TR). 4th, Text Revision ed. Washington DC: American Psychiatric Association; 2000.

2. World Health Organization. The ICD-10 Classification of Mental and Behavioural Disorders. Clinical descriptions and diagnostic guidelines. Geneva: World Health Organization; 1992.

3. Ferrari AJ, Norman RE, Freedman G, et al. The burden attributable to mental and substance use disorders as risk factors for suicide: findings from the Global Burden of Disease Study 2010. *PloS one* 2014; **9**(4): e91936.

4. Ferrari AJ, Saha S, McGrath JJ, et al. Health states for schizophrenia and bipolar disorder within the Global Burden of Disease 2010 Study. *Population Health Metrics* 2012; **10**(1): 16.

Major Depressive Disorder

Flowchart

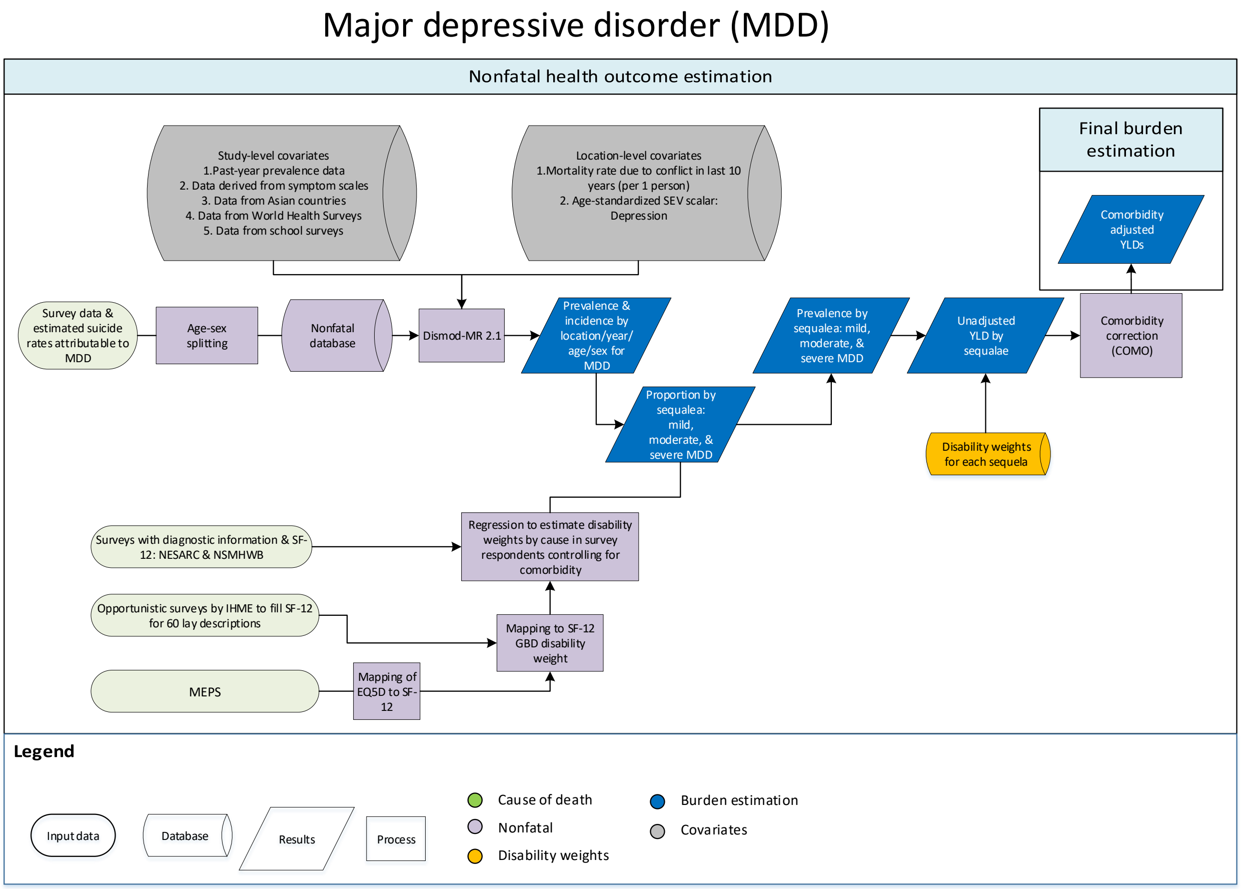

Case Definition

Major depressive disorder (MDD) is an episodic mood disorder involving the experience of one or more major depressive episode(s). Included in GBD disease modelling were cases meeting diagnostic criteria for MDD according to the Diagnostic and Statistical Manual of Mental Disorders (DSM) or the equivalent diagnosis of recurrent depression in the International Classification of Diseases (ICD). These were identified by the following codes: DSM-IV-TR: 296.21–24, 296.31–34; ICD-10: F32.0–9, F33.0–9; excluding those cases due to a general medical condition or substance induced cases.[1,2]

According to DSM-IV-TR criteria, MDD involves the presence of at least one major depressive episode, which is the experience of depressed mood almost all day, every day, for at least two weeks. Mood must represent a change from the person's baseline and impaired functioning must be observed across social, occupational, and educational domains. Additionally, a total of five out nine criteria must be met to make a diagnosis and at least one of the five criteria should either be:

- "depressed mood" for most of every day; or

- “loss of interest in nearly all activities” for most of every day.

The other seven criteria are:

- change in eating, appetite, or weight;
- excessive sleeping or insomnia;
- agitated or slow motor activity;
- fatigue;
- feeling worthless or inappropriately guilty;
- trouble concentrating; and
- repeated thoughts about death

MDD was modelled as an episodic disorder with the average length of a major depressive episode (ie, duration) specified. This was consistent with previously proposed methodology for the modelling of MDD for burden of disease purposes.[3-5]

Input data

Model inputs

For GBD 2010, a systematic review of the literature was conducted to capture studies of prevalence, incidence, duration, and excess mortality associated with MDD. In summary, the search was conducted in three stages involving electronic searches of the peer-reviewed literature (via PsycInfo, Embase and PubMed), the grey literature, and expert consultation. The agreed-upon approach for mental and substance use disorders was to conduct electronic database searches on a rolling basis. All three stages of GBD 2010’s literature review were repeated for GBD 2013 and GBD 2016. In GBD 2017, stages two and three of the literature review were conducted. Additionally, two targeted systematic reviews were conducted to further supplement the MDD dataset. The first review captured studies reporting on the epidemiology of MDD within Maori versus non-Maori populations (as opposed to New Zealand more broadly), given the inclusion of these two sub-groups in GBD 2017. The second review searched for studies on the epidemiology of MDD in China using primarily the China National Knowledge Infrastructure database. The focus was to search for studies published in Chinese journals that would not typically be captured in mainstream databases such as PsycInfo, Embase, and PubMed.

The inclusion criteria stipulated that: (1) the publication year must be from 1980 onward; (2) “caseness” must be based on clinical threshold as established by the DSM or ICD; (3) sufficient information must be provided on study method and sample characteristics to assess the quality of the study; and (4) study samples must be representative of the general population (ie, inpatient or pharmacological treatment samples, case studies, veterans, or refugee samples were excluded). No limitation was set on the language of publication. Methods used for this systematic review have been reported in greater detail elsewhere.[6,7]

The table below summarizes data inputs for MDD by parameter.

	Other	Prevalence
**Site-years (total)**	25	494
**Number of countries with data**	14	97
**Number of GBD regions with data (out of 21 regions)**	5	20
**Number of GBD super-regions with data (out of 7 super-regions)**	2	7

Age- and sex-splitting

Reported estimates of prevalence were split by age and sex where possible. First, if studies reported prevalence for broad age groups by sex (eg, prevalence in 15 to 65 year old males and females separately), and also by specific age groups but for both sexes combined (eg, prevalence in 15- to 30-year-olds, then in 31- to 65-year-olds, for males and females combined); age-specific estimates were split by sex using the reported sex ratio and bounds of uncertainty. Second, where studies reported estimates across age groups spanning 20 years or more, these were split into five-year age groups using the prevalence age pattern estimated by DisMod-MR 2.1.

Attributable suicide estimates

Given that MDD is an established risk factor for suicide,[8] we supplemented the available data on excess mortality with estimated suicide rates (by age, sex, year, and location) attributable to MDD. These were estimated using GBD's comparative risk assessment methodology whereby the current health status was compared with a theoretical-minimum-risk exposure defined as the counterfactual status of the absence of MDD in the population. Population attributable fractions (PAFs) were estimated using this established formula:

$$PAF = \frac{p\ (RR - 1)}{p\ (RR - 1) + 1}$$

P referred to the exposure distribution, which in this case was the DisMod-MR 2.1 prevalence rates of MDD by age, sex, location and year. RR referred to the pooled relative-risk of suicide due to MDD obtained from an existing systematic review and meta-analysis.[8] Age, sex, year, and location-specific PAFs were multiplied by their corresponding GBD suicide rate to estimate the proportion of suicide cases attributable to MDD. These were entered as cause-specific mortality rates in our epidemiological model for MDD.

Severity splits

The basis of the GBD disability weight survey assessments are lay descriptions of sequelae highlighting major functional consequences and symptoms. The lay descriptions and disability weights for MDD severity levels are shown below.

Severity level	Lay description	DW (95% CI)
Mild	Feels persistent sadness and has lost interest in usual activities. The person sometimes sleeps badly, feels tired,	0.145 (0.099–0.209)

	or has trouble concentrating but still manages to function in daily life with extra effort.	
Moderate	Has constant sadness and has lost interest in usual activities. The person has some difficulty in daily life, sleeps badly, has trouble concentrating, and sometimes thinks about harming himself (or herself).	0.396 (0.267–0.531)
Severe	Has overwhelming, constant sadness and cannot function in daily life. The person sometimes loses touch with reality and wants to harm or kill himself (or herself).	0.658 (0.477–0.807)

To determine the proportion of people with MDD within each of the severity levels, the US National Epidemiological Survey on Alcohol and Related Conditions (NESARC, conducted in two waves from 2001 to 2002 and 2004 to 2005)[9] and the Australian National Survey of Mental Health and Wellbeing of Adults (NSMHWB, conducted in 1997)[10] were used to estimate the proportion of MDD cases asymptomatic (13%, 10%–17%), mild (59%, 49%–69%), moderate (17%, 13%–22%), and severe (10%, 3%–20%).

Modelling Strategy

The GBD 2017 epidemiological modelling strategy for MDD made use of DisMod-MR 2.1. Data across all epidemiological parameters were initially included in the modelling process. However, given that the few incidence data points available typically excluded cases of MDD at baseline, new major depressive episodes in people with previous episodes were not counted and incidence was underestimated. For this reason, we chose to exclude all raw incidence data in the final model and instead allowed Dismod-MR 2.1 to calculate incidence based on data from other parameters. We assumed no incidence and prevalence before age 3. This minimum age of onset was corroborated with expert feedback and existing MDD literature.[6] An average remission rate for a major depressive episode of 1.45 (1.3–1.6) was used. This was derived from the four longitudinal studies[11-14] fitting a lognormal curve with least squared differences to data on the proportion of incident cases still fulfilling the case definition for major depression at intervals over a one-year period. As data were only available for a follow-up of one year, a decision had to be made about the maximum allowable duration of an episode. Setting this at 40 years, the average duration implied by the lognormal fit was 0.65 (0.59–0.70) of a year.[15]

Study-level covariates were used to accommodate between-study variability in the raw prevalence data. A cv_past year recall covariate adjusted all data points derived from past year prevalence toward the level they would have been if the study had captured point/past-month prevalence. The latter prevalence period is less affected by recall bias. A cv_symptom scale covariate adjusted all data points derived using a symptom scale toward the level they would have been if the scale had strictly adhered to DSM or ICD thresholds for MDD. A cv_asian data points covariate was used to adjust all estimates derived using diagnostic interviews from East Asia, Southeast Asia, and Asia Pacific high-income using a ratio based on a study in China. Phillips and collaborators[16] reported that the prevalence of MDD in China was 2.07% while the prevalence of mood disorders not otherwise specified (NOS) was 2.06%. Of the 808 individuals diagnosed with mood disorders NOS, 467 (58%) met criteria for minor depression (defined by DSM-IV-TR as two to four of nine symptoms of depression lasting for ≥2 weeks). There is evidence to suggest that these reported cases of minor depression are likely misdiagnosed cases MDD as DSM/ICD diagnostic criteria are not sensitive to cross-cultural presentations of MDD in Asia.[16-19] Based on this, a ratio of MDD + minor depression: MDD only (1.53, 1.45–1.63) was derived from data presented by Phillips and

collaborators and used to adjust prevalence estimates from Asia in the model. The aim of this adjustment was not to capture sub-syndromal depression but instead, to pick up on diagnoses of MDD where there is evidence to suggest that the use of Western-based criteria has underestimated prevalence. A cv_school survey covariate adjusted estimates derived from school surveys downward, to the level they would have been had the study conducted a fully representative population survey. A cv_World Health Survey covariate was used to adjust all World Health Survey data downward. The World Health Surveys are surveys conducted by the World Health Organization in close to 70 countries. While these surveys capture useful information on the prevalence of depression, they make use of a symptom scale which does not fully meet DSM and ICD criteria for MDD. This covariate works in essentially the same way as the symptom scale covariate in adjusting World Health Survey estimates downward toward the level they would have been had the study strictly adhered to DSM or ICD thresholds for MDD.

Location-level covariates were also included in the MDD model. A covariate identifying, for each GBD location, the mean mortality rate in the previous ten years due to war and terrorism informed the estimation of prevalence given existing evidence to show a positive association between conflict status and the prevalence of MDD.[20,21] An age-standardised SEV scalar was also included. This made use of the fraction of MDD burden caused by its relevant risk factors combined to inform the estimation of prevalence. Intimate partner violence and childhood sexual violence are the two established risk factors of MDD for which attributable burden is estimated in GBD studies. Betas and exponentiated values (which can be interpreted as an odds ratio) for each study- and country-level covariate are shown in the table below:

Study/country covariate	Parameter	beta	Exponentiated beta
cv_asian datapoints	Prevalence	-0.42 (-0.48 to -0.37)	0.66 (0.62–0.69)
cv_past year recall	Prevalence	0.67 (0.63–0.72)	1.96 (1.88–2.05)
cv_symptom scale	Prevalence	1.09 (1.04–1.15)	2.98 (2.82–3.15)
cv_school survey	Prevalence	0.27 (0.17–0.38)	1.32 (1.18–1.46)
cv_world health survey	Prevalence	0.84 (0.77–0.92)	2.31 (2.15–2.51)
Mean war mortality rate in the previous ten years	Prevalence	0.49 (0.022–0.97)	1.63 (1.02–2.65)
Age-standardised SEV scalar: Depression	Prevalence	1.15 (0.93–1.25)	3.16 (2.53–3.48)

References

1. American Psychiatric Association. Diagnostic and Statistical Manual of Mental Disorders (DSM). Washington: American Psychiatric Association, 1952.
2. World Health Organization. The ICD-10 Classification of Mental and Behavioural Disorders. Clinical descriptions and diagnostic guidelines. Geneva: World Health Organization; 1992.
3. Vos T, Mathers C, Herrman H, et al. The burden of mental disorders in Victoria, 1996. *Soc Psychiatry Psychiatr Epidemiol* 2001; **36**(2): 53-62.
4. Vos T, Mathers CD. The burden of mental disorders: a comparison of methods between the Australian burden of disease studies and the Global Burden of Disease study. *Bull World Health Organ* 2000; **78**(4): 427-38.
5. Ustun TB, Kessler RC. Global burden of depressive disorders: the issue of duration. *Br J Psychiatry* 2002; **181**: 181-3.

6. Ferrari AJ, Somerville AJ, Baxter AJ, et al. Global variation in the prevalence and incidence of major depressive disorder: a systematic review of the epidemiological literature. *Psychol Med* 2013; **43**(3): 471-81.
7. Ferrari AJ, Charlson FJ, Norman RE, et al. Burden of depressive disorders by country, sex, age, and year: findings from the global burden of disease study 2010. *PLoS Med* 2013; **10**(11): e1001547.
8. Ferrari AJ, Norman RE, Freedman G, et al. The burden attributable to mental and substance use disorders as risk factors for suicide: findings from the Global Burden of Disease Study 2010. *PloS one* 2014; **9**(4): e91936.
9. Introduction to the National Epidemiologic Survey on Alcohol and Related Conditions [http://pubs.niaaa.nih.gov/publications/arh29-2/74-78.htm]. Access date 1 December 2014.
10. Australian Bureau of Statistics. National Survey of Mental Health and Wellbeing of Adults 1997. Canberra: Australian Bureau of Statistics.
11. Kendler KS, Walters EE, Kessler RC. The prediction of length of major depressive episodes: results from an epidemiological sample of female twins. *Psychol Med* 1997; **27**(1): 107-17.
12. Lewinsohn PM, Clarke GN, Seeley JR, Rohde P. Major depression in community adolescents: age at onset, episode duration, and time to recurrence. *J Am Acad Child Adolesc Psychiatry* 1994; **33**(6): 809-18.
13. Spijker J, de Graaf R, Bijl RV, Beekman AT, Ormel J, Nolen WA. Duration of major depressive episodes in the general population: results from The Netherlands Mental Health Survey and Incidence Study (NEMESIS). *Br J Psychiatry* 2002; **181**: 208-13.
14. McLeod JD, Kessler RC, Landis KR. Speed of recovery from major depressive episodes in a community sample of married men and women. *J Abnorm Psychol* 1992; **101**(2): 277-86.
15. Vos T, Haby MM, Barendregt JJ, Kruijshaar M, Corry J, Andrews G. The burden of major depression avoidable by longer-term treatment strategies. *Archives of General Psychiatry* 2004; **61**(11): 1097-103.
16. Phillips MR, Zhang J, Shi Q, et al. Prevalence, treatment, and associated disability of mental disorders in four provinces in China during 2001–05: an epidemiological survey. *The Lancet* 2009; **373**: 2041–53.
17. Bromet E, Andrade LH, Hwang I, et al. Cross-national epidemiology of DSM-IV major depressive episode. *BMC medicine* 2011; **9**: 90.
18. Wang PS, Aguilar-Gaxiola S, Alonso J, et al. Use of mental health services for anxiety, mood, and substance disorders in 17 countries in the WHO world mental health surveys. *The Lancet* 2007; **370**(9590): 841-50.
19. Simon GE, Goldberg D, Von Korff M, Ustun T. Understanding cross-national differences in depression prevalence. *Psychological Medicine* 2002; **32**(4): 585–94.
20. Karam E, Bou GM. Psychosocial consequences of war among civilian populations. *Current Opinion in Psychiatry* 2013; **16**(413–419).
21. Steel Z, Chey T, Silove D, Marnane C, Bryant RA, van Ommeren M. Association of torture and other potentially traumatic events with mental health outcomes among populations exposed to mass conflict and displacement: a systematic review and meta-analysis. *JAMA* 2009; **302**(5): 537-49.

Dysthymia

Flowchart

Dysthymia

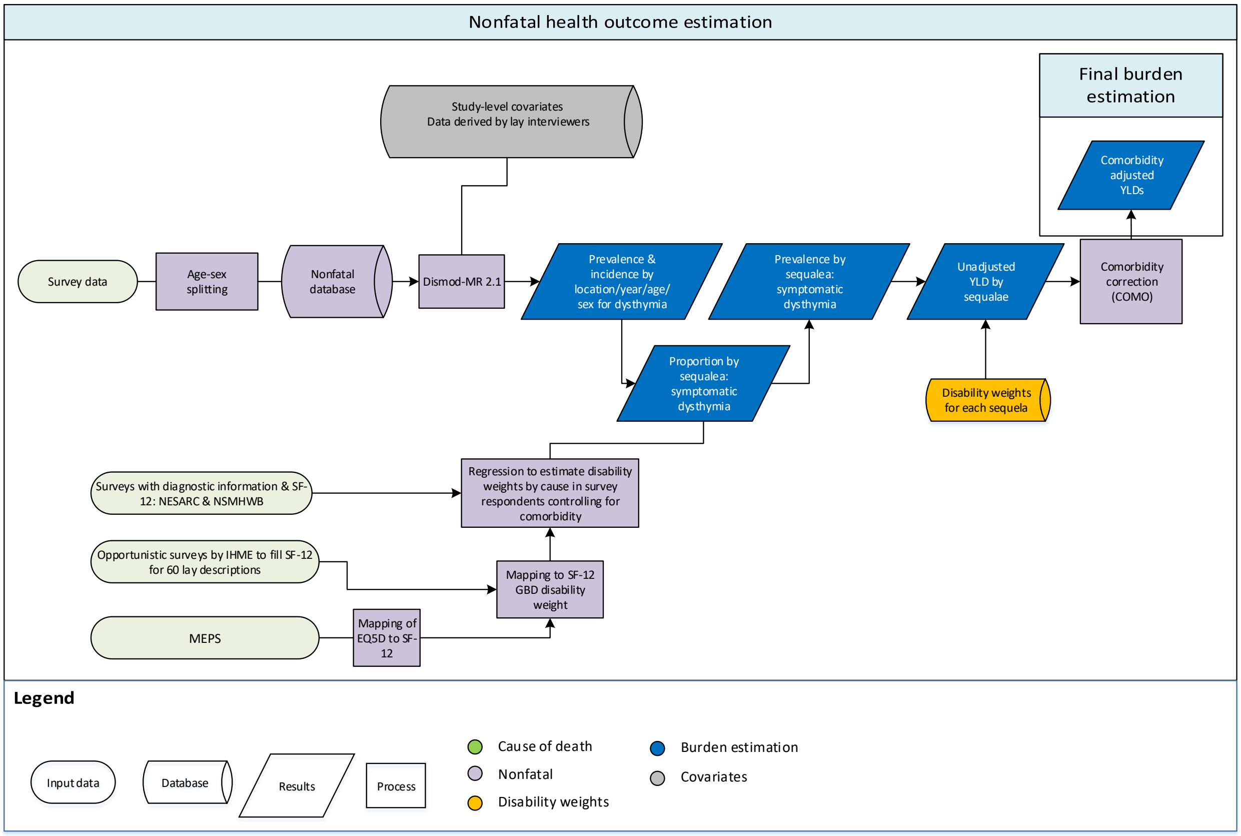

Case Definition

Dysthymia is a mood disorder consisting of chronic depression, demonstrating less severe but longer-lasting symptoms than major depressive disorder. Included in GBD disease modelling were cases meeting diagnostic criteria for dysthymia according to the Diagnostic and Statistical Manual of Mental Disorders (DSM), or the equivalent diagnosis in the International Classification of Diseases (ICD). These were identified by the following codes: DSM-IV-TR: 300.4, ICD-10: F34.1; excluding those cases due to a general medical condition or substance-induced cases.[1,2]

According to DSM-IV TR criteria, dysthymia involves the experience of chronically depressed mood for most of the day, most days that not, for at least two years (or at least one year in children and adolescents). During this period, at least two of the following symptoms must also be experienced:

- poor appetite or overeating;
- insomnia or hypersomnia;
- low energy or fatigue;
- low self-esteem;

- poor concentration or indecisiveness; and
- feelings of hopelessness

Input data

Model inputs

For GBD 2010, a systematic review of the literature was conducted to capture studies of prevalence, incidence, duration, and excess mortality associated with dysthymia. In summary, the search was conducted in three stages involving electronic searches of the peer-reviewed literature (via PsycInfo, Embase, and PubMed), the grey literature, and expert consultation. The agreed-upon approach for mental and substance use disorders was to conduct electronic database searches on a rolling basis. All three stages of GBD 2010's literature review were repeated for GBD 2013 and GBD 2016. In GBD 2017, stages two and three of the literature review were conducted. Additionally, two targeted systematic reviews were conducted to further supplement the dysthymia dataset. The first review captured studies reporting on the epidemiology of dysthymia within Maori versus non-Maori populations (as opposed to New Zealand more broadly), given the inclusion of these two sub-groups in GBD 2017. The second review searched for studies on the epidemiology of dysthymia in China using primarily the China National Knowledge Infrastructure database. The focus was to search for studies published in Chinese journals that would not typically be captured in mainstream databases such as PsycInfo, Embase, and PubMed.

The inclusion criteria stipulated that (1) the publication year must be from 1980 onward; (2) "caseness" must be based on clinical threshold as established by the DSM or ICD; (3) sufficient information must be provided on study method and sample characteristics to assess the quality of the study; and (4) study samples must be representative of the general population (ie, inpatient or pharmacological treatment samples, case studies, veterans, or refugee samples were excluded). No limitation was set on the language of publication. Methods used for this systematic review have been reported in greater detail elsewhere.[3,4] The table below summarises data inputs for dysthymia by parameter.

	**Prevalence**	**Remission**
**Site-years (total)**	69	2
**Number of countries with data**	31	2
**Number of GBD regions with data (out of 21 regions)**	12	2
**Number of GBD super-regions with data (out of 7 super-regions)**	7	1

Age- and sex-splitting

Reported estimates of prevalence were split by age and sex where possible. First, if studies reported prevalence for broad age groups by sex (eg, prevalence in 15- to 65-year-old males and females separately), and also by specific age groups but for both sexes combined (eg, prevalence in 15- to 30-year-olds, then in 31- to 65-year-olds, for males and females combined), age-specific estimates were split by sex using the reported sex ratio and bounds of uncertainty. Second, where studies reported estimates across age groups spanning 20 years or more, these were split into five-year age groups using the prevalence age pattern estimated by DisMod-MR 2.1.

Severity splits

The basis of the GBD disability weight survey assessments are lay descriptions of sequelae highlighting major functional consequences and symptoms. The lay description and disability weight for a symptomatic state of dysthymia are shown below. Given the milder and more stable presentation of dysthymia, it was assigned the same disability weight as that for mild major depressive disorder.

Severity level	Lay description	DW (95% CI)
Symptomatic dysthymia	Feels persistent sadness and has lost interest in usual activities. The person sometimes sleeps badly, feels tired, or has trouble concentrating but still manages to function in daily life with extra effort.	0.145 (0.099–0.209)

To determine the proportion of people with symptomatic and asymptomatic dysthymia, the US National Epidemiological Survey on Alcohol and Related Conditions (NESARC, conducted in two waves from 2001 to 2002 and 2004 to 2005)[5] and the Australian National Survey of Mental Health and Wellbeing of Adults (NSMHWB, conducted in 1997)[6] were used to estimate the proportion of dysthymia cases asymptomatic (29%, 23%–36%) and symptomatic (71%, 64%–77%).

Modelling Strategy

The GBD 2017 epidemiological modelling strategy for dysthymia made use of DisMod-MR 2.1. Data across all epidemiological parameters were initially included in the modelling process. The incidence studies reported estimates which were very low relative to the prevalence data. As prevalence studies contributed much greater world coverage than incidence studies, we excluded the incidence data, relying instead on data from the other parameters. We assumed no incidence and prevalence before age 3. This minimum age of onset was corroborated with expert feedback and was consistent with the available data. Excess-mortality was set to 0 as there is no epidemiological evidence to suggest that dysthymia is associated with a statistically significant risk of mortality.[3,4]

Study-level covariates were used to accommodate between-study variability in the raw prevalence data. A cv_lay interviewer covariate created a crosswalk between prevalence derived from clinically trained interviewers (desirable) and prevalence derived from lay-interviewers. A cv_past year prevalence covariate was originally included to adjust all data points derived from past year prevalence toward the level they would have been if the study had captured point/past-month prevalence. As the effect of this covariate was not statistically significant, it was excluded from the final model. Given that dysthymia is being modelled as a chronic disorder with a long duration of between six and 10 years, it was not surprising that we did not detect significant variation between point and past-year prevalence.

Betas and exponentiated values (which can be interpreted as an odds ratio) for each study level covariate are shown in the table below:

Study covariate	Parameter	beta	Exponentiated beta
cv_lay interviewer	Prevalence	-0.37 (-0.51 to -0.24)	0.69 (0.60–0.78)

Given that there was an overall paucity of epidemiological data available for dysthymia, and the data available were very heterogeneous given differences in the data collection methodology used between studies, we applied a restriction on location random-effects of -0.3 to 0.3 to further guide the estimation of prevalence.

References

1. American Psychiatric Association. Diagnostic and Statistical Manual of Mental Disorders (DSM-IV-TR). 4th, Text Revision ed. Washington DC: American Psychiatric Association; 2000.
2. World Health Organization. The ICD-10 Classification of Mental and Behavioural Disorders. Clinical descriptions and diagnostic guidelines. Geneva: World Health Organization; 1992.
3. Charlson FJ, Ferrari AJ, Flaxman AD, Whiteford HA. The epidemiological modelling of dysthymia: application for the Global Burden of Disease Study 2010. *J Affect Disord* 2013; **151**(1): 111-20.
4. Ferrari AJ, Charlson FJ, Norman RE, et al. Burden of depressive disorders by country, sex, age, and year: findings from the global burden of disease study 2010. *PLoS Med* 2013; **10**(11): e1001547.
5. Introduction to the National Epidemiologic Survey on Alcohol and Related Conditions [http://pubs.niaaa.nih.gov/publications/arh29-2/74-78.htm]. Access date 1 December 2014.
6. Australian Bureau of Statistics. National Survey of Mental Health and Wellbeing of Adults 1997. Canberra: Australian Bureau of Statistics.

Bipolar disorder

Flowchart

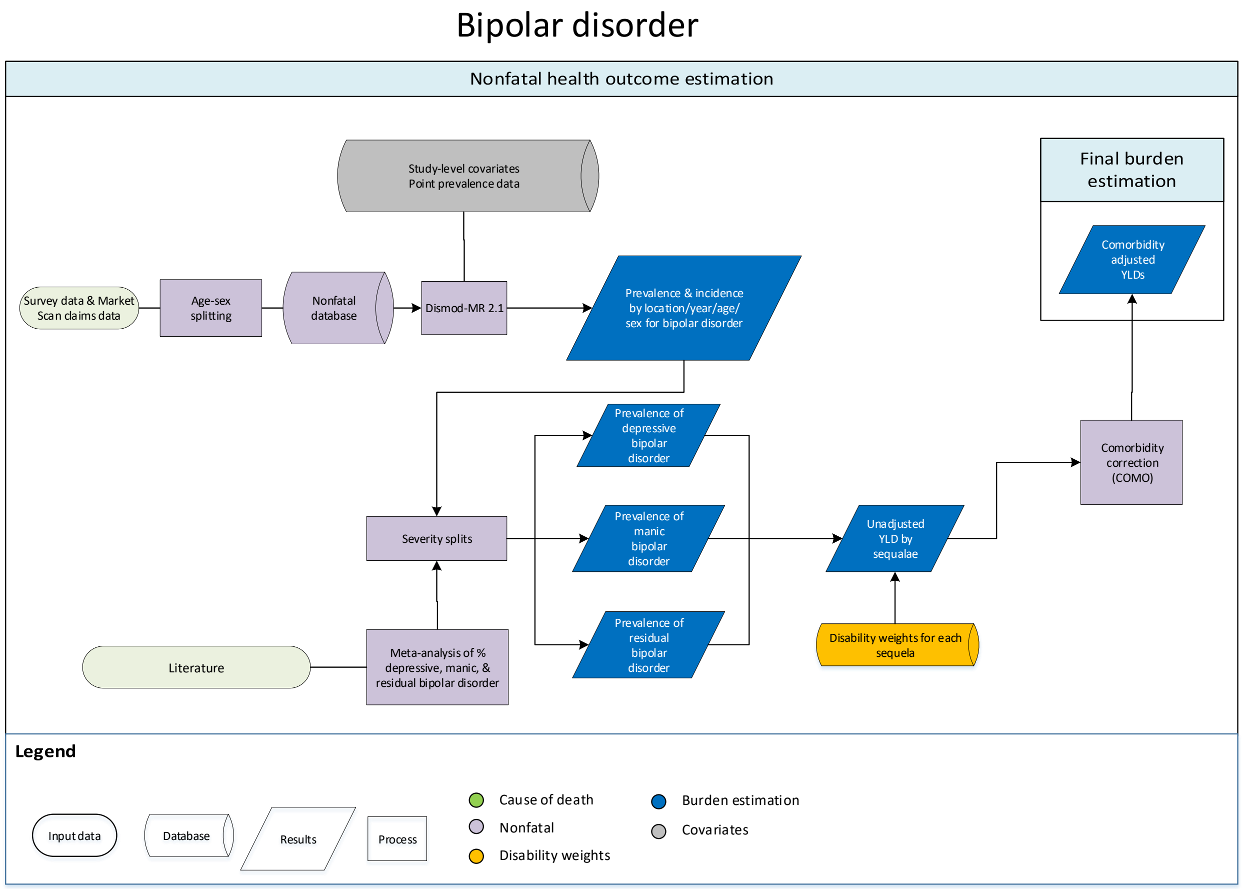

Case Definition

Bipolar disorder is a chronic mood disorder with little or no complete remission. Included in GBD disease modelling were cases meeting diagnostic criteria for bipolar disorder according to the Diagnostic and Statistical Manual of Mental Disorders (DSM), or the equivalent diagnosis in the International Classification of Diseases (ICD).[1,2] These were identified by the following codes: DSM-IV-TR: 296.0–296.8, 296.89, 301.13; ICD-10: F31.0–F31.6, F31.8–F31.9, F34.0–F34.1, excluding those cases due to a general medical condition or substance-induced cases. A diagnosis of bipolar disorder involves the experience of one or more manic or hypomanic episode(s), which can be accompanied by a major depressive episode.

According to DSM-IV-TR a manic episode involves the experience of elevated, expansive, or irritable mood lasting for at least one week. During this period, at least three (or four if mood is only irritable) of the following symptoms must also be experienced;

- inflated self-esteem or grandiosity;
- decreased need for sleep;
- more talkative;

- flight of ideas or experience that thoughts are racing;
- distractibility;
- increase in goal-directed activity; and
- excessive involvement in pleasurable activities with high potential for painful consequences.

A hypomanic episode involves the experience of elevated, expansive, or irritable mood lasting for at least four days. During this period, at least three (or four if mood is only irritable) of the symptoms previously listed for a manic episode must also be experienced.

A major depressive episode involves the experience of depressed mood almost all day, every day, for at least two weeks. A total of five of nine criteria must be met to make a diagnosis and at least one of the five criteria should either be:

- "depressed mood" for most of every day; or
- "loss of interest in nearly all activities" for most of every day.

The other seven criteria are:

- change in eating, appetite, or weight;
- excessive sleeping or insomnia;
- agitated or slow motor activity;
- fatigue;
- feeling worthless or inappropriately guilty;
- trouble concentrating; and
- repeated thoughts about death.

Different subtypes of bipolar disorder can be diagnosed depending on the combination of symptoms experienced. Bipolar I is characterised by at least one manic episode, which can also alternate with a major depressive episode. Bipolar II is characterised by hypomanic episodes alternating with major depressive episodes. Cyclothymia is characterised by subsyndromal hypomanic and major depressive episode episodes. Bipolar disorder not otherwise specified is characterised by clinically significant symptoms of bipolar disorder which do not meet criteria for the other diagnoses.[1,2] In GBD 2017 we estimated burden for the entire spectrum of bipolar disorder simultaneously, rather than individually for each subtype of the disorder. At a minimum, epidemiological studies needed to report on bipolar I and bipolar II combined to be included in analyses.

Input data

Model inputs

For GBD 2010, a systematic review of the literature was conducted to capture studies of prevalence, incidence, duration, and excess mortality associated with bipolar disorder. In summary, the search was conducted in three stages involving electronic searches of the peer-reviewed literature (via PsycInfo, Embase, and PubMed), the grey literature, and expert consultation. The agreed-upon approach for mental and substance use disorders was to conduct electronic database searches on a rolling basis. All three stages of GBD 2010's literature review were repeated for GBD 2013 and GBD 2016. In GBD 2017, stages two and three of the literature review were conducted. Additionally, two targeted systematic

reviews were conducted to further supplement the bipolar disorder dataset. The first review captured studies reporting on the epidemiology of bipolar disorder within Maori versus non-Maori populations (as opposed to New Zealand more broadly), given the inclusion of these two sub-groups in GBD 2017. The second review searched for studies on the epidemiology of bipolar disorder in China using primarily the China National Knowledge Infrastructure database. The focus was to search for studies published in Chinese journals that would not typically be captured in mainstream databases such as PsycInfo, Embase, and PubMed.

The inclusion criteria stipulated that: (1) the publication year must be from 1980 onward; (2) "caseness" must be based on clinical threshold as established by the DSM or ICD; (3) sufficient information must be provided on study method and sample characteristics to assess the quality of the study; and (4) study samples must be representative of the general population (ie, inpatient or pharmacological treatment samples, case studies, veterans, or refugee samples were excluded). No limitation was set on the language of publication. Methods used for this systematic review have been reported in greater detail elsewhere.[3] As previously explained, burden was estimated for the entire spectrum of bipolar disorder simultaneously. Combined estimates of all subtypes of bipolar disorders were required. Studies reporting separate estimates for bipolar I, bipolar II, cyclothymia, and/or bipolar not otherwise specified were accepted if sufficient information was available to sum the disorder-specific estimates. At a minimum, studies needed to report on bipolar I and bipolar II. The table below summarises data inputs for bipolar disorder by parameter.

	**Other**	**Prevalence**
**Site-years (total)**	15	312
**Number of countries with data**	8	28
**Number of GBD regions with data (out of 21 regions)**	4	11
**Number of GBD super-regions with data (out of 7 super-regions)**	2	6

Age- and sex-splitting

Reported estimates of prevalence were split by age and sex where possible. First, if studies reported prevalence for broad age groups by sex (eg, prevalence in 15- to 65-year-old males and females separately) and by specific age groups but for both sexes combined (eg, prevalence in 15- to 30-year-olds, then in 31- to 65-year-olds, for males and females combined), age-specific estimates were split by sex using the reported sex ratio and bounds of uncertainty. Second, where studies reported estimates across age groups spanning 20 years or more, these were split into five-year age groups using the prevalence age pattern estimated by DisMod-MR 2.1.

MarketScan data

We made use of United States (US) MarketScan data in our prevalence dataset. These were prevalence data for bipolar disorder derived from claims information in a database of private and public insurance schemes. Given the sparseness of the bipolar disorder prevalence dataset, this allowed us to incorporate detailed prevalence estimates by state, sex, and age in our modelling. Evaluation of the age-pattern of MarketScan data revealed that it was consistent to what can be observed in population-representative survey estimates; however, given that this data source only captures a subset of the population, the

actual levels of prevalence, and the sex difference in prevalence, were not comparable and had to be adjusted accordingly.

We compared each year of MarketScan estimates against corresponding prevalence data from the National Comorbidity Survey Replication (NCS-R), a survey representative of the general US population. The resulting prevalence ratios were used to adjust all MarketScan estimates before they were entered into the bipolar disorder model. The NCS-R : MarketScan ratios are presented in the table below.

MarketScan year	Male	Female
2000	3.4 (SE: 0.6)	2.6 (SE: 0.4)
2010	2.2 (SE: 0.4)	1.5 (SE: 0.3)
2011	2.1 (SE: 0.4)	1.5 (SE: 0.2)
2012	2.1 (SE: 0.4)	1.5 (SE: 0.2)
2014	2.1 (SE: 0.4)	1.4 (SE: 0.2)

Note. SE: Standard error

Severity splits inputs

The basis of the GBD disability weight survey assessments are lay descriptions of sequelae highlighting major functional consequences and symptoms. The lay descriptions and disability weights for bipolar disorder severity levels are shown below.

Severity level	Lay description	DW (95% CI)
Manic	Is hyperactive, hears and believes things that are not real, and engages in impulsive and aggressive behavior that endanger the person and others.	0.492 (0.341–0.646)
Depressive*	Has constant sadness and has lost interest in usual activities. The person has some difficulty in daily life, sleeps badly, has trouble concentrating, and sometimes thinks about harming himself (or herself).	0.396 (0.267–0.531)
Residual	Has mild mood swings, irritability, and some difficulty with daily activities.	0.032 (0.018–0.051)

*Note.*Equivalent to the disability weight estimated for moderate major depressive disorder*

Information on the distribution of manic, depressive, and residual states of bipolar disorder was obtained from a separate systematic review of the literature.[5] Meta-XL (a Microsoft Excel add-in for meta-analysis) was used to pool estimates across all studies to calculate the overall proportion of bipolar cases in each health state. Six studies provided information on the proportion of bipolar disorder cases in a manic (21%, 12%–33%), depressive (23%, 10%–39%), or residual state (52%, 28%–77%).

Modelling strategy

The GBD 2017 epidemiological modelling strategy for bipolar disorder made use of DisMod-MR 2.1. Data across all epidemiological parameters were initially included in the modelling process. The two studies on incidence reported 0% and 0.1% incidence of bipolar disorder and were low relative to the prevalence

data. They were excluded from the final model where incidence was estimated using data from other parameters. We assumed no incidence and prevalence before age 10. Remission was set to a maximum of 0.05 in agreement with literature and expert advice suggesting no or very little complete remission from bipolar disorder.[6,7]

Study-level covariates were used to accommodate for between-study variability in the raw prevalence data. A cv_point recall covariate adjusted all data points derived from point/past-month prevalence toward the level they would have been if the study had captured 12-month prevalence. We set 12-month prevalence as the desirable level due to the episodic nature of bipolar disorder. Estimates of point prevalence surveying symptoms experienced in the past 30 days or less may fail to diagnose cases of bipolar disorder in a residual state, thereby underestimating prevalence.

The corresponding beta and exponentiated value (which can be interpreted as an odds ratio) is shown in the table below:

Study covariate	Parameter	beta	Exponentiated beta
cv_point recall	Prevalence	-0.86 (-1.17 to -0.61)	0.42 (0.31–0.54)

Given that there was an overall paucity in epidemiological data available for bipolar disorder, and the data available were very heterogeneous given differences in the data-collection methodology used between studies, we applied a restriction on location random-effects of -0.3 to 0.3 to further guide the estimation of prevalence.

References

1. American Psychiatric Association. Diagnostic and Statistical Manual of Mental Disorders (DSM). Washington: American Psychiatric Association, 1952.
2. World Health Organization. The ICD-10 Classification of Mental and Behavioural Disorders. Clinical descriptions and diagnostic guidelines. Geneva: World Health Organization; 1992.
3. Ferrari AJ, Baxter AJ, Whiteford HA. A systematic review of the global distribution and availability of prevalence data for bipolar disorder. *J Affect Disord* 2011; **134**(1-3): 1-13.
4. Ferrari AJ, Norman RE, Freedman G, et al. The burden attributable to mental and substance use disorders as risk factors for suicide: findings from the Global Burden of Disease Study 2010. *PLoS One* 2014; **9**(4): e91936.
5. Ferrari AJ, Saha S, McGrath JJ, et al. Health states for schizophrenia and bipolar disorder within the Global Burden of Disease 2010 Study. *Population health metrics* 2012; **10**(1): 16.
6. Angst J, Sellaro R. Historical perspectives and natural history of bipolar disorder. *Biological Psychiatry* 2000; **48**(6): 445–57.
7. Colom F, Vieta E. The road to DSM-V. Bipolar disorder episode and course specifiers. *Psychopathology* 2009; **42**(4): 209–18.

Anxiety Disorders

Flowchart

Anxiety disorders

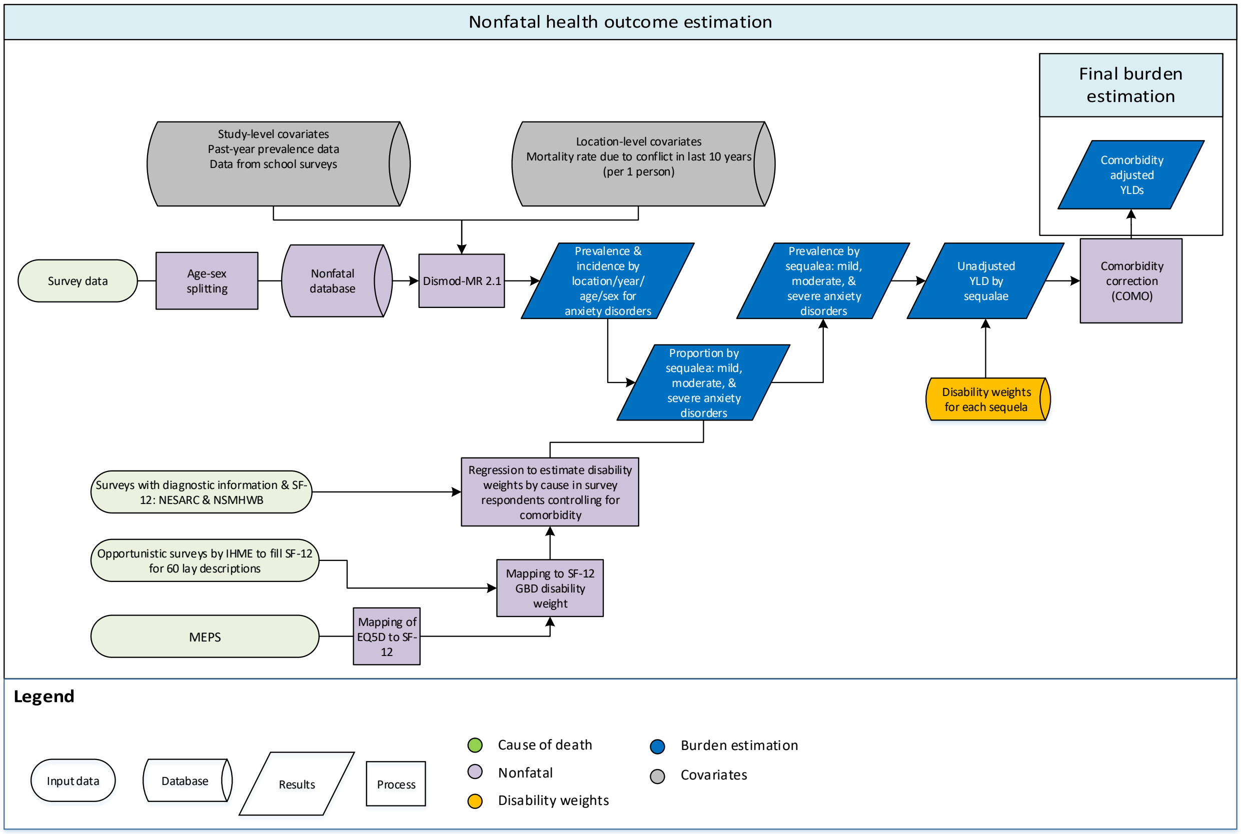

Case Definition

Anxiety disorders are characterised by experiences of intense of fear and distress, typically in combination with other physiological symptoms. We aimed to capture all cases of anxiety disorders reaching diagnostic threshold defined by the Diagnostic and Statistical Manual of Mental Disorders (DSM) or the WHO International Classification of Diseases (ICD-10).[1,2] Included disorders are listed below and can be identified by the DSM-IV-TR and ICD-10 coding systems as DSM IV TR: 299.8, 300.0-300.3, 309.21, 309.81 and ICD-10: F40-42, F43.1, F93.0-93.2, F93.8. Excluded were anxiety disorders due to a general medical condition and substance-induced anxiety disorder.

- panic disorder;
- agoraphobia;
- specific phobia;
- social phobia;
- obsessive-compulsive disorder;
- post-traumatic stress disorder;

- acute stress disorder;
- generalised anxiety disorder;
- separation anxiety disorder; and
- anxiety disorder not otherwise specified

As specific anxiety disorders frequently co-occur, anxiety disorders were modelled as a single cause for "any" anxiety disorder in GBD 2017 to avoid the double-counting of individuals meeting criteria for more than one anxiety disorder. Epidemiological estimates reporting an outcome for "any" or "total" anxiety disorders were included in analyses.

Input data

Model inputs

For GBD 2010, a systematic review of the literature was conducted to capture studies of prevalence, incidence, duration, and excess mortality associated with anxiety disorders. In summary, the search was conducted in three stages involving electronic searches of the peer-reviewed literature (via PsycInfo, Embase, and PubMed), the grey literature, and expert consultation. The agreed-upon approach for mental and substance use disorders was to conduct electronic database searches on a rolling basis. All three stages of GBD 2010's literature review were repeated for GBD 2013 and GBD 2016. In GBD 2017, stages two and three of the literature review were conducted. Additionally, two targeted systematic reviews were conducted to further supplement the anxiety disorders dataset. The first review captured studies reporting on the epidemiology of anxiety disorders within Maori versus non-Maori populations (as opposed to New Zealand more broadly), given the inclusion of these two sub-groups in GBD 2017. The second review searched for studies on the epidemiology of anxiety disorders in China using primarily the China National Knowledge Infrastructure database. The focus was to search for studies published in Chinese journals that would not typically be captured in mainstream databases such as PsycInfo, Embase, and PubMed.

The inclusion criteria stipulated that: (1) the publication year must be from 1980 onward; (2) "caseness" must be based on clinical threshold as established by the DSM or ICD; (3) sufficient information must be provided on study method and sample characteristics to assess the quality of the study; and (4) study samples must be representative of the general population (ie, inpatient or pharmacological treatment samples, case studies, veterans, or refugee samples were excluded). No limitation was set on the language of publication. Methods used for this systematic review have been reported in greater detail elsewhere.[3-5] The table below summarises data inputs for anxiety disorders by parameter.

	**Prevalence**	**Remission**
**Site-years (total)**	168	3
**Number of countries with data**	45	3
**Number of GBD regions with data (out of 21 regions)**	18	2
**Number of GBD super-regions with data (out of 7 super-regions)**	7	1

Age- and sex-splitting

Reported estimates of prevalence were split by age and sex where possible. First, if studies reported prevalence for broad age groups by sex (eg, prevalence in 15- to 65-year-old males and females separately), and also by specific age groups but for both sexes combined (eg, prevalence in 15- to 30-year-olds, then in 31- to 65-year-olds, for males and females combined), age-specific estimates were split by sex using the reported sex ratio and bounds of uncertainty. Second, where studies reported estimates across age groups spanning 20 years or more, these were split into five-year age groups using the prevalence age pattern estimated by DisMod-MR 2.1.

Severity splits

The basis of the GBD disability weight survey assessments are lay descriptions of sequelae highlighting major functional consequences and symptoms. The lay descriptions and disability weights for anxiety disorder severity levels are shown below.

Severity level	Lay description	DW (95% CI)
Mild	Feels mildly anxious and worried, which makes it slightly difficult to concentrate, remember things, and sleep. The person tires easily but is able to perform daily activities.	0.03 (0.018–0.046)
Moderate	Feels anxious and worried, which makes it difficult to concentrate, remember things, and sleep. The person tires easily and finds it difficult to perform daily activities.	0.133 (0.091–0.186)
Severe	Constantly feels very anxious and worried, which makes it difficult to concentrate, remember things, and sleep. The person has lost pleasure in life and thinks about suicide.	0.523 (0.362–0.677)

To determine the proportion of people with anxiety disorders within each of the severity levels, the US National Epidemiological Survey on Alcohol and Related Conditions (NESARC, conducted in two waves from 2001 to 2002 and 2004 to 2005)[7], and the Australian National Survey of Mental Health and Wellbeing of Adults (NSMHWB, conducted in 1997)[8] were used to estimate the proportion of anxiety disorder cases asymptomatic (28.8%, 27.5%–30.1%), mild (39.3%, 34.2%–44.2%), moderate (19.1%, 15.8%–22.7%) and severe (12.7%, 9.2%–16.7%).

Modelling strategy

The GBD 2017 epidemiological modelling strategy for anxiety disorders made use of DisMod-MR 2.1. Data across all epidemiological parameters were initially included in the modelling process. The incidence studies reported estimates which were very low relative to the prevalence data. As prevalence studies contributed much greater world coverage than incidence studies, we excluded the incidence data, relying instead on data from the other parameters. We assumed no incidence and prevalence before age 2 and after age 95. This minimum age of onset was corroborated with expert feedback and existing literature on anxiety disorders. Remission was set to a maximum of 0.2, consistent with the data points available.

Study-level covariates were used to accommodate for between-study variability in the raw prevalence data. A cv_past year recall covariate adjusted all data points derived from past year prevalence toward the level they would have been if the study had captured point/past-month prevalence. The latter prevalence period is less affected by recall bias. A cv_school survey covariate adjusted estimates derived from school surveys downward to the level they would have been had the study conducted a fully representative population survey. A country-level covariate identifying for each GBD location the mean mortality rate in the previous ten years due to war and terrorism was also included in the anxiety disorders model. This informed the estimation of prevalence given existing evidence to show a positive association between conflict status and the prevalence for anxiety disorders.[9,10] Betas and exponentiated values (which can be interpreted as an odds ratio) for each study-level covariate are shown in the table below:

Study/country covariate	Parameter	beta	Exponentiated beta
cv_past year recall	Prevalence	0.39 (0.34–0.45)	1.48 (1.41–1.56)
cv_school survey	Prevalence	0.43 (0.31–0.56)	1.54 (1.36–1.75)
Mean war mortality rate in the previous 10 years	Prevalence	0.50 (0.027–0.97)	1.65 (1.03–2.65)

References

1. American Psychiatric Association. Diagnostic and Statistical Manual of Mental Disorders (DSM-IV-TR). Fourth Edition, Text Revision ed. Washington DC: American Psychiatric Association; 2000.
2. World Health Organization. The ICD-10 Classification of Mental and Behavioural Disorders. Clinical descriptions and diagnostic guidelines. Geneva: World Health Organization; 1992.
3. Baxter AJ, Scott KM, Vos T, Whiteford HA. Global prevalence of anxiety disorders: a systematic review and meta-regression. *Psychological Medicine* 2013; **43**(05): 897-910.
4. Baxter AJ, Vos T, Scott KM, Ferrari AJ, Whiteford HA. The global burden of anxiety disorders in 2010. *Psychological Medicine* 2014; **44**(11): 2363-74.
5. Ferrari AJ, Norman RE, Freedman G, et al. The burden attributable to mental and substance use disorders as risk factors for suicide: findings from the Global Burden of Disease Study 2010. *PLoS One* 2014; **9**(4): e91936.
6. Agency for Healthcare Research and Quality. United States Medical Expenditure Panel Survey. Rockville, United States: Agency for Healthcare Research and Quality.
7. Introduction to the National Epidemiologic Survey on Alcohol and Related Conditions [http://pubs.niaaa.nih.gov/publications/arh29-2/74-78.htm]. Access date 1 December 2014.
8. Australian Bureau of Statistics. National Survey of Mental Health and Wellbeing of Adults 1997. Canberra: Australian Bureau of Statistics.
9. Karam E, Bou GM. Psychosocial consequences of war among civilian populations. *Current Opinion in Psychiatry* 2013; **16**(413–419).
10. Steel Z, Chey T, Silove D, Marnane C, Bryant RA, van Ommeren M. Association of torture and other potentially traumatic events with mental health outcomes among populations exposed to mass conflict and displacement: a systematic review and meta-analysis. *JAMA* 2009; **302**(5): 537-49.

Anorexia nervosa

Flowchart

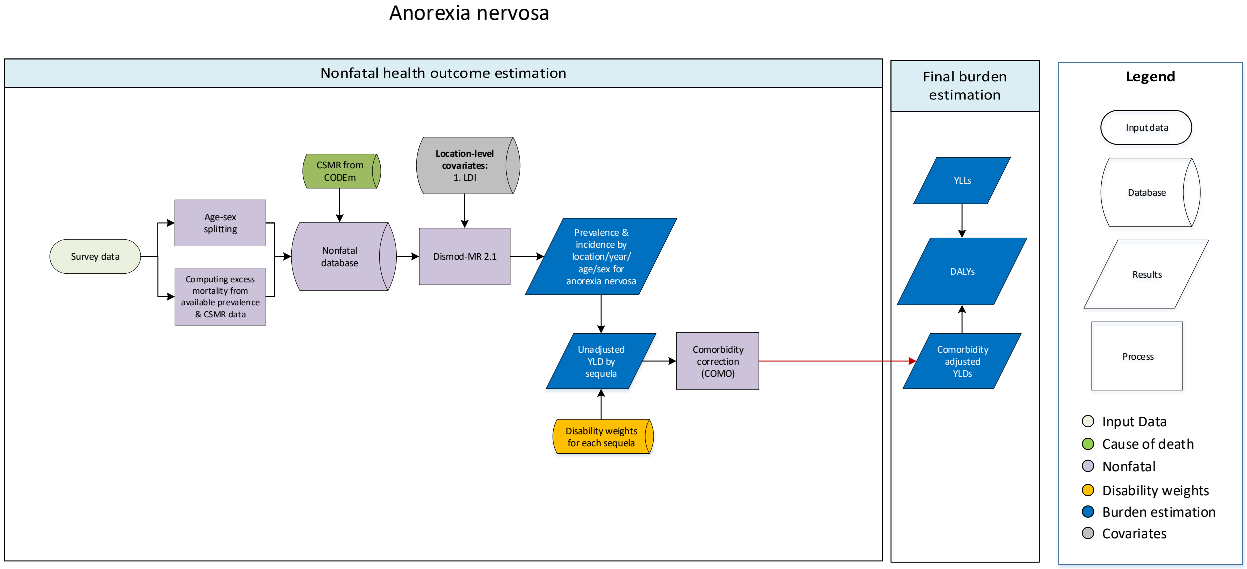

Case definition

According to the Diagnostic and Statistical Manual of Mental Disorders fourth edition, text revision (DSM-IV-TR),[1] anorexia nervosa (AN) is an eating disorder characterised by:

a) Refusal to maintain body weight at or above a minimally normal weight for age and height (eg, weight loss leading to maintenance of body weight less than 85% of that expected; or failure to make expected weight gain during period of growth, leading to body weight less than 85% of that expected).
b) Intense fear of gaining weight or becoming fat, even though underweight (expanded to include any behaviour that interferes with weight gain in DSM-5[2]).
c) Disturbance in the way in which one's body weight or shape is experienced, undue influence of body weight or shape on self-evaluation, or denial of the seriousness of the current low body weight.
d) In postmenarcheal females, amenorrhoea, ie, the absence of at least three consecutive menstrual cycles (this criterion was removed in DSM-5[2]).

Included in GBD were cases meeting diagnostic criteria according to DSM[1] or the International Classification of Diseases (ICD).[3] These were identified by the following codes: 307.1 (DSM-IV-TR) and F50.0-50.1 (ICD-10). Different versions of DSM (DSM-III, DSM-III-R, DSM-IV, DSM-IV-TR, and DSM-5) and ICD (ICD-9 and ICD-10) were accepted.

Input data

Model inputs

A series of systematic literature reviews were conducted to capture studies reporting the prevalence, incidence, remission, and excess mortality of AN. The reviews incorporated searches of peer-reviewed literature via electronic databases, investigations of grey literature, and consultation with experts. In order for a study to be included, it must have been published during or after 1980, use DSM or ICD criteria to define cases, provide sufficient details on study methodology and sample characteristics to determine study quality, and be representative of the general population rather than a special population, eg, prison inmates. No limitation was set on the language of publication. Detailed descriptions of this methodology have been published elsewhere.[4] This methodology was utilised in GBD 2010, GBD 2013, GBD 2015, and GBD 2017. GBD 2016 included additional sources identified by GBD experts and microdata where available. Additionally, in GBD 2017, two targeted systematic reviews were conducted to further supplement the AN dataset. The first review captured studies reporting on the epidemiology of AN within Maori versus non-Maori populations (as opposed to New Zealand more broadly), given the inclusion of these two sub-groups in GBD 2017. The second review utilised the China National Knowledge Infrastructure database to find studies that would not typically be captured in PubMed, Embase, and PsycINFO.

Data counts

	Incidence	Other	Prevalence	Remission
Site-years (total)	7	20	48	21
Number of countries with data	6	9	23	11
Number of GBD regions with data (out of 21 regions)	2	4	10	4
Number of GBD super-regions with data (out of 7 super-regions)	1	3	5	2

Disability weight

No severity splits were applied to AN. The lay description and disability weight for AN are shown in the table below.

Lay description	DW (95% CI)
Feels an overwhelming need to starve and exercises excessively to lose weight. The person is very thin, weak, and anxious.	0.224 (0.150–0.312)

Modelling strategy

We assumed no incidence prior to age 5 or from 50 years onward. These settings are in line with those placed on the corresponding cause of death model for anorexia nervosa. A cap of 0.6 was placed on remission in order to obtain a more plausible fit of the model. We used the function in DisMod-MR 2.1 to

pull in cause-specific mortality rate (CSMR) data from our CODEm and CoDCorrect analyses. As such, other mortality data (standardised mortality ratios and relative risks) were excluded. We also used these CSMR data to estimate priors on excess mortality rates (EMR) by matching them with prevalence data points for the same geography and study year and dividing CSMR by prevalence. A country-level covariate, lagged distributed income (LDI), was included. This covariate represents a moving average of gross domestic product (GDP) over time. The limits placed on this covariate meant that prevalence was assumed to increase with rising GDP. LDI was also applied to excess mortality data in order to better inform regional distribution. The table below illustrates the covariates, parameters, beta and exponentiated beta values for AN.

Covariate	Parameter	beta	Exponentiated beta
LDI ($ per capita)	Prevalence	0.42 (0.23–0.50)	1.52 (1.26–1.64)
LDI ($ per capita)	Excess mortality	-0.26 (-0.48 to -0.11)	0.77 (0.62–0.90)

References

1. American Psychiatric Association. Diagnostic and Statistical Manual of Mental Disorders (DSM-IV-TR). 4th, Text Revision ed. Washington DC: American Psychiatric Association; 2000.
2. American Psychiatric Association. Diagnostic and Statistical Manual of Mental Disorders, Fifth Edition. Arlington, VA: American Psychiatric Association; 2013.
3. World Health Organisation. ICD-10 Classification of Mental and Behavioural Disorders: Clinical descriptions and diagnostic guidelines. Geneva: World Health Organisation; 1992.
4. Whiteford HA, Degenhardt L, Rehm J, et al. Global burden of disease attributable to mental and substance use disorders: findings from the Global Burden of Disease Study 2010. *The Lancet* 2013; **382**(9904): 1575-86.

Bulimia nervosa

Flowchart

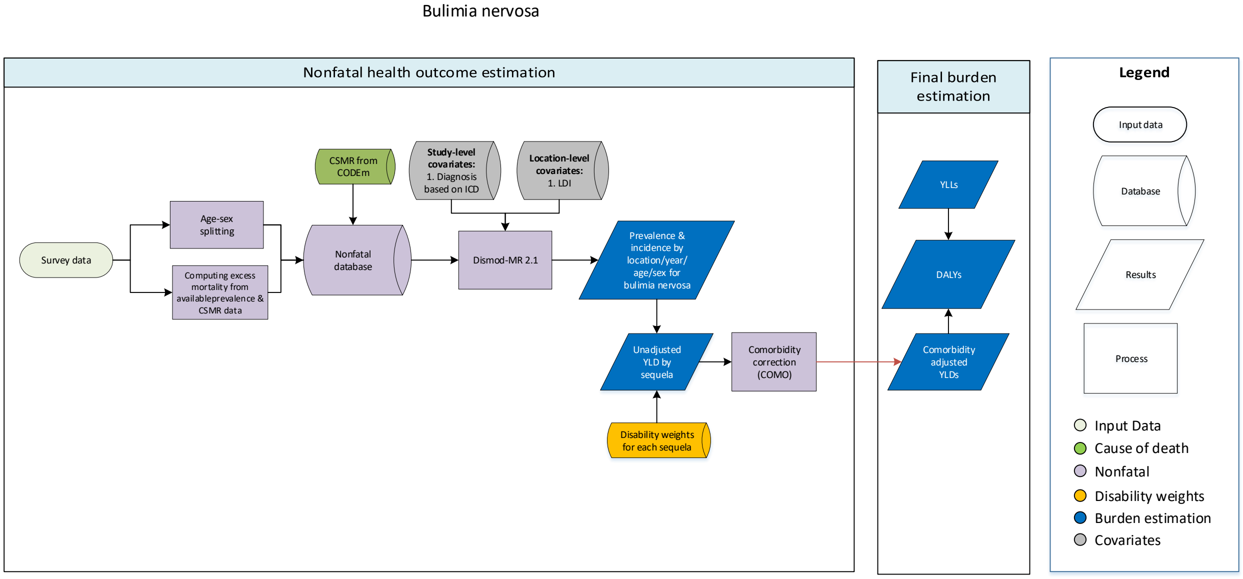

Case definition

According to the Diagnostic and Statistical Manual of Mental Disorders fourth edition, text revision (DSM-IV-TR),[1] bulimia nervosa (BN) is an eating disorder characterised by:

a) Recurrent episodes of binge eating. An episode of binge eating is characterised by both of the following:
   1) eating, in a discrete period of time (eg, within any two-hour period), an amount of food that is definitely larger than most people would eat during a similar period of time and under similar circumstances
   2) a sense of lack of control over eating during the episode (eg, a feeling that one cannot stop eating or control what or how much one is eating)
b) Recurrent inappropriate compensatory behavior in order to prevent weight gain, such as self-induced vomiting; misuse of laxatives, diuretics, enemas, or other medications; fasting; or excessive exercise.
c) The binge eating and inappropriate compensatory behaviors both occur, on average, at least twice a week for three months (changed to once a week for three months in DSM-5[2]).
d) Self-evaluation is unduly influenced by body shape and weight.
e) The disturbance does not occur exclusively during episodes of anorexia nervosa.

Included in GBD were cases meeting diagnostic criteria according to DSM[1] or the International Classification of Diseases (ICD).[3] These were identified by the following codes: 307.51 (DSM-IV-TR) and F50.2 (ICD-10). Different versions of DSM (DSM-III, DSM-III-R, DSM-IV, DSM-IV-TR, and DSM-5) and ICD (ICD-9 and ICD-10) were accepted.

Input data

Model inputs

A series of systematic literature reviews were conducted to capture studies reporting the prevalence, incidence, remission, and excess mortality of BN. The reviews incorporated searches of peer-reviewed literature via electronic databases, investigations of grey literature, and consultation with experts. In order for a study to be included, it must have been published during or after 1980, use DSM or ICD criteria to define cases, provide sufficient details on study methodology and sample characteristics to determine study quality, and be representative of the general population rather than a special population, eg, prison inmates. No limitation was set on the language of publication. Detailed descriptions of this methodology have been published elsewhere.[4] This methodology was utilised in GBD 2010, GBD 2013, GBD 2015, and GBD 2017. GBD 2016 included additional sources identified by GBD experts and microdata where available. Additionally, in GBD 2017, two targeted systematic reviews were conducted to further supplement the BN dataset. The first review captured studies reporting on the epidemiology of BN within Maori versus non-Maori populations (as opposed to New Zealand more broadly), given the inclusion of these two sub-groups in GBD 2017. The second review utilised the China National Knowledge Infrastructure database to find studies that would not typically be captured in PubMed, Embase, and PsycINFO.

Data counts

	Incidence	Other	Prevalence	Remission
Site-years (total)	5	12	58	14
Number of countries with data	4	7	27	7
Number of GBD regions with data (out of 21 regions)	1	3	10	3
Number of GBD super-regions with data (out of 7 super-regions)	1	2	5	1

Disability weight

No severity splits were applied to BN. The lay description and disability weight for BN is shown in the table below.

Lay description	DW (95% CI)
Has uncontrolled overeating followed by guilt, starving, and vomiting to lose weight.	0.223 (0.149–0.311)

Modelling strategy

We assumed no incidence prior to 10 years of age or onward from 40 years of age. We used the function in DisMod-MR 2.1 to pull in cause-specific mortality rate (CSMR) data from our CODEm and CODcorrect analyses. As such, other mortality data (standardised mortality ratios and relative risks) were excluded. We also used CSMR data to estimate priors on excess mortality rates (EMR) by matching them with prevalence data points for the same geography and study year and dividing CSMR by prevalence. A study-

level covariate was applied which adjusted estimates based on ICD criteria toward those based on DSM criteria. A country-level covariate, lagged distributed income (LDI), was also included. This covariate represents a moving average of gross domestic product (GDP) over time. The limits placed on this covariate meant that prevalence was assumed to increase with rising GDP. LDI was also applied to excess mortality data in order to better inform regional distribution. The table below illustrates the covariates, parameters, beta and exponentiated beta values for BN.

Covariate	Parameter	beta	Exponentiated beta
ICD classification	Prevalence	-0.17 (-0.67–0.39)	0.84 (0.51–1.48)
LDI	Prevalence	0.39 (0.15–0.50)	1.48 (1.16–1.64)
LDI	Excess mortality	-0.3 (-0.49 to -0.11)	0.74 (0.61–0.90)

References

1. American Psychiatric Association. Diagnostic and Statistical Manual of Mental Disorders (DSM-IV-TR). 4th, Text Revision ed. Washington DC: American Psychiatric Association; 2000.
2. American Psychiatric Association. Diagnostic and Statistical Manual of Mental Disorders, Fifth Edition. Arlington, VA: American Psychiatric Association; 2013.
3. World Health Organisation. ICD-10 Classification of Mental and Behavioural Disorders: Clinical descriptions and diagnostic guidelines. Geneva: World Health Organisation; 1992.
4. Whiteford HA, Degenhardt L, Rehm J, et al. Global burden of disease attributable to mental and substance use disorders: findings from the Global Burden of Disease Study 2010. *The Lancet* 2013; **382**(9904): 1575-86.

Autism spectrum disorders

Flowchart

Autism Spectrum Disorders

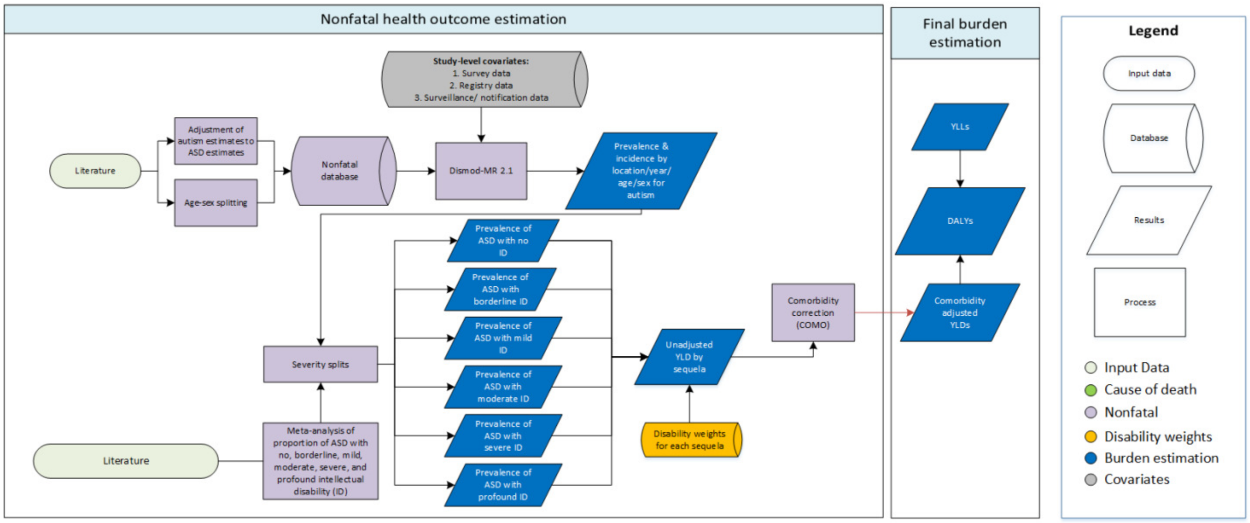

Case definition

Autism spectrum disorder (ASD; also known as pervasive developmental disorder) is a neurodevelopmental disorder with onset occurring in early childhood. It is characterised by pervasive impairment in several areas of development, including social interaction and communication skills, along with restricted and repetitive patterns of behaviours and/or interests.

ASD was an umbrella for five sub-disorders according to the Diagnostic and Statistical Manual of Mental Disorders fourth edition, text revision[2] (DSM-IV-TR): Autistic disorder (299.00), Pervasive Developmental Disorder, Not Otherwise Specified (299.80), Rett's disorder (299.8), and Childhood Disintegrative Disorder (299.10). ASD is still an umbrella for eight sub-disorders according to the International Statistical Classification of Diseases and Related Health Problems 10th Revision[1] (ICD10): Childhood autism (F84.0), Atypical autism (F84.1), Rett syndrome (F84.2), Other childhood disintegrative disorder (F84.3), Overactive disorder associated with mental retardation and stereotyped movements (F84.4), Asperger syndrome (F84.5), Other pervasive developmental disorders (F84.8), and Pervasive disorder unspecified (F84.9). However, it has been amalgamated into a single disorder in the Diagnostic and Statistical Manual for Mental Disorders 5th edition[3] (DSM-5). A diagnosis of ASD according to the DSM-5[3] requires the following criteria to be met:

Persistent deficits in social communication and social interaction across multiple contexts, as manifested by all of the following, currently or by history:

1. *Deficits in social-emotional reciprocity, ranging, for example, from abnormal social approach and failure of normal back-and-forth conversation; to reduced sharing of interests, emotions, or affect; to failure to initiate or respond to social interactions.*
2. *Deficits in nonverbal communicative behaviors used for social interaction, ranging, for example, from poorly integrated verbal and nonverbal communication; to abnormalities in eye*

contact and body language or deficits in understanding and use of gestures; to a total lack of facial expressions and nonverbal communication.

3. *Deficits in developing, maintaining, and understanding relationships, ranging, for example, from difficulties adjusting behavior to suit various social contexts; to difficulties in sharing imaginative play or in making friends; to absence of interest in peers.*

Restricted, repetitive patterns of behaviour, interests, or activities, as manifested by at least two of the following, currently or by history:

1. *Stereotyped or repetitive motor movements, use of objects, or speech (eg, simple motor stereotypies, lining up toys or flipping objects, echolalia, idiosyncratic phrases).*
2. *Insistence on sameness, inflexible adherence to routines, or ritualised patterns of verbal or nonverbal behavior (eg, extreme distress at small changes, difficulties with transitions, rigid thinking patterns, greeting rituals, need to take same route or eat same food every day).*
3. *Highly restricted, fixated interests that are abnormal in intensity or focus (eg, strong attachment to or preoccupation with unusual objects, excessively circumscribed or perseverative interests).*
4. *Hyper- or hyporeactivity to sensory input or unusual interest in sensory aspects of the environment (eg, apparent indifference to pain/temperature, adverse response to specific sounds or textures, excessive smelling or touching of objects, visual fascination with lights or movement).*

The symptoms must be present in the early developmental period, cause clinically significant impairment, and not be better explained by intellectual impairment or global developmental delay.

Input data

Model inputs

A systematic literature review was conducted to capture studies reporting the prevalence, incidence, remission, and excess mortality of ASD. The reviews incorporated searches of peer-reviewed literature via electronic databases, investigations of grey literature, and consultation with experts. In order for a study to be included, it must have been published during or after 1980, provide sufficient details on study methodology and sample characteristics to determine study quality, and be representative of the general population rather than a special population (eg, prison inmates). Cases must have been based on DSM, ICD, Chinese Classification of Mental Disorders (CCMD), or diagnosed by a clinician using established tools. No limitation was set on the language of publication. Due to insufficient data on ASD, estimates of the prevalence of the DSM-IV-TR sub-disorder Autistic disorder (299.00), ICD-10 Childhood autism (F84.0), and their DSM-III, DSM-II-R, DSM-IV, ICD9, and CCMD equivalents were also included with an adjustment so that they reflected what these estimates would be if the data represented ASD.

Prevalence estimates were split by age and sex where possible outside of DisMod-MR 2.1. First, if studies reported prevalence for broad age groups by sex (eg, prevalence in 15- to 65-year-old males and females separately), and also by specific age groups but for both sexes combined (eg, prevalence in 15- to 30-year-olds, then in 31- to 65-year-olds, for males and females combined); age-specific estimates were split by sex using the reported sex ratio and bounds of uncertainty. Studies that only reported the prevalence of autism rather than ASD were included but adjusted up by a factor of 2.31 (se = 0.20) based on 18

studies that used gold-standard sampling methodology and reported prevalence for both ASD and autism. The table below summarises data inputs for ASD by parameter.

	Other	Prevalence	Remission
**Site-years (total)**	3	320	3
**Number of countries with data**	2	33	3
**Number of GBD regions with data (out of 21 regions)**	1	13	2
**Number of GBD super-regions with data (out of 7 super-regions)**	1	7	2

Severity split inputs

ASD is one of the causes that contribute to the intellectual disability (ID) envelope. As such, a gradation of ASD by level of severity was needed.

Meta-analyses were conducted using data from 19 studies that used gold-standard sampling methodology and reported information on the IQ level in those with ASD in order to calculate the severity splits by six sequelae: ASD with 1) no ID, 2) borderline ID, 3) mild ID, 4) moderate ID, 5) severe ID, and 6) profound ID.

The disability weights for each sequela of ASD were calculated using the disability weights for the health states Autism, Asperger's syndrome & other autism spectrum disorders, borderline ID, mild ID, moderate ID, severe ID, and profound ID. These disability weights and their lay descriptions are presented in the table below.

Health state	Lay description	DW (95% CI)
Autism	Has severe problems interacting with others and difficulty understanding simple questions or directions. The person has great difficulty with basic daily activities and becomes distressed by any change in routine.	0.262 (0.176–0.365)
Asperger's syndrome & other autism spectrum disorders	Has difficulty interacting with other people and is slow to understand or respond to questions. The person is often preoccupied with one thing and has some difficulty with basic daily activities.	0.104 (0.071–0.147)
ID, borderline	Is slow in learning at school. As an adult, the person has some difficulty doing complex or unfamiliar tasks but otherwise functions independently.	0.011 (0.005–0.020)
ID, mild	Has low intelligence and is slow in learning at school. As an adult, the person can live independently, but often needs help to raise children and can only work at simple supervised jobs.	0.043 (0.026–0.064)
ID, moderate	Has low intelligence, and is slow in learning to speak and to do even simple tasks. As an adult, the person requires a lot of support to live independently and raise children. The person can only work at the simplest supervised jobs.	0.100 (0.066–0.142)

ID, severe	Has very low intelligence and cannot speak more than a few words, needs constant supervision and help with most daily activities, and can do only the simplest tasks.	0.160 (0.107–0.226)
ID, profound	Has very low intelligence, has almost no language, and does not understand even the most basic requests or instructions. The person requires constant supervision and help for all activities.	0.200 (0.133–0.283)

To estimate the disability weights for each sequela of ASD, the following steps were conducted, each step pulling 1,000 draws of each input:

1. A pooled disability weight for ASD was estimated:
$$DW_{ASD} = DW_{Autism} \times A + DW_{Asperger} \times (1 - A)$$
Where *DW* is disability weight and *A* is the inverse of the autism-to-ASD adjustment described earlier (*A* = 0.43, *se* = 0.04).
2. The disability weight for ASD without ID was estimated:
$$DW_{ASD\ no\ ID} = \frac{DW_{ASD} - \sum_{k=Bord.ID}^{Prof.ID}(P_k \times DW_k)}{P_{ASD\ no\ ID} + \sum_{k=Bord.ID}^{Prof.ID}(P_k \times (1 - DW_k))}$$
Where *DW* is disability weight and *P* is the severity proportion estimated from the meta-analysis.
3. The disability weight for ASD and each remaining level of ID was estimated:
$$DW_{ASD+ID} = 1 - (1 - DW_{ASD\ no\ ID}) \times (1 - DW_{ID})$$

The severity proportions from the meta-analysis used in the above process and the resulting disability weights for each sequela are presented in the table below.

**Sequela**	**Severity proportion (95% UI)**	**DW (95% UI)**
ASD without ID	0.428 (0.369–0.491)	0.143 (0.094–0.202)
ASD with borderline ID	0.187 (0.144–0.236)	0.152 (0.103–0.212)
ASD with mild ID	0.180 (0.134–0.231)	0.179 (0.125–0.245)
ASD with moderate ID	0.133 (0.094–0.177)	0.228 (0.160–0.310)
ASD with severe ID	0.057 (0.034–0.091)	0.279 (0.195–0.378)
ASD with profound ID	0.014 (0.006–0.025)	0.313 (0.215–0.422)

Modelling strategy

We assumed all incidence of ASD occurred at birth. A small setting was placed on excess mortality to help DisMod follow the mortality estimates. Remission was set to 0 after expert consultation revealed we would not expect remission for ASD. Three study-level covariates were applied to adjust estimates with suboptimal sampling methodologies:

1. Survey: Studies that conduct household or school surveys but do not conduct additional active case-finding (such as reviewing special education records) to find cases likely to be missed by survey methodology.

2. Registry data: Studies where prevalence of ASD is estimated from diagnoses within a clinical or educational registry where no population screening procedure is in place.
3. Surveillance/notification data: Studies where researchers review notes of high-risk populations from one or more data sources records (eg, clinical/education records) and determine prevalence based on notes without confirming the diagnosis via clinical evaluation.

Our systematic review revealed four studies that used gold-standard sampling methodology to estimate prevalence and also reported the proportion of their cases of ASD that were captured by registries. The pooled proportion was 0.71 (*se* = 0.05), and this was set as the prior for the study-level covariate for registry data.

Study covariate	Parameter	beta	Exponentiated beta
Survey	Prevalence	-0.14 (-0.36–0.11)	0.87 (0.70–1.11)
Registry data	Prevalence	-0.34 (-0.34 to -0.34)	0.71 (0.71–0.71)
Surveillance / notification data	Prevalence	0.39 (0.21–0.58)	1.48 (1.23–1.78)

References

1. World Health Organisation. ICD-10 Classification of Mental and Behavioural Disorders: Clinical descriptions and diagnostic guidelines. Geneva: World Health Organisation; 1992.
2. American Psychiatric Association. Diagnostic and Statistical Manual of Mental Disorders (DSM-IV-TR). 4th, Text Revision ed. Washington DC: American Psychiatric Association; 2000.
3. American Psychiatric Association. Diagnostic and statistical manual of mental disorders : DSM-5. Arlington, VA: American Psychiatric Publishing; 2013.
4. Baxter AJ, Brugha TS, Erskine HE, Scheurer RW, Vos T, Scott JG. The epidemiology and global burden of autism spectrum disorders. *Psychological Medicine* 2014; **45**(3): 601-13.

Attention-deficit/hyperactivity disorder (ADHD)

Flowchart

Attention-deficit/hyperactivity disorder

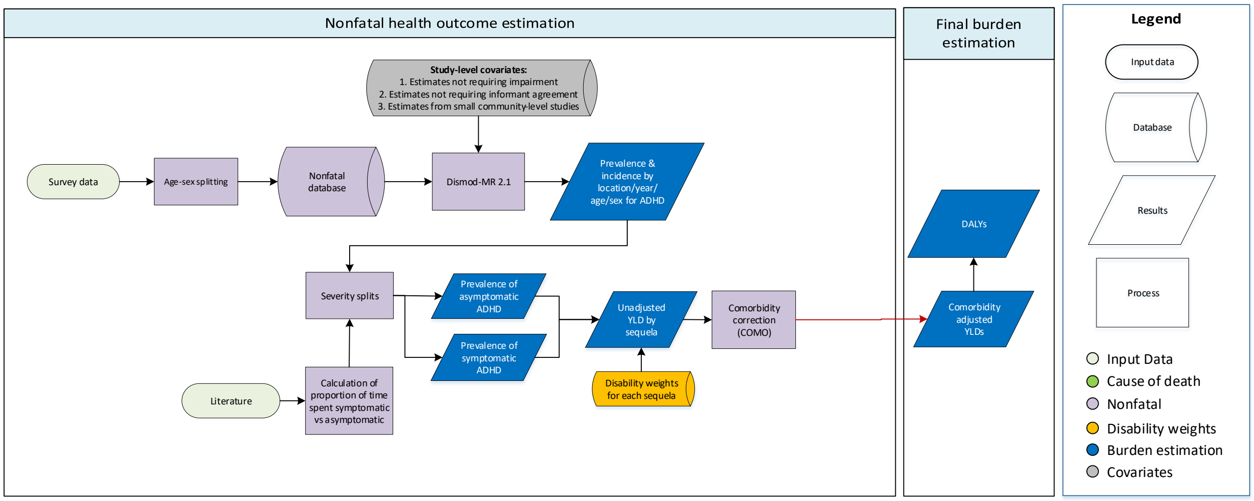

Case definition

Attention-deficit/hyperactivity disorder (ADHD) is an externalising behaviour disorder characterised by persistent inattention and/or hyperactivity-impulsivity. As per criteria set by the Diagnostic and Statistical Manual of Mental Disorders fourth edition, text revision (DSM-IV-TR)[1], diagnosis requires six or more symptoms of inattention or hyperactivity-impulsivity to have persisted for at least six months in two or more settings causing significant impairment to functioning, with at least some impairing symptoms being present prior to 7 years of age (12 years of age in DSM-5[2]). Recognised symptoms include:

Inattention:

- often fails to give close attention to details or makes careless mistakes in schoolwork, work, or other activities
- often has difficulty sustaining attention in tasks or play activities
- often does not seem to listen when spoken to directly
- often does not follow through on instructions and fails to finish schoolwork, chores, or duties in the workplace (not due to oppositional behavior or failure to understand instructions)
- often has difficulty organising tasks and activities
- often avoids, dislikes, or is reluctant to engage in tasks that require sustained mental effort (such as schoolwork or homework)
- often loses things necessary for tasks or activities (eg, toys, school assignments, pencils, books, or tools)
- is often easily distracted by extraneous stimuli
- is often forgetful in daily activities

Hyperactivity

- often fidgets with hands or feet or squirms in seat

- often leaves seat in classroom or in other situations in which remaining seated is expected
- often runs about or climbs excessively in situations in which it is inappropriate (in adolescents or adults, may be limited to subjective feelings of restlessness)
- often has difficulty playing or engaging in leisure activities quietly
- is often "on the go" or often acts as if "driven by a motor"
- often talks excessively

Impulsivity

- often blurts out answers before questions have been completed
- often has difficulty awaiting turn
- often interrupts or intrudes on others (eg, butts into conversations or games)

Included in GBD were cases meeting diagnostic criteria according to DSM[1] or the International Classification of Diseases (ICD)[3] (called "hyperkinetic disorder" in ICD). These were identified by the following codes: 314.0, 314.01 (DSM-IV-TR) and F90 (ICD-10). Different versions of DSM (DSM-III, DSM-III-R, DSM-IV, DSM-IV-TR, and DSM-5) and ICD (ICD-9 and ICD-10) were accepted.

Input data

Model inputs

A series of systematic literature reviews were conducted to capture studies reporting the prevalence, incidence, remission, and excess mortality of ADHD. The reviews incorporated searches of peer-reviewed literature via electronic databases (PubMed, Embase, and PsycINFO), investigations of grey literature, and consultation with experts. In order for a study to be included, it must have been published during or after 1980, use DSM or ICD criteria to define cases, provide sufficient details on study methodology and sample characteristics to determine study quality, and be representative of the general population rather than a special population, eg, prison inmates. No limitation was set on the language of publication. Detailed descriptions of this methodology have been published elsewhere.[4] This methodology was utilised in GBD 2010, GBD 2013, and GBD 2016. GBD 2015 and GBD 2017 included additional sources identified by GBD experts and microdata where available. Additionally, in GBD 2017, two targeted systematic reviews were conducted to further supplement the ADHD dataset. The first review captured studies reporting on the epidemiology of ADHD within Maori versus non-Maori populations (as opposed to New Zealand more broadly), given the inclusion of these two sub-groups in GBD 2017. The second review utilised the China National Knowledge Infrastructure database to find studies that would not typically be captured in PubMed, Embase, and PsycINFO.

Reported estimates of prevalence were split by age and sex where possible. If studies reported prevalence for broad age groups by sex (eg, prevalence in 5-18 year old males and females separately) and by specific age groups but for both sexes combined (eg, prevalence for 5-12 year olds and 13-18 year olds, for males and females combined), age-specific estimates were split by sex using the reported sex ratio and bounds of uncertainty. Also, where studies reported estimates across age groups spanning 20 years or more, these were split into five-year age groups using the prevalence age pattern estimated by DisMod-MR 2.1.

Data counts

	Incidence	Other	Prevalence	Remission
Site-years (total)	2	2	129	13
Number of countries with data	1	2	37	6
Number of GBD regions with data (out of 21 regions)	1	2	13	3
Number of GBD super-regions with data (out of 7 super-regions)	1	1	6	2

Severity splits and disability weight

A severity split for the proportion of time spent symptomatic versus asymptomatic was based on data from the Great Smoky Mountains Study which assessed the levels of disability found in children and adolescents with mental disorders.[5] Of those with ADHD, 48% reported disability while 20% of individuals with no diagnosis reported disability at the time of survey. Using these as estimates of the proportion of time with disability in the "average case," the proportion of disability in children without a diagnosis was subtracted from the proportion with disability for ADHD, giving an adjusted proportion of 28%. Detailed descriptions of this methodology have been published elsewhere.[6] The lay description and disability weight for ADHD is shown in the table below.

Lay description	DW (95% CI)
Is hyperactive and has difficulty concentrating, remembering things, and completing tasks	0.045 (0.028–0.066)

Modelling strategy

We assumed no incidence prior to 3 years of age or onward from 12 years of age. The minimum age of onset was set in consultation with experts and based on current literature, while the upper age limit on incidence was set in line with the latest DSM-5 criteria. Remission was set to zero prior to 12 years, in line with the restriction on incidence. Excess mortality was set to zero given only three estimates were found for this parameter. Three covariates were included in the model. The first covariate was an informant covariate which adjusted estimates not requiring agreement between informants (eg, diagnosis made if either a teacher or parent indicates ADHD) toward estimates which required informant agreement. The second covariate adjusted estimates not requiring impairment (or those not specifying whether impairment was required) for diagnosis toward those which required impairment. The third covariate adjusted studies using small, community samples toward studies representative of entire regions or countries. Bounds for these covariates were calculated from the epidemiological data and applied in DisMod-MR 2.1.

Study covariate	Parameter	beta	Exponentiated beta
No informant agreement	Prevalence	0.49 (0.45–0.57)	1.63 (1.57–1.78)
No impairment	Prevalence	0.039 (0.0034–0.13)	1.04 (1.00–1.13)
Small, community-level studies	Prevalence	0.52 (0.33–0.74)	1.68 (1.38–2.09)

References

1. American Psychiatric Association. Diagnostic and Statistical Manual of Mental Disorders (DSM-IV-TR). 4th, Text Revision ed. Washington DC: American Psychiatric Association; 2000.
2. American Psychiatric Association. Diagnostic and Statistical Manual of Mental Disorders, Fifth Edition. Arlington, VA: American Psychiatric Association; 2013.
3. World Health Organisation. ICD-10 Classification of Mental and Behavioural Disorders: Clinical descriptions and diagnostic guidelines. Geneva: World Health Organisation; 1992.
4. Erskine HE, Ferrari AJ, Nelson P, et al. Research Review: Epidemiological modelling of attention-deficit/hyperactivity disorder and conduct disorder for the Global Burden of Disease Study 2010. *Journal of Child Psychology and Psychiatry* 2013; **54**(12): 1263-74.
5. Ezpeleta L, Keeler G, Erkanli A, Costello EJ, Angold A. Epidemiology of Psychiatric Disability in Childhood and Adolescence. *J Child Psychol Psychiatry* 2001; **42**(7): 901-14.
6. Erskine HE, Ferrari AJ, Polanczyk GV, et al. The global burden of conduct disorder and attention-deficit/hyperactivity disorder in 2010. *J Child Psychol Psychiatry* 2014; **55**(4): 328-36.

Conduct disorder

Flowchart

Conduct disorder

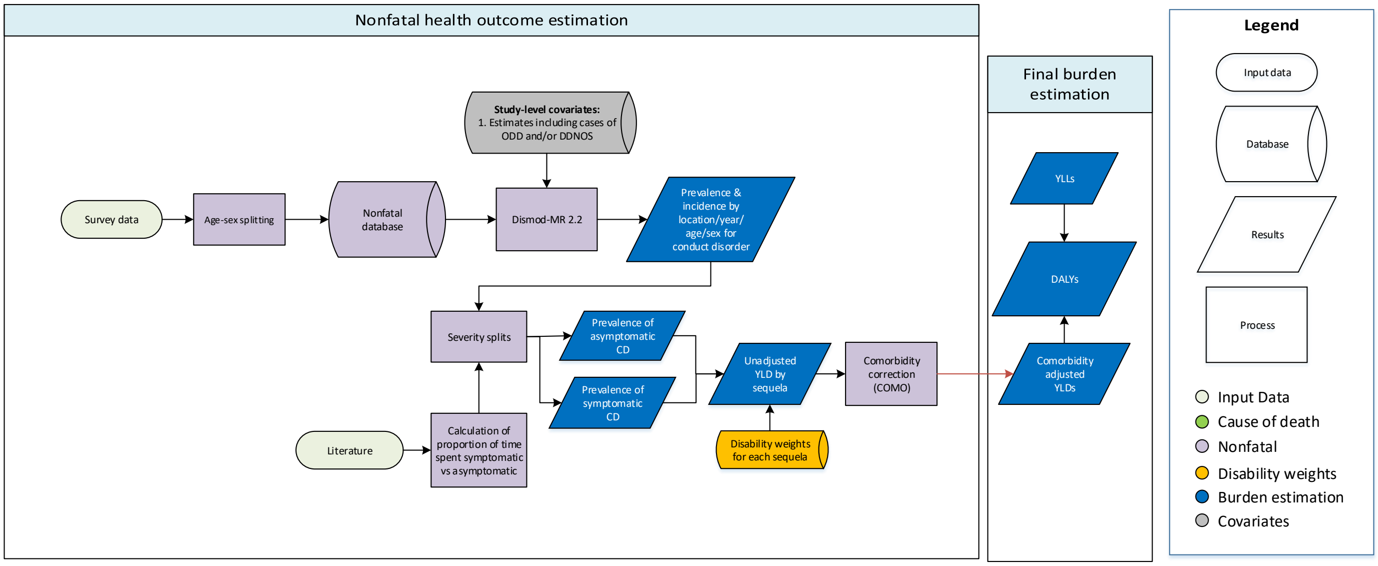

Case definition

Conduct disorder (CD) is an externalising behaviour disorder characterised by a pattern of antisocial behavior that violates the basic rights of others or major age-appropriate societal norms. As per criteria set by the Diagnostic and Statistical Manual of Mental Disorders fourth edition, text revision (DSM-IV-TR),[1] diagnosis requires three or more of the following symptoms to be present in the past 12 months (with at least one present in the last six months) and cause significant impairment in functioning. Symptoms include:

Aggression to people and animals

- often bullies, threatens, or intimidates others
- often initiates physical fights
- has used a weapon that can cause serious physical harm to others (eg, a bat, brick, broken bottle, knife, gun)
- has been physically cruel to people
- has been physically cruel to animals
- has stolen while confronting a victim (eg, mugging, purse snatching, extortion, armed robbery)
- has forced someone into sexual activity

Destruction of property

- has deliberately engaged in fire setting with the intention of causing serious damage
- has deliberately destroyed others' property (other than by fire setting)

Deceitfulness or theft

- has broken into someone else's house, building, or car

- often lies to obtain goods or favors or to avoid obligations (ie, "cons" others)
- has stolen items of nontrivial value without confronting a victim (eg, shoplifting, but without breaking and entering; forgery)

Serious violations of rules

- often stays out at night despite parental prohibitions, beginning before age 13 years
- has run away from home overnight at least twice while living in parental or parental surrogate home (or once without returning for a lengthy period)
- is often truant from school, beginning before age 13 years

CD is considered a disorder of childhood but can be diagnosed in adults who display such behaviors yet do not meet the criteria for antisocial personality disorder. However, there are almost no studies measuring adult CD as existing studies in this area tend to measure adult antisocial behavior rather than adult CD.[2] As such, only childhood CD (ie, cases prior to 18 years of age) was modelled in GBD.

Included in GBD were cases meeting diagnostic criteria according to DSM[1] or the International Classification of Diseases (ICD).[3] These were identified by the following codes: 312 (DSM-IV-TR) and F91 (ICD-10). Different versions of DSM (DSM-III, DSM-III-R, DSM-IV, DSM-IV-TR, and DSM-5) and ICD (ICD-9 and ICD-10) were accepted. Estimates also including oppositional defiant disorder (ODD; DSM-IV-TR: 313.81, ICD-10: F91.3) or disruptive behavior disorder not otherwise specified (DDNOS, DSM-IV-TR: 312.9, ICD-10: 91.9) were accepted and adjusted with a covariate during the modelling process.

Input data

Model inputs

A series of systematic literature reviews were conducted to capture studies reporting the prevalence, incidence, remission, and excess mortality of CD. The reviews incorporated searches of peer-reviewed literature via electronic databases, investigations of grey literature, and consultation with experts. In order for a study to be included, it must have been published during or after 1980, use DSM or ICD criteria to define cases, provide sufficient details on study methodology and sample characteristics to determine study quality, and be representative of the general population rather than a special population, eg, prison inmates. No limitation was set on the language of publication. Detailed descriptions of this methodology have been published elsewhere.[2] This methodology was utilised in GBD 2010, GBD 2013, and GBD 2016. GBD 2015 and GBD 2017 included additional sources identified by GBD experts and microdata where available. Additionally, in GBD 2017, two targeted systematic reviews were conducted to further supplement the CD dataset. The first review captured studies reporting on the epidemiology of CD within Maori versus non-Maori populations (as opposed to New Zealand more broadly), given the inclusion of these two sub-groups in GBD 2017. The second review utilised the China National Knowledge Infrastructure database to find studies that would not typically be captured in PubMed, Embase, and PsycINFO.

Reported estimates of prevalence were split by age and sex where possible. If studies reported prevalence for broad age groups by sex (eg, prevalence in 5-18 year old males and females separately) and by specific age groups but for both sexes combined (eg, prevalence for 5-12 year olds and 13-18 year olds, for males and females combined), age-specific estimates were split by sex using the reported sex ratio and bounds of uncertainty.

Data counts

	Incidence	Prevalence	Remission
Site-years (total)	6	63	6
Number of countries with data	3	27	3
Number of GBD regions with data (out of 21 regions)	2	14	2
Number of GBD super-regions with data (out of 7 super-regions)	1	6	1

Severity splits and disability weight

A severity split for the proportion of time spent symptomatic versus asymptomatic was based on data from the Great Smoky Mountains Study which assessed the levels of disability found in children and adolescents with mental disorders.[4] Of those with CD, 72% reported disability while 20% of individuals with no diagnosis reported disability at the time of survey. Using these as estimates of the proportion of time with disability in the "average case," the proportion of disability in children without a diagnosis was subtracted from the proportion with disability for CD, giving an adjusted proportion of 52%. Detailed descriptions of this methodology have been published elsewhere.[5] The lay description and disability weight for CD is shown in the table below.

Lay description	DW (95% CI)
Has frequent behaviour problems, which are sometimes violent. The person often has difficulty interacting with other people and feels irritable	0.241 (0.159–0.341)

Modelling strategy

We assumed no incidence or prevalence prior to 5 years of age or after 18 years of age. The minimum age of onset was set in consultation with experts while the upper age limit was set in line with DSM criteria. Excess mortality was set to zero given the absence of data demonstrating an association between CD and an increased risk of death. Remission and incidence were capped between ages 4 and 17 years in order to gain more plausible output. A covariate was used to adjust any prevalence estimates which also included cases of oppositional defiant disorder and/or disruptive behaviour disorder not otherwise specified toward those including CD only.

Covariate	Parameter	beta	Exponentiated beta
Identifies estimates also containing ODD &/or DDNOS cases	Prevalence	0.63 (0.39 — 0.84)	1.88 (1.48 — 2.32)

References

1. American Psychiatric Association. Diagnostic and Statistical Manual of Mental Disorders (DSM-IV-TR). 4th, Text Revision ed. Washington DC: American Psychiatric Association; 2000.
2. Erskine HE, Ferrari AJ, Nelson P, et al. Research Review: Epidemiological modelling of attention-deficit/hyperactivity disorder and conduct disorder for the Global Burden of Disease Study 2010. *Journal of Child Psychology and Psychiatry* 2013; **54**(12): 1263-74.
3. World Health Organisation. ICD-10 Classification of Mental and Behavioural Disorders: Clinical descriptions and diagnostic guidelines. Geneva: World Health Organisation; 1992.
4. Ezpeleta L, Keeler G, Erkanli A, Costello EJ, Angold A. Epidemiology of Psychiatric Disability in Childhood and Adolescence. *J Child Psychol Psychiatry* 2001; **42**(7): 901-14.
5. Erskine HE, Ferrari AJ, Polanczyk GV, et al. The global burden of conduct disorder and attention-deficit/hyperactivity disorder in 2010. *Journal of Child Psychology and Psychiatry* 2014; **55**(4): 328-36.

Other mental disorders

Flowchart

Other mental disorders: Personality disorders

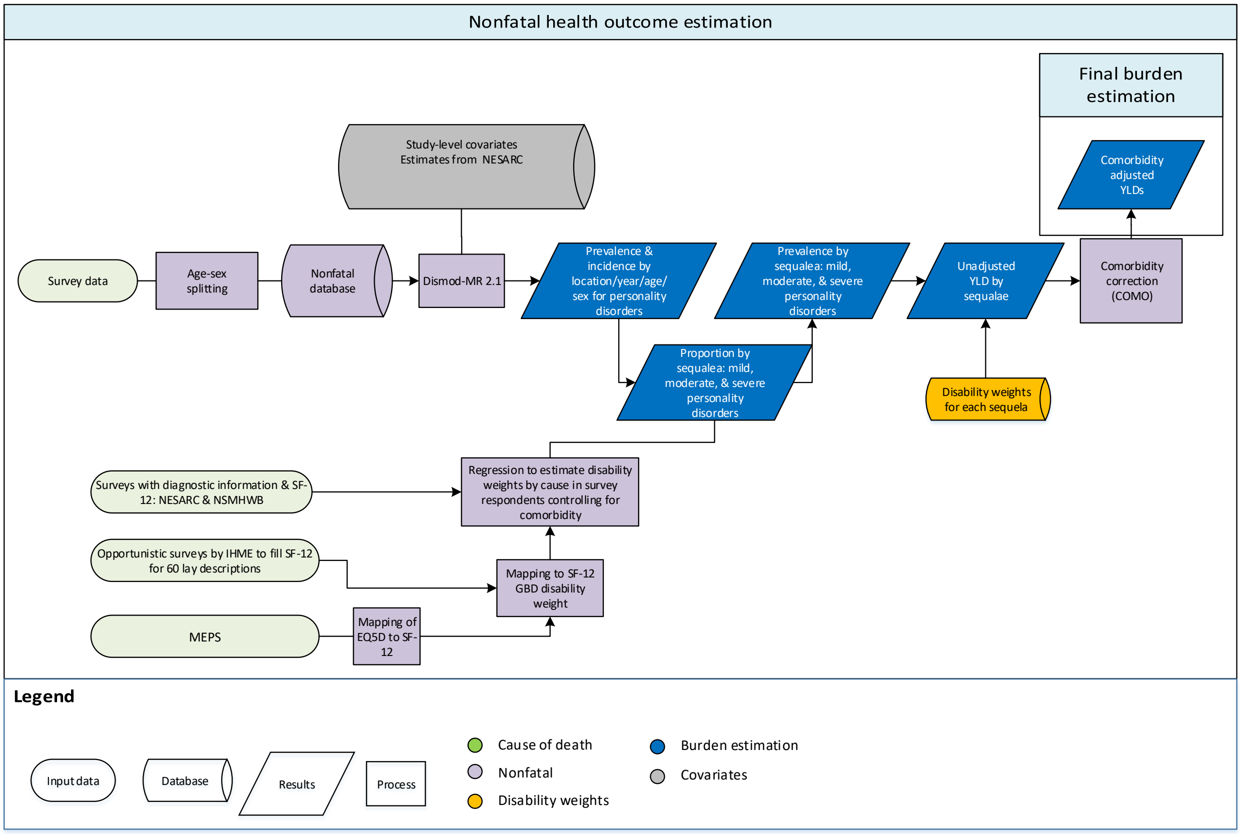

Case Definition

In addition to the individual mental disorders for which we estimate burden, we also estimate the non-fatal burden attributable to a residual cause of "other mental disorders." This is made up of an aggregate group of personality disorders. Personality disorders are characterised by pervasive, inflexible and maladaptive patterns of behaviour and inner experience which are markedly different from what is considered to be acceptable in the individual's culture. These disorders tend to be chronic and are associated with significant distress or disability. Included in GBD 2017 were cases meeting diagnostic criteria for personality disorders according to the Diagnostic and Statistical Manual of Mental Disorders (DSM), or the equivalent diagnosis in the International Classification of Diseases (ICD).[1,2] The aggregated group of personality disorders used in GBD 2017 captured any of the following;

- Paranoid personality disorder
- Schizoid personality disorder
- Schizotypal personality disorder

- Antisocial personality disorder
- Borderline personality disorder
- Histrionic personality disorder
- Narcissistic personality disorder
- Avoidant personality disorder
- Dependent personality disorder
- Obsessive-compulsive personality disorder
- Personality disorder not otherwise specified

Input data

Model inputs

Prevalence estimates for the above personality disorders were obtained from the US National Epidemiological Survey on Alcohol and Related Conditions (NESARC, conducted in two waves from 2001–2002 and 2004–2005)[3] and the Australian National Survey of Mental Health and Wellbeing of Adults (NSMHWB, conducted in 1997).[4] Given that personality disorders often co-occur with other mental and substance use disorders, an adjustment for comorbidity is important so as not to overestimate the overall burden attributable to mental and substance use disorders. Participants meeting criteria for any type of personality disorders from the NESARC and NSMHWB surveys were counted as a prevalent case only if they did not simultaneously meet criteria for another mental and substance use disorder featured in GBD 2017. The table below summarises data inputs by parameter.

	**Prevalence**
**Site-years (total)**	3
**Number of countries with data**	2
**Number of GBD regions with data (out of 21 regions)**	2
**Number of GBD super-regions with data (out of 7 super-regions)**	1

Severity splits

The basis of the GBD disability weight survey assessments are lay descriptions of sequelae highlighting major functional consequences and symptoms. The lay descriptions and disability weights applied to the personality disorders within this residual group are shown below and were those estimated for anxiety disorders.

Severity level	Lay description	DW (95% CI)
Mild	Feels mildly anxious and worried, which makes it slightly difficult to concentrate, remember things, and sleep. The person tires easily but is able to perform daily activities.	0.03 (0.018–0.046)
Moderate	Feels anxious and worried, which makes it difficult to concentrate, remember things, and sleep. The person tires easily and finds it difficult to perform daily activities.	0.133 (0.091–0.186)
Severe	Constantly feels very anxious and worried, which makes it difficult to concentrate, remember things, and sleep. The	0.523 (0.362–0.677)

	person has lost pleasure in life and thinks about suicide.	

To determine the proportion of people with personality disorders within each of the severity levels, the NSMHWB survey was used to estimate the proportion of cases asymptomatic (30%, 28%–32%), mild (41%, 33%–47%), moderate (15%, 11%–20%) and severe (14%, 10%–18%).

Modelling Strategy

The GBD 2017 epidemiological modelling strategy made use of DisMod-MR 2.1. As we only had prevalence data available, a number of expert priors were used in order to run a full-parameter model. We assumed no incidence and prevalence before age 14. This minimum age of onset was corroborated with expert feedback and DSM criteria highlighting the fact that personality disorders typically become recognisable during adolescence and early adulthood. Remission was set to a maximum of 0.01, given that these are understood to be chronic disorders with little or no complete remission. Excess mortality was set to 0 in this model, in the absence of mortality data required for DisMod-MR 2.1 modelling purposes.

Study-level covariates were used to accommodate for between-study variability in the raw prevalence data. A cv_NESARC covariate adjusted all data points derived from NESARC toward the level of data points from the NSMHWB. The latter survey was made up of a more representative list of personality disorders and produced estimates along the levels of what we would expect for personality disorders. The corresponding beta and exponentiated value (which can be interpreted as an odds ratio) is shown in the table below:

Study covariate	Parameter	beta	Exponentiated beta
cv_NESARC	Prevalence	0.65 (0.35–0.98)	1.92 (1.42–2.67)

In this model, global prevalence was exclusively estimated using prevalence estimates from two surveys from the United States and Australia where we had unit record data available to estimate the prevalence of personality disorders, excluding those not simultaneously meeting criteria for another mental or substance use disorder. Given the sparsity of data, we applied a restriction on location random-effects of -0.1 to 0.1 to further guide prevalence estimation. We are currently undertaking a literature review of population-survey data on the epidemiology of personality disorders across low-, middle-, and high-income countries with the aim of providing more robust and globally representative burden estimates for personality disorders in future GBD studies.

References

1. American Psychiatric Association. Diagnostic and Statistical Manual of Mental Disorders (DSM). Washington: American Psychiatric Association, 1952.
2. World Health Organization. The ICD-10 Classification of Mental and Behavioural Disorders. Clinical descriptions and diagnostic guidelines. Geneva: World Health Organization; 1992.
3. Introduction to the National Epidemiologic Survey on Alcohol and Related Conditions [http://pubs.niaaa.nih.gov/publications/arh29-2/74-78.htm]. Access date 1 December 2014.

4. Australian Bureau of Statistics. National Survey of Mental Health and Wellbeing of Adults 1997. Canberra: Australian Bureau of Statistics.

Alcohol use disorders

Flowchart

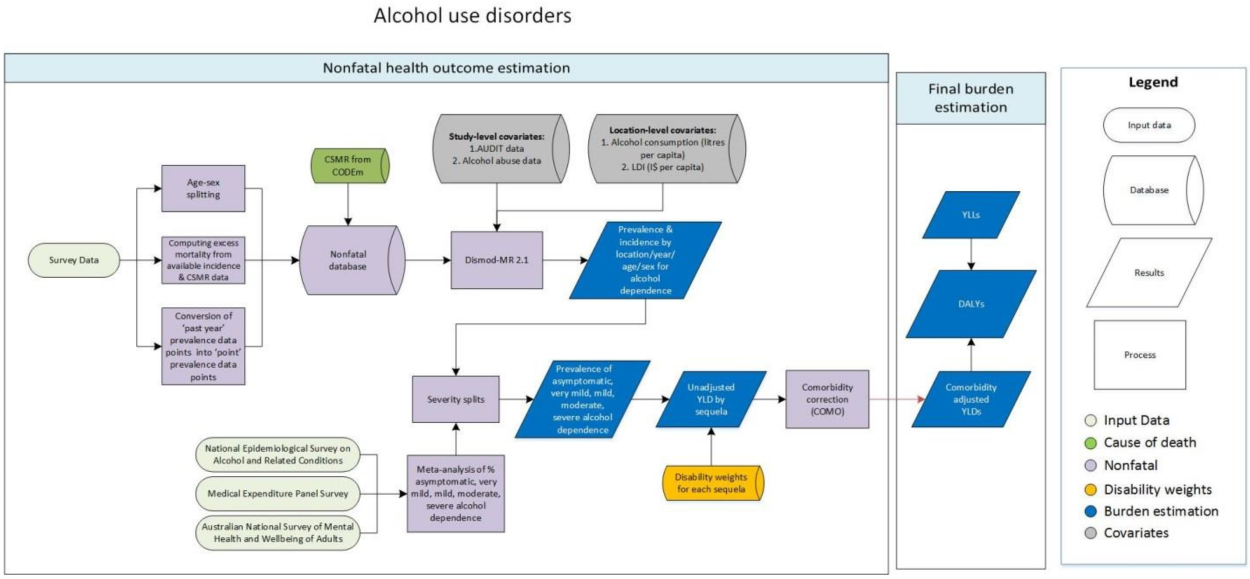

Case definition

Alcohol dependence is a substance-related disorder involving a dysfunctional pattern of alcohol use. According to the Diagnostic and Statistical Manual of Mental Disorders (DSM-IV) criteria for alcohol dependence, at least three out of seven of the following criteria must be manifested during a 12-month period:

- Tolerance
- Withdrawal symptoms or clinically defined alcohol withdrawal syndrome
- Use in larger amounts or for longer periods than intended
- Persistent desire or unsuccessful efforts to cut down on alcohol use
- Time is spent obtaining alcohol or recovering from effects
- Social, occupational, and recreational pursuits are given up or reduced because of alcohol use
- Use is continued despite knowledge of alcohol-related harm (physical or psychological)

The DSM-IV codes for alcohol dependence is 303.90, and the corresponding International Classification of Diseases (ICD-10) codes are F10.1 and F10.2.[1,2]

Input data

Model inputs

In GBD 2013 and GBD 2016, systematic reviews of literature were conducted to capture studies of prevalence, incidence, remission, duration, and excess mortality associated with alcohol dependence. In summary, the search was conducted in three stages involving searches of the peer-reviewed literature (via Medline, Embase, and PubMed), the grey literature, and expert consultation. Updates to systematic reviews are performed on an ongoing schedule across all GBD causes and an update for alcohol dependence will be performed in the next one to two iterations.

The inclusion criteria stipulated that (1) "caseness" must be based on clinical threshold as established by the DSM and ICD; (2) sufficient information must be provided on study method and sample characteristics to assess the quality of the study; and (3) study samples must be representative of the general population (ie, inpatient or pharmacological treatment samples [accepted for estimates of mortality], case studies, and veterans or refugee samples were excluded). Due to insufficient data on alcohol dependence in some regions, estimates using the Alcohol Use Disorder Identification Test (AUDIT) and estimates of alcohol use disorders (alcohol abuse + alcohol dependence) and are included in the epidemiological modelling of alcohol dependence. These estimates are adjusted with study-level covariates to reflect what these estimates would be if the data represented estimates of alcohol dependence.

An adjustment was made outside of DisMod-MR 2.1 to adjust past-year prevalence estimates of alcohol dependence toward the level they would have been had the study measured point prevalence, as the latter is less susceptible to recall bias. Given that remission from alcohol dependence (and hence, average disease duration) vary considerably with age, we also applied an age pattern to this adjustment that cannot be replicated within DisMod-MR 2.1 by use of covariates.

The first step was to estimate the average duration by taking the inverse of remission. Next, we applied an adjustment factor from one-year to point prevalence using the following formula where average duration is expressed in years:

$$\text{adjustment factor} = \frac{\text{average duration}}{\text{average duration} + 1}$$

Age-specific adjustment factors were applied to all one-year prevalence estimates propagating sampling uncertainty around the prevalence and remission input data through to the final adjusted prevalence estimates.

Prevalence estimates were split by age and sex where possible outside of DisMod-MR 2.1. First, if studies reported prevalence for broad age groups by sex (eg, prevalence in 15- to 65-year-old males and females separately), and also by specific age groups but for both sexes combined (eg, prevalence in 15- to 30-year-olds, then in 31- to 65-year-olds, for males and females combined); age-specific estimates were split by sex using the reported sex ratio and bounds of uncertainty. Second, where studies reported estimates across age groups spanning 20 years or more, these were split into five-year age groups using the regional prevalence age pattern estimated by DisMod-MR 2.1.

The table below summarises data inputs for alcohol dependence by parameter.

	Incidence	Other	Prevalence	Remission
**Site-years (total)**	3	37	412	3
**Number of countries with data**	3	15	57	3
**Number of GBD regions with data (out of 21 regions)**	3	8	18	3
**Number of GBD super-regions with data (out of 7 super-regions)**	2	3	7	2

Severity splits & disability weights

The basis of the GBD disability weight survey assessments are lay descriptions of sequelae highlighting major functional consequences and symptoms. The lay descriptions and disability weights for alcohol dependence severity levels are shown below.

Severity level	Lay description	DW (95% CI)
Very mild	Drinks alcohol daily and has difficulty controlling the urge to drink. When sober, the person functions normally.	0.123 (0.082–0.177)
Mild	Drinks a lot of alcohol and sometimes has difficulty controlling the urge to drink. While intoxicated, the person has difficulty performing daily activities.	0.235 (0.16–0.327)
Moderate	Drinks a lot, gets drunk almost every week and has great difficulty controlling the urge to drink. Drinking and recovering cause great difficulty in daily activities, sleep loss, and fatigue.	0.373 (0.248–0.508)
Severe	Gets drunk almost every day and is unable to control the urge to drink. Drinking and recovering replace most daily activities. The person has difficulty thinking, remembering and communicating, and feels constant pain and fatigue.	0.57 (0.396–0.732)

**asymptomatic cases carried no disability weight*

Severity splits used in GBD 2017 were consistent with those used in GBD 2016. The United States' Medical Expenditure Panel Survey (MEPS, conducted in annual waves since 1996)[3], the US National Epidemiological Survey on Alcohol and Related Conditions (NESARC, conducted in two waves from 2001–2002 and 2004–2005)[4], and the Australian National Survey of Mental Health and Wellbeing of Adults (NSMHWB, conducted in 1997)[5] were used to estimate the proportion of alcohol dependence cases in the asymptomatic 40.9% (38.4%–43.3%); very mild 46.9% (43.7%–50.0%); mild 4.0% (1.8%–5.8%); moderate 3.4% (2.3%–4.5%); and severe 4.8% (3.0%–7.0%) disease categories.

Modelling strategy

The GBD 2017 epidemiological modelling strategy for alcohol dependence made use of DisMod-MR 2.1 to estimate prevalence by age, sex, year, and location. Standardised mortality ratio and relative risk data were excluded in the modelling process. Instead we pulled in cause-specific mortality rate (CSMR) data from our CODEm and CODcorrect analyses and matched it with prevalence data points for the same geography and study year to estimate priors on excess mortality rates (by dividing CSMR by prevalence). We assumed no incidence and mortality before age 10. An upper limit of 0.6 was placed on remission (in line with data from the US National Epidemiological Survey on Alcohol and Related Conditions (NESARC) as well as a declining trend with age to restrict DisMod-MR 2.1 from straying too far from the data inputs.

Within DisMod-MR 2.1, study-level covariates were used to accommodate for other sources of between-study variability in the raw prevalence data. Combined abuse and dependence prevalence estimates were crosswalked down toward dependence-only estimates. Similarly, prevalence estimates using AUDIT were crosswalked down toward prevalence estimates from diagnostic (non-AUDIT) measures.

Country-level covariates were also included. The LDI covariate represents a moving average of gross domestic product (GDP) over time. LDI was also applied to excess mortality data with a negative relationship assumed. Alcohol consumption was also represented by a covariate representing this in terms of litres of alcohol per capita.

Study/country covariate	Parameter	beta	Exponentiated beta
Abuse and dependence	Prevalence	0.68 (0.59–0.80)	1.98 (1.80–2.23)
AUDIT	Prevalence	1.34 (1.24–1.43)	3.81 (3.46–4.19)
Alcohol (litres per capita)	Prevalence	0.51 (0.00–1.00)	1.65 (1.00–2.71)
LDI (I$ per capita)	Excess mortality	-0.1 (-0.11 to -0.1)	0.90 (0.90–0.90)

References

1. American Psychiatric Association. Diagnostic and Statistical Manual of Mental Disorders (DSM-IV-TR). 4th, Text Revision ed. Washington DC: American Psychiatric Association; 2000.
2. World Health Organization. The ICD-10 Classification of Mental and Behavioural Disorders. Clinical descriptions and diagnostic guidelines. Geneva: World Health Organization; 1992.
3. Agency for Healthcare Research and Quality. United States Medical Expenditure Panel Survey. Rockville, United States: Agency for Healthcare Research and Quality.
4. Introduction to the National Epidemiologic Survey on Alcohol and Related Conditions [http://pubs.niaaa.nih.gov/publications/arh29-2/74-78.htm]. Access date 1 December 2014.
5. Australian Bureau of Statistics. National Survey of Mental Health and Wellbeing of Adults 1997. Canberra: Australian Bureau of Statistics.

Fetal alcohol syndrome

Flowchart

Fetal alcohol syndrome (FAS)

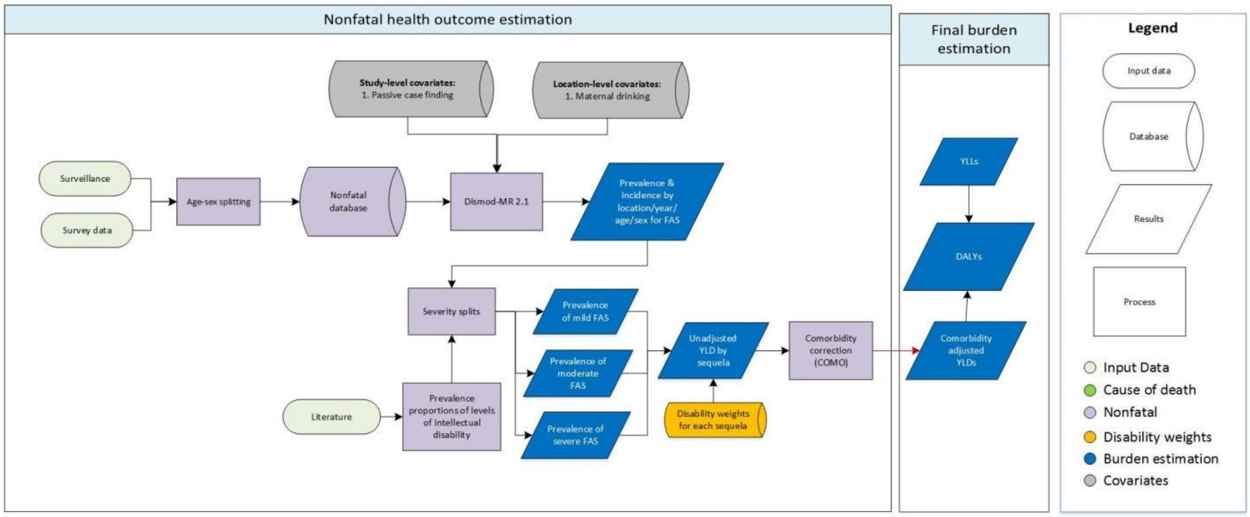

Input data and methodological summary

Case definition

Fetal alcohol syndrome (FAS; ICD-10: Q86.0) is a disorder caused by maternal drinking during pregnancy and is the most severe form of fetal alcohol spectrum disorder (FASD). In GBD, only FAS cases were included in the model. Other manifestations of FASD including partial fetal alcohol syndrome, alcohol-related neurodevelopmental disorder, and alcohol-related birth defects were not included. FAS is characterised by maternal alcohol exposure which results in certain patterns of facial anomalies such as short palpebral fissures and abnormalities in the premaxillary zone (eg, flat upper lip, flattened philtrum, and flat midface), growth retardation (eg, decelerating weight over time not due to nutrition), and central nervous system neurodevelopmental abnormalities (eg, decreased cranial size at birth) in the offspring.[1] Cases were defined according to diagnostic guidelines set by the USA Institute of Medicine, the British Paediatric Association, and other recognised bodies in the area.

Input data

Model inputs

A series of systematic literature reviews were conducted to capture studies reporting the prevalence, incidence, remission, and excess mortality of FAS. The reviews incorporated searches of peer-reviewed literature via electronic databases and consultation with experts. In order for a study to be included, it must use recognised classifications of FAS (eg, the USA Institute of Medicine) and provide sufficient details on study methodology and sample characteristics to determine study quality. No limitation was set on the language of publication. Data from the European Surveillance of Congenital Anomalies (EUROCAT) were also included and updated where relevant. This methodology was utilised in GBD 2015.

Updates to systematic reviews are performed on an ongoing schedule across all GBD causes, and an update for FAS will be performed in the next one to two iterations. The table below summarises data inputs for FAS by parameter.

	**Other**	**Prevalence**
**Site-years (total)**	5	121
**Number of countries with data**	4	31
**Number of GBD regions with data (out of 21 regions)**	3	10
**Number of GBD super-regions with data (out of 7 super-regions)**	1	5

Severity split inputs

There were no data available which gave prevalence of FAS by severity. As such, severity splits for FAS were calculated by matching FAS severity to categories of IQ in children for which prevalence data are available. Severe FAS was matched to an IQ of less than 50, moderate FAS to an IQ of 50 to 69, mild FAS to an IQ of 74 to 84, and asymptomatic FAS to an IQ of 85 or higher. Prevalence data for these IQ levels were then used to calculate severity splits for FAS.

Severity level	Lay description	DW (95% CI)
Mild	Is a little slow in developing physically and mentally, which causes some difficulty in learning but no other difficulties in daily activities.	0.016 (0.008–0.03)
Moderate	Is slow in developing physically and mentally, which causes some difficulty in daily activities.	0.056 (0.035–0.083)
Severe	Is very slow in developing physically and mentally, which causes great difficulty in daily activities.	0.179 (0.119–0.257)

Modelling strategy

Prevalence was set to begin from birth. Incidence was set to zero given cases cannot manifest after birth (despite the fact they may not be diagnosed immediately at birth). Remission was also set to zero. A covariate was included in the model which addressed the heterogeneity introduced by different case-finding methods, ie, active versus passive case-finding. Estimates from known high-drinking populations (eg, indigenous populations) were not considered representative of the general population and were excluded. A country-level covariate was included for GBD 2016 representing the log proportion of pregnant women who drink during their pregnancy, estimated from a meta-analysis.[2]

The table below illustrates the covariate, parameter, beta and exponentiated beta values for the model.

Study/country covariate	Parameter	beta	Exponentiated beta
Passive case finding	Prevalence	0.15 (0.01–0.68)	1.17 (1.01–1.698)
Maternal drinking	Prevalence	0.40 (0.02–0.90)	1.49 (1.02–2.47)

References

1. Stratton K, Howe C, Battaglia F, editors. Fetal alcohol syndrome. Diagnosis, epidemiology, prevention, and treatment. Washington DC: National Academy Press; 1996.
2. Popova S, Lange S, Probst C, Gmel G, Rehm J. Estimation of national, regional, and global prevalence of alcohol use during pregnancy and fetal alcohol syndrome: a systematic review and meta-analysis. *The Lancet Global Health* 2017.

Opioid Use Disorders

Flowchart

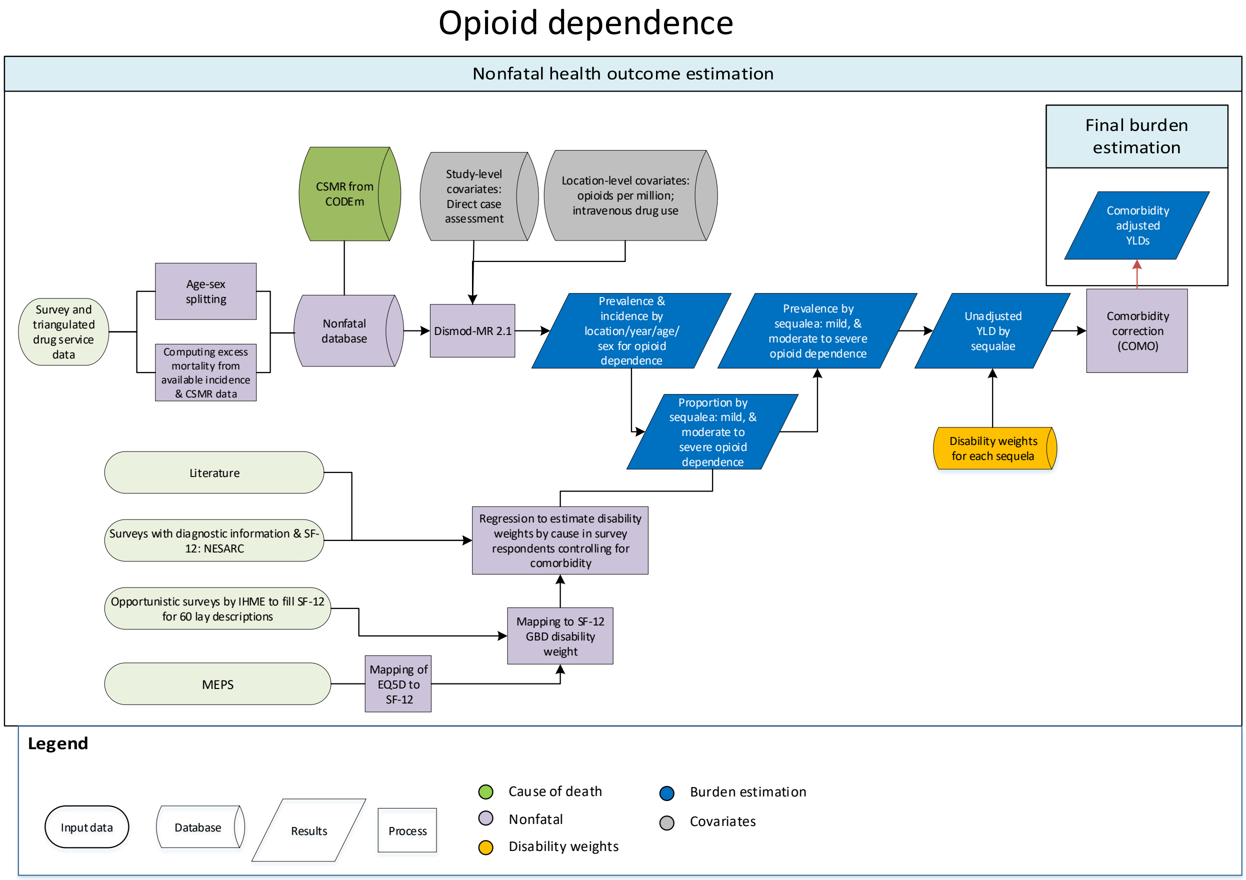

Case Definition

Opioid dependence is a substance-related disorder involving a dysfunctional pattern of opioid use. Included in the GBD disease modelling were cases meeting the Diagnostic and Statistical Manual of Mental Disorders (DSM-IV-TR) or the International Classification of Diseases (ICD-10) diagnostic criteria for opioid dependence (DSM: 304.00; ICD: F11.2), excluding those cases due to a general medical condition.[1,2] According to DSM-IV TR criteria, dependence involves a maladaptive pattern of substance use leading to clinically significant impairment or distress. At least three of the following symptoms must be experienced within the same 12-month period:

- Tolerance, characterised by either
    - a need for increased amounts of the substance to achieve intoxication; or
    - markedly diminished effect with continued use of the same amount of the substance;
- Withdrawal, characterised by either
    - Withdrawal symptoms characteristic to dependence; or
    - the same (or similar) substance is taken to avoid withdrawal symptoms;

- Substance taken in progressively larger amounts or for longer period;
- Persistent desire or unsuccessful efforts to reduce substance use;
- Disproportionate time dedicated to obtaining the substance;
- Other important activities are given up because of the substance use; and
- Substance use is continued despite knowledge of physical or psychological problems occurring as a result of the substance.

Input data

Model inputs

For GBD 2010, a systematic review of the literature was conducted to capture studies of prevalence, incidence, duration, and excess mortality associated with MDD. In summary, the search was conducted in three stages involving electronic searches of the peer-reviewed literature (via PsycInfo, Embase, and PubMed), the grey literature, and expert consultation. The agreed-upon approach for mental and substance use disorders was to conduct electronic database searches on a rolling basis. All three stages of GBD 2010's literature review were repeated for GBD 2013 and GBD 2016. For GBD 2017, literature updates focused on data sources captured within the Global Heath Data Exchange (http://ghdx.healthdata.org/).

Additionally, two targeted systematic reviews were conducted to further supplement the dataset. The first review captured studies reporting on the epidemiology of opioid use disorders within Maori versus non-Maori populations (as opposed to New Zealand more broadly), given the inclusion of these two sub-groups in GBD 2017. The second review searched for studies on the epidemiology of opioid use disorders in China using primarily the China National Knowledge Infrastructure database. The focus was to search for studies published in Chinese journals that would not typically be captured in mainstream databases such as PsycInfo, Embase, and PubMed.

The inclusion criteria stipulated that 1) the publication year must be from 1980 onward; 2) "caseness" must be based on clinical threshold as established by the DSM or ICD; 3) sufficient information must be provided on study method and sample characteristics to assess the quality of the study; and (4) study samples must be representative of the general population (ie, inpatient or pharmacological treatment samples, case studies, veterans or refugee samples were excluded). No limitation was set on the language of publication. Methods used for this systematic review have been reported in greater detail elsewhere.[3,4]

The table below shows the number of site-years of data represented in the GBD 2017 model, as well as the number of countries and GBD world regions represented.

	Prevalence	Remission	Other
Site-years (total)	587	26	45
Number of countries with data	31	6	17
Number of GBD regions with data (out	9	5	6

of 21 regions)			
Number of GBD super-regions with data (out of 7 super-regions)	5	3	5

Age and sex splitting

In GBD 2017, reported estimates of prevalence were split by age and sex where possible. First, if studies reported prevalence for broad age groups by sex (eg, prevalence in 15- to 65-year-old males and females separately), and also by specific age groups but for both sexes combined (eg, prevalence in 15- to 30-year-olds, then in 31- to 65-year-olds, for males and females combined); age-specific estimates were split by sex using the reported sex ratio and bounds of uncertainty. Second, where studies reported estimates across age groups spanning 20 years or more, these were split into five-year age groups using the prevalence age pattern estimated by DisMod-MR.

Severity splits

The basis of the GBD disability weight survey assessments are lay descriptions of sequelae highlighting major functional consequences and symptoms. The lay descriptions and disability weights for opioid dependence severity levels are shown below.

Severity level	Lay description	DW (95% CI)
Mild	Uses heroin (or methadone) daily and has difficulty controlling the habit. When not using, the person functions normally.	0.335 (0.221–0.473)
Moderate to severe	Uses heroin daily and has difficulty controlling the habit. When the effects wear off, the person feels severe nausea, agitation, vomiting, and fever. The person has a lot of difficulty in daily activities.	0.697 (0.510–0.843)

The proportion of people with opioid dependence within each of the severity levels was determined based on available data from US National Epidemiological Survey on Alcohol and Related Conditions (NESARC), conducted in two waves from 2001–2002 and 2004–2005,[5] and the Comorbidity and Trauma study conducted in 2005-2008.[6,7] The estimated distribution of opioid dependent cases by severity were asymptomatic (16%, 13%–19%), mild (37%, 20%–55%), and moderate/severe (47%, 29%–64%).

Modelling Strategy

We ran a DisMod-MR model to produce estimates by age, sex, year, and country. We assumed no incidence and excess mortality before age 15. This minimum age of onset was corroborated with expert feedback and existing literature on opioid dependence. We also assumed no incidence after age 64 as

supported by data from various sources including the European Monitoring Centre for Drugs and Drug Addiction.[8] An upper limit of 0.2 was placed on remission consistent with limits in the dataset. Cause-specific mortality rates (CSMR) from the GBD 2016 cause of death model for opioid use disorders were included as data points in the DisMod-MR model.

The prevalence dataset included data points using “direct” or “indirect” survey methods. “Direct” methods of measuring opioid dependence predominantly involve surveys of the general population that ask if respondents use or are dependent on opioids. Surveys tend to underestimate the prevalence of the most harmful and stigmatised forms of illicit drug use in ways that probably vary between countries and cultures.[9] “Indirect” methods are considered superior; they use different sources of data to indirectly estimate the total number of drug users (methods include “multiplier methods,” back-projection and capture-recapture methods). Due to insufficient data on dependence from indirect survey methods (considered to be the gold standard for GBD purposes), estimates derived from direct survey methods were included in the modelling. The cv_direct covariate was then used to adjust for whether a direct or indirect survey method was used. A crosswalk was estimated to convert all dependence estimates obtained via direct methods in the dataset, into its equivalent value if the study had measured dependence estimates obtained via indirect methods. A direct:indirect dependence ratio of 0.34 (0.33–0.37) was calculated by DisMod-MR based on comparable direct and indirect dependence estimates in the dataset.

In GBD 2017, we introduced two new country-level covariates. Age-standardised prevalence of intravenous drug use was used to inform estimates for countries where there were sparse data. Additionally, estimates of defined daily doses for statistical purposes (SDDD; consumption per day per million population) of opioid analgesics were modelled via spatiotemporal Gaussian process regression (ST-GPR) using data supplied by the International Narcotics Control Board (INCB). No uncertainty was provided around the data provided and so uncertainty was estimated assuming a linear relationship between the coefficient of variation (CoV) and the Healthcare Access and Quality (HAQ) index for the estimate, with the lowest HAQ index having a CoV of 1.0 and the highest HAQ index having a CoV of 0.2. Subnational estimates for the USA were estimated by crosswalking national estimates with the state/national ratios of opioid prescriptions per 100 persons supplied by the Centers for Disease Control and Prevention.

Study covariate	beta	Exponentiated beta
Intravenous drug use (age-standardised proportion)	0.2 (0.0062–0.69)	1.22 (1.01–1.99)
Opioids per million population per day (10-year lag)	0.059 (0.046–0.075)	1.06 (1.05–1.08)

Citations

1. American Psychiatric Association. Diagnostic and Statistical Manual of Mental Disorders (DSM-IV-TR). 4th, Text Revision ed Washington DC: American Psychiatric Association; 2000.

2. World Health Organization. The ICD-10 Classification of Mental and Behavioural Disorders. Clinical descriptions and diagnostic guidelines Geneva: World Health Organization; 1992.
3. Degenhardt L, Bucello C, Calabria B, Nelson P, Roberts A, Hall W, et al. What data are available on the extent of illicit drug use and dependence globally? Results of four systematic reviews. Drug and alcohol dependence. 2011.
4. Calabria B, Degenhardt L, Briegleb C, Vos T, Hall W, Lynskey M, et al. Systematic review of prospective studies investigating "remission" from amphetamine, cannabis, cocaine or opioid dependence. Addictive Behaviors. 2010.
5. Grant BF, Dawson DA. National Institute on Alcohol Abuse and Alcoholism. Alcohol Health & Research World. 2006; 29(2): p. 74.
6. Shand FL, Slade T, Degenhardt L, Baillie A, Nelson EC. Opioid dependence latent structure: two classes with differing severity? Addiction. 2011; 106(3): p. 590-8.
7. Shand FL, Degenhardt L, Slade T, Nelson EC. Sex differences amongst dependent heroin users: Histories, clinical characteristics and predictors of other substance dependence. Addictive behaviors. 2011; 36(1): p. 27-36.
8. European Montioring Centre for Drugs and Drug Addiction. Lisbon, Portugal; 2014.
9. Reuter P, Trautmann F. A Report on Global Illicit Drugs Markets 1998-2007. Utrecht; 2009.

Cocaine use disorders

Flowchart

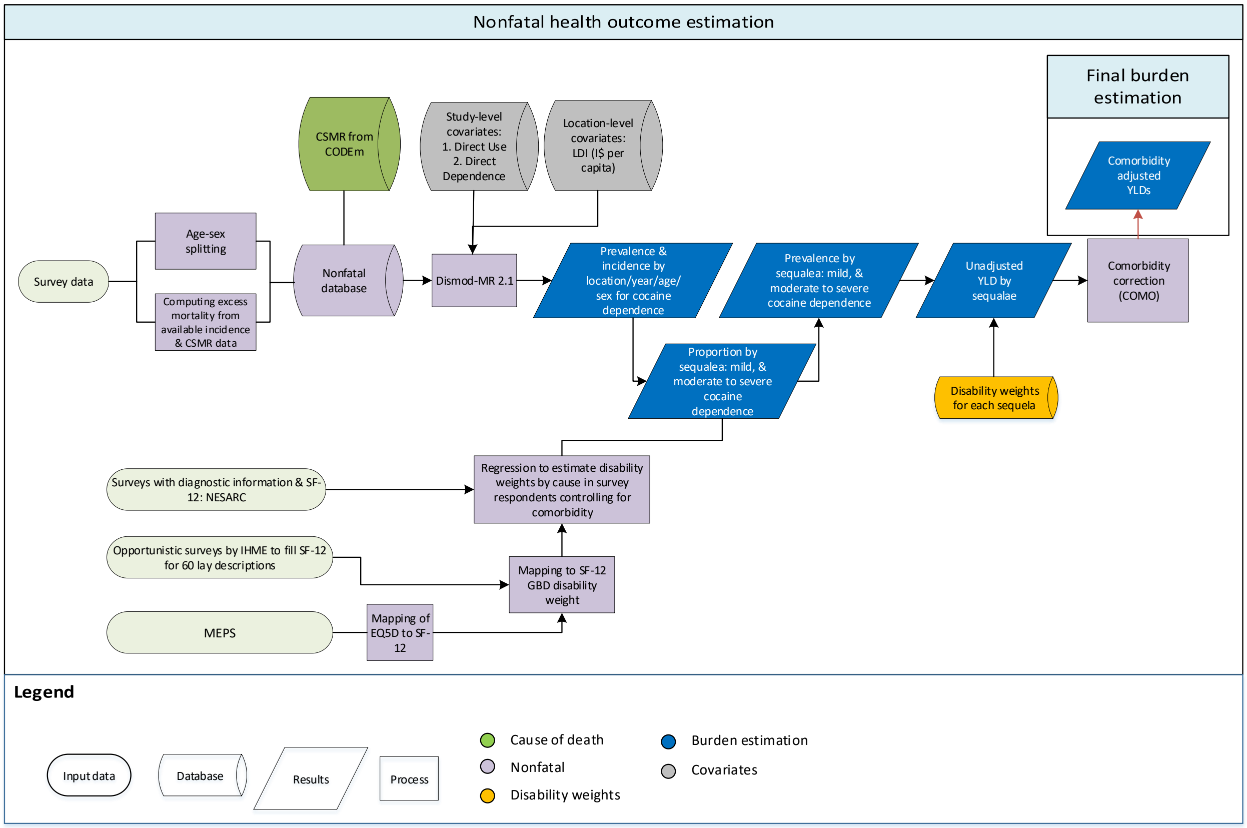

Case definition

Cocaine dependence is a substance-related disorder involving a dysfunctional pattern of cocaine use. Included in the GBD disease modelling were cases meeting the Diagnostic and Statistical Manual of Mental Disorders (DSM-IV-TR) or the International Classification of Diseases (ICD-10) diagnostic criteria for cocaine dependence (DSM: 304.20; ICD: F14.2), excluding those cases due to a general medical condition.[1,2] According to DSM-IV TR criteria, dependence involves a maladaptive pattern of substance use leading to clinically significant impairment or distress. At least three of the following symptoms must be experienced within the same 12-month period:

- Tolerance, characterised by either
  - a need for increased amounts of the substance to achieve intoxication; or
  - markedly diminished effect with continued use of the same amount of the substance;
- Withdrawal, characterised by either
  - Withdrawal symptoms characteristic to dependence; or
  - the same (or similar) substance is taken to avoid withdrawal symptoms;
- Substance taken in progressively larger amounts or for a longer period;

- Persistent desire or unsuccessful efforts to reduce substance use;
- Disproportionate time dedicated to obtaining the substance;
- Other important activities are given up because of the substance use; and
- Substance use is continued despite knowledge of physical or psychological problems occurring as a result of the substance.

Input data

Model inputs

A systematic review of the literature was conducted to capture studies of prevalence, incidence, remission, and excess mortality associated with cocaine dependence. In summary, the search was conducted in three stages involving searches of the peer-reviewed literature (via Medline, Embase, and PubMed), the grey literature, and expert consultation. The agreed-upon approach for mental and substance use disorders was to conduct electronic database searches on a rolling basis. All three stages of GBD 2010's literature review were repeated for GBD 2013 to capture additional data published up to 2013. For GBD 2015, stages 2 and 3 of the literature review were updated, and in GBD 2016, the peer-reviewed database search (stage 1) was conducted via Medline, Embase, and Psycinfo to capture studies published from 2013 to 2016. GBD 2017 included additional sources identified by GBD experts and microdata where available. Additionally, in GBD 2017, two targeted systematic reviews were conducted to further supplement the dataset. The first review captured studies within Maori versus non-Maori populations (as opposed to New Zealand more broadly), given the inclusion of these two sub-groups in GBD 2017. The second review utilised the China National Knowledge Infrastructure database to find studies that would not typically be captured in PubMed, Embase, and PsycINFO.

The inclusion criteria stipulated that 1) the publication year must be from 1980 onward; 2) "caseness" must be based on clinical threshold as established by the DSM or ICD; 3) sufficient information must be provided on study method and sample characteristics to assess the quality of the study; and (4) study samples must be representative of the general population (ie, inpatient or pharmacological treatment samples, case studies, veterans or refugee samples were excluded). No limitation was set on the language of publication. Methods used for this systematic review have been reported in greater detail elsewhere.[3,4]

Age and sex splitting

In GBD 2017, reported estimates of prevalence were split by age and sex where possible. First, if studies reported prevalence for broad age groups by sex (eg, prevalence in 15- to 65-year-old males and females separately), and also by specific age groups but for both sexes combined (eg, prevalence in 15- to 30-year-olds, then in 31- to 65-year-olds, for males and females combined); age-specific estimates were split by sex using the reported sex ratio and bounds of uncertainty. Second, where studies reported estimates across age groups spanning 20 years or more, these were split into five-year age groups using the prevalence age pattern estimated by DisMod-MR 2.1.

Data counts

	Other	Prevalence	Remission
Site-years (total)	8	478	3
Number of countries with data	7	68	2
Number of GBD regions with data (out of 21 regions)	5	18	2
Number of GBD super-regions with data (out of 7 super-regions)	4	7	2

Severity splits and disability weights

The basis of the GBD disability weight survey assessments are lay descriptions of sequelae highlighting major functional consequences and symptoms. The lay descriptions and disability weights for cocaine dependence severity levels are shown below.

Severity level	Lay description	DW (95% CI)
Mild	Uses cocaine at least once a week and has some difficulty controlling the habit. When not using, the person functions normally.	0.116 (0.074–0.165)
Moderate to severe	Uses cocaine and has difficulty controlling the habit. The person sometimes has mood swings, anxiety, paranoia, hallucinations and sleep problems, and has some difficulty in daily activities.	0.479 (0.324–0.634)

The proportion of people with cocaine dependence within each of the severity levels were determined based on available data from US National Epidemiological Survey on Alcohol and Related Conditions (NESARC), conducted in two waves from 2001 to 2002 and 2004 to 2005[5]. The estimated distribution of cocaine dependent cases by severity were asymptomatic (50%, 37%–64%), mild (25%, 18%–33%), and moderate/severe (25%, 17%–33%).

Modelling strategy

We ran a DisMod-MR 2.1 model to produce estimates by age, sex, year, and country. We assumed no incidence, remission, and excess mortality before age 15, and an upper limit of 0.2 on remission. The minimum age of onset was corroborated with expert feedback and existing literature from various sources including the European Monitoring Centre for Drugs and Drug Addiction.[6] Cause-specific mortality rates (CSMR) from the GBD 2017 cause of death model for cocaine use disorders were included as data in the DisMod-MR 2.1 model.

The prevalence dataset included data points of both use and dependence estimated using “direct” or “indirect” survey methods. “Direct” methods of measuring amphetamine dependence predominantly involve surveys of the general population that ask if respondents use or are dependent on amphetamine. Surveys tend to underestimate the prevalence of the most harmful and stigmatised forms of illicit drug

use in ways that probably vary between countries and cultures.[7] "Indirect" methods are considered superior; they use different sources of data to indirectly estimate the total number of drug users (methods include "multiplier methods," back-projection and capture-recapture methods). Due to the lack of data available on cocaine dependence from indirect survey methods (considered to be the gold standard for GBD purposes), estimates of use and/or estimates from direct survey methods were included in the modelling. Study-level covariates were then used to accommodate for between-study variability in the raw prevalence data. The cv_direct use covariate was used to adjust for whether direct or indirect survey methods were used. This converted all use estimates obtained via direct methods in the dataset into their equivalent value if the study had measured dependence estimates obtained via indirect methods. A ratio of direct use:indirect dependence was calculated by comparing similar direct use and indirect dependence estimates in the dataset. To allow for meaningful comparisons, paired direct use and indirect dependence estimates needed to be similar in terms of the country they were from, year, age group, sex, and prevalence type. To maximise the number of data points available for this ratio, paired estimates for psychostimulants (ie, both cocaine and amphetamine) were used. Once a dataset was set up with paired direct use and indirect dependence estimates, MetaXL (a meta-analysis add-in for Microsoft Excel) was utilised to estimate a ratio of direct use:indirect dependence, whereby direct use estimates were found to be 3.6 (2.6–5.2) times higher than indirect dependence estimates. This ratio was used in DisMod-MR to adjust all use estimates in the dataset downward, toward the level they would have been had the study reported indirect dependence. A similar method was used to adjust prevalence estimates of cocaine dependence obtained via direct methods toward the level they would have been had the study measured cocaine dependence using indirect methods. The estimated ratio of direct dependence: indirect dependence was 0.5 (0.2–1.1).

Betas and exponentiated values (which can be interpreted as an odds ratio) for each study-level covariate are shown in the table below:

Study covariate	Parameter	beta	Exponentiated beta
cv_direct use	Prevalence	1.29 (1.29–1.29)	3.63 (3.63–3.63)
cv_direct dependence	Prevalence	-0.68 (-0.68 to -0.68)	0.51 (0.51–0.51)

References

1. American Psychiatric Association. Diagnostic and Statistical Manual of Mental Disorders (DSM-IV-TR). 4th, Text Revision ed Washington DC: American Psychiatric Association. 2000.
2. World Health Organization. The ICD-10 Classification of Mental and Behavioural Disorders. Clinical descriptions and diagnostic guidelines Geneva: World Health Organization. 1992.
3. Degenhardt L, Bucello C, Calabria B, Nelson P, Roberts A, Hall W, et al. What data are available on the extent of illicit drug use and dependence globally? Results of four systematic reviews. Drug and alcohol dependence. 2011.
4. Calabria B, Degenhardt L, Briegleb C, Vos T, Hall W, Lynskey M, et al. Systematic review of prospective studies investigating "remission" from amphetamine, cannabis, cocaine or opioid dependence. Addictive Behaviors. 2010.
5. Grant BF, Dawson DA. National Institute on Alcohol Abuse and Alcoholism. Alcohol Health & Research World. 2006; 29(2): p. 74.
6. European Montioring Centre for Drugs and Drug Addiction. Lisbon, Portugal 2014.
7. Reuter P, Trautmann F. A Report on Global Illicit Drugs Markets 1998-2007. Utrecht. 2009.

Amphetamine use disorders

Flowchart

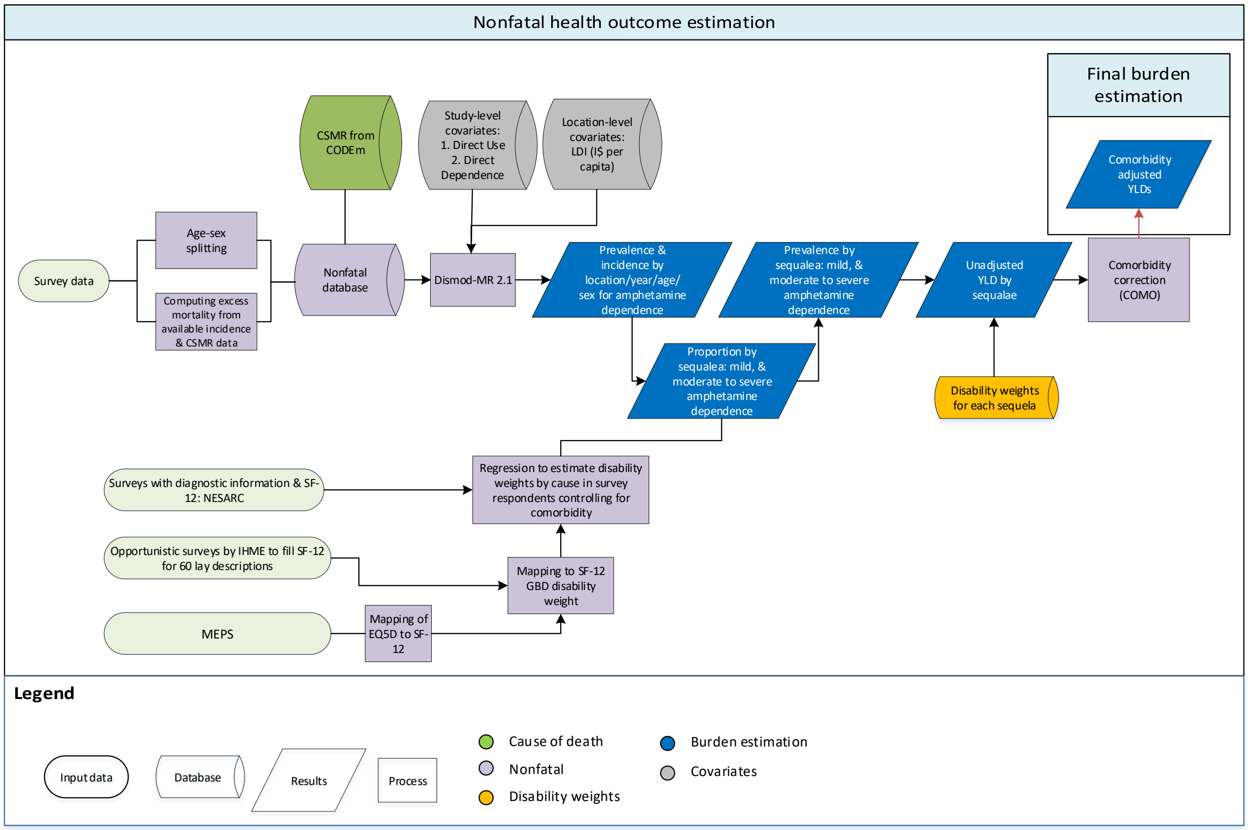

Case definition

Amphetamine dependence is a substance-related disorder involving a dysfunctional pattern of amphetamine use. Included in the GBD disease modelling were cases meeting the Diagnostic and Statistical Manual of Mental Disorders (DSM-IV-TR) or the International Classification of Diseases (ICD-10) diagnostic criteria for amphetamine dependence (DSM: 304.40; ICD: F15.2), excluding those cases due to a general medical condition.[1,2] According to DSM-IV TR criteria, dependence involves a maladaptive pattern of substance use, leading to clinically significant impairment or distress. At least three of the following symptoms must be experienced within the same 12-month period:

- Tolerance, characterised by either
  - a need for increased amounts of the substance to achieve intoxication; or
  - markedly diminished effect with continued use of the same amount of the substance;
- Withdrawal, characterised by either
  - Withdrawal symptoms characteristic to dependence; or
  - the same (or similar) substance is taken to avoid withdrawal symptoms;
- Substance taken in progressively larger amounts or for longer periods;

- Persistent desire or unsuccessful efforts to reduce substance use;
- Disproportionate time dedicated to obtaining the substance;
- Other important activities are given up because of the substance use; and
- Substance use is continued despite knowledge of physical or psychological problems occurring as a result of the substance.

Input data

Model inputs

A systematic review of the literature was conducted to capture studies of prevalence, incidence, remission, and excess mortality associated with amphetamine dependence. In summary, the search was conducted in three stages involving searches of the peer-reviewed literature (via Medline, Embase, and PubMed), the grey literature, and expert consultation. The agreed-upon approach for mental and substance use disorders was to conduct electronic database searches on a rolling basis. All three stages of GBD 2010's literature review were repeated for GBD 2013 to capture additional data published up to 2013. For GBD 2015, stages 2 and 3 of the literature review were updated and in GBD 2016, the peer-reviewed database search (stage 1) was conducted via Medline, Embase, and Psycinfo to capture studies published from 2013 to 2016. GBD 2017 included additional sources identified by GBD experts and microdata where available. Additionally, in GBD 2017, two targeted systematic reviews were conducted to further supplement the dataset. The first review captured studies within Maori versus non-Maori populations (as opposed to New Zealand more broadly), given the inclusion of these two sub-groups in GBD 2017. The second review utilised the China National Knowledge Infrastructure database to find studies that would not typically be captured in PubMed, Embase, and PsycINFO.

The inclusion criteria stipulated that: 1) the publication year must be from 1980 onward; 2) "caseness" must be based on clinical threshold as established by the DSM or ICD; 3) sufficient information must be provided on study method and sample characteristics to assess the quality of the study; and (4) study samples must be representative of the general population (ie, inpatient or pharmacological treatment samples, case studies, veterans, or refugee samples were excluded). No limitation was set on the language of publication. Methods used for this systematic review have been reported in greater detail elsewhere.[3,4]

Age and sex splitting

In GBD 2017, reported estimates of prevalence were split by age and sex where possible. First, if studies reported prevalence for broad age groups by sex (eg, prevalence in 15-to 65-year-old males and females separately), and also by specific age groups but for both sexes combined (eg, prevalence in 15- to 30-year-olds, then in 31- to 65-year-olds, for males and females combined); age-specific estimates were split by sex using the reported sex ratio and bounds of uncertainty. Second, where studies reported estimates across age groups spanning 20 years or more, these were split into five-year age groups using the prevalence age pattern estimated by DisMod-MR.

Data counts

	Other	Prevalence	Remission
Site-years (total)	6	411	1
Number of countries with data	5	58	1
Number of GBD regions with data (out of 21 regions)	3	14	1
Number of GBD super-regions with data (out of 7 super-regions)	3	7	1

Severity splits and disability weights

The basis of the GBD disability weight survey assessments are lay descriptions of sequelae highlighting major functional consequences and symptoms. The lay descriptions and disability weights for amphetamine dependence severity levels are shown below.

Severity level	Lay description	DW (95% CI)
Mild	Uses stimulants (drugs) at least once a week and has some difficulty controlling the habit. When not using, the person functions normally.	0.079 (0.051–0.114)
Moderate to severe	Uses stimulants (drugs) and has difficulty controlling the habit. The person sometimes has depression, hallucinations, and mood swings, and has difficulty in daily activities.	0.486 (0.329–0.637)

The proportion of people with amphetamine dependence within each of the severity levels was determined based on available data from US National Epidemiological Survey on Alcohol and Related Conditions (NESARC), conducted in two waves from 2001 to 2002 and 2004 to 2005.[5] The estimated distribution of amphetamine dependent cases by severity were asymptomatic (55%, 40%–71%), mild (19%, 12%–27%), and moderate/severe (26%, 16%–35%).

Modelling strategy

We ran a DisMod-MR 2.1 model to produce estimates by age, sex, year, and country. We assumed no incidence, remission, and excess mortality before age 15, and an upper limit of 0.35 on remission. The minimum age of onset was corroborated with expert feedback and existing literature from various sources including the European Monitoring Centre for Drugs and Drug Addiction.[6] Cause-specific mortality rates (CSMR) from the GBD 2017 cause of death model for amphetamine use disorders were included as data in the DisMod-MR 2.1 model.

The prevalence dataset included data-points of both use and dependence estimated using “direct” or “indirect” survey methods. “Direct” methods of measuring amphetamine dependence predominantly involve surveys of the general population that ask if respondents use or are dependent on amphetamine. Surveys tend to underestimate the prevalence of the most harmful and stigmatized forms of illicit drug

use in ways that probably vary between countries and cultures.[7] “Indirect” methods are considered superior; they use different sources of data to indirectly estimate the total number of drug users (methods include “multiplier methods,” back-projection and capture-recapture methods). Due to the lack of data available on amphetamine dependence from indirect survey methods (considered to be the gold standard for GBD purposes), estimates of use and/or estimates from direct survey methods were included in the modelling. Study-level covariates were then used to accommodate for between-study variability in the raw prevalence data. The cv_direct use covariate was used to adjust for whether direct or indirect survey methods were used. This converted all use estimates obtained via direct methods in the dataset, into its equivalent value if the study had measured dependence estimates obtained via indirect methods. A ratio of direct use:indirect dependence was calculated by comparing similar direct use and indirect dependence estimates in the dataset. To allow for meaningful comparisons, paired direct use and indirect dependence estimates needed to be similar in terms of the country they were from, year, age group, sex and, prevalence type. To maximise the number of data points available for this ratio paired estimates for psychostimulants (ie, both cocaine and amphetamine) were used. Once a dataset was set up with paired direct use and indirect dependence estimates, MetaXL (a meta-analysis add-in for Microsoft Excel) was utilised to estimate a ratio of direct use:indirect dependence, whereby direct use estimates were found to be 3.6 (2.6–5.2) times higher than indirect dependence estimates. This ratio was used in DisMod-MR to adjust all use estimates in the dataset downward, toward the level they would have been had the study reported indirect dependence. A similar method was used to adjust prevalence estimates of amphetamine dependence obtained via direct methods toward the level they would have been had the study measured amphetamine dependence using indirect methods. The estimated ratio of direct dependence: indirect dependence was 0.5 (0.2–1.1).

Betas and exponentiated values (which can be interpreted as an odds ratio) for each study level covariate are shown in the table below:

Study covariate	Parameter	beta	Exponentiated beta
cv_direct use	Prevalence	1.29 (1.29–1.29)	3.63 (3.63–3.63)
cv_direct dependence	Prevalence	-0.68 (-0.68 to -0.68)	0.51 (0.51–0.51)

References

1. American Psychiatric Association. Diagnostic and Statistical Manual of Mental Disorders (DSM-IV-TR). 4th, Text Revision ed Washington DC: American Psychiatric Association. 2000.
2. World Health Organization. The ICD-10 Classification of Mental and Behavioural Disorders. Clinical descriptions and diagnostic guidelines Geneva: World Health Organization. 1992.
3. Degenhardt L, Bucello C, Calabria B, Nelson P, Roberts A, Hall W, et al. What data are available on the extent of illicit drug use and dependence globally? Results of four systematic reviews. Drug and alcohol dependence. 2011.
4. Calabria B, Degenhardt L, Briegleb C, Vos T, Hall W, Lynskey M, et al. Systematic review of prospective studies investigating “remission” from amphetamine, cannabis, cocaine or opioid dependence. Addictive Behaviors. 2010.
5. Grant BF, Dawson DA. National Institute on Alcohol Abuse and Alcoholism. Alcohol Health & Research World. 2006; 29(2): p. 74.
6. European Montioring Centre for Drugs and Drug Addiction. Lisbon, Portugal 2014.
7. Reuter P, Trautmann F. A Report on Global Illicit Drugs Markets 1998-2007. Utrecht. 2009.

Cannabis use disorders

Flowchart

Cannabis dependence

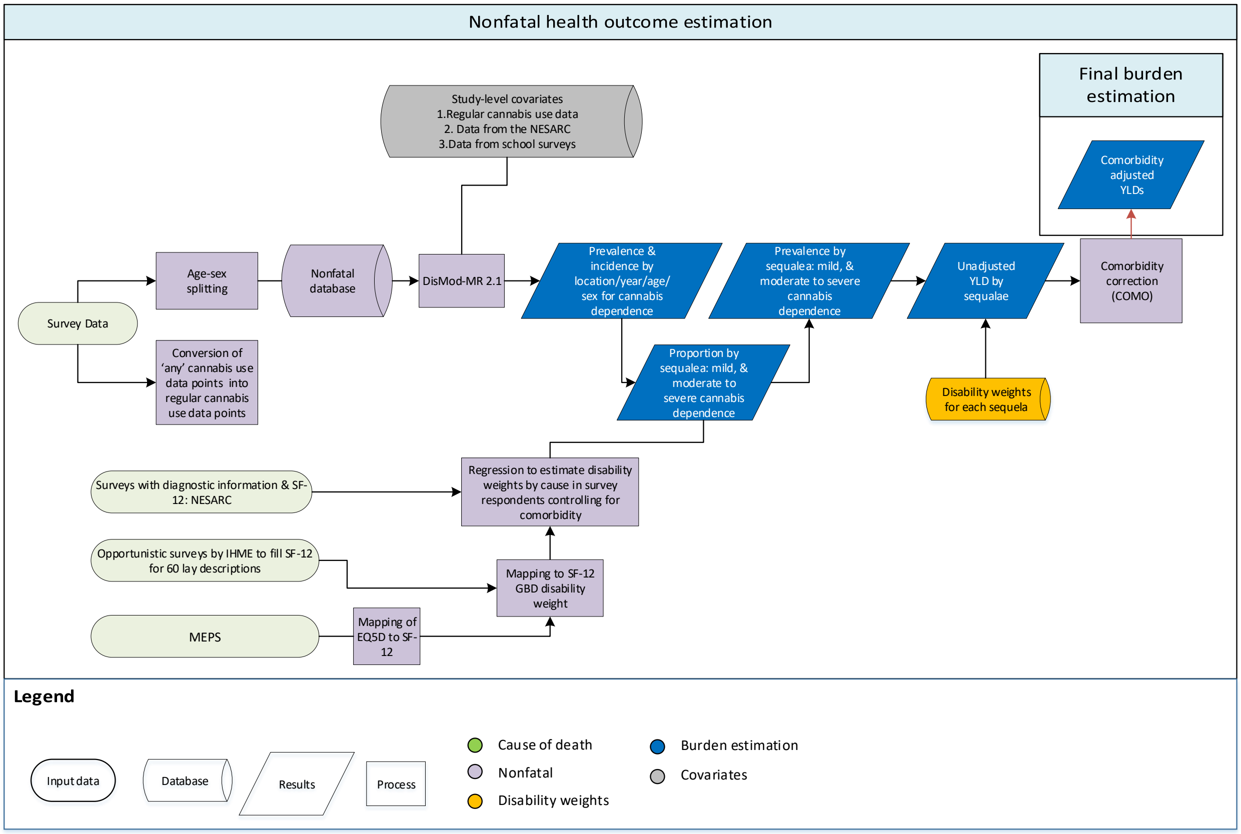

Case Definition

Cannabis dependence is a substance-related disorder involving a dysfunctional pattern of cannabis use. Included in GBD disease modelling were cases meeting diagnostic criteria for cannabis dependence according to the Diagnostic and Statistical Manual of Mental Disorders (DSM), or the equivalent diagnosis in the International Classification of Diseases (ICD). These were identified by the following codes: DSM:304.30, ICD:F12.2; excluding those cases due to a general medical condition.[1,2]

According to DSM-IV-TR criteria, cannabis dependence involves a maladaptive pattern of cannabis use, leading to clinically significant impairment or distress. At least three of the following symptoms must be experienced within the same 12-month period:

- Tolerance, characterised by either
  - a need for increased amounts of the substance to achieve intoxication; or
  - markedly diminished effect with continued use of the same amount of the substance;
- Withdrawal, characterised by either
  - Withdrawal symptoms characteristic to cannabis dependence; or

- the same (or similar) substance is taken to avoid withdrawal symptoms;
- substance taken in progressively larger amounts or for longer periods;
- persistent desire or unsuccessful efforts to reduce substance use;
- disproportionate time dedicated to obtaining the substance;
- other important activities are given up because of the substance use; and
- substance use is continued despite knowledge of physical or psychological problems occurring as a result of the substance.

Input data

Model inputs

For GBD 2010, a systematic review of the literature was conducted to capture studies of prevalence, incidence, duration, and excess mortality associated with cannabis dependence. In summary, the search was conducted in three stages involving electronic searches of the peer-reviewed literature (via PsycInfo, Embase and PubMed), the grey literature and, expert consultation. The agreed-upon approach for mental and substance use disorders was to conduct electronic database searches on a rolling basis. All three stages of GBD 2010's literature review were repeated for GBD 2013 and GBD 2016. In GBD 2017, stages two and three of the literature review were conducted. Additionally, two targeted systematic reviews were conducted to further supplement the cannabis dependence dataset. The first review captured studies reporting on the epidemiology of cannabis dependence within Maori versus non-Maori populations (as opposed to New Zealand more broadly), given the inclusion of these two sub-groups in GBD 2017. The second review searched for studies on the epidemiology of cannabis dependence in China using primarily the China National Knowledge Infrastructure database. The focus was to search for studies published in Chinese journals that would not typically be captured in mainstream databases such as PsycInfo, Embase, and PubMed.

The inclusion criteria stipulated that: (1) the publication year must be from 1980 onward; (2) "caseness" must be based on clinical threshold as established by the DSM or ICD; (3) sufficient information must be provided on study method and sample characteristics to assess the quality of the study; and (4) study samples must be representative of the general population (ie, inpatient or pharmacological treatment samples, case studies, veterans or refugee samples were excluded). No limitation was set on the language of publication. Methods used for this systematic review have been reported in greater detail elsewhere.[3-6] The table below summarises data inputs for cannabis dependence by parameter.

	**Prevalence**	**Remission**
**Site-years (total)**	1101	3
**Number of countries with data**	121	3
**Number of GBD regions with data (out of 21 regions)**	20	3
**Number of GBD super-regions with data (out of 7 super-regions)**	7	1

Age and sex splitting

Reported estimates of prevalence were split by age and sex where possible. First, if studies reported prevalence for broad age groups by sex (eg, prevalence in 15- to 65-year-old males and females separately), and also by specific age groups but for both sexes combined (eg, prevalence in 15- to 30-year-olds, then in 31- to 65-year-olds, for males and females combined); age-specific estimates were split by sex using the reported sex ratio and bounds of uncertainty. Second, where studies reported estimates across age groups spanning 20 years or more, these were split into five-year age groups using the prevalence age pattern estimated by DisMod-MR.

Severity splits

The basis of the GBD disability weight survey assessments are lay descriptions of sequelae highlighting major functional consequences and symptoms. The lay descriptions and disability weights for cannabis dependence severity levels are shown below.

Severity level	Lay description	DW (95% CI)
Mild	Uses marijuana at least once a week and has some difficulty controlling the habit. When not using, the person functions normally.	0.039 (0.024–0.06)
Moderate to severe	Uses marijuana daily and has difficulty controlling the habit. The person sometimes has mood swings, anxiety, and hallucinations, and has some difficulty in daily activities.	0.266 (0.178–0.364)

The US National Epidemiological Survey on Alcohol and Related Conditions (NESARC, conducted in two waves from 2001 to 2002 and 2004 to 2005)[7] was used to estimate the proportion of cannabis dependence cases asymptomatic (58%, 51%–63%), mild (36%, 31%–42%) and moderate to severe (6%, 4%–8%).

Modelling Strategy

The epidemiological modelling strategy for cannabis dependence made use of DisMod-MR 2.1. Due to insufficient data, estimates of any cannabis use and regular (ie, weekly) cannabis use were included in the disease modelling of cannabis dependence in a two-step process. At step 1, a crosswalk was estimated to convert estimates of any use in the dataset into its equivalent value if the study had measured regular use. To do this a ratio of use:regular use was calculated by comparing similar regular use and use estimates in the dataset. To allow for meaningful comparisons, paired regular use and use estimates needed to be similar in terms of the country they were from, year, age group, sex, and prevalence type. Once a dataset was set up with paired regular use and use estimates, MetaXL (a meta-analysis add-in for Microsoft Excel) was used to estimate a ratio of use: regular use whereby use estimates were found to be 2.9 (2.5–3.3) times higher than regular use estimates. This ratio was used to adjust all use estimates in the dataset downward, toward the level they would have been had the study reported regular cannabis use. Step 2 involved the DisMod-MR modelling of the regular cannabis use (from step 1) and cannabis dependence data. This cannabis regular use/dependence dataset was modelled using a study-level covariate which adjusted estimates of regular cannabis use toward the desirable which were estimates of cannabis dependence.

Study-level covariates were used to accommodate for between-study variability in the raw prevalence data. As mentioned previously, a cv_regular use covariate adjusted all regular use estimates toward the level they would have been if the study had measured cannabis dependence. This covariate was informed by a cannabis regular use: dependence ratio (4.1, 3.9–4.6) estimated outside of DisMod-MR using the same methodology outlined above for the use:regular use ratio. Based on expert advice, a cv_nesarc covariate adjusted all estimates derived from the NESARC upward toward the level they would have been if they had been derived by other surveys. Drug use disorders are not well-captured in household surveys. This is especially an issue in NESARC as the sampling strategy used was biased toward less severe cases of drug use disorders. A cv_school survey covariate adjusted estimates derived from school surveys downward to the level they would have been had the study conducted a fully representative population survey. Betas and exponentiated values (which can be interpreted as an odds ratio) for each study-level covariate are shown in the table below.

Study covariate	Parameter	beta	Exponentiated beta
cv_regular use	Prevalence	1.40 (1.40–1.40)	4.06 (4.06–4.06)
cv_nesarc	Prevalence	-0.68 (-0.83 to -0.52)	0.51 (0.44–0.60)
cv_school survey	Prevalence	0.28 (0.24–0.32)	1.32 (1.27–1.38)

References

1. American Psychiatric Association. Diagnostic and Statistical Manual of Mental Disorders (DSM-IV-TR). 4th, Text Revision ed. Washington DC: American Psychiatric Association; 2000.
2. World Health Organization. The ICD-10 Classification of Mental and Behavioural Disorders. Clinical descriptions and diagnostic guidelines. Geneva: World Health Organization; 1992.
3. Calabria B, Degenhardt L, Briegleb C, et al. Systematic review of prospective studies investigating "remission" from amphetamine, cannabis, cocaine or opioid dependence. *Addictive Behaviors* 2010; **35**(8): 741-9.
4. Calabria B, Degenhardt L, Hall W, Lynskey M. Does cannabis use increase the risk of death? Systematic review of epidemiological evidence on adverse effects of cannabis use. *Drug Alcohol Rev* 2010; **29**(3): 318-30.
5. Calabria B, Degenhardt L, Nelson P, et al. What do we know about the extent of cannabis use and dependence? Results of a global systematic review. Sydney: National Drug and Alcohol Research Centre, University of NSW, 2010.
6. Degenhardt L, Ferrari AJ, Calabria B, et al. The global epidemiology and contribution of cannabis use and dependence to the global burden of disease: results from the GBD 2010 study. *PloS one* 2013; **8**(10): e76635.
7. Introduction to the National Epidemiologic Survey on Alcohol and Related Conditions [http://pubs.niaaa.nih.gov/publications/arh29-2/74-78.htm]. Access date 1 December 2014.

Other drug use disorders

Flowchart

Other drug use disorders

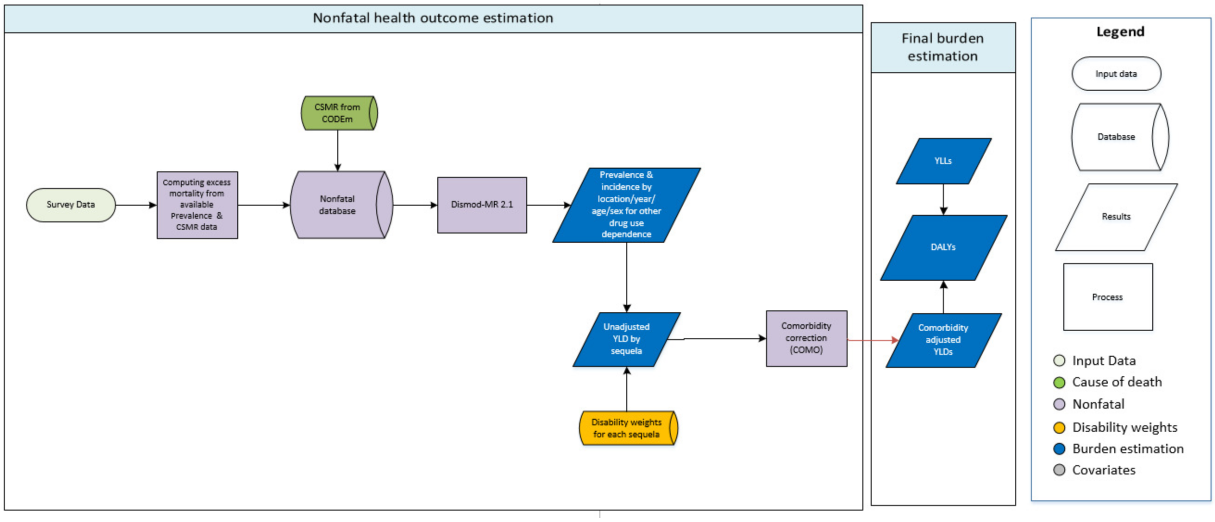

Case definition

In addition to the four drug use disorders for which we specifically estimate non-fatal burden (opioid, cocaine, amphetamine, and cannabis dependence), we also estimate the burden attributable to a residual cause of "other drug use disorders." This is made up of an aggregate group of other forms of drug dependence. Included in the GBD disease modelling were cases meeting the Diagnostic and Statistical Manual of Mental Disorders (DSM-IV-TR)[1] or the International Classification of Diseases (ICD-10)[2] diagnostic criteria for:

- Hallucinogen dependence
- Inhalant or solvent dependence
- Sedative dependence
- Tranquiliser dependence
- Other medicines, drugs, substance dependence

According to DSM-IV TR criteria, dependence involves a maladaptive pattern of substance use, leading to clinically significant impairment or distress. At least three of the following symptoms must be experienced within the same 12-month period:

- Tolerance, characterised by either
    - a need for increased amounts of the substance to achieve intoxication; or
    - markedly diminished effect with continued use of the same amount of the substance;
- Withdrawal, characterised by either
    - Withdrawal symptoms characteristic to dependence; or

- the same (or similar) substance is taken to avoid withdrawal symptoms;
- Substance taken in progressively larger amounts or for longer periods;
- Persistent desire or unsuccessful efforts to reduce substance use;
- Disproportionate time dedicated to obtaining the substance;
- Other important activities are given up because of the substance use; and
- Substance use is continued despite knowledge of physical or psychological problems occurring as a result of the substance.

Input data

Model inputs

Prevalence estimates were obtained from the Australian National Survey of Mental Health and Wellbeing (NSMHWB) conducted in 1997[3],and the US National Epidemiological Survey on Alcohol and Related Conditions (NESARC), conducted in two waves in 2001–2002[4] and 2004–2005.[5] Given that other forms of drug dependence often co-occur with the four types of drug dependence for which we estimate non-fatal burden (opioid, cocaine, amphetamine, and cannabis dependence), an adjustment for co-morbidity is important so as not to overestimate the overall burden attributable to drug dependence. Participants meeting criteria for any other form of drug dependence from each of the surveys used were counted as a prevalent case only if they did not simultaneously meet criteria for opioid, cocaine, amphetamine, or cannabis dependence. The table below summarises data inputs by parameter.

	**Prevalence**
**Site-years (total)**	3
**Number of countries with data**	2
**Number of GBD regions with data (out of 21 regions)**	2
**Number of GBD super-regions with data (out of 7 super-regions)**	1

Severity splits

The basis of the GBD disability weight survey assessments are lay descriptions of sequelae highlighting major functional consequences and symptoms. The average disability weight estimated for cocaine and amphetamine dependence was applied to all cases in this residual group of other drug use disorders. The cocaine and amphetamine lay descriptions and disability weights are shown below:

Severity level	Lay description	DW (95% CI)
**Amphetamine dependence**		
Mild	Uses stimulants (drugs) at least once a week and has some difficulty controlling the habit. When not using, the person functions normally.	0.079 (0.051–0.114)
Moderate to severe	Uses stimulants (drugs) and has difficulty controlling the habit. The person sometimes has depression, hallucinations, and mood swings, and has difficulty in daily activities.	0.486 (0.329–0.637)
**Cocaine dependence**		
Mild	Uses cocaine at least once a week and has some difficulty	0.116 (0.074–0.165)

	controlling the habit. When not using, the person functions normally.	
Moderate to severe	Uses cocaine and has difficulty controlling the habit. The person sometimes has mood swings, anxiety, paranoia, hallucinations, and sleep problems, and has some difficulty in daily activities.	0.479 (0.324–0.634)

Modelling Strategy

The GBD 2017 epidemiological modelling strategy made use of DisMod-MR 2.1. A number of additional expert priors were used in order to run a full parameter model. We assumed no incidence before age 14, a maximum of 0.0004 on incidence from the age of 60 years onward, and a maximum remission of 0.2. These priors were corroborated with expert feedback and existing literature on drug use disorders including the European Monitoring Centre for Drugs and Drug Addiction.[6] Finally, cause-specific mortality rates (CSMR) from the GBD 2017 cause of death model for other drug use disorders were included as data-points in the DisMod-MR model.

References

1. American Psychiatric Association. Diagnostic and Statistical Manual of Mental Disorders (DSM-IV-TR). 4th, Text Revision ed. Washington DC: 2000.

2. World Health Organization. The ICD-10 Classification of Mental and Behavioural Disorders. Clinical descriptions and diagnostic guidelines. Geneva: 1992.

3. Australia Bureau of Statistics. Australia National Survey of Mental Health and Wellbeing 1997. Canberra: 1997.

4. National Institute on Alcohol Abuse and Alcoholism (NIAAA), National Institutes of Health (NIH). United States National Epidemiologic Survey on Alcohol and Related Conditions 2001-2002. 2002.

5. National Institute on Alcohol Abuse and Alcoholism (NIAAA), National Institutes of Health (NIH). United States National Epidemiologic Survey on Alcohol and Related Conditions 2004-2005. 2005.

6. European Monitoring Centre for Drugs and Drug Addiction. Lisbon, Portugal: 2014.

Diabetes Mellitus

Diabetes Mellitus prevalence is estimated for overall Diabetes Mellitus, Diabetes Mellitus Type 1, and Diabetes Mellitus Type 2 in GBD 2017.

Flowchart

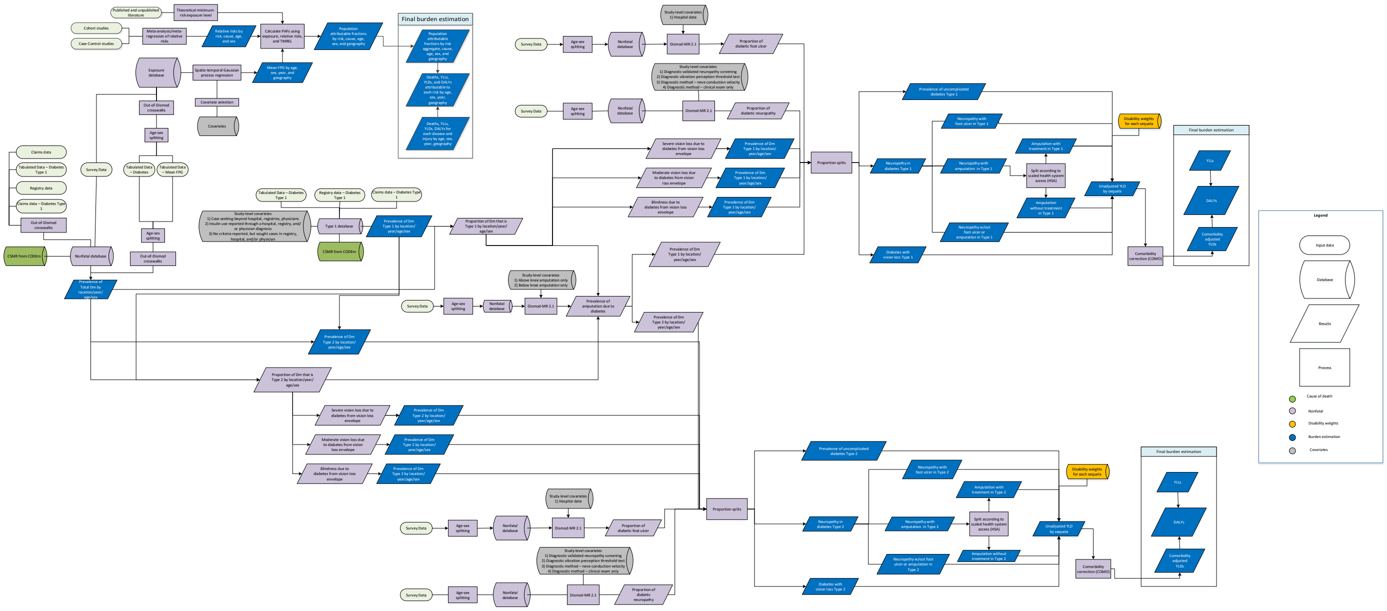

Case definition

The case definitions and diagnostic criteria for overall diabetes mellitus, type 1 diabetes mellitus, and type 2 diabetes mellitus are presented in the table below.

<u>Overall diabetes mellitus</u>

Criterion	Definition
1. Overall diabetes mellitus	Diabetes mellitus (DM) is defined as fasting plasma glucose (FPG) > 126 mg/dL (7 mmol/L) or being on treatment for diabetes.

<u>Diabetes mellitus type 1</u>

Criterion	Definition
1. Overall diabetes mellitus type 1	Cases of DM that are on insulin or diagnosed with a biomarker (eg, c-peptide levels) that is not fasting plasma glucose
2. Uncomplicated diabetes mellitus type 1	Cases of DM that do not have any of the following complications: neuropathy, foot ulcer, leg amputation, or vision loss

3. Diabetic neuropathy among diabetes mellitus type 1	Cases of DM that experience diagnosable neuropathy
4. Diabetic foot due to neuropathy among diabetes mellitus type 1	Cases of DM that currently have a foot ulcer
5. Diabetic neuropathy and amputation with treatment among diabetes mellitus type 1	Cases of DM that have had a leg amputation above or below the knee, with treatment consisting of a prosthetic limb
6. Diabetic neuropathy and amputation without treatment among diabetes mellitus type 1	Cases of DM that have had a leg amputation above or below the knee, with no prosthetic limb
7. Moderate vision impairment due to diabetes mellitus type 1	Cases of DM that have moderate vision loss due to diabetic retinopathy
8. Severe vision impairment due to diabetes mellitus type 1	Cases of DM that have severe vision loss due to diabetic retinopathy
9. Blindness due to diabetes mellitus type 1	Cases of DM that have blindness due to diabetic retinopathy

Diabetes mellitus type 2

Criterion	Definition
1. Overall diabetes mellitus type 2	Cases of diabetes mellitus (DM) type 2 are not reported as type 1 diabetes mellitus.
2. Uncomplicated diabetes mellitus type 2	Cases of DM type 2 that do not have any of the following complications: neuropathy, foot ulcer, leg amputation, or vision loss
3. Diabetic neuropathy among diabetes mellitus type 2	Cases of DM type 2 that experience diagnosable neuropathy
4. Diabetic foot due to neuropathy among diabetes mellitus type 2	Cases of DM type 2 that currently have a foot ulcer
5. Diabetic neuropathy and amputation with treatment among diabetes mellitus type 2	Cases of DM type 2 that have had a leg amputation above or below the knee, with treatment consisting of a prosthetic limb
6. Diabetic neuropathy and amputation without treatment among diabetes mellitus type 2	Cases of DM type 2 that have had a leg amputation above or below the knee, with no prosthetic limb
7. Moderate vision impairment due to diabetes mellitus type 2	Cases of DM type 2 that have moderate vision loss due to diabetic retinopathy
8. Severe vision impairment due to diabetes mellitus type 2	Cases of DM type 2 that have severe vision loss due to diabetic retinopathy
9. Blindness due to diabetes mellitus type 2	Cases of DM type 2 that have blindness due to diabetic retinopathy

Diabetes mellitus, diabetes mellitus type 1, diabetes mellitus type 2:

Data seeking

1. A systematic review of the literature was done for GBD 2017 with the following search terms:

Diabetes Mellitus search string: (diabetes[TI] AND (prevalence[TIAB] OR incidence[TIAB])) OR ('Diabetes Mellitus'[MeSH Terms] AND 'epidemiology'[MeSH Terms]) OR (diabetes[TI] AND 'epidemiology'[MeSH Terms]) NOT gestational[All Fields] NOT ('neoplasms'[MeSH Terms] OR 'neoplasms'[All Fields] OR 'cancer'[All Fields]) NOT ('mice'[MeSH Terms] OR 'mice'[All Fields]) NOT ('schizophrenia'[MeSH Terms] OR 'schizophrenia'[All Fields]) NOT ('emigrants and immigrants'[MeSH Terms] OR ('emigrants'[All Fields] AND 'immigrants'[All Fields]) OR 'emigrants and immigrants'[All Fields] OR 'immigrants'[All Fields]) NOT ('pregnancy'[MeSH Terms] OR 'pregnancy'[All Fields] OR 'gestation'[All Fields]) NOT ('rats'[MeSH Terms] OR 'rats'[All Fields] OR 'rat'[All Fields]) NOT ('kidney'[MeSH Terms] OR 'kidney'[All Fields]) NOT renal[All Fields] NOT ('vitamins'[Pharmacological Action] OR 'vitamins'[MeSH Terms] OR 'vitamins'[All Fields] OR 'vitamin'[All Fields])

And

FPG search string: ((“glucose”[Mesh] OR “hyperglycemia”[Mesh] OR “prediabetic state”[Mesh]) AND "Geographic Locations"[Mesh] NOT "United States"[Mesh]) AND ("humans"[Mesh] AND "adult"[MeSH]) AND ("Data Collection"[Mesh] OR "Health Services Research"[Mesh] OR "Population Surveillance"[Mesh] OR "Vital statistics"[Mesh] OR "Population"[Mesh] OR "Epidemiology"[Mesh] OR "surve*"[TiAb]) NOT Comment[ptyp] NOT Case Reports[ptyp]) NOT "hospital"[TiAb]

Search date: October 15, 2017

The search took place for the following dates: 12/31/2016– 10/15/2017. The number of studies returned was 555, and the number of studies extracted was 58.

2. We systematically searched the Global Health Data Exchange (GHDx) for multi-country survey programs, national surveys, and longitudinal studies that were tagged with either fasting plasma glucose (FPG) or diabetes mellitus.

3. To capture any remaining sources not identified in the GHDx or in PubMed, we looked to other leaders in the field to ensure our datasets were as comprehensive as possible. These included data sources used by other research groups that report on the global burden of diabetes, microdata from not-yet-published national studies, and publications that were not captured in the PubMed search string.

Figure 1: PRISMA diagram of data sources used in GBD 2017 diabetes mellitus model

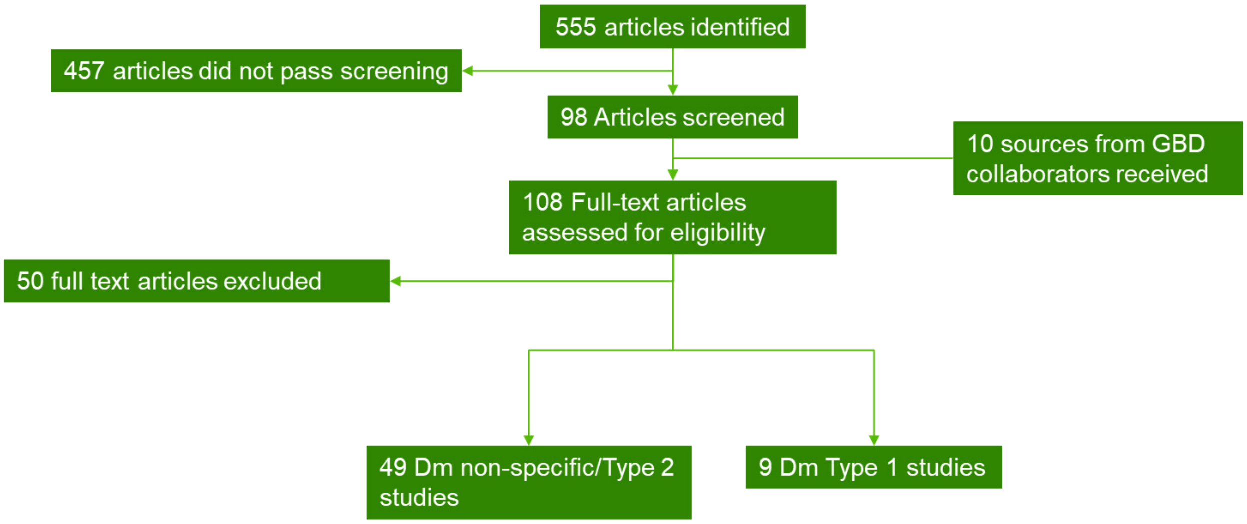

Data inputs

Overall diabetes mellitus

Purpose:

To incorporate all available data related to population-representative estimates of diabetes, we accepted other measures of blood sugar (glycated hemoglobin A1c, oral glucose tolerance test, post-prandial glucose test) to define diabetes and mean fasting plasma glucose (FPG) in a population when data on diabetes were not available as data inputs.

Data:

1. Data inputs come from four types of sources:
   - Estimates of diabetes in a representative population
   - Estimates of mean FPG in a representative population
   - Individual-level data of fasting plasma glucose measured from surveys
   - MarketScan, which are insurance claims data from the United States

When a study reported both mean fasting plasma glucose (FPG) and prevalence of diabetes, we used the prevalence of diabetes. Where possible, individual-level data from a cohort superseded any data described in a study. Individual-level data were collapsed and aggregated to produce estimates for each age group, sex, location, and year a survey is conducted. In addition, data from USA claims data for 2000 and 2010–2014 by state and Taiwan claims data from 2016 were included. The table below shows the number of literature studies included in GBD 2017 for diabetes mellitus, as well as the number of countries or subnational units and GBD world regions represented.

Overall diabetes mellitus

	Incidence	Relative risk	Prevalence
**Site-years (total)**	822	2	2340
**Number of countries with data**	66	2	138
**Number of GBD regions with data (out of 21 regions)**	13	2	21
**Number of GBD super-regions with data (out of 7 super-regions)**	5	2	7

Diabetes type 1

Purpose:

To incorporate all available data related to population-representative estimates of type 1 diabetes mellitus, we accepted data that reported diabetes mellitus type 1, juvenile-onset diabetes, and insulin-dependent diabetes.

Data:

Data inputs come from two types of sources:

- Estimates of type 1 diabetes mellitus in a representative population
- Diabetic registries

Individual-level data were collapsed and aggregated to produce estimates for each age group, sex, location, and year a survey is conducted. In addition, data from USA claims data for 2000 and 2010–2014 by state and Taiwan claims data from 2016 were included. The table below shows the number of literature studies included in GBD 2017 for type 1 diabetes mellitus, as well as the number of countries or subnational units and GBD world regions represented.

Type 1 diabetes mellitus

	Incidence	Standardised mortality ratio	Prevalence
**Site-years (total)**	1093	8	307
**Number of countries with data**	68	3	2
**Number of GBD regions with data (out of 21 regions)**	14	1	2
**Number of GBD super-regions with data (out of 7 super-regions)**	6	1	2

Diabetes type 2

Studies that reported type 2 diabetes mellitus were used in the overall diabetes mellitus model only. We did not directly model estimates of diabetes mellitus type 2 (please see *Modeling strategy* for more information).

Data processing

Overall diabetes mellitus

We perform several processing steps to the data in order to address sampling and measurement inconsistencies that will ensure the data are comparable across data sources and between high fasting plasma glucose modelling efforts.

1. *Small sample size:* Estimates in a sex and age group with a sample size <30 persons were considered a small sample size. In order to avoid small sample size problems that may bias estimates, data were collapsed into the next age group in the same study until the sample size reached at least 30 persons. The intent of collapsing the data is to preserve as much granularity between age groups as possible. If the entire study sample consisted of <30 persons and did not include a population weight, the study was excluded from the modelling process.

2. *Time, age, and sex splitting*

    a. Time: Prior to modelling in DisMod, any study period that spanned more than five years was duplicated.
    b. Age: Prior to modelling in DisMod, data provided in age groups wider than the GBD five-year age groups were split using the global age pattern of diabetes mellitus from data that were in age groups less than 20-year age groups. Uncertainty was propagated by multiplying the standard error of the data performed by the square root of the number of splits performed.
    c. Sex: Prior to modelling in DisMod, data that do not differentiate gender were split into male and female according to the global male to female ratio from data with sex-specific data. Uncertainty was propagated by multiplying the standard error of the data performed by the square root of the number of splits performed.

3. *Mean FPG processing:* For more details on how data points on mean FPG were processed, please see the High Fasting Plasma Glucose capstone appendix in the GBD 2017 Risk Factors Capstone, *Global, regional, and national comparative risk assessment of 84 behavioural, environmental and occupational, and metabolic risks or clusters of risks, 1990–2017: a systematic analysis for the Global Burden of Disease Study 2017*.

4. *Crosswalks*
    1. Case definition
    We performed adjustments (crosswalks) to data points to standardise data to a reference definition: fasting plasma glucose (FPG) >126 mg/dL (7 mmol/L) or on treatment.
        a. Prevalence
            i. Single-component
            Single component case definitions consisted of diabetes defined based on the level of only one biomarker (eg, FPG, HbA1c).
                1. FPG
                We used an ensemble distribution to standardise the case definition of diabetes in surveys by estimating the prevalence of diabetes under different thresholds of FPG. We used individual-level measures of FPG in surveys of a representative population. This allowed us to capture the non-systematic

change in the proportion of population above different levels of FPG. We adjusted the data point by applying the ratio between FPG above 126 mg/dL and the case-definition used in the study. For more details on the approach used in the ensemble distribution, please see the GBD 2017 Risk Factors Capstone, *Global, regional, and national comparative risk assessment of 84 behavioural, environmental and occupational, and metabolic risks or clusters of risks, 1990–2017: a systematic analysis for the Global Burden of Disease Study 2017*.

2. HbA1c

We assumed that HbA1c >6.5% was equivalent to FPG >126 mg/dL.

ii. Multi-component

Multi-component case definition consisted of studies where more than one glucose test was used in the study to identify different segments of the population (eg, FPG and PPG).

1. Multi-component that includes FPG >126 mg/dL

Multi-component case definitions that consisted of FPG >126 mg/dL were assumed to be equivalent to the reference case definition FPG >126 mg/dL or treatment.

2. Multi-component that does not include FPG >126 mg/dL

Case definitions that did not include FPG >126 mg/dL were excluded from the model.

b. Non-prevalence measure

Data from studies with non-prevalence measures (eg, incidence, relative risk, excess mortality) were marked with the case definition and adjusted to the reference case definition within DisMod.

2. MarketScan

Data from MarketScan were included in the model, and a study-level covariate was included in the model to adjust them. These data points were adjusted to the reference case definition within DisMod.

3. Estimate prevalence of diabetes from mean FPG

We also used the ensemble distribution to estimate the prevalence of diabetes based on mean FPG in locations where data on prevalence of diabetes were not available. For more details on the approach used in the ensemble distribution, please see the GBD 2017 Risk Factors Capstone, *Global, regional, and national comparative risk assessment of 84 behavioural, environmental and occupational, and metabolic risks or clusters of risks, 1990–2017: a systematic analysis for the Global Burden of Disease Study 2017*.

Diabetes type 1

Purpose:

To incorporate all available data related to population-representative estimates of diabetes type 1, we accepted data that reported diabetes mellitus type 1, juvenile-onset diabetes, and insulin-dependent diabetes.

Crosswalks

We reviewed the estimates from different iterations of the data source and determined that data sources could be grouped into three categories. We performed adjustments (crosswalks) of data in DisMod from sources that used a different case definition or ascertainment technique from the reference definition. The following list are the case definition/ascertainment methods.

1. Sources that used non-glucose biomarker as part of their criteria. Examples included c-peptide, GAD, islet cell antibodies, ketosis. (reference case definition)
2. Sources that used FPG or OGTT to identify cases.
3. Sources that were capture-recapture.

Modelling Strategy

Overall diabetes mellitus

For GBD 2017, we estimated the overall prevalence of diabetes using DisMod-MR 2.1, a Bayesian meta-regression tool. DisMod-MR produces estimates of the prevalence of diabetes for each age, sex, geographic location, and year. We used data that reported prevalence, incidence, relative risk, and standardised mortality ratio for diabetes mellitus, diabetes mellitus type 1, and diabetes mellitus type 2.

<u>Model parameters and estimates</u>

- We set a value prior of 0 for remission for ages 0 to 14
- We set a value prior of a maximum value of 0.01 for remission for ages 15 to 100
- We set a value prior of a maximum value of 0.15 for excess mortality for all ages
- We set a value prior of 0 for incidence for ages 0 to 1
- We set a value prior of a maximum value of 0.1 for incidence for ages 1 to 100

Study covariate	Parameter	beta	Exponentiated beta
USA Claims data – 2000	Prevalence	-0.72 (-0.77 to -0.66)	0.49 (0.46–0.52)
USA Claims data – 2010–2014	Prevalence	-0.43 (-0.48 to -0.38)	0.65 (0.62–0.68)
Age-standardised prevalence of obesity	Prevalence	1.80 (1.58–1.99)	6.06 (4.84–7.32)
Type 2 diabetes	Prevalence	-0.44 (-0.56 to -0.33)	0.64 (0.57–0.72)

<u>Comparison with other groups</u>

Our estimate of the age-standardised global prevalence of diabetes is slightly higher than the estimates reported previously by the NCD Risk Factor Collaboration (NCD-RisC) and International Diabetes Federation (IDF) (NCD-Risc: http://www.sciencedirect.com/science/article/pii/S0140673616006188 IDF: https://www.idf.org/component/attachments/attachments.html?id=1093&task=download). IDF reported a prevalence for the year 2013 of 8.3% (7.2–11.3) at ages 20 to 80, compared to our estimate for 2017 of 8.9% (7.7–10.1) for the same age range. The NCD-RisC estimates of prevalence for ages over 18 for the year 2014 were 9.0% (7.2–11.1) in males and 7.9% (6.7–9.7) in females, compared to our 2017 estimates of 9.7% (8.4–11.0) for males and 8.9% (7.7–10.2) for females. Several factors can explain the difference in estimates. We include a greater number of data sources. We also define the whole distribution of fasting plasma glucose (FPG) and thus have a more accurate way of including surveys that report on FPG only in our diabetes disease model.

Diabetes mellitus type 1

For GBD 2017, we estimated the overall prevalence of diabetes using DisMod-MR 2.1, a Bayesian meta-regression tool. We used data that reported incidence and standardised mortality ratio for diabetes mellitus type 1.

<u>Model parameters and estimates</u>

- We set a value prior of 0 for remission for all ages

Study covariate	Parameter	beta	Exponentiated beta
Proportion of livebirths in women 35+ years	Incidence	2.00 (1.98–2.00)	7.36 (7.26–7.39)
Capture-recapture	Incidence	-0.00086 (-0.053 to 0.045)	1.00 (0.95–1.05)
FPG or OGTT testing	Incidence	0.12 (0.055–0.20)	1.13 (1.06–1.22)

Diabetes type 2

While we are confident that estimates of type 1 diabetes mellitus are more accurate because of the need for medical care and early onset, we are less confident in sources that describe studies as representing type 2 diabetes, particularly in adults. The diagnostic criteria in the methodological sections are not sufficiently specific to confidently identify the population as type 2 diabetics. Thus, we calculated estimates of diabetes mellitus type 2 by subtracting the estimates of diabetes mellitus type 1 from estimates overall diabetes mellitus for each age, sex, and location from 1990 to 2017. We assumed that prevalence of diabetes mellitus type 2 could not occur before 10 years.

Outcomes

<u>Data seeking</u>

Amputation due to diabetes mellitus

A systematic review of the literature was performed for GBD 2017 with the following search terms:

(‘Diabetes Mellitus’[MeSH Terms] OR (‘diabetes’[All Fields] AND ‘mellitus’[All Fields]) OR ‘Diabetes Mellitus’[All Fields]) AND ‘amputation’[All Fields] AND (proportion OR prevalence OR incidence) NOT gestational NOT cancer NOT mice NOT schizophrenia NOT immigrants NOT gestation NOT rat NOT kidney NOT renal NOT vitamin

- Dates of search: 12/31/16-10/17/2017
- Number of studies returned: 16
- Number of studies extracted: 1

The table below indicates the data inputs for the GBD 2017 estimation of amputation due to diabetes mellitus.

	Incidence	Other	Prevalence
**Site-years (total)**	66	6	29
**Number of countries with data**	15	5	22
**Number of GBD regions with data (out of 21 regions)**	7	5	10
**Number of GBD super-regions with data (out of 7 super-regions)**	4	4	5

Diabetic neuropathy

A systematic review of the literature was performed for GBD 2017 with the following search terms:

(“Diabetes Mellitus”[MeSH Terms] OR (“diabetes”[All Fields] AND “mellitus”[All Fields]) OR “Diabetes Mellitus”[All Fields]) AND neuropathy[All Fields] AND (proportion OR prevalence OR incidence) NOT gestational NOT cancer NOT mice NOT schizophrenia NOT immigrants NOT gestation NOT rat NOT kidney NOT renal NOT vitamin

- Dates: 12/31/16-10/17/2017
- Number of studies returned: 170
- Number of studies extracted: 1

The table below illustrates the model inputs for the GBD 2017 estimation process:

	Proportion
**Site-years (total)**	87
**Number of countries with data**	44
**Number of GBD regions with data (out of 21 regions)**	13
**Number of GBD super-regions with data (out of 7 super-regions)**	7

Diabetic foot ulcer

A systematic review of the literature was performed for GBD 2017 with the following search terms:

((("Diabetes Mellitus"[MeSH Terms] OR ("diabetes"[All Fields] AND "mellitus"[All Fields]) OR "Diabetes Mellitus"[All Fields] OR "diabetes"[All Fields]) AND ("foot"[MeSH Terms] OR "foot"[All Fields]) AND ("ulcer"[MeSH Terms] OR "ulcer"[All Fields])) NOT ("neoplasms"[MeSH Terms] OR "neoplasms"[All Fields] OR "cancer"[All Fields]) NOT ("mice"[MeSH Terms] OR "mice"[All Fields]) NOT ("emigrants and immigrants"[MeSH Terms] OR ("emigrants"[All Fields] AND "immigrants"[All Fields]) OR "emigrants and immigrants"[All Fields] OR "immigrants"[All Fields]) NOT ("pregnancy"[MeSH Terms] OR "pregnancy"[All Fields] OR "gestation"[All Fields]) NOT ("vitamins"[Pharmacological Action] OR "vitamins"[MeSH Terms] OR "vitamins"[All Fields] OR "vitamin"[All Fields]) NOT renal[All Fields] NOT ("kidney"[MeSH Terms] OR "kidney"[All Fields]) AND (proportion[All Fields] OR "incidence"[All Fields] OR "prevalence"[All Fields]) NOT ("schizophrenia"[MeSH Terms] OR "schizophrenia"[All Fields]) NOT ("rats"[MeSH Terms] OR "rats"[All Fields] OR "rat"[All Fields]))

- Dates: 12/31/16-10/17/2017
- Number of studies returned: 48
- Number of studies extracted: 0

The table below illustrates the data inputs used for the GBD 2017 modelling process.

	Proportion
**Site-years (total)**	29
**Number of countries with data**	21
**Number of GBD regions with data (out of 21 regions)**	8
**Number of GBD super-regions with data (out of 7 super-regions)**	6

Modelling strategy

For GBD 2017, we estimated amputation due to diabetes mellitus, diabetic neuropathy, and diabetic foot ulcer for diabetes mellitus type 1 and diabetes mellitus type 2 using DisMod-MR 2.1, a Bayesian meta-regression tool. DisMod-MR produces estimates of the prevalence of diabetes for each age, sex, geographic location, and year. We then multiply all proportion draws from neuropathy/foot ulcer/amputation models by the parent diabetes model so that all estimates are in the same population-space.

Next, we ensure that at least 10% of diabetes cases are uncomplicated for all draws. We ensure that the sum of the prevalence for neuropathy due to diabetes mellitus, moderate vision loss[1] due to diabetes mellitus, severe vision loss due to diabetes mellitus, and blindness due to diabetes mellitus does not exceed 90% of the prevalence of all diabetes mellitus. If the sum exceeds 90% then we rescale the individual outcomes to ensure that the sum does not exceed 90%.

Then, we ensure that at least 10% of diabetic neuropathy cases do not have foot ulcer or amputation for all draws. We perform the same check to ensure that the prevalence of amputation due to diabetes

[1] Moderate vision loss due to diabetes mellitus, severe vision loss due to diabetes mellitus, and blindness due to diabetes mellitus are obtained from vision loss impairment

mellitus and prevalence of foot ulcer due to diabetes mellitus does not exceed 90% of the prevalence of neuropathy due to Diabetes mellitus. If the sum exceeds 90% then we rescale the individual outcomes to ensure that the sum does not exceed 90%. This treats foot ulcer and amputation as mutually exclusive categories by assuming a patient will not have both simultaneously.

From here, we calculate uncomplicated diabetes as the remainder of diabetes cases exclusive of neuropathy and vision loss. In addition, we estimate the prevalence of amputation due to diabetes is split into with and without treatment using scaled health systems access (HSA) values. For diabetic amputation, we calculated a distribution of treated versus untreated amputation, defined as receiving a prosthetic or not. We first rescaled the IHME HSA estimates to be between 0 and 0.9, under the assumption that 10% of amputees will not receive a prosthetic, even in high-income countries. We based this assumption on the retrospective study by Moore and colleagues, which found that about 80% of patients following major lower extremity amputation were fitted with prostheses in the authors' institutions from 1978 to 1986 in the USA. We then performed a population-weighted average of this country-specific value to obtain a proxy for the proportion of amputees that receive a prosthetic by super-region. Because these are rough estimates based on large assumptions, we applied confidence intervals of +/- 50% of the value to reflect our uncertainty.

<u>Model parameters and estimates</u>

Amputation due to diabetes

- We set a value prior of 0 for incidence for ages 0 to 15
- We set a value prior of 0 for remission for all ages
- We crosswalked the incidence of either above- or below-knee amputation only to the incidence of all amputations

DisMod model

	Parameter	beta	Exponentiated beta
Above-knee amputation only	Incidence	-0.31 (-0.55 to -0.065)	0.74 (0.58–0.94)
Below-knee amputation only	Incidence	-0.41 (-0.69 to -0.12)	0.66 (0.50–0.88)

Diabetic neuropathy

- We set a value prior on the proportion of 0 from ages 0 to 1
- We crosswalked data from studies using alternate diagnostic criteria using as reference studies which used the monofilament test as their diagnostic criteria

DisMod model

Study covariate	Parameter	Beta	Exponentiated beta
Diagnostic vibration perception threshold test	Proportion	-0.037 (-0.11 to 0.031)	0.96 (0.90–1.03)
Diagnostic method – nerve conduction velocity	Proportion	0.11 (0.040–0.19)	1.12 (1.04–1.21)

Diagnostic method – clinical exam only	Proportion	-0.16 (-0.22 to -0.088)	0.85 (0.80–0.92)
Diagnostic validated neuropathy scoring	Proportion	0.052 (-0.00041 to 0.10)	1.05 (1.00–1.11)

Diabetic foot ulcer

- We set a value prior on the proportion of 0 from ages 0 to 10.
- We crosswalked data from studies investigating hospitalised patients only using as reference studies which captured all diabetic foot ulcers.

DisMod model

Study covariate	Parameter	beta	Exponentiated beta
Hospital data	Proportion	0.018 (-0.25 to 0.24)	1.02 (0.78–1.27)
Healthcare Access and Quality index	Proportion	-0.0058 (-0.013 to 0.0024)	0.99 (0.99–1.00)

Severity split inputs

Severity splits and disability weights were determined by the GBD disability weight survey assessment for diabetes mellitus. The table below illustrates the severity levels, lay descriptions, and associated disability weights applicable for outcomes related to diabetes mellitus type 1 and diabetes mellitus type 2:

Severity level	Lay description	DW (95% CI)
Uncomplicated diabetes mellitus	Has a chronic disease that requires medication every day and causes some worry, but minimal interference with daily activities.	0.049 (0.031–0.072)
Diabetic neuropathy	Has pain, tingling, and numbness in the arms, legs, hands, and feet. The person sometimes gets cramps and muscle weakness.	0.133 (0.089–0.187)
Diabetic neuropathy with diabetic foot	Has a sore on the foot that is swollen and causes some difficulty in walking.	[a]
Diabetic neuropathy with treated amputation	Has lost part of one leg, leaving pain and tingling in the stump. The person has an artificial leg that helps in moving around.	[a]

Diabetic neuropathy with untreated amputation	Has lost part of one leg, leaving pain and tingling in the stump. The person does not have an artificial leg, has frequent sores, and uses crutches.	[a]
Moderate vision loss due to diabetes mellitus	Has vision problems that make it difficult to recognize faces or objects across a room.	0.031 (0.019–0.049)
Severe vision loss due to diabetes mellitus	Has severe vision loss, which causes difficulty in daily activities, some emotional impact (for example worry), and some difficulty going outside the home without assistance.	0.184 (0.125–0.259)
Blindness due to diabetes mellitus	Is completely blind, which causes great difficulty in some daily activities, worry and anxiety, and great difficulty going outside the home without assistance.	0.187 (0.124–0.26)

[a] *The disability weights are produced from a combination of two health states: neuropathy and diabetic foot/amputation*

References

Moore TJ, Barron J, Hutchinson F 3rd, Golden C, Ellis C, Humphries D. **Prosthetic usage following major lower extremity amputation.** *Clin Orthop Relat Res.* 1989 Jan;(238):219-24.

Chronic Kidney Disease

Flowchart

Chronic Kidney Disease (CKD)

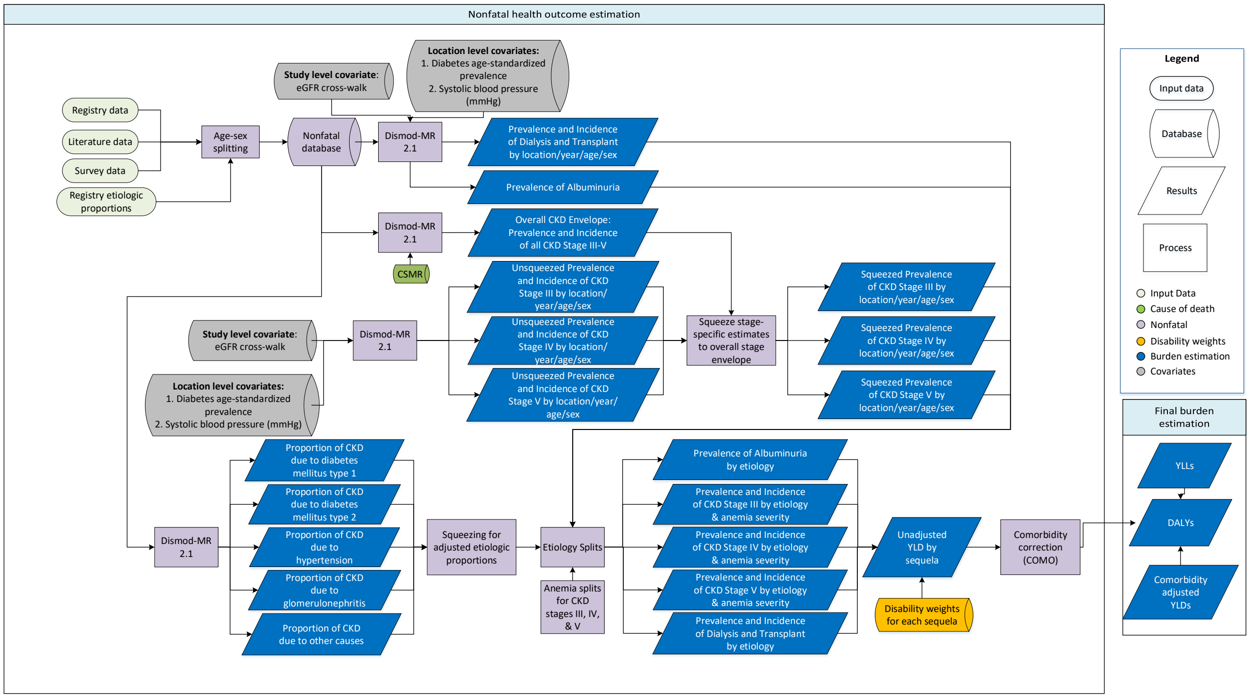

Case definition

Chronic kidney disease (CKD) is defined as a permanent loss of renal function as indicated by estimated glomerular filtration rate (eGFR) and urinary albumin to creatinine ratio (ACR). The GBD study considers six stages of CKD as defined by degree of loss of renal function or receipt of renal replacement therapy: Albuminuria (eGFR > 60ml/min/1.73m^2 and ACR > 30 mg/g), CKD Stage III (eGFR 30-60ml/min/1.73m^2), CKD Stage IV (eGFR 15-30ml/min/1.73m^2), CKD Stage V (eGFR <15ml/min/1.73m^2, not on renal replacement therapy), maintenance dialysis, and renal transplantation.[1] The ICD-10 codes associated with CKD include N18.1-N18.9.

Input data

Model inputs

For GBD 2010, a systematic review of the prevalence of CKD throughout the world was conducted. This search was updated for GBD 2013, GBD 2015, and GBD 2016. For GBD 2017, this literature search was repeated using PubMed search terms ((((("chronic kidney disease"[Title/Abstract]) AND prevalen*[Title/Abstract]) AND ("1980/1/1"[Date - Publication] : "3000"[Date - Publication])) NOT ((animals[MeSH] NOT humans[MeSH])))).

The exclusion criteria were:

1. Studies clearly not representative of the national population
2. Studies that did not provide primary data on epidemiological parameters, eg, a commentary piece
3. Studies of a specific aetiology of CKD

This literature search was augmented by identification of population-based surveys that measured renal function. For maintenance dialysis and renal transplantation, data were largely obtained from renal registry reports.

The tables below show the geographical coverage of data by measure in GBD 2017.

Stages III-V

	Incidence	Prevalence
**Site-years (total)**	3	222
**Number of countries with data**	3	59
**Number of GBD regions with data (out of 21 regions)**	3	20
**Number of GBD super-regions with data (out of 7 super-regions)**	2	7

Stage III

	Incidence	Prevalence
**Site-years (total)**	1	180
**Number of countries with data**	1	54
**Number of GBD regions with data (out of 21 regions)**	1	19
**Number of GBD super-regions with data (out of 7 super-regions)**	1	7

Stage IV

	Incidence	Prevalence
**Site-years (total)**	1	145
**Number of countries with data**	1	49
**Number of GBD regions with data (out of 21 regions)**	1	19
**Number of GBD super-regions with data (out of 7 super-regions)**	1	7

Stage V

	Incidence	Prevalence
**Site-years (total)**	1	141
**Number of countries with data**	1	49
**Number of GBD regions with data (out of 21 regions)**	1	18
**Number of GBD super-regions with data (out of 7 super-regions)**	1	7

End-stage renal disease after transplant

	Incidence	Prevalence
**Site-years (total)**	2363	1525
**Number of countries with data**	82	88
**Number of GBD regions with data (out of 21 regions)**	16	19
**Number of GBD super-regions with data (out of 7 super-regions)**	6	7

End-stage renal disease on dialysis

	Excess mortality rate	Incidence	Prevalence
**Site-years (total)**	55	1484	2592
**Number of countries with data**	13	69	109
**Number of GBD regions with data (out of 21 regions)**	1	15	20
**Number of GBD super-regions with data (out of 7 super-regions)**	1	6	7

Severity splits & disability weights

Estimates of prevalence and incidence are split using CKD aetiology proportion models, resulting in CKD estimates by stage and aetiology. Then a portion of each aetiology split for CKD stages III, IV, and V is attributed a disability weight associated with mild, moderate, or severe anaemia.[2]

Severity level	Lay description	Disability weight (95% CI)
Albuminuria	Asymptomatic	--
CKD stage III without anaemia	Asymptomatic	--
CKD stage III with mild anaemia	Feels slightly tired and weak at times, but this does not interfere with normal daily activities.	0.004 (0.001–0.008)
CKD stage III with moderate anaemia	Feels moderate fatigue, weakness, and shortness of breath after exercise, making daily activities more difficult.	0.052 (0.034–0.076)
CKD stage III with severe anaemia	Feels very weak, tired, and short of breath, and has problems with activities that require physical effort or deep concentration.	0.149 (0.101–0.21)
CKD stage IV without anaemia	Tires easily, has nausea, reduced appetite, and difficulty sleeping.	0.104 (0.07–0.147)
CKD stage IV with mild anaemia		0.108 (0.072–0.151)
CKD stage IV with moderate anaemia		0.15 (0.103–0.207)
CKD stage IV with severe anaemia		0.237 (0.165–0.324)
CKD stage V without anaemia	Has lost a lot of weight and has constant pain. The person has no appetite, feels nauseated, and needs to spend most of the day in bed.	0.569 (0.389–0.727)

CKD stage V with mild anaemia		0.570 (0.391–0.727)
CKD stage V with moderate anaemia		0.591 (0.414–0.743)
CKD stage V with severe anaemia		0.631 (0.456–0.782)
End-stage renal disease, on dialysis	Is tired and has itching, cramps, headache, joint pains, and shortness of breath. The person needs intensive medical care every other day lasting about half a day.	0.571 (0.397–0.725)
End-stage renal disease, with kidney transplant	Sometimes feels tired and down, and has some difficulty with daily activities.	0.024 (0.014–0.039)

Aetiology proportion models are informed by data from end-stage kidney disease registries and the Geisinger Health System in Pennsylvania provided by the Chronic Kidney Disease Prognosis Consortium (CKDPC) at the Johns Hopkins Bloomberg School of Public Health. Aetiologies included in the GBD study include diabetes mellitus type 1, diabetes mellitus type 2, hypertension, glomerulonephritis, and other and unknown causes.

Modelling strategy

CKD Stage Models

We ran a DisMod-MR 2.1 model to produce estimates by age, sex, year, and country for each stage of CKD. To account for progression of individuals from stage III to stage IV, and stage IV to stage V, we informed remission for stages III and IV. Remission data for stage IV were calculated as the ratio of the incidence of stage V and prevalence of stage IV at the gender-, age-, and country-matched level. Remission data for stage III were calculated as the ratio of resulting stage IV incidence and stage III prevalence at the gender-, age-, and country-matched level. Remission was set to 0 for stage V and the excess mortality parameter was used to account for progression to end-stage renal disease and mortality due to CKD stage V. Bounds on excess mortality were informed using a meta-analysis of survival analyses of individuals with untreated CKD stage V.

We used data from sources reporting the prevalence of stage III, IV, and V CKD combined (eGFR < 60 ml/min/1.73m^2) to model the prevalence of stage III-V CKD. We ran a DisMod-MR 2.1 model to produce estimates by age, sex, year, and country for aggregate stage III-V CKD. We used the function in DisMod-MR 2.1 to pull in cause-specific mortality rate (CSMR) data from our chronic kidney disease CODEm and CODcorrect analyses and match them with prevalence data points for the same geography and study year to estimate priors on excess mortality rates (by dividing CSMR by prevalence). In order to enforce more consistency between stage models, prevalence of CKD stage III, IV, and V were then scaled to sum to the prevalence and incidence of the stage III-V CKD model, at the gender-, age-, and country-matched level.

A description of priors and covariates included in each model can be found in the table below:

	Priors (min,max)	Study-level covariate	Country-level covariate

CKD stage III	Remission (0, 0.75)<br>Excess mortality (0, 0.05)	Adjust for estimating equation	Diabetes age-standardised prevalence
			Mean systolic blood pressure
CKD stage IV	Remission (0, 0.75)<br>Excess mortality (0, 0.05)	Adjust for estimating equation	Diabetes age-standardised prevalence
			Mean systolic blood pressure
CKD stage V	Remission (0, 0)<br>Incidence (0, 0.001), age 0-20<br>Excess mortality (0.29, 0.54)	Adjust for estimating equation	Diabetes age-standardised prevalence
			Mean systolic blood pressure
CKD stage III-V	Remission (0, 0)<br>Excess mortality (0, 0.54)	Adjust for estimating equation	Diabetes age-standardised prevalence
			Mean systolic blood pressure

We crosswalked data reporting glomerular filtration rate (GFR) estimated with the Modification of Diet in Renal Disease (MDRD) equation to data reported using the CKD-Epi equation as our reference. GFR reported for children was estimated using the Schwartz equation as the reference among the paediatric population. Bounds on the MDRD-CKD-Epi crosswalk were informed using a meta-analysis of studies reporting prevalence of CKD by stage using both the MDRD and CKD-Epi equations. Betas and exponentiated values for this crosswalk are shown in the table below:

	Study covariate	Parameter	beta	Exponentiated beta
Stage III	eGFR calculated with MDRD equation	Prevalence	0.27 (0.26–0.28)	1.31 (1.30–1.32)
Stage IV	eGFR calculated with MDRD equation	Prevalence	-0.011 (-0.026 to 0.011)	0.99 (0.97–1.01)
Stage V	eGFR calculated with MDRD equation	Prevalence	-0.091 (-0.14 to -0.034)	0.91 (0.87–0.97)
Stage III-V	eGFR calculated with MDRD equation	Prevalence	0.24 (0.23–0.25)	1.27 (1.26–1.28)

In order to obtain an appropriate age-pattern with which to age-split dialysis input data, we first ran a DisMod-MR 2.1 model containing only age-specific dialysis data. We then used age-pattern by super-region from this model to age-split dialysis input data, thereby allowing for variation in the age-pattern by location. After age-splitting, we ran a model on all processed data, including age-split data and age-specific data, to obtain final estimates of dialysis incidence and prevalence by location, year, age, and sex. Remission data for dialysis were calculated as the ratio of the incidence of renal transplantation to prevalence of dialysis at the gender-, age-, and country-matched level.

Given the paucity of age-specific data on renal transplantation, we did not age-split input data for this model. Socio-demographic Index was used as a covariate on incidence. Betas and exponentiated values for SDI are as follows:

	Study covariate	Parameter	beta	Exponentiated beta

ESRD Transplant	Socio-demographic Index	Incidence	1.78 (1.13–2.00)	5.91 (3.08–7.38)

CKD aetiology proportion models

For GBD 2017 we implemented stage-specific aetiology splits to allow for differential aetiologic composition of CKD across stages for disease progression. In order to obtain age-sex-stage-specific aetiology proportions, we utilised data from the Geisinger Health System in Pennsylvania to identify patients with CKD. Analysis of this dataset was conducted by the CKDPC. For each individual with CKD, we scanned their history of recorded ICD codes to identify ICD codes for primary renal diseases. We used this information to map individuals to GBD aetiologies by stage of CKD; individuals with CKD but with no history of a primary renal disease ICD code were classified as having CKD of unknown aetiology. We ran a multinomial logistic regression including sex and a non-linear term for age to predict the probability of each aetiology by age and sex for each stage of CKD (albuminuria, stage 3 CKD, and stage 4/5 CKD combined). For each stage, aetiology, age, and sex, we converted this probability into the proportion of CKD due to the given aetiology, and applied these proportions to the prevalence of CKD for the same stage, age, and sex category to estimate the prevalence of each stage of CKD by aetiology, age, and sex. The ICD to GBD aetiology map utilised in this analysis is as follows:

CKD Aetiology	ICD 9 Codes	ICD 10 Codes
Type 1 diabetes	250.41, 250.43	E10.2, E10.21, E10.22, E10.29
Type 2 diabetes	250.40, 250.42	E11.2, E11.21, E11.22, E11.29
Glomerulonephritis	581, 581.0, 581.1, 581.2, 581.3, 581.8, 581.81, 581.89, 581.9, 582, 582.0, 582.1, 582.2, 582.4, 582.8, 582.81, 582.89, 582.9, 583, 583.0, 583.1, 583.2, 583.4, 583.6, 583.7, 583.8, 583.81, 583.89, 583.9	N02, N02.0, N02.1, N02.2, N02.3, N02.4, N02.5, N02.6, N02.7, N02.8, N02.9, N03, N03.0, N03.1, N03.2, N03.3, N03.4, N03.5, N03.6, N03.7, N03.8, N03.9, N04, N04.0, N04.1, N04.2, N04.3, N04.4, N04.5, N04.6, N04.7, N04.8, N04.9, N05, N05.0, N05.1, N05.2, N05.3, N05.4, N05.5, N05.6, N05.7, N05.8, N05.9, N06, N06.0, N06.1, N06.2, N06.3, N06.4, N06.5, N06.6, N06.7, N06.8, N06.9
Hypertension	403, 403.0, 403.00, 403.01, 403.1, 403.10, 403.11, 403.6, 403.9, 403.90, 403.91, 404, 404.0, 404.00, 404.01, 404.02, 404.03, 404.1, 404.10, 404.11, 404.12, 404.13, 404.9, 404.90, 404.91, 404.92, 404.93	I12, I12.0, I12.1, I12.2, I12.9, I13, I13.0, I13.1, I13.10, I13.11, I13.2, I13.9

Other	589, 589.0, 589.1, 589.9, 753.0, 753.1, 753.10, 753.11, 753.12, 753.13, 753.14, 753.15, 753.16, 753.17, 753.19, 753.2, 753.20, 753.21, 753.22, 753.23, 753.29, 753.3, 283.11, 710.0, 753.0, 753.21, 753.22, 753.29	N07, N07.0, N07.1, N07.2, N07.3, N07.4, N07.5, N07.6, N07.7, N07.8, N07.9, N08, N08.0, N08.1, N08.2, N08.3, N08.4, N08.5, N08.8, N15.0, Q61, Q61.0, Q61.00, Q61.01, Q61.02, Q61.1, Q61.11, Q61.19, Q61.2, Q61.3, Q61.4, Q61.5, Q61.8, Q61.9, Q62, Q62.0, Q62.1, Q62.10, Q62.11, Q62.12, Q62.2, Q62.3, Q62.31, Q62.32, Q62.39, Q62.4, Q62.5, Q62.6, Q62.60, Q62.61, Q62.62, Q62.63, Q62.69, Q62.7, Q62.8, D59.3, M31.31, M32.14, M32.15, N11.9, N13.70, N13.8, Q60.2, Q63.8, N14.0, N14.1, N14.3, N25.89, N26.9, N28.0

In order to maintain consistency between GBD estimates of type 1 diabetes prevalence estimates and CKD due to type 1 diabetes prevalence estimates and generalize the results of the Geisiger analysis to all locations, we performed a location-specific correction for the proportion of CKD due to type 1 and type 2 diabetes, as type 1 diabetes makes up a much larger proportion of total diabetes in the United States than it does in other locations. For each diabetic subtype (e) for a given location (l), age (a), and sex (g) the ratio of subtype-specific diabetes prevalence to total diabetes prevalence (r) is calculated as:

$$r_{e,l,a,g} = \frac{prevalence_{e,l,a,g}}{prevalence_{dm1,l,a,g} + prevalence_{dm2,l,a,g}}$$

Where e represents either type 1 or type 2 diabetes, dm1 represents type 1 diabetes, and dm2 represents type 2 diabetes.

This ratio is used to adjust the proportion of CKD due to a given diabetic subtype (p) for a given CKD stage (s), l, a, and g by scaling the predicted proportion of CKD due to that subtype (k) by the ratio of total DM due to e in l to the ratio of total DM due to e in the United States (USA).

$$p_{s,e,l,a,g} = k_{s,a,g} \times \frac{r_{e,l,a,s}}{r_{e,USA,a,s}}$$

The stage-specific approach utilised to estimate the prevalence albuminuria, stage 3, 4, and 5 CKD Is limited by the use of data from a single geographic region.

For end-stage renal disease on dialysis and end-stage renal disease after transplant, we ran DisMod-MR 2.1 models to obtain estimates of proportions for each subtype by location, year, age, and sex. Data for CKD due to overall DM were more widely available than data by type of DM. Models for the proportion of CKD due to hypertension and diabetes included covariates for mean systolic blood pressure and the age-standardised prevalence of diabetes, respectively. Coefficient values from these models are as follows:

Model	Covariate	Value	Exponentiated
CKD proportion due to diabetes mellitus	Diabetes age-standardised prevalence	0.72 (0.66–0.78)	2.05 (1.93–2.18)
CKD proportion due to hypertension	Mean systolic blood pressure	0.034 (0.00076–0.12)	1.03 (1.00–1.13)

In order to make use of all available data, we modelled the proportion of CKD due to overall DM, DM type 1, and DM type 2. Proportion of CKD due to DM type 1 and DM type 2 were then scaled to sum to the proportion of overall DM at the gender, age, and country-matched level. The results from all subtype-specific models were adjusted so that estimates across the subtypes equaled 1 at each of 1,000 draws. These adjusted proportions were applied to the DisMod models for dialysis and transplant to obtain estimates of each of these entities by aetiology.

Major changes to the chronic kidney disease estimation process for GBD 2017 include addition of albuminuria as a sequela of chronic kidney disease as well as updated aetiological attribution of CKD to diabetes mellitus type 1 and diabetes mellitus type 2 instead of the combined category of all diabetes mellitus, and implementation of a stage-specific aetiology estimation method to capture differences in CKD severity by aetiology.

Acute Glomerulonephritis

Flowchart

Acute Glomerulonephritis

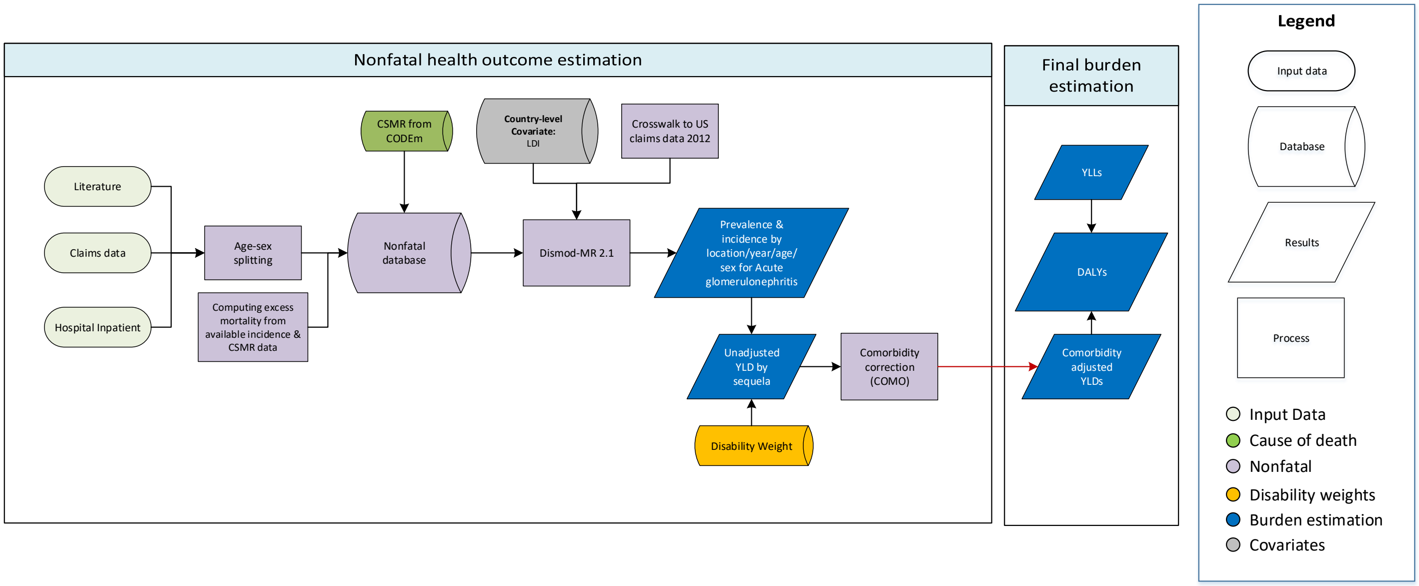

Case Definition

Acute glomerulonephritis (AG) (or post-infectious glomerulonephritis) is an acute episode of haematuria, oedema, hypertension, and acute kidney injury that typically follows infection with specific strains of group A beta-hemolytic *Streptococcus*. As used in the GBD study, this term is synonymous with post-streptococcal or post-staphylococcal glomerulonephritis. This disease is typically seen in children but can demonstrate a bimodal distribution as early life immunity wanes within older years. ICD codes include N00, N00.0, N00.1, N00.2, N00.3, N00.4, N00.5, N00.6, N00.7, N00.8, N00.9, N01, N01.1, N01.2, N01.3, N01.4, N01.5, N01.6, N01.7, N01.8, and N01.9.

Input data

Model inputs

For GBD 2010, a systematic review of the prevalence of AG throughout the world was conducted. This search was updated for GBD 2013, 2015, and 2016. The PubMed search was conducted using the following search terms: (Acute Glomerulonephritis [Title/Abstract] OR GN [Title/Abstract]) AND (Prevalen*[Title/Abstract] OR Inciden*[Title/Abstract]) AND ("2015/01/01"[PDAT] : "3000"[PDAT]) AND "humans"[MeSH Terms].

The exclusion criteria were:

1. Studies clearly not representative of the national population
2. Studies that did not provide primary data on epidemiological parameters, eg, a commentary piece
3. Studies that describe non-infectious glomerulonephritis epidemiology

The most recent literature source dates from 2013. Twenty-one articles have been included in total.

Claims data for the USA, Philippines, Taiwan, and New Zealand were included. Hospital inpatient data were also included. Inpatient data points with an age-standardised incidence rate greater than 1.5 median absolute deviations from the median of the age-standardised incidence rate for all inpatient data were marked as outliers and excluded from analysis.

The table below shows the geographical coverage for AG in GBD 2017.

	**Incidence**
**Site-years (total)**	1336
**Number of countries with data**	47
**Number of GBD regions with data (out of 21 regions)**	16
**Number of GBD super-regions with data (out of 7 super-regions)**	7

Severity split & disability weight

The basis of the GBD disability weight assessment is lay descriptions of sequelae highlighting major functional consequences and symptoms. Disability weighting (DW) for AG associates with systemic symptoms of fever, aches, weakness, and some difficulty with daily activities. The lay description and disability weight for acute glomerulonephritis are shown below.

Cause	Lay description	DW (95% CI)
Acute glomerulonephritis	Has a fever and aches, and feels weak, which causes some difficulty with daily activities.	0.051 (0.032–0.074)

Modelling strategy

We ran a DisMod-MR 2.1 model to produce estimates by age, sex, year, and country. Prior settings in the DisMod model included setting remission of three to four weeks. It was assumed that no one was born with AG.

We applied a crosswalk to USA claims data from the year 2000 to USA claims data for 2010–2014 in order to adjust for the smaller sample included in the 2000 data. Inpatient data were adjusted to account for multiple admissions and multiple diagnoses, based on data from New Zealand, MarketScan, the Philippines, and Taiwan. Inpatient data were crosswalked to claims data in DisMod. We used the function in DisMod-MR 2.1 to pull in cause-specific mortality rate (CSMR) data from our CODEm and CODcorrect analyses and match them with incidence data points for the same geography and study year to estimate priors on excess mortality rates (by dividing CSMR by incidence).

We included the covariate lagged distributed income (LDI) as a country-level covariate to inform excess mortality, with bounds of -0.5, -0.1.

Betas and exponentiated values (which can be interpreted as an odds ratio) are shown in the table below:

Covariate	Parameter	beta	Exponentiated beta
MarketScan data – 2000	Incidence	-0.0.058 (-0.08 to -0.039)	0.94 (0.92–0.96)
Hospital data	Incidence	-0.49 (-0.56 to -0.47)	0.61 (0.57–0.63)

Compared to GBD 2016, changes include inclusion of inpatient data from a greater number of geographies.

Dermatitis

Flowcharts for Atopic dermatitis, Contact dermatitis, & Seborrheic dermatitis

Atopic dermatitis

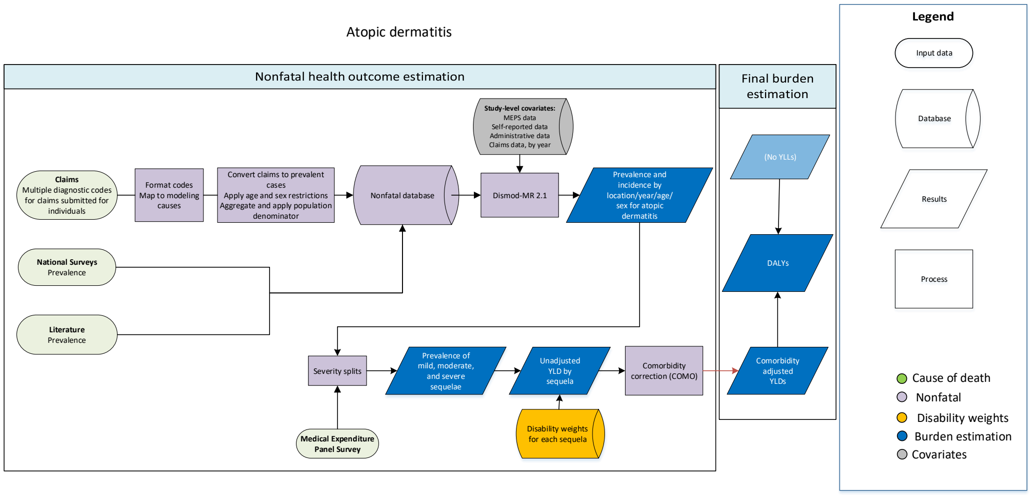

Contact dermatitis

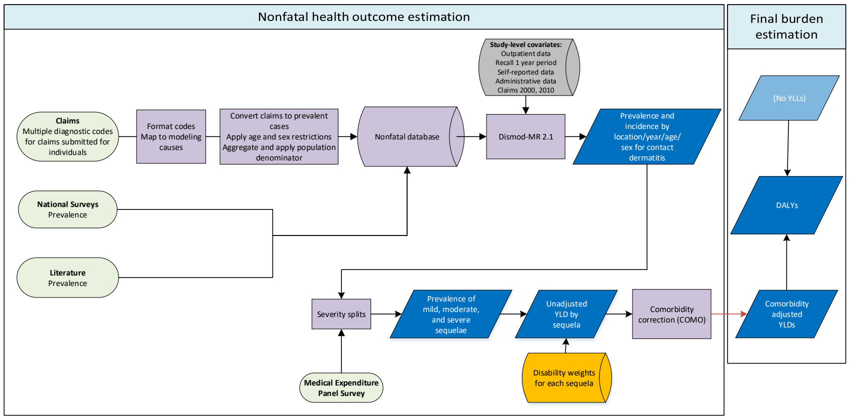

Seborrheic dermatitis

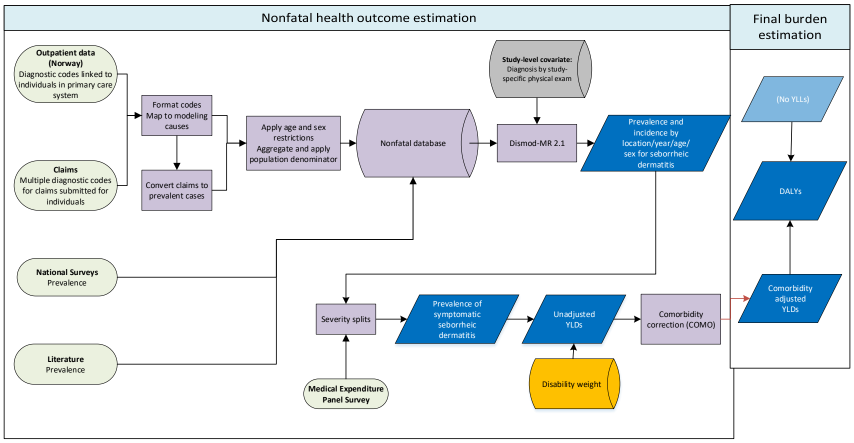

Case definition

Dermatitis, or eczema, refers to inflammation of the dermal layer of the skin, with disruption of the epidermal barrier. This inflammation leads to rashes that are commonly red, scaly, or flaky. Atopic dermatitis is a relapsing dermatitis associated with elevated serum immunoglobulin E and some degree of immune dysregulation; it can be localised or widespread and is commonly characterised by itching that can be extreme (ICD-10: L20). Contact dermatitis is a localised dermatitis caused by direct contact with allergens or irritants; it can be asymptomatic or characterised by itching, stinging or pain (ICD: 10: L22-26). Seborrhoeic dermatitis is a dermatitis affecting the sebaceous-gland-rich areas of skin, and may be itchy (ICD-10: L21). We estimate burden separately for atopic dermatitis, contact dermatitis, and seborrhoeic dermatitis in order to accommodate differences in the epidemiology and burden between these subtypes.

Input data

Model inputs

Data for dermatitis came from scientific literature and claims submitted for individuals to USA commercial insurance. The seborrhoeic dermatitis model additionally incorporated data from a primary database in Norway and from claims in Taiwan. A literature review was conducted in GBD 2016 for studies of the incidence and prevalence of dermatitis, the details of which are described in the appendix to GBD 2016, and the results of this review were used in GBD 2017. Inpatient data were regarded as inappropriate for this chronic, non-fatal condition that is primarily cared for in non-acute settings. Data from the Medical Expenditure Panel Survey (MEPS) in the United States in 2000–2009 (2) were included to inform the age pattern of the prevalence output. Data from the NHANES study and the NHIS study (both from the USA) were not extracted, as questions regarding dermatitis were too broad (ie, asked whether a respondent had experienced eczema or any other rash). The data for dermatitis were expanded based on recommendations of research articles and reviews by the skin expert group.

Data from outpatient encounters in the United States and Sweden were considered for inclusion but were found to violate established age patterns and regional trends and were excluded. Additional data were marked as outliers and excluded if we found them unreasonable when compared to regional, super-regional, and global rates. See descriptions of individual modelling approaches for more information.

		Prevalence	Incidence
Atopic dermatitis*	Site-years (total)	743	0
	Number of countries with data	94	0
	Number of GBD regions with data (out of 21 regions)	19	0
	Number of GBD super-regions with data (out of 7 super-regions)	7	0
Contact dermatitis*	Site-years (total)	179	8
	Number of countries with data	13	1
	Number of GBD regions with data (out of 21 regions)	6	1

	Number of GBD super-regions with data (out of 7 super-regions)	5	1
Seborrhoeic dermatitis**	Site-years (total)	371	7
	Number of countries with data	22	1
	Number of GBD regions with data (out of 21 regions)	11	1
	Number of GBD super-regions with data (out of 7 super-regions)	7	1

*Based on claims data refresh 3 ** Based on refresh 4 + TWN data from refresh 8

Severity splits and disability weights

The basis of the GBD disability weight survey assessments are lay descriptions of sequelae highlighting major functional consequences and symptoms. Severity was split into three levels of disfigurement with pain/itch. See below for a lay descriptions of the severity levels.

Sequela	Severity level	Lay description	DW (95% CI)
Mild atopic dermatitis	Disfigurement, level 1 with itch/pain	The person has a slight, visible physical deformity that is sometimes sore or itchy. Others notice the deformity, which causes some worry and discomfort.	0.027 (0.015–0.042)
Moderate atopic dermatitis	Disfigurement, level 2, with itch/pain	The person has a visible physical deformity that is sore and itchy. Other people stare and comment, which causes the person to worry. The person has trouble sleeping and concentrating.	0.188 (0.124–0.267)
Severe atopic dermatitis	Disfigurement, level 3, with itch/pain	The person has an obvious physical deformity that is very painful and itchy. The physical deformity makes others uncomfortable, which causes the person to avoid social contact, feel worried, sleep poorly, and think about suicide.	0.576 (0.401–0.731)

Mild contact dermatitis	Disfigurement, level 1 with itch/pain	The person has a slight, visible physical deformity that is sometimes sore or itchy. Others notice the deformity, which causes some worry and discomfort.	0.027 (0.015–0.042)
Moderate contact dermatitis	Disfigurement, level 2, with itch/pain	The person has a visible physical deformity that is sore and itchy. Other people stare and comment, which causes the person to worry. The person has trouble sleeping and concentrating.	0.188 (0.124–0.267)
Symptomatic seborrhoeic dermatitis	Disfigurement, level 1 with itch/pain	The person has a slight, visible physical deformity that is sometimes sore or itchy. Others notice the deformity, which causes some worry and discomfort.	0.027 (0.015–0.042)

Modelling strategy

DisMod-MR 2.1 was used to estimate prevalence by age, sex, year, and geography (subnational [select countries], country, region, super-region) for atopic dermatitis, contact dermatitis, and seborrhoeic dermatitis. Separate models were run for each cause.

Model parameters

<u>Atopic dermatitis</u>

Since our available data mostly contained information on prevalence, we specified additional expert priors to further inform analyses. The prior value on excess mortality was set to zero, and the prior value on remission was bounded to 0-0.2 (equivalent to five years to life time duration). Study-level covariates were used to adjust prevalence estimates from the Medical Expenditure Panel Survey (MEPS), USA claims data for 2000, 2010, and 2012, self-reported data, and administrative data toward the reference data, which were data from scientific literature based on clinical examination. To improve regional and global estimates, the minimum coefficient of variation was set at 0.4 and location random effects for Paraguay, Sweden, and England were restricted to [-0.25, 0.25], [-0.25, 0.25], and [-0.5, 0.5], respectively. A time window of ten years was used to determine which data points were used for a particular year of fit.

<u>Contact dermatitis</u>

Similar to atopic dermatitis, mostly prevalence data were available for contact dermatitis. Per expert advice, the remission parameter was set from 0.1 to 4, excess mortality was set to zero, and incidence was set to zero prior to age 6. Study-level covariates were used to adjust data from the Medical Expenditure Panel Survey (MEPS), outpatient data, administrative data, self-reported data, data based on a one-year recall, and claims data for 2000 and 2010 toward the values in the 2012 claims data. In order to improve model estimates, location random effects were added for Sweden [-2, 2], Norway [-2, 2], Denmark [0, 0.5], Italy [-2, 2], Germany [-2, 2], and France

[-0.5, 0]. A time window of 25 years was used to determine which data points were used for a particular year of fit.

Seborrhoeic dermatitis:

As with contact dermatitis, the available data were mostly prevalence estimates. Per expert advice, settings were placed on incidence as follows: 0-4 years = 0-0.1, and 60-100 = 0-0.01. Excess mortality was set to zero while a setting of 0.1-12 was placed on remission, implying a duration of one month to ten years. Prevalence estimates from USA claims data for 2000 and 2010, MEPS, and published studies that ascertained cases from administrative data were similar and used as a single reference case; published studies that ascertained cases by clinical exam were marked with a study-level covariate and adjusted toward the reference data.

	Study covariate	Parameter	Beta	Exp(beta)
Atopic dermatitis<br>Based on model #235847	MEPS	Prevalence	-1.09 (-1.23 to -0.96)	0.34 (0.29 to 0.38)
	Self-reported	Prevalence	0.41 (0.34 to 0.48)	1.51 (1.41 to 1.61)
	Administrative data	Prevalence	-0.32 (-0.55 to -0.09)	0.73 (0.58 to 0.91)
	Claims data - 2000	Prevalence	-1.9 (-1.99 to -1.85)	0.15 (0.14 to 0.16)
	Claims data - 2010	Prevalence	-1.51 (-1.6 to -1.45)	0.22 (0.20 to 0.23)
	Claims data - 2012	Prevalence	-1.47 (-1.57 to -1.41)	0.23 (0.21 to 0.24)
Contact dermatitis<br>Based on model #235850	MEPS	Prevalence	-0.35 (-0.78 to -0.021)	0.71 (0.46 to 0.98)
	Outpatient	Prevalence	-0.66 (-1.78 to -0.02)	0.52 (0.17 to 0.98)
	Recall 1 year	Prevalence	-0.17 (-0.99 to 0.71)	0.84 (0.37 to 2.04)
	Self-reported	Prevalence	1.68 (1.08 to 1.99)	5.38 (2.95 to 7.29)
	Administrative data	Prevalence	1.20 (-0.15 to 1.95)	3.33 (0.86 to 7.03)
	Claims data - 2000	Prevalence	0.32 (0.24 to 0.39)	1.37 (1.27 to 1.48)
	Claims data - 2010	Prevalence	0.53 (0.46 to 0.60)	1.70 (1.59 to 1.83)
Seborrhoeic dermatitis<br>Based on model #350792	Diagnosis physical exam	Prevalence	3.00 (2.99 to 3.00)	20.0 (19.8 to 20.1)

Summary of changes

Models of atopic dermatitis and contact dermatitis were substantially similar between GBD 2016 and GBD 2017. The model for seborrhoeic dermatitis was previously unstable due to sparse data. In GBD 2017, the model for seborrhoeic dermatitis added primary care data from Norway and claims data from Taiwan. Additionally, case-definitions in the literature were reviewed, and studies previously not marked with study-level covariates (and therefore treated as reference case by default in DisMod) were marked to indicate their case ascertainment by self-report, administrative data, or study-specific clinical exam. After incorporating new facilities and claims data and marking all literature studies with covariates, it was noted that studies using clinical exam were systematically higher than all other data sources in the same or nearby locations, and included implausibly high values inconsistent with clinically significant seborrhoeic dermatitis in a general population. Thus, claims, primary care, and administrative-data-based literature were used as a pooled reference case and literature using clinical exam were adjusted.

Psoriasis

Flowchart

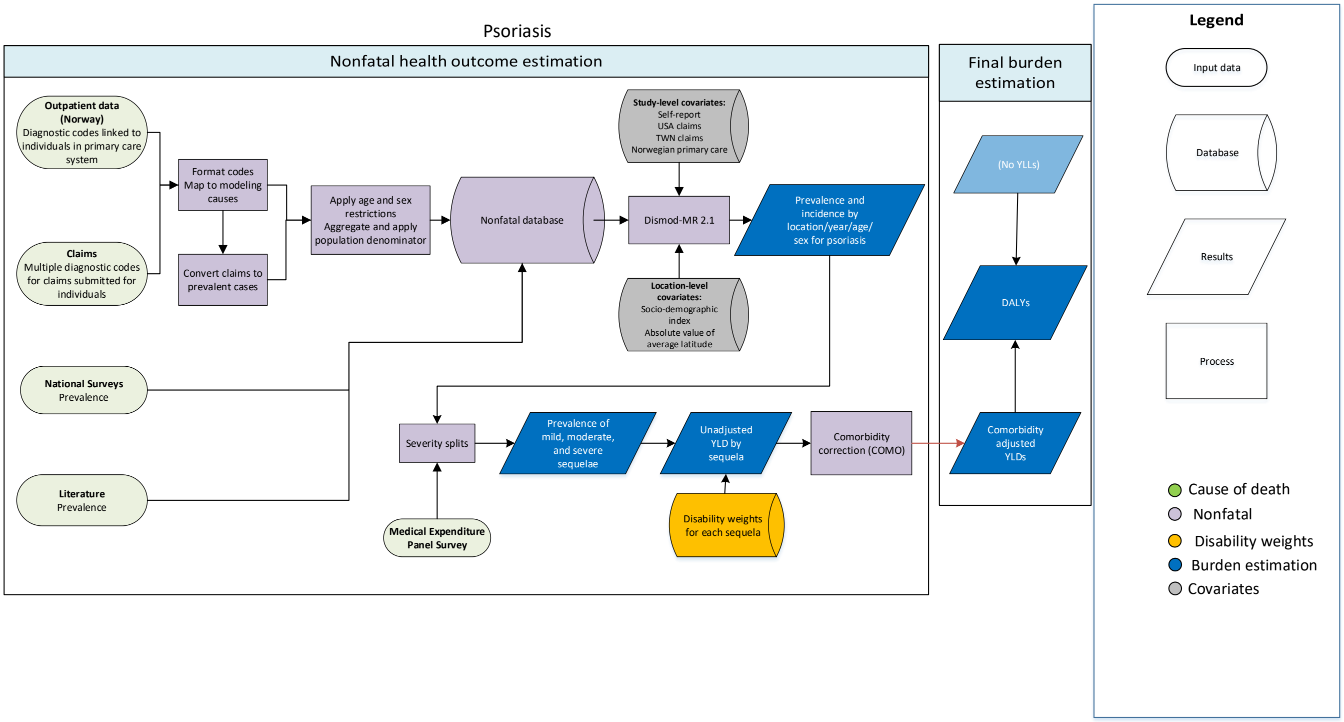

Case definition

Psoriasis is an autoimmune disease characterised by areas of raised, red skin with silvery scales, which may be itchy (ICD-10: L40, L41). It is an immune-mediated disease that involves inflammation and excess growth and abnormal behavior of certain skin cells.

Input data

Model inputs

The data for the psoriasis model come from scientific literature, several large, national surveys, claims data from the United States and Taiwan, and primary care data from Norway.

The literature used has been described in greater detail in previous GBD appendices. In brief, in the GBD 2010 study, a systematic review of the literature was conducted using PubMed and Google Scholar to capture epidemiological data for psoriasis. In GBD 2013, the 2010 search strategy was replicated to capture studies from 2012 to 2014, and it was repeated again in GBD 2016 to capture studies through October 1, 2016. The inclusion criteria stipulated that studies (1) must provide data on the incidence or prevalence of psoriasis; (2) must use samples representative of the general population (ie, samples derived from the experimental arm of clinical trials or based in dermatology clinics were excluded); (3) must use a sample size larger than 100; and (4) must provide sufficient information on study method and sample characteristics to assess the quality of the study.

Surveys used include the Medical Expenditure Panel Survey (MEPS) in the United States for 2000–2009, the Australian National Health Survey 1995–1996, 2001, 2004–2005, 2007–2008, and the USA National Health and Nutrition Examination Survey (NHANES) in 2002 and 2005.

Claims data from the United States and Taiwan link claims for multiple inpatient and outpatient encounters to a single individual. An individual was extracted as a prevalent case if they had one or more inpatient or outpatient encounter with a psoriasis ICD code as any encounter diagnosis. Norwegian primary care data had diagnoses linked to individuals, which were extracted as prevalent cases.

Data from outpatient encounters from facilities in the United States and Sweden were considered for inclusion in the psoriasis database, but these data violated established regional trends and age distributions and were excluded. Data were further considered for exclusion if relatively high values in young age groups led to overestimation of subnational pseudo-random effects and poor model fit, or if we found them unreasonable when compared to regional, super-regional, and global rates, but no data for these models met these criteria for exclusion.

The tables below show the number of studies included in GBD 2017, as well as the number of countries and GBD world regions represented.

	Prevalence	Incidence
Site-years (total)	651	72
Number of countries with data	30	4
Number of GBD regions with data (out of 21 regions)	15	2
Number of GBD super-regions with data (out of 7 super-regions)	7	1

Based on facilities and claims data refresh 8

Severity splits and disability weights

The basis of the GBD disability weight survey assessments are lay descriptions of sequelae highlighting major functional consequences and symptoms. As was the case in GBD 2010, 2013, 2015, and 2016, disability weights used were for disfigurement with itch/pain, levels 1, 2, and 3.

Sequela	Severity level	Lay description	DW (95% CI)
Mild psoriasis	Disfigurement, level 1 with itch/pain	The individual has a slight, visible physical deformity that is sometimes sore or itchy. Others notice the deformity, which causes some worry and discomfort.	0.027 (0.015–0.042)
Moderate psoriasis	Disfigurement, level 2, with itch/pain	The individual has a visible physical deformity that is sore and itchy. Other people stare and comment, which causes the person to worry. The person has trouble sleeping and concentrating.	0.188 (0.124–0.267)
Severe psoriasis	Disfigurement, level 3, with itch/pain	The individual has an obvious physical deformity that is very painful and itchy. The physical	0.576 (0.401–0.731)

		deformity makes others uncomfortable, which causes the person to avoid social contact, feel worried, sleep poorly, and think about suicide.	

Modelling strategy

DisMod-MR 2.1, a Bayesian meta-regression tool, was used to estimate prevalence by age, sex, year, and geography (subnational [select countries], country, region, super-region) for psoriasis.

Data inputs for the psoriasis DisMod model include incidence and prevalence data as described above. Reference data for prevalence were from literature studies where cases were ascertained using administrative data or by study-specific physical examination. Study-level covariates were used to mark data obtained by self-report, primary care data from Norway, and claims data from the United States and Taiwan.

Psoriasis was modelled with remission set between 0.05 and 0.15, implying a duration between 6.6 and 20 years. This was in line with the available epidemiological data, expert opinion, and previous GBD work. Excess mortality was assumed to be zero. The datasets for psoriasis were sufficiently large to make use of a relatively short time window of ten years to determine which data points were used for a particular year of fit. Socio-demographic Index and absolute value of average latitude were used as location-level covariates to guide estimates for countries with few or no data.

Betas and exponentiated values (which can be interpreted as an odds ratio) are shown in the tables below for study-level covariates and location-level covariates.

Covariate type	Covariate	Parameter	Beta	Exp(beta)
Study-level	Self-report	Prevalence	-0.053 (-0.29 to 0.13)	0.95 (0.94–0.96)
Study-level	USA claims	Prevalence	-1.39 (-1.68 to -1.17)	0.15 (0.14–0.18)
Study-level	TWN claims	Prevalence	0.30 (0.19–0.41)	1.35 (1.20–1.51)
Study-level	Norwegian primary care data	Prevalence	-1.88 (-2 to -1.7)	0.15 (0.14–0.18)
Location-level	Socio-demographic Index	Prevalence	2.00 (1.99–2.00)	7.37 (7.29–7.39)
Location-level	Absolute value of average latitude	Prevalence	0.0030 (0.0019–0.0041)	1.00 (1.00–1.00)

Based on model 341099

Summary of changes

Compared to GBD 2016, the psoriasis model for GBD 2017 differs by adding additional years of United States claims data, adding Taiwan claims data, and adding primary care data from Norway. Fewer data were marked as outliers, and study-level covariates were marked on literature data that allowed adjustment toward the reference standard of extreme values previously marked as outliers.

Cellulitis

Flowchart

Cellulitis

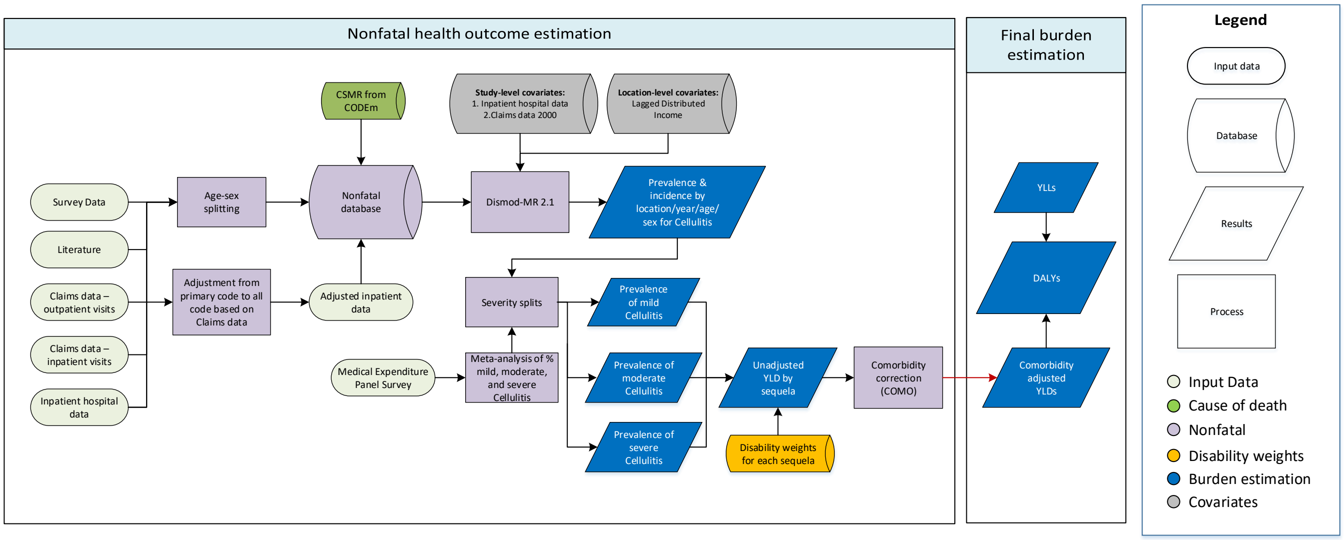

Case definition

Cellulitis was included in the GBD 2017 cause group of skin and subcutaneous conditions. Cellulitis is a skin disease marked by a bacterial infection that affects and spreads through the skin and soft tissues. Symptoms of cellulitis include pain, tenderness, and reddening in the affected area, fever, chills, and lymphadenopathy (ICD-10: L03) (1).

Input data

Model inputs

In the GBD 2010 study, a systematic review of the literature was conducted using PubMed and Google Scholar to capture epidemiological data for cellulitis. Due to lack of published data on the epidemiology of cellulitis, the literature search also included relevant incidence data from national inpatient or outpatient records in Europe, North America, and Latin America. When years in the national data from the hospital records overlapped, inpatient and outpatient data were summed together in an effort to better estimate the population incidence of cellulitis. The final dataset also includes USA claims data and cause-specific mortality rates for cellulitis estimated by CODEm.

The inclusion criteria stipulated that studies (1) must be published between 1980 and 2012; (2) must provide data on the incidence or prevalence of cellulitis; (3) must use samples representative of the general population (ie, samples derived from the experimental arm of clinical trials or based in dermatology clinics were excluded); (4) must use a sample size larger than 100; and (5) must provide sufficient information on study method and sample characteristics to assess the quality of the study. For GBD 2013, the GBD 2010 search strategy was replicated to capture epidemiological studies published between 2012 and 2013. In addition, hospital inpatient data, data from USA claims for 2000 and 2010 through 2014 by state, and Taiwan claims data for 2016 were included in GBD

2017. Data were outliered or excluded if we found them unreasonable when compared to regional, super-regional, and global rates.

Table 1. Site-years of data by different measures and geographical coverage

	Incidence
Site-years (total)	1382
Number of countries with data	42
Number of GBD regions with data (out of 21 regions)	16
Number of GBD super-regions with data (out of 7 super-regions	7

Severity splits

The basis of the GBD disability weight survey assessments are lay descriptions of sequelae highlighting major functional consequences and symptoms. Severity splits for cellulitis were calculated via the Medical Expenditure Panel Survey (MEPS) regression and outlined in the table below.

Table 2. Severity level and lay description.

Sequela	Severity level	Lay description	DW (95% CI)
Mild cellulitis	Infectious disease, acute episode, mild	This person has a low fever and mild discomfort, but no difficulty with daily activities.	0.006 (0.002–0.012)
Moderate cellulitis	Infectious disease, acute episode, moderate	This person has a fever and aches, and feels weak, which causes some difficulty with daily activities.	0.051 (0.032–0.074)
Severe cellulitis	Infectious disease, acute episode, severe	This person has a high fever and pain, and feels very weak, which causes great difficulty with daily activities.	0.133 (0.088–0.19)

Modelling strategy

DisMod-MR 2.1, a Bayesian meta-regression tool, was used to estimate cellulitis prevalence by age, sex, year, and geography (subnational [select countries], country, region, super-region). Cellulitis was modelled with remission set between 12 and 30, implying a duration of 12 days to one month. This was in line with the available epidemiological data, expert opinion, and previous GBD work. The cellulitis dataset was sufficiently large to make use of a relatively short time window of five years to determine which data points were used for a particular year of fit.

Study-level covariates were used to adjust incidence derived from USA claims data for 2000 toward the level of other incidence data points, which were more representative of the general population. USA claims data for 2000 were set at a maximum of zero. Log-transformed lagged distributed income (LDI) was used as a country-level

covariate to guide estimates for locations with few or no data. LDI was restricted to a range of -0.5 to -0.1. We restricted location random effects to (-0.5, 0.5) across all 7 GBD super-regions.

Table 3. Study-level beta and exponentiated values

Covariate	Parameter	Beta	Exp(beta)
Claims data – 2000	Incidence	-0.43 (-0.45 to -0.4)	0.65 (0.64–0.67)

Table 4. Location-level beta and exponentiated values

Covariate	Parameter	Beta	Exp(beta)
Log (LDI)	Excess mortality rate	-0.5 (-0.5 to -0.5)	0.61 (0.61–0.61)

Pyoderma

Flowchart

Impetigo

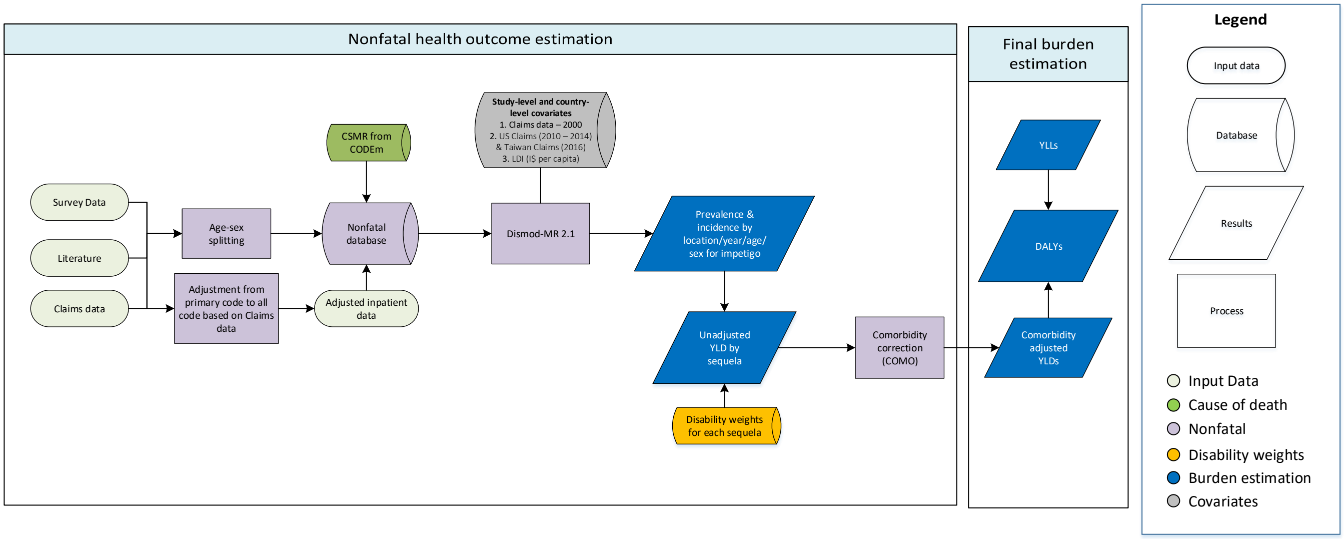

Abscess and Other Bacterial Skin Infections

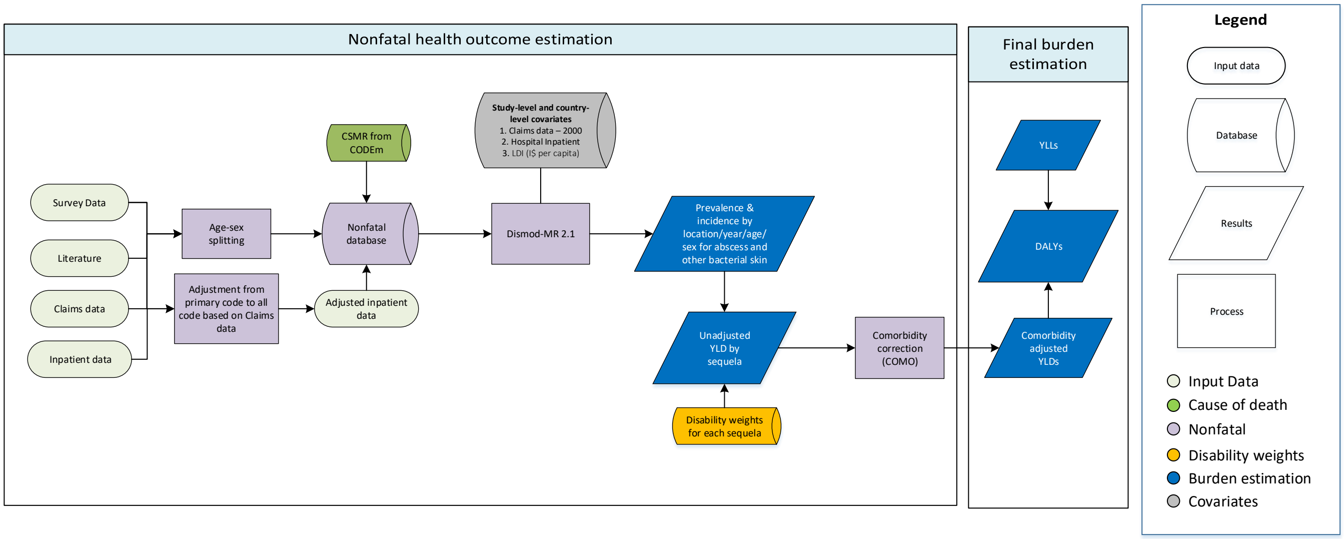

Case definition

Pyoderma refers to any skin disease that is pyogenic, ie, involves the development of pus. These include superficial bacterial conditions such as impetigo, furuncles, ulcers, and abscesses. In line with GBD 2016, for GBD 2017, pyoderma was modelled as two separate groups: impetigo, and abscess and other bacterial skin diseases. Impetigo is a highly contagious bacterial skin infection often characterized by red sores, which eventually leak pus or fluid (ICD-10: L01). An abscess is a collection of pus that builds up within the tissue of the body, with carbuncles and furuncles being examples of specific types of abscess. The abscess and other bacterial skin diseases group included all bacterial skin diseases except impetigo (ICD-10: L00, L02, L04, L05, L08).

Input data

Model inputs

For both impetigo and abscess and other bacterial skin diseases in GBD 2010, a literature review was conducted using PubMed and Google Scholar. The inclusion criteria were studies which were published between 1980 and 2010 and provided data on relevant disease incidence or prevalence. Exclusion criteria were studies with no incidence or prevalence data provided, not community- or population-based, outside of year range, sample size smaller than 100, experimental arm of clinical trial, papers that provided estimates rather than data, and studies that were based in dermatology clinics. The agreed-upon approach for pyoderma disease was to undertake a literature review every two years. For GBD 2016, the GBD 2013 search strategy was replicated to capture epidemiological studies published between 2014 and 2016. Hospital inpatient data were used as model inputs for abscesses and other bacterial skin diseases, but were omitted for impetigo, as the adjustment factor from primary diagnoses codes to all diagnoses codes were found to be implausible.

Table 1. Site-years of data by different measures and geographic coverage

Cause		Prevalence	Incidence
Impetigo	Site-years (total)	8	318
	Number of countries with data	6	4
	Number of GBD regions with data (out of 21 regions)	5	3
	Number of GBD super-regions with data (out of 7 super-regions	4	2
Abscess and other bacterial skin diseases	Site-years (total)	1387	2
	Number of countries with data	43	2
	Number of GBD regions with data (out of 21 regions)	16	2
	Number of GBD super-regions with data (out of 7 super-regions	7	1

Severity splits and disability weights

Information on the distribution of cases of impetigo and abscess and other bacterial skin diseases, asymptomatic, and within disfigurement levels 1 and 2, were obtained from the Medical Expenditures Panel Survey. The symptomatic cases were assigned the disability weight of a mild acute infectious disease case.

Table 2. Severity level and lay description

Sequela	Severity level	Lay description	DW (95% CI)
Impetigo	Infectious disease, acute episode, mild	The person has a low fever and mild discomfort, but no difficulty with daily activities.	0.006 (0.002–0.012)
Abscesses and other bacterial skin diseases	Infectious disease, acute episode, mild	The person has a low fever and mild discomfort, but no difficulty with daily activities.	0.006 (0.002–0.012)

Modelling strategy

DisMod-MR 2.1 was used to estimate prevalence by age, sex, year, and geography (country, region, super-region) for impetigo and abscess and other bacterial skin diseases. Separate models were run for each disease.

Impetigo: Per expert advice, we assumed a remission of 17 to 20, equating to a duration between approximately two and three weeks. A value prior was also placed on incidence, restricting the range between zero and one. In addition, study-level covariates were placed on incidence to adjust USA claims data for 2000 and 2010 through 2014 and Taiwan claims data for 2016 toward the reference literature data. A country-level covariate, log transformed lagged distributed income (I$ per capita), which represents a moving average of gross domestic product (GDP) over time, was also included to inform prevalence and excess mortality estimates. We also used the cause-specific mortality rates for pyoderma estimated using CODEm. We used a time window of five years to determine which data points were used for a particular year of fit.

Abscess and other bacterial skin diseases: Per expert advice, a remission setting of 17 to 30 was applied, which equated to a duration of two to six weeks. Study-level covariates adjusted incidence estimates from USA claims data for 2000 and hospital inpatient data toward the reference literature data, USA 2010–2014 claims data, and Taiwan 2016 claims data. We also used the cause-specific mortality rates for pyoderma estimated using CODEm. In addition, we used a log transformed lagged distributed income (I$ per capita) country covariate on excess mortality. We used a time window of five years to determine which data points were used for a particular year of fit and limited random effects to (-0.5, 0.5) for certain GBD regions and super-regions (South Asia, Central Asia, Latin America & Caribbean, North Africa & Middle East, and high-income) to improve model estimates.

Table 3. Beta and exponentiated values

Impetigo

Covariate type	Covariate	Parameter	beta	Exponentiated beta
Study-level covariate	Claims data – 2000	Incidence	0.026 (-1.98 to 2.00)	1.03 (0.14–7.39)
Study-level covariate	US Claims (2010 – 2014) & Taiwan Claims (2016)	Incidence	0.026 (-1.98 to 2.00)	1.03 (0.14–7.39)
Country covariate	LDI (I$ per capita)	Prevalence	0.054 (0.0011–0.21)	1.06 (1.00–1.23)
Country covariate	LDI (I$ per capita)	Excess mortality rate	-0.2 (-0.2 to -0.2)	0.82 (0.82–0.82)

Abscesses and other bacterial skin diseases

Covariate type	Covariate	Parameter	beta	Exponentiated beta
Study-level covariate	Hospital Inpatient	Incidence	-2.4 (-2.4 to -2.4)	0.091 (0.091–0.091)
Study-level covariate	Claims data - 2000	Incidence	-0.31 (-0.34 to -0.29)	0.73 (0.71–0.75)
Country covariate	LDI (I$ per capita)	Excess mortality rate	-0.5 (-0.54 to -0.46)	0.61 (0.58–0.63)

Scabies

Flowchart

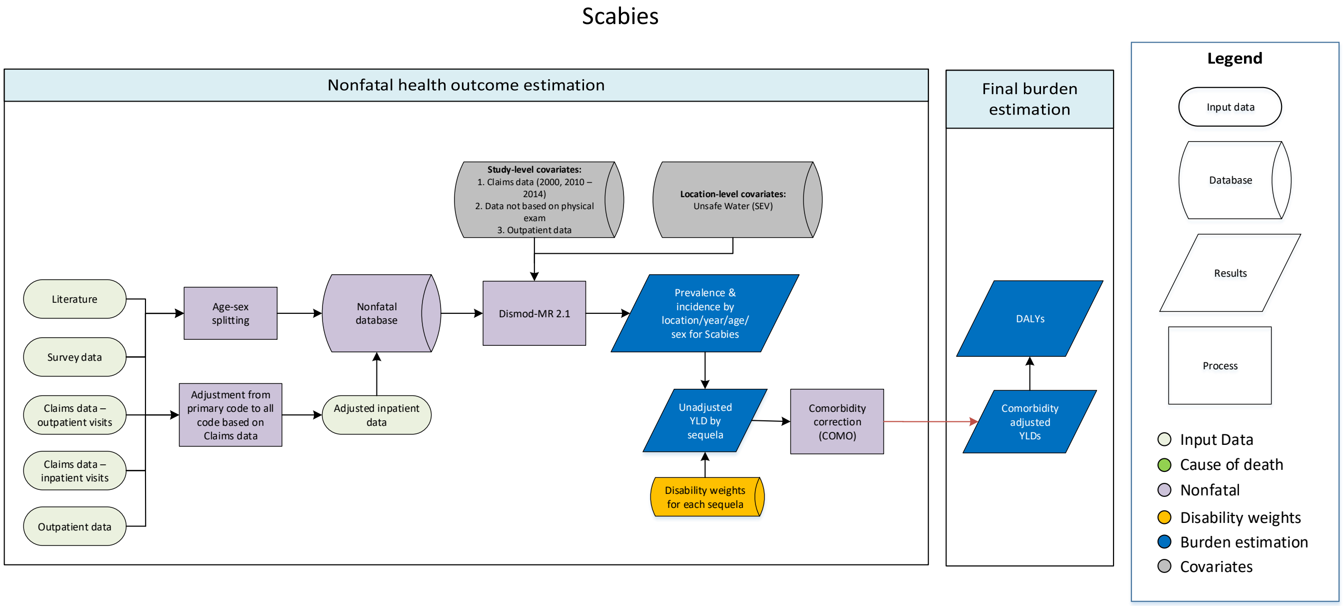

Case definition

Scabies was included in the GBD 2017 cause group of skin and subcutaneous conditions. According to the International Classification of Diseases (ICD-10), scabies is a skin disease caused by the microscopic mite *Sarcoptes scabiei*. The main symptom is an itchy, pimple-like rash (ICD-10: B86) (1).

Input data

Model inputs

In the GBD 2010 study, a systematic review of the literature was conducted using PubMed and Google Scholar to capture epidemiological data for scabies. The inclusion criteria stipulated that studies (1) must be published between 1980 and 2012; (2) must provide data on the incidence or prevalence of scabies; (3) must use samples representative of the general population (ie, samples derived from the experimental arm of clinical trials or based in dermatology clinics were excluded); (4) must use a sample size larger than 100; and (5) must provide sufficient information on study method and sample characteristics to assess the quality of the study. For GBD 2013, the GBD 2010 search strategy was replicated to capture epidemiological studies published between 2011 and 2013. The agreed-upon approach for scabies was to undertake a literature review every two years. Therefore, we updated the systematic review through October 6, 2016, for GBD 2016. Additionally, USA claims data from 2000 and 2010 through 2014 were included in GBD 2017. Data were outliered or excluded if we found them unreasonable when compared to regional, super-regional, and global rates.

Table 1. Site-years of data by different measures and geographical coverage

	Prevalence	Incidence
Site-years (total)	381	20
Number of countries with data	30	4
Number of GBD regions with data (out of 21 regions)	17	3
Number of GBD super-regions with data (out of 7 super-regions	7	2

Scabies was assigned the disability weight for disfigurement level 1. The disability weights used for GBD 2016 were also used for GBD 2017.

Table 2. Severity level and lay descriptions

Severity level	Lay description	DW (95% CI)
Disfigurement, level 1 with itch/pain	The individual has a slight, visible physical deformity that is sometimes sore or itchy. Others notice the deformity, which causes some worry and discomfort.	0.027 (0.015–0.042)

Modelling strategy

DisMod-MR 2.1, a Bayesian meta-regression tool, was used to estimate scabies prevalence by age, sex, year, and geography (subnational [select countries], country, region, super-region).

Scabies was modelled with remission set between 2.5 and 3.5, implying four to five months of duration, and excess mortality was assumed to be zero. This was in line with the available epidemiological data, expert opinion, and previous GBD work.

The datasets for scabies were sufficiently large to make use of a relatively short time window of five years to determine which data points were used for a particular year of fit. Additionally, to improve estimation across all regions, we restricted location random effects to (-0.25, 0.25) in Cambodia, Mali, Nepal, Fiji, Timor-Leste, Vanuatu, the Oceania, Southeast Asia, and East Asia GBD regions, and the corresponding super-region. We also restricted the random effect in Kenya (0, 0.5). We used the unsafe water SEV (summary exposure value) as a location-level covariate and set the minimum coefficient of variation at 0.4.

Study-level covariates were used to adjust prevalence derived from outpatient data, USA claims data for 2000 and 2010 through 2014, and data not based on clinical exams toward the level of other prevalence and incidence data points, which were more representative of the general population.

Table 3. Study-level beta and exponentiated values

Covariate	Parameter	Beta	Exp(beta)
Claims data - 2000	Prevalence	-1.84 (-1.95 to -1.78)	0.16 (0.14–0.17)
Claims data – 2010 - 2014	Prevalence	-1.29 (-1.39 to -1.23)	0.28 (0.25–0.29)
Data were not based on physical examinations	Prevalence	-1.54 (-1.92 to -1.21)	0.21 (0.15–0.30)
Outpatient	Prevalence	1.92 (1.80–2.00)	6.82 (6.05–7.35)

Table 4. Location-level beta and exponentiated values

Covariate	Parameter	Beta	Exp(beta)
Unsafe water (SEV)	Prevalence	1.96 (1.85–2.00)	7.11 (6.37–7.37)

Fungal skin diseases

Flowchart

Tinea Capitis

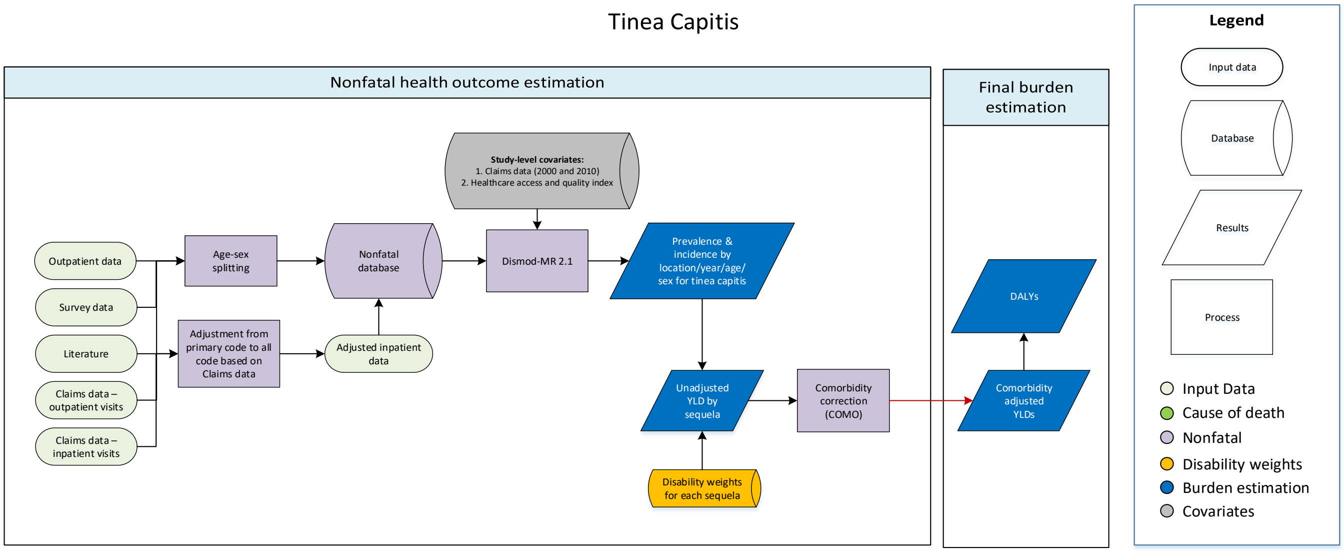

Other Fungal Skin Diseases

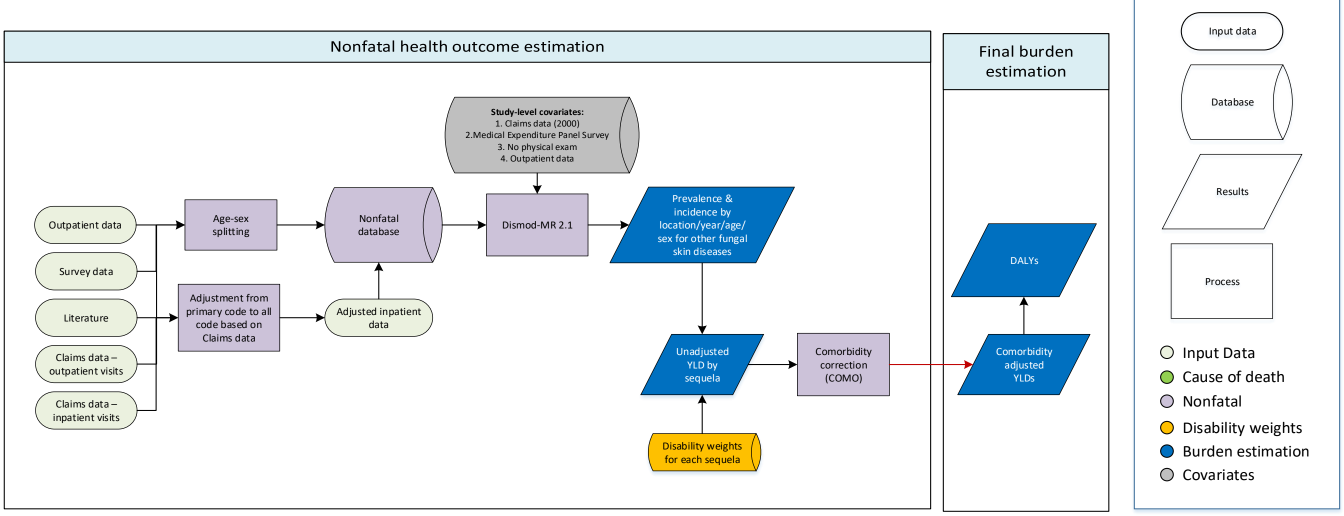

Case definition

Fungal diseases were included in the GBD 2017 cause group of skin and subcutaneous conditions and consisted of tinea capitis and a residual group of "any" other fungal disease. Similar to GBD 2016, tinea capitis was modelled separately from the other fungal skin diseases. This was done to better accommodate differences in burden between tinea capitis and other subtypes of fungal skin diseases.

Tinea capitis is a fungal infection of the scalp and associated hair. It is characterised by the appearance of thickened scaly swellings or as expanding raised red rings (ringworm), mainly caused by species of *Microsporum*, *Trichophyton,* and *Epidermophyton* (ICD-10: B35.0) (1).

The residual group of "any" other fungal skin disease included any fungal skin disease that was specifically not tinea capitis or onychomycosis (ie, fungal nail infection). The ICD-10 (1) list of other fungal skin diseases includes tinea manuum (ICD-10: B35.2), or hand ringworm; tinea pedis (ICD-10: B35.3), or athlete's foot; tinea corporis (ICD-10:B35.4), or ringworm of the body; tinea imbricata (ICD-10:B35.5), a superficial fungal infection limited to parts of Asia and Central America; tinea cruris (ICD-10:B35.6), also known as dhobi itch, groin ringworm, or jock itch. In GBD 2016, we added dermatophytosis (ICD-10:B35.9).

Input data

Model inputs

For GBD 2010, a systematic review of the literature using PubMed and Google Scholar was conducted to capture epidemiological data for fungal skin diseases. The literature search also included any relevant data from the Medical Expenditure Panel Survey (MEPS) in the United States in 2000–2009. The inclusion criteria stipulated that studies (1) must be published between 1980 and 2012; (2) must provide data on the incidence or prevalence of fungal skin diseases; (3) must use samples representative of the general population (ie, samples derived from the experimental arm of clinical trials or based in dermatology clinics were excluded); (4) must use a sample size larger than 100; and (5) must provide sufficient information on study method and sample characteristics to assess the quality of the study. For GBD 2013, the GBD 2010 search strategy was replicated to capture epidemiological studies published between 2012 and 2013. For GBD 2017, the GBD 2010 search strategy was replicated in PubMed to capture epidemiological studies published between 2013 and 2017. Data were outliered or excluded if we found them unreasonable when compared to regional, super-regional, and global rates.

Table 1. Site-years of data by different measures and geographic coverage

Cause		Prevalence
Tinea capitis	Site-years (total)	348
	Number of countries with data	20
	Number of GBD regions with data (out of 21 regions)	8
	Number of GBD super-regions with data (out of 7 super-regions	4
Other Fungal Skin Diseases	Site-years (total)	356
	Number of countries with data	24
	Number of GBD regions with data (out of 21 regions)	12
	Number of GBD super-regions with data (out of 7 super-regions	6

In addition, data from USA claims for 2000 and 2010 through 2014 by state were included for both tinea capitis and other fungal skin diseases. We also used hospital outpatient data for other fungal skin diseases but decided not to use it for tinea capitis because we decided it would not be representative of true prevalence, and variation between countries in the proportion of true prevalent cases captured in hospital inpatient and outpatient data would likely vary more than can be captured by a single crosswalk in DisMod-MR 2.1. For tinea capitis, we compared the rates in the outpatient data from Norway, Sweden, Canada, and the USA and found implausibly large differences with the rates from the claims data.

Severity splits

The basis of the GBD disability weight survey assessments are lay descriptions of sequelae highlighting major functional consequences and symptoms. The same disability weight was used for both tinea capitis and other fungal skin diseases.

Table 2. Severity level and lay description

Severity level	Lay description	DW (95% CI)
Infectious disease, acute episode, mild	The person has a low fever and mild discomfort but no difficulty with daily activities.	0.006 (0.002–0.012)

Modelling strategy

DisMod-MR 2.1, a Bayesian meta-regression tool, was used to estimate tinea capitis and other fungal skin diseases prevalence by age, sex, year, and geography (subnational [select countries], country, region, super-region). Separate models were run for tinea capitis and other fungal skin diseases.

Tinea capitis. To help inform the distribution of tinea capitis across the lifespan, excess mortality was set at zero, remission was set at 0.5 to 4, and incidence was set at 0 to 0.02 between 20 and 100 years. This was in agreement with the available prevalence data and expert advice. We made use of a relatively long time window of 20 years to determine which data points were used for a particular year of fit. This means that for the year 2000, for instance, DisMod-MR 2.1 incorporated all data points ranging from 1980 to present to estimate prevalence. Study-level covariates in the final model included adjustments for USA claims data for 2000. In addition, we limited random effects for sub-Saharan Africa (-2,3) and Western Europe (-0.1, 1) to improve model estimates.

Other fungal skin diseases. The modelling strategy was similar to that for tinea capitis, with remission set between 0.33 and 4. As Medical Expenditure Panel Survey (MEPS) data points were included in this model, we used a study-level covariate to adjust estimates derived from MEPS, outpatient data, and USA claims data for 2000 toward the level of prevalence observed in USA claims data from 2012 and the literature. We also included a covariate to add uncertainty for data that did not arise from physical examination and limited random effects for Nigeria (-0.5, 0.5) and Ethiopia (-0.5, 0.5) to improve model estimates.

Table 3. Study-level beta and exponentiated values

Cause	Covariate	Covariate Type	Parameter	Beta	Exp(beta)

Tinea capitis	Claims data – 2000	Study-level	Prevalence	-0.13 (-0.18 to -0.086)	0.88 (0.84–0.92)
	Healthcare Access and Quality index	Country-level	Prevalence	-0.087 (-0.1 to -0.064)	0.92 (0.91–0.94)
Other fungal skin diseases	MEPS	Study-level	Prevalence	-0.97 (-1.11 to -0.84)	0.38 (0.33–0.43)
	Outpatient	Study-level	Prevalence	-0.99 (-1.98 to 0)	0.37 (0.14–1.00)
	Claims data – 2000	Study-level	Prevalence	-0.21 (-0.22 to -0.19)	0.81 (0.80–0.83)
	Data were not based on physical examinations*	Study-level	Prevalence	1.40 (0.69 to 1.97)	4.05 (2.00–7.19)

** Covariate on the variance.*

Viral skin diseases

Flowchart

Viral Skin Diseases

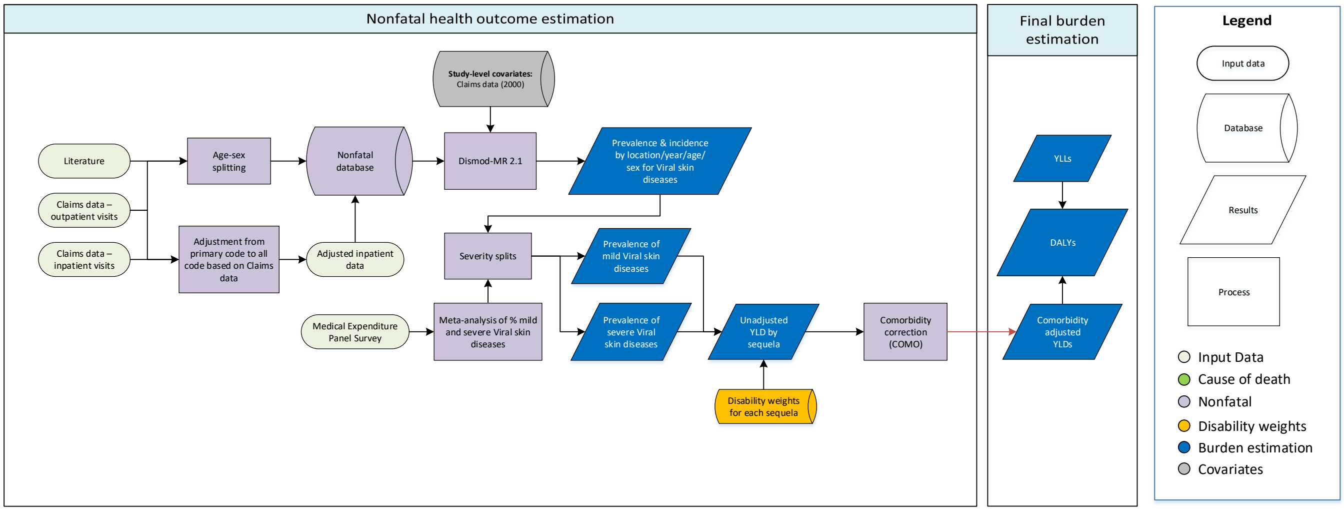

Case definition

Viral skin diseases consist of viral warts and molluscum contagiosum. Viral warts are raised growths on the surface of the skin caused by an infection with the human papillomavirus (ICD-10: B07). Molluscum contagiosum is a viral infection of the skin or occasionally of the mucous membranes characterised by the appearance of waxy, dome-shaped nodules. It is caused by a DNA poxvirus called the molluscum contagiosum virus (ICD-10: B08.1) (1). In GBD 2017, we modelled viral warts and molluscum contagiosum separately in order to better accommodate differences in burden between the subtypes of viral skin diseases.

Input data

Model inputs

In the GBD 2010 study, a systematic review of the literature was conducted using PubMed and Google Scholar to capture epidemiological data for viral skin diseases. Due to lack of published data on the epidemiology of viral skin diseases, the literature search also included relevant incidence data from national inpatient or outpatient records in the USA. The inclusion criteria stipulated that studies (1) must be published between 1980 and 2012; (2) must provide data on the incidence or prevalence of viral warts or molluscum contagiosum; (3) must use samples representative of the general population (ie, samples derived from the experimental arm of clinical trials or based in dermatology clinics were excluded); (4) must use a sample size larger than 100; and (5) must provide sufficient information on study method and sample characteristics to assess the quality of the study. For GBD 2013, the GBD 2010 search strategy was replicated to capture epidemiological studies published between 2012 and 2013. For GBD 2017, the GBD 2010 search strategy was replicated to capture epidemiological studies published between 2013 and 2017. Data were outliered or excluded if we found them unreasonable when compared to regional, super-regional, and global rates.

Table 1. Site-years of data by different measures and geographic coverage

Cause		Prevalence	Incidence
Viral warts	Site-years (total)	336	16
	Number of countries with data	23	4
	Number of GBD regions with data (out of 21 regions)	11	2
	Number of GBD super-regions with data (out of 7 super-regions	6	1
Molluscum contagiosum	Site-years (total)	311	5
	Number of countries with data	5	2
	Number of GBD regions with data (out of 21 regions)	4	1
	Number of GBD super-regions with data (out of 7 super-regions	4	1

Data from USA claims for 2000 and 2010 through 2014 by state were included in GBD 2017, where appropriate. See descriptions of individual modelling approaches for more information.

Severity splits

In GBD 2017, cases of both disorders were allocated a distribution between mild acute infectious disease and disfigurement level 2. The severity splits and disability weights used in GBD 2016 were also applied in GBD 2017.

Table 2. Sequela and disability weight

Sequela	Severity level	Lay description	DW (95% CI)
Mild viral warts	Infectious disease, acute episode, mild	The person has a low fever and mild discomfort, but no difficulty with daily activities.	0.006 (0.002–0.012)

Severe viral warts	Disfigurement, level 2	The person has a visible physical deformity that causes others to stare and comment. As a result, the person is worried and has trouble sleeping and concentrating.	0.067 (0.044–0.096)
Mild molluscum contagiosum	Infectious disease, acute episode, mild	The person has a low fever and mild discomfort but no difficulty with daily activities.	0.006 (0.002–0.012)
Severe molluscum contagiosum	Disfigurement, level 2	The person has a visible physical deformity that causes others to stare and comment. As a result, the person is worried and has trouble sleeping and concentrating.	0.067 (0.044–0.096)

Modelling strategy

For GBD 2017, DisMod-MR 2.1 was used to estimate prevalence, by age, sex, year, and geography (subnational [select countries], country, region, super-region) for viral warts and molluscum contagiosum. Separate models were run for each disease, as illustrated throughout this cause write-up.

Viral warts. Viral warts were modelled with excess mortality set to 0 and remission set between 0.25 and 2, implying a duration of 0.5 to 4 years. This was in line with the levels of prevalence and incidence data, as well as expert opinion. A number of additional settings were used to ensure that DisMod-MR 2.1 sufficiently followed available data points. Incidence was restricted to a maximum of 0.1, and we made use of a relatively long time window of 25 years to determine which data points were used for a particular year of fit. We limited the prevalence random effects for Andean Latin America (-0.2, 0.2) in order to improve model fit. Study-level covariates were used to adjust USA claims data for 2000 toward other data points.

Molluscum contagiosum. As available data only contained information on prevalence and incidence, we specified additional expert priors to further inform analyses. Molluscum contagiosum was modelled with excess mortality set to 0 and remission set between 0.5 and 2, implying a duration of 0.5 to 2 years. This was in line with the available epidemiological data, expert opinion, and previous GBD work. We used a time window of 25 years to determine which data points to include for a particular year of fit. Due to data heterogeneity, we restricted the location random effects to between -0.5 and 0.5 for the Netherlands and select GBD regions and super-regions (Southern Latin America, sub-Saharan Africa, high-income, South Asia, and Southeast Asia, East Asia, and Oceania). Study-level covariates were used to adjust data USA claims data for 2000 toward other data points.

Table 3. Study-level beta and exponentiated values

	Covariate	Parameter	Beta	Exp(beta)
Viral warts	Claims data – 2000	Prevalence	-0.1 (-0.19 to 0.031)	0.90 (0.83–1.03)
Molluscum contagiosum	Claims data – 2000	Prevalence	-1.66 (-1.68 to -1.64)	0.19 (0.19–0.19)

Acne vulgaris

Flowchart

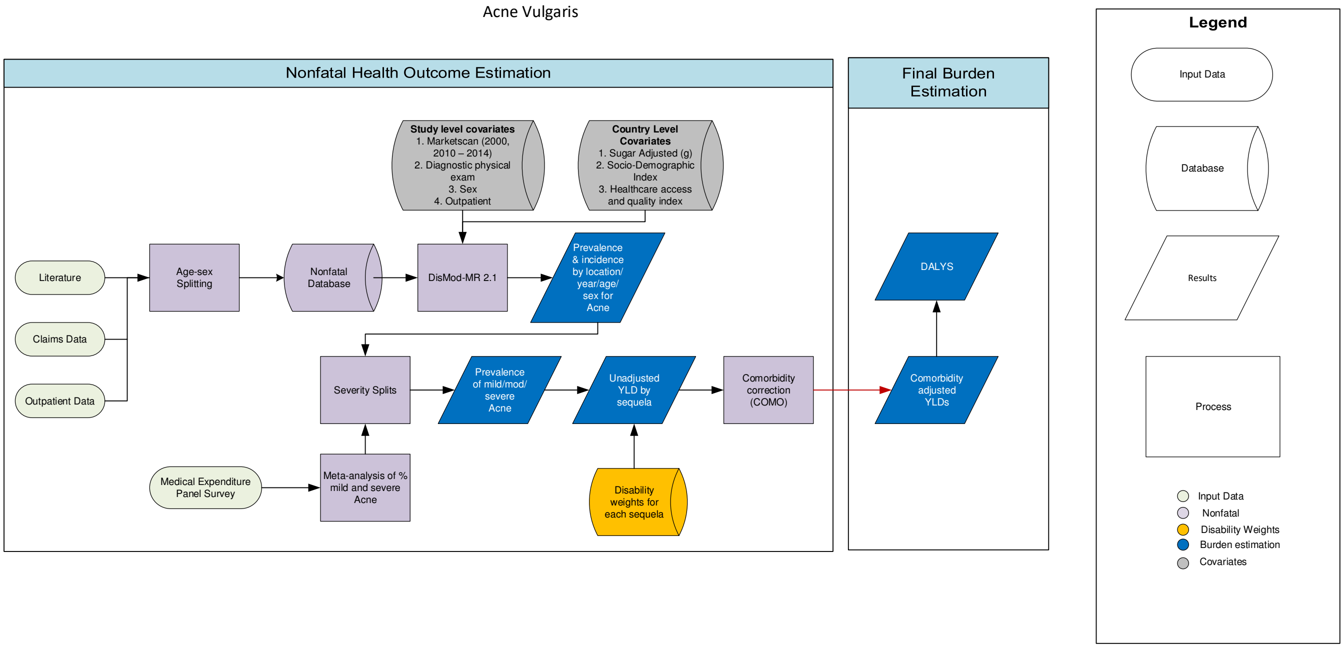

Case definition

Acne vulgaris was included in the GBD 2017 cause group of skin and subcutaneous conditions. Acne vulgaris (or acne) is a chronic inflammatory disease of the pilosebaceous unit associated with an increase in sebum secretion. Included in the GBD 2017 modelling were cases meeting ICD-10 diagnostic criteria for acne vulgaris (ICD-10: L70, excluding L70.4).

Input data

Model inputs

In the GBD 2010 study, a systematic review of the literature was conducted using PubMed and Google Scholar to capture epidemiological data for acne vulgaris. The inclusion criteria stipulated that studies (1) must be published between 1980 and 2012; (2) must provide data on the incidence or prevalence of acne vulgaris; (3) must use samples representative of the general population (ie, samples derived from the experimental arm of clinical trials or based in dermatology clinics were excluded); (4) must use a sample size larger than 100; and (5) must provide sufficient information on study method and sample characteristics to assess the quality of the study. The agreed-upon approach for acne vulgaris was to undertake a literature review every two years. For GBD 2016, the GBD 2010 search strategy was replicated in PubMed to capture epidemiological studies published between 2013 and 2016. An additional literature search was carried out for GBD 2017 for USA data to better inform the DisMod crosswalk from USA claims data to literature data and capture any studies missed in previous literature searches.

This literature search also replicated the GBD 2010 search strategy and captured studies published between 1980 and 2017.

USA claims data from 2000 and 2010 through 2014 are included in this model, along with outpatient data from both Norway. USA outpatient data were not used due to implausibly high adjusted values.

Table 1. Site-years of data by different measures and geographic coverage

	Prevalence
Site-years (total)	541
Number of countries with data	33
Number of GBD regions with data (out of 21 regions)	16
Number of GBD super-regions with data (out of 7 super-regions	7

Severity splits

The table below illustrates the severity level, lay description, and disability weight for acne. In GBD 2016, we added two additional severity levels – disfigurement 2 and disfigurement 3. The disability weight of each severity of acne was applied across 40% of the total prevalence cases to account for biases in outpatient utilisation. The remaining 60% of prevalence cases were considered mild cases (disfigurement level 1). These proportions were generated using the ratio of patients seeking care captured from claims data, to all individuals captured in literature surveying the general population.

For GBD 2017, we performed a meta-analysis of five literature studies that gave the proportion of people seeking care for acne to replace the estimate from claims data. This was done to get a more geographically diverse estimate of care-seeking acne behavior. The disability weight of mild was applied to those who did not seek care and a small fraction of those seeking care and moderate and severe were applied to those seeking care.

Table 2. Severity level and lay description

Sequela	Severity level	Lay description	DW (95% CI)
Mild acne vulgaris	Disfigurement, level 1	The individual has a slight, visible physical deformity that others notice, which causes some worry and discomfort.	0.011 (0.005–0.021)
Moderate acne vulgaris	Disfigurement, level 2	The individual has a visible physical deformity that causes others to stare and comment. As a result, the person is worried and has trouble sleeping and concentrating.	0.067 (0.044–0.096)

Severe acne vulgaris	Disfigurement, level 3	The individual has an obvious physical deformity that makes others uncomfortable, which causes the person to avoid social contact, feel worried, sleep poorly, and think about suicide.	0.405 (0.275–0.546)

Modelling strategy

DisMod-MR 2.1 was used to estimate prevalence by age, sex, year, and country for acne vulgaris.

Since our available data only contained information on prevalence, we specified additional expert priors to further inform analyses. We assumed zero excess mortality and remission from 0.38 to 0.6, implying a duration of approximately two to three years. This was in line with the available epidemiological data, expert opinion, and previous GBD work. A value prior of zero was set for incidence between the ages of 0 and 6, and 61 and 100. We used a time window of five years to determine which data points were used for a particular year of fit.

Study-level covariates were used to adjust prevalence from USA claims data for 2000 and 2010 through 2014 toward the level of other prevalence data points, which were more representative of the general population. A study-level covariate was applied to data that were not based on physical examinations.

In addition, sociodemographic status, sugar consumption, and the Healthcare Access and Quality index were used as country-level covariates to guide estimates for countries with few or no data.

The table below indicates the study covariates, parameters, beta, and exponentiated beta values used in GBD 2017.

Table 3. Beta and exponentiated values

**Covariate type**	**Covariate**	**Parameter**	**beta**	**Exponentiated beta**
Study-level covariate	Outpatient	Prevalence	-1.96 (-2 to -1.84)	0.14 (0.14–0.16)
Study-level covariate	Diagnostic physical exam	Prevalence	1.72 (0.78–2.00)	5.60 (2.18–7.39)
Study-level covariate	All MarketScan, year 2000	Prevalence	-0.55 (-1.29 to 0.13)	0.58 (0.28–1.13)
Study-level covariate	All MarketScan, years 2010–2014	Prevalence	-0.43 (-1.04 to 0.15)	0.65 (0.35–1.16)
Country covariate	Socio-demographic Index	Prevalence	0.98 (0.92–1.00)	2.66 (2.50–2.72)
Country covariate	sugar adjusted (g)	Prevalence	0.00030 (-0.0053 to 0.0072)	1.00 (0.99–1.01)

Country covariate	Healthcare Access and Quality index	Prevalence	0.018 (-0.008 to 0.028)	1.02 (0.99–1.03)

Alopecia areata

Flowchart

Alopecia Areata

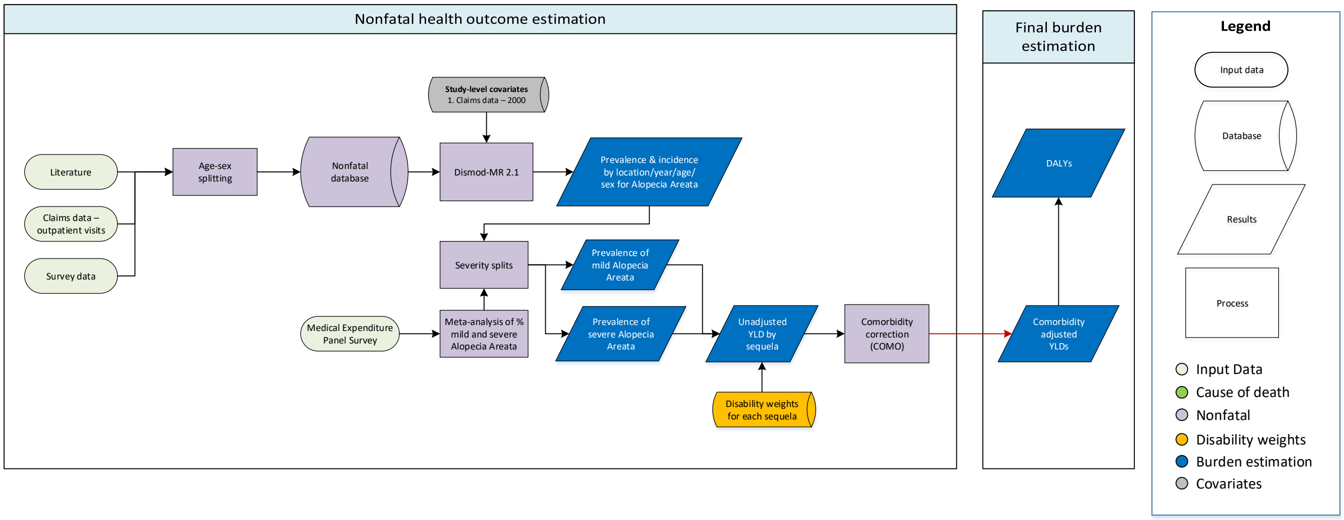

Case definition

Alopecia areata was included in the GBD 2017 cause group of skin and subcutaneous conditions. Alopecia areata is an autoimmune disease that results in hair loss on the scalp and other parts of the body. Included in the GBD disease modelling were cases meeting ICD-10 diagnostic criteria for alopecia (ICD-10: L63).

Input data

Model inputs

In the GBD 2016 study, a systematic review of the literature was conducted using PubMed to expand the GBD dataset (1980–2014) with new epidemiological data for Alopecia areata between 2014 and 2016. The inclusion criteria stipulated that studies (1) must provide data on the incidence or prevalence of alopecia areata; (2) must use samples representative of the general population (ie, samples derived from the experimental arm of clinical trials or based in dermatology clinics were excluded); (3) must use a sample size larger than 100; and (4) must provide sufficient information on study method and sample characteristics to assess the quality of the study. The agreed-upon approach for alopecia areata was to undertake a literature review every two years. Additionally, USA claims data from 2000 and 2010 through 2014 are included in this model.

Table 1. Site-years of data by different measures and geographical coverage

	Prevalence	Incidence
Site-years (total)	329	1
Number of countries with data	14	1
Number of GBD regions with data (out of 21 regions)	10	1
Number of GBD super-regions with data (out of 7 super-regions	6	1

Severity splits & disability weights

The table below illustrates the sequelae, severity level, lay description, and disability weights associated with Alopecia areata.

Table 2. Severity level and lay description

Sequela	Severity level	Lay description	DW (95% CI)
Mild alopecia areata	Disfigurement, level 1	The individual has a slight, visible physical deformity that others notice, which causes some worry and discomfort.	0.011 (0.005–0.021)
Severe alopecia areata	Disfigurement, level 2	The individual has a visible physical deformity that causes others to stare and comment. As a result, the person is worried and has trouble sleeping and concentrating.	0.067 (0.044–0.096)

Modelling strategy

DisMod-MR 2.1 was used to estimate prevalence by age, sex, year, and country for alopecia areata. We assumed zero excess mortality and remission priors implying a minimum duration of seven months. This was in line with the available epidemiological data, expert opinion, and previous GBD work. We used a time window of 20 years to determine which data points were used for a particular year of fit.

Study-level covariates were used to adjust prevalence derived from US claims data for 2000 toward the level of other prevalence data, which were more representative of the general population. To improve estimation across all regions, the minimum global coefficient of variation was set at 0.1. In addition, significant sex differences were observed in the USA claims data, resulting in a higher prevalence in females compared to males, likely due to

more females seeking health consultations for alopecia areata compared to males. To minimise this effect, we set the sex covariate to zero, but this had minimal impact on the global estimates.

Table 3. Beta and exponentiated values

Covariate type	Covariate	Parameter	beta	Exponentiated beta
Study-level covariate	Claims data – 2000	Prevalence	-0.72 (-0.76 to -0.69)	0.48 (0.47–0.50)

Pruritus

Flowchart

Pruritus

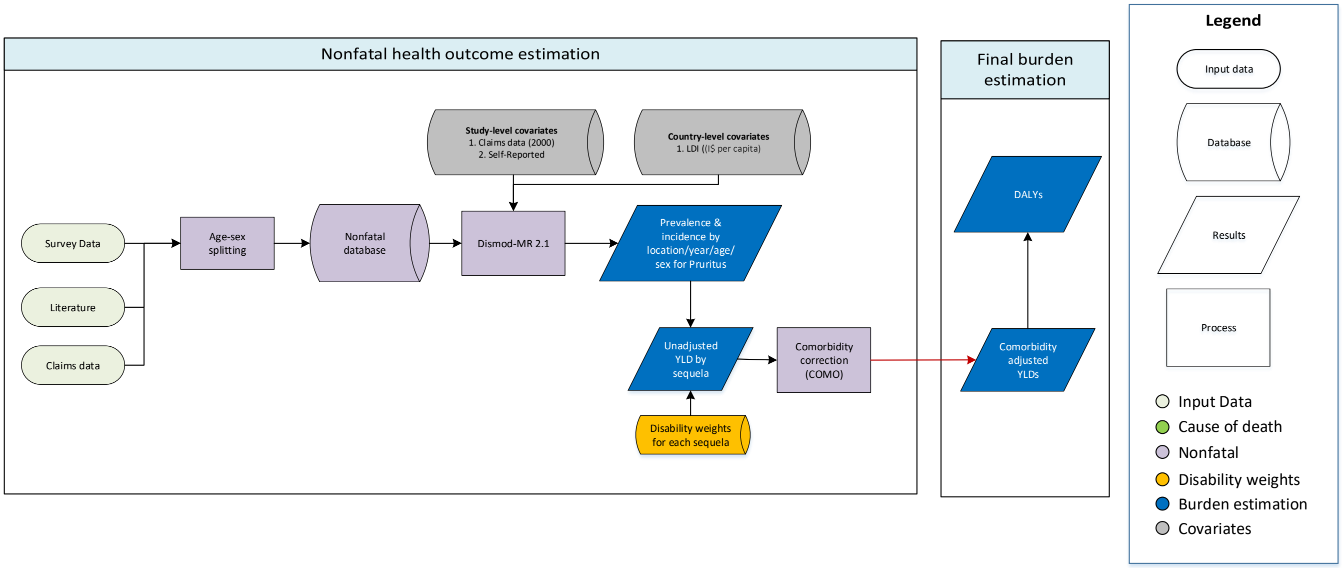

Case definition

Pruritus was included in the GBD 2017 cause group of skin and subcutaneous conditions. Pruritus (or itching) can be a symptom of a condition or disease. Included in the GBD disease modelling were cases meeting ICD-10 diagnostic criteria for pruritus (ICD-10: L29).

Input data

Model inputs

In the GBD 2010 study, a systematic review of the literature was conducted using PubMed and Google Scholar to capture epidemiological data for pruritus. The inclusion criteria stipulated that studies (1) must be published between 1980 and 2012; (2) must provide data on the incidence or prevalence of pruritus; (3) must use samples representative of the general population (ie, samples derived from the experimental arm of clinical trials or based in dermatology clinics were excluded); (4) must use a sample size larger than 100; and (5) must provide sufficient information on study method and sample characteristics to assess the quality of the study. The agreed-upon approach for pruritus was to undertake a literature review every two years. For GBD 2016, the GBD 2010 search strategy was replicated in PubMed to capture epidemiological studies published between 2013 and 2016. Additionally, USA claims data from 2000 and 2010 through 2014 were included.

Table 1. Site-years of data by different measures and geographic coverage

	Prevalence
Site-years (total)	311
Number of countries with data	5
Number of GBD regions with data (out of 21 regions)	3
Number of GBD super-regions with data (out of 7 super-regions	2

Severity splits

The table below illustrates the severity level, lay description, and disability weight for pruritus.

Table 2. Severity level and lay description

Sequela	Severity level	Lay description	DW (95% CI)
Pruritus	Disfigurement, level 1	The individual has a slight, visible physical deformity that others notice, which causes some worry and discomfort.	0.011 (0.005–0.021)

Modelling strategy

DisMod-MR 2.1 was used to estimate prevalence by age, sex, year, and country for pruritus.

Per expert advice, remission was set from 0.2 to 1, implying a duration of three months to one years. We used a time window of 25 years to determine which data points were used for a particular year of fit.

Study-level covariates were used to adjust prevalence derived from self-reported literature and USA claims data for 2000 toward the level of USA claims data 2010 through 2014, which were more representative of the general population.

The data were extremely heterogeneous. Therefore, the random effects were constrained to (-0.2, 0.2) and lagged distributed income was used as a country-level covariate to guide estimates for countries with few or no data.

Table 3. Beta and exponentiated values

**Covariate type**	**Covariate**	**Parameter**	**beta**	**Exponentiated beta**
Study-level covariate	Self-reported	Prevalence	1.62 (0.93–1.99)	5.04 (2.53–7.34)

Study-level covariate	Claims data – 2000	Prevalence	-0.74 (-0.77 to -0.71)	0.48 (0.46–0.49)
Country covariate	LDI (I$ per capita)	Prevalence	0.073 (-0.08 to 0.17)	1.08 (0.92–1.19)

Urticaria

Flowchart

Urticaria

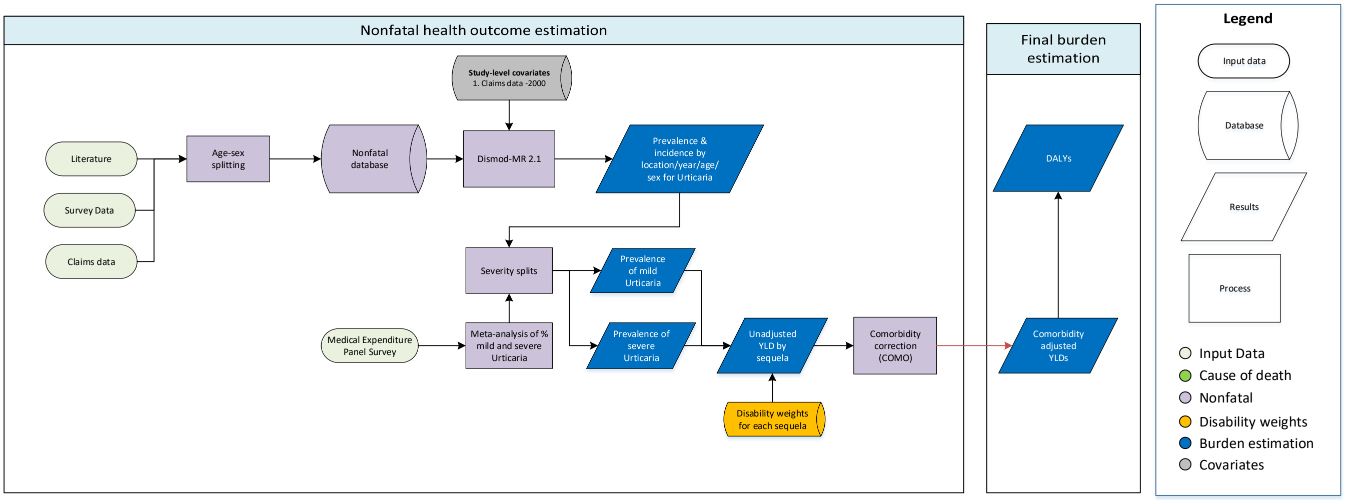

Case definition

Urticaria was included in the GBD 2017 cause group of skin and subcutaneous conditions. Urticaria (hives) refers to a skin reaction that causes itchy, raised bumps. Included in the GBD disease modelling were cases meeting ICD-10 diagnostic criteria for urticaria (ICD-10: L50).

Input data

Model inputs

In the GBD 2010 study, a systematic review of the literature was conducted using PubMed and Google Scholar to capture epidemiological data for urticaria. The inclusion criteria stipulated that studies (1) must be published between 1980 and 2012; (2) must provide data on the incidence or prevalence of urticaria; (3) must use samples representative of the general population (ie, samples derived from the experimental arm of clinical trials or based in dermatology clinics were excluded); (4) must use a sample size larger than 100; and (5) must provide sufficient information on study method and sample characteristics to assess the quality of the study.

The agreed-upon approach for urticaria was to undertake a literature review every two years. For GBD 2016, the GBD 2010 search strategy was replicated to capture epidemiological studies published between 2013 and 2016. Additionally, USA claims data from 2000 and 2010 through 2014 were included in the data used for GBD 2017.

The table below illustrates the data inputs used in GBD 2017 by number of studies, geographic location, and prevalence/incidence.

Table 1. Site-years of data by different measures and geographic coverage

	Prevalence	Incidence
Site-years (total)	334	7
Number of countries with data	20	1
Number of GBD regions with data (out of 21 regions)	9	1
Number of GBD super-regions with data (out of 7 super-regions	6	1

Severity splits & disability weights

The table below illustrates the severity level, lay description, and disability weight for urticaria.

Table 2. Severity level and lay descriptions

Sequela	Severity level	Lay description	DW (95% CI)
Mild urticaria	Disfigurement, level 1 with itch/pain	The person has a slight, visible physical deformity that is sometimes sore or itchy. Others notice the deformity, which causes some worry and discomfort.	0.027 (0.015–0.042)
Severe urticaria	Disfigurement, level 2, with itch/pain	The person has a visible physical deformity that is sore and itchy. Other people stare and comment, which causes the person to worry. The person has trouble sleeping and concentrating.	0.188 (0.124–0.267)

Modelling strategy

DisMod-MR 2.1 was used to estimate prevalence by age, sex, year, and country for urticaria.

The available data were mainly composed of prevalence estimates with a few incidence data points. For GBD 2017, we made both prevalence and incidence estimates. We used a time window set to 25 years. We set excess mortality to zero and remission between 0.5 to 2, implying a duration between ½ and ⅔ years. In addition, location random effects were constrained to (-0.3, 0.3). USA claims data from year 2000 were adjusted toward

the most complete USA 2012 set. Specific data points were outliered if they were overestimates or underestimates in comparison to country, regional, and global patterns.

The table below illustrates the covariate used in the modelling process, as well as associated parameters, beta, and exponentiated beta (confidence interval) values for GBD 2017.

Table 3. Study covariate

**Covariate type**	**Covariate**	**Parameter**	**beta**	**Exponentiated beta**
Study-level covariate	Claims data – 2000	Prevalence	-0.22 (-0.24 to -0.2)	0.80 (0.79–0.82)

Decubitus ulcer

Flowchart

Decubitus Ulcer

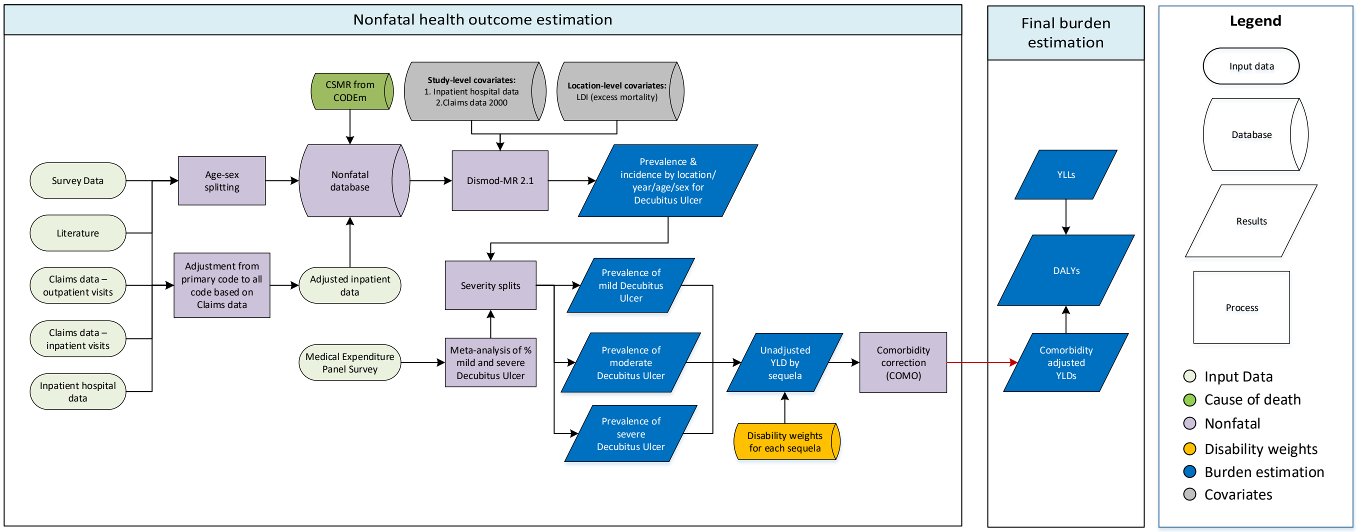

Case definition

Decubitus ulcer was included in the GBD 2017 cause group of skin and subcutaneous conditions. Decubitus ulcer, also known as pressure ulcer/sore, is an injury to the skin and underlying tissue resulting from an obstruction of blood flow due to pressure on the skin. Included in the GBD modelling were cases meeting ICD-10 criteria for decubitus ulcer (ICD-10: L89) (1).

Input data

Model inputs

In the GBD 2010 study, a systematic review of the literature was conducted using PubMed and Google Scholar to capture epidemiological data for decubitus ulcer. The inclusion criteria stipulated that studies (1) must be published between 1980 and 2012; (2) must provide data on the incidence or prevalence of decubitus ulcer; (3) must use samples representative of the general population (ie, samples derived from the experimental arm of clinical trials or based in dermatology clinics were excluded); (4) must use a sample size larger than 100; and (5) must provide sufficient information on study method and sample characteristics to assess the quality of the study. The data from literature were sparse but contained both prevalence and incidence estimates. For GBD 2013, the GBD 2010 search strategy was replicated to capture epidemiological studies published between 2012 and 2013. The available data were from high-income countries. Hospital inpatient, USA claims data from 2000 and 2010 through 2014, and Taiwan claims data for 2016 were also used for GBD 2017. The final dataset also included cause-specific mortality rates for decubitus ulcer estimated by CODEm. Data were outliered or excluded if we found them unreasonable when compared to regional, super-regional, and global rates.

Table 1. Site-years of data by different measures and geographic coverage

	Incidence
Site-years (total)	1429
Number of countries with data	41
Number of GBD regions with data (out of 21 regions)	15
Number of GBD super-regions with data (out of 7 super-regions	7

Severity splits

In line with GBD 2016, decubitus ulcer was assigned the disability weight, disfigurement with itch/pain, levels 1, 2, and 3. The disability weights used for GBD 2015 were based on disfigurement only.

Table 2. Severity level and lay descriptions

Sequela	Severity level	Lay description	DW (95% CI)
Mild decubitus ulcer	Disfigurement, level 1 with itch/pain	The person has a slight, visible physical deformity that is sometimes sore or itchy. Others notice the deformity, which causes some worry and discomfort.	0.027 (0.015–0.042)
Moderate decubitus ulcer	Disfigurement, level 2, with itch/pain	The person has a visible physical deformity that is sore and itchy. Other people stare and comment, which causes the person to worry. The person has trouble sleeping and concentrating.	0.188 (0.124–0.267)
Severe decubitus ulcer	Disfigurement, level 3, with itch/pain	The person has an obvious physical deformity that is very painful and itchy. The physical deformity makes others uncomfortable, which causes the person to avoid social contact, feel worried, sleep poorly, and think about suicide.	0.576 (0.401–0.731)

Modelling strategy

DisMod-MR 2.1 was used to estimate prevalence by age, sex, year, and geography (subnational [select countries], country, region, super-region) for decubitus ulcer. Per expert advice, remission was set from 3 to 4, implying a duration of three to four months. This was based on the assumption that remission does not change with treatment. These values were also in line with the available epidemiological data, expert opinion, and previous GBD work. The decubitus ulcer dataset was sufficiently large to make use of a relatively short time window of five years to determine which data points were used for a particular year of fit.

Study-level covariates were used to adjust incidence derived from hospital inpatient data and USA claims data for 2000 toward the level of other incidence data points, which were more representative of the general population. We excluded estimates less than 10E-6. Log-transformed lagged distributed income (LDI) was used as a country - level covariate on excess mortality to guide estimates for countries with few or no data.

Table 3. Beta and exponentiated values

Study covariate	Parameter	Beta	Exp(beta)
Hospital Inpatient	Incidence	-0.011 (-0.091 to -0.000029)	0.99 (0.91–1.00)
Claims data – 2000	Incidence	-0.26 (-0.29 to -0.22)	0.77 (0.75–0.81)

Table 4. Location-level beta and exponentiated values

Country-level covariate	Parameter	Beta	Exp(beta)
LDI (log-transformed)	Excess mortality rate	-0.5 (-0.5 to -0.5)	0.61 (0.61–0.61)

Other skin and subcutaneous diseases

Flowchart

Other Skin and Subcutaneous Diseases (OSSD)

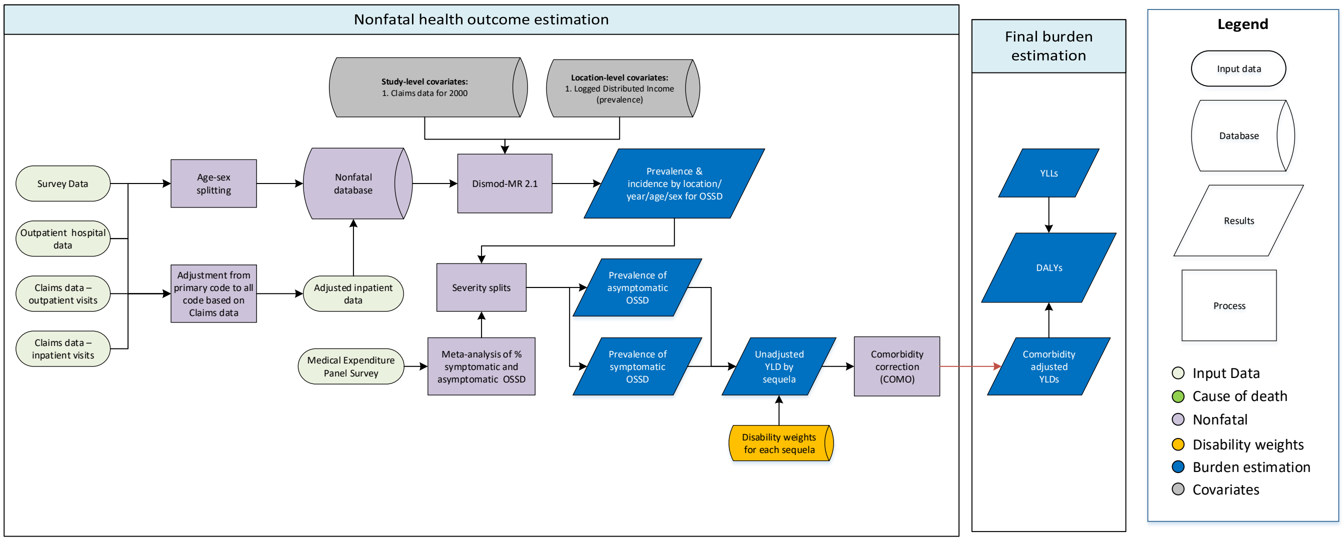

Case definition

The other skin and subcutaneous diseases category encompassed a large group of skin conditions not captured in the other skin categories. We included cases meeting the following ICD-10 diagnostic criteria: other viral infections characterized by skin and mucous membrane lesions, not elsewhere classified (B08), unspecified viral infection characterized by skin and mucous membrane lesions (B09), pediculosis and phthiriasis (B85), myiasis (B87), other infestations (B88), sarcoidosis of skin (D86.3), porphyria cutanea tarda (E80.1), other and unspecified porphyria (E80.2), pemphigus (L10), other acantholytic disorders (L11), pemphigoid (L12), other bullous disorders (L13), bullous disorders in diseases classified elsewhere (L14), lichen simplex chronicus and prurigo (L28), pityriasis rosea (L42), lichen planus (L43), other papulosquamous disorders (L44), papulosquamous disorders in diseases classified elsewhere (L45), exfoliation due to erythematous conditions according to extent of body surface involved (L49), erythema multiforme (L51), erythema nodosum (L52), other erythematous conditions (L53), erythema in diseases classified elsewhere (L54), other acute skin changes due to ultraviolet radiation (L56), skin changes due to chronic exposure to nonionising radiation (L57), other disorders of skin and subcutaneous tissue related to radiation (L59), nail disorders (L60), nail disorders in diseases classified elsewhere (L62), androgenic alopecia (L64), other nonscarring hair loss (L65), cicatricial alopecia [scarring hair loss] (L66), hair color and hair shaft abnormalities (L67), hypertrichosis (L68), rosacea (L71), follicular cysts of skin and subcutaneous tissue (L72), other follicular disorders (L73), eccrine sweat disorders (L74), apocrine sweat disorders (L75), vitiligo (L80), other disorders of pigmentation (L81), seborrhoeic keratosis (L82), acanthosis nigricans (L83), corns and callosities (L84), other epidermal thickening (L85), keratoderma in diseases classified elsewhere (L86), transepidermal elimination disorders (L87), atrophic disorders of skin (L90), hypertrophic disorders of skin (L91),

granulomatous disorders of skin and subcutaneous tissue (L92), other localised connective tissue disorders (L94), vasculitis limited to skin, not elsewhere classified (L95), and other disorders of skin and subcutaneous tissue in diseases classified elsewhere (L99).

Input data

Model inputs

In the GBD 2010 study, a systematic review of the literature was conducted using PubMed and Google Scholar to capture epidemiological data for skin diseases not captured in the other skin categories. The inclusion criteria stipulated that studies (1) must be published between 1980 and 2012; (2) must provide data on the incidence or prevalence; (3) must use samples representative of the general population (ie, samples derived from the experimental arm of clinical trials or based in dermatology clinics were excluded); (4) must use a sample size larger than 100; and (5) must provide sufficient information on study method and sample characteristics to assess the quality of the study. For GBD 2013, the GBD 2010 search strategy was replicated to capture epidemiological studies published between 2012 and 2013. Data from USA claims for 2000 and 2010 through 2014 by US state were included in GBD 2017 as well. Data were outliered or excluded if we found them unreasonable when compared to regional, super-regional, and global rates.

Table 1. Site-years of data by different measures and geographic coverage

	Prevalence
Site-years (total)	306
Number of countries with data	1
Number of GBD regions with data (out of 21 regions)	1
Number of GBD super-regions with data (out of 7 super-regions	1

Severity split & disability weight

Skin and other subcutaneous diseases were assigned the disability weight for disfigurement level 1.

Table 2. Severity level and lay description

Sequela	Severity level	Lay description	DW (95% CI)
Asymptomatic other skin and subcutaneous diseases	Asymptomatic		0
Symptomatic other skin and subcutaneous diseases	Disfigurement, level 1	The person has a slight, visible physical deformity that others notice, which causes some worry and discomfort.	0.011 (0.005–0.021)

Modelling strategy

DisMod-MR 2.1, a Bayesian meta-regression tool, was used to estimate prevalence by age, sex, year, and geography (subnational [select countries], country, region, super-region) for skin and other subcutaneous diseases.

We assumed remission of one, implying a duration of 12 months. Similar to GBD 2016, we used a time window of 25 years to determine which data points were used for a particular year of fit.

Study-level covariates, which were used to adjust prevalence, were derived from USA claims data for 2000 toward the level of other prevalence data, which were more representative of the general population. In addition, log-transformed lagged distributed income was used as a country-level covariate to guide estimates for countries with few or no data.

Table 3. Study-level beta and exponentiated values

Covariate	Parameter	Beta	Exp(beta)
Claims data – 2000	Prevalence	-0.28 (-0.29 to -0.27)	0.76 (0.75–0.77)

Table 4. Location-level beta and exponentiated values

Covariate	Parameter	Beta	Exp(beta)
LDI (log-transformed)	Prevalence	0.28 (0.25–0.30)	1.33 (1.28–1.35)

Other sense organ diseases

Flowchart

Other sense organ diseases

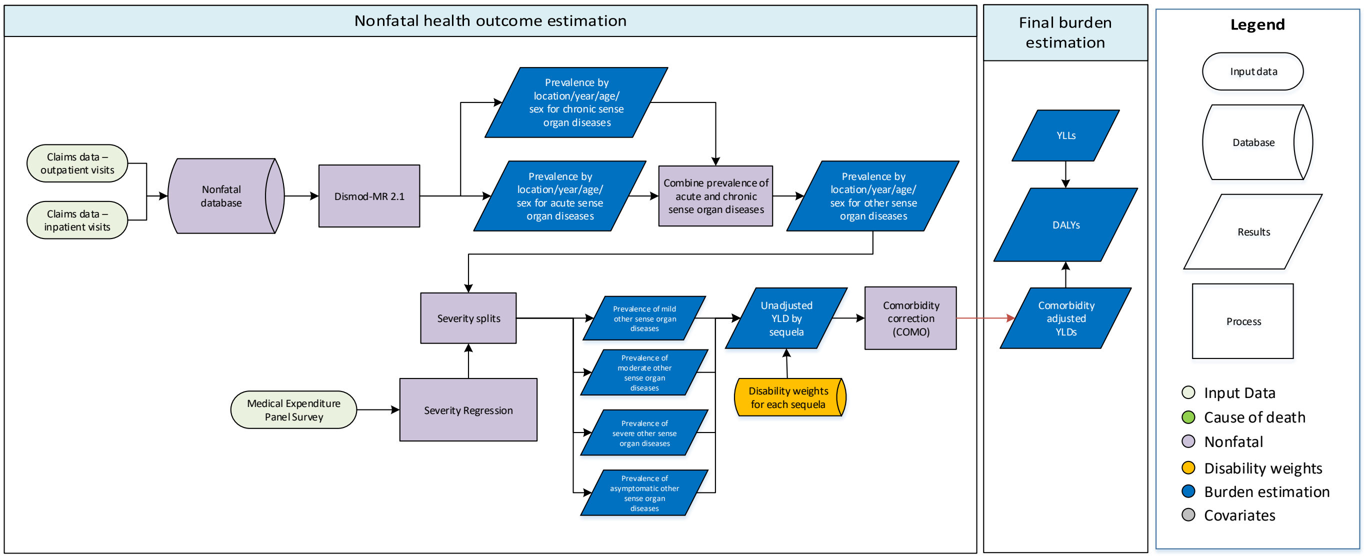

Case definition

Other sense organ disease is a residual cause capturing both acute and chronic conditions that do not map to other causes, but lead to non-trivial morbidity. These include the following ICD codes: 077, 360, 364, 370-77, 379, 380, 386, and 388, which encompass a plethora of eye and ear disorders and conditions.

077	Other diseases of conjunctiva due to viruses and chlamydiae
360	Disorders of the globe
364	Disorders of iris and ciliary body
370-77	Keratitis, Corneal opacity and other disorders of cornea, Disorders of conjunctiva, Inflammation of eyelids, Other disorders of eyelids, Disorders of lacrimal system, Disorders of the orbit, Disorders of optic nerve and visual pathways
379	Other disorders of eye
380	Disorders of external ear
386	Vertiginous syndromes and other disorders of vestibular system
388	Other disorders of ear

Input data

Model inputs

For GBD 2017, we used USA claims data to model other sense organ diseases, since these conditions would not appear in inpatient hospital data. ICD-9 codes were assigned at the five-digit level to either acute or chronic conditions as listed elsewhere in the Appendix table 4.

The tables below show the number of site-years of data included in GBD 2017, as well as the number of countries, regions, and super-regions represented.

**Acute other sense organ disease**	
	**Incidence**
**Site-years (total)**	357
**Number of countries with data**	1
**Number of GBD regions with data (out of 21 regions)**	1
**Number of GBD super-regions with data (out of 7 super-regions)**	1

**Chronic other sense organ disease**	
	**Prevalence**
**Site-years (total)**	357
**Number of countries with data**	1
**Number of GBD regions with data (out of 21 regions)**	1
**Number of GBD super-regions with data (out of 7 super-regions)**	1

Changes to input data for GBD 2017

Changes in how we processed MarketScan data for GBD 2017 had a significant effect on chronic other sense organ disease. For GBD 2017, a symptomatic case of chronic other sense organ disease was defined as a person who had at least one inpatient visit or two visits (either outpatient or inpatient) that were coded to other sense organ disease. In GBD 2016, one visit (either outpatient or inpatient) would have sufficed to define a case of chronic other sense organ disease. The changes led to a significant drop in the prevalence, and therefore the burden, of chronic other sense organ disease in GBD 2017 versus GBD 2016.

Severity splits & disability weights

The basis of the GBD disability weight survey assessments are lay descriptions of sequelae highlighting major functional consequences and symptoms. Severity splits for other sense organ diseases were calculated via the Medical Expenditure Panel Survey (MEPS) regression and outlined in the table below. For GBD 2017, we changed “severe chronic other sense organ diseases” to be “moderate chronic other sense organ diseases” and “moderate chronic other sense organ diseases” to be “mild chronic other sense organ diseases” as we felt that “mild” and “moderate” more accurately described the health states of the chronic other sense organ disease sequela.

<u>Chronic:</u>

Severity	Proportion	Health state	Disability weight
Mild	0.37 (0.30–0.42)	This person has slight physical deformity which	0.011 (0.005–0.021)

		causes some worry and discomfort	
Moderate	0.21 (0.15–0.28)	Vertigo	0.113 (0.074–0.158)
Asymptomatic	0.42 (0.41–0.44)	Asymptomatic	N/A

<u>**Acute**</u>

Severity	Proportion	Health state	Disability weight
Mild	0.30 (0.23–0.37)	This person has low fever and mild discomfort but no difficulty with daily activities	0.006 (0.002–0.012)
Moderate	0.25 (0.18–0.32)	This person has slight physical deformity which causes some worry and discomfort	0.011 (0.005–0.021)
Asymptomatic	0.45 (0.43–0.46)	Asymptomatic	N/A

Modelling strategy

For GBD 2017, hospital data were extracted separately for the chronic and acute conditions included in other sense organ diseases. The chronic data were extracted as prevalence, and acute as incidence. We then ran two separate DisMod-MR 2.1 models. In both models, to correct for systematically lower data from 2000 MarketScan claims, we used a study-level covariate to crosswalk the 2000 data. Since the only data source is from the United States, we did not use any country-level covariates in this model.

We then aggregated chronic and acute prevalence outputs, resulting in the prevalence of other sense organ diseases by country, age, year, and sex.

Rheumatoid arthritis

Flowchart

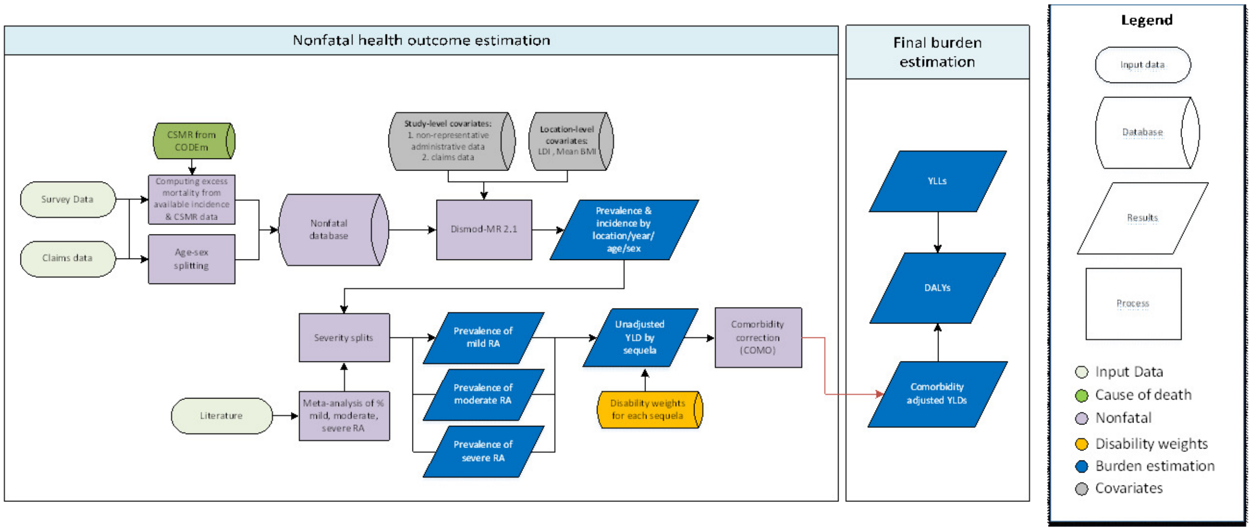

Case definition

Rheumatoid arthritis (RA) is a systemic autoimmune disorder that causes pain and swelling of the joints. While RA is known to affect internal organs in addition to the joints, these extra-articular effects are not factored into the disability weights (DW) used in GBD. The reference case definition for rheumatoid arthritis is based on the 1987 criteria by the American College of Rheumatology (ACR 1987)[1] which stipulate seven diagnostic criteria, of which four need to be satisfied for a diagnosis. Criteria 1 through 4 must have been present for at least six weeks (see table below). For RA, ICD-10 codes are M05, M06, and M08, and ICD-9 codes are 714.0–714.9.

Input data

Model inputs

For GBD 2010, a systematic review of the prevalence of RA throughout the world was conducted. Ovid MEDLINE, EMBASE, CINAHL, CAB abstracts, WHOLIS, and SIGLE databases were searched using the following search terms: (rheumatoid arthritis OR rheumatic disease* OR rheumatism) AND (prevalen* OR inciden* OR cross-sectional OR cross sectional OR epidemiol* OR survey OR population-based OR population based OR population study OR population sample OR cohort OR follow-up OR follow up OR longitudinal OR regist* OR data collection). Opportunistically, we added scientific literatures and population surveys encountered for GBD 2015 and GBD 2016. The most recent PubMed search was conducted in GBD 2017 using the following search terms: ("Arthritis, Rheumatoid"[Mesh] AND ("Prevalence"[Mesh] OR "Incidence"[Mesh])) NOT (Meta-Analysis[ptyp] OR Letter[ptyp] OR Editorial[ptyp] OR Case Reports[ptyp] OR Review[ptyp] OR Controlled Clinical Trial[ptyp]) AND ("2013/01/01"[PDAT] : "2018/1/10"[PDAT]. An age and sex split were applied to extracted data.

The exclusion criteria were:

1. Studies clearly not representative of the national population

2. Studies that were not population-based, eg, hospital or clinic-based studies
3. Studies that did not provide primary data on epidemiological parameters, eg, a commentary piece
4. Studies of a specific type of RA, eg, seropositive RA
5. Studies with a sample size of less than 150
6. Reviews

The table below shows the number site-years of literature studies included in GBD 2017, as well as the number of countries, GBD regions, and GBD super-regions represented.

	Prevalence	Incidence	Other
Site-years (total)	499	151	20
Number of countries with data	42	14	9
Number of GBD regions with data (out of 21 regions)	16	6	5
Number of GBD super-regions with data (out of 7 super-regions)	7	4	3

In addition, data from US claims data for 2000 and 2010–2014 by US state and Taiwan claims for 2016 were included. We decided not to use hospital inpatient data as we considered they would not be representative of true prevalence and that variation between countries in the proportion of true prevalent cases captured in hospital inpatient data systems would likely vary more than can be captured by a single crosswalk in DisMod-MR 2.1. We compared the rates of RA in the outpatient data from Norway, Sweden, Canada, and the USA and found implausibly large differences with the rates from the claims data. The USA outpatient rates were half the value of the claims data and those for the other countries much lower still. For those reasons we decided not to use the outpatient data.

Severity splits

The basis of the GBD disability weight survey assessments are lay descriptions of sequelae highlighting major functional consequences and symptoms. The lay descriptions and disability weights for RA severity levels are shown below.

Severity level	Lay description	DW (95% CI)
Mild	This person has moderate pain and stiffness in the arms and hands which causes difficulty lifting, carrying, and holding things, and trouble sleeping because of the pain.	0.117 (0.080–0.163)
Moderate	This person has pain and deformity in most joints, causing difficulty moving around, getting up and down, and using the hands for lifting and carrying. The person often feels fatigue.	0.317 (0.216–0.440)
Severe	This person has severe, constant pain, and deformity in most joints, causing difficulty moving around, getting up and down, eating, dressing, lifting, carrying, and using the hands. The person often feels sadness, anxiety, and extreme fatigue.	0.581 (0.403–0.739)

The severity distributions are derived from an analysis of the Medical Expenditure Panel Surveys (MEPS) in the USA. MEPS is an overlapping continuous panel survey of the United States non-institutionalised population whose primary purpose is to collect information on how they use health services and cost of health care. Panels are two years long and in five rounds, which are conducted every five to six months. A new panel begins annually, while the last panel is in its second year (http://www.meps.ahrq.gov/survey_comp/hc_data_collection.jsp). Each panel typically contains about 30,000 to 35,000 individual respondents.

MEPS was initiated in 1996 but only began collecting health status data in the form of 12-Item Short Form Survey (SF-12) responses in 2000. For GBD 2016 we used data from 2000–2014. Respondents self-administer the SF-12 twice per panel, at rounds two and four, typically about a year apart. Only adults 18 years and older completed the SF-12. MEPS also usually collects information on diagnoses based on self-report of reasons for encounters with health services. In addition, diagnoses are derived through additional questions on "problems that bother you" or conditions that led to "disability days," ie, days out of role due to illness. Professional coders translate the verbatim text into three-digit ICD-9 codes. The main reason for other MSK being measured in MEPS relates to health care contact.

In order to derive a crosswalk of SF-12 values into a scale comparable with that used by the GBD disability weights, small studies on convenience samples were conducted asking respondents to fill in SF-12 to reflect 62 lay descriptions of diverse severity that were used to derive the GBD disability weights. From these responses a relationship between SF-12 summary score and the GBD DWs was derived. With regression methods, average disability weights were calculated for each of 156 conditions for which there were corresponding diagnoses in MEPS, while controlling for any comorbid other condition by adding dummy variables for each condition.

Modelling strategy

Prior settings in the DisMod model included setting remission to 0 – 0.02 for ages up to 65 and 0 – 0.05 for ages 65+. It was assumed that there was no incidence or prevalence of RA before the age of 5 years.

Data from all sources were re-extracted to better reflect the range of case definitions. We set the American College of Rheumatology (ACR) 1987 criteria[1] as the reference. We marked studies using the Rome 1961,[2] American Rheumatology Association (ARA) 1958,[3] or European League against Rheumatism (EULAR)[4] criteria with a single study covariate "non-ACR_1987" as there were inadequate studies with each alternative classification system to do separate crosswalks.

Additional study covariates were created for studies using administrative health system data sources; for studies covering regional rather than (sub)-nationally representative populations; and for USA MarketScan data in 2000.

We used the function in DisMod-MR 2.1 to pull in cause-specific mortality rate (CSMR) data from our CODEm and CODcorrect analyses and match them with prevalence data points for the same geography and study year to estimate priors on excess mortality rates (by dividing CSMR by prevalence). In GBD 2017, CSMR data include the age groups 80-84, 85-89, 90-94, and 95+ years.

We retained just two RA diagnosis criteria and MarketScan data in 2000 as x-cov (ie, based on a significant coefficient indicating evidence of a systematic bias). Betas and exponentiated values (which can be interpreted as an odds ratio) for these covariates are shown in the table below:

Study covariate	Parameter	beta	Exponentiated beta
RA criteria other than ACR 1987	Prevalence	0.093	1.10 (0.85–1.27)
RA diagnosis from admin data	Prevalence	-0.07	0.93 (0.72–1.07)
MarketScan-2000	Prevalence	-0.31	0.73 (0.72–0.75)

The non-representative covariates were used as z-cov, meaning that DisMod estimates a value that gets added to the standard deviation of data points to reflect that these were not estimated according to our reference case definition/study method.

References

1 Arnett FC, Edworthy SM, Bloch DA, *et al.* The American Rheumatism Association 1987 revised criteria for the classification of rheumatoid arthritis. *Arthritis Rheum* 1988; **31**: 315–24.

2 Kellgren JH. Diagnostic criteria for population studies. *Bull Rheum Dis* 1962; **13**: 291–2.

3 Bennett GA, Cobb S, Jacox R, Jessar RA, Ropes MW. Proposed diagnostic criteria for rheumatoid arthritis. *Bull Rheum Dis* 1956; **7**: 121–4.

4 Vonkeman HE, van de Laar MAFJ. The new European League Against Rheumatism/American College of Rheumatology diagnostic criteria for rheumatoid arthritis: how are they performing? *Curr Opin Rheumatol* 2013; **25**: 354–9.

Osteoarthritis

Flowchart

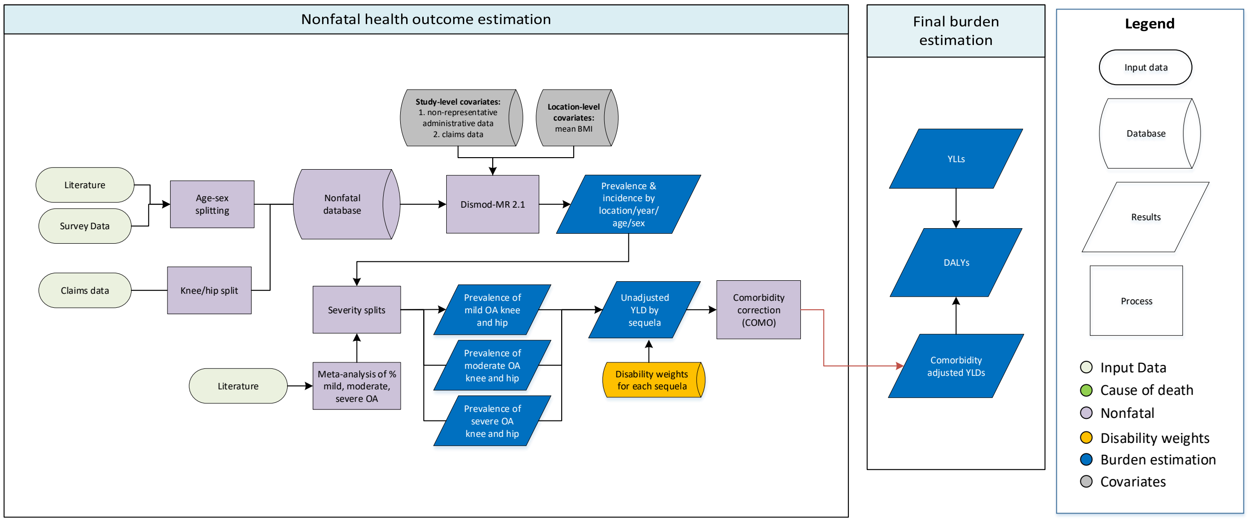

Case definition

The osteoarthritis (OA) reference case definition is symptomatic osteoarthritis of the hip or knee radiologically confirmed as Kellgren-Lawrence grade 2-4. Grade 2 symptomatic requires one defined osteophyte in hip or knee and pain for at least one month out of the last 12. Grade 3-4 symptomatic requires osteophytes and joint space narrowing in hip or knee with deformity also present for grade 4, and pain for at least one month out of the last 12 months.

OA is the most common form of arthritis, involving inflammation and breakdown of joints. For the purposes of OA estimates for this GBD study, only hip and knee sites were reviewed. The hip and knee are the common sites of OA in the larger joints and are considered to produce the greatest disability. Failure of these joints can lead to need for joint replacement surgery, if available, and thus contributes to a significant proportion of the high direct health care costs attributable to arthritis. OA of the spine is also common; however, it was considered that any symptoms and disability related to the cervical and/or lumbar spine would be captured in the estimates of low back pain and neck pain. Hand OA involving the fingers and thumbs is another common site for OA, but as it often overlaps with knee OA and could also be captured in the "Other musculoskeletal disorders" category, it was not considered as a separate entity in these GBD OA estimates.

ICD-10 codes for OA of the hip and knee are M16 and M17, respectively. The ICD-9 code for OA is 715, without specific codes for hip and knee sites.

Input data

Model inputs

A systematic review of the prevalence, incidence, and mortality of OA was performed for the years 1980 to 2009 on MEDLINE, EMBASE, CINAHL, CAB Abstracts, WHO Library (WHOLIS) and OpenSIGLE for GBD2010. For prevalence and incidence, the following search terms were used: (osteoarth* OR gonarthr*) AND (prevalen* OR inciden* OR cross-sectional OR cross sectional OR epidemiol* OR survey OR population-based OR population based OR population study OR population sample OR cohort OR follow-up OR follow up OR longitudinal OR regist*) AND (list of names of all GBD countries). For mortality, the following search terms were used: (osteoarth* OR gonarthr*) AND (Mortality OR death OR standardised mortality ratio OR standardized mortality ratio OR case fatality OR cross-sectional OR cross sectional OR epidemiol* OR survey OR population-based OR population based OR population study OR population sample OR cohort OR follow-up OR follow up OR longitudinal OR regist*) AND (list of names of all GBD countries).

Exclusion criteria were:

1. Sub-populations clearly not representative of the national population
2. Not a population-based study
3. Low sample size (less than 150)
4. Review rather than original studies

The most recent PubMed search was conducted for studies published between 2013 and 2017 using the above search terms. We identified 1,864 articles and extracted data from 26. These studies were from 19 locations: Australia, Brazil, Canada, China, Ecuador, Egypt, India, Iran, United Kingdom, France, Japan, United States, Mongolia, Portugal, Spain, Mexico, Turkey, Venezuela, and Vietnam.

Figure 1: PRISMA diagram of osteoarthritis systematic review from 2013–2017

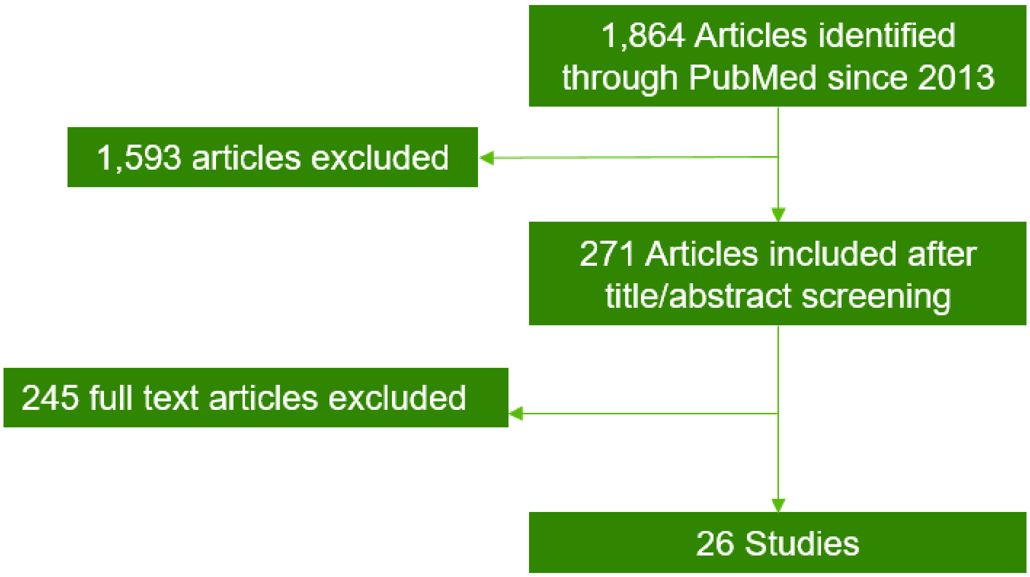

We decided not to use hospital inpatient data as we considered it would not be representative of true prevalence, and that variation between countries in the proportion of true prevalent cases captured in hospital inpatient data system would likely vary more than can be captured by a single crosswalk in DisMod-MR 2.1. In addition, data from USA claims data for 2000 and 2010–2014 by state and Taiwan claims data from 2016 were included. The table below shows the number of literature studies included in GBD 2017 for OA – knee and OA – hip, as well as the number of countries or subnational units and GBD world regions represented.

OA – knee

	Incidence	Prevalence
**Site-years (total)**	5	395
**Number of countries with data**	4	26
**Number of GBD regions with data (out of 21 regions)**	2	12
**Number of GBD super-regions with data (out of 7 super-regions)**	1	7

OA – hip

	Incidence	Relative risk	Prevalence
**Site-years (total)**	5	2	350
**Number of countries with data**	3	2	24
**Number of GBD regions with data (out of 21 regions)**	2	2	9
**Number of GBD super-regions with data (out of 7 super-regions)**	1	2	6

Severity splits

The basis of the GBD disability weight survey assessments are lay descriptions of sequelae highlighting major functional consequences and symptoms. The lay descriptions and disability weights for OA severity levels are shown below.

Severity level	Lay description	DW (95% CI)
Asymptomatic		0
Mild	This person has pain in the leg, which causes some difficulty running, walking long distances, and getting up and down.	0.023 (0.013–0.037)
Moderate	This person has moderate pain in the leg, which makes the person limp, and causes some difficulty walking, standing, lifting and carrying heavy things, getting up and down, and sleeping.	0.079 (0.054–0.110)
Severe	This person has severe pain in the leg, which makes the person limp and causes a lot of difficulty walking, standing, lifting and carrying heavy things, getting up and down, and sleeping.	0.165 (0.112–0.232)

To determine the proportion of people with OA within each of the severity levels, four studies from three regions provided information on the severity of OA. Severity was classified based on the Western Ontario and McMaster Universities Arthritis Index (WOMAC) with scores 0-5 taken as mild, 6-13 as moderate, and 14 and higher as severe. Estimates were pooled across studies using a random effects meta-analysis model. The pooled percentages were mild 47.0% (42.2–51.9), moderate 35.9% (31.3–40.7), and severe 17.1% (12.9–21.6) pooled between patient and physician ratings in a study from Bangladesh, which we apply to low- and middle-income countries. The pooled proportions from three high-income countries were mild 74.3% (64.8–82.7), moderate 24.3% (16.4–33.1), and severe 1.1% (0.6–1.7). After streaming out 1,000 draws assuming a binomial distribution, percentages were scaled to sum to 1 at each draw.

Data processing

MarketScan data reported all osteoarthritis in 2000 and 2010–2014. We used data from NHANES I to estimate the proportion of osteoarthritis that is hip and knee, by sex. NHANES I was the only NHANES cycle that diagnosed and reported osteoarthritis by hip and knee using radiologically confirmed diagnoses and self-reported pain. The figure below shows the fraction of osteoarthritis cases that are knee and hip. We applied this fraction to the MarketScan data to calculate the prevalence of osteoarthritis of hip and osteoarthritis of knee.

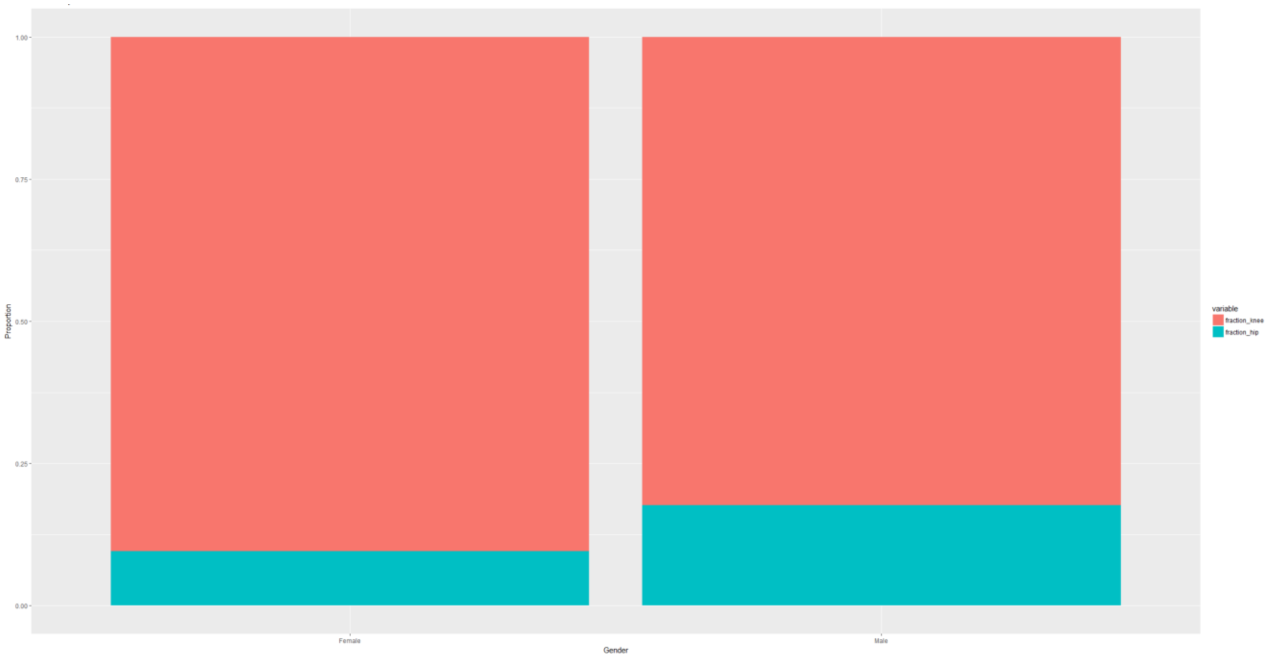

Modelling strategy

Prior settings in the DisMod model included setting remission to 0, and it was assumed that there was no incidence or prevalence of OA before the age of 30 years. We assumed in the final model that excess mortality is zero. While there are some data on excess mortality risk, the values of hazard ratios or standardised mortality ratios are close to one, with some studies reporting mean estimates less than one.

We used study covariates for studies that reported on X-rays only, self-reported OA with pain, or self-reported OA with no information on pain. There were studies that identified cases of osteoarthritis through a review of medical charts. We assumed that these cases were diagnosed by X-ray. For each of these covariates we estimated the crosswalk prior to DisMod comparing like geographies, which had data

according to the reference case definition and the alternative. In DisMod we set bounds with an upper and lower limit. We added covariates for each of three years of claims data in the USA. Betas and exponentiated values (which can be interpreted as an odds ratio) for these covariates are shown in the table below:

Study covariate	Parameter	OA hip		OA knee	
		beta	Exponentiated beta	beta	Exponentiated beta
Self-reported OA with pain	Prevalence	1.08 (1.08–1.08)	2.94 (2.94–2.94)	0.87 (0.87–0.87)	2.38 (2.38–2.38)
Radiography only	Prevalence	1.44 (1.44–1.44)	4.22 (4.22–4.22)	0.62 (0.62–0.62)	1.87 (1.87–1.87)
USA Claims data – 2000	Prevalence	-1.94 (-2 to -1.83)	0.14 (0.14–0.16)	-1.7 (-1.93 to -1.51)	0.18 (0.15–0.22)
USA Claims data – 2010–2014	Prevalence	-1.42 (-1.69 to -1.31)	0.24 (0.18–0.27)	-1.13 (-1.35 to -0.94)	0.32 (0.26–0.39)
Taiwan Claims data – 2016	Prevalence	-2 (-2 to -1.98)	0.14 (0.14–0.14)	-2 (-2 to -1.99)	0.14 (0.14–0.14)
Mean BMI	Prevalence	0.91 (0.75–1.00)	2.49 (2.11–2.71)	0.99 (0.95–1.00)	2.68 (2.59–2.72)
Radiography only	Incidence	0.90 (0.80–1.00)	2.46 (2.23–2.72)	0.62 (0.62–0.62)	1.87 (1.87–1.87)

Low back pain (LBP)

Flowchart

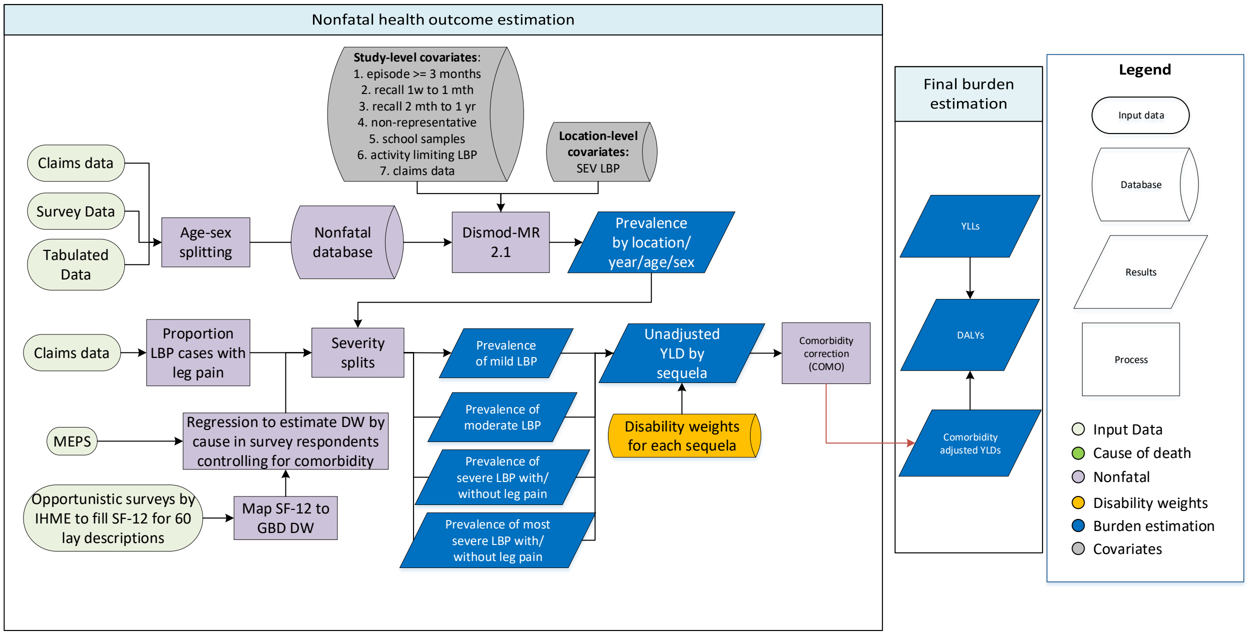

Case definition

Low back pain (LBP) is defined as low back pain (with or without pain referred into one or both lower limbs) that lasts for at least one day. The "low back" is defined as the area on the posterior aspect of the body from the lower margin of the twelfth ribs to the lower gluteal folds.

ICD-10 codes for LBP are M54.3, M54.4 and M54.5. The ICD-9 code is 724.

Input data

Model inputs

Ovid Medline, EMBase, and CINAHL electronic databases were searched in PUBMED through October 2017 for GBD 2017. There were no age, sex, or language restrictions. The terms "back pain," "lumbar pain," "back ache," "backache," and "lumbago" were used individually and combined with each of the following: "prevalence," "incidence," "cross-sectional," and "epidemiology."

Exclusion criteria were:

1. Sub-populations clearly not representative of the national population
2. Not a population-based study
3. Low sample size (less than 150)
4. Review rather than original studies

Figure 1: PRISMA diagram of low back pain systematic review from 2016–2017

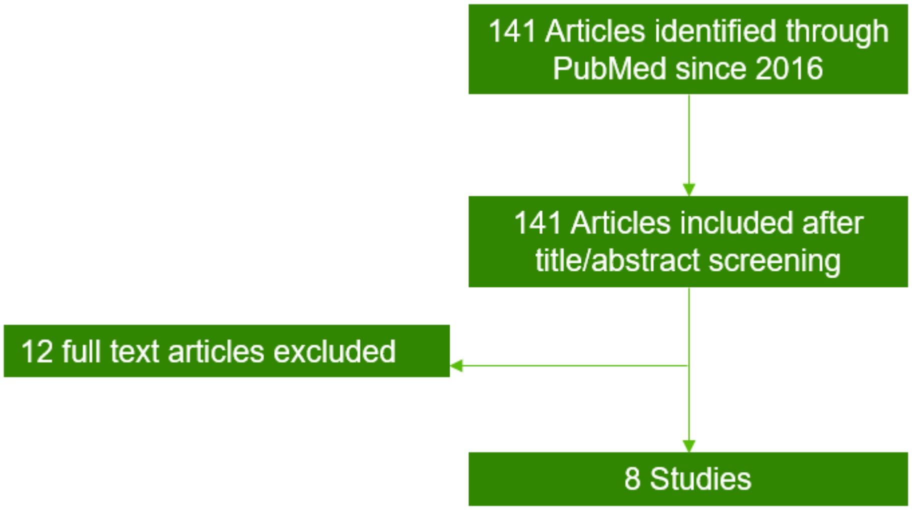

Additional information was derived from unit record data of surveys in the GHDx, GBD's repository of population health data including the World Health surveys and national health surveys. Opportunistically, additional studies encountered during data review were added for GBD 2017. In addition, data from USA claims data for 2000, 2010–2012, and 2014 by state and Taiwan claims data from 2016 were included. The table below shows the number of literature studies included in GBD 2017, as well as the number of countries and GBD world regions represented.

	Incidence	Prevalence	Remission
Site-years (total)	4	741	4
Number of countries with data	2	102	2
Number of GBD regions with data (out of 21 regions)	1	20	1
Number of GBD super-regions with data (out of 7 super-regions)	1	7	1

Severity splits

The basis of the GBD disability weight survey assessments are lay descriptions of sequelae highlighting major functional consequences and symptoms. The lay descriptions and disability weights for LBP severity levels are shown below.

Severity level	Lay description	DW (95% CI)
Low back pain, mild	This person has mild back pain, which causes some difficulty dressing, standing, and lifting things.	0.020 (0.011–0.035)
Low back pain, moderate	This person has moderate back pain, which causes difficulty dressing, sitting, standing, walking, and lifting things.	0.054 (0.035–0.079)

Low back pain, severe without leg pain	This person has severe back pain, which causes difficulty dressing, sitting, standing, walking, and lifting things. The person sleeps poorly and feels worried.	0.272 (0.182–0.373)
Low back pain, severe with leg pain	This person has severe back and leg pain, which causes difficulty dressing, sitting, standing, walking, and lifting things. The person sleeps poorly and feels worried.	0.325 0.219–0.446)
Low back pain, most severe without leg pain	This person has constant back pain, which causes difficulty dressing, sitting, standing, walking, and lifting things. The person sleeps poorly, is worried, and has lost some enjoyment in life.	0.372 (0.250–0.506)
Low back pain, most severe with leg pain	This person has constant back and leg pain, which causes difficulty dressing, sitting, standing, walking, and lifting things. The person sleeps poorly, is worried, and has lost some enjoyment in life.	0.384 (0.256–0.518)

The severity distributions are derived from an analysis of the Medical Expenditure Panel Surveys (MEPS) in the USA. MEPS is an overlapping continuous panel survey of the United States non-institutionalised population whose primary purpose is to collect information on the use and cost of health care. Panels are two years long and are conducted in five rounds, which are conducted every five to six months. A new panel begins annually, while the last panel is in its second year. Each panel typically contains about 30,000 to 35,000 individual respondents. (http://www.meps.ahrq.gov/survey_comp/hc_data_collection.jsp)

MEPS was initiated in 1996 but only began collecting health status data in the form of SF-12 responses in 2000. For GBD 2017 we used data from 2000–2014. Respondents self-administer the SF-12 twice per panel, at rounds 2 and 4, typically about a year apart. Only adults 18 years and older completed the SF-12. MEPS also usually collects information on diagnoses based on self-report of reasons for encounters with health services. In addition, diagnoses are derived through additional questions on "problems that bother you" or conditions that led to "disability days," ie, days out of role due to illness. Professional coders translate the verbatim text into three-digit ICD-9 codes. The main reason for LBP being measured in MEPS relates to health care contact. From MEPS, the severity distribution for LBP without leg pain and with leg pain were derived as shown in the below table.

Severity level	Distribution without leg pain	Distribution with leg pain
Low back pain, mild	0.41 (0.31–0.53)	0.27 (0.19–0.37)
Low back pain, moderate	0.35 (0.25–0.44)	0.36 (0.28–0.43)
Low back pain, severe	0.10 (0.08–0.12)	0.14 (0.10–0.16)
Low back pain, most severe	0.14 (0.09–0.20)	0.23 (0.15–0.32)

We used USA claims data (2012) to derive the proportion of cases with low back pain who report leg pain. The proportions were different by age group as shown in Figure 1. The proportion in each severity level in each age group is calculated by multiplying the proportion in the severity level and the proportion with or without leg pain.

Figure 1: Proportion of LBP with leg pain

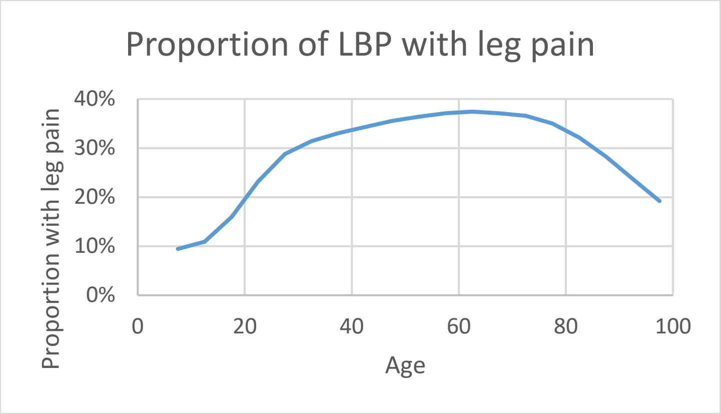

Age (years)	Proportion with leg pain
5–9	9.4 (9.1–9.8) %
10–14	10.9 (10.7–11.1) %
15–19	15.9 (15.8–16.1) %
20–24	23.2 (23.0–23.4) %
25–29	28.8 (28.6–28.9) %
30–34	31.4 (31.3–31.6) %
35–39	33.1 (32.9–33.2) %
40–44	34.3 (34.2–34.4) %
45–49	35.5 (35.4–35.6) %
50–54	36.4 (36.3–36.5) %
55–59	37.1 (37.0–37.2) %
60–64	37.4 (37.3–37.5) %
65–69	37.1 (36.9–37.3) %
70–74	36.5 (36.4–36.7) %
75–79	35.0 (34.8–35.2) %
80–84	32.1 (31.9–32.4) %
85–89	28.3 (28.0–28.5) %
90–94	23.7 (23.2–24.2) %
95–100	19.2 (18.2–20.2) %

Modelling Strategy

Prior settings in the DisMod model included setting excess mortality to 0, and it was assumed that there was no incidence or prevalence of low back pain before the age of 5 years.

We used study covariates for studies that reported a too broad anatomical region, episode duration of greater than three months, recall periods of one week to one month, recall periods between two months and one year, activity-limiting LBP, and studies conducted among schoolchildren or otherwise non-

representative samples as studies covering regional rather than (sub)-nationally representative populations. The mean and standard error for the coefficient of the covariate of studies among schoolchildren population were calculated and used for a constraint in DisMod. The other covariates have their coefficients constraint by reasonable ranges in the direction whether they increased or decreased the estimates. We added covariates for each of three years of claims data in the USA. Betas and exponentiated values (which can be interpreted as an odds ratio) for these two covariates are shown in the table below:

Study covariate	Parameter	beta	Exponentiated beta[1]
Anatomical region too broad	Prevalence	0.0010 (0.00033–0.0026)	1.00 (1.00–1.00)
Episode duration >= 3 months	Prevalence	-0.75 (-0.81 to -0.69)	0.47 (0.45–0.50)
Recall periods of 1 week to 1 month	Prevalence	0.54 (0.51–0.58)	1.72 (1.66–1.79)
Recall periods between 2 months and one year	Prevalence	0.66 (0.58–0.71)	1.93 (1.78–2.03)
Studies among school children	Prevalence	0.49 (0.45–0.54)	1.64 (1.57–1.72)
Activity-limiting LBP	Prevalence	-0.92 (-0.97 to -0.88)	0.40 (0.38–0.42)
USA claims data – 2000	Prevalence	-0.85 (-1.02 to -0.77)	0.43 (0.36–0.46)
USA claims data – 2010–2012, 2014	Prevalence	-0.31 (-0.49 to -0.23)	0.74 (0.61–0.80)

[1] interpretation examples: in DisMod-MR 2.1 activity limiting LBP data points showed a systematic bias downward and were adjusted up by dividing by 0.40, while data points of recall greater than two months were adjusted downward dividing by 1.93.

We included the SEV scalar for low back pain as a country covariate. This combines the exposure measures for risks estimated to impinge on LBP in GBD: occupational ergonomic exposure and increased BMI. We set bounds of 0.75 to 1.25 as the SEV is constructed in a way that if our risk estimates are accurate the value should be 1.

Neck pain (NP)

Flowchart

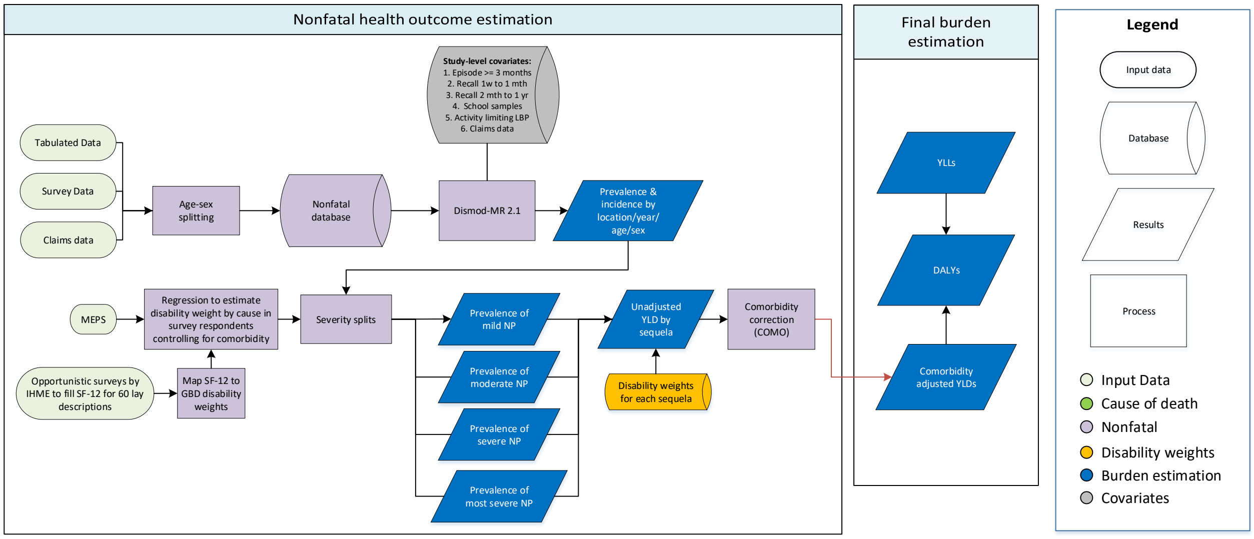

Case definition

Neck pain (NP) was defined as*: neck pain (+/- pain referred into the upper limb(s)) that lasts for at least one day*.

ICD-10 code for neck pain is M54.2. The ICD-9 code is 723.1.

Input data

Model inputs

Ovid MEDLINE, EMBASE, CINAHL, CAB abstracts, WHOLIS, and SIGLE databases were searched for GBD 2010 and PUBMED was searched through October 2017 for GBD 2017. There were no age, sex, or language restrictions. The terms *neck pain, neck ache, neckache, and cervical pain* individually and combined with each of the following terms: *prevalen*, inciden*, cross-sectional, cross sectional, epidemiol*, survey, population-based, population based, population study, population sample*.

Exclusion criteria were:

1. Sub-populations clearly not representative of the national population
2. Not a population-based study
3. Studies on a specific type of neck pain (eg, following neck fracture)
4. Low sample size (less than 150)
5. Review rather than original studies

Figure 1: PRISMA diagram of neck pain systematic review from 2016–2017

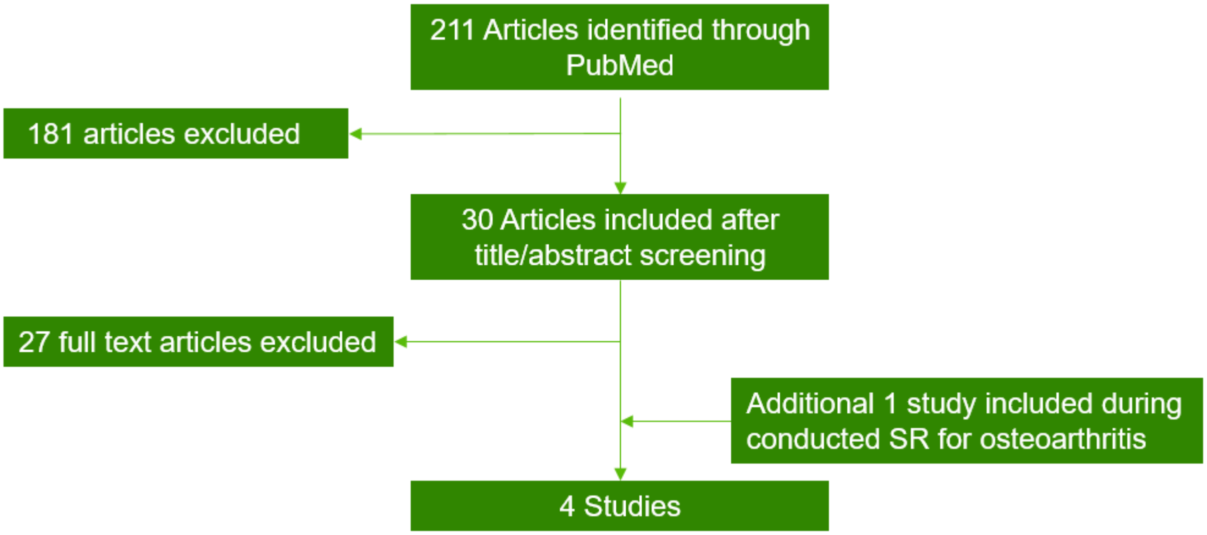

Additional information was derived from unit record data of surveys in the GHDx, GBD's repository of population health data including National Health and Nutrition Examination Survey (NHANES) and National Health Interview Survey (NHIS) in the USA. In addition, data from USA claims data for 2000 and 2010–2014 by state and Taiwan claims data from 2016 were included. The table below shows the number of studies and surveys included in GBD 2017, as well as the number of countries and GBD world regions represented.

	Prevalence	Remission
**Site-years (total)**	388	1
**Number of countries with data**	23	1
**Number of GBD regions with data (out of 21 regions)**	11	1
**Number of GBD super-regions with data (out of 7 super-regions)**	6	1

Severity splits

The basis of the GBD disability weight survey assessments are lay descriptions of sequelae highlighting major functional consequences and symptoms. The lay descriptions and disability weights for neck pain severity levels are shown below.

Severity level	Lay description	DW (95% CI)	Proportions
Neck pain, mild	This person has neck pain, and has difficulty turning the head and lifting things	0.052 (0.036–0.074)	0.67 (0.57–0.75)
Neck pain, moderate	This person has constant neck pain, and has difficulty turning the head, holding arms up, and lifting things	0.112 (0.079–0.162)	0.12 (0.08–0.19)

Neck pain, severe	This person has severe neck pain, and difficulty turning the head and lifting things. The person gets headaches and arm pain, sleeps poorly, and feels tired and worried	0.226 (0.147–0.323)	0.06 (0.05–0.07)
Neck pain, most severe	This person has constant neck pain and arm pain, and difficulty turning the head, holding arms up, and lifting things. The person gets headaches, sleeps poorly, and feels tired and worried	0.300 0.199–0.434)	0.15 (0.11–0.20)

The severity distributions are derived from an analysis of the Medical Expenditure Panel Surveys (MEPS) in the USA. MEPS is an overlapping continuous panel survey of the United States non-institutionalised population whose primary purpose is to collect information on the use and cost of health care. Panels are two years long and are conducted in five rounds, which are conducted every five to six months. A new panel begins annually, while the last panel is in its second year. Each panel typically contains about 30,000 to 35,000 individual respondents. (http://www.meps.ahrq.gov/survey_comp/hc_data_collection.jsp)

MEPS was initiated in 1996 but only began collecting health status data in the form of SF-12 responses in 2000. For GBD 2016 we used data from 2000–2014. Respondents self-administer the SF-12 twice per panel, at rounds two and four, typically about a year apart. Only adults 18 years and older completed the SF-12. MEPS also usually collects information on diagnoses based on self-report of reasons for encounters with health services. In addition, diagnoses are derived through additional questions on "problems that bother you" or conditions that led to "disability days," ie, days out of role due to illness. Professional coders translate the verbatim text into three-digit ICD-9 codes. The main reason for neck pain being measured in MEPS relates to health care contact.

In order to derive a crosswalk of SF-12 values into a scale comparable with that used by the GBD disability weights, small studies on convenience samples were conducted asking respondents to fill in SF-12 to reflect 62 lay descriptions of diverse severity that were used to derive the GBD disability weights. From these responses a relationship between SF-12 summary score and the GBD DWs was derived. With regression methods, average disability weights were calculated for each of 156 conditions for which there were corresponding diagnoses in MEPS, while controlling for any co-morbid other condition by adding dummy variables for each condition. As our case definition is for point prevalence of neck pain, we ignored the proportion of MEPS respondents with a neck pain diagnosis for whom in our regression we found no disability attributable to neck pain. For the remaining cases we binned the amount of DW attributed to neck pain across the four health states assuming thresholds at the midpoints between DW values.

Modelling strategy

Prior settings in the DisMod model included setting excess mortality to 0, and it was assumed that there was no incidence or prevalence of neck pain before the age of 5 years.

We used study covariates for studies that reported a too broad anatomical region, episode duration of greater than three months, recall periods of one week to one month, recall periods between two months and one year, activity-limiting neck pain, and studies conducted among schoolchildren. We added covariates for each of three years of claims data in the USA.

With exceptions for broad anatomical region, studies among schoolchildren population, and activity-limiting neck pain, whose coefficients were consistent with their reasonable ranges, the means and the upper and lower bounds for the covariates were calculated by crosswalking with NHANES data as a baseline. The table below shows the prior values for those covariates' coefficients.

Study covariate	Lower bound	Mean	Upper bound
Anatomical region too broad	0		2
Episode duration >= 3 months	-0.81	-0.74	-0.67
Recall periods of 1 week to 1 month	0.67	0.77	0.87
Recall periods between 2 months and one year	0.77	0.87	0.97
Studies among schoolchildren	0		3
Activity-limiting neck pain	-3		0
Claims data – 2000	-2.20	-2.08	-1.96
Claims data – 2010	-1.73	-1.57	-1.41
Claims data – 2012	-1.65	-1.50	-1.35

Betas and exponentiated values (which can be interpreted as an odds ratio) for these two covariates are shown in the table below:

Study covariate	Parameter	beta	Exponentiated beta[1]
Anatomical region too broad	Prevalence	0.12 (0.028–0.23)	1.13 (1.03–1.26)
Episode duration >= 3 months	Prevalence	-0.7 (-0.77 to -0.67)	0.50 (0.46–0.51)
Young population	Prevalence	1.64 (1.46–1.82)	5.14 (4.31–6.18)
Recall periods of 1 week to 1 month	Prevalence	0.85 (0.81–0.87)	2.35 (2.25–2.39)
Recall periods of 6 months to 12 months	Prevalence	1.07 (0.89–1.25)	2.91 (2.45–3.50)
Recall periods 1 year	Prevalence	0.87 (0.77–0.96)	2.38 (2.17–2.62)
Studies among school children	Prevalence	2.17 (1.96–2.40)	8.78 (7.06–11.03)
Activity-limiting neck pain	Prevalence	-1.34 (-1.56 to -1.13)	0.26 (0.21 — 0.32)
USA Claims data – 2000	Prevalence	-2.11 (-2.2 to -2.01)	0.12 (0.11–0.13)
USA Claims data – 2010–2014	Prevalence	-1.42 (-1.52 to -1.32)	0.24 (0.22–0.27)
Taiwan Claims data – 2016	Prevalence	-0.22 (-1.48 to 0.47)	0.80 (0.23–1.60)

[1] interpretation examples: in DisMod-MR 2.1 activity-limiting NP data points showed a systematic bias downward and were adjusted up by dividing by 0.26 while data points of recall of 6 to 12 months were adjusted downward dividing by 2.91.

Gout

Flowchart

Gout

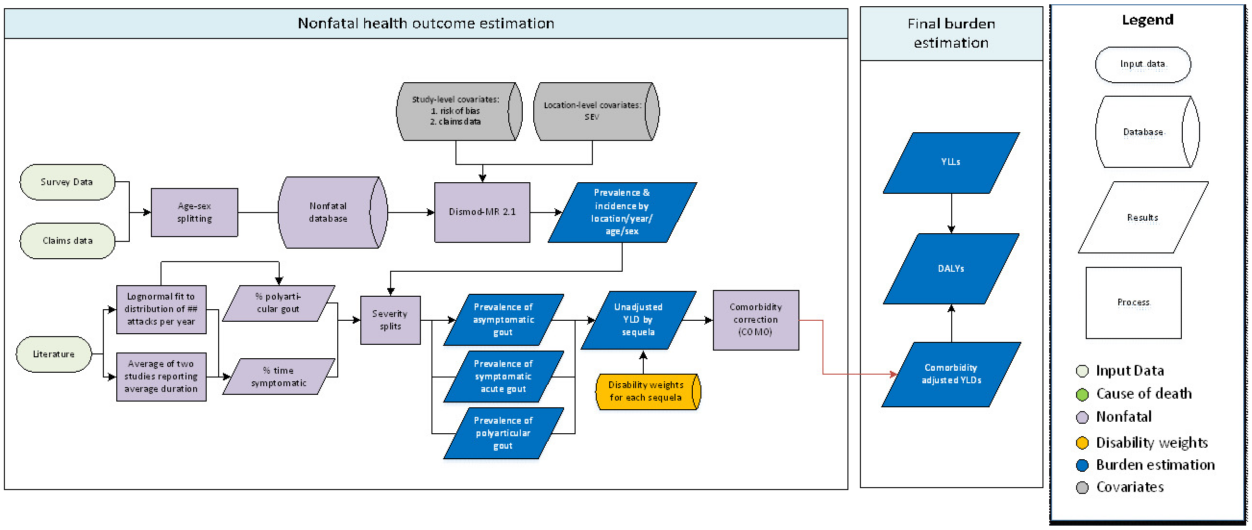

Case definition

Gout is a rheumatic disease that is characterised by formation of monosodium urate (MSU) crystals in the synovial fluid of joints and in other tissues, causing inflammation. The crystal formation is caused by elevated urate levels in extracellular fluids. It is more common in men. GBD uses the case definition of primary gout given by the American College of Rheumatology, generally referred to as ARA 1977 survey criteria requiring the presence of MSU crystals in joint fluid or the presence of a tophus proven to contain MSU crystals and at least six of 12 gout symptoms or findings (>1 attack of acute arthritis, development of maximal inflammation within a day, attack of monoarticular arthritis, observation of joint erythema, pain or swelling in the first MTP joint, unilateral attack involving the first MTP joint, unilateral attack involving tarsal joint, suspected tophus, hyperuricemia, asymmetrical swelling within a joint on X-ray and negative culture of joint fluid for microorganisms during attack of joint inflammation) to make a diagnosis. The ICD-10 code for gout is M10 and the ICD9 code is 274.

Input data

Model Inputs

The most recent PubMed search was conducted in GBD 2013 were performed for years 1980 to 2009 using the following search terms on MEDLINE, EMBASE, CINAHL, CAB Abstracts, WHO Library (WHOLIS), and OpenSIGLE. For prevalence and incidence, the following search terms were used: (gout* OR hyperuricemia) AND (prevalen* OR inciden* OR cross-sectional OR cross sectional OR epidemiol* OR survey OR population-based OR population based OR population study OR population sample OR cohort OR follow-up OR follow up OR longitudinal OR regist*) AND (list of names of all GBD countries). For mortality, the following search terms were used: (gout* OR hyperuricemia) AND (Mortality OR death OR

standardised mortality ratio OR standardized mortality ratio OR case fatality OR cross-sectional OR cross sectional OR epidemiol* OR survey OR population-based OR population based OR population study OR population sample OR cohort OR follow-up OR follow up OR longitudinal OR regist*) AND (list of names of all GBD countries).

Exclusion criteria were:

- Sub-populations clearly not representative of the national population
- Not a population-based study
- Low sample size (less than 150)
- Review rather than original studies

Opportunistically, additional studies encountered during data review were added for GBD 2017. In addition, data from USA claims data for 2000 and 2010–2014 by state and Taiwan claims data from 2016 were included. The table below shows the number of literature studies included in GBD 2017, as well as the number of countries and GBD world regions represented.

	Incidence	Relative risk	Prevalence
Site-years (total)	105	3	507
Number of countries with data	6	2	29
Number of GBD regions with data (out of 21 regions)	4	1	13
Number of GBD super-regions with data (out of 7 super-regions)	3	1	6

Severity splits

The basis of the GBD disability weight (DW) survey assessments are lay descriptions of sequelae highlighting major functional consequences and symptoms. The lay descriptions and disability weights for gout severity levels are shown below.

Severity level	Lay description	DW (95% CI)
Gout, acute	This person has severe pain and swelling in the leg, making it very difficult to get up and down, stand, walk, lift, and carry heavy things. The person has trouble sleeping because of the pain.	0.295 (0.196–0.409)
Polyarticular gout (same as for severe RA)	This person has severe, constant pain and deformity in most joints, causing difficulty moving around, getting up and down, eating, dressing, lifting, carrying, and using the hands. The person often feels sadness, anxiety, and extreme fatigue.	0.581 (0.403–0.739)
Asymptomatic gout		0

We used three studies on the distribution of the number of gout attacks per year and fitted a lognormal curve using a least squared differences method. In the absence of data on the proportion of gout cases who have chronic polyarticular gout, we assumed the proportion is equal to those who would have 52 attacks a year (ie, weekly) or more as implied by the lognormal curve.

The average number of attacks was estimated from the lognormal fit: 5.66 (5.14–6.18). From two studies we derived an average duration of attacks of 6.1 (5.4–6.8) days by simple averaging. The resulting proportion of time symptomatic for acute gout was taken as the multiplication of these two estimates divided by the number of days in a year: 9.4% (8.0–10.9%).

Figure 1: Distribution of cases by frequency

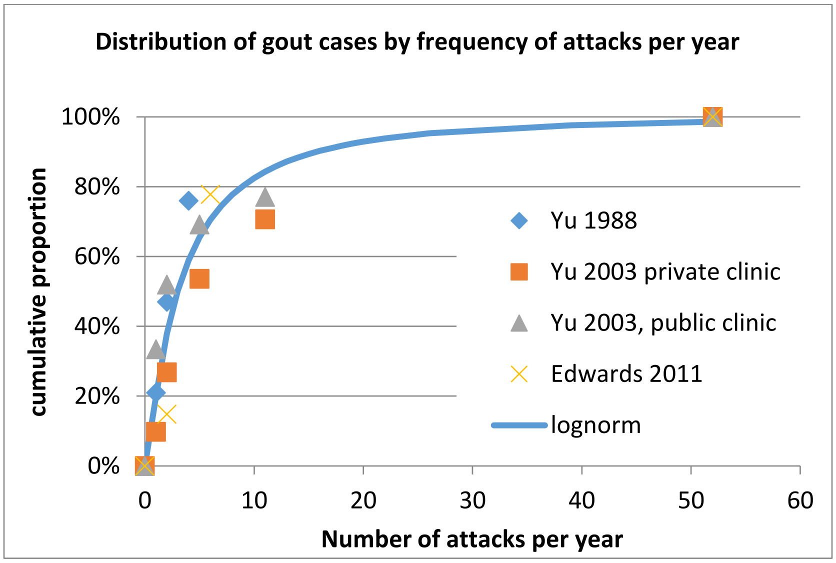

Modelling strategy

Initially we set remission to zero but found this made incidence and prevalence inconsistent. As the ratio of prevalence and incidence in similar locations was in the order of 10:1 we decided to allow remission to range between 0 and 0.2 and that made incidence and prevalence more consistent. We assumed that there was no incidence or prevalence of gout before the age of 15 years.

We created three study-level covariates: for the 2000 USA claims data, the 2010–2014 USA claims data, and 2016 Taiwan claims data. Studies relying on self-reported diagnoses or not stating diagnostic criteria were flagged with a covariate for a risk of bias. However, the risk of bias covariate was found to be insignificant; thus, it was used as a covariate that affects only uncertainty but not mean estimates.

Betas and exponentiated values (which can be interpreted as an odds ratio) for the claims covariate are shown in the table below:

Study covariate	Parameter	beta	Exponentiated beta
USA claims data – 2000	Prevalence	-1.99 (-2 to -1.96)	0.14 (0.14–0.14)
USA claims data – 2010–2014	Prevalence	-1.05 (-1.07 to -1.02)	0.35 (0.34–0.36)
Taiwan claims data – 2016	Prevalence	1.28 (1.02–1.60)	3.58 (2.76–4.94)

We added the summary exposure variable (SEV) scalar for gout which summarises exposure to risks estimated in GBD to impinge on gout, ie, low glomerular filtration rate as a country covariate. We set bounds of 0.75 to 1.25 as the SEV is constructed in a way that if our risk estimates are accurate the value should be 1.

Other musculoskeletal disorders (MSK)

Flowchart

Other musculoskeletal disorders

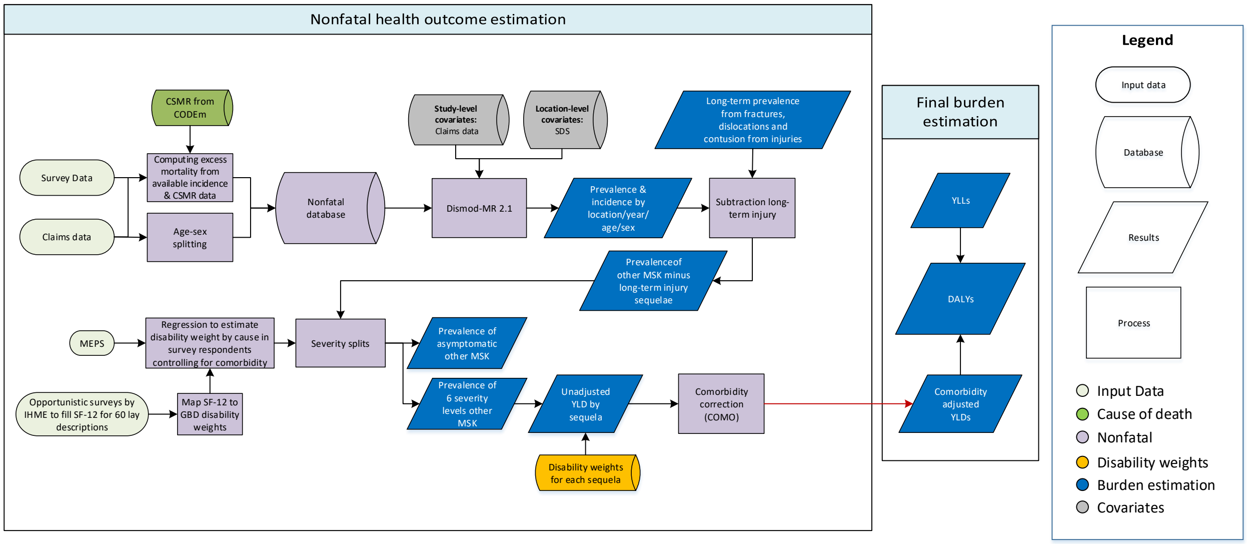

Case definition

Other musculoskeletal (MSK) disorders is a heterogeneous rest category comprising a wide range of disorders of muscles, bones, and ligaments that are not included in the five GBD defined musculoskeletal diseases rheumatoid arthritis, osteoarthritis, low back and neck pain, and gout, and are not captured as long-term sequelae of injuries.

The table below provides detail of the ICD-10 and ICD-9 codes included in this category.

ICD-10 codes	ICD-9 codes
L93—Lupus erythematosus	710.0
M00-M02—Infectious arthropathies	711
M08, M11-M13—Inflammatory polyarthropathies	712–713
M20-M25—Other joint disorders	716–719
M30-M35—Systemic connective tissue disorders	710.1-710.9
M40-M43—Deforming dorsopathies	737
M45-M46—Spondylopathies	720–721
M60 -M63—Disorders of muscles	725
M65-M68—Disorders of synovium and tendon	726–728
M70- M73, M75-M79—Other soft tissue disorders	729
M80-M85—Disorders of bone density and structure	733.0-2
M86—Osteomyelitis	730.1-730.3, 730.7-9
M87-M90—Other osteopathies	731, 733.3-9
M91-M94—Chondropathies	732

M95-M99—Other disorders of the MSK system and connective tissue	734–736, 738–739

Input data

Model Inputs

The above ICD codes were used to extract other MSK prevalence from USA claims data for 2000 and 2010–2014 by state. The systematic review concentrated on finding health surveys that measured an overall amount of musculoskeletal disorders and complaints and reported information to distinguish a rest category that was not OA, RA, gout, or low back or neck pain. These data sources are based on self-reported musculoskeletal conditions or symptoms and not on the listed ICD codes.

The table below shows the number of studies and surveys included in GBD 2017, as well as the number of countries or subnational units and GBD world regions represented.

	**Prevalence**
**Site-years (total)**	348
**Number of countries with data**	18
**Number of GBD regions with data (out of 21 regions)**	10
**Number of GBD super-regions with data (out of 7 super-regions)**	5

Severity splits

The basis of the GBD disability weight survey assessments are lay descriptions of sequelae highlighting major functional consequences and symptoms. The lay descriptions and disability weights for other MSK severity levels are shown below. They include the three levels of health states that are used for osteoarthritis and rheumatoid arthritis, each.

Severity level	Lay description	DW (95% CI)	Proportions
Asymptomatic			0.28 (0.27–0.29)
Musculoskeletal problems, lower limbs, mild	This person has pain in the leg, which causes some difficulty running, walking long distances, and getting up and down.	0.023 (0.013–0.040)	0.22 (0.15–0.30)
Musculoskeletal problems, upper limbs, mild	This person has mild pain and stiffness in the arms and hands. The person has some difficulty lifting, carrying, and holding things.	0.028 (0.017–0.046)	0.20 (0.15–0.29)
Musculoskeletal problems, upper limbs, moderate	This person has moderate pain and stiffness in the arms and hands, which causes difficulty lifting, carrying, and holding things, and trouble sleeping because of the pain.	0.115 (0.079–0.163)	0.10 (0.06–0.15)

Musculoskeletal problems, lower limbs, severe	This person has severe pain in the leg, which makes the person limp and causes a lot of difficulty walking, standing, lifting and carrying heavy things, getting up and down, and sleeping.	0.163 (0.109–0.224)	0.06 (0.04–0.07)
Musculoskeletal problems, generalised, moderate	This person has pain and deformity in most joints, causing difficulty moving around, getting up and down, and using the hands for lifting and carrying. The person often feels fatigue.	0.312 (0.201–0.438)	0.07 (0.06–0.08)
Musculoskeletal problems, generalised, severe	This person has severe, constant pain and deformity in most joints, causing difficulty moving around, getting up and down, eating, dressing, lifting, carrying, and using the hands. The person often feels sadness, anxiety, and extreme fatigue.	0.572 (0.370–0.758)	0.07 (0.07–0.08)

The severity distributions are derived from an analysis of the Medical Expenditure Panel Surveys (MEPS) in the USA. MEPS is an overlapping continuous panel survey of the United States non-institutionalised population whose primary purpose is to collect information on the use and cost of health care. Panels are two years long and are conducted in five rounds, which are conducted every five to six months. A new panel begins annually, while the last panel is in its second year (http://www.meps.ahrq.gov/survey_comp/hc_data_collection.jsp). Each panel typically contains about 30,000 to 35,000 individual respondents.

MEPS was initiated in 1996, but only began collecting health status data in the form of 12-Item Short Form Survey (SF-12) responses in 2000. For GBD 2016 we used data from 2000–2014. Respondents self-administer the SF-12 twice per panel, at rounds two and four, typically about a year apart. Only adults 18 years and older completed the SF-12. MEPS also usually collects information on diagnoses based on self-report of reasons for encounters with health services. In addition, diagnoses are derived through additional questions on “problems that bother you” or conditions that led to “disability days,” ie, days out of role due to illness. Professional coders translate the verbatim text into three-digit ICD-9 codes. The main reason for other MSK being measured in MEPS relates to health care contact.

In order to derive a crosswalk of SF-12 values into a scale comparable with that used by the GBD disability weights, small studies on convenience samples were conducted asking respondents to fill in SF-12 to reflect 62 lay descriptions of diverse severity that were used to derive the GBD disability weights. From these responses a relationship between SF-12 summary score and the GBD DWs was derived. With regression methods, average disability weights were calculated for each of 156 conditions for which there were corresponding diagnoses in MEPS, while controlling for any comorbid other condition by adding dummy variables for each condition. We binned the amount of DW attributed to other MSK across the seven health states assuming thresholds at the midpoints between DW values.

Modelling Strategy

Prior settings in the DisMod model included setting excess mortality to 0, and it was assumed that there was no incidence or prevalence of other MSK before the age of 10 years. In the absence of any meaningful data on incidence and remission for such a heterogeneous category of disorders, we made a rather arbitrary decision of remission of 0.5–1, ie, an average duration of 1-2 years. We included cause-specific mortality rate (CSMR) data for other MSK and estimated priors on excess mortality rate by dividing all prevalence data points by the corresponding CSMR. In GBD 2017, CSMR data included older age groups, ie, 80-85, 85-90, 90-95, and 95+ years.

We used study covariates for MarketScan 2000 and MarketScan data in general. Betas and exponentiated values (which can be interpreted as an odds ratio) for these two covariates are shown in the table below:

Study covariate	Parameter	beta	Exponentiated beta
Sex	Prevalence	-0.31 (-0.32 to -0.29)	0.74 (0.73–0.75)
MarketScan	Prevalence	0.60 (0.52–0.76)	1.82 (1.69–2.14)
All MarketScan, year 2000	Prevalence	0.064 (0.00042–0.22)	1.07 (1.00–1.24)

We allow positive coefficients on claims data as all our other data sources are based on other MSK disorders in the absence of low back pain, neck pain, OA, RA, and gout, while claims data reflect the one-year prevalence of having an ICD-coded other MSK condition mentioned. As there are multiple other MSK conditions that last less than a year, it is not surprising to find higher one-year prevalence in claims data then the point prevalence estimates derived from surveys.

We use the GBD Socio-demographic Index (SDI) scalar variable as a covariate with a small coefficient of 0.17 estimated by DisMod-MR 2.1.

In order to avoid double counting, we subtract the long-term sequelae of fractures, dislocations, and contusions due to injuries from other MSK, as the surveys from which we derive prevalence estimates make no distinction between cases with other MSK problems that are or are not due to injuries.

Congenital anomalies

This write-up covers the following causes: congenital heart defects, neural tube defects, cleft lip and cleft palate, congenital anomalies of the urogenital system, congenital anomalies of the gastrointestinal tract, musculoskeletal congenital anomalies, congenital chromosomal birth defects (Down Syndrome, Turner Syndrome, Klinefelter Syndrome, and other chromosomal abnormalities, genetic syndromes and micro-deletions)

Flowchart

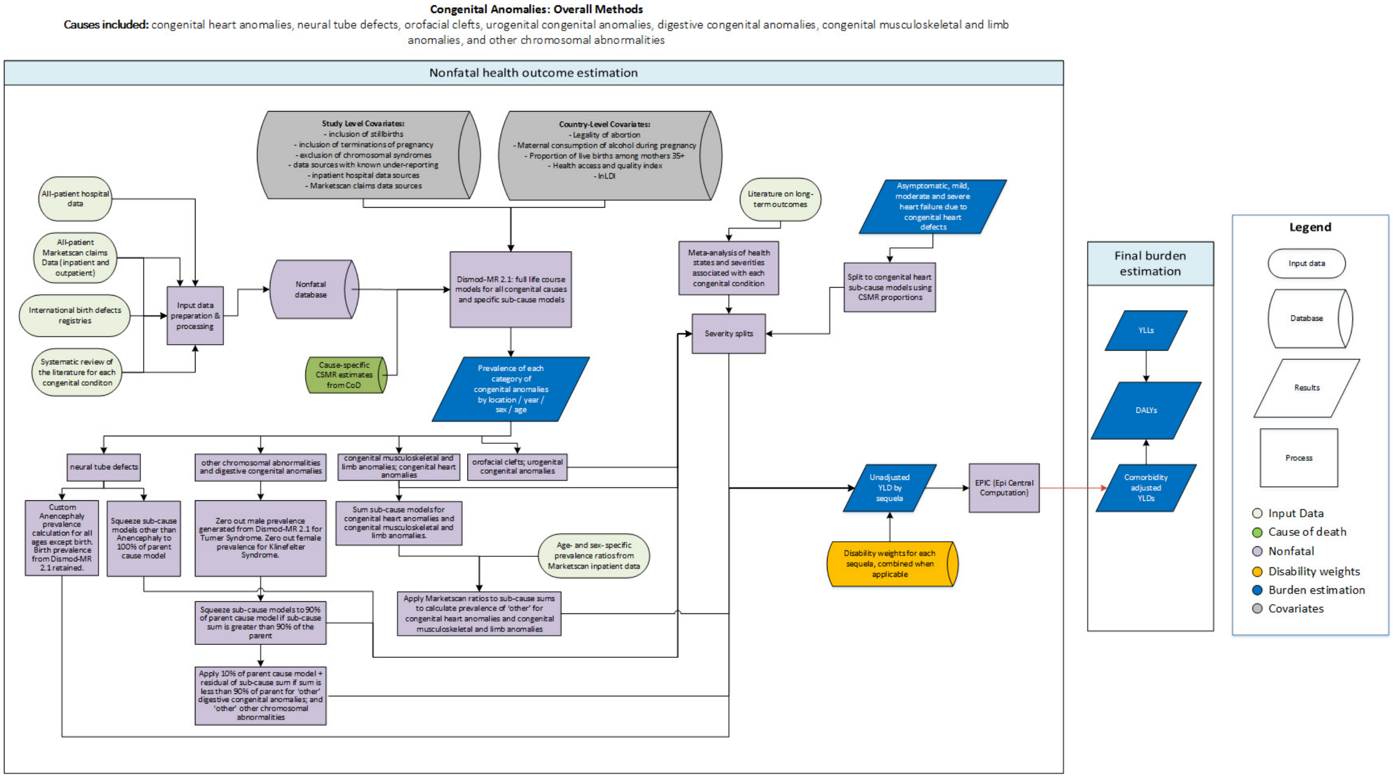

Case Definitions and Introduction

The GBD case definition of congenital anomalies includes any condition present at birth that is a result of abnormalities of embryonic development, excluding those that are directly the result of infections or substance abuse (e.g. fetal alcohol syndrome, congenital syphilis) and excludes minor anomalies as they are defined by EUROCAT. Further, our GBD case definition includes only live births and excludes all terminations of pregnancy following prenatal diagnosis and stillbirths.

We have estimated the prevalence and associated disability of the following categories of congenital birth defects

1. Neural tube defects
    a. Anencephaly
    b. Encephalocele
    c. Spina bifida
2. Congenital heart defects
    a. Single ventricle and single ventricle pathway defects

   b. Severe congenital heart defects excluding single ventricle and single ventricle pathway defects
   c. Critical malformations of great vessels, congenital valvular heart disease and patent ductus arteriosis
   d. Ventricular septal defect and atrial septal defect
   e. Other congenital cardiovascular anomalies
3. **Orofacial clefts: Cleft lip and cleft palate**
4. **Total chromosomal congenital birth defects**
   a. **Down Syndrome**
   b. **Turner Syndrome**
   c. **Klinefelter Syndrome**
   d. **Other chromosomal abnormalities, genetic syndromes, and micro-deletions**
   e. **Edwards Syndrome and Patau Syndrome**
5. **Congenital anomalies of the urogenital system**
   a. Congenital urinary anomalies
   b. Congenital genital anomalies
6. **Congenital anomalies of the digestive system**
   a. Congenital diaphragmatic hernia
   b. Congenital malformations of the abdominal wall
   c. Congenital atresia and/or stenosis of the gastrointestinal tract
   d. Other congenital malformations of the gastrointestinal tract
7. **Musculoskeletal congenital anomalies**
   a. Polydactyly and syndactyly
   b. Limb reduction defects
   c. Other musculoskeletal congenital anomalies
8. **Other congenital anomalies: all birth defects (excluding minor anomalies) not contained in the other categories.**

Total chromosomal birth defects are all new causes estimated for the first time in GBD 2017.

This appendix will first describe the input data sources and aspects of the modelling strategy that are common to all sub-types of congenital anomalies. We will then provide a description of the case definitions, ICD-10 codes, and health states associated with each of the component congenital causes, as well as the specific modelling strategies employed in each congenital cause, including the model settings, study-level and country-level covariates, and other modelling decisions made.

Input Data Sources

Several types of data sources are used in the estimation of congenital anomalies: literature prevalence, with-condition mortality and excess mortality data, birth prevalence and neonatal with-condition mortality data from a number of international birth defects registries and surveillance systems, inpatient hospital and Marketscan claims data prepared internally by the GBD research team, and cause-specific mortality estimates produced by the causes of death analysis.

We conducted a systematic review of the available literature for all types of congenital anomalies by constructing search strings designed to capture information on the prevalence, associated mortality and long-term health outcomes associated with each sub-category of congenital anomalies for GBD 2017 and are continuing to use this for GBD 2017. All results were screened – first abstracts, then full-text screenings – to ensure the availability of required information and the representativeness of the reported population, and the exclusion of duplicate data also reported as part of the birth registry data inputs.

We extracted data from a number of international birth defects registries. The International Clearinghouse for Birth Defects Surveillance and Research (ICBDSR) reports birth prevalence from a number of international member registries. The World Atlas Report also published birth prevalence estimates from these international registries prior to the publication of ICBDSR reports. The European Surveillance of Congenital Anomalies (EUROCAT) reports the birth prevalence of anomalies as for a variety of locations in Western Europe as reported by participating member registries. China's Maternal and Child Health Surveillance survey (MCHS) reports birth prevalence and early neonatal mortality data for all subnational locations of China. The National Birth Defects Prevention Network (NBDPN) reports birth prevalence estimates as compiled by a number of subnational registries within the United States. The Birth Defects Registry of India (BDRI) reports congenital anomalies from participating hospitals within India.

Inpatient hospital and Marketscan claims data for all congenital anomalies causes and sub-cause models was prepped centrally by the GBD research team. The inpatient hospital data was adjusted for multiple inpatient visits by individuals and for the correction of primary diagnoses to all diagnoses including the congenital ICD codes. For more information on the preparation of these data sources, see elsewhere in this appendix.

Modelling Strategy: Overview

All available input data was utilized in a series DisMod-MR models in order to estimate the prevalence of each category of congenital anomalies across the full life course for each location/age/sex combination. Incidence was set to 0 for all congenital models, as congenital conditions occur at the time of birth and by definition there are no incident cases after birth. Remission was allowed only in the models of a select subset of causes for which surgical intervention or spontaneous remission can completely eliminate the disability due to that congenital condition: namely, cleft lip and/or palate, polydactyly and syndactyly, and ventricular septal defect and atrial septal defect. Cause-specific priors and slope priors were used to guide biologically plausible DisMod-MR estimates of excess mortality and remission where applicable. For a subset of conditions, a decreasing slope prior on excess mortality rate was applied to capture the highest risk of mortality from congenital conditions in the neonatal age groups and a subsequent decreasing risk of mortality from congenital conditions later in life. Excess mortality was set to zero after 70 years of age in all models, in keeping with the GBD cause of death estimates for all congenital causes.

For each of congenital heart, musculoskeletal and gastrointestinal anomalies, we used as DisMod-MR model to estimate the total prevalence of all conditions within that cause category. We then squeezed the sum of the specific sub-cause prevalence estimates to these total prevalence estimates in order to ensure internal consistency of our cause-level and sub-cause estimates. The prevalence of other other heart, musculoskeletal, and gastrointestinal anomalies was derived by reducing the total envelope model for each cause by its sub-causes to derive the difference that was attributable to other anomalies in that category.

Study-level Covariates

A number of the input data sources used for the estimation of congenital birth defects are known to have biases leading to under-reporting or over-reporting relative to the true prevalence of congenital anomalies among live births and all subsequent age groups. We used study-level covariates in the each of the DisMod-MR models to adjust for these under-reporting and over-reporting biases.

Where necessary, we used a study-level covariate to adjust for the inclusion of stillbirths in the reported birth prevalence estimates in literature and registry data sources, as stillbirths are not included in our case definition of prevalence among live births. We also used a study-level covariate to adjust for data

sources that included terminations of pregnancy in their birth prevalence estimates. In all models except for the chromosomal conditions, we also used a study-level covariate to adjust for under-reporting in data sources that were extracted to exclude co-occurring chromosomal conditions from the reported prevalence estimates.

For a subset of congenital causes, particularly the congenital heart defects, we noted substantial differences in the lists of case definitions being reported to the various congenital registries. Across all types of congenital heart defects, the National Birth Defects Prevention Network (NBDPN) had the most complete list of reported case definitions – ie, the highest case ascertainment – and was considered the gold standard among all birth registry data sources. We used registry-specific crosswalks to adjust all other birth defects registries to match the case ascertainment seen in the NBDPN.

We also included a series of study-level covariates to adjust for under-reporting in the inpatient hospital data and Marketscan claims data. This included one study-level covariate used specifically to adjust the 2010 inpatient Marketscan data and another used specifically to adjust the 2012 Marketscan data to the reference literature and registry data sources.

Country-level Covariates

Country-level covariates were used in each of the congenital DisMod-MR models based on literature information about the risk factors for these birth defects. Folic acid availability was used as a covariate on prevalence for all neural tube defects models and a subset of the congenital musculoskeletal anomalies models. The legality of abortion was used as a covariate on prevalence for conditions in which prenatal diagnosis is commonly available and the prognosis is severe enough to cause high rate of termination of pregnancy following prenatal diagnosis: these include all chromosomal conditions and a subset of the congenital heart defects. Maternal consumption of alcohol during pregnancy, as a proportion of all pregnancies, was used as a covariate on prevalence for all congenital heart defects. The proportion of live births by mothers age 35+ was used as a covariate on all chromosomal models. A folic acid fortification covariate was used in the neural tube defects and cleft models, which was modelled based on data from the Global Fortification Data Exchange.

Across many of the congenital models, the Health Access and Quality Index covariate was used to guide the global pattern of with-condition mortality and excess mortality, as was the natural log of the lag-distributed income per capita (lnLDI). For most of the severe congenital conditions, the mortality associated with the condition is highly dependent on access to adequate surgical interventions and other medical care during the first hours, weeks, and years of life.

Assigning health states and sequelae for long-term outcomes

To determine the distribution of health outcomes associated with the congenital causes, we performed a review of available literature on the long-term health outcomes of survivors in cohorts born with each type of congenital malformation. For conditions requiring surgical intervention shortly after birth to ensure survival, the health states included in the disability weight calculations correspond to the post-surgery outcomes reported in cohorts of individuals born with these life-threatening congenital conditions. Where data was available on from multiple cohorts, we pooled these cohorts together to calculate the proportion of individuals with each health state. Where data on the joint distribution of the long-term health outcomes was not available, we assumed independence of each long-term health outcome. Combined disability weights were calculated for all necessary combinations of existing disability weights.

Determining outliers and data thresholds

In order to employ a systematic approach to outliering of data, we used Median Absolute Deviation (MAD). We determined lower and upper thresholds where the data could plausibly fall for each model. Any data outside of these bounds were outliered for ages under 1 year. Data for older age groups were outliered if the corresponding under 1 year old data point for the given age/sex/location/source was outside of the MAD bounds. For most models, these bounds were determined to be ½ of the median and +3 MADs around the median of the data for the given model. This was determined based on the right skewed distribution observed in most of the congenital data. For most models, we calculated the MADs using only the EUROCAT data, which we found to be the most reliable source for prevalence of congenital disorders. We then applied those MADs to all data. For neural tube defects, we calculated the MADs on all data for children under age 1 year, including registry data, hospital data, and insurance claims data. For congenital urinary birth defects, we calculated these bounds using EUROCAT data as well as United States insurance claims data for children under 1 year of age. For some of the musculoskeletal disorders, we used only United States insurance claims data for children under 1 year of age to calculate these bounds, as it was the most reliable source. For chromosomal conditions, given the high volume of zeroes in the data, we calculated the MADs slightly differently. For Down Syndrome, we used all data for children under 1 to establish bounds. For Edward Syndrome and Patau Syndrome we used all non-zero EUROCAT data to establish MAD bounds. For Turner and Klinefelter, due to small numbers and issues in data quality, we used EUROCAT data to calculate a logged mean absolute deviation and exponentiated this to determine bounds for these data. In any case where the lower bound (-1 MAD) was negative, we used a threshold of 0. This strategy is still in development, but allows for consistent and empirical outliering of implausibly high or implausibly low data.

Case definitions and modelling strategy specifics

Neural tube defects

Case definitions

Neural tube defects occur when neural tube fails to close completely during development. The GBD 2017 case definition includes spina bifida, in which part of the spinal cord and/or meninges are uncovered by skin; encephalocele, a congenital defect characterized by sac-like protrusions of the brain and meninges through openings in the skull; and anencephaly, the absence of a major portion of the brain, skull, and scalp. Spina bifida occulta, a much less severe form of spina bifida where the defect in vertebral column remains covered by skin, is excluded from the GBD case definition of spina bifida. All infants born with anencephaly die during the first few weeks of life, as there is no remission and no cure for this condition. Infants born with spina bifida or encephalocele typically require surgical intervention during the first few weeks of life, and thereafter may experience a range of neural and motor complications. Our case definitions of spina bifida and encephalocele do not consider surgical intervention for either condition as remission. In GBD 2017, spina bifida, encephalocele, and anencephaly are each modelled separately and then fit to a total model of all neural tube defects. Spina bifida corresponds to the ICD-10 codes Q05.0, Q05.4,Q05.6, Q05.7, Q05.8, and Q05.9. Encephalocele corresponds to the ICD-10 codes Q01.2, Q01.8, and Q01.9. Anencephaly corresponds to the ICD-10 codes Q00.0 and Q00.2.

Health states associated with neural tube defects

All infants with anencephaly are assigned the health state of severe motor and cognitive impairment. Cases of spina bifida and encephalocele are split into every combination of mild, moderate and severe motor impairment, all severities of intellectual disability, and urinary incontinence. These proportions

were calculated using a pooled analysis of available literature on the long-term outcomes in cohorts of individuals born with each sub-type of neural tube defects. The distribution of health states associated with encephalocele[i ii iii] was derived separately from the distribution of health states associated with spina bifida[iv v] , although these two categories of neural tube defects are associated with the same list of long-term outcome sequela.

Neural tube defects overall modelling strategy and model settings

In order to ensure internal consistency of the estimates of each sub-type of neural tube defects, we developed a model of the total prevalence of neural tube defects and used these location, year, sex and age-specific prevalence estimates to scale the estimates of anencephaly, encephalocele and spina bifida prevalence. This modelling strategy allowed us to incorporate the cause-specific mortality estimates from the GBD Cause of Death analysis and also allowed us to use literature data where the prevalence and mortality estimates were reported for the total of all neural tube defects only.

The DisMod-MR model of total neural tube defects used cause-specific mortality (CSMR) estimates from the GBD cause of death analysis for neural tube defects. This model had a minimum excess mortality of 3.0 for the first week of age and a decreasing slope prior on excess mortality rate for all ages as the risk of death due to neural tube defects is greatest shortly after birth. The model also used an increased smoothness (maximum xi=1) on excess mortality rate in order to allow high excess mortality in the early neonatal age group. Random effects on prevalence were limited to +- 0.5 in order to limit geographic variation in the estimated birth prevalence.

Cause	Covariate name	Type	Measure	Beta value	Exponentiated value
Total neural tube congenital anomalies	Composite fortification standard and folic acid inclusion	Country covariate	Prevalence	-0.040 (-0.091 - -0.001)	0.961 (0.913 - 0.999)
Total neural tube congenital anomalies	Folic acid unadjusted (ug)	Country covariate	Prevalence	-0.001 (-0.001 - -0.001)	0.999 (0.999 - 0.999)
Total neural tube congenital anomalies	LDI (I$ per capita)	Country covariate	Excess mortality rate	-0.101 (-0.102 - -0.100)	0.904 (0.903 - 0.905)
Total neural tube congenital anomalies	All MarketScan, year 2010	Study-level x-covariate	Prevalence	-0.215 (-0.281 - -0.141)	0.807 (0.755 - 0.868)
Total neural tube congenital anomalies	All MarketScan, year 2012	Study-level x-covariate	Prevalence	-0.187 (-0.255 - -0.121)	0.830 (0.775 - 0.886)
Total neural tube congenital anomalies	Chromosomal diagnoses excluded	Study-level x-covariate	Prevalence	-0.187 (-0.224 - -0.152)	0.829 (0.799 - 0.859)
Total neural tube congenital anomalies	Hospital data for ages over 1 year only	Study-level x-covariate	Prevalence	-0.354 (-0.442 - -0.272)	0.702 (0.643 - 0.762)
Total neural tube congenital anomalies	Hospital data for the under-1 year age group	Study-level x-covariate	Prevalence	0.225 (0.174 - 0.249)	1.253 (1.190 - 1.283)
Total neural tube congenital anomalies	Stillbirths included as cases	Study-level x-covariate	Prevalence	0.003 (0.000 - 0.012)	1.003 (1.000 - 1.012)
Total neural tube congenital anomalies	Terminations of pregnancy included as cases	Study-level x-covariate	Prevalence	0.001 (0.000 - 0.004)	1.001 (1.000 - 1.004)
Total neural tube congenital anomalies	Under Reported	Study-level x-covariate	Prevalence	-0.043 (-0.112 - -0.002)	0.958 (0.894 - 0.998)

	Prevalence
Site-years (total)	2002
Number of countries with data	75
Number of GBD regions with data (out of 21 regions)	19
Number of GBD super-regions with data (out of 7 super-regions)	7

Anencephaly modelling strategy and model settings

The life expectancy for infants born with anencephaly is on the order of hours or days; none of these infants survive past the neonatal age period. Because of the extremely high excess mortality associated with this condition and the short age range over which the prevalence varies, we used a custom modelling process to estimate the prevalence of anencephaly. We first used DisMod-MR to model the prevalence of anencephaly at birth for every location, year, age and sex combination. We then used literature data on outcomes largest available cohort of infants born with anencephaly[vi] [vii], using the precise time of death information from this cohort to create a life table that applied the high excess mortality rates to all cases of anencephaly at birth.

We applied these mortality rates to both sex and all locations, generating the time lived by infants with anencephaly during the early and late neonatal age groups by location, year and sex. We then used GBB 2017 mortality estimates to calculate the time lived by all infants during the early and late neonatal age groups by location, year and sex, and used these two values to calculate the prevalence of anencephaly in the early and late neonatal age groups; after one month of age, all available literature indicates that no infants born with anencephaly are still alive.

The DisMod-MR model for the birth prevalence of anencephaly has random effects on prevalence limited to +- 1. As this model was designed to estimate only the prevalence at birth, incidence, remission and excess mortality were set to zero for all ages, and the only age mesh points were 0 and 100 years of age.

Cause	Covariate name	Type	Measure	Beta value	Exponentiated value
Anencephaly	Composite fortification standard and folic acid inclusion	Country covariate	Prevalence	-0.288 (-1.996 - -0.041)	0.750 (0.136 - 0.959)
Anencephaly	Folic acid unadjusted (ug)	Country covariate	Prevalence	-0.002 (-0.021 - -0.000)	0.998 (0.980 - 1.000)
Anencephaly	Legality of Abortion	Country covariate	Prevalence	-0.003 (-0.008 - -0.000)	0.997 (0.992 - 1.000)
Anencephaly	Chromosomal diagnoses excluded	Study-level x-covariate	Prevalence	-0.013 (-0.061 - -0.001)	0.987 (0.941 - 0.999)
Anencephaly	Hospital Inpatient	Study-level x-covariate	Prevalence	-0.010 (-0.032 - -0.000)	0.990 (0.968 - 1.000)
Anencephaly	MarketScan	Study-level x-covariate	Prevalence	-1.199 (-1.357 - -1.049)	0.302 (0.257 - 0.350)
Anencephaly	Stillbirths included as cases	Study-level x-covariate	Prevalence	0.571 (0.043 - 0.701)	1.770 (1.044 - 2.016)
Anencephaly	Terminations of pregnancy included as cases	Study-level x-covariate	Prevalence	0.912 (0.233 - 0.997)	2.489 (1.262 - 2.710)

	Prevalence
Site-years (total)	973
Number of countries with data	51
Number of GBD regions with data (out of 21 regions)	17
Number of GBD super-regions with data (out of 7 super-regions)	7

Encephalocele modelling strategy and model settings

The DisMod-MR model for encephalocele had a minimum excess mortality of 5.0 for the first week of age and a decreasing slope prior on excess mortality rate for all ages. The model also used an increased smoothness on excess mortality rate (maximum xi=1). Random effects on prevalence were limited to +- 0.5 as we expect limited geographic variation in the birth prevalence of encephalocele.

Cause	Covariate name	Type	Measure	Beta value	Exponentiated value
Encephalocele	Composite fortification standard and folic acid inclusion	Country covariate	Prevalence	-0.317 (-0.428 - -0.231)	0.728 (0.652 - 0.794)
Encephalocele	Folic acid unadjusted (ug)	Country covariate	Prevalence	-0.000 (-0.001 - -0.000)	1.000 (0.999 - 1.000)
Encephalocele	LDI (I$ per capita)	Country covariate	Excess mortality rate	-0.624 (-1.000 - -0.253)	0.536 (0.368 - 0.777)
Encephalocele	Legality of Abortion	Country covariate	Prevalence	-0.009 (-0.011 - -0.006)	0.991 (0.989 - 0.994)
Encephalocele	Chromosomal diagnoses excluded	Study-level x-covariate	Prevalence	-0.249 (-0.292 - -0.195)	0.780 (0.747 - 0.823)
Encephalocele	Hospital data for ages over 1 year only	Study-level x-covariate	Prevalence	-0.005 (-0.021 - -0.000)	0.995 (0.980 - 1.000)
Encephalocele	Hospital data for the under-1 year age group	Study-level x-covariate	Prevalence	-0.024 (-0.090 - -0.001)	0.976 (0.914 - 0.999)
Encephalocele	MarketScan	Study-level x-covariate	Prevalence	-0.052 (-0.136 - -0.002)	0.950 (0.873 - 0.998)
Encephalocele	Stillbirths included as cases	Study-level x-covariate	Prevalence	0.286 (0.257 - 0.299)	1.331 (1.292 - 1.349)
Encephalocele	Terminations of pregnancy included as cases	Study-level x-covariate	Prevalence	0.142 (0.017 - 0.276)	1.153 (1.017 - 1.318)

	Prevalence
Site-years (total)	1508
Number of countries with data	54
Number of GBD regions with data (out of 21 regions)	16
Number of GBD super-regions with data (out of 7 super-regions)	6

Spina bifida modelling strategy and model settings

The DisMod-MR model for spina bifida had a minimum excess mortality of 3.0 for the first week of age, a decreasing slope prior on excess mortality rate for all ages, and a maximum smoothness on excess mortality rate of xi=1. Random effects on prevalence were also limited to +- 0.5.

Cause	Covariate name	Type	Measure	Beta value	Exponentiated value
Spina bifida	Composite fortification standard and folic acid inclusion	Country covariate	Prevalence	-0.144 (-0.220 - -0.083)	0.866 (0.803 - 0.921)
Spina bifida	Folic acid unadjusted (ug)	Country covariate	Prevalence	-0.000 (-0.000 - -0.000)	1.000 (1.000 - 1.000)
Spina bifida	Healthcare access and quality index	Country covariate	With-condition mortality rate	-0.501 (-1.000 - 0.000)	0.606 (0.368 - 1.000)
Spina bifida	LDI (I$ per capita)	Country covariate	Excess mortality rate	-0.625 (-1.000 - -0.250)	0.535 (0.368 - 0.779)
Spina bifida	Legality of Abortion	Country covariate	Prevalence	-0.008 (-0.009 - -0.006)	0.992 (0.991 - 0.994)
Spina bifida	Chromosomal diagnoses excluded	Study-level x-covariate	Prevalence	-0.022 (-0.054 - -0.001)	0.978 (0.948 - 0.999)

Spina bifida	Hospital data for ages over 1 year only	Study-level x-covariate	Prevalence	-0.039 (-0.114 - -0.002)	0.962 (0.892 - 0.998)
Spina bifida	Hospital data for the under-1 year age group	Study-level x-covariate	Prevalence	-0.012 (-0.042 - -0.000)	0.988 (0.959 - 1.000)
Spina bifida	MarketScan	Study-level x-covariate	Prevalence	-0.257 (-0.336 - -0.176)	0.773 (0.714 - 0.838)
Spina bifida	Stillbirths included as cases	Study-level x-covariate	Prevalence	0.267 (0.230 - 0.298)	1.306 (1.258 - 1.347)
Spina bifida	Terminations of pregnancy included as cases	Study-level x-covariate	Prevalence	0.027 (0.001 - 0.085)	1.027 (1.001 - 1.088)

	**Prevalence**
Site-years (total)	1890
Number of countries with data	66
Number of GBD regions with data (out of 21 regions)	19
Number of GBD super-regions with data (out of 7 super-regions)	7

Congenital heart anomalies

Case definitions

There are many distinct types of congenital heart anomalies with a range of anatomical patterns, severities, and requirements for medical treatment. For the purposes of estimating nonfatal outcomes, in GBD 2017 congenital heart anomalies were split into five-sub categories based on both the anatomical characteristics and the treatment requirements of each condition. This remains our strategy in GBD 2017.

The first sub-cause category, single ventricle and single ventricle pathway defects include tricuspid atresia, hypoplastic left heart syndrome, mitral valve atresia, single left ventricle, double outlet right ventricle, and pulmonary atresia; the corresponding ICD-10 codes are Q20.1, Q20.2, Q20.4, Q22.4, Q22.6 and Q23.4. Each of the single ventricle and single ventricle pathway conditions requires surgical intervention shortly after birth to ensure infant survival.

The second sub-cause category, severe congenital heart defects excluding single ventricle and single ventricle pathway defects, includes common arterial trunk, common truncus, discordant ventriculoaterial connection, transposition of great vessels, atrioventricular septal defect, endocardial cushion defect, Tetralogy of fallot, aortopulmonary septal defect, pulmonary valve atresia, congenital stenosis of aortic valve, and total anomalous pulmonary venous connection. This category of severe congenital heart defects includes ICD-10 codes Q20.0; Q20.3; Q21.2; Q21.3; Q21.4; Q22.0; Q23.0 and Q26.2.

The third sub-cause category is critical malformations of great vessels, congenital valvular heart disease and patent ductus arteriosis. The malformations of vessels and valves in this sub-cause category include Ebstein's anomaly, congenital pulmonary valve stenosis, pulmonary valve insufficiency, other malformations of the pulmonary valve, malformations of the tricuspid valve, tricuspid atresia or stenosis, insufficiency of the aortic valve, mitral stenosis or insufficiency, and other malformations of aortic and mitral valves. Patent ductus arteriosis cases are only included among infants of >37 weeks gestational age, as premature infants often have minor patent ductus arteriosis that closes shortly after birth. The ICD-10 codes corresponding to the critical malformations of great vessels category include Q22.1, Q22.2, Q22.3, Q22.5, Q22.8, Q22.9, Q23.1, Q23.2, Q23.3, Q23.8, Q23, Q25.1, Q25.2, Q25.3, Q25.4, Q25.5, and Q25.0. The majority of these conditions require medical attention shortly within the first few weeks of life.

The fourth sub-cause category, ventricular septal defects and atrial septal defects, includes holes in the walls separating the chambers of the heart. Many of these septal defects close spontaneously, while

other require surgical care. The ICD-10 codes corresponding to ventricular septal defect and atrial septal defect are Q21.0 and Q21.1, respectively.

The fifth and final sub-cause category of congenital heart defects is other congenital cardiovascular anomalies, which correspond to ICD-10 codes Q27, Q27.1, Q27.2, Q27.3, Q27.30, Q27.31, Q27.32, Q27.33, Q27.34, Q27.39, Q27.4, Q27.8, Q27.9, Q28, Q28.0, Q28.1, Q28.2, Q28.3, Q28.8 and Q28.9.

Health states associated with congenital heart anomalies

Every case of congenital heart defects was associated with a health state of congenital heart disease, except for a proportion of ventricular and atrial septal defects which are considered asymptomatic. All congenital heart defects cases were split into a proportion without intellectual disability and a proportion with every severity from borderline to profound intellectual disability. The proportion of congenital heart anomalies cases experiencing each severity of intellectual disability were calculated using available literature sources on the prevalence and severity of intellectual disability in congenital heart defect populations[viii] [ix] [x] . The proportion of VSD/ASD cases attributed to the asymptomatic category was derived from literature sources on the long-term outcomes of patients diagnosed with septal defects at birth[xi] [xii] [xiii]. GBD estimates of congenital heart failure were assigned to the congenital heart defect categories according to the proportion of total congenital heart cause-specific mortality assigned to each category of congenital heart defects.

Modelling strategy and model settings

In order to ensure internally consistent estimates of the prevalence of congenital heart anomalies, we developed a model of the total prevalence of congenital heart anomalies and fit the estimates of each sub-type of congenital heart anomalies proportionally to these total envelope estimates. The prevalence estimates of other congenital heart anomalies were derived by reducing the total envelope model for each cause by its sub-causes to derive the difference that was attributable to other anomalies in that category.

.

The National Birth Defects Prevention Network (NBDPN), the United States state-level birth defects reporting system, includes the most comprehensive case reporting of any registry data source. As reported in the tables below, the congenital heart models each used registry-specific study-level covariate crosswalks to adjust input data from all other birth registries upward according to the composition of cases included in the NBDPN. All hospital and Marketscan data were also adjusted using study-level covariates with the NBDPN and literature prevalence values as the reference data.

Total congenital heart anomalies model settings

In the DisMod model of total congenital heart anomalies, random effects on prevalence were limited to +- 0.5 in order to limit geographic variation in the estimates of birth prevalence. The model included a decreasing slope prior on excess mortality rate for all ages, as the risk of death due to congenital heart anomalies is greatest shortly after birth. The minimum excess mortality rate for the neonatal age range was set to 5.0. The smoothness on excess mortality rate was increased to Xi=5.0 in order to allow high excess mortality in the neonatal age groups and lower excess mortality rates in older ages.

During model development, all registry prevalence values below the threshold of 3 per 1,000 were marked with a study-level covariate to indicate under-reporting, regardless of whether a comprehensive list of ICD codes or cases was included in the input data. These under-reported data sources were then adjusted upwards to the reference data.

Cause	Covariate name	Type	Measure	Beta value	Exponentiated value
Total cardiovascular congenital anomalies	Healthcare access and quality index	Country covariate	Prevalence	0.015 (0.013 - 0.017)	1.015 (1.013 - 1.017)
Total cardiovascular congenital anomalies	LDI (I$ per capita)	Country covariate	Excess mortality rate	-0.999 (-1.250 - -0.750)	0.368 (0.287 - 0.472)
Total cardiovascular congenital anomalies	Maternal alcohol consumption during pregnancy (proportion)	Country covariate	Prevalence	0.299 (0.007 - 0.493)	1.349 (1.007 - 1.637)
Total cardiovascular congenital anomalies	Chromosomal diagnoses excluded	Study-level x-covariate	Prevalence	-0.075 (-0.108 - -0.042)	0.927 (0.897 - 0.959)
Total cardiovascular congenital anomalies	EUROCAT to NBDPN registry case composition adjustment	Study-level x-covariate	Prevalence	-0.003 (-0.008 - -0.000)	0.997 (0.992 - 1.000)
Total cardiovascular congenital anomalies	Hospital data for ages over 1 year only	Study-level x-covariate	Prevalence	-1.618 (-1.828 - -0.626)	0.198 (0.161 - 0.535)
Total cardiovascular congenital anomalies	Hospital data for the under-1 year age group	Study-level x-covariate	Prevalence	0.013 (-0.124 - 0.200)	1.013 (0.883 - 1.221)
Total cardiovascular congenital anomalies	ICDBSR to NBDPN registry case composition adjustment	Study-level x-covariate	Prevalence	-1.400 (-1.554 - -1.318)	0.247 (0.211 - 0.268)
Total cardiovascular congenital anomalies	MarketScan	Study-level x-covariate	Prevalence	-0.700 (-0.824 - -0.042)	0.496 (0.439 - 0.959)
Total cardiovascular congenital anomalies	Stillbirths included as cases	Study-level x-covariate	Prevalence	0.084 (0.037 - 0.099)	1.088 (1.038 - 1.105)
Total cardiovascular congenital anomalies	Terminations of pregnancy included as cases	Study-level x-covariate	Prevalence	0.097 (0.089 - 0.100)	1.102 (1.093 - 1.105)

	Prevalence
Site-years (total)	1618
Number of countries with data	64
Number of GBD regions with data (out of 21 regions)	16
Number of GBD super-regions with data (out of 7 super-regions)	7

Single ventricle and single ventricle pathway defects model settings

In the DisMod model of single ventricle and single ventricle pathway heart defects, random effects on prevalence were limited to +- 0.5 in order to limit the estimated geographic variation in birth prevalence. A minimum excess mortality rate of 10.0 was set for the early neonatal period in order to capture the high mortality risk, based on expert priors and a review available literature on the mortality risk among infants born with single ventricle and single ventricle pathway heart defects. The smoothness on excess mortality rate was set to 5.0 in order to fit steep changes in the excess mortality rate during the first

weeks of life, and a decreasing slope prior on excess mortality rate was applied to all ages, as the risk of death due to these congenital heart anomalies is greatest shortly after birth and diminishes over the life course.

Cause	Covariate name	Type	Measure	Beta value	Exponentiated value
Single ventricle and single ventricle pathway heart defects	LDI (I$ per capita)	Country covariate	Excess mortality rate	-0.747 (-0.998 - -0.500)	0.474 (0.369 - 0.607)
Single ventricle and single ventricle pathway heart defects	Maternal alcohol consumption during pregnancy (proportion)	Country covariate	Prevalence	0.139 (0.005 - 0.436)	1.149 (1.005 - 1.547)
Single ventricle and single ventricle pathway heart defects	Chromosomal diagnoses excluded	Study-level x-covariate	Prevalence	-0.046 (-0.089 - -0.008)	0.955 (0.915 - 0.992)
Single ventricle and single ventricle pathway heart defects	EUROCAT to NBDPN registry case composition adjustment	Study-level x-covariate	Prevalence	-0.035 (-0.100 - -0.001)	0.966 (0.904 - 0.999)
Single ventricle and single ventricle pathway heart defects	Hospital data for ages over 1 year only	Study-level x-covariate	Prevalence	-0.014 (-0.049 - -0.000)	0.986 (0.953 - 1.000)
Single ventricle and single ventricle pathway heart defects	Hospital data for the under-1 year age group	Study-level x-covariate	Prevalence	-0.014 (-0.045 - -0.000)	0.986 (0.956 - 1.000)
Single ventricle and single ventricle pathway heart defects	MarketScan	Study-level x-covariate	Prevalence	-0.001 (-0.003 - -0.000)	0.999 (0.997 - 1.000)
Single ventricle and single ventricle pathway heart defects	Stillbirths included as cases	Study-level x-covariate	Prevalence	0.002 (0.000 - 0.009)	1.002 (1.000 - 1.009)
Single ventricle and single ventricle pathway heart defects	Terminations of pregnancy included as cases	Study-level x-covariate	Prevalence	0.012 (0.000 - 0.046)	1.012 (1.000 - 1.047)
Single ventricle and single ventricle pathway heart defects	Under Reported	Study-level x-covariate	Prevalence	-0.317 (-0.412 - -0.222)	0.728 (0.662 - 0.801)

	Prevalence
Site-years (total)	1332
Number of countries with data	48
Number of GBD regions with data (out of 21 regions)	16
Number of GBD super-regions with data (out of 7 super-regions)	7

Severe congenital heart defects excluding single ventricle and single ventricle pathway defects model settings

In the DisMod model of congenital heart defects excluding single ventricle and single ventricle pathway defects, random effects on prevalence were limited to +- 0.5. A minimum excess mortality rate of 5.0 for the early neonatal period was enforced in order to capture the high risk of mortality associated with these conditions, and a decreasing slope prior on excess mortality rate was applied for all ages. The

smoothness on excess mortality rate was set to Xi = 1.0 in order to allow the model to fit steep changes in the mortality rate of these conditions in the neonatal age period.

Cause	Covariate name	Type	Measure	Beta value	Exponentiated value
Severe congenital heart defects excluding single ventricle and single ventricle pathway	LDI (I$ per capita)	Country covariate	Excess mortality rate	-0.621 (-1.000 - -0.250)	0.537 (0.368 - 0.779)
Severe congenital heart defects excluding single ventricle and single ventricle pathway	Maternal alcohol consumption during pregnancy (proportion)	Country covariate	Prevalence	0.365 (0.094 - 0.497)	1.441 (1.099 - 1.644)
Severe congenital heart defects excluding single ventricle and single ventricle pathway	Chromosomal diagnoses excluded	Study-level x-covariate	Prevalence	-0.204 (-0.231 - -0.180)	0.815 (0.794 - 0.835)
Severe congenital heart defects excluding single ventricle and single ventricle pathway	EUROCAT to NBDPN registry case composition adjustment	Study-level x-covariate	Prevalence	-0.309 (-0.544 - -0.145)	0.734 (0.580 - 0.865)
Severe congenital heart defects excluding single ventricle and single ventricle pathway	Hospital data for ages over 1 year only	Study-level x-covariate	Prevalence	-0.068 (-0.235 - -0.002)	0.935 (0.790 - 0.998)
Severe congenital heart defects excluding single ventricle and single ventricle pathway	Hospital data for the under-1 year age group	Study-level x-covariate	Prevalence	-0.137 (-0.287 - -0.020)	0.872 (0.751 - 0.980)
Severe congenital heart defects excluding single ventricle and single ventricle pathway	ICDBSR to NBDPN registry case composition adjustment	Study-level x-covariate	Prevalence	-0.781 (-0.827 - -0.729)	0.458 (0.437 - 0.483)
Severe congenital heart defects excluding single ventricle and single ventricle pathway	Stillbirths included as cases	Study-level x-covariate	Prevalence	0.004 (0.001 - 0.013)	1.004 (1.001 - 1.013)
Severe congenital heart defects excluding single ventricle and single ventricle pathway	Terminations of pregnancy included as cases	Study-level x-covariate	Prevalence	0.072 (0.009 - 0.135)	1.074 (1.009 - 1.145)
Severe congenital heart defects excluding single ventricle and single ventricle pathway	Under Reported	Study-level x-covariate	Prevalence	-0.174 (-0.344 - -0.044)	0.840 (0.709 - 0.957)

Severe congenital heart defects excluding single ventricle and single ventricle pathway	World Atlas to NBDPN registry case composition adjustment	Study-level x-covariate	Prevalence	-0.826 (-0.881 - -0.766)	0.438 (0.414 - 0.465)

	Prevalence
Site-years (total)	1344
Number of countries with data	45
Number of GBD regions with data (out of 21 regions)	15
Number of GBD super-regions with data (out of 7 super-regions)	6

Critical malformations of great vessels, congenital valvular heart disease and patent ductus arteriosis model settings

In the DisMod model of critical malformations of great vessels, congenital valvular heart disease and patent ductus arteriosis, random effects on prevalence were limited to +- 0.5. A minimum excess mortality rate of 5.0 was set for the early neonatal period in order to capture the high mortality risk associated with these conditions. A decreasing slope prior was applied to excess mortality rate for all ages, as the risk of death due to congenital heart anomalies is highest shortly after birth. The smoothness on excess mortality was increased to Xi = 3.0 in order to fit steep changes in the mortality associated with these conditions during and after the neonatal period.

Cause	Covariate name	Type	Measure	Beta value	Exponentiated value
Critical malformations of great vessels, congenital valvular heart disease and patent ductus arteriosis	LDI (I$ per capita)	Country covariate	Excess mortality rate	-0.275 (-0.500 - -0.056)	0.760 (0.607 - 0.946)
Critical malformations of great vessels, congenital valvular heart disease and patent ductus arteriosis	Maternal alcohol consumption during pregnancy (proportion)	Country covariate	Prevalence	0.130 (0.004 - 0.453)	1.139 (1.004 - 1.573)
Critical malformations of great vessels, congenital valvular heart disease and patent ductus arteriosis	Chromosomal diagnoses excluded	Study-level x-covariate	Prevalence	-0.068 (-0.100 - -0.032)	0.934 (0.905 - 0.969)
Critical malformations of great vessels, congenital valvular heart disease and patent ductus arteriosis	EUROCAT to NBDPN registry case composition adjustment	Study-level x-covariate	Prevalence	-0.169 (-0.386 - -0.021)	0.845 (0.680 - 0.979)
Critical malformations of great vessels, congenital valvular heart disease and	Hospital data for ages over 1 year only	Study-level x-covariate	Prevalence	-0.274 (-0.670 - -0.002)	0.760 (0.512 - 0.998)

patent ductus arteriosis					
Critical malformations of great vessels, congenital valvular heart disease and patent ductus arteriosis	Hospital data for the under-1 year age group	Study-level x-covariate	Prevalence	-0.090 (-0.387 - -0.003)	0.914 (0.679 - 0.997)
Critical malformations of great vessels, congenital valvular heart disease and patent ductus arteriosis	ICDBSR to NBDPN registry case composition adjustment	Study-level x-covariate	Prevalence	-0.504 (-0.752 - -0.396)	0.604 (0.472 - 0.673)
Critical malformations of great vessels, congenital valvular heart disease and patent ductus arteriosis	MarketScan	Study-level x-covariate	Prevalence	-0.059 (-0.225 - -0.001)	0.942 (0.799 - 0.999)
Critical malformations of great vessels, congenital valvular heart disease and patent ductus arteriosis	Stillbirths included as cases	Study-level x-covariate	Prevalence	0.009 (0.000 - 0.033)	1.009 (1.000 - 1.034)
Critical malformations of great vessels, congenital valvular heart disease and patent ductus arteriosis	Terminations of pregnancy included as cases	Study-level x-covariate	Prevalence	0.150 (0.000 - 0.296)	1.162 (1.000 - 1.345)
Critical malformations of great vessels, congenital valvular heart disease and patent ductus arteriosis	Under Reported	Study-level x-covariate	Prevalence	-0.315 (-0.423 - -0.187)	0.730 (0.655 - 0.830)

	**Prevalence**
Site-years (total)	1618
Number of countries with data	64
Number of GBD regions with data (out of 21 regions)	16
Number of GBD super-regions with data (out of 7 super-regions)	7

Ventricular septal defects and atrial septal defects model settings

In the DisMod model of ventricular septal defects and atrial septal defects (VSD/ASD), remission was set to zero for all ages. Cases of septal defects that spontaneously close over time were considered as part of the asymptomatic proportion of VSD/ASD rather than remitted cases. Random effects on prevalence were limited to +- 0.3 in order to limit the random geographic variation in the estimated birth prevalence. No minimum excess mortality rate was set in this model, as VSD/ASD cases are not associated with excess mortality rates as high as the other subtypes of congenital heart defects. The smoothness on excess mortality rate was set to Xi=1.0, and a decreasing slope prior was set on excess mortality rate for all ages.

Cause	Covariate name	Type	Measure	Beta value	Exponentiated value
Ventricular septal defect and atrial septal defect	Healthcare access and quality index	Country covariate	With-condition mortality rate	-0.089 (-0.154 - -0.045)	0.915 (0.858 - 0.956)
Ventricular septal defect and atrial septal defect	LDI (I$ per capita)	Country covariate	Excess mortality rate	-0.025 (-0.050 - -0.000)	0.975 (0.952 - 1.000)
Ventricular septal defect and atrial septal defect	Maternal alcohol consumption during pregnancy (proportion)	Country covariate	Prevalence	0.229 (0.039 - 0.442)	1.257 (1.040 - 1.556)
Ventricular septal defect and atrial septal defect	Hospital data for ages over 1 year only	Study-level x-covariate	Prevalence	-1.558 (-1.684 - -1.440)	0.211 (0.186 - 0.237)
Ventricular septal defect and atrial septal defect	Hospital data for the under-1 year age group	Study-level x-covariate	Prevalence	-0.118 (-0.265 - -0.010)	0.889 (0.767 - 0.991)
Ventricular septal defect and atrial septal defect	MarketScan	Study-level x-covariate	Prevalence	-1.052 (-1.197 - -0.948)	0.349 (0.302 - 0.387)
Ventricular septal defect and atrial septal defect	Stillbirths included as cases	Study-level x-covariate	Prevalence	0.041 (0.002 - 0.113)	1.042 (1.002 - 1.120)
Ventricular septal defect and atrial septal defect	Terminations of pregnancy included as cases	Study-level x-covariate	Prevalence	0.128 (0.007 - 0.281)	1.137 (1.007 - 1.325)
Ventricular septal defect and atrial septal defect	Under Reported	Study-level x-covariate	Prevalence	-0.536 (-0.669 - -0.400)	0.585 (0.512 - 0.670)

	Prevalence
Site-years (total)	1142
Number of countries with data	44
Number of GBD regions with data (out of 21 regions)	16
Number of GBD super-regions with data (out of 7 super-regions)	7

Other congenital cardiovascular birth defects

Other congenital cardiovascular anomalies are modeled by applying the ratio of other congenital digestive anomalies to total congenital digestive anomalies as it is reflected in Marketscan data (a trusted data source), to the sum of the sub-causes of congenital cardiovascular anomalies. The result is prevalence of other congenital cardiovascular anomalies by age/year/sex/location.

Cleft lip & cleft palate (orofacial clefts)

Case definition and associated health states

Orofacial clefts include isolated cleft lip, isolated cleft palate, and combined cleft lip and cleft palate. Cleft lip is an opening in the upper lip that may extend into the nose, and with cleft palate, the roof of the mouth contains an opening into the nose. Both conditions are the result of the tissues of the face not joining properly during development. These conditions can be successfully treated by surgery, which is typically done during the first few months or years of life but may occasionally be completed later in life. The sequelae associated with orofacial clefts are disfigurement level 1, disfigurement level 2, and disfigurement level 2 with speech problems. Additionally, a proportion of the population with orofacial clefts is considered to be asymptomatic. The proportion of cleft cases with associated speech problems was calculated following a review of available literature on orofacial cleft health outcomes.

The GBD case definition of orofacial clefts includes isolated cleft palate, which corresponds to ICD-10 codes Q35.2, Q35.3, Q35.5, Q35.6, Q35.7, Q35.8, and Q35.9, and cleft palate with or without cleft lip, which corresponds to ICD-10 codes Q36.0, Q36.1, Q36.9, Q37.1, Q37.5, Q37.8, and Q37.9.

Modelling strategy and model settings

The DisMod-MR model of orofacial clefts had random effects on prevalence limited to +- 0.5, as we expected limited variation in birth prevalence of orofacial clefts. The model settings allow increased smoothness on both excess mortality rate and remission (maximum Xi = 5.0) in order to fit steep changes in the rates mortality and remission during the first few years of life.

Incidence was set to zero for all ages. Remission was set to zero for the first three months of life, as cleft lip and/or palate are rarely corrected in the first few months of life. A maximum remission of 0.8 was set for ages three months to two years, the age range in which cleft repair is most commonly performed, allowing up to 75% of cleft cases to be repaired between three months and 2 years of age. Remission was bounded from 0 to 0.07 for ages 2 to 5 years, 0 to 0.004 for ages 5 to 20 years, then bounded from 0 to 0.002 for ages 20 to 50 years, and set at 0 for ages 50 years +. These limits on remission reflect our priors that up to 20% of remaining cleft cases are repaired between 2 and 5 years of age, another 5% may be repaired between 5 and 20 years of age, and a maximum 5% of remaining cases are surgically repaired between ages 20 and 50 years.

Priors on excess mortality rate were set at a maximum of 2.5 for the early neonatal period, 0.9 for the late neonatal period, 0.24 for the rest of the first year of life, 0.05 for ages 1 to 5 years, and was set to 0 for ages after 5 years. These limits on excess mortality reflect our priors that up to 5% of individuals with orofacial clefts die in the first week of life, up to 5% die in the following three weeks, up to 20% die in the next 11 months, another maximum of 20% before 5 years of ages, and a maximum of 5% of the remaining individuals die between ages 5 and 10 years.

During model development, all birth registry prevalence values below 2 per 10,000 were excluded as outliers, as these data are considered low enough to indicate severe under-reporting in the input data.

Cause	Covariate name	Type	Measure	Beta value	Exponentiated value
Orofacial clefts	Composite fortification standard and folic acid inclusion	Country covariate	Prevalence	-0.109 (-0.140 - -0.075)	0.897 (0.869 - 0.928)
Orofacial clefts	Folic acid unadjusted (ug)	Country covariate	Prevalence	-0.000 (-0.000 - -0.000)	1.000 (1.000 - 1.000)
Orofacial clefts	Healthcare access and quality index	Country covariate	Prevalence	-0.005 (-0.005 - -0.004)	0.995 (0.995 - 0.996)
Orofacial clefts	LDI (I$ per capita)	Country covariate	Excess mortality rate	-0.749 (-0.750 - -0.746)	0.473 (0.472 - 0.474)
Orofacial clefts	Chromosomal diagnoses excluded	Study-level x-covariate	Prevalence	-0.117 (-0.135 - -0.098)	0.889 (0.874 - 0.906)
Orofacial clefts	Hospital data for ages over 1 year only	Study-level x-covariate	Prevalence	-0.519 (-0.617 - -0.420)	0.595 (0.540 - 0.657)
Orofacial clefts	Hospital data for the under-1 year age group	Study-level x-covariate	Prevalence	-0.**241** (-0.326 - -0.154)	0.786 (0.722 - 0.857)
Orofacial clefts	MarketScan	Study-level x-covariate	Prevalence	-0.230 (-0.271 - -0.189)	0.795 (0.762 - 0.828)
Orofacial clefts	Stillbirths included as cases	Study-level x-covariate	Prevalence	0.071 (0.043 - 0.099)	1.074 (1.044 - 1.104)
Orofacial clefts	Under Reported	Study-level x-covariate	Prevalence	-0.399 (-0.545 - -0.259)	0.671 (0.580 - 0.772)
Orofacial clefts	Under Reported	Study-level z-covariate	Prevalence	0.116 (0.046 - 0.215)	1.123 (1.047 - 1.239)

	Prevalence
Site-years (total)	1701
Number of countries with data	65
Number of GBD regions with data (out of 21 regions)	18
Number of GBD super-regions with data (out of 7 super-regions)	7

Chromosomal Anomalies

In addition to Down syndrome, Turner syndrome, and Klinefelter syndrome, hundreds of different types of chromosomal abnormalities and other genetic syndromes have been identified, described, and categorized. Commonalties between genetic syndromes include the predisposition of affected persons to have dysmorphic body features, congenital heart disease, endocrine problems, and neurodevelopmental abnormalities that can lead to intellectual disability. Many of those with chromosomal abnormalities can be readily recognized or suspected by such features. While each has hallmark physical features and diagnostic criteria, most also require sophisticated laboratory facilities to confirm diagnosis, which means that especially in lower resource settings, a large number of cases are diagnosed as having "unspecified chromosomal abnormalities" which is an ICD code that corresponds to the GBD cause of "other chromosomal abnormalities." Additionally, most congenital birth defects registries have only limited comprehensiveness as they only track a subset of genetic syndromes.

Total chromosomal anomalies

In order to maximize the data basis for estimating chromosomal abnormalities and genetic syndromes, we therefore completed an analysis of all chromosomal abnormalities together, leveraging cause-specific mortality results from the GBD COD analysis (for Down syndrome plus "other chromosomal abnormalities"), all prevalence data from registries, and clinical administrative data (hospital and claims). This modelled estimate of total chromosomal abnormalities was run in DisMod-MR 2.1 and served as the basis for scaling the remaining specific causes (Down, Klinefelter, Turner, Edward/Patau) and estimating the remainder.

Cause	Covariate name	Type	Measure	Beta value	Exponentiated value
Total chromosomal congenital anomalies	LDI (I$ per capita)	Country covariate	Excess mortality rate	-0.334 (-1.204 - -0.000)	0.716 (0.300 - 1.000)
Total chromosomal congenital anomalies	Legality of Abortion	Country covariate	Prevalence	-0.006 (-0.010 - -0.002)	0.994 (0.990 - 0.998)
Total chromosomal congenital anomalies	Live Births 35+ (proportion)	Country covariate	Prevalence	0.211 (0.133 - 0.276)	1.235 (1.142 - 1.318)
Total chromosomal congenital anomalies	Hospital data for ages over 1 year only	Study-level x-covariate	Prevalence	-0.807 (-0.994 - -0.466)	0.446 (0.370 - 0.628)
Total chromosomal congenital anomalies	Hospital data for the under-1 year age group	Study-level x-covariate	Prevalence	-0.114 (-0.459 - 0.285)	0.892 (0.632 - 1.330)

Total chromosomal congenital anomalies	MarketScan	Study-level x-covariate	Prevalence	-0.499 (-0.653 - -0.282)	0.607 (0.521 - 0.754)

	Prevalence
Site-years (total)	597
Number of countries with data	31
Number of GBD regions with data (out of 21 regions)	11
Number of GBD super-regions with data (out of 7 super-regions)	5

Down Syndrome

Case definition and associated health states

Down syndrome, also known as Trisomy 21, is the presence of a third copy of chromosome 21, typically caused by nondisjunction during the production of gametes. Down syndrome is associated with several specific physical characteristics, including decreased muscle tone, flat facial features, an upward slant to the eyes, abnormally shaped ears, a single deep crease across the center of the palm, folded skin on the inner corners of the eyes, and ability to extend joints beyond the usual, among others. The GBD case definition of Down syndrome includes ICD-10 codes Q90.0, Q90.1, Q90.2, and Q90.9.

Individuals with Down syndrome may have several combinations of sequelae: those included in the GBD sequelae list are intellectual disability, congenital heart disease, and dementia. The joint distribution of intellectual disability, congenital heart disease, and dementia associated with cases of Down Syndrome was derived from a review of literature on long-term outcomes in cohorts of Down Syndrome individuals. To calculate the severity distribution of intellectual disability due to Down Syndrome, we used literature values for the IQ distribution of individuals with Down Syndrome[xiv] and calculated the area under the curve. We obtained age-specific proportions of individuals with Down Syndrome and dementia, and thus global age patterns were modelled to calculate the proportion of the population with each combination of sequelae for each of the following age ranges: 0-44 years, 45-49 years, 50-54 years, 55-69 years, 70-79 years, and 80+ years.

Modelling strategy and model settings

The DisMod-MR model of Down Syndrome excluded all data with a prevalence of zero as outliers, as we expect that these low values are indicative of under-reporting in the data sources. The DisMod model used cause-specific mortality rate data from the corresponding Down Syndrome model in the GBD Cause of Death analysis, and converted these data to excess mortality rate estimates where matching prevalence data is available. Random effects on prevalence were limited to +- 0.5 and random effects on excess mortality rate are limited to +- 1.0 in order to limit the geographic variation in birth prevalence allowed in the model. The maximum smoothness on excess mortality rate was increased to x= 2.0 in order to fit the observed steep decline in the mortality risk associated with Down Syndrome after the neonatal age range.

Of note, the use of cause-specific mortality data in the nonfatal model of Down Syndrome is a substantial change in the modelling strategy as compared to the previous iterations of the GBD, and results in much better-informed excess mortality estimates driving the Down Syndrome prevalence estimates across the life course.

Cause	Covariate name	Type	Measure	Beta value	Exponentiated value
Down syndrome	LDI (I$ per capita)	Country covariate	Excess mortality rate	-0.272 (-0.310 - -0.230)	0.762 (0.733 - 0.794)
Down syndrome	Legality of Abortion	Country covariate	Prevalence	-0.005 (-0.007 - -0.003)	0.995 (0.993 - 0.997)
Down syndrome	Live Births 35+ (proportion)	Country covariate	Prevalence	0.026 (0.002 - 0.067)	1.026 (1.002 - 1.069)
Down syndrome	Hospital data for ages over 1 year only	Study-level x-covariate	Prevalence	-0.310 (-0.562 - -0.057)	0.734 (0.570 - 0.945)
Down syndrome	Hospital data for the under-1 year age group	Study-level x-covariate	Prevalence	0.234 (0.080 - 0.381)	1.263 (1.083 - 1.464)
Down syndrome	MarketScan	Study-level x-covariate	Prevalence	-0.157 (-0.201 - -0.118)	0.854 (0.818 - 0.888)
Down syndrome	Stillbirths included as cases	Study-level x-covariate	Prevalence	0.020 (-0.032 - 0.071)	1.020 (0.968 - 1.073)
Down syndrome	Terminations of pregnancy included as cases	Study-level x-covariate	Prevalence	0.006 (-0.048 - 0.108)	1.006 (0.953 - 1.114)

	Prevalence
Site-years (total)	1585
Number of countries with data	61
Number of GBD regions with data (out of 21 regions)	17
Number of GBD super-regions with data (out of 7 super-regions)	7

Turner Syndrome

Case definitions and associated health states

Turner syndrome, also known as 45 XO, is a condition in which a female is partly or completely missing an X chromosome. Turner syndrome can lead to a variety of medical and developmental problems, including short height, failure to start puberty, infertility, heart defects, learning disabilities, and difficulty with social adjustment. The GBD case definition of Turner syndrome includes ICD-10 codes Q96.0, Q96.3, and Q96.9. The sequelae associated with Turner syndrome are congenital heart disease, infertility, and the combination of both congenital heart disease and infertility; additionally, a subset of individuals with Turner syndrome are asymptomatic. The distribution of these sequelae was determined by a review of existing literature on the long-term health consequences of Turner Syndrome.

Modelling strategy and model settings

One of the known limitations to the use of birth prevalence data on Turner Syndrome is that individuals with Turner Syndrome are commonly diagnosed later in life rather than prenatally or at birth. Thus, we implemented a correction factor to account for under-diagnosis in all birth registry data sources, using available literature on the trends in age pattern of Turner Syndrome diagnosis over time[xv] ; although improvements in diagnoses have occurred over time, only between 15% and 30% of all diagnosed Turner Syndrome cases are diagnosed before one year of age. Additionally, the reported denominators from all birth registries – the number of live births in each registry catchment area – were adjusted to include only female births using the GBD fertility estimates of the age, year, and location-specific proportion of total live births that are female. Furthermore, all prevalence data with values of zero were excluded as outliers, as these low values indicate severe under-reporting in the input data. These modelling strategy changes

address known causes of under-reporting of Turner Syndrome in the previous iterations of the GBD and resulted in higher estimates of Turner Syndrome than were reported previously.

The DisMod-MR model of Turner Syndrome had an excess mortality rate capped at 0.1 (slightly higher than the highest available literature estimate of excess mortality rate?). The model did not have a slope prior set on excess mortality rate as the risk of mortality associated with Turner Syndrome is not specific to the neonatal ages. This model also allows an increased maximum smoothness on excess mortality rate and random effects on prevalence limited to +- 0.5 in order to limit random geographic variation in the estimated birth prevalence of Turner Syndrome.

Cause	Covariate name	Type	Measure	Beta value	Exponentiated value
Turner syndrome	Healthcare access and quality index	Country covariate	Excess mortality rate	-0.124 (-0.250 - 0.000)	0.883 (0.779 - 1.000)
Turner syndrome	LDI (I$ per capita)	Country covariate	Excess mortality rate	-0.149 (-0.300 - 0.000)	0.862 (0.741 - 1.000)
Turner syndrome	Live Births 35+ (proportion)	Country covariate	Prevalence	-0.126 (-0.285 - -0.005)	0.882 (0.752 - 0.995)
Turner syndrome	Hospital data for ages over 1 year only	Study-level x-covariate	Prevalence	-0.925 (-1.091 - -0.750)	0.397 (0.336 - 0.472)
Turner syndrome	Hospital data for the under-1 year age group	Study-level x-covariate	Prevalence	-0.296 (-0.653 - -0.016)	0.744 (0.521 - 0.984)
Turner syndrome	MarketScan	Study-level x-covariate	Prevalence	-1.126 (-1.229 - -1.010)	0.324 (0.293 - 0.364)

	Prevalence
Site-years (total)	1336
Number of countries with data	46
Number of GBD regions with data (out of 21 regions)	13
Number of GBD super-regions with data (out of 7 super-regions)	5

Klinefelter Syndrome

Case definitions and associated health states

Klinefelter syndrome, also known as 47 XXY, is a condition in which a male is born with an extra X chromosome in all or some of his cells. We also include other genotypes with supranumary X chromosomes, e.g. XXXY, XXXXY, etc. The primary feature of Klinefelter syndrome is sterility, but it can cause a variety of other conditions, including weaker muscles, increased height, poor coordination abilities, smaller genitals, breast growth, and reduced sexual drive as a result of lower testosterone levels. The GBD case definition of Klinefelter syndrome includes ICD-10 codes Q98.0, Q98.5, and Q99.8. The sequelae associated with Klinefelter syndrome are borderline intellectual disability, mild intellectual disability, primary infertility, the combination of borderline intellectual disability and infertility, and the combination of mild intellectual disability and infertility. In addition, a subset of individuals with Klinefelter syndrome are asymptomatic. The distribution of these sequelae was determined by a review of existing literature on the long-term health consequences of Turner Syndrome.

Modelling strategy and model settings

As discussed above for Turner Syndrome, one limitation to the use of birth registry data for the estimation of Klinefelter Syndrome is that many individuals with Klinefelter Syndrome are not diagnosed

prenatally or at birth. To correct this systematic under-reporting in the birth registry data, we applied a correction factor to all birth registry input data using available literature on the age pattern of Klinefelter Syndrome diagnosis[xvi]. We also adjusted the both-sex live birth denominators provided in registry data using location, age, and year-specific proportions of all live births that were male. Furthermore, all prevalence data with values of zero were excluded as outliers, as these low values indicate severe under-reporting in the input data. These modelling strategy changes address known causes of under-reporting in the previous iterations of the GBD and resulted in higher estimates of Klinefelter Syndrome than were reported previously.

The DisMod-MR model of Klinefelter Syndrome had an excess mortality rate maximum limit of 0.075, allowing the model to fit estimates of excess mortality up to slightly higher than the highest reported literature values. The model did not have a slope prior set on excess mortality and allowed an increased smoothness on excess mortality rate. As with several of the other models of chromosomal conditions, random effects on prevalence were limited to +- 0.5 in order to limit random geographic variation in the estimates of birth prevalence.

Cause	Covariate name	Type	Measure	Beta value	Exponentiated value
Klinefelter syndrome	LDI (I$ per capita)	Country covariate	Excess mortality rate	-0.151 (-0.295 - -0.002)	0.860 (0.744 - 0.998)
Klinefelter syndrome	Legality of Abortion	Country covariate	Prevalence	-0.000 (-0.002 - -0.000)	1.000 (0.998 - 1.000)
Klinefelter syndrome	Live Births 35+ (proportion)	Country covariate	Prevalence	0.263 (0.168 - 0.299)	1.301 (1.183 - 1.349)
Klinefelter syndrome	Hospital data for ages over 1 year only	Study-level x-covariate	Prevalence	-1.823 (-1.989 - -1.620)	0.162 (0.137 - 0.198)
Klinefelter syndrome	Hospital data for the under-1 year age group	Study-level x-covariate	Prevalence	-0.536 (-0.838 - -0.198)	0.585 (0.432 - 0.821)
Klinefelter syndrome	MarketScan	Study-level x-covariate	Prevalence	-2.700 (-3.077 - -2.266)	0.067 (0.046 - 0.104)

	Prevalence
Site-years (total)	864
Number of countries with data	36
Number of GBD regions with data (out of 21 regions)	11
Number of GBD super-regions with data (out of 7 super-regions)	5

Other chromosomal abnormalities, genetic syndromes, and microdeletions

Case definitions and associated health states

Unbalanced chromosomal rearrangements are genetic anomalies that typically occur due to meiotic nondisjunction, when homologous chromosomes do not separate normally in nuclear division during gamete formation. The GBD case definition of other chromosomal rearrangements includes 47,XXX (Triple X syndrome), other meiotic nondisjunction events, other female sex chromosome abnormalities, and other unspecified chromosomal abnormalities. The GBD case definition corresponds to the ICD-10 codes Q92.0, Q97.0, Q97.8, and Q99.9. Excluded from this definition are the chromosomal abnormalities of Down syndrome, Turner syndrome, Klinefelter syndrome, Edward syndrome and Patau syndrome, which are each modelled separately. The sequelae associated with other chromosomal rearrangements include intellectual disability, intellectual disability with dementia, intellectual disability with congenital heart disease and dementia, and intellectual disability with congenital heart disease. Additionally, a

proportion of the individuals with unbalanced chromosomal rearrangements are asymptomatic. In the absence of available literature on the long-term health outcomes among individuals with other chromosomal conditions, the severity distributions associated with Down Syndrome were used for the sequela associated with other chromosomal anomalies.

Edwards Syndrome, also known as Trisomy 18, is the condition in which infants are born with a third copy of chromosome 18. Patau syndrome, also known as Trisomy 13, is the condition in which infants are born with a third copy of chromosome 13. The GBD estimates the combined prevalence of these two conditions in a single model as they present similarly and are associated with similar rates of excess mortality. Infants with Edwards syndrome typically have low birthweights and a range of associated conditions including a small head and jaw, limb abnormalities, and severe intellectual disability. Infants with Patau syndrome have a range of associated defects including musculoskeletal anomalies, developmental abnormalities of the nervous system such as microcephaly, congenital heart defects and severe intellectual disability. The ICD-10 code for Edwards syndrome is Q91.3 and the ICD-10 code for Patau syndrome is Q91.7. In the GBD 2017, all cases of Edwards and Patau syndrome are assigned the sequela of severe motor and cognitive impairment, and a proportion of these cases are also associated with congenital heart disease. The proportion of cases with associated congenital heart disease was 0.775, derived by pooling estimates from available literature on the health states associated with the two trisomies[xvii] [xviii]. This continues to be the case for GBD 2017.

Modelling strategy and model settings

Other chromosomal anomalies were calculated based on reducing the model of total chromosomal anomalies by each of the chromosomal sub-causes, and the remaining prevalence was attributed to other chromosomal anomalies.

In the DisMod-MR model of Edwards Syndrome and Patau Syndrome, random effects on prevalence were limited to +- 0.5, reflecting the expectation of limited geographic variation in the birth prevalence of Edwards Syndrome and Patau Syndrome. An increasing slope prior was set on excess mortality rate for all ages, as individuals with these trisomies generally die within the first few years of life. The model allowed a maximum smoothness of Xi = 2.0 in order to fit high excess mortality in the early age groups.

Cause	Covariate name	Type	Measure	Beta value	Exponentiated value
Edward Syndrome and Patau Syndrome	Healthcare access and quality index	Country covariate	Excess mortality rate	-0.125 (-0.248 - -0.000)	0.883 (0.780 - 1.000)
Edward Syndrome and Patau Syndrome	LDI (I$ per capita)	Country covariate	Excess mortality rate	-0.025 (-0.050 - -0.000)	0.975 (0.952 - 1.000)
Edward Syndrome and Patau Syndrome	Legality of Abortion	Country covariate	Prevalence	-0.002 (-0.004 - -0.000)	0.998 (0.996 - 1.000)
Edward Syndrome and Patau Syndrome	Live Births 35+ (proportion)	Country covariate	Prevalence	0.061 (0.002 - 0.221)	1.063 (1.002 - 1.248)
Edward Syndrome and Patau Syndrome	Hospital data	Study-level x-covariate	Prevalence	0.576 (0.099 - 0.919)	1.778 (1.104 - 2.506)
Edward Syndrome and Patau Syndrome	MarketScan	Study-level x-covariate	Prevalence	-0.040 (-0.163 - -0.001)	0.961 (0.849 - 0.999)
Edward Syndrome and Patau Syndrome	Stillbirths included as cases	Study-level x-covariate	Prevalence	0.136 (0.039 - 0.243)	1.145 (1.039 - 1.275)

Edward Syndrome and Patau Syndrome	Terminations of pregnancy included as cases	Study-level x-covariate	Prevalence	0.229 (0.098 - 0.298)	1.257 (1.102 - 1.348)
Edward Syndrome and Patau Syndrome	Under Reported	Study-level x-covariate	Prevalence	-0.833 (-0.993 - -0.567)	0.435 (0.370 - 0.567)

	Prevalence
Site-years (total)	1094
Number of countries with data	44
Number of GBD regions with data (out of 21 regions)	13
Number of GBD super-regions with data (out of 7 super-regions)	4

All input data with birth prevalence values of zero were excluded as outliers, as these values represent under-reporting and low case ascertainment in the input data rather than a true lack of these chromosomal conditions in the corresponding locations.

Musculoskeletal congenital anomalies

Case definitions and associated health states

The GBD definition of musculoskeletal congenital anomalies includes any anomalies of the muscles or skeletal system present at birth that are not caused by a defined chromosomal syndrome. Within the range of congenital musculoskeletal anomalies, we explicitly model three sub-categories: polydactyly and syndactyly, limb reduction defects, and all other congenital musculoskeletal anomalies.

Polydactyly is the condition of being born with at least one extra digit on either the hand or the foot, while syndactyly is absence of at least one digit. Our case definition of polydactyly corresponds to ICD-10 code Q69, and syndactyly corresponds to Q70. The sequela associated with all cases of polydactyly and syndactyly is level 1 disfigurement. Limb reduction defects are the condition where a part or all of the arm or limb of a fetus fails to form during development, so that the limb is either reduced from its normal size or missing entirely. The GBD case definition of limb reduction defects corresponds with ICD-10 codes Q71 (all three-digit codes under Q71), Q72 (all three-digit codes), Q73.0, Q73.1 and Q73.8.

The other congenital musculoskeletal anomalies included within the total estimate of congenital musculoskeletal anomalies includes clubfoot, skeletal dysplasias, congenital deformities of the spine, congenital dysplasia of the hip, and other congenital musculoskeletal anomalies. This “other” category corresponds to ICD-10 codes Q65, Q65.0, Q65.00, Q65.01, Q65.02, Q65.1; Q65.2; Q65.8; Q65.81; Q65.82; Q65.89; Q65.9; Q66; Q66.0; Q66.1; Q68; Q68.1; Q68.2; Q68.6; Q68.8; Q74; Q74.1; Q74.2; Q74.3; Q74.9; Q75; Q75.0; Q75.5; Q75.9; Q79.8; Q79.9, Q76; Q76.1; Q76.2; Q76.3; Q76.4; Q76.41; Q76.411; Q76.412; Q76.413; Q76.414; Q76.415; Q76.419; Q76.42; Q76.425; Q76.426; Q76.427; Q76.428; Q76.429; Q76.49; Q76.8; Q76.9, Q77; Q77.0; Q77.1; Q77.2; Q77.3; Q77.4; Q77.5; Q77.6; Q77.7; Q77.8; Q77.9; Q78; Q78.0; Q78.1; Q78.2; Q78.3; Q78.4; Q78.5; Q78.6; Q78.8, and Q78.9.

All cases of polydactyly and syndactyly are assigned the health state of level 1 disfigurement. Remission is allowed in the model of polydactyly and syndactyly, as individuals born with these conditions may have them surgically corrected and are then no longer considered within our case definition. However, remission is not included in the models of limb reduction defects or other congenital musculoskeletal defects, as these conditions typically cannot be fully surgically corrected. All cases of limb reduction

defects are associated with level 2 disfigurement. A proportion of limb reduction defect cases are associated with no motor impairment, mild motor impairment with and without pain, and moderate motor impairment with and without pain. The distribution of health states associated with congenital limb reduction was derived from an analysis of available literature on the long-term outcomes among individuals with congenital limb reductions[xix] [xx] .

In the absence comprehensive literature on the long-term outcomes associated with the category of other congenital musculoskeletal anomalies, prevalence estimates of other congenital musculoskeletal anomalies were assigned health states using the proportions derived for limb reduction defects.

Modelling strategy and model settings

As with other categories of congenital anomalies, a model of total musculoskeletal anomalies was used as an envelope model and the sub-categories of congenital musculoskeletal anomalies were squeezed proportionally to these total estimates. The prevalence of other congenital musculoskeletal anomalies was derived from reducing the total musculoskeletal prevalence by polydactyly/syndactyly and limb reduction deficits prevalence.

Total musculoskeletal birth defects

The DisMod model of total musculoskeletal anomalies used cause-specific mortality estimates from the corresponding model in the GBD Causes of Death analysis, and converted these data to excess mortality estimates where corresponding prevalence data were available. Random effects on prevalence were limited to +- 0.5 in order to limit geographic variation in the birth prevalence of congenital musculoskeletal anomalies. Smoothness on excess mortality rate was increased to Xi= 2.0 to allow the model to fit a steep decrease in excess mortality rate after the earliest age groups. The model also included a decreasing slope prior on excess mortality rate for all ages, as the risk of mortality from congenital musculoskeletal anomalies is greatest shortly after birth and decreases over age.

Cause	Covariate name	Type	Measure	Beta value	Exponentiated value
Total musculoskeletal congenital anomalies	LDI (I$ per capita)	Country covariate	Excess mortality rate	-0.498 (-0.500 - -0.495)	0.607 (0.607 - 0.610)
Total musculoskeletal congenital anomalies	Legality of Abortion	Country covariate	Prevalence	-0.001 (-0.002 - -0.000)	0.999 (0.998 - 1.000)
Total musculoskeletal congenital anomalies	Chromosomal diagnoses excluded	Study-level x-covariate	Prevalence	-0.153 (-0.300 - -0.004)	0.858 (0.741 - 0.996)
Total musculoskeletal congenital anomalies	Hospital data for ages over 1 year only	Study-level x-covariate	Prevalence	-0.758 (-1.061 - -0.516)	0.469 (0.346 - 0.597)
Total musculoskeletal congenital anomalies	Hospital data for the under-1 year age group	Study-level x-covariate	Prevalence	0.457 (0.340 - 0.499)	1.580 (1.405 - 1.647)
Total musculoskeletal congenital anomalies	Literature	Study-level x-covariate	Prevalence	-0.066 (-0.221 - -0.003)	0.936 (0.802 - 0.997)

Total musculoskeletal congenital anomalies	MarketScan	Study-level x-covariate	Prevalence	-0.060 (-0.215 - -0.002)	0.941 (0.806 - 0.998)
Total musculoskeletal congenital anomalies	Registry data	Study-level x-covariate	Prevalence	-0.121 (-0.432 - -0.004)	0.886 (0.650 - 0.996)
Total musculoskeletal congenital anomalies	Stillbirths included as cases	Study-level x-covariate	Prevalence	0.248 (0.111 - 0.298)	1.281 (1.117 - 1.348)
Total musculoskeletal congenital anomalies	Terminations of pregnancy included as cases	Study-level x-covariate	Prevalence	0.147 (0.000 - 0.296)	1.158 (1.000 - 1.344)

	Prevalence
Site-years (total)	274
Number of countries with data	16
Number of GBD regions with data (out of 21 regions)	8
Number of GBD super-regions with data (out of 7 super-regions)	5

Limb reduction defects, club feet, and hip dysplasia

In the DisMod model of limb reduction defects, random effects on prevalence were limited to +- 0.3 in order to limit geographic variation in the estimated birth prevalence. The excess mortality rate was set to a maximum of 0.5 before 70 years of age in to reflect the relatively low mortality risk of congenital limb anomalies, and a decreasing slope prior on excess mortality rate was set for all ages as the risk of mortality is highest in the earliest age groups.

Cause	Covariate name	Type	Measure	Beta value	Exponentiated value
Limb reduction deficits	LDI (I$ per capita)	Country covariate	Excess mortality rate	-0.050 (-0.100 - -0.002)	0.951 (0.905 - 0.998)
Limb reduction deficits	Legality of Abortion	Country covariate	Prevalence	-0.000 (-0.002 - -0.000)	1.000 (0.998 - 1.000)
Limb reduction deficits	Chromosomal diagnoses excluded	Study-level x-covariate	Prevalence	-0.147 (-0.291 - -0.007)	0.864 (0.748 - 0.993)
Limb reduction deficits	Hospital data for ages over 1 year only	Study-level x-covariate	Prevalence	-0.901 (-0.996 - -0.726)	0.406 (0.370 - 0.484)
Limb reduction deficits	Hospital data for the under-1 year age group	Study-level x-covariate	Prevalence	-0.347 (-0.586 - -0.129)	0.707 (0.557 - 0.879)
Limb reduction deficits	ICDBSR to NBDPN registry case composition adjustment	Study-level x-covariate	Prevalence	0.372 (0.016 - 0.939)	1.450 (1.016 - 2.558)
Limb reduction deficits	MarketScan	Study-level x-covariate	Prevalence	-0.090 (-0.411 - -0.000)	0.914 (0.663 - 1.000)
Limb reduction deficits	Stillbirths included as cases	Study-level x-covariate	Prevalence	0.129 (0.005 - 0.289)	1.137 (1.005 - 1.335)
Limb reduction deficits	Terminations of pregnancy included as cases	Study-level x-covariate	Prevalence	0.120 (0.004 - 0.286)	1.128 (1.004 - 1.331)
Limb reduction deficits	Under Reported	Study-level x-covariate	Prevalence	-0.647 (-0.984 - -0.123)	0.523 (0.374 - 0.884)

	Prevalence

Site-years (total)	330
Number of countries with data	24
Number of GBD regions with data (out of 21 regions)	8
Number of GBD super-regions with data (out of 7 super-regions)	4

Polydactyly and syndactyly

The DisMod model of polydactly and syndactyly limited random effects on prevalence to +- 0.5, as we expected limited geographic variation in the birth prevalence estimates. Excess mortality was set to 0 for all ages. The remission rate was bounded from 0 to 0.02 for the first three months of life, as surgical correction of polydactyly or syndactyly rarely occurs in the first few months of life. Remission was bounded between 0 and 0.5 for ages 2 to 5 years, the ages during which surgical correction is most likely to occur, then set to a maximum of 0.02 after 5 years of age. The smoothness on remission was set to Xi= 1.5 in order to facilitate steep changes in remission rates during the first few years of life.

Cause	Covariate name	Type	Measure	Beta value	Exponentiated value
Polydactyly and syndactyly	LDI (I$ per capita)	Country covariate	Remission	1.001 (0.506 - 1.500)	2.720 (1.659 - 4.482)
Polydactyly and syndactyly	Chromosomal diagnoses excluded	Study-level x-covariate	Prevalence	-0.041 (-0.063 - -0.018)	0.960 (0.939 - 0.982)
Polydactyly and syndactyly	Crosswalk ICBDSR to EUROCAT data	Study-level x-covariate	Prevalence	-0.225 (-0.332 - -0.105)	0.798 (0.717 - 0.900)
Polydactyly and syndactyly	Hospital data for ages over 1 year only	Study-level x-covariate	Prevalence	0.993 (0.978 - 1.000)	2.701 (2.660 - 2.717)
Polydactyly and syndactyly	Hospital data for the under-1 year age group	Study-level x-covariate	Prevalence	0.185 (-0.086 - 0.525)	1.204 (0.917 - 1.690)
Polydactyly and syndactyly	MarketScan	Study-level x-covariate	Prevalence	-0.043 (-0.235 - -0.002)	0.958 (0.790 - 0.998)
Polydactyly and syndactyly	Stillbirths included as cases	Study-level x-covariate	Prevalence	0.003 (0.000 - 0.011)	1.003 (1.000 - 1.011)
Polydactyly and syndactyly	Terminations of pregnancy included as cases	Study-level x-covariate	Prevalence	0.095 (0.006 - 0.219)	1.099 (1.006 - 1.245)
Polydactyly and syndactyly	Under Reported	Study-level x-covariate	Prevalence	-0.179 (-0.295 - -0.051)	0.836 (0.744 - 0.950)

	Prevalence
Site-years (total)	1406
Number of countries with data	52
Number of GBD regions with data (out of 21 regions)	14
Number of GBD super-regions with data (out of 7 super-regions)	6

Other congenital musculoskeletal birth defects

Other congenital musculoskeletal anomalies are modeled by applying the ratio of other congenital digestive anomalies to total congenital digestive anomalies as it is reflected in Marketscan data (a trusted data source), to the sum of the sub-causes of congenital musculoskeletal anomalies. The result is prevalence of other congenital musculoskeletal anomalies by age/year/sex/location.

Urogenital congenital anomalies

Case definitions and associated health states

The GBD case definition of urogenital congenital anomalies include anomalies of the genitals and the urinary system that are present at birth. While some types of urogenital congenital anomalies encompass both the urinary and genital systems, we have assigned each congenital condition as a malformation of either the urinary or the genital system and model anomalies of the urinary and genital systems separately. Urinary anomalies include congenital malformation of the collecting system, ureter, bladder, and kidney, as well as bladder exstrophy and epispadias. The ICD-10 codes included in the category of urinary anomalies are Q64.0, Q64.1, Q60-Q61 and Q62-Q63. Genital anomalies include hypospadias, ambiguous or indeterminate sex, other congenital abnormalities of the male genitalia, and a variety of female genital malformations. ICD-10 codes Q50-Q52, Q54, Q56, and Q55 (excluding Q55.20-Q55.21) are included in the case definition of congenital genital anomalies. Undescended testicles are excluded from the case definition of genital anomalies, as this is not considered a severe condition. Remission is not permitted in the models or either urinary or genital anomalies, as individuals who receive surgical corrections of these conditions after birth typically continue to experience health consequences as a result of these congenital conditions.

The total prevalence of congenital urinary anomalies was split into proportions with and without each of the following health states: urinary incontinence, impotence, recurrent urinary tract infections and other recurring abdominal issues, and atypical genitalia (corresponding to disfigurement, level 1 in the GBD Disability Weights Study). Cases of congenital genital anomalies was split into proportions with and without primary infertility, impotence, recurrent urinary tract infections and other recurring abdominal issues, and atypical genitalia. Estimates were produced for the prevalence of every possible combination of those long-term sequela, assuming independence between the outcomes.

The distribution of these long-term outcomes was derived from a review of available literature on the long-term outcomes experienced cohorts of individuals born with a range of congenital urogenital anomalies[xxi] [xxii] [xxiii] [xxiv] [xxv] [xxvi].

Modelling strategy and model settings

Congenital urogenital anomalies were modelled as two distinct categories, with distinct model specifications: urinary congenital anomalies and genital congenital anomalies.

Congenital urinary anomalies

In the DisMod model of congenital urinary anomalies, random effects on random effects on prevalence were limited to +- 0.5 and random effects on with-condition mortality were limited to +- 1.0. The maximum excess mortality rate was set to 5.0 for the first year of life and 2.0 for ages 1 year to 70 years. The excess mortality rate set to 0 for ages 70+ years, consistent with the GBD Cause of Death models which exclude congenital urogenital anomalies as a cause of death after 70 years of age. The smoothness on excess mortality rate was set to Xi = 0.6 in order to fit changes in the excess mortality rate during the neonatal period. Cause-Specific Mortality Rate (CSMR) was also pulled in from our Cause of Death model of congenital urogenital anomalies. As we assume no death due to congenital genital anomalies, this model represents deaths associated with exclusively congenital urinary anomalies.

Cause	Covariate name	Type	Measure	Beta value	Exponentiated value
Congenital urinary anomalies	LDI (I$ per capita)	Country covariate	Excess mortality rate	-0.750 (-0.750 - -0.749)	0.472 (0.472 - 0.473)

Congenital urinary anomalies	Chromosomal diagnoses excluded	Study-level x-covariate	Prevalence	-0.012 (-0.035 - -0.001)	0.988 (0.966 - 0.999)
Congenital urinary anomalies	Hospital data for ages over 1 year only	Study-level x-covariate	Prevalence	-0.003 (-0.010 - -0.000)	0.997 (0.990 - 1.000)
Congenital urinary anomalies	Hospital data for the under-1 year age group	Study-level x-covariate	Prevalence	-0.007 (-0.020 - -0.001)	0.993 (0.980 - 0.999)
Congenital urinary anomalies	MarketScan	Study-level x-covariate	Prevalence	-0.438 (-0.551 - -0.315)	0.646 (0.576 - 0.730)
Congenital urinary anomalies	Stillbirths included as cases	Study-level x-covariate	Prevalence	0.187 (0.065 - 0.290)	1.206 (1.067 - 1.336)
Congenital urinary anomalies	Terminations of pregnancy included as cases	Study-level x-covariate	Prevalence	0.202 (0.029 - 0.296)	1.224 (1.029 - 1.345)
Congenital urinary anomalies	Under Reported	Study-level x-covariate	Prevalence	-0.015 (-0.059 - -0.000)	0.985 (0.943 - 1.000)

	Prevalence
Site-years (total)	1207
Number of countries with data	55
Number of GBD regions with data (out of 21 regions)	15
Number of GBD super-regions with data (out of 7 super-regions)	7

Congenital genital anomalies

In the DisMod model of congenital genital anomalies, random effects on prevalence were limited to +- 0.5 in order to limit random geographic variation in the estimates of birth prevalence, and random effects on with-condition mortality were limited to +- 1.0. The maximum excess mortality rate was set to 0.5 for all ages < 70 years, according to maximum observed excess mortality rates in available literature data on congenital genital anomalies. The excess mortality rate set to 0 for ages 70+ years, consistent with the GBD Cause of Death models which exclude congenital urogenital anomalies as a cause of death after 70 years of age.

Cause	Covariate name	Type	Measure	Beta value	Exponentiated value
Congenital genital anomalies	LDI (I$ per capita)	Country covariate	Remission	0.553 (0.101 - 1.000)	1.738 (1.106 - 2.718)
Congenital genital anomalies	Chromosomal diagnoses excluded	Study-level x-covariate	Prevalence	-0.015 (-0.036 - -0.001)	0.985 (0.965 - 0.999)
Congenital genital anomalies	EUROCAT to NBDPN registry case composition adjustment	Study-level x-covariate	Prevalence	-0.308 (-0.392 - -0.151)	0.735 (0.676 - 0.860)
Congenital genital anomalies	Hospital data for ages over 1 year only	Study-level x-covariate	Prevalence	-0.043 (-0.220 - 0.178)	0.958 (0.803 - 1.195)
Congenital genital anomalies	Hospital data for the under-1 year age group	Study-level x-covariate	Prevalence	-0.385 (-0.493 - -0.218)	0.680 (0.611 - 0.804)
Congenital genital anomalies	ICDBSR to NBDPN registry case composition adjustment	Study-level x-covariate	Prevalence	-0.389 (-0.473 - -0.296)	0.678 (0.623 - 0.743)
Congenital genital anomalies	MarketScan	Study-level x-covariate	Prevalence	-0.896 (-0.986 - -0.801)	0.408 (0.373 - 0.449)
Congenital genital anomalies	Stillbirths included as cases	Study-level x-covariate	Prevalence	0.011 (0.001 - 0.041)	1.011 (1.001 - 1.042)
Congenital genital anomalies	Terminations of pregnancy included as cases	Study-level x-covariate	Prevalence	0.065 (0.001 - 0.211)	1.067 (1.001 - 1.235)
Congenital genital anomalies	Under Reported	Study-level x-covariate	Prevalence	-0.068 (-0.175 - -0.002)	0.934 (0.840 - 0.998)

Congenital genital anomalies	Under Reported	Study-level z-covariate	Prevalence	0.139 (0.119 - 0.161)	1.150 (1.126 - 1.175)

	Prevalence
Site-years (total)	1543
Number of countries with data	57
Number of GBD regions with data (out of 21 regions)	16
Number of GBD super-regions with data (out of 7 super-regions)	7

Congenital anomalies of the digestive system

Case definitions

Congenital anomalies of the digestive system include any anomalies of the gastrointestinal tract present at birth as the result of abnormal embryonic development. As with the other congenital causes, this variety of digestive system abnormalities is split into four sub-cause categories. Congenital diaphragmatic hernia, a life-threatening malformation of the diaphragm that allows the abdominal organs to push into the chest cavity and obstructs proper formation of the lungs, is modelled separately from all other congenital malformations of the digestive system. Congenital diaphragmatic hernia corresponds to ICD-10 code Q79.0.

All congenital malformations of the abdominal wall are modelled together as a distinct sub-category. The primary diagnoses in this category are gastroschisis, omphalocele, and prune belly syndrome, corresponding to ICD-10 codes Q79.3, Q79.2, and Q79.4, respectively. All variations of atresia and/or stenosis of the digestive tract are modelled together as the third distinct sub-category of digestive congenital anomalies. This includes biliary atresia, esophageal atresia and/or stenosis with and without tracheoesophageal fistula, and atresia and stenosis of the small intestine, large intestine, rectum and anus. The ICD-10 codes included in the atresia and stenosis sub-cause category are Q42.0; Q42.1; Q42.2; Q42.3; Q42.4; Q42.8; Q42.9, Q42.8; Q42.9, Q42.0; Q42.1; Q42.2; Q42.3; Q42.4; Q41 (Q41.0; Q41.1; Q41.2; Q41.8; Q41.9;), Q44.2, Q39.0; Q39.1 and Q39.2. The final category of digestive congenital anomalies estimated in the GBD is other congenital malformations and diseases of the digestive system. This includes ICD-10 codes Q38 (Q38.0; Q38.3; Q38.4; Q38.6; Q38.7; Q38.8); Q39(Q39.3; Q39.4; Q39.5; Q39.6; Q39.8; Q39.9); Q40(Q40.0; Q40.1; Q40.2; Q40.3; Q40.8; Q40.9); Q43(Q43.1; Q43.2; Q43.3; Q43.4; Q43.5; Q43.6; Q43.7; Q43.8; Q43.9); Q44(Q44.0; Q44.1; Q44.3; Q44.4; Q44.5; Q44.6; Q44.7); Q45(Q45.0; Q45.1; Q45.2; Q45.3; Q45.8; Q45.9); Q79.1, and Q79.5(Q79.51; Q79.59). Inguinal hernias present at birth are excluded from the case definition of gastrointestinal congenital anomalies and are modelled separately as part of the estimation of inguinal hernias.

Most congenital anomalies of the gastrointestinal tract require surgical correction early in life in order to ensure infant survival. Following surgical intervention, individuals born with these congenital anomalies may experience a range of long-term outcomes as a result of their congenital conditions. Thus, we do not permit remission in the models of any congenital digestive anomalies and the health states associated with the various types of digestive anomalies reflect the long-term outcomes experienced by individuals with surgically corrected congenital malformations.

Health states associated with congenital anomalies of the digestive system

The health outcomes associated with congenital diaphragmatic hernia include every combination of disfigurement, chronic abdominal pain, mild chronic respiratory problems and breathlessness, mild

intellectual disability, and a proportion of patients who are asymptomatic. The distribution of these long-term health outcomes was derived from a pooled analysis of available literature on the long-term outcomes in surviving patients born with congenital diaphragmatic hernias[xxvii xxviii xxix].

The health outcomes associated with congenital malformations of the abdominal wall include every combination of constipation, chronic abdominal pain, and disfigurement and concern about scars. The distribution of these outcomes was calculated from a pooled analysis of literature sources on the long-term outcomes among surviving individuals born with congenital malformations of the abdominal wall[xxx xxxi]. Similarly, the outcomes associated with congenital atresia and/or stenosis of the abdominal tract include every combination of dysphagia, acid reflux, chronic abdominal pain and/or nausea, and chronic respiratory problems; the distribution of these long-term outcomes was also derived from available long-term follow-up studies[xxxii xxxiii].

The distribution of health outcomes associated with other congenital anomalies of the gastrointestinal tract was considered to be the same as the health outcomes associated with atresia and/or stenosis of the abdominal tract.

Modelling strategy and model settings

In order to ensure internal consistency in the estimates of each sub-type of congenital digestive anomalies, we generated a model to estimate the total prevalence and associated mortality due to all congenital digestive anomalies, then fit the estimates of each sub-type of congenital digestive anomalies proportionally to the envelope of this total model. The prevalence estimates of other congenital digestive anomalies were derived by reducing the total envelope model for each cause by its sub-causes to derive the difference that was attributable to other anomalies in that category. This modelling strategy allowed us to utilize the GBD Cause of Death estimates as input to the total congenital digestive anomalies estimates and also allowed us to incorporate literature data that reported only the total prevalence of all digestive anomalies.

The DisMod model of total congenital digestive anomalies used cause-specific mortality estimates from the corresponding GBD Cause of Death model of congenital digestive anomalies, and these data were converted to excess mortality estimates where corresponding cause-specific mortality estimates were available. The model had random effects on prevalence limited to +- 0.5 and a decreasing slope prior on excess mortality rate was set for all ages. The smoothness on excess mortality rate was increased to Xi = 1.0 in order to fit steep changes in excess mortality rate during the neonatal age period.

Cause	Covariate name	Type	Measure	Beta value	Exponentiated value
Total digestive congenital anomalies	LDI (I$ per capita)	Country covariate	Excess mortality rate	-0.255 (-0.284 - -0.226)	0.775 (0.753 - 0.798)
Total digestive congenital anomalies	Chromosomal diagnoses excluded	Study-level x-covariate	Prevalence	-0.014 (-0.044 - -0.000)	0.986 (0.957 - 1.000)
Total digestive congenital anomalies	EUROCAT to NBDPN registry case composition adjustment	Study-level x-covariate	Prevalence	-0.002 (-0.010 - -0.000)	0.998 (0.990 - 1.000)
Total digestive congenital anomalies	Hospital data for ages over 1 year only	Study-level x-covariate	Prevalence	-1.989 (-2.000 - -1.956)	0.137 (0.135 - 0.141)
Total digestive congenital anomalies	Hospital data for the under-1 year age group	Study-level x-covariate	Prevalence	0.492 (0.470 - 0.500)	1.635 (1.601 - 1.649)

Total digestive congenital anomalies	MarketScan	Study-level x-covariate	Prevalence	-0.603 (-0.719 - -0.483)	0.547 (0.487 - 0.617)
Total digestive congenital anomalies	Registry data	Study-level x-covariate	Prevalence	-0.003 (-0.011 - -0.000)	0.997 (0.989 - 1.000)
Total digestive congenital anomalies	Stillbirths included as cases	Study-level x-covariate	Prevalence	0.298 (0.291 - 0.300)	1.347 (1.338 - 1.350)
Total digestive congenital anomalies	Terminations of pregnancy included as cases	Study-level x-covariate	Prevalence	0.297 (0.287 - 0.300)	1.345 (1.333 - 1.350)
Total digestive congenital anomalies	Under Reported	Study-level x-covariate	Prevalence	-0.502 (-1.000 - -0.004)	0.605 (0.368 - 0.996)

	Prevalence
Site-years (total)	1143
Number of countries with data	34
Number of GBD regions with data (out of 21 regions)	9
Number of GBD super-regions with data (out of 7 super-regions)	5

Congenital diaphragmatic hernia

In the DisMod model of congenital diaphragmatic hernia, random effects on prevalence were set to +- 0.5 and random effects on with-condition mortality were set to +- 1.0. The minimum excess mortality for the early neonatal age period was set to 2.0. A decreasing slope prior on excess mortality rate was set for all ages, as the risk of mortality due to congenital diaphragmatic hernia is highest shortly after birth and diminishes over the life course following surgical correction of the condition. Smoothness on excess mortality rate was increased to Xi= 1.0 in order to fit steep changes in excess mortality rate during the first weeks of life.

Cause	Covariate name	Type	Measure	Beta value	Exponentiated value
Congenital diaphragmatic hernia	LDI (I$ per capita)	Country covariate	Excess mortality rate	-0.623 (-1.000 - -0.250)	0.536 (0.368 - 0.779)
Congenital diaphragmatic hernia	Chromosomal diagnoses excluded	Study-level x-covariate	Prevalence	-0.034 (-0.082 - -0.002)	0.966 (0.921 - 0.998)
Congenital diaphragmatic hernia	Hospital data	Study-level x-covariate	Prevalence	-0.049 (-0.161 - -0.001)	0.952 (0.851 - 0.999)
Congenital diaphragmatic hernia	Stillbirths included as cases	Study-level x-covariate	Prevalence	0.257 (0.198 - 0.297)	1.293 (1.219 - 1.346)
Congenital diaphragmatic hernia	Terminations of pregnancy included as cases	Study-level x-covariate	Prevalence	0.210 (0.042 - 0.297)	1.234 (1.043 - 1.346)

	Prevalence
Site-years (total)	1774
Number of countries with data	48
Number of GBD regions with data (out of 21 regions)	14
Number of GBD super-regions with data (out of 7 super-regions)	5

Congenital malformations of the abdominal wall

The DisMod model of congenital malformations of the abdominal wall had random effects on prevalence limited to +- 0.5 and random effects on with-condition mortality were limited to +- 1.0. The minimum excess mortality rate was set to 3.0 in the early neonatal period according to the range of excess mortality estimates observed in literature data. A decreasing slope prior on excess mortality rate was set for all ages, and the smoothness on excess mortality rate was set to Xi = 0.8, allowing the model to fit a steep decrease in the excess mortality rate after the neonatal age period.

Cause	Covariate name	Type	Measure	Beta value	Exponentiated value
Congenital malformations of the abdominal wall	LDI (I$ per capita)	Country covariate	Excess mortality rate	-0.425 (-0.750 - -0.102)	0.654 (0.472 - 0.903)
Congenital malformations of the abdominal wall	Chromosomal diagnoses excluded	Study-level x-covariate	Prevalence	-0.012 (-0.038 - -0.000)	0.988 (0.963 - 1.000)
Congenital malformations of the abdominal wall	Hospital data for ages over 1 year only	Study-level x-covariate	Prevalence	-2.726 (-2.967 - -2.453)	0.065 (0.051 - 0.086)
Congenital malformations of the abdominal wall	Hospital data for the under-1 year age group	Study-level x-covariate	Prevalence	-0.012 (-0.045 - -0.001)	0.988 (0.956 - 0.999)
Congenital malformations of the abdominal wall	Stillbirths included as cases	Study-level x-covariate	Prevalence	0.007 (0.000 - 0.025)	1.007 (1.000 - 1.025)
Congenital malformations of the abdominal wall	Terminations of pregnancy included as cases	Study-level x-covariate	Prevalence	0.069 (0.002 - 0.181)	1.071 (1.002 - 1.199)
Congenital malformations of the abdominal wall	Under Reported	Study-level x-covariate	Prevalence	-0.346 (-0.412 - -0.287)	0.708 (0.662 - 0.751)

	Prevalence
Site-years (total)	1543
Number of countries with data	57
Number of GBD regions with data (out of 21 regions)	16
Number of GBD super-regions with data (out of 7 super-regions)	7

Congenital atresia and/or stenosis of the digestive tract

In the DisMod model of congenital atresia and/or stenosis of the digestive tract, random effects on prevalence were set to +- 0.5 and random effects on with-condition mortality were set to +- 1.0. A decreasing slope prior on excess mortality rate was set for all ages, as the risk of mortality due to these congenital digestive anomalies highest shortly after birth. The smoothness on excess mortality rate was increased to Xi = 1.0 in order to fit steep changes in excess mortality rate during the first weeks of life.

Cause	Covariate name	Type	Measure	Beta value	Exponentiated value

Congenital atresia and/or stenosis of the digestive tract	Healthcare access and quality index	Country covariate	Excess mortality rate	-0.249 (-0.499 - 0.000)	0.780 (0.607 - 1.000)
Congenital atresia and/or stenosis of the digestive tract	LDI (I$ per capita)	Country covariate	Excess mortality rate	-0.050 (-0.100 - 0.000)	0.952 (0.905 - 1.000)
Congenital atresia and/or stenosis of the digestive tract	Chromosomal diagnoses excluded	Study-level x-covariate	Prevalence	-0.067 (-0.102 - -0.039)	0.935 (0.903 - 0.962)
Congenital atresia and/or stenosis of the digestive tract	Hospital data for ages over 1 year only	Study-level x-covariate	Prevalence	-0.040 (-0.140 - -0.000)	0.961 (0.870 - 1.000)
Congenital atresia and/or stenosis of the digestive tract	Hospital data for the under-1 year age group	Study-level x-covariate	Prevalence	-0.008 (-0.030 - -0.000)	0.992 (0.971 - 1.000)
Congenital atresia and/or stenosis of the digestive tract	MarketScan	Study-level x-covariate	Prevalence	-0.001 (-0.003 - -0.000)	0.999 (0.997 - 1.000)
Congenital atresia and/or stenosis of the digestive tract	Stillbirths included as cases	Study-level x-covariate	Prevalence	0.002 (0.000 - 0.004)	1.002 (1.000 - 1.004)
Congenital atresia and/or stenosis of the digestive tract	Terminations of pregnancy included as cases	Study-level x-covariate	Prevalence	0.036 (0.001 - 0.117)	1.036 (1.001 - 1.124)
Congenital atresia and/or stenosis of the digestive tract	Under Reported	Study-level x-covariate	Prevalence	-0.366 (-0.494 - -0.061)	0.694 (0.610 - 0.941)

	**Prevalence**
Site-years (total)	1749
Number of countries with data	57
Number of GBD regions with data (out of 21 regions)	16
Number of GBD super-regions with data (out of 7 super-regions)	6

Other congenital digestive anomalies

Other congenital digestive anomalies are calculated by summing all of the sub-causes of congenital digestive anomalies and subtracting this sum from the total congenital digestive model (by age/sex/year/location). This residual is the prevalence of other congenital digestive anomalies. If this residual is less than 10% of the total congenital digestive anomalies model, the other sub-causes are squeezed down and other congenital digestive anomalies becomes 10% of the total congenital digestive anomalies model.

Other congenital anomalies

In addition, of the specific types of congenital anomalies outlined in the preceding pages, there are a number of other types of defects that may be present at birth. These other congenital defects include anomalies of the ears, eyes, face and neck, respiratory malformation and diseases, skin disorders, phakomatoses and other neurological disorders that are not included in the case definition of neural tube defects. Estimates of the YLDs attributable to these other congenital anomalies are derived from a YLL:YLD ratio using YLLs from the cause of death (COD) estimates for other congenital anomalies.

[i] Lanton AP. The Characteristics of Patients with Encephaloceles. *Z Kinderchir*. 1990; 45(Suppl 1): 18-9.

[ii] Da Silva SL, Jeelani Y, Dang H, Krieger MD, McComb JG. Risk factors for hydrocephalus and neurological deficit in children born with an encephalocele. *J Neurosurg Pediatr*. 2015; 15(4): 392–8.

[iii] Lo BWY, Kulkarni AV, Rutka JT, Jea A, Drake JM, Lamberti-Pasculli M, Dirks PB, Thabane L. Clinical predictors of developmental outcome in patients with cephaloceles. *J Neurosurg Pediatr*. 2008; 2(4): 254–7.

[iv] Moeini Naghani I, Hashemi Zonouz T, Shahjouei S, Homayoun AA, Nejat F, El Khashab M. Congenital cardiac anomalies in myelomeningocele patients. *Acta Med Acad*. 2014; 43(2): 160–4.

[v] Oakeshott P, Hunt GM, Poulton A, Reid F. Open spina bifida: birth findings predict long-term outcome. *Arch Dis Child*. 2012; 97(5): 474–6.

[vi] Jaquier M. Anencephaly Online Survey. Anencephaly.info [Internet]. 2006.

[vii] Jaquier M, Klein A, Boltshauser E. Spontaneous pregnancy outcome after prenatal diagnosis of anencephaly. *BJOG*. 2006; 113(8): 951-3.

[viii] Riehle-Colarusso T, Autry A, Razzaghi H, Boyle CA, Mahle WT, Van Naarden Braun K, Correa A. Congenital Heart Defects and Receipt of Special Education Services. *Pediatrics*. 2015; 136(3): 496–504.

[ix] Menting ME, Cuypers JAAE, Opić P, Utens EMWJ, Witsenburg M, van den Bosch AE, van Domburg RT, Meijboom FJ, Boersma E, Bogers AJJC, Roos-Hesselink JW. The unnatural history of the ventricular septal defect: outcome up to 40 years after surgical closure. *J Am Coll Cardiol*. 2015; 65(18): 1941–51.

[x] Gaynor JW, Stopp C, Wypij D, Andropoulos DB, Atallah J, Atz AM, Beca J, Donofrio MT, Duncan K, Ghanayem NS, Goldberg CS, Hövels-Gürich H, Ichida F, Jacobs JP, Justo R, Latal B, Li JS, Mahle WT, McQuillen PS, Menon SC, Pemberton VL, Pike NA, Pizarro C, Shekerdemian LS, Synnes A, Williams I, Bellinger DC, Newburger JW, International Cardiac Collaborative on Neurodevelopment (ICCON) Investigators. Neurodevelopmental outcomes after cardiac surgery in infancy. *Pediatrics*. 2015; 135(5): 816–25.

[xi] Wren C, O'Sullivan JJ. Survival with congenital heart disease and need for follow up in adult life. *Heart*. 2001; 85(4): 438–43.

[xii] Gabriel HM, Heger M, Innerhofer P, Zehetgruber M, Mundigler G, Wimmer M, Maurer G, Baumgartner H. Long-term outcome of patients with ventricular septal defect considered not to require surgical closure during childhood. *J Am Coll Cardiol*. 2002; 39(6): 1066–71.

[xiii] Neumayer U, Stone S, Somerville J. Small ventricular septal defects in adults. *Eur Heart J*. 1998; 19(10): 1573–82.

[xiv] Epstein CJ. Down syndrome (trisomy 21). In: Scriver CR, Beaudet AL, Sly WS, Valle D, eds. The Metabolic Basis of Inherited Disease. 7th ed. New York, United States: McGraw Hill Inc., 1995.

[xv] Massa G, Verlinde F, De Schepper J, Thomas M, Bourguignon JP, Craen M, de Zegher F, Francois I, Du Caju M, Maes M, Heinrichs C, in collaboration with the Belgian Study Group for Paediatric Endocrinology. Trends in age at diagnosis of Turner syndrome. *Arch Dis Child*. 2005; 90(3): 267-8.

[xvi] Bojesen A, Juul S, Gravholt CH. Prenatal and Postnatal Prevalence of Klinefelter Syndrome: A National Registry Study. *J Clin Endocrinol Metab*. 2003; 88(2): 622-6.

[xvii] Petry P, Polli JB, Mattos VF, Rosa RCM, Zen PRG, Graziadio C, Paskulin GA, Rosa RFM. Clinical features and prognosis of a sample of patients with trisomy 13 (Patau syndrome) from Brazil. *Am J Med Genet A*. 2013; 161A(6): 1278–83.

[xviii] Polli JB, Groff D de P, Petry P, Mattos VF, Rosa RCM, Zen PRG, Graziadio C, Paskulin GA, Rosa RFM. Trisomy 13 (Patau syndrome) and congenital heart defects. *Am J Med Genet A*. 2014; 164A(1): 272–5.

[xix] Johansen H, Østlie K, Andersen LØ, Rand-Hendriksen S. Adults with congenital limb deficiency in Norway: demographic and clinical features, pain and the use of health care and welfare services. A cross-sectional study. *Disabil Rehabil*. 2015; 37(22): 2076–82.

[xx] Johansen H, Dammann B, Oinæs Andersen L, Andresen I-L. Children with congenital limb deficiency in Norway: issues related to school life and health-related quality of life. A cross-sectional study. *Disabil Rehabil*. 2016; 38(18): 1803–10.

[xxi] Ching CB, Wood HM, Ross JH, Gao T, Angermeier KW. The Cleveland Clinic experience with adult hypospadias patients undergoing repair: their presentation and a new classification system. *BJU Int*. 2011; 107(7): 1142–6.

[xxii] Davies MC, Liao L-M, Wilcox DT, Woodhouse CRJ, Creighton SM. Anorectal malformations: what happens in adulthood?. *BJU Int*. 2010; 106(3): 398–404.

[xxiii] Rintala RJ. Congenital cloaca: Long-term follow-up results with emphasis on outcomes beyond childhood. *Semin Pediatr Surg*. 2016; 25(2): 112–6.

[xxiv] Sircili MHP, e Silva FA de Q, Costa EMF, Brito VN, Arnhold IJP, Dénes FT, Inacio M, de Mendonca BB. Long-term surgical outcome of masculinizing genitoplasty in large cohort of patients with disorders of sex development. *J Urol*. 2010; 184(3): 1122–7.

[xxv] van der Zwan YG, Callens N, van Kuppenveld J, Kwak K, Drop SLS, Kortmann B, Dessens AB, Wolffenbuttel KP, Dutch Study Group on DSD. Long-term outcomes in males with disorders of sex development. *J Urol*. 2013; 190(3): 1038–42.

[xxvi] Warne SA, Wilcox DT, Creighton S, Ransley PG. Long-term gynecological outcome of patients with persistent cloaca. *J Urol*. 2003; 170(4 Pt 2): 1493–6.

[xxvii] Crankson SJ, Al Jadaan SA, Namshan MA, Al-Rabeeah AA, Oda O. The immediate and long-term outcomes of newborns with congenital diaphragmatic hernia. *Pediatr Surg Int*. 2006; 22(4): 335-40.

[xxviii] Öst E, Joelsson MÖ, Burgos CM, Frenckner B. Self-assessed physical health among children with congenital diaphragmatic hernia. *Pediatr Surg Int*. 2016; 32(5): 493–503.

[xxix] Rocha GM, Bianchi RF, Severo M, Rodrigues MM, Baptista MJ, Correia-Pinto J, Guimarães HA. Congenital Diaphragmatic Hernia. The Post-neonatal period. Part II. *Eur J Pediatr Surg*. 2008; 18(5): 307–12.

[xxx] van Eijck FC, Wijnen RMH, van Goor H. The incidence and morbidity of adhesions after treatment of neonates with gastroschisis and omphalocele: a 30-year review. *J Pediatr Surg*. 2008; 43(3): 479–83.

[xxxi] Harris EL, Minutillo C, Hart S, Warner TM, Ravikumara M, Nathan EA, Dickinson JE. The long term physical consequences of gastroschisis. *J Pediatr Surg*. 2014; 49(10): 1466–70.

[xxxii] Dingemann C, Meyer A, Kircher G, Boemers TM, Vaske B, Till H, Ure BM. Long-term health-related quality of life after complex and/or complicated esophageal atresia in adults and children registered in a German patient support group. *J Pediatr Surg*. 2014; 49(4): 631–8.

[xxxiii] Lilja HE, Wester T. Outcome in neonates with esophageal atresia treated over the last 20 years. *Pediatr Surg Int*. 2008; 24(5): 531–6.

Urinary Tract Infection

Flowchart

Urinary Tract Infection (UTI)

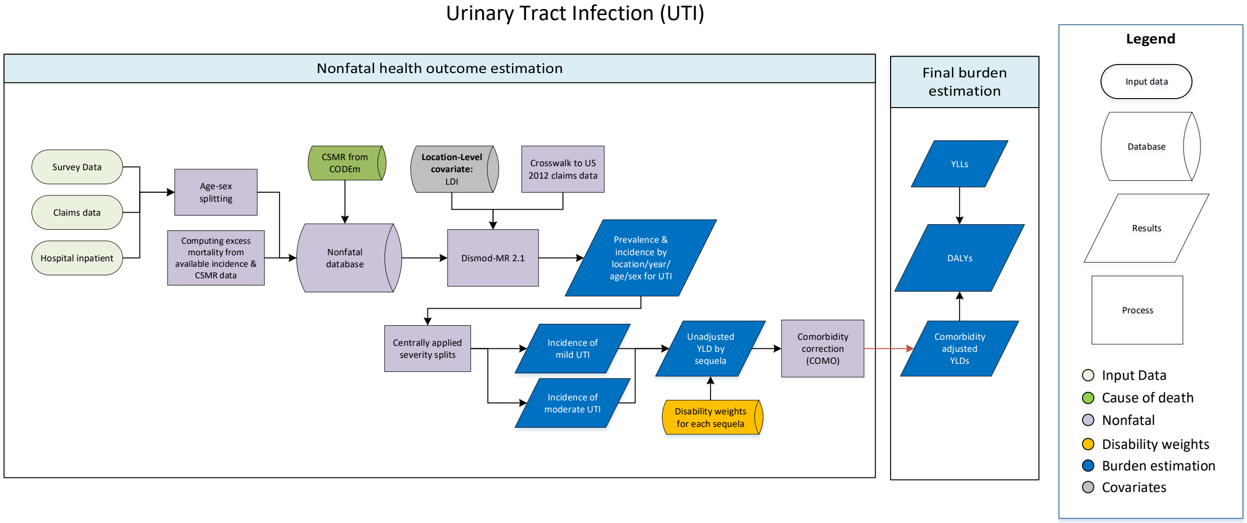

Case definition

Urinary tract infection (UTI) is defined as a kidney infection that can lead to systemic symptoms such as fever and weakness and can cause discomfort and difficulty with daily activities.[1] ICD codes include N10, N10.0, N10.9, N11, N11.0, N11.1, N11.8, N11.9, N12, N12.0, and N12.9.

Input data

Model inputs

The UTI model is informed by survey data, claims data, and global hospital inpatient data. For GBD 2016, a systematic review of the prevalence of UTI throughout the world was conducted using the following search terms: (((Interstitial Nephritis[Title/Abstract]) OR (Urinary Tract Infection[Title/Abstract]))AND (Inciden*[Title/Abstract])) AND ("2013/01/01"[Date - Publication] : "3000"[Date - Publication]) NOT (animals[MeSH] NOT humans[MeSH]).

The exclusion criteria were:

1. Studies clearly not representative of the national population
2. Studies that did not provide primary data on epidemiological parameters, eg, a commentary piece
3. Studies of a specific type of interstitial nephritis

The table below shows the number of countries and GBD world regions represented.

	Incidence
**Site-years (total)**	1412
**Number of countries with data**	44
**Number of GBD regions with data (out of 21 regions)**	16
**Number of GBD super-regions with data (out of 7 super-regions)**	7

Claims data for the USA, Philippines, Taiwan, and New Zealand were included. Hospital inpatient data was also included. Inpatient data points with an age-standardised incidence rate greater than two median absolute deviations from the median of the mean age-standardised incidence rate for all inpatient data were marked as outliers and excluded from analysis.

Severity splits & disability weights

The basis of the GBD disability weight survey assessments is lay descriptions of sequelae highlighting major functional consequences and symptoms. UTI is split into mild and moderate severity. Mild severity is associated with a disability weight that correlates with low fever and mild discomfort, but no difficulty with daily activities. Moderate discomfort is associated with a disability weight that correlates with systemic symptoms of fever, aches, weakness, and some difficulty with daily activities. The lay descriptions and disability weights for UTI are shown below.

Severity level	Lay description	DW (95% CI)
Urinary tract infection, mild	Has a low fever and mild discomfort, but no difficulty with daily activities.	0.006 (0.002, 0.012)
Urinary tract infection, moderate	Has a fever and aches, and feels weak, which causes some difficulty with daily activities.	0.051 (0.032, 0.074)

The severity distribution of UTI was derived from analysis of the Medical Expenditure Panel Surveys (MEPS). MEPS is an overlapping panel survey of the non-institutionalised USA population that collects data on respondents' health service interactions. Panels are initiated every year. Each panel is two years long and consists of five rounds. In 2000, MEPS began using 12-Item Short Form Surveys (SF-12) to collect data on functional health status. The SF-12 survey is administered twice per panel (about once per year).

In order to translate SF-12 scores into GBD disability weights, 62 lay descriptions for conditions representing the full range of disability weight values (from most mild to most severe) were selected. A convenience sample of respondents was then asked to complete an SF-12 form for an individual with the health state described in the lay descriptions of these conditions. Composite mental and physical SF-12 score was regressed on GBD disability weight to derive the relationship between disability weight and SF-12 score. Individual respondent scores were then regressed on reported conditions to obtain a comorbidity-corrected condition-specific disability weight. The distribution of these condition-specific weights was used to derive the proportion of individuals with the conditions that fall within each GBD severity category.

Severity	Distribution
Mild UTI	0.362 (0.258, 0.478)
Moderate UTI	0.638 (0.522, 0.742)

Modelling strategy

We ran a DisMod MR-2.1 model to produce estimates by age, sex, year, and location. Prior settings in the IN DisMod 2.1 model included remission after one week between ages 0 and 100. We used the function in DisMod-MR 2.1 to pull in cause-specific mortality rate (CSMR) data from our CODEm and CODcorrect analyses and match them with incidence data points for the same geography and study year to estimate priors on excess mortality rates (by dividing CSMR by incidence).

We applied a crosswalk to USA claims data from the year 2000 to USA claims data for 2010–2014 in order to adjust for the smaller sample included in the 2000 data. Inpatient data were corrected for multiple admissions and multiple diagnoses based on claims data. Inpatient data were also adjusted to be comparable to claims data (inpatient and outpatient visits) using a correction factor derived from claims datasets.

We included the covariate Lagged Distributed Income (LDI) as a country-level covariate to inform excess mortality, with bounds of -0.5, -0.1. We used this covariate based on the assumption of a higher likelihood of mortality based on the developmental status of a country.

Betas and exponentiated values (which can be interpreted as an odds ratio) are shown in the table below:

Study covariate	Parameter	beta	Exponentiated beta
Claims data – 2000	Incidence	-0.0026 (-0.0099 to -0.000092)	1.00 (0.99–1.00)

Compared to GBD 2016, major changes include inpatient data from a greater number of geographies.

Acute Urolithiasis

Flowchart

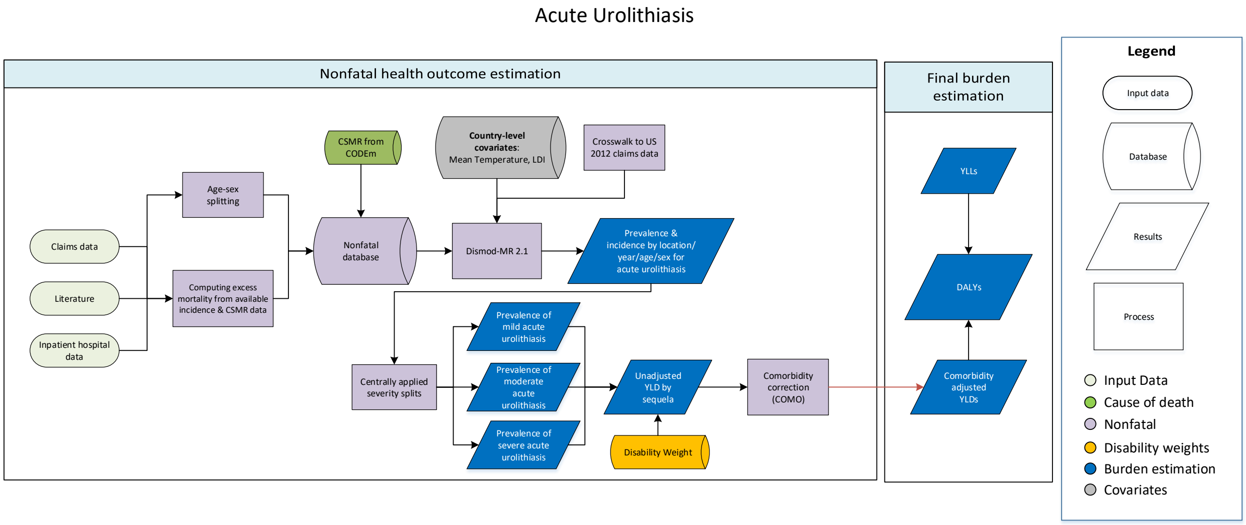

Case definition

Acute urolithiasis (AU) is an acute and usually symptomatic episode of urolithiasis, defined as stone formation located anywhere along the genitourinary tract.[1] Associated ICD codes include N20, N20.0, N20.1, N20.2, N20.9, N21, N21.1, N21.8, N21.9, N22, N22.0, N22.8, N23, and N23.0.

Input data

Model inputs

For GBD 2010, a systematic review of the prevalence of AU throughout the world was conducted. This search was updated for GBD 2013 and again for GBD 2016. A PubMed search was conducted using the following search terms: (Urolithiasis[Title/Abstract] OR Kidney Stones[Title/Abstract]) AND (Prevalence[Title/Abstract] OR Incidence[Title/Abstract]) AND ("2013/01/01"[PDAT] : "3000"[PDAT]) AND "humans"[MeSH Terms].

The exclusion criteria were:

1. Studies clearly not representative of the national population
2. Studies that did not provide primary data on epidemiological parameters, eg, a commentary piece
3. Studies of a specific type of urolithiasis

Four new studies were added based on this systematic review. The table below shows the geographical coverage of urolithiasis data by measure in GBD 2017.

	Incidence	Prevalence
**Site-years (total)**	1420	5
**Number of countries with data**	45	4
**Number of GBD regions with data (out of 21 regions)**	16	4
**Number of GBD super-regions with data (out of 7 super-regions)**	7	3

Claims data for the USA, Philippines, Taiwan, and New Zealand were included. Hospital inpatient data were also included. Inpatient data points with an age-standardised incidence rate greater than two median absolute deviations from the median of the age-standardised incidence rate for all inpatient data were marked as outliers and excluded from analysis.

Severity splits & disability weights

The basis of the GBD disability weight survey assessments is lay descriptions of sequelae highlighting major functional consequences and symptoms. Urolithiasis is split into mild, moderate, and severe categories. The lay descriptions and disability weights for urolithiasis are shown below.

Severity level	Lay description	DW (95% CI)
Mild acute urolithiasis	Has some pain in the belly that causes nausea but does not interfere with daily activities.	0.011 (0.005, 0.021)
Moderate acute urolithiasis	Has pain in the belly and feels nauseous. The person has difficulties with daily activities.	0.114 (0.078, 0.159)
Severe acute urolithiasis	Has severe pain in the belly and feels nauseous. The person is anxious and unable to carry out daily activities.	0.324 (0.220, 0.442)

The severity distribution of urolithiasis was derived from analysis of the Medical Expenditure Panel Surveys (MEPS). MEPS is an overlapping panel survey of the non-institutionalised USA population that collects data on respondents’ health service interactions. Panels are initiated every year. Each panel is two years long and consists of five rounds. In 2000, MEPS began using 12-Item Short Form Surveys (SF-12) to collect data on functional health status. The SF-12 survey is administered twice per panel (about once per year).

In order to translate SF-12 scores into GBD disability weights, 62 lay descriptions for conditions representing the full range of disability weight values (from most mild to most severe) were selected. A convenience sample of respondents was then asked to complete an SF-12 form for an individual with the health state described in the lay descriptions of these conditions. Composite mental and physical SF-12 score was regressed on GBD disability weight to derive the relationship between disability weight and SF-12 score. Individual respondent scores were then regressed on reported conditions to obtain a comorbidity-corrected condition-specific disability weight. The distribution of these condition-specific weights was used to derive the proportion of individuals with the conditions that fall within each GBD severity category.

Severity	Distribution
Mild acute urolithiasis	0.642 (0.536, 0.734)
Moderate acute urolithiasis	0.217 (0.149, 0.296)
Severe acute urolithiasis	0.141 (0.108, 0.178)

Modelling strategy

For GBD 2017, we modelled AU using DisMod MR 2.1, a Bayesian meta-regression modelling program. Prior settings in the DisMod model included setting remission of two weeks. We applied a crosswalk to all USA claims data and hospital inpatient data to adjust to 2012 USA claims data. We used the function in DisMod-MR 2.1 to pull in cause-specific mortality rate (CSMR) data from our CODEm and CODcorrect analyses and match them with incidence data points for the same geography and study year to estimate priors on excess mortality rates (by dividing CSMR by incidence). We set bounds on location random effects for incidence due to sparse data.

We applied a crosswalk to USA claims data from the year 2000 to USA claims data for 2010–2014 in order to adjust for the smaller sample included in the 2000 data. Inpatient data were adjusted to account for multiple admissions and multiple diagnoses, based on data from New Zealand, MarketScan, the Philippines, and Taiwan. Inpatient data were crosswalked to claims data in DisMod.

Betas and exponentiated values (which can be interpreted as an odds ratio) are shown in the table below:

Study covariate	Parameter	beta	Exponentiated beta
Claims data – 2000	Incidence	-0.59 (-0.61 to -0.57)	0.55 (0.54–0.57)
Hospital, inpatient	Incidence	-2.3 (-2.6 to -2.22)	0.10 (0.074–0.11)

Compared to GBD 2016, major changes include inclusion of inpatient data from a greater number of geographies.

Benign Prostatic Hypertrophy (BPH)

Flowchart

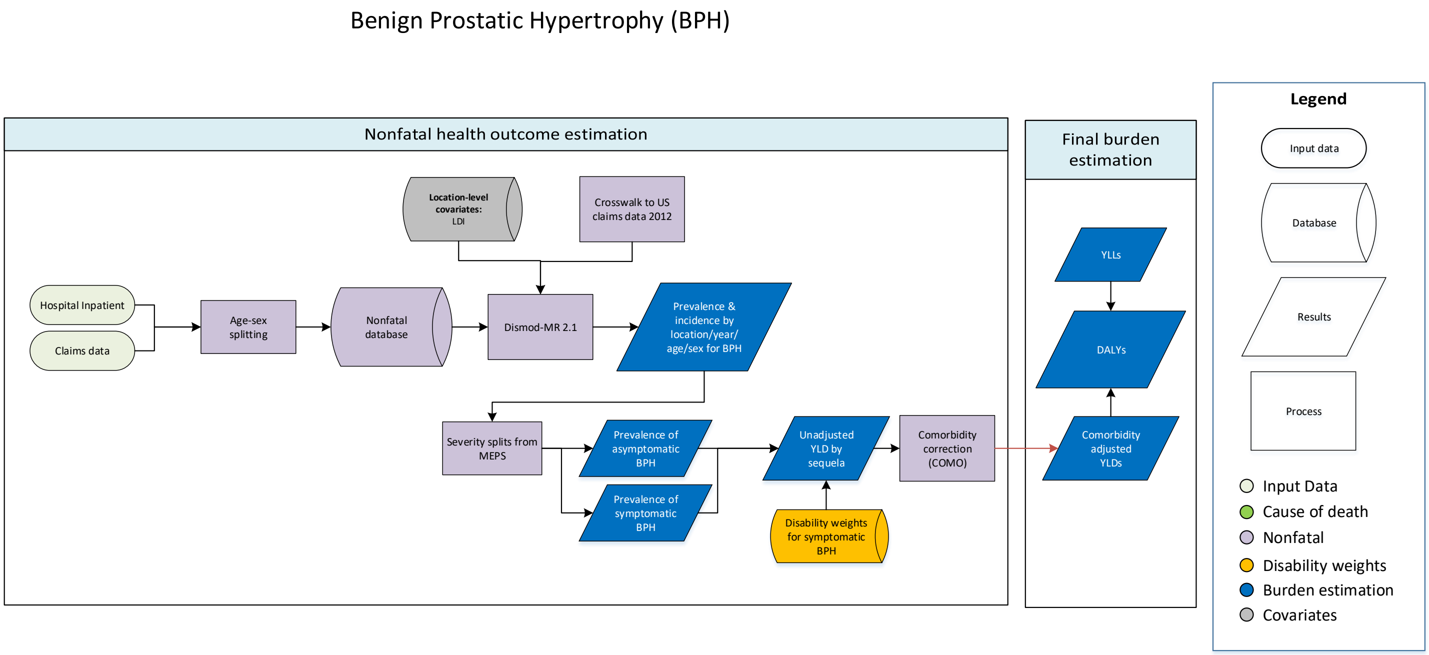

Case definition

Benign prostatic hypertrophy (BPH) is defined as a benign proliferation of prostatic tissue, often leading to symptoms such as urinary retention, bladder outlet obstruction, or urinary tract infection.[1,2] The ICD codes for BPH include N40, N40.0, N40.1, N40.2, N40.3, and N40.9.

Input data

Model inputs

The BPH model is informed by claims data from the USA, Philippines, Taiwan, and New Zealand as well as hospital inpatient data from around the globe. Inpatient data points with an age-standardised mean prevalence greater than two median absolute deviations from the median of the age-standardised mean prevalence for all inpatient data were marked as outliers and excluded from analysis.

	Prevalence
Site-years (total)	1409
Number of countries with data	45
Number of GBD regions with data (out of 21 regions)	16
Number of GBD super-regions with data (out of 7 super-regions)	7

Severity splits

The basis of the GBD disability weight survey assessments are lay descriptions of sequelae highlighting major functional consequences and symptoms of a given cause. BPH is split into symptomatic and asymptomatic types. There is no disability weight (DW) assigned to asymptomatic cases of BPH. The DW associated with symptomatic BPH regards urinary frequency that is sometimes associated with pain – as seen in the table below, which offers further information.

Severity level	Lay description	DW (95% CI)
Asymptomatic	N/A	0
Symptomatic	Feels the urge to urinate frequently, but when passing urine it comes out slowly and sometimes is painful.	0.067 (0.043–0.097)

The proportions symptomatic and asymptomatic were derived from analysis of the Medical Expenditure Panel Surveys (MEPS). MEPS is an overlapping panel survey of the non-institutionalised USA population that collects data on respondents' health service interactions. Panels are initiated every year. Each panel is two years long and consists of five rounds. In 2000, MEPS began using 12-Item Short Form Surveys (SF-12) to collect data on functional health status. The SF-12 survey is administered twice per panel (about once per year).

In order to translate SF-12 scores into GBD disability weights, 62 lay descriptions for conditions representing the full range of disability weight values (from most mild to most severe) were selected. A convenience sample of respondents was then asked to complete an SF-12 form for an individual with the health state described in the lay descriptions of these conditions. Composite mental and physical SF-12 score was regressed on GBD disability weight to derive the relationship between disability weight and SF-12 score. Individual respondent scores were then regressed on reported conditions to obtain a comorbidity-corrected condition-specific disability weight. The distribution of these condition-specific weights was used to derive the proportion of individuals with the conditions that fall within each GBD severity category.

Severity	Distribution
Asymptomatic BPH	0.472 (0.459–0.487)
Symptomatic BPH	0.528 (0.513–0.541)

Modelling strategy

We ran a DisMod 2.1 model to produce estimates by age, sex, year, and location. Prior settings in the BPH DisMod 2.1 model include setting incidence and remission prior to age 40 years to 0. We set an upper bound on remission after age 40 to 0.1, corresponding to a maximum duration of 10 years. We also determined that there was no excess mortality related to BPH.

We applied a crosswalk to USA claims data from the year 2000 to USA claims data for 2010–2014 in order to adjust for the smaller sample included in the 2000 data. Inpatient data were adjusted to account for multiple admissions and multiple diagnoses, based on claims data, and an age-specific crosswalk was applied to adjust inpatient hospital data to USA claims data. This crosswalk was applied by comparing the ratio of prevalence indicated by USA claims data to that indicated by USA inpatient data and applying

these age-specific adjustment factors to global inpatient hospital data. Adjustment factors and their associated standard errors are presented in the table below.

Age start	Age end	Adjustment factor	Standard error
40	44	73.83	0.23
45	49	45.02	0.10
50	54	35.00	0.06
55	59	22.89	0.03
60	64	17.06	0.02
65	69	10.15	0.01
70	74	9.34	0.01
75	79	7.56	0.01
80	84	5.39	0.01
85	89	4.04	0.01
90	94	3.74	0.02
95	99	2.52	0.03

Betas and exponentiated values (which can be interpreted as an odds ratio) are shown in the table below:

Study covariate	Parameter	beta	Exponentiated beta
Claims data – 2000	Prevalence	-0.0023 (-0.0082 to -0.00013)	1.00 (0.99–1.00)
Mean BMI	Prevalence	-0.015 (-0.023 to -0.098)	1.02 (0.98–1.10)

Compared to GBD 2016, the primary change is inclusion of inpatient data from a greater number of geographies.

Other urinary diseases

In addition to the urinary diseases described above, there are many diverse types of urinary diseases, with a range of severities and associated sequelae. Because these urinary diseases are diverse in their underlying causes and risk factors as well as in their associated health outcomes, modelling them together in a DisMod-MR model would not produce reliable estimates of prevalence or excess mortality. Instead, we calculated the YLDs caused by other congenital disorders directly using a YLD/YLL ratio.

We calculated the ratio of YLDs to YLLs across the specified urinary diseases for which non-fatal outcomes were modelled, using YLL estimates from the GBD 2017 cause of death (CoD) analysis. We then multiplied this YLD/YLL ratio by the YLL estimates for other urinary diseases from the GBD 2017 CoD analysis, providing us with an estimate of the YLDs associated with other urinary diseases.

Gynaecologic conditions: uterine fibroids, polycystic ovarian syndrome, endometriosis, genital prolapse, premenstrual disorder, and other gynaecologic conditions (menstrual and non-menstrual disorders)

Uterine Fibroids

Flowchart

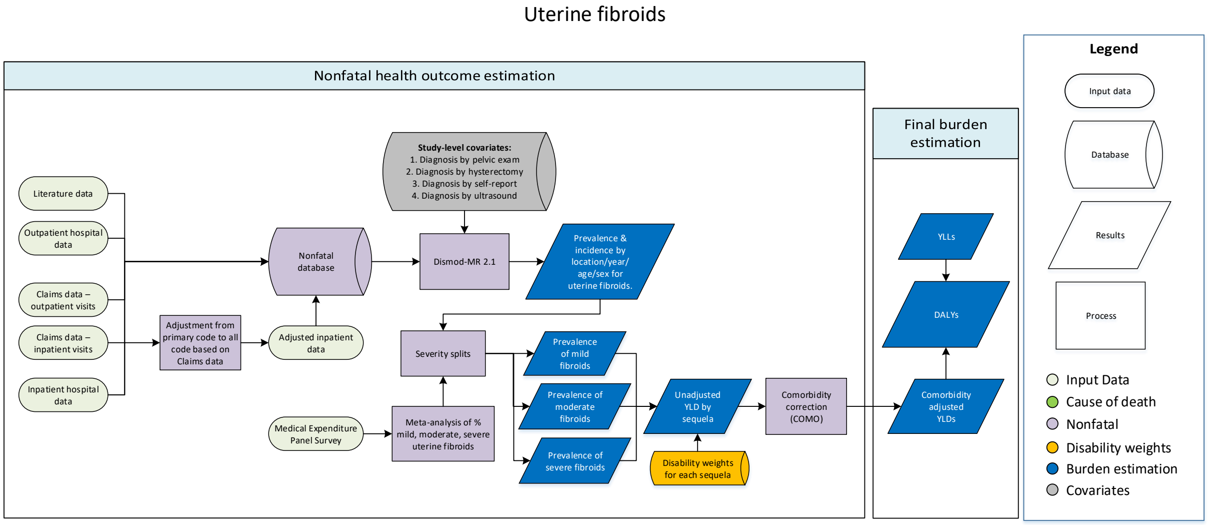

Case definition

Uterine fibroids, also called uterine myomas or leiomyomas, are non-cancerous, compact tumors that occur in the uterus. Fibroids can be diagnosed in a number of ways, including pelvic exam, ultrasound, and hysterectomy. Our reference definition is diagnosis by pelvic exam or ultrasound because it is the most common. However, we incorporate studies that include diagnosis by self-report, pelvic exam only, ultrasound only, hysterectomy only, and all combinations of the three.

Input data

For GBD 2010, a systematic review of uterine fibroids throughout the world was conducted. Ovid MEDLINE, EMBASE, CINAHL, CAB abstracts, WHOLIS, and ISGLE database were searched. The agreed-upon approach for uterine fibroids was to conduct a PubMed literature search every three years. A PubMed search was conducted as part of the initial review in 2010 and is planned for update during the next GBD cycle. The Pubmed search string was as follows:

> ("Leiomyoma"[Mesh] OR fibroid OR fibroids OR leiomyoma OR leiomyomas OR leimyoma OR leimyomas OR leyomyoma OR leyomyomas OR fibromyoma OR fibromyomas OR fibroma OR fibromas OR myoma OR myomas) AND ("Genitalia, Female"[Mesh] OR "Gynecology"[Mesh] OR "Uterus"[Mesh] OR genital OR genitals OR genitalia OR gynecology OR gynaecology OR gynecologic OR gynecological OR gynaecologic OR gynaecological OR uterine OR uterus OR hysterectomy) AND ("Prevalence"[Mesh] OR prevalence OR prevalences)

The EMBASE search string was:

> ('uterus myoma'/exp OR fibroid OR fibroids OR leiomyoma OR leiomyomas OR leimyoma OR leimyomas OR leyomyoma OR leyomyomas OR fibromyoma OR fibromyomas OR fibroma OR fibromas OR myoma OR myomas) AND ('uterus'/exp OR 'gynecology'/exp OR 'female genital system'/exp OR genital OR genitals OR genitalia OR gynecology OR gynaecology OR gynecologic OR gynecological OR gynaecologic OR gynaecological OR uterine OR uterus OR hysterectomy) AND (prevalence/exp OR prevalence OR prevalences)

Exclusion criteria for the initial systematic review were:

1. Studies that did not provide primary data on epidemiological parameters (eg, commentary)
2. Reviews
3. Clearly non-representative studies (eg, only high-risk pregnant women)

Diagnosis by either ultrasound or pelvic exam was set as the reference category. In addition to literature, claims data from the USA (MarketScan), Philippines, and Taiwan were included, along with hospital administrative data that were corrected using a scalar that adjusts for inpatient and outpatient care, converting from inpatient primary admissions to inpatient all diagnoses of individuals based on MarketScan data. The amount of data included in our model increased significantly with GBD 2016 due to the addition of clinical administrative data. The table below shows the dataset contents included in GBD 2017, as well as the number of countries or subnational units and GBD world regions represented.

	Incidence	Prevalence
Site-years (total)	15	1292
Number of countries with data	1	42
Number of GBD regions with data (out of 21 regions)	1	14
Number of GBD super-regions with data (out of 7 super-regions)	1	6

Modelling strategy

We modelled fibroids in DisMod-MR 2.1 to capture the prevalence and incidence of uterine fibroids. There was one important change from prior GBD estimates. This was because, with changes to the hospital and claims administrative data-processing algorithms in GBD 2017, most notably the addition of a requirement that two outpatient visits coded to a cause are required for a person to count as “a case” of a given disease, the inpatient-to-outpatient corrected administrative data became much more variable. This is hypothesised to be due to differences in care-seeking and health-care provision patterns for women with fibroids, including differences between countries in whether women who have procedures for fibroids are categorised as inpatients or outpatients. We therefore used only inpatient hospital and claims data, estimated only symptomatic cases, and then applied an asymptomatic proportion (as described below) to calculate total fibroids cases.

As in previous GBD iterations, incidence was set to zero prior to 15 years of age and after 49. We assume no excess mortality from uterine fibroids. Study-level covariates for diagnosis by hysterectomy only and self-report were included. No country-level covariates were included in this model given the lack of established population-level risk factors. The table below contains the covariates used in this model and their corresponding coefficients:

Covariate name	Type	Measure	Beta value	Exponentiated value
Hospital inpatient	Study covariate	Prevalence	-0.406 (-1.102 to 0.421)	0.666 (0.332–1.523)
Hysterectomy only	Study covariate	Prevalence	-1.415 (-1.593 to -1.252)	0.243 (0.203–0.286)
MarketScan	Study covariate	Prevalence	-0.926 (-1.058 to -0.750)	0.396 (0.347–0.472)

Severity splits and disability weights

The DisMod-MR 2.1 estimates only include those cases that were symptomatic enough to warrant medical care. Symptomatic cases were therefore combined with the asymptomatic proportion of fibroids to calculate total cases.[1] Symptomatic cases were all assumed to have abdominal discomfort, and a subset of all cases also have anaemia due to fibroids. Anaemia causal attribution approaches combined prevalence of fibroids and the quantitative effect of fibroids on haemoglobin concentration (described in detail in anaemia supplementary methods appendix). It should be noted that anaemia alone is not ascribed to fibroids, but only in conjunction with mild abdominal pain with the assumption that more severe, symptomatic cases would be more likely to cause anaemia. Disability weights for each sequela are listed below for reference.

Severity	Lay description	DW (95% CI)
Abdominopelvic problem, mild	has some pain in the belly that causes nausea but does not interfere with daily activities.	0.011 (0.005–0.021)
Anemia, mild	feels slightly tired and weak at times, but this does not interfere with normal daily activities.	0.004 (0.001–0.008)
Anemia, moderate	feels moderate fatigue, weakness, and shortness of breath after exercise, making daily activities more difficult.	0.052 (0.034–0.076)
Anemia, severe	feels very weak, tired and short of breath, and has problems with activities that require physical effort or deep concentration.	0.149 (0.101–0.21)

Polycystic ovarian syndrome (PCOS)

Flowchart

Polycystic ovarian syndrome

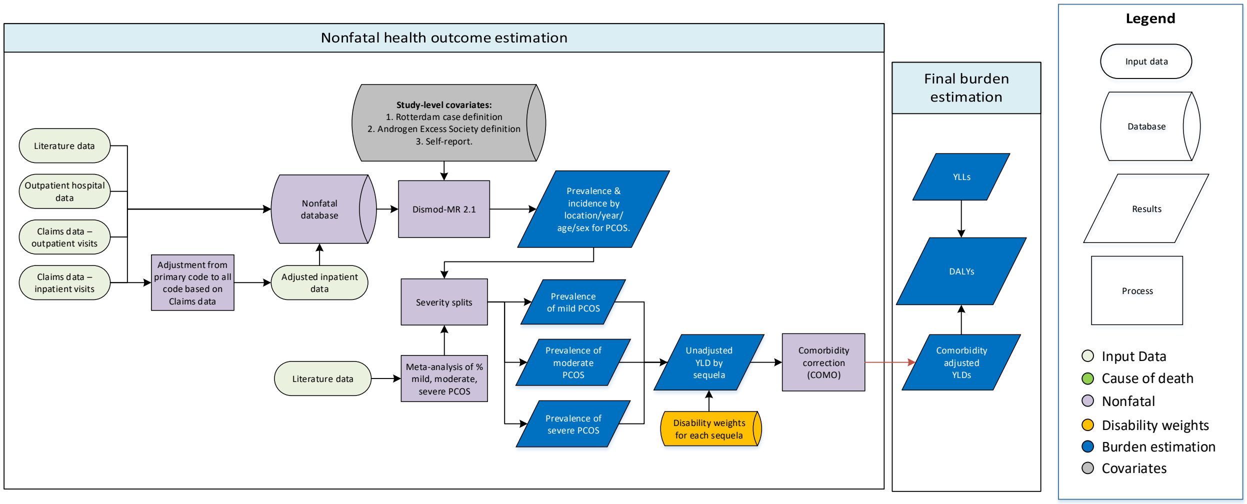

Case definition

Polycystic ovarian syndrome (PCOS) is a gynaecological condition that affects women's ovaries and can lead to a variety of symptoms. Women with PCOS often have enlarged ovaries that contain pockets of fluid, and symptoms include infrequent menstruation, excess hair growth, acne, and obesity.

Input data

For GBD 2010, a systematic review of PCOS throughout the world was conducted. Ovid MEDLINE, EMBASE, CINAHL, CAB abstracts, WHOLIS, and ISGLE database were searched. The agreed-upon approach for PCOS was to conduct a PubMed literature search every three years. A PubMed search was conducted as part of the initial review in 2010 and is planned for update during the next GBD cycle. The Pubmed search string was as follows:

> ("Polycystic Ovary Syndrome"[Mesh] OR "Polycystic Ovary Syndrome" OR "Sclerocystic Ovary Syndrome" OR "Sclerocystic Ovarian Degeneration" OR "Stein-Leventhal Syndrome" OR "Stein Leventhal Syndrome" OR "Sclerocystic Ovaries" OR "Sclerocystic Ovary") AND ("Incidence"[Mesh] OR Incidence OR Incidences OR "Prevalence"[Mesh] OR Prevalence OR Prevalences)

The EMBASE search string was as follows:

> ('ovary polycystic disease'/exp OR "cystic ovary" OR "micropolycystic ovary" OR "multiple follicle cyst" OR "ovary polycystic syndrome" OR "ovary, micropolycystic" OR "ovary, polycystic" OR "polycystic ovarian disease" OR "polycystic ovary" OR "polycystic ovary disease" OR "polycystic ovary syndrome") AND ('incidence'/exp OR incidence OR incidences OR 'prevalence'/exp OR prevalence OR prevalences)

Exclusion criteria for the initial systematic review were:

1. Studies that did not provide primary data on epidemiological parameters (eg, commentary)

2. Reviews
3. Clearly non-representative studies (eg, only high-risk pregnant women)

We use as our reference definition the NIH/NICHD criteria, for which three signs must be present: clinical or biochemical evidence of hyperandrogenism, oligomenorrhoea, and the exclusion of other disorders. In addition to literature, claims data from the USA (MarketScan), Philippines, and Taiwan were included, along with hospital administrative data that were corrected using a scalar that adjusts for inpatient and outpatient care, converting from inpatient primary admissions to inpatient and outpatient all diagnoses for individuals based on MarketScan data. The amount of data included in our model increased significantly with GBD 2016 due to the addition of clinical administrative data. The table below shows the dataset contents included in GBD 2017, as well as the number of countries or subnational units and GBD world regions represented.

	Prevalence
Site-years (total)	367
Number of countries with data	23
Number of GBD regions with data (out of 21 regions)	12
Number of GBD super-regions with data (out of 7 super-regions)	6

Modelling strategy

We modelled PCOS using DisMod-MR 2.1. Incidence was set to zero prior to 10 years of age and after 55 years of age to reflect that women are only susceptible between menarche and menopause. Remission until age 45 was bounded to have a maximum value of 1 per 10,000 person-years and was set to have a maximum duration of 2-13 months after age 55 (remission rate range = 0.9-5). Excess mortality rate among prevalent cases was bounded to have a maximum value of 3 per 10,000 person-years.

Case definitions for PCOS vary according to types of symptoms and duration of symptomology required for diagnosis. Study covariates were used to crosswalk alternate definitions to the reference definitions. Acceptable alternate definitions included the Rotterdam definition, Androgen Excess Society (AES) definition, and self-report. Cause-specific mortality rate (CSMR) from the GBD 2017 cause-specific mortality analysis was used in the model, with Healthcare Access and Quality (HAQ) index as a country covariate on the excess mortality parameter. The table below shows the coefficients for the study- and country-level covariates used in the PCOS model.

Covariate name	Type	Measure	Beta value	Exponentiated value
Healthcare Access and Quality index	Country covariate	Excess mortality rate	-0.005 (-0.018 to 0.000)	0.995 (0.982–1.000)
Androgen Excess Society definition	Study covariate	Prevalence	0.548 (0.102–0.973)	1.730 (1.107–2.645)
Hospital inpatient	Study covariate	Prevalence	-0.292 (-0.414 to -0.142)	0.747 (0.661–0.868)
MarketScan	Study covariate	Prevalence	-1.546 (-1.598 to -1.430)	0.213 (0.202–0.239)
Rotterdam definition	Study covariate	Prevalence	0.339 (0.221–0.398)	1.404 (1.247–1.489)
Self-reported	Study covariate	Prevalence	0.536 (0.082–0.983)	1.710 (1.085–2.672)

Severity splits

The basis of the GBD disability weight (DW) survey assessment are lay descriptions of sequelae highlighting major functional consequences and symptoms. Unfortunately, no health states specific to PCOS were included in the GBD disability weights survey. The main sequelae of PCOS are infertility and hyperandrogenism/hirsutism, the latter of which was approximated with the health state of "disfigurement, level 1." In the NIH definition, which we designated as the reference case definition, all cases of endometriosis have hyperandrogenism and hirsutism, and therefore we assumed that 100% of all PCOS cases would experience this sequela.

Severity	Lay description	DW (95% CI)
Disfigurement, level 1	has a slight, visible physical deformity that others notice, which causes some worry and discomfort.	0.011 (0.005–0.021)
Infertility, primary	wants to have a child and has a fertile partner, but the couple cannot conceive.	0.008 (0.003–0.015)
Infertility, secondary	has at least one child and wants to have more children. The person has a fertile partner, but the couple cannot conceive.	0.005 (0.002–0.011)

Endometriosis

Flowchart

Endometriosis

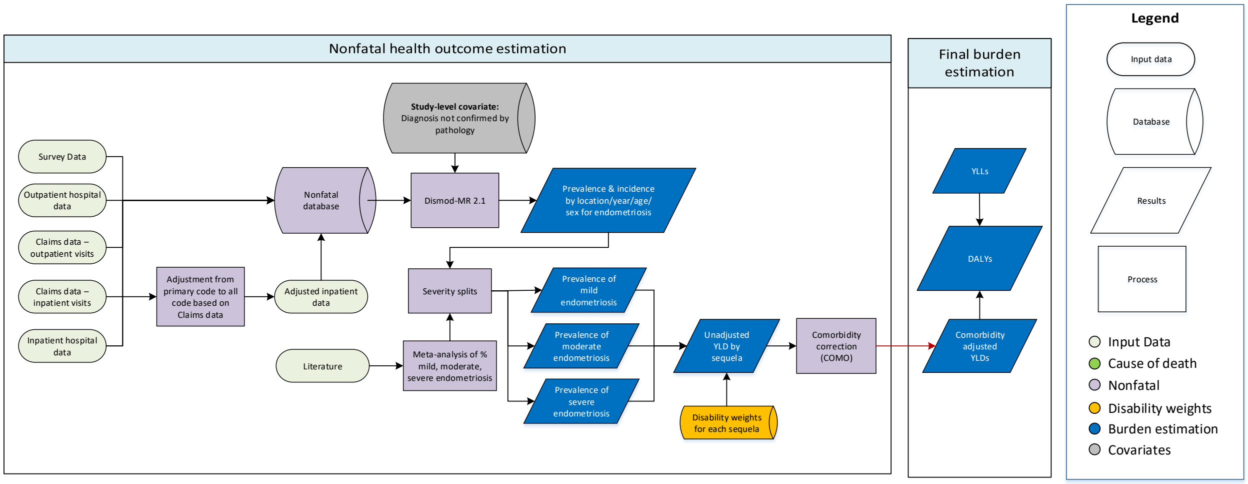

Case definition

Endometriosis is defined as growth of tissue that usually lies inside the uterus outside of it. Common symptoms include chronic pain and infertility. Our reference case definition of endometriosis is diagnosis accompanied by pathological confirmation.

Input data

For GBD 2010, a systematic review of endometriosis throughout the world was conducted. Ovid MEDLINE, EMBASE, CINAHL, CAB abstracts, WHOLIS, and ISGLE database were searched. The agreed-upon approach for endometriosis was to conduct a PubMed literature search every three years. A PubMed search was conducted as part of the initial review in 2010. An updated systematic review is planned for the next GBD cycle. The search string for PubMed was as follows:

("Endometriosis"[Mesh] OR Endometriosis OR Endometrioses OR Endometrioma OR Endometriomas OR Adenomyosis) AND (“Incidence”[Mesh] OR Incidence OR Incidences OR “Prevalence”[Mesh] OR Prevalence OR Prevalences)

The search string for EMBASE was as follows:

(‘endometriosis’/exp OR endometriosis OR endometrioses OR endometrioma OR endometriomas OR adenomyosis) AND (‘incidence’/exp OR incidence OR incidences OR ‘prevalence'/exp OR prevalence OR prevalences)

Exclusion criteria for the initial systematic review were:

4. Studies that did not provide primary data on epidemiological parameters (eg, commentary)
5. Reviews
6. Clearly non-representative studies (eg, only high-risk pregnant women)

Diagnosis confirmed via laparoscopy followed by histologic pathology from literature studies was set as the reference definition. In addition to literature, claims data from the USA (MarketScan), Philippines, and Taiwan were included, along with hospital administrative data that were corrected using a scalar that adjusts for inpatient and outpatient care, converting from inpatient primary admissions to inpatient and outpatient all diagnoses for individuals based on MarketScan data. The table below shows the dataset contents included in GBD 2017, as well as the number of countries or subnational units and GBD world regions represented.

	Incidence	Prevalence
Site-years (total)	32	1164
Number of countries with data	7	36
Number of GBD regions with data (out of 21 regions)	2	13
Number of GBD super-regions with data (out of 7 super-regions)	1	6

Modelling strategy

We used DisMod-MR 2.1, a Bayesian meta-regression epidemiological model, to generate estimates for endometriosis by age, sex, year, and country. As in previous GBD iterations, incidence was assumed to be zero except between the ages of 15 years and 50 years. This is because a woman must enter puberty before she can get endometriosis, and the condition remits spontaneously after the onset of menopause. Remission was bounded to be a maximum of 0.2 before the age of 50 years and was set to be equal to 0.2 (1/remission = duration = 5 years).

In addition to study-level covariate on diagnosis not confirmed by pathology, study-level covariates were added for each year of USA claims data as well as inpatient and outpatient administrative hospital data. We used the Heathcare Access and Quality (HAQ) index as the lone country-level covariate on excess-mortality rate to inform the model. The table below list the study-level and country-level covariates used in the Endometriosis model and the corresponding betas and exponentiated values.

Covariate name	Type	Measure	Beta value	Exponentiated value
Healthcare Access and Quality index	Country covariate	Excess mortality rate	-0.010 (-0.020 to 0.000)	0.990 (0.980–1.000)
All MarketScan, year 2000	Study covariate	Prevalence	-0.498 (-2.000 to 1.000)	0.608 (0.135–2.718)

Endometriosis not confirmed by pathology	Study covariate	Incidence	-0.534 (-0.859 to -0.199)	0.587 (0.423–0.820)
Hospital inpatient	Study covariate	Prevalence	-1.267 (-1.532 to -1.002)	0.282 (0.216–0.367)
MarketScan	Study covariate	Prevalence	-1.908 (-1.999 to -1.714)	0.148 (0.135–0.180)

Severity splits & disability weights

The basis of the GBD disability weight (DW) survey assessment are lay descriptions of sequelae highlighting major functional consequences and symptoms. The GBD 2010 systematic literature review identified three studies that were combined to inform the severity distribution of those with endometriosis. Only one study reported on the proportion of endometriosis cases with chronic abdominal pain,[2] and another was found to contain data on the distribution of pain severity.[3] Data from each study were combined to calculate a pooled proportion of 69.4% (95% CI 66.5–72.4%) of women with endometriosis who have abdominal pain and, of those who suffer pain, 8.2% (7.3–9.1%) with mild pain; 75.1% (73.6–76.5%) with moderate pain; and 16.8% (15.5–18.0%) with severe pain. No information was available on the proportion of time spent with pain. From the Australian Longitudinal Women's Health Study (ALWHS) we were able to derive an estimate of the proportion of women who have endometriosis and long-term infertility.[4] The excess risk of being permanently infertile with endometriosis (relative to no endometriosis) was calculated as the difference in risk of being infertile with and without endometriosis. This excess risk was 6.2% (95% CI: 4.3–8.3%).

Severity	Lay description	DW (95% CI)
Abdominopelvic problem, mild	has some pain in the belly that causes nausea but does not interfere with daily activities.	0.011 (0.005–0.021)
Abdominopelvic problem, moderate	has pain in the belly and feels nauseous. The person has difficulties with daily activities.	0.114 (0.078–0.159)
Abdominopelvic problem, severe	has severe pain in the belly and feels nauseous. The person is anxious and unable to carry out daily activities.	0.324 (0.219–0.442)
Infertility, primary	wants to have a child and has a fertile partner, but the couple cannot conceive.	0.008 (0.003–0.015)
Infertility, secondary	has at least one child and wants to have more children. The person has a fertile partner, but the couple cannot conceive.	0.005 (0.002–0.011)

Genital prolapse

Flowchart

Genital prolapse

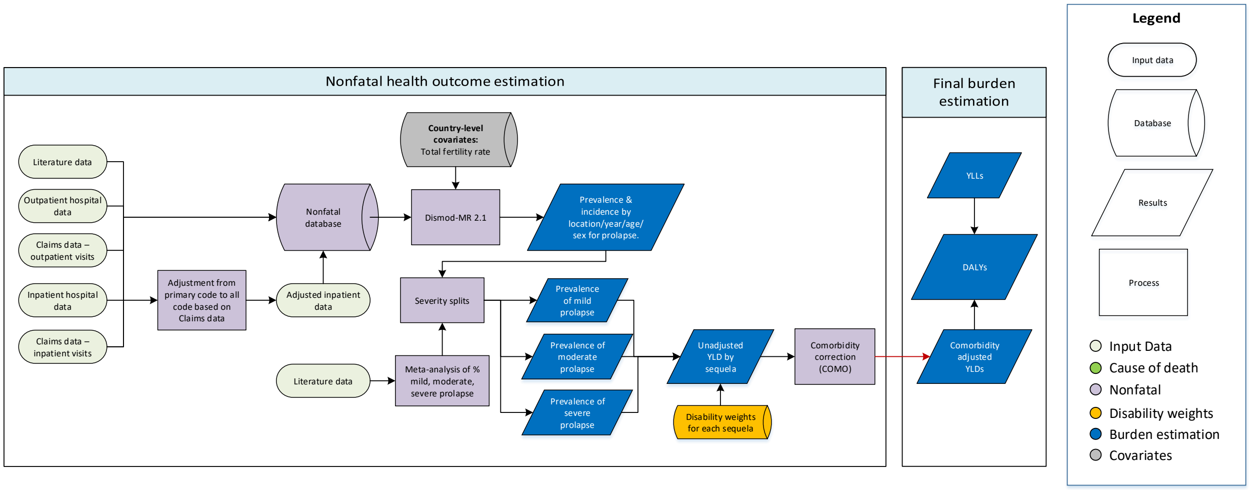

Case definition

Genital prolapse, also called female pelvic organ prolapse, is the clinically relevant descent of one of more of the pelvic structures, including the uterus, bladder, rectum, small or large bowl, or vagina. Risk of prolapse increases with age and can be exacerbated by vaginal childbirth or physical strain. ICD codes associated with genital prolapse include: N81.

Input data

For GBD 2010, a systematic review of genital prolapse throughout the world was conducted. Ovid MEDLINE, EMBASE, CINAHL, CAB abstracts, WHOLIS, and ISGLE database were searched. The agreed-upon approach for genital prolapse was to conduct a PubMed literature search every three years. A PubMed search was conducted as part of the initial review in 2010 and is next due for GBD 2018. The search string used in Pubmed was as follows:

> (("genital prolapse" OR "genital prolapses" OR "vaginal prolapse" OR "vaginal prolapses" OR "uterine prolapse" OR "uterine prolapses" OR "uterovaginal prolapse" OR "uterus prolapse" OR "pelvic organ prolapse" OR "urogenital prolapse" OR "vaginal vault prolapse" OR cystocele OR cystoceles OR “Vaginal enterocele” OR “urethrocele” OR “urethroceles”) AND (prevalence OR prevalences OR epidemiology OR incidence OR incidences)) OR ((“Uterine prolapse”[MeSH] OR “Pelvic organ prolapse”[MeSH] OR “cystocele”[MeSH]) AND (“Prevalence”[MeSH] OR “Epidemiology”[MeSH])

The search string for EMBASE was as follows:

> (("genital prolapse" OR "genital prolapses" OR "vaginal prolapse" OR "vaginal prolapses" OR "uterine prolapse" OR "uterine prolapses" OR "uterovaginal prolapse" OR "uterus prolapse" OR "pelvic organ prolapse" OR "urogenital prolapse" OR "vaginal vault prolapse" OR cystocele OR cystoceles OR “Vaginal enterocele” OR “urethrocele” OR “urethroceles”) AND (‘incidence’/exp OR incidence OR incidences OR ‘prevalence'/exp OR prevalence OR prevalences)) OR ((‘Uterus

prolapse'/exp, 'Pelvic organ prolapse'/exp, 'Cystocele'/exp, 'Enterocele'/exp) AND ('incidence'/exp OR incidence OR incidences OR 'prevalence'/exp OR prevalence OR prevalences))

Exclusion criteria for the initial systematic review were:

1. Studies that did not provide primary data on epidemiological parameters (eg, commentary)
2. Reviews
3. Clearly non-representative studies (eg, only high-risk pregnant women)

Outpatient diagnosis from literature studies was set as the reference definition. In addition to literature, claims data from the USA (MarketScan), Philippines, and Taiwan were included, along with hospital administrative data that were corrected using a scalar that adjusts for inpatient and outpatient care, converting from inpatient primary admissions to inpatient and outpatient all diagnoses for individuals based on MarketScan data. The table below shows the dataset contents included in GBD 2017, as well as the number of countries or subnational units and GBD world regions represented.

	Prevalence
Site-years (total)	1255
Number of countries with data	44
Number of GBD regions with data (out of 21 regions)	17
Number of GBD super-regions with data (out of 7 super-regions)	7

Modelling strategy

We used DisMod-MR 2.1 to run models for genital prolapse. As in previous GBD iterations, incidence was set to zero prior to 15 years of age. This is because it is highly unlikely a woman would experience genital prolapse before entering her reproductive years. We used log-transformed total fertility rate as a country covariate as multiparity is a recognized risk factor for prolapse. To crosswalk data collected from non-reference settings (reference = outpatient literature study), we added study covariates for MarketScan and inpatient-only data. The table below illustrates covariates, measures, parameters, beta, and exponentiated beta values.

Covariate name	Type	Measure	Beta value	Exponentiated value
Total fertility rate	Country covariate	Prevalence	0.923 (0.848–0.979)	2.516 (2.334–2.663)
Hospital inpatient	Study covariate	Prevalence	-1.971 (-2.269 to -1.809)	0.139 (0.103–0.164)
MarketScan	Study covariate	Prevalence	-1.995 (-2.000 to -1.984)	0.136 (0.135–0.138)

Severity splits

The basis of the GBD disability weight (DW) survey assessment are lay descriptions of sequelae highlighting major functional consequences and symptoms. To determine the proportion of people within each domain of disability, several studies from the systematic review were identified to contain information on the proportion of persons with symptoms. These data were pooled and applied to prevalence estimates. Two studies included information on the proportion of women with prolapse who experience a bulging sensation (pooled proportion = 11.7% [95% CI 6.8–19.4%]),[5,6] three that reported on

the proportion with stress incontinence (pooled proportion = 52.8% [40.1–65.1%]),[7–9] and one that reported on the frequency (measured as proportion of the year) of incontinence symptoms (pooled proportion = 7.9% [4.6–13.6%]).[10] Percentages were combined to calculate the proportion of women who fall into both stress incontinence and bulging sensation categories. The lay descriptions and disability weights for genital prolapse are shown below.

Severity	Lay description	DW (95% CI)
Stress incontinence	loses small amounts of urine without meaning to when coughing, sneezing, laughing or during physical exercise.	0.02 (0.011–0.035)
Abdominopelvic problem, mild	has some pain in the belly that causes nausea but does not interfere with daily activities.	0.011 (0.005–0.021)

Premenstrual syndrome (PMS)

Flowchart

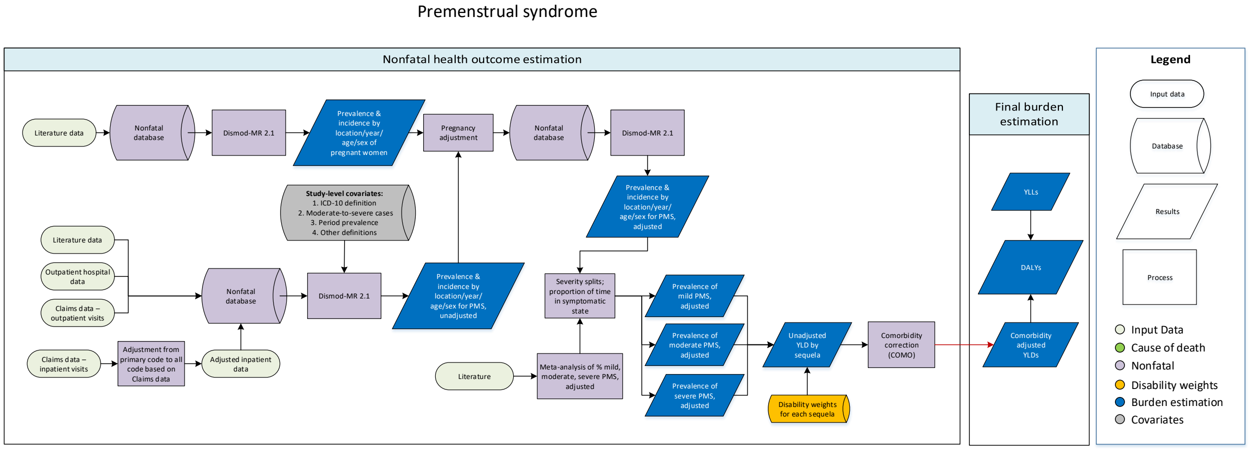

Case definition

Premenstrual syndrome refers to psychological and physical symptoms that occur in the weeks leading up to a woman's period in her menstrual cycle. Symptoms are extremely varied in nature and severity, but include tenderness, bloating, irritability, fatigue, abdominal pain, and altered mental states. Symptoms cease when a woman is pregnant and once she reaches menopause. The ICD-10 code is N94.3.

Input data

For GBD 2010, a systematic review of premenstrual syndrome throughout the world was conducted. Ovid MEDLINE, EMBASE, CINAHL, CAB abstracts, WHOLIS, and ISGLE database were searched. The agreed-upon approach for premenstrual syndrome was to conduct a PubMed literature search every three years. A PubMed search was conducted as part of the initial review in 2010 and is next planned for the upcoming cycle of GBD. The PubMed search string was as follows:

> "Premenstrual Syndrome"[Mesh] OR (premenstrual AND syndrome) OR (premenstrual AND syndrome) OR (premenstrual AND tension) OR (premenstrual AND tensions) OR (premenstrual AND stress) OR "premenstrual dysphoric disorder" OR "premenstrual dysphoric disorders" OR

(menstrual AND distress) AND (("Incidence"[Mesh] OR incidence OR incidences OR onset OR occurrence) OR ("Prevalence"[Mesh] OR prevalence OR prevalences)

The EMBASE search string was as follows:

'premenstrual syndrome'/exp OR 'premenstrual dysphoric disorder'/exp OR (premenstrual AND syndrome) OR (premenstrual AND syndromes) OR (premenstrual AND tension) OR (premenstrual AND tensions) OR (premenstrual AND stress) OR "premenstrual dysphoric disorder" OR "premenstrual dysphoric disorders" OR (menstrual AND distress) AND (('incidence'/exp OR incidence OR incidences OR onset OR occurrence) OR (prevalence/exp OR prevalence OR prevalences)

Exclusion criteria for the initial systematic review were:

1. Studies that did not provide primary data on epidemiological parameters (eg, commentary)
2. Reviews
3. Clearly non-representative studies (eg, only high-risk pregnant women)

The table below shows the number of literature studies included in GBD 2017, as well as the number of countries or subnational units and GBD world regions represented. Inpatient hospital data were not incorporated, as we believed that the likelihood that women with PMS would seek care in the medical system would be far more variable than the true epidemiologic variation.

	Prevalence
Site-years (total)	47
Number of countries with data	23
Number of GBD regions with data (out of 21 regions)	11
Number of GBD super-regions with data (out of 7 super-regions)	6

Modelling strategy

As in previous GBD iterations, incidence was set to zero prior to 15 years of age and after 49 years for GBD 2017. This is because a woman must enter puberty before she is by definition only susceptible between menarche and menopause. We assumed no excess mortality from PMS and further assumed that the duration of the condition is between 3.3 and 5 years (remission rate = 0.2–0.3 per person-year).

Case definitions for PMS vary widely, including varying rosters of symptoms over various time periods. We use as our reference definition the American College of Obstetricians and Gynecologists (ACOG) criteria, which states that the patient reports at least one of each of the following affective and somatic symptoms during the five days before their menses and appear in three consecutive cycles: depression, angry outbursts, irritability, anxiety, confusion, social withdrawal; breast tenderness, abdominal bloating, headache, or swelling of extremities. We include study-level covariates for other common case definitions, including the ICD-10 definition of having at least one premenstrual symptom during period of assessment, and the Premenstrual Symptoms Screening Tool (PSST) definition. We also include covariates for studies that only examine moderate-to-severe PMS, or period prevalence of PMS. We used the Socio-demographic Index (SDI) as a country-level covariate.

The model below shows the coefficients for the country and study level covariates that were used in the PMS model.

Covariate name	Type	Measure	Beta value	Exponentiated value
Socio-demographic Index	Country covariate	Prevalence	-0.450 (-0.979 to 0.419)	0.638 (0.376–1.520)
ICD10 definition of PMS	Study covariate	Prevalence	1.154 (0.834–1.472)	3.170 (2.303–4.358)
Misc. other definitions of PMS	Study covariate	Prevalence	0.432 (0.085–1.201)	1.540 (1.089–3.323)
Moderate-to-severe or severe PMS	Study covariate	Prevalence	0.209 (0.053–0.390)	1.232 (1.054–1.477)
Period prevalence of PMS	Study covariate	Prevalence	0.790 (0.257–1.653)	2.204 (1.293–5.223)

Severity splits and disability weights

Studies on the prevalence of PMS consistently excluded women who were not regularly menstruating. A post-DisMod adjustment was made by dividing DisMod estimates with the age-specific fertility rate (ASFR) from GBD 2017 estimates.

The basis of the GBD disability weight (DW) survey assessment are lay descriptions of sequelae highlighting major functional consequences and symptoms. Unfortunately, no specific disability weights for PMS were estimated during the GBD Disability Weight Measurement Survey. Instead, we identified two health states – abdominopelvic problem (mild) and major depression (mild) – as the closest approximations of the symptoms associated with PMS. To determine the proportion of people within each of these severity levels, five studies were consulted. Three of the prevalence studies in the systematic review provided information on the proportion of PMS cases who feel depressed.[11–13] The pooled proportion was 74.2% (95% CI 69.6–78.3%). Two other studies addressed the proportion of women with PMS who experience abdominal pain.[14,15] The pooled proportion was 41.1% (31.7–51.3%). The lay descriptions and disability weights for premenstrual syndrome are shown below.

Severity	Lay description	DW (95% CI)
Major depressive disorder, mild episode	feels persistent sadness and has lost interest in usual activities. The person sometimes sleeps badly, feels tired, or has trouble concentrating but still manages to function in daily life with extra effort.	0.145 (0.099–0.209)
Abdominopelvic problem, mild	has some pain in the belly that causes nausea but does not interfere with daily activities.	0.011 (0.005–0.021)

Other gynaecological conditions (menstrual and non-menstrual disorders)

Other non-menstrual disorders

Flowchart

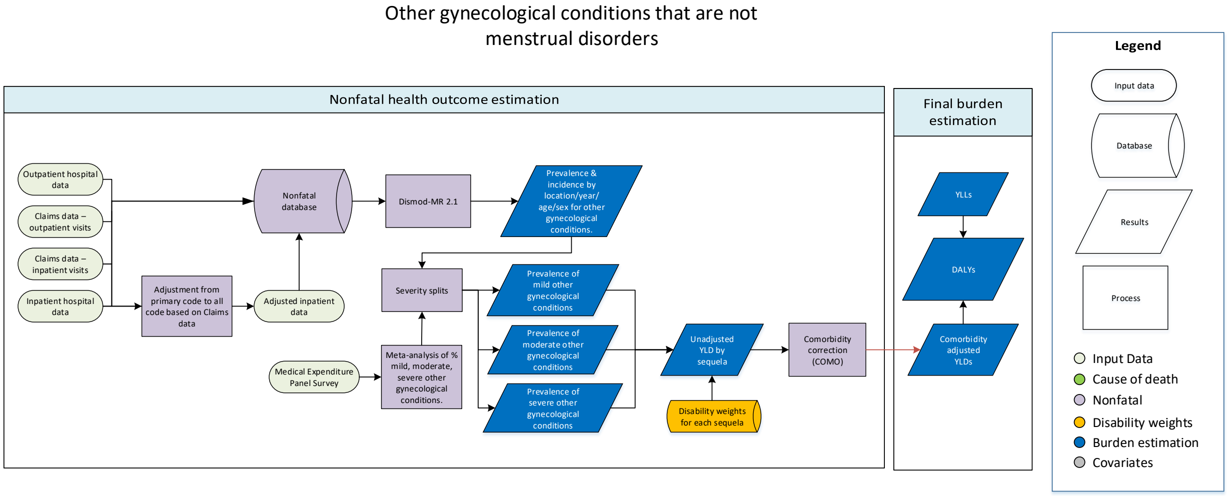

Case definition

Other gynaecological conditions encompasses all disorders that are not menstruation- or bleeding-related that do not fall under the heading of any of the other gynaecological causes. They only affect women. This round of GBD, we are also estimating menstrual disorders, which are written up below.

Input data

No literature data are used to inform models of other gynaecological conditions. We used outpatient hospital data as the reference category. Claims data from the USA (MarketScan), Philippines, and Taiwan were included, along with hospital administrative data that were corrected using a scalar that adjusts for inpatient and outpatient care, converting from inpatient primary admissions to inpatient and outpatient all diagnoses individuals based on MarketScan data. The table below shows the data used in this model:

	Prevalence
Site-years (total)	1160
Number of countries with data	40
Number of GBD regions with data (out of 21 regions)	16
Number of GBD super-regions with data (out of 7 super-regions)	7

Modelling strategy

We ran a DisMod-MR 2.1 model to estimate the global burden of gynaecological diseases. The amount of data included in our model increased significantly with GBD 2015 due to the addition of USA claims data and in GBD 2016 with the addition of hospital data.

As in previous GBD iterations, incidence was set to zero prior to 15 years of age. We assume no excess mortality from other gynaecological conditions over the same age range. We consider the outpatient data as our reference category and crosswalk the inpatient and claims data.

The coefficients for the covariates used in the model are shown in the table below, along with measures, parameters, beta, and exponentiated beta values.

Covariate name	Type	Measure	Beta value	Exponentiated value
Hospital inpatient	Study covariate	Prevalence	-0.741 (-0.918 to -0.615)	0.477 (0.399–0.540)

Severity splits & disability weights

The basis of the GBD disability weight (DW) survey assessment are lay descriptions of sequelae highlighting major functional consequences and symptoms. To determine the proportion of women with other gynaecological conditions who fall into each severity level of abdominopelvic problem, data from the Medical Expenditure Panel Survey (MEPS) were used as described elsewhere in the appendix. The lay descriptions and disability weights for other gynaecological conditions are shown below.

Severity	Lay description	DW (95% CI)
Abdominopelvic problem, mild	has some pain in the belly that causes nausea but does not interfere with daily activities.	0.011 (0.005–0.021)
Abdominopelvic problem, moderate	has pain in the belly and feels nauseous. The person has difficulties with daily activities.	0.114 (0.078–0.159)
Abdominopelvic problem, severe	has severe pain in the belly and feels nauseous. The person is anxious and unable to carry out daily activities.	0.324 (0.219–0.442)

Other menstrual disorders

Flowchart

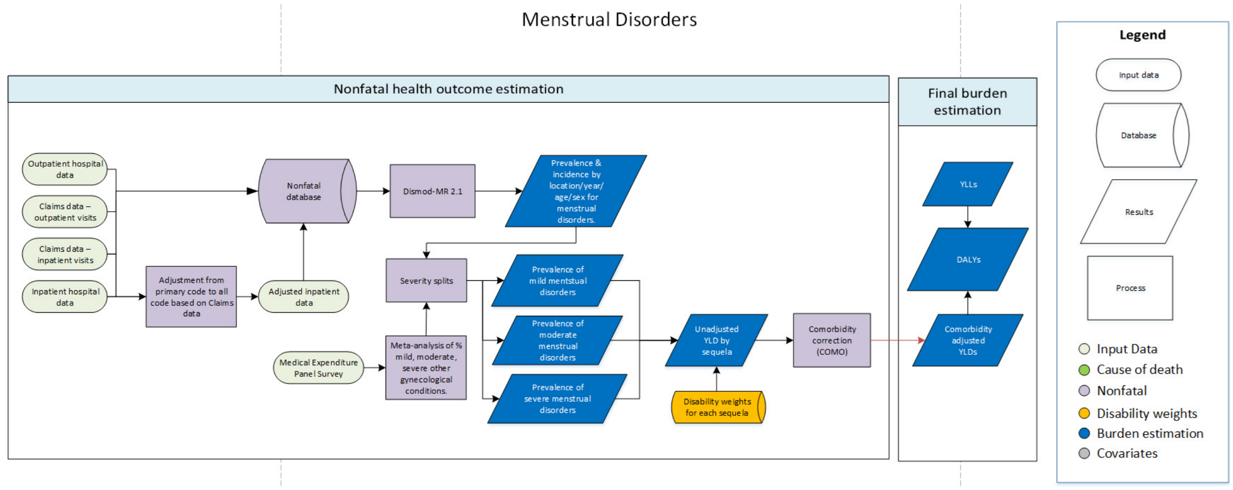

Case definition

Menstrual disorders encompasses all disorders that are menstruation- or bleeding-related that do not fall under the heading of any of the other gynaecological causes. They only affect women.

Input data

This cause was added to the GBD list for GBD 2017. No literature data are used to inform models of menstrual disorders. A systematic literature review is planned for the next GBD cycle. We used outpatient hospital data as the reference category. Claims data from the USA (MarketScan), Philippines, and Taiwan were included, along with hospital administrative data that were corrected using a scalar that adjusts for inpatient and outpatient care, converting from inpatient primary admissions to inpatient and outpatient all diagnoses individuals based on MarketScan data. The table below shows the data used in this model:

	Prevalence
Site-years (total)	1322
Number of countries with data	36
Number of GBD regions with data (out of 21 regions)	14
Number of GBD super-regions with data (out of 7 super-regions)	5

Modelling strategy

We ran a DisMod-MR 2.1 model to estimate the global burden of menstrual disorders. Incidence was set to zero prior to 10 years of age and after 55 years. We assume no excess mortality from other gynaecological conditions over the same age range and crosswalked inpatient and claims data. We used mean BMI and pelvic inflammatory disease prevalence as country-level covariates. The coefficients for the covariates used in the model are shown in the table below.

Covariate name	Type	Measure	Beta value	Exponentiated value
Mean BMI	Country covariate	Prevalence	0.315 (0.301–0.332)	1.371 (1.351–1.394)

Pelvic inflammatory disease age-standardised prevalence	Country covariate	Prevalence	0.490 (0.022–0.970)	1.633 (1.022–2.638)
Hospital inpatient	Study covariate	Prevalence	-1.574 (-1.635 to -1.516)	0.207 (0.195–0.220)
MarketScan	Study covariate	Prevalence	1.171 (1.107–1.219)	3.226 (3.025–3.384)

Severity splits & disability weights

The basis of the GBD disability weight (DW) survey assessment are lay descriptions of sequelae highlighting major functional consequences and symptoms. To determine the proportion of women with other gynaecological conditions who fall into each severity level of abdominopelvic problem, data from the Medical Expenditure Panel Survey (MEPS) were used as described elsewhere in the appendix. Anaemia causal attribution analysis used prevalence of menstrual disorders and information on the quantitative effect of menstrual disorders on haemoglobin level as to estimate the proportion of overall anaemia by severity that is due to menstrual disorders (described in more detail in the anaemia supplementary methods appendix). This addition replaced anaemia cases being assigned to "other gynaecologic disorders" as a residual category in the anaemia analysis. The lay descriptions and disability weights for other gynecological conditions are shown below.

Severity	Lay description	DW (95% CI)
Abdominopelvic problem, mild	has some pain in the belly that causes nausea but does not interfere with daily activities.	0.011 (0.005–0.021)
Abdominopelvic problem, moderate	has pain in the belly and feels nauseous. The person has difficulties with daily activities.	0.114 (0.078–0.159)
Abdominopelvic problem, severe	has severe pain in the belly and feels nauseous. The person is anxious and unable to carry out daily activities.	0.324 (0.219–0.442)
Anaemia, mild	feels slightly tired and weak at times, but this does not interfere with normal daily activities.	0.004 (0.001–0.008)
Anaemia, moderate	feels moderate fatigue, weakness, and shortness of breath after exercise, making daily activities more difficult.	0.052 (0.034–0.076)
Anaemia, severe	feels very weak, tired and short of breath, and has problems with activities that require physical effort or deep concentration.	0.149 (0.101–0.21)

References

1 Divakar H. Asymptomatic uterine fibroids. *Best Pract Res Clin Obstet Gynaecol* 2008; **22**: 643–54.

2 Sinaii N, Plumb K, Cotton L, *et al.* Differences in characteristics among 1,000 women with endometriosis based on extent of disease. *Fertil Steril* 2008; **89**: 538–45.

3 Sinaii N, Cleary SD, Ballweg ML, Nieman LK, Stratton P. High rates of autoimmune and endocrine disorders, fibromyalgia, chronic fatigue syndrome and atopic diseases among women with endometriosis: a survey analysis. *Hum Reprod* 2002; **17**: 2715–24.

4 Loxton D, Dobson A, Byles J, Tooth L. Australian Longitudinal Study on Women's Health (ALSWH). http://www.alswh.org.au/.

5 Slieker-ten Hove MCP, Pool-Goudzwaard AL, Eijkemans MJC, Steegers-Theunissen RPM, Burger CW, Vierhout ME. Symptomatic pelvic organ prolapse and possible risk factors in a general population. *Am J Obstet Gynecol* 2009; **200**: 184.e1-7.

6 Scherf C, Morison L, Fiander A, Ekpo G, Walraven G. Epidemiology of pelvic organ prolapse in rural Gambia, West Africa. *BJOG* 2002; **109**: 431–6.

7 Lawrence JM, Lukacz ES, Nager CW, Hsu J-WY, Luber KM. Prevalence and co-occurrence of pelvic floor disorders in community-dwelling women. *Obstet Gynecol* 2008; **111**: 678–85.

8 Gomman HM, Nossier SA, Fotohi EM, Kholeif AE. Prevalence and factors associated with genital prolapse: a hospital-based study in Alexandria (Part I). *J Egypt Public Health Assoc* 2001; **76**: 313–35.

9 Chuenchompoonut V, Bunyavejchevin S, Wisawasukmongchol W, Taechakraichana N. Prevalence of genital prolapse in Thai menopausal women (using new standardization classification). *J Med Assoc Thai* 2005; **88**: 1–4.

10 Townsend MK, Danforth KN, Lifford KL, *et al.* Incidence and remission of urinary incontinence in middle-aged women. *Am J Obstet Gynecol* 2007; **197**: 167.e1-5.

11 Nisar N, Zehra N, Haider G, Munir AA, Sohoo NA. Frequency, intensity and impact of premenstrual syndrome in medical students. *J Coll Physicians Surg Pak* 2008; **18**: 481–4.

12 Tabassum S, Afridi B, Aman Z, Tabassum W, Durrani R. Premenstrual syndrome: frequency and severity in young college girls. *J Pak Med Assoc* 2005; **55**: 546–9.

13 Steiner M, Macdougall M, Brown E. The premenstrual symptoms screening tool (PSST) for clinicians. *Arch Womens Ment Health* 2003; **6**: 203–9.

14 Choi D, Lee D-Y, Lehert P, Lee IS, Kim SH, Dennerstein L. The impact of premenstrual symptoms on activities of daily life in Korean women. *J Psychosom Obstet Gynaecol* 2010; **31**: 10–5.

15 Deuster PA, Adera T, South-Paul J. Biological, social, and behavioral factors associated with premenstrual syndrome. *Arch Fam Med* 1999; **8**: 122–8.

Haemoglobinopathies and haemolytic anaemias

Sickle cell disorders, thalassaemias, glucose-6-phosphate dehydrogenase (G6PD) deficiency, sickle cell trait, thalassaemia trait, hemizygous G6PD deficiency, other haemoglobinopathies and haemolytic anaemias

Flowchart

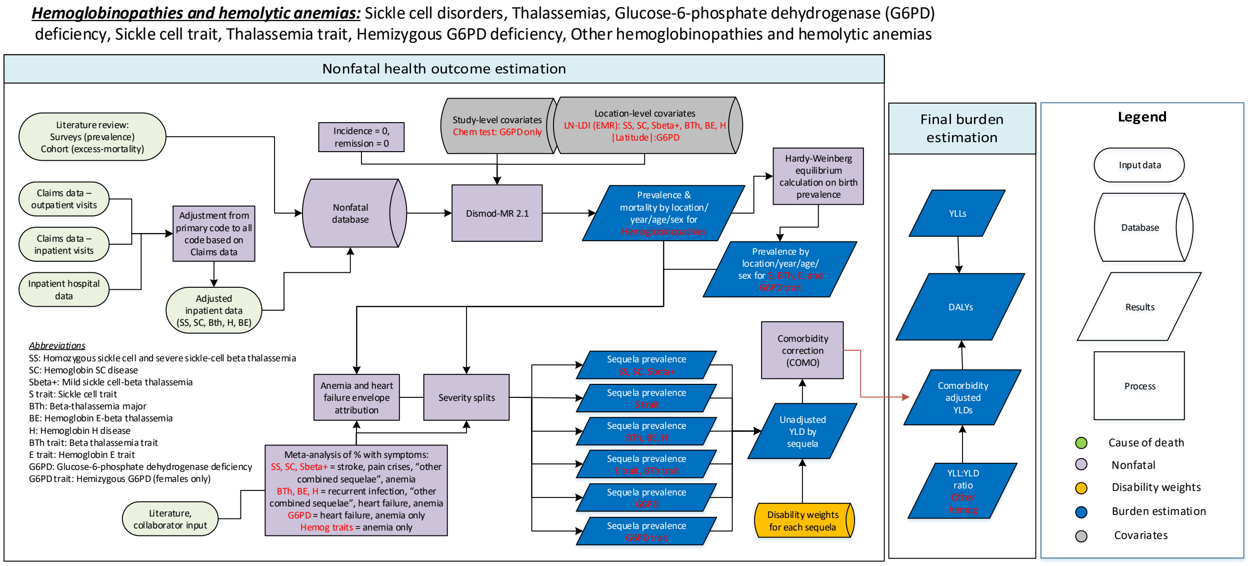

Input data and methodological summary

Case definition

Haemoglobinopathies and haemolytic anaemias span four GBD causes: thalassaemias, sickle cell disorders, glucose-6-phosphate dehydrogenase (G6PD) deficiency, and other haemoglobinopathies. ICD-9 and ICD-10 codes for each are contained in Table 1. Within each category, several unique combinations of genetic mutations lead to distinct phenotypes with different natural history, which has led us to estimate several distinct subtypes of thalassaemias and sickle cell disorders. The three thalassaemia models included 1) beta-thalassaemia major, 2) hemoglobin E/beta-thalassaemia, and 3) hemoglobin H disease. Sickle cell models included 1) homozygous sickle cell and severe sickle cell/beta-thalassaemia, 2) haemoglobin SC disease, and 3) "mild" sickle cell-beta thalassaemia. We also estimated the burden of glucose-6-phosphate dehydrogenase (G6PD) deficiency. Finally, we estimated prevalence and YLDs due to other haemoglobinopathies and haemolytic anaemias assuming the YLD-to-YLL ratio for each age, sex, location, and year was similar to that of the aggregate of sickle cell, thalassaemias, and G6PD deficiency.

TABLE 1. International classification of diseases codes for haemoglobinopathies and haemolytic anaemias in GBD 2015 cause of death analysis		
Condition	ICD-10 code	ICD-9 code
Total	D55-D59	282.0-282.1, 282.7-285.8, 282.2-282.3, 282.5-282.6, 282.4
Thalassaemias	D56	282.4
Sickle cell disorders	D57	282.5-282.6
G6PD deficiency	D55	282.2-282.3
Other haemoglobinopathies and haemolytic anaemias	D58-D64.8	282.0-282.1, 282.7-285.8

Input data

Model inputs

Systematic literature reviews were completed for GBD 2010 and GBD 2013. These were updated on May 1, 2015, using the following search strings:

(thalassemias[Title/Abstract] AND (prevalence[Title/Abstract] OR survival[Title/Abstract] OR mortality[Title/Abstract]) AND ("2008"[PDAT] : "2013"[PDAT])) AND "humans"[MeSH Terms]

(sickle cell[Title/Abstract] AND (mortality[Title/Abstract] OR survival[Title/Abstract] OR prevalence[Title/Abstract]) AND ("2008"[PDAT] : "2013"[PDAT])) AND "humans"[MeSH Terms]

(G6PD[Title/Abstract] OR G6PD deficiency[Title/Abstract] OR glucose-6 phosphate dehydrogenase[Title/Abstract] OR glucose-6-phosphate dehydrogenase deficiency[Title/Abstract] AND (survival[Title/Abstract] OR mortality[Title/Abstract]) AND ("2008"[PDAT] : "2013"[PDAT])) AND "humans"[MeSH Terms]

Of note, upon the recommendation from multiple GBD collaborators, we identified and re-extracted all primary data that had been used in GBD 2013. In some situations, this process identified cases of sickle cell or thalassaemia that had been assigned to incorrect subtypes, but mostly the data were correct and verified. We excluded any data where the results presented in a study were themselves the result of modelling exercises. The most significant change as a result of re-extraction was when we identified that much of the literature data used in GBD 2013 from females with G6PD deficiency actually did not correspond to our case definition of homozygous disease, but rather included combined case counts for homozygotes and hemizygotes. We only included homozygous disease in our datasets for GBD 2017, which has led to much lower estimates of G6PD deficiency in females.

We extracted prevalence data from population-level and community surveys as well as with-condition mortality and excess-mortality data from cohort studies. Age-specific survival proportions were converted to with-condition mortality rates as needed. We also included data from hospital and claims data for a subset of haemoglobinopathy models, including beta-thalassaemia major, haemoglobin E/beta-thalassaemia, homozygous sickle cell and severe sickle cell/beta-thalassaemia, haemoglobin SC disease, and mild sickle cell/beta-thalassaemia. The extraction and processing of hospital and claims data is described separately. Composition of final datasets are shown below for each of the different haemoglobinopathies and haemolytic anaemias models.

<u>Data availability</u>

Homozygous sickle cell and severe sickle cell/beta-thalassaemia (2097):

	**Prevalence**	**Excess mortality rate**	**With condition mortality**
**Site-years (total)**	1749	23	18
**Number of countries with data**	95	9	11
**Number of GBD regions with data (out of 21 regions)**	20	7	8
**Number of GBD super-regions with data (out of 7 super-regions)**	7	5	4

Hemoglobin SC disease (2100):

	**Prevalence**	**Excess mortality rate**	**With condition mortality**
**Site-years (total)**	2327	8	7
**Number of countries with data**	79	3	4
**Number of GBD regions with data (out of 21 regions)**	20	3	4
**Number of GBD super-regions with data (out of 7 super-regions)**	7	2	3

Mild sickle cell/beta-thalassaemia (2103):

	**Prevalence**	**Excess mortality rate**	**With condition mortality**
**Site-years (total)**	1469	8	3
**Number of countries with data**	65	3	2
**Number of GBD regions with data (out of 21 regions)**	16	3	2
**Number of GBD super-regions with data (out of 7 super-regions)**	7	2	1

Beta-thalassaemia major (2085):

	**Prevalence**	**Excess mortality rate**
**Site-years (total)**	736	29
**Number of countries with data**	88	6
**Number of GBD regions with data (out of 21 regions)**	18	3
**Number of GBD super-regions with data (out of 7 super-regions)**	7	2

Hemoglobin E/beta-thalassaemia (2087):

	Prevalence	With condition mortality
**Site-years (total)**	340	1
**Number of countries with data**	28	1
**Number of GBD regions with data (out of 21 regions)**	10	1
**Number of GBD super-regions with data (out of 7 super-regions)**	5	1

Hemoglobin H disease (2089):

	Prevalence
**Site-years (total)**	60
**Number of countries with data**	21
**Number of GBD regions with data (out of 21 regions)**	11
**Number of GBD super-regions with data (out of 7 super-regions)**	6

G6PD deficiency (2112):

	Prevalence
**Site-years (total)**	368
**Number of countries with data**	92
**Number of GBD regions with data (out of 21 regions)**	18
**Number of GBD super-regions with data (out of 7 super-regions)**	7

Modelling strategy

Besides data re-extraction and addition of hospital and claims data, we have made no substantive changes to the estimation strategy since GBD 2016. We estimated the non-fatal burden of haemoglobinopathies in three parts.

First, we used the datasets described above to estimate prevalence for each age-sex-location-year in the GBD 2017 location hierarchy using DisMod-MR 2.1. For mild sickle cell/beta-thalassaemia models, study-level covariates were used to identify and crosswalk those data from MarketScan in the years 2000, 2010, and 2012, which were systematically lower than later years. In all sickle cell models and beta-thalassaemia major, the natural log of lag-distributed income per capita (LN-LDI) was used as a sole country covariate on excess mortality, meant to reflect the profound impact that health system financial resources can have on survival from these conditions. For G6PD deficiency, data where diagnosis was made only on the basis of chemical or reagent testing was crosswalked to the reference definition of genetic G6PD deficiency; absolute value of latitude was the sole country covariate for this model.

Second, we calculated prevalence of haemoglobinopathy traits (sickle cell trait, haemoglobin E trait, haemoglobin beta trait, G6PD trait) by back-calculating from birth prevalence estimates from

corresponding DisMod-MR 2.1 models, assuming Hardy-Weinberg equilibrium, and no excess mortality. Third, age-specific prevalence for all subtypes of haemoglobinopathies were paired with estimated sequelae distributions from a series of cohort studies and clinical data in GBD 2010 and GBD 2013. This included consideration of the burden of anaemia associated with homozygous and heterozygous persons and ensuring the estimates of haemoglobinopathy-induced anaemia were internally consistent with overall estimates for each condition, including prevention of double counting. The anaemia estimation process is described separately.

Third, and finally, we found the ratio of YLD to YLL ratio for all haemoglobinopathies and then applied it to YLLs estimated for other haemoglobinopathies and haemolytic anaemias in our cause-specific mortality analysis. Quantitative crosswalk results for each model are shown below.

Covariate, parameter, beta, and exponentiated beta values

Homozygous sickle cell and severe sickle cell/beta-thalassaemia (2097):

Study-level covariate	Parameter	Geography level	Beta	Exponentiated beta
LDI (I$ per capita)	Excess mortality rate	Global	-0.49	0.61 (0.45–0.90)
Malaria Lysenko PfPR (2 Highest Endemicity)	Prevalence	Global	0.47	1.60 (1.42–1.65)

Haemoglobin SC disease (2100):

Study-level covariate	Parameter	Geography level	Beta	Exponentiated beta
LDI (I$ per capita)	Excess mortality rate	Global	-0.03	0.97 (0.95–1.00)
All MarketScan	Prevalence	Global	2.00	7.38 (7.37–7.39)

Mild sickle cell/beta-thalassaemia (2103):

Study-level covariate	Parameter	Geography level	Beta	Exponentiated beta
LDI (I$ per capita)	Excess mortality rate	Global	-0.17	0.85 (0.74–0.99)
All MarketScan, year 2000	Prevalence	Global	1.99	7.3 (7.01–7.39)
All MarketScan, year 2010	Prevalence	Global	1.96	7.1 (5.13–7.37)

All MarketScan, year 2012	Prevalence	Global	1.96	7.1 (5.65–7.38)

Beta-thalassaemia major (2085):

Study-level covariate	Parameter	Geography level	Beta	Exponentiated beta
LDI (I$ per capita)	Excess mortality rate	Global	-0.55	0.58 (0.55–0.67)

Haemoglobin E/beta-thalassaemia (2087):

Study-level covariate	Parameter	Geography level	Beta	Exponentiated beta
Year	Prevalence	Global	0.02	0.98 (0.98–0.98)

Haemoglobin H disease (2089):

Study-level covariate	Parameter	Geography level	Beta	Exponentiated beta
Year	Prevalence	Global	-0.01	0.99 (0.98–1.01)

G6PD deficiency (2112):

Study-level covariate	Parameter	Geography level	Beta	Exponentiated beta
Absolute value of average latitude	Prevalence	Super-region	-0.01	0.99 (0.99–1.00)
Diagnostic modality based on chemical/reagent testing	Prevalence	Super-region	0.13	1.14 (1.04–1.24)
Year	Prevalence	Super-region	-0.01	1.00 (1.00–1.00)

Sequelae

With the exception of anaemia, only homozygous individuals were considered to experience disability. Estimated sequelae of thalassaemias included anaemia (described separately), heart failure (described separately), and periodic severe infection. Another series of common, but not universal, sequelae also occur in those with thalassaemias, including splenomegaly, skeletal deformity, delayed growth/puberty, diabetes, hypothyroidism, and leg ulcers. Given sparse data on the occurrence of these sequelae, they were approximated with a health state named "other combined sequelae of thalassaemia," for which we used the disability weight corresponding to a health state of "generic uncomplicated disease, anxiety about diagnosis and daily medication" which, of note, was also used to approximate the disability for

those with cancer in remission. For sickle cell disorders, we similarly estimated YLDs for anaemia (described separately), stroke, and pain crises separately and approximated the myriad additional complications of sickle cell disease with the health state "other combined sequelae of sickle cell disease." The only sequelae estimated for G6PD deficiency were anaemia (described separately) and heart failure (described separately). Notably, however, G6PD deficiency is considered to be asymptomatic for a vast majority of those with the condition, with only a very small subset of around 1 in 1,000,000 having chronic haemolysis (Class I disease) and approximately 1% having periodic haemolytic episodes (Class II disease) with exposure to environmental, pharmaceutical, or food products. Females heterozygous for G6PD deficiency exhibit chimerism, as one X chromosome becomes dominant in each of the red blood cells, so we estimated half as many heterozygous females will be symptomatic as homozygous females.

Uncertainty and model selection

For all haemoglobinopathies estimates, uncertainty bounds include uncertainty due to input data, crosswalks from non-reference definitions in study covariates above, uncertainty in numerical solutions (posteriors) of each DisMod-MR 2.1 model, and proportion of all persons with each type of symptom.

In consultation with GBD researchers and collaborators, final models were selected on a combination of qualitative and quantitative goodness of fit to input data, plausibility of geographic and temporal trends, consistency of age pattern, and, when available, comparison with other published studies on haemoglobinopathy epidemiology. Directionality, magnitude, and plausibility of study-level and country-level covariates was also considered in the process of model development. Of note, due to the nature of statistical modelling, final results do not always cover the values reported in input data.

No other significant changes were made to the modelling strategy.

Endocrine, Metabolic, Blood, and Immune Disorders

Flowchart

Endocrine, Metabolic, Blood, and Immune Disorders (EMBID)

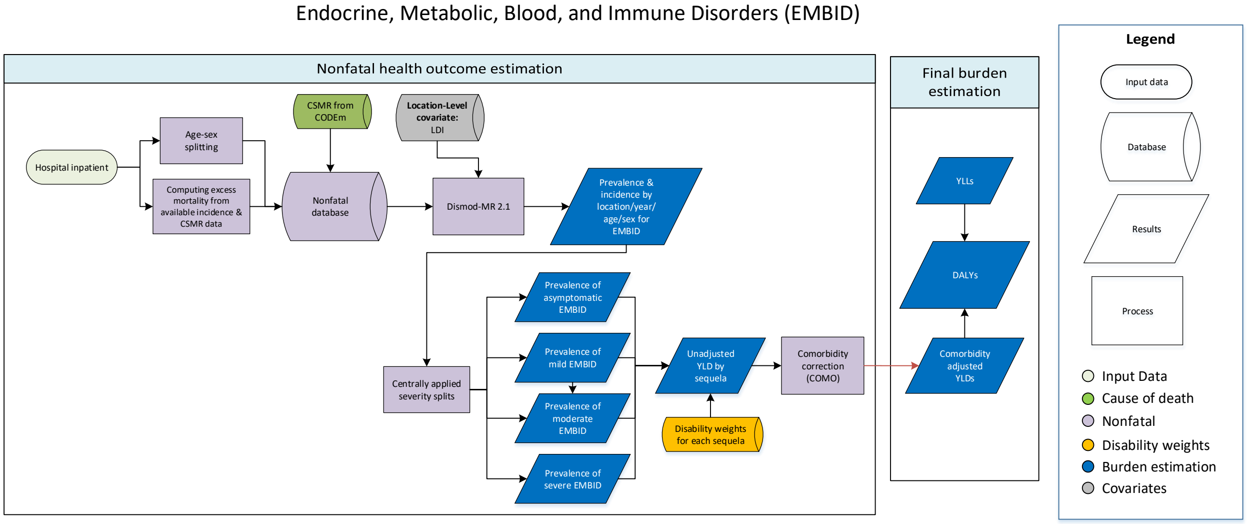

Case definition

Endocrine, metabolic, blood, and immune disorders (EMBID) is a residual cause consisting of conditions that do not map to other causes within the diabetes, urogenital, blood, and endocrine disease hierarchy. This residual group consists mainly of thyroid disorders, rare metabolic and immune disorders, and blood disorders not resulting in anaemia. From the ICD chapter on Endocrine, Metabolic, and Immune Disorders (the E chapter) GBD's definition of EMBID excludes the codes for nutritional deficiencies, diabetes and anaemia which are modelled as separate causes; as well as those for obesity and hypercholesterolemia which are modelled as risks, not causes.

ICD 10 codes for EMBID include: D64.4, D64.8, D68-D68.6, D68.8-D68.9, D69-D69.4, D69.6, D69.8, D70-D70.4, D70.8-D70.9, D72-D72.1, D72.8-D72.9, D73- D73.5, D73.8-D73.9, D74.0, D74.8-D74.9, D75-D75.2, D75.8-D75.9, D76-D76.3, D80-D80.9, D81-D81.9, D82-D82.4, D82.8-D82.9, D83-D83.2, D83.8-D83.9, D84-D84.1, D84.8-D84.9, D86.8, D89-D89.3, D89.8-D89.9, E03-E03.1, E03.3-E03.5, E03.8-E03.9, E04-E04.2, E04.8-E04.9, E05-E05.5, E05.8-E05.9, E06-E06.3, E06.5, E06.9, E07-E07.1, E07.8-E07.9, E16.1-E16.4, E16.8-E16.9, E20-E20.1, E20.8-E20.9, E21-E21.5, E22-E22.2, E22.8-E22.9, E23.0, E23.2-E23.3, E23.6-E23.7, E24-E24.1, E24.3-E24.4, E24.8-E24.9, E25.0, E25.8-E25.9, E26-E26.1, E26.8-E26.9, E27-E27.2, E27.4-E27.5, E27.8-E27.9, E28-E28.1, E28.3, E28.8-E28.9, E29-E29.1, E29.8-E29.9, E30-E30.1, E30.8-E30.9, E31-E31.2, E31.8-E31.9, E32-E32.1, E32.8-E32.9, E34-E34.5, E34.8-E34.9, E67-E67.3, E67.8, E70-E70.5, E70.8-E70.9, E71-E71.5, E72-E72.5, E72.8-E72.9, E73-E73.1, E73.8-E73.9, E74-E74.4, E74.8-E74.9, E75-E75.6, E76-E76.3, E76.8-E76.9, E77-E77.1, E77.8-E77.9, E79-E79.2, E79.8-E79.9, E80-E80.7, E83-E83.9, E84-E84.9, E85-E85.9, E88-E88.9.

Input data

Model inputs

Data from US claims data for 2000, 2010, and 2012 by US state were included. Hospital inpatient data was also included. Inpatient data points with an age-standardised mean prevalence greater than 1.5 median absolute deviations from the median of the age-standardised mean prevalence rate for all inpatient data were marked as outliers and excluded from analysis.

The table below shows the number of countries and GBD world regions represented.

	Prevalence
**Site-years (total)**	1438
**Number of countries with data**	46
**Number of GBD regions with data (out of 21 regions)**	16
**Number of GBD super-regions with data (out of 7 super-regions)**	7

Severity splits & disability weights

The basis of the GBD disability weight survey assessments is lay descriptions of sequelae highlighting major functional consequences and symptoms. EMBID is split into asymptomatic, mild, moderate, and severe categories. The lay descriptions and disability weights for EMBID are shown below.

Severity level	Lay description	DW (95% CI)
Asymptomatic endocrine, metabolic, blood, and immune disorders	--	--
Mild endocrine, metabolic, blood, and immune disorders	Has low energy and feels cold.	0.019 (0.01–0.032)
Moderate endocrine, metabolic, blood, and immune disorders	Feels nervous, has palpitations, sweats a lot, and has difficulty sleeping.	0.145 (0.096–0.202)
Severe endocrine, metabolic, blood, and immune disorders	Easily bruises and sometimes bleeds from the gums and nose; feels weak and has some difficulty with daily activities.	0.159 (0.106–0.226)

The severity distribution of EMBID was derived from analysis of the Medical Expenditure Panel Surveys (MEPS). MEPS is an overlapping panel survey of the non-institutionalised USA population that collects data on respondents' health service interactions. Panels are initiated every year. Each panel is two years long and consists of five rounds. In 2000, MEPS began using 12-Item Short Form Surveys (SF-12) to collect data on functional health status. The SF-12 survey is administered twice per panel (about once per year).

In order to translate SF-12 scores into GBD disability weights, 62 lay descriptions for conditions representing the full range of disability weight values (from most mild to most severe) were selected. A convenience sample of respondents was then asked to complete an SF-12 form for an individual with the

health state described in the lay descriptions of these conditions. Composite mental and physical SF-12 score was regressed on GBD disability weight to derive the relationship between disability weight and SF-12 score. Individual respondent scores were then regressed on reported conditions to obtain a comorbidity-corrected condition-specific disability weight. The distribution of these condition-specific weights was used derive the proportion of individuals with the conditions that fall within each GBD severity category.

Severity	Distribution
Asymptomatic endocrine, metabolic, blood, and immune disorders	0.410 (0.398, 0.423)
Mild endocrine, metabolic, blood, and immune disorders	0.387 (0.328, 0.430)
Moderate endocrine, metabolic, blood, and immune disorders	0.061 (0.042, 0.060)
Severe endocrine, metabolic, blood, and immune disorders	0.142 (0.115, 0.173)

Modelling strategy

We ran a DisMod MR-2.1 model to produce estimates by age, sex, year, and location. Prior settings in the EMBID DisMod model include an upper bound on remission of 0.25, corresponding to a maximum duration of four years. We used the function in DisMod-MR 2.1 to pull in cause-specific mortality rate (CSMR) data from our CODEm and CODcorrect analyses and match them with prevalence data points for the same geography and study year to estimate priors on excess mortality rates (by dividing CSMR by prevalence).

We included the covariate Lagged Distributed Income (LDI) as a country-level covariate to inform excess mortality, with bounds of -0.5, -0.1.

Compared to GBD 2016, major changes include inclusion of inpatient data from a greater number of geographies.

Oral disorders: *Edentulism, Caries of deciduous teeth, Caries of permanent teeth, Chronic periodontal disease, and Other oral disorders*

Edentulism

Flowchart for edentulism

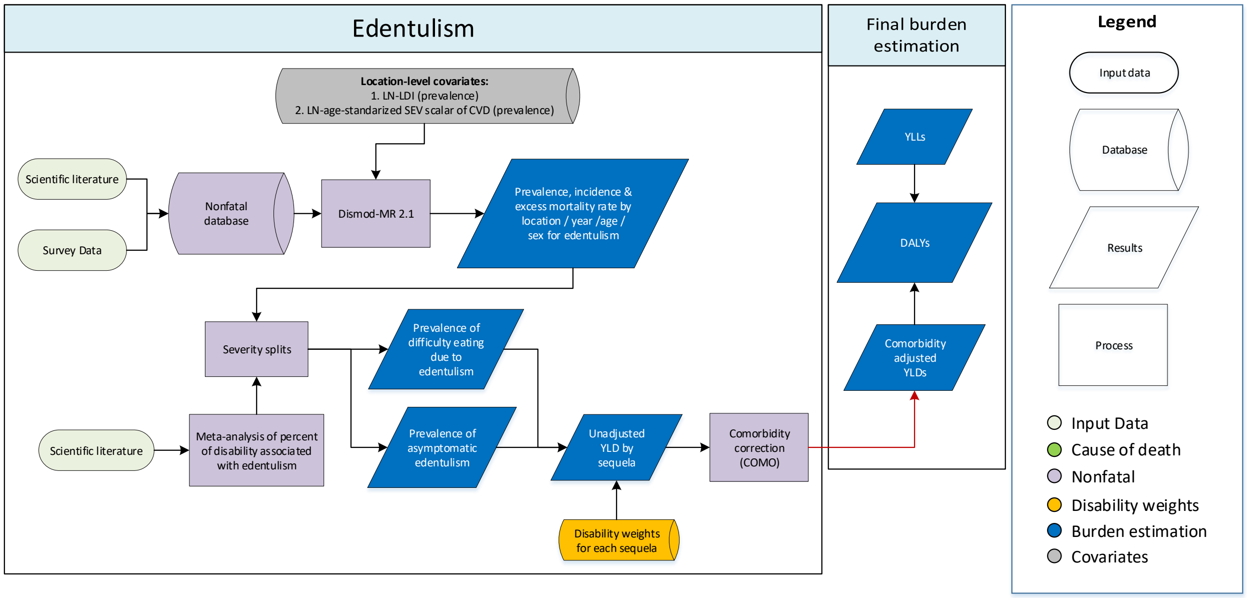

Figure 1: Modelling flowchart for edentulism

Case definition

The case definition of edentulism includes any individual with zero remaining permanent teeth; toothlessness of infancy is not included. The assessment of this disease includes quantification of the prevalence of the disease as well as estimation of the major sequelae: asymptomatic toothlessness and symptomatic toothlessness leading to "great difficulty in eating meat, fruits, and vegetables." A small body of evidence has begun to emerge that implicates edentulousness as predisposing individuals to increased risk for ischaemic cardiovascular events including myocardial infarction and stroke. These data are sparse but have been included in models estimating the excess mortality of those with complete tooth loss. Given that the association is believed to be ecological rather than causal, however, edentulism has not been estimated as an underlying cause of death and it is not included in the risk factor analysis for cardiovascular diseases.

Input data

An initial literature review was done by the Expert Group for GBD 2010, including published articles as well as the results of national and subnational reports. A new systematic review was completed for GBD 2017. The search terms for this systematic review included: (Edentulism[Title/Abstract]) OR (edentulous[Title/Abstract]) OR (endentulousness[Title/Abstract]) OR (severe tooth loss[Title/Abstract]) OR (total tooth loss[Title/Abstract]) OR (complete tooth loss[Title/Abstract]) AND (prevalence[Title/Abstract]) AND ("2010"[Date - Publication] : "2017"[Date - Publication]).

While an additional literature review was not performed for GBD 2015, new World Health Survey data were added for 47 countries. Additional data identified by GBD collaborators has continued to be added as it has been identified. Updates to systematic reviews are performed on an ongoing schedule across all GBD causes.

Bias in the dataset was considered to be negligible. Diagnostic criteria for this condition are very clear (zero remaining teeth). Additionally, all included studies are considered representative of the study population. Thus, covariates to account for excess variability were not deemed necessary. Few data points were marked as outliers during the modelling process. The table below illustrates data availability.

	Incidence	RR	Prevalence
Site-years (total)	11	9	602
Number of countries with data	4	5	88
Number of GBD regions with data (out of 21 regions)	4	3	21
Number of GBD super-regions with data (out of 7 super-regions)	2	1	7

Table 1: Data availability for edentulism by parameter type

Modelling strategy

First, estimates for the prevalence of edentulism and severe toothlessness were calculated for each location/year/sex/age using DisMod-MR 2.1. Then, estimates of the proportion of the population with access to dentures were generated for each location, and the disability weight for "great difficulty in eating meat, fruits, and vegetables" was applied to the proportion of the population with edentulism and no access to dentures.

DisMod model development

As would be expected for an irreversible condition, remission was fixed at zero for all ages. Mortality and relative risk were both fixed at zero before age 30, as any excess cardiovascular events resulting from severe tooth loss would not be expected at younger ages. We also assigned incidence and prevalence to be zero during childhood. Incidence was allowed to rise beginning at age 15, which was chosen based on the age at which the permanent dentition is expected to have fully formed in all individuals. The random effect limits for all locations were bounded at +/- 1.

As mentioned above, the criteria for diagnosis of edentulism are straightforward, and bias in the dataset was considered negligible. Thus, no study-level covariates were used in modelling the prevalence of edentulism. We included two location-level covariates in the model: 1) Log-transformed lag-distributed income (LDI) with a minimum beta value of 0.02 and 2) Log-transformed age-standardised summary exposure value (SEV) scalar of cardiovascular disease (CVD) in recognition of the common risk factors between CVD and tooth loss.

Models were vetted based on the plausibility of the results, the extent to which estimates fit the data, and the plausibility of the range of estimates across location hierarchies. We have made no substantive changes in the modelling strategy in GBD 2017.

Severity distributions and disability weights

The disability weight used for symptomatic toothlessness leading to "great difficulty in eating meats, fruits, and vegetables" is 0.067 (0.045–0.095) as determined by the GBD disability survey. We considered all those with severe tooth loss and no access to dentures to experience this disability.

However, the proportion of those with edentulism and severe tooth loss who have dentures has not been studied extensively.

In order to estimate the proportion of edentulous individuals with no access to dentures, we completed a supplemental literature review of dentures prevalence for GBD 2010. Only six systematic surveys of dentures prevalence were identified, all in high- and middle-income countries. All were completed since 2000. After extracting the data from the studies, we performed linear regressions of denture presence and denture absence against health system access (HSA), a standardised covariate of treatment availability used in many disease estimation models. From the results of the regression, the prevalence of no dentures was calculated for all super-regions. We then completed a population-weighted average of all countries in the super-region based on 2003 populations, the average year of the dentures studies. Uncertainties for the prevalence of dentures were calculated by finding the standard deviation and standard error of the calculated prevalence values.

The estimated prevalence of dentures in each location was used to calculate the proportion of individuals with asymptomatic edentulism and severe tooth loss (eg, those who have access to dentures) and difficulty eating due to edentulism and severe tooth loss (eg, those without access to dentures). This latter sequela was included as a cause of years lost due to disability (YLDs).

Caries of permanent teeth and caries of deciduous teeth

Flowchart for caries of deciduous teeth

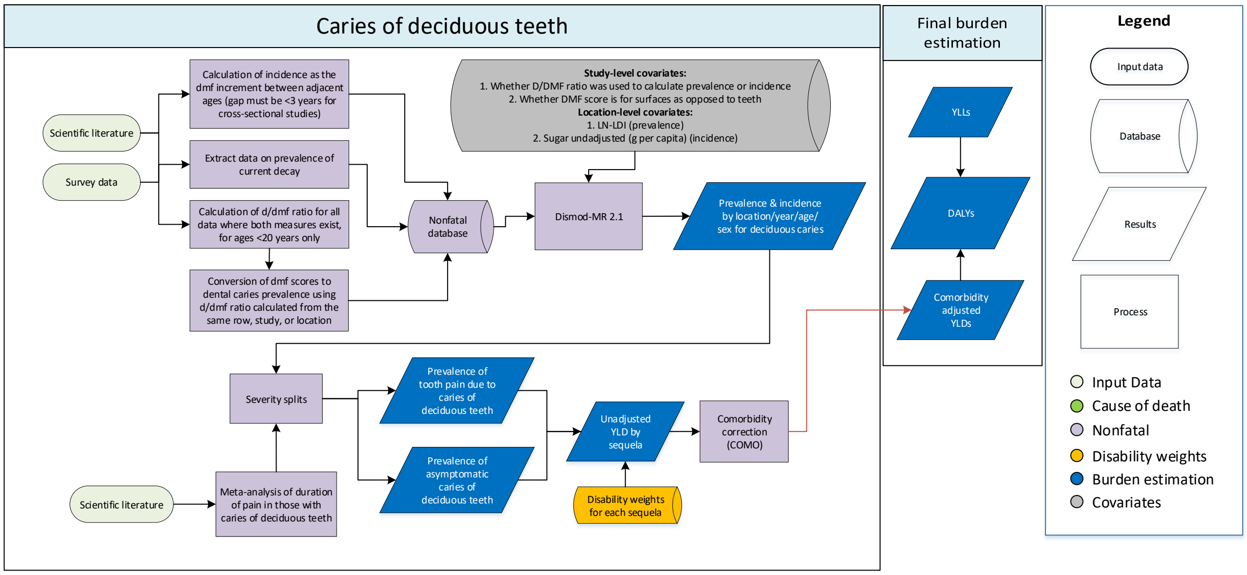

Figure 3: Modelling flowchart for caries of deciduous teeth

Flowchart for caries of permanent teeth

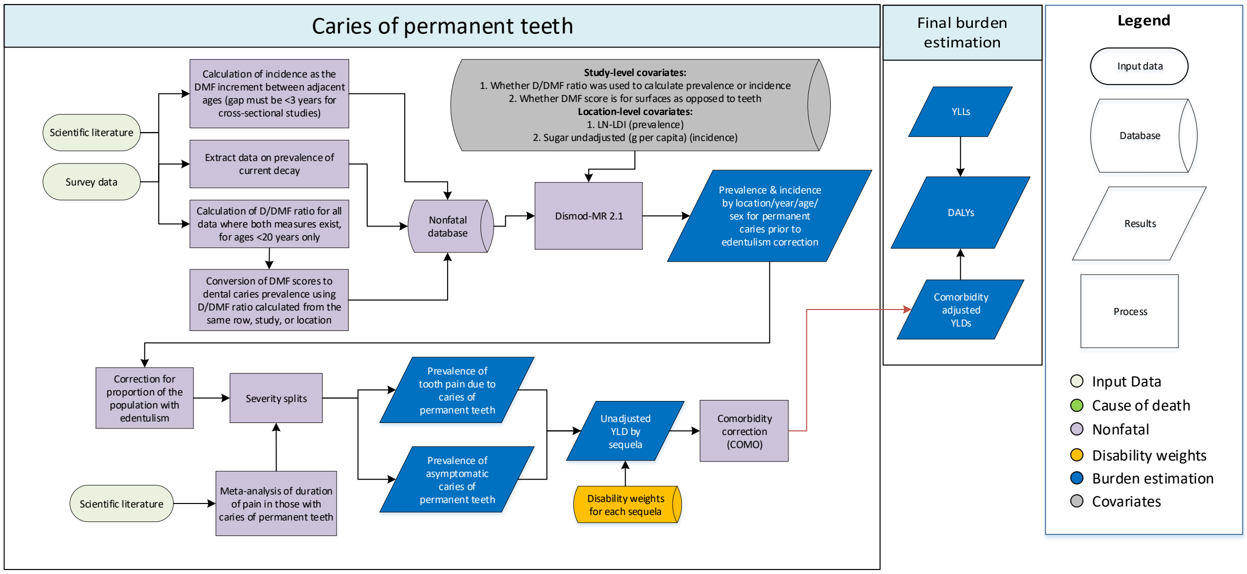

Figure 4: Modelling flowchart for caries of permanent teeth

Case definition

The case definition for dental caries is “teeth with unmistakable coronal cavity at dentin level, root cavity in cementum that feel soft or leathery to probing, temporary or permanent restorations, or missing teeth extracted due to a caries lesion.” This definition corresponds to an ICD-9 code of 521.0 and an ICD-10 code of K02.3 – K02.9. Most caries are subclinical in the sense that they do not cause symptoms a majority of the time. Once a carious lesion develops, it will occasionally recede without intervention. Generally, however, it worsens with time and eventually requires either filling or extraction. The major sequela associated with the condition is symptomatic caries, which is defined as “a toothache, which causes some difficulty eating.”

Public health dentists commonly measure dental caries using the dmft/DMFT index, which is an incremental measure of the proportion of unhealthy teeth and is also a measure of an individual’s lifetime prevalence of caries. Lowercase letters (dmft) are used for deciduous dentition and uppercase letters (DMFT) for permanent dentition. D is for decayed, M for missing, F for filled, and T for teeth. The maximum dmft score is 20 and the maximum DMFT score is 32. Furthermore, some dentists prefer to measure dental caries in terms of tooth surfaces, rather than number of teeth, and report their results using an analogous dmfs/DMFS index. The maximum dmfs score is 88, and the maximum DMFS score is 128 or 148 depending on whether the third molars are counted.

The DMFT index is easy to measure and inter-rater reliability is high. However, the primary shortcoming of the DMFT is that it does not discriminate well between current and past caries. Strategies we employed to maximally utilise dmf/DMF data for estimating the prevalence of burden due to permanent caries are described below.

Input data

A literature review was conducted by the expert group for GBD 2010, and an additional systematic review was performed for GBD 2013 and updated for GBD 2017. The search terms used in the literature

review for permanent caries were (Permanent caries[Title/Abstract]) OR (caries prevalence[Title/Abstract]) OR (dental health[Title/Abstract]) OR (oral health[Title/Abstract]) AND (prevalence[Title/Abstract]) AND (“2010”[Date - Publication] : “2013”[Date - Publication]). The search string for deciduous caries was (Deciduous caries[Title/Abstract]) OR (milk caries[Title/Abstract]) OR (baby caries[Title/Abstract]) OR (caries[Title/Abstract]) OR (dental health[Title/Abstract]) OR (oral health[Title/Abstract]) AND (prevalence[Title/Abstract]) AND (“2010”[Date - Publication] : “2017”[Date - Publication]).

The reference definition for this model was presence of one or more teeth with current decay (for prevalence) whereas each additional carious tooth was counted as a separate incident event. Many of the studies presented dmft or DMFT scores, which represent lifetime prevalence and were often described as “caries experience.” For the purposes of measuring the burden of disability from dental caries, we converted lifetime prevalence data to current prevalence for individuals aged 20 years and less. We did this by multiplying the observed lifetime prevalence by the ratio of d/D to dmf/DMF. When d/dmf or D/DMF information was available from the same study, this ratio was applied. When not available from the same study, the pooled ratio from the closest matching GBD geography was used for the multiplication (country, region, super-region, global). If dmf/DMF data were from surfaces rather than teeth, these data were used but were converted during the modelling process to be equivalent to if a per-tooth measure had been used by applying a study-level covariate and crosswalk in DisMod-MR 2.1.

Calculation of incidence from dmft/DMFT increment

Whereas in the deciduous dentition, a vast majority of the dmf index is accounted for by caries, tooth loss is a major contributor to the DMF index for the permanent dentition. Caries of permanent teeth may not necessarily be the primary driver of this tooth loss, as other factors such as periodontal disease and trauma may contribute significantly. Thus, we performed the conversions of incremental dmf/DMF scores to incidence values for permanent caries only in individuals ages 20 years or less and for all ages in the case of deciduous caries. For longitudinal studies, the difference between the dmf/DMF score in the initial versus subsequent examination was taken to be equivalent to the number of incident caries over that time period. This assumes a negligible proportion of dmf/DMF increment is due to trauma in children. For cross-sectional studies examining children of different ages, we only calculated incidence when the gap in age was three years or fewer given the propensity for strong cohort effects in caries epidemiology. The final dataset contents are shown in the tables below.

	Incidence	Prevalence
Site-years (total)	220	857
Number of countries with data	31	79
Number of GBD regions with data (out of 21 regions)	16	20
Number of GBD super-regions with data (out of 7 super-regions)	7	7

Table 3: Data availability for caries of deciduous teeth by parameter type

	Incidence	Prevalence
Site-years (total)	65	441
Number of countries with data	30	81
Number of GBD regions with data (out of 21 regions)	14	20
Number of GBD super-regions with data (out of 7 super-regions)	7	7

Table 4: Data availability for caries of permanent teeth by parameter type

Modelling strategy

Separate estimates of caries of deciduous teeth and caries of permanent teeth

The natural histories of deciduous and permanent caries share many similarities, but they also share some important differences. Age patterns of decay in permanent and deciduous dentition are distinct, and duration of a carious lesion in deciduous teeth also tends to be shorter than an untreated episode of permanent caries. Sugar consumption and feeding with formula are both associated with development of deciduous caries, but not with permanent caries. Finally, it is unclear whether the gender patterns and regional differences are the same for both deciduous and permanent caries. For all of these reasons, we elected to model deciduous caries and permanent caries as separate entities and then add the estimates together for an overall estimation of the global burden of dental caries. This is the modelling approach which has been taken in each iteration since GBD 2010.

DisMod model development

Serious health consequences of caries were also assumed to be uncommon and death very rare. We therefore assigned excess mortality to be zero from age 0 to 100. For both types of caries, most of the model settings were similar. Several study-level covariates were used to identify data points that were systematically different than the reference of current decay. These include if d/dmf ratio was used to convert lifetime prevalence, if dmf increment was used to calculate incidence data, and if the dmf score was based on number of affected surfaces rather than number of affected teeth. Each study covariate acts as a conversion factor where the original value is divided by the exponentiated beta value of the covariate. Location-level covariates were assigned separately on prevalence and incidence. Sugar availability in food from the GBD diet analysis was used as a covariate on incidence with a positive beta, while prevalence was assigned log-transformed LDI with a negative beta to reflect the association with access to dental care.

The primary difference between the two models was in value priors. We assumed zero incident caries in infants under 1 year old and similarly zero incident deciduous caries from age 11 onward. For permanent caries, we assumed zero incident cases in children under 5 years old.

Although studies were screened carefully during data extraction to ensure that they specified whether they were measuring permanent or deciduous caries, some data points were marked as outliers during modelling due to their high prevalence values in young ages, as it was deemed likely that some of these studies were reporting deciduous in addition to permanent caries.

As with deciduous caries, models for permanent caries were vetted based on the plausibility of the results, the extent to which estimates fit the data, and the plausibility of the range of estimates across location hierarchies.

Correction for edentulism

One systematic source of bias in the literature was the exclusion of edentate individuals from the study populations, which leads to systematic overestimation of caries prevalence when modelled over the entire population. To account for this bias, we used our GBD estimates of edentulism prevalence to adjust YLD estimates for caries of permanent teeth. Final DisMod-MR 2.1 estimates of edentulism prevalence were paired with the corresponding results for caries of permanent teeth by age group, sex,

location, and year to adjust for the proportion of the population that was excluded from the denominator of permanent caries models. No adjustment was made to the estimates of caries of deciduous teeth.

Severity distributions and disability weights

As described above, the GBD definition of disability associated with symptomatic dental caries is "this person has a toothache, which causes some difficulty eating." The disability weight associated with this condition is 0.01 (0.005–0.019), as derived from the GBD disability weights study.

Not all those with dental caries experience this disability all the time. We considered only those with active dentinal decay to experience symptomatic tooth pain. Those with deciduous caries who had undergone exfoliation or had their cavities filled were considered to have no disability. Likewise, those with permanent caries who had received fillings, had their cavities extracted, or lost a carious tooth altogether were considered to have no disability. Thus, two additional pieces of information are required to complete the calculation of years of life lived with disability (YLDs): proportion with symptoms and duration of disability.

To determine which segment of the population has ongoing tooth pain and the proportion of time spent with tooth pain, we considered several different options. First, we examined the data on dental caries symptoms and disability from the Medical Expenditure Panel Survey (MEPS) conducted by the USA Department of Health and Human Services in 2000–2009. MEPS data were widely used in GBD 2010 analyses. Respondents to the survey are asked about all medical conditions. Conditions for which provider care was sought are reported by the respondents at every round, and respondents also report problems for which they did not see a provider if the symptoms were "bothering" them. Conditions can be added to the condition roster if 1) they are reported as a reason for a medical event, 2) the condition was reported as the reason for one or more disability days, or 3) the condition was "bothering" the person during the reference period. Conditions are then recorded as verbatim text and coded to ICD-9CM 3rd digit codes by professional medical coders. These ICD9 codes were mapped to GBD causes, including dental caries. From the MEPS, symptomatic caries in the previous year were reported by 48.4% (95% CI 44.3–52.9) of the respondents. This number is in agreement with our DisMod 3 estimates of 1-2 years duration in North America, high-income for permanent caries if we consider people to only have symptoms at the end of a course of caries. The two primary shortcomings of using this approach are 1) it does not provide enough detail to differentiate between the experiences of those with deciduous versus permanent caries, and 2) it indicates the proportion of those with caries who were symptomatic during the previous year, but it does not provide information on the amount of time during that year spent with symptoms (ie, one day versus 12 months). The approach described below addresses both issues.

To determine duration, we adapted the method employed by the Australian Burden of Disease (AusBoD) Study in 1996. For total duration, we used the posterior estimates of duration from final DisMod models. For those with symptoms, we split this total duration into two distinct phases of caries disability. The "initial" phase is characterised by *periodic* pain that we assigned to occur an average of one hour per day. The "terminal" phase is a period of *constant* symptoms at the end of an episode. The length of the terminal phase was determined by literature review as described by the AusBoD group. For deciduous caries we used a study by Mason and colleagues of children in the UK presenting to a casualty ward with tooth pain [1]. The length of time each child had been experiencing tooth pain was recorded. Based on the distribution of time courses, a log-normal distribution was plotted that approximated the

average duration of *constant* symptoms at 27.6 days leading up to seeking care. For permanent caries, a similar study of the tooth pain experience of adults in New Zealand who presented to hospital dental departments and an emergency clinic [2] resulted in an estimated 55.2 days spent in the terminal phase of caries. For those with severe disease, the length of time spent in the terminal phase was subtracted from the total duration to determine the amount of time spent in the initial phase. For those with mild disease, we considered the entire duration to be spent in the initial phase. These calculations were last completed as part of the GBD 2013 analysis.

To determine proportion with symptoms, we completed a supplemental literature review of tooth pain and caries. We identified a total of 21 studies with data about the prevalence of pain. The studies were grouped according to the type of dentition studied (deciduous or permanent) and the location of the study group (high-income or low- and middle-income countries). We extracted data on the proportion in each group that described symptoms of pain related to their caries as well as a subset who described their symptoms as being severe. The proportions in each group were weighted according to sample size to give estimates of the relative sizes of three groups: asymptomatic, mild, and severe. The results of this meta-analysis are illustrated in the table below.

We considered asymptomatic individuals to experience no disability. Those with mild disease spent the entire duration in the initial phase of disease (one hour of pain per day). Those with severe disease spent a majority of the duration in the initial phase followed by a period of time in the terminal phase (constant pain). YLDs were calculated by multiplying the prevalence, duration, proportion, and disability weight for each age, country, sex, and year.

	# of studies	% symptomatic of total	% severe among symptomatic	% mild of total	% severe of total	% asymptomatic of total
			**Deciduous caries**			
**Data-rich**	5	0.35	0.257	0.26	0.09	0.65
**All others**	4	0.555	0.438	0.312	0.243	0.445
			**Permanent caries**			
**Data-rich**	6	0.602	0.315	0.412	0.189	0.398
**All others**	6	0.954	0.548	0.432	0.521	0.046
			**Duration of phases**			
			**Initial phase**			1 hour per day
			**Terminal phase (deciduous caries)**			27.6 days
			**Terminal phase (permanent caries)**			55.2 days

Table 6: Duration and distribution of severity for tooth pain due to caries of deciduous and permanent teeth

References

1. C Mason, SR Porter, G Madland, J Parry. Early management of dental pain in children and adolescents. *J Dent.* Jan 1997; 25(1): 31-4.
2. RA Whyman, ET Treasure, KM Ayers. Dental disease levels and reasons for emergency clinic attendance in patients seeking relief of pain in Auckland. *NZ Dent J.* Dec 1996; 92(410): 114-7

Chronic Periodontal Disease

Flowchart for chronic periodontal disease

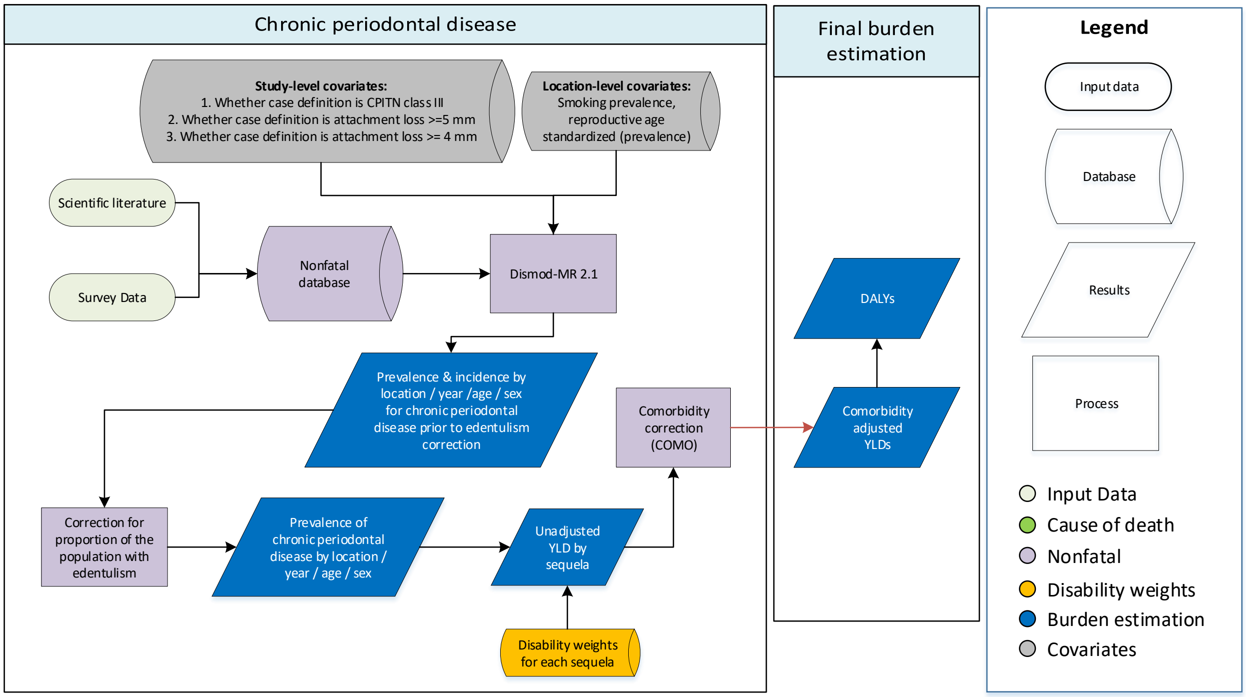

Figure 7: Modelling flowchart for chronic periodontal disease

Case definition

Chronic periodontal disease is caused by chronic bacterial infection around the teeth. Symptoms of gingivitis, the mildest form of the disease, include swelling, redness, and propensity of the gums to bleed when perturbed. If the infection is not treated appropriately, it will eventually spread below the gum line, leading to a chronic inflammatory state of the periodontal tissues. Over time, there will be loss of gingival tissue and alveolar bone destruction. Teeth will become loose and may need to be extracted.

The GBD definition of disability associated with symptomatic severe periodontal disease is "bad breath, a bad taste in the mouth, and gums that bleed a little from time to time, but which does not interfere with daily activities." The ICD-10 codes for periodontal disease are K05.0 – K05.6, and the ICD-9 codes are 523.0 – 523.9.

Defining periodontal disease in a meaningful, reproducible manner has been an ongoing challenge for public health dentists. Attachment loss (AL) and pocket depth (PD) have emerged as the most common metrics of periodontal health measurement. Attachment loss (AL) is measured as the difference between the distance from the gingival margin to the bottom of the pocket and the distance from the cemento-enamel junction to the bottom of the pocket.

The Community Periodontal Index of Treatment Needs (CPITN) is a classification system that was developed by WHO as a standardised method of periodontal health measurement. CPITN classification is based on quantifying the probing depth between teeth and gums. The mouth is divided into six sections, called sextants. Sextants with fewer than two teeth are excluded. Multiple teeth in each sextant are examined. A standard-sized probe is used, with depth markings from 3.5 to 5.5 mm. The probe is inserted into the sulcus between a tooth and the gingiva until it meets resistance. The

surrounding area is then explored with the probe to determine the maximum depth of the pocket. Multiple areas around each tooth are probed. Scores range from 0 to 4 in order of increasing severity. When the CPITN method was employed, we considered those with Class 4 only. We excluded studies in which the study population was reported as the number of sextants rather than the number of individuals surveyed.

In 2007, a new CDC proposal for gold standard diagnosis of severe, chronic periodontitis was published [1]. This standard specified that a stricter definition of the condition should be implemented. This more exclusive definition of chronic periodontal disease includes $\geq$ 2 interproximal sites with AL $\geq$ 6 mm ***AND*** $\geq$ 1 interproximal site with PD $\geq$ 5 mm.

A small body of evidence has begun to emerge that implicates chronic periodontal disease as predisposing individuals to increased risk for ischaemic cardiovascular events including myocardial infarction and stroke. These data are sparse but have been included in models estimating the excess mortality of those with chronic periodontal disease. Given that the association is believed to be ecological rather than causal, however, periodontal disease has not been estimated as an underlying cause of death and it is not included in the risk factor analysis for cardiovascular diseases.

Input data

For GBD 2010, a review of the literature on periodontal disease prevalence was conducted by the Expert Group and it was updated for GBD 2013 and GBD 2017. The literature review used the following search terms: (Periodontal disease[Title/Abstract]) OR (periodontitis[Title/Abstract]) OR (periodontal[Title/Abstract]) AND (prevalence[Title/Abstract]) AND (“2010”[Date - Publication] : “2017”[Date - Publication]). Additional data identified by collaborators from scientific studies or from health surveys has been extracted and included in models on an ongoing basis.

We implemented a hierarchical preference for case definitions. We included the following definitions of severe periodontal disease commonly found in the literature:

1. Community Periodontal Index of Treatment Needs (CPITN) – Class 4 only
2. Clinical Attachment Loss (AL) > 6mm
3. Clinical Attachment Loss (AL) > 5mm
4. Clinical Attachment Loss (AL) > 4mm
5. Gingival Pocket Depth (PD) > 5mm

If more than one type of data was included in a study, our first preference was for CPITN = 4, followed by AL >6 mm, and PD >5. All were considered equivalently as reference definitions with no additional crosswalking performed. All definitions were extracted for each datum as available this time, and a series of study covariates were used to crosswalk non-standard definitions to the reference standard of CPITN stage 4. The table below illustrates data availability for chronic periodontal disease models.

	Incidence	Prevalence
Site-years (total)	65	441
Number of countries with data	30	81
Number of GBD regions with data (out of 21 regions)	14	20
Number of GBD super-regions with data (out of 7 super-regions)	7	7

Table 7: Data availability for chronic periodontal disease by parameter type

Modelling strategy

First, estimates for the prevalence of chronic periodontal disease were generated for each location/year/sex/age using DisMod-MR 2.1. Then, these prevalence estimates were adjusted for the prevalence of edentulism given that edentate individuals are systematically excluded from surveys of periodontal disease. Final estimates were multiplied by the disability weight for "bad breath, a bad taste in the mouth, and gums that bleed a little from time to time, but which does not interfere with daily activities" to calculate years of life lived with disability (YLDs).

DisMod model development

Mortality was fixed to zero, and relative risk was fixed to 1.0 before age 30, as any excess cardiovascular events that occur in those with severe tooth loss would not be expected at young ages. Incidence and prevalence were assigned to be zero until age 8, as periodontal disease is largely considered to be a disease of adulthood. Incidence was allowed to rise beginning at age 9, based on the youngest age at which there was a non-zero point estimate for prevalence in the dataset. Additional bounds were assigned for incidence, remission, and excess mortality to improve plausibility in the DisMod estimates. Remission was bounded 0 to 0.05, excess mortality rate from 0 to 0.0001, and incidence from 0 to 0.05. We considered these bounds to reasonably reflect the natural history of the disease.

Study-level covariates were used to identify data that were not from the reference category. Non-standard definition data in the model included 1) CPITN class III and 2) AL >5mm. Reproductive age-standardised smoking prevalence and the log-transformed age-standardised SEV scalar for CVD were both used as location-level covariates.

Models were vetted based on the plausibility of the results, the extent to which estimates fit the data, and the plausibility of the range of estimates across location hierarchies.

Correction for edentulism

One systematic source of bias in the literature was the exclusion of edentate individuals from the study populations, which leads to systematic overestimation of periodontal disease prevalence when modelled over the entire population. To account for this bias, we used our GBD estimates of edentulism prevalence to adjust YLD estimates for chronic periodontal disease. Final DisMod-MR 2.1 estimates of edentulism prevalence were paired with the corresponding results for caries of permanent teeth by age group, sex, location, and year to adjust for the proportion of the population that was excluded from the denominator of permanent caries models. No adjustment was made to the estimates of caries of deciduous teeth.

Severity distributions and disability weights

We considered all estimated prevalent cases of chronic periodontal disease to experience the disability described by "bad breath, a bad taste in the mouth, and gums that bleed a little from time to time, but this does not interfere with daily activities." The GBD disability survey differentiated between those who experience pain and those who do not, but the calculated disability weight was the same for both forms of the condition, 0.007 (0.003–0.014).

References

1. RC Page and PI Eke. Case Definitions for Use in Population-Based Surveillance of Periodontitis. *J Periodont*. Jul 2007. 78(7 Suppl): 1387-99

Other Oral Disorders

Other oral disorders encompass a wide variety of dental, tongue, and jaw disorders and malformations, including all oral disorders that are not included in the case definitions of permanent or deciduous dental caries, periodontal disease, or edentulism and severe tooth loss. All data on the prevalence of other oral disorders were obtained from the United States Medical Expenditure Panel Surveys (MEPS), a nationally representative survey conducted yearly from 1996 to 2011 by the US Agency for Healthcare Research and Quality. These data were modelled in DisMod-MR 2.1 using a prevalence-only model. Disability weights and severity distribution for these causes were also derived from MEPS.

Injuries

Flowchart

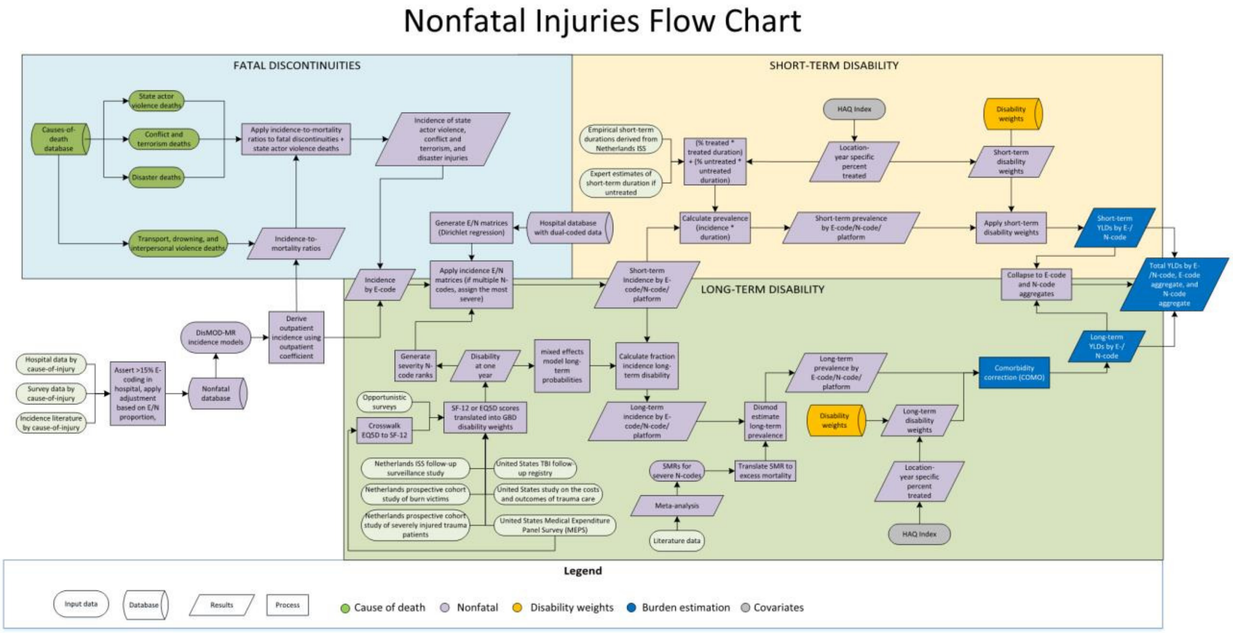

Case definition

For GBD 2017, the Injuries estimation process for non-fatal health outcomes encompasses a range of 30 causes, including transport injuries, falls, drowning, self-harm, interpersonal violence, and animal contact. Injury incidence is defined using ICD-9 codes E000-E999 and ICD-10 chapters V to Y. For non-fatal estimation, Chapters S and T in ICD-10 and codes 800-999 in ICD9 are used to estimate morbidity. Each of these 30 causes can result in a variety of physical injury sequelae (eg, traumatic brain injury), which we call the "nature of injury." Though the initial DisMod models are at the "cause of injury" level (eg, drowning), each cause of injury is broken out into cause-nature pairs. We report incidence, prevalence, and YLDs due to injuries at the cause-nature pair level.

We make additional distinctions between inpatient and outpatient injuries and between short-term and long-term injuries. Inpatient injuries are defined as injuries that led to hospitalisation, whereas outpatient injuries are ones that were treated in outpatient settings or emergency care. We define short-term injuries as injuries lasting less than one year and long-term injuries as those lasting longer than one year, at which point we assume lifelong disability.

Input data

Model inputs

To estimate morbidity from injuries, we used data from hospital records, emergency department records, insurance claims, and surveys to produce years lost to disability (YLDs) by country, year, sex, age, external cause of injury, and nature-of-injury category.

Many countries report their data using a mix of cause-of-injury and nature-of-injury codes. In order to retain as much of the data as possible, we included all datasets that had at least 15% of cases coded to the cause of injury. In GBD 2015, we chose 45% as the threshold but have since lowered the threshold to 15%. We made this distinction after assessing the proportions of major injury causes (road injury and falls) in each of the data sources. We concluded that there were no obvious differences between country data with 15%–45% coverage of external cause codes and those with more than 45% coverage. Below the 15% threshold, the cause of nature coding became more disproportionate when compared to sources with higher cause of nature coding. We assessed the raw hospital data to make sure that there was no disproportionate coding to certain causes in the 15%–45% cause-of-injury coding range. We increased the cause-specific injury cases from these datasets proportionately to sum to the total number of injury cases.

Conflict, war and executions, and police conflict data were obtained from the Uppsala Conflict Data Program [1], the International Institute for Strategic Studies [2], the Armed Conflict Location and Event Dataset [3], the Social Conflict Analysis Database [4], and vital registration systems. Disaster data were obtained from the International Disaster Database from the Center for Research on the Epidemiology of Disasters [5].

Data searches

For GBD 2017, hospital and emergency department records were supplemented with more recent and available site-years. A hospital utilisation envelope that gave reliable denominators for hospital data allowed for the use of more data sources. We applied correction factors to account for repeat hospital visits within a three-month time window (derived from USA claims data) to the incidence estimates to avoid double-counting multiple health service contacts for the same injury.

Additionally, prior to estimation, we reviewed other types of data that could be incorporated into non-fatal estimates of injuries. We incorporated injury claims data from the Accident Compensation Corporation in New Zealand into the transport, self-harm, and animal contact incidence models. These claims data span ten years (2008–2017) and provide detailed information on age and ethnicity (Maori/non-Maori). National survey data from China, Ghana, India, Mexico, Russia, and South Africa from the World Health Organization's Study on Global AGEing and Adult Health were included in the estimation of injuries due to road accidents and falls.

Infrequently, data points were marked as outliers. Reasons for this were that the data point did not follow the age or time pattern as expected and/or if the incidence rate of people sustaining an injury from a certain cause of injury was not plausible. Table 1 contains information about data coverage for each cause of injury.

Table 1 Incidence data density by cause of injury

Cause of Injury	Site-years (total)	Number of countries with data	Number of GBD regions with data (out of 21 regions)	Number of GBD super-regions with data (out of 7 super-regions)
Road injuries	739	76	19	7
Pedestrian road injuries by road vehicle	345	20	12	6
Cyclist road injuries	344	20	12	6
Motorcyclist road injuries	338	20	12	6

Motor vehicle road injuries	335	21	12	6
Other road injuries	329	18	12	6
Other transport injuries	350	20	12	6
Falls	895	41	18	7
Drowning	557	24	13	7
Fire, heat, and hot substances	587	35	15	7
Poisonings	628	33	13	7
Poisoning by carbon monoxide	308	17	10	5
Poisoning by other means	309	18	11	6
Exposure to mechanical forces	628	30	14	7
Unintentional firearm injuries	575	19	12	6
Other exposure to mechanical forces	617	22	12	6
Adverse effects of medical treatment	1485	45	16	7
Animal contact	657	34	16	7
Venomous animal contact	543	21	12	6
Non-venomous animal contact	612	21	12	6
Pulmonary aspiration and foreign body in airway	513	20	12	6
Foreign body in eyes	548	19	10	4
Foreign body in other body part	636	22	12	6
Environmental heat and cold exposure	343	20	11	6
Other unintentional injuries	411	21	12	6
Self-harm	667	28	14	7
Self-harm by firearm	307	15	9	4
Self-harm by other specified means	327	19	11	6
Interpersonal violence	681	36	16	7
Assault by firearm	474	19	11	5
Assault by sharp object	622	20	12	6
Assault by other means	617	22	12	6

Modelling strategy

As in previous GBD iterations, two categories of injury severity were separately modelled: injuries warranting inpatient care and injuries warranting other health care. Injuries warranting inpatient care refer to injury cases of sufficient severity to require inpatient care, if there are no restrictions in access to health care. Injuries warranting other health care refer to injury cases of sufficient severity to require health care attention but not hospitalisation. This category includes emergency department visits. In order to best measure the burden of injuries, the GBD 2017 estimates excluded trivial injuries by restricting morbidity analysis to cases warranting some form of health care in a system with full access to health care. We intended to include cases with injuries that did not receive care in areas with restricted access to health care, but that would have warranted some type of health care in a system with full access to health care. In some surveys, after asking about recall of injuries in the past month or year, respondents were further probed about whether they sought care and if they did not what the reasons were. This allowed us to include cases who cited financial or geographic barriers as reasons for not seeking care.

Cause-of-injury incidence

The list of unique (ie, not counting aggregate categories like road injuries or interpersonal violence) cause-of-injury categories has changed from 29 to 30 for GBD 2017 following the addition of “poisoning by carbon monoxide” and “poisoning by other means” and the re-specification of “unintentional suffocation,” which now falls under the cause “pulmonary aspiration and foreign body in airways.” The fatal discontinuities cause-of-injury categories include “conflict and terrorism” and “exposure to forces of nature, disaster.” We treat executions and police conflict (“state actor violence”) as a typical cause of

injury rather than as a fatal discontinuity; however, the cause is modelled using the fatal discontinuity estimation strategy using incidence-to-mortality ratios because we do not have incidence data for state actor violence.

The majority of incidence data exist at the external cause-of-injury level. Incidence for cause-of-injury categories was modelled using DisMod-MR 2.1. DisMod-MR 2.1 is a descriptive epidemiological meta-regression tool that produces simultaneous estimates of disease incidence, prevalence, remission, and mortality. Multiple datasets from hospital and emergency/outpatient departments, insurance claims, and surveys were fed into these incidence models. Table 1 contains information about data coverage for each cause of injury. We separately estimated two categories of injury severity: inpatient and outpatient injuries. For GBD 2017, we used two covariates in each DisMod-MR model as a multiplier from inpatient to outpatient incidence, namely covariates "outpatient" and "in- and outpatient."

Due to the sporadic nature of the incidence of injuries and a lack of time trend that results from fatal discontinuities, DisMod-MR 2.1 was not used to model incidence due to fatal discontinuities, including state actor violence, exposure to forces of nature (ie, natural disaster), and conflict and terrorism. Instead, incidence-to-mortality ratios were averaged over super-region, year, and sex to limit the variability in the ratios applied to fatal discontinuities. For disaster incidence, the incidence-to-mortality ratio was calculated as an average of road injuries and drowning if there was a water-related natural disaster in that specific country-year noted in the International Disaster Database [5]. For conflict and terrorism, the incidence-to-mortality ratio was calculated as an average of the road injuries and interpersonal violence causes. We treated executions and police conflict as similar to the fatal discontinuities in that we imputed the incidence using the incidence-to-mortality ratio of interpersonal violence.

Follow-up studies

Similar to GBD 2016, we used follow-up data obtained from a pooled dataset of six follow-up studies from China, the Netherlands, and the USA (see Table 2). These studies followed patients for at least one year after the injury. We also used the Medical Expenditure Panel Survey (MEPS) [6]. MEPS is a large-scale overlapping continuous panel survey of the USA non-institutionalised population that collects information on use and cost of health care and SF-12 responses. SF-12 responses are elicited twice over the two-year period that any individual is part of the study. Thus, MEPS offered the benefit of including health state measures of non-injured and destined to be injured and the benefit of having pre-injury and post-injury SF-12 responses. We pooled all available MEPS data over a 19-year span.

The follow-up studies used different patient-reported outcome measures to assess health status, namely the SF-36, Version 1 SF-12, and the EQ-5D. To enable comparison across the six datasets, it was necessary to analyse the data in a standardised patient-reported outcome measure. First, we mapped all patient-reported outcome measures to Version 2 SF-12 (SF-12v2). Second, we normalised the health status measurements by mapping the SF-12 scores to a corresponding disability weight based on several opportunistic surveys asking respondents to score SF-12 based on the lay descriptions for a selection of 60 GBD health states. We ran a regression of logit-transformed disability weight on nature-of-injury category and age group and never-injured status. The pooled dataset informed both the nature-of-injury category hierarchy and the long-term probability of injuries, discussed below.

Table 2 Details of injury follow-up surveys used in GBD 2017

Dataset	Year	Type of data collected	Type of patients	Setting	Sample size* and response
Guangdong follow-up survey, China[#]	2006–2007	Follow-up survey among sample of ISS patients	Patients (15+ years) who were hospitalised that had been injured by road traffic injury, fall, or blunt or penetrating trauma	Based on three national injury surveillance hospitals in Zhuhai, Guangdong Province in China	998 (response 87%)
LIS follow-up survey, Netherlands[1]	2001–2002	Follow-up survey among stratified sample of ISS patients (oversampling less common, severe injuries)	Patients (15+ years) who visited the emergency department of a hospital and were discharged to the home environment and patients who were admitted to hospital	Based on 17 public hospitals in the Netherlands	8,564 (response 37%)
LIS follow-up survey, Netherlands[2]	2007–2008	Follow-up survey among stratified sample of ISS patients (oversampling less common, severe injuries)	Patients (15+ years) who visited the emergency department of a hospital and were discharged to the home environment and patients who were admitted to hospital	Based on 15 public hospitals in the Netherlands	8,057 (response 36%)
NSCOT – National study on Costs and Outcomes of Trauma, USA[4]	2001–2002	A prospective cohort study was conducted among a sample of adult trauma patients treated at Level I trauma centers and non-trauma center hospitals	Patients treated for a moderate to severe injury (as defined by at least one injury of an Abbreviated Injury Scale (AIS) score of 3 or greater	Based on 69 hospitals in 12 states in the USA	5,191 (response 61%)
SCTBIFR – South Carolina Traumatic Brain injury Follow-up Registry, USA[5]	1999–2002	A prospective cohort study was conducted among injured inpatients with a traumatic brain injury-related injury	Patients (15+ years) who were admitted to hospitals and met the CDC case definition of TBI – trauma to the head associated with altered consciousness, amnesia, neurological abnormalities, skull fracture, intracranial lesion, or death	Discharged from all non-federal in-state acute care hospitals	7,613 (response 28%)

Dataset	Year	Type of data collected	Type of patients	Setting	Sample size* and response
Burns outcome study, Netherlands[6]	2003–2006	A multicenter prospective cohort was conducted among adult (severe) burn patients	Injury patients who sustained severe burns	Three public hospitals with specialised burn units.	311 (response 78%)

*number of patients that met the inclusion criteria; response rate = percentage of patients who responded to the follow-up survey (in case of multiple follow-up times the response rate of the first follow-up moment is reported).

[#] data from CDC China, jointly analysed by study authors from IHME and China CDC

Nature-of-injury category hierarchy

Multiple injuries can occur in one individual. For GBD 2017, a nature-of-injuries severity hierarchy was developed to establish a one-to-one relationship between cause-of-injury and nature-of-injury category. This means that in the case of multiple injuries the nature-of-injury category that was likely to be responsible for the largest burden was selected. To construct the hierarchy, we used data from the pooled dataset of follow-up studies. The output of the regression of logit-transformed disability weight on nature-of-injury category and individual characteristics of the follow-up studies were used to calculate the mean long-term disability attributable to each nature-of-injury category. The ranking of nature-of-injury categories by their long-term disability weights formed the basis of our severity hierarchy. Hierarchies were developed separately for injuries warranting inpatient care and injuries warranting other health care.

Table 3 Nature-of-injury hierarchies: combination of empirical hierarchies estimated from pooled follow-up studies and expert adjustments, for inpatient and outpatient injuries

Rank	Inpatient hierarchy	Outpatient hierarchy
1	Spinal cord lesion below neck level	Fracture of pelvis
2	Amputation of lower limbs, bilateral	Fracture of patella, tibia, or fibula, or ankle
3	Amputation of upper limbs, bilateral	Fracture of hip
4	Spinal cord lesion at neck level	Fracture of skull
5	Fracture of hip	Amputation of thumb
6	Fracture of femur, other than femoral neck	Fracture of vertebral column
7	Amputation of upper limb, unilateral	Multiple fractures, dislocations, crashes, wounds, sprains, and strains
8	Amputation of lower limb, unilateral	Internal haemorrhage in abdomen and pelvis
9	Multiple fractures, dislocations, crashes, wounds, sprains, and strains	Fracture of femur, other than femoral neck
10	Effect of different environmental factors	Dislocation of hip
11	Fracture of patella, tibia, or fibula, or ankle	Amputation of toe/toes
12	Moderate-severe traumatic brain injury	Fracture of hand (wrist and other distal part of hand)
13	Fracture of foot bones except ankle	Amputation of fingers (excluding thumb)
14	Internal haemorrhage in abdomen and pelvis	Burns, <20% of total burned surface area without lower airway burns
15	Crush injury	Dislocation of knee
16	Minor traumatic brain injury	Contusion in any part of the body

17	Fracture of pelvis	Minor traumatic brain injury
18	Nerve injury	Foreign body in respiratory system
19	Severe chest injury	Severe chest injury
20	Dislocation of hip	Drowning and non-fatal submersion
21	Burns, >= 20% total burned surface area or >= 10% burned surface are if head/neck or hands/wrist involved w/o lower airway burns	Asphyxiation
22	Lower airway burns	Poisoning requiring urgent care
23	Fracture of skull	Effect of different environmental factors
24	Amputation of thumb	Foreign body in GI and urogenital system
25	Fracture of hand (wrist and other distal part of hand)	Fracture of sternum and/or fracture of one or more ribs
26	Fracture of vertebral column	Nerve injury
27	Contusion in any part of the body	Fracture of face bones
28	Open wound(s)	Dislocation of shoulder
29	Amputation of toe/toes	Injury to eyes
30	Dislocation of knee	Fracture of clavicle, scapula, or humerus
31	Amputation of fingers (excluding thumb)	Fracture of radius and/or ulna
32	Drowning and non-fatal submersion	Fracture of foot bones except ankle
33	Asphyxiation	Foreign body in ear
34	Burns, <20% total burned surface area without lower airway burns	Muscle and tendon injuries, including sprains and strains lesser dislocations
35	Muscle and tendon injuries, including sprains and strains lesser dislocations	Superficial injury of any part of the body
36	Fracture of face bones	Open wound(s)
37	Foreign body in respiratory system	Complications following therapeutic procedures
38	Poisoning requiring urgent care	
39	Foreign body in GI and urogenital system	
40	Fracture of sternum and/or fracture of one or more ribs	
41	Dislocation of shoulder	
42	Injury to eyes	
43	Fracture of clavicle, scapula, or humerus	
44	Fracture of radius and/or ulna	
45	Foreign body in ear	
46	Superficial injury of any part of the body	
47	Complications following therapeutic procedures	

Cause-nature matrices

Because injury disability is linked more to the nature of injury than to the cause of injury, matrices were generated to map the proportion of each cause-of-injury category that results in a particular nature-of-injury category. These matrices are based on a collection of dual-coded (ie, both cause-of-injury and nature-of-injury coded) hospital and emergency department datasets. The data for this step came from inpatient, outpatient, and emergency room discharge data from Argentina, Bulgaria, China, Colombia, Cyprus, Czech Republic, Denmark, Egypt, Estonia, Hungary, Iceland, Iran, Italy, Latvia, Malta, Mauritius, Mexico, Mozambique, Netherlands, Norway, Portugal, Slovenia, Spain, Sweden, Macedonia, Uganda, United States, and Zambia. We applied our nature-of-injury severity hierarchy above to assert that every observation had one cause of injury and one nature of injury.

Dirichlet models were used to estimate all of the nature-of-injury category proportions for one cause of injury simultaneously. These models allow for consistent borrowing of information across age, sex, inpatient/outpatient, and high/low-income countries and assert that the nature-of-injury proportions within a cause-of-injury category must add up to 1. One cause-nature matrix was created for each combination of injury warranting hospital admission versus injury warranting other health care, high/low-income countries, male/female, and age category. Applying these matrices to our cause-of-injury incidence from DisMod-MR, we produced cases of injury warranting hospital admission and incidence of injury warranting other health care by cause and nature of injury.

Probability of permanent health loss

Disability due to injury was assumed to affect all cases in the short term with a proportion having long-term (permanent) outcomes. The probability of long-term outcomes was needed to estimate the incidence and subsequently the prevalence of cases with permanent health loss. In our conceptual model, individuals who suffer a non-fatal injury will, in the long term, return to either full or partial health. If one-year post-injury patients return to a health status with more disability than their pre-injury health status, injury patients are assumed to have permanent disability from their injury. The difference between the pre-injury health states and health status one year after injury is assumed to be their permanent level of injury-related disability. We assessed the probability of developing permanent health loss using the pooled dataset of follow-up studies and the MEPS that were also used to generate the nature-of-injury hierarchy. To assess the probability of permanent health loss, we estimated the effects using a logit-linear mixed effects regression:

$$\begin{aligned} Logit(DW)_{im} = {} & \alpha + \beta(injuries_{im}) + \beta(never\ injured_i) + \beta(never\ injured_i * age_i) \\ & + \beta(fracture\ of\ pelvis_i) + \beta(fracture\ of\ pelvis_i * age_i) + \beta(poisoning_i * age_i) \\ & + \beta(moderate\ to\ severe\ TBI_i * age_i) + RE_c + RE_i \end{aligned}$$

where we included dummies for all the nature-of-injury categories $(injuries_{im})$, with the reference category being no injury (from MEPS dataset). We also included a dummy for never injured prior to the current injury, age, interactions between age and never-injured status, and interactions with three long-term nature-of-injury categories that were found to significantly vary with age: pelvis fractures, poisonings, and moderate/severe traumatic brain injuries. In notation, subscript m refers to patient-reported outcome measure, i refers to individual, and c refers to country. Random effects (RE) were included to control for variation between countries and individuals.

After predicting overall disability at one-year follow-up, we estimated a counterfactual by setting all observations to "no injury," the reference group for $\beta(injuries_{im})$ in our model. The disability attributable to the nature of injury at one year was assumed to be the difference between our counterfactual of no injury and predicted disability with injury. The probability of treated long-term outcomes was estimated via the ratio of this attributable disability relative to the long-term disability weight for that injury.

$$Probability\ of\ long-term\ disability = \frac{with\ injury\ disability_{im} - counterfactual\ disability_{im}}{DW_m}$$

We developed estimates of the probability of permanent health loss by nature-of-injury category, injury severity level (injuries warranting inpatient admission and injuries warranting other health care), and age.

These probabilities are shown in Figure 1 for three selected age groups (25-30, 50-55, 75-80) and selected nature-of-injury categories by inpatient and outpatient. Moderate-severe TBI and spinal cord lesions only have inpatient injury long-term probabilities, and nerve injury, open wounds, and severe chest injury have long-term probabilities of zero for outpatient cases.

Figure 1. Long-term probabilities derived from the MEPS data for selected nature of injuries and age groups

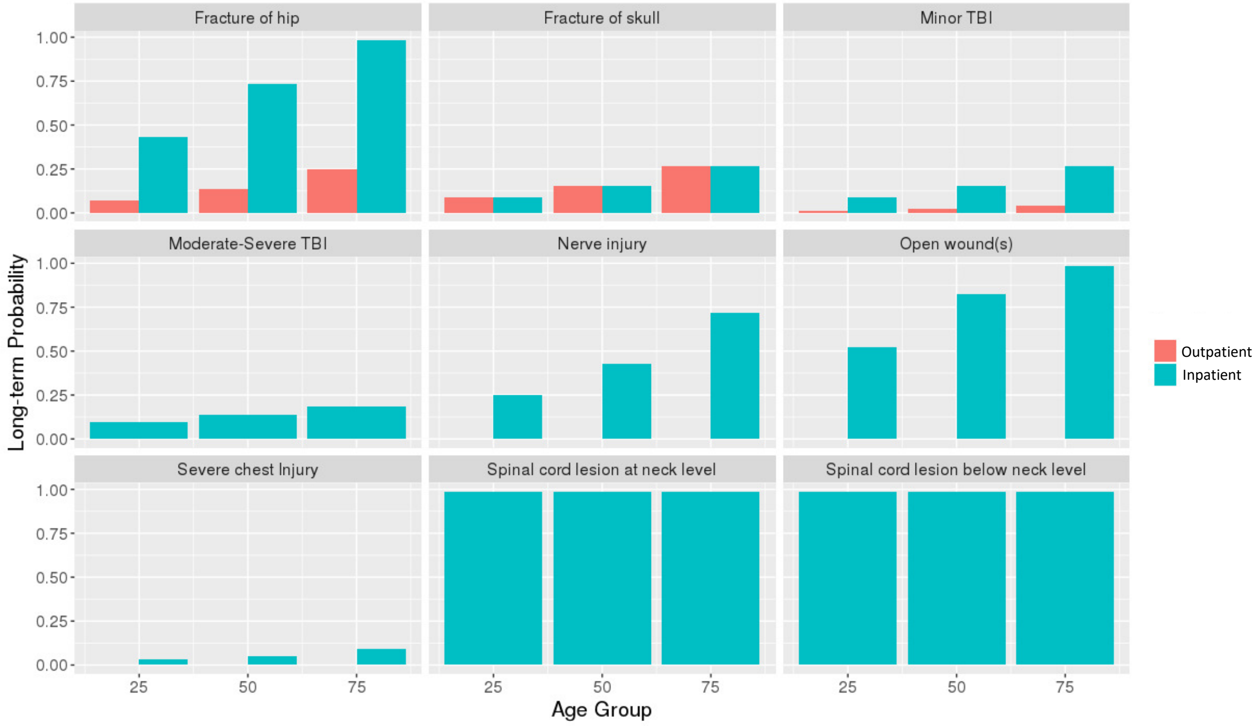

Disability associated with treated and untreated cases

For many nature-of-injury categories, GBD 2017 has a separate disability weight for treated and untreated cases. To estimate the percent treated for injuries in a given location-year, we used the Healthcare Access and Quality (HAQ) Index with the same strategy described for the probability of permanent health loss. We chose a reasonable cutoff for the HAQ Index (75 on a scale of 0–100) as the threshold at and above which 100% of injuries were treated. This value captured most OECD countries for all years back to 1980. We then scaled all remaining location-years between 10% and 100% treated based on their HAQ Index value and used that as the percent treated in a given location-year. This was done at the draw level to propagate uncertainty. We made the decision to ignore any long-term disability from injuries with implausibly high estimates of long-term disability.

Duration of short-term health loss

To determine the duration for treated cases of short-term injury, we analysed patient responses from two Dutch Injury Surveillance System follow-up studies conducted from 2001–2003 and 2007–2009 [7]. These studies collected data at 2.5, 5, 9, and 12 months post-injury to determine whether injury patients were still experiencing problems due to their injury. If not, the patients were asked how many days they had experienced problems. The injury patients that still reported having problems one year after the

injury were assumed to be captured in our analysis of permanent disability. The duration for treated cases of short-term injury was estimated for injuries warranting inpatient admission and injuries warranting other health care separately. The estimates were supplemented by expert-driven estimates of short-term duration for nature-of-injury categories that did not appear in the Dutch dataset and untreated injuries.

Calculation of prevalence from incidence data – short-term injury

For short-term injury outcomes, which were assumed to be less than one year in duration, the prevalence for each cause-of-injury/nature-of-injury/severity-level grouping was approximated by the incidence for that grouping multiplied by the associated nature-of-injury/severity-level-specific duration.

Calculation of prevalence from incidence data – permanent health loss

For permanent health loss, we assumed no remission and thus integrated incidence over time to arrive at prevalence estimates. We used DisMod ODE (ie, the “engine” of DisMod-MR 2.1) to carry out this integration for each combination of cause of injury and nature of injury by country, year, and sex. For this step we used random effects meta-analysis to pool data on standardised mortality ratios derived from literature reviews for spinal cord injury, burns covering more than 20% of the body, moderate to severe traumatic brain injury, hip fracture, and multiple significant injuries. Here we include examples of these meta-analyses: hip fractures and traumatic brain injuries.

Figure 2. Meta-analyses of standardised mortality ratios derived from literature reviews: hip fractures and traumatic brain injury

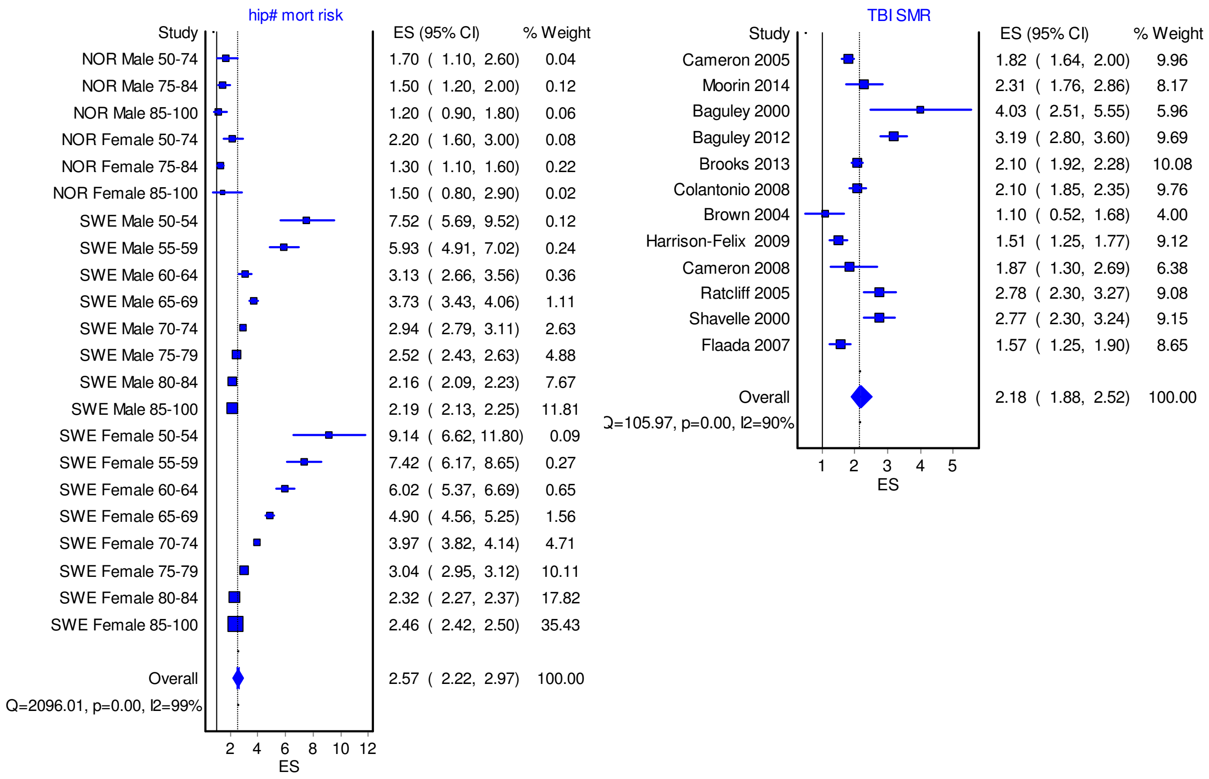

For all other nature-of-injury categories, we assumed no long-term excess mortality. For the incidence estimates derived from fatal discontinuities – “exposure to forces of nature” and “conflict and terrorism” – we did not use DisMod, as discontinuities by definition violate the assumption of a steady state in DisMod to estimate prevalence from incidence. For these two cause-of-injury categories, we coded the differential equations from DisMod ODE that determine the relationship between incidence, remission, mortality risk, and prevalence into Python and streamed out the prevalence from the incidence in the years of war or disaster by integrating over one year at a time.

No significant changes were made to the injuries modelling process for GBD 2017.

References

1. Department of Peace and Conflict Research, Uppsala University. UCDP Nonstate Conflict Dataset. Uppsala, Sweden: Department of Peace and Conflict Research, Uppsala University.

2. International Institute for Strategic Studies. International Institute for Strategic Studies Armed Conflict Database. London, United Kingdom: International Institute for Strategic Studies.

3. Climate Change and African Political Stability Project (CCAPS). Armed Conflict Location and Event Dataset, Realtime - Robert S. Strauss Center as referenced in Raleigh, Clionadh, Andrew Linke, Havard Hegre and Joakim Karlsen. 2010. Introducing ACLED-Armed Conflict Location and Event Data. Journal of Peace Research 47(5), 1-10.

4. Salehyan I, Hendrix CS, Hamner J, Case C, Linebarger C, Stull E, Williams J, Robert S. Strauss Center for International Security and Law. Social Conflict in Africa: A New Database. Int Interact. 2012; 38(4): 503-511.

5. Centre for Research on the Epidemiology of Disasters (CRED). EM-DAT: The OFDA/CRED International Disaster Database. Brussels, Belgium: Catholic University of Leuven.

6. Agency for Healthcare Research and Quality. United States Medical Expenditure Panel Survey. Rockville, United States: Agency for Healthcare Research and Quality.

7. Consumer Safety Institute (Netherlands). Netherlands Injury Surveillance System.

Sexual Violence

Flowchart

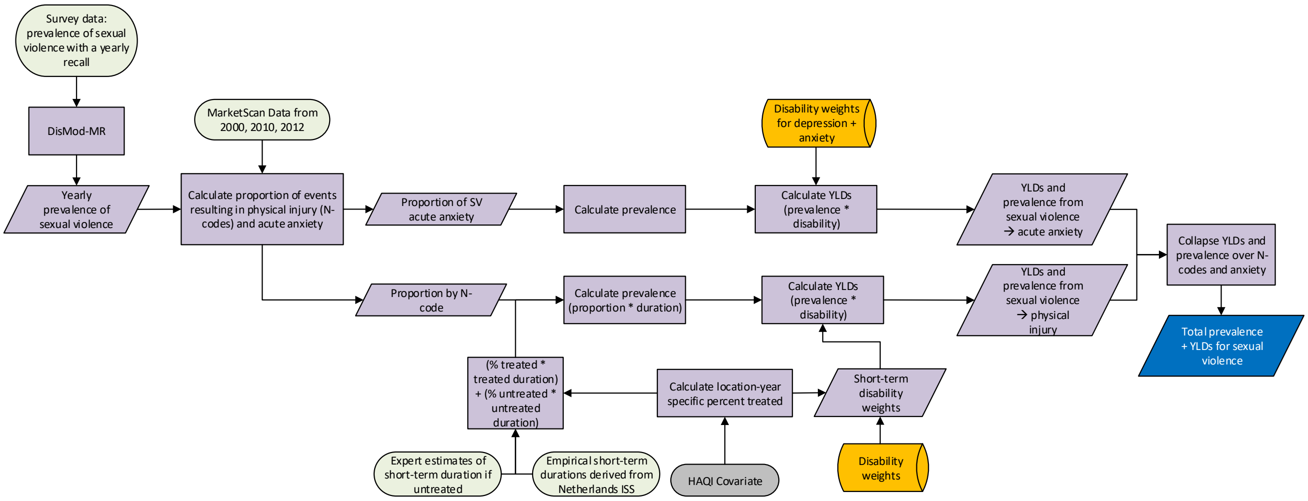

Case definition

For the sexual violence cause, we estimate the yearly prevalence of sexual violence, ie, the proportion of the population that experienced at least one event of sexual violence in the last year. We define sexual violence as any sexual assault, including both penetrative sexual violence (rape) and non-penetrative sexual violence (other forms of unwanted sexual touching).

Input data

Model inputs

The majority of the data for sexual violence comes from various health and demographic surveys. We include many Demographic and Health Surveys (DHS) and Reproductive Health Surveys (RHS). Other survey series include the USA Behavioral Risk Factor Surveillance Survey (BRFSS) and the British Crime Surveys. For GBD 2017, we added survey data from Australia, Botswana, China, England, and South Africa. The China Health and Family Life Survey from 1999–2000 asks about lifetime prevalence of sexual assault; however, we were able to extract yearly prevalence by pairing a respondent's current age with the reported age of when the sexual assault occurred. Table 1 contains information about our input data for the sexual violence modelling process. Table 2 provides more information about data coverage in the seven Global Burden of Disease super-regions.

Table 1. Data inputs for sexual violence modelling

	**Prevalence**
Site-years (total)	263
Number of countries with data	93
Number of GBD regions with data (out of 21 regions)	19
Number of GBD super-regions with data (out of 7 super-regions)	7

Table 2. Unique location-sources for data on sexual violence yearly prevalence used in the DisMod model by Global Burden of Disease super-region

Super-region name	Unique number of sources
Southeast Asia, East Asia, and Oceania	11
Central Europe, Eastern Europe, and Central Asia	23
High-income	57
Latin America and Caribbean	26
North Africa and Middle East	7
South Asia	91
Sub-Saharan Africa	48

Many other non-survey data sources exist for sexual violence. We explored the use of the United Nations Office on Drugs and Crime (UNODC) Statistics [1] that covers a wide range of geographies from 2003 to 2014. However, these estimates are based only on police reports, and their incidence is about 20 times lower than the incidence seen in the same location-years from survey data. Although we could include a covariate in our models to adjust for this underreporting, we deemed the source unusable because of the magnitude of the difference between the police reports and survey data. Survey data typically range between 1% and 10% of individuals experiencing sexual violence in the last year. Figure 1 shows the incidence estimates from the UNODC data, where most of the estimates are below about 0.05%. The geographic pattern is the opposite of what we see in survey data, with higher-income countries having higher estimates in the UNODC data. Additionally, the reports were not age-sex-specific, and the definition for what constitutes sexual violence varies across countries.

Figure 1 United Nations Office of Drugs and Crime Statistics: estimates of sexual violence (incidence per person), color by Global Burden of Disease super-regions

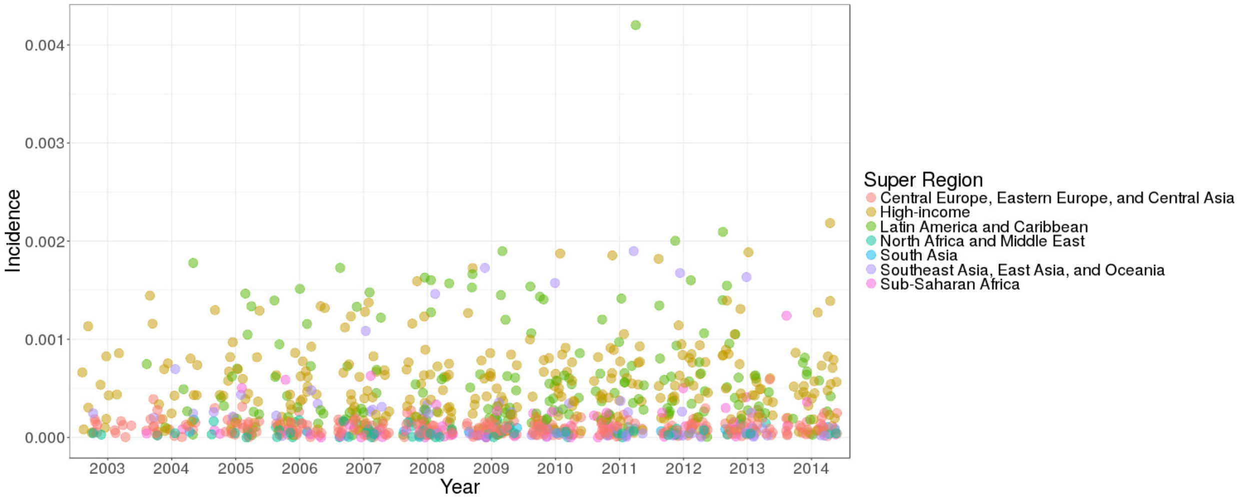

We also chose not to include the Centers for Disease Control non-fatal injury reports of sexual violence. Although this data source includes age- and sex-specific estimates for sexual violence in the United States, only sexual violence cases which resulted in physical injury are reported. These estimates are also systematically lower than the survey data, to the degree that any adjustment with covariates would be unreliable. Lastly, we excluded a source from the United States Federal Bureau of Investigation: The Uniform Crime Reporting (UCR) program. The FBI estimates are produced at the state level for the United States and are meant to be comparable across states. However, police report data for sexual violence are systematically lower, similar in magnitude to the UNODC data, so we chose to exclude them.

Data searches

To find large data sources for sexual violence, we searched through the Global Health Data Exchange (GHDx) to identify survey series with relevant questions and reviewed surveys that were being used for intimate partner violence (IPV) already. We identified 107 sources with relevant data that were being used for IPV and 33 additional surveys with sexual violence questions. We excluded sources that only asked about lifetime prevalence of sexual violence because our case definition is specific to the past year. We extracted data on the perpetrator of sexual violence where possible (partner versus non-partner).

We completed a systematic review in PubMed to identify additional sources. We identified 415 sources and excluded 314 based on title/abstract screening. Of the 111 sources remaining after title/abstract screening, 84 did not have usable data, were of non-representative samples, or referenced data that were already being captured from the GHDx. Samples were non-representative if they were only taken among high-risk populations (war-afflicted, sex workers, intravenous drug users, etc.), sexually abused individuals, or women suffering intimate partner violence. We also excluded studies that only asked about sexual violence in the context of alcohol. After full-text screening, 18 sources remained, only two of which had yearly recall prevalence.

Modelling strategy

Prevalence of sexual violence

To produce estimates of the yearly prevalence of sexual violence, we used DisMod-MR 2.1. DisMod-MR 2.1 is a descriptive epidemiological meta-regression tool that uses the integrative systems modelling approach to produce simultaneous estimates of disease incidence, prevalence, remission, and mortality. To preserve variation between male- and female-specific estimates, we have separate models for men and women.

We make various assumptions within DisMod-MR 2.1, including no excess mortality due to sexual violence and no incidence between 0–2 and 98–100 years of age. Because of the different ways that questions about sexual violence in the last year can be asked, we include multiple study-level covariates (for coefficient estimates, see Table 3). We bounded the covariates at logical values to minimise the effect of collinearity between the covariates, ie, we expect studies that ask about penetrative sexual violence only to have lower estimates of sexual violence overall, so that covariate has an upper bound of 1.

Table 3 Study- and country-level covariates for DisMod-MR 2.1 yearly recall prevalence models for sexual violence

Covariate	Covariate Bounds	Exponentiated Value
Study-level covariates		
Physically forced sexual violence only	Upper: 1	0.94 (0.82–1.00)
Ever-partnered people only	None	1.68 (1.25–2.48)
Ever-married people only	None	2.73 (1.89–4.23)
Specifies specifically degrading or humiliating sex acts	Upper: 1	0.91 (0.70–1.00)
Ever had sex	None	1.40 (1.20–1.63)
Ever married or lived with a partner	None	2.10 (1.63–2.87)
Does not include coerced or feared sex in definition	Upper: 1	0.94 (0.85–1.00)
Penetrative sexual violence only	Upper: 1	0.80 (0.68–0.95)
Does not include non-partner non-penetrative	Upper: 1	0.99 (0.95–1.00)
Only includes partner sexual violence	Upper: 1	0.84 (0.61–0.99)
Includes attempted sexual violence	Lower: 1	1.35 (1.02–1.96)
Country-level covariates		
Alcohol (litres per capita)		1.11 (1.08–1.13)

Years lived with disability (YLDs) due to sexual violence

To calculate the years lived with disability (YLDs) due to having experienced sexual violence in the past year, we utilised claims data from the United States from the years 2000, 2010, and 2012 to assess sexual violence sequelae. We searched through the claims database for the following ICD9 diagnosis codes: 995.53 (child sexual abuse), 995.83 (adult sexual abuse), and E960.1 (rape). We considered sequelae relating to both physical injuries and mental health consequences, in the short-term.

In this process of calculating of years lived with disability due to sexual violence, we currently measure only the short-term physical and psychological effects of sexual violence. In future GBD iterations, we plan to include sexual violence as a risk factor including both sexual violence in the last year and lifetime exposure to sexual violence (independent from, and in interaction with, intimate partner violence) in order to capture the long-term mental health consequences of sexual violence.

Physical injury

For the physical injury sequelae, we looked for any nature-of-injury ICD9 code on the same date of contact with medical service providers for a sexual violence ICD9 code (above) and categorised the nature-of-injury codes as we do for the general injuries non-fatal modelling process (see appendix: nonfatal injuries). We calculate the proportion of individuals with any sexual violence code that result in each of the physical injuries categories. This strategy is similar to the strategy that we use for the cause-nature of injury matrices in the general injuries modelling process, but we have an additional category for no physical injury result as the majority of sexual violence incidents do not result in physical injury in the claims database. Additionally, because we only have one data source, we do not model these proportions with Dirichlet regression like we do for the injuries cause-nature of injury matrices but just compute them

directly from the claims data. To estimate the physical injuries component of YLDs, we multiply the DisMod estimates of yearly prevalence of sexual violence by these proportions and then multiply by each physical injuries' respective short-term duration and disability weight that we use in the general injuries process (see appendix: non-fatal injuries).

Acute anxiety and/or reaction to stress

For the mental and psychological sequelae of sexual violence, we searched an individual being coded to any of the following ICD9 codes at any point *after* a sexual violence incident was noted. The codes are meant to reflect conditions relating to an "acute anxiety and/or reaction to stress" condition following a traumatic incident, displayed in Table 4.

Table 4 ICD9 codes included in the "acute anxiety and/or reaction to stress" condition as a sequela for sexual violence

ICD9 code	Condition description
**308**	Acute reaction to stress
**308**	Predominant disturbance of emotions
**308.1**	Predominant disturbance of consciousness
**308.2**	Predominant psychomotor disturbance
**308.3**	Other acute reactions to stress
**308.4**	Mixed disorders as reaction to stress
**308.9**	Unspecified acute reaction to stress
**309**	Adjustment reaction
**309**	Adjustment disorder with depressed mood
**309.1**	Prolonged depressive reaction
**309.2**	Adjustment reaction with predominant disturbance of other emotions
**309.21**	Separation anxiety disorder
**309.22**	Emancipation disorder of adolescence and early adult life
**309.23**	Specific academic or work inhibition
**309.24**	Adjustment disorder with anxiety
**309.28**	Adjustment disorder with mixed anxiety and depressed mood
**309.29**	Other adjustment reactions with predominant disturbance of other emotions
**309.3**	Adjustment disorder with disturbance of conduct
**309.4**	Adjustment disorder with mixed disturbance of emotions and conduct
**309.8**	Other specified adjustment reactions
**309.81**	Posttraumatic stress disorder
**309.82**	Adjustment reaction with physical symptoms
**309.83**	Adjustment reaction with withdrawal
**309.89**	Other specified adjustment reactions
**309.9**	Unspecified adjustment reaction

It is possible that the appearance of one of these ICD9 codes is entirely unrelated to the sexual violence incident. Additionally, the appearance of one of these codes could be related instead to underlying depression and anxiety. To control for these confounding factors, we also searched for these ICD9 codes among individuals that were not victims of sexual violence in the past year. We used Poisson regression with robust standard errors to model the relative risk of the "acute anxiety and/or reaction to stress" comparing individuals with and without sexual violence within the year, controlling for underlying diagnoses of depression and anxiety:

$$\log(\lambda) = \beta_0 + \beta_1(sexual\ violence) + \beta_2(depression) + \beta_3(anxiety) + \beta_4(female) + \beta_5(age)$$

where λ is the risk of "acute anxiety and/or reaction to stress," and e^{β_1} is the relative risk of "acute anxiety and/or reaction to stress" comparing those experiencing at least one sexual violence incident to those with no sexual violence incidence, holding underlying depression, anxiety, sex, and age constant. We can approximate the risk of "acute anxiety and/or reaction to stress" for each age and sex experiencing sexual violence by:

$$\lambda_{age,sex} = e^{\beta_1} * (e^{\beta_0} * e^{sex*\beta_4+age*\beta_5}) - (e^{\beta_0} * e^{sex*\beta_4+age*\beta_5})$$

The claims data had n = 70,6707,63 observations (n = 8,331 sexual violence cases). Using the equation above, the transformed coefficients and transformed robust standard errors (transformations were performed with the Delta method) are shown in Table 5.

Table 5 Estimates of the risk of "acute anxiety and/or reaction to stress" ($\lambda_{age,sex}$) among people experiencing sexual violence over a year time-period, specific to age and sex

Age	Male		Female	
	Estimate	*Standard error*	*Estimate*	*Standard error*
**0-4**	0.0967	0.0023	0.1205	0.0028
**5-9**	0.0933	0.0021	0.1162	0.0027
**10-14**	0.0899	0.0021	0.1120	0.0026
**15-19**	0.0867	0.0020	0.1080	0.0025
**20-24**	0.0836	0.0020	0.1042	0.0024
**25-29**	0.0806	0.0019	0.1004	0.0024
**30-34**	0.0777	0.0018	0.0968	0.0023
**35-39**	0.0749	0.0018	0.0934	0.0022
**40-44**	0.0722	0.0017	0.0900	0.0021
**45-49**	0.0697	0.0016	0.0868	0.0020
**50-54**	0.0672	0.0016	0.0837	0.0020
**55-59**	0.0648	0.0015	0.0807	0.0019
**60-64**	0.0624	0.0015	0.0778	0.0018
**65-69**	0.0602	0.0014	0.0750	0.0018
**70-74**	0.0581	0.0014	0.0723	0.0017
**75-79**	0.0560	0.0013	0.0697	0.0016
**80-84**	0.0540	0.0013	0.0672	0.0016
**85-89**	0.0520	0.0012	0.0648	0.0015

**90-94**	0.0502	0.0012	0.0625	0.0015
**95-99**	0.0484	0.0011	0.0603	0.0014

We multiplied the prevalence of yearly sexual violence by $\lambda_{age,sex}$ to get the prevalence of "acute anxiety and/or reaction to stress" due exclusively to sexual violence. To estimate YLDs for this sexual violence sequela, we used the average of the disability weights for mild depression and anxiety. For simplicity, we assume a duration of one year; thus, the YLDs for the mental and psychological stress component of sexual violence is the product of the residual probability of "acute anxiety and/or reaction to stress" and the disability weight.

Due to data limitations, we are currently unable to capture the long-term disability from sexual violence. However, in future GBD iterations, we plan to address this issue.

References

1. United Nations Office on Drugs and Crime (UNODC). United Nations Office on Drugs and Crime Global Study on Homicide. Vienna, Austria: United Nations Office on Drugs and Crime (UNODC).

Anaemia Impairment

Flowchart

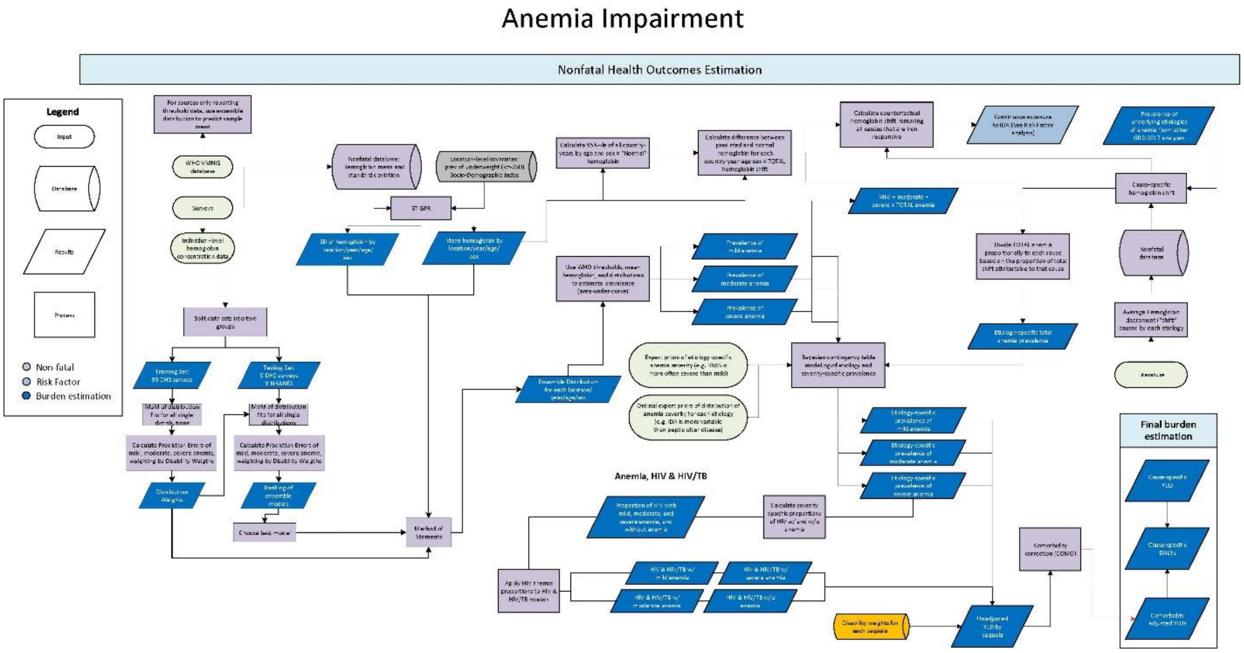

Input data and methodological summary

Case definition

The prevalence of anaemia is defined by the following WHO thresholds for haemoglobin in g/L.

Severity definitions used to calculate GBD 2016 anaemia envelope			
	Severity of anaemia		
	Mild	Moderate	Severe
Age < 1 month			
Males	130 - 149 g/L	90 - 129 g/L	< 90 g/L
Females	130 - 149 g/L	90 - 129 g/L	< 90 g/L
Age 1 month - 4 years			
Males	100 - 109 g/L	70 - 99 g/L	< 70 g/L
Females	100 - 109 g/L	70 - 99 g/L	< 70 g/L
Age 5 – 14 years			
Males	110 - 114 g/L	70 - 99 g/L	< 70 g/L
Females	110 - 114 g/L	70 - 99 g/L	< 70 g/L

Age 15+ years			
Males<br>Females, non-pregnant<br>Females, pregnant	110 – 129 g/L<br>110 - 119 g/L<br>100 – 109 g/L	80 - 109 g/L<br>80 - 109 g/L<br>70 - 99 g/L	< 80 g/L<br>< 80 g/L<br>< 70 g/L

Input data

Model inputs

The envelope approach to the anaemia impairment utilises data from a variety of sources. Population-based surveys of haemoglobin concentration were the primary input to our analytic dataset. Examples include the Demographic and Health Survey (DHS) and Multiple Indicator Cluster Survey (MICS) series, along with other national and subnational surveys that completed haemoglobin testing. We supplemented with pertinent sources downloaded from the WHO Vitamin and Mineral Nutrition Information System (VMNIS) available at http://www.who.int/vmnis/database/anaemia/countries/en/. A full source list is available elsewhere in this appendix. Most used a HemoCue test, adjusted for altitude, and excluded those with terminal or acute medical conditions. Inclusion, exclusion, and diagnostic criteria for other studies were similar and can be found in each study. Any data that were presented as both sexes combined or an age range encompassing more than one GBD age group were split by age and/or sex prior to modelling.

Data availability

	Hemoglobin Mean	Hemoglobin Standard Deviation
**Site-years (total)**	1070	858
**Number of countries with data**	118	99
**Number of GBD regions with data (out of 21 regions)**	21	21
**Number of GBD super-regions with data (out of 7 super-regions)**	7	7

Modelling strategy

Anaemia Impairment Envelope

1) Estimation of population mean and standard deviation of haemoglobin

We ran two ST-GPR models – one for mean haemoglobin and one for standard deviation of haemoglobin. In both models, we included fixed effects on underweight (proportion of children under 5 <2SD weight for age), and Socio-demographic Index (SDI). Mean haemoglobin was used as a fixed effect in the standard deviation model. The models were run with the following parameters:

	Haemoglobin	Haemoglobin SD
**predict_re**	0	0
**custom_hyperparameters**	1	0
**density_cutoffs**	5,10,15	

**st_lambdaa**	2,2,1.5,1	
**st_omega**	2,2,2,2	
**st_zeta**	.7,.8,.9,.99	
**gpr_scale**	15,15,15,10	
**amp_factor**	1	1
**level_4_to_3_agg**	6, 130, 163, 179, 180	6, 130, 163, 179, 180
**level_5_to_4_agg**	6, 163, 180	6, 163, 180
**add_nsv**	1	1

2) Estimation of prevalence of anaemia by severity

We modelled the full distribution of haemoglobin for each population (location/age/year/sex), from which we applied the WHO thresholds to calculate prevalence of each severity of anaemia. In GBD 2015, a Weibull distribution was fit using shape and scale parameters estimated from mean haemoglobin. For GBD 2017, as with GBD 2016, we combine multiple two-parameter distributions to create a more precise and unbiased ensemble distribution.

First, we created a training and testing set of individual-level haemoglobin measurements. The training set consisted of 90 DHS surveys, providing 290 group-specific samples of microdata from children <5, males 15-45, pregnant females 15-45, and non-pregnant females 15-45 (not all groups were sampled in each DHS). A set of two-parameter distributions (gamma, mirror gamma, Weibull, mirror lognormal, and mirror gumbel) were fit to the sample's haemoglobin mean and variance. These distributions were combined using weights optimised by a loss function of severity-specific prediction error weighted by the ratio of the severity's disability weight (DW) to mild anaemia DW. Weights were constrained to be positive and sum to 1, so that the resultant ensemble distribution is a proper probability density function. All permutations of the five distributions were tested (ie, we optimised weights for both a mix of all five distributions as well as a gamma-Weibull two-way combination).

The loss function is

$$\sum_{i=1}^{n_i}\sum_{j=1}^{n_j}\sum_{k=1}^{n_k} r_j|p_{ijk} - \hat{p}_{ijk}|$$

Where

$$\hat{p}_{ijk} = \sum_{z=1}^{n_z} w_z \int_{t1_{jk}}^{t2_{jk}} PDF_{ijz}$$

n_i is a list of surveys (in either the training or testing set)

n_j is the list of groups: children <5, males 15-45, pregnant females 15-45, non-pregnant females 15-45, males >45, and females >45

n_k is the list of severities (mild, moderate, severe)

n_z is the list of distributions (gamma, mirror gamma, Weibull, mirror lognormal, and mirror gumbel)

r is the ratio of the severity j disability weight to that of mild anaemia

r_k = 13 for moderate and r_k = 40 for severe

PDF is a probability density function fit to the sample mean and variance

t1 and t2 are the lower and upper bounds to the WHO anaemia definition for the group

w is the set of distribution weights (each constrained to be positive) such that

$\sum_{z=1}^{n_z} w_z = 1$ and all $w_z > 0$

Therefore $\sum_{z=1}^{n_z} w_z * PDF_z$ describes the ensemble probability density function that can be integrated to calculate prevalence for any severity

The testing set consisted of nine NHANES and nine DHS surveys not included in the training data. Inclusion of NHANES as half the testing set ensured out of sample predictive validity by challenging the global weights, as it provided the ensemble distribution with high-income data (DHS is from LMIC countries) and data from adults >45 (DHS did not take blood tests from the elderly). We selected the combination of distributions (including all individual component distributions) that minimised the loss function.

With a set of component distributions and global weights, we then modelled the distribution of haemoglobin in each location/year/age/sex by fitting each component distribution using modelled mean and standard deviation, then weighting to create the ensemble distribution $\sum_{z=1}^{n_z} w_z * PDF_z$. We integrated area under the curve for each group-specific WHO threshold to calculate prevalence of anaemia by severity.

Because anaemia thresholds depend on pregnancy, we separately modelled the distribution of pregnant and non-pregnant females. The method for fitting the ensemble distribution to pregnant women was identical to that of non-pregnant but used the mean and variance from the two DisMod models adjusted by the estimated beta on the pregnancy status fixed effect. The prevalence of anaemia in pregnant and non-pregnant women were weighted by the pregnancy rate and combined to estimate population prevalence of anaemia. The pregnancy rate for each age is estimated as

$$pregnancy = (ASFR + SB) * 46/52$$

Where

ASFR is the location- and age-specific fertility rate

SB is the location-specific stillbirth rate

Below are some examples of the haemoglobin distributions for various locations and groups, combining the sample distribution (histogram), individual distributions, and ensemble distribution.

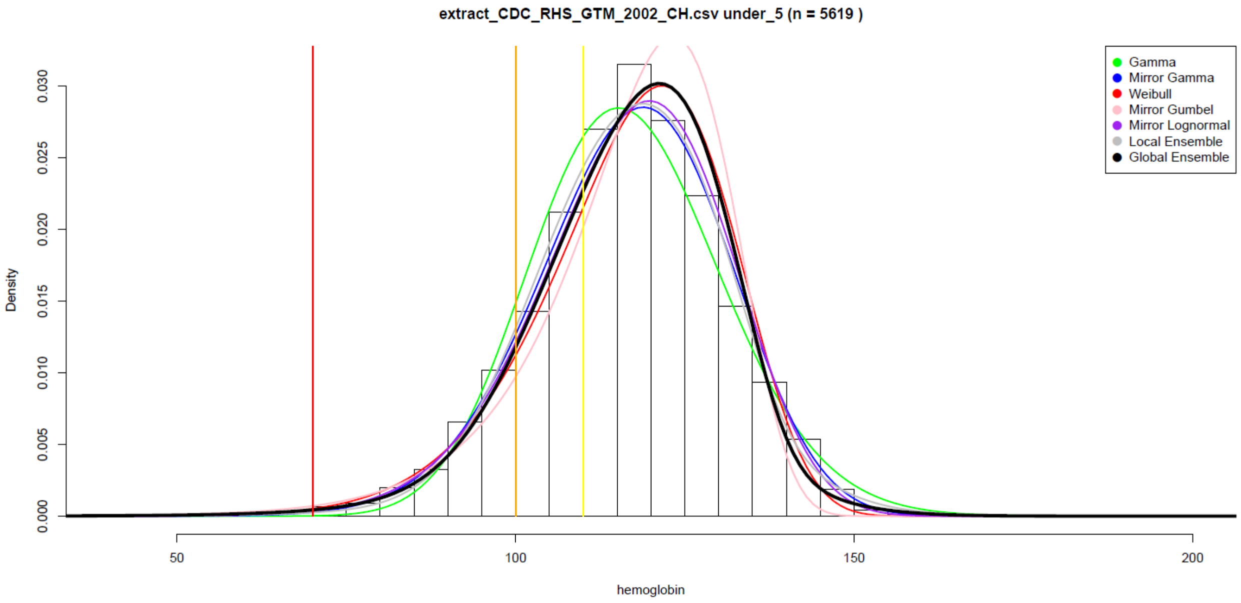
extract_CDC_RHS_GTM_2002_CH.csv under_5 (n = 5619)
Gamma
Mirror Gamma
Weibull
Mirror Gumbel
Mirror Lognormal
Local Ensemble
Global Ensemble
Density
0.000
0.005
0.010
0.015
0.020
0.025
0.030
50
100
150
200
hemoglobin

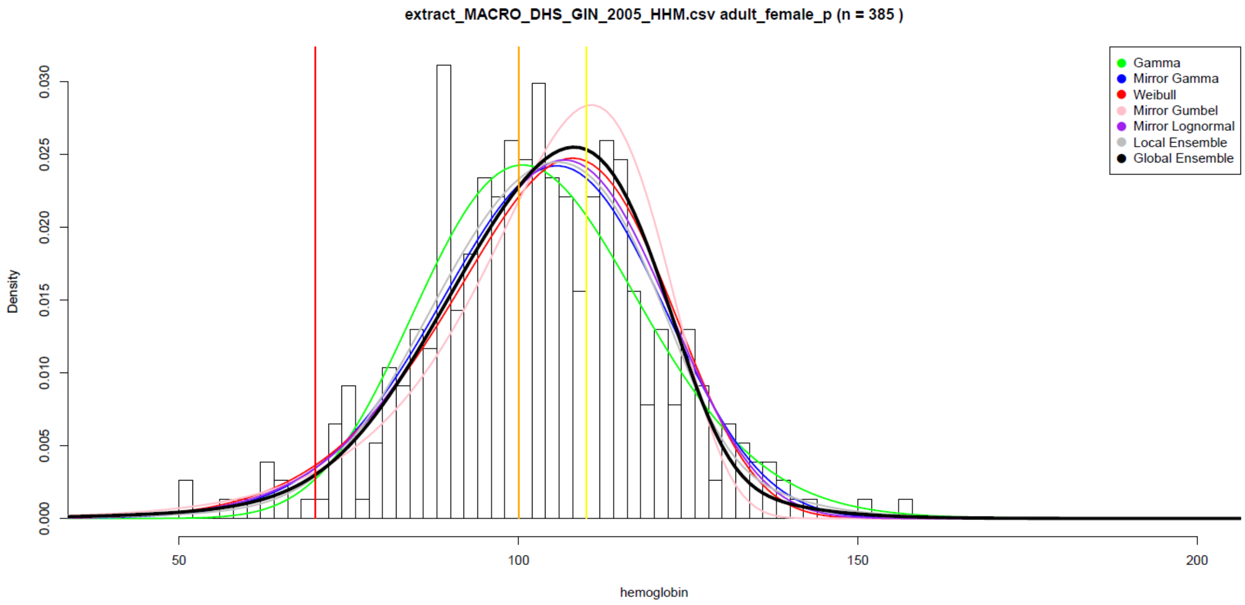
extract_MACRO_DHS_GIN_2005_HHM.csv adult_female_p (n = 385)
Gamma
Mirror Gamma
Weibull
Mirror Gumbel
Mirror Lognormal
Local Ensemble
Global Ensemble
Density
0.000
0.005
0.010
0.015
0.020
0.025
0.030
50
100
150
200
hemoglobin

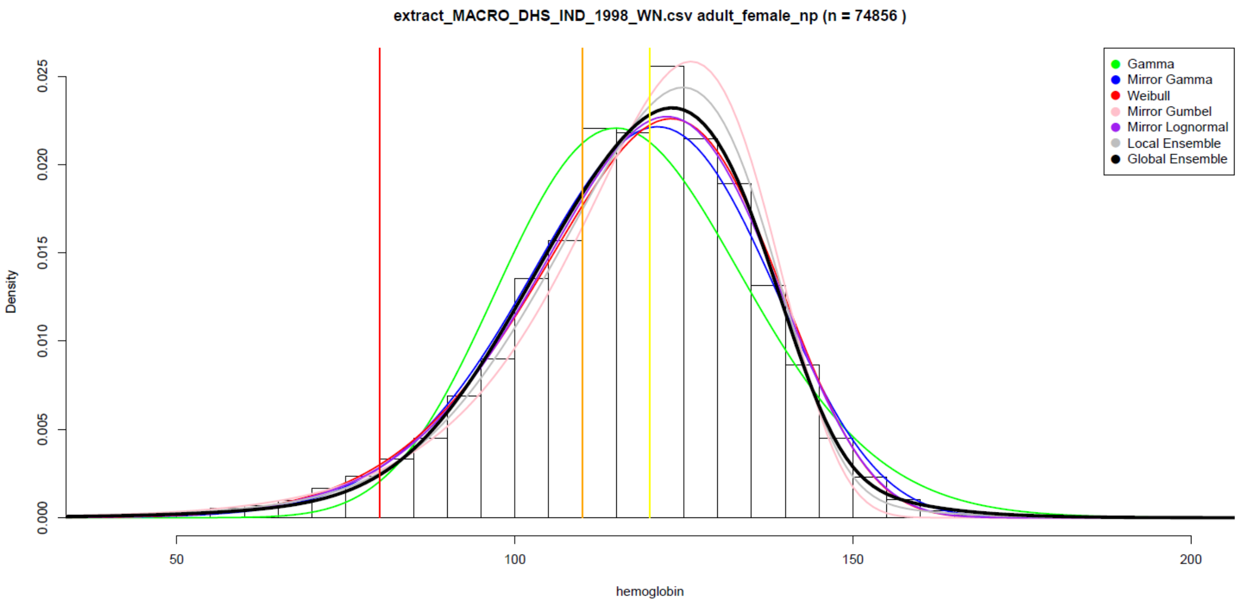
extract_MACRO_DHS_IND_1998_WN.csv adult_female_np (n = 74856)
Gamma
Mirror Gamma
Weibull
Mirror Gumbel
Mirror Lognormal
Local Ensemble
Global Ensemble
Density
0.000
0.005
0.010
0.015
0.020
0.025
50
100
150
200
hemoglobin

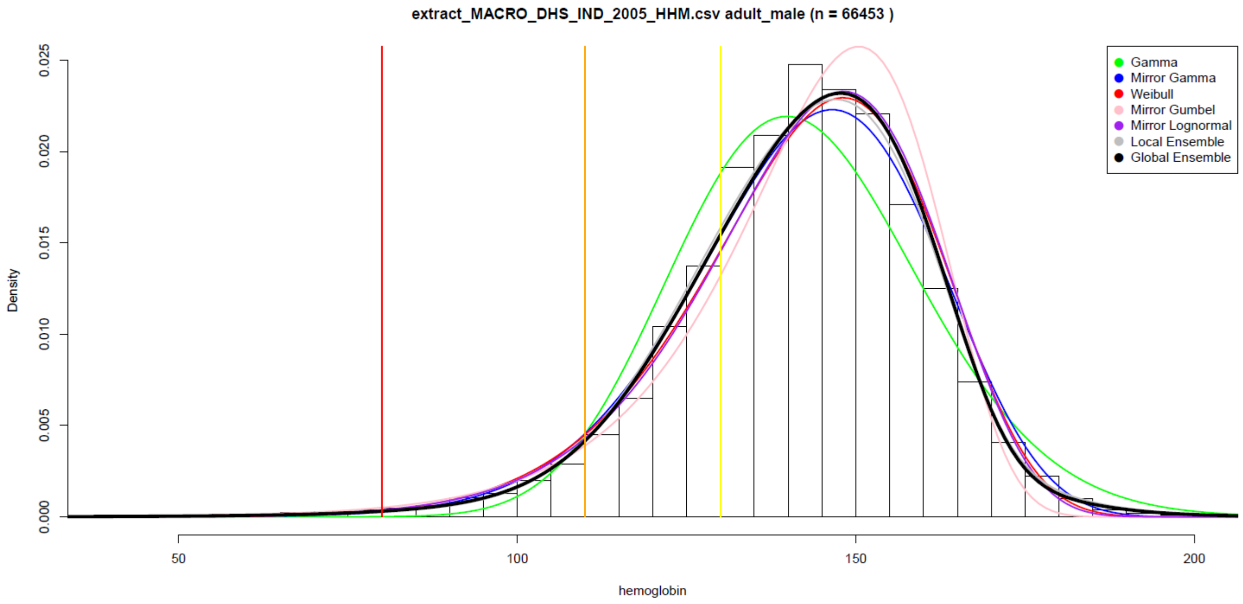
extract_MACRO_DHS_IND_2005_HHM.csv adult_male (n = 66453)
Gamma
Mirror Gamma
Weibull
Mirror Gumbel
Mirror Lognormal
Local Ensemble
Global Ensemble
Density
0.000
0.005
0.010
0.015
0.020
0.025
50
100
150
200
hemoglobin

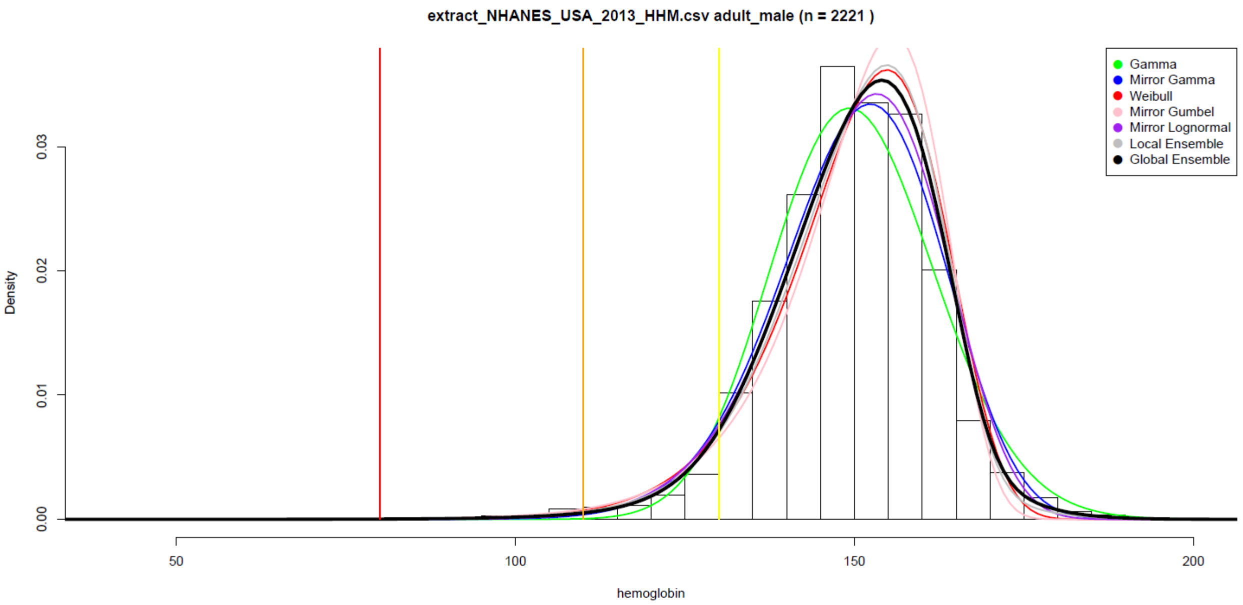
extract_NHANES_USA_2013_HHM.csv adult_male (n = 2221)
Gamma
Mirror Gamma
Weibull
Mirror Gumbel
Mirror Lognormal
Local Ensemble
Global Ensemble
Density
0.00
0.01
0.02
0.03
50
100
150
200
hemoglobin

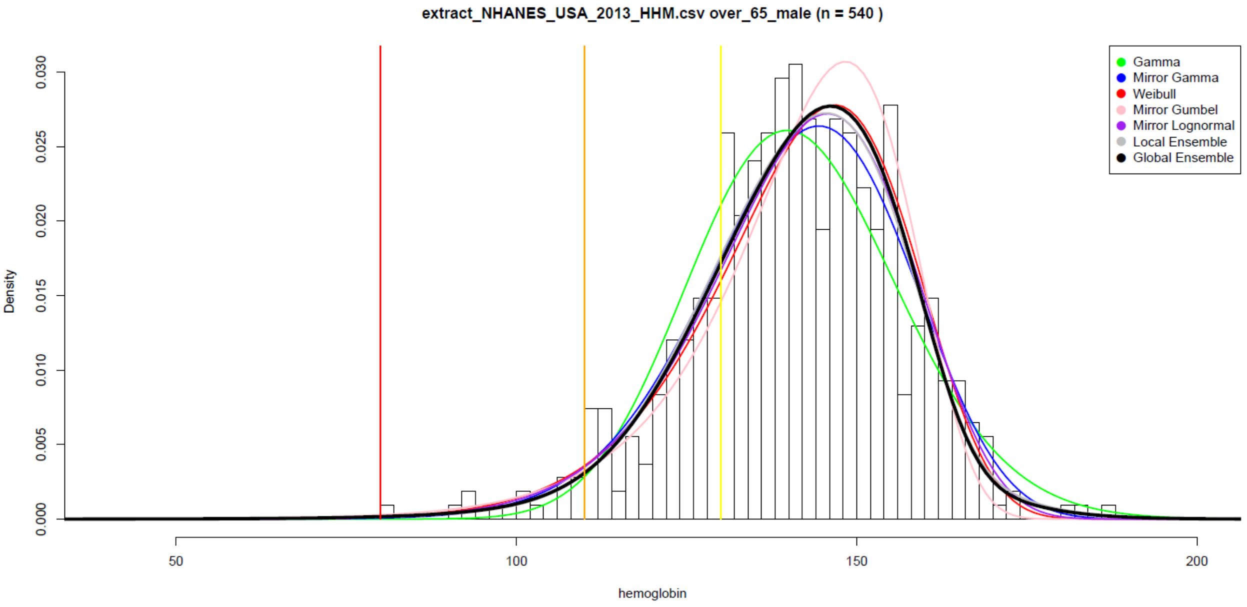
extract_NHANES_USA_2013_HHM.csv over_65_male (n = 540)
Gamma
Mirror Gamma
Weibull
Mirror Gumbel
Mirror Lognormal
Local Ensemble
Global Ensemble
Density
0.000
0.005
0.010
0.015
0.020
0.025
0.030
50
100
150
200
hemoglobin

Due to instability in our prevalence estimates in early life, we copied the prevalence estimates for the post-neonatal age group over the early and late neonatal estimates for each location, year, and sex.

Causal Attribution

We performed cause-specific attribution on the anaemia envelope using information on cause-specific prevalence and haemoglobin shift, which uses the same overall method as in the GBD 2010 with the addition of a number of causes and updates to haemoglobin shifts for inputs to causal attribution.

Total "haemoglobin shift" was determined as the difference between the normal and predicted mean haemoglobin levels for each population group. We denoted the normal haemoglobin level as the global 95th percentile of the distribution of mean haemoglobin within each age group, sex, and year. We then determined a total shift for each country in the corresponding age group, sex, and year by finding the difference between the global "normal" and the country-specific predicted mean haemoglobin. Our model of attribution followed that, because the shift is a disease state experienced by 100% of the population, then the sum of cause-specific haemoglobin shifts times the prevalence of each contributing cause should add up to the total. We summed shift times prevalence estimates from all causes, compared to the total predicted haemoglobin shift, and proportionally assigned. We distributed the residual envelope among five remaining causes.

Of note, our iron-deficiency anaemia (IDA) estimates include acute and chronic haemorrhagic states for which supplementation may be helpful, but poor nutritional intake is not the only underlying problem. A few causes in this category – hookworm, schistosomiasis, upper gastrointestinal bleeding, and gynaecologic diseases – were considered separately from IDA because there were enough data from GBD prevalence estimation processes to do so. Distribution of anaemia burden to IDA only after assignment to "known" causes avoided double counting of these cases. Most other causes of anaemia not specifically considered were included in the "other" categories.

For all causes with specific population-specific prevalence estimates, we enforced a condition where the sum of mild, moderate, and severe anaemia would not exceed the total prevalence within each population. Additionally, because inherent in our method of determining "normal" haemoglobin is the fact that 5% of population groups will have zero, or negative, total shift, we assigned a minimum of 10% of all anaemia to be assigned to residual causes based on review of findings from National Health and Nutrition Examination Survey (NHANES) in the United States[5,6].

We again used Bayesian contingency table modelling methods developed for GBD 2013 to disaggregate marginal estimates of anaemia severity and aetiology into a complete set of prevalence estimates for aetiology/severity pairs. Marginal estimates of column sums (total anaemia prevalence by severity [mild, moderate, severe]) and row sums (total aetiology prevalence for each cause) were paired with priors on the aetiology-specific haemoglobin shifts (the same as were used for overall aetiologic attribution) and rank order of variation of severity (eg, malaria-induced anaemia severity is highly variable, while that due to homozygous sickle cell disease is less so). Nonlinear optimisation methods were then used to populate a complete matrix of aetiology-severity estimates from the marginal estimates and distribution priors. We found the maximum a posteriori (MAP) point estimate for five samples from estimated posterior distributions independently for each population group, then scaled the results to ensure row sums were non-zero and column sums matched the original draws. We then took the mean of the scaled posteriors

for each population group. To estimate uncertainty for each scaled posterior mean, we first calculated the ratio of each draw to the mean of all draws for the anaemia envelope. For non-residual causes, we also calculated the ratio of each draw to the mean of all draws. We then multiplied the scaled posterior mean by these ratios.

The anaemia causal attribution process produces estimates for mild, moderate, and severe anaemia due to HIV. Using these estimates, we calculated proportions of HIV with mild, moderate, severe, and no anaemia for each demographic group. GBD produces estimates for seven HIV sub-causes: early HIV, symptomatic HIV, AIDS with antiretroviral treatment, AIDS without antiretroviral treatment, drug-sensitive HIV/AIDS–tuberculosis, multidrug-resistant HIV/AIDS–tuberculosis, and extensively drug-resistant HIV/AIDS–tuberculosis. We assumed the anaemia severity proportions were equivalent across the seven sub-causes and estimated the anaemia severity levels for each by multiplying the HIV sub-causes by the anaemia proportions.

For GBD 2017, we made several updates to the anaemia cause list. This year, we removed "other gynaecological disorders" from the cause list, and instead added "menstrual disorders," which is a directly modelled, non-residual cause. We also added cirrhosis, Crohn's disease, ulcerative colitis, and Vitamin A deficiency to the list. Because reviewed studies provided insufficient evidence of vitamin A deficiency causing any haemoglobin shift in adults[7], we age-restricted the vitamin A deficiency input model, such that the haemoglobin shift was only applicable to age groups under 15 years. Per updates to the GBD 2017 diabetes modelling strategy, we updated the CKD due to diabetes aetiologies to differentiate between type 1 and type 2 diabetes. We also added the differentiation between clinical malaria, *P. falciparum* parasitemia without clinical malaria, and *P. vivax* parasitemia without clinical malaria.

Priors: Largest*

Anaemia subtype	Expected largest
P. falciparum parasitaemia without clinical malaria	2
P. vivax parasitaemia without clinical malaria	2
Clinical malaria	2
Schistosomiasis	0
Hookworm disease	0
Other neglected tropical diseases	0
Maternal haemorrhage	1
Iron deficiency	0
Vitamin A deficiency	0
Other infectious diseases	0
Peptic ulcer disease	2
Gastritis	2
Stage III chronic kidney disease due to diabetes mellitus type 1	1
Stage IV chronic kidney disease due to diabetes mellitus type 1	1
Stage V chronic kidney disease due to diabetes mellitus type 1	2
Stage III chronic kidney disease due to diabetes mellitus type 2	1
Stage IV chronic kidney disease due to diabetes mellitus type 2	1
Stage V chronic kidney disease due to diabetes mellitus type 2	2

Stage III chronic kidney disease due to hypertension	1
Stage IV chronic kidney disease due to hypertension	1
Stage V chronic kidney disease due to hypertension	2
Stage III chronic kidney disease due to glomerulonephritis	1
Stage IV chronic kidney disease due to glomerulonephritis	1
Stage V chronic kidney disease due to glomerulonephritis	2
Stage III chronic kidney disease due to other and unspecified causes	1
Stage IV chronic kidney disease due to other and unspecified causes	1
Stage V chronic kidney disease due to other and unspecified causes	2
Uterine fibroids	0
Menstrual disorders	0
Other haemoglobinopathies and haemolytic anemias	1
Other endocrine, nutrition, blood, and immune disorders	0
G6PD deficiency	1
Hemizygous G6PD deficiency	1
Beta-thalassaemia major	2
Beta-thalassaemia trait	1
Haemoglobin E trait	0
Haemoglobin E/beta-thalassaemia	2
Haemoglobin H disease	2
Homozygous sickle cell and severe sickle cell/beta-thalassaemia parent	2
Hemoglobin SC disease	2
Mild sickle cell/beta-thalassaemia	2
Sickle cell trait	0
HIV	0
Cirrhosis and other chronic liver diseases, decompensated	1
Ulcerative colitis	0
Crohn's disease	0

*0 = mild anaemia; 1 = moderate anaemia; 2 = severe anaemia

Priors: Severity

Anemia subtype	Expected variation
P. falciparum parasitaemia without clinical malaria	1
P. vivax parasitaemia without clinical malaria	2
Clinical malaria	3
Gastritis	4
Peptic ulcer disease	5
Crohn's disease	6
Ulcerative colitis	7
Maternal haemorrhage	8

Other endocrine, blood, and immune disorders	9
Menstrual disorders	10
Uterine fibroids	11
Iron deficiency	12
Vitamin A deficiency	13
Other infectious diseases	14
Other neglected tropical diseases	15
HIV	16
Stage III chronic kidney disease due to other and unspecified causes	17
Stage III chronic kidney disease due to hypertension	18
Stage III chronic kidney disease due to diabetes mellitus type 1	19
Stage III chronic kidney disease due to diabetes mellitus type 2	20
Stage III chronic kidney disease due to glomerulonephritis	21
Stage IV chronic kidney disease due to other and unspecified causes	22
Stage IV chronic kidney disease due to hypertension	23
Stage IV chronic kidney disease due to diabetes mellitus type 1	24
Stage IV chronic kidney disease due to diabetes mellitus type 2	25
Stage IV chronic kidney disease due to glomerulonephritis	26
Cirrhosis and other chronic liver diseases, decompensated	27
Stage V chronic kidney disease due to other and unspecified causes	28
Stage V chronic kidney disease due to hypertension	29
Stage V chronic kidney disease due to diabetes mellitus type 1	30
Stage V chronic kidney disease due to diabetes mellitus type 2	31
Stage V chronic kidney disease due to glomerulonephritis	32
Haemoglobin E trait	33
Sickle cell trait	34
Beta-thalassaemia trait	35
G6PD deficiency	36
Hemizygous G6PD deficiency	37
Haemoglobin SC disease	38
Schistosomiasis	39
Haemoglobin H disease	40
Other haemoglobinopathies and haemolytic anaemias	41
Mild sickle cell/beta-thalassaemia	42
Hookworm disease	43
Haemoglobin E/beta-thalassaemia	44
Homozygous sickle cell and severe sickle cell/beta-thalassaemia	45
Beta-thalassaemia major	46

Causes for which allocation of residual anaemia envelope
was based on fixed proportion redistribution methods*:

Iron-deficiency anaemia (IDA)
Other infectious diseases
Other neglected tropical diseases
Other endocrine, nutrition, blood, and immune disorders
Other haemoglobinopathies and haemolytic anaemias

* A minimum of 10% of all anaemia was assigned to residual categories based on analysis of NHANES-III data from the United States

References

1. Kassebaum NJ. The Global Burden of Anemia. *Hematology/Oncology Clinics* 2016; **30**: 247–308.
2. Kassebaum NJ, Jasrasaria R, Naghavi M, *et al.* A systematic analysis of global anemia burden from 1990 to 2010. *Blood* 2014; **123**: 615–24.
3. WHO | Haemoglobin concentrations for the diagnosis of anaemia and assessment of severity. Geneva: World Health Organization, 2011 http://www.who.int/vmnis/indicators/haemoglobin.pdf.
4. Kates EH, Kates JS. Anemia and polycythemia in the newborn. *Pediatr Rev* 2007; **28**: 33–4.
5. Centers for Disease Control and Prevention (CDC). Iron deficiency--United States, 1999-2000. *MMWR Morb Mortal Wkly Rep* 2002; **51**: 897–9.
6. Looker AC, Dallman PR, Carroll MD, Gunter EW, Johnson CL. Prevalence of iron deficiency in the united states. *JAMA* 1997; **277**: 973–6.
7. Semba RD, Bloem MW. The anemia of vitamin A deficiency: epidemiology and pathogenesis. *European Journal of Clinical Nutriton* 2002; **56**: 271-281.

Epilepsy impairment

Flowchart

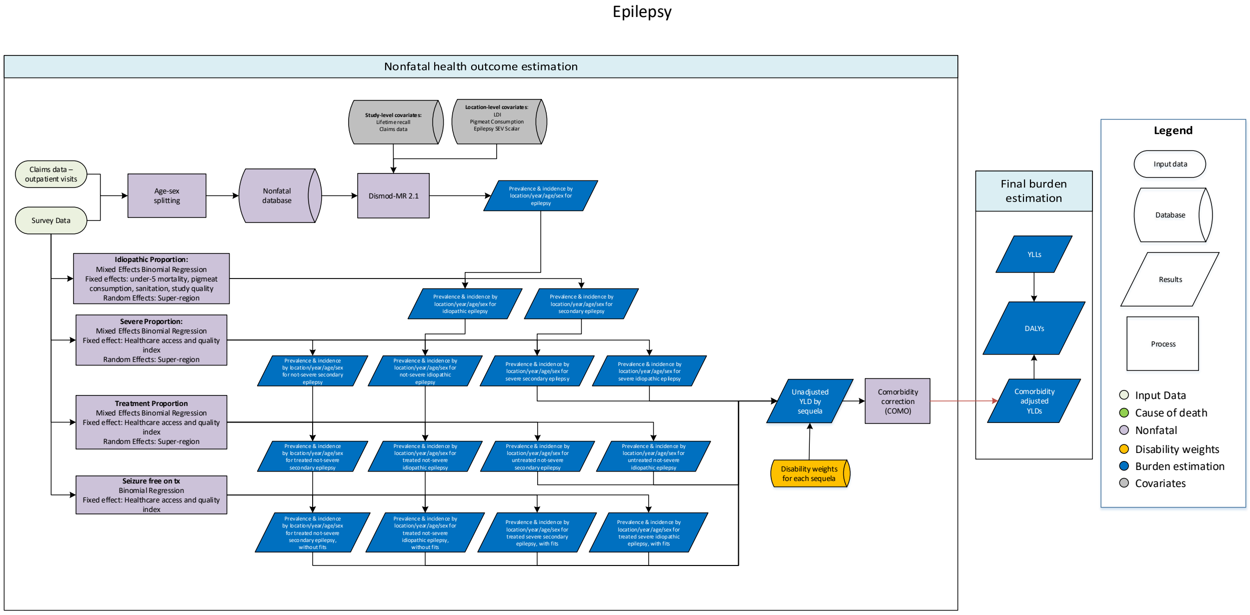

Case definition

Since GBD 2013, we have used the following definitions from the "Guidelines for Epidemiologic Studies on Epilepsy": 1) Epilepsy: a condition characterised by recurrent (two or more) epileptic seizures, unprovoked by any immediate identified cause, and 2) "Active" epilepsy: a prevalent case of active epilepsy is defined as a person with epilepsy who has had at least one epileptic seizure in the previous five years, regardless of antiepileptic drug (AED) treatment. We also use the following ICD-10 codes for epilepsy: G40 (Neuro, epilepsy, total) and G41 (Neuro, epilepsy, status epilepticus). We defined severe epilepsy as having seizures one or more times per month.

Input data

Model inputs

For GBD 2016, we conducted a systematic review covering 10/1/2014 to 10/7/2016 using the following search string:

("2014/10/01"[PDAT] : "2016"[PDAT]) AND ("epilepsy"[MeSH Terms] OR "epilepsy, partial, motor"[MeSH Terms] OR "epilepsy, benign neonatal"[MeSH Terms] OR "epilepsy, reflex"[MeSH Terms] OR "myoclonic epilepsy, juvenile"[MeSH Terms] OR "epilepsy, frontal lobe"[MeSH Terms] OR "epilepsy, complex partial"[MeSH Terms] OR "epilepsy, post-traumatic"[MeSH Terms] OR "epilepsy, temporal lobe"[MeSH Terms] OR "epilepsy, absence"[MeSH Terms] OR "epilepsy, tonic-clonic"[MeSH Terms] OR "epilepsies, myoclonic"[MeSH Terms] OR "epilepsies, partial"[MeSH Terms] OR epilepsy[Title/Abstract]) AND (incidence[Title/Abstract] OR prevalence[Title/Abstract]) NOT(animals[MeSH] NOT humans[MeSH]).

We included representative, population-based surveys that reported on prevalence, incidence, remission rate, excess mortality rate, relative risk of mortality, standardised mortality ratio, or with-condition mortality rate. We excluded studies with no clearly defined sample (eg, among clinic attenders or patient organisation members with non-specific or non-representative catchment area).

This year we also added three additional years of claims data from the USA (2011, 2013, 2014), and one year of claims data from Taiwan (2016). We also changed the algorithm for deriving prevalence from the claims data. Previously, an individual with any inpatient or outpatient visit in a given year would be counted as a previous case. However, this would pick up visits where a doctor may have used a potential or probable diagnosis without confirmation. Therefore, the new algorithm only counts a prevalent case where an individual has one inpatient visit, two outpatient visits, or one outpatient and one inpatient visit. This change to the algorithm resulted in a decrease in prevalence estimates for the United States. The table below details the model inputs used to estimate the epilepsy impairment.

	Prevalence	Incidence	Excess mortality rate	Remission	Other
**Site-years (total)**	638	206	25	5	11
**Number of countries with data**	81	36	14	3	5
**Number of GBD regions with data (out of 21 regions)**	20	15	9	3	4
**Number of GBD super-regions with data (out of 7 super-regions)**	7	7	5	2	4

The inputs for the regressions used to split the epilepsy impairment envelope include data on the proportion of epilepsy that is primary or idiopathic, the proportion of epilepsy that is severe (one or more fits per month), the proportion of epilepsy that is untreated (the treatment gap), and the proportion of treated epilepsy that is treated without fits (no fits reported in the preceding year). For the proportion of epilepsy that is idiopathic, we have 89 unique sources covering 18 of 21 world regions. For the proportion of epilepsy that is severe, we have 29 unique sources covering 12 unique regions. For the proportion of treated epilepsy that has no fits we have ten unique sources covering six regions. Finally, for the proportion of epilepsy that is treated we have 68 unique studies covering 16 unique regions.

Severity splits & disability weights

The table below illustrates the severity levels, descriptions, and disability weights associated with epilepsy. These are calculated using regressions from literature (ie, frequency of seizures).

Severity level	Lay description	Disability weights (95% CI)
severe (seizures >= once per month)	This person has sudden seizures one or more times each month, with violent muscle contractions and stiffness, loss of consciousness, and loss of urine or bowel control. Between seizures the person has memory loss and difficulty concentrating.	0.552 (0.375–0.71)

less severe (seizures < once per month)	This person has sudden seizures two to five times a year, with violent muscle contractions and stiffness, loss of consciousness, and loss of urine or bowel control.	0.263 (0.173–0.367)
Treated without fits	This person has a chronic disease that requires medication every day and causes some worry but minimal interference with daily activities.	0.049 (0.031–0.072)

Modelling strategy

We modelled the prevalence of epilepsy in two steps: first, we created an epilepsy impairment envelope. Second, we split the envelope into primary (or idiopathic) and secondary epilepsies. Each of these were subdivided into "severe" (on average one or more fits per month) and "non-severe." Non-severe cases were subdivided into "treated" and "un-treated." Finally, "treated" cases were divided into "treated cases with fits" (between one and 11 fits on average in the preceding year) and "treated cases without fits" (no fits reported in the preceding year).

In the first step, we used DisMod-MR 2.1 for the epilepsy impairment envelope to model a consistent fit between incidence, prevalence, remission, and standardised mortality ratio data while using meta-regression to correct data points with non-reference study quality characteristics. We found no systematic bias for the covariate "non-standard case definition" indicating studies that did not define "active epilepsy" and additionally the covariate was not significant as a "z-cov", which acts as a multiplier applied to the standard error and thus results in these data points being given less weight in the analysis than the "reference" data points. Therefore, we excluded this covariate from the model. We also included data on lifetime prevalence and therefore added a covariate on lifetime prevalence data points. We added covariates to adjust the USA claims data as well. Because the data from 2000 were systematically lower than the other years, we added a year-specific covariate for 2000, while all other years were adjusted with one covariate. We did not adjust Taiwan claims data because the data aligned well with available literature data from Taiwan.

We also included country-level covariates on prevalence for the SEV epilepsy scalar, which summarises the epilepsy risk exposure level for each country, and pig meat consumption per capita, which is used as a proxy for the level of neurocysticercosis, a common cause of secondary epilepsy. We included cause-specific mortality rate (CSMR) results from the epilepsy mortality model as input data to the DisMod model. Where age-specific prevalence data were available, we calculated excess mortality rate (EMR) from prevalence and CSMR. We included the log of the lag-distributed income (LDI) as a covariate on EMR to account for lower mortality in developed countries. We included Bayesian priors on remission to account for the scarcity of remission data. We set bounds on remission from 0 to 0.25 from age 0-60 and 0 to 0.05 from age 61-100. The table below indicates the covariates used in the estimation process, as well as parameters, betas, and exponentiated betas.

Measure	Variable Name	Beta	Exponentiated
Prevalence	Recall lifetime	0.19	1.21 (1.18–1.24)

Prevalence	All MarketScan, year 2000	-1.44	0.24 (0.22–0.25)
Prevalence	All MarketScan, other years	-0.78	0.46 (0.44–0.48)
Prevalence	Pig meat consumption (kg per capita)	0.0037	1.00 (1.00–1.01)
Prevalence	Log-transformed age-standardised SEV scalar for epilepsy	0.76	2.14 (2.12–2.21)
Excess mortality rate	LDI (I$ per capita)	-0.55	0.58 (0.37–0.90)

In the second step, we used mixed-effects generalised linear models (binomial family) to predict the proportion of idiopathic epilepsy, the proportion of severe epilepsy, the proportion of treated epilepsy and the proportion of epilepsy that is treated without fits.

Because not all of the data on the proportion of idiopathic epilepsy use optimal case finding methods (using CT scans or MRIs in addition to EEGs in order to diagnose secondary epilepsy), we first run an initial linear regression model with a covariate on study quality. We then use the beta from this model to crosswalk studies with non-optimal case finding methods to those with adequate methods. The adjusted data are then used in the regression for the proportion of epilepsy that is idiopathic, with a fixed effect on SDI as well as a random effect on super-region.

We used similar models to predict the proportion of severe epilepsy and treatment gap based on the reported proportions extracted from the systematic review. To predict the proportion of severe epilepsy and the treatment gap, we used mixed-effects models with a fixed effect on the log of HAQ Index and a random effect on super-region.

For the regression to determine the proportion of treated epilepsy cases that have not had a fit in the last year, there is a much smaller dataset, and therefore we cannot use a random effect in the model. Therefore, we use generalised linear model (binomial family) to generate predictions for the proportion of treated epilepsy that is seizure-free with a fixed effect on the log of HAQ Index.

We tested a fixed effect on epilepsy cause-specific mortality, under-5 mortality rate, sanitation, and pig meat consumption as well as random effects on region and country in different models, but they did not improve the models. We generated 1,000 draws of country-specific estimates for each year between 1980 and 2017 for each of the models. The table below shows the betas from these regressions.

Regression	covariate	beta	SE
Idiopathic	Study quality	0.75	0.59
Idiopathic	SDI	1.39	1.12
Severe	HAQ Index	-1.23	1.05
Treatment gap	HAQ Index	-3.54	1.37
Treated without fits	HAQ Index	2.49	1.87

Guillain-Barré syndrome (GBS) impairment

Flowchart

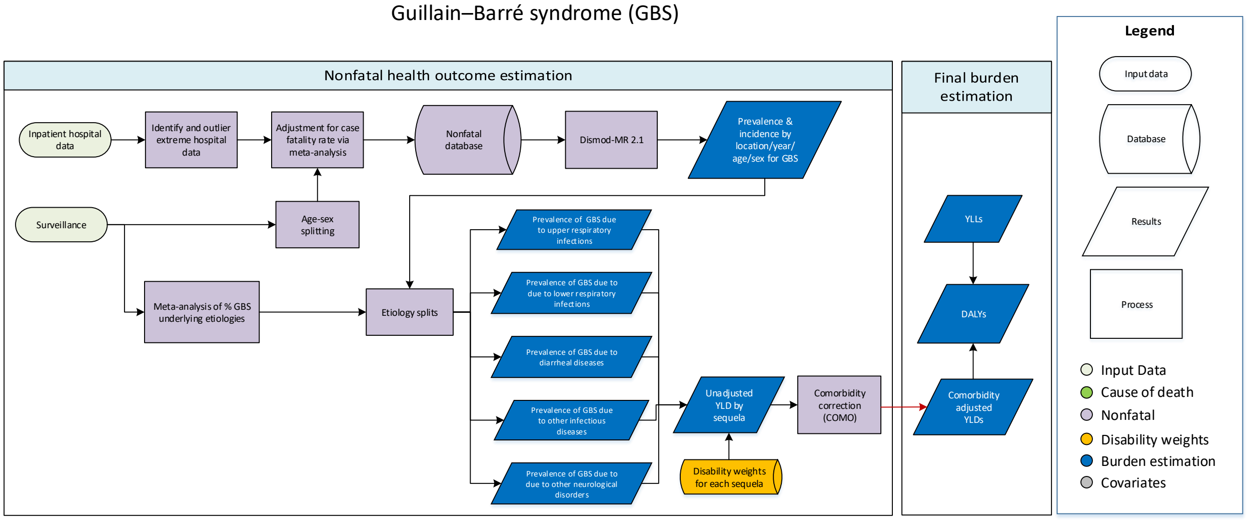

Case definition

Guillain-Barré syndrome is a rare condition that usually occurs as a complication of respiratory or gastrointestinal infection. It is considered an immune-mediated nerve dysfunction with rapid onset of weakness in feet and hands ascending toward the trunk. In the acute phase, about a quarter of cases required mechanical ventilation for survival. The majority of cases fully recover within months to a year. The following ICD codes are used G61.0 (GBS) and 357.0 (Acute infective polyneuritis). These codes are also referenced in Methods Appendix Table 4.

Input data

Model inputs

An updated systematic review was done for GBD 2017 from January 2008 to September 2017 using the search string ((((((("guillain barre syndrome"[MeSH Terms] OR ("guillain"[Title/Abstract] AND ("barre"[Title/Abstract] OR "barre"[Title/Abstract]) AND Title/Abstract[All Fields] AND "syndrome"[Title/Abstract])) OR "guillain-barre syndrome"[Title/Abstract]) OR "guillain-barre syndrome"[Title/Abstract]) OR "polyradiculoneuropathy"[Title/Abstract]) OR "Guillain-Barre syndrome"[Title/Abstract]) AND ("prevalence"[Title/Abstract] OR "incidence"[Title/Abstract] OR "epidemiology"[Title/Abstract] OR "remission"[Title/Abstract]))) AND ("2008/01/01"[Date - Publication] : "2017/09/26"[Date - Publication]). This search yielded 436 hits with 25 sources marked for extraction. A flowchart documenting this review is displayed below.

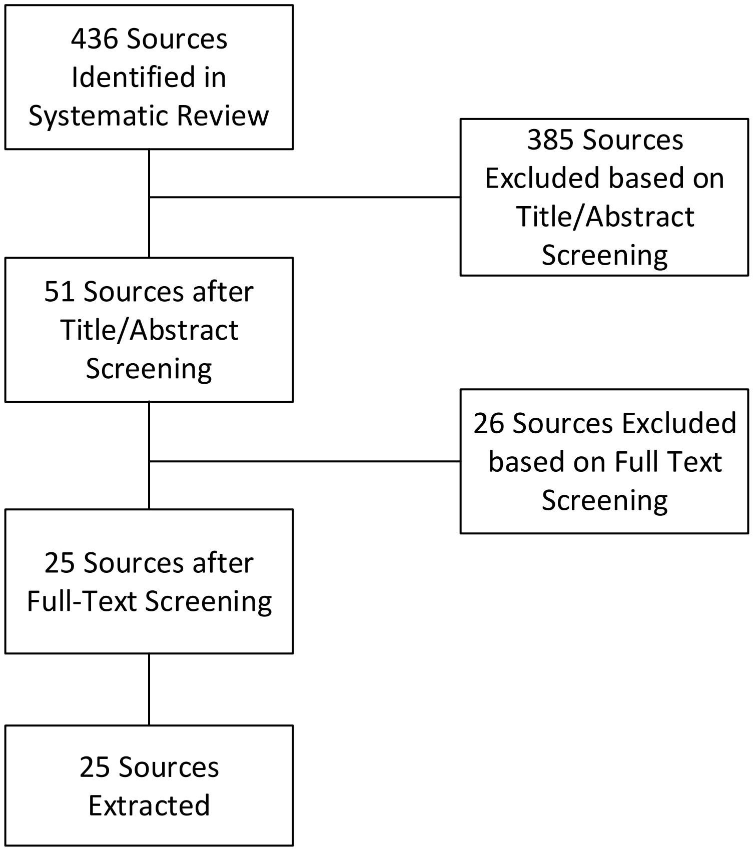

An additional search was undertaken for more information on remission and duration of GBS. As the remission and duration did not vary significantly across the studies and there were time constraints, instead of doing a systematic search for GBS mortality and remission, a general search was carried out. We extracted remission from four studies.

	Incidence	Remission
Site-years (total)	827	4
Number of countries with data	41	4
Number of GBD regions with data (out of 21 regions)	15	3
Number of GBD super-regions with data (out of 7 super-regions)	7	2

Inpatient hospital data were extracted using the ICD codes listed above. Only primary diagnoses were considered, with the reasoning that Guillain-Barré syndrome should appear as a primary diagnosis and we do not wish to include follow-up visits that may be listed as secondary or tertiary codes. This year we also added three additional years of claims data from the USA (2011, 2013, 2014) and one year of claims data from Taiwan (2016).

Aetiology splits

Information on aetiology splits come from a systematic review and meta-analysis of the literature completed for GBD 2010. This review searched for articles providing information on the proportion of Guillain-Barré cases with any described aetiological cause, the proportion of Guillain-Barré cases

attributed to influenza, the proportion of Guillain-Barré cases attributed to upper respiratory infections, the proportion of Guillain-Barré cases attributed to diarrhoeal diseases and the proportion of Guillain-Barré cases attributed to other infections. This review yielded 35 articles; a breakdown of how many articles inform each proportion contributing to the split is provided below:

Split	Number of sources
All specified aetiologies	31
Influenza	3
Upper respiratory infections	26
Diarrhoeal diseases	25
Other infectious diseases	14

First the envelope for Guillain-Barré cases due to all specified aetiologies is established by doing a meta-analysis on the proportions reported in the studies included. Then, the proportions for each of the other splits are squeezed to fit the envelope created in the all specified meta-analysis. Finally, the difference between all specified and 100% is attributed to other neurological disorders. The final results of these aetiology splits are shown below:

Aetiology	Mean	Lower	Upper
Other neurological disorders	0.382	0.331	0.669
Influenza	0.119	0.071	0.192
Upper respiratory infections	0.319	0.27	0.372
Diarrhoeal diseases	0.109	0.086	0.135
Other infectious diseases	0.071	0.054	0.093

Disability weights

One health state was used for all Guillain-Barré cases. It is described as paralysed from the waist down, cannot feel or move the legs, and has difficulties with urine and bowel control. The person uses a wheelchair to move around. The disability weight is 0.296 (0.198–0.414).

Modelling strategy

All data, from both the literature review and hospital extraction, were corrected for the survival rate. A random effects meta-analysis calculated a 95% case fatality rate (95% CI 93–98%). A forest plot showing the results of this meta-analysis is displayed below. As mortality mainly occurs during the acute phase of the disease (usually within four weeks of onset), the pooled survival rate was used to get the incidence of the people surviving after the acute phase of the GBS. Where surveillance data were reported at age groups of over 20 years, we applied the age pattern derived from USA MarketScan claims data to age-split the data.

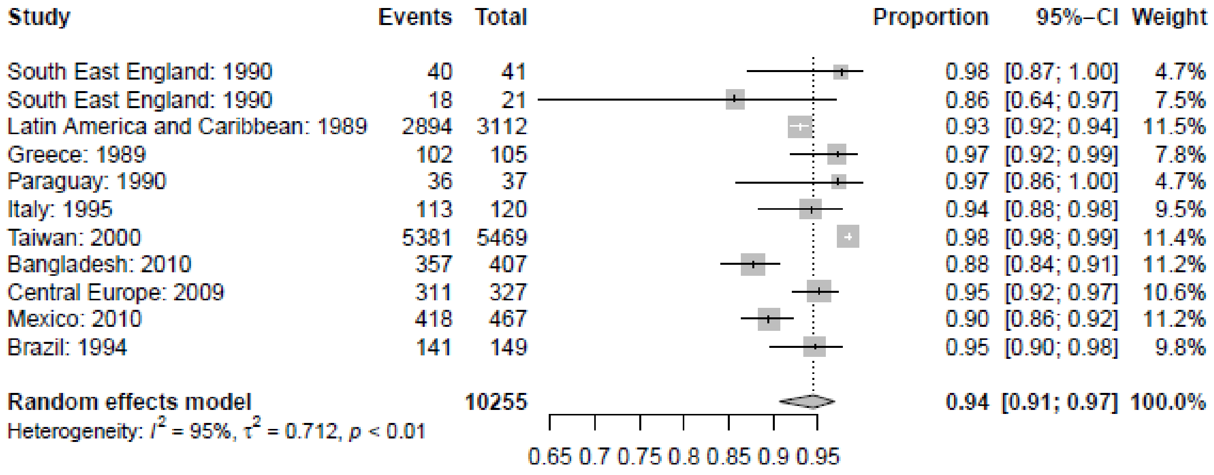

Dismod-MR 2.1 was used to estimate prevalence of Guillain-Barré syndrome for every location, year, age, and sex. For this round we decided not to adjust the hospital data as they were fairly similar to literature data and we would expect almost all cases of GBS to be captured in hospital admissions records. We then split the overall prevalence of the impairment by underlying aetiology (upper respiratory infections, influenza, diarrhoeal diseases, other infections, and other neurological causes). We used random effects meta-analysis to pool these proportions. We squeeze the proportions for influenza, diarrhoeal diseases, upper respiratory infections, and other infectious diseases to add to the proportion for all identified infectious underlying diseases. We assigned the complement to one of the proportion with any underlying infectious disease to a rest category of “idiopathic Guillain-Barre syndrome” that is classified under neurological disorders.

Hearing impairment

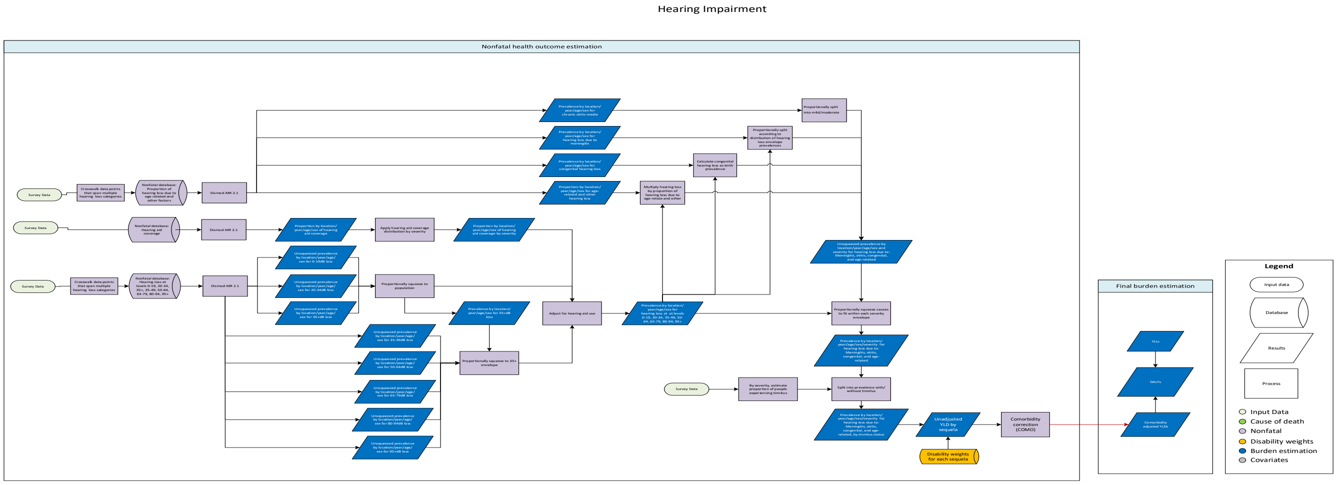

Case definition

For GBD 2017, we modelled the following severities of hearing loss:

Severity thresholds of interest for hearing loss	
**Severity**	**Threshold (in decibels)**
None	0–19
Mild	20–34
Moderate	35–49
Moderately severe	50–64
Severe	65–79
Profound	80–94
Complete	95+

We modelled the following causes of hearing loss: congenital, meningitis, otitis, and age-related and other hearing loss. Hearing loss due to meningitis and otitis are modelled as part of their underlying cause as described in their respective sections. Congenital hearing loss is defined as hearing loss present at birth. Age-related and other hearing loss includes causes not identified as meningitis, otitis, or congenital. This includes presbycusis, the gradual increase in hearing loss over age frequently caused by the natural breakdown of neurons in the inner ear. For all causes, we estimate hearing loss with and without tinnitus, the perception of noise or ringing in the ears.

Input data

Model inputs

For the estimation of the severity-specific envelopes, we used a series of systematic reviews and survey extraction. Data sources up to 2008 were identified by a published systematic review (http://www.ncbi.nlm.nih.gov/pubmed/19444763). A systematic review covering 2008–2013 was conducted with the following search terms:

> (hearing impairment[Title/Abstract] OR deafness[Title/Abstract] OR hearing loss[Title/Abstract]) AND (prevalence[Title/Abstract]) AND ("2008"[PDAT] : "3000"[PDAT]) AND (cross sectional OR survey)

In addition, we extracted hearing loss measurement from the United States National Health and Examination Surveys (NHANES) and the Health Survey for England (HSE). Self-reported data, from both the literature and surveys, were excluded. This includes censuses in the Integrated Public Use Microdata Series (IPUMS), the WHO Studies on Global Ageing and Adult Health (SAGE), and the WHO Multi-Country Survey Study on Health and Responsiveness (MCSS). Systematic reviews and self-reported survey data (including MCSS, SAGE, and NHANES) were used to estimate hearing aid coverage.

For GBD 2016, we conducted a systematic review on November 30, 2016, using the following search terms:

(hearing impairment[Title/Abstract] OR deafness[Title/Abstract] OR hearing loss[Title/Abstract] OR audiometry[Title/Abstract]) AND (prevalence[Title/Abstract]) AND ("2008/11/26"[PDAT] : "3000"[PDAT]) AND (cross sectional OR survey)

This returned 239 results, of which 17 were accepted.

Where studies reported hearing loss spanning multiple thresholds (eg, 80+, rather than 80-94 and 95+), we crosswalked using ratios predicted by a linear regression on age, using NHANES microdata. Where studies reported severity categories that did not align with GBD thresholds, we crosswalked using NHANES microdata to the nearest GBD severity category.

The table below shows the number of site-years of data included in severity envelope models for GBD 2017, as well as the number of countries, regions, and super-regions represented.

**0-19 dB**	
	**Prevalence**
**Site-years (total)**	186
**Number of countries with data**	17
**Number of GBD regions with data (out of 21 regions)**	10
**Number of GBD super-regions with data (out of 7 super-regions)**	5

**20-34 dB**	
	**Prevalence**
**Site-years (total)**	184
**Number of countries with data**	15
**Number of GBD regions with data (out of 21 regions)**	11
**Number of GBD super-regions with data (out of 7 super-regions)**	6

35-49 dB	
	Prevalence
Site-years (total)	38
Number of countries with data	17
Number of GBD regions with data (out of 21 regions)	10
Number of GBD super-regions with data (out of 7 super-regions)	5

50-64 dB	
	Prevalence
Site-years (total)	53
Number of countries with data	18
Number of GBD regions with data (out of 21 regions)	11
Number of GBD super-regions with data (out of 7 super-regions)	5

65-79 dB	
	Prevalence
Site-years (total)	38
Number of countries with data	19
Number of GBD regions with data (out of 21 regions)	12
Number of GBD super-regions with data (out of 7 super-regions)	5

80-94 dB	
	Prevalence
Site-years (total)	39
Number of countries with data	10
Number of GBD regions with data (out of 21 regions)	8
Number of GBD super-regions with data (out of 7 super-regions)	5

95+ dB	
	Prevalence
Site-years (total)	44
Number of countries with data	17
Number of GBD regions with data (out of 21 regions)	10
Number of GBD super-regions with data (out of 7 super-regions)	6

Health states and disability weights

Health state name	Health state description	Disability weight

Hearing loss, mild	has great difficulty hearing and understanding another person talking in a noisy place (for example, on an urban street).	0.01 (0.004–0.019)
Hearing loss, mild, with ringing	has great difficulty hearing and understanding another person talking in a noisy place (for example, on an urban street), and sometimes has annoying ringing in the ears.	0.021 (0.012–0.036)
Hearing loss, moderate	is unable to hear and understand another person talking in a noisy place (for example, on an urban street), and has difficulty hearing another person talking even in a quiet place or on the phone.	0.027 (0.015–0.042)
Hearing loss, moderate, with ringing	is unable to hear and understand another person talking in a noisy place (for example, on an urban street), and has difficulty hearing another person talking even in a quiet place or on the phone, and has annoying ringing in the ears for more than 5 minutes at a time, almost every day.	0.074 (0.048–0.107)
Hearing loss, moderately severe	(custom DW from hearing loss impairment envelope)	0.092 (0.064–0.129)
Hearing loss, moderately severe, with ringing	(custom DW from hearing loss impairment envelope)	0.167 (0.114–0.231)
Hearing loss, severe	is unable to hear and understand another person talking, even in a quiet place, and unable to take part in a phone conversation. Difficulties with communicating and relating to others cause emotional impact at times (for example worry or depression).	0.158 (0.104–0.227)
Hearing loss, severe, with ringing	is unable to hear and understand another person talking, even in a quiet place, and unable to take part in a phone conversation, and has annoying ringing in the ears for more than 5 minutes at a time, almost every day. Difficulties with communicating and relating to others cause emotional impact at times (for example worry or depression).	0.261 (0.174–0.361)
Hearing loss, profound	is unable to hear and understand another person talking, even in a quiet place, is unable to take part in a phone conversation, and has great difficulty hearing anything in any other situation. Difficulties with communicating and relating to others often cause worry, depression, and loneliness.	0.204 (0.134–0.288)
Hearing loss, profound, with ringing	is unable to hear and understand another person talking, even in a quiet place, is unable to take part in a phone conversation, has great difficulty hearing anything in any other situation, and has annoying ringing in the ears for more than 5 minutes at a time, several times a day. Difficulties with communicating and relating to others often cause worry, depression, or loneliness.	0.277 (0.182–0.388)
Hearing loss, complete	cannot hear at all in any situation, including even the loudest sounds, and cannot communicate verbally or use a phone. Difficulties with communicating and relating to others often cause worry, depression or loneliness.	0.215 (0.143–0.307)
Hearing loss, complete, with ringing	cannot hear at all in any situation, including even the loudest sounds, and cannot communicate verbally or use a phone, and has very annoying ringing in the ears for more than half of the day. Difficulties with communicating and relating to others often cause worry, depression or loneliness.	0.316 (0.211–0.436)

Modelling strategy

We modelled the prevalence of hearing loss over five steps. First, we ran three DisMod-MR 2.1 models to estimate the total prevalence estimates of hearing loss: normal hearing (0–19dB), mild hearing loss (20–34dB), and moderate hearing loss and above (35+ dB). We squeezed the prevalence estimates from these DisMod-MR 2.1 models to fit within the entire population of each country. We estimated prevalence of normal hearing for this squeezing purpose only, and hence it did not form part of further analysis. Betas and exponentiated values, which can be interpreted as an odds ratio, are shown in the table below for each covariate.

Model	Covariate name	Type	Measure	Beta value	Exponentiated value
Hearing loss impairment at 35+ dB	Socio-demographic Index	Country covariate	Prevalence	-0.23 (-0.64 to -0.011)	0.79 (0.53–0.99)

Hearing loss impairment at 95+ dB	Socio-demographic Index	Country covariate	Prevalence	-0.84 (-1.88 to -0.037)	0.43 (0.15–0.96)
Hearing aids (proportion of total hearing loss)	LDI (I$ per capita)	Country covariate	Prevalence	0.76 (0.42–1.00)	2.14 (1.51 to 2.72)
Hearing loss impairment at 0-19 dB	Socio-demographic Index	Country covariate	Prevalence	0.18 (0.066–0.31)	1.19 (1.07–1.37)

Second, we ran five additional DisMod-MR 2.1 models for each severity levels of hearing loss above mild: moderate (35–49dB), moderately severe (50–64dB), severe (65–79dB), profound (80–94dB), and complete (95+). We then squeezed the prevalence estimates from these models to fit within the prevalence that were estimated for 35+dB in the first step. By the end of the second step, we had estimated prevalence of six severity levels of hearing loss, including mild (20–34dB). We also ran a DisMod-MR 2.1 model for the coverage of hearing aids, using (logged) lag distributed income (LDI) as a covariate.

Third, we adjusted the prevalence of each severity level by accounting for hearing aids. We assumed the use of hearing aids reduced the severity by one level. Data obtained from the HUNT-2 study in Norway and NHANES provided detailed information on people with hearing aids, which was used to estimate the proportion of hearing aids for each severity level. We ran a logistic regression on age with binary indicators for severity levels and sex. We calculated country-specific hearing aid coverage by multiplying the severity-specific coverage in Norway by the ratio of hearing aid coverage in a given country to that of Norway for each age-sex. We shifted the identified fraction of people in each severity level a level below, except for profound and complete hearing loss, which we assumed were not correctable by hearing aids. This provided the adjusted prevalence of six severity levels of all-cause hearing loss.

Fourth, we estimated the prevalence of hearing loss due to multiple causes: otitis media, congenital, meningitis (pneumococcal, *H influenzae* type B meningitis, meningococcal, and other bacterial), and age-related and other causes not classified elsewhere. For congenital hearing loss, we assumed that all hearing losses occurring at the time of birth are of congenital nature. We assumed that all hearing loss due to otitis media is at the mild or moderate level. Up to the age of 20, we implemented proportional squeezes to scale cause-specific hearing loss prevalence to the total prevalence of each severity level. Above age 20, we subtracted the prevalence of congenital hearing loss, meningitis, and otitis from the total and called any remainder age-related and other hearing loss. Limitations in the model and underlying data for age-related and other hearing loss required such a step. Since we ensured that congenital prevalence was constant in each age group for every location, year, and sex combination after conducting the proportional squeeze, the sum of the prevalence of all hearing loss aetiologies sometimes exceeded the total prevalence of some severity levels.

Finally, we estimated the percent of people experiencing tinnitus. The data used to model tinnitus were markedly different in GBD 2017 versus GBD 2016. Instead of using a consistent definition of a tinnitus case for each severity level as was done last year (ie, in GBD 2016, anyone experiencing ringing for at least five minutes per day was considered to be suffering from tinnitus, regardless of severity level), we used severity-specific definitions of tinnitus, which is more consistent with the health state descriptions. We determined the proportion of people suffering from tinnitus using data from NHANES years that

asked about the frequency each survey respondent heard ringing, roaring, and/or buzzing (1999, 2001, 2003, and 2011–2012). We labeled anyone with mild hearing loss and ringing, roaring, or buzzing "at least once a month" as a mild hearing loss with tinnitus case. Anyone with moderate hearing through to severe hearing loss and ringing, roaring, or buzzing "at least once a day" was labelled as a moderate hearing loss with tinnitus case. Anyone with complete hearing loss who responded that they "almost always" had hearing loss was labelled as a complete hearing loss with tinnitus case. The effect of using the new definitions unsurprisingly increased the estimated proportion of tinnitus among those with mild hearing loss and decreased the estimated proportion of tinnitus among those with complete hearing loss. Using the data from NHANES, we calculated confidence intervals assuming a binomial distribution. We assumed the same distribution of tinnitus across all aetiologies of hearing loss.

Heart failure impairment

Flowcharts

Overall modelling strategy

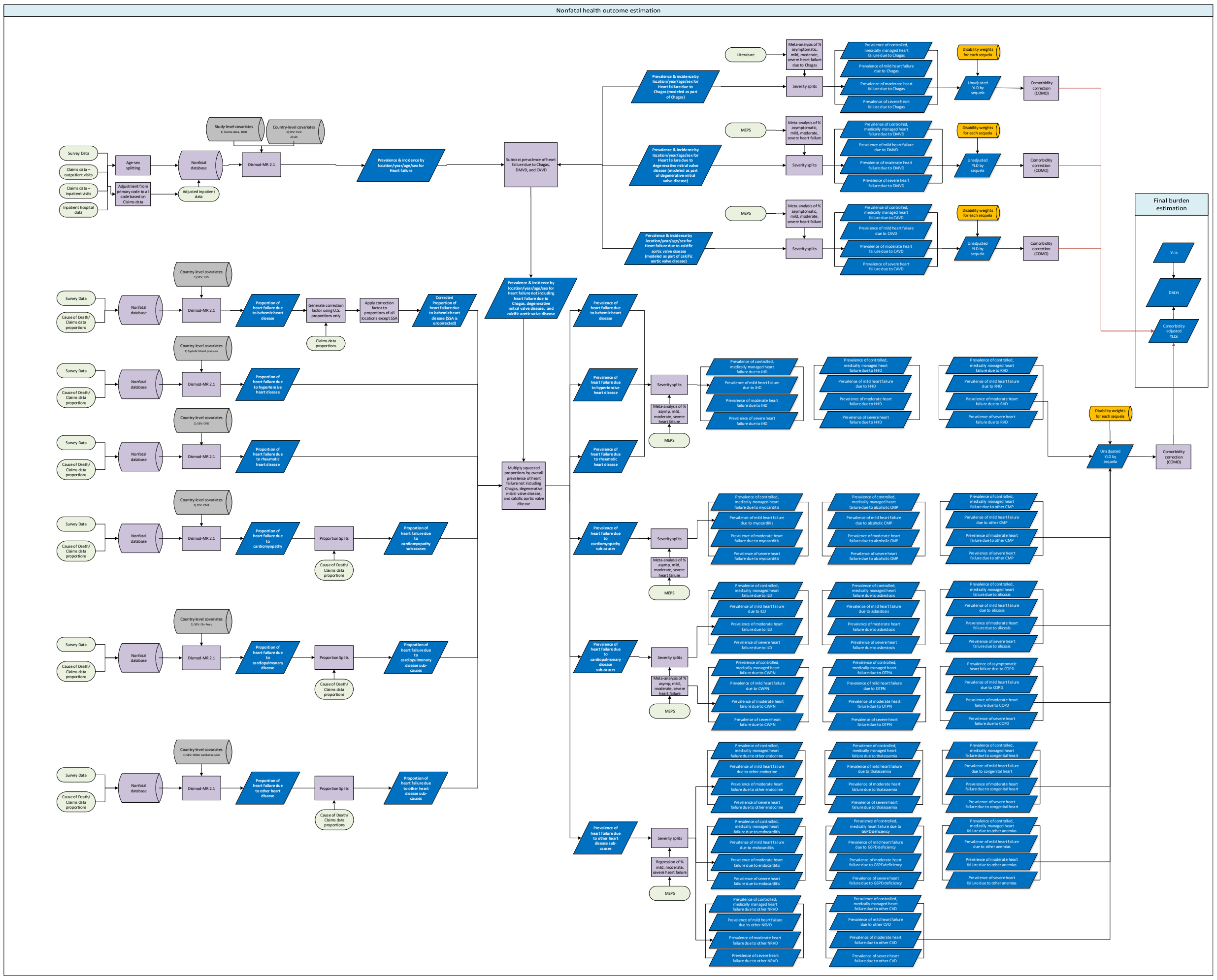

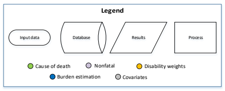

Abbreviations

DMVD: Degenerative mitral valve disease; CAVD: Calcific aortic valve disease; IHD: Ischaemic heart disease; CMP: Cardiomyopathy and myocarditis; HHD: Hypertensive heart disease; ILD: Interstitial lung disease; CWPN: Coal workers pneumoconiosis; OTPN: Other pneumoconiosis; COPD: Chronic obstructive pulmonary disease; RHD: Rheumatic heart disease; CVD: Cardiovascular disease; NRVD: Non-rheumatic valve disease

Proportion splits and correction factor estimation

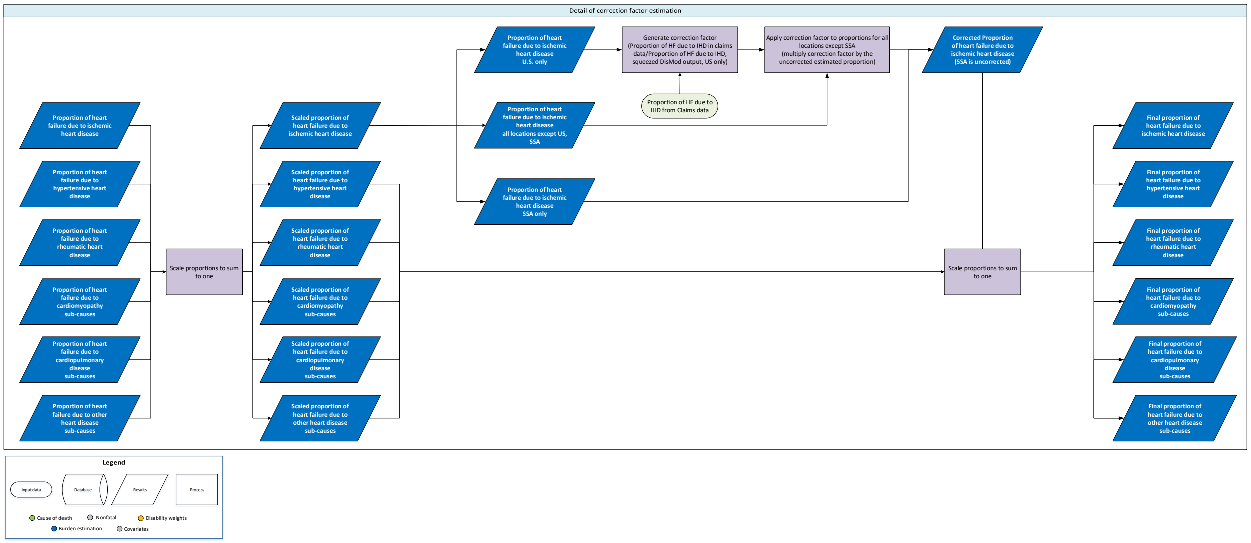

Case definition

Heart failure was diagnosed clinically using structured criteria such as the Framingham or European Society of Cardiology criteria. Previous iterations of GBD modelled symptomatic (ie, NYHA Class II and above) episodes of HF only. Beginning in GBD 2016, we used ACC/AHA Stage C and above to capture both persons who are currently symptomatic and those who have been diagnosed with heart failure but are currently asymptomatic.

Framingham Criteria (1): Must fulfill two major criteria or one major and two minor criteria.
Major criteria: Paroxysmal nocturnal dyspnoea, neck vein distention, rales, radiographic cardiomegaly, acute pulmonary oedema, S3 gallop, increased central venous pressure (>16 cm H_2O at right atrium), hepatojugular reflux; weight loss >4.5 kg in 5 days in response to treatment
Minor criteria: bilateral ankle oedema, nocturnal cough, dyspnoea on ordinary exertion, hepatomegaly, pleural effusion, decrease in vital capacity by one-third from maximum recorded, tachycardia (heart rate>120 beats/min).

European Society of Cardiology (2):
Typical signs (elevated jugular venous pressure, pulmonary crackles and peripheral oedema) and symptoms (eg, breathlessness, ankle swelling, and fatigue) caused by a structural and/or functional cardiac abnormality, resulting in a reduced cardiac output and/or elevated intracardiac pressures at rest or during stress.

Input data

Model inputs

A systematic review was not performed for GBD 2017, but was for GBD 2016. The search terms used were: "heart failure"[TIAB] AND (epidemiology[MeSH Terms] OR prevalence[TIAB] OR incidence[TIAB] OR mortality[TIAB]) AND ("1990/01/01"[PDAT] : "2016/09/02"[PDAT]) NOT “animal model” NOT rat NOT mice NOT diabetes[TIAB] NOT “renal transplant”[TIAB]. The dates of the search were 01/01/1990 through 09/02/2016. 37,891 initial hits were returned, and 57 sources were added. An unstructured review of the

literature yielded an additional 30 sources, of which six were extracted. Prior to GBD 2016, a systematic review of the literature had not been performed since the GBD 2010 study.

The tables below show the number of site-years and types of data included in the estimation process.

Overall heart failure prevalence

	Case fatality rate	Incidence	Mortality risk	Prevalence
**Site-years (total)**	58	46	115	1234
**Number of countries with data**	18	14	24	38
**Number of GBD regions with data (out of 21 regions)**	8	6	11	12
**Number of GBD super-regions with data (out of 7 super-regions)**	5	4	7	6

Heart failure due to ischaemic heart disease

	Proportion
**Site-years (total)**	74
**Number of countries with data**	45
**Number of GBD regions with data (out of 21 regions)**	16
**Number of GBD super-regions with data (out of 7 super-regions)**	7

Heart failure due to hypertensive heart disease

	Proportion
**Site-years (total)**	52
**Number of countries with data**	33
**Number of GBD regions with data (out of 21 regions)**	16
**Number of GBD super-regions with data (out of 7 super-regions)**	7

Heart failure due to rheumatic heart disease

	Proportion
**Site-years (total)**	11
**Number of countries with data**	9
**Number of GBD regions with data (out of 21 regions)**	6
**Number of GBD super-regions with data (out of 7 super-regions)**	4

Heart failure due to cardiomyopathy

	Proportion
**Site-years (total)**	53
**Number of countries with data**	39
**Number of GBD regions with data (out of 21 regions)**	15
**Number of GBD super-regions with data (out of 7 super-regions)**	7

Heart failure due to cardiopulmonary disease

	Proportion
**Site-years (total)**	63
**Number of countries with data**	45
**Number of GBD regions with data (out of 21 regions)**	16
**Number of GBD super-regions with data (out of 7 super-regions)**	7

Severity split inputs

The table below includes lay descriptions and disability weights for the severity levels of heart failure for GBD 2016.

Severity level	Lay description	DW (95% CI)
Controlled, medically managed	Has been diagnosed with clinical heart failure, a chronic disease that requires medication every day and causes some worry but minimal interference with daily activities.	0.012 (0.006-0.023)
Mild	Is short of breath and easily tires with moderate physical activity, such as walking uphill or more than a quarter-mile on level ground. The person feels comfortable at rest or during activities requiring less effort.	0.041 (0.026–0.062)
Moderate	Is short of breath and easily tires with minimal physical activity, such as walking only a short distance. The person feels comfortable at rest but avoids moderate activity.	0.072 (0.047–0.103)
Severe	Is short of breath and feels tired when at rest. The person avoids any physical activity, for fear of worsening the breathing problems.	0.179 (0.122–0.251)

Modelling strategy

The general analytical strategy included estimating the overall prevalence of heart failure using DisMod-MR 2.1, followed by a multi-step analysis of published literature, claims data, and cause of death data to estimate the aetiological fraction for each cause of heart failure. The latter process includes an initial assessment of the fraction of heart failure cases attributable to each of the high-level parent cause groupings, followed by further division into the detailed causes within each of these groupings, and finally a correction factor applied to adjust these proportions. The selection for aetiological causes was based on a review of the literature and expert opinion regarding diseases that lead to congestive heart failure.

We first estimated an overall prevalence of AHA/ACC stage C or D heart failure using literature data, hospital data, and claims data. Inpatient hospital data were corrected for readmission, primary to any diagnosis, and inpatient to outpatient utilisation ratios using adjustment factors calculated from individual-level claims data. We included covariates in DisMod to adjust US claims data and ICPC-coded data from Norway, using as reference literature data and inpatient hospital data.

We set a prior of no remission and capped excess mortality at 1.

Estimates for the prevalence of heart failure due to Chagas, degenerative mitral valve disease, and calcific aortic valve disease were generated separately as part of the modelling strategy for those causes. We subtracted the prevalence of heart failure due to these causes from the overall heart failure estimates to give an adjusted prevalence of heart failure due to all other aetiologies.

Our estimation of the aetiological causes of heart failure makes several assumptions and has several limitations. First, we assume that each case of heart failure only has one cause. Second, we rely on claims data from the United States, the only country where detailed person-level claims data were available, to assess the association between heart failure and underlying aetiologies. Third, we rely on mortality estimates due to these underlying causes (regardless of heart failure) to estimate their contribution to heart failure deaths in countries where detailed person-level claims data are not yet available. Fourth, we utilise the association between claims data and death certificate data in the United States to adjust for the differences in coding for ischaemic heart disease, the most common cause of heart failure globally, in claims data versus death certificates. This approach allows us to produce estimates for all locations and can be updated to include more detailed health record and claims data from additional locations as they become available.

To estimate the aetiological fractions for each cause, we calculated the proportion of people with a diagnosis of one of the underlying causes of heart failure who also had a diagnosis of heart failure listed using claims data from the United States. These proportions were then multiplied by age-, sex-, and location-specific deaths (post CoDCorrect) to yield an overall number for each underlying cause of heart failure. We then divided these cause-specific totals by the sum of deaths for all aetiologies in order to yield a proportion of deaths due to heart failure for each cause. These proportions, along with literature data, were used to inform DisMod-MR 2.1 models for the six broadest and mutually exclusive and collectively exhaustive cause groupings: ischaemic heart disease, hypertensive heart disease, cardiomyopathy and myocarditis, rheumatic heart disease, cardiopulmonary disease, and other cardiovascular and circulatory diseases. An exception to this approach was made for sub-Saharan Africa, where we excluded the proportion estimates generated from claims and death data, relying instead on published literature to determine the proportions of heart failure aetiologies. This decision was based on expert opinion that local patterns differed significantly from what would have been determined from claims and death data. The THESUS-HF study, a large-scale, prospective, echocardiographic study of heart failure aetiologies in multiple African countries, provided these proportions (3). The results of these six proportion models were scaled to sum to one.

For heart failure due to cardiopulmonary disease, heart failure due to cardiomyopathy and myocarditis, and heart failure due to other causes, we calculated the proportion for each sub-cause according to the proportion of that cause within each larger aggregate group.

After the initial splitting and scaling steps, we compared the estimated proportions for the United States with the proportions originally calculated from the USA claims data. The estimated proportion of HF due to IHD was much higher than the proportion of HF due to IHD in the claims data. This difference was due to the large number of IHD deaths not related to heart failure. In order to correct this over-estimation, we generated a correction factor for HF due to IHD by taking the proportion of HF due to IHD from the claims data and dividing it by the proportion of HF due to IHD from the scaled DisMod results. We then multiplied the proportion of HF due to IHD from the scaled DisMod results by this correction factor for all locations except sub-Saharan Africa. Since echocardiographic evidence rather than claims data were used

to model HF aetiology proportions in sub-Saharan Africa, we did not adjust the estimated proportion of HF due to IHD for this super-region. In the next step, after the proportion of HF due to IHD has been corrected, the proportions were all rescaled to sum to one. These final scaled proportions were then multiplied by the overall HF envelope (after adjustment) to yield prevalence estimates of HF due to all aetiologies.

These estimates were then split into asymptomatic, mild, moderate, and severe heart failure based on an analysis of MEPS data, with the exception of Chagas disease. For that aetiology, we based the severity splits on a meta-analysis of NYHA class among persons diagnosed with heart failure due to Chagas disease in areas where Chagas is endemic.

Models were evaluated based on expert opinion, comparison of results with other rounds of GBD, and model fit.

Overall heart failure impairment envelope

Study covariate	Parameter	beta	Exponentiated beta
MarketScan (USA claims)	Prevalence	-0.4 (-0.43 to -0.4)	0.67 (0.65–0.67)
Outpatient (ICPC, Norway)	Prevalence	-1 (-1 to -1)	0.37 (0.37–0.37)
Log-transformed age-standardised SEV scalar: CVD	Prevalence	0.75 (0.75–0.77)	2.38 (2.21–2.53)
LDI (I$ per capita)	Excess mortality rate	-0.3 (-0.5 to -0.1)	0.74 (0.61–0.90)

Six main sub-cause proportion envelopes

Sub-cause	Covariate	Parameter	beta	Exponentiated beta
Heart failure due to cardiomyopathy impairment envelope	Log-transformed age-standardised SEV scalar: CMP	Proportion	0.75 (0.75–0.75)	2.12 (2.12–2.12)
Heart failure due to cardiopulmonary disease impairment envelope	Log-transformed age-standardised SEV scalar: Chr Resp	Proportion	0.75 (0.75–0.77)	2.13 (2.12–2.15)
Heart failure due to hypertensive heart disease impairment envelope	Systolic blood pressure (mmHg)	Proportion	2.6E-5 (3.E-6 to 4.7E-5)	1.00 (1.00–1.00)
Heart failure due to ischaemic heart disease impairment envelope	Log-transformed age-standardised SEV scalar: IHD	Proportion	0.75 (0.75–0.75)	2.12 (2.12–2.12)
Heart failure due to other causes impairment envelope	Log-transformed SEV scalar: Oth Cardio	Proportion	0.75 (0.75–0.75)	2.12 (2.12–2.13)

Heart failure due to valvular heart disease impairment envelope	Log-transformed age-standardised SEV scalar: CVD	Proportion	0.75 (0.75–0.75)	2.12 (2.12–2.12)

For GBD 2017, we estimated the prevalence of heart failure due to degenerative mitral valve disease, calcific aortic valve disease, and other non-rheumatic valve disease as distinct aetiologies. In previous iterations, these had been collapsed as part of the other cardiovascular disease category. We eliminated iron-deficiency anaemia and iodine deficiency as aetiologies of heart failure after review of the scientific literature. No other significant changes were made to the modelling strategy for GBD 2016.

References

1) http://www.framinghamheartstudy.org/share/protocols/soe0_03s_protocol.pdf
2) 2016 ESC Guidelines for the diagnosis and treatment of acute and chronic heart failure: The Task Force for the diagnosis and treatment of acute and chronic heart failure of the European Society of Cardiology (ESC) Developed with the special contribution of the Heart Failure Association (HFA) of the ESC. Eur Heart J 2016; 37 (27): 2129-2200.
3) Reitsma, Marissa B., et al. "Smoking prevalence and attributable disease burden in 195 countries and territories, 1990–2015: a systematic analysis from the Global Burden of Disease Study 2015." The Lancet.
4) Damasceno A, Mayosi BM, Sani M, Ogah OS, Mondo C, Ojji D, Dzudie A, Kouam CK, Suliman A, Schrueder N, Yonga G, Ba SA, Maru F, Alemayehu B, Edwards C, Davison BA, Cotter G, Sliwa K. The Causes, Treatment, and Outcome of Acute Heart Failure in 1006 Africans From 9 Countries. Results of the Sub-Saharan Africa Survey of Heart Failure. Arch Intern Med. 2012;172(18):1386-1394.

Infertility impairment

Flowchart

Infertility

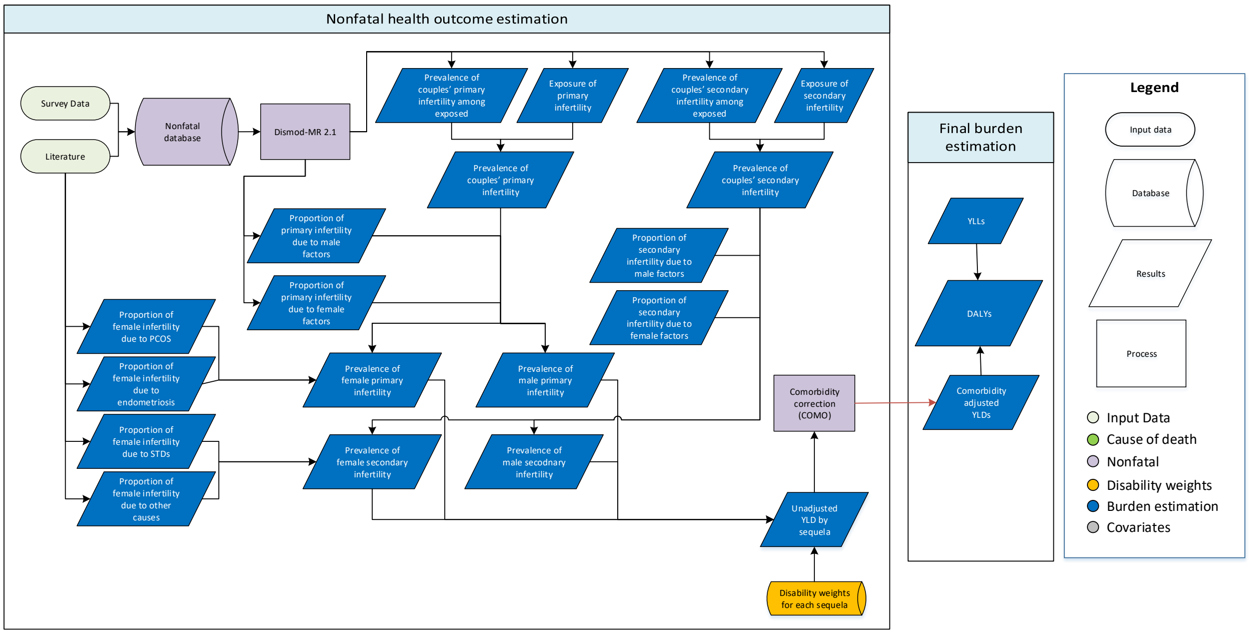

Case definition and modelling summary

For GBD 2017, the following case definitions were used for infertility:

1. Primary infertility is defined as a couple who have not had a livebirth, who wish a child, and have been in a union for more than five years without using contraceptives.

2. Secondary infertility is defined in a couple who wish a child and have been in a union for more than five years without using contraceptives since the last livebirth.

Estimation is completed in three steps. First, we estimate total *primary* (unable to have any child) and *secondary* (unable to have an additional child) infertility in *couples*. This is accomplished by first quantifying the rate of infertility among survey respondents who are *married* (the subset to whom such questions are directed) and then quantifying how the married population relates to the overall population. Second, we model which proportion of primary and secondary infertility is due to female and male factors, respectively, to estimate four "envelopes" of infertility: male primary infertility, male secondary infertility, female primary infertility, and female secondary infertility. Third, we execute a "causal attribution" process to assign cases of each envelope to likely underlying causes and assign the remainder to idiopathic infertility (ie, unknown causes).

Input data

Our primary data sources are population surveys. The datasets were last updated for GBD 2015. Data extraction included data for women in five-year age groups between 15 and 49 from population-based surveys including the Demographic and Health Surveys (DHS), World Fertility Surveys (WFS), Reproductive

Health Surveys (RHS), Family and Fertility Survey (FFS), and others (EUR, NSF, PCD, PFM). Such surveys only ask fertility-related questions to married women. Even though only women are interviewed, we treated the responses as a proxy for the infertility of couples in unions because the questions are not structured in a way that it is possible to determine which partner is the cause of the couples' inability to conceive a child. The desire to have a child is the crucial determinant of whether a couple is labeled as infertile (ie, if no child is wanted, infertility is not present).

The combination of variables in surveys that were used to construct each of the four datasets (primary "impairment" and "exposure" and secondary "impairment" and "exposure") are illustrated in the table below. As described below, overall primary and secondary infertility are estimated by multiplying prevalence among those with the "impairment" of infertility (married women who desire a[nother] child) by the prevalence of the "exposure" (being married for 5+ years, not using contraception for 5+ years).

Model name	Infertility type	Numerator	Denominator
Primary (impairment)	Exposure to primary infertility among married women	Married 5+ years; no contraception for 5+ years prior to survey; no previous births; desires a child.	Married 5+ years
Primary (exposure)	Prevalence of exposure	Married 5+ years; no contraception for 5+ plus years prior to survey	All women
Secondary (impairment)	Exposure to secondary infertility among married women	Married 5+ years; no contraception for 5+ years prior to survey; last birth 5+ years ago; desires a child.	Married 5+ years; 1+ children
Secondary (exposure)	Prevalence of exposure to secondary infertility	married 5+ years; no contraception for 5+ years prior to survey; 1+ children	All women

The table below illustrates the extent of data coverage for the infertility envelope models for GBD 2017.

Primary infertility impairment	Prevalence
Site-years (total)	325
Number of countries with data	113
Number of GBD regions with data (out of 21 regions)	20
Number of GBD super-regions with data (out of 7 super-regions)	7

Primary infertility exposure	Prevalence
Site-years (total)	274
Number of countries with data	101
Number of GBD regions with data (out of 21 regions)	17
Number of GBD super-regions with data (out of 7 super-regions)	7

Secondary infertility impairment	Prevalence
Site-years (total)	327
Number of countries with data	112
Number of GBD regions with data (out of 21 regions)	19
Number of GBD super-regions with data (out of 7 super-regions)	7

Secondary infertility exposure	Prevalence
Site-years (total)	274
Number of countries with data	101
Number of GBD regions with data (out of 21 regions)	17
Number of GBD super-regions with data (out of 7 super-regions)	7

The second set of four datasets informed estimates of which component of primary and secondary infertility were due to female and male factors, respectively. To obtain data on the sex and cause breakdown for infertility, we systematically searched the literature in GBD 2010 using the following search string:

causes[Title/abstract] AND infertility[Title] NOT mouse NOT murine NOT rat NOT rodent

We received 626 hits from PubMed and excluded studies according to the following exclusion criteria:

1. studies not representative of the national population;
2. studies that provide no raw data,
3. studies that provide only estimates;
4. studies performed before 1970;
5. case studies or studies with sample size less than 50;
6. studies that provide no data on the sex of the partner responsible for infertility among couples.

The majority of excluded studies were excluded because of the latter criterion. In total, 15 studies were included in our analysis for the sex breakdown among infertile couples. Infertility among couples was reported as due to one of the following causes: male factor, female factor, both, or unknown. Couples with infertility due to both partners were allocated to both male factor and female factor, and couples with infertility of unknown cause were allocated to male and female factors based on the proportion observed in other couples in the study. We estimated the proportion of couples' infertility due to male factors and female factors separately in DisMod-MR 2.1. The quantity modelled was the proportion of couples' infertility due to each sex for each of primary and secondary infertility. The table below shows the dataset contents for these four models, each of which used the same set of sources.

Proportion sex-specific primary and secondary infertility	Proportion
Site-years (total)	19
Number of countries with data	15
Number of GBD regions with data (out of 21 regions)	8
Number of GBD super-regions with data (out of 7 super-regions)	6

Modelling strategy

For GBD 2017, we estimated the prevalence of primary and secondary infertility by sex and cause in three steps: 1) estimation of couples' infertility [four DisMod-MR 2.1 models], 2) estimation of infertility by sex [four DisMod-MR 2.1 models], and 3) causal attribution of infertility. We assumed zero infertility prior to age 15 or after age 50 years as fertility is not expected to be desired outside these age ranges in women; an assumption that was therefore carried over to men as well. All DisMod-MR 2.1 models were run as single parameter models. No study or country covariates were used in any models.

Estimation of couples' infertility

To estimate the prevalence of primary and secondary infertility among couples, we first run four DisMod-MR 2.1 models to estimate the four parameters detailed above, prevalence of primary infertility (1), prevalence of primary infertility exposure (2), prevalence of secondary infertility (3), and prevalence of secondary infertility exposure (4). For prevalence of infertility (models 1 and 3), we tried using the natural log of the age-standardised death rate (lnASDR) of sexually transmitted infections (STIs), but it was not statistically significant so we did not use it in the final model. We did not use any study- or country-level covariates for these models. Next, we estimated primary and secondary couples' infertility from DisMod-

MR 2.1 models by multiplying the estimates for prevalence of infertility among exposed women by the prevalence of exposure to infertility to obtain prevalence of infertility among all women and all men.

Estimation of infertility by sex

After running the four models estimating overall infertility, described above, we ran four DisMod-MR 2.1 models to estimate the proportion of primary and secondary infertility by sex, proportion of primary female infertility, proportion of secondary female infertility, proportion of primary male infertility, and proportion of secondary male infertility. We model sex-specific infertility as a proportion. Because infertility in some couples is attributable to both partners rather than just one, the sum of the proportions due to each partner is greater than one when both partners are infertile. When the sum of the proportions is lower than one, we scale it to be equal to one through custom code. Again, we tried using lnASDR of STIs as a covariate, but it was not statistically significant so we did not use it in the final model. We did not use any study- or country- level covariates for these models. We multiplied our prevalence of primary and secondary infertility derived in step 1 by the proportion due to male and female factors to estimate primary and secondary infertility by sex.

Causal attribution

There are seven identified causes of female infertility in the GBD 2017 cause list: pelvic inflammatory disease (PID) due to chlamydia, PID due to gonorrhoea, PID due to other sexually transmitted diseases, maternal sepsis, polycystic ovarian syndrome, endometriosis, and Turner syndrome. For each of these diseases, we determined the prevalence of infertility by a literature review of the probability of becoming infertile due to that disease. For STIs, we applied a proportion with infertility derived from Westrom and colleagues[1] to incident cases of PID and used DisMod-MR 2.1 to calculate corresponding prevalence for each subsequent age group through the fertile years, assuming zero remission or excess mortality. For the others, we added all the disease-specific estimates of prevalence and assigned the remaining proportion to categories of "female primary infertility due to other causes" and "female secondary infertility due to other causes." We assumed all infertility from Turner syndrome is primary infertility and all infertility following maternal sepsis is secondary infertility. The only recognised cause of male infertility in the GBD 2017 cause list is Klinefelter syndrome. We assigned all other male infertility to "male infertility due to other causes."

Sequelae/disability weights

Every person with infertility was assumed to experience the health state as determined from the GBD disability weights survey. The lay descriptions of primary and secondary are listed below.

Health state name	Health state description	Disability weight
**Infertility, primary**	This person wants to have a child and has a fertile partner, but the couple cannot conceive.	0.008 (0.003–0.015)
**Infertility, secondary**	This person has at least one child, and wants to have more children. The person has a fertile partner, but the couple cannot conceive.	0.005 (0.002–0.011)

References

1 Weström L, Joesoef R, Reynolds G, Hagdu A, Thompson SE. Pelvic inflammatory disease and fertility. A cohort study of 1,844 women with laparoscopically verified disease and 657 control women with normal laparoscopic results. *Sex Transm Dis* 1992; **19**: 185–92.

Developmental intellectual disability

Flowchart

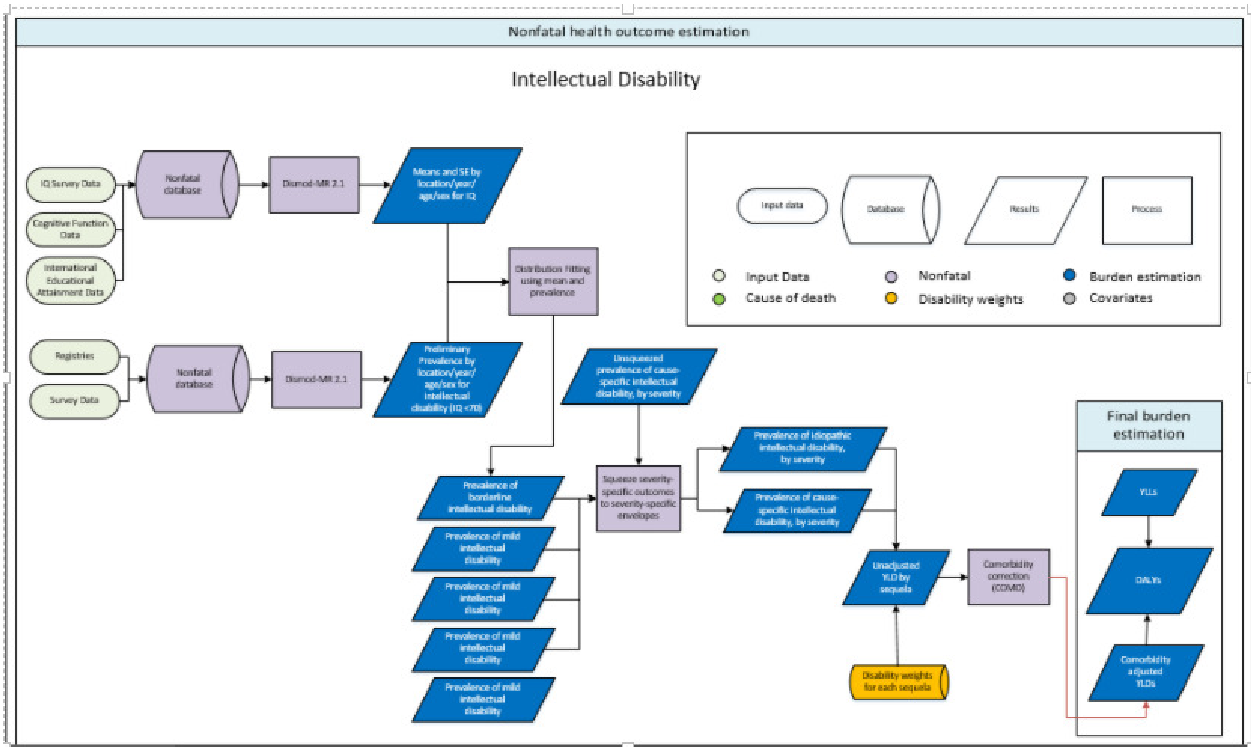

Case definition

Developmental intellectual disability (ID) is a condition of below-average intelligence or mental ability. Consistent with the American Association on Intellectual and Developmental Disabilities, we define developmental intellectual disability as a condition originating before age 18 (thus, does not include impairment due to stroke, Alzheimer's disease, or other conditions). We model the severities shown in Table 1, as measured by score on intelligence quotient (IQ) tests, which are standardised to have a mean of 100.

Table 1. ID severity definitions

Severity of intellectual disability	IQ score
Profound	0 to 19
Severe	20 to 34
Moderate	35 to 49
Mild	50 to 69
Borderline	70 to 85

Input data

Model inputs

The prevalence of intellectual disability (IQ score <70) came from a systematic review starting on January 1, 1990, using the following search string: *(((intellectual disability[MeSH Terms]) AND prevalence[Title/Abstract]) AND ('1990'[Date - Publication] : '3000'[Date - Publication]))*.

For GBD 2015, this search had 2,115 hits, of which 13 were extracted that had not been previously included in the GBD. We included studies that estimate the general population prevalence of intellectual disability. We excluded studies that did not use a case definition based on intelligence quotient (IQ) and studies that investigated non-representative groups, like hospital patients or people of a specific ethnicity. This systematic review was updated for GBD 2016.

Counts of unique input data sources for the intellectual disability envelope model are shown in Table 2.

Table 2. Input source counts

	**Prevalence**
**Site-years (total)**	86
**Number of countries with data**	31
**Number of GBD regions with data (out of 21 regions)**	10
**Number of GBD super-regions with data (out of 7 super-regions)**	6

Severity splits – disability weights

Table 3. Intellectual disability severity disability weights

Health state	Description	Disability weight
Borderline intellectual functioning	This person is slow in learning at school. As an adult, the person has some difficulty doing complex or unfamiliar tasks but otherwise functions independently.	0.011 (0.005–0.02)
Intellectual disability/mental retardation, mild	This person has low intelligence and is slow in learning at school. As an adult, the person can live independently, but often needs help to raise children and can only work at simple supervised jobs.	0.043 (0.026–0.064)
Intellectual disability/mental retardation, moderate	This person has low intelligence, and is slow in learning to speak and to do even simple tasks. As an adult, the person requires a lot of support to live independently and raise children. The person can only work at the simplest supervised jobs.	0.1 (0.066–0.142)
Intellectual disability/mental retardation, severe	This person has very low intelligence and cannot speak more than a few words, needs constant supervision and help with most daily activities, and can do only the simplest tasks.	0.16 (0.107–0.226)
Intellectual disability/mental retardation, profound	This person has very low intelligence, has almost no language, and does not understand even the most basic requests or instructions.	0.2 (0.133–0.283)

	The person requires constant supervision and help for all activities.	

Modelling strategy

We modelled the prevalence of intellectual disability (ID), both aetiology-specific IDs and idiopathic ID, over multiple steps.

First, we ran a DisMod-MR 2.1 model to estimate the total prevalence of intellectual disability of level IQ <70. We included lag-distributed income and education in the model as predictive covariates. Table 4 shows raw and exponentiated model coefficients for the covariates used in the estimation process for the DisMod model. Exponentiated coefficients can be interpreted as odds ratios.

Table 4. Model coefficient values (raw and exponentiated)

Covariate	Parameter	Coefficient (95% CI)	Exponentiated coefficient (95% CI)
Lagged distributed income (LDI) per capita	Prevalence	-0.25 (-0.44 to -0.068)	0.78 (0.65–0.93)
Underweight (proportion less than 2 SD below the mean weight for age in children under 5)	Prevalence	1.27 (0.09–2.85)	3.57 (1.09–17.34)
Sex	Prevalence	0.28 (0.12–0.44)	1.32 (1.13–1.55)

Second, we split the total prevalence of idiopathic into four severity levels: mild (IQ 50-69), moderate (IQ 35-49), severe (IQ 20-34), and profound (IQ below 20). We pooled a subset of studies that distinguished intellectual disability by these severity levels. We used cumulative severity levels (ie, IQ <50, IQ <35, and IQ <20) to maximise the number of sources. We estimated these cumulative severities' proportion of the <70 envelope via random effects meta-analyses stratified by two levels of income status (high-income versus low- and middle-income). These proportions were used to estimate discrete severities from the overall intellectual disability (IQ <70) prevalence. We estimated borderline disability (IQ 70-84) via another random-effects meta-analysis of the ratio of IQ 70-84 to IQ <70. The uncertainty of the pooled fractions and ratios were propagated throughout our calculations using 1,000 draws from a normal distribution with mean and standard error estimated by the meta-analysis. The results of the meta-analysis are shown in Table 5.

Table 5. Proportion intellectual disability cases by severity

Severity	Mean	Standard error
None	0.161	0.034
Borderline	0.161	0.034
Mild	0.375	0.037
Moderate	0.190	0.031

Severe	0.090	0.177
Profound	0.024	0.134

Third, we estimated prevalence of each aetiology-specific intellectual disability using models of the following parent causes. Since we are modelling only developmental intellectual disability, causes such as stroke and Alzheimer's disease are not included in the causal attribution process.

Parent causes included in causal attribution:

- Neonatal preterm birth complications (<28w, 28-32w, 32-36w)
- Neonatal encephalopathy due to birth asphyxia and trauma
- Congenital birth defects (diaphragmatic hernia, cardiovascular anomalies)
- Haemolytic disease and other neonatal jaundice
- Meningitis (pneumococcal, *H influenzae* type B, meningococcal, other bacterial)
- Encephalitis
- Malaria
- Neonatal tetanus
- Neonatal sepsis and other neonatal infections
- Iodine deficiency
- African trypanosomiasis
- Down syndrome
- Klinefelter syndrome
- Chromosomal abnormalities (unbalanced rearrangements, Down syndrome, Edwards syndrome, Patau syndrome, other chromosomal abnormalities)
- Neural tube defects (eg, spina bifida, encephalocele)
- Hypertensive disorders of pregnancy (eclampsia, preeclampsia)
- Autism spectrum disorders (ASD)
- Fetal alcohol syndrome

For autism spectrum disorders (ASD), we identified six studies reporting severity of intellectual disability. We conducted a meta-analysis to produce a severity distribution which we applied to the prevalence of autism to produce severity-specific ID due to autism.

We calculated the prevalence of idiopathic ID by subtracting all severity- and aetiology-specific ID from the severity-specific envelope assuming the residuals to represent idiopathic disability. If the residual was less than 5% of the severity-specific envelope, the prevalence of all aetiology-specific ID was proportionally squeezed to fit within 95% of the envelope, leaving 5% for idiopathic ID.

As we estimated the prevalence of individual aetiology-specific ID by models from the respective parent causes, the squeezing may result in a distorted balance of prevalence estimates within their parent causes. With the aim to maintain consistencies of prevalence within each of the parent causes, we added the difference between the original and the squeezed prevalence estimates to the "motor impairment" sequela if the squeezed sequela represented "motor and cognitive impairment." For autism, we obtained the fraction of cases that result in ID from literature (0.29; 95% CI 0.27–0.30) and applied this fraction to

the subtraction and squeezing processes. We assume all ID cases due to iodine deficiency (cretinism) to result in either severe or profound disability, and Klinefelter syndrome cases that result in ID will have either borderline or mild severity.

In GBD 2013, all aetiology-specific models were squeezed into the overall (IQ <70) envelope. In GBD 2015, 2016, and 2017, we squeezed each model into its discrete severity envelope.

Pelvic inflammatory disease (PID) impairment

Flowchart

Pelvic Inflammatory Disease

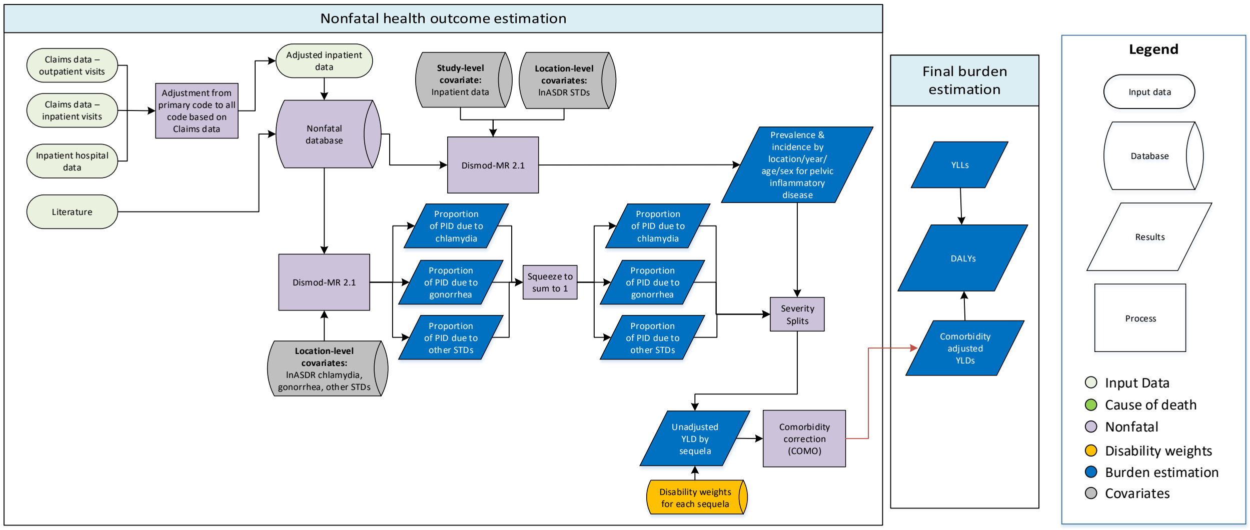

Case definition

Pelvic inflammatory disease (PID) is an infection of the female reproductive organs presenting as a complication of infection by a sexually transmitted disease. It can irreversibly damage the uterus, fallopian tubes, or other parts of the female reproductive system, leading to infertility.

Input data

Model inputs

A systematic review was completed for GBD 2013 on October 28, 2013, using the following search terms:

- (("pelvic inflammatory disease"[Title/Abstract] OR "salpingitis"[Title/Abstract]) AND ("1994"[Date – Publication] : "2013"[Date – Publication]))

In addition, we included studies with confirmed clinical diagnosis in general population.

No systematic review was conducted for GBD 2017. Updates to systematic reviews are performed on an ongoing schedule across all GBD causes, and an update for PID will be performed in the next one to two iterations.

Inpatient and outpatient MarketScan data were extracted as incidence rates, as per the ICD codes above. We also extracted inpatient hospital data and employed two crosswalks derived from MarketScan data. First, we adjusted hospital inpatient data using a ratio of primary diagnoses for PID to all diagnoses, since the sexually transmitted disease (rather than PID, the consequent syndrome) may appear as the primary diagnosis. Second, we adjusted using a ratio of inpatient/outpatient diagnoses, since not all PID must be treated in an inpatient facility. The result is an estimate of total PID incidence.

PID incidence input data

The table below illustrates the data sources used in GBD 2017:

	Incidence
**Site-years (total)**	1270
**Number of countries with data**	33
**Number of GBD regions with data (out of 21 regions)**	12
**Number of GBD super-regions with data (out of 7 super-regions)**	5

A subset of the studies from the systematic review reported the underlying aetiology of PID, allowing us to estimate the proportion of PID due to chlamydia, gonorrhoea, and other sexually transmitted diseases.

Proportion of PID due to	Site-years (total)	Number of countries with data	Number of GBD regions with data (out of 21 regions)	Number of GBD super-regions with data (out of 7 super-regions)
Chlamydia	16	8	6	3
Gonorrhoea	13	6	4	2
Other STDs	18	8	6	3

Health states and disability weights

Health state name	Health state description	Disability weight
Abdominopelvic problem, severe	This person has severe pain in the belly and feels nauseated. The person is anxious and unable to carry out daily activities.	0.324 (0.219–0.442)
Abdominopelvic problem, moderate	This person has pain in the belly and feels nauseated. The person has difficulties with daily activities.	0.114 (0.078–0.159)

Modelling strategy

First, we estimated the total incidence and prevalence of pelvic inflammatory disease using a DisMod-MR 2.1. We used Bayesian priors on remission (13–17) and excess mortality rate (0–0.02).

Covariate	Parameter	beta	Exponentiated beta
All MarketScan, year 2000	Incidence	0.21 (0.16–0.25)	1.23 (1.17–1.29)
lnASDR STDs (location)	Incidence	0.013 (0.0014–0.027)	1.01 (1.00–1.03)

Second, we ran three separate DisMod models for the proportion of PID due to the following three causes: chlamydia, gonorrhoea, and other STDs. For each model, we used the natural log of the age-standardised death rate (lnASDR) of the underlying STD.

Covariate	Parameter	Exponentiated beta
lnASDR gonorrhoea	Proportion	1.14 (1.00–1.33)
lnASDR STDs	Proportion	1.00 (1.00–1.01)

DisMod estimates for the proportions due to each aetiology were proportionally squeezed to sum to 1.

We extracted MarketScan claims data for the first time in GBD 2015 and continue to do so. This allowed us to make two crosswalks on inpatient hospital data (from primary diagnosis to all diagnoses, and from inpatient to all diagnoses) that we had not previously. Additionally, in order to correct for outpatient cases, we applied an age-specific correction factor based on the ratio of MarketScan data to Healthcare Cost and Utilization Project (HCUP) data in USA states where there was overlap in year and location in the data sources. A graph of the ratios across all age groups is shown below, with an overall mean of 2.52.

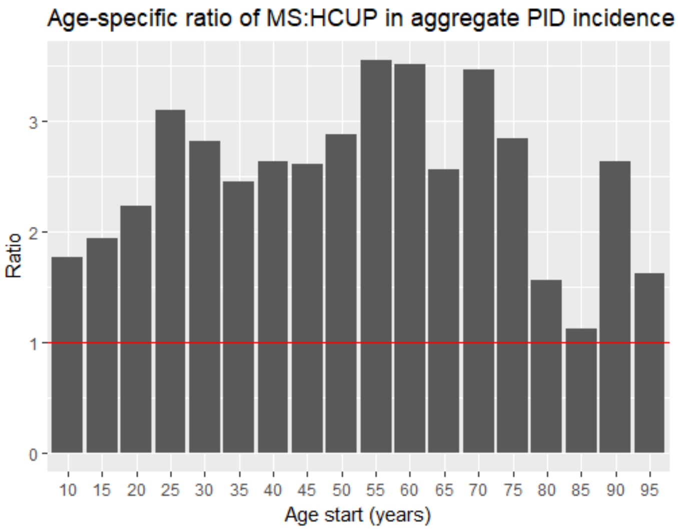

A mean absolute deviation (MAD) outliering process was used to identify hospital data that were unreasonably high or low. No additional changes were made to the estimation process for GBD 2017.

Blindness and vision impairment

Flowcharts

Vision impairment

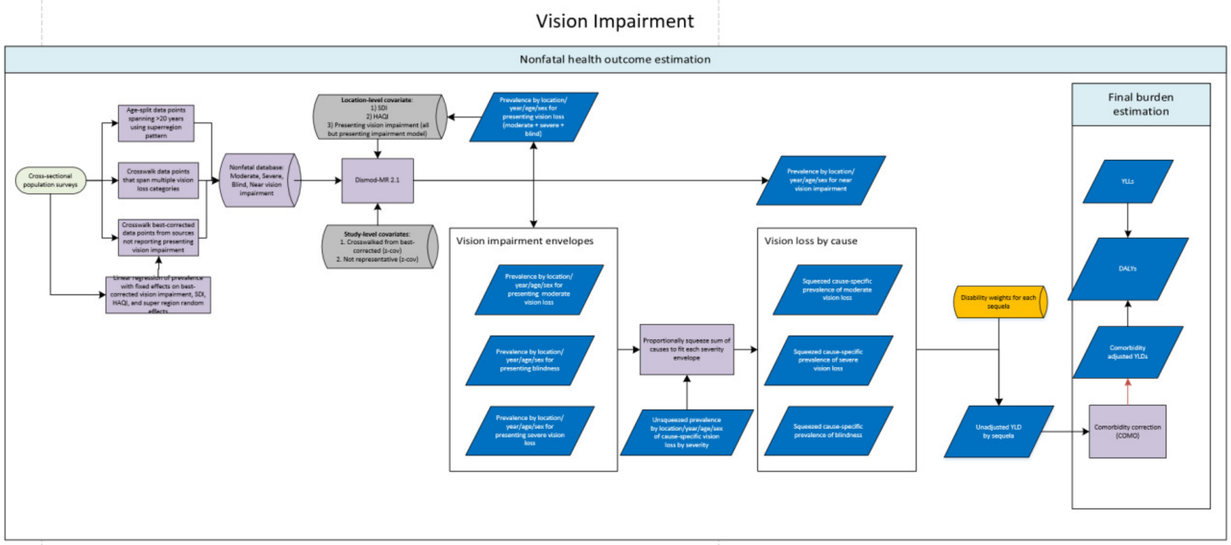

Diabetic retinopathy

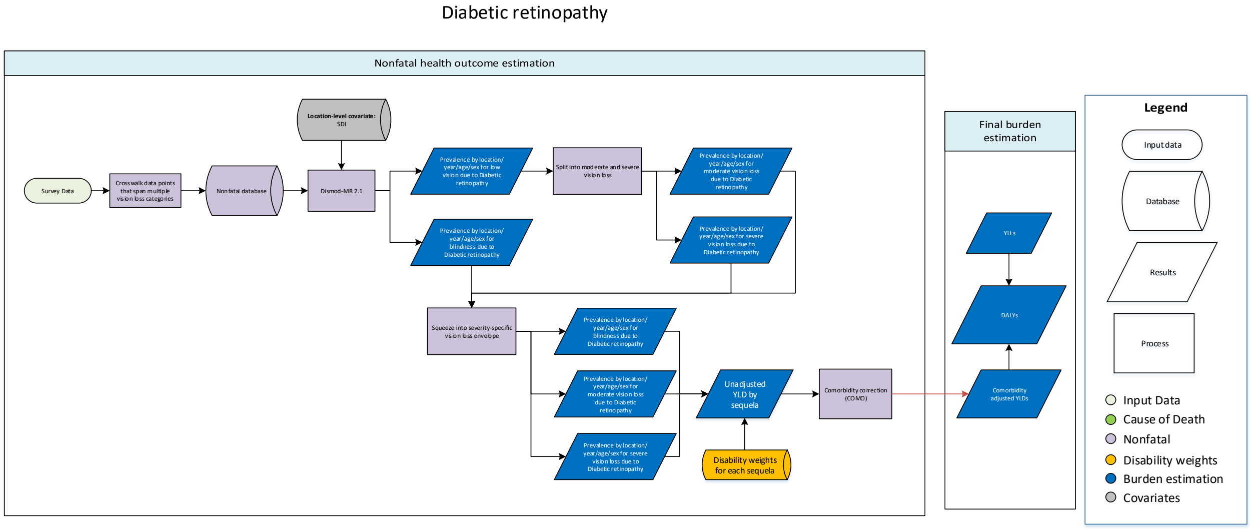

Macular degeneration

Macular degeneration

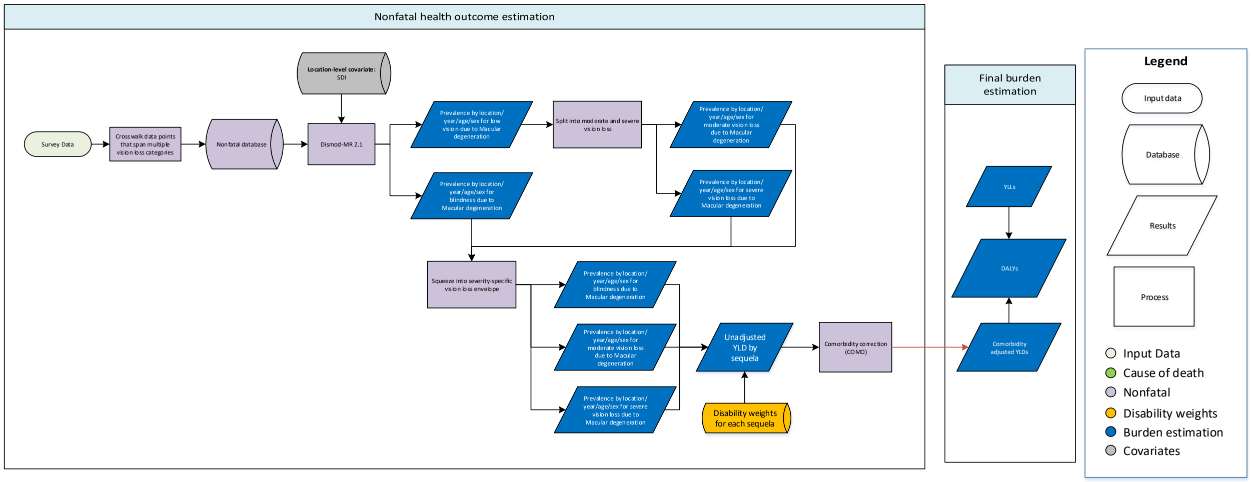

Cataract

Cataract

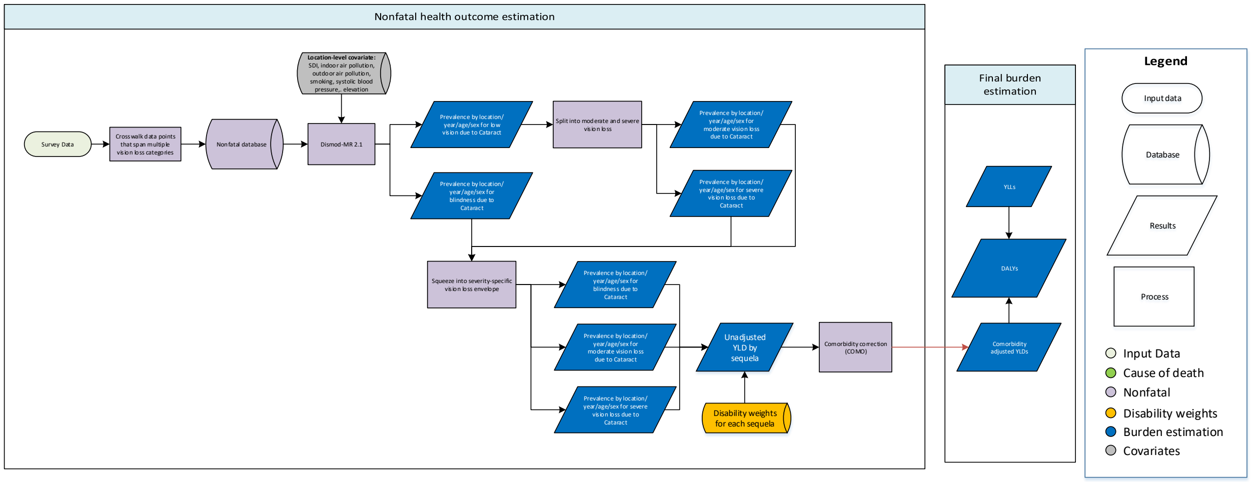

Glaucoma

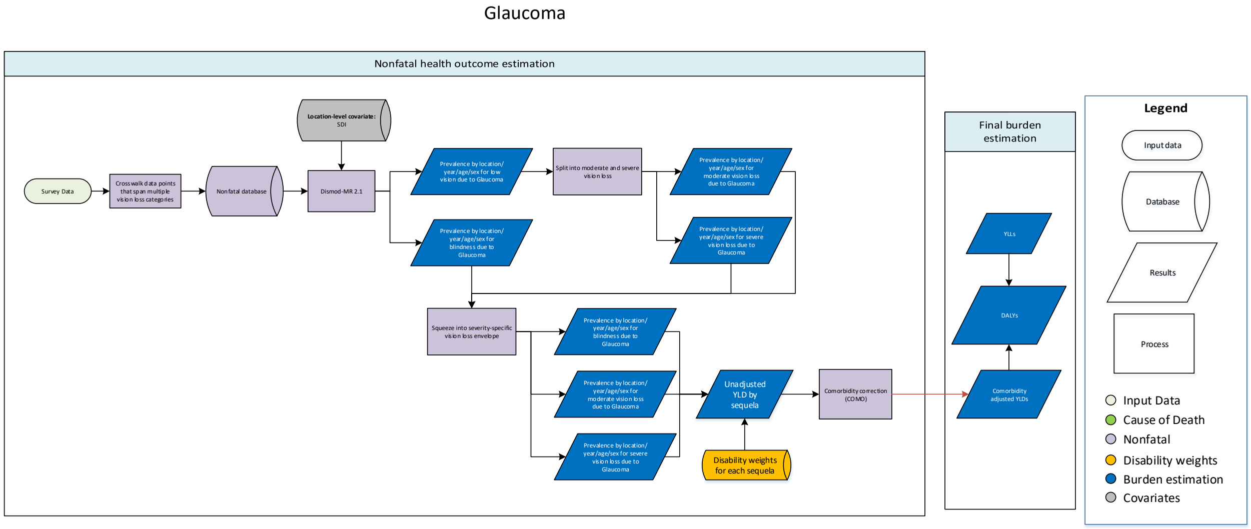

Trachoma

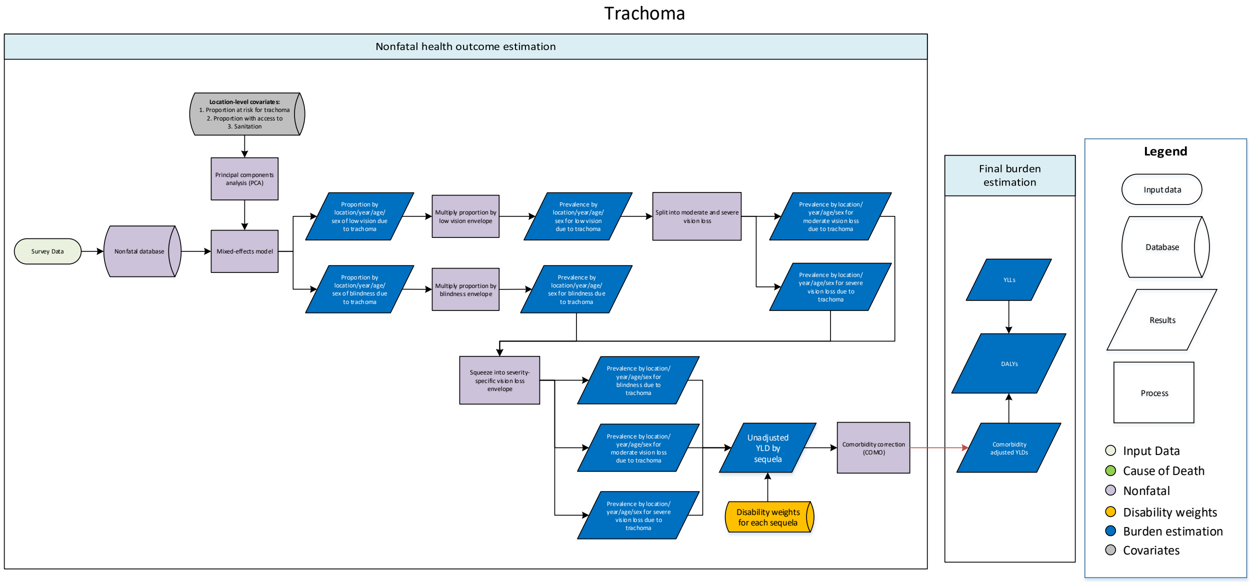

Other vision loss

Other vision loss

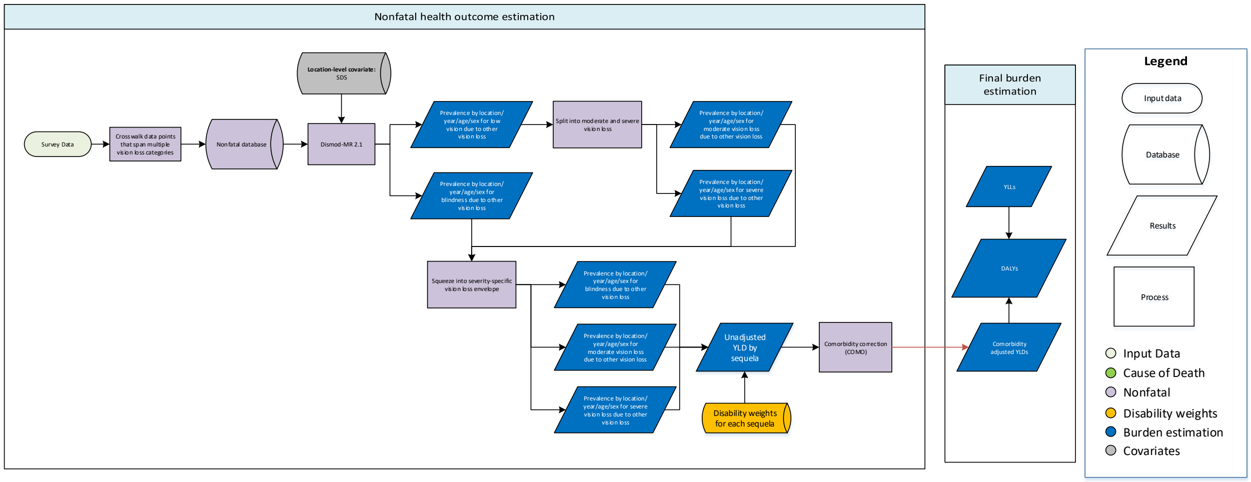

Case definition

We model vision impairment as visual acuity <6/18 according to the Snellen chart. The following impairments are modeled:

Condition	Case definition
Blindness	Visual acuity of <3/60 or <10% visual field around central fixation
Severe vision impairment	≥3/60 and <6/60
Moderate vision impairment	≥6/60 and <6/18
Near vision impairment envelope	Near visual acuity of <6/18 distance equivalent

Near vision impairment describes the progressive inability to focus on near objects as individuals age. This impairs the ability to read. The majority of presbyopia can be corrected by the use of reading glasses, contact lenses, or refractive surgery.

We model vision impairment due to the following causes: uncorrected refractive error, cataract, glaucoma, macular degeneration, diabetic retinopathy, trachoma, vitamin A deficiency, retinopathy of prematurity, meningitis, encephalitis, onchocerciasis, and other vision loss. Vision loss due to vitamin A deficiency, retinopathy of prematurity, meningitis, encephalitis, and onchocerciasis are modelled as part of their underlying cause as described in their respective sections.

Refractive error is blurry vision due to the lens's inability to focus. The blurriness caused by refractive error can be addressed through the use of contact lenses, glasses, or refractive surgery. Cataract is

clouding of the lens of the eye due to protein buildup that impairs vision. Glaucoma is a condition with increased intraocular pressure which can lead to damage of the optic nerve. Macular degeneration is a deterioration of the macula, leading to central vision loss. Diabetic retinopathy is damage to the retina caused by damaged blood vessels that can leak blood into the retina and cause scarring of the retina. Trachoma results from a conjunctival bacterial infection (*Chlamydia trachomatis*) that produces inflammation and scarring which leads to an inversion of the eyelids and eyelashes scratching the cornea, which eventually leads to scarring of the cornea and vision impairment or blindness.

Input data

Model inputs

Data on overall vision impairment come from surveys measuring visual acuity in representative population-based studies, either from publications in peer-reviewed and grey literature or surveys for which we had the unit record data. Data were excluded if no test was used of visual acuity that can be converted to the Snellen scale, and if a study did not assess "presenting" or "best-corrected" vision. A subset of these studies that reported vision loss by cause were used to estimate the prevalence of vision loss due to cataract, glaucoma, macular degeneration, diabetic retinopathy, and other causes.

For GBD 2015, we conducted a systematic review for new sources since GBD 2013 (covering 1/1/2013 – 5/20/2015), using the following search string:

((((glaucoma[Title/Abstract] OR cataract[Title/Abstract] OR macular[Title/Abstract] OR 'refractive error'[Title/Abstract] OR presbyopia[Title/Abstract]) OR (('blindness'[MeSH Terms] OR 'blindness'[All Fields]) OR 'vision, low'[MeSH Terms])) AND ('2013'[PDAT] : '3000'[PDAT])) AND 'humans'[MeSH Terms]) AND (prevalence[Title/Abstract] OR incidence[Title/Abstract] OR epidemiology[Title/Abstract])

This yielded 1,169 results, of which we extracted 20 sources. Furthermore, we extracted from the following nationally representative surveys measuring visual acuity: the WHO Studies on Global Ageing and Adult Health (SAGE) and the United States National Health and Examination Surveys (NHANES).

For GBD 2016 and GBD 2017, we did a comprehensive extraction of the Rapid Assessment of Avoidable Blindness (RAAB) repository (http://raabdata.info/), a database of vision impairment studies in developing settings across the world. There are 266 site-years of data, the majority of which have publicly available reports or publications of the data. A standardised methodology was used by all sources in the repository. This allowed us to use all 185 available reports, 70 of which were newly included for GBD 2017. In addition, we extracted two state-level national surveys from India.

Due to the sparse literature reporting measured near-vision visual acuity, we also extracted data from the following nationally representative studies measuring self-reported near vision loss: SAGE; NHANES; the Surveys of Health, Ageing, and Retirement in Europe (SHARE); the Multi-Country Survey Study on Health and Responsiveness (MCSS); and the World Health Surveys (WHS).

Several adjustments were made to raw data.

1) Where studies reported visual acuity spanning multiple thresholds (eg, <6/60, rather than separate severe and blind estimates), we crosswalked using ratios predicted by a linear regression on age, using data from studies reporting vision loss by each severity.
2) Some studies reported best-corrected vision impairment, but not presenting vision impairment (PVI). We crosswalked these data points using a linear regression of logit-transformed PVI prevalence with fixed effects on best-corrected VI, age, and per capita lag-distributed income (LDI) and super-region random effects. This gave us a predicted PVI data points for these studies not explicitly reporting PVI. These crosswalked data points were flagged with a study-level covariate that increased standard error in DisMod.
3) Where data points spanned more than 20 years of age, we age-split using an algorithm that applies the age-pattern of the super-region to split the data to five-year age groups.

Whereas other vision impairment aetiologies are modelled based on prevalence data, vision impairment due to trachoma is modelled as a proportion of the overall best-corrected vision impairment envelope, a strategy that was chosen based on the nature of available data.

The table below shows the number of site-years of data included in envelope models for GBD 2017, as well as the number of countries, regions, and super-regions represented.

**Blindness impairment envelope**	
	**Prevalence**
**Site-years (total)**	415
**Number of countries with data**	100
**Number of GBD regions with data (out of 21 regions)**	20
**Number of GBD super-regions with data (out of 7 super-regions)**	7

**Severe vision impairment envelope**	
	**Prevalence**
**Site-years (total)**	402
**Number of countries with data**	97
**Number of GBD regions with data (out of 21 regions)**	19
**Number of GBD super-regions with data (out of 7 super-regions)**	7

**Moderate vision impairment envelope**	
	**Prevalence**
**Site-years (total)**	378
**Number of countries with data**	94
**Number of GBD regions with data (out of 21 regions)**	19
**Number of GBD super-regions with data (out of 7 super-regions)**	7

Health states and disability weights

Health state name	Health state description	Disability weight

Distance vision, severe impairment	This person has severe vision loss, which causes difficulty in daily activities, some emotional impact (for example, worry), and some difficulty going outside the home without assistance.	0.184 (0.125–0.259)
Distance vision, moderate impairment	This person has vision problems that make it difficult to recognise faces or objects across a room.	0.031 (0.019–0.049)
Distance vision blindness	This person is completely blind, which causes great difficulty in some daily activities, worry and anxiety, and great difficulty going outside the home without assistance.	0.187 (0.124–0.26)
Near vision loss	This person has difficulty seeing things that are nearer than 3 feet, but has no difficulty with seeing things at a distance.	0.011 (0.005–0.02)

Modelling strategy

We modelled the prevalence of vision loss in two steps. In the first step, we estimated the total prevalence estimates of presenting vision loss: moderate vision impairment, severe vision impairment, blindness, and near vision impairment (presbyopia). We directly derived prevalence of near vision impairment from this step, whereas the remaining three models that reflect different severity levels of distance vision loss continued to the next step.

1) Estimate severity-specific vision impairment (the "envelopes")

First, we ran five DisMod-MR 2.1 models to estimate the total prevalence estimates of presenting vision loss: moderate vision impairment, severe vision impairment, blindness, near vision impairment (presbyopia), and presenting vision impairment (moderate + severe + blindness). The presenting vision impairment model was used as a covariate in the severity-specific models to improve consistency across severities.

Betas and exponentiated values, which can be interpreted as an odds ratio, are shown in the table below for each covariate. The best-corrected covariate indicates whether the test measures visual acuity with the level of correction the patient presents with (best_corrected = 0) or the ophthalmologist provides additional correction via pinhole (best_corrected = 1). Rapid-assessment corrects for potential biases in cause-specific vision loss from studies using expedited visual acuity measurement. Socio-demographic Index (SDI) and Healthcare Access and Quality (HAQ) index are used as location covariates as a proxy measure of access to eye care such as cataract surgery. Data points that were crosswalked from best-corrected visual acuity are flagged with a z-cov to adjust uncertainty in the crosswalk process. For near vision impairment, non-standard severity definition is used to crosswalk between the self-report questionnaire of SHARE (nonstandard) and the other surveys. All self-reported data are crosswalked to examination data based on whether the survey question asked about reading at a comfortable distance or recognising an object at arm's length.

Model	Covariate name	Type	Measure	Beta value	Exponentiated value
Vision impairment due to glaucoma unsqueezed	Socio-demographic Index	Country covariate	Prevalence	-0.45 (-0.94 to -0.039)	0.64 (0.39–0.96)
Blindness due to glaucoma unsqueezed	Socio-demographic Index	Country covariate	Prevalence	-1.04 (-1.59 to -0.39)	0.35 (0.20–0.67)

Vision impairment due to cataract unsqueezed	Socio-demographic Index	Country covariate	Prevalence	-0.36 (-0.67 to -0.033)	0.70 (0.51–0.97)
Blindness due to cataract unsqueezed	Socio-demographic Index	Country covariate	Prevalence	-2.9 (-3 to -2.64)	0.055 (0.050–0.072)
Vision impairment due to macular degeneration unsqueezed	Socio-demographic Index	Country covariate	Prevalence	0.50 (-0.21 to 0.97)	1.65 (0.81–2.65)
Blindness due to macular degeneration unsqueezed	Socio-demographic Index	Country covariate	Prevalence	0.027 (-0.85 to 0.91)	1.03 (0.43–2.48)
Near vision loss	Socio-demographic Index	Country covariate	Prevalence	-0.8 (-1.37 to -0.2)	0.45 (0.25–0.82)
Near vision loss	Non-standard severity definition	Study-level x-covariate	Prevalence	-0.19 (-0.2 to -0.17)	0.83 (0.82–0.85)
Near vision loss	Comfortable reading distance	Study-level x-covariate	Prevalence	-0.49 (-0.5 to -0.46)	0.61 (0.61–0.63)
Near vision loss	Recognise object at arm's length	Study-level x-covariate	Prevalence	0.035 (-0.021 to 0.094)	1.04 (0.98–1.10)
Vision impairment due to other vision loss unsqueezed	Socio-demographic Index	Country covariate	Prevalence	-0.064 (-0.23 to -0.0031)	0.94 (0.79–1.00)
Blindness due to other vision loss unsqueezed	Socio-demographic Index	Country covariate	Prevalence	-0.81 (-0.99 to -0.47)	0.45 (0.37–0.63)
Vision impairment envelope	Socio-demographic Index	Country covariate	Prevalence	-0.29 (-0.52 to -0.065)	0.75 (0.59–0.94)
Blindness impairment envelope	Healthcare Access and Quality index	Country covariate	Prevalence	-0.018 (-0.022 to -0.016)	0.98 (0.98–0.98)
Blindness impairment envelope	Presenting vision impairment	Country covariate	Prevalence	0.33 (0.17–0.48)	1.40 (1.18–1.62)
Blindness impairment envelope	Socio-demographic Index	Country covariate	Prevalence	-0.077 (-0.25 to -0.0038)	0.93 (0.78–1.00)
Blindness impairment envelope	Best-corrected crosswalk	Study-level z-covariate	Prevalence	0.0025 (0.00020–0.0070)	1.00 (1.00–1.01)
Moderate vision impairment envelope	Presenting vision impairment	Country covariate	Prevalence	0.74 (0.65–0.83)	2.09 (1.92–2.29)

Moderate vision impairment envelope	Socio-demographic Index	Country covariate	Prevalence	-0.021 (-0.082 to -0.000018)	0.98 (0.92–1.00)
Moderate vision impairment envelope	Best-corrected crosswalk	Study-level z-covariate	Prevalence	0.10 (0.070–0.13)	1.11 (1.07–1.14)
Severe vision impairment envelope	Presenting vision impairment	Country covariate	Prevalence	0.35 (0.24–0.45)	1.42 (1.28–1.58)
Severe vision impairment envelope	Socio-demographic Index	Country covariate	Prevalence	-0.018 (-0.072 to -0.0014)	0.98 (0.93–1.00)
Severe vision impairment envelope	Best-corrected crosswalk	Study-level z-covariate	Prevalence	0.032 (0.0073–0.062)	1.03 (1.01–1.06)
Vision impairment due to diabetes mellitus	Diabetes age-standardised prevalence (proportion)	Country covariate	Prevalence	1.25 (0.85–1.66)	3.50 (2.35–5.24)
Vision impairment due to diabetes mellitus	Socio-demographic Index	Country covariate	Prevalence	-0.066 (-0.24 to -0.00064)	0.94 (0.79–1.00)
Blindness due to diabetes mellitus unsqueezed	Diabetes age-standardised prevalence (proportion)	Country covariate	Prevalence	3.79 (3.19–4.00)	44.40 (24.41–54.33)
Blindness due to diabetes mellitus unsqueezed	Socio-demographic Index	Country covariate	Prevalence	-1.58 (-1.99 to -0.6)	0.20 (0.14–0.55)
Moderate vision impairment due to uncorrected refractive error unsqueezed	Socio-demographic Index	Country covariate	Prevalence	-0.95 (-1 to -0.82)	0.39 (0.37–0.44)
Severe vision impairment due to uncorrected refractive error unsqueezed	Socio-demographic Index	Country covariate	Prevalence	-0.95 (-1 to -0.83)	0.39 (0.37–0.44)
Blindness due to uncorrected refractive error unsqueezed	Socio-demographic Index	Country covariate	Prevalence	-0.98 (-1 to -0.93)	0.37 (0.37–0.40)

2) Estimate cause-specific vision impairment

In the second step, we estimated the prevalence of vision loss due to multiple causes: refractive error, cataract, glaucoma, macular degeneration, diabetic retinopathy, retinopathy due to prematurity, trachoma, vitamin A deficiency, onchocerciasis, meningitis, and other causes not classified elsewhere. The vision loss due to retinopathy of prematurity, vitamin A deficiency, onchocerciasis, meningitis, tetanus,

and neonatal conditions was modelled as part of these underlying causes. Vision loss due to trachoma is modelled as a proportion of the envelope, with separate proportion models for vision impairment and blindness. For each of cataract, glaucoma, macular degeneration, diabetic retinopathy, and other vision loss, we ran two DisMod-MR 2.1 models: one for the combined category of moderate and severe vision loss due to the cause, and one for blindness due to the cause. Moderate and severe vision loss were modelled together because input data were mostly available for the aggregate. Refractive error was modelled in three models, one for each severity. We used the following age restrictions:

Cause	Minimum age
Cataracts	20
Glaucoma	45
Macular degeneration	45
Diabetic retinopathy	20
Trachoma	15
Other vision loss	0

We estimated the proportions of low vision and blindness due to trachoma using custom mixed-effects models. For consistency, the two models (blindness and low vision) were parameterised identically and differ only in their input data. Our model included fixed effects on age (using cubic splines with knots at 0, 40, and 100 years of age), sex, and a covariate derived from a principal components analysis of the proportion of the population at risk for trachoma and the proportion of the population with access to sanitation. We included nested random effects on super-region, region, and country. Finally, we applied geographic and age restrictions to ensure that we estimate zero proportions in non-endemic locations and among those younger than 15 year of age (as scarring of the cornea due to trachoma takes decades to develop). The prevalence of trachoma at each severity level was calculated by multiplying the proportion of vision loss (vision impairment or blindness) due to trachoma by the corresponding best-corrected vision loss envelope.

We split the moderate plus severe vision loss estimates for each cause into moderate and severe using the ratio of presenting moderate and severe vision loss envelopes. As exceptions, onchocerciasis and retinopathy of prematurity were modelled for moderate and severe vision loss as part of the estimation process of these causes.

We scaled the cause-specific vision loss prevalence to the total prevalence of the best-corrected vision loss envelopes for each of the three severity levels. The final result is prevalence of vision loss due to each cause by severity.

The following changes have been implemented since GBD 2016:

- We added an additional 70 RAAB sources
- Several data points from previous rounds have been reevaluated and then re-extracted or determined to not meet our criteria and were taken out of the model
- We elevated presbyopia and refractive error from the sequela-level to the cause-level within the GBD hierarchy
- We crosswalk self-report near vision data by survey question

Figure 1a. Analytical flowchart for the estimation of cause-specific YLDs by location, age, sex, and year for GBD 2015

Ovals represent data inputs, square boxes represent analytical steps, cylinders represent databases, and parallelograms represent intermediate and final results. The flowchart is color-coded by major estimation component: raw data sources, in pink; data adjustments, in yellow; DisMod-MR 2.1 estimation, in purple; alternative modelling strategies, in light green; injury modeling strategy, in dark green; estimation of impairments and underlying causes, in brown; severity distributions and comorbidity correction, in blue; disability weights in orange; and cause of death and demographic inputs, in grey. GBD = Global Burden of Disease; TB=tuberculosis; HIV =human immunodeficiency virus; AIDS=acquired immunodeficiency syndrome; SF-12=Short Form 12 questions; MEPS=Medical Expenditure Panel Surveys; CSMR=cause-specific mortality rate; SMR=standardized mortality ratio; YLDs=years lived with disability; YLLs=years of life lost.

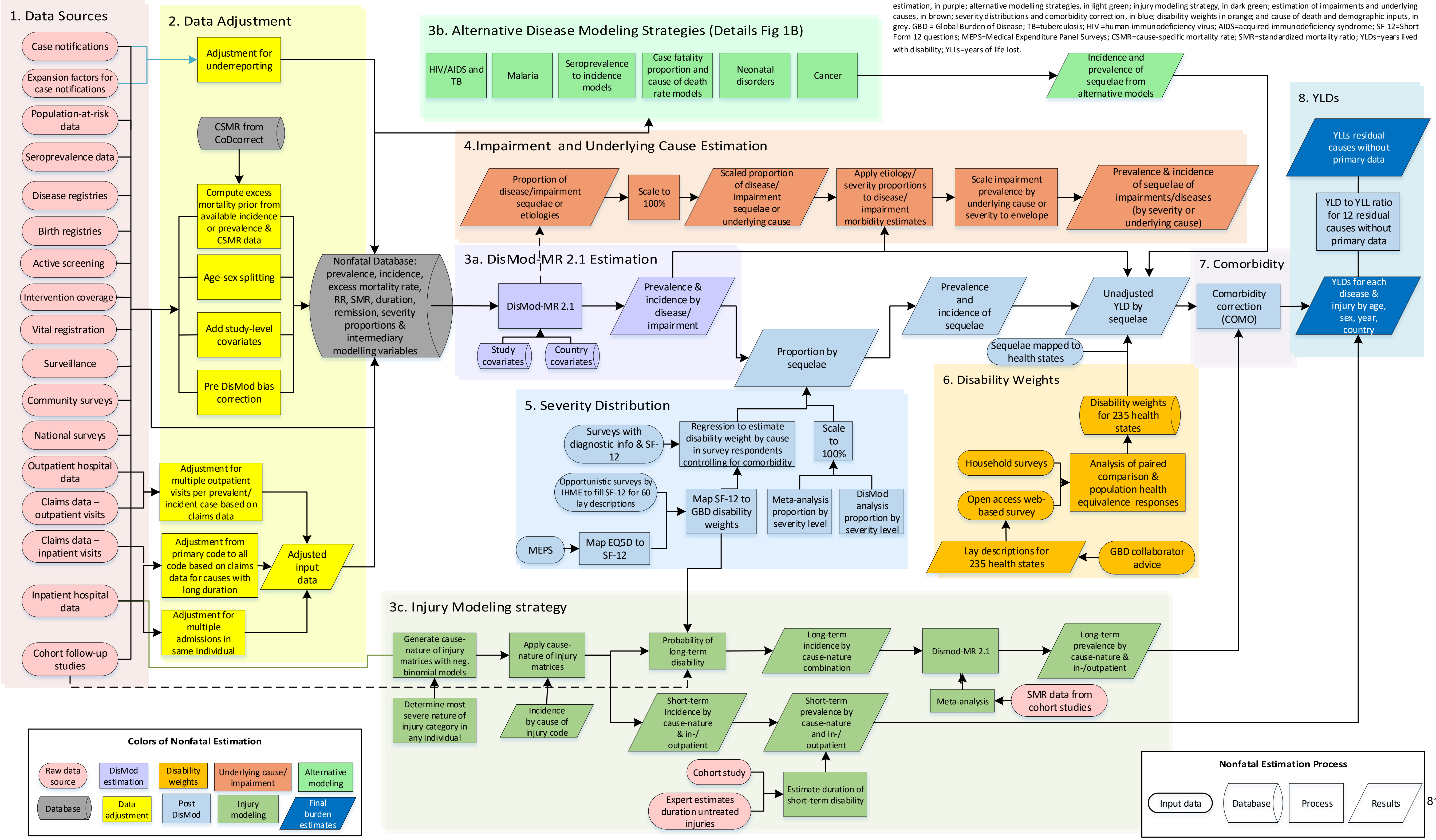

Figure 1b. Analytical flowchart for modeling strategies other than DisMod 2.1 modelling strategies and injuries, GBD 2017

Ovals represent data inputs, square boxes represent analytical steps, cylinders represent databases, and parallelograms represent intermediate and final results. The flowchart is color-coded by major estimation component: raw data sources, in pink; data adjustments, in yellow; DisMod-MR 2.1 estimation, in purple; alternative modelling strategies, in light green; injury modeling strategy, in dark green; estimation of impairments and underlying causes, in brown; severity distributions and comorbidity correction, in blue; disability weights in orange; and cause of death and demographic inputs, in grey. GBD = Global Burden of Disease; TB=tuberculosis; HIV =human immunodeficiency virus; AIDS=acquired immunodeficiency syndrome; SF-12=Short Form 12 questions; MEPS=Medical Expenditure Panel Surveys; CSMR=cause-specific mortality rate; SMR=standardized mortality ratio; YLDs=years lived with disability; YLLs=years of life lost.

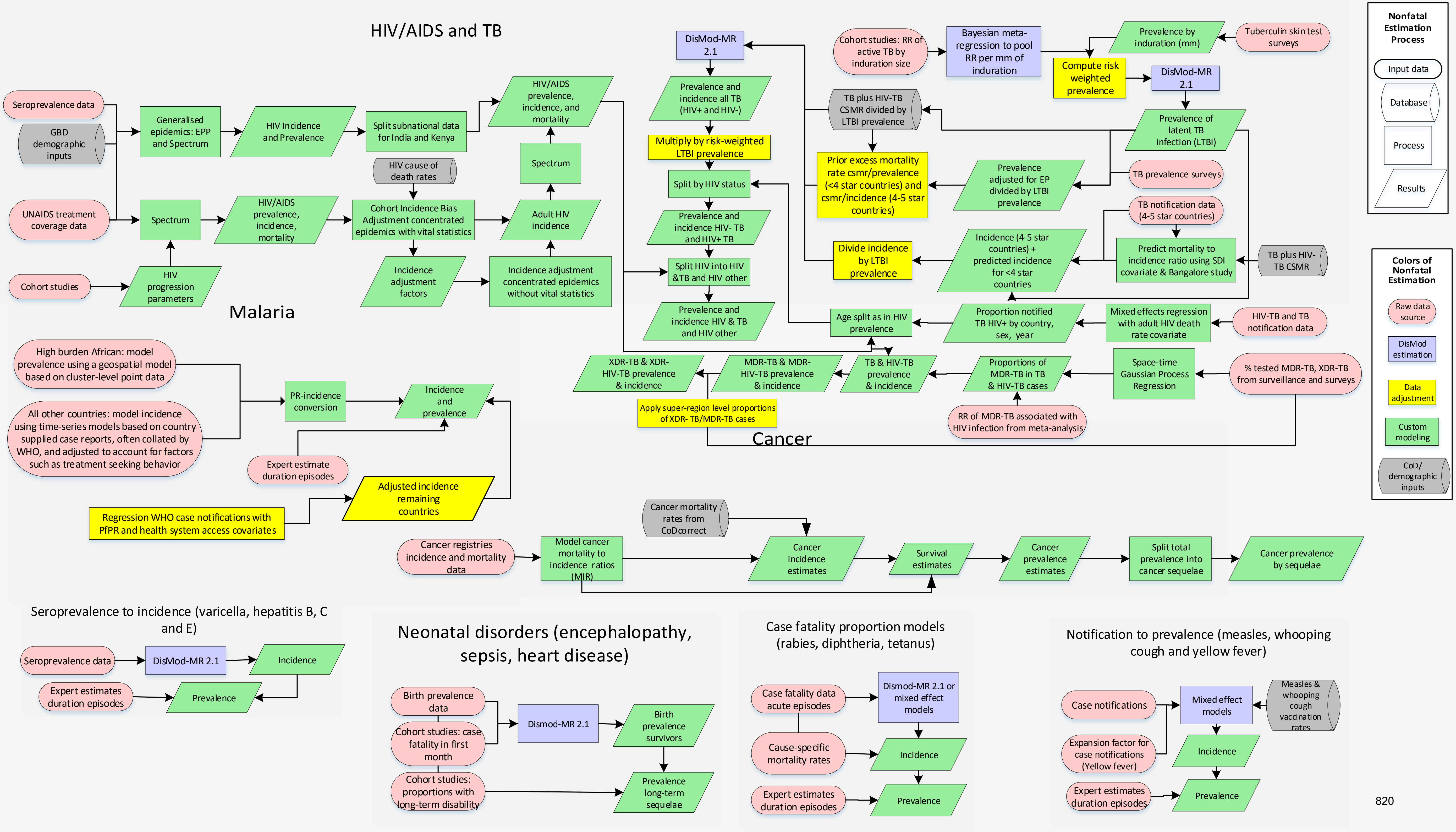

Appendix Figure 2. GBD 2017 DisMod-MR 2.1 analytical cascade

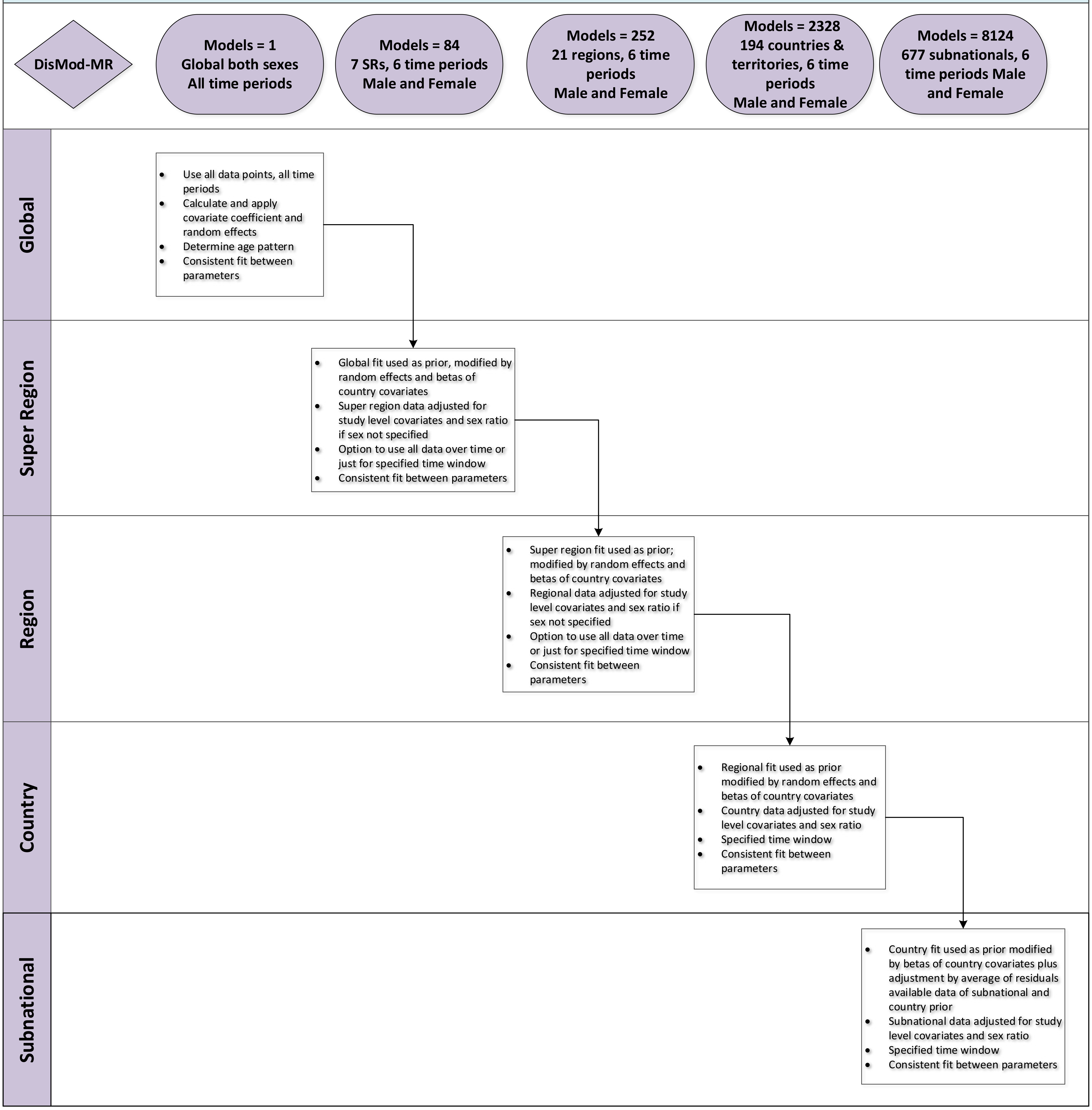

Methods Figure 3: Number of most detailed GBD causes with any non–fatal data by location, 1990 to 2017

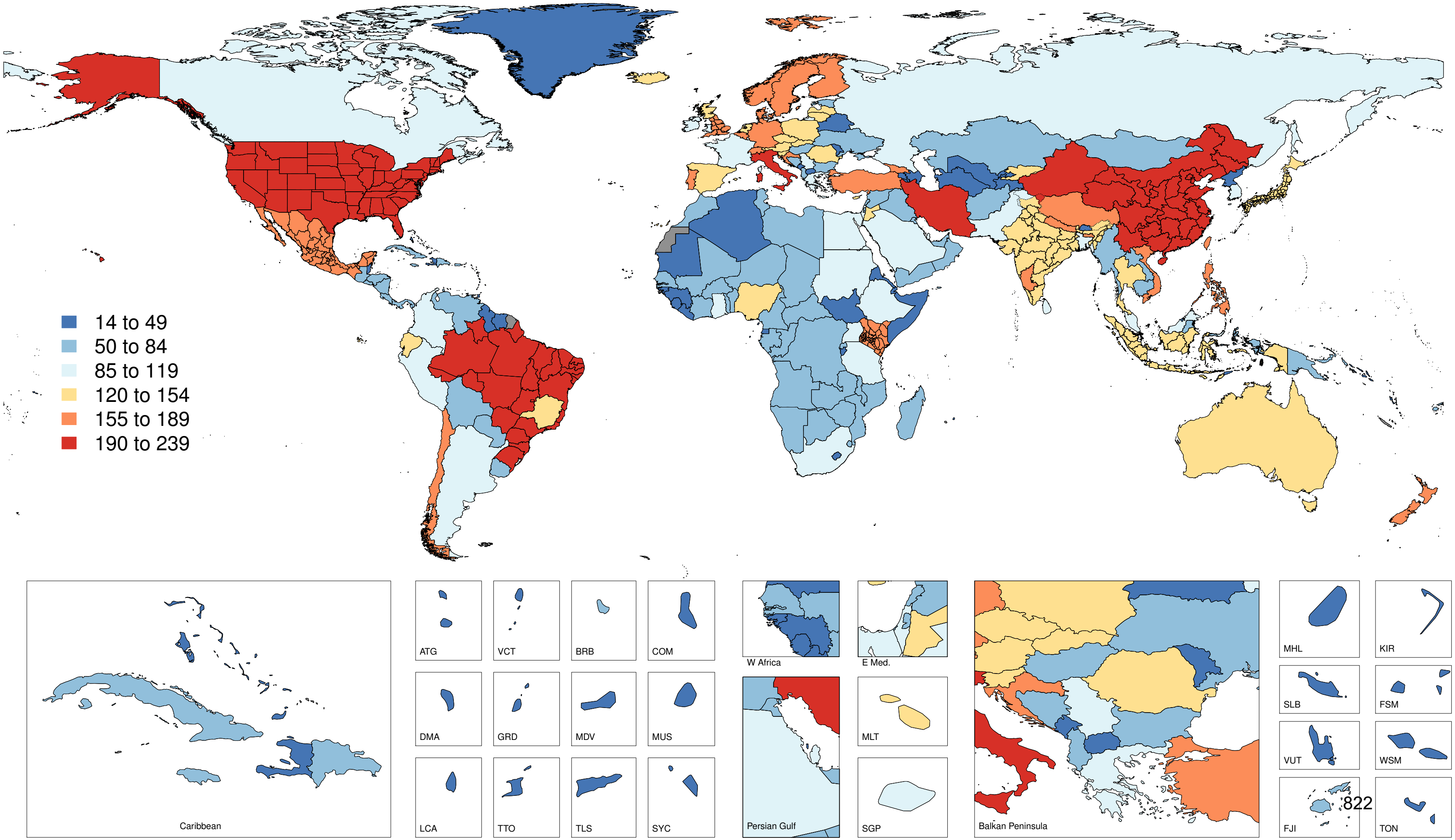

Appendix Table 1. GBD location hierarchy with levels

Geography	Level
Global	0
Low SDI	1
Low-middle SDI	1
Middle SDI	1
High-middle SDI	1
High SDI	1
Central Europe, Eastern Europe, and Central Asia	1
Central Asia	2
Armenia	3
Azerbaijan	3
Georgia	3
Kazakhstan	3
Kyrgyzstan	3
Mongolia	3
Tajikistan	3
Turkmenistan	3
Uzbekistan	3
Central Europe	2
Albania	3
Bosnia and Herzegovina	3
Bulgaria	3
Croatia	3
Czech Republic	3
Hungary	3
Macedonia	3
Montenegro	3
Poland	3
Romania	3
Serbia	3
Slovakia	3
Slovenia	3
Eastern Europe	2
Belarus	3
Estonia	3
Latvia	3
Lithuania	3
Moldova	3
Russian Federation	3
Ukraine	3
High-income	1
Australasia	2
Australia	3
New Zealand	3
High-income Asia-Pacific	2
Brunei	3
Japan	3

Appendix Table 1. GBD location hierarchy with levels	
**Geography**	**Level**
Aichi	4
Akita	4
Aomori	4
Chiba	4
Ehime	4
Fukui	4
Fukuoka	4
Fukushima	4
Gifu	4
Gunma	4
Hiroshima	4
Hokkaidō	4
Hyōgo	4
Ibaraki	4
Ishikawa	4
Iwate	4
Kagawa	4
Kagoshima	4
Kanagawa	4
Kōchi	4
Kumamoto	4
Kyōto	4
Mie	4
Miyagi	4
Miyazaki	4
Nagano	4
Nagasaki	4
Nara	4
Niigata	4
Ōita	4
Okayama	4
Okinawa	4
Ōsaka	4
Saga	4
Saitama	4
Shiga	4
Shimane	4
Shizuoka	4
Tochigi	4
Tokushima	4
Tōkyō	4
Tottori	4
Toyama	4
Wakayama	4
Yamagata	4
Yamaguchi	4

Appendix Table 1. GBD location hierarchy with levels

Geography	Level
Yamanashi	4
South Korea	3
Singapore	3
High-income North America	2
Canada	3
Greenland	3
USA	3
Alabama	4
Alaska	4
Arizona	4
Arkansas	4
California	4
Colorado	4
Connecticut	4
Delaware	4
Washington, DC	4
Florida	4
Georgia	4
Hawaii	4
Idaho	4
Illinois	4
Indiana	4
Iowa	4
Kansas	4
Kentucky	4
Louisiana	4
Maine	4
Maryland	4
Massachusetts	4
Michigan	4
Minnesota	4
Mississippi	4
Missouri	4
Montana	4
Nebraska	4
Nevada	4
New Hampshire	4
New Jersey	4
New Mexico	4
New York	4
North Carolina	4
North Dakota	4
Ohio	4
Oklahoma	4
Oregon	4
Pennsylvania	4

Appendix Table 1. GBD location hierarchy with levels

Geography	Level
Rhode Island	4
South Carolina	4
South Dakota	4
Tennessee	4
Texas	4
Utah	4
Vermont	4
Virginia	4
Washington	4
West Virginia	4
Wisconsin	4
Wyoming	4
Southern Latin America	2
Argentina	3
Chile	3
Uruguay	3
Western Europe	2
Andorra	3
Austria	3
Belgium	3
Cyprus	3
Denmark	3
Finland	3
France	3
Germany	3
Greece	3
Iceland	3
Ireland	3
Israel	3
Italy	3
Luxembourg	3
Malta	3
Netherlands	3
Norway	3
Portugal	3
Spain	3
Sweden	3
Stockholm	4
Sweden except Stockholm	4
Switzerland	3
United Kingdom	3
England	4
East Midlands	5
Derby	6
Derbyshire	6
Leicester	6

Appendix Table 1. GBD location hierarchy with levels	
**Geography**	**Level**
Leicestershire	6
Lincolnshire	6
Northamptonshire	6
Nottingham	6
Nottinghamshire	6
Rutland	6
East of England	5
Bedford	6
Cambridgeshire	6
Central Bedfordshire	6
Essex	6
Hertfordshire	6
Luton	6
Norfolk	6
Peterborough	6
Southend-on-Sea	6
Suffolk	6
Thurrock	6
Greater London	5
Barking and Dagenham	6
Barnet	6
Bexley	6
Brent	6
Bromley	6
Camden	6
Croydon	6
Ealing	6
Enfield	6
Greenwich	6
Hackney	6
Hammersmith and Fulham	6
Haringey	6
Harrow	6
Havering	6
Hillingdon	6
Hounslow	6
Islington	6
Kensington and Chelsea	6
Kingston upon Thames	6
Lambeth	6
Lewisham	6
Merton	6
Newham	6
Redbridge	6
Richmond upon Thames	6
Southwark	6

Appendix Table 1. GBD location hierarchy with levels	
**Geography**	**Level**
Sutton	6
Tower Hamlets	6
Waltham Forest	6
Wandsworth	6
Westminster	6
North East England	5
County Durham	6
Darlington	6
Gateshead	6
Hartlepool	6
Middlesbrough	6
Newcastle upon Tyne	6
North Tyneside	6
Northumberland	6
Redcar and Cleveland	6
South Tyneside	6
Stockton-on-Tees	6
Sunderland	6
North West England	5
Blackburn with Darwen	6
Blackpool	6
Bolton	6
Bury	6
Cheshire East	6
Cheshire West and Chester	6
Cumbria	6
Halton	6
Knowsley	6
Lancashire	6
Liverpool	6
Manchester	6
Oldham	6
Rochdale	6
Salford	6
Sefton	6
St Helens	6
Stockport	6
Tameside	6
Trafford	6
Warrington	6
Wigan	6
Wirral	6
South East England	5
Bracknell Forest	6
Brighton and Hove	6
Buckinghamshire	6

Appendix Table 1. GBD location hierarchy with levels

Geography	Level
East Sussex	6
Hampshire	6
Isle of Wight	6
Kent	6
Medway	6
Milton Keynes	6
Oxfordshire	6
Portsmouth	6
Reading	6
Slough	6
Southampton	6
Surrey	6
West Berkshire	6
West Sussex	6
Windsor and Maidenhead	6
Wokingham	6
South West England	5
Bath and North East Somerset	6
Bournemouth	6
Bristol, City of	6
Cornwall	6
Devon	6
Dorset	6
Gloucestershire	6
North Somerset	6
Plymouth	6
Poole	6
Somerset	6
South Gloucestershire	6
Swindon	6
Torbay	6
Wiltshire	6
West Midlands	5
Birmingham	6
Coventry	6
Dudley	6
Herefordshire, County of	6
Sandwell	6
Shropshire	6
Solihull	6
Staffordshire	6
Stoke-on-Trent	6
Telford and Wrekin	6
Walsall	6
Warwickshire	6
Wolverhampton	6

Appendix Table 1. GBD location hierarchy with levels

Geography	Level
Worcestershire	6
Yorkshire and the Humber	5
Barnsley	6
Bradford	6
Calderdale	6
Doncaster	6
East Riding of Yorkshire	6
Kingston upon Hull, City of	6
Kirklees	6
Leeds	6
North East Lincolnshire	6
North Lincolnshire	6
North Yorkshire	6
Rotherham	6
Sheffield	6
Wakefield	6
York	6
Northern Ireland	4
Scotland	4
Wales	4
Latin America and Caribbean	1
Andean Latin America	2
Bolivia	3
Ecuador	3
Peru	3
Caribbean	2
Antigua and Barbuda	3
The Bahamas	3
Barbados	3
Belize	3
Bermuda	3
Cuba	3
Dominica	3
Dominican Republic	3
Grenada	3
Guyana	3
Haiti	3
Jamaica	3
Puerto Rico	3
Saint Lucia	3
Saint Vincent and the Grenadines	3
Suriname	3
Trinidad and Tobago	3
Virgin Islands	3
Central Latin America	2
Colombia	3

Appendix Table 1. GBD location hierarchy with levels

Geography	Level
Costa Rica	3
El Salvador	3
Guatemala	3
Honduras	3
Mexico	3
Aguascalientes	4
Baja California	4
Baja California Sur	4
Campeche	4
Chiapas	4
Chihuahua	4
Coahuila	4
Colima	4
Distrito Federal	4
Durango	4
Guanajuato	4
Guerrero	4
Hidalgo	4
Jalisco	4
México	4
Michoacán de Ocampo	4
Morelos	4
Nayarit	4
Nuevo León	4
Oaxaca	4
Puebla	4
Querétaro	4
Quintana Roo	4
San Luis Potosí	4
Sinaloa	4
Sonora	4
Tabasco	4
Tamaulipas	4
Tlaxcala	4
Veracruz de Ignacio de la Llave	4
Yucatán	4
Zacatecas	4
Nicaragua	3
Panama	3
Venezuela	3
Tropical Latin America	2
Brazil	3
Acre	4
Alagoas	4
Amapá	4
Amazonas	4

Appendix Table 1. GBD location hierarchy with levels	
**Geography**	**Level**
Bahia	4
Ceará	4
Distrito Federal	4
Espírito Santo	4
Goiás	4
Maranhão	4
Mato Grosso	4
Mato Grosso do Sul	4
Minas Gerais	4
Pará	4
Paraíba	4
Paraná	4
Pernambuco	4
Piauí	4
Rio de Janeiro	4
Rio Grande do Norte	4
Rio Grande do Sul	4
Rondônia	4
Roraima	4
Santa Catarina	4
São Paulo	4
Sergipe	4
Tocantins	4
Paraguay	3
North Africa and Middle East	1
North Africa and Middle East	2
Afghanistan	3
Algeria	3
Bahrain	3
Egypt	3
Iran	3
Iraq	3
Jordan	3
Kuwait	3
Lebanon	3
Libya	3
Morocco	3
Palestine	3
Oman	3
Qatar	3
Saudi Arabia	3
Sudan	3
Syria	3
Tunisia	3
Turkey	3
United Arab Emirates	3

Appendix Table 1. GBD location hierarchy with levels

Geography	Level
Yemen	3
South Asia	1
South Asia	2
Bangladesh	3
Bhutan	3
India	3
Andhra Pradesh	4
Arunachal Pradesh	4
Assam	4
Bihar	4
Chhattisgarh	4
Delhi	4
Goa	4
Gujarat	4
Haryana	4
Himachal Pradesh	4
Jammu and Kashmir	4
Jharkhand	4
Karnataka	4
Kerala	4
Madhya Pradesh	4
Maharashtra	4
Manipur	4
Meghalaya	4
Mizoram	4
Nagaland	4
Odisha	4
Punjab	4
Rajasthan	4
Sikkim	4
Tamil Nadu	4
Telangana	4
Tripura	4
Uttar Pradesh	4
Uttarakhand	4
West Bengal	4
Union Territories other than Delhi	4
Nepal	3
Pakistan	3
Southeast Asia, East Asia, and Oceania	1
East Asia	2
China	3
North Korea	3
Taiwan (Province of China)	3
Oceania	2
American Samoa	3

Appendix Table 1. GBD location hierarchy with levels

Geography	Level
Federated States of Micronesia	3
Fiji	3
Guam	3
Kiribati	3
Marshall Islands	3
Northern Mariana Islands	3
Papua New Guinea	3
Samoa	3
Solomon Islands	3
Tonga	3
Vanuatu	3
Southeast Asia	2
Cambodia	3
Indonesia	3
Laos	3
Malaysia	3
Maldives	3
Mauritius	3
Myanmar	3
Philippines	3
Sri Lanka	3
Seychelles	3
Thailand	3
Timor-Leste	3
Vietnam	3
Sub-Saharan Africa	1
Central sub-Saharan Africa	2
Angola	3
Central African Republic	3
Congo (Brazzaville)	3
DR Congo	3
Equatorial Guinea	3
Gabon	3
Eastern sub-Saharan Africa	2
Burundi	3
Comoros	3
Djibouti	3
Eritrea	3
Ethiopia	3
Kenya	3
Baringo	4
Bomet	4
Bungoma	4
Busia	4
Elgeyo Marakwet	4
Embu	4

Appendix Table 1. GBD location hierarchy with levels

Geography	Level
Garissa	4
Homa Bay	4
Isiolo	4
Kajiado	4
Kakamega	4
Kericho	4
Kiambu	4
Kilifi	4
Kirinyaga	4
Kisii	4
Kisumu	4
Kitui	4
Kwale	4
Laikipia	4
Lamu	4
Machakos	4
Makueni	4
Mandera	4
Marsabit	4
Meru	4
Migori	4
Mombasa	4
Murang'a	4
Nairobi	4
Nakuru	4
Nandi	4
Narok	4
Nyamira	4
Nyandarua	4
Nyeri	4
Samburu	4
Siaya	4
Taita Taveta	4
Tana River	4
Tharaka Nithi	4
Trans Nzoia	4
Turkana	4
Uasin Gishu	4
Vihiga	4
Wajir	4
West Pokot	4
Madagascar	3
Malawi	3
Mozambique	3
Rwanda	3
Somalia	3

Appendix Table 1. GBD location hierarchy with levels	
**Geography**	**Level**
South Sudan	3
Tanzania	3
Uganda	3
Zambia	3
Southern sub-Saharan Africa	2
Botswana	3
Lesotho	3
Namibia	3
South Africa	3
Eastern Cape	4
Free State	4
Gauteng	4
KwaZulu-Natal	4
Limpopo	4
Mpumalanga	4
North West	4
Northern Cape	4
Western Cape	4
Swaziland	3
Zimbabwe	3
Western sub-Saharan Africa	2
Benin	3
Burkina Faso	3
Cameroon	3
Cape Verde	3
Chad	3
Cote d'Ivoire	3
The Gambia	3
Ghana	3
Guinea	3
Guinea-Bissau	3
Liberia	3
Mali	3
Mauritania	3
Niger	3
Nigeria	3
Sao Tome and Principe	3
Senegal	3
Sierra Leone	3
Togo	3

Appendix Table 2. GATHER checklist of information that should be included in reports of global health estimates, with description of compliance and location of information for GBD 2017

#	GATHER checklist item	Description of compliance	Reference
**Objectives and funding**			
1	Define the indicators, populations, and time periods for which estimates were made.	Narrative provided in paper and methods appendix describing indicators, definitions, and populations	Main text (Methods) and methods appendix
2	List the funding sources for the work.	Funding sources listed in paper	Main text (Summary)
**Data Inputs**			
For all data inputs from multiple sources that are synthesized as part of the study:			
3	Describe how the data were identified and how the data were accessed.	Narrative provided in paper and methods appendix describing data seeking methods	Main text (Methods) and methods appendix
4	Specify the inclusion and exclusion criteria. Identify all ad-hoc exclusions.	Narrative provided in paper and methods appendix describing inclusion and exclusion criteria by data type	Main text (Methods) and methods appendix
5	Provide information on all included data sources and their main characteristics. For each data source used, report reference information or contact name/institution, population represented, data collection method, year(s) of data collection, sex and age range, diagnostic criteria or measurement method, and sample size, as relevant.	Metadata for data sources by component, geography, cause, risk, or impairment is available through an interactive, online data source tool	Online data citation tool [link to be added upon acceptance]
6	Identify and describe any categories of input data that have potentially important biases (e.g., based on characteristics listed in item 5).	Summary of known biases by cause included in methods appendix	Methods appendix
For data inputs that contribute to the analysis but were not synthesized as part of the study:			
7	Describe and give sources for any other data inputs.	Included in online data source tool	Online data citation tool [link to be added upon acceptance]
For all data inputs:			
8	Provide all data inputs in a file format from which data can be efficiently extracted (e.g., a spreadsheet as opposed to a PDF), including all relevant meta-data listed in item 5. For any data inputs that cannot be shared due to ethical or legal reasons, such as third-party ownership, provide a contact	Downloads of input data are available through online tools, including data visualization	Online data visualization tools, data query tools, and the Global Health Data Exchange [link to be

	name or the name of the institution that retains the right to the data.		added upon acceptance]
**Data analysis**			
9	Provide a conceptual overview of the data analysis method. A diagram may be helpful.	Flow diagrams of the overall methodological processes, as well as cause-specific modelling processes, have been provided	Main text (Methods) and methods appendix
10	Provide a detailed description of all steps of the analysis, including mathematical formulae. This description should cover, as relevant, data cleaning, data pre-processing, data adjustments and weighting of data sources, and mathematical or statistical model(s).	Flow diagrams and corresponding methodological write-ups for each cause, as well as the demographics and cause of death databases and modelling processes, have been provided	Main text (Methods) and methods appendix
11	Describe how candidate models were evaluated and how the final model(s) were selected.	Details on model evaluation and finalization have been provided	Methods appendix
12	Provide the results of an evaluation of model performance, if done, as well as the results of any relevant sensitivity analysis.	Details on evaluation of model performance have been provided	Methods appendix
13	Describe methods for calculating uncertainty of the estimates. State which sources of uncertainty were, and were not, accounted for in the uncertainty analysis.	Details on uncertainty calculations have been provided	Methods appendix
14	State how analytic or statistical source code used to generate estimates can be accessed.	Access statement provided	Code is provided in an online repository [link to be added upon acceptance]
**Results and Discussion**			
15	Provide published estimates in a file format from which data can be efficiently extracted.	GBD 2017 results are available through online data visualization tools, the Global Health Data Exchange, and the online data query tool	Online data tools [links to be added upon acceptance]
16	Report a quantitative measure of the uncertainty of the estimates (e.g. uncertainty intervals).	Uncertainty intervals are provided with all results	Main text, methods appendix, and online data tools [links to be added upon acceptance]

17	Interpret results in light of existing evidence. If updating a previous set of estimates, describe the reasons for changes in estimates.	Discussion of methodological changes between GBD 2016 and GBD 2017 was provided	Main text (Methods and Discussion) and methods appendix
18	Discuss limitations of the estimates. Include a discussion of any modelling assumptions or data limitations that affect interpretation of the estimates.	Discussion of limitations was provided	Main text (Limitations) and methods appendix

Appendix Table 3: GBD 2017 Cause and Sequela Hierarchy

Level 5 Sequelae are nested under causes. Most detailed sequelae are nested under their parent sequela where they exist

Cause or Sequela Name (Causes in bold)	GBD ID	Level
**All causes**	294	0
**Communicable, maternal, neonatal, and nutritional diseases**	295	1
**HIV/AIDS and sexually transmitted infections**	955	2
**HIV/AIDS**	298	3
**HIV/AIDS - Drug-susceptible Tuberculosis**	948	4
HIV/AIDS - Drug-susceptible Tuberculosis	22682	5
HIV/AIDS - Drug-susceptible Tuberculosis without anemia	5108	6
HIV/AIDS - Drug-susceptible Tuberculosis with mild anemia	5099	6
HIV/AIDS - Drug-susceptible Tuberculosis with moderate anemia	5102	6
HIV/AIDS - Drug-susceptible Tuberculosis with severe anemia	5105	6
**HIV/AIDS - Multidrug-resistant Tuberculosis without extensive drug resistance**	949	4
HIV/AIDS - Multidrug-resistant Tuberculosis without extensive drug resistance	6482	5
HIV/AIDS - Multidrug-resistant Tuberculosis without extensive drug resistance without anemia	5120	6
HIV/AIDS - Multidrug-resistant Tuberculosis without extensive drug resistance with mild anemia	5111	6

GBD 2017 Cause and Sequela Hierarchy<br>Level 5 Sequelae are nested under causes. Most detailed sequelae are nested under their parent sequela where they exist		
**Cause or Sequela Name (Causes in bold)**	**GBD ID**	**Level**
HIV/AIDS - Multidrug-resistant Tuberculosis without extensive drug resistance with moderate anemia	5114	6
HIV/AIDS - Multidrug-resistant Tuberculosis without extensive drug resistance with severe anemia	5117	6
**HIV/AIDS - Extensively drug-resistant Tuberculosis**	950	4
HIV/AIDS - Extensively drug-resistant Tuberculosis	6479	5
HIV/AIDS - Extensively drug-resistant Tuberculosis without anemia	5132	6
HIV/AIDS - Extensively drug-resistant Tuberculosis with mild anemia	5123	6
HIV/AIDS - Extensively drug-resistant Tuberculosis with moderate anemia	5126	6
HIV/AIDS - Extensively drug-resistant Tuberculosis with severe anemia	5129	6
**HIV/AIDS resulting in other diseases**	300	4
HIV/AIDS not on antiretroviral treatment without Tuberculosis	6485	5
AIDS with mild anemia	5087	6
AIDS with moderate anemia	5090	6
AIDS with severe anemia	5093	6
AIDS without anemia	5096	6

GBD 2017 Cause and Sequela Hierarchy<br>Level 5 Sequelae are nested under causes. Most detailed sequelae are nested under their parent sequela where they exist		
**Cause or Sequela Name (Causes in bold)**	**GBD ID**	**Level**
Early HIV with mild anemia	5051	6
Early HIV with moderate anemia	5054	6
Early HIV with severe anemia	5057	6
Early HIV without anemia	5060	6
Symptomatic HIV with mild anemia	5063	6
Symptomatic HIV with moderate anemia	5066	6
Symptomatic HIV with severe anemia	5069	6
Symptomatic HIV without anemia	5072	6
HIV/AIDS on antiretroviral treatment without Tuberculosis	6488	5
HIV/AIDS with antiretroviral treatment with mild anemia	5075	6
HIV/AIDS with antiretroviral treatment with moderate anemia	5078	6
HIV/AIDS with antiretroviral treatment with severe anemia	5081	6
HIV/AIDS with antiretroviral treatment without anemia	5084	6
**Sexually transmitted infections excluding HIV**	393	3

GBD 2017 Cause and Sequela Hierarchy

Level 5 Sequelae are nested under causes. Most detailed sequelae are nested under their parent sequela where they exist

Cause or Sequela Name (Causes in bold)	GBD ID	Level
**Syphilis**	394	4
Early syphilis	6326	5
Asymptomatic early syphilis infection	1202	6
Mild early syphilis infection	1203	6
Tertiary syphilis	6872	5
Asymptomatic adult tertiary syphilis	5156	6
Cardiovascular complications due to adult tertiary syphilis	5153	6
Neurological problems and cardiovascular complications due to adult tertiary syphilis	2048	6
Neurological problems due to adult tertiary syphilis	5150	6
Severe disfigurement and cardiovascular complications due to adult tertiary syphilis	2051	6
Severe disfigurement and neurological problems due to adult tertiary syphilis	2054	6
Severe disfigurement due to adult tertiary syphilis	5147	6
Severe disfigurement, neurological problems, and cardiovascular complications due to adult tertiary syphilis	2057	6
**Chlamydial infection**	395	4

GBD 2017 Cause and Sequela Hierarchy<br>Level 5 Sequelae are nested under causes. Most detailed sequelae are nested under their parent sequela where they exist		
**Cause or Sequela Name (Causes in bold)**	**GBD ID**	**Level**
Chlamydia episode	6239	5
Asymptomatic chlamydial infection	918	6
Mild chlamydial infection	917	6
Chlamydial infection complications	6242	5
Epididymo-orchitis due to chlamydial infection	214	6
Moderate pelvic inflammatory diseases due to chlamydial infection	215	6
Severe pelvic inflammatory diseases due to chlamydial infection	216	6
Primary infertility due to chlamydial infection	217	6
Secondary infertility due to chlamydial infection	218	6
**Gonococcal infection**	396	4
Gonococcal infection complications	6398	5
Epididymo-orchitis due to gonococcal infection	219	6
Moderate pelvic inflammatory diseases due to gonococcal infection	220	6
Severe pelvic inflammatory diseases due to gonococcal infection	221	6

Cause or Sequela Name (Causes in bold)	GBD ID	Level
Gonorrhoea episode	6401	5
Asymptomatic gonococcal infection	920	6
Mild gonococcal infection	919	6
Primary infertility due to gonococcal infection	222	6
Secondary infertility due to gonococcal infection	223	6
**Trichomoniasis**	397	4
Trichomoniasis	6893	5
Asymptomatic trichomoniasis infection	922	6
Acute trichomoniasis infection	921	6
**Genital herpes**	398	4
Genital herpes	6392	5
Asymptomatic genital herpes	831	6
Symptomatic genital herpes	788	6
Moderate infection due to initial genital herpes episode	942	6

GBD 2017 Cause and Sequela Hierarchy<br>Level 5 Sequelae are nested under causes. Most detailed sequelae are nested under their parent sequela where they exist		
Cause or Sequela Name (Causes in bold)	GBD ID	Level
**Other sexually transmitted infections**	399	4
Other sexually transmitted diseases	22847	5
Primary infertility due to other sexually transmitted diseases	226	6
Secondary infertility due to other sexually transmitted diseases	227	6
Moderate pelvic inflammatory diseases due to other sexually transmitted diseases	224	6
Severe pelvic inflammatory diseases due to other sexually transmitted diseases	225	6
Other sexually transmitted diseases residual	808	6
**Respiratory infections and tuberculosis**	956	2
**Tuberculosis**	297	3
**Latent tuberculosis infection**	954	4
Latent tuberculosis infection	2192	5
**Drug-susceptible tuberculosis**	934	4
Drug-susceptible tuberculosis	1493	5
**Multidrug-resistant tuberculosis without extensive drug resistance**	946	4

GBD 2017 Cause and Sequela Hierarchy<br>Level 5 Sequelae are nested under causes. Most detailed sequelae are nested under their parent sequela where they exist		
**Cause or Sequela Name (Causes in bold)**	**GBD ID**	**Level**
Multidrug-resistant tuberculosis without extensive drug resistance	1496	5
**Extensively drug-resistant tuberculosis**	947	4
Extensively drug-resistant tuberculosis	1499	5
**Lower respiratory infections**	322	3
Lower respiratory infection episode	6569	5
Moderate lower respiratory infections	669	6
Severe lower respiratory infections	670	6
Guillain-Barré syndrome due to lower respiratory infections	671	5
**Upper respiratory infections**	328	3
Upper respiratory infection episode	6926	5
Mild upper respiratory infections	15	6
Moderate upper respiratory infections	16	6
Guillain-Barré syndrome due to upper respiratory infections	17	5
**Otitis media**	329	3

GBD 2017 Cause and Sequela Hierarchy		
Level 5 Sequelae are nested under causes. Most detailed sequelae are nested under their parent sequela where they exist		
**Cause or Sequela Name (Causes in bold)**	**GBD ID**	**Level**
Acute otitis media	18	5
Chronic otitis media	6263	5
Mild hearing loss due to chronic otitis media	1054	6
Mild hearing loss with ringing due to chronic otitis media	1055	6
Moderate hearing loss due to chronic otitis media	1104	6
Moderate hearing loss with ringing due to chronic otitis media	1105	6
Severe infectious complications due to chronic otitis media	829	6
Vertigo with mild hearing loss and ringing due to chronic otitis media	973	6
Vertigo with mild hearing loss due to chronic otitis media	972	6
Vertigo with moderate hearing loss and ringing due to chronic otitis media	1103	6
Vertigo with moderate hearing loss due to chronic otitis media	1102	6
**Enteric infections**	957	2
**Diarrheal diseases**	302	3
Diarrheal disease episode	6308	5

GBD 2017 Cause and Sequela Hierarchy

Level 5 Sequelae are nested under causes. Most detailed sequelae are nested under their parent sequela where they exist

Cause or Sequela Name (Causes in bold)	GBD ID	Level
Mild diarrheal diseases	665	6
Moderate diarrheal diseases	666	6
Severe diarrheal diseases	667	6
Guillain-Barré syndrome due to diarrheal diseases	668	5
**Typhoid and paratyphoid**	958	3
**Typhoid fever**	319	4
Typhoid fever episode	6911	5
Acute typhoid infection	7	6
Severe typhoid fever	8	6
Typhoid fever complications	6908	5
Gastrointestinal bleeding due to typhoid	971	6
Intestinal perforation due to typhoid	9	6
**Paratyphoid fever**	320	4
Paratyphoid fever episode	6734	5

GBD 2017 Cause and Sequela Hierarchy<br>Level 5 Sequelae are nested under causes. Most detailed sequelae are nested under their parent sequela where they exist		
Cause or Sequela Name (Causes in bold)	GBD ID	Level
Acute paratyphoid infection	10	6
Moderate paratyphoid fever	11	6
Severe paratyphoid fever	12	6
Intestinal perforation due to paratyphoid	13	5
**Invasive Non-typhoidal Salmonella (iNTS)**	959	3
Severe acute iNTS	5555	5
**Other intestinal infectious diseases**	321	3
Other intestinal infectious diseases	14	5
**Neglected tropical diseases and malaria**	344	2
**Malaria**	345	3
Malaria parasitemia	22685	5
Asymptomatic malaria parasitemia (PfPR)	1056	6
Mild anemia due to malaria parasitemia (PfPR)	1057	6
Moderate anemia due to malaria parasitemia (PfPR)	1058	6

GBD 2017 Cause and Sequela Hierarchy

Level 5 Sequelae are nested under causes. Most detailed sequelae are nested under their parent sequela where they exist

Cause or Sequela Name (Causes in bold)	GBD ID	Level
Severe anemia due to malaria parasitemia (PfPR)	1059	6
Asymptomatic malaria vivax (PvPR)	5615	6
Mild anemia due to malaria vivax (PvPR)	5606	6
Moderate anemia due to malaria vivax (PvPR)	5609	6
Severe anemia due to malaria vivax (PvPR)	5612	6
Malaria episode	6581	5
Mild malaria	121	6
Mild malaria with mild anemia	5027	6
Mild malaria with moderate anemia	5030	6
Mild malaria with severe anemia	5033	6
Moderate malaria	122	6
Moderate malaria with mild anemia	5036	6
Moderate malaria with moderate anemia	5039	6
Moderate malaria with severe anemia	5042	6

GBD 2017 Cause and Sequela Hierarchy

Level 5 Sequelae are nested under causes. Most detailed sequelae are nested under their parent sequela where they exist

Cause or Sequela Name (Causes in bold)	GBD ID	Level
Severe malaria	123	6
Severe malaria with mild anemia	5018	6
Severe malaria with moderate anemia	5021	6
Severe malaria with severe anemia	5024	6
Malaria complications	6578	5
Moderate motor impairment due to malaria	884	6
Moderate motor impairment with blindness and epilepsy due to malaria	887	6
Moderate motor impairment with blindness due to malaria	885	6
Moderate motor impairment with epilepsy due to malaria	886	6
Moderate motor plus cognitive impairment with blindness and epilepsy due to malaria	890	6
Moderate motor plus cognitive impairment with blindness due to malaria	888	6
Moderate motor plus cognitive impairment with epilepsy due to malaria	889	6
Severe motor impairment due to malaria	891	6
Severe motor impairment with blindness and epilepsy due to malaria	894	6

GBD 2017 Cause and Sequela Hierarchy

Level 5 Sequelae are nested under causes. Most detailed sequelae are nested under their parent sequela where they exist

Cause or Sequela Name (Causes in bold)	GBD ID	Level
Severe motor impairment with blindness due to malaria	892	6
Severe motor impairment with epilepsy due to malaria	893	6
Severe motor plus cognitive impairment with blindness and epilepsy due to malaria	897	6
Severe motor plus cognitive impairment with blindness due to malaria	895	6
Severe motor plus cognitive impairment with epilepsy due to malaria	896	6
**Chagas disease**	346	3
Asymptomatic Chagas disease	935	5
Acute Chagas disease	124	5
Symptomatic chronic Chagas infection	6866	5
Atrial fibrillation and flutter due to Chagas disease	125	6
Mild chronic digestive disease due to Chagas disease	126	6
Moderate chronic digestive disease due to Chagas disease	127	6
Controlled, medically managed heart failure due to Chagas disease	5735	6
Mild heart failure due to Chagas disease	128	6

GBD 2017 Cause and Sequela Hierarchy

Level 5 Sequelae are nested under causes. Most detailed sequelae are nested under their parent sequela where they exist

Cause or Sequela Name (Causes in bold)	GBD ID	Level
Moderate heart failure due to Chagas disease	129	6
Severe heart failure due to Chagas disease	130	6
**Leishmaniasis**	347	3
**Visceral leishmaniasis**	348	4
Visceral leishmaniasis	6953	5
Moderate visceral leishmaniasis	131	6
Severe visceral leishmaniasis	132	6
**Cutaneous and mucocutaneous leishmaniasis**	349	4
Cutaneous and mucocutaneous leishmaniasis	133	5
**African trypanosomiasis**	350	3
Trypanosomiasis Gambiense	6899	5
Skin disfigurement due to Trypanosoma brucei gambiense	1454	6
Sleeping sickness due to Trypanosoma brucei gambiense	1457	6
Trypanosomiasis Rhodesiense	6902	5

GBD 2017 Cause and Sequela Hierarchy		
Level 5 Sequelae are nested under causes. Most detailed sequelae are nested under their parent sequela where they exist		
**Cause or Sequela Name (Causes in bold)**	**GBD ID**	**Level**
Skin disfigurement due to Trypanosoma brucei rhodesiense	1463	6
Sleeping sickness due to Trypanosoma brucei rhodesiense	1460	6
**Schistosomiasis**	351	3
Mild schistosomiasis	136	5
Anemia due to schistosomiasis	6161	5
Mild anemia due to schistosomiasis	144	6
Moderate anemia due to schistosomiasis	145	6
Severe anemia due to schistosomiasis	146	6
Schistosomiasis complications	6779	5
Ascites due to schistosomiasis	140	6
Bladder pathology due to schistosomiasis	142	6
Dysuria due to schistosomiasis	141	6
Hematemesis due to schistosomiasis	138	6
Hepatomegaly due to schistosomiasis	139	6

GBD 2017 Cause and Sequela Hierarchy

Level 5 Sequelae are nested under causes. Most detailed sequelae are nested under their parent sequela where they exist

Cause or Sequela Name (Causes in bold)	GBD ID	Level
Hydronephrosis due to schistosomiasis	143	6
Mild diarrhea due to schistosomiasis	137	6
**Cysticercosis**	352	3
Neurocysticercosis with epilepsy	689	5
**Cystic echinococcosis**	353	3
Cystic echinococcosis	22688	5
Abdominal problems due to cystic echinococcosis	147	6
Chronic respiratory disease due to cystic echinococcosis	148	6
Epilepsy due to echinococcosis	787	6
**Lymphatic filariasis**	354	3
Prevalence of detectable microfiliaria due to lymphatic filariasis	149	5
Lymphatic filariasis complications	6572	5
Acute adenolymphangitis due to lymphatic filariasis	4946	6
Hydrocele due to lymphatic filariasis	151	6

GBD 2017 Cause and Sequela Hierarchy<br>Level 5 Sequelae are nested under causes. Most detailed sequelae are nested under their parent sequela where they exist		
**Cause or Sequela Name (Causes in bold)**	**GBD ID**	**Level**
Lymphedema due to lymphatic filariasis	150	6
**Onchocerciasis**	355	3
Asymptomatic onchocerciasis	956	5
Skin disease due to onchocerciasis	6803	5
Mild skin disease due to onchocerciasis	152	6
Mild skin disease without itch due to onchocerciasis	673	6
Moderate skin disease due to onchocerciasis	153	6
Severe skin disease due to onchocerciasis	658	6
Severe skin disease without itch due to onchocerciasis	674	6
Vision loss due to onchocerciasis	6965	5
Blindness due to onchocerciasis	875	6
Moderate vision impairment due to onchocerciasis	876	6
Severe vision impairment due to onchocerciasis	877	6
**Trachoma**	356	3

GBD 2017 Cause and Sequela Hierarchy

Level 5 Sequelae are nested under causes. Most detailed sequelae are nested under their parent sequela where they exist

Cause or Sequela Name (Causes in bold)	GBD ID	Level
Vision impairment due to trachoma	22691	5
Moderate vision impairment due to trachoma	154	6
Severe vision impairment due to trachoma	155	6
Blindness due to trachoma	156	6
**Dengue**	357	3
Dengue episode	6299	5
Moderate dengue	157	6
Severe dengue	158	6
Post-dengue chronic fatigue syndrome	159	5
**Yellow fever**	358	3
Asymptomatic yellow fever	1112	5
Yellow fever episode	6977	5
Moderate yellow fever	160	6
Severe yellow fever	161	6

GBD 2017 Cause and Sequela Hierarchy

Level 5 Sequelae are nested under causes. Most detailed sequelae are nested under their parent sequela where they exist

Cause or Sequela Name (Causes in bold)	GBD ID	Level
**Rabies**	359	3
Rabies	162	5
**Intestinal nematode infections**	360	3
**Ascariasis**	361	4
Asymptomatic ascariasis	957	5
Ascariasis complications	6176	5
Heavy infestation of ascariasis	163	6
Mild abdominopelvic problems due to ascariasis	164	6
Severe wasting due to ascariasis	165	6
**Trichuriasis**	362	4
Asymptomatic trichuriasis	958	5
Trichuriasis complications	6896	5
Heavy infestation of trichuriasis	166	6
Mild abdominopelvic problems due to trichuriasis	167	6

GBD 2017 Cause and Sequela Hierarchy

Level 5 Sequelae are nested under causes. Most detailed sequelae are nested under their parent sequela where they exist

Cause or Sequela Name (Causes in bold)	GBD ID	Level
Severe wasting due to trichuriasis	168	6
**Hookworm disease**	363	4
Asymptomatic hookworm disease	974	5
Anemia due to hookworm disease	6149	5
Mild anemia due to hookworm disease	172	6
Moderate anemia due to hookworm disease	173	6
Severe anemia due to hookworm disease	174	6
Hookworm disease complications	6494	5
Heavy infestation of hookworm	169	6
Mild abdominopelvic problems due to hookworm disease	170	6
Severe wasting due to hookworm disease	171	6
**Food-borne trematodiases**	364	3
Asymptomatic food-borne tremodiases	6182	5
Asymptomatic clonorchiasis	959	6

GBD 2017 Cause and Sequela Hierarchy

Level 5 Sequelae are nested under causes. Most detailed sequelae are nested under their parent sequela where they exist

Cause or Sequela Name (Causes in bold)	GBD ID	Level
Asymptomatic fascioliasis	960	6
Asymptomatic intestinal fluke infection	961	6
Asymptomatic opisthorchiasis	962	6
Asymptomatic paragonimiasis	963	6
Food-borne tremodiases complications	6371	5
Cerebral paragonimiasis	175	6
Heavy clonorchiasis due to food-borne trematodiases	908	6
Heavy fascioliasis due to food-borne trematodiases	909	6
Heavy intestinal fluke infection due to food-borne trematodiases	910	6
Heavy opisthorchiasis due to food-borne trematodiases	911	6
Mild paragonimiasis due to food-borne trematodiases	1341	6
Moderate paragonimiasis due to food-borne trematodiases	1342	6
Severe paragonimiasis due to food-borne trematodiases	1343	6
**Leprosy**	405	3

Cause or Sequela Name (Causes in bold)	GBD ID	Level
Leprosy	22694	5
Disfigurement level 1 due to leprosy	237	6
Disfigurement level 2 due to leprosy	238	6
**Ebola**	843	3
Ebola cases	1184	5
Post-Ebola chronic fatigue syndrome	1185	5
**Zika virus**	935	3
Zika virus episode	6983	5
Asymptomatic Zika infection	1520	6
Acute Zika infection	1511	6
Zika virus complications	6980	5
Congenital Zika syndrome	1517	6
Guillain–Barré syndrome due to Zika infection	1514	6
**Guinea worm disease**	936	3

GBD 2017 Cause and Sequela Hierarchy

Level 5 Sequelae are nested under causes. Most detailed sequelae are nested under their parent sequela where they exist

Cause or Sequela Name (Causes in bold)	GBD ID	Level
Guinea worm disease complications	6407	5
Mild pain due to Guinea worm emergence	2204	6
Moderate pain and limited mobility due to guinea worm	5537	5
**Other neglected tropical diseases**	365	3
Acute infection due to other neglected tropical diseases	176	5
Anemia due to other neglected tropical diseases	6158	5
Mild anemia due to other neglected tropical diseases	177	6
Moderate anemia due to other neglected tropical diseases	178	6
Severe anemia due to other neglected tropical diseases	179	6
**Other infectious diseases**	961	2
**Meningitis**	332	3
**Pneumococcal meningitis**	333	4
Acute pneumococcal meningitis	19	5
Pneumococcal meningitis complications	6998	5

GBD 2017 Cause and Sequela Hierarchy		
Level 5 Sequelae are nested under causes. Most detailed sequelae are nested under their parent sequela where they exist		
**Cause or Sequela Name (Causes in bold)**	**GBD ID**	**Level**
Mild hearing loss due to pneumococcal meningitis	30	6
Mild hearing loss with ringing due to pneumococcal meningitis	31	6
Moderate hearing loss due to pneumococcal meningitis	32	6
Moderate hearing loss with ringing due to pneumococcal meningitis	33	6
Moderately severe hearing loss due to pneumococcal meningitis	34	6
Moderately severe hearing loss with ringing due to pneumococcal meningitis	35	6
Severe hearing loss due to pneumococcal meningitis	36	6
Severe hearing loss with ringing due to pneumococcal meningitis	37	6
Profound hearing loss due to pneumococcal meningitis	38	6
Profound hearing loss with ringing due to pneumococcal meningitis	39	6
Complete hearing loss due to pneumococcal meningitis	40	6
Complete hearing loss with ringing due to pneumococcal meningitis	41	6
Monocular distance vision loss due to pneumococcal meningitis	640	6
Moderate vision impairment due to pneumococcol meningitis	1143	6

GBD 2017 Cause and Sequela Hierarchy		
Level 5 Sequelae are nested under causes. Most detailed sequelae are nested under their parent sequela where they exist		
**Cause or Sequela Name (Causes in bold)**	**GBD ID**	**Level**
Severe vision impairment due to pneumococcol meningitis	1144	6
Blindness due to pneumococcal meningitis	850	6
Borderline intellectual disability due to pneumococcal meningitis	23	6
Mild intellectual disability due to pneumococcal meningitis	24	6
Epilepsy due to pneumococcal meningitis	29	6
Mild behavioral problems due to pneumococcal meningitis	20	6
Mild motor impairment due to long term due to pneumococcal meningitis	21	6
Mild motor plus cognitive impairments due to pneumococcal meningitis	22	6
Moderate motor impairment due to pneumococcal meningitis	25	6
Moderate motor plus cognitive impairments due to pneumococcal meningitis	27	6
Severe motor impairment due to pneumococcal meningitis	26	6
Severe motor plus cognitive impairments due to pneumococcal meningitis	28	6
**H influenzae type B meningitis**	334	4
Acute H influenzae type B meningitis	42	5

GBD 2017 Cause and Sequela Hierarchy

Level 5 Sequelae are nested under causes. Most detailed sequelae are nested under their parent sequela where they exist

Cause or Sequela Name (Causes in bold)	GBD ID	Level
H Influenza type B meningitis complications	6989	5
Mild hearing loss due to H influenzae type B meningitis	53	6
Mild hearing loss with ringing due to H influenzae type B meningitis	54	6
Moderate hearing loss due to H influenzae type B meningitis	55	6
Moderate hearing loss with ringing due to H influenzae type B meningitis	56	6
Moderately severe hearing loss due to H influenzae type B meningitis	57	6
Moderately severe hearing loss with ringing due to H influenzae type B meningitis	58	6
Severe hearing loss due to H influenzae type B meningitis	59	6
Severe hearing loss with ringing due to H influenzae type B meningitis	60	6
Profound hearing loss due to H influenzae type B meningitis	61	6
Profound hearing loss with ringing due to H influenzae type B meningitis	62	6
Complete hearing loss due to H influenzae type B meningitis	63	6
Complete hearing loss with ringing due to H influenzae type B meningitis	64	6
Monocular distance vision loss due to H influenzae type B meningitis	641	6

GBD 2017 Cause and Sequela Hierarchy<br>Level 5 Sequelae are nested under causes. Most detailed sequelae are nested under their parent sequela where they exist		
**Cause or Sequela Name (Causes in bold)**	**GBD ID**	**Level**
Moderate vision impairment due to H influenza type B meningitis	1145	6
Severe vision impairment due to H influenza type B meningitis	1146	6
Blindness due to H influenzae type B meningitis	847	6
Borderline intellectual disability due to H influenzae type B meningitis	46	6
Mild intellectual disability due to H influenzae type B meningitis	47	6
Epilepsy due to H influenzae type B meningitis	52	6
Mild behavioral problems due to H influenzae type B meningitis	43	6
Mild motor impairment due to long term due to H influenzae type B meningitis	44	6
Mild motor plus cognitive impairments due to H influenzae type B meningitis	45	6
Moderate motor impairment due to H influenzae type B meningitis	48	6
Moderate motor plus cognitive impairments due to H influenzae type B meningitis	50	6
Severe motor impairment due to H influenzae type B meningitis	49	6
Severe motor plus cognitive impairments due to H influenzae type B meningitis	51	6
**Meningococcal meningitis**	335	4

GBD 2017 Cause and Sequela Hierarchy

Level 5 Sequelae are nested under causes. Most detailed sequelae are nested under their parent sequela where they exist

Cause or Sequela Name (Causes in bold)	GBD ID	Level
Acute meningococcal meningitis	65	5
Meningococcal meningitis complications	6992	5
Mild hearing loss due to meningococcal meningitis	76	6
Mild hearing loss with ringing due to meningococcal meningitis	77	6
Moderate hearing loss due to meningococcal meningitis	78	6
Moderate hearing loss with ringing due to meningococcal meningitis	79	6
Moderately severe hearing loss due to meningococcal meningitis	80	6
Moderately severe hearing loss with ringing due to meningococcal meningitis	81	6
Severe hearing loss due to meningococcal meningitis	82	6
Severe hearing loss with ringing due to meningococcal meningitis	83	6
Profound hearing loss due to meningococcal meningitis	84	6
Profound hearing loss with ringing due to meningococcal meningitis	85	6
Complete hearing loss due to meningococcal meningitis	86	6
Complete hearing loss with ringing due to meningococcal meningitis	87	6

GBD 2017 Cause and Sequela Hierarchy

Level 5 Sequelae are nested under causes. Most detailed sequelae are nested under their parent sequela where they exist

Cause or Sequela Name (Causes in bold)	GBD ID	Level
Monocular distance vision loss due to meningococcal meningitis	642	6
Moderate vision impairment due to meningococcal meningitis	1147	6
Severe vision impairment due to meningococcal meningitis	1148	6
Blindness due to meningococcal meningitis	848	6
Borderline intellectual disability due to meningococcal meningitis	69	6
Epilepsy due to meningococcal meningitis	75	6
Mild behavioral problems due to meningococcal meningitis	66	6
Mild intellectual disability due to meningococcal meningitis	70	6
Mild motor impairment due to long term due to meningococcal meningitis	67	6
Mild motor plus cognitive impairments due to meningococcal meningitis	68	6
Moderate motor impairment due to meningococcal meningitis	71	6
Moderate motor plus cognitive impairments due to meningococcal meningitis	73	6
Severe motor impairment due to meningococcal meningitis	72	6
Severe motor plus cognitive impairments due to meningococcal meningitis	74	6

GBD 2017 Cause and Sequela Hierarchy

Level 5 Sequelae are nested under causes. Most detailed sequelae are nested under their parent sequela where they exist

Cause or Sequela Name (Causes in bold)	GBD ID	Level
**Other meningitis**	336	4
Other acute bacterial meningitis	88	5
Other bacterial meningitis complications	6995	5
Mild hearing loss due to other bacterial meningitis	99	6
Mild hearing loss due with ringing to other bacterial meningitis	100	6
Moderate hearing loss due to other bacterial meningitis	101	6
Moderate hearing loss with ringing due to other bacterial meningitis	102	6
Moderately severe hearing loss due to other bacterial meningitis	103	6
Moderately severe hearing loss with ringing due to other bacterial meningitis	104	6
Severe hearing loss due to other bacterial meningitis	105	6
Severe hearing loss with ringing due to other bacterial meningitis	106	6
Profound hearing loss due to other bacterial meningitis	107	6
Profound hearing loss with ringing due to other bacterial meningitis	108	6
Complete hearing loss due to other bacterial meningitis	109	6

Cause or Sequela Name (Causes in bold)	GBD ID	Level
Complete hearing loss with ringing due to other bacterial meningitis	110	6
Monocular distance vision loss due to other bacterial meningitis	643	6
Moderate vision impairment due to other bacterial meningitis	1149	6
Severe vision impairment due to other bacterial meningitis	1150	6
Blindness due to other bacterial meningitis	849	6
Borderline intellectual disability due to other bacterial meningitis	92	6
Epilepsy due to other meningitis	98	6
Mild behavioral problems due to other bacterial meningitis	89	6
Mild intellectual disability due to other bacterial meningitis	93	6
Mild motor impairment due to long term due to other bacterial meningitis	90	6
Mild motor plus cognitive impairments due to other bacterial meningitis	91	6
Moderate motor impairment due to other bacterial meningitis	94	6
Moderate motor plus cognitive impairments due to other bacterial meningitis	96	6
Severe motor impairment due to other bacterial meningitis	95	6

GBD 2017 Cause and Sequela Hierarchy

Level 5 Sequelae are nested under causes. Most detailed sequelae are nested under their parent sequela where they exist

Cause or Sequela Name (Causes in bold)	GBD ID	Level
Severe motor plus cognitive impairments due to other bacterial meningitis	97	6
Acute viral meningitis	111	5
**Encephalitis**	337	3
Acute encephalitis	112	5
Encephalitis complications	6986	5
Monocular distance vision loss due to encephalitis	797	6
Moderate vision impairment due to encephalitis	1151	6
Severe vision impairment due to encephalitis	1152	6
Blindness due to encephalitis	874	6
Borderline intellectual disability due to encephalitis	796	6
Epilepsy due to encephalitis	803	6
Mild behavioral problems due to encephalitis	793	6
Mild intellectual disability due to encephalitis	798	6
Mild motor impairment due to long term due to encephalitis	794	6

GBD 2017 Cause and Sequela Hierarchy

Level 5 Sequelae are nested under causes. Most detailed sequelae are nested under their parent sequela where they exist

Cause or Sequela Name (Causes in bold)	GBD ID	Level
Mild motor plus cognitive impairments due to encephalitis	795	6
Moderate motor impairment due to encephalitis	799	6
Moderate motor plus cognitive impairments due to encephalitis	801	6
Severe motor impairment due to encephalitis	800	6
Severe motor plus cognitive impairments due to encephalitis	802	6
**Diphtheria**	338	3
Diphtheria episodes	22697	5
Moderate diphtheria	113	6
Severe diphtheria	114	6
**Whooping cough**	339	3
Whooping cough	115	5
**Tetanus**	340	3
Severe tetanus	116	5
Neonatal tetanus complications	6653	5

GBD 2017 Cause and Sequela Hierarchy

Level 5 Sequelae are nested under causes. Most detailed sequelae are nested under their parent sequela where they exist

Cause or Sequela Name (Causes in bold)	GBD ID	Level
Mild motor impairment due to neonatal tetanus	785	6
Mild motor plus cognitive impairments due to neonatal tetanus	786	6
Moderate motor impairment due to neonatal tetanus	737	6
Moderate motor impairment with blindness and epilepsy due to neonatal tetanus	754	6
Moderate motor impairment with blindness due to neonatal tetanus	746	6
Moderate motor impairment with epilepsy due to neonatal tetanus	756	6
Moderate motor plus cognitive impairment with blindness and epilepsy due to neonatal tetanus	710	6
Moderate motor plus cognitive impairment with blindness due to neonatal tetanus	706	6
Moderate motor plus cognitive impairment with epilepsy due to neonatal tetanus	714	6
Severe motor impairment due to neonatal tetanus	766	6
Severe motor impairment with blindness and epilepsy due to neonatal tetanus	773	6
Severe motor impairment with blindness due to neonatal tetanus	768	6
Severe motor impairment with epilepsy due to neonatal tetanus	781	6
Severe motor plus cognitive impairment with blindness and epilepsy due to neonatal tetanus	730	6

GBD 2017 Cause and Sequela Hierarchy

Level 5 Sequelae are nested under causes. Most detailed sequelae are nested under their parent sequela where they exist

Cause or Sequela Name (Causes in bold)	GBD ID	Level
Severe motor plus cognitive impairment with blindness due to neonatal tetanus	719	6
Severe motor plus cognitive impairment with epilepsy due to neonatal tetanus	736	6
**Measles**	341	3
Measles episodes	22700	5
Moderate measles	117	6
Severe measles	118	6
**Varicella and herpes zoster**	342	3
Chickenpox	119	5
Herpes zoster	120	5
**Acute hepatitis**	400	3
**Acute hepatitis A**	401	4
Acute Hepatitis A episode	6101	5
Asymptomatic acute hepatitis A	862	6
Moderate acute hepatitis A	229	6

GBD 2017 Cause and Sequela Hierarchy

Level 5 Sequelae are nested under causes. Most detailed sequelae are nested under their parent sequela where they exist

Cause or Sequela Name (Causes in bold)	GBD ID	Level
Severe acute hepatitis A	230	6
**Acute hepatitis B**	402	4
Acute Hepatitis B episode	6104	5
Asymptomatic acute hepatitis B	863	6
Moderate acute hepatitis B	231	6
Severe acute hepatitis B	232	6
**Acute hepatitis C**	403	4
Acute Hepatitis C episode	6107	5
Asymptomatic acute hepatitis C	864	6
Moderate acute hepatitis C	233	6
Severe acute hepatitis C	234	6
**Acute hepatitis E**	404	4
Acute Hepatitis E episode	6110	5
Asymptomatic acute hepatitis E	865	6

GBD 2017 Cause and Sequela Hierarchy

Level 5 Sequelae are nested under causes. Most detailed sequelae are nested under their parent sequela where they exist

Cause or Sequela Name (Causes in bold)	GBD ID	Level
Moderate acute hepatitis E	235	6
Severe acute hepatitis E	236	6
**Other unspecified infectious diseases**	408	3
Other infectious diseases	5159	5
Anemia due to other infectious diseases	6155	5
Mild anemia due to other infectious diseases	240	6
Moderate anemia due to other infectious diseases	241	6
Severe anemia due to other infectious diseases	242	6
Guillain-Barré syndrome due to other infectious diseases	243	5
**Maternal and neonatal disorders**	962	2
**Maternal disorders**	366	3
**Maternal hemorrhage**	367	4
Maternal hemorrhage episode	6593	5
Maternal hemorrhage (< 1L blood lost)	180	6

GBD 2017 Cause and Sequela Hierarchy

Level 5 Sequelae are nested under causes. Most detailed sequelae are nested under their parent sequela where they exist

Cause or Sequela Name (Causes in bold)	GBD ID	Level
Maternal hemorrhage (> 1L blood lost)	181	6
Maternal hemorrhage complications	6590	5
Mild anemia due to maternal hemorrhage	182	6
Moderate anemia due to maternal hemorrhage	183	6
Severe anemia due to maternal hemorrhage	184	6
**Maternal sepsis and other maternal infections**	368	4
Maternal sepsis and other maternal infection episode	6608	5
Other maternal infections	938	6
Puerperal sepsis	937	6
Infertility due to puerperal sepsis	675	5
**Maternal hypertensive disorders**	369	4
Maternal hypertensive disorder episode	6599	5
Severe pre-eclampsia	185	6
Eclampsia	186	6

GBD 2017 Cause and Sequela Hierarchy<br>Level 5 Sequelae are nested under causes. Most detailed sequelae are nested under their parent sequela where they exist		
**Cause or Sequela Name (Causes in bold)**	**GBD ID**	**Level**
Other hypertensive disorders of pregnancy	676	6
Maternal hypertensive disorder complications	6596	5
Long term sequelae of severe pre-eclampsia	187	6
Long term sequelae of eclampsia	677	6
**Maternal obstructed labor and uterine rupture**	370	4
Obstructed labor, acute event	188	5
Maternal obstructed labor complications	6602	5
Rectovaginal fistula	189	6
Vesicovaginal fistula	190	6
**Maternal abortion and miscarriage**	995	4
Maternal abortive outcome	191	5
**Ectopic pregnancy**	374	4
Ectopic Pregnancy	5165	5
**Indirect maternal deaths**	375	4

GBD 2017 Cause and Sequela Hierarchy		
Level 5 Sequelae are nested under causes. Most detailed sequelae are nested under their parent sequela where they exist		
**Cause or Sequela Name (Causes in bold)**	**GBD ID**	**Level**
**Late maternal deaths**	376	4
**Maternal deaths aggravated by HIV/AIDS**	741	4
**Other maternal disorders**	379	4
Other maternal disorders	192	5
**Neonatal disorders**	380	3
**Neonatal preterm birth**	381	4
Uncomplicated preterm birth	6914	5
Asymptomatic neonatal preterm birth 28-<32 wks	1247	6
Asymptomatic neonatal preterm birth 32-<37wks	1248	6
Asymptomatic neonatal preterm birth <28 weeks	1246	6
Preterm birth complications	6767	5
Mild motor impairment due to neonatal preterm birth complications 28-32wks	869	6
Mild motor impairment due to neonatal preterm birth complications 32-36wks	870	6
Mild motor impairment due to neonatal preterm birth complications <28wks	868	6

Cause or Sequela Name (Causes in bold)	GBD ID	Level
Mild motor plus cognitive impairments due to neonatal preterm birth complications 28-32wks	872	6
Mild motor plus cognitive impairments due to neonatal preterm birth complications 32-36wks	873	6
Mild motor plus cognitive impairments due to neonatal preterm birth complications <28wks	871	6
Moderate motor impairment due to neonatal preterm birth complications 28-32wks	739	6
Moderate motor impairment due to neonatal preterm birth complications 32-36wks	742	6
Moderate motor impairment due to neonatal preterm birth complications <28wks	741	6
Moderate motor impairment with blindness and epilepsy due to neonatal preterm birth complications 28-32wks	752	6
Moderate motor impairment with blindness and epilepsy due to neonatal preterm birth complications 32-36wks	751	6
Moderate motor impairment with blindness and epilepsy due to neonatal preterm birth complications <28wks	753	6
Moderate motor impairment with blindness due to neonatal preterm birth complications 28-32wks	744	6
Moderate motor impairment with blindness due to neonatal preterm birth complications 32-36wks	745	6
Moderate motor impairment with blindness due to neonatal preterm birth complications <28wks	743	6
Moderate motor impairment with epilepsy due to neonatal preterm birth complications 28-32wks	757	6
Moderate motor impairment with epilepsy due to neonatal preterm birth complications 32-36wks	755	6

GBD 2017 Cause and Sequela Hierarchy

Level 5 Sequelae are nested under causes. Most detailed sequelae are nested under their parent sequela where they exist

Cause or Sequela Name (Causes in bold)	GBD ID	Level
Moderate motor impairment with epilepsy due to neonatal preterm birth complications <28wks	759	6
Moderate motor plus cognitive impairment with blindness and epilepsy due to neonatal preterm birth complications 28-32wks	709	6
Moderate motor plus cognitive impairment with blindness and epilepsy due to neonatal preterm birth complications 32-36wks	707	6
Moderate motor plus cognitive impairment with blindness and epilepsy due to neonatal preterm birth complications <28wks	708	6
Moderate motor plus cognitive impairment with blindness due to neonatal preterm birth complications 28-32wks	702	6
Moderate motor plus cognitive impairment with blindness due to neonatal preterm birth complications 32-36wks	704	6
Moderate motor plus cognitive impairment with blindness due to neonatal preterm birth complications <28wks	703	6
Moderate motor plus cognitive impairment with epilepsy due to neonatal preterm birth complications 28-32wks	718	6
Moderate motor plus cognitive impairment with epilepsy due to neonatal preterm birth complications 32-36wks	713	6
Moderate motor plus cognitive impairment with epilepsy due to neonatal preterm birth complications <28wks	717	6
Severe motor impairment due to neonatal preterm birth complications 28-32wks	765	6
Severe motor impairment due to neonatal preterm birth complications 32-36wks	764	6
Severe motor impairment due to neonatal preterm birth complications <28wks	763	6
Severe motor impairment with blindness and epilepsy due to neonatal preterm birth complications 28-32wks	775	6

GBD 2017 Cause and Sequela Hierarchy

Level 5 Sequelae are nested under causes. Most detailed sequelae are nested under their parent sequela where they exist

Cause or Sequela Name (Causes in bold)	GBD ID	Level
Severe motor impairment with blindness and epilepsy due to neonatal preterm birth complications 32-36wks	777	6
Severe motor impairment with blindness and epilepsy due to neonatal preterm birth complications <28wks	774	6
Severe motor impairment with blindness due to neonatal preterm birth complications 28-32wks	767	6
Severe motor impairment with blindness due to neonatal preterm birth complications 32-36wks	771	6
Severe motor impairment with blindness due to neonatal preterm birth complications <28wks	770	6
Severe motor impairment with epilepsy due to neonatal preterm birth complications 28-32wks	783	6
Severe motor impairment with epilepsy due to neonatal preterm birth complications 32-36wks	780	6
Severe motor impairment with epilepsy due to neonatal preterm birth complications <28wks	782	6
Severe motor plus cognitive impairment with blindness and epilepsy due to neonatal preterm birth complications 28-32wks	725	6
Severe motor plus cognitive impairment with blindness and epilepsy due to neonatal preterm birth complications 32-36wks	729	6
Severe motor plus cognitive impairment with blindness and epilepsy due to neonatal preterm birth complications <28wks	726	6
Severe motor plus cognitive impairment with blindness due to neonatal preterm birth complications 28-32wks	720	6
Severe motor plus cognitive impairment with blindness due to neonatal preterm birth complications 32-36wks	721	6
Severe motor plus cognitive impairment with blindness due to neonatal preterm birth complications <28wks	724	6

GBD 2017 Cause and Sequela Hierarchy

Level 5 Sequelae are nested under causes. Most detailed sequelae are nested under their parent sequela where they exist

Cause or Sequela Name (Causes in bold)	GBD ID	Level
Severe motor plus cognitive impairment with epilepsy due to neonatal preterm birth complications 28-32wks	735	6
Severe motor plus cognitive impairment with epilepsy due to neonatal preterm birth complications 32-36wks	734	6
Severe motor plus cognitive impairment with epilepsy due to neonatal preterm birth complications <28wks	731	6
Retinopathy of prematurity	6773	5
Asymptomatic retinopathy of prematurity	866	6
Blindness due to retinopathy of prematurity	867	6
Mild vision impairment due to retinopathy of prematurity	193	6
Moderate vision impairment due to retinopathy of prematurity	915	6
Severe vision impairment due to retinopathy of prematurity	916	6
**Neonatal encephalopathy due to birth asphyxia and trauma**	382	4
Neonatal encephalopathy due to birth asphyxia and trauma	6644	5
Mild motor plus cognitive impairments due to neonatal encephalopathy due to birth asphyxia and trauma	194	6
Mild motor impairment due to neonatal encephalopathy due to birth asphyxia and trauma	195	6
Moderate motor plus cognitive impairment with blindness due to neonatal encephalopathy due to birth asphyxia and trauma	705	6

GBD 2017 Cause and Sequela Hierarchy

Level 5 Sequelae are nested under causes. Most detailed sequelae are nested under their parent sequela where they exist

Cause or Sequela Name (Causes in bold)	GBD ID	Level
Moderate motor plus cognitive impairment with blindness and epilepsy due to neonatal encephalopathy due to birth asphyxia and trauma	712	6
Moderate motor plus cognitive impairment with epilepsy due to neonatal encephalopathy due to birth asphyxia and trauma	716	6
Severe motor plus cognitive impairment with blindness due to neonatal encephalopathy due to birth asphyxia and trauma	723	6
Severe motor plus cognitive impairment with blindness and epilepsy due to neonatal encephalopathy due to birth asphyxia and trauma	728	6
Severe motor plus cognitive impairment with epilepsy due to neonatal encephalopathy due to birth asphyxia and trauma	732	6
Moderate motor impairment due to neonatal encephalopathy due to birth asphyxia and trauma	740	6
Moderate motor impairment with blindness due to neonatal encephalopathy due to birth asphyxia and trauma	747	6
Moderate motor impairment with blindness and epilepsy due to neonatal encephalopathy due to birth asphyxia and trauma	750	6
Moderate motor impairment with epilepsy due to neonatal encephalopathy due to birth asphyxia and trauma	758	6
Severe motor impairment due to neonatal encephalopathy due to birth asphyxia and trauma	761	6
Severe motor impairment with blindness due to neonatal encephalopathy due to birth asphyxia and trauma	772	6
Severe motor impairment with blindness and epilepsy due to neonatal encephalopathy due to birth asphyxia and trauma	778	6
Severe motor impairment with epilepsy due to neonatal encephalopathy due to birth asphyxia and trauma	779	6
Asymptomatic neonatal encephalopathy due to birth asphyxia and trauma	1249	6

GBD 2017 Cause and Sequela Hierarchy<br>Level 5 Sequelae are nested under causes. Most detailed sequelae are nested under their parent sequela where they exist		
**Cause or Sequela Name (Causes in bold)**	**GBD ID**	**Level**
**Neonatal sepsis and other neonatal infections**	383	4
Neonatal sepsis and other neonatal infection episode	6650	5
Neonatal sepsis and other neonatal infection complications	6647	5
Mild motor impairment due to neonatal sepsis and other neonatal infections	1252	6
Mild motor plus cognitive impairments due to neonatal sepsis and other neonatal infections	1251	6
Moderate motor impairment due to neonatal sepsis and other neonatal infections	1253	6
Moderate motor impairment with blindness and epilepsy due to neonatal sepsis and other neonatal infections	1256	6
Moderate motor impairment with blindness due to neonatal sepsis and other neonatal infections	1254	6
Moderate motor impairment with epilepsy due to neonatal sepsis and other neonatal infections	1255	6
Moderate motor plus cognitive impairment with blindness and epilepsy due to neonatal sepsis and other neonatal infections	1259	6
Moderate motor plus cognitive impairment with blindness due to neonatal sepsis and other neonatal infections	1257	6
Moderate motor plus cognitive impairment with epilepsy due to neonatal sepsis and other neonatal infections	1258	6
Severe motor impairment due to neonatal sepsis and other neonatal infections	1260	6
Severe motor impairment with blindness and epilepsy due to neonatal sepsis and other neonatal infections	1263	6

GBD 2017 Cause and Sequela Hierarchy

Level 5 Sequelae are nested under causes. Most detailed sequelae are nested under their parent sequela where they exist

Cause or Sequela Name (Causes in bold)	GBD ID	Level
Severe motor impairment with blindness due to neonatal sepsis and other neonatal infections	1261	6
Severe motor impairment with epilepsy due to neonatal sepsis and other neonatal infections	1262	6
Severe motor plus cognitive impairment with blindness and epilepsy due to neonatal sepsis and other neonatal infections	1266	6
Severe motor plus cognitive impairment with blindness due to neonatal sepsis and other neonatal infections	1264	6
Severe motor plus cognitive impairment with epilepsy due to neonatal sepsis and other neonatal infections	1265	6
Asymptomatic neonatal sepsis and other neonatal infections	1250	6
**Hemolytic disease and other neonatal jaundice**	384	4
Hemolytic disease and other neonatal jaundice	6470	5
Moderate motor impairment due to hemolytic disease and other neonatal jaundice	738	6
Moderate motor impairment with blindness due to hemolytic disease and other neonatal jaundice	748	6
Moderate motor impairment with blindness and epilepsy due to hemolytic disease and other neonatal jaundice	749	6
Moderate motor impairment with epilepsy due to hemolytic disease and other neonatal jaundice	760	6
Moderate motor plus cognitive impairment with blindness due to hemolytic disease and other neonatal jaundice	701	6
Moderate motor plus cognitive impairment with blindness and epilepsy due to hemolytic disease and other neonatal jaundice	711	6

GBD 2017 Cause and Sequela Hierarchy

Level 5 Sequelae are nested under causes. Most detailed sequelae are nested under their parent sequela where they exist

Cause or Sequela Name (Causes in bold)	GBD ID	Level
Moderate motor plus cognitive impairment with epilepsy due to hemolytic disease and other neonatal jaundice	715	6
Severe motor plus cognitive impairment with blindness due to hemolytic disease and other neonatal jaundice	722	6
Severe motor plus cognitive impairment with blindness and epilepsy due to hemolytic disease and other neonatal jaundice	727	6
Severe motor plus cognitive impairment with epilepsy due to hemolytic disease and other neonatal jaundice	733	6
Severe motor impairment severe due to hemolytic disease and other neonatal jaundice	762	6
Severe motor impairment with blindness due to hemolytic disease and other neonatal jaundice	769	6
Severe motor impairment with blindness and epilepsy due to hemolytic disease and other neonatal jaundice	776	6
Severe motor impairment with epilepsy due to hemolytic disease and other neonatal jaundice	784	6
Extreme hyperbilirubinemia due to hemolytic disease and other neonatal jaundice, without kernicterus	7223	6
**Other neonatal disorders**	385	4
Other neonatal disorders	197	5
**Nutritional deficiencies**	386	2
**Protein-energy malnutrition**	387	3
Protein-energy malnutrition	6473	5

GBD 2017 Cause and Sequela Hierarchy<br>Level 5 Sequelae are nested under causes. Most detailed sequelae are nested under their parent sequela where they exist		
**Cause or Sequela Name (Causes in bold)**	**GBD ID**	**Level**
Moderate wasting with edema	198	6
Severe wasting without edema	199	6
Moderate wasting without edema	2033	6
Severe wasting with edema	2036	6
**Iodine deficiency**	388	3
Visible goiter without symptoms	200	5
Visible goiter with complications	22703	5
Visible goiter with profound intellectual disability due to iodine deficiency	202	6
Visible goiter with severe intellectual disability due to iodine deficiency	201	6
**Vitamin A deficiency**	389	3
Asymptomatic vitamin A deficiency	2189	5
Vitamin A deficiency complications	6971	5
Blindness due to vitamin A deficiency	205	6
Moderate vision impairment loss due to vitamin A deficiency	203	6

GBD 2017 Cause and Sequela Hierarchy

Level 5 Sequelae are nested under causes. Most detailed sequelae are nested under their parent sequela where they exist

Cause or Sequela Name (Causes in bold)	GBD ID	Level
Severe vision impairment loss due to vitamin A deficiency	204	6
Vitamin A deficiency with anemia	6974	5
Vitamin A deficiency with mild anemia	5393	6
Vitamin A deficiency with moderate anemia	5396	6
Vitamin A deficiency with severe anemia	5399	6
**Dietary iron deficiency**	390	3
Iron-deficiency anemia	6530	5
Mild iron-deficiency anemia	206	6
Moderate iron-deficiency anemia	207	6
Severe iron-deficiency anemia	208	6
**Other nutritional deficiencies**	391	3
Other nutritional deficiencies	212	5
**Non-communicable diseases**	409	1
**Neoplasms**	410	2

GBD 2017 Cause and Sequela Hierarchy

Level 5 Sequelae are nested under causes. Most detailed sequelae are nested under their parent sequela where they exist

Cause or Sequela Name (Causes in bold)	GBD ID	Level
**Lip and oral cavity cancer**	444	3
Diagnosis and primary therapy phase of mouth cancer	301	5
Controlled phase of mouth cancer	302	5
Metastatic phase of mouth cancer	303	5
Terminal phase of mouth cancer	304	5
**Nasopharynx cancer**	447	3
Diagnosis and primary therapy phase of nasopharynx cancer	305	5
Controlled phase of nasopharynx cancer	306	5
Metastatic phase of nasopharynx cancer	307	5
Terminal phase of nasopharynx cancer	308	5
**Other pharynx cancer**	450	3
Diagnosis and primary therapy phase of other pharynx cancer	309	5
Controlled phase of other pharynx cancer	310	5
Metastatic phase of other pharynx cancer	311	5

GBD 2017 Cause and Sequela Hierarchy		
Level 5 Sequelae are nested under causes. Most detailed sequelae are nested under their parent sequela where they exist		
**Cause or Sequela Name (Causes in bold)**	**GBD ID**	**Level**
Terminal phase of other pharynx cancer	312	5
**Esophageal cancer**	411	3
Diagnosis and primary therapy phase of esophageal cancer	244	5
Controlled phase of esophageal cancer	245	5
Metastatic phase of esophageal cancer	246	5
Terminal phase of esophageal cancer	247	5
**Stomach cancer**	414	3
Diagnosis and primary therapy phase of stomach cancer	248	5
Controlled phase of stomach cancer	249	5
Metastatic phase of stomach cancer	250	5
Terminal phase of stomach cancer	251	5
**Colon and rectum cancer**	441	3
Diagnosis and primary therapy phase of colon and rectum cancers	296	5
Controlled phase of colon and rectum cancers	297	5

GBD 2017 Cause and Sequela Hierarchy

Level 5 Sequelae are nested under causes. Most detailed sequelae are nested under their parent sequela where they exist

Cause or Sequela Name (Causes in bold)	GBD ID	Level
Controlled phase of colon and rectum cancers, with stoma	5519	6
Controlled phase of colon and rectum cancers, without stoma	5522	6
Metastatic phase of colon and rectum cancers	298	5
Terminal phase of colon and rectum cancers	299	5
Stoma from colon and rectum cancers, beyond 10 years	5525	5
**Liver cancer**	417	3
**Liver cancer due to hepatitis B**	418	4
Diagnosis and primary therapy phase of liver cancer due to hepatitis B	252	5
Controlled phase of liver cancer due to hepatitis B	253	5
Metastatic phase of liver cancer due to hepatitis B	254	5
Terminal phase of liver cancer due to hepatitis B	255	5
**Liver cancer due to hepatitis C**	419	4
Diagnosis and primary therapy phase of liver cancer due to hepatitis C	256	5
Controlled phase of liver cancer due to hepatitis C	257	5

GBD 2017 Cause and Sequela Hierarchy

Level 5 Sequelae are nested under causes. Most detailed sequelae are nested under their parent sequela where they exist

Cause or Sequela Name (Causes in bold)	GBD ID	Level
Metastatic phase of liver cancer due to hepatitis C	258	5
Terminal phase of liver cancer due to hepatitis C	259	5
**Liver cancer due to alcohol use**	420	4
Diagnosis and primary therapy phase of liver cancer due to alcohol use	260	5
Controlled phase of liver cancer due to alcohol use	261	5
Metastatic phase of liver cancer due to alcohol use	262	5
Terminal phase of liver cancer due to alcohol use	263	5
**Liver cancer due to NASH**	996	4
Diagnosis and primary therapy phase of liver cancer due to NASH	5417	5
Controlled phase of liver cancer due to NASH	5420	5
Metastatic phase of liver cancer due to NASH	5423	5
Terminal phase of liver cancer due to NASH	5426	5
**Liver cancer due to other causes**	421	4
Diagnosis and primary therapy phase of liver cancer due to other causes	264	5

GBD 2017 Cause and Sequela Hierarchy<br>Level 5 Sequelae are nested under causes. Most detailed sequelae are nested under their parent sequela where they exist		
**Cause or Sequela Name (Causes in bold)**	**GBD ID**	**Level**
Controlled phase of liver cancer due to other causes	265	5
Metastatic phase of liver cancer due to other causes	266	5
Terminal phase of liver cancer due to other causes	267	5
**Gallbladder and biliary tract cancer**	453	3
Diagnosis and primary therapy phase of gallbladder and biliary tract cancer	313	5
Controlled phase of gallbladder and biliary tract cancer	314	5
Metastatic phase of gallbladder and biliary tract cancer	315	5
Terminal phase of gallbladder and biliary tract cancer	316	5
**Pancreatic cancer**	456	3
Diagnosis and primary therapy phase of pancreatic cancer	317	5
Controlled phase of pancreatic cancer	318	5
Metastatic phase of pancreatic cancer	319	5
Terminal phase of pancreatic cancer	320	5
**Larynx cancer**	423	3

GBD 2017 Cause and Sequela Hierarchy		
Level 5 Sequelae are nested under causes. Most detailed sequelae are nested under their parent sequela where they exist		
**Cause or Sequela Name (Causes in bold)**	**GBD ID**	**Level**
Diagnosis and primary therapy phase of larynx cancer	268	5
Controlled phase of larynx cancer	269	5
Controlled phase of larynx cancer, with laryngectomy	5510	6
Controlled phase of larynx cancer, without laryngectomy	5513	6
Metastatic phase of larynx cancer	270	5
Terminal phase of larynx cancer	271	5
Laryngectomy from larynx cancer, beyond 10 years	5516	5
**Tracheal, bronchus, and lung cancer**	426	3
Diagnosis and primary therapy phase of lung, bronchus, and trachea cancer	273	5
Controlled phase of lung, bronchus, and trachea cancer	274	5
Metastatic phase of lung, bronchus, and trachea cancer	275	5
Terminal phase of lung, bronchus, and trachea cancer	276	5
**Malignant skin melanoma**	459	3
Diagnosis and primary therapy phase of malignant skin melanoma	321	5

GBD 2017 Cause and Sequela Hierarchy		
Level 5 Sequelae are nested under causes. Most detailed sequelae are nested under their parent sequela where they exist		
**Cause or Sequela Name (Causes in bold)**	**GBD ID**	**Level**
Controlled phase of malignant skin melanoma	322	5
Metastatic phase of malignant skin melanoma	323	5
Terminal phase of malignant skin melanoma	324	5
**Non-melanoma skin cancer**	462	3
**Non-melanoma skin cancer (squamous-cell carcinoma)**	849	4
Squamous cell carcinoma	6809	5
Mild disfigurement due to squamous cell carcinoma	2039	6
Moderate disfigurement due to squamous cell carcinoma	2042	6
Severe disfigurement due to squamous cell carcinoma	2045	6
**Non-melanoma skin cancer (basal-cell carcinoma)**	850	4
Basal cell carcinoma	6209	5
Disfigurement due to basal cell carcinoma	657	6
Basal cell carcinoma without disfigurement	7226	6
**Breast cancer**	429	3

GBD 2017 Cause and Sequela Hierarchy		
Level 5 Sequelae are nested under causes. Most detailed sequelae are nested under their parent sequela where they exist		
**Cause or Sequela Name (Causes in bold)**	**GBD ID**	**Level**
Diagnosis and primary therapy phase of breast cancer	277	5
Controlled phase of breast cancer	278	5
Controlled phase of breast cancer, with mastectomy	5486	6
Controlled phase of breast cancer, without mastectomy	5489	6
Metastatic phase of breast cancer	279	5
Terminal phase of breast cancer	280	5
Mastectomy from breast cancer, beyond 10 years	5492	5
**Cervical cancer**	432	3
Diagnosis and primary therapy phase of cervical cancer	282	5
Controlled phase of cervical cancer	283	5
Metastatic phase of cervical cancer	284	5
Terminal phase of cervical cancer	285	5
**Uterine cancer**	435	3
Diagnosis and primary therapy phase of uterine cancer	286	5

Cause or Sequela Name (Causes in bold)	GBD ID	Level
Controlled phase of uterine cancer	287	5
Metastatic phase of uterine cancer	288	5
Terminal phase of uterine cancer	289	5
**Ovarian cancer**	465	3
Diagnosis and primary therapy phase of ovarian cancer	329	5
Controlled phase of ovarian cancer	330	5
Metastatic phase of ovarian cancer	331	5
Terminal phase of ovarian cancer	332	5
**Prostate cancer**	438	3
Diagnosis and primary therapy phase of prostate cancer	290	5
Controlled phase of prostate cancer	291	5
Controlled phase of prostate cancer, with impotence	5495	6
Controlled phase of prostate cancer, with incontinence	5498	6
Controlled phase of prostate cancer, without impotence or incontinence	5501	6

GBD 2017 Cause and Sequela Hierarchy

Level 5 Sequelae are nested under causes. Most detailed sequelae are nested under their parent sequela where they exist

Cause or Sequela Name (Causes in bold)	GBD ID	Level
Metastatic phase of prostate cancer	292	5
Terminal phase of prostate cancer	293	5
Impotence and Incontinence after 10-year survival from prostate cancer	6503	5
Impotence from prostate cancer, beyond 10 years	5504	6
Incontinence from prostate cancer, beyond 10 years	5507	6
**Testicular cancer**	468	3
Diagnosis and primary therapy phase of testicular cancer	333	5
Controlled phase of testicular cancer	334	5
Metastatic phase of testicular cancer	335	5
Terminal phase of testicular cancer	336	5
**Kidney cancer**	471	3
Diagnosis and primary therapy phase of kidney cancer	337	5
Controlled phase of kidney cancer	338	5
Metastatic phase of kidney cancer	339	5

GBD 2017 Cause and Sequela Hierarchy

Level 5 Sequelae are nested under causes. Most detailed sequelae are nested under their parent sequela where they exist

Cause or Sequela Name (Causes in bold)	GBD ID	Level
Terminal phase of kidney cancer	340	5
**Bladder cancer**	474	3
Diagnosis and primary therapy phase of bladder cancer	341	5
Controlled phase of bladder cancer	342	5
Controlled phase of bladder cancer, with incontinence	5528	6
Controlled phase of bladder cancer, without incontinence	5531	6
Metastatic phase of bladder cancer	343	5
Terminal phase of bladder cancer	344	5
Incontinence from bladder cancer, beyond 10 years	5534	5
**Brain and nervous system cancer**	477	3
Diagnosis and primary therapy phase of brain and nervous system cancers	346	5
Controlled phase of brain and nervous system cancers	347	5
Metastatic phase of brain and nervous system cancers	348	5
Terminal phase of brain and nervous system cancers	349	5

GBD 2017 Cause and Sequela Hierarchy

Level 5 Sequelae are nested under causes. Most detailed sequelae are nested under their parent sequela where they exist

Cause or Sequela Name (Causes in bold)	GBD ID	Level
**Thyroid cancer**	480	3
Diagnosis and primary therapy phase of thyroid cancer	350	5
Controlled phase of thyroid cancer	351	5
Metastatic phase of thyroid cancer	352	5
Terminal phase of thyroid cancer	353	5
**Mesothelioma**	483	3
Diagnosis and primary therapy phase of mesothelioma	354	5
Controlled phase of mesothelioma	355	5
Metastatic phase of mesothelioma	356	5
Terminal phase of mesothelioma	357	5
**Hodgkin lymphoma**	484	3
Diagnosis and primary therapy phase of Hodgkin disease	358	5
Controlled phase of Hodgkin disease	359	5
Metastatic phase of Hodgkin disease	360	5

GBD 2017 Cause and Sequela Hierarchy

Level 5 Sequelae are nested under causes. Most detailed sequelae are nested under their parent sequela where they exist

Cause or Sequela Name (Causes in bold)	GBD ID	Level
Terminal phase of Hodgkin disease	361	5
**Non-Hodgkin lymphoma**	485	3
Diagnosis and primary therapy phase of non-Hodgkin lymphoma	362	5
Controlled phase of non-Hodgkin lymphoma	363	5
Metastatic phase of non-Hodgkin lymphoma	364	5
Terminal phase of non-Hodgkin lymphoma	365	5
**Multiple myeloma**	486	3
Diagnosis and primary therapy phase of multiple myeloma	366	5
Controlled phase of multiple myeloma	367	5
Metastatic phase of multiple myeloma	368	5
Terminal phase of multiple myeloma	369	5
**Leukemia**	487	3
**Acute lymphoid leukemia**	845	4
Diagnosis and primary therapy phase of acute lymphoid leukemia	1186	5

GBD 2017 Cause and Sequela Hierarchy

Level 5 Sequelae are nested under causes. Most detailed sequelae are nested under their parent sequela where they exist

Cause or Sequela Name (Causes in bold)	GBD ID	Level
Controlled phase of acute lymphoid leukemia	1190	5
Metastatic phase of acute lymphoid leukemia	1194	5
Terminal phase of acute lymphoid leukemia	1198	5
**Chronic lymphoid leukemia**	846	4
Diagnosis and primary therapy phase of chronic lymphoid leukemia	1188	5
Controlled phase of chronic lymphoid leukemia	1192	5
Metastatic phase of chronic lymphoid leukemia	1196	5
Terminal phase of chronic lymphoid leukemia	1200	5
**Acute myeloid leukemia**	847	4
Diagnosis and primary therapy phase of acute myeloid leukemia	1187	5
Controlled phase of acute myeloid leukemia	1191	5
Metastatic phase of acute myeloid leukemia	1195	5
Terminal phase of acute myeloid leukemia	1199	5
**Chronic myeloid leukemia**	848	4

GBD 2017 Cause and Sequela Hierarchy

Level 5 Sequelae are nested under causes. Most detailed sequelae are nested under their parent sequela where they exist

Cause or Sequela Name (Causes in bold)	GBD ID	Level
Diagnosis and primary therapy phase of chronic myeloid leukemia	1189	5
Controlled phase of chronic myeloid leukemia	1193	5
Metastatic phase of chronic myeloid leukemia	1197	5
Terminal phase of chronic myeloid leukemia	1201	5
**Other leukemia**	943	4
Metastatic phase of other leukemia	1481	5
Diagnosis and primary therapy phase of other leukemia	1484	5
Terminal phase of other leukemia	1487	5
Controlled phase of other leukemia	1490	5
**Other malignant neoplasms**	489	3
Diagnosis and primary therapy phase of other malignant neoplasms	5864	5
Controlled phase of other malignant neoplasms	5867	5
Metastatic phase of other malignant neoplasms	5870	5
Terminal phase of other malignant neoplasms	5873	5

GBD 2017 Cause and Sequela Hierarchy

Level 5 Sequelae are nested under causes. Most detailed sequelae are nested under their parent sequela where they exist

Cause or Sequela Name (Causes in bold)	GBD ID	Level
**Other neoplasms**	490	3
**Myelodysplastic, myeloproliferative, and other hematopoietic neoplasms**	964	4
Myelodysplastic, myeloproliferative, and other hematopoietic neoplasms	5405	5
**Benign and in situ intestinal neoplasms**	965	4
Benign and in situ intestinal neoplasms	5408	5
**Benign and in situ cervical and uterine neoplasms**	966	4
Benign and in situ cervical and uterine neoplasms	5411	5
**Other benign and in situ neoplasms**	967	4
Other benign and in situ neoplasms	5414	5
**Cardiovascular diseases**	491	2
**Rheumatic heart disease**	492	3
Rheumatic heart disease, without heart failure	1116	5
Heart failure due to rheumatic heart disease	6461	5
Controlled, medically managed heart failure due to rheumatic heart disease	5768	6

Cause or Sequela Name (Causes in bold)	GBD ID	Level
Mild heart failure due to rheumatic heart disease	1117	6
Moderate heart failure due to rheumatic heart disease	1118	6
Severe heart failure due to rheumatic heart disease	1119	6
**Ischemic heart disease**	493	3
Myocardial infarction due to ischemic heart disease	6635	5
Acute myocardial infarction 3 to 28 days	379	6
Acute myocardial infarction first 2 days	378	6
Angina due to ischemic heart disease	6167	5
Asymptomatic angina due to ischemic heart disease	953	6
Mild angina due to ischemic heart disease	380	6
Moderate angina due to ischemic heart disease	381	6
Severe angina due to ischemic heart disease	382	6
Heart failure due to ischemic heart disease	6443	5
Controlled, medically managed heart failure due ischemic heart disease	5726	6

GBD 2017 Cause and Sequela Hierarchy

Level 5 Sequelae are nested under causes. Most detailed sequelae are nested under their parent sequela where they exist

Cause or Sequela Name (Causes in bold)	GBD ID	Level
Mild heart failure due to ischemic heart disease	383	6
Moderate heart failure due to ischemic heart disease	384	6
Severe heart failure due to ischemic heart disease	385	6
Asymptomatic ischemic heart disease following myocardial infarction	1040	5
**Stroke**	494	3
**Ischemic stroke**	495	4
Acute ischemic stroke	6116	5
Acute ischemic stroke severity level 1	386	6
Acute ischemic stroke severity level 2	387	6
Acute ischemic stroke severity level 3	389	6
Acute ischemic stroke severity level 4	388	6
Acute ischemic stroke severity level 5	390	6
Chronic ischemic stroke	6248	5
Asymptomatic chronic ischemic stroke	946	6

GBD 2017 Cause and Sequela Hierarchy

Level 5 Sequelae are nested under causes. Most detailed sequelae are nested under their parent sequela where they exist

Cause or Sequela Name (Causes in bold)	GBD ID	Level
Chronic ischemic stroke severity level 1	391	6
Chronic ischemic stroke severity level 2	392	6
Chronic ischemic stroke severity level 3	394	6
Chronic ischemic stroke severity level 4	393	6
Chronic ischemic stroke severity level 5	395	6
**Intracerebral hemorrhage**	496	4
Acute intracerebral hemorrhage	6113	5
Acute intracerebral hemorrhage severity level 1	396	6
Acute intracerebral hemorrhage severity level 2	397	6
Acute intracerebral hemorrhage severity level 3	399	6
Acute intracerebral hemorrhage severity level 4	398	6
Acute intracerebral hemorrhage severity level 5	400	6
Chronic intracerebral hemorrhage	6245	5
Asymptomatic chronic intracerebral hemorrhage	947	6

GBD 2017 Cause and Sequela Hierarchy

Level 5 Sequelae are nested under causes. Most detailed sequelae are nested under their parent sequela where they exist

Cause or Sequela Name (Causes in bold)	GBD ID	Level
Chronic intracerebral hemorrhage severity level 1	401	6
Chronic intracerebral hemorrhage severity level 2	402	6
Chronic intracerebral hemorrhage severity level 3	404	6
Chronic intracerebral hemorrhage severity level 4	403	6
Chronic intracerebral hemorrhage severity level 5	405	6
**Subarachnoid hemorrhage**	497	4
Acute subarachnoid hemorrhage	6131	5
Acute subarachnoid hemorrhage severity level 1	5168	6
Acute subarachnoid hemorrhage severity level 2	5171	6
Acute subarachnoid hemorrhage severity level 3	5174	6
Acute subarachnoid hemorrhage severity level 4	5177	6
Acute subarachnoid hemorrhage severity level 5	5180	6
Chronic subarachnoid hemorrhage	6266	5
Asymptomatic chronic subarachnoid hemorrhage	5183	6

GBD 2017 Cause and Sequela Hierarchy<br>Level 5 Sequelae are nested under causes. Most detailed sequelae are nested under their parent sequela where they exist		
**Cause or Sequela Name (Causes in bold)**	**GBD ID**	**Level**
Chronic subarachnoid hemorrhage severity level 1	5186	6
Chronic subarachnoid hemorrhage severity level 2	5189	6
Chronic subarachnoid hemorrhage severity level 3	5192	6
Chronic subarachnoid hemorrhage severity level 4	5195	6
Chronic subarachnoid hemorrhage severity level 5	5198	6
**Hypertensive heart disease**	498	3
Heart failure due to hypertensive heart disease	6434	5
Controlled, medically managed heart failure due to hypertensive heart disease	5750	6
Mild heart failure due to hypertensive heart disease	406	6
Moderate heart failure due to hypertensive heart disease	407	6
Severe heart failure due to hypertensive heart disease	408	6
**Non-rheumatic valvular heart disease**	504	3
**Non-rheumatic calcific aortic valve disease**	968	4
Heart failure due to calcific aortic valve disease	6419	5

Cause or Sequela Name (Causes in bold)	GBD ID	Level
Controlled, medically managed heart failure due to calcific aortic valve disease	5732	6
Mild heart failure due to calcific aortic valve disease	5291	6
Moderate heart failure due to calcific aortic valve disease	5294	6
Severe heart failure due to calcific aortic valve disease	5297	6
Non-rheumatic calcific aortic valve disease	6662	5
Asymptomatic calcific aortic valve disease	5288	6
Calcific aortic valve disease after valve intervention	5285	6
**Non-rheumatic degenerative mitral valve disease**	969	4
Heart failure due to degenerative mitral valve disease	6425	5
Controlled, medically managed heart failure due to degenerative mitral valve disease	5738	6
Mild heart failure due to degenerative mitral valve disease	5306	6
Moderate heart failure due to degenerative mitral valve disease	5309	6
Severe heart failure due to degenerative mitral valve disease	5312	6
Non-rheumatic degenerative mitral valve disease	6665	5

GBD 2017 Cause and Sequela Hierarchy<br>Level 5 Sequelae are nested under causes. Most detailed sequelae are nested under their parent sequela where they exist		
Cause or Sequela Name (Causes in bold)	GBD ID	Level
Asymptomatic degenerative mitral valve disease	5303	6
Degenerative mitral valve disease after valve intervention	5300	6
**Other non-rheumatic valve diseases**	970	4
Controlled, medically managed heart failure due to other non-rheumatic valve disease	5765	5
Mild heart failure due to other non-rheumatic valve disease	5321	5
Moderate heart failure due to other non-rheumatic valve disease	5324	5
Severe heart failure due to other non-rheumatic valve disease	5327	5
**Cardiomyopathy and myocarditis**	499	3
**Myocarditis**	942	4
Acute myocarditis	409	5
Heart failure due to myocarditis	6446	5
Controlled, medically managed heart failure due to myocarditis	5753	6
Mild heart failure due to myocarditis	1523	6
Moderate heart failure due to myocarditis	1526	6

GBD 2017 Cause and Sequela Hierarchy

Level 5 Sequelae are nested under causes. Most detailed sequelae are nested under their parent sequela where they exist

Cause or Sequela Name (Causes in bold)	GBD ID	Level
Severe heart failure due to myocarditis	1529	6
**Alcoholic cardiomyopathy**	938	4
Heart failure due to alcoholic cardiomyopathy	6413	5
Controlled, medically managed heart failure due to alcoholic cardiomyopathy	5729	6
Mild heart failure due to alcoholic cardiomyopathy	1532	6
Moderate heart failure due to alcoholic cardiomyopathy	1535	6
Severe heart failure due to alcoholic cardiomyopathy	1538	6
**Other cardiomyopathy**	944	4
Heart failure due to other cardiomyopathy	6449	5
Controlled, medically managed heart failure due to other cardiomyopathy	5756	6
Mild heart failure due to other cardiomyopathy	1541	6
Moderate heart failure due to other cardiomyopathy	1544	6
Severe heart failure due to other cardiomyopathy	1547	6
**Atrial fibrillation and flutter**	500	3

GBD 2017 Cause and Sequela Hierarchy

Level 5 Sequelae are nested under causes. Most detailed sequelae are nested under their parent sequela where they exist

Cause or Sequela Name (Causes in bold)	GBD ID	Level
Asymptomatic atrial fibrillation and flutter	809	5
Symptomatic atrial fibrillation and flutter	913	5
**Aortic aneurysm**	501	3
**Peripheral artery disease**	502	3
Asymptomatic peripheral arterial disease	964	5
Symptomatic claudication due to peripheral arterial disease	1041	5
**Endocarditis**	503	3
Acute endocarditis	6098	5
Moderate endocarditis	413	6
Severe endocarditis	414	6
Heart failure due to endocarditis	6428	5
Controlled, medically managed heart failure due to endocarditis	5741	6
Mild heart failure due to endocarditis	415	6
Moderate heart failure due to endocarditis	416	6

GBD 2017 Cause and Sequela Hierarchy

Level 5 Sequelae are nested under causes. Most detailed sequelae are nested under their parent sequela where they exist

Cause or Sequela Name (Causes in bold)	GBD ID	Level
Severe heart failure due to endocarditis	417	6
**Other cardiovascular and circulatory diseases**	507	3
Other cardiovascular and circulatory disease episodes	6677	5
Asymptomatic other cardiovascular diseases	839	6
Mild other cardiovascular diseases	840	6
Moderate other cardiovascular diseases	841	6
Severe other cardiovascular diseases	842	6
Heart failure due to other cardiovascular diseases	6452	5
Controlled, medically managed heart failure due to other cardiovascular disease	5759	6
Mild heart failure due to other cardiovascular diseases	418	6
Moderate heart failure due to other cardiovascular diseases	419	6
Severe heart failure due to other cardiovascular diseases	420	6
**Chronic respiratory diseases**	508	2
**Chronic obstructive pulmonary disease**	509	3

GBD 2017 Cause and Sequela Hierarchy		
Level 5 Sequelae are nested under causes. Most detailed sequelae are nested under their parent sequela where they exist		
**Cause or Sequela Name (Causes in bold)**	**GBD ID**	**Level**
Chronic obstructive pulmonary disease without heart failure	6260	5
Asymptomatic chronic obstructive pulmonary disease	929	6
Mild chronic obstructive pulmonary disease	421	6
Moderate chronic obstructive pulmonary disease	422	6
Severe chronic obstructive pulmonary disease without heart failure	983	6
Chronic obstructive pulmonary disease with heart failure	6257	5
Controlled, medically managed heart failure due to severe chronic obstructive pulmonary disease	5774	6
Mild heart failure due to severe chronic obstructive pulmonary disease	980	6
Moderate heart failure due to severe chronic obstructive pulmonary disease	981	6
Severe heart failure due to severe chronic obstructive pulmonary disease	982	6
**Pneumoconiosis**	510	3
**Silicosis**	511	4
Silicosis without heart failure	6797	5
Asymptomatic silicosis	948	6

GBD 2017 Cause and Sequela Hierarchy		
Level 5 Sequelae are nested under causes. Most detailed sequelae are nested under their parent sequela where they exist		
**Cause or Sequela Name (Causes in bold)**	**GBD ID**	**Level**
Mild silicosis	423	6
Moderate silicosis	424	6
Severe silicosis without heart failure	987	6
Heart failure due to silicosis	6464	5
Controlled, medically managed heart failure due to severe silicosis	5786	6
Mild heart failure due to severe silicosis	984	6
Moderate heart failure due to severe silicosis	985	6
Severe heart failure due to severe silicosis	986	6
**Asbestosis**	512	4
Asbestosis without heart failure	6173	5
Asymptomatic asbestosis	949	6
Mild asbestosis	425	6
Moderate asbestosis	426	6
Severe asbestosis without heart failure	991	6

Cause or Sequela Name (Causes in bold)	GBD ID	Level
Heart failure due to asbestosis	6416	5
Controlled, medically managed heart failure due to severe asbestosis	5771	6
Mild heart failure due to severe asbestosis	988	6
Moderate heart failure due to severe asbestosis	989	6
Severe heart failure due to severe asbestosis	990	6
**Coal workers pneumoconiosis**	513	4
Coal workers pneumoconiosis without heart failure	6275	5
Asymptomatic coal workers pneumoconiosis	950	6
Mild coal workers pneumoconiosis	427	6
Moderate coal workers pneumoconiosis	428	6
Severe coal workers pneumoconiosis without heart failure	995	6
Heart failure due to Coal workers pneumoconiosis	6422	5
Controlled, medically managed heart failure due to severe coal workers pneumoconiosis	5777	6
Mild heart failure due to severe coal workers pneumoconiosis	992	6

GBD 2017 Cause and Sequela Hierarchy

Level 5 Sequelae are nested under causes. Most detailed sequelae are nested under their parent sequela where they exist

Cause or Sequela Name (Causes in bold)	GBD ID	Level
Moderate heart failure due to severe coal workers pneumoconiosis	993	6
Severe heart failure due to severe coal workers pneumoconiosis	994	6
**Other pneumoconiosis**	514	4
Other pneumoconiosis without heart failure	6716	5
Asymptomatic other pneumoconiosis	951	6
Mild other pneumoconiosis	429	6
Moderate other pneumoconiosis	430	6
Severe other pneumoconiosis without heart failure	999	6
Heart failure due to other pneumoconiosis	6458	5
Controlled, medically managed heart failure due to severe other pneumoconiosis	5783	6
Mild heart failure due to severe other pneumoconiosis	996	6
Moderate heart failure due to severe other pneumoconiosis	997	6
Severe heart failure due to severe other pneumoconiosis	998	6
**Asthma**	515	3

GBD 2017 Cause and Sequela Hierarchy

Level 5 Sequelae are nested under causes. Most detailed sequelae are nested under their parent sequela where they exist

Cause or Sequela Name (Causes in bold)	GBD ID	Level
Asymptomatic asthma	830	5
Symptomatic asthma	6863	5
Controlled asthma	431	6
Partially controlled asthma	432	6
Uncontrolled asthma	433	6
**Interstitial lung disease and pulmonary sarcoidosis**	516	3
Interstitial lung disease and pulmonary sarcoidosis without heart failure	6521	5
Asymptomatic interstitial lung disease and pulmonary sarcoidosis	952	6
Mild interstitial lung disease and pulmonary sarcoidosis	434	6
Moderate interstitial lung disease and pulmonary sarcoidosis	435	6
Severe interstitial lung disease and pulmonary sarcoidosis without heart failure	1003	6
Heart failure due to interstitial lung disease and pulmonary sarcoidosis	6437	5
Controlled, medically managed heart failure due to severe interstitial lung disease and pulmonary sarcoidosis	5780	6
Mild heart failure due to severe interstitial lung disease and pulmonary sarcoidosis	1000	6

GBD 2017 Cause and Sequela Hierarchy

Level 5 Sequelae are nested under causes. Most detailed sequelae are nested under their parent sequela where they exist

Cause or Sequela Name (Causes in bold)	GBD ID	Level
Moderate heart failure due to severe interstitial lung disease and pulmonary sarcoidosis	1001	6
Severe heart failure due to severe interstitial lung disease and pulmonary sarcoidosis	1002	6
**Other chronic respiratory diseases**	520	3
Other chronic respiratory diseases	436	5
**Digestive diseases**	526	2
**Cirrhosis and other chronic liver diseases**	521	3
**Cirrhosis and other chronic liver diseases due to hepatitis B**	522	4
Chronic hepatitis B without cirrhosis	902	5
Cirrhosis and other chronic liver diseases due to hepatitis B, compensated	5135	5
Cirrhosis and other chronic liver diseases due to hepatitis B, decompensated	835	5
**Cirrhosis and other chronic liver diseases due to hepatitis C**	523	4
Chronic hepatitis C without cirrhosis	903	5
Cirrhosis and other chronic liver diseases due to hepatitis C, compensated	5138	5
Cirrhosis and other chronic liver diseases due to hepatitis C, decompensated	836	5

GBD 2017 Cause and Sequela Hierarchy

Level 5 Sequelae are nested under causes. Most detailed sequelae are nested under their parent sequela where they exist

Cause or Sequela Name (Causes in bold)	GBD ID	Level
**Cirrhosis and other chronic liver diseases due to alcohol use**	524	4
Cirrhosis and other chronic liver diseases due to alcohol, compensated	5141	5
Cirrhosis and other chronic liver diseases due to alcohol, decompensated	834	5
**Cirrhosis due to NASH**	971	4
Non-alcoholic fatty liver disease (NAFLD) / Non-alcoholic steatohepatitis (NASH)	5477	5
Cirrhosis and other chronic liver diseases due to NASH	6269	5
Cirrhosis and other chronic liver diseases due to NASH, compensated	5387	6
Cirrhosis and other chronic liver diseases due to NASH, decompensated	5390	6
**Cirrhosis and other chronic liver diseases due to other causes**	525	4
Cirrhosis and other chronic liver diseases due to other cause, compensated	5144	5
Cirrhosis and other chronic liver diseases due to other cause, decompensated	837	5
**Upper digestive system diseases**	992	3
**Peptic ulcer disease**	527	4
Acute peptic ulcer disease	22706	5

GBD 2017 Cause and Sequela Hierarchy		
Level 5 Sequelae are nested under causes. Most detailed sequelae are nested under their parent sequela where they exist		
**Cause or Sequela Name (Causes in bold)**	**GBD ID**	**Level**
Acute peptic ulcer disease, with no anemia	7199	6
Acute peptic ulcer disease, with mild anemia	7202	6
Acute peptic ulcer disease, with moderate anemia	7205	6
Acute peptic ulcer disease, with severe anemia	7208	6
Chronic peptic ulcer disease	22709	5
Asymptomatic peptic ulcer disease without anemia	5045	6
Asymptomatic peptic ulcer disease with mild anemia	4976	6
Asymptomatic peptic ulcer disease with moderate anemia	4979	6
Asymptomatic peptic ulcer disease with severe anemia	4982	6
Mild symptomatic peptic ulcer disease without anemia	4970	6
Mild symptomatic peptic ulcer disease with mild anemia	4952	6
Mild symptomatic peptic ulcer disease with moderate anemia	4958	6
Mild symptomatic peptic ulcer disease with severe anemia	4964	6
Moderate symptomatic peptic ulcer disease without anemia	4973	6

GBD 2017 Cause and Sequela Hierarchy

Level 5 Sequelae are nested under causes. Most detailed sequelae are nested under their parent sequela where they exist

Cause or Sequela Name (Causes in bold)	GBD ID	Level
Moderate symptomatic peptic ulcer disease with mild anemia	4955	6
Moderate symptomatic peptic ulcer disease with moderate anemia	4961	6
Moderate symptomatic peptic ulcer disease with severe anemia	4967	6
Complicated peptic ulcer disease with no anemia	5624	6
Complicated peptic ulcer disease with mild anemia	5627	6
Complicated peptic ulcer disease with moderate anemia	5630	6
Complicated peptic ulcer disease with severe anemia	5633	6
**Gastritis and duodenitis**	528	4
Acute gastritis and duodenitis	22712	5
Acute gastritis and duodenitis, with no anemia	7211	6
Acute gastritis and duodenitis, with mild anemia	7214	6
Acute gastritis and duodenitis, with moderate anemia	7217	6
Acute gastritis and duodenitis, with severe anemia	7220	6
Chronic gastritis and duodenitis	22715	5

GBD 2017 Cause and Sequela Hierarchy

Level 5 Sequelae are nested under causes. Most detailed sequelae are nested under their parent sequela where they exist

Cause or Sequela Name (Causes in bold)	GBD ID	Level
Asymptomatic gastritis and duodenitis without anemia	5048	6
Asymptomatic gastritis and duodenitis with mild anemia	5009	6
Asymptomatic gastritis and duodenitis with moderate anemia	5012	6
Asymptomatic gastritis and duodenitis with severe anemia	5015	6
Mild symptomatic gastritis and duodenitis without anemia	5003	6
Mild symptomatic gastritis and duodenitis with mild anemia	4985	6
Mild symptomatic gastritis and duodenitis with moderate anemia	4991	6
Mild symptomatic gastritis and duodenitis with severe anemia	4997	6
Moderate symptomatic gastritis and duodenitis without anemia	5006	6
Moderate symptomatic gastritis and duodenitis with mild anemia	4988	6
Moderate symptomatic gastritis and duodenitis with moderate anemia	4994	6
Moderate symptomatic gastritis and duodenitis with severe anemia	5000	6
Complicated gastritis and duodenitis with no anemia	5675	6
Complicated gastritis and duodenitis with mild anemia	5678	6

GBD 2017 Cause and Sequela Hierarchy		
Level 5 Sequelae are nested under causes. Most detailed sequelae are nested under their parent sequela where they exist		
**Cause or Sequela Name (Causes in bold)**	**GBD ID**	**Level**
Complicated gastritis and duodenitis with moderate anemia	5681	6
Complicated gastritis and duodenitis with severe anemia	5684	6
**Gastroesophageal reflux disease**	536	4
Gastroesophageal reflux disease	22718	5
Mild to moderate GERD, asymptomatic days	7187	6
Mild to moderate GERD, symptomatic days	7184	6
Severe GERD, asymptomatic days	7193	6
Severe GERD, symptomatic days	7190	6
**Appendicitis**	529	3
Appendicitis	445	5
**Paralytic ileus and intestinal obstruction**	530	3
Paralytic ileus and intestinal obstruction	446	5
**Inguinal, femoral, and abdominal hernia**	531	3
Inguinal, femoral and abdominal hernia	22721	5

GBD 2017 Cause and Sequela Hierarchy		
Level 5 Sequelae are nested under causes. Most detailed sequelae are nested under their parent sequela where they exist		
**Cause or Sequela Name (Causes in bold)**	**GBD ID**	**Level**
Mild symptomatic inguinal, femoral and abdominal hernia	4595	6
Moderate symptomatic inguinal, femoral and abdominal hernia	4598	6
Severe symptomatic inguinal, femoral and abdominal hernia	4601	6
**Inflammatory bowel disease**	532	3
Ulcerative colitis	954	5
Asymptomatic ulcerative colitis	4604	6
Ulcerative colitis without anemia	5576	6
Ulcerative colitis with mild anemia	5567	6
Ulcerative colitis with moderate anemia	5570	6
Ulcerative colitis with severe anemia	5573	6
Crohn's disease	955	5
Asymptomatic Crohn's disease	4607	6
Crohn's disease without anemia	5588	6
Crohn's disease with mild anemia	5579	6

GBD 2017 Cause and Sequela Hierarchy

Level 5 Sequelae are nested under causes. Most detailed sequelae are nested under their parent sequela where they exist

Cause or Sequela Name (Causes in bold)	GBD ID	Level
Crohn's disease with moderate anemia	5582	6
Crohn's disease with severe anemia	5585	6
**Vascular intestinal disorders**	533	3
Vascular intestinal disorders	448	5
**Gallbladder and biliary diseases**	534	3
Gallbladder and biliary diseases	449	5
Asymptomatic gallbladder and biliary diseases	1135	6
Mild symptomatic episodes gallbladder and biliary diseases	4586	6
Moderate symptomatic episodes gallbladder and biliary diseases	4589	6
Severe symptomatic episodes gallbladder and biliary diseases	4592	6
**Pancreatitis**	535	3
Acute pancreatitis	5621	5
Chronic pancreatitis	22724	5
Asymptomatic chronic pancreatitis	7196	6

GBD 2017 Cause and Sequela Hierarchy<br>Level 5 Sequelae are nested under causes. Most detailed sequelae are nested under their parent sequela where they exist		
**Cause or Sequela Name (Causes in bold)**	**GBD ID**	**Level**
Mild chronic pancreatitis	4610	6
Moderate chronic pancreatitis	4613	6
Severe chronic pancreatitis	4616	6
**Other digestive diseases**	541	3
Other digestive diseases	451	5
**Neurological disorders**	542	2
**Alzheimer's disease and other dementias**	543	3
Alzheimer's disease and other dementias	22727	5
Mild Alzheimer's disease and other dementias	452	6
Moderate Alzheimer's disease and other dementias	453	6
Severe Alzheimer's disease and other dementias	454	6
**Parkinson's disease**	544	3
Parkinson's disease	22730	5
Mild Parkinson's disease	455	6

GBD 2017 Cause and Sequela Hierarchy

Level 5 Sequelae are nested under causes. Most detailed sequelae are nested under their parent sequela where they exist

Cause or Sequela Name (Causes in bold)	GBD ID	Level
Moderate Parkinson's disease	456	6
Severe Parkinson's disease	457	6
**Epilepsy**	545	3
Idiopathic epilepsy	6500	5
Idiopathic, seizure-free, treated epilepsy	458	6
Idiopathic, less severe epilepsy	459	6
Idiopathic, severe epilepsy	460	6
**Multiple sclerosis**	546	3
Multiple sclerosis	6632	5
Asymptomatic multiple sclerosis	4949	6
Mild multiple sclerosis	461	6
Moderate multiple sclerosis	462	6
Severe multiple sclerosis	463	6
**Motor neuron disease**	554	3

Cause or Sequela Name (Causes in bold)	GBD ID	Level
Motor neuron disease	6626	5
Diagnosis of motor neuron disease	1183	6
Mild motor impairment due to motor neuron disease	1173	6
Mild motor impairment and speech problems due to motor neuron disease	1156	6
Mild motor impairment and mild respiratory problems due to motor neuron disease	1163	6
Mild motor impairment and moderate respiratory problems due to motor neuron disease	22733	6
Mild motor impairment and severe respiratory problems due to motor neuron disease	1164	6
Mild motor impairment, mild respiratory problems, and speech problems due to motor neuron disease	1153	6
Mild motor impairment, moderate respiratory problems, and speech problems due to motor neuron disease	1154	6
Mild motor impairment, severe respiratory problems and speech problems due to motor neuron disease	1155	6
Moderate motor impairment due to motor neuron disease	1174	6
Moderate motor impairment and speech problems due to motor neuron disease	1171	6
Moderate motor impairment and mild respiratory problems due to motor neuron disease	1165	6
Moderate motor impairment and moderate respiratory problems due to motor neuron disease	1166	6

GBD 2017 Cause and Sequela Hierarchy

Level 5 Sequelae are nested under causes. Most detailed sequelae are nested under their parent sequela where they exist

Cause or Sequela Name (Causes in bold)	GBD ID	Level
Moderate motor impairment and severe respiratory problems due to motor neuron disease	1167	6
Moderate motor impairment, mild respiratory problems, and speech problems due to motor neuron disease	1157	6
Moderate motor impairment, moderate respiratory problems, and speech problems due to motor neuron disease	1158	6
Moderate motor impairment, severe respiratory problems, and speech problems due to motor neuron disease	1159	6
Severe motor impairment due to motor neuron disease	1175	6
Severe motor impairment and speech problems due to motor neuron disease	1172	6
Severe motor impairment and mild respiratory problems due to motor neuron disease	1168	6
Severe motor impairment and moderate respiratory problems due to motor neuron disease	1169	6
Severe motor impairment and severe respiratory problems due to motor neuron disease	1170	6
Severe motor impairment, mild respiratory problems, and speech problems due to motor neuron disease	1160	6
Severe motor impairment, moderate respiratory problems, and speech problems due to motor neuron disease	1161	6
Severe motor impairment, severe respiratory problems, and speech problems due to motor neuron disease	1162	6
Mild respiratory problems due to motor neuron disease	1179	6
Moderate respiratory problems due to motor neuron disease	1180	6

GBD 2017 Cause and Sequela Hierarchy

Level 5 Sequelae are nested under causes. Most detailed sequelae are nested under their parent sequela where they exist

Cause or Sequela Name (Causes in bold)	GBD ID	Level
Severe respiratory problems due to motor neuron disease	1181	6
Mild respiratory problems and speech problems due to motor neuron disease	1176	6
Moderate respiratory problems and speech problems due to motor neuron disease	1177	6
Severe respiratory problems and speech problems due to motor neuron disease	1178	6
Speech problems due to motor neuron disease	1182	6
**Headache disorders**	972	3
**Migraine**	547	4
Migraine	6623	5
Symptomatic probable migraine	7166	6
Asymptomatic probable migraine	7169	6
Symptomatic definite migraine	7172	6
Asymptomatic definite migraine	7175	6
Medication overuse headache due to migraine	6614	5
Asymptomatic medication overuse headache due to migraine	1346	6

GBD 2017 Cause and Sequela Hierarchy

Level 5 Sequelae are nested under causes. Most detailed sequelae are nested under their parent sequela where they exist

Cause or Sequela Name (Causes in bold)	GBD ID	Level
Symptomatic medication overuse headache due to migraine	1349	6
**Tension-type headache**	548	4
Tension-type headache	6869	5
Asymptomatic tension-type headache	828	6
Symptomatic tension-type headache	827	6
Medication overuse headache due to tension-type headache	6617	5
Asymptomatic medication overuse headache due to tension-type headache	1352	6
Symptomatic medication overuse headache due to tension-type headache	1355	6
**Other neurological disorders**	557	3
Other neurological disorders	464	5
Guillain-Barré syndrome due to other neurological disorders	465	5
**Mental disorders**	558	2
**Schizophrenia**	559	3
Schizophrenia	6785	5

GBD 2017 Cause and Sequela Hierarchy

Level 5 Sequelae are nested under causes. Most detailed sequelae are nested under their parent sequela where they exist

Cause or Sequela Name (Causes in bold)	GBD ID	Level
Schizophrenia acute state	466	6
Schizophrenia residual state	467	6
**Depressive disorders**	567	3
**Major depressive disorder**	568	4
Major depressive disorder	6575	5
Major depressive disorder, currently without symptoms	822	6
Mild major depressive disorder	475	6
Moderate major depressive disorder	476	6
Severe major depressive disorder	477	6
**Dysthymia**	569	4
Dysthymia	6323	5
Dysthymia, currently without symptoms	821	6
Symptomatic dysthymia	820	6
**Bipolar disorder**	570	3

GBD 2017 Cause and Sequela Hierarchy

Level 5 Sequelae are nested under causes. Most detailed sequelae are nested under their parent sequela where they exist

Cause or Sequela Name (Causes in bold)	GBD ID	Level
Bipolar disorder	6215	5
Bipolar disorder manic state	478	6
Bipolar disorder residual state	479	6
Bipolar disorder depressive state	644	6
**Anxiety disorders**	571	3
Anxiety disorders	6170	5
Anxiety disorders, currently without symptoms	814	6
Mild anxiety disorders	480	6
Moderate anxiety disorders	481	6
Severe anxiety disorders	482	6
**Eating disorders**	572	3
**Anorexia nervosa**	573	4
Anorexia nervosa	483	5
**Bulimia nervosa**	574	4

GBD 2017 Cause and Sequela Hierarchy

Level 5 Sequelae are nested under causes. Most detailed sequelae are nested under their parent sequela where they exist

Cause or Sequela Name (Causes in bold)	GBD ID	Level
Bulimia nervosa	484	5
**Autism spectrum disorders**	575	3
Autism spectrum disorders	6206	5
Autism spectrum disorders without intellectual disability	1327	6
Autism spectrum disorders with borderline intellectual disability	1328	6
Autism spectrum disorders with mild intellectual disability	1329	6
Autism spectrum disorders with moderate intellectual disability	1330	6
Autism spectrum disorders with severe intellectual disability	1331	6
Autism spectrum disorders with profound intellectual disability	1332	6
**Attention-deficit/hyperactivity disorder**	578	3
Attention-deficit/hyperactivity disorder	6203	5
Attention-deficit/hyperactivity disorder, currently without symptoms	811	6
Symptomatic attention-deficit/hyperactivity disorder	810	6
**Conduct disorder**	579	3

GBD 2017 Cause and Sequela Hierarchy

Level 5 Sequelae are nested under causes. Most detailed sequelae are nested under their parent sequela where they exist

Cause or Sequela Name (Causes in bold)	GBD ID	Level
Conduct disorder	6284	5
Conduct disorder, currently without symptoms	816	6
Symptomatic conduct disorder	815	6
**Idiopathic developmental intellectual disability**	582	3
Idiopathic developmental intellectual disability	6497	5
Borderline idiopathic developmental intellectual disability	487	6
Mild idiopathic developmental intellectual disability	488	6
Moderate idiopathic developmental intellectual disability	489	6
Severe idiopathic developmental intellectual disability	490	6
Profound idiopathic developmental intellectual disability	491	6
**Other mental disorders**	585	3
Other mental disorders	6701	5
Other mental disorders, currently without symptoms	851	6
Mild other mental disorders	492	6

GBD 2017 Cause and Sequela Hierarchy

Level 5 Sequelae are nested under causes. Most detailed sequelae are nested under their parent sequela where they exist

Cause or Sequela Name (Causes in bold)	GBD ID	Level
Moderate other mental disorders	680	6
Severe other mental disorders	681	6
**Substance use disorders**	973	2
**Alcohol use disorders**	560	3
Alcohol dependence	6137	5
Asymptomatic alcohol dependence	812	6
Very mild alcohol dependence	678	6
Mild alcohol dependence	468	6
Moderate alcohol dependence	469	6
Severe alcohol dependence	470	6
Fetal alcohol syndrome	6368	5
Asymptomatic fetal alcohol syndrome	813	6
Mild fetal alcohol syndrome	471	6
Moderate fetal alcohol syndrome	472	6

GBD 2017 Cause and Sequela Hierarchy

Level 5 Sequelae are nested under causes. Most detailed sequelae are nested under their parent sequela where they exist

Cause or Sequela Name (Causes in bold)	GBD ID	Level
Severe fetal alcohol syndrome	473	6
**Drug use disorders**	561	3
**Opioid use disorders**	562	4
Opioid dependence	22736	5
Asymptomatic opioid dependence	818	6
Mild opioid dependence	690	6
Severe opioid dependence	804	6
**Cocaine use disorders**	563	4
Cocaine dependence	22739	5
Asymptomatic cocaine dependence	819	6
Mild cocaine dependence	687	6
Severe cocaine dependence	805	6
**Amphetamine use disorders**	564	4
Amphetamine dependence	22742	5

Cause or Sequela Name (Causes in bold)	GBD ID	Level
Asymptomatic amphetamine dependence	833	6
Mild amphetamine dependence	679	6
Severe amphetamine dependence	806	6
**Cannabis use disorders**	565	4
Cannabis dependence	22745	5
Asymptomatic cannabis dependence	817	6
Mild cannabis dependence	686	6
Severe cannabis dependence	807	6
**Other drug use disorders**	566	4
Other drug use disorders	474	5
**Diabetes and kidney diseases**	974	2
**Diabetes mellitus**	587	3
**Diabetes mellitus type 1**	975	4
Uncomplicated diabetes mellitus type 1	5441	5

Cause or Sequela Name (Causes in bold)	GBD ID	Level
Vision loss due to diabetes mellitus type 1 retinopathy	22748	5
Moderate vision impairment due to diabetes mellitus type 1 retinopathy	5444	6
Severe vision impairment due to diabetes mellitus type 1 retinopathy	5447	6
Blindness due to diabetes mellitus type 1 retinopathy	5450	6
Diabetic neuropathy due to diabetes mellitus type 1	22853	5
Diabetic neuropathy due to diabetes mellitus type 1, without diabetic foot or amputation	5432	6
Diabetic foot due to neuropathy due to diabetes mellitus type 1	5429	6
Diabetic neuropathy and amputation with treatment due to diabetes mellitus type 1	5435	6
Diabetic neuropathy and amputation without treatment due to diabetes mellitus type 1	5438	6
**Diabetes mellitus type 2**	976	4
Uncomplicated diabetes mellitus type 2	5465	5
Vision loss due to diabetes mellitus type 2 retinopathy	22751	5
Moderate vision impairment due to diabetes mellitus type 2 retinopathy	5468	6
Severe vision impairment due to diabetes mellitus type 2 retinopathy	5471	6

GBD 2017 Cause and Sequela Hierarchy

Level 5 Sequelae are nested under causes. Most detailed sequelae are nested under their parent sequela where they exist

Cause or Sequela Name (Causes in bold)	GBD ID	Level
Blindness due to diabetes mellitus type 2 retinopathy	5474	6
Diabetic neuropathy due to diabetes mellitus type 2	22856	5
Diabetic neuropathy due to diabetes mellitus type 2, without diabetic foot or amputation	5456	6
Diabetic foot due to neuropathy due to diabetes mellitus type 2	5453	6
Diabetic neuropathy and amputation with treatment due to diabetes mellitus type 2	5459	6
Diabetic neuropathy and amputation without treatment due to diabetes mellitus type 2	5462	6
**Chronic kidney disease**	589	3
**Chronic kidney disease due to diabetes mellitus type 1**	997	4
Albuminuria with preserved GFR due to type 1 diabetes mellitus	5540	5
Stage III chronic kidney disease due to diabetes mellitus type 1	6812	5
Stage III chronic kidney disease and mild anemia due to type 1 diabetes mellitus	5225	6
Stage III chronic kidney disease and moderate anemia due to type 1 diabetes mellitus	5219	6
Stage III chronic kidney disease and severe anemia due to type 1 diabetes mellitus	5213	6
Stage III chronic kidney disease without anemia due to type 1 diabetes mellitus	5231	6

Cause or Sequela Name (Causes in bold)	GBD ID	Level
Stage IV chronic kidney disease due to diabetes mellitus type 1	6827	5
Stage IV chronic kidney disease untreated and mild anemia due to type 1 diabetes mellitus	5249	6
Stage IV chronic kidney disease untreated and moderate anemia due to type 1 diabetes mellitus	5243	6
Stage IV chronic kidney disease untreated and severe anemia due to type 1 diabetes mellitus	5237	6
Stage IV chronic kidney disease untreated without anemia due to type 1 diabetes mellitus	5255	6
Stage V chronic kidney disease due to diabetes mellitus type 1	6842	5
Stage V chronic kidney disease untreated and mild anemia due to type 1 diabetes mellitus	5273	6
Stage V chronic kidney disease untreated and moderate anemia due to type 1 diabetes mellitus	5267	6
Stage V chronic kidney disease untreated and severe anemia due to type 1 diabetes mellitus	5261	6
Stage V chronic kidney disease untreated without anemia due to type 1 diabetes mellitus	5279	6
End-stage chronic kidney disease due to diabetes mellitus type 1	6341	5
End-stage renal disease after transplant due to type 1 diabetes mellitus	5201	6
End-stage renal disease on dialysis due to type 1 diabetes mellitus	5207	6
**Chronic kidney disease due to diabetes mellitus type 2**	998	4

GBD 2017 Cause and Sequela Hierarchy

Level 5 Sequelae are nested under causes. Most detailed sequelae are nested under their parent sequela where they exist

Cause or Sequela Name (Causes in bold)	GBD ID	Level
Albuminuria with preserved GFR due to type 2 diabetes mellitus	5543	5
Stage III chronic kidney disease due to diabetes mellitus type 2	6815	5
Stage III chronic kidney disease and mild anemia due to type 2 diabetes mellitus	5228	6
Stage III chronic kidney disease and moderate anemia due to type 2 diabetes mellitus	5222	6
Stage III chronic kidney disease and severe anemia due to type 2 diabetes mellitus	5216	6
Stage III chronic kidney disease without anemia due to type 2 diabetes mellitus	5234	6
Stage IV chronic kidney disease due to diabetes mellitus type 2	6830	5
Stage IV chronic kidney disease untreated and mild anemia due to type 2 diabetes mellitus	5252	6
Stage IV chronic kidney disease untreated and moderate anemia due to type 2 diabetes mellitus	5246	6
Stage IV chronic kidney disease untreated and severe anemia due to type 2 diabetes mellitus	5240	6
Stage IV chronic kidney disease untreated without anemia due to type 2 diabetes mellitus	5258	6
Stage V chronic kidney disease due to diabetes mellitus type 2	6845	5
Stage V chronic kidney disease untreated and mild anemia due to type 2 diabetes mellitus	5276	6
Stage V chronic kidney disease untreated and moderate anemia due to type 2 diabetes mellitus	5270	6

GBD 2017 Cause and Sequela Hierarchy

Level 5 Sequelae are nested under causes. Most detailed sequelae are nested under their parent sequela where they exist

Cause or Sequela Name (Causes in bold)	GBD ID	Level
Stage V chronic kidney disease untreated and severe anemia due to type 2 diabetes mellitus	5264	6
Stage V chronic kidney disease untreated without anemia due to type 2 diabetes mellitus	5282	6
End-stage chronic kidney disease due to diabetes mellitus type 2	6344	5
End-stage renal disease after transplant due to type 2 diabetes mellitus	5204	6
End-stage renal disease on dialysis due to type 2 diabetes mellitus	5210	6
**Chronic kidney disease due to hypertension**	591	4
Albuminuria with preserved GFR due to hypertension	5546	5
Stage III chronic kidney disease due to hypertension	6821	5
Stage III chronic kidney disease and mild anemia due to hypertension	1016	6
Stage III chronic kidney disease and moderate anemia due to hypertension	1017	6
Stage III chronic kidney disease and severe anemia due to hypertension	1018	6
Stage III chronic kidney disease without anemia due to hypertension	1019	6
Stage IV chronic kidney disease due to hypertension	6836	5
Stage IV chronic kidney disease untreated and mild anemia due to hypertension	1421	6

GBD 2017 Cause and Sequela Hierarchy

Level 5 Sequelae are nested under causes. Most detailed sequelae are nested under their parent sequela where they exist

Cause or Sequela Name (Causes in bold)	GBD ID	Level
Stage IV chronic kidney disease untreated and moderate anemia due to hypertension	1424	6
Stage IV chronic kidney disease untreated and severe anemia due to hypertension	1427	6
Stage IV chronic kidney disease untreated without anemia due to hypertension	1418	6
Stage V chronic kidney disease due to hypertension	6851	5
Stage V chronic kidney disease untreated and mild anemia due to hypertension	1373	6
Stage V chronic kidney disease untreated and moderate anemia due to hypertension	1376	6
Stage V chronic kidney disease untreated and severe anemia due to hypertension	1379	6
Stage V chronic kidney disease untreated without anemia due to hypertension	1370	6
End-stage chronic kidney disease due to hypertension	6350	5
End-stage renal disease after transplant due to hypertension	501	6
End-stage renal disease on dialysis due to hypertension	502	6
**Chronic kidney disease due to glomerulonephritis**	592	4
Albuminuria with preserved GFR due to glomerulonephritis	5549	5
Stage III chronic kidney disease due to glomerulonephritis	6818	5

GBD 2017 Cause and Sequela Hierarchy

Level 5 Sequelae are nested under causes. Most detailed sequelae are nested under their parent sequela where they exist

Cause or Sequela Name (Causes in bold)	GBD ID	Level
Stage III chronic kidney disease and mild anemia due to glomerulonephritis	1024	6
Stage III chronic kidney disease and moderate anemia due to glomerulonephritis	1025	6
Stage III chronic kidney disease and severe anemia due to glomerulonephritis	1026	6
Stage III chronic kidney disease without anemia due to glomerulonephritis	1027	6
Stage IV chronic kidney disease due to glomerulonephritis	6833	5
Stage IV chronic kidney disease untreated and mild anemia due to glomerulonephritis	1433	6
Stage IV chronic kidney disease untreated and moderate anemia due to glomerulonephritis	1436	6
Stage IV chronic kidney disease untreated and severe anemia due to glomerulonephritis	1439	6
Stage IV chronic kidney disease untreated without anemia due to glomerulonephritis	1430	6
Stage V chronic kidney disease due to glomerulonephritis	6848	5
Stage V chronic kidney disease untreated and mild anemia due to glomerulonephritis	1385	6
Stage V chronic kidney disease untreated and moderate anemia due to glomerulonephritis	1388	6
Stage V chronic kidney disease untreated and severe anemia due to glomerulonephritis	1391	6
Stage V chronic kidney disease untreated without anemia due to glomerulonephritis	1382	6

GBD 2017 Cause and Sequela Hierarchy<br>Level 5 Sequelae are nested under causes. Most detailed sequelae are nested under their parent sequela where they exist		
**Cause or Sequela Name (Causes in bold)**	**GBD ID**	**Level**
End-stage chronic kidney disease due to glomerulonephritis	6347	5
End-stage renal disease after transplant due to glomerulonephritis	504	6
End-stage renal disease on dialysis due to glomerulonephritis	505	6
**Chronic kidney disease due to other and unspecified causes**	593	4
Albuminuria with preserved GFR due to other and unspecified causes	5552	5
Stage III chronic kidney disease due to other causes	6824	5
Stage III chronic kidney disease and mild anemia due to other and unspecified causes	1032	6
Stage III chronic kidney disease and moderate anemia due to other and unspecified causes	1033	6
Stage III chronic kidney disease and severe anemia due to other and unspecified causes	1034	6
Stage III chronic kidney disease without anemia due to other and unspecified causes	1035	6
Stage IV chronic kidney disease due to other causes	6839	5
Stage IV chronic kidney disease untreated and mild anemia due to other and unspecified causes	1445	6
Stage IV chronic kidney disease untreated and moderate anemia due to other and unspecified causes	1448	6
Stage IV chronic kidney disease untreated and severe anemia due to other and unspecified causes	1451	6

GBD 2017 Cause and Sequela Hierarchy

Level 5 Sequelae are nested under causes. Most detailed sequelae are nested under their parent sequela where they exist

Cause or Sequela Name (Causes in bold)	GBD ID	Level
Stage IV chronic kidney disease untreated without anemia due to other and unspecified causes	1442	6
Stage V chronic kidney disease due to other causes	6854	5
Stage V chronic kidney disease untreated and mild anemia due to other and unspecified causes	1397	6
Stage V chronic kidney disease untreated and moderate anemia due to other and unspecified causes	1400	6
Stage V chronic kidney disease untreated and severe anemia due to other and unspecified causes	1403	6
Stage V chronic kidney disease untreated without anemia due to other and unspecified causes	1394	6
End-stage chronic kidney disease due to other causes	6353	5
End-stage renal disease after transplant due to other and unspecified causes	507	6
End-stage renal disease on dialysis due to other and unspecified causes	508	6
**Acute glomerulonephritis**	588	3
Acute glomerulonephritis	497	5
**Skin and subcutaneous diseases**	653	2
**Dermatitis**	654	3
**Atopic dermatitis**	977	4

GBD 2017 Cause and Sequela Hierarchy

Level 5 Sequelae are nested under causes. Most detailed sequelae are nested under their parent sequela where they exist

Cause or Sequela Name (Causes in bold)	GBD ID	Level
Atopic dermatitis	22754	5
Mild atopic dermatitis	588	6
Moderate atopic dermatitis	589	6
Severe atopic dermatitis	590	6
**Contact dermatitis**	978	4
Contact dermatitis	22757	5
Mild contact dermatitis	659	6
Moderate contact dermatitis	660	6
Asymptomatic contact dermatitis	1047	6
**Seborrhoeic dermatitis**	979	4
Seborrhoeic dermatitis	22760	5
Symptomatic seborrhoeic dermatitis	661	6
Asymptomatic seborrhoeic dermatitis	1046	6
**Psoriasis**	655	3

GBD 2017 Cause and Sequela Hierarchy

Level 5 Sequelae are nested under causes. Most detailed sequelae are nested under their parent sequela where they exist

Cause or Sequela Name (Causes in bold)	GBD ID	Level
Psoriasis	22763	5
Mild psoriasis	591	6
Moderate psoriasis	592	6
Severe psoriasis	593	6
**Bacterial skin diseases**	980	3
**Cellulitis**	656	4
Cellulitis	22766	5
Mild cellulitis	969	6
Moderate cellulitis	4628	6
Severe cellulitis	970	6
**Pyoderma**	657	4
Impetigo	594	5
Abscess and other bacterial skin diseases	595	5
**Scabies**	658	3

GBD 2017 Cause and Sequela Hierarchy

Level 5 Sequelae are nested under causes. Most detailed sequelae are nested under their parent sequela where they exist

Cause or Sequela Name (Causes in bold)	GBD ID	Level
Scabies	596	5
**Fungal skin diseases**	659	3
Tinea capitis	597	5
Other fungal skin diseases	672	5
**Viral skin diseases**	660	3
Viral warts	7004	5
Mild viral warts	598	6
Severe viral warts	599	6
Molluscum contagiosum	7007	5
Mild molluscum contagiosum	900	6
Severe molluscum contagiosum	901	6
**Acne vulgaris**	661	3
Acne vulgaris	6092	5
Non-disabling symptomatic acne	5618	6

GBD 2017 Cause and Sequela Hierarchy<br>Level 5 Sequelae are nested under causes. Most detailed sequelae are nested under their parent sequela where they exist		
**Cause or Sequela Name (Causes in bold)**	**GBD ID**	**Level**
Mild acne vulgaris	4619	6
Moderate acne vulgaris	4622	6
Severe acne vulgaris	4625	6
**Alopecia areata**	662	3
Alopecia areata	22769	5
Mild alopecia areata	601	6
Severe alopecia areata	602	6
**Pruritus**	663	3
Pruritus	603	5
**Urticaria**	664	3
Urticaria	22772	5
Mild urticaria	604	6
Severe urticaria	605	6
**Decubitus ulcer**	665	3

GBD 2017 Cause and Sequela Hierarchy

Level 5 Sequelae are nested under causes. Most detailed sequelae are nested under their parent sequela where they exist

Cause or Sequela Name (Causes in bold)	GBD ID	Level
Decubitus ulcer	22775	5
Mild decubitus ulcer	606	6
Moderate decubitus ulcer	607	6
Severe decubitus ulcer	608	6
**Other skin and subcutaneous diseases**	668	3
Other skin and subcutaneous diseases	22778	5
Asymptomatic other skin and subcutaneous diseases	898	6
Symptomatic other skin and subcutaneous diseases	899	6
**Sense organ diseases**	669	2
**Blindness and vision impairment**	981	3
**Glaucoma**	670	4
Vision impairment due to glaucoma	22781	5
Moderate vision impairment due to glaucoma	609	6
Severe vision impairment due to glaucoma	610	6

GBD 2017 Cause and Sequela Hierarchy

Level 5 Sequelae are nested under causes. Most detailed sequelae are nested under their parent sequela where they exist

Cause or Sequela Name (Causes in bold)	GBD ID	Level
Blindness due to glaucoma	611	6
**Cataract**	671	4
Vision impairment due to cataract	22784	5
Moderate vision impairment due to cataract	612	6
Severe vision impairment due to cataract	613	6
Blindness due to cataract	614	6
**Age-related macular degeneration**	672	4
Vision impairment due to macular degeneration	22787	5
Moderate vision impairment due to macular degeneration	615	6
Severe vision impairment due to macular degeneration	616	6
Blindness due to macular degeneration	617	6
**Refraction disorders**	999	4
Vision impairment due to uncorrected refractive error	22790	5
Moderate vision impairment due to uncorrected refractive error	618	6

GBD 2017 Cause and Sequela Hierarchy

Level 5 Sequelae are nested under causes. Most detailed sequelae are nested under their parent sequela where they exist

Cause or Sequela Name (Causes in bold)	GBD ID	Level
Severe vision impairment due to uncorrected refractive error	619	6
Blindness due to uncorrected refractive error	620	6
**Near vision loss**	1000	4
Near vision loss	5483	5
**Other vision loss**	675	4
Vision impairment due to other vision loss	22793	5
Moderate vision impairment due to other vision loss	634	6
Severe vision impairment due to other vision loss	635	6
Blindness due to other vision loss	636	6
**Age-related and other hearing loss**	674	3
Age-related and other hearing loss	6134	5
Mild hearing loss due to age-related and other hearing loss	621	6
Mild hearing loss with ringing due to age-related and other hearing loss	622	6
Moderate hearing loss due to age-related and other hearing loss	623	6

GBD 2017 Cause and Sequela Hierarchy		
Level 5 Sequelae are nested under causes. Most detailed sequelae are nested under their parent sequela where they exist		
**Cause or Sequela Name (Causes in bold)**	**GBD ID**	**Level**
Moderate hearing loss with ringing due to age-related and other hearing loss	624	6
Moderately severe hearing loss due to age-related and other hearing loss	625	6
Moderately severe hearing loss with ringing due to age-related and other hearing loss	626	6
Severe hearing loss with ringing due to age-related and other hearing loss	627	6
Severe hearing loss due to age-related and other hearing loss	628	6
Profound hearing loss due to age-related and other hearing loss	629	6
Profound hearing loss with ringing due to age-related and other hearing loss	630	6
Complete hearing loss due to age-related and other hearing loss	631	6
Complete hearing loss with ringing due to age-related and other hearing loss	632	6
**Other sense organ diseases**	679	3
Chronic other sense organ diseases	2660	5
Asymptomatic chronic other sense organ diseases	1337	6
Mild chronic other sense organ diseases	1339	6
Moderate chronic other sense organ diseases	1340	6

GBD 2017 Cause and Sequela Hierarchy<br>Level 5 Sequelae are nested under causes. Most detailed sequelae are nested under their parent sequela where they exist		
**Cause or Sequela Name (Causes in bold)**	**GBD ID**	**Level**
Acute other sense organ diseases	6125	5
Asymptomatic acute other sense organ diseases	1333	6
Mild acute other sense organ diseases	1334	6
Moderate acute other sense organ diseases	1335	6
**Musculoskeletal disorders**	626	2
**Rheumatoid arthritis**	627	3
Rheumatoid arthritis	6776	5
Asymptomatic rheumatoid arthritis	7163	6
Mild rheumatoid arthritis	543	6
Moderate rheumatoid arthritis	544	6
Severe rheumatoid arthritis	545	6
**Osteoarthritis**	628	3
Osteoarthritis of the hip	6671	5
Asymptomatic osteoarthritis of the hip	7181	6

GBD 2017 Cause and Sequela Hierarchy		
Level 5 Sequelae are nested under causes. Most detailed sequelae are nested under their parent sequela where they exist		
**Cause or Sequela Name (Causes in bold)**	**GBD ID**	**Level**
Mild osteoarthritis of the hip	546	6
Moderate osteoarthritis of the hip	547	6
Severe osteoarthritis of the hip	548	6
Osteoarthritis of the knee	6674	5
Asymptomatic osteoarthritis of the knee	7178	6
Mild osteoarthritis of the knee	549	6
Moderate osteoarthritis of the knee	550	6
Severe osteoarthritis of the knee	551	6
**Low back pain**	630	3
Low back pain with leg pain	6563	5
Mild low back pain with leg pain	682	6
Moderate low back pain with leg pain	684	6
Most severe low back pain with leg pain	554	6
Severe low back pain with leg pain	552	6

GBD 2017 Cause and Sequela Hierarchy

Level 5 Sequelae are nested under causes. Most detailed sequelae are nested under their parent sequela where they exist

Cause or Sequela Name (Causes in bold)	GBD ID	Level
Low back pain without leg pain	6566	5
Mild low back pain without leg pain	683	6
Moderate low back pain without leg pain	685	6
Most severe low back pain without leg pain	555	6
Severe low back pain without leg pain	553	6
**Neck pain**	631	3
Neck pain	6641	5
Mild neck pain	556	6
Severe neck pain	557	6
Moderate neck pain	558	6
Most severe neck pain	559	6
**Gout**	632	3
Gout	6404	5
Asymptomatic gout	824	6

Cause or Sequela Name (Causes in bold)	GBD ID	Level
Symptomatic episodes of gout	823	6
Polyarticular gout	560	6
**Other musculoskeletal disorders**	639	3
Other musculoskeletal disorders	6704	5
Asymptomatic other musculoskeletal disorders	968	6
Other musculoskeletal disorders severity level 1	565	6
Other musculoskeletal disorders severity level 2	561	6
Other musculoskeletal disorders severity level 3	562	6
Other musculoskeletal disorders severity level 4	566	6
Other musculoskeletal disorders severity level 5	563	6
Other musculoskeletal disorders severity level 6	564	6
**Other non-communicable diseases**	640	2
**Congenital birth defects**	641	3
**Neural tube defects**	642	4

GBD 2017 Cause and Sequela Hierarchy

Level 5 Sequelae are nested under causes. Most detailed sequelae are nested under their parent sequela where they exist

Cause or Sequela Name (Causes in bold)	GBD ID	Level
Encephalocoele due to neural tube defects	6338	5
Asymptomatic encephalocele following treatment	1907	6
Incontinence due to encephalocele	1925	6
Borderline intellectual disability due to encephalocele	1910	6
Borderline intellectual disability and incontinence due to encephalocele	1928	6
Mild intellectual disability due to encephalocele	1913	6
Mild intellectual disability and incontinence due to encephalocele	1931	6
Moderate intellectual disability and incontinence due to encephalocele	1934	6
Moderate intellectual disability due to encephalocele	1916	6
Severe intellectual disability due to encephalocele	1919	6
Severe intellectual disability and incontinence due to encephalocele	1937	6
Profound intellectual disability due to encephalocele	1922	6
Profound intellectual disability and incontinence due to encephalocele	1940	6
Mild motor impairment due to encephalocele	2078	6

Cause or Sequela Name (Causes in bold)	GBD ID	Level
Mild motor impairment and incontinence due to encephalocele	2087	6
Mild motor impairment and borderline intellectual disability due to encephalocele	2096	6
Mild motor impairment and mild intellectual disability due to encephalocele	2105	6
Mild motor impairment and moderate intellectual disability due to encephalocele	2114	6
Mild motor impairment and severe intellectual disability due to encephalocele	2123	6
Mild motor impairment and profound intellectual disability due to encephalocele	2132	6
Mild motor impairment, borderline intellectual disability and incontinence due to encephalocele	2141	6
Mild motor impairment, mild intellectual disability and incontinence due to encephalocele	2150	6
Mild motor impairment, moderate intellectual disability and incontinence due to encephalocele	2159	6
Mild motor impairment, severe intellectual disability and incontinence due to encephalocele	2168	6
Mild motor impairment, profound intellectual disability and incontinence due to encephalocele	2177	6
Moderate motor impairment due to encephalocele	2081	6
Moderate motor impairment and incontinence due to encephalocele	2090	6
Moderate motor impairment and borderline intellectual disability due to encephalocele	2099	6

GBD 2017 Cause and Sequela Hierarchy		
Level 5 Sequelae are nested under causes. Most detailed sequelae are nested under their parent sequela where they exist		
**Cause or Sequela Name (Causes in bold)**	**GBD ID**	**Level**
Moderate motor impairment and mild intellectual disability due to encephalocele	2108	6
Moderate motor impairment and moderate intellectual disability due to encephalocele	2117	6
Moderate motor impairment and severe intellectual disability due to encephalocele	2126	6
Moderate motor impairment and profound intellectual disability due to encephalocele	2135	6
Moderate motor impairment, borderline intellectual disability and incontinence due to encephalocele	2144	6
Moderate motor impairment, mild intellectual disability and incontinence due to encephalocele	2153	6
Moderate motor impairment, moderate intellectual disability and incontinence due to encephalocele	2162	6
Moderate motor impairment, severe intellectual disability and incontinence due to encephalocele	2171	6
Moderate motor impairment, profound intellectual disability and incontinence due to encephalocele	2180	6
Severe motor impairment due to encephalocele	2084	6
Severe motor impairment and incontinence due to encephalocele	2093	6
Severe motor impairment and borderline intellectual disability due to encephalocele	2102	6
Severe motor impairment and mild intellectual disability due to encephalocele	2111	6
Severe motor impairment and moderate intellectual disability due to encephalocele	2120	6

GBD 2017 Cause and Sequela Hierarchy

Level 5 Sequelae are nested under causes. Most detailed sequelae are nested under their parent sequela where they exist

Cause or Sequela Name (Causes in bold)	GBD ID	Level
Severe motor impairment and severe intellectual disability due to encephalocele	2129	6
Severe motor impairment and profound intellectual disability due to encephalocele	2138	6
Severe motor impairment, borderline intellectual disability and incontinence due to encephalocele	2147	6
Severe motor impairment, mild intellectual disability and incontinence due to encephalocele	2156	6
Severe motor impairment, moderate intellectual disability and incontinence due to encephalocele	2165	6
Severe motor impairment, severe intellectual disability and incontinence due to encephalocele	2174	6
Severe motor impairment, profound intellectual disability and incontinence due to encephalocele	2183	6
Spina bifida due to neural tube defects	6806	5
Mild motor impairment due to spina bifida	1943	6
Mild motor impairment and incontinence due to spina bifida	2024	6
Mild motor impairment and borderline intellectual disability due to spina bifida	1952	6
Mild motor impairment and mild intellectual disability due to spina bifida	1961	6
Mild motor impairment and moderate intellectual disability due to spina bifida	1970	6
Mild motor impairment and severe intellectual disability due to spina bifida	1979	6

GBD 2017 Cause and Sequela Hierarchy

Level 5 Sequelae are nested under causes. Most detailed sequelae are nested under their parent sequela where they exist

Cause or Sequela Name (Causes in bold)	GBD ID	Level
Mild motor impairment and profound intellectual disability due to spina bifida	2060	6
Mild motor impairment, borderline intellectual disability and incontinence due to spina bifida	1988	6
Mild motor impairment, mild intellectual disability and incontinence due to spina bifida	1997	6
Mild motor impairment, moderate intellectual disability and incontinence due to spina bifida	2006	6
Mild motor impairment, severe intellectual disability and incontinence due to spina bifida	2015	6
Mild motor impairment, profound intellectual disability and incontinence due to spina bifida	2069	6
Moderate motor impairment due to spina bifida	1946	6
Moderate motor impairment and incontinence due to spina bifida	2027	6
Moderate motor impairment and borderline intellectual disability due to spina bifida	1955	6
Moderate motor impairment and mild intellectual disability due to spina bifida	1964	6
Moderate motor impairment and moderate intellectual disability due to spina bifida	1973	6
Moderate motor impairment and severe intellectual disability due to spina bifida	1982	6
Moderate motor impairment and profound intellectual disability due to spina bifida	2063	6
Moderate motor impairment, borderline intellectual disability and incontinence due to spina bifida	1991	6

GBD 2017 Cause and Sequela Hierarchy

Level 5 Sequelae are nested under causes. Most detailed sequelae are nested under their parent sequela where they exist

Cause or Sequela Name (Causes in bold)	GBD ID	Level
Moderate motor impairment, mild intellectual disability and incontinence due to spina bifida	2000	6
Moderate motor impairment, moderate intellectual disability and incontinence due to spina bifida	2009	6
Moderate motor impairment, severe intellectual disability and incontinence due to spina bifida	2018	6
Moderate motor impairment, profound intellectual disability and incontinence due to spina bifida	2072	6
Severe motor impairment due to spina bifida	1949	6
Severe motor impairment and incontinence due to spina bifida	2030	6
Severe motor impairment and borderline intellectual disability due to spina bifida	1958	6
Severe motor impairment and mild intellectual disability due to spina bifida	1967	6
Severe motor impairment and moderate intellectual disability due to spina bifida	1976	6
Severe motor impairment and severe intellectual disability due to spina bifida	1985	6
Severe motor impairment and profound intellectual disability due to spina bifida	2066	6
Severe motor impairment, borderline intellectual disability and incontinence due to spina bifida	1994	6
Severe motor impairment, mild intellectual disability and incontinence due to spina bifida	2003	6
Severe motor impairment, moderate intellectual disability and incontinence due to spina bifida	2012	6

GBD 2017 Cause and Sequela Hierarchy

Level 5 Sequelae are nested under causes. Most detailed sequelae are nested under their parent sequela where they exist

Cause or Sequela Name (Causes in bold)	GBD ID	Level
Severe motor impairment, severe intellectual disability and incontinence due to spina bifida	2021	6
Severe motor impairment, profound intellectual disability and incontinence due to spina bifida	2075	6
Severe motor and cognitive impairment due to anencephaly	1904	5
**Congenital heart anomalies**	643	4
Critical malformations of great vessels, congenital valvular heart disease and patent ductus arteriosus	6290	5
Congenital heart disease and borderline intellectual disability without heart failure due to critical malformations of great vessels, congenital valvular heart disease and patent ductus arteriosus	4805	6
Congenital heart disease and controlled, medically managed heart failure without intellectual disability due to critical malformations of great vessels, congenital valvular heart disease and patent ductus arteriosus	5792	6
Congenital heart disease and mild heart failure without intellectual disability due to critical malformations of great vessels, congenital valvular heart disease and patent ductus arteriosus	4820	6
Congenital heart disease and mild intellectual disability without heart failure due to critical malformations of great vessels, congenital valvular heart disease and patent ductus arteriosus	4808	6
Congenital heart disease and moderate heart failure without intellectual disability due to critical malformations of great vessels, congenital valvular heart disease and patent ductus arteriosus	4838	6
Congenital heart disease and moderate intellectual disability without heart failure due to critical malformations of great vessels, congenital valvular heart disease and patent ductus arteriosus	4811	6
Congenital heart disease and profound intellectual disability without heart failure due to critical malformations of great vessels, congenital valvular heart disease and patent ductus arteriosus	4817	6
Congenital heart disease and severe heart failure without intellectual disability due to critical malformations of great vessels, congenital valvular heart disease and patent ductus arteriosus	4856	6
Congenital heart disease and severe intellectual disability without heart failure due to critical malformations of great vessels, congenital valvular heart disease and patent ductus arteriosus	4814	6

Cause or Sequela Name (Causes in bold)	GBD ID	Level
Congenital heart disease without heart failure or intellectual disability due to critical malformations of great vessels, congenital valvular heart disease and patent ductus arteriosus	4802	6
Congenital heart disease, boderline intellectual disability and controlled, medically managed heart failure due to critical malformations of great vessels, congenital valvular heart disease and patent ductus arteriosus	5804	6
Congenital heart disease, boderline intellectual disability and mild heart failure due to critical malformations of great vessels, congenital valvular heart disease and patent ductus arteriosus	4823	6
Congenital heart disease, borderline intellectual disability and moderate heart failure due to critical malformations of great vessels, congenital valvular heart disease and patent ductus arteriosus	4841	6
Congenital heart disease, borderline intellectual disability and severe heart failure due to critical malformations of great vessels, congenital valvular heart disease and patent ductus arteriosus	4859	6
Congenital heart disease, mild intellectual disability and controlled, medically managed heart failure due to critical malformations of great vessels, congenital valvular heart disease and patent ductus arteriosus	5816	6
Congenital heart disease, mild intellectual disability and mild heart failure due to critical malformations of great vessels, congenital valvular heart disease and patent ductus arteriosus	4826	6
Congenital heart disease, mild intellectual disability and moderate heart failure due to critical malformations of great vessels, congenital valvular heart disease and patent ductus arteriosus	4844	6
Congenital heart disease, mild intellectual disability and severe heart failure due to critical malformations of great vessels, congenital valvular heart disease and patent ductus arteriosus	4862	6
Congenital heart disease, moderate intellectual disability and controlled, medically managed heart failure due to critical malformations of great vessels, congenital valvular heart disease and patent ductus arteriosus	5828	6
Congenital heart disease, moderate intellectual disability and mild heart failure due to critical malformations of great vessels, congenital valvular heart disease and patent ductus arteriosus	4829	6
Congenital heart disease, moderate intellectual disability and moderate heart failure due to critical malformations of great vessels, congenital valvular heart disease and patent ductus arteriosus	4847	6
Congenital heart disease, moderate intellectual disability and severe heart failure due to critical malformations of great vessels, congenital valvular heart disease and patent ductus arteriosus	4865	6
Congenital heart disease, profound intellectual disability and controlled, medically managed heart failure due to critical malformations of great vessels, congenital valvular heart disease and patent ductus arteriosus	5849	6

Cause or Sequela Name (Causes in bold)	GBD ID	Level
Congenital heart disease, profound intellectual disability and mild heart failure due to critical malformations of great vessels, congenital valvular heart disease and patent ductus arteriosus	4835	6
Congenital heart disease, profound intellectual disability and moderate heart failure due to critical malformations of great vessels, congenital valvular heart disease and patent ductus arteriosus	4853	6
Congenital heart disease, profound intellectual disability and severe heart failure due to critical malformations of great vessels, congenital valvular heart disease and patent ductus arteriosus	4871	6
Congenital heart disease, severe intellectual disability and controlled, medically managed heart failure due to critical malformations of great vessels, congenital valvular heart disease and patent ductus arteriosus	5852	6
Congenital heart disease, severe intellectual disability and mild heart failure due to critical malformations of great vessels, congenital valvular heart disease and patent ductus arteriosus	4832	6
Congenital heart disease, severe intellectual disability and moderate heart failure due to critical malformations of great vessels, congenital valvular heart disease and patent ductus arteriosus	4850	6
Congenital heart disease, severe intellectual disability and severe heart failure due to critical malformations of great vessels, congenital valvular heart disease and patent ductus arteriosus	4868	6
Other congenital heart anomalies	6683	5
Congenital heart disease and borderline intellectual disability without heart failure due to other congenital cardiovascular anomalies	4643	6
Congenital heart disease and mild intellectual disability without heart failure due to other congenital cardiovascular anomalies	4646	6
Congenital heart disease and moderate intellectual disability without heart failure due to other congenital cardiovascular anomalies	4649	6
Congenital heart disease and profound intellectual disability without heart failure due to other congenital cardiovascular anomalies	4655	6
Congenital heart disease and severe intellectual disability without heart failure due to other congenital cardiovascular anomalies	4652	6
Congenital heart disease without intellectual disability or heart failure due to other congenital cardiovascular anomalies	4640	6

GBD 2017 Cause and Sequela Hierarchy

Level 5 Sequelae are nested under causes. Most detailed sequelae are nested under their parent sequela where they exist

Cause or Sequela Name (Causes in bold)	GBD ID	Level
Severe congenital heart anomalies excluding single ventricle heart defects	6788	5
Congenital heart disease and borderline intellectual disability without heart failure due to severe congenital heart anomalies excluding single ventricle heart defects	4733	6
Congenital heart disease and controlled, medically managed heart failure without intellectual disability due to severe congenital heart anomalies excluding single ventricle heart defects	5795	6
Congenital heart disease and mild heart failure without intellectual disability due to severe congenital heart anomalies excluding single ventricle heart defects	4748	6
Congenital heart disease and mild intellectual disability without heart failure due to severe congenital heart anomalies excluding single ventricle heart defects	4736	6
Congenital heart disease and moderate heart failure without intellectual disability due to severe congenital heart anomalies excluding single ventricle heart defects	4766	6
Congenital heart disease and moderate intellectual disability without heart failure due to severe congenital heart anomalies excluding single ventricle heart defects	4739	6
Congenital heart disease and profound intellectual disability without heart failure due to severe congenital heart anomalies excluding single ventricle heart defects	4745	6
Congenital heart disease and severe heart failure without intellectual disability due to severe congenital heart anomalies excluding single ventricle heart defects	4784	6
Congenital heart disease and severe intellectual disability without heart failure due to severe congenital heart anomalies excluding single ventricle heart defects	4742	6
Congenital heart disease without intellectual disability or heart failure due to severe congenital heart anomalies excluding single ventricle heart defects	4730	6
Congenital heart disease, boderline intellectual disability and controlled, medically managed heart failure due to severe congenital heart anomalies excluding single ventricle heart defects	5807	6
Congenital heart disease, boderline intellectual disability and mild heart failure due to severe congenital heart anomalies excluding single ventricle heart defects	4751	6
Congenital heart disease, borderline intellectual disability and moderate heart failure due to severe congenital heart anomalies excluding single ventricle heart defects	4769	6

Cause or Sequela Name (Causes in bold)	GBD ID	Level
Congenital heart disease, borderline intellectual disability and severe heart failure due to severe congenital heart anomalies excluding single ventricle heart defects	4787	6
Congenital heart disease, mild intellectual disability and controlled, medically managed heart failure due to severe congenital heart anomalies excluding single ventricle heart defects	5819	6
Congenital heart disease, mild intellectual disability and mild heart failure due to severe congenital heart anomalies excluding single ventricle heart defects	4754	6
Congenital heart disease, mild intellectual disability and moderate heart failure due to severe congenital heart anomalies excluding single ventricle heart defects	4772	6
Congenital heart disease, mild intellectual disability and severe heart failure due to severe congenital heart anomalies excluding single ventricle heart defects	4790	6
Congenital heart disease, moderate intellectual disability and controlled, medically managed heart failure due to severe congenital heart anomalies excluding single ventricle heart defects	5831	6
Congenital heart disease, moderate intellectual disability and mild heart failure due to severe congenital heart anomalies excluding single ventricle heart defects	4757	6
Congenital heart disease, moderate intellectual disability and moderate heart failure due to severe congenital heart anomalies excluding single ventricle heart defects	4775	6
Congenital heart disease, moderate intellectual disability and severe heart failure due to severe congenital heart anomalies excluding single ventricle heart defects	4793	6
Congenital heart disease, profound intellectual disability and controlled, medically managed heart failure due to severe congenital heart anomalies excluding single ventricle heart defects	5840	6
Congenital heart disease, profound intellectual disability and mild heart failure due to severe congenital heart anomalies excluding single ventricle heart defects	4763	6
Congenital heart disease, profound intellectual disability and moderate heart failure due to severe congenital heart anomalies excluding single ventricle heart defects	4781	6
Congenital heart disease, profound intellectual disability and severe heart failure due to severe congenital heart anomalies excluding single ventricle heart defects	4799	6
Congenital heart disease, severe intellectual disability and controlled, medically managed heart failure due to severe congenital heart anomalies excluding single ventricle heart defects	5855	6

Cause or Sequela Name (Causes in bold)	GBD ID	Level
Congenital heart disease, severe intellectual disability and mild heart failure due to severe congenital heart anomalies excluding single ventricle heart defects	4760	6
Congenital heart disease, severe intellectual disability and moderate heart failure due to severe congenital heart anomalies excluding single ventricle heart defects	4778	6
Congenital heart disease, severe intellectual disability and severe heart failure due to severe congenital heart anomalies excluding single ventricle heart defects	4796	6
Single ventricle and single ventricle pathway congenital heart anomalies	6800	5
Congenital heart disease and borderline intellectual disability without heart failure due to single ventricle and single ventricle pathway heart defects	4661	6
Congenital heart disease and controlled, medically managed heart failure without intellectual disability due to single ventricle and single ventricle pathway heart defects	5798	6
Congenital heart disease and mild heart failure without intellectual disability due to single ventricle and single ventricle pathway heart defects	4676	6
Congenital heart disease and mild intellectual disability without heart failure due to single ventricle and single ventricle pathway heart defects	4664	6
Congenital heart disease and moderate heart failure without intellectual disability due to single ventricle and single ventricle pathway heart defects	4694	6
Congenital heart disease and moderate intellectual disability without heart failure due to single ventricle and single ventricle pathway heart defects	4667	6
Congenital heart disease and profound intellectual disability without heart failure due to single ventricle and single ventricle pathway heart defects	4673	6
Congenital heart disease and severe heart failure without intellectual disability due to single ventricle and single ventricle pathway heart defects	4712	6
Congenital heart disease and severe intellectual disability without heart failure due to single ventricle and single ventricle pathway heart defects	4670	6
Congenital heart disease without intellectual disability or heart failure due to single ventricle and single ventricle pathway heart defects	4658	6

GBD 2017 Cause and Sequela Hierarchy<br>Level 5 Sequelae are nested under causes. Most detailed sequelae are nested under their parent sequela where they exist		
**Cause or Sequela Name (Causes in bold)**	**GBD ID**	**Level**
Congenital heart disease, boderline intellectual disability and controlled, medically managed heart failure due to single ventricle and single ventricle pathway heart defects	5810	6
Congenital heart disease, boderline intellectual disability and mild heart failure due to single ventricle and single ventricle pathway heart defects	4679	6
Congenital heart disease, borderline intellectual disability and moderate heart failure due to single ventricle and single ventricle pathway heart defects	4697	6
Congenital heart disease, borderline intellectual disability and severe heart failure due to single ventricle and single ventricle pathway heart defects	4715	6
Congenital heart disease, mild intellectual disability and controlled, medically managed heart failure due to single ventricle and single ventricle pathway heart defects	5822	6
Congenital heart disease, mild intellectual disability and mild heart failure due to single ventricle and single ventricle pathway heart defects	4682	6
Congenital heart disease, mild intellectual disability and moderate heart failure due to single ventricle and single ventricle pathway heart defects	4700	6
Congenital heart disease, mild intellectual disability and severe heart failure due to single ventricle and single ventricle pathway heart defects	4718	6
Congenital heart disease, moderate intellectual disability and controlled, medically managed heart failure due to single ventricle and single ventricle pathway heart defects	5834	6
Congenital heart disease, moderate intellectual disability and mild heart failure due to single ventricle and single ventricle pathway heart defects	4685	6
Congenital heart disease, moderate intellectual disability and moderate heart failure due to single ventricle and single ventricle pathway heart defects	4703	6
Congenital heart disease, moderate intellectual disability and severe heart failure due to single ventricle and single ventricle pathway heart defects	4721	6
Congenital heart disease, profound intellectual disability and controlled, medically managed heart failure due to single ventricle and single ventricle pathway heart defects	5843	6
Congenital heart disease, profound intellectual disability and mild heart failure due to single ventricle and single ventricle pathway heart defects	4691	6

Cause or Sequela Name (Causes in bold)	GBD ID	Level
Congenital heart disease, profound intellectual disability and moderate heart failure due to single ventricle and single ventricle pathway heart defects	4709	6
Congenital heart disease, profound intellectual disability and severe heart failure due to single ventricle and single ventricle pathway heart defects	4727	6
Congenital heart disease, severe intellectual disability and controlled, medically managed heart failure due to single ventricle and single ventricle pathway heart defects	5858	6
Congenital heart disease, severe intellectual disability and mild heart failure due to single ventricle and single ventricle pathway heart defects	4688	6
Congenital heart disease, severe intellectual disability and moderate heart failure due to single ventricle and single ventricle pathway heart defects	4706	6
Congenital heart disease, severe intellectual disability and severe heart failure due to single ventricle and single ventricle pathway heart defects	4724	6
Ventricular septal defect and atrial septal defect	6950	5
Asymptomatic ventricular septal defect and atrial septal defect	1757	6
Congenital heart disease and borderline intellectual disability without heart failure due to ventricular septal defect and atrial septal defect	4877	6
Congenital heart disease and controlled, medically managed heart failure without intellectual disability due to ventricular septal defect and atrial septal defect	5801	6
Congenital heart disease and mild heart failure without intellectual disability due to ventricular septal defect and atrial septal defect	4892	6
Congenital heart disease and mild intellectual disability without heart failure due to ventricular septal defect and atrial septal defect	4880	6
Congenital heart disease and moderate heart failure without intellectual disability due to ventricular septal defect and atrial septal defect	4910	6
Congenital heart disease and moderate intellectual disability without heart failure due to ventricular septal defect and atrial septal defect	4883	6

GBD 2017 Cause and Sequela Hierarchy<br>Level 5 Sequelae are nested under causes. Most detailed sequelae are nested under their parent sequela where they exist		
**Cause or Sequela Name (Causes in bold)**	**GBD ID**	**Level**
Congenital heart disease and profound intellectual disability without heart failure due to ventricular septal defect and atrial septal defect	4889	6
Congenital heart disease and severe heart without intellectual disability due to ventricular septal defect and atrial septal defect	4928	6
Congenital heart disease and severe intellectual disability without heart failure due to ventricular septal defect and atrial septal defect	4886	6
Congenital heart disease without heart failure or intellectual disability due to ventricular septal defect and atrial septal defect	4874	6
Congenital heart disease, boderline intellectual disability and controlled, medically managed heart failure due to ventricular septal defect and atrial septal defect	5813	6
Congenital heart disease, boderline intellectual disability and mild heart failure due to ventricular septal defect and atrial septal defect	4895	6
Congenital heart disease, borderline intellectual disability and moderate heart failure due to ventricular septal defect and atrial septal defect	4913	6
Congenital heart disease, borderline intellectual disability and severe heart failure due to ventricular septal defect and atrial septal defect	4931	6
Congenital heart disease, mild intellectual disability and controlled, medically managed heart failure due to ventricular septal defect and atrial septal defect	5825	6
Congenital heart disease, mild intellectual disability and mild heart failure due to ventricular septal defect and atrial septal defect	4898	6
Congenital heart disease, mild intellectual disability and moderate heart failure due to ventricular septal defect and atrial septal defect	4916	6
Congenital heart disease, mild intellectual disability and severe heart failure due to ventricular septal defect and atrial septal defect	4934	6
Congenital heart disease, moderate intellectual disability and controlled, medically managed heart failure due to ventricular septal defect and atrial septal defect	5837	6
Congenital heart disease, moderate intellectual disability and mild heart failure due to ventricular septal defect and atrial septal defect	4901	6

GBD 2017 Cause and Sequela Hierarchy

Level 5 Sequelae are nested under causes. Most detailed sequelae are nested under their parent sequela where they exist

Cause or Sequela Name (Causes in bold)	GBD ID	Level
Congenital heart disease, moderate intellectual disability and moderate heart failure due to ventricular septal defect and atrial septal defect	4919	6
Congenital heart disease, moderate intellectual disability and severe heart failure due to ventricular septal defect and atrial septal defect	4937	6
Congenital heart disease, profound intellectual disability and controlled, medically managed heart failure due to ventricular septal defect and atrial septal defect	5846	6
Congenital heart disease, profound intellectual disability and mild heart failure due to ventricular septal defect and atrial septal defect	4907	6
Congenital heart disease, profound intellectual disability and moderate heart failure due to ventricular septal defect and atrial septal defect	4925	6
Congenital heart disease, profound intellectual disability and severe heart failure due to ventricular septal defect and atrial septal defect	4943	6
Congenital heart disease, severe intellectual disability and controlled, medically managed heart failure due to ventricular septal defect and atrial septal defect	5861	6
Congenital heart disease, severe intellectual disability and mild heart failure due to ventricular septal defect and atrial septal defect	4904	6
Congenital heart disease, severe intellectual disability and moderate heart failure due to ventricular septal defect and atrial septal defect	4922	6
Congenital heart disease, severe intellectual disability and severe heart failure due to ventricular septal defect and atrial septal defect	4940	6
**Orofacial clefts**	644	4
Orofacial clefts	22796	5
Asymptomatic orofacial clefts	926	6
Disfigurement level 1 due to orofacial clefts	923	6

GBD 2017 Cause and Sequela Hierarchy

Level 5 Sequelae are nested under causes. Most detailed sequelae are nested under their parent sequela where they exist

Cause or Sequela Name (Causes in bold)	GBD ID	Level
Disfigurement level 2 due to orofacial clefts	924	6
Disfigurement level 2 and speech problems due to orofacial clefts	925	6
**Down syndrome**	645	4
Down syndrome	6320	5
Asymptomatic Down syndrome	930	6
Isolated congenital heart disease due to Down syndrome	838	6
Borderline intellectual disability due to Down syndrome	1212	6
Mild intellectual disability due to Down syndrome	1213	6
Moderate intellectual disability due to Down syndrome	1214	6
Severe intellectual disability due to Down syndrome	1215	6
Profound intellectual disability due to Down syndrome	1216	6
Borderline intellectual disability with congenital heart disease due to Down syndrome	1217	6
Mild intellectual disability with congenital heart disease due to Down syndrome	1218	6
Moderate intellectual disability with congenital heart disease due to Down syndrome	1219	6

GBD 2017 Cause and Sequela Hierarchy

Level 5 Sequelae are nested under causes. Most detailed sequelae are nested under their parent sequela where they exist

Cause or Sequela Name (Causes in bold)	GBD ID	Level
Severe intellectual disability with congenital heart disease due to Down syndrome	1220	6
Profound intellectual disability with congenital heart disease due to Down syndrome	1221	6
Mild dementia due to Down syndrome	1234	6
Moderate dementia due to Down syndrome	1235	6
Severe dementia due to Down syndrome	1236	6
Congenital heart disease and mild dementia due to Down syndrome	1237	6
Congenital heart disease and moderate dementia due to Down syndrome	1238	6
Congenital heart disease and severe dementia due to Down syndrome	1239	6
Borderline intellectual disability, mild dementia, and congenital heart disease due to Down syndrome	1267	6
Mild intellectual disability, mild dementia, and congenital heart disease due to Down syndrome	1268	6
Moderate intellectual disability, mild dementia, and congenital heart disease due to Down syndrome	1269	6
Severe intellectual disability, mild dementia, and congenital heart disease due to Down syndrome	1270	6
Profound intellectual disability, mild dementia, and congenital heart disease due to Down syndrome	1271	6
Borderline intellectual disability, moderate dementia, and congenital heart disease due to Down syndrome	1272	6

Cause or Sequela Name (Causes in bold)	GBD ID	Level
Mild intellectual disability, moderate dementia, and congenital heart disease due to Down syndrome	1273	6
Moderate intellectual disability, moderate dementia, and congenital heart disease due to Down syndrome	1274	6
Severe intellectual disability, moderate dementia, and congenital heart disease due to Down syndrome	1275	6
Profound intellectual disability, moderate dementia, and congenital heart disease due to Down syndrome	1276	6
Borderline intellectual disability, severe dementia, and congenital heart disease due to Down syndrome	1277	6
Mild intellectual disability, severe dementia, and congenital heart disease due to Down syndrome	1278	6
Moderate intellectual disability, severe dementia, and congenital heart disease due to Down syndrome	1279	6
Severe intellectual disability, severe dementia, and congenital heart disease due to Down syndrome	1280	6
Profound intellectual disability, severe dementia, and congenital heart disease due to Down syndrome	1281	6
Profound intellectual disability and mild dementia due to Down syndrome	1297	6
Severe intellectual disability and mild dementia due to Down syndrome	1298	6
Moderate intellectual disability and mild dementia due to Down syndrome	1299	6
Mild intellectual disability and mild dementia due to Down syndrome	1300	6
Borderline intellectual disability and mild dementia due to Down syndrome	1301	6

Cause or Sequela Name (Causes in bold)	GBD ID	Level
Profound intellectual disability and moderate dementia due to Down syndrome	1302	6
Severe intellectual disability and moderate dementia due to Down syndrome	1303	6
Moderate intellectual disability and moderate dementia due to Down syndrome	1304	6
Mild intellectual disability and moderate dementia due to Down syndrome	1305	6
Borderline intellectual disability and moderate dementia due to Down syndrome	1306	6
Profound intellectual disability and severe dementia due to Down syndrome	1307	6
Severe intellectual disability and severe dementia due to Down syndrome	1308	6
Moderate intellectual disability and severe dementia due to Down syndrome	1309	6
Mild intellectual disability and severe dementia due to Down syndrome	1310	6
Borderline intellectual disability and severe dementia due to Down syndrome	1311	6
**Turner syndrome**	646	4
Turner syndrome	6905	5
Asymptomatic Turner syndrome	914	6
Congenital heart disease due to Turner syndrome	570	6

GBD 2017 Cause and Sequela Hierarchy

Level 5 Sequelae are nested under causes. Most detailed sequelae are nested under their parent sequela where they exist

Cause or Sequela Name (Causes in bold)	GBD ID	Level
Primary infertility due to Turner syndrome	571	6
Congenital heart disease with infertility due to Turner syndrome	688	6
**Klinefelter syndrome**	647	4
Klinefelter syndrome	6536	5
Asymptomatic Klinefelter syndrome	933	6
Borderline intellectual disability due to Klinefelter syndrome	572	6
Mild intellectual disability due to Klinefelter syndrome	573	6
Mild intellectual disability with infertility due to Klinefelter syndrome	574	6
Borderline intellectual disability with infertility due to Klinefelter syndrome	691	6
Primary infertility due to Klinefelter syndrome	932	6
**Other chromosomal abnormalities**	648	4
Other chromosomal abnormalities	6680	5
Asymptomatic other chromosomal abnormalities	931	6
Isolated congenital heart disease due to other chromosomal abnormalities	934	6

GBD 2017 Cause and Sequela Hierarchy

Level 5 Sequelae are nested under causes. Most detailed sequelae are nested under their parent sequela where they exist

Cause or Sequela Name (Causes in bold)	GBD ID	Level
Borderline intellectual disability due to other chromosomal abnormalities	1223	6
Mild intellectual disability due to other chromosomal abnormalities	1224	6
Moderate intellectual disability due to other chromosomal abnormalities	1225	6
Severe intellectual disability due to other chromosomal abnormalities	1226	6
Profound intellectual disability due to other chromosomal abnormalities	1227	6
Borderline intellectual disability with congenital heart disease due to other chromosomal abnormalities	1228	6
Mild intellectual disability with congenital heart disease due to other chromosomal abnormalities	1229	6
Moderate intellectual disability with congenital heart disease due to other chromosomal abnormalities	1230	6
Severe intellectual disability with congenital heart disease due to other chromosomal abnormalities	1231	6
Profound intellectual disability with congenital heart disease due to other chromosomal abnormalities	1232	6
Mild dementia due to other chromosomal abnormalities	1240	6
Moderate dementia due to other chromosomal abnormalities	1241	6
Severe dementia due to other chromosomal abnormalities	1242	6
Congenital heart disease and mild dementia due to other chromosomal abnormalities	1243	6

GBD 2017 Cause and Sequela Hierarchy

Level 5 Sequelae are nested under causes. Most detailed sequelae are nested under their parent sequela where they exist

Cause or Sequela Name (Causes in bold)	GBD ID	Level
Congenital heart disease and moderate dementia due to other chromosomal abnormalities	1244	6
Congenital heart disease and severe dementia due to other chromosomal abnormalities	1245	6
Borderline intellectual disability, mild dementia, and congenital heart disease due to other chromosomal abnormalities	1282	6
Mild intellectual disability, mild dementia, and congenital heart disease due to other chromosomal abnormalities	1283	6
Moderate intellectual disability, mild dementia, and congenital heart disease due to other chromosomal abnormalities	1284	6
Severe intellectual disability, mild dementia, and congenital heart disease due to other chromosomal abnormalities	1285	6
Profound intellectual disability, mild dementia, and congenital heart disease due to other chromosomal abnormalities	1286	6
Borderline intellectual disability, moderate dementia, and congenital heart disease due to other chromosomal abnormalities	1287	6
Mild intellectual disability, moderate dementia, and congenital heart disease due to other chromosomal abnormalities	1288	6
Moderate intellectual disability, moderate dementia, and congenital heart disease due to other chromosomal abnormalities	1289	6
Severe intellectual disability, moderate dementia, and congenital heart disease due to other chromosomal abnormalities	1290	6
Profound intellectual disability, moderate dementia, and congenital heart disease due to other chromosomal abnormalities	1291	6
Borderline intellectual disability, severe dementia, and congenital heart disease due to other chromosomal abnormalities	1292	6
Mild intellectual disability, severe dementia, and congenital heart disease due to other chromosomal abnormalities	1293	6

GBD 2017 Cause and Sequela Hierarchy

Level 5 Sequelae are nested under causes. Most detailed sequelae are nested under their parent sequela where they exist

Cause or Sequela Name (Causes in bold)	GBD ID	Level
Moderate intellectual disability, severe dementia, and congenital heart disease due to other chromosomal abnormalities	1294	6
Severe intellectual disability, severe dementia, and congenital heart disease due to other chromosomal abnormalities	1295	6
Profound intellectual disability, severe dementia, and congenital heart disease due to other chromosomal abnormalities	1296	6
Profound intellectual disability and mild dementia due to other chromosomal abnormalities	1312	6
Severe intellectual disability and mild dementia due to other chromosomal abnormalities	1313	6
Moderate intellectual disability and mild dementia due to other chromosomal abnormalities	1314	6
Mild intellectual disability and mild dementia due to other chromosomal abnormalities	1315	6
Borderline intellectual disability and mild dementia due to other chromosomal abnormalities	1316	6
Profound intellectual disability and moderate dementia due to other chromosomal abnormalities	1317	6
Severe intellectual disability and moderate dementia due to other chromosomal abnormalities	1318	6
Moderate intellectual disability and moderate dementia due to other chromosomal abnormalities	1319	6
Mild intellectual disability and moderate dementia due to other chromosomal abnormalities	1320	6
Borderline intellectual disability and moderate dementia due to other chromosomal abnormalities	1321	6
Profound intellectual disability and severe dementia due to other chromosomal abnormalities	1322	6

GBD 2017 Cause and Sequela Hierarchy

Level 5 Sequelae are nested under causes. Most detailed sequelae are nested under their parent sequela where they exist

Cause or Sequela Name (Causes in bold)	GBD ID	Level
Severe intellectual disability and severe dementia due to other chromosomal abnormalities	1323	6
Moderate intellectual disability and severe dementia due to other chromosomal abnormalities	1324	6
Mild intellectual disability and severe dementia due to other chromosomal abnormalities	1325	6
Borderline intellectual disability and severe dementia due to other chromosomal abnormalities	1326	6
Severe motor and cognitive impairment due to Edward Syndrome or Patau Syndrome	1898	6
Severe motor and cognitive impairment with congenital heart disease due to Edward Syndrome or Patau Syndrome	1901	6
**Congenital musculoskeletal and limb anomalies**	649	4
Polydactyly and syndactyly	22799	5
Disfigurement level 1 due to polydactyly and syndactyly	1550	6
Congenital limb deficiency	22802	5
Disfigurement level 2 due to congenital limb deficiency	1553	6
Disfigurement level 2 with pain due to congenital limb deficiency	1556	6
Disfigurement level 2 and mild motor impairment due to congenital limb deficiency	1565	6
Disfigurement level 2 and moderate motor impairment due to congenital limb deficiency	1568	6

Cause or Sequela Name (Causes in bold)	GBD ID	Level
Disfigurement level 2 with pain and mild motor impairment due to congenital limb deficiency	1571	6
Disfigurement level 2 with pain and moderate motor impairment due to congenital limb deficiency	1574	6
Other congenital musculoskeletal anomalies	22805	5
Disfigurement level 2 due to other congenital musculoskeletal anomalies	1577	6
Disfigurement level 2 with pain due to other congenital musculoskeletal anomalies	1580	6
Disfigurement level 2 and mild motor impairment due to other congenital musculoskeletal anomalies	1589	6
Disfigurement level 2 and moderate motor impairment due to other congenital musculoskeletal anomalies	1592	6
Disfigurement level 2 with pain and mild motor impairment due to other congenital musculoskeletal anomalies	1595	6
Disfigurement level 2 with pain and moderate motor impairment due to other congenital musculoskeletal anomalies	1598	6
**Urogenital congenital anomalies**	650	4
Congenital genital anomalies	22808	5
Asymptomatic congenital genital anomalies	1709	6
Atypical genitalia due to congenital genital anomalies	1712	6
Primary infertility due to congenital genital anomalies	1715	6

GBD 2017 Cause and Sequela Hierarchy

Level 5 Sequelae are nested under causes. Most detailed sequelae are nested under their parent sequela where they exist

Cause or Sequela Name (Causes in bold)	GBD ID	Level
Impotence due to congenital genital anomalies	1718	6
Recurrent urinary tract infections or other abdominal issues due to congenital genital anomalies	1721	6
Atypical genital and recurrent urinary tract infections and other abdominal issues due to congenital genital anomalies	1724	6
Atypical genitalia and primary infertility due to congenital genital anomalies	1727	6
Atypical genitalia and impotence due to congenital genital anomalies	1730	6
Primary infertility and recurrent urinary tract infections or other abdominal issues due to congenital genital anomalies	1733	6
Impotence and recurrent urinary tract infections or other abdominal issues due to congenital genital anomalies	1736	6
Infertility and impotence due to congenital genital anomalies	1739	6
Atypical genitalia, recurrent urinary tract infections or other abdominal issues and infertility due to congenital genital anomalies	1742	6
Atypical genitalia, recurrent urinary tract infections or other abdominal issues and impotence due to congenital genital anomalies	1745	6
Atypical genitalia, infertility and impotence due to congenital genital anomalies	1748	6
Infertility, impotence, and recurrent urinary tract infections or other abdominal issues and impotence due to congenital genital anomalies	1751	6
Atypical genitalia, infertility, impotence, and recurrent urinary tract infections or other abdominal issues and impotence due to congenital genital anomalies	1754	6
Asymptomatic congenital anomalies of the urinary tract	1850	6

GBD 2017 Cause and Sequela Hierarchy

Level 5 Sequelae are nested under causes. Most detailed sequelae are nested under their parent sequela where they exist

Cause or Sequela Name (Causes in bold)	GBD ID	Level
Atypical genitalia due to congenital anomalies of the urinary tract	1853	6
Incontinence due to congenital anomalies of the urinary tract	1856	6
Impotence due to congenital anomalies of the urinary tract	1859	6
Recurrent urinary tract infections or other abdominal issues due to congenital anomalies of the urinary tract	1862	6
Atypical genital and recurrent urinary tract infections and other abdominal issues due to congenital anomalies of the urinary tract	1865	6
Atypical genitalia and incontinence due to congenital anomalies of the urinary tract	1868	6
Atypical genitalia and impotence due to congenital anomalies of the urinary tract	1871	6
Incontinence and recurrent urinary tract infections or other abdominal issues due to congenital anomalies of the urinary tract	1874	6
Impotence and recurrent urinary tract infections or other abdominal issues due to congenital anomalies of the urinary tract	1877	6
Incontinence and impotence due to congenital anomalies of the urinary tract	1880	6
Atypical genitalia, recurrent urinary tract infections or other abdominal issues and incontinence due to congenital anomalies of the urinary tract	1883	6
Atypical genitalia, recurrent urinary tract infections or other abdominal issues and impotence due to congenital anomalies of the urinary tract	1886	6
Atypical genitalia, incontinence and impotence due to congenital anomalies of the urinary tract	1889	6
Incontinence, impotence, and recurrent urinary tract infections or other abdominal issues and impotence due to congenital anomalies of the urinary tract	1892	6

GBD 2017 Cause and Sequela Hierarchy

Level 5 Sequelae are nested under causes. Most detailed sequelae are nested under their parent sequela where they exist

Cause or Sequela Name (Causes in bold)	GBD ID	Level
Atypical genitalia, incontinence, impotence, and recurrent urinary tract infections or other abdominal issues and impotence due to congenital anomalies of the urinary tract	1895	6
**Digestive congenital anomalies**	651	4
Congenital diaphragmatic hernia	22811	5
Asymptomatic congenital diaphragmatic hernia	1601	6
Chronic abdominal pain due to congenital diaphragmatic hernia	1604	6
Disfigurement due to congenital diaphragmatic hernia	1607	6
Mild chronic respiratory problems and breathlessness due to congenital diaphragmatic hernia	1610	6
Developmental delay or mild intellectual disability due to congenital diaphragmatic hernia	1613	6
Chronic abdominal pain and disfigurement due to congenital diaphragmatic hernia	1616	6
Chronic abdominal pain and mild chronic respiratory problems due to congenital diaphragmatic hernia	1619	6
Chronic abdominal pain and developmental delay due to congenital diaphragmatic hernia	1622	6
Disfigurement and mild chronic respiratory problems due to congenital diaphragmatic hernia	1625	6
Disfigurement and developmental delay due to congenital diaphragmatic hernia	1628	6
Mild chronic respiratory problems and developmental delay due to congenital diaphragmatic hernia	1631	6

GBD 2017 Cause and Sequela Hierarchy

Level 5 Sequelae are nested under causes. Most detailed sequelae are nested under their parent sequela where they exist

Cause or Sequela Name (Causes in bold)	GBD ID	Level
Chronic abdominal pain, disfigurement and chronic respiratory problems due to congenital diaphragmatic hernia	1634	6
Chronic abdominal pain, disfigurement and developmental delay due to congenital diaphragmatic hernia	1637	6
Chronic abdominal pain, chronic respiratory problems and developmental delay due to congenital diaphragmatic hernia	1640	6
Disfigurement, chronic respiratory problems and developmental delay due to congenital diaphragmatic hernia	1643	6
Chronic abdominal pain, disfigurement, developmental delay and chronic respiratory problems due to congenital diaphragmatic hernia	1646	6
Congenital atresia and/or stenosis of the digestive tract	22814	5
Asymptomatic congenital atresia and/or stenosis of the digestive tract	1649	6
Dysphagia or acid reflux due to congenital atresia and/or stenosis of the digestive tract	1652	6
Chronic abdominal pain due to congenital atresia and/or stenosis of the digestive tract	1655	6
Chronic respiratory problems including difficulty breaking and recurrent upper respiratory infections due to atresia and/or stenosis of the digestive tract	1658	6
Chronic respiratory problems and dysphagia or acid reflux due to congenital atresia and/or stenosis of the digestive tract	1661	6
Chronic respiratory problems and abdominal pain due to congenital atresia and/or stenosis of the digestive tract	1664	6
Dysphagia or acid reflux and chronic abdominal pain due to congenital atresia and/or stenosis of the digestive tract	1667	6
Dysphagia or acid reflux, chronic abdominal pain and chronic respiratory problems due to congenital atresia and/or stenosis of the digestive tract	1670	6

GBD 2017 Cause and Sequela Hierarchy

Level 5 Sequelae are nested under causes. Most detailed sequelae are nested under their parent sequela where they exist

Cause or Sequela Name (Causes in bold)	GBD ID	Level
Congenital malformations of the abdominal wall after treatment	22817	5
Asymptomatic congenital malformations of the abdominal wall after treatment	1673	6
Disfigurement from scars following treatment for congenital malformations of the abdominal wall	1676	6
Constipation due to congenital malformations of the abdominal wall	1679	6
Chronic abdominal pain due to congenital malformations of the abdominal wall	1682	6
Constipation and concern about scars due to congenital malformations of the abdominal wall	1685	6
Chronic abdominal pain and concern about scars due to congenital malformations of the abdominal wall	1688	6
Constipation and chronic abdominal pain due to congenital malformations of the abdominal wall	1691	6
Constipation, chronic abdominal pain and concern about scars due to congenital malformations of the abdominal wall	1694	6
Other congenital malformations of the digestive tract	22820	5
Asymptomatic other congenital malformations of the digestive tract	1697	6
Acid reflux, dyspahgia, and/or constipation due to other congenital malformations of the digestive tract	1700	6
Chronic abdominal pain and/or nausea due to other congenital malformations of the digestive tract	1703	6
Chronic abdominal pain and/or nausea with acid reflux, dyspahgia, and/or constipation due to other congenital malformations of the digestive tract	1706	6

GBD 2017 Cause and Sequela Hierarchy

Level 5 Sequelae are nested under causes. Most detailed sequelae are nested under their parent sequela where they exist

Cause or Sequela Name (Causes in bold)	GBD ID	Level
**Other congenital birth defects**	652	4
Hearing loss due to other congenital anomalies	6410	5
Mild hearing loss due to other congenital anomalies	576	6
Mild hearing loss with ringing due to other congenital anomalies	577	6
Moderate hearing loss due to other congenital anomalies	578	6
Moderate hearing loss with ringing due to other congenital anomalies	579	6
Moderately severe hearing loss due to other congenital anomalies	584	6
Moderately severe hearing loss with ringing due to other congenital anomalies	585	6
Severe hearing loss due to other congenital anomalies	581	6
Severe hearing loss with ringing due to other congenital anomalies	580	6
Profound hearing loss due to other congenital anomalies	586	6
Profound hearing loss with ringing due to other congenital anomalies	587	6
Complete hearing loss due to other congenital anomalies	582	6
Complete hearing loss with ringing due to other congenital anomalies	583	6

GBD 2017 Cause and Sequela Hierarchy

Level 5 Sequelae are nested under causes. Most detailed sequelae are nested under their parent sequela where they exist

Cause or Sequela Name (Causes in bold)	GBD ID	Level
Other congenital birth defects	5162	5
**Urinary diseases and male infertility**	594	3
**Urinary tract infections**	595	4
Urinary tract infections	6929	5
Mild urinary tract infections	510	6
Moderate urinary tract infections	511	6
**Urolithiasis**	596	4
Urolithiasis episodes	22823	5
Mild urolithiasis episodes	4631	6
Moderate urolithiasis episodes	4634	6
Severe urolithiasis episodes	4637	6
**Benign prostatic hyperplasia**	597	4
Benign prostatic hyperplasia	6212	5
Asymptomatic benign prostatic hyperplasia	965	6

GBD 2017 Cause and Sequela Hierarchy

Level 5 Sequelae are nested under causes. Most detailed sequelae are nested under their parent sequela where they exist

Cause or Sequela Name (Causes in bold)	GBD ID	Level
Symptomatic benign prostatic hyperplasia	966	6
**Male infertility**	598	4
Male infertility	22826	5
Idiopathic primary male infertility	514	6
Idiopathic secondary male infertility	515	6
**Other urinary diseases**	602	4
Other urinary diseases	516	5
**Gynecological diseases**	603	3
**Uterine fibroids**	604	4
Uterine fibroids	22829	5
Asymptomatic uterine fibroids	967	6
Mild abdominal pain due to uterine fibroids, without anemia	1109	6
Mild abdominal pain due to uterine fibroids, with mild anemia	1106	6
Mild abdominal pain due to uterine fibroids, with moderate anemia	1107	6

GBD 2017 Cause and Sequela Hierarchy

Level 5 Sequelae are nested under causes. Most detailed sequelae are nested under their parent sequela where they exist

Cause or Sequela Name (Causes in bold)	GBD ID	Level
Mild abdominal pain due to uterine fibroids, with severe anemia	1108	6
**Polycystic ovarian syndrome**	605	4
Polycystic ovarian syndrome	22832	5
Asymptomatic polycystic ovarian syndrome	855	6
Hirsutism and primary infertility due to polycystic ovarian syndrome	857	6
Hirsutism and secondary infertility due to polycystic ovarian syndrome	939	6
Hirsutism due to polycystic ovarian syndrome	856	6
Primary infertility due to polycystic ovarian syndrome	517	6
Secondary infertility due to polycystic ovarian syndrome	940	6
**Female infertility**	606	4
Female infertility	22835	5
Idiopathic primary female infertility	518	6
Idiopathic secondary female infertility	519	6
**Endometriosis**	607	4

GBD 2017 Cause and Sequela Hierarchy

Level 5 Sequelae are nested under causes. Most detailed sequelae are nested under their parent sequela where they exist

Cause or Sequela Name (Causes in bold)	GBD ID	Level
Endometriosis	22838	5
Asymptomatic endometriosis	832	6
Mild abdominal pain due to endometriosis	520	6
Moderate abdominal pain due to endometriosis	521	6
Severe abdominal pain due to endometriosis	941	6
Mild abdominal pain and primary infertility due to endometriosis	878	6
Mild abdominal pain and secondary infertility due to endometriosis	881	6
Moderate abdominal pain and primary infertility due to endometriosis	879	6
Moderate abdominal pain and secondary infertility due to endometriosis	882	6
Primary infertility due to endometriosis	522	6
Secondary infertility due to endometriosis	523	6
Severe abdominal pain and primary infertility due to endometriosis	880	6
Severe abdominal pain and secondary infertility due to endometriosis	883	6
**Genital prolapse**	608	4

GBD 2017 Cause and Sequela Hierarchy

Level 5 Sequelae are nested under causes. Most detailed sequelae are nested under their parent sequela where they exist

Cause or Sequela Name (Causes in bold)	GBD ID	Level
Genital prolapse	6395	5
Asymptomatic genital prolapse	858	6
Abdominal pain due to genital prolapse	859	6
Stress incontinence due to genital prolapse	860	6
Abdominal pain and stress incontinence due to genital prolapse	861	6
**Premenstrual syndrome**	609	4
Premenstrual syndrome	6764	5
Abdominal pain due to premenstrual syndrome	524	6
Depression due to premenstrual syndrome	700	6
Asymptomatic premenstrual syndrome	853	6
Abdominal pain and depression due to premenstrual syndrome	854	6
**Other gynecological diseases**	612	4
Other gynecological diseases	6686	5
Asymptomatic other gynecological disorders	843	6

GBD 2017 Cause and Sequela Hierarchy

Level 5 Sequelae are nested under causes. Most detailed sequelae are nested under their parent sequela where they exist

Cause or Sequela Name (Causes in bold)	GBD ID	Level
Menstrual disorders without anemia	5558	6
Mild other gynecological disorders	844	6
Moderate other gynecological disorders	845	6
Severe other gynecological disorders	846	6
Other gynecological diseases with anemia	6689	5
Mild anemia due to menstrual disorders	525	6
Moderate anemia due to menstrual disorders	526	6
Severe anemia due to menstrual disorders	527	6
**Hemoglobinopathies and hemolytic anemias**	613	3
**Thalassemias**	614	4
Thalassemias	6881	5
Mild heart failure due to thalassemias	528	6
Moderate heart failure due to thalassemias	529	6
Severe heart failure due to thalassemias	530	6

GBD 2017 Cause and Sequela Hierarchy

Level 5 Sequelae are nested under causes. Most detailed sequelae are nested under their parent sequela where they exist

Cause or Sequela Name (Causes in bold)	GBD ID	Level
Beta-thalassemia major, severe infection with severe anemia	1060	6
Beta-thalassemia major, with mild anemia	1061	6
Beta-thalassemia major, with moderate anemia	1062	6
Beta-thalassemia major, with severe anemia	1063	6
Hemoglobin E/beta-thalassemia, severe infection with severe anemia	1064	6
Hemoglobin E/beta-thalassemia, with mild anemia	1065	6
Hemoglobin E/beta-thalassemia, with moderate anemia	1066	6
Hemoglobin E/beta-thalassemia, with severe anemia	1067	6
Hemoglobin H disease, severe infection with severe anemia	1068	6
Hemoglobin H disease, with mild anemia	1069	6
Hemoglobin H disease, with moderate anemia	1070	6
Hemoglobin H disease, with severe anemia	1071	6
Hemoglobin H disease, without anemia	1113	6
Beta-thalassemia major, without anemia	1114	6

GBD 2017 Cause and Sequela Hierarchy

Level 5 Sequelae are nested under causes. Most detailed sequelae are nested under their parent sequela where they exist

Cause or Sequela Name (Causes in bold)	GBD ID	Level
Hemoglobin E/Beta-thalassemia, without anemia	1115	6
Controlled, medically managed heart failure due to thalassemias	5789	6
**Thalassemias trait**	837	4
Thalassemia trait	22841	5
Asymptomatic B-thalassemia trait	1050	6
Mild anemia due to B-thalassemia trait	645	6
Moderate anemia due to B-thalassemia trait	646	6
Severe anemia due to B-thalassemia trait	647	6
Asymptomatic hemoglobin E trait	1051	6
Mild anemia due to hemoglobin E trait	648	6
Moderate anemia due to hemoglobin E trait	649	6
Severe anemia due to hemoglobin E trait	650	6
**Sickle cell disorders**	615	4
Sickle cell disorders	6791	5

GBD 2017 Cause and Sequela Hierarchy

Level 5 Sequelae are nested under causes. Most detailed sequelae are nested under their parent sequela where they exist

Cause or Sequela Name (Causes in bold)	GBD ID	Level
Homozygous sickle cell and severe sickle cell/beta-thalassemia, without anemia	1072	6
Homozygous sickle cell and severe sickle cell/beta-thalassemia, with vaso-occlusive crisis, without anemia	1073	6
Homozygous sickle cell and severe sickle cell/beta-thalassemia, with vaso-occlusive crisis and severe anemia	1074	6
Homozygous sickle cell and severe sickle cell/beta-thalassemia, with stroke and severe anemia	1075	6
Homozygous sickle cell and severe sickle cell/beta-thalassemia, with stroke, without anemia	1076	6
Homozygous sickle cell and severe sickle cell/beta-thalassemia, with vaso-occlusive crisis, stroke, and severe anemia	1077	6
Homozygous sickle cell and severe sickle cell/beta-thalassemia, with vaso-occlusive crisis and stroke, without anemia	1078	6
Homozygous sickle cell and severe sickle cell/beta-thalassemia, with mild anemia	1079	6
Homozygous sickle cell and severe sickle cell/beta-thalassemia, with moderate anemia	1080	6
Homozygous sickle cell and severe sickle cell/beta-thalassemia, with severe anemia	1081	6
Hemoglobin SC disease, without anemia	1082	6
Hemoglobin SC disease, with vaso-occlusive crisis and severe anemia	1083	6
Hemoglobin SC disease, with vaso-occlusive crisis, without anemia	1084	6
Hemoglobin SC disease, with stroke and severe anemia	1085	6

GBD 2017 Cause and Sequela Hierarchy

Level 5 Sequelae are nested under causes. Most detailed sequelae are nested under their parent sequela where they exist

Cause or Sequela Name (Causes in bold)	GBD ID	Level
Hemoglobin SC disease, with stroke, without anemia	1086	6
Hemoglobin SC disease, with vaso-occlusive crisis, stroke, and severe anemia	1087	6
Hemoglobin SC disease, with vaso-occlusive crisis and stroke, without anemia	1088	6
Hemoglobin SC disease, with mild anemia	1089	6
Hemoglobin SC disease, with moderate anemia	1090	6
Hemoglobin SC disease, with severe anemia	1091	6
Mild sickle cell/beta-thalassemia, without anemia	1092	6
Mild sickle cell/beta-thalassemia, with vaso-occlusive crisis and severe anemia	1093	6
Mild sickle cell/beta-thalassemia, with vaso-occlusive crisis, without anemia	1094	6
Mild sickle cell/beta-thalassemia, with stroke and severe anemia	1095	6
Mild sickle cell/beta-thalassemia, with stroke, without anemia	1096	6
Mild sickle cell/beta-thalassemia, with vaso-occlusive crisis, stroke, and severe anemia	1097	6
Mild sickle cell/beta-thalassemia, with vaso-occlusive crisis and stroke, without anemia	1098	6
Mild sickle cell/beta-thalassemia, with mild anemia	1099	6

GBD 2017 Cause and Sequela Hierarchy

Level 5 Sequelae are nested under causes. Most detailed sequelae are nested under their parent sequela where they exist

Cause or Sequela Name (Causes in bold)	GBD ID	Level
Mild sickle cell/beta-thalassemia, with moderate anemia	1100	6
Mild sickle cell/beta-thalassemia, with severe anemia	1101	6
**Sickle cell trait**	838	4
Sickle cell trait	6794	5
Asymptomatic sickle cell trait	1052	6
Mild anemia due to sickle cell trait	651	6
Moderate anemia due to sickle cell trait	652	6
Severe anemia due to sickle cell trait	653	6
**G6PD deficiency**	616	4
G6PD deficiency	6374	5
Asymptomatic G6PD deficiency	1126	6
Mild anemia due to G6PD deficiency	1120	6
Moderate anemia due to G6PD deficiency	1121	6
Severe anemia due to G6PD deficiency	1122	6

GBD 2017 Cause and Sequela Hierarchy

Level 5 Sequelae are nested under causes. Most detailed sequelae are nested under their parent sequela where they exist

Cause or Sequela Name (Causes in bold)	GBD ID	Level
Controlled, medically managed heart failure due to G6PD deficiency	5747	6
Mild heart failure due to G6PD deficiency	1123	6
Moderate heart failure due to G6PD deficiency	1124	6
Severe heart failure due to G6PD deficiency	1125	6
**G6PD trait**	839	4
G6PD trait	6377	5
Asymptomatic hemizygous G6PD trait	1053	6
Mild anemia due to hemizygous G6PD trait	654	6
Moderate anemia due to hemizygous G6PD trait	655	6
Severe anemia due to hemizygous G6PD trait	656	6
**Other hemoglobinopathies and hemolytic anemias**	618	4
Other hemoglobinopathies and hemolytic anemias	5876	5
Controlled, medically managed heart failure due to other hemoglobinopathies and hemolytic anemias	5762	6
Mild anemia due to other hemoglobinopathies and hemolytic anemias	531	6

GBD 2017 Cause and Sequela Hierarchy

Level 5 Sequelae are nested under causes. Most detailed sequelae are nested under their parent sequela where they exist

Cause or Sequela Name (Causes in bold)	GBD ID	Level
Moderate anemia due to other hemoglobinopathies and hemolytic anemias	532	6
Severe anemia due to other hemoglobinopathies and hemolytic anemias	533	6
Mild heart failure due to other hemoglobinopathies and hemolytic anemias	534	6
Moderate heart failure due to other hemoglobinopathies and hemolytic anemias	535	6
Severe heart failure due to other hemoglobinopathies and hemolytic anemias	536	6
Other hemoglobinopathies and hemolytic anemias residual	789	6
**Endocrine, metabolic, blood, and immune disorders**	619	3
Anemia due to endocrine, metabolic, blood, and immune disorders	6146	5
Mild anemia due to endocrine, metabolic, blood, and immune disorders	537	6
Moderate anemia due to endocrine, metabolic, blood, and immune disorders	538	6
Severe anemia due to endocrine, metabolic, blood, and immune disorders	539	6
Endocrine, metabolic, blood, and immune disorders	6356	5
Asymptomatic endocrine, metabolic, blood, and immune disorders	904	6
Mild endocrine, metabolic, blood, and immune disorders	905	6

GBD 2017 Cause and Sequela Hierarchy

Level 5 Sequelae are nested under causes. Most detailed sequelae are nested under their parent sequela where they exist

Cause or Sequela Name (Causes in bold)	GBD ID	Level
Moderate endocrine, metabolic, blood, and immune disorders	906	6
Severe endocrine, metabolic, blood, and immune disorders	907	6
Heart failure due to endocrine, metabolic, blood, and immune disorders	6431	5
Controlled, medically managed heart failure due to endocrine, metabolic, blood, and immune disorders	5744	6
Mild heart failure due to endocrine, metabolic, blood, and immune disorders	540	6
Moderate heart failure due to endocrine, metabolic, blood, and immune disorders	541	6
Severe heart failure due to endocrine, metabolic, blood, and immune disorders	542	6
**Oral disorders**	680	3
**Caries of deciduous teeth**	681	4
Caries of deciduous teeth	6230	5
Asymptomatic caries of deciduous teeth	943	6
Pain due to caries of deciduous teeth	662	6
**Caries of permanent teeth**	682	4
Caries of permanent teeth	6233	5

GBD 2017 Cause and Sequela Hierarchy

Level 5 Sequelae are nested under causes. Most detailed sequelae are nested under their parent sequela where they exist

Cause or Sequela Name (Causes in bold)	GBD ID	Level
Asymptomatic caries of permanent teeth	944	6
Pain due to caries of permanent teeth	663	6
**Periodontal diseases**	683	4
Chronic periodontal diseases	936	5
**Edentulism and severe tooth loss**	684	4
Edentulism and severe tooth loss	6335	5
Asymptomatic edentulism and severe tooth loss	945	6
Difficulty eating due to edentulism and severe tooth loss	664	6
**Other oral disorders**	685	4
Other oral disorders	6710	5
Mild other oral disorders	1048	6
Severe other oral disorders	1049	6
**Sudden infant death syndrome**	686	3
**Injuries**	687	1

GBD 2017 Cause and Sequela Hierarchy

Level 5 Sequelae are nested under causes. Most detailed sequelae are nested under their parent sequela where they exist

Cause or Sequela Name (Causes in bold)	GBD ID	Level
**Transport injuries**	688	2
**Road injuries**	689	3
**Pedestrian road injuries**	690	4
**Cyclist road injuries**	691	4
**Motorcyclist road injuries**	692	4
**Motor vehicle road injuries**	693	4
**Other road injuries**	694	4
**Other transport injuries**	695	3
**Unintentional injuries**	696	2
**Falls**	697	3
**Drowning**	698	3
**Fire, heat, and hot substances**	699	3
**Poisonings**	700	3
**Poisoning by carbon monoxide**	701	4

GBD 2017 Cause and Sequela Hierarchy

Level 5 Sequelae are nested under causes. Most detailed sequelae are nested under their parent sequela where they exist

Cause or Sequela Name (Causes in bold)	GBD ID	Level
**Poisoning by other means**	703	4
**Exposure to mechanical forces**	704	3
**Unintentional firearm injuries**	705	4
**Other exposure to mechanical forces**	707	4
**Adverse effects of medical treatment**	708	3
**Animal contact**	709	3
**Venomous animal contact**	710	4
**Non-venomous animal contact**	711	4
**Foreign body**	712	3
**Pulmonary aspiration and foreign body in airway**	713	4
**Foreign body in eyes**	714	4
**Foreign body in other body part**	715	4
**Environmental heat and cold exposure**	842	3
**Exposure to forces of nature**	729	3

GBD 2017 Cause and Sequela Hierarchy

Level 5 Sequelae are nested under causes. Most detailed sequelae are nested under their parent sequela where they exist

Cause or Sequela Name (Causes in bold)	GBD ID	Level
**Other unintentional injuries**	716	3
**Self-harm and interpersonal violence**	717	2
**Self-harm**	718	3
**Self-harm by firearm**	721	4
**Self-harm by other specified means**	723	4
**Interpersonal violence**	724	3
**Physical violence by firearm**	725	4
**Physical violence by sharp object**	726	4
**Sexual violence**	941	4
**Physical violence by other means**	727	4
**Conflict and terrorism**	945	3
**Executions and police conflict**	854	3

Appendix Table #4: List of International Classification of Diseases (ICD) codes mapped to the Global Burden of Disease cause list

Cause	ICD10	ICD10 Used in Hospital/Claims Analyses	ICD9	ICD9 Used in Hospital/Claims Analyses
HIV/AIDS	B20-B23.8, B24-B24.0, B97.81, C46-C46.52, C46.7-C46.9, O98.7-O98.73, Z11.4, Z20.6, Z21, Z22.6, Z83.0		042-044.9, 176-176.9, V08	
HIV/AIDS - Drug-susceptible Tuberculosis	B20.0			
HIV/AIDS - Multidrug-resistant Tuberculosis without extensive drug resistance				
HIV/AIDS - Extensively drug-resistant Tuberculosis				
HIV/AIDS resulting in other diseases	B20.1-B23.8, B24-B24.0, B97.81, C46-C46.52, C46.7-C46.9		176-176.9	
Sexually transmitted infections excluding HIV	A50-A60.9, A63-A64.0, B63, I98.0, K67.0-K67.2, M73.0-M73.8, N70-N71.9, N73-N74, N74.2-N74.8, Z11.3, Z20.2, Z22.4		054.1, 054.11-054.19, 090-099.9, 131-131.9, 613-615.9, V01.6, V02.7-V02.9, V73.8, V73.88, V73.9-V73.98, V74.5-	
Syphilis	A50-A53.9, I98.0, K67.2, M73.1-M73.8	A51-A52.9, I98.0	090-097.9	091-095.9
Chlamydial infection	A55-A56.8, K67.0, N74.4		099.41, 099.5	
Gonococcal infection	A54-A54.9, K67.1, M73.0, N74.3		098-098.9	
Trichomoniasis	A59-A59.9		131-131.9	
Genital herpes	A60-A60.9		054.1, 054.11-054.19	
Other sexually transmitted infections	A57-A58, A63-A64.0, B63, N70-N71.9, N73-N74, N74.2, N74.8		099-099.40, 099.49, 099.50-099.9, 613-615.9	
Tuberculosis	A10-A14, A15 -A18.89, A19-A19.9, B90-B90.9, K67.3, K93.0, M49.0, N74.0-N74.1, P37.0, U84.3, Z03.0, Z11.1, Z20.1,		010-019.9, 137-137.9, 320.4, 730.4-730.6, V01.1, V03.2, V12.01, V74.1	
Latent tuberculosis infection				
Drug-susceptible tuberculosis	A10-A14, A15 -A18.89, A19-A19.9, B90-B90.9, K67.3, K93.0, M49.0, N74.0-N74.1, P37.0		010-019.9, 137-137.9, 320.4, 730.4-730.6	
Multidrug-resistant tuberculosis without extensive drug resistance	U84.3			
Extensively drug-resistant tuberculosis				
Lower respiratory infections	A48.1, A70, B96.0-B96.1, B97.21, B97.4-B97.6, J09-J18.2, J18.8-J18.9, J19.6-J22.9, J85.1, P23-P23.9, U04 -U04.9, Z25.1	A48.1, A70, B96.0-B97.6, J09-J22.9, J85.1, P23-P23.9, U04 -U04.9	079.82, 466-469, 470.0, 480-484, 484.1-490.9, 510-511.9, 513.0-513.9, 770.0, V01.82, V04.7, V04.81, V12.61	079.82, 466-470.0, 480-484, 484.1-490.9, 510-513.9, 770.0, V12.61
Upper respiratory infections				
Otitis media	H65-H70.93	H65-H71.93	381-383.9	381.0-382.3, 385.3-385.82
Diarrheal diseases	A00-A00.9, A02-A07, A07.2-A07.4, A08-A08.8, A09, Z22.1, Z23.0	A00-A00.9, A02-A09	001-001.9, 003.8-009.9, V01.0, V01.83, V02.0, V02.2-V02.3, V03.0, V74.0	001-001.9, 003.8-009.9
Typhoid fever	A01.0-A01.09		002.0	
Paratyphoid fever	A01.1-A01.4		002.1-002.9	
Other intestinal infectious diseases				
Malaria	B50-B50.0, B50.8-B52.0, B52.8-B53.1, B53.8-B54.0, P37.3-P37.4		084-084.9, V12.03, V75.1	
Chagas disease	B57-B57.5, K93.1		086-086.2, 425.6	
Leishmaniasis	B55-B55.9, Z26.0		085-085.9, V05.2, V75.2	
Visceral leishmaniasis	B55.0		085.0	
Cutaneous and mucocutaneous leishmaniasis	B55.1-B55.2		085.1-085.5	
African trypanosomiasis	B56-B56.9		086.3-086.9, V75.3	
Schistosomiasis	B65-B65.9		120-120.9, V75.5	
Cysticercosis	B69-B69.9		123.1	
Cystic echinococcosis	B67-B67.4, B67.8-B67.99		122-122.4, 122.8-122.9	
Lymphatic filariasis	B74-B74.2		125.0-125.2	
Onchocerciasis	B73-B73.1		125.3	
Trachoma	A71-A71.9, A74.0, B94.0		076-076.9, V73.6	
Dengue	A90-A91.0		061-061.8	
Yellow fever	A95-A95.9, Z24.3		060-060.9, V04.4, V73.4	
Rabies	A82-A82.9, Z20.3, Z24.2		071-071.9, V01.5, V04.5	
Intestinal nematode infections	B76-B77.9, B79, Z11.6		126-126.9, 127.0, 127.3, V75.7	
Ascariasis	B77-B77.9		127.0	
Trichuriasis	B79		127.3	
Hookworm disease	B76-B76.9		126-126.9	
Food-borne trematodiases	B66-B66.9, B72.0		121-121.9, V75.6	
Leprosy	A30-A30.9, B92		030-030.9, V74.2	
Ebola	A98.4			
Zika virus	U06-U06.9		066.3	
Guinea worm disease	B72			
Other neglected tropical diseases	A68-A68.9, A69.2-A69.29, A69.8-A69.9, A75-A75.9, A77-A79.9, A92-A94.0, A96-A96.9, A98-A98.3, A98.5-A99.0, B33.0-B33.1, B60-B60.8, B64, B67.5-B67.7, B68-B68.9, B70-B71.9, B74.3-B75, B78-B78.9, B80-B83.9, B89, P37.1		065-066.2, 066.4-066.9, 080-083.9, 087-088.9, 122.5-122.7, 123-123.0, 123.2-125, 125.4-125.9, 127, 127.1-127.2, 127.4-129.0, V73.5, V75.8	
Meningitis	A39-A39.9, A87-A87.9, D86.81, G00-G03.9, G06-G09.9, Z20.811, Z22.31	A39-A39.9, A87-A87.9, G00-G03.9	036-036.9, 047-049.9, 054.72, 320-320.3, 320.5-322.9, 324-326.9, V01.84	036-036.9, 047-049.9, 054.72, 320-320.3, 320.5-322.9
Pneumococcal meningitis	G00.1		320.1	
H influenzae type B meningitis	G00.0		320.0	
Meningococcal meningitis	A39-A39.9		036-036.9, 320.5	
Other meningitis	A87-A87.9, D86.81, G03-G03.9		047-049.9, 054.72, 321-322.9	
[truncated: 634,458 more chars]
