# Supplementary material for: Global, regional, and national incidence, prevalence, and years lived with disability for 354 diseases and injuries for 195 countries and territories, 1990–2017: a systematic analysis for the Global Burden of Disease Study 2017
Source: Lancet. 2018 Nov 10;392(10159):1789–858. doi: 10.1016/S0140-6736(18)32279-7 (PMC6227754; doi:10.1016/S0140-6736(18)32279-7)
Supplement: Supplementary appendix 2 [file mmc2.pdf]

THE LANCET

# Supplementary appendix 2

This appendix formed part of the original submission and has been peer reviewed. We post it as supplied by the authors.

Supplement to: GBD 2017 Disease and Injury Incidence and Prevalence Collaborators. Global, regional, and national incidence, prevalence, and years lived with disability for 354 diseases and injuries for 195 countries and territories, 1990–2017: a systematic analysis for the Global Burden of Disease Study 2017. *Lancet* 2018; **392:** 1789–858.

# Supplementary Results for “Global, regional, and national incidence, prevalence, and YLDs for 354 diseases and injuries for 195 countries, 1990–2017: a systematic analysis for the Global Burden of Disease Study 2017”

This supplement provides additional figures and more detailed results causes of non-fatal health loss and years lived with disability.

Complete results from the Global Burden of Disease Study (GBD 2017) are now measured in terabytes. These results are available in an interactive data downloading tool in the Global Health Data exchange (GHDx). The tool contains the complete set of results from all GBD 2017 papers; however, specialized tables from the papers will be available as separate entries in the GHDx, as was made available in GBD 2016. Access to the results and tables through the GHDx are available at http://ghdx.healthdata.org/gbd−2017.

## Supplementary Results Figures and Tables:


SR Figure 1. Leading GBD Level 3 causes of age-standardised YLDs rates by location for a) females and b) males, 2017 ........ **1**

SR Figure 2. Leading GBD Level 3 causes of age-standardised prevalence rates by location for a) females and b) males, 2017 ........ **3**

SR Figure 3. Global distribution of total YLDs by 23 GBD age groups for Level 2 causes of non-fatal burden in 1990 and 2017 for a) females and b) males ........ **5**

SR Figure 4. Trends of age-standardised YLD rates per 100,000 for the top eight Level 3 causes of nonfatal burden in 2017 by SDI quintile for both sexes, from 1990 to 2017 ........ **7**

SR Figure 5. Trends of age−standardised YLD rates per 100,000 for the leading six impairments in 2017 by SDI quintile for both sexes, from 1990 to 2017 ........ **8**

SR Table 1: Global prevalence and YLDs for 2017, percentage change of YLD counts and percentage change of age-standardised YLD rates between 2007 and 2017 of anemia by cause, all ages and both sexes combined ........ **9**

SR Table 2: Global prevalence and YLDs for 2017, percentage change of YLD counts and percentage change of age-standardised YLD rates between 2007 and 2017 of epilepsy by cause, all ages and both sexes combined ........ **11**

SR Table 3. Global prevalence YLDs for 2017, percentage change of YLD counts and percentage change of age-standardised YLD rates between 2007 and 2017 of Guillain-Barre syndrome by cause, all ages and both sexes combined ........ **12**

SR Table 4. Global prevalence and YLDs for 2017, percentage change of YLD counts and percentage change of age-standardised YLD rates between 2007 and 2017 of hearing loss by cause, all ages and both sexes combined ..... **13**

SR Table 5. Global prevalence and YLDs for 2017, percentage change of YLD counts and percentage change of age-standardised YLD rates between 2007 and 2017 of heart failure by cause, all ages and both sexes combined ..... **14**

SR Table 6. Global prevalence and YLDs for 2017, percentage change of YLD counts and percentage change of age-standardised YLD rates between 2007 and 2017 of infertility by cause, all ages and both sexes combined ..... **15**

SR Table 7. Global prevalence and YLDs for 2017, percentage change of YLD counts and percentage change of age-standardised YLD rates between 2007 and 2017 of developmental intellectual disability by cause, all ages and both sexes combined ..... **16**

SR Table 8. Global prevalence and YLDs for 2017, percentage change of YLD counts and percentage change of age-standardised YLD rates between 2007 and 2017 of pelvic inflammatory disease by cause, all ages and both sexes combined ..... **17**

SR Table 9. Global prevalence and YLDs for 2017, percentage change of YLD counts and percentage change of age-standardised YLD rates between 2007 and 2017 of blindness and vision impairment by cause, all ages and both sexes combined ..... **18**

SR Table 10. Global prevalence, incidence and YLDs for 2017, percentage change of YLDs counts, and percentage change of YLD rates between 2007 and 2017 by cause and by age ..... **19**

SR Table 11. Prevalence, incidence and YLDs for 2017, percentage change of YLDs counts, and percentage change of age-standardised YLD rates between 2007 and 2017 by cause and GBD location ..... **318**

## SR Figure 1a–b. Leading GBD 2017 Level 3 Causes of Age–Standardised YLD rates by location for a) Females, 2017

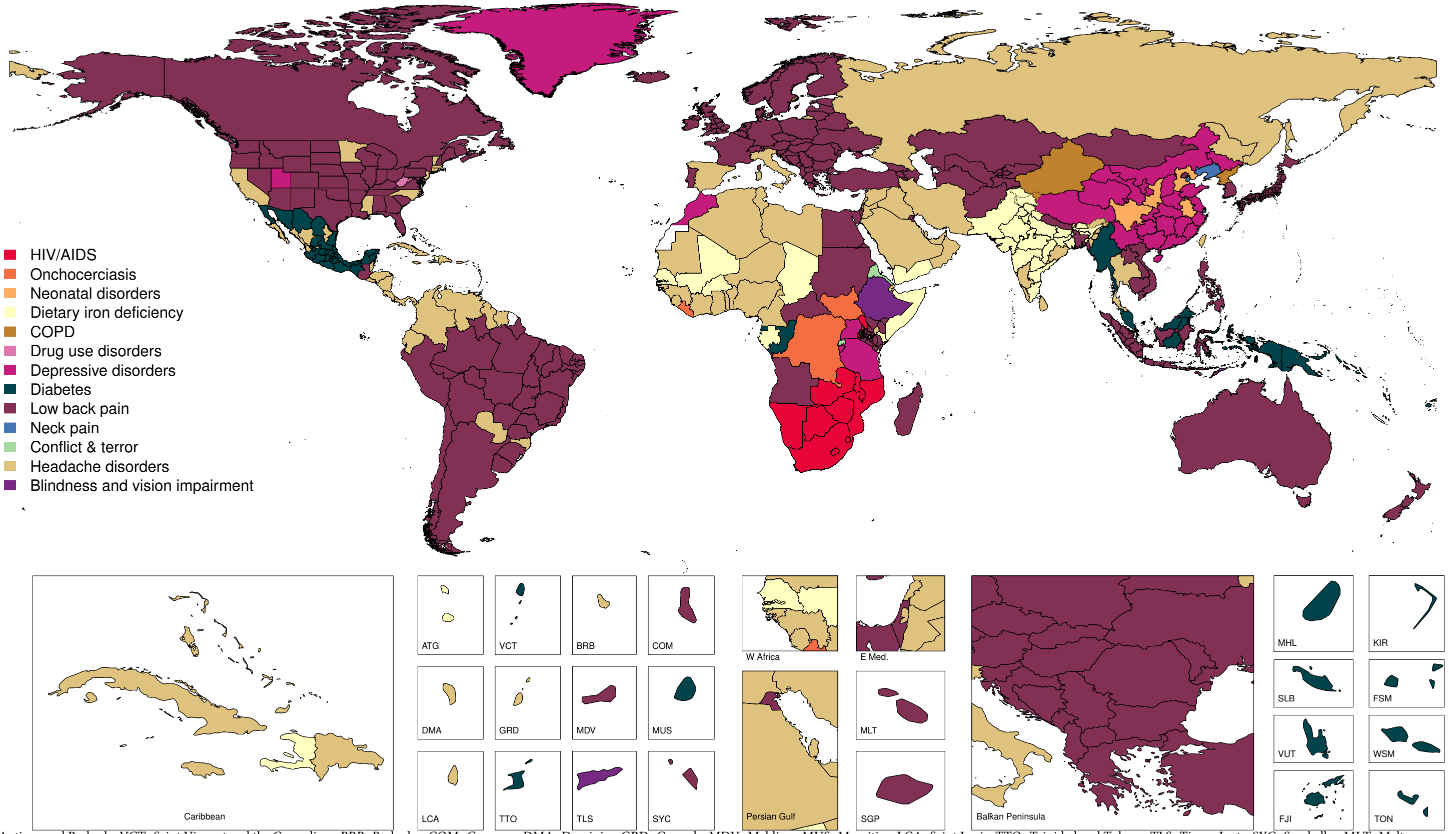


ATG=Antigua and Barbuda. VCT=Saint Vincent and the Grenadines. BRB=Barbados. COM=Comoros. DMA=Dominica. GRD=Grenada. MDV=Maldives. MUS=Mauritius. LCA=Saint Lucia. TTO=Trinidad and Tobago. TLS=Timor-Leste. SYC=Seychelles. MLT=Malta. SGP=Singapore. MHL=Marshall Islands. KIR=Kiribati. SLB=Solomon Islands. FSM=Federated States of Micronesia. VUT=Vanuatu. WSM=Samoa. FJI=Fiji. TON=Tonga.

## SR Figure 1a–b. Leading GBD 2017 Level 3 Causes of Age–Standardised YLD rates by location for b) Males, 2017

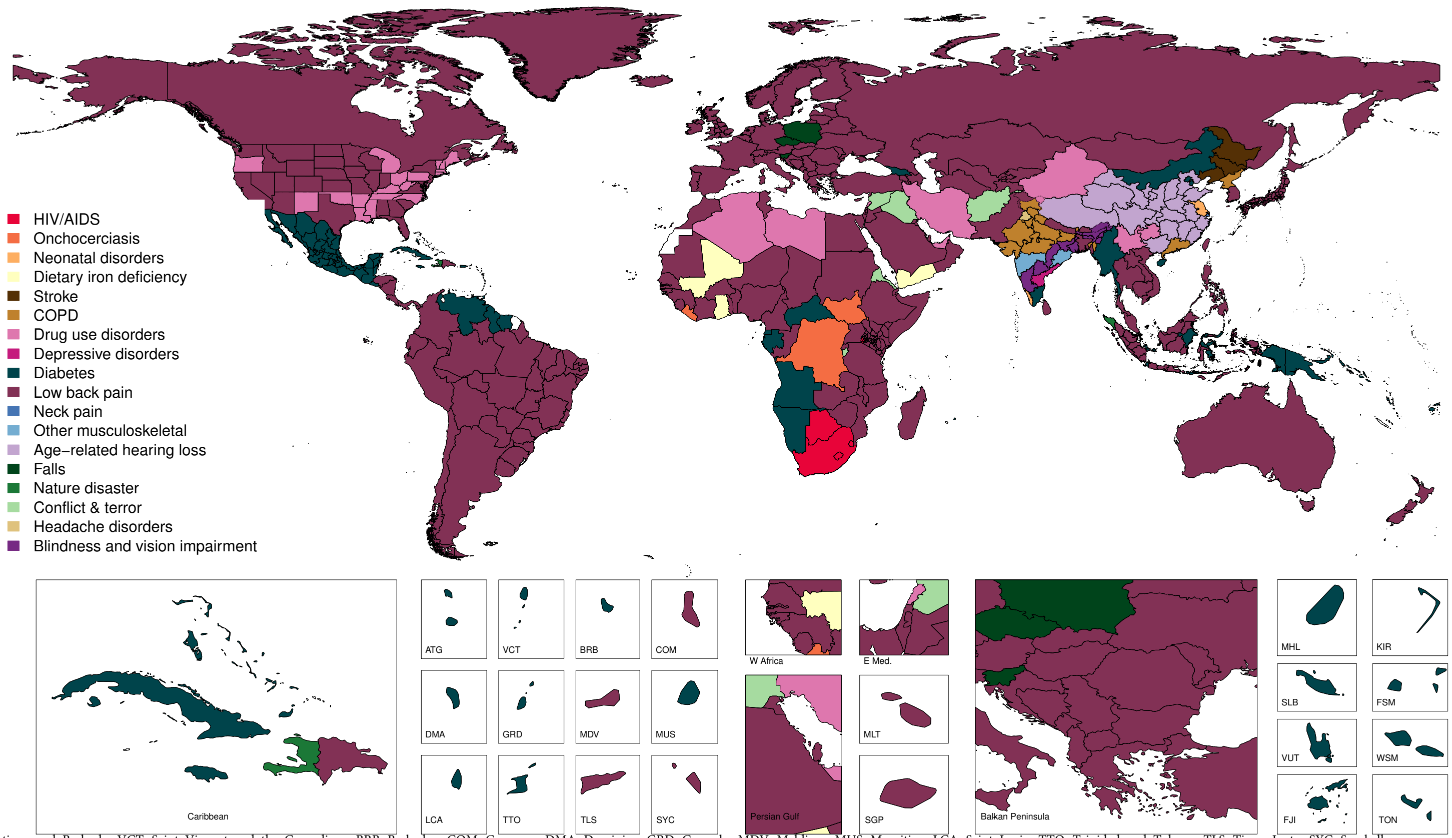


ATG=Antigua and Barbuda. VCT=Saint Vincent and the Grenadines. BRB=Barbados. COM=Comoros. DMA=Dominica. GRD=Grenada. MDV=Maldives. MUS=Mauritius. LCA=Saint Lucia. TTO=Trinidad and Tobago. TLS=Timor-Leste. SYC=Seychelles. MLT=Malta. SGP=Singapore. MHL=Marshall Islands. KIR=Kiribati. SLB=Solomon Islands. FSM=Federated States of Micronesia. VUT=Vanuatu. WSM=Samoa. FJI=Fiji. TON=Tonga.

**Figure 2a–b. Leading GBD 2017 Level 3 Causes of Age-Standardised Prevalence rates by location for a) Females, 2017**

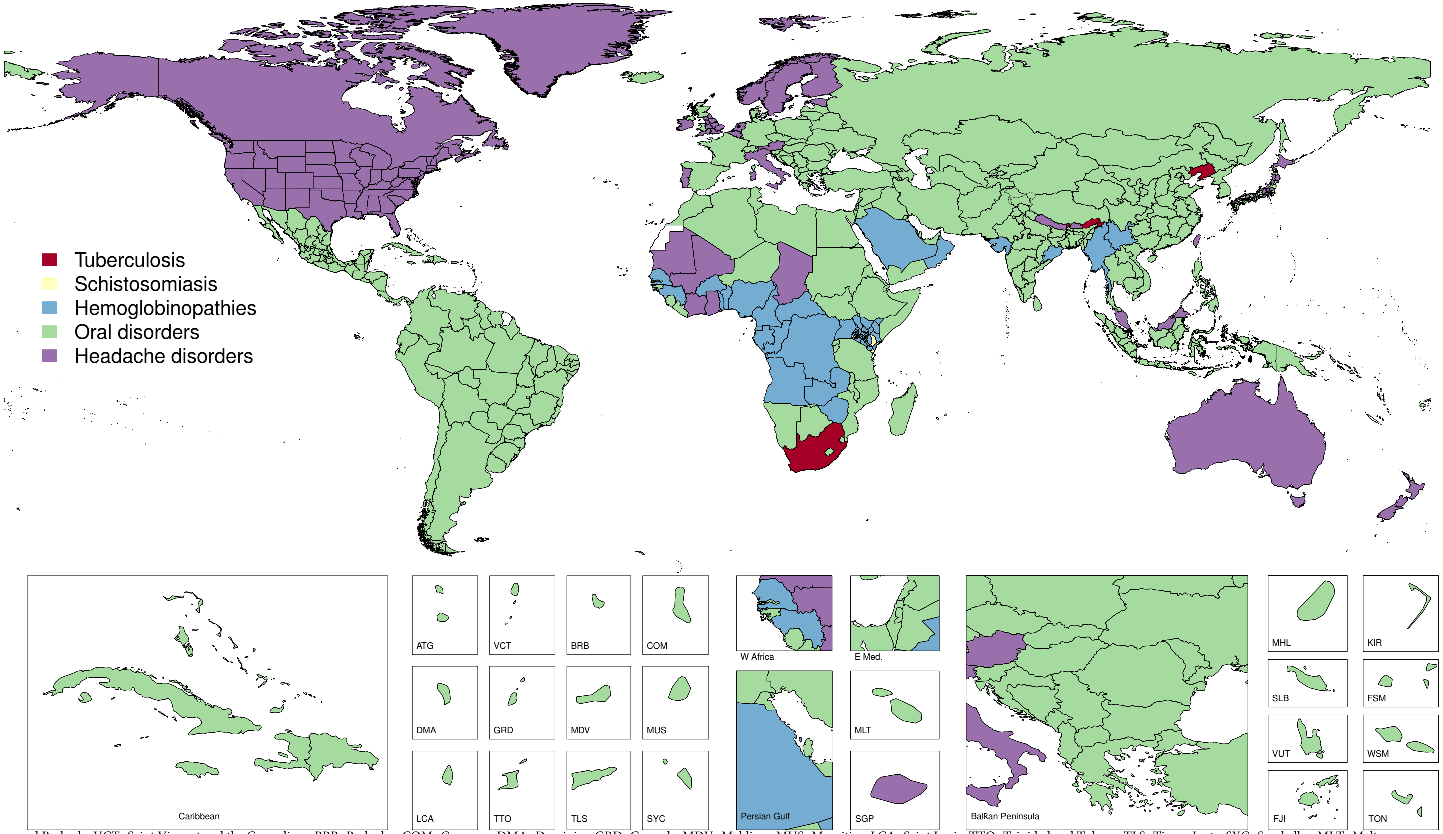


ATG=Antigua and Barbuda. VCT=Saint Vincent and the Grenadines. BRB=Barbados. COM=Comoros. DMA=Dominica. GRD=Grenada. MDV=Maldives. MUS=Mauritius. LCA=Saint Lucia. TTO=Trinidad and Tobago. TLS=Timor-Leste. SYC=Seychelles. MLT=Malta. SGP=Singapore. MHL=Marshall Islands. KIR=Kiribati. SLB=Solomon Islands. FSM=Federated States of Micronesia. VUT=Vanuatu. WSM=Samoa. FJI=Fiji. TON=Tonga.

**Figure 2a–b. Leading GBD 2017 Level 3 Causes of Age-Standardised Prevalence rates by location for b) Males, 2017**

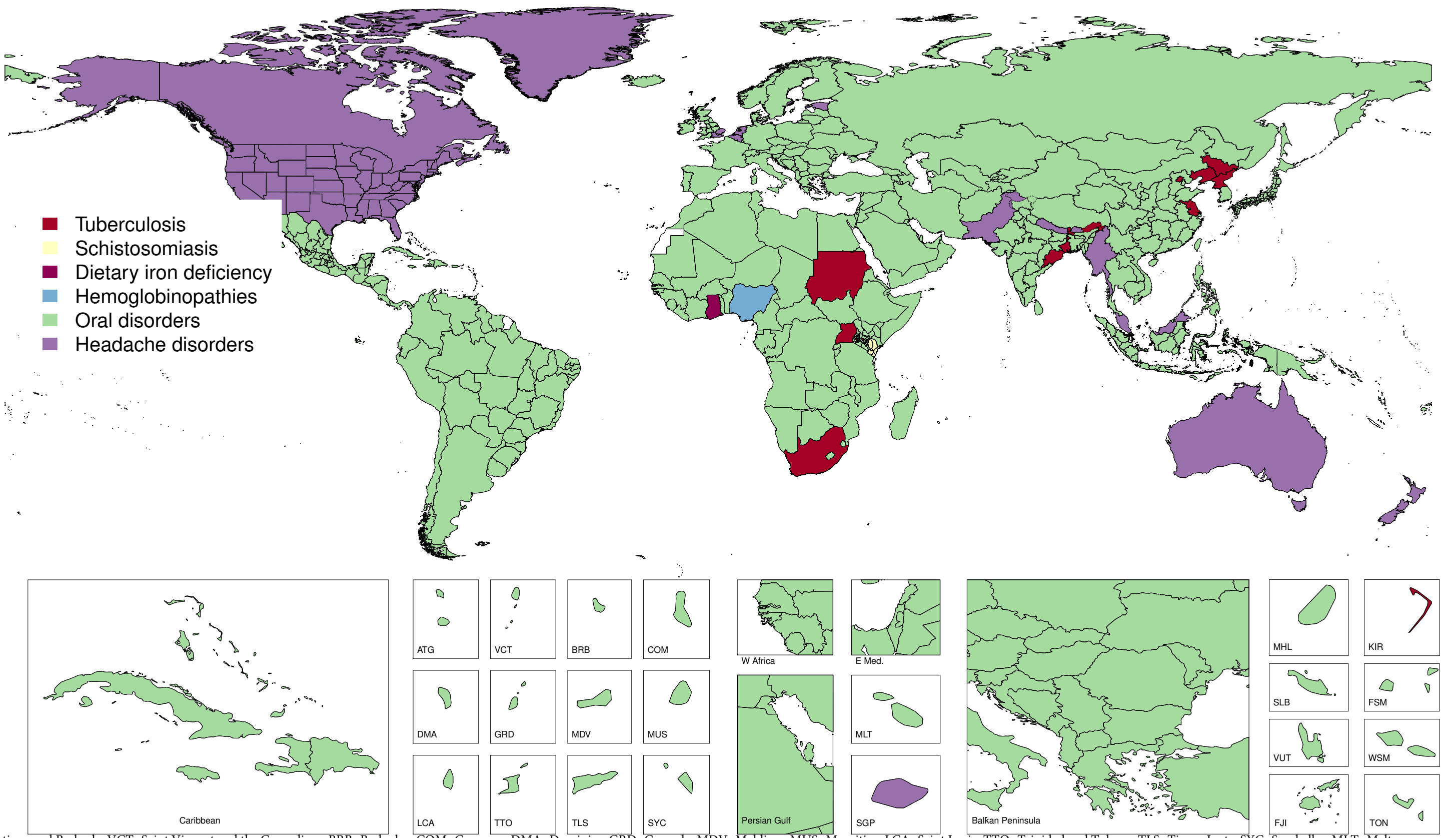


ATG=Antigua and Barbuda. VCT=Saint Vincent and the Grenadines. BRB=Barbados. COM=Comoros. DMA=Dominica. GRD=Grenada. MDV=Maldives. MUS=Mauritius. LCA=Saint Lucia. TTO=Trinidad and Tobago. TLS=Timor-Leste. SYC=Seychelles. MLT=Malta. SGP=Singapore. MHL=Marshall Islands. KIR=Kiribati. SLB=Solomon Islands. FSM=Federated States of Micronesia. VUT=Vanuatu. WSM=Samoa. FJI=Fiji. TON=Tonga.

SR Figure 3a. Global distribution of total YLDs by 23 GBD age groups for Level 2 causes of non-fatal burden in 1990 and 2017, females

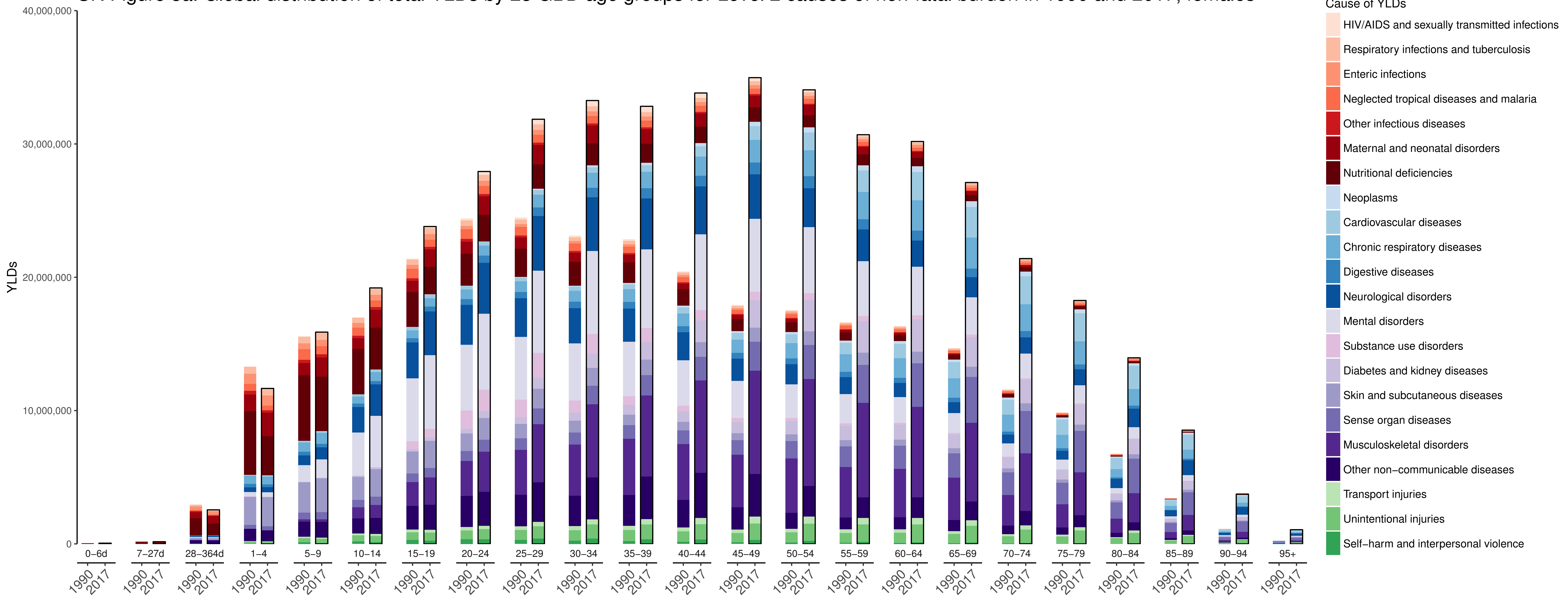


Composition of Level 2 causes of disability globally for females by age group showing difference in composition between 1990 and 2017. Number of total YLDs due to Level 2 causes is indicated by height of bar; causes are colour−coded to highlight the relative number of total YLDs due to a specific cause. YLD=years lived with disability.

SR Figure 3b. Global distribution of total YLDs by 23 GBD age groups for Level 2 causes of non-fatal burden in 1990 and 2017, males

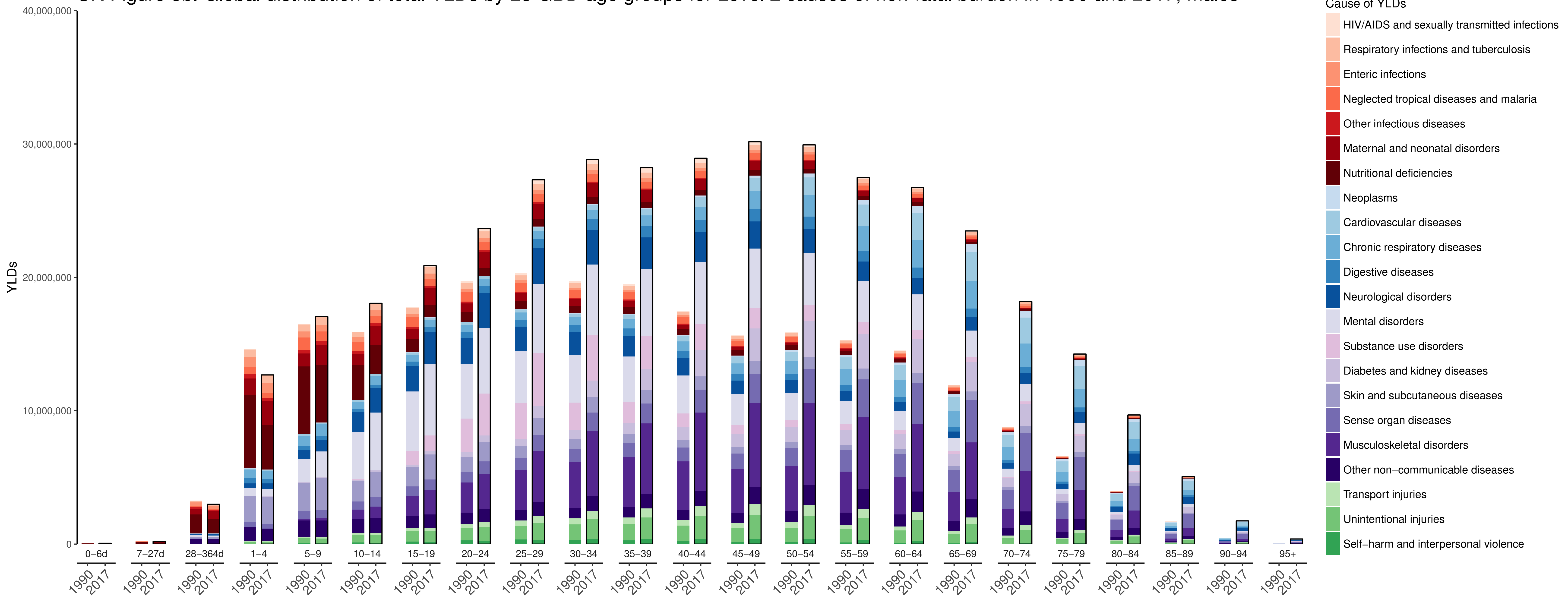


Composition of Level 2 causes of disability globally for males by age group showing difference in composition between 1990 and 2017. Number of total YLDs due to Level 2 causes is indicated by height of bar; causes are colour−coded to highlight the relative number of total YLDs due to a specific cause. YLD=years lived with disability.

**SR Figure 4. Trends of age-standardised YLD rates per 100,000 for the top eight Level 3 causes of non−fatal burden in 2017 by SDI quintile for both sexes, from 1990 to 2017.**

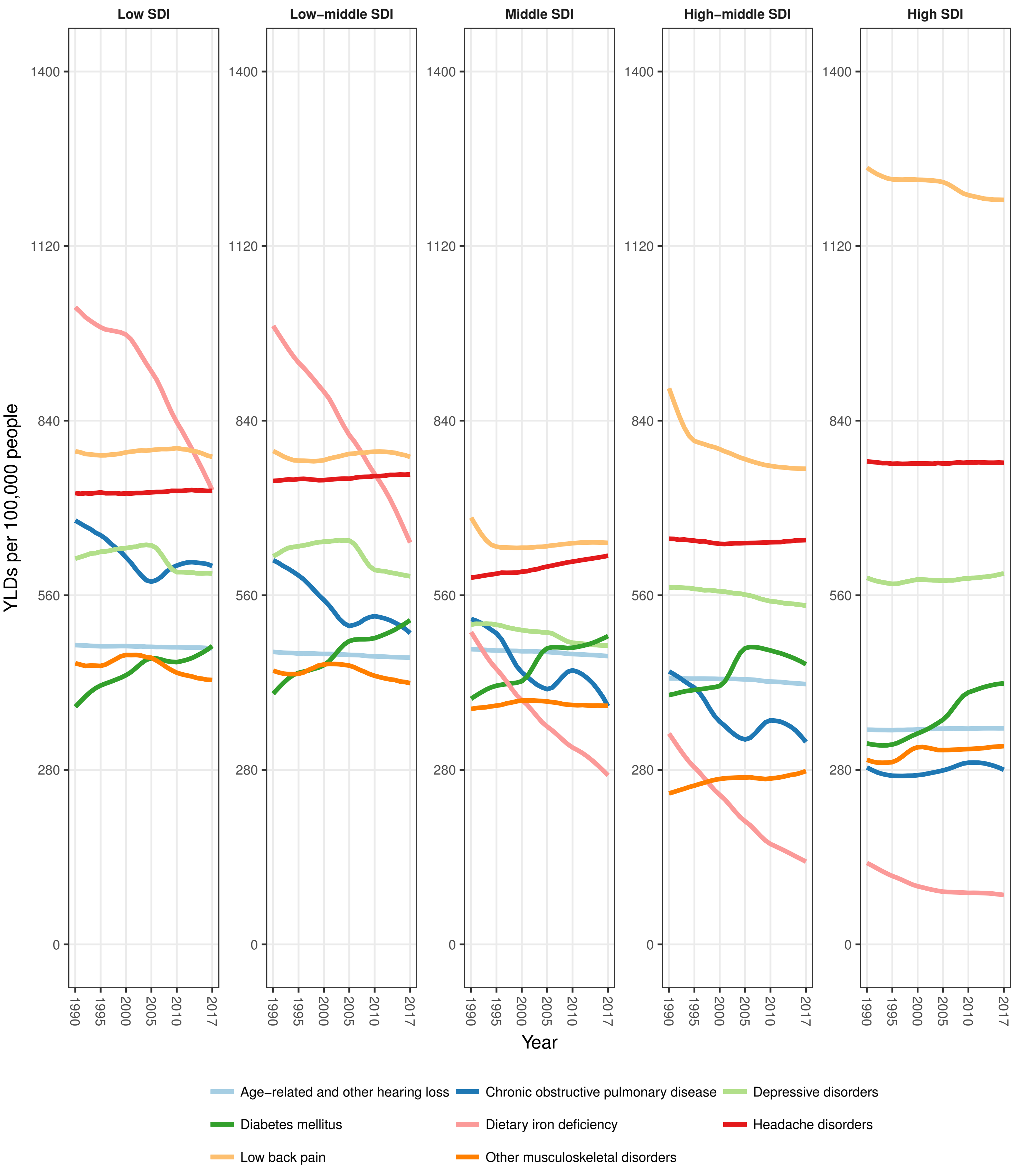


GBD=Global Burden of Disease. YLDs=years lived disability. SDI=Socio−demographic Index. Mean estimates are shown

**SR Figure 5. Trends of age−standardised YLD rates per 100,000 for the leading six impairments in 2017 by SDI quintile for both sexes, from 1990 to 2017**

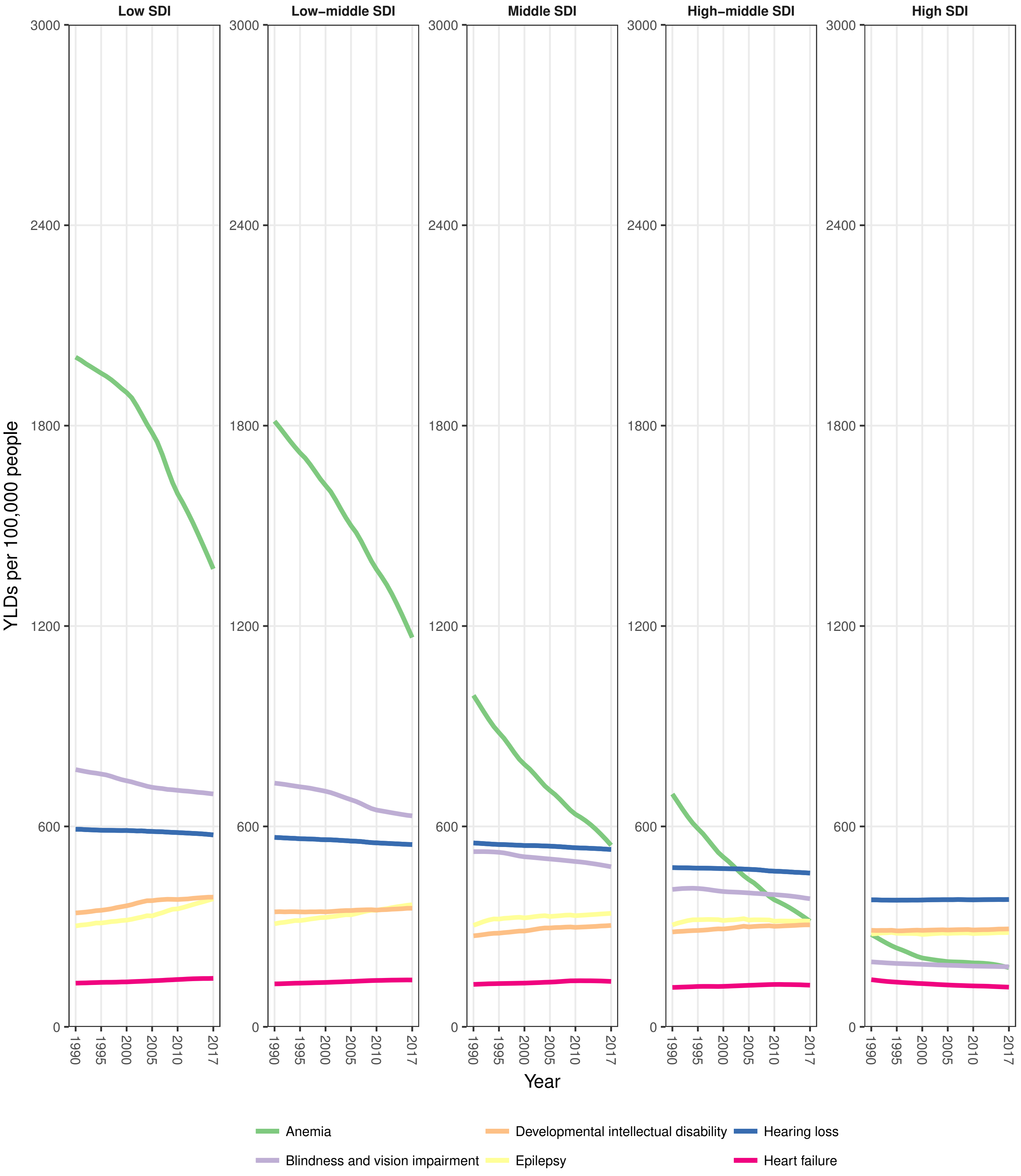


GBD=Global Burden of Disease. YLDs=years lived disability. SDI=Socio−demographic Index.

| SR Table 1: Global prevalence and YLDs for 2017, percentage change of YLD counts and percentage change of age-standardised YLD rates between 2007 and 2017 of anemia by cause, all ages and both sexes combined | | | | |
|---|---|---|---|---|
| Cause | Prevalence in thousands (95% uncertainty interval) | YLDs in thousands (95% uncertainty interval) | | |
| | 2017 | 2017 | Percentage change in counts between 2007 and 2017 | Percentage change in age-standardised rates between 2007 and 2017 |
| Dietary iron deficiency | 36372.4<br>(1  7517.2 to 1  56232.4) | 30013.9<br>(20323.3 to 43628.0) | -5.2<br>(-8.3 to -  .9) | -14.3<br>(-17.2 to -  .4) |
| Vitamin A deficiency | 212971.7<br>(189376.4 to 240436.1) | 8009.0<br>(5  96.4 to 1  716.1) | -18.1<br>(-21.8 to -  4.3) | -23.0<br>(-26.5 to -  9.5) |
| Gastritis and duodenitis | 38256.8<br>(34364.0 to 42488.0) | 2278.7<br>(1509.4 to 3245.1) | 5.8<br>(3.2 to 8.4) | -9.9<br>(-12.2 to -7.7) |
| Endocrine, metabolic, blood, and immune disorders | 81387.3<br>(80146.9 to 82729.4) | 2177.1<br>(1481.4 to 3  30.1) | -4.9<br>(-8.1 to -  .5) | -14.3<br>(-17.2 to -  .3) |
| Chronic kidney disease due to other and unspecified causes | 57243.2<br>(5  465.6 to 64073.9) | 955.3<br>(1409.0 to 2602.1) | 5.7<br>(10.1 to 22.0) | -6.8<br>(-1  .4 to -  .9) |
| Thalassaemias trait | 81  60.5<br>(78372.9 to 84177.2) | 908.8<br>(1282.8 to 2781.8) | -12.1<br>(-16.1 to -7.7) | -2  .6<br>(-25.1 to -  8.0) |
| Other hemoglobinopathies and hemolytic anaemias | 791  4.8<br>(77558.5 to 80769.9) | 834.3<br>(1234.2 to 2673.3) | -3.0<br>(-6.4 to 0.6) | -13.8<br>(-16.8 to -  0.5) |
| Other neglected tropical diseases | 52797.1<br>(5  667.9 to 54034.5) | 517.9<br>(1018.7 to 2  85.7) | -6.2<br>(-10.2 to -2.0) | -13.7<br>(-17.6 to -9.8) |
| Other unspecified infectious diseases | 53636.3<br>(52678.9 to 54662.5) | 434.7<br>(967.8 to 2073.7) | -5.7<br>(-9.1 to -2.2) | -13.9<br>(-17.2 to -  0.6) |
| Sickle cell trait | 43157.3<br>(42347.6 to 44070.2) | 203.4<br>(8  2.3 to 1731.4) | -1.3<br>(-5.1 to 2.9) | -10.3<br>(-13.9 to -6.4) |
| Malaria | 26206.7<br>(23984.2 to 28815.4) | 988.6<br>(641.0 to 1449.3) | -32.2<br>(-38.0 to -25.0) | -37.1<br>(-42.5 to -30.3) |
| Uterine fibroids | 27774.6<br>(26777.8 to 28832.2) | 977.7<br>(6  9.5 to 1523.2) | -0.1<br>(-4.5 to 4.6) | -14.5<br>(-18.2 to -  0.6) |
| HIV/AIDS resulting in other diseases | 5594.1<br>(5359.6 to 5841.1) | 648.8<br>(464.1 to 867.5) | -1  .0<br>(-25.8 to 6.1) | -22.1<br>(-34.9 to -7.4) |
| Chronic kidney disease due to hypertension | 7243.9<br>(6478.8 to 8  77.3) | 575.2<br>(421.6 to 742.1) | 6.8<br>(10.3 to 23.5) | -6.6<br>(-1  .8 to -  .3) |
| Chronic kidney disease due to diabetes mellitus type 2 | 9808.6<br>(8715.6 to 1  64.2) | 566.2<br>(4  5.2 to 732.6) | 20.0<br>(12.6 to 28.3) | -7.4<br>(-13.1 to -  .3) |
| Inflammatory bowel disease | 2284.1<br>(2099.3 to 2481.4) | 489.0<br>(340.8 to 679.3) | 4.3<br>(-1.8 to 10.6) | -13.5<br>(-18.4 to -8.3) |
| Chronic kidney disease due to glomerulonephritis | 3527.7<br>(3078.0 to 4068.2) | 431.9<br>(307.8 to 566.7) | 0.5<br>(3.9 to 17.7) | -4.5<br>(-10.2 to 1.6) |
| Peptic ulcer disease | 4245.2<br>(3854.8 to 4659.9) | 279.4<br>(189.6 to 394.0) | 6.9<br>(2.8 to 10.4) | -1  .0<br>(-14.4 to -8.2) |
| Hookworm disease | 9536.1<br>(8764.4 to 10362.8) | 245.9<br>(164.0 to 360.4) | -35.4<br>(-41.8 to -28.5) | -4  .3<br>(-47.1 to -34.9) |
| Schistosomiasis | 7618.2<br>(6901.9 to 8321.6) | 80.6<br>(1  9.1 to 268.6) | -30.4<br>(-35.1 to -25.9) | -38.6<br>(-42.8 to -34.6) |
| Sickle cell disorders | 2865.4<br>(2635.7 to 3  9.3) | 65.8<br>(1  0.0 to 241.0) | 2.5<br>(-6.9 to 1  .0) | -3.5<br>(-12.3 to 4.3) |
| Other gynecological diseases | 4562.4<br>(4423.1 to 4703.7) | 99.6<br>(66.3 to 146.8) | 6.6<br>(1.1 to 1  .8) | -4.2<br>(-8.9 to 0.6) |
| HIV/AIDS - Drug-susceptible Tuberculosis | 73.9<br>(148.6 to 205.9) | 71.3<br>(48.6 to 98.1) | -22.4<br>(-28.2 to -  5.7) | -3  .8<br>(-37.0 to -25.8) |
| Chronic kidney disease due to diabetes mellitus type 1 | 621.3<br>(535.3 to 726.6) | 67.8<br>(47.8 to 9  .5) | 8.4<br>(1  .4 to 25.8) | -0.0<br>(-5.9 to 6.2) |
| Maternal haemorrhage | 506.9<br>(1412.6 to 1601.5) | 35.5<br>(23.6 to 5  .8) | -6.7<br>(-14.7 to 1.1) | -14.6<br>(-21.7 to -7.5) |
| G6PD deficiency | 435.2<br>(4  9.2 to 453.7) | 22.8<br>(15.7 to 3  .7) | 0.6<br>(-6.8 to 7.2) | -9.5<br>(-16.2 to -3.4) |
| Thalassemias | 231.4<br>(2  3.6 to 251.3) | 4.7<br>(9.9 to 2  .2) | -24.4<br>(-30.3 to -  8.9) | -28.6<br>(-34.2 to -23.4) |
| HIV/AIDS - Multidrug-resistant Tuberculosis without extensive drug resistance | 6.2<br>(4.2 to 9.1) | 2.6<br>(1.5 to 4.2) | -26.6<br>(-49.8 to 8.7) | -35.7<br>(-55.9 to -4.3) |

| Cause | Prevalence in thousands (95% uncertainty interval) | YLDs in thousands (95% uncertainty interval) | | |
|---|---|---|---|---|
| | 2017 | 2017 | Percentage change in counts between 2007 and 2017 | Percentage change in age-standardised rates between 2007 and 2017 |
| G6PD trait | 6.1<br>(5.9 to 6.3) | 0.4<br>(0.3 to 0.6) | 3.0<br>(-1.7 to 7.8) | -8.1<br>(-12.2 to -3.9) |
| HIV/AIDS - Extensively drug-resistant Tuberculosis | 0.2<br>(0.1 to 0.4) | 0.1<br>(0.1 to 0.2) | 24.8<br>(-24.2 to 102.5) | 8.7<br>(-33.7 to 77.8) |
| **All causes** | **1950345.6<br>(1909153.0 to 1990086.8)** | **58197.1<br>(39535.4 to 83046.0)** | **-6.7<br>(-9.5 to -3.8)** | **-16.2<br>(-18.8 to -13.5)** |

| SR Table 2: Global prevalence and YLDs for 2017, percentage change of YLD counts and percentage change of age-standardised YLD rates between 2007 and 2017 of epilepsy by cause, all ages and both sexes combined | | | | |
|---|---|---|---|---|
| **Cause** | **Prevalence in thousands (95% uncertainty interval)** | **YLDs in thousands (95% uncertainty interval)** | | |
| | **2017** | **2017** | **Percentage change in counts between 2007 and 2017** | **Percentage change in age-standardised rates between 2007 and 2017** |
| Epilepsy | 27288.3 (21576.0 to 33443.8) | 8561.9 (5380.6 to 12551.5) | 15.9 (3.5 to 31.2) | 3.4 (-7.8 to 16.8) |
| Neonatal preterm birth | 13044.2 (9861.9 to 16385.6) | 6753.7 (4603.9 to 9297.2) | 19.2 (5.1 to 33.8) | 9.0 (-3.8 to 22.3) |
| Neonatal encephalopathy due to birth asphyxia and trauma | 10710.6 (6431.6 to 16469.3) | 5451.7 (2916.8 to 8825.3) | 17.7 (11.8 to 23.5) | 6.5 (1.3 to 11.5) |
| Neonatal sepsis and other neonatal infections | 5098.1 (2535.7 to 8571.0) | 2564.7 (1244.9 to 4594.8) | 15.7 (8.8 to 21.0) | 3.9 (-2.3 to 8.7) |
| Cysticercosis | 5417.9 (4662.0 to 6190.3) | 1568.5 (1015.3 to 2181.0) | 8.5 (3.7 to 12.8) | -9.1 (-13.1 to -5.6) |
| Hemolytic disease and other neonatal jaundice | 1030.1 (862.6 to 1210.6) | 564.5 (401.7 to 739.8) | 15.6 (9.8 to 20.7) | 5.0 (-0.4 to 9.8) |
| Malaria | 434.3 (383.7 to 491.3) | 239.7 (170.5 to 311.3) | 25.4 (19.6 to 31.9) | 16.2 (10.7 to 22.2) |
| Encephalitis | 249.2 (222.5 to 275.3) | 81.8 (53.5 to 111.7) | -4.2 (-9.1 to 0.5) | -16.3 (-20.5 to -12.0) |
| Other meningitis | 180.4 (154.6 to 214.7) | 63.0 (40.1 to 90.9) | 45.7 (34.2 to 57.3) | 33.3 (22.5 to 44.1) |
| Pneumococcal meningitis | 154.3 (126.2 to 192.9) | 53.2 (33.8 to 79.1) | -33.1 (-38.4 to -27.6) | -39.0 (-44.1 to -33.8) |
| Food-borne trematodiases | 94.1 (25.7 to 201.6) | 25.2 (6.1 to 59.0) | -22.5 (-33.7 to -11.4) | -33.4 (-42.9 to -24.2) |
| H influenzae type B meningitis | 45.4 (35.3 to 59.2) | 16.4 (10.3 to 24.6) | -50.9 (-54.7 to -47.4) | -54.4 (-58.2 to -51.0) |
| Meningococcal infection | 30.7 (25.2 to 38.5) | 10.9 (6.9 to 16.1) | -15.1 (-19.1 to -11.2) | -22.7 (-26.6 to -18.9) |
| Cystic echinococcosis | 5.3 (1.1 to 13.5) | 1.7 (0.3 to 4.4) | 12.1 (6.2 to 17.8) | -2.5 (-6.8 to 0.5) |
| Zika virus disease | 0.7 (0.5 to 1.3) | 0.4 (0.3 to 0.7) | < 0.05 | < 0.05 |
| Tetanus | < 0.05 | < 0.05 | -12.8 (-23.5 to -1.4) | -15.9 (-26.2 to -4.9) |
| **All causes** | **63783.6 (55496.5 to 73199.5)** | **25957.3 (20369.4 to 32900.4)** | **16.3 (10.6 to 22.1)** | **4.5 (-0.7 to 9.8)** |

| SR Table 3: Global prevalence and YLDs for 2017, percentage change of YLD counts and percentage change of age-standardised YLD rates between 2007 and 2017 of Guillain-Barré syndrome by cause, all ages and both sexes combined | | | | |
|---|---|---|---|---|
| **Cause** | **Prevalence in thousands (95% uncertainty interval)** | **YLDs in thousands (95% uncertainty interval)** | | |
| | **2017** | **2017** | **Percentage change in counts between 2007 and 2017** | **Percentage change in age-standardised rates between 2007 and 2017** |
| Other neurological disorders | 38.9 (25.6 to 53.2) | 11.5 (6.4 to 18.1) | 18.2 (15.8 to 20.8) | 3.1 (2.0 to 4.3) |
| Upper respiratory infections | 33.4 (24.4 to 44.7) | 9.9 (5.8 to 15.5) | 17.9 (15.5 to 20.6) | 3.1 (1.9 to 4.2) |
| Lower respiratory infections | 12.3 (6.9 to 19.9) | 3.6 (1.7 to 6.6) | 17.9 (15.5 to 20.6) | 3.1 (1.9 to 4.2) |
| Diarrhoeal diseases | 11.4 (7.8 to 15.7) | 3.4 (2.0 to 5.4) | 17.9 (15.5 to 20.6) | 3.1 (2.0 to 4.2) |
| Other unspecified infectious diseases | 7.4 (5.0 to 10.7) | 2.2 (1.2 to 3.6) | 18.0 (15.5 to 20.6) | 3.1 (2.0 to 4.2) |
| Zika virus disease | 0.3 (0.2 to 0.4) | 0.1 (0.0 to 0.1) | < 0.05 | < 0.05 |
| **All causes** | **103.7 (82.7 to 127.2)** | **30.7 (19.3 to 44.4)** | **18.3 (15.9 to 21.0)** | **3.4 (2.2 to 4.5)** |

| SR Table 4: Global prevalence and YLDs for 2017, percentage change of YLD counts and percentage change of age-standardised YLD rates between 2007 and 2017 of hearing loss by cause, all ages and both sexes combined | | | | |
|---|---|---|---|---|
| **Cause** | **Prevalence in thousands (95% uncertainty interval)** | **YLDs in thousands (95% uncertainty interval)** | | |
| | **2017** | **2017** | **Percentage change in counts between 2007 and 2017** | **Percentage change in age-standardised rates between 2007 and 2017** |
| Age-related and other hearing loss | 1317431.7 (1276962.6 to 1356229.1) | 34229.6 (23176.8 to 48684.1) | 24.9 (23.9 to 26.0) | -0.2 (-1.0 to 0.5) |
| Other congenital anomalies | 25911.9 (22431.7 to 30006.9) | 3353.0 (2227.2 to 4697.0) | 11.2 (8.4 to 13.9) | -0.4 (-2.8 to 2.1) |
| Otitis media | 83474.1 (75143.3 to 92173.0) | 1795.5 (1107.2 to 2820.6) | 3.9 (1.0 to 7.0) | -5.2 (-7.8 to -2.5) |
| Other meningitis | 772.1 (458.8 to 1029.2) | 36.4 (7.7 to 56.4) | 42.2 (34.5 to 51.1) | 28.1 (21.0 to 36.2) |
| Pneumococcal meningitis | 551.9 (325.4 to 749.0) | 25.1 (5.4 to 40.1) | -30.9 (-36.2 to -25.2) | -38.3 (-43.1 to -33.1) |
| Meningococcal infection | 170.4 (100.6 to 244.5) | 8.0 (1.7 to 13.3) | -15.0 (-21.2 to -8.4) | -23.8 (-29.4 to -17.8) |
| H influenzae type B meningitis | 138.0 (78.3 to 203.2) | 6.7 (1.4 to 11.2) | -49.6 (-54.6 to -44.3) | -54.4 (-59.1 to -49.4) |
| **All causes** | **1428450.1 (1388276.9 to 1465389.9)** | **39454.3 (27100.6 to 55310.2)** | **22.4 (21.4 to 23.3)** | **-0.5 (-1.2 to 0.1)** |

| SR Table 5: Global prevalence and YLDs for 2017, percentage change of YLD counts and percentage change of age-standardised YLD rates between 2007 and 2017 of heart failure by cause, all ages and both sexes combined | | | | |
|---|---|---|---|---|
| **Cause** | **Prevalence in thousands (95% uncertainty interval)** | **YLDs in thousands (95% uncertainty interval)** | | |
| | **2017** | **2017** | **Percentage change in counts between 2007 and 2017** | **Percentage change in age-standardised rates between 2007 and 2017** |
| Chronic obstructive pulmonary disease | 14890.7 (12183.6 to 17715.3) | 5673.6 (4163.8 to 7049.6) | 36.1 (32.1 to 39.1) | 1.3 (-1.1 to 3.4) |
| Hypertensive heart disease | 17067.7 (14426.9 to 19874.3) | 1408.0 (966.6 to 1982.4) | 34.9 (33.2 to 36.6) | 1.9 (0.7 to 2.9) |
| Ischaemic heart disease | 17007.2 (14512.0 to 19849.5) | 1388.9 (951.7 to 1947.2) | 25.9 (24.3 to 27.5) | -6.4 (-7.6 to -5.1) |
| Other cardiomyopathy | 4212.7 (3634.6 to 4869.9) | 353.3 (237.9 to 493.9) | 25.7 (23.4 to 27.9) | -2.5 (-3.9 to -1.0) |
| Interstitial lung disease and pulmonary sarcoidosis | 416.8 (322.3 to 505.6) | 169.8 (114.4 to 236.1) | 31.4 (28.6 to 34.2) | -1.2 (-3.3 to 0.9) |
| Non-rheumatic degenerative mitral valve disease | 1723.6 (1111.4 to 2494.0) | 139.9 (81.5 to 217.1) | 26.7 (15.5 to 38.6) | -6.8 (-15.0 to 2.0) |
| Alcoholic cardiomyopathy | 1621.8 (1370.5 to 1902.4) | 139.1 (95.1 to 196.1) | 15.5 (12.9 to 18.1) | -9.5 (-11.2 to -7.7) |
| Other cardiovascular and circulatory diseases | 1562.0 (1279.4 to 1882.1) | 129.6 (86.6 to 185.3) | 30.0 (27.5 to 32.6) | -2.0 (-3.8 to -0.2) |
| Non-rheumatic calcific aortic valve disease | 1386.0 (896.6 to 2029.9) | 111.0 (63.7 to 173.8) | 30.6 (13.2 to 47.6) | -6.0 (-19.0 to 6.2) |
| Rheumatic heart disease | 1199.9 (1006.1 to 1403.8) | 103.1 (69.7 to 145.1) | 27.2 (24.4 to 30.2) | -0.1 (-2.2 to 1.9) |
| Myocarditis | 1063.7 (914.1 to 1215.2) | 95.7 (63.9 to 133.5) | 14.8 (12.3 to 17.3) | -2.1 (-3.4 to -0.8) |
| Congenital heart anomalies | 532.7 (418.5 to 671.9) | 51.4 (32.6 to 76.4) | 3.5 (2.5 to 4.6) | -3.4 (-4.2 to -2.7) |
| Endocarditis | 566.2 (480.9 to 655.8) | 48.9 (33.3 to 68.7) | 24.5 (21.8 to 27.4) | -3.2 (-5.1 to -1.3) |
| Endocrine, metabolic, blood, and immune disorders | 390.1 (322.2 to 465.9) | 32.9 (21.8 to 46.4) | 28.1 (24.7 to 31.5) | -4.6 (-6.9 to -2.1) |
| Other hemoglobinopathies and hemolytic anaemias | 328.1 (268.8 to 392.8) | 28.5 (18.9 to 41.0) | 30.0 (26.9 to 33.1) | -1.4 (-3.6 to 1.1) |
| Chagas disease | 296.2 (196.1 to 427.4) | 16.0 (9.1 to 26.3) | 5.5 (1.9 to 9.6) | -16.9 (-19.7 to -13.8) |
| Silicosis | 11.0 (9.1 to 13.1) | 5.0 (3.4 to 6.9) | 31.0 (26.8 to 35.3) | -1.6 (-4.8 to 1.9) |
| Coal workers pneumoconiosis | 7.0 (5.9 to 8.2) | 3.2 (2.2 to 4.3) | 27.5 (23.0 to 32.1) | -3.1 (-6.6 to 0.4) |
| Asbestosis | 5.6 (4.7 to 6.5) | 2.5 (1.7 to 3.5) | 27.6 (23.8 to 31.8) | -6.0 (-8.9 to -3.0) |
| Other non-rheumatic valve diseases | 26.9 (22.1 to 32.2) | 2.5 (1.6 to 3.6) | 37.7 (34.1 to 41.1) | 6.1 (4.1 to 8.1) |
| G6PD deficiency | 20.3 (16.7 to 24.6) | 1.9 (1.2 to 2.7) | 29.1 (26.0 to 32.1) | 2.2 (0.5 to 3.8) |
| Other pneumoconiosis | 4.1 (3.4 to 4.7) | 1.9 (1.3 to 2.6) | 27.8 (23.5 to 31.9) | -4.5 (-7.5 to -1.9) |
| Thalassemias | 3.6 (2.8 to 4.6) | 0.3 (0.2 to 0.5) | -5.9 (-8.2 to -3.4) | -8.8 (-10.7 to -6.7) |
| **All causes** | **64343.9 (57187.0 to 71648.3)** | **9906.9 (7261.9 to 12443.6)** | **32.6 (30.3 to 34.6)** | **-0.5 (-2.0 to 0.7)** |

| SR Table 6: Global prevalence and YLDs for 2017, percentage change of YLD counts and percentage change of age-standardised YLD rates between 2007 and 2017 of infertility by cause, all ages and both sexes combined | | | | |
|---|---|---|---|---|
| **Cause** | **Prevalence in thousands (95% uncertainty interval)** | **YLDs in thousands (95% uncertainty interval)** | | |
| | **2017** | **2017** | **Percentage change in counts between 2007 and 2017** | **Percentage change in age-standardised rates between 2007 and 2017** |
| Female infertility | 61238.4 (43321.2 to 82668.2) | 342.6 (129.5 to 723.4) | 27.5 (22.2 to 32.7) | 16.5 (12.0 to 20.8) |
| Endometriosis | 2281.6 (1664.3 to 3116.9) | 225.1 (137.3 to 349.0) | 7.6 (4.9 to 10.4) | -2.6 (-5.0 to -0.0) |
| Male infertility | 30432.7 (24686.0 to 36812.7) | 180.9 (74.8 to 373.9) | 17.0 (13.7 to 20.1) | 6.9 (4.1 to 9.7) |
| Polycystic ovarian syndrome | 9535.3 (6365.9 to 13862.9) | 92.0 (36.6 to 197.9) | 10.8 (8.4 to 12.8) | 1.6 (-0.3 to 3.3) |
| Other sexually transmitted infections | 11118.0 (10399.8 to 11996.9) | 61.4 (25.1 to 124.6) | 15.6 (13.9 to 17.5) | 4.0 (2.5 to 5.4) |
| Chlamydial infection | 4347.4 (4062.7 to 4680.3) | 24.3 (9.9 to 49.7) | 19.0 (16.9 to 21.2) | 7.3 (5.4 to 9.1) |
| Maternal sepsis and other pregnancy related infections | 2073.0 (1839.6 to 2335.1) | 10.4 (3.9 to 21.8) | 21.8 (19.2 to 24.3) | 6.6 (4.3 to 8.8) |
| Gonococcal infection | 1209.3 (1122.9 to 1301.5) | 6.7 (2.8 to 13.9) | 16.6 (13.3 to 19.7) | 5.0 (2.0 to 7.7) |
| Turner syndrome | 294.0 (256.9 to 335.3) | 6.2 (3.3 to 10.1) | 8.5 (6.2 to 10.7) | 1.0 (-1.2 to 3.0) |
| Klinefelter syndrome | 374.4 (323.5 to 430.4) | 3.8 (1.7 to 7.7) | 9.0 (5.3 to 12.9) | -1.5 (-4.8 to 2.0) |
| Urogenital congenital anomalies | 180.9 (86.2 to 305.4) | 3.4 (1.1 to 8.7) | 2.2 (1.1 to 3.4) | -3.7 (-4.8 to -2.6) |
| **All causes** | **123084.9 (99543.3 to 150556.1)** | **956.9 (487.4 to 1791.9)** | **17.3 (13.6 to 20.5)** | **6.8 (3.5 to 9.8)** |

| SR Table 7: Global prevalence and YLDs for 2017, percentage change of YLD counts and percentage change of age-standardised YLD rates between 2007 and 2017 of developmental intellectual disability by cause, all ages and both sexes combined | | | | |
|---|---|---|---|---|
| Cause | Prevalence in thousands (95% uncertainty interval) | YLDs in thousands (95% uncertainty interval) | | |
| | 2017 | 2017 | Percentage change in counts between 2007 and 2017 | Percentage change in age-standardised rates between 2007 and 2017 |
| Neonatal preterm birth | 21451.7 (17169.3 to 26176.2) | 6205.7 (4422.2 to 8281.8) | 19.8 (6.4 to 33.8) | 9.2 (-3.0 to 22.0) |
| Neonatal encephalopathy due to birth asphyxia and trauma | 17180.6 (12325.2 to 23314.3) | 5062.2 (2860.4 to 8079.1) | 18.0 (12.4 to 23.9) | 6.5 (1.6 to 11.4) |
| Idiopathic developmental intellectual disability | 100572.2 (58404.5 to 143878.6) | 4046.4 (1940.2 to 6859.5) | 0.0 (-2.8 to 1.5) | -8.7 (-11.1 to -7.5) |
| Neonatal sepsis and other neonatal infections | 10533.7 (6569.2 to 16023.8) | 3971.4 (2074.7 to 6743.9) | 19.1 (15.2 to 22.3) | 7.1 (3.7 to 9.9) |
| Autism spectrum disorders | 17797.3 (15470.0 to 20170.9) | 3081.6 (2075.0 to 4263.5) | 11.4 (10.6 to 12.3) | -0.3 (-1.0 to 0.4) |
| Iodine deficiency | 2628.8 (1500.7 to 3684.3) | 848.9 (445.5 to 1286.3) | -8.1 (-15.8 to -3.1) | -17.3 (-24.2 to -12.8) |
| Other chromosomal abnormalities | 6034.6 (4786.9 to 7405.1) | 576.9 (377.5 to 815.9) | 19.6 (16.8 to 22.4) | 7.2 (4.7 to 9.6) |
| Hemolytic disease and other neonatal jaundice | 850.3 (709.9 to 1004.0) | 491.2 (357.2 to 633.3) | 16.2 (10.4 to 21.2) | 5.3 (-0.0 to 10.0) |
| Down's syndrome | 3345.4 (2923.9 to 3798.8) | 318.8 (217.5 to 442.9) | 11.7 (9.0 to 14.6) | -0.2 (-2.6 to 2.4) |
| Malaria | 363.1 (319.0 to 414.1) | 208.7 (153.6 to 265.4) | 25.0 (18.6 to 31.8) | 15.7 (9.8 to 22.1) |
| Neural tube defects | 561.6 (394.8 to 784.2) | 187.1 (110.8 to 291.1) | 17.1 (14.3 to 19.9) | 5.1 (2.6 to 7.7) |
| Encephalitis | 2530.9 (858.4 to 4830.3) | 106.4 (61.1 to 170.9) | -4.0 (-10.8 to 1.5) | -15.0 (-20.8 to -10.1) |
| Other meningitis | 1411.3 (737.8 to 2139.7) | 54.7 (30.6 to 88.6) | 30.5 (14.6 to 51.1) | 18.3 (4.0 to 37.2) |
| Congenital heart anomalies | 1315.6 (1166.9 to 1476.6) | 49.1 (31.5 to 72.2) | -4.5 (-9.0 to -0.8) | -9.9 (-14.1 to -6.3) |
| Pneumococcal meningitis | 924.9 (682.0 to 1255.7) | 48.8 (29.8 to 75.8) | -38.7 (-43.9 to -32.5) | -44.5 (-49.2 to -38.9) |
| Meningococcal infection | 279.2 (125.9 to 448.0) | 13.0 (7.1 to 21.6) | -24.6 (-33.3 to -13.8) | -31.6 (-39.5 to -21.7) |
| H influenzae type B meningitis | 288.9 (160.9 to 458.0) | 12.2 (6.8 to 20.2) | -63.9 (-69.5 to -57.3) | -67.0 (-72.0 to -60.9) |
| Alcohol use disorders | 254.1 (199.1 to 316.5) | 11.1 (6.6 to 17.0) | -4.7 (-10.1 to -0.2) | -14.7 (-19.4 to -10.6) |
| Klinefelter syndrome | 178.9 (130.7 to 234.3) | 2.9 (1.4 to 5.5) | 6.1 (2.9 to 9.6) | -2.0 (-4.9 to 1.2) |
| Digestive congenital anomalies | 56.6 (45.3 to 70.0) | 2.9 (1.7 to 4.7) | 6.5 (5.6 to 7.3) | -1.8 (-2.6 to -1.0) |
| Tetanus | 24.8 (15.2 to 33.5) | 0.8 (0.4 to 1.3) | 6.5 (3.6 to 9.2) | -3.8 (-6.4 to -1.3) |
| Zika virus disease | 0.7 (0.5 to 1.3) | 0.4 (0.3 to 0.7) | < 0.05 | < 0.05 |
| **All causes** | **188585.1 (145641.8 to 230402.8)** | **25301.2 (19706.5 to 31400.2)** | **12.9 (10.0 to 16.1)** | **2.1 (-0.5 to 5.1)** |

| SR Table 8: Global prevalence and YLDs for 2017, percentage change of YLD counts and percentage change of age-standardised YLD rates between 2007 and 2017 of pelvic inflammatory disease by cause, all ages and both sexes combined | | | | |
|---|---|---|---|---|
| **Cause** | **Prevalence in thousands (95% uncertainty interval)** | **YLDs in thousands (95% uncertainty interval)** | | |
| | **2017** | **2017** | **Percentage change in counts between 2007 and 2017** | **Percentage change in age-standardised rates between 2007 and 2017** |
| Other sexually transmitted infections | 742.5<br>(626.5 to 875.6) | 97.9<br>(65.1 to 136.4) | 15.5<br>(12.6 to 18.8) | 1.8<br>(-0.7 to 4.5) |
| Chlamydial infection | 261.5<br>(220.4 to 308.8) | 34.8<br>(22.8 to 48.1) | 18.1<br>(13.3 to 23.1) | 4.8<br>(0.6 to 9.2) |
| Gonococcal infection | 60.2<br>(48.5 to 73.6) | 8.3<br>(5.4 to 11.9) | 13.0<br>(11.3 to 14.7) | 0.9<br>(-0.5 to 2.4) |
| **All causes** | **1064.1**<br>**(906.7 to 1249.0)** | **141.0**<br>**(94.8 to 197.2)** | **16.0**<br>**(13.6 to 18.7)** | **2.5**<br>**(0.4 to 4.7)** |

| SR Table 9: Global prevalence and YLDs for 2017, percentage change of YLD counts and percentage change of age-standardised YLD rates between 2007 and 2017 of blindness and vision impairment by cause, all ages and both sexes combined | | | | |
|---|---|---|---|---|
| **Cause** | **Prevalence in thousands (95% uncertainty interval)** | **YLDs in thousands (95% uncertainty interval)** | | |
| | **2017** | **2017** | **Percentage change in counts between 2007 and 2017** | **Percentage change in age-standardised rates between 2007 and 2017** |
| Near vision loss | 969669.5<br>(932613.8 to 1009067.7) | 9802.9<br>(4669.6 to 17922.5) | 24.6<br>(24.0 to 25.2) | 0.5<br>(0.1 to 0.9) |
| Cataract | 107987.7<br>(95775.3 to 122319.3) | 8005.8<br>(5579.2 to 10820.9) | 29.6<br>(27.4 to 31.7) | -2.0<br>(-3.6 to -0.5) |
| Refraction disorders | 185392.8<br>(165673.9 to 208575.2) | 7984.3<br>(5353.0 to 11487.0) | 15.3<br>(13.6 to 17.0) | -3.1<br>(-4.2 to -1.9) |
| Other vision loss | 34607.5<br>(30956.5 to 38397.4) | 2885.3<br>(2013.7 to 3884.0) | 21.0<br>(19.4 to 22.6) | -3.7<br>(-4.8 to -2.6) |
| Neonatal preterm birth | 8767.9<br>(7198.0 to 10479.8) | 1608.1<br>(1167.6 to 2101.4) | 17.4<br>(9.4 to 26.5) | 3.8<br>(-3.0 to 11.4) |
| Neonatal encephalopathy due to birth asphyxia and trauma | 1853.4<br>(1085.0 to 2841.4) | 921.1<br>(497.8 to 1463.0) | 25.7<br>(20.0 to 32.8) | 9.9<br>(5.0 to 15.8) |
| Glaucoma | 5993.5<br>(5157.5 to 6984.8) | 686.1<br>(462.9 to 948.8) | 27.9<br>(26.4 to 29.4) | -4.7<br>(-5.8 to -3.6) |
| Age-related macular degeneration | 6758.8<br>(5612.6 to 8183.0) | 530.7<br>(363.6 to 728.6) | 30.7<br>(28.6 to 32.9) | -3.7<br>(-5.3 to -2.1) |
| Neonatal sepsis and other neonatal infections | 811.6<br>(407.0 to 1386.6) | 409.1<br>(197.5 to 712.3) | 20.5<br>(15.0 to 25.3) | 4.5<br>(-0.4 to 8.7) |
| Diabetes mellitus type 2 | 4509.0<br>(3835.7 to 5282.0) | 362.5<br>(252.6 to 500.2) | 21.1<br>(18.2 to 23.6) | -5.7<br>(-7.7 to -3.8) |
| Vitamin A deficiency | 5403.4<br>(4329.4 to 6754.9) | 304.0<br>(195.6 to 442.0) | 13.4<br>(9.9 to 17.1) | 3.7<br>(0.6 to 7.1) |
| Trachoma | 3818.9<br>(2842.6 to 5135.2) | 302.9<br>(201.7 to 425.1) | -5.5<br>(-13.1 to 2.0) | -28.2<br>(-33.8 to -22.5) |
| Onchocerciasis | 1152.1<br>(829.0 to 1703.6) | 96.1<br>(60.6 to 141.5) | 7.0<br>(-4.8 to 21.4) | -15.0<br>(-24.3 to -3.8) |
| Hemolytic disease and other neonatal jaundice | 159.3<br>(131.8 to 190.6) | 81.8<br>(61.8 to 105.3) | 18.8<br>(14.2 to 22.8) | 4.9<br>(0.7 to 8.6) |
| Encephalitis | 1361.0<br>(555.7 to 2549.1) | 42.5<br>(23.8 to 71.2) | 8.7<br>(6.7 to 11.3) | -8.2<br>(-9.7 to -6.4) |
| Malaria | 76.4<br>(64.3 to 88.5) | 39.7<br>(30.5 to 50.3) | 17.8<br>(13.7 to 21.6) | 7.9<br>(3.7 to 11.6) |
| Other meningitis | 823.9<br>(480.3 to 1220.6) | 29.8<br>(18.2 to 46.5) | 45.7<br>(37.3 to 54.6) | 28.1<br>(21.1 to 36.2) |
| Meningococcal infection | 230.6<br>(140.2 to 340.5) | 12.2<br>(7.3 to 19.4) | -13.5<br>(-19.7 to -7.6) | -24.3<br>(-29.9 to -18.9) |
| Pneumococcal meningitis | 397.6<br>(247.7 to 605.0) | 11.5<br>(6.8 to 18.6) | -30.3<br>(-35.6 to -24.9) | -38.9<br>(-43.7 to -34.0) |
| Diabetes mellitus type 1 | 82.5<br>(65.3 to 102.7) | 6.9<br>(4.5 to 10.1) | 4.0<br>(-0.9 to 9.0) | -15.1<br>(-19.2 to -11.1) |
| H influenzae type B meningitis | 112.9<br>(48.1 to 196.9) | 2.1<br>(0.8 to 3.9) | -50.1<br>(-55.3 to -44.7) | -55.4<br>(-60.1 to -50.4) |
| Tetanus | < 0.05 | < 0.05 | -26.5<br>(-33.1 to -20.2) | -29.1<br>(-35.5 to -23.0) |
| **All causes** | **1339970.3<br>(1291077.0 to 1393811.0)** | **34125.4<br>(23206.6 to 48924.6)** | **22.2<br>(21.2 to 23.3)** | **-1.5<br>(-2.3 to -0.7)** |

| Cause | Prevalence in thousands (95% UI) | Incidence in thousands (95% UI) | YLDs in thousands (95% UI) | | |
|---|---|---|---|---|---|
| SR Table 10a: Global prevalence, incidence and YLDs for 2017, percentage change of YLD counts and percentage change of YLD rates between 2007 and 2017 by cause, age 0 to 6 days | | | | | |
| | 2017 counts | 2017 counts | 2017 counts | Percentage change in counts between 2007 and 2017 | Percentage change in rates between 2007 and 2017 |
| **All causes** | **2031.1 (1939.1 to 2116.0)** | **20977.0 (18395.1 to 23858.7)** | **119.4 (86.6 to 158.1)** | **-6.2* (-9.1 to -2.8)** | **-8.6* (-11.4 to -5.3)** |
| **Communicable, maternal, neonatal, and nutritional disorders** | **1695.1 (1550.7 to 1825.7)** | **19032.8 (16443.5 to 21908.4)** | **90.2 (64.7 to 120.9)** | **-10.0* (-13.5 to -5.8)** | **-12.3* (-15.7 to -8.2)** |
| **HIV/AIDS and sexually transmitted infections** | **1.6 (1.4 to 1.8)** | **2.8 (2.4 to 3.2)** | **0.2 (0.2 to 0.3)** | **-56.5* (-60.9 to -52.1)** | **-57.7* (-61.9 to -53.3)** |
| HIV/AIDS | 2.0 (1.8 to 2.2) | 2.8^ (2.4 to 3.2) | 0.2 (0.2 to 0.3) | -56.5* (-60.9 to -52.1) | -57.7* (-61.9 to -53.3) |
| HIV/AIDS - Drug-susceptible Tuberculosis | -- | -- | -- | -- | -- |
| HIV/AIDS - Multidrug-resistant Tuberculosis without extensive drug resistance | -- | -- | -- | -- | -- |
| HIV/AIDS - Extensively drug-resistant Tuberculosis | -- | -- | -- | -- | -- |
| HIV/AIDS resulting in other diseases | 2.0 (1.8 to 2.2) | 2.8 (2.4 to 3.2) | 0.2 (0.2 to 0.3) | -56.5* (-60.9 to -52.1) | -57.7* (-61.9 to -53.3) |
| HIV/AIDS not on antiretroviral treatment without Tuberculosis | 1.4 (1.2 to 1.5) | 2.8 (2.4 to 3.2) | 0.2 (0.1 to 0.2) | -63.2* (-67.6 to -58.8) | -64.1* (-68.4 to -59.9) |
| HIV/AIDS on antiretroviral treatment without Tuberculosis | 0.6 (0.5 to 0.6) | -- | 0.1 (0.0 to 0.1) | 30.8* (5.0 to 68.1) | 27.4* (2.2 to 63.8) |
| Sexually transmitted infections excluding HIV | -- | -- | -- | -- | -- |
| Syphilis | -- | -- | -- | -- | -- |
| Early syphilis | -- | -- | -- | -- | -- |
| Tertiary syphilis | -- | -- | -- | -- | -- |
| Chlamydial infection | -- | -- | -- | -- | -- |
| Chlamydia episode | -- | -- | -- | -- | -- |
| Chlamydial infection complications | -- | -- | -- | -- | -- |
| Gonococcal infection | -- | -- | -- | -- | -- |
| Gonococcal infection complications | -- | -- | -- | -- | -- |
| Gonorrhoea episode | -- | -- | -- | -- | -- |
| Trichomoniasis | -- | -- | -- | -- | -- |
| Genital herpes | -- | -- | -- | -- | -- |
| Other sexually transmitted infections | -- | -- | -- | -- | -- |
| Other sexually transmitted diseases residual | -- | -- | -- | -- | -- |
| Other sexually transmitted diseases | -- | -- | -- | -- | -- |
| **Respiratory infections and tuberculosis** | **352.6 (287.9 to 431.6)** | **10126.4 (7973.6 to 12499.3)** | **4.4 (2.7 to 6.8)** | **-13.8* (-22.6 to -4.7)** | **-16.0* (-24.6 to -7.2)** |
| Tuberculosis | 259.7 (196.5 to 342.2) | 3.6 (3.0 to 4.3) | -- | -14.4* (-21.0 to -7.5) | -16.6* (-23.1 to -9.9) |
| Latent tuberculosis infection | 259.6 (196.5 to 342.2) | -- | -- | -- | -- |
| Drug-susceptible tuberculosis | -- | 3.5 (2.9 to 4.1) | -- | -14.1* (-21.1 to -7.0) | -16.3* (-23.2 to -9.4) |
| Multidrug-resistant tuberculosis without extensive drug resistance | -- | 0.1 (0.1 to 0.2) | -- | -22.4 (-49.0 to 15.9) | -24.4 (-50.3 to 12.9) |
| Extensively drug-resistant tuberculosis | -- | -- | -- | 3.1 (-34.5 to 71.1) | 0.4 (-36.2 to 66.7) |
| Lower respiratory infections | 48.7 (36.2 to 64.6) | 309.3 (234.8 to 394.5) | 3.0 (1.7 to 4.6) | -20.5* (-32.3 to -8.3) | -22.6* (-34.1 to -10.7) |
| Guillain-Barré syndrome due to lower respiratory infections | -- | -- | -- | 2.4* (0.4 to 4.3) | -0.3 (-2.2 to 1.6) |
| Lower respiratory infection episode | 48.7 (36.2 to 64.6) | 309.3 (234.8 to 394.5) | 3.0 (1.7 to 4.6) | -20.5* (-32.3 to -8.3) | -22.6* (-34.1 to -10.7) |
| Upper respiratory infections | 51.0 (38.7 to 64.4) | 9152.4 (6958.6 to 11569.7) | 1.3 (0.7 to 2.1) | 6.2* (4.2 to 8.2) | 3.5* (1.5 to 5.4) |
| Guillain-Barré syndrome due to upper respiratory infections | -- | -- | -- | 2.4* (0.4 to 4.4) | -0.3 (-2.2 to 1.7) |
| Upper respiratory infection episode | 51.0 (38.7 to 64.4) | 9152.4 (6958.6 to 11569.7) | 1.3 (0.7 to 2.1) | 6.2* (4.2 to 8.2) | 3.5* (1.5 to 5.4) |
| Otitis media | 5.9 (4.1 to 8.1) | 661.1 (448.8 to 942.4) | 0.1 (0.0 to 0.2) | 0.4 (-2.7 to 3.5) | -2.2 (-5.2 to 0.8) |
| Acute otitis media | 5.3 (3.5 to 7.5) | 661.0 (448.7 to 942.3) | 0.1 (0.0 to 0.1) | 1.3 (-2.2 to 4.9) | -1.4 (-4.7 to 2.2) |
| Chronic otitis media | 0.6 (0.5 to 0.8) | 0.1 (0.0 to 0.2) | -- | -4.0 (-8.6 to 1.0) | -6.4* (-11.0 to -1.6) |
| **Enteric infections** | **37.6 (30.8 to 45.2)** | **6746.4 (5477.7 to 8157.6)** | **4.4 (2.9 to 6.3)** | **-9.4* (-18.1 to -0.5)** | **-11.7* (-20.2 to -3.1)** |
| Diarrhoeal diseases | 37.8 (30.8 to 45.5) | 6746.4 (5477.7 to 8157.6) | 4.4 (2.9 to 6.3) | -9.4* (-18.1 to -0.5) | -11.7* (-20.2 to -3.1) |
| Guillain-Barré syndrome due to diarrheal diseases | -- | -- | -- | 2.4* (0.4 to 4.3) | -0.3 (-2.2 to 1.6) |
| Diarrheal disease episode | 37.8 (30.8 to 45.5) | 6746.4 (5477.7 to 8157.6) | 4.4 (2.9 to 6.3) | -9.4* (-18.1 to -0.5) | -11.7* (-20.2 to -3.1) |
| Typhoid and paratyphoid | -- | -- | -- | -- | -- |
| Typhoid fever | -- | -- | -- | -- | -- |
| Typhoid fever complications | -- | -- | -- | -- | -- |
| Typhoid fever episode | -- | -- | -- | -- | -- |
| Paratyphoid fever | -- | -- | -- | -- | -- |
| Intestinal perforation due to paratyphoid | -- | -- | -- | -- | -- |
| Paratyphoid fever episode | -- | -- | -- | -- | -- |
| iNTS | -- | -- | -- | -- | -- |
| Other intestinal infectious diseases | -- | -- | -- | -- | -- |
| **Neglected tropical diseases and malaria** | **67.2 (61.9 to 71.7)** | **1.1 (0.8 to 1.7)** | **0.6 (0.4 to 0.9)** | **-30.2* (-39.6 to -17.9)** | **-32.0* (-41.2 to -20.0)** |
| Malaria | 68.7 (63.1 to 73.4) | -- | 0.6 (0.4 to 0.9) | -31.0* (-40.5 to -18.8) | -32.8* (-42.0 to -20.9) |
| Malaria complications | -- | -- | -- | 10.2* (4.8 to 16.2) | 7.3* (2.1 to 13.2) |
| Malaria episode | -- | -- | -- | -- | -- |

| | | | | | |
|---|---|---|---|---|---|
| Malaria parasitemia | 68.7 (63.1 to 73.4) | -- | 0.6 (0.4 to 0.9) | -31.0* (-40.5 to -18.8) | -32.8* (-42.0 to -20.9) |
| Chagas disease | 0.1 (0.1 to 0.2) | 0.1 (0.0 to 0.1) | -- | -30.0* (-36.7 to -23.8) | -31.8* (-38.4 to -25.8) |
| Acute Chagas disease | -- | 0.1 (0.0 to 0.1) | -- | -30.5* (-37.5 to -24.2) | -32.3* (-39.1 to -26.2) |
| Asymptomatic Chagas disease | 0.1 (0.1 to 0.2) | -- | -- | -- | -- |
| Symptomatic chronic Chagas infection | -- | -- | -- | -9.9* (-15.1 to -4.7) | -12.2* (-17.3 to -7.2) |
| Leishmaniasis | -- | -- | -- | -- | -- |
| Visceral leishmaniasis | -- | -- | -- | -- | -- |
| Cutaneous and mucocutaneous leishmaniasis | -- | -- | -- | -- | -- |
| African trypanosomiasis | -- | -- | -- | -- | -- |
| Trypanosomiasis Gambiense | -- | -- | -- | -- | -- |
| Trypanosomiasis Rhodesiense | -- | -- | -- | -- | -- |
| Schistosomiasis | -- | -- | -- | -- | -- |
| Mild schistosomiasis | -- | -- | -- | -- | -- |
| Anemia due to schistosomiasis | -- | -- | -- | -- | -- |
| Schistosomiasis complications | -- | -- | -- | -- | -- |
| Cysticercosis | -- | -- | -- | -- | -- |
| Cystic echinococcosis | -- | -- | -- | -- | -- |
| Lymphatic filariasis | -- | -- | -- | -- | -- |
| Prevalence of detectable microfiliaria due to lymphatic filariasis | -- | -- | -- | -- | -- |
| Lymphatic filariasis complications | -- | -- | -- | -- | -- |
| Onchocerciasis | -- | -- | -- | -- | -- |
| Asymptomatic onchocerciasis | -- | -- | -- | -- | -- |
| Skin disease due to onchocerciasis | -- | -- | -- | -- | -- |
| Vision loss due to onchocerciasis | -- | -- | -- | -- | -- |
| Trachoma | -- | -- | -- | -- | -- |
| Dengue | -- | -- | -- | -- | -- |
| Post-dengue chronic fatigue syndrome | -- | -- | -- | -- | -- |
| Dengue episode | -- | -- | -- | -- | -- |
| Yellow fever | -- | -- | -- | -- | -- |
| Asymptomatic yellow fever | -- | -- | -- | -- | -- |
| Yellow fever episode | -- | -- | -- | -- | -- |
| Rabies | -- | -- | -- | -- | -- |
| Intestinal nematode infections | -- | -- | -- | -- | -- |
| Ascariasis | -- | -- | -- | -- | -- |
| Asymptomatic ascariasis | -- | -- | -- | -- | -- |
| Ascariasis complications | -- | -- | -- | -- | -- |
| Trichuriasis | -- | -- | -- | -- | -- |
| Asymptomatic trichuriasis | -- | -- | -- | -- | -- |
| Trichuriasis complications | -- | -- | -- | -- | -- |
| Hookworm disease | -- | -- | -- | -- | -- |
| Asymptomatic hookworm disease | -- | -- | -- | -- | -- |
| Anemia due to hookworm disease | -- | -- | -- | -- | -- |
| Hookworm disease complications | -- | -- | -- | -- | -- |
| Food-borne trematodiases | -- | -- | -- | -- | -- |
| Asymptomatic food-borne tremodiases | -- | -- | -- | -- | -- |
| Food-borne tremodiases complications | -- | -- | -- | -- | -- |
| Leprosy | -- | -- | -- | -- | -- |
| Ebola virus disease | -- | -- | -- | -- | -- |
| Ebola cases | -- | -- | -- | -- | -- |
| Post-Ebola chronic fatigue syndrome | -- | -- | -- | -- | -- |
| Zika virus disease | -- | 1.0 (0.7 to 1.6) | -- | -- | -- |
| Zika virus complications | -- | 0.6 (0.4 to 1.2) | -- | -- | -- |
| Zika virus episode | -- | 0.5 (0.3 to 0.9) | -- | -- | -- |
| Guinea worm disease | -- | -- | -- | -- | -- |
| Other neglected tropical diseases | -- | -- | -- | -- | -- |
| Acute infection due to other neglected tropical diseases | -- | -- | -- | -- | -- |
| Anemia due to other neglected tropical diseases | -- | -- | -- | -- | -- |
| **Other infectious diseases** | **94.9 (81.1 to 108.5)** | **1428.3 (1170.1 to 1745.6)** | **1.0 (0.7 to 1.5)** | **-28.4* (-36.8 to -20.5)** | **-30.2* (-38.4 to -22.6)** |
| Meningitis | 0.3 (0.3 to 0.4) | 40.6 (32.1 to 50.7) | 0.0 (0.0 to 0.1) | -20.4* (-31.2 to -9.2) | -22.4* (-32.9 to -11.5) |
| Pneumococcal meningitis | -- | 2.3 (1.6 to 3.1) | -- | -48.6* (-53.6 to -43.6) | -49.9* (-54.8 to -45.0) |

| | | | | | |
|---|---|---|---|---|---|
| Acute pneumococcal meningitis | -- | 2.3 (1.6 to 3.1) | -- | -48.0* (-54.7 to -41.1) | -49.3* (-55.9 to -42.6) |
| Pneumococcal meningitis complications | -- | -- | -- | -50.0* (-52.7 to -47.5) | -51.3* (-54.0 to -48.8) |
| H influenzae type B meningitis | -- | 2.3 (1.6 to 3.1) | -- | -47.1* (-53.7 to -41.1) | -48.5* (-54.9 to -42.6) |
| Acute H influenzae type B meningitis | -- | 2.3 (1.6 to 3.1) | -- | -46.3* (-54.0 to -39.2) | -47.7* (-55.1 to -40.7) |
| H Influenza type B meningitis complications | -- | -- | -- | -51.3* (-54.0 to -48.9) | -52.5* (-55.2 to -50.2) |
| Meningococcal infection | -- | 2.4 (1.7 to 3.2) | -- | -45.5* (-51.6 to -39.1) | -46.9* (-52.9 to -40.7) |
| Acute meningococcal meningitis | -- | 2.4 (1.7 to 3.2) | -- | -44.9* (-52.1 to -37.6) | -46.4* (-53.3 to -39.2) |
| Meningococcal meningitis complications | -- | -- | -- | -48.2* (-51.0 to -45.3) | -49.5* (-52.3 to -46.7) |
| Other meningitis | 0.3 (0.2 to 0.3) | 33.6 (26.5 to 42.0) | 0.0 (0.0 to 0.1) | -9.2 (-23.9 to 7.0) | -11.6 (-25.8 to 4.3) |
| Other acute bacterial meningitis | 0.1 (0.1 to 0.2) | 20.1 (15.7 to 25.3) | -- | -1.7 (-24.4 to 24.4) | -4.3 (-26.4 to 21.2) |
| Acute viral meningitis | 0.1 (0.1 to 0.1) | 13.5 (10.7 to 16.9) | -- | -18.4 (-37.2 to 4.4) | -20.5 (-38.8 to 1.7) |
| Other bacterial meningitis complications | -- | -- | -- | -9.3* (-12.5 to -6.1) | -11.7* (-14.7 to -8.5) |
| Encephalitis | -- | 5.4 (5.2 to 5.7) | -- | 1.0* (0.3 to 1.6) | -1.6* (-2.3 to -1.0) |
| Acute encephalitis | -- | 5.4 (5.2 to 5.7) | -- | 2.7* (2.2 to 3.4) | 0.1 (-0.4 to 0.8) |
| Encephalitis complications | -- | -- | -- | -13.2* (-15.3 to -11.0) | -15.4* (-17.5 to -13.3) |
| Diphtheria | -- | -- | -- | -- | -- |
| Whooping cough | -- | -- | -- | -- | -- |
| Tetanus | 0.7 (0.5 to 1.0) | 12.1 (8.6 to 17.8) | 0.1 (0.0 to 0.1) | -72.2* (-81.0 to -57.5) | -72.9* (-81.5 to -58.6) |
| Severe tetanus | 0.7 (0.5 to 1.0) | 12.1 (8.6 to 17.8) | 0.1 (0.0 to 0.1) | -72.3* (-81.2 to -57.7) | -73.0* (-81.7 to -58.8) |
| Neonatal tetanus complications | -- | -- | -- | -16.0* (-18.5 to -12.3) | -18.1* (-20.6 to -14.6) |
| Measles | -- | -- | -- | -- | -- |
| Varicella and herpes zoster | 11.7 (8.0 to 16.6) | 611.0 (419.7 to 864.4) | 0.1 (0.0 to 0.2) | -2.8* (-5.1 to -0.7) | -5.3* (-7.6 to -3.2) |
| Chickenpox | 11.7 (8.0 to 16.6) | 611.0 (419.6 to 864.4) | 0.1 (0.0 to 0.2) | -2.8* (-5.1 to -0.7) | -5.3* (-7.6 to -3.2) |
| Herpes zoster | -- | -- | -- | 2.7* (1.8 to 3.6) | 0.1 (-0.8 to 0.9) |
| Acute hepatitis | 59.9 (46.6 to 73.0) | 759.2 (586.6 to 927.9) | -- | -10.9 (-24.1 to 4.3) | -13.2 (-26.1 to 1.6) |
| Acute hepatitis A | 54.4 (40.9 to 67.4) | 707.3 (531.6 to 875.7) | -- | 2.2 (-33.3 to 52.9) | -0.4 (-35.1 to 48.9) |
| Acute hepatitis B | 3.6 (2.2 to 5.6) | 31.3 (18.7 to 48.9) | -- | 6.0 (-27.6 to 52.8) | 3.3 (-29.5 to 48.9) |
| Acute hepatitis C | 1.0 (0.6 to 1.5) | 8.8 (5.5 to 12.9) | -- | -13.5 (-28.8 to 2.2) | -15.7* (-30.6 to -0.5) |
| Acute hepatitis E | 0.9 (0.6 to 1.2) | 11.7 (8.1 to 16.2) | -- | 3.8 (-34.0 to 55.9) | 1.1 (-35.7 to 51.9) |
| Other unspecified infectious diseases | 23.9 (22.3 to 25.5) | -- | 0.8 (0.5 to 1.2) | -17.0* (-25.6 to -7.8) | -19.1* (-27.5 to -10.1) |
| Guillain-Barré syndrome due to other infectious diseases | -- | -- | -- | 2.4* (0.4 to 4.3) | -0.3 (-2.2 to 1.6) |
| Other infectious diseases | -- | -- | 0.1 (0.0 to 0.1) | -15.7* (-24.6 to -6.8) | -17.9* (-26.5 to -9.2) |
| Anemia due to other infectious diseases | 23.9 (22.3 to 25.5) | -- | 0.7 (0.5 to 1.1) | -17.1* (-26.6 to -7.1) | -19.2* (-28.5 to -9.5) |
| **Maternal and neonatal disorders** | **377.6 (350.8 to 403.3)** | **--** | **29.7 (21.8 to 39.5)** | **2.4 (-4.3 to 10.7)** | **-0.2 (-6.7 to 7.8)** |
| Maternal disorders | -- | -- | -- | -- | -- |
| Maternal haemorrhage | -- | -- | -- | -- | -- |
| Maternal hemorrhage complications | -- | -- | -- | -- | -- |
| Maternal hemorrhage episode | -- | -- | -- | -- | -- |
| Maternal sepsis and other pregnancy related infections | -- | -- | -- | -- | -- |
| Infertility due to puerperal sepsis | -- | -- | -- | -- | -- |
| Maternal sepsis and other maternal infection episode | -- | -- | -- | -- | -- |
| Maternal hypertensive disorders | -- | -- | -- | -- | -- |
| Maternal hypertensive disorder complications | -- | -- | -- | -- | -- |
| Maternal hypertensive disorder episode | -- | -- | -- | -- | -- |
| Maternal obstructed labour and uterine rupture | -- | -- | -- | -- | -- |
| Obstructed labor, acute event | -- | -- | -- | -- | -- |
| Maternal obstructed labor complications | -- | -- | -- | -- | -- |
| Maternal abortion and miscarriage | -- | -- | -- | -- | -- |
| Ectopic pregnancy | -- | -- | -- | -- | -- |
| Other maternal disorders | -- | -- | -- | -- | -- |
| Neonatal disorders | 377.6 (350.8 to 403.3) | -- | 29.7 (21.8 to 39.5) | 2.4 (-4.3 to 10.7) | -0.2 (-6.7 to 7.8) |
| Neonatal preterm birth | 326.6 (304.5 to 348.1) | -- | 12.2 (8.3 to 17.0) | 2.6 (-12.1 to 19.2) | -0.0 (-14.3 to 16.1) |
| Preterm birth complications | 54.9 (42.4 to 72.0) | -- | 12.0 (8.1 to 16.8) | 2.8 (-12.3 to 19.7) | 0.1 (-14.5 to 16.6) |
| Retinopathy of prematurity | 4.1 (3.0 to 5.2) | -- | 0.2 (0.1 to 0.3) | -4.8 (-12.0 to 3.0) | -7.2 (-14.2 to 0.3) |
| Uncomplicated preterm birth | 267.7 (249.1 to 280.8) | -- | -- | -- | -- |
| Neonatal encephalopathy due to birth asphyxia and trauma | 45.4 (34.5 to 58.9) | -- | 6.2 (3.8 to 9.4) | 3.4 (-0.3 to 7.7) | 0.7 (-2.8 to 5.0) |
| Neonatal sepsis and other neonatal infections | 13.8 (10.5 to 18.2) | -- | 1.9 (1.0 to 3.1) | 1.7 (-2.5 to 6.3) | -0.9 (-5.0 to 3.5) |
| Neonatal sepsis and other neonatal infection complications | 7.5 (4.5 to 11.5) | -- | 1.9 (1.0 to 3.1) | 1.7 (-2.5 to 6.3) | -0.9 (-5.0 to 3.5) |
| Neonatal sepsis and other neonatal infection episode | 6.3 (5.0 to 7.9) | -- | -- | -- | -- |
| Hemolytic disease and other neonatal jaundice | 12.5 (5.2 to 23.2) | -- | 3.9 (1.6 to 7.9) | -0.4 (-4.2 to 5.0) | -2.9 (-6.7 to 2.3) |

| | | | | | |
|---|---|---|---|---|---|
| Other neonatal disorders | -- | -- | 5.6 (4.1 to 7.5) | 3.0 (-3.7 to 11.3) | 0.4 (-6.2 to 8.4) |
| **Nutritional deficiencies** | **1311.6 (1110.9 to 1497.8)** | **727.8 (620.9 to 846.6)** | **49.8 (32.1 to 72.6)** | **-14.7* (-19.0 to -9.9)** | **-16.9* (-21.1 to -12.3)** |
| Protein-energy malnutrition | 298.4 (292.5 to 305.0) | 340.3 (334.8 to 346.3) | 11.2 (7.3 to 16.0) | -13.6* (-17.0 to -10.3) | -15.8* (-19.1 to -12.6) |
| Iodine deficiency | 13.4 (9.8 to 18.2) | -- | 0.2 (0.1 to 0.3) | -45.2* (-48.2 to -42.0) | -46.6* (-49.5 to -43.5) |
| Visible goiter without symptoms | 13.4 (9.8 to 18.2) | -- | 0.1 (0.1 to 0.3) | -46.6* (-49.8 to -43.6) | -48.0* (-51.1 to -45.1) |
| Visible goiter with complications | -- | -- | -- | -7.6 (-18.6 to 1.4) | -10.0* (-20.7 to -1.2) |
| Vitamin A deficiency | 1119.0 (772.6 to 1442.5) | 387.6 (280.5 to 504.7) | 26.5 (15.0 to 41.8) | -14.3* (-19.7 to -8.8) | -16.5* (-21.8 to -11.1) |
| Asymptomatic vitamin A deficiency | 381.6 (260.5 to 492.3) | 387.6 (280.5 to 504.7) | -- | -- | -- |
| Vitamin A deficiency complications | -- | -- | -- | -- | -- |
| Vitamin A deficiency with anemia | 737.8 (509.5 to 963.3) | -- | 26.5 (15.0 to 41.8) | -14.3* (-19.7 to -8.8) | -16.5* (-21.8 to -11.1) |
| Dietary iron deficiency | 379.4 (354.2 to 404.8) | -- | 12.0 (7.8 to 17.7) | -15.9* (-24.9 to -6.1) | -18.1* (-26.9 to -8.5) |
| Other nutritional deficiencies | -- | -- | -- | -- | -- |
| **Non-communicable diseases** | **983.1 (953.5 to 1011.8)** | **1803.1 (1603.2 to 2037.5)** | **28.3 (20.5 to 37.5)** | **8.1* (4.0 to 13.5)** | **5.3* (1.3 to 10.6)** |
| **Neoplasms** | **70.2 (52.5 to 93.0)** | **3.4 (3.1 to 3.7)** | **4.0 (2.4 to 6.1)** | **107.5* (55.1 to 182.0)** | **102.1* (51.1 to 174.7)** |
| Lip and oral cavity cancer | -- | -- | -- | -- | -- |
| Diagnosis and primary therapy phase of mouth cancer | -- | -- | -- | -- | -- |
| Controlled phase of mouth cancer | -- | -- | -- | -- | -- |
| Metastatic phase of mouth cancer | -- | -- | -- | -- | -- |
| Terminal phase of mouth cancer | -- | -- | -- | -- | -- |
| Nasopharynx cancer | -- | -- | -- | -- | -- |
| Diagnosis and primary therapy phase of nasopharynx cancer | -- | -- | -- | -- | -- |
| Controlled phase of nasopharynx cancer | -- | -- | -- | -- | -- |
| Metastatic phase of nasopharynx cancer | -- | -- | -- | -- | -- |
| Terminal phase of nasopharynx cancer | -- | -- | -- | -- | -- |
| Other pharynx cancer | -- | -- | -- | -- | -- |
| Diagnosis and primary therapy phase of other pharynx cancer | -- | -- | -- | -- | -- |
| Controlled phase of other pharynx cancer | -- | -- | -- | -- | -- |
| Metastatic phase of other pharynx cancer | -- | -- | -- | -- | -- |
| Terminal phase of other pharynx cancer | -- | -- | -- | -- | -- |
| Oesophageal cancer | -- | -- | -- | -- | -- |
| Diagnosis and primary therapy phase of esophageal cancer | -- | -- | -- | -- | -- |
| Controlled phase of esophageal cancer | -- | -- | -- | -- | -- |
| Metastatic phase of esophageal cancer | -- | -- | -- | -- | -- |
| Terminal phase of esophageal cancer | -- | -- | -- | -- | -- |
| Stomach cancer | -- | -- | -- | -- | -- |
| Diagnosis and primary therapy phase of stomach cancer | -- | -- | -- | -- | -- |
| Controlled phase of stomach cancer | -- | -- | -- | -- | -- |
| Metastatic phase of stomach cancer | -- | -- | -- | -- | -- |
| Terminal phase of stomach cancer | -- | -- | -- | -- | -- |
| Colon and rectum cancer | -- | -- | -- | -- | -- |
| Diagnosis and primary therapy phase of colon and rectum cancers | -- | -- | -- | -- | -- |
| Controlled phase of colon and rectum cancers | -- | -- | -- | -- | -- |
| Metastatic phase of colon and rectum cancers | -- | -- | -- | -- | -- |
| Terminal phase of colon and rectum cancers | -- | -- | -- | -- | -- |
| Stoma from colon and rectum cancers, beyond 10 years | -- | -- | -- | -- | -- |
| Liver cancer | -- | -- | -- | -- | -- |
| Liver cancer due to hepatitis B | -- | -- | -- | -- | -- |
| Diagnosis and primary therapy phase of liver cancer due to hepatitis B | -- | -- | -- | -- | -- |
| Controlled phase of liver cancer due to hepatitis B | -- | -- | -- | -- | -- |
| Metastatic phase of liver cancer due to hepatitis B | -- | -- | -- | -- | -- |
| Terminal phase of liver cancer due to hepatitis B | -- | -- | -- | -- | -- |
| Liver cancer due to hepatitis C | -- | -- | -- | -- | -- |
| Diagnosis and primary therapy phase of liver cancer due to hepatitis C | -- | -- | -- | -- | -- |
| Controlled phase of liver cancer due to hepatitis C | -- | -- | -- | -- | -- |
| Metastatic phase of liver cancer due to hepatitis C | -- | -- | -- | -- | -- |
| Terminal phase of liver cancer due to hepatitis C | -- | -- | -- | -- | -- |
| Liver cancer due to alcohol use | -- | -- | -- | -- | -- |
| Diagnosis and primary therapy phase of liver cancer due to alcohol use | -- | -- | -- | -- | -- |
| Controlled phase of liver cancer due to alcohol use | -- | -- | -- | -- | -- |
| Metastatic phase of liver cancer due to alcohol use | -- | -- | -- | -- | -- |

| | | | | | |
|---|---|---|---|---|---|
| Terminal phase of liver cancer due to alcohol use | -- | -- | -- | -- | -- |
| Liver cancer due to NASH | -- | -- | -- | -- | -- |
| Diagnosis and primary therapy phase of liver cancer due to NASH | -- | -- | -- | -- | -- |
| Controlled phase of liver cancer due to NASH | -- | -- | -- | -- | -- |
| Metastatic phase of liver cancer due to NASH | -- | -- | -- | -- | -- |
| Terminal phase of liver cancer due to NASH | -- | -- | -- | -- | -- |
| Liver cancer due to other causes | -- | -- | -- | -- | -- |
| Diagnosis and primary therapy phase of liver cancer due to other causes | -- | -- | -- | -- | -- |
| Controlled phase of liver cancer due to other causes | -- | -- | -- | -- | -- |
| Metastatic phase of liver cancer due to other causes | -- | -- | -- | -- | -- |
| Terminal phase of liver cancer due to other causes | -- | -- | -- | -- | -- |
| Gallbladder and biliary tract cancer | -- | -- | -- | -- | -- |
| Diagnosis and primary therapy phase of gallbladder and biliary tract cancer | -- | -- | -- | -- | -- |
| Controlled phase of gallbladder and biliary tract cancer | -- | -- | -- | -- | -- |
| Metastatic phase of gallbladder and biliary tract cancer | -- | -- | -- | -- | -- |
| Terminal phase of gallbladder and biliary tract cancer | -- | -- | -- | -- | -- |
| Pancreatic cancer | -- | -- | -- | -- | -- |
| Diagnosis and primary therapy phase of pancreatic cancer | -- | -- | -- | -- | -- |
| Controlled phase of pancreatic cancer | -- | -- | -- | -- | -- |
| Metastatic phase of pancreatic cancer | -- | -- | -- | -- | -- |
| Terminal phase of pancreatic cancer | -- | -- | -- | -- | -- |
| Larynx cancer | -- | -- | -- | -- | -- |
| Diagnosis and primary therapy phase of larynx cancer | -- | -- | -- | -- | -- |
| Controlled phase of larynx cancer | -- | -- | -- | -- | -- |
| Metastatic phase of larynx cancer | -- | -- | -- | -- | -- |
| Terminal phase of larynx cancer | -- | -- | -- | -- | -- |
| Laryngectomy from larynx cancer, beyond 10 years | -- | -- | -- | -- | -- |
| Tracheal, bronchus, and lung cancer | -- | -- | -- | -- | -- |
| Diagnosis and primary therapy phase of lung, bronchus, and trachea cancer | -- | -- | -- | -- | -- |
| Controlled phase of lung, bronchus, and trachea cancer | -- | -- | -- | -- | -- |
| Metastatic phase of lung, bronchus, and trachea cancer | -- | -- | -- | -- | -- |
| Terminal phase of lung, bronchus, and trachea cancer | -- | -- | -- | -- | -- |
| Malignant skin melanoma | -- | -- | -- | -- | -- |
| Diagnosis and primary therapy phase of malignant skin melanoma | -- | -- | -- | -- | -- |
| Controlled phase of malignant skin melanoma | -- | -- | -- | -- | -- |
| Metastatic phase of malignant skin melanoma | -- | -- | -- | -- | -- |
| Terminal phase of malignant skin melanoma | -- | -- | -- | -- | -- |
| Non-melanoma skin cancer | -- | -- | -- | -- | -- |
| Non-melanoma skin cancer (squamous-cell carcinoma) | -- | -- | -- | -- | -- |
| Non-melanoma skin cancer (basal-cell carcinoma) | -- | -- | -- | -- | -- |
| Breast cancer | -- | -- | -- | -- | -- |
| Diagnosis and primary therapy phase of breast cancer | -- | -- | -- | -- | -- |
| Controlled phase of breast cancer | -- | -- | -- | -- | -- |
| Metastatic phase of breast cancer | -- | -- | -- | -- | -- |
| Terminal phase of breast cancer | -- | -- | -- | -- | -- |
| Mastectomy from breast cancer, beyond 10 years | -- | -- | -- | -- | -- |
| Cervical cancer | -- | -- | -- | -- | -- |
| Diagnosis and primary therapy phase of cervical cancer | -- | -- | -- | -- | -- |
| Controlled phase of cervical cancer | -- | -- | -- | -- | -- |
| Metastatic phase of cervical cancer | -- | -- | -- | -- | -- |
| Terminal phase of cervical cancer | -- | -- | -- | -- | -- |
| Uterine cancer | -- | -- | -- | -- | -- |
| Diagnosis and primary therapy phase of uterine cancer | -- | -- | -- | -- | -- |
| Controlled phase of uterine cancer | -- | -- | -- | -- | -- |
| Metastatic phase of uterine cancer | -- | -- | -- | -- | -- |
| Terminal phase of uterine cancer | -- | -- | -- | -- | -- |
| Ovarian cancer | -- | -- | -- | -- | -- |
| Diagnosis and primary therapy phase of ovarian cancer | -- | -- | -- | -- | -- |
| Controlled phase of ovarian cancer | -- | -- | -- | -- | -- |
| Metastatic phase of ovarian cancer | -- | -- | -- | -- | -- |

| | | | | | |
|---|---|---|---|---|---|
| Terminal phase of ovarian cancer | -- | -- | -- | -- | -- |
| Prostate cancer | -- | -- | -- | -- | -- |
| Diagnosis and primary therapy phase of prostate cancer | -- | -- | -- | -- | -- |
| Controlled phase of prostate cancer | -- | -- | -- | -- | -- |
| Metastatic phase of prostate cancer | -- | -- | -- | -- | -- |
| Terminal phase of prostate cancer | -- | -- | -- | -- | -- |
| Impotence and Incontinence after 10-year survival from prostate cancer | -- | -- | -- | -- | -- |
| Testicular cancer | -- | -- | -- | -- | -- |
| Diagnosis and primary therapy phase of testicular cancer | -- | -- | -- | -- | -- |
| Controlled phase of testicular cancer | -- | -- | -- | -- | -- |
| Metastatic phase of testicular cancer | -- | -- | -- | -- | -- |
| Terminal phase of testicular cancer | -- | -- | -- | -- | -- |
| Kidney cancer | -- | -- | -- | -- | -- |
| Diagnosis and primary therapy phase of kidney cancer | -- | -- | -- | -- | -- |
| Controlled phase of kidney cancer | -- | -- | -- | -- | -- |
| Metastatic phase of kidney cancer | -- | -- | -- | -- | -- |
| Terminal phase of kidney cancer | -- | -- | -- | -- | -- |
| Bladder cancer | -- | -- | -- | -- | -- |
| Diagnosis and primary therapy phase of bladder cancer | -- | -- | -- | -- | -- |
| Controlled phase of bladder cancer | -- | -- | -- | -- | -- |
| Metastatic phase of bladder cancer | -- | -- | -- | -- | -- |
| Terminal phase of bladder cancer | -- | -- | -- | -- | -- |
| Incontinence from bladder cancer, beyond 10 years | -- | -- | -- | -- | -- |
| Brain and nervous system cancer | 4.0<br>(3.1 to 4.8) | 0.5<br>(0.4 to 0.6) | 0.3<br>(0.2 to 0.4) | 10.1<br>(-6.8 to 40.3) | 7.3<br>(-9.2 to 36.7) |
| Diagnosis and primary therapy phase of brain and nervous system cancers | 0.2<br>(0.1 to 0.2) | 0.5<br>(0.4 to 0.6) | 0.0<br>(0.0 to 0.1) | 14.3<br>(-6.4 to 50.2) | 11.3<br>(-8.8 to 46.3) |
| Controlled phase of brain and nervous system cancers | 3.8<br>(2.9 to 4.5) | -- | 0.2<br>(0.1 to 0.3) | 14.8<br>(-3.5 to 48.3) | 11.8<br>(-6.0 to 44.5) |
| Metastatic phase of brain and nervous system cancers | 0.0<br>(0.0 to 0.1) | -- | -- | -17.7*<br>(-26.8 to -7.3) | -19.9*<br>(-28.7 to -9.7) |
| Terminal phase of brain and nervous system cancers | -- | -- | -- | -17.6*<br>(-27.4 to -7.0) | -19.7*<br>(-29.3 to -9.4) |
| Thyroid cancer | -- | -- | -- | -- | -- |
| Diagnosis and primary therapy phase of thyroid cancer | -- | -- | -- | -- | -- |
| Controlled phase of thyroid cancer | -- | -- | -- | -- | -- |
| Metastatic phase of thyroid cancer | -- | -- | -- | -- | -- |
| Terminal phase of thyroid cancer | -- | -- | -- | -- | -- |
| Mesothelioma | -- | -- | -- | -- | -- |
| Diagnosis and primary therapy phase of mesothelioma | -- | -- | -- | -- | -- |
| Controlled phase of mesothelioma | -- | -- | -- | -- | -- |
| Metastatic phase of mesothelioma | -- | -- | -- | -- | -- |
| Terminal phase of mesothelioma | -- | -- | -- | -- | -- |
| Hodgkin lymphoma | -- | -- | -- | -- | -- |
| Diagnosis and primary therapy phase of Hodgkin disease | -- | -- | -- | -- | -- |
| Controlled phase of Hodgkin disease | -- | -- | -- | -- | -- |
| Metastatic phase of Hodgkin disease | -- | -- | -- | -- | -- |
| Terminal phase of Hodgkin disease | -- | -- | -- | -- | -- |
| Non-Hodgkin's lymphoma | -- | -- | -- | -- | -- |
| Diagnosis and primary therapy phase of non-Hodgkin lymphoma | -- | -- | -- | -- | -- |
| Controlled phase of non-Hodgkin lymphoma | -- | -- | -- | -- | -- |
| Metastatic phase of non-Hodgkin lymphoma | -- | -- | -- | -- | -- |
| Terminal phase of non-Hodgkin lymphoma | -- | -- | -- | -- | -- |
| Multiple myeloma | -- | -- | -- | -- | -- |
| Diagnosis and primary therapy phase of multiple myeloma | -- | -- | -- | -- | -- |
| Controlled phase of multiple myeloma | -- | -- | -- | -- | -- |
| Metastatic phase of multiple myeloma | -- | -- | -- | -- | -- |
| Terminal phase of multiple myeloma | -- | -- | -- | -- | -- |
| Leukaemia | 0.3<br>(0.2 to 0.3) | 0.1<br>(0.1 to 0.2) | -- | -2.6<br>(-19.5 to 20.0) | -5.1<br>(-21.6 to 16.9) |
| Acute lymphoid leukaemia | -- | -- | -- | -- | -- |
| Diagnosis and primary therapy phase of acute lymphoid leukemia | -- | -- | -- | -- | -- |
| Controlled phase of acute lymphoid leukemia | -- | -- | -- | -- | -- |
| Metastatic phase of acute lymphoid leukemia | -- | -- | -- | -- | -- |
| Terminal phase of acute lymphoid leukemia | -- | -- | -- | -- | -- |
| Chronic lymphoid leukaemia | -- | -- | -- | -- | -- |

| | | | | | |
|---|---|---|---|---|---|
| Diagnosis and primary therapy phase of chronic lymphoid leukemia | -- | -- | -- | -- | -- |
| Controlled phase of chronic lymphoid leukemia | -- | -- | -- | -- | -- |
| Metastatic phase of chronic lymphoid leukemia | -- | -- | -- | -- | -- |
| Terminal phase of chronic lymphoid leukemia | -- | -- | -- | -- | -- |
| Acute myeloid leukaemia | 0.3<br>(0.2 to 0.3) | 0.1<br>(0.1 to 0.2) | -- | -2.6<br>(-19.5 to 20.0) | -5.1<br>(-21.6 to 16.9) |
| Diagnosis and primary therapy phase of acute myeloid leukemia | -- | 0.1<br>(0.1 to 0.2) | -- | -- | -- |
| Controlled phase of acute myeloid leukemia | 0.2<br>(0.2 to 0.2) | -- | -- | -0.3<br>(-20.3 to 26.7) | -2.9<br>(-22.3 to 23.4) |
| Metastatic phase of acute myeloid leukemia | -- | -- | -- | -3.6<br>(-20.9 to 19.6) | -6.1<br>(-22.9 to 16.5) |
| Terminal phase of acute myeloid leukemia | -- | -- | -- | -3.5<br>(-20.2 to 17.9) | -6.0<br>(-22.3 to 14.8) |
| Chronic myeloid leukaemia | -- | -- | -- | -- | -- |
| Diagnosis and primary therapy phase of chronic myeloid leukemia | -- | -- | -- | -- | -- |
| Controlled phase of chronic myeloid leukemia | -- | -- | -- | -- | -- |
| Metastatic phase of chronic myeloid leukemia | -- | -- | -- | -- | -- |
| Terminal phase of chronic myeloid leukemia | -- | -- | -- | -- | -- |
| Other leukaemia | -- | -- | -- | -- | -- |
| Metastatic phase of other leukemia | -- | -- | -- | -- | -- |
| Diagnosis and primary therapy phase of other leukemia | -- | -- | -- | -- | -- |
| Terminal phase of other leukemia | -- | -- | -- | -- | -- |
| Controlled phase of other leukemia | -- | -- | -- | -- | -- |
| Other malignant cancers | 66.9<br>(49.1 to 90.2) | 2.8<br>(2.5 to 3.1) | 3.7<br>(2.2 to 5.7) | 123.8*<br>(62.1 to 211.9) | 118.0*<br>(57.9 to 203.8) |
| Diagnosis and primary therapy phase of other malignant neoplasms | 1.3<br>(0.9 to 2.0) | 2.8<br>(2.5 to 3.1) | 0.4<br>(0.2 to 0.6) | 147.5*<br>(68.2 to 282.8) | 141.1*<br>(63.9 to 272.9) |
| Controlled phase of other malignant neoplasms | 71.4<br>(48.4 to 109.6) | -- | 3.2<br>(1.8 to 5.1) | 132.4*<br>(67.8 to 221.3) | 126.4*<br>(63.5 to 213.0) |
| Metastatic phase of other malignant neoplasms | 0.2<br>(0.2 to 0.2) | -- | 0.1<br>(0.1 to 0.1) | -5.5<br>(-24.4 to 17.2) | -7.9<br>(-26.4 to 14.2) |
| Terminal phase of other malignant neoplasms | -- | -- | -- | -5.8<br>(-14.9 to 5.5) | -8.2<br>(-17.1 to 2.8) |
| Other neoplasms | -- | -- | -- | -- | -- |
| Myelodysplastic, myeloproliferative, and other hematopoietic neoplasms | -- | -- | -- | -- | -- |
| Benign and in situ intestinal neoplasms | -- | -- | -- | -- | -- |
| Benign and in situ cervical and uterine neoplasms | -- | -- | -- | -- | -- |
| Other benign and in situ neoplasms | -- | -- | -- | -- | -- |
| **Cardiovascular diseases** | **--** | **1.1<br>(0.9 to 1.3)** | **--** | **-46.0*<br>(-50.9 to -41.3)** | **-47.4*<br>(-52.2 to -42.8)** |
| Rheumatic heart disease | -- | -- | -- | -- | -- |
| Rheumatic heart disease, without heart failure | -- | -- | -- | -- | -- |
| Heart failure due to rheumatic heart disease | -- | -- | -- | -- | -- |
| Ischaemic heart disease | -- | -- | -- | -- | -- |
| Asymptomatic ischemic heart disease following myocardial infarction | -- | -- | -- | -- | -- |
| Angina due to ischemic heart disease | -- | -- | -- | -- | -- |
| Heart failure due to ischemic heart disease | -- | -- | -- | -- | -- |
| Myocardial infarction due to ischemic heart disease | -- | -- | -- | -- | -- |
| Stroke | -- | 0.3‡<br>(0.2 to 0.4) | -- | -54.2*<br>(-58.8 to -49.5) | -55.4*<br>(-59.8 to -50.8) |
| Ischaemic stroke | -- | 0.2<br>(0.2 to 0.3) | -- | -55.8*<br>(-60.7 to -50.9) | -57.0*<br>(-61.7 to -52.1) |
| Acute ischemic stroke | -- | 0.2<br>(0.2 to 0.3) | -- | -56.3*<br>(-63.1 to -49.4) | -57.4*<br>(-64.0 to -50.7) |
| Chronic ischemic stroke | -- | -- | -- | -54.8*<br>(-59.8 to -49.3) | -56.0*<br>(-60.8 to -50.6) |
| Intracerebral hemorrhage | -- | -- | -- | -- | -- |
| Acute intracerebral hemorrhage | -- | -- | -- | -- | -- |
| Chronic intracerebral hemorrhage | -- | -- | -- | -- | -- |
| Subarachnoid hemorrhage | -- | 0.0<br>(0.0 to 0.1) | -- | -35.9*<br>(-59.9 to -21.3) | -37.6*<br>(-61.0 to -23.3) |
| Acute subarachnoid hemorrhage | -- | 0.0<br>(0.0 to 0.1) | -- | -42.3*<br>(-65.7 to -24.8) | -43.8*<br>(-66.6 to -26.7) |
| Chronic subarachnoid hemorrhage | -- | -- | -- | -15.6*<br>(-25.9 to -7.4) | -17.8*<br>(-27.8 to -9.8) |
| Hypertensive heart disease | -- | -- | -- | -- | -- |
| Non-rheumatic valvular heart disease | -- | -- | -- | -- | -- |
| Non-rheumatic calcific aortic valve disease | -- | -- | -- | -- | -- |
| Heart failure due to calcific aortic valve disease | -- | -- | -- | -- | -- |
| Non-rheumatic calcific aortic valve disease | -- | -- | -- | -- | -- |
| Non-rheumatic degenerative mitral valve disease | -- | -- | -- | -- | -- |
| Heart failure due to degenerative mitral valve disease | -- | -- | -- | -- | -- |
| Non-rheumatic degenerative mitral valve disease | -- | -- | -- | -- | -- |
| Other non-rheumatic valve diseases | -- | -- | -- | -- | -- |
| Mild heart failure due to other non-rheumatic valve disease | -- | -- | -- | -- | -- |
| Moderate heart failure due to other non-rheumatic valve disease | -- | -- | -- | -- | -- |
| Severe heart failure due to other non-rheumatic valve disease | -- | -- | -- | -- | -- |

| | | | | | |
|---|---|---|---|---|---|
| Controlled, medically managed heart failure due to other non-rheumatic valve disease | -- | -- | -- | -- | -- |
| Cardiomyopathy and myocarditis | -- | -- | -- | -- | -- |
| Myocarditis | -- | -- | -- | -- | -- |
| Acute myocarditis | -- | -- | -- | -- | -- |
| Heart failure due to myocarditis | -- | -- | -- | -- | -- |
| Alcoholic cardiomyopathy | -- | -- | -- | -- | -- |
| Other cardiomyopathy | -- | -- | -- | -- | -- |
| Atrial fibrillation and flutter | -- | -- | -- | -- | -- |
| Asymptomatic atrial fibrillation and flutter | -- | -- | -- | -- | -- |
| Symptomatic atrial fibrillation and flutter | -- | -- | -- | -- | -- |
| Peripheral vascular disease | -- | -- | -- | -- | -- |
| Asymptomatic peripheral arterial disease | -- | -- | -- | -- | -- |
| Symptomatic claudication due to peripheral arterial disease | -- | -- | -- | -- | -- |
| Endocarditis | -- | 0.8<br>(0.7 to 1.0) | -- | 10.1*<br>(2.3 to 16.7) | 7.3<br>(-0.3 to 13.7) |
| Acute endocarditis | -- | 0.8<br>(0.7 to 1.0) | -- | 10.0*<br>(1.8 to 16.7) | 7.2<br>(-0.8 to 13.7) |
| Heart failure due to endocarditis | -- | -- | -- | 15.5*<br>(10.3 to 20.0) | 12.5*<br>(7.4 to 16.9) |
| Other cardiovascular and circulatory diseases | -- | -- | -- | -- | -- |
| Heart failure due to other cardiovascular diseases | -- | -- | -- | -- | -- |
| Other cardiovascular and circulatory disease episodes | -- | -- | -- | -- | -- |
| **Chronic respiratory diseases** | **--** | **--** | **--** | **--** | **--** |
| Chronic obstructive pulmonary disease | -- | -- | -- | -- | -- |
| Chronic obstructive pulmonary disease with heart failure | -- | -- | -- | -- | -- |
| Chronic obstructive pulmonary disease without heart failure | -- | -- | -- | -- | -- |
| Pneumoconiosis | -- | -- | -- | -- | -- |
| Silicosis | -- | -- | -- | -- | -- |
| Heart failure due to silicosis | -- | -- | -- | -- | -- |
| Silicosis without heart failure | -- | -- | -- | -- | -- |
| Asbestosis | -- | -- | -- | -- | -- |
| Asbestosis without heart failure | -- | -- | -- | -- | -- |
| Heart failure due to asbestosis | -- | -- | -- | -- | -- |
| Coal workers pneumoconiosis | -- | -- | -- | -- | -- |
| Coal workers pneumoconiosis without heart failure | -- | -- | -- | -- | -- |
| Heart failure due to Coal workers pneumoconiosis | -- | -- | -- | -- | -- |
| Other pneumoconiosis | -- | -- | -- | -- | -- |
| Heart failure due to other pneumoconiosis | -- | -- | -- | -- | -- |
| Other pneumoconiosis without heart failure | -- | -- | -- | -- | -- |
| Asthma | -- | -- | -- | -- | -- |
| Asymptomatic asthma | -- | -- | -- | -- | -- |
| Symptomatic asthma | -- | -- | -- | -- | -- |
| Interstitial lung disease and pulmonary sarcoidosis | -- | -- | -- | -- | -- |
| Heart failure due to interstitial lung disease and pulmonary sarcoidosis | -- | -- | -- | -- | -- |
| Interstitial lung disease and pulmonary sarcoidosis without heart failure | -- | -- | -- | -- | -- |
| Other chronic respiratory diseases | -- | -- | -- | -- | -- |
| **Digestive diseases** | **65.2<br>(48.9 to 82.0)** | **12.4<br>(10.8 to 14.4)** | **1.4<br>(0.8 to 2.1)** | **2.4<br>(-1.2 to 5.6)** | **-0.2<br>(-3.8 to 2.9)** |
| Cirrhosis and other chronic liver diseases | 53.0<br>(37.6 to 69.2) | 5.6<br>(4.8 to 6.4) | -- | 4.7*<br>(4.0 to 5.3) | 2.0*<br>(1.3 to 2.6) |
| Cirrhosis and other chronic liver diseases due to hepatitis B | 52.9<br>(37.5 to 69.1) | -- | -- | -7.0*<br>(-11.5 to -0.5) | -9.4*<br>(-13.8 to -3.1) |
| Cirrhosis and other chronic liver diseases due to hepatitis B, decompensated | -- | -- | -- | -7.0*<br>(-11.5 to -0.5) | -9.4*<br>(-13.8 to -3.1) |
| Chronic hepatitis B without cirrhosis | 52.9<br>(37.5 to 69.1) | -- | -- | -- | -- |
| Cirrhosis and other chronic liver diseases due to hepatitis B, compensated | -- | -- | -- | -- | -- |
| Cirrhosis and other chronic liver diseases due to hepatitis C | 0.1<br>(0.0 to 0.1) | -- | -- | 2.6<br>(-0.5 to 6.0) | -0.1<br>(-3.1 to 3.3) |
| Cirrhosis and other chronic liver diseases due to hepatitis C, decompensated | -- | -- | -- | 2.6<br>(-0.5 to 6.0) | -0.1<br>(-3.1 to 3.3) |
| Chronic hepatitis C without cirrhosis | 0.1<br>(0.0 to 0.1) | -- | -- | -- | -- |
| Cirrhosis and other chronic liver diseases due to hepatitis C, compensated | -- | -- | -- | -- | -- |
| Cirrhosis and other chronic liver diseases due to alcohol use | -- | -- | -- | -- | -- |
| Cirrhosis and other chronic liver diseases due to alcohol, decompensated | -- | -- | -- | -- | -- |
| Cirrhosis and other chronic liver diseases due to alcohol, compensated | -- | -- | -- | -- | -- |
| Cirrhosis due to NASH | -- | -- | -- | -- | -- |
| Non-alcoholic fatty liver disease (NAFLD) / Non-alcoholic steatohepatitis (NASH) | -- | -- | -- | -- | -- |
| Cirrhosis and other chronic liver diseases due to NASH | -- | -- | -- | -- | -- |
| Cirrhosis and other chronic liver diseases due to other causes | 0.1<br>(0.0 to 0.1) | 5.6<br>(4.8 to 6.4) | -- | 4.7*<br>(4.0 to 5.3) | 2.0*<br>(1.3 to 2.6) |

| | | | | | |
|---|---|---|---|---|---|
| Cirrhosis and other chronic liver diseases due to other cause, decompensated | -- | 1.4 (1.3 to 1.4) | -- | 4.7* (4.0 to 5.3) | 2.0* (1.3 to 2.6) |
| Cirrhosis and other chronic liver diseases due to other cause, compensated | -- | 4.2 (3.4 to 5.0) | -- | -- | -- |
| Upper digestive system diseases | -- | -- | -- | -- | -- |
| Peptic ulcer disease | -- | -- | -- | -- | -- |
| Acute peptic ulcer disease | -- | -- | -- | -- | -- |
| Chronic peptic ulcer disease | -- | -- | -- | -- | -- |
| Gastritis and duodenitis | -- | -- | -- | -- | -- |
| Acute gastritis and duodenitis | -- | -- | -- | -- | -- |
| Chronic gastritis and duodenitis | -- | -- | -- | -- | -- |
| GERD | -- | -- | -- | -- | -- |
| Appendicitis | -- | -- | -- | -- | -- |
| Paralytic ileus and intestinal obstruction | 0.3 (0.2 to 0.4) | 1.9 (1.4 to 2.4) | 0.1 (0.0 to 0.1) | -14.5 (-31.8 to 2.7) | -16.7 (-33.5 to 0.0) |
| Inguinal, femoral, and abdominal hernia | 12.4 (8.3 to 18.4) | 5.0 (3.6 to 6.8) | 1.3 (0.7 to 2.0) | 3.7* (0.5 to 6.6) | 1.0 (-2.1 to 3.8) |
| Inflammatory bowel disease | -- | -- | -- | -- | -- |
| Ulcerative colitis | -- | -- | -- | -- | -- |
| Crohn's disease | -- | -- | -- | -- | -- |
| Vascular intestinal disorders | -- | -- | -- | -- | -- |
| Gallbladder and biliary diseases | -- | -- | -- | -- | -- |
| Pancreatitis | -- | -- | -- | -- | -- |
| Acute pancreatitis | -- | -- | -- | -- | -- |
| Chronic pancreatitis | -- | -- | -- | -- | -- |
| Other digestive diseases | -- | -- | -- | -- | -- |
| **Neurological disorders** | **7.0 (4.6 to 9.9)** | **2.2 (1.5 to 3.0)** | **2.4 (1.4 to 3.9)** | **8.4 (-9.3 to 28.8)** | **5.6 (-11.6 to 25.4)** |
| Alzheimer's disease and other dementias | -- | -- | -- | -- | -- |
| Parkinson's disease | -- | -- | -- | -- | -- |
| Epilepsy | 7.0 (4.6 to 10.0) | 2.1 (1.4 to 2.9) | 2.4 (1.4 to 3.9) | 8.4 (-9.3 to 28.8) | 5.6 (-11.6 to 25.4) |
| Multiple sclerosis | -- | -- | -- | -- | -- |
| Motor neuron disease | -- | 0.1 (0.1 to 0.1) | -- | -7.6* (-9.8 to -5.1) | -10.0* (-12.1 to -7.5) |
| Headache disorders | -- | -- | -- | -- | -- |
| Migraine | -- | -- | -- | -- | -- |
| Medication overuse headache due to migraine | -- | -- | -- | -- | -- |
| Migraine | -- | -- | -- | -- | -- |
| Tension-type headache | -- | -- | -- | -- | -- |
| Medication overuse headache due to tension-type headache | -- | -- | -- | -- | -- |
| Tension-type headache | -- | -- | -- | -- | -- |
| Other neurological disorders | -- | -- | -- | -- | -- |
| Other neurological disorders | -- | -- | -- | -- | -- |
| Guillain-Barré syndrome due to other neurological disorders | -- | -- | -- | -- | -- |
| **Mental disorders** | **53.3 (35.8 to 72.3)** | **--** | **3.7 (2.4 to 5.5)** | **-7.5* (-9.6 to -4.7)** | **-9.9* (-11.9 to -7.2)** |
| Schizophrenia | -- | -- | -- | -- | -- |
| Depressive disorders | -- | -- | -- | -- | -- |
| Major depressive disorder | -- | -- | -- | -- | -- |
| Dysthymia | -- | -- | -- | -- | -- |
| Bipolar disorder | -- | -- | -- | -- | -- |
| Anxiety disorders | -- | -- | -- | -- | -- |
| Eating disorders | -- | -- | -- | -- | -- |
| Anorexia nervosa | -- | -- | -- | -- | -- |
| Bulimia nervosa | -- | -- | -- | -- | -- |
| Autism spectrum disorders | 13.0 (11.6 to 14.3) | -- | 2.0 (1.4 to 2.8) | 3.2* (1.1 to 5.3) | 0.6 (-1.5 to 2.6) |
| Attention-deficit/hyperactivity disorder | -- | -- | -- | -- | -- |
| Conduct disorder | -- | -- | -- | -- | -- |
| Idiopathic developmental intellectual disability | 41.3 (23.2 to 60.9) | -- | 1.7 (0.8 to 3.0) | -17.6* (-21.3 to -15.2) | -19.7* (-23.3 to -17.4) |
| Other mental disorders | -- | -- | -- | -- | -- |
| **Substance use disorders** | **--** | **--** | **--** | **-11.0* (-17.0 to -5.2)** | **-13.3* (-19.2 to -7.7)** |
| Alcohol use disorders | 0.1 (0.1 to 0.2) | -- | -- | -11.0* (-17.0 to -5.2) | -13.3* (-19.2 to -7.7) |
| Alcohol dependence | -- | -- | -- | -- | -- |
| Fetal alcohol syndrome | 0.1 (0.1 to 0.2) | -- | -- | -11.0* (-17.0 to -5.2) | -13.3* (-19.2 to -7.7) |
| Drug use disorders | -- | -- | -- | -- | -- |
| Opioid use disorders | -- | -- | -- | -- | -- |
| Cocaine use disorders | -- | -- | -- | -- | -- |

| | | | | | |
|---|---|---|---|---|---|
| Amphetamine use disorders | -- | -- | -- | -- | -- |
| Cannabis use disorders | -- | -- | -- | -- | -- |
| Other drug use disorders | -- | -- | -- | -- | -- |
| **Diabetes and kidney diseases** | **--** | **2.0 (1.4 to 2.7)** | **--** | **-3.6 (-17.3 to 11.8)** | **-6.1 (-19.4 to 8.9)** |
| Diabetes mellitus | -- | 0.1 (0.1 to 0.2) | -- | -- | -- |
| Diabetes mellitus type 1 | -- | 0.1 (0.1 to 0.2) | -- | -- | -- |
| Uncomplicated diabetes mellitus type 1 | -- | 0.1 (0.1 to 0.2) | -- | -- | -- |
| Vision loss due to diabetes mellitus type 1 retinopathy | -- | -- | -- | -- | -- |
| Diabetic neuropathy due to diabetes mellitus type 1 | -- | -- | -- | -- | -- |
| Diabetes mellitus type 2 | -- | -- | -- | -- | -- |
| Uncomplicated diabetes mellitus type 2 | -- | -- | -- | -- | -- |
| Vision loss due to diabetes mellitus type 2 retinopathy | -- | -- | -- | -- | -- |
| Diabetic neuropathy due to diabetes mellitus type 2 | -- | -- | -- | -- | -- |
| Chronic kidney disease | -- | 1.7 (1.1 to 2.5) | -- | -3.5 (-17.6 to 12.4) | -6.0 (-19.8 to 9.5) |
| Chronic kidney disease due to diabetes mellitus type 1 | -- | 0.1 (0.1 to 0.2) | -- | -3.5 (-19.6 to 13.1) | -6.0 (-21.7 to 10.2) |
| Albuminuria with preserved GFR due to type 1 diabetes mellitus | -- | -- | -- | -- | -- |
| End-stage chronic kidney disease due to diabetes mellitus type 1 | -- | -- | -- | 1.0 (-1.6 to 3.6) | -1.6 (-4.1 to 1.0) |
| Stage III chronic kidney disease due to diabetes mellitus type 1 | -- | 0.1 (0.1 to 0.2) | -- | 0.4 (-18.3 to 20.2) | -2.2 (-20.4 to 17.1) |
| Stage IV chronic kidney disease due to diabetes mellitus type 1 | -- | -- | -- | -- | -- |
| Stage V chronic kidney disease due to diabetes mellitus type 1 | -- | -- | -- | -5.0 (-20.2 to 10.7) | -7.4 (-22.2 to 7.9) |
| Chronic kidney disease due to diabetes mellitus type 2 | -- | -- | -- | -3.5 (-18.5 to 13.1) | -6.0 (-20.6 to 10.2) |
| Albuminuria with preserved GFR due to type 2 diabetes mellitus | -- | -- | -- | -- | -- |
| End-stage chronic kidney disease due to diabetes mellitus type 2 | -- | -- | -- | 0.9 (-2.0 to 3.7) | -1.7 (-4.5 to 1.0) |
| Stage III chronic kidney disease due to diabetes mellitus type 2 | -- | -- | -- | -0.3 (-17.4 to 19.2) | -2.9 (-19.6 to 16.1) |
| Stage IV chronic kidney disease due to diabetes mellitus type 2 | -- | -- | -- | -- | -- |
| Stage V chronic kidney disease due to diabetes mellitus type 2 | -- | -- | -- | -4.4 (-19.7 to 12.3) | -6.8 (-21.8 to 9.4) |
| Chronic kidney disease due to hypertension | -- | 0.4 (0.3 to 0.6) | -- | -4.6 (-18.9 to 11.2) | -7.0 (-21.0 to 8.3) |
| Albuminuria with preserved GFR due to hypertension | -- | -- | -- | -- | -- |
| End-stage chronic kidney disease due to hypertension | -- | -- | -- | 2.6 (-0.7 to 5.8) | -0.0 (-3.2 to 3.1) |
| Stage III chronic kidney disease due to hypertension | -- | 0.4 (0.3 to 0.6) | -- | 0.1 (-18.0 to 19.6) | -2.5 (-20.1 to 16.6) |
| Stage IV chronic kidney disease due to hypertension | -- | -- | -- | -- | -- |
| Stage V chronic kidney disease due to hypertension | -- | -- | -- | -4.7 (-19.1 to 11.1) | -7.2 (-21.2 to 8.2) |
| Chronic kidney disease due to glomerulonephritis | -- | 0.1 (0.0 to 0.1) | -- | -3.5 (-18.1 to 13.3) | -6.0 (-20.2 to 10.4) |
| Albuminuria with preserved GFR due to glomerulonephritis | -- | -- | -- | -- | -- |
| End-stage chronic kidney disease due to glomerulonephritis | -- | -- | -- | 0.5 (-1.7 to 2.5) | -2.1* (-4.2 to -0.2) |
| Stage III chronic kidney disease due to glomerulonephritis | -- | 0.1 (0.0 to 0.1) | -- | 2.5 (-16.5 to 23.6) | -0.1 (-18.6 to 20.5) |
| Stage IV chronic kidney disease due to glomerulonephritis | -- | -- | -- | -- | -- |
| Stage V chronic kidney disease due to glomerulonephritis | -- | -- | -- | -4.6 (-19.9 to 12.3) | -7.1 (-21.9 to 9.4) |
| Chronic kidney disease due to other and unspecified causes | -- | 1.1 (0.7 to 1.6) | -- | -3.3 (-17.4 to 12.4) | -5.7 (-19.5 to 9.5) |
| Albuminuria with preserved GFR due to other and unspecified causes | -- | -- | -- | -- | -- |
| End-stage chronic kidney disease due to other causes | -- | -- | -- | -0.8 (-3.2 to 1.2) | -3.4* (-5.7 to -1.4) |
| Stage III chronic kidney disease due to other causes | -- | 1.1 (0.7 to 1.6) | -- | -0.2 (-17.9 to 19.5) | -2.8 (-20.0 to 16.4) |
| Stage IV chronic kidney disease due to other causes | -- | -- | -- | -- | -- |
| Stage V chronic kidney disease due to other causes | -- | -- | -- | -4.2 (-19.3 to 12.4) | -6.6 (-21.3 to 9.5) |
| Acute glomerulonephritis | -- | 0.1 (0.1 to 0.2) | -- | -7.0* (-10.5 to -4.7) | -9.4* (-12.8 to -7.1) |
| **Skin and subcutaneous diseases** | **15.4 (13.5 to 17.6)** | **1719.5 (1521.4 to 1948.5)** | **0.2 (0.1 to 0.3)** | **-1.7 (-5.8 to 3.1)** | **-4.2 (-8.2 to 0.4)** |
| Dermatitis | -- | -- | -- | -- | -- |
| Atopic dermatitis | -- | -- | -- | -- | -- |
| Contact dermatitis | -- | -- | -- | -- | -- |
| Seborrhoeic dermatitis | -- | -- | -- | -- | -- |
| Psoriasis | -- | -- | -- | -- | -- |
| Bacterial skin diseases | 1.6 (1.5 to 1.7) | 220.9 (209.3 to 231.7) | -- | 5.5* (1.4 to 10.1) | 2.7 (-1.2 to 7.3) |
| Cellulitis | 0.1 (0.1 to 0.1) | 11.0 (9.3 to 12.8) | -- | -0.2 (-1.0 to 0.6) | -2.8* (-3.5 to -2.0) |
| Pyoderma | 1.7 (1.6 to 1.8) | 209.9 (198.7 to 220.7) | -- | 8.9* (2.4 to 16.0) | 6.1 (-0.2 to 13.0) |
| Impetigo | 0.7 (0.6 to 0.7) | 85.7 (78.9 to 92.0) | -- | 9.7 (-0.3 to 21.0) | 6.9 (-2.8 to 17.9) |
| Abscess and other bacterial skin diseases | 1.0 (0.9 to 1.1) | 124.1 (115.8 to 133.0) | -- | 8.4* (0.1 to 17.6) | 5.6 (-2.4 to 14.5) |
| Scabies | 1.6 (1.2 to 2.0) | 170.1 (130.2 to 219.1) | 0.0 (0.0 to 0.1) | 1.9 (-3.9 to 8.3) | -0.7 (-6.4 to 5.5) |
| Fungal skin diseases | 8.9 (7.2 to 11.0) | 957.1 (768.5 to 1179.3) | 0.1 (0.0 to 0.1) | -14.0* (-16.8 to -11.1) | -16.2* (-19.0 to -13.4) |
| Tinea capitis | 6.6 (4.9 to 8.7) | 711.2 (528.8 to 937.6) | 0.0 (0.0 to 0.1) | -18.9* (-21.7 to -15.8) | -21.0* (-23.7 to -18.0) |
| Other fungal skin diseases | 2.3 (1.7 to 3.0) | 245.9 (184.1 to 318.6) | -- | 4.2 (-1.5 to 10.4) | 1.5 (-4.0 to 7.6) |

| | | | | | |
|---|---|---|---|---|---|
| Viral skin diseases | -- | -- | -- | -- | -- |
| Viral warts | -- | -- | -- | -- | -- |
| Molluscum contagiosum | -- | -- | -- | -- | -- |
| Acne vulgaris | -- | -- | -- | -- | -- |
| Alopecia areata | -- | -- | -- | -- | -- |
| Pruritus | 0.1 (0.1 to 0.2) | 11.7 (8.6 to 16.2) | -- | 3.6* (2.7 to 4.6) | 0.9* (0.0 to 1.9) |
| Urticaria | 1.4 (1.1 to 1.8) | 147.9 (112.5 to 192.9) | 0.1 (0.1 to 0.1) | 2.9 (-6.7 to 12.5) | 0.3 (-9.1 to 9.6) |
| Decubitus ulcer | -- | -- | -- | -- | -- |
| Other skin and subcutaneous diseases | 2.0 (1.9 to 2.2) | 211.8 (195.4 to 227.0) | -- | 7.0 (-0.2 to 14.4) | 4.2 (-2.8 to 11.5) |
| **Sense organ diseases** | **30.0 (22.8 to 38.9)** | **--** | **1.6 (1.1 to 2.4)** | **-2.6 (-6.4 to 2.0)** | **-5.1* (-8.8 to -0.6)** |
| Blindness and vision impairment | 17.0 (9.8 to 26.2) | -- | 0.8 (0.4 to 1.3) | -6.6* (-11.4 to -2.6) | -9.0* (-13.6 to -5.1) |
| Glaucoma | -- | -- | -- | -- | -- |
| Cataract | -- | -- | -- | -- | -- |
| Age-related macular degeneration | -- | -- | -- | -- | -- |
| Refraction disorders | 2.0 (1.1 to 3.2) | -- | 0.1 (0.0 to 0.1) | -12.4* (-20.0 to -6.0) | -14.6* (-22.1 to -8.4) |
| Near vision loss | -- | -- | -- | -- | -- |
| Other vision loss | 15.1 (8.8 to 23.5) | -- | 0.7 (0.4 to 1.2) | -6.0* (-10.7 to -1.9) | -8.5* (-13.0 to -4.4) |
| Age-related and other hearing loss | 4.7 (4.0 to 5.5) | -- | 0.7 (0.5 to 0.9) | 0.3 (-7.7 to 9.3) | -2.3 (-10.1 to 6.5) |
| Other sense organ diseases | 9.2 (8.5 to 9.9) | -- | 0.2 (0.1 to 0.3) | 4.8 (-0.1 to 9.9) | 2.1 (-2.7 to 7.1) |
| Chronic other sense organ diseases | 5.1 (4.5 to 5.7) | -- | 0.1 (0.1 to 0.2) | 5.2 (-1.4 to 11.9) | 2.5 (-3.9 to 9.1) |
| Acute other sense organ diseases | 4.1 (3.8 to 4.4) | -- | 0.1 (0.0 to 0.1) | 3.8 (-2.9 to 11.4) | 1.2 (-5.4 to 8.5) |
| **Musculoskeletal disorders** | **--** | **--** | **--** | **--** | **--** |
| Rheumatoid arthritis | -- | -- | -- | -- | -- |
| Osteoarthritis | -- | -- | -- | -- | -- |
| Osteoarthritis of the hip | -- | -- | -- | -- | -- |
| Osteoarthritis of the knee | -- | -- | -- | -- | -- |
| Low back pain | -- | -- | -- | -- | -- |
| Low back pain with leg pain | -- | -- | -- | -- | -- |
| Low back pain without leg pain | -- | -- | -- | -- | -- |
| Neck pain | -- | -- | -- | -- | -- |
| Gout | -- | -- | -- | -- | -- |
| Other musculoskeletal disorders | -- | -- | -- | -- | -- |
| **Other non-communicable diseases** | **820.9 (803.4 to 840.3)** | **62.5 (50.2 to 77.2)** | **15.0 (10.7 to 19.7)** | **1.4 (-0.7 to 3.7)** | **-1.2 (-3.2 to 1.0)** |
| Congenital anomalies | 105.8 (99.5 to 113.4) | -- | 11.9 (8.6 to 15.7) | 4.4* (2.8 to 5.9) | 1.7* (0.2 to 3.1) |
| Neural tube defects | 2.8 (2.5 to 3.2) | -- | 0.8 (0.6 to 1.1) | 10.6* (4.2 to 16.7) | 7.8* (1.5 to 13.7) |
| Severe motor and cognitive impairment due to anencephaly | 0.1 (0.1 to 0.1) | -- | 0.0 (0.0 to 0.1) | -6.5* (-8.7 to -4.4) | -8.9* (-11.1 to -6.9) |
| Encephalocoele due to neural tube defects | 0.5 (0.4 to 0.7) | -- | 0.1 (0.1 to 0.1) | 2.8 (-1.1 to 6.9) | 0.1 (-3.6 to 4.1) |
| Spina bifida due to neural tube defects | 2.2 (1.9 to 2.5) | -- | 0.7 (0.5 to 0.9) | 13.1* (5.5 to 20.4) | 10.1* (2.7 to 17.3) |
| Congenital heart anomalies | 46.9 (41.6 to 52.5) | -- | 2.1 (1.0 to 3.5) | 1.1 (-2.3 to 3.4) | -1.5 (-4.8 to 0.8) |
| Critical malformations of great vessels, congenital valvular heart disease and patent ductus arteriosus | 9.9 (7.8 to 12.4) | -- | 0.5 (0.2 to 0.9) | 4.4* (0.3 to 8.2) | 1.7 (-2.3 to 5.4) |
| Other congenital heart anomalies | 7.5 (6.7 to 8.4) | -- | 0.4 (0.2 to 0.6) | 1.1 (-3.1 to 4.8) | -1.5 (-5.6 to 2.1) |
| Severe congenital heart anomalies excluding single ventricle heart defects | 10.7 (8.6 to 13.2) | -- | 0.6 (0.3 to 0.9) | -0.5 (-4.9 to 3.5) | -3.1 (-7.3 to 0.8) |
| Single ventricle and single ventricle pathway congenital heart anomalies | 4.6 (3.2 to 6.2) | -- | 0.2 (0.1 to 0.4) | -2.9 (-8.2 to 2.7) | -5.4 (-10.5 to 0.1) |
| Ventricular septal defect and atrial septal defect | 14.2 (11.4 to 17.5) | -- | 0.4 (0.2 to 0.7) | 1.6 (-2.3 to 5.1) | -1.1 (-4.8 to 2.4) |
| Orofacial clefts | 6.8 (5.7 to 8.2) | -- | 0.2 (0.1 to 0.4) | 2.0 (-6.5 to 11.5) | -0.6 (-8.9 to 8.6) |
| Down's syndrome | 2.1 (1.8 to 2.5) | -- | 0.2 (0.1 to 0.3) | 9.7* (4.5 to 16.0) | 6.9* (1.8 to 13.0) |
| Turner syndrome | 0.4 (0.3 to 0.4) | -- | -- | 1.6 (-7.2 to 11.7) | -1.1 (-9.6 to 8.8) |
| Klinefelter syndrome | 0.5 (0.5 to 0.6) | -- | -- | -1.6 (-9.6 to 7.5) | -4.2 (-11.9 to 4.7) |
| Other chromosomal abnormalities | 5.8 (4.5 to 7.1) | -- | 0.7 (0.4 to 0.9) | 8.3* (2.4 to 14.0) | 5.5 (-0.2 to 11.1) |
| Congenital musculoskeletal and limb anomalies | 29.7 (25.6 to 34.3) | -- | 4.0 (2.6 to 5.5) | 2.8* (1.1 to 4.6) | 0.1 (-1.5 to 1.9) |
| Polydactyly and syndactyly | 4.2 (3.1 to 5.7) | -- | 0.0 (0.0 to 0.1) | 4.7 (-0.5 to 9.6) | 2.0 (-3.1 to 6.7) |
| Congenital limb deficiency | 7.5 (6.6 to 8.7) | -- | 1.2 (0.8 to 1.6) | 2.1 (-0.8 to 5.1) | -0.5 (-3.4 to 2.4) |
| Other congenital musculoskeletal anomalies | 17.9 (15.5 to 20.8) | -- | 2.8 (1.8 to 3.9) | 3.0* (0.7 to 5.3) | 0.4 (-1.9 to 2.6) |
| Urogenital congenital anomalies | 4.8 (4.2 to 5.5) | -- | 0.1 (0.1 to 0.2) | 9.1* (5.4 to 13.1) | 6.3* (2.7 to 10.2) |
| Digestive congenital anomalies | 6.8 (6.1 to 7.6) | -- | 0.4 (0.3 to 0.6) | 2.3 (-2.3 to 7.0) | -0.4 (-4.8 to 4.2) |
| Congenital diaphragmatic hernia | 0.3 (0.2 to 0.3) | -- | -- | -0.9 (-2.6 to 0.8) | -3.5* (-5.2 to -1.8) |
| Congenital atresia and/or stenosis of the digestive tract | 2.6 (1.8 to 3.3) | -- | 0.2 (0.1 to 0.3) | 4.9 (-1.5 to 11.9) | 2.2 (-4.0 to 9.0) |
| Congenital malformations of the abdominal wall after treatment | 1.6 (1.4 to 1.9) | -- | 0.1 (0.0 to 0.1) | 0.7 (-1.7 to 2.8) | -1.9 (-4.2 to 0.2) |
| Other congenital malformations of the digestive tract | 2.3 (1.7 to 3.3) | -- | 0.1 (0.1 to 0.2) | -1.2 (-10.9 to 8.9) | -3.7 (-13.2 to 6.1) |
| Other congenital anomalies | 9.9 (8.6 to 11.5) | -- | 3.4 (2.3 to 4.6) | 6.2* (2.2 to 10.0) | 3.4 (-0.5 to 7.2) |

| | | | | | |
|---|---|---|---|---|---|
| Other congenital birth defects | -- | -- | 2.0 (1.2 to 3.0) | 12.1* (9.6 to 14.5) | 9.2* (6.7 to 11.5) |
| Hearing loss due to other congenital anomalies | 9.9 (8.6 to 11.5) | -- | 1.4 (0.9 to 1.9) | -1.5 (-8.3 to 6.2) | -4.1 (-10.7 to 3.5) |
| Urinary diseases and male infertility | 0.2 (0.1 to 0.2) | 62.5 (50.2 to 77.2) | 0.1 (0.0 to 0.1) | -13.2* (-20.9 to -5.3) | -15.4* (-22.9 to -7.8) |
| Urinary tract infections | 0.4 (0.3 to 0.5) | 62.5 (50.2 to 77.2) | -- | -2.5 (-11.2 to 6.3) | -5.0 (-13.4 to 3.6) |
| Urolithiasis | -- | -- | -- | -- | -- |
| Benign prostatic hyperplasia | -- | -- | -- | -- | -- |
| Male infertility | -- | -- | -- | -- | -- |
| Other urinary diseases | -- | -- | 0.0 (0.0 to 0.1) | -15.8* (-23.3 to -8.2) | -18.0* (-25.3 to -10.6) |
| Gynecological diseases | -- | -- | -- | -- | -- |
| Uterine fibroids | -- | -- | -- | -- | -- |
| Polycystic ovarian syndrome | -- | -- | -- | -- | -- |
| Female infertility | -- | -- | -- | -- | -- |
| Endometriosis | -- | -- | -- | -- | -- |
| Genital prolapse | -- | -- | -- | -- | -- |
| Premenstrual syndrome | -- | -- | -- | -- | -- |
| Other gynecological diseases | -- | -- | -- | -- | -- |
| Other gynecological diseases | -- | -- | -- | -- | -- |
| Other gynecological diseases with anemia | -- | -- | -- | -- | -- |
| Hemoglobinopathies and hemolytic anaemias | 732.2 (714.4 to 751.7) | -- | 2.5 (1.6 to 3.6) | -6.4 (-13.2 to 0.7) | -8.8* (-15.5 to -1.9) |
| Thalassemias | 2.1 (2.0 to 2.3) | -- | 0.1 (0.1 to 0.1) | -13.1* (-21.8 to -5.1) | -15.4* (-23.8 to -7.5) |
| Thalassaemias trait | 96.3 (92.9 to 100.0) | -- | 0.8 (0.5 to 1.2) | -8.7 (-17.8 to 0.3) | -11.1* (-19.9 to -2.3) |
| Sickle cell disorders | 5.5 (5.0 to 6.0) | -- | 0.3 (0.2 to 0.5) | 1.6 (-9.2 to 12.0) | -1.0 (-11.6 to 9.1) |
| Sickle cell trait | 207.1 (189.2 to 227.0) | -- | 0.8 (0.5 to 1.2) | 0.7 (-8.7 to 10.1) | -1.9 (-11.1 to 7.2) |
| G6PD deficiency | 150.9 (144.5 to 157.7) | -- | -- | 7.5 (-12.3 to 33.6) | 4.8 (-14.5 to 30.2) |
| G6PD trait | 347.0 (341.0 to 353.0) | -- | -- | -3.6 (-12.7 to 6.8) | -6.0 (-15.0 to 4.0) |
| Other hemoglobinopathies and hemolytic anaemias | 13.8 (12.8 to 14.8) | -- | 0.4 (0.3 to 0.6) | -17.8* (-27.7 to -7.0) | -19.9* (-29.6 to -9.4) |
| Endocrine, metabolic, blood, and immune disorders | 17.6 (16.4 to 18.8) | -- | 0.5 (0.3 to 0.8) | -16.5* (-26.3 to -6.2) | -18.7* (-28.2 to -8.6) |
| Anemia due to endocrine, metabolic, blood, and immune disorders | 17.5 (16.3 to 18.6) | -- | 0.5 (0.3 to 0.8) | -16.6* (-26.5 to -6.2) | -18.8* (-28.4 to -8.6) |
| Endocrine, metabolic, blood, and immune disorders | 0.1 (0.1 to 0.2) | -- | -- | -5.7 (-13.3 to 3.4) | -8.2 (-15.5 to 0.7) |
| Heart failure due to endocrine, metabolic, blood, and immune disorders | -- | -- | -- | 0.7 (-0.8 to 2.1) | -1.9* (-3.4 to -0.5) |
| Oral disorders | -- | -- | -- | -- | -- |
| Caries of deciduous teeth | -- | -- | -- | -- | -- |
| Caries of permanent teeth | -- | -- | -- | -- | -- |
| Periodontal disease | -- | -- | -- | -- | -- |
| Edentulism and severe tooth loss | -- | -- | -- | -- | -- |
| Other oral disorders | -- | -- | -- | -- | -- |
| **Injuries** | **12.9 (10.9 to 15.2)** | **141.1 (124.8 to 159.0)** | **0.9 (0.6 to 1.2)** | **4.2* (0.2 to 7.9)** | **1.5 (-2.4 to 5.1)** |
| **Transport injuries** | **1.3 (0.9 to 1.8)** | **11.6 (8.4 to 15.3)** | **0.1 (0.1 to 0.2)** | **2.7 (-5.9 to 10.9)** | **0.1 (-8.3 to 8.0)** |
| Road injuries | 0.1 (0.1 to 0.1) | 0.8 (0.5 to 1.2) | -- | 16.3 (-4.9 to 36.5) | 13.3 (-7.4 to 33.0) |
| Pedestrian road injuries | -- | 0.2 (0.1 to 0.3) | -- | 2.1 (-19.9 to 23.8) | -0.5 (-21.9 to 20.6) |
| Cyclist road injuries | -- | -- | -- | -- | -- |
| Motorcyclist road injuries | 0.0 (0.0 to 0.1) | 0.3 (0.2 to 0.5) | -- | 16.5 (-7.0 to 37.8) | 13.5 (-9.4 to 34.2) |
| Motor vehicle road injuries | -- | 0.2 (0.1 to 0.3) | -- | 20.5 (-2.7 to 43.0) | 17.4 (-5.2 to 39.3) |
| Other road injuries | -- | 0.1 (0.1 to 0.2) | -- | 52.6* (23.2 to 78.4) | 48.7* (20.0 to 73.8) |
| Other transport injuries | 1.2 (0.9 to 1.7) | 10.8 (7.6 to 14.5) | 0.1 (0.1 to 0.2) | 2.0 (-7.0 to 10.7) | -0.6 (-9.4 to 7.8) |
| **Unintentional injuries** | **10.6 (8.8 to 12.8)** | **118.6 (102.7 to 136.1)** | **0.7 (0.4 to 0.9)** | **3.4 (-1.1 to 7.8)** | **0.8 (-3.7 to 5.0)** |
| Falls | 3.7 (2.6 to 5.1) | 34.7 (25.0 to 46.6) | 0.2 (0.1 to 0.3) | 5.6 (-5.4 to 16.4) | 2.9 (-7.8 to 13.4) |
| Drowning | -- | 0.2 (0.1 to 0.2) | -- | -12.5* (-22.2 to -2.4) | -14.7* (-24.2 to -4.9) |
| Fire, heat, and hot substances | 0.1 (0.1 to 0.2) | 2.2 (1.5 to 3.2) | -- | 14.3* (0.3 to 27.2) | 11.4 (-2.3 to 23.9) |
| Poisonings | 0.0 (0.0 to 0.1) | 2.7 (1.8 to 3.7) | -- | 12.7* (3.1 to 20.4) | 9.8* (0.5 to 17.3) |
| Poisoning by carbon monoxide | -- | 0.4 (0.2 to 0.7) | -- | 13.1 (-4.8 to 28.0) | 10.2 (-7.3 to 24.7) |
| Poisoning by other means | -- | 2.3 (1.6 to 3.2) | -- | 12.6* (3.4 to 20.4) | 9.7* (0.7 to 17.3) |
| Exposure to mechanical forces | 2.6 (1.8 to 3.6) | 27.6 (20.5 to 38.0) | 0.1 (0.1 to 0.2) | -1.8 (-12.2 to 8.2) | -4.3 (-14.4 to 5.4) |
| Unintentional firearm injuries | 0.2 (0.1 to 0.3) | 1.7 (1.0 to 2.8) | -- | 1.9 (-15.1 to 17.5) | -0.8 (-17.3 to 14.4) |
| Other exposure to mechanical forces | 2.4 (1.7 to 3.4) | 25.9 (19.1 to 36.0) | 0.1 (0.1 to 0.2) | -2.4 (-13.0 to 7.2) | -4.9 (-15.2 to 4.4) |
| Adverse effects of medical treatment | 0.5 (0.4 to 0.7) | 6.9 (5.0 to 8.8) | 0.1 (0.0 to 0.1) | 1.1 (-9.4 to 11.0) | -1.5 (-11.7 to 8.1) |
| Animal contact | 1.1 (0.7 to 1.6) | 18.3 (12.5 to 26.5) | 0.1 (0.0 to 0.1) | -5.1 (-15.0 to 5.2) | -7.6 (-17.2 to 2.5) |
| Venomous animal contact | 0.2 (0.1 to 0.3) | 6.5 (4.2 to 10.0) | -- | -4.0 (-14.6 to 6.8) | -6.5 (-16.8 to 4.1) |
| Non-venomous animal contact | 0.9 (0.6 to 1.3) | 11.8 (8.0 to 17.3) | 0.0 (0.0 to 0.1) | -6.0 (-16.3 to 4.6) | -8.4 (-18.4 to 1.9) |
| Foreign body | 0.9 (0.4 to 1.5) | 9.3 (6.9 to 12.2) | 0.1 (0.0 to 0.1) | 6.2* (2.4 to 10.3) | 3.4 (-0.3 to 7.4) |

| | | | | | |
|---|---|---|---|---|---|
| Pulmonary aspiration and foreign body in airway | 0.0 (0.0 to 0.1) | 1.0 (0.6 to 1.6) | -- | -9.3 (-24.4 to 3.8) | -11.7 (-26.3 to 1.1) |
| Foreign body in eyes | 0.6 (0.2 to 1.2) | 4.6 (2.8 to 7.2) | 0.0 (0.0 to 0.1) | 5.6* (3.8 to 8.0) | 2.9* (1.2 to 5.2) |
| Foreign body in other body part | 0.2 (0.1 to 0.3) | 3.6 (2.5 to 5.0) | -- | 12.9* (2.7 to 21.3) | 10.0* (0.1 to 18.1) |
| Environmental heat and cold exposure | 0.4 (0.3 to 0.6) | 4.2 (2.8 to 6.0) | 0.0 (0.0 to 0.1) | 3.2 (-7.9 to 14.2) | 0.6 (-10.3 to 11.3) |
| Exposure to forces of nature | -- | -- | -- | -65.3* (-66.6 to -63.9) | -66.2* (-67.4 to -64.9) |
| Other unintentional injuries | 1.2 (0.8 to 1.8) | 12.4 (8.5 to 17.4) | 0.1 (0.0 to 0.1) | 11.2 (-2.0 to 22.6) | 8.3 (-4.6 to 19.4) |
| **Self-harm and interpersonal violence** | **1.0 (0.7 to 1.3)** | **11.0 (8.4 to 14.1)** | **0.1 (0.1 to 0.1)** | **12.5* (1.4 to 24.1)** | **9.6 (-1.2 to 20.9)** |
| Self-harm | -- | -- | -- | -- | -- |
| Self-harm by firearm | -- | -- | -- | -- | -- |
| Self-harm by other specified means | -- | -- | -- | -- | -- |
| Interpersonal violence | 0.7 (0.5 to 1.0) | 8.4 (6.0 to 11.5) | 0.1 (0.0 to 0.1) | 1.1 (-13.0 to 16.0) | -1.5 (-15.2 to 13.0) |
| Assault by firearm | -- | 0.2 (0.1 to 0.3) | -- | 8.5 (-14.1 to 32.3) | 5.7 (-16.3 to 28.8) |
| Assault by sharp object | 0.1 (0.1 to 0.2) | 1.2 (0.7 to 1.9) | -- | 8.6 (-11.0 to 27.3) | 5.8 (-13.3 to 24.0) |
| Sexual violence | -- | -- | -- | -- | -- |
| Assault by other means | 0.6 (0.4 to 0.9) | 7.1 (5.0 to 9.7) | 0.1 (0.0 to 0.1) | -0.7 (-15.1 to 14.4) | -3.3 (-17.3 to 11.4) |
| Conflict and terrorism | 0.2 (0.2 to 0.3) | 2.5 (1.9 to 3.4) | -- | 57.8* (51.5 to 65.2) | 53.8* (47.6 to 60.9) |
| Executions and police conflict | -- | -- | -- | -- | -- |
| **Nature of injury aggregates** | | | | | |
| Amputations | -- | 2.7 (2.2 to 3.3) | -- | -6.0* (-9.3 to -2.8) | -8.4* (-11.6 to -5.3) |
| Burns | 0.3 (0.2 to 0.4) | 5.3 (4.4 to 6.4) | 0.1 (0.0 to 0.1) | 6.2* (1.2 to 11.2) | 3.4 (-1.5 to 8.3) |
| Fractures | 4.4 (3.6 to 5.3) | 28.7 (24.4 to 33.6) | 0.2 (0.2 to 0.4) | 5.1* (0.2 to 10.0) | 2.4 (-2.3 to 7.1) |
| Head Injuries | 0.6 (0.5 to 0.7) | 5.7 (4.7 to 6.7) | 0.1 (0.1 to 0.2) | 3.3 (-1.7 to 8.1) | 0.6 (-4.2 to 5.3) |
| Spinal Injuries | -- | 0.2 (0.2 to 0.3) | -- | 5.1 (-0.0 to 10.8) | 2.4 (-2.6 to 8.0) |
| Minor Injuries | 4.4 (3.3 to 5.7) | 54.9 (46.8 to 64.2) | 0.0 (0.0 to 0.1) | 1.3 (-3.6 to 6.4) | -1.3 (-6.1 to 3.7) |
| Other Injuries | 3.1 (2.3 to 4.1) | 43.6 (38.6 to 49.3) | 0.4 (0.3 to 0.6) | 3.8* (0.2 to 7.4) | 1.1 (-2.4 to 4.6) |
| **Impairments** | | | | | |
| Anaemia | 1246.4 (1010.6 to 1475.3) | -- | 42.8 (26.7 to 64.2) | -14.8* (-20.6 to -8.5) | -17.0* (-22.6 to -10.9) |
| Epilepsy | 20.3 (14.7 to 27.0) | -- | 9.6 (6.5 to 13.5) | 7.4 (-2.0 to 16.7) | 4.6 (-4.6 to 13.7) |
| Guillain-Barré syndrome | -- | -- | -- | 11.2* (5.5 to 24.3) | 8.4* (2.8 to 21.1) |
| Hearing loss | 15.2 (13.3 to 17.5) | -- | 2.1 (1.4 to 2.9) | -1.0 (-6.3 to 5.2) | -3.5 (-8.8 to 2.5) |
| Heart failure | -- | -- | -- | 0.3 (-0.8 to 1.3) | -2.3* (-3.4 to -1.3) |
| Infertility | 0.1 (0.1 to 0.2) | -- | -- | 7.0* (5.2 to 8.8) | 4.3* (2.5 to 6.0) |
| Developmental intellectual disability | 89.9 (69.3 to 110.3) | -- | 11.7 (9.0 to 14.9) | 0.8 (-3.2 to 5.4) | -1.8 (-5.7 to 2.7) |
| Pelvic inflammatory disease | -- | -- | -- | -- | -- |
| Vision loss | 20.6 (13.4 to 29.6) | -- | 1.6 (1.1 to 2.2) | -5.3* (-7.7 to -3.0) | -7.7* (-10.1 to -5.5) |

We did not calculate incidence for the nine impairments and certain NTDs. Blank cells mean that no estimate is available or that the estimate has a magnitude less than 50.
Table presents prevalence and incidence counts in thousands.
* Percentage changes that are statistically significant
‡ Incidence estimates for stroke represent first-ever stroke only.
^ Incidence of HIV/AIDS represents new infections of HIV only and does not include new infections of Tuberculosis in HIV positive cases.

| SR Table 10b: Global prevalence, incidence and YLDs for 2017, percentage change of YLD counts and percentage change of YLD rates between 2007 and 2017 by cause, age 7 to 27 days | | | | | |
|---|---|---|---|---|---|
| Cause | Prevalence in thousands (95% UI) | Incidence in thousands (95% UI) | YLDs in thousands (95% UI) | | |
| | 2017 counts | 2017 counts | 2017 counts | Percentage change in counts between 2007 and 2017 | Percentage change in rates between 2007 and 2017 |
| **All causes** | **6053.2 (5807.3 to 6287.5)** | **62550.5 (55601.6 to 70422.3)** | **358.2 (261.2 to 475.3)** | **-7.3* (-10.2 to -4.3)** | **-10.0* (-12.8 to -7.1)** |
| **Communicable, maternal, neonatal, and nutritional disorders** | **5101.8 (4707.5 to 5460.2)** | **56841.6 (49732.9 to 64773.6)** | **282.1 (202.5 to 377.2)** | **-9.6* (-12.9 to -6.0)** | **-12.2* (-15.4 to -8.7)** |
| **HIV/AIDS and sexually transmitted infections** | **4.7 (4.2 to 5.2)** | **8.2 (7.2 to 9.4)** | **0.7 (0.5 to 0.9)** | **-54.4* (-58.5 to -49.8)** | **-55.7* (-59.7 to -51.3)** |
| HIV/AIDS | 5.8 (5.2 to 6.3) | 8.2^ (7.2 to 9.4) | 0.7 (0.5 to 0.9) | -54.4* (-58.5 to -49.8) | -55.7* (-59.7 to -51.3) |
| HIV/AIDS - Drug-susceptible Tuberculosis | -- | -- | -- | -- | -- |
| HIV/AIDS - Multidrug-resistant Tuberculosis without extensive drug resistance | -- | -- | -- | -- | -- |
| HIV/AIDS - Extensively drug-resistant Tuberculosis | -- | -- | -- | -- | -- |
| HIV/AIDS resulting in other diseases | 5.8 (5.2 to 6.3) | 8.2 (7.2 to 9.4) | 0.7 (0.5 to 0.9) | -54.4* (-58.5 to -49.8) | -55.7* (-59.7 to -51.3) |
| HIV/AIDS not on antiretroviral treatment without Tuberculosis | 4.1 (3.6 to 4.5) | 8.2 (7.2 to 9.4) | 0.5 (0.4 to 0.7) | -61.3* (-65.5 to -56.7) | -62.5* (-66.5 to -58.0) |
| HIV/AIDS on antiretroviral treatment without Tuberculosis | 1.7 (1.5 to 1.8) | -- | 0.1 (0.1 to 0.2) | 36.0* (11.2 to 68.1) | 32.0* (7.9 to 63.3) |
| Sexually transmitted infections excluding HIV | -- | -- | -- | -- | -- |
| Syphilis | -- | -- | -- | -- | -- |
| Early syphilis | -- | -- | -- | -- | -- |
| Tertiary syphilis | -- | -- | -- | -- | -- |
| Chlamydial infection | -- | -- | -- | -- | -- |
| Chlamydia episode | -- | -- | -- | -- | -- |
| Chlamydial infection complications | -- | -- | -- | -- | -- |
| Gonococcal infection | -- | -- | -- | -- | -- |
| Gonococcal infection complications | -- | -- | -- | -- | -- |
| Gonorrhoea episode | -- | -- | -- | -- | -- |
| Trichomoniasis | -- | -- | -- | -- | -- |
| Genital herpes | -- | -- | -- | -- | -- |
| Other sexually transmitted infections | -- | -- | -- | -- | -- |
| Other sexually transmitted diseases residual | -- | -- | -- | -- | -- |
| Other sexually transmitted diseases | -- | -- | -- | -- | -- |
| **Respiratory infections and tuberculosis** | **1160.0 (966.1 to 1404.8)** | **29513.5 (23502.1 to 36129.2)** | **12.5 (7.6 to 19.4)** | **-1.0 (-5.0 to 2.5)** | **-3.9* (-7.7 to -0.5)** |
| Tuberculosis | 772.7 (585.0 to 1016.6) | 10.7 (9.0 to 12.7) | 0.1 (0.1 to 0.2) | 16.6 (-5.5 to 44.5) | 13.2 (-8.2 to 40.3) |
| Latent tuberculosis infection | 772.4 (584.7 to 1016.3) | -- | -- | -- | -- |
| Drug-susceptible tuberculosis | 0.3 (0.3 to 0.4) | 10.2 (8.6 to 12.2) | 0.1 (0.1 to 0.2) | 16.9 (-5.7 to 45.8) | 13.5 (-8.5 to 41.6) |
| Multidrug-resistant tuberculosis without extensive drug resistance | -- | 0.4 (0.3 to 0.6) | -- | 10.2 (-27.7 to 65.8) | 7.0 (-29.8 to 61.0) |
| Extensively drug-resistant tuberculosis | -- | -- | -- | 25.4 (-17.5 to 107.2) | 21.8 (-19.9 to 101.2) |
| Lower respiratory infections | 55.2 (44.6 to 68.3) | 934.6 (717.7 to 1177.8) | 3.4 (2.1 to 5.0) | -16.0* (-25.4 to -6.0) | -18.4* (-27.6 to -8.7) |
| Guillain-Barré syndrome due to lower respiratory infections | -- | -- | -- | 2.7* (0.7 to 4.6) | -0.3 (-2.2 to 1.6) |
| Lower respiratory infection episode | 55.2 (44.6 to 68.3) | 934.6 (717.7 to 1177.8) | 3.4 (2.1 to 5.0) | -16.0* (-25.4 to -6.0) | -18.4* (-27.6 to -8.7) |
| Upper respiratory infections | 322.5 (247.8 to 402.0) | 26609.4 (20629.5 to 33357.3) | 8.2 (4.6 to 13.4) | 6.5* (4.6 to 8.5) | 3.4* (1.6 to 5.3) |
| Guillain-Barré syndrome due to upper respiratory infections | -- | -- | -- | 2.7* (0.7 to 4.7) | -0.2 (-2.2 to 1.6) |
| Upper respiratory infection episode | 322.5 (247.8 to 402.0) | 26609.4 (20629.5 to 33357.3) | 8.2 (4.6 to 13.4) | 6.5* (4.6 to 8.5) | 3.4* (1.6 to 5.3) |
| Otitis media | 60.6 (43.7 to 82.3) | 1958.8 (1383.5 to 2713.3) | 0.9 (0.4 to 1.6) | 1.0 (-1.6 to 3.6) | -2.0 (-4.4 to 0.6) |
| Acute otitis media | 54.3 (37.5 to 76.3) | 1958.7 (1383.2 to 2713.3) | 0.7 (0.3 to 1.4) | 1.7 (-1.3 to 4.9) | -1.3 (-4.1 to 1.8) |
| Chronic otitis media | 6.3 (5.1 to 7.6) | 0.2 (0.0 to 0.6) | 0.1 (0.1 to 0.2) | -2.4 (-7.9 to 2.8) | -5.2* (-10.6 to -0.2) |
| **Enteric infections** | **237.8 (196.7 to 286.3)** | **19539.6 (16112.2 to 23341.8)** | **27.8 (18.5 to 39.5)** | **-7.4 (-16.2 to 1.4)** | **-10.1* (-18.6 to -1.6)** |
| Diarrhoeal diseases | 240.0 (198.0 to 290.6) | 19536.3 (16108.4 to 23338.9) | 27.8 (18.5 to 39.5) | -7.4 (-16.1 to 1.4) | -10.0* (-18.6 to -1.5) |
| Guillain-Barré syndrome due to diarrheal diseases | -- | -- | -- | 2.7* (0.7 to 4.6) | -0.2 (-2.2 to 1.6) |
| Diarrheal disease episode | 240.0 (198.0 to 290.6) | 19536.3 (16108.4 to 23338.9) | 27.8 (18.5 to 39.5) | -7.4 (-16.1 to 1.4) | -10.0* (-18.6 to -1.5) |
| Typhoid and paratyphoid | -- | 0.0 (0.0 to 0.1) | -- | -15.5 (-34.7 to 6.9) | -17.9 (-36.6 to 3.8) |
| Typhoid fever | -- | -- | -- | -16.3 (-36.9 to 7.2) | -18.8 (-38.7 to 4.1) |
| Typhoid fever complications | -- | -- | -- | -16.3 (-46.7 to 27.5) | -18.8 (-48.2 to 23.8) |
| Typhoid fever episode | -- | -- | -- | -16.3 (-39.7 to 15.6) | -18.7 (-41.5 to 12.3) |
| Paratyphoid fever | -- | -- | -- | -1.3 (-21.4 to 26.9) | -4.1 (-23.7 to 23.2) |
| Intestinal perforation due to paratyphoid | -- | -- | -- | -1.0 (-30.1 to 42.5) | -3.9 (-32.1 to 38.4) |
| Paratyphoid fever episode | -- | -- | -- | -1.3 (-22.6 to 28.4) | -4.2 (-24.8 to 24.6) |
| iNTS | 0.1 (0.1 to 0.2) | 3.2 (2.5 to 4.8) | -- | -35.7* (-56.0 to -6.9) | -37.6* (-57.2 to -9.6) |
| Other intestinal infectious diseases | -- | -- | -- | -- | -- |
| **Neglected tropical diseases and malaria** | **200.9 (185.1 to 215.1)** | **451.7 (292.9 to 639.8)** | **2.7 (1.7 to 4.0)** | **-33.6* (-41.6 to -23.2)** | **-35.5* (-43.2 to -25.5)** |
| Malaria | 207.9 (190.9 to 221.9) | 450.1 (291.3 to 638.3) | 2.6 (1.7 to 4.0) | -34.2* (-42.4 to -24.0) | -36.1* (-44.1 to -26.2) |
| Malaria complications | -- | -- | -- | 10.5* (5.1 to 16.5) | 7.3* (2.1 to 13.1) |
| Malaria episode | 26.2 (14.2 to 41.9) | 450.1 (291.3 to 638.3) | 1.1 (0.5 to 1.9) | -29.7* (-45.1 to -15.7) | -31.8* (-46.7 to -18.2) |

| | | | | | |
|---|---|---|---|---|---|
| Malaria parasitemia | 181.7 (161.0 to 201.3) | -- | 1.6 (1.0 to 2.3) | -37.0* (-45.5 to -26.5) | -38.8* (-47.1 to -28.7) |
| Chagas disease | 0.4 (0.3 to 0.5) | 0.2 (0.1 to 0.3) | -- | -29.8* (-36.6 to -23.6) | -31.9* (-38.4 to -25.8) |
| Acute Chagas disease | -- | 0.2 (0.1 to 0.3) | -- | -30.3* (-37.4 to -24.0) | -32.3* (-39.2 to -26.3) |
| Asymptomatic Chagas disease | 0.4 (0.3 to 0.5) | -- | -- | -- | -- |
| Symptomatic chronic Chagas infection | -- | -- | -- | -10.2* (-15.3 to -4.9) | -12.8* (-17.8 to -7.7) |
| Leishmaniasis | -- | -- | -- | -- | -- |
| Visceral leishmaniasis | -- | -- | -- | -- | -- |
| Cutaneous and mucocutaneous leishmaniasis | -- | -- | -- | -- | -- |
| African trypanosomiasis | -- | -- | -- | -- | -- |
| Trypanosomiasis Gambiense | -- | -- | -- | -- | -- |
| Trypanosomiasis Rhodesiense | -- | -- | -- | -- | -- |
| Schistosomiasis | -- | -- | -- | -- | -- |
| Mild schistosomiasis | -- | -- | -- | -- | -- |
| Anemia due to schistosomiasis | -- | -- | -- | -- | -- |
| Schistosomiasis complications | -- | -- | -- | -- | -- |
| Cysticercosis | -- | -- | -- | -- | -- |
| Cystic echinococcosis | -- | -- | -- | -- | -- |
| Lymphatic filariasis | -- | -- | -- | -- | -- |
| Prevalence of detectable microfiliaria due to lymphatic filariasis | -- | -- | -- | -- | -- |
| Lymphatic filariasis complications | -- | -- | -- | -- | -- |
| Onchocerciasis | -- | -- | -- | -- | -- |
| Asymptomatic onchocerciasis | -- | -- | -- | -- | -- |
| Skin disease due to onchocerciasis | -- | -- | -- | -- | -- |
| Vision loss due to onchocerciasis | -- | -- | -- | -- | -- |
| Trachoma | -- | -- | -- | -- | -- |
| Dengue | -- | -- | -- | -- | -- |
| Post-dengue chronic fatigue syndrome | -- | -- | -- | -- | -- |
| Dengue episode | -- | -- | -- | -- | -- |
| Yellow fever | -- | 0.0 (0.0 to 0.1) | -- | -27.2 (-49.9 to 4.3) | -29.4 (-51.3 to 1.3) |
| Asymptomatic yellow fever | -- | 0.0 (0.0 to 0.1) | -- | -- | -- |
| Yellow fever episode | -- | 0.0 (0.0 to 0.1) | -- | -27.2 (-49.9 to 4.3) | -29.4 (-51.3 to 1.3) |
| Rabies | -- | -- | -- | -- | -- |
| Intestinal nematode infections | -- | -- | -- | -- | -- |
| Ascariasis | -- | -- | -- | -- | -- |
| Asymptomatic ascariasis | -- | -- | -- | -- | -- |
| Ascariasis complications | -- | -- | -- | -- | -- |
| Trichuriasis | -- | -- | -- | -- | -- |
| Asymptomatic trichuriasis | -- | -- | -- | -- | -- |
| Trichuriasis complications | -- | -- | -- | -- | -- |
| Hookworm disease | -- | -- | -- | -- | -- |
| Asymptomatic hookworm disease | -- | -- | -- | -- | -- |
| Anemia due to hookworm disease | -- | -- | -- | -- | -- |
| Hookworm disease complications | -- | -- | -- | -- | -- |
| Food-borne trematodiases | -- | -- | -- | -- | -- |
| Asymptomatic food-borne tremodiases | -- | -- | -- | -- | -- |
| Food-borne tremodiases complications | -- | -- | -- | -- | -- |
| Leprosy | -- | -- | -- | -- | -- |
| Ebola virus disease | -- | -- | -- | -- | -- |
| Ebola cases | -- | -- | -- | -- | -- |
| Post-Ebola chronic fatigue syndrome | -- | -- | -- | -- | -- |
| Zika virus disease | 0.1 (0.0 to 0.1) | 1.4 (0.8 to 2.8) | -- | -- | -- |
| Zika virus complications | 0.0 (0.0 to 0.1) | -- | -- | -- | -- |
| Zika virus episode | -- | 1.4 (0.8 to 2.8) | -- | -- | -- |
| Guinea worm disease | -- | -- | -- | -- | -- |
| Other neglected tropical diseases | -- | -- | -- | -16.6 (-29.9 to 0.4) | -19.0* (-31.9 to -2.5) |
| Acute infection due to other neglected tropical diseases | -- | -- | -- | -16.6 (-29.9 to 0.4) | -19.0* (-31.9 to -2.5) |
| Anemia due to other neglected tropical diseases | -- | -- | -- | -- | -- |
| **Other infectious diseases** | **353.3 (295.8 to 408.1)** | **5149.0 (4262.7 to 6125.2)** | **3.2 (2.1 to 4.6)** | **-8.3* (-16.0 to -0.2)** | **-10.9* (-18.5 to -3.1)** |
| Meningitis | 3.1 (2.6 to 3.8) | 112.8 (89.6 to 140.3) | 0.4 (0.3 to 0.6) | -19.5* (-26.9 to -11.5) | -21.8* (-29.1 to -14.1) |
| Pneumococcal meningitis | 0.3 (0.2 to 0.3) | 6.7 (4.8 to 8.8) | -- | -47.3* (-54.4 to -39.7) | -48.8* (-55.8 to -41.4) |

| | | | | | |
|---|---|---|---|---|---|
| Acute pneumococcal meningitis | 0.1 (0.1 to 0.2) | 6.7 (4.8 to 8.8) | -- | -46.4* (-57.6 to -33.0) | -47.9* (-58.9 to -34.9) |
| Pneumococcal meningitis complications | 0.1 (0.1 to 0.1) | -- | -- | -48.5* (-51.1 to -46.2) | -50.0* (-52.6 to -47.7) |
| H influenzae type B meningitis | 0.2 (0.1 to 0.3) | 6.6 (4.8 to 9.1) | -- | -46.5* (-55.9 to -36.2) | -48.0* (-57.2 to -38.1) |
| Acute H influenzae type B meningitis | 0.1 (0.1 to 0.2) | 6.6 (4.8 to 9.1) | -- | -45.3* (-57.7 to -31.1) | -46.9* (-58.9 to -33.1) |
| H Influenza type B meningitis complications | 0.0 (0.0 to 0.1) | -- | -- | -50.0* (-52.5 to -47.7) | -51.5* (-53.9 to -49.2) |
| Meningococcal infection | 0.2 (0.2 to 0.3) | 6.8 (4.8 to 9.2) | -- | -44.3* (-53.5 to -33.1) | -45.9* (-54.8 to -35.1) |
| Acute meningococcal meningitis | 0.2 (0.1 to 0.2) | 6.8 (4.8 to 9.2) | -- | -43.6* (-55.3 to -29.1) | -45.3* (-56.6 to -31.2) |
| Meningococcal meningitis complications | 0.1 (0.0 to 0.1) | -- | -- | -46.6* (-49.4 to -43.5) | -48.2* (-50.8 to -45.2) |
| Other meningitis | 2.5 (2.0 to 3.0) | 92.6 (73.8 to 114.9) | 0.3 (0.2 to 0.5) | -7.3 (-17.5 to 2.9) | -10.0* (-19.9 to -0.1) |
| Other acute bacterial meningitis | 1.3 (1.0 to 1.6) | 57.1 (44.7 to 71.3) | 0.2 (0.1 to 0.3) | 0.6 (-14.7 to 18.7) | -2.3 (-17.2 to 15.3) |
| Acute viral meningitis | 0.8 (0.6 to 1.0) | 35.6 (28.3 to 44.2) | 0.1 (0.1 to 0.2) | -17.9* (-32.3 to -2.6) | -20.3* (-34.3 to -5.4) |
| Other bacterial meningitis complications | 0.4 (0.3 to 0.5) | -- | 0.0 (0.0 to 0.1) | -6.6* (-9.6 to -3.5) | -9.3* (-12.2 to -6.3) |
| Encephalitis | 0.4 (0.4 to 0.5) | 15.2 (14.6 to 15.8) | 0.1 (0.0 to 0.1) | 1.3 (-7.7 to 11.2) | -1.6 (-10.4 to 8.0) |
| Acute encephalitis | 0.4 (0.4 to 0.4) | 15.2 (14.6 to 15.8) | 0.0 (0.0 to 0.1) | 3.4 (-7.2 to 15.6) | 0.4 (-9.9 to 12.2) |
| Encephalitis complications | 0.1 (0.0 to 0.1) | -- | -- | -10.6* (-12.6 to -8.4) | -13.2* (-15.1 to -11.1) |
| Diphtheria | -- | -- | -- | -- | -- |
| Whooping cough | -- | -- | -- | -- | -- |
| Tetanus | 0.8 (0.5 to 1.2) | 13.7 (8.7 to 20.0) | 0.1 (0.1 to 0.2) | -62.8* (-75.5 to -41.8) | -63.9* (-76.2 to -43.5) |
| Severe tetanus | 0.8 (0.5 to 1.1) | 13.7 (8.7 to 20.0) | 0.1 (0.1 to 0.2) | -64.1* (-77.1 to -42.6) | -65.2* (-77.8 to -44.3) |
| Neonatal tetanus complications | 0.1 (0.1 to 0.1) | -- | -- | -15.9* (-18.5 to -12.3) | -18.4* (-20.9 to -14.9) |
| Measles | -- | -- | -- | -- | -- |
| Varicella and herpes zoster | 33.8 (23.9 to 47.2) | 1761.7 (1249.0 to 2463.5) | 0.2 (0.1 to 0.4) | -2.2* (-4.5 to -0.0) | -5.1* (-7.3 to -2.9) |
| Chickenpox | 33.8 (23.9 to 47.2) | 1761.6 (1248.8 to 2463.4) | 0.2 (0.1 to 0.4) | -2.2* (-4.5 to -0.0) | -5.1* (-7.3 to -2.9) |
| Herpes zoster | -- | 0.1 (0.1 to 0.1) | -- | 3.1* (2.1 to 3.9) | 0.1 (-0.9 to 0.9) |
| Acute hepatitis | 254.1 (197.8 to 308.6) | 3245.6 (2506.1 to 3957.8) | 0.1 (0.1 to 0.2) | -1.7 (-9.4 to 6.3) | -4.5 (-12.0 to 3.2) |
| Acute hepatitis A | 238.0 (180.9 to 292.7) | 3094.5 (2351.4 to 3804.5) | -- | 0.9 (-34.5 to 52.9) | -2.0 (-36.4 to 48.5) |
| Acute hepatitis B | 10.4 (6.3 to 16.0) | 90.0 (54.3 to 138.4) | 0.1 (0.0 to 0.1) | 6.3 (-2.0 to 16.2) | 3.2 (-4.9 to 12.8) |
| Acute hepatitis C | 3.0 (1.9 to 4.4) | 26.0 (16.4 to 38.0) | 0.0 (0.0 to 0.1) | -13.3 (-27.5 to 1.7) | -15.8* (-29.6 to -1.3) |
| Acute hepatitis E | 2.7 (1.9 to 3.7) | 35.1 (24.5 to 48.5) | -- | 4.9 (-33.7 to 61.5) | 1.9 (-35.6 to 56.8) |
| Other unspecified infectious diseases | 68.7 (64.1 to 73.5) | -- | 2.3 (1.5 to 3.4) | -0.3 (-10.8 to 11.3) | -3.2 (-13.4 to 8.0) |
| Guillain-Barré syndrome due to other infectious diseases | -- | -- | -- | 2.7* (0.8 to 4.7) | -0.2 (-2.2 to 1.6) |
| Other infectious diseases | -- | -- | 0.2 (0.1 to 0.3) | 10.0* (5.1 to 14.2) | 6.8* (2.0 to 10.9) |
| Anemia due to other infectious diseases | 68.7 (64.1 to 73.5) | -- | 2.1 (1.3 to 3.1) | -1.2 (-12.7 to 11.2) | -4.1 (-15.2 to 8.0) |
| **Maternal and neonatal disorders** | **1110.5 (1031.7 to 1183.7)** | **--** | **87.3 (64.2 to 115.5)** | **3.3 (-2.8 to 10.7)** | **0.3 (-5.7 to 7.5)** |
| Maternal disorders | -- | -- | -- | -- | -- |
| Maternal haemorrhage | -- | -- | -- | -- | -- |
| Maternal hemorrhage complications | -- | -- | -- | -- | -- |
| Maternal hemorrhage episode | -- | -- | -- | -- | -- |
| Maternal sepsis and other pregnancy related infections | -- | -- | -- | -- | -- |
| Infertility due to puerperal sepsis | -- | -- | -- | -- | -- |
| Maternal sepsis and other maternal infection episode | -- | -- | -- | -- | -- |
| Maternal hypertensive disorders | -- | -- | -- | -- | -- |
| Maternal hypertensive disorder complications | -- | -- | -- | -- | -- |
| Maternal hypertensive disorder episode | -- | -- | -- | -- | -- |
| Maternal obstructed labour and uterine rupture | -- | -- | -- | -- | -- |
| Obstructed labor, acute event | -- | -- | -- | -- | -- |
| Maternal obstructed labor complications | -- | -- | -- | -- | -- |
| Maternal abortion and miscarriage | -- | -- | -- | -- | -- |
| Ectopic pregnancy | -- | -- | -- | -- | -- |
| Other maternal disorders | -- | -- | -- | -- | -- |
| Neonatal disorders | 1110.5 (1031.7 to 1183.7) | -- | 87.3 (64.2 to 115.5) | 3.3 (-2.8 to 10.7) | 0.3 (-5.7 to 7.5) |
| Neonatal preterm birth | 958.8 (896.3 to 1018.2) | -- | 33.5 (23.3 to 46.1) | 4.0 (-10.1 to 19.1) | 1.0 (-12.7 to 15.7) |
| Preterm birth complications | 151.0 (118.5 to 194.4) | -- | 33.0 (22.9 to 45.5) | 4.2 (-10.2 to 19.4) | 1.2 (-12.8 to 16.0) |
| Retinopathy of prematurity | 12.0 (9.0 to 15.3) | -- | 0.6 (0.4 to 0.8) | -4.5 (-11.6 to 3.0) | -7.3* (-14.2 to -0.0) |
| Uncomplicated preterm birth | 795.7 (740.8 to 834.7) | -- | -- | -- | -- |
| Neonatal encephalopathy due to birth asphyxia and trauma | 133.2 (101.0 to 172.2) | -- | 18.1 (10.8 to 27.5) | 4.7* (1.1 to 8.8) | 1.6 (-1.9 to 5.6) |
| Neonatal sepsis and other neonatal infections | 41.0 (31.1 to 53.9) | -- | 5.6 (3.0 to 9.3) | 2.2 (-2.2 to 6.9) | -0.7 (-5.0 to 3.8) |
| Neonatal sepsis and other neonatal infection complications | 22.2 (13.3 to 34.0) | -- | 5.6 (3.0 to 9.3) | 2.2 (-2.2 to 6.9) | -0.7 (-5.0 to 3.8) |
| Neonatal sepsis and other neonatal infection episode | 18.8 (14.8 to 23.6) | -- | -- | -- | -- |
| Hemolytic disease and other neonatal jaundice | 37.1 (15.5 to 68.8) | -- | 11.5 (4.7 to 23.4) | 0.0 (-4.0 to 5.1) | -2.9 (-6.8 to 2.1) |

| | | | | | |
|---|---|---|---|---|---|
| Other neonatal disorders | -- | -- | 18.6 (13.7 to 24.6) | 2.8 (-3.2 to 10.2) | -0.2 (-6.0 to 7.0) |
| **Nutritional deficiencies** | **3809.4 (3243.8 to 4334.7)** | **2179.5 (1868.5 to 2511.3)** | **147.9 (95.3 to 214.1)** | **-15.8* (-19.9 to -11.4)** | **-18.2* (-22.3 to -13.9)** |
| Protein-energy malnutrition | 869.8 (841.6 to 899.7) | 971.6 (941.2 to 1004.0) | 33.8 (22.1 to 48.0) | -19.3* (-22.9 to -15.6) | -21.6* (-25.1 to -18.0) |
| Iodine deficiency | 40.1 (29.4 to 54.2) | 0.1 (0.0 to 0.1) | 0.6 (0.3 to 0.9) | -41.0* (-45.2 to -35.0) | -42.7* (-46.8 to -36.9) |
| Visible goiter without symptoms | 39.8 (29.0 to 53.9) | 0.1 (0.0 to 0.1) | 0.4 (0.2 to 0.8) | -46.3* (-49.4 to -43.2) | -47.8* (-50.8 to -44.8) |
| Visible goiter with complications | 0.4 (0.2 to 0.5) | -- | 0.1 (0.1 to 0.2) | -10.5 (-27.2 to 5.9) | -13.1 (-29.4 to 2.8) |
| Vitamin A deficiency | 3187.4 (2224.4 to 4107.5) | 1207.8 (897.3 to 1538.5) | 79.4 (45.7 to 125.3) | -19.1* (-23.8 to -13.9) | -21.4* (-26.0 to -16.4) |
| Asymptomatic vitamin A deficiency | 971.3 (662.2 to 1259.5) | 1207.8 (897.3 to 1538.5) | -- | -- | -- |
| Vitamin A deficiency complications | -- | -- | -- | -- | -- |
| Vitamin A deficiency with anemia | 2216.7 (1560.5 to 2852.7) | -- | 79.4 (45.7 to 125.3) | -19.1* (-23.8 to -13.9) | -21.4* (-26.0 to -16.4) |
| Dietary iron deficiency | 1087.7 (1013.6 to 1162.9) | -- | 34.2 (22.2 to 50.8) | -1.8 (-12.7 to 10.4) | -4.6 (-15.2 to 7.2) |
| Other nutritional deficiencies | -- | -- | -- | -- | -- |
| **Non-communicable diseases** | **2909.3 (2839.8 to 2981.3)** | **5287.9 (4720.3 to 5935.9)** | **73.4 (53.1 to 97.6)** | **2.0 (-0.4 to 4.7)** | **-0.9 (-3.3 to 1.6)** |
| **Neoplasms** | **41.8 (30.8 to 60.4)** | **2.0 (1.8 to 2.2)** | **2.4 (1.4 to 3.8)** | **106.6* (53.4 to 187.5)** | **100.6* (48.9 to 179.2)** |
| Lip and oral cavity cancer | -- | -- | -- | -- | -- |
| Diagnosis and primary therapy phase of mouth cancer | -- | -- | -- | -- | -- |
| Controlled phase of mouth cancer | -- | -- | -- | -- | -- |
| Metastatic phase of mouth cancer | -- | -- | -- | -- | -- |
| Terminal phase of mouth cancer | -- | -- | -- | -- | -- |
| Nasopharynx cancer | -- | -- | -- | -- | -- |
| Diagnosis and primary therapy phase of nasopharynx cancer | -- | -- | -- | -- | -- |
| Controlled phase of nasopharynx cancer | -- | -- | -- | -- | -- |
| Metastatic phase of nasopharynx cancer | -- | -- | -- | -- | -- |
| Terminal phase of nasopharynx cancer | -- | -- | -- | -- | -- |
| Other pharynx cancer | -- | -- | -- | -- | -- |
| Diagnosis and primary therapy phase of other pharynx cancer | -- | -- | -- | -- | -- |
| Controlled phase of other pharynx cancer | -- | -- | -- | -- | -- |
| Metastatic phase of other pharynx cancer | -- | -- | -- | -- | -- |
| Terminal phase of other pharynx cancer | -- | -- | -- | -- | -- |
| Oesophageal cancer | -- | -- | -- | -- | -- |
| Diagnosis and primary therapy phase of esophageal cancer | -- | -- | -- | -- | -- |
| Controlled phase of esophageal cancer | -- | -- | -- | -- | -- |
| Metastatic phase of esophageal cancer | -- | -- | -- | -- | -- |
| Terminal phase of esophageal cancer | -- | -- | -- | -- | -- |
| Stomach cancer | -- | -- | -- | -- | -- |
| Diagnosis and primary therapy phase of stomach cancer | -- | -- | -- | -- | -- |
| Controlled phase of stomach cancer | -- | -- | -- | -- | -- |
| Metastatic phase of stomach cancer | -- | -- | -- | -- | -- |
| Terminal phase of stomach cancer | -- | -- | -- | -- | -- |
| Colon and rectum cancer | -- | -- | -- | -- | -- |
| Diagnosis and primary therapy phase of colon and rectum cancers | -- | -- | -- | -- | -- |
| Controlled phase of colon and rectum cancers | -- | -- | -- | -- | -- |
| Metastatic phase of colon and rectum cancers | -- | -- | -- | -- | -- |
| Terminal phase of colon and rectum cancers | -- | -- | -- | -- | -- |
| Stoma from colon and rectum cancers, beyond 10 years | -- | -- | -- | -- | -- |
| Liver cancer | -- | -- | -- | -- | -- |
| Liver cancer due to hepatitis B | -- | -- | -- | -- | -- |
| Diagnosis and primary therapy phase of liver cancer due to hepatitis B | -- | -- | -- | -- | -- |
| Controlled phase of liver cancer due to hepatitis B | -- | -- | -- | -- | -- |
| Metastatic phase of liver cancer due to hepatitis B | -- | -- | -- | -- | -- |
| Terminal phase of liver cancer due to hepatitis B | -- | -- | -- | -- | -- |
| Liver cancer due to hepatitis C | -- | -- | -- | -- | -- |
| Diagnosis and primary therapy phase of liver cancer due to hepatitis C | -- | -- | -- | -- | -- |
| Controlled phase of liver cancer due to hepatitis C | -- | -- | -- | -- | -- |
| Metastatic phase of liver cancer due to hepatitis C | -- | -- | -- | -- | -- |
| Terminal phase of liver cancer due to hepatitis C | -- | -- | -- | -- | -- |
| Liver cancer due to alcohol use | -- | -- | -- | -- | -- |
| Diagnosis and primary therapy phase of liver cancer due to alcohol use | -- | -- | -- | -- | -- |
| Controlled phase of liver cancer due to alcohol use | -- | -- | -- | -- | -- |
| Metastatic phase of liver cancer due to alcohol use | -- | -- | -- | -- | -- |

| | | | | | |
|---|---|---|---|---|---|
| Terminal phase of liver cancer due to alcohol use | -- | -- | -- | -- | -- |
| Liver cancer due to NASH | -- | -- | -- | -- | -- |
| Diagnosis and primary therapy phase of liver cancer due to NASH | -- | -- | -- | -- | -- |
| Controlled phase of liver cancer due to NASH | -- | -- | -- | -- | -- |
| Metastatic phase of liver cancer due to NASH | -- | -- | -- | -- | -- |
| Terminal phase of liver cancer due to NASH | -- | -- | -- | -- | -- |
| Liver cancer due to other causes | -- | -- | -- | -- | -- |
| Diagnosis and primary therapy phase of liver cancer due to other causes | -- | -- | -- | -- | -- |
| Controlled phase of liver cancer due to other causes | -- | -- | -- | -- | -- |
| Metastatic phase of liver cancer due to other causes | -- | -- | -- | -- | -- |
| Terminal phase of liver cancer due to other causes | -- | -- | -- | -- | -- |
| Gallbladder and biliary tract cancer | -- | -- | -- | -- | -- |
| Diagnosis and primary therapy phase of gallbladder and biliary tract cancer | -- | -- | -- | -- | -- |
| Controlled phase of gallbladder and biliary tract cancer | -- | -- | -- | -- | -- |
| Metastatic phase of gallbladder and biliary tract cancer | -- | -- | -- | -- | -- |
| Terminal phase of gallbladder and biliary tract cancer | -- | -- | -- | -- | -- |
| Pancreatic cancer | -- | -- | -- | -- | -- |
| Diagnosis and primary therapy phase of pancreatic cancer | -- | -- | -- | -- | -- |
| Controlled phase of pancreatic cancer | -- | -- | -- | -- | -- |
| Metastatic phase of pancreatic cancer | -- | -- | -- | -- | -- |
| Terminal phase of pancreatic cancer | -- | -- | -- | -- | -- |
| Larynx cancer | -- | -- | -- | -- | -- |
| Diagnosis and primary therapy phase of larynx cancer | -- | -- | -- | -- | -- |
| Controlled phase of larynx cancer | -- | -- | -- | -- | -- |
| Metastatic phase of larynx cancer | -- | -- | -- | -- | -- |
| Terminal phase of larynx cancer | -- | -- | -- | -- | -- |
| Laryngectomy from larynx cancer, beyond 10 years | -- | -- | -- | -- | -- |
| Tracheal, bronchus, and lung cancer | -- | -- | -- | -- | -- |
| Diagnosis and primary therapy phase of lung, bronchus, and trachea cancer | -- | -- | -- | -- | -- |
| Controlled phase of lung, bronchus, and trachea cancer | -- | -- | -- | -- | -- |
| Metastatic phase of lung, bronchus, and trachea cancer | -- | -- | -- | -- | -- |
| Terminal phase of lung, bronchus, and trachea cancer | -- | -- | -- | -- | -- |
| Malignant skin melanoma | -- | -- | -- | -- | -- |
| Diagnosis and primary therapy phase of malignant skin melanoma | -- | -- | -- | -- | -- |
| Controlled phase of malignant skin melanoma | -- | -- | -- | -- | -- |
| Metastatic phase of malignant skin melanoma | -- | -- | -- | -- | -- |
| Terminal phase of malignant skin melanoma | -- | -- | -- | -- | -- |
| Non-melanoma skin cancer | -- | -- | -- | -- | -- |
| Non-melanoma skin cancer (squamous-cell carcinoma) | -- | -- | -- | -- | -- |
| Non-melanoma skin cancer (basal-cell carcinoma) | -- | -- | -- | -- | -- |
| Breast cancer | -- | -- | -- | -- | -- |
| Diagnosis and primary therapy phase of breast cancer | -- | -- | -- | -- | -- |
| Controlled phase of breast cancer | -- | -- | -- | -- | -- |
| Metastatic phase of breast cancer | -- | -- | -- | -- | -- |
| Terminal phase of breast cancer | -- | -- | -- | -- | -- |
| Mastectomy from breast cancer, beyond 10 years | -- | -- | -- | -- | -- |
| Cervical cancer | -- | -- | -- | -- | -- |
| Diagnosis and primary therapy phase of cervical cancer | -- | -- | -- | -- | -- |
| Controlled phase of cervical cancer | -- | -- | -- | -- | -- |
| Metastatic phase of cervical cancer | -- | -- | -- | -- | -- |
| Terminal phase of cervical cancer | -- | -- | -- | -- | -- |
| Uterine cancer | -- | -- | -- | -- | -- |
| Diagnosis and primary therapy phase of uterine cancer | -- | -- | -- | -- | -- |
| Controlled phase of uterine cancer | -- | -- | -- | -- | -- |
| Metastatic phase of uterine cancer | -- | -- | -- | -- | -- |
| Terminal phase of uterine cancer | -- | -- | -- | -- | -- |
| Ovarian cancer | -- | -- | -- | -- | -- |
| Diagnosis and primary therapy phase of ovarian cancer | -- | -- | -- | -- | -- |
| Controlled phase of ovarian cancer | -- | -- | -- | -- | -- |
| Metastatic phase of ovarian cancer | -- | -- | -- | -- | -- |

| | | | | | |
|---|---|---|---|---|---|
| Terminal phase of ovarian cancer | -- | -- | -- | -- | -- |
| Prostate cancer | -- | -- | -- | -- | -- |
| Diagnosis and primary therapy phase of prostate cancer | -- | -- | -- | -- | -- |
| Controlled phase of prostate cancer | -- | -- | -- | -- | -- |
| Metastatic phase of prostate cancer | -- | -- | -- | -- | -- |
| Terminal phase of prostate cancer | -- | -- | -- | -- | -- |
| Impotence and Incontinence after 10-year survival from prostate cancer | -- | -- | -- | -- | -- |
| Testicular cancer | -- | -- | -- | -- | -- |
| Diagnosis and primary therapy phase of testicular cancer | -- | -- | -- | -- | -- |
| Controlled phase of testicular cancer | -- | -- | -- | -- | -- |
| Metastatic phase of testicular cancer | -- | -- | -- | -- | -- |
| Terminal phase of testicular cancer | -- | -- | -- | -- | -- |
| Kidney cancer | -- | -- | -- | -- | -- |
| Diagnosis and primary therapy phase of kidney cancer | -- | -- | -- | -- | -- |
| Controlled phase of kidney cancer | -- | -- | -- | -- | -- |
| Metastatic phase of kidney cancer | -- | -- | -- | -- | -- |
| Terminal phase of kidney cancer | -- | -- | -- | -- | -- |
| Bladder cancer | -- | -- | -- | -- | -- |
| Diagnosis and primary therapy phase of bladder cancer | -- | -- | -- | -- | -- |
| Controlled phase of bladder cancer | -- | -- | -- | -- | -- |
| Metastatic phase of bladder cancer | -- | -- | -- | -- | -- |
| Terminal phase of bladder cancer | -- | -- | -- | -- | -- |
| Incontinence from bladder cancer, beyond 10 years | -- | -- | -- | -- | -- |
| Brain and nervous system cancer | 2.9<br>(2.3 to 3.6) | 0.4<br>(0.3 to 0.5) | 0.2<br>(0.1 to 0.3) | 7.1<br>(-9.0 to 33.6) | 4.0<br>(-11.7 to 29.7) |
| Diagnosis and primary therapy phase of brain and nervous system cancers | 0.1<br>(0.1 to 0.2) | 0.4<br>(0.3 to 0.5) | 0.0<br>(0.0 to 0.1) | 12.4<br>(-5.5 to 39.9) | 9.1<br>(-8.2 to 35.9) |
| Controlled phase of brain and nervous system cancers | 2.8<br>(2.2 to 3.4) | -- | 0.1<br>(0.1 to 0.2) | 12.3<br>(-5.8 to 40.1) | 9.1<br>(-8.5 to 36.1) |
| Metastatic phase of brain and nervous system cancers | -- | -- | -- | -24.8*<br>(-35.0 to -9.8) | -27.0*<br>(-36.9 to -12.4) |
| Terminal phase of brain and nervous system cancers | -- | -- | -- | -24.8*<br>(-35.0 to -9.8) | -27.0*<br>(-36.9 to -12.4) |
| Thyroid cancer | -- | -- | -- | -- | -- |
| Diagnosis and primary therapy phase of thyroid cancer | -- | -- | -- | -- | -- |
| Controlled phase of thyroid cancer | -- | -- | -- | -- | -- |
| Metastatic phase of thyroid cancer | -- | -- | -- | -- | -- |
| Terminal phase of thyroid cancer | -- | -- | -- | -- | -- |
| Mesothelioma | -- | -- | -- | -- | -- |
| Diagnosis and primary therapy phase of mesothelioma | -- | -- | -- | -- | -- |
| Controlled phase of mesothelioma | -- | -- | -- | -- | -- |
| Metastatic phase of mesothelioma | -- | -- | -- | -- | -- |
| Terminal phase of mesothelioma | -- | -- | -- | -- | -- |
| Hodgkin lymphoma | -- | -- | -- | -- | -- |
| Diagnosis and primary therapy phase of Hodgkin disease | -- | -- | -- | -- | -- |
| Controlled phase of Hodgkin disease | -- | -- | -- | -- | -- |
| Metastatic phase of Hodgkin disease | -- | -- | -- | -- | -- |
| Terminal phase of Hodgkin disease | -- | -- | -- | -- | -- |
| Non-Hodgkin's lymphoma | -- | -- | -- | -- | -- |
| Diagnosis and primary therapy phase of non-Hodgkin lymphoma | -- | -- | -- | -- | -- |
| Controlled phase of non-Hodgkin lymphoma | -- | -- | -- | -- | -- |
| Metastatic phase of non-Hodgkin lymphoma | -- | -- | -- | -- | -- |
| Terminal phase of non-Hodgkin lymphoma | -- | -- | -- | -- | -- |
| Multiple myeloma | -- | -- | -- | -- | -- |
| Diagnosis and primary therapy phase of multiple myeloma | -- | -- | -- | -- | -- |
| Controlled phase of multiple myeloma | -- | -- | -- | -- | -- |
| Metastatic phase of multiple myeloma | -- | -- | -- | -- | -- |
| Terminal phase of multiple myeloma | -- | -- | -- | -- | -- |
| Leukaemia | 0.2<br>(0.2 to 0.3) | 0.1<br>(0.1 to 0.1) | -- | -6.2<br>(-24.3 to 16.1) | -8.9<br>(-26.5 to 12.7) |
| Acute lymphoid leukaemia | -- | -- | -- | -- | -- |
| Diagnosis and primary therapy phase of acute lymphoid leukemia | -- | -- | -- | -- | -- |
| Controlled phase of acute lymphoid leukemia | -- | -- | -- | -- | -- |
| Metastatic phase of acute lymphoid leukemia | -- | -- | -- | -- | -- |
| Terminal phase of acute lymphoid leukemia | -- | -- | -- | -- | -- |
| Chronic lymphoid leukaemia | -- | -- | -- | -- | -- |

| | | | | | |
|---|---|---|---|---|---|
| Diagnosis and primary therapy phase of chronic lymphoid leukemia | -- | -- | -- | -- | -- |
| Controlled phase of chronic lymphoid leukemia | -- | -- | -- | -- | -- |
| Metastatic phase of chronic lymphoid leukemia | -- | -- | -- | -- | -- |
| Terminal phase of chronic lymphoid leukemia | -- | -- | -- | -- | -- |
| Acute myeloid leukaemia | 0.2 (0.2 to 0.3) | 0.1 (0.1 to 0.1) | -- | -6.2 (-24.3 to 16.1) | -8.9 (-26.5 to 12.7) |
| Diagnosis and primary therapy phase of acute myeloid leukemia | -- | 0.1 (0.1 to 0.1) | -- | -- | -- |
| Controlled phase of acute myeloid leukemia | 0.2 (0.2 to 0.2) | -- | -- | -3.7 (-22.7 to 20.2) | -6.5 (-25.0 to 16.7) |
| Metastatic phase of acute myeloid leukemia | -- | -- | -- | -7.2 (-25.2 to 15.3) | -9.9 (-27.4 to 12.0) |
| Terminal phase of acute myeloid leukemia | -- | -- | -- | -7.2 (-25.2 to 15.3) | -9.9 (-27.3 to 12.0) |
| Chronic myeloid leukaemia | -- | -- | -- | -- | -- |
| Diagnosis and primary therapy phase of chronic myeloid leukemia | -- | -- | -- | -- | -- |
| Controlled phase of chronic myeloid leukemia | -- | -- | -- | -- | -- |
| Metastatic phase of chronic myeloid leukemia | -- | -- | -- | -- | -- |
| Terminal phase of chronic myeloid leukemia | -- | -- | -- | -- | -- |
| Other leukaemia | -- | -- | -- | -- | -- |
| Metastatic phase of other leukemia | -- | -- | -- | -- | -- |
| Diagnosis and primary therapy phase of other leukemia | -- | -- | -- | -- | -- |
| Terminal phase of other leukemia | -- | -- | -- | -- | -- |
| Controlled phase of other leukemia | -- | -- | -- | -- | -- |
| Other malignant cancers | 39.5 (28.3 to 58.2) | 1.5 (1.3 to 1.6) | 2.1 (1.2 to 3.5) | 129.6* (64.5 to 230.5) | 122.9* (59.7 to 220.9) |
| Diagnosis and primary therapy phase of other malignant neoplasms | 0.7 (0.5 to 1.0) | 1.5 (1.3 to 1.6) | 0.2 (0.1 to 0.3) | 138.4* (68.0 to 251.2) | 131.5* (63.1 to 241.0) |
| Controlled phase of other malignant neoplasms | 38.8 (27.7 to 57.3) | -- | 1.9 (1.0 to 3.2) | 140.0* (69.2 to 244.5) | 133.0* (64.3 to 234.5) |
| Metastatic phase of other malignant neoplasms | 0.1 (0.1 to 0.1) | -- | 0.0 (0.0 to 0.1) | -9.4 (-23.9 to 3.1) | -12.0 (-26.1 to 0.1) |
| Terminal phase of other malignant neoplasms | -- | -- | -- | -9.4 (-23.9 to 3.1) | -12.0 (-26.1 to 0.1) |
| Other neoplasms | 0.1 (0.1 to 0.1) | 6.8 (6.5 to 7.2) | -- | -37.9* (-43.5 to -34.1) | -39.7* (-45.1 to -36.0) |
| Myelodysplastic, myeloproliferative, and other hematopoietic neoplasms | -- | 0.1 (0.1 to 0.1) | -- | -37.9* (-43.5 to -34.1) | -39.7* (-45.1 to -36.0) |
| Benign and in situ intestinal neoplasms | -- | 0.1 (0.1 to 0.1) | -- | -- | -- |
| Benign and in situ cervical and uterine neoplasms | -- | -- | -- | -- | -- |
| Other benign and in situ neoplasms | 0.3 (0.3 to 0.3) | 6.6 (6.3 to 7.0) | -- | -- | -- |
| **Cardiovascular diseases** | **--** | **3.2 (2.7 to 3.8)** | **--** | **-46.4* (-50.9 to -42.1)** | **-48.0* (-52.3 to -43.7)** |
| Rheumatic heart disease | -- | -- | -- | -- | -- |
| Rheumatic heart disease, without heart failure | -- | -- | -- | -- | -- |
| Heart failure due to rheumatic heart disease | -- | -- | -- | -- | -- |
| Ischaemic heart disease | -- | -- | -- | -- | -- |
| Asymptomatic ischemic heart disease following myocardial infarction | -- | -- | -- | -- | -- |
| Angina due to ischemic heart disease | -- | -- | -- | -- | -- |
| Heart failure due to ischemic heart disease | -- | -- | -- | -- | -- |
| Myocardial infarction due to ischemic heart disease | -- | -- | -- | -- | -- |
| Stroke | -- | 0.8‡ (0.6 to 1.1) | -- | -53.6* (-58.0 to -49.2) | -55.0* (-59.3 to -50.7) |
| Ischaemic stroke | 0.1 (0.0 to 0.1) | 0.7 (0.5 to 1.0) | -- | -55.3* (-60.0 to -50.6) | -56.6* (-61.1 to -52.1) |
| Acute ischemic stroke | -- | 0.7 (0.5 to 1.0) | -- | -55.8* (-62.6 to -48.8) | -57.1* (-63.7 to -50.3) |
| Chronic ischemic stroke | -- | -- | -- | -54.6* (-59.7 to -48.9) | -55.9* (-60.8 to -50.4) |
| Intracerebral hemorrhage | -- | -- | -- | -- | -- |
| Acute intracerebral hemorrhage | -- | -- | -- | -- | -- |
| Chronic intracerebral hemorrhage | -- | -- | -- | -- | -- |
| Subarachnoid hemorrhage | -- | 0.1 (0.1 to 0.2) | -- | -33.2* (-57.5 to -19.4) | -35.1* (-58.8 to -21.7) |
| Acute subarachnoid hemorrhage | -- | 0.1 (0.1 to 0.2) | -- | -41.5* (-65.0 to -24.3) | -43.2* (-66.0 to -26.4) |
| Chronic subarachnoid hemorrhage | -- | -- | -- | -15.2* (-25.5 to -7.2) | -17.7* (-27.6 to -9.9) |
| Hypertensive heart disease | -- | -- | -- | -- | -- |
| Non-rheumatic valvular heart disease | -- | -- | -- | -- | -- |
| Non-rheumatic calcific aortic valve disease | -- | -- | -- | -- | -- |
| Heart failure due to calcific aortic valve disease | -- | -- | -- | -- | -- |
| Non-rheumatic calcific aortic valve disease | -- | -- | -- | -- | -- |
| Non-rheumatic degenerative mitral valve disease | -- | -- | -- | -- | -- |
| Heart failure due to degenerative mitral valve disease | -- | -- | -- | -- | -- |
| Non-rheumatic degenerative mitral valve disease | -- | -- | -- | -- | -- |
| Other non-rheumatic valve diseases | -- | -- | -- | -- | -- |
| Mild heart failure due to other non-rheumatic valve disease | -- | -- | -- | -- | -- |
| Moderate heart failure due to other non-rheumatic valve disease | -- | -- | -- | -- | -- |
| Severe heart failure due to other non-rheumatic valve disease | -- | -- | -- | -- | -- |

| | | | | | |
|---|---|---|---|---|---|
| Controlled, medically managed heart failure due to other non-rheumatic valve disease | -- | -- | -- | -- | -- |
| Cardiomyopathy and myocarditis | -- | -- | -- | -- | -- |
| Myocarditis | -- | -- | -- | -- | -- |
| Acute myocarditis | -- | -- | -- | -- | -- |
| Heart failure due to myocarditis | -- | -- | -- | -- | -- |
| Alcoholic cardiomyopathy | -- | -- | -- | -- | -- |
| Other cardiomyopathy | -- | -- | -- | -- | -- |
| Atrial fibrillation and flutter | -- | -- | -- | -- | -- |
| Asymptomatic atrial fibrillation and flutter | -- | -- | -- | -- | -- |
| Symptomatic atrial fibrillation and flutter | -- | -- | -- | -- | -- |
| Peripheral vascular disease | -- | -- | -- | -- | -- |
| Asymptomatic peripheral arterial disease | -- | -- | -- | -- | -- |
| Symptomatic claudication due to peripheral arterial disease | -- | -- | -- | -- | -- |
| Endocarditis | 0.1 (0.1 to 0.1) | 2.4 (1.9 to 2.9) | -- | 10.8* (3.2 to 17.3) | 7.6* (0.2 to 13.9) |
| Acute endocarditis | 0.1 (0.1 to 0.1) | 2.4 (1.9 to 2.9) | -- | 10.5* (2.4 to 17.3) | 7.3 (-0.6 to 13.9) |
| Heart failure due to endocarditis | -- | -- | -- | 15.1* (10.0 to 19.6) | 11.8* (6.8 to 16.1) |
| Other cardiovascular and circulatory diseases | -- | -- | -- | -- | -- |
| Heart failure due to other cardiovascular diseases | -- | -- | -- | -- | -- |
| Other cardiovascular and circulatory disease episodes | -- | -- | -- | -- | -- |
| **Chronic respiratory diseases** | **--** | **--** | **--** | **--** | **--** |
| Chronic obstructive pulmonary disease | -- | -- | -- | -- | -- |
| Chronic obstructive pulmonary disease with heart failure | -- | -- | -- | -- | -- |
| Chronic obstructive pulmonary disease without heart failure | -- | -- | -- | -- | -- |
| Pneumoconiosis | -- | -- | -- | -- | -- |
| Silicosis | -- | -- | -- | -- | -- |
| Heart failure due to silicosis | -- | -- | -- | -- | -- |
| Silicosis without heart failure | -- | -- | -- | -- | -- |
| Asbestosis | -- | -- | -- | -- | -- |
| Asbestosis without heart failure | -- | -- | -- | -- | -- |
| Heart failure due to asbestosis | -- | -- | -- | -- | -- |
| Coal workers pneumoconiosis | -- | -- | -- | -- | -- |
| Coal workers pneumoconiosis without heart failure | -- | -- | -- | -- | -- |
| Heart failure due to Coal workers pneumoconiosis | -- | -- | -- | -- | -- |
| Other pneumoconiosis | -- | -- | -- | -- | -- |
| Heart failure due to other pneumoconiosis | -- | -- | -- | -- | -- |
| Other pneumoconiosis without heart failure | -- | -- | -- | -- | -- |
| Asthma | -- | -- | -- | -- | -- |
| Asymptomatic asthma | -- | -- | -- | -- | -- |
| Symptomatic asthma | -- | -- | -- | -- | -- |
| Interstitial lung disease and pulmonary sarcoidosis | -- | -- | -- | -- | -- |
| Heart failure due to interstitial lung disease and pulmonary sarcoidosis | -- | -- | -- | -- | -- |
| Interstitial lung disease and pulmonary sarcoidosis without heart failure | -- | -- | -- | -- | -- |
| Other chronic respiratory diseases | -- | -- | -- | -- | -- |
| **Digestive diseases** | **195.8 (147.5 to 244.8)** | **36.7 (31.9 to 42.5)** | **3.8 (2.3 to 6.1)** | **3.0 (-0.1 to 5.8)** | **0.0 (-3.0 to 2.7)** |
| Cirrhosis and other chronic liver diseases | 161.6 (115.1 to 208.7) | 16.2 (13.9 to 18.5) | -- | 5.1* (4.4 to 5.7) | 2.0* (1.4 to 2.7) |
| Cirrhosis and other chronic liver diseases due to hepatitis B | 159.9 (113.4 to 207.0) | -- | -- | -6.6* (-11.1 to -0.2) | -9.3* (-13.7 to -3.1) |
| Cirrhosis and other chronic liver diseases due to hepatitis B, decompensated | -- | -- | -- | -6.6* (-11.1 to -0.2) | -9.3* (-13.7 to -3.1) |
| Chronic hepatitis B without cirrhosis | 159.9 (113.4 to 207.0) | -- | -- | -- | -- |
| Cirrhosis and other chronic liver diseases due to hepatitis B, compensated | -- | -- | -- | -- | -- |
| Cirrhosis and other chronic liver diseases due to hepatitis C | 0.9 (0.6 to 1.4) | -- | -- | 3.0 (-0.1 to 6.3) | -0.0 (-3.0 to 3.3) |
| Cirrhosis and other chronic liver diseases due to hepatitis C, decompensated | -- | -- | -- | 3.0 (-0.1 to 6.3) | -0.0 (-3.0 to 3.3) |
| Chronic hepatitis C without cirrhosis | 0.9 (0.6 to 1.4) | -- | -- | -- | -- |
| Cirrhosis and other chronic liver diseases due to hepatitis C, compensated | -- | -- | -- | -- | -- |
| Cirrhosis and other chronic liver diseases due to alcohol use | -- | -- | -- | -- | -- |
| Cirrhosis and other chronic liver diseases due to alcohol, decompensated | -- | -- | -- | -- | -- |
| Cirrhosis and other chronic liver diseases due to alcohol, compensated | -- | -- | -- | -- | -- |
| Cirrhosis due to NASH | -- | -- | -- | -- | -- |
| Non-alcoholic fatty liver disease (NAFLD) / Non-alcoholic steatohepatitis (NASH) | -- | -- | -- | -- | -- |
| Cirrhosis and other chronic liver diseases due to NASH | -- | -- | -- | -- | -- |
| Cirrhosis and other chronic liver diseases due to other causes | 0.8 (0.7 to 0.9) | 16.2 (13.9 to 18.5) | -- | 5.1* (4.4 to 5.7) | 2.0* (1.4 to 2.7) |

| | | | | | |
|---|---|---|---|---|---|
| Cirrhosis and other chronic liver diseases due to other cause, decompensated | 0.2 (0.2 to 0.2) | 3.9 (3.7 to 4.2) | -- | 5.1* (4.4 to 5.7) | 2.0* (1.4 to 2.7) |
| Cirrhosis and other chronic liver diseases due to other cause, compensated | 0.6 (0.5 to 0.7) | 12.2 (10.0 to 14.5) | -- | -- | -- |
| Upper digestive system diseases | -- | -- | -- | -- | -- |
| Peptic ulcer disease | -- | -- | -- | -- | -- |
| Acute peptic ulcer disease | -- | -- | -- | -- | -- |
| Chronic peptic ulcer disease | -- | -- | -- | -- | -- |
| Gastritis and duodenitis | -- | -- | -- | -- | -- |
| Acute gastritis and duodenitis | -- | -- | -- | -- | -- |
| Chronic gastritis and duodenitis | -- | -- | -- | -- | -- |
| GERD | -- | -- | -- | -- | -- |
| Appendicitis | -- | -- | -- | -- | -- |
| Paralytic ileus and intestinal obstruction | 0.4 (0.3 to 0.5) | 5.5 (4.2 to 7.0) | 0.1 (0.1 to 0.2) | -9.8 (-23.5 to 3.7) | -12.4 (-25.7 to 0.7) |
| Inguinal, femoral, and abdominal hernia | 35.8 (24.4 to 52.3) | 15.0 (11.0 to 20.2) | 3.7 (2.2 to 5.9) | 3.5* (0.4 to 6.3) | 0.5 (-2.5 to 3.3) |
| Inflammatory bowel disease | -- | -- | -- | -- | -- |
| Ulcerative colitis | -- | -- | -- | -- | -- |
| Crohn's disease | -- | -- | -- | -- | -- |
| Vascular intestinal disorders | -- | -- | -- | -- | -- |
| Gallbladder and biliary diseases | -- | -- | -- | -- | -- |
| Pancreatitis | -- | -- | -- | -- | -- |
| Acute pancreatitis | -- | -- | -- | -- | -- |
| Chronic pancreatitis | -- | -- | -- | -- | -- |
| Other digestive diseases | -- | -- | -- | -- | -- |
| **Neurological disorders** | **21.0 (13.8 to 29.8)** | **6.5 (4.5 to 8.8)** | **7.3 (4.3 to 11.6)** | **8.8 (-8.9 to 29.3)** | **5.7 (-11.6 to 25.6)** |
| Alzheimer's disease and other dementias | -- | -- | -- | -- | -- |
| Parkinson's disease | -- | -- | -- | -- | -- |
| Epilepsy | 21.0 (13.9 to 29.7) | 6.2 (4.2 to 8.5) | 7.3 (4.3 to 11.6) | 8.8 (-8.9 to 29.3) | 5.7 (-11.6 to 25.6) |
| Multiple sclerosis | -- | -- | -- | -- | -- |
| Motor neuron disease | -- | 0.3 (0.2 to 0.3) | -- | -6.1* (-8.1 to -3.7) | -8.8* (-10.7 to -6.5) |
| Headache disorders | -- | -- | -- | -- | -- |
| Migraine | -- | -- | -- | -- | -- |
| Medication overuse headache due to migraine | -- | -- | -- | -- | -- |
| Migraine | -- | -- | -- | -- | -- |
| Tension-type headache | -- | -- | -- | -- | -- |
| Medication overuse headache due to tension-type headache | -- | -- | -- | -- | -- |
| Tension-type headache | -- | -- | -- | -- | -- |
| Other neurological disorders | -- | -- | -- | -- | -- |
| Other neurological disorders | -- | -- | -- | -- | -- |
| Guillain-Barré syndrome due to other neurological disorders | -- | -- | -- | -- | -- |
| **Mental disorders** | **160.0 (108.1 to 216.7)** | **--** | **11.2 (7.1 to 16.4)** | **-7.2* (-9.2 to -4.4)** | **-9.8* (-11.9 to -7.2)** |
| Schizophrenia | -- | -- | -- | -- | -- |
| Depressive disorders | -- | -- | -- | -- | -- |
| Major depressive disorder | -- | -- | -- | -- | -- |
| Dysthymia | -- | -- | -- | -- | -- |
| Bipolar disorder | -- | -- | -- | -- | -- |
| Anxiety disorders | -- | -- | -- | -- | -- |
| Eating disorders | -- | -- | -- | -- | -- |
| Anorexia nervosa | -- | -- | -- | -- | -- |
| Bulimia nervosa | -- | -- | -- | -- | -- |
| Autism spectrum disorders | 38.6 (34.6 to 42.5) | -- | 6.0 (4.1 to 8.4) | 3.5* (1.3 to 5.6) | 0.5 (-1.6 to 2.5) |
| Attention-deficit/hyperactivity disorder | -- | -- | -- | -- | -- |
| Conduct disorder | -- | -- | -- | -- | -- |
| Idiopathic developmental intellectual disability | 124.3 (69.7 to 182.8) | -- | 5.2 (2.4 to 9.0) | -17.1* (-20.9 to -14.6) | -19.5* (-23.2 to -17.0) |
| Other mental disorders | -- | -- | -- | -- | -- |
| **Substance use disorders** | **0.1 (0.0 to 0.1)** | **--** | **--** | **-10.6* (-16.7 to -5.0)** | **-13.2* (-19.1 to -7.8)** |
| Alcohol use disorders | 0.4 (0.3 to 0.4) | -- | -- | -10.6* (-16.7 to -5.0) | -13.2* (-19.1 to -7.8) |
| Alcohol dependence | -- | -- | -- | -- | -- |
| Fetal alcohol syndrome | 0.4 (0.3 to 0.4) | -- | -- | -10.6* (-16.7 to -5.0) | -13.2* (-19.1 to -7.8) |
| Drug use disorders | -- | -- | -- | -- | -- |
| Opioid use disorders | -- | -- | -- | -- | -- |
| Cocaine use disorders | -- | -- | -- | -- | -- |

| | | | | | |
|---|---|---|---|---|---|
| Amphetamine use disorders | -- | -- | -- | -- | -- |
| Cannabis use disorders | -- | -- | -- | -- | -- |
| Other drug use disorders | -- | -- | -- | -- | -- |
| **Diabetes and kidney diseases** | **--** | **5.8 (4.0 to 8.0)** | **--** | **-3.5 (-16.9 to 12.1)** | **-6.3 (-19.3 to 8.9)** |
| Diabetes mellitus | -- | 0.3 (0.2 to 0.5) | -- | -- | -- |
| Diabetes mellitus type 1 | -- | 0.3 (0.2 to 0.5) | -- | -- | -- |
| Uncomplicated diabetes mellitus type 1 | -- | 0.3 (0.2 to 0.5) | -- | -- | -- |
| Vision loss due to diabetes mellitus type 1 retinopathy | -- | -- | -- | -- | -- |
| Diabetic neuropathy due to diabetes mellitus type 1 | -- | -- | -- | -- | -- |
| Diabetes mellitus type 2 | -- | -- | -- | -- | -- |
| Uncomplicated diabetes mellitus type 2 | -- | -- | -- | -- | -- |
| Vision loss due to diabetes mellitus type 2 retinopathy | -- | -- | -- | -- | -- |
| Diabetic neuropathy due to diabetes mellitus type 2 | -- | -- | -- | -- | -- |
| Chronic kidney disease | 0.2 (0.2 to 0.3) | 5.0 (3.3 to 7.2) | -- | -3.4 (-17.3 to 12.5) | -6.2 (-19.7 to 9.2) |
| Chronic kidney disease due to diabetes mellitus type 1 | -- | 0.3 (0.2 to 0.5) | -- | -4.5 (-19.8 to 11.6) | -7.3 (-22.2 to 8.4) |
| Albuminuria with preserved GFR due to type 1 diabetes mellitus | -- | -- | -- | -- | -- |
| End-stage chronic kidney disease due to diabetes mellitus type 1 | -- | -- | -- | 1.3 (-1.4 to 3.9) | -1.7 (-4.2 to 0.9) |
| Stage III chronic kidney disease due to diabetes mellitus type 1 | -- | 0.3 (0.2 to 0.5) | -- | -6.2 (-23.0 to 11.7) | -8.9 (-25.2 to 8.5) |
| Stage IV chronic kidney disease due to diabetes mellitus type 1 | -- | -- | -- | -- | -- |
| Stage V chronic kidney disease due to diabetes mellitus type 1 | -- | -- | -- | -3.9 (-19.0 to 12.0) | -6.7 (-21.4 to 8.8) |
| Chronic kidney disease due to diabetes mellitus type 2 | -- | 0.0 (0.0 to 0.1) | -- | -3.6 (-17.9 to 12.4) | -6.4 (-20.3 to 9.1) |
| Albuminuria with preserved GFR due to type 2 diabetes mellitus | -- | -- | -- | -- | -- |
| End-stage chronic kidney disease due to diabetes mellitus type 2 | -- | -- | -- | 1.2 (-1.7 to 4.0) | -1.8 (-4.5 to 1.0) |
| Stage III chronic kidney disease due to diabetes mellitus type 2 | -- | 0.0 (0.0 to 0.1) | -- | -4.8 (-20.4 to 12.6) | -7.6 (-22.7 to 9.3) |
| Stage IV chronic kidney disease due to diabetes mellitus type 2 | -- | -- | -- | -- | -- |
| Stage V chronic kidney disease due to diabetes mellitus type 2 | -- | -- | -- | -3.3 (-18.6 to 13.4) | -6.1 (-20.9 to 10.1) |
| Chronic kidney disease due to hypertension | 0.0 (0.0 to 0.1) | 1.2 (0.8 to 1.7) | -- | -3.8 (-17.8 to 12.0) | -6.6 (-20.2 to 8.7) |
| Albuminuria with preserved GFR due to hypertension | -- | -- | -- | -- | -- |
| End-stage chronic kidney disease due to hypertension | -- | -- | -- | 2.9 (-0.4 to 6.1) | -0.1 (-3.3 to 3.0) |
| Stage III chronic kidney disease due to hypertension | 0.0 (0.0 to 0.1) | 1.2 (0.8 to 1.7) | -- | -4.6 (-20.1 to 13.4) | -7.4 (-22.4 to 10.1) |
| Stage IV chronic kidney disease due to hypertension | -- | -- | -- | -- | -- |
| Stage V chronic kidney disease due to hypertension | -- | -- | -- | -3.7 (-17.9 to 12.1) | -6.5 (-20.3 to 8.8) |
| Chronic kidney disease due to glomerulonephritis | -- | 0.2 (0.1 to 0.2) | -- | -3.8 (-18.0 to 12.5) | -6.6 (-20.4 to 9.3) |
| Albuminuria with preserved GFR due to glomerulonephritis | -- | -- | -- | -- | -- |
| End-stage chronic kidney disease due to glomerulonephritis | -- | -- | -- | 0.7 (-1.5 to 2.8) | -2.2* (-4.3 to -0.2) |
| Stage III chronic kidney disease due to glomerulonephritis | -- | 0.2 (0.1 to 0.2) | -- | -6.2 (-23.2 to 11.7) | -8.9 (-25.4 to 8.5) |
| Stage IV chronic kidney disease due to glomerulonephritis | -- | -- | -- | -- | -- |
| Stage V chronic kidney disease due to glomerulonephritis | -- | -- | -- | -3.6 (-18.6 to 13.5) | -6.4 (-20.9 to 10.2) |
| Chronic kidney disease due to other and unspecified causes | 0.2 (0.1 to 0.2) | 3.3 (2.2 to 4.7) | -- | -3.1 (-16.7 to 12.7) | -5.9 (-19.1 to 9.4) |
| Albuminuria with preserved GFR due to other and unspecified causes | -- | -- | -- | -- | -- |
| End-stage chronic kidney disease due to other causes | -- | -- | -- | -0.5 (-2.9 to 1.4) | -3.4* (-5.8 to -1.5) |
| Stage III chronic kidney disease due to other causes | 0.1 (0.1 to 0.2) | 3.3 (2.2 to 4.7) | -- | -3.6 (-19.5 to 14.1) | -6.4 (-21.9 to 10.8) |
| Stage IV chronic kidney disease due to other causes | -- | -- | -- | -- | -- |
| Stage V chronic kidney disease due to other causes | -- | -- | -- | -3.1 (-17.9 to 13.6) | -5.9 (-20.3 to 10.3) |
| Acute glomerulonephritis | -- | 0.4 (0.3 to 0.6) | -- | -7.0* (-10.3 to -4.5) | -9.7* (-12.9 to -7.3) |
| **Skin and subcutaneous diseases** | **201.3 (177.6 to 228.4)** | **5041.5 (4482.6 to 5699.4)** | **2.7 (1.6 to 4.2)** | **-1.3 (-3.9 to 1.9)** | **-4.2* (-6.7 to -1.1)** |
| Dermatitis | -- | -- | -- | -- | -- |
| Atopic dermatitis | -- | -- | -- | -- | -- |
| Contact dermatitis | -- | -- | -- | -- | -- |
| Seborrhoeic dermatitis | -- | -- | -- | -- | -- |
| Psoriasis | -- | -- | -- | -- | -- |
| Bacterial skin diseases | 17.1 (16.1 to 18.0) | 651.1 (618.2 to 683.1) | 0.2 (0.1 to 0.3) | 5.8* (3.0 to 9.0) | 2.8* (0.0 to 5.8) |
| Cellulitis | 1.0 (0.8 to 1.1) | 33.1 (28.4 to 38.1) | 0.1 (0.0 to 0.1) | 0.2 (-0.8 to 1.2) | -2.7* (-3.7 to -1.7) |
| Pyoderma | 17.0 (16.2 to 17.9) | 618.0 (587.2 to 648.6) | 0.1 (0.0 to 0.2) | 9.4* (5.5 to 13.8) | 6.2* (2.4 to 10.5) |
| Impetigo | 7.1 (6.5 to 7.6) | 254.1 (235.1 to 271.2) | 0.0 (0.0 to 0.1) | 10.2* (4.4 to 16.4) | 7.0* (1.3 to 13.0) |
| Abscess and other bacterial skin diseases | 9.9 (9.3 to 10.6) | 363.9 (341.0 to 388.7) | 0.1 (0.0 to 0.1) | 8.9* (3.8 to 14.2) | 5.7* (0.8 to 10.9) |
| Scabies | 21.0 (16.2 to 26.5) | 507.2 (389.8 to 645.1) | 0.5 (0.3 to 0.9) | 2.5 (-0.6 to 5.6) | -0.5 (-3.5 to 2.5) |
| Fungal skin diseases | 116.8 (95.0 to 142.9) | 2792.1 (2265.8 to 3392.7) | 0.7 (0.3 to 1.4) | -13.7* (-15.6 to -11.5) | -16.2* (-18.0 to -14.1) |
| Tinea capitis | 86.6 (65.7 to 112.8) | 2060.8 (1555.4 to 2671.0) | 0.5 (0.2 to 1.0) | -18.6* (-20.6 to -16.9) | -21.0* (-22.9 to -19.3) |
| Other fungal skin diseases | 30.2 (22.7 to 38.4) | 731.3 (555.5 to 937.8) | 0.2 (0.1 to 0.4) | 4.6* (2.0 to 7.2) | 1.5 (-1.0 to 4.1) |

| | | | | | |
|---|---|---|---|---|---|
| Viral skin diseases | -- | -- | -- | -- | -- |
| Viral warts | -- | -- | -- | -- | -- |
| Molluscum contagiosum | -- | -- | -- | -- | -- |
| Acne vulgaris | -- | -- | -- | -- | -- |
| Alopecia areata | -- | -- | -- | -- | -- |
| Pruritus | 1.6<br>(1.2 to 2.2) | 34.9<br>(25.8 to 47.6) | -- | 4.1<br>(-6.0 to 15.4) | 1.0<br>(-8.7 to 12.1) |
| Urticaria | 19.1<br>(14.5 to 24.6) | 438.0<br>(334.3 to 565.8) | 1.2<br>(0.7 to 1.8) | 3.1<br>(-1.3 to 7.7) | 0.1<br>(-4.1 to 4.5) |
| Decubitus ulcer | -- | -- | -- | -- | -- |
| Other skin and subcutaneous diseases | 27.9<br>(25.7 to 29.8) | 618.3<br>(571.1 to 660.7) | 0.2<br>(0.1 to 0.3) | 7.5*<br>(3.9 to 11.1) | 4.4*<br>(0.9 to 7.9) |
| **Sense organ diseases** | **106.5<br>(85.7 to 132.5)** | **--** | **5.1<br>(3.3 to 7.3)** | **-1.7<br>(-5.2 to 2.5)** | **-4.5*<br>(-8.0 to -0.5)** |
| Blindness and vision impairment | 51.8<br>(31.3 to 78.3) | -- | 2.4<br>(1.3 to 3.9) | -5.9*<br>(-10.3 to -2.2) | -8.7*<br>(-12.9 to -5.0) |
| Glaucoma | -- | -- | -- | -- | -- |
| Cataract | -- | -- | -- | -- | -- |
| Age-related macular degeneration | -- | -- | -- | -- | -- |
| Refraction disorders | 19.0<br>(11.0 to 30.1) | -- | 0.6<br>(0.3 to 1.1) | -11.4*<br>(-17.3 to -6.8) | -14.0*<br>(-19.7 to -9.5) |
| Near vision loss | -- | -- | -- | -- | -- |
| Other vision loss | 33.4<br>(20.2 to 51.9) | -- | 1.7<br>(0.9 to 2.8) | -3.8<br>(-8.5 to 0.3) | -6.6*<br>(-11.1 to -2.6) |
| Age-related and other hearing loss | 11.6<br>(9.9 to 13.5) | -- | 1.9<br>(1.2 to 2.6) | 1.3<br>(-7.1 to 10.2) | -1.7<br>(-9.8 to 7.0) |
| Other sense organ diseases | 45.9<br>(43.2 to 48.9) | -- | 0.8<br>(0.5 to 1.3) | 4.8*<br>(1.1 to 8.6) | 1.8<br>(-1.8 to 5.5) |
| Chronic other sense organ diseases | 15.4<br>(13.7 to 17.3) | -- | 0.4<br>(0.2 to 0.6) | 5.5<br>(-0.9 to 12.3) | 2.5<br>(-3.8 to 9.0) |
| Acute other sense organ diseases | 30.5<br>(28.5 to 32.8) | -- | 0.4<br>(0.3 to 0.7) | 4.1<br>(-0.0 to 8.4) | 1.1<br>(-2.9 to 5.3) |
| **Musculoskeletal disorders** | **--** | **--** | **--** | **--** | **--** |
| Rheumatoid arthritis | -- | -- | -- | -- | -- |
| Osteoarthritis | -- | -- | -- | -- | -- |
| Osteoarthritis of the hip | -- | -- | -- | -- | -- |
| Osteoarthritis of the knee | -- | -- | -- | -- | -- |
| Low back pain | -- | -- | -- | -- | -- |
| Low back pain with leg pain | -- | -- | -- | -- | -- |
| Low back pain without leg pain | -- | -- | -- | -- | -- |
| Neck pain | -- | -- | -- | -- | -- |
| Gout | -- | -- | -- | -- | -- |
| Other musculoskeletal disorders | -- | -- | -- | -- | -- |
| **Other non-communicable diseases** | **2432.1<br>(2378.9 to 2489.3)** | **185.3<br>(149.1 to 228.7)** | **40.9<br>(29.5 to 53.5)** | **1.4<br>(-0.8 to 3.6)** | **-1.6<br>(-3.7 to 0.6)** |
| Congenital anomalies | 304.4<br>(285.5 to 326.1) | -- | 31.7<br>(23.0 to 41.5) | 4.1*<br>(2.5 to 5.8) | 1.1<br>(-0.4 to 2.7) |
| Neural tube defects | 7.2<br>(6.3 to 8.1) | -- | 2.1<br>(1.4 to 2.8) | 12.9*<br>(6.5 to 20.2) | 9.7*<br>(3.4 to 16.7) |
| Severe motor and cognitive impairment due to anencephaly | -- | -- | -- | -6.5*<br>(-7.2 to -5.8) | -9.2*<br>(-9.9 to -8.5) |
| Encephalocoele due to neural tube defects | 1.3<br>(1.0 to 1.7) | -- | 0.2<br>(0.1 to 0.3) | 4.2*<br>(0.5 to 8.1) | 1.2<br>(-2.4 to 5.0) |
| Spina bifida due to neural tube defects | 5.9<br>(5.1 to 6.7) | -- | 1.8<br>(1.3 to 2.5) | 14.1*<br>(7.0 to 22.3) | 10.8*<br>(3.9 to 18.8) |
| Congenital heart anomalies | 130.8<br>(116.1 to 147.0) | -- | 5.8<br>(2.8 to 9.7) | 1.6<br>(-1.5 to 4.2) | -1.3<br>(-4.4 to 1.2) |
| Critical malformations of great vessels, congenital valvular heart disease and patent ductus arteriosus | 28.1<br>(22.0 to 35.1) | -- | 1.4<br>(0.6 to 2.5) | 4.9*<br>(0.4 to 8.8) | 1.9<br>(-2.5 to 5.6) |
| Other congenital heart anomalies | 21.0<br>(18.6 to 23.6) | -- | 1.1<br>(0.5 to 1.8) | 1.7<br>(-2.6 to 5.5) | -1.3<br>(-5.4 to 2.4) |
| Severe congenital heart anomalies excluding single ventricle heart defects | 30.2<br>(24.3 to 37.3) | -- | 1.6<br>(0.8 to 2.7) | 0.0<br>(-4.3 to 3.8) | -2.9<br>(-7.1 to 0.8) |
| Single ventricle and single ventricle pathway congenital heart anomalies | 9.7<br>(7.0 to 13.1) | -- | 0.5<br>(0.2 to 0.9) | -2.7<br>(-8.2 to 2.5) | -5.5*<br>(-10.9 to -0.4) |
| Ventricular septal defect and atrial septal defect | 41.8<br>(33.5 to 51.7) | -- | 1.2<br>(0.6 to 2.1) | 1.9<br>(-2.1 to 5.3) | -1.0<br>(-4.9 to 2.2) |
| Orofacial clefts | 20.2<br>(16.7 to 24.1) | -- | 0.7<br>(0.4 to 1.1) | 2.5<br>(-6.9 to 12.3) | -0.5<br>(-9.6 to 9.1) |
| Down's syndrome | 6.1<br>(5.2 to 7.2) | -- | 0.5<br>(0.3 to 0.8) | 10.1*<br>(4.8 to 16.3) | 6.9*<br>(1.7 to 12.9) |
| Turner syndrome | 1.1<br>(1.0 to 1.3) | -- | -- | 2.2<br>(-6.0 to 11.7) | -0.7<br>(-8.8 to 8.4) |
| Klinefelter syndrome | 1.6<br>(1.4 to 1.9) | -- | -- | -1.3<br>(-10.2 to 7.9) | -4.2<br>(-12.8 to 4.8) |
| Other chromosomal abnormalities | 17.0<br>(13.3 to 20.9) | -- | 1.9<br>(1.3 to 2.6) | 8.7*<br>(3.7 to 13.6) | 5.6*<br>(0.7 to 10.3) |
| Congenital musculoskeletal and limb anomalies | 88.2<br>(76.2 to 102.0) | -- | 11.8<br>(7.7 to 16.5) | 3.2*<br>(1.2 to 5.2) | 0.2<br>(-1.7 to 2.1) |
| Polydactyly and syndactyly | 12.6<br>(9.2 to 17.0) | -- | 0.1<br>(0.1 to 0.3) | 5.0<br>(-0.1 to 9.7) | 1.9<br>(-3.0 to 6.5) |
| Congenital limb deficiency | 22.3<br>(19.6 to 25.7) | -- | 3.4<br>(2.2 to 4.7) | 2.5<br>(-0.3 to 5.5) | -0.5<br>(-3.2 to 2.5) |
| Other congenital musculoskeletal anomalies | 53.3<br>(46.0 to 61.7) | -- | 8.2<br>(5.4 to 11.7) | 3.4*<br>(1.1 to 6.0) | 0.4<br>(-1.8 to 2.9) |
| Urogenital congenital anomalies | 14.2<br>(12.4 to 16.3) | -- | 0.4<br>(0.2 to 0.6) | 9.5*<br>(6.1 to 13.5) | 6.3*<br>(3.0 to 10.2) |
| Digestive congenital anomalies | 19.5<br>(17.6 to 21.8) | -- | 1.1<br>(0.7 to 1.6) | 2.8<br>(-1.7 to 7.2) | -0.2<br>(-4.6 to 4.1) |
| Congenital diaphragmatic hernia | 0.8<br>(0.7 to 0.9) | -- | -- | 0.0<br>(-1.4 to 1.7) | -2.9*<br>(-4.2 to -1.2) |
| Congenital atresia and/or stenosis of the digestive tract | 6.8<br>(4.8 to 8.8) | -- | 0.5<br>(0.3 to 0.8) | 4.9<br>(-1.5 to 11.2) | 1.8<br>(-4.3 to 8.0) |
| Congenital malformations of the abdominal wall after treatment | 4.7<br>(4.1 to 5.5) | -- | 0.2<br>(0.1 to 0.3) | 1.3<br>(-0.6 to 3.4) | -1.7<br>(-3.5 to 0.4) |
| Other congenital malformations of the digestive tract | 7.1<br>(5.2 to 9.9) | -- | 0.3<br>(0.2 to 0.5) | 0.8<br>(-8.8 to 10.9) | -2.1<br>(-11.4 to 7.7) |
| Other congenital anomalies | 29.4<br>(25.5 to 34.0) | -- | 7.4<br>(5.1 to 9.8) | 4.1<br>(-0.9 to 8.8) | 1.0<br>(-3.8 to 5.6) |

| | | | | | |
|---|---|---|---|---|---|
| Other congenital birth defects | -- | -- | 3.3 (1.9 to 4.9) | 11.4* (9.0 to 14.2) | 8.2* (5.8 to 10.9) |
| Hearing loss due to other congenital anomalies | 29.4 (25.5 to 34.0) | -- | 4.1 (2.7 to 5.8) | -1.2 (-9.1 to 6.9) | -4.1 (-11.7 to 3.8) |
| Urinary diseases and male infertility | 2.7 (2.2 to 3.4) | 185.3 (149.1 to 228.7) | 0.2 (0.1 to 0.4) | -17.7* (-25.2 to -9.9) | -20.1* (-27.4 to -12.5) |
| Urinary tract infections | 2.9 (2.3 to 3.6) | 185.3 (149.1 to 228.7) | 0.1 (0.1 to 0.2) | -1.8 (-10.8 to 7.6) | -4.7 (-13.3 to 4.5) |
| Urolithiasis | -- | -- | -- | -- | -- |
| Benign prostatic hyperplasia | -- | -- | -- | -- | -- |
| Male infertility | -- | -- | -- | -- | -- |
| Other urinary diseases | -- | -- | 0.1 (0.1 to 0.2) | -25.8* (-32.6 to -18.7) | -28.0* (-34.5 to -21.1) |
| Gynecological diseases | -- | -- | -- | -- | -- |
| Uterine fibroids | -- | -- | -- | -- | -- |
| Polycystic ovarian syndrome | -- | -- | -- | -- | -- |
| Female infertility | -- | -- | -- | -- | -- |
| Endometriosis | -- | -- | -- | -- | -- |
| Genital prolapse | -- | -- | -- | -- | -- |
| Premenstrual syndrome | -- | -- | -- | -- | -- |
| Other gynecological diseases | -- | -- | -- | -- | -- |
| Other gynecological diseases | -- | -- | -- | -- | -- |
| Other gynecological diseases with anemia | -- | -- | -- | -- | -- |
| Hemoglobinopathies and hemolytic anaemias | 2174.2 (2121.7 to 2231.8) | -- | 7.4 (4.8 to 10.8) | -8.0* (-14.7 to -1.3) | -10.7* (-17.2 to -4.1) |
| Thalassemias | 6.2 (5.8 to 6.7) | -- | 0.2 (0.2 to 0.4) | -13.1* (-21.3 to -4.9) | -15.6* (-23.6 to -7.7) |
| Thalassaemias trait | 286.8 (276.5 to 297.9) | -- | 2.5 (1.6 to 3.7) | -11.4* (-20.1 to -2.9) | -14.0* (-22.4 to -5.7) |
| Sickle cell disorders | 16.4 (14.7 to 17.9) | -- | 0.9 (0.6 to 1.4) | -5.0 (-14.3 to 4.3) | -7.7 (-16.8 to 1.2) |
| Sickle cell trait | 613.5 (560.3 to 672.3) | -- | 2.5 (1.6 to 3.7) | -8.0 (-15.9 to 0.0) | -10.7* (-18.4 to -2.9) |
| G6PD deficiency | 447.6 (428.4 to 467.7) | -- | 0.1 (0.0 to 0.1) | -4.7 (-21.0 to 16.9) | -7.5 (-23.2 to 13.6) |
| G6PD trait | 1031.9 (1014.2 to 1049.9) | -- | -- | -1.6 (-9.4 to 8.0) | -4.4 (-12.0 to 4.9) |
| Other hemoglobinopathies and hemolytic anaemias | 39.6 (36.7 to 42.5) | -- | 1.2 (0.8 to 1.7) | -1.6 (-13.5 to 12.0) | -4.5 (-16.0 to 8.8) |
| Endocrine, metabolic, blood, and immune disorders | 51.9 (48.5 to 55.5) | -- | 1.6 (1.0 to 2.3) | -1.0 (-12.5 to 11.6) | -3.9 (-15.1 to 8.3) |
| Anemia due to endocrine, metabolic, blood, and immune disorders | 50.0 (46.6 to 53.6) | -- | 1.5 (1.0 to 2.2) | -0.8 (-12.7 to 12.4) | -3.7 (-15.2 to 9.2) |
| Endocrine, metabolic, blood, and immune disorders | 1.9 (1.5 to 2.3) | -- | 0.1 (0.0 to 0.1) | -4.5 (-12.9 to 5.1) | -7.3 (-15.4 to 2.0) |
| Heart failure due to endocrine, metabolic, blood, and immune disorders | -- | -- | -- | 2.0* (0.7 to 3.3) | -1.0 (-2.2 to 0.3) |
| Oral disorders | -- | -- | -- | -- | -- |
| Caries of deciduous teeth | -- | -- | -- | -- | -- |
| Caries of permanent teeth | -- | -- | -- | -- | -- |
| Periodontal disease | -- | -- | -- | -- | -- |
| Edentulism and severe tooth loss | -- | -- | -- | -- | -- |
| Other oral disorders | -- | -- | -- | -- | -- |
| **Injuries** | **38.9 (33.1 to 45.7)** | **421.0 (373.4 to 473.0)** | **2.7 (1.8 to 3.6)** | **5.2* (1.3 to 8.8)** | **2.1 (-1.6 to 5.7)** |
| **Transport injuries** | **3.9 (2.9 to 5.2)** | **34.3 (25.1 to 45.1)** | **0.4 (0.3 to 0.6)** | **3.4 (-4.9 to 11.2)** | **0.4 (-7.7 to 8.0)** |
| Road injuries | 0.3 (0.2 to 0.4) | 2.4 (1.5 to 3.8) | -- | 16.0 (-4.7 to 35.7) | 12.6 (-7.4 to 31.7) |
| Pedestrian road injuries | 0.1 (0.0 to 0.1) | 0.6 (0.3 to 1.0) | -- | 1.8 (-19.2 to 22.7) | -1.2 (-21.5 to 19.2) |
| Cyclist road injuries | -- | -- | -- | -- | -- |
| Motorcyclist road injuries | 0.1 (0.1 to 0.2) | 0.9 (0.5 to 1.6) | -- | 16.4 (-6.6 to 37.2) | 13.0 (-9.3 to 33.3) |
| Motor vehicle road injuries | 0.1 (0.0 to 0.1) | 0.5 (0.3 to 0.9) | -- | 20.1 (-2.4 to 42.3) | 16.6 (-5.2 to 38.2) |
| Other road injuries | 0.0 (0.0 to 0.1) | 0.4 (0.2 to 0.7) | -- | 51.8* (23.7 to 76.5) | 47.4* (20.1 to 71.3) |
| Other transport injuries | 3.7 (2.6 to 5.0) | 31.8 (22.7 to 42.7) | 0.4 (0.2 to 0.6) | 2.7 (-6.2 to 11.2) | -0.3 (-8.9 to 7.9) |
| **Unintentional injuries** | **31.9 (26.4 to 38.2)** | **352.5 (306.7 to 404.2)** | **1.9 (1.3 to 2.7)** | **4.0 (-0.6 to 8.2)** | **0.9 (-3.5 to 5.1)** |
| Falls | 11.3 (8.0 to 15.2) | 103.6 (75.4 to 138.6) | 0.6 (0.4 to 0.9) | 6.3 (-4.4 to 16.9) | 3.2 (-7.2 to 13.5) |
| Drowning | 0.0 (0.0 to 0.1) | 0.5 (0.4 to 0.7) | -- | -12.4* (-21.7 to -2.5) | -14.9* (-24.0 to -5.3) |
| Fire, heat, and hot substances | 0.4 (0.3 to 0.6) | 6.7 (4.6 to 9.7) | 0.1 (0.0 to 0.1) | 14.8* (1.5 to 27.5) | 11.5 (-1.4 to 23.8) |
| Poisonings | 0.1 (0.1 to 0.2) | 8.2 (5.5 to 11.2) | -- | 12.9* (4.4 to 20.0) | 9.7* (1.4 to 16.5) |
| Poisoning by carbon monoxide | -- | 1.2 (0.6 to 2.0) | -- | 13.0 (-2.9 to 27.0) | 9.7 (-5.7 to 23.3) |
| Poisoning by other means | 0.1 (0.1 to 0.1) | 7.0 (4.7 to 9.7) | -- | 12.9* (4.9 to 19.9) | 9.6* (1.8 to 16.4) |
| Exposure to mechanical forces | 7.7 (5.6 to 10.7) | 81.6 (60.4 to 112.2) | 0.3 (0.2 to 0.5) | -1.0 (-11.4 to 8.7) | -3.9 (-13.9 to 5.5) |
| Unintentional firearm injuries | 0.5 (0.3 to 0.8) | 4.9 (2.8 to 8.1) | 0.1 (0.0 to 0.1) | 2.4 (-14.1 to 17.7) | -0.6 (-16.6 to 14.2) |
| Other exposure to mechanical forces | 7.2 (5.2 to 10.1) | 76.7 (56.8 to 105.9) | 0.3 (0.2 to 0.5) | -1.6 (-12.1 to 7.8) | -4.5 (-14.7 to 4.6) |
| Adverse effects of medical treatment | 1.6 (1.1 to 2.1) | 20.3 (14.7 to 26.1) | 0.2 (0.1 to 0.3) | 1.6 (-8.9 to 11.3) | -1.4 (-11.5 to 8.1) |
| Animal contact | 3.3 (2.1 to 4.8) | 54.2 (37.0 to 77.6) | 0.2 (0.1 to 0.3) | -4.4 (-14.1 to 5.7) | -7.2 (-16.6 to 2.7) |
| Venomous animal contact | 0.6 (0.4 to 1.0) | 19.2 (12.3 to 29.2) | 0.1 (0.0 to 0.1) | -3.3 (-13.5 to 7.2) | -6.1 (-16.0 to 4.1) |
| Non-venomous animal contact | 2.6 (1.6 to 4.0) | 35.0 (23.8 to 51.0) | 0.1 (0.1 to 0.2) | -5.3 (-15.5 to 5.1) | -8.1 (-17.9 to 2.1) |
| Foreign body | 2.6 (1.3 to 4.4) | 27.7 (20.8 to 36.1) | 0.2 (0.1 to 0.3) | 6.5* (2.8 to 10.4) | 3.4 (-0.2 to 7.2) |

| | | | | | |
|---|---|---|---|---|---|
| Pulmonary aspiration and foreign body in airway | 0.1 (0.1 to 0.2) | 3.1 (1.9 to 4.7) | -- | -9.6 (-24.0 to 3.1) | -12.2 (-26.2 to 0.1) |
| Foreign body in eyes | 1.8 (0.5 to 3.5) | 13.8 (8.4 to 21.1) | 0.1 (0.0 to 0.2) | 6.0* (4.3 to 8.3) | 2.9* (1.2 to 5.2) |
| Foreign body in other body part | 0.7 (0.4 to 1.0) | 10.8 (7.4 to 14.8) | 0.1 (0.0 to 0.1) | 13.4* (3.6 to 21.5) | 10.2* (0.6 to 18.0) |
| Environmental heat and cold exposure | 1.2 (0.8 to 1.8) | 12.5 (8.4 to 17.8) | 0.1 (0.1 to 0.2) | 3.9 (-7.0 to 14.7) | 0.9 (-9.7 to 11.3) |
| Exposure to forces of nature | -- | 0.2 (0.1 to 0.3) | -- | -62.5* (-64.0 to -60.8) | -63.6* (-65.1 to -61.9) |
| Other unintentional injuries | 3.7 (2.5 to 5.3) | 36.9 (25.6 to 51.7) | 0.2 (0.1 to 0.4) | 11.6 (-0.8 to 22.8) | 8.4 (-3.7 to 19.3) |
| **Self-harm and interpersonal violence** | **3.0 (2.3 to 3.9)** | **34.2 (26.8 to 43.6)** | **0.3 (0.2 to 0.4)** | **17.3* (6.1 to 28.8)** | **13.9* (3.0 to 25.1)** |
| Self-harm | -- | -- | -- | -- | -- |
| Self-harm by firearm | -- | -- | -- | -- | -- |
| Self-harm by other specified means | -- | -- | -- | -- | -- |
| Interpersonal violence | 2.2 (1.5 to 3.1) | 25.0 (17.7 to 33.7) | 0.2 (0.1 to 0.3) | 1.6 (-12.1 to 16.4) | -1.3 (-14.7 to 13.0) |
| Assault by firearm | 0.0 (0.0 to 0.1) | 0.5 (0.2 to 0.8) | -- | 9.1 (-12.9 to 32.6) | 5.9 (-15.4 to 28.8) |
| Assault by sharp object | 0.3 (0.2 to 0.5) | 3.5 (1.9 to 5.5) | 0.0 (0.0 to 0.1) | 9.0 (-10.3 to 27.6) | 5.9 (-12.9 to 23.9) |
| Sexual violence | -- | -- | -- | -- | -- |
| Assault by other means | 1.8 (1.3 to 2.6) | 21.0 (14.8 to 28.4) | 0.2 (0.1 to 0.2) | -0.2 (-14.4 to 14.5) | -3.1 (-16.9 to 11.2) |
| Conflict and terrorism | 0.8 (0.5 to 1.1) | 9.3 (6.9 to 12.7) | 0.1 (0.1 to 0.2) | 74.1* (70.2 to 77.9) | 69.0* (65.2 to 72.7) |
| Executions and police conflict | -- | -- | -- | -- | -- |
| **Nature of injury aggregates** | | | | | |
| Amputations | 0.4 (0.3 to 0.5) | 8.2 (6.7 to 10.0) | -- | -5.2* (-8.4 to -2.0) | -7.9* (-11.1 to -4.8) |
| Burns | 1.0 (0.8 to 1.4) | 15.9 (13.1 to 19.2) | 0.2 (0.1 to 0.3) | 6.9* (2.1 to 11.9) | 3.8 (-0.8 to 8.7) |
| Fractures | 13.1 (10.8 to 15.7) | 85.8 (72.8 to 100.2) | 0.7 (0.5 to 1.1) | 6.4* (1.5 to 11.2) | 3.3 (-1.4 to 7.9) |
| Head Injuries | 1.8 (1.4 to 2.2) | 16.9 (14.2 to 20.0) | 0.3 (0.2 to 0.5) | 4.0 (-0.8 to 8.9) | 1.0 (-3.7 to 5.7) |
| Spinal Injuries | 0.0 (0.0 to 0.1) | 0.6 (0.5 to 0.8) | -- | 6.3* (1.2 to 13.0) | 3.2 (-1.7 to 9.7) |
| Minor Injuries | 13.2 (9.9 to 17.0) | 163.2 (139.6 to 189.9) | 0.1 (0.0 to 0.2) | 2.1 (-2.8 to 7.1) | -0.9 (-5.6 to 4.0) |
| Other Injuries | 9.4 (6.9 to 12.1) | 130.4 (115.7 to 146.9) | 1.3 (0.8 to 1.8) | 4.8* (1.2 to 8.3) | 1.8 (-1.7 to 5.1) |
| **Impairments** | | | | | |
| Anaemia | 3693.9 (3007.7 to 4357.0) | -- | 127.1 (79.4 to 190.6) | -14.4* (-19.8 to -8.4) | -16.9* (-22.1 to -11.0) |
| Epilepsy | 60.7 (44.1 to 80.1) | -- | 28.6 (19.5 to 39.9) | 7.7 (-1.5 to 17.1) | 4.6 (-4.3 to 13.7) |
| Guillain-Barré syndrome | -- | -- | -- | 4.7* (2.2 to 8.1) | 1.7 (-0.7 to 5.0) |
| Hearing loss | 47.3 (41.6 to 54.0) | -- | 6.1 (4.1 to 8.6) | -0.6 (-6.3 to 5.4) | -3.4 (-9.0 to 2.3) |
| Heart failure | 0.1 (0.1 to 0.2) | -- | -- | 0.7 (-0.4 to 1.7) | -2.2* (-3.3 to -1.3) |
| Infertility | 0.4 (0.2 to 0.7) | -- | -- | 7.3* (5.5 to 9.0) | 4.2* (2.4 to 5.8) |
| Developmental intellectual disability | 266.6 (205.7 to 327.1) | -- | 34.9 (26.6 to 44.3) | 1.1 (-3.0 to 6.1) | -1.8 (-5.8 to 3.1) |
| Pelvic inflammatory disease | -- | -- | -- | -- | -- |
| Vision loss | 62.9 (41.8 to 89.3) | -- | 4.7 (3.3 to 6.6) | -4.7* (-7.0 to -2.4) | -7.5* (-9.7 to -5.3) |

We did not calculate incidence for the nine impairments and certain NTDs. Blank cells mean that no estimate is available or that the estimate has a magnitude less than 50.
Table presents prevalence and incidence counts in thousands.
* Percentage changes that are statistically significant
‡ Incidence estimates for stroke represent first-ever stroke only.
^ Incidence of HIV/AIDS represents new infections of HIV only and does not include new infections of Tuberculosis in HIV positive cases.

**SR Table 10c: Global prevalence, incidence and YLDs for 2017, percentage change of YLD counts and percentage change of YLD rates between 2007 and 2017 by cause, age 28 to 364 days**

| Cause | Prevalence in thousands (95% UI) | Incidence in thousands (95% UI) | YLDs in thousands (95% UI) | | |
|---|---|---|---|---|---|
| | 2017 counts | 2017 counts | 2017 counts | Percentage change in counts between 2007 and 2017 | Percentage change in rates between 2007 and 2017 |
| **All causes** | **97934.1 (96007.0 to 99984.2)** | **951695.1 (841275.5 to 1071082.0)** | **5545.2 (4008.9 to 7348.8)** | **-5.5* (-8.3 to -2.6)** | **-8.9* (-11.6 to -6.2)** |
| **Communicable, maternal, neonatal, and nutritional disorders** | **78556.0 (75316.9 to 81974.1)** | **869644.2 (759426.9 to 991594.0)** | **4115.0 (2984.4 to 5466.4)** | **-8.0* (-11.2 to -4.4)** | **-11.3* (-14.4 to -7.9)** |
| **HIV/AIDS and sexually transmitted infections** | **75.8 (67.1 to 84.5)** | **128.6 (112.3 to 146.5)** | **11.7 (8.4 to 15.7)** | **-53.4* (-57.1 to -49.0)** | **-55.0* (-58.7 to -50.9)** |
| HIV/AIDS | 93.3 (84.9 to 101.6) | 128.6^ (112.3 to 146.5) | 11.7 (8.4 to 15.7) | -53.4* (-57.1 to -49.0) | -55.0* (-58.7 to -50.9) |
| HIV/AIDS - Drug-susceptible Tuberculosis | 2.9 (2.7 to 3.2) | 4.4 (4.0 to 4.8) | 1.2 (0.8 to 1.6) | -67.9* (-71.9 to -63.2) | -69.1* (-72.9 to -64.6) |
| HIV/AIDS - Multidrug-resistant Tuberculosis without extensive drug resistance | 0.1 (0.1 to 0.2) | 0.2 (0.1 to 0.3) | 0.0 (0.0 to 0.1) | -66.7* (-77.6 to -51.1) | -67.9* (-78.4 to -52.9) |
| HIV/AIDS - Extensively drug-resistant Tuberculosis | -- | -- | -- | -33.3 (-57.6 to 7.6) | -35.7 (-59.1 to 3.7) |
| HIV/AIDS resulting in other diseases | 90.2 (81.7 to 98.5) | 128.6 (112.3 to 146.5) | 10.4 (7.5 to 14.0) | -50.7* (-55.1 to -45.4) | -52.5* (-56.7 to -47.4) |
| HIV/AIDS not on antiretroviral treatment without Tuberculosis | 63.9 (56.8 to 70.5) | 128.6 (112.3 to 146.5) | 8.2 (5.9 to 11.0) | -58.1* (-62.7 to -52.6) | -59.6* (-64.0 to -54.3) |
| HIV/AIDS on antiretroviral treatment without Tuberculosis | 26.4 (24.2 to 28.7) | -- | 2.2 (1.5 to 3.2) | 40.5* (17.0 to 77.1) | 35.4* (12.8 to 70.7) |
| Sexually transmitted infections excluding HIV | -- | -- | -- | -- | -- |
| Syphilis | -- | -- | -- | -- | -- |
| Early syphilis | -- | -- | -- | -- | -- |
| Tertiary syphilis | -- | -- | -- | -- | -- |
| Chlamydial infection | -- | -- | -- | -- | -- |
| Chlamydia episode | -- | -- | -- | -- | -- |
| Chlamydial infection complications | -- | -- | -- | -- | -- |
| Gonococcal infection | -- | -- | -- | -- | -- |
| Gonococcal infection complications | -- | -- | -- | -- | -- |
| Gonorrhoea episode | -- | -- | -- | -- | -- |
| Trichomoniasis | -- | -- | -- | -- | -- |
| Genital herpes | -- | -- | -- | -- | -- |
| Other sexually transmitted infections | -- | -- | -- | -- | -- |
| Other sexually transmitted diseases residual | -- | -- | -- | -- | -- |
| Other sexually transmitted diseases | -- | -- | -- | -- | -- |
| **Respiratory infections and tuberculosis** | **19857.6 (16917.3 to 23208.1)** | **473038.2 (380237.8 to 579746.9)** | **217.2 (134.4 to 339.8)** | **1.9 (-0.8 to 4.1)** | **-1.8 (-4.4 to 0.4)** |
| Tuberculosis | 12585.4 (9721.1 to 15852.2) | 160.0 (136.6 to 188.5) | 13.1 (8.4 to 18.6) | -21.3* (-28.6 to -13.7) | -24.1* (-31.1 to -16.8) |
| Latent tuberculosis infection | 12544.8 (9679.5 to 15808.5) | -- | -- | -- | -- |
| Drug-susceptible tuberculosis | 39.1 (32.3 to 47.3) | 153.7 (131.0 to 180.8) | 12.5 (8.1 to 17.9) | -21.5* (-29.2 to -13.6) | -24.3* (-31.8 to -16.7) |
| Multidrug-resistant tuberculosis without extensive drug resistance | 1.5 (1.0 to 2.4) | 6.2 (4.1 to 9.3) | 0.5 (0.3 to 0.8) | -16.3 (-43.3 to 25.4) | -19.3 (-45.3 to 20.9) |
| Extensively drug-resistant tuberculosis | 0.0 (0.0 to 0.1) | 0.2 (0.1 to 0.3) | -- | 22.0 (-17.9 to 97.5) | 17.6 (-20.9 to 90.4) |
| Lower respiratory infections | 391.0 (328.0 to 458.9) | 15589.4 (12369.2 to 19445.6) | 24.3 (15.4 to 35.5) | -11.3* (-18.8 to -3.9) | -14.5* (-21.7 to -7.4) |
| Guillain-Barré syndrome due to lower respiratory infections | 0.2 (0.1 to 0.3) | -- | 0.1 (0.0 to 0.1) | 3.3* (1.3 to 5.2) | -0.4 (-2.4 to 1.4) |
| Lower respiratory infection episode | 390.9 (327.9 to 458.7) | 15589.4 (12369.2 to 19445.6) | 24.2 (15.3 to 35.5) | -11.4* (-18.8 to -3.9) | -14.6* (-21.7 to -7.4) |
| Upper respiratory infections | 5753.7 (4531.2 to 7071.8) | 423517.8 (330821.8 to 526287.7) | 145.8 (83.6 to 239.8) | 7.5* (5.4 to 9.4) | 3.6* (1.6 to 5.5) |
| Guillain-Barré syndrome due to upper respiratory infections | 0.5 (0.3 to 0.7) | -- | 0.1 (0.1 to 0.2) | 3.3* (1.3 to 5.2) | -0.4 (-2.4 to 1.4) |
| Upper respiratory infection episode | 5753.2 (4530.7 to 7071.2) | 423517.8 (330821.8 to 526287.7) | 145.7 (83.5 to 239.7) | 7.5* (5.4 to 9.4) | 3.6* (1.6 to 5.5) |
| Otitis media | 2140.2 (1692.5 to 2696.2) | 33771.0 (23762.4 to 45503.9) | 34.1 (18.3 to 58.4) | 1.3 (-1.1 to 3.8) | -2.4 (-4.7 to 0.0) |
| Acute otitis media | 1611.1 (1169.1 to 2174.2) | 33768.6 (23762.1 to 45499.7) | 21.2 (9.6 to 38.1) | 1.9 (-0.6 to 4.7) | -1.7 (-4.2 to 0.9) |
| Chronic otitis media | 529.2 (471.0 to 595.6) | 2.5 (0.1 to 9.5) | 12.8 (7.8 to 19.9) | 0.2 (-4.3 to 4.8) | -3.4 (-7.8 to 1.0) |
| **Enteric infections** | **4110.8 (3494.2 to 4831.8)** | **287243.6 (233085.9 to 352468.7)** | **481.6 (320.1 to 686.6)** | **-5.0 (-12.4 to 2.7)** | **-8.5* (-15.5 to -1.0)** |
| Diarrhoeal diseases | 4152.6 (3515.5 to 4888.3) | 287172.6 (233006.3 to 352380.6) | 480.8 (319.6 to 685.9) | -5.0 (-12.3 to 2.9) | -8.4* (-15.5 to -0.9) |
| Guillain-Barré syndrome due to diarrheal diseases | 0.2 (0.1 to 0.3) | -- | 0.0 (0.0 to 0.1) | 3.3* (1.3 to 5.2) | -0.4 (-2.4 to 1.4) |
| Diarrheal disease episode | 4152.4 (3515.3 to 4888.1) | 287172.6 (233006.3 to 352380.6) | 480.8 (319.6 to 685.9) | -5.0 (-12.3 to 2.9) | -8.4* (-15.5 to -0.9) |
| Typhoid and paratyphoid | -- | 18.9 (15.3 to 23.2) | 0.2 (0.1 to 0.2) | -15.3 (-33.3 to 8.7) | -18.3 (-35.7 to 4.8) |
| Typhoid fever | 1.0 (0.7 to 1.3) | 15.1 (12.1 to 18.6) | 0.1 (0.1 to 0.2) | -16.2 (-35.2 to 8.7) | -19.2 (-37.5 to 4.8) |
| Typhoid fever complications | 0.2 (0.1 to 0.3) | 2.6 (1.9 to 3.4) | 0.1 (0.0 to 0.1) | -16.0 (-46.6 to 26.1) | -19.1 (-48.5 to 21.6) |
| Typhoid fever episode | 0.8 (0.5 to 1.0) | 12.5 (10.0 to 15.4) | 0.1 (0.0 to 0.1) | -16.4 (-39.2 to 16.2) | -19.4 (-41.4 to 12.0) |
| Paratyphoid fever | 0.2 (0.1 to 0.2) | 3.8 (2.8 to 5.1) | -- | 0.5 (-18.5 to 26.9) | -3.1 (-21.5 to 22.3) |
| Intestinal perforation due to paratyphoid | -- | 0.2 (0.1 to 0.3) | -- | 0.8 (-28.2 to 43.9) | -2.9 (-30.8 to 38.7) |
| Paratyphoid fever episode | 0.2 (0.1 to 0.2) | 3.6 (2.7 to 4.8) | -- | 0.5 (-19.8 to 28.2) | -3.1 (-22.7 to 23.6) |
| iNTS | 2.0 (1.4 to 3.2) | 52.1 (38.2 to 78.8) | 0.3 (0.1 to 0.4) | -34.6* (-55.5 to -1.0) | -36.9* (-57.1 to -4.5) |
| Other intestinal infectious diseases | -- | -- | 0.3 (0.2 to 0.5) | -49.6* (-60.3 to -35.3) | -51.4* (-61.7 to -37.6) |
| **Neglected tropical diseases and malaria** | **7768.3 (7451.5 to 8078.4)** | **12601.8 (8746.2 to 17457.8)** | **143.6 (95.4 to 208.8)** | **-16.1* (-22.4 to -8.6)** | **-19.2* (-25.2 to -11.9)** |
| Malaria | 3464.0 (3182.7 to 3699.0) | 11022.1 (7136.6 to 15628.4) | 45.2 (28.3 to 69.0) | -36.6* (-44.9 to -26.4) | -38.9* (-46.9 to -29.1) |
| Malaria complications | 1.6 (1.4 to 1.9) | -- | 0.5 (0.4 to 0.7) | 10.7* (5.0 to 16.6) | 6.7* (1.2 to 12.4) |
| Malaria episode | 640.2 (348.1 to 1024.3) | 11022.1 (7136.6 to 15628.4) | 24.0 (11.6 to 42.7) | -32.5* (-47.1 to -19.5) | -35.0* (-49.0 to -22.4) |

| | | | | | |
|---|---|---|---|---|---|
| Malaria parasitemia | 2822.2 (2394.2 to 3200.5) | -- | 20.7 (13.5 to 30.5) | -41.4* (-49.9 to -31.4) | -43.5* (-51.7 to -33.9) |
| Chagas disease | 7.5 (5.4 to 10.4) | 2.9 (1.9 to 4.1) | -- | -29.3* (-35.8 to -23.1) | -31.8* (-38.1 to -25.8) |
| Acute Chagas disease | -- | 2.9 (1.9 to 4.1) | -- | -30.0* (-37.3 to -23.7) | -32.6* (-39.5 to -26.5) |
| Asymptomatic Chagas disease | 7.5 (5.4 to 10.4) | -- | -- | -- | -- |
| Symptomatic chronic Chagas infection | -- | -- | -- | -14.3* (-19.1 to -8.6) | -17.4* (-22.0 to -11.9) |
| Leishmaniasis | 1.2 (0.7 to 1.9) | 4.3 (3.3 to 5.7) | 0.1 (0.1 to 0.2) | -39.0* (-56.1 to -13.2) | -41.2* (-57.7 to -16.3) |
| Visceral leishmaniasis | 0.4 (0.3 to 0.6) | 1.5 (1.0 to 2.3) | -- | -73.3* (-81.5 to -56.2) | -74.3* (-82.1 to -57.8) |
| Cutaneous and mucocutaneous leishmaniasis | 1.6 (1.1 to 2.3) | 2.8 (1.9 to 4.0) | 0.1 (0.1 to 0.2) | -11.0 (-40.6 to 37.5) | -14.2 (-42.7 to 32.6) |
| African trypanosomiasis | -- | -- | -- | -- | -- |
| Trypanosomiasis Gambiense | -- | -- | -- | -- | -- |
| Trypanosomiasis Rhodesiense | -- | -- | -- | -- | -- |
| Schistosomiasis | 0.2 (0.1 to 0.2) | -- | -- | 14.0* (4.4 to 27.4) | 9.9* (0.6 to 22.8) |
| Mild schistosomiasis | 0.2 (0.1 to 0.2) | -- | -- | 16.2* (11.1 to 25.3) | 12.0* (7.0 to 20.8) |
| Anemia due to schistosomiasis | -- | -- | -- | 10.9 (-6.7 to 35.8) | 6.9 (-10.0 to 30.9) |
| Schistosomiasis complications | -- | -- | -- | -- | -- |
| Cysticercosis | -- | -- | -- | -- | -- |
| Cystic echinococcosis | -- | -- | -- | -- | -- |
| Lymphatic filariasis | -- | -- | -- | -- | -- |
| Prevalence of detectable microfiliaria due to lymphatic filariasis | -- | -- | -- | -- | -- |
| Lymphatic filariasis complications | -- | -- | -- | -- | -- |
| Onchocerciasis | -- | -- | -- | -- | -- |
| Asymptomatic onchocerciasis | -- | -- | -- | -- | -- |
| Skin disease due to onchocerciasis | -- | -- | -- | -- | -- |
| Vision loss due to onchocerciasis | -- | -- | -- | -- | -- |
| Trachoma | -- | -- | -- | -- | -- |
| Dengue | 92.6 (50.5 to 156.7) | 1549.1 (943.7 to 2347.6) | 15.5 (6.8 to 29.0) | 36.4* (16.4 to 113.8) | 31.5* (12.2 to 106.0) |
| Post-dengue chronic fatigue syndrome | 65.3 (30.5 to 119.4) | -- | 13.9 (5.7 to 26.6) | 36.4* (15.7 to 114.6) | 31.5* (11.6 to 106.9) |
| Dengue episode | 27.3 (16.5 to 41.0) | 1549.1 (943.7 to 2347.6) | 1.6 (0.8 to 2.9) | 36.2* (16.9 to 106.4) | 31.3* (12.7 to 98.9) |
| Yellow fever | 0.0 (0.0 to 0.1) | 0.9 (0.3 to 2.4) | -- | -27.9 (-48.1 to 5.7) | -30.5 (-50.0 to 1.9) |
| Asymptomatic yellow fever | -- | 0.5 (0.1 to 1.4) | -- | -- | -- |
| Yellow fever episode | -- | 0.4 (0.1 to 1.1) | -- | -27.9 (-48.1 to 5.7) | -30.5 (-50.0 to 1.9) |
| Rabies | -- | 0.2 (0.1 to 0.3) | -- | -53.0* (-67.2 to -30.0) | -54.7* (-68.4 to -32.6) |
| Intestinal nematode infections | 2428.4 (2276.3 to 2605.4) | -- | 4.8 (2.9 to 7.6) | -35.4* (-40.0 to -29.8) | -37.7* (-42.1 to -32.3) |
| Ascariasis | 1471.0 (1325.5 to 1639.1) | -- | 2.1 (1.1 to 3.6) | -39.5* (-46.4 to -31.7) | -41.6* (-48.3 to -34.1) |
| Asymptomatic ascariasis | 1352.9 (1220.0 to 1509.0) | -- | -- | -- | -- |
| Ascariasis complications | 118.1 (105.6 to 131.9) | -- | 2.1 (1.1 to 3.6) | -39.5* (-46.4 to -31.7) | -41.6* (-48.3 to -34.1) |
| Trichuriasis | 437.0 (393.5 to 490.4) | -- | 0.2 (0.1 to 0.3) | -35.3* (-44.9 to -24.2) | -37.6* (-46.9 to -26.9) |
| Asymptomatic trichuriasis | 426.0 (382.8 to 478.3) | -- | -- | -- | -- |
| Trichuriasis complications | 10.9 (10.0 to 11.9) | -- | 0.2 (0.1 to 0.3) | -35.3* (-44.9 to -24.2) | -37.6* (-46.9 to -26.9) |
| Hookworm disease | 561.3 (527.7 to 598.8) | -- | 2.5 (1.6 to 3.7) | -31.6* (-38.2 to -23.4) | -34.1* (-40.4 to -26.2) |
| Asymptomatic hookworm disease | 478.9 (449.7 to 510.8) | -- | -- | -- | -- |
| Anemia due to hookworm disease | 24.9 (22.7 to 27.2) | -- | 1.3 (0.9 to 1.9) | -30.7* (-40.9 to -18.6) | -33.2* (-43.0 to -21.5) |
| Hookworm disease complications | 57.5 (54.1 to 61.1) | -- | 1.2 (0.6 to 1.9) | -32.6* (-38.9 to -25.9) | -35.0* (-41.1 to -28.6) |
| Food-borne trematodiases | -- | -- | -- | -- | -- |
| Asymptomatic food-borne tremodiases | -- | -- | -- | -- | -- |
| Food-borne tremodiases complications | -- | -- | -- | -- | -- |
| Leprosy | -- | -- | -- | -- | -- |
| Ebola virus disease | -- | -- | -- | -95.0* (-95.4 to -93.8) | -95.2* (-95.6 to -94.0) |
| Ebola cases | -- | -- | -- | -95.0* (-95.1 to -94.9) | -95.2* (-95.3 to -95.0) |
| Post-Ebola chronic fatigue syndrome | -- | -- | -- | -95.0* (-95.5 to -93.7) | -95.2* (-95.6 to -94.0) |
| Zika virus disease | 0.9 (0.6 to 1.4) | 22.3 (12.4 to 40.5) | 0.4 (0.2 to 0.6) | -- | -- |
| Zika virus complications | 0.5 (0.4 to 1.1) | -- | 0.3 (0.2 to 0.6) | -- | -- |
| Zika virus episode | 0.4 (0.2 to 0.7) | 22.3 (12.4 to 40.5) | -- | -- | -- |
| Guinea worm disease | -- | -- | -- | -- | -- |
| Other neglected tropical diseases | 2205.0 (2090.8 to 2314.8) | -- | 77.6 (51.3 to 112.1) | -4.1 (-11.9 to 4.8) | -7.5 (-15.1 to 1.0) |
| Acute infection due to other neglected tropical diseases | -- | -- | 0.9 (0.5 to 1.6) | 71.2* (47.6 to 150.8) | 65.0* (42.3 to 141.7) |
| Anemia due to other neglected tropical diseases | 2205.0 (2090.8 to 2314.8) | -- | 76.7 (50.7 to 111.1) | -4.6 (-12.5 to 4.4) | -8.0 (-15.7 to 0.7) |
| **Other infectious diseases** | **5209.3 (4732.4 to 5681.1)** | **61365.8 (53321.5 to 70568.2)** | **128.7 (84.3 to 187.3)** | **-14.2* (-20.4 to -9.1)** | **-17.3* (-23.2 to -12.4)** |
| Meningitis | 101.7 (85.3 to 120.9) | 1053.8 (836.4 to 1316.5) | 12.4 (8.5 to 17.1) | -15.3* (-20.6 to -10.4) | -18.4* (-23.5 to -13.7) |
| Pneumococcal meningitis | 16.2 (12.9 to 20.2) | 76.2 (52.1 to 107.8) | 1.9 (1.3 to 2.7) | -41.4* (-46.4 to -36.6) | -43.5* (-48.3 to -38.8) |

| | | | | | |
|---|---|---|---|---|---|
| Acute pneumococcal meningitis | 2.8 (1.9 to 3.9) | 76.2 (52.1 to 107.8) | 0.4 (0.2 to 0.6) | -41.1* (-58.5 to -17.9) | -43.2* (-60.0 to -20.8) |
| Pneumococcal meningitis complications | 13.4 (10.7 to 16.7) | -- | 1.6 (1.1 to 2.2) | -41.5* (-45.1 to -37.7) | -43.6* (-47.1 to -40.0) |
| H influenzae type B meningitis | 8.3 (6.5 to 10.9) | 71.9 (48.8 to 105.5) | 1.0 (0.7 to 1.5) | -44.7* (-50.5 to -38.3) | -46.7* (-52.3 to -40.6) |
| Acute H influenzae type B meningitis | 2.6 (1.8 to 3.9) | 71.9 (48.8 to 105.5) | 0.3 (0.2 to 0.6) | -43.5* (-58.0 to -24.8) | -45.6* (-59.6 to -27.6) |
| H Influenza type B meningitis complications | 5.7 (4.3 to 7.5) | -- | 0.7 (0.5 to 1.0) | -45.2* (-48.2 to -42.4) | -47.2* (-50.1 to -44.4) |
| Meningococcal infection | 7.9 (5.8 to 10.5) | 76.4 (51.2 to 109.4) | 0.9 (0.6 to 1.3) | -37.5* (-44.4 to -29.5) | -39.7* (-46.4 to -32.0) |
| Acute meningococcal meningitis | 2.8 (1.9 to 4.0) | 76.4 (51.2 to 109.4) | 0.4 (0.2 to 0.6) | -35.5* (-52.2 to -14.8) | -37.9* (-53.9 to -17.8) |
| Meningococcal meningitis complications | 5.1 (3.7 to 6.9) | -- | 0.6 (0.4 to 0.8) | -38.7* (-41.5 to -35.9) | -40.9* (-43.7 to -38.2) |
| Other meningitis | 69.3 (56.3 to 83.1) | 829.4 (656.4 to 1044.8) | 8.5 (5.9 to 11.9) | 6.3 (-2.1 to 14.7) | 2.5 (-5.6 to 10.6) |
| Other acute bacterial meningitis | 18.7 (14.8 to 23.1) | 508.8 (400.3 to 638.2) | 2.4 (1.5 to 3.7) | 16.1 (-2.8 to 36.8) | 11.9 (-6.3 to 31.9) |
| Acute viral meningitis | 11.7 (9.4 to 14.4) | 320.6 (254.5 to 400.6) | 1.5 (1.0 to 2.4) | -9.9 (-26.3 to 7.4) | -13.1 (-29.0 to 3.5) |
| Other bacterial meningitis complications | 38.9 (30.1 to 47.9) | -- | 4.6 (3.1 to 6.3) | 7.9* (0.2 to 16.3) | 4.0 (-3.4 to 12.1) |
| Encephalitis | 12.0 (9.5 to 15.4) | 104.9 (100.6 to 109.2) | 1.4 (1.0 to 1.9) | 1.4 (-0.2 to 2.9) | -2.3* (-3.8 to -0.8) |
| Acute encephalitis | 5.4 (5.2 to 5.6) | 104.9 (100.6 to 109.2) | 0.7 (0.5 to 1.0) | 3.9* (3.5 to 4.4) | 0.1 (-0.3 to 0.6) |
| Encephalitis complications | 6.7 (4.1 to 10.1) | -- | 0.7 (0.5 to 0.9) | -1.1 (-3.9 to 1.9) | -4.7* (-7.4 to -1.8) |
| Diphtheria | 0.2 (0.1 to 0.3) | 2.3 (1.3 to 3.7) | -- | -38.0 (-64.3 to 8.7) | -40.2 (-65.6 to 4.8) |
| Whooping cough | 858.4 (662.9 to 1082.5) | 6266.3 (4839.5 to 7902.6) | 42.7 (25.2 to 67.0) | -10.3* (-12.3 to -8.5) | -13.6* (-15.4 to -11.8) |
| Tetanus | 1.5 (1.4 to 1.7) | 5.5 (3.6 to 9.2) | 0.1 (0.1 to 0.1) | -48.5* (-62.9 to -27.2) | -50.4* (-64.2 to -29.9) |
| Severe tetanus | 0.3 (0.2 to 0.5) | 5.5 (3.6 to 9.2) | 0.0 (0.0 to 0.1) | -65.8* (-80.4 to -35.5) | -67.0* (-81.1 to -37.8) |
| Neonatal tetanus complications | 1.2 (1.1 to 1.2) | -- | 0.1 (0.0 to 0.1) | -13.7* (-16.1 to -11.1) | -16.8* (-19.2 to -14.3) |
| Measles | 130.5 (46.4 to 288.9) | 4764.7 (1695.4 to 10543.8) | 11.7 (3.9 to 26.6) | -48.8* (-53.4 to -43.3) | -50.6* (-55.1 to -45.4) |
| Varicella and herpes zoster | 367.8 (283.3 to 467.6) | 19128.1 (14715.0 to 24344.0) | 2.2 (0.9 to 4.5) | 0.9 (-1.8 to 3.6) | -2.7* (-5.4 to -0.2) |
| Chickenpox | 366.3 (281.7 to 466.3) | 19114.5 (14699.0 to 24330.6) | 2.1 (0.8 to 4.4) | 0.8 (-2.1 to 3.5) | -2.9* (-5.6 to -0.2) |
| Herpes zoster | 1.5 (1.1 to 2.0) | 13.7 (10.4 to 18.1) | 0.1 (0.0 to 0.1) | 3.8* (2.7 to 4.9) | 0.1 (-1.1 to 1.1) |
| Acute hepatitis | 2392.1 (2001.9 to 2789.7) | 30040.1 (24840.7 to 35256.6) | 2.3 (1.3 to 3.7) | 2.2 (-7.9 to 13.6) | -1.5 (-11.2 to 9.5) |
| Acute hepatitis A | 2102.2 (1701.4 to 2523.2) | 27328.6 (22117.8 to 32801.9) | 0.3 (0.1 to 0.5) | 2.1 (-36.2 to 56.3) | -1.6 (-38.5 to 50.7) |
| Acute hepatitis B | 200.5 (129.6 to 288.1) | 1737.5 (1123.0 to 2497.3) | 1.4 (0.7 to 2.5) | 8.1 (-4.5 to 23.2) | 4.2 (-7.9 to 18.8) |
| Acute hepatitis C | 43.4 (30.0 to 58.9) | 375.9 (260.3 to 510.7) | 0.6 (0.3 to 1.2) | -9.4 (-23.9 to 5.9) | -12.7 (-26.7 to 2.1) |
| Acute hepatitis E | 46.0 (32.6 to 62.7) | 598.1 (424.2 to 814.7) | -- | 4.0 (-32.7 to 57.0) | 0.3 (-35.1 to 51.3) |
| Other unspecified infectious diseases | 1540.2 (1462.3 to 1616.0) | -- | 55.9 (36.6 to 80.8) | -5.2 (-12.5 to 2.6) | -8.6* (-15.7 to -1.1) |
| Guillain-Barré syndrome due to other infectious diseases | 0.1 (0.1 to 0.2) | -- | 0.0 (0.0 to 0.1) | 3.3* (1.3 to 5.2) | -0.4 (-2.3 to 1.4) |
| Other infectious diseases | -- | -- | 3.5 (2.2 to 5.4) | 25.1* (18.5 to 30.2) | 20.5* (14.2 to 25.5) |
| Anemia due to other infectious diseases | 1540.1 (1462.2 to 1615.9) | -- | 52.3 (34.4 to 76.0) | -6.7 (-14.4 to 1.3) | -10.1* (-17.5 to -2.3) |
| **Maternal and neonatal disorders** | **16678.3 (15548.2 to 17659.4)** | **--** | **1105.2 (838.6 to 1405.1)** | **10.8* (4.3 to 18.5)** | **6.8* (0.5 to 14.2)** |
| Maternal disorders | -- | -- | -- | -- | -- |
| Maternal haemorrhage | -- | -- | -- | -- | -- |
| Maternal hemorrhage complications | -- | -- | -- | -- | -- |
| Maternal hemorrhage episode | -- | -- | -- | -- | -- |
| Maternal sepsis and other pregnancy related infections | -- | -- | -- | -- | -- |
| Infertility due to puerperal sepsis | -- | -- | -- | -- | -- |
| Maternal sepsis and other maternal infection episode | -- | -- | -- | -- | -- |
| Maternal hypertensive disorders | -- | -- | -- | -- | -- |
| Maternal hypertensive disorder complications | -- | -- | -- | -- | -- |
| Maternal hypertensive disorder episode | -- | -- | -- | -- | -- |
| Maternal obstructed labour and uterine rupture | -- | -- | -- | -- | -- |
| Obstructed labor, acute event | -- | -- | -- | -- | -- |
| Maternal obstructed labor complications | -- | -- | -- | -- | -- |
| Maternal abortion and miscarriage | -- | -- | -- | -- | -- |
| Ectopic pregnancy | -- | -- | -- | -- | -- |
| Other maternal disorders | -- | -- | -- | -- | -- |
| Neonatal disorders | 16678.3 (15548.2 to 17659.4) | -- | 1105.2 (838.6 to 1405.1) | 10.8* (4.3 to 18.5) | 6.8* (0.5 to 14.2) |
| Neonatal preterm birth | 14821.3 (13891.0 to 15607.0) | -- | 444.6 (318.6 to 598.9) | 7.4 (-5.6 to 21.3) | 3.5 (-9.0 to 16.9) |
| Preterm birth complications | 1976.5 (1603.8 to 2437.3) | -- | 435.6 (311.8 to 587.7) | 7.7 (-5.5 to 21.8) | 3.8 (-9.0 to 17.4) |
| Retinopathy of prematurity | 192.7 (144.1 to 244.2) | -- | 9.0 (5.8 to 13.0) | -5.1 (-12.6 to 2.3) | -8.5* (-15.7 to -1.4) |
| Uncomplicated preterm birth | 12652.2 (11785.1 to 13267.8) | -- | -- | -- | -- |
| Neonatal encephalopathy due to birth asphyxia and trauma | 1929.5 (1449.6 to 2467.5) | -- | 252.5 (151.8 to 387.6) | 11.8* (6.7 to 16.5) | 7.8* (2.9 to 12.3) |
| Neonatal sepsis and other neonatal infections | 631.9 (483.6 to 824.7) | -- | 88.0 (47.2 to 146.8) | 5.7* (0.9 to 10.9) | 1.9 (-2.7 to 6.9) |
| Neonatal sepsis and other neonatal infection complications | 333.2 (201.5 to 509.5) | -- | 88.0 (47.2 to 146.8) | 5.7* (0.9 to 10.9) | 1.9 (-2.7 to 6.9) |
| Neonatal sepsis and other neonatal infection episode | 298.7 (234.6 to 374.0) | -- | -- | -- | -- |
| Hemolytic disease and other neonatal jaundice | 78.6 (66.6 to 91.7) | -- | 24.7 (18.4 to 32.0) | 4.8 (-1.9 to 12.3) | 1.1 (-5.5 to 8.3) |

| | | | | | |
|---|---|---|---|---|---|
| Other neonatal disorders | -- | -- | 295.5 (224.2 to 375.6) | 17.7* (10.8 to 25.8) | 13.4* (6.8 to 21.3) |
| **Nutritional deficiencies** | **53993.2 (48968.2 to 58805.7)** | **35261.7 (31646.2 to 38963.9)** | **2027.0 (1354.5 to 2892.5)** | **-15.8* (-19.9 to -11.1)** | **-18.8* (-22.8 to -14.3)** |
| Protein-energy malnutrition | 10314.9 (9981.7 to 10688.3) | 9592.8 (9268.7 to 9948.0) | 310.2 (201.3 to 438.4) | -21.2* (-25.3 to -17.1) | -24.1* (-28.0 to -20.0) |
| Iodine deficiency | 657.7 (483.8 to 885.4) | 9.3 (7.4 to 11.1) | 15.1 (9.1 to 24.0) | -36.1* (-42.9 to -28.0) | -38.4* (-44.9 to -30.6) |
| Visible goiter without symptoms | 633.0 (463.7 to 855.3) | 9.3 (7.4 to 11.1) | 6.9 (3.1 to 13.0) | -45.7* (-48.9 to -42.6) | -47.7* (-50.7 to -44.7) |
| Visible goiter with complications | 24.7 (16.2 to 34.1) | -- | 8.2 (4.5 to 12.6) | -24.9* (-37.4 to -13.7) | -27.6* (-39.7 to -16.8) |
| Vitamin A deficiency | 35545.3 (27140.9 to 43547.7) | 25659.6 (21971.4 to 29269.3) | 852.2 (526.8 to 1286.7) | -20.6* (-24.9 to -15.8) | -23.5* (-27.6 to -18.8) |
| Asymptomatic vitamin A deficiency | 10576.0 (7855.8 to 13203.4) | 25659.6 (21971.4 to 29269.3) | -- | -- | -- |
| Vitamin A deficiency complications | 381.4 (252.6 to 555.5) | -- | 19.0 (10.5 to 30.5) | -2.0 (-6.8 to 3.0) | -5.5* (-10.2 to -0.7) |
| Vitamin A deficiency with anemia | 24588.6 (18927.4 to 29996.5) | -- | 833.1 (516.0 to 1262.3) | -21.0* (-25.4 to -16.0) | -23.8* (-28.1 to -19.0) |
| Dietary iron deficiency | 24457.6 (23259.9 to 25608.6) | -- | 839.4 (552.3 to 1225.2) | -6.9 (-13.9 to 0.8) | -10.2* (-17.0 to -2.8) |
| Other nutritional deficiencies | -- | -- | 10.2 (6.6 to 14.4) | -33.5* (-36.9 to -29.9) | -35.9* (-39.2 to -32.4) |
| **Non-communicable diseases** | **55223.0 (53804.8 to 56725.2)** | **75641.1 (66496.5 to 87602.4)** | **1382.8 (989.3 to 1855.4)** | **2.1 (-0.4 to 5.3)** | **-1.6 (-4.0 to 1.5)** |
| **Neoplasms** | **614.9 (471.2 to 857.4)** | **31.9 (27.8 to 35.5)** | **32.8 (19.5 to 53.5)** | **103.5* (53.8 to 175.7)** | **96.2* (48.2 to 165.8)** |
| Lip and oral cavity cancer | -- | -- | -- | -- | -- |
| Diagnosis and primary therapy phase of mouth cancer | -- | -- | -- | -- | -- |
| Controlled phase of mouth cancer | -- | -- | -- | -- | -- |
| Metastatic phase of mouth cancer | -- | -- | -- | -- | -- |
| Terminal phase of mouth cancer | -- | -- | -- | -- | -- |
| Nasopharynx cancer | -- | -- | -- | -- | -- |
| Diagnosis and primary therapy phase of nasopharynx cancer | -- | -- | -- | -- | -- |
| Controlled phase of nasopharynx cancer | -- | -- | -- | -- | -- |
| Metastatic phase of nasopharynx cancer | -- | -- | -- | -- | -- |
| Terminal phase of nasopharynx cancer | -- | -- | -- | -- | -- |
| Other pharynx cancer | -- | -- | -- | -- | -- |
| Diagnosis and primary therapy phase of other pharynx cancer | -- | -- | -- | -- | -- |
| Controlled phase of other pharynx cancer | -- | -- | -- | -- | -- |
| Metastatic phase of other pharynx cancer | -- | -- | -- | -- | -- |
| Terminal phase of other pharynx cancer | -- | -- | -- | -- | -- |
| Oesophageal cancer | -- | -- | -- | -- | -- |
| Diagnosis and primary therapy phase of esophageal cancer | -- | -- | -- | -- | -- |
| Controlled phase of esophageal cancer | -- | -- | -- | -- | -- |
| Metastatic phase of esophageal cancer | -- | -- | -- | -- | -- |
| Terminal phase of esophageal cancer | -- | -- | -- | -- | -- |
| Stomach cancer | -- | -- | -- | -- | -- |
| Diagnosis and primary therapy phase of stomach cancer | -- | -- | -- | -- | -- |
| Controlled phase of stomach cancer | -- | -- | -- | -- | -- |
| Metastatic phase of stomach cancer | -- | -- | -- | -- | -- |
| Terminal phase of stomach cancer | -- | -- | -- | -- | -- |
| Colon and rectum cancer | -- | -- | -- | -- | -- |
| Diagnosis and primary therapy phase of colon and rectum cancers | -- | -- | -- | -- | -- |
| Controlled phase of colon and rectum cancers | -- | -- | -- | -- | -- |
| Metastatic phase of colon and rectum cancers | -- | -- | -- | -- | -- |
| Terminal phase of colon and rectum cancers | -- | -- | -- | -- | -- |
| Stoma from colon and rectum cancers, beyond 10 years | -- | -- | -- | -- | -- |
| Liver cancer | -- | -- | -- | -- | -- |
| Liver cancer due to hepatitis B | -- | -- | -- | -- | -- |
| Diagnosis and primary therapy phase of liver cancer due to hepatitis B | -- | -- | -- | -- | -- |
| Controlled phase of liver cancer due to hepatitis B | -- | -- | -- | -- | -- |
| Metastatic phase of liver cancer due to hepatitis B | -- | -- | -- | -- | -- |
| Terminal phase of liver cancer due to hepatitis B | -- | -- | -- | -- | -- |
| Liver cancer due to hepatitis C | -- | -- | -- | -- | -- |
| Diagnosis and primary therapy phase of liver cancer due to hepatitis C | -- | -- | -- | -- | -- |
| Controlled phase of liver cancer due to hepatitis C | -- | -- | -- | -- | -- |
| Metastatic phase of liver cancer due to hepatitis C | -- | -- | -- | -- | -- |
| Terminal phase of liver cancer due to hepatitis C | -- | -- | -- | -- | -- |
| Liver cancer due to alcohol use | -- | -- | -- | -- | -- |
| Diagnosis and primary therapy phase of liver cancer due to alcohol use | -- | -- | -- | -- | -- |
| Controlled phase of liver cancer due to alcohol use | -- | -- | -- | -- | -- |
| Metastatic phase of liver cancer due to alcohol use | -- | -- | -- | -- | -- |

| | | | | | |
|---|---|---|---|---|---|
| Terminal phase of liver cancer due to alcohol use | -- | -- | -- | -- | -- |
| Liver cancer due to NASH | -- | -- | -- | -- | -- |
| Diagnosis and primary therapy phase of liver cancer due to NASH | -- | -- | -- | -- | -- |
| Controlled phase of liver cancer due to NASH | -- | -- | -- | -- | -- |
| Metastatic phase of liver cancer due to NASH | -- | -- | -- | -- | -- |
| Terminal phase of liver cancer due to NASH | -- | -- | -- | -- | -- |
| Liver cancer due to other causes | -- | -- | -- | -- | -- |
| Diagnosis and primary therapy phase of liver cancer due to other causes | -- | -- | -- | -- | -- |
| Controlled phase of liver cancer due to other causes | -- | -- | -- | -- | -- |
| Metastatic phase of liver cancer due to other causes | -- | -- | -- | -- | -- |
| Terminal phase of liver cancer due to other causes | -- | -- | -- | -- | -- |
| Gallbladder and biliary tract cancer | -- | -- | -- | -- | -- |
| Diagnosis and primary therapy phase of gallbladder and biliary tract cancer | -- | -- | -- | -- | -- |
| Controlled phase of gallbladder and biliary tract cancer | -- | -- | -- | -- | -- |
| Metastatic phase of gallbladder and biliary tract cancer | -- | -- | -- | -- | -- |
| Terminal phase of gallbladder and biliary tract cancer | -- | -- | -- | -- | -- |
| Pancreatic cancer | -- | -- | -- | -- | -- |
| Diagnosis and primary therapy phase of pancreatic cancer | -- | -- | -- | -- | -- |
| Controlled phase of pancreatic cancer | -- | -- | -- | -- | -- |
| Metastatic phase of pancreatic cancer | -- | -- | -- | -- | -- |
| Terminal phase of pancreatic cancer | -- | -- | -- | -- | -- |
| Larynx cancer | -- | -- | -- | -- | -- |
| Diagnosis and primary therapy phase of larynx cancer | -- | -- | -- | -- | -- |
| Controlled phase of larynx cancer | -- | -- | -- | -- | -- |
| Metastatic phase of larynx cancer | -- | -- | -- | -- | -- |
| Terminal phase of larynx cancer | -- | -- | -- | -- | -- |
| Laryngectomy from larynx cancer, beyond 10 years | -- | -- | -- | -- | -- |
| Tracheal, bronchus, and lung cancer | -- | -- | -- | -- | -- |
| Diagnosis and primary therapy phase of lung, bronchus, and trachea cancer | -- | -- | -- | -- | -- |
| Controlled phase of lung, bronchus, and trachea cancer | -- | -- | -- | -- | -- |
| Metastatic phase of lung, bronchus, and trachea cancer | -- | -- | -- | -- | -- |
| Terminal phase of lung, bronchus, and trachea cancer | -- | -- | -- | -- | -- |
| Malignant skin melanoma | -- | -- | -- | -- | -- |
| Diagnosis and primary therapy phase of malignant skin melanoma | -- | -- | -- | -- | -- |
| Controlled phase of malignant skin melanoma | -- | -- | -- | -- | -- |
| Metastatic phase of malignant skin melanoma | -- | -- | -- | -- | -- |
| Terminal phase of malignant skin melanoma | -- | -- | -- | -- | -- |
| Non-melanoma skin cancer | -- | -- | -- | -- | -- |
| Non-melanoma skin cancer (squamous-cell carcinoma) | -- | -- | -- | -- | -- |
| Non-melanoma skin cancer (basal-cell carcinoma) | -- | -- | -- | -- | -- |
| Breast cancer | -- | -- | -- | -- | -- |
| Diagnosis and primary therapy phase of breast cancer | -- | -- | -- | -- | -- |
| Controlled phase of breast cancer | -- | -- | -- | -- | -- |
| Metastatic phase of breast cancer | -- | -- | -- | -- | -- |
| Terminal phase of breast cancer | -- | -- | -- | -- | -- |
| Mastectomy from breast cancer, beyond 10 years | -- | -- | -- | -- | -- |
| Cervical cancer | -- | -- | -- | -- | -- |
| Diagnosis and primary therapy phase of cervical cancer | -- | -- | -- | -- | -- |
| Controlled phase of cervical cancer | -- | -- | -- | -- | -- |
| Metastatic phase of cervical cancer | -- | -- | -- | -- | -- |
| Terminal phase of cervical cancer | -- | -- | -- | -- | -- |
| Uterine cancer | -- | -- | -- | -- | -- |
| Diagnosis and primary therapy phase of uterine cancer | -- | -- | -- | -- | -- |
| Controlled phase of uterine cancer | -- | -- | -- | -- | -- |
| Metastatic phase of uterine cancer | -- | -- | -- | -- | -- |
| Terminal phase of uterine cancer | -- | -- | -- | -- | -- |
| Ovarian cancer | -- | -- | -- | -- | -- |
| Diagnosis and primary therapy phase of ovarian cancer | -- | -- | -- | -- | -- |
| Controlled phase of ovarian cancer | -- | -- | -- | -- | -- |
| Metastatic phase of ovarian cancer | -- | -- | -- | -- | -- |

| | | | | | |
|---|---|---|---|---|---|
| Terminal phase of ovarian cancer | -- | -- | -- | -- | -- |
| Prostate cancer | -- | -- | -- | -- | -- |
| Diagnosis and primary therapy phase of prostate cancer | -- | -- | -- | -- | -- |
| Controlled phase of prostate cancer | -- | -- | -- | -- | -- |
| Metastatic phase of prostate cancer | -- | -- | -- | -- | -- |
| Terminal phase of prostate cancer | -- | -- | -- | -- | -- |
| Impotence and Incontinence after 10-year survival from prostate cancer | -- | -- | -- | -- | -- |
| Testicular cancer | -- | -- | -- | -- | -- |
| Diagnosis and primary therapy phase of testicular cancer | -- | -- | -- | -- | -- |
| Controlled phase of testicular cancer | -- | -- | -- | -- | -- |
| Metastatic phase of testicular cancer | -- | -- | -- | -- | -- |
| Terminal phase of testicular cancer | -- | -- | -- | -- | -- |
| Kidney cancer | -- | -- | -- | -- | -- |
| Diagnosis and primary therapy phase of kidney cancer | -- | -- | -- | -- | -- |
| Controlled phase of kidney cancer | -- | -- | -- | -- | -- |
| Metastatic phase of kidney cancer | -- | -- | -- | -- | -- |
| Terminal phase of kidney cancer | -- | -- | -- | -- | -- |
| Bladder cancer | -- | -- | -- | -- | -- |
| Diagnosis and primary therapy phase of bladder cancer | -- | -- | -- | -- | -- |
| Controlled phase of bladder cancer | -- | -- | -- | -- | -- |
| Metastatic phase of bladder cancer | -- | -- | -- | -- | -- |
| Terminal phase of bladder cancer | -- | -- | -- | -- | -- |
| Incontinence from bladder cancer, beyond 10 years | -- | -- | -- | -- | -- |
| Brain and nervous system cancer | 44.8 (36.0 to 56.4) | 5.7 (4.6 to 6.9) | 2.9 (1.8 to 4.1) | 20.0 (-1.0 to 50.6) | 15.7 (-4.6 to 45.2) |
| Diagnosis and primary therapy phase of brain and nervous system cancers | 1.9 (1.6 to 2.5) | 5.7 (4.6 to 6.9) | 0.6 (0.4 to 0.8) | 26.0* (3.4 to 59.4) | 21.4 (-0.3 to 53.6) |
| Controlled phase of brain and nervous system cancers | 42.3 (33.9 to 53.5) | -- | 2.1 (1.2 to 3.2) | 25.9* (3.5 to 57.8) | 21.3 (-0.3 to 52.1) |
| Metastatic phase of brain and nervous system cancers | 0.4 (0.3 to 0.5) | -- | 0.2 (0.1 to 0.3) | -19.5 (-31.5 to 1.9) | -22.4* (-34.0 to -1.8) |
| Terminal phase of brain and nervous system cancers | 0.1 (0.1 to 0.1) | -- | 0.0 (0.0 to 0.1) | -19.5 (-31.5 to 1.9) | -22.4* (-34.0 to -1.8) |
| Thyroid cancer | -- | -- | -- | -- | -- |
| Diagnosis and primary therapy phase of thyroid cancer | -- | -- | -- | -- | -- |
| Controlled phase of thyroid cancer | -- | -- | -- | -- | -- |
| Metastatic phase of thyroid cancer | -- | -- | -- | -- | -- |
| Terminal phase of thyroid cancer | -- | -- | -- | -- | -- |
| Mesothelioma | -- | -- | -- | -- | -- |
| Diagnosis and primary therapy phase of mesothelioma | -- | -- | -- | -- | -- |
| Controlled phase of mesothelioma | -- | -- | -- | -- | -- |
| Metastatic phase of mesothelioma | -- | -- | -- | -- | -- |
| Terminal phase of mesothelioma | -- | -- | -- | -- | -- |
| Hodgkin lymphoma | 3.6 (2.7 to 4.3) | 0.4 (0.3 to 0.5) | 0.2 (0.1 to 0.3) | 7.8 (-10.0 to 31.0) | 4.0 (-13.2 to 26.3) |
| Diagnosis and primary therapy phase of Hodgkin disease | 0.1 (0.1 to 0.1) | 0.4 (0.3 to 0.5) | -- | 9.3 (-4.3 to 26.7) | 5.3 (-7.7 to 22.1) |
| Controlled phase of Hodgkin disease | 3.4 (2.6 to 4.1) | -- | 0.2 (0.1 to 0.3) | 9.8 (-11.2 to 35.8) | 5.8 (-14.4 to 30.9) |
| Metastatic phase of Hodgkin disease | -- | -- | -- | -18.0 (-34.0 to 7.5) | -21.0 (-36.4 to 3.6) |
| Terminal phase of Hodgkin disease | -- | -- | -- | -18.0 (-34.0 to 7.5) | -21.0 (-36.4 to 3.6) |
| Non-Hodgkin's lymphoma | -- | -- | -- | -- | -- |
| Diagnosis and primary therapy phase of non-Hodgkin lymphoma | -- | -- | -- | -- | -- |
| Controlled phase of non-Hodgkin lymphoma | -- | -- | -- | -- | -- |
| Metastatic phase of non-Hodgkin lymphoma | -- | -- | -- | -- | -- |
| Terminal phase of non-Hodgkin lymphoma | -- | -- | -- | -- | -- |
| Multiple myeloma | -- | -- | -- | -- | -- |
| Diagnosis and primary therapy phase of multiple myeloma | -- | -- | -- | -- | -- |
| Controlled phase of multiple myeloma | -- | -- | -- | -- | -- |
| Metastatic phase of multiple myeloma | -- | -- | -- | -- | -- |
| Terminal phase of multiple myeloma | -- | -- | -- | -- | -- |
| Leukaemia | 167.5 (127.1 to 199.1) | 14.8 (11.4 to 17.4) | 8.8 (5.5 to 13.5) | 27.1 (-0.8 to 53.5) | 22.5 (-4.4 to 48.0) |
| Acute lymphoid leukaemia | 61.9 (44.3 to 78.5) | 1.7 (1.4 to 1.9) | 3.4 (2.0 to 5.3) | 76.1* (17.1 to 129.6) | 69.8* (12.8 to 121.3) |
| Diagnosis and primary therapy phase of acute lymphoid leukemia | 1.3 (1.1 to 1.5) | 1.7 (1.4 to 1.9) | 0.4 (0.2 to 0.6) | 5.2 (-20.6 to 27.0) | 1.4 (-23.4 to 22.4) |
| Controlled phase of acute lymphoid leukemia | 60.4 (42.8 to 76.8) | -- | 2.9 (1.6 to 4.7) | 103.7* (35.2 to 167.6) | 96.4* (30.3 to 157.9) |
| Metastatic phase of acute lymphoid leukemia | 0.1 (0.1 to 0.2) | -- | 0.1 (0.0 to 0.1) | -35.2* (-49.3 to -16.9) | -37.5* (-51.1 to -19.9) |
| Terminal phase of acute lymphoid leukemia | -- | -- | -- | -35.2* (-49.3 to -16.9) | -37.5* (-51.1 to -19.9) |
| Chronic lymphoid leukaemia | -- | -- | -- | -- | -- |

| | | | | | |
|---|---|---|---|---|---|
| Diagnosis and primary therapy phase of chronic lymphoid leukemia | -- | -- | -- | -- | -- |
| Controlled phase of chronic lymphoid leukemia | -- | -- | -- | -- | -- |
| Metastatic phase of chronic lymphoid leukemia | -- | -- | -- | -- | -- |
| Terminal phase of chronic lymphoid leukemia | -- | -- | -- | -- | -- |
| Acute myeloid leukaemia | 3.2 (2.5 to 3.7) | 1.6 (1.2 to 1.8) | 0.4 (0.3 to 0.6) | -1.8 (-27.6 to 26.7) | -5.3 (-30.2 to 22.1) |
| Diagnosis and primary therapy phase of acute myeloid leukemia | -- | 1.6 (1.2 to 1.8) | -- | -- | -- |
| Controlled phase of acute myeloid leukemia | 2.6 (2.1 to 3.0) | -- | 0.1 (0.1 to 0.2) | 1.2 (-25.6 to 30.4) | -2.5 (-28.3 to 25.7) |
| Metastatic phase of acute myeloid leukemia | 0.5 (0.4 to 0.6) | -- | 0.2 (0.1 to 0.3) | -3.1 (-28.1 to 25.5) | -6.6 (-30.7 to 20.9) |
| Terminal phase of acute myeloid leukemia | 0.1 (0.1 to 0.1) | -- | 0.1 (0.0 to 0.1) | -3.1 (-28.1 to 25.5) | -6.6 (-30.7 to 20.9) |
| Chronic myeloid leukaemia | -- | -- | -- | -- | -- |
| Diagnosis and primary therapy phase of chronic myeloid leukemia | -- | -- | -- | -- | -- |
| Controlled phase of chronic myeloid leukemia | -- | -- | -- | -- | -- |
| Metastatic phase of chronic myeloid leukemia | -- | -- | -- | -- | -- |
| Terminal phase of chronic myeloid leukemia | -- | -- | -- | -- | -- |
| Other leukaemia | 102.4 (77.0 to 124.1) | 11.5 (8.7 to 14.0) | 5.0 (3.0 to 7.8) | 9.1 (-16.0 to 36.4) | 5.1 (-19.1 to 31.5) |
| Metastatic phase of other leukemia | 0.1 (0.1 to 0.1) | -- | -- | -40.5* (-51.4 to -23.2) | -42.6* (-53.2 to -26.0) |
| Diagnosis and primary therapy phase of other leukemia | -- | 11.5 (8.7 to 14.0) | -- | -- | -- |
| Terminal phase of other leukemia | -- | -- | -- | -40.5* (-51.4 to -23.2) | -42.6* (-53.2 to -26.0) |
| Controlled phase of other leukemia | 102.3 (77.0 to 124.0) | -- | 5.0 (3.0 to 7.8) | 9.8 (-15.5 to 37.8) | 5.9 (-18.5 to 32.8) |
| Other malignant cancers | 388.8 (257.3 to 627.0) | 11.0 (9.7 to 12.4) | 20.9 (11.2 to 37.8) | 219.1* (113.0 to 383.5) | 207.6* (105.3 to 366.0) |
| Diagnosis and primary therapy phase of other malignant neoplasms | 6.8 (4.5 to 11.0) | 11.0 (9.7 to 12.4) | 1.9 (1.0 to 3.3) | 228.6* (115.6 to 414.5) | 216.7* (107.8 to 395.9) |
| Controlled phase of other malignant neoplasms | 381.6 (252.1 to 615.2) | -- | 18.6 (9.7 to 34.4) | 232.1* (121.8 to 407.2) | 220.1* (113.8 to 388.9) |
| Metastatic phase of other malignant neoplasms | 0.7 (0.6 to 0.8) | -- | 0.3 (0.2 to 0.4) | 2.2 (-17.9 to 24.9) | -1.5 (-20.9 to 20.4) |
| Terminal phase of other malignant neoplasms | 0.1 (0.1 to 0.2) | -- | 0.1 (0.0 to 0.1) | 2.2 (-17.9 to 24.9) | -1.5 (-20.9 to 20.4) |
| Other neoplasms | 35.8 (33.2 to 38.6) | 106.6 (101.7 to 112.4) | 0.0 (0.0 to 0.1) | -33.6* (-39.0 to -29.6) | -36.0* (-41.2 to -32.1) |
| Myelodysplastic, myeloproliferative, and other hematopoietic neoplasms | 0.7 (0.5 to 0.9) | 1.5 (1.1 to 2.0) | 0.0 (0.0 to 0.1) | -33.6* (-39.0 to -29.6) | -36.0* (-41.2 to -32.1) |
| Benign and in situ intestinal neoplasms | 0.8 (0.7 to 0.8) | 1.9 (1.8 to 2.0) | -- | -- | -- |
| Benign and in situ cervical and uterine neoplasms | -- | 0.1 (0.0 to 0.1) | -- | -- | -- |
| Other benign and in situ neoplasms | 40.1 (38.1 to 42.2) | 103.1 (98.3 to 108.8) | -- | -- | -- |
| **Cardiovascular diseases** | **--** | **48.9 (41.3 to 57.7)** | **1.3 (0.9 to 1.7)** | **-47.0* (-50.6 to -43.2)** | **-48.9* (-52.3 to -45.3)** |
| Rheumatic heart disease | -- | -- | -- | -- | -- |
| Rheumatic heart disease, without heart failure | -- | -- | -- | -- | -- |
| Heart failure due to rheumatic heart disease | -- | -- | -- | -- | -- |
| Ischaemic heart disease | -- | -- | -- | -- | -- |
| Asymptomatic ischemic heart disease following myocardial infarction | -- | -- | -- | -- | -- |
| Angina due to ischemic heart disease | -- | -- | -- | -- | -- |
| Heart failure due to ischemic heart disease | -- | -- | -- | -- | -- |
| Myocardial infarction due to ischemic heart disease | -- | -- | -- | -- | -- |
| Stroke | -- | 12.5‡ (9.4 to 16.6) | 1.1 (0.8 to 1.5) | -50.8* (-54.7 to -46.8) | -52.6* (-56.3 to -48.8) |
| Ischaemic stroke | 6.2 (5.1 to 7.5) | 10.9 (8.0 to 15.0) | 1.0 (0.7 to 1.3) | -53.0* (-57.2 to -48.7) | -54.7* (-58.7 to -50.6) |
| Acute ischemic stroke | 0.7 (0.5 to 1.0) | 10.9 (8.0 to 15.0) | 0.2 (0.1 to 0.3) | -53.9* (-60.7 to -47.2) | -55.5* (-62.2 to -49.1) |
| Chronic ischemic stroke | 5.5 (4.4 to 6.8) | -- | 0.8 (0.5 to 1.1) | -52.8* (-57.5 to -47.4) | -54.5* (-59.0 to -49.3) |
| Intracerebral hemorrhage | -- | -- | -- | -- | -- |
| Acute intracerebral hemorrhage | -- | -- | -- | -- | -- |
| Chronic intracerebral hemorrhage | -- | -- | -- | -- | -- |
| Subarachnoid hemorrhage | 0.8 (0.6 to 1.0) | 1.5 (1.0 to 2.6) | 0.1 (0.1 to 0.2) | -19.9* (-37.3 to -10.4) | -22.8* (-39.6 to -13.7) |
| Acute subarachnoid hemorrhage | 0.1 (0.0 to 0.1) | 1.5 (1.0 to 2.6) | -- | -38.7* (-63.2 to -22.1) | -40.9* (-64.5 to -24.9) |
| Chronic subarachnoid hemorrhage | 0.7 (0.5 to 0.9) | -- | 0.1 (0.1 to 0.1) | -13.8* (-23.3 to -6.3) | -16.9* (-26.1 to -9.7) |
| Hypertensive heart disease | -- | -- | -- | -- | -- |
| Non-rheumatic valvular heart disease | -- | -- | -- | -- | -- |
| Non-rheumatic calcific aortic valve disease | -- | -- | -- | -- | -- |
| Heart failure due to calcific aortic valve disease | -- | -- | -- | -- | -- |
| Non-rheumatic calcific aortic valve disease | -- | -- | -- | -- | -- |
| Non-rheumatic degenerative mitral valve disease | -- | -- | -- | -- | -- |
| Heart failure due to degenerative mitral valve disease | -- | -- | -- | -- | -- |
| Non-rheumatic degenerative mitral valve disease | -- | -- | -- | -- | -- |
| Other non-rheumatic valve diseases | -- | -- | -- | -- | -- |
| Mild heart failure due to other non-rheumatic valve disease | -- | -- | -- | -- | -- |
| Moderate heart failure due to other non-rheumatic valve disease | -- | -- | -- | -- | -- |
| Severe heart failure due to other non-rheumatic valve disease | -- | -- | -- | -- | -- |

| | | | | | |
|---|---|---|---|---|---|
| Controlled, medically managed heart failure due to other non-rheumatic valve disease | -- | -- | -- | -- | -- |
| Cardiomyopathy and myocarditis | -- | -- | -- | -- | -- |
| Myocarditis | -- | -- | -- | -- | -- |
| Acute myocarditis | -- | -- | -- | -- | -- |
| Heart failure due to myocarditis | -- | -- | -- | -- | -- |
| Alcoholic cardiomyopathy | -- | -- | -- | -- | -- |
| Other cardiomyopathy | -- | -- | -- | -- | -- |
| Atrial fibrillation and flutter | -- | -- | -- | -- | -- |
| Asymptomatic atrial fibrillation and flutter | -- | -- | -- | -- | -- |
| Symptomatic atrial fibrillation and flutter | -- | -- | -- | -- | -- |
| Peripheral vascular disease | -- | -- | -- | -- | -- |
| Asymptomatic peripheral arterial disease | -- | -- | -- | -- | -- |
| Symptomatic claudication due to peripheral arterial disease | -- | -- | -- | -- | -- |
| Endocarditis | 2.5 (2.0 to 2.9) | 36.4 (29.5 to 44.7) | 0.2 (0.1 to 0.2) | 10.4* (5.5 to 14.9) | 6.4* (1.7 to 10.8) |
| Acute endocarditis | 1.7 (1.4 to 2.1) | 36.4 (29.5 to 44.7) | 0.1 (0.1 to 0.1) | 12.0* (3.7 to 19.2) | 7.9 (-0.1 to 14.9) |
| Heart failure due to endocarditis | 0.8 (0.5 to 1.1) | -- | 0.1 (0.0 to 0.1) | 8.3* (6.1 to 10.3) | 4.4* (2.2 to 6.4) |
| Other cardiovascular and circulatory diseases | -- | -- | -- | -- | -- |
| Heart failure due to other cardiovascular diseases | -- | -- | -- | -- | -- |
| Other cardiovascular and circulatory disease episodes | -- | -- | -- | -- | -- |
| **Chronic respiratory diseases** | **--** | **--** | **--** | **--** | **--** |
| Chronic obstructive pulmonary disease | -- | -- | -- | -- | -- |
| Chronic obstructive pulmonary disease with heart failure | -- | -- | -- | -- | -- |
| Chronic obstructive pulmonary disease without heart failure | -- | -- | -- | -- | -- |
| Pneumoconiosis | -- | -- | -- | -- | -- |
| Silicosis | -- | -- | -- | -- | -- |
| Heart failure due to silicosis | -- | -- | -- | -- | -- |
| Silicosis without heart failure | -- | -- | -- | -- | -- |
| Asbestosis | -- | -- | -- | -- | -- |
| Asbestosis without heart failure | -- | -- | -- | -- | -- |
| Heart failure due to asbestosis | -- | -- | -- | -- | -- |
| Coal workers pneumoconiosis | -- | -- | -- | -- | -- |
| Coal workers pneumoconiosis without heart failure | -- | -- | -- | -- | -- |
| Heart failure due to Coal workers pneumoconiosis | -- | -- | -- | -- | -- |
| Other pneumoconiosis | -- | -- | -- | -- | -- |
| Heart failure due to other pneumoconiosis | -- | -- | -- | -- | -- |
| Other pneumoconiosis without heart failure | -- | -- | -- | -- | -- |
| Asthma | -- | -- | -- | -- | -- |
| Asymptomatic asthma | -- | -- | -- | -- | -- |
| Symptomatic asthma | -- | -- | -- | -- | -- |
| Interstitial lung disease and pulmonary sarcoidosis | -- | -- | -- | -- | -- |
| Heart failure due to interstitial lung disease and pulmonary sarcoidosis | -- | -- | -- | -- | -- |
| Interstitial lung disease and pulmonary sarcoidosis without heart failure | -- | -- | -- | -- | -- |
| Other chronic respiratory diseases | -- | -- | -- | -- | -- |
| **Digestive diseases** | **3757.0 (2943.0 to 4472.6)** | **519.2 (441.8 to 617.6)** | **53.3 (33.8 to 80.9)** | **2.7 (-0.4 to 6.1)** | **-1.0 (-4.0 to 2.3)** |
| Cirrhosis and other chronic liver diseases | 3315.0 (2543.0 to 4030.8) | 171.3 (154.5 to 190.6) | 4.2 (2.9 to 5.9) | 6.3 (-2.6 to 15.9) | 2.5 (-6.1 to 11.7) |
| Cirrhosis and other chronic liver diseases due to hepatitis B | 3051.1 (2297.1 to 3755.0) | 0.8 (0.6 to 0.9) | -- | -4.6 (-8.9 to 1.4) | -8.0* (-12.2 to -2.2) |
| Cirrhosis and other chronic liver diseases due to hepatitis B, decompensated | 0.1 (0.1 to 0.1) | 0.2 (0.2 to 0.2) | -- | -4.6 (-8.9 to 1.4) | -8.0* (-12.2 to -2.2) |
| Chronic hepatitis B without cirrhosis | 3050.6 (2296.5 to 3754.5) | -- | -- | -- | -- |
| Cirrhosis and other chronic liver diseases due to hepatitis B, compensated | 0.4 (0.3 to 0.5) | 0.6 (0.5 to 0.7) | -- | -- | -- |
| Cirrhosis and other chronic liver diseases due to hepatitis C | 157.2 (105.3 to 218.6) | 0.5 (0.4 to 0.6) | -- | 4.5* (1.6 to 7.5) | 0.7 (-2.0 to 3.6) |
| Cirrhosis and other chronic liver diseases due to hepatitis C, decompensated | 0.1 (0.1 to 0.1) | 0.1 (0.1 to 0.1) | -- | 4.5* (1.6 to 7.5) | 0.7 (-2.0 to 3.6) |
| Chronic hepatitis C without cirrhosis | 156.9 (105.0 to 218.3) | -- | -- | -- | -- |
| Cirrhosis and other chronic liver diseases due to hepatitis C, compensated | 0.2 (0.2 to 0.3) | 0.4 (0.3 to 0.5) | -- | -- | -- |
| Cirrhosis and other chronic liver diseases due to alcohol use | -- | -- | -- | -- | -- |
| Cirrhosis and other chronic liver diseases due to alcohol, decompensated | -- | -- | -- | -- | -- |
| Cirrhosis and other chronic liver diseases due to alcohol, compensated | -- | -- | -- | -- | -- |
| Cirrhosis due to NASH | -- | -- | -- | -- | -- |
| Non-alcoholic fatty liver disease (NAFLD) / Non-alcoholic steatohepatitis (NASH) | -- | -- | -- | -- | -- |
| Cirrhosis and other chronic liver diseases due to NASH | -- | -- | -- | -- | -- |
| Cirrhosis and other chronic liver diseases due to other causes | 106.7 (95.3 to 119.1) | 170.0 (153.4 to 189.2) | 4.2 (2.8 to 5.8) | 6.4 (-2.6 to 16.1) | 2.5 (-6.2 to 11.9) |

| | | | | | |
|---|---|---|---|---|---|
| Cirrhosis and other chronic liver diseases due to other cause, decompensated | 23.8 (22.6 to 25.1) | 36.8 (35.1 to 38.6) | 4.2 (2.8 to 5.8) | 6.4 (-2.6 to 16.1) | 2.5 (-6.2 to 11.9) |
| Cirrhosis and other chronic liver diseases due to other cause, compensated | 82.9 (71.5 to 95.3) | 133.2 (116.6 to 152.7) | -- | -- | -- |
| Upper digestive system diseases | -- | -- | -- | -- | -- |
| Peptic ulcer disease | -- | -- | -- | -- | -- |
| Acute peptic ulcer disease | -- | -- | -- | -- | -- |
| Chronic peptic ulcer disease | -- | -- | -- | -- | -- |
| Gastritis and duodenitis | -- | -- | -- | -- | -- |
| Acute gastritis and duodenitis | -- | -- | -- | -- | -- |
| Chronic gastritis and duodenitis | -- | -- | -- | -- | -- |
| GERD | -- | -- | -- | -- | -- |
| Appendicitis | -- | -- | -- | -- | -- |
| Paralytic ileus and intestinal obstruction | 3.4 (2.7 to 4.0) | 80.4 (62.9 to 99.3) | 1.1 (0.7 to 1.6) | 1.4 (-3.7 to 6.2) | -2.2 (-7.1 to 2.4) |
| Inguinal, femoral, and abdominal hernia | 468.0 (348.9 to 629.9) | 267.5 (193.8 to 363.7) | 48.0 (29.9 to 73.6) | 2.4 (-1.0 to 6.0) | -1.3 (-4.6 to 2.2) |
| Inflammatory bowel disease | -- | -- | -- | -- | -- |
| Ulcerative colitis | -- | -- | -- | -- | -- |
| Crohn's disease | -- | -- | -- | -- | -- |
| Vascular intestinal disorders | -- | -- | -- | -- | -- |
| Gallbladder and biliary diseases | -- | -- | -- | -- | -- |
| Pancreatitis | -- | -- | -- | -- | -- |
| Acute pancreatitis | -- | -- | -- | -- | -- |
| Chronic pancreatitis | -- | -- | -- | -- | -- |
| Other digestive diseases | -- | -- | -- | -- | -- |
| **Neurological disorders** | **350.8 (242.9 to 488.3)** | **101.8 (69.3 to 143.0)** | **181.0 (108.5 to 284.4)** | **13.1 (-3.8 to 33.2)** | **9.0 (-7.3 to 28.4)** |
| Alzheimer's disease and other dementias | -- | -- | -- | -- | -- |
| Parkinson's disease | -- | -- | -- | -- | -- |
| Epilepsy | 352.0 (243.7 to 488.6) | 99.0 (66.3 to 140.4) | 121.8 (73.0 to 191.4) | 8.7 (-7.6 to 28.0) | 4.8 (-10.9 to 23.4) |
| Multiple sclerosis | -- | -- | -- | -- | -- |
| Motor neuron disease | 1.0 (0.8 to 1.2) | 2.8 (2.3 to 3.3) | 0.2 (0.1 to 0.3) | 0.8 (-1.2 to 2.7) | -2.9* (-4.7 to -1.0) |
| Headache disorders | -- | -- | -- | -- | -- |
| Migraine | -- | -- | -- | -- | -- |
| Medication overuse headache due to migraine | -- | -- | -- | -- | -- |
| Migraine | -- | -- | -- | -- | -- |
| Tension-type headache | -- | -- | -- | -- | -- |
| Medication overuse headache due to tension-type headache | -- | -- | -- | -- | -- |
| Tension-type headache | -- | -- | -- | -- | -- |
| Other neurological disorders | -- | -- | 59.0 (35.4 to 92.7) | 23.4* (5.0 to 45.4) | 19.0* (1.2 to 40.2) |
| Other neurological disorders | -- | -- | 59.0 (35.4 to 92.7) | 23.4* (5.0 to 45.4) | 19.0* (1.2 to 40.2) |
| Guillain-Barré syndrome due to other neurological disorders | -- | -- | -- | -- | -- |
| **Mental disorders** | **2647.7 (1765.7 to 3557.3)** | **--** | **181.2 (113.3 to 264.5)** | **-6.0* (-8.0 to -3.4)** | **-9.4* (-11.4 to -6.9)** |
| Schizophrenia | -- | -- | -- | -- | -- |
| Depressive disorders | -- | -- | -- | -- | -- |
| Major depressive disorder | -- | -- | -- | -- | -- |
| Dysthymia | -- | -- | -- | -- | -- |
| Bipolar disorder | -- | -- | -- | -- | -- |
| Anxiety disorders | -- | -- | -- | -- | -- |
| Eating disorders | -- | -- | -- | -- | -- |
| Anorexia nervosa | -- | -- | -- | -- | -- |
| Bulimia nervosa | -- | -- | -- | -- | -- |
| Autism spectrum disorders | 611.8 (549.0 to 674.4) | -- | 95.5 (65.2 to 132.5) | 4.3* (2.2 to 6.5) | 0.5 (-1.5 to 2.7) |
| Attention-deficit/hyperactivity disorder | -- | -- | -- | -- | -- |
| Conduct disorder | -- | -- | -- | -- | -- |
| Idiopathic developmental intellectual disability | 2082.7 (1173.3 to 3016.7) | -- | 85.7 (40.5 to 148.3) | -15.3* (-18.9 to -13.0) | -18.4* (-21.9 to -16.2) |
| Other mental disorders | -- | -- | -- | -- | -- |
| **Substance use disorders** | **0.9 (0.6 to 1.2)** | **--** | **0.2 (0.1 to 0.3)** | **-9.3* (-16.3 to -2.8)** | **-12.6* (-19.3 to -6.3)** |
| Alcohol use disorders | 5.8 (4.6 to 7.3) | -- | 0.2 (0.1 to 0.3) | -9.3* (-16.3 to -2.8) | -12.6* (-19.3 to -6.3) |
| Alcohol dependence | -- | -- | -- | -- | -- |
| Fetal alcohol syndrome | 5.8 (4.6 to 7.3) | -- | 0.2 (0.1 to 0.3) | -9.3* (-16.3 to -2.8) | -12.6* (-19.3 to -6.3) |
| Drug use disorders | -- | -- | -- | -- | -- |
| Opioid use disorders | -- | -- | -- | -- | -- |
| Cocaine use disorders | -- | -- | -- | -- | -- |

| | | | | | |
|---|---|---|---|---|---|
| Amphetamine use disorders | -- | -- | -- | -- | -- |
| Cannabis use disorders | -- | -- | -- | -- | -- |
| Other drug use disorders | -- | -- | -- | -- | -- |
| **Diabetes and kidney diseases** | **10.7 (7.3 to 15.0)** | **79.9 (58.1 to 106.5)** | **3.3 (1.8 to 5.3)** | **2.3 (-10.3 to 17.6)** | **-1.4 (-13.6 to 13.3)** |
| Diabetes mellitus | -- | 5.9 (4.1 to 8.0) | -- | -- | -- |
| Diabetes mellitus type 1 | -- | 5.9 (4.1 to 8.0) | -- | -- | -- |
| Uncomplicated diabetes mellitus type 1 | -- | 5.9 (4.1 to 8.0) | -- | -- | -- |
| Vision loss due to diabetes mellitus type 1 retinopathy | -- | -- | -- | -- | -- |
| Diabetic neuropathy due to diabetes mellitus type 1 | -- | -- | -- | -- | -- |
| Diabetes mellitus type 2 | -- | -- | -- | -- | -- |
| Uncomplicated diabetes mellitus type 2 | -- | -- | -- | -- | -- |
| Vision loss due to diabetes mellitus type 2 retinopathy | -- | -- | -- | -- | -- |
| Diabetic neuropathy due to diabetes mellitus type 2 | -- | -- | -- | -- | -- |
| Chronic kidney disease | 35.6 (24.5 to 50.4) | 66.8 (45.3 to 93.1) | 3.3 (1.8 to 5.3) | 2.4 (-10.4 to 17.7) | -1.3 (-13.6 to 13.5) |
| Chronic kidney disease due to diabetes mellitus type 1 | 2.2 (1.5 to 3.2) | 4.5 (2.9 to 6.4) | 0.1 (0.1 to 0.2) | 1.6 (-12.7 to 17.8) | -2.1 (-15.9 to 13.5) |
| Albuminuria with preserved GFR due to type 1 diabetes mellitus | -- | -- | -- | -- | -- |
| End-stage chronic kidney disease due to diabetes mellitus type 1 | -- | -- | -- | 1.8 (-1.0 to 4.5) | -1.9 (-4.6 to 0.8) |
| Stage III chronic kidney disease due to diabetes mellitus type 1 | 2.0 (1.4 to 3.0) | 4.5 (2.9 to 6.4) | 0.0 (0.0 to 0.1) | -1.4 (-17.1 to 17.3) | -5.0 (-20.1 to 13.1) |
| Stage IV chronic kidney disease due to diabetes mellitus type 1 | -- | -- | -- | 4.0 (-10.5 to 20.9) | 0.3 (-13.7 to 16.5) |
| Stage V chronic kidney disease due to diabetes mellitus type 1 | 0.1 (0.1 to 0.2) | -- | 0.1 (0.0 to 0.1) | 2.9 (-11.4 to 18.7) | -0.8 (-14.6 to 14.4) |
| Chronic kidney disease due to diabetes mellitus type 2 | 0.3 (0.2 to 0.4) | 0.6 (0.4 to 0.9) | -- | 2.4 (-11.4 to 18.2) | -1.3 (-14.6 to 13.9) |
| Albuminuria with preserved GFR due to type 2 diabetes mellitus | -- | -- | -- | -- | -- |
| End-stage chronic kidney disease due to diabetes mellitus type 2 | -- | -- | -- | 1.6 (-1.4 to 4.4) | -2.1 (-4.9 to 0.7) |
| Stage III chronic kidney disease due to diabetes mellitus type 2 | 0.3 (0.2 to 0.4) | 0.6 (0.4 to 0.9) | -- | -1.4 (-16.3 to 16.0) | -4.9 (-19.3 to 11.8) |
| Stage IV chronic kidney disease due to diabetes mellitus type 2 | -- | -- | -- | 4.3 (-10.3 to 21.8) | 0.6 (-13.6 to 17.4) |
| Stage V chronic kidney disease due to diabetes mellitus type 2 | -- | -- | -- | 3.5 (-11.0 to 19.0) | -0.3 (-14.2 to 14.7) |
| Chronic kidney disease due to hypertension | 6.4 (4.4 to 9.1) | 15.8 (10.8 to 22.3) | 0.5 (0.2 to 0.8) | 2.9 (-10.6 to 19.1) | -0.8 (-13.8 to 14.8) |
| Albuminuria with preserved GFR due to hypertension | -- | -- | -- | -- | -- |
| End-stage chronic kidney disease due to hypertension | -- | -- | -- | 3.4* (0.0 to 6.5) | -0.3 (-3.6 to 2.6) |
| Stage III chronic kidney disease due to hypertension | 5.6 (3.7 to 8.0) | 15.8 (10.8 to 22.3) | -- | -1.3 (-16.4 to 16.9) | -4.8 (-19.4 to 12.7) |
| Stage IV chronic kidney disease due to hypertension | -- | -- | -- | 4.0 (-10.3 to 21.5) | 0.2 (-13.5 to 17.1) |
| Stage V chronic kidney disease due to hypertension | 0.8 (0.4 to 1.2) | -- | 0.5 (0.2 to 0.8) | 3.1 (-10.4 to 19.0) | -0.7 (-13.6 to 14.7) |
| Chronic kidney disease due to glomerulonephritis | 3.9 (2.6 to 5.5) | 2.0 (1.4 to 2.9) | 0.9 (0.5 to 1.4) | 2.4 (-10.4 to 18.3) | -1.3 (-13.7 to 14.0) |
| Albuminuria with preserved GFR due to glomerulonephritis | -- | -- | -- | -- | -- |
| End-stage chronic kidney disease due to glomerulonephritis | 0.1 (0.1 to 0.2) | -- | 0.0 (0.0 to 0.1) | 1.3 (-1.1 to 3.4) | -2.4* (-4.7 to -0.3) |
| Stage III chronic kidney disease due to glomerulonephritis | 2.6 (1.8 to 3.7) | 2.0 (1.4 to 2.9) | 0.1 (0.1 to 0.2) | -1.4 (-18.1 to 16.9) | -5.0 (-21.0 to 12.7) |
| Stage IV chronic kidney disease due to glomerulonephritis | -- | -- | -- | 4.1 (-10.5 to 22.1) | 0.4 (-13.8 to 17.7) |
| Stage V chronic kidney disease due to glomerulonephritis | 1.2 (0.7 to 1.9) | -- | 0.7 (0.3 to 1.2) | 3.2 (-10.8 to 19.0) | -0.5 (-14.0 to 14.7) |
| Chronic kidney disease due to other and unspecified causes | 22.8 (15.8 to 32.2) | 43.8 (29.8 to 61.0) | 1.8 (1.0 to 2.9) | 2.3 (-10.1 to 17.3) | -1.4 (-13.3 to 13.0) |
| Albuminuria with preserved GFR due to other and unspecified causes | -- | -- | -- | -- | -- |
| End-stage chronic kidney disease due to other causes | 0.4 (0.3 to 0.5) | -- | 0.1 (0.1 to 0.2) | 0.0 (-2.4 to 2.0) | -3.6* (-6.0 to -1.7) |
| Stage III chronic kidney disease due to other causes | 20.2 (13.9 to 28.9) | 43.8 (29.8 to 61.0) | 0.4 (0.2 to 0.5) | -1.5 (-16.6 to 16.3) | -5.1 (-19.6 to 12.1) |
| Stage IV chronic kidney disease due to other causes | 0.0 (0.0 to 0.1) | -- | -- | 4.4 (-10.0 to 21.9) | 0.7 (-13.3 to 17.5) |
| Stage V chronic kidney disease due to other causes | 2.2 (1.2 to 3.6) | -- | 1.3 (0.6 to 2.2) | 3.7 (-9.8 to 19.7) | -0.0 (-13.1 to 15.3) |
| Acute glomerulonephritis | 0.4 (0.3 to 0.6) | 7.2 (5.3 to 9.5) | -- | -6.6* (-10.6 to -3.7) | -10.0* (-13.8 to -7.2) |
| **Skin and subcutaneous diseases** | **16636.1 (14689.5 to 18756.0)** | **71919.8 (62734.1 to 83787.4)** | **257.9 (159.6 to 399.6)** | **-0.1 (-2.3 to 2.2)** | **-3.8* (-5.8 to -1.5)** |
| Dermatitis | -- | -- | -- | -- | -- |
| Atopic dermatitis | -- | -- | -- | -- | -- |
| Contact dermatitis | -- | -- | -- | -- | -- |
| Seborrhoeic dermatitis | -- | -- | -- | -- | -- |
| Psoriasis | -- | -- | -- | -- | -- |
| Bacterial skin diseases | 439.7 (414.8 to 464.1) | 9561.2 (8998.7 to 10131.5) | 4.3 (2.4 to 7.5) | 6.1* (3.5 to 8.4) | 2.3 (-0.3 to 4.5) |
| Cellulitis | 31.2 (26.3 to 36.4) | 595.7 (504.3 to 704.2) | 1.8 (1.2 to 2.7) | 1.1 (-1.5 to 3.7) | -2.5* (-5.0 to -0.0) |
| Pyoderma | 431.2 (407.6 to 453.5) | 8965.5 (8399.4 to 9538.6) | 2.5 (1.0 to 5.1) | 10.2* (7.2 to 13.1) | 6.2* (3.3 to 9.0) |
| Impetigo | 197.9 (181.8 to 214.5) | 4051.2 (3652.5 to 4447.7) | 1.1 (0.5 to 2.3) | 10.9* (6.6 to 15.3) | 6.9* (2.7 to 11.1) |
| Abscess and other bacterial skin diseases | 233.3 (217.4 to 248.8) | 4914.3 (4575.0 to 5270.9) | 1.3 (0.5 to 2.9) | 9.6* (5.8 to 13.9) | 5.7* (2.0 to 9.8) |
| Scabies | 1892.1 (1481.1 to 2367.1) | 8428.1 (6378.8 to 10754.5) | 49.7 (26.0 to 85.3) | 3.4* (1.8 to 4.8) | -0.4 (-1.9 to 1.0) |
| Fungal skin diseases | 9740.0 (7753.8 to 11980.5) | 39156.0 (30234.3 to 49720.4) | 55.9 (21.0 to 120.0) | -13.2* (-15.0 to -11.2) | -16.4* (-18.1 to -14.4) |
| Tinea capitis | 7061.6 (5313.0 to 9197.2) | 27158.5 (19393.0 to 37674.1) | 40.5 (14.9 to 88.0) | -18.7* (-20.2 to -17.1) | -21.6* (-23.1 to -20.1) |
| Other fungal skin diseases | 2678.5 (2005.8 to 3514.3) | 11997.5 (8649.1 to 15739.6) | 15.4 (5.7 to 33.4) | 5.4* (4.2 to 6.6) | 1.6* (0.4 to 2.7) |

| | | | | | |
|---|---|---|---|---|---|
| Viral skin diseases | -- | -- | -- | -- | -- |
| Viral warts | -- | -- | -- | -- | -- |
| Molluscum contagiosum | -- | -- | -- | -- | -- |
| Acne vulgaris | -- | -- | -- | -- | -- |
| Alopecia areata | -- | -- | -- | -- | -- |
| Pruritus | 230.0 (174.9 to 301.8) | 558.4 (408.6 to 747.7) | 2.5 (1.1 to 4.9) | 5.0* (1.3 to 8.6) | 1.2 (-2.3 to 4.7) |
| Urticaria | 2079.8 (1602.2 to 2587.1) | 6670.0 (5123.6 to 8413.0) | 127.6 (80.2 to 191.3) | 3.9* (2.2 to 5.7) | 0.1 (-1.5 to 1.8) |
| Decubitus ulcer | -- | -- | -- | -- | -- |
| Other skin and subcutaneous diseases | 3157.2 (2967.6 to 3329.5) | 7546.1 (7047.8 to 8005.6) | 17.9 (8.5 to 32.9) | 8.5* (7.3 to 9.9) | 4.6* (3.4 to 5.9) |
| **Sense organ diseases** | **1826.4 (1601.4 to 2103.6)** | **--** | **72.8 (48.7 to 103.3)** | **2.0 (-1.2 to 5.6)** | **-1.7 (-4.8 to 1.8)** |
| Blindness and vision impairment | 820.6 (606.1 to 1094.5) | -- | 30.5 (18.1 to 47.9) | -3.1* (-6.2 to -0.2) | -6.6* (-9.6 to -3.8) |
| Glaucoma | -- | -- | -- | -- | -- |
| Cataract | -- | -- | -- | -- | -- |
| Age-related macular degeneration | -- | -- | -- | -- | -- |
| Refraction disorders | 635.8 (449.1 to 869.7) | -- | 22.0 (12.4 to 35.9) | -4.4* (-7.8 to -1.3) | -7.9* (-11.2 to -4.8) |
| Near vision loss | 61.1 (56.2 to 66.7) | -- | 0.6 (0.3 to 1.2) | -1.8 (-8.6 to 5.1) | -5.4 (-11.9 to 1.3) |
| Other vision loss | 137.7 (94.5 to 196.3) | -- | 7.8 (4.5 to 12.2) | 0.7 (-4.6 to 5.9) | -3.0 (-8.0 to 2.1) |
| Age-related and other hearing loss | 192.7 (161.1 to 225.3) | -- | 26.3 (16.5 to 38.9) | 6.3 (-1.7 to 14.7) | 2.5 (-5.2 to 10.6) |
| Other sense organ diseases | 863.4 (818.1 to 907.3) | -- | 16.0 (9.8 to 23.7) | 5.4* (1.9 to 9.3) | 1.6 (-1.8 to 5.4) |
| Chronic other sense organ diseases | 293.5 (271.4 to 318.8) | -- | 8.0 (4.8 to 11.9) | 5.7 (-0.4 to 11.6) | 1.9 (-4.0 to 7.6) |
| Acute other sense organ diseases | 569.9 (533.8 to 605.6) | -- | 8.0 (4.8 to 12.4) | 5.2* (1.1 to 9.1) | 1.4 (-2.5 to 5.2) |
| **Musculoskeletal disorders** | **--** | **--** | **--** | **--** | **--** |
| Rheumatoid arthritis | -- | -- | -- | -- | -- |
| Osteoarthritis | -- | -- | -- | -- | -- |
| Osteoarthritis of the hip | -- | -- | -- | -- | -- |
| Osteoarthritis of the knee | -- | -- | -- | -- | -- |
| Low back pain | -- | -- | -- | -- | -- |
| Low back pain with leg pain | -- | -- | -- | -- | -- |
| Low back pain without leg pain | -- | -- | -- | -- | -- |
| Neck pain | -- | -- | -- | -- | -- |
| Gout | -- | -- | -- | -- | -- |
| Other musculoskeletal disorders | -- | -- | -- | -- | -- |
| **Other non-communicable diseases** | **38618.1 (37776.2 to 39506.0)** | **2833.1 (2311.6 to 3453.0)** | **599.1 (428.8 to 796.1)** | **0.2 (-1.9 to 2.5)** | **-3.4* (-5.4 to -1.2)** |
| Congenital anomalies | 4120.5 (3863.2 to 4422.2) | -- | 432.0 (311.8 to 571.9) | 4.2* (2.6 to 5.8) | 0.4 (-1.1 to 2.0) |
| Neural tube defects | 88.5 (78.8 to 99.5) | -- | 26.0 (18.1 to 35.2) | 11.3* (4.9 to 18.3) | 7.3* (1.1 to 14.0) |
| Severe motor and cognitive impairment due to anencephaly | -- | -- | -- | -- | -- |
| Encephalocoele due to neural tube defects | 11.8 (9.3 to 14.8) | -- | 2.0 (1.3 to 3.0) | 2.9 (-0.2 to 6.0) | -0.8 (-3.8 to 2.2) |
| Spina bifida due to neural tube defects | 76.6 (68.1 to 86.8) | -- | 23.9 (16.6 to 32.2) | 12.0* (5.1 to 19.5) | 8.0* (1.3 to 15.2) |
| Congenital heart anomalies | 1523.1 (1349.4 to 1721.0) | -- | 65.5 (32.8 to 108.6) | 2.6 (-0.5 to 4.8) | -1.1 (-4.1 to 1.0) |
| Critical malformations of great vessels, congenital valvular heart disease and patent ductus arteriosus | 307.1 (243.5 to 382.4) | -- | 16.1 (7.4 to 27.2) | 5.5* (1.3 to 9.4) | 1.7 (-2.3 to 5.5) |
| Other congenital heart anomalies | 243.3 (215.6 to 275.0) | -- | 12.5 (6.1 to 21.3) | 2.6 (-1.4 to 6.4) | -1.1 (-4.9 to 2.6) |
| Severe congenital heart anomalies excluding single ventricle heart defects | 322.8 (262.9 to 393.0) | -- | 16.9 (8.5 to 27.9) | 0.4 (-3.8 to 4.1) | -3.2 (-7.2 to 0.4) |
| Single ventricle and single ventricle pathway congenital heart anomalies | 52.8 (41.6 to 66.4) | -- | 2.9 (1.5 to 4.7) | -1.8 (-8.2 to 5.2) | -5.3 (-11.5 to 1.4) |
| Ventricular septal defect and atrial septal defect | 597.1 (478.4 to 740.5) | -- | 17.0 (8.0 to 29.9) | 2.7 (-1.2 to 6.1) | -1.0 (-4.8 to 2.3) |
| Orofacial clefts | 307.9 (255.0 to 365.4) | -- | 10.6 (6.3 to 16.2) | 3.0 (-6.3 to 12.3) | -0.7 (-9.6 to 8.3) |
| Down's syndrome | 82.3 (71.4 to 96.6) | -- | 7.1 (4.7 to 10.0) | 8.6* (4.2 to 13.7) | 4.7* (0.4 to 9.6) |
| Turner syndrome | 17.1 (14.8 to 20.0) | -- | 0.2 (0.1 to 0.4) | 2.7 (-3.3 to 9.5) | -1.0 (-6.8 to 5.5) |
| Klinefelter syndrome | 25.4 (21.7 to 29.4) | -- | 0.1 (0.0 to 0.1) | -0.9 (-9.2 to 7.9) | -4.4 (-12.5 to 4.0) |
| Other chromosomal abnormalities | 239.9 (190.2 to 292.5) | -- | 25.5 (17.4 to 34.8) | 9.8* (5.5 to 14.2) | 5.8* (1.6 to 10.0) |
| Congenital musculoskeletal and limb anomalies | 1356.8 (1179.8 to 1563.8) | -- | 182.5 (119.6 to 254.0) | 3.8* (1.9 to 5.6) | 0.0 (-1.8 to 1.8) |
| Polydactyly and syndactyly | 187.1 (137.2 to 252.3) | -- | 2.0 (0.9 to 3.9) | 5.6* (0.2 to 10.2) | 1.8 (-3.4 to 6.3) |
| Congenital limb deficiency | 349.9 (308.4 to 403.6) | -- | 54.0 (34.9 to 74.9) | 3.1* (0.3 to 6.0) | -0.6 (-3.3 to 2.2) |
| Other congenital musculoskeletal anomalies | 819.7 (712.5 to 945.8) | -- | 126.4 (83.2 to 177.9) | 4.0* (1.7 to 6.3) | 0.2 (-2.0 to 2.5) |
| Urogenital congenital anomalies | 215.0 (185.5 to 247.8) | -- | 5.6 (3.3 to 9.3) | 9.7* (6.5 to 13.0) | 5.7* (2.6 to 8.9) |
| Digestive congenital anomalies | 287.9 (257.8 to 322.5) | -- | 14.5 (9.6 to 20.5) | 3.1 (-1.3 to 7.4) | -0.6 (-4.8 to 3.5) |
| Congenital diaphragmatic hernia | 13.2 (11.4 to 15.0) | -- | 0.3 (0.2 to 0.4) | 1.2 (-0.1 to 2.4) | -2.4* (-3.7 to -1.3) |
| Congenital atresia and/or stenosis of the digestive tract | 47.4 (33.9 to 62.7) | -- | 3.8 (2.2 to 6.0) | 2.7 (-2.4 to 8.4) | -1.1 (-5.9 to 4.5) |
| Congenital malformations of the abdominal wall after treatment | 62.1 (54.3 to 71.4) | -- | 2.7 (1.7 to 3.9) | 2.3* (1.0 to 3.7) | -1.4* (-2.6 to -0.1) |
| Other congenital malformations of the digestive tract | 165.2 (133.9 to 201.7) | -- | 7.8 (4.8 to 11.5) | 3.7 (-3.9 to 11.3) | -0.0 (-7.4 to 7.3) |
| Other congenital anomalies | 466.3 (404.7 to 540.1) | -- | 94.4 (65.4 to 127.8) | 2.5 (-3.1 to 7.8) | -1.2 (-6.6 to 3.9) |

| | | | | | |
|---|---|---|---|---|---|
| Other congenital birth defects | -- | -- | 29.8 (18.1 to 44.4) | 9.8* (7.3 to 12.3) | 5.9* (3.4 to 8.3) |
| Hearing loss due to other congenital anomalies | 466.3 (404.7 to 540.1) | -- | 64.7 (42.6 to 92.1) | -0.6 (-8.2 to 6.8) | -4.2 (-11.5 to 2.9) |
| Urinary diseases and male infertility | 52.1 (42.0 to 65.4) | 2833.1 (2311.6 to 3453.0) | 2.5 (1.4 to 3.9) | -10.2* (-19.2 to -2.0) | -13.4* (-22.1 to -5.6) |
| Urinary tract infections | 54.3 (44.1 to 67.2) | 2833.1 (2311.6 to 3453.0) | 1.8 (1.1 to 2.9) | -0.9 (-10.8 to 8.1) | -4.4 (-14.0 to 4.2) |
| Urolithiasis | -- | -- | -- | -- | -- |
| Benign prostatic hyperplasia | -- | -- | -- | -- | -- |
| Male infertility | -- | -- | -- | -- | -- |
| Other urinary diseases | -- | -- | 0.7 (0.4 to 1.0) | -29.0* (-36.1 to -22.6) | -31.6* (-38.4 to -25.4) |
| Gynecological diseases | -- | -- | -- | -- | -- |
| Uterine fibroids | -- | -- | -- | -- | -- |
| Polycystic ovarian syndrome | -- | -- | -- | -- | -- |
| Female infertility | -- | -- | -- | -- | -- |
| Endometriosis | -- | -- | -- | -- | -- |
| Genital prolapse | -- | -- | -- | -- | -- |
| Premenstrual syndrome | -- | -- | -- | -- | -- |
| Other gynecological diseases | -- | -- | -- | -- | -- |
| Other gynecological diseases | -- | -- | -- | -- | -- |
| Other gynecological diseases with anemia | -- | -- | -- | -- | -- |
| Hemoglobinopathies and hemolytic anaemias | 34650.0 (33805.4 to 35543.0) | -- | 118.2 (77.6 to 172.7) | -9.9* (-16.1 to -3.3) | -13.1* (-19.1 to -6.8) |
| Thalassemias | 86.5 (81.1 to 92.3) | -- | 3.3 (2.1 to 5.2) | -12.4* (-20.6 to -4.6) | -15.6* (-23.5 to -8.0) |
| Thalassaemias trait | 4592.1 (4426.4 to 4768.9) | -- | 36.7 (24.1 to 53.4) | -12.3* (-20.6 to -3.5) | -15.5* (-23.4 to -6.9) |
| Sickle cell disorders | 236.2 (214.2 to 257.7) | -- | 12.4 (8.0 to 18.8) | -7.1 (-17.7 to 2.9) | -10.5* (-20.7 to -0.8) |
| Sickle cell trait | 9675.2 (8834.2 to 10606.2) | -- | 35.3 (23.4 to 51.8) | -9.9* (-17.0 to -2.4) | -13.2* (-20.0 to -5.9) |
| G6PD deficiency | 7080.0 (6777.0 to 7398.2) | -- | 0.7 (0.5 to 1.0) | -4.3 (-21.3 to 18.4) | -7.8 (-24.1 to 14.1) |
| G6PD trait | 16379.9 (16099.2 to 16665.7) | -- | -- | -6.1 (-14.0 to 3.0) | -9.5* (-17.1 to -0.8) |
| Other hemoglobinopathies and hemolytic anaemias | 888.4 (844.2 to 933.6) | -- | 29.7 (19.5 to 43.5) | -7.6 (-15.4 to 0.7) | -11.0* (-18.4 to -3.0) |
| Endocrine, metabolic, blood, and immune disorders | 1345.4 (1275.8 to 1415.9) | -- | 46.4 (31.3 to 67.2) | -5.4 (-11.7 to 1.7) | -8.8* (-14.9 to -2.0) |
| Anemia due to endocrine, metabolic, blood, and immune disorders | 1125.4 (1069.2 to 1179.2) | -- | 38.0 (24.8 to 55.5) | -6.9 (-14.5 to 1.2) | -10.2* (-17.6 to -2.4) |
| Endocrine, metabolic, blood, and immune disorders | 219.9 (178.5 to 258.5) | -- | 8.4 (5.4 to 12.3) | 1.8 (-7.0 to 11.5) | -1.9 (-10.3 to 7.4) |
| Heart failure due to endocrine, metabolic, blood, and immune disorders | 0.1 (0.1 to 0.1) | -- | -- | 0.5 (-0.8 to 1.7) | -3.1* (-4.4 to -2.0) |
| Oral disorders | -- | -- | -- | -- | -- |
| Caries of deciduous teeth | -- | -- | -- | -- | -- |
| Caries of permanent teeth | -- | -- | -- | -- | -- |
| Periodontal disease | -- | -- | -- | -- | -- |
| Edentulism and severe tooth loss | -- | -- | -- | -- | -- |
| Other oral disorders | -- | -- | -- | -- | -- |
| **Injuries** | **740.4 (655.0 to 837.7)** | **6409.8 (5691.1 to 7203.4)** | **47.3 (32.1 to 65.9)** | **9.7* (6.9 to 12.4)** | **5.7* (3.1 to 8.3)** |
| **Transport injuries** | **83.8 (67.8 to 104.6)** | **525.1 (381.9 to 699.7)** | **8.1 (5.3 to 11.8)** | **6.8* (1.6 to 11.5)** | **2.9 (-2.0 to 7.5)** |
| Road injuries | 8.6 (6.2 to 11.8) | 62.1 (40.8 to 91.0) | 0.7 (0.4 to 1.2) | 8.3 (-2.8 to 20.6) | 4.4 (-6.3 to 16.3) |
| Pedestrian road injuries | 2.4 (1.6 to 3.4) | 16.5 (9.6 to 25.6) | 0.2 (0.1 to 0.3) | -5.6 (-17.7 to 7.8) | -9.0 (-20.7 to 3.9) |
| Cyclist road injuries | -- | -- | -- | -- | -- |
| Motorcyclist road injuries | 3.3 (2.2 to 4.7) | 21.7 (12.5 to 34.2) | 0.3 (0.2 to 0.5) | 11.6 (-1.2 to 25.2) | 7.6 (-4.8 to 20.7) |
| Motor vehicle road injuries | 1.7 (1.1 to 2.6) | 13.9 (8.0 to 22.3) | 0.2 (0.1 to 0.3) | 11.8 (-2.5 to 27.3) | 7.8 (-6.0 to 22.7) |
| Other road injuries | 1.2 (0.7 to 1.9) | 9.9 (5.0 to 16.9) | 0.1 (0.0 to 0.1) | 36.6* (19.4 to 54.5) | 31.7* (15.1 to 48.9) |
| Other transport injuries | 75.3 (59.6 to 96.0) | 463.1 (313.1 to 636.5) | 7.3 (4.8 to 10.9) | 6.6* (1.0 to 11.7) | 2.8 (-2.6 to 7.7) |
| **Unintentional injuries** | **608.4 (529.6 to 703.3)** | **5441.8 (4781.8 to 6203.3)** | **35.1 (23.4 to 49.0)** | **9.9* (6.7 to 13.2)** | **5.9* (2.8 to 9.1)** |
| Falls | 216.5 (168.9 to 275.7) | 1658.7 (1209.1 to 2187.4) | 11.6 (7.2 to 17.5) | 13.4* (5.9 to 20.9) | 9.3* (2.1 to 16.5) |
| Drowning | 1.1 (0.9 to 1.4) | 8.7 (6.7 to 11.3) | 0.1 (0.1 to 0.1) | -15.9* (-21.2 to -9.9) | -18.9* (-24.1 to -13.2) |
| Fire, heat, and hot substances | 17.2 (13.4 to 21.8) | 120.2 (83.7 to 173.5) | 1.6 (1.0 to 2.4) | 16.1* (7.1 to 25.2) | 11.9* (3.2 to 20.7) |
| Poisonings | 4.0 (3.2 to 5.0) | 138.3 (91.1 to 188.1) | 0.5 (0.3 to 0.8) | 15.1* (8.2 to 21.8) | 11.0* (4.3 to 17.4) |
| Poisoning by carbon monoxide | 0.6 (0.4 to 0.8) | 21.8 (11.7 to 35.5) | 0.1 (0.0 to 0.1) | 11.3* (2.8 to 18.8) | 7.3 (-0.9 to 14.5) |
| Poisoning by other means | 3.5 (2.7 to 4.4) | 116.4 (76.1 to 162.7) | 0.5 (0.3 to 0.7) | 15.8* (8.5 to 22.8) | 11.6* (4.5 to 18.4) |
| Exposure to mechanical forces | 143.0 (110.8 to 188.2) | 1198.1 (893.2 to 1661.0) | 5.9 (3.7 to 8.9) | 6.9 (-0.9 to 14.3) | 3.0 (-4.5 to 10.2) |
| Unintentional firearm injuries | 8.8 (6.0 to 12.9) | 64.7 (37.0 to 104.6) | 0.8 (0.4 to 1.3) | 6.0 (-6.0 to 18.4) | 2.2 (-9.4 to 14.1) |
| Other exposure to mechanical forces | 134.1 (103.6 to 176.9) | 1133.4 (845.5 to 1568.9) | 5.1 (3.2 to 7.8) | 7.0 (-1.2 to 14.6) | 3.1 (-4.7 to 10.4) |
| Adverse effects of medical treatment | 22.1 (16.0 to 30.8) | 289.0 (212.9 to 378.1) | 2.9 (1.7 to 4.7) | 6.6 (-2.0 to 14.7) | 2.8 (-5.5 to 10.6) |
| Animal contact | 56.2 (40.1 to 77.6) | 792.6 (544.8 to 1136.6) | 2.8 (1.8 to 4.4) | 2.2 (-4.7 to 8.8) | -1.5 (-8.2 to 4.8) |
| Venomous animal contact | 13.3 (9.4 to 18.3) | 283.0 (187.1 to 423.1) | 1.4 (0.9 to 2.2) | 3.0 (-3.8 to 9.7) | -0.7 (-7.3 to 5.8) |
| Non-venomous animal contact | 43.0 (29.4 to 60.8) | 509.6 (351.4 to 730.8) | 1.4 (0.8 to 2.3) | 1.4 (-6.7 to 9.4) | -2.2 (-10.1 to 5.4) |
| Foreign body | 48.9 (25.8 to 79.2) | 453.6 (348.8 to 584.0) | 3.2 (1.7 to 5.3) | 6.8* (4.2 to 9.4) | 3.0* (0.4 to 5.4) |

| | | | | | |
|---|---|---|---|---|---|
| Pulmonary aspiration and foreign body in airway | 4.0 (2.9 to 5.2) | 63.2 (40.5 to 89.9) | 0.4 (0.3 to 0.6) | -10.3* (-17.9 to -3.0) | -13.6* (-20.9 to -6.5) |
| Foreign body in eyes | 29.9 (8.8 to 57.8) | 226.3 (141.6 to 339.6) | 1.7 (0.5 to 3.4) | 6.4* (4.9 to 8.4) | 2.6* (1.1 to 4.5) |
| Foreign body in other body part | 14.9 (11.3 to 19.4) | 164.1 (114.0 to 223.0) | 1.1 (0.7 to 1.6) | 16.1* (10.6 to 20.9) | 11.9* (6.6 to 16.5) |
| Environmental heat and cold exposure | 27.0 (20.9 to 34.3) | 195.1 (137.6 to 266.7) | 2.2 (1.4 to 3.1) | 8.2* (1.1 to 14.7) | 4.3 (-2.6 to 10.6) |
| Exposure to forces of nature | 0.2 (0.2 to 0.4) | 2.7 (1.8 to 3.9) | 0.0 (0.0 to 0.1) | -64.6* (-65.8 to -63.2) | -65.8* (-67.1 to -64.6) |
| Other unintentional injuries | 72.1 (54.2 to 94.0) | 584.8 (413.7 to 795.6) | 4.2 (2.5 to 6.3) | 16.2* (8.2 to 24.2) | 12.0* (4.3 to 19.8) |
| **Self-harm and interpersonal violence** | **48.2 (38.2 to 60.7)** | **442.9 (331.4 to 571.8)** | **4.2 (2.7 to 6.1)** | **13.7* (4.8 to 22.6)** | **9.6* (1.1 to 18.1)** |
| Self-harm | -- | -- | -- | -- | -- |
| Self-harm by firearm | -- | -- | -- | -- | -- |
| Self-harm by other specified means | -- | -- | -- | -- | -- |
| Interpersonal violence | 42.4 (32.6 to 54.3) | 374.3 (264.5 to 505.6) | 3.5 (2.2 to 5.2) | 6.3 (-3.1 to 16.7) | 2.4 (-6.6 to 12.5) |
| Assault by firearm | 0.9 (0.6 to 1.4) | 6.6 (3.6 to 11.6) | 0.1 (0.1 to 0.2) | 14.7* (0.2 to 31.3) | 10.5 (-3.5 to 26.6) |
| Assault by sharp object | 7.2 (5.1 to 10.2) | 57.1 (33.2 to 89.9) | 0.6 (0.3 to 1.1) | 12.2 (-1.4 to 25.8) | 8.1 (-4.9 to 21.3) |
| Sexual violence | -- | -- | -- | -- | -- |
| Assault by other means | 34.2 (26.0 to 44.7) | 310.6 (218.1 to 423.2) | 2.7 (1.7 to 4.1) | 4.6 (-4.9 to 14.9) | 0.9 (-8.3 to 10.8) |
| Conflict and terrorism | 5.3 (3.6 to 7.6) | 62.3 (46.1 to 87.1) | 0.6 (0.4 to 1.1) | 72.2* (68.5 to 76.9) | 66.0* (62.4 to 70.5) |
| Executions and police conflict | 0.5 (0.2 to 1.1) | 6.3 (2.1 to 13.3) | 0.1 (0.0 to 0.1) | 128.0 (-24.0 to 517.6) | 119.8 (-26.7 to 495.3) |
| **Nature of injury aggregates** | | | | | |
| Amputations | 69.2 (60.7 to 77.9) | 121.3 (99.1 to 149.7) | 1.5 (1.0 to 2.1) | -1.1 (-4.9 to 3.0) | -4.7* (-8.3 to -0.8) |
| Burns | 42.1 (36.5 to 48.2) | 254.5 (209.3 to 311.9) | 4.6 (3.0 to 6.5) | 7.5* (3.7 to 11.7) | 3.6 (-0.1 to 7.7) |
| Fractures | 218.4 (184.7 to 257.1) | 1315.0 (1116.7 to 1533.5) | 12.2 (7.7 to 17.6) | 11.6* (8.0 to 15.3) | 7.6* (4.1 to 11.1) |
| Head Injuries | 32.1 (26.8 to 38.4) | 256.1 (213.6 to 307.3) | 5.6 (3.6 to 8.2) | 10.6* (7.2 to 13.9) | 6.6* (3.3 to 9.8) |
| Spinal Injuries | 4.7 (4.2 to 5.3) | 9.2 (7.5 to 11.2) | 1.8 (1.3 to 2.4) | 8.7* (7.2 to 10.3) | 4.8* (3.3 to 6.3) |
| Minor Injuries | 222.3 (172.3 to 278.7) | 2479.4 (2136.8 to 2886.2) | 1.6 (0.7 to 3.3) | 9.3* (5.5 to 13.2) | 5.4* (1.7 to 9.2) |
| Other Injuries | 151.6 (113.5 to 198.4) | 1974.3 (1751.2 to 2222.3) | 20.0 (13.2 to 28.0) | 9.8* (6.9 to 12.5) | 5.8* (3.1 to 8.4) |
| **Impairments** | | | | | |
| Anaemia | 58258.7 (52382.9 to 64778.5) | -- | 2000.4 (1309.8 to 2948.8) | -14.3* (-19.5 to -8.4) | -17.4* (-22.4 to -11.7) |
| Epilepsy | 1014.3 (784.1 to 1288.0) | -- | 476.5 (335.8 to 639.9) | 7.8 (-0.5 to 15.9) | 3.9 (-4.0 to 11.7) |
| Guillain-Barré syndrome | 0.9 (0.5 to 1.4) | -- | 0.3 (0.1 to 0.5) | 3.6* (1.6 to 5.6) | -0.1 (-2.1 to 1.8) |
| Hearing loss | 1192.5 (1089.4 to 1309.1) | -- | 104.7 (72.4 to 145.4) | 0.9 (-3.9 to 6.0) | -2.7 (-7.4 to 2.1) |
| Heart failure | 17.4 (11.9 to 23.8) | -- | 1.7 (1.0 to 2.7) | 2.0* (1.0 to 2.8) | -1.7* (-2.7 to -0.9) |
| Infertility | 6.4 (3.1 to 10.9) | -- | 0.1 (0.0 to 0.3) | 8.6* (6.9 to 10.3) | 4.7* (3.0 to 6.3) |
| Developmental intellectual disability | 4209.6 (3247.9 to 5160.7) | -- | 561.8 (429.7 to 702.8) | 1.7 (-1.8 to 6.1) | -2.0 (-5.3 to 2.3) |
| Pelvic inflammatory disease | -- | -- | -- | -- | -- |
| Vision loss | 1393.3 (1082.5 to 1782.7) | -- | 87.6 (61.2 to 119.3) | -2.3* (-4.2 to -0.4) | -5.8* (-7.7 to -4.0) |

We did not calculate incidence for the nine impairments and certain NTDs. Blank cells mean that no estimate is available or that the estimate has a magnitude less than 50.
Table presents prevalence and incidence counts in thousands.
* Percentage changes that are statistically significant
‡ Incidence estimates for stroke represent first-ever stroke only.
^ Incidence of HIV/AIDS represents new infections of HIV only and does not include new infections of Tuberculosis in HIV positive cases.

**SR Table 10d: Global prevalence, incidence and YLDs for 2017, percentage change of YLD counts and percentage change of YLD rates between 2007 and 2017 by cause, age 1 to 4 years**

| Cause | Prevalence in thousands (95% UI) | Incidence in thousands (95% UI) | YLDs in thousands (95% UI) | | |
|---|---|---|---|---|---|
| | 2017 counts | 2017 counts | 2017 counts | Percentage change in counts between 2007 and 2017 | Percentage change in rates between 2007 and 2017 |
| **All causes** | **481960.3 (471375.0 to 493797.8)** | **3718431.1 (3253997.7 to 4260040.8)** | **24347.7 (17597.1 to 32322.0)** | **-1.5 (-3.8 to 0.8)** | **-7.4* (-9.6 to -5.3)** |
| **Communicable, maternal, neonatal, and nutritional disorders** | **315081.0 (303553.9 to 328040.7)** | **3028875.5 (2594712.4 to 3559755.3)** | **13598.8 (9917.5 to 17982.4)** | **-6.7* (-10.0 to -3.1)** | **-12.4* (-15.5 to -9.0)** |
| **HIV/AIDS and sexually transmitted infections** | **316.6 (281.3 to 353.6)** | **--** | **48.7 (34.5 to 65.2)** | **-50.3* (-54.5 to -45.5)** | **-53.3* (-57.3 to -48.8)** |
| HIV/AIDS | 388.8 (353.2 to 424.2) | -- | 48.7 (34.5 to 65.2) | -50.3* (-54.5 to -45.5) | -53.3* (-57.3 to -48.8) |
| HIV/AIDS - Drug-susceptible Tuberculosis | 12.8 (11.8 to 13.9) | 18.3 (16.7 to 20.2) | 5.3 (3.5 to 7.2) | -64.6* (-68.7 to -60.0) | -66.7* (-70.6 to -62.4) |
| HIV/AIDS - Multidrug-resistant Tuberculosis without extensive drug resistance | 0.4 (0.3 to 0.7) | 0.7 (0.5 to 1.0) | 0.2 (0.1 to 0.3) | -64.5* (-76.1 to -47.6) | -66.6* (-77.5 to -50.7) |
| HIV/AIDS - Extensively drug-resistant Tuberculosis | -- | -- | -- | -29.4 (-55.8 to 14.6) | -33.7 (-58.4 to 7.6) |
| HIV/AIDS resulting in other diseases | 375.5 (339.8 to 410.8) | -- | 43.2 (30.9 to 57.9) | -47.6* (-52.6 to -41.8) | -50.8* (-55.5 to -45.3) |
| HIV/AIDS not on antiretroviral treatment without Tuberculosis | 266.0 (236.9 to 294.3) | -- | 34.1 (24.2 to 46.0) | -55.5* (-60.6 to -49.5) | -58.2* (-63.0 to -52.5) |
| HIV/AIDS on antiretroviral treatment without Tuberculosis | 109.5 (100.3 to 119.5) | -- | 9.2 (6.1 to 13.1) | 53.2* (26.2 to 89.9) | 44.0* (18.6 to 78.4) |
| Sexually transmitted infections excluding HIV | -- | -- | -- | -- | -- |
| Syphilis | -- | -- | -- | -- | -- |
| Early syphilis | -- | -- | -- | -- | -- |
| Tertiary syphilis | -- | -- | -- | -- | -- |
| Chlamydial infection | -- | -- | -- | -- | -- |
| Chlamydia episode | -- | -- | -- | -- | -- |
| Chlamydial infection complications | -- | -- | -- | -- | -- |
| Gonococcal infection | -- | -- | -- | -- | -- |
| Gonococcal infection complications | -- | -- | -- | -- | -- |
| Gonorrhoea episode | -- | -- | -- | -- | -- |
| Trichomoniasis | -- | -- | -- | -- | -- |
| Genital herpes | -- | -- | -- | -- | -- |
| Other sexually transmitted infections | -- | -- | -- | -- | -- |
| Other sexually transmitted diseases residual | -- | -- | -- | -- | -- |
| Other sexually transmitted diseases | -- | -- | -- | -- | -- |
| **Respiratory infections and tuberculosis** | **95625.1 (79484.2 to 118771.2)** | **1891469.0 (1466553.1 to 2391593.3)** | **1048.0 (647.2 to 1623.2)** | **4.4* (1.3 to 7.1)** | **-2.0 (-4.9 to 0.6)** |
| Tuberculosis | 60529.6 (43704.1 to 83807.1) | 514.5 (424.5 to 616.3) | 76.3 (49.8 to 112.9) | -20.2* (-26.1 to -13.8) | -25.0* (-30.5 to -19.0) |
| Latent tuberculosis infection | 60292.4 (43457.0 to 83575.3) | -- | -- | -- | -- |
| Drug-susceptible tuberculosis | 227.8 (182.1 to 280.1) | 493.5 (407.3 to 590.3) | 73.2 (47.9 to 108.4) | -20.4* (-26.5 to -13.8) | -25.2* (-31.0 to -19.0) |
| Multidrug-resistant tuberculosis without extensive drug resistance | 9.2 (5.6 to 14.2) | 20.4 (13.2 to 31.2) | 3.0 (1.7 to 5.0) | -14.4 (-43.7 to 29.7) | -19.6 (-47.1 to 21.9) |
| Extensively drug-resistant tuberculosis | 0.3 (0.2 to 0.4) | 0.6 (0.4 to 0.9) | 0.1 (0.0 to 0.2) | 29.5 (-11.4 to 99.8) | 21.7 (-16.8 to 87.7) |
| Lower respiratory infections | 1480.3 (1175.3 to 1843.8) | 66190.4 (51443.2 to 83343.3) | 92.0 (57.8 to 138.8) | -11.7* (-19.2 to -3.7) | -17.1* (-24.0 to -9.5) |
| Guillain-Barré syndrome due to lower respiratory infections | 1.2 (0.5 to 2.2) | -- | 0.4 (0.1 to 0.7) | 7.4* (5.3 to 9.4) | 0.9 (-1.1 to 2.7) |
| Lower respiratory infection episode | 1479.1 (1174.2 to 1841.7) | 66190.4 (51443.2 to 83343.3) | 91.6 (57.3 to 138.4) | -11.8* (-19.3 to -3.7) | -17.1* (-24.2 to -9.5) |
| Upper respiratory infections | 23456.0 (17763.3 to 29977.1) | 1688480.2 (1283320.6 to 2177147.3) | 594.0 (329.9 to 998.6) | 11.5* (8.8 to 14.0) | 4.8* (2.2 to 7.1) |
| Guillain-Barré syndrome due to upper respiratory infections | 3.2 (1.8 to 4.9) | -- | 1.0 (0.5 to 1.7) | 7.4* (5.3 to 9.3) | 0.9 (-1.1 to 2.7) |
| Upper respiratory infection episode | 23452.8 (17759.8 to 29973.3) | 1688480.2 (1283320.6 to 2177147.3) | 593.0 (328.9 to 998.0) | 11.5* (8.8 to 14.0) | 4.8* (2.2 to 7.1) |
| Otitis media | 15877.1 (13187.1 to 19011.4) | 136284.0 (94754.6 to 191274.2) | 285.8 (168.6 to 471.1) | 5.1* (2.4 to 7.7) | -1.3 (-3.8 to 1.2) |
| Acute otitis media | 7799.1 (5330.4 to 10791.3) | 136277.0 (94746.7 to 191263.8) | 102.7 (46.7 to 195.6) | 6.5* (3.0 to 9.7) | 0.1 (-3.2 to 3.1) |
| Chronic otitis media | 8078.0 (7121.3 to 9133.2) | 6.9 (0.3 to 26.0) | 183.1 (110.6 to 285.3) | 4.3* (0.8 to 7.9) | -2.0 (-5.3 to 1.4) |
| **Enteric infections** | **12968.2 (10826.1 to 15433.6)** | **796347.5 (643160.0 to 982414.5)** | **1522.1 (981.9 to 2209.8)** | **0.3 (-6.4 to 6.8)** | **-5.8 (-12.1 to 0.3)** |
| Diarrhoeal diseases | 13021.2 (10811.1 to 15486.3) | 794378.3 (641078.4 to 980547.1) | 1506.5 (972.5 to 2186.9) | 0.8 (-6.0 to 7.2) | -5.3 (-11.7 to 0.7) |
| Guillain-Barré syndrome due to diarrheal diseases | 1.1 (0.6 to 1.8) | -- | 0.3 (0.2 to 0.6) | 7.4* (5.3 to 9.4) | 0.9 (-1.1 to 2.7) |
| Diarrheal disease episode | 13020.1 (10810.1 to 15485.7) | 794378.3 (641078.4 to 980547.1) | 1506.2 (972.4 to 2186.2) | 0.8 (-6.0 to 7.2) | -5.3 (-11.7 to 0.7) |
| Typhoid and paratyphoid | 55.3 (33.5 to 86.6) | 1791.1 (1157.2 to 2696.4) | 14.5 (8.0 to 26.1) | -32.0* (-45.7 to -15.1) | -36.1* (-49.0 to -20.2) |
| Typhoid fever | 88.2 (55.4 to 136.0) | 1389.0 (880.3 to 2081.3) | 13.4 (7.2 to 24.2) | -32.9* (-47.2 to -15.1) | -36.9* (-50.4 to -20.3) |
| Typhoid fever complications | 18.4 (10.7 to 30.9) | 239.5 (149.9 to 371.6) | 5.9 (2.9 to 10.6) | -33.0* (-55.5 to -1.4) | -37.0* (-58.2 to -7.4) |
| Typhoid fever episode | 69.8 (42.3 to 106.9) | 1149.5 (734.1 to 1715.9) | 7.6 (3.9 to 13.5) | -32.8* (-50.5 to -10.5) | -36.8* (-53.5 to -15.9) |
| Paratyphoid fever | 17.6 (10.5 to 29.8) | 402.1 (247.4 to 664.3) | 1.1 (0.6 to 2.0) | -18.9 (-33.0 to 0.2) | -23.8* (-37.0 to -5.9) |
| Intestinal perforation due to paratyphoid | 0.8 (0.5 to 1.3) | 20.6 (12.5 to 35.2) | 0.1 (0.0 to 0.2) | -19.1 (-38.2 to 7.5) | -24.0 (-42.0 to 1.0) |
| Paratyphoid fever episode | 16.9 (10.1 to 28.5) | 381.5 (234.9 to 631.0) | 1.0 (0.5 to 1.9) | -18.9 (-33.6 to 1.8) | -23.8* (-37.6 to -4.4) |
| iNTS | 6.8 (4.0 to 11.9) | 178.1 (111.2 to 297.6) | 0.9 (0.4 to 1.7) | -22.3 (-43.8 to 8.1) | -27.0 (-47.2 to 1.6) |
| Other intestinal infectious diseases | -- | -- | 0.2 (0.1 to 0.4) | -54.8* (-63.9 to -43.5) | -57.5* (-66.1 to -47.0) |
| **Neglected tropical diseases and malaria** | **93934.0 (89504.5 to 98874.7)** | **71760.6 (48962.5 to 99666.0)** | **723.3 (475.6 to 1063.1)** | **-23.8* (-28.9 to -18.7)** | **-28.4* (-33.2 to -23.6)** |
| Malaria | 21791.5 (20129.6 to 23205.7) | 65519.8 (42625.8 to 92657.9) | 262.1 (164.5 to 395.7) | -34.1* (-42.4 to -24.7) | -38.1* (-45.8 to -29.3) |
| Malaria complications | 33.5 (28.0 to 39.4) | -- | 13.9 (10.1 to 18.4) | 16.8* (13.6 to 19.8) | 9.7* (6.7 to 12.6) |
| Malaria episode | 3806.6 (2071.7 to 6080.7) | 65519.8 (42625.8 to 92657.9) | 134.6 (64.5 to 239.7) | -30.3* (-44.7 to -17.8) | -34.5* (-48.1 to -22.8) |

| | | | | | |
|---|---|---|---|---|---|
| Malaria parasitemia | 17951.3 (15390.0 to 20223.0) | -- | 113.6 (74.8 to 172.2) | -41.1* (-49.2 to -31.7) | -44.6* (-52.3 to -35.8) |
| Chagas disease | 63.6 (48.4 to 81.2) | 12.7 (8.8 to 17.0) | -- | -24.7* (-30.4 to -19.0) | -29.3* (-34.6 to -23.9) |
| Acute Chagas disease | 0.1 (0.0 to 0.1) | 12.7 (8.8 to 17.0) | -- | -27.5* (-34.9 to -21.5) | -31.9* (-38.9 to -26.3) |
| Asymptomatic Chagas disease | 63.5 (48.4 to 81.0) | -- | -- | -- | -- |
| Symptomatic chronic Chagas infection | 0.1 (0.0 to 0.1) | -- | -- | -21.1* (-26.3 to -15.6) | -25.9* (-30.8 to -20.7) |
| Leishmaniasis | 68.2 (52.2 to 86.1) | 59.5 (44.2 to 79.0) | 4.9 (3.1 to 7.2) | -3.3 (-20.8 to 18.1) | -9.1 (-25.6 to 11.0) |
| Visceral leishmaniasis | 2.8 (2.0 to 4.4) | 11.1 (8.2 to 17.7) | 0.2 (0.1 to 0.3) | -73.1* (-81.0 to -56.9) | -74.7* (-82.2 to -59.5) |
| Cutaneous and mucocutaneous leishmaniasis | 71.0 (55.4 to 89.1) | 48.5 (33.5 to 67.5) | 4.7 (2.9 to 7.0) | 8.6 (-12.0 to 35.8) | 2.0 (-17.3 to 27.6) |
| African trypanosomiasis | 0.2 (0.0 to 0.8) | 0.1 (0.0 to 0.4) | 0.0 (0.0 to 0.2) | -82.4* (-98.0 to -5.8) | -83.5* (-98.2 to -11.5) |
| Trypanosomiasis Gambiense | 0.2 (0.0 to 0.8) | 0.1 (0.0 to 0.4) | 0.0 (0.0 to 0.2) | -81.7 (-98.2 to 2.0) | -82.8* (-98.3 to -4.2) |
| Trypanosomiasis Rhodesiense | -- | -- | -- | -93.4* (-99.4 to -46.0) | -93.8* (-99.4 to -49.2) |
| Schistosomiasis | 54.1 (43.3 to 66.1) | -- | 0.5 (0.3 to 0.9) | -26.1* (-35.3 to -16.1) | -30.6* (-39.3 to -21.2) |
| Mild schistosomiasis | 49.2 (38.8 to 60.8) | -- | 0.3 (0.1 to 0.6) | -22.6* (-36.3 to -6.6) | -27.2* (-40.1 to -12.3) |
| Anemia due to schistosomiasis | 2.9 (2.3 to 3.6) | -- | 0.2 (0.1 to 0.3) | -28.1* (-38.7 to -14.6) | -32.5* (-42.4 to -19.8) |
| Schistosomiasis complications | 2.0 (0.9 to 4.0) | -- | 0.0 (0.0 to 0.1) | -45.4* (-49.7 to -40.1) | -48.7* (-52.7 to -43.7) |
| Cysticercosis | -- | -- | -- | -- | -- |
| Cystic echinococcosis | 0.7 (0.4 to 1.3) | 0.5 (0.3 to 0.9) | 0.1 (0.0 to 0.1) | 19.6 (-8.1 to 63.5) | 12.3 (-13.7 to 53.6) |
| Lymphatic filariasis | 1345.9 (1005.0 to 1721.2) | -- | -- | -- | -- |
| Prevalence of detectable microfiliaria due to lymphatic filariasis | 1345.9 (1005.0 to 1721.2) | -- | -- | -- | -- |
| Lymphatic filariasis complications | -- | -- | -- | -- | -- |
| Onchocerciasis | -- | -- | -- | -- | -- |
| Asymptomatic onchocerciasis | -- | -- | -- | -- | -- |
| Skin disease due to onchocerciasis | -- | -- | -- | -- | -- |
| Vision loss due to onchocerciasis | -- | -- | -- | -- | -- |
| Trachoma | -- | -- | -- | -- | -- |
| Dengue | 338.3 (184.3 to 573.1) | 5656.9 (3441.0 to 8581.3) | 56.7 (24.7 to 106.7) | 44.4* (24.1 to 125.3) | 35.7* (16.6 to 111.6) |
| Post-dengue chronic fatigue syndrome | 238.5 (111.3 to 436.1) | -- | 50.7 (20.9 to 98.1) | 44.4* (22.8 to 127.0) | 35.7* (15.4 to 113.2) |
| Dengue episode | 99.8 (60.3 to 149.9) | 5656.9 (3441.0 to 8581.3) | 6.0 (3.1 to 10.4) | 44.2* (22.1 to 115.1) | 35.4* (14.7 to 102.1) |
| Yellow fever | 0.3 (0.1 to 0.7) | 9.2 (2.6 to 25.4) | -- | -21.8 (-45.7 to 9.1) | -26.6 (-49.0 to 2.5) |
| Asymptomatic yellow fever | 0.1 (0.0 to 0.4) | 5.1 (1.2 to 14.5) | -- | -- | -- |
| Yellow fever episode | 0.1 (0.0 to 0.3) | 4.1 (1.1 to 10.9) | -- | -21.8 (-45.7 to 9.1) | -26.6 (-49.0 to 2.5) |
| Rabies | 0.1 (0.0 to 0.1) | 1.5 (0.9 to 2.7) | -- | -36.4 (-58.2 to 5.1) | -40.2* (-60.8 to -1.3) |
| Intestinal nematode infections | 68656.2 (64373.3 to 73727.6) | -- | 121.2 (69.6 to 200.8) | -36.3* (-40.3 to -32.1) | -40.1* (-44.0 to -36.2) |
| Ascariasis | 43864.6 (39312.0 to 49063.4) | -- | 62.3 (33.3 to 108.4) | -37.1* (-43.8 to -30.2) | -40.9* (-47.2 to -34.4) |
| Asymptomatic ascariasis | 40325.7 (36165.9 to 45104.0) | -- | -- | -- | -- |
| Ascariasis complications | 3538.9 (3155.7 to 3964.1) | -- | 62.3 (33.3 to 108.4) | -37.1* (-43.8 to -30.2) | -40.9* (-47.2 to -34.4) |
| Trichuriasis | 12961.0 (11664.0 to 14548.3) | -- | 6.1 (3.4 to 10.2) | -32.8* (-39.7 to -25.0) | -36.9* (-43.3 to -29.5) |
| Asymptomatic trichuriasis | 12632.6 (11354.7 to 14218.7) | -- | -- | -- | -- |
| Trichuriasis complications | 328.4 (300.4 to 359.2) | -- | 6.1 (3.4 to 10.2) | -32.8* (-39.7 to -25.0) | -36.9* (-43.3 to -29.5) |
| Hookworm disease | 16171.8 (15196.9 to 17237.7) | -- | 52.8 (32.2 to 83.6) | -35.6* (-40.6 to -30.3) | -39.5* (-44.2 to -34.5) |
| Asymptomatic hookworm disease | 13854.2 (13016.0 to 14781.9) | -- | -- | -- | -- |
| Anemia due to hookworm disease | 666.9 (616.6 to 721.4) | -- | 19.8 (12.9 to 29.1) | -42.7* (-49.3 to -35.3) | -46.2* (-52.3 to -39.2) |
| Hookworm disease complications | 1650.7 (1553.7 to 1755.3) | -- | 33.0 (18.1 to 54.9) | -30.3* (-35.2 to -25.3) | -34.5* (-39.1 to -29.9) |
| Food-borne trematodiases | 995.7 (762.3 to 1289.3) | 396.8 (317.2 to 496.4) | 7.8 (4.2 to 12.9) | 4.6 (-7.6 to 17.5) | -1.7 (-13.2 to 10.4) |
| Asymptomatic food-borne tremodiases | 926.5 (706.8 to 1201.4) | 367.1 (292.0 to 461.2) | -- | -- | -- |
| Food-borne tremodiases complications | 69.2 (42.8 to 104.8) | 29.6 (18.6 to 45.0) | 7.8 (4.2 to 12.9) | 4.6 (-7.6 to 17.5) | -1.7 (-13.2 to 10.4) |
| Leprosy | 0.2 (0.2 to 0.3) | 0.1 (0.1 to 0.2) | -- | -37.5* (-38.7 to -36.3) | -41.3* (-42.4 to -40.2) |
| Ebola virus disease | -- | -- | -- | -96.2* (-96.8 to -95.1) | -96.4* (-97.0 to -95.4) |
| Ebola cases | -- | -- | -- | -98.5* (-98.8 to -98.3) | -98.6* (-98.9 to -98.4) |
| Post-Ebola chronic fatigue syndrome | -- | -- | -- | -95.9* (-96.4 to -94.8) | -96.2* (-96.6 to -95.1) |
| Zika virus disease | 1.8 (1.1 to 3.2) | 103.6 (59.9 to 187.1) | 0.1 (0.1 to 0.2) | -- | -- |
| Zika virus complications | 0.1 (0.1 to 0.2) | -- | 0.1 (0.0 to 0.1) | -- | -- |
| Zika virus episode | 1.7 (1.0 to 3.1) | 103.6 (59.9 to 187.1) | 0.0 (0.0 to 0.1) | -- | -- |
| Guinea worm disease | -- | -- | -- | -99.4* (-99.6 to -99.2) | -99.5* (-99.6 to -99.3) |
| Other neglected tropical diseases | 8606.4 (8155.1 to 9111.2) | -- | 269.9 (179.0 to 393.5) | -12.5* (-19.9 to -5.4) | -17.7* (-24.8 to -11.1) |
| Acute infection due to other neglected tropical diseases | -- | -- | 2.7 (1.4 to 4.7) | 101.8* (70.5 to 184.0) | 89.6* (60.2 to 166.8) |
| Anemia due to other neglected tropical diseases | 8606.4 (8155.1 to 9111.2) | -- | 267.2 (177.5 to 390.0) | -13.0* (-20.4 to -5.9) | -18.2* (-25.2 to -11.6) |
| **Other infectious diseases** | **13773.4 (13047.4 to 14457.6)** | **119977.0 (107798.9 to 136533.7)** | **398.5 (271.8 to 558.2)** | **-14.6* (-20.1 to -9.7)** | **-19.8* (-24.9 to -15.2)** |
| Meningitis | 693.6 (574.1 to 827.6) | 1203.8 (883.8 to 1591.8) | 81.9 (55.9 to 112.0) | -8.5* (-14.2 to -3.0) | -14.0* (-19.4 to -8.9) |
| Pneumococcal meningitis | 177.8 (141.7 to 219.2) | 137.0 (78.7 to 219.9) | 21.9 (14.4 to 30.3) | -32.6* (-40.1 to -24.9) | -36.7* (-43.7 to -29.5) |

| | | | | | |
|---|---|---|---|---|---|
| Acute pneumococcal meningitis | 6.4<br>(3.7 to 10.2) | 137.0<br>(78.7 to 219.9) | 0.9<br>(0.4 to 1.5) | -29.8*<br>(-39.2 to -21.1) | -34.1*<br>(-42.9 to -25.8) |
| Pneumococcal meningitis complications | 171.3<br>(136.5 to 212.7) | -- | 21.1<br>(13.8 to 29.2) | -32.7*<br>(-40.5 to -24.7) | -36.8*<br>(-44.1 to -29.2) |
| H influenzae type B meningitis | 68.6<br>(51.2 to 90.7) | 98.3<br>(57.4 to 158.5) | 8.9<br>(6.0 to 12.3) | -45.1*<br>(-49.7 to -40.4) | -48.4*<br>(-52.8 to -44.0) |
| Acute H influenzae type B meningitis | 4.6<br>(2.6 to 7.4) | 98.3<br>(57.4 to 158.5) | 0.6<br>(0.3 to 1.1) | -45.8*<br>(-53.1 to -37.9) | -49.0*<br>(-55.9 to -41.6) |
| H Influenza type B meningitis complications | 64.0<br>(47.4 to 84.7) | -- | 8.2<br>(5.5 to 11.5) | -45.0*<br>(-49.9 to -39.9) | -48.4*<br>(-53.0 to -43.5) |
| Meningococcal infection | 64.0<br>(45.3 to 85.5) | 123.8<br>(69.1 to 203.2) | 7.3<br>(4.9 to 10.2) | -18.5*<br>(-22.9 to -14.0) | -23.4*<br>(-27.6 to -19.2) |
| Acute meningococcal meningitis | 5.8<br>(3.3 to 9.3) | 123.8<br>(69.1 to 203.2) | 0.8<br>(0.4 to 1.4) | -3.8<br>(-16.2 to 9.2) | -9.6<br>(-21.2 to 2.6) |
| Meningococcal meningitis complications | 58.2<br>(41.1 to 77.2) | -- | 6.5<br>(4.4 to 9.1) | -19.9*<br>(-24.5 to -15.6) | -24.8*<br>(-29.1 to -20.8) |
| Other meningitis | 383.2<br>(295.0 to 479.6) | 844.8<br>(621.5 to 1147.3) | 43.8<br>(29.8 to 60.1) | 37.4*<br>(25.7 to 49.5) | 29.1*<br>(18.1 to 40.4) |
| Other acute bacterial meningitis | 20.6<br>(15.2 to 27.6) | 435.0<br>(312.7 to 599.2) | 2.7<br>(1.6 to 4.4) | 81.3*<br>(52.6 to 115.1) | 70.3*<br>(43.4 to 102.1) |
| Acute viral meningitis | 19.3<br>(14.6 to 25.2) | 409.8<br>(300.9 to 542.0) | 2.5<br>(1.5 to 4.1) | 6.4<br>(-13.1 to 29.5) | -0.0<br>(-18.4 to 21.7) |
| Other bacterial meningitis complications | 343.3<br>(258.8 to 434.9) | -- | 38.5<br>(26.5 to 52.9) | 37.6*<br>(25.6 to 50.9) | 29.3*<br>(18.0 to 41.8) |
| Encephalitis | 125.9<br>(79.6 to 188.4) | 263.2<br>(249.5 to 277.3) | 12.5<br>(8.8 to 16.5) | 0.1<br>(-2.2 to 2.3) | -6.0*<br>(-8.1 to -3.9) |
| Acute encephalitis | 13.6<br>(12.8 to 14.3) | 263.2<br>(249.5 to 277.3) | 1.8<br>(1.2 to 2.6) | 5.2*<br>(4.6 to 5.7) | -1.2*<br>(-1.7 to -0.7) |
| Encephalitis complications | 112.3<br>(66.4 to 174.8) | -- | 10.7<br>(7.5 to 14.0) | -0.8<br>(-3.3 to 1.7) | -6.8*<br>(-9.1 to -4.4) |
| Diphtheria | 0.6<br>(0.3 to 1.2) | 8.2<br>(4.1 to 16.1) | 0.0<br>(0.0 to 0.1) | -29.0<br>(-67.4 to 47.3) | -33.3<br>(-69.4 to 38.4) |
| Whooping cough | 971.6<br>(750.5 to 1225.4) | 7092.9<br>(5479.0 to 8945.8) | 48.2<br>(28.6 to 76.3) | -6.8*<br>(-10.4 to -3.4) | -12.4*<br>(-15.8 to -9.2) |
| Tetanus | 5.0<br>(4.8 to 5.4) | 4.4<br>(1.9 to 8.9) | 0.1<br>(0.1 to 0.2) | -25.3*<br>(-40.8 to -10.3) | -29.8*<br>(-44.4 to -15.7) |
| Severe tetanus | 0.2<br>(0.1 to 0.5) | 4.4<br>(1.9 to 8.9) | 0.0<br>(0.0 to 0.1) | -55.7*<br>(-77.5 to -21.7) | -58.4*<br>(-78.8 to -26.4) |
| Neonatal tetanus complications | 4.8<br>(4.6 to 5.0) | -- | 0.1<br>(0.1 to 0.2) | -2.4*<br>(-4.5 to -0.5) | -8.3*<br>(-10.2 to -6.5) |
| Measles | 356.2<br>(126.8 to 789.0) | 13002.3<br>(4627.7 to 28798.7) | 31.9<br>(10.9 to 73.2) | -46.6*<br>(-51.8 to -40.7) | -49.8*<br>(-54.7 to -44.2) |
| Varicella and herpes zoster | 564.9<br>(479.5 to 661.8) | 27078.5<br>(22779.9 to 32000.7) | 5.9<br>(3.2 to 10.3) | 6.2*<br>(2.7 to 10.2) | -0.2<br>(-3.5 to 3.5) |
| Chickenpox | 512.8<br>(431.0 to 607.4) | 26755.3<br>(22489.7 to 31694.2) | 2.9<br>(1.2 to 6.2) | 6.2*<br>(1.3 to 10.8) | -0.2<br>(-4.8 to 4.1) |
| Herpes zoster | 52.1<br>(39.0 to 70.0) | 323.2<br>(244.9 to 428.5) | 3.0<br>(1.6 to 4.8) | 6.3*<br>(0.8 to 12.5) | -0.2<br>(-5.3 to 5.7) |
| Acute hepatitis | 5798.4<br>(5328.5 to 6301.5) | 71323.8<br>(65487.6 to 77157.2) | 22.0<br>(13.0 to 33.6) | 6.2<br>(-17.0 to 37.4) | -0.2<br>(-22.0 to 29.1) |
| Acute hepatitis A | 4609.2<br>(4206.3 to 5017.6) | 59919.6<br>(54682.1 to 65228.2) | 14.0<br>(7.7 to 22.7) | 6.2<br>(-27.6 to 54.2) | -0.3<br>(-32.0 to 44.8) |
| Acute hepatitis B | 812.8<br>(605.7 to 1071.1) | 7044.4<br>(5249.5 to 9283.0) | 5.8<br>(3.5 to 9.4) | 8.8<br>(-10.0 to 30.9) | 2.2<br>(-15.4 to 23.0) |
| Acute hepatitis C | 123.0<br>(90.5 to 160.4) | 1065.7<br>(784.7 to 1390.0) | 1.7<br>(0.8 to 3.4) | -1.5<br>(-17.7 to 16.1) | -7.5<br>(-22.7 to 9.1) |
| Acute hepatitis E | 253.4<br>(169.6 to 350.7) | 3294.1<br>(2204.8 to 4559.3) | 0.5<br>(0.2 to 0.9) | 6.9<br>(-30.4 to 61.2) | 0.4<br>(-34.6 to 51.5) |
| Other unspecified infectious diseases | 6112.6<br>(5820.5 to 6438.4) | -- | 195.9<br>(130.7 to 285.8) | -13.6*<br>(-20.0 to -7.1) | -18.8*<br>(-24.8 to -12.7) |
| Guillain-Barré syndrome due to other infectious diseases | 0.7<br>(0.4 to 1.2) | -- | 0.2<br>(0.1 to 0.4) | 7.4*<br>(5.3 to 9.4) | 0.9<br>(-1.0 to 2.7) |
| Other infectious diseases | -- | -- | 11.3<br>(6.7 to 17.6) | 40.1*<br>(33.2 to 46.7) | 31.7*<br>(25.1 to 37.8) |
| Anemia due to other infectious diseases | 6111.9<br>(5819.8 to 6437.8) | -- | 184.4<br>(122.3 to 267.9) | -15.6*<br>(-22.1 to -9.1) | -20.7*<br>(-26.8 to -14.6) |
| **Maternal and neonatal disorders** | **12277.1<br>(10526.0 to 14255.3)** | **--** | **3587.9<br>(2733.7 to 4625.7)** | **18.0*<br>(11.0 to 26.3)** | **10.9*<br>(4.3 to 18.7)** |
| Maternal disorders | -- | -- | -- | -- | -- |
| Maternal haemorrhage | -- | -- | -- | -- | -- |
| Maternal hemorrhage complications | -- | -- | -- | -- | -- |
| Maternal hemorrhage episode | -- | -- | -- | -- | -- |
| Maternal sepsis and other pregnancy related infections | -- | -- | -- | -- | -- |
| Infertility due to puerperal sepsis | -- | -- | -- | -- | -- |
| Maternal sepsis and other maternal infection episode | -- | -- | -- | -- | -- |
| Maternal hypertensive disorders | -- | -- | -- | -- | -- |
| Maternal hypertensive disorder complications | -- | -- | -- | -- | -- |
| Maternal hypertensive disorder episode | -- | -- | -- | -- | -- |
| Maternal obstructed labour and uterine rupture | -- | -- | -- | -- | -- |
| Obstructed labor, acute event | -- | -- | -- | -- | -- |
| Maternal obstructed labor complications | -- | -- | -- | -- | -- |
| Maternal abortion and miscarriage | -- | -- | -- | -- | -- |
| Ectopic pregnancy | -- | -- | -- | -- | -- |
| Other maternal disorders | -- | -- | -- | -- | -- |
| Neonatal disorders | 12277.1<br>(10526.0 to 14255.3) | -- | 3587.9<br>(2733.7 to 4625.7) | 18.0*<br>(11.0 to 26.3) | 10.9*<br>(4.3 to 18.7) |
| Neonatal preterm birth | 7154.7<br>(6015.5 to 8560.6) | -- | 1398.5<br>(995.5 to 1880.4) | 17.8*<br>(3.3 to 33.2) | 10.7<br>(-2.9 to 25.1) |
| Preterm birth complications | 6305.4<br>(5139.7 to 7735.4) | -- | 1356.2<br>(959.5 to 1827.1) | 18.6*<br>(3.5 to 34.7) | 11.5<br>(-2.8 to 26.6) |
| Retinopathy of prematurity | 849.3<br>(647.5 to 1068.1) | -- | 42.4<br>(27.5 to 60.3) | -3.8<br>(-11.1 to 3.8) | -9.6*<br>(-16.5 to -2.4) |
| Uncomplicated preterm birth | -- | -- | -- | -- | -- |
| Neonatal encephalopathy due to birth asphyxia and trauma | 4436.3<br>(3160.2 to 5955.5) | -- | 882.6<br>(524.4 to 1364.8) | 22.0*<br>(16.0 to 28.5) | 14.6*<br>(9.0 to 20.7) |
| Neonatal sepsis and other neonatal infections | 1224.2<br>(747.6 to 1875.5) | -- | 359.1<br>(192.7 to 612.5) | 16.8*<br>(11.8 to 22.0) | 9.7*<br>(5.1 to 14.6) |
| Neonatal sepsis and other neonatal infection complications | 1224.2<br>(747.6 to 1875.5) | -- | 359.1<br>(192.7 to 612.5) | 16.8*<br>(11.8 to 22.0) | 9.7*<br>(5.1 to 14.6) |
| Neonatal sepsis and other neonatal infection episode | -- | -- | -- | -- | -- |
| Hemolytic disease and other neonatal jaundice | 245.8<br>(211.4 to 281.8) | -- | 88.7<br>(67.8 to 111.3) | 11.1*<br>(5.2 to 17.4) | 4.4<br>(-1.2 to 10.3) |

| | | | | | |
|---|---|---|---|---|---|
| Other neonatal disorders | -- | -- | 859.0 (654.5 to 1107.4) | 15.8* (8.9 to 23.9) | 8.8* (2.3 to 16.4) |
| **Nutritional deficiencies** | **199194.7 (186470.4 to 213355.5)** | **149302.3 (135181.9 to 161960.4)** | **6270.2 (4188.4 to 8823.0)** | **-16.5* (-20.9 to -12.1)** | **-21.5* (-25.6 to -17.5)** |
| Protein-energy malnutrition | 40744.2 (40108.1 to 41451.7) | 23375.8 (23011.1 to 23790.3) | 1078.5 (699.9 to 1523.9) | -3.3 (-7.1 to 0.3) | -9.1* (-12.7 to -5.8) |
| Iodine deficiency | 3226.8 (2495.4 to 4172.9) | 221.3 (178.6 to 264.1) | 76.9 (47.1 to 120.1) | -36.2* (-40.8 to -31.0) | -40.1* (-44.4 to -35.2) |
| Visible goiter without symptoms | 3096.7 (2376.8 to 3995.1) | 221.3 (178.6 to 264.1) | 33.7 (15.4 to 62.3) | -40.2* (-42.9 to -37.5) | -43.8* (-46.4 to -41.3) |
| Visible goiter with complications | 130.1 (83.7 to 175.0) | -- | 43.3 (24.9 to 66.2) | -32.7* (-40.7 to -24.6) | -36.8* (-44.3 to -29.2) |
| Vitamin A deficiency | 109919.0 (91974.8 to 130139.9) | 125705.2 (111782.3 to 138412.0) | 2097.3 (1319.1 to 3157.7) | -22.9* (-28.1 to -18.0) | -27.5* (-32.5 to -23.0) |
| Asymptomatic vitamin A deficiency | 40167.8 (32901.0 to 48770.3) | 125705.2 (111782.3 to 138412.0) | -- | -- | -- |
| Vitamin A deficiency complications | 1337.6 (945.2 to 1914.1) | -- | 66.0 (37.7 to 102.9) | 5.6* (0.4 to 10.9) | -0.8 (-5.7 to 4.2) |
| Vitamin A deficiency with anemia | 68413.8 (57127.7 to 81752.8) | -- | 2031.3 (1276.9 to 3067.5) | -23.5* (-28.9 to -18.5) | -28.2* (-33.2 to -23.5) |
| Dietary iron deficiency | 97062.1 (92434.9 to 102283.2) | -- | 2979.4 (1981.3 to 4345.7) | -14.9* (-21.1 to -8.4) | -20.1* (-25.9 to -14.0) |
| Other nutritional deficiencies | -- | -- | 38.1 (24.7 to 53.8) | -22.8* (-25.8 to -19.9) | -27.4* (-30.3 to -24.8) |
| **Non-communicable diseases** | **407741.4 (384147.1 to 433678.1)** | **662788.5 (542827.3 to 784990.7)** | **10344.4 (7202.3 to 14281.8)** | **5.7* (3.8 to 7.7)** | **-0.7 (-2.5 to 1.2)** |
| **Neoplasms** | **2040.1 (1689.1 to 2638.4)** | **121.9 (108.2 to 133.5)** | **98.7 (61.1 to 152.7)** | **67.1* (25.7 to 132.5)** | **57.0* (18.1 to 118.4)** |
| Lip and oral cavity cancer | -- | -- | -- | -- | -- |
| Diagnosis and primary therapy phase of mouth cancer | -- | -- | -- | -- | -- |
| Controlled phase of mouth cancer | -- | -- | -- | -- | -- |
| Metastatic phase of mouth cancer | -- | -- | -- | -- | -- |
| Terminal phase of mouth cancer | -- | -- | -- | -- | -- |
| Nasopharynx cancer | -- | -- | -- | -- | -- |
| Diagnosis and primary therapy phase of nasopharynx cancer | -- | -- | -- | -- | -- |
| Controlled phase of nasopharynx cancer | -- | -- | -- | -- | -- |
| Metastatic phase of nasopharynx cancer | -- | -- | -- | -- | -- |
| Terminal phase of nasopharynx cancer | -- | -- | -- | -- | -- |
| Other pharynx cancer | -- | -- | -- | -- | -- |
| Diagnosis and primary therapy phase of other pharynx cancer | -- | -- | -- | -- | -- |
| Controlled phase of other pharynx cancer | -- | -- | -- | -- | -- |
| Metastatic phase of other pharynx cancer | -- | -- | -- | -- | -- |
| Terminal phase of other pharynx cancer | -- | -- | -- | -- | -- |
| Oesophageal cancer | -- | -- | -- | -- | -- |
| Diagnosis and primary therapy phase of esophageal cancer | -- | -- | -- | -- | -- |
| Controlled phase of esophageal cancer | -- | -- | -- | -- | -- |
| Metastatic phase of esophageal cancer | -- | -- | -- | -- | -- |
| Terminal phase of esophageal cancer | -- | -- | -- | -- | -- |
| Stomach cancer | -- | -- | -- | -- | -- |
| Diagnosis and primary therapy phase of stomach cancer | -- | -- | -- | -- | -- |
| Controlled phase of stomach cancer | -- | -- | -- | -- | -- |
| Metastatic phase of stomach cancer | -- | -- | -- | -- | -- |
| Terminal phase of stomach cancer | -- | -- | -- | -- | -- |
| Colon and rectum cancer | -- | -- | -- | -- | -- |
| Diagnosis and primary therapy phase of colon and rectum cancers | -- | -- | -- | -- | -- |
| Controlled phase of colon and rectum cancers | -- | -- | -- | -- | -- |
| Metastatic phase of colon and rectum cancers | -- | -- | -- | -- | -- |
| Terminal phase of colon and rectum cancers | -- | -- | -- | -- | -- |
| Stoma from colon and rectum cancers, beyond 10 years | -- | -- | -- | -- | -- |
| Liver cancer | -- | -- | -- | -- | -- |
| Liver cancer due to hepatitis B | -- | -- | -- | -- | -- |
| Diagnosis and primary therapy phase of liver cancer due to hepatitis B | -- | -- | -- | -- | -- |
| Controlled phase of liver cancer due to hepatitis B | -- | -- | -- | -- | -- |
| Metastatic phase of liver cancer due to hepatitis B | -- | -- | -- | -- | -- |
| Terminal phase of liver cancer due to hepatitis B | -- | -- | -- | -- | -- |
| Liver cancer due to hepatitis C | -- | -- | -- | -- | -- |
| Diagnosis and primary therapy phase of liver cancer due to hepatitis C | -- | -- | -- | -- | -- |
| Controlled phase of liver cancer due to hepatitis C | -- | -- | -- | -- | -- |
| Metastatic phase of liver cancer due to hepatitis C | -- | -- | -- | -- | -- |
| Terminal phase of liver cancer due to hepatitis C | -- | -- | -- | -- | -- |
| Liver cancer due to alcohol use | -- | -- | -- | -- | -- |
| Diagnosis and primary therapy phase of liver cancer due to alcohol use | -- | -- | -- | -- | -- |
| Controlled phase of liver cancer due to alcohol use | -- | -- | -- | -- | -- |
| Metastatic phase of liver cancer due to alcohol use | -- | -- | -- | -- | -- |

| | | | | | |
|---|---|---|---|---|---|
| Terminal phase of liver cancer due to alcohol use | -- | -- | -- | -- | -- |
| Liver cancer due to NASH | -- | -- | -- | -- | -- |
| Diagnosis and primary therapy phase of liver cancer due to NASH | -- | -- | -- | -- | -- |
| Controlled phase of liver cancer due to NASH | -- | -- | -- | -- | -- |
| Metastatic phase of liver cancer due to NASH | -- | -- | -- | -- | -- |
| Terminal phase of liver cancer due to NASH | -- | -- | -- | -- | -- |
| Liver cancer due to other causes | -- | -- | -- | -- | -- |
| Diagnosis and primary therapy phase of liver cancer due to other causes | -- | -- | -- | -- | -- |
| Controlled phase of liver cancer due to other causes | -- | -- | -- | -- | -- |
| Metastatic phase of liver cancer due to other causes | -- | -- | -- | -- | -- |
| Terminal phase of liver cancer due to other causes | -- | -- | -- | -- | -- |
| Gallbladder and biliary tract cancer | -- | -- | -- | -- | -- |
| Diagnosis and primary therapy phase of gallbladder and biliary tract cancer | -- | -- | -- | -- | -- |
| Controlled phase of gallbladder and biliary tract cancer | -- | -- | -- | -- | -- |
| Metastatic phase of gallbladder and biliary tract cancer | -- | -- | -- | -- | -- |
| Terminal phase of gallbladder and biliary tract cancer | -- | -- | -- | -- | -- |
| Pancreatic cancer | -- | -- | -- | -- | -- |
| Diagnosis and primary therapy phase of pancreatic cancer | -- | -- | -- | -- | -- |
| Controlled phase of pancreatic cancer | -- | -- | -- | -- | -- |
| Metastatic phase of pancreatic cancer | -- | -- | -- | -- | -- |
| Terminal phase of pancreatic cancer | -- | -- | -- | -- | -- |
| Larynx cancer | -- | -- | -- | -- | -- |
| Diagnosis and primary therapy phase of larynx cancer | -- | -- | -- | -- | -- |
| Controlled phase of larynx cancer | -- | -- | -- | -- | -- |
| Metastatic phase of larynx cancer | -- | -- | -- | -- | -- |
| Terminal phase of larynx cancer | -- | -- | -- | -- | -- |
| Laryngectomy from larynx cancer, beyond 10 years | -- | -- | -- | -- | -- |
| Tracheal, bronchus, and lung cancer | -- | -- | -- | -- | -- |
| Diagnosis and primary therapy phase of lung, bronchus, and trachea cancer | -- | -- | -- | -- | -- |
| Controlled phase of lung, bronchus, and trachea cancer | -- | -- | -- | -- | -- |
| Metastatic phase of lung, bronchus, and trachea cancer | -- | -- | -- | -- | -- |
| Terminal phase of lung, bronchus, and trachea cancer | -- | -- | -- | -- | -- |
| Malignant skin melanoma | -- | -- | -- | -- | -- |
| Diagnosis and primary therapy phase of malignant skin melanoma | -- | -- | -- | -- | -- |
| Controlled phase of malignant skin melanoma | -- | -- | -- | -- | -- |
| Metastatic phase of malignant skin melanoma | -- | -- | -- | -- | -- |
| Terminal phase of malignant skin melanoma | -- | -- | -- | -- | -- |
| Non-melanoma skin cancer | -- | -- | -- | -- | -- |
| Non-melanoma skin cancer (squamous-cell carcinoma) | -- | -- | -- | -- | -- |
| Non-melanoma skin cancer (basal-cell carcinoma) | -- | -- | -- | -- | -- |
| Breast cancer | -- | -- | -- | -- | -- |
| Diagnosis and primary therapy phase of breast cancer | -- | -- | -- | -- | -- |
| Controlled phase of breast cancer | -- | -- | -- | -- | -- |
| Metastatic phase of breast cancer | -- | -- | -- | -- | -- |
| Terminal phase of breast cancer | -- | -- | -- | -- | -- |
| Mastectomy from breast cancer, beyond 10 years | -- | -- | -- | -- | -- |
| Cervical cancer | -- | -- | -- | -- | -- |
| Diagnosis and primary therapy phase of cervical cancer | -- | -- | -- | -- | -- |
| Controlled phase of cervical cancer | -- | -- | -- | -- | -- |
| Metastatic phase of cervical cancer | -- | -- | -- | -- | -- |
| Terminal phase of cervical cancer | -- | -- | -- | -- | -- |
| Uterine cancer | -- | -- | -- | -- | -- |
| Diagnosis and primary therapy phase of uterine cancer | -- | -- | -- | -- | -- |
| Controlled phase of uterine cancer | -- | -- | -- | -- | -- |
| Metastatic phase of uterine cancer | -- | -- | -- | -- | -- |
| Terminal phase of uterine cancer | -- | -- | -- | -- | -- |
| Ovarian cancer | -- | -- | -- | -- | -- |
| Diagnosis and primary therapy phase of ovarian cancer | -- | -- | -- | -- | -- |
| Controlled phase of ovarian cancer | -- | -- | -- | -- | -- |
| Metastatic phase of ovarian cancer | -- | -- | -- | -- | -- |

| | | | | | |
|---|---|---|---|---|---|
| Terminal phase of ovarian cancer | -- | -- | -- | -- | -- |
| Prostate cancer | -- | -- | -- | -- | -- |
| Diagnosis and primary therapy phase of prostate cancer | -- | -- | -- | -- | -- |
| Controlled phase of prostate cancer | -- | -- | -- | -- | -- |
| Metastatic phase of prostate cancer | -- | -- | -- | -- | -- |
| Terminal phase of prostate cancer | -- | -- | -- | -- | -- |
| Impotence and Incontinence after 10-year survival from prostate cancer | -- | -- | -- | -- | -- |
| Testicular cancer | -- | -- | -- | -- | -- |
| Diagnosis and primary therapy phase of testicular cancer | -- | -- | -- | -- | -- |
| Controlled phase of testicular cancer | -- | -- | -- | -- | -- |
| Metastatic phase of testicular cancer | -- | -- | -- | -- | -- |
| Terminal phase of testicular cancer | -- | -- | -- | -- | -- |
| Kidney cancer | 137.2 (120.5 to 154.7) | 15.1 (13.3 to 17.1) | 6.6 (4.1 to 10.0) | -6.1 (-26.6 to 25.0) | -11.7 (-31.1 to 17.4) |
| Diagnosis and primary therapy phase of kidney cancer | -- | 15.1 (13.3 to 17.1) | -- | -- | -- |
| Controlled phase of kidney cancer | 137.2 (120.5 to 154.7) | -- | 6.6 (4.1 to 10.0) | -6.1 (-26.6 to 25.0) | -11.7 (-31.1 to 17.4) |
| Metastatic phase of kidney cancer | -- | -- | -- | -- | -- |
| Terminal phase of kidney cancer | -- | -- | -- | -- | -- |
| Bladder cancer | -- | -- | -- | -- | -- |
| Diagnosis and primary therapy phase of bladder cancer | -- | -- | -- | -- | -- |
| Controlled phase of bladder cancer | -- | -- | -- | -- | -- |
| Metastatic phase of bladder cancer | -- | -- | -- | -- | -- |
| Terminal phase of bladder cancer | -- | -- | -- | -- | -- |
| Incontinence from bladder cancer, beyond 10 years | -- | -- | -- | -- | -- |
| Brain and nervous system cancer | 151.9 (128.5 to 182.4) | 20.4 (17.5 to 24.1) | 9.9 (6.6 to 14.2) | 16.3 (-5.8 to 49.1) | 9.2 (-11.5 to 40.0) |
| Diagnosis and primary therapy phase of brain and nervous system cancers | 6.6 (5.5 to 7.9) | 20.4 (17.5 to 24.1) | 1.9 (1.2 to 2.8) | 21.4 (-0.8 to 53.8) | 14.1 (-6.8 to 44.5) |
| Controlled phase of brain and nervous system cancers | 143.0 (120.7 to 172.1) | -- | 6.9 (4.3 to 10.7) | 22.1 (-1.7 to 55.7) | 14.7 (-7.6 to 46.3) |
| Metastatic phase of brain and nervous system cancers | 2.0 (1.7 to 2.3) | -- | 0.9 (0.6 to 1.2) | -15.5 (-31.3 to 12.8) | -20.6 (-35.5 to 6.0) |
| Terminal phase of brain and nervous system cancers | 0.4 (0.3 to 0.5) | -- | 0.2 (0.1 to 0.3) | -15.5 (-31.3 to 12.8) | -20.7 (-35.5 to 6.0) |
| Thyroid cancer | -- | -- | -- | -- | -- |
| Diagnosis and primary therapy phase of thyroid cancer | -- | -- | -- | -- | -- |
| Controlled phase of thyroid cancer | -- | -- | -- | -- | -- |
| Metastatic phase of thyroid cancer | -- | -- | -- | -- | -- |
| Terminal phase of thyroid cancer | -- | -- | -- | -- | -- |
| Mesothelioma | -- | -- | -- | -- | -- |
| Diagnosis and primary therapy phase of mesothelioma | -- | -- | -- | -- | -- |
| Controlled phase of mesothelioma | -- | -- | -- | -- | -- |
| Metastatic phase of mesothelioma | -- | -- | -- | -- | -- |
| Terminal phase of mesothelioma | -- | -- | -- | -- | -- |
| Hodgkin lymphoma | 14.0 (10.5 to 17.4) | 2.1 (1.5 to 2.7) | 0.9 (0.6 to 1.4) | 6.6 (-18.8 to 44.0) | 0.1 (-23.7 to 35.3) |
| Diagnosis and primary therapy phase of Hodgkin disease | 0.4 (0.3 to 0.5) | 2.1 (1.5 to 2.7) | 0.1 (0.1 to 0.2) | 9.4 (-9.2 to 36.0) | 2.8 (-14.7 to 27.8) |
| Controlled phase of Hodgkin disease | 13.2 (10.0 to 16.4) | -- | 0.6 (0.4 to 1.0) | 8.9 (-20.7 to 49.5) | 2.3 (-25.5 to 40.4) |
| Metastatic phase of Hodgkin disease | 0.3 (0.2 to 0.4) | -- | 0.1 (0.1 to 0.2) | -4.1 (-33.6 to 49.6) | -9.9 (-37.6 to 40.5) |
| Terminal phase of Hodgkin disease | 0.1 (0.0 to 0.1) | -- | -- | -4.1 (-33.6 to 49.6) | -9.9 (-37.6 to 40.5) |
| Non-Hodgkin's lymphoma | 63.0 (54.5 to 74.6) | 10.2 (8.7 to 12.3) | 4.3 (2.9 to 6.3) | 13.6 (-12.4 to 49.5) | 6.7 (-17.7 to 40.5) |
| Diagnosis and primary therapy phase of non-Hodgkin lymphoma | 2.0 (1.7 to 2.3) | 10.2 (8.7 to 12.3) | 0.6 (0.4 to 0.8) | 17.2 (-6.7 to 49.3) | 10.1 (-12.3 to 40.3) |
| Controlled phase of non-Hodgkin lymphoma | 59.1 (51.2 to 69.9) | -- | 2.9 (1.7 to 4.4) | 17.5 (-10.2 to 57.0) | 10.4 (-15.7 to 47.5) |
| Metastatic phase of non-Hodgkin lymphoma | 1.6 (1.3 to 2.0) | -- | 0.7 (0.5 to 1.0) | 0.7 (-24.7 to 40.0) | -5.4 (-29.3 to 31.6) |
| Terminal phase of non-Hodgkin lymphoma | 0.3 (0.3 to 0.4) | -- | 0.2 (0.1 to 0.2) | 0.7 (-24.7 to 40.0) | -5.4 (-29.3 to 31.6) |
| Multiple myeloma | -- | -- | -- | -- | -- |
| Diagnosis and primary therapy phase of multiple myeloma | -- | -- | -- | -- | -- |
| Controlled phase of multiple myeloma | -- | -- | -- | -- | -- |
| Metastatic phase of multiple myeloma | -- | -- | -- | -- | -- |
| Terminal phase of multiple myeloma | -- | -- | -- | -- | -- |
| Leukaemia | 393.7 (328.1 to 458.6) | 37.8 (31.9 to 44.6) | 22.1 (14.1 to 31.9) | 14.4 (-12.8 to 39.5) | 7.5 (-18.1 to 31.1) |
| Acute lymphoid leukaemia | 175.4 (136.4 to 210.7) | 7.5 (6.4 to 8.9) | 10.2 (6.5 to 15.0) | 39.4 (-2.6 to 74.3) | 30.9 (-8.5 to 63.7) |
| Diagnosis and primary therapy phase of acute lymphoid leukemia | 5.5 (4.7 to 6.5) | 7.5 (6.4 to 8.9) | 1.6 (1.0 to 2.3) | -1.0 (-30.2 to 21.4) | -7.0 (-34.4 to 14.0) |
| Controlled phase of acute lymphoid leukemia | 168.9 (131.1 to 203.5) | -- | 8.2 (4.7 to 12.5) | 59.2* (12.0 to 99.6) | 49.6* (5.2 to 87.5) |
| Metastatic phase of acute lymphoid leukemia | 0.8 (0.7 to 1.0) | -- | 0.4 (0.2 to 0.5) | -22.8 (-45.6 to 0.2) | -27.5* (-48.9 to -5.8) |
| Terminal phase of acute lymphoid leukemia | 0.2 (0.1 to 0.2) | -- | 0.1 (0.1 to 0.1) | -22.8 (-45.6 to 0.2) | -27.5* (-48.9 to -5.8) |
| Chronic lymphoid leukaemia | -- | -- | -- | -- | -- |

| | | | | | |
|---|---|---|---|---|---|
| Diagnosis and primary therapy phase of chronic lymphoid leukemia | -- | -- | -- | -- | -- |
| Controlled phase of chronic lymphoid leukemia | -- | -- | -- | -- | -- |
| Metastatic phase of chronic lymphoid leukemia | -- | -- | -- | -- | -- |
| Terminal phase of chronic lymphoid leukemia | -- | -- | -- | -- | -- |
| Acute myeloid leukaemia | 13.8 (11.0 to 16.0) | 6.9 (5.4 to 8.0) | 1.8 (1.2 to 2.4) | 7.5 (-28.1 to 47.8) | 1.0 (-32.5 to 38.8) |
| Diagnosis and primary therapy phase of acute myeloid leukemia | -- | 6.9 (5.4 to 8.0) | -- | -- | -- |
| Controlled phase of acute myeloid leukemia | 11.2 (9.0 to 13.0) | -- | 0.5 (0.3 to 0.8) | 9.1 (-26.4 to 48.1) | 2.5 (-30.8 to 39.1) |
| Metastatic phase of acute myeloid leukemia | 2.2 (1.7 to 2.5) | -- | 1.0 (0.6 to 1.3) | 6.8 (-28.8 to 47.3) | 0.4 (-33.1 to 38.3) |
| Terminal phase of acute myeloid leukemia | 0.5 (0.4 to 0.5) | -- | 0.3 (0.2 to 0.3) | 6.8 (-28.8 to 47.3) | 0.3 (-33.1 to 38.3) |
| Chronic myeloid leukaemia | -- | -- | -- | -- | -- |
| Diagnosis and primary therapy phase of chronic myeloid leukemia | -- | -- | -- | -- | -- |
| Controlled phase of chronic myeloid leukemia | -- | -- | -- | -- | -- |
| Metastatic phase of chronic myeloid leukemia | -- | -- | -- | -- | -- |
| Terminal phase of chronic myeloid leukemia | -- | -- | -- | -- | -- |
| Other leukaemia | 204.6 (162.9 to 249.2) | 23.5 (18.8 to 28.4) | 10.1 (6.1 to 15.3) | -2.2 (-27.7 to 30.9) | -8.1 (-32.1 to 23.0) |
| Metastatic phase of other leukemia | 0.3 (0.2 to 0.4) | -- | 0.2 (0.1 to 0.2) | -31.3* (-50.1 to -0.5) | -35.4* (-53.1 to -6.6) |
| Diagnosis and primary therapy phase of other leukemia | -- | 23.5 (18.8 to 28.4) | -- | -- | -- |
| Terminal phase of other leukemia | 0.1 (0.0 to 0.1) | -- | -- | -31.3* (-50.1 to -0.5) | -35.4* (-53.1 to -6.6) |
| Controlled phase of other leukemia | 204.2 (162.5 to 248.9) | -- | 9.9 (6.0 to 15.1) | -1.4 (-27.1 to 31.4) | -7.4 (-31.5 to 23.5) |
| Other malignant cancers | 1006.8 (686.1 to 1586.2) | 36.2 (31.3 to 40.8) | 54.3 (30.2 to 95.9) | 188.6* (88.0 to 358.4) | 171.1* (76.7 to 330.7) |
| Diagnosis and primary therapy phase of other malignant neoplasms | 17.6 (12.0 to 27.8) | 36.2 (31.3 to 40.8) | 5.0 (2.7 to 9.0) | 199.2* (88.8 to 403.4) | 181.1* (77.4 to 373.0) |
| Controlled phase of other malignant neoplasms | 986.0 (671.3 to 1555.4) | -- | 47.8 (25.3 to 86.2) | 203.2* (97.1 to 383.4) | 184.9* (85.2 to 354.2) |
| Metastatic phase of other malignant neoplasms | 2.7 (2.2 to 3.2) | -- | 1.2 (0.8 to 1.7) | 8.4 (-20.5 to 37.5) | 1.8 (-25.3 to 29.2) |
| Terminal phase of other malignant neoplasms | 0.5 (0.4 to 0.6) | -- | 0.3 (0.2 to 0.4) | 8.3 (-20.5 to 37.5) | 1.8 (-25.3 to 29.2) |
| Other neoplasms | 399.0 (373.3 to 426.4) | 373.1 (357.4 to 390.2) | 0.5 (0.3 to 0.9) | -23.6* (-29.7 to -18.7) | -28.2* (-33.9 to -23.7) |
| Myelodysplastic, myeloproliferative, and other hematopoietic neoplasms | 11.2 (8.4 to 14.2) | 5.0 (3.7 to 6.5) | 0.5 (0.3 to 0.9) | -23.6* (-29.7 to -18.7) | -28.2* (-33.9 to -23.7) |
| Benign and in situ intestinal neoplasms | 8.0 (7.5 to 8.6) | 6.7 (6.3 to 7.1) | -- | -- | -- |
| Benign and in situ cervical and uterine neoplasms | 0.4 (0.3 to 0.6) | 0.3 (0.2 to 0.4) | -- | -- | -- |
| Other benign and in situ neoplasms | 397.6 (376.2 to 418.4) | 361.0 (345.6 to 378.2) | -- | -- | -- |
| **Cardiovascular diseases** | **873.4 (618.8 to 1244.1)** | **393.9 (362.0 to 430.8)** | **86.7 (58.3 to 126.0)** | **-15.6* (-21.6 to -10.0)** | **-20.7* (-26.3 to -15.4)** |
| Rheumatic heart disease | 249.3 (233.3 to 264.0) | 142.1 (132.8 to 150.5) | 12.1 (7.8 to 17.6) | 10.2* (3.2 to 17.5) | 3.6 (-3.0 to 10.4) |
| Rheumatic heart disease, without heart failure | 248.1 (232.1 to 262.8) | 142.1 (132.8 to 150.5) | 11.9 (7.7 to 17.5) | 10.3* (3.2 to 17.7) | 3.6 (-3.1 to 10.6) |
| Heart failure due to rheumatic heart disease | 1.2 (0.7 to 1.7) | -- | 0.1 (0.1 to 0.2) | 6.4* (4.3 to 8.2) | -0.1 (-2.0 to 1.6) |
| Ischaemic heart disease | -- | -- | -- | -- | -- |
| Asymptomatic ischemic heart disease following myocardial infarction | -- | -- | -- | -- | -- |
| Angina due to ischemic heart disease | -- | -- | -- | -- | -- |
| Heart failure due to ischemic heart disease | -- | -- | -- | -- | -- |
| Myocardial infarction due to ischemic heart disease | -- | -- | -- | -- | -- |
| Stroke | 28.4 (23.0 to 34.4) | 52.4‡ (39.4 to 70.8) | 30.5 (21.1 to 40.2) | -40.1* (-44.5 to -35.9) | -43.8* (-47.9 to -39.8) |
| Ischaemic stroke | 123.5 (102.4 to 147.8) | 31.1 (20.3 to 47.5) | 18.0 (12.1 to 24.6) | -45.4* (-50.6 to -40.3) | -48.7* (-53.6 to -43.9) |
| Acute ischemic stroke | 2.5 (1.7 to 3.7) | 31.1 (20.3 to 47.5) | 0.7 (0.4 to 1.2) | -45.6* (-54.6 to -36.0) | -48.9* (-57.4 to -39.9) |
| Chronic ischemic stroke | 121.0 (99.6 to 145.4) | -- | 17.3 (11.7 to 23.7) | -45.4* (-50.9 to -40.1) | -48.7* (-53.9 to -43.7) |
| Intracerebral hemorrhage | 69.0 (55.9 to 84.1) | 15.9 (10.1 to 24.1) | 10.2 (6.9 to 14.2) | -33.9* (-41.9 to -25.0) | -37.9* (-45.4 to -29.6) |
| Acute intracerebral hemorrhage | 0.9 (0.6 to 1.3) | 15.9 (10.1 to 24.1) | 0.3 (0.2 to 0.5) | -57.1* (-66.2 to -47.1) | -59.6* (-68.2 to -50.3) |
| Chronic intracerebral hemorrhage | 68.0 (55.0 to 83.1) | -- | 9.9 (6.7 to 13.8) | -32.9* (-41.5 to -23.8) | -36.9* (-45.0 to -28.4) |
| Subarachnoid hemorrhage | 15.7 (11.5 to 19.9) | 5.5 (3.4 to 8.0) | 2.3 (1.4 to 3.3) | -10.1* (-18.2 to -4.0) | -15.5* (-23.1 to -9.8) |
| Acute subarachnoid hemorrhage | 0.3 (0.2 to 0.4) | 5.5 (3.4 to 8.0) | 0.1 (0.1 to 0.1) | -25.1* (-55.2 to -8.4) | -29.7* (-57.9 to -14.0) |
| Chronic subarachnoid hemorrhage | 15.4 (11.1 to 19.6) | -- | 2.2 (1.4 to 3.1) | -9.3* (-16.9 to -3.5) | -14.8* (-21.9 to -9.4) |
| Hypertensive heart disease | -- | -- | -- | -- | -- |
| Non-rheumatic valvular heart disease | -- | -- | -- | -- | -- |
| Non-rheumatic calcific aortic valve disease | -- | -- | -- | -- | -- |
| Heart failure due to calcific aortic valve disease | -- | -- | -- | -- | -- |
| Non-rheumatic calcific aortic valve disease | -- | -- | -- | -- | -- |
| Non-rheumatic degenerative mitral valve disease | -- | -- | -- | -- | -- |
| Heart failure due to degenerative mitral valve disease | -- | -- | -- | -- | -- |
| Non-rheumatic degenerative mitral valve disease | -- | -- | -- | -- | -- |
| Other non-rheumatic valve diseases | -- | -- | -- | -- | -- |
| Mild heart failure due to other non-rheumatic valve disease | -- | -- | -- | -- | -- |
| Moderate heart failure due to other non-rheumatic valve disease | -- | -- | -- | -- | -- |
| Severe heart failure due to other non-rheumatic valve disease | -- | -- | -- | -- | -- |

| | | | | | |
|---|---|---|---|---|---|
| Controlled, medically managed heart failure due to other non-rheumatic valve disease | -- | -- | -- | -- | -- |
| Cardiomyopathy and myocarditis | 1.5<br>(0.8 to 2.3) | 82.5<br>(66.2 to 101.9) | 5.9<br>(3.6 to 9.2) | 8.8*<br>(7.6 to 10.2) | 2.3*<br>(1.1 to 3.5) |
| Myocarditis | 44.8<br>(35.0 to 56.4) | 82.5<br>(66.2 to 101.9) | 3.3<br>(2.1 to 5.0) | 8.2*<br>(6.5 to 10.0) | 1.7*<br>(0.1 to 3.4) |
| Acute myocarditis | 19.9<br>(15.7 to 25.1) | 82.5<br>(66.2 to 101.9) | 1.0<br>(0.6 to 1.6) | 9.2*<br>(4.4 to 14.2) | 2.6<br>(-1.9 to 7.3) |
| Heart failure due to myocarditis | 24.9<br>(16.7 to 34.9) | -- | 2.3<br>(1.3 to 3.7) | 7.8*<br>(6.7 to 9.1) | 1.3*<br>(0.2 to 2.5) |
| Alcoholic cardiomyopathy | -- | -- | -- | -- | -- |
| Other cardiomyopathy | 28.5<br>(19.0 to 40.0) | -- | 2.6<br>(1.5 to 4.2) | 9.6*<br>(8.3 to 11.1) | 3.0*<br>(1.8 to 4.4) |
| Atrial fibrillation and flutter | -- | -- | -- | -- | -- |
| Asymptomatic atrial fibrillation and flutter | -- | -- | -- | -- | -- |
| Symptomatic atrial fibrillation and flutter | -- | -- | -- | -- | -- |
| Peripheral vascular disease | -- | -- | -- | -- | -- |
| Asymptomatic peripheral arterial disease | -- | -- | -- | -- | -- |
| Symptomatic claudication due to peripheral arterial disease | -- | -- | -- | -- | -- |
| Endocarditis | 28.2<br>(21.1 to 36.6) | 116.9<br>(95.2 to 141.4) | 2.4<br>(1.5 to 3.7) | 9.9*<br>(7.7 to 12.1) | 3.3*<br>(1.2 to 5.3) |
| Acute endocarditis | 6.7<br>(5.4 to 8.2) | 116.9<br>(95.2 to 141.4) | 0.4<br>(0.2 to 0.6) | 10.4*<br>(2.6 to 17.0) | 3.7<br>(-3.6 to 10.0) |
| Heart failure due to endocarditis | 21.5<br>(14.7 to 30.0) | -- | 2.0<br>(1.2 to 3.1) | 9.9*<br>(7.7 to 12.0) | 3.2*<br>(1.1 to 5.2) |
| Other cardiovascular and circulatory diseases | 643.9<br>(376.8 to 1063.2) | -- | 35.8<br>(18.9 to 65.6) | 7.9*<br>(1.6 to 14.9) | 1.4<br>(-4.5 to 7.9) |
| Heart failure due to other cardiovascular diseases | 13.3<br>(9.1 to 18.3) | -- | 1.2<br>(0.7 to 1.9) | 7.6*<br>(6.5 to 8.8) | 1.1*<br>(0.1 to 2.2) |
| Other cardiovascular and circulatory disease episodes | 630.6<br>(366.7 to 1045.9) | -- | 34.6<br>(18.2 to 64.1) | 7.9*<br>(1.4 to 15.2) | 1.4<br>(-4.7 to 8.2) |
| **Chronic respiratory diseases** | **27632.0<br>(18756.8 to 37870.1)** | **15644.3<br>(10853.9 to 21163.1)** | **1206.7<br>(685.9 to 1884.3)** | **12.8*<br>(1.0 to 23.0)** | **5.9<br>(-5.1 to 15.6)** |
| Chronic obstructive pulmonary disease | 238.7<br>(169.6 to 287.6) | 80.2<br>(58.0 to 96.4) | 29.3<br>(20.2 to 37.5) | 3.7<br>(-5.6 to 13.4) | -2.6<br>(-11.3 to 6.5) |
| Chronic obstructive pulmonary disease with heart failure | -- | -- | -- | -- | -- |
| Chronic obstructive pulmonary disease without heart failure | 238.7<br>(169.6 to 287.6) | 80.2<br>(58.0 to 96.4) | 29.3<br>(20.2 to 37.5) | 3.7<br>(-5.6 to 13.4) | -2.6<br>(-11.3 to 6.5) |
| Pneumoconiosis | -- | -- | -- | -- | -- |
| Silicosis | -- | -- | -- | -- | -- |
| Heart failure due to silicosis | -- | -- | -- | -- | -- |
| Silicosis without heart failure | -- | -- | -- | -- | -- |
| Asbestosis | -- | -- | -- | -- | -- |
| Asbestosis without heart failure | -- | -- | -- | -- | -- |
| Heart failure due to asbestosis | -- | -- | -- | -- | -- |
| Coal workers pneumoconiosis | -- | -- | -- | -- | -- |
| Coal workers pneumoconiosis without heart failure | -- | -- | -- | -- | -- |
| Heart failure due to Coal workers pneumoconiosis | -- | -- | -- | -- | -- |
| Other pneumoconiosis | -- | -- | -- | -- | -- |
| Heart failure due to other pneumoconiosis | -- | -- | -- | -- | -- |
| Other pneumoconiosis without heart failure | -- | -- | -- | -- | -- |
| Asthma | 28245.5<br>(18915.1 to 39157.3) | 15563.1<br>(10773.9 to 21085.3) | 1139.3<br>(622.3 to 1812.3) | 12.9*<br>(0.3 to 23.8) | 6.1<br>(-5.8 to 16.3) |
| Asymptomatic asthma | 10262.0<br>(6847.2 to 14313.3) | 5640.8<br>(3859.0 to 7729.9) | -- | -- | -- |
| Symptomatic asthma | 17983.6<br>(11909.2 to 25363.0) | 9922.3<br>(6784.3 to 13588.3) | 1139.3<br>(622.3 to 1812.3) | 12.9*<br>(0.3 to 23.8) | 6.1<br>(-5.8 to 16.3) |
| Interstitial lung disease and pulmonary sarcoidosis | 0.3<br>(0.1 to 0.4) | 1.0<br>(0.6 to 1.5) | -- | 9.1*<br>(6.8 to 11.6) | 2.5*<br>(0.3 to 4.8) |
| Heart failure due to interstitial lung disease and pulmonary sarcoidosis | -- | -- | -- | -- | -- |
| Interstitial lung disease and pulmonary sarcoidosis without heart failure | 0.3<br>(0.1 to 0.4) | 1.0<br>(0.6 to 1.5) | -- | 9.1*<br>(6.8 to 11.6) | 2.5*<br>(0.3 to 4.8) |
| Other chronic respiratory diseases | -- | -- | 38.1<br>(26.3 to 48.8) | 15.7*<br>(5.2 to 26.4) | 8.7<br>(-1.1 to 18.8) |
| **Digestive diseases** | **29602.9<br>(25367.6 to 33593.5)** | **5890.1<br>(4906.5 to 7067.8)** | **531.7<br>(345.5 to 759.8)** | **5.9*<br>(3.4 to 8.6)** | **-0.5<br>(-2.8 to 2.0)** |
| Cirrhosis and other chronic liver diseases | 23706.2<br>(19450.1 to 27678.2) | 230.4<br>(190.0 to 274.9) | 35.8<br>(24.5 to 50.5) | 10.1*<br>(2.4 to 17.8) | 3.4<br>(-3.8 to 10.7) |
| Cirrhosis and other chronic liver diseases due to hepatitis B | 19533.7<br>(15405.2 to 23430.0) | 4.7<br>(3.6 to 5.9) | 0.9<br>(0.6 to 1.3) | -8.8*<br>(-13.9 to -3.9) | -14.3*<br>(-19.2 to -9.7) |
| Cirrhosis and other chronic liver diseases due to hepatitis B, decompensated | 5.0<br>(4.3 to 5.9) | 0.6<br>(0.5 to 0.7) | 0.9<br>(0.6 to 1.3) | -8.8*<br>(-13.9 to -3.9) | -14.3*<br>(-19.2 to -9.7) |
| Chronic hepatitis B without cirrhosis | 19505.3<br>(15379.4 to 23400.1) | -- | -- | -- | -- |
| Cirrhosis and other chronic liver diseases due to hepatitis B, compensated | 23.4<br>(18.5 to 28.6) | 4.1<br>(3.0 to 5.3) | -- | -- | -- |
| Cirrhosis and other chronic liver diseases due to hepatitis C | 2946.4<br>(2181.1 to 3874.6) | 3.7<br>(2.7 to 4.9) | 0.6<br>(0.4 to 0.9) | 6.8*<br>(4.3 to 9.1) | 0.3<br>(-2.0 to 2.5) |
| Cirrhosis and other chronic liver diseases due to hepatitis C, decompensated | 3.4<br>(2.8 to 4.3) | 0.5<br>(0.3 to 0.6) | 0.6<br>(0.4 to 0.9) | 6.8*<br>(4.3 to 9.1) | 0.3<br>(-2.0 to 2.5) |
| Chronic hepatitis C without cirrhosis | 2925.3<br>(2157.0 to 3856.8) | -- | -- | -- | -- |
| Cirrhosis and other chronic liver diseases due to hepatitis C, compensated | 17.7<br>(13.5 to 22.4) | 3.3<br>(2.3 to 4.4) | -- | -- | -- |
| Cirrhosis and other chronic liver diseases due to alcohol use | -- | -- | -- | -- | -- |
| Cirrhosis and other chronic liver diseases due to alcohol, decompensated | -- | -- | -- | -- | -- |
| Cirrhosis and other chronic liver diseases due to alcohol, compensated | -- | -- | -- | -- | -- |
| Cirrhosis due to NASH | -- | -- | -- | -- | -- |
| Non-alcoholic fatty liver disease (NAFLD) / Non-alcoholic steatohepatitis (NASH) | -- | -- | -- | -- | -- |
| Cirrhosis and other chronic liver diseases due to NASH | -- | -- | -- | -- | -- |
| Cirrhosis and other chronic liver diseases due to other causes | 1226.0<br>(1091.7 to 1363.7) | 222.0<br>(182.4 to 265.4) | 34.3<br>(23.4 to 48.4) | 10.8*<br>(2.7 to 18.7) | 4.1<br>(-3.5 to 11.6) |

| | | | | | |
|---|---|---|---|---|---|
| Cirrhosis and other chronic liver diseases due to other cause, decompensated | 197.8 (188.2 to 208.0) | 28.7 (25.8 to 31.6) | 34.3 (23.4 to 48.4) | 10.8* (2.7 to 18.7) | 4.1 (-3.5 to 11.6) |
| Cirrhosis and other chronic liver diseases due to other cause, compensated | 1028.2 (894.7 to 1172.0) | 193.3 (153.9 to 235.5) | -- | -- | -- |
| Upper digestive system diseases | 4319.8 (3187.6 to 5751.2) | 3428.8 (2597.0 to 4499.6) | 202.5 (122.4 to 312.5) | 4.8* (1.5 to 7.8) | -1.6 (-4.7 to 1.3) |
| Peptic ulcer disease | -- | -- | -- | -- | -- |
| Acute peptic ulcer disease | -- | -- | -- | -- | -- |
| Chronic peptic ulcer disease | -- | -- | -- | -- | -- |
| Gastritis and duodenitis | 4370.3 (3211.2 to 5810.1) | 3428.8 (2597.0 to 4499.6) | 202.5 (122.4 to 312.5) | 4.8* (1.5 to 7.8) | -1.6 (-4.7 to 1.3) |
| Acute gastritis and duodenitis | -- | -- | -- | -- | -- |
| Chronic gastritis and duodenitis | 4370.3 (3211.2 to 5810.1) | 3428.8 (2597.0 to 4499.6) | 202.5 (122.4 to 312.5) | 4.8* (1.5 to 7.8) | -1.6 (-4.7 to 1.3) |
| GERD | -- | -- | -- | -- | -- |
| Appendicitis | 20.0 (16.6 to 24.4) | 562.3 (459.6 to 687.3) | 6.5 (4.2 to 9.3) | 6.4* (5.1 to 7.5) | -0.1 (-1.2 to 1.0) |
| Paralytic ileus and intestinal obstruction | 9.0 (6.7 to 12.0) | 235.4 (172.9 to 314.5) | 2.9 (1.8 to 4.4) | 8.5* (5.1 to 12.2) | 2.0 (-1.3 to 5.4) |
| Inguinal, femoral, and abdominal hernia | 1964.5 (1502.2 to 2524.3) | 1350.8 (973.0 to 1812.0) | 201.2 (126.5 to 305.6) | 7.3* (2.8 to 12.1) | 0.8 (-3.5 to 5.4) |
| Inflammatory bowel disease | 4.9 (3.9 to 6.1) | 21.8 (17.9 to 26.1) | 0.8 (0.5 to 1.2) | -10.4* (-14.5 to -6.6) | -15.8* (-19.7 to -12.2) |
| Ulcerative colitis | 1.7 (1.2 to 2.2) | 10.8 (8.1 to 13.9) | 0.3 (0.2 to 0.4) | -19.5* (-23.2 to -16.1) | -24.3* (-27.9 to -21.2) |
| Crohn's disease | 3.3 (2.3 to 4.3) | 11.1 (8.0 to 14.6) | 0.5 (0.3 to 0.8) | -5.3* (-10.7 to -0.9) | -11.0* (-16.1 to -6.9) |
| Vascular intestinal disorders | 4.6 (2.9 to 7.0) | 17.2 (12.0 to 23.4) | 1.5 (0.8 to 2.5) | 10.2* (8.1 to 12.4) | 3.5* (1.6 to 5.6) |
| Gallbladder and biliary diseases | 191.8 (135.3 to 259.3) | 29.3 (17.2 to 45.3) | 0.2 (0.1 to 0.3) | 1.5 (-2.2 to 4.4) | -4.7* (-8.1 to -1.9) |
| Pancreatitis | 25.1 (20.0 to 32.1) | 14.0 (10.6 to 18.0) | 1.7 (0.8 to 2.9) | 10.0* (8.7 to 11.2) | 3.4* (2.1 to 4.5) |
| Acute pancreatitis | 0.6 (0.4 to 0.8) | 7.9 (4.9 to 11.7) | 0.2 (0.1 to 0.4) | 3.2* (2.1 to 4.2) | -3.0* (-4.1 to -2.1) |
| Chronic pancreatitis | 24.6 (19.5 to 31.5) | 6.1 (4.7 to 7.9) | 1.4 (0.6 to 2.6) | 11.2* (10.0 to 12.5) | 4.5* (3.4 to 5.7) |
| Other digestive diseases | -- | -- | 78.6 (51.2 to 112.9) | 3.6* (0.9 to 6.4) | -2.7* (-5.2 to -0.1) |
| **Neurological disorders** | **1805.5 (1330.0 to 2387.6)** | **373.2 (252.8 to 522.4)** | **801.0 (495.1 to 1230.5)** | **14.0 (-2.4 to 33.6)** | **7.1 (-8.3 to 25.5)** |
| Alzheimer's disease and other dementias | -- | -- | -- | -- | -- |
| Parkinson's disease | -- | -- | -- | -- | -- |
| Epilepsy | 1808.9 (1328.1 to 2377.2) | 369.5 (248.7 to 518.5) | 625.6 (386.7 to 961.7) | 12.3 (-4.0 to 31.5) | 5.5 (-9.8 to 23.6) |
| Multiple sclerosis | -- | -- | -- | -- | -- |
| Motor neuron disease | 7.9 (6.1 to 9.9) | 3.7 (2.7 to 5.0) | 1.7 (1.1 to 2.4) | 6.7* (5.3 to 8.0) | 0.2 (-1.1 to 1.5) |
| Headache disorders | -- | -- | -- | -- | -- |
| Migraine | -- | -- | -- | -- | -- |
| Medication overuse headache due to migraine | -- | -- | -- | -- | -- |
| Migraine | -- | -- | -- | -- | -- |
| Tension-type headache | -- | -- | -- | -- | -- |
| Medication overuse headache due to tension-type headache | -- | -- | -- | -- | -- |
| Tension-type headache | -- | -- | -- | -- | -- |
| Other neurological disorders | 3.8 (2.1 to 6.4) | -- | 173.7 (107.2 to 266.3) | 21.1* (3.7 to 41.8) | 13.8 (-2.6 to 33.2) |
| Other neurological disorders | -- | -- | 172.6 (106.7 to 265.3) | 21.2* (3.6 to 42.0) | 13.9 (-2.6 to 33.4) |
| Guillain-Barré syndrome due to other neurological disorders | 3.8 (2.1 to 6.4) | -- | 1.1 (0.6 to 2.0) | 7.4* (5.3 to 9.3) | 0.9 (-1.1 to 2.7) |
| **Mental disorders** | **13886.7 (9896.3 to 18019.9)** | **1741.9 (1502.7 to 1997.1)** | **888.9 (567.2 to 1281.1)** | **-0.3 (-2.1 to 1.8)** | **-6.4* (-8.0 to -4.4)** |
| Schizophrenia | -- | -- | -- | -- | -- |
| Depressive disorders | -- | 19.8 (9.8 to 37.5) | 1.5 (0.7 to 3.0) | 7.8* (6.8 to 9.0) | 1.3* (0.3 to 2.4) |
| Major depressive disorder | 5.4 (2.0 to 11.2) | 15.4 (5.8 to 32.5) | 1.2 (0.4 to 2.7) | 8.2* (7.1 to 9.6) | 1.7* (0.6 to 3.0) |
| Dysthymia | 2.9 (1.1 to 5.7) | 4.4 (1.7 to 8.6) | 0.3 (0.1 to 0.6) | 6.3* (4.0 to 8.8) | -0.1 (-2.3 to 2.2) |
| Bipolar disorder | -- | -- | -- | -- | -- |
| Anxiety disorders | 650.7 (561.5 to 749.9) | 685.7 (593.0 to 785.4) | 65.2 (44.1 to 92.6) | 6.2 (-1.3 to 14.6) | -0.3 (-7.3 to 7.6) |
| Eating disorders | -- | -- | -- | -- | -- |
| Anorexia nervosa | -- | -- | -- | -- | -- |
| Bulimia nervosa | -- | -- | -- | -- | -- |
| Autism spectrum disorders | 2570.0 (2307.6 to 2832.7) | -- | 400.6 (271.0 to 555.6) | 7.1* (5.3 to 9.1) | 0.7 (-1.1 to 2.5) |
| Attention-deficit/hyperactivity disorder | 1078.9 (874.6 to 1321.1) | 1036.4 (840.0 to 1269.3) | 13.2 (7.9 to 21.7) | 8.4* (2.3 to 14.7) | 1.9 (-3.9 to 7.7) |
| Conduct disorder | -- | -- | -- | -- | -- |
| Idiopathic developmental intellectual disability | 9837.2 (5763.1 to 13931.4) | -- | 408.3 (196.5 to 693.2) | -7.8* (-11.0 to -5.8) | -13.4* (-16.4 to -11.5) |
| Other mental disorders | -- | -- | -- | -- | -- |
| **Substance use disorders** | **3.8 (2.6 to 5.1)** | **--** | **0.9 (0.6 to 1.4)** | **-7.8* (-14.7 to -0.9)** | **-13.4* (-19.8 to -6.9)** |
| Alcohol use disorders | 24.5 (19.7 to 30.4) | -- | 0.9 (0.6 to 1.4) | -7.8* (-14.7 to -0.9) | -13.4* (-19.8 to -6.9) |
| Alcohol dependence | -- | -- | -- | -- | -- |
| Fetal alcohol syndrome | 24.5 (19.7 to 30.4) | -- | 0.9 (0.6 to 1.4) | -7.8* (-14.7 to -0.9) | -13.4* (-19.8 to -6.9) |
| Drug use disorders | -- | -- | -- | -- | -- |
| Opioid use disorders | -- | -- | -- | -- | -- |
| Cocaine use disorders | -- | -- | -- | -- | -- |

| | | | | | |
|---|---|---|---|---|---|
| Amphetamine use disorders | -- | -- | -- | -- | -- |
| Cannabis use disorders | -- | -- | -- | -- | -- |
| Other drug use disorders | -- | -- | -- | -- | -- |
| **Diabetes and kidney diseases** | **414.1 (281.3 to 563.5)** | **286.6 (203.8 to 381.8)** | **41.9 (24.6 to 65.4)** | **16.4* (5.5 to 28.9)** | **9.4 (-0.8 to 21.1)** |
| Diabetes mellitus | 35.1 (20.3 to 55.8) | 39.6 (28.5 to 55.3) | 1.7 (0.8 to 3.2) | 2.8 (-5.6 to 10.3) | -3.4 (-11.3 to 3.6) |
| Diabetes mellitus type 1 | 35.1 (20.3 to 55.8) | 39.6 (28.5 to 55.3) | 1.7 (0.8 to 3.2) | 2.8 (-5.6 to 10.3) | -3.4 (-11.3 to 3.6) |
| Uncomplicated diabetes mellitus type 1 | 35.1 (20.3 to 55.8) | 39.6 (28.5 to 55.3) | 1.7 (0.8 to 3.2) | 2.8 (-5.6 to 10.3) | -3.4 (-11.3 to 3.6) |
| Vision loss due to diabetes mellitus type 1 retinopathy | -- | -- | -- | -- | -- |
| Diabetic neuropathy due to diabetes mellitus type 1 | -- | -- | -- | 3.2 (-1.5 to 6.3) | -3.0* (-7.4 to -0.2) |
| Diabetes mellitus type 2 | -- | -- | -- | -- | -- |
| Uncomplicated diabetes mellitus type 2 | -- | -- | -- | -- | -- |
| Vision loss due to diabetes mellitus type 2 retinopathy | -- | -- | -- | -- | -- |
| Diabetic neuropathy due to diabetes mellitus type 2 | -- | -- | -- | -- | -- |
| Chronic kidney disease | 636.5 (433.5 to 859.7) | 206.6 (128.9 to 299.2) | 40.0 (23.0 to 62.9) | 17.1* (6.0 to 29.9) | 10.0 (-0.4 to 22.0) |
| Chronic kidney disease due to diabetes mellitus type 1 | 39.3 (25.9 to 54.6) | 13.7 (8.3 to 20.3) | 1.4 (0.8 to 2.3) | 16.7* (2.9 to 31.1) | 9.6 (-3.4 to 23.2) |
| Albuminuria with preserved GFR due to type 1 diabetes mellitus | 0.8 (0.3 to 1.8) | -- | -- | -- | -- |
| End-stage chronic kidney disease due to diabetes mellitus type 1 | 0.0 (0.0 to 0.1) | -- | -- | 6.2* (3.2 to 9.1) | -0.2 (-3.0 to 2.5) |
| Stage III chronic kidney disease due to diabetes mellitus type 1 | 36.8 (24.0 to 51.6) | 13.7 (8.3 to 20.3) | 0.5 (0.3 to 0.9) | 10.4 (-5.8 to 28.0) | 3.7 (-11.5 to 20.3) |
| Stage IV chronic kidney disease due to diabetes mellitus type 1 | 0.1 (0.1 to 0.3) | -- | -- | 29.9* (13.3 to 50.5) | 22.0* (6.4 to 41.4) |
| Stage V chronic kidney disease due to diabetes mellitus type 1 | 1.5 (0.9 to 2.4) | -- | 0.9 (0.4 to 1.5) | 20.8* (8.2 to 35.8) | 13.5* (1.7 to 27.6) |
| Chronic kidney disease due to diabetes mellitus type 2 | 7.3 (4.9 to 10.1) | 1.9 (1.2 to 2.8) | 0.3 (0.2 to 0.5) | 18.0* (5.8 to 31.8) | 10.9 (-0.6 to 23.8) |
| Albuminuria with preserved GFR due to type 2 diabetes mellitus | 1.8 (0.6 to 3.9) | -- | -- | -- | -- |
| End-stage chronic kidney disease due to diabetes mellitus type 2 | 0.0 (0.0 to 0.1) | -- | -- | 6.3* (2.9 to 9.2) | -0.1 (-3.3 to 2.6) |
| Stage III chronic kidney disease due to diabetes mellitus type 2 | 5.1 (3.4 to 7.0) | 1.9 (1.2 to 2.8) | 0.1 (0.0 to 0.1) | 11.6 (-3.3 to 27.6) | 4.9 (-9.1 to 19.9) |
| Stage IV chronic kidney disease due to diabetes mellitus type 2 | 0.0 (0.0 to 0.1) | -- | -- | 30.1* (12.8 to 52.1) | 22.2* (6.0 to 42.9) |
| Stage V chronic kidney disease due to diabetes mellitus type 2 | 0.3 (0.2 to 0.5) | -- | 0.2 (0.1 to 0.3) | 21.6* (8.6 to 35.3) | 14.2* (2.0 to 27.1) |
| Chronic kidney disease due to hypertension | 114.1 (76.2 to 158.4) | 48.6 (29.8 to 71.2) | 5.3 (2.8 to 8.8) | 20.6* (8.5 to 34.5) | 13.3* (1.9 to 26.4) |
| Albuminuria with preserved GFR due to hypertension | 0.1 (0.0 to 0.2) | -- | -- | -- | -- |
| End-stage chronic kidney disease due to hypertension | 0.1 (0.0 to 0.1) | -- | 0.0 (0.0 to 0.1) | 8.1* (4.7 to 11.3) | 1.5 (-1.6 to 4.6) |
| Stage III chronic kidney disease due to hypertension | 104.7 (69.8 to 146.0) | 48.6 (29.8 to 71.2) | 0.3 (0.1 to 0.4) | 11.6 (-3.9 to 27.4) | 4.8 (-9.7 to 19.7) |
| Stage IV chronic kidney disease due to hypertension | 0.8 (0.3 to 1.6) | -- | 0.1 (0.0 to 0.2) | 29.6* (12.5 to 50.7) | 21.8* (5.7 to 41.6) |
| Stage V chronic kidney disease due to hypertension | 8.4 (4.8 to 12.9) | -- | 4.9 (2.5 to 8.2) | 21.1* (8.8 to 35.2) | 13.8* (2.2 to 27.0) |
| Chronic kidney disease due to glomerulonephritis | 59.6 (39.9 to 81.3) | 6.3 (3.8 to 9.1) | 10.7 (6.1 to 16.8) | 17.4* (6.1 to 30.3) | 10.3 (-0.3 to 22.4) |
| Albuminuria with preserved GFR due to glomerulonephritis | 0.4 (0.1 to 0.9) | -- | -- | -- | -- |
| End-stage chronic kidney disease due to glomerulonephritis | 2.7 (1.8 to 3.9) | -- | 1.1 (0.6 to 1.7) | 6.6* (4.0 to 8.9) | 0.2 (-2.3 to 2.3) |
| Stage III chronic kidney disease due to glomerulonephritis | 42.5 (27.6 to 59.0) | 6.3 (3.8 to 9.1) | 2.0 (1.1 to 3.2) | 9.9 (-5.3 to 27.3) | 3.3 (-11.0 to 19.6) |
| Stage IV chronic kidney disease due to glomerulonephritis | 1.2 (0.5 to 2.4) | -- | 0.2 (0.1 to 0.3) | 29.7* (12.7 to 50.9) | 21.8* (5.9 to 41.8) |
| Stage V chronic kidney disease due to glomerulonephritis | 12.8 (7.4 to 19.9) | -- | 7.5 (3.8 to 12.5) | 21.2* (9.5 to 35.2) | 13.9* (2.8 to 27.0) |
| Chronic kidney disease due to other and unspecified causes | 416.3 (286.7 to 562.7) | 136.2 (85.2 to 198.1) | 22.3 (13.1 to 34.7) | 16.2* (5.1 to 28.4) | 9.2 (-1.3 to 20.6) |
| Albuminuria with preserved GFR due to other and unspecified causes | 15.8 (5.2 to 34.1) | -- | -- | -- | -- |
| End-stage chronic kidney disease due to other causes | 8.1 (6.5 to 9.7) | -- | 3.2 (2.1 to 4.4) | 5.6* (3.0 to 7.9) | -0.8 (-3.2 to 1.4) |
| Stage III chronic kidney disease due to other causes | 365.8 (242.6 to 500.6) | 136.2 (85.2 to 198.1) | 4.6 (2.5 to 7.3) | 7.6 (-10.3 to 26.6) | 1.1 (-15.7 to 19.0) |
| Stage IV chronic kidney disease due to other causes | 2.2 (1.0 to 4.6) | -- | 0.3 (0.1 to 0.7) | 30.2* (13.4 to 51.7) | 22.3* (6.5 to 42.6) |
| Stage V chronic kidney disease due to other causes | 24.3 (14.2 to 37.8) | -- | 14.2 (7.2 to 23.6) | 21.8* (9.8 to 35.5) | 14.5* (3.1 to 27.3) |
| Acute glomerulonephritis | 2.7 (1.8 to 3.7) | 40.3 (26.5 to 56.8) | 0.1 (0.1 to 0.2) | -1.2 (-5.1 to 1.4) | -7.2* (-10.9 to -4.7) |
| **Skin and subcutaneous diseases** | **153222.9 (141112.4 to 167812.9)** | **310966.5 (271982.2 to 361488.8)** | **4268.2 (2664.6 to 6448.9)** | **5.8* (4.4 to 7.2)** | **-0.6 (-1.9 to 0.7)** |
| Dermatitis | 39373.3 (35925.4 to 43297.3) | 10794.9 (9492.4 to 12193.4) | 1766.3 (924.9 to 3008.8) | 8.5* (7.2 to 9.9) | 2.0* (0.7 to 3.2) |
| Atopic dermatitis | 39391.1 (35890.5 to 43325.7) | 9244.5 (7944.4 to 10622.6) | 1762.1 (921.9 to 3001.9) | 8.5* (7.2 to 9.9) | 2.0* (0.7 to 3.2) |
| Contact dermatitis | -- | -- | -- | -- | -- |
| Seborrhoeic dermatitis | 300.2 (240.8 to 364.8) | 1550.4 (1258.5 to 1898.9) | 4.2 (2.3 to 6.9) | 5.8 (-1.2 to 12.9) | -0.6 (-7.2 to 6.0) |
| Psoriasis | 758.6 (695.8 to 825.3) | 306.6 (277.3 to 342.1) | 69.8 (49.3 to 95.3) | 9.9* (4.8 to 15.4) | 3.3 (-1.6 to 8.4) |
| Bacterial skin diseases | 1753.4 (1626.2 to 1878.9) | 35768.5 (33413.6 to 38205.1) | 18.0 (10.3 to 31.0) | 10.6* (7.5 to 13.1) | 3.9* (1.0 to 6.2) |
| Cellulitis | 140.0 (113.3 to 168.1) | 2599.0 (2137.8 to 3064.4) | 8.3 (5.2 to 12.5) | 5.3* (2.4 to 8.2) | -1.0 (-3.8 to 1.6) |
| Pyoderma | 1705.2 (1580.2 to 1824.6) | 33169.5 (30834.7 to 35537.3) | 9.8 (3.9 to 20.1) | 15.4* (12.2 to 18.5) | 8.4* (5.4 to 11.4) |
| Impetigo | 1086.5 (982.2 to 1190.1) | 20626.9 (18627.2 to 22597.5) | 6.2 (2.5 to 12.9) | 16.3* (12.2 to 20.5) | 9.3* (5.4 to 13.2) |
| Abscess and other bacterial skin diseases | 618.8 (563.7 to 677.3) | 12542.6 (11260.0 to 13917.2) | 3.6 (1.4 to 7.6) | 13.8* (8.6 to 19.4) | 6.9* (2.0 to 12.2) |
| Scabies | 12795.1 (9268.5 to 16643.1) | 39121.1 (27950.0 to 51354.4) | 335.7 (173.7 to 578.5) | 3.6* (2.3 to 4.8) | -2.6* (-3.9 to -1.6) |
| Fungal skin diseases | 61313.9 (47379.4 to 79380.4) | 155237.1 (118193.4 to 203488.1) | 353.0 (132.7 to 787.4) | -9.4* (-11.6 to -6.9) | -14.9* (-17.0 to -12.5) |
| Tinea capitis | 43028.5 (30481.7 to 59497.9) | 100242.7 (68536.7 to 144301.1) | 248.2 (90.9 to 551.0) | -15.3* (-17.1 to -13.4) | -20.4* (-22.2 to -18.6) |
| Other fungal skin diseases | 18285.4 (12586.2 to 25422.2) | 54994.4 (37545.7 to 74397.9) | 104.8 (38.7 to 229.1) | 8.2* (7.2 to 9.1) | 1.7* (0.7 to 2.5) |

| | | | | | |
|---|---|---|---|---|---|
| Viral skin diseases | 23763.7 (21891.8 to 25584.1) | 25927.2 (23725.3 to 28026.7) | 741.7 (466.3 to 1104.9) | 6.6* (5.7 to 7.6) | 0.1 (-0.7 to 1.0) |
| Viral warts | 3257.2 (2937.4 to 3615.1) | 3315.7 (2959.4 to 3686.7) | 101.8 (64.0 to 152.3) | 5.3* (2.9 to 7.5) | -1.1 (-3.4 to 1.0) |
| Molluscum contagiosum | 20506.5 (18737.5 to 22268.2) | 22611.5 (20383.3 to 24743.2) | 639.9 (403.7 to 959.8) | 6.8* (5.8 to 7.8) | 0.3 (-0.6 to 1.3) |
| Acne vulgaris | -- | -- | -- | -- | -- |
| Alopecia areata | 312.7 (282.2 to 346.6) | 575.5 (512.6 to 636.4) | 10.5 (6.5 to 16.3) | 6.2 (-1.2 to 14.4) | -0.2 (-7.2 to 7.5) |
| Pruritus | 2610.9 (1895.8 to 3495.4) | 2305.5 (1600.1 to 3150.3) | 28.4 (12.5 to 55.0) | 7.8* (5.6 to 10.0) | 1.3 (-0.7 to 3.4) |
| Urticaria | 13633.4 (10302.4 to 17474.8) | 23305.8 (17048.1 to 30086.8) | 835.4 (517.1 to 1283.1) | 6.9* (5.6 to 8.3) | 0.4 (-0.8 to 1.7) |
| Decubitus ulcer | 11.8 (8.1 to 16.5) | 42.3 (29.1 to 60.1) | 2.1 (1.2 to 3.3) | 5.7* (4.5 to 6.8) | -0.7 (-1.8 to 0.3) |
| Other skin and subcutaneous diseases | 18978.4 (17490.3 to 20591.7) | 17582.0 (16063.0 to 19307.7) | 107.3 (50.1 to 200.2) | 12.0* (10.8 to 13.3) | 5.2* (4.1 to 6.4) |
| **Sense organ diseases** | **16068.0 (14038.9 to 18360.8)** | **--** | **631.9 (421.2 to 896.2)** | **6.0* (3.3 to 8.6)** | **-0.4 (-3.0 to 2.0)** |
| Blindness and vision impairment | 9634.7 (7669.9 to 11890.2) | -- | 318.8 (193.6 to 489.0) | 4.8* (2.5 to 7.0) | -1.5 (-3.7 to 0.5) |
| Glaucoma | -- | -- | -- | -- | -- |
| Cataract | -- | -- | -- | -- | -- |
| Age-related macular degeneration | -- | -- | -- | -- | -- |
| Refraction disorders | 7517.4 (5652.9 to 9652.7) | -- | 273.1 (163.8 to 422.2) | 5.1* (2.6 to 7.3) | -1.3 (-3.7 to 0.8) |
| Near vision loss | 1733.4 (1594.5 to 1892.3) | -- | 18.2 (8.4 to 34.1) | 2.3 (-0.4 to 5.2) | -3.9* (-6.4 to -1.1) |
| Other vision loss | 467.4 (337.5 to 616.0) | -- | 27.5 (17.1 to 40.6) | 3.8 (-2.9 to 10.1) | -2.4 (-8.8 to 3.5) |
| Age-related and other hearing loss | 3121.5 (2420.2 to 3900.9) | -- | 245.3 (158.8 to 361.4) | 7.0* (0.9 to 13.2) | 0.5 (-5.2 to 6.3) |
| Other sense organ diseases | 3527.3 (3288.4 to 3778.7) | -- | 67.9 (41.8 to 102.3) | 8.0* (4.8 to 11.3) | 1.5 (-1.5 to 4.6) |
| Chronic other sense organ diseases | 1402.7 (1259.7 to 1561.6) | -- | 38.0 (22.9 to 57.1) | 8.0* (3.4 to 13.1) | 1.5 (-2.9 to 6.3) |
| Acute other sense organ diseases | 2124.6 (1945.0 to 2336.7) | -- | 29.9 (17.9 to 46.2) | 8.0* (4.0 to 12.3) | 1.4 (-2.3 to 5.5) |
| **Musculoskeletal disorders** | **--** | **--** | **--** | **--** | **--** |
| Rheumatoid arthritis | -- | -- | -- | -- | -- |
| Osteoarthritis | -- | -- | -- | -- | -- |
| Osteoarthritis of the hip | -- | -- | -- | -- | -- |
| Osteoarthritis of the knee | -- | -- | -- | -- | -- |
| Low back pain | -- | -- | -- | -- | -- |
| Low back pain with leg pain | -- | -- | -- | -- | -- |
| Low back pain without leg pain | -- | -- | -- | -- | -- |
| Neck pain | -- | -- | -- | -- | -- |
| Gout | -- | -- | -- | -- | -- |
| Other musculoskeletal disorders | -- | -- | -- | -- | -- |
| **Other non-communicable diseases** | **319867.4 (280999.9 to 360688.0)** | **326997.1 (212959.5 to 437014.2)** | **1787.7 (1280.5 to 2374.7)** | **-0.4 (-2.9 to 2.2)** | **-6.4* (-8.8 to -4.0)** |
| Congenital anomalies | 10285.3 (9708.7 to 10930.9) | -- | 1123.7 (812.3 to 1492.6) | 7.3* (5.0 to 9.6) | 0.8 (-1.4 to 2.9) |
| Neural tube defects | 288.6 (255.8 to 323.6) | -- | 86.5 (60.4 to 115.9) | 13.0* (6.5 to 19.9) | 6.2* (0.1 to 12.7) |
| Severe motor and cognitive impairment due to anencephaly | -- | -- | -- | -- | -- |
| Encephalocoele due to neural tube defects | 26.9 (21.0 to 33.6) | -- | 4.6 (3.0 to 6.6) | 6.3* (3.2 to 9.5) | -0.2 (-3.0 to 2.8) |
| Spina bifida due to neural tube defects | 261.7 (231.7 to 294.1) | -- | 81.9 (57.0 to 109.9) | 13.4* (6.6 to 20.6) | 6.5* (0.2 to 13.3) |
| Congenital heart anomalies | 3575.4 (3189.9 to 4005.2) | -- | 157.7 (85.1 to 252.2) | 5.8* (3.0 to 8.3) | -0.6 (-3.3 to 1.7) |
| Critical malformations of great vessels, congenital valvular heart disease and patent ductus arteriosus | 617.3 (508.8 to 743.4) | -- | 35.6 (19.6 to 56.9) | 7.1* (3.4 to 11.3) | 0.6 (-2.9 to 4.6) |
| Other congenital heart anomalies | 619.5 (550.5 to 695.4) | -- | 32.0 (16.1 to 54.3) | 5.9* (1.3 to 10.4) | -0.5 (-4.9 to 3.7) |
| Severe congenital heart anomalies excluding single ventricle heart defects | 631.9 (537.7 to 740.6) | -- | 36.0 (20.0 to 55.9) | 4.8* (0.4 to 9.3) | -1.6 (-5.7 to 2.6) |
| Single ventricle and single ventricle pathway congenital heart anomalies | 104.6 (83.1 to 130.6) | -- | 6.1 (3.4 to 9.7) | 5.1 (-2.4 to 13.5) | -1.2 (-8.3 to 6.6) |
| Ventricular septal defect and atrial septal defect | 1602.1 (1288.9 to 1939.4) | -- | 48.0 (24.8 to 81.0) | 5.6* (1.6 to 9.2) | -0.8 (-4.5 to 2.6) |
| Orofacial clefts | 964.4 (866.0 to 1070.6) | -- | 32.0 (20.8 to 47.6) | 5.6 (-1.7 to 13.7) | -0.8 (-7.6 to 6.9) |
| Down's syndrome | 292.5 (256.1 to 333.3) | -- | 25.2 (17.0 to 35.4) | 9.6* (6.8 to 12.6) | 3.0* (0.3 to 5.8) |
| Turner syndrome | 67.1 (58.1 to 77.3) | -- | 0.9 (0.4 to 1.7) | 6.7* (4.2 to 9.4) | 0.3 (-2.1 to 2.7) |
| Klinefelter syndrome | 102.3 (87.6 to 118.3) | -- | 0.3 (0.1 to 0.5) | 2.4 (-5.5 to 11.6) | -3.7 (-11.2 to 4.9) |
| Other chromosomal abnormalities | 760.3 (612.4 to 922.0) | -- | 69.0 (46.0 to 96.2) | 14.6* (9.8 to 19.5) | 7.7* (3.2 to 12.3) |
| Congenital musculoskeletal and limb anomalies | 2138.3 (1901.1 to 2433.8) | -- | 310.7 (205.3 to 428.3) | 7.2* (4.7 to 9.5) | 0.7 (-1.6 to 2.9) |
| Polydactyly and syndactyly | 132.2 (99.9 to 173.0) | -- | 1.4 (0.6 to 2.7) | 9.2 (-0.1 to 18.5) | 2.6 (-6.1 to 11.3) |
| Congenital limb deficiency | 1403.0 (1242.1 to 1611.7) | -- | 216.1 (142.7 to 300.6) | 7.1* (4.2 to 9.9) | 0.6 (-2.1 to 3.3) |
| Other congenital musculoskeletal anomalies | 603.2 (536.3 to 686.5) | -- | 93.2 (60.7 to 129.2) | 7.3* (3.1 to 11.7) | 0.8 (-3.1 to 4.9) |
| Urogenital congenital anomalies | 833.5 (715.1 to 962.5) | -- | 20.4 (11.7 to 34.4) | 9.8* (6.6 to 12.7) | 3.2* (0.2 to 5.9) |
| Digestive congenital anomalies | 1164.0 (1045.2 to 1304.8) | -- | 53.6 (35.1 to 76.4) | 6.5* (1.6 to 11.9) | 0.1 (-4.6 to 5.1) |
| Congenital diaphragmatic hernia | 51.0 (44.2 to 58.1) | -- | 1.0 (0.6 to 1.7) | 4.1* (2.9 to 5.1) | -2.2* (-3.3 to -1.2) |
| Congenital atresia and/or stenosis of the digestive tract | 35.2 (25.3 to 46.8) | -- | 2.8 (1.7 to 4.5) | 4.5* (1.9 to 7.1) | -1.9 (-4.3 to 0.6) |
| Congenital malformations of the abdominal wall after treatment | 207.7 (182.0 to 238.6) | -- | 8.9 (5.8 to 13.0) | 5.7* (4.6 to 6.7) | -0.7 (-1.8 to 0.2) |
| Other congenital malformations of the digestive tract | 870.0 (749.9 to 1012.6) | -- | 40.8 (26.2 to 59.5) | 6.9* (0.3 to 13.9) | 0.5 (-5.7 to 7.0) |
| Other congenital anomalies | 2000.5 (1738.4 to 2314.4) | -- | 367.4 (254.5 to 501.7) | 5.5 (-0.8 to 11.2) | -0.9 (-6.8 to 4.5) |

| | | | | | |
|---|---|---|---|---|---|
| Other congenital birth defects | -- | -- | 89.7 (58.9 to 125.8) | 13.4* (10.5 to 16.2) | 6.5* (3.8 to 9.2) |
| Hearing loss due to other congenital anomalies | 2000.5 (1738.4 to 2314.4) | -- | 277.7 (181.9 to 396.4) | 3.1 (-4.5 to 10.4) | -3.1 (-10.2 to 3.7) |
| Urinary diseases and male infertility | 176.9 (142.0 to 220.6) | 9805.1 (7753.8 to 12108.7) | 15.5 (9.2 to 23.8) | -6.7 (-13.7 to 0.3) | -12.3* (-19.0 to -5.7) |
| Urinary tract infections | 193.9 (156.1 to 238.3) | 9584.3 (7536.9 to 11885.9) | 6.6 (3.8 to 10.4) | 4.1 (-4.6 to 12.7) | -2.2 (-10.3 to 5.8) |
| Urolithiasis | 7.8 (5.2 to 11.4) | 220.8 (142.6 to 324.7) | 0.6 (0.3 to 1.0) | 8.4* (7.3 to 9.7) | 1.8* (0.8 to 3.0) |
| Benign prostatic hyperplasia | -- | -- | -- | -- | -- |
| Male infertility | -- | -- | -- | -- | -- |
| Other urinary diseases | -- | -- | 8.3 (4.9 to 12.8) | -14.6* (-21.0 to -8.2) | -19.7* (-25.8 to -13.7) |
| Gynecological diseases | -- | -- | -- | -- | -- |
| Uterine fibroids | -- | -- | -- | -- | -- |
| Polycystic ovarian syndrome | -- | -- | -- | -- | -- |
| Female infertility | -- | -- | -- | -- | -- |
| Endometriosis | -- | -- | -- | -- | -- |
| Genital prolapse | -- | -- | -- | -- | -- |
| Premenstrual syndrome | -- | -- | -- | -- | -- |
| Other gynecological diseases | -- | -- | -- | -- | -- |
| Other gynecological diseases | -- | -- | -- | -- | -- |
| Other gynecological diseases with anemia | -- | -- | -- | -- | -- |
| Hemoglobinopathies and hemolytic anaemias | 147498.6 (143862.5 to 151380.2) | -- | 393.2 (261.1 to 579.6) | -14.5* (-21.0 to -8.3) | -19.7* (-25.8 to -13.9) |
| Thalassemias | 183.9 (171.9 to 198.0) | -- | 7.0 (4.6 to 10.4) | -22.0* (-30.0 to -14.2) | -26.7* (-34.2 to -19.4) |
| Thalassaemias trait | 18977.7 (18298.7 to 19707.3) | -- | 113.1 (72.0 to 166.1) | -18.8* (-28.0 to -9.3) | -23.7* (-32.4 to -14.8) |
| Sickle cell disorders | 756.5 (683.8 to 831.5) | -- | 36.4 (23.4 to 55.1) | -3.7 (-14.7 to 7.7) | -9.5 (-19.9 to 1.2) |
| Sickle cell trait | 41776.6 (38045.1 to 45914.7) | -- | 128.1 (84.1 to 187.8) | -11.8* (-19.4 to -3.3) | -17.2* (-24.2 to -9.1) |
| G6PD deficiency | 30121.8 (28836.3 to 31467.7) | -- | 2.7 (1.7 to 3.8) | -3.6 (-20.8 to 19.8) | -9.4 (-25.6 to 12.6) |
| G6PD trait | 70269.6 (69065.7 to 71488.8) | -- | -- | -1.7 (-9.0 to 6.5) | -7.7 (-14.5 to 0.1) |
| Other hemoglobinopathies and hemolytic anaemias | 3524.4 (3347.3 to 3717.0) | -- | 105.9 (70.1 to 154.8) | -15.9* (-22.7 to -8.3) | -21.0* (-27.3 to -13.9) |
| Endocrine, metabolic, blood, and immune disorders | 6098.1 (5689.4 to 6499.3) | -- | 196.4 (132.4 to 280.2) | -8.4* (-14.4 to -2.7) | -13.9* (-19.6 to -8.6) |
| Anemia due to endocrine, metabolic, blood, and immune disorders | 4465.5 (4251.1 to 4704.0) | -- | 134.3 (88.8 to 194.6) | -15.2* (-22.4 to -8.1) | -20.3* (-27.1 to -13.7) |
| Endocrine, metabolic, blood, and immune disorders | 1631.4 (1296.1 to 1978.0) | -- | 62.0 (39.5 to 92.6) | 10.9* (3.9 to 18.6) | 4.2 (-2.3 to 11.4) |
| Heart failure due to endocrine, metabolic, blood, and immune disorders | 1.3 (0.9 to 1.8) | -- | 0.1 (0.1 to 0.2) | 3.3* (2.3 to 4.2) | -3.0* (-3.9 to -2.1) |
| Oral disorders | 224032.8 (169208.4 to 281065.1) | 317192.0 (203211.1 to 428136.8) | 58.9 (23.9 to 119.3) | 4.9* (3.1 to 6.6) | -1.5 (-3.2 to 0.2) |
| Caries of deciduous teeth | 226643.8 (170641.5 to 285260.4) | 317192.0 (203211.1 to 428136.8) | 58.9 (23.9 to 119.3) | 4.9* (3.1 to 6.6) | -1.5 (-3.2 to 0.2) |
| Caries of permanent teeth | -- | -- | -- | -- | -- |
| Periodontal disease | -- | -- | -- | -- | -- |
| Edentulism and severe tooth loss | -- | -- | -- | -- | -- |
| Other oral disorders | -- | -- | -- | -- | -- |
| **Injuries** | **7603.9 (6825.7 to 8390.3)** | **26767.0 (23257.0 to 30901.9)** | **404.5 (286.3 to 546.0)** | **17.6* (15.1 to 20.2)** | **10.5* (8.1 to 13.0)** |
| **Transport injuries** | **953.1 (737.5 to 1231.9)** | **2322.0 (1677.8 to 3132.7)** | **76.4 (51.2 to 107.2)** | **8.4* (5.0 to 11.8)** | **1.9 (-1.3 to 5.0)** |
| Road injuries | 227.4 (184.5 to 281.3) | 940.7 (617.4 to 1403.5) | 18.0 (11.7 to 26.6) | 1.3 (-5.2 to 8.1) | -4.8 (-11.0 to 1.5) |
| Pedestrian road injuries | 63.7 (46.7 to 85.5) | 226.6 (129.7 to 379.4) | 5.2 (3.2 to 7.8) | -11.8* (-18.1 to -4.9) | -17.1* (-23.0 to -10.6) |
| Cyclist road injuries | 34.1 (23.3 to 48.4) | 180.7 (95.7 to 314.4) | 2.5 (1.5 to 3.8) | 2.2 (-5.5 to 10.3) | -4.0 (-11.2 to 3.6) |
| Motorcyclist road injuries | 63.0 (47.3 to 82.3) | 193.9 (108.6 to 334.2) | 5.0 (3.2 to 7.5) | 8.6* (2.2 to 15.5) | 2.0 (-4.0 to 8.5) |
| Motor vehicle road injuries | 41.6 (28.5 to 59.1) | 211.7 (120.4 to 351.6) | 3.7 (2.2 to 5.9) | 7.4 (-1.4 to 16.6) | 0.9 (-7.4 to 9.6) |
| Other road injuries | 24.9 (16.5 to 36.1) | 127.9 (67.0 to 222.7) | 1.7 (1.0 to 2.7) | 15.8* (7.1 to 24.4) | 8.8* (0.6 to 16.8) |
| Other transport injuries | 725.7 (512.5 to 991.0) | 1381.3 (863.4 to 2013.5) | 58.4 (38.2 to 83.1) | 10.8* (7.3 to 14.6) | 4.1* (0.8 to 7.6) |
| **Unintentional injuries** | **5468.8 (4820.8 to 6265.7)** | **22348.3 (19074.4 to 26282.3)** | **279.3 (195.5 to 383.6)** | **16.8* (13.9 to 19.7)** | **9.8* (7.0 to 12.4)** |
| Falls | 2025.9 (1578.1 to 2629.0) | 7989.0 (5294.3 to 11267.8) | 104.0 (69.8 to 149.0) | 20.8* (17.2 to 24.6) | 13.5* (10.1 to 17.0) |
| Drowning | 16.0 (11.9 to 21.4) | 34.9 (26.6 to 45.7) | 1.3 (0.9 to 2.0) | -20.0* (-23.7 to -16.5) | -24.9* (-28.3 to -21.5) |
| Fire, heat, and hot substances | 422.0 (314.4 to 571.0) | 666.6 (444.0 to 1002.1) | 23.3 (15.8 to 33.5) | 10.6* (3.4 to 16.9) | 3.9 (-2.9 to 9.8) |
| Poisonings | 65.1 (48.3 to 82.6) | 607.2 (384.1 to 882.5) | 8.5 (5.1 to 12.9) | 22.9* (16.9 to 29.1) | 15.5* (9.8 to 21.3) |
| Poisoning by carbon monoxide | 9.1 (6.2 to 12.7) | 104.8 (50.7 to 186.5) | 1.2 (0.7 to 1.9) | 17.4* (8.2 to 26.9) | 10.3* (1.7 to 19.2) |
| Poisoning by other means | 56.0 (40.8 to 73.6) | 502.4 (315.4 to 738.5) | 7.4 (4.3 to 11.4) | 23.8* (17.9 to 29.8) | 16.3* (10.7 to 21.9) |
| Exposure to mechanical forces | 1125.0 (864.2 to 1434.6) | 4248.8 (2773.3 to 6132.7) | 41.7 (27.6 to 61.1) | 24.2* (19.0 to 29.6) | 16.7* (11.8 to 21.7) |
| Unintentional firearm injuries | 72.0 (48.0 to 113.1) | 175.0 (84.6 to 311.1) | 4.7 (2.9 to 7.1) | 16.7* (10.9 to 23.3) | 9.6* (4.2 to 15.8) |
| Other exposure to mechanical forces | 1053.0 (800.0 to 1353.2) | 4073.7 (2652.5 to 5911.9) | 37.0 (24.3 to 54.5) | 25.2* (20.2 to 30.7) | 17.6* (12.9 to 22.8) |
| Adverse effects of medical treatment | 67.5 (44.5 to 98.6) | 882.0 (625.5 to 1248.7) | 9.0 (5.0 to 14.9) | 20.2* (14.0 to 26.9) | 13.0* (7.1 to 19.2) |
| Animal contact | 396.6 (289.8 to 524.7) | 2965.0 (1925.5 to 4106.6) | 19.7 (12.4 to 28.3) | 13.3* (7.1 to 18.7) | 6.5* (0.7 to 11.5) |
| Venomous animal contact | 136.2 (98.3 to 186.3) | 1118.7 (699.1 to 1654.1) | 13.1 (7.9 to 19.5) | 14.9* (6.6 to 22.5) | 7.9* (0.2 to 15.1) |
| Non-venomous animal contact | 260.4 (173.3 to 377.7) | 1846.2 (1196.1 to 2559.2) | 6.6 (3.9 to 10.5) | 10.4* (5.0 to 15.3) | 3.7 (-1.4 to 8.3) |
| Foreign body | 356.1 (244.8 to 514.7) | 1859.9 (1355.5 to 2541.1) | 22.5 (14.0 to 34.2) | 13.0* (11.0 to 15.2) | 6.1* (4.3 to 8.3) |

| | | | | | |
|---|---|---|---|---|---|
| Pulmonary aspiration and foreign body in airway | 51.8 (34.7 to 72.6) | 238.3 (144.9 to 362.5) | 4.2 (2.7 to 5.9) | 3.6 (-1.0 to 8.0) | -2.7 (-7.0 to 1.5) |
| Foreign body in eyes | 140.6 (42.8 to 278.0) | 1052.9 (628.2 to 1644.8) | 8.0 (2.4 to 16.6) | 10.7* (9.3 to 12.6) | 4.0* (2.7 to 5.8) |
| Foreign body in other body part | 163.7 (118.1 to 215.4) | 568.7 (369.2 to 816.1) | 10.3 (6.8 to 14.4) | 19.3* (16.6 to 22.0) | 12.1* (9.6 to 14.6) |
| Environmental heat and cold exposure | 308.6 (220.5 to 411.5) | 734.4 (488.8 to 1058.3) | 18.8 (12.3 to 26.8) | 13.8* (9.4 to 17.8) | 7.0* (2.8 to 10.7) |
| Exposure to forces of nature | 14.1 (8.4 to 23.0) | 26.2 (16.3 to 40.8) | 0.9 (0.5 to 1.4) | -83.5* (-85.9 to -80.9) | -84.5* (-86.8 to -82.0) |
| Other unintentional injuries | 671.8 (496.3 to 921.6) | 2334.3 (1521.1 to 3315.7) | 29.7 (19.2 to 43.3) | 27.5* (22.8 to 31.8) | 19.8* (15.4 to 23.8) |
| **Self-harm and interpersonal violence** | **1182.1 (956.7 to 1444.2)** | **2096.7 (1600.4 to 2750.8)** | **48.8 (33.9 to 67.6)** | **41.6* (33.0 to 51.9)** | **33.1* (25.0 to 42.7)** |
| Self-harm | -- | -- | -- | -- | -- |
| Self-harm by firearm | -- | -- | -- | -- | -- |
| Self-harm by other specified means | -- | -- | -- | -- | -- |
| Interpersonal violence | 890.1 (698.4 to 1110.4) | 1112.0 (716.1 to 1664.1) | 27.5 (19.5 to 37.7) | 18.5* (13.7 to 23.7) | 11.3* (6.9 to 16.2) |
| Assault by firearm | 8.2 (5.9 to 11.3) | 18.0 (8.6 to 34.3) | 0.7 (0.4 to 1.1) | 27.7* (20.7 to 35.6) | 20.0* (13.4 to 27.4) |
| Assault by sharp object | 77.5 (52.0 to 110.9) | 213.3 (114.6 to 359.6) | 4.2 (2.6 to 6.4) | 22.7* (15.8 to 29.7) | 15.3* (8.8 to 21.9) |
| Sexual violence | 561.1 (391.3 to 751.1) | -- | 5.9 (3.3 to 9.2) | 6.9* (5.4 to 8.3) | 0.4 (-1.0 to 1.8) |
| Assault by other means | 243.2 (170.8 to 359.2) | 880.8 (565.5 to 1348.7) | 16.7 (11.1 to 24.3) | 21.6* (15.5 to 28.3) | 14.3* (8.5 to 20.5) |
| Conflict and terrorism | 278.3 (177.6 to 441.4) | 925.0 (632.7 to 1322.9) | 20.4 (11.5 to 32.5) | 85.4* (73.6 to 100.3) | 74.2* (63.1 to 88.2) |
| Executions and police conflict | 13.7 (8.0 to 21.1) | 59.7 (36.8 to 88.7) | 0.9 (0.5 to 1.5) | 282.3* (216.1 to 338.8) | 259.2* (197.0 to 312.2) |
| **Nature of injury aggregates** | | | | | |
| Amputations | 1697.3 (1452.9 to 2012.6) | 513.4 (411.6 to 637.0) | 35.4 (23.6 to 51.2) | 10.1* (4.0 to 16.5) | 3.5 (-2.3 to 9.4) |
| Burns | 1005.5 (846.5 to 1202.6) | 1155.7 (939.8 to 1440.8) | 61.2 (44.2 to 83.7) | 6.6 (-1.0 to 14.2) | 0.1 (-7.0 to 7.3) |
| Fractures | 1626.2 (1410.8 to 1869.0) | 5819.1 (4720.0 to 7091.0) | 92.3 (59.7 to 132.2) | 21.9* (19.0 to 25.2) | 14.5* (11.8 to 17.6) |
| Head Injuries | 258.3 (227.8 to 293.9) | 1055.1 (856.0 to 1281.6) | 42.6 (27.8 to 60.0) | 21.9* (16.3 to 27.7) | 14.5* (9.2 to 20.0) |
| Spinal Injuries | 122.0 (103.2 to 144.3) | 40.1 (31.1 to 51.5) | 42.2 (29.1 to 57.2) | 20.0* (12.8 to 30.4) | 12.7* (6.0 to 22.5) |
| Minor Injuries | 1459.0 (1223.4 to 1741.2) | 10131.9 (8428.1 to 12040.5) | 11.6 (5.1 to 23.1) | 21.7* (18.5 to 24.8) | 14.4* (11.3 to 17.3) |
| Other Injuries | 874.5 (706.2 to 1065.3) | 8051.7 (7095.1 to 9175.9) | 113.4 (76.5 to 159.2) | 21.1* (18.2 to 24.0) | 13.8* (11.1 to 16.5) |
| **Impairments** | | | | | |
| Anaemia | 204579.4 (190484.2 to 220510.8) | -- | 6317.8 (4177.9 to 9065.5) | -18.8* (-24.3 to -13.5) | -23.7* (-28.9 to -18.7) |
| Epilepsy | 4704.7 (3906.0 to 5688.8) | -- | 2161.8 (1622.6 to 2784.0) | 13.9* (7.0 to 21.1) | 7.0* (0.5 to 13.8) |
| Guillain-Barré syndrome | 10.0 (5.9 to 15.5) | -- | 3.0 (1.6 to 5.1) | 7.6* (5.4 to 9.5) | 1.1 (-0.9 to 2.9) |
| Hearing loss | 13244.9 (11950.5 to 14667.0) | -- | 711.8 (499.5 to 978.4) | 4.6* (0.7 to 8.1) | -1.8 (-5.4 to 1.6) |
| Heart failure | 286.7 (197.1 to 394.6) | -- | 28.2 (16.8 to 44.5) | 5.9* (5.0 to 6.9) | -0.5 (-1.4 to 0.5) |
| Infertility | 26.6 (12.7 to 45.1) | -- | 0.5 (0.2 to 1.3) | 10.2* (8.3 to 11.8) | 3.5* (1.7 to 5.1) |
| Developmental intellectual disability | 18008.4 (13909.1 to 22145.0) | -- | 2349.7 (1817.7 to 2946.0) | 7.6* (4.0 to 11.7) | 1.1 (-2.3 to 5.0) |
| Pelvic inflammatory disease | -- | -- | -- | -- | -- |
| Vision loss | 11902.5 (9685.4 to 14340.2) | -- | 549.0 (378.7 to 770.5) | 4.7* (3.0 to 6.3) | -1.7* (-3.2 to -0.2) |

We did not calculate incidence for the nine impairments and certain NTDs. Blank cells mean that no estimate is available or that the estimate has a magnitude less than 50.
Table presents prevalence and incidence counts in thousands.
* Percentage changes that are statistically significant
‡ Incidence estimates for stroke represent first-ever stroke only.
^ Incidence of HIV/AIDS represents new infections of HIV only and does not include new infections of Tuberculosis in HIV positive cases.

| SR Table 10e: Global prevalence, incidence and YLDs for 2017, percentage change of YLD counts and percentage change of YLD rates between 2007 and 2017 by cause, age 5 to 9 years | | | | | |
|---|---|---|---|---|---|
| Cause | Prevalence in thousands (95% UI) | Incidence in thousands (95% UI) | YLDs in thousands (95% UI) | | |
| | 2017 counts | 2017 counts | 2017 counts | Percentage change in counts between 2007 and 2017 | Percentage change in rates between 2007 and 2017 |
| **All causes** | **609889.2 (599321.5 to 620030.7)** | **3901714.2 (3346080.3 to 4528117.1)** | **32937.4 (23541.5 to 43762.8)** | **0.9 (-1.7 to 3.6)** | **-6.4* (-8.9 to -3.9)** |
| **Communicable, maternal, neonatal, and nutritional disorders** | **378724.5 (360717.9 to 400527.2)** | **2629134.6 (2138224.1 to 3224795.2)** | **15343.6 (10853.3 to 20873.1)** | **-7.0* (-11.3 to -1.8)** | **-13.7* (-17.8 to -8.9)** |
| **HIV/AIDS and sexually transmitted infections** | **657.5 (601.9 to 709.8)** | **1.3 (0.3 to 3.4)** | **100.0 (72.7 to 130.1)** | **-25.1* (-30.5 to -19.5)** | **-30.6* (-35.6 to -25.3)** |
| HIV/AIDS | 736.5 (686.8 to 787.7) | -- | 100.0 (72.7 to 130.1) | -25.1* (-30.5 to -19.5) | -30.6* (-35.6 to -25.3) |
| HIV/AIDS - Drug-susceptible Tuberculosis | 21.4 (19.6 to 23.3) | 34.0 (30.9 to 37.5) | 8.7 (5.9 to 12.1) | -40.9* (-48.0 to -33.8) | -45.2* (-51.8 to -38.6) |
| HIV/AIDS - Multidrug-resistant Tuberculosis without extensive drug resistance | 0.8 (0.6 to 1.1) | 1.4 (1.0 to 1.9) | 0.3 (0.2 to 0.5) | -34.0* (-54.7 to -7.2) | -38.8* (-58.0 to -13.9) |
| HIV/AIDS - Extensively drug-resistant Tuberculosis | -- | -- | -- | 17.3 (-18.7 to 71.2) | 8.8 (-24.6 to 58.8) |
| HIV/AIDS resulting in other diseases | 714.2 (664.2 to 765.1) | -- | 90.9 (66.2 to 118.3) | -23.1* (-29.2 to -17.0) | -28.7* (-34.3 to -23.1) |
| HIV/AIDS not on antiretroviral treatment without Tuberculosis | 423.6 (388.2 to 460.4) | -- | 66.8 (48.3 to 86.1) | -40.6* (-44.7 to -36.3) | -44.9* (-48.7 to -40.9) |
| HIV/AIDS on antiretroviral treatment without Tuberculosis | 290.6 (269.6 to 311.3) | -- | 24.1 (16.2 to 34.1) | 313.0* (244.2 to 414.0) | 283.0* (219.3 to 376.7) |
| Sexually transmitted infections excluding HIV | -- | 1.3 (0.3 to 3.4) | -- | 4.1 (-1.9 to 8.8) | -3.4 (-9.0 to 0.9) |
| Syphilis | 0.6 (0.2 to 1.5) | 1.3 (0.3 to 3.4) | -- | 4.1 (-1.9 to 8.8) | -3.4 (-9.0 to 0.9) |
| Early syphilis | 0.6 (0.2 to 1.5) | 1.3 (0.3 to 3.4) | -- | 4.1 (-1.9 to 8.8) | -3.4 (-9.0 to 0.9) |
| Tertiary syphilis | -- | -- | -- | -- | -- |
| Chlamydial infection | -- | -- | -- | -- | -- |
| Chlamydia episode | -- | -- | -- | -- | -- |
| Chlamydial infection complications | -- | -- | -- | -- | -- |
| Gonococcal infection | -- | -- | -- | -- | -- |
| Gonococcal infection complications | -- | -- | -- | -- | -- |
| Gonorrhoea episode | -- | -- | -- | -- | -- |
| Trichomoniasis | -- | -- | -- | -- | -- |
| Genital herpes | -- | -- | -- | -- | -- |
| Other sexually transmitted infections | -- | -- | -- | -- | -- |
| Other sexually transmitted diseases residual | -- | -- | -- | -- | -- |
| Other sexually transmitted diseases | -- | -- | -- | -- | -- |
| **Respiratory infections and tuberculosis** | **128842.7 (100415.0 to 164085.5)** | **1874265.0 (1383155.8 to 2433847.0)** | **1109.4 (666.1 to 1699.0)** | **8.3* (5.8 to 10.4)** | **0.5 (-1.9 to 2.4)** |
| Tuberculosis | 92472.2 (62410.6 to 129888.7) | 361.6 (265.9 to 472.5) | 60.8 (36.7 to 93.2) | -13.6* (-21.3 to -5.1) | -19.9* (-27.0 to -12.0) |
| Latent tuberculosis infection | 92282.6 (62207.1 to 129675.4) | -- | -- | -- | -- |
| Drug-susceptible tuberculosis | 181.7 (133.3 to 238.3) | 346.2 (254.9 to 453.2) | 58.2 (35.1 to 89.5) | -13.8* (-21.9 to -5.2) | -20.0* (-27.6 to -12.1) |
| Multidrug-resistant tuberculosis without extensive drug resistance | 7.6 (4.3 to 12.7) | 14.8 (8.8 to 23.5) | 2.5 (1.2 to 4.4) | -11.3 (-42.6 to 32.9) | -17.7 (-46.8 to 23.2) |
| Extensively drug-resistant tuberculosis | 0.3 (0.2 to 0.5) | 0.6 (0.4 to 0.9) | 0.1 (0.0 to 0.2) | 43.9 (-1.1 to 128.6) | 33.4 (-8.3 to 112.0) |
| Lower respiratory infections | 1350.1 (1031.1 to 1709.3) | 59906.5 (46189.2 to 75756.8) | 83.8 (50.8 to 129.9) | 9.9* (0.7 to 18.9) | 1.9 (-6.6 to 10.3) |
| Guillain-Barré syndrome due to lower respiratory infections | 1.3 (0.6 to 2.5) | -- | 0.4 (0.1 to 0.8) | 9.1* (6.6 to 11.3) | 1.2 (-1.1 to 3.2) |
| Lower respiratory infection episode | 1348.9 (1030.0 to 1707.3) | 59906.5 (46189.2 to 75756.8) | 83.5 (50.6 to 129.6) | 9.9* (0.7 to 18.9) | 1.9 (-6.6 to 10.3) |
| Upper respiratory infections | 24305.5 (17350.6 to 32285.0) | 1739349.0 (1256245.3 to 2292860.6) | 614.0 (322.9 to 1018.7) | 12.4* (10.0 to 14.7) | 4.2* (2.1 to 6.4) |
| Guillain-Barré syndrome due to upper respiratory infections | 3.4 (1.8 to 5.9) | -- | 1.0 (0.5 to 1.8) | 9.1* (6.6 to 11.3) | 1.2 (-1.2 to 3.2) |
| Upper respiratory infection episode | 24302.0 (17347.6 to 32282.0) | 1739349.0 (1256245.3 to 2292860.6) | 613.0 (321.6 to 1017.9) | 12.4* (10.1 to 14.7) | 4.2* (2.1 to 6.4) |
| Otitis media | 17931.7 (15275.3 to 20914.6) | 74648.0 (47762.6 to 107130.6) | 350.8 (204.8 to 570.8) | 5.9* (2.9 to 8.8) | -1.8 (-4.6 to 0.9) |
| Acute otitis media | 4694.2 (3014.3 to 6863.3) | 74644.1 (47756.0 to 107127.8) | 62.0 (26.9 to 124.4) | 8.2* (3.7 to 12.7) | 0.4 (-3.8 to 4.5) |
| Chronic otitis media | 13237.5 (11150.6 to 15517.9) | 3.9 (0.1 to 14.4) | 288.8 (176.1 to 443.9) | 5.4* (1.9 to 8.9) | -2.3 (-5.5 to 1.0) |
| **Enteric infections** | **7956.8 (6273.6 to 9923.2)** | **526550.8 (395229.8 to 665533.0)** | **932.4 (592.4 to 1376.7)** | **3.7 (-3.7 to 10.8)** | **-3.8 (-10.7 to 2.7)** |
| Diarrhoeal diseases | 7844.8 (6091.6 to 9819.2) | 522875.8 (392087.9 to 662081.9) | 903.3 (572.4 to 1337.2) | 5.6 (-2.1 to 12.5) | -2.1 (-9.2 to 4.4) |
| Guillain-Barré syndrome due to diarrheal diseases | 1.2 (0.6 to 2.1) | -- | 0.3 (0.2 to 0.6) | 9.1* (6.6 to 11.3) | 1.2 (-1.1 to 3.2) |
| Diarrheal disease episode | 7843.6 (6091.0 to 9818.1) | 522875.8 (392087.9 to 662081.9) | 902.9 (572.2 to 1336.7) | 5.6 (-2.1 to 12.5) | -2.1 (-9.2 to 4.4) |
| Typhoid and paratyphoid | 143.7 (99.4 to 199.1) | 3591.4 (2694.2 to 4717.3) | 28.6 (17.5 to 44.9) | -33.7* (-46.0 to -18.7) | -38.5* (-49.9 to -24.6) |
| Typhoid fever | 173.7 (122.5 to 240.3) | 2749.9 (1994.6 to 3649.8) | 26.3 (15.7 to 42.1) | -34.6* (-47.6 to -18.0) | -39.3* (-51.4 to -24.0) |
| Typhoid fever complications | 36.1 (23.9 to 53.0) | 472.4 (337.9 to 648.3) | 11.4 (6.4 to 19.3) | -34.4* (-54.8 to -7.1) | -39.2* (-58.0 to -13.8) |
| Typhoid fever episode | 137.6 (95.6 to 194.1) | 2277.5 (1658.9 to 3028.9) | 14.9 (8.6 to 23.8) | -34.7* (-50.6 to -14.3) | -39.4* (-54.2 to -20.5) |
| Paratyphoid fever | 36.9 (24.0 to 52.3) | 841.5 (559.5 to 1186.5) | 2.3 (1.2 to 3.8) | -21.9* (-38.3 to -1.8) | -27.6* (-42.8 to -8.9) |
| Intestinal perforation due to paratyphoid | 1.6 (1.1 to 2.4) | 43.0 (27.6 to 62.1) | 0.2 (0.1 to 0.3) | -22.6* (-38.9 to -0.3) | -28.3* (-43.4 to -7.5) |
| Paratyphoid fever episode | 35.2 (22.9 to 50.1) | 798.4 (529.6 to 1127.5) | 2.1 (1.1 to 3.5) | -21.9* (-39.3 to -0.3) | -27.6* (-43.7 to -7.5) |
| iNTS | 3.2 (1.7 to 5.3) | 83.6 (46.4 to 132.5) | 0.4 (0.2 to 0.8) | -11.4 (-20.2 to 2.4) | -17.8* (-25.9 to -5.0) |
| Other intestinal infectious diseases | -- | -- | 0.0 (0.0 to 0.1) | -46.3* (-56.3 to -34.2) | -50.2* (-59.5 to -38.9) |
| **Neglected tropical diseases and malaria** | **146006.1 (138881.1 to 154380.7)** | **46142.2 (34500.0 to 64575.0)** | **1321.1 (887.7 to 1871.5)** | **-20.1* (-25.2 to -14.4)** | **-25.9* (-30.6 to -20.6)** |
| Malaria | 25903.4 (23997.6 to 27568.5) | 32917.3 (23514.5 to 51478.8) | 309.2 (209.8 to 442.5) | -27.1* (-35.2 to -17.8) | -32.4* (-39.9 to -23.7) |
| Malaria complications | 96.0 (82.6 to 111.0) | -- | 39.6 (28.9 to 52.0) | 24.2* (9.6 to 40.0) | 15.2* (1.6 to 29.8) |
| Malaria episode | 1913.4 (1064.4 to 3172.4) | 32917.3 (23514.5 to 51478.8) | 76.0 (35.6 to 140.3) | -20.3* (-30.6 to -5.3) | -26.1* (-35.6 to -12.2) |

| | | | | | |
|---|---|---|---|---|---|
| Malaria parasitemia | 23894.0 (21743.8 to 25852.5) | -- | 193.6 (126.3 to 282.9) | -34.8* (-43.6 to -24.0) | -39.5* (-47.7 to -29.5) |
| Chagas disease | 145.0 (110.4 to 182.9) | 15.1 (10.8 to 19.6) | 0.1 (0.1 to 0.2) | -26.6* (-36.2 to -17.7) | -31.9* (-40.8 to -23.7) |
| Acute Chagas disease | 0.1 (0.0 to 0.2) | 15.1 (10.8 to 19.6) | -- | -25.7* (-32.6 to -20.6) | -31.1* (-37.5 to -26.3) |
| Asymptomatic Chagas disease | 143.0 (108.8 to 180.0) | -- | -- | -- | -- |
| Symptomatic chronic Chagas infection | 2.0 (1.4 to 2.7) | -- | 0.1 (0.1 to 0.2) | -26.6* (-36.5 to -17.4) | -32.0* (-41.1 to -23.4) |
| Leishmaniasis | 245.6 (203.4 to 293.5) | 108.7 (75.5 to 149.9) | 16.5 (10.5 to 23.8) | 13.4 (-3.1 to 33.6) | 5.2 (-10.1 to 23.9) |
| Visceral leishmaniasis | 2.4 (1.8 to 3.7) | 9.6 (7.2 to 15.0) | 0.2 (0.1 to 0.3) | -72.7* (-80.7 to -56.3) | -74.7* (-82.1 to -59.5) |
| Cutaneous and mucocutaneous leishmaniasis | 249.2 (210.0 to 296.0) | 99.1 (65.9 to 140.2) | 16.3 (10.3 to 23.6) | 17.3 (-0.5 to 38.9) | 8.8 (-7.7 to 28.9) |
| African trypanosomiasis | 0.5 (0.1 to 2.1) | 0.3 (0.1 to 1.2) | 0.1 (0.0 to 0.6) | -81.7 (-97.7 to 3.7) | -83.0* (-97.8 to -3.9) |
| Trypanosomiasis Gambiense | 0.5 (0.1 to 2.1) | 0.3 (0.1 to 1.2) | 0.1 (0.0 to 0.6) | -80.9 (-97.8 to 10.3) | -82.3 (-97.9 to 2.3) |
| Trypanosomiasis Rhodesiense | -- | 0.0 (0.0 to 0.1) | -- | -92.7* (-99.0 to -56.1) | -93.2* (-99.1 to -59.3) |
| Schistosomiasis | 1337.8 (1103.5 to 1619.6) | -- | 11.2 (5.7 to 20.4) | -30.6* (-38.0 to -20.8) | -35.6* (-42.5 to -26.6) |
| Mild schistosomiasis | 1161.6 (927.9 to 1423.7) | -- | 6.6 (2.6 to 14.0) | -29.2* (-33.8 to -24.5) | -34.3* (-38.6 to -30.0) |
| Anemia due to schistosomiasis | 70.9 (53.3 to 92.8) | -- | 3.5 (2.0 to 5.5) | -28.3* (-48.0 to -1.3) | -33.5* (-51.8 to -8.5) |
| Schistosomiasis complications | 105.3 (52.2 to 174.5) | -- | 1.1 (0.4 to 2.5) | -42.6* (-48.6 to -35.4) | -46.8* (-52.3 to -40.1) |
| Cysticercosis | 0.7 (0.2 to 1.4) | -- | 0.2 (0.1 to 0.5) | 5.5 (-0.8 to 11.5) | -2.2 (-8.0 to 3.4) |
| Cystic echinococcosis | 6.0 (2.8 to 11.9) | 3.2 (1.2 to 7.3) | 0.5 (0.2 to 1.1) | 19.3* (1.8 to 41.2) | 10.6 (-5.6 to 31.0) |
| Lymphatic filariasis | 4987.0 (4122.0 to 5854.3) | -- | 84.5 (42.9 to 141.0) | -41.0* (-50.8 to -31.0) | -45.3* (-54.4 to -36.0) |
| Prevalence of detectable microfiliaria due to lymphatic filariasis | 4255.7 (3461.8 to 5074.8) | -- | -- | -- | -- |
| Lymphatic filariasis complications | 731.2 (443.8 to 1129.6) | -- | 84.5 (42.9 to 141.0) | -41.0* (-50.8 to -31.0) | -45.3* (-54.4 to -36.0) |
| Onchocerciasis | 1388.3 (750.8 to 2215.4) | -- | 98.8 (36.6 to 188.6) | -3.5 (-27.8 to 14.5) | -10.5 (-33.1 to 6.2) |
| Asymptomatic onchocerciasis | 55.1 (0.0 to 477.1) | -- | -- | -- | -- |
| Skin disease due to onchocerciasis | 1332.2 (726.6 to 2086.0) | -- | 98.8 (36.4 to 188.5) | -3.6 (-28.0 to 14.4) | -10.6 (-33.2 to 6.1) |
| Vision loss due to onchocerciasis | 1.0 (0.6 to 1.9) | -- | 0.1 (0.0 to 0.2) | 706.6* (531.4 to 1000.0) | 648.1* (485.6 to 920.2) |
| Trachoma | -- | -- | -- | -- | -- |
| Dengue | 736.6 (401.1 to 1248.3) | 12316.6 (7488.6 to 18688.9) | 122.8 (54.4 to 229.4) | 46.2* (26.5 to 124.5) | 35.6* (17.3 to 108.2) |
| Post-dengue chronic fatigue syndrome | 519.2 (242.4 to 949.5) | -- | 109.9 (46.2 to 208.8) | 46.2* (26.2 to 125.1) | 35.6* (17.1 to 108.8) |
| Dengue episode | 217.4 (131.2 to 326.4) | 12316.6 (7488.6 to 18688.9) | 12.9 (6.6 to 22.8) | 45.9* (24.7 to 120.1) | 35.4* (15.7 to 104.1) |
| Yellow fever | 0.5 (0.1 to 1.3) | 18.5 (5.1 to 51.5) | -- | -13.3 (-38.8 to 25.4) | -19.6 (-43.2 to 16.3) |
| Asymptomatic yellow fever | 0.3 (0.1 to 0.8) | 10.4 (2.6 to 29.7) | -- | -- | -- |
| Yellow fever episode | 0.2 (0.1 to 0.6) | 8.2 (2.3 to 21.8) | -- | -13.3 (-38.8 to 25.4) | -19.6 (-43.2 to 16.3) |
| Rabies | 0.1 (0.0 to 0.1) | 1.8 (1.2 to 2.6) | -- | -50.7* (-64.9 to -28.9) | -54.3* (-67.5 to -34.1) |
| Intestinal nematode infections | 113729.5 (106566.2 to 122249.1) | -- | 241.7 (139.2 to 392.3) | -37.9* (-42.3 to -33.5) | -42.4* (-46.5 to -38.4) |
| Ascariasis | 74150.4 (66162.9 to 83495.8) | -- | 120.5 (65.1 to 211.2) | -39.9* (-46.8 to -32.2) | -44.3* (-50.7 to -37.2) |
| Asymptomatic ascariasis | 67772.8 (60497.6 to 76317.7) | -- | -- | -- | -- |
| Ascariasis complications | 6377.6 (5609.9 to 7235.0) | -- | 120.5 (65.1 to 211.2) | -39.9* (-46.8 to -32.2) | -44.3* (-50.7 to -37.2) |
| Trichuriasis | 23393.7 (20971.6 to 26367.1) | -- | 16.8 (9.4 to 28.7) | -39.8* (-46.1 to -32.3) | -44.1* (-50.0 to -37.2) |
| Asymptomatic trichuriasis | 22531.7 (20183.3 to 25416.9) | -- | -- | -- | -- |
| Trichuriasis complications | 861.9 (778.7 to 958.3) | -- | 16.8 (9.4 to 28.7) | -39.8* (-46.1 to -32.3) | -44.1* (-50.0 to -37.2) |
| Hookworm disease | 26544.7 (24892.0 to 28398.8) | -- | 104.3 (64.4 to 161.4) | -35.1* (-41.8 to -28.9) | -39.8* (-46.0 to -34.0) |
| Asymptomatic hookworm disease | 22490.5 (21059.9 to 24095.6) | -- | -- | -- | -- |
| Anemia due to hookworm disease | 1036.1 (942.6 to 1141.7) | -- | 42.4 (27.8 to 60.8) | -41.1* (-49.3 to -32.4) | -45.4* (-53.0 to -37.3) |
| Hookworm disease complications | 3018.1 (2837.9 to 3224.1) | -- | 61.9 (33.9 to 102.0) | -30.2* (-34.9 to -25.3) | -35.3* (-39.6 to -30.7) |
| Food-borne trematodiases | 2327.6 (1839.7 to 2877.2) | 617.9 (496.0 to 749.5) | 20.2 (11.6 to 32.2) | -4.3 (-17.0 to 7.1) | -11.3* (-23.0 to -0.7) |
| Asymptomatic food-borne tremodiases | 2149.6 (1696.2 to 2672.2) | 560.6 (439.3 to 688.7) | -- | -- | -- |
| Food-borne tremodiases complications | 178.0 (118.5 to 265.0) | 57.2 (29.6 to 112.2) | 20.2 (11.6 to 32.2) | -4.3 (-17.0 to 7.1) | -11.3* (-23.0 to -0.7) |
| Leprosy | 0.8 (0.7 to 0.9) | 0.4 (0.3 to 0.5) | 0.0 (0.0 to 0.1) | -36.7* (-38.0 to -35.6) | -41.3* (-42.5 to -40.3) |
| Ebola virus disease | -- | -- | -- | -98.3* (-98.6 to -97.9) | -98.5* (-98.7 to -98.1) |
| Ebola cases | -- | -- | -- | -98.6* (-98.7 to -98.5) | -98.7* (-98.8 to -98.6) |
| Post-Ebola chronic fatigue syndrome | -- | -- | -- | -98.3* (-98.6 to -97.9) | -98.5* (-98.7 to -98.1) |
| Zika virus disease | 2.4 (1.3 to 4.2) | 142.4 (81.0 to 257.6) | 0.0 (0.0 to 0.1) | -- | -- |
| Zika virus complications | -- | -- | -- | -- | -- |
| Zika virus episode | 2.3 (1.3 to 4.2) | 142.4 (81.0 to 257.6) | 0.0 (0.0 to 0.1) | -- | -- |
| Guinea worm disease | -- | -- | -- | -99.4* (-99.6 to -99.2) | -99.5* (-99.6 to -99.3) |
| Other neglected tropical diseases | 9597.9 (8894.9 to 10427.0) | -- | 415.0 (269.6 to 598.1) | -9.0 (-17.7 to 1.5) | -15.6* (-23.6 to -5.8) |
| Acute infection due to other neglected tropical diseases | -- | -- | 1.4 (0.7 to 2.4) | 147.6* (111.5 to 257.8) | 129.7* (96.1 to 231.8) |
| Anemia due to other neglected tropical diseases | 9597.9 (8894.9 to 10427.0) | -- | 413.6 (269.0 to 596.5) | -9.2 (-17.8 to 1.3) | -15.8* (-23.8 to -6.0) |
| **Other infectious diseases** | **11156.3 (10620.7 to 11743.5)** | **52894.0 (47696.1 to 58818.5)** | **524.9 (361.3 to 712.7)** | **-7.5* (-12.6 to -1.1)** | **-14.2* (-18.9 to -8.3)** |
| Meningitis | 1053.0 (861.5 to 1275.8) | 423.7 (292.1 to 586.9) | 110.2 (74.4 to 153.5) | -3.9 (-9.3 to 2.2) | -10.9* (-15.9 to -5.2) |
| Pneumococcal meningitis | 320.2 (252.3 to 403.1) | 52.0 (31.1 to 81.2) | 35.4 (23.6 to 49.9) | -27.3* (-34.0 to -20.4) | -32.6* (-38.8 to -26.2) |

| | | | | | |
|---|---|---|---|---|---|
| Acute pneumococcal meningitis | 2.5<br>(1.5 to 4.2) | 52.0<br>(31.1 to 81.2) | 0.3<br>(0.2 to 0.6) | -23.3*<br>(-34.4 to -12.5) | -28.9*<br>(-39.2 to -18.9) |
| Pneumococcal meningitis complications | 317.7<br>(249.5 to 400.4) | -- | 35.0<br>(23.3 to 49.4) | -27.4*<br>(-34.1 to -20.3) | -32.7*<br>(-38.8 to -26.1) |
| H influenzae type B meningitis | 106.3<br>(76.9 to 144.3) | 25.6<br>(14.3 to 42.8) | 12.0<br>(8.0 to 17.1) | -45.1*<br>(-49.0 to -41.0) | -49.1*<br>(-52.7 to -45.3) |
| Acute H influenzae type B meningitis | 1.2<br>(0.7 to 2.1) | 25.6<br>(14.3 to 42.8) | 0.2<br>(0.1 to 0.3) | -51.8*<br>(-59.2 to -44.1) | -55.3*<br>(-62.2 to -48.1) |
| H Influenza type B meningitis complications | 105.1<br>(76.2 to 143.2) | -- | 11.9<br>(7.8 to 16.9) | -45.0*<br>(-49.0 to -40.9) | -49.0*<br>(-52.7 to -45.2) |
| Meningococcal infection | 103.2<br>(71.2 to 139.5) | 42.6<br>(24.3 to 69.1) | 10.8<br>(7.1 to 15.2) | -5.7*<br>(-10.3 to -0.6) | -12.5*<br>(-16.8 to -7.8) |
| Acute meningococcal meningitis | 2.1<br>(1.2 to 3.5) | 42.6<br>(24.3 to 69.1) | 0.3<br>(0.1 to 0.5) | 20.9*<br>(7.2 to 35.8) | 12.1<br>(-0.6 to 26.0) |
| Meningococcal meningitis complications | 101.1<br>(69.2 to 137.1) | -- | 10.5<br>(6.9 to 14.9) | -6.2*<br>(-10.9 to -1.2) | -13.0*<br>(-17.4 to -8.4) |
| Other meningitis | 523.2<br>(390.1 to 677.2) | 303.5<br>(207.4 to 421.3) | 52.0<br>(35.0 to 71.6) | 59.2*<br>(45.6 to 74.7) | 47.6*<br>(35.0 to 62.0) |
| Other acute bacterial meningitis | 4.8<br>(3.1 to 7.2) | 99.2<br>(65.6 to 142.3) | 0.6<br>(0.3 to 1.1) | 123.6*<br>(102.8 to 145.7) | 107.4*<br>(88.0 to 127.9) |
| Acute viral meningitis | 10.0<br>(6.5 to 14.5) | 204.3<br>(140.9 to 283.0) | 1.3<br>(0.7 to 2.2) | 9.3*<br>(0.4 to 18.4) | 1.3<br>(-6.9 to 9.8) |
| Other bacterial meningitis complications | 508.4<br>(376.1 to 660.2) | -- | 50.0<br>(33.6 to 69.0) | 60.5*<br>(46.5 to 76.8) | 48.9*<br>(35.9 to 64.0) |
| Encephalitis | 308.2<br>(188.7 to 468.8) | 289.3<br>(275.3 to 304.5) | 30.6<br>(21.5 to 40.7) | -3.4*<br>(-6.0 to -0.5) | -10.4*<br>(-12.8 to -7.7) |
| Acute encephalitis | 16.2<br>(15.3 to 17.1) | 289.3<br>(275.3 to 304.5) | 2.2<br>(1.4 to 3.1) | 5.2*<br>(4.4 to 5.8) | -2.5*<br>(-3.2 to -1.9) |
| Encephalitis complications | 292.0<br>(172.1 to 452.8) | -- | 28.4<br>(19.9 to 37.6) | -4.0*<br>(-6.7 to -1.0) | -10.9*<br>(-13.5 to -8.2) |
| Diphtheria | 0.1<br>(0.1 to 0.2) | 1.3<br>(0.8 to 2.2) | -- | -40.5*<br>(-63.8 to -2.9) | -44.8*<br>(-66.4 to -9.9) |
| Whooping cough | 97.7<br>(75.5 to 123.2) | 713.2<br>(551.1 to 899.6) | 4.8<br>(2.8 to 7.8) | -4.7<br>(-15.4 to 7.2) | -11.6*<br>(-21.5 to -0.5) |
| Tetanus | 6.1<br>(5.8 to 6.4) | 2.9<br>(1.5 to 5.3) | 0.1<br>(0.1 to 0.2) | -15.3*<br>(-26.9 to -6.5) | -21.5*<br>(-32.2 to -13.2) |
| Severe tetanus | 0.2<br>(0.1 to 0.3) | 2.9<br>(1.5 to 5.3) | -- | -53.8*<br>(-70.8 to -27.8) | -57.2*<br>(-72.9 to -33.0) |
| Neonatal tetanus complications | 5.9<br>(5.7 to 6.2) | -- | 0.1<br>(0.1 to 0.2) | -0.1<br>(-2.2 to 1.7) | -7.4*<br>(-9.2 to -5.7) |
| Measles | 57.5<br>(20.5 to 127.5) | 2098.8<br>(746.8 to 4654.4) | 5.2<br>(1.8 to 11.8) | -44.6*<br>(-52.1 to -35.8) | -48.6*<br>(-55.6 to -40.5) |
| Varicella and herpes zoster | 348.7<br>(257.5 to 451.5) | 9929.3<br>(5877.9 to 14191.7) | 11.0<br>(6.1 to 17.6) | 7.9<br>(-0.2 to 16.3) | 0.1<br>(-7.4 to 7.9) |
| Chickenpox | 171.4<br>(94.3 to 251.5) | 8945.4<br>(4919.4 to 13125.2) | 1.0<br>(0.3 to 2.4) | 11.2*<br>(2.1 to 22.5) | 3.2<br>(-5.3 to 13.6) |
| Herpes zoster | 177.3<br>(133.2 to 235.8) | 984.0<br>(744.6 to 1303.9) | 10.0<br>(5.5 to 16.0) | 7.6<br>(-0.9 to 17.0) | -0.2<br>(-8.0 to 8.5) |
| Acute hepatitis | 3368.3<br>(3077.8 to 3692.7) | 39435.4<br>(36617.8 to 42433.2) | 50.0<br>(30.6 to 74.5) | 8.1<br>(-12.6 to 33.6) | 0.2<br>(-19.0 to 23.9) |
| Acute hepatitis A | 2039.7<br>(1918.8 to 2107.0) | 26516.3<br>(24944.2 to 27390.6) | 38.5<br>(23.0 to 58.5) | 8.2<br>(-17.9 to 39.8) | 0.3<br>(-23.9 to 29.7) |
| Acute hepatitis B | 924.3<br>(677.6 to 1232.8) | 8010.6<br>(5872.8 to 10684.1) | 6.6<br>(4.0 to 10.5) | 8.9<br>(-17.0 to 44.9) | 1.0<br>(-23.0 to 34.4) |
| Acute hepatitis C | 80.2<br>(49.6 to 112.4) | 694.7<br>(429.7 to 973.9) | 1.1<br>(0.4 to 2.2) | 9.2*<br>(1.2 to 18.0) | 1.3<br>(-6.1 to 9.4) |
| Acute hepatitis E | 324.1<br>(231.2 to 425.1) | 4213.8<br>(3006.1 to 5526.6) | 3.8<br>(2.1 to 6.5) | 5.4<br>(-25.4 to 48.8) | -2.3<br>(-30.8 to 38.0) |
| Other unspecified infectious diseases | 7015.3<br>(6548.9 to 7547.7) | -- | 312.9<br>(203.2 to 452.0) | -10.5*<br>(-18.5 to -0.9) | -17.0*<br>(-24.4 to -8.1) |
| Guillain-Barré syndrome due to other infectious diseases | 0.8<br>(0.4 to 1.3) | -- | 0.2<br>(0.1 to 0.4) | 9.1*<br>(6.6 to 11.3) | 1.2<br>(-1.1 to 3.2) |
| Other infectious diseases | -- | -- | 14.2<br>(8.3 to 22.2) | 22.6*<br>(17.0 to 27.8) | 13.7*<br>(8.5 to 18.5) |
| Anemia due to other infectious diseases | 7014.6<br>(6548.1 to 7547.4) | -- | 298.5<br>(193.8 to 431.6) | -11.7*<br>(-19.8 to -1.8) | -18.1*<br>(-25.6 to -9.0) |
| **Maternal and neonatal disorders** | **13572.8<br>(11669.5 to 15753.8)** | **--** | **2960.1<br>(2234.5 to 3863.6)** | **21.6*<br>(14.5 to 30.1)** | **12.8*<br>(6.2 to 20.7)** |
| Maternal disorders | -- | -- | -- | -- | -- |
| Maternal haemorrhage | -- | -- | -- | -- | -- |
| Maternal hemorrhage complications | -- | -- | -- | -- | -- |
| Maternal hemorrhage episode | -- | -- | -- | -- | -- |
| Maternal sepsis and other pregnancy related infections | -- | -- | -- | -- | -- |
| Infertility due to puerperal sepsis | -- | -- | -- | -- | -- |
| Maternal sepsis and other maternal infection episode | -- | -- | -- | -- | -- |
| Maternal hypertensive disorders | -- | -- | -- | -- | -- |
| Maternal hypertensive disorder complications | -- | -- | -- | -- | -- |
| Maternal hypertensive disorder episode | -- | -- | -- | -- | -- |
| Maternal obstructed labour and uterine rupture | -- | -- | -- | -- | -- |
| Obstructed labor, acute event | -- | -- | -- | -- | -- |
| Maternal obstructed labor complications | -- | -- | -- | -- | -- |
| Maternal abortion and miscarriage | -- | -- | -- | -- | -- |
| Ectopic pregnancy | -- | -- | -- | -- | -- |
| Other maternal disorders | -- | -- | -- | -- | -- |
| Neonatal disorders | 13572.8<br>(11669.5 to 15753.8) | -- | 2960.1<br>(2234.5 to 3863.6) | 21.6*<br>(14.5 to 30.1) | 12.8*<br>(6.2 to 20.7) |
| Neonatal preterm birth | 7913.5<br>(6692.3 to 9454.2) | -- | 1481.0<br>(1045.6 to 1998.0) | 21.7*<br>(5.9 to 38.4) | 12.8<br>(-1.8 to 28.3) |
| Preterm birth complications | 6863.8<br>(5635.4 to 8401.9) | -- | 1426.9<br>(993.6 to 1945.7) | 22.8*<br>(6.3 to 40.1) | 13.9<br>(-1.4 to 29.9) |
| Retinopathy of prematurity | 1049.8<br>(810.3 to 1310.1) | -- | 54.1<br>(35.5 to 76.1) | -1.8<br>(-9.3 to 6.5) | -8.9*<br>(-15.9 to -1.2) |
| Uncomplicated preterm birth | -- | -- | -- | -- | -- |
| Neonatal encephalopathy due to birth asphyxia and trauma | 4972.3<br>(3575.6 to 6583.8) | -- | 966.0<br>(575.5 to 1499.8) | 22.6*<br>(17.1 to 28.7) | 13.7*<br>(8.6 to 19.4) |
| Neonatal sepsis and other neonatal infections | 1367.9<br>(838.3 to 2088.0) | -- | 415.0<br>(219.4 to 709.8) | 20.7*<br>(14.9 to 26.4) | 11.9*<br>(6.6 to 17.2) |
| Neonatal sepsis and other neonatal infection complications | 1367.9<br>(838.3 to 2088.0) | -- | 415.0<br>(219.4 to 709.8) | 20.7*<br>(14.9 to 26.4) | 11.9*<br>(6.6 to 17.2) |
| Neonatal sepsis and other neonatal infection episode | -- | -- | -- | -- | -- |
| Hemolytic disease and other neonatal jaundice | 262.1<br>(225.7 to 298.9) | -- | 98.1<br>(74.1 to 123.1) | 14.2*<br>(7.0 to 21.2) | 5.9<br>(-0.7 to 12.4) |

| | | | | | |
|---|---|---|---|---|---|
| Other neonatal disorders | -- | -- | -- | -- | -- |
| **Nutritional deficiencies** | **209103.9 (194491.5 to 227565.6)** | **129245.8 (115742.3 to 143943.3)** | **8395.8 (5509.2 to 12053.5)** | **-14.2* (-20.6 to -6.9)** | **-20.4* (-26.4 to -13.6)** |
| Protein-energy malnutrition | 3354.8 (2723.7 to 4144.8) | 2096.5 (1674.4 to 2536.7) | 57.6 (35.2 to 85.6) | -7.7 (-17.9 to 2.2) | -14.4* (-23.9 to -5.2) |
| Iodine deficiency | 6085.5 (5231.7 to 7159.1) | 659.2 (533.8 to 784.2) | 137.2 (87.3 to 205.5) | -28.6* (-33.4 to -23.7) | -33.8* (-38.3 to -29.2) |
| Visible goiter without symptoms | 5863.3 (5038.5 to 6909.3) | 659.2 (533.8 to 784.2) | 63.6 (30.0 to 117.4) | -29.5* (-32.1 to -27.0) | -34.7* (-37.0 to -32.3) |
| Visible goiter with complications | 222.2 (134.5 to 291.2) | -- | 73.5 (40.3 to 112.2) | -27.7* (-36.8 to -18.9) | -33.0* (-41.4 to -24.8) |
| Vitamin A deficiency | 129678.0 (110837.4 to 153885.0) | 126490.1 (113041.3 to 140891.5) | 3408.5 (2180.6 to 5041.2) | -16.8* (-22.3 to -10.6) | -22.8* (-28.0 to -17.0) |
| Asymptomatic vitamin A deficiency | 50476.3 (42046.3 to 61359.9) | 126490.1 (113041.3 to 140891.5) | -- | -- | -- |
| Vitamin A deficiency complications | 957.8 (612.0 to 1447.5) | -- | 49.1 (28.8 to 81.3) | 10.1* (3.1 to 18.0) | 2.1 (-4.4 to 9.4) |
| Vitamin A deficiency with anemia | 78244.8 (66609.3 to 93184.3) | -- | 3359.4 (2144.8 to 4975.6) | -17.1* (-22.7 to -10.8) | -23.1* (-28.3 to -17.3) |
| Dietary iron deficiency | 111292.1 (103856.2 to 119935.2) | -- | 4783.6 (3111.4 to 6934.2) | -11.7* (-19.4 to -2.5) | -18.1* (-25.2 to -9.6) |
| Other nutritional deficiencies | -- | -- | 9.0 (5.5 to 13.3) | -29.5* (-37.3 to -21.9) | -34.6* (-41.8 to -27.6) |
| **Non-communicable diseases** | **548557.7 (527624.4 to 569645.6)** | **1239141.3 (1038394.0 to 1448393.0)** | **16585.3 (11897.8 to 22266.5)** | **8.5* (6.9 to 10.4)** | **0.7 (-0.8 to 2.4)** |
| **Neoplasms** | **1617.8 (1339.1 to 2139.3)** | **78.5 (71.4 to 84.7)** | **84.8 (53.3 to 131.1)** | **101.7* (63.1 to 164.1)** | **87.1* (51.3 to 145.0)** |
| Lip and oral cavity cancer | -- | -- | -- | -- | -- |
| Diagnosis and primary therapy phase of mouth cancer | -- | -- | -- | -- | -- |
| Controlled phase of mouth cancer | -- | -- | -- | -- | -- |
| Metastatic phase of mouth cancer | -- | -- | -- | -- | -- |
| Terminal phase of mouth cancer | -- | -- | -- | -- | -- |
| Nasopharynx cancer | 4.3 (3.8 to 5.0) | 0.7 (0.6 to 0.8) | 0.3 (0.2 to 0.5) | -8.4 (-22.1 to 9.1) | -15.0 (-27.7 to 1.2) |
| Diagnosis and primary therapy phase of nasopharynx cancer | 0.2 (0.2 to 0.3) | 0.7 (0.6 to 0.8) | 0.1 (0.0 to 0.1) | -6.2 (-20.0 to 11.2) | -13.0 (-25.8 to 3.1) |
| Controlled phase of nasopharynx cancer | 3.9 (3.4 to 4.5) | -- | 0.2 (0.1 to 0.3) | -5.8 (-19.8 to 11.2) | -12.6 (-25.6 to 3.1) |
| Metastatic phase of nasopharynx cancer | 0.2 (0.1 to 0.2) | -- | 0.1 (0.0 to 0.1) | -15.1 (-29.0 to 2.8) | -21.3* (-34.1 to -4.6) |
| Terminal phase of nasopharynx cancer | -- | -- | -- | -18.4* (-32.1 to -0.8) | -24.3* (-37.0 to -8.0) |
| Other pharynx cancer | -- | -- | -- | -- | -- |
| Diagnosis and primary therapy phase of other pharynx cancer | -- | -- | -- | -- | -- |
| Controlled phase of other pharynx cancer | -- | -- | -- | -- | -- |
| Metastatic phase of other pharynx cancer | -- | -- | -- | -- | -- |
| Terminal phase of other pharynx cancer | -- | -- | -- | -- | -- |
| Oesophageal cancer | -- | -- | -- | -- | -- |
| Diagnosis and primary therapy phase of esophageal cancer | -- | -- | -- | -- | -- |
| Controlled phase of esophageal cancer | -- | -- | -- | -- | -- |
| Metastatic phase of esophageal cancer | -- | -- | -- | -- | -- |
| Terminal phase of esophageal cancer | -- | -- | -- | -- | -- |
| Stomach cancer | -- | -- | -- | -- | -- |
| Diagnosis and primary therapy phase of stomach cancer | -- | -- | -- | -- | -- |
| Controlled phase of stomach cancer | -- | -- | -- | -- | -- |
| Metastatic phase of stomach cancer | -- | -- | -- | -- | -- |
| Terminal phase of stomach cancer | -- | -- | -- | -- | -- |
| Colon and rectum cancer | -- | -- | -- | -- | -- |
| Diagnosis and primary therapy phase of colon and rectum cancers | -- | -- | -- | -- | -- |
| Controlled phase of colon and rectum cancers | -- | -- | -- | -- | -- |
| Metastatic phase of colon and rectum cancers | -- | -- | -- | -- | -- |
| Terminal phase of colon and rectum cancers | -- | -- | -- | -- | -- |
| Stoma from colon and rectum cancers, beyond 10 years | -- | -- | -- | -- | -- |
| Liver cancer | -- | 1.1 (1.0 to 1.3) | 0.2 (0.2 to 0.3) | -4.1 (-14.3 to 12.1) | -11.0 (-20.6 to 4.0) |
| Liver cancer due to hepatitis B | 0.7 (0.6 to 0.8) | 0.7 (0.6 to 0.8) | 0.2 (0.1 to 0.2) | -5.8 (-16.1 to 9.5) | -12.6 (-22.2 to 1.6) |
| Diagnosis and primary therapy phase of liver cancer due to hepatitis B | 0.2 (0.1 to 0.2) | 0.7 (0.6 to 0.8) | 0.0 (0.0 to 0.1) | -4.8 (-15.4 to 11.0) | -11.7 (-21.5 to 3.0) |
| Controlled phase of liver cancer due to hepatitis B | 0.3 (0.3 to 0.4) | -- | -- | -2.2 (-12.6 to 12.6) | -9.3 (-19.0 to 4.4) |
| Metastatic phase of liver cancer due to hepatitis B | 0.1 (0.1 to 0.2) | -- | 0.1 (0.0 to 0.1) | -6.9 (-17.1 to 8.4) | -13.6 (-23.1 to 0.5) |
| Terminal phase of liver cancer due to hepatitis B | 0.1 (0.0 to 0.1) | -- | -- | -6.9 (-17.1 to 8.4) | -13.6 (-23.1 to 0.5) |
| Liver cancer due to hepatitis C | -- | -- | -- | 0.3 (-14.1 to 20.1) | -7.0 (-20.3 to 11.4) |
| Diagnosis and primary therapy phase of liver cancer due to hepatitis C | -- | -- | -- | 1.3 (-13.0 to 21.3) | -6.1 (-19.3 to 12.5) |
| Controlled phase of liver cancer due to hepatitis C | -- | -- | -- | -0.2 (-14.5 to 19.3) | -7.5 (-20.7 to 10.7) |
| Metastatic phase of liver cancer due to hepatitis C | -- | -- | -- | -0.1 (-14.6 to 20.1) | -7.3 (-20.7 to 11.4) |
| Terminal phase of liver cancer due to hepatitis C | -- | -- | -- | -0.1 (-14.6 to 20.1) | -7.3 (-20.7 to 11.4) |
| Liver cancer due to alcohol use | -- | -- | -- | -- | -- |
| Diagnosis and primary therapy phase of liver cancer due to alcohol use | -- | -- | -- | -- | -- |
| Controlled phase of liver cancer due to alcohol use | -- | -- | -- | -- | -- |
| Metastatic phase of liver cancer due to alcohol use | -- | -- | -- | -- | -- |

| | | | | | |
|---|---|---|---|---|---|
| Terminal phase of liver cancer due to alcohol use | -- | -- | -- | -- | -- |
| Liver cancer due to NASH | -- | -- | -- | -- | -- |
| Diagnosis and primary therapy phase of liver cancer due to NASH | -- | -- | -- | -- | -- |
| Controlled phase of liver cancer due to NASH | -- | -- | -- | -- | -- |
| Metastatic phase of liver cancer due to NASH | -- | -- | -- | -- | -- |
| Terminal phase of liver cancer due to NASH | -- | -- | -- | -- | -- |
| Liver cancer due to other causes | 0.4<br>(0.3 to 0.4) | 0.4<br>(0.3 to 0.5) | 0.1<br>(0.1 to 0.1) | -1.2<br>(-12.3 to 16.0) | -8.3<br>(-18.6 to 7.6) |
| Diagnosis and primary therapy phase of liver cancer due to other causes | 0.1<br>(0.1 to 0.1) | 0.4<br>(0.3 to 0.5) | -- | -0.3<br>(-11.6 to 17.1) | -7.6<br>(-18.0 to 8.6) |
| Controlled phase of liver cancer due to other causes | 0.2<br>(0.2 to 0.2) | -- | -- | 0.9<br>(-10.4 to 17.5) | -6.4<br>(-16.9 to 9.0) |
| Metastatic phase of liver cancer due to other causes | 0.1<br>(0.1 to 0.1) | -- | -- | -2.0<br>(-12.9 to 15.3) | -9.1<br>(-19.2 to 6.9) |
| Terminal phase of liver cancer due to other causes | -- | -- | -- | -2.0<br>(-12.9 to 15.3) | -9.1<br>(-19.2 to 6.9) |
| Gallbladder and biliary tract cancer | -- | -- | -- | -- | -- |
| Diagnosis and primary therapy phase of gallbladder and biliary tract cancer | -- | -- | -- | -- | -- |
| Controlled phase of gallbladder and biliary tract cancer | -- | -- | -- | -- | -- |
| Metastatic phase of gallbladder and biliary tract cancer | -- | -- | -- | -- | -- |
| Terminal phase of gallbladder and biliary tract cancer | -- | -- | -- | -- | -- |
| Pancreatic cancer | -- | -- | -- | -- | -- |
| Diagnosis and primary therapy phase of pancreatic cancer | -- | -- | -- | -- | -- |
| Controlled phase of pancreatic cancer | -- | -- | -- | -- | -- |
| Metastatic phase of pancreatic cancer | -- | -- | -- | -- | -- |
| Terminal phase of pancreatic cancer | -- | -- | -- | -- | -- |
| Larynx cancer | -- | -- | -- | -- | -- |
| Diagnosis and primary therapy phase of larynx cancer | -- | -- | -- | -- | -- |
| Controlled phase of larynx cancer | -- | -- | -- | -- | -- |
| Metastatic phase of larynx cancer | -- | -- | -- | -- | -- |
| Terminal phase of larynx cancer | -- | -- | -- | -- | -- |
| Laryngectomy from larynx cancer, beyond 10 years | -- | -- | -- | -- | -- |
| Tracheal, bronchus, and lung cancer | -- | -- | -- | -- | -- |
| Diagnosis and primary therapy phase of lung, bronchus, and trachea cancer | -- | -- | -- | -- | -- |
| Controlled phase of lung, bronchus, and trachea cancer | -- | -- | -- | -- | -- |
| Metastatic phase of lung, bronchus, and trachea cancer | -- | -- | -- | -- | -- |
| Terminal phase of lung, bronchus, and trachea cancer | -- | -- | -- | -- | -- |
| Malignant skin melanoma | -- | -- | -- | -- | -- |
| Diagnosis and primary therapy phase of malignant skin melanoma | -- | -- | -- | -- | -- |
| Controlled phase of malignant skin melanoma | -- | -- | -- | -- | -- |
| Metastatic phase of malignant skin melanoma | -- | -- | -- | -- | -- |
| Terminal phase of malignant skin melanoma | -- | -- | -- | -- | -- |
| Non-melanoma skin cancer | -- | -- | -- | -- | -- |
| Non-melanoma skin cancer (squamous-cell carcinoma) | -- | -- | -- | -- | -- |
| Non-melanoma skin cancer (basal-cell carcinoma) | -- | -- | -- | -- | -- |
| Breast cancer | -- | -- | -- | -- | -- |
| Diagnosis and primary therapy phase of breast cancer | -- | -- | -- | -- | -- |
| Controlled phase of breast cancer | -- | -- | -- | -- | -- |
| Metastatic phase of breast cancer | -- | -- | -- | -- | -- |
| Terminal phase of breast cancer | -- | -- | -- | -- | -- |
| Mastectomy from breast cancer, beyond 10 years | -- | -- | -- | -- | -- |
| Cervical cancer | -- | -- | -- | -- | -- |
| Diagnosis and primary therapy phase of cervical cancer | -- | -- | -- | -- | -- |
| Controlled phase of cervical cancer | -- | -- | -- | -- | -- |
| Metastatic phase of cervical cancer | -- | -- | -- | -- | -- |
| Terminal phase of cervical cancer | -- | -- | -- | -- | -- |
| Uterine cancer | -- | -- | -- | -- | -- |
| Diagnosis and primary therapy phase of uterine cancer | -- | -- | -- | -- | -- |
| Controlled phase of uterine cancer | -- | -- | -- | -- | -- |
| Metastatic phase of uterine cancer | -- | -- | -- | -- | -- |
| Terminal phase of uterine cancer | -- | -- | -- | -- | -- |
| Ovarian cancer | -- | -- | -- | -- | -- |
| Diagnosis and primary therapy phase of ovarian cancer | -- | -- | -- | -- | -- |
| Controlled phase of ovarian cancer | -- | -- | -- | -- | -- |
| Metastatic phase of ovarian cancer | -- | -- | -- | -- | -- |

| | | | | | |
|---|---|---|---|---|---|
| Terminal phase of ovarian cancer | -- | -- | -- | -- | -- |
| Prostate cancer | -- | -- | -- | -- | -- |
| Diagnosis and primary therapy phase of prostate cancer | -- | -- | -- | -- | -- |
| Controlled phase of prostate cancer | -- | -- | -- | -- | -- |
| Metastatic phase of prostate cancer | -- | -- | -- | -- | -- |
| Terminal phase of prostate cancer | -- | -- | -- | -- | -- |
| Impotence and Incontinence after 10-year survival from prostate cancer | -- | -- | -- | -- | -- |
| Testicular cancer | -- | -- | -- | -- | -- |
| Diagnosis and primary therapy phase of testicular cancer | -- | -- | -- | -- | -- |
| Controlled phase of testicular cancer | -- | -- | -- | -- | -- |
| Metastatic phase of testicular cancer | -- | -- | -- | -- | -- |
| Terminal phase of testicular cancer | -- | -- | -- | -- | -- |
| Kidney cancer | 45.1 (40.7 to 49.4) | 5.5 (4.9 to 6.0) | 2.3 (1.5 to 3.4) | -0.1 (-12.8 to 13.2) | -7.3 (-19.1 to 4.9) |
| Diagnosis and primary therapy phase of kidney cancer | -- | 5.5 (4.9 to 6.0) | -- | -- | -- |
| Controlled phase of kidney cancer | 44.8 (40.5 to 49.1) | -- | 2.2 (1.4 to 3.3) | -0.0 (-12.9 to 13.6) | -7.3 (-19.2 to 5.3) |
| Metastatic phase of kidney cancer | 0.2 (0.2 to 0.3) | -- | 0.1 (0.1 to 0.1) | -1.6 (-12.3 to 10.1) | -8.7 (-18.7 to 2.2) |
| Terminal phase of kidney cancer | -- | -- | -- | -1.6 (-12.3 to 10.2) | -8.7 (-18.7 to 2.2) |
| Bladder cancer | -- | -- | -- | -- | -- |
| Diagnosis and primary therapy phase of bladder cancer | -- | -- | -- | -- | -- |
| Controlled phase of bladder cancer | -- | -- | -- | -- | -- |
| Metastatic phase of bladder cancer | -- | -- | -- | -- | -- |
| Terminal phase of bladder cancer | -- | -- | -- | -- | -- |
| Incontinence from bladder cancer, beyond 10 years | -- | -- | -- | -- | -- |
| Brain and nervous system cancer | 101.2 (86.3 to 116.0) | 18.2 (15.6 to 21.0) | 8.1 (5.5 to 11.2) | 32.1* (17.4 to 49.6) | 22.5* (8.9 to 38.8) |
| Diagnosis and primary therapy phase of brain and nervous system cancers | 5.2 (4.4 to 6.0) | 18.2 (15.6 to 21.0) | 1.5 (1.0 to 2.1) | 46.7* (33.2 to 63.2) | 36.1* (23.6 to 51.3) |
| Controlled phase of brain and nervous system cancers | 91.3 (77.8 to 104.7) | -- | 4.4 (2.8 to 6.7) | 54.6* (36.4 to 76.7) | 43.4* (26.5 to 63.8) |
| Metastatic phase of brain and nervous system cancers | 3.9 (3.4 to 4.6) | -- | 1.8 (1.2 to 2.5) | -3.1 (-12.6 to 10.5) | -10.2 (-18.9 to 2.5) |
| Terminal phase of brain and nervous system cancers | 0.7 (0.6 to 0.8) | -- | 0.4 (0.3 to 0.5) | -5.5 (-14.8 to 8.0) | -12.4 (-21.0 to 0.2) |
| Thyroid cancer | -- | -- | -- | -- | -- |
| Diagnosis and primary therapy phase of thyroid cancer | -- | -- | -- | -- | -- |
| Controlled phase of thyroid cancer | -- | -- | -- | -- | -- |
| Metastatic phase of thyroid cancer | -- | -- | -- | -- | -- |
| Terminal phase of thyroid cancer | -- | -- | -- | -- | -- |
| Mesothelioma | -- | -- | -- | -- | -- |
| Diagnosis and primary therapy phase of mesothelioma | -- | -- | -- | -- | -- |
| Controlled phase of mesothelioma | -- | -- | -- | -- | -- |
| Metastatic phase of mesothelioma | -- | -- | -- | -- | -- |
| Terminal phase of mesothelioma | -- | -- | -- | -- | -- |
| Hodgkin lymphoma | 18.7 (14.1 to 22.7) | 3.0 (2.2 to 3.8) | 1.3 (0.8 to 1.9) | 14.0* (0.7 to 31.2) | 5.7 (-6.6 to 21.7) |
| Diagnosis and primary therapy phase of Hodgkin disease | 0.6 (0.4 to 0.7) | 3.0 (2.2 to 3.8) | 0.2 (0.1 to 0.2) | 25.2* (14.1 to 39.6) | 16.1* (5.8 to 29.4) |
| Controlled phase of Hodgkin disease | 17.6 (13.3 to 21.2) | -- | 0.9 (0.5 to 1.3) | 25.1* (10.3 to 42.8) | 16.0* (2.3 to 32.5) |
| Metastatic phase of Hodgkin disease | 0.5 (0.3 to 0.6) | -- | 0.2 (0.1 to 0.3) | -15.6 (-29.0 to 6.5) | -21.7* (-34.2 to -1.2) |
| Terminal phase of Hodgkin disease | 0.1 (0.1 to 0.1) | -- | 0.1 (0.0 to 0.1) | -15.6 (-29.0 to 6.5) | -21.8* (-34.2 to -1.2) |
| Non-Hodgkin's lymphoma | 39.6 (34.9 to 44.6) | 7.6 (6.6 to 8.8) | 3.0 (2.0 to 4.2) | 20.0* (2.8 to 39.0) | 11.3 (-4.7 to 28.9) |
| Diagnosis and primary therapy phase of non-Hodgkin lymphoma | 1.2 (1.1 to 1.4) | 7.6 (6.6 to 8.8) | 0.4 (0.2 to 0.5) | 27.1* (12.2 to 42.3) | 17.9* (4.1 to 32.0) |
| Controlled phase of non-Hodgkin lymphoma | 36.5 (32.2 to 41.0) | -- | 1.8 (1.1 to 2.7) | 27.2* (7.8 to 48.2) | 18.0 (-0.0 to 37.4) |
| Metastatic phase of non-Hodgkin lymphoma | 1.5 (1.3 to 1.8) | -- | 0.7 (0.4 to 1.0) | 5.4 (-11.4 to 24.5) | -2.3 (-17.8 to 15.5) |
| Terminal phase of non-Hodgkin lymphoma | 0.3 (0.3 to 0.4) | -- | 0.2 (0.1 to 0.2) | 5.2 (-11.6 to 24.4) | -2.5 (-18.0 to 15.4) |
| Multiple myeloma | -- | -- | -- | -- | -- |
| Diagnosis and primary therapy phase of multiple myeloma | -- | -- | -- | -- | -- |
| Controlled phase of multiple myeloma | -- | -- | -- | -- | -- |
| Metastatic phase of multiple myeloma | -- | -- | -- | -- | -- |
| Terminal phase of multiple myeloma | -- | -- | -- | -- | -- |
| Leukaemia | 188.9 (160.2 to 211.2) | 28.2 (24.1 to 32.1) | 14.7 (10.1 to 20.0) | 17.3* (2.9 to 31.1) | 8.8 (-4.6 to 21.6) |
| Acute lymphoid leukaemia | 80.1 (66.2 to 92.0) | 8.2 (7.0 to 9.5) | 6.3 (4.4 to 8.8) | 23.5 (-0.0 to 46.9) | 14.6 (-7.3 to 36.3) |
| Diagnosis and primary therapy phase of acute lymphoid leukemia | 4.2 (3.6 to 4.7) | 8.2 (7.0 to 9.5) | 1.2 (0.8 to 1.7) | 1.7 (-16.1 to 18.1) | -5.6 (-22.2 to 9.5) |
| Controlled phase of acute lymphoid leukemia | 72.4 (59.7 to 83.5) | -- | 3.5 (2.2 to 5.3) | 42.2* (14.9 to 71.1) | 31.9* (6.5 to 58.7) |
| Metastatic phase of acute lymphoid leukemia | 2.9 (2.4 to 3.4) | -- | 1.3 (0.9 to 1.8) | 10.0 (-9.3 to 30.2) | 2.0 (-15.9 to 20.8) |
| Terminal phase of acute lymphoid leukemia | 0.6 (0.5 to 0.6) | -- | 0.3 (0.2 to 0.4) | 8.5 (-10.6 to 28.5) | 0.7 (-17.0 to 19.2) |
| Chronic lymphoid leukaemia | -- | -- | -- | -- | -- |

| | | | | | |
|---|---|---|---|---|---|
| Diagnosis and primary therapy phase of chronic lymphoid leukemia | -- | -- | -- | -- | -- |
| Controlled phase of chronic lymphoid leukemia | -- | -- | -- | -- | -- |
| Metastatic phase of chronic lymphoid leukemia | -- | -- | -- | -- | -- |
| Terminal phase of chronic lymphoid leukemia | -- | -- | -- | -- | -- |
| Acute myeloid leukaemia | 5.1 (4.2 to 6.0) | 4.1 (3.3 to 4.8) | 1.0 (0.7 to 1.3) | 6.8 (-15.0 to 31.0) | -1.0 (-21.2 to 21.5) |
| Diagnosis and primary therapy phase of acute myeloid leukemia | -- | 4.1 (3.3 to 4.8) | -- | -- | -- |
| Controlled phase of acute myeloid leukemia | 3.4 (2.8 to 4.0) | -- | 0.2 (0.1 to 0.3) | 5.8 (-16.2 to 29.5) | -1.9 (-22.3 to 20.1) |
| Metastatic phase of acute myeloid leukemia | 1.4 (1.2 to 1.7) | -- | 0.7 (0.4 to 0.9) | 7.0 (-14.9 to 31.3) | -0.8 (-21.1 to 21.8) |
| Terminal phase of acute myeloid leukemia | 0.3 (0.3 to 0.4) | -- | 0.2 (0.1 to 0.2) | 7.0 (-14.9 to 31.3) | -0.8 (-21.1 to 21.8) |
| Chronic myeloid leukaemia | -- | -- | -- | -- | -- |
| Diagnosis and primary therapy phase of chronic myeloid leukemia | -- | -- | -- | -- | -- |
| Controlled phase of chronic myeloid leukemia | -- | -- | -- | -- | -- |
| Metastatic phase of chronic myeloid leukemia | -- | -- | -- | -- | -- |
| Terminal phase of chronic myeloid leukemia | -- | -- | -- | -- | -- |
| Other leukaemia | 103.7 (85.5 to 120.7) | 15.9 (13.2 to 18.5) | 7.4 (4.9 to 10.1) | 13.8 (-1.3 to 32.0) | 5.6 (-8.5 to 22.4) |
| Metastatic phase of other leukemia | 5.3 (4.4 to 6.2) | -- | 2.4 (1.6 to 3.3) | 2.4 (-10.8 to 17.0) | -5.1 (-17.3 to 8.5) |
| Diagnosis and primary therapy phase of other leukemia | -- | 15.9 (13.2 to 18.5) | -- | -- | -- |
| Terminal phase of other leukemia | 0.4 (0.4 to 0.5) | -- | 0.2 (0.2 to 0.3) | -15.0* (-25.8 to -1.7) | -21.1* (-31.2 to -8.9) |
| Controlled phase of other leukemia | 98.0 (80.9 to 114.3) | -- | 4.7 (2.9 to 7.1) | 22.7* (4.9 to 45.1) | 13.8 (-2.7 to 34.6) |
| Other malignant cancers | 960.2 (690.6 to 1477.5) | 14.2 (12.4 to 15.7) | 53.9 (30.1 to 90.6) | 243.5* (139.0 to 417.5) | 218.6* (121.7 to 379.9) |
| Diagnosis and primary therapy phase of other malignant neoplasms | 17.6 (12.7 to 27.1) | 14.2 (12.4 to 15.7) | 5.0 (2.8 to 8.8) | 259.5* (137.3 to 457.7) | 233.5* (120.1 to 417.3) |
| Controlled phase of other malignant neoplasms | 934.8 (671.3 to 1440.3) | -- | 45.3 (23.6 to 77.2) | 275.7* (154.3 to 476.1) | 248.5* (135.8 to 434.3) |
| Metastatic phase of other malignant neoplasms | 6.8 (5.5 to 8.8) | -- | 3.0 (1.9 to 4.6) | 61.7* (29.8 to 111.1) | 50.0* (20.3 to 95.8) |
| Terminal phase of other malignant neoplasms | 1.0 (0.8 to 1.3) | -- | 0.5 (0.4 to 0.8) | 52.1* (24.6 to 94.2) | 41.0* (15.6 to 80.2) |
| Other neoplasms | 445.5 (417.3 to 475.3) | 286.2 (267.2 to 305.6) | 1.0 (0.6 to 1.5) | -15.4* (-21.3 to -9.4) | -21.5* (-27.0 to -16.0) |
| Myelodysplastic, myeloproliferative, and other hematopoietic neoplasms | 19.9 (15.7 to 24.7) | 2.9 (2.2 to 3.6) | 1.0 (0.6 to 1.5) | -15.4* (-21.3 to -9.4) | -21.5* (-27.0 to -16.0) |
| Benign and in situ intestinal neoplasms | 8.9 (8.3 to 9.7) | 5.1 (4.5 to 5.8) | -- | -- | -- |
| Benign and in situ cervical and uterine neoplasms | 0.7 (0.6 to 0.9) | 0.4 (0.3 to 0.6) | -- | -- | -- |
| Other benign and in situ neoplasms | 447.2 (422.2 to 474.7) | 277.7 (258.8 to 297.2) | -- | -- | -- |
| **Cardiovascular diseases** | **2544.1 (2147.2 to 3014.8)** | **473.6 (431.4 to 519.4)** | **210.7 (147.6 to 290.7)** | **-6.9* (-11.5 to -2.9)** | **-13.6* (-18.0 to -10.0)** |
| Rheumatic heart disease | 1376.0 (1292.0 to 1453.6) | 267.4 (253.0 to 280.9) | 66.3 (42.8 to 97.0) | 11.4* (8.0 to 15.3) | 3.4* (0.1 to 6.9) |
| Rheumatic heart disease, without heart failure | 1373.0 (1288.4 to 1450.6) | 267.4 (253.0 to 280.9) | 66.0 (42.6 to 96.6) | 11.5* (8.0 to 15.3) | 3.4* (0.2 to 7.0) |
| Heart failure due to rheumatic heart disease | 3.0 (1.7 to 4.7) | -- | 0.3 (0.1 to 0.5) | 6.1* (4.3 to 7.9) | -1.6 (-3.3 to 0.1) |
| Ischaemic heart disease | -- | -- | -- | -- | -- |
| Asymptomatic ischemic heart disease following myocardial infarction | -- | -- | -- | -- | -- |
| Angina due to ischemic heart disease | -- | -- | -- | -- | -- |
| Heart failure due to ischemic heart disease | -- | -- | -- | -- | -- |
| Myocardial infarction due to ischemic heart disease | -- | -- | -- | -- | -- |
| Stroke | 239.3 (194.6 to 290.0) | 38.8‡ (22.3 to 60.5) | 78.2 (52.7 to 106.7) | -26.8* (-32.3 to -21.6) | -32.1* (-37.3 to -27.3) |
| Ischaemic stroke | 331.5 (259.0 to 412.2) | 23.1 (8.9 to 42.2) | 48.1 (31.6 to 67.7) | -30.5* (-38.1 to -23.3) | -35.6* (-42.6 to -28.8) |
| Acute ischemic stroke | 1.9 (0.7 to 3.4) | 23.1 (8.9 to 42.2) | 0.5 (0.2 to 1.1) | 5.6 (-8.2 to 12.5) | -2.0 (-14.8 to 4.3) |
| Chronic ischemic stroke | 329.7 (256.9 to 408.7) | -- | 47.5 (31.3 to 67.1) | -30.8* (-38.6 to -23.4) | -35.8* (-43.1 to -29.0) |
| Intracerebral hemorrhage | 161.7 (126.6 to 203.4) | 10.4 (3.6 to 20.2) | 24.0 (15.6 to 34.0) | -23.4* (-31.3 to -15.2) | -29.0* (-36.3 to -21.4) |
| Acute intracerebral hemorrhage | 0.7 (0.2 to 1.3) | 10.4 (3.6 to 20.2) | 0.2 (0.1 to 0.4) | -5.4 (-23.7 to 3.0) | -12.2* (-29.2 to -4.5) |
| Chronic intracerebral hemorrhage | 161.0 (125.9 to 202.7) | -- | 23.8 (15.4 to 33.6) | -23.5* (-31.5 to -15.3) | -29.1* (-36.5 to -21.4) |
| Subarachnoid hemorrhage | 42.4 (31.1 to 56.5) | 5.2 (3.1 to 7.8) | 6.1 (3.9 to 9.0) | -3.2 (-8.9 to 1.1) | -10.2* (-15.5 to -6.2) |
| Acute subarachnoid hemorrhage | 0.3 (0.2 to 0.4) | 5.2 (3.1 to 7.8) | 0.1 (0.0 to 0.1) | 7.9 (-2.5 to 14.8) | 0.1 (-9.5 to 6.5) |
| Chronic subarachnoid hemorrhage | 42.1 (30.7 to 56.2) | -- | 6.0 (3.8 to 8.9) | -3.4 (-9.2 to 1.0) | -10.4* (-15.8 to -6.3) |
| Hypertensive heart disease | -- | -- | -- | -- | -- |
| Non-rheumatic valvular heart disease | -- | -- | -- | -- | -- |
| Non-rheumatic calcific aortic valve disease | -- | -- | -- | -- | -- |
| Heart failure due to calcific aortic valve disease | -- | -- | -- | -- | -- |
| Non-rheumatic calcific aortic valve disease | -- | -- | -- | -- | -- |
| Non-rheumatic degenerative mitral valve disease | -- | -- | -- | -- | -- |
| Heart failure due to degenerative mitral valve disease | -- | -- | -- | -- | -- |
| Non-rheumatic degenerative mitral valve disease | -- | -- | -- | -- | -- |
| Other non-rheumatic valve diseases | -- | -- | -- | -- | -- |
| Mild heart failure due to other non-rheumatic valve disease | -- | -- | -- | -- | -- |
| Moderate heart failure due to other non-rheumatic valve disease | -- | -- | -- | -- | -- |
| Severe heart failure due to other non-rheumatic valve disease | -- | -- | -- | -- | -- |

| | | | | | |
|---|---|---|---|---|---|
| Controlled, medically managed heart failure due to other non-rheumatic valve disease | -- | -- | -- | -- | -- |
| Cardiomyopathy and myocarditis | 7.4 (4.5 to 10.9) | 94.1 (66.8 to 130.3) | 11.7 (6.9 to 18.3) | 7.4* (4.8 to 9.8) | -0.4 (-2.8 to 1.8) |
| Myocarditis | 96.9 (68.7 to 132.8) | 94.1 (66.8 to 130.3) | 8.0 (4.8 to 12.5) | 5.7* (2.2 to 9.1) | -1.9 (-5.2 to 1.2) |
| Acute myocarditis | 22.9 (16.6 to 31.1) | 94.1 (66.8 to 130.3) | 1.2 (0.6 to 1.9) | 7.6* (6.7 to 8.5) | -0.2 (-1.0 to 0.6) |
| Heart failure due to myocarditis | 74.0 (47.4 to 109.1) | -- | 6.8 (3.9 to 11.1) | 5.4* (1.4 to 9.4) | -2.2 (-6.0 to 1.5) |
| Alcoholic cardiomyopathy | -- | -- | -- | -- | -- |
| Other cardiomyopathy | 39.6 (25.3 to 58.1) | -- | 3.7 (2.1 to 5.9) | 11.0* (9.9 to 12.1) | 3.0* (2.0 to 4.0) |
| Atrial fibrillation and flutter | -- | -- | -- | -- | -- |
| Asymptomatic atrial fibrillation and flutter | -- | -- | -- | -- | -- |
| Symptomatic atrial fibrillation and flutter | -- | -- | -- | -- | -- |
| Peripheral vascular disease | -- | -- | -- | -- | -- |
| Asymptomatic peripheral arterial disease | -- | -- | -- | -- | -- |
| Symptomatic claudication due to peripheral arterial disease | -- | -- | -- | -- | -- |
| Endocarditis | 5.3 (4.2 to 6.7) | 73.3 (56.7 to 94.1) | 0.3 (0.2 to 0.5) | 11.7* (5.9 to 16.3) | 3.6 (-1.8 to 7.8) |
| Acute endocarditis | 5.3 (4.2 to 6.7) | 73.3 (56.7 to 94.1) | 0.3 (0.2 to 0.5) | 11.7* (5.9 to 16.3) | 3.6 (-1.8 to 7.8) |
| Heart failure due to endocarditis | -- | -- | -- | -- | -- |
| Other cardiovascular and circulatory diseases | 987.8 (595.1 to 1518.7) | -- | 54.3 (29.3 to 91.1) | 11.4* (5.0 to 17.0) | 3.3 (-2.6 to 8.6) |
| Heart failure due to other cardiovascular diseases | 8.6 (5.4 to 12.5) | -- | 0.8 (0.4 to 1.3) | 11.1* (9.8 to 12.6) | 3.1* (1.8 to 4.4) |
| Other cardiovascular and circulatory disease episodes | 979.2 (590.2 to 1506.5) | -- | 53.5 (28.8 to 90.0) | 11.4* (4.9 to 17.1) | 3.3 (-2.7 to 8.6) |
| **Chronic respiratory diseases** | **36556.4 (25758.7 to 49436.1)** | **5134.0 (3270.8 to 8113.8)** | **1681.5 (1059.4 to 2615.2)** | **22.0* (12.0 to 32.0)** | **13.2* (3.9 to 22.4)** |
| Chronic obstructive pulmonary disease | 728.3 (558.6 to 859.2) | 97.8 (69.2 to 116.1) | 87.9 (64.4 to 109.1) | 5.6 (-3.8 to 15.0) | -2.0 (-10.7 to 6.7) |
| Chronic obstructive pulmonary disease with heart failure | -- | -- | -- | -- | -- |
| Chronic obstructive pulmonary disease without heart failure | 728.3 (558.6 to 859.2) | 97.8 (69.2 to 116.1) | 87.9 (64.4 to 109.1) | 5.6 (-3.8 to 15.0) | -2.0 (-10.7 to 6.7) |
| Pneumoconiosis | -- | -- | -- | -- | -- |
| Silicosis | -- | -- | -- | -- | -- |
| Heart failure due to silicosis | -- | -- | -- | -- | -- |
| Silicosis without heart failure | -- | -- | -- | -- | -- |
| Asbestosis | -- | -- | -- | -- | -- |
| Asbestosis without heart failure | -- | -- | -- | -- | -- |
| Heart failure due to asbestosis | -- | -- | -- | -- | -- |
| Coal workers pneumoconiosis | -- | -- | -- | -- | -- |
| Coal workers pneumoconiosis without heart failure | -- | -- | -- | -- | -- |
| Heart failure due to Coal workers pneumoconiosis | -- | -- | -- | -- | -- |
| Other pneumoconiosis | -- | -- | -- | -- | -- |
| Heart failure due to other pneumoconiosis | -- | -- | -- | -- | -- |
| Other pneumoconiosis without heart failure | -- | -- | -- | -- | -- |
| Asthma | 36867.0 (25578.2 to 50471.8) | 5034.8 (3165.3 to 8012.2) | 1480.3 (871.1 to 2384.0) | 23.7* (12.2 to 34.6) | 14.8* (4.1 to 24.9) |
| Asymptomatic asthma | 13389.4 (9242.4 to 18313.0) | 1825.6 (1128.2 to 2893.5) | -- | -- | -- |
| Symptomatic asthma | 23477.6 (16169.4 to 32660.9) | 3209.2 (2021.9 to 5169.5) | 1480.3 (871.1 to 2384.0) | 23.7* (12.2 to 34.6) | 14.8* (4.1 to 24.9) |
| Interstitial lung disease and pulmonary sarcoidosis | 0.6 (0.3 to 1.1) | 1.5 (0.8 to 2.4) | 0.1 (0.0 to 0.1) | 14.1* (11.1 to 17.4) | 5.8* (3.0 to 8.9) |
| Heart failure due to interstitial lung disease and pulmonary sarcoidosis | -- | -- | -- | -- | -- |
| Interstitial lung disease and pulmonary sarcoidosis without heart failure | 0.6 (0.3 to 1.1) | 1.5 (0.8 to 2.4) | 0.1 (0.0 to 0.1) | 14.1* (11.1 to 17.4) | 5.8* (3.0 to 8.9) |
| Other chronic respiratory diseases | -- | -- | 113.1 (82.9 to 140.3) | 14.7* (4.5 to 24.9) | 6.4 (-3.0 to 15.8) |
| **Digestive diseases** | **45006.1 (39369.6 to 50215.2)** | **6203.0 (5052.2 to 7488.9)** | **588.4 (382.9 to 819.8)** | **8.6* (5.8 to 11.6)** | **0.8 (-1.9 to 3.5)** |
| Cirrhosis and other chronic liver diseases | 38892.8 (33352.8 to 44058.6) | 82.7 (61.3 to 107.8) | 44.1 (30.8 to 62.1) | 13.3* (6.6 to 20.8) | 5.1 (-1.1 to 12.0) |
| Cirrhosis and other chronic liver diseases due to hepatitis B | 30370.6 (25020.2 to 35211.0) | 3.9 (2.7 to 5.3) | 2.6 (1.7 to 3.8) | -7.0 (-19.6 to 7.5) | -13.7* (-25.4 to -0.3) |
| Cirrhosis and other chronic liver diseases due to hepatitis B, decompensated | 14.8 (12.9 to 17.1) | 0.2 (0.1 to 0.2) | 2.6 (1.7 to 3.8) | -7.0 (-19.6 to 7.5) | -13.7* (-25.4 to -0.3) |
| Chronic hepatitis B without cirrhosis | 30258.3 (24907.9 to 35095.5) | -- | -- | -- | -- |
| Cirrhosis and other chronic liver diseases due to hepatitis B, compensated | 97.5 (79.5 to 117.8) | 3.7 (2.6 to 5.2) | -- | -- | -- |
| Cirrhosis and other chronic liver diseases due to hepatitis C | 6603.5 (5102.3 to 8397.3) | 3.3 (2.2 to 4.6) | 1.9 (1.2 to 2.8) | 10.5* (6.7 to 14.1) | 2.5 (-1.0 to 5.8) |
| Cirrhosis and other chronic liver diseases due to hepatitis C, decompensated | 10.9 (8.9 to 13.1) | 0.1 (0.1 to 0.2) | 1.9 (1.2 to 2.8) | 10.5* (6.7 to 14.1) | 2.5 (-1.0 to 5.8) |
| Chronic hepatitis C without cirrhosis | 6514.2 (5014.8 to 8317.0) | -- | -- | -- | -- |
| Cirrhosis and other chronic liver diseases due to hepatitis C, compensated | 78.5 (62.0 to 97.7) | 3.1 (2.1 to 4.4) | -- | -- | -- |
| Cirrhosis and other chronic liver diseases due to alcohol use | -- | -- | -- | -- | -- |
| Cirrhosis and other chronic liver diseases due to alcohol, decompensated | -- | -- | -- | -- | -- |
| Cirrhosis and other chronic liver diseases due to alcohol, compensated | -- | -- | -- | -- | -- |
| Cirrhosis due to NASH | -- | -- | -- | -- | -- |
| Non-alcoholic fatty liver disease (NAFLD) / Non-alcoholic steatohepatitis (NASH) | -- | -- | -- | -- | -- |
| Cirrhosis and other chronic liver diseases due to NASH | -- | -- | -- | -- | -- |
| Cirrhosis and other chronic liver diseases due to other causes | 1918.7 (1717.2 to 2144.8) | 75.6 (55.9 to 98.8) | 39.5 (27.6 to 55.5) | 15.1* (7.5 to 23.7) | 6.7 (-0.3 to 14.7) |

| | | | | | |
|---|---|---|---|---|---|
| Cirrhosis and other chronic liver diseases due to other cause, decompensated | 228.8 (217.0 to 241.0) | 3.5 (2.5 to 4.6) | 39.5 (27.6 to 55.5) | 15.1* (7.5 to 23.7) | 6.7 (-0.3 to 14.7) |
| Cirrhosis and other chronic liver diseases due to other cause, compensated | 1689.9 (1488.4 to 1918.1) | 72.0 (52.1 to 95.4) | -- | -- | -- |
| Upper digestive system diseases | 4852.1 (3305.6 to 6703.3) | 3140.2 (2165.7 to 4262.4) | 242.5 (140.0 to 376.0) | 5.1* (1.0 to 8.8) | -2.5 (-6.4 to 0.9) |
| Peptic ulcer disease | 73.3 (47.2 to 106.8) | 61.9 (41.7 to 92.6) | 4.4 (2.7 to 6.7) | 3.5 (-6.2 to 13.7) | -4.0 (-13.0 to 5.5) |
| Acute peptic ulcer disease | -- | -- | -- | 6.2* (3.1 to 9.2) | -1.5 (-4.3 to 1.2) |
| Chronic peptic ulcer disease | 73.2 (47.1 to 106.8) | 61.9 (41.7 to 92.6) | 4.4 (2.7 to 6.7) | 3.5 (-6.2 to 13.7) | -4.0 (-13.0 to 5.5) |
| Gastritis and duodenitis | 4930.2 (3319.5 to 6804.3) | 3078.3 (2099.8 to 4205.0) | 238.1 (137.0 to 372.2) | 5.2* (0.9 to 8.8) | -2.5 (-6.4 to 0.9) |
| Acute gastritis and duodenitis | 0.3 (0.1 to 0.6) | -- | 0.1 (0.0 to 0.2) | 7.4* (4.9 to 10.2) | -0.3 (-2.7 to 2.3) |
| Chronic gastritis and duodenitis | 4929.9 (3319.1 to 6804.0) | 3078.3 (2099.8 to 4205.0) | 238.0 (136.9 to 372.1) | 5.2* (0.9 to 8.8) | -2.5 (-6.4 to 0.9) |
| GERD | -- | -- | -- | -- | -- |
| Appendicitis | 53.6 (44.2 to 63.7) | 1515.8 (1232.0 to 1828.9) | 17.1 (10.8 to 24.6) | 9.1 (-2.5 to 21.6) | 1.1 (-9.6 to 12.8) |
| Paralytic ileus and intestinal obstruction | 6.7 (4.4 to 9.6) | 169.9 (111.7 to 250.8) | 2.2 (1.2 to 3.4) | 10.3* (7.1 to 13.3) | 2.3 (-0.7 to 5.0) |
| Inguinal, femoral, and abdominal hernia | 2164.0 (1590.7 to 2833.8) | 1143.2 (777.5 to 1583.7) | 221.1 (132.2 to 332.9) | 7.0* (1.7 to 12.6) | -0.8 (-5.7 to 4.4) |
| Inflammatory bowel disease | 27.8 (23.1 to 33.4) | 94.6 (77.7 to 112.8) | 4.5 (2.9 to 6.4) | -7.0* (-12.7 to -0.2) | -13.8* (-19.0 to -7.5) |
| Ulcerative colitis | 10.0 (7.7 to 12.8) | 44.8 (33.1 to 57.4) | 1.6 (1.0 to 2.3) | -13.9* (-20.2 to -6.9) | -20.1* (-26.0 to -13.7) |
| Crohn's disease | 17.7 (13.8 to 22.6) | 49.8 (38.3 to 63.0) | 2.9 (1.8 to 4.3) | -2.8 (-11.1 to 5.7) | -9.9* (-17.6 to -2.0) |
| Vascular intestinal disorders | 1.9 (1.0 to 3.2) | 9.3 (5.9 to 13.8) | 0.6 (0.3 to 1.1) | 10.0* (8.2 to 11.9) | 2.0* (0.3 to 3.8) |
| Gallbladder and biliary diseases | 155.2 (103.3 to 224.8) | 31.5 (17.6 to 50.7) | 0.2 (0.1 to 0.3) | 3.3 (-0.4 to 6.4) | -4.2* (-7.7 to -1.3) |
| Pancreatitis | 53.0 (42.7 to 65.4) | 15.8 (11.2 to 22.1) | 3.4 (1.6 to 5.8) | 10.5* (8.8 to 11.8) | 2.5* (0.9 to 3.7) |
| Acute pancreatitis | 0.8 (0.5 to 1.2) | 12.2 (7.8 to 18.4) | 0.3 (0.2 to 0.5) | 1.6* (0.2 to 2.8) | -5.8* (-7.1 to -4.6) |
| Chronic pancreatitis | 52.1 (41.7 to 64.5) | 3.6 (2.2 to 5.2) | 3.0 (1.4 to 5.5) | 11.6* (10.1 to 13.0) | 3.5* (2.1 to 4.8) |
| Other digestive diseases | -- | -- | 52.8 (34.1 to 74.8) | 35.4* (31.5 to 39.4) | 25.6* (22.0 to 29.3) |
| **Neurological disorders** | **93908.1 (68985.2 to 123239.4)** | **80927.9 (58434.6 to 108533.4)** | **1724.8 (1115.0 to 2511.3)** | **15.1* (5.2 to 27.6)** | **6.8 (-2.4 to 18.3)** |
| Alzheimer's disease and other dementias | -- | -- | -- | -- | -- |
| Parkinson's disease | -- | -- | -- | -- | -- |
| Epilepsy | 2556.3 (1805.7 to 3462.0) | 325.4 (206.3 to 483.5) | 878.0 (519.4 to 1371.1) | 15.1 (-0.4 to 32.6) | 6.7 (-7.6 to 23.0) |
| Multiple sclerosis | 0.4 (0.2 to 0.7) | 0.3 (0.1 to 0.4) | 0.1 (0.1 to 0.2) | 3.4 (-1.9 to 6.3) | -4.1* (-9.1 to -1.4) |
| Motor neuron disease | 11.1 (8.7 to 13.8) | 0.6 (0.3 to 0.9) | 2.4 (1.5 to 3.3) | 8.1* (7.1 to 9.2) | 0.3 (-0.7 to 1.3) |
| Headache disorders | 91711.8 (66805.8 to 121142.6) | 80601.7 (58042.8 to 108185.7) | 607.8 (356.6 to 948.3) | 7.5* (3.8 to 10.9) | -0.3 (-3.7 to 2.9) |
| Migraine | 15581.4 (12392.2 to 19080.5) | 9920.8 (7855.2 to 12190.4) | 470.9 (240.7 to 800.9) | 7.2* (2.7 to 11.7) | -0.5 (-4.7 to 3.6) |
| Medication overuse headache due to migraine | 169.4 (104.5 to 244.3) | -- | 19.5 (9.9 to 32.9) | 10.2 (-1.2 to 23.1) | 2.2 (-8.4 to 14.1) |
| Migraine | 15412.0 (12219.1 to 18888.4) | 9920.8 (7855.2 to 12190.4) | 451.4 (225.8 to 779.6) | 7.1* (2.3 to 11.9) | -0.6 (-5.1 to 3.7) |
| Tension-type headache | 78667.8 (52848.9 to 108921.5) | 70680.8 (48042.6 to 98739.2) | 136.9 (56.4 to 269.5) | 8.2* (6.0 to 10.5) | 0.4 (-1.7 to 2.5) |
| Medication overuse headache due to tension-type headache | 63.1 (34.4 to 101.5) | -- | 7.5 (3.3 to 14.6) | 10.0* (9.3 to 10.8) | 2.0* (1.4 to 2.7) |
| Tension-type headache | 78604.6 (52791.2 to 108866.8) | 70680.8 (48042.6 to 98739.2) | 129.4 (49.8 to 263.7) | 8.1* (5.8 to 10.5) | 0.3 (-1.9 to 2.5) |
| Other neurological disorders | 4.1 (2.0 to 7.2) | -- | 236.5 (140.0 to 368.0) | 41.3* (22.5 to 62.6) | 31.1* (13.6 to 50.8) |
| Other neurological disorders | -- | -- | 235.3 (139.2 to 367.5) | 41.5* (22.5 to 63.1) | 31.2* (13.6 to 51.3) |
| Guillain-Barré syndrome due to other neurological disorders | 4.1 (2.0 to 7.2) | -- | 1.2 (0.5 to 2.3) | 9.1* (6.6 to 11.3) | 1.2 (-1.1 to 3.2) |
| **Mental disorders** | **44152.3 (38029.2 to 50447.1)** | **14050.5 (11676.5 to 16751.4)** | **3312.4 (2287.2 to 4623.6)** | **6.5* (4.9 to 8.2)** | **-1.2 (-2.7 to 0.4)** |
| Schizophrenia | -- | -- | -- | -- | -- |
| Depressive disorders | 809.6 (565.0 to 1162.8) | 1575.2 (1038.0 to 2413.6) | 169.1 (95.9 to 279.6) | 14.6* (9.6 to 20.0) | 6.3* (1.7 to 11.3) |
| Major depressive disorder | 686.5 (429.4 to 1078.3) | 1429.7 (894.3 to 2289.6) | 147.0 (78.8 to 256.7) | 15.6* (10.4 to 22.1) | 7.2* (2.4 to 13.2) |
| Dysthymia | 219.6 (136.0 to 319.3) | 145.6 (87.9 to 217.2) | 22.0 (12.0 to 36.7) | 8.2 (-1.5 to 18.8) | 0.4 (-8.7 to 10.2) |
| Bipolar disorder | -- | -- | -- | -- | -- |
| Anxiety disorders | 9256.4 (7963.2 to 10660.8) | 4039.2 (3498.2 to 4617.7) | 924.3 (628.6 to 1297.5) | 7.6* (4.8 to 10.3) | -0.2 (-2.8 to 2.3) |
| Eating disorders | 15.7 (7.2 to 27.1) | 65.6 (39.2 to 101.8) | 10.7 (4.9 to 19.7) | 15.2* (9.9 to 21.0) | 6.8* (2.0 to 12.2) |
| Anorexia nervosa | 44.3 (20.3 to 76.8) | 39.1 (18.4 to 69.4) | 9.9 (4.2 to 18.7) | 15.0* (9.3 to 21.3) | 6.7* (1.4 to 12.5) |
| Bulimia nervosa | 3.8 (1.6 to 7.5) | 26.5 (12.1 to 51.0) | 0.8 (0.3 to 1.8) | 16.9* (15.0 to 18.7) | 8.4* (6.7 to 10.1) |
| Autism spectrum disorders | 3051.7 (2740.2 to 3364.0) | -- | 474.3 (320.5 to 660.0) | 8.7* (6.6 to 10.8) | 0.8 (-1.1 to 2.8) |
| Attention-deficit/hyperactivity disorder | 10988.6 (8920.1 to 13438.7) | 2090.4 (1700.6 to 2549.0) | 134.4 (81.1 to 218.0) | 9.8* (7.1 to 12.4) | 1.8 (-0.7 to 4.3) |
| Conduct disorder | 8884.6 (6023.1 to 12566.0) | 6280.0 (4146.4 to 8699.9) | 1076.4 (607.5 to 1763.1) | 9.9* (7.3 to 12.2) | 2.0 (-0.5 to 4.1) |
| Idiopathic developmental intellectual disability | 12641.0 (7756.2 to 17546.1) | -- | 523.2 (268.4 to 868.8) | -5.8* (-9.1 to -3.7) | -12.6* (-15.7 to -10.7) |
| Other mental disorders | -- | -- | -- | -- | -- |
| **Substance use disorders** | **4.9 (3.4 to 6.5)** | **--** | **1.2 (0.7 to 1.8)** | **-6.2 (-14.0 to 1.6)** | **-13.0* (-20.2 to -5.7)** |
| Alcohol use disorders | 29.2 (23.6 to 36.0) | -- | 1.2 (0.7 to 1.8) | -6.2 (-14.0 to 1.6) | -13.0* (-20.2 to -5.7) |
| Alcohol dependence | -- | -- | -- | -- | -- |
| Fetal alcohol syndrome | 29.2 (23.6 to 36.0) | -- | 1.2 (0.7 to 1.8) | -6.2 (-14.0 to 1.6) | -13.0* (-20.2 to -5.7) |
| Drug use disorders | -- | -- | -- | -- | -- |
| Opioid use disorders | -- | -- | -- | -- | -- |
| Cocaine use disorders | -- | -- | -- | -- | -- |

| | | | | | |
|---|---|---|---|---|---|
| Amphetamine use disorders | -- | -- | -- | -- | -- |
| Cannabis use disorders | -- | -- | -- | -- | -- |
| Other drug use disorders | -- | -- | -- | -- | -- |
| **Diabetes and kidney diseases** | **2128.2<br>(1628.6 to 2705.8)** | **443.1<br>(325.4 to 592.0)** | **99.8<br>(64.6 to 142.9)** | **17.5*<br>(9.7 to 26.1)** | **9.0*<br>(1.7 to 17.0)** |
| Diabetes mellitus | 487.7<br>(350.8 to 667.1) | 70.3<br>(51.9 to 91.2) | 23.4<br>(12.9 to 38.7) | 8.4*<br>(2.6 to 14.4) | 0.6<br>(-4.9 to 6.1) |
| Diabetes mellitus type 1 | 487.7<br>(350.8 to 667.1) | 70.3<br>(51.9 to 91.2) | 23.4<br>(12.9 to 38.7) | 8.4*<br>(2.6 to 14.4) | 0.6<br>(-4.9 to 6.1) |
| Uncomplicated diabetes mellitus type 1 | 487.6<br>(350.8 to 667.0) | 70.3<br>(51.9 to 91.2) | 23.4<br>(12.9 to 38.7) | 8.4*<br>(2.6 to 14.4) | 0.6<br>(-4.9 to 6.1) |
| Vision loss due to diabetes mellitus type 1 retinopathy | -- | -- | -- | -- | -- |
| Diabetic neuropathy due to diabetes mellitus type 1 | 0.1<br>(0.0 to 0.4) | -- | 0.0<br>(0.0 to 0.1) | 8.9*<br>(4.2 to 12.4) | 1.0<br>(-3.4 to 4.2) |
| Diabetes mellitus type 2 | -- | -- | -- | -- | -- |
| Uncomplicated diabetes mellitus type 2 | -- | -- | -- | -- | -- |
| Vision loss due to diabetes mellitus type 2 retinopathy | -- | -- | -- | -- | -- |
| Diabetic neuropathy due to diabetes mellitus type 2 | -- | -- | -- | -- | -- |
| Chronic kidney disease | 2030.1<br>(1463.1 to 2697.6) | 301.4<br>(185.5 to 454.4) | 76.1<br>(45.9 to 114.2) | 20.6*<br>(10.6 to 31.8) | 11.9*<br>(2.6 to 22.2) |
| Chronic kidney disease due to diabetes mellitus type 1 | 124.2<br>(84.7 to 170.6) | 19.9<br>(12.0 to 30.7) | 3.1<br>(1.8 to 4.8) | 18.8*<br>(6.8 to 32.6) | 10.2<br>(-1.0 to 23.0) |
| Albuminuria with preserved GFR due to type 1 diabetes mellitus | 9.3<br>(4.4 to 17.9) | -- | -- | -- | -- |
| End-stage chronic kidney disease due to diabetes mellitus type 1 | 0.1<br>(0.1 to 0.2) | -- | 0.1<br>(0.0 to 0.1) | 18.1*<br>(11.9 to 24.6) | 9.5*<br>(3.8 to 15.5) |
| Stage III chronic kidney disease due to diabetes mellitus type 1 | 111.6<br>(75.2 to 154.0) | 19.9<br>(12.0 to 30.7) | 1.5<br>(0.9 to 2.5) | 13.3<br>(-1.2 to 31.6) | 5.1<br>(-8.4 to 22.0) |
| Stage IV chronic kidney disease due to diabetes mellitus type 1 | 0.8<br>(0.4 to 1.6) | -- | 0.1<br>(0.0 to 0.2) | 31.1*<br>(13.6 to 55.8) | 21.6*<br>(5.3 to 44.5) |
| Stage V chronic kidney disease due to diabetes mellitus type 1 | 2.4<br>(1.4 to 4.0) | -- | 1.4<br>(0.7 to 2.4) | 24.5*<br>(13.4 to 36.3) | 15.5*<br>(5.2 to 26.4) |
| Chronic kidney disease due to diabetes mellitus type 2 | 36.7<br>(24.2 to 55.8) | 2.8<br>(1.7 to 4.2) | 0.6<br>(0.4 to 0.9) | 21.1*<br>(9.8 to 33.2) | 12.3*<br>(1.9 to 23.6) |
| Albuminuria with preserved GFR due to type 2 diabetes mellitus | 20.4<br>(10.0 to 39.6) | -- | -- | -- | -- |
| End-stage chronic kidney disease due to diabetes mellitus type 2 | 0.1<br>(0.0 to 0.1) | -- | 0.0<br>(0.0 to 0.1) | 18.0*<br>(11.8 to 24.3) | 9.5*<br>(3.7 to 15.3) |
| Stage III chronic kidney disease due to diabetes mellitus type 2 | 15.5<br>(10.6 to 21.4) | 2.8<br>(1.7 to 4.2) | 0.2<br>(0.1 to 0.4) | 15.2*<br>(0.6 to 31.3) | 6.9<br>(-6.7 to 21.8) |
| Stage IV chronic kidney disease due to diabetes mellitus type 2 | 0.2<br>(0.1 to 0.3) | -- | -- | 31.6*<br>(13.9 to 55.6) | 22.1*<br>(5.6 to 44.3) |
| Stage V chronic kidney disease due to diabetes mellitus type 2 | 0.5<br>(0.3 to 0.9) | -- | 0.3<br>(0.2 to 0.5) | 25.4*<br>(14.6 to 37.0) | 16.3*<br>(6.3 to 27.0) |
| Chronic kidney disease due to hypertension | 338.6<br>(236.1 to 465.7) | 70.1<br>(43.7 to 105.6) | 9.5<br>(5.1 to 15.7) | 24.4*<br>(13.5 to 36.0) | 15.4*<br>(5.3 to 26.1) |
| Albuminuria with preserved GFR due to hypertension | 1.1<br>(0.5 to 2.2) | -- | -- | -- | -- |
| End-stage chronic kidney disease due to hypertension | 0.2<br>(0.1 to 0.4) | -- | 0.1<br>(0.0 to 0.2) | 18.0*<br>(12.0 to 23.7) | 9.5*<br>(3.9 to 14.7) |
| Stage III chronic kidney disease due to hypertension | 318.9<br>(221.7 to 438.8) | 70.1<br>(43.7 to 105.6) | 0.8<br>(0.4 to 1.2) | 15.6*<br>(1.2 to 32.6) | 7.2<br>(-6.1 to 23.0) |
| Stage IV chronic kidney disease due to hypertension | 4.7<br>(2.1 to 8.6) | -- | 0.6<br>(0.3 to 1.2) | 31.1*<br>(13.4 to 55.3) | 21.6*<br>(5.2 to 44.1) |
| Stage V chronic kidney disease due to hypertension | 13.7<br>(7.9 to 21.8) | -- | 8.0<br>(4.1 to 13.6) | 24.9*<br>(14.2 to 36.0) | 15.8*<br>(5.9 to 26.1) |
| Chronic kidney disease due to glomerulonephritis | 165.5<br>(116.0 to 224.5) | 9.1<br>(5.6 to 13.7) | 21.0<br>(12.3 to 31.8) | 21.3*<br>(11.1 to 32.8) | 12.5*<br>(3.0 to 23.2) |
| Albuminuria with preserved GFR due to glomerulonephritis | 4.5<br>(2.1 to 8.8) | -- | -- | -- | -- |
| End-stage chronic kidney disease due to glomerulonephritis | 6.2<br>(4.0 to 9.1) | -- | 2.5<br>(1.3 to 4.0) | 18.4*<br>(12.4 to 24.2) | 9.8*<br>(4.3 to 15.2) |
| Stage III chronic kidney disease due to glomerulonephritis | 126.9<br>(85.6 to 174.5) | 9.1<br>(5.6 to 13.7) | 5.4<br>(3.1 to 8.8) | 13.7<br>(-3.1 to 31.9) | 5.4<br>(-10.1 to 22.4) |
| Stage IV chronic kidney disease due to glomerulonephritis | 7.1<br>(3.2 to 13.4) | -- | 0.9<br>(0.4 to 1.8) | 31.2*<br>(13.7 to 55.0) | 21.7*<br>(5.5 to 43.7) |
| Stage V chronic kidney disease due to glomerulonephritis | 20.8<br>(12.1 to 33.1) | -- | 12.2<br>(6.3 to 20.3) | 25.0*<br>(14.2 to 36.4) | 15.9*<br>(5.9 to 26.5) |
| Chronic kidney disease due to other and unspecified causes | 1365.0<br>(994.1 to 1802.5) | 199.5<br>(123.8 to 298.4) | 41.9<br>(25.8 to 62.2) | 19.6*<br>(9.5 to 31.0) | 10.9*<br>(1.5 to 21.5) |
| Albuminuria with preserved GFR due to other and unspecified causes | 181.9<br>(88.5 to 352.6) | -- | -- | -- | -- |
| End-stage chronic kidney disease due to other causes | 14.4<br>(11.1 to 17.8) | -- | 5.7<br>(3.7 to 8.0) | 16.3*<br>(10.4 to 22.0) | 7.9*<br>(2.4 to 13.1) |
| Stage III chronic kidney disease due to other causes | 1115.3<br>(769.3 to 1530.0) | 199.5<br>(123.8 to 298.4) | 11.1<br>(6.2 to 18.3) | 8.5<br>(-8.8 to 28.8) | 0.7<br>(-15.4 to 19.5) |
| Stage IV chronic kidney disease due to other causes | 13.6<br>(6.2 to 25.0) | -- | 1.8<br>(0.7 to 3.5) | 31.7*<br>(14.2 to 55.5) | 22.1*<br>(5.9 to 44.2) |
| Stage V chronic kidney disease due to other causes | 39.9<br>(23.3 to 62.4) | -- | 23.3<br>(12.1 to 38.7) | 25.7*<br>(15.3 to 37.0) | 16.6*<br>(6.9 to 27.0) |
| Acute glomerulonephritis | 4.8<br>(3.3 to 7.0) | 71.4<br>(49.0 to 103.6) | 0.2<br>(0.1 to 0.4) | 5.5*<br>(2.5 to 8.3) | -2.2<br>(-5.0 to 0.5) |
| **Skin and subcutaneous diseases** | **180361.0<br>(162793.1 to 199393.2)** | **332090.4<br>(285372.9 to 386001.1)** | **4929.6<br>(3065.5 to 7436.0)** | **7.8*<br>(6.3 to 9.2)** | **-0.0<br>(-1.4 to 1.3)** |
| Dermatitis | 41405.6<br>(37276.8 to 45994.4) | 8240.0<br>(6777.2 to 10022.4) | 1820.0<br>(979.9 to 3069.1) | 11.5*<br>(9.9 to 13.2) | 3.4*<br>(2.0 to 5.0) |
| Atopic dermatitis | 39665.0<br>(35441.9 to 44218.1) | 3312.5<br>(2629.8 to 4037.4) | 1768.1<br>(926.3 to 3001.8) | 11.7*<br>(10.0 to 13.4) | 3.6*<br>(2.1 to 5.1) |
| Contact dermatitis | 1850.0<br>(1201.5 to 2694.7) | 3484.9<br>(2191.5 to 4915.3) | 47.8<br>(24.9 to 80.6) | 7.6*<br>(1.9 to 13.4) | -0.2<br>(-5.5 to 5.2) |
| Seborrhoeic dermatitis | 294.8<br>(231.3 to 367.6) | 1442.6<br>(1129.4 to 1781.8) | 4.1<br>(2.2 to 7.0) | 3.0<br>(-3.4 to 9.8) | -4.4<br>(-10.4 to 1.8) |
| Psoriasis | 1948.2<br>(1771.2 to 2135.4) | 375.8<br>(331.8 to 423.8) | 177.6<br>(124.4 to 240.7) | 12.4*<br>(7.6 to 17.3) | 4.3<br>(-0.2 to 8.8) |
| Bacterial skin diseases | 1748.6<br>(1594.3 to 1915.2) | 34958.2<br>(32424.6 to 37738.4) | 17.1<br>(9.5 to 29.7) | 14.8*<br>(11.1 to 18.2) | 6.5*<br>(3.1 to 9.6) |
| Cellulitis | 123.2<br>(100.5 to 152.3) | 2540.4<br>(2083.0 to 3044.2) | 7.3<br>(4.6 to 10.9) | 7.2*<br>(4.2 to 10.2) | -0.6<br>(-3.4 to 2.2) |
| Pyoderma | 1713.9<br>(1565.0 to 1883.8) | 32417.8<br>(29874.9 to 35225.6) | 9.8<br>(3.9 to 20.8) | 21.2*<br>(17.6 to 24.8) | 12.4*<br>(9.0 to 15.8) |
| Impetigo | 1380.9<br>(1239.0 to 1533.8) | 24811.9<br>(22532.4 to 27108.6) | 7.9<br>(3.2 to 17.2) | 22.9*<br>(18.5 to 27.1) | 13.9*<br>(9.9 to 17.8) |
| Abscess and other bacterial skin diseases | 332.9<br>(289.2 to 382.3) | 7605.9<br>(6571.8 to 8680.7) | 1.9<br>(0.8 to 4.1) | 14.6*<br>(7.5 to 21.9) | 6.3<br>(-0.3 to 13.1) |
| Scabies | 17335.7<br>(12098.3 to 24169.7) | 52310.4<br>(36247.2 to 73070.6) | 453.1<br>(227.7 to 817.8) | 4.9*<br>(3.6 to 6.1) | -2.7*<br>(-3.9 to -1.6) |
| Fungal skin diseases | 73836.4<br>(53971.6 to 97073.2) | 164601.3<br>(124055.5 to 212745.3) | 423.5<br>(162.5 to 925.7) | -6.2*<br>(-9.2 to -2.6) | -13.0*<br>(-15.8 to -9.7) |
| Tinea capitis | 46154.7<br>(30998.8 to 67324.6) | 82580.7<br>(54761.9 to 114674.1) | 265.1<br>(96.7 to 602.1) | -14.2*<br>(-16.4 to -11.6) | -20.4*<br>(-22.4 to -18.0) |
| Other fungal skin diseases | 27681.7<br>(18432.7 to 39380.4) | 82020.6<br>(55678.6 to 118792.6) | 158.4<br>(57.0 to 370.9) | 11.1*<br>(10.2 to 12.2) | 3.1*<br>(2.2 to 4.0) |

| | | | | | |
|---|---|---|---|---|---|
| Viral skin diseases | 37022.5 (33804.0 to 40508.1) | 31555.6 (28768.3 to 34404.1) | 1152.8 (732.1 to 1705.3) | 7.4* (6.5 to 8.3) | -0.4 (-1.2 to 0.4) |
| Viral warts | 6818.1 (6071.2 to 7763.6) | 3232.6 (2909.2 to 3604.4) | 212.7 (134.0 to 326.0) | 4.0* (2.3 to 5.7) | -3.6* (-5.2 to -2.0) |
| Molluscum contagiosum | 30204.4 (27085.3 to 33677.0) | 28323.0 (25601.9 to 31113.0) | 940.1 (592.7 to 1403.7) | 8.2* (7.1 to 9.2) | 0.4 (-0.6 to 1.3) |
| Acne vulgaris | 1128.5 (756.1 to 1501.2) | 1497.5 (855.2 to 2057.1) | 24.7 (13.5 to 42.6) | 21.5* (18.0 to 25.2) | 12.7* (9.4 to 16.1) |
| Alopecia areata | 416.9 (371.8 to 462.8) | 716.4 (630.6 to 803.5) | 14.0 (8.7 to 21.2) | 7.5* (0.2 to 14.8) | -0.3 (-7.0 to 6.5) |
| Pruritus | 3519.3 (2451.3 to 4964.3) | 2642.2 (1863.0 to 3663.7) | 38.3 (16.7 to 78.1) | 9.1* (6.8 to 11.4) | 1.2 (-0.9 to 3.3) |
| Urticaria | 11695.0 (8036.4 to 15529.6) | 19161.5 (13415.7 to 25551.6) | 714.3 (396.8 to 1095.4) | 8.3* (6.7 to 10.0) | 0.4 (-1.0 to 2.1) |
| Decubitus ulcer | 16.5 (11.0 to 24.1) | 58.3 (39.0 to 85.8) | 2.9 (1.6 to 4.7) | 9.0* (7.8 to 10.1) | 1.1 (-0.0 to 2.1) |
| Other skin and subcutaneous diseases | 16189.4 (14485.9 to 17956.0) | 15973.3 (14235.2 to 17781.2) | 91.4 (42.1 to 174.7) | 13.7* (12.2 to 15.3) | 5.5* (4.0 to 6.9) |
| **Sense organ diseases** | **30645.9 (26321.9 to 35598.9)** | **--** | **1169.0 (781.3 to 1681.2)** | **7.8* (5.2 to 10.4)** | **0.0 (-2.4 to 2.4)** |
| Blindness and vision impairment | 18337.9 (14622.5 to 22858.8) | -- | 564.2 (334.6 to 901.9) | 8.0* (6.0 to 9.9) | 0.1 (-1.7 to 2.0) |
| Glaucoma | -- | -- | -- | -- | -- |
| Cataract | -- | -- | -- | -- | -- |
| Age-related macular degeneration | -- | -- | -- | -- | -- |
| Refraction disorders | 12583.7 (8998.9 to 16893.0) | -- | 470.8 (278.8 to 749.2) | 8.6* (6.4 to 10.7) | 0.7 (-1.4 to 2.6) |
| Near vision loss | 5344.0 (4914.4 to 5832.3) | -- | 55.9 (25.4 to 104.6) | 4.5* (2.6 to 6.3) | -3.1* (-4.9 to -1.4) |
| Other vision loss | 586.7 (396.6 to 839.3) | -- | 37.5 (22.9 to 58.4) | 5.5 (-1.9 to 12.5) | -2.1 (-9.0 to 4.4) |
| Age-related and other hearing loss | 9676.1 (7505.5 to 12130.8) | -- | 542.7 (358.9 to 768.7) | 7.6* (2.5 to 12.5) | -0.2 (-5.0 to 4.4) |
| Other sense organ diseases | 3158.8 (2872.8 to 3451.2) | -- | 62.0 (37.7 to 93.1) | 9.0* (4.6 to 13.3) | 1.1 (-3.0 to 5.1) |
| Chronic other sense organ diseases | 1361.2 (1187.3 to 1551.1) | -- | 36.8 (21.7 to 55.7) | 9.0* (2.4 to 15.4) | 1.1 (-5.0 to 7.0) |
| Acute other sense organ diseases | 1797.7 (1593.3 to 2021.2) | -- | 25.2 (14.9 to 39.2) | 9.1* (4.0 to 14.7) | 1.2 (-3.5 to 6.4) |
| **Musculoskeletal disorders** | **3073.1 (2030.8 to 4234.6)** | **2933.8 (1945.8 to 4077.4)** | **357.2 (207.3 to 560.8)** | **7.7* (4.5 to 10.9)** | **-0.1 (-3.0 to 2.9)** |
| Rheumatoid arthritis | 23.3 (15.0 to 32.6) | 14.0 (9.0 to 19.5) | 3.4 (1.9 to 5.4) | 10.7* (8.1 to 13.3) | 2.7* (0.2 to 5.1) |
| Osteoarthritis | -- | -- | -- | -- | -- |
| Osteoarthritis of the hip | -- | -- | -- | -- | -- |
| Osteoarthritis of the knee | -- | -- | -- | -- | -- |
| Low back pain | 2916.6 (1823.1 to 4119.5) | 2665.3 (1682.0 to 3784.1) | 316.9 (178.0 to 514.9) | 7.9* (4.3 to 11.6) | 0.1 (-3.3 to 3.5) |
| Low back pain with leg pain | 276.3 (164.8 to 410.3) | 252.3 (154.8 to 374.3) | 43.0 (22.8 to 72.0) | 7.8* (2.2 to 14.6) | 0.0 (-5.2 to 6.3) |
| Low back pain without leg pain | 2640.3 (1639.8 to 3733.0) | 2413.0 (1516.3 to 3425.7) | 273.9 (154.1 to 436.9) | 7.9* (4.0 to 12.2) | 0.1 (-3.6 to 4.1) |
| Neck pain | 345.1 (203.4 to 529.4) | 254.6 (147.2 to 396.3) | 36.9 (18.7 to 61.3) | 5.8* (1.6 to 10.3) | -1.9 (-5.8 to 2.3) |
| Gout | -- | -- | -- | -- | -- |
| Other musculoskeletal disorders | -- | -- | -- | -- | -- |
| **Other non-communicable diseases** | **428066.0 (389326.5 to 467290.4)** | **796520.2 (601785.4 to 986832.0)** | **2426.0 (1716.3 to 3295.3)** | **0.6 (-2.3 to 4.0)** | **-6.7* (-9.4 to -3.5)** |
| Congenital anomalies | 9751.2 (9231.2 to 10296.7) | -- | 1152.3 (832.2 to 1536.8) | 9.1* (6.4 to 12.0) | 1.1 (-1.3 to 3.9) |
| Neural tube defects | 297.0 (264.7 to 331.1) | -- | 89.7 (62.2 to 121.7) | 14.9* (8.2 to 21.2) | 6.6* (0.3 to 12.5) |
| Severe motor and cognitive impairment due to anencephaly | -- | -- | -- | -- | -- |
| Encephalocoele due to neural tube defects | 23.0 (18.1 to 28.1) | -- | 4.0 (2.6 to 5.6) | 7.2* (4.3 to 10.3) | -0.6 (-3.3 to 2.3) |
| Spina bifida due to neural tube defects | 274.1 (244.4 to 305.9) | -- | 85.7 (58.9 to 115.7) | 15.3* (8.3 to 22.0) | 6.9* (0.5 to 13.1) |
| Congenital heart anomalies | 2147.7 (1938.2 to 2389.0) | -- | 107.1 (48.9 to 184.1) | 8.3* (5.7 to 11.1) | 0.5 (-2.0 to 3.0) |
| Critical malformations of great vessels, congenital valvular heart disease and patent ductus arteriosus | 355.0 (297.6 to 418.2) | -- | 22.5 (10.7 to 38.0) | 8.1* (2.7 to 14.0) | 0.3 (-4.8 to 5.7) |
| Other congenital heart anomalies | 409.0 (366.4 to 455.8) | -- | 24.3 (10.0 to 42.9) | 8.6* (2.7 to 14.5) | 0.7 (-4.8 to 6.2) |
| Severe congenital heart anomalies excluding single ventricle heart defects | 420.0 (361.9 to 483.1) | -- | 25.6 (11.8 to 43.2) | 8.2* (2.4 to 14.9) | 0.3 (-5.0 to 6.6) |
| Single ventricle and single ventricle pathway congenital heart anomalies | 66.6 (54.2 to 81.5) | -- | 4.3 (2.1 to 7.2) | 9.8 (-1.0 to 21.0) | 1.8 (-8.2 to 12.2) |
| Ventricular septal defect and atrial septal defect | 897.0 (739.3 to 1067.8) | -- | 30.4 (13.5 to 53.7) | 8.3* (3.5 to 13.3) | 0.4 (-4.0 to 5.1) |
| Orofacial clefts | 951.4 (870.7 to 1031.6) | -- | 30.7 (20.1 to 44.6) | 6.7 (-1.9 to 15.7) | -1.0 (-9.1 to 7.3) |
| Down's syndrome | 345.6 (302.2 to 393.6) | -- | 29.7 (20.1 to 41.5) | 10.9* (8.0 to 13.9) | 2.9* (0.2 to 5.7) |
| Turner syndrome | 77.5 (67.1 to 89.2) | -- | 1.1 (0.4 to 1.9) | 8.8* (5.7 to 11.9) | 0.9 (-2.0 to 3.8) |
| Klinefelter syndrome | 118.2 (101.4 to 136.2) | -- | 0.3 (0.1 to 0.6) | 4.9 (-3.1 to 14.0) | -2.7 (-10.1 to 5.7) |
| Other chromosomal abnormalities | 742.7 (592.9 to 910.1) | -- | 63.3 (41.8 to 89.8) | 17.8* (12.9 to 23.3) | 9.2* (4.7 to 14.4) |
| Congenital musculoskeletal and limb anomalies | 2169.8 (1928.2 to 2487.7) | -- | 331.4 (219.6 to 459.8) | 9.2* (6.4 to 12.2) | 1.2 (-1.3 to 4.0) |
| Polydactyly and syndactyly | 14.7 (11.8 to 17.9) | -- | 0.2 (0.1 to 0.3) | 9.3* (7.1 to 11.7) | 1.4 (-0.7 to 3.6) |
| Congenital limb deficiency | 1552.7 (1377.2 to 1783.8) | -- | 238.3 (158.0 to 331.2) | 9.1* (5.6 to 12.6) | 1.2 (-2.1 to 4.4) |
| Other congenital musculoskeletal anomalies | 602.4 (535.4 to 690.6) | -- | 92.9 (60.6 to 130.3) | 9.2* (4.7 to 14.3) | 1.3 (-2.9 to 6.0) |
| Urogenital congenital anomalies | 880.3 (757.7 to 1019.2) | -- | 20.5 (11.8 to 34.4) | 10.3* (7.3 to 13.3) | 2.3 (-0.5 to 5.1) |
| Digestive congenital anomalies | 1408.8 (1266.8 to 1580.4) | -- | 64.1 (42.0 to 92.3) | 8.2* (3.0 to 14.3) | 0.3 (-4.5 to 6.0) |
| Congenital diaphragmatic hernia | 51.1 (44.4 to 58.1) | -- | 1.0 (0.6 to 1.7) | 5.1* (4.0 to 6.1) | -2.6* (-3.6 to -1.6) |
| Congenital atresia and/or stenosis of the digestive tract | 9.3 (6.6 to 12.8) | -- | 0.7 (0.5 to 1.2) | 16.7* (12.8 to 20.7) | 8.2* (4.6 to 12.0) |
| Congenital malformations of the abdominal wall after treatment | 205.4 (180.3 to 235.6) | -- | 8.8 (5.7 to 12.9) | 6.7* (5.7 to 7.7) | -1.0* (-2.0 to -0.1) |
| Other congenital malformations of the digestive tract | 1143.0 (998.6 to 1313.2) | -- | 53.5 (34.6 to 77.2) | 8.3* (2.1 to 15.7) | 0.5 (-5.3 to 7.3) |
| Other congenital anomalies | 2442.6 (2123.4 to 2824.6) | -- | 414.3 (283.0 to 571.9) | 6.9* (0.6 to 14.0) | -0.9 (-6.7 to 5.7) |

| | | | | | |
|---|---|---|---|---|---|
| Other congenital birth defects | -- | -- | 77.6 (51.0 to 109.4) | 15.8* (12.3 to 19.6) | 7.4* (4.2 to 10.9) |
| Hearing loss due to other congenital anomalies | 2442.6 (2123.4 to 2824.6) | -- | 336.7 (218.8 to 479.3) | 5.0 (-2.3 to 13.6) | -2.6 (-9.4 to 5.4) |
| Urinary diseases and male infertility | 132.4 (93.5 to 183.4) | 9002.1 (6585.5 to 12117.5) | 11.2 (6.5 to 18.3) | -5.5 (-12.2 to 1.6) | -12.3* (-18.6 to -5.8) |
| Urinary tract infections | 166.8 (119.0 to 229.1) | 8540.0 (6117.5 to 11710.5) | 5.7 (3.0 to 9.7) | 6.4 (-2.9 to 16.1) | -1.3 (-10.0 to 7.7) |
| Urolithiasis | 17.0 (11.5 to 24.0) | 462.1 (303.2 to 649.0) | 1.3 (0.8 to 2.1) | 8.4* (7.5 to 9.4) | 0.5 (-0.3 to 1.5) |
| Benign prostatic hyperplasia | -- | -- | -- | -- | -- |
| Male infertility | -- | -- | -- | -- | -- |
| Other urinary diseases | -- | -- | 4.2 (2.4 to 6.9) | -20.6* (-26.3 to -14.7) | -26.4* (-31.6 to -20.9) |
| Gynecological diseases | -- | -- | -- | -- | -- |
| Uterine fibroids | -- | -- | -- | -- | -- |
| Polycystic ovarian syndrome | -- | -- | -- | -- | -- |
| Female infertility | -- | -- | -- | -- | -- |
| Endometriosis | -- | -- | -- | -- | -- |
| Genital prolapse | -- | -- | -- | -- | -- |
| Premenstrual syndrome | -- | -- | -- | -- | -- |
| Other gynecological diseases | -- | -- | -- | -- | -- |
| Other gynecological diseases | -- | -- | -- | -- | -- |
| Other gynecological diseases with anemia | -- | -- | -- | -- | -- |
| Hemoglobinopathies and hemolytic anaemias | 178538.0 (174177.0 to 183209.5) | -- | 650.2 (430.2 to 923.6) | -11.0* (-17.5 to -3.5) | -17.4* (-23.5 to -10.5) |
| Thalassemias | 74.0 (66.8 to 81.9) | -- | 3.4 (2.2 to 5.0) | -25.6* (-35.5 to -15.4) | -31.0* (-40.1 to -21.5) |
| Thalassaemias trait | 23166.1 (22312.6 to 24062.9) | -- | 200.7 (132.8 to 288.9) | -18.4* (-26.4 to -8.6) | -24.3* (-31.8 to -15.2) |
| Sickle cell disorders | 694.9 (615.2 to 785.4) | -- | 58.8 (39.5 to 86.0) | 13.2* (1.3 to 26.5) | 5.0 (-6.1 to 17.4) |
| Sickle cell trait | 50437.2 (45865.6 to 55560.1) | -- | 211.2 (139.9 to 299.5) | -7.3 (-16.0 to 2.3) | -14.0* (-22.1 to -5.1) |
| G6PD deficiency | 36360.1 (34831.4 to 37975.5) | -- | 3.0 (2.0 to 4.3) | -2.4 (-20.0 to 20.7) | -9.5 (-25.8 to 11.9) |
| G6PD trait | 85447.0 (83980.5 to 86915.9) | -- | -- | 2.0 (-7.5 to 13.0) | -5.4 (-14.2 to 4.8) |
| Other hemoglobinopathies and hemolytic anaemias | 4043.1 (3770.4 to 4343.1) | -- | 173.1 (112.1 to 248.8) | -12.2* (-20.4 to -2.3) | -18.6* (-26.2 to -9.4) |
| Endocrine, metabolic, blood, and immune disorders | 6573.2 (6092.6 to 7097.7) | -- | 273.6 (182.1 to 388.5) | -7.0 (-13.7 to 1.2) | -13.7* (-19.9 to -6.2) |
| Anemia due to endocrine, metabolic, blood, and immune disorders | 5126.8 (4780.4 to 5522.4) | -- | 218.7 (142.7 to 317.5) | -11.0* (-19.3 to -1.2) | -17.5* (-25.1 to -8.3) |
| Endocrine, metabolic, blood, and immune disorders | 1445.0 (1137.6 to 1793.6) | -- | 54.8 (33.7 to 80.9) | 13.6* (7.3 to 19.6) | 5.4 (-0.5 to 10.9) |
| Heart failure due to endocrine, metabolic, blood, and immune disorders | 1.4 (0.9 to 1.9) | -- | 0.1 (0.1 to 0.2) | 5.4* (4.3 to 6.4) | -2.3* (-3.3 to -1.3) |
| Oral disorders | 330442.7 (276375.6 to 385006.0) | 787518.1 (594291.7 to 977725.8) | 338.7 (196.9 to 548.2) | 6.4* (5.1 to 7.8) | -1.4 (-2.6 to 0.0) |
| Caries of deciduous teeth | 291833.7 (235726.1 to 356694.9) | 672304.9 (492690.7 to 854777.7) | 76.4 (33.0 to 154.2) | 5.4* (3.1 to 7.2) | -2.2* (-4.4 to -0.5) |
| Caries of permanent teeth | 71785.7 (44931.9 to 98825.4) | 115213.2 (65248.9 to 166887.5) | 49.6 (20.0 to 97.8) | 1.1 (-2.5 to 3.8) | -6.2* (-9.5 to -3.7) |
| Periodontal disease | -- | -- | -- | -- | -- |
| Edentulism and severe tooth loss | -- | -- | -- | -- | -- |
| Other oral disorders | 7180.3 (6187.4 to 8209.4) | -- | 212.7 (130.4 to 328.7) | 8.0* (6.2 to 10.0) | 0.2 (-1.5 to 2.0) |
| **Injuries** | **22765.6 (20476.7 to 25488.1)** | **33438.3 (28167.4 to 39812.0)** | **1008.5 (743.3 to 1326.1)** | **14.7* (12.0 to 17.3)** | **6.4* (3.9 to 8.8)** |
| **Transport injuries** | **2542.0 (2070.1 to 3120.7)** | **3265.8 (2300.9 to 4459.7)** | **191.6 (130.4 to 260.1)** | **7.8* (4.8 to 11.0)** | **0.0 (-2.8 to 3.0)** |
| Road injuries | 972.7 (812.1 to 1140.9) | 2284.7 (1478.2 to 3328.0) | 73.3 (50.3 to 101.3) | 2.7 (-1.5 to 7.6) | -4.7* (-8.7 to -0.2) |
| Pedestrian road injuries | 294.8 (214.8 to 398.5) | 523.8 (287.8 to 861.7) | 22.6 (14.6 to 33.4) | -9.7* (-14.0 to -5.0) | -16.3* (-20.2 to -11.9) |
| Cyclist road injuries | 182.2 (133.4 to 245.9) | 593.5 (311.6 to 959.3) | 12.9 (8.1 to 18.4) | 1.8 (-3.1 to 8.0) | -5.6 (-10.1 to 0.2) |
| Motorcyclist road injuries | 222.1 (166.1 to 303.2) | 322.7 (171.3 to 540.2) | 16.7 (10.8 to 24.8) | 11.1* (6.6 to 16.0) | 3.0 (-1.2 to 7.6) |
| Motor vehicle road injuries | 178.5 (131.3 to 245.9) | 547.5 (315.9 to 867.3) | 15.0 (9.7 to 22.6) | 12.6* (6.6 to 20.1) | 4.5 (-1.1 to 11.4) |
| Other road injuries | 95.1 (63.5 to 132.7) | 297.1 (147.6 to 525.8) | 6.2 (3.9 to 9.2) | 14.6* (8.8 to 21.1) | 6.3* (0.9 to 12.3) |
| Other transport injuries | 1569.3 (1131.9 to 2101.4) | 981.0 (548.3 to 1529.1) | 118.3 (78.6 to 165.1) | 11.3* (8.0 to 14.6) | 3.2* (0.1 to 6.3) |
| **Unintentional injuries** | **14230.7 (12584.7 to 16305.9)** | **28140.2 (22957.9 to 34181.7)** | **680.5 (491.1 to 917.7)** | **15.0* (11.7 to 17.8)** | **6.7* (3.6 to 9.3)** |
| Falls | 5515.0 (4080.9 to 7271.5) | 11573.9 (7587.3 to 17137.0) | 276.2 (189.0 to 384.4) | 19.2* (16.3 to 22.0) | 10.6* (7.9 to 13.2) |
| Drowning | 42.5 (31.3 to 55.2) | 35.4 (26.0 to 47.8) | 3.4 (2.2 to 4.8) | -19.4* (-23.6 to -15.7) | -25.3* (-29.1 to -21.9) |
| Fire, heat, and hot substances | 1548.8 (1143.3 to 2235.7) | 963.9 (579.7 to 1429.3) | 81.6 (54.4 to 114.1) | 9.7* (3.9 to 14.5) | 1.7 (-3.6 to 6.2) |
| Poisonings | 176.1 (126.7 to 228.0) | 625.5 (356.0 to 1009.0) | 22.6 (13.9 to 34.6) | 28.0* (22.7 to 33.7) | 18.7* (13.8 to 24.0) |
| Poisoning by carbon monoxide | 24.9 (17.9 to 35.5) | 107.6 (45.6 to 203.4) | 3.1 (1.9 to 5.0) | 26.9* (20.2 to 33.9) | 17.7* (11.5 to 24.1) |
| Poisoning by other means | 151.1 (103.8 to 199.8) | 517.9 (285.9 to 849.2) | 19.5 (11.8 to 30.1) | 28.1* (22.4 to 34.0) | 18.9* (13.5 to 24.3) |
| Exposure to mechanical forces | 2730.5 (2002.3 to 3621.1) | 4993.2 (2912.3 to 7566.5) | 91.2 (61.6 to 132.3) | 28.9* (24.6 to 32.6) | 19.5* (15.6 to 22.9) |
| Unintentional firearm injuries | 147.4 (106.8 to 205.9) | 147.9 (62.9 to 301.9) | 8.3 (5.5 to 12.2) | 20.6* (16.1 to 25.3) | 11.8* (7.7 to 16.2) |
| Other exposure to mechanical forces | 2583.2 (1868.4 to 3489.1) | 4845.2 (2809.0 to 7308.4) | 82.9 (54.7 to 120.8) | 29.8* (25.5 to 33.5) | 20.3* (16.4 to 23.8) |
| Adverse effects of medical treatment | 57.7 (33.3 to 88.9) | 753.5 (452.2 to 1136.3) | 7.7 (3.9 to 13.2) | 16.8* (12.1 to 20.8) | 8.3* (4.0 to 12.0) |
| Animal contact | 874.8 (680.6 to 1107.5) | 3657.0 (2308.1 to 5544.6) | 41.2 (26.2 to 58.1) | 12.2* (6.2 to 18.4) | 4.1 (-1.5 to 9.8) |
| Venomous animal contact | 341.0 (252.4 to 448.4) | 1543.7 (933.5 to 2395.2) | 30.3 (18.2 to 45.3) | 13.3* (5.7 to 21.4) | 5.1 (-2.0 to 12.5) |
| Non-venomous animal contact | 533.8 (380.7 to 726.4) | 2113.3 (1301.7 to 3176.5) | 10.8 (6.4 to 17.2) | 9.3* (4.5 to 13.6) | 1.3 (-3.1 to 5.3) |
| Foreign body | 670.3 (482.5 to 908.1) | 1954.0 (1322.7 to 2814.3) | 39.9 (25.8 to 59.8) | 15.9* (13.0 to 18.8) | 7.5* (4.8 to 10.2) |

| | | | | | |
|---|---|---|---|---|---|
| Pulmonary aspiration and foreign body in airway | 99.2 (66.9 to 136.2) | 100.9 (58.2 to 157.2) | 7.2 (4.6 to 10.2) | 10.3* (5.2 to 15.9) | 2.3 (-2.4 to 7.5) |
| Foreign body in eyes | 191.3 (60.3 to 379.2) | 1406.1 (826.1 to 2194.8) | 10.9 (3.5 to 23.7) | 13.2* (11.7 to 15.0) | 5.0* (3.6 to 6.7) |
| Foreign body in other body part | 379.9 (271.7 to 532.6) | 447.0 (239.3 to 720.2) | 21.7 (14.5 to 30.6) | 19.4* (15.5 to 22.8) | 10.7* (7.1 to 13.9) |
| Environmental heat and cold exposure | 806.6 (563.3 to 1139.4) | 734.9 (410.2 to 1159.6) | 43.0 (28.3 to 60.0) | 14.5* (10.7 to 18.0) | 6.2* (2.6 to 9.4) |
| Exposure to forces of nature | 167.8 (109.8 to 252.0) | 88.8 (57.0 to 133.2) | 8.6 (5.4 to 13.3) | -68.7* (-73.2 to -63.8) | -71.0* (-75.1 to -66.4) |
| Other unintentional injuries | 1640.6 (1235.6 to 2187.7) | 2760.2 (1684.6 to 4318.7) | 65.2 (42.9 to 95.8) | 29.3* (25.5 to 32.9) | 19.9* (16.4 to 23.3) |
| **Self-harm and interpersonal violence** | **5992.9 (4686.4 to 7371.0)** | **2032.3 (1508.2 to 2662.6)** | **136.4 (99.1 to 181.2)** | **23.9* (17.5 to 31.7)** | **14.9* (9.0 to 22.1)** |
| Self-harm | -- | -- | -- | -- | -- |
| Self-harm by firearm | -- | -- | -- | -- | -- |
| Self-harm by other specified means | -- | -- | -- | -- | -- |
| Interpersonal violence | 5159.5 (3922.2 to 6486.6) | 853.6 (547.5 to 1328.5) | 84.0 (61.9 to 112.0) | 15.3* (12.0 to 19.1) | 6.9* (3.9 to 10.5) |
| Assault by firearm | 17.0 (12.8 to 23.6) | 13.9 (6.0 to 27.5) | 1.3 (0.9 to 1.8) | 31.3* (24.4 to 38.4) | 21.8* (15.3 to 28.4) |
| Assault by sharp object | 175.1 (127.1 to 233.3) | 204.8 (100.5 to 356.9) | 8.0 (5.4 to 11.4) | 25.2* (20.0 to 30.4) | 16.1* (11.3 to 20.9) |
| Sexual violence | 4492.4 (3258.5 to 5756.5) | -- | 45.8 (27.0 to 71.0) | 6.5* (5.1 to 8.0) | -1.2 (-2.5 to 0.1) |
| Assault by other means | 475.1 (349.2 to 642.5) | 634.9 (386.1 to 1008.9) | 29.0 (19.7 to 41.0) | 28.4* (23.4 to 33.7) | 19.1* (14.4 to 24.0) |
| Conflict and terrorism | 804.8 (509.9 to 1301.2) | 1116.1 (773.7 to 1585.2) | 50.8 (29.5 to 84.0) | 39.3* (23.0 to 56.6) | 29.2* (14.1 to 45.2) |
| Executions and police conflict | 28.6 (17.3 to 45.1) | 62.6 (37.7 to 94.7) | 1.6 (0.9 to 2.5) | 120.3* (98.2 to 145.0) | 104.3* (83.8 to 127.2) |
| **Nature of injury aggregates** | | | | | |
| Amputations | 5401.6 (4640.9 to 6372.0) | 637.7 (492.5 to 821.6) | 107.8 (73.1 to 158.3) | 12.0* (5.4 to 18.0) | 3.9 (-2.3 to 9.5) |
| Burns | 3293.9 (2812.0 to 3957.9) | 1451.2 (1114.8 to 1837.6) | 181.9 (130.2 to 244.2) | 3.8 (-2.7 to 9.8) | -3.7 (-9.8 to 1.9) |
| Fractures | 3806.7 (3302.3 to 4400.3) | 7730.7 (6209.9 to 9826.1) | 215.1 (141.7 to 307.3) | 19.1* (15.9 to 22.3) | 10.5* (7.5 to 13.4) |
| Head Injuries | 621.2 (561.3 to 692.1) | 1335.8 (1082.3 to 1653.2) | 99.3 (67.9 to 137.8) | 21.0* (15.7 to 26.4) | 12.2* (7.3 to 17.3) |
| Spinal Injuries | 385.8 (332.6 to 466.4) | 49.3 (37.2 to 66.6) | 129.4 (88.6 to 179.6) | 19.0* (10.5 to 27.4) | 10.3* (2.5 to 18.2) |
| Minor Injuries | 3149.1 (2734.9 to 3643.3) | 12876.7 (10362.9 to 15988.6) | 26.4 (12.2 to 51.4) | 21.6* (18.5 to 24.4) | 12.7* (9.9 to 15.3) |
| Other Injuries | 1614.9 (1379.4 to 1896.4) | 9356.9 (8048.8 to 11025.2) | 202.8 (138.4 to 279.8) | 18.0* (14.5 to 21.5) | 9.4* (6.1 to 12.7) |
| **Impairments** | | | | | |
| Anaemia | 234137.3 (216563.3 to 255323.1) | -- | 10162.5 (6690.6 to 14609.5) | -14.2* (-20.8 to -6.6) | -20.4* (-26.6 to -13.3) |
| Epilepsy | 5951.2 (4780.6 to 7281.8) | -- | 2661.5 (1961.2 to 3478.3) | 15.6* (8.1 to 23.2) | 7.2* (0.2 to 14.3) |
| Guillain-Barré syndrome | 10.8 (6.0 to 17.8) | -- | 3.2 (1.6 to 5.6) | 9.3* (6.8 to 11.5) | 1.4 (-0.9 to 3.4) |
| Hearing loss | 25468.8 (22734.5 to 28377.2) | -- | 1177.6 (826.2 to 1586.0) | 6.1* (2.9 to 9.6) | -1.6 (-4.5 to 1.7) |
| Heart failure | 222.1 (145.3 to 316.6) | -- | 20.6 (11.9 to 33.3) | 6.7* (5.1 to 8.1) | -1.1 (-2.5 to 0.3) |
| Infertility | 29.5 (14.2 to 49.8) | -- | 0.6 (0.2 to 1.5) | 11.8* (10.5 to 13.1) | 3.7* (2.4 to 4.9) |
| Developmental intellectual disability | 21463.9 (16582.7 to 26376.3) | -- | 2754.6 (2118.8 to 3466.4) | 8.8* (5.0 to 13.1) | 0.9 (-2.6 to 4.9) |
| Pelvic inflammatory disease | -- | -- | -- | -- | -- |
| Vision loss | 20584.6 (16619.6 to 25502.0) | -- | 818.3 (546.9 to 1207.7) | 8.0* (6.4 to 9.5) | 0.2 (-1.3 to 1.6) |

We did not calculate incidence for the nine impairments and certain NTDs. Blank cells mean that no estimate is available or that the estimate has a magnitude less than 50.
Table presents prevalence and incidence counts in thousands.
* Percentage changes that are statistically significant
‡ Incidence estimates for stroke represent first-ever stroke only.
^ Incidence of HIV/AIDS represents new infections of HIV only and does not include new infections of Tuberculosis in HIV positive cases.

| SR Table 10f: Global prevalence, incidence and YLDs for 2017, percentage change of YLD counts and percentage change of YLD rates between 2007 and 2017 by cause, age 10 to 14 years | | | | | |
|---|---|---|---|---|---|
| Cause | Prevalence in thousands (95% UI) | Incidence in thousands (95% UI) | YLDs in thousands (95% UI) | | |
| | 2017 counts | 2017 counts | 2017 counts | Percentage change in counts between 2007 and 2017 | Percentage change in rates between 2007 and 2017 |
| **All causes** | **588881.1 (579699.6 to 598096.1)** | **3044898.1 (2537895.5 to 3586430.3)** | **37259.9 (27265.8 to 48590.9)** | **2.9* (1.3 to 4.5)** | **-1.2 (-2.7 to 0.4)** |
| **Communicable, maternal, neonatal, and nutritional disorders** | **337680.4 (315177.3 to 363244.8)** | **2244730.3 (1728029.4 to 2765554.8)** | **11421.3 (8428.6 to 15114.6)** | **-1.9 (-6.1 to 2.7)** | **-5.8* (-9.8 to -1.4)** |
| **HIV/AIDS and sexually transmitted infections** | **8034.5 (6757.2 to 9483.6)** | **11233.2 (8925.1 to 14130.6)** | **145.2 (108.7 to 186.6)** | **25.2* (16.3 to 34.0)** | **20.3* (11.6 to 28.7)** |
| HIV/AIDS | 791.9 (743.9 to 846.1) | -- | 132.3 (96.4 to 173.1) | 27.3* (17.2 to 38.4) | 22.3* (12.5 to 32.9) |
| HIV/AIDS - Drug-susceptible Tuberculosis | 21.2 (19.3 to 23.1) | 34.6 (31.4 to 38.1) | 8.6 (5.7 to 11.7) | -0.3 (-10.7 to 11.6) | -4.2 (-14.2 to 7.2) |
| HIV/AIDS - Multidrug-resistant Tuberculosis without extensive drug resistance | 0.8 (0.6 to 1.2) | 1.4 (1.0 to 2.0) | 0.3 (0.2 to 0.5) | 20.8 (-16.7 to 72.2) | 16.0 (-20.1 to 65.4) |
| HIV/AIDS - Extensively drug-resistant Tuberculosis | -- | -- | -- | 113.5* (47.5 to 209.8) | 105.0* (41.7 to 197.5) |
| HIV/AIDS resulting in other diseases | 769.8 (722.1 to 823.8) | -- | 123.4 (90.1 to 161.2) | 29.8* (18.6 to 42.3) | 24.7* (13.9 to 36.6) |
| HIV/AIDS not on antiretroviral treatment without Tuberculosis | 528.6 (491.8 to 568.6) | -- | 103.8 (74.7 to 135.1) | 12.2* (3.8 to 20.9) | 7.7 (-0.3 to 16.1) |
| HIV/AIDS on antiretroviral treatment without Tuberculosis | 241.2 (221.8 to 260.0) | -- | 19.6 (13.0 to 28.1) | 677.0* (487.6 to 947.7) | 646.1* (464.2 to 906.1) |
| Sexually transmitted infections excluding HIV | 7328.2 (6057.0 to 8794.2) | 11233.2 (8925.1 to 14130.6) | 12.9 (6.5 to 24.5) | 7.2* (2.1 to 12.2) | 2.9 (-1.9 to 7.8) |
| Syphilis | 672.3 (434.5 to 985.6) | 534.0 (347.0 to 782.8) | 0.2 (0.0 to 0.4) | 8.9 (-2.7 to 21.9) | 4.5 (-6.6 to 17.0) |
| Early syphilis | 672.3 (434.5 to 985.6) | 534.0 (347.0 to 782.8) | 0.2 (0.0 to 0.4) | 8.9 (-2.7 to 21.9) | 4.5 (-6.6 to 17.0) |
| Tertiary syphilis | -- | -- | -- | -- | -- |
| Chlamydial infection | 378.7 (226.2 to 643.0) | 1102.1 (640.8 to 1889.0) | 1.2 (0.7 to 2.2) | 8.0* (0.9 to 16.1) | 3.7 (-3.1 to 11.5) |
| Chlamydia episode | 373.7 (220.9 to 639.5) | 1102.1 (640.8 to 1889.0) | 0.6 (0.2 to 1.4) | 4.0 (-6.3 to 15.8) | -0.1 (-10.1 to 11.2) |
| Chlamydial infection complications | 5.0 (3.8 to 6.6) | -- | 0.7 (0.4 to 1.0) | 11.7* (3.6 to 22.3) | 7.3 (-0.5 to 17.4) |
| Gonococcal infection | 1417.9 (768.1 to 2336.2) | 3735.1 (2006.8 to 6210.8) | 5.0 (2.3 to 10.6) | 5.1 (-3.9 to 13.1) | 0.9 (-7.7 to 8.6) |
| Gonococcal infection complications | 9.9 (6.1 to 15.4) | -- | 1.3 (0.7 to 2.1) | 6.4 (-12.1 to 24.8) | 2.1 (-15.5 to 19.8) |
| Gonorrhoea episode | 1408.0 (759.6 to 2324.4) | 3735.1 (2006.8 to 6210.8) | 3.7 (1.2 to 9.1) | 4.6 (-5.7 to 12.1) | 0.5 (-9.5 to 7.7) |
| Trichomoniasis | 767.0 (469.0 to 1167.6) | 863.4 (523.9 to 1331.3) | 1.4 (0.5 to 3.2) | 13.4* (5.6 to 21.8) | 8.9* (1.4 to 17.0) |
| Genital herpes | 4197.5 (3222.1 to 5451.3) | 4998.7 (3838.3 to 6459.0) | 2.3 (0.8 to 5.0) | 8.8* (0.3 to 17.9) | 4.5 (-3.7 to 13.2) |
| Other sexually transmitted infections | 3.9 (2.7 to 5.3) | -- | 2.8 (1.5 to 5.2) | 6.2* (0.4 to 12.1) | 2.0 (-3.6 to 7.6) |
| Other sexually transmitted diseases residual | -- | -- | 2.3 (1.1 to 4.7) | 5.9 (-1.4 to 13.1) | 1.6 (-5.3 to 8.7) |
| Other sexually transmitted diseases | 3.9 (2.7 to 5.3) | -- | 2.8 (1.5 to 5.2) | 6.2* (0.4 to 12.1) | 2.0 (-3.6 to 7.6) |
| **Respiratory infections and tuberculosis** | **142800.0 (103871.0 to 188523.4)** | **1555894.3 (1048917.5 to 2079377.5)** | **908.4 (546.9 to 1397.5)** | **2.8* (0.9 to 4.5)** | **-1.2 (-3.1 to 0.4)** |
| Tuberculosis | 115837.4 (76309.9 to 163790.3) | 353.7 (255.6 to 468.0) | 65.8 (37.2 to 100.3) | -12.6* (-19.9 to -5.4) | -16.1* (-23.1 to -9.2) |
| Latent tuberculosis infection | 115632.1 (76132.7 to 163603.0) | -- | -- | -- | -- |
| Drug-susceptible tuberculosis | 196.7 (139.5 to 271.3) | 338.5 (245.0 to 449.8) | 63.0 (35.8 to 95.2) | -12.6* (-20.4 to -4.6) | -16.0* (-23.5 to -8.4) |
| Multidrug-resistant tuberculosis without extensive drug resistance | 8.3 (4.5 to 14.4) | 14.6 (8.5 to 23.5) | 2.7 (1.3 to 5.3) | -15.4 (-47.2 to 29.0) | -18.8 (-49.3 to 23.9) |
| Extensively drug-resistant tuberculosis | 0.3 (0.2 to 0.6) | 0.6 (0.4 to 0.9) | 0.1 (0.1 to 0.2) | 29.7 (-16.0 to 113.0) | 24.5 (-19.3 to 104.6) |
| Lower respiratory infections | 1154.9 (857.6 to 1479.1) | 51449.3 (38823.0 to 66056.0) | 71.4 (43.7 to 108.1) | 16.4* (5.8 to 26.8) | 11.8* (1.6 to 21.8) |
| Guillain-Barré syndrome due to lower respiratory infections | 1.0 (0.4 to 2.1) | -- | 0.3 (0.1 to 0.6) | 7.8* (5.3 to 10.1) | 3.5* (1.2 to 5.7) |
| Lower respiratory infection episode | 1154.0 (856.4 to 1478.5) | 51449.3 (38823.0 to 66056.0) | 71.1 (43.5 to 107.3) | 16.4* (5.7 to 26.9) | 11.8* (1.5 to 21.8) |
| Upper respiratory infections | 20434.3 (13626.6 to 27421.6) | 1484310.0 (976229.7 to 2005010.3) | 515.0 (271.4 to 865.6) | 4.1* (2.8 to 5.6) | 0.0 (-1.3 to 1.4) |
| Guillain-Barré syndrome due to upper respiratory infections | 2.7 (1.3 to 5.2) | -- | 0.8 (0.3 to 1.6) | 7.8* (5.4 to 10.1) | 3.5* (1.2 to 5.8) |
| Upper respiratory infection episode | 20431.7 (13624.2 to 27416.5) | 1484310.0 (976229.7 to 2005010.3) | 514.2 (270.7 to 864.6) | 4.1* (2.8 to 5.6) | 0.0 (-1.3 to 1.4) |
| Otitis media | 12271.0 (10033.6 to 14552.4) | 19781.3 (13476.1 to 27898.4) | 256.2 (149.0 to 408.4) | 1.6 (-2.0 to 4.9) | -2.4 (-5.8 to 0.8) |
| Acute otitis media | 1190.2 (848.1 to 1661.7) | 19779.1 (13468.7 to 27897.1) | 15.6 (7.5 to 29.2) | 5.7* (1.2 to 11.0) | 1.5 (-2.8 to 6.6) |
| Chronic otitis media | 11080.9 (8999.5 to 13300.8) | 2.2 (0.1 to 8.5) | 240.6 (142.0 to 382.9) | 1.3 (-2.3 to 4.8) | -2.7 (-6.2 to 0.7) |
| **Enteric infections** | **7263.3 (6284.8 to 8443.5)** | **494865.8 (418079.2 to 579744.4)** | **848.6 (566.4 to 1191.2)** | **9.8* (4.9 to 15.2)** | **5.5* (0.8 to 10.6)** |
| Diarrhoeal diseases | 7200.5 (6225.6 to 8411.8) | 492210.2 (415781.9 to 577310.4) | 827.5 (552.8 to 1164.3) | 11.4* (6.3 to 16.6) | 7.0* (2.1 to 12.0) |
| Guillain-Barré syndrome due to diarrheal diseases | 0.9 (0.4 to 1.7) | -- | 0.3 (0.1 to 0.6) | 7.8* (5.3 to 10.1) | 3.5* (1.1 to 5.7) |
| Diarrheal disease episode | 7199.6 (6224.9 to 8410.5) | 492210.2 (415781.9 to 577310.4) | 827.3 (552.6 to 1164.0) | 11.4* (6.3 to 16.6) | 7.0* (2.1 to 12.0) |
| Typhoid and paratyphoid | 91.8 (63.0 to 127.1) | 2618.4 (1951.6 to 3494.2) | 20.9 (12.1 to 32.2) | -29.4* (-42.4 to -14.3) | -32.2* (-44.7 to -17.7) |
| Typhoid fever | 126.4 (88.4 to 176.2) | 1993.6 (1462.6 to 2729.6) | 19.2 (11.0 to 30.2) | -30.2* (-43.7 to -13.7) | -33.0* (-46.0 to -17.2) |
| Typhoid fever complications | 26.2 (17.5 to 39.1) | 343.7 (245.2 to 478.7) | 8.3 (4.5 to 13.9) | -30.7* (-52.3 to -2.1) | -33.4* (-54.1 to -6.0) |
| Typhoid fever episode | 100.2 (68.9 to 141.2) | 1649.9 (1207.6 to 2249.3) | 10.9 (5.9 to 17.7) | -29.9* (-46.6 to -10.3) | -32.7* (-48.7 to -13.9) |
| Paratyphoid fever | 27.4 (17.9 to 40.4) | 624.8 (412.1 to 932.9) | 1.7 (0.9 to 2.9) | -17.9 (-34.2 to 1.9) | -21.2* (-36.8 to -2.2) |
| Intestinal perforation due to paratyphoid | 1.2 (0.8 to 1.9) | 32.2 (21.0 to 49.6) | 0.1 (0.1 to 0.2) | -18.0 (-35.9 to 4.8) | -21.3 (-38.5 to 0.6) |
| Paratyphoid fever episode | 26.2 (17.1 to 38.2) | 592.6 (389.5 to 886.6) | 1.6 (0.9 to 2.6) | -17.9 (-35.2 to 2.7) | -21.2* (-37.7 to -1.4) |
| iNTS | 1.4 (0.7 to 2.3) | 37.2 (20.3 to 59.7) | 0.2 (0.1 to 0.3) | -12.5* (-18.8 to -6.7) | -16.0* (-22.0 to -10.4) |
| Other intestinal infectious diseases | -- | -- | -- | -40.0* (-51.1 to -27.2) | -42.4* (-53.1 to -30.1) |
| **Neglected tropical diseases and malaria** | **126528.8 (120551.6 to 133369.6)** | **39397.6 (29630.2 to 53777.0)** | **1043.6 (709.0 to 1467.6)** | **-15.5* (-21.5 to -8.7)** | **-18.9* (-24.6 to -12.3)** |
| Malaria | 20403.4 (18921.8 to 21779.3) | 25955.4 (18751.6 to 40185.2) | 234.4 (160.7 to 343.7) | -15.5* (-26.8 to -0.9) | -18.8* (-29.7 to -4.9) |
| Malaria complications | 127.1 (114.9 to 141.1) | -- | 52.4 (39.7 to 66.4) | 27.4* (12.8 to 45.0) | 22.3* (8.3 to 39.2) |
| Malaria episode | 1513.4 (857.8 to 2487.6) | 25955.4 (18751.6 to 40185.2) | 54.2 (24.9 to 101.4) | -16.6* (-28.2 to -0.0) | -19.9* (-31.0 to -4.0) |

| | | | | | |
|---|---|---|---|---|---|
| Malaria parasitemia | 18762.9 (17042.6 to 20288.9) | -- | 127.7 (77.2 to 198.2) | -25.4* (-40.0 to -6.0) | -28.3* (-42.4 to -9.7) |
| Chagas disease | 218.6 (167.0 to 276.0) | 14.8 (10.6 to 19.7) | 0.6 (0.3 to 0.9) | -29.8* (-43.4 to -13.4) | -32.6* (-45.6 to -16.8) |
| Acute Chagas disease | 0.1 (0.0 to 0.2) | 14.8 (10.6 to 19.7) | -- | -24.8* (-29.6 to -21.1) | -27.8* (-32.4 to -24.2) |
| Asymptomatic Chagas disease | 208.1 (158.6 to 263.1) | -- | -- | -- | -- |
| Symptomatic chronic Chagas infection | 10.4 (7.5 to 13.8) | -- | 0.5 (0.3 to 0.9) | -29.9* (-43.6 to -13.3) | -32.7* (-45.8 to -16.8) |
| Leishmaniasis | 368.8 (316.0 to 430.9) | 98.1 (65.1 to 142.0) | 24.5 (15.8 to 35.8) | 22.0* (6.3 to 39.6) | 17.2* (2.1 to 34.0) |
| Visceral leishmaniasis | 1.3 (0.9 to 2.0) | 5.1 (3.6 to 7.8) | 0.1 (0.1 to 0.2) | -71.8* (-79.8 to -57.3) | -73.0* (-80.6 to -59.0) |
| Cutaneous and mucocutaneous leishmaniasis | 372.8 (322.1 to 434.6) | 92.9 (59.5 to 137.2) | 24.4 (15.8 to 35.6) | 23.6* (7.3 to 41.3) | 18.7* (3.1 to 35.7) |
| African trypanosomiasis | 0.6 (0.1 to 2.1) | 0.4 (0.1 to 1.5) | 0.2 (0.0 to 0.6) | -79.9* (-97.0 to -6.4) | -80.7* (-97.1 to -10.1) |
| Trypanosomiasis Gambiense | 0.5 (0.1 to 2.1) | 0.4 (0.1 to 1.4) | 0.1 (0.0 to 0.6) | -79.0 (-97.1 to 6.8) | -79.8 (-97.2 to 2.6) |
| Trypanosomiasis Rhodesiense | -- | 0.0 (0.0 to 0.1) | -- | -92.6* (-99.0 to -57.6) | -92.9* (-99.0 to -59.3) |
| Schistosomiasis | 6454.9 (5451.1 to 7671.3) | -- | 51.0 (24.8 to 95.2) | -30.7* (-36.4 to -25.8) | -33.5* (-38.9 to -28.7) |
| Mild schistosomiasis | 5376.8 (4381.9 to 6486.1) | -- | 30.7 (12.0 to 64.0) | -25.7* (-29.2 to -22.1) | -28.7* (-32.0 to -25.2) |
| Anemia due to schistosomiasis | 300.7 (218.2 to 402.1) | -- | 11.9 (6.9 to 19.2) | -34.5* (-49.2 to -16.4) | -37.1* (-51.2 to -19.8) |
| Schistosomiasis complications | 777.3 (511.8 to 1107.1) | -- | 8.4 (3.6 to 16.7) | -40.4* (-43.2 to -37.4) | -42.8* (-45.5 to -39.9) |
| Cysticercosis | 26.8 (14.9 to 45.3) | -- | 9.1 (4.4 to 16.9) | 1.7 (-11.1 to 16.8) | -2.4 (-14.7 to 12.2) |
| Cystic echinococcosis | 21.6 (8.9 to 45.4) | 9.5 (3.5 to 23.5) | 1.8 (0.7 to 4.1) | 3.8 (-7.4 to 18.3) | -0.4 (-11.1 to 13.6) |
| Lymphatic filariasis | 6354.1 (5291.9 to 7413.6) | -- | 107.9 (57.6 to 176.8) | -34.5* (-45.9 to -23.3) | -37.1* (-48.1 to -26.3) |
| Prevalence of detectable microfiliaria due to lymphatic filariasis | 5407.8 (4445.0 to 6430.1) | -- | -- | -- | -- |
| Lymphatic filariasis complications | 946.3 (615.4 to 1404.1) | -- | 107.9 (57.6 to 176.8) | -34.5* (-45.9 to -23.3) | -37.1* (-48.1 to -26.3) |
| Onchocerciasis | 2204.7 (1299.5 to 3761.9) | -- | 135.3 (54.8 to 256.6) | 1.6 (-21.6 to 20.2) | -2.4 (-24.7 to 15.4) |
| Asymptomatic onchocerciasis | 408.8 (0.0 to 1821.9) | -- | -- | -- | -- |
| Skin disease due to onchocerciasis | 1794.9 (1189.3 to 2591.3) | -- | 135.1 (54.7 to 256.4) | 1.5 (-22.0 to 20.1) | -2.5 (-25.1 to 15.3) |
| Vision loss due to onchocerciasis | 1.0 (0.5 to 1.9) | -- | 0.2 (0.1 to 0.4) | 750.5* (230.2 to 1735.3) | 716.7* (217.1 to 1662.4) |
| Trachoma | -- | -- | -- | -- | -- |
| Dengue | 735.4 (400.4 to 1246.3) | 12295.4 (7474.7 to 18658.0) | 122.6 (53.6 to 230.6) | 47.1* (26.1 to 126.0) | 41.3* (21.1 to 117.0) |
| Post-dengue chronic fatigue syndrome | 518.4 (242.1 to 948.0) | -- | 109.7 (45.7 to 210.3) | 47.2* (25.7 to 127.5) | 41.3* (20.7 to 118.5) |
| Dengue episode | 217.0 (131.0 to 325.8) | 12295.4 (7474.7 to 18658.0) | 12.9 (6.6 to 22.5) | 46.5* (24.6 to 121.3) | 40.7* (19.7 to 112.5) |
| Yellow fever | 0.4 (0.1 to 1.1) | 14.9 (4.1 to 38.0) | -- | -15.2 (-40.5 to 24.2) | -18.6 (-42.9 to 19.3) |
| Asymptomatic yellow fever | 0.2 (0.1 to 0.6) | 8.3 (2.1 to 22.2) | -- | -- | -- |
| Yellow fever episode | 0.2 (0.0 to 0.5) | 6.6 (1.9 to 16.8) | -- | -15.2 (-40.5 to 24.2) | -18.6 (-42.9 to 19.3) |
| Rabies | 0.0 (0.0 to 0.1) | 1.1 (0.8 to 1.7) | -- | -43.9* (-59.4 to -21.4) | -46.1* (-61.0 to -24.5) |
| Intestinal nematode infections | 95784.0 (89813.7 to 102816.5) | -- | 174.3 (98.7 to 285.6) | -35.7* (-39.9 to -31.2) | -38.2* (-42.3 to -34.0) |
| Ascariasis | 54272.6 (48192.8 to 61281.9) | -- | 73.8 (39.5 to 128.3) | -39.1* (-46.2 to -31.1) | -41.5* (-48.4 to -33.8) |
| Asymptomatic ascariasis | 50391.5 (44767.4 to 57049.1) | -- | -- | -- | -- |
| Ascariasis complications | 3881.1 (3411.6 to 4398.1) | -- | 73.8 (39.5 to 128.3) | -39.1* (-46.2 to -31.1) | -41.5* (-48.4 to -33.8) |
| Trichuriasis | 28692.0 (25593.7 to 32469.5) | -- | 20.6 (11.6 to 34.4) | -38.2* (-44.1 to -31.2) | -40.6* (-46.3 to -34.0) |
| Asymptomatic trichuriasis | 27662.6 (24641.4 to 31336.8) | -- | -- | -- | -- |
| Trichuriasis complications | 1029.3 (934.0 to 1141.3) | -- | 20.6 (11.6 to 34.4) | -38.2* (-44.1 to -31.2) | -40.6* (-46.3 to -34.0) |
| Hookworm disease | 20668.1 (19326.7 to 22145.9) | -- | 79.9 (47.5 to 123.2) | -31.4* (-37.7 to -24.5) | -34.1* (-40.2 to -27.5) |
| Asymptomatic hookworm disease | 17462.5 (16273.9 to 18793.9) | -- | -- | -- | -- |
| Anemia due to hookworm disease | 680.6 (576.4 to 810.6) | -- | 27.2 (17.1 to 41.9) | -34.5* (-45.6 to -20.9) | -37.1* (-47.8 to -24.0) |
| Hookworm disease complications | 2525.1 (2368.8 to 2708.4) | -- | 52.7 (29.1 to 86.4) | -29.6* (-34.7 to -24.5) | -32.4* (-37.3 to -27.5) |
| Food-borne trematodiases | 3171.0 (2544.3 to 3868.8) | 848.7 (692.5 to 1029.5) | 42.9 (25.8 to 65.2) | -23.7* (-32.9 to -13.7) | -26.7* (-35.6 to -17.1) |
| Asymptomatic food-borne tremodiases | 2796.5 (2224.1 to 3435.1) | 723.4 (492.6 to 937.8) | -- | -- | -- |
| Food-borne tremodiases complications | 374.5 (272.7 to 498.1) | 125.3 (39.8 to 336.2) | 42.9 (25.8 to 65.2) | -23.7* (-32.9 to -13.7) | -26.7* (-35.6 to -17.1) |
| Leprosy | 2.5 (2.2 to 2.8) | 1.4 (1.2 to 1.6) | 0.1 (0.1 to 0.2) | -35.4* (-36.8 to -34.2) | -37.9* (-39.3 to -36.9) |
| Ebola virus disease | -- | -- | -- | -95.6* (-98.1 to -89.1) | -95.8* (-98.2 to -89.5) |
| Ebola cases | -- | -- | -- | -98.2* (-98.3 to -98.1) | -98.2* (-98.4 to -98.1) |
| Post-Ebola chronic fatigue syndrome | -- | -- | -- | -95.5* (-98.1 to -88.7) | -95.7* (-98.2 to -89.2) |
| Zika virus disease | 2.6 (1.6 to 4.7) | 158.0 (94.2 to 283.5) | 0.1 (0.0 to 0.1) | -- | -- |
| Zika virus complications | -- | -- | -- | -- | -- |
| Zika virus episode | 2.6 (1.5 to 4.7) | 158.0 (94.2 to 283.5) | 0.0 (0.0 to 0.1) | -- | -- |
| Guinea worm disease | -- | -- | -- | -99.4* (-99.6 to -99.2) | -99.4* (-99.6 to -99.2) |
| Other neglected tropical diseases | 3268.0 (2964.1 to 3641.4) | -- | 138.9 (91.2 to 201.3) | -3.3 (-12.4 to 8.1) | -7.1 (-15.9 to 3.8) |
| Acute infection due to other neglected tropical diseases | -- | -- | 1.0 (0.5 to 1.8) | 137.7* (103.1 to 250.8) | 128.3* (95.1 to 236.8) |
| Anemia due to other neglected tropical diseases | 3268.0 (2964.1 to 3641.4) | -- | 137.9 (90.5 to 200.1) | -3.7 (-12.8 to 7.6) | -7.5 (-16.3 to 3.4) |
| **Other infectious diseases** | **6947.8 (6433.2 to 7587.7)** | **32768.7 (29210.8 to 37028.3)** | **382.4 (267.6 to 516.3)** | **-3.2 (-7.6 to 1.5)** | **-7.1* (-11.2 to -2.6)** |
| Meningitis | 1073.0 (883.5 to 1287.0) | 282.4 (188.2 to 389.2) | 106.0 (72.4 to 144.9) | -4.4 (-9.8 to 0.9) | -8.2* (-13.3 to -3.1) |
| Pneumococcal meningitis | 347.6 (280.2 to 435.3) | 29.0 (17.6 to 45.0) | 36.4 (24.0 to 50.4) | -27.3* (-34.0 to -20.2) | -30.2* (-36.6 to -23.3) |

| | | | | | |
|---|---|---|---|---|---|
| Acute pneumococcal meningitis | 1.4 (0.8 to 2.3) | 29.0 (17.6 to 45.0) | 0.2 (0.1 to 0.3) | -28.3* (-38.7 to -17.5) | -31.1* (-41.2 to -20.8) |
| Pneumococcal meningitis complications | 346.2 (278.5 to 433.4) | -- | 36.2 (23.8 to 50.2) | -27.3* (-34.0 to -20.1) | -30.2* (-36.6 to -23.3) |
| H influenzae type B meningitis | 105.9 (77.1 to 142.4) | 11.4 (6.2 to 19.5) | 11.0 (7.3 to 15.3) | -47.1* (-50.4 to -43.9) | -49.2* (-52.4 to -46.1) |
| Acute H influenzae type B meningitis | 0.6 (0.3 to 1.0) | 11.4 (6.2 to 19.5) | 0.1 (0.0 to 0.1) | -57.6* (-64.4 to -50.5) | -59.3* (-65.8 to -52.5) |
| H Influenza type B meningitis complications | 105.4 (76.4 to 141.8) | -- | 10.9 (7.3 to 15.2) | -47.0* (-50.3 to -43.8) | -49.1* (-52.3 to -46.0) |
| Meningococcal infection | 106.4 (75.1 to 144.1) | 23.7 (13.4 to 37.7) | 10.7 (7.2 to 15.1) | -3.2 (-7.7 to 1.5) | -7.0* (-11.4 to -2.5) |
| Acute meningococcal meningitis | 1.2 (0.7 to 1.9) | 23.7 (13.4 to 37.7) | 0.2 (0.1 to 0.3) | 12.0 (-2.2 to 25.7) | 7.6 (-6.0 to 20.7) |
| Meningococcal meningitis complications | 105.2 (73.7 to 143.2) | -- | 10.6 (7.0 to 14.9) | -3.4 (-8.0 to 1.4) | -7.2* (-11.6 to -2.6) |
| Other meningitis | 513.1 (379.3 to 666.1) | 218.3 (146.3 to 302.4) | 47.9 (32.6 to 65.2) | 65.6* (52.4 to 80.9) | 59.0* (46.3 to 73.7) |
| Other acute bacterial meningitis | 2.6 (1.6 to 4.0) | 53.2 (34.4 to 77.4) | 0.3 (0.2 to 0.6) | 104.1* (82.3 to 126.0) | 96.0* (75.1 to 117.0) |
| Acute viral meningitis | 8.2 (5.3 to 12.1) | 165.0 (110.0 to 227.3) | 1.1 (0.6 to 1.8) | 2.4 (-7.3 to 12.5) | -1.7 (-10.9 to 8.0) |
| Other bacterial meningitis complications | 502.3 (369.0 to 654.0) | -- | 46.4 (31.6 to 63.4) | 67.8* (54.2 to 83.4) | 61.1* (48.0 to 76.1) |
| Encephalitis | 413.2 (247.7 to 636.3) | 194.4 (183.7 to 205.3) | 40.1 (28.3 to 53.1) | -4.7 (-9.9 to 0.9) | -8.5* (-13.5 to -3.1) |
| Acute encephalitis | 10.9 (10.3 to 11.6) | 194.4 (183.7 to 205.3) | 1.5 (1.0 to 2.1) | 2.8* (1.9 to 3.8) | -1.3* (-2.1 to -0.4) |
| Encephalitis complications | 402.2 (236.8 to 625.3) | -- | 38.6 (27.2 to 51.2) | -5.0 (-10.3 to 0.9) | -8.8* (-13.9 to -3.2) |
| Diphtheria | 0.0 (0.0 to 0.1) | 0.5 (0.3 to 0.9) | -- | -37.6* (-58.6 to -5.1) | -40.1* (-60.3 to -8.9) |
| Whooping cough | 18.3 (14.2 to 23.1) | 133.9 (103.5 to 168.9) | 0.9 (0.5 to 1.5) | -5.4* (-5.9 to -4.9) | -9.2* (-9.7 to -8.7) |
| Tetanus | 5.9 (5.6 to 6.1) | 2.5 (1.4 to 4.5) | 0.1 (0.1 to 0.2) | -11.1* (-21.8 to -3.4) | -14.7* (-24.9 to -7.3) |
| Severe tetanus | 0.1 (0.1 to 0.3) | 2.5 (1.4 to 4.5) | -- | -50.9* (-66.7 to -26.4) | -52.8* (-68.0 to -29.4) |
| Neonatal tetanus complications | 5.7 (5.5 to 6.0) | -- | 0.1 (0.1 to 0.2) | 2.7* (0.7 to 4.5) | -1.4 (-3.3 to 0.3) |
| Measles | 15.2 (5.4 to 33.7) | 553.1 (196.7 to 1229.1) | 1.4 (0.5 to 3.1) | -44.0* (-48.3 to -39.4) | -46.2* (-50.4 to -41.8) |
| Varicella and herpes zoster | 297.2 (232.5 to 380.0) | 4035.7 (2805.9 to 5626.0) | 14.2 (7.9 to 22.6) | 3.7 (-3.3 to 10.6) | -0.4 (-7.2 to 6.2) |
| Chickenpox | 52.6 (28.9 to 83.2) | 2742.0 (1505.8 to 4343.5) | 0.3 (0.1 to 0.8) | 10.6 (-4.3 to 27.6) | 6.2 (-8.1 to 22.5) |
| Herpes zoster | 244.6 (187.6 to 325.2) | 1293.7 (1038.5 to 1602.2) | 13.9 (7.6 to 22.2) | 3.6 (-3.6 to 10.5) | -0.5 (-7.4 to 6.1) |
| Acute hepatitis | 2507.4 (2173.7 to 2956.0) | 27566.2 (24387.7 to 31556.1) | 50.9 (32.0 to 75.0) | 3.3 (-8.9 to 16.6) | -0.8 (-12.5 to 12.0) |
| Acute hepatitis A | 1117.6 (996.6 to 1208.0) | 14528.8 (12955.6 to 15704.3) | 37.2 (23.0 to 55.0) | 3.6 (-10.7 to 19.8) | -0.5 (-14.3 to 15.0) |
| Acute hepatitis B | 1109.5 (812.7 to 1558.7) | 9615.3 (7043.8 to 13508.4) | 7.9 (4.6 to 12.9) | 3.2 (-27.2 to 47.4) | -0.9 (-30.1 to 41.5) |
| Acute hepatitis C | 51.4 (28.3 to 84.0) | 445.7 (245.7 to 727.7) | 0.7 (0.3 to 1.6) | 7.0* (2.7 to 11.8) | 2.8 (-1.4 to 7.4) |
| Acute hepatitis E | 228.9 (151.9 to 326.1) | 2976.3 (1975.2 to 4239.2) | 5.1 (2.7 to 8.7) | 0.9 (-25.1 to 30.9) | -3.1 (-28.1 to 25.7) |
| Other unspecified infectious diseases | 3672.4 (3361.7 to 4056.1) | -- | 168.9 (112.2 to 241.4) | -4.0 (-11.7 to 5.2) | -7.8 (-15.2 to 1.0) |
| Guillain-Barré syndrome due to other infectious diseases | 0.6 (0.3 to 1.1) | -- | 0.2 (0.1 to 0.3) | 7.8* (5.4 to 10.1) | 3.5* (1.2 to 5.8) |
| Other infectious diseases | -- | -- | 13.6 (8.0 to 21.4) | 14.9* (11.1 to 18.2) | 10.3* (6.7 to 13.6) |
| Anemia due to other infectious diseases | 3671.8 (3361.2 to 4055.6) | -- | 155.1 (102.9 to 221.3) | -5.3 (-13.5 to 4.6) | -9.1 (-17.0 to 0.4) |
| **Maternal and neonatal disorders** | **12641.0 (10890.9 to 14713.4)** | **216.1 (180.0 to 257.7)** | **2747.5 (2058.3 to 3579.5)** | **15.4* (8.6 to 23.2)** | **10.9* (4.3 to 18.3)** |
| Maternal disorders | 0.1 (0.0 to 0.1) | 216.1 (180.0 to 257.7) | 1.6 (1.0 to 2.4) | -1.2 (-10.3 to 10.6) | -5.2 (-13.9 to 6.2) |
| Maternal haemorrhage | 1.7 (1.5 to 2.1) | 12.1 (8.3 to 17.2) | 0.1 (0.1 to 0.2) | -2.7 (-18.2 to 17.6) | -6.5 (-21.4 to 12.9) |
| Maternal hemorrhage complications | 1.5 (1.3 to 1.7) | -- | 0.1 (0.1 to 0.1) | -3.5 (-20.2 to 17.8) | -7.4 (-23.4 to 13.1) |
| Maternal hemorrhage episode | 0.3 (0.2 to 0.4) | 12.1 (8.3 to 17.2) | 0.0 (0.0 to 0.1) | -0.7 (-31.1 to 42.9) | -4.7 (-33.8 to 37.2) |
| Maternal sepsis and other pregnancy related infections | 5.0 (3.1 to 7.7) | 40.0 (26.9 to 58.9) | 0.2 (0.1 to 0.3) | 7.4 (-37.0 to 74.4) | 3.2 (-39.5 to 67.5) |
| Infertility due to puerperal sepsis | 2.0 (1.3 to 3.2) | 0.8 (0.5 to 1.4) | -- | 25.4* (21.4 to 30.0) | 20.4* (16.6 to 24.8) |
| Maternal sepsis and other maternal infection episode | 3.0 (1.4 to 5.5) | 39.1 (26.2 to 58.2) | 0.2 (0.1 to 0.3) | 6.4 (-40.6 to 77.6) | 2.2 (-42.9 to 70.6) |
| Maternal hypertensive disorders | 3.6 (2.0 to 5.7) | 26.3 (20.8 to 32.7) | 0.2 (0.1 to 0.3) | -7.3 (-35.9 to 28.6) | -10.9 (-38.5 to 23.5) |
| Maternal hypertensive disorder complications | 0.2 (0.2 to 0.3) | 4.5 (3.9 to 5.3) | -- | 20.6* (18.1 to 23.4) | 15.8* (13.4 to 18.5) |
| Maternal hypertensive disorder episode | 3.3 (1.8 to 5.5) | 21.8 (16.1 to 28.0) | 0.2 (0.1 to 0.3) | -9.2 (-40.7 to 29.3) | -12.8 (-43.0 to 24.1) |
| Maternal obstructed labour and uterine rupture | 2.2 (1.2 to 3.6) | 19.9 (14.0 to 27.0) | 0.8 (0.4 to 1.4) | -5.9 (-13.2 to 1.4) | -9.6* (-16.7 to -2.6) |
| Obstructed labor, acute event | 0.2 (0.1 to 0.4) | 17.2 (11.6 to 23.6) | 0.1 (0.0 to 0.1) | 1.9 (-40.0 to 63.2) | -2.2 (-42.4 to 56.7) |
| Maternal obstructed labor complications | 2.0 (1.0 to 3.4) | 2.8 (1.4 to 5.1) | 0.7 (0.3 to 1.3) | -6.7* (-12.4 to -1.0) | -10.4* (-15.9 to -4.9) |
| Maternal abortion and miscarriage | 0.5 (0.3 to 0.7) | 55.2 (38.9 to 78.7) | 0.1 (0.0 to 0.1) | 5.4 (-32.4 to 53.8) | 1.2 (-35.1 to 47.7) |
| Ectopic pregnancy | 0.5 (0.3 to 0.8) | 62.6 (39.1 to 92.1) | 0.1 (0.0 to 0.1) | 26.3 (-20.1 to 88.1) | 21.3 (-23.3 to 80.7) |
| Other maternal disorders | -- | -- | 0.2 (0.1 to 0.3) | 10.9* (0.3 to 24.1) | 6.5 (-3.6 to 19.2) |
| Neonatal disorders | 12640.9 (10890.8 to 14713.3) | -- | 2745.9 (2057.0 to 3577.7) | 15.5* (8.6 to 23.2) | 10.9* (4.3 to 18.3) |
| Neonatal preterm birth | 7386.7 (6276.0 to 8784.9) | -- | 1367.1 (973.2 to 1854.7) | 16.9* (2.3 to 32.7) | 12.3 (-1.8 to 27.5) |
| Preterm birth complications | 6376.2 (5257.0 to 7767.6) | -- | 1314.4 (922.1 to 1797.2) | 17.9* (2.3 to 34.1) | 13.2 (-1.8 to 28.7) |
| Retinopathy of prematurity | 1010.5 (783.6 to 1257.1) | -- | 52.7 (35.2 to 75.0) | -2.8 (-10.7 to 4.4) | -6.6 (-14.3 to 0.3) |
| Uncomplicated preterm birth | -- | -- | -- | -- | -- |
| Neonatal encephalopathy due to birth asphyxia and trauma | 4662.6 (3352.8 to 6185.9) | -- | 900.3 (536.7 to 1415.8) | 14.7* (9.9 to 19.4) | 10.1* (5.5 to 14.6) |
| Neonatal sepsis and other neonatal infections | 1263.3 (776.1 to 1936.8) | -- | 387.3 (202.8 to 653.1) | 13.2* (8.3 to 18.0) | 8.7* (4.0 to 13.3) |
| Neonatal sepsis and other neonatal infection complications | 1263.3 (776.1 to 1936.8) | -- | 387.3 (202.8 to 653.1) | 13.2* (8.3 to 18.0) | 8.7* (4.0 to 13.3) |
| Neonatal sepsis and other neonatal infection episode | -- | -- | -- | -- | -- |
| Hemolytic disease and other neonatal jaundice | 241.3 (207.3 to 276.0) | -- | 91.2 (69.5 to 115.0) | 11.8* (5.1 to 17.6) | 7.4* (0.9 to 13.0) |

| | | | | | |
|---|---|---|---|---|---|
| Other neonatal disorders | -- | -- | -- | -- | -- |
| **Nutritional deficiencies** | **146183.3 (137803.3 to 155945.4)** | **110318.6 (99082.5 to 122867.4)** | **5345.6 (3552.9 to 7748.1)** | **-8.8* (-15.3 to -1.3)** | **-12.4* (-18.6 to -5.2)** |
| Protein-energy malnutrition | 1986.2 (1695.4 to 2309.8) | 2378.3 (1999.8 to 2782.3) | 28.0 (17.5 to 39.9) | 19.7* (9.8 to 31.1) | 15.0* (5.4 to 25.9) |
| Iodine deficiency | 9271.6 (8194.2 to 10489.6) | 628.3 (515.1 to 742.0) | 190.5 (114.8 to 292.7) | -20.1* (-24.9 to -14.8) | -23.2* (-27.9 to -18.2) |
| Visible goiter without symptoms | 8991.2 (7952.7 to 10190.5) | 628.3 (515.1 to 742.0) | 97.6 (47.4 to 183.1) | -21.1* (-23.6 to -18.9) | -24.2* (-26.6 to -22.1) |
| Visible goiter with complications | 280.4 (161.3 to 380.4) | -- | 92.8 (48.4 to 141.9) | -19.0* (-29.2 to -8.5) | -22.2* (-32.0 to -12.1) |
| Vitamin A deficiency | 77449.6 (68625.3 to 86985.3) | 107312.1 (95929.5 to 119818.1) | 1710.4 (1108.9 to 2484.0) | -10.9* (-17.9 to -3.1) | -14.4* (-21.1 to -6.9) |
| Asymptomatic vitamin A deficiency | 38121.2 (33119.2 to 43934.3) | 107312.1 (95929.5 to 119818.1) | -- | -- | -- |
| Vitamin A deficiency complications | 558.6 (357.6 to 813.8) | -- | 31.1 (18.2 to 49.7) | 11.1* (2.9 to 20.2) | 6.7 (-1.2 to 15.4) |
| Vitamin A deficiency with anemia | 38770.0 (33548.9 to 44724.0) | -- | 1679.3 (1084.9 to 2443.7) | -11.2* (-18.2 to -3.4) | -14.7* (-21.4 to -7.3) |
| Dietary iron deficiency | 79647.6 (73012.8 to 87642.0) | -- | 3410.6 (2264.3 to 4926.4) | -7.1 (-14.5 to 1.7) | -10.8* (-17.9 to -2.3) |
| Other nutritional deficiencies | -- | -- | 6.0 (3.7 to 8.6) | -19.5* (-26.2 to -11.9) | -22.7* (-29.1 to -15.4) |
| **Non-communicable diseases** | **542753.1 (525526.6 to 559824.5)** | **765842.6 (646208.2 to 917975.5)** | **24253.9 (17711.6 to 32004.6)** | **5.1* (4.1 to 6.0)** | **0.9 (-0.0 to 1.8)** |
| **Neoplasms** | **757.5 (668.2 to 897.4)** | **64.0 (60.0 to 67.6)** | **47.0 (31.7 to 66.2)** | **62.1* (39.5 to 95.1)** | **55.7* (34.0 to 87.3)** |
| Lip and oral cavity cancer | -- | -- | -- | -- | -- |
| Diagnosis and primary therapy phase of mouth cancer | -- | -- | -- | -- | -- |
| Controlled phase of mouth cancer | -- | -- | -- | -- | -- |
| Metastatic phase of mouth cancer | -- | -- | -- | -- | -- |
| Terminal phase of mouth cancer | -- | -- | -- | -- | -- |
| Nasopharynx cancer | 11.4 (8.9 to 14.5) | 2.0 (1.6 to 2.6) | 1.0 (0.7 to 1.5) | 0.9 (-22.7 to 33.4) | -3.1 (-25.8 to 28.1) |
| Diagnosis and primary therapy phase of nasopharynx cancer | 0.6 (0.5 to 0.8) | 2.0 (1.6 to 2.6) | 0.2 (0.1 to 0.3) | 3.5 (-19.1 to 36.2) | -0.6 (-22.3 to 30.8) |
| Controlled phase of nasopharynx cancer | 9.9 (7.8 to 12.6) | -- | 0.5 (0.3 to 0.8) | 3.2 (-20.7 to 36.3) | -0.9 (-23.9 to 30.9) |
| Metastatic phase of nasopharynx cancer | 0.7 (0.6 to 0.9) | -- | 0.3 (0.2 to 0.5) | -2.3 (-26.1 to 31.9) | -6.2 (-29.0 to 26.6) |
| Terminal phase of nasopharynx cancer | 0.1 (0.1 to 0.1) | -- | 0.0 (0.0 to 0.1) | -7.2 (-31.7 to 27.1) | -10.9 (-34.4 to 22.0) |
| Other pharynx cancer | -- | -- | -- | -- | -- |
| Diagnosis and primary therapy phase of other pharynx cancer | -- | -- | -- | -- | -- |
| Controlled phase of other pharynx cancer | -- | -- | -- | -- | -- |
| Metastatic phase of other pharynx cancer | -- | -- | -- | -- | -- |
| Terminal phase of other pharynx cancer | -- | -- | -- | -- | -- |
| Oesophageal cancer | -- | -- | -- | -- | -- |
| Diagnosis and primary therapy phase of esophageal cancer | -- | -- | -- | -- | -- |
| Controlled phase of esophageal cancer | -- | -- | -- | -- | -- |
| Metastatic phase of esophageal cancer | -- | -- | -- | -- | -- |
| Terminal phase of esophageal cancer | -- | -- | -- | -- | -- |
| Stomach cancer | -- | -- | -- | -- | -- |
| Diagnosis and primary therapy phase of stomach cancer | -- | -- | -- | -- | -- |
| Controlled phase of stomach cancer | -- | -- | -- | -- | -- |
| Metastatic phase of stomach cancer | -- | -- | -- | -- | -- |
| Terminal phase of stomach cancer | -- | -- | -- | -- | -- |
| Colon and rectum cancer | -- | -- | -- | -- | -- |
| Diagnosis and primary therapy phase of colon and rectum cancers | -- | -- | -- | -- | -- |
| Controlled phase of colon and rectum cancers | -- | -- | -- | -- | -- |
| Metastatic phase of colon and rectum cancers | -- | -- | -- | -- | -- |
| Terminal phase of colon and rectum cancers | -- | -- | -- | -- | -- |
| Stoma from colon and rectum cancers, beyond 10 years | -- | -- | -- | -- | -- |
| Liver cancer | -- | 1.0 (0.9 to 1.1) | 0.2 (0.2 to 0.3) | -2.0 (-11.4 to 7.4) | -5.9 (-14.9 to 3.1) |
| Liver cancer due to hepatitis B | 0.7 (0.6 to 0.8) | 0.7 (0.6 to 0.8) | 0.2 (0.1 to 0.2) | -3.4 (-13.4 to 6.4) | -7.3 (-16.9 to 2.2) |
| Diagnosis and primary therapy phase of liver cancer due to hepatitis B | 0.2 (0.1 to 0.2) | 0.7 (0.6 to 0.8) | 0.0 (0.0 to 0.1) | -2.5 (-12.8 to 7.7) | -6.4 (-16.3 to 3.4) |
| Controlled phase of liver cancer due to hepatitis B | 0.4 (0.3 to 0.4) | -- | -- | -2.1 (-11.6 to 8.0) | -6.0 (-15.1 to 3.7) |
| Metastatic phase of liver cancer due to hepatitis B | 0.1 (0.1 to 0.2) | -- | 0.1 (0.0 to 0.1) | -4.1 (-14.2 to 5.7) | -7.9 (-17.6 to 1.5) |
| Terminal phase of liver cancer due to hepatitis B | 0.1 (0.1 to 0.1) | -- | -- | -4.1 (-14.2 to 5.7) | -7.9 (-17.6 to 1.5) |
| Liver cancer due to hepatitis C | -- | -- | -- | 2.4 (-7.9 to 15.9) | -1.6 (-11.6 to 11.3) |
| Diagnosis and primary therapy phase of liver cancer due to hepatitis C | -- | -- | -- | 3.5 (-7.1 to 17.1) | -0.7 (-10.8 to 12.5) |
| Controlled phase of liver cancer due to hepatitis C | -- | -- | -- | -0.2 (-9.5 to 12.0) | -4.2 (-13.0 to 7.6) |
| Metastatic phase of liver cancer due to hepatitis C | -- | -- | -- | 2.4 (-8.1 to 16.0) | -1.7 (-11.8 to 11.4) |
| Terminal phase of liver cancer due to hepatitis C | -- | -- | -- | 2.4 (-8.1 to 16.0) | -1.7 (-11.8 to 11.4) |
| Liver cancer due to alcohol use | -- | -- | -- | -- | -- |
| Diagnosis and primary therapy phase of liver cancer due to alcohol use | -- | -- | -- | -- | -- |
| Controlled phase of liver cancer due to alcohol use | -- | -- | -- | -- | -- |
| Metastatic phase of liver cancer due to alcohol use | -- | -- | -- | -- | -- |

| | | | | | |
|---|---|---|---|---|---|
| Terminal phase of liver cancer due to alcohol use | -- | -- | -- | -- | -- |
| Liver cancer due to NASH | -- | -- | -- | -- | -- |
| Diagnosis and primary therapy phase of liver cancer due to NASH | -- | -- | -- | -- | -- |
| Controlled phase of liver cancer due to NASH | -- | -- | -- | -- | -- |
| Metastatic phase of liver cancer due to NASH | -- | -- | -- | -- | -- |
| Terminal phase of liver cancer due to NASH | -- | -- | -- | -- | -- |
| Liver cancer due to other causes | 0.2<br>(0.2 to 0.3) | 0.2<br>(0.2 to 0.3) | 0.1<br>(0.0 to 0.1) | 2.2<br>(-6.4 to 12.2) | -1.8<br>(-10.1 to 7.7) |
| Diagnosis and primary therapy phase of liver cancer due to other causes | 0.1<br>(0.0 to 0.1) | 0.2<br>(0.2 to 0.3) | -- | 3.2<br>(-5.6 to 13.2) | -0.9<br>(-9.3 to 8.7) |
| Controlled phase of liver cancer due to other causes | 0.1<br>(0.1 to 0.1) | -- | -- | 2.6<br>(-5.3 to 12.0) | -1.5<br>(-9.1 to 7.5) |
| Metastatic phase of liver cancer due to other causes | 0.0<br>(0.0 to 0.1) | -- | -- | 1.7<br>(-7.0 to 11.6) | -2.3<br>(-10.6 to 7.1) |
| Terminal phase of liver cancer due to other causes | -- | -- | -- | 1.7<br>(-7.0 to 11.6) | -2.3<br>(-10.6 to 7.1) |
| Gallbladder and biliary tract cancer | -- | -- | -- | -- | -- |
| Diagnosis and primary therapy phase of gallbladder and biliary tract cancer | -- | -- | -- | -- | -- |
| Controlled phase of gallbladder and biliary tract cancer | -- | -- | -- | -- | -- |
| Metastatic phase of gallbladder and biliary tract cancer | -- | -- | -- | -- | -- |
| Terminal phase of gallbladder and biliary tract cancer | -- | -- | -- | -- | -- |
| Pancreatic cancer | -- | -- | -- | -- | -- |
| Diagnosis and primary therapy phase of pancreatic cancer | -- | -- | -- | -- | -- |
| Controlled phase of pancreatic cancer | -- | -- | -- | -- | -- |
| Metastatic phase of pancreatic cancer | -- | -- | -- | -- | -- |
| Terminal phase of pancreatic cancer | -- | -- | -- | -- | -- |
| Larynx cancer | -- | -- | -- | -- | -- |
| Diagnosis and primary therapy phase of larynx cancer | -- | -- | -- | -- | -- |
| Controlled phase of larynx cancer | -- | -- | -- | -- | -- |
| Metastatic phase of larynx cancer | -- | -- | -- | -- | -- |
| Terminal phase of larynx cancer | -- | -- | -- | -- | -- |
| Laryngectomy from larynx cancer, beyond 10 years | -- | -- | -- | -- | -- |
| Tracheal, bronchus, and lung cancer | -- | -- | -- | -- | -- |
| Diagnosis and primary therapy phase of lung, bronchus, and trachea cancer | -- | -- | -- | -- | -- |
| Controlled phase of lung, bronchus, and trachea cancer | -- | -- | -- | -- | -- |
| Metastatic phase of lung, bronchus, and trachea cancer | -- | -- | -- | -- | -- |
| Terminal phase of lung, bronchus, and trachea cancer | -- | -- | -- | -- | -- |
| Malignant skin melanoma | -- | -- | -- | -- | -- |
| Diagnosis and primary therapy phase of malignant skin melanoma | -- | -- | -- | -- | -- |
| Controlled phase of malignant skin melanoma | -- | -- | -- | -- | -- |
| Metastatic phase of malignant skin melanoma | -- | -- | -- | -- | -- |
| Terminal phase of malignant skin melanoma | -- | -- | -- | -- | -- |
| Non-melanoma skin cancer | -- | -- | -- | -- | -- |
| Non-melanoma skin cancer (squamous-cell carcinoma) | -- | -- | -- | -- | -- |
| Non-melanoma skin cancer (basal-cell carcinoma) | -- | -- | -- | -- | -- |
| Breast cancer | -- | -- | -- | -- | -- |
| Diagnosis and primary therapy phase of breast cancer | -- | -- | -- | -- | -- |
| Controlled phase of breast cancer | -- | -- | -- | -- | -- |
| Metastatic phase of breast cancer | -- | -- | -- | -- | -- |
| Terminal phase of breast cancer | -- | -- | -- | -- | -- |
| Mastectomy from breast cancer, beyond 10 years | -- | -- | -- | -- | -- |
| Cervical cancer | -- | -- | -- | -- | -- |
| Diagnosis and primary therapy phase of cervical cancer | -- | -- | -- | -- | -- |
| Controlled phase of cervical cancer | -- | -- | -- | -- | -- |
| Metastatic phase of cervical cancer | -- | -- | -- | -- | -- |
| Terminal phase of cervical cancer | -- | -- | -- | -- | -- |
| Uterine cancer | -- | -- | -- | -- | -- |
| Diagnosis and primary therapy phase of uterine cancer | -- | -- | -- | -- | -- |
| Controlled phase of uterine cancer | -- | -- | -- | -- | -- |
| Metastatic phase of uterine cancer | -- | -- | -- | -- | -- |
| Terminal phase of uterine cancer | -- | -- | -- | -- | -- |
| Ovarian cancer | -- | -- | -- | -- | -- |
| Diagnosis and primary therapy phase of ovarian cancer | -- | -- | -- | -- | -- |
| Controlled phase of ovarian cancer | -- | -- | -- | -- | -- |
| Metastatic phase of ovarian cancer | -- | -- | -- | -- | -- |

| | | | | | |
|---|---|---|---|---|---|
| Terminal phase of ovarian cancer | -- | -- | -- | -- | -- |
| Prostate cancer | -- | -- | -- | -- | -- |
| Diagnosis and primary therapy phase of prostate cancer | -- | -- | -- | -- | -- |
| Controlled phase of prostate cancer | -- | -- | -- | -- | -- |
| Metastatic phase of prostate cancer | -- | -- | -- | -- | -- |
| Terminal phase of prostate cancer | -- | -- | -- | -- | -- |
| Impotence and Incontinence after 10-year survival from prostate cancer | -- | -- | -- | -- | -- |
| Testicular cancer | -- | -- | -- | -- | -- |
| Diagnosis and primary therapy phase of testicular cancer | -- | -- | -- | -- | -- |
| Controlled phase of testicular cancer | -- | -- | -- | -- | -- |
| Metastatic phase of testicular cancer | -- | -- | -- | -- | -- |
| Terminal phase of testicular cancer | -- | -- | -- | -- | -- |
| Kidney cancer | 15.4<br>(14.1 to 16.5) | 2.2<br>(2.0 to 2.4) | 0.9<br>(0.6 to 1.3) | 8.8<br>(-0.0 to 16.7) | 4.5<br>(-4.0 to 12.1) |
| Diagnosis and primary therapy phase of kidney cancer | -- | 2.2<br>(2.0 to 2.4) | -- | -- | -- |
| Controlled phase of kidney cancer | 15.0<br>(13.7 to 16.1) | -- | 0.7<br>(0.5 to 1.1) | 8.8<br>(-0.0 to 16.8) | 4.5<br>(-4.0 to 12.2) |
| Metastatic phase of kidney cancer | 0.3<br>(0.3 to 0.4) | -- | 0.2<br>(0.1 to 0.2) | 8.5*<br>(0.0 to 16.4) | 4.2<br>(-4.0 to 11.8) |
| Terminal phase of kidney cancer | 0.1<br>(0.1 to 0.1) | -- | -- | 8.5*<br>(0.0 to 16.4) | 4.2<br>(-3.9 to 11.8) |
| Bladder cancer | -- | -- | -- | -- | -- |
| Diagnosis and primary therapy phase of bladder cancer | -- | -- | -- | -- | -- |
| Controlled phase of bladder cancer | -- | -- | -- | -- | -- |
| Metastatic phase of bladder cancer | -- | -- | -- | -- | -- |
| Terminal phase of bladder cancer | -- | -- | -- | -- | -- |
| Incontinence from bladder cancer, beyond 10 years | -- | -- | -- | -- | -- |
| Brain and nervous system cancer | 65.3<br>(57.6 to 73.8) | 13.3<br>(11.7 to 15.2) | 6.1<br>(4.3 to 8.3) | 41.2*<br>(29.0 to 53.7) | 35.6*<br>(23.9 to 47.6) |
| Diagnosis and primary therapy phase of brain and nervous system cancers | 3.9<br>(3.4 to 4.4) | 13.3<br>(11.7 to 15.2) | 1.1<br>(0.7 to 1.6) | 55.9*<br>(42.6 to 69.8) | 49.7*<br>(37.0 to 63.0) |
| Controlled phase of brain and nervous system cancers | 56.7<br>(50.0 to 63.9) | -- | 2.7<br>(1.7 to 4.1) | 62.3*<br>(43.9 to 81.7) | 55.9*<br>(38.2 to 74.5) |
| Metastatic phase of brain and nervous system cancers | 4.1<br>(3.6 to 4.7) | -- | 1.8<br>(1.2 to 2.5) | 17.5*<br>(8.6 to 26.2) | 12.8*<br>(4.3 to 21.2) |
| Terminal phase of brain and nervous system cancers | 0.7<br>(0.6 to 0.8) | -- | 0.4<br>(0.3 to 0.5) | 12.9*<br>(4.2 to 21.0) | 8.4*<br>(0.1 to 16.2) |
| Thyroid cancer | 15.6<br>(14.0 to 17.8) | 1.8<br>(1.6 to 2.0) | 0.9<br>(0.6 to 1.3) | 19.0*<br>(1.0 to 44.3) | 14.3<br>(-3.0 to 38.5) |
| Diagnosis and primary therapy phase of thyroid cancer | 0.4<br>(0.4 to 0.5) | 1.8<br>(1.6 to 2.0) | 0.1<br>(0.1 to 0.2) | 20.1*<br>(9.3 to 36.0) | 15.3*<br>(4.9 to 30.6) |
| Controlled phase of thyroid cancer | 15.1<br>(13.5 to 17.2) | -- | 0.7<br>(0.5 to 1.1) | 19.9<br>(-0.9 to 50.4) | 15.2<br>(-4.8 to 44.4) |
| Metastatic phase of thyroid cancer | 0.1<br>(0.1 to 0.1) | -- | 0.1<br>(0.0 to 0.1) | 7.8<br>(-3.8 to 24.0) | 3.5<br>(-7.6 to 19.0) |
| Terminal phase of thyroid cancer | -- | -- | -- | 1.8<br>(-10.0 to 18.9) | -2.3<br>(-13.6 to 14.2) |
| Mesothelioma | -- | -- | -- | -- | -- |
| Diagnosis and primary therapy phase of mesothelioma | -- | -- | -- | -- | -- |
| Controlled phase of mesothelioma | -- | -- | -- | -- | -- |
| Metastatic phase of mesothelioma | -- | -- | -- | -- | -- |
| Terminal phase of mesothelioma | -- | -- | -- | -- | -- |
| Hodgkin lymphoma | 20.5<br>(16.6 to 24.0) | 3.4<br>(2.7 to 4.0) | 1.6<br>(1.1 to 2.2) | 20.8*<br>(9.5 to 32.6) | 16.0*<br>(5.2 to 27.3) |
| Diagnosis and primary therapy phase of Hodgkin disease | 0.7<br>(0.5 to 0.8) | 3.4<br>(2.7 to 4.0) | 0.2<br>(0.1 to 0.3) | 29.6*<br>(17.5 to 43.0) | 24.5*<br>(12.8 to 37.3) |
| Controlled phase of Hodgkin disease | 18.8<br>(15.2 to 22.0) | -- | 0.9<br>(0.5 to 1.4) | 29.4*<br>(14.8 to 45.4) | 24.3*<br>(10.2 to 39.6) |
| Metastatic phase of Hodgkin disease | 0.9<br>(0.7 to 1.1) | -- | 0.4<br>(0.3 to 0.6) | 6.3<br>(-3.4 to 15.6) | 2.1<br>(-7.3 to 11.0) |
| Terminal phase of Hodgkin disease | 0.1<br>(0.1 to 0.1) | -- | 0.1<br>(0.0 to 0.1) | -3.6<br>(-13.8 to 7.7) | -7.5<br>(-17.2 to 3.5) |
| Non-Hodgkin's lymphoma | 26.4<br>(24.6 to 28.2) | 5.6<br>(5.1 to 6.1) | 2.2<br>(1.6 to 3.0) | 22.1*<br>(12.4 to 32.5) | 17.3*<br>(7.9 to 27.3) |
| Diagnosis and primary therapy phase of non-Hodgkin lymphoma | 0.9<br>(0.9 to 1.0) | 5.6<br>(5.1 to 6.1) | 0.3<br>(0.2 to 0.4) | 28.1*<br>(19.4 to 36.6) | 23.0*<br>(14.7 to 31.2) |
| Controlled phase of non-Hodgkin lymphoma | 23.7<br>(22.2 to 25.3) | -- | 1.2<br>(0.7 to 1.7) | 28.3*<br>(17.1 to 41.4) | 23.2*<br>(12.4 to 35.7) |
| Metastatic phase of non-Hodgkin lymphoma | 1.5<br>(1.3 to 1.6) | -- | 0.7<br>(0.4 to 0.9) | 12.9*<br>(2.4 to 23.8) | 8.4<br>(-1.7 to 18.9) |
| Terminal phase of non-Hodgkin lymphoma | 0.3<br>(0.2 to 0.3) | -- | 0.1<br>(0.1 to 0.2) | 11.5*<br>(0.8 to 22.7) | 7.1<br>(-3.2 to 17.8) |
| Multiple myeloma | -- | -- | -- | -- | -- |
| Diagnosis and primary therapy phase of multiple myeloma | -- | -- | -- | -- | -- |
| Controlled phase of multiple myeloma | -- | -- | -- | -- | -- |
| Metastatic phase of multiple myeloma | -- | -- | -- | -- | -- |
| Terminal phase of multiple myeloma | -- | -- | -- | -- | -- |
| Leukaemia | 82.5<br>(71.6 to 90.7) | 17.3<br>(15.1 to 19.3) | 8.7<br>(6.1 to 11.5) | 3.5<br>(-2.7 to 10.5) | -0.6<br>(-6.6 to 6.1) |
| Acute lymphoid leukaemia | 36.0<br>(30.9 to 39.5) | 5.5<br>(4.7 to 6.2) | 3.5<br>(2.5 to 4.7) | 15.1*<br>(3.2 to 27.2) | 10.5<br>(-0.9 to 22.2) |
| Diagnosis and primary therapy phase of acute lymphoid leukemia | 2.6<br>(2.3 to 2.9) | 5.5<br>(4.7 to 6.2) | 0.7<br>(0.5 to 1.1) | 1.9<br>(-8.7 to 11.7) | -2.1<br>(-12.3 to 7.2) |
| Controlled phase of acute lymphoid leukemia | 30.7<br>(26.4 to 33.6) | -- | 1.5<br>(0.9 to 2.2) | 24.4*<br>(8.7 to 40.3) | 19.4*<br>(4.4 to 34.7) |
| Metastatic phase of acute lymphoid leukemia | 2.3<br>(1.9 to 2.5) | -- | 1.0<br>(0.7 to 1.4) | 13.8*<br>(2.3 to 26.7) | 9.3<br>(-1.8 to 21.7) |
| Terminal phase of acute lymphoid leukemia | 0.4<br>(0.4 to 0.5) | -- | 0.2<br>(0.2 to 0.3) | 13.0*<br>(1.6 to 26.1) | 8.5<br>(-2.5 to 21.0) |
| Chronic lymphoid leukaemia | -- | -- | -- | -- | -- |

| | | | | | |
|---|---|---|---|---|---|
| Diagnosis and primary therapy phase of chronic lymphoid leukemia | -- | -- | -- | -- | -- |
| Controlled phase of chronic lymphoid leukemia | -- | -- | -- | -- | -- |
| Metastatic phase of chronic lymphoid leukemia | -- | -- | -- | -- | -- |
| Terminal phase of chronic lymphoid leukemia | -- | -- | -- | -- | -- |
| Acute myeloid leukaemia | 4.0 (3.4 to 4.6) | 3.6 (3.1 to 4.1) | 0.9 (0.6 to 1.2) | 2.2 (-10.1 to 15.0) | -1.8 (-13.7 to 10.4) |
| Diagnosis and primary therapy phase of acute myeloid leukemia | -- | 3.6 (3.1 to 4.1) | -- | -- | -- |
| Controlled phase of acute myeloid leukemia | 2.5 (2.1 to 2.8) | -- | 0.1 (0.1 to 0.2) | 0.4 (-12.1 to 12.2) | -3.6 (-15.6 to 7.8) |
| Metastatic phase of acute myeloid leukemia | 1.3 (1.1 to 1.5) | -- | 0.6 (0.4 to 0.8) | 2.5 (-9.8 to 15.4) | -1.5 (-13.4 to 10.8) |
| Terminal phase of acute myeloid leukemia | 0.3 (0.2 to 0.3) | -- | 0.2 (0.1 to 0.2) | 2.5 (-9.8 to 15.4) | -1.5 (-13.4 to 10.8) |
| Chronic myeloid leukaemia | -- | -- | -- | -- | -- |
| Diagnosis and primary therapy phase of chronic myeloid leukemia | -- | -- | -- | -- | -- |
| Controlled phase of chronic myeloid leukemia | -- | -- | -- | -- | -- |
| Metastatic phase of chronic myeloid leukemia | -- | -- | -- | -- | -- |
| Terminal phase of chronic myeloid leukemia | -- | -- | -- | -- | -- |
| Other leukaemia | 42.4 (36.1 to 48.9) | 8.2 (7.0 to 9.4) | 4.3 (3.0 to 5.8) | -4.1 (-13.5 to 11.6) | -7.9 (-16.9 to 7.2) |
| Metastatic phase of other leukemia | 5.2 (4.4 to 5.8) | -- | 2.3 (1.5 to 3.2) | -6.2 (-14.5 to 7.7) | -9.9 (-17.9 to 3.4) |
| Diagnosis and primary therapy phase of other leukemia | -- | 8.2 (7.0 to 9.4) | -- | -- | -- |
| Terminal phase of other leukemia | 0.3 (0.3 to 0.4) | -- | 0.2 (0.1 to 0.2) | -15.9* (-23.6 to -4.2) | -19.2* (-26.6 to -8.0) |
| Controlled phase of other leukemia | 36.9 (31.3 to 42.9) | -- | 1.8 (1.1 to 2.7) | 0.3 (-14.0 to 20.3) | -3.7 (-17.5 to 15.6) |
| Other malignant cancers | 315.7 (231.4 to 460.3) | 17.4 (15.7 to 18.9) | 24.4 (15.3 to 37.9) | 163.8* (91.4 to 279.1) | 153.3* (83.8 to 264.0) |
| Diagnosis and primary therapy phase of other malignant neoplasms | 7.2 (5.3 to 10.6) | 17.4 (15.7 to 18.9) | 2.1 (1.2 to 3.3) | 191.6* (106.3 to 329.8) | 180.0* (98.1 to 312.7) |
| Controlled phase of other malignant neoplasms | 290.3 (211.8 to 425.4) | -- | 14.0 (7.8 to 23.8) | 207.9* (116.0 to 352.0) | 195.7* (107.4 to 334.0) |
| Metastatic phase of other malignant neoplasms | 16.0 (12.3 to 22.7) | -- | 7.1 (4.3 to 11.0) | 110.3* (59.7 to 185.8) | 102.0* (53.4 to 174.4) |
| Terminal phase of other malignant neoplasms | 2.2 (1.8 to 3.2) | -- | 1.2 (0.7 to 1.8) | 98.3* (52.3 to 171.8) | 90.4* (46.3 to 161.0) |
| Other neoplasms | 383.4 (352.4 to 419.2) | 222.9 (205.4 to 240.5) | 0.9 (0.5 to 1.4) | -11.9* (-18.2 to -5.2) | -15.4* (-21.5 to -9.0) |
| Myelodysplastic, myeloproliferative, and other hematopoietic neoplasms | 18.7 (14.9 to 22.8) | 1.0 (0.5 to 1.7) | 0.9 (0.5 to 1.4) | -11.9* (-18.2 to -5.2) | -15.4* (-21.5 to -9.0) |
| Benign and in situ intestinal neoplasms | 8.3 (7.4 to 9.3) | 5.4 (4.8 to 6.0) | -- | -- | -- |
| Benign and in situ cervical and uterine neoplasms | 2.8 (2.5 to 3.1) | 2.3 (2.1 to 2.5) | -- | -- | -- |
| Other benign and in situ neoplasms | 384.1 (355.1 to 418.1) | 214.2 (196.8 to 231.9) | -- | -- | -- |
| **Cardiovascular diseases** | **3898.0 (3436.9 to 4546.6)** | **411.1 (371.6 to 454.9)** | **300.4 (212.4 to 415.1)** | **-5.7* (-9.6 to -2.0)** | **-9.4* (-13.2 to -5.9)** |
| Rheumatic heart disease | 2488.3 (2357.7 to 2610.1) | 229.4 (220.4 to 238.5) | 119.7 (77.6 to 173.7) | 8.3* (5.9 to 10.8) | 4.0* (1.7 to 6.4) |
| Rheumatic heart disease, without heart failure | 2484.3 (2353.6 to 2605.7) | 229.4 (220.4 to 238.5) | 119.3 (77.4 to 173.4) | 8.4* (5.9 to 10.9) | 4.1* (1.7 to 6.5) |
| Heart failure due to rheumatic heart disease | 4.0 (2.4 to 6.4) | -- | 0.4 (0.2 to 0.6) | 3.9* (1.9 to 6.0) | -0.2 (-2.2 to 1.7) |
| Ischaemic heart disease | -- | -- | -- | -- | -- |
| Asymptomatic ischemic heart disease following myocardial infarction | -- | -- | -- | -- | -- |
| Angina due to ischemic heart disease | -- | -- | -- | -- | -- |
| Heart failure due to ischemic heart disease | -- | -- | -- | -- | -- |
| Myocardial infarction due to ischemic heart disease | -- | -- | -- | -- | -- |
| Stroke | 442.5 (370.1 to 530.9) | 38.8‡ (24.0 to 61.7) | 111.6 (76.7 to 151.3) | -21.7* (-26.8 to -17.0) | -24.9* (-29.7 to -20.3) |
| Ischaemic stroke | 475.8 (382.4 to 583.8) | 22.7 (9.8 to 43.7) | 69.5 (46.8 to 98.8) | -23.7* (-30.9 to -17.0) | -26.7* (-33.7 to -20.3) |
| Acute ischemic stroke | 1.8 (0.8 to 3.6) | 22.7 (9.8 to 43.7) | 0.5 (0.2 to 1.1) | 7.2 (-1.1 to 13.0) | 3.0 (-5.0 to 8.5) |
| Chronic ischemic stroke | 473.9 (380.4 to 582.4) | -- | 69.0 (46.3 to 97.8) | -23.9* (-31.1 to -17.2) | -26.9* (-33.9 to -20.4) |
| Intracerebral hemorrhage | 219.4 (175.8 to 268.3) | 10.9 (5.0 to 19.0) | 33.0 (21.8 to 46.0) | -21.5* (-28.6 to -14.6) | -24.7* (-31.5 to -18.0) |
| Acute intracerebral hemorrhage | 0.8 (0.3 to 1.4) | 10.9 (5.0 to 19.0) | 0.2 (0.1 to 0.4) | -14.8* (-31.2 to -4.9) | -18.1* (-33.9 to -8.7) |
| Chronic intracerebral hemorrhage | 218.6 (175.0 to 267.8) | -- | 32.7 (21.6 to 45.8) | -21.6* (-28.7 to -14.6) | -24.7* (-31.6 to -18.0) |
| Subarachnoid hemorrhage | 62.1 (46.7 to 81.5) | 5.2 (3.4 to 7.3) | 9.1 (6.0 to 13.3) | -4.0* (-7.9 to -0.5) | -7.8* (-11.6 to -4.5) |
| Acute subarachnoid hemorrhage | 0.3 (0.2 to 0.4) | 5.2 (3.4 to 7.3) | 0.1 (0.0 to 0.1) | 0.4 (-3.1 to 2.5) | -3.6* (-7.0 to -1.6) |
| Chronic subarachnoid hemorrhage | 61.8 (46.4 to 81.2) | -- | 9.0 (5.9 to 13.2) | -4.0* (-8.0 to -0.5) | -7.8* (-11.7 to -4.5) |
| Hypertensive heart disease | -- | -- | -- | -- | -- |
| Non-rheumatic valvular heart disease | -- | -- | -- | -- | -- |
| Non-rheumatic calcific aortic valve disease | -- | -- | -- | -- | -- |
| Heart failure due to calcific aortic valve disease | -- | -- | -- | -- | -- |
| Non-rheumatic calcific aortic valve disease | -- | -- | -- | -- | -- |
| Non-rheumatic degenerative mitral valve disease | -- | -- | -- | -- | -- |
| Heart failure due to degenerative mitral valve disease | -- | -- | -- | -- | -- |
| Non-rheumatic degenerative mitral valve disease | -- | -- | -- | -- | -- |
| Other non-rheumatic valve diseases | -- | -- | -- | -- | -- |
| Mild heart failure due to other non-rheumatic valve disease | -- | -- | -- | -- | -- |
| Moderate heart failure due to other non-rheumatic valve disease | -- | -- | -- | -- | -- |
| Severe heart failure due to other non-rheumatic valve disease | -- | -- | -- | -- | -- |

| | | | | | |
|---|---|---|---|---|---|
| Controlled, medically managed heart failure due to other non-rheumatic valve disease | -- | -- | -- | -- | -- |
| Cardiomyopathy and myocarditis | 5.5 (3.2 to 8.5) | 107.4 (79.1 to 145.0) | 12.3 (7.1 to 19.4) | 1.4 (-0.7 to 3.6) | -2.7* (-4.7 to -0.6) |
| Myocarditis | 91.2 (66.4 to 120.5) | 107.4 (79.1 to 145.0) | 7.4 (4.3 to 11.5) | -0.7 (-4.0 to 2.6) | -4.7* (-7.8 to -1.5) |
| Acute myocarditis | 25.4 (18.1 to 35.0) | 107.4 (79.1 to 145.0) | 1.3 (0.7 to 2.1) | 2.2* (1.3 to 3.1) | -1.9* (-2.8 to -1.0) |
| Heart failure due to myocarditis | 65.8 (42.4 to 95.0) | -- | 6.1 (3.4 to 9.8) | -1.3 (-5.3 to 2.7) | -5.2* (-9.0 to -1.4) |
| Alcoholic cardiomyopathy | -- | -- | -- | -- | -- |
| Other cardiomyopathy | 52.9 (34.1 to 77.0) | -- | 4.9 (2.8 to 7.9) | 4.7* (3.5 to 5.9) | 0.5 (-0.6 to 1.7) |
| Atrial fibrillation and flutter | -- | -- | -- | -- | -- |
| Asymptomatic atrial fibrillation and flutter | -- | -- | -- | -- | -- |
| Symptomatic atrial fibrillation and flutter | -- | -- | -- | -- | -- |
| Peripheral vascular disease | -- | -- | -- | -- | -- |
| Asymptomatic peripheral arterial disease | -- | -- | -- | -- | -- |
| Symptomatic claudication due to peripheral arterial disease | -- | -- | -- | -- | -- |
| Endocarditis | 22.6 (14.4 to 33.9) | 35.7 (22.1 to 55.5) | 2.0 (1.1 to 3.3) | 5.8* (3.7 to 7.9) | 1.6 (-0.5 to 3.6) |
| Acute endocarditis | 2.9 (1.8 to 4.6) | 35.7 (22.1 to 55.5) | 0.2 (0.1 to 0.3) | 8.0* (5.7 to 10.4) | 3.7* (1.5 to 6.0) |
| Heart failure due to endocarditis | 19.7 (11.5 to 31.0) | -- | 1.8 (0.9 to 3.1) | 5.6* (3.2 to 7.8) | 1.4 (-0.9 to 3.5) |
| Other cardiovascular and circulatory diseases | 999.6 (570.5 to 1627.5) | -- | 54.9 (27.1 to 96.6) | 6.5* (0.4 to 12.5) | 2.3 (-3.6 to 8.0) |
| Heart failure due to other cardiovascular diseases | 5.3 (3.1 to 8.2) | -- | 0.5 (0.3 to 0.8) | 6.8* (5.0 to 8.6) | 2.6* (0.8 to 4.3) |
| Other cardiovascular and circulatory disease episodes | 994.3 (567.4 to 1618.0) | -- | 54.4 (26.9 to 95.8) | 6.5* (0.3 to 12.5) | 2.3 (-3.6 to 8.1) |
| **Chronic respiratory diseases** | **24667.8 (19388.1 to 30521.5)** | **4205.7 (2763.7 to 5955.1)** | **1325.1 (925.4 to 1825.5)** | **12.9* (7.6 to 17.8)** | **8.4* (3.3 to 13.1)** |
| Chronic obstructive pulmonary disease | 1314.7 (1047.7 to 1519.9) | 181.2 (137.2 to 227.5) | 157.5 (121.3 to 189.8) | 2.6 (-3.3 to 8.4) | -1.5 (-7.2 to 4.1) |
| Chronic obstructive pulmonary disease with heart failure | -- | -- | -- | -- | -- |
| Chronic obstructive pulmonary disease without heart failure | 1314.7 (1047.7 to 1519.9) | 181.2 (137.2 to 227.5) | 157.5 (121.3 to 189.8) | 2.6 (-3.3 to 8.4) | -1.5 (-7.2 to 4.1) |
| Pneumoconiosis | -- | -- | -- | 2.8 (-1.2 to 6.9) | -1.3 (-5.1 to 2.7) |
| Silicosis | -- | -- | -- | 5.1 (-1.9 to 13.2) | 0.9 (-5.8 to 8.7) |
| Heart failure due to silicosis | -- | -- | -- | -- | -- |
| Silicosis without heart failure | -- | -- | -- | 5.1 (-1.9 to 13.2) | 0.9 (-5.8 to 8.7) |
| Asbestosis | -- | -- | -- | 3.9 (-4.2 to 11.5) | -0.2 (-8.0 to 7.1) |
| Asbestosis without heart failure | -- | -- | -- | 3.9 (-4.2 to 11.5) | -0.2 (-8.0 to 7.1) |
| Heart failure due to asbestosis | -- | -- | -- | -- | -- |
| Coal workers pneumoconiosis | -- | -- | -- | -4.3 (-9.2 to 1.9) | -8.1* (-12.8 to -2.1) |
| Coal workers pneumoconiosis without heart failure | -- | -- | -- | -4.3 (-9.2 to 1.9) | -8.1* (-12.8 to -2.1) |
| Heart failure due to Coal workers pneumoconiosis | -- | -- | -- | -- | -- |
| Other pneumoconiosis | -- | -- | -- | -1.9 (-7.1 to 1.1) | -5.8* (-10.8 to -2.9) |
| Heart failure due to other pneumoconiosis | -- | -- | -- | -- | -- |
| Other pneumoconiosis without heart failure | -- | -- | -- | -1.9 (-7.1 to 1.1) | -5.8* (-10.8 to -2.9) |
| Asthma | 23813.6 (18376.9 to 29957.8) | 4022.4 (2564.0 to 5764.6) | 955.0 (576.4 to 1445.9) | 14.2* (7.1 to 20.1) | 9.6* (2.8 to 15.4) |
| Asymptomatic asthma | 8650.7 (6493.4 to 11102.2) | 1457.3 (921.1 to 2114.3) | -- | -- | -- |
| Symptomatic asthma | 15162.9 (11471.7 to 19190.4) | 2565.1 (1608.3 to 3697.5) | 955.0 (576.4 to 1445.9) | 14.2* (7.1 to 20.1) | 9.6* (2.8 to 15.4) |
| Interstitial lung disease and pulmonary sarcoidosis | 2.3 (1.3 to 3.5) | 2.1 (1.1 to 3.3) | 0.3 (0.1 to 0.5) | 7.1* (5.2 to 9.5) | 2.9* (1.0 to 5.1) |
| Heart failure due to interstitial lung disease and pulmonary sarcoidosis | -- | -- | -- | -- | -- |
| Interstitial lung disease and pulmonary sarcoidosis without heart failure | 2.3 (1.3 to 3.5) | 2.1 (1.1 to 3.3) | 0.3 (0.1 to 0.5) | 7.1* (5.2 to 9.5) | 2.9* (1.0 to 5.1) |
| Other chronic respiratory diseases | -- | -- | 212.4 (163.7 to 256.1) | 15.7* (9.1 to 22.3) | 11.1* (4.8 to 17.4) |
| **Digestive diseases** | **48524.8 (43399.3 to 53441.8)** | **7129.8 (5923.8 to 8543.9)** | **497.4 (326.7 to 706.5)** | **1.0 (-1.5 to 3.5)** | **-3.0* (-5.4 to -0.6)** |
| Cirrhosis and other chronic liver diseases | 42998.4 (38327.9 to 47677.7) | 105.5 (75.5 to 137.7) | 38.6 (26.3 to 54.0) | 8.1* (1.4 to 15.1) | 3.8 (-2.6 to 10.5) |
| Cirrhosis and other chronic liver diseases due to hepatitis B | 32856.6 (28319.3 to 37097.2) | 9.5 (6.5 to 12.7) | 3.9 (2.6 to 5.7) | -12.2* (-21.2 to -2.2) | -15.7* (-24.3 to -6.1) |
| Cirrhosis and other chronic liver diseases due to hepatitis B, decompensated | 21.8 (18.9 to 25.0) | 0.3 (0.2 to 0.4) | 3.9 (2.6 to 5.7) | -12.2* (-21.2 to -2.2) | -15.7* (-24.3 to -6.1) |
| Chronic hepatitis B without cirrhosis | 32644.8 (28109.6 to 36860.6) | -- | -- | -- | -- |
| Cirrhosis and other chronic liver diseases due to hepatitis B, compensated | 190.0 (155.1 to 229.0) | 9.2 (6.2 to 12.4) | -- | -- | -- |
| Cirrhosis and other chronic liver diseases due to hepatitis C | 8176.7 (6421.8 to 10087.4) | 7.1 (4.9 to 9.8) | 2.9 (1.9 to 4.4) | 5.0 (-2.9 to 13.9) | 0.9 (-6.8 to 9.4) |
| Cirrhosis and other chronic liver diseases due to hepatitis C, decompensated | 16.5 (13.7 to 19.9) | 0.2 (0.1 to 0.3) | 2.9 (1.9 to 4.4) | 5.0 (-2.9 to 13.9) | 0.9 (-6.8 to 9.4) |
| Chronic hepatitis C without cirrhosis | 8005.5 (6261.7 to 9937.5) | -- | -- | -- | -- |
| Cirrhosis and other chronic liver diseases due to hepatitis C, compensated | 154.6 (123.9 to 190.9) | 6.9 (4.7 to 9.6) | -- | -- | -- |
| Cirrhosis and other chronic liver diseases due to alcohol use | -- | -- | -- | -- | -- |
| Cirrhosis and other chronic liver diseases due to alcohol, decompensated | -- | -- | -- | -- | -- |
| Cirrhosis and other chronic liver diseases due to alcohol, compensated | -- | -- | -- | -- | -- |
| Cirrhosis due to NASH | -- | -- | -- | -- | -- |
| Non-alcoholic fatty liver disease (NAFLD) / Non-alcoholic steatohepatitis (NASH) | -- | -- | -- | -- | -- |
| Cirrhosis and other chronic liver diseases due to NASH | -- | -- | -- | -- | -- |
| Cirrhosis and other chronic liver diseases due to other causes | 1965.1 (1758.7 to 2173.7) | 88.9 (63.5 to 116.9) | 31.8 (21.5 to 44.3) | 11.6* (3.6 to 20.4) | 7.2 (-0.5 to 15.6) |

| | | | | | |
|---|---|---|---|---|---|
| Cirrhosis and other chronic liver diseases due to other cause, decompensated | 184.3 (174.1 to 194.8) | 3.2 (2.2 to 4.2) | 31.8 (21.5 to 44.3) | 11.6* (3.6 to 20.4) | 7.2 (-0.5 to 15.6) |
| Cirrhosis and other chronic liver diseases due to other cause, compensated | 1780.8 (1575.7 to 1992.0) | 85.8 (60.4 to 113.7) | -- | -- | -- |
| Upper digestive system diseases | 5435.3 (3784.6 to 7558.8) | 3426.7 (2384.4 to 4707.3) | 266.1 (166.2 to 408.2) | 1.7 (-1.3 to 4.8) | -2.4 (-5.2 to 0.6) |
| Peptic ulcer disease | 337.5 (235.5 to 465.1) | 164.6 (114.1 to 232.2) | 18.7 (11.3 to 28.1) | 5.7 (-2.2 to 13.7) | 1.5 (-6.1 to 9.2) |
| Acute peptic ulcer disease | 0.1 (0.0 to 0.1) | -- | -- | -2.2 (-4.6 to 0.2) | -6.1* (-8.4 to -3.7) |
| Chronic peptic ulcer disease | 337.4 (235.4 to 465.1) | 164.6 (114.1 to 232.2) | 18.7 (11.2 to 28.0) | 5.7 (-2.2 to 13.7) | 1.5 (-6.1 to 9.2) |
| Gastritis and duodenitis | 5326.1 (3667.4 to 7498.7) | 3262.1 (2222.6 to 4563.0) | 247.4 (153.7 to 385.2) | 1.4 (-1.7 to 4.6) | -2.6 (-5.6 to 0.4) |
| Acute gastritis and duodenitis | 1.0 (0.6 to 1.6) | -- | 0.3 (0.2 to 0.6) | 5.1* (3.4 to 6.9) | 1.0 (-0.7 to 2.6) |
| Chronic gastritis and duodenitis | 5325.1 (3666.3 to 7497.5) | 3262.1 (2222.6 to 4563.0) | 247.1 (153.4 to 384.7) | 1.4 (-1.7 to 4.6) | -2.7 (-5.6 to 0.4) |
| GERD | -- | -- | -- | -- | -- |
| Appendicitis | 95.3 (78.9 to 112.5) | 2589.4 (2116.5 to 3079.7) | 29.8 (18.8 to 42.2) | 3.4 (-7.8 to 15.3) | -0.7 (-11.4 to 10.7) |
| Paralytic ileus and intestinal obstruction | 5.2 (3.2 to 7.7) | 134.7 (83.1 to 199.4) | 1.7 (0.9 to 2.8) | 7.8* (4.4 to 10.6) | 3.5* (0.3 to 6.2) |
| Inguinal, femoral, and abdominal hernia | 1043.1 (746.6 to 1420.7) | 618.0 (396.6 to 882.1) | 106.5 (62.3 to 167.4) | -8.8* (-13.9 to -3.5) | -12.4* (-17.3 to -7.4) |
| Inflammatory bowel disease | 74.3 (62.6 to 87.5) | 183.6 (151.3 to 220.8) | 11.9 (7.8 to 17.0) | -3.7 (-10.2 to 2.7) | -7.6* (-13.8 to -1.4) |
| Ulcerative colitis | 24.4 (18.8 to 31.9) | 85.0 (62.2 to 114.0) | 3.8 (2.3 to 5.6) | -8.0* (-15.8 to -0.7) | -11.6* (-19.2 to -4.6) |
| Crohn's disease | 49.9 (40.4 to 61.8) | 98.6 (75.7 to 125.3) | 8.1 (5.2 to 11.9) | -1.6 (-10.2 to 5.8) | -5.6 (-13.8 to 1.6) |
| Vascular intestinal disorders | 1.2 (0.6 to 2.1) | 6.9 (4.2 to 10.1) | 0.4 (0.2 to 0.7) | 2.6* (0.8 to 4.4) | -1.5 (-3.2 to 0.3) |
| Gallbladder and biliary diseases | 197.1 (130.7 to 281.4) | 42.9 (25.7 to 65.2) | 0.2 (0.1 to 0.4) | 1.1 (-1.4 to 3.3) | -2.9* (-5.3 to -0.8) |
| Pancreatitis | 68.3 (55.2 to 82.8) | 22.1 (15.1 to 30.5) | 4.4 (2.2 to 7.6) | 6.4* (4.9 to 7.6) | 2.2* (0.8 to 3.4) |
| Acute pancreatitis | 1.3 (0.8 to 1.9) | 18.4 (12.0 to 26.7) | 0.5 (0.3 to 0.8) | -0.1 (-1.2 to 1.3) | -4.1* (-5.2 to -2.7) |
| Chronic pancreatitis | 67.1 (54.0 to 81.3) | 3.7 (2.2 to 5.9) | 3.9 (1.8 to 7.0) | 7.4* (6.0 to 8.7) | 3.1* (1.8 to 4.3) |
| Other digestive diseases | -- | -- | 37.9 (24.9 to 54.7) | 23.1* (19.7 to 26.3) | 18.2* (15.0 to 21.3) |
| **Neurological disorders** | **266264.9 (224125.7 to 317173.1)** | **103905.4 (81233.2 to 129445.3)** | **4186.0 (2809.2 to 5866.1)** | **8.1* (3.7 to 13.4)** | **3.8 (-0.4 to 8.9)** |
| Alzheimer's disease and other dementias | -- | -- | -- | -- | -- |
| Parkinson's disease | -- | -- | -- | -- | -- |
| Epilepsy | 2571.4 (1822.9 to 3423.7) | 237.7 (156.4 to 356.3) | 878.6 (516.3 to 1352.6) | 10.0 (-4.6 to 25.8) | 5.6 (-8.4 to 20.8) |
| Multiple sclerosis | 3.8 (2.6 to 5.5) | 1.3 (0.8 to 2.1) | 1.1 (0.6 to 1.6) | 0.7 (-2.1 to 3.1) | -3.3* (-6.0 to -1.0) |
| Motor neuron disease | 10.9 (8.6 to 13.5) | 0.1 (0.1 to 0.3) | 2.3 (1.5 to 3.3) | 2.5* (1.5 to 3.4) | -1.6* (-2.5 to -0.7) |
| Headache disorders | 264766.4 (222183.3 to 315597.4) | 103666.3 (81041.6 to 129219.9) | 2998.8 (1893.7 to 4367.3) | 4.8* (2.9 to 6.8) | 0.6 (-1.2 to 2.6) |
| Migraine | 72543.0 (60963.6 to 84913.7) | 15397.4 (11577.4 to 19480.0) | 2554.3 (1546.7 to 3813.6) | 4.7* (2.5 to 7.1) | 0.6 (-1.5 to 2.9) |
| Medication overuse headache due to migraine | 1835.6 (1307.2 to 2443.6) | -- | 210.4 (117.8 to 344.6) | 6.8* (3.3 to 10.2) | 2.6 (-0.8 to 5.9) |
| Migraine | 70707.4 (59067.5 to 83117.6) | 15397.4 (11577.4 to 19480.0) | 2343.9 (1397.7 to 3544.8) | 4.5* (2.2 to 7.2) | 0.4 (-1.9 to 2.9) |
| Tension-type headache | 223244.7 (175807.0 to 283072.2) | 88268.9 (66036.1 to 113466.6) | 444.5 (216.6 to 798.1) | 5.1* (3.6 to 6.8) | 0.9 (-0.5 to 2.6) |
| Medication overuse headache due to tension-type headache | 683.7 (417.8 to 1033.0) | -- | 77.7 (36.8 to 143.3) | 6.9* (0.8 to 12.9) | 2.7 (-3.2 to 8.4) |
| Tension-type headache | 222561.0 (174787.2 to 282526.6) | 88268.9 (66036.1 to 113466.6) | 366.8 (145.2 to 708.4) | 4.7* (3.6 to 5.9) | 0.5 (-0.5 to 1.7) |
| Other neurological disorders | 3.1 (1.5 to 6.3) | -- | 305.2 (179.7 to 470.8) | 47.5* (27.9 to 68.5) | 41.6* (22.9 to 61.8) |
| Other neurological disorders | -- | -- | 304.3 (178.9 to 468.4) | 47.6* (28.1 to 68.8) | 41.8* (23.0 to 62.1) |
| Guillain-Barré syndrome due to other neurological disorders | 3.1 (1.5 to 6.3) | -- | 0.9 (0.4 to 2.0) | 7.8* (5.3 to 10.1) | 3.5* (1.1 to 5.7) |
| **Mental disorders** | **79898.0 (71805.3 to 88865.8)** | **27051.0 (22646.7 to 32617.8)** | **8143.2 (5653.0 to 11509.6)** | **5.1* (4.0 to 6.1)** | **0.9 (-0.1 to 1.8)** |
| Schizophrenia | 35.7 (20.4 to 55.5) | 21.7 (12.4 to 33.5) | 25.3 (13.4 to 40.8) | 3.0* (1.5 to 4.2) | -1.1 (-2.5 to 0.1) |
| Depressive disorders | 7312.9 (5610.2 to 9676.9) | 10961.7 (7835.1 to 15143.6) | 1376.4 (858.5 to 2111.4) | 7.2* (4.9 to 9.4) | 3.0* (0.8 to 5.1) |
| Major depressive disorder | 5698.8 (4085.9 to 7970.5) | 10236.8 (7182.1 to 14215.0) | 1211.1 (727.4 to 1913.1) | 7.5* (5.0 to 9.9) | 3.2* (0.8 to 5.5) |
| Dysthymia | 1649.9 (1169.2 to 2238.0) | 724.9 (479.5 to 1060.5) | 165.3 (97.1 to 262.6) | 5.5* (1.8 to 9.1) | 1.3 (-2.3 to 4.7) |
| Bipolar disorder | 1706.5 (1284.6 to 2193.5) | 942.0 (709.6 to 1221.9) | 363.9 (212.1 to 568.5) | 6.2* (2.3 to 10.1) | 2.0 (-1.8 to 5.7) |
| Anxiety disorders | 21867.7 (18785.2 to 25157.1) | 5085.2 (4531.3 to 5654.0) | 2175.1 (1489.5 to 3049.9) | 3.8* (1.9 to 5.7) | -0.3 (-2.1 to 1.5) |
| Eating disorders | 604.8 (427.1 to 879.6) | 1209.0 (748.1 to 1896.9) | 131.1 (77.7 to 208.9) | 12.4* (7.8 to 17.0) | 7.9* (3.5 to 12.3) |
| Anorexia nervosa | 284.2 (194.7 to 392.7) | 194.6 (130.4 to 272.6) | 61.7 (36.4 to 97.7) | 12.0* (5.6 to 18.9) | 7.5* (1.4 to 14.1) |
| Bulimia nervosa | 321.0 (171.7 to 561.9) | 1014.4 (559.0 to 1723.2) | 69.5 (32.1 to 130.1) | 12.8* (6.0 to 19.4) | 8.3* (1.8 to 14.7) |
| Autism spectrum disorders | 2842.0 (2554.7 to 3138.2) | -- | 441.3 (299.5 to 612.6) | 4.6* (2.6 to 6.7) | 0.5 (-1.5 to 2.5) |
| Attention-deficit/hyperactivity disorder | 14072.5 (11428.5 to 17030.5) | 175.9 (143.3 to 213.9) | 172.1 (103.2 to 280.0) | 3.9* (1.6 to 6.2) | -0.3 (-2.5 to 2.0) |
| Conduct disorder | 24030.3 (17883.4 to 31368.3) | 8655.5 (6022.6 to 11391.9) | 2920.9 (1676.2 to 4834.3) | 6.6* (4.7 to 8.6) | 2.3* (0.6 to 4.3) |
| Idiopathic developmental intellectual disability | 11775.1 (7090.2 to 16420.9) | -- | 477.3 (239.4 to 803.6) | -4.9* (-7.6 to -2.9) | -8.7* (-11.3 to -6.7) |
| Other mental disorders | 762.5 (636.0 to 905.9) | -- | 59.8 (37.7 to 85.8) | 4.4 (-2.2 to 11.0) | 0.2 (-6.1 to 6.5) |
| **Substance use disorders** | **740.0 (531.9 to 1030.4)** | **963.3 (707.0 to 1328.7)** | **69.6 (40.9 to 115.6)** | **1.5 (-1.8 to 4.4)** | **-2.6 (-5.7 to 0.2)** |
| Alcohol use disorders | 498.6 (313.0 to 800.7) | 474.3 (295.3 to 772.0) | 50.9 (26.9 to 93.1) | 2.3 (-1.6 to 6.1) | -1.7 (-5.5 to 1.9) |
| Alcohol dependence | 471.6 (286.4 to 772.4) | 474.3 (295.3 to 772.0) | 49.9 (26.1 to 92.0) | 2.6 (-1.3 to 6.6) | -1.5 (-5.2 to 2.3) |
| Fetal alcohol syndrome | 27.1 (21.8 to 33.3) | -- | 1.1 (0.6 to 1.6) | -9.0* (-16.6 to -1.8) | -12.6* (-20.0 to -5.7) |
| Drug use disorders | 320.8 (200.2 to 486.9) | 489.0 (321.8 to 724.5) | 18.7 (10.7 to 29.4) | -0.9 (-5.2 to 3.2) | -4.8* (-9.0 to -0.9) |
| Opioid use disorders | -- | -- | -- | -- | -- |
| Cocaine use disorders | -- | -- | -- | -- | -- |

| | | | | | |
|---|---|---|---|---|---|
| Amphetamine use disorders | 36.1 (20.6 to 56.3) | 72.2 (41.2 to 112.8) | 5.0 (2.5 to 8.8) | -0.8 (-7.6 to 5.5) | -4.7 (-11.3 to 1.3) |
| Cannabis use disorders | 349.3 (214.2 to 541.8) | 416.7 (257.1 to 643.4) | 10.4 (5.2 to 18.3) | -1.0 (-7.3 to 4.3) | -4.9 (-11.0 to 0.2) |
| Other drug use disorders | 0.0 (0.0 to 0.1) | -- | 3.3 (1.6 to 5.7) | -0.8 (-8.1 to 6.1) | -4.7 (-11.8 to 1.9) |
| **Diabetes and kidney diseases** | **6285.4 (5116.4 to 7609.6)** | **969.2 (746.5 to 1215.0)** | **210.2 (141.3 to 297.0)** | **10.4* (5.4 to 15.6)** | **6.0* (1.3 to 11.1)** |
| Diabetes mellitus | 2103.6 (1604.4 to 2629.3) | 482.8 (330.0 to 675.0) | 108.1 (62.6 to 167.5) | 9.4* (5.5 to 13.5) | 5.1* (1.3 to 9.0) |
| Diabetes mellitus type 1 | 668.6 (548.6 to 808.5) | 62.3 (41.3 to 83.3) | 34.5 (21.0 to 51.7) | 5.4* (1.0 to 9.9) | 1.2 (-3.0 to 5.5) |
| Uncomplicated diabetes mellitus type 1 | 641.8 (527.8 to 777.3) | 62.3 (41.3 to 83.3) | 30.9 (18.5 to 47.3) | 5.5* (0.7 to 10.6) | 1.3 (-3.3 to 6.2) |
| Vision loss due to diabetes mellitus type 1 retinopathy | -- | -- | -- | -- | -- |
| Diabetic neuropathy due to diabetes mellitus type 1 | 26.8 (20.4 to 34.2) | -- | 3.6 (2.2 to 5.4) | 4.5 (-1.3 to 9.8) | 0.4 (-5.3 to 5.4) |
| Diabetes mellitus type 2 | 1435.0 (947.1 to 1938.8) | 420.5 (265.1 to 612.8) | 73.6 (38.9 to 116.5) | 11.4* (5.9 to 17.2) | 7.0* (1.7 to 12.5) |
| Uncomplicated diabetes mellitus type 2 | 1377.3 (910.4 to 1861.7) | 420.5 (265.1 to 612.8) | 66.1 (34.5 to 106.9) | 11.5* (5.7 to 17.1) | 7.0* (1.5 to 12.5) |
| Vision loss due to diabetes mellitus type 2 retinopathy | -- | -- | -- | -- | -- |
| Diabetic neuropathy due to diabetes mellitus type 2 | 57.7 (37.4 to 83.0) | -- | 7.5 (4.0 to 12.2) | 10.8 (-6.1 to 29.1) | 6.4 (-9.9 to 24.0) |
| Chronic kidney disease | 4664.8 (3671.7 to 5952.9) | 410.7 (271.9 to 576.0) | 101.9 (62.0 to 154.4) | 11.5* (1.8 to 21.9) | 7.1 (-2.2 to 17.0) |
| Chronic kidney disease due to diabetes mellitus type 1 | 128.0 (89.1 to 175.4) | 11.7 (6.8 to 18.0) | 2.9 (1.7 to 4.4) | 13.0 (-1.6 to 56.0) | 8.5 (-5.5 to 49.8) |
| Albuminuria with preserved GFR due to type 1 diabetes mellitus | 25.0 (14.3 to 40.7) | -- | -- | -- | -- |
| End-stage chronic kidney disease due to diabetes mellitus type 1 | 0.2 (0.1 to 0.3) | -- | 0.1 (0.0 to 0.1) | 16.8* (6.8 to 27.1) | 12.2* (2.5 to 22.0) |
| Stage III chronic kidney disease due to diabetes mellitus type 1 | 100.0 (66.4 to 141.3) | 11.7 (6.8 to 18.0) | 1.8 (1.1 to 2.9) | 10.9 (-5.7 to 58.3) | 6.5 (-9.4 to 52.0) |
| Stage IV chronic kidney disease due to diabetes mellitus type 1 | 1.3 (0.6 to 2.4) | -- | 0.2 (0.1 to 0.3) | 9.8 (-6.6 to 50.4) | 5.4 (-10.3 to 44.5) |
| Stage V chronic kidney disease due to diabetes mellitus type 1 | 1.5 (0.9 to 2.5) | -- | 0.9 (0.5 to 1.5) | 17.7* (4.4 to 56.6) | 13.0* (0.2 to 50.4) |
| Chronic kidney disease due to diabetes mellitus type 2 | 362.9 (196.0 to 822.8) | 13.1 (5.8 to 37.0) | 2.0 (0.9 to 5.3) | -47.4 (-82.7 to 12.6) | -49.5 (-83.4 to 8.1) |
| Albuminuria with preserved GFR due to type 2 diabetes mellitus | 258.6 (137.2 to 540.5) | -- | -- | -- | -- |
| End-stage chronic kidney disease due to diabetes mellitus type 2 | 0.1 (0.0 to 0.2) | -- | 0.0 (0.0 to 0.1) | 16.7* (6.6 to 27.1) | 12.0* (2.4 to 22.0) |
| Stage III chronic kidney disease due to diabetes mellitus type 2 | 99.9 (48.0 to 300.4) | 13.1 (5.8 to 37.0) | 0.4 (0.2 to 0.6) | 9.0 (-5.8 to 29.1) | 4.7 (-9.6 to 24.0) |
| Stage IV chronic kidney disease due to diabetes mellitus type 2 | 2.0 (0.7 to 6.1) | -- | 0.2 (0.1 to 0.7) | -54.5 (-85.2 to 10.8) | -56.3 (-85.8 to 6.4) |
| Stage V chronic kidney disease due to diabetes mellitus type 2 | 2.3 (0.9 to 6.5) | -- | 1.3 (0.5 to 4.1) | -53.6 (-84.9 to 13.3) | -55.4 (-85.5 to 8.8) |
| Chronic kidney disease due to hypertension | 692.0 (508.0 to 933.3) | 96.4 (63.1 to 136.2) | 13.7 (7.6 to 22.1) | 17.7* (5.2 to 60.5) | 13.0* (1.1 to 54.1) |
| Albuminuria with preserved GFR due to hypertension | 5.3 (2.9 to 8.8) | -- | -- | -- | -- |
| End-stage chronic kidney disease due to hypertension | 0.4 (0.2 to 0.7) | -- | 0.1 (0.1 to 0.3) | 15.1* (6.6 to 23.7) | 10.5* (2.4 to 18.8) |
| Stage III chronic kidney disease due to hypertension | 652.8 (478.0 to 886.1) | 96.4 (63.1 to 136.2) | 1.2 (0.7 to 1.8) | 10.8 (-5.0 to 44.1) | 6.4 (-8.8 to 38.4) |
| Stage IV chronic kidney disease due to hypertension | 15.4 (7.9 to 26.9) | -- | 1.9 (0.9 to 3.5) | 12.2 (-3.5 to 53.5) | 7.7 (-7.4 to 47.4) |
| Stage V chronic kidney disease due to hypertension | 18.0 (10.8 to 28.7) | -- | 10.5 (5.4 to 17.3) | 19.6* (7.0 to 65.3) | 14.9* (2.7 to 58.8) |
| Chronic kidney disease due to glomerulonephritis | 294.7 (222.4 to 388.6) | 12.6 (8.0 to 18.1) | 27.9 (17.0 to 42.1) | 16.0* (3.9 to 54.5) | 11.4 (-0.3 to 48.3) |
| Albuminuria with preserved GFR due to glomerulonephritis | 21.9 (12.6 to 35.0) | -- | -- | -- | -- |
| End-stage chronic kidney disease due to glomerulonephritis | 7.0 (4.5 to 10.1) | -- | 2.3 (1.3 to 3.7) | 16.9* (7.8 to 26.6) | 12.2* (3.5 to 21.6) |
| Stage III chronic kidney disease due to glomerulonephritis | 215.1 (156.0 to 291.8) | 12.6 (8.0 to 18.1) | 6.8 (4.0 to 10.7) | 9.4 (-10.6 to 56.0) | 5.1 (-14.2 to 49.8) |
| Stage IV chronic kidney disease due to glomerulonephritis | 23.4 (12.0 to 40.6) | -- | 2.9 (1.3 to 5.6) | 12.2 (-3.5 to 51.4) | 7.8 (-7.3 to 45.4) |
| Stage V chronic kidney disease due to glomerulonephritis | 27.3 (16.4 to 43.1) | -- | 15.9 (8.3 to 26.3) | 19.7* (7.4 to 63.8) | 14.9* (3.1 to 57.3) |
| Chronic kidney disease due to other and unspecified causes | 3187.2 (2441.8 to 4121.6) | 276.9 (180.7 to 390.9) | 55.4 (33.8 to 83.2) | 12.2* (0.8 to 38.7) | 7.7 (-3.2 to 33.2) |
| Albuminuria with preserved GFR due to other and unspecified causes | 892.0 (516.5 to 1416.7) | -- | -- | -- | -- |
| End-stage chronic kidney disease due to other causes | 14.4 (11.0 to 18.3) | -- | 5.1 (3.2 to 7.4) | 13.4* (5.6 to 21.7) | 8.9* (1.4 to 16.8) |
| Stage III chronic kidney disease due to other causes | 2183.7 (1628.0 to 2873.1) | 276.9 (180.7 to 390.9) | 14.3 (8.4 to 23.2) | -1.2 (-17.6 to 23.0) | -5.1 (-20.9 to 18.1) |
| Stage IV chronic kidney disease due to other causes | 44.6 (22.9 to 76.7) | -- | 5.6 (2.5 to 10.5) | 12.0 (-3.4 to 44.2) | 7.5 (-7.2 to 38.5) |
| Stage V chronic kidney disease due to other causes | 52.4 (31.7 to 83.0) | -- | 30.4 (15.9 to 50.2) | 19.6* (7.2 to 54.8) | 14.8* (3.0 to 48.6) |
| Acute glomerulonephritis | 5.3 (3.4 to 7.7) | 75.6 (49.3 to 107.2) | 0.3 (0.1 to 0.5) | 3.9* (0.6 to 7.2) | -0.2 (-3.4 to 3.0) |
| **Skin and subcutaneous diseases** | **157256.0 (144980.9 to 172637.8)** | **293171.3 (256702.7 to 336318.3)** | **3991.9 (2536.5 to 5937.3)** | **5.5* (4.2 to 6.7)** | **1.3* (0.1 to 2.5)** |
| Dermatitis | 31534.6 (28294.8 to 35354.4) | 15989.9 (12020.2 to 21329.1) | 1303.6 (741.5 to 2133.5) | 7.0* (4.9 to 8.9) | 2.7* (0.7 to 4.5) |
| Atopic dermatitis | 25999.8 (23252.3 to 29292.2) | 2193.1 (1744.1 to 2731.8) | 1155.5 (623.0 to 1972.9) | 7.4* (5.3 to 9.6) | 3.1* (1.1 to 5.2) |
| Contact dermatitis | 5501.4 (3774.6 to 7832.3) | 12277.9 (8370.7 to 17608.3) | 142.0 (78.5 to 238.5) | 4.0* (1.0 to 6.9) | -0.1 (-3.0 to 2.6) |
| Seborrhoeic dermatitis | 435.3 (345.4 to 550.9) | 1518.8 (1214.2 to 1885.2) | 6.0 (3.2 to 10.0) | -7.0* (-11.6 to -2.1) | -10.7* (-15.1 to -6.0) |
| Psoriasis | 2548.4 (2338.5 to 2786.4) | 380.7 (329.8 to 429.8) | 231.2 (158.1 to 308.8) | 8.4* (4.2 to 13.0) | 4.1* (0.0 to 8.5) |
| Bacterial skin diseases | 1196.6 (1079.1 to 1315.2) | 24439.6 (22413.4 to 26539.4) | 13.0 (7.5 to 22.0) | 11.5* (6.9 to 15.9) | 7.0* (2.6 to 11.3) |
| Cellulitis | 107.9 (86.4 to 132.9) | 2286.1 (1843.6 to 2779.8) | 6.4 (4.0 to 9.6) | 2.6 (-0.4 to 5.9) | -1.5 (-4.4 to 1.7) |
| Pyoderma | 1168.5 (1056.9 to 1283.6) | 22153.5 (20186.4 to 24137.4) | 6.7 (2.7 to 14.2) | 21.4* (17.1 to 25.7) | 16.6* (12.4 to 20.7) |
| Impetigo | 891.0 (781.5 to 1003.1) | 15573.4 (13809.4 to 17356.6) | 5.1 (2.1 to 11.0) | 24.5* (19.5 to 29.5) | 19.5* (14.8 to 24.4) |
| Abscess and other bacterial skin diseases | 277.4 (239.6 to 316.6) | 6580.1 (5620.6 to 7487.1) | 1.6 (0.6 to 3.4) | 12.6* (5.3 to 21.2) | 8.2* (1.1 to 16.4) |
| Scabies | 18681.8 (12850.3 to 26134.0) | 56789.2 (39432.2 to 80329.3) | 487.6 (243.3 to 859.8) | 0.9 (-0.3 to 2.2) | -3.1* (-4.2 to -1.8) |
| Fungal skin diseases | 54587.6 (41835.7 to 71363.5) | 125265.2 (96622.3 to 160491.3) | 312.9 (119.4 to 688.4) | -3.8* (-7.4 to -0.2) | -7.6* (-11.0 to -4.2) |
| Tinea capitis | 22429.7 (14039.7 to 35249.8) | 32465.7 (20323.5 to 48319.2) | 128.5 (44.8 to 309.7) | -17.3* (-20.6 to -14.0) | -20.6* (-23.7 to -17.4) |
| Other fungal skin diseases | 32157.9 (22758.6 to 44116.6) | 92799.4 (66951.2 to 126001.1) | 184.4 (68.7 to 404.5) | 8.5* (7.4 to 9.5) | 4.1* (3.2 to 5.2) |

| | | | | | |
|---|---|---|---|---|---|
| Viral skin diseases | 18392.4<br>(17066.2 to 19914.3) | 15236.5<br>(13738.0 to 16705.0) | 572.8<br>(369.0 to 865.7) | 1.7*<br>(0.5 to 2.8) | -2.4*<br>(-3.5 to -1.3) |
| Viral warts | 7869.9<br>(7068.5 to 8719.8) | 2520.2<br>(2214.2 to 2842.0) | 245.4<br>(153.5 to 368.5) | -1.6*<br>(-3.2 to -0.0) | -5.5*<br>(-7.0 to -4.0) |
| Molluscum contagiosum | 10522.5<br>(9325.1 to 11770.6) | 12716.3<br>(11236.7 to 14172.9) | 327.3<br>(208.3 to 501.7) | 4.3*<br>(2.8 to 5.9) | 0.1<br>(-1.3 to 1.7) |
| Acne vulgaris | 20092.0<br>(16183.6 to 24860.8) | 17618.4<br>(12747.6 to 24600.8) | 433.7<br>(252.4 to 713.8) | 19.5*<br>(17.0 to 22.3) | 14.8*<br>(12.4 to 17.4) |
| Alopecia areata | 511.8<br>(458.8 to 568.6) | 978.8<br>(871.8 to 1092.5) | 17.1<br>(10.5 to 25.7) | 3.6<br>(-2.8 to 10.5) | -0.5<br>(-6.7 to 6.2) |
| Pruritus | 3343.6<br>(2358.4 to 4725.0) | 2590.4<br>(1842.1 to 3507.5) | 36.4<br>(15.2 to 72.2) | 5.4*<br>(3.1 to 7.8) | 1.2<br>(-1.0 to 3.5) |
| Urticaria | 7728.7<br>(5296.9 to 11065.9) | 13121.7<br>(9182.2 to 18486.1) | 471.2<br>(282.3 to 740.1) | 4.3*<br>(2.2 to 6.3) | 0.2<br>(-1.8 to 2.1) |
| Decubitus ulcer | 18.4<br>(11.6 to 26.4) | 66.0<br>(43.0 to 92.9) | 3.3<br>(1.8 to 5.3) | 6.4*<br>(5.5 to 7.3) | 2.2*<br>(1.3 to 3.0) |
| Other skin and subcutaneous diseases | 19287.1<br>(17488.4 to 21068.4) | 20694.9<br>(18797.2 to 22562.4) | 109.0<br>(51.0 to 204.4) | 8.8*<br>(7.5 to 10.0) | 4.4*<br>(3.3 to 5.6) |
| **Sense organ diseases** | **36220.9<br>(32051.5 to 41246.9)** | **--** | **1333.5<br>(905.8 to 1892.2)** | **4.5*<br>(1.8 to 7.1)** | **0.3<br>(-2.3 to 2.9)** |
| Blindness and vision impairment | 20659.7<br>(17192.4 to 25182.5) | -- | 589.9<br>(367.7 to 933.3) | 4.3*<br>(2.6 to 6.0) | 0.2<br>(-1.5 to 1.8) |
| Glaucoma | -- | -- | -- | -- | -- |
| Cataract | -- | -- | -- | -- | -- |
| Age-related macular degeneration | -- | -- | -- | -- | -- |
| Refraction disorders | 11753.7<br>(8449.7 to 16200.4) | -- | 456.8<br>(281.0 to 725.0) | 4.8*<br>(2.8 to 6.8) | 0.6<br>(-1.2 to 2.6) |
| Near vision loss | 8504.0<br>(7817.2 to 9277.1) | -- | 89.0<br>(41.1 to 166.7) | 3.5*<br>(1.8 to 5.1) | -0.6<br>(-2.2 to 0.9) |
| Other vision loss | 626.1<br>(439.9 to 864.5) | -- | 44.2<br>(27.7 to 67.3) | 1.2<br>(-5.5 to 8.1) | -2.8<br>(-9.3 to 3.8) |
| Age-related and other hearing loss | 13770.5<br>(11161.2 to 16720.4) | -- | 690.8<br>(458.0 to 980.0) | 4.6<br>(-0.0 to 9.5) | 0.4<br>(-4.0 to 5.2) |
| Other sense organ diseases | 2529.2<br>(2303.6 to 2769.4) | -- | 52.8<br>(31.4 to 78.0) | 5.0*<br>(0.9 to 9.2) | 0.8<br>(-3.1 to 4.9) |
| Chronic other sense organ diseases | 1338.4<br>(1149.8 to 1545.2) | -- | 36.1<br>(20.8 to 54.8) | 5.0<br>(-0.6 to 10.5) | 0.8<br>(-4.5 to 6.1) |
| Acute other sense organ diseases | 1190.8<br>(1065.5 to 1325.0) | -- | 16.7<br>(10.0 to 25.9) | 5.0<br>(-0.6 to 11.1) | 0.8<br>(-4.5 to 6.7) |
| **Musculoskeletal disorders** | **16763.5<br>(12941.0 to 20594.9)** | **8989.9<br>(6515.2 to 11805.6)** | **1834.5<br>(1186.9 to 2582.3)** | **1.0<br>(-1.0 to 2.7)** | **-3.0*<br>(-4.9 to -1.4)** |
| Rheumatoid arthritis | 136.0<br>(97.7 to 181.5) | 30.8<br>(23.8 to 39.0) | 19.7<br>(11.6 to 30.2) | 7.6*<br>(2.3 to 12.3) | 3.3<br>(-1.8 to 7.9) |
| Osteoarthritis | -- | -- | -- | -- | -- |
| Osteoarthritis of the hip | -- | -- | -- | -- | -- |
| Osteoarthritis of the knee | -- | -- | -- | -- | -- |
| Low back pain | 14202.3<br>(10405.8 to 18150.9) | 8359.2<br>(5974.0 to 11143.2) | 1540.9<br>(947.9 to 2230.1) | 2.6*<br>(0.7 to 4.3) | -1.5<br>(-3.3 to 0.2) |
| Low back pain with leg pain | 1549.5<br>(1073.6 to 2081.6) | 913.1<br>(611.2 to 1296.9) | 236.9<br>(139.0 to 367.0) | 2.6<br>(-1.5 to 6.6) | -1.5<br>(-5.4 to 2.4) |
| Low back pain without leg pain | 12652.9<br>(9246.3 to 16231.3) | 7446.1<br>(5329.3 to 9917.4) | 1304.0<br>(799.4 to 1927.5) | 2.6*<br>(0.6 to 4.3) | -1.5<br>(-3.4 to 0.2) |
| Neck pain | 1640.7<br>(1140.3 to 2290.5) | 599.9<br>(374.9 to 897.4) | 172.1<br>(98.8 to 270.3) | -0.3<br>(-4.0 to 3.8) | -4.2*<br>(-7.9 to -0.3) |
| Gout | -- | -- | -- | -- | -- |
| Other musculoskeletal disorders | 1122.7<br>(651.5 to 1698.5) | -- | 101.7<br>(51.9 to 172.0) | -17.6*<br>(-26.0 to -10.8) | -20.9*<br>(-28.9 to -14.4) |
| **Other non-communicable diseases** | **334731.0<br>(299940.5 to 375002.0)** | **318759.1<br>(214276.3 to 466050.0)** | **2315.0<br>(1624.7 to 3167.1)** | **-0.0<br>(-3.1 to 3.3)** | **-4.0*<br>(-7.0 to -0.8)** |
| Congenital anomalies | 8633.4<br>(8166.1 to 9167.2) | -- | 1049.7<br>(739.3 to 1413.5) | 5.8*<br>(2.9 to 8.5) | 1.6<br>(-1.2 to 4.2) |
| Neural tube defects | 264.9<br>(236.2 to 294.8) | -- | 80.1<br>(56.2 to 108.0) | 11.1*<br>(4.9 to 17.4) | 6.6*<br>(0.7 to 12.8) |
| Severe motor and cognitive impairment due to anencephaly | -- | -- | -- | -- | -- |
| Encephalocoele due to neural tube defects | 19.6<br>(15.7 to 23.8) | -- | 3.4<br>(2.2 to 4.7) | 3.1*<br>(0.4 to 5.9) | -1.0<br>(-3.6 to 1.6) |
| Spina bifida due to neural tube defects | 245.3<br>(219.1 to 273.7) | -- | 76.7<br>(53.6 to 103.3) | 11.4*<br>(5.0 to 18.0) | 7.0*<br>(0.9 to 13.4) |
| Congenital heart anomalies | 1206.3<br>(1098.9 to 1325.7) | -- | 62.6<br>(28.6 to 106.3) | 5.1*<br>(1.8 to 8.6) | 0.9<br>(-2.2 to 4.2) |
| Critical malformations of great vessels, congenital valvular heart disease and patent ductus arteriosus | 202.0<br>(171.4 to 236.3) | -- | 13.0<br>(6.5 to 21.4) | 3.3<br>(-2.6 to 9.6) | -0.8<br>(-6.5 to 5.2) |
| Other congenital heart anomalies | 252.9<br>(230.3 to 278.6) | -- | 15.0<br>(6.2 to 26.9) | 5.2<br>(-1.4 to 12.6) | 1.1<br>(-5.3 to 8.2) |
| Severe congenital heart anomalies excluding single ventricle heart defects | 295.3<br>(255.8 to 336.8) | -- | 18.1<br>(8.5 to 30.6) | 6.9*<br>(0.3 to 13.4) | 2.6<br>(-3.7 to 8.9) |
| Single ventricle and single ventricle pathway congenital heart anomalies | 36.5<br>(30.0 to 44.1) | -- | 2.4<br>(1.3 to 3.9) | 9.2*<br>(6.9 to 11.3) | 4.8*<br>(2.7 to 6.8) |
| Ventricular septal defect and atrial septal defect | 419.6<br>(351.6 to 496.3) | -- | 14.1<br>(6.1 to 24.8) | 3.6<br>(-2.4 to 10.0) | -0.5<br>(-6.3 to 5.6) |
| Orofacial clefts | 870.4<br>(797.3 to 942.2) | -- | 27.7<br>(18.0 to 41.3) | 4.6<br>(-4.1 to 13.5) | 0.4<br>(-7.9 to 9.0) |
| Down's syndrome | 329.8<br>(288.5 to 375.2) | -- | 28.4<br>(19.2 to 39.6) | 5.8*<br>(3.3 to 8.8) | 1.6<br>(-0.8 to 4.5) |
| Turner syndrome | 72.7<br>(62.9 to 83.4) | -- | 1.0<br>(0.4 to 1.8) | 7.2*<br>(4.4 to 9.9) | 3.0*<br>(0.2 to 5.6) |
| Klinefelter syndrome | 109.8<br>(94.1 to 126.5) | -- | 0.3<br>(0.1 to 0.5) | 1.1<br>(-6.8 to 9.8) | -2.9<br>(-10.5 to 5.4) |
| Other chromosomal abnormalities | 628.5<br>(494.5 to 776.1) | -- | 53.5<br>(34.2 to 76.6) | 16.1*<br>(11.2 to 21.3) | 11.4*<br>(6.8 to 16.5) |
| Congenital musculoskeletal and limb anomalies | 2298.5<br>(2042.4 to 2637.2) | -- | 351.1<br>(229.2 to 487.6) | 5.0*<br>(2.4 to 7.7) | 0.9<br>(-1.7 to 3.4) |
| Polydactyly and syndactyly | 10.5<br>(8.3 to 12.8) | -- | 0.1<br>(0.1 to 0.2) | 2.8*<br>(0.9 to 4.7) | -1.3<br>(-3.1 to 0.5) |
| Congenital limb deficiency | 1401.5<br>(1242.5 to 1610.6) | -- | 214.9<br>(140.2 to 299.0) | 5.0*<br>(1.8 to 8.2) | 0.9<br>(-2.2 to 3.9) |
| Other congenital musculoskeletal anomalies | 886.4<br>(787.8 to 1017.1) | -- | 136.1<br>(88.1 to 189.0) | 5.0*<br>(1.3 to 9.2) | 0.9<br>(-2.7 to 4.8) |
| Urogenital congenital anomalies | 733.6<br>(628.4 to 851.9) | -- | 16.6<br>(9.4 to 28.8) | 2.4<br>(-0.6 to 5.2) | -1.6<br>(-4.5 to 1.0) |
| Digestive congenital anomalies | 1347.4<br>(1210.8 to 1512.1) | -- | 61.4<br>(40.6 to 88.7) | 2.6<br>(-2.3 to 7.8) | -1.5<br>(-6.2 to 3.5) |
| Congenital diaphragmatic hernia | 39.9<br>(34.9 to 45.5) | -- | 0.8<br>(0.5 to 1.3) | 0.9<br>(-0.0 to 1.8) | -3.1*<br>(-4.0 to -2.2) |
| Congenital atresia and/or stenosis of the digestive tract | 3.6<br>(2.5 to 4.9) | -- | 0.3<br>(0.2 to 0.5) | 15.8*<br>(12.7 to 19.2) | 11.2*<br>(8.2 to 14.5) |
| Congenital malformations of the abdominal wall after treatment | 158.2<br>(139.0 to 181.0) | -- | 6.8<br>(4.4 to 9.9) | 2.6*<br>(1.7 to 3.5) | -1.4*<br>(-2.3 to -0.7) |
| Other congenital malformations of the digestive tract | 1145.6<br>(1007.0 to 1308.9) | -- | 53.5<br>(35.0 to 77.3) | 2.5<br>(-2.9 to 8.5) | -1.6<br>(-6.8 to 4.2) |
| Other congenital anomalies | 2357.5<br>(2051.7 to 2728.0) | -- | 367.1<br>(249.0 to 507.9) | 5.0<br>(-1.9 to 12.0) | 0.8<br>(-5.8 to 7.6) |

| | | | | | |
|---|---|---|---|---|---|
| Other congenital birth defects | -- | -- | 44.4 (30.4 to 60.1) | 11.5* (7.5 to 15.7) | 7.1* (3.3 to 11.1) |
| Hearing loss due to other congenital anomalies | 2357.5 (2051.7 to 2728.0) | -- | 322.7 (213.3 to 454.8) | 4.2 (-3.5 to 12.0) | 0.0 (-7.3 to 7.5) |
| Urinary diseases and male infertility | 181.4 (133.8 to 237.0) | 12246.8 (9496.7 to 15323.0) | 13.9 (8.6 to 21.2) | -9.9* (-15.3 to -4.4) | -13.4* (-18.6 to -8.2) |
| Urinary tract infections | 206.3 (153.0 to 266.3) | 11385.5 (8587.1 to 14422.3) | 7.0 (3.9 to 11.4) | -1.2 (-8.8 to 7.2) | -5.1 (-12.4 to 2.9) |
| Urolithiasis | 30.8 (21.2 to 41.5) | 861.3 (576.2 to 1182.4) | 2.4 (1.4 to 3.6) | 4.4* (3.4 to 5.5) | 0.3 (-0.8 to 1.3) |
| Benign prostatic hyperplasia | -- | -- | -- | -- | -- |
| Male infertility | -- | -- | -- | -- | -- |
| Other urinary diseases | -- | -- | 4.6 (2.8 to 6.9) | -25.2* (-29.7 to -20.7) | -28.2* (-32.5 to -23.9) |
| Gynecological diseases | 688.4 (397.6 to 1016.7) | 404.9 (231.0 to 602.0) | 6.0 (2.3 to 12.1) | 4.5 (-0.2 to 9.4) | 0.4 (-4.2 to 5.0) |
| Uterine fibroids | -- | -- | -- | -- | -- |
| Polycystic ovarian syndrome | 688.4 (392.7 to 1023.7) | 404.9 (231.0 to 602.0) | 6.0 (2.3 to 12.1) | 4.5 (-0.2 to 9.4) | 0.4 (-4.2 to 5.0) |
| Female infertility | -- | -- | -- | -- | -- |
| Endometriosis | -- | -- | -- | -- | -- |
| Genital prolapse | -- | -- | -- | -- | -- |
| Premenstrual syndrome | -- | -- | -- | -- | -- |
| Other gynecological diseases | -- | -- | -- | -- | -- |
| Other gynecological diseases | -- | -- | -- | -- | -- |
| Other gynecological diseases with anemia | -- | -- | -- | -- | -- |
| Hemoglobinopathies and hemolytic anaemias | 171872.4 (167797.9 to 176351.5) | -- | 608.0 (410.4 to 868.6) | -6.8 (-14.4 to 2.4) | -10.5* (-17.8 to -1.7) |
| Thalassemias | 27.5 (23.7 to 31.8) | -- | 1.2 (0.8 to 1.8) | -19.4* (-29.6 to -7.2) | -22.6* (-32.4 to -10.9) |
| Thalassaemias trait | 22862.8 (22028.6 to 23754.7) | -- | 153.7 (100.7 to 225.6) | -18.5* (-28.1 to -7.9) | -21.8* (-31.0 to -11.5) |
| Sickle cell disorders | 464.2 (398.9 to 541.7) | -- | 39.6 (26.7 to 57.5) | 20.8* (6.5 to 35.9) | 16.0* (2.3 to 30.5) |
| Sickle cell trait | 47304.2 (42919.6 to 52263.1) | -- | 163.6 (108.6 to 236.4) | 1.5 (-9.0 to 12.5) | -2.5 (-12.6 to 8.0) |
| G6PD deficiency | 34403.8 (32960.0 to 35918.1) | -- | 2.2 (1.4 to 3.3) | 1.1 (-18.1 to 28.7) | -2.9 (-21.3 to 23.6) |
| G6PD trait | 81846.2 (80441.3 to 83231.6) | -- | -- | 0.2 (-10.1 to 12.5) | -3.7 (-13.7 to 8.0) |
| Other hemoglobinopathies and hemolytic anaemias | 5908.4 (5304.8 to 6644.1) | -- | 247.7 (163.4 to 356.1) | -7.0 (-16.2 to 4.2) | -10.7 (-19.5 to 0.1) |
| Endocrine, metabolic, blood, and immune disorders | 6934.1 (6445.9 to 7542.5) | -- | 297.8 (202.2 to 423.5) | -6.1 (-12.5 to 0.8) | -9.9* (-15.9 to -3.2) |
| Anemia due to endocrine, metabolic, blood, and immune disorders | 6061.3 (5625.9 to 6595.7) | -- | 264.6 (179.1 to 378.5) | -7.4 (-14.4 to 0.4) | -11.1* (-17.8 to -3.6) |
| Endocrine, metabolic, blood, and immune disorders | 871.6 (684.5 to 1120.8) | -- | 33.1 (20.6 to 49.8) | 5.4 (-0.0 to 10.7) | 1.2 (-4.0 to 6.3) |
| Heart failure due to endocrine, metabolic, blood, and immune disorders | 1.2 (0.7 to 1.7) | -- | 0.1 (0.1 to 0.2) | 0.5 (-0.8 to 2.0) | -3.4* (-4.8 to -2.0) |
| Oral disorders | 214382.0 (166692.4 to 270332.5) | 306107.4 (202957.2 to 454367.3) | 339.4 (189.0 to 554.2) | 2.4* (1.0 to 3.7) | -1.7* (-3.0 to -0.4) |
| Caries of deciduous teeth | 13324.2 (2980.0 to 26311.3) | 68037.4 (3009.9 to 210552.7) | 3.5 (0.6 to 9.6) | -5.3 (-27.9 to 1.5) | -9.1* (-30.7 to -2.5) |
| Caries of permanent teeth | 205536.9 (154877.2 to 264408.8) | 238070.0 (182011.5 to 309241.2) | 138.8 (58.8 to 274.4) | -0.1 (-2.5 to 1.8) | -4.1* (-6.3 to -2.2) |
| Periodontal disease | -- | -- | -- | -- | -- |
| Edentulism and severe tooth loss | -- | -- | -- | -- | -- |
| Other oral disorders | 6646.6 (5694.6 to 7701.6) | -- | 197.0 (120.8 to 299.0) | 4.4* (2.7 to 6.1) | 0.3 (-1.4 to 1.9) |
| **Injuries** | **38397.4 (34621.4 to 43158.4)** | **34325.2 (28747.9 to 41453.1)** | **1584.8 (1182.7 to 2062.1)** | **6.1* (3.5 to 8.5)** | **1.9 (-0.6 to 4.2)** |
| **Transport injuries** | **4096.3 (3455.8 to 4829.7)** | **3756.3 (2618.7 to 5414.7)** | **301.9 (213.0 to 411.0)** | **3.8* (1.3 to 6.2)** | **-0.4 (-2.7 to 2.0)** |
| Road injuries | 2039.8 (1741.8 to 2360.5) | 3021.1 (1938.9 to 4569.7) | 148.0 (104.9 to 201.9) | -0.2 (-3.2 to 3.0) | -4.2* (-7.0 to -1.1) |
| Pedestrian road injuries | 631.4 (453.9 to 860.2) | 653.3 (351.7 to 1099.4) | 46.2 (30.3 to 69.2) | -10.7* (-13.8 to -7.5) | -14.2* (-17.3 to -11.2) |
| Cyclist road injuries | 422.0 (305.3 to 583.2) | 815.5 (448.7 to 1347.3) | 28.7 (18.9 to 41.2) | -2.1 (-5.5 to 2.1) | -6.0* (-9.3 to -2.0) |
| Motorcyclist road injuries | 432.5 (330.4 to 571.3) | 446.5 (216.9 to 783.9) | 31.5 (20.9 to 45.1) | 7.4* (4.0 to 11.1) | 3.2 (-0.2 to 6.7) |
| Motor vehicle road injuries | 375.9 (286.4 to 491.2) | 767.7 (418.0 to 1274.7) | 30.2 (20.0 to 43.6) | 8.9* (4.6 to 13.6) | 4.6* (0.5 to 9.1) |
| Other road injuries | 177.9 (130.1 to 246.1) | 338.1 (169.0 to 620.1) | 11.4 (7.2 to 16.8) | 11.0* (6.9 to 15.3) | 6.6* (2.6 to 10.7) |
| Other transport injuries | 2056.6 (1559.4 to 2685.0) | 735.2 (429.5 to 1220.8) | 153.9 (104.3 to 217.5) | 7.9* (5.3 to 10.6) | 3.6* (1.1 to 6.2) |
| **Unintentional injuries** | **22290.7 (19786.5 to 25444.5)** | **27798.4 (22552.9 to 34731.4)** | **1051.9 (762.7 to 1408.5)** | **9.3* (6.8 to 11.7)** | **5.0* (2.5 to 7.2)** |
| Falls | 8875.4 (6958.9 to 11605.5) | 11341.8 (6861.3 to 17709.1) | 446.2 (307.2 to 609.4) | 10.5* (8.3 to 12.9) | 6.1* (4.0 to 8.4) |
| Drowning | 63.4 (49.0 to 78.6) | 28.3 (20.3 to 37.6) | 4.9 (3.2 to 6.7) | -19.3* (-22.7 to -16.3) | -22.5* (-25.7 to -19.7) |
| Fire, heat, and hot substances | 2454.5 (1886.9 to 3470.5) | 892.4 (510.2 to 1392.1) | 132.1 (88.2 to 184.7) | 7.4* (2.1 to 11.9) | 3.1 (-1.9 to 7.4) |
| Poisonings | 239.3 (181.9 to 303.8) | 431.7 (243.6 to 708.3) | 30.3 (19.3 to 45.3) | 21.7* (17.6 to 25.7) | 16.8* (12.9 to 20.7) |
| Poisoning by carbon monoxide | 36.1 (26.9 to 48.1) | 88.0 (37.4 to 168.1) | 4.4 (2.8 to 6.9) | 24.3* (18.4 to 30.1) | 19.4* (13.7 to 24.9) |
| Poisoning by other means | 203.1 (144.6 to 266.2) | 343.6 (190.8 to 583.9) | 25.8 (16.2 to 39.2) | 21.2* (16.8 to 25.3) | 16.4* (12.2 to 20.3) |
| Exposure to mechanical forces | 4293.4 (3270.5 to 5656.5) | 5239.2 (3280.7 to 7648.1) | 135.0 (90.9 to 190.9) | 22.2* (19.4 to 24.8) | 17.3* (14.6 to 19.9) |
| Unintentional firearm injuries | 222.2 (156.6 to 310.8) | 137.7 (54.9 to 282.1) | 11.5 (7.9 to 16.5) | 17.1* (14.0 to 20.2) | 12.5* (9.5 to 15.5) |
| Other exposure to mechanical forces | 4071.3 (3041.6 to 5446.9) | 5101.5 (3204.7 to 7436.8) | 123.5 (82.4 to 175.9) | 22.7* (19.7 to 25.4) | 17.8* (14.9 to 20.4) |
| Adverse effects of medical treatment | 49.7 (28.1 to 80.0) | 649.8 (378.8 to 1015.2) | 6.6 (3.2 to 11.6) | 3.7 (-0.2 to 7.4) | -0.5 (-4.1 to 3.1) |
| Animal contact | 1317.7 (1023.8 to 1636.1) | 3754.0 (2450.7 to 5610.2) | 59.0 (39.4 to 83.3) | 6.8* (2.1 to 11.4) | 2.5 (-2.0 to 7.0) |
| Venomous animal contact | 567.3 (433.4 to 731.3) | 1802.7 (1133.3 to 2765.6) | 45.4 (28.7 to 67.2) | 7.7* (1.8 to 13.6) | 3.4 (-2.2 to 9.1) |
| Non-venomous animal contact | 750.4 (529.9 to 1038.1) | 1951.4 (1220.1 to 2955.9) | 13.6 (7.8 to 21.7) | 3.7* (0.4 to 6.8) | -0.4 (-3.6 to 2.5) |
| Foreign body | 787.3 (583.1 to 1053.0) | 1707.3 (1080.5 to 2485.1) | 46.7 (30.8 to 66.7) | 12.2* (9.9 to 14.5) | 7.8* (5.6 to 9.9) |

| | | | | | |
|---|---|---|---|---|---|
| Pulmonary aspiration and foreign body in airway | 111.7 (81.7 to 147.2) | 45.8 (26.2 to 78.1) | 7.8 (5.0 to 11.0) | 8.0* (4.0 to 12.7) | 3.7 (-0.2 to 8.2) |
| Foreign body in eyes | 190.1 (65.3 to 375.4) | 1368.0 (766.2 to 2128.7) | 11.1 (3.7 to 22.7) | 11.5* (10.0 to 13.2) | 7.1* (5.6 to 8.7) |
| Foreign body in other body part | 485.5 (350.2 to 677.1) | 293.5 (153.5 to 507.5) | 27.9 (18.9 to 38.5) | 13.7* (10.7 to 16.5) | 9.2* (6.3 to 11.8) |
| Environmental heat and cold exposure | 1141.0 (820.8 to 1562.3) | 664.3 (376.4 to 1067.2) | 60.0 (40.3 to 82.9) | 9.9* (7.0 to 12.5) | 5.5* (2.7 to 8.0) |
| Exposure to forces of nature | 642.9 (451.1 to 889.2) | 137.4 (90.5 to 204.7) | 35.3 (22.9 to 52.9) | -37.5* (-48.1 to -24.9) | -40.0* (-50.2 to -27.9) |
| Other unintentional injuries | 2426.2 (1897.5 to 3147.9) | 2952.3 (1810.9 to 4593.0) | 95.8 (63.8 to 136.2) | 20.5* (17.7 to 23.3) | 15.7* (13.0 to 18.4) |
| **Self-harm and interpersonal violence** | **12010.3 (9153.5 to 15444.5)** | **2770.6 (2107.4 to 3570.6)** | **230.9 (168.7 to 308.7)** | **-3.9 (-11.1 to 3.2)** | **-7.7* (-14.7 to -0.9)** |
| Self-harm | 27.3 (20.0 to 37.4) | 142.6 (80.8 to 228.7) | 2.5 (1.6 to 3.7) | -5.6* (-9.5 to -1.9) | -9.4* (-13.1 to -5.8) |
| Self-harm by firearm | 0.7 (0.5 to 1.0) | 2.0 (0.6 to 4.7) | 0.1 (0.0 to 0.1) | -7.5* (-13.2 to -0.7) | -11.2* (-16.6 to -4.7) |
| Self-harm by other specified means | 26.6 (19.3 to 36.5) | 140.6 (80.0 to 225.8) | 2.5 (1.6 to 3.6) | -5.6* (-9.5 to -1.9) | -9.3* (-13.1 to -5.8) |
| Interpersonal violence | 10761.1 (7898.1 to 14063.3) | 1109.9 (689.7 to 1606.5) | 152.2 (106.0 to 211.9) | 7.2* (4.6 to 10.6) | 3.0* (0.5 to 6.2) |
| Assault by firearm | 25.0 (19.7 to 33.5) | 19.5 (8.3 to 38.4) | 1.8 (1.2 to 2.5) | 25.1* (19.9 to 31.0) | 20.2* (15.2 to 25.8) |
| Assault by sharp object | 271.6 (201.2 to 376.7) | 264.8 (135.8 to 466.0) | 12.1 (8.2 to 17.3) | 17.0* (12.5 to 21.4) | 12.3* (8.0 to 16.6) |
| Sexual violence | 9799.7 (6959.2 to 13132.6) | -- | 98.1 (56.2 to 153.7) | 0.8 (-0.7 to 2.1) | -3.2* (-4.6 to -2.0) |
| Assault by other means | 664.7 (510.1 to 867.0) | 825.6 (485.7 to 1215.9) | 40.2 (27.8 to 56.1) | 22.5* (18.6 to 26.3) | 17.6* (13.9 to 21.3) |
| Conflict and terrorism | 1179.3 (739.5 to 1825.0) | 1417.2 (949.7 to 1982.4) | 73.8 (44.5 to 122.5) | -21.7* (-34.6 to -6.5) | -24.8* (-37.2 to -10.2) |
| Executions and police conflict | 42.6 (25.3 to 66.3) | 100.9 (58.8 to 156.0) | 2.4 (1.5 to 3.8) | 83.4* (56.6 to 112.3) | 76.1* (50.3 to 103.9) |
| **Nature of injury aggregates** | | | | | |
| Amputations | 8933.3 (7732.9 to 10457.6) | 678.7 (525.5 to 871.9) | 175.4 (119.4 to 256.3) | 5.1 (-0.4 to 9.5) | 0.9 (-4.4 to 5.2) |
| Burns | 5213.9 (4531.4 to 6208.1) | 1386.8 (1053.8 to 1796.8) | 290.8 (207.6 to 384.1) | -0.8 (-7.2 to 5.2) | -4.7 (-10.9 to 1.0) |
| Fractures | 5953.6 (5242.1 to 6891.1) | 8143.4 (6450.0 to 10568.2) | 338.6 (228.0 to 480.1) | 8.7* (4.2 to 11.8) | 4.4* (0.1 to 7.4) |
| Head Injuries | 984.2 (899.5 to 1093.3) | 1420.8 (1128.0 to 1779.9) | 155.3 (106.8 to 212.0) | 12.6* (8.5 to 16.4) | 8.1* (4.2 to 11.8) |
| Spinal Injuries | 645.8 (563.2 to 764.2) | 51.3 (38.0 to 72.4) | 214.8 (149.0 to 293.8) | 7.1* (1.4 to 13.0) | 2.9 (-2.6 to 8.5) |
| Minor Injuries | 4673.0 (4117.8 to 5396.2) | 13302.8 (10692.6 to 16383.3) | 40.1 (18.6 to 75.7) | 13.5* (11.1 to 15.6) | 9.0* (6.7 to 11.0) |
| Other Injuries | 2193.9 (1945.0 to 2468.3) | 9341.3 (7986.4 to 10860.4) | 271.7 (188.9 to 371.1) | 8.4* (4.0 to 11.8) | 4.1 (-0.1 to 7.4) |
| **Impairments** | | | | | |
| Anaemia | 152060.1 (139541.6 to 167273.5) | -- | 6608.5 (4399.0 to 9473.3) | -8.6* (-15.4 to -0.5) | -12.2* (-18.8 to -4.5) |
| Epilepsy | 5825.6 (4699.6 to 7042.9) | -- | 2576.8 (1943.9 to 3419.3) | 10.0* (2.7 to 16.9) | 5.6 (-1.3 to 12.2) |
| Guillain-Barré syndrome | 8.3 (4.4 to 15.9) | -- | 2.4 (1.2 to 4.9) | 8.1* (5.6 to 10.4) | 3.8* (1.5 to 6.0) |
| Hearing loss | 27358.6 (24627.5 to 30360.8) | -- | 1264.7 (880.7 to 1742.5) | 3.7* (0.6 to 7.1) | -0.4 (-3.4 to 2.8) |
| Heart failure | 210.6 (138.7 to 298.9) | -- | 19.6 (11.3 to 32.1) | 1.3 (-0.3 to 2.8) | -2.7* (-4.2 to -1.3) |
| Infertility | 27.4 (14.1 to 44.4) | -- | 0.5 (0.2 to 1.2) | 3.3* (2.2 to 4.4) | -0.8 (-1.8 to 0.3) |
| Developmental intellectual disability | 20163.9 (15573.9 to 24783.0) | -- | 2603.9 (2016.1 to 3243.6) | 5.4* (2.1 to 9.2) | 1.2 (-1.9 to 4.8) |
| Pelvic inflammatory disease | 7.0 (4.9 to 9.4) | -- | 1.0 (0.6 to 1.5) | 8.2* (6.7 to 10.0) | 3.9* (2.5 to 5.6) |
| Vision loss | 22554.4 (18878.4 to 27433.9) | -- | 829.4 (566.2 to 1214.0) | 4.7* (3.4 to 6.1) | 0.6 (-0.7 to 1.9) |

We did not calculate incidence for the nine impairments and certain NTDs. Blank cells mean that no estimate is available or that the estimate has a magnitude less than 50.
Table presents prevalence and incidence counts in thousands.
* Percentage changes that are statistically significant
‡ Incidence estimates for stroke represent first-ever stroke only.
^ Incidence of HIV/AIDS represents new infections of HIV only and does not include new infections of Tuberculosis in HIV positive cases.

| SR Table 10g: Global prevalence, incidence and YLDs for 2017, percentage change of YLD counts and percentage change of YLD rates between 2007 and 2017 by cause, age 15 to 19 years | | | | | |
|---|---|---|---|---|---|
| Cause | Prevalence in thousands (95% UI) | Incidence in thousands (95% UI) | YLDs in thousands (95% UI) | | |
| | 2017 counts | 2017 counts | 2017 counts | Percentage change in counts between 2007 and 2017 | Percentage change in rates between 2007 and 2017 |
| **All causes** | **592266.5 (587080.6 to 597983.9)** | **2995283.4 (2555606.0 to 3515029.9)** | **44686.9 (33250.6 to 57876.0)** | **-2.2* (-3.1 to -1.3)** | **0.1 (-0.8 to 1.0)** |
| **Communicable, maternal, neonatal, and nutritional disorders** | **385039.0 (363838.4 to 413370.4)** | **2171398.4 (1734342.7 to 2690560.6)** | **8959.2 (6619.4 to 11787.3)** | **-4.0* (-6.9 to -0.4)** | **-1.7 (-4.7 to 2.0)** |
| **HIV/AIDS and sexually transmitted infections** | **52734.9 (44908.7 to 61337.0)** | **81136.8 (64468.3 to 100526.0)** | **223.8 (147.2 to 334.1)** | **9.8* (4.5 to 17.2)** | **12.4* (7.0 to 20.0)** |
| HIV/AIDS | 1018.2 (921.3 to 1133.2) | 165.0^ (134.9 to 202.1) | 112.6 (78.6 to 157.1) | 24.0* (12.2 to 36.4) | 26.9* (14.8 to 39.7) |
| HIV/AIDS - Drug-susceptible Tuberculosis | 42.0 (38.4 to 45.8) | 50.6 (46.2 to 55.7) | 16.8 (11.4 to 23.3) | -7.4 (-16.0 to 1.4) | -5.2 (-14.0 to 3.9) |
| HIV/AIDS - Multidrug-resistant Tuberculosis without extensive drug resistance | 1.2 (0.8 to 1.7) | 1.7 (1.2 to 2.5) | 0.5 (0.3 to 0.7) | -28.1 (-51.2 to 6.8) | -26.4 (-50.0 to 9.3) |
| HIV/AIDS - Extensively drug-resistant Tuberculosis | 0.0 (0.0 to 0.1) | 0.0 (0.0 to 0.1) | -- | -5.1 (-48.6 to 65.7) | -2.9 (-47.4 to 69.6) |
| HIV/AIDS resulting in other diseases | 975.0 (877.8 to 1088.1) | 165.0 (134.9 to 202.1) | 95.3 (64.9 to 136.1) | 32.4* (17.1 to 48.2) | 35.5* (19.9 to 51.7) |
| HIV/AIDS not on antiretroviral treatment without Tuberculosis | 627.9 (545.2 to 729.8) | 165.0 (134.9 to 202.1) | 67.8 (44.5 to 101.7) | -3.6 (-20.7 to 14.3) | -1.4 (-18.8 to 17.0) |
| HIV/AIDS on antiretroviral treatment without Tuberculosis | 347.0 (312.8 to 385.1) | -- | 27.6 (18.5 to 39.3) | 1520.9* (1236.1 to 1881.0) | 1559.4* (1267.8 to 1928.0) |
| Sexually transmitted infections excluding HIV | 51982.6 (44097.7 to 60677.6) | 80971.8 (64297.0 to 100358.8) | 111.1 (56.9 to 211.7) | -1.5 (-5.4 to 1.6) | 0.8 (-3.2 to 4.0) |
| Syphilis | 3503.3 (2657.8 to 4541.6) | 1713.2 (1189.6 to 2323.4) | 0.9 (0.3 to 2.2) | 1.7 (-8.7 to 9.9) | 4.1 (-6.5 to 12.5) |
| Early syphilis | 3502.8 (2657.3 to 4541.1) | 1713.2 (1189.6 to 2323.4) | 0.8 (0.2 to 2.1) | 5.0 (-5.1 to 15.7) | 7.5 (-2.9 to 18.4) |
| Tertiary syphilis | 0.5 (0.4 to 0.6) | -- | 0.1 (0.1 to 0.1) | -22.7* (-24.7 to -20.9) | -20.9* (-23.0 to -19.0) |
| Chlamydial infection | 8205.7 (5674.8 to 11263.2) | 33846.5 (22796.5 to 47358.2) | 21.1 (11.2 to 39.6) | -7.4* (-13.2 to -2.3) | -5.2* (-11.2 to -0.0) |
| Chlamydia episode | 8064.7 (5532.1 to 11129.4) | 33846.5 (22796.5 to 47358.2) | 13.3 (5.0 to 30.0) | -5.5* (-7.6 to -3.2) | -3.3* (-5.4 to -0.9) |
| Chlamydial infection complications | 141.0 (122.0 to 164.3) | -- | 7.9 (4.6 to 12.3) | -10.5 (-22.6 to 1.7) | -8.4 (-20.8 to 4.1) |
| Gonococcal infection | 8920.8 (5370.1 to 13793.6) | 22483.1 (13404.1 to 35943.7) | 29.9 (14.1 to 60.2) | -0.3 (-7.1 to 5.9) | 2.1 (-4.9 to 8.4) |
| Gonococcal infection complications | 73.5 (56.4 to 93.6) | -- | 7.1 (4.1 to 11.2) | 3.6 (-14.3 to 21.7) | 6.0 (-12.2 to 24.6) |
| Gonorrhoea episode | 8847.3 (5305.6 to 13720.6) | 22483.1 (13404.1 to 35943.7) | 22.8 (8.1 to 52.1) | -1.5 (-9.2 to 3.2) | 0.9 (-7.1 to 5.6) |
| Trichomoniasis | 8284.5 (5801.0 to 11280.5) | 9691.5 (6322.8 to 14357.2) | 14.7 (5.7 to 31.2) | 7.7* (5.0 to 10.5) | 10.3* (7.5 to 13.1) |
| Genital herpes | 25657.3 (19874.3 to 32986.1) | 13237.4 (10356.3 to 16850.8) | 9.6 (3.5 to 20.5) | 2.0 (-2.6 to 6.6) | 4.4 (-0.3 to 9.2) |
| Other sexually transmitted infections | 99.3 (86.7 to 111.1) | -- | 34.9 (18.6 to 65.8) | -3.3 (-7.9 to 0.7) | -1.0 (-5.7 to 3.0) |
| Other sexually transmitted diseases residual | -- | -- | 32.0 (16.1 to 62.1) | -4.0 (-9.1 to 0.3) | -1.7 (-7.0 to 2.7) |
| Other sexually transmitted diseases | 99.3 (86.7 to 111.1) | -- | 34.9 (18.6 to 65.8) | -3.3 (-7.9 to 0.7) | -1.0 (-5.7 to 3.0) |
| **Respiratory infections and tuberculosis** | **163308.6 (119531.9 to 221272.8)** | **1455699.3 (1030933.5 to 1978151.5)** | **937.3 (594.7 to 1424.0)** | **-3.9* (-5.5 to -2.4)** | **-1.6* (-3.2 to -0.1)** |
| Tuberculosis | 139695.9 (93581.7 to 202077.7) | 806.1 (618.7 to 1037.5) | 160.5 (98.5 to 246.2) | -10.4* (-14.7 to -5.7) | -8.2* (-12.7 to -3.5) |
| Latent tuberculosis infection | 139193.0 (93226.8 to 201576.0) | -- | -- | -- | -- |
| Drug-susceptible tuberculosis | 480.8 (364.2 to 631.6) | 770.1 (585.8 to 988.8) | 153.2 (94.6 to 235.7) | -10.2* (-15.5 to -4.8) | -8.0* (-13.5 to -2.6) |
| Multidrug-resistant tuberculosis without extensive drug resistance | 21.2 (10.9 to 35.9) | 34.6 (20.3 to 58.6) | 7.0 (3.2 to 12.9) | -14.8 (-50.0 to 37.9) | -12.8 (-48.8 to 41.2) |
| Extensively drug-resistant tuberculosis | 0.9 (0.5 to 1.6) | 1.4 (0.9 to 2.1) | 0.3 (0.1 to 0.5) | 8.5 (-30.6 to 79.9) | 11.1 (-29.0 to 84.2) |
| Lower respiratory infections | 1030.9 (870.0 to 1212.2) | 45797.1 (38252.1 to 54021.9) | 63.5 (41.4 to 93.7) | 19.3* (10.0 to 28.6) | 22.2* (12.6 to 31.7) |
| Guillain-Barré syndrome due to lower respiratory infections | 0.8 (0.4 to 1.5) | -- | 0.2 (0.1 to 0.5) | 6.0* (3.5 to 8.7) | 8.5* (5.9 to 11.2) |
| Lower respiratory infection episode | 1030.1 (868.8 to 1210.7) | 45797.1 (38252.1 to 54021.9) | 63.3 (41.0 to 93.4) | 19.4* (10.0 to 28.7) | 22.2* (12.6 to 31.7) |
| Upper respiratory infections | 19214.1 (13322.5 to 26392.0) | 1399497.4 (975899.8 to 1924477.1) | 483.7 (250.6 to 839.5) | -3.7* (-4.9 to -2.4) | -1.4* (-2.6 to -0.1) |
| Guillain-Barré syndrome due to upper respiratory infections | 2.2 (1.2 to 3.9) | -- | 0.6 (0.3 to 1.2) | 6.0* (3.5 to 8.7) | 8.5* (5.9 to 11.2) |
| Upper respiratory infection episode | 19212.0 (13320.8 to 26390.4) | 1399497.4 (975899.8 to 1924477.1) | 483.0 (250.3 to 838.9) | -3.7* (-4.9 to -2.4) | -1.4* (-2.7 to -0.1) |
| Otitis media | 11173.8 (8938.2 to 13583.5) | 9598.7 (6826.8 to 12576.2) | 229.6 (131.2 to 375.2) | -4.5* (-7.6 to -1.8) | -2.2 (-5.4 to 0.6) |
| Acute otitis media | 572.4 (422.2 to 756.4) | 9597.3 (6825.3 to 12574.5) | 7.5 (3.7 to 14.0) | 1.5 (-3.1 to 6.6) | 4.0 (-0.8 to 9.2) |
| Chronic otitis media | 10601.4 (8349.5 to 12950.0) | 1.4 (0.0 to 5.5) | 222.1 (126.4 to 360.9) | -4.7* (-7.8 to -1.9) | -2.4 (-5.7 to 0.5) |
| **Enteric infections** | **6690.6 (5774.3 to 7757.1)** | **462874.3 (385114.1 to 553304.0)** | **777.6 (521.5 to 1089.9)** | **18.1* (12.1 to 25.0)** | **20.9* (14.7 to 28.0)** |
| Diarrhoeal diseases | 6669.6 (5755.2 to 7729.2) | 461053.1 (383119.3 to 551653.7) | 763.1 (512.2 to 1068.5) | 19.3* (13.2 to 26.3) | 22.1* (15.9 to 29.3) |
| Guillain-Barré syndrome due to diarrheal diseases | 0.7 (0.4 to 1.3) | -- | 0.2 (0.1 to 0.4) | 6.0* (3.5 to 8.7) | 8.5* (5.9 to 11.2) |
| Diarrheal disease episode | 6668.8 (5754.3 to 7728.7) | 461053.1 (383119.3 to 551653.7) | 762.9 (512.0 to 1068.3) | 19.3* (13.2 to 26.3) | 22.1* (15.9 to 29.3) |
| Typhoid and paratyphoid | 49.9 (33.0 to 69.9) | 1797.4 (1347.7 to 2349.7) | 14.4 (8.5 to 21.9) | -21.7* (-36.5 to -2.8) | -19.8* (-35.0 to -0.5) |
| Typhoid fever | 86.6 (62.2 to 115.9) | 1363.1 (996.9 to 1800.3) | 13.2 (7.7 to 20.3) | -22.6* (-38.0 to -2.1) | -20.8 (-36.5 to 0.2) |
| Typhoid fever complications | 18.0 (11.8 to 25.7) | 233.6 (162.5 to 314.7) | 5.7 (3.1 to 9.3) | -22.9 (-46.3 to 9.0) | -21.1 (-45.0 to 11.6) |
| Typhoid fever episode | 68.6 (48.1 to 94.8) | 1129.5 (828.0 to 1507.1) | 7.5 (4.2 to 12.0) | -22.3 (-41.7 to 2.7) | -20.5 (-40.3 to 5.1) |
| Paratyphoid fever | 19.0 (12.4 to 27.2) | 434.3 (286.5 to 617.6) | 1.2 (0.6 to 2.0) | -9.9 (-24.9 to 8.0) | -7.8 (-23.1 to 10.6) |
| Intestinal perforation due to paratyphoid | 0.9 (0.5 to 1.3) | 22.2 (14.2 to 32.7) | 0.1 (0.1 to 0.2) | -9.5 (-29.4 to 16.1) | -7.4 (-27.7 to 18.8) |
| Paratyphoid fever episode | 18.2 (11.8 to 26.0) | 412.1 (271.9 to 587.5) | 1.1 (0.6 to 1.8) | -9.9 (-25.7 to 9.2) | -7.8 (-23.9 to 11.8) |
| iNTS | 0.9 (0.5 to 1.5) | 23.7 (12.4 to 38.4) | 0.1 (0.1 to 0.2) | -15.8* (-22.2 to -10.7) | -13.8* (-20.3 to -8.5) |
| Other intestinal infectious diseases | -- | -- | -- | -39.0* (-50.6 to -24.4) | -37.6* (-49.4 to -22.6) |
| **Neglected tropical diseases and malaria** | **141188.0 (134761.6 to 148617.6)** | **32103.1 (24934.2 to 40347.6)** | **1096.5 (750.3 to 1561.2)** | **-17.3* (-22.7 to -11.2)** | **-15.4* (-20.8 to -9.0)** |
| Malaria | 14841.8 (13794.0 to 15845.7) | 19458.7 (14562.8 to 25381.0) | 139.7 (102.8 to 190.1) | -17.2* (-28.0 to -5.8) | -15.2* (-26.3 to -3.5) |
| Malaria complications | 122.5 (111.5 to 134.6) | -- | 50.5 (38.8 to 63.1) | 24.9* (12.7 to 37.7) | 27.9* (15.4 to 41.0) |
| Malaria episode | 1132.1 (720.9 to 1661.1) | 19458.7 (14562.8 to 25381.0) | 34.1 (17.0 to 60.1) | -9.0 (-18.6 to 2.8) | -6.8 (-16.6 to 5.2) |

| | | | | | |
|---|---|---|---|---|---|
| Malaria parasitemia | 13587.3 (12458.4 to 14835.4) | -- | 55.1 (34.2 to 86.4) | -39.3* (-51.8 to -25.5) | -37.9* (-50.6 to -23.8) |
| Chagas disease | 303.8 (237.5 to 376.4) | 14.9 (10.5 to 20.0) | 1.2 (0.8 to 1.9) | -26.0* (-37.3 to -14.3) | -24.3* (-35.8 to -12.2) |
| Acute Chagas disease | 0.1 (0.0 to 0.2) | 14.9 (10.5 to 20.0) | -- | -19.9* (-24.0 to -16.4) | -18.0* (-22.2 to -14.4) |
| Asymptomatic Chagas disease | 280.1 (220.2 to 346.0) | -- | -- | -- | -- |
| Symptomatic chronic Chagas infection | 23.6 (17.3 to 31.3) | -- | 1.2 (0.7 to 1.9) | -26.1* (-37.3 to -14.2) | -24.3* (-35.8 to -12.2) |
| Leishmaniasis | 412.1 (362.1 to 469.2) | 68.9 (47.5 to 95.6) | 27.1 (17.7 to 39.3) | 48.7* (31.9 to 66.2) | 52.2* (35.0 to 70.2) |
| Visceral leishmaniasis | 0.8 (0.6 to 1.3) | 3.2 (2.4 to 5.0) | 0.1 (0.0 to 0.1) | -71.5* (-79.5 to -56.4) | -70.8* (-79.0 to -55.3) |
| Cutaneous and mucocutaneous leishmaniasis | 416.2 (367.3 to 472.2) | 65.7 (43.9 to 92.6) | 27.1 (17.6 to 39.2) | 50.0* (32.9 to 68.1) | 53.6* (36.0 to 72.0) |
| African trypanosomiasis | 0.5 (0.1 to 2.2) | 0.4 (0.1 to 1.2) | 0.1 (0.0 to 0.6) | -80.9 (-97.5 to 23.6) | -80.5 (-97.5 to 26.5) |
| Trypanosomiasis Gambiense | 0.5 (0.1 to 2.2) | 0.4 (0.1 to 1.1) | 0.1 (0.0 to 0.6) | -80.2 (-97.6 to 29.7) | -79.7 (-97.6 to 32.8) |
| Trypanosomiasis Rhodesiense | 0.0 (0.0 to 0.1) | 0.0 (0.0 to 0.1) | -- | -91.5* (-99.1 to -40.3) | -91.3* (-99.1 to -38.9) |
| Schistosomiasis | 13481.2 (11180.7 to 15883.4) | -- | 107.4 (50.2 to 208.0) | -27.8* (-31.6 to -24.5) | -26.1* (-30.0 to -22.7) |
| Mild schistosomiasis | 9329.8 (7132.0 to 11447.8) | -- | 53.2 (21.0 to 115.7) | -29.5* (-33.3 to -26.1) | -27.9* (-31.7 to -24.4) |
| Anemia due to schistosomiasis | 671.2 (529.0 to 831.1) | -- | 14.7 (8.7 to 24.0) | -44.4* (-54.7 to -32.5) | -43.1* (-53.6 to -30.9) |
| Schistosomiasis complications | 3480.3 (2601.2 to 4363.1) | -- | 39.4 (18.7 to 78.0) | -15.6* (-21.5 to -10.1) | -13.6* (-19.7 to -7.9) |
| Cysticercosis | 191.6 (128.9 to 277.7) | -- | 62.4 (35.3 to 101.0) | -4.2 (-13.1 to 5.0) | -1.9 (-11.0 to 7.5) |
| Cystic echinococcosis | 45.9 (16.7 to 107.0) | 14.1 (4.8 to 31.0) | 3.9 (1.2 to 9.5) | -7.2 (-18.7 to 3.0) | -5.0 (-16.8 to 5.4) |
| Lymphatic filariasis | 6598.4 (5742.6 to 7559.0) | -- | 108.8 (59.0 to 173.6) | -34.7* (-46.6 to -23.0) | -33.1* (-45.4 to -21.2) |
| Prevalence of detectable microfiliaria due to lymphatic filariasis | 5638.5 (4836.1 to 6487.4) | -- | -- | -- | -- |
| Lymphatic filariasis complications | 959.9 (632.3 to 1397.9) | -- | 108.8 (59.0 to 173.6) | -34.7* (-46.6 to -23.0) | -33.1* (-45.4 to -21.2) |
| Onchocerciasis | 2601.9 (1530.0 to 4670.6) | -- | 149.4 (62.2 to 275.1) | 4.1 (-18.4 to 22.2) | 6.6 (-16.5 to 25.1) |
| Asymptomatic onchocerciasis | 686.8 (0.0 to 2546.9) | -- | -- | -- | -- |
| Skin disease due to onchocerciasis | 1912.7 (1333.4 to 2675.4) | -- | 149.2 (62.0 to 274.9) | 4.1 (-18.5 to 22.3) | 6.6 (-16.5 to 25.2) |
| Vision loss due to onchocerciasis | 2.3 (1.6 to 3.5) | -- | 0.2 (0.1 to 0.4) | 17.7 (-24.4 to 78.4) | 20.5 (-22.6 to 82.6) |
| Trachoma | 3.8 (1.3 to 9.6) | -- | 0.4 (0.2 to 0.9) | -50.4* (-71.6 to -16.7) | -49.3* (-70.9 to -14.7) |
| Dengue | 668.5 (363.8 to 1133.1) | 11173.5 (6790.4 to 16958.8) | 110.8 (48.1 to 208.8) | 52.0* (30.7 to 133.7) | 55.6* (33.8 to 139.3) |
| Post-dengue chronic fatigue syndrome | 471.3 (220.0 to 861.8) | -- | 99.1 (40.9 to 188.9) | 52.0* (29.8 to 136.0) | 55.6* (32.8 to 141.6) |
| Dengue episode | 197.2 (119.0 to 296.2) | 11173.5 (6790.4 to 16958.8) | 11.7 (6.0 to 20.8) | 52.5* (30.8 to 128.4) | 56.1* (33.9 to 133.8) |
| Yellow fever | 0.3 (0.1 to 0.8) | 11.8 (3.2 to 31.5) | -- | -16.6 (-42.1 to 19.9) | -14.6 (-40.7 to 22.8) |
| Asymptomatic yellow fever | 0.2 (0.0 to 0.5) | 6.6 (1.6 to 18.7) | -- | -- | -- |
| Yellow fever episode | 0.1 (0.0 to 0.4) | 5.2 (1.4 to 14.2) | -- | -16.6 (-42.1 to 19.9) | -14.6 (-40.7 to 22.8) |
| Rabies | -- | 0.8 (0.6 to 1.2) | -- | -36.3* (-52.2 to -13.9) | -34.7* (-51.0 to -11.9) |
| Intestinal nematode infections | 109447.1 (102723.5 to 116949.2) | -- | 209.5 (120.2 to 340.3) | -32.7* (-36.4 to -29.1) | -31.1* (-34.9 to -27.5) |
| Ascariasis | 48113.3 (42420.0 to 54842.1) | -- | 62.5 (33.5 to 107.7) | -36.8* (-43.2 to -29.9) | -35.3* (-41.8 to -28.2) |
| Asymptomatic ascariasis | 44771.0 (39435.2 to 51028.3) | -- | -- | -- | -- |
| Ascariasis complications | 3342.4 (2976.1 to 3743.5) | -- | 62.5 (33.5 to 107.7) | -36.8* (-43.2 to -29.9) | -35.3* (-41.8 to -28.2) |
| Trichuriasis | 43585.4 (38705.3 to 49554.3) | -- | 36.6 (20.4 to 60.5) | -26.9* (-33.2 to -20.1) | -25.2* (-31.6 to -18.2) |
| Asymptomatic trichuriasis | 41770.8 (36996.2 to 47760.3) | -- | -- | -- | -- |
| Trichuriasis complications | 1814.6 (1657.9 to 1973.2) | -- | 36.6 (20.4 to 60.5) | -26.9* (-33.2 to -20.1) | -25.2* (-31.6 to -18.2) |
| Hookworm disease | 29384.9 (27376.0 to 31605.7) | -- | 110.4 (65.6 to 177.2) | -32.1* (-37.5 to -26.3) | -30.4* (-36.0 to -24.6) |
| Asymptomatic hookworm disease | 24217.0 (22546.8 to 26107.9) | -- | -- | -- | -- |
| Anemia due to hookworm disease | 1216.7 (1099.2 to 1344.6) | -- | 26.6 (17.0 to 40.2) | -40.8* (-49.0 to -31.5) | -39.4* (-47.8 to -29.9) |
| Hookworm disease complications | 3951.2 (3703.3 to 4232.4) | -- | 83.7 (46.2 to 137.6) | -28.7* (-33.4 to -23.3) | -27.0* (-31.8 to -21.5) |
| Food-borne trematodiases | 4364.0 (3633.8 to 5149.4) | 1174.5 (930.0 to 1467.9) | 67.4 (37.2 to 111.8) | -37.2* (-44.2 to -28.0) | -35.7* (-42.9 to -26.3) |
| Asymptomatic food-borne tremodiases | 3780.2 (3121.2 to 4518.6) | 989.1 (616.8 to 1339.0) | -- | -- | -- |
| Food-borne tremodiases complications | 583.8 (382.7 to 895.3) | 185.4 (47.6 to 511.8) | 67.4 (37.2 to 111.8) | -37.2* (-44.2 to -28.0) | -35.7* (-42.9 to -26.3) |
| Leprosy | 6.1 (5.5 to 6.8) | 2.7 (2.4 to 3.1) | 0.4 (0.2 to 0.5) | -34.5* (-38.3 to -30.5) | -33.0* (-36.8 to -28.8) |
| Ebola virus disease | -- | -- | -- | -96.1* (-96.5 to -95.3) | -96.0* (-96.4 to -95.2) |
| Ebola cases | -- | -- | -- | -97.4* (-97.6 to -97.2) | -97.3* (-97.6 to -97.1) |
| Post-Ebola chronic fatigue syndrome | -- | -- | -- | -96.1* (-96.4 to -95.2) | -96.0* (-96.3 to -95.1) |
| Zika virus disease | 3.0 (1.8 to 5.7) | 182.8 (106.2 to 346.9) | 0.1 (0.0 to 0.1) | -- | -- |
| Zika virus complications | -- | -- | -- | -- | -- |
| Zika virus episode | 3.0 (1.7 to 5.7) | 182.8 (106.2 to 346.9) | 0.1 (0.0 to 0.1) | -- | -- |
| Guinea worm disease | -- | -- | -- | -99.4* (-99.6 to -99.0) | -99.4* (-99.6 to -99.0) |
| Other neglected tropical diseases | 4888.8 (4560.3 to 5259.6) | -- | 107.9 (69.9 to 160.9) | -7.0 (-14.9 to 2.6) | -4.8 (-12.9 to 5.1) |
| Acute infection due to other neglected tropical diseases | -- | -- | 0.8 (0.4 to 1.5) | 115.4* (84.7 to 219.2) | 120.6* (89.1 to 226.8) |
| Anemia due to other neglected tropical diseases | 4888.8 (4560.3 to 5259.6) | -- | 107.0 (69.2 to 159.9) | -7.4 (-15.4 to 2.3) | -5.2 (-13.3 to 4.7) |
| **Other infectious diseases** | **8634.6 (8042.3 to 9376.0)** | **26715.9 (22563.3 to 32037.7)** | **340.7 (238.8 to 466.4)** | **-7.4* (-10.8 to -3.4)** | **-5.2* (-8.6 to -1.1)** |
| Meningitis | 1031.6 (855.7 to 1242.1) | 255.1 (171.6 to 363.0) | 96.6 (66.4 to 131.8) | -7.4* (-12.0 to -2.4) | -5.2* (-9.9 to -0.1) |
| Pneumococcal meningitis | 345.1 (281.2 to 429.8) | 20.8 (12.5 to 31.9) | 34.2 (23.0 to 47.0) | -29.2* (-35.1 to -23.5) | -27.6* (-33.6 to -21.7) |

| | | | | | |
|---|---|---|---|---|---|
| Acute pneumococcal meningitis | 1.0 (0.6 to 1.6) | 20.8 (12.5 to 31.9) | 0.1 (0.1 to 0.2) | -32.0* (-40.7 to -23.4) | -30.4* (-39.3 to -21.6) |
| Pneumococcal meningitis complications | 344.0 (280.0 to 428.8) | -- | 34.1 (23.0 to 46.8) | -29.2* (-35.2 to -23.5) | -27.5* (-33.6 to -21.7) |
| H influenzae type B meningitis | 98.2 (70.9 to 131.3) | 7.8 (4.3 to 13.5) | 9.5 (6.4 to 13.0) | -49.5* (-52.7 to -46.0) | -48.3* (-51.6 to -44.8) |
| Acute H influenzae type B meningitis | 0.4 (0.2 to 0.6) | 7.8 (4.3 to 13.5) | 0.1 (0.0 to 0.1) | -59.0* (-64.5 to -53.5) | -58.0* (-63.6 to -52.4) |
| H Influenza type B meningitis complications | 97.8 (70.6 to 131.0) | -- | 9.4 (6.3 to 13.0) | -49.4* (-52.6 to -46.0) | -48.2* (-51.5 to -44.7) |
| Meningococcal infection | 102.4 (72.2 to 138.8) | 17.9 (10.6 to 28.9) | 10.0 (6.7 to 13.8) | -6.5* (-10.6 to -2.4) | -4.3* (-8.5 to -0.1) |
| Acute meningococcal meningitis | 0.9 (0.6 to 1.4) | 17.9 (10.6 to 28.9) | 0.1 (0.1 to 0.2) | 0.1 (-10.5 to 10.9) | 2.5 (-8.4 to 13.5) |
| Meningococcal meningitis complications | 101.5 (71.3 to 137.3) | -- | 9.9 (6.6 to 13.6) | -6.6* (-10.8 to -2.5) | -4.3* (-8.6 to -0.2) |
| Other meningitis | 486.0 (358.8 to 626.3) | 208.5 (143.2 to 300.3) | 42.9 (29.2 to 58.1) | 61.9* (50.4 to 74.4) | 65.8* (53.9 to 78.5) |
| Other acute bacterial meningitis | 2.2 (1.5 to 3.2) | 45.4 (30.2 to 65.4) | 0.3 (0.2 to 0.5) | 76.7* (59.9 to 93.9) | 80.8* (63.7 to 98.5) |
| Acute viral meningitis | 8.1 (5.7 to 11.3) | 163.1 (109.8 to 232.3) | 1.1 (0.6 to 1.7) | -1.4 (-9.2 to 5.6) | 1.0 (-7.0 to 8.1) |
| Other bacterial meningitis complications | 475.7 (348.4 to 615.5) | -- | 41.5 (28.3 to 56.6) | 64.6* (52.4 to 77.7) | 68.5* (56.0 to 82.0) |
| Encephalitis | 473.2 (280.4 to 734.2) | 143.3 (137.3 to 149.6) | 44.5 (31.3 to 58.8) | -9.1* (-15.0 to -2.9) | -7.0* (-13.0 to -0.5) |
| Acute encephalitis | 8.0 (7.7 to 8.4) | 143.3 (137.3 to 149.6) | 1.1 (0.7 to 1.5) | -0.2 (-0.7 to 0.4) | 2.2* (1.6 to 2.7) |
| Encephalitis complications | 465.1 (272.6 to 726.3) | -- | 43.5 (30.5 to 57.4) | -9.3* (-15.3 to -2.9) | -7.2* (-13.3 to -0.6) |
| Diphtheria | -- | 0.2 (0.1 to 0.3) | -- | -40.3* (-59.5 to -11.7) | -38.9* (-58.6 to -9.6) |
| Whooping cough | 6.8 (5.3 to 8.6) | 49.6 (38.3 to 62.6) | 0.3 (0.2 to 0.5) | -7.2* (-7.4 to -7.0) | -5.0* (-5.2 to -4.8) |
| Tetanus | 5.7 (5.4 to 6.0) | 4.0 (2.2 to 7.0) | 0.1 (0.1 to 0.2) | -13.5* (-25.4 to -3.1) | -11.4* (-23.7 to -0.8) |
| Severe tetanus | 0.2 (0.1 to 0.4) | 4.0 (2.2 to 7.0) | 0.0 (0.0 to 0.1) | -48.8* (-63.9 to -25.2) | -47.6* (-63.0 to -23.4) |
| Neonatal tetanus complications | 5.5 (5.3 to 5.7) | -- | 0.1 (0.1 to 0.2) | 6.4* (4.7 to 8.0) | 8.9* (7.2 to 10.5) |
| Measles | 5.4 (1.9 to 12.0) | 196.6 (69.9 to 437.7) | 0.5 (0.2 to 1.1) | -42.6* (-47.0 to -38.0) | -41.3* (-45.8 to -36.5) |
| Varicella and herpes zoster | 266.4 (214.0 to 325.2) | 2520.1 (1744.5 to 3451.6) | 13.8 (8.1 to 21.6) | -3.0 (-8.8 to 3.7) | -0.7 (-6.7 to 6.2) |
| Chickenpox | 23.0 (9.7 to 40.2) | 1200.7 (506.6 to 2098.8) | 0.1 (0.0 to 0.4) | 7.5* (2.8 to 13.2) | 10.1* (5.2 to 15.9) |
| Herpes zoster | 243.4 (193.3 to 300.1) | 1319.3 (1034.1 to 1636.2) | 13.7 (8.1 to 21.4) | -3.0 (-8.9 to 3.7) | -0.7 (-6.7 to 6.1) |
| Acute hepatitis | 2270.2 (1835.1 to 2869.0) | 23547.1 (19447.5 to 28637.5) | 44.2 (28.5 to 65.8) | -5.9 (-16.3 to 6.3) | -3.7 (-14.3 to 8.8) |
| Acute hepatitis A | 738.3 (638.3 to 819.9) | 9598.4 (8298.4 to 10658.5) | 29.6 (18.9 to 44.7) | -5.0 (-14.0 to 4.1) | -2.8 (-12.0 to 6.5) |
| Acute hepatitis B | 1339.0 (922.3 to 1929.2) | 11604.5 (7993.2 to 16719.9) | 9.7 (5.4 to 16.6) | -8.9 (-41.0 to 38.6) | -6.7 (-39.6 to 41.9) |
| Acute hepatitis C | 37.6 (20.5 to 59.1) | 325.7 (177.9 to 512.1) | 0.5 (0.2 to 1.1) | -0.1 (-3.8 to 5.6) | 2.3 (-1.5 to 8.1) |
| Acute hepatitis E | 155.3 (112.8 to 207.2) | 2018.5 (1466.6 to 2693.8) | 4.4 (2.3 to 7.2) | -6.1 (-24.9 to 15.6) | -3.8 (-23.1 to 18.3) |
| Other unspecified infectious diseases | 5573.8 (5237.1 to 5942.9) | -- | 140.5 (92.2 to 210.8) | -7.4 (-13.7 to 0.1) | -5.3 (-11.7 to 2.4) |
| Guillain-Barré syndrome due to other infectious diseases | 0.5 (0.2 to 0.8) | -- | 0.1 (0.1 to 0.3) | 6.0* (3.5 to 8.6) | 8.5* (5.9 to 11.2) |
| Other infectious diseases | -- | -- | 19.8 (11.4 to 32.5) | 0.7 (-1.6 to 2.9) | 3.1* (0.7 to 5.4) |
| Anemia due to other infectious diseases | 5573.3 (5236.8 to 5942.6) | -- | 120.5 (78.0 to 181.1) | -8.7* (-16.0 to -0.1) | -6.5 (-14.0 to 2.2) |
| **Maternal and neonatal disorders** | **12351.2 (10713.7 to 14238.4)** | **8444.2 (7339.2 to 9790.9)** | **2601.5 (1963.2 to 3358.3)** | **5.3 (-0.6 to 12.3)** | **7.8* (1.7 to 15.0)** |
| Maternal disorders | 493.6 (383.2 to 637.8) | 8444.2 (7339.2 to 9790.9) | 54.4 (36.8 to 76.8) | -13.6* (-24.8 to -1.6) | -11.5 (-23.0 to 0.8) |
| Maternal haemorrhage | 118.4 (106.6 to 131.3) | 577.5 (401.9 to 781.9) | 5.2 (3.3 to 7.3) | -20.7* (-33.6 to -4.8) | -18.8* (-32.0 to -2.5) |
| Maternal hemorrhage complications | 105.8 (95.0 to 117.5) | -- | 3.0 (2.0 to 4.4) | -22.8* (-34.5 to -8.7) | -20.9* (-33.0 to -6.5) |
| Maternal hemorrhage episode | 12.6 (7.7 to 19.3) | 577.5 (401.9 to 781.9) | 2.1 (1.1 to 3.5) | -17.5 (-43.1 to 20.4) | -15.6 (-41.7 to 23.3) |
| Maternal sepsis and other pregnancy related infections | 144.1 (88.9 to 226.2) | 1483.3 (1083.4 to 2003.4) | 5.9 (2.9 to 11.4) | -11.3 (-49.2 to 50.3) | -9.2 (-48.0 to 53.9) |
| Infertility due to puerperal sepsis | 32.9 (24.9 to 42.2) | 16.3 (11.7 to 21.7) | 0.2 (0.1 to 0.4) | 14.8 (-3.7 to 37.5) | 17.6 (-1.4 to 40.8) |
| Maternal sepsis and other maternal infection episode | 111.2 (55.9 to 190.7) | 1467.0 (1063.3 to 1985.5) | 5.7 (2.7 to 11.2) | -11.9 (-51.4 to 51.4) | -9.8 (-50.3 to 55.0) |
| Maternal hypertensive disorders | 210.1 (119.4 to 340.7) | 1278.7 (962.3 to 1666.4) | 10.8 (5.4 to 19.0) | -24.8 (-49.4 to 5.9) | -23.0 (-48.2 to 8.5) |
| Maternal hypertensive disorder complications | 7.3 (6.7 to 8.0) | 141.3 (129.6 to 154.9) | 0.5 (0.3 to 0.8) | 9.4* (5.7 to 13.6) | 11.9* (8.2 to 16.3) |
| Maternal hypertensive disorder episode | 202.8 (112.2 to 333.6) | 1137.3 (821.3 to 1523.0) | 10.3 (5.0 to 18.4) | -25.9 (-51.0 to 5.8) | -24.1 (-49.8 to 8.3) |
| Maternal obstructed labour and uterine rupture | 67.4 (51.2 to 88.8) | 828.3 (590.7 to 1170.2) | 22.3 (13.8 to 33.4) | -11.2 (-25.1 to 3.7) | -9.1 (-23.3 to 6.2) |
| Obstructed labor, acute event | 10.7 (6.3 to 17.7) | 783.7 (544.1 to 1128.1) | 3.4 (1.7 to 6.1) | -13.4 (-49.9 to 43.9) | -11.3 (-48.7 to 47.3) |
| Maternal obstructed labor complications | 56.7 (41.6 to 76.5) | 44.7 (31.7 to 59.6) | 18.9 (11.5 to 29.3) | -10.8 (-24.7 to 4.5) | -8.6 (-22.9 to 7.0) |
| Maternal abortion and miscarriage | 17.7 (10.8 to 27.2) | 2162.4 (1571.2 to 2855.1) | 2.0 (1.0 to 3.3) | -12.3 (-43.0 to 31.1) | -10.3 (-41.6 to 34.2) |
| Ectopic pregnancy | 17.3 (10.1 to 27.5) | 2114.0 (1405.5 to 2902.8) | 1.9 (0.9 to 3.4) | 10.6 (-37.8 to 82.3) | 13.2 (-36.4 to 86.6) |
| Other maternal disorders | -- | -- | 6.4 (4.3 to 9.1) | -0.1 (-13.9 to 14.9) | 2.2 (-11.9 to 17.6) |
| Neonatal disorders | 11866.8 (10223.2 to 13727.8) | -- | 2547.1 (1923.9 to 3297.7) | 5.8 (-0.3 to 12.9) | 8.3* (2.1 to 15.6) |
| Neonatal preterm birth | 6932.9 (5911.2 to 8244.5) | -- | 1252.0 (891.9 to 1696.8) | 10.2 (-4.0 to 24.0) | 12.8 (-1.7 to 26.9) |
| Preterm birth complications | 5959.6 (4911.4 to 7230.9) | -- | 1200.8 (845.1 to 1642.2) | 11.0 (-4.1 to 25.3) | 13.6 (-1.8 to 28.2) |
| Retinopathy of prematurity | 973.3 (759.6 to 1207.3) | -- | 51.3 (33.7 to 72.1) | -5.2 (-12.0 to 1.9) | -2.9 (-10.0 to 4.4) |
| Uncomplicated preterm birth | -- | -- | -- | -- | -- |
| Neonatal encephalopathy due to birth asphyxia and trauma | 4432.2 (3194.1 to 5882.9) | -- | 847.9 (504.0 to 1333.2) | 1.1 (-2.5 to 4.5) | 3.5 (-0.2 to 7.0) |
| Neonatal sepsis and other neonatal infections | 1187.8 (731.2 to 1821.9) | -- | 362.7 (191.7 to 619.7) | 2.2 (-2.4 to 6.5) | 4.6 (-0.0 to 9.0) |
| Neonatal sepsis and other neonatal infection complications | 1187.8 (731.2 to 1821.9) | -- | 362.7 (191.7 to 619.7) | 2.2 (-2.4 to 6.5) | 4.6 (-0.0 to 9.0) |
| Neonatal sepsis and other neonatal infection episode | -- | -- | -- | -- | -- |
| Hemolytic disease and other neonatal jaundice | 222.6 (191.5 to 254.1) | -- | 84.5 (64.9 to 106.1) | 9.6* (3.8 to 15.6) | 12.2* (6.3 to 18.4) |

| | | | | | |
|---|---|---|---|---|---|
| Other neonatal disorders | -- | -- | -- | -- | -- |
| **Nutritional deficiencies** | **174563.0 (167009.2 to 182902.8)** | **104372.5 (94048.3 to 115518.4)** | **2981.9 (1958.2 to 4393.5)** | **-10.4* (-16.4 to -3.4)** | **-8.3* (-14.4 to -1.1)** |
| Protein-energy malnutrition | 2257.4 (1988.6 to 2555.6) | 2660.9 (2320.1 to 3035.9) | 29.4 (18.6 to 42.0) | 21.2* (11.6 to 31.5) | 24.1* (14.3 to 34.6) |
| Iodine deficiency | 10853.6 (9566.5 to 12249.7) | 243.4 (208.7 to 280.7) | 214.7 (129.3 to 332.1) | -13.6* (-18.5 to -8.0) | -11.6* (-16.6 to -5.8) |
| Visible goiter without symptoms | 10550.1 (9288.3 to 11918.5) | 243.4 (208.7 to 280.7) | 114.4 (55.1 to 212.8) | -16.8* (-18.8 to -14.9) | -14.8* (-16.9 to -12.9) |
| Visible goiter with complications | 303.5 (176.6 to 422.7) | -- | 100.3 (51.7 to 155.0) | -9.8 (-20.4 to 1.1) | -7.6 (-18.5 to 3.5) |
| Vitamin A deficiency | 69388.2 (61830.2 to 77719.9) | 101468.1 (91034.7 to 112579.1) | 23.2 (14.0 to 36.3) | 10.3* (2.2 to 19.3) | 12.9* (4.6 to 22.1) |
| Asymptomatic vitamin A deficiency | 69000.4 (61458.6 to 77318.2) | 101468.1 (91034.7 to 112579.1) | -- | -- | -- |
| Vitamin A deficiency complications | 387.8 (263.7 to 547.5) | -- | 23.2 (14.0 to 36.3) | 10.3* (2.2 to 19.3) | 12.9* (4.6 to 22.1) |
| Vitamin A deficiency with anemia | -- | -- | -- | -- | -- |
| Dietary iron deficiency | 121018.7 (113857.5 to 128760.4) | -- | 2703.7 (1758.3 to 4031.3) | -10.5* (-17.3 to -2.7) | -8.4* (-15.3 to -0.4) |
| Other nutritional deficiencies | -- | -- | 10.8 (6.9 to 15.5) | -11.7* (-18.7 to -4.2) | -9.6* (-16.7 to -1.9) |
| **Non-communicable diseases** | **557312.7 (544852.2 to 570240.8)** | **787557.2 (710992.9 to 869745.2)** | **33456.8 (24752.0 to 43859.4)** | **-1.8* (-2.5 to -1.2)** | **0.5 (-0.2 to 1.1)** |
| **Neoplasms** | **776.2 (721.2 to 861.0)** | **90.4 (78.6 to 123.0)** | **46.9 (33.1 to 63.9)** | **14.0* (5.3 to 26.4)** | **16.7* (7.8 to 29.4)** |
| Lip and oral cavity cancer | 11.0 (8.9 to 14.2) | 2.2 (1.8 to 2.9) | 1.0 (0.7 to 1.5) | 17.4 (-8.4 to 56.5) | 20.2 (-6.2 to 60.2) |
| Diagnosis and primary therapy phase of mouth cancer | 0.6 (0.5 to 0.8) | 2.2 (1.8 to 2.9) | 0.2 (0.1 to 0.3) | 20.6 (-1.3 to 52.0) | 23.4* (1.1 to 55.6) |
| Controlled phase of mouth cancer | 9.6 (7.8 to 12.4) | -- | 0.5 (0.3 to 0.8) | 22.1 (-19.6 to 87.8) | 25.0 (-17.7 to 92.3) |
| Metastatic phase of mouth cancer | 0.7 (0.6 to 0.9) | -- | 0.3 (0.2 to 0.4) | 10.9 (-8.9 to 39.1) | 13.6 (-6.8 to 42.4) |
| Terminal phase of mouth cancer | 0.1 (0.1 to 0.1) | -- | 0.1 (0.0 to 0.1) | 9.5 (-10.8 to 38.1) | 12.1 (-8.7 to 41.4) |
| Nasopharynx cancer | 6.6 (5.9 to 7.4) | 1.2 (1.0 to 1.3) | 0.6 (0.4 to 0.8) | -15.0* (-24.1 to -4.1) | -13.0* (-22.3 to -1.9) |
| Diagnosis and primary therapy phase of nasopharynx cancer | 0.4 (0.3 to 0.4) | 1.2 (1.0 to 1.3) | 0.1 (0.1 to 0.2) | -14.0* (-23.2 to -3.1) | -11.9* (-21.4 to -0.8) |
| Controlled phase of nasopharynx cancer | 5.8 (5.2 to 6.4) | -- | 0.3 (0.2 to 0.4) | -14.0* (-23.1 to -3.2) | -12.0* (-21.3 to -1.0) |
| Metastatic phase of nasopharynx cancer | 0.4 (0.4 to 0.5) | -- | 0.2 (0.1 to 0.3) | -16.5* (-25.4 to -5.6) | -14.5* (-23.7 to -3.3) |
| Terminal phase of nasopharynx cancer | 0.0 (0.0 to 0.1) | -- | -- | -18.5* (-27.5 to -7.7) | -16.5* (-25.8 to -5.6) |
| Other pharynx cancer | 1.2 (1.0 to 1.4) | 0.5 (0.4 to 0.6) | 0.2 (0.1 to 0.2) | -4.3 (-14.7 to 8.1) | -2.0 (-12.7 to 10.6) |
| Diagnosis and primary therapy phase of other pharynx cancer | 0.1 (0.1 to 0.1) | 0.5 (0.4 to 0.6) | -- | -1.8 (-12.0 to 9.7) | 0.5 (-9.9 to 12.3) |
| Controlled phase of other pharynx cancer | 0.9 (0.8 to 1.0) | -- | 0.0 (0.0 to 0.1) | -1.7 (-11.9 to 9.6) | 0.6 (-9.8 to 12.2) |
| Metastatic phase of other pharynx cancer | 0.2 (0.2 to 0.2) | -- | 0.1 (0.1 to 0.1) | -5.6 (-16.4 to 7.2) | -3.4 (-14.4 to 9.7) |
| Terminal phase of other pharynx cancer | -- | -- | -- | -6.0 (-16.8 to 6.9) | -3.8 (-14.8 to 9.4) |
| Oesophageal cancer | 0.6 (0.5 to 0.6) | 0.3 (0.3 to 0.3) | 0.1 (0.1 to 0.1) | -19.8* (-26.4 to -12.7) | -17.9* (-24.7 to -10.7) |
| Diagnosis and primary therapy phase of esophageal cancer | 0.0 (0.0 to 0.1) | 0.3 (0.3 to 0.3) | -- | -16.6* (-23.6 to -8.8) | -14.6* (-21.8 to -6.6) |
| Controlled phase of esophageal cancer | 0.4 (0.4 to 0.4) | -- | -- | -15.1* (-22.7 to -7.2) | -13.1* (-20.9 to -5.0) |
| Metastatic phase of esophageal cancer | 0.1 (0.1 to 0.1) | -- | 0.0 (0.0 to 0.1) | -22.0* (-28.2 to -14.7) | -20.1* (-26.5 to -12.7) |
| Terminal phase of esophageal cancer | -- | -- | -- | -22.0* (-28.2 to -14.7) | -20.1* (-26.5 to -12.7) |
| Stomach cancer | 2.5 (2.3 to 2.8) | 1.0 (1.0 to 1.1) | 0.3 (0.2 to 0.4) | -16.0* (-21.0 to -9.5) | -14.0* (-19.1 to -7.4) |
| Diagnosis and primary therapy phase of stomach cancer | 0.2 (0.2 to 0.2) | 1.0 (1.0 to 1.1) | 0.1 (0.0 to 0.1) | -11.0* (-16.9 to -3.9) | -8.9* (-15.0 to -1.7) |
| Controlled phase of stomach cancer | 2.0 (1.8 to 2.1) | -- | 0.1 (0.1 to 0.1) | -2.1 (-9.5 to 7.2) | 0.3 (-7.4 to 9.7) |
| Metastatic phase of stomach cancer | 0.3 (0.3 to 0.3) | -- | 0.1 (0.1 to 0.2) | -24.0* (-27.7 to -19.7) | -22.1* (-26.0 to -17.8) |
| Terminal phase of stomach cancer | 0.1 (0.1 to 0.1) | -- | -- | -24.0* (-27.7 to -19.7) | -22.1* (-26.0 to -17.8) |
| Colon and rectum cancer | 8.0 (7.4 to 8.6) | 1.6 (1.4 to 1.7) | 0.9 (0.6 to 1.2) | -4.5 (-12.9 to 4.1) | -2.2 (-10.8 to 6.5) |
| Diagnosis and primary therapy phase of colon and rectum cancers | 0.3 (0.3 to 0.3) | 1.6 (1.4 to 1.7) | 0.1 (0.1 to 0.1) | -0.8 (-8.8 to 7.9) | 1.6 (-6.6 to 10.4) |
| Controlled phase of colon and rectum cancers | 7.1 (6.6 to 7.6) | -- | 0.5 (0.3 to 0.7) | -3.5 (-11.5 to 4.6) | -1.2 (-9.4 to 7.1) |
| Metastatic phase of colon and rectum cancers | 0.5 (0.4 to 0.5) | -- | 0.2 (0.1 to 0.3) | -7.3 (-15.9 to 2.0) | -5.1 (-13.9 to 4.5) |
| Terminal phase of colon and rectum cancers | 0.1 (0.1 to 0.1) | -- | 0.0 (0.0 to 0.1) | -9.0 (-17.7 to 0.6) | -6.8 (-15.8 to 3.0) |
| Stoma from colon and rectum cancers, beyond 10 years | 0.0 (0.0 to 0.1) | -- | -- | -10.3* (-14.2 to -7.7) | -8.1* (-12.2 to -5.5) |
| Liver cancer | -- | 1.4 (1.3 to 1.5) | 0.3 (0.2 to 0.4) | -15.5* (-22.4 to -7.8) | -13.5* (-20.6 to -5.6) |
| Liver cancer due to hepatitis B | 1.1 (1.0 to 1.2) | 1.0 (0.9 to 1.1) | 0.2 (0.2 to 0.3) | -18.7* (-25.7 to -10.5) | -16.8* (-23.9 to -8.4) |
| Diagnosis and primary therapy phase of liver cancer due to hepatitis B | 0.2 (0.2 to 0.3) | 1.0 (0.9 to 1.1) | 0.1 (0.0 to 0.1) | -17.7* (-24.8 to -9.2) | -15.7* (-23.0 to -7.0) |
| Controlled phase of liver cancer due to hepatitis B | 0.6 (0.5 to 0.7) | -- | -- | -20.9* (-27.6 to -13.8) | -19.0* (-25.9 to -11.7) |
| Metastatic phase of liver cancer due to hepatitis B | 0.2 (0.2 to 0.2) | -- | 0.1 (0.1 to 0.1) | -18.8* (-25.8 to -10.5) | -16.8* (-24.0 to -8.4) |
| Terminal phase of liver cancer due to hepatitis B | 0.1 (0.1 to 0.1) | -- | 0.0 (0.0 to 0.1) | -18.7* (-25.8 to -10.5) | -16.8* (-24.0 to -8.4) |
| Liver cancer due to hepatitis C | -- | -- | -- | -5.8 (-15.0 to 6.2) | -3.6 (-12.9 to 8.7) |
| Diagnosis and primary therapy phase of liver cancer due to hepatitis C | -- | -- | -- | -4.5 (-14.0 to 7.6) | -2.3 (-12.0 to 10.1) |
| Controlled phase of liver cancer due to hepatitis C | -- | -- | -- | -11.0 (-19.3 to 0.6) | -8.8 (-17.4 to 2.9) |
| Metastatic phase of liver cancer due to hepatitis C | -- | -- | -- | -5.3 (-14.7 to 6.9) | -3.0 (-12.6 to 9.4) |
| Terminal phase of liver cancer due to hepatitis C | -- | -- | -- | -5.3 (-14.7 to 6.9) | -3.0 (-12.6 to 9.4) |
| Liver cancer due to alcohol use | -- | -- | -- | -4.3 (-12.2 to 4.4) | -2.1 (-10.1 to 6.8) |
| Diagnosis and primary therapy phase of liver cancer due to alcohol use | -- | -- | -- | -3.2 (-11.3 to 6.3) | -0.9 (-9.2 to 8.8) |
| Controlled phase of liver cancer due to alcohol use | -- | -- | -- | -9.5* (-16.7 to -2.2) | -7.3 (-14.7 to 0.2) |
| Metastatic phase of liver cancer due to alcohol use | -- | -- | -- | -3.7 (-11.8 to 5.4) | -1.5 (-9.7 to 7.9) |

| | | | | | |
|---|---|---|---|---|---|
| Terminal phase of liver cancer due to alcohol use | -- | -- | -- | -3.7 (-11.8 to 5.4) | -1.5 (-9.7 to 7.9) |
| Liver cancer due to NASH | 0.1 (0.1 to 0.2) | 0.1 (0.1 to 0.2) | -- | -1.9 (-9.9 to 6.4) | 0.4 (-7.8 to 9.0) |
| Diagnosis and primary therapy phase of liver cancer due to NASH | -- | 0.1 (0.1 to 0.2) | -- | -1.0 (-9.2 to 7.5) | 1.3 (-7.0 to 10.1) |
| Controlled phase of liver cancer due to NASH | 0.1 (0.1 to 0.1) | -- | -- | -5.0 (-12.8 to 2.8) | -2.7 (-10.8 to 5.2) |
| Metastatic phase of liver cancer due to NASH | -- | -- | -- | -1.7 (-9.8 to 6.9) | 0.7 (-7.6 to 9.4) |
| Terminal phase of liver cancer due to NASH | -- | -- | -- | -1.7 (-9.8 to 6.9) | 0.7 (-7.6 to 9.4) |
| Liver cancer due to other causes | 0.3 (0.2 to 0.3) | 0.3 (0.2 to 0.3) | 0.1 (0.0 to 0.1) | -10.1* (-17.1 to -1.7) | -8.0 (-15.2 to 0.6) |
| Diagnosis and primary therapy phase of liver cancer due to other causes | 0.1 (0.0 to 0.1) | 0.3 (0.2 to 0.3) | -- | -9.0* (-16.1 to -0.4) | -6.9 (-14.1 to 1.9) |
| Controlled phase of liver cancer due to other causes | 0.2 (0.1 to 0.2) | -- | -- | -13.3* (-19.9 to -5.3) | -11.2* (-18.0 to -3.0) |
| Metastatic phase of liver cancer due to other causes | 0.0 (0.0 to 0.1) | -- | -- | -9.9* (-17.0 to -1.4) | -7.8 (-15.0 to 0.9) |
| Terminal phase of liver cancer due to other causes | -- | -- | -- | -9.9* (-17.0 to -1.4) | -7.8 (-15.0 to 0.9) |
| Gallbladder and biliary tract cancer | 0.2 (0.1 to 0.2) | 0.1 (0.1 to 0.1) | -- | -5.7 (-12.2 to 2.3) | -3.5 (-10.1 to 4.7) |
| Diagnosis and primary therapy phase of gallbladder and biliary tract cancer | -- | 0.1 (0.1 to 0.1) | -- | -4.8 (-11.0 to 2.6) | -2.5 (-8.9 to 5.1) |
| Controlled phase of gallbladder and biliary tract cancer | 0.1 (0.1 to 0.1) | -- | -- | 6.7* (2.0 to 11.6) | 9.3* (4.4 to 14.2) |
| Metastatic phase of gallbladder and biliary tract cancer | -- | -- | -- | -8.0 (-15.1 to 0.4) | -5.9 (-13.1 to 2.8) |
| Terminal phase of gallbladder and biliary tract cancer | -- | -- | -- | -8.0 (-15.1 to 0.4) | -5.9 (-13.1 to 2.8) |
| Pancreatic cancer | 0.1 (0.1 to 0.2) | 0.2 (0.2 to 0.2) | 0.0 (0.0 to 0.1) | -6.5 (-11.5 to 0.1) | -4.3 (-9.4 to 2.5) |
| Diagnosis and primary therapy phase of pancreatic cancer | -- | 0.2 (0.2 to 0.2) | -- | -6.5 (-11.5 to 0.2) | -4.2 (-9.4 to 2.6) |
| Controlled phase of pancreatic cancer | -- | -- | -- | -11.1* (-15.2 to -5.8) | -9.0* (-13.2 to -3.5) |
| Metastatic phase of pancreatic cancer | -- | -- | -- | -6.1 (-11.2 to 0.8) | -3.9 (-9.0 to 3.2) |
| Terminal phase of pancreatic cancer | -- | -- | -- | -6.1 (-11.2 to 0.8) | -3.9 (-9.0 to 3.2) |
| Larynx cancer | 2.1 (2.0 to 2.2) | 0.2 (0.2 to 0.3) | 0.2 (0.1 to 0.2) | -4.2 (-8.6 to 1.0) | -2.0 (-6.5 to 3.4) |
| Diagnosis and primary therapy phase of larynx cancer | 0.1 (0.1 to 0.1) | 0.2 (0.2 to 0.3) | -- | -0.2 (-6.3 to 6.8) | 2.2 (-4.0 to 9.3) |
| Controlled phase of larynx cancer | 1.0 (0.9 to 1.1) | -- | 0.1 (0.0 to 0.1) | -2.6 (-8.1 to 3.7) | -0.3 (-5.9 to 6.2) |
| Metastatic phase of larynx cancer | 0.1 (0.1 to 0.1) | -- | -- | -7.2 (-13.8 to 1.3) | -5.0 (-11.8 to 3.7) |
| Terminal phase of larynx cancer | -- | -- | -- | -9.4* (-16.4 to -0.7) | -7.3 (-14.4 to 1.6) |
| Laryngectomy from larynx cancer, beyond 10 years | 1.0 (0.9 to 1.1) | -- | 0.0 (0.0 to 0.1) | -4.8* (-5.4 to -4.3) | -2.6* (-3.1 to -2.1) |
| Tracheal, bronchus, and lung cancer | 1.2 (1.1 to 1.2) | 0.9 (0.9 to 1.0) | 0.2 (0.2 to 0.3) | -14.5* (-19.8 to -8.6) | -12.4* (-17.9 to -6.4) |
| Diagnosis and primary therapy phase of lung, bronchus, and trachea cancer | 0.1 (0.1 to 0.1) | 0.9 (0.9 to 1.0) | -- | -12.5* (-17.8 to -6.7) | -10.5* (-15.9 to -4.5) |
| Controlled phase of lung, bronchus, and trachea cancer | 0.7 (0.6 to 0.7) | -- | -- | -9.4* (-14.9 to -3.3) | -7.2* (-12.8 to -1.0) |
| Metastatic phase of lung, bronchus, and trachea cancer | 0.3 (0.3 to 0.4) | -- | 0.2 (0.1 to 0.2) | -15.5* (-20.8 to -9.7) | -13.5* (-18.9 to -7.6) |
| Terminal phase of lung, bronchus, and trachea cancer | 0.1 (0.1 to 0.1) | -- | 0.0 (0.0 to 0.1) | -15.5* (-20.8 to -9.7) | -13.5* (-18.9 to -7.6) |
| Malignant skin melanoma | 14.3 (12.3 to 18.4) | 1.7 (1.5 to 2.2) | 0.8 (0.5 to 1.3) | 4.8 (-6.7 to 18.3) | 7.3 (-4.4 to 21.1) |
| Diagnosis and primary therapy phase of malignant skin melanoma | 0.4 (0.3 to 0.5) | 1.7 (1.5 to 2.2) | 0.1 (0.1 to 0.2) | 5.2 (-2.0 to 15.6) | 7.6* (0.4 to 18.4) |
| Controlled phase of malignant skin melanoma | 13.8 (11.8 to 17.8) | -- | 0.7 (0.4 to 1.1) | 5.5 (-7.9 to 20.9) | 8.0 (-5.7 to 23.8) |
| Metastatic phase of malignant skin melanoma | 0.1 (0.1 to 0.2) | -- | 0.1 (0.0 to 0.1) | -1.9 (-11.3 to 7.3) | 0.5 (-9.2 to 9.9) |
| Terminal phase of malignant skin melanoma | -- | -- | -- | -2.4 (-12.2 to 6.9) | -0.1 (-10.1 to 9.5) |
| Non-melanoma skin cancer | -- | 12.6 (2.2 to 45.4) | 0.1 (0.0 to 0.2) | 15.9* (8.6 to 20.6) | 18.7* (11.2 to 23.5) |
| Non-melanoma skin cancer (squamous-cell carcinoma) | 1.7 (0.4 to 4.7) | 2.2 (0.5 to 6.0) | 0.1 (0.0 to 0.2) | 16.1* (8.4 to 21.0) | 18.9* (11.0 to 23.8) |
| Non-melanoma skin cancer (basal-cell carcinoma) | 1.0 (0.1 to 4.7) | 10.5 (0.9 to 43.6) | -- | 12.2 (-10.6 to 17.6) | 14.9 (-8.5 to 20.4) |
| Breast cancer | 13.4 (10.1 to 15.9) | 1.6 (1.2 to 2.0) | 1.1 (0.7 to 1.6) | 21.9* (1.2 to 47.2) | 24.8* (3.6 to 50.7) |
| Diagnosis and primary therapy phase of breast cancer | 0.4 (0.3 to 0.4) | 1.6 (1.2 to 2.0) | 0.1 (0.1 to 0.2) | 25.7* (9.0 to 48.1) | 28.7* (11.6 to 51.6) |
| Controlled phase of breast cancer | 12.3 (9.2 to 14.7) | -- | 0.8 (0.4 to 1.2) | 22.5 (-1.4 to 52.3) | 25.4* (1.0 to 55.9) |
| Metastatic phase of breast cancer | 0.4 (0.3 to 0.5) | -- | 0.2 (0.1 to 0.3) | 20.4* (4.6 to 42.2) | 23.3* (7.1 to 45.5) |
| Terminal phase of breast cancer | -- | -- | -- | 15.8 (-0.9 to 38.5) | 18.5* (1.5 to 41.7) |
| Mastectomy from breast cancer, beyond 10 years | 0.2 (0.2 to 0.3) | -- | -- | -12.5* (-14.1 to -10.9) | -10.4* (-12.1 to -8.7) |
| Cervical cancer | 9.6 (8.6 to 10.6) | 1.3 (1.1 to 1.4) | 0.7 (0.5 to 1.0) | -12.4* (-18.5 to -4.4) | -10.3* (-16.6 to -2.2) |
| Diagnosis and primary therapy phase of cervical cancer | 0.5 (0.4 to 0.5) | 1.3 (1.1 to 1.4) | 0.1 (0.1 to 0.2) | -12.0* (-17.9 to -4.1) | -9.9* (-15.9 to -1.8) |
| Controlled phase of cervical cancer | 8.8 (7.9 to 9.7) | -- | 0.4 (0.3 to 0.7) | -11.6* (-17.8 to -3.8) | -9.5* (-15.9 to -1.5) |
| Metastatic phase of cervical cancer | 0.3 (0.2 to 0.3) | -- | 0.1 (0.1 to 0.2) | -14.7* (-20.6 to -6.8) | -12.7* (-18.7 to -4.6) |
| Terminal phase of cervical cancer | -- | -- | -- | -16.2* (-22.1 to -8.4) | -14.2* (-20.2 to -6.2) |
| Uterine cancer | 2.8 (2.6 to 3.0) | 0.4 (0.3 to 0.4) | 0.2 (0.1 to 0.3) | -0.4 (-7.4 to 7.3) | 2.0 (-5.2 to 9.8) |
| Diagnosis and primary therapy phase of uterine cancer | 0.1 (0.1 to 0.1) | 0.4 (0.3 to 0.4) | -- | 0.2 (-6.8 to 7.8) | 2.6 (-4.6 to 10.4) |
| Controlled phase of uterine cancer | 2.6 (2.4 to 2.8) | -- | 0.1 (0.1 to 0.2) | 0.3 (-6.8 to 7.8) | 2.7 (-4.6 to 10.4) |
| Metastatic phase of uterine cancer | 0.1 (0.1 to 0.1) | -- | -- | -3.6 (-11.1 to 5.2) | -1.4 (-9.0 to 7.7) |
| Terminal phase of uterine cancer | -- | -- | -- | -6.5 (-14.3 to 3.4) | -4.3 (-12.3 to 5.9) |
| Ovarian cancer | 26.0 (22.7 to 31.9) | 3.7 (3.2 to 4.6) | 3.1 (2.2 to 4.3) | 10.2 (-6.8 to 37.1) | 12.8 (-4.6 to 40.4) |
| Diagnosis and primary therapy phase of ovarian cancer | 1.0 (0.8 to 1.2) | 3.7 (3.2 to 4.6) | 0.3 (0.2 to 0.4) | 10.8 (-4.3 to 34.7) | 13.4 (-2.1 to 37.9) |
| Controlled phase of ovarian cancer | 21.2 (18.5 to 25.9) | -- | 1.0 (0.6 to 1.6) | 10.4 (-14.1 to 44.4) | 13.1 (-12.1 to 47.8) |
| Metastatic phase of ovarian cancer | 3.7 (3.2 to 4.6) | -- | 1.7 (1.1 to 2.4) | 10.0 (-5.6 to 34.7) | 12.6 (-3.4 to 37.9) |

| | | | | | |
|---|---|---|---|---|---|
| Terminal phase of ovarian cancer | 0.1 (0.1 to 0.2) | -- | 0.1 (0.1 to 0.1) | 9.7 (-6.1 to 34.6) | 12.3 (-3.9 to 37.8) |
| Prostate cancer | 4.1 (3.6 to 5.2) | 0.5 (0.4 to 0.6) | 0.3 (0.2 to 0.5) | 14.3* (8.2 to 20.0) | 17.0* (10.7 to 22.9) |
| Diagnosis and primary therapy phase of prostate cancer | 0.1 (0.1 to 0.2) | 0.5 (0.4 to 0.6) | 0.0 (0.0 to 0.1) | 15.5* (9.1 to 21.3) | 18.3* (11.7 to 24.1) |
| Controlled phase of prostate cancer | 3.9 (3.4 to 5.0) | -- | 0.3 (0.2 to 0.4) | 14.9* (8.8 to 20.9) | 17.7* (11.4 to 23.7) |
| Metastatic phase of prostate cancer | -- | -- | -- | 0.1 (-5.4 to 5.3) | 2.5 (-3.2 to 7.7) |
| Terminal phase of prostate cancer | -- | -- | -- | -6.5* (-11.9 to -1.5) | -4.2 (-9.8 to 0.8) |
| Impotence and Incontinence after 10-year survival from prostate cancer | -- | -- | -- | -- | -- |
| Testicular cancer | 23.0 (21.4 to 25.4) | 2.8 (2.6 to 3.1) | 1.4 (1.0 to 2.0) | 8.1 (-3.2 to 21.4) | 10.6 (-0.9 to 24.3) |
| Diagnosis and primary therapy phase of testicular cancer | 0.7 (0.7 to 0.8) | 2.8 (2.6 to 3.1) | 0.2 (0.1 to 0.3) | 10.4* (3.2 to 18.9) | 13.0* (5.6 to 21.7) |
| Controlled phase of testicular cancer | 21.9 (20.4 to 24.2) | -- | 1.1 (0.7 to 1.6) | 10.4 (-3.9 to 27.9) | 13.0 (-1.6 to 30.9) |
| Metastatic phase of testicular cancer | 0.3 (0.3 to 0.3) | -- | 0.1 (0.1 to 0.2) | -7.7 (-14.5 to 0.7) | -5.5 (-12.4 to 3.1) |
| Terminal phase of testicular cancer | -- | -- | -- | -14.4* (-21.4 to -5.5) | -12.4* (-19.5 to -3.3) |
| Kidney cancer | 10.5 (9.7 to 11.0) | 1.5 (1.4 to 1.6) | 0.6 (0.4 to 0.9) | -5.4 (-10.8 to 0.3) | -3.1 (-8.7 to 2.7) |
| Diagnosis and primary therapy phase of kidney cancer | -- | 1.5 (1.4 to 1.6) | -- | -- | -- |
| Controlled phase of kidney cancer | 10.2 (9.5 to 10.7) | -- | 0.5 (0.3 to 0.7) | -5.3 (-10.8 to 0.3) | -3.1 (-8.7 to 2.7) |
| Metastatic phase of kidney cancer | 0.2 (0.2 to 0.2) | -- | 0.1 (0.1 to 0.1) | -5.5 (-10.8 to 0.1) | -3.2 (-8.7 to 2.5) |
| Terminal phase of kidney cancer | -- | -- | -- | -5.5 (-10.8 to 0.1) | -3.3 (-8.7 to 2.4) |
| Bladder cancer | 4.2 (3.9 to 4.6) | 0.6 (0.5 to 0.6) | 0.4 (0.3 to 0.5) | 4.2 (-3.3 to 13.3) | 6.7 (-1.0 to 16.0) |
| Diagnosis and primary therapy phase of bladder cancer | 0.2 (0.2 to 0.2) | 0.6 (0.5 to 0.6) | 0.1 (0.0 to 0.1) | 5.8 (-2.0 to 15.1) | 8.3* (0.3 to 17.8) |
| Controlled phase of bladder cancer | 3.9 (3.6 to 4.3) | -- | 0.3 (0.2 to 0.4) | 4.5 (-2.9 to 13.5) | 7.0 (-0.6 to 16.2) |
| Metastatic phase of bladder cancer | 0.1 (0.1 to 0.1) | -- | -- | 0.1 (-8.7 to 10.6) | 2.4 (-6.5 to 13.2) |
| Terminal phase of bladder cancer | -- | -- | -- | -0.6 (-9.3 to 10.2) | 1.8 (-7.2 to 12.8) |
| Incontinence from bladder cancer, beyond 10 years | -- | -- | -- | -3.9* (-5.1 to -2.7) | -1.7* (-2.8 to -0.4) |
| Brain and nervous system cancer | 41.9 (35.9 to 48.2) | 8.8 (7.5 to 10.0) | 4.0 (2.7 to 5.4) | 11.5* (1.2 to 22.9) | 14.2* (3.7 to 25.8) |
| Diagnosis and primary therapy phase of brain and nervous system cancers | 2.5 (2.2 to 2.9) | 8.8 (7.5 to 10.0) | 0.7 (0.5 to 1.0) | 18.0* (7.6 to 29.6) | 20.8* (10.1 to 32.6) |
| Controlled phase of brain and nervous system cancers | 36.2 (31.1 to 41.7) | -- | 1.7 (1.1 to 2.6) | 21.6* (6.2 to 38.7) | 24.5* (8.7 to 42.0) |
| Metastatic phase of brain and nervous system cancers | 2.7 (2.3 to 3.1) | -- | 1.2 (0.8 to 1.7) | -0.4 (-8.4 to 7.9) | 1.9 (-6.3 to 10.5) |
| Terminal phase of brain and nervous system cancers | 0.5 (0.4 to 0.5) | -- | 0.2 (0.2 to 0.3) | -2.7 (-10.5 to 6.0) | -0.4 (-8.4 to 8.5) |
| Thyroid cancer | 31.8 (26.8 to 40.0) | 3.6 (3.0 to 4.5) | 1.8 (1.2 to 2.8) | 12.9 (-10.9 to 43.9) | 15.6 (-8.8 to 47.3) |
| Diagnosis and primary therapy phase of thyroid cancer | 0.8 (0.7 to 1.1) | 3.6 (3.0 to 4.5) | 0.2 (0.2 to 0.4) | 13.3 (-3.6 to 39.8) | 16.0 (-1.3 to 43.1) |
| Controlled phase of thyroid cancer | 30.7 (25.8 to 38.6) | -- | 1.5 (0.9 to 2.4) | 14.0 (-12.3 to 48.6) | 16.7 (-10.2 to 52.2) |
| Metastatic phase of thyroid cancer | 0.2 (0.2 to 0.3) | -- | 0.1 (0.1 to 0.1) | 0.3 (-15.0 to 24.3) | 2.7 (-13.0 to 27.2) |
| Terminal phase of thyroid cancer | -- | -- | -- | -2.8 (-18.8 to 22.2) | -0.5 (-16.8 to 25.1) |
| Mesothelioma | 0.3 (0.2 to 0.3) | 0.1 (0.1 to 0.1) | 0.1 (0.0 to 0.1) | -11.6* (-20.3 to -3.1) | -9.5* (-18.4 to -0.8) |
| Diagnosis and primary therapy phase of mesothelioma | -- | 0.1 (0.1 to 0.1) | -- | -12.0* (-20.8 to -3.5) | -9.9* (-18.9 to -1.2) |
| Controlled phase of mesothelioma | 0.2 (0.2 to 0.2) | -- | -- | -11.7* (-20.4 to -3.2) | -9.6* (-18.5 to -0.9) |
| Metastatic phase of mesothelioma | 0.1 (0.1 to 0.1) | -- | -- | -11.5* (-20.2 to -3.0) | -9.5* (-18.3 to -0.7) |
| Terminal phase of mesothelioma | -- | -- | -- | -11.4* (-20.0 to -2.6) | -9.3* (-18.1 to -0.2) |
| Hodgkin lymphoma | 38.2 (32.7 to 47.8) | 5.7 (4.9 to 7.0) | 2.8 (1.9 to 3.9) | -3.8 (-12.3 to 6.3) | -1.5 (-10.3 to 8.8) |
| Diagnosis and primary therapy phase of Hodgkin disease | 1.3 (1.1 to 1.6) | 5.7 (4.9 to 7.0) | 0.4 (0.2 to 0.5) | -2.4 (-10.6 to 6.1) | -0.1 (-8.5 to 8.7) |
| Controlled phase of Hodgkin disease | 35.2 (30.2 to 44.3) | -- | 1.7 (1.1 to 2.6) | -2.3 (-13.3 to 10.5) | 0.0 (-11.3 to 13.1) |
| Metastatic phase of Hodgkin disease | 1.5 (1.3 to 1.8) | -- | 0.7 (0.4 to 0.9) | -7.2 (-14.9 to 0.5) | -5.0 (-12.9 to 2.8) |
| Terminal phase of Hodgkin disease | 0.2 (0.1 to 0.2) | -- | 0.1 (0.1 to 0.1) | -10.3* (-19.2 to -2.1) | -8.2 (-17.3 to 0.2) |
| Non-Hodgkin's lymphoma | 30.5 (28.9 to 32.4) | 6.1 (5.7 to 6.5) | 2.5 (1.7 to 3.4) | 6.3 (-1.2 to 14.6) | 8.8* (1.2 to 17.3) |
| Diagnosis and primary therapy phase of non-Hodgkin lymphoma | 1.1 (1.0 to 1.1) | 6.1 (5.7 to 6.5) | 0.3 (0.2 to 0.4) | 9.4* (3.1 to 16.5) | 12.0* (5.6 to 19.2) |
| Controlled phase of non-Hodgkin lymphoma | 27.5 (26.1 to 29.3) | -- | 1.3 (0.8 to 2.0) | 9.5* (0.5 to 19.9) | 12.1* (2.9 to 22.7) |
| Metastatic phase of non-Hodgkin lymphoma | 1.6 (1.5 to 1.7) | -- | 0.7 (0.5 to 0.9) | 0.8 (-6.9 to 9.2) | 3.2 (-4.7 to 11.8) |
| Terminal phase of non-Hodgkin lymphoma | 0.3 (0.3 to 0.3) | -- | 0.1 (0.1 to 0.2) | -0.1 (-8.0 to 8.3) | 2.2 (-5.9 to 10.9) |
| Multiple myeloma | 1.2 (0.9 to 1.3) | 0.4 (0.3 to 0.4) | 0.2 (0.2 to 0.3) | 25.3* (5.2 to 41.8) | 28.2* (7.7 to 45.2) |
| Diagnosis and primary therapy phase of multiple myeloma | 0.1 (0.1 to 0.1) | 0.4 (0.3 to 0.4) | -- | 34.9* (12.7 to 52.8) | 38.1* (15.3 to 56.4) |
| Controlled phase of multiple myeloma | 0.7 (0.5 to 0.8) | -- | 0.0 (0.0 to 0.1) | 35.0* (12.7 to 52.9) | 38.2* (15.3 to 56.6) |
| Metastatic phase of multiple myeloma | 0.4 (0.3 to 0.4) | -- | 0.2 (0.1 to 0.2) | 24.0* (4.4 to 40.2) | 26.9* (6.9 to 43.5) |
| Terminal phase of multiple myeloma | -- | -- | -- | 6.7 (-8.2 to 19.5) | 9.3 (-6.0 to 22.4) |
| Leukaemia | 54.8 (48.5 to 60.8) | 14.1 (12.5 to 15.7) | 6.3 (4.5 to 8.3) | -16.7* (-22.3 to -10.7) | -14.8* (-20.5 to -8.6) |
| Acute lymphoid leukaemia | 21.0 (18.6 to 23.3) | 4.1 (3.7 to 4.7) | 2.2 (1.6 to 3.0) | -2.7 (-11.3 to 5.4) | -0.4 (-9.2 to 7.9) |
| Diagnosis and primary therapy phase of acute lymphoid leukemia | 1.9 (1.7 to 2.1) | 4.1 (3.7 to 4.7) | 0.5 (0.4 to 0.8) | -8.5* (-16.3 to -1.6) | -6.4 (-14.3 to 0.7) |
| Controlled phase of acute lymphoid leukemia | 17.4 (15.4 to 19.2) | -- | 0.9 (0.5 to 1.3) | 0.3 (-9.6 to 9.6) | 2.7 (-7.5 to 12.2) |
| Metastatic phase of acute lymphoid leukemia | 1.5 (1.3 to 1.7) | -- | 0.7 (0.4 to 0.9) | -1.6 (-10.4 to 7.0) | 0.7 (-8.3 to 9.6) |
| Terminal phase of acute lymphoid leukemia | 0.3 (0.2 to 0.3) | -- | 0.1 (0.1 to 0.2) | -1.7 (-10.5 to 6.9) | 0.6 (-8.3 to 9.4) |
| Chronic lymphoid leukaemia | 3.9 (3.2 to 4.5) | 0.5 (0.4 to 0.6) | 0.5 (0.3 to 0.6) | 0.4 (-14.5 to 17.7) | 2.7 (-12.5 to 20.5) |

| | | | | | |
|---|---|---|---|---|---|
| Diagnosis and primary therapy phase of chronic lymphoid leukemia | 0.2 (0.2 to 0.2) | 0.5 (0.4 to 0.6) | 0.1 (0.0 to 0.1) | 1.4 (-13.4 to 18.5) | 3.8 (-11.3 to 21.3) |
| Controlled phase of chronic lymphoid leukemia | 3.1 (2.6 to 3.6) | -- | 0.2 (0.1 to 0.2) | 1.2 (-13.3 to 17.8) | 3.7 (-11.2 to 20.6) |
| Metastatic phase of chronic lymphoid leukemia | 0.5 (0.4 to 0.6) | -- | 0.2 (0.2 to 0.3) | 0.1 (-15.6 to 17.7) | 2.5 (-13.6 to 20.5) |
| Terminal phase of chronic lymphoid leukemia | -- | -- | -- | -10.8 (-24.1 to 4.5) | -8.7 (-22.3 to 6.9) |
| Acute myeloid leukaemia | 4.1 (3.7 to 4.7) | 3.6 (3.2 to 4.1) | 0.9 (0.6 to 1.1) | -4.8 (-14.1 to 4.8) | -2.6 (-12.0 to 7.3) |
| Diagnosis and primary therapy phase of acute myeloid leukemia | -- | 3.6 (3.2 to 4.1) | -- | -- | -- |
| Controlled phase of acute myeloid leukemia | 2.5 (2.3 to 2.9) | -- | 0.1 (0.1 to 0.2) | -7.6 (-16.6 to 2.2) | -5.4 (-14.6 to 4.6) |
| Metastatic phase of acute myeloid leukemia | 1.3 (1.1 to 1.5) | -- | 0.6 (0.4 to 0.8) | -4.3 (-13.4 to 5.6) | -2.1 (-11.4 to 8.1) |
| Terminal phase of acute myeloid leukemia | 0.3 (0.2 to 0.3) | -- | 0.2 (0.1 to 0.2) | -4.3 (-13.4 to 5.6) | -2.1 (-11.4 to 8.1) |
| Chronic myeloid leukaemia | 1.8 (1.6 to 2.1) | 0.6 (0.5 to 0.7) | 0.2 (0.1 to 0.3) | -19.6* (-27.3 to -10.9) | -17.7* (-25.6 to -8.8) |
| Diagnosis and primary therapy phase of chronic myeloid leukemia | 0.1 (0.1 to 0.1) | 0.6 (0.5 to 0.7) | 0.0 (0.0 to 0.1) | -18.1* (-25.4 to -9.8) | -16.1* (-23.7 to -7.6) |
| Controlled phase of chronic myeloid leukemia | 1.5 (1.3 to 1.7) | -- | 0.1 (0.0 to 0.1) | -17.8* (-25.3 to -9.4) | -15.8* (-23.5 to -7.3) |
| Metastatic phase of chronic myeloid leukemia | 0.2 (0.1 to 0.2) | -- | 0.1 (0.1 to 0.1) | -21.4* (-29.3 to -12.2) | -19.6* (-27.6 to -10.1) |
| Terminal phase of chronic myeloid leukemia | -- | -- | -- | -21.4* (-29.3 to -12.2) | -19.6* (-27.6 to -10.1) |
| Other leukaemia | 24.0 (20.4 to 27.7) | 5.3 (4.5 to 6.1) | 2.6 (1.8 to 3.4) | -30.3* (-37.1 to -20.9) | -28.6* (-35.6 to -19.0) |
| Metastatic phase of other leukemia | 3.1 (2.7 to 3.6) | -- | 1.4 (0.9 to 1.9) | -30.8* (-37.4 to -21.7) | -29.2* (-35.9 to -19.8) |
| Diagnosis and primary therapy phase of other leukemia | -- | 5.3 (4.5 to 6.1) | -- | -- | -- |
| Terminal phase of other leukemia | 0.3 (0.2 to 0.3) | -- | 0.1 (0.1 to 0.2) | -30.2* (-36.6 to -21.4) | -28.6* (-35.1 to -19.5) |
| Controlled phase of other leukemia | 20.6 (17.5 to 23.8) | -- | 1.0 (0.6 to 1.5) | -29.5* (-38.6 to -17.9) | -27.8* (-37.2 to -16.0) |
| Other malignant cancers | 193.1 (147.7 to 271.6) | 15.3 (13.7 to 16.6) | 15.6 (10.2 to 23.5) | 64.9* (28.6 to 115.4) | 68.8* (31.7 to 120.5) |
| Diagnosis and primary therapy phase of other malignant neoplasms | 4.4 (3.4 to 6.2) | 15.3 (13.7 to 16.6) | 1.3 (0.8 to 2.0) | 83.3* (37.5 to 152.4) | 87.6* (40.8 to 158.4) |
| Controlled phase of other malignant neoplasms | 175.9 (133.0 to 249.2) | -- | 8.5 (4.9 to 13.7) | 93.8* (44.0 to 160.8) | 98.4* (47.4 to 167.0) |
| Metastatic phase of other malignant neoplasms | 11.2 (9.2 to 14.3) | -- | 5.0 (3.2 to 7.2) | 34.2* (8.3 to 71.3) | 37.4* (10.9 to 75.3) |
| Terminal phase of other malignant neoplasms | 1.6 (1.4 to 2.1) | -- | 0.9 (0.6 to 1.2) | 28.6* (5.5 to 58.7) | 31.7* (8.0 to 62.5) |
| Other neoplasms | 497.1 (467.9 to 526.5) | 301.6 (280.7 to 323.3) | 0.9 (0.6 to 1.4) | -15.1* (-21.0 to -8.6) | -13.1* (-19.1 to -6.4) |
| Myelodysplastic, myeloproliferative, and other hematopoietic neoplasms | 18.8 (15.4 to 22.6) | 0.9 (0.5 to 1.6) | 0.9 (0.6 to 1.4) | -15.1* (-21.0 to -8.6) | -13.1* (-19.1 to -6.4) |
| Benign and in situ intestinal neoplasms | 15.2 (14.1 to 16.4) | 9.6 (8.7 to 10.6) | -- | -- | -- |
| Benign and in situ cervical and uterine neoplasms | 13.6 (12.6 to 14.7) | 6.1 (5.7 to 6.7) | -- | -- | -- |
| Other benign and in situ neoplasms | 476.3 (447.9 to 505.1) | 284.9 (264.1 to 306.8) | -- | -- | -- |
| **Cardiovascular diseases** | **6621.8 (5713.6 to 7826.4)** | **437.0 (391.6 to 491.4)** | **471.6 (331.1 to 659.5)** | **-8.4* (-12.0 to -5.5)** | **-6.3* (-9.9 to -3.2)** |
| Rheumatic heart disease | 3343.1 (3201.8 to 3490.3) | 195.1 (184.3 to 205.5) | 160.3 (103.8 to 234.1) | 1.5 (-0.3 to 3.5) | 3.9* (2.0 to 5.9) |
| Rheumatic heart disease, without heart failure | 3335.5 (3193.3 to 3483.2) | 195.1 (184.3 to 205.5) | 159.6 (103.4 to 233.2) | 1.5 (-0.3 to 3.5) | 3.9* (2.0 to 5.9) |
| Heart failure due to rheumatic heart disease | 7.6 (5.1 to 10.9) | -- | 0.7 (0.4 to 1.2) | -2.2* (-4.1 to -0.2) | 0.1 (-1.8 to 2.2) |
| Ischaemic heart disease | 86.8 (70.3 to 104.4) | 12.1 (2.7 to 29.3) | 4.4 (2.8 to 6.6) | -4.4* (-5.3 to -3.4) | -2.1* (-3.1 to -1.2) |
| Asymptomatic ischemic heart disease following myocardial infarction | 14.0 (8.6 to 20.5) | -- | -- | -- | -- |
| Angina due to ischemic heart disease | 71.8 (56.9 to 88.0) | -- | 4.3 (2.7 to 6.4) | -4.5* (-5.5 to -3.6) | -2.3* (-3.2 to -1.3) |
| Heart failure due to ischemic heart disease | 0.5 (0.2 to 1.1) | -- | 0.0 (0.0 to 0.1) | 5.7 (-1.6 to 9.6) | 8.2* (0.7 to 12.2) |
| Myocardial infarction due to ischemic heart disease | 0.5 (0.1 to 1.3) | 12.1 (2.7 to 29.3) | 0.1 (0.0 to 0.1) | -3.8 (-10.9 to 0.9) | -1.5 (-8.8 to 3.3) |
| Stroke | 662.8 (566.3 to 776.0) | 41.4‡ (25.2 to 63.0) | 144.9 (102.1 to 193.0) | -25.6* (-30.2 to -21.4) | -23.8* (-28.5 to -19.5) |
| Ischaemic stroke | 606.4 (503.1 to 736.2) | 22.5 (7.9 to 42.7) | 89.0 (61.0 to 123.6) | -26.1* (-32.5 to -20.0) | -24.3* (-30.9 to -18.1) |
| Acute ischemic stroke | 1.8 (0.7 to 3.4) | 22.5 (7.9 to 42.7) | 0.5 (0.2 to 1.0) | -1.0 (-7.6 to 3.3) | 1.4 (-5.4 to 5.8) |
| Chronic ischemic stroke | 604.6 (501.3 to 734.5) | -- | 88.5 (60.6 to 123.2) | -26.2* (-32.6 to -20.0) | -24.5* (-31.0 to -18.1) |
| Intracerebral hemorrhage | 284.1 (234.1 to 340.4) | 12.7 (6.2 to 21.0) | 43.3 (29.1 to 59.9) | -28.1* (-34.2 to -21.9) | -26.4* (-32.7 to -20.1) |
| Acute intracerebral hemorrhage | 0.9 (0.5 to 1.5) | 12.7 (6.2 to 21.0) | 0.3 (0.1 to 0.5) | -36.6* (-52.0 to -25.8) | -35.1* (-50.9 to -24.0) |
| Chronic intracerebral hemorrhage | 283.2 (233.1 to 339.9) | -- | 43.0 (28.9 to 59.7) | -28.1* (-34.2 to -21.8) | -26.4* (-32.6 to -20.0) |
| Subarachnoid hemorrhage | 84.6 (66.5 to 107.1) | 6.1 (3.7 to 9.3) | 12.5 (8.4 to 17.7) | -10.0* (-13.2 to -7.2) | -7.8* (-11.1 to -5.0) |
| Acute subarachnoid hemorrhage | 0.4 (0.2 to 0.5) | 6.1 (3.7 to 9.3) | 0.1 (0.1 to 0.2) | -6.9* (-11.1 to -4.6) | -4.7* (-9.0 to -2.4) |
| Chronic subarachnoid hemorrhage | 84.2 (66.2 to 106.7) | -- | 12.4 (8.3 to 17.5) | -10.0* (-13.2 to -7.2) | -7.9* (-11.1 to -5.0) |
| Hypertensive heart disease | 0.4 (0.2 to 0.7) | -- | 0.0 (0.0 to 0.1) | -5.8* (-8.1 to -3.3) | -3.6* (-5.9 to -1.0) |
| Non-rheumatic valvular heart disease | -- | 12.3 (11.8 to 12.9) | 0.1 (0.1 to 0.2) | -6.7* (-8.1 to -5.2) | -4.5* (-5.9 to -2.9) |
| Non-rheumatic calcific aortic valve disease | 2.3 (1.9 to 2.8) | 2.3 (1.9 to 2.8) | -- | -3.4 (-9.8 to 2.6) | -1.1 (-7.7 to 5.1) |
| Heart failure due to calcific aortic valve disease | -- | -- | -- | -8.2 (-27.5 to 13.6) | -6.0 (-25.8 to 16.3) |
| Non-rheumatic calcific aortic valve disease | 2.3 (1.9 to 2.8) | 2.3 (1.9 to 2.8) | -- | 3.5 (-12.3 to 21.6) | 5.9 (-10.2 to 24.4) |
| Non-rheumatic degenerative mitral valve disease | 10.0 (9.6 to 10.4) | 10.0 (9.6 to 10.4) | -- | -2.0 (-7.5 to 3.7) | 0.3 (-5.3 to 6.2) |
| Heart failure due to degenerative mitral valve disease | 0.0 (0.0 to 0.1) | -- | -- | -5.8 (-20.4 to 10.8) | -3.6 (-18.5 to 13.4) |
| Non-rheumatic degenerative mitral valve disease | 10.0 (9.6 to 10.4) | 10.0 (9.6 to 10.4) | -- | 9.0 (-13.8 to 35.0) | 11.6 (-11.8 to 38.3) |
| Other non-rheumatic valve diseases | 1.0 (0.6 to 1.4) | -- | 0.1 (0.0 to 0.1) | -6.9* (-8.4 to -5.4) | -4.7* (-6.2 to -3.2) |
| Mild heart failure due to other non-rheumatic valve disease | 0.2 (0.1 to 0.3) | -- | -- | -6.9* (-8.4 to -5.4) | -4.7* (-6.3 to -3.2) |
| Moderate heart failure due to other non-rheumatic valve disease | 0.1 (0.1 to 0.2) | -- | -- | -6.9* (-8.4 to -5.4) | -4.7* (-6.2 to -3.2) |
| Severe heart failure due to other non-rheumatic valve disease | 0.3 (0.2 to 0.5) | -- | 0.1 (0.0 to 0.1) | -6.9* (-8.4 to -5.4) | -4.7* (-6.2 to -3.2) |

| | | | | | |
|---|---|---|---|---|---|
| Controlled, medically managed heart failure due to other non-rheumatic valve disease | 0.4 (0.2 to 0.5) | -- | -- | -6.9* (-8.4 to -5.4) | -4.7* (-6.3 to -3.2) |
| Cardiomyopathy and myocarditis | 10.9 (7.4 to 15.5) | 142.0 (104.4 to 188.5) | 16.0 (9.8 to 24.6) | -6.8* (-9.3 to -4.2) | -4.6* (-7.2 to -1.9) |
| Myocarditis | 115.1 (90.5 to 145.0) | 142.0 (104.4 to 188.5) | 9.2 (5.8 to 13.8) | -10.0* (-14.1 to -5.8) | -7.9* (-12.0 to -3.6) |
| Acute myocarditis | 34.1 (25.1 to 45.8) | 142.0 (104.4 to 188.5) | 1.7 (1.0 to 2.8) | -5.2* (-8.5 to -2.0) | -2.9 (-6.3 to 0.3) |
| Heart failure due to myocarditis | 81.0 (59.4 to 108.4) | -- | 7.5 (4.5 to 11.7) | -11.1* (-16.0 to -5.9) | -9.0* (-14.0 to -3.7) |
| Alcoholic cardiomyopathy | 4.5 (3.3 to 6.0) | -- | 0.4 (0.2 to 0.6) | -9.6* (-10.6 to -8.5) | -7.4* (-8.5 to -6.3) |
| Other cardiomyopathy | 68.6 (50.7 to 91.5) | -- | 6.4 (3.8 to 9.9) | -1.5* (-2.5 to -0.6) | 0.8 (-0.1 to 1.8) |
| Atrial fibrillation and flutter | -- | -- | -- | -- | -- |
| Asymptomatic atrial fibrillation and flutter | -- | -- | -- | -- | -- |
| Symptomatic atrial fibrillation and flutter | -- | -- | -- | -- | -- |
| Peripheral vascular disease | -- | -- | -- | -- | -- |
| Asymptomatic peripheral arterial disease | -- | -- | -- | -- | -- |
| Symptomatic claudication due to peripheral arterial disease | -- | -- | -- | -- | -- |
| Endocarditis | 13.1 (9.4 to 17.6) | 34.2 (20.9 to 48.2) | 1.1 (0.6 to 1.7) | 0.1 (-1.2 to 1.5) | 2.5* (1.1 to 3.9) |
| Acute endocarditis | 2.8 (1.7 to 3.8) | 34.2 (20.9 to 48.2) | 0.2 (0.1 to 0.3) | -0.7 (-3.0 to 1.2) | 1.7 (-0.7 to 3.7) |
| Heart failure due to endocarditis | 10.4 (6.7 to 14.9) | -- | 1.0 (0.5 to 1.6) | 0.3 (-1.3 to 1.9) | 2.7* (1.1 to 4.3) |
| Other cardiovascular and circulatory diseases | 2657.0 (1709.7 to 3808.6) | -- | 144.7 (80.6 to 240.6) | 3.8* (0.1 to 7.3) | 6.3* (2.5 to 9.9) |
| Heart failure due to other cardiovascular diseases | 8.3 (5.4 to 11.9) | -- | 0.8 (0.4 to 1.2) | 3.7* (2.0 to 5.4) | 6.2* (4.4 to 7.9) |
| Other cardiovascular and circulatory disease episodes | 2648.7 (1703.9 to 3795.7) | -- | 143.9 (80.2 to 239.4) | 3.8* (0.1 to 7.3) | 6.3* (2.4 to 9.9) |
| **Chronic respiratory diseases** | **20130.4 (15864.4 to 24877.8)** | **3213.7 (2369.7 to 4169.6)** | **1136.5 (807.4 to 1533.2)** | **-0.2 (-4.6 to 3.6)** | **2.1 (-2.3 to 6.1)** |
| Chronic obstructive pulmonary disease | 2565.7 (2066.3 to 3082.6) | 344.2 (226.2 to 474.6) | 301.8 (232.8 to 375.7) | -5.8* (-11.3 to -0.4) | -3.6 (-9.2 to 1.9) |
| Chronic obstructive pulmonary disease with heart failure | 0.1 (0.0 to 0.2) | -- | 0.0 (0.0 to 0.1) | -12.9* (-15.0 to -11.0) | -10.9* (-13.0 to -8.9) |
| Chronic obstructive pulmonary disease without heart failure | 2565.6 (2066.2 to 3082.5) | 344.2 (226.2 to 474.6) | 301.7 (232.8 to 375.6) | -5.8* (-11.3 to -0.4) | -3.6 (-9.2 to 1.9) |
| Pneumoconiosis | 0.7 (0.4 to 1.0) | 0.3 (0.2 to 0.5) | 0.1 (0.1 to 0.2) | -3.1 (-6.9 to 0.9) | -0.8 (-4.7 to 3.3) |
| Silicosis | 0.3 (0.1 to 0.5) | 0.1 (0.1 to 0.2) | 0.0 (0.0 to 0.1) | -1.3 (-7.7 to 6.0) | 1.0 (-5.5 to 8.5) |
| Heart failure due to silicosis | -- | -- | -- | -- | -- |
| Silicosis without heart failure | 0.3 (0.1 to 0.5) | 0.1 (0.1 to 0.2) | 0.0 (0.0 to 0.1) | -1.3 (-7.7 to 6.0) | 1.0 (-5.5 to 8.5) |
| Asbestosis | 0.2 (0.1 to 0.5) | 0.1 (0.0 to 0.2) | 0.0 (0.0 to 0.1) | -0.6 (-7.0 to 8.1) | 1.8 (-4.8 to 10.6) |
| Asbestosis without heart failure | 0.2 (0.1 to 0.5) | 0.1 (0.0 to 0.2) | 0.0 (0.0 to 0.1) | -0.6 (-7.0 to 8.1) | 1.8 (-4.8 to 10.6) |
| Heart failure due to asbestosis | -- | -- | -- | -- | -- |
| Coal workers pneumoconiosis | 0.1 (0.0 to 0.1) | 0.0 (0.0 to 0.1) | -- | -11.4* (-16.3 to -6.0) | -9.2* (-14.3 to -3.8) |
| Coal workers pneumoconiosis without heart failure | 0.1 (0.0 to 0.1) | 0.0 (0.0 to 0.1) | -- | -11.4* (-16.3 to -6.0) | -9.2* (-14.3 to -3.8) |
| Heart failure due to Coal workers pneumoconiosis | -- | -- | -- | -- | -- |
| Other pneumoconiosis | 0.1 (0.0 to 0.2) | 0.0 (0.0 to 0.1) | -- | -8.0* (-12.7 to -4.7) | -5.8* (-10.6 to -2.4) |
| Heart failure due to other pneumoconiosis | -- | -- | -- | -- | -- |
| Other pneumoconiosis without heart failure | 0.1 (0.0 to 0.2) | 0.0 (0.0 to 0.1) | -- | -8.0* (-12.7 to -4.7) | -5.8* (-10.6 to -2.4) |
| Asthma | 17897.0 (13707.4 to 22639.8) | 2865.6 (2029.9 to 3816.3) | 714.5 (429.6 to 1086.9) | 2.1 (-3.7 to 6.8) | 4.6 (-1.4 to 9.4) |
| Asymptomatic asthma | 6502.3 (4874.9 to 8539.2) | 1038.3 (728.7 to 1398.5) | -- | -- | -- |
| Symptomatic asthma | 11394.7 (8566.8 to 14611.0) | 1827.3 (1291.2 to 2457.6) | 714.5 (429.6 to 1086.9) | 2.1 (-3.7 to 6.8) | 4.6 (-1.4 to 9.4) |
| Interstitial lung disease and pulmonary sarcoidosis | 9.1 (5.5 to 13.5) | 3.6 (2.3 to 5.5) | 1.0 (0.6 to 1.8) | -7.4* (-9.0 to -6.0) | -5.2* (-6.9 to -3.7) |
| Heart failure due to interstitial lung disease and pulmonary sarcoidosis | 0.0 (0.0 to 0.1) | -- | -- | -7.7* (-9.1 to -6.1) | -5.6* (-7.0 to -3.9) |
| Interstitial lung disease and pulmonary sarcoidosis without heart failure | 9.1 (5.5 to 13.5) | 3.6 (2.3 to 5.5) | 1.0 (0.5 to 1.8) | -7.4* (-9.1 to -5.9) | -5.2* (-6.9 to -3.7) |
| Other chronic respiratory diseases | -- | -- | 119.1 (91.9 to 148.3) | 0.9 (-5.0 to 6.7) | 3.3 (-2.7 to 9.2) |
| **Digestive diseases** | **98362.0 (91003.4 to 106948.5)** | **22521.7 (17999.8 to 28246.9)** | **718.7 (474.4 to 1029.9)** | **-4.7* (-6.6 to -2.9)** | **-2.4* (-4.4 to -0.6)** |
| Cirrhosis and other chronic liver diseases | 74790.7 (69317.6 to 80185.3) | 219.5 (167.0 to 276.8) | 38.2 (26.3 to 53.0) | -2.5 (-8.4 to 2.8) | -0.2 (-6.2 to 5.2) |
| Cirrhosis and other chronic liver diseases due to hepatitis B | 33875.1 (29980.2 to 38044.2) | 35.4 (25.8 to 47.1) | 5.3 (3.4 to 7.9) | -24.1* (-32.7 to -15.6) | -22.3* (-31.2 to -13.6) |
| Cirrhosis and other chronic liver diseases due to hepatitis B, decompensated | 30.1 (25.1 to 35.6) | 1.7 (1.3 to 2.1) | 5.3 (3.4 to 7.9) | -24.1* (-32.7 to -15.6) | -22.3* (-31.2 to -13.6) |
| Chronic hepatitis B without cirrhosis | 33476.5 (29619.6 to 37647.0) | -- | -- | -- | -- |
| Cirrhosis and other chronic liver diseases due to hepatitis B, compensated | 368.5 (295.4 to 452.3) | 33.7 (24.3 to 45.2) | -- | -- | -- |
| Cirrhosis and other chronic liver diseases due to hepatitis C | 9118.0 (7226.5 to 11059.7) | 22.2 (15.7 to 30.3) | 4.1 (2.6 to 6.1) | -4.6 (-12.8 to 4.4) | -2.4 (-10.7 to 6.8) |
| Cirrhosis and other chronic liver diseases due to hepatitis C, decompensated | 23.3 (18.8 to 28.7) | 1.2 (0.9 to 1.6) | 4.1 (2.6 to 6.1) | -4.6 (-12.8 to 4.4) | -2.4 (-10.7 to 6.8) |
| Chronic hepatitis C without cirrhosis | 8804.7 (6893.2 to 10742.2) | -- | -- | -- | -- |
| Cirrhosis and other chronic liver diseases due to hepatitis C, compensated | 290.0 (224.7 to 368.3) | 21.0 (14.6 to 29.0) | -- | -- | -- |
| Cirrhosis and other chronic liver diseases due to alcohol use | 27.3 (12.8 to 43.7) | 2.2 (1.0 to 3.7) | 0.4 (0.2 to 0.7) | -9.3* (-12.0 to -6.4) | -7.2* (-9.9 to -4.2) |
| Cirrhosis and other chronic liver diseases due to alcohol, decompensated | 2.1 (1.0 to 3.3) | 0.1 (0.1 to 0.2) | 0.4 (0.2 to 0.7) | -9.3* (-12.0 to -6.4) | -7.2* (-9.9 to -4.2) |
| Cirrhosis and other chronic liver diseases due to alcohol, compensated | 25.2 (11.8 to 40.4) | 2.1 (0.9 to 3.5) | -- | -- | -- |
| Cirrhosis due to NASH | 29581.5 (26349.5 to 33361.5) | 6.6 (4.7 to 8.9) | 1.1 (0.7 to 1.7) | -0.2 (-3.0 to 2.4) | 2.2 (-0.7 to 4.9) |
| Non-alcoholic fatty liver disease (NAFLD) / Non-alcoholic steatohepatitis (NASH) | 29494.9 (26264.6 to 33257.5) | -- | -- | -- | -- |
| Cirrhosis and other chronic liver diseases due to NASH | 86.6 (68.1 to 109.0) | 6.6 (4.7 to 8.9) | 1.1 (0.7 to 1.7) | -0.2 (-3.0 to 2.4) | 2.2 (-0.7 to 4.9) |
| Cirrhosis and other chronic liver diseases due to other causes | 2188.8 (1928.7 to 2444.1) | 153.1 (115.5 to 195.0) | 27.2 (18.3 to 37.9) | 3.5 (-4.6 to 11.6) | 6.0 (-2.3 to 14.3) |

| | | | | | |
|---|---|---|---|---|---|
| Cirrhosis and other chronic liver diseases due to other cause, decompensated | 158.8 (146.3 to 171.4) | 9.4 (7.9 to 11.0) | 27.2 (18.3 to 37.9) | 3.5 (-4.6 to 11.6) | 6.0 (-2.3 to 14.3) |
| Cirrhosis and other chronic liver diseases due to other cause, compensated | 2030.1 (1773.3 to 2278.8) | 143.7 (106.3 to 185.8) | -- | -- | -- |
| Upper digestive system diseases | 29039.3 (22574.1 to 37162.7) | 18183.1 (13657.4 to 23720.3) | 514.1 (304.2 to 804.2) | -4.0* (-6.0 to -2.2) | -1.7 (-3.7 to 0.1) |
| Peptic ulcer disease | 679.4 (476.5 to 944.6) | 286.2 (199.5 to 388.0) | 35.3 (21.2 to 54.5) | 0.0 (-5.8 to 6.0) | 2.4 (-3.6 to 8.6) |
| Acute peptic ulcer disease | 0.1 (0.1 to 0.2) | -- | 0.0 (0.0 to 0.1) | -7.9* (-9.6 to -6.5) | -5.7* (-7.5 to -4.3) |
| Chronic peptic ulcer disease | 679.2 (476.4 to 944.5) | 286.2 (199.5 to 388.0) | 35.2 (21.2 to 54.4) | 0.0 (-5.8 to 6.1) | 2.4 (-3.6 to 8.6) |
| Gastritis and duodenitis | 6228.6 (4113.4 to 9266.1) | 4131.0 (2794.6 to 5932.7) | 279.5 (163.9 to 446.8) | -6.3* (-9.5 to -3.3) | -4.0* (-7.3 to -1.0) |
| Acute gastritis and duodenitis | 1.7 (1.0 to 2.6) | -- | 0.6 (0.3 to 0.9) | 3.8* (2.2 to 5.6) | 6.2* (4.6 to 8.1) |
| Chronic gastritis and duodenitis | 6226.9 (4111.7 to 9264.0) | 4131.0 (2794.6 to 5932.7) | 278.9 (163.6 to 446.3) | -6.3* (-9.5 to -3.3) | -4.1* (-7.3 to -1.0) |
| GERD | 22924.8 (16807.2 to 30815.7) | 13766.0 (9505.1 to 19444.7) | 199.3 (95.4 to 356.3) | -1.3 (-3.1 to 0.2) | 1.0 (-0.8 to 2.6) |
| Appendicitis | 113.7 (94.1 to 135.2) | 2961.7 (2472.6 to 3483.6) | 35.3 (23.0 to 50.1) | -0.0 (-9.8 to 11.0) | 2.4 (-7.6 to 13.7) |
| Paralytic ileus and intestinal obstruction | 5.0 (3.3 to 6.9) | 132.7 (87.8 to 181.9) | 1.6 (0.9 to 2.6) | 4.5* (1.1 to 7.4) | 7.0* (3.5 to 10.0) |
| Inguinal, femoral, and abdominal hernia | 667.8 (469.3 to 910.5) | 614.7 (412.0 to 822.1) | 67.7 (38.8 to 104.0) | -18.0* (-23.2 to -13.0) | -16.0* (-21.4 to -11.0) |
| Inflammatory bowel disease | 155.4 (134.6 to 178.1) | 282.3 (234.6 to 338.6) | 25.3 (16.6 to 35.5) | -13.6* (-21.4 to -5.5) | -11.5* (-19.5 to -3.3) |
| Ulcerative colitis | 55.5 (45.1 to 67.0) | 136.7 (104.3 to 176.3) | 8.9 (5.6 to 12.8) | -14.3* (-22.9 to -5.5) | -12.3* (-21.1 to -3.3) |
| Crohn's disease | 99.9 (82.2 to 120.7) | 145.6 (112.5 to 183.2) | 16.5 (10.5 to 23.8) | -13.1* (-22.7 to -3.3) | -11.1* (-20.9 to -1.0) |
| Vascular intestinal disorders | 1.2 (0.7 to 2.2) | 7.7 (5.0 to 11.0) | 0.4 (0.2 to 0.7) | -3.2* (-4.9 to -1.3) | -0.9 (-2.7 to 1.0) |
| Gallbladder and biliary diseases | 437.8 (301.3 to 597.4) | 84.4 (54.4 to 123.9) | 0.4 (0.2 to 0.7) | -3.7* (-5.3 to -2.1) | -1.5 (-3.1 to 0.2) |
| Pancreatitis | 91.5 (75.3 to 110.8) | 35.6 (26.6 to 46.5) | 6.0 (3.0 to 10.3) | 1.1 (-0.7 to 2.5) | 3.5* (1.6 to 4.9) |
| Acute pancreatitis | 2.0 (1.4 to 2.8) | 29.1 (20.4 to 39.3) | 0.8 (0.5 to 1.3) | -7.7* (-8.7 to -6.7) | -5.6* (-6.5 to -4.5) |
| Chronic pancreatitis | 89.5 (73.2 to 108.8) | 6.5 (4.0 to 10.0) | 5.2 (2.3 to 9.3) | 2.7* (1.3 to 3.9) | 5.1* (3.7 to 6.4) |
| Other digestive diseases | -- | -- | 29.7 (19.6 to 43.0) | 23.8* (20.4 to 27.1) | 26.7* (23.3 to 30.1) |
| **Neurological disorders** | **280631.7 (230702.2 to 340699.9)** | **88799.7 (62923.0 to 120965.5)** | **5703.9 (3745.4 to 8075.1)** | **2.0 (-0.6 to 4.9)** | **4.4* (1.7 to 7.4)** |
| Alzheimer's disease and other dementias | -- | -- | -- | -- | -- |
| Parkinson's disease | -- | -- | -- | -- | -- |
| Epilepsy | 2475.2 (1780.6 to 3261.5) | 197.1 (135.5 to 284.0) | 833.1 (485.0 to 1247.1) | 3.3 (-10.1 to 17.1) | 5.8 (-8.0 to 19.9) |
| Multiple sclerosis | 16.1 (11.9 to 21.9) | 4.1 (2.7 to 5.4) | 4.5 (2.9 to 6.6) | -2.7* (-5.7 to -0.2) | -0.4 (-3.5 to 2.2) |
| Motor neuron disease | 10.5 (8.4 to 13.0) | 0.1 (0.0 to 0.2) | 2.2 (1.5 to 3.2) | -7.0* (-7.8 to -6.2) | -4.8* (-5.6 to -4.0) |
| Headache disorders | 279276.2 (228986.2 to 339718.0) | 88598.5 (62773.7 to 120776.4) | 4640.8 (2971.6 to 6766.9) | 1.1 (-0.4 to 2.6) | 3.5* (2.0 to 5.0) |
| Migraine | 111551.8 (91520.0 to 133387.4) | 14329.0 (9943.9 to 18879.0) | 4058.1 (2458.7 to 6098.8) | 1.3 (-0.3 to 3.1) | 3.8* (2.1 to 5.5) |
| Medication overuse headache due to migraine | 5509.8 (4013.0 to 7252.4) | -- | 627.6 (361.2 to 1007.5) | -0.3 (-2.0 to 1.6) | 2.1* (0.3 to 4.0) |
| Migraine | 106042.0 (86333.0 to 127651.6) | 14329.0 (9943.9 to 18879.0) | 3430.5 (2010.3 to 5327.3) | 1.7 (-0.3 to 3.7) | 4.1* (2.0 to 6.1) |
| Tension-type headache | 215217.5 (155828.7 to 288769.1) | 74269.5 (48706.8 to 107087.2) | 582.7 (310.0 to 965.4) | -0.8 (-2.2 to 0.7) | 1.6* (0.1 to 3.1) |
| Medication overuse headache due to tension-type headache | 2051.2 (1295.9 to 3050.8) | -- | 231.4 (111.9 to 435.1) | -0.3 (-3.6 to 2.9) | 2.1 (-1.3 to 5.3) |
| Tension-type headache | 213166.3 (154167.5 to 286346.4) | 74269.5 (48706.8 to 107087.2) | 351.3 (137.5 to 691.3) | -1.1 (-2.2 to 0.1) | 1.3* (0.1 to 2.5) |
| Other neurological disorders | 2.6 (1.3 to 4.5) | -- | 223.2 (130.2 to 333.8) | 19.4* (4.0 to 35.2) | 22.2* (6.5 to 38.4) |
| Other neurological disorders | -- | -- | 222.5 (129.7 to 332.9) | 19.5* (4.0 to 35.3) | 22.3* (6.5 to 38.5) |
| Guillain-Barré syndrome due to other neurological disorders | 2.6 (1.3 to 4.5) | -- | 0.8 (0.3 to 1.4) | 6.0* (3.4 to 8.6) | 8.5* (5.9 to 11.2) |
| **Mental disorders** | **90519.2 (83075.6 to 98634.4)** | **30244.3 (25583.4 to 35636.3)** | **10854.3 (7812.5 to 14608.1)** | **-1.1* (-2.0 to -0.3)** | **1.2* (0.3 to 2.1)** |
| Schizophrenia | 301.4 (222.2 to 402.3) | 114.7 (71.9 to 166.9) | 202.6 (130.9 to 288.4) | -6.6* (-12.0 to -1.3) | -4.4 (-9.9 to 1.0) |
| Depressive disorders | 16597.7 (13561.8 to 20386.3) | 19860.3 (15772.4 to 25037.5) | 3031.9 (1984.5 to 4436.0) | -2.0* (-4.0 to -0.3) | 0.3 (-1.7 to 2.1) |
| Major depressive disorder | 12233.7 (9210.4 to 15642.7) | 18651.8 (14449.5 to 23847.9) | 2578.0 (1652.1 to 3880.7) | -2.5* (-4.8 to -0.4) | -0.2 (-2.5 to 1.9) |
| Dysthymia | 4560.0 (3298.1 to 6295.4) | 1208.6 (904.7 to 1590.6) | 453.9 (276.3 to 704.0) | 0.9 (-1.5 to 3.0) | 3.3* (0.9 to 5.4) |
| Bipolar disorder | 5376.5 (4114.1 to 6841.6) | 919.5 (694.6 to 1146.5) | 1137.1 (682.3 to 1750.1) | 1.8* (0.0 to 3.7) | 4.2* (2.4 to 6.1) |
| Anxiety disorders | 25846.6 (22638.3 to 29270.4) | 4008.2 (3390.7 to 4594.8) | 2550.0 (1772.4 to 3495.2) | -2.5* (-4.2 to -1.0) | -0.2 (-1.9 to 1.4) |
| Eating disorders | 2107.4 (1418.9 to 3061.4) | 3004.0 (1744.6 to 4691.4) | 453.4 (256.7 to 733.0) | 6.9* (4.5 to 9.2) | 9.4* (7.0 to 11.8) |
| Anorexia nervosa | 673.6 (443.5 to 961.8) | 276.8 (175.3 to 400.8) | 145.1 (81.8 to 233.3) | 5.1* (0.9 to 9.4) | 7.6* (3.3 to 12.0) |
| Bulimia nervosa | 1436.2 (828.1 to 2341.8) | 2727.2 (1514.8 to 4407.5) | 308.2 (148.3 to 553.8) | 7.7* (4.7 to 10.6) | 10.3* (7.2 to 13.2) |
| Autism spectrum disorders | 2659.9 (2387.3 to 2934.6) | -- | 410.8 (277.5 to 567.8) | -1.9 (-3.9 to 0.2) | 0.4 (-1.6 to 2.5) |
| Attention-deficit/hyperactivity disorder | 11005.1 (9081.5 to 13375.9) | -- | 134.2 (80.0 to 215.4) | -5.9* (-8.0 to -3.8) | -3.6* (-5.8 to -1.5) |
| Conduct disorder | 17446.3 (12528.5 to 22950.5) | 2337.6 (1287.1 to 3496.1) | 2103.8 (1197.2 to 3399.6) | 0.3 (-1.9 to 2.8) | 2.7* (0.4 to 5.2) |
| Idiopathic developmental intellectual disability | 11072.3 (6650.9 to 15532.7) | -- | 443.7 (217.4 to 737.4) | -3.2* (-5.6 to -1.3) | -0.9 (-3.3 to 1.1) |
| Other mental disorders | 5003.7 (4171.2 to 5941.6) | -- | 386.8 (243.1 to 551.9) | -1.8 (-4.5 to 1.1) | 0.5 (-2.2 to 3.5) |
| **Substance use disorders** | **12293.4 (10278.5 to 14614.0)** | **5595.3 (4506.3 to 6780.0)** | **1838.8 (1261.1 to 2483.0)** | **-8.0* (-10.6 to -5.2)** | **-5.9* (-8.5 to -3.0)** |
| Alcohol use disorders | 4843.4 (3479.5 to 6559.8) | 3365.1 (2316.7 to 4484.3) | 498.0 (299.4 to 774.8) | -5.1* (-7.7 to -2.5) | -2.8* (-5.5 to -0.2) |
| Alcohol dependence | 4818.0 (3451.4 to 6538.4) | 3365.1 (2316.7 to 4484.3) | 497.0 (298.5 to 772.9) | -5.1* (-7.6 to -2.5) | -2.8* (-5.5 to -0.2) |
| Fetal alcohol syndrome | 25.3 (20.4 to 31.1) | -- | 1.0 (0.6 to 1.5) | -14.9* (-20.9 to -8.3) | -12.9* (-19.0 to -6.1) |
| Drug use disorders | 7569.8 (6123.8 to 9442.7) | 2230.2 (1875.7 to 2657.5) | 1340.7 (910.9 to 1831.6) | -9.1* (-12.5 to -5.5) | -6.9* (-10.5 to -3.3) |
| Opioid use disorders | 1676.8 (1315.2 to 2146.3) | 784.9 (588.9 to 1015.7) | 717.6 (462.3 to 995.7) | -10.2* (-16.2 to -4.2) | -8.1* (-14.2 to -1.9) |
| Cocaine use disorders | 711.8 (599.4 to 849.0) | 207.8 (174.2 to 248.4) | 98.9 (63.4 to 143.3) | 1.9 (-1.5 to 5.9) | 4.3* (0.8 to 8.4) |

| | | | | | |
|---|---|---|---|---|---|
| Amphetamine use disorders | 1585.0 (969.8 to 2406.3) | 425.2 (268.8 to 628.2) | 211.8 (108.7 to 364.1) | -11.8* (-15.6 to -7.9) | -9.7* (-13.6 to -5.7) |
| Cannabis use disorders | 3750.0 (2568.1 to 5419.2) | 812.3 (604.9 to 1110.7) | 109.8 (60.9 to 181.0) | -7.6* (-11.7 to -4.2) | -5.4* (-9.6 to -1.9) |
| Other drug use disorders | 9.8 (6.1 to 14.8) | -- | 202.7 (122.1 to 320.2) | -7.8* (-11.6 to -3.3) | -5.7* (-9.5 to -1.0) |
| **Diabetes and kidney diseases** | **15432.3 (13164.4 to 17982.2)** | **1555.6 (1197.5 to 1969.4)** | **504.2 (328.9 to 721.4)** | **4.8* (0.7 to 8.8)** | **7.3* (3.1 to 11.4)** |
| Diabetes mellitus | 6195.8 (4687.0 to 7945.6) | 1105.1 (756.1 to 1472.3) | 352.9 (208.6 to 535.2) | 5.8* (1.1 to 10.3) | 8.3* (3.5 to 12.9) |
| Diabetes mellitus type 1 | 944.0 (781.2 to 1119.1) | 44.2 (28.8 to 61.7) | 54.4 (34.0 to 80.5) | 1.7 (-1.8 to 5.4) | 4.1* (0.5 to 7.9) |
| Uncomplicated diabetes mellitus type 1 | 833.2 (691.9 to 985.1) | 44.2 (28.8 to 61.7) | 40.0 (23.9 to 60.2) | 1.8 (-1.8 to 5.5) | 4.2* (0.5 to 8.0) |
| Vision loss due to diabetes mellitus type 1 retinopathy | -- | -- | -- | -- | -- |
| Diabetic neuropathy due to diabetes mellitus type 1 | 110.8 (85.5 to 138.2) | -- | 14.4 (9.1 to 21.6) | 1.2 (-7.5 to 10.4) | 3.6 (-5.3 to 13.0) |
| Diabetes mellitus type 2 | 5251.9 (3770.8 to 6991.0) | 1060.9 (714.2 to 1428.5) | 298.5 (168.5 to 468.9) | 6.6* (0.8 to 11.7) | 9.1* (3.2 to 14.4) |
| Uncomplicated diabetes mellitus type 2 | 4685.5 (3376.4 to 6270.8) | 1060.9 (714.2 to 1428.5) | 224.9 (126.3 to 358.4) | 6.5* (1.0 to 11.5) | 9.0* (3.4 to 14.1) |
| Vision loss due to diabetes mellitus type 2 retinopathy | -- | -- | -- | -- | -- |
| Diabetic neuropathy due to diabetes mellitus type 2 | 566.4 (384.5 to 795.4) | -- | 73.7 (41.6 to 114.7) | 6.8 (-1.2 to 14.6) | 9.3* (1.2 to 17.3) |
| Chronic kidney disease | 9963.4 (8123.9 to 12059.3) | 392.7 (250.7 to 560.4) | 151.0 (97.1 to 222.1) | 2.7 (-4.5 to 10.7) | 5.2 (-2.2 to 13.4) |
| Chronic kidney disease due to diabetes mellitus type 1 | 163.7 (124.6 to 213.5) | 7.5 (4.4 to 11.8) | 3.4 (2.2 to 5.1) | 7.9 (-4.5 to 44.5) | 10.4 (-2.2 to 47.9) |
| Albuminuria with preserved GFR due to type 1 diabetes mellitus | 62.2 (41.9 to 87.6) | -- | -- | -- | -- |
| End-stage chronic kidney disease due to diabetes mellitus type 1 | 0.5 (0.3 to 0.9) | -- | 0.2 (0.1 to 0.4) | 4.7 (-1.1 to 10.8) | 7.1* (1.2 to 13.5) |
| Stage III chronic kidney disease due to diabetes mellitus type 1 | 97.6 (70.2 to 133.7) | 7.5 (4.4 to 11.8) | 2.1 (1.3 to 3.3) | 4.5 (-11.0 to 43.5) | 7.0 (-8.9 to 46.9) |
| Stage IV chronic kidney disease due to diabetes mellitus type 1 | 1.8 (0.9 to 2.9) | -- | 0.2 (0.1 to 0.4) | 9.9 (-6.0 to 48.6) | 12.5 (-3.8 to 52.1) |
| Stage V chronic kidney disease due to diabetes mellitus type 1 | 1.5 (0.9 to 2.3) | -- | 0.9 (0.5 to 1.5) | 16.8* (4.5 to 57.7) | 19.6* (7.0 to 61.4) |
| Chronic kidney disease due to diabetes mellitus type 2 | 979.7 (644.0 to 1608.5) | 8.4 (4.7 to 14.8) | 2.5 (1.5 to 4.4) | -51.4* (-85.5 to -6.2) | -50.3* (-85.2 to -4.0) |
| Albuminuria with preserved GFR due to type 2 diabetes mellitus | 869.2 (556.9 to 1393.4) | -- | -- | -- | -- |
| End-stage chronic kidney disease due to diabetes mellitus type 2 | 0.2 (0.1 to 0.4) | -- | 0.1 (0.0 to 0.2) | 5.0 (-1.0 to 11.2) | 7.4* (1.4 to 13.8) |
| Stage III chronic kidney disease due to diabetes mellitus type 2 | 105.1 (68.1 to 179.1) | 8.4 (4.7 to 14.8) | 0.8 (0.5 to 1.1) | -2.1 (-13.7 to 11.2) | 0.2 (-11.7 to 13.8) |
| Stage IV chronic kidney disease due to diabetes mellitus type 2 | 2.8 (1.5 to 5.4) | -- | 0.3 (0.1 to 0.6) | -60.1* (-88.4 to -9.7) | -59.2* (-88.1 to -7.5) |
| Stage V chronic kidney disease due to diabetes mellitus type 2 | 2.4 (1.3 to 4.3) | -- | 1.4 (0.7 to 2.7) | -61.5* (-88.8 to -10.1) | -60.6* (-88.5 to -8.0) |
| Chronic kidney disease due to hypertension | 1031.5 (824.8 to 1322.2) | 92.8 (59.5 to 132.5) | 22.6 (13.7 to 34.8) | 8.4 (-1.3 to 44.9) | 11.0* (1.0 to 48.4) |
| Albuminuria with preserved GFR due to hypertension | 21.8 (14.5 to 31.2) | -- | -- | -- | -- |
| End-stage chronic kidney disease due to hypertension | 1.2 (0.7 to 2.0) | -- | 0.5 (0.2 to 0.8) | 7.3* (2.3 to 12.4) | 9.8* (4.7 to 15.0) |
| Stage III chronic kidney disease due to hypertension | 949.0 (750.9 to 1219.1) | 92.8 (59.5 to 132.5) | 2.1 (1.3 to 3.2) | 1.8 (-11.3 to 23.6) | 4.2 (-9.2 to 26.6) |
| Stage IV chronic kidney disease due to hypertension | 32.3 (18.6 to 52.2) | -- | 4.2 (2.0 to 7.4) | 4.6 (-9.8 to 39.4) | 7.0 (-7.7 to 42.7) |
| Stage V chronic kidney disease due to hypertension | 27.2 (17.7 to 39.9) | -- | 15.9 (9.4 to 24.9) | 10.5* (0.5 to 51.2) | 13.1* (2.9 to 54.7) |
| Chronic kidney disease due to glomerulonephritis | 578.0 (464.6 to 714.7) | 12.3 (7.8 to 17.6) | 41.2 (26.2 to 60.6) | 6.9 (-2.2 to 36.3) | 9.5* (0.1 to 39.5) |
| Albuminuria with preserved GFR due to glomerulonephritis | 89.0 (59.4 to 126.6) | -- | -- | -- | -- |
| End-stage chronic kidney disease due to glomerulonephritis | 11.8 (7.7 to 16.3) | -- | 4.0 (2.3 to 5.9) | 6.1* (0.6 to 11.8) | 8.6* (3.0 to 14.4) |
| Stage III chronic kidney disease due to glomerulonephritis | 387.0 (300.1 to 497.8) | 12.3 (7.8 to 17.6) | 6.9 (4.0 to 11.4) | -1.9 (-20.5 to 23.5) | 0.5 (-18.7 to 26.5) |
| Stage IV chronic kidney disease due to glomerulonephritis | 49.0 (28.5 to 79.1) | -- | 6.3 (3.1 to 11.2) | 4.7 (-10.1 to 38.4) | 7.2 (-7.9 to 41.7) |
| Stage V chronic kidney disease due to glomerulonephritis | 41.2 (27.3 to 60.7) | -- | 24.0 (14.0 to 38.3) | 10.5* (0.5 to 50.3) | 13.2* (2.9 to 53.9) |
| Chronic kidney disease due to other and unspecified causes | 7210.5 (5836.7 to 8817.7) | 271.7 (173.0 to 385.8) | 81.3 (52.4 to 119.5) | 2.6 (-6.0 to 21.7) | 5.0 (-3.8 to 24.6) |
| Albuminuria with preserved GFR due to other and unspecified causes | 3630.4 (2505.5 to 4981.3) | -- | -- | -- | -- |
| End-stage chronic kidney disease due to other causes | 19.8 (14.6 to 24.9) | -- | 7.4 (4.6 to 10.6) | 7.3* (2.7 to 12.1) | 9.9* (5.2 to 14.7) |
| Stage III chronic kidney disease due to other causes | 3387.9 (2654.0 to 4329.4) | 271.7 (173.0 to 385.8) | 16.3 (9.5 to 26.6) | -16.5* (-27.7 to -3.4) | -14.5* (-26.0 to -1.1) |
| Stage IV chronic kidney disease due to other causes | 93.6 (53.9 to 149.9) | -- | 12.0 (5.8 to 21.2) | 4.2 (-11.5 to 29.7) | 6.7 (-9.4 to 32.8) |
| Stage V chronic kidney disease due to other causes | 78.9 (52.0 to 115.8) | -- | 45.8 (26.9 to 72.4) | 10.2* (0.0 to 43.0) | 12.8* (2.4 to 46.4) |
| Acute glomerulonephritis | 4.1 (2.5 to 6.1) | 57.9 (36.9 to 86.4) | 0.2 (0.1 to 0.4) | -6.1* (-10.0 to -2.3) | -3.9 (-7.9 to 0.1) |
| **Skin and subcutaneous diseases** | **159459.3 (146824.2 to 173855.2)** | **283049.6 (244921.4 to 327715.2)** | **3897.2 (2535.7 to 5748.3)** | **0.4 (-0.9 to 1.9)** | **2.8* (1.5 to 4.3)** |
| Dermatitis | 24834.7 (21678.3 to 28804.0) | 22605.8 (16100.3 to 30916.7) | 968.5 (546.4 to 1568.6) | 1.4 (-0.5 to 3.4) | 3.8* (1.8 to 5.8) |
| Atopic dermatitis | 17748.7 (15523.8 to 20244.5) | 1489.4 (1140.8 to 1868.3) | 784.6 (425.8 to 1350.0) | 2.8* (0.5 to 4.9) | 5.2* (2.9 to 7.4) |
| Contact dermatitis | 6844.3 (4688.9 to 9952.3) | 19439.3 (13034.2 to 27712.8) | 176.0 (99.5 to 293.4) | -2.8* (-5.4 to -0.2) | -0.5 (-3.1 to 2.2) |
| Seborrhoeic dermatitis | 562.9 (448.0 to 691.1) | 1677.0 (1313.9 to 2075.8) | 7.8 (4.2 to 12.8) | -25.8* (-29.6 to -22.6) | -24.1* (-27.9 to -20.7) |
| Psoriasis | 2990.2 (2742.5 to 3257.3) | 419.2 (361.1 to 480.2) | 268.9 (186.5 to 360.1) | 2.1 (-1.2 to 5.6) | 4.5* (1.1 to 8.1) |
| Bacterial skin diseases | 830.8 (755.7 to 905.0) | 18474.0 (17062.6 to 19990.2) | 11.6 (7.0 to 18.4) | 3.2 (-0.6 to 6.9) | 5.6* (1.7 to 9.5) |
| Cellulitis | 120.2 (95.8 to 147.4) | 2507.0 (2034.4 to 3057.2) | 7.1 (4.5 to 10.7) | -2.6 (-5.6 to 0.6) | -0.3 (-3.4 to 3.0) |
| Pyoderma | 796.2 (727.0 to 867.5) | 15967.0 (14699.3 to 17407.5) | 4.5 (1.8 to 9.5) | 13.7* (9.7 to 17.8) | 16.4* (12.3 to 20.6) |
| Impetigo | 435.8 (381.2 to 496.6) | 7413.7 (6489.1 to 8393.8) | 2.5 (1.0 to 5.3) | 19.6* (13.2 to 26.0) | 22.5* (15.8 to 29.0) |
| Abscess and other bacterial skin diseases | 360.4 (320.4 to 400.6) | 8553.3 (7585.2 to 9622.4) | 2.1 (0.8 to 4.3) | 7.2* (1.6 to 13.2) | 9.8* (4.0 to 15.9) |
| Scabies | 20779.0 (14429.4 to 29405.2) | 62987.2 (42880.8 to 88252.3) | 541.3 (274.8 to 937.3) | -8.4* (-9.4 to -7.3) | -6.2* (-7.3 to -5.1) |
| Fungal skin diseases | 43619.5 (32284.9 to 57953.2) | 109783.6 (81719.0 to 143385.8) | 249.6 (92.7 to 524.5) | -4.4* (-7.1 to -2.4) | -2.1* (-4.9 to -0.1) |
| Tinea capitis | 11494.8 (7569.6 to 17808.1) | 13546.8 (8792.1 to 20466.6) | 65.8 (23.0 to 155.9) | -17.0* (-20.7 to -13.1) | -15.0* (-18.8 to -11.0) |
| Other fungal skin diseases | 32124.7 (21750.9 to 45594.8) | 96236.8 (67584.5 to 129268.8) | 183.8 (66.6 to 396.7) | 1.1 (-0.0 to 2.1) | 3.5* (2.4 to 4.5) |

| | | | | | |
|---|---|---|---|---|---|
| Viral skin diseases | 10522.5 (9651.7 to 11395.0) | 9048.2 (8284.8 to 9871.5) | 326.6 (208.5 to 497.3) | -8.6* (-9.9 to -7.4) | -6.4* (-7.8 to -5.2) |
| Viral warts | 5982.7 (5295.7 to 6732.0) | 2088.0 (1776.5 to 2389.7) | 185.7 (117.8 to 283.5) | -12.8* (-14.5 to -11.3) | -10.8* (-12.4 to -9.2) |
| Molluscum contagiosum | 4539.8 (4065.7 to 5090.2) | 6960.2 (6266.1 to 7705.6) | 140.9 (89.4 to 213.8) | -2.4* (-4.4 to -0.5) | -0.1 (-2.2 to 1.9) |
| Acne vulgaris | 43821.6 (35533.8 to 53861.7) | 20665.2 (16900.0 to 25477.7) | 940.8 (545.3 to 1535.4) | 11.3* (9.5 to 13.1) | 13.9* (12.1 to 15.8) |
| Alopecia areata | 901.8 (812.6 to 994.5) | 1687.2 (1506.8 to 1859.4) | 30.1 (19.1 to 45.3) | -2.9 (-6.7 to 1.0) | -0.6 (-4.5 to 3.4) |
| Pruritus | 3698.0 (2608.0 to 5207.4) | 2912.9 (2065.5 to 3979.1) | 40.1 (17.3 to 77.0) | -1.3 (-3.0 to 0.4) | 1.1 (-0.7 to 2.8) |
| Urticaria | 6334.0 (4489.0 to 8843.3) | 10915.6 (7682.5 to 14847.6) | 384.9 (217.8 to 608.2) | -2.4* (-4.2 to -0.4) | -0.1 (-2.0 to 1.9) |
| Decubitus ulcer | 22.0 (14.6 to 30.8) | 78.6 (50.3 to 108.7) | 3.9 (2.2 to 6.1) | 2.8* (1.9 to 3.7) | 5.3* (4.3 to 6.2) |
| Other skin and subcutaneous diseases | 23245.4 (21293.6 to 25330.6) | 23472.2 (21488.0 to 25575.5) | 130.9 (61.3 to 249.2) | 1.7* (0.8 to 2.7) | 4.1* (3.2 to 5.1) |
| **Sense organ diseases** | **47321.4 (42817.9 to 52144.6)** | **--** | **1522.4 (1039.2 to 2187.3)** | **-1.6 (-4.0 to 0.8)** | **0.7 (-1.8 to 3.2)** |
| Blindness and vision impairment | 22576.3 (19467.0 to 26131.1) | -- | 606.0 (383.8 to 932.6) | -1.1 (-2.8 to 0.6) | 1.3 (-0.5 to 2.9) |
| Glaucoma | -- | -- | -- | -- | -- |
| Cataract | -- | -- | -- | -- | -- |
| Age-related macular degeneration | -- | -- | -- | -- | -- |
| Refraction disorders | 10752.5 (7939.5 to 14136.3) | -- | 432.9 (272.5 to 656.2) | -1.0 (-3.1 to 1.0) | 1.4 (-0.8 to 3.4) |
| Near vision loss | 11380.5 (10456.7 to 12415.5) | -- | 118.8 (54.1 to 222.3) | 0.9 (-0.4 to 2.2) | 3.2* (2.0 to 4.6) |
| Other vision loss | 709.6 (510.5 to 952.8) | -- | 54.3 (33.7 to 81.0) | -5.9* (-11.8 to -0.4) | -3.7 (-9.7 to 1.9) |
| Age-related and other hearing loss | 23543.6 (20069.5 to 27169.6) | -- | 858.2 (593.8 to 1197.5) | -2.0 (-5.8 to 2.3) | 0.4 (-3.6 to 4.8) |
| Other sense organ diseases | 2618.6 (2359.1 to 2873.1) | -- | 58.2 (35.4 to 88.1) | -1.8 (-5.6 to 2.0) | 0.5 (-3.4 to 4.4) |
| Chronic other sense organ diseases | 1677.0 (1462.9 to 1889.5) | -- | 45.0 (27.5 to 67.7) | -1.8 (-6.6 to 2.8) | 0.5 (-4.4 to 5.2) |
| Acute other sense organ diseases | 941.5 (835.8 to 1044.4) | -- | 13.2 (7.7 to 20.5) | -1.7 (-7.0 to 4.0) | 0.6 (-4.8 to 6.5) |
| **Musculoskeletal disorders** | **35412.2 (28060.3 to 43128.8)** | **12827.7 (9131.7 to 16734.3)** | **3869.6 (2544.2 to 5432.2)** | **-6.0* (-7.7 to -4.5)** | **-3.8* (-5.5 to -2.2)** |
| Rheumatoid arthritis | 286.0 (222.3 to 357.3) | 37.5 (25.2 to 51.2) | 41.1 (25.7 to 59.2) | 3.5 (-1.1 to 8.3) | 6.0* (1.3 to 10.8) |
| Osteoarthritis | -- | -- | -- | -- | -- |
| Osteoarthritis of the hip | -- | -- | -- | -- | -- |
| Osteoarthritis of the knee | -- | -- | -- | -- | -- |
| Low back pain | 25615.4 (18221.4 to 33596.1) | 11527.7 (7904.6 to 15410.7) | 2823.4 (1713.5 to 4198.9) | -3.7* (-5.5 to -2.0) | -1.4 (-3.3 to 0.3) |
| Low back pain with leg pain | 4073.5 (2722.3 to 5598.5) | 1846.8 (1184.7 to 2585.5) | 617.0 (361.9 to 937.0) | -3.7* (-6.2 to -1.3) | -1.4 (-4.0 to 1.0) |
| Low back pain without leg pain | 21541.9 (15330.3 to 28561.6) | 9680.9 (6663.4 to 13079.5) | 2206.4 (1328.0 to 3345.4) | -3.7* (-5.6 to -2.0) | -1.4 (-3.3 to 0.4) |
| Neck pain | 3447.3 (2367.8 to 4910.2) | 1241.0 (722.9 to 1893.8) | 358.6 (217.5 to 554.0) | -11.4* (-14.4 to -8.7) | -9.3* (-12.4 to -6.6) |
| Gout | 30.6 (17.3 to 49.1) | 21.5 (12.1 to 34.5) | 1.1 (0.5 to 1.9) | -4.3* (-5.9 to -3.1) | -2.0* (-3.7 to -0.8) |
| Other musculoskeletal disorders | 7194.0 (4742.8 to 9919.8) | -- | 645.4 (369.8 to 1021.7) | -12.9* (-17.1 to -9.3) | -10.8* (-15.1 to -7.2) |
| **Other non-communicable diseases** | **362694.4 (329824.7 to 399290.4)** | **338920.5 (277986.4 to 400986.6)** | **2893.0 (2009.1 to 4015.0)** | **-4.7* (-6.6 to -2.6)** | **-2.4* (-4.4 to -0.3)** |
| Congenital anomalies | 8473.6 (8006.4 to 9019.1) | -- | 1057.8 (742.0 to 1424.1) | 0.7 (-1.6 to 3.2) | 3.1* (0.7 to 5.7) |
| Neural tube defects | 244.2 (217.6 to 272.3) | -- | 73.4 (51.1 to 98.5) | 4.1 (-1.6 to 10.0) | 6.5* (0.7 to 12.6) |
| Severe motor and cognitive impairment due to anencephaly | -- | -- | -- | -- | -- |
| Encephalocoele due to neural tube defects | 19.4 (15.6 to 23.5) | -- | 3.3 (2.2 to 4.7) | -1.8 (-4.6 to 1.0) | 0.6 (-2.3 to 3.4) |
| Spina bifida due to neural tube defects | 224.8 (200.9 to 250.9) | -- | 70.1 (48.7 to 93.8) | 4.3 (-1.6 to 10.6) | 6.8* (0.7 to 13.2) |
| Congenital heart anomalies | 783.6 (718.6 to 854.3) | -- | 41.8 (19.0 to 70.7) | -0.9 (-4.9 to 2.9) | 1.4 (-2.6 to 5.3) |
| Critical malformations of great vessels, congenital valvular heart disease and patent ductus arteriosus | 129.8 (111.0 to 151.6) | -- | 8.3 (4.2 to 13.7) | -5.9 (-13.2 to 2.0) | -3.6 (-11.1 to 4.4) |
| Other congenital heart anomalies | 183.6 (168.1 to 200.8) | -- | 10.9 (4.4 to 18.9) | -0.8 (-7.5 to 6.7) | 1.6 (-5.3 to 9.2) |
| Severe congenital heart anomalies excluding single ventricle heart defects | 225.7 (196.2 to 256.5) | -- | 13.8 (6.4 to 23.4) | 3.5 (-2.9 to 10.8) | 5.9 (-0.6 to 13.5) |
| Single ventricle and single ventricle pathway congenital heart anomalies | 21.9 (18.1 to 26.2) | -- | 1.4 (0.7 to 2.3) | 6.0* (2.8 to 8.3) | 8.5* (5.2 to 10.9) |
| Ventricular septal defect and atrial septal defect | 222.6 (186.5 to 262.4) | -- | 7.4 (3.1 to 13.3) | -4.3 (-12.3 to 3.4) | -2.0 (-10.3 to 5.8) |
| Orofacial clefts | 835.4 (767.6 to 903.6) | -- | 26.2 (17.2 to 38.6) | 0.2 (-7.4 to 8.4) | 2.6 (-5.2 to 11.0) |
| Down's syndrome | 318.5 (278.0 to 362.4) | -- | 27.3 (18.4 to 38.2) | -1.8 (-4.2 to 0.9) | 0.6 (-2.0 to 3.3) |
| Turner syndrome | 68.1 (59.0 to 78.3) | -- | 1.4 (0.8 to 2.4) | 3.2 (-2.0 to 8.2) | 5.6* (0.3 to 10.7) |
| Klinefelter syndrome | 102.5 (88.2 to 117.2) | -- | 0.3 (0.1 to 0.5) | -3.0 (-11.0 to 6.2) | -0.7 (-8.9 to 8.7) |
| Other chromosomal abnormalities | 554.1 (431.4 to 687.3) | -- | 47.1 (30.3 to 67.8) | 11.1* (6.5 to 15.9) | 13.7* (9.1 to 18.7) |
| Congenital musculoskeletal and limb anomalies | 2767.9 (2457.6 to 3176.7) | -- | 420.8 (274.7 to 587.8) | -1.0 (-3.2 to 1.3) | 1.4 (-0.9 to 3.7) |
| Polydactyly and syndactyly | 9.8 (7.7 to 11.8) | -- | 0.1 (0.0 to 0.2) | -3.7* (-5.2 to -2.1) | -1.4 (-2.9 to 0.2) |
| Congenital limb deficiency | 1293.9 (1147.8 to 1486.7) | -- | 197.3 (130.1 to 275.9) | -0.9 (-4.0 to 2.2) | 1.5 (-1.7 to 4.7) |
| Other congenital musculoskeletal anomalies | 1464.2 (1300.1 to 1680.8) | -- | 223.4 (146.1 to 311.8) | -1.1 (-3.8 to 1.8) | 1.3 (-1.5 to 4.2) |
| Urogenital congenital anomalies | 620.0 (529.8 to 716.2) | -- | 13.8 (7.9 to 23.8) | -9.8* (-12.3 to -7.1) | -7.6* (-10.2 to -4.8) |
| Digestive congenital anomalies | 1307.6 (1174.0 to 1466.8) | -- | 59.5 (39.4 to 85.7) | -5.9* (-10.8 to -0.7) | -3.7 (-8.7 to 1.6) |
| Congenital diaphragmatic hernia | 31.8 (27.7 to 36.2) | -- | 0.6 (0.4 to 1.0) | -5.2* (-6.1 to -4.4) | -3.0* (-3.8 to -2.2) |
| Congenital atresia and/or stenosis of the digestive tract | 1.9 (1.3 to 2.5) | -- | 0.1 (0.1 to 0.2) | 9.7* (6.7 to 13.0) | 12.3* (9.2 to 15.7) |
| Congenital malformations of the abdominal wall after treatment | 124.3 (109.2 to 141.7) | -- | 5.3 (3.4 to 7.8) | -4.0* (-4.8 to -3.3) | -1.7* (-2.5 to -1.0) |
| Other congenital malformations of the digestive tract | 1149.7 (1014.3 to 1307.3) | -- | 53.4 (34.9 to 77.4) | -6.1* (-11.5 to -0.3) | -3.9 (-9.4 to 2.0) |
| Other congenital anomalies | 2272.0 (1977.3 to 2627.8) | -- | 346.0 (232.6 to 480.5) | 3.0 (-3.5 to 10.1) | 5.5 (-1.2 to 12.7) |

| | | | | | |
|---|---|---|---|---|---|
| Other congenital birth defects | -- | -- | 39.0 (27.4 to 52.2) | 16.4* (12.0 to 20.9) | 19.1* (14.6 to 23.7) |
| Hearing loss due to other congenital anomalies | 2272.0 (1977.3 to 2627.8) | -- | 307.0 (202.3 to 433.6) | 1.5 (-5.4 to 9.3) | 3.9 (-3.2 to 11.9) |
| Urinary diseases and male infertility | 1610.0 (1118.3 to 2215.9) | 20531.1 (15828.2 to 26229.4) | 30.1 (18.4 to 47.2) | -1.7 (-5.9 to 3.0) | 0.7 (-3.7 to 5.5) |
| Urinary tract infections | 341.2 (260.6 to 444.7) | 18477.7 (13987.6 to 24084.3) | 11.5 (6.7 to 18.5) | -6.4* (-12.5 to -0.1) | -4.2 (-10.4 to 2.3) |
| Urolithiasis | 72.4 (49.6 to 100.4) | 2053.4 (1419.9 to 2838.6) | 5.6 (3.3 to 8.8) | 3.8* (2.3 to 5.8) | 6.2* (4.7 to 8.3) |
| Benign prostatic hyperplasia | -- | -- | -- | -- | -- |
| Male infertility | 1270.1 (782.0 to 1858.9) | -- | 8.4 (3.2 to 18.5) | 11.9* (4.2 to 20.9) | 14.5* (6.7 to 23.8) |
| Other urinary diseases | -- | -- | 4.7 (3.0 to 7.0) | -14.9* (-19.0 to -10.9) | -12.9* (-17.0 to -8.8) |
| Gynecological diseases | 60185.4 (55082.0 to 65517.8) | 33243.0 (30586.7 to 35930.2) | 640.1 (428.5 to 933.2) | -2.7* (-4.7 to -1.0) | -0.4 (-2.4 to 1.3) |
| Uterine fibroids | 273.4 (196.7 to 364.8) | 52.8 (29.1 to 80.4) | 3.4 (2.1 to 5.3) | -7.5 (-16.1 to 1.4) | -5.3 (-14.1 to 3.8) |
| Polycystic ovarian syndrome | 4527.0 (3353.0 to 5889.9) | 967.6 (664.5 to 1409.6) | 41.6 (17.9 to 80.9) | -0.4 (-2.2 to 1.4) | 2.0* (0.1 to 3.8) |
| Female infertility | 1361.6 (509.2 to 2469.3) | -- | 8.5 (2.3 to 21.2) | 28.6* (11.8 to 55.9) | 31.7* (14.5 to 59.6) |
| Endometriosis | 1031.0 (757.2 to 1404.2) | 708.0 (513.9 to 935.3) | 97.3 (59.5 to 151.2) | -3.5 (-7.4 to 0.5) | -1.2 (-5.2 to 2.8) |
| Genital prolapse | 317.0 (215.5 to 454.8) | 226.8 (154.6 to 317.7) | 1.0 (0.4 to 2.1) | -6.8 (-18.4 to 6.1) | -4.5 (-16.5 to 8.6) |
| Premenstrual syndrome | 53618.5 (48314.4 to 59085.8) | 29130.7 (26552.0 to 31756.1) | 454.8 (288.1 to 691.3) | -3.4* (-5.8 to -1.3) | -1.1 (-3.6 to 1.0) |
| Other gynecological diseases | 1895.7 (1748.4 to 2060.6) | 2157.2 (1953.7 to 2391.3) | 33.5 (21.0 to 52.0) | 0.0 (-4.7 to 4.9) | 2.4 (-2.4 to 7.4) |
| Other gynecological diseases | 1610.7 (1463.5 to 1773.4) | 2157.2 (1953.7 to 2391.3) | 27.1 (16.6 to 42.4) | 6.9* (1.7 to 12.1) | 9.5* (4.1 to 14.8) |
| Other gynecological diseases with anemia | 285.0 (259.1 to 311.8) | -- | 6.4 (4.0 to 9.8) | -21.4* (-32.6 to -9.3) | -19.5* (-31.0 to -7.1) |
| Hemoglobinopathies and hemolytic anaemias | 167215.8 (163162.9 to 171475.4) | -- | 455.5 (299.6 to 675.2) | -16.8* (-23.3 to -9.3) | -14.8* (-21.5 to -7.1) |
| Thalassemias | 14.1 (12.1 to 16.2) | -- | 0.8 (0.6 to 1.2) | -22.6* (-30.3 to -14.1) | -20.7* (-28.7 to -12.1) |
| Thalassaemias trait | 23020.0 (22106.3 to 23972.8) | -- | 133.6 (82.0 to 210.0) | -34.3* (-44.0 to -22.8) | -32.7* (-42.7 to -21.0) |
| Sickle cell disorders | 387.7 (335.7 to 444.9) | -- | 38.4 (24.7 to 56.5) | 23.5* (8.9 to 38.0) | 26.4* (11.5 to 41.3) |
| Sickle cell trait | 44323.0 (40102.8 to 49150.3) | -- | 98.0 (63.0 to 146.4) | -9.6 (-18.4 to 0.2) | -7.5 (-16.4 to 2.6) |
| G6PD deficiency | 32342.4 (31007.9 to 33744.4) | -- | 2.1 (1.4 to 3.0) | -5.4 (-19.7 to 13.0) | -3.2 (-17.8 to 15.6) |
| G6PD trait | 79044.8 (77718.9 to 80365.2) | -- | 0.0 (0.0 to 0.1) | -15.7* (-27.8 to -0.9) | -13.7 (-26.1 to 1.5) |
| Other hemoglobinopathies and hemolytic anaemias | 8951.7 (8328.9 to 9624.6) | -- | 182.7 (117.7 to 272.6) | -9.2* (-16.4 to -0.1) | -7.1 (-14.4 to 2.3) |
| Endocrine, metabolic, blood, and immune disorders | 9934.7 (9388.8 to 10485.9) | -- | 252.5 (165.7 to 367.6) | -11.2* (-17.1 to -4.5) | -9.0* (-15.1 to -2.2) |
| Anemia due to endocrine, metabolic, blood, and immune disorders | 9241.8 (8737.6 to 9760.2) | -- | 226.3 (147.5 to 331.8) | -11.9* (-18.7 to -4.4) | -9.8* (-16.8 to -2.1) |
| Endocrine, metabolic, blood, and immune disorders | 691.5 (526.8 to 893.2) | -- | 26.1 (15.9 to 38.9) | -4.3 (-8.9 to 0.4) | -2.1 (-6.8 to 2.7) |
| Heart failure due to endocrine, metabolic, blood, and immune disorders | 1.3 (0.9 to 1.8) | -- | 0.1 (0.1 to 0.2) | -8.1* (-9.2 to -7.0) | -5.9* (-7.0 to -4.7) |
| Oral disorders | 226863.3 (176688.0 to 284254.3) | 285146.4 (224899.6 to 346755.1) | 456.9 (257.0 to 756.5) | -1.6* (-2.7 to -0.6) | 0.7 (-0.4 to 1.8) |
| Caries of deciduous teeth | -- | -- | -- | -- | -- |
| Caries of permanent teeth | 223001.7 (170230.2 to 284670.0) | 283557.0 (222821.9 to 345564.9) | 151.3 (63.0 to 306.2) | -2.8* (-4.4 to -1.4) | -0.5 (-2.2 to 0.9) |
| Periodontal disease | 5312.0 (3081.0 to 8601.2) | 1589.4 (883.8 to 2570.1) | 35.5 (11.9 to 82.5) | 10.1* (8.0 to 12.9) | 12.7* (10.6 to 15.6) |
| Edentulism and severe tooth loss | -- | -- | -- | -- | -- |
| Other oral disorders | 9151.8 (8159.4 to 10233.1) | -- | 270.1 (167.1 to 411.3) | -2.3* (-3.6 to -1.0) | 0.0 (-1.3 to 1.4) |
| **Injuries** | **61604.1 (54340.2 to 70249.3)** | **36327.8 (30891.1 to 42193.6)** | **2270.9 (1714.0 to 2910.1)** | **-0.6 (-2.5 to 1.0)** | **1.8 (-0.1 to 3.4)** |
| **Transport injuries** | **6116.0 (5284.6 to 7024.1)** | **4944.2 (3651.4 to 6691.0)** | **439.2 (310.0 to 593.6)** | **-4.3* (-6.1 to -2.5)** | **-2.0* (-3.9 to -0.2)** |
| Road injuries | 3599.8 (3160.3 to 4029.8) | 4280.2 (3036.0 to 6048.0) | 255.6 (182.5 to 344.7) | -8.2* (-10.1 to -6.4) | -6.0* (-8.0 to -4.2) |
| Pedestrian road injuries | 1030.7 (783.5 to 1304.7) | 829.5 (500.6 to 1287.5) | 73.9 (50.0 to 104.9) | -14.8* (-16.9 to -12.7) | -12.8* (-14.9 to -10.6) |
| Cyclist road injuries | 684.7 (520.4 to 882.4) | 936.1 (538.0 to 1487.2) | 45.4 (30.2 to 63.4) | -8.4* (-10.6 to -5.9) | -6.2* (-8.5 to -3.6) |
| Motorcyclist road injuries | 862.1 (680.3 to 1065.7) | 854.1 (525.2 to 1319.2) | 61.9 (41.4 to 87.1) | -3.7* (-6.1 to -1.3) | -1.4 (-3.9 to 1.0) |
| Motor vehicle road injuries | 736.3 (567.4 to 929.9) | 1279.3 (824.1 to 1911.0) | 56.8 (38.5 to 80.8) | -6.2* (-9.7 to -3.1) | -4.0* (-7.5 to -0.8) |
| Other road injuries | 286.0 (213.0 to 385.7) | 381.2 (202.1 to 668.8) | 17.6 (11.7 to 26.0) | 1.8 (-0.9 to 4.9) | 4.2* (1.5 to 7.3) |
| Other transport injuries | 2516.2 (1936.6 to 3265.3) | 664.0 (408.0 to 974.1) | 183.6 (125.0 to 257.4) | 1.8 (-0.6 to 4.1) | 4.2* (1.8 to 6.6) |
| **Unintentional injuries** | **31087.1 (27921.9 to 34785.0)** | **26799.8 (21662.0 to 32571.9)** | **1415.7 (1034.4 to 1894.9)** | **4.4* (2.9 to 5.8)** | **6.9* (5.3 to 8.3)** |
| Falls | 12120.2 (9561.7 to 14989.9) | 10037.4 (6173.6 to 15164.9) | 598.4 (423.5 to 814.9) | 1.9* (0.2 to 3.4) | 4.3* (2.6 to 5.8) |
| Drowning | 82.5 (66.1 to 101.5) | 24.4 (17.9 to 32.8) | 6.1 (4.2 to 8.4) | -21.1* (-23.9 to -18.7) | -19.2* (-22.1 to -16.7) |
| Fire, heat, and hot substances | 3772.3 (2934.4 to 4871.1) | 843.9 (509.5 to 1279.0) | 191.3 (130.7 to 253.5) | 3.5 (-0.3 to 7.3) | 6.0* (2.1 to 9.9) |
| Poisonings | 274.4 (220.4 to 337.0) | 291.6 (158.2 to 454.2) | 33.7 (21.7 to 48.8) | 12.5* (8.8 to 16.3) | 15.2* (11.4 to 19.1) |
| Poisoning by carbon monoxide | 42.9 (33.3 to 54.6) | 72.7 (30.0 to 143.2) | 5.1 (3.2 to 7.6) | 14.6* (9.5 to 19.7) | 17.3* (12.1 to 22.5) |
| Poisoning by other means | 231.5 (175.3 to 292.4) | 218.9 (115.8 to 347.1) | 28.6 (18.1 to 42.3) | 12.1* (8.3 to 16.0) | 14.8* (10.8 to 18.8) |
| Exposure to mechanical forces | 6020.9 (4627.5 to 7729.6) | 5720.2 (3568.1 to 8226.0) | 181.1 (123.4 to 254.1) | 11.2* (9.1 to 13.2) | 13.8* (11.7 to 15.9) |
| Unintentional firearm injuries | 296.9 (214.1 to 388.3) | 164.2 (68.1 to 314.0) | 14.8 (10.4 to 21.0) | 9.7* (7.3 to 12.2) | 12.3* (9.8 to 14.8) |
| Other exposure to mechanical forces | 5724.0 (4329.4 to 7450.4) | 5556.0 (3437.8 to 8024.4) | 166.3 (112.8 to 236.8) | 11.3* (9.2 to 13.5) | 14.0* (11.8 to 16.2) |
| Adverse effects of medical treatment | 53.1 (30.7 to 85.6) | 693.4 (413.0 to 1091.2) | 7.1 (3.5 to 12.1) | -9.1* (-13.6 to -5.3) | -7.0* (-11.6 to -3.0) |
| Animal contact | 1746.8 (1410.2 to 2165.0) | 3773.2 (2358.5 to 5881.7) | 72.6 (47.2 to 102.7) | 4.8* (0.9 to 9.0) | 7.2* (3.3 to 11.6) |
| Venomous animal contact | 779.1 (593.3 to 1001.6) | 1960.3 (1166.9 to 3118.8) | 56.0 (36.1 to 81.4) | 6.6* (1.7 to 11.7) | 9.1* (4.1 to 14.3) |
| Non-venomous animal contact | 967.7 (700.4 to 1291.4) | 1812.9 (1090.6 to 2880.6) | 16.5 (9.8 to 26.7) | -1.0 (-3.6 to 1.3) | 1.3 (-1.4 to 3.8) |
| Foreign body | 899.1 (707.8 to 1143.5) | 1609.7 (1048.6 to 2307.4) | 52.1 (35.1 to 73.1) | 5.8* (3.8 to 7.7) | 8.3* (6.3 to 10.3) |

| | | | | | |
|---|---|---|---|---|---|
| Pulmonary aspiration and foreign body in airway | 118.9<br>(87.3 to 152.5) | 34.6<br>(16.6 to 59.7) | 8.0<br>(5.2 to 11.2) | 2.2<br>(-1.6 to 6.6) | 4.6*<br>(0.7 to 9.1) |
| Foreign body in eyes | 189.4<br>(67.0 to 362.3) | 1320.1<br>(814.9 to 2016.7) | 11.3<br>(4.0 to 22.6) | 8.0*<br>(6.5 to 9.6) | 10.6*<br>(9.0 to 12.2) |
| Foreign body in other body part | 590.8<br>(444.4 to 767.8) | 255.0<br>(132.5 to 464.5) | 32.8<br>(22.7 to 44.9) | 6.0*<br>(3.7 to 8.3) | 8.5*<br>(6.2 to 10.9) |
| Environmental heat and cold exposure | 1473.5<br>(1105.5 to 1936.2) | 639.2<br>(388.8 to 1001.5) | 75.4<br>(50.2 to 103.1) | 3.7*<br>(1.7 to 5.5) | 6.1*<br>(4.1 to 8.0) |
| Exposure to forces of nature | 1157.3<br>(828.3 to 1559.2) | 56.4<br>(40.8 to 77.2) | 64.8<br>(43.3 to 93.0) | -1.7<br>(-13.4 to 10.2) | 0.6<br>(-11.3 to 12.8) |
| Other unintentional injuries | 3487.0<br>(2747.8 to 4644.7) | 3110.3<br>(1936.8 to 4669.5) | 133.3<br>(90.3 to 189.0) | 12.3*<br>(9.9 to 14.5) | 14.9*<br>(12.6 to 17.3) |
| **Self-harm and interpersonal violence** | **24401.0<br>(17666.4 to 32648.8)** | **4583.9<br>(3650.9 to 5630.7)** | **415.9<br>(302.1 to 558.8)** | **-11.4*<br>(-16.0 to -7.1)** | **-9.3*<br>(-14.0 to -4.9)** |
| Self-harm | 119.3<br>(87.2 to 162.8) | 408.0<br>(268.2 to 596.3) | 9.9<br>(6.4 to 14.1) | -15.2*<br>(-17.8 to -12.4) | -13.1*<br>(-15.9 to -10.4) |
| Self-harm by firearm | 2.2<br>(1.6 to 3.1) | 2.9<br>(1.0 to 7.3) | 0.1<br>(0.1 to 0.2) | -15.3*<br>(-19.3 to -10.8) | -13.3*<br>(-17.3 to -8.7) |
| Self-harm by other specified means | 117.1<br>(84.8 to 160.7) | 405.1<br>(266.2 to 593.3) | 9.8<br>(6.3 to 13.9) | -15.1*<br>(-17.8 to -12.4) | -13.1*<br>(-15.8 to -10.3) |
| Interpersonal violence | 22390.7<br>(15675.5 to 30726.9) | 2026.9<br>(1337.3 to 2824.8) | 290.3<br>(196.2 to 417.0) | -7.3*<br>(-9.0 to -5.3) | -5.1*<br>(-6.9 to -3.1) |
| Assault by firearm | 47.4<br>(37.3 to 58.0) | 58.0<br>(30.6 to 97.5) | 3.3<br>(2.3 to 4.5) | 4.8*<br>(0.7 to 9.6) | 7.3*<br>(3.1 to 12.2) |
| Assault by sharp object | 440.3<br>(341.1 to 562.8) | 466.2<br>(263.7 to 757.7) | 19.3<br>(13.2 to 27.4) | 0.0<br>(-4.0 to 3.8) | 2.4<br>(-1.7 to 6.3) |
| Sexual violence | 20864.7<br>(14115.8 to 29318.2) | -- | 207.4<br>(118.2 to 330.7) | -11.0*<br>(-13.0 to -9.4) | -8.9*<br>(-11.0 to -7.2) |
| Assault by other means | 1038.3<br>(818.0 to 1342.1) | 1502.6<br>(943.6 to 2154.8) | 60.3<br>(41.6 to 82.4) | 4.5*<br>(0.9 to 8.0) | 7.0*<br>(3.3 to 10.6) |
| Conflict and terrorism | 1771.0<br>(1189.8 to 2628.0) | 1803.3<br>(1366.5 to 2422.8) | 108.8<br>(68.9 to 167.3) | -23.1*<br>(-32.3 to -10.5) | -21.2*<br>(-30.7 to -8.4) |
| Executions and police conflict | 120.0<br>(77.7 to 184.4) | 345.6<br>(236.7 to 492.8) | 6.9<br>(4.4 to 10.4) | 129.4*<br>(99.4 to 164.0) | 134.9*<br>(104.1 to 170.3) |
| **Nature of injury aggregates** | | | | | |
| Amputations | 12746.4<br>(11241.0 to 14719.8) | 747.2<br>(592.3 to 940.3) | 246.0<br>(168.0 to 356.3) | 0.3<br>(-4.0 to 3.7) | 2.7<br>(-1.7 to 6.2) |
| Burns | 7917.7<br>(6984.5 to 9093.5) | 1354.2<br>(1049.8 to 1696.6) | 411.5<br>(294.9 to 536.3) | -1.5<br>(-6.8 to 2.7) | 0.8<br>(-4.6 to 5.2) |
| Fractures | 8503.8<br>(7621.2 to 9514.8) | 8665.8<br>(7011.9 to 10597.7) | 486.1<br>(332.9 to 687.8) | 0.7<br>(-2.4 to 3.1) | 3.1<br>(-0.1 to 5.6) |
| Head Injuries | 1415.5<br>(1305.0 to 1553.0) | 1610.1<br>(1309.6 to 1948.0) | 221.4<br>(155.2 to 303.4) | 3.0*<br>(0.2 to 6.0) | 5.5*<br>(2.6 to 8.5) |
| Spinal Injuries | 915.3<br>(802.6 to 1080.2) | 56.0<br>(42.3 to 74.6) | 300.7<br>(208.2 to 408.9) | 0.3<br>(-5.3 to 4.7) | 2.6<br>(-3.1 to 7.2) |
| Minor Injuries | 6425.0<br>(5760.3 to 7228.4) | 14034.4<br>(11445.5 to 16707.7) | 55.8<br>(26.1 to 106.5) | 4.3*<br>(2.6 to 6.0) | 6.8*<br>(5.0 to 8.5) |
| Other Injuries | 2815.6<br>(2542.3 to 3113.4) | 9860.2<br>(8472.6 to 11420.5) | 342.0<br>(241.7 to 471.8) | 1.3<br>(-2.8 to 4.4) | 3.7<br>(-0.5 to 6.8) |
| **Impairments** | | | | | |
| Anaemia | 169198.1<br>(159238.0 to 180239.5) | -- | 3950.8<br>(2575.1 to 5869.7) | -12.6*<br>(-18.7 to -5.0) | -10.5*<br>(-16.8 to -2.8) |
| Epilepsy | 5674.4<br>(4683.0 to 6831.6) | -- | 2456.3<br>(1856.8 to 3223.4) | 2.5<br>(-3.6 to 8.8) | 4.9<br>(-1.3 to 11.4) |
| Guillain-Barré syndrome | 6.8<br>(3.8 to 11.7) | -- | 2.0<br>(1.0 to 3.7) | 6.3*<br>(3.9 to 9.1) | 8.8*<br>(6.3 to 11.7) |
| Hearing loss | 36577.2<br>(33265.8 to 40257.2) | -- | 1395.9<br>(982.7 to 1908.9) | -1.7<br>(-4.8 to 1.4) | 0.6<br>(-2.6 to 3.8) |
| Heart failure | 224.3<br>(167.1 to 298.0) | -- | 20.8<br>(12.7 to 31.8) | -7.7*<br>(-9.8 to -5.7) | -5.5*<br>(-7.6 to -3.4) |
| Infertility | 3857.6<br>(2450.4 to 5611.0) | -- | 35.1<br>(16.8 to 70.0) | 8.7*<br>(3.1 to 14.8) | 11.3*<br>(5.5 to 17.5) |
| Developmental intellectual disability | 19007.5<br>(14683.2 to 23427.6) | -- | 2437.3<br>(1889.4 to 3031.4) | 0.6<br>(-2.2 to 3.6) | 2.9*<br>(0.2 to 6.0) |
| Pelvic inflammatory disease | 30.5<br>(21.2 to 42.3) | -- | 4.2<br>(2.4 to 6.5) | 5.6*<br>(4.0 to 7.5) | 8.1*<br>(6.4 to 10.1) |
| Vision loss | 24339.7<br>(21125.0 to 28083.2) | -- | 842.7<br>(582.4 to 1198.1) | -1.0<br>(-2.3 to 0.4) | 1.3*<br>(0.0 to 2.8) |

We did not calculate incidence for the nine impairments and certain NTDs. Blank cells mean that no estimate is available or that the estimate has a magnitude less than 50.

Table presents prevalence and incidence counts in thousands.

* Percentage changes that are statistically significant

‡ Incidence estimates for stroke represent first-ever stroke only.

^ Incidence of HIV/AIDS represents new infections of HIV only and does not include new infections of Tuberculosis in HIV positive cases.

| SR Table 10h: Global prevalence, incidence and YLDs for 2017, percentage change of YLD counts and percentage change of YLD rates between 2007 and 2017 by cause, age 20 to 24 years | | | | | |
|---|---|---|---|---|---|
| Cause | Prevalence in thousands (95% UI) | Incidence in thousands (95% UI) | YLDs in thousands (95% UI) | | |
| | 2017 counts | 2017 counts | 2017 counts | Percentage change in counts between 2007 and 2017 | Percentage change in rates between 2007 and 2017 |
| **All causes** | **588515.1 (584692.5 to 592146.9)** | **3044714.4 (2595614.1 to 3543370.2)** | **51609.3 (38571.4 to 65975.1)** | **1.1* (0.4 to 1.9)** | **-0.8* (-1.6 to -0.1)** |
| **Communicable, maternal, neonatal, and nutritional disorders** | **400624.2 (374555.4 to 429435.7)** | **2182743.8 (1730597.2 to 2682687.1)** | **8792.7 (6497.1 to 11703.6)** | **-1.6 (-4.7 to 1.9)** | **-3.4* (-6.5 to -0.0)** |
| **HIV/AIDS and sexually transmitted infections** | **109756.9 (96239.7 to 125054.7)** | **149859.2 (124174.5 to 182822.9)** | **460.2 (315.0 to 694.8)** | **-9.7* (-18.4 to -2.0)** | **-11.4* (-19.9 to -3.9)** |
| HIV/AIDS | 2488.6 (2241.8 to 2750.5) | 362.2^ (306.4 to 432.3) | 248.7 (175.8 to 334.5) | -17.8* (-27.6 to -5.7) | -19.4* (-29.0 to -7.5) |
| HIV/AIDS - Drug-susceptible Tuberculosis | 100.6 (91.7 to 110.0) | 118.0 (107.6 to 129.8) | 39.5 (26.7 to 53.2) | -34.3* (-38.5 to -30.0) | -35.5* (-39.6 to -31.3) |
| HIV/AIDS - Multidrug-resistant Tuberculosis without extensive drug resistance | 2.9 (1.9 to 4.2) | 4.1 (2.9 to 5.8) | 1.2 (0.7 to 1.9) | -48.7* (-66.2 to -24.5) | -49.7* (-66.9 to -26.0) |
| HIV/AIDS - Extensively drug-resistant Tuberculosis | 0.1 (0.1 to 0.2) | 0.1 (0.1 to 0.1) | 0.0 (0.0 to 0.1) | -19.8 (-52.9 to 37.7) | -21.3 (-53.8 to 35.0) |
| HIV/AIDS resulting in other diseases | 2385.1 (2135.4 to 2651.8) | 362.2 (306.4 to 432.3) | 207.9 (146.3 to 283.4) | -13.3 (-25.7 to 2.4) | -15.0 (-27.1 to 0.5) |
| HIV/AIDS not on antiretroviral treatment without Tuberculosis | 1664.8 (1475.1 to 1896.7) | 362.2 (306.4 to 432.3) | 151.2 (104.5 to 206.6) | -35.4* (-46.9 to -19.6) | -36.7* (-47.9 to -21.1) |
| HIV/AIDS on antiretroviral treatment without Tuberculosis | 720.2 (636.0 to 810.8) | -- | 56.7 (37.5 to 80.4) | 888.4* (720.7 to 1115.0) | 869.5* (705.0 to 1091.7) |
| Sexually transmitted infections excluding HIV | 108242.6 (94656.8 to 123707.7) | 149497.0 (123800.8 to 182018.9) | 211.6 (108.5 to 413.0) | 2.2 (-0.9 to 4.8) | 0.2 (-2.8 to 2.8) |
| Syphilis | 6611.9 (4719.1 to 8855.8) | 2152.8 (1523.8 to 2974.8) | 2.2 (1.1 to 4.6) | -2.3 (-10.7 to 4.8) | -4.2 (-12.4 to 2.8) |
| Early syphilis | 6608.4 (4715.9 to 8852.2) | 2152.8 (1523.8 to 2974.8) | 1.6 (0.5 to 4.0) | 5.5 (-1.3 to 13.0) | 3.5 (-3.2 to 10.8) |
| Tertiary syphilis | 3.5 (2.7 to 4.4) | -- | 0.6 (0.4 to 0.9) | -17.7* (-19.9 to -15.6) | -19.3* (-21.5 to -17.3) |
| Chlamydial infection | 19087.2 (14477.1 to 24880.9) | 74306.5 (55777.9 to 99300.9) | 52.8 (30.1 to 97.5) | -1.9 (-7.2 to 2.8) | -3.7 (-9.0 to 0.8) |
| Chlamydia episode | 18702.0 (14103.4 to 24481.9) | 74306.5 (55777.9 to 99300.9) | 31.0 (12.0 to 69.2) | -0.3 (-1.8 to 1.2) | -2.2* (-3.7 to -0.8) |
| Chlamydial infection complications | 385.2 (339.2 to 435.9) | -- | 21.8 (13.7 to 31.4) | -4.0 (-14.0 to 5.5) | -5.9 (-15.6 to 3.5) |
| Gonococcal infection | 14253.8 (8923.3 to 21923.2) | 35508.9 (21570.9 to 53203.1) | 50.6 (24.6 to 105.7) | 3.6 (-2.9 to 9.2) | 1.6 (-4.7 to 7.1) |
| Gonococcal infection complications | 159.2 (127.7 to 192.6) | -- | 13.8 (8.3 to 20.8) | 8.5 (-6.0 to 23.5) | 6.4 (-7.8 to 21.1) |
| Gonorrhoea episode | 14094.5 (8762.6 to 21765.4) | 35508.9 (21570.9 to 53203.1) | 36.8 (13.3 to 88.6) | 1.9 (-5.1 to 5.9) | -0.1 (-6.9 to 3.9) |
| Trichomoniasis | 21527.8 (14732.0 to 29423.1) | 22750.8 (15757.1 to 31772.8) | 38.4 (14.2 to 83.5) | 6.8* (4.7 to 8.9) | 4.7* (2.7 to 6.8) |
| Genital herpes | 57830.6 (46094.5 to 72280.0) | 14778.0 (12486.2 to 17415.0) | 17.7 (6.2 to 39.0) | 4.5* (1.5 to 7.2) | 2.5 (-0.5 to 5.2) |
| Other sexually transmitted infections | 414.6 (373.8 to 456.2) | -- | 49.8 (28.4 to 94.4) | 1.3 (-2.4 to 5.0) | -0.6 (-4.2 to 3.0) |
| Other sexually transmitted diseases residual | -- | -- | 40.9 (21.7 to 81.0) | 0.6 (-4.0 to 4.4) | -1.3 (-5.9 to 2.4) |
| Other sexually transmitted diseases | 414.6 (373.8 to 456.2) | -- | 49.8 (28.4 to 94.4) | 1.3 (-2.4 to 5.0) | -0.6 (-4.2 to 3.0) |
| **Respiratory infections and tuberculosis** | **181368.8 (130272.1 to 247284.0)** | **1432055.1 (1014068.3 to 1960010.9)** | **948.5 (610.7 to 1448.8)** | **0.2 (-1.5 to 2.0)** | **-1.7 (-3.4 to 0.0)** |
| Tuberculosis | 161466.1 (107882.0 to 228446.1) | 983.1 (730.0 to 1277.4) | 243.4 (146.7 to 372.4) | -0.3 (-4.6 to 4.9) | -2.2 (-6.4 to 2.9) |
| Latent tuberculosis infection | 160697.8 (107226.8 to 227790.9) | -- | -- | -- | -- |
| Drug-susceptible tuberculosis | 733.0 (522.7 to 999.7) | 937.0 (695.9 to 1223.2) | 231.9 (137.5 to 359.1) | -0.0 (-5.3 to 5.8) | -1.9 (-7.1 to 3.8) |
| Multidrug-resistant tuberculosis without extensive drug resistance | 33.7 (16.9 to 61.5) | 44.2 (24.7 to 74.1) | 10.9 (4.8 to 21.2) | -6.9 (-48.0 to 55.5) | -8.7 (-49.0 to 52.5) |
| Extensively drug-resistant tuberculosis | 1.6 (0.9 to 2.9) | 2.0 (1.3 to 3.1) | 0.5 (0.3 to 1.0) | 15.3 (-26.7 to 96.9) | 13.1 (-28.1 to 93.1) |
| Lower respiratory infections | 877.2 (698.2 to 1075.8) | 38815.4 (30578.3 to 47270.0) | 53.9 (33.8 to 81.3) | 25.9* (13.2 to 38.4) | 23.5* (11.0 to 35.8) |
| Guillain-Barré syndrome due to lower respiratory infections | 0.7 (0.3 to 1.4) | -- | 0.2 (0.1 to 0.4) | 8.2* (5.4 to 11.3) | 6.2* (3.4 to 9.2) |
| Lower respiratory infection episode | 876.5 (697.7 to 1074.9) | 38815.4 (30578.3 to 47270.0) | 53.7 (33.7 to 81.1) | 26.0* (13.2 to 38.6) | 23.6* (11.0 to 35.9) |
| Upper respiratory infections | 18933.7 (13108.2 to 25709.3) | 1387228.3 (963415.5 to 1912084.4) | 476.5 (244.8 to 829.2) | 0.4 (-0.8 to 1.7) | -1.5* (-2.7 to -0.3) |
| Guillain-Barré syndrome due to upper respiratory infections | 1.9 (1.0 to 3.5) | -- | 0.6 (0.3 to 1.2) | 8.2* (5.4 to 11.2) | 6.2* (3.4 to 9.1) |
| Upper respiratory infection episode | 18931.7 (13105.8 to 25706.2) | 1387228.3 (963415.5 to 1912084.4) | 476.0 (244.4 to 828.5) | 0.4 (-0.9 to 1.7) | -1.5* (-2.8 to -0.3) |
| Otitis media | 8210.3 (5789.7 to 11163.1) | 5028.3 (3189.6 to 7457.3) | 174.7 (94.7 to 303.5) | -5.6* (-9.8 to -1.2) | -7.4* (-11.5 to -3.1) |
| Acute otitis media | 272.4 (170.8 to 412.9) | 5027.4 (3186.3 to 7456.8) | 3.6 (1.6 to 7.2) | 4.2 (-2.9 to 12.0) | 2.2 (-4.7 to 9.8) |
| Chronic otitis media | 7937.9 (5555.4 to 10827.0) | 0.9 (0.0 to 3.4) | 171.1 (93.0 to 297.6) | -5.8* (-10.2 to -1.3) | -7.6* (-11.9 to -3.1) |
| **Enteric infections** | **6173.6 (5131.2 to 7434.4)** | **432128.9 (349070.0 to 531349.3)** | **714.1 (475.5 to 1011.9)** | **28.0* (21.3 to 35.3)** | **25.5* (18.9 to 32.7)** |
| Diarrhoeal diseases | 6185.8 (5131.7 to 7421.1) | 430858.8 (347821.4 to 530282.3) | 703.9 (468.1 to 998.6) | 28.8* (21.8 to 36.2) | 26.4* (19.5 to 33.6) |
| Guillain-Barré syndrome due to diarrheal diseases | 0.7 (0.3 to 1.2) | -- | 0.2 (0.1 to 0.4) | 8.2* (5.4 to 11.3) | 6.2* (3.4 to 9.2) |
| Diarrheal disease episode | 6185.1 (5131.1 to 7420.6) | 430858.8 (347821.4 to 530282.3) | 703.7 (468.0 to 998.4) | 28.8* (21.8 to 36.2) | 26.4* (19.5 to 33.6) |
| Typhoid and paratyphoid | 21.8 (14.7 to 30.0) | 1248.6 (970.2 to 1525.2) | 10.1 (6.2 to 15.1) | -11.1 (-28.6 to 8.7) | -12.8 (-29.9 to 6.6) |
| Typhoid fever | 60.0 (43.3 to 78.6) | 946.5 (715.9 to 1189.2) | 9.2 (5.6 to 13.9) | -11.9 (-30.2 to 9.6) | -13.6 (-31.6 to 7.5) |
| Typhoid fever complications | 12.6 (8.6 to 17.8) | 163.9 (120.2 to 216.0) | 4.1 (2.3 to 6.4) | -11.5 (-38.0 to 23.8) | -13.2 (-39.1 to 21.4) |
| Typhoid fever episode | 47.4 (33.9 to 62.9) | 782.6 (593.4 to 989.4) | 5.2 (3.1 to 8.1) | -12.3 (-34.9 to 17.1) | -14.0 (-36.2 to 14.9) |
| Paratyphoid fever | 13.2 (8.7 to 18.8) | 302.2 (200.3 to 421.9) | 0.8 (0.5 to 1.3) | -0.0 (-16.1 to 19.6) | -1.9 (-17.7 to 17.3) |
| Intestinal perforation due to paratyphoid | 0.6 (0.4 to 0.9) | 15.5 (10.1 to 22.2) | 0.1 (0.0 to 0.1) | 0.8 (-21.4 to 29.7) | -1.2 (-22.9 to 27.3) |
| Paratyphoid fever episode | 12.6 (8.3 to 18.0) | 286.7 (189.6 to 400.7) | 0.8 (0.4 to 1.2) | -0.1 (-17.3 to 20.6) | -2.0 (-18.8 to 18.3) |
| iNTS | 0.8 (0.5 to 1.3) | 21.4 (12.4 to 34.6) | 0.1 (0.1 to 0.2) | -20.7* (-26.4 to -15.1) | -22.3* (-27.8 to -16.7) |
| Other intestinal infectious diseases | -- | -- | -- | -37.1* (-49.5 to -23.2) | -38.3* (-50.5 to -24.6) |
| **Neglected tropical diseases and malaria** | **134482.1 (128035.0 to 141589.8)** | **26308.8 (20770.6 to 32881.1)** | **1187.1 (823.7 to 1653.8)** | **-15.3* (-20.1 to -10.1)** | **-17.0* (-21.6 to -11.8)** |
| Malaria | 11179.2 (10397.2 to 11915.5) | 14569.2 (11103.6 to 18676.4) | 109.3 (79.9 to 149.2) | -15.5* (-25.2 to -2.2) | -17.1* (-26.7 to -4.0) |
| Malaria complications | 101.0 (92.0 to 110.7) | -- | 41.7 (32.0 to 52.3) | 22.3* (10.2 to 35.5) | 20.0* (8.1 to 32.9) |
| Malaria episode | 849.2 (540.4 to 1231.9) | 14569.2 (11103.6 to 18676.4) | 25.6 (13.0 to 44.5) | -11.2* (-20.4 to -0.4) | -12.9* (-21.9 to -2.3) |

| | | | | | |
|---|---|---|---|---|---|
| Malaria parasitemia | 10229.0 (9383.1 to 11150.7) | -- | 42.0 (26.6 to 66.1) | -36.8* (-49.0 to -18.0) | -38.0* (-50.0 to -19.6) |
| Chagas disease | 386.4 (309.7 to 469.3) | 14.7 (10.4 to 19.4) | 2.0 (1.2 to 2.9) | -20.0* (-29.9 to -8.9) | -21.5* (-31.2 to -10.6) |
| Acute Chagas disease | 0.1 (0.0 to 0.1) | 14.7 (10.4 to 19.4) | -- | -16.3* (-20.4 to -12.4) | -17.9* (-21.9 to -14.1) |
| Asymptomatic Chagas disease | 349.0 (276.8 to 423.0) | -- | -- | -- | -- |
| Symptomatic chronic Chagas infection | 37.3 (27.7 to 49.2) | -- | 1.9 (1.2 to 2.9) | -20.0* (-29.9 to -8.8) | -21.5* (-31.2 to -10.6) |
| Leishmaniasis | 415.5 (362.6 to 478.1) | 50.5 (34.6 to 70.7) | 27.1 (17.8 to 38.7) | 61.6* (44.7 to 81.6) | 58.5* (41.9 to 78.1) |
| Visceral leishmaniasis | 0.6 (0.4 to 0.9) | 2.2 (1.6 to 3.4) | 0.0 (0.0 to 0.1) | -72.5* (-79.1 to -59.5) | -73.1* (-79.5 to -60.3) |
| Cutaneous and mucocutaneous leishmaniasis | 419.0 (366.4 to 479.6) | 48.2 (32.5 to 68.4) | 27.1 (17.8 to 38.6) | 62.7* (45.6 to 82.9) | 59.6* (42.8 to 79.4) |
| African trypanosomiasis | 0.5 (0.1 to 2.4) | 0.4 (0.1 to 1.4) | 0.1 (0.0 to 0.7) | -79.5 (-97.3 to 6.6) | -79.9 (-97.4 to 4.6) |
| Trypanosomiasis Gambiense | 0.5 (0.1 to 2.4) | 0.3 (0.1 to 1.3) | 0.1 (0.0 to 0.7) | -78.7 (-97.4 to 21.2) | -79.1 (-97.5 to 18.9) |
| Trypanosomiasis Rhodesiense | -- | 0.0 (0.0 to 0.1) | -- | -92.3* (-99.0 to -54.2) | -92.5* (-99.1 to -55.1) |
| Schistosomiasis | 17090.9 (14414.2 to 19946.8) | -- | 134.7 (64.5 to 264.5) | -26.0* (-29.6 to -22.3) | -27.4* (-31.0 to -23.7) |
| Mild schistosomiasis | 12368.0 (9978.4 to 14687.4) | -- | 70.5 (28.1 to 152.9) | -28.2* (-31.4 to -25.5) | -29.5* (-32.7 to -26.9) |
| Anemia due to schistosomiasis | 855.8 (670.7 to 1092.4) | -- | 19.1 (11.5 to 31.2) | -39.3* (-52.1 to -22.7) | -40.5* (-53.0 to -24.2) |
| Schistosomiasis complications | 3867.0 (3076.2 to 4807.1) | -- | 45.2 (23.0 to 85.6) | -14.1* (-18.7 to -8.7) | -15.7* (-20.3 to -10.5) |
| Cysticercosis | 398.5 (289.2 to 540.6) | -- | 126.7 (75.8 to 197.1) | -5.7 (-13.5 to 2.3) | -7.5 (-15.1 to 0.4) |
| Cystic echinococcosis | 63.1 (24.2 to 135.6) | 15.1 (6.0 to 33.1) | 5.3 (1.8 to 12.0) | 2.1 (-8.4 to 11.0) | 0.2 (-10.1 to 8.9) |
| Lymphatic filariasis | 6293.2 (5409.2 to 7306.6) | -- | 106.0 (57.2 to 167.5) | -37.6* (-48.8 to -26.6) | -38.8* (-49.8 to -28.0) |
| Prevalence of detectable microfiliaria due to lymphatic filariasis | 5353.2 (4495.3 to 6302.5) | -- | -- | -- | -- |
| Lymphatic filariasis complications | 940.0 (626.3 to 1363.5) | -- | 106.0 (57.2 to 167.5) | -37.6* (-48.8 to -26.6) | -38.8* (-49.8 to -28.0) |
| Onchocerciasis | 2642.5 (1487.2 to 4897.9) | -- | 142.8 (60.7 to 255.5) | 2.1 (-20.6 to 20.0) | 0.2 (-22.1 to 17.7) |
| Asymptomatic onchocerciasis | 866.1 (8.7 to 2908.0) | -- | -- | -- | -- |
| Skin disease due to onchocerciasis | 1769.0 (1264.9 to 2425.1) | -- | 142.3 (59.7 to 254.9) | 2.4 (-20.3 to 20.2) | 0.4 (-21.8 to 17.9) |
| Vision loss due to onchocerciasis | 7.4 (4.8 to 11.8) | -- | 0.6 (0.3 to 0.9) | -36.7* (-46.2 to -26.4) | -37.9* (-47.2 to -27.8) |
| Trachoma | 8.3 (3.8 to 17.1) | -- | 1.2 (0.5 to 2.3) | -46.9* (-67.0 to -16.8) | -47.9* (-67.6 to -18.4) |
| Dengue | 589.3 (321.2 to 997.8) | 9846.5 (5991.8 to 14930.9) | 97.3 (43.1 to 183.7) | 56.3* (35.6 to 140.8) | 53.3* (33.0 to 136.2) |
| Post-dengue chronic fatigue syndrome | 415.5 (194.2 to 759.7) | -- | 87.0 (36.2 to 165.5) | 56.3* (34.9 to 141.3) | 53.3* (32.4 to 136.7) |
| Dengue episode | 173.8 (105.0 to 260.8) | 9846.5 (5991.8 to 14930.9) | 10.3 (5.4 to 18.0) | 56.3* (32.5 to 136.9) | 53.4* (30.0 to 132.4) |
| Yellow fever | 0.3 (0.1 to 0.7) | 9.3 (2.6 to 25.2) | -- | -16.2 (-41.9 to 20.8) | -17.8 (-43.0 to 18.5) |
| Asymptomatic yellow fever | 0.1 (0.0 to 0.4) | 5.2 (1.3 to 14.4) | -- | -- | -- |
| Yellow fever episode | 0.1 (0.0 to 0.3) | 4.1 (1.1 to 11.1) | -- | -16.2 (-41.9 to 20.8) | -17.8 (-43.0 to 18.5) |
| Rabies | -- | 0.9 (0.6 to 1.2) | -- | -29.6* (-50.3 to -2.1) | -30.9* (-51.3 to -4.0) |
| Intestinal nematode infections | 101948.8 (95507.2 to 108911.0) | -- | 192.4 (111.4 to 314.9) | -30.5* (-34.2 to -26.7) | -31.9* (-35.4 to -28.1) |
| Ascariasis | 41402.1 (36462.0 to 47202.2) | -- | 53.8 (28.8 to 92.7) | -34.2* (-40.8 to -26.6) | -35.4* (-42.0 to -28.0) |
| Asymptomatic ascariasis | 38518.8 (33898.2 to 43914.5) | -- | -- | -- | -- |
| Ascariasis complications | 2883.3 (2563.1 to 3234.4) | -- | 53.8 (28.8 to 92.7) | -34.2* (-40.8 to -26.6) | -35.4* (-42.0 to -28.0) |
| Trichuriasis | 43255.8 (38304.9 to 49199.4) | -- | 35.3 (19.8 to 59.5) | -22.6* (-29.1 to -15.2) | -24.0* (-30.4 to -16.8) |
| Asymptomatic trichuriasis | 41497.6 (36619.2 to 47282.8) | -- | -- | -- | -- |
| Trichuriasis complications | 1758.2 (1607.0 to 1911.5) | -- | 35.3 (19.8 to 59.5) | -22.6* (-29.1 to -15.2) | -24.0* (-30.4 to -16.8) |
| Hookworm disease | 27896.4 (25987.5 to 30026.1) | -- | 103.4 (61.1 to 166.4) | -31.0* (-36.3 to -25.0) | -32.3* (-37.5 to -26.4) |
| Asymptomatic hookworm disease | 23068.7 (21446.0 to 24847.2) | -- | -- | -- | -- |
| Anemia due to hookworm disease | 1105.5 (984.3 to 1240.1) | -- | 24.9 (16.1 to 38.0) | -38.8* (-47.4 to -28.7) | -39.9* (-48.4 to -30.0) |
| Hookworm disease complications | 3722.1 (3480.3 to 3988.8) | -- | 78.5 (43.4 to 129.2) | -28.1* (-32.8 to -22.6) | -29.4* (-34.1 to -24.1) |
| Food-borne trematodiases | 6570.9 (5533.7 to 7748.8) | 1597.8 (1320.2 to 1917.0) | 150.6 (82.4 to 256.9) | -16.8* (-21.6 to -11.0) | -18.4* (-23.1 to -12.7) |
| Asymptomatic food-borne tremodiases | 5260.8 (4303.0 to 6331.6) | 1323.6 (903.1 to 1701.4) | -- | -- | -- |
| Food-borne tremodiases complications | 1310.1 (844.4 to 2052.0) | 274.1 (82.9 to 642.2) | 150.6 (82.4 to 256.9) | -16.8* (-21.6 to -11.0) | -18.4* (-23.1 to -12.7) |
| Leprosy | 12.2 (11.0 to 13.4) | 4.2 (3.8 to 4.8) | 0.7 (0.5 to 1.1) | -35.2* (-40.7 to -29.0) | -36.5* (-41.9 to -30.4) |
| Ebola virus disease | -- | -- | -- | -97.6* (-98.3 to -95.6) | -97.6* (-98.3 to -95.7) |
| Ebola cases | -- | -- | -- | -98.3* (-98.3 to -98.3) | -98.3* (-98.4 to -98.3) |
| Post-Ebola chronic fatigue syndrome | -- | -- | -- | -97.5* (-98.3 to -95.6) | -97.6* (-98.3 to -95.6) |
| Zika virus disease | 3.3 (2.0 to 5.9) | 200.2 (117.7 to 358.9) | 0.1 (0.0 to 0.1) | -- | -- |
| Zika virus complications | -- | -- | -- | -- | -- |
| Zika virus episode | 3.3 (1.9 to 5.9) | 200.2 (117.7 to 358.9) | 0.1 (0.0 to 0.1) | -- | -- |
| Guinea worm disease | -- | -- | -- | -99.4* (-99.6 to -99.1) | -99.4* (-99.6 to -99.1) |
| Other neglected tropical diseases | 3991.5 (3705.3 to 4318.2) | -- | 90.6 (59.8 to 137.2) | -7.1 (-15.7 to 2.1) | -8.9 (-17.3 to 0.2) |
| Acute infection due to other neglected tropical diseases | -- | -- | 0.7 (0.4 to 1.2) | 98.7* (69.0 to 195.0) | 94.9* (65.7 to 189.4) |
| Anemia due to other neglected tropical diseases | 3991.5 (3705.3 to 4318.2) | -- | 89.9 (59.4 to 136.2) | -7.5 (-16.1 to 1.7) | -9.3* (-17.7 to -0.2) |
| **Other infectious diseases** | **7766.2 (7114.6 to 8632.1)** | **25719.3 (20832.3 to 32851.5)** | **308.4 (216.2 to 421.2)** | **-5.6* (-9.3 to -1.3)** | **-7.4* (-11.0 to -3.2)** |
| Meningitis | 966.6 (803.8 to 1150.9) | 225.6 (146.7 to 330.3) | 87.7 (60.4 to 118.6) | -8.0* (-12.6 to -3.2) | -9.7* (-14.3 to -5.0) |
| Pneumococcal meningitis | 330.4 (269.8 to 411.3) | 15.7 (8.9 to 25.6) | 31.7 (21.3 to 43.8) | -29.3* (-34.6 to -23.4) | -30.7* (-35.8 to -24.8) |

| | | | | | |
|---|---|---|---|---|---|
| Acute pneumococcal meningitis | 0.8 (0.4 to 1.3) | 15.7 (8.9 to 25.6) | 0.1 (0.1 to 0.2) | -33.1* (-42.0 to -24.7) | -34.4* (-43.1 to -26.1) |
| Pneumococcal meningitis complications | 329.7 (269.1 to 410.2) | -- | 31.6 (21.3 to 43.6) | -29.3* (-34.6 to -23.3) | -30.7* (-35.8 to -24.8) |
| H influenzae type B meningitis | 88.4 (63.7 to 118.2) | 5.5 (2.9 to 9.7) | 8.1 (5.5 to 11.1) | -51.0* (-54.4 to -47.5) | -52.0* (-55.3 to -48.5) |
| Acute H influenzae type B meningitis | 0.3 (0.1 to 0.5) | 5.5 (2.9 to 9.7) | 0.0 (0.0 to 0.1) | -59.2* (-64.8 to -53.7) | -59.9* (-65.5 to -54.6) |
| H Influenza type B meningitis complications | 88.2 (63.4 to 117.9) | -- | 8.1 (5.5 to 11.1) | -51.0* (-54.4 to -47.5) | -51.9* (-55.3 to -48.5) |
| Meningococcal infection | 97.5 (68.6 to 132.1) | 14.4 (8.5 to 23.1) | 9.3 (6.2 to 12.9) | -7.7* (-11.7 to -4.0) | -9.5* (-13.4 to -5.8) |
| Acute meningococcal meningitis | 0.7 (0.4 to 1.2) | 14.4 (8.5 to 23.1) | 0.1 (0.0 to 0.2) | -2.7 (-13.3 to 7.6) | -4.6 (-15.0 to 5.5) |
| Meningococcal meningitis complications | 96.7 (67.7 to 131.0) | -- | 9.2 (6.1 to 12.8) | -7.8* (-11.8 to -4.0) | -9.6* (-13.5 to -5.8) |
| Other meningitis | 450.3 (330.2 to 573.8) | 189.9 (123.3 to 281.0) | 38.6 (26.2 to 52.0) | 62.0* (49.9 to 74.4) | 58.9* (47.0 to 71.0) |
| Other acute bacterial meningitis | 1.9 (1.2 to 2.8) | 38.8 (24.6 to 59.0) | 0.3 (0.1 to 0.4) | 65.7* (48.0 to 81.4) | 62.6* (45.1 to 78.0) |
| Acute viral meningitis | 7.5 (4.8 to 10.7) | 151.2 (98.2 to 221.4) | 1.0 (0.5 to 1.6) | -1.2 (-10.9 to 5.9) | -3.1 (-12.6 to 3.9) |
| Other bacterial meningitis complications | 440.9 (323.3 to 564.9) | -- | 37.3 (25.5 to 50.2) | 64.8* (52.4 to 77.8) | 61.6* (49.4 to 74.4) |
| Encephalitis | 505.2 (295.3 to 790.4) | 113.4 (107.5 to 119.3) | 45.7 (32.1 to 60.6) | -4.8 (-11.0 to 2.0) | -6.6 (-12.7 to 0.0) |
| Acute encephalitis | 6.2 (5.9 to 6.6) | 113.4 (107.5 to 119.3) | 0.8 (0.6 to 1.2) | 5.8* (5.2 to 6.5) | 3.8* (3.2 to 4.5) |
| Encephalitis complications | 499.0 (289.1 to 784.3) | -- | 44.9 (31.5 to 59.5) | -5.0 (-11.2 to 1.9) | -6.8* (-12.9 to -0.0) |
| Diphtheria | -- | 0.2 (0.1 to 0.2) | -- | -37.3* (-56.2 to -6.8) | -38.5* (-57.0 to -8.6) |
| Whooping cough | 5.1 (4.0 to 6.5) | 37.5 (29.0 to 47.3) | 0.3 (0.2 to 0.4) | -5.1* (-5.5 to -4.8) | -7.0* (-7.3 to -6.6) |
| Tetanus | 5.2 (4.9 to 5.4) | 3.0 (1.5 to 5.3) | 0.1 (0.1 to 0.2) | -11.9* (-24.3 to -2.0) | -13.6* (-25.7 to -3.8) |
| Severe tetanus | 0.2 (0.1 to 0.3) | 3.0 (1.5 to 5.3) | -- | -51.8* (-66.3 to -29.6) | -52.7* (-66.9 to -30.9) |
| Neonatal tetanus complications | 5.0 (4.8 to 5.2) | -- | 0.1 (0.1 to 0.2) | 8.3* (6.7 to 9.9) | 6.3* (4.6 to 7.8) |
| Measles | 2.8 (1.0 to 6.3) | 103.0 (36.6 to 228.4) | 0.3 (0.1 to 0.6) | -40.2* (-45.1 to -35.0) | -41.4* (-46.2 to -36.2) |
| Varicella and herpes zoster | 261.5 (193.6 to 334.9) | 2163.9 (1524.1 to 2944.5) | 13.9 (7.9 to 23.1) | 1.5 (-4.9 to 8.2) | -0.4 (-6.7 to 6.2) |
| Chickenpox | 15.3 (7.0 to 27.9) | 798.7 (363.5 to 1456.5) | 0.1 (0.0 to 0.2) | 11.9* (8.2 to 17.1) | 9.8* (6.1 to 14.8) |
| Herpes zoster | 246.2 (178.5 to 317.3) | 1365.2 (950.2 to 1838.1) | 13.8 (7.8 to 23.0) | 1.5 (-5.0 to 8.2) | -0.5 (-6.9 to 6.2) |
| Acute hepatitis | 2330.6 (1790.3 to 3092.8) | 23072.7 (18326.7 to 29865.1) | 40.4 (25.8 to 61.4) | -2.0 (-16.9 to 19.0) | -3.9 (-18.5 to 16.7) |
| Acute hepatitis A | 553.1 (477.2 to 616.3) | 7190.5 (6203.6 to 8011.6) | 23.2 (14.6 to 34.4) | 0.1 (-7.9 to 8.6) | -1.8 (-9.6 to 6.5) |
| Acute hepatitis B | 1636.8 (1103.7 to 2399.0) | 14186.0 (9565.4 to 20791.3) | 13.4 (7.2 to 22.6) | -5.8 (-41.5 to 55.6) | -7.6 (-42.6 to 52.6) |
| Acute hepatitis C | 30.5 (11.4 to 51.1) | 264.2 (99.1 to 443.1) | 0.4 (0.1 to 1.0) | 2.5 (-1.8 to 9.6) | 0.5 (-3.6 to 7.5) |
| Acute hepatitis E | 110.2 (72.7 to 154.5) | 1432.0 (945.3 to 2008.2) | 3.4 (1.8 to 5.6) | -0.9 (-20.0 to 21.8) | -2.8 (-21.6 to 19.5) |
| Other unspecified infectious diseases | 4616.9 (4339.6 to 4966.8) | -- | 120.1 (79.3 to 181.7) | -5.8 (-12.2 to 1.4) | -7.6* (-13.9 to -0.6) |
| Guillain-Barré syndrome due to other infectious diseases | 0.4 (0.2 to 0.8) | -- | 0.1 (0.1 to 0.3) | 8.2* (5.4 to 11.2) | 6.2* (3.4 to 9.1) |
| Other infectious diseases | -- | -- | 17.1 (9.6 to 29.0) | 3.8* (1.3 to 6.3) | 1.9 (-0.7 to 4.3) |
| Anemia due to other infectious diseases | 4616.5 (4339.2 to 4966.5) | -- | 102.8 (67.7 to 154.6) | -7.3 (-14.6 to 0.9) | -9.1* (-16.2 to -1.0) |
| **Maternal and neonatal disorders** | **13088.9 (11479.3 to 14937.0)** | **19862.9 (17028.3 to 22987.0)** | **2578.8 (1936.3 to 3306.1)** | **10.0* (4.1 to 16.4)** | **7.9* (2.1 to 14.2)** |
| Maternal disorders | 1727.4 (1352.4 to 2192.1) | 19862.9 (17028.3 to 22987.0) | 171.0 (116.6 to 240.1) | -11.2 (-22.2 to 1.0) | -12.9* (-23.7 to -0.9) |
| Maternal haemorrhage | 363.3 (314.6 to 423.7) | 1630.5 (1108.4 to 2275.8) | 14.0 (9.0 to 20.2) | -22.4* (-35.5 to -4.4) | -23.9* (-36.8 to -6.2) |
| Maternal hemorrhage complications | 327.7 (281.7 to 382.5) | -- | 8.1 (5.2 to 12.1) | -24.1* (-37.9 to -3.7) | -25.6* (-39.1 to -5.6) |
| Maternal hemorrhage episode | 35.6 (20.4 to 56.4) | 1630.5 (1108.4 to 2275.8) | 5.9 (3.2 to 9.6) | -20.0 (-44.7 to 16.1) | -21.6 (-45.8 to 13.9) |
| Maternal sepsis and other pregnancy related infections | 402.3 (270.5 to 613.3) | 3325.2 (2261.1 to 4697.9) | 13.7 (6.5 to 27.5) | -8.9 (-48.0 to 51.8) | -10.6 (-49.0 to 48.9) |
| Infertility due to puerperal sepsis | 147.8 (117.5 to 183.3) | 29.1 (22.4 to 37.5) | 0.7 (0.3 to 1.5) | 12.9* (2.6 to 22.9) | 10.8* (0.6 to 20.5) |
| Maternal sepsis and other maternal infection episode | 254.5 (125.4 to 445.1) | 3296.1 (2234.3 to 4663.0) | 13.0 (5.9 to 26.6) | -9.9 (-50.1 to 53.5) | -11.6 (-51.1 to 50.6) |
| Maternal hypertensive disorders | 750.7 (423.0 to 1204.1) | 4166.3 (2986.1 to 5629.1) | 37.7 (18.0 to 66.8) | -14.6 (-43.4 to 23.8) | -16.2 (-44.5 to 21.5) |
| Maternal hypertensive disorder complications | 22.4 (20.7 to 24.0) | 388.0 (357.1 to 416.8) | 1.5 (0.9 to 2.2) | 9.7 (-3.5 to 26.0) | 7.6 (-5.4 to 23.6) |
| Maternal hypertensive disorder episode | 728.3 (400.2 to 1182.1) | 3778.4 (2584.3 to 5240.8) | 36.2 (17.1 to 64.8) | -15.3 (-44.6 to 24.1) | -17.0 (-45.7 to 21.7) |
| Maternal obstructed labour and uterine rupture | 223.4 (168.4 to 289.8) | 2293.0 (1461.9 to 3399.9) | 73.1 (45.6 to 110.7) | -13.4* (-22.0 to -3.7) | -15.1* (-23.5 to -5.6) |
| Obstructed labor, acute event | 30.4 (16.4 to 51.6) | 2226.2 (1399.0 to 3339.7) | 9.5 (4.3 to 17.7) | -13.3 (-48.0 to 40.9) | -14.9 (-49.0 to 38.2) |
| Maternal obstructed labor complications | 193.0 (138.6 to 256.5) | 66.7 (50.8 to 86.5) | 63.6 (38.6 to 97.5) | -13.5* (-21.0 to -5.1) | -15.1* (-22.5 to -7.0) |
| Maternal abortion and miscarriage | 40.8 (24.6 to 64.2) | 4975.3 (3499.2 to 6922.4) | 4.5 (2.3 to 7.8) | -9.6 (-43.8 to 36.3) | -11.3 (-44.9 to 33.7) |
| Ectopic pregnancy | 28.4 (16.5 to 46.0) | 3472.6 (2226.9 to 4955.8) | 3.1 (1.5 to 5.3) | 10.0 (-32.9 to 70.2) | 7.9 (-34.1 to 67.0) |
| Other maternal disorders | -- | -- | 25.0 (16.9 to 35.2) | 7.4 (-6.4 to 23.3) | 5.4 (-8.2 to 20.9) |
| Neonatal disorders | 11394.4 (9835.1 to 13145.7) | -- | 2407.7 (1799.9 to 3141.0) | 11.9* (5.5 to 19.4) | 9.7* (3.5 to 17.2) |
| Neonatal preterm birth | 6612.8 (5651.7 to 7825.8) | -- | 1157.6 (817.0 to 1581.3) | 17.3* (2.9 to 31.6) | 15.0* (0.9 to 29.1) |
| Preterm birth complications | 5682.9 (4718.2 to 6880.6) | -- | 1107.9 (781.8 to 1526.4) | 18.3* (3.1 to 33.5) | 16.1* (1.1 to 30.9) |
| Retinopathy of prematurity | 929.9 (730.2 to 1145.2) | -- | 49.7 (33.1 to 70.3) | -2.9 (-9.4 to 4.8) | -4.7 (-11.1 to 2.8) |
| Uncomplicated preterm birth | -- | -- | -- | -- | -- |
| Neonatal encephalopathy due to birth asphyxia and trauma | 4342.8 (3149.4 to 5752.2) | -- | 823.0 (492.3 to 1284.6) | 7.7* (4.0 to 11.3) | 5.7* (2.0 to 9.2) |
| Neonatal sepsis and other neonatal infections | 1153.4 (711.7 to 1773.9) | -- | 349.6 (187.1 to 589.7) | 5.1* (0.7 to 9.5) | 3.1 (-1.3 to 7.4) |
| Neonatal sepsis and other neonatal infection complications | 1153.4 (711.7 to 1773.9) | -- | 349.6 (187.1 to 589.7) | 5.1* (0.7 to 9.5) | 3.1 (-1.3 to 7.4) |
| Neonatal sepsis and other neonatal infection episode | -- | -- | -- | -- | -- |
| Hemolytic disease and other neonatal jaundice | 205.2 (176.8 to 234.7) | -- | 77.6 (59.0 to 97.3) | 13.8* (8.5 to 18.7) | 11.6* (6.4 to 16.4) |

| | | | | | |
|---|---|---|---|---|---|
| Other neonatal disorders | -- | -- | -- | -- | -- |
| **Nutritional deficiencies** | **153977.4 (147179.6 to 161827.6)** | **96687.5 (87179.7 to 106719.8)** | **2595.6 (1728.0 to 3870.2)** | **-8.8* (-14.7 to -1.8)** | **-10.5* (-16.4 to -3.7)** |
| Protein-energy malnutrition | 2568.4 (2315.9 to 2839.2) | 2976.0 (2649.0 to 3321.6) | 29.0 (18.2 to 41.4) | 20.3* (11.4 to 30.1) | 18.0* (9.2 to 27.6) |
| Iodine deficiency | 10646.2 (9391.6 to 11990.7) | 72.2 (57.6 to 90.1) | 204.7 (125.1 to 320.9) | -10.4* (-15.0 to -5.5) | -12.1* (-16.6 to -7.3) |
| Visible goiter without symptoms | 10364.7 (9146.0 to 11683.7) | 72.2 (57.6 to 90.1) | 112.1 (54.4 to 209.4) | -13.4* (-15.3 to -11.8) | -15.1* (-17.0 to -13.5) |
| Visible goiter with complications | 281.6 (160.5 to 395.2) | -- | 92.6 (47.7 to 141.5) | -6.4 (-16.7 to 3.4) | -8.2 (-18.2 to 1.5) |
| Vitamin A deficiency | 62737.0 (56220.5 to 69676.4) | 93639.4 (84089.4 to 103571.1) | 18.5 (10.8 to 29.2) | 12.8* (3.3 to 22.4) | 10.7* (1.3 to 20.0) |
| Asymptomatic vitamin A deficiency | 62444.0 (55939.8 to 69334.4) | 93639.4 (84089.4 to 103571.1) | -- | -- | -- |
| Vitamin A deficiency complications | 293.0 (193.9 to 416.1) | -- | 18.5 (10.8 to 29.2) | 12.8* (3.3 to 22.4) | 10.7* (1.3 to 20.0) |
| Vitamin A deficiency with anemia | -- | -- | -- | -- | -- |
| Dietary iron deficiency | 101038.3 (95083.8 to 108083.4) | -- | 2329.6 (1529.4 to 3498.5) | -9.1* (-15.9 to -1.2) | -10.8* (-17.5 to -3.1) |
| Other nutritional deficiencies | -- | -- | 13.8 (8.7 to 19.7) | -3.9 (-11.1 to 3.9) | -5.7 (-12.8 to 1.9) |
| **Non-communicable diseases** | **561160.7 (552315.9 to 569688.8)** | **823019.4 (760173.9 to 890651.8)** | **39811.9 (29525.2 to 51509.9)** | **1.7* (1.1 to 2.3)** | **-0.3 (-0.9 to 0.3)** |
| **Neoplasms** | **1199.0 (1125.5 to 1288.5)** | **162.9 (135.7 to 208.3)** | **72.4 (51.4 to 97.3)** | **15.0* (7.7 to 24.0)** | **12.8* (5.7 to 21.6)** |
| Lip and oral cavity cancer | 23.9 (19.5 to 30.9) | 4.6 (3.7 to 5.9) | 2.1 (1.4 to 3.1) | 25.1 (-2.0 to 64.1) | 22.7 (-3.9 to 61.0) |
| Diagnosis and primary therapy phase of mouth cancer | 1.3 (1.1 to 1.7) | 4.6 (3.7 to 5.9) | 0.4 (0.2 to 0.5) | 28.3* (3.9 to 60.4) | 25.8* (1.9 to 57.3) |
| Controlled phase of mouth cancer | 21.0 (17.0 to 27.1) | -- | 1.0 (0.6 to 1.7) | 28.9 (-7.8 to 80.8) | 26.4 (-9.5 to 77.3) |
| Metastatic phase of mouth cancer | 1.4 (1.2 to 1.8) | -- | 0.6 (0.4 to 0.9) | 19.1 (-3.5 to 48.8) | 16.8 (-5.4 to 45.9) |
| Terminal phase of mouth cancer | 0.2 (0.2 to 0.3) | -- | 0.1 (0.1 to 0.2) | 17.3 (-5.5 to 47.2) | 15.0 (-7.3 to 44.4) |
| Nasopharynx cancer | 11.5 (10.2 to 12.8) | 1.9 (1.7 to 2.2) | 1.0 (0.7 to 1.4) | -8.5 (-19.7 to 4.5) | -10.3 (-21.3 to 2.5) |
| Diagnosis and primary therapy phase of nasopharynx cancer | 0.6 (0.6 to 0.7) | 1.9 (1.7 to 2.2) | 0.2 (0.1 to 0.3) | -7.5 (-19.0 to 5.9) | -9.3 (-20.5 to 3.9) |
| Controlled phase of nasopharynx cancer | 10.0 (8.9 to 11.2) | -- | 0.5 (0.3 to 0.8) | -7.8 (-19.3 to 5.9) | -9.6 (-20.8 to 3.8) |
| Metastatic phase of nasopharynx cancer | 0.7 (0.6 to 0.8) | -- | 0.3 (0.2 to 0.4) | -9.7 (-20.9 to 3.3) | -11.4 (-22.4 to 1.3) |
| Terminal phase of nasopharynx cancer | 0.1 (0.1 to 0.1) | -- | 0.0 (0.0 to 0.1) | -12.6* (-23.3 to -0.5) | -14.3* (-24.8 to -2.4) |
| Other pharynx cancer | 2.0 (1.6 to 2.3) | 0.8 (0.7 to 1.0) | 0.3 (0.2 to 0.4) | 3.4 (-6.8 to 15.5) | 1.4 (-8.6 to 13.3) |
| Diagnosis and primary therapy phase of other pharynx cancer | 0.1 (0.1 to 0.1) | 0.8 (0.7 to 1.0) | -- | 5.8 (-4.1 to 17.6) | 3.7 (-5.9 to 15.3) |
| Controlled phase of other pharynx cancer | 1.5 (1.2 to 1.8) | -- | 0.1 (0.0 to 0.1) | 6.2 (-3.7 to 17.6) | 4.1 (-5.6 to 15.3) |
| Metastatic phase of other pharynx cancer | 0.3 (0.3 to 0.4) | -- | 0.1 (0.1 to 0.2) | 1.8 (-8.7 to 14.6) | -0.1 (-10.4 to 12.4) |
| Terminal phase of other pharynx cancer | 0.1 (0.0 to 0.1) | -- | -- | 1.6 (-9.0 to 14.3) | -0.4 (-10.7 to 12.2) |
| Oesophageal cancer | 1.5 (1.4 to 1.6) | 0.7 (0.6 to 0.7) | 0.2 (0.2 to 0.3) | -13.7* (-19.1 to -7.4) | -15.4* (-20.6 to -9.1) |
| Diagnosis and primary therapy phase of esophageal cancer | 0.1 (0.1 to 0.1) | 0.7 (0.6 to 0.7) | -- | -12.3* (-17.7 to -5.7) | -14.0* (-19.2 to -7.5) |
| Controlled phase of esophageal cancer | 1.1 (1.0 to 1.2) | -- | 0.1 (0.0 to 0.1) | -11.5* (-17.0 to -5.0) | -13.2* (-18.6 to -6.8) |
| Metastatic phase of esophageal cancer | 0.2 (0.2 to 0.3) | -- | 0.1 (0.1 to 0.1) | -15.0* (-20.4 to -8.4) | -16.6* (-21.9 to -10.2) |
| Terminal phase of esophageal cancer | 0.0 (0.0 to 0.1) | -- | -- | -15.0* (-20.4 to -8.4) | -16.6* (-21.9 to -10.2) |
| Stomach cancer | 7.2 (6.8 to 7.6) | 2.8 (2.7 to 3.0) | 0.9 (0.7 to 1.2) | -12.7* (-17.2 to -8.0) | -14.3* (-18.7 to -9.8) |
| Diagnosis and primary therapy phase of stomach cancer | 0.6 (0.6 to 0.7) | 2.8 (2.7 to 3.0) | 0.2 (0.1 to 0.3) | -8.7* (-13.5 to -3.9) | -10.5* (-15.2 to -5.7) |
| Controlled phase of stomach cancer | 5.6 (5.3 to 5.9) | -- | 0.3 (0.2 to 0.4) | -1.9 (-7.4 to 3.7) | -3.8 (-9.1 to 1.7) |
| Metastatic phase of stomach cancer | 0.8 (0.7 to 0.8) | -- | 0.3 (0.2 to 0.5) | -19.3* (-23.3 to -15.6) | -20.9* (-24.8 to -17.2) |
| Terminal phase of stomach cancer | 0.2 (0.2 to 0.2) | -- | 0.1 (0.1 to 0.1) | -19.3* (-23.3 to -15.6) | -20.9* (-24.8 to -17.2) |
| Colon and rectum cancer | 24.3 (22.4 to 25.8) | 4.5 (4.1 to 4.7) | 2.5 (1.7 to 3.4) | 4.9 (-3.6 to 13.1) | 2.9 (-5.4 to 11.0) |
| Diagnosis and primary therapy phase of colon and rectum cancers | 1.0 (0.9 to 1.1) | 4.5 (4.1 to 4.7) | 0.3 (0.2 to 0.4) | 9.0* (0.3 to 17.3) | 6.9 (-1.7 to 15.0) |
| Controlled phase of colon and rectum cancers | 21.6 (19.9 to 22.9) | -- | 1.5 (1.0 to 2.1) | 6.5 (-2.2 to 14.6) | 4.4 (-4.1 to 12.4) |
| Metastatic phase of colon and rectum cancers | 1.3 (1.2 to 1.4) | -- | 0.6 (0.4 to 0.8) | 1.0 (-8.1 to 9.6) | -1.0 (-9.9 to 7.5) |
| Terminal phase of colon and rectum cancers | 0.2 (0.2 to 0.2) | -- | 0.1 (0.1 to 0.1) | -1.5 (-10.7 to 7.4) | -3.4 (-12.4 to 5.3) |
| Stoma from colon and rectum cancers, beyond 10 years | 0.2 (0.1 to 0.3) | -- | -- | -5.9* (-8.5 to -3.7) | -7.7* (-10.2 to -5.6) |
| Liver cancer | -- | 3.0 (2.7 to 3.3) | 0.7 (0.5 to 0.9) | -10.6* (-19.1 to -1.2) | -12.3* (-20.7 to -3.0) |
| Liver cancer due to hepatitis B | 2.7 (2.4 to 3.0) | 2.1 (1.9 to 2.4) | 0.5 (0.3 to 0.7) | -12.8* (-21.3 to -2.9) | -14.5* (-22.8 to -4.8) |
| Diagnosis and primary therapy phase of liver cancer due to hepatitis B | 0.5 (0.5 to 0.6) | 2.1 (1.9 to 2.4) | 0.1 (0.1 to 0.2) | -11.0 (-20.0 to 0.2) | -12.7* (-21.5 to -1.7) |
| Controlled phase of liver cancer due to hepatitis B | 1.6 (1.5 to 1.7) | -- | 0.1 (0.0 to 0.1) | -19.5* (-25.7 to -12.3) | -21.0* (-27.1 to -14.0) |
| Metastatic phase of liver cancer due to hepatitis B | 0.4 (0.4 to 0.5) | -- | 0.2 (0.1 to 0.3) | -11.7* (-20.7 to -0.8) | -13.4* (-22.2 to -2.7) |
| Terminal phase of liver cancer due to hepatitis B | 0.2 (0.1 to 0.2) | -- | 0.1 (0.1 to 0.1) | -11.7* (-20.7 to -0.8) | -13.4* (-22.2 to -2.7) |
| Liver cancer due to hepatitis C | 0.2 (0.1 to 0.2) | 0.1 (0.1 to 0.2) | -- | -4.1 (-14.2 to 9.0) | -6.0 (-15.9 to 7.0) |
| Diagnosis and primary therapy phase of liver cancer due to hepatitis C | -- | 0.1 (0.1 to 0.2) | -- | -2.5 (-12.9 to 11.1) | -4.4 (-14.6 to 8.9) |
| Controlled phase of liver cancer due to hepatitis C | 0.1 (0.1 to 0.1) | -- | -- | -10.9 (-20.2 to 1.3) | -12.6* (-21.7 to -0.6) |
| Metastatic phase of liver cancer due to hepatitis C | -- | -- | -- | -3.0 (-13.4 to 10.5) | -4.8 (-15.0 to 8.4) |
| Terminal phase of liver cancer due to hepatitis C | -- | -- | -- | -3.0 (-13.4 to 10.5) | -4.8 (-15.0 to 8.4) |
| Liver cancer due to alcohol use | 0.1 (0.1 to 0.2) | 0.1 (0.1 to 0.1) | -- | 1.1 (-9.6 to 10.0) | -0.9 (-11.3 to 7.9) |
| Diagnosis and primary therapy phase of liver cancer due to alcohol use | -- | 0.1 (0.1 to 0.1) | -- | 2.3 (-8.3 to 11.6) | 0.3 (-10.0 to 9.5) |
| Controlled phase of liver cancer due to alcohol use | 0.1 (0.1 to 0.1) | -- | -- | -4.7 (-14.7 to 3.9) | -6.5 (-16.3 to 1.9) |
| Metastatic phase of liver cancer due to alcohol use | -- | -- | -- | 2.0 (-8.7 to 11.3) | 0.1 (-10.5 to 9.1) |

| | | | | | |
|---|---|---|---|---|---|
| Terminal phase of liver cancer due to alcohol use | -- | -- | -- | 2.0 (-8.7 to 11.3) | 0.1 (-10.5 to 9.1) |
| Liver cancer due to NASH | 0.3 (0.2 to 0.3) | 0.2 (0.2 to 0.3) | 0.1 (0.0 to 0.1) | 0.9 (-8.9 to 10.2) | -1.0 (-10.6 to 8.1) |
| Diagnosis and primary therapy phase of liver cancer due to NASH | 0.1 (0.0 to 0.1) | 0.2 (0.2 to 0.3) | -- | 2.2 (-8.0 to 11.8) | 0.3 (-9.7 to 9.6) |
| Controlled phase of liver cancer due to NASH | 0.2 (0.1 to 0.2) | -- | -- | -4.2 (-13.0 to 5.2) | -6.0 (-14.6 to 3.2) |
| Metastatic phase of liver cancer due to NASH | 0.0 (0.0 to 0.1) | -- | -- | 1.8 (-8.4 to 11.3) | -0.2 (-10.1 to 9.2) |
| Terminal phase of liver cancer due to NASH | -- | -- | -- | 1.8 (-8.4 to 11.3) | -0.2 (-10.1 to 9.2) |
| Liver cancer due to other causes | 0.6 (0.5 to 0.6) | 0.4 (0.4 to 0.5) | 0.1 (0.1 to 0.1) | -9.1 (-17.3 to 0.7) | -10.8* (-18.9 to -1.3) |
| Diagnosis and primary therapy phase of liver cancer due to other causes | 0.1 (0.1 to 0.1) | 0.4 (0.4 to 0.5) | -- | -7.3 (-16.2 to 3.2) | -9.1 (-17.8 to 1.3) |
| Controlled phase of liver cancer due to other causes | 0.3 (0.3 to 0.4) | -- | -- | -15.6* (-22.6 to -7.2) | -17.2* (-24.1 to -9.0) |
| Metastatic phase of liver cancer due to other causes | 0.1 (0.1 to 0.1) | -- | 0.0 (0.0 to 0.1) | -8.0 (-16.8 to 2.5) | -9.7 (-18.4 to 0.5) |
| Terminal phase of liver cancer due to other causes | -- | -- | -- | -8.0 (-16.8 to 2.5) | -9.7 (-18.4 to 0.5) |
| Gallbladder and biliary tract cancer | 0.4 (0.3 to 0.4) | 0.3 (0.2 to 0.3) | 0.1 (0.0 to 0.1) | -4.6 (-13.7 to 7.0) | -6.4 (-15.3 to 4.9) |
| Diagnosis and primary therapy phase of gallbladder and biliary tract cancer | 0.0 (0.0 to 0.1) | 0.3 (0.2 to 0.3) | -- | -4.1 (-13.0 to 7.2) | -5.9 (-14.6 to 5.1) |
| Controlled phase of gallbladder and biliary tract cancer | 0.2 (0.2 to 0.3) | -- | -- | 0.6 (-7.4 to 10.0) | -1.3 (-9.2 to 7.9) |
| Metastatic phase of gallbladder and biliary tract cancer | 0.1 (0.1 to 0.1) | -- | -- | -5.9 (-15.4 to 6.2) | -7.7 (-17.0 to 4.2) |
| Terminal phase of gallbladder and biliary tract cancer | -- | -- | -- | -6.0 (-15.4 to 6.2) | -7.8 (-17.0 to 4.2) |
| Pancreatic cancer | 0.5 (0.5 to 0.5) | 0.4 (0.4 to 0.4) | 0.1 (0.1 to 0.1) | -3.0 (-7.7 to 3.4) | -4.9 (-9.5 to 1.4) |
| Diagnosis and primary therapy phase of pancreatic cancer | 0.1 (0.1 to 0.1) | 0.4 (0.4 to 0.4) | -- | -3.0 (-7.7 to 3.6) | -4.8 (-9.5 to 1.6) |
| Controlled phase of pancreatic cancer | 0.3 (0.3 to 0.3) | -- | -- | -4.5 (-8.8 to 1.3) | -6.3* (-10.6 to -0.7) |
| Metastatic phase of pancreatic cancer | 0.1 (0.1 to 0.1) | -- | 0.0 (0.0 to 0.1) | -2.6 (-7.5 to 4.0) | -4.5 (-9.3 to 2.0) |
| Terminal phase of pancreatic cancer | -- | -- | -- | -2.6 (-7.5 to 4.0) | -4.5 (-9.3 to 2.0) |
| Larynx cancer | 3.0 (2.8 to 3.3) | 0.4 (0.4 to 0.5) | 0.3 (0.2 to 0.4) | -2.6 (-12.8 to 8.3) | -4.5 (-14.5 to 6.2) |
| Diagnosis and primary therapy phase of larynx cancer | 0.1 (0.1 to 0.2) | 0.4 (0.4 to 0.5) | 0.0 (0.0 to 0.1) | -0.0 (-11.5 to 12.3) | -1.9 (-13.2 to 10.1) |
| Controlled phase of larynx cancer | 2.0 (1.8 to 2.2) | -- | 0.1 (0.1 to 0.2) | -1.0 (-11.2 to 10.0) | -2.9 (-12.9 to 7.9) |
| Metastatic phase of larynx cancer | 0.2 (0.1 to 0.2) | -- | 0.1 (0.0 to 0.1) | -6.1 (-19.5 to 8.6) | -7.9 (-21.0 to 6.5) |
| Terminal phase of larynx cancer | -- | -- | -- | -8.1 (-21.7 to 7.0) | -9.8 (-23.2 to 5.0) |
| Laryngectomy from larynx cancer, beyond 10 years | 0.7 (0.7 to 0.8) | -- | 0.0 (0.0 to 0.1) | -1.2* (-1.8 to -0.7) | -3.1* (-3.7 to -2.6) |
| Tracheal, bronchus, and lung cancer | 3.0 (2.9 to 3.2) | 2.1 (2.0 to 2.2) | 0.6 (0.4 to 0.7) | -6.9 (-13.0 to 1.0) | -8.7* (-14.6 to -1.0) |
| Diagnosis and primary therapy phase of lung, bronchus, and trachea cancer | 0.2 (0.2 to 0.2) | 2.1 (2.0 to 2.2) | 0.1 (0.0 to 0.1) | -5.0 (-11.2 to 2.9) | -6.8 (-12.9 to 0.9) |
| Controlled phase of lung, bronchus, and trachea cancer | 1.9 (1.8 to 2.1) | -- | 0.1 (0.1 to 0.1) | -2.4 (-8.6 to 5.3) | -4.3 (-10.3 to 3.3) |
| Metastatic phase of lung, bronchus, and trachea cancer | 0.7 (0.7 to 0.8) | -- | 0.3 (0.2 to 0.4) | -8.1* (-14.1 to -0.2) | -9.8* (-15.7 to -2.2) |
| Terminal phase of lung, bronchus, and trachea cancer | 0.2 (0.2 to 0.2) | -- | 0.1 (0.1 to 0.1) | -8.1* (-14.1 to -0.2) | -9.8* (-15.7 to -2.2) |
| Malignant skin melanoma | 42.3 (36.8 to 57.8) | 4.9 (4.3 to 6.7) | 2.4 (1.5 to 3.7) | 8.4 (-1.0 to 21.5) | 6.3 (-2.9 to 19.1) |
| Diagnosis and primary therapy phase of malignant skin melanoma | 1.1 (1.0 to 1.5) | 4.9 (4.3 to 6.7) | 0.3 (0.2 to 0.5) | 9.0* (1.3 to 20.1) | 6.9 (-0.6 to 17.8) |
| Controlled phase of malignant skin melanoma | 40.9 (35.5 to 55.8) | -- | 2.0 (1.2 to 3.1) | 8.8 (-1.7 to 22.9) | 6.7 (-3.6 to 20.5) |
| Metastatic phase of malignant skin melanoma | 0.3 (0.3 to 0.4) | -- | 0.1 (0.1 to 0.2) | 2.6 (-6.1 to 13.2) | 0.7 (-7.9 to 11.1) |
| Terminal phase of malignant skin melanoma | 0.1 (0.0 to 0.1) | -- | -- | 2.3 (-7.1 to 13.1) | 0.3 (-8.8 to 11.0) |
| Non-melanoma skin cancer | -- | 37.3 (11.1 to 82.8) | 0.1 (0.0 to 0.3) | 23.8* (16.2 to 29.1) | 21.5* (14.0 to 26.7) |
| Non-melanoma skin cancer (squamous-cell carcinoma) | 2.1 (0.6 to 5.6) | 2.6 (0.8 to 6.4) | 0.1 (0.0 to 0.3) | 25.2* (17.3 to 30.7) | 22.8* (15.0 to 28.2) |
| Non-melanoma skin cancer (basal-cell carcinoma) | 3.4 (0.8 to 10.9) | 34.7 (8.6 to 78.5) | 0.0 (0.0 to 0.1) | 15.4* (3.7 to 20.3) | 13.2* (1.7 to 18.0) |
| Breast cancer | 48.9 (41.8 to 56.6) | 5.8 (4.9 to 6.8) | 3.9 (2.5 to 5.6) | 20.8* (2.8 to 43.8) | 18.5* (0.9 to 41.0) |
| Diagnosis and primary therapy phase of breast cancer | 1.3 (1.1 to 1.5) | 5.8 (4.9 to 6.8) | 0.4 (0.2 to 0.5) | 23.5* (7.6 to 42.5) | 21.1* (5.6 to 39.8) |
| Controlled phase of breast cancer | 44.8 (38.1 to 51.9) | -- | 2.7 (1.7 to 4.2) | 21.4* (1.0 to 48.3) | 19.1 (-1.0 to 45.4) |
| Metastatic phase of breast cancer | 1.5 (1.2 to 1.7) | -- | 0.7 (0.4 to 0.9) | 19.8* (3.6 to 39.6) | 17.5* (1.6 to 36.9) |
| Terminal phase of breast cancer | 0.1 (0.1 to 0.1) | -- | 0.1 (0.0 to 0.1) | 16.1 (-1.5 to 37.2) | 13.9 (-3.3 to 34.6) |
| Mastectomy from breast cancer, beyond 10 years | 1.2 (1.1 to 1.4) | -- | 0.0 (0.0 to 0.1) | -8.1* (-9.7 to -6.7) | -9.9* (-11.4 to -8.5) |
| Cervical cancer | 53.0 (47.2 to 59.2) | 6.8 (6.0 to 7.6) | 3.9 (2.6 to 5.3) | -10.8 (-21.1 to 1.1) | -12.5* (-22.6 to -0.9) |
| Diagnosis and primary therapy phase of cervical cancer | 2.7 (2.4 to 3.0) | 6.8 (6.0 to 7.6) | 0.8 (0.5 to 1.1) | -10.5* (-18.1 to -0.1) | -12.2* (-19.7 to -2.0) |
| Controlled phase of cervical cancer | 48.6 (43.3 to 54.3) | -- | 2.3 (1.4 to 3.6) | -9.9 (-22.9 to 5.4) | -11.6 (-24.4 to 3.4) |
| Metastatic phase of cervical cancer | 1.5 (1.3 to 1.7) | -- | 0.7 (0.4 to 0.9) | -13.7* (-21.3 to -3.4) | -15.3* (-22.8 to -5.3) |
| Terminal phase of cervical cancer | 0.2 (0.1 to 0.2) | -- | 0.1 (0.1 to 0.1) | -14.9* (-22.5 to -4.8) | -16.5* (-24.0 to -6.6) |
| Uterine cancer | 7.2 (6.6 to 8.2) | 0.9 (0.8 to 1.0) | 0.5 (0.3 to 0.7) | -5.6 (-16.1 to 8.6) | -7.4 (-17.7 to 6.5) |
| Diagnosis and primary therapy phase of uterine cancer | 0.3 (0.3 to 0.4) | 0.9 (0.8 to 1.0) | 0.1 (0.1 to 0.1) | -5.1 (-13.6 to 7.6) | -6.9 (-15.2 to 5.5) |
| Controlled phase of uterine cancer | 6.7 (6.1 to 7.6) | -- | 0.3 (0.2 to 0.5) | -5.0 (-17.2 to 9.8) | -6.8 (-18.8 to 7.7) |
| Metastatic phase of uterine cancer | 0.1 (0.1 to 0.2) | -- | 0.1 (0.0 to 0.1) | -8.5 (-17.7 to 5.6) | -10.3 (-19.3 to 3.5) |
| Terminal phase of uterine cancer | -- | -- | -- | -10.9 (-20.2 to 4.6) | -12.6 (-21.7 to 2.6) |
| Ovarian cancer | 48.2 (42.2 to 58.6) | 6.8 (5.9 to 8.3) | 5.6 (3.8 to 8.1) | 13.4 (-8.9 to 45.7) | 11.3 (-10.7 to 42.9) |
| Diagnosis and primary therapy phase of ovarian cancer | 1.8 (1.6 to 2.2) | 6.8 (5.9 to 8.3) | 0.5 (0.3 to 0.8) | 14.1 (-1.4 to 36.4) | 11.9 (-3.3 to 33.8) |
| Controlled phase of ovarian cancer | 39.3 (34.5 to 47.7) | -- | 1.9 (1.1 to 2.9) | 14.1 (-7.2 to 44.5) | 11.9 (-9.0 to 41.7) |
| Metastatic phase of ovarian cancer | 6.8 (5.9 to 8.3) | -- | 3.0 (1.9 to 4.6) | 12.9 (-18.2 to 61.0) | 10.8 (-19.7 to 57.9) |

| | | | | | |
|---|---|---|---|---|---|
| Terminal phase of ovarian cancer | 0.3 (0.2 to 0.3) | -- | 0.1 (0.1 to 0.2) | 12.8 (-3.1 to 36.1) | 10.6 (-4.9 to 33.5) |
| Prostate cancer | 8.6 (7.4 to 10.8) | 1.0 (0.8 to 1.2) | 0.7 (0.5 to 1.1) | 22.4* (17.1 to 30.2) | 20.1* (14.9 to 27.7) |
| Diagnosis and primary therapy phase of prostate cancer | 0.3 (0.3 to 0.4) | 1.0 (0.8 to 1.2) | 0.1 (0.1 to 0.1) | 23.5* (17.8 to 31.8) | 21.1* (15.6 to 29.2) |
| Controlled phase of prostate cancer | 8.3 (7.1 to 10.3) | -- | 0.6 (0.4 to 1.0) | 23.0* (17.7 to 31.0) | 20.7* (15.4 to 28.5) |
| Metastatic phase of prostate cancer | 0.1 (0.1 to 0.1) | -- | -- | 9.7* (5.0 to 15.5) | 7.6* (3.0 to 13.3) |
| Terminal phase of prostate cancer | -- | -- | -- | 3.0 (-1.4 to 8.5) | 1.0 (-3.3 to 6.5) |
| Impotence and Incontinence after 10-year survival from prostate cancer | -- | -- | -- | -- | -- |
| Testicular cancer | 78.2 (72.8 to 84.9) | 9.3 (8.7 to 10.1) | 4.8 (3.2 to 6.7) | 16.3* (6.2 to 28.1) | 14.1* (4.1 to 25.6) |
| Diagnosis and primary therapy phase of testicular cancer | 2.5 (2.4 to 2.8) | 9.3 (8.7 to 10.1) | 0.7 (0.5 to 1.0) | 17.7* (7.5 to 29.3) | 15.4* (5.5 to 26.8) |
| Controlled phase of testicular cancer | 74.7 (69.6 to 81.1) | -- | 3.6 (2.2 to 5.4) | 18.2* (6.9 to 32.3) | 15.9* (4.8 to 29.8) |
| Metastatic phase of testicular cancer | 0.9 (0.8 to 1.0) | -- | 0.4 (0.3 to 0.5) | 2.6 (-4.7 to 10.7) | 0.7 (-6.5 to 8.6) |
| Terminal phase of testicular cancer | 0.1 (0.1 to 0.1) | -- | 0.1 (0.0 to 0.1) | -5.9 (-13.3 to 3.2) | -7.7 (-14.9 to 1.2) |
| Kidney cancer | 17.4 (16.0 to 18.4) | 2.5 (2.3 to 2.7) | 1.0 (0.7 to 1.5) | -0.5 (-6.9 to 5.9) | -2.4 (-8.7 to 3.8) |
| Diagnosis and primary therapy phase of kidney cancer | -- | 2.5 (2.3 to 2.7) | -- | -- | -- |
| Controlled phase of kidney cancer | 17.0 (15.6 to 17.9) | -- | 0.8 (0.5 to 1.2) | -0.4 (-6.9 to 5.9) | -2.3 (-8.7 to 3.9) |
| Metastatic phase of kidney cancer | 0.4 (0.4 to 0.4) | -- | 0.2 (0.1 to 0.2) | -0.7 (-6.9 to 5.6) | -2.6 (-8.7 to 3.5) |
| Terminal phase of kidney cancer | 0.1 (0.1 to 0.1) | -- | 0.0 (0.0 to 0.1) | -0.7 (-6.9 to 5.6) | -2.6 (-8.7 to 3.6) |
| Bladder cancer | 8.0 (7.1 to 8.8) | 1.0 (0.9 to 1.1) | 0.7 (0.5 to 1.0) | 8.0 (-3.4 to 20.8) | 5.9 (-5.3 to 18.5) |
| Diagnosis and primary therapy phase of bladder cancer | 0.4 (0.4 to 0.5) | 1.0 (0.9 to 1.1) | 0.1 (0.1 to 0.2) | 9.6* (0.8 to 19.9) | 7.5 (-1.1 to 17.6) |
| Controlled phase of bladder cancer | 7.4 (6.6 to 8.1) | -- | 0.5 (0.3 to 0.7) | 8.5 (-4.6 to 24.5) | 6.4 (-6.4 to 22.1) |
| Metastatic phase of bladder cancer | 0.1 (0.1 to 0.2) | -- | 0.1 (0.0 to 0.1) | 3.9 (-4.9 to 14.8) | 1.9 (-6.8 to 12.6) |
| Terminal phase of bladder cancer | -- | -- | -- | 3.2 (-5.6 to 14.2) | 1.3 (-7.4 to 12.0) |
| Incontinence from bladder cancer, beyond 10 years | 0.1 (0.1 to 0.1) | -- | -- | 0.2 (-1.0 to 1.4) | -1.7* (-2.9 to -0.5) |
| Brain and nervous system cancer | 58.9 (48.9 to 71.2) | 11.3 (9.4 to 13.3) | 5.3 (3.6 to 7.3) | 23.1* (9.7 to 38.7) | 20.8* (7.6 to 36.0) |
| Diagnosis and primary therapy phase of brain and nervous system cancers | 3.5 (2.9 to 4.2) | 11.3 (9.4 to 13.3) | 1.0 (0.7 to 1.5) | 29.9* (16.6 to 44.8) | 27.4* (14.4 to 42.0) |
| Controlled phase of brain and nervous system cancers | 51.5 (42.7 to 62.6) | -- | 2.5 (1.5 to 3.8) | 34.3* (15.3 to 56.1) | 31.7* (13.1 to 53.1) |
| Metastatic phase of brain and nervous system cancers | 3.4 (2.8 to 3.8) | -- | 1.5 (1.0 to 2.1) | 8.3 (-1.0 to 19.2) | 6.2 (-2.9 to 16.9) |
| Terminal phase of brain and nervous system cancers | 0.6 (0.5 to 0.6) | -- | 0.3 (0.2 to 0.4) | 5.4 (-3.6 to 15.8) | 3.4 (-5.4 to 13.6) |
| Thyroid cancer | 54.2 (45.9 to 69.6) | 6.1 (5.2 to 7.8) | 3.1 (2.0 to 4.7) | 14.4 (-5.8 to 44.7) | 12.2 (-7.6 to 41.9) |
| Diagnosis and primary therapy phase of thyroid cancer | 1.4 (1.2 to 1.8) | 6.1 (5.2 to 7.8) | 0.4 (0.3 to 0.6) | 15.0 (-1.2 to 40.7) | 12.8 (-3.1 to 38.0) |
| Controlled phase of thyroid cancer | 52.3 (44.3 to 67.2) | -- | 2.5 (1.5 to 4.0) | 15.2 (-7.1 to 47.4) | 13.0 (-8.9 to 44.6) |
| Metastatic phase of thyroid cancer | 0.4 (0.3 to 0.5) | -- | 0.2 (0.1 to 0.3) | 4.1 (-10.8 to 28.6) | 2.1 (-12.5 to 26.1) |
| Terminal phase of thyroid cancer | 0.0 (0.0 to 0.1) | -- | -- | 0.9 (-14.1 to 26.7) | -1.0 (-15.7 to 24.3) |
| Mesothelioma | 0.5 (0.4 to 0.6) | 0.2 (0.2 to 0.3) | 0.1 (0.1 to 0.1) | -10.0 (-19.5 to 0.3) | -11.7* (-21.1 to -1.6) |
| Diagnosis and primary therapy phase of mesothelioma | 0.0 (0.0 to 0.1) | 0.2 (0.2 to 0.3) | -- | -9.3 (-18.5 to 0.4) | -11.0* (-20.1 to -1.5) |
| Controlled phase of mesothelioma | 0.3 (0.3 to 0.4) | -- | -- | -10.1 (-19.7 to 0.1) | -11.8* (-21.3 to -1.8) |
| Metastatic phase of mesothelioma | 0.1 (0.1 to 0.1) | -- | 0.1 (0.0 to 0.1) | -10.1 (-19.6 to 0.3) | -11.8* (-21.2 to -1.6) |
| Terminal phase of mesothelioma | -- | -- | -- | -10.4 (-20.2 to 0.2) | -12.1* (-21.7 to -1.7) |
| Hodgkin lymphoma | 73.7 (64.7 to 91.5) | 10.1 (8.9 to 12.4) | 5.3 (3.6 to 7.4) | -4.7 (-12.2 to 3.5) | -6.6 (-13.9 to 1.5) |
| Diagnosis and primary therapy phase of Hodgkin disease | 2.4 (2.1 to 3.0) | 10.1 (8.9 to 12.4) | 0.7 (0.5 to 1.0) | -3.8 (-10.9 to 4.6) | -5.7 (-12.6 to 2.6) |
| Controlled phase of Hodgkin disease | 68.5 (60.1 to 85.1) | -- | 3.3 (2.0 to 5.0) | -3.7 (-12.6 to 6.2) | -5.5 (-14.3 to 4.1) |
| Metastatic phase of Hodgkin disease | 2.5 (2.2 to 3.1) | -- | 1.1 (0.8 to 1.6) | -7.6* (-14.1 to -0.4) | -9.4* (-15.8 to -2.3) |
| Terminal phase of Hodgkin disease | 0.2 (0.2 to 0.3) | -- | 0.1 (0.1 to 0.2) | -9.9* (-17.7 to -2.2) | -11.7* (-19.3 to -4.1) |
| Non-Hodgkin's lymphoma | 34.4 (32.5 to 36.7) | 7.5 (7.0 to 8.1) | 3.0 (2.1 to 4.0) | 7.0 (-1.0 to 16.1) | 5.0 (-2.9 to 13.9) |
| Diagnosis and primary therapy phase of non-Hodgkin lymphoma | 1.2 (1.1 to 1.3) | 7.5 (7.0 to 8.1) | 0.3 (0.2 to 0.5) | 10.1* (3.6 to 17.4) | 8.0* (1.6 to 15.2) |
| Controlled phase of non-Hodgkin lymphoma | 30.8 (29.1 to 32.8) | -- | 1.5 (1.0 to 2.2) | 10.1 (-0.0 to 22.9) | 8.0 (-1.9 to 20.6) |
| Metastatic phase of non-Hodgkin lymphoma | 2.1 (1.9 to 2.2) | -- | 0.9 (0.6 to 1.2) | 2.4 (-5.2 to 11.4) | 0.5 (-7.0 to 9.3) |
| Terminal phase of non-Hodgkin lymphoma | 0.4 (0.3 to 0.4) | -- | 0.2 (0.1 to 0.3) | 1.7 (-6.1 to 10.8) | -0.2 (-7.9 to 8.7) |
| Multiple myeloma | 2.0 (1.7 to 2.2) | 0.5 (0.4 to 0.6) | 0.4 (0.3 to 0.5) | 26.8* (8.5 to 40.8) | 24.4* (6.4 to 38.1) |
| Diagnosis and primary therapy phase of multiple myeloma | 0.1 (0.1 to 0.1) | 0.5 (0.4 to 0.6) | 0.0 (0.0 to 0.1) | 32.4* (13.4 to 47.2) | 29.9* (11.3 to 44.4) |
| Controlled phase of multiple myeloma | 1.2 (1.0 to 1.4) | -- | 0.1 (0.0 to 0.1) | 32.6* (13.6 to 47.4) | 30.1* (11.4 to 44.6) |
| Metastatic phase of multiple myeloma | 0.6 (0.5 to 0.7) | -- | 0.3 (0.2 to 0.4) | 25.9* (7.8 to 40.0) | 23.5* (5.7 to 37.3) |
| Terminal phase of multiple myeloma | -- | -- | -- | 12.5 (-3.6 to 24.8) | 10.3 (-5.5 to 22.4) |
| Leukaemia | 49.9 (44.2 to 55.5) | 13.5 (12.0 to 15.0) | 5.8 (4.1 to 7.6) | -11.6* (-17.3 to -4.4) | -13.3* (-18.9 to -6.3) |
| Acute lymphoid leukaemia | 14.4 (12.8 to 16.2) | 3.6 (3.3 to 4.1) | 1.6 (1.2 to 2.2) | 0.1 (-8.6 to 8.1) | -1.8 (-10.4 to 6.1) |
| Diagnosis and primary therapy phase of acute lymphoid leukemia | 1.7 (1.5 to 1.9) | 3.6 (3.3 to 4.1) | 0.5 (0.3 to 0.7) | -1.1 (-9.7 to 6.8) | -3.0 (-11.4 to 4.7) |
| Controlled phase of acute lymphoid leukemia | 11.5 (10.2 to 12.9) | -- | 0.6 (0.4 to 0.8) | 0.8 (-8.1 to 8.7) | -1.2 (-9.9 to 6.6) |
| Metastatic phase of acute lymphoid leukemia | 1.0 (0.9 to 1.2) | -- | 0.5 (0.3 to 0.6) | 0.6 (-8.5 to 8.9) | -1.3 (-10.3 to 6.8) |
| Terminal phase of acute lymphoid leukemia | 0.2 (0.2 to 0.2) | -- | 0.1 (0.1 to 0.1) | 0.6 (-8.6 to 8.9) | -1.3 (-10.3 to 6.8) |
| Chronic lymphoid leukaemia | 6.3 (5.3 to 7.4) | 0.8 (0.7 to 1.0) | 0.7 (0.5 to 1.0) | 37.7* (19.4 to 59.8) | 35.0* (17.1 to 56.7) |

| | | | | | |
|---|---|---|---|---|---|
| Diagnosis and primary therapy phase of chronic lymphoid leukemia | 0.3 (0.3 to 0.4) | 0.8 (0.7 to 1.0) | 0.1 (0.1 to 0.1) | 37.7* (20.2 to 59.5) | 35.0* (17.9 to 56.5) |
| Controlled phase of chronic lymphoid leukemia | 5.1 (4.3 to 6.0) | -- | 0.2 (0.2 to 0.4) | 36.7* (17.9 to 60.6) | 34.1* (15.6 to 57.5) |
| Metastatic phase of chronic lymphoid leukemia | 0.8 (0.7 to 1.0) | -- | 0.4 (0.2 to 0.5) | 38.8* (19.7 to 61.3) | 36.2* (17.4 to 58.2) |
| Terminal phase of chronic lymphoid leukemia | -- | -- | -- | 24.4* (7.6 to 44.0) | 22.0* (5.5 to 41.3) |
| Acute myeloid leukaemia | 5.8 (5.2 to 6.6) | 3.8 (3.3 to 4.3) | 0.9 (0.7 to 1.3) | -1.0 (-10.4 to 7.4) | -2.9 (-12.1 to 5.4) |
| Diagnosis and primary therapy phase of acute myeloid leukemia | -- | 3.8 (3.3 to 4.3) | -- | -- | -- |
| Controlled phase of acute myeloid leukemia | 4.2 (3.8 to 4.8) | -- | 0.2 (0.1 to 0.3) | 0.3 (-9.6 to 9.4) | -1.6 (-11.4 to 7.3) |
| Metastatic phase of acute myeloid leukemia | 1.3 (1.1 to 1.5) | -- | 0.6 (0.4 to 0.8) | -1.4 (-10.7 to 7.0) | -3.3 (-12.5 to 5.0) |
| Terminal phase of acute myeloid leukemia | 0.3 (0.2 to 0.3) | -- | 0.2 (0.1 to 0.2) | -1.4 (-10.7 to 7.0) | -3.3 (-12.5 to 5.0) |
| Chronic myeloid leukaemia | 3.1 (2.7 to 3.5) | 0.9 (0.8 to 1.1) | 0.3 (0.2 to 0.5) | -16.9* (-24.0 to -7.6) | -18.5* (-25.5 to -9.4) |
| Diagnosis and primary therapy phase of chronic myeloid leukemia | 0.2 (0.2 to 0.2) | 0.9 (0.8 to 1.1) | 0.1 (0.0 to 0.1) | -15.3* (-22.2 to -7.0) | -17.0* (-23.7 to -8.8) |
| Controlled phase of chronic myeloid leukemia | 2.5 (2.2 to 2.9) | -- | 0.1 (0.1 to 0.2) | -15.2* (-21.9 to -6.9) | -16.8* (-23.4 to -8.6) |
| Metastatic phase of chronic myeloid leukemia | 0.3 (0.2 to 0.3) | -- | 0.1 (0.1 to 0.2) | -18.8* (-26.7 to -8.6) | -20.3* (-28.1 to -10.4) |
| Terminal phase of chronic myeloid leukemia | 0.1 (0.1 to 0.1) | -- | -- | -18.8* (-26.7 to -8.6) | -20.3* (-28.1 to -10.4) |
| Other leukaemia | 20.3 (16.8 to 22.9) | 4.2 (3.5 to 4.8) | 2.1 (1.5 to 2.9) | -29.3* (-35.6 to -19.4) | -30.6* (-36.9 to -21.0) |
| Metastatic phase of other leukemia | 2.6 (2.2 to 3.0) | -- | 1.2 (0.8 to 1.6) | -29.9* (-36.0 to -20.1) | -31.2* (-37.2 to -21.6) |
| Diagnosis and primary therapy phase of other leukemia | -- | 4.2 (3.5 to 4.8) | -- | -- | -- |
| Terminal phase of other leukemia | 0.2 (0.2 to 0.2) | -- | 0.1 (0.1 to 0.1) | -28.9* (-34.8 to -19.3) | -30.3* (-36.1 to -20.8) |
| Controlled phase of other leukemia | 17.5 (14.5 to 19.7) | -- | 0.9 (0.5 to 1.3) | -28.4* (-36.0 to -17.7) | -29.8* (-37.2 to -19.3) |
| Other malignant cancers | 197.8 (150.9 to 277.6) | 15.7 (14.3 to 17.0) | 16.0 (10.2 to 23.9) | 74.0* (37.6 to 128.1) | 70.7* (35.0 to 123.8) |
| Diagnosis and primary therapy phase of other malignant neoplasms | 4.6 (3.5 to 6.3) | 15.7 (14.3 to 17.0) | 1.3 (0.8 to 2.0) | 93.6* (49.8 to 158.3) | 89.9* (47.0 to 153.4) |
| Controlled phase of other malignant neoplasms | 180.1 (137.2 to 254.1) | -- | 8.7 (4.9 to 13.9) | 105.5* (57.3 to 180.3) | 101.5* (54.3 to 175.0) |
| Metastatic phase of other malignant neoplasms | 11.5 (9.3 to 14.8) | -- | 5.1 (3.2 to 7.4) | 41.0* (14.8 to 80.5) | 38.3* (12.6 to 77.1) |
| Terminal phase of other malignant neoplasms | 1.7 (1.4 to 2.1) | -- | 0.9 (0.6 to 1.2) | 35.4* (12.4 to 66.1) | 32.8* (10.3 to 62.9) |
| Other neoplasms | 689.7 (644.2 to 738.3) | 391.4 (368.9 to 413.1) | 1.0 (0.6 to 1.6) | -5.9 (-12.6 to 1.8) | -7.7* (-14.3 to -0.2) |
| Myelodysplastic, myeloproliferative, and other hematopoietic neoplasms | 21.3 (17.5 to 26.0) | 1.2 (0.7 to 1.8) | 1.0 (0.6 to 1.6) | -5.9 (-12.6 to 1.8) | -7.7* (-14.3 to -0.2) |
| Benign and in situ intestinal neoplasms | 24.7 (22.7 to 26.6) | 14.1 (13.1 to 15.2) | -- | -- | -- |
| Benign and in situ cervical and uterine neoplasms | 62.3 (59.0 to 65.9) | 31.0 (29.5 to 32.6) | -- | -- | -- |
| Other benign and in situ neoplasms | 611.5 (569.0 to 657.6) | 345.1 (323.3 to 365.8) | -- | -- | -- |
| **Cardiovascular diseases** | **6653.0 (6019.8 to 7490.8)** | **516.0 (462.8 to 572.0)** | **499.9 (356.0 to 674.8)** | **-4.6* (-7.7 to -1.9)** | **-6.4* (-9.5 to -3.8)** |
| Rheumatic heart disease | 3952.3 (3795.5 to 4112.9) | 154.5 (143.2 to 165.5) | 189.2 (122.1 to 275.1) | 5.5* (3.8 to 7.3) | 3.5* (1.8 to 5.3) |
| Rheumatic heart disease, without heart failure | 3938.6 (3782.2 to 4101.7) | 154.5 (143.2 to 165.5) | 188.0 (121.5 to 274.2) | 5.5* (3.8 to 7.4) | 3.5* (1.8 to 5.3) |
| Heart failure due to rheumatic heart disease | 13.7 (9.6 to 18.4) | -- | 1.3 (0.8 to 2.0) | 5.5* (3.9 to 7.3) | 3.5* (1.9 to 5.3) |
| Ischaemic heart disease | 302.2 (263.6 to 342.6) | 29.6 (12.2 to 56.7) | 17.1 (11.0 to 24.6) | -0.4 (-7.7 to 7.6) | -2.3 (-9.5 to 5.6) |
| Asymptomatic ischemic heart disease following myocardial infarction | 19.2 (11.1 to 29.2) | -- | -- | -- | -- |
| Angina due to ischemic heart disease | 268.0 (230.0 to 308.0) | -- | 15.7 (10.1 to 23.0) | -0.8 (-8.6 to 7.9) | -2.7 (-10.4 to 5.8) |
| Heart failure due to ischemic heart disease | 13.4 (9.5 to 18.3) | -- | 1.2 (0.7 to 1.9) | 5.4* (3.4 to 7.4) | 3.4* (1.4 to 5.4) |
| Myocardial infarction due to ischemic heart disease | 1.5 (0.6 to 3.1) | 29.6 (12.2 to 56.7) | 0.2 (0.1 to 0.3) | -0.1 (-5.0 to 2.4) | -2.0 (-6.9 to 0.5) |
| Stroke | 953.4 (827.6 to 1087.7) | 54.9‡ (39.6 to 76.0) | 187.6 (132.4 to 245.2) | -16.4* (-21.1 to -11.7) | -18.0* (-22.6 to -13.4) |
| Ischaemic stroke | 754.9 (629.4 to 899.6) | 26.7 (13.6 to 46.3) | 112.2 (77.4 to 151.0) | -16.1* (-23.0 to -9.3) | -17.7* (-24.4 to -11.0) |
| Acute ischemic stroke | 2.1 (1.0 to 3.8) | 26.7 (13.6 to 46.3) | 0.6 (0.3 to 1.1) | 4.6 (-1.0 to 9.3) | 2.6 (-2.9 to 7.2) |
| Chronic ischemic stroke | 752.8 (627.2 to 897.0) | -- | 111.6 (77.0 to 150.4) | -16.2* (-23.1 to -9.3) | -17.8* (-24.5 to -11.0) |
| Intracerebral hemorrhage | 373.4 (309.4 to 447.2) | 18.1 (11.5 to 26.5) | 57.8 (39.8 to 79.1) | -20.3* (-27.1 to -13.5) | -21.8* (-28.5 to -15.1) |
| Acute intracerebral hemorrhage | 1.3 (0.8 to 1.9) | 18.1 (11.5 to 26.5) | 0.4 (0.2 to 0.6) | -36.5* (-48.0 to -27.0) | -37.7* (-49.0 to -28.4) |
| Chronic intracerebral hemorrhage | 372.2 (308.2 to 445.9) | -- | 57.4 (39.6 to 78.5) | -20.2* (-27.1 to -13.3) | -21.7* (-28.5 to -15.0) |
| Subarachnoid hemorrhage | 116.4 (92.4 to 142.1) | 10.1 (7.4 to 13.5) | 17.7 (11.9 to 24.2) | -3.5* (-6.9 to -0.0) | -5.3* (-8.7 to -1.9) |
| Acute subarachnoid hemorrhage | 0.6 (0.4 to 0.8) | 10.1 (7.4 to 13.5) | 0.2 (0.1 to 0.3) | -2.0 (-7.3 to 0.6) | -3.8* (-9.0 to -1.3) |
| Chronic subarachnoid hemorrhage | 115.7 (91.8 to 141.5) | -- | 17.5 (11.7 to 24.0) | -3.5* (-7.0 to -0.0) | -5.3* (-8.8 to -1.9) |
| Hypertensive heart disease | 46.4 (32.2 to 65.1) | -- | 4.3 (2.6 to 6.8) | -5.0* (-7.7 to -2.4) | -6.8* (-9.5 to -4.2) |
| Non-rheumatic valvular heart disease | 45.4 (40.6 to 50.3) | 82.9 (79.4 to 86.9) | 0.1 (0.0 to 0.2) | -1.9 (-4.3 to 0.8) | -3.7* (-6.1 to -1.1) |
| Non-rheumatic calcific aortic valve disease | 15.8 (13.2 to 18.7) | 15.8 (13.2 to 18.7) | -- | -0.3 (-6.9 to 5.6) | -2.2 (-8.6 to 3.6) |
| Heart failure due to calcific aortic valve disease | 0.1 (0.0 to 0.1) | -- | -- | -6.5 (-25.6 to 15.4) | -8.3 (-27.0 to 13.2) |
| Non-rheumatic calcific aortic valve disease | 15.8 (13.2 to 18.7) | 15.8 (13.2 to 18.7) | -- | 8.6 (-7.2 to 26.7) | 6.5 (-9.0 to 24.3) |
| Non-rheumatic degenerative mitral valve disease | 67.1 (64.6 to 69.7) | 67.1 (64.6 to 69.7) | 0.0 (0.0 to 0.1) | 1.0 (-4.5 to 6.4) | -1.0 (-6.3 to 4.4) |
| Heart failure due to degenerative mitral valve disease | 0.3 (0.1 to 0.9) | -- | 0.0 (0.0 to 0.1) | -4.0 (-18.0 to 12.1) | -5.8 (-19.6 to 10.0) |
| Non-rheumatic degenerative mitral valve disease | 66.8 (64.2 to 69.5) | 67.1 (64.6 to 69.7) | -- | 15.3 (-7.2 to 41.0) | 13.1 (-8.9 to 38.3) |
| Other non-rheumatic valve diseases | 0.6 (0.4 to 0.9) | -- | 0.1 (0.0 to 0.1) | -4.1* (-5.4 to -2.6) | -5.9* (-7.3 to -4.4) |
| Mild heart failure due to other non-rheumatic valve disease | 0.1 (0.1 to 0.2) | -- | -- | -4.1* (-5.4 to -2.6) | -5.9* (-7.2 to -4.5) |
| Moderate heart failure due to other non-rheumatic valve disease | 0.1 (0.0 to 0.1) | -- | -- | -4.1* (-5.4 to -2.6) | -5.9* (-7.2 to -4.5) |
| Severe heart failure due to other non-rheumatic valve disease | 0.2 (0.1 to 0.3) | -- | 0.0 (0.0 to 0.1) | -4.1* (-5.4 to -2.6) | -5.9* (-7.3 to -4.5) |

| | | | | | |
|---|---|---|---|---|---|
| Controlled, medically managed heart failure due to other non-rheumatic valve disease | 0.2 (0.1 to 0.3) | -- | -- | -4.1* (-5.4 to -2.6) | -5.9* (-7.2 to -4.4) |
| Cardiomyopathy and myocarditis | 9.1 (6.8 to 11.6) | 157.5 (120.0 to 204.3) | 16.5 (10.5 to 24.7) | -0.3 (-1.6 to 0.9) | -2.3* (-3.4 to -1.0) |
| Myocarditis | 104.9 (85.0 to 127.4) | 157.5 (120.0 to 204.3) | 8.1 (5.2 to 11.9) | -1.1 (-2.6 to 0.5) | -3.0* (-4.5 to -1.5) |
| Acute myocarditis | 39.0 (28.5 to 51.9) | 157.5 (120.0 to 204.3) | 2.0 (1.1 to 3.2) | 0.1 (-3.0 to 2.8) | -1.8 (-4.9 to 0.9) |
| Heart failure due to myocarditis | 65.8 (49.2 to 85.3) | -- | 6.1 (3.7 to 9.3) | -1.5 (-3.2 to 0.3) | -3.4* (-5.1 to -1.6) |
| Alcoholic cardiomyopathy | 9.3 (7.0 to 11.9) | -- | 0.9 (0.5 to 1.3) | -11.2* (-12.4 to -10.2) | -12.9* (-14.0 to -11.9) |
| Other cardiomyopathy | 81.8 (61.8 to 105.2) | -- | 7.6 (4.5 to 11.5) | 1.9* (0.1 to 3.9) | -0.0 (-1.8 to 2.0) |
| Atrial fibrillation and flutter | -- | -- | -- | -- | -- |
| Asymptomatic atrial fibrillation and flutter | -- | -- | -- | -- | -- |
| Symptomatic atrial fibrillation and flutter | -- | -- | -- | -- | -- |
| Peripheral vascular disease | -- | -- | -- | -- | -- |
| Asymptomatic peripheral arterial disease | -- | -- | -- | -- | -- |
| Symptomatic claudication due to peripheral arterial disease | -- | -- | -- | -- | -- |
| Endocarditis | 12.9 (9.2 to 18.0) | 36.5 (24.1 to 51.2) | 1.1 (0.6 to 1.7) | 3.0* (1.6 to 4.3) | 1.0 (-0.3 to 2.3) |
| Acute endocarditis | 2.9 (1.8 to 4.1) | 36.5 (24.1 to 51.2) | 0.2 (0.1 to 0.3) | 4.2* (1.8 to 6.1) | 2.2 (-0.2 to 4.0) |
| Heart failure due to endocarditis | 10.0 (6.6 to 15.0) | -- | 0.9 (0.5 to 1.6) | 2.8* (1.2 to 4.2) | 0.8 (-0.8 to 2.2) |
| Other cardiovascular and circulatory diseases | 1549.0 (999.1 to 2351.0) | -- | 84.0 (46.4 to 139.8) | 4.2 (-0.0 to 8.3) | 2.2 (-1.9 to 6.2) |
| Heart failure due to other cardiovascular diseases | 6.6 (4.3 to 9.8) | -- | 0.6 (0.3 to 1.0) | 4.2* (2.8 to 5.5) | 2.2* (0.8 to 3.5) |
| Other cardiovascular and circulatory disease episodes | 1542.5 (994.8 to 2341.4) | -- | 83.4 (46.1 to 138.9) | 4.2 (-0.0 to 8.3) | 2.2 (-1.9 to 6.2) |
| **Chronic respiratory diseases** | **17975.9 (14615.2 to 21402.1)** | **2416.5 (1649.7 to 3262.4)** | **1269.7 (947.5 to 1639.9)** | **0.7 (-4.2 to 5.3)** | **-1.3 (-6.0 to 3.3)** |
| Chronic obstructive pulmonary disease | 4528.0 (3488.1 to 5648.5) | 442.1 (330.8 to 573.0) | 520.0 (385.6 to 673.0) | -3.0 (-9.5 to 3.0) | -4.9 (-11.2 to 1.1) |
| Chronic obstructive pulmonary disease with heart failure | 0.6 (0.4 to 0.9) | -- | 0.3 (0.2 to 0.4) | -6.8* (-8.2 to -5.3) | -8.6* (-10.0 to -7.1) |
| Chronic obstructive pulmonary disease without heart failure | 4527.4 (3487.5 to 5648.0) | 442.1 (330.8 to 573.0) | 519.7 (385.4 to 672.8) | -3.0 (-9.5 to 3.1) | -4.9 (-11.2 to 1.1) |
| Pneumoconiosis | 3.1 (2.1 to 4.4) | 0.6 (0.4 to 0.8) | 0.5 (0.3 to 0.8) | -0.1 (-3.2 to 3.2) | -2.0 (-5.0 to 1.2) |
| Silicosis | 1.3 (0.7 to 2.2) | 0.2 (0.1 to 0.4) | 0.2 (0.1 to 0.4) | 1.3 (-4.3 to 6.8) | -0.6 (-6.1 to 4.7) |
| Heart failure due to silicosis | -- | -- | -- | -- | -- |
| Silicosis without heart failure | 1.3 (0.7 to 2.2) | 0.2 (0.1 to 0.4) | 0.2 (0.1 to 0.4) | 1.3 (-4.3 to 6.8) | -0.6 (-6.1 to 4.7) |
| Asbestosis | 1.0 (0.4 to 1.9) | 0.2 (0.1 to 0.3) | 0.2 (0.1 to 0.3) | 3.4 (-2.2 to 11.8) | 1.4 (-4.0 to 9.7) |
| Asbestosis without heart failure | 1.0 (0.4 to 1.9) | 0.2 (0.1 to 0.3) | 0.2 (0.1 to 0.3) | 3.4 (-2.2 to 11.8) | 1.4 (-4.0 to 9.7) |
| Heart failure due to asbestosis | -- | -- | -- | -- | -- |
| Coal workers pneumoconiosis | 0.3 (0.1 to 0.6) | 0.1 (0.0 to 0.1) | 0.1 (0.0 to 0.1) | -7.9* (-13.4 to -3.4) | -9.6* (-15.1 to -5.3) |
| Coal workers pneumoconiosis without heart failure | 0.3 (0.1 to 0.6) | 0.1 (0.0 to 0.1) | 0.1 (0.0 to 0.1) | -7.9* (-13.4 to -3.4) | -9.6* (-15.1 to -5.3) |
| Heart failure due to Coal workers pneumoconiosis | -- | -- | -- | -- | -- |
| Other pneumoconiosis | 0.5 (0.2 to 0.8) | 0.1 (0.1 to 0.2) | 0.1 (0.0 to 0.1) | -5.1* (-8.4 to -2.7) | -6.9* (-10.1 to -4.6) |
| Heart failure due to other pneumoconiosis | -- | -- | -- | -- | -- |
| Other pneumoconiosis without heart failure | 0.5 (0.2 to 0.8) | 0.1 (0.1 to 0.2) | 0.1 (0.0 to 0.1) | -5.1* (-8.4 to -2.7) | -6.9* (-10.1 to -4.6) |
| Asthma | 13734.4 (10607.9 to 17285.3) | 1967.1 (1217.6 to 2767.2) | 545.0 (333.3 to 816.4) | 1.3 (-4.7 to 6.8) | -0.7 (-6.6 to 4.8) |
| Asymptomatic asthma | 4990.6 (3786.5 to 6453.5) | 713.2 (434.5 to 1013.3) | -- | -- | -- |
| Symptomatic asthma | 8743.8 (6691.8 to 11023.0) | 1254.0 (780.4 to 1785.6) | 545.0 (333.3 to 816.4) | 1.3 (-4.7 to 6.8) | -0.7 (-6.6 to 4.8) |
| Interstitial lung disease and pulmonary sarcoidosis | 29.3 (20.6 to 42.2) | 6.6 (4.5 to 9.3) | 3.3 (1.9 to 5.5) | -4.0* (-5.3 to -2.9) | -5.9* (-7.1 to -4.7) |
| Heart failure due to interstitial lung disease and pulmonary sarcoidosis | 0.3 (0.2 to 0.4) | -- | 0.1 (0.1 to 0.2) | -2.6* (-3.8 to -1.0) | -4.4* (-5.7 to -2.9) |
| Interstitial lung disease and pulmonary sarcoidosis without heart failure | 29.1 (20.4 to 42.0) | 6.6 (4.5 to 9.3) | 3.2 (1.9 to 5.4) | -4.1* (-5.4 to -2.9) | -5.9* (-7.2 to -4.8) |
| Other chronic respiratory diseases | -- | -- | 200.8 (149.1 to 259.2) | 9.8* (2.6 to 16.7) | 7.7* (0.6 to 14.5) |
| **Digestive diseases** | **138027.8 (125681.0 to 152730.0)** | **32790.9 (25586.5 to 41925.8)** | **1051.9 (660.1 to 1561.2)** | **2.5* (0.6 to 4.0)** | **0.5 (-1.3 to 2.0)** |
| Cirrhosis and other chronic liver diseases | 93576.2 (86861.4 to 101249.0) | 399.7 (315.5 to 481.0) | 44.1 (30.1 to 61.9) | 1.8 (-2.8 to 6.4) | -0.1 (-4.6 to 4.4) |
| Cirrhosis and other chronic liver diseases due to hepatitis B | 34937.4 (31260.2 to 38819.2) | 143.0 (112.6 to 176.9) | 13.5 (9.1 to 19.3) | -9.0 (-17.7 to 0.5) | -10.8* (-19.3 to -1.4) |
| Cirrhosis and other chronic liver diseases due to hepatitis B, decompensated | 78.0 (69.0 to 88.0) | 8.8 (7.3 to 10.4) | 13.5 (9.1 to 19.3) | -9.0 (-17.7 to 0.5) | -10.8* (-19.3 to -1.4) |
| Chronic hepatitis B without cirrhosis | 33536.8 (29955.5 to 37448.7) | -- | -- | -- | -- |
| Cirrhosis and other chronic liver diseases due to hepatitis B, compensated | 1322.7 (1136.4 to 1540.5) | 134.2 (103.5 to 167.3) | -- | -- | -- |
| Cirrhosis and other chronic liver diseases due to hepatitis C | 9968.8 (8224.6 to 11895.9) | 82.2 (62.1 to 103.7) | 9.7 (6.3 to 14.0) | 4.2 (-3.5 to 12.4) | 2.2 (-5.4 to 10.3) |
| Cirrhosis and other chronic liver diseases due to hepatitis C, decompensated | 55.7 (47.4 to 64.5) | 5.8 (4.8 to 7.1) | 9.7 (6.3 to 14.0) | 4.2 (-3.5 to 12.4) | 2.2 (-5.4 to 10.3) |
| Chronic hepatitis C without cirrhosis | 9024.2 (7258.2 to 10901.5) | -- | -- | -- | -- |
| Cirrhosis and other chronic liver diseases due to hepatitis C, compensated | 888.9 (733.5 to 1074.9) | 76.3 (56.8 to 97.6) | -- | -- | -- |
| Cirrhosis and other chronic liver diseases due to alcohol use | 432.6 (347.8 to 526.9) | 41.5 (31.6 to 53.2) | 4.6 (2.9 to 6.7) | 2.5 (-4.1 to 9.0) | 0.5 (-5.9 to 6.9) |
| Cirrhosis and other chronic liver diseases due to alcohol, decompensated | 25.8 (21.5 to 30.4) | 3.0 (2.4 to 3.6) | 4.6 (2.9 to 6.7) | 2.5 (-4.1 to 9.0) | 0.5 (-5.9 to 6.9) |
| Cirrhosis and other chronic liver diseases due to alcohol, compensated | 406.8 (326.7 to 496.5) | 38.5 (28.7 to 50.0) | -- | -- | -- |
| Cirrhosis due to NASH | 46839.0 (41471.2 to 53024.0) | 20.6 (15.5 to 26.6) | 2.3 (1.5 to 3.4) | 11.1* (8.9 to 13.0) | 9.0* (6.9 to 10.8) |
| Non-alcoholic fatty liver disease (NAFLD) / Non-alcoholic steatohepatitis (NASH) | 46614.6 (41247.9 to 52771.4) | -- | -- | -- | -- |
| Cirrhosis and other chronic liver diseases due to NASH | 224.4 (181.1 to 275.9) | 20.6 (15.5 to 26.6) | 2.3 (1.5 to 3.4) | 11.1* (8.9 to 13.0) | 9.0* (6.9 to 10.8) |
| Cirrhosis and other chronic liver diseases due to other causes | 1398.3 (1141.7 to 1684.2) | 112.5 (84.1 to 147.4) | 14.1 (9.1 to 20.2) | 11.0* (2.2 to 20.0) | 8.9* (0.2 to 17.7) |

| | | | | | |
|---|---|---|---|---|---|
| Cirrhosis and other chronic liver diseases due to other cause, decompensated | 82.0 (68.5 to 96.8) | 9.0 (7.3 to 11.1) | 14.1 (9.1 to 20.2) | 11.0* (2.2 to 20.0) | 8.9* (0.2 to 17.7) |
| Cirrhosis and other chronic liver diseases due to other cause, compensated | 1316.3 (1070.4 to 1591.3) | 103.5 (75.8 to 137.2) | -- | -- | -- |
| Upper digestive system diseases | 55950.4 (43258.2 to 72656.1) | 28279.4 (21103.6 to 37581.6) | 819.0 (472.5 to 1291.6) | 3.5* (1.8 to 5.2) | 1.5 (-0.2 to 3.2) |
| Peptic ulcer disease | 1053.9 (752.9 to 1469.7) | 373.1 (260.6 to 512.1) | 52.4 (31.2 to 81.6) | 4.7 (-1.0 to 10.1) | 2.7 (-2.9 to 8.0) |
| Acute peptic ulcer disease | 0.2 (0.1 to 0.3) | -- | 0.1 (0.0 to 0.1) | -8.8* (-10.7 to -7.2) | -10.6* (-12.4 to -9.0) |
| Chronic peptic ulcer disease | 1053.8 (752.8 to 1469.5) | 373.1 (260.6 to 512.1) | 52.4 (31.2 to 81.5) | 4.7 (-1.0 to 10.1) | 2.7 (-2.9 to 8.0) |
| Gastritis and duodenitis | 7375.8 (4987.9 to 10601.2) | 4985.0 (3391.4 to 7091.4) | 333.5 (196.6 to 552.1) | 3.1 (-0.4 to 6.3) | 1.2 (-2.3 to 4.3) |
| Acute gastritis and duodenitis | 2.1 (1.2 to 3.2) | -- | 0.7 (0.4 to 1.2) | 6.2* (4.6 to 7.8) | 4.1* (2.6 to 5.7) |
| Chronic gastritis and duodenitis | 7373.7 (4985.3 to 10599.5) | 4985.0 (3391.4 to 7091.4) | 332.8 (196.2 to 551.0) | 3.1 (-0.4 to 6.3) | 1.2 (-2.3 to 4.3) |
| GERD | 49967.9 (36642.9 to 68132.4) | 22921.3 (16190.4 to 31549.3) | 433.0 (216.1 to 771.3) | 3.7* (2.1 to 5.3) | 1.7* (0.1 to 3.3) |
| Appendicitis | 98.4 (80.0 to 120.0) | 2513.0 (2053.1 to 3025.3) | 30.4 (19.3 to 44.7) | 3.4 (-6.7 to 15.3) | 1.4 (-8.5 to 13.1) |
| Paralytic ileus and intestinal obstruction | 5.2 (3.6 to 7.1) | 138.9 (96.7 to 191.7) | 1.7 (1.0 to 2.6) | 9.0* (4.6 to 12.2) | 6.9* (2.6 to 10.1) |
| Inguinal, femoral, and abdominal hernia | 838.1 (589.4 to 1152.2) | 864.6 (612.4 to 1199.3) | 84.4 (49.3 to 130.5) | -7.1* (-11.8 to -2.0) | -8.9* (-13.5 to -3.9) |
| Inflammatory bowel disease | 227.9 (196.2 to 263.2) | 359.8 (300.2 to 426.7) | 36.6 (24.3 to 51.6) | -4.4 (-13.3 to 7.0) | -6.2 (-15.0 to 4.9) |
| Ulcerative colitis | 97.4 (79.3 to 120.3) | 195.5 (149.5 to 252.5) | 15.3 (9.7 to 22.2) | -5.6 (-15.6 to 5.7) | -7.4 (-17.3 to 3.7) |
| Crohn's disease | 130.5 (108.2 to 157.7) | 164.3 (129.1 to 202.5) | 21.3 (13.9 to 30.8) | -3.5 (-14.1 to 8.6) | -5.3 (-15.7 to 6.5) |
| Vascular intestinal disorders | 1.5 (0.9 to 2.5) | 9.2 (6.2 to 13.4) | 0.5 (0.3 to 0.9) | 2.9* (1.0 to 4.9) | 1.0 (-1.0 to 2.9) |
| Gallbladder and biliary diseases | 952.5 (673.1 to 1319.4) | 170.0 (116.7 to 236.6) | 0.8 (0.4 to 1.3) | 1.8* (0.2 to 3.5) | -0.1 (-1.7 to 1.5) |
| Pancreatitis | 132.7 (110.3 to 161.4) | 56.4 (42.5 to 72.9) | 8.8 (4.4 to 15.3) | 4.7* (2.6 to 6.5) | 2.7* (0.6 to 4.5) |
| Acute pancreatitis | 3.3 (2.4 to 4.6) | 44.7 (31.9 to 60.2) | 1.4 (0.9 to 2.1) | -3.5* (-4.4 to -2.7) | -5.3* (-6.2 to -4.5) |
| Chronic pancreatitis | 129.3 (106.6 to 157.6) | 11.8 (7.6 to 16.8) | 7.5 (3.3 to 13.7) | 6.3* (4.3 to 8.3) | 4.3* (2.3 to 6.3) |
| Other digestive diseases | -- | -- | 25.7 (16.9 to 38.8) | 15.1* (12.1 to 18.1) | 12.9* (9.9 to 15.9) |
| **Neurological disorders** | **291001.4 (250114.4 to 339075.9)** | **90621.4 (64498.1 to 116998.5)** | **6461.6 (4223.1 to 9311.7)** | **4.6* (2.5 to 6.9)** | **2.6* (0.6 to 4.9)** |
| Alzheimer's disease and other dementias | -- | -- | -- | -- | -- |
| Parkinson's disease | 1.7 (0.7 to 3.0) | 1.0 (0.4 to 1.7) | 0.3 (0.1 to 0.5) | -0.8 (-4.8 to 3.4) | -2.7 (-6.6 to 1.5) |
| Epilepsy | 2327.9 (1716.1 to 3040.5) | 165.5 (106.7 to 242.5) | 766.8 (466.6 to 1136.0) | 5.5 (-6.8 to 20.1) | 3.5 (-8.6 to 17.8) |
| Multiple sclerosis | 47.8 (38.1 to 60.1) | 8.7 (6.3 to 11.3) | 13.2 (8.8 to 18.7) | 1.9 (-3.0 to 6.5) | -0.1 (-4.9 to 4.5) |
| Motor neuron disease | 10.4 (8.3 to 12.6) | 0.1 (0.0 to 0.2) | 2.2 (1.5 to 3.1) | -0.1 (-0.9 to 0.8) | -2.0* (-2.8 to -1.2) |
| Headache disorders | 289789.9 (248745.0 to 337796.3) | 90446.0 (64345.7 to 116852.0) | 5526.8 (3507.7 to 8195.1) | 4.1* (2.9 to 5.3) | 2.1* (0.9 to 3.3) |
| Migraine | 138701.4 (117779.2 to 160059.6) | 14232.1 (10129.8 to 18996.6) | 4901.3 (3039.4 to 7465.1) | 4.3* (2.9 to 5.6) | 2.3* (0.9 to 3.6) |
| Medication overuse headache due to migraine | 6767.2 (4708.1 to 9460.3) | -- | 765.2 (429.7 to 1256.2) | 2.0* (0.4 to 3.6) | 0.0 (-1.5 to 1.6) |
| Migraine | 131934.2 (111648.8 to 154269.2) | 14232.1 (10129.8 to 18996.6) | 4136.2 (2493.1 to 6410.3) | 4.7* (3.1 to 6.4) | 2.7* (1.2 to 4.3) |
| Tension-type headache | 211097.7 (160598.7 to 275250.7) | 76214.0 (51271.1 to 102664.2) | 625.4 (338.7 to 1031.1) | 2.5* (0.8 to 3.8) | 0.5 (-1.1 to 1.8) |
| Medication overuse headache due to tension-type headache | 2516.3 (1541.3 to 3892.0) | -- | 282.6 (129.5 to 534.0) | 2.0 (-0.9 to 4.8) | 0.0 (-2.8 to 2.8) |
| Tension-type headache | 208581.4 (157954.1 to 272363.8) | 76214.0 (51271.1 to 102664.2) | 342.9 (132.3 to 651.4) | 2.8* (1.8 to 4.1) | 0.9 (-0.2 to 2.2) |
| Other neurological disorders | 2.3 (1.1 to 4.2) | -- | 152.4 (93.0 to 225.0) | 20.4* (6.6 to 36.7) | 18.1* (4.5 to 34.1) |
| Other neurological disorders | -- | -- | 151.7 (92.6 to 224.1) | 20.4* (6.5 to 36.8) | 18.1* (4.5 to 34.2) |
| Guillain-Barré syndrome due to other neurological disorders | 2.3 (1.1 to 4.2) | -- | 0.7 (0.3 to 1.3) | 8.2* (5.5 to 11.3) | 6.2* (3.4 to 9.2) |
| **Mental disorders** | **83958.5 (77962.7 to 90806.7)** | **28131.5 (23214.9 to 33617.4)** | **10599.9 (7639.0 to 13867.6)** | **1.2* (0.2 to 2.1)** | **-0.7 (-1.7 to 0.1)** |
| Schizophrenia | 1140.4 (829.3 to 1508.6) | 235.4 (166.4 to 315.6) | 760.1 (499.5 to 1063.4) | -1.4 (-4.7 to 1.6) | -3.3* (-6.5 to -0.4) |
| Depressive disorders | 20396.9 (16700.2 to 24753.6) | 21677.7 (17086.1 to 27441.0) | 3588.6 (2363.8 to 5199.2) | -1.1 (-3.2 to 0.7) | -3.0* (-5.0 to -1.2) |
| Major depressive disorder | 13983.0 (10732.1 to 18187.0) | 20357.0 (15902.4 to 26133.7) | 2923.0 (1848.0 to 4387.7) | -2.3* (-5.0 to -0.0) | -4.2* (-6.8 to -1.9) |
| Dysthymia | 6716.3 (5127.3 to 8603.9) | 1320.7 (946.9 to 1782.7) | 665.7 (417.6 to 1042.6) | 4.6* (2.6 to 7.0) | 2.6* (0.6 to 4.9) |
| Bipolar disorder | 5167.3 (3984.8 to 6541.1) | 279.3 (159.1 to 435.5) | 1084.8 (658.8 to 1662.3) | 4.2* (2.4 to 6.1) | 2.2* (0.4 to 4.0) |
| Anxiety disorders | 25223.5 (21730.6 to 28792.5) | 3589.6 (2890.1 to 4246.6) | 2468.8 (1721.9 to 3397.1) | 1.4 (-0.1 to 3.0) | -0.5 (-2.0 to 1.0) |
| Eating disorders | 2646.2 (1788.8 to 3919.0) | 2349.6 (1305.3 to 4125.4) | 564.3 (322.5 to 926.3) | 11.9* (9.5 to 14.2) | 9.8* (7.4 to 12.1) |
| Anorexia nervosa | 700.5 (475.9 to 992.6) | 205.1 (143.1 to 280.2) | 149.8 (85.9 to 240.2) | 9.6* (5.4 to 13.7) | 7.5* (3.4 to 11.5) |
| Bulimia nervosa | 1949.6 (1137.3 to 3173.1) | 2144.4 (1072.2 to 3913.9) | 414.5 (203.8 to 741.9) | 12.7* (10.0 to 15.4) | 10.6* (7.8 to 13.1) |
| Autism spectrum disorders | 2528.6 (2271.9 to 2792.3) | -- | 389.0 (263.4 to 533.8) | 2.2* (0.2 to 4.2) | 0.2 (-1.7 to 2.2) |
| Attention-deficit/hyperactivity disorder | 8383.8 (6944.4 to 10091.8) | -- | 101.9 (60.1 to 162.7) | -0.6 (-3.0 to 1.7) | -2.5* (-4.9 to -0.2) |
| Conduct disorder | 2867.2 (1866.8 to 4090.4) | -- | 344.1 (182.8 to 566.1) | 4.1 (-0.6 to 9.0) | 2.1 (-2.5 to 6.9) |
| Idiopathic developmental intellectual disability | 9831.9 (5880.7 to 13904.6) | -- | 393.3 (191.7 to 658.6) | -1.3 (-3.8 to 0.6) | -3.2* (-5.7 to -1.3) |
| Other mental disorders | 11773.4 (9903.3 to 13938.6) | -- | 905.0 (572.8 to 1290.7) | 2.6* (0.7 to 4.5) | 0.6 (-1.2 to 2.5) |
| **Substance use disorders** | **24887.6 (20914.1 to 29118.3)** | **7703.5 (6191.6 to 9393.3)** | **4755.0 (3280.2 to 6360.0)** | **2.0 (-0.6 to 4.6)** | **0.0 (-2.5 to 2.6)** |
| Alcohol use disorders | 12365.7 (8843.8 to 16448.4) | 6245.6 (4774.0 to 7900.8) | 1265.3 (766.8 to 1949.5) | -2.3* (-4.9 to -0.0) | -4.2* (-6.7 to -1.9) |
| Alcohol dependence | 12341.2 (8818.7 to 16425.0) | 6245.6 (4774.0 to 7900.8) | 1264.3 (765.9 to 1948.2) | -2.3 (-4.9 to 0.0) | -4.2* (-6.7 to -1.9) |
| Fetal alcohol syndrome | 24.4 (19.6 to 30.0) | -- | 0.9 (0.6 to 1.4) | -11.4* (-17.5 to -4.9) | -13.1* (-19.0 to -6.7) |
| Drug use disorders | 12961.8 (11091.0 to 15243.8) | 1457.9 (1166.1 to 1779.9) | 3489.7 (2358.4 to 4762.2) | 3.7* (0.2 to 6.9) | 1.7 (-1.7 to 4.8) |
| Opioid use disorders | 6339.5 (4775.0 to 8172.6) | 1049.7 (796.9 to 1361.8) | 2700.0 (1744.0 to 3754.8) | 5.3* (0.8 to 9.2) | 3.3 (-1.1 to 7.1) |
| Cocaine use disorders | 818.4 (703.9 to 950.5) | 13.3 (7.0 to 21.0) | 113.0 (72.9 to 164.1) | 5.8* (2.2 to 9.3) | 3.8* (0.2 to 7.2) |

| | | | | | |
|---|---|---|---|---|---|
| Amphetamine use disorders | 2208.7 (1448.4 to 3187.2) | 113.9 (59.6 to 197.0) | 292.9 (161.3 to 480.5) | -4.1* (-8.1 to -0.4) | -6.0* (-9.9 to -2.3) |
| Cannabis use disorders | 3760.9 (2870.7 to 5029.3) | 281.1 (178.9 to 421.4) | 109.3 (66.3 to 166.5) | -1.1 (-5.1 to 2.6) | -2.9 (-6.9 to 0.6) |
| Other drug use disorders | 100.1 (76.2 to 130.8) | -- | 274.6 (168.4 to 415.8) | -1.6 (-5.2 to 2.3) | -3.4 (-7.0 to 0.3) |
| **Diabetes and kidney diseases** | **30396.8 (26017.1 to 35085.3)** | **1865.7 (1390.9 to 2360.1)** | **1002.8 (645.7 to 1439.2)** | **6.3* (1.2 to 10.8)** | **4.2 (-0.8 to 8.7)** |
| Diabetes mellitus | 12306.6 (9507.1 to 15160.9) | 1488.2 (1045.0 to 1939.0) | 786.3 (465.0 to 1176.2) | 6.5* (0.5 to 11.8) | 4.5 (-1.4 to 9.7) |
| Diabetes mellitus type 1 | 1109.6 (944.3 to 1290.6) | 34.5 (20.9 to 49.4) | 70.7 (46.0 to 103.3) | 4.4* (1.0 to 7.8) | 2.4 (-0.9 to 5.7) |
| Uncomplicated diabetes mellitus type 1 | 893.6 (756.4 to 1041.9) | 34.5 (20.9 to 49.4) | 42.6 (26.2 to 64.4) | 4.7* (1.3 to 8.0) | 2.7 (-0.6 to 5.9) |
| Vision loss due to diabetes mellitus type 1 retinopathy | 0.7 (0.4 to 1.2) | -- | 0.0 (0.0 to 0.1) | -11.6* (-17.1 to -6.7) | -13.3* (-18.7 to -8.5) |
| Diabetic neuropathy due to diabetes mellitus type 1 | 215.3 (171.1 to 263.5) | -- | 28.0 (17.7 to 41.7) | 3.9 (-3.3 to 10.8) | 1.9 (-5.2 to 8.6) |
| Diabetes mellitus type 2 | 11197.1 (8433.0 to 14089.1) | 1453.7 (1006.6 to 1902.7) | 715.5 (415.6 to 1083.8) | 6.8* (0.1 to 12.6) | 4.7 (-1.8 to 10.4) |
| Uncomplicated diabetes mellitus type 2 | 9030.6 (6776.6 to 11450.1) | 1453.7 (1006.6 to 1902.7) | 432.5 (242.6 to 675.7) | 6.7 (-0.1 to 12.8) | 4.6 (-2.0 to 10.6) |
| Vision loss due to diabetes mellitus type 2 retinopathy | 4.9 (3.2 to 7.2) | -- | 0.4 (0.2 to 0.6) | -0.9 (-6.9 to 4.6) | -2.8 (-8.7 to 2.6) |
| Diabetic neuropathy due to diabetes mellitus type 2 | 2161.5 (1560.0 to 2900.4) | -- | 282.6 (164.7 to 432.9) | 6.9 (-0.7 to 14.3) | 4.8 (-2.6 to 12.1) |
| Chronic kidney disease | 19385.2 (15758.1 to 23762.2) | 335.2 (195.2 to 500.5) | 216.4 (142.6 to 313.9) | 5.3 (-2.2 to 13.6) | 3.3 (-4.1 to 11.4) |
| Chronic kidney disease due to diabetes mellitus type 1 | 218.6 (170.8 to 280.3) | 4.8 (2.6 to 7.8) | 3.7 (2.4 to 5.3) | 4.7 (-5.0 to 14.9) | 2.7 (-6.8 to 12.7) |
| Albuminuria with preserved GFR due to type 1 diabetes mellitus | 122.5 (87.0 to 172.4) | -- | -- | -- | -- |
| End-stage chronic kidney disease due to diabetes mellitus type 1 | 2.1 (1.1 to 3.5) | -- | 0.8 (0.4 to 1.5) | 1.2 (-5.5 to 9.3) | -0.7 (-7.3 to 7.3) |
| Stage III chronic kidney disease due to diabetes mellitus type 1 | 90.2 (66.7 to 121.2) | 4.8 (2.6 to 7.8) | 1.7 (1.0 to 2.5) | -2.3 (-17.4 to 13.1) | -4.2 (-19.0 to 10.9) |
| Stage IV chronic kidney disease due to diabetes mellitus type 1 | 2.1 (1.2 to 3.4) | -- | 0.3 (0.1 to 0.5) | 15.8* (0.6 to 35.3) | 13.6 (-1.4 to 32.8) |
| Stage V chronic kidney disease due to diabetes mellitus type 1 | 1.7 (1.1 to 2.5) | -- | 1.0 (0.6 to 1.5) | 18.7* (8.7 to 31.9) | 16.4* (6.6 to 29.4) |
| Chronic kidney disease due to diabetes mellitus type 2 | 2220.2 (1544.9 to 3211.1) | 6.0 (3.3 to 9.6) | 3.3 (2.1 to 5.1) | -21.5 (-52.1 to 2.3) | -23.0 (-53.0 to 0.4) |
| Albuminuria with preserved GFR due to type 2 diabetes mellitus | 2101.9 (1440.7 to 3083.6) | -- | -- | -- | -- |
| End-stage chronic kidney disease due to diabetes mellitus type 2 | 0.7 (0.3 to 1.3) | -- | 0.3 (0.1 to 0.5) | 1.6 (-5.1 to 9.9) | -0.4 (-6.9 to 7.8) |
| Stage III chronic kidney disease due to diabetes mellitus type 2 | 110.9 (79.7 to 165.5) | 6.0 (3.3 to 9.6) | 0.9 (0.6 to 1.3) | 0.7 (-12.0 to 17.9) | -1.2 (-13.7 to 15.7) |
| Stage IV chronic kidney disease due to diabetes mellitus type 2 | 3.8 (2.2 to 6.4) | -- | 0.4 (0.2 to 0.8) | -26.8 (-59.9 to 3.7) | -28.2 (-60.7 to 1.8) |
| Stage V chronic kidney disease due to diabetes mellitus type 2 | 2.9 (1.9 to 4.6) | -- | 1.7 (1.0 to 2.8) | -30.8 (-61.0 to 0.3) | -32.1* (-61.7 to -1.7) |
| Chronic kidney disease due to hypertension | 1365.7 (1121.7 to 1681.2) | 77.9 (44.4 to 118.1) | 33.7 (21.3 to 50.2) | 8.5* (0.1 to 18.4) | 6.4 (-1.8 to 16.1) |
| Albuminuria with preserved GFR due to hypertension | 60.7 (42.8 to 84.4) | -- | -- | -- | -- |
| End-stage chronic kidney disease due to hypertension | 3.4 (2.1 to 4.9) | -- | 1.3 (0.7 to 2.1) | 6.7* (1.0 to 12.8) | 4.6 (-1.0 to 10.6) |
| Stage III chronic kidney disease due to hypertension | 1209.6 (977.7 to 1506.1) | 77.9 (44.4 to 118.1) | 2.4 (1.5 to 3.6) | -1.2 (-15.7 to 16.5) | -3.1 (-17.3 to 14.3) |
| Stage IV chronic kidney disease due to hypertension | 51.7 (31.4 to 81.1) | -- | 6.5 (3.4 to 11.4) | 7.5 (-6.5 to 24.0) | 5.4 (-8.3 to 21.6) |
| Stage V chronic kidney disease due to hypertension | 40.3 (27.8 to 57.6) | -- | 23.4 (14.5 to 36.1) | 10.0* (1.8 to 20.2) | 7.9 (-0.1 to 17.9) |
| Chronic kidney disease due to glomerulonephritis | 868.4 (721.5 to 1058.9) | 10.6 (6.0 to 16.0) | 59.7 (39.8 to 86.8) | 6.6 (-1.3 to 16.2) | 4.6 (-3.2 to 14.0) |
| Albuminuria with preserved GFR due to glomerulonephritis | 247.9 (179.2 to 343.8) | -- | -- | -- | -- |
| End-stage chronic kidney disease due to glomerulonephritis | 20.1 (13.4 to 28.2) | -- | 7.1 (4.0 to 10.8) | 4.1 (-1.8 to 11.7) | 2.1 (-3.7 to 9.5) |
| Stage III chronic kidney disease due to glomerulonephritis | 460.8 (364.5 to 582.0) | 10.6 (6.0 to 16.0) | 7.4 (4.5 to 11.7) | -5.9 (-22.5 to 14.2) | -7.7 (-23.9 to 12.0) |
| Stage IV chronic kidney disease due to glomerulonephritis | 78.5 (47.3 to 123.1) | -- | 9.8 (5.0 to 17.1) | 7.5 (-7.3 to 24.5) | 5.5 (-9.0 to 22.1) |
| Stage V chronic kidney disease due to glomerulonephritis | 61.1 (42.1 to 87.4) | -- | 35.4 (22.1 to 54.5) | 9.9* (1.3 to 20.1) | 7.8 (-0.6 to 17.8) |
| Chronic kidney disease due to other and unspecified causes | 14712.3 (11911.5 to 18195.2) | 236.0 (137.7 to 351.9) | 116.0 (76.4 to 166.6) | 4.8 (-3.5 to 14.7) | 2.8 (-5.3 to 12.5) |
| Albuminuria with preserved GFR due to other and unspecified causes | 10115.5 (7525.9 to 13548.3) | -- | -- | -- | -- |
| End-stage chronic kidney disease due to other causes | 27.4 (19.6 to 35.6) | -- | 10.6 (6.4 to 15.5) | 6.1* (0.1 to 13.6) | 4.1 (-1.8 to 11.5) |
| Stage III chronic kidney disease due to other causes | 4302.0 (3465.3 to 5352.0) | 236.0 (137.7 to 351.9) | 20.5 (12.5 to 32.9) | -10.9 (-24.4 to 3.0) | -12.6 (-25.8 to 1.0) |
| Stage IV chronic kidney disease due to other causes | 150.6 (91.3 to 236.7) | -- | 18.4 (9.4 to 32.3) | 7.1 (-7.4 to 22.6) | 5.1 (-9.2 to 20.2) |
| Stage V chronic kidney disease due to other causes | 116.9 (81.3 to 167.4) | -- | 66.4 (40.5 to 102.5) | 9.9* (0.3 to 21.1) | 7.8 (-1.6 to 18.7) |
| Acute glomerulonephritis | 2.9 (1.8 to 4.4) | 42.3 (26.5 to 62.1) | 0.1 (0.1 to 0.3) | -7.4* (-10.8 to -3.5) | -9.2* (-12.5 to -5.3) |
| **Skin and subcutaneous diseases** | **131657.1 (123219.9 to 141451.7)** | **262191.5 (234956.8 to 294433.2)** | **3066.4 (1975.1 to 4565.8)** | **2.9* (2.1 to 4.0)** | **1.0* (0.1 to 2.1)** |
| Dermatitis | 19792.8 (16988.2 to 23245.3) | 23398.6 (16938.7 to 32044.2) | 733.3 (420.3 to 1167.6) | 3.8* (2.1 to 5.8) | 1.8* (0.2 to 3.7) |
| Atopic dermatitis | 12418.4 (10798.1 to 14385.8) | 1164.7 (915.9 to 1464.5) | 546.5 (291.2 to 936.7) | 5.1* (2.9 to 7.2) | 3.0* (0.9 to 5.2) |
| Contact dermatitis | 6923.8 (4602.8 to 9609.4) | 20311.7 (13943.5 to 28892.9) | 177.2 (99.1 to 294.2) | 1.4 (-0.9 to 3.8) | -0.6 (-2.8 to 1.8) |
| Seborrhoeic dermatitis | 692.1 (555.0 to 872.1) | 1922.2 (1519.8 to 2411.1) | 9.6 (5.1 to 16.2) | -14.8* (-18.7 to -10.8) | -16.4* (-20.3 to -12.5) |
| Psoriasis | 3512.2 (3249.9 to 3805.9) | 503.5 (437.3 to 578.5) | 314.1 (215.4 to 424.2) | 5.9* (2.7 to 9.1) | 3.8* (0.8 to 7.0) |
| Bacterial skin diseases | 614.3 (564.0 to 670.2) | 15207.1 (13911.6 to 16497.5) | 10.6 (6.4 to 16.7) | 4.3* (1.3 to 7.4) | 2.3 (-0.7 to 5.4) |
| Cellulitis | 122.5 (96.8 to 151.1) | 2501.9 (2057.9 to 3050.0) | 7.2 (4.4 to 10.8) | 1.5 (-1.6 to 4.7) | -0.4 (-3.4 to 2.7) |
| Pyoderma | 594.8 (545.0 to 647.0) | 12705.2 (11544.3 to 13943.3) | 3.4 (1.3 to 7.2) | 10.7* (6.2 to 15.1) | 8.6* (4.2 to 12.9) |
| Impetigo | 178.9 (156.1 to 202.2) | 3065.1 (2702.4 to 3429.4) | 1.0 (0.4 to 2.2) | 17.3* (9.3 to 25.5) | 15.0* (7.2 to 23.1) |
| Abscess and other bacterial skin diseases | 415.9 (373.0 to 462.2) | 9640.1 (8546.7 to 10821.3) | 2.4 (0.9 to 5.0) | 8.1* (2.9 to 13.1) | 6.1* (1.0 to 10.9) |
| Scabies | 20614.3 (14730.1 to 28366.1) | 60435.6 (43069.7 to 82264.7) | 536.8 (274.6 to 927.6) | -3.5* (-4.4 to -2.5) | -5.4* (-6.3 to -4.4) |
| Fungal skin diseases | 38275.9 (31022.4 to 46673.6) | 108073.7 (87371.7 to 132915.9) | 218.4 (82.2 to 458.6) | 2.3* (1.2 to 3.2) | 0.3 (-0.7 to 1.2) |
| Tinea capitis | 5268.6 (3575.9 to 7338.4) | 6313.8 (4368.2 to 8130.1) | 30.0 (11.7 to 66.9) | -7.6* (-11.3 to -4.0) | -9.4* (-13.0 to -5.8) |
| Other fungal skin diseases | 33007.3 (25879.8 to 41191.3) | 101759.8 (81265.9 to 126602.6) | 188.4 (71.5 to 394.4) | 4.1* (3.3 to 4.8) | 2.1* (1.3 to 2.8) |

| | | | | | |
|---|---|---|---|---|---|
| Viral skin diseases | 7588.6 (6984.3 to 8263.5) | 7672.4 (7084.1 to 8322.3) | 234.6 (149.8 to 349.3) | -2.4* (-3.8 to -1.0) | -4.3* (-5.6 to -2.9) |
| Viral warts | 4226.2 (3770.1 to 4762.5) | 2468.4 (2178.1 to 2757.2) | 130.7 (82.6 to 198.3) | -5.7* (-7.5 to -3.7) | -7.5* (-9.3 to -5.6) |
| Molluscum contagiosum | 3362.4 (2980.9 to 3788.4) | 5204.0 (4666.0 to 5793.6) | 103.9 (66.2 to 154.4) | 2.0 (-0.0 to 4.3) | 0.0 (-1.9 to 2.3) |
| Acne vulgaris | 20553.0 (17376.8 to 24094.0) | 6242.3 (4936.1 to 7761.5) | 439.0 (262.4 to 708.6) | 12.0* (10.0 to 14.1) | 9.8* (7.9 to 11.9) |
| Alopecia areata | 1471.3 (1340.1 to 1621.3) | 2765.0 (2498.8 to 3051.5) | 48.9 (31.2 to 73.8) | 1.4 (-1.6 to 4.6) | -0.5 (-3.5 to 2.6) |
| Pruritus | 4356.0 (3052.3 to 6109.7) | 3447.5 (2410.1 to 4812.3) | 47.4 (20.6 to 99.6) | 3.2* (1.6 to 4.9) | 1.3 (-0.3 to 2.9) |
| Urticaria | 5696.7 (3995.0 to 7821.9) | 9932.0 (6909.4 to 14184.6) | 344.3 (199.3 to 554.9) | 1.7 (-0.3 to 3.8) | -0.2 (-2.2 to 1.8) |
| Decubitus ulcer | 25.0 (16.5 to 35.2) | 88.8 (58.2 to 126.1) | 4.4 (2.5 to 7.2) | 4.7* (3.7 to 5.5) | 2.7* (1.8 to 3.5) |
| Other skin and subcutaneous diseases | 23973.8 (21648.9 to 26318.6) | 24424.8 (22144.9 to 26877.7) | 134.7 (62.2 to 251.1) | 6.4* (5.4 to 7.4) | 4.4* (3.4 to 5.3) |
| **Sense organ diseases** | **70423.4 (64610.8 to 77285.8)** | **--** | **1853.5 (1229.8 to 2735.5)** | **2.5* (0.3 to 4.7)** | **0.5 (-1.6 to 2.7)** |
| Blindness and vision impairment | 30847.5 (27581.2 to 34592.8) | -- | 704.8 (439.5 to 1096.2) | 1.9* (0.4 to 3.4) | -0.0 (-1.5 to 1.4) |
| Glaucoma | -- | -- | -- | -- | -- |
| Cataract | 47.5 (32.5 to 67.4) | -- | 5.5 (3.1 to 8.8) | -12.0* (-23.2 to -0.0) | -13.7* (-24.6 to -1.9) |
| Age-related macular degeneration | -- | -- | -- | -- | -- |
| Refraction disorders | 10149.2 (7393.6 to 13310.2) | -- | 419.4 (262.5 to 647.2) | 2.3* (0.2 to 4.4) | 0.3 (-1.7 to 2.4) |
| Near vision loss | 20252.9 (18669.3 to 22027.7) | -- | 211.0 (99.7 to 389.2) | 3.2* (2.4 to 4.2) | 1.3* (0.4 to 2.2) |
| Other vision loss | 852.1 (624.7 to 1146.6) | -- | 68.9 (43.5 to 101.4) | -2.9 (-7.9 to 2.1) | -4.8 (-9.7 to 0.1) |
| Age-related and other hearing loss | 39856.9 (34305.2 to 46241.9) | -- | 1080.2 (723.1 to 1547.0) | 2.8 (-0.7 to 6.5) | 0.9 (-2.6 to 4.5) |
| Other sense organ diseases | 2949.9 (2671.9 to 3246.5) | -- | 68.4 (41.7 to 102.7) | 2.7 (-1.0 to 6.9) | 0.7 (-2.9 to 4.8) |
| Chronic other sense organ diseases | 2134.7 (1875.0 to 2424.6) | -- | 57.0 (34.9 to 86.3) | 2.7 (-1.5 to 7.2) | 0.8 (-3.4 to 5.2) |
| Acute other sense organ diseases | 815.2 (728.2 to 907.8) | -- | 11.3 (6.7 to 17.6) | 2.6 (-3.4 to 8.9) | 0.7 (-5.3 to 6.8) |
| **Musculoskeletal disorders** | **51257.4 (42466.3 to 61311.3)** | **15731.1 (11226.5 to 20582.4)** | **5641.6 (3774.2 to 7915.8)** | **-0.9 (-2.2 to 0.3)** | **-2.8* (-4.1 to -1.6)** |
| Rheumatoid arthritis | 456.3 (354.0 to 565.7) | 44.3 (32.0 to 57.1) | 64.8 (41.1 to 92.6) | 7.9* (2.8 to 13.2) | 5.9* (0.8 to 11.1) |
| Osteoarthritis | -- | -- | -- | -- | -- |
| Osteoarthritis of the hip | -- | -- | -- | -- | -- |
| Osteoarthritis of the knee | -- | -- | -- | -- | -- |
| Low back pain | 30581.5 (21913.1 to 40822.8) | 13454.0 (8858.8 to 18451.6) | 3470.9 (2117.5 to 5273.9) | 1.1 (-0.3 to 2.4) | -0.8 (-2.2 to 0.4) |
| Low back pain with leg pain | 7091.4 (4954.4 to 9806.9) | 3137.0 (2064.5 to 4464.2) | 1065.1 (632.2 to 1650.5) | 1.1 (-1.0 to 2.9) | -0.9 (-2.9 to 0.9) |
| Low back pain without leg pain | 23490.1 (16788.4 to 31580.3) | 10316.9 (6727.2 to 14244.7) | 2405.8 (1461.2 to 3796.3) | 1.1 (-0.2 to 2.5) | -0.8 (-2.2 to 0.5) |
| Neck pain | 7007.8 (4838.3 to 9635.0) | 2140.2 (1371.5 to 3138.4) | 725.2 (415.7 to 1163.9) | -3.1* (-5.4 to -0.6) | -5.0* (-7.2 to -2.5) |
| Gout | 219.8 (161.3 to 295.1) | 92.6 (71.7 to 116.6) | 7.7 (4.6 to 11.8) | 2.5* (1.2 to 3.7) | 0.5 (-0.7 to 1.7) |
| Other musculoskeletal disorders | 15400.1 (11255.9 to 19701.3) | -- | 1372.9 (832.5 to 2048.8) | -4.9* (-8.5 to -2.0) | -6.8* (-10.2 to -3.9) |
| **Other non-communicable diseases** | **381850.8 (355096.3 to 416009.7)** | **380497.3 (323808.6 to 427239.9)** | **3537.1 (2428.8 to 4898.4)** | **-0.3 (-2.1 to 1.5)** | **-2.2* (-4.0 to -0.5)** |
| Congenital anomalies | 7643.4 (7208.2 to 8122.7) | -- | 955.6 (672.6 to 1277.4) | 4.1* (1.5 to 6.9) | 2.1 (-0.4 to 4.8) |
| Neural tube defects | 228.6 (203.8 to 254.9) | -- | 68.4 (47.7 to 91.3) | 7.3* (2.2 to 13.0) | 5.2* (0.3 to 10.8) |
| Severe motor and cognitive impairment due to anencephaly | -- | -- | -- | -- | -- |
| Encephalocoele due to neural tube defects | 20.0 (16.3 to 24.2) | -- | 3.4 (2.3 to 4.9) | 2.3 (-0.7 to 5.1) | 0.3 (-2.6 to 3.1) |
| Spina bifida due to neural tube defects | 208.6 (186.2 to 233.0) | -- | 65.0 (45.6 to 86.9) | 7.6* (2.3 to 13.5) | 5.5* (0.3 to 11.3) |
| Congenital heart anomalies | 565.5 (518.9 to 613.9) | -- | 30.9 (14.1 to 52.8) | 3.6 (-0.7 to 7.4) | 1.6 (-2.6 to 5.4) |
| Critical malformations of great vessels, congenital valvular heart disease and patent ductus arteriosus | 92.2 (78.9 to 107.6) | -- | 5.9 (3.0 to 9.6) | -0.8 (-8.4 to 6.6) | -2.7 (-10.1 to 4.6) |
| Other congenital heart anomalies | 148.8 (136.6 to 162.1) | -- | 8.8 (3.7 to 15.4) | 3.9 (-3.8 to 12.2) | 1.9 (-5.7 to 10.1) |
| Severe congenital heart anomalies excluding single ventricle heart defects | 180.0 (157.7 to 202.9) | -- | 10.9 (4.9 to 18.9) | 6.8* (0.1 to 14.1) | 4.8 (-1.9 to 11.9) |
| Single ventricle and single ventricle pathway congenital heart anomalies | 14.0 (11.7 to 16.8) | -- | 0.9 (0.5 to 1.5) | 9.5* (6.4 to 11.8) | 7.5* (4.3 to 9.7) |
| Ventricular septal defect and atrial septal defect | 130.4 (108.7 to 154.8) | -- | 4.3 (1.8 to 7.6) | 0.6 (-6.7 to 8.0) | -1.3 (-8.5 to 6.0) |
| Orofacial clefts | 820.0 (753.0 to 886.5) | -- | 25.2 (16.6 to 37.0) | 5.0 (-2.7 to 13.8) | 3.0 (-4.6 to 11.6) |
| Down's syndrome | 313.8 (273.8 to 356.5) | -- | 26.9 (18.2 to 37.6) | 2.0 (-0.8 to 4.8) | 0.1 (-2.7 to 2.8) |
| Turner syndrome | 62.0 (53.7 to 71.4) | -- | 1.3 (0.7 to 2.2) | 5.4* (0.6 to 10.2) | 3.4 (-1.3 to 8.1) |
| Klinefelter syndrome | 95.4 (82.3 to 109.4) | -- | 1.0 (0.4 to 1.9) | 1.5 (-6.4 to 9.5) | -0.5 (-8.2 to 7.4) |
| Other chromosomal abnormalities | 504.7 (391.2 to 630.0) | -- | 42.8 (27.4 to 61.6) | 13.6* (9.1 to 18.0) | 11.5* (7.0 to 15.7) |
| Congenital musculoskeletal and limb anomalies | 2410.7 (2143.6 to 2774.6) | -- | 364.1 (237.0 to 503.6) | 2.4* (0.2 to 4.5) | 0.4 (-1.7 to 2.5) |
| Polydactyly and syndactyly | 9.1 (7.2 to 11.1) | -- | 0.1 (0.0 to 0.2) | -0.1 (-1.6 to 1.6) | -2.0* (-3.5 to -0.3) |
| Congenital limb deficiency | 1214.8 (1078.1 to 1399.7) | -- | 184.1 (119.8 to 253.7) | 2.3 (-0.7 to 5.2) | 0.4 (-2.6 to 3.2) |
| Other congenital musculoskeletal anomalies | 1186.8 (1055.3 to 1366.1) | -- | 179.9 (117.2 to 250.3) | 2.4 (-0.6 to 5.6) | 0.4 (-2.5 to 3.6) |
| Urogenital congenital anomalies | 549.2 (467.8 to 637.4) | -- | 12.2 (6.9 to 20.9) | -5.3* (-8.3 to -2.1) | -7.1* (-10.0 to -3.9) |
| Digestive congenital anomalies | 1302.5 (1170.5 to 1459.8) | -- | 59.3 (39.6 to 84.8) | -2.8 (-7.4 to 2.0) | -4.7 (-9.2 to 0.0) |
| Congenital diaphragmatic hernia | 25.9 (22.7 to 29.5) | -- | 0.5 (0.3 to 0.8) | -0.3 (-1.1 to 0.4) | -2.2* (-3.0 to -1.5) |
| Congenital atresia and/or stenosis of the digestive tract | 1.2 (0.9 to 1.6) | -- | 0.1 (0.1 to 0.1) | 18.6* (15.2 to 22.2) | 16.3* (13.0 to 19.9) |
| Congenital malformations of the abdominal wall after treatment | 100.5 (88.0 to 114.3) | -- | 4.3 (2.8 to 6.3) | 0.6 (-0.2 to 1.3) | -1.3* (-2.1 to -0.6) |
| Other congenital malformations of the digestive tract | 1174.9 (1041.3 to 1332.8) | -- | 54.4 (35.9 to 78.1) | -3.1 (-8.2 to 2.0) | -5.0 (-10.0 to 0.1) |
| Other congenital anomalies | 2164.5 (1882.6 to 2506.0) | -- | 323.5 (218.1 to 447.8) | 6.2 (-0.7 to 13.5) | 4.2 (-2.6 to 11.3) |

| | | | | | |
|---|---|---|---|---|---|
| Other congenital birth defects | -- | -- | 36.1 (25.6 to 47.8) | 21.9* (17.6 to 26.5) | 19.6* (15.4 to 24.1) |
| Hearing loss due to other congenital anomalies | 2164.5 (1882.6 to 2506.0) | -- | 287.4 (191.3 to 405.6) | 4.5 (-3.1 to 12.6) | 2.5 (-5.0 to 10.4) |
| Urinary diseases and male infertility | 5594.3 (4106.6 to 7590.0) | 28415.2 (23229.4 to 34408.8) | 66.1 (38.4 to 114.0) | 6.8* (2.0 to 12.1) | 4.8 (-0.0 to 10.0) |
| Urinary tract infections | 463.5 (364.2 to 585.2) | 24686.8 (19555.5 to 30610.1) | 15.5 (9.1 to 24.4) | -1.9 (-7.3 to 3.2) | -3.8 (-9.1 to 1.3) |
| Urolithiasis | 136.8 (98.9 to 181.8) | 3728.4 (2659.0 to 4980.7) | 10.6 (6.4 to 16.0) | 7.7* (6.2 to 9.5) | 5.6* (4.1 to 7.4) |
| Benign prostatic hyperplasia | -- | -- | -- | -- | -- |
| Male infertility | 5090.6 (3563.0 to 7099.8) | -- | 33.4 (13.1 to 73.0) | 18.2* (11.7 to 24.2) | 15.9* (9.5 to 21.8) |
| Other urinary diseases | -- | -- | 6.6 (4.4 to 9.5) | -17.3* (-20.1 to -14.6) | -18.9* (-21.7 to -16.2) |
| Gynecological diseases | 86492.9 (80382.5 to 93221.3) | 22646.8 (20426.1 to 24902.7) | 1228.4 (849.7 to 1717.7) | 0.6 (-1.3 to 2.6) | -1.3 (-3.2 to 0.7) |
| Uterine fibroids | 1264.7 (911.0 to 1701.3) | 243.1 (164.7 to 350.3) | 11.5 (6.5 to 19.2) | -5.6 (-14.5 to 3.5) | -7.4 (-16.1 to 1.5) |
| Polycystic ovarian syndrome | 7252.2 (5633.1 to 9407.0) | 184.6 (85.7 to 307.3) | 66.9 (29.2 to 130.3) | 3.1* (1.5 to 4.7) | 1.1 (-0.5 to 2.7) |
| Female infertility | 9062.8 (5542.4 to 13598.9) | -- | 56.1 (21.5 to 127.5) | 28.5* (19.6 to 37.0) | 26.0* (17.3 to 34.4) |
| Endometriosis | 4676.2 (3460.6 to 6031.9) | 1210.7 (841.8 to 1652.8) | 438.1 (267.3 to 641.9) | -1.3 (-3.5 to 1.0) | -3.1* (-5.3 to -0.9) |
| Genital prolapse | 2065.7 (1451.3 to 2781.5) | 587.7 (423.9 to 758.5) | 6.6 (2.9 to 12.6) | -9.7* (-16.2 to -3.7) | -11.4* (-17.8 to -5.5) |
| Premenstrual syndrome | 66425.7 (60284.0 to 73394.0) | 15918.7 (13718.5 to 18150.1) | 560.6 (357.3 to 853.9) | -0.8 (-4.1 to 1.7) | -2.7* (-6.0 to -0.3) |
| Other gynecological diseases | 4609.7 (4225.5 to 4984.9) | 4501.9 (4165.7 to 4836.6) | 88.6 (58.1 to 132.3) | 5.9* (1.0 to 10.0) | 3.9 (-0.9 to 7.9) |
| Other gynecological diseases | 4176.8 (3799.9 to 4552.9) | 4501.9 (4165.7 to 4836.6) | 78.7 (51.1 to 118.9) | 5.8* (0.9 to 9.9) | 3.8 (-1.0 to 7.8) |
| Other gynecological diseases with anemia | 432.9 (396.3 to 472.3) | -- | 9.8 (6.3 to 14.8) | 6.4 (-8.7 to 24.2) | 4.4 (-10.5 to 21.8) |
| Hemoglobinopathies and hemolytic anaemias | 159264.9 (155410.8 to 163263.9) | -- | 400.9 (268.4 to 595.4) | -12.2* (-21.1 to -3.1) | -13.9* (-22.6 to -4.9) |
| Thalassemias | 7.0 (6.1 to 8.0) | -- | 0.4 (0.3 to 0.6) | -17.2* (-25.9 to -7.0) | -18.8* (-27.3 to -8.8) |
| Thalassaemias trait | 23375.3 (22446.3 to 24375.7) | -- | 140.8 (90.5 to 213.0) | -23.2* (-37.6 to -6.2) | -24.7* (-38.8 to -8.0) |
| Sickle cell disorders | 243.4 (209.6 to 285.2) | -- | 25.8 (16.7 to 37.0) | 24.4* (9.5 to 40.6) | 22.0* (7.4 to 37.9) |
| Sickle cell trait | 40348.1 (36461.5 to 44787.8) | -- | 81.8 (53.8 to 124.5) | -5.8 (-15.2 to 6.1) | -7.6 (-16.9 to 4.1) |
| G6PD deficiency | 30247.1 (29004.0 to 31539.5) | -- | 1.5 (1.0 to 2.3) | -9.0 (-25.4 to 18.0) | -10.7 (-26.8 to 15.8) |
| G6PD trait | 76984.1 (75695.9 to 78250.0) | -- | 0.0 (0.0 to 0.1) | -8.9 (-19.9 to 5.1) | -10.7 (-21.4 to 3.0) |
| Other hemoglobinopathies and hemolytic anaemias | 7096.4 (6587.8 to 7742.0) | -- | 150.4 (97.7 to 229.5) | -8.0 (-15.8 to 2.3) | -9.8 (-17.4 to 0.4) |
| Endocrine, metabolic, blood, and immune disorders | 8901.4 (8443.3 to 9445.0) | -- | 230.0 (154.0 to 332.9) | -9.1* (-14.8 to -3.2) | -10.8* (-16.4 to -5.0) |
| Anemia due to endocrine, metabolic, blood, and immune disorders | 8189.0 (7771.3 to 8685.4) | -- | 203.2 (133.8 to 296.9) | -10.2* (-16.7 to -3.7) | -12.0* (-18.3 to -5.6) |
| Endocrine, metabolic, blood, and immune disorders | 710.8 (536.7 to 932.4) | -- | 26.7 (15.7 to 40.5) | 0.6 (-4.5 to 6.5) | -1.3 (-6.4 to 4.4) |
| Heart failure due to endocrine, metabolic, blood, and immune disorders | 1.6 (1.1 to 2.3) | -- | 0.1 (0.1 to 0.2) | -3.3* (-4.5 to -2.2) | -5.1* (-6.3 to -4.0) |
| Oral disorders | 248299.6 (203436.1 to 303696.8) | 329435.3 (273711.8 to 376719.6) | 656.0 (378.9 to 1067.6) | 3.2* (2.3 to 4.2) | 1.2* (0.3 to 2.2) |
| Caries of deciduous teeth | -- | -- | -- | -- | -- |
| Caries of permanent teeth | 237367.8 (188038.2 to 299828.3) | 325715.7 (270199.1 to 372637.9) | 163.4 (69.2 to 306.8) | -1.0 (-2.3 to 0.3) | -2.9* (-4.1 to -1.6) |
| Periodontal disease | 14943.8 (9495.0 to 22455.4) | 3263.7 (1872.2 to 5030.9) | 99.4 (36.5 to 215.8) | 11.0* (9.8 to 12.6) | 8.9* (7.7 to 10.5) |
| Edentulism and severe tooth loss | 2099.0 (1478.1 to 2837.1) | 455.9 (329.0 to 616.1) | 60.9 (35.1 to 96.1) | 10.4* (6.9 to 13.9) | 8.2* (4.8 to 11.7) |
| Other oral disorders | 11296.8 (10212.9 to 12412.1) | -- | 332.3 (204.2 to 507.2) | 2.0* (0.8 to 3.2) | 0.1 (-1.1 to 1.2) |
| **Injuries** | **84348.2 (74491.1 to 96002.7)** | **38951.1 (33751.8 to 44792.3)** | **3004.8 (2273.3 to 3882.6)** | **1.8* (0.3 to 3.2)** | **-0.1 (-1.6 to 1.2)** |
| **Transport injuries** | **8947.7 (7814.9 to 10005.0)** | **6142.3 (4403.0 to 8265.5)** | **619.6 (435.2 to 837.9)** | **-2.1* (-3.8 to -0.6)** | **-4.0* (-5.6 to -2.5)** |
| Road injuries | 5968.3 (5302.8 to 6591.7) | 5510.6 (3831.2 to 7739.3) | 408.9 (286.7 to 553.3) | -5.3* (-7.1 to -3.5) | -7.1* (-8.9 to -5.4) |
| Pedestrian road injuries | 1614.7 (1267.1 to 1984.0) | 999.1 (556.3 to 1634.8) | 112.4 (75.5 to 157.7) | -10.0* (-12.1 to -8.1) | -11.7* (-13.8 to -9.9) |
| Cyclist road injuries | 1051.8 (827.7 to 1333.0) | 1020.8 (567.9 to 1691.4) | 67.3 (45.4 to 92.6) | -2.6* (-4.7 to -0.3) | -4.5* (-6.5 to -2.2) |
| Motorcyclist road injuries | 1583.6 (1244.6 to 1916.3) | 1271.8 (815.2 to 1914.2) | 108.1 (72.5 to 149.8) | -2.6* (-5.0 to -0.1) | -4.5* (-6.8 to -2.0) |
| Motor vehicle road injuries | 1286.1 (1031.9 to 1634.1) | 1784.0 (1124.2 to 2640.9) | 95.2 (64.9 to 136.3) | -7.6* (-10.4 to -5.0) | -9.4* (-12.1 to -6.8) |
| Other road injuries | 432.1 (324.0 to 554.5) | 434.9 (210.6 to 739.1) | 26.0 (17.3 to 37.4) | 9.3* (6.7 to 11.8) | 7.2* (4.7 to 9.7) |
| Other transport injuries | 2979.4 (2273.7 to 3763.8) | 631.8 (388.1 to 969.0) | 210.7 (144.7 to 291.2) | 4.8* (2.6 to 7.0) | 2.8* (0.6 to 5.0) |
| **Unintentional injuries** | **40000.4 (36233.0 to 44153.4)** | **26933.9 (22218.6 to 32108.0)** | **1760.5 (1287.0 to 2365.2)** | **6.3* (5.0 to 7.4)** | **4.2* (3.0 to 5.3)** |
| Falls | 15810.4 (12908.2 to 19498.0) | 9126.8 (6057.9 to 13197.2) | 771.1 (547.1 to 1083.8) | 3.7* (2.4 to 4.9) | 1.7* (0.4 to 2.9) |
| Drowning | 101.5 (83.8 to 123.1) | 22.7 (16.4 to 31.0) | 7.2 (5.0 to 10.0) | -13.6* (-16.3 to -11.0) | -15.3* (-17.9 to -12.7) |
| Fire, heat, and hot substances | 4693.7 (3754.2 to 6053.4) | 868.1 (555.0 to 1246.8) | 222.1 (154.8 to 303.1) | 3.5 (-0.1 to 6.9) | 1.5 (-2.0 to 4.9) |
| Poisonings | 302.2 (248.4 to 366.6) | 252.2 (141.6 to 388.2) | 36.1 (23.4 to 51.5) | 14.7* (11.8 to 17.8) | 12.5* (9.7 to 15.6) |
| Poisoning by carbon monoxide | 49.6 (39.6 to 61.9) | 73.1 (30.8 to 143.6) | 5.6 (3.6 to 8.4) | 17.3* (13.0 to 21.9) | 15.1* (10.8 to 19.5) |
| Poisoning by other means | 252.6 (196.5 to 315.5) | 179.1 (95.6 to 284.7) | 30.4 (19.4 to 44.7) | 14.2* (11.1 to 17.4) | 12.1* (8.9 to 15.2) |
| Exposure to mechanical forces | 8063.7 (6251.1 to 9843.9) | 6479.8 (4240.8 to 9263.2) | 235.7 (163.3 to 326.4) | 10.3* (8.3 to 12.3) | 8.2* (6.3 to 10.2) |
| Unintentional firearm injuries | 392.0 (294.8 to 489.3) | 199.6 (91.8 to 369.8) | 19.0 (13.3 to 26.5) | 11.5* (9.3 to 13.7) | 9.3* (7.2 to 11.5) |
| Other exposure to mechanical forces | 7671.7 (5798.2 to 9481.1) | 6280.2 (4102.3 to 9005.8) | 216.7 (148.1 to 301.7) | 10.3* (8.1 to 12.3) | 8.1* (6.1 to 10.2) |
| Adverse effects of medical treatment | 64.5 (39.5 to 98.0) | 842.2 (538.6 to 1224.4) | 8.6 (4.5 to 14.4) | -6.0* (-11.5 to -1.5) | -7.8* (-13.2 to -3.4) |
| Animal contact | 2144.8 (1735.8 to 2635.9) | 3642.9 (2244.8 to 5431.0) | 81.5 (53.9 to 114.8) | 6.8* (3.7 to 9.9) | 4.8* (1.8 to 7.8) |
| Venomous animal contact | 975.7 (741.2 to 1288.1) | 1935.4 (1158.3 to 3003.3) | 62.2 (40.5 to 89.8) | 8.5* (4.6 to 12.4) | 6.4* (2.6 to 10.3) |
| Non-venomous animal contact | 1169.1 (880.4 to 1522.8) | 1707.5 (961.1 to 2751.9) | 19.3 (11.7 to 30.7) | 1.7 (-0.7 to 3.7) | -0.2 (-2.6 to 1.7) |
| Foreign body | 1039.8 (838.6 to 1281.6) | 1724.9 (1131.2 to 2489.2) | 58.6 (40.3 to 82.8) | 7.1* (5.3 to 8.7) | 5.1* (3.3 to 6.6) |

| | | | | | |
|---|---|---|---|---|---|
| Pulmonary aspiration and foreign body in airway | 131.2 (100.7 to 165.4) | 50.6 (24.5 to 84.5) | 8.4 (5.6 to 11.6) | 5.9* (2.7 to 9.3) | 3.9* (0.8 to 7.2) |
| Foreign body in eyes | 208.2 (82.0 to 392.4) | 1428.2 (836.6 to 2159.1) | 12.6 (4.8 to 25.4) | 8.3* (7.0 to 9.6) | 6.3* (4.9 to 7.5) |
| Foreign body in other body part | 700.3 (538.7 to 879.1) | 246.0 (142.7 to 411.2) | 37.6 (26.1 to 50.9) | 7.0* (4.8 to 9.1) | 4.9* (2.8 to 7.0) |
| Environmental heat and cold exposure | 1768.6 (1390.5 to 2212.5) | 626.8 (370.1 to 985.2) | 88.7 (61.7 to 122.3) | 5.5* (3.7 to 7.4) | 3.5* (1.7 to 5.3) |
| Exposure to forces of nature | 1582.8 (1112.8 to 2216.3) | 53.0 (37.1 to 73.0) | 84.2 (57.1 to 117.6) | 16.3* (7.7 to 25.0) | 14.0* (5.7 to 22.6) |
| Other unintentional injuries | 4428.5 (3546.0 to 5732.4) | 3294.4 (2149.1 to 4737.2) | 166.7 (115.8 to 234.4) | 12.1* (9.9 to 14.3) | 10.0* (7.8 to 12.1) |
| **Self-harm and interpersonal violence** | **35400.1 (26543.6 to 45347.0)** | **5874.9 (4758.8 to 7291.0)** | **624.7 (453.3 to 827.1)** | **-5.6* (-9.5 to -2.3)** | **-7.4* (-11.2 to -4.1)** |
| Self-harm | 284.2 (203.4 to 397.5) | 549.7 (367.5 to 794.7) | 21.6 (14.0 to 30.8) | -11.9* (-14.3 to -9.8) | -13.6* (-15.9 to -11.6) |
| Self-harm by firearm | 3.5 (2.7 to 4.9) | 2.2 (0.6 to 5.5) | 0.2 (0.1 to 0.3) | -10.7* (-13.8 to -7.3) | -12.4* (-15.5 to -9.1) |
| Self-harm by other specified means | 280.7 (200.2 to 394.0) | 547.6 (366.0 to 792.8) | 21.4 (13.8 to 30.5) | -11.9* (-14.3 to -9.8) | -13.6* (-16.0 to -11.5) |
| Interpersonal violence | 32146.2 (23423.7 to 42106.1) | 2786.6 (1858.7 to 3952.3) | 417.2 (283.6 to 594.2) | -1.3 (-2.8 to 0.1) | -3.1* (-4.6 to -1.8) |
| Assault by firearm | 96.4 (73.5 to 134.1) | 89.6 (49.7 to 146.2) | 5.8 (4.0 to 8.3) | 3.3 (-0.5 to 7.2) | 1.3 (-2.4 to 5.2) |
| Assault by sharp object | 688.4 (521.0 to 938.3) | 603.5 (345.5 to 962.5) | 28.6 (19.4 to 40.8) | -2.0 (-5.2 to 1.1) | -3.9* (-7.0 to -0.9) |
| Sexual violence | 29744.0 (20900.5 to 39747.7) | -- | 293.7 (175.1 to 463.2) | -1.7* (-3.7 to -0.1) | -3.6* (-5.6 to -2.0) |
| Assault by other means | 1617.5 (1273.0 to 2111.0) | 2093.4 (1361.4 to 3069.3) | 89.1 (61.5 to 119.8) | 0.2 (-2.5 to 3.3) | -1.7 (-4.4 to 1.3) |
| Conflict and terrorism | 2770.7 (1904.3 to 4000.9) | 2142.5 (1696.5 to 2720.3) | 175.6 (111.3 to 274.5) | -15.8* (-22.5 to -7.6) | -17.4* (-24.0 to -9.4) |
| Executions and police conflict | 199.0 (130.3 to 300.7) | 396.1 (275.5 to 559.8) | 10.4 (6.7 to 15.7) | 72.2* (52.3 to 94.7) | 68.9* (49.4 to 91.0) |
| **Nature of injury aggregates** | | | | | |
| Amputations | 17377.5 (15521.6 to 19774.8) | 826.2 (666.4 to 1023.4) | 325.2 (222.2 to 475.8) | 1.9 (-1.6 to 4.8) | -0.1 (-3.5 to 2.8) |
| Burns | 9913.8 (8806.7 to 11291.0) | 1376.5 (1086.6 to 1705.7) | 493.9 (361.4 to 656.5) | -0.9 (-5.4 to 2.9) | -2.8 (-7.2 to 1.0) |
| Fractures | 11881.0 (10795.0 to 13227.8) | 9337.5 (7827.8 to 11029.8) | 679.6 (461.9 to 960.6) | 2.2 (-0.3 to 3.9) | 0.2 (-2.2 to 1.9) |
| Head Injuries | 2006.8 (1857.4 to 2163.9) | 1808.5 (1473.2 to 2185.2) | 311.3 (218.7 to 426.0) | 5.2* (3.1 to 7.3) | 3.2* (1.1 to 5.3) |
| Spinal Injuries | 1253.1 (1108.0 to 1480.4) | 61.5 (46.4 to 82.5) | 406.6 (285.1 to 556.0) | 3.0 (-1.8 to 6.7) | 1.1 (-3.7 to 4.6) |
| Minor Injuries | 8594.4 (7801.8 to 9548.1) | 14882.0 (12501.7 to 17527.6) | 75.3 (35.4 to 143.5) | 5.7* (4.3 to 7.0) | 3.7* (2.3 to 5.0) |
| Other Injuries | 3577.7 (3257.4 to 3911.4) | 10658.9 (9270.6 to 12234.9) | 419.2 (291.1 to 576.6) | 2.8 (-0.5 to 5.4) | 0.8 (-2.4 to 3.4) |
| **Impairments** | | | | | |
| Anaemia | 144477.1 (135841.6 to 154719.5) | -- | 3554.2 (2361.4 to 5279.5) | -10.3* (-16.7 to -3.0) | -12.1* (-18.3 to -4.9) |
| Epilepsy | 5520.9 (4595.5 to 6669.4) | -- | 2328.2 (1784.5 to 3045.5) | 5.5 (-0.7 to 11.6) | 3.5 (-2.6 to 9.5) |
| Guillain-Barré syndrome | 6.0 (3.2 to 10.5) | -- | 1.8 (0.8 to 3.5) | 8.7* (5.8 to 11.8) | 6.6* (3.8 to 9.7) |
| Hearing loss | 50112.5 (45086.1 to 56072.1) | -- | 1545.5 (1069.9 to 2166.2) | 2.0 (-0.6 to 5.0) | 0.1 (-2.5 to 3.0) |
| Heart failure | 282.6 (220.2 to 358.3) | -- | 26.4 (16.6 to 39.4) | -1.5* (-2.6 to -0.5) | -3.4* (-4.4 to -2.4) |
| Infertility | 16948.9 (11919.3 to 23120.4) | -- | 142.2 (72.8 to 278.2) | 14.6* (8.0 to 20.1) | 12.4* (5.9 to 17.8) |
| Developmental intellectual disability | 17340.6 (13419.3 to 21311.4) | -- | 2257.9 (1753.1 to 2825.7) | 4.2* (1.5 to 7.3) | 2.2 (-0.5 to 5.2) |
| Pelvic inflammatory disease | 80.1 (63.6 to 99.5) | -- | 10.8 (6.8 to 15.3) | 6.1 (-2.1 to 14.9) | 4.1 (-4.0 to 12.7) |
| Vision loss | 32721.5 (29243.7 to 36632.8) | -- | 950.6 (643.1 to 1374.0) | 2.1* (0.9 to 3.3) | 0.2 (-1.0 to 1.3) |

We did not calculate incidence for the nine impairments and certain NTDs. Blank cells mean that no estimate is available or that the estimate has a magnitude less than 50.
Table presents prevalence and incidence counts in thousands.
* Percentage changes that are statistically significant
‡ Incidence estimates for stroke represent first-ever stroke only.
^ Incidence of HIV/AIDS represents new infections of HIV only and does not include new infections of Tuberculosis in HIV positive cases.

| SR Table 10i: Global prevalence, incidence and YLDs for 2017, percentage change of YLD counts and percentage change of YLD rates between 2007 and 2017 by cause, age 25 to 29 years | | | | | |
|---|---|---|---|---|---|
| Cause | Prevalence in thousands (95% UI) | Incidence in thousands (95% UI) | YLDs in thousands (95% UI) | | |
| | 2017 counts | 2017 counts | 2017 counts | Percentage change in counts between 2007 and 2017 | Percentage change in rates between 2007 and 2017 |
| **All causes** | **603731.2 (600615.4 to 606933.3)** | **3064568.2 (2620032.7 to 3590547.7)** | **59171.2 (44409.5 to 76255.3)** | **14.2* (13.3 to 15.0)** | **-2.1* (-2.8 to -1.4)** |
| **Communicable, maternal, neonatal, and nutritional disorders** | **408817.4 (382187.2 to 442981.5)** | **2176147.3 (1747477.4 to 2703230.5)** | **8719.8 (6490.1 to 11423.2)** | **8.1* (4.7 to 12.2)** | **-7.3* (-10.2 to -3.8)** |
| **HIV/AIDS and sexually transmitted infections** | **136206.6 (120548.6 to 153599.2)** | **147453.2 (116762.8 to 178128.6)** | **680.9 (475.5 to 946.0)** | **-14.9* (-25.4 to -4.6)** | **-27.0* (-36.0 to -18.2)** |
| HIV/AIDS | 4420.6 (4078.7 to 4777.8) | 359.4^ (299.0 to 429.5) | 453.5 (328.3 to 604.7) | -25.0* (-34.2 to -16.3) | -35.7* (-43.5 to -28.2) |
| HIV/AIDS - Drug-susceptible Tuberculosis | 148.9 (133.0 to 165.3) | 184.6 (167.5 to 203.8) | 58.0 (38.8 to 79.1) | -33.1* (-37.0 to -29.2) | -42.7* (-46.0 to -39.3) |
| HIV/AIDS - Multidrug-resistant Tuberculosis without extensive drug resistance | 4.9 (3.3 to 7.0) | 7.0 (5.1 to 9.5) | 2.0 (1.2 to 3.1) | -40.1* (-59.8 to -11.0) | -48.6* (-65.5 to -23.7) |
| HIV/AIDS - Extensively drug-resistant Tuberculosis | 0.2 (0.1 to 0.3) | 0.2 (0.2 to 0.3) | 0.1 (0.0 to 0.1) | 4.5 (-35.5 to 65.6) | -10.4 (-44.7 to 42.0) |
| HIV/AIDS resulting in other diseases | 4266.7 (3920.7 to 4623.2) | 359.4 (299.0 to 429.5) | 393.5 (279.8 to 533.6) | -23.6* (-34.5 to -13.2) | -34.5* (-43.8 to -25.6) |
| HIV/AIDS not on antiretroviral treatment without Tuberculosis | 2530.9 (2261.9 to 2864.5) | 359.4 (299.0 to 429.5) | 257.0 (177.8 to 356.8) | -48.1* (-54.3 to -41.9) | -55.5* (-60.8 to -50.1) |
| HIV/AIDS on antiretroviral treatment without Tuberculosis | 1735.8 (1545.8 to 1929.5) | -- | 136.5 (90.8 to 194.3) | 599.3* (523.7 to 686.2) | 499.7* (434.8 to 574.2) |
| Sexually transmitted infections excluding HIV | 133696.3 (117916.4 to 151189.1) | 147093.8 (116409.3 to 177771.3) | 227.3 (125.2 to 408.1) | 16.6* (13.7 to 19.9) | -0.0 (-2.5 to 2.8) |
| Syphilis | 6823.3 (4907.6 to 9372.6) | 1780.3 (1141.9 to 2586.2) | 3.7 (2.3 to 6.1) | 13.7* (10.3 to 17.2) | -2.5 (-5.4 to 0.5) |
| Early syphilis | 6811.7 (4893.2 to 9361.6) | 1780.3 (1141.9 to 2586.2) | 1.6 (0.5 to 4.3) | 14.3* (6.9 to 21.7) | -2.0 (-8.3 to 4.3) |
| Tertiary syphilis | 11.5 (9.0 to 14.2) | -- | 2.1 (1.4 to 2.9) | 13.2* (10.4 to 16.3) | -2.9* (-5.3 to -0.2) |
| Chlamydial infection | 21608.7 (15102.9 to 29637.3) | 72214.9 (49400.0 to 99715.5) | 64.9 (36.5 to 117.0) | 16.9* (11.3 to 22.8) | 0.2 (-4.5 to 5.3) |
| Chlamydia episode | 20920.9 (14376.9 to 28947.3) | 72214.9 (49400.0 to 99715.5) | 35.6 (12.8 to 81.2) | 16.1* (14.4 to 17.7) | -0.5 (-1.9 to 1.0) |
| Chlamydial infection complications | 687.7 (629.9 to 756.6) | -- | 29.3 (18.6 to 41.2) | 17.9* (6.4 to 29.8) | 1.1 (-8.7 to 11.3) |
| Gonococcal infection | 9754.4 (5505.6 to 15571.4) | 28842.0 (16371.5 to 44400.7) | 40.2 (20.2 to 79.7) | 17.4* (9.0 to 25.5) | 0.7 (-6.5 to 7.6) |
| Gonococcal infection complications | 240.7 (208.8 to 280.6) | -- | 14.5 (8.9 to 22.5) | 22.7* (4.8 to 39.7) | 5.2 (-10.1 to 19.8) |
| Gonorrhoea episode | 9513.7 (5267.1 to 15339.3) | 28842.0 (16371.5 to 44400.7) | 25.7 (8.6 to 62.9) | 14.7* (6.5 to 19.5) | -1.7 (-8.7 to 2.5) |
| Trichomoniasis | 22152.7 (14590.9 to 32592.0) | 31465.5 (21208.1 to 43307.7) | 38.9 (14.5 to 86.6) | 15.0* (12.7 to 17.1) | -1.4 (-3.4 to 0.4) |
| Genital herpes | 86269.7 (72246.3 to 103013.2) | 12791.0 (9481.9 to 17263.5) | 24.1 (8.0 to 55.0) | 16.8* (14.4 to 19.3) | 0.2 (-1.8 to 2.3) |
| Other sexually transmitted infections | 1168.0 (1081.1 to 1264.6) | -- | 55.6 (34.3 to 91.0) | 16.8* (13.0 to 20.7) | 0.2 (-3.1 to 3.5) |
| Other sexually transmitted diseases residual | -- | -- | 34.1 (18.7 to 61.3) | 17.2* (12.3 to 22.7) | 0.5 (-3.7 to 5.2) |
| Other sexually transmitted diseases | 1168.0 (1081.1 to 1264.6) | -- | 55.6 (34.3 to 91.0) | 16.8* (13.0 to 20.7) | 0.2 (-3.1 to 3.5) |
| **Respiratory infections and tuberculosis** | **193542.2 (139168.3 to 271533.7)** | **1451426.0 (1032358.3 to 1961168.9)** | **880.8 (553.4 to 1326.3)** | **14.4* (12.4 to 16.4)** | **-1.9* (-3.6 to -0.2)** |
| Tuberculosis | 175606.7 (118007.3 to 256358.9) | 822.6 (606.2 to 1084.0) | 229.8 (137.4 to 349.7) | 15.4* (10.0 to 21.6) | -1.0 (-5.7 to 4.3) |
| Latent tuberculosis infection | 174876.9 (117406.0 to 255670.4) | -- | -- | -- | -- |
| Drug-susceptible tuberculosis | 695.0 (496.0 to 956.9) | 779.5 (576.6 to 1027.9) | 218.5 (131.4 to 336.4) | 15.6* (9.4 to 23.2) | -0.9 (-6.2 to 5.7) |
| Multidrug-resistant tuberculosis without extensive drug resistance | 33.1 (16.3 to 61.2) | 40.6 (22.3 to 69.7) | 10.7 (4.9 to 21.8) | 10.8 (-39.0 to 87.2) | -5.0 (-47.7 to 60.5) |
| Extensively drug-resistant tuberculosis | 1.8 (1.0 to 3.5) | 2.4 (1.6 to 3.6) | 0.6 (0.3 to 1.2) | 53.2 (-3.2 to 165.7) | 31.4 (-17.0 to 127.8) |
| Lower respiratory infections | 738.1 (616.7 to 866.1) | 32674.2 (27436.9 to 38188.2) | 45.2 (29.1 to 66.8) | 33.6* (22.9 to 44.5) | 14.6* (5.4 to 23.9) |
| Guillain-Barré syndrome due to lower respiratory infections | 0.7 (0.3 to 1.3) | -- | 0.2 (0.1 to 0.4) | 16.6* (14.1 to 19.4) | -0.0 (-2.1 to 2.4) |
| Lower respiratory infection episode | 737.4 (615.9 to 865.5) | 32674.2 (27436.9 to 38188.2) | 45.0 (28.8 to 66.5) | 33.7* (23.0 to 44.6) | 14.6* (5.4 to 24.0) |
| Upper respiratory infections | 19428.7 (13548.4 to 26593.8) | 1412268.1 (995068.3 to 1923253.3) | 486.5 (249.3 to 851.3) | 14.8* (13.5 to 16.2) | -1.5* (-2.7 to -0.4) |
| Guillain-Barré syndrome due to upper respiratory infections | 1.9 (1.1 to 3.3) | -- | 0.6 (0.3 to 1.1) | 16.6* (14.1 to 19.3) | -0.0 (-2.1 to 2.3) |
| Upper respiratory infection episode | 19426.8 (13546.9 to 26591.2) | 1412268.1 (995068.3 to 1923253.3) | 486.0 (248.8 to 850.7) | 14.8* (13.5 to 16.2) | -1.5* (-2.7 to -0.4) |
| Otitis media | 5656.4 (4177.4 to 7513.7) | 5661.1 (3864.0 to 7676.8) | 119.3 (65.1 to 204.8) | 5.3* (0.4 to 10.2) | -9.7* (-13.9 to -5.5) |
| Acute otitis media | 300.2 (217.5 to 405.4) | 5660.5 (3863.5 to 7676.4) | 3.9 (1.8 to 7.3) | 15.7* (8.5 to 22.8) | -0.8 (-6.9 to 5.3) |
| Chronic otitis media | 5356.2 (3897.1 to 7166.3) | 0.6 (0.0 to 2.4) | 115.4 (62.8 to 198.0) | 4.9 (-0.0 to 10.1) | -10.0* (-14.3 to -5.6) |
| **Enteric infections** | **5984.0 (5171.2 to 6961.6)** | **417136.4 (352671.8 to 492535.7)** | **686.9 (465.5 to 961.9)** | **36.8* (30.7 to 43.2)** | **17.3* (12.1 to 22.8)** |
| Diarrhoeal diseases | 6006.6 (5186.3 to 6968.3) | 416283.4 (351830.5 to 491721.8) | 680.1 (459.7 to 953.7) | 37.6* (31.4 to 43.9) | 18.0* (12.7 to 23.4) |
| Guillain-Barré syndrome due to diarrheal diseases | 0.7 (0.4 to 1.1) | -- | 0.2 (0.1 to 0.4) | 16.6* (14.1 to 19.3) | -0.0 (-2.1 to 2.3) |
| Diarrheal disease episode | 6005.9 (5185.6 to 6967.8) | 416283.4 (351830.5 to 491721.8) | 679.9 (459.6 to 953.6) | 37.6* (31.4 to 43.9) | 18.0* (12.7 to 23.4) |
| Typhoid and paratyphoid | 9.7 (6.2 to 13.9) | 830.5 (688.7 to 975.9) | 6.7 (4.2 to 9.7) | -12.3 (-28.5 to 6.5) | -24.8* (-38.7 to -8.7) |
| Typhoid fever | 39.6 (29.8 to 50.4) | 629.0 (512.7 to 763.5) | 6.1 (3.8 to 9.0) | -13.0 (-30.3 to 7.9) | -25.4* (-40.2 to -7.4) |
| Typhoid fever complications | 8.3 (5.8 to 11.4) | 108.1 (84.4 to 137.4) | 2.7 (1.6 to 4.0) | -13.1 (-37.6 to 20.0) | -25.5 (-46.5 to 2.9) |
| Typhoid fever episode | 31.3 (23.2 to 40.5) | 520.9 (420.8 to 633.8) | 3.4 (2.0 to 5.3) | -12.8 (-34.5 to 16.7) | -25.3 (-43.9 to 0.1) |
| Paratyphoid fever | 8.8 (6.0 to 12.0) | 201.5 (137.6 to 270.7) | 0.6 (0.3 to 0.9) | -4.9 (-19.4 to 13.2) | -18.4* (-30.8 to -2.9) |
| Intestinal perforation due to paratyphoid | 0.4 (0.3 to 0.5) | 10.3 (6.7 to 14.1) | 0.0 (0.0 to 0.1) | -4.5 (-25.2 to 23.8) | -18.1 (-35.8 to 6.1) |
| Paratyphoid fever episode | 8.4 (5.7 to 11.4) | 191.2 (130.8 to 255.9) | 0.5 (0.3 to 0.8) | -4.9 (-20.3 to 14.5) | -18.5* (-31.6 to -1.8) |
| iNTS | 0.9 (0.5 to 1.4) | 22.5 (12.6 to 36.3) | 0.1 (0.1 to 0.2) | -23.7* (-29.3 to -18.2) | -34.6* (-39.4 to -29.9) |
| Other intestinal infectious diseases | -- | -- | -- | -30.5* (-43.3 to -15.6) | -40.4* (-51.4 to -27.6) |
| **Neglected tropical diseases and malaria** | **116398.5 (110570.8 to 122693.9)** | **22341.7 (17895.9 to 27908.5)** | **1169.4 (811.6 to 1612.1)** | **-8.1* (-14.0 to -1.7)** | **-21.2* (-26.2 to -15.7)** |
| Malaria | 8717.9 (8117.2 to 9263.5) | 11084.2 (8636.2 to 13919.6) | 86.1 (64.5 to 115.3) | -14.7* (-24.0 to -2.8) | -26.8* (-34.8 to -16.7) |
| Malaria complications | 81.7 (74.4 to 89.8) | -- | 33.7 (26.2 to 42.1) | 23.5* (11.2 to 37.0) | 5.9 (-4.6 to 17.4) |
| Malaria episode | 647.2 (415.7 to 930.1) | 11084.2 (8636.2 to 13919.6) | 19.5 (10.0 to 33.0) | -12.9* (-21.2 to -4.1) | -25.3* (-32.5 to -17.8) |

| | | | | | |
|---|---|---|---|---|---|
| Malaria parasitemia | 7989.1 (7352.7 to 8694.0) | -- | 32.9 (21.1 to 50.0) | -35.7* (-47.6 to -19.2) | -44.9* (-55.0 to -30.7) |
| Chagas disease | 455.0 (366.3 to 549.9) | 14.0 (9.9 to 19.0) | 2.6 (1.6 to 4.0) | -15.8* (-24.3 to -6.5) | -27.8* (-35.1 to -19.8) |
| Acute Chagas disease | 0.1 (0.0 to 0.1) | 14.0 (9.9 to 19.0) | -- | -14.5* (-18.6 to -10.3) | -26.6* (-30.2 to -23.1) |
| Asymptomatic Chagas disease | 405.0 (324.1 to 489.4) | -- | -- | -- | -- |
| Symptomatic chronic Chagas infection | 49.9 (37.5 to 63.9) | -- | 2.6 (1.6 to 4.0) | -15.8* (-24.3 to -6.4) | -27.8* (-35.1 to -19.8) |
| Leishmaniasis | 340.5 (291.5 to 397.9) | 37.6 (27.2 to 50.8) | 22.1 (14.6 to 32.0) | 28.6* (13.1 to 46.7) | 10.3 (-3.0 to 25.8) |
| Visceral leishmaniasis | 0.5 (0.4 to 0.8) | 2.0 (1.5 to 3.1) | 0.0 (0.0 to 0.1) | -72.5* (-79.0 to -58.5) | -76.4* (-82.0 to -64.4) |
| Cutaneous and mucocutaneous leishmaniasis | 344.2 (297.0 to 400.5) | 35.6 (25.2 to 48.5) | 22.1 (14.6 to 32.0) | 29.4* (13.7 to 47.9) | 11.0 (-2.5 to 26.8) |
| African trypanosomiasis | 0.5 (0.1 to 2.0) | 0.3 (0.1 to 1.1) | 0.1 (0.0 to 0.6) | -79.4 (-97.4 to 9.5) | -82.3* (-97.8 to -6.1) |
| Trypanosomiasis Gambiense | 0.5 (0.1 to 2.0) | 0.3 (0.1 to 1.1) | 0.1 (0.0 to 0.6) | -78.6 (-97.5 to 22.2) | -81.6 (-97.8 to 4.8) |
| Trypanosomiasis Rhodesiense | -- | 0.0 (0.0 to 0.1) | -- | -92.1* (-99.0 to -53.8) | -93.2* (-99.1 to -60.4) |
| Schistosomiasis | 18126.5 (15236.5 to 21111.7) | -- | 141.9 (70.3 to 272.1) | -21.9* (-25.4 to -18.0) | -33.1* (-36.1 to -29.7) |
| Mild schistosomiasis | 13897.4 (11353.3 to 16583.9) | -- | 78.9 (31.1 to 166.6) | -24.8* (-28.4 to -21.7) | -35.5* (-38.6 to -32.8) |
| Anemia due to schistosomiasis | 981.5 (788.8 to 1207.5) | -- | 22.9 (14.4 to 35.4) | -29.2* (-41.4 to -15.1) | -39.3* (-49.7 to -27.2) |
| Schistosomiasis complications | 3248.3 (2590.5 to 3920.9) | -- | 40.1 (20.9 to 72.6) | -9.8* (-14.5 to -3.4) | -22.7* (-26.7 to -17.2) |
| Cysticercosis | 484.4 (345.4 to 644.0) | -- | 150.3 (88.8 to 225.8) | -0.9 (-8.8 to 6.2) | -15.0* (-21.7 to -8.9) |
| Cystic echinococcosis | 70.3 (32.5 to 145.2) | 15.2 (7.7 to 29.0) | 5.9 (2.3 to 12.8) | 24.2* (11.8 to 34.9) | 6.5 (-4.2 to 15.7) |
| Lymphatic filariasis | 5953.8 (5323.8 to 6680.3) | -- | 113.8 (61.2 to 183.5) | -40.1* (-50.8 to -29.6) | -48.7* (-57.8 to -39.6) |
| Prevalence of detectable microfiliaria due to lymphatic filariasis | 4940.2 (4397.5 to 5527.1) | -- | -- | -- | -- |
| Lymphatic filariasis complications | 1013.6 (675.2 to 1461.9) | -- | 113.8 (61.2 to 183.5) | -40.1* (-50.8 to -29.6) | -48.7* (-57.8 to -39.6) |
| Onchocerciasis | 2399.4 (1344.1 to 4486.0) | -- | 130.7 (56.4 to 234.4) | 4.8 (-18.8 to 23.4) | -10.1 (-30.3 to 5.8) |
| Asymptomatic onchocerciasis | 831.9 (8.9 to 2727.1) | -- | -- | -- | -- |
| Skin disease due to onchocerciasis | 1548.8 (1115.7 to 2090.8) | -- | 129.2 (55.6 to 232.9) | 5.7 (-17.9 to 24.4) | -9.4 (-29.6 to 6.7) |
| Vision loss due to onchocerciasis | 18.7 (12.9 to 28.3) | -- | 1.5 (0.9 to 2.3) | -40.1* (-52.6 to -26.6) | -48.7* (-59.3 to -37.1) |
| Trachoma | 11.3 (5.9 to 19.9) | -- | 1.8 (0.9 to 3.2) | -42.1* (-62.2 to -14.0) | -50.3* (-67.6 to -26.2) |
| Dengue | 526.4 (287.0 to 891.2) | 8795.4 (5352.9 to 13335.6) | 86.5 (38.0 to 163.0) | 65.5* (43.0 to 153.6) | 41.9* (22.6 to 117.5) |
| Post-dengue chronic fatigue syndrome | 371.2 (173.5 to 678.7) | -- | 77.3 (32.3 to 146.5) | 65.5* (41.9 to 155.6) | 41.9* (21.7 to 119.2) |
| Dengue episode | 155.2 (93.8 to 232.9) | 8795.4 (5352.9 to 13335.6) | 9.2 (4.7 to 16.1) | 65.3* (41.7 to 148.2) | 41.8* (21.5 to 112.9) |
| Yellow fever | 0.2 (0.1 to 0.5) | 7.6 (2.0 to 20.0) | -- | -14.9 (-39.1 to 20.1) | -27.0 (-47.8 to 3.0) |
| Asymptomatic yellow fever | 0.1 (0.0 to 0.3) | 4.2 (1.0 to 11.6) | -- | -- | -- |
| Yellow fever episode | 0.1 (0.0 to 0.2) | 3.4 (0.9 to 8.9) | -- | -14.9 (-39.1 to 20.1) | -27.0 (-47.8 to 3.0) |
| Rabies | -- | 0.4 (0.3 to 0.6) | -- | -30.6* (-47.9 to -12.0) | -40.5* (-55.3 to -24.6) |
| Intestinal nematode infections | 81451.4 (75770.2 to 87927.4) | -- | 140.0 (80.0 to 230.5) | -25.7* (-29.8 to -21.6) | -36.3* (-39.8 to -32.7) |
| Ascariasis | 35849.2 (31547.5 to 40905.5) | -- | 46.3 (25.0 to 79.2) | -30.8* (-38.1 to -23.3) | -40.7* (-46.9 to -34.2) |
| Asymptomatic ascariasis | 33357.9 (29315.3 to 38215.1) | -- | -- | -- | -- |
| Ascariasis complications | 2491.3 (2218.2 to 2802.6) | -- | 46.3 (25.0 to 79.2) | -30.8* (-38.1 to -23.3) | -40.7* (-46.9 to -34.2) |
| Trichuriasis | 34292.6 (30015.8 to 39478.5) | -- | 26.3 (14.4 to 43.7) | -17.3* (-24.3 to -9.4) | -29.1* (-35.1 to -22.3) |
| Asymptomatic trichuriasis | 32975.4 (28776.2 to 38097.4) | -- | -- | -- | -- |
| Trichuriasis complications | 1317.2 (1201.4 to 1433.3) | -- | 26.3 (14.4 to 43.7) | -17.3* (-24.3 to -9.4) | -29.1* (-35.1 to -22.3) |
| Hookworm disease | 17886.2 (16597.0 to 19283.3) | -- | 67.4 (39.9 to 108.0) | -24.9* (-30.9 to -19.0) | -35.6* (-40.7 to -30.6) |
| Asymptomatic hookworm disease | 14750.9 (13672.7 to 15903.4) | -- | -- | -- | -- |
| Anemia due to hookworm disease | 766.2 (682.9 to 861.6) | -- | 17.6 (11.3 to 26.5) | -27.5* (-37.8 to -16.7) | -37.8* (-46.7 to -28.6) |
| Hookworm disease complications | 2369.1 (2207.2 to 2541.8) | -- | 49.8 (27.6 to 82.2) | -24.0* (-29.2 to -18.6) | -34.8* (-39.3 to -30.2) |
| Food-borne trematodiases | 8807.2 (7569.9 to 10300.3) | 2175.9 (1866.6 to 2538.8) | 205.3 (107.5 to 369.5) | 21.2* (14.6 to 27.7) | 4.0 (-1.7 to 9.5) |
| Asymptomatic food-borne tremodiases | 7021.6 (5753.2 to 8478.0) | 1814.7 (1231.1 to 2287.9) | -- | -- | -- |
| Food-borne tremodiases complications | 1785.5 (1068.9 to 2954.7) | 361.1 (98.3 to 893.5) | 205.3 (107.5 to 369.5) | 21.2* (14.6 to 27.7) | 4.0 (-1.7 to 9.5) |
| Leprosy | 19.3 (17.6 to 21.1) | 3.8 (3.5 to 4.2) | 1.2 (0.8 to 1.7) | -31.1* (-38.7 to -22.2) | -40.9* (-47.4 to -33.2) |
| Ebola virus disease | -- | -- | -- | -96.1* (-96.5 to -95.2) | -96.6* (-97.0 to -95.9) |
| Ebola cases | -- | -- | -- | -96.9* (-97.0 to -96.8) | -97.3* (-97.5 to -97.2) |
| Post-Ebola chronic fatigue syndrome | -- | -- | -- | -96.1* (-96.4 to -95.1) | -96.6* (-96.9 to -95.8) |
| Zika virus disease | 3.4 (2.0 to 5.8) | 207.2 (122.4 to 353.1) | 0.1 (0.0 to 0.1) | -- | -- |
| Zika virus complications | 0.0 (0.0 to 0.1) | -- | -- | -- | -- |
| Zika virus episode | 3.4 (2.0 to 5.8) | 207.2 (122.4 to 353.1) | 0.1 (0.0 to 0.1) | -- | -- |
| Guinea worm disease | -- | -- | -- | -99.4* (-99.6 to -99.2) | -99.5* (-99.7 to -99.3) |
| Other neglected tropical diseases | 3596.0 (3362.6 to 3874.7) | -- | 81.0 (52.5 to 119.9) | -1.9 (-10.3 to 6.7) | -15.9* (-23.1 to -8.5) |
| Acute infection due to other neglected tropical diseases | -- | -- | 0.9 (0.5 to 1.6) | 118.3* (87.3 to 222.8) | 87.2* (60.6 to 176.9) |
| Anemia due to other neglected tropical diseases | 3596.0 (3362.6 to 3874.7) | -- | 80.1 (51.9 to 118.4) | -2.5 (-10.9 to 6.2) | -16.4* (-23.6 to -8.9) |
| **Other infectious diseases** | **7601.4 (6863.9 to 8661.1)** | **26438.0 (20934.9 to 35438.1)** | **299.5 (209.6 to 409.8)** | **3.8 (-1.9 to 9.7)** | **-11.0* (-15.8 to -6.0)** |
| Meningitis | 894.0 (742.7 to 1057.9) | 206.5 (144.5 to 300.6) | 79.2 (54.8 to 107.6) | -4.5 (-9.0 to 0.6) | -18.1* (-22.0 to -13.7) |
| Pneumococcal meningitis | 310.5 (253.6 to 382.9) | 13.2 (7.6 to 21.1) | 29.0 (19.7 to 39.8) | -26.4* (-31.8 to -20.4) | -36.9* (-41.5 to -31.8) |

| | | | | | |
|---|---|---|---|---|---|
| Acute pneumococcal meningitis | 0.6 (0.4 to 1.0) | 13.2 (7.6 to 21.1) | 0.1 (0.0 to 0.2) | -29.7* (-38.3 to -20.9) | -39.7* (-47.1 to -32.2) |
| Pneumococcal meningitis complications | 309.9 (252.8 to 382.2) | -- | 28.9 (19.7 to 39.7) | -26.4* (-31.8 to -20.4) | -36.9* (-41.5 to -31.8) |
| H influenzae type B meningitis | 77.8 (55.8 to 103.8) | 4.3 (2.5 to 7.3) | 6.9 (4.7 to 9.5) | -50.5* (-53.8 to -47.1) | -57.5* (-60.4 to -54.6) |
| Acute H influenzae type B meningitis | 0.2 (0.1 to 0.4) | 4.3 (2.5 to 7.3) | 0.0 (0.0 to 0.1) | -58.1* (-63.3 to -52.7) | -64.1* (-68.5 to -59.5) |
| H Influenza type B meningitis complications | 77.6 (55.7 to 103.6) | -- | 6.8 (4.6 to 9.5) | -50.5* (-53.8 to -47.1) | -57.5* (-60.4 to -54.6) |
| Meningococcal infection | 91.3 (64.5 to 122.2) | 12.6 (7.5 to 20.4) | 8.6 (5.8 to 12.1) | -4.0* (-8.1 to -0.2) | -17.7* (-21.2 to -14.4) |
| Acute meningococcal meningitis | 0.6 (0.4 to 1.0) | 12.6 (7.5 to 20.4) | 0.1 (0.0 to 0.2) | 3.8 (-7.0 to 15.1) | -11.0* (-20.2 to -1.3) |
| Meningococcal meningitis complications | 90.7 (63.8 to 121.6) | -- | 8.6 (5.7 to 12.0) | -4.1* (-8.1 to -0.2) | -17.8* (-21.2 to -14.4) |
| Other meningitis | 414.4 (305.0 to 528.3) | 176.4 (122.8 to 255.8) | 34.7 (23.7 to 47.3) | 68.3* (57.2 to 80.4) | 44.3* (34.8 to 54.7) |
| Other acute bacterial meningitis | 1.7 (1.1 to 2.5) | 34.7 (23.1 to 50.6) | 0.2 (0.1 to 0.4) | 65.1* (49.3 to 81.8) | 41.6* (28.0 to 55.9) |
| Acute viral meningitis | 6.9 (4.7 to 10.2) | 141.8 (99.2 to 206.2) | 0.9 (0.5 to 1.6) | 3.9 (-4.6 to 13.0) | -10.9* (-18.2 to -3.1) |
| Other bacterial meningitis complications | 405.8 (297.2 to 519.3) | -- | 33.5 (23.0 to 45.8) | 71.3* (59.5 to 84.1) | 46.9* (36.8 to 57.9) |
| Encephalitis | 550.4 (315.5 to 865.7) | 108.1 (103.0 to 113.2) | 47.5 (33.1 to 62.5) | 9.2* (2.0 to 16.4) | -6.4* (-12.5 to -0.2) |
| Acute encephalitis | 5.9 (5.6 to 6.2) | 108.1 (103.0 to 113.2) | 0.8 (0.5 to 1.1) | 17.9* (17.3 to 18.5) | 1.1* (0.6 to 1.6) |
| Encephalitis complications | 544.5 (309.3 to 859.7) | -- | 46.8 (32.6 to 61.5) | 9.0* (1.8 to 16.4) | -6.5* (-12.7 to -0.2) |
| Diphtheria | -- | 0.2 (0.1 to 0.2) | -- | -35.1* (-52.9 to -7.3) | -44.3* (-59.6 to -20.5) |
| Whooping cough | 3.4 (2.7 to 4.3) | 25.0 (19.4 to 31.6) | 0.2 (0.1 to 0.3) | 0.8* (0.0 to 1.5) | -13.6* (-14.2 to -12.9) |
| Tetanus | 4.7 (4.5 to 4.9) | 2.5 (1.3 to 4.1) | 0.1 (0.1 to 0.2) | -5.5 (-16.6 to 4.9) | -18.9* (-28.5 to -10.1) |
| Severe tetanus | 0.1 (0.1 to 0.2) | 2.5 (1.3 to 4.1) | -- | -49.4* (-62.3 to -26.6) | -56.6* (-67.7 to -37.1) |
| Neonatal tetanus complications | 4.5 (4.4 to 4.7) | -- | 0.1 (0.0 to 0.1) | 14.8* (12.9 to 16.5) | -1.6* (-3.2 to -0.1) |
| Measles | 1.6 (0.6 to 3.5) | 57.7 (20.6 to 128.1) | 0.1 (0.0 to 0.3) | -32.8* (-39.1 to -26.4) | -42.4* (-47.7 to -36.9) |
| Varicella and herpes zoster | 287.1 (209.3 to 373.1) | 2238.7 (1628.9 to 2995.6) | 15.4 (8.9 to 25.9) | 16.9* (9.9 to 23.7) | 0.3 (-5.8 to 6.1) |
| Chickenpox | 13.3 (6.1 to 24.6) | 695.3 (318.4 to 1286.0) | 0.1 (0.0 to 0.2) | 22.5* (18.2 to 27.5) | 5.1* (1.4 to 9.4) |
| Herpes zoster | 273.7 (197.4 to 356.2) | 1543.4 (1110.4 to 2021.5) | 15.3 (8.8 to 25.7) | 16.9* (9.8 to 23.7) | 0.3 (-5.9 to 6.1) |
| Acute hepatitis | 2469.6 (1835.7 to 3479.6) | 23799.3 (18288.8 to 32610.1) | 45.8 (28.4 to 69.3) | 15.2 (-10.9 to 53.6) | -1.2 (-23.6 to 31.7) |
| Acute hepatitis A | 459.3 (399.6 to 508.9) | 5970.4 (5195.4 to 6615.2) | 19.3 (12.3 to 29.1) | 18.1* (8.6 to 28.5) | 1.3 (-6.9 to 10.2) |
| Acute hepatitis B | 1887.2 (1250.2 to 2897.5) | 16355.7 (10835.0 to 25111.6) | 23.2 (12.3 to 39.7) | 12.7 (-34.1 to 90.4) | -3.3 (-43.5 to 63.3) |
| Acute hepatitis C | 29.5 (11.4 to 52.9) | 255.5 (98.7 to 458.2) | 0.4 (0.1 to 1.0) | 12.1* (5.4 to 17.2) | -3.8 (-9.6 to 0.5) |
| Acute hepatitis E | 93.7 (64.5 to 135.1) | 1217.8 (838.9 to 1755.8) | 2.9 (1.6 to 5.0) | 16.6 (-4.9 to 42.1) | -0.0 (-18.4 to 21.8) |
| Other unspecified infectious diseases | 4277.2 (4031.3 to 4564.3) | -- | 111.1 (71.8 to 165.9) | 2.2 (-4.3 to 8.6) | -12.4* (-17.9 to -6.9) |
| Guillain-Barré syndrome due to other infectious diseases | 0.4 (0.2 to 0.7) | -- | 0.1 (0.1 to 0.2) | 16.6* (14.1 to 19.3) | -0.0 (-2.1 to 2.3) |
| Other infectious diseases | -- | -- | 17.2 (9.6 to 29.2) | 22.2* (18.9 to 25.6) | 4.8* (1.9 to 7.7) |
| Anemia due to other infectious diseases | 4276.7 (4030.7 to 4563.8) | -- | 93.8 (61.1 to 138.4) | -0.8 (-7.9 to 6.8) | -15.0* (-21.1 to -8.4) |
| **Maternal and neonatal disorders** | **13570.9 (11920.9 to 15465.8)** | **19989.5 (17145.8 to 23146.7)** | **2616.2 (1976.5 to 3351.0)** | **30.5* (22.9 to 39.2)** | **11.9* (5.4 to 19.4)** |
| Maternal disorders | 1974.9 (1607.8 to 2424.2) | 19989.5 (17145.8 to 23146.7) | 187.7 (131.0 to 256.4) | -1.1 (-11.8 to 10.8) | -15.2* (-24.4 to -5.0) |
| Maternal haemorrhage | 472.7 (416.7 to 533.6) | 1921.0 (1320.9 to 2632.5) | 16.7 (10.9 to 24.1) | 6.1 (-10.7 to 25.9) | -9.0 (-23.4 to 8.0) |
| Maternal hemorrhage complications | 430.7 (379.0 to 494.3) | -- | 9.7 (6.1 to 14.5) | 6.3 (-7.9 to 25.7) | -8.9 (-21.1 to 7.8) |
| Maternal hemorrhage episode | 42.1 (25.3 to 66.5) | 1921.0 (1320.9 to 2632.5) | 7.0 (3.8 to 11.5) | 5.9 (-26.6 to 50.6) | -9.1 (-37.1 to 29.2) |
| Maternal sepsis and other pregnancy related infections | 504.9 (376.1 to 688.5) | 3139.7 (2183.0 to 4289.5) | 13.5 (6.5 to 25.3) | 12.5 (-33.5 to 81.3) | -3.5 (-43.0 to 55.5) |
| Infertility due to puerperal sepsis | 263.8 (215.6 to 318.6) | 26.0 (18.3 to 34.0) | 1.3 (0.5 to 2.8) | 16.3* (9.0 to 23.2) | -0.3 (-6.5 to 5.6) |
| Maternal sepsis and other maternal infection episode | 241.2 (124.3 to 420.6) | 3113.7 (2163.1 to 4265.5) | 12.2 (5.7 to 23.0) | 12.1 (-38.7 to 89.3) | -3.8 (-47.5 to 62.3) |
| Maternal hypertensive disorders | 756.2 (445.3 to 1181.3) | 4165.8 (3138.4 to 5503.3) | 37.9 (18.8 to 67.6) | 1.0 (-32.0 to 45.4) | -13.4 (-41.7 to 24.7) |
| Maternal hypertensive disorder complications | 28.0 (25.8 to 30.3) | 433.6 (400.0 to 468.9) | 1.8 (1.1 to 2.8) | 17.2 (-0.3 to 35.7) | 0.5 (-14.5 to 16.4) |
| Maternal hypertensive disorder episode | 728.2 (416.9 to 1151.4) | 3732.2 (2714.5 to 5083.2) | 36.1 (17.4 to 64.9) | 0.3 (-34.1 to 46.4) | -14.0 (-43.5 to 25.6) |
| Maternal obstructed labour and uterine rupture | 260.5 (207.2 to 323.9) | 2222.1 (1493.0 to 3086.5) | 84.6 (54.8 to 121.6) | -12.0* (-19.7 to -3.5) | -24.5* (-31.1 to -17.3) |
| Obstructed labor, acute event | 29.6 (17.4 to 47.6) | 2172.5 (1433.4 to 3039.3) | 9.3 (4.6 to 16.5) | 10.0 (-35.6 to 79.6) | -5.7 (-44.7 to 54.0) |
| Maternal obstructed labor complications | 230.9 (181.0 to 292.5) | 49.6 (38.3 to 63.7) | 75.3 (47.8 to 112.6) | -14.1* (-20.8 to -6.7) | -26.3* (-32.1 to -20.0) |
| Maternal abortion and miscarriage | 38.0 (22.7 to 59.8) | 4638.9 (3161.0 to 6709.7) | 4.2 (2.1 to 7.6) | 3.5 (-34.4 to 55.7) | -11.2 (-43.7 to 33.5) |
| Ectopic pregnancy | 31.9 (18.2 to 50.1) | 3902.0 (2697.0 to 5524.9) | 3.5 (1.7 to 6.1) | 13.1 (-31.3 to 78.3) | -3.0 (-41.1 to 52.9) |
| Other maternal disorders | -- | -- | 27.3 (19.1 to 37.6) | 28.2* (13.7 to 44.4) | 9.9 (-2.5 to 23.9) |
| Neonatal disorders | 11633.2 (10051.4 to 13505.5) | -- | 2428.5 (1806.8 to 3159.7) | 33.8* (25.5 to 43.3) | 14.7* (7.6 to 22.9) |
| Neonatal preterm birth | 6691.1 (5716.0 to 7919.6) | -- | 1148.6 (809.7 to 1572.8) | 38.6* (20.0 to 57.7) | 18.9* (2.9 to 35.2) |
| Preterm birth complications | 5778.1 (4791.7 to 6983.3) | -- | 1098.8 (768.9 to 1512.1) | 40.5* (20.7 to 60.7) | 20.5* (3.5 to 37.8) |
| Retinopathy of prematurity | 913.0 (720.7 to 1115.9) | -- | 49.9 (33.4 to 69.8) | 6.6 (-0.6 to 14.0) | -8.6* (-14.8 to -2.2) |
| Uncomplicated preterm birth | -- | -- | -- | -- | -- |
| Neonatal encephalopathy due to birth asphyxia and trauma | 4541.7 (3341.3 to 5993.4) | -- | 849.6 (513.1 to 1324.0) | 33.1* (27.5 to 39.6) | 14.1* (9.4 to 19.7) |
| Neonatal sepsis and other neonatal infections | 1192.4 (734.1 to 1826.3) | -- | 357.0 (191.8 to 598.6) | 23.1* (17.8 to 28.5) | 5.6* (1.0 to 10.2) |
| Neonatal sepsis and other neonatal infection complications | 1192.4 (734.1 to 1826.3) | -- | 357.0 (191.8 to 598.6) | 23.1* (17.8 to 28.5) | 5.6* (1.0 to 10.2) |
| Neonatal sepsis and other neonatal infection episode | -- | -- | -- | -- | -- |
| Hemolytic disease and other neonatal jaundice | 196.4 (169.3 to 225.0) | -- | 73.2 (55.9 to 91.6) | 26.0* (20.7 to 31.5) | 8.1* (3.5 to 12.7) |

| | | | | | |
|---|---|---|---|---|---|
| Other neonatal disorders | -- | -- | -- | -- | -- |
| **Nutritional deficiencies** | **144366.1 (137860.6 to 151033.3)** | **91170.8 (82342.0 to 100640.0)** | **2386.1 (1576.7 to 3539.0)** | **-1.7 (-7.7 to 4.5)** | **-15.7* (-20.8 to -10.4)** |
| Protein-energy malnutrition | 3006.4 (2761.0 to 3279.1) | 3421.9 (3099.2 to 3766.1) | 28.9 (18.4 to 41.0) | 24.8* (15.3 to 34.7) | 7.0 (-1.1 to 15.5) |
| Iodine deficiency | 10393.6 (9234.8 to 11626.1) | 74.2 (59.8 to 91.4) | 190.7 (115.5 to 301.9) | -3.1 (-7.4 to 1.3) | -16.9* (-20.6 to -13.2) |
| Visible goiter without symptoms | 10145.3 (9053.2 to 11360.0) | 74.2 (59.8 to 91.4) | 109.4 (53.3 to 202.8) | -4.7* (-6.5 to -3.1) | -18.3* (-19.8 to -16.9) |
| Visible goiter with complications | 248.3 (138.9 to 353.9) | -- | 81.3 (41.7 to 125.3) | -0.8 (-11.7 to 8.3) | -15.0* (-24.3 to -7.1) |
| Vitamin A deficiency | 57464.2 (51749.4 to 63572.6) | 87674.7 (78806.6 to 97079.0) | 16.4 (9.9 to 25.8) | 21.4* (12.8 to 31.4) | 4.1 (-3.3 to 12.7) |
| Asymptomatic vitamin A deficiency | 57207.3 (51452.3 to 63256.7) | 87674.7 (78806.6 to 97079.0) | -- | -- | -- |
| Vitamin A deficiency complications | 256.8 (181.4 to 350.3) | -- | 16.4 (9.9 to 25.8) | 21.4* (12.8 to 31.4) | 4.1 (-3.3 to 12.7) |
| Vitamin A deficiency with anemia | -- | -- | -- | -- | -- |
| Dietary iron deficiency | 93631.6 (88577.2 to 99579.6) | -- | 2134.5 (1404.3 to 3151.8) | -2.2 (-8.7 to 4.8) | -16.1* (-21.7 to -10.1) |
| Other nutritional deficiencies | -- | -- | 15.7 (10.0 to 22.3) | 20.3* (11.1 to 29.8) | 3.2 (-4.7 to 11.3) |
| **Non-communicable diseases** | **582365.3 (574503.4 to 589416.8)** | **847297.0 (777052.1 to 921152.9)** | **46692.4 (34878.1 to 60577.2)** | **15.4* (14.7 to 16.1)** | **-1.0* (-1.7 to -0.4)** |
| **Neoplasms** | **2470.2 (2311.2 to 2690.0)** | **321.3 (272.2 to 406.4)** | **156.3 (112.4 to 212.4)** | **49.3* (39.4 to 63.6)** | **28.0* (19.5 to 40.3)** |
| Lip and oral cavity cancer | 40.5 (34.7 to 48.8) | 7.5 (6.4 to 9.0) | 3.6 (2.5 to 4.9) | 34.0* (11.5 to 63.0) | 14.9 (-4.4 to 39.8) |
| Diagnosis and primary therapy phase of mouth cancer | 2.2 (1.9 to 2.7) | 7.5 (6.4 to 9.0) | 0.6 (0.4 to 0.9) | 37.9* (16.8 to 63.7) | 18.2* (0.1 to 40.4) |
| Controlled phase of mouth cancer | 35.5 (30.5 to 42.9) | -- | 1.7 (1.1 to 2.6) | 37.9* (8.4 to 77.1) | 18.2 (-7.0 to 51.9) |
| Metastatic phase of mouth cancer | 2.4 (2.0 to 2.8) | -- | 1.1 (0.7 to 1.5) | 27.7* (8.2 to 50.1) | 9.5 (-7.3 to 28.7) |
| Terminal phase of mouth cancer | 0.3 (0.3 to 0.4) | -- | 0.2 (0.1 to 0.3) | 25.2* (5.2 to 48.5) | 7.3 (-9.8 to 27.3) |
| Nasopharynx cancer | 18.2 (16.5 to 20.0) | 3.0 (2.7 to 3.2) | 1.6 (1.1 to 2.2) | 21.8* (8.9 to 37.5) | 4.5 (-6.6 to 17.9) |
| Diagnosis and primary therapy phase of nasopharynx cancer | 1.0 (0.9 to 1.1) | 3.0 (2.7 to 3.2) | 0.3 (0.2 to 0.4) | 24.2* (11.4 to 38.1) | 6.5 (-4.5 to 18.4) |
| Controlled phase of nasopharynx cancer | 16.0 (14.5 to 17.6) | -- | 0.8 (0.5 to 1.2) | 24.0* (6.3 to 45.8) | 6.3 (-8.8 to 25.1) |
| Metastatic phase of nasopharynx cancer | 1.1 (1.0 to 1.2) | -- | 0.5 (0.3 to 0.7) | 18.3* (6.4 to 31.9) | 1.5 (-8.8 to 13.1) |
| Terminal phase of nasopharynx cancer | 0.1 (0.1 to 0.1) | -- | 0.1 (0.0 to 0.1) | 12.8* (2.0 to 25.2) | -3.3 (-12.6 to 7.4) |
| Other pharynx cancer | 3.4 (2.9 to 3.8) | 1.4 (1.2 to 1.6) | 0.5 (0.3 to 0.6) | 17.4* (5.6 to 29.3) | 0.7 (-9.4 to 10.9) |
| Diagnosis and primary therapy phase of other pharynx cancer | 0.2 (0.2 to 0.2) | 1.4 (1.2 to 1.6) | 0.1 (0.0 to 0.1) | 20.9* (9.1 to 32.4) | 3.6 (-6.4 to 13.6) |
| Controlled phase of other pharynx cancer | 2.6 (2.2 to 2.9) | -- | 0.1 (0.1 to 0.2) | 21.2* (9.4 to 32.8) | 4.0 (-6.2 to 13.9) |
| Metastatic phase of other pharynx cancer | 0.5 (0.4 to 0.6) | -- | 0.2 (0.2 to 0.3) | 15.4* (3.5 to 27.5) | -1.1 (-11.3 to 9.3) |
| Terminal phase of other pharynx cancer | 0.1 (0.1 to 0.1) | -- | 0.1 (0.0 to 0.1) | 15.0* (3.1 to 27.1) | -1.4 (-11.6 to 9.0) |
| Oesophageal cancer | 3.4 (3.2 to 3.6) | 1.4 (1.3 to 1.5) | 0.5 (0.3 to 0.6) | 16.0* (8.5 to 24.0) | -0.5 (-6.9 to 6.3) |
| Diagnosis and primary therapy phase of esophageal cancer | 0.3 (0.3 to 0.3) | 1.4 (1.3 to 1.5) | 0.1 (0.1 to 0.1) | 23.4* (14.8 to 31.9) | 5.8 (-1.6 to 13.1) |
| Controlled phase of esophageal cancer | 2.6 (2.4 to 2.7) | -- | 0.1 (0.1 to 0.2) | 26.4* (17.6 to 35.4) | 8.4* (0.9 to 16.1) |
| Metastatic phase of esophageal cancer | 0.5 (0.4 to 0.5) | -- | 0.2 (0.1 to 0.3) | 9.7* (2.9 to 16.9) | -5.9 (-11.8 to 0.3) |
| Terminal phase of esophageal cancer | 0.1 (0.1 to 0.1) | -- | 0.1 (0.0 to 0.1) | 9.7* (2.9 to 16.9) | -5.9 (-11.8 to 0.3) |
| Stomach cancer | 22.5 (21.6 to 23.5) | 8.0 (7.7 to 8.3) | 2.7 (1.9 to 3.6) | 31.7* (23.5 to 40.2) | 12.9* (5.9 to 20.2) |
| Diagnosis and primary therapy phase of stomach cancer | 1.9 (1.8 to 2.0) | 8.0 (7.7 to 8.3) | 0.6 (0.4 to 0.8) | 41.7* (34.9 to 49.0) | 21.5* (15.7 to 27.8) |
| Controlled phase of stomach cancer | 18.0 (17.2 to 18.8) | -- | 0.9 (0.5 to 1.3) | 58.5* (41.0 to 77.1) | 36.0* (20.9 to 51.8) |
| Metastatic phase of stomach cancer | 2.1 (2.0 to 2.2) | -- | 1.0 (0.6 to 1.3) | 14.5* (9.5 to 19.8) | -1.8 (-6.1 to 2.7) |
| Terminal phase of stomach cancer | 0.5 (0.5 to 0.6) | -- | 0.3 (0.2 to 0.4) | 14.5* (9.5 to 19.8) | -1.8 (-6.1 to 2.7) |
| Colon and rectum cancer | 68.0 (64.4 to 71.0) | 11.4 (10.7 to 11.9) | 6.5 (4.6 to 8.7) | 40.0* (31.6 to 48.7) | 20.1* (12.9 to 27.5) |
| Diagnosis and primary therapy phase of colon and rectum cancers | 2.8 (2.7 to 2.9) | 11.4 (10.7 to 11.9) | 0.8 (0.5 to 1.1) | 50.9* (44.0 to 57.9) | 29.4* (23.5 to 35.5) |
| Controlled phase of colon and rectum cancers | 61.1 (57.9 to 63.8) | -- | 3.9 (2.6 to 5.6) | 42.5* (31.9 to 54.1) | 22.2* (13.1 to 32.2) |
| Metastatic phase of colon and rectum cancers | 3.2 (3.0 to 3.3) | -- | 1.4 (1.0 to 1.9) | 32.1* (24.7 to 38.9) | 13.3* (6.9 to 19.2) |
| Terminal phase of colon and rectum cancers | 0.4 (0.4 to 0.5) | -- | 0.2 (0.2 to 0.3) | 25.7* (18.3 to 32.5) | 7.8* (1.4 to 13.6) |
| Stoma from colon and rectum cancers, beyond 10 years | 0.5 (0.4 to 0.6) | -- | 0.0 (0.0 to 0.1) | 10.7* (8.6 to 12.9) | -5.0* (-6.9 to -3.2) |
| Liver cancer | 0.9 (0.3 to 1.7) | 8.5 (7.8 to 9.1) | 2.1 (1.5 to 2.8) | 40.0* (26.2 to 53.2) | 20.0* (8.2 to 31.4) |
| Liver cancer due to hepatitis B | 9.6 (8.6 to 10.5) | 6.0 (5.4 to 6.6) | 1.5 (1.0 to 2.0) | 43.9* (28.5 to 59.0) | 23.4* (10.2 to 36.4) |
| Diagnosis and primary therapy phase of liver cancer due to hepatitis B | 1.5 (1.4 to 1.7) | 6.0 (5.4 to 6.6) | 0.4 (0.3 to 0.6) | 43.2* (28.3 to 57.1) | 22.8* (10.1 to 34.7) |
| Controlled phase of liver cancer due to hepatitis B | 6.5 (5.8 to 7.1) | -- | 0.3 (0.2 to 0.5) | 67.0* (44.6 to 93.1) | 43.2* (24.0 to 65.6) |
| Metastatic phase of liver cancer due to hepatitis B | 1.1 (1.0 to 1.2) | -- | 0.5 (0.3 to 0.7) | 36.4* (22.2 to 49.7) | 17.0* (4.8 to 28.4) |
| Terminal phase of liver cancer due to hepatitis B | 0.5 (0.4 to 0.5) | -- | 0.2 (0.2 to 0.3) | 36.4* (22.2 to 49.7) | 17.0* (4.8 to 28.4) |
| Liver cancer due to hepatitis C | 0.8 (0.6 to 1.0) | 0.5 (0.4 to 0.6) | 0.1 (0.1 to 0.2) | 28.2* (16.5 to 41.7) | 10.0 (-0.1 to 21.5) |
| Diagnosis and primary therapy phase of liver cancer due to hepatitis C | 0.1 (0.1 to 0.2) | 0.5 (0.4 to 0.6) | 0.0 (0.0 to 0.1) | 27.8* (16.3 to 41.3) | 9.6 (-0.2 to 21.1) |
| Controlled phase of liver cancer due to hepatitis C | 0.5 (0.4 to 0.6) | -- | -- | 43.2* (29.2 to 59.0) | 22.8* (10.8 to 36.4) |
| Metastatic phase of liver cancer due to hepatitis C | 0.1 (0.1 to 0.1) | -- | 0.0 (0.0 to 0.1) | 23.1* (12.1 to 35.5) | 5.6 (-3.9 to 16.2) |
| Terminal phase of liver cancer due to hepatitis C | -- | -- | -- | 23.1* (12.1 to 35.5) | 5.6 (-3.9 to 16.2) |
| Liver cancer due to alcohol use | 0.7 (0.5 to 0.9) | 0.4 (0.3 to 0.6) | 0.1 (0.1 to 0.2) | 15.6* (2.9 to 24.4) | -0.8 (-11.7 to 6.7) |
| Diagnosis and primary therapy phase of liver cancer due to alcohol use | 0.1 (0.1 to 0.1) | 0.4 (0.3 to 0.6) | -- | 15.5* (2.8 to 24.3) | -1.0 (-11.9 to 6.6) |
| Controlled phase of liver cancer due to alcohol use | 0.4 (0.3 to 0.6) | -- | -- | 21.5* (8.8 to 31.2) | 4.2 (-6.7 to 12.5) |
| Metastatic phase of liver cancer due to alcohol use | 0.1 (0.1 to 0.1) | -- | 0.0 (0.0 to 0.1) | 13.5* (0.9 to 22.4) | -2.6 (-13.5 to 5.0) |

| | | | | | |
|---|---|---|---|---|---|
| Terminal phase of liver cancer due to alcohol use | -- | -- | -- | 13.6* (0.9 to 22.4) | -2.6 (-13.5 to 5.0) |
| Liver cancer due to NASH | 0.7 (0.5 to 0.8) | 0.4 (0.3 to 0.6) | 0.1 (0.1 to 0.2) | 34.7* (21.1 to 45.3) | 15.5* (3.9 to 24.6) |
| Diagnosis and primary therapy phase of liver cancer due to NASH | 0.1 (0.1 to 0.1) | 0.4 (0.3 to 0.6) | -- | 34.4* (20.9 to 44.8) | 15.2* (3.7 to 24.2) |
| Controlled phase of liver cancer due to NASH | 0.5 (0.4 to 0.6) | -- | -- | 49.4* (34.8 to 61.5) | 28.1* (15.6 to 38.5) |
| Metastatic phase of liver cancer due to NASH | 0.1 (0.1 to 0.1) | -- | 0.0 (0.0 to 0.1) | 30.0* (16.8 to 39.7) | 11.4* (0.2 to 19.8) |
| Terminal phase of liver cancer due to NASH | -- | -- | -- | 30.0* (16.8 to 39.7) | 11.5* (0.2 to 19.8) |
| Liver cancer due to other causes | 1.7 (1.4 to 2.1) | 1.1 (0.9 to 1.3) | 0.3 (0.2 to 0.4) | 38.5* (25.9 to 51.3) | 18.8* (8.0 to 29.7) |
| Diagnosis and primary therapy phase of liver cancer due to other causes | 0.3 (0.2 to 0.3) | 1.1 (0.9 to 1.3) | 0.1 (0.0 to 0.1) | 37.9* (25.0 to 50.3) | 18.3* (7.2 to 28.9) |
| Controlled phase of liver cancer due to other causes | 1.1 (0.9 to 1.4) | -- | 0.1 (0.0 to 0.1) | 58.4* (43.1 to 73.3) | 35.9* (22.7 to 48.6) |
| Metastatic phase of liver cancer due to other causes | 0.2 (0.2 to 0.2) | -- | 0.1 (0.1 to 0.1) | 31.9* (19.8 to 43.7) | 13.1* (2.7 to 23.2) |
| Terminal phase of liver cancer due to other causes | 0.1 (0.1 to 0.1) | -- | 0.0 (0.0 to 0.1) | 31.9* (19.8 to 43.7) | 13.1* (2.7 to 23.2) |
| Gallbladder and biliary tract cancer | 0.9 (0.8 to 1.0) | 0.6 (0.5 to 0.7) | 0.2 (0.1 to 0.2) | 12.7* (4.5 to 25.0) | -3.4 (-10.4 to 7.2) |
| Diagnosis and primary therapy phase of gallbladder and biliary tract cancer | 0.1 (0.1 to 0.1) | 0.6 (0.5 to 0.7) | -- | 13.6* (5.6 to 25.4) | -2.6 (-9.4 to 7.6) |
| Controlled phase of gallbladder and biliary tract cancer | 0.6 (0.5 to 0.7) | -- | -- | 21.6* (13.6 to 32.3) | 4.3 (-2.6 to 13.5) |
| Metastatic phase of gallbladder and biliary tract cancer | 0.2 (0.1 to 0.2) | -- | 0.1 (0.0 to 0.1) | 9.9* (1.7 to 22.4) | -5.7 (-12.8 to 5.0) |
| Terminal phase of gallbladder and biliary tract cancer | 0.0 (0.0 to 0.1) | -- | -- | 9.9* (1.7 to 22.4) | -5.7 (-12.8 to 5.0) |
| Pancreatic cancer | 1.5 (1.5 to 1.6) | 1.1 (1.1 to 1.1) | 0.3 (0.2 to 0.4) | 21.8* (16.7 to 26.6) | 4.5* (0.1 to 8.6) |
| Diagnosis and primary therapy phase of pancreatic cancer | 0.3 (0.3 to 0.3) | 1.1 (1.1 to 1.1) | 0.1 (0.1 to 0.1) | 21.9* (16.8 to 26.8) | 4.5* (0.2 to 8.7) |
| Controlled phase of pancreatic cancer | 1.0 (0.9 to 1.0) | -- | 0.0 (0.0 to 0.1) | 19.8* (15.1 to 24.4) | 2.7 (-1.3 to 6.7) |
| Metastatic phase of pancreatic cancer | 0.2 (0.2 to 0.2) | -- | 0.1 (0.1 to 0.1) | 22.4* (17.2 to 27.4) | 5.0* (0.5 to 9.2) |
| Terminal phase of pancreatic cancer | 0.1 (0.1 to 0.1) | -- | 0.0 (0.0 to 0.1) | 22.4* (17.2 to 27.4) | 5.0* (0.5 to 9.2) |
| Larynx cancer | 4.7 (4.4 to 5.1) | 0.8 (0.7 to 0.9) | 0.5 (0.3 to 0.6) | 16.6* (7.8 to 26.0) | 0.0 (-7.5 to 8.1) |
| Diagnosis and primary therapy phase of larynx cancer | 0.2 (0.2 to 0.3) | 0.8 (0.7 to 0.9) | 0.1 (0.0 to 0.1) | 22.2* (12.3 to 32.5) | 4.8 (-3.7 to 13.6) |
| Controlled phase of larynx cancer | 3.6 (3.4 to 3.9) | -- | 0.2 (0.1 to 0.3) | 18.8* (10.5 to 28.0) | 1.9 (-5.3 to 9.8) |
| Metastatic phase of larynx cancer | 0.3 (0.3 to 0.3) | -- | 0.1 (0.1 to 0.2) | 12.0* (1.1 to 23.1) | -3.9 (-13.3 to 5.6) |
| Terminal phase of larynx cancer | -- | -- | -- | 8.9 (-2.0 to 20.0) | -6.6 (-15.9 to 2.9) |
| Laryngectomy from larynx cancer, beyond 10 years | 0.6 (0.5 to 0.6) | -- | -- | 14.0* (13.3 to 14.6) | -2.3* (-2.8 to -1.8) |
| Tracheal, bronchus, and lung cancer | 10.9 (10.3 to 11.5) | 5.6 (5.3 to 5.9) | 1.7 (1.2 to 2.2) | 33.6* (25.0 to 42.3) | 14.6* (7.2 to 22.0) |
| Diagnosis and primary therapy phase of lung, bronchus, and trachea cancer | 0.7 (0.6 to 0.7) | 5.6 (5.3 to 5.9) | 0.2 (0.1 to 0.3) | 44.2* (35.2 to 53.4) | 23.7* (16.0 to 31.5) |
| Controlled phase of lung, bronchus, and trachea cancer | 7.9 (7.4 to 8.4) | -- | 0.4 (0.2 to 0.6) | 54.6* (44.6 to 65.8) | 32.6* (24.0 to 42.2) |
| Metastatic phase of lung, bronchus, and trachea cancer | 1.9 (1.8 to 2.0) | -- | 0.9 (0.6 to 1.1) | 25.9* (17.9 to 34.0) | 8.0* (1.1 to 14.9) |
| Terminal phase of lung, bronchus, and trachea cancer | 0.4 (0.4 to 0.4) | -- | 0.2 (0.2 to 0.3) | 25.9* (17.9 to 34.0) | 8.0* (1.1 to 14.9) |
| Malignant skin melanoma | 106.1 (89.7 to 138.0) | 12.1 (10.3 to 15.7) | 6.0 (3.9 to 8.9) | 21.8* (12.5 to 34.0) | 4.5 (-3.5 to 14.9) |
| Diagnosis and primary therapy phase of malignant skin melanoma | 2.8 (2.3 to 3.6) | 12.1 (10.3 to 15.7) | 0.8 (0.5 to 1.2) | 22.1* (13.8 to 32.3) | 4.8 (-2.4 to 13.5) |
| Controlled phase of malignant skin melanoma | 102.5 (86.7 to 133.5) | -- | 4.9 (3.0 to 7.4) | 22.5* (12.3 to 35.9) | 5.1 (-3.7 to 16.6) |
| Metastatic phase of malignant skin melanoma | 0.6 (0.6 to 0.8) | -- | 0.3 (0.2 to 0.4) | 12.7* (5.6 to 21.5) | -3.4 (-9.5 to 4.2) |
| Terminal phase of malignant skin melanoma | 0.1 (0.1 to 0.1) | -- | 0.1 (0.0 to 0.1) | 11.4* (4.0 to 20.3) | -4.5 (-10.8 to 3.2) |
| Non-melanoma skin cancer | 0.2 (0.0 to 0.6) | 67.4 (21.4 to 151.5) | 0.2 (0.1 to 0.4) | 32.9* (25.8 to 40.0) | 14.0* (7.9 to 20.0) |
| Non-melanoma skin cancer (squamous-cell carcinoma) | 3.4 (1.2 to 9.1) | 3.5 (1.2 to 9.1) | 0.2 (0.1 to 0.4) | 34.4* (26.5 to 41.5) | 15.3* (8.5 to 21.4) |
| Non-melanoma skin cancer (basal-cell carcinoma) | 6.1 (1.7 to 13.9) | 63.8 (17.7 to 150.1) | 0.0 (0.0 to 0.1) | 24.5* (15.8 to 28.7) | 6.8 (-0.7 to 10.4) |
| Breast cancer | 238.9 (213.3 to 260.5) | 28.3 (25.2 to 31.0) | 19.1 (12.9 to 26.6) | 39.2* (24.5 to 53.9) | 19.4* (6.8 to 32.0) |
| Diagnosis and primary therapy phase of breast cancer | 6.6 (5.9 to 7.2) | 28.3 (25.2 to 31.0) | 1.9 (1.3 to 2.7) | 42.8* (30.5 to 54.7) | 22.5* (11.9 to 32.7) |
| Controlled phase of breast cancer | 222.9 (198.9 to 243.1) | -- | 13.7 (8.5 to 20.3) | 39.7* (23.6 to 57.0) | 19.8* (6.0 to 34.7) |
| Metastatic phase of breast cancer | 7.0 (6.2 to 7.6) | -- | 3.1 (2.1 to 4.2) | 36.7* (24.0 to 49.8) | 17.2* (6.3 to 28.5) |
| Terminal phase of breast cancer | 0.5 (0.4 to 0.5) | -- | 0.3 (0.2 to 0.3) | 31.1* (18.1 to 45.5) | 12.4* (1.3 to 24.8) |
| Mastectomy from breast cancer, beyond 10 years | 2.0 (1.7 to 2.2) | -- | 0.1 (0.0 to 0.1) | 9.0* (7.8 to 10.2) | -6.5* (-7.6 to -5.5) |
| Cervical cancer | 216.0 (194.6 to 235.7) | 27.4 (24.7 to 30.0) | 15.5 (10.6 to 21.4) | 7.4 (-1.7 to 17.3) | -7.9 (-15.7 to 0.6) |
| Diagnosis and primary therapy phase of cervical cancer | 10.9 (9.8 to 11.9) | 27.4 (24.7 to 30.0) | 3.1 (2.0 to 4.3) | 8.1 (-1.2 to 18.5) | -7.3 (-15.3 to 1.6) |
| Controlled phase of cervical cancer | 198.6 (178.9 to 216.6) | -- | 9.4 (5.9 to 14.2) | 8.9 (-1.8 to 20.3) | -6.6 (-15.8 to 3.2) |
| Metastatic phase of cervical cancer | 5.9 (5.3 to 6.4) | -- | 2.7 (1.8 to 3.5) | 2.5 (-6.1 to 11.7) | -12.1* (-19.4 to -4.2) |
| Terminal phase of cervical cancer | 0.6 (0.6 to 0.7) | -- | 0.3 (0.2 to 0.5) | 0.8 (-7.2 to 10.0) | -13.5* (-20.4 to -5.7) |
| Uterine cancer | 23.8 (22.1 to 25.4) | 2.9 (2.7 to 3.1) | 1.6 (1.1 to 2.3) | 32.9* (16.9 to 48.9) | 14.0* (0.2 to 27.7) |
| Diagnosis and primary therapy phase of uterine cancer | 1.1 (1.0 to 1.1) | 2.9 (2.7 to 3.1) | 0.3 (0.2 to 0.4) | 35.0* (25.4 to 45.0) | 15.8* (7.5 to 24.4) |
| Controlled phase of uterine cancer | 22.2 (20.7 to 23.7) | -- | 1.1 (0.7 to 1.6) | 34.1* (12.9 to 57.3) | 15.0 (-3.2 to 34.9) |
| Metastatic phase of uterine cancer | 0.5 (0.4 to 0.5) | -- | 0.2 (0.1 to 0.3) | 26.1* (17.1 to 35.9) | 8.2* (0.4 to 16.5) |
| Terminal phase of uterine cancer | 0.0 (0.0 to 0.1) | -- | -- | 20.4* (10.9 to 31.0) | 3.2 (-4.9 to 12.4) |
| Ovarian cancer | 70.7 (64.3 to 81.7) | 10.1 (9.2 to 11.7) | 8.2 (5.8 to 11.2) | 25.1* (6.4 to 49.1) | 7.3 (-8.7 to 27.9) |
| Diagnosis and primary therapy phase of ovarian cancer | 2.6 (2.4 to 3.0) | 10.1 (9.2 to 11.7) | 0.7 (0.5 to 1.1) | 26.4* (14.7 to 44.2) | 8.4 (-1.6 to 23.6) |
| Controlled phase of ovarian cancer | 57.5 (52.3 to 66.4) | -- | 2.7 (1.7 to 4.2) | 26.4* (7.9 to 51.4) | 8.4 (-7.5 to 29.9) |
| Metastatic phase of ovarian cancer | 10.2 (9.2 to 11.8) | -- | 4.5 (2.9 to 6.5) | 24.2 (-2.1 to 57.9) | 6.5 (-16.1 to 35.4) |

| | | | | | |
|---|---|---|---|---|---|
| Terminal phase of ovarian cancer | 0.4 (0.4 to 0.5) | -- | 0.2 (0.1 to 0.3) | 24.3* (12.1 to 43.6) | 6.6 (-3.9 to 23.2) |
| Prostate cancer | 15.1 (13.2 to 18.8) | 1.7 (1.5 to 2.1) | 1.3 (0.8 to 1.8) | 41.3* (35.1 to 50.7) | 21.2* (15.8 to 29.2) |
| Diagnosis and primary therapy phase of prostate cancer | 0.5 (0.5 to 0.7) | 1.7 (1.5 to 2.1) | 0.2 (0.1 to 0.2) | 43.0* (36.5 to 52.6) | 22.6* (17.1 to 30.9) |
| Controlled phase of prostate cancer | 14.3 (12.5 to 17.9) | -- | 1.0 (0.7 to 1.6) | 42.3* (35.8 to 52.1) | 22.1* (16.4 to 30.4) |
| Metastatic phase of prostate cancer | 0.1 (0.1 to 0.1) | -- | 0.1 (0.0 to 0.1) | 23.8* (18.8 to 29.8) | 6.1* (1.9 to 11.3) |
| Terminal phase of prostate cancer | -- | -- | -- | 17.6* (13.2 to 23.1) | 0.9 (-2.9 to 5.6) |
| Impotence and Incontinence after 10-year survival from prostate cancer | 0.1 (0.1 to 0.1) | -- | -- | 11.4* (10.6 to 12.1) | -4.5* (-5.2 to -3.8) |
| Testicular cancer | 132.1 (123.4 to 142.7) | 15.6 (14.6 to 16.8) | 8.0 (5.5 to 11.3) | 27.0* (15.9 to 38.9) | 8.9 (-0.6 to 19.1) |
| Diagnosis and primary therapy phase of testicular cancer | 4.3 (4.0 to 4.7) | 15.6 (14.6 to 16.8) | 1.2 (0.8 to 1.7) | 29.1* (17.5 to 41.6) | 10.8* (0.7 to 21.4) |
| Controlled phase of testicular cancer | 126.2 (117.8 to 136.3) | -- | 6.0 (3.8 to 9.0) | 29.2* (16.9 to 42.4) | 10.8* (0.3 to 22.1) |
| Metastatic phase of testicular cancer | 1.4 (1.3 to 1.6) | -- | 0.6 (0.4 to 0.9) | 9.6* (2.2 to 18.3) | -6.0 (-12.3 to 1.4) |
| Terminal phase of testicular cancer | 0.2 (0.2 to 0.2) | -- | 0.1 (0.1 to 0.1) | -0.9 (-8.2 to 8.2) | -15.0* (-21.2 to -7.2) |
| Kidney cancer | 34.4 (31.9 to 36.4) | 4.9 (4.6 to 5.2) | 2.0 (1.4 to 2.9) | 24.8* (15.4 to 36.0) | 7.0 (-1.0 to 16.7) |
| Diagnosis and primary therapy phase of kidney cancer | -- | 4.9 (4.6 to 5.2) | -- | -- | -- |
| Controlled phase of kidney cancer | 33.5 (31.1 to 35.5) | -- | 1.6 (1.0 to 2.4) | 25.1* (15.2 to 37.1) | 7.3 (-1.2 to 17.6) |
| Metastatic phase of kidney cancer | 0.8 (0.7 to 0.8) | -- | 0.3 (0.2 to 0.5) | 23.6* (16.3 to 32.9) | 6.0 (-0.3 to 14.0) |
| Terminal phase of kidney cancer | 0.1 (0.1 to 0.2) | -- | 0.1 (0.1 to 0.1) | 23.6* (16.3 to 32.9) | 6.0 (-0.3 to 14.0) |
| Bladder cancer | 16.0 (14.6 to 17.4) | 2.0 (1.8 to 2.2) | 1.4 (1.0 to 2.0) | 25.3* (13.5 to 37.3) | 7.5 (-2.6 to 17.7) |
| Diagnosis and primary therapy phase of bladder cancer | 0.8 (0.8 to 0.9) | 2.0 (1.8 to 2.2) | 0.2 (0.2 to 0.3) | 28.4* (18.0 to 39.0) | 10.1* (1.2 to 19.2) |
| Controlled phase of bladder cancer | 14.7 (13.4 to 16.0) | -- | 1.0 (0.6 to 1.4) | 25.9* (13.0 to 39.8) | 8.0 (-3.1 to 19.9) |
| Metastatic phase of bladder cancer | 0.3 (0.2 to 0.3) | -- | 0.1 (0.1 to 0.2) | 18.8* (8.6 to 29.4) | 1.8 (-6.9 to 10.9) |
| Terminal phase of bladder cancer | 0.0 (0.0 to 0.1) | -- | -- | 17.8* (7.6 to 28.5) | 1.0 (-7.7 to 10.2) |
| Incontinence from bladder cancer, beyond 10 years | 0.2 (0.1 to 0.2) | -- | -- | 18.0* (16.6 to 19.5) | 1.2 (-0.0 to 2.5) |
| Brain and nervous system cancer | 107.7 (91.3 to 123.9) | 19.7 (16.9 to 22.4) | 9.4 (6.5 to 12.9) | 75.1* (57.4 to 92.6) | 50.2* (35.0 to 65.1) |
| Diagnosis and primary therapy phase of brain and nervous system cancers | 6.4 (5.5 to 7.3) | 19.7 (16.9 to 22.4) | 1.8 (1.2 to 2.6) | 91.3* (72.0 to 111.2) | 64.1* (47.5 to 81.1) |
| Controlled phase of brain and nervous system cancers | 94.7 (80.1 to 109.3) | -- | 4.5 (2.8 to 6.9) | 100.3* (74.5 to 127.1) | 71.8* (49.7 to 94.8) |
| Metastatic phase of brain and nervous system cancers | 5.7 (4.9 to 6.4) | -- | 2.6 (1.7 to 3.5) | 42.8* (29.4 to 54.7) | 22.5* (11.0 to 32.6) |
| Terminal phase of brain and nervous system cancers | 0.9 (0.8 to 1.0) | -- | 0.5 (0.4 to 0.7) | 36.4* (23.6 to 46.9) | 17.0* (6.0 to 26.0) |
| Thyroid cancer | 92.9 (79.9 to 113.0) | 10.6 (9.1 to 12.9) | 5.4 (3.5 to 7.9) | 24.3* (5.4 to 47.0) | 6.6 (-9.6 to 26.0) |
| Diagnosis and primary therapy phase of thyroid cancer | 2.5 (2.1 to 3.0) | 10.6 (9.1 to 12.9) | 0.7 (0.5 to 1.0) | 26.0* (3.6 to 52.8) | 8.1 (-11.2 to 31.1) |
| Controlled phase of thyroid cancer | 89.6 (77.1 to 109.0) | -- | 4.3 (2.6 to 6.6) | 25.2* (5.0 to 50.2) | 7.4 (-9.9 to 28.8) |
| Metastatic phase of thyroid cancer | 0.7 (0.6 to 0.9) | -- | 0.3 (0.2 to 0.5) | 13.1 (-1.9 to 34.3) | -3.0 (-15.9 to 15.2) |
| Terminal phase of thyroid cancer | 0.1 (0.1 to 0.1) | -- | 0.0 (0.0 to 0.1) | 9.0 (-6.6 to 30.8) | -6.5 (-19.9 to 12.2) |
| Mesothelioma | 0.9 (0.8 to 1.0) | 0.4 (0.4 to 0.5) | 0.2 (0.1 to 0.2) | 5.4 (-6.4 to 15.5) | -9.6* (-19.7 to -0.9) |
| Diagnosis and primary therapy phase of mesothelioma | 0.1 (0.1 to 0.1) | 0.4 (0.4 to 0.5) | -- | 6.2 (-5.4 to 16.0) | -8.9* (-18.9 to -0.5) |
| Controlled phase of mesothelioma | 0.6 (0.5 to 0.6) | -- | -- | 4.4 (-7.3 to 14.8) | -10.4* (-20.5 to -1.6) |
| Metastatic phase of mesothelioma | 0.2 (0.2 to 0.2) | -- | 0.1 (0.1 to 0.1) | 5.5 (-6.3 to 15.7) | -9.5* (-19.7 to -0.8) |
| Terminal phase of mesothelioma | -- | -- | -- | 5.1 (-7.0 to 15.6) | -9.9* (-20.2 to -0.8) |
| Hodgkin lymphoma | 85.7 (75.6 to 109.5) | 11.9 (10.5 to 15.0) | 6.1 (4.1 to 8.7) | 14.6* (5.2 to 24.9) | -1.7 (-9.8 to 7.1) |
| Diagnosis and primary therapy phase of Hodgkin disease | 2.8 (2.5 to 3.6) | 11.9 (10.5 to 15.0) | 0.8 (0.5 to 1.2) | 18.0* (9.1 to 28.3) | 1.2 (-6.4 to 10.0) |
| Controlled phase of Hodgkin disease | 79.5 (70.1 to 101.6) | -- | 3.8 (2.3 to 5.8) | 18.2* (7.2 to 31.0) | 1.3 (-8.1 to 12.3) |
| Metastatic phase of Hodgkin disease | 3.0 (2.7 to 3.7) | -- | 1.4 (0.9 to 1.9) | 6.1 (-1.5 to 14.0) | -9.0* (-15.5 to -2.3) |
| Terminal phase of Hodgkin disease | 0.3 (0.2 to 0.3) | -- | 0.2 (0.1 to 0.2) | -2.0 (-9.9 to 4.7) | -16.0* (-22.8 to -10.2) |
| Non-Hodgkin's lymphoma | 60.4 (57.8 to 63.5) | 11.7 (11.2 to 12.4) | 4.9 (3.5 to 6.5) | 32.7* (24.3 to 42.7) | 13.8* (6.6 to 22.4) |
| Diagnosis and primary therapy phase of non-Hodgkin lymphoma | 2.1 (2.1 to 2.2) | 11.7 (11.2 to 12.4) | 0.6 (0.4 to 0.9) | 38.2* (31.8 to 45.4) | 18.5* (13.0 to 24.7) |
| Controlled phase of non-Hodgkin lymphoma | 54.8 (52.5 to 57.5) | -- | 2.6 (1.7 to 3.9) | 38.8* (26.6 to 53.1) | 19.0* (8.6 to 31.3) |
| Metastatic phase of non-Hodgkin lymphoma | 3.0 (2.8 to 3.2) | -- | 1.3 (0.9 to 1.8) | 22.4* (15.3 to 30.2) | 4.9 (-1.1 to 11.6) |
| Terminal phase of non-Hodgkin lymphoma | 0.5 (0.5 to 0.6) | -- | 0.3 (0.2 to 0.4) | 20.7* (13.6 to 28.6) | 3.5 (-2.6 to 10.3) |
| Multiple myeloma | 3.8 (3.1 to 4.1) | 0.9 (0.7 to 1.0) | 0.7 (0.5 to 1.0) | 75.5* (49.4 to 91.3) | 50.5* (28.1 to 64.0) |
| Diagnosis and primary therapy phase of multiple myeloma | 0.2 (0.2 to 0.3) | 0.9 (0.7 to 1.0) | 0.1 (0.0 to 0.1) | 84.3* (57.1 to 101.0) | 58.0* (34.7 to 72.4) |
| Controlled phase of multiple myeloma | 2.3 (1.9 to 2.6) | -- | 0.1 (0.1 to 0.2) | 84.7* (57.3 to 101.5) | 58.4* (34.9 to 72.8) |
| Metastatic phase of multiple myeloma | 1.1 (0.9 to 1.2) | -- | 0.5 (0.3 to 0.7) | 74.0* (48.5 to 89.4) | 49.2* (27.4 to 62.5) |
| Terminal phase of multiple myeloma | 0.1 (0.0 to 0.1) | -- | -- | 51.1* (30.8 to 64.6) | 29.6* (12.1 to 41.2) |
| Leukaemia | 68.2 (56.6 to 73.5) | 18.1 (15.3 to 19.4) | 7.8 (5.4 to 10.6) | 23.9* (14.3 to 32.4) | 6.2 (-1.9 to 13.6) |
| Acute lymphoid leukaemia | 13.0 (10.6 to 14.2) | 4.0 (3.3 to 4.4) | 1.6 (1.1 to 2.1) | 22.3* (9.9 to 33.5) | 4.9 (-5.7 to 14.5) |
| Diagnosis and primary therapy phase of acute lymphoid leukemia | 1.8 (1.5 to 1.9) | 4.0 (3.3 to 4.4) | 0.5 (0.3 to 0.7) | 19.6* (8.6 to 29.6) | 2.6 (-6.9 to 11.2) |
| Controlled phase of acute lymphoid leukemia | 10.1 (8.2 to 11.0) | -- | 0.5 (0.3 to 0.7) | 23.9* (10.8 to 35.5) | 6.3 (-5.0 to 16.2) |
| Metastatic phase of acute lymphoid leukemia | 1.0 (0.8 to 1.1) | -- | 0.5 (0.3 to 0.6) | 23.4* (10.4 to 35.3) | 5.8 (-5.3 to 16.0) |
| Terminal phase of acute lymphoid leukemia | 0.2 (0.2 to 0.2) | -- | 0.1 (0.1 to 0.1) | 23.5* (10.6 to 35.4) | 5.9 (-5.2 to 16.1) |
| Chronic lymphoid leukaemia | 11.9 (9.4 to 13.5) | 1.6 (1.2 to 1.8) | 1.4 (0.9 to 1.9) | 154.9* (104.6 to 196.6) | 118.6* (75.5 to 154.3) |

| | | | | | |
|---|---|---|---|---|---|
| Diagnosis and primary therapy phase of chronic lymphoid leukemia | 0.6 (0.5 to 0.7) | 1.6 (1.2 to 1.8) | 0.2 (0.1 to 0.3) | 155.7* (106.9 to 195.4) | 119.3* (77.5 to 153.4) |
| Controlled phase of chronic lymphoid leukemia | 9.6 (7.7 to 10.9) | -- | 0.5 (0.3 to 0.7) | 154.7* (104.6 to 199.8) | 118.4* (75.4 to 157.1) |
| Metastatic phase of chronic lymphoid leukemia | 1.5 (1.2 to 1.8) | -- | 0.7 (0.5 to 1.0) | 156.0* (104.6 to 200.8) | 119.5* (75.4 to 158.0) |
| Terminal phase of chronic lymphoid leukemia | 0.0 (0.0 to 0.1) | -- | -- | 126.7* (83.1 to 165.5) | 94.4* (57.0 to 127.7) |
| Acute myeloid leukaemia | 8.6 (7.6 to 9.6) | 4.9 (4.3 to 5.4) | 1.3 (0.9 to 1.7) | 11.9* (5.2 to 19.5) | -4.0 (-9.8 to 2.5) |
| Diagnosis and primary therapy phase of acute myeloid leukemia | -- | 4.9 (4.3 to 5.4) | -- | -- | -- |
| Controlled phase of acute myeloid leukemia | 6.6 (5.9 to 7.3) | -- | 0.3 (0.2 to 0.5) | 12.6* (5.9 to 20.7) | -3.4 (-9.2 to 3.5) |
| Metastatic phase of acute myeloid leukemia | 1.6 (1.4 to 1.8) | -- | 0.7 (0.5 to 1.0) | 11.7* (4.6 to 19.1) | -4.2 (-10.3 to 2.2) |
| Terminal phase of acute myeloid leukemia | 0.4 (0.3 to 0.4) | -- | 0.2 (0.1 to 0.3) | 11.7* (4.6 to 19.1) | -4.2 (-10.3 to 2.2) |
| Chronic myeloid leukaemia | 6.2 (5.4 to 6.9) | 1.8 (1.6 to 2.0) | 0.7 (0.5 to 0.9) | -0.0 (-6.8 to 7.9) | -14.3* (-20.1 to -7.4) |
| Diagnosis and primary therapy phase of chronic myeloid leukemia | 0.4 (0.4 to 0.5) | 1.8 (1.6 to 2.0) | 0.1 (0.1 to 0.2) | 3.5 (-3.0 to 11.3) | -11.3* (-16.8 to -4.6) |
| Controlled phase of chronic myeloid leukemia | 5.1 (4.5 to 5.8) | -- | 0.3 (0.2 to 0.4) | 4.3 (-2.2 to 12.2) | -10.6* (-16.2 to -3.8) |
| Metastatic phase of chronic myeloid leukemia | 0.5 (0.4 to 0.6) | -- | 0.2 (0.1 to 0.3) | -4.9 (-12.3 to 3.4) | -18.5* (-24.8 to -11.3) |
| Terminal phase of chronic myeloid leukemia | 0.1 (0.1 to 0.1) | -- | 0.1 (0.0 to 0.1) | -4.9 (-12.3 to 3.4) | -18.5* (-24.8 to -11.3) |
| Other leukaemia | 28.7 (21.8 to 31.5) | 5.9 (4.5 to 6.4) | 3.0 (2.0 to 4.1) | 9.4 (-0.9 to 20.4) | -6.2 (-15.0 to 3.2) |
| Metastatic phase of other leukemia | 3.7 (2.8 to 4.0) | -- | 1.7 (1.1 to 2.2) | 7.8 (-0.8 to 17.2) | -7.5 (-14.9 to 0.5) |
| Diagnosis and primary therapy phase of other leukemia | -- | 5.9 (4.5 to 6.4) | -- | -- | -- |
| Terminal phase of other leukemia | 0.3 (0.2 to 0.3) | -- | 0.1 (0.1 to 0.2) | 10.4 (-0.4 to 20.4) | -5.3 (-14.6 to 3.3) |
| Controlled phase of other leukemia | 24.7 (18.9 to 27.2) | -- | 1.2 (0.7 to 1.9) | 11.5 (-2.6 to 27.2) | -4.4 (-16.5 to 9.1) |
| Other malignant cancers | 502.3 (368.0 to 717.7) | 26.3 (23.6 to 28.2) | 37.0 (23.0 to 57.0) | 211.1* (129.0 to 337.0) | 166.8* (96.4 to 274.8) |
| Diagnosis and primary therapy phase of other malignant neoplasms | 11.3 (8.3 to 16.2) | 26.3 (23.6 to 28.2) | 3.2 (1.9 to 5.1) | 241.4* (145.9 to 389.3) | 192.7* (110.8 to 319.7) |
| Controlled phase of other malignant neoplasms | 465.3 (340.1 to 669.7) | -- | 22.2 (12.6 to 36.6) | 270.4* (167.9 to 427.3) | 217.7* (129.8 to 352.2) |
| Metastatic phase of other malignant neoplasms | 22.6 (17.4 to 30.7) | -- | 9.9 (6.1 to 14.8) | 135.3* (79.5 to 229.4) | 101.8* (53.9 to 182.5) |
| Terminal phase of other malignant neoplasms | 3.2 (2.5 to 4.2) | -- | 1.7 (1.1 to 2.5) | 125.3* (77.6 to 203.1) | 93.2* (52.3 to 159.9) |
| Other neoplasms | 989.8 (939.6 to 1041.0) | 492.3 (460.6 to 524.9) | 1.3 (0.8 to 2.0) | 17.7* (9.0 to 27.5) | 0.9 (-6.5 to 9.3) |
| Myelodysplastic, myeloproliferative, and other hematopoietic neoplasms | 27.3 (22.9 to 32.7) | 1.7 (0.9 to 2.8) | 1.3 (0.8 to 2.0) | 17.7* (9.0 to 27.5) | 0.9 (-6.5 to 9.3) |
| Benign and in situ intestinal neoplasms | 40.4 (37.6 to 43.1) | 19.3 (17.4 to 21.0) | -- | -- | -- |
| Benign and in situ cervical and uterine neoplasms | 156.3 (148.0 to 164.5) | 80.8 (76.1 to 85.6) | -- | -- | -- |
| Other benign and in situ neoplasms | 800.3 (753.7 to 847.8) | 390.5 (358.3 to 422.5) | -- | -- | -- |
| **Cardiovascular diseases** | **8364.7 (7659.1 to 9193.3)** | **669.9 (600.4 to 737.6)** | **635.5 (461.1 to 845.0)** | **12.0* (9.2 to 14.4)** | **-4.0* (-6.4 to -1.9)** |
| Rheumatic heart disease | 4468.2 (4294.3 to 4645.5) | 114.9 (107.4 to 122.3) | 213.5 (139.1 to 312.3) | 19.8* (18.1 to 21.7) | 2.7* (1.2 to 4.3) |
| Rheumatic heart disease, without heart failure | 4445.4 (4270.5 to 4623.5) | 114.9 (107.4 to 122.3) | 211.4 (137.2 to 308.6) | 19.7* (18.0 to 21.7) | 2.7* (1.2 to 4.3) |
| Heart failure due to rheumatic heart disease | 22.8 (14.9 to 33.2) | -- | 2.1 (1.2 to 3.5) | 23.5* (21.6 to 25.9) | 5.9* (4.3 to 8.0) |
| Ischaemic heart disease | 684.7 (625.4 to 747.8) | 41.6 (17.7 to 74.2) | 38.7 (26.2 to 55.0) | 12.3* (6.4 to 18.2) | -3.7 (-8.8 to 1.4) |
| Asymptomatic ischemic heart disease following myocardial infarction | 45.5 (25.7 to 69.1) | -- | -- | -- | -- |
| Angina due to ischemic heart disease | 590.1 (536.9 to 644.9) | -- | 34.1 (22.6 to 48.4) | 11.5* (4.9 to 18.2) | -4.4 (-10.0 to 1.4) |
| Heart failure due to ischemic heart disease | 46.9 (31.3 to 65.5) | -- | 4.3 (2.5 to 6.8) | 19.1* (17.6 to 20.5) | 2.1* (0.9 to 3.4) |
| Myocardial infarction due to ischemic heart disease | 2.2 (1.0 to 3.9) | 41.6 (17.7 to 74.2) | 0.2 (0.1 to 0.4) | 12.4* (6.2 to 15.1) | -3.6* (-9.0 to -1.3) |
| Stroke | 1397.1 (1240.0 to 1558.9) | 82.3‡ (58.3 to 117.3) | 259.5 (183.0 to 338.0) | 5.4* (0.1 to 10.4) | -9.6* (-14.2 to -5.3) |
| Ischaemic stroke | 984.1 (835.9 to 1137.4) | 36.3 (16.3 to 64.6) | 149.3 (103.1 to 199.7) | 7.4* (0.1 to 14.9) | -7.9* (-14.1 to -1.4) |
| Acute ischemic stroke | 2.9 (1.3 to 5.1) | 36.3 (16.3 to 64.6) | 0.8 (0.3 to 1.5) | 22.0* (17.8 to 26.3) | 4.6* (1.0 to 8.4) |
| Chronic ischemic stroke | 981.2 (832.1 to 1133.4) | -- | 148.5 (102.3 to 198.5) | 7.3* (0.0 to 14.9) | -8.0* (-14.2 to -1.4) |
| Intracerebral hemorrhage | 521.6 (440.7 to 610.7) | 28.4 (17.8 to 42.2) | 82.5 (57.1 to 110.9) | -0.6 (-8.9 to 7.8) | -14.7* (-21.9 to -7.5) |
| Acute intracerebral hemorrhage | 1.9 (1.2 to 2.8) | 28.4 (17.8 to 42.2) | 0.5 (0.3 to 0.8) | -21.4* (-34.7 to -10.2) | -32.6* (-44.0 to -22.9) |
| Chronic intracerebral hemorrhage | 519.7 (438.8 to 608.6) | -- | 82.0 (56.7 to 110.2) | -0.4 (-8.7 to 8.1) | -14.6* (-21.7 to -7.3) |
| Subarachnoid hemorrhage | 176.2 (145.2 to 209.2) | 17.6 (11.5 to 25.0) | 27.7 (19.0 to 37.3) | 14.4* (9.7 to 19.6) | -1.9 (-5.9 to 2.6) |
| Acute subarachnoid hemorrhage | 1.1 (0.7 to 1.6) | 17.6 (11.5 to 25.0) | 0.3 (0.2 to 0.5) | 12.6* (5.1 to 16.1) | -3.5* (-9.9 to -0.5) |
| Chronic subarachnoid hemorrhage | 175.1 (144.2 to 208.1) | -- | 27.4 (18.7 to 36.9) | 14.4* (9.7 to 19.7) | -1.9 (-6.0 to 2.7) |
| Hypertensive heart disease | 148.7 (102.2 to 210.1) | -- | 13.5 (8.2 to 21.0) | 17.6* (9.7 to 26.9) | 0.8 (-6.0 to 8.9) |
| Non-rheumatic valvular heart disease | 204.5 (190.5 to 218.1) | 227.7 (217.7 to 238.5) | 0.2 (0.1 to 0.5) | 10.1* (6.4 to 13.6) | -5.5* (-8.8 to -2.6) |
| Non-rheumatic calcific aortic valve disease | 44.0 (36.7 to 52.1) | 44.0 (36.7 to 52.1) | 0.0 (0.0 to 0.1) | 8.8* (1.8 to 14.9) | -6.7* (-12.7 to -1.4) |
| Heart failure due to calcific aortic valve disease | 0.2 (0.0 to 0.5) | -- | -- | 1.5 (-19.1 to 24.5) | -13.0 (-30.6 to 6.7) |
| Non-rheumatic calcific aortic valve disease | 43.9 (36.7 to 52.0) | 44.0 (36.7 to 52.1) | -- | 19.1* (2.2 to 37.8) | 2.1 (-12.4 to 18.2) |
| Non-rheumatic degenerative mitral valve disease | 183.6 (176.8 to 190.7) | 183.6 (176.8 to 190.7) | 0.1 (0.0 to 0.4) | 11.2* (5.4 to 16.9) | -4.7 (-9.7 to 0.3) |
| Heart failure due to degenerative mitral valve disease | 1.1 (0.3 to 3.1) | -- | 0.1 (0.0 to 0.3) | 3.8 (-10.6 to 20.5) | -11.0 (-23.4 to 3.4) |
| Non-rheumatic degenerative mitral valve disease | 182.6 (175.5 to 189.9) | 183.6 (176.8 to 190.7) | 0.0 (0.0 to 0.1) | 32.1* (8.3 to 58.7) | 13.3 (-7.2 to 36.1) |
| Other non-rheumatic valve diseases | 0.5 (0.3 to 0.8) | -- | 0.0 (0.0 to 0.1) | 8.1* (6.5 to 9.7) | -7.3* (-8.7 to -5.9) |
| Mild heart failure due to other non-rheumatic valve disease | 0.1 (0.1 to 0.1) | -- | -- | 8.1* (6.5 to 9.7) | -7.3* (-8.7 to -5.9) |
| Moderate heart failure due to other non-rheumatic valve disease | 0.1 (0.0 to 0.1) | -- | -- | 8.1* (6.5 to 9.7) | -7.3* (-8.7 to -5.9) |
| Severe heart failure due to other non-rheumatic valve disease | 0.2 (0.1 to 0.3) | -- | -- | 8.1* (6.4 to 9.7) | -7.3* (-8.7 to -5.9) |

| | | | | | |
|---|---|---|---|---|---|
| Controlled, medically managed heart failure due to other non-rheumatic valve disease | 0.2 (0.1 to 0.3) | -- | -- | 8.1* (6.5 to 9.7) | -7.3* (-8.7 to -5.9) |
| Cardiomyopathy and myocarditis | 29.9 (21.9 to 39.3) | 159.5 (108.1 to 214.4) | 17.4 (10.5 to 27.1) | 12.4* (9.5 to 15.1) | -3.6* (-6.1 to -1.3) |
| Myocarditis | 92.2 (71.4 to 115.6) | 159.5 (108.1 to 214.4) | 6.8 (4.3 to 10.5) | 16.0* (12.9 to 19.0) | -0.6 (-3.2 to 2.1) |
| Acute myocarditis | 39.6 (28.4 to 52.4) | 159.5 (108.1 to 214.4) | 2.0 (1.2 to 3.2) | 15.1* (12.0 to 18.1) | -1.3 (-3.9 to 1.3) |
| Heart failure due to myocarditis | 52.6 (36.2 to 72.9) | -- | 4.9 (2.8 to 7.7) | 16.3* (12.1 to 20.9) | -0.2 (-3.8 to 3.7) |
| Alcoholic cardiomyopathy | 38.4 (27.4 to 51.9) | -- | 3.5 (2.1 to 5.6) | 4.7 (-0.9 to 10.5) | -10.2* (-15.0 to -5.2) |
| Other cardiomyopathy | 76.8 (53.2 to 105.9) | -- | 7.1 (4.1 to 11.1) | 13.0* (8.3 to 17.5) | -3.1 (-7.1 to 0.8) |
| Atrial fibrillation and flutter | -- | -- | -- | -- | -- |
| Asymptomatic atrial fibrillation and flutter | -- | -- | -- | -- | -- |
| Symptomatic atrial fibrillation and flutter | -- | -- | -- | -- | -- |
| Peripheral vascular disease | -- | -- | -- | -- | -- |
| Asymptomatic peripheral arterial disease | -- | -- | -- | -- | -- |
| Symptomatic claudication due to peripheral arterial disease | -- | -- | -- | -- | -- |
| Endocarditis | 17.7 (12.5 to 24.6) | 43.9 (29.6 to 62.0) | 1.5 (0.9 to 2.3) | 12.3* (10.7 to 13.8) | -3.7* (-5.0 to -2.4) |
| Acute endocarditis | 3.4 (2.4 to 4.7) | 43.9 (29.6 to 62.0) | 0.2 (0.1 to 0.3) | 18.1* (14.1 to 20.9) | 1.3 (-2.1 to 3.7) |
| Heart failure due to endocarditis | 14.2 (9.2 to 20.8) | -- | 1.3 (0.7 to 2.1) | 11.5* (9.7 to 13.2) | -4.4* (-5.9 to -3.0) |
| Other cardiovascular and circulatory diseases | 1691.1 (1099.4 to 2493.8) | -- | 91.2 (49.9 to 144.0) | 13.9* (9.6 to 18.1) | -2.4 (-6.0 to 1.3) |
| Heart failure due to other cardiovascular diseases | 6.3 (4.1 to 9.2) | -- | 0.6 (0.3 to 0.9) | 13.7* (12.2 to 15.2) | -2.5* (-3.8 to -1.2) |
| Other cardiovascular and circulatory disease episodes | 1684.7 (1095.3 to 2484.9) | -- | 90.6 (49.6 to 143.1) | 13.9* (9.6 to 18.1) | -2.4 (-6.0 to 1.3) |
| **Chronic respiratory diseases** | **20096.4 (16831.4 to 23549.4)** | **2071.8 (1551.2 to 2646.9)** | **1547.0 (1188.0 to 1941.7)** | **13.0* (7.8 to 18.2)** | **-3.1 (-7.5 to 1.4)** |
| Chronic obstructive pulmonary disease | 7011.6 (5579.3 to 8611.7) | 489.4 (320.0 to 727.4) | 787.4 (592.5 to 1009.5) | 12.3* (4.9 to 19.2) | -3.7 (-10.0 to 2.2) |
| Chronic obstructive pulmonary disease with heart failure | 1.6 (1.0 to 2.3) | -- | 0.7 (0.4 to 1.2) | 14.5* (12.9 to 16.4) | -1.8* (-3.2 to -0.2) |
| Chronic obstructive pulmonary disease without heart failure | 7010.0 (5577.5 to 8610.5) | 489.4 (320.0 to 727.4) | 786.7 (591.8 to 1008.9) | 12.3* (4.9 to 19.2) | -3.7 (-10.0 to 2.2) |
| Pneumoconiosis | 6.2 (4.6 to 8.3) | 0.7 (0.4 to 1.0) | 1.0 (0.6 to 1.5) | 13.0* (10.0 to 15.8) | -3.1* (-5.7 to -0.7) |
| Silicosis | 2.5 (1.5 to 3.8) | 0.2 (0.1 to 0.5) | 0.4 (0.2 to 0.7) | 14.2* (8.9 to 19.2) | -2.1 (-6.6 to 2.3) |
| Heart failure due to silicosis | -- | -- | -- | -- | -- |
| Silicosis without heart failure | 2.5 (1.5 to 3.8) | 0.2 (0.1 to 0.5) | 0.4 (0.2 to 0.7) | 14.2* (8.9 to 19.2) | -2.1 (-6.6 to 2.3) |
| Asbestosis | 1.8 (0.9 to 3.3) | 0.2 (0.1 to 0.4) | 0.3 (0.1 to 0.6) | 16.2* (10.6 to 22.1) | -0.3 (-5.1 to 4.7) |
| Asbestosis without heart failure | 1.8 (0.9 to 3.3) | 0.2 (0.1 to 0.4) | 0.3 (0.1 to 0.6) | 16.2* (10.6 to 22.1) | -0.3 (-5.1 to 4.7) |
| Heart failure due to asbestosis | -- | -- | -- | -- | -- |
| Coal workers pneumoconiosis | 0.8 (0.4 to 1.3) | 0.1 (0.0 to 0.2) | 0.1 (0.1 to 0.2) | 9.7* (2.7 to 14.9) | -6.0* (-11.9 to -1.5) |
| Coal workers pneumoconiosis without heart failure | 0.8 (0.4 to 1.3) | 0.1 (0.0 to 0.2) | 0.1 (0.1 to 0.2) | 9.7* (2.7 to 14.9) | -6.0* (-11.9 to -1.5) |
| Heart failure due to Coal workers pneumoconiosis | -- | -- | -- | -- | -- |
| Other pneumoconiosis | 1.2 (0.7 to 1.9) | 0.2 (0.1 to 0.3) | 0.2 (0.1 to 0.3) | 7.8* (4.4 to 10.7) | -7.5* (-10.5 to -5.1) |
| Heart failure due to other pneumoconiosis | -- | -- | -- | -- | -- |
| Other pneumoconiosis without heart failure | 1.2 (0.7 to 1.9) | 0.2 (0.1 to 0.3) | 0.2 (0.1 to 0.3) | 7.8* (4.4 to 10.7) | -7.5* (-10.5 to -5.1) |
| Asthma | 13422.4 (10429.0 to 16811.0) | 1570.1 (1099.5 to 2113.9) | 530.0 (325.3 to 806.2) | 10.8* (4.3 to 17.2) | -5.0 (-10.6 to 0.5) |
| Asymptomatic asthma | 4877.6 (3742.8 to 6244.9) | 569.3 (390.3 to 775.7) | -- | -- | -- |
| Symptomatic asthma | 8544.8 (6639.0 to 10673.9) | 1000.8 (698.8 to 1356.9) | 530.0 (325.3 to 806.2) | 10.8* (4.3 to 17.2) | -5.0 (-10.6 to 0.5) |
| Interstitial lung disease and pulmonary sarcoidosis | 73.9 (56.1 to 96.2) | 11.6 (8.0 to 15.8) | 8.3 (5.1 to 12.7) | 12.9* (8.4 to 17.4) | -3.2 (-7.0 to 0.7) |
| Heart failure due to interstitial lung disease and pulmonary sarcoidosis | 0.9 (0.6 to 1.4) | -- | 0.4 (0.3 to 0.7) | 9.9* (8.3 to 11.5) | -5.7* (-7.1 to -4.4) |
| Interstitial lung disease and pulmonary sarcoidosis without heart failure | 73.0 (55.2 to 95.3) | 11.6 (8.0 to 15.8) | 7.9 (4.7 to 12.3) | 13.1* (8.3 to 17.8) | -3.0 (-7.1 to 1.1) |
| Other chronic respiratory diseases | -- | -- | 220.2 (166.4 to 282.0) | 21.6* (13.7 to 29.0) | 4.3 (-2.5 to 10.6) |
| **Digestive diseases** | **180256.0 (163949.5 to 200541.9)** | **38773.6 (29922.1 to 49801.1)** | **1340.3 (861.1 to 2013.3)** | **15.9* (14.2 to 17.5)** | **-0.6 (-2.1 to 0.8)** |
| Cirrhosis and other chronic liver diseases | 126289.0 (116842.9 to 136859.6) | 644.4 (524.1 to 763.0) | 60.0 (41.5 to 83.4) | 22.4* (17.7 to 27.7) | 4.9* (0.9 to 9.5) |
| Cirrhosis and other chronic liver diseases due to hepatitis B | 37533.4 (34198.0 to 41565.2) | 270.7 (217.6 to 324.1) | 22.9 (15.5 to 32.7) | 19.2* (10.3 to 29.2) | 2.2 (-5.4 to 10.8) |
| Cirrhosis and other chronic liver diseases due to hepatitis B, decompensated | 134.0 (118.6 to 150.7) | 20.1 (17.1 to 23.2) | 22.9 (15.5 to 32.7) | 19.2* (10.3 to 29.2) | 2.2 (-5.4 to 10.8) |
| Chronic hepatitis B without cirrhosis | 34592.7 (31232.4 to 38626.8) | -- | -- | -- | -- |
| Cirrhosis and other chronic liver diseases due to hepatitis B, compensated | 2806.7 (2448.6 to 3219.4) | 250.6 (197.7 to 303.0) | -- | -- | -- |
| Cirrhosis and other chronic liver diseases due to hepatitis C | 11124.0 (9304.5 to 13152.2) | 158.8 (122.7 to 196.2) | 15.6 (10.2 to 22.3) | 23.0* (13.1 to 32.0) | 5.5 (-3.0 to 13.2) |
| Cirrhosis and other chronic liver diseases due to hepatitis C, decompensated | 91.1 (76.8 to 105.1) | 13.0 (10.5 to 15.8) | 15.6 (10.2 to 22.3) | 23.0* (13.1 to 32.0) | 5.5 (-3.0 to 13.2) |
| Chronic hepatitis C without cirrhosis | 9310.8 (7545.5 to 11345.2) | -- | -- | -- | -- |
| Cirrhosis and other chronic liver diseases due to hepatitis C, compensated | 1722.2 (1431.9 to 2040.3) | 145.8 (110.6 to 182.6) | -- | -- | -- |
| Cirrhosis and other chronic liver diseases due to alcohol use | 1031.1 (855.6 to 1231.3) | 97.6 (77.0 to 121.4) | 9.2 (6.0 to 13.3) | 21.7* (13.6 to 30.1) | 4.4 (-2.6 to 11.5) |
| Cirrhosis and other chronic liver diseases due to alcohol, decompensated | 53.0 (44.4 to 61.9) | 8.3 (6.8 to 10.1) | 9.2 (6.0 to 13.3) | 21.7* (13.6 to 30.1) | 4.4 (-2.6 to 11.5) |
| Cirrhosis and other chronic liver diseases due to alcohol, compensated | 978.1 (809.3 to 1170.7) | 89.3 (69.5 to 111.6) | -- | -- | -- |
| Cirrhosis due to NASH | 75640.3 (67038.0 to 85080.7) | 38.3 (28.6 to 49.9) | 3.7 (2.4 to 5.6) | 34.1* (29.9 to 38.0) | 15.0* (11.4 to 18.3) |
| Non-alcoholic fatty liver disease (NAFLD) / Non-alcoholic steatohepatitis (NASH) | 75208.0 (66504.3 to 84596.1) | -- | -- | -- | -- |
| Cirrhosis and other chronic liver diseases due to NASH | 432.3 (341.4 to 537.9) | 38.3 (28.6 to 49.9) | 3.7 (2.4 to 5.6) | 34.1* (29.9 to 38.0) | 15.0* (11.4 to 18.3) |
| Cirrhosis and other chronic liver diseases due to other causes | 960.2 (743.5 to 1216.8) | 79.0 (56.4 to 104.5) | 8.5 (5.5 to 12.7) | 26.1* (16.5 to 37.2) | 8.2 (-0.1 to 17.7) |

| | | | | | |
|---|---|---|---|---|---|
| Cirrhosis and other chronic liver diseases due to other cause, decompensated | 49.1 (38.5 to 60.7) | 6.8 (5.2 to 8.6) | 8.5 (5.5 to 12.7) | 26.1* (16.5 to 37.2) | 8.2 (-0.1 to 17.7) |
| Cirrhosis and other chronic liver diseases due to other cause, compensated | 911.1 (702.1 to 1157.0) | 72.2 (50.8 to 97.3) | -- | -- | -- |
| Upper digestive system diseases | 73851.3 (55499.6 to 96347.9) | 34109.7 (25334.5 to 45022.1) | 1038.9 (610.1 to 1652.2) | 16.1* (14.4 to 17.7) | -0.4 (-1.9 to 1.0) |
| Peptic ulcer disease | 1243.8 (869.6 to 1675.7) | 406.9 (291.7 to 543.5) | 61.2 (35.7 to 94.1) | 16.4* (10.7 to 21.7) | -0.1 (-5.1 to 4.4) |
| Acute peptic ulcer disease | 0.2 (0.1 to 0.3) | -- | 0.1 (0.0 to 0.1) | -2.7* (-5.3 to -0.6) | -16.6* (-18.8 to -14.7) |
| Chronic peptic ulcer disease | 1243.6 (869.3 to 1675.4) | 406.9 (291.7 to 543.5) | 61.2 (35.6 to 94.0) | 16.5* (10.7 to 21.8) | -0.1 (-5.0 to 4.4) |
| Gastritis and duodenitis | 8610.0 (6029.6 to 12392.2) | 5945.9 (4227.9 to 8060.0) | 385.7 (230.3 to 622.8) | 14.8* (11.3 to 17.9) | -1.6 (-4.5 to 1.1) |
| Acute gastritis and duodenitis | 2.3 (1.4 to 3.8) | -- | 0.8 (0.4 to 1.4) | 10.2* (8.2 to 12.1) | -5.5* (-7.2 to -3.9) |
| Chronic gastritis and duodenitis | 8607.7 (6027.7 to 12390.2) | 5945.9 (4227.9 to 8060.0) | 384.9 (229.2 to 621.9) | 14.8* (11.3 to 18.0) | -1.6 (-4.5 to 1.2) |
| GERD | 68171.4 (48110.5 to 93569.0) | 27757.0 (19055.2 to 38161.8) | 591.9 (285.6 to 1151.0) | 17.0* (15.1 to 19.0) | 0.4 (-1.3 to 2.1) |
| Appendicitis | 74.2 (61.1 to 90.1) | 1875.5 (1552.9 to 2275.3) | 22.9 (14.6 to 33.7) | 12.2 (-0.5 to 25.5) | -3.8 (-14.7 to 7.6) |
| Paralytic ileus and intestinal obstruction | 5.6 (3.9 to 7.9) | 148.8 (103.6 to 211.2) | 1.8 (1.0 to 2.8) | 20.7* (15.9 to 24.9) | 3.5 (-0.6 to 7.1) |
| Inguinal, femoral, and abdominal hernia | 1115.7 (798.1 to 1505.7) | 1207.6 (889.0 to 1574.9) | 111.5 (68.3 to 173.5) | 8.6* (3.7 to 14.2) | -6.8* (-11.1 to -2.1) |
| Inflammatory bowel disease | 325.8 (284.2 to 376.0) | 408.5 (337.4 to 497.7) | 52.2 (33.6 to 73.2) | 14.6* (2.8 to 30.4) | -1.7 (-11.8 to 11.8) |
| Ulcerative colitis | 163.3 (134.2 to 198.8) | 254.2 (192.3 to 329.5) | 25.7 (16.4 to 37.4) | 15.6* (3.1 to 31.4) | -0.9 (-11.6 to 12.7) |
| Crohn's disease | 162.4 (134.5 to 196.2) | 154.4 (119.9 to 195.8) | 26.6 (17.0 to 37.7) | 13.6* (0.4 to 30.9) | -2.6 (-13.9 to 12.2) |
| Vascular intestinal disorders | 1.9 (1.1 to 3.3) | 11.1 (7.2 to 16.2) | 0.6 (0.3 to 1.2) | 15.0* (12.3 to 17.3) | -1.4 (-3.7 to 0.6) |
| Gallbladder and biliary diseases | 1643.0 (1203.8 to 2195.0) | 280.4 (195.4 to 380.4) | 1.3 (0.8 to 2.1) | 14.7* (12.8 to 16.6) | -1.6 (-3.2 to 0.0) |
| Pancreatitis | 205.1 (171.4 to 252.6) | 87.4 (68.1 to 111.2) | 13.6 (7.1 to 24.0) | 17.0* (13.1 to 20.7) | 0.3 (-3.0 to 3.5) |
| Acute pancreatitis | 5.2 (3.8 to 7.0) | 67.7 (49.9 to 90.0) | 2.2 (1.3 to 3.3) | 13.5* (12.3 to 14.5) | -2.7* (-3.7 to -1.8) |
| Chronic pancreatitis | 199.9 (165.4 to 246.9) | 19.7 (13.8 to 27.4) | 11.5 (5.1 to 21.3) | 17.7* (13.2 to 22.2) | 0.9 (-3.0 to 4.8) |
| Other digestive diseases | -- | -- | 37.4 (24.2 to 54.9) | 27.5* (24.5 to 30.6) | 9.4* (6.8 to 12.0) |
| **Neurological disorders** | **313455.1 (263933.7 to 368191.5)** | **100233.1 (70265.0 to 137720.6)** | **6768.3 (4385.3 to 9685.0)** | **15.6* (13.6 to 17.6)** | **-0.9 (-2.6 to 0.9)** |
| Alzheimer's disease and other dementias | -- | -- | -- | -- | -- |
| Parkinson's disease | 12.0 (5.5 to 21.4) | 3.1 (1.4 to 5.5) | 2.0 (0.8 to 3.8) | 18.2* (13.5 to 22.3) | 1.3 (-2.7 to 4.9) |
| Epilepsy | 2141.9 (1594.4 to 2815.3) | 145.7 (94.8 to 209.9) | 687.6 (415.7 to 1012.3) | 16.1* (3.0 to 32.4) | -0.4 (-11.7 to 13.5) |
| Multiple sclerosis | 104.6 (85.7 to 126.3) | 12.3 (9.2 to 15.2) | 28.3 (19.1 to 38.9) | 11.3* (5.3 to 17.8) | -4.5 (-9.7 to 1.1) |
| Motor neuron disease | 10.6 (8.6 to 12.9) | 0.3 (0.1 to 0.4) | 2.3 (1.5 to 3.2) | 20.4* (18.9 to 21.7) | 3.2* (2.0 to 4.4) |
| Headache disorders | 312393.0 (262240.7 to 367183.0) | 100071.8 (70141.1 to 137510.3) | 5942.6 (3714.3 to 8706.5) | 15.3* (14.0 to 16.5) | -1.1* (-2.3 to -0.1) |
| Migraine | 152852.7 (131763.8 to 177978.2) | 12009.8 (7788.5 to 16181.1) | 5279.3 (3240.2 to 7886.5) | 15.2* (13.7 to 16.6) | -1.2* (-2.5 to -0.0) |
| Medication overuse headache due to migraine | 7051.3 (4901.6 to 9810.9) | -- | 792.6 (439.1 to 1263.6) | 15.9* (13.9 to 17.6) | -0.6 (-2.3 to 0.9) |
| Migraine | 145801.4 (125877.8 to 170301.4) | 12009.8 (7788.5 to 16181.1) | 4486.7 (2579.2 to 6978.7) | 15.1* (13.3 to 16.6) | -1.3 (-2.8 to 0.0) |
| Tension-type headache | 229291.0 (164756.5 to 304891.4) | 88062.0 (58265.6 to 125840.3) | 663.4 (362.5 to 1107.8) | 16.0* (14.6 to 17.5) | -0.5 (-1.7 to 0.8) |
| Medication overuse headache due to tension-type headache | 2617.4 (1627.1 to 4134.8) | -- | 292.3 (143.9 to 552.2) | 15.8* (12.9 to 18.8) | -0.7 (-3.1 to 1.9) |
| Tension-type headache | 226673.6 (161302.3 to 302467.7) | 88062.0 (58265.6 to 125840.3) | 371.1 (142.8 to 743.9) | 16.2* (15.1 to 17.3) | -0.4 (-1.3 to 0.6) |
| Other neurological disorders | 2.3 (1.2 to 3.8) | -- | 105.5 (65.0 to 153.9) | 32.5* (18.1 to 50.3) | 13.6* (1.3 to 28.9) |
| Other neurological disorders | -- | -- | 104.8 (64.4 to 153.0) | 32.6* (18.1 to 50.4) | 13.7* (1.3 to 29.0) |
| Guillain-Barré syndrome due to other neurological disorders | 2.3 (1.2 to 3.8) | -- | 0.7 (0.3 to 1.2) | 16.6* (14.1 to 19.3) | -0.0 (-2.1 to 2.3) |
| **Mental disorders** | **86248.9 (80163.1 to 92754.1)** | **27693.6 (23206.3 to 32888.4)** | **11351.1 (8378.5 to 14671.5)** | **14.0* (13.0 to 15.0)** | **-2.2* (-3.1 to -1.4)** |
| Schizophrenia | 2170.9 (1721.3 to 2713.9) | 224.5 (152.3 to 316.9) | 1436.4 (989.7 to 1907.8) | 17.4* (14.5 to 20.3) | 0.6 (-1.8 to 3.1) |
| Depressive disorders | 21928.6 (18537.0 to 25674.0) | 21703.0 (17206.2 to 26811.6) | 3721.9 (2495.2 to 5316.8) | 10.8* (8.6 to 12.8) | -5.0* (-6.8 to -3.3) |
| Major depressive disorder | 13978.5 (11253.2 to 17098.4) | 20290.3 (15768.8 to 25427.3) | 2903.1 (1917.3 to 4244.0) | 9.0* (6.2 to 11.4) | -6.5* (-8.9 to -4.4) |
| Dysthymia | 8293.7 (6363.5 to 10753.7) | 1412.7 (1134.1 to 1756.1) | 818.9 (513.5 to 1274.3) | 17.9* (15.4 to 20.5) | 1.1 (-1.0 to 3.4) |
| Bipolar disorder | 4677.4 (3746.0 to 5763.1) | 261.8 (162.3 to 374.2) | 975.0 (590.4 to 1501.7) | 14.6* (12.4 to 16.7) | -1.8 (-3.6 to 0.1) |
| Anxiety disorders | 25834.7 (22429.7 to 29478.4) | 3837.1 (3387.2 to 4308.6) | 2512.1 (1759.9 to 3436.7) | 13.7* (11.8 to 15.4) | -2.5* (-4.1 to -1.0) |
| Eating disorders | 2887.6 (2121.0 to 3958.1) | 1667.2 (1172.0 to 2329.9) | 611.8 (369.8 to 937.7) | 25.2* (22.7 to 27.5) | 7.3* (5.2 to 9.4) |
| Anorexia nervosa | 595.2 (433.3 to 799.5) | 137.0 (106.0 to 173.8) | 126.4 (74.0 to 192.8) | 22.8* (17.6 to 28.1) | 5.3* (0.8 to 9.8) |
| Bulimia nervosa | 2296.3 (1566.9 to 3309.9) | 1530.2 (1035.1 to 2186.4) | 485.4 (275.0 to 779.9) | 25.8* (22.9 to 28.3) | 7.9* (5.4 to 10.1) |
| Autism spectrum disorders | 2504.7 (2253.3 to 2768.6) | -- | 383.5 (259.7 to 527.6) | 16.1* (13.9 to 18.4) | -0.4 (-2.3 to 1.5) |
| Attention-deficit/hyperactivity disorder | 7048.8 (5766.4 to 8453.6) | -- | 85.3 (51.1 to 135.2) | 17.4* (14.7 to 20.4) | 0.7 (-1.6 to 3.3) |
| Conduct disorder | -- | -- | -- | -- | -- |
| Idiopathic developmental intellectual disability | 8684.5 (5068.8 to 12388.8) | -- | 347.3 (167.1 to 586.3) | 6.0* (2.3 to 8.1) | -9.1* (-12.2 to -7.3) |
| Other mental disorders | 16714.8 (14289.1 to 19446.1) | -- | 1277.8 (827.5 to 1789.8) | 16.7* (15.1 to 18.2) | 0.1 (-1.3 to 1.4) |
| **Substance use disorders** | **28566.2 (25067.5 to 32635.0)** | **8906.9 (7017.6 to 10992.9)** | **5825.9 (4119.0 to 7779.2)** | **21.0* (17.2 to 24.5)** | **3.8* (0.5 to 6.7)** |
| Alcohol use disorders | 15226.8 (12266.5 to 18919.3) | 7941.2 (6097.3 to 9930.9) | 1547.9 (1015.7 to 2234.9) | 10.2* (7.4 to 12.6) | -5.5* (-7.9 to -3.4) |
| Alcohol dependence | 15202.2 (12244.8 to 18896.6) | 7941.2 (6097.3 to 9930.9) | 1547.0 (1015.0 to 2234.0) | 10.2* (7.5 to 12.6) | -5.5* (-7.8 to -3.4) |
| Fetal alcohol syndrome | 24.6 (19.7 to 30.2) | -- | 0.9 (0.5 to 1.4) | -1.1 (-8.5 to 6.5) | -15.2* (-21.5 to -8.7) |
| Drug use disorders | 13907.8 (11825.8 to 16267.2) | 965.7 (751.6 to 1203.9) | 4278.0 (2934.5 to 5734.6) | 25.4* (20.9 to 29.4) | 7.6* (3.7 to 10.9) |
| Opioid use disorders | 8599.8 (6760.8 to 10872.7) | 635.3 (452.6 to 861.0) | 3639.9 (2402.8 to 5030.6) | 27.0* (21.9 to 31.6) | 8.9* (4.5 to 12.8) |
| Cocaine use disorders | 694.0 (601.3 to 796.1) | 9.3 (6.0 to 13.4) | 95.4 (61.5 to 138.9) | 14.1* (9.9 to 18.1) | -2.2 (-5.8 to 1.3) |

| | | | | | |
|---|---|---|---|---|---|
| Amphetamine use disorders | 1664.8 (1185.4 to 2290.7) | 84.3 (50.6 to 125.3) | 219.7 (122.7 to 350.4) | 19.1* (13.0 to 24.7) | 2.1 (-3.1 to 6.9) |
| Cannabis use disorders | 2915.0 (2112.5 to 3870.8) | 236.8 (147.6 to 344.9) | 84.4 (50.4 to 129.1) | 13.8* (9.1 to 18.4) | -2.4 (-6.5 to 1.5) |
| Other drug use disorders | 307.5 (235.6 to 382.2) | -- | 238.5 (153.3 to 346.4) | 17.5* (12.9 to 22.2) | 0.8 (-3.2 to 4.8) |
| **Diabetes and kidney diseases** | **50978.1 (43234.9 to 58271.5)** | **1839.7 (1354.7 to 2440.7)** | **1693.1 (1106.9 to 2414.4)** | **21.9* (16.3 to 27.3)** | **4.5 (-0.2 to 9.2)** |
| Diabetes mellitus | 19785.6 (15683.9 to 24138.2) | 1444.8 (998.2 to 2017.3) | 1389.6 (873.3 to 2025.0) | 23.1* (16.5 to 29.4) | 5.6 (-0.1 to 11.0) |
| Diabetes mellitus type 1 | 1246.0 (1082.6 to 1424.6) | 28.3 (18.1 to 39.8) | 87.4 (58.3 to 125.7) | 13.7* (10.5 to 17.2) | -2.5 (-5.3 to 0.5) |
| Uncomplicated diabetes mellitus type 1 | 907.9 (776.1 to 1050.1) | 28.3 (18.1 to 39.8) | 43.2 (26.7 to 65.1) | 14.7* (10.7 to 18.5) | -1.7 (-5.1 to 1.6) |
| Vision loss due to diabetes mellitus type 1 retinopathy | 3.4 (2.0 to 5.3) | -- | 0.2 (0.1 to 0.4) | -5.0 (-11.3 to 0.8) | -18.5* (-23.9 to -13.5) |
| Diabetic neuropathy due to diabetes mellitus type 1 | 334.8 (270.4 to 405.5) | -- | 44.0 (28.0 to 64.7) | 12.9* (6.5 to 19.4) | -3.1 (-8.7 to 2.4) |
| Diabetes mellitus type 2 | 18539.6 (14446.7 to 22887.0) | 1416.5 (966.1 to 1984.1) | 1302.2 (807.3 to 1903.7) | 23.8* (16.7 to 30.4) | 6.1* (0.0 to 11.8) |
| Uncomplicated diabetes mellitus type 2 | 13558.4 (10408.3 to 16976.7) | 1416.5 (966.1 to 1984.1) | 646.9 (379.2 to 1008.3) | 24.9* (17.6 to 31.3) | 7.1* (0.8 to 12.6) |
| Vision loss due to diabetes mellitus type 2 retinopathy | 35.2 (23.5 to 50.5) | -- | 2.9 (1.7 to 4.5) | 7.3 (-0.7 to 14.7) | -8.0* (-14.9 to -1.7) |
| Diabetic neuropathy due to diabetes mellitus type 2 | 4946.0 (3659.8 to 6413.3) | -- | 652.4 (405.5 to 963.6) | 22.7* (15.0 to 30.4) | 5.3 (-1.4 to 11.8) |
| Chronic kidney disease | 33667.3 (26992.4 to 41140.7) | 356.4 (206.5 to 567.8) | 303.4 (204.1 to 429.9) | 16.7* (8.6 to 24.7) | 0.1 (-6.9 to 6.9) |
| Chronic kidney disease due to diabetes mellitus type 1 | 252.9 (199.9 to 308.7) | 3.7 (2.1 to 6.0) | 7.3 (4.8 to 10.3) | 13.9* (6.4 to 21.1) | -2.3 (-8.8 to 3.8) |
| Albuminuria with preserved GFR due to type 1 diabetes mellitus | 160.7 (115.1 to 212.0) | -- | -- | -- | -- |
| End-stage chronic kidney disease due to diabetes mellitus type 1 | 8.4 (5.1 to 13.2) | -- | 3.2 (1.7 to 5.2) | 11.7* (4.5 to 19.8) | -4.2 (-10.4 to 2.8) |
| Stage III chronic kidney disease due to diabetes mellitus type 1 | 76.3 (58.8 to 98.5) | 3.7 (2.1 to 6.0) | 1.7 (1.0 to 2.5) | 4.9 (-9.9 to 20.7) | -10.0 (-22.8 to 3.5) |
| Stage IV chronic kidney disease due to diabetes mellitus type 1 | 4.2 (2.5 to 6.6) | -- | 0.6 (0.3 to 1.0) | 24.4* (9.5 to 41.8) | 6.6 (-6.1 to 21.6) |
| Stage V chronic kidney disease due to diabetes mellitus type 1 | 3.2 (2.2 to 4.5) | -- | 1.9 (1.2 to 2.8) | 23.9* (14.3 to 34.6) | 6.2 (-2.0 to 15.4) |
| Chronic kidney disease due to diabetes mellitus type 2 | 4733.7 (3554.5 to 6197.0) | 9.7 (5.5 to 15.5) | 8.3 (5.4 to 12.1) | 10.8* (1.8 to 19.9) | -5.0 (-12.7 to 2.8) |
| Albuminuria with preserved GFR due to type 2 diabetes mellitus | 4510.6 (3335.0 to 5983.9) | -- | -- | -- | -- |
| End-stage chronic kidney disease due to diabetes mellitus type 2 | 2.1 (0.9 to 3.9) | -- | 0.8 (0.3 to 1.6) | 13.0* (5.7 to 21.1) | -3.1 (-9.4 to 3.9) |
| Stage III chronic kidney disease due to diabetes mellitus type 2 | 202.7 (158.6 to 259.2) | 9.7 (5.5 to 15.5) | 1.8 (1.2 to 2.7) | 6.7 (-4.4 to 19.6) | -8.5 (-18.0 to 2.6) |
| Stage IV chronic kidney disease due to diabetes mellitus type 2 | 10.7 (6.7 to 17.5) | -- | 1.3 (0.7 to 2.2) | 11.8 (-2.6 to 27.6) | -4.1 (-16.4 to 9.4) |
| Stage V chronic kidney disease due to diabetes mellitus type 2 | 7.6 (5.2 to 10.8) | -- | 4.4 (2.7 to 6.8) | 11.9* (0.8 to 23.3) | -4.0 (-13.5 to 5.7) |
| Chronic kidney disease due to hypertension | 1393.5 (1173.3 to 1674.7) | 61.8 (35.0 to 97.6) | 49.7 (32.1 to 72.4) | 18.3* (10.0 to 27.1) | 1.5 (-5.7 to 9.0) |
| Albuminuria with preserved GFR due to hypertension | 139.8 (99.0 to 184.1) | -- | -- | -- | -- |
| End-stage chronic kidney disease due to hypertension | 7.8 (5.2 to 11.1) | -- | 3.1 (1.8 to 4.8) | 16.6* (10.8 to 22.7) | -0.0 (-4.9 to 5.2) |
| Stage III chronic kidney disease due to hypertension | 1106.7 (909.7 to 1348.5) | 61.8 (35.0 to 97.6) | 2.9 (1.8 to 4.4) | 2.9 (-11.4 to 18.1) | -11.8 (-24.0 to 1.3) |
| Stage IV chronic kidney disease due to hypertension | 81.3 (51.1 to 124.2) | -- | 10.2 (5.5 to 17.2) | 17.8* (2.4 to 33.6) | 1.0 (-12.1 to 14.5) |
| Stage V chronic kidney disease due to hypertension | 58.0 (41.2 to 80.6) | -- | 33.5 (20.8 to 49.9) | 20.2* (11.1 to 29.9) | 3.1 (-4.7 to 11.4) |
| Chronic kidney disease due to glomerulonephritis | 1218.7 (1026.3 to 1443.2) | 13.3 (7.8 to 20.9) | 85.7 (56.9 to 123.1) | 17.0* (8.6 to 26.2) | 0.4 (-6.9 to 8.3) |
| Albuminuria with preserved GFR due to glomerulonephritis | 485.8 (354.8 to 627.7) | -- | -- | -- | -- |
| End-stage chronic kidney disease due to glomerulonephritis | 31.9 (21.9 to 43.1) | -- | 11.5 (6.7 to 17.5) | 14.2* (7.2 to 22.6) | -2.0 (-8.0 to 5.1) |
| Stage III chronic kidney disease due to glomerulonephritis | 484.5 (397.3 to 594.2) | 13.3 (7.8 to 20.9) | 7.4 (4.5 to 11.5) | 3.2 (-12.8 to 19.8) | -11.5 (-25.2 to 2.7) |
| Stage IV chronic kidney disease due to glomerulonephritis | 126.3 (79.9 to 195.7) | -- | 15.6 (8.6 to 26.3) | 17.8* (2.0 to 34.6) | 1.0 (-12.5 to 15.5) |
| Stage V chronic kidney disease due to glomerulonephritis | 90.1 (64.2 to 125.3) | -- | 51.2 (31.7 to 76.4) | 19.8* (9.4 to 31.3) | 2.7 (-6.2 to 12.6) |
| Chronic kidney disease due to other and unspecified causes | 26068.6 (20789.7 to 31857.8) | 267.9 (155.7 to 423.4) | 152.3 (103.9 to 212.0) | 16.6* (8.0 to 25.6) | -0.0 (-7.4 to 7.7) |
| Albuminuria with preserved GFR due to other and unspecified causes | 20214.3 (14902.0 to 25890.3) | -- | -- | -- | -- |
| End-stage chronic kidney disease due to other causes | 39.4 (27.7 to 51.7) | -- | 15.2 (9.1 to 22.2) | 16.2* (6.9 to 25.2) | -0.4 (-8.3 to 7.3) |
| Stage III chronic kidney disease due to other causes | 5456.9 (4490.2 to 6661.7) | 267.9 (155.7 to 423.4) | 28.9 (17.6 to 45.0) | 5.4 (-6.4 to 17.9) | -9.6 (-19.7 to 1.1) |
| Stage IV chronic kidney disease due to other causes | 209.9 (132.5 to 326.8) | -- | 25.5 (13.8 to 42.9) | 18.6* (3.0 to 35.4) | 1.7 (-11.6 to 16.1) |
| Stage V chronic kidney disease due to other causes | 148.0 (104.5 to 206.0) | -- | 82.8 (52.0 to 122.8) | 20.5* (9.9 to 31.6) | 3.3 (-5.8 to 12.9) |
| Acute glomerulonephritis | 2.6 (1.7 to 3.9) | 38.5 (24.3 to 57.7) | 0.1 (0.1 to 0.2) | 2.8 (-0.6 to 6.7) | -11.8* (-14.8 to -8.5) |
| **Skin and subcutaneous diseases** | **125427.8 (115767.5 to 134984.6)** | **262733.4 (227237.2 to 298473.2)** | **2770.9 (1825.8 to 4122.8)** | **17.4* (16.5 to 18.2)** | **0.7 (-0.1 to 1.4)** |
| Dermatitis | 17744.4 (15166.7 to 20920.5) | 23515.9 (17136.4 to 30875.9) | 632.9 (369.9 to 993.6) | 15.2* (13.4 to 16.8) | -1.2 (-2.7 to 0.2) |
| Atopic dermatitis | 10137.1 (8872.1 to 11621.5) | 1181.5 (950.8 to 1463.6) | 444.2 (233.2 to 754.3) | 14.9* (12.6 to 17.1) | -1.5 (-3.5 to 0.4) |
| Contact dermatitis | 6909.5 (4702.6 to 9870.6) | 19935.2 (13591.7 to 27134.5) | 176.2 (96.9 to 289.4) | 15.7* (13.3 to 18.2) | -0.8 (-2.8 to 1.4) |
| Seborrhoeic dermatitis | 912.6 (720.8 to 1156.3) | 2399.1 (1909.0 to 2979.4) | 12.6 (6.6 to 20.5) | 19.6* (14.6 to 24.7) | 2.6 (-1.7 to 6.9) |
| Psoriasis | 4391.2 (4036.1 to 4759.3) | 625.7 (542.9 to 714.1) | 390.4 (271.0 to 521.9) | 19.2* (16.0 to 22.5) | 2.2 (-0.6 to 5.1) |
| Bacterial skin diseases | 485.8 (441.6 to 534.3) | 13345.7 (12174.7 to 14641.2) | 10.2 (6.3 to 15.9) | 12.7* (9.3 to 15.8) | -3.4* (-6.2 to -0.7) |
| Cellulitis | 127.1 (103.6 to 154.5) | 2627.5 (2175.8 to 3137.5) | 7.4 (4.6 to 11.3) | 10.6* (7.0 to 14.3) | -5.1* (-8.2 to -2.0) |
| Pyoderma | 480.2 (434.2 to 526.9) | 10718.2 (9690.7 to 11877.2) | 2.7 (1.1 to 5.7) | 18.6* (13.8 to 23.9) | 1.7 (-2.4 to 6.3) |
| Impetigo | 96.7 (84.1 to 110.2) | 1726.2 (1522.2 to 1953.2) | 0.6 (0.2 to 1.2) | 23.2* (14.6 to 33.5) | 5.6 (-1.7 to 14.4) |
| Abscess and other bacterial skin diseases | 383.5 (341.3 to 430.2) | 8992.0 (7995.7 to 10196.2) | 2.2 (0.9 to 4.6) | 17.4* (11.9 to 23.3) | 0.7 (-4.0 to 5.7) |
| Scabies | 17265.0 (11658.2 to 23744.2) | 49785.2 (34334.6 to 68252.7) | 448.6 (220.5 to 784.8) | 16.2* (15.0 to 17.4) | -0.4 (-1.4 to 0.7) |
| Fungal skin diseases | 39917.2 (30792.2 to 50170.9) | 117370.0 (86131.1 to 149846.8) | 227.4 (85.4 to 490.9) | 15.2* (14.1 to 16.0) | -1.2* (-2.1 to -0.5) |
| Tinea capitis | 4527.4 (3167.0 to 6186.8) | 6328.1 (4539.3 to 7896.0) | 25.8 (9.5 to 55.9) | 2.2 (-1.2 to 5.4) | -12.4* (-15.3 to -9.6) |
| Other fungal skin diseases | 35389.9 (26450.8 to 45571.3) | 111041.9 (79395.4 to 143117.9) | 201.6 (75.6 to 436.8) | 17.1* (16.5 to 17.7) | 0.4 (-0.1 to 0.9) |

| | | | | | |
|---|---|---|---|---|---|
| Viral skin diseases | 5935.6<br>(5447.2 to 6469.5) | 5941.3<br>(5432.7 to 6419.9) | 182.9<br>(117.0 to 273.5) | 16.5*<br>(14.4 to 18.5) | -0.1<br>(-1.9 to 1.6) |
| Viral warts | 3773.0<br>(3353.2 to 4287.0) | 2779.0<br>(2460.9 to 3095.7) | 116.3<br>(72.9 to 173.1) | 16.2*<br>(13.7 to 18.7) | -0.4<br>(-2.5 to 1.8) |
| Molluscum contagiosum | 2162.7<br>(1902.6 to 2446.5) | 3162.3<br>(2783.4 to 3559.7) | 66.6<br>(42.4 to 100.7) | 17.0*<br>(13.6 to 20.5) | 0.3<br>(-2.5 to 3.4) |
| Acne vulgaris | 11203.7<br>(9663.0 to 12990.4) | 4562.7<br>(3791.4 to 5349.8) | 238.4<br>(140.7 to 380.9) | 24.0*<br>(21.2 to 27.0) | 6.4*<br>(4.0 to 8.9) |
| Alopecia areata | 2109.0<br>(1925.9 to 2303.1) | 3770.1<br>(3431.7 to 4100.2) | 70.0<br>(44.2 to 106.9) | 15.3*<br>(12.4 to 18.6) | -1.1<br>(-3.7 to 1.7) |
| Pruritus | 5138.3<br>(3625.3 to 7180.8) | 4045.9<br>(2651.3 to 5786.2) | 55.8<br>(24.2 to 115.2) | 18.7*<br>(16.9 to 20.5) | 1.8*<br>(0.2 to 3.3) |
| Urticaria | 5827.4<br>(4047.6 to 8235.6) | 10290.0<br>(7094.6 to 14632.6) | 350.8<br>(198.6 to 573.3) | 16.8*<br>(14.7 to 19.1) | 0.2<br>(-1.6 to 2.1) |
| Decubitus ulcer | 28.0<br>(18.2 to 39.8) | 99.4<br>(64.5 to 140.1) | 5.0<br>(2.8 to 8.0) | 13.8*<br>(12.7 to 14.7) | -2.4*<br>(-3.4 to -1.6) |
| Other skin and subcutaneous diseases | 28324.3<br>(25848.6 to 30910.2) | 29381.6<br>(26862.4 to 32007.5) | 158.6<br>(74.6 to 296.2) | 22.3*<br>(21.2 to 23.4) | 4.8*<br>(3.9 to 5.8) |
| **Sense organ diseases** | **103576.1<br>(96423.7 to 110991.9)** | **--** | **2380.3<br>(1543.4 to 3600.2)** | **16.1*<br>(14.0 to 18.0)** | **-0.4<br>(-2.2 to 1.2)** |
| Blindness and vision impairment | 46238.3<br>(41411.2 to 51470.1) | -- | 918.5<br>(567.8 to 1448.4) | 13.8*<br>(12.4 to 15.0) | -2.4*<br>(-3.6 to -1.4) |
| Glaucoma | -- | -- | -- | -- | -- |
| Cataract | 265.9<br>(186.5 to 363.5) | -- | 30.4<br>(17.7 to 47.5) | -2.1<br>(-9.4 to 4.8) | -16.1*<br>(-22.3 to -10.1) |
| Age-related macular degeneration | -- | -- | -- | -- | -- |
| Refraction disorders | 10383.2<br>(7952.7 to 13116.3) | -- | 434.1<br>(277.6 to 653.1) | 14.9*<br>(12.7 to 17.0) | -1.5<br>(-3.4 to 0.3) |
| Near vision loss | 35291.4<br>(31123.5 to 39685.3) | -- | 367.0<br>(175.5 to 677.9) | 15.0*<br>(14.1 to 16.0) | -1.3*<br>(-2.2 to -0.5) |
| Other vision loss | 1075.8<br>(793.0 to 1379.3) | -- | 87.0<br>(53.9 to 129.2) | 10.2*<br>(5.1 to 15.2) | -5.5*<br>(-9.9 to -1.2) |
| Age-related and other hearing loss | 60399.9<br>(53809.9 to 67327.4) | -- | 1369.4<br>(895.9 to 2023.8) | 17.5*<br>(14.2 to 20.9) | 0.8<br>(-2.1 to 3.7) |
| Other sense organ diseases | 3874.8<br>(3541.0 to 4228.7) | -- | 92.4<br>(55.4 to 136.9) | 17.9*<br>(14.3 to 21.6) | 1.1<br>(-2.0 to 4.2) |
| Chronic other sense organ diseases | 3033.2<br>(2715.0 to 3392.6) | -- | 80.8<br>(48.6 to 120.5) | 17.9*<br>(13.7 to 22.0) | 1.1<br>(-2.5 to 4.6) |
| Acute other sense organ diseases | 841.6<br>(761.1 to 931.0) | -- | 11.7<br>(7.0 to 18.1) | 17.9*<br>(11.2 to 25.1) | 1.1<br>(-4.7 to 7.3) |
| **Musculoskeletal disorders** | **74052.3<br>(61049.5 to 89281.6)** | **20850.1<br>(15430.6 to 27460.6)** | **8199.3<br>(5477.6 to 11760.1)** | **12.7*<br>(11.2 to 14.2)** | **-3.3*<br>(-4.6 to -2.1)** |
| Rheumatoid arthritis | 663.5<br>(523.1 to 802.4) | 53.2<br>(36.1 to 73.9) | 93.0<br>(58.5 to 132.9) | 22.0*<br>(16.4 to 27.3) | 4.6<br>(-0.2 to 9.2) |
| Osteoarthritis | -- | -- | -- | -- | -- |
| Osteoarthritis of the hip | -- | -- | -- | -- | -- |
| Osteoarthritis of the knee | -- | -- | -- | -- | -- |
| Low back pain | 38085.2<br>(27805.3 to 50998.7) | 16890.5<br>(11565.9 to 23300.6) | 4402.9<br>(2699.0 to 6866.5) | 10.8*<br>(9.5 to 12.1) | -5.0*<br>(-6.1 to -3.9) |
| Low back pain with leg pain | 11010.8<br>(7653.8 to 15224.2) | 4884.2<br>(3231.9 to 6981.9) | 1654.4<br>(981.6 to 2686.5) | 10.8*<br>(9.0 to 12.4) | -5.0*<br>(-6.6 to -3.6) |
| Low back pain without leg pain | 27074.4<br>(19544.8 to 36120.3) | 12006.3<br>(8216.7 to 16733.3) | 2748.5<br>(1669.7 to 4290.2) | 10.8*<br>(9.4 to 12.1) | -5.0*<br>(-6.2 to -3.8) |
| Neck pain | 12210.9<br>(8785.2 to 16890.3) | 3708.7<br>(2416.6 to 5641.2) | 1258.7<br>(764.4 to 1934.1) | 17.1*<br>(14.3 to 19.7) | 0.4<br>(-2.0 to 2.6) |
| Gout | 688.8<br>(526.1 to 877.7) | 197.7<br>(140.2 to 263.3) | 23.5<br>(14.5 to 35.0) | 19.3*<br>(10.6 to 28.8) | 2.3<br>(-5.1 to 10.4) |
| Other musculoskeletal disorders | 27297.6<br>(19101.9 to 36945.1) | -- | 2421.2<br>(1446.7 to 3770.9) | 13.7*<br>(9.4 to 17.4) | -2.5<br>(-6.2 to 0.7) |
| **Other non-communicable diseases** | **389397.2<br>(361217.4 to 420472.1)** | **382711.3<br>(334989.1 to 429611.0)** | **4024.5<br>(2772.8 to 5615.2)** | **13.2*<br>(11.5 to 14.9)** | **-2.9*<br>(-4.4 to -1.5)** |
| Congenital anomalies | 7430.6<br>(7002.7 to 7916.0) | -- | 912.2<br>(635.6 to 1225.1) | 15.9*<br>(13.3 to 18.6) | -0.6<br>(-2.9 to 1.7) |
| Neural tube defects | 222.9<br>(198.6 to 249.0) | -- | 66.4<br>(46.6 to 88.3) | 20.8*<br>(15.2 to 26.6) | 3.6<br>(-1.2 to 8.6) |
| Severe motor and cognitive impairment due to anencephaly | -- | -- | -- | -- | -- |
| Encephalocoele due to neural tube defects | 21.4<br>(17.2 to 25.5) | -- | 3.7<br>(2.5 to 5.1) | 15.2*<br>(11.9 to 18.3) | -1.2<br>(-4.0 to 1.4) |
| Spina bifida due to neural tube defects | 201.5<br>(179.7 to 224.8) | -- | 62.7<br>(43.9 to 83.7) | 21.2*<br>(15.3 to 27.3) | 3.9<br>(-1.1 to 9.1) |
| Congenital heart anomalies | 443.4<br>(407.0 to 480.5) | -- | 24.7<br>(11.4 to 42.4) | 17.1*<br>(12.5 to 22.0) | 0.4<br>(-3.6 to 4.6) |
| Critical malformations of great vessels, congenital valvular heart disease and patent ductus arteriosus | 73.2<br>(62.7 to 85.3) | -- | 4.7<br>(2.4 to 7.8) | 16.9*<br>(9.9 to 24.9) | 0.3<br>(-5.7 to 7.1) |
| Other congenital heart anomalies | 125.5<br>(115.1 to 136.7) | -- | 7.4<br>(3.0 to 13.2) | 17.0*<br>(7.6 to 27.5) | 0.4<br>(-7.7 to 9.4) |
| Severe congenital heart anomalies excluding single ventricle heart defects | 152.1<br>(134.0 to 171.4) | -- | 9.2<br>(4.3 to 15.6) | 17.4*<br>(9.3 to 26.4) | 0.7<br>(-6.3 to 8.4) |
| Single ventricle and single ventricle pathway congenital heart anomalies | 9.8<br>(8.2 to 11.8) | -- | 0.7<br>(0.4 to 1.0) | 22.0*<br>(19.6 to 24.1) | 4.6*<br>(2.6 to 6.4) |
| Ventricular septal defect and atrial septal defect | 82.9<br>(68.7 to 99.0) | -- | 2.8<br>(1.2 to 4.8) | 15.2*<br>(7.3 to 23.4) | -1.2<br>(-8.0 to 5.8) |
| Orofacial clefts | 838.4<br>(768.8 to 906.9) | -- | 25.1<br>(16.5 to 36.7) | 19.8*<br>(10.5 to 29.9) | 2.8<br>(-5.2 to 11.4) |
| Down's syndrome | 322.2<br>(281.1 to 365.6) | -- | 27.6<br>(18.7 to 38.5) | 16.4*<br>(13.2 to 19.6) | -0.2<br>(-2.9 to 2.6) |
| Turner syndrome | 52.7<br>(45.9 to 60.4) | -- | 1.1<br>(0.6 to 1.8) | 14.3*<br>(8.8 to 20.1) | -2.0<br>(-6.7 to 3.0) |
| Klinefelter syndrome | 87.8<br>(75.5 to 100.8) | -- | 0.9<br>(0.4 to 1.8) | 13.5*<br>(5.2 to 23.0) | -2.6<br>(-9.8 to 5.5) |
| Other chromosomal abnormalities | 490.9<br>(380.7 to 613.6) | -- | 41.6<br>(26.3 to 59.5) | 24.7*<br>(20.0 to 29.3) | 6.9*<br>(2.9 to 10.9) |
| Congenital musculoskeletal and limb anomalies | 2371.4<br>(2110.1 to 2727.9) | -- | 356.2<br>(231.3 to 494.0) | 15.3*<br>(12.9 to 17.8) | -1.1<br>(-3.2 to 1.0) |
| Polydactyly and syndactyly | 8.7<br>(6.9 to 10.6) | -- | 0.1<br>(0.0 to 0.2) | 12.4*<br>(10.3 to 14.4) | -3.6*<br>(-5.4 to -1.9) |
| Congenital limb deficiency | 1183.7<br>(1052.2 to 1361.8) | -- | 178.4<br>(116.8 to 248.8) | 15.3*<br>(11.9 to 18.5) | -1.1<br>(-4.1 to 1.6) |
| Other congenital musculoskeletal anomalies | 1179.0<br>(1049.1 to 1356.2) | -- | 177.7<br>(116.4 to 246.0) | 15.3*<br>(11.7 to 18.7) | -1.1<br>(-4.2 to 1.8) |
| Urogenital congenital anomalies | 510.6<br>(431.3 to 601.3) | -- | 11.4<br>(6.4 to 19.2) | 11.2*<br>(7.7 to 15.0) | -4.6*<br>(-7.6 to -1.4) |
| Digestive congenital anomalies | 1355.4<br>(1219.9 to 1519.4) | -- | 61.6<br>(41.2 to 88.7) | 10.5*<br>(4.7 to 15.7) | -5.3*<br>(-10.2 to -0.7) |
| Congenital diaphragmatic hernia | 22.2<br>(19.4 to 25.2) | -- | 0.4<br>(0.3 to 0.7) | 15.3*<br>(14.3 to 16.2) | -1.1*<br>(-2.0 to -0.3) |
| Congenital atresia and/or stenosis of the digestive tract | 0.9<br>(0.6 to 1.2) | -- | 0.1<br>(0.0 to 0.1) | 36.4*<br>(31.8 to 40.9) | 17.0*<br>(13.1 to 20.9) |
| Congenital malformations of the abdominal wall after treatment | 85.9<br>(75.4 to 97.5) | -- | 3.7<br>(2.4 to 5.4) | 15.8*<br>(14.9 to 16.6) | -0.7<br>(-1.5 to 0.0) |
| Other congenital malformations of the digestive tract | 1246.3<br>(1110.7 to 1411.5) | -- | 57.4<br>(38.0 to 82.8) | 10.1*<br>(4.0 to 15.7) | -5.6*<br>(-10.8 to -0.8) |
| Other congenital anomalies | 2124.4<br>(1840.5 to 2461.5) | -- | 295.7<br>(199.5 to 410.0) | 15.3*<br>(8.5 to 23.4) | -1.1<br>(-7.0 to 5.8) |

| | | | | | |
|---|---|---|---|---|---|
| Other congenital birth defects | -- | -- | 20.1 (14.5 to 26.7) | 22.9* (18.4 to 27.6) | 5.4* (1.5 to 9.4) |
| Hearing loss due to other congenital anomalies | 2124.4 (1840.5 to 2461.5) | -- | 275.6 (183.6 to 386.2) | 14.8* (7.5 to 23.6) | -1.5 (-7.8 to 6.0) |
| Urinary diseases and male infertility | 6405.0 (4468.8 to 8977.4) | 36291.8 (27897.7 to 45589.1) | 79.4 (46.1 to 133.5) | 17.4* (12.3 to 22.5) | 0.6 (-3.7 to 5.1) |
| Urinary tract infections | 583.9 (435.3 to 754.5) | 30946.3 (22652.1 to 40028.0) | 19.5 (11.2 to 31.7) | 13.8* (7.8 to 19.5) | -2.4 (-7.6 to 2.4) |
| Urolithiasis | 199.4 (143.5 to 270.3) | 5345.6 (3803.1 to 7320.5) | 15.4 (9.2 to 23.7) | 17.6* (15.0 to 20.4) | 0.8 (-1.3 to 3.3) |
| Benign prostatic hyperplasia | -- | -- | -- | -- | -- |
| Male infertility | 5733.3 (3827.0 to 8302.5) | -- | 35.3 (13.3 to 76.4) | 29.2* (22.3 to 34.2) | 10.8* (4.9 to 15.1) |
| Other urinary diseases | -- | -- | 9.2 (6.0 to 13.5) | -9.0* (-11.9 to -6.4) | -21.9* (-24.4 to -19.7) |
| Gynecological diseases | 96322.2 (89485.4 to 103743.3) | 26406.8 (24392.3 to 28551.8) | 1521.6 (1046.6 to 2117.8) | 14.6* (12.6 to 16.6) | -1.7* (-3.4 to -0.0) |
| Uterine fibroids | 4514.1 (3485.5 to 5683.0) | 689.6 (459.9 to 982.2) | 43.1 (24.3 to 71.3) | 12.9* (4.4 to 21.6) | -3.2 (-10.5 to 4.3) |
| Polycystic ovarian syndrome | 8139.6 (6408.1 to 10496.3) | 76.2 (38.9 to 123.9) | 73.0 (32.3 to 141.7) | 16.3* (14.7 to 17.9) | -0.3 (-1.7 to 1.1) |
| Female infertility | 11572.4 (6016.9 to 19663.7) | -- | 66.9 (22.5 to 152.2) | 44.9* (34.5 to 53.6) | 24.3* (15.3 to 31.7) |
| Endometriosis | 6823.3 (5207.3 to 8545.9) | 968.5 (660.6 to 1327.1) | 636.0 (409.7 to 934.3) | 13.5* (11.3 to 15.8) | -2.6* (-4.5 to -0.7) |
| Genital prolapse | 4145.0 (3247.7 to 5259.9) | 728.0 (506.7 to 1039.6) | 13.3 (6.0 to 25.9) | 0.2 (-5.0 to 4.5) | -14.1* (-18.5 to -10.4) |
| Premenstrual syndrome | 67552.2 (61236.2 to 73782.7) | 18245.8 (16303.4 to 20076.7) | 567.3 (358.0 to 853.0) | 12.9* (8.9 to 16.1) | -3.2* (-6.6 to -0.5) |
| Other gynecological diseases | 5884.2 (5345.3 to 6496.2) | 5698.6 (5079.7 to 6429.8) | 122.0 (81.9 to 177.4) | 16.7* (12.7 to 20.4) | 0.0 (-3.4 to 3.3) |
| Other gynecological diseases | 5432.3 (4894.4 to 6050.4) | 5698.6 (5079.7 to 6429.8) | 112.2 (74.8 to 163.8) | 16.3* (12.0 to 20.4) | -0.2 (-4.0 to 3.2) |
| Other gynecological diseases with anemia | 451.9 (419.8 to 485.7) | -- | 9.9 (6.3 to 15.1) | 20.7* (5.5 to 35.9) | 3.5 (-9.5 to 16.5) |
| Hemoglobinopathies and hemolytic anaemias | 156816.3 (153350.7 to 160620.9) | -- | 396.7 (260.3 to 581.8) | 2.8 (-6.0 to 12.0) | -11.9* (-19.4 to -4.0) |
| Thalassemias | 4.1 (3.7 to 4.5) | -- | 0.3 (0.2 to 0.4) | 1.8 (-9.2 to 14.9) | -12.7* (-22.1 to -1.5) |
| Thalassaemias trait | 24979.0 (24016.2 to 26021.5) | -- | 160.7 (101.2 to 246.6) | 1.4 (-14.3 to 19.1) | -13.0 (-26.5 to 2.2) |
| Sickle cell disorders | 146.8 (125.6 to 171.7) | -- | 17.0 (11.3 to 24.8) | 28.8* (14.6 to 45.3) | 10.5 (-1.7 to 24.6) |
| Sickle cell trait | 37151.4 (33556.2 to 41243.1) | -- | 80.3 (53.1 to 119.1) | 7.2 (-2.0 to 17.2) | -8.1 (-16.0 to 0.5) |
| G6PD deficiency | 29225.0 (28050.6 to 30440.0) | -- | 1.5 (1.0 to 2.2) | 5.2 (-10.5 to 25.6) | -9.8 (-23.3 to 7.7) |
| G6PD trait | 76725.6 (75457.4 to 77970.5) | -- | 0.0 (0.0 to 0.1) | 22.5* (3.2 to 59.1) | 5.1 (-11.5 to 36.5) |
| Other hemoglobinopathies and hemolytic anaemias | 6543.0 (6101.0 to 7086.8) | -- | 136.9 (89.8 to 204.4) | -0.7 (-8.4 to 7.5) | -14.8* (-21.5 to -7.8) |
| Endocrine, metabolic, blood, and immune disorders | 8572.2 (8103.5 to 9062.2) | -- | 221.5 (148.3 to 320.1) | -0.9 (-6.6 to 5.3) | -15.0* (-19.9 to -9.7) |
| Anemia due to endocrine, metabolic, blood, and immune disorders | 7637.0 (7260.3 to 8055.4) | -- | 186.6 (123.9 to 274.5) | -3.5 (-10.2 to 3.3) | -17.2* (-23.0 to -11.4) |
| Endocrine, metabolic, blood, and immune disorders | 933.6 (708.1 to 1200.8) | -- | 34.8 (20.9 to 52.4) | 15.7* (9.8 to 21.9) | -0.8 (-5.8 to 4.5) |
| Heart failure due to endocrine, metabolic, blood, and immune disorders | 1.7 (1.1 to 2.4) | -- | 0.2 (0.1 to 0.2) | 9.4* (7.9 to 10.9) | -6.2* (-7.5 to -4.9) |
| Oral disorders | 252162.6 (208219.8 to 302871.5) | 320012.7 (271425.7 to 366308.7) | 893.0 (511.3 to 1420.4) | 16.9* (16.0 to 17.9) | 0.3 (-0.5 to 1.1) |
| Caries of deciduous teeth | -- | -- | -- | -- | -- |
| Caries of permanent teeth | 227199.1 (178330.8 to 283861.3) | 314197.6 (265088.9 to 360475.2) | 158.3 (63.9 to 303.2) | 11.0* (9.6 to 12.4) | -4.8* (-6.0 to -3.6) |
| Periodontal disease | 31327.0 (21586.8 to 43657.6) | 5335.7 (3308.1 to 8316.7) | 208.1 (77.9 to 442.5) | 19.9* (19.0 to 20.9) | 2.8* (2.0 to 3.7) |
| Edentulism and severe tooth loss | 4494.1 (3354.5 to 5873.4) | 479.4 (310.4 to 684.0) | 129.9 (78.5 to 200.9) | 20.5* (17.9 to 23.0) | 3.4* (1.1 to 5.5) |
| Other oral disorders | 13527.9 (12121.0 to 15141.6) | -- | 396.6 (242.1 to 595.6) | 16.8* (15.6 to 18.0) | 0.1 (-0.9 to 1.2) |
| **Injuries** | **105840.3 (95895.1 to 117537.6)** | **41123.9 (35179.7 to 47929.8)** | **3759.0 (2850.7 to 4904.7)** | **13.6* (10.9 to 15.6)** | **-2.6* (-4.9 to -0.8)** |
| **Transport injuries** | **12689.5 (11408.7 to 14097.0)** | **6821.8 (4906.0 to 9512.8)** | **849.2 (608.4 to 1136.6)** | **13.4* (11.6 to 15.3)** | **-2.8* (-4.3 to -1.1)** |
| Road injuries | 9175.6 (8299.5 to 10088.6) | 6214.2 (4306.9 to 8869.9) | 607.1 (434.2 to 820.2) | 11.4* (9.5 to 13.4) | -4.5* (-6.1 to -2.8) |
| Pedestrian road injuries | 2398.6 (1910.2 to 3094.3) | 1124.3 (606.9 to 1778.1) | 160.9 (108.6 to 224.7) | 7.4* (5.0 to 9.7) | -7.9* (-10.0 to -5.9) |
| Cyclist road injuries | 1541.0 (1238.5 to 1951.4) | 1170.5 (635.7 to 1911.3) | 96.0 (64.7 to 130.5) | 16.8* (14.2 to 19.7) | 0.1 (-2.0 to 2.6) |
| Motorcyclist road injuries | 2500.4 (2068.2 to 3001.7) | 1397.9 (837.0 to 2198.9) | 165.2 (113.1 to 228.2) | 12.3* (9.9 to 15.1) | -3.7* (-5.8 to -1.3) |
| Motor vehicle road injuries | 2095.5 (1681.8 to 2693.6) | 2012.7 (1306.4 to 2982.3) | 147.6 (103.8 to 206.6) | 6.9* (4.1 to 9.5) | -8.3* (-10.7 to -6.1) |
| Other road injuries | 640.0 (477.3 to 856.7) | 508.8 (261.5 to 898.3) | 37.4 (24.9 to 53.8) | 33.9* (30.8 to 37.1) | 14.8* (12.2 to 17.6) |
| Other transport injuries | 3513.9 (2719.7 to 4399.7) | 607.6 (375.4 to 899.9) | 242.1 (169.2 to 334.0) | 18.8* (15.9 to 21.6) | 1.9 (-0.6 to 4.3) |
| **Unintentional injuries** | **51869.6 (47292.4 to 57289.7)** | **29056.5 (23731.7 to 34953.8)** | **2165.8 (1585.4 to 2928.6)** | **17.7* (15.9 to 19.4)** | **0.9 (-0.6 to 2.4)** |
| Falls | 20404.8 (16775.0 to 24968.3) | 9540.4 (6102.2 to 13919.5) | 974.7 (689.9 to 1357.0) | 18.2* (16.5 to 19.7) | 1.3 (-0.1 to 2.6) |
| Drowning | 131.5 (109.2 to 158.5) | 23.3 (16.7 to 32.1) | 8.9 (6.2 to 12.2) | 5.7* (2.1 to 9.2) | -9.4* (-12.5 to -6.3) |
| Fire, heat, and hot substances | 6272.4 (5129.3 to 7685.2) | 888.8 (547.5 to 1313.3) | 266.8 (186.6 to 361.7) | 12.2* (7.6 to 16.9) | -3.8 (-7.7 to 0.3) |
| Poisonings | 342.7 (284.9 to 408.5) | 293.3 (179.8 to 470.0) | 39.3 (25.5 to 55.5) | 29.4* (25.9 to 32.8) | 10.9* (8.0 to 13.9) |
| Poisoning by carbon monoxide | 59.6 (48.6 to 72.9) | 94.7 (44.3 to 174.0) | 6.3 (4.1 to 9.3) | 34.4* (29.9 to 39.3) | 15.3* (11.4 to 19.4) |
| Poisoning by other means | 283.1 (223.3 to 348.5) | 198.7 (117.1 to 317.1) | 32.9 (21.0 to 48.0) | 28.4* (24.7 to 32.1) | 10.1* (7.0 to 13.3) |
| Exposure to mechanical forces | 11105.9 (8918.1 to 13225.5) | 7348.0 (4805.9 to 10493.6) | 310.7 (215.4 to 437.7) | 22.5* (20.4 to 24.6) | 5.1* (3.2 to 6.8) |
| Unintentional firearm injuries | 545.9 (423.2 to 663.1) | 213.6 (103.6 to 382.6) | 24.3 (17.1 to 33.4) | 22.8* (20.4 to 25.1) | 5.3* (3.3 to 7.2) |
| Other exposure to mechanical forces | 10560.0 (8295.4 to 12750.8) | 7134.4 (4680.9 to 10206.9) | 286.4 (196.2 to 407.8) | 22.5* (20.3 to 24.7) | 5.1* (3.1 to 6.9) |
| Adverse effects of medical treatment | 82.9 (50.5 to 127.2) | 1083.2 (668.3 to 1609.1) | 11.1 (5.8 to 19.2) | 9.2* (3.5 to 14.0) | -6.4* (-11.2 to -2.2) |
| Animal contact | 2491.6 (2112.2 to 2970.5) | 3473.3 (2161.1 to 5337.6) | 86.0 (57.7 to 120.5) | 15.6* (12.0 to 19.2) | -0.8 (-3.9 to 2.2) |
| Venomous animal contact | 1116.1 (875.3 to 1445.8) | 1871.1 (1108.2 to 2896.8) | 63.9 (41.9 to 92.0) | 16.7* (12.2 to 21.5) | 0.1 (-3.8 to 4.2) |
| Non-venomous animal contact | 1375.5 (1068.7 to 1735.7) | 1602.2 (930.6 to 2559.1) | 22.1 (13.0 to 35.0) | 12.6* (9.5 to 15.1) | -3.5* (-6.1 to -1.3) |
| Foreign body | 1259.0 (1025.0 to 1549.1) | 1979.6 (1307.5 to 2871.3) | 68.0 (48.3 to 94.9) | 20.5* (18.7 to 22.2) | 3.3* (1.8 to 4.8) |

| | | | | | |
|---|---|---|---|---|---|
| Pulmonary aspiration and foreign body in airway | 167.9 (128.8 to 210.6) | 127.8 (66.0 to 204.3) | 10.0 (6.8 to 13.7) | 24.0* (20.5 to 27.5) | 6.4* (3.4 to 9.4) |
| Foreign body in eyes | 236.8 (96.6 to 445.5) | 1593.4 (932.5 to 2513.9) | 14.5 (5.8 to 28.1) | 18.6* (17.1 to 19.8) | 1.7* (0.4 to 2.7) |
| Foreign body in other body part | 854.3 (673.4 to 1043.2) | 258.4 (151.1 to 407.6) | 43.5 (30.6 to 58.4) | 20.3* (17.9 to 22.5) | 3.1* (1.1 to 5.1) |
| Environmental heat and cold exposure | 2194.8 (1739.2 to 2730.8) | 646.0 (379.1 to 1048.4) | 104.4 (71.5 to 142.7) | 19.6* (16.8 to 22.1) | 2.6* (0.1 to 4.7) |
| Exposure to forces of nature | 1638.8 (1176.6 to 2233.4) | 57.3 (39.3 to 83.2) | 79.9 (55.6 to 111.9) | 2.8 (-3.8 to 9.9) | -11.8* (-17.5 to -5.7) |
| Other unintentional injuries | 5945.3 (4862.6 to 7346.5) | 3723.1 (2235.5 to 5813.6) | 216.0 (149.9 to 305.4) | 20.4* (18.1 to 22.6) | 3.2* (1.3 to 5.1) |
| **Self-harm and interpersonal violence** | **41281.2 (32251.1 to 52970.3)** | **5245.6 (4180.9 to 6407.8)** | **743.9 (561.2 to 976.1)** | **3.3 (-4.3 to 10.5)** | **-11.4* (-18.0 to -5.2)** |
| Self-harm | 448.2 (341.1 to 593.2) | 497.7 (315.8 to 712.9) | 30.7 (20.4 to 43.3) | -1.3 (-3.8 to 0.9) | -15.4* (-17.5 to -13.5) |
| Self-harm by firearm | 4.8 (3.8 to 6.4) | 1.7 (0.5 to 4.5) | 0.3 (0.2 to 0.3) | 1.7 (-1.8 to 5.0) | -12.8* (-15.8 to -9.9) |
| Self-harm by other specified means | 443.5 (336.6 to 587.4) | 496.0 (314.4 to 710.7) | 30.5 (20.1 to 43.0) | -1.4 (-3.8 to 0.9) | -15.4* (-17.5 to -13.5) |
| Interpersonal violence | 37304.0 (28527.1 to 48882.3) | 2999.2 (2057.8 to 4145.6) | 499.2 (357.9 to 694.4) | 21.5* (18.6 to 24.1) | 4.2* (1.7 to 6.4) |
| Assault by firearm | 152.4 (116.0 to 200.2) | 82.4 (44.6 to 140.7) | 8.6 (6.0 to 11.8) | 13.0* (9.7 to 16.7) | -3.1 (-5.9 to 0.1) |
| Assault by sharp object | 1020.2 (762.2 to 1477.7) | 611.6 (336.1 to 969.8) | 38.8 (26.3 to 55.3) | 10.3* (7.2 to 13.5) | -5.4* (-8.1 to -2.6) |
| Sexual violence | 33659.3 (24842.1 to 44999.0) | -- | 325.8 (190.9 to 508.5) | 26.6* (24.1 to 29.0) | 8.6* (6.4 to 10.6) |
| Assault by other means | 2472.1 (1916.1 to 3349.0) | 2305.2 (1560.3 to 3247.7) | 126.1 (84.6 to 172.8) | 13.7* (11.3 to 16.5) | -2.5* (-4.6 to -0.1) |
| Conflict and terrorism | 3278.1 (2278.5 to 4691.6) | 1435.6 (1111.1 to 1873.0) | 202.3 (130.7 to 314.3) | -25.0* (-30.4 to -18.7) | -35.7* (-40.3 to -30.3) |
| Executions and police conflict | 250.8 (167.5 to 386.6) | 313.1 (214.8 to 451.2) | 11.6 (7.7 to 17.5) | 39.6* (25.3 to 58.2) | 19.7* (7.4 to 35.6) |
| **Nature of injury aggregates** | | | | | |
| Amputations | 22787.2 (20548.2 to 25626.3) | 859.6 (687.0 to 1067.9) | 409.3 (276.0 to 598.9) | 10.7* (5.5 to 15.0) | -5.0* (-9.5 to -1.4) |
| Burns | 13061.6 (11676.0 to 14624.6) | 1363.2 (1063.4 to 1683.6) | 577.7 (417.7 to 765.7) | 4.3 (-1.8 to 10.0) | -10.6* (-15.8 to -5.6) |
| Fractures | 16160.2 (14748.1 to 17748.8) | 9963.1 (8109.4 to 12135.1) | 913.8 (620.6 to 1293.4) | 13.8* (9.8 to 16.3) | -2.4* (-5.8 to -0.3) |
| Head Injuries | 2759.9 (2575.4 to 2947.2) | 1924.7 (1570.8 to 2368.4) | 424.9 (298.8 to 581.6) | 20.5* (18.2 to 22.8) | 3.4* (1.4 to 5.3) |
| Spinal Injuries | 1637.8 (1457.0 to 1908.3) | 62.7 (48.2 to 81.2) | 519.4 (364.5 to 698.1) | 13.7* (5.3 to 19.9) | -2.5 (-9.7 to 2.8) |
| Minor Injuries | 11364.9 (10389.5 to 12534.5) | 15747.6 (12888.5 to 18836.5) | 100.3 (47.4 to 191.4) | 19.6* (17.4 to 21.2) | 2.6* (0.7 to 4.0) |
| Other Injuries | 4409.2 (4061.7 to 4791.9) | 11202.9 (9742.6 to 12873.4) | 487.8 (340.6 to 663.8) | 12.8* (8.1 to 16.5) | -3.3* (-7.3 to -0.1) |
| **Impairments** | | | | | |
| Anaemia | 136808.9 (129668.3 to 145065.9) | -- | 3438.0 (2311.0 to 5030.3) | -2.5 (-8.8 to 3.9) | -16.4* (-21.8 to -10.9) |
| Epilepsy | 5318.9 (4429.3 to 6337.9) | -- | 2206.0 (1683.9 to 2889.5) | 18.8* (11.9 to 26.1) | 1.9 (-4.0 to 8.1) |
| Guillain-Barré syndrome | 6.0 (3.5 to 9.8) | -- | 1.8 (0.9 to 3.2) | 17.1* (14.7 to 19.8) | 0.4 (-1.7 to 2.7) |
| Hearing loss | 68014.0 (61544.4 to 74848.0) | -- | 1765.4 (1197.8 to 2551.3) | 16.1* (13.1 to 18.9) | -0.4 (-3.0 to 1.9) |
| Heart failure | 442.8 (327.3 to 577.4) | -- | 41.3 (25.8 to 63.1) | 14.8* (11.8 to 18.0) | -1.5 (-4.1 to 1.2) |
| Infertility | 21584.3 (14250.7 to 32155.4) | -- | 174.8 (85.8 to 333.5) | 27.9* (20.6 to 34.1) | 9.7* (3.4 to 15.0) |
| Developmental intellectual disability | 16051.1 (12411.5 to 19625.5) | -- | 2163.1 (1688.6 to 2728.5) | 18.2* (14.9 to 22.3) | 1.4 (-1.5 to 4.9) |
| Pelvic inflammatory disease | 165.9 (119.5 to 218.7) | -- | 22.1 (13.6 to 33.2) | 16.3* (9.6 to 23.1) | -0.3 (-6.0 to 5.6) |
| Vision loss | 48511.2 (43433.0 to 53938.4) | -- | 1200.7 (817.1 to 1798.8) | 14.9* (13.7 to 16.1) | -1.5* (-2.5 to -0.4) |

We did not calculate incidence for the nine impairments and certain NTDs. Blank cells mean that no estimate is available or that the estimate has a magnitude less than 50.
Table presents prevalence and incidence counts in thousands.
* Percentage changes that are statistically significant
‡ Incidence estimates for stroke represent first-ever stroke only.
^ Incidence of HIV/AIDS represents new infections of HIV only and does not include new infections of Tuberculosis in HIV positive cases.

| SR Table 10j: Global prevalence, incidence and YLDs for 2017, percentage change of YLD counts and percentage change of YLD rates between 2007 and 2017 by cause, age 30 to 34 years | | | | | |
|---|---|---|---|---|---|
| Cause | Prevalence in thousands (95% UI) | Incidence in thousands (95% UI) | YLDs in thousands (95% UI) | | |
| | 2017 counts | 2017 counts | 2017 counts | Percentage change in counts between 2007 and 2017 | Percentage change in rates between 2007 and 2017 |
| All causes | 577272.7 (574761.4 to 579555.9) | 2828257.8 (2419632.4 to 3308711.5) | 62106.5 (46803.0 to 80241.6) | 14.9* (14.0 to 15.7) | -0.8* (-1.6 to -0.1) |
| Communicable, maternal, neonatal, and nutritional disorders | 390307.1 (363193.8 to 422632.5) | 2009145.3 (1604652.8 to 2470256.1) | 8190.9 (6088.5 to 10770.9) | 8.3* (4.2 to 12.3) | -6.5* (-10.1 to -3.1) |
| HIV/AIDS and sexually transmitted infections | 141496.9 (124996.3 to 158902.1) | 110530.3 (91251.2 to 134877.5) | 786.3 (558.4 to 1069.5) | -12.2 (-29.5 to 2.7) | -24.2* (-39.1 to -11.3) |
| HIV/AIDS | 5618.6 (5218.9 to 6065.9) | 327.4^ (272.3 to 381.9) | 581.1 (418.6 to 794.6) | -19.4* (-35.9 to -3.7) | -30.4* (-44.7 to -16.9) |
| HIV/AIDS - Drug-susceptible Tuberculosis | 166.6 (150.1 to 183.6) | 210.6 (186.2 to 237.1) | 64.4 (42.8 to 87.0) | -26.8* (-30.4 to -22.4) | -36.8* (-39.9 to -33.0) |
| HIV/AIDS - Multidrug-resistant Tuberculosis without extensive drug resistance | 5.9 (4.0 to 8.5) | 8.5 (6.1 to 11.6) | 2.4 (1.4 to 3.8) | -30.6 (-52.5 to 2.6) | -40.1* (-59.0 to -11.4) |
| HIV/AIDS - Extensively drug-resistant Tuberculosis | 0.2 (0.2 to 0.4) | 0.3 (0.2 to 0.5) | 0.1 (0.1 to 0.2) | 29.6 (-14.0 to 86.5) | 11.9 (-25.8 to 61.0) |
| HIV/AIDS resulting in other diseases | 5445.9 (5048.8 to 5892.5) | 327.4 (272.3 to 381.9) | 514.3 (361.6 to 711.7) | -18.3* (-37.1 to -0.0) | -29.5* (-45.7 to -13.7) |
| HIV/AIDS not on antiretroviral treatment without Tuberculosis | 2677.1 (2386.5 to 3014.7) | 327.4 (272.3 to 381.9) | 297.5 (198.6 to 456.6) | -50.2* (-57.9 to -43.5) | -57.0* (-63.6 to -51.2) |
| HIV/AIDS on antiretroviral treatment without Tuberculosis | 2768.8 (2482.0 to 3063.6) | -- | 216.8 (144.7 to 304.8) | 571.3* (475.9 to 663.3) | 479.5* (397.2 to 559.0) |
| Sexually transmitted infections excluding HIV | 138385.9 (121762.9 to 155989.0) | 110202.8 (90894.6 to 134544.9) | 205.2 (111.9 to 369.2) | 17.4* (15.1 to 20.8) | 1.4 (-0.7 to 4.3) |
| Syphilis | 5455.4 (3956.5 to 7448.6) | 1324.2 (859.6 to 1833.3) | 5.2 (3.5 to 7.6) | 13.2* (9.3 to 17.7) | -2.3 (-5.6 to 1.7) |
| Early syphilis | 5433.6 (3933.2 to 7431.4) | 1324.2 (859.6 to 1833.3) | 1.3 (0.4 to 3.3) | 15.5* (8.2 to 23.6) | -0.2 (-6.6 to 6.7) |
| Tertiary syphilis | 21.8 (17.2 to 26.7) | -- | 3.9 (2.6 to 5.6) | 12.4* (7.9 to 17.5) | -2.9 (-6.8 to 1.5) |
| Chlamydial infection | 17938.6 (13059.2 to 24434.4) | 45491.1 (31980.9 to 63744.5) | 53.9 (29.7 to 96.4) | 16.7* (12.0 to 22.9) | 0.8 (-3.3 to 6.1) |
| Chlamydia episode | 16974.1 (12073.6 to 23468.3) | 45491.1 (31980.9 to 63744.5) | 29.0 (10.3 to 65.2) | 14.7* (13.1 to 16.5) | -1.0 (-2.4 to 0.5) |
| Chlamydial infection complications | 964.5 (888.1 to 1044.5) | -- | 24.9 (16.2 to 36.4) | 19.1* (9.7 to 30.1) | 2.8 (-5.3 to 12.3) |
| Gonococcal infection | 5226.1 (3152.4 to 8634.9) | 18263.9 (10860.8 to 27174.4) | 25.2 (13.5 to 48.0) | 19.3* (11.1 to 28.7) | 3.0 (-4.1 to 11.1) |
| Gonococcal infection complications | 319.1 (287.0 to 354.5) | -- | 11.6 (7.1 to 18.0) | 25.3* (9.7 to 41.4) | 8.2 (-5.3 to 22.1) |
| Gonorrhoea episode | 4907.0 (2829.5 to 8278.3) | 18263.9 (10860.8 to 27174.4) | 13.5 (4.7 to 32.8) | 14.6* (7.1 to 19.9) | -1.1 (-7.5 to 3.5) |
| Trichomoniasis | 18122.4 (11639.4 to 26567.6) | 34959.1 (24194.1 to 50095.2) | 30.9 (11.1 to 67.9) | 16.8* (14.5 to 19.1) | 0.8 (-1.1 to 2.9) |
| Genital herpes | 102429.2 (85922.0 to 120902.9) | 10164.4 (7401.3 to 13813.5) | 27.3 (8.9 to 63.8) | 18.4* (16.6 to 20.2) | 2.2* (0.7 to 3.7) |
| Other sexually transmitted infections | 2176.3 (2004.6 to 2348.9) | -- | 62.7 (38.4 to 103.1) | 17.5* (14.4 to 21.6) | 1.5 (-1.2 to 5.0) |
| Other sexually transmitted diseases residual | -- | -- | 31.5 (16.6 to 57.9) | 17.2* (12.6 to 24.1) | 1.2 (-2.8 to 7.1) |
| Other sexually transmitted diseases | 2176.3 (2004.6 to 2348.9) | -- | 62.7 (38.4 to 103.1) | 17.5* (14.4 to 21.6) | 1.5 (-1.2 to 5.0) |
| Respiratory infections and tuberculosis | 189218.7 (131528.9 to 257493.2) | 1358213.7 (953886.9 to 1812678.1) | 804.9 (520.2 to 1198.4) | 13.6* (11.7 to 15.5) | -1.9* (-3.6 to -0.3) |
| Tuberculosis | 173782.5 (113418.7 to 245296.0) | 715.7 (514.2 to 961.3) | 237.4 (138.8 to 367.9) | 12.7* (7.9 to 18.2) | -2.7 (-6.8 to 2.1) |
| Latent tuberculosis infection | 173024.0 (112578.4 to 244602.1) | -- | -- | -- | -- |
| Drug-susceptible tuberculosis | 720.9 (516.4 to 969.9) | 674.8 (483.1 to 904.8) | 225.4 (132.2 to 350.2) | 12.7* (6.5 to 18.7) | -2.7 (-8.1 to 2.4) |
| Multidrug-resistant tuberculosis without extensive drug resistance | 35.6 (17.6 to 66.4) | 38.3 (20.7 to 67.1) | 11.4 (4.9 to 23.0) | 11.8 (-39.6 to 88.2) | -3.5 (-47.8 to 62.5) |
| Extensively drug-resistant tuberculosis | 2.0 (1.1 to 3.6) | 2.6 (1.7 to 3.9) | 0.7 (0.3 to 1.3) | 57.4* (4.7 to 154.9) | 35.9 (-9.6 to 120.0) |
| Lower respiratory infections | 606.0 (531.6 to 685.6) | 26816.6 (23457.9 to 30542.3) | 37.0 (24.1 to 53.6) | 37.1* (27.2 to 46.5) | 18.4* (9.8 to 26.5) |
| Guillain-Barré syndrome due to lower respiratory infections | 0.7 (0.3 to 1.3) | -- | 0.2 (0.1 to 0.4) | 17.9* (15.2 to 20.7) | 1.8 (-0.6 to 4.2) |
| Lower respiratory infection episode | 605.3 (531.0 to 685.0) | 26816.6 (23457.9 to 30542.3) | 36.8 (23.9 to 53.3) | 37.2* (27.3 to 46.7) | 18.5* (9.9 to 26.7) |
| Upper respiratory infections | 18125.5 (12728.8 to 24400.8) | 1325101.4 (918806.5 to 1779773.8) | 450.3 (242.6 to 754.2) | 14.2* (13.1 to 15.5) | -1.4* (-2.4 to -0.3) |
| Guillain-Barré syndrome due to upper respiratory infections | 2.0 (1.1 to 3.3) | -- | 0.6 (0.3 to 1.1) | 17.9* (15.1 to 20.7) | 1.8 (-0.6 to 4.2) |
| Upper respiratory infection episode | 18123.5 (12727.1 to 24397.7) | 1325101.4 (918806.5 to 1779773.8) | 449.7 (242.3 to 753.9) | 14.2* (13.0 to 15.5) | -1.4* (-2.4 to -0.3) |
| Otitis media | 3843.9 (2690.6 to 5459.1) | 5580.1 (3926.2 to 7615.4) | 80.1 (42.5 to 142.3) | 4.4 (-0.4 to 9.5) | -9.8* (-14.0 to -5.5) |
| Acute otitis media | 301.6 (210.2 to 424.5) | 5579.6 (3925.5 to 7614.8) | 3.9 (1.9 to 7.4) | 17.0* (10.6 to 23.9) | 1.0 (-4.5 to 6.9) |
| Chronic otitis media | 3542.3 (2363.8 to 5159.2) | 0.5 (0.0 to 1.8) | 76.2 (40.0 to 135.8) | 3.9 (-1.2 to 9.1) | -10.3* (-14.7 to -5.8) |
| Enteric infections | 5752.0 (5152.3 to 6491.5) | 398881.2 (349443.8 to 456501.6) | 655.2 (451.5 to 908.9) | 40.4* (34.9 to 45.7) | 21.2* (16.5 to 25.8) |
| Diarrhoeal diseases | 5776.7 (5155.5 to 6509.8) | 398284.0 (348860.8 to 455975.2) | 650.5 (447.1 to 902.7) | 41.1* (35.6 to 46.4) | 21.8* (17.1 to 26.4) |
| Guillain-Barré syndrome due to diarrheal diseases | 0.7 (0.4 to 1.2) | -- | 0.2 (0.1 to 0.4) | 17.9* (15.1 to 20.7) | 1.8 (-0.6 to 4.2) |
| Diarrheal disease episode | 5776.0 (5154.7 to 6509.3) | 398284.0 (348860.8 to 455975.2) | 650.3 (446.9 to 902.5) | 41.1* (35.6 to 46.4) | 21.8* (17.1 to 26.4) |
| Typhoid and paratyphoid | 4.9 (2.7 to 7.5) | 574.1 (454.1 to 713.3) | 4.6 (2.8 to 7.0) | -15.7 (-30.3 to 1.9) | -27.2* (-39.9 to -12.0) |
| Typhoid fever | 27.3 (20.0 to 36.4) | 433.5 (330.7 to 547.7) | 4.2 (2.5 to 6.4) | -15.9 (-31.8 to 3.2) | -27.4* (-41.1 to -10.9) |
| Typhoid fever complications | 5.7 (4.0 to 7.9) | 74.7 (55.4 to 98.2) | 1.9 (1.0 to 3.0) | -15.7 (-38.0 to 15.2) | -27.2* (-46.4 to -0.6) |
| Typhoid fever episode | 21.5 (15.6 to 29.0) | 358.8 (276.2 to 452.8) | 2.4 (1.4 to 3.9) | -16.0 (-36.7 to 10.4) | -27.5* (-45.3 to -4.7) |
| Paratyphoid fever | 6.2 (4.1 to 8.7) | 140.6 (94.3 to 195.9) | 0.4 (0.2 to 0.6) | -13.8 (-26.5 to 1.1) | -25.6* (-36.5 to -12.7) |
| Intestinal perforation due to paratyphoid | 0.3 (0.2 to 0.4) | 7.2 (4.7 to 10.2) | 0.0 (0.0 to 0.1) | -14.1 (-32.2 to 9.8) | -25.8* (-41.5 to -5.2) |
| Paratyphoid fever episode | 5.9 (3.9 to 8.3) | 133.4 (89.5 to 185.4) | 0.4 (0.2 to 0.6) | -13.8 (-27.0 to 2.0) | -25.6* (-37.0 to -11.9) |
| iNTS | 0.9 (0.5 to 1.4) | 23.1 (14.5 to 35.5) | 0.1 (0.1 to 0.2) | -23.8* (-28.6 to -18.9) | -34.2* (-38.4 to -30.0) |
| Other intestinal infectious diseases | -- | -- | -- | -27.8* (-40.3 to -12.8) | -37.7* (-48.5 to -24.7) |
| Neglected tropical diseases and malaria | 101116.9 (96051.0 to 106705.9) | 18818.4 (15271.0 to 23175.5) | 1127.3 (774.9 to 1551.0) | -8.9* (-14.8 to -3.1) | -21.4* (-26.5 to -16.3) |
| Malaria | 6957.6 (6481.3 to 7386.6) | 8248.2 (6540.1 to 10205.0) | 72.4 (53.2 to 96.8) | -12.2 (-25.4 to 1.3) | -24.2* (-35.6 to -12.6) |
| Malaria complications | 66.3 (60.3 to 73.1) | -- | 27.3 (21.1 to 34.1) | 27.9* (15.3 to 40.4) | 10.4 (-0.4 to 21.3) |
| Malaria episode | 481.8 (311.6 to 685.9) | 8248.2 (6540.1 to 10205.0) | 15.2 (7.8 to 26.0) | -11.8* (-21.0 to -1.9) | -23.9* (-31.8 to -15.3) |

| | | | | | |
|---|---|---|---|---|---|
| Malaria parasitemia | 6409.5 (5923.7 to 6948.5) | -- | 29.9 (18.7 to 45.9) | -31.8* (-47.8 to -12.0) | -41.1* (-55.0 to -24.0) |
| Chagas disease | 509.4 (412.6 to 609.3) | 13.3 (9.2 to 18.5) | 3.3 (2.0 to 4.8) | -9.6* (-17.6 to -1.9) | -22.0* (-28.9 to -15.3) |
| Acute Chagas disease | 0.1 (0.0 to 0.1) | 13.3 (9.2 to 18.5) | -- | -7.9* (-12.4 to -3.6) | -20.5* (-24.4 to -16.8) |
| Asymptomatic Chagas disease | 448.4 (362.1 to 537.4) | -- | -- | -- | -- |
| Symptomatic chronic Chagas infection | 61.0 (46.3 to 77.1) | -- | 3.3 (2.0 to 4.8) | -9.6* (-17.6 to -1.9) | -22.0* (-28.9 to -15.3) |
| Leishmaniasis | 313.0 (262.4 to 376.8) | 34.6 (25.1 to 46.3) | 20.2 (13.0 to 29.8) | 3.6 (-11.6 to 23.1) | -10.5 (-23.7 to 6.3) |
| Visceral leishmaniasis | 0.5 (0.3 to 0.7) | 1.8 (1.3 to 2.8) | 0.0 (0.0 to 0.1) | -72.3* (-78.5 to -58.6) | -76.1* (-81.5 to -64.3) |
| Cutaneous and mucocutaneous leishmaniasis | 315.7 (265.5 to 378.6) | 32.7 (23.4 to 44.6) | 20.1 (13.0 to 29.8) | 4.1 (-11.3 to 24.0) | -10.1 (-23.4 to 7.1) |
| African trypanosomiasis | 0.5 (0.1 to 2.1) | 0.3 (0.1 to 1.0) | 0.1 (0.0 to 0.5) | -77.7 (-97.4 to 18.3) | -80.8 (-97.8 to 2.1) |
| Trypanosomiasis Gambiense | 0.5 (0.1 to 2.1) | 0.3 (0.1 to 1.0) | 0.1 (0.0 to 0.5) | -76.9 (-97.4 to 36.5) | -80.1 (-97.8 to 17.8) |
| Trypanosomiasis Rhodesiense | -- | 0.0 (0.0 to 0.1) | -- | -91.7* (-99.0 to -47.6) | -92.8* (-99.1 to -54.7) |
| Schistosomiasis | 17490.4 (15181.6 to 19965.1) | -- | 136.2 (67.5 to 262.1) | -18.9* (-22.9 to -14.5) | -30.0* (-33.5 to -26.2) |
| Mild schistosomiasis | 13876.0 (11802.8 to 16128.7) | -- | 78.5 (31.1 to 169.2) | -20.8* (-24.0 to -17.8) | -31.6* (-34.4 to -29.1) |
| Anemia due to schistosomiasis | 1004.9 (820.0 to 1226.7) | -- | 23.4 (14.3 to 36.5) | -29.4* (-43.7 to -11.3) | -39.0* (-51.4 to -23.4) |
| Schistosomiasis complications | 2614.4 (1996.0 to 3274.8) | -- | 34.2 (18.6 to 61.6) | -3.7 (-10.0 to 4.0) | -16.8* (-22.3 to -10.2) |
| Cysticercosis | 481.1 (332.6 to 647.4) | -- | 147.1 (83.8 to 221.7) | -0.3 (-8.3 to 7.0) | -13.9* (-20.8 to -7.6) |
| Cystic echinococcosis | 65.3 (33.5 to 122.7) | 13.6 (7.1 to 25.8) | 5.4 (2.3 to 11.0) | 29.4* (17.6 to 40.4) | 11.7* (1.5 to 21.2) |
| Lymphatic filariasis | 5762.3 (5209.1 to 6387.1) | -- | 129.0 (70.7 to 206.5) | -40.7* (-51.7 to -30.4) | -48.8* (-58.3 to -39.9) |
| Prevalence of detectable microfiliaria due to lymphatic filariasis | 4608.5 (4220.7 to 5032.9) | -- | -- | -- | -- |
| Lymphatic filariasis complications | 1153.8 (762.9 to 1654.5) | -- | 129.0 (70.7 to 206.5) | -40.7* (-51.7 to -30.4) | -48.8* (-58.3 to -39.9) |
| Onchocerciasis | 1981.5 (1130.1 to 3677.8) | -- | 116.2 (51.1 to 215.5) | 5.0 (-17.9 to 24.2) | -9.4 (-29.1 to 7.2) |
| Asymptomatic onchocerciasis | 628.2 (3.0 to 2171.8) | -- | -- | -- | -- |
| Skin disease due to onchocerciasis | 1311.1 (952.4 to 1758.5) | -- | 112.8 (47.6 to 211.1) | 6.4 (-17.5 to 26.0) | -8.1 (-28.8 to 8.7) |
| Vision loss due to onchocerciasis | 42.2 (29.0 to 68.5) | -- | 3.4 (2.0 to 5.3) | -27.7* (-41.3 to -11.5) | -37.5* (-49.3 to -23.6) |
| Trachoma | 15.6 (8.2 to 26.8) | -- | 2.4 (1.2 to 4.2) | -37.7* (-55.7 to -10.9) | -46.2* (-61.7 to -23.1) |
| Dengue | 455.7 (248.3 to 771.6) | 7614.2 (4632.6 to 11547.1) | 74.4 (32.6 to 138.8) | 68.4* (44.8 to 159.4) | 45.4* (25.0 to 124.0) |
| Post-dengue chronic fatigue syndrome | 321.3 (150.2 to 587.4) | -- | 66.5 (27.6 to 126.2) | 68.5* (44.4 to 161.5) | 45.5* (24.6 to 125.7) |
| Dengue episode | 134.4 (81.2 to 201.7) | 7614.2 (4632.6 to 11547.1) | 7.9 (4.1 to 13.9) | 67.7* (44.7 to 152.4) | 44.8* (24.9 to 117.9) |
| Yellow fever | 0.2 (0.0 to 0.5) | 6.1 (1.8 to 17.0) | -- | -15.7 (-39.2 to 17.3) | -27.3 (-47.5 to 1.3) |
| Asymptomatic yellow fever | 0.1 (0.0 to 0.3) | 3.4 (0.9 to 9.4) | -- | -- | -- |
| Yellow fever episode | 0.1 (0.0 to 0.2) | 2.7 (0.8 to 7.1) | -- | -15.7 (-39.2 to 17.3) | -27.3 (-47.5 to 1.3) |
| Rabies | -- | 0.6 (0.5 to 0.9) | -- | -33.1* (-49.1 to -13.0) | -42.2* (-56.1 to -24.9) |
| Intestinal nematode infections | 67528.3 (62631.5 to 73075.5) | -- | 115.3 (65.9 to 187.5) | -25.6* (-29.9 to -21.5) | -35.8* (-39.5 to -32.2) |
| Ascariasis | 31444.8 (27605.0 to 36107.1) | -- | 40.1 (21.5 to 69.0) | -29.8* (-37.3 to -21.7) | -39.4* (-45.8 to -32.4) |
| Asymptomatic ascariasis | 29277.8 (25623.7 to 33726.4) | -- | -- | -- | -- |
| Ascariasis complications | 2167.0 (1922.3 to 2450.3) | -- | 40.1 (21.5 to 69.0) | -29.8* (-37.3 to -21.7) | -39.4* (-45.8 to -32.4) |
| Trichuriasis | 26385.9 (22990.6 to 30550.9) | -- | 19.6 (10.9 to 32.7) | -14.9* (-22.1 to -6.7) | -26.6* (-32.8 to -19.4) |
| Asymptomatic trichuriasis | 25401.1 (22078.3 to 29541.0) | -- | -- | -- | -- |
| Trichuriasis complications | 984.8 (897.6 to 1072.9) | -- | 19.6 (10.9 to 32.7) | -14.9* (-22.1 to -6.7) | -26.6* (-32.8 to -19.4) |
| Hookworm disease | 14549.3 (13465.3 to 15716.1) | -- | 55.6 (33.0 to 88.5) | -25.7* (-32.1 to -18.8) | -35.8* (-41.3 to -29.9) |
| Asymptomatic hookworm disease | 11956.3 (11054.1 to 12920.2) | -- | -- | -- | -- |
| Anemia due to hookworm disease | 677.6 (603.1 to 763.8) | -- | 15.6 (10.2 to 23.0) | -29.5* (-40.1 to -16.7) | -39.2* (-48.3 to -28.1) |
| Hookworm disease complications | 1915.4 (1779.6 to 2056.6) | -- | 40.0 (22.3 to 66.5) | -24.1* (-29.4 to -18.3) | -34.5* (-39.0 to -29.5) |
| Food-borne trematodiases | 8889.2 (7634.6 to 10212.3) | 2674.8 (2257.5 to 3157.2) | 232.2 (129.0 to 397.7) | 11.8* (7.1 to 17.6) | -3.5 (-7.5 to 1.6) |
| Asymptomatic food-borne tremodiases | 6842.4 (5517.2 to 8236.7) | 2103.7 (1396.9 to 2701.4) | -- | -- | -- |
| Food-borne tremodiases complications | 2046.8 (1325.6 to 3172.9) | 571.2 (143.7 to 1256.5) | 232.2 (129.0 to 397.7) | 11.8* (7.1 to 17.6) | -3.5 (-7.5 to 1.6) |
| Leprosy | 27.7 (25.4 to 30.4) | 3.5 (3.2 to 3.8) | 1.7 (1.1 to 2.6) | -22.6* (-31.5 to -13.2) | -33.2* (-40.8 to -25.0) |
| Ebola virus disease | -- | -- | -- | -96.5* (-96.9 to -95.7) | -97.0* (-97.3 to -96.3) |
| Ebola cases | -- | -- | -- | -97.5* (-97.7 to -97.3) | -97.8* (-98.0 to -97.7) |
| Post-Ebola chronic fatigue syndrome | -- | -- | -- | -96.5* (-96.8 to -95.6) | -96.9* (-97.2 to -96.2) |
| Zika virus disease | 3.5 (2.0 to 6.3) | 209.2 (120.1 to 379.4) | 0.1 (0.0 to 0.1) | -- | -- |
| Zika virus complications | 0.0 (0.0 to 0.1) | -- | -- | -- | -- |
| Zika virus episode | 3.4 (2.0 to 6.2) | 209.2 (120.1 to 379.4) | 0.1 (0.0 to 0.1) | -- | -- |
| Guinea worm disease | -- | -- | -- | -99.5* (-99.7 to -99.2) | -99.5* (-99.7 to -99.3) |
| Other neglected tropical diseases | 3239.4 (3028.8 to 3483.5) | -- | 71.4 (46.4 to 105.6) | 0.5 (-8.1 to 8.8) | -13.2* (-20.6 to -6.1) |
| Acute infection due to other neglected tropical diseases | -- | -- | 0.8 (0.4 to 1.4) | 126.3* (95.5 to 233.2) | 95.4* (68.7 to 187.6) |
| Anemia due to other neglected tropical diseases | 3239.4 (3028.8 to 3483.5) | -- | 70.6 (45.9 to 104.3) | -0.1 (-8.6 to 8.3) | -13.8* (-21.1 to -6.5) |
| **Other infectious diseases** | **6923.1 (6257.0 to 7966.5)** | **22943.1 (17811.5 to 30886.6)** | **285.6 (198.7 to 392.5)** | **4.4 (-2.4 to 13.3)** | **-9.8* (-15.7 to -2.2)** |
| Meningitis | 826.6 (689.4 to 976.9) | 190.9 (130.6 to 265.9) | 71.4 (48.8 to 95.9) | -2.3 (-6.9 to 2.7) | -15.7* (-19.6 to -11.3) |
| Pneumococcal meningitis | 291.2 (239.5 to 356.7) | 11.8 (7.1 to 18.4) | 26.4 (18.1 to 36.1) | -23.8* (-29.4 to -18.1) | -34.2* (-39.0 to -29.3) |

| | | | | | |
|---|---|---|---|---|---|
| Acute pneumococcal meningitis | 0.6 (0.3 to 0.9) | 11.8 (7.1 to 18.4) | 0.1 (0.0 to 0.1) | -25.9* (-35.6 to -16.4) | -36.0* (-44.4 to -27.8) |
| Pneumococcal meningitis complications | 290.6 (239.0 to 356.2) | -- | 26.3 (18.0 to 36.0) | -23.8* (-29.4 to -18.1) | -34.2* (-39.1 to -29.3) |
| H influenzae type B meningitis | 69.5 (50.0 to 92.5) | 3.7 (2.1 to 6.0) | 5.9 (4.0 to 8.2) | -50.2* (-53.6 to -46.8) | -57.0* (-59.9 to -54.1) |
| Acute H influenzae type B meningitis | 0.2 (0.1 to 0.3) | 3.7 (2.1 to 6.0) | -- | -56.7* (-62.4 to -50.9) | -62.6* (-67.5 to -57.6) |
| H Influenza type B meningitis complications | 69.3 (49.8 to 92.3) | -- | 5.9 (4.0 to 8.2) | -50.2* (-53.5 to -46.8) | -57.0* (-59.9 to -54.1) |
| Meningococcal infection | 85.3 (60.4 to 113.8) | 11.1 (6.2 to 18.1) | 7.9 (5.4 to 11.0) | -0.1 (-4.4 to 3.9) | -13.7* (-17.5 to -10.3) |
| Acute meningococcal meningitis | 0.5 (0.3 to 0.9) | 11.1 (6.2 to 18.1) | 0.1 (0.0 to 0.1) | 11.3 (-1.2 to 23.8) | -3.9 (-14.7 to 6.9) |
| Meningococcal meningitis complications | 84.8 (59.9 to 113.3) | -- | 7.9 (5.3 to 10.9) | -0.2 (-4.5 to 3.9) | -13.8* (-17.5 to -10.3) |
| Other meningitis | 380.6 (280.5 to 486.4) | 164.4 (113.7 to 230.7) | 31.2 (21.2 to 42.1) | 67.2* (55.7 to 79.0) | 44.3* (34.5 to 54.5) |
| Other acute bacterial meningitis | 1.5 (0.9 to 2.2) | 31.2 (21.1 to 44.0) | 0.2 (0.1 to 0.3) | 64.5* (48.6 to 81.4) | 42.0* (28.3 to 56.6) |
| Acute viral meningitis | 6.3 (4.1 to 9.3) | 133.2 (91.2 to 185.6) | 0.8 (0.5 to 1.4) | 8.2 (-0.8 to 18.0) | -6.6 (-14.3 to 1.9) |
| Other bacterial meningitis complications | 372.8 (273.3 to 478.4) | -- | 30.1 (20.4 to 40.8) | 69.8* (57.9 to 82.0) | 46.6* (36.4 to 57.1) |
| Encephalitis | 557.0 (316.2 to 882.1) | 104.5 (100.0 to 109.0) | 46.5 (32.7 to 61.6) | 7.1* (1.0 to 14.1) | -7.6* (-12.8 to -1.5) |
| Acute encephalitis | 5.7 (5.4 to 5.9) | 104.5 (100.0 to 109.0) | 0.8 (0.5 to 1.1) | 18.0* (17.2 to 18.7) | 1.8* (1.2 to 2.4) |
| Encephalitis complications | 551.3 (310.7 to 876.5) | -- | 45.8 (32.1 to 60.7) | 6.9* (0.8 to 14.0) | -7.7* (-13.0 to -1.6) |
| Diphtheria | -- | 0.2 (0.1 to 0.2) | -- | -35.2* (-53.3 to -8.2) | -44.1* (-59.7 to -20.8) |
| Whooping cough | 2.7 (2.1 to 3.4) | 19.8 (15.3 to 25.0) | 0.1 (0.1 to 0.2) | 0.2 (-0.5 to 0.9) | -13.5* (-14.1 to -12.9) |
| Tetanus | 4.3 (4.1 to 4.5) | 2.4 (1.4 to 3.8) | 0.1 (0.1 to 0.2) | -4.1 (-15.8 to 5.4) | -17.2* (-27.3 to -9.0) |
| Severe tetanus | 0.1 (0.1 to 0.2) | 2.4 (1.4 to 3.8) | -- | -45.3* (-57.8 to -23.7) | -52.8* (-63.6 to -34.2) |
| Neonatal tetanus complications | 4.1 (4.0 to 4.3) | -- | 0.1 (0.0 to 0.1) | 14.7* (13.0 to 16.3) | -1.0 (-2.5 to 0.4) |
| Measles | 1.0 (0.4 to 2.3) | 37.9 (13.5 to 84.2) | 0.1 (0.0 to 0.2) | -32.1* (-38.1 to -25.8) | -41.3* (-46.6 to -35.9) |
| Varicella and herpes zoster | 312.7 (252.4 to 378.9) | 2333.1 (1825.3 to 2930.4) | 16.8 (10.0 to 27.0) | 14.8* (8.9 to 21.1) | -0.9 (-6.0 to 4.6) |
| Chickenpox | 12.4 (6.0 to 21.7) | 646.4 (313.7 to 1131.1) | 0.1 (0.0 to 0.2) | 26.5* (22.2 to 31.4) | 9.2* (5.5 to 13.5) |
| Herpes zoster | 300.4 (241.8 to 365.1) | 1686.8 (1361.6 to 2095.3) | 16.7 (9.9 to 26.9) | 14.8* (8.9 to 21.1) | -0.9 (-6.0 to 4.5) |
| Acute hepatitis | 2123.5 (1533.9 to 3073.4) | 20254.2 (15100.8 to 28446.3) | 49.5 (29.7 to 78.4) | 10.3 (-25.3 to 67.0) | -4.8 (-35.5 to 44.2) |
| Acute hepatitis A | 355.5 (309.0 to 398.5) | 4621.4 (4017.0 to 5181.0) | 15.0 (9.5 to 22.2) | 17.2* (8.1 to 27.6) | 1.2 (-6.7 to 10.2) |
| Acute hepatitis B | 1670.2 (1089.1 to 2630.2) | 14475.3 (9438.7 to 22795.2) | 31.9 (16.6 to 57.6) | 7.1 (-41.8 to 95.6) | -7.6 (-49.7 to 68.9) |
| Acute hepatitis C | 26.3 (10.8 to 46.6) | 227.6 (93.6 to 403.8) | 0.4 (0.1 to 0.9) | 10.8* (4.9 to 14.6) | -4.3* (-9.4 to -1.1) |
| Acute hepatitis E | 71.5 (43.3 to 111.7) | 929.9 (563.4 to 1451.5) | 2.3 (1.1 to 4.0) | 14.3 (-4.4 to 35.3) | -1.4 (-17.5 to 16.8) |
| Other unspecified infectious diseases | 3917.7 (3679.7 to 4170.8) | -- | 101.0 (66.2 to 149.9) | 4.2 (-2.9 to 10.7) | -10.1* (-16.1 to -4.5) |
| Guillain-Barré syndrome due to other infectious diseases | 0.4 (0.2 to 0.8) | -- | 0.1 (0.1 to 0.3) | 17.9* (15.2 to 20.7) | 1.8 (-0.5 to 4.2) |
| Other infectious diseases | -- | -- | 17.1 (9.9 to 27.6) | 21.5* (17.0 to 26.1) | 4.9* (1.0 to 8.8) |
| Anemia due to other infectious diseases | 3917.2 (3679.2 to 4170.2) | -- | 83.7 (55.4 to 123.4) | 1.2 (-6.6 to 8.8) | -12.6* (-19.4 to -6.1) |
| **Maternal and neonatal disorders** | **12528.1 (11044.7 to 14241.1)** | **15906.4 (13860.9 to 18133.6)** | **2383.7 (1808.2 to 3062.6)** | **29.6* (23.0 to 37.3)** | **11.9* (6.2 to 18.5)** |
| Maternal disorders | 1729.6 (1434.8 to 2074.6) | 15906.4 (13860.9 to 18133.6) | 160.9 (113.3 to 216.5) | 4.6 (-5.9 to 16.7) | -9.7 (-18.8 to 0.7) |
| Maternal haemorrhage | 390.7 (349.8 to 438.5) | 1556.3 (1130.4 to 2061.2) | 13.7 (9.1 to 19.8) | 7.5 (-8.8 to 29.1) | -7.2 (-21.2 to 11.5) |
| Maternal hemorrhage complications | 356.6 (319.3 to 401.5) | -- | 8.1 (5.0 to 12.0) | 2.4 (-12.7 to 20.1) | -11.6 (-24.6 to 3.7) |
| Maternal hemorrhage episode | 34.1 (21.0 to 53.7) | 1556.3 (1130.4 to 2061.2) | 5.6 (3.1 to 9.2) | 15.6 (-20.3 to 68.7) | -0.2 (-31.2 to 45.6) |
| Maternal sepsis and other pregnancy related infections | 501.4 (406.0 to 627.4) | 2147.7 (1531.3 to 2927.2) | 9.9 (5.0 to 18.1) | 19.9 (-26.8 to 88.0) | 3.5 (-36.8 to 62.3) |
| Infertility due to puerperal sepsis | 338.7 (287.6 to 392.7) | 19.9 (14.3 to 26.4) | 1.7 (0.6 to 3.6) | 21.2* (15.3 to 27.1) | 4.6 (-0.4 to 9.7) |
| Maternal sepsis and other maternal infection episode | 162.7 (82.3 to 282.2) | 2127.8 (1512.3 to 2904.8) | 8.2 (3.9 to 15.1) | 19.7 (-34.6 to 105.0) | 3.3 (-43.5 to 77.0) |
| Maternal hypertensive disorders | 624.2 (367.9 to 944.0) | 3371.5 (2462.5 to 4335.7) | 31.1 (16.0 to 53.9) | 18.0 (-21.1 to 66.9) | 1.8 (-31.9 to 44.1) |
| Maternal hypertensive disorder complications | 21.9 (19.9 to 24.1) | 327.9 (299.9 to 358.6) | 1.4 (0.9 to 2.3) | 23.1* (8.6 to 39.5) | 6.3 (-6.3 to 20.4) |
| Maternal hypertensive disorder episode | 602.3 (345.8 to 921.8) | 3043.6 (2131.2 to 4008.0) | 29.6 (14.9 to 52.0) | 17.7 (-23.0 to 69.2) | 1.6 (-33.5 to 46.1) |
| Maternal obstructed labour and uterine rupture | 238.9 (192.0 to 293.1) | 1512.2 (1068.0 to 2133.6) | 77.1 (49.3 to 109.3) | -8.8* (-16.0 to -1.0) | -21.3* (-27.5 to -14.5) |
| Obstructed labor, acute event | 20.2 (11.7 to 32.3) | 1479.2 (1031.1 to 2096.0) | 6.3 (3.1 to 11.3) | 16.9 (-31.2 to 86.2) | 1.0 (-40.6 to 60.8) |
| Maternal obstructed labor complications | 218.7 (173.7 to 273.6) | 33.0 (22.9 to 45.0) | 70.8 (44.3 to 101.6) | -10.6* (-17.9 to -3.3) | -22.8* (-29.1 to -16.5) |
| Maternal abortion and miscarriage | 32.5 (19.7 to 49.0) | 3967.9 (2843.4 to 5457.0) | 3.6 (1.9 to 6.0) | 17.4 (-25.0 to 77.2) | 1.4 (-35.2 to 53.0) |
| Ectopic pregnancy | 27.4 (15.9 to 42.4) | 3350.7 (2350.5 to 4528.4) | 3.0 (1.5 to 5.0) | 18.8 (-26.3 to 87.3) | 2.6 (-36.3 to 61.7) |
| Other maternal disorders | -- | -- | 22.6 (15.8 to 30.5) | 37.9* (23.5 to 54.3) | 19.0* (6.6 to 33.2) |
| Neonatal disorders | 10829.5 (9376.9 to 12503.3) | -- | 2222.8 (1666.3 to 2868.1) | 31.9* (24.5 to 40.6) | 13.9* (7.5 to 21.4) |
| Neonatal preterm birth | 6223.0 (5322.1 to 7316.6) | -- | 1038.2 (729.4 to 1416.5) | 38.1* (21.0 to 55.4) | 19.2* (4.5 to 34.2) |
| Preterm birth complications | 5358.7 (4453.3 to 6438.1) | -- | 990.0 (688.3 to 1362.7) | 40.0* (21.6 to 58.4) | 20.9* (5.0 to 36.7) |
| Retinopathy of prematurity | 864.3 (685.3 to 1055.8) | -- | 48.2 (32.4 to 67.3) | 7.9* (1.4 to 15.3) | -6.8* (-12.4 to -0.4) |
| Uncomplicated preterm birth | -- | -- | -- | -- | -- |
| Neonatal encephalopathy due to birth asphyxia and trauma | 4268.1 (3148.5 to 5635.4) | -- | 782.7 (479.0 to 1235.6) | 27.3* (22.5 to 32.8) | 9.9* (5.7 to 14.6) |
| Neonatal sepsis and other neonatal infections | 1138.7 (695.9 to 1743.1) | -- | 335.4 (181.4 to 556.1) | 25.8* (20.5 to 31.2) | 8.6* (4.1 to 13.3) |
| Neonatal sepsis and other neonatal infection complications | 1138.7 (695.9 to 1743.1) | -- | 335.4 (181.4 to 556.1) | 25.8* (20.5 to 31.2) | 8.6* (4.1 to 13.3) |
| Neonatal sepsis and other neonatal infection episode | -- | -- | -- | -- | -- |
| Hemolytic disease and other neonatal jaundice | 179.4 (154.4 to 205.3) | -- | 66.4 (50.6 to 83.6) | 27.5* (22.5 to 32.4) | 10.1* (5.7 to 14.3) |

| | | | | | |
|---|---|---|---|---|---|
| Other neonatal disorders | -- | -- | -- | -- | -- |
| **Nutritional deficiencies** | **132692.6 (127233.3 to 138750.6)** | **83632.8 (75515.2 to 92335.7)** | **2147.8 (1427.3 to 3140.1)** | **0.3 (-6.1 to 6.6)** | **-13.4* (-18.9 to -8.0)** |
| Protein-energy malnutrition | 3224.4 (2993.5 to 3474.8) | 3602.8 (3306.3 to 3912.3) | 29.0 (18.5 to 40.9) | 28.2* (18.0 to 37.6) | 10.7* (1.9 to 18.8) |
| Iodine deficiency | 10010.0 (8942.7 to 11153.0) | 67.2 (54.7 to 82.1) | 177.5 (104.9 to 280.7) | -0.8 (-5.4 to 3.4) | -14.4* (-18.3 to -10.7) |
| Visible goiter without symptoms | 9787.2 (8743.1 to 10922.2) | 67.2 (54.7 to 82.1) | 105.2 (51.3 to 195.1) | -1.7* (-3.5 to -0.2) | -15.1* (-16.7 to -13.8) |
| Visible goiter with complications | 222.8 (122.1 to 318.6) | -- | 72.3 (35.9 to 112.0) | 0.4 (-10.7 to 9.4) | -13.3* (-22.9 to -5.6) |
| Vitamin A deficiency | 51779.4 (46835.8 to 56955.5) | 79962.8 (71914.2 to 88611.7) | 14.5 (8.7 to 22.6) | 22.2* (13.5 to 31.7) | 5.5 (-2.0 to 13.7) |
| Asymptomatic vitamin A deficiency | 51556.0 (46623.1 to 56772.7) | 79962.8 (71914.2 to 88611.7) | -- | -- | -- |
| Vitamin A deficiency complications | 223.4 (152.8 to 312.0) | -- | 14.5 (8.7 to 22.6) | 22.2* (13.5 to 31.7) | 5.5 (-2.0 to 13.7) |
| Vitamin A deficiency with anemia | -- | -- | -- | -- | -- |
| Dietary iron deficiency | 85700.8 (80764.4 to 91087.0) | -- | 1912.9 (1268.4 to 2803.3) | -0.2 (-7.3 to 6.7) | -13.8* (-20.0 to -7.8) |
| Other nutritional deficiencies | -- | -- | 14.0 (8.9 to 19.7) | 27.6* (17.5 to 37.0) | 10.2* (1.4 to 18.3) |
| **Non-communicable diseases** | **562073.8 (556517.9 to 567423.1)** | **778996.1 (715650.9 to 844584.9)** | **49564.2 (37132.0 to 63992.1)** | **15.9* (15.2 to 16.6)** | **0.1 (-0.5 to 0.7)** |
| **Neoplasms** | **3649.9 (3410.4 to 3971.8)** | **483.7 (424.4 to 582.3)** | **237.2 (167.3 to 318.7)** | **49.8* (40.3 to 63.3)** | **29.4* (21.2 to 41.0)** |
| Lip and oral cavity cancer | 59.8 (53.9 to 67.4) | 11.4 (10.2 to 12.9) | 5.4 (3.8 to 7.3) | 37.7* (22.0 to 56.3) | 18.9* (5.3 to 35.0) |
| Diagnosis and primary therapy phase of mouth cancer | 3.3 (3.0 to 3.7) | 11.4 (10.2 to 12.9) | 1.0 (0.6 to 1.3) | 41.0* (26.9 to 58.1) | 21.7* (9.6 to 36.5) |
| Controlled phase of mouth cancer | 52.3 (47.0 to 59.0) | -- | 2.5 (1.5 to 3.8) | 41.7* (20.1 to 67.9) | 22.3* (3.7 to 44.9) |
| Metastatic phase of mouth cancer | 3.7 (3.3 to 4.1) | -- | 1.7 (1.1 to 2.2) | 31.9* (18.0 to 48.5) | 13.9* (1.8 to 28.2) |
| Terminal phase of mouth cancer | 0.5 (0.5 to 0.6) | -- | 0.3 (0.2 to 0.4) | 29.8* (15.5 to 46.9) | 12.1 (-0.3 to 26.8) |
| Nasopharynx cancer | 32.9 (29.1 to 37.0) | 5.3 (4.7 to 6.0) | 2.9 (2.1 to 4.0) | 21.4* (3.6 to 41.7) | 4.8 (-10.6 to 22.3) |
| Diagnosis and primary therapy phase of nasopharynx cancer | 1.9 (1.7 to 2.1) | 5.3 (4.7 to 6.0) | 0.5 (0.4 to 0.8) | 23.7* (6.7 to 42.1) | 6.8 (-7.9 to 22.6) |
| Controlled phase of nasopharynx cancer | 28.8 (25.5 to 32.4) | -- | 1.4 (0.9 to 2.1) | 23.4* (2.1 to 48.3) | 6.5 (-11.8 to 28.0) |
| Metastatic phase of nasopharynx cancer | 2.0 (1.8 to 2.3) | -- | 0.9 (0.6 to 1.2) | 18.2* (1.8 to 36.7) | 2.0 (-12.1 to 18.0) |
| Terminal phase of nasopharynx cancer | 0.2 (0.2 to 0.2) | -- | 0.1 (0.1 to 0.1) | 12.8 (-1.8 to 29.0) | -2.6 (-15.2 to 11.4) |
| Other pharynx cancer | 6.1 (4.9 to 6.7) | 2.5 (2.0 to 2.7) | 0.8 (0.6 to 1.1) | 29.5* (16.8 to 40.4) | 11.8* (0.8 to 21.2) |
| Diagnosis and primary therapy phase of other pharynx cancer | 0.3 (0.3 to 0.4) | 2.5 (2.0 to 2.7) | 0.1 (0.1 to 0.1) | 32.5* (20.0 to 42.9) | 14.4* (3.6 to 23.3) |
| Controlled phase of other pharynx cancer | 4.7 (3.8 to 5.1) | -- | 0.2 (0.1 to 0.3) | 32.8* (18.0 to 46.4) | 14.7* (1.9 to 26.4) |
| Metastatic phase of other pharynx cancer | 0.9 (0.8 to 1.0) | -- | 0.4 (0.3 to 0.6) | 27.6* (14.7 to 38.4) | 10.1 (-0.9 to 19.5) |
| Terminal phase of other pharynx cancer | 0.2 (0.1 to 0.2) | -- | 0.1 (0.1 to 0.1) | 27.3* (14.4 to 38.1) | 9.9 (-1.2 to 19.2) |
| Oesophageal cancer | 6.1 (5.8 to 6.5) | 2.7 (2.6 to 2.8) | 0.9 (0.6 to 1.2) | 17.5* (11.1 to 24.6) | 1.4 (-4.1 to 7.6) |
| Diagnosis and primary therapy phase of esophageal cancer | 0.5 (0.5 to 0.5) | 2.7 (2.6 to 2.8) | 0.1 (0.1 to 0.2) | 25.3* (18.0 to 33.3) | 8.1* (1.9 to 15.1) |
| Controlled phase of esophageal cancer | 4.5 (4.3 to 4.8) | -- | 0.2 (0.1 to 0.3) | 28.8* (21.1 to 37.5) | 11.2* (4.5 to 18.7) |
| Metastatic phase of esophageal cancer | 0.9 (0.9 to 1.0) | -- | 0.4 (0.3 to 0.6) | 11.4* (5.7 to 18.1) | -3.8 (-8.7 to 1.9) |
| Terminal phase of esophageal cancer | 0.2 (0.2 to 0.2) | -- | 0.1 (0.1 to 0.1) | 11.4* (5.7 to 18.1) | -3.8 (-8.7 to 1.9) |
| Stomach cancer | 44.5 (42.8 to 46.3) | 15.2 (14.7 to 15.8) | 5.2 (3.7 to 6.9) | 21.6* (14.9 to 29.5) | 5.0 (-0.8 to 11.8) |
| Diagnosis and primary therapy phase of stomach cancer | 3.8 (3.6 to 3.9) | 15.2 (14.7 to 15.8) | 1.1 (0.7 to 1.5) | 29.6* (23.7 to 35.6) | 11.9* (6.8 to 17.1) |
| Controlled phase of stomach cancer | 35.7 (34.3 to 37.2) | -- | 1.7 (1.1 to 2.5) | 40.6* (23.7 to 58.0) | 21.4* (6.8 to 36.4) |
| Metastatic phase of stomach cancer | 4.0 (3.8 to 4.1) | -- | 1.8 (1.2 to 2.4) | 7.9* (3.5 to 12.3) | -6.9* (-10.6 to -3.0) |
| Terminal phase of stomach cancer | 1.0 (1.0 to 1.1) | -- | 0.6 (0.4 to 0.7) | 7.9* (3.5 to 12.3) | -6.9* (-10.6 to -3.0) |
| Colon and rectum cancer | 123.0 (117.7 to 127.8) | 21.0 (20.1 to 21.8) | 11.4 (8.1 to 15.3) | 38.0* (29.2 to 46.6) | 19.2* (11.5 to 26.6) |
| Diagnosis and primary therapy phase of colon and rectum cancers | 5.1 (4.9 to 5.3) | 21.0 (20.1 to 21.8) | 1.5 (1.0 to 2.0) | 48.5* (41.8 to 55.1) | 28.2* (22.4 to 33.9) |
| Controlled phase of colon and rectum cancers | 110.1 (105.4 to 114.5) | -- | 6.7 (4.3 to 9.7) | 40.6* (29.6 to 52.4) | 21.4* (11.9 to 31.5) |
| Metastatic phase of colon and rectum cancers | 6.0 (5.7 to 6.2) | -- | 2.7 (1.8 to 3.6) | 30.7* (23.1 to 37.4) | 12.8* (6.2 to 18.6) |
| Terminal phase of colon and rectum cancers | 0.8 (0.8 to 0.9) | -- | 0.5 (0.3 to 0.6) | 24.8* (16.8 to 31.5) | 7.7* (0.8 to 13.6) |
| Stoma from colon and rectum cancers, beyond 10 years | 1.0 (0.8 to 1.2) | -- | 0.1 (0.1 to 0.1) | 7.3* (5.8 to 8.6) | -7.4* (-8.6 to -6.2) |
| Liver cancer | 6.2 (3.7 to 9.0) | 16.5 (15.0 to 18.1) | 4.2 (2.9 to 5.6) | 29.2* (16.7 to 44.1) | 11.5* (0.8 to 24.4) |
| Liver cancer due to hepatitis B | 18.3 (16.0 to 20.7) | 11.3 (10.1 to 12.6) | 2.8 (2.0 to 3.9) | 29.8* (16.2 to 46.9) | 12.1* (0.3 to 26.8) |
| Diagnosis and primary therapy phase of liver cancer due to hepatitis B | 2.8 (2.5 to 3.2) | 11.3 (10.1 to 12.6) | 0.8 (0.5 to 1.2) | 28.6* (15.8 to 42.9) | 11.1 (-0.0 to 23.4) |
| Controlled phase of liver cancer due to hepatitis B | 12.5 (10.8 to 14.2) | -- | 0.6 (0.4 to 0.9) | 64.8* (28.6 to 108.5) | 42.2* (11.1 to 80.0) |
| Metastatic phase of liver cancer due to hepatitis B | 2.1 (1.9 to 2.4) | -- | 1.0 (0.7 to 1.3) | 19.7* (8.5 to 32.0) | 3.3 (-6.4 to 13.9) |
| Terminal phase of liver cancer due to hepatitis B | 0.9 (0.8 to 0.9) | -- | 0.5 (0.3 to 0.6) | 19.7* (8.5 to 32.0) | 3.3 (-6.4 to 13.9) |
| Liver cancer due to hepatitis C | 2.1 (1.7 to 2.5) | 1.3 (1.0 to 1.5) | 0.3 (0.2 to 0.4) | 27.3* (14.7 to 41.5) | 9.9 (-1.0 to 22.1) |
| Diagnosis and primary therapy phase of liver cancer due to hepatitis C | 0.3 (0.3 to 0.4) | 1.3 (1.0 to 1.5) | 0.1 (0.1 to 0.1) | 26.4* (14.8 to 39.2) | 9.1 (-0.9 to 20.2) |
| Controlled phase of liver cancer due to hepatitis C | 1.4 (1.2 to 1.7) | -- | 0.1 (0.0 to 0.1) | 55.4* (25.5 to 98.8) | 34.1* (8.4 to 71.6) |
| Metastatic phase of liver cancer due to hepatitis C | 0.2 (0.2 to 0.3) | -- | 0.1 (0.1 to 0.1) | 18.5* (8.3 to 29.7) | 2.3 (-6.5 to 12.0) |
| Terminal phase of liver cancer due to hepatitis C | 0.1 (0.1 to 0.1) | -- | 0.1 (0.0 to 0.1) | 18.5* (8.3 to 29.7) | 2.3 (-6.5 to 12.0) |
| Liver cancer due to alcohol use | 2.0 (1.6 to 2.5) | 1.3 (1.0 to 1.6) | 0.3 (0.2 to 0.5) | 18.9* (8.7 to 27.6) | 2.6 (-6.1 to 10.1) |
| Diagnosis and primary therapy phase of liver cancer due to alcohol use | 0.3 (0.3 to 0.4) | 1.3 (1.0 to 1.6) | 0.1 (0.1 to 0.1) | 18.3* (7.6 to 26.5) | 2.1 (-7.1 to 9.2) |
| Controlled phase of liver cancer due to alcohol use | 1.3 (1.1 to 1.7) | -- | 0.1 (0.0 to 0.1) | 32.6* (20.5 to 43.5) | 14.5* (4.0 to 23.9) |
| Metastatic phase of liver cancer due to alcohol use | 0.2 (0.2 to 0.3) | -- | 0.1 (0.1 to 0.2) | 14.5* (4.4 to 22.3) | -1.2 (-9.9 to 5.6) |

| | | | | | |
|---|---|---|---|---|---|
| Terminal phase of liver cancer due to alcohol use | 0.1 (0.1 to 0.1) | -- | 0.1 (0.0 to 0.1) | 14.5* (4.4 to 22.3) | -1.2 (-9.9 to 5.6) |
| Liver cancer due to NASH | 1.2 (0.9 to 1.5) | 0.8 (0.6 to 0.9) | 0.2 (0.1 to 0.3) | 33.3* (21.9 to 44.7) | 15.1* (5.3 to 24.9) |
| Diagnosis and primary therapy phase of liver cancer due to NASH | 0.2 (0.1 to 0.2) | 0.8 (0.6 to 0.9) | 0.1 (0.0 to 0.1) | 32.3* (20.8 to 43.2) | 14.2* (4.3 to 23.6) |
| Controlled phase of liver cancer due to NASH | 0.8 (0.6 to 1.0) | -- | 0.0 (0.0 to 0.1) | 60.8* (45.0 to 76.0) | 38.8* (25.2 to 52.0) |
| Metastatic phase of liver cancer due to NASH | 0.1 (0.1 to 0.2) | -- | 0.1 (0.0 to 0.1) | 25.3* (15.0 to 35.1) | 8.2 (-0.7 to 16.6) |
| Terminal phase of liver cancer due to NASH | 0.1 (0.0 to 0.1) | -- | -- | 25.3* (15.0 to 35.1) | 8.2 (-0.7 to 16.6) |
| Liver cancer due to other causes | 3.1 (2.4 to 3.8) | 1.9 (1.5 to 2.3) | 0.5 (0.3 to 0.7) | 32.8* (20.5 to 46.2) | 14.6* (4.0 to 26.2) |
| Diagnosis and primary therapy phase of liver cancer due to other causes | 0.5 (0.4 to 0.6) | 1.9 (1.5 to 2.3) | 0.1 (0.1 to 0.2) | 31.5* (19.4 to 44.9) | 13.5* (3.1 to 25.1) |
| Controlled phase of liver cancer due to other causes | 2.1 (1.6 to 2.6) | -- | 0.1 (0.1 to 0.2) | 67.0* (49.4 to 86.7) | 44.2* (29.0 to 61.2) |
| Metastatic phase of liver cancer due to other causes | 0.4 (0.3 to 0.4) | -- | 0.2 (0.1 to 0.2) | 22.7* (12.1 to 34.4) | 5.9 (-3.2 to 16.0) |
| Terminal phase of liver cancer due to other causes | 0.1 (0.1 to 0.2) | -- | 0.1 (0.1 to 0.1) | 22.7* (12.1 to 34.4) | 5.9 (-3.2 to 16.0) |
| Gallbladder and biliary tract cancer | 2.0 (1.7 to 2.2) | 1.3 (1.1 to 1.5) | 0.3 (0.2 to 0.5) | 17.9* (10.3 to 28.2) | 1.8 (-4.7 to 10.6) |
| Diagnosis and primary therapy phase of gallbladder and biliary tract cancer | 0.2 (0.2 to 0.3) | 1.3 (1.1 to 1.5) | 0.1 (0.0 to 0.1) | 19.2* (11.8 to 29.4) | 2.9 (-3.5 to 11.7) |
| Controlled phase of gallbladder and biliary tract cancer | 1.3 (1.1 to 1.4) | -- | 0.1 (0.0 to 0.1) | 30.3* (21.6 to 40.7) | 12.5* (5.0 to 21.5) |
| Metastatic phase of gallbladder and biliary tract cancer | 0.4 (0.3 to 0.4) | -- | 0.2 (0.1 to 0.2) | 14.4* (7.3 to 24.6) | -1.2 (-7.3 to 7.6) |
| Terminal phase of gallbladder and biliary tract cancer | 0.1 (0.1 to 0.1) | -- | 0.1 (0.0 to 0.1) | 14.4* (7.3 to 24.6) | -1.2 (-7.3 to 7.6) |
| Pancreatic cancer | 2.9 (2.8 to 3.0) | 2.4 (2.3 to 2.5) | 0.6 (0.4 to 0.8) | 24.3* (18.6 to 29.4) | 7.4* (2.4 to 11.7) |
| Diagnosis and primary therapy phase of pancreatic cancer | 0.6 (0.6 to 0.6) | 2.4 (2.3 to 2.5) | 0.2 (0.1 to 0.2) | 24.4* (18.6 to 29.5) | 7.4* (2.4 to 11.8) |
| Controlled phase of pancreatic cancer | 1.6 (1.6 to 1.7) | -- | 0.1 (0.1 to 0.1) | 22.1* (16.6 to 27.2) | 5.4* (0.6 to 9.8) |
| Metastatic phase of pancreatic cancer | 0.5 (0.5 to 0.5) | -- | 0.2 (0.2 to 0.3) | 24.9* (19.1 to 29.9) | 7.8* (2.8 to 12.2) |
| Terminal phase of pancreatic cancer | 0.2 (0.2 to 0.2) | -- | 0.1 (0.1 to 0.1) | 24.9* (19.1 to 29.9) | 7.8* (2.8 to 12.2) |
| Larynx cancer | 7.7 (7.4 to 8.2) | 1.4 (1.4 to 1.5) | 0.8 (0.6 to 1.1) | 18.7* (12.6 to 25.1) | 2.5 (-2.8 to 8.0) |
| Diagnosis and primary therapy phase of larynx cancer | 0.4 (0.4 to 0.5) | 1.4 (1.4 to 1.5) | 0.1 (0.1 to 0.2) | 23.2* (16.9 to 30.0) | 6.4* (0.9 to 12.2) |
| Controlled phase of larynx cancer | 6.3 (6.0 to 6.7) | -- | 0.4 (0.3 to 0.6) | 21.7* (15.9 to 27.7) | 5.1* (0.1 to 10.3) |
| Metastatic phase of larynx cancer | 0.5 (0.5 to 0.5) | -- | 0.2 (0.2 to 0.3) | 13.2* (6.6 to 20.2) | -2.3 (-8.0 to 3.8) |
| Terminal phase of larynx cancer | 0.1 (0.1 to 0.1) | -- | 0.0 (0.0 to 0.1) | 10.2* (3.6 to 17.3) | -4.8 (-10.6 to 1.3) |
| Laryngectomy from larynx cancer, beyond 10 years | 0.4 (0.4 to 0.4) | -- | -- | 14.0* (13.3 to 14.6) | -1.6* (-2.2 to -1.0) |
| Tracheal, bronchus, and lung cancer | 18.5 (17.6 to 19.5) | 10.4 (10.0 to 10.9) | 3.0 (2.1 to 3.9) | 26.8* (20.0 to 34.6) | 9.5* (3.6 to 16.2) |
| Diagnosis and primary therapy phase of lung, bronchus, and trachea cancer | 1.1 (1.1 to 1.2) | 10.4 (10.0 to 10.9) | 0.3 (0.2 to 0.5) | 41.2* (33.9 to 48.8) | 21.9* (15.6 to 28.5) |
| Controlled phase of lung, bronchus, and trachea cancer | 13.0 (12.3 to 13.7) | -- | 0.6 (0.4 to 0.9) | 57.0* (43.9 to 74.0) | 35.5* (24.2 to 50.3) |
| Metastatic phase of lung, bronchus, and trachea cancer | 3.6 (3.5 to 3.8) | -- | 1.6 (1.1 to 2.2) | 17.8* (11.9 to 23.9) | 1.7 (-3.4 to 7.0) |
| Terminal phase of lung, bronchus, and trachea cancer | 0.8 (0.8 to 0.8) | -- | 0.4 (0.3 to 0.6) | 17.9* (11.9 to 23.9) | 1.7 (-3.4 to 7.0) |
| Malignant skin melanoma | 138.2 (118.2 to 186.1) | 15.9 (13.7 to 21.4) | 7.9 (5.0 to 11.8) | 26.2* (15.6 to 39.5) | 8.9 (-0.2 to 20.4) |
| Diagnosis and primary therapy phase of malignant skin melanoma | 3.6 (3.1 to 4.9) | 15.9 (13.7 to 21.4) | 1.0 (0.7 to 1.6) | 26.8* (18.0 to 39.1) | 9.5* (1.9 to 20.1) |
| Controlled phase of malignant skin melanoma | 133.5 (114.2 to 179.8) | -- | 6.3 (3.8 to 9.9) | 27.1* (15.3 to 41.7) | 9.7 (-0.4 to 22.4) |
| Metastatic phase of malignant skin melanoma | 0.9 (0.8 to 1.2) | -- | 0.4 (0.3 to 0.6) | 15.0* (8.3 to 24.1) | -0.7 (-6.5 to 7.2) |
| Terminal phase of malignant skin melanoma | 0.2 (0.1 to 0.2) | -- | 0.1 (0.1 to 0.1) | 13.0* (6.2 to 22.1) | -2.4 (-8.3 to 5.4) |
| Non-melanoma skin cancer | 2.6 (1.1 to 5.3) | 100.6 (42.8 to 202.5) | 0.3 (0.1 to 0.6) | 29.2* (20.7 to 37.5) | 11.6* (4.2 to 18.7) |
| Non-melanoma skin cancer (squamous-cell carcinoma) | 5.5 (2.4 to 11.5) | 5.5 (2.6 to 10.3) | 0.3 (0.1 to 0.5) | 29.9* (20.0 to 39.0) | 12.2* (3.6 to 20.0) |
| Non-melanoma skin cancer (basal-cell carcinoma) | 9.0 (3.4 to 18.2) | 95.1 (37.8 to 196.8) | 0.0 (0.0 to 0.1) | 24.9* (16.1 to 30.4) | 7.8* (0.2 to 12.6) |
| Breast cancer | 502.0 (464.4 to 538.0) | 60.6 (56.2 to 65.2) | 40.5 (27.4 to 56.1) | 34.6* (23.5 to 45.0) | 16.2* (6.6 to 25.2) |
| Diagnosis and primary therapy phase of breast cancer | 13.9 (12.8 to 14.9) | 60.6 (56.2 to 65.2) | 4.0 (2.7 to 5.5) | 38.2* (27.9 to 47.4) | 19.3* (10.4 to 27.3) |
| Controlled phase of breast cancer | 468.0 (432.4 to 501.6) | -- | 29.0 (18.4 to 42.7) | 35.4* (24.2 to 46.7) | 16.9* (7.2 to 26.7) |
| Metastatic phase of breast cancer | 15.1 (14.0 to 16.2) | -- | 6.7 (4.6 to 9.1) | 30.9* (17.4 to 45.6) | 13.0* (1.3 to 25.7) |
| Terminal phase of breast cancer | 1.1 (1.0 to 1.2) | -- | 0.6 (0.4 to 0.7) | 25.2* (14.6 to 35.8) | 8.1 (-1.1 to 17.2) |
| Mastectomy from breast cancer, beyond 10 years | 4.1 (3.7 to 4.4) | -- | 0.1 (0.1 to 0.2) | 9.3* (8.2 to 10.4) | -5.7* (-6.6 to -4.7) |
| Cervical cancer | 432.4 (389.0 to 463.9) | 55.5 (50.0 to 59.6) | 31.0 (21.5 to 41.7) | 11.8* (3.1 to 20.1) | -3.5 (-11.0 to 3.7) |
| Diagnosis and primary therapy phase of cervical cancer | 21.9 (19.8 to 23.6) | 55.5 (50.0 to 59.6) | 6.1 (4.1 to 8.5) | 12.6 (-0.0 to 26.5) | -2.8 (-13.7 to 9.2) |
| Controlled phase of cervical cancer | 396.9 (357.0 to 425.8) | -- | 18.7 (11.9 to 27.5) | 13.7* (5.3 to 23.1) | -1.9 (-9.1 to 6.2) |
| Metastatic phase of cervical cancer | 12.2 (11.0 to 13.2) | -- | 5.4 (3.6 to 7.2) | 5.8 (-4.2 to 16.1) | -8.6 (-17.3 to 0.3) |
| Terminal phase of cervical cancer | 1.3 (1.2 to 1.5) | -- | 0.7 (0.5 to 0.9) | 3.9 (-4.5 to 12.6) | -10.3* (-17.6 to -2.8) |
| Uterine cancer | 45.1 (42.5 to 48.1) | 5.5 (5.2 to 5.9) | 3.0 (2.1 to 4.2) | 30.4* (17.5 to 45.1) | 12.6* (1.4 to 25.3) |
| Diagnosis and primary therapy phase of uterine cancer | 2.0 (1.9 to 2.1) | 5.5 (5.2 to 5.9) | 0.6 (0.4 to 0.8) | 31.6* (23.6 to 40.8) | 13.6* (6.8 to 21.6) |
| Controlled phase of uterine cancer | 42.1 (39.7 to 44.9) | -- | 2.0 (1.3 to 3.0) | 32.0* (14.6 to 52.1) | 13.9 (-1.0 to 31.4) |
| Metastatic phase of uterine cancer | 0.9 (0.8 to 0.9) | -- | 0.4 (0.3 to 0.5) | 23.0* (15.2 to 31.8) | 6.2 (-0.5 to 13.8) |
| Terminal phase of uterine cancer | 0.1 (0.1 to 0.1) | -- | 0.0 (0.0 to 0.1) | 17.4* (9.5 to 26.7) | 1.3 (-5.4 to 9.4) |
| Ovarian cancer | 75.8 (70.9 to 81.9) | 11.2 (10.4 to 12.1) | 9.0 (6.4 to 11.9) | 25.7* (11.5 to 42.4) | 8.5 (-3.8 to 22.9) |
| Diagnosis and primary therapy phase of ovarian cancer | 2.8 (2.6 to 3.0) | 11.2 (10.4 to 12.1) | 0.8 (0.5 to 1.1) | 27.2* (17.6 to 39.5) | 9.8* (1.5 to 20.4) |
| Controlled phase of ovarian cancer | 61.1 (57.1 to 66.0) | -- | 2.9 (1.8 to 4.3) | 27.1* (11.5 to 45.2) | 9.7 (-3.7 to 25.3) |
| Metastatic phase of ovarian cancer | 11.5 (10.7 to 12.4) | -- | 5.1 (3.4 to 7.0) | 24.7* (4.4 to 48.3) | 7.7 (-9.8 to 28.0) |

| | | | | | |
|---|---|---|---|---|---|
| Terminal phase of ovarian cancer | 0.5 (0.4 to 0.5) | -- | 0.3 (0.2 to 0.3) | 24.2* (14.6 to 37.4) | 7.2 (-1.1 to 18.7) |
| Prostate cancer | 20.4 (17.3 to 24.6) | 2.2 (1.9 to 2.7) | 1.6 (1.0 to 2.4) | 48.4* (40.6 to 58.7) | 28.1* (21.4 to 37.0) |
| Diagnosis and primary therapy phase of prostate cancer | 0.7 (0.6 to 0.8) | 2.2 (1.9 to 2.7) | 0.2 (0.1 to 0.3) | 52.0* (45.0 to 63.1) | 31.2* (25.2 to 40.8) |
| Controlled phase of prostate cancer | 19.0 (16.1 to 23.1) | -- | 1.3 (0.8 to 2.0) | 49.9* (41.3 to 61.0) | 29.4* (22.0 to 39.0) |
| Metastatic phase of prostate cancer | 0.2 (0.1 to 0.2) | -- | 0.1 (0.0 to 0.1) | 31.7* (25.7 to 40.0) | 13.7* (8.5 to 20.9) |
| Terminal phase of prostate cancer | -- | -- | -- | 24.2* (18.8 to 30.7) | 7.2* (2.5 to 12.8) |
| Impotence and Incontinence after 10-year survival from prostate cancer | 0.5 (0.5 to 0.5) | -- | -- | 13.2* (12.5 to 13.9) | -2.3* (-2.9 to -1.7) |
| Testicular cancer | 120.1 (112.4 to 128.7) | 14.3 (13.4 to 15.2) | 7.3 (4.8 to 10.1) | 29.8* (18.8 to 42.6) | 12.0* (2.6 to 23.1) |
| Diagnosis and primary therapy phase of testicular cancer | 3.9 (3.7 to 4.2) | 14.3 (13.4 to 15.2) | 1.1 (0.7 to 1.6) | 31.4* (16.3 to 49.7) | 13.4* (0.4 to 29.3) |
| Controlled phase of testicular cancer | 114.7 (107.3 to 122.9) | -- | 5.5 (3.4 to 7.9) | 32.0* (19.8 to 46.8) | 14.0* (3.4 to 26.7) |
| Metastatic phase of testicular cancer | 1.4 (1.3 to 1.4) | -- | 0.6 (0.4 to 0.8) | 13.7* (6.3 to 22.1) | -1.9 (-8.2 to 5.4) |
| Terminal phase of testicular cancer | 0.2 (0.1 to 0.2) | -- | 0.1 (0.1 to 0.1) | 4.1 (-3.2 to 12.5) | -10.1* (-16.5 to -2.8) |
| Kidney cancer | 45.6 (42.3 to 48.1) | 6.6 (6.1 to 7.0) | 2.7 (1.8 to 3.9) | 23.5* (14.6 to 34.6) | 6.6 (-1.0 to 16.2) |
| Diagnosis and primary therapy phase of kidney cancer | -- | 6.6 (6.1 to 7.0) | -- | -- | -- |
| Controlled phase of kidney cancer | 44.4 (41.2 to 46.9) | -- | 2.1 (1.3 to 3.2) | 23.7* (14.2 to 36.1) | 6.8 (-1.4 to 17.5) |
| Metastatic phase of kidney cancer | 1.0 (0.9 to 1.1) | -- | 0.5 (0.3 to 0.6) | 22.4* (15.3 to 30.8) | 5.7 (-0.4 to 12.9) |
| Terminal phase of kidney cancer | 0.2 (0.2 to 0.2) | -- | 0.1 (0.1 to 0.1) | 22.4* (15.4 to 30.8) | 5.7 (-0.4 to 12.9) |
| Bladder cancer | 31.2 (28.0 to 34.1) | 4.0 (3.6 to 4.3) | 2.7 (1.9 to 3.8) | 31.0* (20.5 to 45.2) | 13.1* (4.0 to 25.4) |
| Diagnosis and primary therapy phase of bladder cancer | 1.6 (1.4 to 1.8) | 4.0 (3.6 to 4.3) | 0.5 (0.3 to 0.7) | 35.2* (25.4 to 47.6) | 16.7* (8.2 to 27.4) |
| Controlled phase of bladder cancer | 28.6 (25.7 to 31.3) | -- | 1.9 (1.2 to 2.8) | 31.2* (19.1 to 46.9) | 13.3* (2.8 to 26.9) |
| Metastatic phase of bladder cancer | 0.5 (0.5 to 0.6) | -- | 0.2 (0.2 to 0.3) | 25.4* (16.0 to 37.5) | 8.3* (0.2 to 18.7) |
| Terminal phase of bladder cancer | 0.1 (0.1 to 0.1) | -- | 0.1 (0.0 to 0.1) | 24.5* (15.1 to 36.2) | 7.5 (-0.6 to 17.6) |
| Incontinence from bladder cancer, beyond 10 years | 0.3 (0.3 to 0.4) | -- | 0.0 (0.0 to 0.1) | 18.0* (17.0 to 19.2) | 1.9* (1.0 to 2.9) |
| Brain and nervous system cancer | 129.5 (111.9 to 148.7) | 24.2 (20.9 to 27.3) | 11.5 (8.0 to 15.5) | 74.3* (58.1 to 91.6) | 50.5* (36.5 to 65.4) |
| Diagnosis and primary therapy phase of brain and nervous system cancers | 7.7 (6.7 to 8.9) | 24.2 (20.9 to 27.3) | 2.2 (1.4 to 3.2) | 90.4* (73.0 to 109.7) | 64.4* (49.3 to 81.0) |
| Controlled phase of brain and nervous system cancers | 113.5 (98.1 to 130.7) | -- | 5.4 (3.4 to 8.1) | 98.3* (74.8 to 124.7) | 71.2* (50.9 to 94.0) |
| Metastatic phase of brain and nervous system cancers | 7.1 (6.1 to 8.0) | -- | 3.2 (2.1 to 4.3) | 43.9* (31.9 to 56.0) | 24.2* (13.9 to 34.7) |
| Terminal phase of brain and nervous system cancers | 1.2 (1.0 to 1.3) | -- | 0.6 (0.4 to 0.8) | 37.5* (26.4 to 48.6) | 18.7* (9.1 to 28.3) |
| Thyroid cancer | 161.0 (147.5 to 179.7) | 17.8 (16.3 to 19.9) | 9.1 (6.0 to 13.1) | 40.0* (26.3 to 55.6) | 20.8* (9.1 to 34.4) |
| Diagnosis and primary therapy phase of thyroid cancer | 4.3 (3.9 to 4.8) | 17.8 (16.3 to 19.9) | 1.2 (0.8 to 1.7) | 40.9* (22.7 to 60.4) | 21.7* (5.9 to 38.5) |
| Controlled phase of thyroid cancer | 155.5 (142.5 to 173.6) | -- | 7.4 (4.6 to 11.0) | 40.8* (26.0 to 57.8) | 21.5* (8.8 to 36.2) |
| Metastatic phase of thyroid cancer | 1.1 (1.0 to 1.2) | -- | 0.5 (0.3 to 0.7) | 28.3* (18.3 to 40.8) | 10.7* (2.1 to 21.6) |
| Terminal phase of thyroid cancer | 0.1 (0.1 to 0.1) | -- | 0.1 (0.0 to 0.1) | 26.2* (16.3 to 39.0) | 9.0* (0.4 to 20.0) |
| Mesothelioma | 1.1 (1.0 to 1.2) | 0.5 (0.4 to 0.5) | 0.2 (0.1 to 0.3) | 10.5 (-2.6 to 21.4) | -4.6 (-15.9 to 4.8) |
| Diagnosis and primary therapy phase of mesothelioma | 0.1 (0.1 to 0.1) | 0.5 (0.4 to 0.5) | -- | 12.3 (-0.3 to 22.9) | -3.1 (-13.9 to 6.1) |
| Controlled phase of mesothelioma | 0.7 (0.6 to 0.8) | -- | 0.0 (0.0 to 0.1) | 10.1 (-3.2 to 21.1) | -5.0 (-16.4 to 4.6) |
| Metastatic phase of mesothelioma | 0.3 (0.2 to 0.3) | -- | 0.1 (0.1 to 0.2) | 10.3 (-2.9 to 21.4) | -4.7 (-16.2 to 4.8) |
| Terminal phase of mesothelioma | -- | -- | -- | 9.4 (-4.0 to 20.6) | -5.6 (-17.1 to 4.1) |
| Hodgkin lymphoma | 80.1 (69.3 to 100.9) | 11.0 (9.5 to 13.7) | 5.7 (3.9 to 8.1) | 28.2* (17.3 to 39.5) | 10.7* (1.3 to 20.5) |
| Diagnosis and primary therapy phase of Hodgkin disease | 2.7 (2.3 to 3.3) | 11.0 (9.5 to 13.7) | 0.8 (0.5 to 1.1) | 32.7* (22.0 to 43.5) | 14.6* (5.3 to 23.9) |
| Controlled phase of Hodgkin disease | 74.4 (64.4 to 93.7) | -- | 3.5 (2.2 to 5.4) | 32.5* (18.5 to 48.6) | 14.4* (2.3 to 28.3) |
| Metastatic phase of Hodgkin disease | 2.8 (2.4 to 3.5) | -- | 1.3 (0.8 to 1.8) | 17.6* (9.4 to 25.9) | 1.6 (-5.5 to 8.7) |
| Terminal phase of Hodgkin disease | 0.3 (0.2 to 0.3) | -- | 0.1 (0.1 to 0.2) | 6.6 (-0.5 to 13.4) | -8.0* (-14.1 to -2.1) |
| Non-Hodgkin's lymphoma | 88.5 (84.7 to 92.4) | 15.6 (15.0 to 16.3) | 6.8 (4.8 to 9.2) | 44.7* (35.2 to 54.9) | 24.9* (16.7 to 33.7) |
| Diagnosis and primary therapy phase of non-Hodgkin lymphoma | 3.2 (3.0 to 3.3) | 15.6 (15.0 to 16.3) | 0.9 (0.6 to 1.3) | 51.1* (44.0 to 59.1) | 30.4* (24.3 to 37.4) |
| Controlled phase of non-Hodgkin lymphoma | 81.0 (77.5 to 84.6) | -- | 3.9 (2.4 to 5.7) | 50.9* (37.6 to 66.1) | 30.3* (18.8 to 43.4) |
| Metastatic phase of non-Hodgkin lymphoma | 3.7 (3.5 to 3.9) | -- | 1.7 (1.1 to 2.2) | 32.1* (25.9 to 39.0) | 14.1* (8.7 to 20.0) |
| Terminal phase of non-Hodgkin lymphoma | 0.6 (0.6 to 0.7) | -- | 0.3 (0.2 to 0.4) | 29.7* (23.2 to 36.7) | 12.0* (6.4 to 18.0) |
| Multiple myeloma | 5.3 (4.7 to 5.9) | 1.3 (1.2 to 1.5) | 1.0 (0.7 to 1.4) | 54.6* (41.1 to 65.9) | 33.4* (21.8 to 43.2) |
| Diagnosis and primary therapy phase of multiple myeloma | 0.3 (0.3 to 0.4) | 1.3 (1.2 to 1.5) | 0.1 (0.1 to 0.1) | 61.0* (47.4 to 73.2) | 39.0* (27.3 to 49.5) |
| Controlled phase of multiple myeloma | 3.3 (2.9 to 3.6) | -- | 0.2 (0.1 to 0.2) | 61.2* (47.6 to 73.4) | 39.2* (27.4 to 49.7) |
| Metastatic phase of multiple myeloma | 1.6 (1.4 to 1.8) | -- | 0.7 (0.5 to 1.0) | 53.6* (40.3 to 64.8) | 32.6* (21.1 to 42.3) |
| Terminal phase of multiple myeloma | 0.1 (0.1 to 0.1) | -- | 0.0 (0.0 to 0.1) | 37.4* (25.7 to 47.7) | 18.6* (8.5 to 27.5) |
| Leukaemia | 72.6 (61.5 to 77.8) | 18.6 (15.9 to 19.7) | 8.4 (6.0 to 11.3) | 26.6* (18.8 to 34.6) | 9.3* (2.6 to 16.2) |
| Acute lymphoid leukaemia | 12.9 (10.8 to 14.0) | 3.0 (2.5 to 3.2) | 1.4 (1.0 to 2.0) | 31.4* (18.6 to 43.3) | 13.5* (2.4 to 23.7) |
| Diagnosis and primary therapy phase of acute lymphoid leukemia | 1.3 (1.1 to 1.4) | 3.0 (2.5 to 3.2) | 0.4 (0.3 to 0.5) | 22.1* (13.1 to 30.9) | 5.4 (-2.4 to 13.0) |
| Controlled phase of acute lymphoid leukemia | 10.4 (8.6 to 11.3) | -- | 0.5 (0.3 to 0.8) | 34.4* (20.6 to 46.9) | 16.1* (4.1 to 26.8) |
| Metastatic phase of acute lymphoid leukemia | 1.0 (0.8 to 1.1) | -- | 0.5 (0.3 to 0.6) | 35.8* (20.8 to 49.5) | 17.2* (4.3 to 29.1) |
| Terminal phase of acute lymphoid leukemia | 0.2 (0.2 to 0.2) | -- | 0.1 (0.1 to 0.1) | 36.0* (21.0 to 49.8) | 17.4* (4.5 to 29.3) |
| Chronic lymphoid leukaemia | 14.8 (12.1 to 16.8) | 1.9 (1.6 to 2.2) | 1.7 (1.2 to 2.4) | 134.2* (99.6 to 170.9) | 102.2* (72.3 to 133.9) |

| | | | | | |
|---|---|---|---|---|---|
| Diagnosis and primary therapy phase of chronic lymphoid leukemia | 0.8 (0.7 to 0.9) | 1.9 (1.6 to 2.2) | 0.2 (0.2 to 0.3) | 137.4* (107.0 to 171.4) | 105.0* (78.7 to 134.3) |
| Controlled phase of chronic lymphoid leukemia | 12.0 (9.9 to 13.7) | -- | 0.6 (0.4 to 0.9) | 136.4* (96.6 to 182.1) | 104.1* (69.7 to 143.6) |
| Metastatic phase of chronic lymphoid leukemia | 1.9 (1.6 to 2.2) | -- | 0.9 (0.6 to 1.2) | 133.1* (100.1 to 170.9) | 101.3* (72.8 to 133.9) |
| Terminal phase of chronic lymphoid leukemia | 0.1 (0.0 to 0.1) | -- | -- | 105.1* (76.6 to 137.6) | 77.1* (52.5 to 105.1) |
| Acute myeloid leukaemia | 7.1 (6.3 to 7.9) | 5.1 (4.5 to 5.6) | 1.3 (0.9 to 1.7) | 18.5* (11.2 to 25.3) | 2.3 (-4.0 to 8.2) |
| Diagnosis and primary therapy phase of acute myeloid leukemia | -- | 5.1 (4.5 to 5.6) | -- | -- | -- |
| Controlled phase of acute myeloid leukemia | 4.9 (4.4 to 5.5) | -- | 0.2 (0.1 to 0.4) | 18.9* (11.4 to 26.7) | 2.7 (-3.9 to 9.4) |
| Metastatic phase of acute myeloid leukemia | 1.8 (1.6 to 2.0) | -- | 0.8 (0.5 to 1.1) | 18.4* (10.8 to 25.3) | 2.3 (-4.3 to 8.1) |
| Terminal phase of acute myeloid leukemia | 0.4 (0.3 to 0.4) | -- | 0.2 (0.1 to 0.3) | 18.4* (10.8 to 25.3) | 2.3 (-4.3 to 8.1) |
| Chronic myeloid leukaemia | 7.0 (6.1 to 7.6) | 2.0 (1.8 to 2.2) | 0.7 (0.5 to 1.0) | 4.9 (-1.5 to 12.0) | -9.5* (-15.0 to -3.3) |
| Diagnosis and primary therapy phase of chronic myeloid leukemia | 0.5 (0.4 to 0.5) | 2.0 (1.8 to 2.2) | 0.1 (0.1 to 0.2) | 8.1* (1.6 to 15.2) | -6.6* (-12.3 to -0.5) |
| Controlled phase of chronic myeloid leukemia | 5.8 (5.1 to 6.3) | -- | 0.3 (0.2 to 0.4) | 8.9* (2.3 to 16.0) | -6.0 (-11.7 to 0.1) |
| Metastatic phase of chronic myeloid leukemia | 0.6 (0.5 to 0.6) | -- | 0.3 (0.2 to 0.3) | 0.3 (-6.4 to 7.4) | -13.4* (-19.2 to -7.3) |
| Terminal phase of chronic myeloid leukemia | 0.1 (0.1 to 0.1) | -- | 0.1 (0.0 to 0.1) | 0.3 (-6.4 to 7.4) | -13.4* (-19.2 to -7.3) |
| Other leukaemia | 30.9 (24.4 to 33.7) | 6.5 (5.1 to 7.1) | 3.3 (2.2 to 4.4) | 6.9 (-1.6 to 17.4) | -7.7 (-15.0 to 1.3) |
| Metastatic phase of other leukemia | 4.0 (3.2 to 4.4) | -- | 1.8 (1.2 to 2.5) | 5.3 (-1.7 to 13.8) | -9.1* (-15.2 to -1.8) |
| Diagnosis and primary therapy phase of other leukemia | -- | 6.5 (5.1 to 7.1) | -- | -- | -- |
| Terminal phase of other leukemia | 0.3 (0.2 to 0.3) | -- | 0.2 (0.1 to 0.2) | 8.5* (0.2 to 18.1) | -6.3 (-13.5 to 2.0) |
| Controlled phase of other leukemia | 26.6 (20.9 to 29.0) | -- | 1.3 (0.8 to 1.9) | 9.1 (-4.3 to 25.6) | -5.8 (-17.4 to 8.4) |
| Other malignant cancers | 714.2 (511.4 to 1034.8) | 28.0 (25.5 to 30.0) | 51.3 (32.0 to 80.0) | 238.5* (145.8 to 383.4) | 192.2* (112.2 to 317.4) |
| Diagnosis and primary therapy phase of other malignant neoplasms | 16.1 (11.5 to 23.4) | 28.0 (25.5 to 30.0) | 4.5 (2.7 to 7.3) | 259.7* (146.7 to 428.9) | 210.6* (113.0 to 356.7) |
| Controlled phase of other malignant neoplasms | 664.0 (474.7 to 961.3) | -- | 31.5 (17.8 to 52.6) | 283.6* (173.3 to 464.3) | 231.2* (135.9 to 387.1) |
| Metastatic phase of other malignant neoplasms | 30.0 (22.1 to 41.8) | -- | 13.0 (8.0 to 19.8) | 169.6* (93.8 to 288.6) | 132.8* (67.3 to 235.5) |
| Terminal phase of other malignant neoplasms | 4.1 (3.1 to 5.7) | -- | 2.2 (1.4 to 3.3) | 161.5* (95.0 to 266.0) | 125.8* (68.3 to 216.0) |
| Other neoplasms | 1212.6 (1142.6 to 1298.0) | 565.9 (533.0 to 597.6) | 1.6 (1.0 to 2.4) | 18.6* (10.5 to 27.2) | 2.4 (-4.6 to 9.8) |
| Myelodysplastic, myeloproliferative, and other hematopoietic neoplasms | 32.5 (26.9 to 39.8) | 2.5 (1.7 to 3.6) | 1.6 (1.0 to 2.4) | 18.6* (10.5 to 27.2) | 2.4 (-4.6 to 9.8) |
| Benign and in situ intestinal neoplasms | 66.6 (61.8 to 71.0) | 28.5 (26.8 to 30.4) | -- | -- | -- |
| Benign and in situ cervical and uterine neoplasms | 194.0 (183.0 to 205.4) | 84.8 (80.3 to 89.6) | -- | -- | -- |
| Other benign and in situ neoplasms | 960.0 (890.7 to 1040.6) | 450.1 (418.6 to 481.4) | -- | -- | -- |
| **Cardiovascular diseases** | **10069.3 (9362.0 to 10784.4)** | **957.2 (888.1 to 1027.8)** | **777.3 (566.0 to 1020.3)** | **11.2* (8.7 to 13.5)** | **-4.0* (-6.2 to -2.0)** |
| Rheumatic heart disease | 4495.5 (4325.2 to 4673.7) | 74.8 (69.9 to 79.3) | 214.5 (139.1 to 313.2) | 16.5* (14.9 to 18.1) | 0.6 (-0.8 to 2.0) |
| Rheumatic heart disease, without heart failure | 4461.1 (4287.6 to 4639.5) | 74.8 (69.9 to 79.3) | 211.3 (136.7 to 308.9) | 16.5* (14.9 to 18.1) | 0.6 (-0.8 to 2.0) |
| Heart failure due to rheumatic heart disease | 34.4 (23.3 to 47.4) | -- | 3.2 (1.8 to 5.1) | 16.9* (15.1 to 18.7) | 0.9 (-0.6 to 2.5) |
| Ischaemic heart disease | 1331.9 (1212.3 to 1458.4) | 87.4 (57.3 to 122.0) | 72.1 (49.1 to 102.2) | 12.4* (8.3 to 16.5) | -3.0 (-6.5 to 0.6) |
| Asymptomatic ischemic heart disease following myocardial infarction | 131.0 (81.0 to 189.2) | -- | -- | -- | -- |
| Angina due to ischemic heart disease | 1108.5 (1003.0 to 1219.3) | -- | 63.6 (42.2 to 90.0) | 11.7* (7.2 to 16.3) | -3.6 (-7.4 to 0.4) |
| Heart failure due to ischemic heart disease | 87.9 (60.9 to 117.2) | -- | 8.1 (4.8 to 12.2) | 18.1* (14.7 to 21.7) | 2.0 (-1.0 to 5.0) |
| Myocardial infarction due to ischemic heart disease | 4.5 (2.9 to 6.4) | 87.4 (57.3 to 122.0) | 0.4 (0.3 to 0.7) | 11.8* (9.5 to 13.3) | -3.5* (-5.5 to -2.1) |
| Stroke | 1925.3 (1728.4 to 2128.9) | 146.1‡ (115.6 to 181.5) | 332.3 (234.8 to 432.3) | 6.2* (1.3 to 11.0) | -8.3* (-12.5 to -4.2) |
| Ischaemic stroke | 1219.1 (1059.3 to 1382.3) | 62.9 (38.8 to 94.7) | 184.3 (128.7 to 243.4) | 10.6* (3.4 to 17.7) | -4.5 (-10.7 to 1.6) |
| Acute ischemic stroke | 4.8 (2.8 to 7.4) | 62.9 (38.8 to 94.7) | 1.3 (0.7 to 2.2) | 20.1* (16.4 to 22.9) | 3.7* (0.5 to 6.1) |
| Chronic ischemic stroke | 1214.2 (1055.6 to 1378.6) | -- | 183.0 (127.7 to 241.9) | 10.6* (3.3 to 17.7) | -4.5 (-10.8 to 1.6) |
| Intracerebral hemorrhage | 678.1 (570.9 to 805.4) | 49.8 (37.7 to 63.6) | 106.6 (74.3 to 145.7) | -2.9 (-12.0 to 6.4) | -16.2* (-24.1 to -8.1) |
| Acute intracerebral hemorrhage | 3.1 (2.3 to 4.1) | 49.8 (37.7 to 63.6) | 0.8 (0.5 to 1.2) | -19.0* (-29.0 to -9.9) | -30.1* (-38.7 to -22.2) |
| Chronic intracerebral hemorrhage | 675.0 (568.6 to 802.3) | -- | 105.8 (73.8 to 144.8) | -2.8 (-12.0 to 6.6) | -16.1* (-24.0 to -7.9) |
| Subarachnoid hemorrhage | 263.0 (219.0 to 309.6) | 33.4 (25.8 to 42.1) | 41.4 (28.1 to 55.1) | 13.5* (7.4 to 20.6) | -2.0 (-7.3 to 4.1) |
| Acute subarachnoid hemorrhage | 2.1 (1.6 to 2.7) | 33.4 (25.8 to 42.1) | 0.6 (0.4 to 0.8) | 12.8* (5.3 to 16.2) | -2.6 (-9.1 to 0.3) |
| Chronic subarachnoid hemorrhage | 260.9 (217.2 to 307.8) | -- | 40.8 (27.7 to 54.3) | 13.5* (7.2 to 20.7) | -2.0 (-7.4 to 4.2) |
| Hypertensive heart disease | 243.8 (173.9 to 323.0) | -- | 21.8 (13.6 to 33.5) | 14.5* (7.2 to 21.4) | -1.1 (-7.5 to 4.8) |
| Non-rheumatic valvular heart disease | 392.3 (370.6 to 412.9) | 426.3 (407.7 to 446.8) | 0.5 (0.2 to 1.2) | 11.3* (7.0 to 15.1) | -3.9* (-7.6 to -0.6) |
| Non-rheumatic calcific aortic valve disease | 83.9 (70.1 to 99.4) | 83.9 (70.1 to 99.4) | 0.1 (0.0 to 0.2) | 9.4* (2.6 to 15.3) | -5.6* (-11.4 to -0.4) |
| Heart failure due to calcific aortic valve disease | 0.5 (0.1 to 1.3) | -- | 0.0 (0.0 to 0.1) | 2.7 (-16.8 to 25.0) | -11.3 (-28.2 to 7.9) |
| Non-rheumatic calcific aortic valve disease | 83.4 (69.9 to 98.5) | 83.9 (70.1 to 99.4) | 0.0 (0.0 to 0.1) | 18.9* (2.8 to 36.9) | 2.7 (-11.2 to 18.2) |
| Non-rheumatic degenerative mitral valve disease | 342.5 (329.7 to 355.7) | 342.5 (329.7 to 355.7) | 0.4 (0.1 to 0.9) | 11.7* (6.3 to 17.0) | -3.6 (-8.2 to 1.0) |
| Heart failure due to degenerative mitral valve disease | 2.8 (0.8 to 7.2) | -- | 0.3 (0.1 to 0.7) | 4.8 (-8.9 to 20.4) | -9.5 (-21.3 to 3.9) |
| Non-rheumatic degenerative mitral valve disease | 339.7 (326.3 to 353.7) | 342.5 (329.7 to 355.7) | 0.1 (0.0 to 0.3) | 31.5* (8.9 to 56.8) | 13.5 (-6.0 to 35.4) |
| Other non-rheumatic valve diseases | 0.4 (0.3 to 0.6) | -- | 0.0 (0.0 to 0.1) | 11.4* (9.8 to 13.0) | -3.9* (-5.2 to -2.5) |
| Mild heart failure due to other non-rheumatic valve disease | 0.1 (0.0 to 0.1) | -- | -- | 11.4* (9.8 to 12.9) | -3.9* (-5.2 to -2.5) |
| Moderate heart failure due to other non-rheumatic valve disease | 0.1 (0.0 to 0.1) | -- | -- | 11.4* (9.8 to 12.9) | -3.9* (-5.2 to -2.5) |
| Severe heart failure due to other non-rheumatic valve disease | 0.1 (0.1 to 0.2) | -- | -- | 11.4* (9.8 to 13.0) | -3.9* (-5.2 to -2.5) |

| | | | | | |
|---|---|---|---|---|---|
| Controlled, medically managed heart failure due to other non-rheumatic valve disease | 0.2 (0.1 to 0.2) | -- | -- | 11.4* (9.8 to 12.9) | -3.9* (-5.2 to -2.5) |
| Cardiomyopathy and myocarditis | 46.8 (34.8 to 60.9) | 156.2 (110.8 to 207.6) | 19.3 (12.0 to 29.6) | 13.2* (10.0 to 15.9) | -2.3 (-5.0 to 0.0) |
| Myocarditis | 86.0 (66.9 to 107.4) | 156.2 (110.8 to 207.6) | 6.3 (4.0 to 9.7) | 11.6* (8.7 to 14.5) | -3.7* (-6.1 to -1.1) |
| Acute myocarditis | 38.5 (26.9 to 52.0) | 156.2 (110.8 to 207.6) | 1.9 (1.1 to 3.1) | 14.2* (11.6 to 16.7) | -1.4 (-3.6 to 0.7) |
| Heart failure due to myocarditis | 47.6 (33.0 to 64.7) | -- | 4.4 (2.6 to 6.9) | 10.4* (6.8 to 14.4) | -4.7* (-7.8 to -1.3) |
| Alcoholic cardiomyopathy | 47.3 (34.0 to 61.7) | -- | 4.3 (2.6 to 6.5) | 11.0* (3.8 to 18.4) | -4.2 (-10.3 to 2.2) |
| Other cardiomyopathy | 95.3 (66.5 to 128.8) | -- | 8.7 (5.1 to 13.7) | 15.5* (10.5 to 20.2) | -0.3 (-4.6 to 3.8) |
| Atrial fibrillation and flutter | 404.1 (316.0 to 506.2) | 18.9 (12.6 to 25.3) | 34.2 (21.2 to 51.0) | 15.1* (7.2 to 23.8) | -0.7 (-7.4 to 6.9) |
| Asymptomatic atrial fibrillation and flutter | 242.4 (189.9 to 303.1) | 18.9 (12.6 to 25.3) | -- | -- | -- |
| Symptomatic atrial fibrillation and flutter | 161.6 (124.8 to 202.5) | -- | 34.2 (21.2 to 51.0) | 15.1* (7.2 to 23.8) | -0.7 (-7.4 to 6.9) |
| Peripheral vascular disease | -- | -- | -- | -- | -- |
| Asymptomatic peripheral arterial disease | -- | -- | -- | -- | -- |
| Symptomatic claudication due to peripheral arterial disease | -- | -- | -- | -- | -- |
| Endocarditis | 13.9 (9.9 to 18.5) | 47.4 (33.4 to 63.2) | 1.2 (0.7 to 1.8) | 14.0* (12.4 to 15.6) | -1.6* (-3.0 to -0.2) |
| Acute endocarditis | 3.7 (2.5 to 5.0) | 47.4 (33.4 to 63.2) | 0.2 (0.1 to 0.3) | 19.2* (15.8 to 21.8) | 2.9* (0.0 to 5.2) |
| Heart failure due to endocarditis | 10.3 (6.6 to 14.6) | -- | 1.0 (0.5 to 1.5) | 12.9* (11.3 to 14.5) | -2.5* (-3.9 to -1.1) |
| Other cardiovascular and circulatory diseases | 1517.0 (982.2 to 2183.5) | -- | 81.5 (44.6 to 130.3) | 14.9* (10.9 to 19.2) | -0.8 (-4.3 to 2.9) |
| Heart failure due to other cardiovascular diseases | 8.9 (5.7 to 12.8) | -- | 0.8 (0.4 to 1.3) | 15.2* (13.7 to 16.7) | -0.5 (-1.8 to 0.7) |
| Other cardiovascular and circulatory disease episodes | 1508.0 (976.2 to 2171.2) | -- | 80.6 (44.1 to 128.9) | 14.9* (10.9 to 19.2) | -0.8 (-4.3 to 2.9) |
| **Chronic respiratory diseases** | **22811.7 (19625.5 to 26423.1)** | **1875.9 (1430.5 to 2455.0)** | **1826.0 (1419.5 to 2300.0)** | **14.1* (9.0 to 19.6)** | **-1.5 (-5.9 to 3.3)** |
| Chronic obstructive pulmonary disease | 9156.4 (7393.6 to 11262.3) | 566.8 (371.1 to 793.1) | 1011.7 (769.5 to 1286.0) | 11.4* (4.3 to 18.9) | -3.8 (-10.0 to 2.6) |
| Chronic obstructive pulmonary disease with heart failure | 3.2 (2.1 to 4.5) | -- | 1.5 (0.9 to 2.3) | 8.6* (7.0 to 10.2) | -6.3* (-7.6 to -4.9) |
| Chronic obstructive pulmonary disease without heart failure | 9153.2 (7390.3 to 11258.3) | 566.8 (371.1 to 793.1) | 1010.2 (768.5 to 1285.0) | 11.4* (4.3 to 18.9) | -3.8 (-10.0 to 2.6) |
| Pneumoconiosis | 9.6 (7.3 to 12.7) | 1.0 (0.7 to 1.3) | 1.5 (0.9 to 2.3) | 12.2* (8.6 to 15.4) | -3.2* (-6.2 to -0.3) |
| Silicosis | 3.6 (2.3 to 5.5) | 0.3 (0.2 to 0.6) | 0.6 (0.3 to 1.0) | 15.5* (8.5 to 22.7) | -0.3 (-6.4 to 5.9) |
| Heart failure due to silicosis | -- | -- | -- | -- | -- |
| Silicosis without heart failure | 3.6 (2.3 to 5.5) | 0.3 (0.2 to 0.6) | 0.6 (0.3 to 1.0) | 15.5* (8.5 to 22.7) | -0.3 (-6.4 to 5.9) |
| Asbestosis | 2.6 (1.3 to 4.7) | 0.2 (0.1 to 0.4) | 0.4 (0.2 to 0.8) | 18.9* (13.4 to 25.2) | 2.6 (-2.1 to 8.1) |
| Asbestosis without heart failure | 2.6 (1.3 to 4.7) | 0.2 (0.1 to 0.4) | 0.4 (0.2 to 0.8) | 18.9* (13.4 to 25.2) | 2.6 (-2.1 to 8.1) |
| Heart failure due to asbestosis | -- | -- | -- | -- | -- |
| Coal workers pneumoconiosis | 1.3 (0.7 to 2.2) | 0.2 (0.1 to 0.3) | 0.2 (0.1 to 0.4) | 3.2 (-5.5 to 9.8) | -10.9* (-18.4 to -5.2) |
| Coal workers pneumoconiosis without heart failure | 1.3 (0.7 to 2.2) | 0.2 (0.1 to 0.3) | 0.2 (0.1 to 0.4) | 3.2 (-5.5 to 9.8) | -10.9* (-18.4 to -5.2) |
| Heart failure due to Coal workers pneumoconiosis | -- | -- | -- | -- | -- |
| Other pneumoconiosis | 2.1 (1.3 to 3.5) | 0.3 (0.2 to 0.4) | 0.3 (0.2 to 0.6) | 5.3* (1.3 to 9.2) | -9.0* (-12.6 to -5.7) |
| Heart failure due to other pneumoconiosis | -- | -- | -- | -- | -- |
| Other pneumoconiosis without heart failure | 2.1 (1.3 to 3.5) | 0.3 (0.2 to 0.4) | 0.3 (0.2 to 0.6) | 5.3* (1.3 to 9.2) | -9.0* (-12.6 to -5.7) |
| Asthma | 14068.9 (11415.6 to 17198.5) | 1292.3 (913.8 to 1790.0) | 553.2 (350.5 to 849.9) | 15.2* (9.0 to 21.4) | -0.6 (-5.9 to 4.8) |
| Asymptomatic asthma | 5112.7 (4046.4 to 6471.5) | 468.6 (324.7 to 668.1) | -- | -- | -- |
| Symptomatic asthma | 8956.3 (7175.5 to 10959.0) | 823.8 (576.8 to 1150.3) | 553.2 (350.5 to 849.9) | 15.2* (9.0 to 21.4) | -0.6 (-5.9 to 4.8) |
| Interstitial lung disease and pulmonary sarcoidosis | 136.0 (107.5 to 170.0) | 15.9 (10.9 to 21.8) | 15.1 (9.5 to 22.4) | 12.5* (7.4 to 17.1) | -2.9 (-7.2 to 1.1) |
| Heart failure due to interstitial lung disease and pulmonary sarcoidosis | 1.4 (0.9 to 1.9) | -- | 0.6 (0.4 to 1.0) | 13.0* (11.5 to 14.5) | -2.5* (-3.7 to -1.2) |
| Interstitial lung disease and pulmonary sarcoidosis without heart failure | 134.6 (106.0 to 168.7) | 15.9 (10.9 to 21.8) | 14.5 (9.0 to 21.6) | 12.5* (7.2 to 17.2) | -2.9 (-7.4 to 1.2) |
| Other chronic respiratory diseases | -- | -- | 244.5 (185.8 to 311.0) | 24.0* (16.3 to 32.1) | 7.1* (0.4 to 14.1) |
| **Digestive diseases** | **189895.2 (172474.8 to 210484.5)** | **40809.5 (31890.2 to 52228.8)** | **1505.0 (990.0 to 2258.3)** | **15.8* (14.2 to 17.3)** | **-0.0 (-1.4 to 1.3)** |
| Cirrhosis and other chronic liver diseases | 134280.2 (123965.1 to 145006.1) | 770.6 (646.3 to 912.4) | 81.8 (56.1 to 114.3) | 24.9* (20.5 to 29.5) | 7.9* (4.1 to 11.8) |
| Cirrhosis and other chronic liver diseases due to hepatitis B | 36305.6 (33382.1 to 39981.1) | 296.3 (238.0 to 355.4) | 30.7 (20.9 to 42.8) | 20.2* (12.4 to 28.3) | 3.7 (-3.0 to 10.7) |
| Cirrhosis and other chronic liver diseases due to hepatitis B, decompensated | 180.3 (163.8 to 197.1) | 32.5 (28.3 to 36.6) | 30.7 (20.9 to 42.8) | 20.2* (12.4 to 28.3) | 3.7 (-3.0 to 10.7) |
| Chronic hepatitis B without cirrhosis | 32196.0 (29233.5 to 35955.0) | -- | -- | -- | -- |
| Cirrhosis and other chronic liver diseases due to hepatitis B, compensated | 3929.4 (3523.4 to 4396.2) | 263.8 (206.3 to 324.1) | -- | -- | -- |
| Cirrhosis and other chronic liver diseases due to hepatitis C | 11369.7 (9526.2 to 13349.9) | 209.6 (169.8 to 255.2) | 22.3 (15.1 to 31.4) | 26.5* (18.1 to 35.0) | 9.2* (2.0 to 16.6) |
| Cirrhosis and other chronic liver diseases due to hepatitis C, decompensated | 131.4 (115.9 to 147.7) | 22.8 (19.4 to 26.5) | 22.3 (15.1 to 31.4) | 26.5* (18.1 to 35.0) | 9.2* (2.0 to 16.6) |
| Chronic hepatitis C without cirrhosis | 8638.7 (6764.4 to 10625.4) | -- | -- | -- | -- |
| Cirrhosis and other chronic liver diseases due to hepatitis C, compensated | 2599.6 (2263.6 to 2970.7) | 186.8 (148.3 to 231.7) | -- | -- | -- |
| Cirrhosis and other chronic liver diseases due to alcohol use | 1888.1 (1684.6 to 2123.2) | 151.3 (125.5 to 179.3) | 16.3 (10.9 to 23.1) | 25.7* (17.2 to 34.9) | 8.5* (1.2 to 16.5) |
| Cirrhosis and other chronic liver diseases due to alcohol, decompensated | 95.4 (85.0 to 106.6) | 17.5 (15.0 to 20.1) | 16.3 (10.9 to 23.1) | 25.7* (17.2 to 34.9) | 8.5* (1.2 to 16.5) |
| Cirrhosis and other chronic liver diseases due to alcohol, compensated | 1792.7 (1590.7 to 2020.8) | 133.8 (108.1 to 160.7) | -- | -- | -- |
| Cirrhosis due to NASH | 83936.2 (73958.4 to 94905.2) | 54.7 (43.6 to 67.1) | 6.1 (4.0 to 8.6) | 40.2* (31.7 to 48.9) | 21.0* (13.7 to 28.6) |
| Non-alcoholic fatty liver disease (NAFLD) / Non-alcoholic steatohepatitis (NASH) | 83200.1 (73305.9 to 94164.8) | -- | -- | -- | -- |
| Cirrhosis and other chronic liver diseases due to NASH | 736.1 (624.4 to 853.8) | 54.7 (43.6 to 67.1) | 6.1 (4.0 to 8.6) | 40.2* (31.7 to 48.9) | 21.0* (13.7 to 28.6) |
| Cirrhosis and other chronic liver diseases due to other causes | 780.6 (589.4 to 999.0) | 58.7 (42.1 to 79.6) | 6.5 (4.0 to 10.0) | 28.9* (18.7 to 40.2) | 11.3* (2.5 to 21.0) |

| | | | | | |
|---|---|---|---|---|---|
| Cirrhosis and other chronic liver diseases due to other cause, decompensated | 37.6 (28.8 to 47.9) | 6.3 (4.6 to 8.3) | 6.5 (4.0 to 10.0) | 28.9* (18.7 to 40.2) | 11.3* (2.5 to 21.0) |
| Cirrhosis and other chronic liver diseases due to other cause, compensated | 743.0 (558.9 to 952.4) | 52.4 (37.4 to 71.3) | -- | -- | -- |
| Upper digestive system diseases | 79010.5 (59829.3 to 103345.1) | 36135.9 (27459.3 to 47321.6) | 1142.9 (693.1 to 1829.7) | 15.9* (14.2 to 17.6) | 0.1 (-1.4 to 1.5) |
| Peptic ulcer disease | 1310.2 (964.9 to 1706.3) | 446.9 (318.4 to 596.7) | 64.2 (38.1 to 95.8) | 16.9* (11.9 to 22.1) | 1.0 (-3.4 to 5.4) |
| Acute peptic ulcer disease | 0.3 (0.2 to 0.4) | -- | 0.1 (0.0 to 0.1) | -1.3 (-3.4 to 0.7) | -14.8* (-16.6 to -13.1) |
| Chronic peptic ulcer disease | 1309.9 (964.6 to 1706.1) | 446.9 (318.4 to 596.7) | 64.1 (38.0 to 95.7) | 17.0* (11.9 to 22.1) | 1.0 (-3.4 to 5.4) |
| Gastritis and duodenitis | 10010.0 (6838.9 to 13937.4) | 7055.9 (4953.2 to 9734.0) | 448.4 (262.2 to 700.2) | 12.6* (9.1 to 15.9) | -2.8 (-5.8 to 0.0) |
| Acute gastritis and duodenitis | 2.2 (1.4 to 3.3) | -- | 0.7 (0.4 to 1.2) | 8.4* (6.7 to 9.9) | -6.4* (-7.9 to -5.1) |
| Chronic gastritis and duodenitis | 10007.8 (6837.0 to 13935.4) | 7055.9 (4953.2 to 9734.0) | 447.7 (261.8 to 699.6) | 12.6* (9.1 to 15.9) | -2.8 (-5.8 to 0.0) |
| GERD | 72761.1 (51604.0 to 100951.4) | 28633.0 (20510.8 to 39648.7) | 630.3 (312.0 to 1175.5) | 18.3* (16.6 to 20.0) | 2.1* (0.7 to 3.6) |
| Appendicitis | 52.7 (41.9 to 64.7) | 1339.1 (1078.2 to 1641.9) | 16.3 (10.3 to 24.2) | 16.2* (4.4 to 30.1) | 0.3 (-9.8 to 12.4) |
| Paralytic ileus and intestinal obstruction | 5.9 (4.1 to 7.9) | 158.5 (108.8 to 213.0) | 1.9 (1.1 to 3.0) | 22.6* (18.2 to 26.9) | 5.9* (2.0 to 9.5) |
| Inguinal, femoral, and abdominal hernia | 1340.6 (972.8 to 1833.3) | 1494.4 (1051.1 to 2034.0) | 132.6 (79.8 to 199.5) | 11.1* (6.5 to 16.0) | -4.1 (-8.1 to 0.1) |
| Inflammatory bowel disease | 383.0 (337.1 to 437.4) | 408.2 (334.2 to 497.5) | 60.0 (39.8 to 84.9) | 7.4 (-1.8 to 16.2) | -7.2 (-15.2 to 0.3) |
| Ulcerative colitis | 216.5 (178.2 to 264.0) | 280.1 (209.2 to 365.1) | 33.4 (21.0 to 48.0) | 9.9 (-1.3 to 21.3) | -5.1 (-14.8 to 4.7) |
| Crohn's disease | 166.5 (141.5 to 197.0) | 128.1 (97.9 to 165.2) | 26.7 (17.7 to 37.3) | 4.5 (-6.5 to 15.5) | -9.8* (-19.3 to -0.3) |
| Vascular intestinal disorders | 2.3 (1.2 to 3.7) | 13.4 (8.8 to 19.7) | 0.7 (0.3 to 1.3) | 17.8* (14.9 to 20.4) | 1.7 (-0.8 to 3.9) |
| Gallbladder and biliary diseases | 2184.2 (1569.2 to 2946.3) | 371.0 (265.9 to 507.4) | 1.8 (1.1 to 2.9) | 17.5* (15.5 to 19.6) | 1.4 (-0.3 to 3.3) |
| Pancreatitis | 303.8 (255.5 to 364.2) | 118.4 (92.9 to 148.7) | 19.7 (9.9 to 34.8) | 18.3* (12.6 to 23.7) | 2.2 (-2.8 to 6.8) |
| Acute pancreatitis | 7.1 (5.2 to 9.5) | 88.6 (64.3 to 116.0) | 2.9 (1.8 to 4.3) | 12.4* (11.4 to 13.5) | -3.0* (-3.8 to -2.0) |
| Chronic pancreatitis | 296.7 (248.5 to 356.6) | 29.8 (21.4 to 39.4) | 16.7 (7.4 to 31.1) | 19.4* (12.6 to 25.8) | 3.1 (-2.8 to 8.6) |
| Other digestive diseases | -- | -- | 47.1 (30.5 to 67.6) | 21.2* (18.4 to 23.9) | 4.6* (2.2 to 7.0) |
| **Neurological disorders** | **312089.4 (266032.8 to 364073.4)** | **95346.7 (68875.5 to 130002.7)** | **6627.4 (4347.5 to 9556.5)** | **17.6* (15.9 to 19.4)** | **1.5* (0.1 to 3.1)** |
| Alzheimer's disease and other dementias | -- | -- | -- | -- | -- |
| Parkinson's disease | 31.1 (17.5 to 52.3) | 5.0 (3.2 to 7.2) | 5.2 (2.6 to 9.1) | 14.6* (11.2 to 17.6) | -1.1 (-4.0 to 1.5) |
| Epilepsy | 1832.1 (1333.1 to 2421.3) | 126.4 (81.7 to 181.8) | 577.9 (351.3 to 872.8) | 17.1* (3.8 to 33.2) | 1.1 (-10.3 to 15.0) |
| Multiple sclerosis | 161.6 (139.1 to 184.4) | 11.0 (8.4 to 14.6) | 43.2 (30.3 to 57.5) | 16.8* (11.2 to 22.7) | 0.9 (-4.0 to 5.9) |
| Motor neuron disease | 9.8 (8.0 to 11.8) | 0.7 (0.4 to 1.0) | 2.1 (1.4 to 2.9) | 18.4* (17.1 to 19.5) | 2.2* (1.1 to 3.2) |
| Headache disorders | 311204.7 (264666.6 to 363197.9) | 95203.6 (68760.7 to 129830.6) | 5915.2 (3732.1 to 8717.7) | 17.4* (16.3 to 18.6) | 1.4* (0.4 to 2.4) |
| Migraine | 149747.7 (127530.3 to 172721.0) | 11179.3 (7545.5 to 15289.1) | 5217.9 (3265.7 to 7757.0) | 17.4* (16.2 to 18.7) | 1.4* (0.3 to 2.5) |
| Medication overuse headache due to migraine | 7793.2 (5685.5 to 10429.5) | -- | 869.9 (496.1 to 1355.8) | 18.2* (16.6 to 19.8) | 2.0* (0.6 to 3.5) |
| Migraine | 141954.5 (120331.5 to 164647.8) | 11179.3 (7545.5 to 15289.1) | 4348.0 (2553.7 to 6596.4) | 17.3* (15.8 to 18.8) | 1.3 (-0.0 to 2.5) |
| Tension-type headache | 233913.4 (175156.2 to 304312.1) | 84024.3 (58037.7 to 118075.5) | 697.3 (378.6 to 1153.9) | 17.2* (15.9 to 18.7) | 1.1* (0.0 to 2.5) |
| Medication overuse headache due to tension-type headache | 2895.1 (1814.6 to 4169.1) | -- | 321.0 (154.8 to 576.7) | 18.2* (15.5 to 20.7) | 2.0 (-0.3 to 4.2) |
| Tension-type headache | 231018.2 (171999.4 to 301058.6) | 84024.3 (58037.7 to 118075.5) | 376.2 (145.9 to 734.1) | 16.3* (15.2 to 17.4) | 0.4 (-0.6 to 1.4) |
| Other neurological disorders | 2.3 (1.2 to 3.9) | -- | 83.9 (52.2 to 124.2) | 33.6* (19.8 to 50.0) | 15.3* (3.4 to 29.5) |
| Other neurological disorders | -- | -- | 83.2 (51.6 to 123.3) | 33.7* (19.8 to 50.4) | 15.4* (3.4 to 29.8) |
| Guillain-Barré syndrome due to other neurological disorders | 2.3 (1.2 to 3.9) | -- | 0.7 (0.3 to 1.3) | 17.9* (15.2 to 20.8) | 1.8 (-0.5 to 4.3) |
| **Mental disorders** | **85033.9 (79390.2 to 90843.9)** | **27080.2 (22673.3 to 32208.9)** | **11516.9 (8577.8 to 14833.8)** | **14.6* (13.5 to 15.5)** | **-1.1* (-2.0 to -0.3)** |
| Schizophrenia | 2586.7 (2164.8 to 3152.9) | 152.7 (99.2 to 220.0) | 1698.0 (1237.8 to 2186.3) | 14.8* (12.6 to 17.0) | -0.9 (-2.8 to 1.1) |
| Depressive disorders | 22396.0 (18686.8 to 26389.7) | 21857.9 (17358.0 to 26900.9) | 3727.6 (2460.5 to 5354.3) | 11.7* (9.3 to 14.1) | -3.5* (-5.6 to -1.5) |
| Major depressive disorder | 13807.4 (10670.4 to 17383.6) | 20445.2 (16128.3 to 25453.6) | 2847.4 (1830.2 to 4126.5) | 10.3* (7.1 to 13.4) | -4.8* (-7.6 to -2.1) |
| Dysthymia | 8958.1 (7271.5 to 11295.0) | 1412.8 (1125.3 to 1731.8) | 880.2 (559.3 to 1348.4) | 16.7* (14.2 to 19.2) | 0.7 (-1.4 to 2.9) |
| Bipolar disorder | 4423.2 (3589.4 to 5374.3) | 267.6 (177.6 to 390.3) | 915.1 (566.8 to 1403.0) | 17.3* (15.1 to 19.2) | 1.2 (-0.6 to 2.9) |
| Anxiety disorders | 25695.3 (22877.0 to 28652.4) | 3859.4 (3472.5 to 4272.9) | 2482.3 (1744.3 to 3348.9) | 15.2* (13.5 to 17.0) | -0.5 (-2.0 to 1.0) |
| Eating disorders | 2962.8 (2171.9 to 3929.5) | 942.5 (590.5 to 1372.6) | 623.8 (383.3 to 954.9) | 27.2* (24.7 to 29.4) | 9.8* (7.7 to 11.8) |
| Anorexia nervosa | 418.5 (298.7 to 575.8) | 82.3 (57.2 to 117.0) | 88.4 (51.9 to 144.0) | 21.3* (15.4 to 26.8) | 4.7 (-0.4 to 9.5) |
| Bulimia nervosa | 2547.0 (1750.5 to 3523.2) | 860.2 (504.4 to 1293.4) | 535.5 (321.4 to 848.0) | 28.2* (25.5 to 30.7) | 10.7* (8.3 to 12.8) |
| Autism spectrum disorders | 2337.0 (2101.4 to 2583.8) | -- | 355.8 (243.4 to 488.0) | 15.3* (13.1 to 17.4) | -0.5 (-2.4 to 1.4) |
| Attention-deficit/hyperactivity disorder | 5667.0 (4698.0 to 6822.0) | -- | 68.4 (41.3 to 110.3) | 15.8* (12.8 to 19.1) | -0.0 (-2.6 to 2.9) |
| Conduct disorder | -- | -- | -- | -- | -- |
| Idiopathic developmental intellectual disability | 7605.8 (4373.1 to 10918.2) | -- | 302.9 (145.9 to 512.3) | 6.6* (2.9 to 8.7) | -8.0* (-11.1 to -6.1) |
| Other mental disorders | 17677.3 (15241.9 to 20334.6) | -- | 1343.0 (882.0 to 1868.2) | 15.9* (14.4 to 17.5) | 0.1 (-1.3 to 1.4) |
| **Substance use disorders** | **25187.6 (21470.7 to 29320.4)** | **8647.3 (6969.3 to 10635.4)** | **4861.5 (3397.6 to 6498.1)** | **22.5* (19.1 to 26.2)** | **5.7* (2.9 to 9.0)** |
| Alcohol use disorders | 14949.8 (11592.0 to 18586.8) | 7979.9 (6292.0 to 9947.3) | 1509.9 (990.2 to 2170.9) | 12.6* (9.5 to 15.2) | -2.8* (-5.5 to -0.6) |
| Alcohol dependence | 14927.1 (11568.6 to 18562.3) | 7979.9 (6292.0 to 9947.3) | 1509.1 (989.3 to 2169.8) | 12.6* (9.5 to 15.2) | -2.8* (-5.5 to -0.5) |
| Fetal alcohol syndrome | 22.7 (18.3 to 27.9) | -- | 0.8 (0.5 to 1.2) | -0.8 (-7.6 to 6.6) | -14.4* (-20.3 to -8.0) |
| Drug use disorders | 10735.3 (9102.0 to 12801.9) | 667.4 (488.4 to 903.5) | 3351.6 (2307.6 to 4627.1) | 27.5* (23.0 to 32.1) | 10.1* (6.2 to 14.0) |
| Opioid use disorders | 6990.6 (5440.3 to 8990.0) | 365.5 (207.1 to 589.9) | 2937.0 (1950.5 to 4125.3) | 29.3* (24.2 to 34.4) | 11.6* (7.2 to 16.1) |
| Cocaine use disorders | 581.5 (509.3 to 659.8) | 6.6 (3.4 to 10.9) | 79.4 (51.4 to 115.5) | 18.2* (13.9 to 22.8) | 2.1 (-1.7 to 6.0) |

| | | | | | |
|---|---|---|---|---|---|
| Amphetamine use disorders | 821.5 (551.8 to 1158.8) | 55.6 (27.6 to 93.4) | 107.6 (56.5 to 180.1) | 15.6* (8.3 to 22.3) | -0.2 (-6.5 to 5.6) |
| Cannabis use disorders | 2141.0 (1576.8 to 2783.9) | 239.7 (151.7 to 340.5) | 61.7 (37.0 to 95.2) | 12.8* (7.5 to 17.9) | -2.6 (-7.2 to 1.8) |
| Other drug use disorders | 403.9 (317.9 to 499.0) | -- | 165.9 (108.1 to 234.0) | 16.6* (12.7 to 20.6) | 0.7 (-2.7 to 4.1) |
| **Diabetes and kidney diseases** | **67064.3 (57287.4 to 77359.2)** | **2181.4 (1659.0 to 2722.7)** | **2318.6 (1557.0 to 3241.1)** | **21.1* (15.6 to 26.2)** | **4.5 (-0.2 to 9.0)** |
| Diabetes mellitus | 25647.4 (21271.0 to 30634.9) | 1648.8 (1167.3 to 2159.0) | 1943.3 (1245.4 to 2796.2) | 22.1* (15.6 to 28.0) | 5.4 (-0.2 to 10.5) |
| Diabetes mellitus type 1 | 1287.0 (1131.4 to 1467.1) | 23.7 (14.5 to 34.4) | 97.4 (65.8 to 139.9) | 15.1* (12.2 to 18.1) | -0.6 (-3.2 to 1.9) |
| Uncomplicated diabetes mellitus type 1 | 857.7 (738.5 to 989.8) | 23.7 (14.5 to 34.4) | 40.6 (25.2 to 60.5) | 15.0* (11.4 to 18.4) | -0.7 (-3.8 to 2.2) |
| Vision loss due to diabetes mellitus type 1 retinopathy | 6.6 (4.2 to 9.7) | -- | 0.5 (0.3 to 0.8) | -0.6 (-7.0 to 5.8) | -14.2* (-19.7 to -8.7) |
| Diabetic neuropathy due to diabetes mellitus type 1 | 422.7 (353.5 to 501.7) | -- | 56.3 (37.0 to 80.9) | 15.4* (10.4 to 20.0) | -0.4 (-4.7 to 3.6) |
| Diabetes mellitus type 2 | 24360.4 (19998.2 to 29343.1) | 1625.2 (1145.1 to 2135.8) | 1845.9 (1183.6 to 2662.7) | 22.5* (15.6 to 28.7) | 5.7 (-0.2 to 11.1) |
| Uncomplicated diabetes mellitus type 2 | 16232.7 (13200.8 to 19977.2) | 1625.2 (1145.1 to 2135.8) | 770.9 (464.0 to 1159.3) | 22.8* (15.9 to 29.1) | 6.1* (0.0 to 11.4) |
| Vision loss due to diabetes mellitus type 2 retinopathy | 96.9 (67.3 to 133.8) | -- | 8.1 (4.9 to 12.4) | 12.1* (2.6 to 21.0) | -3.2 (-11.4 to 4.5) |
| Diabetic neuropathy due to diabetes mellitus type 2 | 8030.9 (6299.2 to 10051.9) | -- | 1067.0 (687.4 to 1538.9) | 22.3* (14.8 to 29.1) | 5.6 (-0.9 to 11.5) |
| Chronic kidney disease | 45401.0 (35948.3 to 55694.7) | 492.5 (315.2 to 767.4) | 375.2 (251.0 to 539.2) | 16.1* (8.7 to 23.8) | 0.3 (-6.1 to 6.9) |
| Chronic kidney disease due to diabetes mellitus type 1 | 295.6 (237.6 to 361.7) | 4.4 (2.7 to 6.9) | 15.0 (9.8 to 21.2) | 15.6* (8.7 to 22.1) | -0.2 (-6.2 to 5.4) |
| Albuminuria with preserved GFR due to type 1 diabetes mellitus | 183.7 (133.7 to 244.4) | -- | -- | -- | -- |
| End-stage chronic kidney disease due to diabetes mellitus type 1 | 23.1 (14.9 to 33.9) | -- | 8.8 (5.0 to 14.0) | 11.8* (2.6 to 20.0) | -3.5 (-11.4 to 3.6) |
| Stage III chronic kidney disease due to diabetes mellitus type 1 | 75.6 (60.2 to 94.9) | 4.4 (2.7 to 6.9) | 1.7 (1.1 to 2.6) | 12.1 (-4.7 to 27.3) | -3.2 (-17.7 to 9.9) |
| Stage IV chronic kidney disease due to diabetes mellitus type 1 | 7.4 (4.6 to 11.5) | -- | 1.1 (0.6 to 1.8) | 27.2* (13.0 to 44.9) | 9.8 (-2.4 to 25.1) |
| Stage V chronic kidney disease due to diabetes mellitus type 1 | 5.8 (4.0 to 8.1) | -- | 3.4 (2.2 to 5.2) | 24.9* (15.4 to 34.4) | 7.8 (-0.4 to 16.1) |
| Chronic kidney disease due to diabetes mellitus type 2 | 6939.4 (5263.2 to 8870.7) | 19.5 (12.2 to 30.5) | 17.2 (11.5 to 24.5) | 15.7* (7.9 to 23.6) | -0.1 (-6.8 to 6.7) |
| Albuminuria with preserved GFR due to type 2 diabetes mellitus | 6556.4 (4876.1 to 8491.2) | -- | -- | -- | -- |
| End-stage chronic kidney disease due to diabetes mellitus type 2 | 5.4 (2.5 to 10.0) | -- | 2.1 (0.9 to 4.1) | 12.7* (4.3 to 20.3) | -2.7 (-10.0 to 3.9) |
| Stage III chronic kidney disease due to diabetes mellitus type 2 | 340.1 (278.0 to 412.0) | 19.5 (12.2 to 30.5) | 3.1 (2.0 to 4.5) | 11.6 (-4.6 to 29.9) | -3.6 (-17.7 to 12.2) |
| Stage IV chronic kidney disease due to diabetes mellitus type 2 | 21.5 (13.5 to 32.9) | -- | 2.7 (1.5 to 4.5) | 17.0* (3.8 to 32.2) | 1.0 (-10.4 to 14.2) |
| Stage V chronic kidney disease due to diabetes mellitus type 2 | 15.9 (11.4 to 22.2) | -- | 9.3 (5.7 to 13.9) | 17.5* (9.1 to 26.3) | 1.4 (-5.8 to 9.1) |
| Chronic kidney disease due to hypertension | 1380.0 (1170.8 to 1644.7) | 63.1 (40.0 to 100.4) | 62.8 (40.7 to 92.9) | 19.4* (11.0 to 28.4) | 3.1 (-4.1 to 10.8) |
| Albuminuria with preserved GFR due to hypertension | 228.3 (164.7 to 300.1) | -- | -- | -- | -- |
| End-stage chronic kidney disease due to hypertension | 14.1 (9.7 to 19.5) | -- | 5.7 (3.3 to 8.7) | 17.8* (10.8 to 25.0) | 1.7 (-4.3 to 7.9) |
| Stage III chronic kidney disease due to hypertension | 967.1 (802.0 to 1165.5) | 63.1 (40.0 to 100.4) | 3.1 (2.0 to 4.7) | 1.6 (-15.1 to 18.8) | -12.3 (-26.7 to 2.6) |
| Stage IV chronic kidney disease due to hypertension | 97.4 (62.0 to 148.5) | -- | 12.2 (6.8 to 20.2) | 19.6* (5.0 to 36.5) | 3.3 (-9.4 to 17.8) |
| Stage V chronic kidney disease due to hypertension | 73.1 (52.1 to 99.9) | -- | 41.8 (25.9 to 63.3) | 21.1* (11.4 to 31.3) | 4.6 (-3.8 to 13.4) |
| Chronic kidney disease due to glomerulonephritis | 1486.6 (1255.2 to 1751.9) | 21.4 (13.5 to 33.4) | 103.2 (68.0 to 149.5) | 18.4* (10.1 to 26.9) | 2.2 (-4.9 to 9.6) |
| Albuminuria with preserved GFR due to glomerulonephritis | 658.1 (483.5 to 850.3) | -- | -- | -- | -- |
| End-stage chronic kidney disease due to glomerulonephritis | 42.4 (29.2 to 58.4) | -- | 15.6 (9.0 to 24.8) | 15.6* (7.1 to 24.0) | -0.2 (-7.6 to 7.0) |
| Stage III chronic kidney disease due to glomerulonephritis | 527.6 (434.0 to 637.5) | 21.4 (13.5 to 33.4) | 7.0 (4.4 to 10.9) | 1.5 (-12.8 to 16.6) | -12.3 (-24.8 to 0.7) |
| Stage IV chronic kidney disease due to glomerulonephritis | 147.8 (93.8 to 225.8) | -- | 18.3 (10.1 to 30.1) | 20.6* (5.3 to 37.4) | 4.1 (-9.1 to 18.7) |
| Stage V chronic kidney disease due to glomerulonephritis | 110.8 (78.4 to 152.4) | -- | 62.3 (38.3 to 95.3) | 20.7* (10.5 to 31.3) | 4.2 (-4.6 to 13.3) |
| Chronic kidney disease due to other and unspecified causes | 35299.4 (27723.2 to 43378.4) | 384.2 (245.1 to 595.4) | 176.9 (121.1 to 246.5) | 13.9* (5.6 to 22.0) | -1.7 (-8.8 to 5.3) |
| Albuminuria with preserved GFR due to other and unspecified causes | 28221.7 (20748.5 to 36460.3) | -- | -- | -- | -- |
| End-stage chronic kidney disease due to other causes | 51.6 (35.4 to 68.1) | -- | 20.1 (12.1 to 29.5) | 17.0* (5.1 to 29.2) | 1.0 (-9.2 to 11.5) |
| Stage III chronic kidney disease due to other causes | 6635.1 (5475.3 to 7965.8) | 384.2 (245.1 to 595.4) | 38.2 (23.5 to 60.1) | -4.8 (-14.9 to 5.8) | -17.8* (-26.5 to -8.7) |
| Stage IV chronic kidney disease due to other causes | 225.0 (143.6 to 344.0) | -- | 26.8 (14.9 to 44.6) | 20.2* (6.0 to 36.5) | 3.8 (-8.4 to 17.8) |
| Stage V chronic kidney disease due to other causes | 166.0 (117.8 to 226.4) | -- | 91.8 (56.9 to 139.8) | 21.2* (10.9 to 31.6) | 4.6 (-4.2 to 13.6) |
| Acute glomerulonephritis | 2.7 (1.6 to 3.9) | 40.1 (24.6 to 58.4) | 0.1 (0.1 to 0.2) | 1.5 (-2.9 to 6.4) | -12.4* (-16.2 to -8.1) |
| **Skin and subcutaneous diseases** | **118131.9 (108467.5 to 129174.7)** | **246671.5 (213940.9 to 280888.6)** | **2508.8 (1666.3 to 3634.9)** | **16.3* (15.5 to 17.2)** | **0.4 (-0.3 to 1.2)** |
| Dermatitis | 16364.1 (13869.8 to 19351.5) | 22640.2 (16571.0 to 30270.2) | 573.4 (330.1 to 890.5) | 14.8* (13.1 to 16.5) | -0.9 (-2.4 to 0.6) |
| Atopic dermatitis | 8958.3 (7874.7 to 10237.4) | 1133.8 (922.2 to 1353.8) | 390.6 (202.5 to 663.5) | 15.0* (12.8 to 17.2) | -0.7 (-2.7 to 1.1) |
| Contact dermatitis | 6724.3 (4442.4 to 9518.1) | 19178.7 (13134.1 to 26695.7) | 170.7 (94.2 to 289.4) | 14.8* (12.3 to 17.3) | -0.9 (-3.0 to 1.3) |
| Seborrhoeic dermatitis | 881.3 (692.8 to 1106.6) | 2327.7 (1822.6 to 2918.3) | 12.1 (6.5 to 19.9) | 6.8* (2.4 to 11.4) | -7.8* (-11.6 to -3.8) |
| Psoriasis | 4981.3 (4613.6 to 5408.5) | 663.5 (572.4 to 769.9) | 440.2 (306.9 to 596.2) | 20.5* (17.9 to 23.4) | 4.1* (1.7 to 6.5) |
| Bacterial skin diseases | 451.6 (407.5 to 495.6) | 12518.7 (11397.2 to 13822.5) | 10.6 (6.6 to 16.2) | 15.2* (12.1 to 18.5) | -0.5 (-3.2 to 2.3) |
| Cellulitis | 139.2 (113.1 to 171.9) | 2818.9 (2303.9 to 3390.5) | 8.1 (5.1 to 12.2) | 13.2* (9.7 to 16.9) | -2.3 (-5.3 to 0.9) |
| Pyoderma | 428.8 (387.1 to 471.3) | 9699.8 (8702.6 to 10717.7) | 2.4 (1.0 to 5.0) | 22.7* (17.6 to 27.8) | 5.9* (1.5 to 10.3) |
| Impetigo | 71.0 (61.7 to 81.0) | 1296.1 (1131.0 to 1469.2) | 0.4 (0.2 to 0.9) | 23.9* (14.1 to 34.1) | 7.0 (-1.5 to 15.8) |
| Abscess and other bacterial skin diseases | 357.8 (318.4 to 401.3) | 8403.7 (7394.6 to 9442.3) | 2.0 (0.8 to 4.2) | 22.5* (16.7 to 28.1) | 5.7* (0.8 to 10.6) |
| Scabies | 12341.3 (8231.1 to 17126.8) | 36085.6 (23876.1 to 50066.1) | 319.0 (159.8 to 548.4) | 10.8* (9.5 to 12.2) | -4.4* (-5.5 to -3.1) |
| Fungal skin diseases | 39668.2 (29874.0 to 50224.0) | 118662.6 (87715.2 to 150328.4) | 225.4 (84.6 to 494.4) | 14.8* (13.8 to 15.6) | -0.9* (-1.7 to -0.2) |
| Tinea capitis | 4005.5 (2710.8 to 5590.3) | 5755.3 (3974.8 to 7356.0) | 22.7 (8.2 to 50.3) | 1.7 (-1.5 to 5.0) | -12.2* (-15.0 to -9.4) |
| Other fungal skin diseases | 35662.7 (26091.2 to 46554.5) | 112907.3 (81519.7 to 144349.5) | 202.7 (76.0 to 443.5) | 16.4* (15.9 to 17.0) | 0.5* (0.0 to 1.0) |

| | | | | | |
|---|---|---|---|---|---|
| Viral skin diseases | 4689.3 (4246.1 to 5161.8) | 4067.0 (3729.9 to 4421.3) | 143.8 (91.2 to 211.2) | 11.8* (9.8 to 13.9) | -3.5* (-5.2 to -1.7) |
| Viral warts | 3346.3 (2947.5 to 3785.7) | 2152.9 (1895.7 to 2422.7) | 102.7 (65.6 to 151.8) | 10.1* (7.8 to 12.4) | -5.0* (-6.9 to -3.0) |
| Molluscum contagiosum | 1342.9 (1176.2 to 1523.1) | 1914.1 (1704.9 to 2135.7) | 41.1 (26.2 to 61.2) | 16.3* (12.3 to 20.2) | 0.4 (-3.1 to 3.7) |
| Acne vulgaris | 7538.8 (6485.7 to 8719.4) | 3187.7 (2698.3 to 3750.0) | 159.5 (94.0 to 256.1) | 23.6* (20.5 to 26.9) | 6.7* (4.0 to 9.6) |
| Alopecia areata | 2115.3 (1905.6 to 2343.8) | 3680.2 (3327.2 to 4042.9) | 69.8 (43.7 to 103.9) | 14.2* (11.4 to 17.0) | -1.4 (-3.8 to 1.0) |
| Pruritus | 5430.2 (3629.3 to 7679.2) | 4097.1 (2772.3 to 5666.2) | 58.5 (25.7 to 114.5) | 18.1* (16.3 to 19.7) | 1.9* (0.4 to 3.4) |
| Urticaria | 5521.4 (3872.8 to 7665.0) | 9568.7 (6672.1 to 13046.2) | 330.6 (194.0 to 508.0) | 16.5* (14.3 to 18.5) | 0.5 (-1.3 to 2.3) |
| Decubitus ulcer | 30.2 (20.4 to 41.8) | 107.5 (73.6 to 153.8) | 5.3 (3.1 to 8.5) | 19.1* (18.2 to 20.0) | 2.8* (2.0 to 3.6) |
| Other skin and subcutaneous diseases | 30928.7 (28100.7 to 33825.3) | 31392.8 (28438.8 to 34528.7) | 172.6 (82.9 to 321.6) | 21.8* (20.8 to 22.9) | 5.2* (4.3 to 6.1) |
| **Sense organ diseases** | **129130.5 (120391.3 to 137745.0)** | **--** | **2788.4 (1797.0 to 4262.1)** | **15.4* (13.6 to 17.1)** | **-0.4 (-1.9 to 1.1)** |
| Blindness and vision impairment | 62339.8 (57378.3 to 68049.0) | -- | 1140.8 (712.5 to 1791.3) | 14.9* (13.6 to 16.0) | -0.8 (-2.0 to 0.2) |
| Glaucoma | -- | -- | -- | -- | -- |
| Cataract | 651.5 (482.3 to 833.2) | -- | 68.1 (43.1 to 103.3) | 3.7 (-2.1 to 9.4) | -10.4* (-15.5 to -5.5) |
| Age-related macular degeneration | -- | -- | -- | -- | -- |
| Refraction disorders | 10319.8 (7763.1 to 13583.4) | -- | 435.5 (271.8 to 636.7) | 16.0* (13.7 to 18.0) | 0.2 (-1.8 to 1.9) |
| Near vision loss | 51284.4 (46612.2 to 56335.3) | -- | 531.6 (254.9 to 978.6) | 15.9* (15.1 to 16.6) | 0.0 (-0.7 to 0.7) |
| Other vision loss | 1297.5 (985.3 to 1691.0) | -- | 105.6 (67.7 to 155.0) | 13.1* (8.6 to 17.4) | -2.4 (-6.2 to 1.3) |
| Age-related and other hearing loss | 73802.2 (64820.4 to 83148.4) | -- | 1539.0 (983.0 to 2337.4) | 15.7* (12.9 to 18.6) | -0.1 (-2.5 to 2.4) |
| Other sense organ diseases | 4512.3 (4096.0 to 4935.1) | -- | 108.7 (65.8 to 160.7) | 17.0* (13.8 to 19.9) | 1.0 (-1.8 to 3.5) |
| Chronic other sense organ diseases | 3655.6 (3238.1 to 4071.6) | -- | 96.8 (57.7 to 142.7) | 16.9* (13.5 to 20.2) | 1.0 (-2.1 to 3.8) |
| Acute other sense organ diseases | 856.7 (765.6 to 955.0) | -- | 11.8 (7.3 to 18.2) | 17.2* (10.8 to 23.9) | 1.2 (-4.3 to 7.0) |
| **Musculoskeletal disorders** | **95293.1 (80634.6 to 111931.6)** | **25575.5 (19493.2 to 33108.4)** | **10347.0 (7140.9 to 14675.8)** | **14.0* (12.7 to 15.4)** | **-1.6* (-2.7 to -0.3)** |
| Rheumatoid arthritis | 866.4 (702.9 to 1047.3) | 69.0 (52.0 to 89.8) | 120.3 (77.2 to 171.2) | 23.4* (18.2 to 28.6) | 6.6* (2.0 to 11.0) |
| Osteoarthritis | 5055.1 (4302.0 to 5815.8) | 570.2 (442.6 to 706.9) | 168.7 (83.7 to 349.8) | 19.3* (17.1 to 21.7) | 3.0* (1.1 to 5.1) |
| Osteoarthritis of the hip | 687.8 (618.0 to 767.0) | 69.9 (55.0 to 88.2) | 23.2 (11.5 to 48.1) | 22.3* (17.0 to 28.2) | 5.6* (1.0 to 10.7) |
| Osteoarthritis of the knee | 4367.3 (3642.7 to 5140.8) | 500.3 (374.9 to 638.4) | 145.6 (72.5 to 300.0) | 18.9* (16.4 to 21.3) | 2.6* (0.5 to 4.8) |
| Low back pain | 43976.6 (31584.0 to 59902.9) | 19148.9 (13482.2 to 26132.0) | 5096.7 (3200.6 to 7845.5) | 13.3* (12.1 to 14.5) | -2.2* (-3.2 to -1.2) |
| Low back pain with leg pain | 13859.3 (9722.0 to 19389.7) | 6030.0 (4151.9 to 8550.7) | 2057.8 (1211.9 to 3170.8) | 13.3* (11.8 to 14.7) | -2.2* (-3.5 to -0.9) |
| Low back pain without leg pain | 30117.3 (21468.7 to 41354.5) | 13118.9 (9056.4 to 17964.3) | 3038.9 (1874.3 to 4776.9) | 13.3* (12.0 to 14.6) | -2.2* (-3.3 to -1.1) |
| Neck pain | 18770.6 (13784.8 to 25905.6) | 5486.8 (3596.7 to 7903.5) | 1923.9 (1172.2 to 3030.1) | 15.2* (12.9 to 17.5) | -0.6 (-2.6 to 1.4) |
| Gout | 1319.1 (1015.9 to 1679.5) | 300.5 (241.1 to 370.6) | 44.7 (28.4 to 65.1) | 17.7* (10.7 to 24.6) | 1.6 (-4.4 to 7.5) |
| Other musculoskeletal disorders | 33939.2 (23591.6 to 46004.4) | -- | 2992.7 (1746.0 to 4633.3) | 13.8* (9.8 to 17.5) | -1.7 (-5.2 to 1.4) |
| **Other non-communicable diseases** | **379899.4 (356554.2 to 405734.3)** | **328801.3 (283276.9 to 371747.4)** | **4250.2 (2897.7 to 5975.9)** | **12.3* (10.6 to 13.9)** | **-3.0* (-4.5 to -1.7)** |
| Congenital anomalies | 6910.3 (6514.8 to 7360.8) | -- | 852.4 (594.5 to 1144.0) | 16.6* (13.8 to 19.4) | 0.7 (-1.8 to 3.1) |
| Neural tube defects | 206.1 (183.6 to 230.6) | -- | 61.3 (42.4 to 82.3) | 20.4* (14.8 to 25.8) | 4.0 (-0.9 to 8.6) |
| Severe motor and cognitive impairment due to anencephaly | -- | -- | -- | -- | -- |
| Encephalocoele due to neural tube defects | 21.5 (17.3 to 25.8) | -- | 3.7 (2.5 to 5.2) | 15.1* (11.9 to 18.3) | -0.6 (-3.4 to 2.2) |
| Spina bifida due to neural tube defects | 184.5 (164.4 to 206.3) | -- | 57.6 (39.8 to 77.5) | 20.8* (15.0 to 26.4) | 4.3 (-0.7 to 9.2) |
| Congenital heart anomalies | 339.3 (312.1 to 368.5) | -- | 19.1 (8.8 to 32.7) | 16.8* (12.5 to 21.4) | 0.8 (-2.9 to 4.8) |
| Critical malformations of great vessels, congenital valvular heart disease and patent ductus arteriosus | 55.7 (47.8 to 64.3) | -- | 3.5 (1.8 to 5.9) | 14.0* (6.9 to 21.8) | -1.6 (-7.7 to 5.2) |
| Other congenital heart anomalies | 100.4 (92.2 to 109.4) | -- | 5.9 (2.4 to 10.2) | 16.8* (7.6 to 27.3) | 0.8 (-7.1 to 9.9) |
| Severe congenital heart anomalies excluding single ventricle heart defects | 126.3 (111.7 to 142.2) | -- | 7.6 (3.5 to 13.0) | 18.4* (10.9 to 26.7) | 2.2 (-4.3 to 9.4) |
| Single ventricle and single ventricle pathway congenital heart anomalies | 6.8 (5.6 to 8.2) | -- | 0.5 (0.2 to 0.7) | 24.4* (21.3 to 26.9) | 7.4* (4.7 to 9.6) |
| Ventricular septal defect and atrial septal defect | 50.1 (41.2 to 59.9) | -- | 1.7 (0.7 to 3.0) | 13.6* (7.9 to 19.8) | -2.0 (-6.9 to 3.5) |
| Orofacial clefts | 796.0 (730.0 to 861.9) | -- | 23.4 (15.5 to 34.0) | 18.5* (9.7 to 28.1) | 2.3 (-5.3 to 10.6) |
| Down's syndrome | 309.4 (270.3 to 351.2) | -- | 26.5 (18.0 to 37.0) | 16.8* (13.7 to 20.1) | 0.9 (-1.9 to 3.7) |
| Turner syndrome | 39.4 (34.5 to 45.2) | -- | 0.8 (0.4 to 1.4) | 13.3* (7.7 to 19.0) | -2.2 (-7.0 to 2.8) |
| Klinefelter syndrome | 73.1 (62.7 to 83.9) | -- | 0.7 (0.3 to 1.5) | 13.4* (4.4 to 22.8) | -2.1 (-9.9 to 6.0) |
| Other chromosomal abnormalities | 469.1 (362.7 to 582.6) | -- | 39.7 (25.2 to 57.2) | 24.6* (19.9 to 29.1) | 7.6* (3.5 to 11.5) |
| Congenital musculoskeletal and limb anomalies | 2239.5 (1995.1 to 2571.1) | -- | 334.5 (218.2 to 461.3) | 17.1* (14.8 to 19.4) | 1.1 (-0.9 to 3.1) |
| Polydactyly and syndactyly | 7.9 (6.3 to 9.5) | -- | 0.1 (0.0 to 0.2) | 10.2* (8.0 to 12.2) | -4.9* (-6.7 to -3.1) |
| Congenital limb deficiency | 1093.9 (973.2 to 1257.0) | -- | 163.9 (107.1 to 226.0) | 17.2* (13.9 to 20.7) | 1.2 (-1.7 to 4.2) |
| Other congenital musculoskeletal anomalies | 1137.8 (1013.7 to 1306.3) | -- | 170.5 (111.7 to 237.1) | 16.9* (13.7 to 20.4) | 1.0 (-1.8 to 4.0) |
| Urogenital congenital anomalies | 412.9 (349.1 to 487.0) | -- | 9.4 (5.3 to 15.6) | 6.2* (2.6 to 9.7) | -8.3* (-11.4 to -5.3) |
| Digestive congenital anomalies | 1314.7 (1184.2 to 1472.9) | -- | 59.6 (39.8 to 85.7) | 11.4* (6.0 to 16.9) | -3.9 (-8.5 to 0.9) |
| Congenital diaphragmatic hernia | 17.9 (15.7 to 20.3) | -- | 0.4 (0.2 to 0.6) | 14.8* (13.8 to 15.7) | -0.9* (-1.8 to -0.1) |
| Congenital atresia and/or stenosis of the digestive tract | 0.6 (0.5 to 0.9) | -- | 0.1 (0.0 to 0.1) | 37.9* (32.9 to 42.7) | 19.1* (14.8 to 23.2) |
| Congenital malformations of the abdominal wall after treatment | 69.2 (60.7 to 78.3) | -- | 3.0 (1.9 to 4.4) | 15.1* (14.2 to 16.0) | -0.6 (-1.4 to 0.1) |
| Other congenital malformations of the digestive tract | 1227.0 (1094.2 to 1385.3) | -- | 56.2 (37.2 to 80.9) | 11.1* (5.4 to 17.0) | -4.1 (-9.0 to 1.0) |
| Other congenital anomalies | 1986.7 (1719.8 to 2302.0) | -- | 277.4 (186.3 to 385.4) | 15.6* (8.0 to 23.3) | -0.2 (-6.8 to 6.4) |

| | | | | | |
|---|---|---|---|---|---|
| Other congenital birth defects | -- | -- | 22.8 (16.4 to 30.1) | 28.8* (24.2 to 33.1) | 11.2* (7.2 to 14.9) |
| Hearing loss due to other congenital anomalies | 1986.7 (1719.8 to 2302.0) | -- | 254.5 (168.7 to 358.8) | 14.6* (6.5 to 22.7) | -1.1 (-8.1 to 5.9) |
| Urinary diseases and male infertility | 6811.1 (4619.5 to 9515.4) | 37096.6 (29285.3 to 46113.7) | 86.2 (51.4 to 138.1) | 13.9* (9.7 to 17.8) | -1.7 (-5.3 to 1.7) |
| Urinary tract infections | 593.1 (436.5 to 766.8) | 30562.7 (22756.0 to 39163.2) | 19.7 (11.5 to 32.3) | 16.6* (10.9 to 22.2) | 0.6 (-4.3 to 5.5) |
| Urolithiasis | 245.6 (177.6 to 331.8) | 6533.9 (4663.8 to 8831.1) | 18.9 (11.4 to 29.1) | 15.0* (10.5 to 19.7) | -0.7 (-4.6 to 3.4) |
| Benign prostatic hyperplasia | -- | -- | -- | -- | -- |
| Male infertility | 6075.1 (3916.4 to 8800.3) | -- | 35.3 (13.4 to 74.9) | 22.8* (17.0 to 28.0) | 6.0* (1.0 to 10.5) |
| Other urinary diseases | -- | -- | 12.3 (8.0 to 18.5) | -9.6* (-12.4 to -6.7) | -21.9* (-24.4 to -19.4) |
| Gynecological diseases | 103622.4 (96048.3 to 111493.6) | 27124.9 (25226.7 to 29073.9) | 1650.6 (1131.3 to 2306.0) | 13.1* (11.2 to 15.0) | -2.4* (-4.0 to -0.7) |
| Uterine fibroids | 11397.8 (8945.4 to 14142.2) | 1545.9 (1075.2 to 2130.8) | 113.3 (64.9 to 185.3) | 1.5 (-7.0 to 9.1) | -12.4* (-19.7 to -5.8) |
| Polycystic ovarian syndrome | 7972.0 (6276.4 to 10260.0) | 19.7 (7.4 to 42.7) | 70.1 (31.2 to 134.8) | 16.7* (15.1 to 18.3) | 0.8 (-0.6 to 2.2) |
| Female infertility | 13023.3 (6034.1 to 21894.5) | -- | 71.0 (23.0 to 165.7) | 36.1* (25.7 to 44.3) | 17.5* (8.6 to 24.6) |
| Endometriosis | 7201.3 (5391.5 to 9548.3) | 968.0 (669.2 to 1342.9) | 667.9 (423.6 to 1024.4) | 13.5* (11.4 to 15.4) | -2.1* (-3.8 to -0.4) |
| Genital prolapse | 6243.0 (4861.6 to 7974.2) | 975.6 (752.5 to 1220.0) | 20.0 (8.9 to 39.4) | 8.3* (4.3 to 12.3) | -6.5* (-9.9 to -3.0) |
| Premenstrual syndrome | 67476.6 (61135.7 to 73534.5) | 16683.9 (14971.9 to 18380.9) | 563.0 (358.8 to 838.3) | 11.9* (8.0 to 15.1) | -3.4* (-6.7 to -0.7) |
| Other gynecological diseases | 7111.2 (6457.3 to 7887.9) | 6931.7 (6306.5 to 7643.1) | 145.2 (94.6 to 212.9) | 16.2* (11.6 to 20.7) | 0.3 (-3.7 to 4.2) |
| Other gynecological diseases | 6516.4 (5859.1 to 7285.7) | 6931.7 (6306.5 to 7643.1) | 131.9 (85.9 to 195.1) | 16.8* (11.9 to 21.7) | 0.8 (-3.4 to 5.0) |
| Other gynecological diseases with anemia | 594.8 (554.1 to 642.8) | -- | 13.3 (8.6 to 19.7) | 10.9 (-1.5 to 23.9) | -4.3 (-14.9 to 7.0) |
| Hemoglobinopathies and hemolytic anaemias | 146676.1 (143419.2 to 150166.1) | -- | 369.1 (244.5 to 548.5) | -6.5 (-14.0 to 2.2) | -19.3* (-25.7 to -11.8) |
| Thalassemias | 2.4 (2.2 to 2.6) | -- | 0.2 (0.1 to 0.2) | -11.2* (-20.6 to -0.6) | -23.3* (-31.5 to -14.2) |
| Thalassaemias trait | 23728.0 (22799.8 to 24695.9) | -- | 157.7 (99.5 to 238.9) | -16.2* (-27.7 to -1.8) | -27.6* (-37.5 to -15.2) |
| Sickle cell disorders | 87.0 (73.1 to 103.4) | -- | 10.5 (6.6 to 15.3) | 32.3* (13.3 to 55.6) | 14.2 (-2.2 to 34.4) |
| Sickle cell trait | 34030.2 (30713.4 to 37795.4) | -- | 76.7 (50.2 to 112.1) | 1.7 (-8.0 to 12.3) | -12.2* (-20.6 to -3.0) |
| G6PD deficiency | 27321.2 (26239.5 to 28440.1) | -- | 1.6 (1.1 to 2.3) | 2.1 (-14.9 to 17.9) | -11.8 (-26.5 to 1.8) |
| G6PD trait | 72004.4 (70828.3 to 73157.8) | -- | 0.0 (0.0 to 0.1) | -3.8 (-15.1 to 9.5) | -17.0* (-26.7 to -5.5) |
| Other hemoglobinopathies and hemolytic anaemias | 6001.0 (5587.9 to 6436.2) | -- | 122.4 (80.9 to 180.5) | 0.8 (-6.8 to 8.8) | -13.0* (-19.5 to -6.0) |
| Endocrine, metabolic, blood, and immune disorders | 8148.5 (7687.7 to 8657.7) | -- | 210.4 (143.1 to 299.7) | 2.9 (-2.8 to 9.0) | -11.1* (-16.0 to -5.9) |
| Anemia due to endocrine, metabolic, blood, and immune disorders | 6985.3 (6643.0 to 7368.1) | -- | 167.3 (112.0 to 241.1) | -0.2 (-6.9 to 7.3) | -13.8* (-19.6 to -7.4) |
| Endocrine, metabolic, blood, and immune disorders | 1161.2 (874.4 to 1520.1) | -- | 42.9 (26.1 to 64.7) | 17.1* (11.7 to 23.0) | 1.1 (-3.5 to 6.2) |
| Heart failure due to endocrine, metabolic, blood, and immune disorders | 2.0 (1.3 to 2.9) | -- | 0.2 (0.1 to 0.3) | 9.5* (8.1 to 11.0) | -5.4* (-6.7 to -4.2) |
| Oral disorders | 250130.8 (212679.1 to 290949.0) | 264579.8 (219785.4 to 308377.4) | 1081.5 (610.8 to 1778.4) | 17.6* (16.9 to 18.5) | 1.6* (0.9 to 2.3) |
| Caries of deciduous teeth | -- | -- | -- | -- | -- |
| Caries of permanent teeth | 209928.6 (166386.9 to 258312.2) | 257260.0 (212505.6 to 301021.7) | 147.6 (61.6 to 278.6) | 11.0* (9.6 to 12.2) | -4.2* (-5.4 to -3.2) |
| Periodontal disease | 51997.8 (37108.4 to 71732.8) | 6874.6 (3803.3 to 11021.3) | 345.0 (129.4 to 742.6) | 19.7* (18.9 to 20.6) | 3.4* (2.7 to 4.2) |
| Edentulism and severe tooth loss | 6473.8 (4955.9 to 8314.8) | 445.3 (284.8 to 643.9) | 186.3 (114.5 to 285.2) | 23.2* (21.2 to 25.2) | 6.3* (4.6 to 8.1) |
| Other oral disorders | 13786.4 (12403.5 to 15344.3) | -- | 402.7 (244.9 to 603.1) | 16.1* (15.0 to 17.2) | 0.2 (-0.8 to 1.2) |
| **Injuries** | **121034.5 (111005.8 to 133772.6)** | **40116.5 (34710.5 to 46247.4)** | **4351.4 (3244.5 to 5728.8)** | **16.4* (14.3 to 18.2)** | **0.5 (-1.4 to 2.0)** |
| **Transport injuries** | **15881.2 (14583.4 to 17266.8)** | **6324.4 (4531.8 to 8573.7)** | **1024.6 (742.6 to 1366.6)** | **14.6* (13.1 to 16.1)** | **-1.1 (-2.4 to 0.2)** |
| Road injuries | 12018.4 (11104.9 to 13159.2) | 5775.3 (4016.4 to 8041.1) | 767.9 (547.1 to 1028.0) | 13.6* (11.9 to 15.1) | -1.9* (-3.4 to -0.6) |
| Pedestrian road injuries | 3088.8 (2540.3 to 3930.3) | 1046.6 (578.8 to 1661.5) | 198.2 (135.5 to 274.0) | 8.9* (6.9 to 10.6) | -6.0* (-7.7 to -4.5) |
| Cyclist road injuries | 1937.0 (1517.8 to 2445.4) | 1134.4 (633.1 to 1860.7) | 116.9 (78.7 to 163.5) | 18.9* (16.3 to 21.6) | 2.7* (0.4 to 5.0) |
| Motorcyclist road injuries | 3370.5 (2780.1 to 4153.5) | 1217.3 (749.0 to 1889.6) | 214.8 (145.2 to 299.1) | 12.7* (10.6 to 14.8) | -2.7* (-4.5 to -0.9) |
| Motor vehicle road injuries | 2815.8 (2311.7 to 3500.4) | 1894.1 (1155.4 to 2817.6) | 192.0 (133.6 to 258.0) | 12.2* (10.0 to 14.5) | -3.1* (-5.0 to -1.2) |
| Other road injuries | 806.3 (630.7 to 1093.1) | 482.9 (238.4 to 841.0) | 46.0 (31.3 to 66.1) | 35.1* (32.1 to 38.1) | 16.6* (14.0 to 19.2) |
| Other transport injuries | 3862.8 (3029.7 to 4754.0) | 549.1 (331.4 to 846.2) | 256.6 (178.1 to 351.3) | 17.7* (15.4 to 20.2) | 1.6 (-0.4 to 3.8) |
| **Unintentional injuries** | **64356.4 (59092.5 to 70165.1)** | **29310.4 (24427.5 to 35172.1)** | **2535.5 (1828.8 to 3425.3)** | **21.1* (19.5 to 22.6)** | **4.5* (3.1 to 5.8)** |
| Falls | 24319.6 (20387.2 to 28915.0) | 9905.4 (6373.4 to 14468.2) | 1145.6 (805.9 to 1575.0) | 21.2* (19.5 to 22.7) | 4.6* (3.2 to 5.9) |
| Drowning | 149.7 (126.6 to 178.9) | 21.7 (15.2 to 30.6) | 9.8 (6.8 to 13.3) | 7.4* (4.0 to 10.8) | -7.2* (-10.2 to -4.3) |
| Fire, heat, and hot substances | 8927.7 (7364.7 to 10864.4) | 812.2 (475.6 to 1216.4) | 317.1 (221.3 to 436.3) | 14.8* (10.5 to 19.3) | -0.9 (-4.6 to 3.0) |
| Poisonings | 365.7 (306.7 to 433.0) | 300.7 (169.4 to 458.2) | 39.7 (26.3 to 56.3) | 32.6* (29.3 to 36.0) | 14.5* (11.6 to 17.4) |
| Poisoning by carbon monoxide | 66.9 (54.8 to 82.5) | 100.4 (45.1 to 178.1) | 6.5 (4.3 to 9.6) | 36.4* (31.9 to 41.2) | 17.8* (13.9 to 21.9) |
| Poisoning by other means | 298.8 (238.6 to 365.9) | 200.3 (111.6 to 323.6) | 33.1 (21.4 to 48.3) | 31.9* (28.4 to 35.5) | 13.9* (10.9 to 17.0) |
| Exposure to mechanical forces | 13815.9 (11373.0 to 16583.1) | 6976.0 (4638.2 to 10287.0) | 373.4 (257.4 to 528.3) | 24.3* (22.5 to 26.2) | 7.3* (5.7 to 8.9) |
| Unintentional firearm injuries | 670.7 (513.1 to 847.5) | 189.0 (83.8 to 347.5) | 28.1 (19.2 to 39.2) | 24.3* (22.4 to 26.3) | 7.3* (5.7 to 9.1) |
| Other exposure to mechanical forces | 13145.2 (10635.6 to 15979.3) | 6787.0 (4504.1 to 9992.3) | 345.3 (237.0 to 493.1) | 24.3* (22.4 to 26.2) | 7.3* (5.6 to 9.0) |
| Adverse effects of medical treatment | 102.6 (64.5 to 156.2) | 1340.4 (855.7 to 2009.9) | 13.7 (7.2 to 24.2) | 16.0* (10.5 to 21.1) | 0.1 (-4.6 to 4.5) |
| Animal contact | 2831.2 (2416.8 to 3295.7) | 3395.1 (2084.9 to 5140.1) | 88.6 (59.9 to 124.4) | 16.5* (13.5 to 19.3) | 0.6 (-2.0 to 3.0) |
| Venomous animal contact | 1280.4 (1010.3 to 1622.1) | 1844.0 (1094.9 to 2900.5) | 64.1 (42.6 to 91.8) | 18.1* (14.4 to 21.7) | 2.0 (-1.2 to 5.1) |
| Non-venomous animal contact | 1550.8 (1223.4 to 1856.1) | 1551.0 (892.2 to 2455.6) | 24.5 (14.7 to 39.5) | 12.6* (9.8 to 14.7) | -2.8* (-5.2 to -1.0) |
| Foreign body | 1409.3 (1166.8 to 1690.7) | 1996.9 (1308.2 to 2843.7) | 74.1 (52.6 to 102.0) | 20.2* (18.3 to 22.1) | 3.8* (2.2 to 5.4) |

| | | | | | |
|---|---|---|---|---|---|
| Pulmonary aspiration and foreign body in airway | 214.1<br>(167.2 to 264.4) | 170.9<br>(103.2 to 253.2) | 11.7<br>(7.9 to 16.1) | 23.4*<br>(20.0 to 26.7) | 6.6*<br>(3.6 to 9.3) |
| Foreign body in eyes | 242.5<br>(99.9 to 442.0) | 1567.8<br>(919.7 to 2467.1) | 15.1<br>(6.3 to 29.9) | 22.0*<br>(20.1 to 23.3) | 5.3*<br>(3.7 to 6.4) |
| Foreign body in other body part | 952.7<br>(749.2 to 1158.5) | 258.2<br>(151.8 to 421.3) | 47.3<br>(33.4 to 62.7) | 18.8*<br>(16.7 to 21.1) | 2.6*<br>(0.7 to 4.6) |
| Environmental heat and cold exposure | 2661.3<br>(2151.8 to 3246.6) | 604.4<br>(363.2 to 960.1) | 119.2<br>(81.5 to 163.1) | 22.1*<br>(19.6 to 24.4) | 5.4*<br>(3.3 to 7.4) |
| Exposure to forces of nature | 1841.8<br>(1310.2 to 2474.7) | 51.5<br>(35.7 to 71.1) | 85.2<br>(60.2 to 118.2) | 28.5*<br>(18.7 to 40.0) | 10.9*<br>(2.5 to 20.9) |
| Other unintentional injuries | 7931.7<br>(6420.3 to 9671.9) | 3906.1<br>(2527.9 to 5781.2) | 269.3<br>(185.2 to 384.6) | 22.4*<br>(20.3 to 24.4) | 5.7*<br>(3.9 to 7.4) |
| **Self-harm and interpersonal violence** | **40796.8**<br>**(32501.6 to 52479.0)** | **4481.7**<br>**(3466.6 to 5793.6)** | **791.3**<br>**(590.5 to 1016.9)** | **5.5***<br>**(0.2 to 10.4)** | **-9.0***<br>**(-13.5 to -4.7)** |
| Self-harm | 602.5<br>(457.7 to 759.9) | 425.4<br>(291.6 to 620.1) | 36.8<br>(25.3 to 50.5) | 0.7<br>(-1.3 to 2.7) | -13.1*<br>(-14.8 to -11.3) |
| Self-harm by firearm | 5.7<br>(4.6 to 7.3) | 1.7<br>(0.4 to 4.7) | 0.3<br>(0.2 to 0.4) | 2.9*<br>(0.0 to 6.0) | -11.1*<br>(-13.6 to -8.5) |
| Self-harm by other specified means | 596.9<br>(451.8 to 753.9) | 423.7<br>(289.4 to 618.8) | 36.5<br>(25.0 to 50.2) | 0.7<br>(-1.3 to 2.7) | -13.1*<br>(-14.8 to -11.3) |
| Interpersonal violence | 35547.0<br>(27567.9 to 46412.9) | 2707.6<br>(1808.6 to 3968.0) | 496.3<br>(360.0 to 678.7) | 16.0*<br>(14.2 to 17.7) | 0.1<br>(-1.4 to 1.6) |
| Assault by firearm | 203.0<br>(157.9 to 262.1) | 63.1<br>(32.1 to 107.8) | 10.4<br>(7.1 to 14.3) | 15.5*<br>(12.8 to 18.3) | -0.3<br>(-2.6 to 2.2) |
| Assault by sharp object | 1257.6<br>(962.0 to 1917.6) | 507.9<br>(284.5 to 844.3) | 45.1<br>(31.0 to 64.6) | 9.0*<br>(6.4 to 12.0) | -5.9*<br>(-8.1 to -3.3) |
| Sexual violence | 30815.1<br>(22946.6 to 42007.2) | -- | 286.6<br>(172.1 to 451.1) | 18.4*<br>(16.3 to 20.5) | 2.3*<br>(0.4 to 4.1) |
| Assault by other means | 3271.4<br>(2646.3 to 4145.4) | 2136.7<br>(1393.2 to 3126.4) | 154.2<br>(107.9 to 209.5) | 13.7*<br>(11.5 to 16.2) | -1.8<br>(-3.7 to 0.3) |
| Conflict and terrorism | 4341.7<br>(2982.0 to 6172.7) | 1121.4<br>(841.8 to 1450.2) | 245.5<br>(160.5 to 378.1) | -10.7*<br>(-17.7 to -2.4) | -22.9*<br>(-29.0 to -15.8) |
| Executions and police conflict | 305.6<br>(205.8 to 456.3) | 227.3<br>(151.9 to 331.1) | 12.8<br>(8.9 to 18.6) | 14.9*<br>(1.9 to 42.2) | -0.8<br>(-12.0 to 22.8) |
| **Nature of injury aggregates** | | | | | |
| Amputations | 27375.7<br>(24774.5 to 30527.4) | 830.1<br>(670.8 to 1040.3) | 480.6<br>(318.8 to 697.5) | 13.3*<br>(8.5 to 17.3) | -2.2<br>(-6.3 to 1.3) |
| Burns | 18804.8<br>(16698.4 to 21008.9) | 1234.6<br>(949.5 to 1558.2) | 686.8<br>(491.1 to 926.3) | 10.4*<br>(5.6 to 15.5) | -4.7*<br>(-8.8 to -0.3) |
| Fractures | 20112.1<br>(18518.6 to 21853.7) | 9886.5<br>(8157.9 to 11945.3) | 1131.0<br>(768.4 to 1594.5) | 17.4*<br>(14.7 to 19.3) | 1.3<br>(-1.0 to 3.0) |
| Head Injuries | 3360.7<br>(3141.7 to 3564.8) | 1835.1<br>(1508.5 to 2218.1) | 513.0<br>(361.3 to 700.4) | 22.7*<br>(20.5 to 24.7) | 5.9*<br>(4.0 to 7.7) |
| Spinal Injuries | 1916.3<br>(1710.8 to 2232.6) | 60.2<br>(46.7 to 76.8) | 599.8<br>(423.4 to 805.6) | 17.7*<br>(12.0 to 21.6) | 1.6<br>(-3.3 to 5.0) |
| Minor Injuries | 13593.4<br>(12525.1 to 14795.4) | 15148.2<br>(12686.6 to 18098.7) | 120.7<br>(57.9 to 225.6) | 21.1*<br>(19.6 to 22.4) | 4.5*<br>(3.2 to 5.7) |
| Other Injuries | 5056.4<br>(4703.5 to 5463.8) | 11121.8<br>(9672.0 to 12760.2) | 532.9<br>(375.8 to 717.3) | 16.1*<br>(12.2 to 18.8) | 0.2<br>(-3.2 to 2.6) |
| **Impairments** | | | | | |
| Anaemia | 129272.1<br>(122213.1 to 136885.6) | -- | 3300.3<br>(2230.1 to 4746.6) | -2.6<br>(-8.9 to 3.8) | -15.9*<br>(-21.4 to -10.4) |
| Epilepsy | 4706.6<br>(3876.2 to 5664.2) | -- | 1933.7<br>(1467.0 to 2569.0) | 19.2*<br>(12.5 to 25.6) | 2.9<br>(-2.8 to 8.4) |
| Guillain-Barré syndrome | 6.2<br>(3.6 to 10.0) | -- | 1.8<br>(1.0 to 3.3) | 18.4*<br>(15.7 to 21.3) | 2.2<br>(-0.1 to 4.7) |
| Hearing loss | 79452.4<br>(70647.8 to 88673.2) | -- | 1873.9<br>(1253.6 to 2744.4) | 14.9*<br>(12.4 to 17.7) | -0.8<br>(-3.0 to 1.6) |
| Heart failure | 616.8<br>(470.9 to 774.1) | -- | 57.3<br>(36.6 to 84.0) | 14.0*<br>(10.8 to 16.9) | -1.6<br>(-4.3 to 1.0) |
| Infertility | 24820.2<br>(15523.0 to 36388.2) | -- | 186.9<br>(90.9 to 354.3) | 24.3*<br>(19.0 to 29.4) | 7.3*<br>(2.8 to 11.7) |
| Developmental intellectual disability | 14423.5<br>(11191.0 to 17613.2) | -- | 1957.5<br>(1532.0 to 2456.7) | 18.7*<br>(15.3 to 22.4) | 2.5<br>(-0.4 to 5.7) |
| Pelvic inflammatory disease | 207.7<br>(158.7 to 266.4) | -- | 27.6<br>(17.8 to 40.9) | 18.1*<br>(12.7 to 24.0) | 1.9<br>(-2.7 to 7.0) |
| Vision loss | 65113.4<br>(59898.9 to 71243.8) | -- | 1445.0<br>(969.6 to 2128.1) | 15.8*<br>(14.7 to 16.9) | -0.0<br>(-1.0 to 0.9) |

We did not calculate incidence for the nine impairments and certain NTDs. Blank cells mean that no estimate is available or that the estimate has a magnitude less than 50.
Table presents prevalence and incidence counts in thousands.
* Percentage changes that are statistically significant
‡ Incidence estimates for stroke represent first-ever stroke only.
^ Incidence of HIV/AIDS represents new infections of HIV only and does not include new infections of Tuberculosis in HIV positive cases.

| SR Table 10k: Global prevalence, incidence and YLDs for 2017, percentage change of YLD counts and percentage change of YLD rates between 2007 and 2017 by cause, age 35 to 39 years | | | | | |
|---|---|---|---|---|---|
| Cause | Prevalence in thousands (95% UI) | Incidence in thousands (95% UI) | YLDs in thousands (95% UI) | | |
| | 2017 counts | 2017 counts | 2017 counts | Percentage change in counts between 2007 and 2017 | Percentage change in rates between 2007 and 2017 |
| **All causes** | **518917.8 (517075.7 to 520572.5)** | **2497367.5 (2133699.4 to 2955897.9)** | **61049.8 (46026.1 to 78754.6)** | **6.9* (6.2 to 7.6)** | **0.6 (-0.1 to 1.2)** |
| **Communicable, maternal, neonatal, and nutritional disorders** | **355361.3 (331483.5 to 385040.4)** | **1778116.8 (1433678.3 to 2232991.3)** | **7245.0 (5411.0 to 9534.9)** | **0.4 (-3.9 to 4.3)** | **-5.5* (-9.5 to -1.9)** |
| **HIV/AIDS and sexually transmitted infections** | **137034.9 (122010.1 to 153516.2)** | **84369.4 (68551.8 to 103886.3)** | **787.8 (547.5 to 1084.5)** | **-13.8 (-31.4 to 5.6)** | **-18.9* (-35.4 to -0.6)** |
| HIV/AIDS | 5685.5 (5378.2 to 6044.9) | 212.9^ (155.0 to 281.3) | 610.3 (414.7 to 857.2) | -18.6 (-36.0 to 4.4) | -23.4* (-39.8 to -1.8) |
| HIV/AIDS - Drug-susceptible Tuberculosis | 151.0 (135.7 to 166.5) | 188.1 (164.3 to 210.4) | 57.9 (39.1 to 78.4) | -21.3* (-25.2 to -17.0) | -25.9* (-29.6 to -21.9) |
| HIV/AIDS - Multidrug-resistant Tuberculosis without extensive drug resistance | 5.5 (3.7 to 8.0) | 7.7 (5.5 to 10.5) | 2.2 (1.3 to 3.5) | -24.4 (-48.4 to 12.1) | -28.9 (-51.5 to 5.5) |
| HIV/AIDS - Extensively drug-resistant Tuberculosis | 0.2 (0.2 to 0.3) | 0.3 (0.2 to 0.4) | 0.1 (0.1 to 0.1) | 37.7 (-7.2 to 94.6) | 29.6 (-12.7 to 83.1) |
| HIV/AIDS resulting in other diseases | 5528.8 (5213.7 to 5890.2) | 212.9 (155.0 to 281.3) | 550.1 (369.8 to 780.5) | -18.3 (-37.4 to 8.3) | -23.1 (-41.1 to 1.9) |
| HIV/AIDS not on antiretroviral treatment without Tuberculosis | 2215.1 (1925.3 to 2572.9) | 212.9 (155.0 to 281.3) | 293.1 (174.1 to 470.3) | -53.4* (-59.3 to -46.9) | -56.2* (-61.7 to -50.0) |
| HIV/AIDS on antiretroviral treatment without Tuberculosis | 3313.7 (3108.9 to 3534.1) | -- | 256.9 (172.3 to 362.5) | 491.0* (403.4 to 595.7) | 456.1* (373.6 to 554.6) |
| Sexually transmitted infections excluding HIV | 133964.5 (118644.6 to 150691.3) | 84156.6 (68310.9 to 103652.0) | 177.4 (97.5 to 332.8) | 8.2* (5.9 to 10.3) | 1.8 (-0.3 to 3.8) |
| Syphilis | 3964.3 (2806.8 to 5362.7) | 935.3 (584.0 to 1331.7) | 5.8 (4.1 to 8.1) | -3.7 (-7.6 to 0.9) | -9.4* (-13.1 to -5.0) |
| Early syphilis | 3936.9 (2780.3 to 5336.9) | 935.3 (584.0 to 1331.7) | 0.9 (0.3 to 2.5) | 10.1* (2.0 to 18.1) | 3.6 (-4.1 to 11.1) |
| Tertiary syphilis | 27.4 (22.3 to 32.9) | -- | 4.9 (3.3 to 6.9) | -6.0* (-9.4 to -2.1) | -11.6* (-14.8 to -7.9) |
| Chlamydial infection | 14962.4 (10948.9 to 19668.3) | 29476.3 (20885.6 to 39747.2) | 44.6 (24.6 to 81.9) | 6.3* (2.4 to 10.7) | 0.1 (-3.7 to 4.2) |
| Chlamydia episode | 13753.3 (9740.4 to 18543.4) | 29476.3 (20885.6 to 39747.2) | 23.4 (8.9 to 53.1) | 3.2* (1.5 to 4.7) | -2.9* (-4.5 to -1.5) |
| Chlamydial infection complications | 1209.1 (1128.4 to 1291.0) | -- | 21.2 (13.6 to 31.9) | 10.0* (1.8 to 17.9) | 3.5 (-4.2 to 10.9) |
| Gonococcal infection | 3030.7 (1908.2 to 4954.4) | 11370.1 (6655.9 to 17832.8) | 15.8 (8.7 to 27.9) | 12.2* (5.1 to 21.3) | 5.5 (-1.1 to 14.1) |
| Gonococcal infection complications | 367.2 (338.1 to 398.6) | -- | 8.4 (5.1 to 12.9) | 16.6* (3.9 to 30.3) | 9.7 (-2.3 to 22.6) |
| Gonorrhoea episode | 2663.5 (1536.4 to 4577.2) | 11370.1 (6655.9 to 17832.8) | 7.4 (2.4 to 17.6) | 7.5* (1.0 to 11.8) | 1.2 (-5.0 to 5.2) |
| Trichomoniasis | 16320.9 (11137.3 to 24081.0) | 35212.0 (24057.7 to 50812.2) | 27.3 (10.3 to 66.4) | 10.7* (8.6 to 12.7) | 4.2* (2.2 to 6.0) |
| Genital herpes | 104857.0 (88886.7 to 122439.8) | 7163.0 (5264.8 to 9600.5) | 27.1 (8.6 to 64.5) | 10.1* (8.7 to 11.6) | 3.6* (2.3 to 5.0) |
| Other sexually transmitted infections | 3030.5 (2844.3 to 3211.4) | -- | 56.7 (33.9 to 94.7) | 7.8* (5.0 to 11.1) | 1.4 (-1.2 to 4.5) |
| Other sexually transmitted diseases residual | -- | -- | 23.2 (12.3 to 44.7) | 4.5* (0.3 to 9.4) | -1.7 (-5.6 to 3.0) |
| Other sexually transmitted diseases | 3030.5 (2844.3 to 3211.4) | -- | 56.7 (33.9 to 94.7) | 7.8* (5.0 to 11.1) | 1.4 (-1.2 to 4.5) |
| **Respiratory infections and tuberculosis** | **179919.6 (127798.0 to 246943.9)** | **1209757.0 (869277.1 to 1672656.2)** | **728.2 (470.4 to 1075.4)** | **3.4* (1.7 to 5.0)** | **-2.7* (-4.3 to -1.2)** |
| Tuberculosis | 166674.2 (113247.6 to 236455.4) | 565.2 (384.6 to 768.2) | 229.3 (139.6 to 354.1) | 0.4 (-3.6 to 4.1) | -5.5* (-9.3 to -2.0) |
| Latent tuberculosis infection | 165936.4 (112441.5 to 235740.4) | -- | -- | -- | -- |
| Drug-susceptible tuberculosis | 700.4 (510.9 to 942.8) | 531.0 (360.6 to 719.8) | 217.5 (133.1 to 330.8) | 0.4 (-5.1 to 5.1) | -5.6* (-10.7 to -1.1) |
| Multidrug-resistant tuberculosis without extensive drug resistance | 35.6 (16.1 to 71.9) | 31.9 (16.4 to 58.0) | 11.3 (4.8 to 24.0) | 0.3 (-49.5 to 74.5) | -5.6 (-52.4 to 64.1) |
| Extensively drug-resistant tuberculosis | 1.8 (1.1 to 3.2) | 2.3 (1.5 to 3.4) | 0.6 (0.3 to 1.1) | 34.3 (-12.5 to 112.0) | 26.4 (-17.7 to 99.5) |
| Lower respiratory infections | 469.0 (400.1 to 541.6) | 20734.4 (17406.0 to 24532.1) | 28.6 (18.6 to 41.8) | 32.2* (21.2 to 43.3) | 24.4* (14.1 to 34.9) |
| Guillain-Barré syndrome due to lower respiratory infections | 0.8 (0.3 to 1.4) | -- | 0.2 (0.1 to 0.4) | 14.2* (11.4 to 16.7) | 7.4* (4.8 to 9.8) |
| Lower respiratory infection episode | 468.2 (398.9 to 541.1) | 20734.4 (17406.0 to 24532.1) | 28.3 (18.4 to 41.5) | 32.4* (21.3 to 43.6) | 24.6* (14.1 to 35.2) |
| Upper respiratory infections | 16258.7 (11616.0 to 22417.1) | 1183619.9 (842059.6 to 1646949.4) | 402.8 (220.1 to 686.9) | 4.7* (3.6 to 5.9) | -1.4* (-2.5 to -0.4) |
| Guillain-Barré syndrome due to upper respiratory infections | 2.0 (1.2 to 3.5) | -- | 0.6 (0.3 to 1.1) | 14.2* (11.4 to 16.7) | 7.4* (4.8 to 9.8) |
| Upper respiratory infection episode | 16256.7 (11614.2 to 22413.5) | 1183619.9 (842059.6 to 1646949.4) | 402.2 (219.5 to 686.2) | 4.7* (3.6 to 5.9) | -1.5* (-2.5 to -0.4) |
| Otitis media | 3254.3 (2420.3 to 4345.8) | 4837.5 (3427.0 to 6683.2) | 67.5 (37.4 to 117.6) | -3.1 (-6.9 to 1.6) | -8.8* (-12.4 to -4.4) |
| Acute otitis media | 264.7 (190.6 to 352.1) | 4837.0 (3426.8 to 6682.9) | 3.4 (1.7 to 6.4) | 10.2* (4.4 to 16.8) | 3.7 (-1.8 to 9.9) |
| Chronic otitis media | 2989.6 (2177.8 to 4051.1) | 0.4 (0.0 to 1.6) | 64.0 (35.5 to 111.6) | -3.7 (-7.8 to 1.3) | -9.4* (-13.3 to -4.7) |
| **Enteric infections** | **5302.6 (4792.6 to 5863.9)** | **365797.6 (326832.9 to 410013.1)** | **599.6 (417.6 to 834.1)** | **36.5* (31.7 to 41.4)** | **28.5* (23.9 to 33.0)** |
| Diarrhoeal diseases | 5326.3 (4822.3 to 5883.1) | 365352.0 (326403.6 to 409558.3) | 596.0 (415.1 to 829.5) | 37.0* (32.1 to 41.9) | 28.9* (24.3 to 33.5) |
| Guillain-Barré syndrome due to diarrheal diseases | 0.7 (0.4 to 1.2) | -- | 0.2 (0.1 to 0.4) | 14.2* (11.4 to 16.6) | 7.4* (4.9 to 9.7) |
| Diarrheal disease episode | 5325.6 (4821.7 to 5882.5) | 365352.0 (326403.6 to 409558.3) | 595.8 (415.0 to 829.3) | 37.0* (32.1 to 41.9) | 28.9* (24.3 to 33.6) |
| Typhoid and paratyphoid | 3.3 (1.7 to 5.4) | 422.8 (342.0 to 513.7) | 3.4 (2.1 to 5.0) | -15.2 (-30.8 to 2.0) | -20.2* (-34.9 to -4.0) |
| Typhoid fever | 20.1 (15.4 to 25.9) | 318.4 (249.4 to 400.9) | 3.1 (1.9 to 4.6) | -14.9 (-31.8 to 3.8) | -19.9* (-35.8 to -2.3) |
| Typhoid fever complications | 4.2 (2.9 to 5.9) | 54.8 (41.7 to 70.5) | 1.4 (0.8 to 2.1) | -15.3 (-38.7 to 15.5) | -20.3 (-42.4 to 8.7) |
| Typhoid fever episode | 15.9 (12.0 to 21.0) | 263.7 (206.7 to 332.0) | 1.7 (1.0 to 2.7) | -14.5 (-36.4 to 13.4) | -19.6 (-40.2 to 6.7) |
| Paratyphoid fever | 4.6 (3.3 to 6.1) | 104.4 (75.1 to 137.0) | 0.3 (0.2 to 0.5) | -18.5* (-29.2 to -5.8) | -23.3* (-33.4 to -11.4) |
| Intestinal perforation due to paratyphoid | 0.2 (0.1 to 0.3) | 5.3 (3.7 to 7.2) | -- | -18.4 (-34.5 to 2.3) | -23.2* (-38.4 to -3.8) |
| Paratyphoid fever episode | 4.4 (3.2 to 5.8) | 99.1 (71.4 to 130.4) | 0.3 (0.2 to 0.4) | -18.5* (-30.1 to -4.7) | -23.3* (-34.2 to -10.3) |
| iNTS | 0.9 (0.5 to 1.4) | 22.8 (13.1 to 36.8) | 0.1 (0.1 to 0.2) | -23.8* (-28.4 to -19.2) | -28.3* (-32.6 to -23.9) |
| Other intestinal infectious diseases | -- | -- | -- | -28.8* (-41.9 to -14.3) | -33.0* (-45.3 to -19.4) |
| **Neglected tropical diseases and malaria** | **87275.3 (82950.5 to 91743.9)** | **16089.3 (13082.6 to 19856.2)** | **1013.3 (703.1 to 1401.9)** | **-15.0* (-20.3 to -10.0)** | **-20.0* (-25.0 to -15.3)** |
| Malaria | 5558.0 (5178.3 to 5911.7) | 6012.3 (4841.9 to 7328.8) | 55.7 (41.3 to 73.5) | -14.6* (-24.1 to -4.1) | -19.6* (-28.5 to -9.8) |
| Malaria complications | 51.9 (47.1 to 57.3) | -- | 21.4 (16.5 to 26.8) | 31.6* (17.4 to 45.6) | 23.8* (10.5 to 37.0) |
| Malaria episode | 350.8 (227.7 to 500.1) | 6012.3 (4841.9 to 7328.8) | 11.3 (5.9 to 19.3) | -16.4* (-24.7 to -7.4) | -21.4* (-29.1 to -12.9) |

| | | | | | |
|---|---|---|---|---|---|
| Malaria parasitemia | 5155.3 (4770.2 to 5582.0) | -- | 23.0 (14.3 to 34.9) | -35.1* (-47.1 to -21.5) | -39.0* (-50.2 to -26.2) |
| Chagas disease | 554.8 (455.2 to 658.5) | 12.5 (8.4 to 17.6) | 3.9 (2.4 to 5.8) | -5.0 (-12.5 to 2.7) | -10.6* (-17.7 to -3.3) |
| Acute Chagas disease | 0.1 (0.0 to 0.1) | 12.5 (8.4 to 17.6) | -- | -2.5 (-7.1 to 2.4) | -8.3* (-12.6 to -3.7) |
| Asymptomatic Chagas disease | 483.8 (394.4 to 574.4) | -- | -- | -- | -- |
| Symptomatic chronic Chagas infection | 71.0 (54.1 to 89.7) | -- | 3.9 (2.4 to 5.7) | -5.0 (-12.5 to 2.7) | -10.6* (-17.7 to -3.3) |
| Leishmaniasis | 316.6 (255.2 to 396.0) | 33.8 (24.4 to 45.8) | 20.2 (12.7 to 30.6) | -0.2 (-15.3 to 21.2) | -6.1 (-20.3 to 14.0) |
| Visceral leishmaniasis | 0.4 (0.3 to 0.5) | 1.5 (1.1 to 2.2) | -- | -72.2* (-78.2 to -60.2) | -73.9* (-79.5 to -62.6) |
| Cutaneous and mucocutaneous leishmaniasis | 319.5 (258.7 to 401.3) | 32.3 (22.8 to 44.6) | 20.2 (12.7 to 30.6) | 0.1 (-15.0 to 21.8) | -5.8 (-20.1 to 14.6) |
| African trypanosomiasis | 0.4 (0.0 to 1.6) | 0.2 (0.1 to 0.8) | 0.1 (0.0 to 0.5) | -78.5 (-97.4 to 17.9) | -79.8 (-97.6 to 10.9) |
| Trypanosomiasis Gambiense | 0.4 (0.0 to 1.6) | 0.2 (0.0 to 0.8) | 0.1 (0.0 to 0.5) | -77.9 (-97.8 to 25.6) | -79.2 (-97.9 to 18.2) |
| Trypanosomiasis Rhodesiense | -- | 0.0 (0.0 to 0.1) | -- | -90.4* (-98.9 to -41.8) | -91.0* (-99.0 to -45.2) |
| Schistosomiasis | 15520.8 (13657.0 to 17555.7) | -- | 119.8 (60.3 to 223.8) | -16.4* (-20.3 to -11.3) | -21.4* (-25.0 to -16.6) |
| Mild schistosomiasis | 12697.2 (10950.9 to 14489.0) | -- | 71.5 (28.6 to 149.9) | -18.8* (-21.9 to -16.3) | -23.6* (-26.5 to -21.2) |
| Anemia due to schistosomiasis | 881.3 (727.7 to 1073.5) | -- | 20.6 (12.4 to 31.3) | -25.0* (-39.4 to -4.9) | -29.4* (-43.0 to -10.5) |
| Schistosomiasis complications | 1947.3 (1566.0 to 2386.2) | -- | 27.6 (15.7 to 46.6) | -0.2 (-6.4 to 6.7) | -6.1 (-11.9 to 0.4) |
| Cysticercosis | 439.1 (312.3 to 595.6) | -- | 132.5 (77.2 to 204.5) | -2.7 (-10.1 to 4.9) | -8.5* (-15.4 to -1.3) |
| Cystic echinococcosis | 54.7 (27.8 to 104.9) | 11.3 (5.1 to 25.6) | 4.5 (2.0 to 9.0) | 22.2* (11.9 to 32.8) | 15.0* (5.3 to 25.0) |
| Lymphatic filariasis | 5491.1 (4932.2 to 6147.3) | -- | 140.5 (77.8 to 220.4) | -40.1* (-51.4 to -29.5) | -43.6* (-54.3 to -33.7) |
| Prevalence of detectable microfiliaria due to lymphatic filariasis | 4224.4 (3840.3 to 4682.9) | -- | -- | -- | -- |
| Lymphatic filariasis complications | 1266.7 (856.8 to 1807.2) | -- | 140.5 (77.8 to 220.4) | -40.1* (-51.4 to -29.5) | -43.6* (-54.3 to -33.7) |
| Onchocerciasis | 1569.4 (935.3 to 2899.3) | -- | 99.2 (45.4 to 185.3) | -1.9 (-22.8 to 16.0) | -7.7 (-27.4 to 9.2) |
| Asymptomatic onchocerciasis | 439.0 (0.4 to 1632.7) | -- | -- | -- | -- |
| Skin disease due to onchocerciasis | 1059.4 (758.0 to 1407.8) | -- | 93.3 (39.9 to 179.3) | -0.8 (-23.2 to 17.4) | -6.7 (-27.8 to 10.5) |
| Vision loss due to onchocerciasis | 71.0 (49.4 to 109.8) | -- | 5.9 (3.6 to 9.3) | -16.1 (-30.7 to 0.5) | -21.1* (-34.7 to -5.4) |
| Trachoma | 24.6 (15.4 to 39.9) | -- | 3.8 (2.1 to 6.2) | -33.6* (-50.0 to -12.8) | -37.5* (-53.0 to -17.9) |
| Dengue | 405.5 (221.1 to 686.6) | 6776.5 (4123.8 to 10275.1) | 65.7 (29.5 to 122.4) | 64.9* (43.2 to 158.5) | 55.1* (34.8 to 143.3) |
| Post-dengue chronic fatigue syndrome | 285.9 (133.7 to 522.8) | -- | 58.7 (24.7 to 110.4) | 64.9* (43.0 to 159.1) | 55.1* (34.5 to 143.8) |
| Dengue episode | 119.6 (72.3 to 179.5) | 6776.5 (4123.8 to 10275.1) | 7.0 (3.6 to 12.4) | 64.7* (41.5 to 146.5) | 55.0* (33.2 to 132.0) |
| Yellow fever | 0.1 (0.0 to 0.4) | 4.9 (1.4 to 13.7) | -- | -14.4 (-39.1 to 20.0) | -19.4 (-42.7 to 12.9) |
| Asymptomatic yellow fever | 0.1 (0.0 to 0.2) | 2.8 (0.7 to 8.1) | -- | -- | -- |
| Yellow fever episode | 0.1 (0.0 to 0.2) | 2.2 (0.6 to 6.1) | -- | -14.4 (-39.1 to 20.0) | -19.4 (-42.7 to 12.9) |
| Rabies | -- | 0.7 (0.5 to 0.9) | -- | -34.1* (-48.8 to -15.3) | -38.0* (-51.8 to -20.3) |
| Intestinal nematode infections | 57663.2 (53650.1 to 62232.3) | -- | 104.3 (60.1 to 170.4) | -26.9* (-31.1 to -22.3) | -31.2* (-35.1 to -26.8) |
| Ascariasis | 27630.2 (24197.4 to 31687.8) | -- | 35.0 (18.9 to 60.4) | -30.5* (-37.9 to -22.3) | -34.6* (-41.5 to -26.9) |
| Asymptomatic ascariasis | 25733.4 (22494.5 to 29578.7) | -- | -- | -- | -- |
| Ascariasis complications | 1896.8 (1682.0 to 2149.3) | -- | 35.0 (18.9 to 60.4) | -30.5* (-37.9 to -22.3) | -34.6* (-41.5 to -26.9) |
| Trichuriasis | 19799.6 (17345.1 to 22761.8) | -- | 14.8 (8.3 to 24.6) | -14.7* (-22.7 to -6.3) | -19.7* (-27.3 to -11.8) |
| Asymptomatic trichuriasis | 19051.4 (16631.7 to 22002.8) | -- | -- | -- | -- |
| Trichuriasis complications | 748.2 (681.0 to 815.2) | -- | 14.8 (8.3 to 24.6) | -14.7* (-22.7 to -6.3) | -19.7* (-27.3 to -11.8) |
| Hookworm disease | 14327.8 (13257.1 to 15488.0) | -- | 54.5 (32.3 to 87.4) | -27.3* (-33.2 to -20.6) | -31.6* (-37.1 to -25.3) |
| Asymptomatic hookworm disease | 11758.8 (10854.1 to 12749.8) | -- | -- | -- | -- |
| Anemia due to hookworm disease | 692.5 (617.6 to 771.5) | -- | 15.5 (10.0 to 22.8) | -30.1* (-39.3 to -19.0) | -34.2* (-42.9 to -23.8) |
| Hookworm disease complications | 1876.6 (1744.1 to 2016.6) | -- | 39.0 (21.8 to 64.3) | -26.2* (-31.8 to -20.0) | -30.5* (-35.8 to -24.8) |
| Food-borne trematodiases | 7775.0 (6777.4 to 8874.0) | 3033.8 (2485.7 to 3643.4) | 200.4 (115.3 to 332.1) | -11.4* (-16.6 to -5.0) | -16.7* (-21.6 to -10.6) |
| Asymptomatic food-borne tremodiases | 5994.9 (4923.1 to 7081.1) | 2387.7 (1526.9 to 3131.6) | -- | -- | -- |
| Food-borne tremodiases complications | 1780.0 (1176.7 to 2700.3) | 646.2 (136.4 to 1439.8) | 200.4 (115.3 to 332.1) | -11.4* (-16.6 to -5.0) | -16.7* (-21.6 to -10.6) |
| Leprosy | 35.8 (32.8 to 39.3) | 3.1 (2.7 to 3.5) | 2.3 (1.5 to 3.3) | -13.3* (-21.8 to -2.9) | -18.5* (-26.5 to -8.6) |
| Ebola virus disease | -- | -- | -- | -97.0* (-97.3 to -96.4) | -97.2* (-97.4 to -96.6) |
| Ebola cases | -- | -- | -- | -95.9* (-96.2 to -95.5) | -96.1* (-96.4 to -95.8) |
| Post-Ebola chronic fatigue syndrome | -- | -- | -- | -97.1* (-97.3 to -96.4) | -97.2* (-97.5 to -96.6) |
| Zika virus disease | 3.3 (2.0 to 5.8) | 200.0 (118.2 to 353.6) | 0.1 (0.0 to 0.1) | -- | -- |
| Zika virus complications | 0.0 (0.0 to 0.1) | -- | -- | -- | -- |
| Zika virus episode | 3.3 (1.9 to 5.8) | 200.0 (118.2 to 353.6) | 0.1 (0.0 to 0.1) | -- | -- |
| Guinea worm disease | -- | -- | -- | -99.5* (-99.7 to -99.2) | -99.5* (-99.7 to -99.3) |
| Other neglected tropical diseases | 2696.9 (2514.6 to 2913.7) | -- | 60.4 (39.5 to 89.2) | -8.4 (-16.3 to 0.5) | -13.8* (-21.3 to -5.4) |
| Acute infection due to other neglected tropical diseases | -- | -- | 0.8 (0.4 to 1.3) | 101.5* (73.7 to 197.7) | 89.6* (63.4 to 180.1) |
| Anemia due to other neglected tropical diseases | 2696.9 (2514.6 to 2913.7) | -- | 59.6 (39.1 to 88.2) | -9.0* (-17.0 to -0.0) | -14.4* (-21.9 to -5.9) |
| **Other infectious diseases** | **5953.3 (5367.2 to 6783.1)** | **18811.0 (14442.6 to 25787.5)** | **255.1 (180.7 to 343.6)** | **-2.6 (-9.3 to 5.6)** | **-8.4* (-14.7 to -0.6)** |
| Meningitis | 741.8 (623.5 to 874.6) | 173.9 (121.1 to 245.4) | 62.3 (43.5 to 84.2) | -2.9 (-7.6 to 1.9) | -8.7* (-13.1 to -4.1) |
| Pneumococcal meningitis | 263.9 (216.8 to 320.9) | 10.8 (6.4 to 17.1) | 23.2 (15.8 to 31.6) | -23.1* (-28.9 to -17.5) | -27.7* (-33.1 to -22.4) |

| | | | | | |
|---|---|---|---|---|---|
| Acute pneumococcal meningitis | 0.5<br>(0.3 to 0.8) | 10.8<br>(6.4 to 17.1) | 0.1<br>(0.0 to 0.1) | -22.9*<br>(-32.4 to -13.7) | -27.5*<br>(-36.4 to -18.8) |
| Pneumococcal meningitis complications | 263.4<br>(216.3 to 320.4) | -- | 23.2<br>(15.7 to 31.6) | -23.1*<br>(-28.9 to -17.5) | -27.7*<br>(-33.1 to -22.3) |
| H influenzae type B meningitis | 61.1<br>(43.8 to 81.8) | 3.3<br>(1.8 to 5.4) | 5.0<br>(3.4 to 6.8) | -50.5*<br>(-53.5 to -47.2) | -53.4*<br>(-56.3 to -50.3) |
| Acute H influenzae type B meningitis | 0.2<br>(0.1 to 0.2) | 3.3<br>(1.8 to 5.4) | -- | -54.8*<br>(-60.7 to -49.7) | -57.4*<br>(-63.0 to -52.7) |
| H Influenza type B meningitis complications | 61.0<br>(43.7 to 81.6) | -- | 5.0<br>(3.4 to 6.7) | -50.5*<br>(-53.5 to -47.1) | -53.4*<br>(-56.2 to -50.3) |
| Meningococcal infection | 77.0<br>(54.7 to 102.3) | 9.7<br>(5.3 to 15.8) | 7.0<br>(4.8 to 9.6) | 0.7<br>(-3.5 to 4.9) | -5.2*<br>(-9.2 to -1.3) |
| Acute meningococcal meningitis | 0.4<br>(0.2 to 0.7) | 9.7<br>(5.3 to 15.8) | 0.1<br>(0.0 to 0.1) | 15.0*<br>(2.3 to 27.7) | 8.2<br>(-3.7 to 20.2) |
| Meningococcal meningitis complications | 76.6<br>(54.2 to 102.0) | -- | 6.9<br>(4.7 to 9.5) | 0.6<br>(-3.6 to 4.8) | -5.3*<br>(-9.3 to -1.4) |
| Other meningitis | 339.8<br>(250.9 to 431.0) | 150.1<br>(104.8 to 212.2) | 27.1<br>(18.8 to 36.4) | 59.8*<br>(48.9 to 70.5) | 50.4*<br>(40.1 to 60.4) |
| Other acute bacterial meningitis | 1.3<br>(0.8 to 1.8) | 27.6<br>(18.7 to 39.4) | 0.2<br>(0.1 to 0.3) | 61.1*<br>(46.3 to 75.0) | 51.6*<br>(37.6 to 64.7) |
| Acute viral meningitis | 5.6<br>(3.7 to 8.0) | 122.4<br>(85.2 to 172.8) | 0.7<br>(0.4 to 1.2) | 9.8*<br>(0.1 to 18.5) | 3.3<br>(-5.9 to 11.5) |
| Other bacterial meningitis complications | 332.9<br>(243.9 to 425.2) | -- | 26.2<br>(18.3 to 35.2) | 61.9*<br>(50.6 to 72.9) | 52.3*<br>(41.7 to 62.7) |
| Encephalitis | 524.1<br>(293.2 to 833.5) | 98.9<br>(95.0 to 103.5) | 42.6<br>(29.9 to 56.4) | -1.6<br>(-7.9 to 4.8) | -7.4*<br>(-13.3 to -1.4) |
| Acute encephalitis | 5.3<br>(5.1 to 5.6) | 98.9<br>(95.0 to 103.5) | 0.7<br>(0.5 to 1.0) | 10.5*<br>(10.0 to 11.0) | 3.9*<br>(3.5 to 4.4) |
| Encephalitis complications | 518.7<br>(287.9 to 828.2) | -- | 41.9<br>(29.3 to 55.5) | -1.8<br>(-8.1 to 4.7) | -7.6*<br>(-13.5 to -1.4) |
| Diphtheria | -- | 0.2<br>(0.1 to 0.3) | -- | -36.0*<br>(-54.2 to -9.6) | -39.8*<br>(-56.9 to -14.9) |
| Whooping cough | 2.0<br>(1.6 to 2.6) | 14.9<br>(11.6 to 18.9) | 0.1<br>(0.1 to 0.2) | -4.0*<br>(-4.3 to -3.7) | -9.7*<br>(-9.9 to -9.4) |
| Tetanus | 3.8<br>(3.6 to 3.9) | 2.4<br>(1.4 to 3.5) | 0.1<br>(0.1 to 0.1) | -7.0<br>(-18.2 to 1.8) | -12.5*<br>(-23.0 to -4.2) |
| Severe tetanus | 0.1<br>(0.1 to 0.2) | 2.4<br>(1.4 to 3.5) | -- | -43.6*<br>(-57.3 to -24.1) | -47.0*<br>(-59.8 to -28.6) |
| Neonatal tetanus complications | 3.6<br>(3.5 to 3.8) | -- | 0.1<br>(0.0 to 0.1) | 10.8*<br>(9.2 to 12.2) | 4.3*<br>(2.8 to 5.5) |
| Measles | 0.6<br>(0.2 to 1.4) | 23.7<br>(8.5 to 52.7) | 0.1<br>(0.0 to 0.1) | -34.5*<br>(-39.9 to -29.0) | -38.3*<br>(-43.4 to -33.2) |
| Varicella and herpes zoster | 314.6<br>(251.1 to 384.3) | 2192.6<br>(1712.5 to 2733.2) | 17.0<br>(9.8 to 27.2) | 3.8<br>(-1.3 to 9.2) | -2.3<br>(-7.1 to 2.7) |
| Chickenpox | 9.4<br>(4.5 to 16.8) | 489.8<br>(234.5 to 877.2) | 0.1<br>(0.0 to 0.1) | 23.3*<br>(19.5 to 28.7) | 16.1*<br>(12.5 to 21.1) |
| Herpes zoster | 305.2<br>(242.7 to 376.0) | 1702.9<br>(1312.8 to 2135.9) | 17.0<br>(9.8 to 27.1) | 3.7<br>(-1.4 to 9.1) | -2.4<br>(-7.2 to 2.7) |
| Acute hepatitis | 1728.4<br>(1222.4 to 2515.7) | 16304.3<br>(11994.0 to 23169.8) | 44.0<br>(26.5 to 70.4) | -3.6<br>(-32.2 to 43.5) | -9.3<br>(-36.2 to 35.0) |
| Acute hepatitis A | 257.5<br>(223.1 to 290.6) | 3346.9<br>(2900.8 to 3778.0) | 10.8<br>(6.7 to 16.1) | 5.4<br>(-3.0 to 14.4) | -0.8<br>(-8.7 to 7.7) |
| Acute hepatitis B | 1401.1<br>(898.5 to 2175.4) | 12142.4<br>(7787.3 to 18853.5) | 31.4<br>(16.9 to 54.5) | -6.8<br>(-44.1 to 59.9) | -12.3<br>(-47.4 to 50.5) |
| Acute hepatitis C | 21.5<br>(8.2 to 38.9) | 186.1<br>(71.4 to 337.0) | 0.3<br>(0.1 to 0.7) | 2.1<br>(-2.3 to 5.6) | -3.9*<br>(-8.1 to -0.6) |
| Acute hepatitis E | 48.4<br>(33.0 to 69.2) | 628.9<br>(428.6 to 899.7) | 1.5<br>(0.8 to 2.6) | 4.6<br>(-13.5 to 24.2) | -1.5<br>(-18.6 to 16.8) |
| Other unspecified infectious diseases | 3361.4<br>(3165.4 to 3588.3) | -- | 89.0<br>(58.3 to 131.3) | -3.5<br>(-9.5 to 2.9) | -9.2*<br>(-14.8 to -3.2) |
| Guillain-Barré syndrome due to other infectious diseases | 0.5<br>(0.2 to 0.8) | -- | 0.1<br>(0.1 to 0.3) | 14.1*<br>(11.4 to 16.7) | 7.4*<br>(4.8 to 9.8) |
| Other infectious diseases | -- | -- | 15.6<br>(8.9 to 25.9) | 8.6*<br>(4.8 to 13.0) | 2.2<br>(-1.4 to 6.4) |
| Anemia due to other infectious diseases | 3360.9<br>(3165.0 to 3587.8) | -- | 73.2<br>(48.3 to 107.0) | -5.7<br>(-12.6 to 1.7) | -11.3*<br>(-17.7 to -4.3) |
| **Maternal and neonatal disorders** | **10445.4<br>(9211.1 to 11834.3)** | **9624.5<br>(8083.0 to 11473.1)** | **1986.8<br>(1496.6 to 2565.1)** | **16.4*<br>(10.7 to 23.0)** | **9.6*<br>(4.2 to 15.7)** |
| Maternal disorders | 1176.8<br>(1008.4 to 1382.8) | 9624.5<br>(8083.0 to 11473.1) | 108.2<br>(75.4 to 144.8) | 4.0<br>(-5.4 to 14.9) | -2.2<br>(-11.0 to 8.1) |
| Maternal haemorrhage | 220.4<br>(198.2 to 244.1) | 881.8<br>(583.7 to 1249.4) | 7.6<br>(4.9 to 11.1) | 9.1<br>(-8.3 to 31.3) | 2.6<br>(-13.7 to 23.5) |
| Maternal hemorrhage complications | 201.1<br>(181.2 to 221.5) | -- | 4.4<br>(2.8 to 6.5) | 7.6<br>(-7.7 to 25.7) | 1.3<br>(-13.2 to 18.3) |
| Maternal hemorrhage episode | 19.3<br>(10.7 to 31.8) | 881.8<br>(583.7 to 1249.4) | 3.2<br>(1.7 to 5.5) | 11.0<br>(-22.9 to 56.6) | 4.5<br>(-27.4 to 47.3) |
| Maternal sepsis and other pregnancy related infections | 451.8<br>(382.2 to 537.1) | 1156.6<br>(773.8 to 1685.5) | 6.2<br>(3.0 to 11.4) | 15.5<br>(-25.3 to 69.5) | 8.7<br>(-29.7 to 59.5) |
| Infertility due to puerperal sepsis | 365.7<br>(320.9 to 416.4) | 12.5<br>(8.4 to 16.8) | 1.8<br>(0.7 to 4.0) | 22.6*<br>(17.4 to 28.1) | 15.3*<br>(10.5 to 20.6) |
| Maternal sepsis and other maternal infection episode | 86.1<br>(40.9 to 150.4) | 1144.2<br>(762.0 to 1675.5) | 4.4<br>(2.0 to 8.0) | 12.8<br>(-38.9 to 90.1) | 6.1<br>(-42.5 to 78.9) |
| Maternal hypertensive disorders | 354.6<br>(205.8 to 541.3) | 1924.0<br>(1441.1 to 2433.4) | 17.7<br>(9.3 to 30.4) | 16.2<br>(-22.4 to 63.5) | 9.4<br>(-26.9 to 53.9) |
| Maternal hypertensive disorder complications | 14.2<br>(12.9 to 15.7) | 198.3<br>(181.5 to 216.9) | 0.9<br>(0.6 to 1.4) | 24.9*<br>(11.3 to 40.3) | 17.5*<br>(4.7 to 32.0) |
| Maternal hypertensive disorder episode | 340.4<br>(191.6 to 527.5) | 1725.8<br>(1248.1 to 2235.1) | 16.8<br>(8.6 to 29.2) | 15.8<br>(-24.4 to 66.5) | 9.0<br>(-28.9 to 56.7) |
| Maternal obstructed labour and uterine rupture | 179.0<br>(145.0 to 218.2) | 747.7<br>(516.0 to 1072.4) | 57.4<br>(36.8 to 81.2) | -7.3<br>(-14.8 to 0.4) | -12.8*<br>(-19.9 to -5.5) |
| Obstructed labor, acute event | 10.0<br>(5.3 to 16.3) | 731.5<br>(501.3 to 1055.9) | 3.2<br>(1.5 to 5.5) | 12.0<br>(-36.0 to 81.0) | 5.4<br>(-39.7 to 70.3) |
| Maternal obstructed labor complications | 169.0<br>(135.0 to 207.9) | 16.3<br>(12.6 to 20.3) | 54.2<br>(34.6 to 77.2) | -8.3*<br>(-15.3 to -0.6) | -13.7*<br>(-20.3 to -6.5) |
| Maternal abortion and miscarriage | 21.5<br>(12.1 to 35.4) | 2625.0<br>(1663.6 to 3894.5) | 2.4<br>(1.2 to 4.2) | 16.2<br>(-25.7 to 74.4) | 9.3<br>(-30.1 to 64.1) |
| Ectopic pregnancy | 18.7<br>(10.6 to 30.1) | 2289.3<br>(1496.3 to 3313.5) | 2.0<br>(1.0 to 3.4) | 18.9<br>(-25.1 to 86.3) | 11.8<br>(-29.6 to 75.3) |
| Other maternal disorders | -- | -- | 14.9<br>(10.4 to 20.0) | 37.8*<br>(24.9 to 52.9) | 29.6*<br>(17.5 to 43.9) |
| Neonatal disorders | 9288.4<br>(8037.3 to 10685.1) | -- | 1878.5<br>(1399.6 to 2427.9) | 17.2*<br>(11.2 to 24.4) | 10.3*<br>(4.7 to 17.0) |
| Neonatal preterm birth | 5361.5<br>(4623.1 to 6282.4) | -- | 863.8<br>(606.3 to 1183.8) | 22.9*<br>(8.1 to 38.4) | 15.6*<br>(1.7 to 30.2) |
| Preterm birth complications | 4582.2<br>(3840.7 to 5486.6) | -- | 819.4<br>(571.7 to 1135.3) | 24.2*<br>(8.3 to 40.4) | 16.9*<br>(1.9 to 32.1) |
| Retinopathy of prematurity | 779.3<br>(621.0 to 945.6) | -- | 44.3<br>(29.3 to 61.4) | 2.6<br>(-3.5 to 9.4) | -3.5<br>(-9.2 to 2.9) |
| Uncomplicated preterm birth | -- | -- | -- | -- | -- |
| Neonatal encephalopathy due to birth asphyxia and trauma | 3672.1<br>(2703.6 to 4855.7) | -- | 664.2<br>(410.0 to 1043.4) | 10.6*<br>(7.0 to 14.3) | 4.1*<br>(0.7 to 7.5) |
| Neonatal sepsis and other neonatal infections | 1002.1<br>(611.8 to 1536.7) | -- | 293.5<br>(156.0 to 484.7) | 16.4*<br>(11.6 to 21.2) | 9.6*<br>(5.0 to 14.0) |
| Neonatal sepsis and other neonatal infection complications | 1002.1<br>(611.8 to 1536.7) | -- | 293.5<br>(156.0 to 484.7) | 16.4*<br>(11.6 to 21.2) | 9.6*<br>(5.0 to 14.0) |
| Neonatal sepsis and other neonatal infection episode | -- | -- | -- | -- | -- |
| Hemolytic disease and other neonatal jaundice | 153.3<br>(131.6 to 175.6) | -- | 57.1<br>(43.9 to 71.3) | 21.6*<br>(17.1 to 26.1) | 14.4*<br>(10.2 to 18.6) |

| | | | | | |
|---|---|---|---|---|---|
| Other neonatal disorders | -- | -- | -- | -- | -- |
| **Nutritional deficiencies** | **115014.7 (110116.4 to 120123.8)** | **73471.9 (66404.7 to 80906.3)** | **1874.4 (1231.9 to 2739.3)** | **-6.3 (-11.8 to 0.1)** | **-11.8* (-17.0 to -5.8)** |
| Protein-energy malnutrition | 3202.9 (2990.3 to 3428.9) | 3513.4 (3251.7 to 3784.1) | 27.8 (17.9 to 39.1) | 27.4* (15.6 to 37.2) | 19.9* (8.8 to 29.1) |
| Iodine deficiency | 9109.0 (8177.6 to 10145.5) | 55.7 (45.6 to 67.7) | 156.7 (94.0 to 250.7) | -3.4 (-7.8 to 1.2) | -9.1* (-13.2 to -4.8) |
| Visible goiter without symptoms | 8919.4 (7995.8 to 9945.4) | 55.7 (45.6 to 67.7) | 95.5 (46.7 to 177.9) | -4.6* (-6.4 to -3.0) | -10.3* (-11.9 to -8.8) |
| Visible goiter with complications | 189.6 (98.9 to 273.4) | -- | 61.2 (29.1 to 97.2) | -1.4 (-13.8 to 8.3) | -7.2 (-18.9 to 1.9) |
| Vitamin A deficiency | 44848.9 (40631.4 to 49205.1) | 69902.8 (62913.4 to 77305.6) | 12.6 (7.7 to 19.0) | 17.7* (8.9 to 27.3) | 10.7* (2.5 to 19.8) |
| Asymptomatic vitamin A deficiency | 44658.9 (40435.5 to 49003.4) | 69902.8 (62913.4 to 77305.6) | -- | -- | -- |
| Vitamin A deficiency complications | 190.0 (138.1 to 255.9) | -- | 12.6 (7.7 to 19.0) | 17.7* (8.9 to 27.3) | 10.7* (2.5 to 19.8) |
| Vitamin A deficiency with anemia | -- | -- | -- | -- | -- |
| Dietary iron deficiency | 73335.3 (69226.6 to 77929.1) | -- | 1664.4 (1101.2 to 2430.8) | -7.2* (-13.4 to -0.3) | -12.7* (-18.5 to -6.2) |
| Other nutritional deficiencies | -- | -- | 12.9 (8.3 to 18.2) | 18.9* (7.9 to 28.0) | 11.9* (1.5 to 20.5) |
| **Non-communicable diseases** | **508519.8 (504457.0 to 512257.3)** | **682945.4 (631217.2 to 737672.2)** | **49258.4 (37202.7 to 64069.3)** | **7.6* (7.0 to 8.2)** | **1.2* (0.6 to 1.8)** |
| **Neoplasms** | **4074.6 (3934.7 to 4268.7)** | **613.8 (525.0 to 811.3)** | **264.9 (187.0 to 351.3)** | **17.6* (12.9 to 22.9)** | **10.6* (6.3 to 15.7)** |
| Lip and oral cavity cancer | 85.2 (78.4 to 94.2) | 16.5 (15.1 to 18.4) | 7.8 (5.6 to 10.5) | 26.3* (13.8 to 40.5) | 18.9* (7.1 to 32.2) |
| Diagnosis and primary therapy phase of mouth cancer | 4.8 (4.4 to 5.3) | 16.5 (15.1 to 18.4) | 1.4 (0.9 to 1.9) | 28.3* (16.8 to 40.7) | 20.7* (9.9 to 32.4) |
| Controlled phase of mouth cancer | 74.2 (68.2 to 82.1) | -- | 3.5 (2.2 to 5.2) | 29.3* (13.6 to 48.1) | 21.7* (6.9 to 39.4) |
| Metastatic phase of mouth cancer | 5.5 (5.0 to 6.1) | -- | 2.5 (1.6 to 3.3) | 22.1* (9.2 to 35.6) | 14.9* (2.7 to 27.6) |
| Terminal phase of mouth cancer | 0.8 (0.7 to 0.9) | -- | 0.4 (0.3 to 0.6) | 21.1* (9.1 to 33.0) | 14.0* (2.6 to 25.1) |
| Nasopharynx cancer | 42.4 (37.6 to 47.3) | 7.1 (6.3 to 7.9) | 3.9 (2.7 to 5.2) | -10.6 (-22.3 to 3.2) | -15.9* (-26.9 to -2.9) |
| Diagnosis and primary therapy phase of nasopharynx cancer | 2.4 (2.1 to 2.7) | 7.1 (6.3 to 7.9) | 0.7 (0.5 to 1.0) | -9.7 (-21.0 to 3.1) | -15.1* (-25.7 to -2.9) |
| Controlled phase of nasopharynx cancer | 37.0 (32.8 to 41.3) | -- | 1.8 (1.1 to 2.7) | -9.7 (-24.2 to 7.5) | -15.0 (-28.7 to 1.1) |
| Metastatic phase of nasopharynx cancer | 2.7 (2.4 to 3.1) | -- | 1.2 (0.8 to 1.7) | -12.0 (-22.6 to 0.4) | -17.2* (-27.2 to -5.5) |
| Terminal phase of nasopharynx cancer | 0.3 (0.3 to 0.3) | -- | 0.2 (0.1 to 0.2) | -14.1* (-23.9 to -3.1) | -19.2* (-28.4 to -8.8) |
| Other pharynx cancer | 10.9 (9.0 to 11.8) | 4.3 (3.5 to 4.7) | 1.5 (1.0 to 2.0) | 25.7* (13.1 to 35.8) | 18.2* (6.4 to 27.8) |
| Diagnosis and primary therapy phase of other pharynx cancer | 0.6 (0.5 to 0.6) | 4.3 (3.5 to 4.7) | 0.2 (0.1 to 0.2) | 26.8* (15.4 to 36.0) | 19.3* (8.5 to 27.9) |
| Controlled phase of other pharynx cancer | 8.4 (6.9 to 9.0) | -- | 0.4 (0.3 to 0.6) | 26.3* (11.3 to 41.0) | 18.8* (4.7 to 32.6) |
| Metastatic phase of other pharynx cancer | 1.6 (1.3 to 1.8) | -- | 0.7 (0.5 to 1.0) | 25.2* (12.7 to 35.4) | 17.8* (6.1 to 27.4) |
| Terminal phase of other pharynx cancer | 0.3 (0.2 to 0.3) | -- | 0.2 (0.1 to 0.2) | 25.1* (12.5 to 35.4) | 17.7* (5.8 to 27.4) |
| Oesophageal cancer | 10.2 (9.7 to 10.7) | 4.6 (4.4 to 4.8) | 1.5 (1.1 to 2.0) | -9.1* (-13.6 to -3.8) | -14.4* (-18.7 to -9.4) |
| Diagnosis and primary therapy phase of esophageal cancer | 0.8 (0.8 to 0.9) | 4.6 (4.4 to 4.8) | 0.2 (0.2 to 0.3) | -6.4* (-11.3 to -0.9) | -11.9* (-16.6 to -6.7) |
| Controlled phase of esophageal cancer | 7.5 (7.1 to 7.9) | -- | 0.4 (0.2 to 0.6) | -5.0 (-11.2 to 1.2) | -10.6* (-16.4 to -4.8) |
| Metastatic phase of esophageal cancer | 1.6 (1.5 to 1.6) | -- | 0.7 (0.5 to 0.9) | -11.3* (-15.5 to -6.2) | -16.5* (-20.5 to -11.8) |
| Terminal phase of esophageal cancer | 0.3 (0.3 to 0.4) | -- | 0.2 (0.1 to 0.2) | -11.3* (-15.5 to -6.2) | -16.5* (-20.5 to -11.8) |
| Stomach cancer | 55.7 (53.6 to 57.9) | 20.3 (19.6 to 21.0) | 6.7 (4.8 to 8.7) | -10.6* (-15.3 to -5.4) | -15.8* (-20.3 to -10.9) |
| Diagnosis and primary therapy phase of stomach cancer | 4.8 (4.6 to 5.0) | 20.3 (19.6 to 21.0) | 1.4 (0.9 to 1.9) | -6.8* (-11.1 to -2.4) | -12.3* (-16.3 to -8.2) |
| Controlled phase of stomach cancer | 44.1 (42.4 to 45.9) | -- | 2.1 (1.3 to 3.1) | -0.7 (-11.3 to 10.8) | -6.5 (-16.5 to 4.3) |
| Metastatic phase of stomach cancer | 5.4 (5.2 to 5.6) | -- | 2.4 (1.6 to 3.2) | -17.5* (-21.0 to -13.8) | -22.3* (-25.6 to -18.9) |
| Terminal phase of stomach cancer | 1.4 (1.3 to 1.4) | -- | 0.7 (0.5 to 1.0) | -17.4* (-21.0 to -13.8) | -22.3* (-25.6 to -18.9) |
| Colon and rectum cancer | 170.2 (164.1 to 175.9) | 28.5 (27.4 to 29.5) | 15.6 (11.0 to 20.8) | 13.7* (6.9 to 20.2) | 7.0* (0.6 to 13.1) |
| Diagnosis and primary therapy phase of colon and rectum cancers | 7.0 (6.8 to 7.3) | 28.5 (27.4 to 29.5) | 2.0 (1.4 to 2.8) | 17.5* (11.8 to 23.1) | 10.6* (5.2 to 15.8) |
| Controlled phase of colon and rectum cancers | 152.1 (146.6 to 157.3) | -- | 9.1 (6.0 to 13.0) | 15.8* (8.4 to 24.1) | 9.0* (2.0 to 16.8) |
| Metastatic phase of colon and rectum cancers | 8.1 (7.8 to 8.3) | -- | 3.6 (2.5 to 4.8) | 9.2* (2.8 to 14.8) | 2.7 (-3.2 to 8.0) |
| Terminal phase of colon and rectum cancers | 1.1 (1.1 to 1.2) | -- | 0.6 (0.4 to 0.8) | 6.2 (-0.4 to 11.8) | -0.1 (-6.3 to 5.2) |
| Stoma from colon and rectum cancers, beyond 10 years | 1.9 (1.7 to 2.2) | -- | 0.2 (0.1 to 0.3) | -6.2* (-7.0 to -5.4) | -11.7* (-12.5 to -11.0) |
| Liver cancer | 11.9 (9.0 to 15.3) | 21.4 (19.9 to 23.2) | 5.5 (3.9 to 7.2) | -13.8* (-20.4 to -4.4) | -18.9* (-25.1 to -10.0) |
| Liver cancer due to hepatitis B | 23.4 (20.8 to 26.3) | 13.5 (12.1 to 15.1) | 3.5 (2.4 to 4.7) | -17.6* (-25.1 to -7.1) | -22.4* (-29.5 to -12.6) |
| Diagnosis and primary therapy phase of liver cancer due to hepatitis B | 3.4 (3.0 to 3.8) | 13.5 (12.1 to 15.1) | 1.0 (0.6 to 1.4) | -19.2* (-27.9 to -6.6) | -24.0* (-32.1 to -12.1) |
| Controlled phase of liver cancer due to hepatitis B | 16.4 (14.5 to 18.6) | -- | 0.8 (0.5 to 1.2) | 3.5 (-17.0 to 31.0) | -2.6 (-21.9 to 23.3) |
| Metastatic phase of liver cancer due to hepatitis B | 2.6 (2.3 to 2.9) | -- | 1.2 (0.8 to 1.6) | -23.9* (-29.8 to -15.9) | -28.4* (-33.9 to -20.9) |
| Terminal phase of liver cancer due to hepatitis B | 1.0 (0.9 to 1.1) | -- | 0.6 (0.4 to 0.7) | -23.9* (-29.8 to -15.9) | -28.4* (-33.9 to -20.9) |
| Liver cancer due to hepatitis C | 3.6 (2.9 to 4.4) | 2.1 (1.7 to 2.6) | 0.5 (0.4 to 0.7) | -6.1 (-13.6 to 3.4) | -11.7* (-18.7 to -2.7) |
| Diagnosis and primary therapy phase of liver cancer due to hepatitis C | 0.5 (0.4 to 0.6) | 2.1 (1.7 to 2.6) | 0.1 (0.1 to 0.2) | -7.7 (-14.3 to 0.8) | -13.1* (-19.3 to -5.2) |
| Controlled phase of liver cancer due to hepatitis C | 2.5 (2.0 to 3.1) | -- | 0.1 (0.1 to 0.2) | 13.2 (-3.0 to 33.8) | 6.6 (-8.8 to 25.9) |
| Metastatic phase of liver cancer due to hepatitis C | 0.4 (0.3 to 0.5) | -- | 0.2 (0.1 to 0.3) | -12.3* (-18.6 to -4.7) | -17.5* (-23.4 to -10.3) |
| Terminal phase of liver cancer due to hepatitis C | 0.2 (0.1 to 0.2) | -- | 0.1 (0.1 to 0.1) | -12.3* (-18.6 to -4.7) | -17.5* (-23.4 to -10.3) |
| Liver cancer due to alcohol use | 4.3 (3.2 to 5.6) | 2.7 (2.0 to 3.4) | 0.7 (0.4 to 1.0) | -2.2 (-8.9 to 5.7) | -8.0* (-14.3 to -0.5) |
| Diagnosis and primary therapy phase of liver cancer due to alcohol use | 0.7 (0.5 to 0.8) | 2.7 (2.0 to 3.4) | 0.2 (0.1 to 0.3) | -3.1 (-9.8 to 4.7) | -8.8* (-15.2 to -1.5) |
| Controlled phase of liver cancer due to alcohol use | 3.0 (2.2 to 3.9) | -- | 0.1 (0.1 to 0.2) | 8.1* (0.6 to 17.3) | 1.7 (-5.4 to 10.3) |
| Metastatic phase of liver cancer due to alcohol use | 0.5 (0.4 to 0.7) | -- | 0.2 (0.1 to 0.3) | -5.6 (-12.2 to 2.1) | -11.1* (-17.4 to -3.9) |

| | | | | | |
|---|---|---|---|---|---|
| Terminal phase of liver cancer due to alcohol use | 0.2 (0.2 to 0.3) | -- | 0.1 (0.1 to 0.2) | -5.6 (-12.2 to 2.1) | -11.1* (-17.4 to -3.9) |
| Liver cancer due to NASH | 1.6 (1.2 to 1.9) | 0.9 (0.8 to 1.2) | 0.2 (0.2 to 0.3) | -2.8 (-9.3 to 5.5) | -8.6* (-14.6 to -0.8) |
| Diagnosis and primary therapy phase of liver cancer due to NASH | 0.2 (0.2 to 0.3) | 0.9 (0.8 to 1.2) | 0.1 (0.0 to 0.1) | -4.1 (-10.5 to 3.6) | -9.8* (-15.7 to -2.5) |
| Controlled phase of liver cancer due to NASH | 1.1 (0.8 to 1.3) | -- | 0.1 (0.0 to 0.1) | 14.9* (5.9 to 25.5) | 8.1 (-0.4 to 18.1) |
| Metastatic phase of liver cancer due to NASH | 0.2 (0.1 to 0.2) | -- | 0.1 (0.1 to 0.1) | -8.2* (-14.1 to -0.8) | -13.6* (-19.2 to -6.7) |
| Terminal phase of liver cancer due to NASH | 0.1 (0.1 to 0.1) | -- | 0.0 (0.0 to 0.1) | -8.2* (-14.1 to -0.8) | -13.6* (-19.2 to -6.7) |
| Liver cancer due to other causes | 3.7 (3.0 to 4.6) | 2.2 (1.7 to 2.7) | 0.6 (0.4 to 0.8) | -12.5* (-19.8 to -2.4) | -17.6* (-24.6 to -8.1) |
| Diagnosis and primary therapy phase of liver cancer due to other causes | 0.5 (0.4 to 0.7) | 2.2 (1.7 to 2.7) | 0.2 (0.1 to 0.2) | -14.0* (-20.5 to -5.4) | -19.0* (-25.2 to -11.0) |
| Controlled phase of liver cancer due to other causes | 2.6 (2.1 to 3.3) | -- | 0.1 (0.1 to 0.2) | 8.0 (-8.4 to 30.4) | 1.6 (-13.8 to 22.7) |
| Metastatic phase of liver cancer due to other causes | 0.4 (0.3 to 0.5) | -- | 0.2 (0.1 to 0.3) | -18.8* (-24.9 to -10.6) | -23.6* (-29.3 to -15.8) |
| Terminal phase of liver cancer due to other causes | 0.2 (0.1 to 0.2) | -- | 0.1 (0.1 to 0.1) | -18.8* (-24.9 to -10.6) | -23.6* (-29.3 to -15.8) |
| Gallbladder and biliary tract cancer | 3.3 (2.9 to 3.6) | 2.1 (1.8 to 2.4) | 0.6 (0.4 to 0.7) | 4.8 (-2.4 to 12.8) | -1.4 (-8.2 to 6.2) |
| Diagnosis and primary therapy phase of gallbladder and biliary tract cancer | 0.4 (0.3 to 0.4) | 2.1 (1.8 to 2.4) | 0.1 (0.1 to 0.2) | 5.5 (-1.8 to 13.6) | -0.7 (-7.6 to 6.9) |
| Controlled phase of gallbladder and biliary tract cancer | 2.1 (1.9 to 2.3) | -- | 0.1 (0.1 to 0.2) | 12.4* (4.4 to 20.9) | 5.7 (-1.7 to 13.7) |
| Metastatic phase of gallbladder and biliary tract cancer | 0.6 (0.5 to 0.6) | -- | 0.3 (0.2 to 0.4) | 2.5 (-4.4 to 10.8) | -3.6 (-10.0 to 4.2) |
| Terminal phase of gallbladder and biliary tract cancer | 0.2 (0.1 to 0.2) | -- | 0.1 (0.1 to 0.1) | 2.5 (-4.4 to 10.8) | -3.6 (-10.0 to 4.2) |
| Pancreatic cancer | 5.1 (4.9 to 5.3) | 4.2 (4.1 to 4.4) | 1.0 (0.7 to 1.3) | 5.9* (1.8 to 10.2) | -0.4 (-4.2 to 3.7) |
| Diagnosis and primary therapy phase of pancreatic cancer | 1.0 (1.0 to 1.0) | 4.2 (4.1 to 4.4) | 0.3 (0.2 to 0.4) | 5.9* (1.8 to 10.2) | -0.4 (-4.3 to 3.7) |
| Controlled phase of pancreatic cancer | 2.9 (2.8 to 3.0) | -- | 0.1 (0.1 to 0.2) | 5.0* (1.0 to 8.9) | -1.2 (-5.0 to 2.5) |
| Metastatic phase of pancreatic cancer | 0.9 (0.8 to 0.9) | -- | 0.4 (0.3 to 0.5) | 6.1* (1.9 to 10.6) | -0.2 (-4.1 to 4.1) |
| Terminal phase of pancreatic cancer | 0.3 (0.3 to 0.4) | -- | 0.2 (0.1 to 0.2) | 6.1* (1.9 to 10.6) | -0.2 (-4.1 to 4.1) |
| Larynx cancer | 14.8 (14.1 to 15.6) | 2.8 (2.7 to 3.0) | 1.6 (1.1 to 2.1) | 6.0* (0.8 to 11.5) | -0.2 (-5.2 to 4.9) |
| Diagnosis and primary therapy phase of larynx cancer | 0.9 (0.8 to 0.9) | 2.8 (2.7 to 3.0) | 0.2 (0.2 to 0.3) | 8.0* (2.6 to 13.7) | 1.6 (-3.5 to 7.0) |
| Controlled phase of larynx cancer | 12.5 (11.9 to 13.1) | -- | 0.8 (0.5 to 1.2) | 8.2* (3.4 to 13.5) | 1.8 (-2.7 to 6.8) |
| Metastatic phase of larynx cancer | 1.0 (0.9 to 1.0) | -- | 0.4 (0.3 to 0.6) | 2.3 (-3.4 to 8.2) | -3.8 (-9.1 to 1.8) |
| Terminal phase of larynx cancer | 0.1 (0.1 to 0.2) | -- | 0.1 (0.1 to 0.1) | 0.5 (-5.4 to 6.7) | -5.5 (-11.0 to 0.4) |
| Laryngectomy from larynx cancer, beyond 10 years | 0.4 (0.4 to 0.4) | -- | -- | 5.1* (4.6 to 5.7) | -1.1* (-1.6 to -0.6) |
| Tracheal, bronchus, and lung cancer | 28.4 (27.4 to 29.6) | 16.2 (15.6 to 16.9) | 4.7 (3.4 to 6.1) | -8.0* (-12.0 to -3.6) | -13.5* (-17.2 to -9.3) |
| Diagnosis and primary therapy phase of lung, bronchus, and trachea cancer | 1.8 (1.7 to 1.8) | 16.2 (15.6 to 16.9) | 0.5 (0.3 to 0.7) | 0.5 (-3.1 to 4.8) | -5.4* (-8.9 to -1.4) |
| Controlled phase of lung, bronchus, and trachea cancer | 19.8 (19.0 to 20.6) | -- | 1.0 (0.6 to 1.4) | 9.6 (-0.5 to 21.5) | 3.1 (-6.4 to 14.3) |
| Metastatic phase of lung, bronchus, and trachea cancer | 5.6 (5.4 to 5.8) | -- | 2.5 (1.7 to 3.4) | -13.4* (-16.7 to -9.9) | -18.5* (-21.6 to -15.2) |
| Terminal phase of lung, bronchus, and trachea cancer | 1.2 (1.2 to 1.3) | -- | 0.7 (0.5 to 0.9) | -13.4* (-16.7 to -9.9) | -18.5* (-21.6 to -15.2) |
| Malignant skin melanoma | 147.0 (124.7 to 196.1) | 17.2 (14.7 to 22.8) | 8.4 (5.4 to 12.6) | 14.1* (5.7 to 23.8) | 7.4 (-0.5 to 16.5) |
| Diagnosis and primary therapy phase of malignant skin melanoma | 3.9 (3.3 to 5.2) | 17.2 (14.7 to 22.8) | 1.1 (0.7 to 1.7) | 14.7* (4.5 to 25.5) | 7.9 (-1.7 to 18.1) |
| Controlled phase of malignant skin melanoma | 141.9 (120.3 to 189.2) | -- | 6.7 (4.1 to 10.4) | 14.8* (5.0 to 25.0) | 8.0 (-1.2 to 17.6) |
| Metastatic phase of malignant skin melanoma | 1.1 (1.0 to 1.4) | -- | 0.5 (0.3 to 0.7) | 6.6* (0.4 to 13.7) | 0.3 (-5.5 to 6.9) |
| Terminal phase of malignant skin melanoma | 0.2 (0.2 to 0.2) | -- | 0.1 (0.1 to 0.1) | 5.4 (-0.8 to 12.4) | -0.8 (-6.6 to 5.8) |
| Non-melanoma skin cancer | 8.5 (4.0 to 15.7) | 135.5 (50.9 to 334.3) | 0.5 (0.2 to 1.1) | 11.3* (4.3 to 17.8) | 4.7 (-1.9 to 10.9) |
| Non-melanoma skin cancer (squamous-cell carcinoma) | 9.4 (3.3 to 20.5) | 8.4 (2.9 to 18.4) | 0.4 (0.1 to 1.0) | 11.0* (2.5 to 18.5) | 4.4 (-3.5 to 11.5) |
| Non-melanoma skin cancer (basal-cell carcinoma) | 12.5 (4.7 to 33.9) | 127.1 (42.4 to 328.2) | 0.1 (0.0 to 0.2) | 14.0* (0.6 to 23.6) | 7.3 (-5.3 to 16.3) |
| Breast cancer | 828.3 (785.1 to 885.0) | 100.2 (94.9 to 107.5) | 66.2 (45.9 to 91.7) | 16.3* (8.2 to 25.5) | 9.5* (1.8 to 18.1) |
| Diagnosis and primary therapy phase of breast cancer | 22.7 (21.5 to 24.3) | 100.2 (94.9 to 107.5) | 6.3 (4.2 to 8.8) | 18.5* (5.5 to 33.9) | 11.5 (-0.7 to 26.0) |
| Controlled phase of breast cancer | 765.5 (725.4 to 818.4) | -- | 47.6 (30.5 to 69.6) | 16.9* (8.7 to 25.3) | 10.0* (2.3 to 17.9) |
| Metastatic phase of breast cancer | 25.4 (24.0 to 27.3) | -- | 10.9 (7.4 to 14.4) | 14.0 (-0.6 to 31.4) | 7.2 (-6.4 to 23.7) |
| Terminal phase of breast cancer | 1.9 (1.7 to 2.0) | -- | 1.0 (0.7 to 1.3) | 11.4* (2.5 to 20.6) | 4.8 (-3.6 to 13.5) |
| Mastectomy from breast cancer, beyond 10 years | 12.9 (11.9 to 13.9) | -- | 0.5 (0.3 to 0.7) | -1.3 (-8.9 to 7.3) | -7.1 (-14.3 to 0.9) |
| Cervical cancer | 501.0 (460.0 to 533.3) | 66.7 (61.3 to 71.2) | 36.3 (25.4 to 48.8) | 2.9 (-3.8 to 10.3) | -3.2 (-9.5 to 3.8) |
| Diagnosis and primary therapy phase of cervical cancer | 25.6 (23.5 to 27.3) | 66.7 (61.3 to 71.2) | 7.0 (4.7 to 9.7) | 3.8 (-9.2 to 17.6) | -2.4 (-14.6 to 10.6) |
| Controlled phase of cervical cancer | 458.1 (420.6 to 487.4) | -- | 21.5 (13.5 to 31.4) | 4.5 (-1.9 to 12.0) | -1.6 (-7.7 to 5.4) |
| Metastatic phase of cervical cancer | 15.5 (14.3 to 16.6) | -- | 6.8 (4.4 to 9.2) | -1.8 (-12.2 to 9.6) | -7.6 (-17.3 to 3.1) |
| Terminal phase of cervical cancer | 1.8 (1.6 to 1.9) | -- | 1.0 (0.7 to 1.2) | -3.5 (-9.6 to 3.2) | -9.2* (-14.9 to -2.9) |
| Uterine cancer | 78.0 (74.0 to 82.5) | 9.5 (9.0 to 10.0) | 5.2 (3.6 to 7.0) | 4.7 (-4.7 to 15.9) | -1.5 (-10.4 to 9.0) |
| Diagnosis and primary therapy phase of uterine cancer | 3.5 (3.3 to 3.7) | 9.5 (9.0 to 10.0) | 1.0 (0.7 to 1.4) | 5.2 (-1.3 to 13.7) | -1.0 (-7.2 to 7.0) |
| Controlled phase of uterine cancer | 72.9 (69.2 to 77.1) | -- | 3.5 (2.2 to 5.0) | 5.6 (-6.4 to 19.2) | -0.7 (-11.9 to 12.2) |
| Metastatic phase of uterine cancer | 1.5 (1.4 to 1.6) | -- | 0.7 (0.5 to 0.9) | 0.6 (-6.2 to 9.5) | -5.4 (-11.7 to 3.0) |
| Terminal phase of uterine cancer | 0.1 (0.1 to 0.2) | -- | 0.1 (0.1 to 0.1) | -2.8 (-9.8 to 6.6) | -8.5 (-15.1 to 0.3) |
| Ovarian cancer | 86.9 (81.4 to 92.9) | 13.3 (12.5 to 14.2) | 10.6 (7.6 to 13.9) | 17.4* (6.1 to 31.6) | 10.4 (-0.1 to 23.8) |
| Diagnosis and primary therapy phase of ovarian cancer | 3.1 (3.0 to 3.4) | 13.3 (12.5 to 14.2) | 0.9 (0.6 to 1.3) | 18.1* (9.7 to 28.0) | 11.2* (3.2 to 20.5) |
| Controlled phase of ovarian cancer | 69.4 (65.1 to 74.1) | -- | 3.3 (2.1 to 4.8) | 18.3* (3.9 to 34.3) | 11.3 (-2.3 to 26.3) |
| Metastatic phase of ovarian cancer | 13.8 (12.9 to 14.7) | -- | 6.1 (4.1 to 8.3) | 16.8* (1.7 to 35.7) | 9.9 (-4.3 to 27.7) |

| | | | | | |
|---|---|---|---|---|---|
| Terminal phase of ovarian cancer | 0.6 (0.5 to 0.6) | -- | 0.3 (0.2 to 0.4) | 16.0* (6.9 to 26.5) | 9.1* (0.6 to 19.0) |
| Prostate cancer | 24.2 (20.4 to 28.6) | 2.6 (2.2 to 3.1) | 1.8 (1.2 to 2.6) | 34.6* (27.1 to 43.7) | 26.7* (19.6 to 35.2) |
| Diagnosis and primary therapy phase of prostate cancer | 0.8 (0.7 to 1.0) | 2.6 (2.2 to 3.1) | 0.2 (0.2 to 0.3) | 38.6* (32.6 to 46.6) | 30.4* (24.7 to 38.0) |
| Controlled phase of prostate cancer | 22.1 (18.5 to 26.4) | -- | 1.4 (0.9 to 2.1) | 36.5* (27.7 to 46.9) | 28.4* (20.2 to 38.2) |
| Metastatic phase of prostate cancer | 0.2 (0.2 to 0.2) | -- | 0.1 (0.1 to 0.1) | 22.7* (17.8 to 28.5) | 15.4* (10.8 to 20.9) |
| Terminal phase of prostate cancer | -- | -- | -- | 15.7* (11.4 to 21.2) | 8.9* (4.8 to 14.1) |
| Impotence and Incontinence after 10-year survival from prostate cancer | 1.1 (1.0 to 1.1) | -- | 0.0 (0.0 to 0.1) | 3.6* (2.9 to 4.2) | -2.6* (-3.2 to -2.0) |
| Testicular cancer | 77.6 (71.6 to 84.2) | 9.3 (8.6 to 10.1) | 4.7 (3.2 to 6.6) | 14.6* (3.7 to 27.1) | 7.9 (-2.4 to 19.6) |
| Diagnosis and primary therapy phase of testicular cancer | 2.5 (2.3 to 2.7) | 9.3 (8.6 to 10.1) | 0.7 (0.5 to 1.0) | 16.1* (6.3 to 26.3) | 9.3* (0.0 to 18.8) |
| Controlled phase of testicular cancer | 74.0 (68.3 to 80.4) | -- | 3.5 (2.2 to 5.2) | 15.8* (2.7 to 30.4) | 8.9 (-3.3 to 22.7) |
| Metastatic phase of testicular cancer | 0.9 (0.9 to 1.0) | -- | 0.4 (0.3 to 0.6) | 5.9 (-0.5 to 13.8) | -0.4 (-6.4 to 7.1) |
| Terminal phase of testicular cancer | 0.1 (0.1 to 0.1) | -- | 0.1 (0.0 to 0.1) | 0.1 (-6.6 to 8.0) | -5.9 (-12.2 to 1.6) |
| Kidney cancer | 69.2 (64.6 to 72.6) | 9.9 (9.2 to 10.4) | 4.1 (2.8 to 5.6) | 9.4* (2.1 to 18.5) | 3.0 (-3.9 to 11.5) |
| Diagnosis and primary therapy phase of kidney cancer | -- | 9.9 (9.2 to 10.4) | -- | -- | -- |
| Controlled phase of kidney cancer | 67.4 (62.9 to 70.7) | -- | 3.2 (2.0 to 4.7) | 9.7* (1.1 to 20.3) | 3.2 (-4.9 to 13.2) |
| Metastatic phase of kidney cancer | 1.5 (1.4 to 1.6) | -- | 0.7 (0.5 to 0.9) | 8.6* (3.0 to 14.3) | 2.2 (-3.1 to 7.6) |
| Terminal phase of kidney cancer | 0.3 (0.3 to 0.3) | -- | 0.2 (0.1 to 0.2) | 8.6* (3.0 to 14.3) | 2.2 (-3.1 to 7.6) |
| Bladder cancer | 50.2 (45.2 to 54.2) | 6.4 (5.7 to 6.9) | 4.4 (3.0 to 6.1) | 14.2* (5.5 to 24.5) | 7.5 (-0.7 to 17.2) |
| Diagnosis and primary therapy phase of bladder cancer | 2.6 (2.3 to 2.8) | 6.4 (5.7 to 6.9) | 0.7 (0.5 to 1.1) | 16.3* (8.0 to 25.1) | 9.4* (1.6 to 17.7) |
| Controlled phase of bladder cancer | 46.0 (41.4 to 49.7) | -- | 3.1 (2.0 to 4.4) | 14.5* (4.2 to 26.0) | 7.8 (-2.0 to 18.6) |
| Metastatic phase of bladder cancer | 0.9 (0.8 to 0.9) | -- | 0.4 (0.3 to 0.5) | 10.8* (3.0 to 19.7) | 4.2 (-3.1 to 12.6) |
| Terminal phase of bladder cancer | 0.2 (0.1 to 0.2) | -- | 0.1 (0.1 to 0.1) | 10.2* (2.5 to 19.1) | 3.7 (-3.6 to 12.1) |
| Incontinence from bladder cancer, beyond 10 years | 0.6 (0.5 to 0.6) | -- | 0.1 (0.1 to 0.1) | 4.6* (3.7 to 5.4) | -1.6* (-2.5 to -0.8) |
| Brain and nervous system cancer | 113.2 (96.6 to 134.2) | 22.5 (19.6 to 25.8) | 10.3 (7.2 to 14.1) | 40.5* (26.2 to 60.4) | 32.2* (18.7 to 50.9) |
| Diagnosis and primary therapy phase of brain and nervous system cancers | 6.8 (5.8 to 8.0) | 22.5 (19.6 to 25.8) | 2.0 (1.3 to 2.8) | 51.4* (35.1 to 74.0) | 42.5* (27.1 to 63.7) |
| Controlled phase of brain and nervous system cancers | 98.5 (83.7 to 117.2) | -- | 4.7 (2.9 to 7.2) | 57.9* (36.6 to 86.4) | 48.6* (28.5 to 75.4) |
| Metastatic phase of brain and nervous system cancers | 6.8 (6.0 to 7.7) | -- | 3.1 (2.1 to 4.1) | 20.0* (10.4 to 32.3) | 12.9* (3.9 to 24.5) |
| Terminal phase of brain and nervous system cancers | 1.1 (1.0 to 1.3) | -- | 0.6 (0.4 to 0.8) | 16.0* (7.1 to 27.3) | 9.2* (0.8 to 19.8) |
| Thyroid cancer | 186.7 (174.2 to 205.4) | 20.7 (19.3 to 22.8) | 10.6 (7.1 to 15.3) | 23.9* (13.5 to 36.6) | 16.6* (6.8 to 28.5) |
| Diagnosis and primary therapy phase of thyroid cancer | 4.9 (4.6 to 5.4) | 20.7 (19.3 to 22.8) | 1.4 (0.9 to 2.0) | 24.5* (8.2 to 41.2) | 17.2* (1.8 to 32.9) |
| Controlled phase of thyroid cancer | 180.3 (168.3 to 198.4) | -- | 8.5 (5.4 to 12.8) | 24.5* (12.8 to 37.9) | 17.1* (6.2 to 29.8) |
| Metastatic phase of thyroid cancer | 1.3 (1.3 to 1.5) | -- | 0.6 (0.4 to 0.8) | 16.7* (8.0 to 26.6) | 9.8* (1.6 to 19.1) |
| Terminal phase of thyroid cancer | 0.1 (0.1 to 0.1) | -- | 0.1 (0.0 to 0.1) | 15.4* (7.0 to 25.5) | 8.5* (0.7 to 18.1) |
| Mesothelioma | 1.7 (1.5 to 1.8) | 0.7 (0.7 to 0.8) | 0.3 (0.2 to 0.4) | 2.1 (-9.8 to 13.2) | -4.0 (-15.1 to 6.5) |
| Diagnosis and primary therapy phase of mesothelioma | 0.2 (0.2 to 0.2) | 0.7 (0.7 to 0.8) | 0.0 (0.0 to 0.1) | 3.2 (-8.1 to 14.0) | -2.9 (-13.5 to 7.2) |
| Controlled phase of mesothelioma | 1.0 (0.9 to 1.2) | -- | 0.1 (0.0 to 0.1) | 2.2 (-9.8 to 13.3) | -3.8 (-15.1 to 6.6) |
| Metastatic phase of mesothelioma | 0.4 (0.4 to 0.4) | -- | 0.2 (0.1 to 0.2) | 1.8 (-10.1 to 13.1) | -4.2 (-15.4 to 6.4) |
| Terminal phase of mesothelioma | 0.1 (0.1 to 0.1) | -- | -- | 1.2 (-11.0 to 12.6) | -4.8 (-16.3 to 5.9) |
| Hodgkin lymphoma | 55.6 (49.0 to 71.0) | 7.9 (6.9 to 9.9) | 4.0 (2.7 to 5.6) | 13.4* (4.1 to 22.7) | 6.7 (-2.0 to 15.4) |
| Diagnosis and primary therapy phase of Hodgkin disease | 1.8 (1.6 to 2.3) | 7.9 (6.9 to 9.9) | 0.5 (0.3 to 0.8) | 16.1* (7.4 to 24.5) | 9.2* (1.1 to 17.2) |
| Controlled phase of Hodgkin disease | 51.5 (45.4 to 65.8) | -- | 2.5 (1.5 to 3.8) | 16.2* (4.7 to 28.5) | 9.3 (-1.5 to 20.9) |
| Metastatic phase of Hodgkin disease | 2.1 (1.8 to 2.6) | -- | 0.9 (0.6 to 1.3) | 7.1* (0.4 to 13.7) | 0.8 (-5.5 to 7.0) |
| Terminal phase of Hodgkin disease | 0.2 (0.2 to 0.2) | -- | 0.1 (0.1 to 0.1) | 0.7 (-5.9 to 6.6) | -5.3 (-11.4 to 0.3) |
| Non-Hodgkin's lymphoma | 86.0 (82.6 to 89.3) | 15.6 (15.1 to 16.2) | 6.7 (4.8 to 8.9) | 21.9* (14.4 to 29.3) | 14.7* (7.7 to 21.7) |
| Diagnosis and primary therapy phase of non-Hodgkin lymphoma | 3.1 (3.0 to 3.2) | 15.6 (15.1 to 16.2) | 0.9 (0.6 to 1.2) | 25.4* (19.2 to 31.1) | 18.0* (12.2 to 23.4) |
| Controlled phase of non-Hodgkin lymphoma | 78.5 (75.3 to 81.4) | -- | 3.7 (2.4 to 5.5) | 25.2* (14.3 to 36.3) | 17.8* (7.5 to 28.2) |
| Metastatic phase of non-Hodgkin lymphoma | 3.8 (3.7 to 4.0) | -- | 1.7 (1.2 to 2.3) | 15.3* (9.1 to 20.6) | 8.5* (2.6 to 13.5) |
| Terminal phase of non-Hodgkin lymphoma | 0.7 (0.6 to 0.7) | -- | 0.4 (0.2 to 0.5) | 14.1* (7.7 to 19.5) | 7.3* (1.4 to 12.4) |
| Multiple myeloma | 8.1 (7.4 to 9.4) | 2.1 (1.9 to 2.4) | 1.6 (1.1 to 2.1) | 26.3* (18.1 to 34.3) | 18.9* (11.1 to 26.4) |
| Diagnosis and primary therapy phase of multiple myeloma | 0.5 (0.5 to 0.6) | 2.1 (1.9 to 2.4) | 0.2 (0.1 to 0.2) | 29.0* (20.8 to 37.0) | 21.3* (13.7 to 28.9) |
| Controlled phase of multiple myeloma | 5.0 (4.5 to 5.8) | -- | 0.2 (0.2 to 0.4) | 29.0* (20.9 to 37.1) | 21.4* (13.7 to 29.0) |
| Metastatic phase of multiple myeloma | 2.5 (2.3 to 2.8) | -- | 1.1 (0.7 to 1.5) | 25.9* (17.6 to 33.9) | 18.5* (10.7 to 26.0) |
| Terminal phase of multiple myeloma | 0.1 (0.1 to 0.2) | -- | 0.1 (0.0 to 0.1) | 18.9* (10.2 to 26.5) | 11.9* (3.7 to 19.1) |
| Leukaemia | 62.2 (55.2 to 68.1) | 16.6 (14.9 to 18.1) | 7.3 (5.2 to 9.8) | -1.5 (-8.2 to 8.6) | -7.3 (-13.6 to 2.2) |
| Acute lymphoid leukaemia | 9.9 (8.6 to 10.8) | 2.6 (2.3 to 2.8) | 1.2 (0.8 to 1.6) | 9.4 (-1.7 to 17.7) | 2.9 (-7.5 to 10.7) |
| Diagnosis and primary therapy phase of acute lymphoid leukemia | 1.1 (1.0 to 1.2) | 2.6 (2.3 to 2.8) | 0.3 (0.2 to 0.5) | 6.0 (-3.1 to 13.2) | -0.3 (-8.8 to 6.5) |
| Controlled phase of acute lymphoid leukemia | 7.8 (6.8 to 8.6) | -- | 0.4 (0.2 to 0.6) | 10.9 (-0.8 to 19.9) | 4.4 (-6.7 to 12.8) |
| Metastatic phase of acute lymphoid leukemia | 0.8 (0.7 to 0.9) | -- | 0.4 (0.2 to 0.5) | 10.5 (-1.0 to 19.4) | 4.0 (-6.9 to 12.4) |
| Terminal phase of acute lymphoid leukemia | 0.2 (0.1 to 0.2) | -- | 0.1 (0.1 to 0.1) | 10.6 (-0.9 to 19.5) | 4.0 (-6.8 to 12.4) |
| Chronic lymphoid leukaemia | 12.3 (10.7 to 14.2) | 1.6 (1.4 to 1.9) | 1.4 (1.0 to 1.9) | 46.6* (29.9 to 66.9) | 38.0* (22.2 to 57.0) |

| | | | | | |
|---|---|---|---|---|---|
| Diagnosis and primary therapy phase of chronic lymphoid leukemia | 0.7 (0.6 to 0.8) | 1.6 (1.4 to 1.9) | 0.2 (0.1 to 0.3) | 48.0* (31.7 to 68.0) | 39.2* (23.9 to 58.1) |
| Controlled phase of chronic lymphoid leukemia | 10.0 (8.7 to 11.5) | -- | 0.5 (0.3 to 0.7) | 47.1* (27.2 to 70.9) | 38.4* (19.7 to 60.8) |
| Metastatic phase of chronic lymphoid leukemia | 1.6 (1.4 to 1.8) | -- | 0.7 (0.5 to 1.0) | 46.6* (30.4 to 66.1) | 38.0* (22.7 to 56.3) |
| Terminal phase of chronic lymphoid leukemia | 0.0 (0.0 to 0.1) | -- | -- | 32.1* (17.7 to 49.5) | 24.3* (10.7 to 40.6) |
| Acute myeloid leukaemia | 6.8 (6.2 to 7.5) | 4.9 (4.4 to 5.4) | 1.2 (0.9 to 1.6) | 10.5* (2.6 to 17.7) | 4.0 (-3.4 to 10.7) |
| Diagnosis and primary therapy phase of acute myeloid leukemia | -- | 4.9 (4.4 to 5.4) | -- | -- | -- |
| Controlled phase of acute myeloid leukemia | 4.7 (4.3 to 5.2) | -- | 0.2 (0.1 to 0.3) | 9.7* (2.0 to 17.6) | 3.3 (-4.0 to 10.7) |
| Metastatic phase of acute myeloid leukemia | 1.7 (1.5 to 1.9) | -- | 0.8 (0.5 to 1.1) | 10.7* (2.5 to 18.1) | 4.2 (-3.5 to 11.1) |
| Terminal phase of acute myeloid leukemia | 0.4 (0.3 to 0.4) | -- | 0.2 (0.1 to 0.3) | 10.7* (2.5 to 18.1) | 4.2 (-3.5 to 11.1) |
| Chronic myeloid leukaemia | 6.9 (6.2 to 7.4) | 2.0 (1.8 to 2.2) | 0.7 (0.5 to 1.0) | -5.7 (-11.6 to 0.5) | -11.2* (-16.9 to -5.4) |
| Diagnosis and primary therapy phase of chronic myeloid leukemia | 0.5 (0.4 to 0.5) | 2.0 (1.8 to 2.2) | 0.1 (0.1 to 0.2) | -4.1 (-10.3 to 2.5) | -9.8* (-15.6 to -3.6) |
| Controlled phase of chronic myeloid leukemia | 5.7 (5.2 to 6.2) | -- | 0.3 (0.2 to 0.4) | -3.6 (-9.9 to 2.9) | -9.3* (-15.2 to -3.1) |
| Metastatic phase of chronic myeloid leukemia | 0.6 (0.5 to 0.6) | -- | 0.3 (0.2 to 0.3) | -8.0* (-14.1 to -2.2) | -13.5* (-19.2 to -8.0) |
| Terminal phase of chronic myeloid leukemia | 0.1 (0.1 to 0.1) | -- | 0.1 (0.0 to 0.1) | -8.0* (-14.1 to -2.2) | -13.5* (-19.2 to -8.0) |
| Other leukaemia | 26.4 (22.2 to 29.5) | 5.5 (4.6 to 6.2) | 2.8 (1.9 to 3.7) | -20.6* (-28.2 to -6.7) | -25.3* (-32.5 to -12.3) |
| Metastatic phase of other leukemia | 3.5 (2.9 to 3.9) | -- | 1.6 (1.0 to 2.1) | -21.5* (-28.3 to -9.0) | -26.2* (-32.5 to -14.3) |
| Diagnosis and primary therapy phase of other leukemia | -- | 5.5 (4.6 to 6.2) | -- | -- | -- |
| Terminal phase of other leukemia | 0.3 (0.2 to 0.3) | -- | 0.1 (0.1 to 0.2) | -21.1* (-27.9 to -8.6) | -25.7* (-32.2 to -14.0) |
| Controlled phase of other leukemia | 22.6 (19.0 to 25.3) | -- | 1.1 (0.7 to 1.7) | -19.3* (-30.8 to -1.5) | -24.0* (-34.9 to -7.3) |
| Other malignant cancers | 402.9 (311.7 to 557.9) | 29.0 (26.4 to 31.6) | 29.8 (19.6 to 44.9) | 117.2* (66.0 to 187.3) | 104.4* (56.2 to 170.3) |
| Diagnosis and primary therapy phase of other malignant neoplasms | 9.1 (7.1 to 12.6) | 29.0 (26.4 to 31.6) | 2.6 (1.6 to 4.1) | 134.5* (72.3 to 217.9) | 120.6* (62.1 to 199.1) |
| Controlled phase of other malignant neoplasms | 372.5 (287.6 to 516.2) | -- | 17.6 (10.6 to 29.1) | 150.4* (85.3 to 240.8) | 135.6* (74.4 to 220.6) |
| Metastatic phase of other malignant neoplasms | 18.7 (15.2 to 24.6) | -- | 8.2 (5.3 to 11.8) | 72.7* (37.2 to 120.9) | 62.5* (29.1 to 107.8) |
| Terminal phase of other malignant neoplasms | 2.6 (2.2 to 3.5) | -- | 1.4 (0.9 to 2.1) | 67.9* (36.4 to 115.7) | 58.0* (28.3 to 102.9) |
| Other neoplasms | 1414.2 (1346.7 to 1488.1) | 630.6 (590.1 to 679.0) | 1.8 (1.1 to 2.8) | 6.6 (-2.3 to 16.9) | 0.3 (-8.1 to 10.0) |
| Myelodysplastic, myeloproliferative, and other hematopoietic neoplasms | 37.7 (31.0 to 45.4) | 3.2 (1.7 to 4.9) | 1.8 (1.1 to 2.8) | 6.6 (-2.3 to 16.9) | 0.3 (-8.1 to 10.0) |
| Benign and in situ intestinal neoplasms | 122.0 (115.2 to 129.6) | 42.5 (39.2 to 46.0) | -- | -- | -- |
| Benign and in situ cervical and uterine neoplasms | 185.4 (176.9 to 194.3) | 46.5 (44.5 to 48.7) | -- | -- | -- |
| Other benign and in situ neoplasms | 1108.0 (1045.5 to 1182.0) | 538.4 (498.0 to 587.0) | -- | -- | -- |
| **Cardiovascular diseases** | **12906.3 (11894.4 to 14122.4)** | **1300.5 (1192.1 to 1410.0)** | **974.0 (704.6 to 1284.8)** | **2.8* (0.6 to 4.9)** | **-3.3* (-5.3 to -1.3)** |
| Rheumatic heart disease | 4069.7 (3916.7 to 4222.8) | 38.3 (34.7 to 42.1) | 193.7 (125.7 to 282.6) | 5.6* (4.1 to 7.1) | -0.7 (-2.0 to 0.7) |
| Rheumatic heart disease, without heart failure | 4029.6 (3879.9 to 4188.5) | 38.3 (34.7 to 42.1) | 190.0 (123.2 to 276.9) | 5.7* (4.3 to 7.3) | -0.5 (-1.9 to 0.9) |
| Heart failure due to rheumatic heart disease | 40.1 (26.8 to 56.9) | -- | 3.7 (2.1 to 6.0) | -2.2 (-6.5 to 1.9) | -8.0* (-12.1 to -4.1) |
| Ischaemic heart disease | 2530.4 (2300.3 to 2775.5) | 155.3 (92.8 to 234.5) | 127.1 (85.4 to 178.3) | 4.0* (1.2 to 6.8) | -2.2 (-4.8 to 0.5) |
| Asymptomatic ischemic heart disease following myocardial infarction | 372.0 (272.3 to 484.7) | -- | -- | -- | -- |
| Angina due to ischemic heart disease | 2043.1 (1824.7 to 2266.2) | -- | 116.5 (78.4 to 164.6) | 3.9* (1.0 to 6.9) | -2.2 (-4.9 to 0.6) |
| Heart failure due to ischemic heart disease | 107.0 (69.6 to 153.8) | -- | 9.7 (5.4 to 15.7) | 4.8 (-0.9 to 11.0) | -1.4 (-6.7 to 4.4) |
| Myocardial infarction due to ischemic heart disease | 8.4 (5.1 to 12.6) | 155.3 (92.8 to 234.5) | 0.8 (0.4 to 1.4) | 3.3* (1.2 to 4.7) | -2.8* (-4.8 to -1.5) |
| Stroke | 2684.3 (2450.7 to 2944.7) | 230.5‡ (179.2 to 289.5) | 429.5 (301.9 to 556.3) | -1.1 (-5.1 to 3.5) | -6.9* (-10.7 to -2.6) |
| Ischaemic stroke | 1570.2 (1380.2 to 1778.6) | 99.4 (56.4 to 154.8) | 233.7 (164.3 to 310.3) | 5.3 (-0.7 to 11.2) | -0.9 (-6.5 to 4.7) |
| Acute ischemic stroke | 7.9 (4.6 to 12.2) | 99.4 (56.4 to 154.8) | 2.1 (1.1 to 3.6) | 9.4* (6.7 to 11.9) | 3.0* (0.4 to 5.3) |
| Chronic ischemic stroke | 1562.4 (1371.5 to 1770.0) | -- | 231.6 (163.2 to 307.2) | 5.3 (-0.7 to 11.3) | -0.9 (-6.6 to 4.7) |
| Intracerebral hemorrhage | 863.9 (732.5 to 1016.0) | 76.5 (54.9 to 100.2) | 133.2 (94.9 to 178.1) | -12.5* (-19.8 to -4.9) | -17.7* (-24.5 to -10.5) |
| Acute intracerebral hemorrhage | 4.6 (3.3 to 6.0) | 76.5 (54.9 to 100.2) | 1.2 (0.8 to 1.8) | -25.1* (-37.1 to -14.5) | -29.5* (-40.8 to -19.5) |
| Chronic intracerebral hemorrhage | 859.3 (727.8 to 1011.8) | -- | 132.0 (94.2 to 176.8) | -12.4* (-19.8 to -4.7) | -17.5* (-24.5 to -10.3) |
| Subarachnoid hemorrhage | 411.1 (352.4 to 471.2) | 54.6 (39.2 to 73.9) | 62.5 (42.8 to 83.1) | 4.4 (-0.8 to 10.3) | -1.8 (-6.7 to 3.8) |
| Acute subarachnoid hemorrhage | 3.7 (2.6 to 5.0) | 54.6 (39.2 to 73.9) | 1.0 (0.6 to 1.5) | 7.5 (-1.3 to 11.8) | 1.1 (-7.1 to 5.2) |
| Chronic subarachnoid hemorrhage | 407.4 (348.7 to 467.6) | -- | 61.5 (42.0 to 81.9) | 4.4 (-1.0 to 10.3) | -1.8 (-6.8 to 3.8) |
| Hypertensive heart disease | 253.2 (173.1 to 344.1) | -- | 22.5 (13.4 to 34.3) | 1.6 (-4.1 to 7.6) | -4.4 (-9.8 to 1.2) |
| Non-rheumatic valvular heart disease | 625.3 (589.5 to 658.8) | 652.2 (623.0 to 683.6) | 1.0 (0.3 to 2.2) | 4.7* (1.0 to 8.1) | -1.5 (-5.0 to 1.7) |
| Non-rheumatic calcific aortic valve disease | 132.9 (111.4 to 157.2) | 132.9 (111.4 to 157.2) | 0.2 (0.1 to 0.4) | 3.2 (-2.8 to 8.5) | -2.9 (-8.5 to 2.1) |
| Heart failure due to calcific aortic valve disease | 1.1 (0.3 to 2.5) | -- | 0.1 (0.0 to 0.2) | -2.2 (-19.8 to 17.6) | -8.0 (-24.6 to 10.7) |
| Non-rheumatic calcific aortic valve disease | 131.9 (110.9 to 155.8) | 132.9 (111.4 to 157.2) | 0.1 (0.0 to 0.2) | 11.1 (-3.5 to 27.0) | 4.5 (-9.2 to 19.5) |
| Non-rheumatic degenerative mitral valve disease | 519.2 (499.9 to 539.3) | 519.2 (499.9 to 539.3) | 0.8 (0.2 to 1.8) | 5.1* (0.6 to 9.7) | -1.1 (-5.3 to 3.2) |
| Heart failure due to degenerative mitral valve disease | 5.8 (1.9 to 13.7) | -- | 0.5 (0.2 to 1.3) | 0.0 (-11.8 to 13.5) | -5.9 (-17.0 to 6.8) |
| Non-rheumatic degenerative mitral valve disease | 513.4 (492.8 to 534.4) | 519.2 (499.9 to 539.3) | 0.2 (0.1 to 0.5) | 19.6* (0.2 to 41.3) | 12.6 (-5.7 to 32.9) |
| Other non-rheumatic valve diseases | 0.5 (0.3 to 0.8) | -- | 0.0 (0.0 to 0.1) | 3.7* (2.1 to 5.4) | -2.5* (-4.0 to -0.8) |
| Mild heart failure due to other non-rheumatic valve disease | 0.1 (0.1 to 0.2) | -- | -- | 3.7* (2.0 to 5.4) | -2.5* (-4.0 to -0.8) |
| Moderate heart failure due to other non-rheumatic valve disease | 0.1 (0.0 to 0.1) | -- | -- | 3.7* (2.1 to 5.4) | -2.5* (-4.0 to -0.8) |
| Severe heart failure due to other non-rheumatic valve disease | 0.2 (0.1 to 0.3) | -- | 0.0 (0.0 to 0.1) | 3.7* (2.1 to 5.4) | -2.5* (-4.0 to -0.8) |

| | | | | | |
|---|---|---|---|---|---|
| Controlled, medically managed heart failure due to other non-rheumatic valve disease | 0.2 (0.1 to 0.3) | -- | -- | 3.7* (2.1 to 5.4) | -2.5* (-4.0 to -0.8) |
| Cardiomyopathy and myocarditis | 83.7 (61.2 to 108.2) | 149.2 (105.3 to 203.4) | 24.6 (14.8 to 37.1) | 5.6* (2.4 to 8.3) | -0.7 (-3.6 to 1.9) |
| Myocarditis | 98.5 (75.9 to 125.2) | 149.2 (105.3 to 203.4) | 7.5 (4.6 to 11.1) | 1.4 (-1.2 to 4.2) | -4.6* (-7.0 to -2.0) |
| Acute myocarditis | 36.6 (26.1 to 49.6) | 149.2 (105.3 to 203.4) | 1.8 (1.0 to 2.9) | 5.4* (2.8 to 7.8) | -0.8 (-3.3 to 1.5) |
| Heart failure due to myocarditis | 61.8 (42.8 to 85.9) | -- | 5.7 (3.3 to 8.8) | 0.1 (-3.1 to 3.6) | -5.8* (-8.8 to -2.5) |
| Alcoholic cardiomyopathy | 70.0 (48.9 to 95.5) | -- | 6.3 (3.7 to 9.6) | 4.4 (-1.7 to 10.6) | -1.8 (-7.5 to 4.1) |
| Other cardiomyopathy | 118.6 (82.2 to 162.9) | -- | 10.8 (6.2 to 16.6) | 9.4* (4.5 to 14.5) | 3.0 (-1.7 to 7.7) |
| Atrial fibrillation and flutter | 478.9 (378.2 to 592.3) | 28.5 (15.0 to 42.6) | 40.3 (25.2 to 59.4) | 6.5* (0.7 to 13.2) | 0.2 (-5.2 to 6.5) |
| Asymptomatic atrial fibrillation and flutter | 287.3 (227.9 to 354.8) | 28.5 (15.0 to 42.6) | -- | -- | -- |
| Symptomatic atrial fibrillation and flutter | 191.5 (149.8 to 236.3) | -- | 40.3 (25.2 to 59.4) | 6.5* (0.7 to 13.2) | 0.2 (-5.2 to 6.5) |
| Peripheral vascular disease | -- | -- | -- | -- | -- |
| Asymptomatic peripheral arterial disease | -- | -- | -- | -- | -- |
| Symptomatic claudication due to peripheral arterial disease | -- | -- | -- | -- | -- |
| Endocarditis | 13.7 (9.7 to 18.3) | 46.5 (32.3 to 63.3) | 1.1 (0.7 to 1.8) | 8.8* (7.2 to 10.4) | 2.3* (0.9 to 3.9) |
| Acute endocarditis | 3.6 (2.5 to 4.9) | 46.5 (32.3 to 63.3) | 0.2 (0.1 to 0.3) | 11.6* (8.2 to 14.6) | 5.0* (1.8 to 7.9) |
| Heart failure due to endocarditis | 10.1 (6.6 to 14.6) | -- | 0.9 (0.5 to 1.5) | 8.2* (6.4 to 10.0) | 1.8* (0.1 to 3.5) |
| Other cardiovascular and circulatory diseases | 2513.1 (1619.6 to 3639.1) | -- | 134.3 (73.4 to 212.5) | 9.5* (6.3 to 12.9) | 3.0* (0.0 to 6.3) |
| Heart failure due to other cardiovascular diseases | 12.4 (8.1 to 18.0) | -- | 1.1 (0.6 to 1.8) | 9.5* (8.0 to 11.0) | 3.0* (1.6 to 4.5) |
| Other cardiovascular and circulatory disease episodes | 2500.6 (1611.6 to 3621.1) | -- | 133.1 (72.7 to 210.7) | 9.5* (6.3 to 13.0) | 3.0* (0.0 to 6.3) |
| **Chronic respiratory diseases** | **24841.6 (21897.4 to 28458.6)** | **1962.2 (1450.6 to 2531.7)** | **2049.3 (1606.6 to 2548.0)** | **7.1* (2.6 to 11.9)** | **0.7 (-3.5 to 5.3)** |
| Chronic obstructive pulmonary disease | 11257.0 (9475.5 to 13409.7) | 787.1 (495.2 to 1170.6) | 1229.6 (950.8 to 1534.2) | 3.2 (-3.1 to 9.4) | -2.9 (-8.8 to 2.9) |
| Chronic obstructive pulmonary disease with heart failure | 5.6 (3.3 to 8.7) | -- | 2.6 (1.4 to 4.4) | -8.8* (-10.6 to -6.9) | -14.2* (-15.9 to -12.4) |
| Chronic obstructive pulmonary disease without heart failure | 11251.4 (9469.9 to 13403.4) | 787.1 (495.2 to 1170.6) | 1227.0 (948.6 to 1531.4) | 3.2 (-3.1 to 9.4) | -2.9 (-8.8 to 3.0) |
| Pneumoconiosis | 13.2 (10.4 to 16.4) | 1.3 (0.8 to 1.9) | 2.1 (1.3 to 3.0) | -1.0 (-4.9 to 2.9) | -6.9* (-10.6 to -3.2) |
| Silicosis | 4.7 (3.0 to 7.1) | 0.5 (0.2 to 0.9) | 0.7 (0.4 to 1.2) | 4.1 (-4.0 to 11.8) | -2.1 (-9.6 to 5.2) |
| Heart failure due to silicosis | -- | -- | -- | -- | -- |
| Silicosis without heart failure | 4.7 (3.0 to 7.1) | 0.5 (0.2 to 0.9) | 0.7 (0.4 to 1.2) | 4.1 (-4.0 to 11.8) | -2.1 (-9.6 to 5.2) |
| Asbestosis | 3.3 (1.7 to 5.6) | 0.2 (0.1 to 0.5) | 0.5 (0.2 to 0.9) | 14.0* (8.7 to 20.9) | 7.3* (2.3 to 13.8) |
| Asbestosis without heart failure | 3.3 (1.7 to 5.6) | 0.2 (0.1 to 0.5) | 0.5 (0.2 to 0.9) | 14.0* (8.7 to 20.9) | 7.3* (2.3 to 13.8) |
| Heart failure due to asbestosis | -- | -- | -- | -- | -- |
| Coal workers pneumoconiosis | 2.0 (1.2 to 3.0) | 0.2 (0.1 to 0.4) | 0.3 (0.2 to 0.5) | -18.8* (-27.0 to -12.5) | -23.6* (-31.3 to -17.6) |
| Coal workers pneumoconiosis without heart failure | 1.9 (1.1 to 2.9) | 0.2 (0.1 to 0.4) | 0.3 (0.1 to 0.5) | -17.3* (-26.1 to -9.7) | -22.2* (-30.5 to -15.0) |
| Heart failure due to Coal workers pneumoconiosis | 0.1 (0.0 to 0.1) | -- | 0.0 (0.0 to 0.1) | -29.1* (-38.0 to -23.3) | -33.3* (-41.6 to -27.8) |
| Other pneumoconiosis | 3.2 (2.2 to 4.6) | 0.3 (0.1 to 0.6) | 0.5 (0.3 to 0.8) | -7.3* (-11.3 to -3.8) | -12.8* (-16.5 to -9.5) |
| Heart failure due to other pneumoconiosis | -- | -- | -- | -20.3* (-22.8 to -17.6) | -25.0* (-27.4 to -22.5) |
| Other pneumoconiosis without heart failure | 3.2 (2.2 to 4.6) | 0.3 (0.1 to 0.6) | 0.5 (0.3 to 0.8) | -7.1* (-11.0 to -3.4) | -12.6* (-16.3 to -9.1) |
| Asthma | 14075.1 (11667.3 to 16943.9) | 1154.8 (763.7 to 1617.7) | 550.3 (353.5 to 831.0) | 10.7* (5.1 to 16.2) | 4.1 (-1.1 to 9.3) |
| Asymptomatic asthma | 5115.0 (4089.4 to 6403.0) | 418.6 (273.4 to 598.0) | -- | -- | -- |
| Symptomatic asthma | 8960.1 (7368.9 to 10898.5) | 736.2 (483.3 to 1032.4) | 550.3 (353.5 to 831.0) | 10.7* (5.1 to 16.2) | 4.1 (-1.1 to 9.3) |
| Interstitial lung disease and pulmonary sarcoidosis | 200.6 (159.0 to 246.9) | 19.1 (13.1 to 26.5) | 22.2 (13.7 to 33.0) | 5.1* (1.0 to 8.9) | -1.1 (-4.9 to 2.4) |
| Heart failure due to interstitial lung disease and pulmonary sarcoidosis | 2.0 (1.2 to 3.0) | -- | 0.9 (0.5 to 1.5) | 7.4* (5.8 to 9.5) | 1.1 (-0.5 to 3.0) |
| Interstitial lung disease and pulmonary sarcoidosis without heart failure | 198.6 (157.3 to 245.1) | 19.1 (13.1 to 26.5) | 21.3 (13.1 to 31.8) | 5.0* (0.7 to 9.0) | -1.2 (-5.2 to 2.5) |
| Other chronic respiratory diseases | -- | -- | 245.2 (189.2 to 305.5) | 21.2* (14.0 to 28.3) | 14.0* (7.3 to 20.7) |
| **Digestive diseases** | **180132.9 (165610.1 to 198943.1)** | **39281.2 (30977.8 to 49436.9)** | **1586.2 (1038.8 to 2415.7)** | **7.9* (6.4 to 9.4)** | **1.5* (0.1 to 2.9)** |
| Cirrhosis and other chronic liver diseases | 126910.7 (118093.2 to 136619.8) | 643.2 (528.6 to 755.7) | 107.4 (73.7 to 148.9) | 14.6* (10.6 to 18.5) | 7.9* (4.0 to 11.5) |
| Cirrhosis and other chronic liver diseases due to hepatitis B | 32373.5 (29925.7 to 35464.0) | 221.7 (174.9 to 272.9) | 37.0 (24.9 to 50.9) | 4.6 (-1.8 to 11.1) | -1.6 (-7.6 to 4.6) |
| Cirrhosis and other chronic liver diseases due to hepatitis B, decompensated | 218.9 (194.0 to 244.5) | 40.3 (34.3 to 46.4) | 37.0 (24.9 to 50.9) | 4.6 (-1.8 to 11.1) | -1.6 (-7.6 to 4.6) |
| Chronic hepatitis B without cirrhosis | 27875.3 (25416.7 to 30981.5) | -- | -- | -- | -- |
| Cirrhosis and other chronic liver diseases due to hepatitis B, compensated | 4279.4 (3769.8 to 4837.0) | 181.4 (136.5 to 230.9) | -- | -- | -- |
| Cirrhosis and other chronic liver diseases due to hepatitis C | 10817.1 (9139.7 to 12659.7) | 185.1 (146.7 to 226.6) | 29.8 (20.0 to 42.7) | 20.0* (12.8 to 26.6) | 12.9* (6.1 to 19.1) |
| Cirrhosis and other chronic liver diseases due to hepatitis C, decompensated | 177.5 (151.5 to 208.2) | 31.8 (26.3 to 38.6) | 29.8 (20.0 to 42.7) | 20.0* (12.8 to 26.6) | 12.9* (6.1 to 19.1) |
| Chronic hepatitis C without cirrhosis | 7414.9 (5661.5 to 9332.8) | -- | -- | -- | -- |
| Cirrhosis and other chronic liver diseases due to hepatitis C, compensated | 3224.7 (2690.1 to 3788.0) | 153.3 (115.7 to 191.8) | -- | -- | -- |
| Cirrhosis and other chronic liver diseases due to alcohol use | 2753.6 (2368.5 to 3135.4) | 147.3 (115.8 to 179.6) | 25.7 (17.1 to 36.1) | 17.9* (11.0 to 26.3) | 11.0* (4.4 to 18.8) |
| Cirrhosis and other chronic liver diseases due to alcohol, decompensated | 152.5 (131.8 to 175.0) | 27.9 (23.6 to 32.5) | 25.7 (17.1 to 36.1) | 17.9* (11.0 to 26.3) | 11.0* (4.4 to 18.8) |
| Cirrhosis and other chronic liver diseases due to alcohol, compensated | 2601.1 (2232.4 to 2963.5) | 119.4 (88.6 to 149.0) | -- | -- | -- |
| Cirrhosis due to NASH | 80332.5 (71981.5 to 89087.1) | 54.7 (40.2 to 70.0) | 9.2 (5.8 to 13.6) | 31.4* (20.4 to 42.8) | 23.6* (13.3 to 34.4) |
| Non-alcoholic fatty liver disease (NAFLD) / Non-alcoholic steatohepatitis (NASH) | 79296.1 (70830.4 to 87983.1) | -- | -- | -- | -- |
| Cirrhosis and other chronic liver diseases due to NASH | 1036.4 (827.4 to 1258.5) | 54.7 (40.2 to 70.0) | 9.2 (5.8 to 13.6) | 31.4* (20.4 to 42.8) | 23.6* (13.3 to 34.4) |
| Cirrhosis and other chronic liver diseases due to other causes | 634.0 (470.5 to 825.0) | 34.3 (24.1 to 46.7) | 5.6 (3.4 to 8.7) | 21.5* (12.1 to 31.4) | 14.3* (5.5 to 23.7) |

| | | | | | |
|---|---|---|---|---|---|
| Cirrhosis and other chronic liver diseases due to other cause, decompensated | 32.7 (24.3 to 42.7) | 5.9 (4.2 to 7.8) | 5.6 (3.4 to 8.7) | 21.5* (12.1 to 31.4) | 14.3* (5.5 to 23.7) |
| Cirrhosis and other chronic liver diseases due to other cause, compensated | 601.3 (445.3 to 785.7) | 28.4 (19.5 to 39.5) | -- | -- | -- |
| Upper digestive system diseases | 76545.5 (58300.2 to 98426.8) | 34768.3 (26460.9 to 44904.8) | 1153.2 (674.9 to 1895.1) | 7.6* (5.7 to 9.3) | 1.2 (-0.5 to 2.8) |
| Peptic ulcer disease | 1314.1 (999.5 to 1740.5) | 451.2 (326.7 to 593.3) | 64.7 (40.0 to 95.3) | 7.5* (2.9 to 12.0) | 1.1 (-3.2 to 5.4) |
| Acute peptic ulcer disease | 0.3 (0.2 to 0.5) | -- | 0.1 (0.1 to 0.2) | -5.3* (-7.0 to -3.8) | -10.9* (-12.5 to -9.5) |
| Chronic peptic ulcer disease | 1313.7 (999.2 to 1740.3) | 451.2 (326.7 to 593.3) | 64.6 (39.9 to 95.2) | 7.5* (2.9 to 12.0) | 1.1 (-3.2 to 5.4) |
| Gastritis and duodenitis | 10811.5 (7123.5 to 15430.0) | 7453.1 (4989.7 to 10155.3) | 486.4 (285.6 to 762.5) | 3.9* (1.0 to 6.6) | -2.2 (-5.0 to 0.3) |
| Acute gastritis and duodenitis | 2.3 (1.4 to 3.5) | -- | 0.7 (0.4 to 1.3) | 2.3* (0.8 to 3.6) | -3.7* (-5.2 to -2.5) |
| Chronic gastritis and duodenitis | 10809.2 (7121.7 to 15427.5) | 7453.1 (4989.7 to 10155.3) | 485.6 (285.0 to 762.1) | 3.9* (1.0 to 6.6) | -2.2 (-5.0 to 0.3) |
| GERD | 69765.2 (49726.8 to 94592.9) | 26864.0 (19081.3 to 36387.7) | 602.2 (291.2 to 1170.7) | 10.8* (9.0 to 12.6) | 4.2* (2.6 to 6.0) |
| Appendicitis | 38.7 (31.1 to 47.5) | 995.1 (806.1 to 1194.7) | 12.0 (7.6 to 17.5) | 10.5 (-0.6 to 24.1) | 3.9 (-6.4 to 16.8) |
| Paralytic ileus and intestinal obstruction | 6.4 (4.5 to 8.7) | 173.3 (119.3 to 234.0) | 2.1 (1.2 to 3.2) | 15.9* (12.0 to 19.0) | 9.1* (5.4 to 12.0) |
| Inguinal, femoral, and abdominal hernia | 1585.6 (1087.6 to 2140.5) | 1745.0 (1231.2 to 2383.9) | 155.8 (92.5 to 239.8) | 7.2* (3.0 to 11.3) | 0.8 (-3.1 to 4.7) |
| Inflammatory bowel disease | 473.6 (413.3 to 548.5) | 371.0 (305.1 to 455.2) | 74.8 (49.3 to 104.9) | -1.7 (-10.8 to 8.4) | -7.5 (-16.0 to 2.0) |
| Ulcerative colitis | 293.8 (241.2 to 354.6) | 263.9 (203.2 to 349.6) | 45.6 (29.4 to 66.0) | -1.2 (-11.7 to 10.2) | -7.1 (-16.9 to 3.7) |
| Crohn's disease | 179.8 (152.4 to 214.7) | 107.1 (82.3 to 141.1) | 29.2 (19.0 to 41.9) | -2.5 (-12.8 to 9.0) | -8.3 (-17.9 to 2.5) |
| Vascular intestinal disorders | 2.6 (1.6 to 4.0) | 15.7 (10.8 to 22.7) | 0.8 (0.4 to 1.5) | 11.5* (8.9 to 14.3) | 4.9* (2.5 to 7.6) |
| Gallbladder and biliary diseases | 2548.7 (1807.9 to 3472.7) | 435.9 (305.1 to 583.5) | 2.1 (1.2 to 3.4) | 10.2* (8.1 to 12.2) | 3.7* (1.7 to 5.6) |
| Pancreatitis | 408.4 (344.4 to 483.5) | 133.8 (105.3 to 166.5) | 25.7 (13.0 to 45.7) | 10.2* (4.4 to 15.6) | 3.7 (-1.8 to 8.8) |
| Acute pancreatitis | 8.5 (6.3 to 11.1) | 100.5 (75.1 to 129.9) | 3.5 (2.2 to 5.1) | -1.9* (-2.8 to -1.0) | -7.7* (-8.5 to -6.9) |
| Chronic pancreatitis | 399.9 (335.6 to 474.1) | 33.3 (23.3 to 44.3) | 22.2 (10.1 to 41.0) | 12.4* (5.6 to 19.3) | 5.7 (-0.6 to 12.2) |
| Other digestive diseases | -- | -- | 52.3 (34.9 to 75.0) | 17.3* (14.8 to 19.8) | 10.4* (8.0 to 12.8) |
| **Neurological disorders** | **279666.0 (236006.2 to 328907.1)** | **83093.1 (59797.9 to 109116.1)** | **6229.1 (4125.0 to 9015.6)** | **11.2* (9.7 to 12.8)** | **4.6* (3.2 to 6.2)** |
| Alzheimer's disease and other dementias | -- | -- | -- | -- | -- |
| Parkinson's disease | 53.5 (34.7 to 77.7) | 6.2 (2.5 to 11.5) | 9.0 (5.1 to 14.1) | 1.9 (-1.1 to 4.7) | -4.1* (-7.0 to -1.4) |
| Epilepsy | 1554.5 (1154.1 to 2047.4) | 108.7 (71.3 to 154.7) | 483.2 (291.5 to 727.3) | 10.8 (-1.1 to 24.7) | 4.3 (-7.0 to 17.4) |
| Multiple sclerosis | 194.1 (170.0 to 218.0) | 6.6 (4.4 to 9.2) | 51.3 (36.7 to 67.5) | 10.8* (5.3 to 16.2) | 4.2 (-0.9 to 9.3) |
| Motor neuron disease | 9.4 (7.4 to 11.6) | 1.4 (0.9 to 2.1) | 2.0 (1.3 to 2.8) | 4.7* (3.9 to 5.6) | -1.5* (-2.2 to -0.7) |
| Headache disorders | 278889.8 (235203.9 to 328435.6) | 82970.3 (59664.9 to 109025.7) | 5600.1 (3630.0 to 8206.0) | 11.1* (9.9 to 12.2) | 4.5* (3.4 to 5.6) |
| Migraine | 142071.4 (122730.6 to 162102.6) | 10327.5 (7449.9 to 13984.8) | 4961.8 (3132.6 to 7451.9) | 11.3* (10.1 to 12.6) | 4.7* (3.6 to 5.9) |
| Medication overuse headache due to migraine | 7638.6 (5429.8 to 10671.4) | -- | 846.2 (492.1 to 1331.5) | 11.1* (9.6 to 12.7) | 4.5* (3.1 to 6.1) |
| Migraine | 134432.8 (115461.9 to 153792.3) | 10327.5 (7449.9 to 13984.8) | 4115.6 (2483.9 to 6308.3) | 11.3* (9.8 to 12.9) | 4.8* (3.4 to 6.3) |
| Tension-type headache | 204466.0 (144196.0 to 270319.1) | 72642.7 (49660.3 to 98681.0) | 638.2 (352.2 to 1054.1) | 9.3* (8.0 to 11.0) | 2.9* (1.6 to 4.5) |
| Medication overuse headache due to tension-type headache | 2837.5 (1764.5 to 4280.1) | -- | 312.0 (145.5 to 560.8) | 11.0* (8.7 to 13.5) | 4.5* (2.3 to 6.8) |
| Tension-type headache | 201628.5 (140618.7 to 267856.9) | 72642.7 (49660.3 to 98681.0) | 326.2 (132.1 to 645.2) | 7.8* (6.6 to 9.0) | 1.4* (0.3 to 2.6) |
| Other neurological disorders | 2.4 (1.3 to 4.0) | -- | 83.5 (52.3 to 123.3) | 23.0* (11.2 to 36.2) | 15.7* (4.6 to 28.2) |
| Other neurological disorders | -- | -- | 82.8 (51.8 to 122.6) | 23.1* (11.2 to 36.4) | 15.8* (4.6 to 28.4) |
| Guillain-Barré syndrome due to other neurological disorders | 2.4 (1.3 to 4.0) | -- | 0.7 (0.3 to 1.3) | 14.2* (11.5 to 16.7) | 7.4* (4.9 to 9.8) |
| **Mental disorders** | **78901.0 (74536.9 to 83794.7)** | **26297.9 (22589.4 to 30811.8)** | **10882.2 (8097.5 to 14025.7)** | **6.2* (5.4 to 7.0)** | **-0.1 (-0.9 to 0.7)** |
| Schizophrenia | 2504.2 (2149.8 to 2986.3) | 106.7 (82.2 to 132.6) | 1629.4 (1193.2 to 2067.1) | 3.1* (1.0 to 5.0) | -3.0* (-4.9 to -1.2) |
| Depressive disorders | 22374.1 (19494.1 to 25667.1) | 21932.7 (18180.9 to 26357.8) | 3712.2 (2562.9 to 5117.6) | 4.2* (2.5 to 5.9) | -1.9* (-3.5 to -0.4) |
| Major depressive disorder | 13918.1 (11644.6 to 16600.1) | 20609.5 (16810.9 to 25058.7) | 2845.5 (1908.1 to 3944.1) | 4.1* (1.8 to 6.1) | -2.1* (-4.2 to -0.2) |
| Dysthymia | 8867.5 (7138.6 to 10909.4) | 1323.2 (962.7 to 1729.5) | 866.6 (548.9 to 1343.5) | 4.8* (2.3 to 7.2) | -1.4 (-3.7 to 0.9) |
| Bipolar disorder | 4173.5 (3450.6 to 5047.3) | 288.1 (179.2 to 415.1) | 856.8 (530.4 to 1302.6) | 11.9* (10.1 to 13.8) | 5.3* (3.6 to 7.1) |
| Anxiety disorders | 24513.4 (22128.7 to 27163.7) | 3658.6 (3194.7 to 4164.9) | 2350.9 (1684.6 to 3145.7) | 7.7* (6.1 to 9.4) | 1.4 (-0.2 to 2.9) |
| Eating disorders | 2300.0 (1645.2 to 3064.5) | 311.9 (201.1 to 439.1) | 481.2 (293.5 to 754.4) | 19.2* (16.7 to 21.7) | 12.1* (9.8 to 14.5) |
| Anorexia nervosa | 295.8 (207.0 to 410.1) | 53.0 (36.9 to 75.4) | 62.0 (36.2 to 101.4) | 11.9* (5.8 to 17.7) | 5.3 (-0.4 to 10.7) |
| Bulimia nervosa | 2006.9 (1354.8 to 2773.6) | 258.9 (151.8 to 389.7) | 419.1 (243.1 to 671.7) | 20.3* (17.7 to 23.1) | 13.2* (10.7 to 15.8) |
| Autism spectrum disorders | 2060.7 (1852.9 to 2281.2) | -- | 312.2 (214.0 to 428.4) | 5.5* (3.7 to 7.3) | -0.7 (-2.5 to 1.0) |
| Attention-deficit/hyperactivity disorder | 4301.8 (3544.7 to 5142.8) | -- | 51.7 (31.1 to 82.8) | 3.8* (0.9 to 6.9) | -2.4 (-5.1 to 0.6) |
| Conduct disorder | -- | -- | -- | -- | -- |
| Idiopathic developmental intellectual disability | 6312.3 (3591.0 to 9099.5) | -- | 249.8 (120.5 to 425.3) | 1.3 (-1.7 to 3.3) | -4.6* (-7.5 to -2.8) |
| Other mental disorders | 16403.5 (14231.5 to 18755.3) | -- | 1238.1 (817.6 to 1718.6) | 6.3* (4.9 to 7.7) | 0.0 (-1.3 to 1.3) |
| **Substance use disorders** | **19721.7 (17283.3 to 22321.5)** | **6917.7 (5576.1 to 8423.2)** | **3535.9 (2471.1 to 4713.2)** | **11.8* (9.1 to 15.2)** | **5.2* (2.7 to 8.4)** |
| Alcohol use disorders | 12716.2 (10445.3 to 15064.4) | 6424.6 (5074.6 to 7885.1) | 1277.8 (832.6 to 1809.7) | 3.7* (1.3 to 5.8) | -2.4* (-4.6 to -0.4) |
| Alcohol dependence | 12696.1 (10428.6 to 15042.9) | 6424.6 (5074.6 to 7885.1) | 1277.1 (831.8 to 1809.0) | 3.7* (1.4 to 5.9) | -2.4* (-4.6 to -0.4) |
| Fetal alcohol syndrome | 20.1 (16.1 to 24.7) | -- | 0.7 (0.4 to 1.1) | -8.5* (-14.8 to -2.7) | -13.9* (-19.8 to -8.5) |
| Drug use disorders | 7367.8 (6211.3 to 8782.3) | 493.1 (370.2 to 635.2) | 2258.1 (1554.6 to 3094.6) | 17.0* (12.8 to 21.1) | 10.1* (6.2 to 14.0) |
| Opioid use disorders | 4763.8 (3700.0 to 6143.2) | 277.0 (177.1 to 405.0) | 1983.6 (1331.8 to 2789.0) | 18.6* (14.0 to 23.1) | 11.6* (7.3 to 15.8) |
| Cocaine use disorders | 483.8 (425.4 to 550.9) | 5.6 (3.3 to 8.4) | 65.6 (42.9 to 95.9) | 12.3* (7.5 to 16.9) | 5.6* (1.2 to 10.0) |

| | | | | | |
|---|---|---|---|---|---|
| Amphetamine use disorders | 400.0<br>(275.7 to 552.5) | 38.5<br>(23.2 to 55.6) | 52.2<br>(27.9 to 87.5) | 3.6<br>(-4.3 to 11.0) | -2.5<br>(-9.9 to 4.5) |
| Cannabis use disorders | 1525.3<br>(1155.0 to 1986.1) | 171.9<br>(105.8 to 250.5) | 43.8<br>(26.3 to 67.4) | 2.4<br>(-2.6 to 7.7) | -3.7<br>(-8.4 to 1.4) |
| Other drug use disorders | 333.5<br>(274.3 to 405.5) | -- | 113.0<br>(75.3 to 154.4) | 7.2*<br>(3.4 to 11.1) | 0.9<br>(-2.7 to 4.6) |
| **Diabetes and kidney diseases** | **75622.6<br>(65422.9 to 87496.8)** | **2749.7<br>(2074.5 to 3491.4)** | **2875.1<br>(1965.9 to 4057.6)** | **12.3*<br>(7.3 to 17.1)** | **5.7*<br>(1.0 to 10.1)** |
| Diabetes mellitus | 30699.1<br>(26067.7 to 35869.2) | 1951.4<br>(1355.2 to 2644.3) | 2451.9<br>(1619.5 to 3528.6) | 12.7*<br>(7.1 to 18.2) | 6.0*<br>(0.8 to 11.2) |
| Diabetes mellitus type 1 | 1243.6<br>(1102.2 to 1410.7) | 18.7<br>(12.8 to 25.8) | 98.8<br>(67.2 to 139.7) | 9.3*<br>(6.5 to 12.1) | 2.8*<br>(0.2 to 5.4) |
| Uncomplicated diabetes mellitus type 1 | 771.3<br>(661.3 to 886.4) | 18.7<br>(12.8 to 25.8) | 36.4<br>(22.2 to 53.8) | 7.5*<br>(3.9 to 10.9) | 1.1<br>(-2.3 to 4.4) |
| Vision loss due to diabetes mellitus type 1 retinopathy | 9.4<br>(6.6 to 13.1) | -- | 0.8<br>(0.5 to 1.2) | -2.0<br>(-8.0 to 4.1) | -7.8*<br>(-13.4 to -2.1) |
| Diabetic neuropathy due to diabetes mellitus type 1 | 462.9<br>(389.0 to 552.9) | -- | 61.6<br>(40.4 to 88.4) | 10.5*<br>(6.2 to 14.9) | 4.0<br>(-0.1 to 8.1) |
| Diabetes mellitus type 2 | 29455.5<br>(24788.0 to 34570.7) | 1932.7<br>(1334.4 to 2626.9) | 2353.0<br>(1549.1 to 3391.0) | 12.9*<br>(7.0 to 18.5) | 6.2*<br>(0.7 to 11.5) |
| Uncomplicated diabetes mellitus type 2 | 18107.8<br>(14948.7 to 21717.7) | 1932.7<br>(1334.4 to 2626.9) | 855.6<br>(522.0 to 1268.2) | 11.1*<br>(4.8 to 17.1) | 4.5<br>(-1.4 to 10.2) |
| Vision loss due to diabetes mellitus type 2 retinopathy | 192.7<br>(146.9 to 246.1) | -- | 16.4<br>(10.6 to 24.1) | 9.0*<br>(0.3 to 17.1) | 2.6<br>(-5.6 to 10.2) |
| Diabetic neuropathy due to diabetes mellitus type 2 | 11155.0<br>(8997.1 to 13631.3) | -- | 1481.1<br>(968.7 to 2187.7) | 13.9*<br>(7.3 to 20.1) | 7.2*<br>(0.9 to 13.0) |
| Chronic kidney disease | 50459.9<br>(40105.7 to 62826.2) | 757.5<br>(459.1 to 1145.1) | 423.1<br>(290.4 to 598.2) | 10.2*<br>(3.6 to 17.6) | 3.7<br>(-2.5 to 10.6) |
| Chronic kidney disease due to diabetes mellitus type 1 | 322.8<br>(262.6 to 395.1) | 6.1<br>(3.6 to 9.4) | 25.1<br>(16.2 to 35.6) | 7.4*<br>(1.1 to 14.3) | 1.1<br>(-4.8 to 7.6) |
| Albuminuria with preserved GFR due to type 1 diabetes mellitus | 179.6<br>(130.1 to 243.3) | -- | -- | -- | -- |
| End-stage chronic kidney disease due to diabetes mellitus type 1 | 42.6<br>(28.0 to 60.3) | -- | 16.4<br>(9.2 to 25.1) | 3.1<br>(-5.1 to 11.1) | -3.0<br>(-10.7 to 4.5) |
| Stage III chronic kidney disease due to diabetes mellitus type 1 | 80.4<br>(64.0 to 101.2) | 6.1<br>(3.6 to 9.4) | 1.8<br>(1.1 to 2.7) | 7.8<br>(-7.1 to 24.5) | 1.4<br>(-12.6 to 17.1) |
| Stage IV chronic kidney disease due to diabetes mellitus type 1 | 11.3<br>(6.8 to 17.2) | -- | 1.6<br>(0.8 to 2.7) | 21.3*<br>(7.8 to 35.3) | 14.2*<br>(1.5 to 27.3) |
| Stage V chronic kidney disease due to diabetes mellitus type 1 | 8.9<br>(6.3 to 12.6) | -- | 5.3<br>(3.2 to 7.8) | 18.6*<br>(9.6 to 27.8) | 11.6*<br>(3.2 to 20.3) |
| Chronic kidney disease due to diabetes mellitus type 2 | 8247.5<br>(6231.4 to 10655.6) | 42.6<br>(25.5 to 63.7) | 29.7<br>(19.9 to 42.0) | 7.9*<br>(0.9 to 15.3) | 1.6<br>(-5.1 to 8.5) |
| Albuminuria with preserved GFR due to type 2 diabetes mellitus | 7592.6<br>(5592.7 to 10034.9) | -- | -- | -- | -- |
| End-stage chronic kidney disease due to diabetes mellitus type 2 | 12.4<br>(6.3 to 21.1) | -- | 5.0<br>(2.3 to 9.0) | 3.0<br>(-3.2 to 8.2) | -3.1<br>(-8.9 to 1.8) |
| Stage III chronic kidney disease due to diabetes mellitus type 2 | 577.4<br>(477.1 to 699.0) | 42.6<br>(25.5 to 63.7) | 3.4<br>(2.1 to 5.2) | -4.5<br>(-20.8 to 14.4) | -10.2<br>(-25.5 to 7.6) |
| Stage IV chronic kidney disease due to diabetes mellitus type 2 | 36.7<br>(22.3 to 55.2) | -- | 4.7<br>(2.5 to 8.0) | 11.3<br>(-1.9 to 25.3) | 4.8<br>(-7.7 to 17.9) |
| Stage V chronic kidney disease due to diabetes mellitus type 2 | 28.5<br>(20.4 to 39.5) | -- | 16.6<br>(10.3 to 24.9) | 11.6*<br>(3.8 to 19.7) | 5.0<br>(-2.4 to 12.7) |
| Chronic kidney disease due to hypertension | 1353.9<br>(1157.9 to 1592.2) | 70.6<br>(42.0 to 107.1) | 70.3<br>(46.4 to 101.6) | 11.1*<br>(3.4 to 19.1) | 4.5<br>(-2.8 to 12.1) |
| Albuminuria with preserved GFR due to hypertension | 290.3<br>(212.4 to 385.1) | -- | -- | -- | -- |
| End-stage chronic kidney disease due to hypertension | 22.0<br>(15.5 to 30.5) | -- | 8.9<br>(5.3 to 13.7) | 9.2*<br>(2.1 to 15.3) | 2.8<br>(-3.9 to 8.5) |
| Stage III chronic kidney disease due to hypertension | 859.4<br>(707.0 to 1043.6) | 70.6<br>(42.0 to 107.1) | 3.4<br>(2.1 to 5.0) | -7.5<br>(-23.2 to 9.9) | -12.9<br>(-27.8 to 3.4) |
| Stage IV chronic kidney disease due to hypertension | 102.5<br>(62.6 to 153.7) | -- | 12.8<br>(6.7 to 21.6) | 12.5<br>(-1.9 to 28.0) | 5.9<br>(-7.7 to 20.5) |
| Stage V chronic kidney disease due to hypertension | 79.8<br>(57.2 to 110.4) | -- | 45.2<br>(28.0 to 68.4) | 12.8*<br>(4.1 to 22.4) | 6.1<br>(-2.1 to 15.2) |
| Chronic kidney disease due to glomerulonephritis | 1593.4<br>(1349.9 to 1873.3) | 37.5<br>(22.5 to 56.4) | 106.6<br>(70.8 to 152.4) | 10.8*<br>(3.6 to 18.7) | 4.2<br>(-2.5 to 11.7) |
| Albuminuria with preserved GFR due to glomerulonephritis | 678.5<br>(504.4 to 898.7) | -- | -- | -- | -- |
| End-stage chronic kidney disease due to glomerulonephritis | 50.3<br>(33.1 to 71.6) | -- | 18.8<br>(10.5 to 30.3) | 7.1<br>(-0.4 to 14.7) | 0.8<br>(-6.3 to 7.9) |
| Stage III chronic kidney disease due to glomerulonephritis | 607.9<br>(499.1 to 740.4) | 37.5<br>(22.5 to 56.4) | 7.3<br>(4.6 to 11.1) | -2.4<br>(-14.7 to 11.2) | -8.2<br>(-19.8 to 4.6) |
| Stage IV chronic kidney disease due to glomerulonephritis | 144.3<br>(88.1 to 215.8) | -- | 17.7<br>(9.4 to 29.2) | 13.0<br>(-1.5 to 28.5) | 6.3<br>(-7.4 to 20.9) |
| Stage V chronic kidney disease due to glomerulonephritis | 112.3<br>(80.5 to 155.5) | -- | 62.7<br>(38.8 to 93.2) | 13.1*<br>(3.8 to 23.1) | 6.4<br>(-2.3 to 15.8) |
| Chronic kidney disease due to other and unspecified causes | 38942.3<br>(30907.7 to 48559.8) | 600.7<br>(365.2 to 914.6) | 191.4<br>(133.5 to 267.0) | 10.3*<br>(3.4 to 18.5) | 3.8<br>(-2.7 to 11.5) |
| Albuminuria with preserved GFR due to other and unspecified causes | 30380.3<br>(22492.9 to 39842.5) | -- | -- | -- | -- |
| End-stage chronic kidney disease due to other causes | 62.0<br>(41.2 to 82.9) | -- | 24.2<br>(14.3 to 36.1) | 8.4<br>(-1.4 to 18.9) | 2.0<br>(-7.2 to 11.9) |
| Stage III chronic kidney disease due to other causes | 8126.2<br>(6716.9 to 9847.7) | 600.7<br>(365.2 to 914.6) | 52.9<br>(32.4 to 81.6) | 5.2<br>(-5.7 to 16.3) | -1.0<br>(-11.3 to 9.4) |
| Stage IV chronic kidney disease due to other causes | 210.5<br>(128.9 to 314.9) | -- | 25.2<br>(13.4 to 41.7) | 12.8<br>(-1.2 to 28.7) | 6.2<br>(-7.0 to 21.1) |
| Stage V chronic kidney disease due to other causes | 163.3<br>(116.9 to 225.9) | -- | 89.1<br>(55.3 to 132.9) | 13.4*<br>(4.6 to 23.6) | 6.7<br>(-1.6 to 16.3) |
| Acute glomerulonephritis | 2.8<br>(1.8 to 4.0) | 40.8<br>(26.8 to 57.8) | 0.1<br>(0.1 to 0.2) | -7.5*<br>(-12.8 to -2.5) | -13.0*<br>(-18.0 to -8.3) |
| **Skin and subcutaneous diseases** | **106919.0<br>(99641.3 to 114788.6)** | **225533.4<br>(202183.2 to 249881.3)** | **2196.8<br>(1481.5 to 3220.3)** | **6.5*<br>(5.6 to 7.6)** | **0.2<br>(-0.6 to 1.2)** |
| Dermatitis | 14347.1<br>(12089.8 to 17061.0) | 20110.9<br>(14662.2 to 26728.9) | 497.8<br>(295.7 to 767.4) | 6.0*<br>(4.2 to 7.8) | -0.3<br>(-2.0 to 1.4) |
| Atopic dermatitis | 7715.7<br>(6732.9 to 8853.1) | 1017.5<br>(815.3 to 1231.6) | 334.4<br>(181.2 to 564.4) | 7.2*<br>(4.9 to 9.6) | 0.9<br>(-1.3 to 3.1) |
| Contact dermatitis | 6104.7<br>(4169.3 to 8665.8) | 17159.9<br>(11753.2 to 23752.1) | 153.7<br>(87.9 to 250.1) | 5.0*<br>(2.5 to 7.4) | -1.2<br>(-3.5 to 1.1) |
| Seborrhoeic dermatitis | 701.7<br>(557.6 to 874.2) | 1933.5<br>(1524.5 to 2397.0) | 9.6<br>(5.3 to 15.9) | -15.9*<br>(-19.2 to -12.0) | -20.8*<br>(-24.0 to -17.2) |
| Psoriasis | 5162.2<br>(4747.2 to 5597.0) | 648.3<br>(554.7 to 750.3) | 452.8<br>(315.3 to 612.0) | 11.9*<br>(9.5 to 14.4) | 5.2*<br>(3.0 to 7.7) |
| Bacterial skin diseases | 415.1<br>(375.8 to 457.2) | 11414.3<br>(10399.7 to 12480.4) | 10.1<br>(6.4 to 15.1) | 10.1*<br>(6.3 to 13.7) | 3.6<br>(-0.0 to 6.9) |
| Cellulitis | 137.4<br>(109.9 to 165.9) | 2762.7<br>(2299.6 to 3293.3) | 8.0<br>(5.1 to 11.6) | 8.1*<br>(3.9 to 12.1) | 1.7<br>(-2.2 to 5.4) |
| Pyoderma | 379.4<br>(342.6 to 416.9) | 8651.6<br>(7733.1 to 9547.8) | 2.1<br>(0.9 to 4.4) | 18.2*<br>(13.6 to 23.5) | 11.3*<br>(6.9 to 16.2) |
| Impetigo | 57.1<br>(50.1 to 64.7) | 1050.9<br>(928.0 to 1177.5) | 0.3<br>(0.1 to 0.7) | 17.3*<br>(9.3 to 26.7) | 10.4*<br>(2.9 to 19.2) |
| Abscess and other bacterial skin diseases | 322.4<br>(286.4 to 358.9) | 7600.7<br>(6696.6 to 8486.4) | 1.8<br>(0.7 to 3.8) | 18.4*<br>(13.1 to 24.6) | 11.4*<br>(6.5 to 17.2) |
| Scabies | 9429.1<br>(6502.9 to 12941.0) | 28139.9<br>(19792.6 to 38938.8) | 242.8<br>(123.8 to 430.1) | -4.1*<br>(-5.3 to -2.8) | -9.8*<br>(-10.9 to -8.5) |
| Fungal skin diseases | 37052.2<br>(30620.4 to 44121.3) | 112507.5<br>(92154.7 to 134026.1) | 209.7<br>(84.9 to 446.1) | 6.7*<br>(5.8 to 7.4) | 0.4<br>(-0.5 to 1.1) |
| Tinea capitis | 3369.6<br>(2306.2 to 4611.6) | 4995.4<br>(3683.3 to 6357.2) | 18.9<br>(7.0 to 41.3) | -3.0<br>(-6.1 to 0.4) | -8.7*<br>(-11.7 to -5.5) |
| Other fungal skin diseases | 33682.5<br>(27499.1 to 40862.7) | 107512.1<br>(87207.3 to 128782.0) | 190.7<br>(76.9 to 412.1) | 7.7*<br>(6.9 to 8.5) | 1.4*<br>(0.6 to 2.1) |

| | | | | | |
|---|---|---|---|---|---|
| Viral skin diseases | 3784.3 (3403.0 to 4198.4) | 3223.8 (2963.4 to 3518.5) | 115.5 (73.6 to 176.3) | -2.2* (-4.0 to -0.1) | -8.0* (-9.6 to -6.0) |
| Viral warts | 2763.7 (2405.4 to 3181.8) | 1662.0 (1437.9 to 1946.0) | 84.4 (53.0 to 128.1) | -5.1* (-7.3 to -2.8) | -10.7* (-12.8 to -8.5) |
| Molluscum contagiosum | 1020.6 (902.4 to 1153.4) | 1561.9 (1403.3 to 1723.2) | 31.1 (19.4 to 48.0) | 6.7* (2.8 to 10.8) | 0.4 (-3.2 to 4.3) |
| Acne vulgaris | 4817.2 (4224.6 to 5533.0) | 2080.8 (1775.0 to 2416.1) | 101.9 (60.7 to 165.3) | 14.7* (11.9 to 17.5) | 7.9* (5.3 to 10.6) |
| Alopecia areata | 1790.7 (1599.9 to 1988.9) | 3097.4 (2787.9 to 3419.7) | 58.8 (37.9 to 87.6) | 4.7* (2.2 to 7.1) | -1.5 (-3.9 to 0.8) |
| Pruritus | 5240.3 (3560.5 to 7229.8) | 3966.8 (2695.8 to 5435.7) | 56.2 (24.1 to 112.4) | 7.9* (6.6 to 9.6) | 1.5* (0.3 to 3.1) |
| Urticaria | 4583.5 (3158.8 to 6514.9) | 7973.6 (5504.8 to 10990.0) | 272.7 (154.9 to 437.3) | 7.3* (5.2 to 9.4) | 0.9 (-1.1 to 2.9) |
| Decubitus ulcer | 31.8 (21.6 to 44.6) | 113.8 (78.6 to 161.1) | 5.6 (3.3 to 9.0) | 15.0* (13.9 to 16.1) | 8.2* (7.1 to 9.2) |
| Other skin and subcutaneous diseases | 31076.8 (28254.9 to 34247.0) | 32256.2 (29386.2 to 35363.1) | 172.8 (80.6 to 319.3) | 11.7* (10.9 to 12.6) | 5.1* (4.3 to 6.0) |
| **Sense organ diseases** | **141049.6 (132730.9 to 149705.6)** | **--** | **3009.4 (1897.7 to 4584.4)** | **6.7* (5.1 to 8.1)** | **0.4 (-1.1 to 1.7)** |
| Blindness and vision impairment | 76029.4 (68477.0 to 83453.1) | -- | 1357.7 (837.6 to 2153.3) | 8.5* (7.3 to 9.6) | 2.1* (0.9 to 3.1) |
| Glaucoma | -- | -- | -- | -- | -- |
| Cataract | 1341.5 (1028.0 to 1707.1) | -- | 126.6 (82.1 to 186.3) | 4.7* (0.3 to 9.2) | -1.5 (-5.6 to 2.8) |
| Age-related macular degeneration | -- | -- | -- | -- | -- |
| Refraction disorders | 10058.8 (7571.1 to 12834.8) | -- | 432.8 (274.4 to 632.5) | 9.1* (6.6 to 11.3) | 2.6* (0.3 to 4.7) |
| Near vision loss | 64971.1 (57127.5 to 72542.9) | -- | 670.9 (317.4 to 1240.6) | 9.1* (8.1 to 10.0) | 2.6* (1.7 to 3.5) |
| Other vision loss | 1514.8 (1159.5 to 1940.4) | -- | 127.3 (84.6 to 187.1) | 7.2* (3.1 to 11.2) | 0.9 (-3.0 to 4.7) |
| Age-related and other hearing loss | 75870.5 (69055.7 to 83182.4) | -- | 1540.8 (976.7 to 2350.3) | 5.2* (2.6 to 7.8) | -1.0 (-3.4 to 1.4) |
| Other sense organ diseases | 4582.0 (4180.0 to 5070.9) | -- | 110.9 (68.0 to 165.6) | 6.9* (4.3 to 9.6) | 0.6 (-1.8 to 3.1) |
| Chronic other sense organ diseases | 3802.8 (3394.7 to 4269.4) | -- | 100.1 (61.5 to 151.6) | 6.8* (4.1 to 9.8) | 0.5 (-2.1 to 3.3) |
| Acute other sense organ diseases | 779.2 (698.1 to 859.0) | -- | 10.7 (6.5 to 16.4) | 7.1* (1.7 to 12.6) | 0.8 (-4.3 to 5.9) |
| **Musculoskeletal disorders** | **104818.5 (89652.3 to 121716.5)** | **29373.6 (22426.9 to 36618.6)** | **11368.8 (7933.8 to 15708.7)** | **6.6* (5.4 to 7.8)** | **0.3 (-0.8 to 1.4)** |
| Rheumatoid arthritis | 1084.9 (920.8 to 1278.5) | 87.9 (61.6 to 121.2) | 149.1 (97.3 to 209.1) | 14.9* (10.6 to 19.6) | 8.1* (4.1 to 12.5) |
| Osteoarthritis | 8695.0 (7342.9 to 10278.8) | 1157.7 (841.1 to 1570.4) | 288.3 (142.3 to 589.9) | 9.1* (7.4 to 10.9) | 2.7* (1.0 to 4.3) |
| Osteoarthritis of the hip | 1095.5 (986.3 to 1219.2) | 126.5 (101.6 to 154.6) | 36.4 (18.6 to 74.4) | 14.9* (10.6 to 19.6) | 8.1* (4.0 to 12.6) |
| Osteoarthritis of the knee | 7599.5 (6274.4 to 9178.5) | 1031.3 (706.5 to 1447.1) | 251.9 (123.6 to 530.7) | 8.3* (6.4 to 10.3) | 1.9* (0.2 to 3.8) |
| Low back pain | 47727.8 (34805.8 to 62804.1) | 21130.5 (14881.5 to 27990.4) | 5513.4 (3548.8 to 8311.2) | 9.0* (7.9 to 10.0) | 2.6* (1.5 to 3.5) |
| Low back pain with leg pain | 15734.2 (11082.0 to 21451.0) | 7009.6 (4847.2 to 9576.4) | 2327.8 (1427.2 to 3579.1) | 9.0* (7.7 to 10.3) | 2.5* (1.3 to 3.8) |
| Low back pain without leg pain | 31993.6 (23086.2 to 42483.4) | 14121.0 (9967.1 to 19187.0) | 3185.6 (2048.9 to 4886.1) | 9.0* (7.9 to 10.2) | 2.6* (1.6 to 3.7) |
| Neck pain | 23862.9 (17311.7 to 32494.8) | 6614.1 (4106.5 to 9968.1) | 2430.4 (1453.0 to 3847.7) | 3.9* (1.7 to 5.9) | -2.3* (-4.3 to -0.4) |
| Gout | 1945.8 (1577.1 to 2437.9) | 383.4 (261.6 to 540.8) | 64.8 (41.1 to 94.4) | 7.3* (2.2 to 12.9) | 1.0 (-3.9 to 6.3) |
| Other musculoskeletal disorders | 33377.5 (23961.6 to 45164.9) | -- | 2922.9 (1741.3 to 4454.5) | 4.0* (0.0 to 7.8) | -2.1 (-5.9 to 1.5) |
| **Other non-communicable diseases** | **358840.5 (338121.8 to 381428.6)** | **265191.6 (229331.9 to 305560.2)** | **4286.7 (2895.9 to 6177.5)** | **4.0* (2.4 to 5.6)** | **-2.1* (-3.7 to -0.7)** |
| Congenital anomalies | 6035.7 (5678.8 to 6423.6) | -- | 742.8 (520.8 to 997.1) | 9.2* (6.7 to 12.0) | 2.8* (0.4 to 5.4) |
| Neural tube defects | 180.6 (160.9 to 202.0) | -- | 53.5 (37.4 to 71.6) | 11.2* (6.1 to 16.6) | 4.7 (-0.2 to 9.7) |
| Severe motor and cognitive impairment due to anencephaly | -- | -- | -- | -- | -- |
| Encephalocoele due to neural tube defects | 20.4 (16.5 to 24.4) | -- | 3.5 (2.3 to 5.0) | 7.0* (3.7 to 10.1) | 0.7 (-2.4 to 3.6) |
| Spina bifida due to neural tube defects | 160.2 (142.6 to 179.1) | -- | 49.9 (34.7 to 67.0) | 11.5* (6.2 to 17.2) | 4.9 (-0.0 to 10.3) |
| Congenital heart anomalies | 257.4 (237.0 to 279.2) | -- | 14.9 (7.0 to 25.3) | 8.6* (4.5 to 12.9) | 2.2 (-1.7 to 6.2) |
| Critical malformations of great vessels, congenital valvular heart disease and patent ductus arteriosus | 42.2 (36.5 to 48.5) | -- | 2.7 (1.4 to 4.5) | 1.5 (-1.7 to 4.6) | -4.5* (-7.5 to -1.6) |
| Other congenital heart anomalies | 77.2 (71.0 to 84.0) | -- | 4.5 (1.8 to 8.0) | 8.9 (-0.3 to 18.0) | 2.4 (-6.2 to 11.1) |
| Severe congenital heart anomalies excluding single ventricle heart defects | 103.5 (92.1 to 116.3) | -- | 6.3 (3.0 to 10.5) | 12.2* (5.0 to 19.8) | 5.6 (-1.2 to 12.7) |
| Single ventricle and single ventricle pathway congenital heart anomalies | 4.8 (4.0 to 5.7) | -- | 0.3 (0.2 to 0.5) | 17.7* (14.5 to 20.3) | 10.8* (7.7 to 13.2) |
| Ventricular septal defect and atrial septal defect | 29.6 (24.2 to 35.3) | -- | 1.0 (0.4 to 1.8) | 3.4 (-3.2 to 10.2) | -2.7 (-8.9 to 3.7) |
| Orofacial clefts | 709.7 (651.0 to 768.8) | -- | 20.6 (13.3 to 29.9) | 8.7* (1.2 to 17.5) | 2.3 (-4.8 to 10.6) |
| Down's syndrome | 279.6 (244.3 to 317.0) | -- | 23.9 (16.1 to 33.4) | 8.4* (5.6 to 11.3) | 2.0 (-0.6 to 4.7) |
| Turner syndrome | 29.2 (25.6 to 33.5) | -- | 0.6 (0.3 to 1.0) | 5.5* (3.1 to 7.9) | -0.7 (-3.0 to 1.5) |
| Klinefelter syndrome | 54.1 (46.6 to 62.3) | -- | 0.6 (0.2 to 1.1) | 5.3 (-2.5 to 13.5) | -0.9 (-8.3 to 6.8) |
| Other chromosomal abnormalities | 431.2 (336.3 to 532.3) | -- | 36.4 (23.3 to 51.8) | 17.4* (13.2 to 21.7) | 10.5* (6.5 to 14.5) |
| Congenital musculoskeletal and limb anomalies | 1962.7 (1746.0 to 2250.5) | -- | 291.4 (190.5 to 402.4) | 10.1* (7.8 to 12.5) | 3.6* (1.4 to 5.8) |
| Polydactyly and syndactyly | 6.7 (5.3 to 8.2) | -- | 0.1 (0.0 to 0.1) | 0.9 (-1.0 to 2.6) | -5.1* (-6.8 to -3.4) |
| Congenital limb deficiency | 955.3 (848.5 to 1096.4) | -- | 142.2 (93.9 to 195.9) | 10.2* (7.1 to 13.2) | 3.7* (0.8 to 6.5) |
| Other congenital musculoskeletal anomalies | 1000.6 (890.1 to 1147.5) | -- | 149.1 (97.1 to 206.5) | 10.0* (6.9 to 13.5) | 3.5* (0.6 to 6.8) |
| Urogenital congenital anomalies | 293.3 (247.0 to 349.6) | -- | 7.1 (4.1 to 11.3) | -6.8* (-10.4 to -3.0) | -12.3* (-15.7 to -8.7) |
| Digestive congenital anomalies | 1204.6 (1084.5 to 1346.0) | -- | 54.4 (36.0 to 77.3) | 3.0 (-2.1 to 8.1) | -3.1 (-7.9 to 1.7) |
| Congenital diaphragmatic hernia | 13.7 (12.1 to 15.5) | -- | 0.3 (0.2 to 0.4) | 5.5* (4.7 to 6.4) | -0.7 (-1.5 to 0.1) |
| Congenital atresia and/or stenosis of the digestive tract | 0.5 (0.3 to 0.6) | -- | 0.0 (0.0 to 0.1) | 27.5* (23.0 to 32.1) | 20.0* (15.7 to 24.3) |
| Congenital malformations of the abdominal wall after treatment | 53.1 (46.6 to 60.4) | -- | 2.3 (1.5 to 3.4) | 5.6* (4.7 to 6.4) | -0.7 (-1.4 to 0.1) |
| Other congenital malformations of the digestive tract | 1137.2 (1016.0 to 1281.2) | -- | 51.9 (34.2 to 74.1) | 2.9 (-2.5 to 8.3) | -3.2 (-8.2 to 1.9) |
| Other congenital anomalies | 1757.3 (1521.2 to 2036.1) | -- | 239.6 (161.4 to 336.4) | 8.8* (1.9 to 16.5) | 2.4 (-4.1 to 9.6) |

| | | | | | |
|---|---|---|---|---|---|
| Other congenital birth defects | -- | -- | 15.2 (10.9 to 19.9) | 25.9* (21.2 to 30.6) | 18.4* (14.0 to 22.9) |
| Hearing loss due to other congenital anomalies | 1757.3 (1521.2 to 2036.1) | -- | 224.4 (148.8 to 316.9) | 7.8* (0.5 to 15.8) | 1.4 (-5.4 to 8.9) |
| Urinary diseases and male infertility | 6779.0 (4618.6 to 9614.0) | 31040.8 (24876.1 to 38689.3) | 80.9 (48.7 to 130.1) | 3.5 (-0.1 to 7.3) | -2.6 (-6.0 to 1.0) |
| Urinary tract infections | 471.2 (360.9 to 614.2) | 24183.8 (18001.9 to 31985.2) | 15.6 (8.9 to 24.1) | 8.8* (3.1 to 14.8) | 2.4 (-3.0 to 8.0) |
| Urolithiasis | 260.8 (185.8 to 352.1) | 6857.0 (4943.5 to 9135.6) | 19.8 (12.1 to 30.9) | 3.3 (-1.0 to 8.0) | -2.8 (-6.9 to 1.6) |
| Benign prostatic hyperplasia | -- | -- | -- | -- | -- |
| Male infertility | 6133.3 (3966.0 to 9029.6) | -- | 34.4 (12.8 to 74.1) | 8.2* (2.4 to 13.1) | 1.8 (-3.7 to 6.4) |
| Other urinary diseases | -- | -- | 11.0 (7.0 to 16.0) | -13.7* (-16.3 to -10.5) | -18.8* (-21.3 to -15.8) |
| Gynecological diseases | 104821.7 (97194.1 to 112811.6) | 25911.0 (24048.5 to 27959.0) | 1749.7 (1198.3 to 2504.6) | 3.1* (1.2 to 5.2) | -3.0* (-4.7 to -1.0) |
| Uterine fibroids | 23321.4 (18667.2 to 28880.4) | 2755.8 (2082.3 to 3535.6) | 240.9 (141.0 to 393.7) | 1.6 (-5.8 to 9.8) | -4.4 (-11.4 to 3.3) |
| Polycystic ovarian syndrome | 7324.5 (5791.5 to 9405.3) | 19.6 (7.7 to 37.2) | 63.2 (27.7 to 121.5) | 9.3* (7.9 to 10.9) | 2.9* (1.6 to 4.3) |
| Female infertility | 13963.4 (6377.8 to 24602.3) | -- | 74.8 (22.5 to 177.6) | 17.3* (5.4 to 24.9) | 10.4 (-0.9 to 17.5) |
| Endometriosis | 7619.8 (5867.4 to 9566.2) | 1178.1 (807.2 to 1641.7) | 703.0 (452.5 to 1058.4) | -0.2 (-2.1 to 1.7) | -6.1* (-7.8 to -4.3) |
| Genital prolapse | 8356.2 (6772.5 to 10024.8) | 1232.7 (849.4 to 1675.5) | 26.6 (12.0 to 51.0) | 7.5* (4.8 to 10.4) | 1.2 (-1.4 to 3.8) |
| Premenstrual syndrome | 57686.3 (53579.1 to 62096.0) | 12771.1 (11200.7 to 14351.4) | 478.2 (305.5 to 707.9) | 3.9* (1.4 to 6.0) | -2.3* (-4.6 to -0.2) |
| Other gynecological diseases | 9064.2 (8502.2 to 9686.7) | 7953.7 (7342.4 to 8607.9) | 163.0 (108.6 to 244.1) | 9.9* (5.1 to 14.1) | 3.4 (-1.1 to 7.4) |
| Other gynecological diseases | 8175.9 (7601.3 to 8776.5) | 7953.7 (7342.4 to 8607.9) | 143.2 (94.5 to 216.2) | 9.8* (4.8 to 14.4) | 3.4 (-1.4 to 7.6) |
| Other gynecological diseases with anemia | 888.3 (823.3 to 956.9) | -- | 19.8 (12.6 to 29.1) | 10.4 (-1.4 to 22.3) | 3.9 (-7.3 to 15.1) |
| Hemoglobinopathies and hemolytic anaemias | 129762.0 (126839.2 to 132922.1) | -- | 330.5 (215.8 to 482.6) | -12.4* (-19.8 to -4.8) | -17.6* (-24.5 to -10.4) |
| Thalassemias | 1.4 (1.4 to 1.5) | -- | 0.1 (0.1 to 0.2) | -18.0* (-29.7 to -6.0) | -22.9* (-33.9 to -11.6) |
| Thalassaemias trait | 20984.8 (20166.6 to 21906.0) | -- | 145.5 (89.5 to 217.2) | -23.3* (-33.2 to -11.6) | -27.8* (-37.1 to -16.9) |
| Sickle cell disorders | 50.4 (41.3 to 59.8) | -- | 6.3 (3.9 to 9.5) | 30.9* (11.2 to 52.7) | 23.2* (4.6 to 43.7) |
| Sickle cell trait | 29860.3 (26917.9 to 33163.0) | -- | 70.3 (45.7 to 103.4) | 4.7 (-4.0 to 14.0) | -1.4 (-9.7 to 7.3) |
| G6PD deficiency | 24123.7 (23161.7 to 25116.7) | -- | 1.5 (1.0 to 2.2) | -1.0 (-14.2 to 13.3) | -6.8 (-19.3 to 6.6) |
| G6PD trait | 63992.3 (62940.6 to 65028.0) | -- | 0.0 (0.0 to 0.1) | 7.8 (-6.5 to 24.8) | 1.5 (-12.0 to 17.5) |
| Other hemoglobinopathies and hemolytic anaemias | 5168.0 (4832.2 to 5563.9) | -- | 106.7 (70.3 to 157.9) | -6.3 (-13.4 to 1.6) | -11.9* (-18.6 to -4.4) |
| Endocrine, metabolic, blood, and immune disorders | 7255.6 (6801.2 to 7802.6) | -- | 192.4 (130.1 to 275.8) | -5.2 (-10.4 to 0.2) | -10.8* (-15.7 to -5.7) |
| Anemia due to endocrine, metabolic, blood, and immune disorders | 5960.0 (5662.3 to 6323.1) | -- | 144.6 (95.7 to 208.7) | -8.6* (-15.2 to -1.7) | -14.0* (-20.2 to -7.6) |
| Endocrine, metabolic, blood, and immune disorders | 1292.9 (965.9 to 1689.2) | -- | 47.5 (28.6 to 72.6) | 7.3* (2.7 to 12.6) | 1.0 (-3.4 to 6.0) |
| Heart failure due to endocrine, metabolic, blood, and immune disorders | 2.7 (1.8 to 3.8) | -- | 0.2 (0.1 to 0.4) | 1.1 (-0.5 to 2.6) | -4.9* (-6.4 to -3.4) |
| Oral disorders | 248905.7 (214494.5 to 286121.1) | 208239.9 (173620.1 to 248198.0) | 1190.4 (650.5 to 2061.9) | 9.7* (8.9 to 10.6) | 3.2* (2.5 to 4.0) |
| Caries of deciduous teeth | -- | -- | -- | -- | -- |
| Caries of permanent teeth | 199710.6 (158440.4 to 246057.1) | 200297.8 (165604.3 to 239833.0) | 141.6 (61.8 to 278.0) | 4.6* (3.5 to 5.7) | -1.6* (-2.6 to -0.5) |
| Periodontal disease | 69573.3 (49929.9 to 95163.0) | 7431.8 (3910.6 to 11338.0) | 460.9 (171.3 to 1008.6) | 10.8* (9.4 to 12.0) | 4.2* (3.0 to 5.3) |
| Edentulism and severe tooth loss | 8070.6 (6457.7 to 9968.0) | 510.2 (322.0 to 777.7) | 231.1 (144.9 to 343.4) | 16.4* (14.7 to 18.1) | 9.5* (7.9 to 11.1) |
| Other oral disorders | 12277.4 (10771.1 to 13819.2) | -- | 356.9 (218.0 to 531.5) | 6.5* (5.3 to 7.6) | 0.2 (-0.9 to 1.2) |
| **Injuries** | **121970.3 (112236.2 to 132199.9)** | **36305.3 (31069.9 to 42208.8)** | **4546.4 (3398.9 to 5986.1)** | **10.6* (9.1 to 12.1)** | **4.0* (2.7 to 5.5)** |
| **Transport injuries** | **17196.5 (15813.6 to 18602.7)** | **5259.5 (3698.1 to 7311.7)** | **1092.2 (786.9 to 1453.9)** | **5.1* (3.9 to 6.3)** | **-1.1 (-2.2 to 0.0)** |
| Road injuries | 13325.5 (12357.2 to 14590.6) | 4748.6 (3205.5 to 6819.3) | 838.4 (603.1 to 1121.9) | 4.3* (3.0 to 5.4) | -1.9* (-3.1 to -0.8) |
| Pedestrian road injuries | 3351.0 (2820.0 to 4228.6) | 880.5 (498.2 to 1429.7) | 212.2 (148.7 to 286.2) | -0.9 (-2.5 to 0.6) | -6.7* (-8.2 to -5.4) |
| Cyclist road injuries | 2132.6 (1684.1 to 2656.3) | 964.5 (527.7 to 1627.6) | 128.2 (86.7 to 176.8) | 8.0* (6.1 to 9.8) | 1.6 (-0.2 to 3.4) |
| Motorcyclist road injuries | 3727.7 (3065.0 to 4459.8) | 956.2 (579.7 to 1475.6) | 234.1 (156.8 to 323.8) | 2.8* (1.1 to 4.4) | -3.3* (-4.9 to -1.8) |
| Motor vehicle road injuries | 3270.2 (2740.3 to 3961.9) | 1552.3 (965.7 to 2420.1) | 216.0 (152.3 to 293.6) | 5.7* (4.1 to 7.4) | -0.5 (-2.0 to 1.0) |
| Other road injuries | 844.1 (665.7 to 1084.5) | 395.1 (184.6 to 720.6) | 47.9 (32.9 to 68.1) | 22.0* (19.7 to 24.2) | 14.8* (12.6 to 16.9) |
| Other transport injuries | 3871.0 (3116.1 to 4693.5) | 511.0 (311.4 to 741.9) | 253.8 (182.0 to 344.1) | 8.0* (6.4 to 9.8) | 1.7* (0.1 to 3.3) |
| **Unintentional injuries** | **69369.1 (63973.7 to 75482.1)** | **27493.9 (22632.6 to 33013.3)** | **2684.3 (1938.3 to 3606.7)** | **15.7* (14.4 to 16.9)** | **8.8* (7.6 to 10.0)** |
| Falls | 26297.8 (22333.4 to 30575.6) | 9772.4 (6487.1 to 14050.8) | 1235.3 (878.8 to 1675.6) | 14.9* (13.5 to 16.2) | 8.1* (6.8 to 9.3) |
| Drowning | 153.4 (130.5 to 182.6) | 18.6 (13.3 to 25.8) | 9.8 (6.8 to 13.2) | -1.1 (-3.7 to 1.6) | -7.0* (-9.4 to -4.4) |
| Fire, heat, and hot substances | 9162.1 (7671.7 to 11072.8) | 679.2 (418.8 to 979.1) | 314.8 (219.0 to 431.2) | 10.0* (6.7 to 13.1) | 3.5* (0.4 to 6.4) |
| Poisonings | 356.0 (298.5 to 420.9) | 240.6 (131.6 to 386.1) | 37.3 (24.6 to 53.0) | 25.8* (22.4 to 29.2) | 18.4* (15.2 to 21.5) |
| Poisoning by carbon monoxide | 66.7 (55.1 to 82.1) | 82.7 (37.2 to 155.3) | 6.1 (4.0 to 8.9) | 26.1* (21.4 to 30.5) | 18.6* (14.2 to 22.8) |
| Poisoning by other means | 289.3 (231.5 to 353.3) | 157.9 (79.1 to 266.0) | 31.1 (20.1 to 45.5) | 25.7* (22.2 to 29.4) | 18.3* (15.0 to 21.8) |
| Exposure to mechanical forces | 15226.5 (12809.5 to 18261.4) | 5733.3 (3839.6 to 8355.7) | 402.2 (283.3 to 576.9) | 16.5* (14.9 to 17.7) | 9.6* (8.1 to 10.8) |
| Unintentional firearm injuries | 702.2 (547.0 to 861.7) | 142.3 (63.0 to 273.8) | 28.8 (19.9 to 39.5) | 18.3* (16.9 to 19.8) | 11.3* (9.9 to 12.8) |
| Other exposure to mechanical forces | 14524.3 (12050.5 to 17533.6) | 5591.0 (3714.3 to 8104.1) | 373.5 (260.3 to 539.7) | 16.3* (14.7 to 17.7) | 9.4* (7.9 to 10.7) |
| Adverse effects of medical treatment | 126.1 (75.8 to 188.4) | 1648.2 (1032.5 to 2400.2) | 16.8 (8.9 to 29.5) | 11.0* (6.0 to 15.4) | 4.5 (-0.3 to 8.6) |
| Animal contact | 3008.0 (2602.3 to 3461.6) | 3353.9 (2042.9 to 5306.2) | 86.9 (59.0 to 120.2) | 12.3* (9.3 to 15.1) | 5.7* (2.9 to 8.3) |
| Venomous animal contact | 1354.5 (1101.1 to 1657.6) | 1775.0 (998.1 to 2840.2) | 60.9 (40.4 to 85.4) | 15.1* (10.9 to 18.8) | 8.3* (4.3 to 11.8) |
| Non-venomous animal contact | 1653.5 (1334.8 to 1958.2) | 1578.9 (911.5 to 2525.2) | 26.0 (16.2 to 40.6) | 6.3* (4.0 to 8.1) | 0.0 (-2.1 to 1.7) |
| Foreign body | 1433.5 (1197.1 to 1685.6) | 1810.5 (1166.5 to 2607.2) | 74.0 (52.3 to 102.1) | 9.6* (7.7 to 11.5) | 3.1* (1.4 to 4.9) |

| | | | | | |
|---|---|---|---|---|---|
| Pulmonary aspiration and foreign body in airway | 228.2 (176.5 to 286.9) | 122.3 (72.8 to 196.3) | 11.9 (8.2 to 16.4) | 4.6* (1.3 to 8.0) | -1.6 (-4.7 to 1.6) |
| Foreign body in eyes | 236.1 (105.0 to 417.9) | 1450.3 (833.1 to 2216.1) | 15.0 (6.7 to 29.3) | 15.1* (13.2 to 16.6) | 8.3* (6.5 to 9.7) |
| Foreign body in other body part | 969.2 (774.3 to 1157.8) | 238.0 (139.1 to 371.6) | 47.1 (33.3 to 63.0) | 9.2* (7.2 to 11.2) | 2.7* (0.9 to 4.6) |
| Environmental heat and cold exposure | 2686.1 (2183.0 to 3294.7) | 521.7 (322.1 to 780.6) | 118.2 (81.4 to 159.6) | 14.9* (13.0 to 16.7) | 8.1* (6.4 to 9.8) |
| Exposure to forces of nature | 1937.4 (1398.4 to 2606.0) | 42.3 (28.6 to 60.8) | 86.2 (61.5 to 120.3) | 57.9* (42.4 to 75.5) | 48.6* (34.0 to 65.2) |
| Other unintentional injuries | 8982.2 (7386.2 to 10894.5) | 3673.3 (2315.4 to 5671.0) | 302.7 (208.6 to 431.0) | 17.6* (15.9 to 19.4) | 10.7* (9.0 to 12.3) |
| **Self-harm and interpersonal violence** | **35405.9 (27749.2 to 43875.1)** | **3551.9 (2714.2 to 4608.2)** | **769.9 (580.1 to 966.6)** | **2.4 (-2.0 to 7.3)** | **-3.7 (-7.8 to 1.0)** |
| Self-harm | 673.5 (535.8 to 822.9) | 387.3 (266.1 to 550.9) | 39.5 (27.0 to 53.5) | -4.1* (-5.7 to -2.5) | -9.7* (-11.3 to -8.2) |
| Self-harm by firearm | 6.1 (5.0 to 7.7) | 1.7 (0.4 to 4.7) | 0.3 (0.2 to 0.4) | -2.7* (-5.3 to -0.1) | -8.5* (-10.9 to -6.0) |
| Self-harm by other specified means | 667.4 (530.3 to 817.0) | 385.6 (264.6 to 548.4) | 39.2 (26.8 to 53.2) | -4.1* (-5.7 to -2.5) | -9.8* (-11.3 to -8.2) |
| Interpersonal violence | 29138.3 (21915.7 to 37322.0) | 2124.4 (1418.9 to 3116.5) | 435.4 (324.8 to 575.3) | 1.5* (0.4 to 2.5) | -4.5* (-5.5 to -3.6) |
| Assault by firearm | 224.3 (177.3 to 282.1) | 48.4 (23.5 to 88.7) | 11.2 (7.8 to 15.5) | 8.6* (6.6 to 10.7) | 2.2* (0.3 to 4.1) |
| Assault by sharp object | 1329.3 (1031.3 to 1949.3) | 373.6 (188.3 to 631.6) | 46.1 (32.0 to 64.5) | -1.0 (-2.9 to 1.2) | -6.8* (-8.6 to -4.8) |
| Sexual violence | 24027.8 (16814.6 to 32322.1) | -- | 213.0 (125.0 to 332.2) | 0.6 (-1.1 to 2.2) | -5.3* (-6.9 to -3.9) |
| Assault by other means | 3556.9 (2854.2 to 4600.0) | 1702.4 (1122.9 to 2525.7) | 165.1 (114.4 to 230.7) | 2.9* (1.3 to 4.6) | -3.2* (-4.7 to -1.6) |
| Conflict and terrorism | 5265.1 (3602.4 to 7634.9) | 867.8 (635.4 to 1184.3) | 281.9 (185.8 to 434.5) | 6.3 (-5.2 to 20.6) | 0.0 (-10.8 to 13.5) |
| Executions and police conflict | 329.0 (221.9 to 481.5) | 172.4 (113.8 to 251.7) | 13.2 (9.3 to 19.3) | -20.6 (-30.6 to 2.8) | -25.3* (-34.7 to -3.2) |
| **Nature of injury aggregates** | | | | | |
| Amputations | 29877.1 (27158.4 to 33159.5) | 735.2 (589.2 to 915.3) | 515.6 (343.8 to 755.0) | 8.1* (4.2 to 11.5) | 1.7 (-1.9 to 4.9) |
| Burns | 19592.7 (17522.0 to 21711.4) | 1024.8 (821.5 to 1279.4) | 688.8 (489.4 to 936.7) | 9.0* (5.4 to 12.4) | 2.5 (-0.8 to 5.7) |
| Fractures | 22732.6 (21085.6 to 24436.2) | 9085.8 (7474.6 to 10944.7) | 1274.8 (868.5 to 1785.8) | 11.5* (9.8 to 12.9) | 4.9* (3.3 to 6.3) |
| Head Injuries | 3669.7 (3457.3 to 3878.4) | 1600.7 (1309.5 to 1948.0) | 555.5 (391.2 to 754.9) | 13.5* (11.8 to 15.2) | 6.8* (5.2 to 8.4) |
| Spinal Injuries | 2020.1 (1802.4 to 2364.1) | 53.9 (42.8 to 68.9) | 626.6 (442.6 to 839.2) | 12.8* (9.8 to 15.9) | 6.1* (3.3 to 9.1) |
| Minor Injuries | 14723.3 (13684.5 to 15868.1) | 13367.4 (10974.7 to 16046.9) | 131.1 (62.6 to 244.1) | 12.7* (11.5 to 13.8) | 6.0* (4.9 to 7.1) |
| Other Injuries | 5328.2 (4960.3 to 5718.8) | 10437.5 (8999.8 to 11977.3) | 541.0 (383.9 to 724.2) | 11.2* (8.8 to 13.5) | 4.7* (2.4 to 6.8) |
| **Impairments** | | | | | |
| Anaemia | 117338.4 (111139.6 to 124361.5) | -- | 3130.7 (2108.8 to 4435.6) | -7.8* (-13.6 to -1.8) | -13.2* (-18.7 to -7.6) |
| Epilepsy | 4018.7 (3310.8 to 4799.7) | -- | 1632.5 (1228.0 to 2139.8) | 11.2* (5.6 to 16.9) | 4.7 (-0.6 to 10.0) |
| Guillain-Barré syndrome | 6.4 (3.9 to 10.3) | -- | 1.9 (1.0 to 3.3) | 14.6* (11.9 to 17.3) | 7.8* (5.3 to 10.4) |
| Hearing loss | 80728.0 (73908.7 to 87890.1) | -- | 1832.9 (1201.2 to 2673.2) | 5.1* (2.9 to 7.5) | -1.1 (-3.2 to 1.2) |
| Heart failure | 728.0 (545.9 to 934.2) | -- | 68.1 (42.0 to 100.6) | 2.7* (0.2 to 5.2) | -3.3* (-5.7 to -1.0) |
| Infertility | 26933.2 (17085.5 to 40563.5) | -- | 195.7 (94.6 to 366.2) | 10.5* (5.6 to 15.1) | 4.0 (-0.7 to 8.3) |
| Developmental intellectual disability | 12273.9 (9540.8 to 14954.7) | -- | 1676.0 (1309.5 to 2092.9) | 10.4* (7.3 to 13.7) | 3.8* (1.0 to 7.0) |
| Pelvic inflammatory disease | 191.7 (148.4 to 246.3) | -- | 25.3 (16.6 to 36.6) | 10.3* (5.3 to 16.0) | 3.8 (-0.9 to 9.2) |
| Vision loss | 79484.6 (71690.9 to 87406.7) | -- | 1666.3 (1099.7 to 2482.7) | 8.9* (7.8 to 9.9) | 2.4* (1.4 to 3.4) |

We did not calculate incidence for the nine impairments and certain NTDs. Blank cells mean that no estimate is available or that the estimate has a magnitude less than 50.
Table presents prevalence and incidence counts in thousands.
* Percentage changes that are statistically significant
‡ Incidence estimates for stroke represent first-ever stroke only.
^ Incidence of HIV/AIDS represents new infections of HIV only and does not include new infections of Tuberculosis in HIV positive cases.

SR Table 10l: Global prevalence, incidence and YLDs for 2017, percentage change of YLD counts and percentage change of YLD rates between 2007 and 2017 by cause, age 40 to 44 years

| Cause | Prevalence in thousands (95% UI) | Incidence in thousands (95% UI) | YLDs in thousands (95% UI) | | |
|---|---|---|---|---|---|
| | 2017 counts | 2017 counts | 2017 counts | Percentage change in counts between 2007 and 2017 | Percentage change in rates between 2007 and 2017 |
| **All causes** | **489126.4 (487785.2 to 490309.6)** | **2265067.3 (1935276.8 to 2681470.7)** | **62757.2 (46793.1 to 80775.9)** | **9.4* (8.7 to 10.1)** | **0.2 (-0.5 to 0.8)** |
| **Communicable, maternal, neonatal, and nutritional disorders** | **333907.7 (308779.7 to 362277.9)** | **1587528.1 (1261707.6 to 1980212.6)** | **6543.6 (4903.2 to 8460.0)** | **5.7* (0.7 to 9.8)** | **-3.1 (-7.7 to 0.5)** |
| **HIV/AIDS and sexually transmitted infections** | **132984.5 (119361.2 to 148092.8)** | **68147.4 (54514.6 to 84041.5)** | **677.3 (449.3 to 964.6)** | **-8.3 (-28.0 to 14.0)** | **-16.0 (-34.1 to 4.4)** |
| HIV/AIDS | 4818.5 (4383.6 to 5309.3) | 125.8^ (91.9 to 162.4) | 522.5 (339.2 to 773.0) | -12.7 (-32.2 to 15.5) | -20.0 (-37.9 to 5.8) |
| HIV/AIDS - Drug-susceptible Tuberculosis | 123.4 (111.2 to 135.9) | 154.8 (137.7 to 173.7) | 46.9 (31.8 to 63.6) | -12.2* (-17.0 to -7.1) | -19.6* (-24.0 to -14.9) |
| HIV/AIDS - Multidrug-resistant Tuberculosis without extensive drug resistance | 4.6 (3.1 to 6.7) | 6.2 (4.5 to 8.5) | 1.9 (1.1 to 3.0) | -14.5 (-42.5 to 25.4) | -21.7 (-47.3 to 14.9) |
| HIV/AIDS - Extensively drug-resistant Tuberculosis | 0.2 (0.1 to 0.3) | 0.2 (0.2 to 0.3) | 0.1 (0.0 to 0.1) | 47.4 (-1.4 to 121.4) | 35.1 (-9.7 to 102.8) |
| HIV/AIDS resulting in other diseases | 4690.3 (4256.8 to 5183.4) | 125.8 (91.9 to 162.4) | 473.6 (298.6 to 711.0) | -12.7 (-33.7 to 20.0) | -20.0 (-39.3 to 10.0) |
| HIV/AIDS not on antiretroviral treatment without Tuberculosis | 1507.4 (1270.6 to 1855.7) | 125.8 (91.9 to 162.4) | 226.9 (120.7 to 397.1) | -54.2* (-59.8 to -47.7) | -58.0* (-63.2 to -52.1) |
| HIV/AIDS on antiretroviral treatment without Tuberculosis | 3182.9 (2911.1 to 3458.8) | -- | 246.8 (165.4 to 346.8) | 417.1* (335.5 to 531.8) | 373.7* (298.9 to 478.7) |
| Sexually transmitted infections excluding HIV | 130397.8 (116573.2 to 145716.8) | 68021.6 (54365.6 to 83936.5) | 154.8 (83.1 to 285.6) | 10.5* (8.6 to 12.4) | 1.2 (-0.5 to 3.0) |
| Syphilis | 3025.0 (2125.5 to 4072.5) | 719.5 (450.8 to 1080.7) | 6.9 (4.8 to 9.2) | 0.7 (-5.9 to 7.6) | -7.8* (-13.8 to -1.4) |
| Early syphilis | 2990.3 (2089.9 to 4038.7) | 719.5 (450.8 to 1080.7) | 0.7 (0.2 to 1.9) | 13.3* (4.6 to 21.7) | 3.8 (-4.2 to 11.5) |
| Tertiary syphilis | 34.6 (28.7 to 40.7) | -- | 6.1 (4.2 to 8.5) | -0.6 (-7.8 to 6.9) | -8.9* (-15.5 to -2.1) |
| Chlamydial infection | 12836.0 (9387.1 to 17030.4) | 20534.2 (14379.7 to 27716.2) | 37.9 (20.5 to 69.3) | 10.0* (6.3 to 14.5) | 0.8 (-2.6 to 4.9) |
| Chlamydia episode | 11418.1 (7947.7 to 15659.9) | 20534.2 (14379.7 to 27716.2) | 19.1 (7.2 to 41.8) | 6.0* (4.2 to 7.7) | -2.9* (-4.5 to -1.3) |
| Chlamydial infection complications | 1417.8 (1316.8 to 1531.7) | -- | 18.8 (11.8 to 28.6) | 14.4* (6.6 to 21.9) | 4.8 (-2.4 to 11.7) |
| Gonococcal infection | 1999.2 (1268.7 to 3166.4) | 7132.0 (4193.6 to 11478.3) | 10.7 (5.9 to 19.2) | 12.9* (6.2 to 21.0) | 3.4 (-2.7 to 10.8) |
| Gonococcal infection complications | 396.3 (366.9 to 430.1) | -- | 6.3 (3.8 to 9.6) | 16.4* (5.9 to 26.8) | 6.7 (-3.0 to 16.1) |
| Gonorrhoea episode | 1602.9 (876.0 to 2756.0) | 7132.0 (4193.6 to 11478.3) | 4.4 (1.5 to 10.4) | 8.3* (1.2 to 13.3) | -0.8 (-7.3 to 3.8) |
| Trichomoniasis | 15374.8 (9991.7 to 22702.5) | 34287.2 (24275.9 to 48379.8) | 25.4 (9.4 to 57.7) | 11.3* (9.1 to 13.4) | 2.0 (-0.1 to 3.9) |
| Genital herpes | 104797.1 (89507.3 to 120973.3) | 5348.8 (3521.4 to 7595.8) | 26.6 (8.4 to 64.2) | 11.2* (9.7 to 12.6) | 1.9* (0.5 to 3.1) |
| Other sexually transmitted infections | 3792.6 (3529.1 to 4059.0) | -- | 47.4 (27.6 to 81.3) | 11.1* (8.3 to 13.9) | 1.8 (-0.8 to 4.3) |
| Other sexually transmitted diseases residual | -- | -- | 13.0 (6.7 to 24.6) | 7.4* (3.0 to 12.8) | -1.6 (-5.6 to 3.3) |
| Other sexually transmitted diseases | 3792.6 (3529.1 to 4059.0) | -- | 47.4 (27.6 to 81.3) | 11.1* (8.3 to 13.9) | 1.8 (-0.8 to 4.3) |
| **Respiratory infections and tuberculosis** | **172602.7 (119524.6 to 232940.9)** | **1086064.8 (753853.6 to 1485019.8)** | **701.7 (463.6 to 1032.6)** | **6.0* (4.2 to 7.8)** | **-2.9* (-4.6 to -1.3)** |
| Tuberculosis | 160537.1 (105077.1 to 222536.9) | 534.8 (375.5 to 742.2) | 245.8 (149.6 to 369.2) | 3.9 (-0.0 to 7.9) | -4.8* (-8.4 to -1.2) |
| Latent tuberculosis infection | 159738.0 (104221.8 to 221708.7) | -- | -- | -- | -- |
| Drug-susceptible tuberculosis | 758.3 (552.4 to 1000.3) | 502.1 (351.7 to 696.4) | 233.1 (141.9 to 350.8) | 3.8 (-1.8 to 9.1) | -4.9* (-10.1 to -0.0) |
| Multidrug-resistant tuberculosis without extensive drug resistance | 38.8 (17.0 to 77.8) | 30.4 (15.7 to 55.0) | 12.1 (4.7 to 25.3) | 4.2 (-49.4 to 87.3) | -4.6 (-53.7 to 71.6) |
| Extensively drug-resistant tuberculosis | 2.0 (1.1 to 3.5) | 2.3 (1.5 to 3.4) | 0.7 (0.3 to 1.2) | 40.9 (-9.1 to 126.1) | 29.1 (-16.8 to 107.1) |
| Lower respiratory infections | 382.4 (319.6 to 453.5) | 17232.9 (14248.6 to 20705.6) | 23.2 (15.1 to 33.9) | 29.1* (17.2 to 40.5) | 18.3* (7.4 to 28.7) |
| Guillain-Barré syndrome due to lower respiratory infections | 0.7 (0.3 to 1.5) | -- | 0.2 (0.1 to 0.5) | 17.1* (14.1 to 20.1) | 7.3* (4.6 to 10.0) |
| Lower respiratory infection episode | 381.6 (319.1 to 452.4) | 17232.9 (14248.6 to 20705.6) | 23.0 (15.0 to 33.5) | 29.3* (17.2 to 40.6) | 18.4* (7.3 to 28.8) |
| Upper respiratory infections | 14671.6 (10010.4 to 20372.7) | 1064047.4 (732448.6 to 1462876.9) | 361.4 (186.5 to 608.1) | 7.5* (6.1 to 8.8) | -1.6* (-2.8 to -0.4) |
| Guillain-Barré syndrome due to upper respiratory infections | 2.0 (1.1 to 3.5) | -- | 0.6 (0.3 to 1.1) | 17.1* (14.1 to 20.0) | 7.3* (4.6 to 9.9) |
| Upper respiratory infection episode | 14669.6 (10008.9 to 20370.5) | 1064047.4 (732448.6 to 1462876.9) | 360.8 (185.7 to 607.8) | 7.5* (6.1 to 8.8) | -1.6* (-2.8 to -0.4) |
| Otitis media | 3437.1 (2452.7 to 4768.0) | 4249.6 (2910.9 to 5923.5) | 71.2 (38.1 to 122.9) | 0.1 (-4.3 to 4.5) | -8.3* (-12.3 to -4.3) |
| Acute otitis media | 234.8 (155.8 to 329.3) | 4249.1 (2910.4 to 5923.1) | 3.0 (1.5 to 5.9) | 11.4* (4.9 to 18.2) | 2.0 (-3.9 to 8.2) |
| Chronic otitis media | 3202.3 (2202.9 to 4523.2) | 0.5 (0.0 to 1.9) | 68.2 (36.5 to 119.2) | -0.4 (-4.9 to 4.2) | -8.7* (-12.9 to -4.5) |
| **Enteric infections** | **4846.6 (4353.8 to 5342.0)** | **332722.8 (293812.1 to 373839.2)** | **543.9 (378.8 to 760.6)** | **37.0* (31.6 to 41.9)** | **25.5* (20.6 to 30.0)** |
| Diarrhoeal diseases | 4870.4 (4360.0 to 5381.8) | 332374.5 (293466.5 to 373494.0) | 541.2 (376.8 to 756.3) | 37.4* (32.0 to 42.3) | 25.9* (20.9 to 30.4) |
| Guillain-Barré syndrome due to diarrheal diseases | 0.7 (0.4 to 1.2) | -- | 0.2 (0.1 to 0.4) | 17.1* (14.1 to 20.0) | 7.3* (4.5 to 9.9) |
| Diarrheal disease episode | 4869.7 (4358.8 to 5381.1) | 332374.5 (293466.5 to 373494.0) | 541.0 (376.5 to 756.0) | 37.4* (32.0 to 42.3) | 25.9* (20.9 to 30.4) |
| Typhoid and paratyphoid | 1.9 (0.7 to 3.3) | 329.1 (255.6 to 408.7) | 2.6 (1.6 to 4.0) | -10.9 (-26.2 to 6.9) | -18.3* (-32.4 to -2.1) |
| Typhoid fever | 15.6 (11.5 to 20.4) | 246.3 (188.1 to 310.7) | 2.4 (1.5 to 3.7) | -10.3 (-27.2 to 9.2) | -17.8 (-33.3 to 0.0) |
| Typhoid fever complications | 3.3 (2.2 to 4.6) | 42.5 (30.9 to 56.1) | 1.1 (0.6 to 1.6) | -10.3 (-34.2 to 23.6) | -17.8 (-39.7 to 13.2) |
| Typhoid fever episode | 12.3 (8.9 to 16.5) | 203.9 (155.2 to 258.1) | 1.4 (0.8 to 2.1) | -10.2 (-31.3 to 19.2) | -17.8 (-37.1 to 9.2) |
| Paratyphoid fever | 3.6 (2.5 to 4.9) | 82.7 (58.6 to 111.2) | 0.2 (0.1 to 0.4) | -16.7* (-27.8 to -3.1) | -23.7* (-33.8 to -11.2) |
| Intestinal perforation due to paratyphoid | 0.2 (0.1 to 0.2) | 4.2 (2.9 to 5.8) | -- | -16.8 (-34.2 to 5.1) | -23.8* (-39.7 to -3.7) |
| Paratyphoid fever episode | 3.5 (2.4 to 4.7) | 78.5 (55.4 to 105.3) | 0.2 (0.1 to 0.3) | -16.7* (-28.4 to -2.2) | -23.7* (-34.4 to -10.4) |
| iNTS | 0.7 (0.4 to 1.2) | 19.2 (11.6 to 31.0) | 0.1 (0.0 to 0.2) | -22.7* (-28.0 to -16.9) | -29.2* (-34.1 to -23.9) |
| Other intestinal infectious diseases | -- | -- | -- | -23.4* (-36.6 to -8.2) | -29.9* (-42.0 to -15.9) |
| **Neglected tropical diseases and malaria** | **74910.2 (71162.7 to 78903.6)** | **13570.1 (11096.7 to 16699.4)** | **944.5 (655.5 to 1301.0)** | **-11.6* (-17.4 to -6.3)** | **-19.0* (-24.3 to -14.1)** |
| Malaria | 4406.5 (4100.9 to 4700.8) | 4303.4 (3521.7 to 5157.9) | 42.4 (31.0 to 55.8) | -14.6* (-24.1 to -4.5) | -21.8* (-30.4 to -12.5) |
| Malaria complications | 38.9 (35.2 to 42.8) | -- | 16.0 (12.4 to 19.9) | 33.3* (19.9 to 48.2) | 22.1* (9.8 to 35.8) |
| Malaria episode | 251.3 (163.2 to 353.3) | 4303.4 (3521.7 to 5157.9) | 8.5 (4.4 to 14.1) | -15.9* (-25.2 to -4.8) | -22.9* (-31.4 to -12.8) |

| | | | | | |
|---|---|---|---|---|---|
| Malaria parasitemia | 4116.3 (3810.9 to 4453.7) | -- | 17.9 (11.3 to 27.0) | -35.0* (-46.4 to -21.9) | -40.5* (-50.9 to -28.4) |
| Chagas disease | 558.0 (460.2 to 661.2) | 10.9 (7.3 to 15.5) | 4.2 (2.7 to 6.2) | -6.5 (-13.3 to 0.3) | -14.3* (-20.6 to -8.1) |
| Acute Chagas disease | 0.1 (0.0 to 0.1) | 10.9 (7.3 to 15.5) | -- | -4.0 (-8.5 to 0.7) | -12.1* (-16.1 to -7.8) |
| Asymptomatic Chagas disease | 481.9 (398.0 to 570.0) | -- | -- | -- | -- |
| Symptomatic chronic Chagas infection | 76.0 (58.5 to 95.6) | -- | 4.2 (2.7 to 6.2) | -6.5 (-13.3 to 0.3) | -14.3* (-20.6 to -8.1) |
| Leishmaniasis | 347.0 (264.6 to 459.0) | 31.3 (22.0 to 42.6) | 21.9 (13.5 to 34.5) | 47.1* (35.8 to 63.8) | 34.8* (24.4 to 50.1) |
| Visceral leishmaniasis | 0.3 (0.2 to 0.4) | 1.1 (0.8 to 1.6) | -- | -71.6* (-77.7 to -60.4) | -74.0* (-79.5 to -63.7) |
| Cutaneous and mucocutaneous leishmaniasis | 348.8 (267.0 to 462.0) | 30.2 (20.9 to 41.6) | 21.9 (13.5 to 34.4) | 47.7* (36.2 to 64.7) | 35.3* (24.8 to 50.8) |
| African trypanosomiasis | 0.3 (0.0 to 1.5) | 0.2 (0.0 to 0.6) | 0.1 (0.0 to 0.5) | -76.3 (-97.3 to 50.6) | -78.3 (-97.6 to 38.0) |
| Trypanosomiasis Gambiense | 0.3 (0.0 to 1.5) | 0.2 (0.0 to 0.6) | 0.1 (0.0 to 0.5) | -75.6 (-97.6 to 55.3) | -77.7 (-97.8 to 42.2) |
| Trypanosomiasis Rhodesiense | -- | 0.0 (0.0 to 0.1) | -- | -89.8* (-99.0 to -29.1) | -90.6* (-99.1 to -35.0) |
| Schistosomiasis | 13021.5 (11261.9 to 14743.8) | -- | 97.9 (48.0 to 186.4) | -17.9* (-21.4 to -14.5) | -24.8* (-28.0 to -21.7) |
| Mild schistosomiasis | 10910.0 (9324.6 to 12508.4) | -- | 61.2 (24.8 to 128.6) | -19.0* (-22.2 to -16.2) | -25.8* (-28.7 to -23.2) |
| Anemia due to schistosomiasis | 704.3 (581.3 to 844.0) | -- | 15.7 (9.5 to 24.3) | -30.2* (-41.6 to -17.7) | -36.0* (-46.5 to -24.6) |
| Schistosomiasis complications | 1410.6 (1083.4 to 1819.8) | -- | 21.0 (12.0 to 35.3) | -0.8 (-8.3 to 7.9) | -9.1* (-16.0 to -1.2) |
| Cysticercosis | 422.4 (297.7 to 572.4) | -- | 125.6 (73.7 to 191.0) | 0.4 (-6.9 to 7.3) | -8.0* (-14.7 to -1.7) |
| Cystic echinococcosis | 47.7 (22.7 to 102.7) | 10.3 (4.8 to 22.7) | 3.9 (1.7 to 8.6) | 16.0* (5.4 to 26.5) | 6.3 (-3.5 to 15.9) |
| Lymphatic filariasis | 5062.7 (4509.0 to 5719.1) | -- | 144.5 (79.9 to 223.9) | -38.1* (-49.6 to -27.4) | -43.3* (-53.8 to -33.5) |
| Prevalence of detectable microfiliaria due to lymphatic filariasis | 3748.6 (3375.5 to 4185.7) | -- | -- | -- | -- |
| Lymphatic filariasis complications | 1314.1 (897.3 to 1861.2) | -- | 144.5 (79.9 to 223.9) | -38.1* (-49.6 to -27.4) | -43.3* (-53.8 to -33.5) |
| Onchocerciasis | 1343.5 (820.5 to 2435.1) | -- | 90.8 (43.2 to 170.4) | 0.1 (-20.7 to 17.7) | -8.3 (-27.3 to 7.8) |
| Asymptomatic onchocerciasis | 337.3 (0.1 to 1322.7) | -- | -- | -- | -- |
| Skin disease due to onchocerciasis | 907.5 (648.1 to 1220.6) | -- | 82.3 (34.6 to 161.6) | 0.5 (-23.8 to 19.3) | -7.9 (-30.2 to 9.3) |
| Vision loss due to onchocerciasis | 98.8 (68.5 to 154.3) | -- | 8.6 (5.0 to 13.2) | -3.7 (-18.5 to 13.1) | -11.8 (-25.4 to 3.6) |
| Trachoma | 45.5 (27.9 to 77.6) | -- | 6.8 (3.9 to 10.9) | -30.8* (-45.9 to -11.0) | -36.6* (-50.4 to -18.4) |
| Dengue | 339.9 (185.2 to 575.7) | 5680.1 (3455.3 to 8615.1) | 54.7 (24.2 to 103.1) | 69.3* (46.5 to 161.5) | 55.1* (34.2 to 139.6) |
| Post-dengue chronic fatigue syndrome | 239.7 (112.0 to 438.2) | -- | 48.9 (20.6 to 93.3) | 69.4* (45.8 to 162.3) | 55.2* (33.5 to 140.2) |
| Dengue episode | 100.3 (60.6 to 150.5) | 5680.1 (3455.3 to 8615.1) | 5.9 (3.0 to 10.2) | 69.0* (45.1 to 153.3) | 54.8* (32.9 to 132.1) |
| Yellow fever | 0.1 (0.0 to 0.3) | 3.9 (1.1 to 10.9) | -- | -13.4 (-39.6 to 22.7) | -20.7 (-44.7 to 12.4) |
| Asymptomatic yellow fever | 0.1 (0.0 to 0.2) | 2.2 (0.5 to 6.0) | -- | -- | -- |
| Yellow fever episode | 0.0 (0.0 to 0.1) | 1.7 (0.5 to 4.8) | -- | -13.4 (-39.6 to 22.7) | -20.7 (-44.7 to 12.4) |
| Rabies | -- | 0.8 (0.6 to 1.0) | -- | -35.9* (-51.0 to -17.1) | -41.3* (-55.1 to -24.1) |
| Intestinal nematode infections | 47667.7 (44266.9 to 51675.4) | -- | 86.8 (50.2 to 141.1) | -25.7* (-30.3 to -20.7) | -32.0* (-36.2 to -27.4) |
| Ascariasis | 22376.4 (19544.2 to 25698.7) | -- | 27.9 (15.0 to 47.8) | -28.7* (-36.3 to -20.4) | -34.7* (-41.6 to -27.1) |
| Asymptomatic ascariasis | 20852.9 (18166.8 to 23941.9) | -- | -- | -- | -- |
| Ascariasis complications | 1523.5 (1348.2 to 1727.5) | -- | 27.9 (15.0 to 47.8) | -28.7* (-36.3 to -20.4) | -34.7* (-41.6 to -27.1) |
| Trichuriasis | 15284.4 (13214.8 to 17858.3) | -- | 10.6 (5.9 to 17.5) | -13.0* (-20.6 to -3.9) | -20.3* (-27.3 to -11.9) |
| Asymptomatic trichuriasis | 14746.6 (12707.8 to 17246.9) | -- | -- | -- | -- |
| Trichuriasis complications | 537.9 (489.6 to 588.4) | -- | 10.6 (5.9 to 17.5) | -13.0* (-20.6 to -3.9) | -20.3* (-27.3 to -11.9) |
| Hookworm disease | 13021.8 (11951.3 to 14181.3) | -- | 48.3 (28.6 to 77.6) | -26.3* (-32.9 to -19.0) | -32.5* (-38.6 to -25.8) |
| Asymptomatic hookworm disease | 10735.9 (9862.0 to 11713.2) | -- | -- | -- | -- |
| Anemia due to hookworm disease | 590.7 (521.6 to 667.9) | -- | 13.2 (8.6 to 19.5) | -33.5* (-42.7 to -23.1) | -39.1* (-47.5 to -29.5) |
| Hookworm disease complications | 1695.2 (1562.8 to 1835.8) | -- | 35.1 (19.7 to 58.9) | -23.2* (-29.3 to -16.5) | -29.7* (-35.2 to -23.5) |
| Food-borne trematodiases | 8093.7 (7079.9 to 9270.2) | 3343.6 (2642.9 to 4067.0) | 208.7 (112.7 to 361.8) | -2.2 (-6.7 to 2.9) | -10.4* (-14.5 to -5.7) |
| Asymptomatic food-borne tremodiases | 6229.9 (5066.0 to 7370.0) | 2397.7 (1226.4 to 3383.1) | -- | -- | -- |
| Food-borne tremodiases complications | 1863.7 (1167.3 to 2927.8) | 945.9 (216.7 to 2088.7) | 208.7 (112.7 to 361.8) | -2.2 (-6.7 to 2.9) | -10.4* (-14.5 to -5.7) |
| Leprosy | 43.2 (39.9 to 47.3) | 4.2 (3.7 to 4.8) | 2.7 (1.8 to 3.8) | -5.1 (-14.0 to 5.0) | -13.0* (-21.2 to -3.8) |
| Ebola virus disease | -- | -- | -- | -95.8* (-97.5 to -91.1) | -96.1* (-97.8 to -91.8) |
| Ebola cases | -- | -- | -- | -97.8* (-97.9 to -97.7) | -98.0* (-98.1 to -97.9) |
| Post-Ebola chronic fatigue syndrome | -- | -- | -- | -95.7* (-97.5 to -90.8) | -96.1* (-97.7 to -91.6) |
| Zika virus disease | 3.0 (1.7 to 5.9) | 181.4 (105.0 to 354.9) | 0.1 (0.0 to 0.1) | -- | -- |
| Zika virus complications | -- | -- | -- | -- | -- |
| Zika virus episode | 3.0 (1.7 to 5.8) | 181.4 (105.0 to 354.9) | 0.1 (0.0 to 0.1) | -- | -- |
| Guinea worm disease | -- | -- | -- | -99.5* (-99.7 to -99.3) | -99.5* (-99.7 to -99.3) |
| Other neglected tropical diseases | 2523.4 (2337.8 to 2738.2) | -- | 53.4 (35.2 to 79.0) | 3.3 (-4.9 to 12.7) | -5.4 (-12.9 to 3.3) |
| Acute infection due to other neglected tropical diseases | -- | -- | 0.7 (0.3 to 1.1) | 106.0* (77.0 to 204.1) | 88.7* (62.1 to 178.6) |
| Anemia due to other neglected tropical diseases | 2523.4 (2337.8 to 2738.2) | -- | 52.8 (34.8 to 78.2) | 2.7 (-5.5 to 12.2) | -6.0 (-13.5 to 2.8) |
| **Other infectious diseases** | **5530.7 (4971.4 to 6211.2)** | **16747.5 (12894.3 to 22367.7)** | **229.6 (160.4 to 312.0)** | **2.5 (-5.3 to 11.1)** | **-6.1 (-13.3 to 1.8)** |
| Meningitis | 654.1 (548.6 to 767.3) | 156.9 (108.3 to 223.8) | 53.3 (37.3 to 71.6) | -1.4 (-6.2 to 3.5) | -9.7* (-14.1 to -5.2) |
| Pneumococcal meningitis | 233.8 (192.3 to 283.2) | 9.9 (5.8 to 15.7) | 19.9 (13.8 to 27.0) | -21.4* (-26.8 to -15.7) | -28.0* (-33.0 to -22.8) |

| | | | | | |
|---|---|---|---|---|---|
| Acute pneumococcal meningitis | 0.4 (0.3 to 0.7) | 9.9 (5.8 to 15.7) | 0.1 (0.0 to 0.1) | -19.2* (-28.6 to -9.5) | -26.0* (-34.6 to -17.1) |
| Pneumococcal meningitis complications | 233.3 (191.8 to 282.9) | -- | 19.9 (13.7 to 26.9) | -21.4* (-26.8 to -15.7) | -28.0* (-33.0 to -22.8) |
| H influenzae type B meningitis | 52.6 (37.5 to 70.9) | 3.1 (1.7 to 4.9) | 4.1 (2.8 to 5.6) | -49.5* (-52.7 to -46.3) | -53.8* (-56.7 to -50.8) |
| Acute H influenzae type B meningitis | 0.1 (0.1 to 0.2) | 3.1 (1.7 to 4.9) | -- | -52.1* (-58.1 to -46.8) | -56.2* (-61.6 to -51.3) |
| H Influenza type B meningitis complications | 52.5 (37.4 to 70.8) | -- | 4.1 (2.8 to 5.6) | -49.5* (-52.7 to -46.2) | -53.8* (-56.7 to -50.7) |
| Meningococcal infection | 67.8 (48.3 to 89.8) | 8.6 (4.8 to 13.9) | 6.0 (4.1 to 8.1) | 1.5 (-2.1 to 5.2) | -7.0* (-10.3 to -3.6) |
| Acute meningococcal meningitis | 0.4 (0.2 to 0.6) | 8.6 (4.8 to 13.9) | 0.1 (0.0 to 0.1) | 16.4* (3.0 to 29.1) | 6.6 (-5.7 to 18.2) |
| Meningococcal meningitis complications | 67.4 (48.0 to 89.5) | -- | 5.9 (4.1 to 8.0) | 1.4 (-2.2 to 5.1) | -7.1* (-10.4 to -3.7) |
| Other meningitis | 299.9 (221.6 to 380.7) | 135.3 (93.3 to 192.6) | 23.3 (16.2 to 31.5) | 58.7* (48.5 to 69.0) | 45.4* (36.1 to 54.8) |
| Other acute bacterial meningitis | 1.1 (0.7 to 1.7) | 25.2 (17.2 to 35.8) | 0.1 (0.1 to 0.2) | 61.8* (46.9 to 76.6) | 48.2* (34.6 to 61.8) |
| Acute viral meningitis | 4.9 (3.1 to 7.3) | 110.2 (76.0 to 157.2) | 0.7 (0.4 to 1.1) | 12.7* (3.0 to 21.9) | 3.3 (-5.6 to 11.7) |
| Other bacterial meningitis complications | 293.9 (215.0 to 373.3) | -- | 22.5 (15.5 to 30.6) | 60.6* (49.8 to 71.2) | 47.1* (37.2 to 56.9) |
| Encephalitis | 525.0 (292.1 to 839.8) | 99.0 (95.3 to 102.7) | 41.0 (28.5 to 54.4) | 3.7 (-2.7 to 10.4) | -5.0 (-10.9 to 1.1) |
| Acute encephalitis | 5.3 (5.1 to 5.5) | 99.0 (95.3 to 102.7) | 0.7 (0.5 to 1.0) | 14.2* (13.6 to 14.7) | 4.6* (4.1 to 5.1) |
| Encephalitis complications | 519.7 (286.7 to 834.6) | -- | 40.3 (27.9 to 53.5) | 3.5 (-3.0 to 10.4) | -5.2 (-11.1 to 1.1) |
| Diphtheria | -- | 0.3 (0.2 to 0.4) | -- | -28.4* (-45.4 to -3.0) | -34.4* (-50.0 to -11.2) |
| Whooping cough | 2.3 (1.7 to 2.8) | 16.5 (12.8 to 20.8) | 0.1 (0.1 to 0.2) | -2.7* (-3.0 to -2.4) | -10.9* (-11.1 to -10.6) |
| Tetanus | 3.4 (3.2 to 3.5) | 3.0 (1.6 to 4.6) | 0.1 (0.0 to 0.1) | -6.9 (-19.5 to 4.3) | -14.7* (-26.2 to -4.4) |
| Severe tetanus | 0.2 (0.1 to 0.3) | 3.0 (1.6 to 4.6) | -- | -38.8* (-52.1 to -16.8) | -44.0* (-56.1 to -23.8) |
| Neonatal tetanus complications | 3.2 (3.1 to 3.3) | -- | 0.1 (0.0 to 0.1) | 14.8* (13.1 to 16.2) | 5.2* (3.6 to 6.5) |
| Measles | 0.5 (0.2 to 1.1) | 17.7 (6.3 to 39.4) | 0.0 (0.0 to 0.1) | -28.6* (-34.8 to -22.5) | -34.6* (-40.3 to -29.0) |
| Varicella and herpes zoster | 333.0 (244.5 to 431.4) | 2134.6 (1559.4 to 2696.9) | 18.1 (10.3 to 29.6) | 7.3* (1.7 to 12.9) | -1.7 (-6.9 to 3.4) |
| Chickenpox | 6.3 (3.0 to 11.1) | 328.1 (154.6 to 579.3) | 0.0 (0.0 to 0.1) | 27.0* (23.7 to 31.3) | 16.4* (13.3 to 20.3) |
| Herpes zoster | 326.7 (238.6 to 424.2) | 1806.5 (1273.7 to 2343.6) | 18.0 (10.3 to 29.4) | 7.3* (1.6 to 12.8) | -1.7 (-6.9 to 3.4) |
| Acute hepatitis | 1535.6 (1100.3 to 2197.9) | 14319.7 (10486.9 to 20054.3) | 39.2 (23.1 to 63.0) | -0.6 (-33.2 to 46.7) | -8.9 (-38.8 to 34.4) |
| Acute hepatitis A | 197.7 (168.2 to 224.5) | 2570.2 (2186.3 to 2918.9) | 8.2 (5.1 to 12.4) | 9.5 (-0.3 to 19.4) | 0.3 (-8.7 to 9.4) |
| Acute hepatitis B | 1282.0 (840.1 to 1936.7) | 11111.0 (7280.6 to 16784.5) | 29.6 (16.1 to 50.8) | -3.4 (-42.0 to 59.5) | -11.5 (-46.9 to 46.1) |
| Acute hepatitis C | 20.1 (7.1 to 38.7) | 174.6 (61.6 to 335.6) | 0.3 (0.1 to 0.7) | 0.9 (-5.3 to 4.2) | -7.5* (-13.3 to -4.6) |
| Acute hepatitis E | 35.7 (23.3 to 51.5) | 464.0 (302.8 to 669.7) | 1.1 (0.6 to 1.9) | 9.6 (-8.4 to 29.8) | 0.4 (-16.1 to 18.9) |
| Other unspecified infectious diseases | 3136.1 (2934.8 to 3344.8) | -- | 77.7 (51.0 to 115.1) | 5.3 (-0.8 to 12.1) | -3.6 (-9.1 to 2.7) |
| Guillain-Barré syndrome due to other infectious diseases | 0.4 (0.2 to 0.8) | -- | 0.1 (0.1 to 0.3) | 17.1* (14.1 to 20.0) | 7.3* (4.5 to 9.9) |
| Other infectious diseases | -- | -- | 12.5 (6.9 to 20.6) | 9.1* (4.7 to 13.1) | -0.1 (-4.1 to 3.6) |
| Anemia due to other infectious diseases | 3135.7 (2934.4 to 3344.4) | -- | 65.1 (43.2 to 95.9) | 4.6 (-2.5 to 12.6) | -4.2 (-10.7 to 3.1) |
| **Maternal and neonatal disorders** | **9239.2 (8071.1 to 10518.9)** | **4408.2 (3805.5 to 5075.4)** | **1770.7 (1329.5 to 2282.8)** | **20.8* (14.9 to 27.8)** | **10.6* (5.3 to 17.1)** |
| Maternal disorders | 676.8 (601.1 to 761.5) | 4408.2 (3805.5 to 5075.4) | 61.1 (43.1 to 80.6) | -0.3 (-8.4 to 8.0) | -8.6* (-16.1 to -1.0) |
| Maternal haemorrhage | 77.2 (69.6 to 85.2) | 333.8 (233.1 to 446.8) | 2.8 (1.9 to 4.1) | 1.8 (-15.9 to 23.1) | -6.8 (-22.9 to 12.8) |
| Maternal hemorrhage complications | 69.9 (63.2 to 77.2) | -- | 1.6 (1.0 to 2.4) | -4.8 (-21.5 to 16.7) | -12.8 (-28.1 to 6.9) |
| Maternal hemorrhage episode | 7.3 (4.4 to 11.7) | 333.8 (233.1 to 446.8) | 1.2 (0.7 to 2.0) | 11.8 (-22.2 to 61.2) | 2.4 (-28.8 to 47.7) |
| Maternal sepsis and other pregnancy related infections | 390.8 (343.0 to 444.2) | 576.3 (437.5 to 773.5) | 3.9 (1.9 to 7.2) | 17.1 (-18.8 to 59.9) | 7.3 (-25.7 to 46.5) |
| Infertility due to puerperal sepsis | 347.6 (306.8 to 392.1) | 6.3 (4.3 to 8.8) | 1.7 (0.7 to 3.7) | 22.8* (17.3 to 28.4) | 12.5* (7.5 to 17.6) |
| Maternal sepsis and other maternal infection episode | 43.2 (22.7 to 72.8) | 570.0 (431.0 to 768.3) | 2.2 (1.1 to 3.9) | 13.1 (-38.4 to 92.6) | 3.6 (-43.6 to 76.5) |
| Maternal hypertensive disorders | 122.6 (69.1 to 187.3) | 715.5 (531.6 to 894.5) | 6.2 (3.1 to 10.9) | 14.7 (-23.1 to 60.0) | 5.0 (-29.6 to 46.6) |
| Maternal hypertensive disorder complications | 6.0 (5.3 to 6.7) | 81.4 (73.2 to 89.2) | 0.4 (0.2 to 0.6) | 26.4* (4.2 to 52.0) | 15.8 (-4.5 to 39.3) |
| Maternal hypertensive disorder episode | 116.6 (63.0 to 181.1) | 634.1 (449.6 to 814.1) | 5.8 (2.8 to 10.3) | 14.0 (-25.9 to 62.0) | 4.4 (-32.1 to 48.4) |
| Maternal obstructed labour and uterine rupture | 113.2 (94.7 to 133.2) | 247.2 (176.2 to 354.0) | 36.1 (24.2 to 50.0) | -5.9 (-14.0 to 2.6) | -13.8* (-21.2 to -6.0) |
| Obstructed labor, acute event | 3.3 (1.8 to 5.3) | 240.9 (169.6 to 348.1) | 1.1 (0.5 to 1.9) | 14.6 (-33.2 to 81.1) | 5.0 (-38.8 to 65.9) |
| Maternal obstructed labor complications | 109.9 (92.0 to 129.6) | 6.3 (4.1 to 8.8) | 35.0 (23.3 to 48.9) | -6.5 (-15.0 to 1.9) | -14.3* (-22.1 to -6.6) |
| Maternal abortion and miscarriage | 10.4 (6.5 to 15.5) | 1274.3 (941.3 to 1673.4) | 1.2 (0.6 to 2.0) | 15.4 (-26.8 to 72.4) | 5.7 (-32.9 to 58.0) |
| Ectopic pregnancy | 10.3 (6.2 to 15.8) | 1261.0 (873.8 to 1718.8) | 1.1 (0.5 to 1.8) | 16.8 (-29.8 to 84.4) | 7.0 (-35.7 to 68.9) |
| Other maternal disorders | -- | -- | 9.8 (6.9 to 13.0) | 4.1 (-4.3 to 12.9) | -4.7 (-12.4 to 3.5) |
| Neonatal disorders | 8573.2 (7392.9 to 9847.4) | -- | 1709.6 (1269.9 to 2208.8) | 21.7* (15.6 to 28.9) | 11.5* (5.9 to 18.1) |
| Neonatal preterm birth | 4873.6 (4204.3 to 5734.4) | -- | 762.4 (533.5 to 1047.1) | 26.2* (11.0 to 41.4) | 15.6* (1.7 to 29.6) |
| Preterm birth complications | 4154.9 (3478.7 to 4961.3) | -- | 720.9 (497.3 to 1003.0) | 27.7* (11.4 to 43.5) | 17.0* (2.1 to 31.5) |
| Retinopathy of prematurity | 718.6 (577.3 to 868.1) | -- | 41.5 (27.8 to 57.6) | 5.6 (-0.5 to 12.8) | -3.2 (-8.9 to 3.4) |
| Uncomplicated preterm birth | -- | -- | -- | -- | -- |
| Neonatal encephalopathy due to birth asphyxia and trauma | 3525.7 (2613.8 to 4673.4) | -- | 630.4 (383.0 to 973.5) | 18.1* (14.1 to 23.0) | 8.2* (4.5 to 12.6) |
| Neonatal sepsis and other neonatal infections | 907.3 (558.4 to 1380.3) | -- | 266.5 (142.7 to 438.4) | 17.3* (11.8 to 22.1) | 7.5* (2.4 to 11.9) |
| Neonatal sepsis and other neonatal infection complications | 907.3 (558.4 to 1380.3) | -- | 266.5 (142.7 to 438.4) | 17.3* (11.8 to 22.1) | 7.5* (2.4 to 11.9) |
| Neonatal sepsis and other neonatal infection episode | -- | -- | -- | -- | -- |
| Hemolytic disease and other neonatal jaundice | 135.3 (116.2 to 155.0) | -- | 50.3 (38.5 to 62.9) | 25.7* (21.1 to 30.0) | 15.1* (11.0 to 19.1) |

| | | | | | |
|---|---|---|---|---|---|
| Other neonatal disorders | -- | -- | -- | -- | -- |
| **Nutritional deficiencies** | **105484.9 (101094.8 to 110233.3)** | **65706.1 (59441.2 to 72251.9)** | **1675.9 (1106.9 to 2443.7)** | **2.6 (-3.2 to 9.0)** | **-6.0* (-11.3 to -0.1)** |
| Protein-energy malnutrition | 3414.3 (3204.3 to 3633.4) | 3674.6 (3407.7 to 3942.1) | 26.1 (17.0 to 36.7) | 30.2* (17.4 to 40.6) | 19.3* (7.5 to 28.8) |
| Iodine deficiency | 8447.1 (7603.6 to 9388.6) | 51.8 (42.7 to 62.4) | 139.2 (82.5 to 224.0) | 0.3 (-4.2 to 5.0) | -8.1* (-12.3 to -3.9) |
| Visible goiter without symptoms | 8288.3 (7452.0 to 9229.4) | 51.8 (42.7 to 62.4) | 88.4 (43.0 to 165.3) | -0.4 (-1.8 to 1.0) | -8.7* (-10.1 to -7.4) |
| Visible goiter with complications | 158.9 (81.6 to 231.6) | -- | 50.8 (23.5 to 81.4) | 1.5 (-12.7 to 11.2) | -7.0 (-20.0 to 1.9) |
| Vitamin A deficiency | 39201.0 (35575.5 to 42838.8) | 61979.7 (55700.9 to 68533.0) | 11.2 (6.8 to 17.0) | 21.1* (11.8 to 30.6) | 11.0* (2.4 to 19.6) |
| Asymptomatic vitamin A deficiency | 39034.0 (35420.3 to 42675.0) | 61979.7 (55700.9 to 68533.0) | -- | -- | -- |
| Vitamin A deficiency complications | 167.0 (118.0 to 236.4) | -- | 11.2 (6.8 to 17.0) | 21.1* (11.8 to 30.6) | 11.0* (2.4 to 19.6) |
| Vitamin A deficiency with anemia | -- | -- | -- | -- | -- |
| Dietary iron deficiency | 68196.3 (64070.9 to 72490.1) | -- | 1485.8 (988.2 to 2176.5) | 2.1 (-4.3 to 9.2) | -6.4 (-12.3 to 0.1) |
| Other nutritional deficiencies | -- | -- | 13.6 (8.9 to 19.2) | 32.4* (19.3 to 42.9) | 21.3* (9.3 to 30.9) |
| **Non-communicable diseases** | **481775.6 (478920.8 to 484519.8)** | **643609.0 (592857.7 to 695112.1)** | **51423.9 (38292.0 to 66565.9)** | **9.5* (8.8 to 10.1)** | **0.3 (-0.3 to 0.9)** |
| **Neoplasms** | **5261.5 (5080.6 to 5477.9)** | **861.0 (759.8 to 1021.3)** | **357.9 (256.7 to 474.9)** | **15.3* (11.3 to 20.0)** | **5.6* (2.0 to 9.9)** |
| Lip and oral cavity cancer | 91.4 (85.2 to 98.6) | 20.0 (18.4 to 21.7) | 8.8 (6.4 to 11.8) | 21.4* (10.1 to 34.2) | 11.2* (0.9 to 22.9) |
| Diagnosis and primary therapy phase of mouth cancer | 5.1 (4.8 to 5.5) | 20.0 (18.4 to 21.7) | 1.5 (1.0 to 2.0) | 22.6* (11.7 to 34.0) | 12.3* (2.3 to 22.7) |
| Controlled phase of mouth cancer | 78.3 (73.0 to 84.4) | -- | 3.7 (2.4 to 5.5) | 23.5* (10.3 to 39.6) | 13.1* (1.0 to 27.9) |
| Metastatic phase of mouth cancer | 6.9 (6.4 to 7.5) | -- | 3.1 (2.1 to 4.3) | 18.8* (3.4 to 38.1) | 8.9 (-5.3 to 26.5) |
| Terminal phase of mouth cancer | 1.1 (1.0 to 1.2) | -- | 0.6 (0.4 to 0.7) | 19.8* (9.1 to 30.5) | 9.8 (-0.1 to 19.5) |
| Nasopharynx cancer | 64.8 (54.5 to 76.3) | 11.6 (9.9 to 13.5) | 6.1 (4.1 to 8.5) | -5.6 (-22.3 to 14.4) | -13.5 (-28.8 to 4.8) |
| Diagnosis and primary therapy phase of nasopharynx cancer | 3.7 (3.1 to 4.4) | 11.6 (9.9 to 13.5) | 1.1 (0.7 to 1.6) | -3.7 (-25.5 to 23.8) | -11.8 (-31.8 to 13.4) |
| Controlled phase of nasopharynx cancer | 56.1 (47.1 to 66.0) | -- | 2.7 (1.7 to 4.2) | -4.8 (-23.6 to 17.4) | -12.8 (-30.0 to 7.6) |
| Metastatic phase of nasopharynx cancer | 4.6 (3.8 to 5.3) | -- | 2.0 (1.4 to 2.8) | -6.9 (-26.0 to 18.0) | -14.7 (-32.3 to 8.1) |
| Terminal phase of nasopharynx cancer | 0.5 (0.4 to 0.6) | -- | 0.3 (0.2 to 0.4) | -10.0 (-23.5 to 5.3) | -17.6* (-29.9 to -3.5) |
| Other pharynx cancer | 22.8 (19.7 to 24.7) | 8.5 (7.2 to 9.3) | 3.0 (2.1 to 4.0) | 23.9* (12.5 to 34.9) | 13.5* (3.1 to 23.6) |
| Diagnosis and primary therapy phase of other pharynx cancer | 1.2 (1.1 to 1.3) | 8.5 (7.2 to 9.3) | 0.4 (0.2 to 0.5) | 22.2* (12.4 to 30.5) | 11.9* (3.0 to 19.6) |
| Controlled phase of other pharynx cancer | 17.8 (15.4 to 19.2) | -- | 0.9 (0.5 to 1.3) | 22.1* (6.3 to 39.5) | 11.9 (-2.7 to 27.7) |
| Metastatic phase of other pharynx cancer | 3.2 (2.7 to 3.5) | -- | 1.4 (1.0 to 1.9) | 25.2* (13.4 to 35.5) | 14.6* (3.9 to 24.1) |
| Terminal phase of other pharynx cancer | 0.6 (0.5 to 0.6) | -- | 0.3 (0.2 to 0.4) | 25.4* (13.4 to 35.9) | 14.9* (3.8 to 24.5) |
| Oesophageal cancer | 22.4 (21.4 to 23.4) | 9.7 (9.3 to 10.1) | 3.2 (2.3 to 4.2) | -17.5* (-22.3 to -12.9) | -24.5* (-28.8 to -20.2) |
| Diagnosis and primary therapy phase of esophageal cancer | 1.8 (1.7 to 1.9) | 9.7 (9.3 to 10.1) | 0.5 (0.4 to 0.7) | -14.4* (-18.6 to -9.9) | -21.6* (-25.5 to -17.5) |
| Controlled phase of esophageal cancer | 16.6 (15.8 to 17.4) | -- | 0.8 (0.5 to 1.2) | -12.7 (-23.5 to 0.5) | -20.0* (-29.9 to -7.9) |
| Metastatic phase of esophageal cancer | 3.3 (3.1 to 3.4) | -- | 1.5 (1.0 to 2.0) | -20.3* (-23.8 to -16.5) | -27.0* (-30.2 to -23.5) |
| Terminal phase of esophageal cancer | 0.7 (0.7 to 0.7) | -- | 0.4 (0.3 to 0.5) | -20.3* (-23.8 to -16.5) | -27.0* (-30.2 to -23.5) |
| Stomach cancer | 87.2 (83.8 to 90.8) | 32.2 (31.1 to 33.3) | 10.5 (7.5 to 13.8) | -8.5* (-13.5 to -3.2) | -16.2* (-20.8 to -11.3) |
| Diagnosis and primary therapy phase of stomach cancer | 7.6 (7.3 to 7.9) | 32.2 (31.1 to 33.3) | 2.2 (1.5 to 3.0) | -3.6 (-8.6 to 1.7) | -11.7* (-16.3 to -6.9) |
| Controlled phase of stomach cancer | 68.8 (66.0 to 71.7) | -- | 3.3 (2.1 to 4.8) | 5.3 (-5.4 to 17.3) | -3.5 (-13.3 to 7.5) |
| Metastatic phase of stomach cancer | 8.6 (8.3 to 8.9) | -- | 3.9 (2.6 to 5.2) | -17.3* (-21.0 to -13.4) | -24.3* (-27.7 to -20.7) |
| Terminal phase of stomach cancer | 2.2 (2.1 to 2.3) | -- | 1.2 (0.8 to 1.5) | -17.4* (-21.1 to -13.5) | -24.3* (-27.7 to -20.8) |
| Colon and rectum cancer | 276.9 (267.5 to 285.2) | 45.3 (43.8 to 46.7) | 25.0 (17.8 to 33.5) | 14.2* (8.3 to 20.0) | 4.6 (-0.8 to 9.9) |
| Diagnosis and primary therapy phase of colon and rectum cancers | 11.4 (11.0 to 11.8) | 45.3 (43.8 to 46.7) | 3.3 (2.2 to 4.6) | 15.9* (10.5 to 21.8) | 6.1* (1.2 to 11.5) |
| Controlled phase of colon and rectum cancers | 247.6 (239.1 to 255.2) | -- | 14.7 (9.6 to 21.0) | 17.1* (10.3 to 24.0) | 7.3* (1.0 to 13.6) |
| Metastatic phase of colon and rectum cancers | 12.7 (12.3 to 13.1) | -- | 5.7 (3.9 to 7.7) | 8.8* (3.3 to 14.2) | -0.3 (-5.4 to 4.6) |
| Terminal phase of colon and rectum cancers | 1.8 (1.7 to 1.8) | -- | 0.9 (0.7 to 1.2) | 6.2* (0.5 to 11.7) | -2.7 (-7.9 to 2.3) |
| Stoma from colon and rectum cancers, beyond 10 years | 3.4 (3.0 to 3.8) | -- | 0.3 (0.2 to 0.5) | -4.3* (-5.1 to -3.5) | -12.3* (-13.0 to -11.6) |
| Liver cancer | 35.6 (29.6 to 42.7) | 39.7 (36.7 to 42.9) | 10.6 (7.5 to 14.2) | 2.5 (-6.9 to 14.3) | -6.1 (-14.7 to 4.7) |
| Liver cancer due to hepatitis B | 49.1 (43.6 to 54.7) | 24.7 (22.0 to 27.3) | 6.6 (4.5 to 8.9) | 0.1 (-11.4 to 14.0) | -8.3 (-18.8 to 4.5) |
| Diagnosis and primary therapy phase of liver cancer due to hepatitis B | 6.3 (5.6 to 6.9) | 24.7 (22.0 to 27.3) | 1.8 (1.1 to 2.6) | -2.5 (-23.2 to 21.4) | -10.7 (-29.6 to 11.2) |
| Controlled phase of liver cancer due to hepatitis B | 36.4 (32.3 to 40.6) | -- | 1.7 (1.1 to 2.6) | 25.7* (4.2 to 50.4) | 15.2 (-4.5 to 37.8) |
| Metastatic phase of liver cancer due to hepatitis B | 4.6 (4.1 to 5.1) | -- | 2.1 (1.4 to 2.8) | -9.0 (-19.1 to 3.1) | -16.7* (-25.9 to -5.6) |
| Terminal phase of liver cancer due to hepatitis B | 1.9 (1.7 to 2.0) | -- | 1.0 (0.7 to 1.3) | -8.8* (-15.8 to -0.3) | -16.5* (-22.9 to -8.6) |
| Liver cancer due to hepatitis C | 8.9 (7.6 to 10.4) | 4.6 (3.9 to 5.4) | 1.2 (0.8 to 1.7) | 7.8 (-1.0 to 18.1) | -1.3 (-9.3 to 8.2) |
| Diagnosis and primary therapy phase of liver cancer due to hepatitis C | 1.2 (1.0 to 1.4) | 4.6 (3.9 to 5.4) | 0.3 (0.2 to 0.5) | 5.9 (-0.9 to 14.2) | -3.0 (-9.2 to 4.6) |
| Controlled phase of liver cancer due to hepatitis C | 6.5 (5.6 to 7.7) | -- | 0.3 (0.2 to 0.5) | 29.3* (6.7 to 55.8) | 18.5 (-2.3 to 42.8) |
| Metastatic phase of liver cancer due to hepatitis C | 0.9 (0.7 to 1.0) | -- | 0.4 (0.3 to 0.5) | -0.3 (-6.6 to 6.9) | -8.7* (-14.4 to -2.1) |
| Terminal phase of liver cancer due to hepatitis C | 0.3 (0.3 to 0.4) | -- | 0.2 (0.1 to 0.3) | -0.3 (-6.6 to 6.9) | -8.7* (-14.4 to -2.1) |
| Liver cancer due to alcohol use | 9.3 (7.0 to 11.9) | 5.0 (3.9 to 6.4) | 1.3 (0.9 to 1.9) | 5.4 (-1.6 to 13.7) | -3.4 (-9.9 to 4.1) |
| Diagnosis and primary therapy phase of liver cancer due to alcohol use | 1.3 (1.0 to 1.6) | 5.0 (3.9 to 6.4) | 0.4 (0.2 to 0.5) | 4.3 (-2.6 to 11.9) | -4.5 (-10.8 to 2.6) |
| Controlled phase of liver cancer due to alcohol use | 6.7 (5.0 to 8.5) | -- | 0.3 (0.2 to 0.5) | 18.3* (6.7 to 31.5) | 8.4 (-2.3 to 20.4) |
| Metastatic phase of liver cancer due to alcohol use | 1.0 (0.7 to 1.2) | -- | 0.4 (0.3 to 0.6) | 0.5 (-6.0 to 7.9) | -7.9* (-13.9 to -1.1) |

| | | | | | |
|---|---|---|---|---|---|
| Terminal phase of liver cancer due to alcohol use | 0.4 (0.3 to 0.5) | -- | 0.2 (0.1 to 0.3) | 0.5 (-6.0 to 7.9) | -7.9* (-13.9 to -1.1) |
| Liver cancer due to NASH | 3.2 (2.6 to 3.9) | 1.7 (1.4 to 2.1) | 0.5 (0.3 to 0.6) | 12.1* (4.6 to 20.9) | 2.7 (-4.2 to 10.8) |
| Diagnosis and primary therapy phase of liver cancer due to NASH | 0.4 (0.3 to 0.5) | 1.7 (1.4 to 2.1) | 0.1 (0.1 to 0.2) | 10.2* (2.9 to 18.5) | 0.9 (-5.7 to 8.6) |
| Controlled phase of liver cancer due to NASH | 2.3 (1.9 to 2.9) | -- | 0.1 (0.1 to 0.2) | 34.2* (24.2 to 46.0) | 22.9* (13.8 to 33.8) |
| Metastatic phase of liver cancer due to NASH | 0.3 (0.3 to 0.4) | -- | 0.1 (0.1 to 0.2) | 4.2 (-2.5 to 11.8) | -4.6 (-10.7 to 2.4) |
| Terminal phase of liver cancer due to NASH | 0.1 (0.1 to 0.2) | -- | 0.1 (0.0 to 0.1) | 4.2 (-2.5 to 11.8) | -4.6 (-10.7 to 2.4) |
| Liver cancer due to other causes | 7.4 (5.8 to 9.3) | 3.7 (3.0 to 4.7) | 1.0 (0.7 to 1.4) | 4.2 (-4.8 to 16.0) | -4.5 (-12.8 to 6.3) |
| Diagnosis and primary therapy phase of liver cancer due to other causes | 0.9 (0.7 to 1.2) | 3.7 (3.0 to 4.7) | 0.3 (0.2 to 0.4) | 2.0 (-5.6 to 12.3) | -6.5 (-13.6 to 2.9) |
| Controlled phase of liver cancer due to other causes | 5.5 (4.3 to 6.9) | -- | 0.3 (0.2 to 0.4) | 29.4* (4.8 to 60.4) | 18.5 (-4.0 to 46.9) |
| Metastatic phase of liver cancer due to other causes | 0.7 (0.6 to 0.9) | -- | 0.3 (0.2 to 0.5) | -5.2 (-12.0 to 4.0) | -13.1* (-19.4 to -4.7) |
| Terminal phase of liver cancer due to other causes | 0.3 (0.2 to 0.3) | -- | 0.2 (0.1 to 0.2) | -5.2 (-12.0 to 4.0) | -13.1* (-19.4 to -4.7) |
| Gallbladder and biliary tract cancer | 5.4 (4.8 to 5.9) | 3.6 (3.1 to 3.9) | 0.9 (0.7 to 1.2) | 4.7 (-1.5 to 12.5) | -4.1 (-9.8 to 3.0) |
| Diagnosis and primary therapy phase of gallbladder and biliary tract cancer | 0.6 (0.5 to 0.7) | 3.6 (3.1 to 3.9) | 0.2 (0.1 to 0.3) | 5.2 (-1.1 to 13.1) | -3.6 (-9.4 to 3.6) |
| Controlled phase of gallbladder and biliary tract cancer | 3.6 (3.2 to 3.9) | -- | 0.2 (0.1 to 0.3) | 9.6* (2.3 to 17.2) | 0.4 (-6.3 to 7.3) |
| Metastatic phase of gallbladder and biliary tract cancer | 1.0 (0.8 to 1.1) | -- | 0.4 (0.3 to 0.6) | 3.2 (-2.7 to 11.0) | -5.5 (-10.9 to 1.7) |
| Terminal phase of gallbladder and biliary tract cancer | 0.3 (0.2 to 0.3) | -- | 0.1 (0.1 to 0.2) | 3.2 (-2.7 to 11.0) | -5.5 (-10.9 to 1.7) |
| Pancreatic cancer | 9.6 (9.3 to 9.9) | 7.8 (7.6 to 8.1) | 1.9 (1.3 to 2.4) | 2.7 (-1.1 to 7.2) | -5.9* (-9.4 to -1.8) |
| Diagnosis and primary therapy phase of pancreatic cancer | 1.9 (1.8 to 1.9) | 7.8 (7.6 to 8.1) | 0.5 (0.4 to 0.7) | 2.8 (-0.9 to 7.4) | -5.8* (-9.2 to -1.6) |
| Controlled phase of pancreatic cancer | 5.5 (5.3 to 5.7) | -- | 0.3 (0.2 to 0.4) | -1.8 (-5.4 to 2.4) | -10.0* (-13.3 to -6.2) |
| Metastatic phase of pancreatic cancer | 1.6 (1.5 to 1.6) | -- | 0.7 (0.5 to 1.0) | 3.8 (-0.1 to 8.5) | -4.9* (-8.5 to -0.6) |
| Terminal phase of pancreatic cancer | 0.6 (0.6 to 0.6) | -- | 0.3 (0.2 to 0.4) | 3.8 (-0.1 to 8.5) | -4.9* (-8.5 to -0.6) |
| Larynx cancer | 31.6 (30.3 to 33.1) | 6.0 (5.7 to 6.3) | 3.3 (2.3 to 4.4) | 3.0 (-2.7 to 9.1) | -5.6* (-10.9 to -0.0) |
| Diagnosis and primary therapy phase of larynx cancer | 1.9 (1.8 to 2.0) | 6.0 (5.7 to 6.3) | 0.5 (0.4 to 0.7) | 2.9 (-2.0 to 8.3) | -5.7* (-10.3 to -0.8) |
| Controlled phase of larynx cancer | 27.0 (25.7 to 28.2) | -- | 1.7 (1.1 to 2.4) | 5.5 (-1.8 to 13.7) | -3.3 (-10.1 to 4.2) |
| Metastatic phase of larynx cancer | 2.0 (1.9 to 2.2) | -- | 0.9 (0.6 to 1.2) | -0.5 (-5.6 to 5.3) | -8.8* (-13.5 to -3.5) |
| Terminal phase of larynx cancer | 0.3 (0.3 to 0.3) | -- | 0.2 (0.1 to 0.2) | -1.6 (-7.0 to 4.3) | -9.9* (-14.8 to -4.4) |
| Laryngectomy from larynx cancer, beyond 10 years | 0.5 (0.4 to 0.5) | -- | -- | 9.5* (8.9 to 10.2) | 0.3 (-0.3 to 0.9) |
| Tracheal, bronchus, and lung cancer | 58.2 (56.1 to 60.3) | 32.6 (31.4 to 33.9) | 9.4 (6.7 to 12.3) | -13.8* (-17.7 to -9.6) | -21.1* (-24.6 to -17.2) |
| Diagnosis and primary therapy phase of lung, bronchus, and trachea cancer | 3.6 (3.5 to 3.7) | 32.6 (31.4 to 33.9) | 1.0 (0.7 to 1.4) | -7.9* (-11.3 to -4.3) | -15.6* (-18.8 to -12.3) |
| Controlled phase of lung, bronchus, and trachea cancer | 40.8 (39.4 to 42.3) | -- | 1.9 (1.2 to 2.8) | -1.0 (-12.5 to 11.5) | -9.3 (-19.8 to 2.2) |
| Metastatic phase of lung, bronchus, and trachea cancer | 11.3 (10.9 to 11.7) | -- | 5.1 (3.5 to 6.8) | -17.9* (-21.6 to -14.2) | -24.8* (-28.1 to -21.4) |
| Terminal phase of lung, bronchus, and trachea cancer | 2.5 (2.4 to 2.6) | -- | 1.3 (0.9 to 1.7) | -18.0* (-21.2 to -14.7) | -24.9* (-27.8 to -21.9) |
| Malignant skin melanoma | 159.2 (130.1 to 203.5) | 18.9 (15.6 to 24.0) | 9.1 (5.8 to 13.5) | 7.9* (1.1 to 15.7) | -1.1 (-7.4 to 6.0) |
| Diagnosis and primary therapy phase of malignant skin melanoma | 4.2 (3.4 to 5.4) | 18.9 (15.6 to 24.0) | 1.2 (0.8 to 1.8) | 8.6 (-0.3 to 18.8) | -0.5 (-8.7 to 8.8) |
| Controlled phase of malignant skin melanoma | 153.4 (125.4 to 196.5) | -- | 7.2 (4.3 to 11.2) | 8.9* (1.2 to 17.6) | -0.3 (-7.3 to 7.7) |
| Metastatic phase of malignant skin melanoma | 1.3 (1.1 to 1.7) | -- | 0.6 (0.4 to 0.8) | -1.5 (-6.7 to 5.0) | -9.7* (-14.5 to -3.8) |
| Terminal phase of malignant skin melanoma | 0.2 (0.2 to 0.3) | -- | 0.1 (0.1 to 0.2) | -2.8 (-8.1 to 4.0) | -10.9* (-15.8 to -4.8) |
| Non-melanoma skin cancer | 21.6 (11.6 to 38.9) | 192.2 (95.2 to 350.8) | 1.0 (0.4 to 2.3) | -2.9 (-14.2 to 5.8) | -11.0* (-21.4 to -3.1) |
| Non-melanoma skin cancer (squamous-cell carcinoma) | 20.1 (8.2 to 41.8) | 19.5 (8.6 to 40.3) | 0.9 (0.3 to 2.1) | -3.5 (-16.8 to 5.8) | -11.6* (-23.8 to -3.1) |
| Non-melanoma skin cancer (basal-cell carcinoma) | 17.2 (6.3 to 38.7) | 172.7 (77.2 to 332.8) | 0.1 (0.0 to 0.2) | 5.6 (-9.7 to 20.5) | -3.3 (-17.3 to 10.4) |
| Breast cancer | 1335.9 (1275.1 to 1421.3) | 159.1 (151.5 to 169.8) | 105.5 (73.8 to 144.6) | 15.2* (8.2 to 22.4) | 5.5 (-0.8 to 12.1) |
| Diagnosis and primary therapy phase of breast cancer | 36.3 (34.6 to 38.6) | 159.1 (151.5 to 169.8) | 9.7 (6.6 to 13.6) | 15.3* (0.7 to 31.4) | 5.6 (-7.8 to 20.4) |
| Controlled phase of breast cancer | 1223.1 (1166.6 to 1301.6) | -- | 76.3 (49.5 to 111.0) | 16.2* (9.7 to 23.2) | 6.5* (0.5 to 12.8) |
| Metastatic phase of breast cancer | 40.7 (38.7 to 43.4) | -- | 16.8 (11.4 to 22.0) | 12.2 (-1.4 to 28.7) | 2.8 (-9.6 to 17.9) |
| Terminal phase of breast cancer | 2.9 (2.7 to 3.1) | -- | 1.6 (1.1 to 2.0) | 11.3* (3.8 to 18.7) | 1.9 (-4.9 to 8.8) |
| Mastectomy from breast cancer, beyond 10 years | 32.9 (31.1 to 34.6) | -- | 1.1 (0.6 to 1.8) | 0.5 (-10.1 to 13.4) | -7.9 (-17.7 to 3.9) |
| Cervical cancer | 527.5 (486.7 to 558.1) | 74.6 (69.1 to 79.1) | 38.9 (27.6 to 51.6) | 7.2* (1.2 to 15.0) | -1.8 (-7.3 to 5.4) |
| Diagnosis and primary therapy phase of cervical cancer | 27.0 (24.9 to 28.6) | 74.6 (69.1 to 79.1) | 7.3 (4.9 to 10.2) | 8.1 (-3.9 to 21.4) | -1.0 (-11.9 to 11.2) |
| Controlled phase of cervical cancer | 479.6 (442.5 to 507.4) | -- | 22.4 (14.3 to 32.5) | 8.9* (2.6 to 15.6) | -0.2 (-6.0 to 5.9) |
| Metastatic phase of cervical cancer | 18.5 (17.2 to 19.7) | -- | 8.0 (5.4 to 10.6) | 3.1 (-7.6 to 16.3) | -5.5 (-15.3 to 6.6) |
| Terminal phase of cervical cancer | 2.3 (2.1 to 2.4) | -- | 1.2 (0.8 to 1.6) | 1.0 (-4.0 to 6.8) | -7.5* (-12.1 to -2.1) |
| Uterine cancer | 132.1 (125.7 to 138.7) | 16.0 (15.2 to 16.8) | 8.8 (6.0 to 11.9) | 1.3 (-6.5 to 10.6) | -7.2 (-14.3 to 1.3) |
| Diagnosis and primary therapy phase of uterine cancer | 5.9 (5.6 to 6.2) | 16.0 (15.2 to 16.8) | 1.7 (1.1 to 2.4) | 2.1 (-4.9 to 10.9) | -6.5 (-12.9 to 1.6) |
| Controlled phase of uterine cancer | 123.4 (117.4 to 129.5) | -- | 5.8 (3.7 to 8.5) | 2.1 (-7.1 to 13.2) | -6.5 (-14.9 to 3.7) |
| Metastatic phase of uterine cancer | 2.6 (2.4 to 2.7) | -- | 1.2 (0.8 to 1.5) | -2.5 (-8.8 to 5.8) | -10.7* (-16.5 to -3.0) |
| Terminal phase of uterine cancer | 0.3 (0.2 to 0.3) | -- | 0.1 (0.1 to 0.2) | -5.4 (-11.7 to 3.0) | -13.3* (-19.1 to -5.6) |
| Ovarian cancer | 120.1 (113.6 to 127.7) | 19.1 (18.0 to 20.3) | 14.7 (10.8 to 19.3) | 17.8* (8.9 to 28.8) | 7.9 (-0.3 to 18.0) |
| Diagnosis and primary therapy phase of ovarian cancer | 4.3 (4.1 to 4.6) | 19.1 (18.0 to 20.3) | 1.2 (0.8 to 1.7) | 18.0* (11.7 to 25.8) | 8.1* (2.3 to 15.3) |
| Controlled phase of ovarian cancer | 95.2 (90.0 to 101.2) | -- | 4.4 (2.8 to 6.5) | 18.3* (7.4 to 30.9) | 8.3 (-1.6 to 19.9) |
| Metastatic phase of ovarian cancer | 19.7 (18.6 to 21.0) | -- | 8.5 (5.8 to 11.3) | 17.5* (4.6 to 31.9) | 7.7 (-4.2 to 20.8) |

| | | | | | |
|---|---|---|---|---|---|
| Terminal phase of ovarian cancer | 0.9 (0.8 to 0.9) | -- | 0.5 (0.3 to 0.6) | 16.0* (9.3 to 24.3) | 6.2* (0.1 to 13.9) |
| Prostate cancer | 41.8 (36.1 to 49.5) | 4.5 (3.9 to 5.4) | 3.0 (2.0 to 4.2) | 20.9* (12.6 to 29.8) | 10.7* (3.1 to 18.9) |
| Diagnosis and primary therapy phase of prostate cancer | 1.4 (1.2 to 1.7) | 4.5 (3.9 to 5.4) | 0.4 (0.3 to 0.6) | 24.3* (17.5 to 32.8) | 13.9* (7.7 to 21.6) |
| Controlled phase of prostate cancer | 38.3 (32.8 to 45.6) | -- | 2.3 (1.4 to 3.4) | 22.2* (13.0 to 32.4) | 12.0* (3.5 to 21.3) |
| Metastatic phase of prostate cancer | 0.4 (0.4 to 0.5) | -- | 0.2 (0.1 to 0.3) | 9.4* (3.9 to 15.9) | 0.3 (-4.9 to 6.2) |
| Terminal phase of prostate cancer | -- | -- | -- | 8.7* (3.4 to 14.6) | -0.4 (-5.2 to 5.0) |
| Impotence and Incontinence after 10-year survival from prostate cancer | 1.7 (1.5 to 1.8) | -- | 0.1 (0.1 to 0.1) | 1.2* (0.5 to 1.8) | -7.3* (-7.9 to -6.7) |
| Testicular cancer | 47.7 (43.7 to 52.2) | 5.8 (5.3 to 6.3) | 2.9 (2.0 to 4.1) | 8.1 (-4.1 to 22.3) | -0.9 (-12.2 to 12.1) |
| Diagnosis and primary therapy phase of testicular cancer | 1.6 (1.4 to 1.7) | 5.8 (5.3 to 6.3) | 0.4 (0.3 to 0.6) | 9.4 (-0.9 to 20.8) | 0.2 (-9.3 to 10.6) |
| Controlled phase of testicular cancer | 45.4 (41.7 to 49.8) | -- | 2.2 (1.4 to 3.2) | 9.6 (-4.5 to 26.6) | 0.4 (-12.5 to 16.0) |
| Metastatic phase of testicular cancer | 0.6 (0.6 to 0.7) | -- | 0.3 (0.2 to 0.4) | -1.7 (-8.8 to 6.1) | -9.9* (-16.5 to -2.8) |
| Terminal phase of testicular cancer | 0.1 (0.1 to 0.1) | -- | 0.0 (0.0 to 0.1) | -5.1 (-12.5 to 2.8) | -13.0* (-19.8 to -5.8) |
| Kidney cancer | 111.2 (105.2 to 116.7) | 15.9 (15.0 to 16.7) | 6.4 (4.4 to 8.9) | 4.1 (-3.0 to 11.8) | -4.7 (-11.1 to 2.4) |
| Diagnosis and primary therapy phase of kidney cancer | -- | 15.9 (15.0 to 16.7) | -- | -- | -- |
| Controlled phase of kidney cancer | 108.2 (102.4 to 113.7) | -- | 5.1 (3.3 to 7.4) | 4.4 (-3.5 to 13.6) | -4.4 (-11.6 to 4.0) |
| Metastatic phase of kidney cancer | 2.5 (2.3 to 2.6) | -- | 1.1 (0.7 to 1.5) | 3.0 (-1.8 to 8.2) | -5.7* (-10.1 to -0.9) |
| Terminal phase of kidney cancer | 0.5 (0.4 to 0.5) | -- | 0.3 (0.2 to 0.3) | 3.0 (-1.8 to 8.3) | -5.7* (-10.1 to -0.8) |
| Bladder cancer | 65.1 (59.8 to 69.2) | 8.6 (7.9 to 9.2) | 5.7 (4.0 to 7.9) | 11.2* (4.2 to 19.3) | 1.9 (-4.6 to 9.3) |
| Diagnosis and primary therapy phase of bladder cancer | 3.4 (3.1 to 3.6) | 8.6 (7.9 to 9.2) | 1.0 (0.6 to 1.3) | 11.9* (5.3 to 19.1) | 2.5 (-3.6 to 9.1) |
| Controlled phase of bladder cancer | 59.2 (54.4 to 63.1) | -- | 3.9 (2.6 to 5.6) | 12.4* (3.5 to 22.3) | 2.9 (-5.2 to 12.1) |
| Metastatic phase of bladder cancer | 1.3 (1.2 to 1.4) | -- | 0.6 (0.4 to 0.8) | 5.8 (-0.4 to 12.8) | -3.1 (-8.7 to 3.3) |
| Terminal phase of bladder cancer | 0.2 (0.2 to 0.3) | -- | 0.1 (0.1 to 0.2) | 5.3 (-0.8 to 12.3) | -3.5 (-9.1 to 2.9) |
| Incontinence from bladder cancer, beyond 10 years | 1.0 (0.9 to 1.1) | -- | 0.1 (0.1 to 0.2) | 5.8* (4.4 to 7.0) | -3.1* (-4.4 to -2.0) |
| Brain and nervous system cancer | 101.2 (87.0 to 121.8) | 22.3 (19.5 to 25.7) | 9.8 (6.7 to 13.3) | 36.6* (22.9 to 59.3) | 25.1* (12.5 to 45.9) |
| Diagnosis and primary therapy phase of brain and nervous system cancers | 6.2 (5.3 to 7.4) | 22.3 (19.5 to 25.7) | 1.8 (1.1 to 2.6) | 51.1* (34.4 to 78.9) | 38.4* (23.2 to 63.9) |
| Controlled phase of brain and nervous system cancers | 86.7 (74.2 to 105.0) | -- | 4.1 (2.5 to 6.2) | 54.8* (35.0 to 86.2) | 41.8* (23.7 to 70.5) |
| Metastatic phase of brain and nervous system cancers | 7.1 (6.3 to 8.2) | -- | 3.2 (2.1 to 4.4) | 17.5* (8.9 to 31.9) | 7.7 (-0.3 to 20.8) |
| Terminal phase of brain and nervous system cancers | 1.2 (1.1 to 1.4) | -- | 0.6 (0.5 to 0.9) | 13.4* (5.5 to 26.2) | 3.9 (-3.4 to 15.6) |
| Thyroid cancer | 210.3 (197.2 to 228.5) | 23.4 (21.9 to 25.4) | 11.9 (8.1 to 16.8) | 22.3* (12.6 to 33.4) | 12.0* (3.1 to 22.2) |
| Diagnosis and primary therapy phase of thyroid cancer | 5.6 (5.2 to 6.1) | 23.4 (21.9 to 25.4) | 1.6 (1.0 to 2.2) | 22.6* (9.3 to 38.4) | 12.3* (0.1 to 26.8) |
| Controlled phase of thyroid cancer | 202.9 (190.2 to 220.5) | -- | 9.5 (6.2 to 14.0) | 23.0* (12.3 to 35.1) | 12.6* (2.9 to 23.7) |
| Metastatic phase of thyroid cancer | 1.7 (1.6 to 1.8) | -- | 0.8 (0.5 to 1.0) | 14.9* (7.5 to 24.3) | 5.3 (-1.5 to 13.9) |
| Terminal phase of thyroid cancer | 0.1 (0.1 to 0.2) | -- | 0.1 (0.1 to 0.1) | 14.2* (6.9 to 23.6) | 4.6 (-2.0 to 13.2) |
| Mesothelioma | 2.7 (2.5 to 3.0) | 1.2 (1.1 to 1.3) | 0.5 (0.4 to 0.7) | 3.0 (-6.3 to 13.0) | -5.6 (-14.2 to 3.5) |
| Diagnosis and primary therapy phase of mesothelioma | 0.3 (0.3 to 0.3) | 1.2 (1.1 to 1.3) | 0.1 (0.1 to 0.1) | 3.4 (-6.0 to 13.6) | -5.3 (-13.9 to 4.0) |
| Controlled phase of mesothelioma | 1.7 (1.5 to 1.8) | -- | 0.1 (0.1 to 0.1) | 3.3 (-6.1 to 13.2) | -5.4 (-14.0 to 3.7) |
| Metastatic phase of mesothelioma | 0.6 (0.6 to 0.7) | -- | 0.3 (0.2 to 0.4) | 2.9 (-6.4 to 13.0) | -5.7 (-14.3 to 3.5) |
| Terminal phase of mesothelioma | 0.1 (0.1 to 0.1) | -- | 0.1 (0.0 to 0.1) | 2.7 (-6.6 to 12.6) | -5.9 (-14.5 to 3.2) |
| Hodgkin lymphoma | 39.7 (33.7 to 48.9) | 5.8 (5.0 to 7.1) | 2.9 (2.0 to 4.1) | 8.6* (0.1 to 17.3) | -0.6 (-8.3 to 7.5) |
| Diagnosis and primary therapy phase of Hodgkin disease | 1.3 (1.1 to 1.6) | 5.8 (5.0 to 7.1) | 0.4 (0.2 to 0.5) | 11.2* (3.3 to 19.6) | 1.9 (-5.4 to 9.6) |
| Controlled phase of Hodgkin disease | 36.6 (31.2 to 45.1) | -- | 1.7 (1.1 to 2.7) | 11.5* (0.3 to 24.2) | 2.2 (-8.1 to 13.7) |
| Metastatic phase of Hodgkin disease | 1.6 (1.4 to 2.0) | -- | 0.7 (0.5 to 1.0) | 2.1 (-3.5 to 8.1) | -6.4* (-11.6 to -0.9) |
| Terminal phase of Hodgkin disease | 0.2 (0.1 to 0.2) | -- | 0.1 (0.1 to 0.1) | -2.5 (-7.6 to 3.2) | -10.7* (-15.4 to -5.5) |
| Non-Hodgkin's lymphoma | 106.6 (103.2 to 110.5) | 19.0 (18.4 to 19.6) | 8.2 (5.8 to 11.0) | 17.8* (10.5 to 25.7) | 7.9* (1.2 to 15.2) |
| Diagnosis and primary therapy phase of non-Hodgkin lymphoma | 3.8 (3.7 to 4.0) | 19.0 (18.4 to 19.6) | 1.1 (0.7 to 1.5) | 20.3* (15.2 to 26.1) | 10.2* (5.6 to 15.5) |
| Controlled phase of non-Hodgkin lymphoma | 97.4 (94.3 to 101.0) | -- | 4.6 (2.9 to 6.7) | 20.3* (10.0 to 30.8) | 10.2* (0.8 to 19.8) |
| Metastatic phase of non-Hodgkin lymphoma | 4.6 (4.4 to 4.7) | -- | 2.1 (1.4 to 2.7) | 12.6* (6.8 to 17.6) | 3.1 (-2.1 to 7.8) |
| Terminal phase of non-Hodgkin lymphoma | 0.8 (0.8 to 0.8) | -- | 0.4 (0.3 to 0.5) | 11.6* (5.8 to 16.8) | 2.3 (-3.1 to 7.0) |
| Multiple myeloma | 15.2 (14.0 to 18.2) | 3.7 (3.4 to 4.3) | 3.0 (2.0 to 4.0) | 20.5* (12.2 to 28.4) | 10.3* (2.7 to 17.6) |
| Diagnosis and primary therapy phase of multiple myeloma | 1.0 (0.9 to 1.2) | 3.7 (3.4 to 4.3) | 0.3 (0.2 to 0.4) | 22.1* (13.6 to 30.7) | 11.8* (4.1 to 19.7) |
| Controlled phase of multiple myeloma | 9.4 (8.6 to 11.3) | -- | 0.5 (0.3 to 0.7) | 22.5* (12.9 to 32.1) | 12.2* (3.4 to 21.1) |
| Metastatic phase of multiple myeloma | 4.6 (4.2 to 5.4) | -- | 2.1 (1.4 to 2.8) | 20.1* (11.9 to 28.1) | 10.0* (2.5 to 17.4) |
| Terminal phase of multiple myeloma | 0.2 (0.2 to 0.3) | -- | 0.1 (0.1 to 0.2) | 15.7* (7.1 to 22.9) | 6.0 (-1.9 to 12.6) |
| Leukaemia | 70.7 (63.8 to 78.3) | 17.9 (16.1 to 19.5) | 8.3 (6.0 to 11.1) | 2.1 (-5.3 to 14.3) | -6.5 (-13.2 to 4.7) |
| Acute lymphoid leukaemia | 10.0 (8.6 to 11.0) | 2.3 (2.0 to 2.5) | 1.1 (0.8 to 1.5) | 13.4* (2.1 to 23.3) | 3.9 (-6.5 to 12.9) |
| Diagnosis and primary therapy phase of acute lymphoid leukemia | 1.0 (0.9 to 1.1) | 2.3 (2.0 to 2.5) | 0.3 (0.2 to 0.4) | 7.9 (-1.5 to 15.5) | -1.1 (-9.7 to 5.8) |
| Controlled phase of acute lymphoid leukemia | 8.0 (6.9 to 8.8) | -- | 0.4 (0.2 to 0.6) | 15.7* (3.2 to 26.6) | 6.0 (-5.5 to 15.9) |
| Metastatic phase of acute lymphoid leukemia | 0.8 (0.7 to 0.9) | -- | 0.4 (0.2 to 0.5) | 15.3* (2.9 to 26.6) | 5.6 (-5.8 to 16.0) |
| Terminal phase of acute lymphoid leukemia | 0.1 (0.1 to 0.2) | -- | 0.1 (0.1 to 0.1) | 15.4* (3.0 to 26.8) | 5.7 (-5.7 to 16.1) |
| Chronic lymphoid leukaemia | 18.2 (16.2 to 21.1) | 2.4 (2.2 to 2.8) | 2.1 (1.5 to 2.9) | 47.1* (30.8 to 69.4) | 34.8* (19.8 to 55.2) |

| | | | | | |
|---|---|---|---|---|---|
| Diagnosis and primary therapy phase of chronic lymphoid leukemia | 1.0 (0.9 to 1.1) | 2.4 (2.2 to 2.8) | 0.3 (0.2 to 0.4) | 48.9* (32.6 to 71.9) | 36.4* (21.5 to 57.5) |
| Controlled phase of chronic lymphoid leukemia | 14.7 (13.1 to 17.0) | -- | 0.7 (0.4 to 1.1) | 47.7* (28.1 to 74.1) | 35.3* (17.4 to 59.5) |
| Metastatic phase of chronic lymphoid leukemia | 2.4 (2.2 to 2.8) | -- | 1.1 (0.7 to 1.5) | 46.7* (30.6 to 68.4) | 34.4* (19.7 to 54.2) |
| Terminal phase of chronic lymphoid leukemia | 0.1 (0.1 to 0.1) | -- | 0.0 (0.0 to 0.1) | 36.2* (21.5 to 56.4) | 24.8* (11.3 to 43.2) |
| Acute myeloid leukaemia | 7.3 (6.7 to 8.2) | 5.2 (4.7 to 5.7) | 1.3 (0.9 to 1.7) | 11.4* (3.5 to 18.7) | 2.0 (-5.2 to 8.8) |
| Diagnosis and primary therapy phase of acute myeloid leukemia | -- | 5.2 (4.7 to 5.7) | -- | -- | -- |
| Controlled phase of acute myeloid leukemia | 5.1 (4.7 to 5.6) | -- | 0.3 (0.2 to 0.4) | 8.2* (0.8 to 15.4) | -0.9 (-7.7 to 5.7) |
| Metastatic phase of acute myeloid leukemia | 1.8 (1.6 to 2.0) | -- | 0.8 (0.6 to 1.1) | 12.2* (4.1 to 20.4) | 2.8 (-4.7 to 10.3) |
| Terminal phase of acute myeloid leukemia | 0.4 (0.4 to 0.4) | -- | 0.2 (0.1 to 0.3) | 12.2* (4.1 to 20.4) | 2.8 (-4.7 to 10.3) |
| Chronic myeloid leukaemia | 7.0 (6.3 to 7.7) | 2.0 (1.8 to 2.2) | 0.7 (0.5 to 1.0) | -6.7* (-12.7 to -0.1) | -14.5* (-20.0 to -8.5) |
| Diagnosis and primary therapy phase of chronic myeloid leukemia | 0.5 (0.4 to 0.5) | 2.0 (1.8 to 2.2) | 0.1 (0.1 to 0.2) | -5.9 (-12.1 to 1.2) | -13.8* (-19.5 to -7.3) |
| Controlled phase of chronic myeloid leukemia | 5.8 (5.2 to 6.4) | -- | 0.3 (0.2 to 0.4) | -5.4 (-11.6 to 1.9) | -13.3* (-19.0 to -6.7) |
| Metastatic phase of chronic myeloid leukemia | 0.6 (0.5 to 0.6) | -- | 0.3 (0.2 to 0.3) | -8.1* (-14.0 to -1.9) | -15.8* (-21.2 to -10.1) |
| Terminal phase of chronic myeloid leukemia | 0.1 (0.1 to 0.1) | -- | 0.1 (0.0 to 0.1) | -8.1* (-14.0 to -1.9) | -15.8* (-21.2 to -10.1) |
| Other leukaemia | 28.2 (24.3 to 32.2) | 5.9 (5.1 to 6.8) | 3.0 (2.1 to 4.0) | -19.3* (-27.6 to -3.9) | -26.0* (-33.6 to -11.9) |
| Metastatic phase of other leukemia | 3.8 (3.2 to 4.3) | -- | 1.7 (1.1 to 2.3) | -20.5* (-28.2 to -5.0) | -27.1* (-34.2 to -12.9) |
| Diagnosis and primary therapy phase of other leukemia | -- | 5.9 (5.1 to 6.8) | -- | -- | -- |
| Terminal phase of other leukemia | 0.3 (0.2 to 0.3) | -- | 0.1 (0.1 to 0.2) | -19.3* (-26.7 to -4.1) | -26.0* (-32.9 to -12.1) |
| Controlled phase of other leukemia | 24.1 (20.9 to 27.5) | -- | 1.2 (0.7 to 1.7) | -17.5* (-28.9 to -0.3) | -24.4* (-34.8 to -8.7) |
| Other malignant cancers | 433.2 (332.7 to 611.9) | 36.1 (32.9 to 39.3) | 32.3 (21.1 to 49.5) | 111.6* (61.4 to 188.3) | 93.8* (47.8 to 164.1) |
| Diagnosis and primary therapy phase of other malignant neoplasms | 9.9 (7.6 to 13.9) | 36.1 (32.9 to 39.3) | 2.8 (1.7 to 4.6) | 131.3* (71.7 to 221.3) | 111.9* (57.3 to 194.3) |
| Controlled phase of other malignant neoplasms | 400.0 (305.5 to 564.8) | -- | 18.9 (11.1 to 31.5) | 149.3* (83.8 to 246.2) | 128.4* (68.4 to 217.1) |
| Metastatic phase of other malignant neoplasms | 20.4 (16.9 to 26.7) | -- | 9.0 (5.7 to 13.3) | 64.4* (31.3 to 110.5) | 50.6* (20.2 to 92.8) |
| Terminal phase of other malignant neoplasms | 2.9 (2.4 to 3.7) | -- | 1.6 (1.0 to 2.3) | 58.8* (27.0 to 101.3) | 45.5* (16.4 to 84.4) |
| Other neoplasms | 1634.9 (1541.5 to 1734.8) | 708.3 (666.7 to 755.5) | 2.3 (1.4 to 3.4) | 9.7 (-0.1 to 20.8) | 0.5 (-8.5 to 10.6) |
| Myelodysplastic, myeloproliferative, and other hematopoietic neoplasms | 47.8 (37.8 to 59.0) | 4.5 (2.9 to 6.2) | 2.3 (1.4 to 3.4) | 9.7 (-0.1 to 20.8) | 0.5 (-8.5 to 10.6) |
| Benign and in situ intestinal neoplasms | 205.6 (193.7 to 218.8) | 56.9 (53.8 to 60.7) | -- | -- | -- |
| Benign and in situ cervical and uterine neoplasms | 163.4 (154.9 to 171.9) | 21.4 (19.7 to 23.3) | -- | -- | -- |
| Other benign and in situ neoplasms | 1255.6 (1166.5 to 1354.4) | 625.5 (584.8 to 672.2) | -- | -- | -- |
| **Cardiovascular diseases** | **24129.8 (21946.5 to 26660.1)** | **2649.0 (2476.5 to 2861.5)** | **1474.9 (1042.0 to 1967.7)** | **8.8* (6.9 to 10.8)** | **-0.3 (-2.0 to 1.5)** |
| Rheumatic heart disease | 3786.1 (3652.7 to 3928.0) | 18.5 (15.3 to 21.8) | 180.2 (116.7 to 263.2) | 9.8* (8.2 to 11.4) | 0.6 (-0.9 to 2.0) |
| Rheumatic heart disease, without heart failure | 3725.6 (3590.1 to 3871.0) | 18.5 (15.3 to 21.8) | 174.7 (113.0 to 254.6) | 10.0* (8.6 to 11.6) | 0.8 (-0.5 to 2.3) |
| Heart failure due to rheumatic heart disease | 60.5 (41.9 to 84.9) | -- | 5.5 (3.1 to 8.6) | 1.8 (-7.0 to 11.1) | -6.7 (-14.8 to 1.8) |
| Ischaemic heart disease | 4865.5 (4414.6 to 5325.0) | 292.1 (220.1 to 375.9) | 238.5 (161.3 to 334.2) | 5.6* (3.6 to 7.8) | -3.2* (-5.1 to -1.2) |
| Asymptomatic ischemic heart disease following myocardial infarction | 771.4 (599.1 to 983.3) | -- | -- | -- | -- |
| Angina due to ischemic heart disease | 3898.3 (3494.9 to 4318.5) | -- | 220.9 (148.9 to 310.1) | 5.6* (3.4 to 7.9) | -3.3* (-5.3 to -1.2) |
| Heart failure due to ischemic heart disease | 180.2 (122.9 to 253.3) | -- | 16.1 (9.1 to 25.3) | 6.5 (-0.3 to 14.2) | -2.5 (-8.7 to 4.6) |
| Myocardial infarction due to ischemic heart disease | 15.6 (11.5 to 20.4) | 292.1 (220.1 to 375.9) | 1.5 (0.9 to 2.4) | 2.2 (-1.2 to 5.4) | -6.4* (-9.5 to -3.5) |
| Stroke | 4071.1 (3728.6 to 4473.7) | 420.4‡ (359.3 to 484.9) | 630.0 (445.3 to 813.0) | 9.0* (4.6 to 13.3) | -0.2 (-4.2 to 3.8) |
| Ischaemic stroke | 2324.7 (2044.4 to 2653.6) | 202.4 (148.0 to 261.4) | 348.1 (243.2 to 459.3) | 17.7* (11.8 to 24.2) | 7.8* (2.4 to 13.8) |
| Acute ischemic stroke | 15.4 (11.3 to 20.2) | 202.4 (148.0 to 261.4) | 4.0 (2.5 to 6.0) | 13.3* (11.1 to 15.4) | 3.8* (1.8 to 5.7) |
| Chronic ischemic stroke | 2309.4 (2028.7 to 2636.0) | -- | 344.1 (240.7 to 454.1) | 17.8* (11.7 to 24.3) | 7.9* (2.3 to 13.9) |
| Intracerebral hemorrhage | 1209.4 (1030.8 to 1437.2) | 136.8 (112.9 to 162.5) | 186.7 (131.5 to 253.3) | -4.2 (-12.0 to 4.7) | -12.2* (-19.4 to -4.1) |
| Acute intracerebral hemorrhage | 7.6 (6.1 to 9.4) | 136.8 (112.9 to 162.5) | 1.9 (1.3 to 2.7) | -18.7* (-28.0 to -9.9) | -25.6* (-34.1 to -17.4) |
| Chronic intracerebral hemorrhage | 1201.7 (1022.3 to 1430.4) | -- | 184.8 (129.9 to 251.2) | -4.0 (-11.9 to 4.9) | -12.1* (-19.3 to -3.9) |
| Subarachnoid hemorrhage | 649.9 (563.0 to 751.1) | 81.2 (65.3 to 101.5) | 95.2 (65.7 to 127.3) | 8.6* (2.1 to 15.8) | -0.5 (-6.5 to 6.1) |
| Acute subarachnoid hemorrhage | 5.8 (4.6 to 7.3) | 81.2 (65.3 to 101.5) | 1.5 (1.0 to 2.1) | 10.7* (3.6 to 13.8) | 1.4 (-5.1 to 4.3) |
| Chronic subarachnoid hemorrhage | 644.1 (557.0 to 745.5) | -- | 93.7 (64.6 to 125.5) | 8.5* (1.8 to 15.9) | -0.6 (-6.7 to 6.1) |
| Hypertensive heart disease | 344.5 (247.3 to 457.5) | -- | 30.2 (18.3 to 45.2) | 7.5* (1.7 to 13.7) | -1.6 (-6.9 to 4.2) |
| Non-rheumatic valvular heart disease | 915.7 (864.1 to 965.7) | 941.8 (903.1 to 982.9) | 1.9 (0.7 to 4.0) | 6.1* (2.5 to 9.6) | -2.8 (-6.1 to 0.4) |
| Non-rheumatic calcific aortic valve disease | 202.5 (174.0 to 234.2) | 202.5 (174.0 to 234.2) | 0.4 (0.1 to 0.8) | 3.7 (-1.8 to 8.8) | -5.0* (-10.0 to -0.3) |
| Heart failure due to calcific aortic valve disease | 2.3 (0.8 to 5.0) | -- | 0.2 (0.1 to 0.5) | -1.9 (-18.0 to 16.3) | -10.1 (-24.9 to 6.6) |
| Non-rheumatic calcific aortic valve disease | 200.2 (172.3 to 231.9) | 202.5 (174.0 to 234.2) | 0.2 (0.1 to 0.4) | 12.3 (-2.3 to 27.8) | 2.9 (-10.5 to 17.1) |
| Non-rheumatic degenerative mitral valve disease | 739.2 (714.8 to 765.6) | 739.2 (714.8 to 765.6) | 1.5 (0.5 to 3.2) | 6.5* (2.0 to 11.0) | -2.4 (-6.5 to 1.6) |
| Heart failure due to degenerative mitral valve disease | 11.4 (4.4 to 24.7) | -- | 1.1 (0.4 to 2.3) | 1.4 (-9.7 to 13.9) | -7.1 (-17.3 to 4.4) |
| Non-rheumatic degenerative mitral valve disease | 727.9 (700.0 to 755.9) | 739.2 (714.8 to 765.6) | 0.4 (0.1 to 1.0) | 21.2* (2.3 to 41.2) | 11.1 (-6.3 to 29.3) |
| Other non-rheumatic valve diseases | 0.7 (0.5 to 1.0) | -- | 0.1 (0.0 to 0.1) | 11.6* (9.9 to 13.4) | 2.2* (0.7 to 3.9) |
| Mild heart failure due to other non-rheumatic valve disease | 0.1 (0.1 to 0.2) | -- | -- | 11.6* (9.8 to 13.4) | 2.2* (0.6 to 3.9) |
| Moderate heart failure due to other non-rheumatic valve disease | 0.1 (0.0 to 0.1) | -- | -- | 11.6* (9.9 to 13.4) | 2.2* (0.7 to 3.9) |
| Severe heart failure due to other non-rheumatic valve disease | 0.2 (0.1 to 0.3) | -- | 0.0 (0.0 to 0.1) | 11.6* (9.9 to 13.4) | 2.2* (0.7 to 3.9) |

| | | | | | |
|---|---|---|---|---|---|
| Controlled, medically managed heart failure due to other non-rheumatic valve disease | 0.2<br>(0.2 to 0.4) | -- | -- | 11.6*<br>(9.9 to 13.4) | 2.2*<br>(0.6 to 3.9) |
| Cardiomyopathy and myocarditis | 146.9<br>(111.0 to 189.5) | 159.9<br>(114.2 to 213.6) | 30.2<br>(17.7 to 44.7) | 8.9*<br>(5.4 to 12.4) | -0.2<br>(-3.5 to 2.9) |
| Myocarditis | 116.6<br>(89.1 to 148.3) | 159.9<br>(114.2 to 213.6) | 9.1<br>(5.4 to 13.3) | 9.0*<br>(5.1 to 13.1) | -0.2<br>(-3.7 to 3.6) |
| Acute myocarditis | 38.7<br>(26.7 to 52.7) | 159.9<br>(114.2 to 213.6) | 2.0<br>(1.1 to 3.2) | 8.2*<br>(3.4 to 12.7) | -0.9<br>(-5.3 to 3.3) |
| Heart failure due to myocarditis | 78.0<br>(54.1 to 106.0) | -- | 7.1<br>(4.0 to 10.8) | 9.2*<br>(4.6 to 14.3) | 0.0<br>(-4.2 to 4.7) |
| Alcoholic cardiomyopathy | 79.5<br>(58.8 to 104.0) | -- | 7.1<br>(4.3 to 10.5) | 1.2<br>(-4.6 to 7.1) | -7.3*<br>(-12.6 to -1.8) |
| Other cardiomyopathy | 156.4<br>(111.3 to 210.6) | -- | 14.0<br>(8.1 to 21.0) | 13.3*<br>(7.8 to 19.4) | 3.8<br>(-1.2 to 9.4) |
| Atrial fibrillation and flutter | 643.9<br>(507.9 to 788.9) | 57.8<br>(41.2 to 76.8) | 53.8<br>(34.4 to 77.7) | 8.4*<br>(3.0 to 13.7) | -0.7<br>(-5.6 to 4.2) |
| Asymptomatic atrial fibrillation and flutter | 386.4<br>(304.6 to 472.7) | 57.8<br>(41.2 to 76.8) | -- | -- | -- |
| Symptomatic atrial fibrillation and flutter | 257.5<br>(203.4 to 317.6) | -- | 53.8<br>(34.4 to 77.7) | 8.4*<br>(3.0 to 13.7) | -0.7<br>(-5.6 to 4.2) |
| Peripheral vascular disease | 4279.0<br>(3387.2 to 5417.3) | 708.5<br>(576.7 to 875.6) | 0.1<br>(0.0 to 0.3) | 2.9*<br>(1.1 to 4.8) | -5.8*<br>(-7.4 to -4.0) |
| Asymptomatic peripheral arterial disease | 4270.3<br>(3380.6 to 5406.2) | 708.5<br>(576.7 to 875.6) | -- | -- | -- |
| Symptomatic claudication due to peripheral arterial disease | 8.7<br>(5.0 to 14.5) | -- | 0.1<br>(0.0 to 0.3) | 2.9*<br>(1.1 to 4.8) | -5.8*<br>(-7.4 to -4.0) |
| Endocarditis | 15.7<br>(11.5 to 20.7) | 50.0<br>(34.3 to 66.1) | 1.3<br>(0.8 to 2.0) | 10.3*<br>(8.5 to 12.1) | 1.0<br>(-0.6 to 2.7) |
| Acute endocarditis | 3.8<br>(2.6 to 5.2) | 50.0<br>(34.3 to 66.1) | 0.2<br>(0.1 to 0.3) | 15.2*<br>(11.3 to 18.4) | 5.5*<br>(2.0 to 8.4) |
| Heart failure due to endocarditis | 11.9<br>(8.1 to 16.8) | -- | 1.1<br>(0.6 to 1.8) | 9.4*<br>(7.4 to 11.4) | 0.2<br>(-1.6 to 2.0) |
| Other cardiovascular and circulatory diseases | 5820.7<br>(3912.7 to 8260.2) | -- | 308.5<br>(174.2 to 497.8) | 10.8*<br>(8.6 to 13.1) | 1.5<br>(-0.6 to 3.6) |
| Heart failure due to other cardiovascular diseases | 19.9<br>(13.4 to 28.1) | -- | 1.8<br>(1.0 to 2.9) | 11.0*<br>(9.5 to 12.5) | 1.7*<br>(0.3 to 3.1) |
| Other cardiovascular and circulatory disease episodes | 5800.9<br>(3898.7 to 8231.5) | -- | 306.7<br>(173.2 to 495.0) | 10.8*<br>(8.5 to 13.1) | 1.5<br>(-0.6 to 3.6) |
| **Chronic respiratory diseases** | **28588.8<br>(24994.9 to 32680.0)** | **2307.0<br>(1782.1 to 2873.6)** | **2473.9<br>(1940.5 to 3040.4)** | **11.4*<br>(6.4 to 16.4)** | **2.0<br>(-2.5 to 6.7)** |
| Chronic obstructive pulmonary disease | 15086.6<br>(12554.9 to 17989.5) | 1114.3<br>(737.3 to 1547.3) | 1634.6<br>(1268.1 to 2035.2) | 8.3*<br>(2.2 to 14.7) | -0.8<br>(-6.4 to 5.1) |
| Chronic obstructive pulmonary disease with heart failure | 18.8<br>(12.6 to 27.6) | -- | 8.7<br>(5.0 to 13.6) | 0.7<br>(-0.9 to 2.4) | -7.7*<br>(-9.2 to -6.2) |
| Chronic obstructive pulmonary disease without heart failure | 15067.8<br>(12539.8 to 17972.3) | 1114.3<br>(737.3 to 1547.3) | 1625.9<br>(1258.4 to 2026.9) | 8.4*<br>(2.2 to 14.8) | -0.7<br>(-6.4 to 5.2) |
| Pneumoconiosis | 18.9<br>(15.0 to 23.5) | 2.1<br>(1.5 to 2.7) | 3.0<br>(1.9 to 4.4) | 3.0<br>(-1.3 to 7.1) | -5.7*<br>(-9.6 to -1.8) |
| Silicosis | 6.4<br>(4.1 to 9.6) | 0.8<br>(0.5 to 1.3) | 1.0<br>(0.6 to 1.6) | 8.8<br>(-1.0 to 17.9) | -0.4<br>(-9.3 to 8.0) |
| Heart failure due to silicosis | -- | -- | -- | -- | -- |
| Silicosis without heart failure | 6.4<br>(4.1 to 9.6) | 0.8<br>(0.5 to 1.3) | 1.0<br>(0.6 to 1.6) | 8.8<br>(-1.0 to 17.9) | -0.4<br>(-9.3 to 8.0) |
| Asbestosis | 4.1<br>(2.2 to 6.9) | 0.3<br>(0.1 to 0.6) | 0.6<br>(0.3 to 1.1) | 17.1*<br>(11.6 to 24.2) | 7.3*<br>(2.2 to 13.8) |
| Asbestosis without heart failure | 4.1<br>(2.2 to 6.9) | 0.3<br>(0.1 to 0.6) | 0.6<br>(0.3 to 1.1) | 17.1*<br>(11.6 to 24.2) | 7.3*<br>(2.2 to 13.8) |
| Heart failure due to asbestosis | -- | -- | -- | -- | -- |
| Coal workers pneumoconiosis | 3.4<br>(2.1 to 5.1) | 0.4<br>(0.2 to 0.6) | 0.5<br>(0.3 to 0.9) | -11.9*<br>(-19.2 to -5.4) | -19.3*<br>(-26.0 to -13.4) |
| Coal workers pneumoconiosis without heart failure | 3.4<br>(2.1 to 5.1) | 0.4<br>(0.2 to 0.6) | 0.5<br>(0.3 to 0.9) | -11.8*<br>(-19.2 to -5.4) | -19.2*<br>(-26.0 to -13.3) |
| Heart failure due to Coal workers pneumoconiosis | -- | -- | -- | -15.5*<br>(-24.5 to -10.2) | -22.6*<br>(-30.8 to -17.7) |
| Other pneumoconiosis | 5.0<br>(3.4 to 7.3) | 0.5<br>(0.3 to 0.8) | 0.8<br>(0.4 to 1.3) | -1.9<br>(-5.7 to 1.7) | -10.1*<br>(-13.6 to -6.8) |
| Heart failure due to other pneumoconiosis | 0.0<br>(0.0 to 0.1) | -- | -- | -5.3*<br>(-7.2 to -3.4) | -13.2*<br>(-15.0 to -11.5) |
| Other pneumoconiosis without heart failure | 5.0<br>(3.4 to 7.2) | 0.5<br>(0.3 to 0.8) | 0.8<br>(0.4 to 1.3) | -1.8<br>(-5.7 to 1.8) | -10.0*<br>(-13.6 to -6.8) |
| Asthma | 14119.6<br>(11565.9 to 17431.3) | 1166.3<br>(815.7 to 1557.5) | 548.5<br>(350.4 to 819.6) | 12.5*<br>(6.5 to 18.6) | 3.1<br>(-2.5 to 8.6) |
| Asymptomatic asthma | 5131.2<br>(4047.6 to 6363.4) | 422.8<br>(286.9 to 578.3) | -- | -- | -- |
| Symptomatic asthma | 8988.4<br>(7188.9 to 11191.9) | 743.5<br>(518.5 to 1006.9) | 548.5<br>(350.4 to 819.6) | 12.5*<br>(6.5 to 18.6) | 3.1<br>(-2.5 to 8.6) |
| Interstitial lung disease and pulmonary sarcoidosis | 286.9<br>(233.6 to 349.8) | 24.3<br>(15.9 to 34.1) | 31.6<br>(19.9 to 46.7) | 8.2*<br>(4.4 to 12.0) | -0.9<br>(-4.3 to 2.6) |
| Heart failure due to interstitial lung disease and pulmonary sarcoidosis | 4.1<br>(2.8 to 5.9) | -- | 1.9<br>(1.1 to 2.9) | 10.6*<br>(9.2 to 12.1) | 1.3*<br>(0.0 to 2.7) |
| Interstitial lung disease and pulmonary sarcoidosis without heart failure | 282.8<br>(230.2 to 345.4) | 24.3<br>(15.9 to 34.1) | 29.7<br>(18.4 to 44.6) | 8.0*<br>(4.0 to 12.1) | -1.0<br>(-4.7 to 2.7) |
| Other chronic respiratory diseases | -- | -- | 256.3<br>(198.6 to 317.9) | 32.6*<br>(25.2 to 40.2) | 21.5*<br>(14.7 to 28.5) |
| **Digestive diseases** | **182141.1<br>(166668.4 to 199783.0)** | **39152.1<br>(30683.1 to 48409.8)** | **1682.0<br>(1115.1 to 2435.1)** | **9.7*<br>(8.0 to 11.3)** | **0.5<br>(-1.1 to 1.9)** |
| Cirrhosis and other chronic liver diseases | 132386.4<br>(122736.6 to 142324.6) | 406.1<br>(325.2 to 492.3) | 144.3<br>(99.6 to 197.2) | 20.2*<br>(16.0 to 24.5) | 10.1*<br>(6.3 to 14.0) |
| Cirrhosis and other chronic liver diseases due to hepatitis B | 31587.7<br>(29189.2 to 34439.5) | 133.5<br>(102.2 to 169.2) | 48.2<br>(32.5 to 65.9) | 13.9*<br>(7.5 to 20.6) | 4.4<br>(-1.5 to 10.5) |
| Cirrhosis and other chronic liver diseases due to hepatitis B, decompensated | 287.0<br>(254.1 to 320.6) | 48.4<br>(41.4 to 55.6) | 48.2<br>(32.5 to 65.9) | 13.9*<br>(7.5 to 20.6) | 4.4<br>(-1.5 to 10.5) |
| Chronic hepatitis B without cirrhosis | 26571.8<br>(24129.9 to 29520.0) | -- | -- | -- | -- |
| Cirrhosis and other chronic liver diseases due to hepatitis B, compensated | 4729.0<br>(4217.5 to 5289.7) | 85.1<br>(55.3 to 118.3) | -- | -- | -- |
| Cirrhosis and other chronic liver diseases due to hepatitis C | 10791.9<br>(9293.7 to 12601.2) | 117.7<br>(93.3 to 147.1) | 39.8<br>(26.9 to 56.4) | 24.7*<br>(17.9 to 32.0) | 14.2*<br>(8.0 to 20.9) |
| Cirrhosis and other chronic liver diseases due to hepatitis C, decompensated | 237.8<br>(206.2 to 274.6) | 41.0<br>(34.3 to 48.8) | 39.8<br>(26.9 to 56.4) | 24.7*<br>(17.9 to 32.0) | 14.2*<br>(8.0 to 20.9) |
| Chronic hepatitis C without cirrhosis | 6908.9<br>(5324.2 to 8705.9) | -- | -- | -- | -- |
| Cirrhosis and other chronic liver diseases due to hepatitis C, compensated | 3645.3<br>(3115.5 to 4191.2) | 76.7<br>(53.5 to 104.1) | -- | -- | -- |
| Cirrhosis and other chronic liver diseases due to alcohol use | 3371.4<br>(2980.5 to 3795.5) | 96.5<br>(77.4 to 117.2) | 36.9<br>(25.0 to 51.9) | 19.6*<br>(13.2 to 26.3) | 9.5*<br>(3.7 to 15.7) |
| Cirrhosis and other chronic liver diseases due to alcohol, decompensated | 220.4<br>(193.9 to 246.7) | 38.5<br>(33.3 to 44.6) | 36.9<br>(25.0 to 51.9) | 19.6*<br>(13.2 to 26.3) | 9.5*<br>(3.7 to 15.7) |
| Cirrhosis and other chronic liver diseases due to alcohol, compensated | 3151.0<br>(2783.3 to 3552.2) | 58.0<br>(40.2 to 77.2) | -- | -- | -- |
| Cirrhosis due to NASH | 86015.2<br>(77240.1 to 95597.6) | 38.7<br>(29.2 to 49.8) | 13.0<br>(8.3 to 18.4) | 34.1*<br>(23.2 to 45.7) | 22.8*<br>(12.8 to 33.5) |
| Non-alcoholic fatty liver disease (NAFLD) / Non-alcoholic steatohepatitis (NASH) | 84746.5<br>(75971.3 to 94287.0) | -- | -- | -- | -- |
| Cirrhosis and other chronic liver diseases due to NASH | 1268.7<br>(1045.9 to 1477.0) | 38.7<br>(29.2 to 49.8) | 13.0<br>(8.3 to 18.4) | 34.1*<br>(23.2 to 45.7) | 22.8*<br>(12.8 to 33.5) |
| Cirrhosis and other chronic liver diseases due to other causes | 620.2<br>(460.4 to 799.9) | 19.7<br>(13.5 to 27.5) | 6.5<br>(4.0 to 9.5) | 20.7*<br>(10.4 to 32.3) | 10.6*<br>(1.1 to 21.2) |

| | | | | | |
|---|---|---|---|---|---|
| Cirrhosis and other chronic liver diseases due to other cause, decompensated | 37.9 (28.0 to 49.0) | 6.7 (4.9 to 8.9) | 6.5 (4.0 to 9.5) | 20.7* (10.4 to 32.3) | 10.6* (1.1 to 21.2) |
| Cirrhosis and other chronic liver diseases due to other cause, compensated | 582.3 (433.3 to 752.6) | 13.0 (8.3 to 19.2) | -- | -- | -- |
| Upper digestive system diseases | 75251.3 (57852.3 to 97956.9) | 34473.1 (25961.6 to 43608.5) | 1170.0 (696.1 to 1827.2) | 9.0* (7.0 to 10.6) | -0.2 (-1.9 to 1.4) |
| Peptic ulcer disease | 1347.6 (1016.5 to 1799.4) | 467.3 (324.3 to 636.4) | 66.8 (40.9 to 100.0) | 9.9* (4.6 to 14.6) | 0.7 (-4.2 to 5.0) |
| Acute peptic ulcer disease | 0.4 (0.3 to 0.6) | -- | 0.1 (0.1 to 0.2) | -3.2* (-4.9 to -1.7) | -11.3* (-12.9 to -9.9) |
| Chronic peptic ulcer disease | 1347.2 (1016.1 to 1799.1) | 467.3 (324.3 to 636.4) | 66.6 (40.8 to 99.9) | 9.9* (4.6 to 14.7) | 0.7 (-4.2 to 5.0) |
| Gastritis and duodenitis | 11683.3 (7976.9 to 16120.4) | 8118.6 (5486.1 to 11222.0) | 523.7 (307.4 to 815.8) | 5.9* (2.4 to 9.2) | -3.0 (-6.2 to 0.1) |
| Acute gastritis and duodenitis | 2.6 (1.6 to 3.8) | -- | 0.8 (0.5 to 1.4) | 0.3 (-1.5 to 1.8) | -8.1* (-9.8 to -6.7) |
| Chronic gastritis and duodenitis | 11680.7 (7975.1 to 16117.9) | 8118.6 (5486.1 to 11222.0) | 522.8 (306.8 to 814.7) | 5.9* (2.4 to 9.3) | -3.0 (-6.2 to 0.1) |
| GERD | 67717.5 (48403.4 to 92979.8) | 25887.1 (17889.0 to 34792.3) | 579.5 (285.2 to 1028.5) | 11.8* (9.9 to 13.5) | 2.4* (0.7 to 4.0) |
| Appendicitis | 30.9 (25.3 to 37.7) | 796.6 (654.3 to 967.0) | 9.7 (6.3 to 14.0) | 12.4* (2.4 to 23.3) | 3.0 (-6.2 to 13.0) |
| Paralytic ileus and intestinal obstruction | 7.3 (5.2 to 9.6) | 197.2 (139.2 to 258.8) | 2.4 (1.4 to 3.5) | 16.4* (12.0 to 19.8) | 6.7* (2.6 to 9.7) |
| Inguinal, femoral, and abdominal hernia | 1801.6 (1296.8 to 2405.4) | 2273.4 (1533.6 to 3184.0) | 176.2 (106.4 to 272.6) | 9.2* (5.3 to 13.2) | 0.0 (-3.5 to 3.7) |
| Inflammatory bowel disease | 571.3 (490.9 to 661.5) | 352.6 (283.7 to 444.1) | 88.4 (59.3 to 124.2) | 1.3 (-7.7 to 10.9) | -7.2 (-15.5 to 1.6) |
| Ulcerative colitis | 390.7 (320.6 to 477.4) | 255.8 (190.6 to 338.1) | 59.4 (38.9 to 84.8) | 4.9 (-6.1 to 16.9) | -3.9 (-14.0 to 7.1) |
| Crohn's disease | 180.6 (152.7 to 213.9) | 96.8 (72.3 to 126.6) | 28.9 (18.8 to 41.2) | -5.3 (-14.6 to 4.8) | -13.3* (-21.7 to -4.0) |
| Vascular intestinal disorders | 3.1 (1.9 to 4.6) | 20.0 (13.9 to 27.4) | 1.0 (0.5 to 1.7) | 13.5* (10.3 to 16.2) | 4.0* (1.1 to 6.4) |
| Gallbladder and biliary diseases | 2902.4 (2100.8 to 3899.1) | 486.7 (346.4 to 661.3) | 2.4 (1.3 to 3.8) | 12.3* (9.6 to 15.4) | 2.9* (0.4 to 5.7) |
| Pancreatitis | 508.6 (432.2 to 597.3) | 146.4 (115.6 to 182.9) | 31.5 (15.7 to 54.8) | 11.4* (5.4 to 17.1) | 2.1 (-3.5 to 7.3) |
| Acute pancreatitis | 10.2 (7.7 to 13.0) | 115.2 (85.3 to 148.6) | 4.2 (2.7 to 6.1) | 0.3 (-0.7 to 1.2) | -8.1* (-9.0 to -7.3) |
| Chronic pancreatitis | 498.3 (420.5 to 588.0) | 31.3 (22.1 to 43.1) | 27.3 (12.5 to 49.7) | 13.3* (6.3 to 20.1) | 3.8 (-2.6 to 10.1) |
| Other digestive diseases | -- | -- | 56.2 (36.1 to 80.6) | 14.8* (12.0 to 17.5) | 5.2* (2.6 to 7.6) |
| **Neurological disorders** | **255272.0 (215527.0 to 299062.2)** | **69020.1 (48377.0 to 94328.0)** | **5823.2 (3858.7 to 8250.0)** | **12.1* (10.6 to 13.5)** | **2.7* (1.3 to 4.0)** |
| Alzheimer's disease and other dementias | 29.1 (15.4 to 47.9) | 17.2 (9.1 to 28.4) | 3.9 (1.7 to 7.1) | 3.0* (0.3 to 4.9) | -5.6* (-8.1 to -3.9) |
| Parkinson's disease | 93.7 (61.2 to 135.5) | 13.4 (8.2 to 19.3) | 15.5 (8.9 to 24.4) | 7.4* (1.6 to 13.3) | -1.6 (-7.0 to 3.8) |
| Epilepsy | 1406.4 (1033.4 to 1840.0) | 100.5 (64.7 to 141.5) | 429.4 (256.6 to 643.0) | 12.3* (0.8 to 25.7) | 2.9 (-7.7 to 15.1) |
| Multiple sclerosis | 198.5 (176.5 to 222.9) | 3.5 (2.2 to 5.2) | 52.2 (37.4 to 69.1) | 6.0* (0.6 to 11.5) | -2.9 (-7.8 to 2.1) |
| Motor neuron disease | 11.4 (8.6 to 14.9) | 2.5 (1.7 to 3.6) | 2.4 (1.6 to 3.6) | 8.5* (7.2 to 9.6) | -0.6 (-1.8 to 0.4) |
| Headache disorders | 254521.2 (214752.9 to 298462.3) | 68883.1 (48245.3 to 94208.9) | 5230.8 (3397.8 to 7621.1) | 12.0* (10.8 to 13.1) | 2.6* (1.5 to 3.6) |
| Migraine | 130953.2 (114410.1 to 148594.4) | 7910.8 (5359.3 to 10962.3) | 4616.6 (2922.3 to 6820.1) | 12.2* (10.8 to 13.3) | 2.7* (1.5 to 3.8) |
| Medication overuse headache due to migraine | 7930.8 (5768.5 to 10761.8) | -- | 871.8 (509.7 to 1365.8) | 11.6* (10.2 to 13.1) | 2.3* (0.9 to 3.6) |
| Migraine | 123022.4 (106453.9 to 140486.3) | 7910.8 (5359.3 to 10962.3) | 3744.8 (2307.2 to 5695.6) | 12.3* (10.7 to 13.7) | 2.9* (1.4 to 4.1) |
| Tension-type headache | 183532.4 (131123.7 to 242924.7) | 60972.3 (40227.6 to 86219.9) | 614.2 (327.6 to 1005.5) | 10.9* (9.5 to 12.4) | 1.6* (0.3 to 2.9) |
| Medication overuse headache due to tension-type headache | 2948.8 (1872.2 to 4425.9) | -- | 322.1 (157.0 to 567.5) | 11.7* (9.3 to 14.2) | 2.3* (0.1 to 4.7) |
| Tension-type headache | 180583.6 (128854.4 to 239876.9) | 60972.3 (40227.6 to 86219.9) | 292.1 (121.7 to 584.7) | 10.0* (8.8 to 11.2) | 0.8 (-0.3 to 1.9) |
| Other neurological disorders | 2.4 (1.3 to 4.2) | -- | 88.9 (57.3 to 130.1) | 20.6* (9.7 to 32.9) | 10.4* (0.5 to 21.8) |
| Other neurological disorders | -- | -- | 88.2 (56.6 to 129.1) | 20.6* (9.7 to 33.0) | 10.5* (0.5 to 21.9) |
| Guillain-Barré syndrome due to other neurological disorders | 2.4 (1.3 to 4.2) | -- | 0.7 (0.3 to 1.3) | 17.1* (14.2 to 20.0) | 7.3* (4.6 to 9.9) |
| **Mental disorders** | **74793.4 (70039.0 to 79690.7)** | **25686.5 (21776.6 to 30287.1)** | **10365.0 (7708.4 to 13300.0)** | **7.7* (6.7 to 8.6)** | **-1.3* (-2.2 to -0.5)** |
| Schizophrenia | 2424.7 (2136.3 to 2793.5) | 81.6 (57.0 to 110.6) | 1563.3 (1153.6 to 1949.8) | 7.6* (5.5 to 9.7) | -1.5 (-3.4 to 0.5) |
| Depressive disorders | 22845.8 (19481.5 to 26971.8) | 21943.4 (17984.5 to 26387.9) | 3741.4 (2569.9 to 5261.9) | 5.4* (3.3 to 7.4) | -3.4* (-5.4 to -1.6) |
| Major depressive disorder | 13992.5 (11228.6 to 17227.5) | 20573.8 (16733.8 to 25016.8) | 2837.2 (1846.5 to 4049.6) | 5.3* (2.4 to 7.8) | -3.6* (-6.2 to -1.3) |
| Dysthymia | 9305.1 (7115.8 to 11679.7) | 1369.6 (1045.4 to 1760.0) | 904.1 (556.4 to 1385.5) | 6.1* (2.9 to 9.4) | -2.8 (-5.7 to 0.2) |
| Bipolar disorder | 4028.4 (3283.1 to 4960.6) | 308.0 (212.4 to 424.8) | 819.7 (504.2 to 1250.1) | 12.6* (10.6 to 14.6) | 3.1* (1.4 to 5.0) |
| Anxiety disorders | 23715.6 (21002.2 to 26738.5) | 3323.4 (2893.8 to 3795.6) | 2256.6 (1569.8 to 3045.2) | 8.1* (6.3 to 10.0) | -1.0 (-2.6 to 0.7) |
| Eating disorders | 1412.0 (1039.8 to 1887.2) | 30.2 (21.0 to 43.0) | 294.7 (177.3 to 461.0) | 20.6* (17.5 to 23.2) | 10.5* (7.6 to 12.9) |
| Anorexia nervosa | 211.5 (151.5 to 286.5) | 30.2 (21.0 to 43.0) | 44.1 (25.9 to 69.5) | 13.4* (7.3 to 19.7) | 3.9 (-1.7 to 9.7) |
| Bulimia nervosa | 1208.4 (855.9 to 1683.4) | -- | 250.6 (149.1 to 409.3) | 22.0* (18.6 to 25.1) | 11.7* (8.7 to 14.6) |
| Autism spectrum disorders | 1899.1 (1704.8 to 2101.2) | -- | 285.9 (195.7 to 391.7) | 7.8* (5.7 to 9.9) | -1.3 (-3.1 to 0.6) |
| Attention-deficit/hyperactivity disorder | 3510.9 (2826.4 to 4227.4) | -- | 42.0 (25.2 to 67.5) | 7.7* (4.3 to 11.3) | -1.3 (-4.5 to 2.0) |
| Conduct disorder | -- | -- | -- | -- | -- |
| Idiopathic developmental intellectual disability | 5267.4 (2919.4 to 7674.2) | -- | 207.7 (97.9 to 356.4) | 3.8* (0.2 to 6.0) | -4.9* (-8.2 to -2.9) |
| Other mental disorders | 15388.3 (13400.4 to 17482.7) | -- | 1153.7 (757.1 to 1591.0) | 9.0* (7.6 to 10.5) | -0.1 (-1.5 to 1.2) |
| **Substance use disorders** | **16161.1 (13572.3 to 18957.2)** | **5745.0 (4463.2 to 7197.5)** | **2702.0 (1834.1 to 3678.5)** | **12.8* (9.5 to 16.5)** | **3.3* (0.3 to 6.7)** |
| Alcohol use disorders | 11117.9 (8653.6 to 13847.2) | 5359.9 (4091.4 to 6865.6) | 1111.0 (706.2 to 1646.8) | 3.8* (0.4 to 6.5) | -4.9* (-8.0 to -2.4) |
| Alcohol dependence | 11099.7 (8635.0 to 13826.8) | 5359.9 (4091.4 to 6865.6) | 1110.4 (705.8 to 1646.0) | 3.8* (0.4 to 6.5) | -4.9* (-8.0 to -2.4) |
| Fetal alcohol syndrome | 18.2 (14.5 to 22.4) | -- | 0.6 (0.4 to 1.0) | -9.6* (-15.4 to -4.4) | -17.2* (-22.5 to -12.4) |
| Drug use disorders | 5316.1 (4497.2 to 6271.4) | 385.1 (276.6 to 522.9) | 1591.0 (1072.0 to 2191.8) | 20.0* (14.9 to 25.3) | 10.0* (5.3 to 14.7) |
| Opioid use disorders | 3359.7 (2570.6 to 4313.4) | 230.1 (133.7 to 362.2) | 1386.7 (914.2 to 1956.1) | 22.3* (16.6 to 28.5) | 12.1* (6.8 to 17.8) |
| Cocaine use disorders | 390.0 (341.0 to 443.2) | 4.8 (2.5 to 7.5) | 52.6 (34.5 to 75.9) | 6.6* (1.8 to 11.2) | -2.4 (-6.7 to 1.9) |

| | | | | | |
|---|---|---|---|---|---|
| Amphetamine use disorders | 236.2 (158.4 to 325.6) | 28.8 (13.3 to 44.9) | 31.0 (17.2 to 50.7) | 7.3 (-1.7 to 16.4) | -1.7 (-10.0 to 6.7) |
| Cannabis use disorders | 1137.0 (856.3 to 1518.5) | 121.3 (77.5 to 173.4) | 32.6 (19.9 to 49.5) | 3.6 (-2.2 to 9.6) | -5.1 (-10.4 to 0.4) |
| Other drug use disorders | 310.5 (240.9 to 393.0) | -- | 88.1 (60.1 to 119.9) | 7.0* (3.0 to 10.9) | -2.0 (-5.7 to 1.6) |
| **Diabetes and kidney diseases** | **84694.7 (74354.9 to 95736.1)** | **3356.4 (2603.9 to 4119.9)** | **3615.3 (2470.3 to 5085.2)** | **14.8* (9.2 to 20.6)** | **5.2* (0.0 to 10.5)** |
| Diabetes mellitus | 37611.9 (31929.1 to 43829.6) | 2235.0 (1686.1 to 2827.9) | 3131.4 (2103.4 to 4471.2) | 15.6* (9.0 to 22.2) | 5.9 (-0.1 to 12.0) |
| Diabetes mellitus type 1 | 1181.7 (1051.8 to 1333.8) | 15.2 (9.8 to 21.5) | 97.6 (65.9 to 137.2) | 9.1* (6.2 to 12.0) | -0.1 (-2.7 to 2.6) |
| Uncomplicated diabetes mellitus type 1 | 682.6 (571.2 to 794.7) | 15.2 (9.8 to 21.5) | 32.1 (19.3 to 48.0) | 6.4* (1.9 to 10.2) | -2.6 (-6.7 to 0.9) |
| Vision loss due to diabetes mellitus type 1 retinopathy | 11.1 (7.7 to 15.6) | -- | 1.0 (0.6 to 1.5) | -3.0 (-9.8 to 4.3) | -11.1* (-17.3 to -4.5) |
| Diabetic neuropathy due to diabetes mellitus type 1 | 488.0 (400.1 to 592.6) | -- | 64.6 (42.3 to 93.3) | 10.7* (5.8 to 15.5) | 1.4 (-3.1 to 5.8) |
| Diabetes mellitus type 2 | 36430.2 (30743.0 to 42683.8) | 2219.8 (1673.5 to 2817.4) | 3033.8 (2032.6 to 4330.4) | 15.8* (9.0 to 22.7) | 6.1 (-0.2 to 12.4) |
| Uncomplicated diabetes mellitus type 2 | 20696.3 (16684.5 to 25182.3) | 2219.8 (1673.5 to 2817.4) | 973.0 (587.4 to 1465.5) | 13.4* (5.7 to 21.0) | 3.9 (-3.2 to 10.8) |
| Vision loss due to diabetes mellitus type 2 retinopathy | 312.9 (239.3 to 410.7) | -- | 27.8 (18.1 to 40.6) | 7.3 (-0.2 to 14.1) | -1.7 (-8.5 to 4.5) |
| Diabetic neuropathy due to diabetes mellitus type 2 | 15420.9 (12123.4 to 19297.5) | -- | 2033.0 (1329.2 to 3010.7) | 17.1* (9.4 to 24.8) | 7.3* (0.2 to 14.3) |
| Chronic kidney disease | 54561.4 (44071.4 to 65257.5) | 1079.1 (694.1 to 1598.5) | 483.8 (340.2 to 671.4) | 10.1* (3.5 to 17.5) | 0.9 (-5.2 to 7.7) |
| Chronic kidney disease due to diabetes mellitus type 1 | 337.6 (279.0 to 406.4) | 7.5 (4.7 to 11.6) | 35.9 (23.0 to 51.8) | 11.4* (3.5 to 19.8) | 2.1 (-5.2 to 9.7) |
| Albuminuria with preserved GFR due to type 1 diabetes mellitus | 158.8 (115.3 to 208.7) | -- | -- | -- | -- |
| End-stage chronic kidney disease due to diabetes mellitus type 1 | 63.5 (41.1 to 89.6) | -- | 24.8 (14.2 to 38.8) | 8.9 (-1.8 to 19.8) | -0.2 (-10.1 to 9.8) |
| Stage III chronic kidney disease due to diabetes mellitus type 1 | 87.0 (67.9 to 111.0) | 7.5 (4.7 to 11.6) | 1.8 (1.1 to 2.7) | 24.8* (7.6 to 42.6) | 14.3 (-1.5 to 30.6) |
| Stage IV chronic kidney disease due to diabetes mellitus type 1 | 16.4 (10.5 to 24.3) | -- | 2.3 (1.3 to 3.8) | 18.1* (3.5 to 33.7) | 8.2 (-5.2 to 22.5) |
| Stage V chronic kidney disease due to diabetes mellitus type 1 | 11.9 (8.4 to 16.7) | -- | 7.0 (4.2 to 10.6) | 15.5* (5.7 to 25.8) | 5.8 (-3.2 to 15.2) |
| Chronic kidney disease due to diabetes mellitus type 2 | 9608.6 (7452.0 to 11891.9) | 83.8 (54.1 to 124.8) | 53.1 (35.4 to 75.8) | 10.1* (3.2 to 17.5) | 0.9 (-5.5 to 7.7) |
| Albuminuria with preserved GFR due to type 2 diabetes mellitus | 8442.4 (6304.5 to 10744.9) | -- | -- | -- | -- |
| End-stage chronic kidney disease due to diabetes mellitus type 2 | 28.4 (16.0 to 45.2) | -- | 11.5 (5.8 to 19.5) | 7.2 (-1.5 to 15.0) | -1.8 (-9.7 to 5.3) |
| Stage III chronic kidney disease due to diabetes mellitus type 2 | 1022.1 (835.2 to 1243.0) | 83.8 (54.1 to 124.8) | 5.4 (3.3 to 8.4) | 4.2 (-9.7 to 20.1) | -4.5 (-17.3 to 10.0) |
| Stage IV chronic kidney disease due to diabetes mellitus type 2 | 67.4 (43.7 to 98.8) | -- | 8.5 (4.5 to 13.8) | 10.3 (-3.9 to 25.1) | 1.0 (-11.9 to 14.6) |
| Stage V chronic kidney disease due to diabetes mellitus type 2 | 48.3 (35.1 to 67.0) | -- | 27.7 (17.0 to 41.5) | 12.6* (4.1 to 22.2) | 3.1 (-4.7 to 12.0) |
| Chronic kidney disease due to hypertension | 1437.2 (1237.7 to 1682.1) | 71.8 (46.0 to 107.2) | 80.6 (54.6 to 115.7) | 11.9* (4.1 to 20.5) | 2.5 (-4.6 to 10.4) |
| Albuminuria with preserved GFR due to hypertension | 347.2 (259.6 to 446.8) | -- | -- | -- | -- |
| End-stage chronic kidney disease due to hypertension | 33.2 (23.4 to 44.7) | -- | 13.4 (7.9 to 20.1) | 9.5* (0.6 to 17.7) | 0.4 (-7.9 to 7.8) |
| Stage III chronic kidney disease due to hypertension | 850.7 (702.5 to 1040.2) | 71.8 (46.0 to 107.2) | 4.2 (2.5 to 6.4) | -3.8 (-18.0 to 13.3) | -11.8 (-24.9 to 3.8) |
| Stage IV chronic kidney disease due to hypertension | 120.3 (77.4 to 176.8) | -- | 14.7 (8.0 to 23.9) | 11.8 (-2.3 to 26.8) | 2.4 (-10.5 to 16.1) |
| Stage V chronic kidney disease due to hypertension | 85.9 (62.0 to 118.0) | -- | 48.3 (29.5 to 72.2) | 14.2* (4.1 to 25.3) | 4.6 (-4.6 to 14.8) |
| Chronic kidney disease due to glomerulonephritis | 1705.2 (1456.8 to 1975.9) | 59.9 (38.7 to 89.2) | 107.6 (71.6 to 152.0) | 11.9* (4.2 to 20.7) | 2.5 (-4.5 to 10.5) |
| Albuminuria with preserved GFR due to glomerulonephritis | 643.6 (484.8 to 825.6) | -- | -- | -- | -- |
| End-stage chronic kidney disease due to glomerulonephritis | 61.5 (39.2 to 88.5) | -- | 23.1 (12.8 to 36.4) | 6.8 (-1.5 to 15.5) | -2.2 (-9.8 to 5.8) |
| Stage III chronic kidney disease due to glomerulonephritis | 743.8 (610.4 to 904.5) | 59.9 (38.7 to 89.2) | 7.0 (4.4 to 10.8) | 7.7 (-5.4 to 23.5) | -1.4 (-13.4 to 13.1) |
| Stage IV chronic kidney disease due to glomerulonephritis | 149.5 (96.6 to 220.4) | -- | 18.1 (9.7 to 29.8) | 12.3 (-1.4 to 27.3) | 2.9 (-9.7 to 16.6) |
| Stage V chronic kidney disease due to glomerulonephritis | 106.8 (77.3 to 145.2) | -- | 59.4 (36.4 to 89.2) | 14.4* (4.2 to 25.5) | 4.8 (-4.5 to 14.9) |
| Chronic kidney disease due to other and unspecified causes | 41472.7 (33476.7 to 49659.8) | 856.1 (551.8 to 1274.6) | 206.5 (146.8 to 286.0) | 8.4* (0.9 to 16.7) | -0.7 (-7.5 to 6.9) |
| Albuminuria with preserved GFR due to other and unspecified causes | 30549.9 (22920.7 to 38947.0) | -- | -- | -- | -- |
| End-stage chronic kidney disease due to other causes | 72.7 (48.2 to 99.7) | -- | 28.1 (16.2 to 42.9) | 9.9 (-1.1 to 21.6) | 0.7 (-9.4 to 11.4) |
| Stage III chronic kidney disease due to other causes | 10468.6 (8642.2 to 12746.0) | 856.1 (551.8 to 1274.6) | 65.7 (40.5 to 101.0) | -1.0 (-10.7 to 9.3) | -9.3 (-18.2 to 0.2) |
| Stage IV chronic kidney disease due to other causes | 221.9 (142.9 to 324.1) | -- | 26.3 (14.5 to 43.1) | 13.1 (-0.8 to 27.7) | 3.6 (-9.1 to 17.0) |
| Stage V chronic kidney disease due to other causes | 159.5 (115.6 to 218.3) | -- | 86.4 (53.2 to 127.7) | 14.7* (4.8 to 25.8) | 5.0 (-4.0 to 15.2) |
| Acute glomerulonephritis | 2.8 (1.9 to 4.2) | 42.3 (27.7 to 63.9) | 0.1 (0.1 to 0.3) | -8.9* (-14.3 to -4.3) | -16.5* (-21.5 to -12.3) |
| **Skin and subcutaneous diseases** | **104864.0 (97745.3 to 112352.3)** | **222278.2 (198027.1 to 248284.0)** | **2083.4 (1419.3 to 3084.1)** | **8.8* (8.0 to 9.8)** | **-0.3 (-1.1 to 0.5)** |
| Dermatitis | 13204.0 (11314.3 to 15932.3) | 19294.2 (14247.1 to 26759.2) | 451.9 (273.0 to 698.7) | 7.6* (5.8 to 9.3) | -1.4 (-3.1 to 0.2) |
| Atopic dermatitis | 6940.2 (6127.9 to 7847.5) | 961.5 (782.4 to 1166.5) | 298.9 (162.5 to 506.2) | 8.4* (6.0 to 10.7) | -0.7 (-2.9 to 1.4) |
| Contact dermatitis | 5731.1 (3951.3 to 8301.7) | 16519.7 (11415.1 to 23932.3) | 143.6 (85.9 to 231.1) | 7.0* (4.5 to 9.3) | -2.0 (-4.3 to 0.2) |
| Seborrhoeic dermatitis | 691.7 (539.9 to 861.0) | 1813.0 (1433.7 to 2236.1) | 9.4 (5.2 to 15.4) | -7.5* (-11.6 to -2.7) | -15.3* (-19.0 to -10.9) |
| Psoriasis | 5371.6 (4891.6 to 5824.5) | 656.7 (562.4 to 751.9) | 467.6 (327.1 to 620.5) | 11.0* (8.5 to 13.4) | 1.7 (-0.6 to 3.9) |
| Bacterial skin diseases | 391.1 (354.7 to 429.1) | 10949.5 (9925.6 to 12071.0) | 9.7 (6.1 to 14.7) | 8.6* (4.7 to 12.3) | -0.5 (-4.1 to 2.9) |
| Cellulitis | 134.6 (109.7 to 164.3) | 2742.9 (2258.4 to 3275.3) | 7.8 (4.9 to 11.5) | 6.5* (2.2 to 10.5) | -2.5 (-6.4 to 1.2) |
| Pyoderma | 351.8 (319.4 to 384.0) | 8206.6 (7313.2 to 9209.7) | 2.0 (0.8 to 4.1) | 18.1* (13.3 to 22.8) | 8.2* (3.8 to 12.5) |
| Impetigo | 46.8 (40.6 to 53.9) | 852.6 (749.3 to 964.9) | 0.3 (0.1 to 0.6) | 17.9* (11.0 to 25.4) | 8.0* (1.7 to 14.9) |
| Abscess and other bacterial skin diseases | 305.0 (272.6 to 338.5) | 7354.0 (6459.4 to 8362.8) | 1.7 (0.7 to 3.5) | 18.1* (12.8 to 23.6) | 8.2* (3.3 to 13.2) |
| Scabies | 9078.8 (6171.9 to 12621.1) | 27317.9 (19105.1 to 37999.7) | 233.0 (118.9 to 409.4) | 1.9* (0.6 to 3.3) | -6.6* (-7.8 to -5.4) |
| Fungal skin diseases | 36222.6 (29827.5 to 43748.3) | 111116.5 (90168.3 to 136436.1) | 204.2 (82.3 to 421.5) | 9.6* (8.8 to 10.3) | 0.4 (-0.4 to 1.1) |
| Tinea capitis | 3025.2 (2037.2 to 4125.5) | 4548.6 (3199.5 to 5824.7) | 17.0 (6.5 to 36.0) | -1.1 (-4.0 to 2.3) | -9.4* (-12.0 to -6.3) |
| Other fungal skin diseases | 33197.4 (27004.9 to 40654.7) | 106567.9 (85348.6 to 131834.8) | 187.2 (75.3 to 389.3) | 10.7* (10.0 to 11.3) | 1.4* (0.8 to 2.0) |

| | | | | | |
|---|---|---|---|---|---|
| Viral skin diseases | 3411.9<br>(3109.3 to 3732.2) | 3164.7<br>(2885.5 to 3453.7) | 103.6<br>(65.4 to 153.6) | 2.3*<br>(0.4 to 4.3) | -6.3*<br>(-8.1 to -4.5) |
| Viral warts | 2603.5<br>(2311.5 to 2924.0) | 1875.2<br>(1633.7 to 2123.1) | 79.1<br>(48.8 to 119.2) | 0.3<br>(-2.0 to 2.6) | -8.2*<br>(-10.3 to -6.1) |
| Molluscum contagiosum | 808.4<br>(723.7 to 908.1) | 1289.6<br>(1169.7 to 1435.8) | 24.5<br>(15.4 to 37.1) | 9.7*<br>(5.2 to 14.5) | 0.5<br>(-3.6 to 4.8) |
| Acne vulgaris | 3383.0<br>(2946.6 to 3840.7) | 1428.8<br>(1227.6 to 1665.6) | 71.7<br>(42.7 to 115.2) | 17.6*<br>(14.8 to 20.5) | 7.7*<br>(5.2 to 10.4) |
| Alopecia areata | 1468.1<br>(1306.1 to 1654.5) | 2483.9<br>(2255.9 to 2747.9) | 48.0<br>(30.3 to 73.9) | 7.0*<br>(4.1 to 9.9) | -2.0<br>(-4.7 to 0.7) |
| Pruritus | 5308.3<br>(3621.5 to 7326.5) | 4054.4<br>(2833.2 to 5543.2) | 56.6<br>(24.3 to 109.5) | 10.8*<br>(9.3 to 12.2) | 1.5*<br>(0.2 to 2.8) |
| Urticaria | 4115.9<br>(2851.0 to 5614.8) | 7148.8<br>(4951.1 to 9705.7) | 243.2<br>(143.2 to 389.2) | 10.1*<br>(7.9 to 12.4) | 0.9<br>(-1.1 to 3.0) |
| Decubitus ulcer | 34.1<br>(22.8 to 48.1) | 121.5<br>(81.0 to 167.6) | 6.0<br>(3.6 to 9.6) | 13.4*<br>(12.3 to 14.5) | 3.9*<br>(2.9 to 4.9) |
| Other skin and subcutaneous diseases | 33908.1<br>(30420.3 to 37305.9) | 34541.3<br>(31209.3 to 38022.6) | 187.8<br>(88.1 to 346.4) | 14.0*<br>(13.2 to 14.9) | 4.4*<br>(3.7 to 5.3) |
| **Sense organ diseases** | **162314.3<br>(153821.0 to 171038.5)** | **--** | **3504.6<br>(2207.7 to 5347.4)** | **9.7*<br>(8.2 to 11.0)** | **0.5<br>(-0.9 to 1.7)** |
| Blindness and vision impairment | 95633.9<br>(88211.8 to 103291.9) | -- | 1699.3<br>(1054.4 to 2698.0) | 10.9*<br>(9.7 to 12.1) | 1.6*<br>(0.5 to 2.7) |
| Glaucoma | -- | -- | -- | -- | -- |
| Cataract | 2295.0<br>(1734.0 to 3009.6) | -- | 206.1<br>(134.3 to 302.5) | 8.5*<br>(4.8 to 12.4) | -0.6<br>(-4.0 to 3.0) |
| Age-related macular degeneration | -- | -- | -- | -- | -- |
| Refraction disorders | 10492.7<br>(7832.5 to 13599.3) | -- | 463.6<br>(298.2 to 700.4) | 11.0*<br>(8.2 to 13.8) | 1.7<br>(-0.9 to 4.2) |
| Near vision loss | 83950.0<br>(76905.5 to 91450.8) | -- | 863.8<br>(406.1 to 1556.6) | 11.8*<br>(11.1 to 12.5) | 2.4*<br>(1.7 to 3.1) |
| Other vision loss | 1906.9<br>(1482.8 to 2496.7) | -- | 165.7<br>(110.1 to 240.4) | 9.2*<br>(5.7 to 12.7) | 0.1<br>(-3.2 to 3.3) |
| Age-related and other hearing loss | 83376.0<br>(74302.4 to 92471.2) | -- | 1679.7<br>(1037.9 to 2571.9) | 8.6*<br>(5.8 to 11.0) | -0.5<br>(-3.1 to 1.7) |
| Other sense organ diseases | 5163.0<br>(4658.3 to 5718.7) | -- | 125.7<br>(76.6 to 186.6) | 9.8*<br>(7.3 to 12.4) | 0.6<br>(-1.7 to 3.0) |
| Chronic other sense organ diseases | 4389.1<br>(3887.5 to 4930.3) | -- | 115.0<br>(69.5 to 170.0) | 9.7*<br>(7.1 to 12.5) | 0.5<br>(-1.9 to 3.1) |
| Acute other sense organ diseases | 773.9<br>(696.7 to 849.6) | -- | 10.6<br>(6.2 to 16.3) | 10.4*<br>(4.1 to 16.3) | 1.1<br>(-4.6 to 6.6) |
| **Musculoskeletal disorders** | **118908.5<br>(102023.5 to 136174.2)** | **31660.5<br>(24829.5 to 39656.6)** | **12784.1<br>(8924.4 to 18090.1)** | **8.5*<br>(7.1 to 10.0)** | **-0.6<br>(-1.8 to 0.7)** |
| Rheumatoid arthritis | 1394.4<br>(1162.6 to 1655.1) | 108.8<br>(82.0 to 138.3) | 189.4<br>(122.0 to 267.1) | 16.6*<br>(12.3 to 20.8) | 6.8*<br>(2.8 to 10.7) |
| Osteoarthritis | 15248.0<br>(12471.2 to 18732.1) | 1737.9<br>(1410.6 to 2107.2) | 502.4<br>(244.7 to 1025.2) | 12.8*<br>(11.4 to 14.4) | 3.3*<br>(2.0 to 4.8) |
| Osteoarthritis of the hip | 1781.2<br>(1589.1 to 2008.8) | 194.1<br>(169.5 to 221.2) | 58.6<br>(30.4 to 117.1) | 16.4*<br>(13.0 to 19.8) | 6.6*<br>(3.6 to 9.8) |
| Osteoarthritis of the knee | 13466.8<br>(10694.2 to 16923.4) | 1543.8<br>(1222.1 to 1907.0) | 443.8<br>(212.6 to 903.1) | 12.3*<br>(10.7 to 14.0) | 2.9*<br>(1.4 to 4.4) |
| Low back pain | 51679.4<br>(38458.9 to 68164.0) | 21693.5<br>(15802.1 to 28994.2) | 5958.6<br>(3859.3 to 8958.5) | 9.6*<br>(8.6 to 10.8) | 0.4<br>(-0.5 to 1.5) |
| Low back pain with leg pain | 17777.2<br>(12932.5 to 23521.5) | 7464.9<br>(5260.2 to 10223.0) | 2602.2<br>(1569.2 to 4015.1) | 9.6*<br>(8.4 to 10.9) | 0.4<br>(-0.7 to 1.6) |
| Low back pain without leg pain | 33902.2<br>(25121.1 to 45176.2) | 14228.6<br>(10169.8 to 19302.4) | 3356.4<br>(2124.7 to 5144.7) | 9.7*<br>(8.5 to 10.8) | 0.5<br>(-0.6 to 1.5) |
| Neck pain | 30300.9<br>(20109.3 to 41682.4) | 7618.1<br>(4792.4 to 11009.0) | 3063.6<br>(1778.1 to 4732.1) | 5.3*<br>(3.1 to 7.8) | -3.5*<br>(-5.5 to -1.3) |
| Gout | 2667.6<br>(2056.0 to 3482.2) | 502.2<br>(375.4 to 643.4) | 87.8<br>(54.9 to 131.9) | 10.1*<br>(4.9 to 15.3) | 0.9<br>(-3.9 to 5.6) |
| Other musculoskeletal disorders | 34324.3<br>(23296.4 to 48051.8) | -- | 2982.4<br>(1725.3 to 4705.9) | 8.3*<br>(3.3 to 12.9) | -0.8<br>(-5.3 to 3.4) |
| **Other non-communicable diseases** | **347620.1<br>(327828.1 to 369355.6)** | **240184.9<br>(204390.8 to 273802.3)** | **4557.6<br>(3034.5 to 6653.5)** | **6.1*<br>(4.6 to 7.7)** | **-2.8*<br>(-4.2 to -1.3)** |
| Congenital anomalies | 5518.1<br>(5195.0 to 5885.0) | -- | 676.1<br>(473.0 to 908.4) | 11.2*<br>(8.7 to 13.8) | 1.8<br>(-0.4 to 4.2) |
| Neural tube defects | 165.0<br>(147.0 to 184.4) | -- | 48.7<br>(33.8 to 64.7) | 14.1*<br>(8.8 to 18.9) | 4.5<br>(-0.3 to 8.9) |
| Severe motor and cognitive impairment due to anencephaly | -- | -- | -- | -- | -- |
| Encephalocoele due to neural tube defects | 19.9<br>(16.2 to 23.7) | -- | 3.4<br>(2.3 to 4.9) | 9.6*<br>(6.2 to 12.8) | 0.4<br>(-2.7 to 3.3) |
| Spina bifida due to neural tube defects | 145.1<br>(129.2 to 162.6) | -- | 45.2<br>(31.2 to 60.3) | 14.4*<br>(8.9 to 19.6) | 4.8<br>(-0.3 to 9.6) |
| Congenital heart anomalies | 206.5<br>(190.1 to 224.5) | -- | 12.2<br>(5.8 to 20.4) | 11.1*<br>(6.8 to 15.3) | 1.8<br>(-2.2 to 5.6) |
| Critical malformations of great vessels, congenital valvular heart disease and patent ductus arteriosus | 35.5<br>(30.8 to 41.0) | -- | 2.3<br>(1.2 to 3.8) | 5.9*<br>(3.8 to 8.0) | -3.0*<br>(-4.9 to -1.1) |
| Other congenital heart anomalies | 61.1<br>(56.0 to 66.5) | -- | 3.6<br>(1.5 to 6.3) | 11.0*<br>(1.5 to 20.7) | 1.7<br>(-7.1 to 10.6) |
| Severe congenital heart anomalies excluding single ventricle heart defects | 87.5<br>(77.9 to 98.0) | -- | 5.4<br>(2.7 to 8.9) | 13.9*<br>(6.2 to 22.1) | 4.3<br>(-2.7 to 11.9) |
| Single ventricle and single ventricle pathway congenital heart anomalies | 3.5<br>(2.9 to 4.2) | -- | 0.3<br>(0.2 to 0.4) | 20.2*<br>(17.7 to 22.6) | 10.1*<br>(7.9 to 12.3) |
| Ventricular septal defect and atrial septal defect | 18.9<br>(15.3 to 22.6) | -- | 0.6<br>(0.3 to 1.1) | 5.7*<br>(3.0 to 7.8) | -3.2*<br>(-5.6 to -1.2) |
| Orofacial clefts | 670.4<br>(614.1 to 726.7) | -- | 19.0<br>(12.5 to 27.6) | 12.4*<br>(3.6 to 21.3) | 3.0<br>(-5.1 to 11.1) |
| Down's syndrome | 261.5<br>(228.5 to 296.9) | -- | 22.3<br>(15.1 to 31.2) | 10.0*<br>(6.9 to 13.1) | 0.8<br>(-2.1 to 3.6) |
| Turner syndrome | 23.4<br>(20.3 to 26.9) | -- | 0.5<br>(0.3 to 0.8) | 7.9*<br>(5.3 to 10.8) | -1.1<br>(-3.5 to 1.5) |
| Klinefelter syndrome | 38.2<br>(32.7 to 44.3) | -- | 0.4<br>(0.2 to 0.8) | 8.1*<br>(0.9 to 16.1) | -0.9<br>(-7.5 to 6.3) |
| Other chromosomal abnormalities | 409.7<br>(322.2 to 506.0) | -- | 34.5<br>(22.3 to 48.8) | 20.7*<br>(16.2 to 25.0) | 10.5*<br>(6.5 to 14.5) |
| Congenital musculoskeletal and limb anomalies | 1811.3<br>(1609.6 to 2070.5) | -- | 266.9<br>(174.9 to 367.0) | 11.7*<br>(9.5 to 14.0) | 2.3*<br>(0.3 to 4.4) |
| Polydactyly and syndactyly | 6.0<br>(4.8 to 7.3) | -- | 0.1<br>(0.0 to 0.1) | 3.4*<br>(1.5 to 5.3) | -5.2*<br>(-7.0 to -3.6) |
| Congenital limb deficiency | 856.4<br>(760.2 to 979.8) | -- | 126.5<br>(82.9 to 174.0) | 11.7*<br>(8.6 to 15.0) | 2.4<br>(-0.5 to 5.3) |
| Other congenital musculoskeletal anomalies | 948.9<br>(843.3 to 1084.8) | -- | 140.4<br>(91.9 to 193.4) | 11.7*<br>(8.6 to 14.9) | 2.3<br>(-0.5 to 5.3) |
| Urogenital congenital anomalies | 205.6<br>(172.9 to 244.3) | -- | 5.3<br>(3.2 to 8.7) | -6.8*<br>(-10.6 to -2.7) | -14.6*<br>(-18.1 to -10.9) |
| Digestive congenital anomalies | 1139.1<br>(1024.0 to 1272.0) | -- | 51.3<br>(34.2 to 72.9) | 3.2<br>(-2.3 to 9.0) | -5.4*<br>(-10.5 to -0.2) |
| Congenital diaphragmatic hernia | 11.3<br>(9.9 to 12.8) | -- | 0.2<br>(0.1 to 0.4) | 9.5*<br>(8.5 to 10.4) | 0.3<br>(-0.6 to 1.2) |
| Congenital atresia and/or stenosis of the digestive tract | 0.4<br>(0.3 to 0.5) | -- | -- | 28.9*<br>(23.7 to 33.8) | 18.1*<br>(13.3 to 22.6) |
| Congenital malformations of the abdominal wall after treatment | 43.6<br>(38.2 to 49.5) | -- | 1.9<br>(1.2 to 2.8) | 9.0*<br>(8.1 to 9.9) | -0.1<br>(-1.0 to 0.7) |
| Other congenital malformations of the digestive tract | 1083.8<br>(969.6 to 1219.4) | -- | 49.1<br>(32.5 to 69.9) | 3.0<br>(-2.8 to 8.9) | -5.7*<br>(-11.0 to -0.2) |
| Other congenital anomalies | 1622.3<br>(1402.5 to 1877.4) | -- | 215.0<br>(145.0 to 298.6) | 11.0*<br>(4.3 to 18.4) | 1.7<br>(-4.4 to 8.5) |

| | | | | | |
|---|---|---|---|---|---|
| Other congenital birth defects | -- | -- | 12.2<br>(8.8 to 16.0) | 27.0*<br>(22.2 to 31.6) | 16.3*<br>(12.0 to 20.5) |
| Hearing loss due to other congenital anomalies | 1622.3<br>(1402.5 to 1877.4) | -- | 202.7<br>(136.3 to 283.1) | 10.2*<br>(3.1 to 17.9) | 1.0<br>(-5.5 to 8.0) |
| Urinary diseases and male infertility | 6838.5<br>(5144.3 to 9117.4) | 28517.9<br>(22577.7 to 35586.0) | 98.6<br>(61.9 to 149.4) | 4.9*<br>(1.8 to 8.0) | -3.9*<br>(-6.8 to -1.1) |
| Urinary tract infections | 401.8<br>(286.8 to 550.2) | 20839.8<br>(15265.6 to 27928.9) | 13.3<br>(7.2 to 20.7) | 12.2*<br>(5.8 to 18.3) | 2.8<br>(-3.1 to 8.4) |
| Urolithiasis | 275.7<br>(198.9 to 365.4) | 7270.3<br>(5245.7 to 9619.4) | 20.8<br>(12.5 to 31.5) | 2.9<br>(-2.1 to 8.6) | -5.7*<br>(-10.4 to -0.5) |
| Benign prostatic hyperplasia | 697.5<br>(530.7 to 893.1) | 407.8<br>(322.2 to 495.5) | 23.7<br>(14.1 to 36.5) | 7.7*<br>(2.7 to 13.0) | -1.3<br>(-5.9 to 3.5) |
| Male infertility | 5558.0<br>(3818.8 to 7801.6) | -- | 30.9<br>(11.8 to 63.1) | 8.5*<br>(3.4 to 12.9) | -0.6<br>(-5.3 to 3.4) |
| Other urinary diseases | -- | -- | 9.9<br>(6.4 to 14.3) | -13.4*<br>(-17.0 to -9.7) | -20.7*<br>(-24.0 to -17.3) |
| Gynecological diseases | 113827.7<br>(107071.9 to 121560.5) | 31922.6<br>(30198.3 to 33665.6) | 1955.2<br>(1336.4 to 2826.2) | 4.2*<br>(2.3 to 6.1) | -4.6*<br>(-6.3 to -2.8) |
| Uterine fibroids | 36283.4<br>(29555.4 to 43751.8) | 2355.8<br>(1761.3 to 3046.4) | 356.0<br>(212.9 to 579.4) | -3.5<br>(-10.3 to 2.3) | -11.6*<br>(-17.8 to -6.3) |
| Polycystic ovarian syndrome | 7061.2<br>(5622.8 to 9052.8) | 58.6<br>(23.0 to 117.7) | 60.3<br>(26.6 to 114.5) | 12.6*<br>(11.0 to 14.2) | 3.2*<br>(1.7 to 4.6) |
| Female infertility | 12141.8<br>(5387.6 to 20495.4) | -- | 64.8<br>(18.9 to 155.1) | 15.9*<br>(2.6 to 23.9) | 6.1<br>(-6.0 to 13.5) |
| Endometriosis | 8426.0<br>(6536.6 to 10613.5) | 935.8<br>(644.2 to 1272.4) | 772.8<br>(502.3 to 1162.0) | 2.8*<br>(1.0 to 4.5) | -5.8*<br>(-7.4 to -4.3) |
| Genital prolapse | 10270.7<br>(8046.1 to 12827.3) | 1329.1<br>(998.9 to 1713.7) | 32.5<br>(14.6 to 63.3) | 10.0*<br>(7.4 to 12.3) | 0.8<br>(-1.6 to 2.9) |
| Premenstrual syndrome | 60375.6<br>(55645.0 to 65335.9) | 20592.1<br>(19153.8 to 22032.4) | 497.1<br>(319.0 to 738.8) | 7.4*<br>(5.4 to 9.4) | -1.6<br>(-3.4 to 0.2) |
| Other gynecological diseases | 10116.0<br>(9444.3 to 10834.2) | 6651.2<br>(6070.5 to 7232.1) | 171.7<br>(107.8 to 261.0) | 11.4*<br>(6.4 to 16.0) | 2.1<br>(-2.5 to 6.3) |
| Other gynecological diseases | 9166.3<br>(8486.6 to 9878.7) | 6651.2<br>(6070.5 to 7232.1) | 150.4<br>(92.7 to 231.4) | 13.7*<br>(8.0 to 19.0) | 4.2<br>(-1.1 to 9.0) |
| Other gynecological diseases with anemia | 949.7<br>(882.4 to 1019.1) | -- | 21.3<br>(13.8 to 31.6) | -2.5<br>(-12.1 to 7.3) | -10.7*<br>(-19.5 to -1.7) |
| Hemoglobinopathies and hemolytic anaemias | 119608.4<br>(116977.6 to 122413.0) | -- | 305.3<br>(199.8 to 457.2) | -9.7*<br>(-18.0 to -0.7) | -17.3*<br>(-24.8 to -9.0) |
| Thalassemias | 1.0<br>(0.9 to 1.0) | -- | 0.1<br>(0.1 to 0.1) | 0.7<br>(-12.9 to 15.7) | -7.7<br>(-20.2 to 6.0) |
| Thalassaemias trait | 20991.7<br>(20160.7 to 21883.5) | -- | 146.1<br>(93.1 to 225.2) | -19.8*<br>(-32.0 to -5.1) | -26.5*<br>(-37.7 to -13.1) |
| Sickle cell disorders | 26.1<br>(21.2 to 31.5) | -- | 3.6<br>(2.3 to 5.5) | 33.4*<br>(16.0 to 51.6) | 22.2*<br>(6.3 to 38.9) |
| Sickle cell trait | 26184.3<br>(23590.1 to 29114.8) | -- | 57.7<br>(37.7 to 84.1) | -1.2<br>(-8.2 to 6.2) | -9.5*<br>(-15.9 to -2.7) |
| G6PD deficiency | 21801.5<br>(20949.4 to 22679.7) | -- | 1.2<br>(0.8 to 1.7) | -2.2<br>(-13.9 to 13.2) | -10.4<br>(-21.1 to 3.7) |
| G6PD trait | 58664.6<br>(57706.7 to 59605.0) | -- | -- | -9.1<br>(-18.5 to 1.1) | -16.7*<br>(-25.3 to -7.4) |
| Other hemoglobinopathies and hemolytic anaemias | 4931.1<br>(4588.6 to 5314.6) | -- | 96.5<br>(63.8 to 143.6) | 3.1<br>(-4.5 to 11.7) | -5.6<br>(-12.5 to 2.3) |
| Endocrine, metabolic, blood, and immune disorders | 6892.1<br>(6384.1 to 7446.2) | -- | 182.9<br>(124.0 to 259.4) | 3.9<br>(-1.1 to 9.6) | -4.8<br>(-9.4 to 0.4) |
| Anemia due to endocrine, metabolic, blood, and immune disorders | 5389.9<br>(5105.7 to 5697.3) | -- | 127.8<br>(84.0 to 184.9) | 1.4<br>(-5.1 to 9.0) | -7.1*<br>(-13.1 to -0.1) |
| Endocrine, metabolic, blood, and immune disorders | 1498.3<br>(1093.7 to 1985.0) | -- | 54.7<br>(33.4 to 83.2) | 10.2*<br>(5.0 to 15.3) | 1.0<br>(-3.9 to 5.6) |
| Heart failure due to endocrine, metabolic, blood, and immune disorders | 3.9<br>(2.6 to 5.5) | -- | 0.4<br>(0.2 to 0.6) | 2.4*<br>(0.8 to 3.9) | -6.2*<br>(-7.6 to -4.8) |
| Oral disorders | 244459.6<br>(210321.7 to 281876.2) | 179744.4<br>(145224.8 to 212061.7) | 1339.7<br>(742.6 to 2309.0) | 11.3*<br>(10.6 to 12.1) | 2.0*<br>(1.3 to 2.7) |
| Caries of deciduous teeth | -- | -- | -- | -- | -- |
| Caries of permanent teeth | 182326.6<br>(142696.2 to 229105.1) | 170898.0<br>(137192.5 to 202438.8) | 129.4<br>(56.6 to 246.4) | 5.8*<br>(4.5 to 7.1) | -3.1*<br>(-4.3 to -1.9) |
| Periodontal disease | 86993.5<br>(63023.6 to 114466.6) | 8079.0<br>(4576.4 to 11376.2) | 575.4<br>(218.6 to 1268.4) | 12.1*<br>(10.8 to 13.3) | 2.7*<br>(1.5 to 3.8) |
| Edentulism and severe tooth loss | 10342.8<br>(8476.6 to 12863.0) | 767.4<br>(502.8 to 1111.6) | 294.7<br>(186.9 to 430.6) | 14.7*<br>(13.1 to 16.2) | 5.1*<br>(3.7 to 6.4) |
| Other oral disorders | 11764.6<br>(10353.5 to 13012.0) | -- | 340.2<br>(208.5 to 507.9) | 9.4*<br>(8.3 to 10.5) | 0.3<br>(-0.8 to 1.2) |
| **Injuries** | **127120.9<br>(117968.2 to 138251.1)** | **33930.2<br>(28866.8 to 39563.5)** | **4789.7<br>(3540.0 to 6303.0)** | **13.8*<br>(12.4 to 15.2)** | **4.2*<br>(3.0 to 5.5)** |
| **Transport injuries** | **19415.9<br>(17885.5 to 21212.2)** | **4786.6<br>(3357.8 to 6594.5)** | **1205.9<br>(869.2 to 1615.1)** | **8.7*<br>(7.6 to 9.8)** | **-0.4<br>(-1.5 to 0.6)** |
| Road injuries | 15267.1<br>(14083.4 to 16721.3) | 4301.2<br>(2894.1 to 6131.3) | 942.3<br>(674.8 to 1267.6) | 7.7*<br>(6.5 to 8.8) | -1.4*<br>(-2.4 to -0.3) |
| Pedestrian road injuries | 3915.1<br>(3317.5 to 4919.2) | 852.5<br>(482.0 to 1371.7) | 242.4<br>(171.6 to 333.4) | 4.0*<br>(2.4 to 5.6) | -4.7*<br>(-6.2 to -3.2) |
| Cyclist road injuries | 2484.8<br>(2034.4 to 3028.1) | 939.8<br>(512.6 to 1572.7) | 146.6<br>(99.6 to 201.6) | 12.8*<br>(10.8 to 14.7) | 3.3*<br>(1.5 to 5.0) |
| Motorcyclist road injuries | 4236.5<br>(3545.9 to 5031.8) | 814.4<br>(468.4 to 1284.2) | 261.6<br>(179.9 to 362.3) | 6.7*<br>(5.1 to 8.1) | -2.3*<br>(-3.8 to -0.9) |
| Motor vehicle road injuries | 3646.6<br>(3083.6 to 4468.2) | 1313.2<br>(815.9 to 2002.5) | 237.2<br>(165.1 to 325.8) | 5.5*<br>(4.1 to 7.1) | -3.3*<br>(-4.7 to -1.9) |
| Other road injuries | 984.0<br>(803.8 to 1232.8) | 381.2<br>(198.3 to 661.9) | 54.4<br>(38.2 to 75.9) | 29.7*<br>(27.2 to 32.1) | 18.9*<br>(16.5 to 21.0) |
| Other transport injuries | 4148.8<br>(3363.6 to 4988.4) | 485.5<br>(308.6 to 692.2) | 263.6<br>(186.8 to 357.1) | 12.5*<br>(10.7 to 14.3) | 3.1*<br>(1.5 to 4.7) |
| **Unintentional injuries** | **73999.8<br>(68297.6 to 81345.4)** | **26372.3<br>(21610.3 to 32013.0)** | **2842.4<br>(2042.6 to 3837.6)** | **18.0*<br>(16.9 to 19.1)** | **8.1*<br>(7.1 to 9.1)** |
| Falls | 29096.9<br>(24828.3 to 33886.7) | 9803.4<br>(6407.5 to 14350.1) | 1368.0<br>(978.6 to 1861.1) | 17.4*<br>(16.1 to 18.7) | 7.6*<br>(6.4 to 8.7) |
| Drowning | 168.7<br>(146.5 to 197.6) | 17.8<br>(12.9 to 25.3) | 10.4<br>(7.3 to 14.3) | 5.8*<br>(2.7 to 8.7) | -3.1*<br>(-5.9 to -0.4) |
| Fire, heat, and hot substances | 9134.2<br>(7697.1 to 10911.1) | 592.2<br>(347.2 to 855.4) | 304.1<br>(212.5 to 421.4) | 13.1*<br>(9.7 to 15.9) | 3.6*<br>(0.5 to 6.2) |
| Poisonings | 355.9<br>(299.5 to 417.3) | 197.8<br>(116.5 to 329.2) | 35.8<br>(23.7 to 50.3) | 26.2*<br>(22.9 to 29.9) | 15.6*<br>(12.5 to 19.0) |
| Poisoning by carbon monoxide | 69.5<br>(58.1 to 84.5) | 69.1<br>(30.4 to 128.5) | 6.0<br>(4.0 to 8.6) | 26.8*<br>(22.2 to 31.5) | 16.2*<br>(11.9 to 20.4) |
| Poisoning by other means | 286.5<br>(229.0 to 347.6) | 128.7<br>(68.7 to 223.1) | 29.8<br>(19.2 to 43.3) | 26.0*<br>(22.3 to 29.8) | 15.5*<br>(12.1 to 18.9) |
| Exposure to mechanical forces | 16540.5<br>(13978.9 to 19588.1) | 5045.8<br>(3119.0 to 7226.0) | 428.8<br>(295.2 to 609.9) | 18.3*<br>(17.0 to 19.5) | 8.4*<br>(7.2 to 9.5) |
| Unintentional firearm injuries | 753.0<br>(600.0 to 900.0) | 112.6<br>(49.0 to 206.0) | 29.2<br>(20.4 to 40.1) | 20.5*<br>(19.0 to 21.9) | 10.3*<br>(9.0 to 11.6) |
| Other exposure to mechanical forces | 15787.5<br>(13238.8 to 18890.4) | 4933.2<br>(3068.7 to 7078.3) | 399.6<br>(272.3 to 577.4) | 18.1*<br>(16.8 to 19.4) | 8.2*<br>(7.0 to 9.4) |
| Adverse effects of medical treatment | 168.3<br>(101.3 to 253.3) | 2199.1<br>(1411.9 to 3205.9) | 22.4<br>(12.0 to 38.2) | 20.4*<br>(15.0 to 25.7) | 10.3*<br>(5.3 to 15.1) |
| Animal contact | 3169.8<br>(2748.3 to 3619.4) | 3125.9<br>(1883.5 to 4710.4) | 85.4<br>(58.5 to 116.5) | 15.5*<br>(12.9 to 17.8) | 5.8*<br>(3.4 to 7.9) |
| Venomous animal contact | 1377.6<br>(1140.8 to 1644.4) | 1607.7<br>(934.2 to 2486.4) | 57.4<br>(38.3 to 80.6) | 18.0*<br>(14.5 to 21.2) | 8.1*<br>(4.9 to 11.0) |
| Non-venomous animal contact | 1792.2<br>(1483.5 to 2165.8) | 1518.2<br>(847.8 to 2404.4) | 28.0<br>(17.1 to 45.0) | 10.7*<br>(8.3 to 12.7) | 1.4<br>(-0.8 to 3.2) |
| Foreign body | 1529.5<br>(1290.8 to 1770.8) | 1594.1<br>(1009.4 to 2372.4) | 76.7<br>(54.9 to 106.0) | 13.6*<br>(11.9 to 15.3) | 4.1*<br>(2.5 to 5.6) |

| | | | | | |
|---|---|---|---|---|---|
| Pulmonary aspiration and foreign body in airway | 259.1 (207.6 to 325.6) | 82.6 (42.7 to 134.6) | 12.7 (8.8 to 17.8) | 9.8* (6.6 to 12.9) | 0.6 (-2.3 to 3.4) |
| Foreign body in eyes | 226.0 (112.2 to 397.5) | 1286.7 (730.5 to 2050.9) | 14.7 (7.2 to 27.3) | 14.5* (12.3 to 16.3) | 4.9* (2.9 to 6.6) |
| Foreign body in other body part | 1044.5 (839.5 to 1219.0) | 224.9 (129.5 to 347.4) | 49.3 (34.4 to 65.5) | 14.3* (12.4 to 16.3) | 4.7* (3.0 to 6.5) |
| Environmental heat and cold exposure | 2840.1 (2327.7 to 3427.9) | 483.9 (296.3 to 756.3) | 122.4 (85.3 to 165.7) | 16.9* (15.1 to 18.6) | 7.1* (5.4 to 8.6) |
| Exposure to forces of nature | 1630.4 (1197.9 to 2178.7) | 34.9 (23.6 to 49.5) | 71.7 (51.4 to 97.9) | 69.1* (50.3 to 92.0) | 54.9* (37.7 to 75.8) |
| Other unintentional injuries | 9365.5 (7702.8 to 11253.1) | 3277.5 (2007.5 to 5018.9) | 316.6 (217.6 to 443.2) | 18.7* (17.2 to 20.1) | 8.7* (7.4 to 10.0) |
| **Self-harm and interpersonal violence** | **33719.7 (26683.7 to 42000.5)** | **2771.3 (2124.7 to 3480.1)** | **741.5 (553.3 to 937.4)** | **7.0* (2.4 to 12.9)** | **-2.0 (-6.2 to 3.4)** |
| Self-harm | 750.6 (610.3 to 909.2) | 350.2 (234.8 to 496.6) | 41.7 (29.2 to 55.7) | -3.3* (-4.7 to -1.9) | -11.4* (-12.7 to -10.2) |
| Self-harm by firearm | 6.8 (5.7 to 8.2) | 1.8 (0.4 to 5.0) | 0.3 (0.2 to 0.4) | -2.3 (-5.0 to 0.5) | -10.5* (-13.0 to -8.0) |
| Self-harm by other specified means | 743.9 (603.6 to 901.9) | 348.4 (233.7 to 494.8) | 41.4 (29.0 to 55.3) | -3.3* (-4.7 to -1.9) | -11.5* (-12.7 to -10.2) |
| Interpersonal violence | 27460.6 (20460.0 to 35455.5) | 1772.7 (1161.3 to 2488.2) | 424.2 (314.2 to 557.0) | 5.3* (4.0 to 6.5) | -3.6* (-4.8 to -2.5) |
| Assault by firearm | 240.3 (191.8 to 299.4) | 36.4 (18.2 to 64.3) | 11.5 (8.0 to 15.8) | 6.5* (4.7 to 8.5) | -2.4* (-4.1 to -0.6) |
| Assault by sharp object | 1428.2 (1128.7 to 2032.4) | 287.8 (153.5 to 480.8) | 47.3 (32.8 to 67.4) | 1.3 (-0.7 to 3.4) | -7.2* (-9.0 to -5.2) |
| Sexual violence | 21854.6 (15059.5 to 30156.7) | -- | 188.3 (104.9 to 299.4) | 7.6* (5.9 to 9.2) | -1.5 (-3.0 to 0.0) |
| Assault by other means | 3937.4 (3182.3 to 4976.1) | 1448.5 (937.4 to 2050.3) | 177.1 (124.5 to 245.3) | 3.9* (2.5 to 5.3) | -4.9* (-6.1 to -3.6) |
| Conflict and terrorism | 5139.4 (3503.4 to 7397.2) | 528.5 (387.3 to 721.3) | 261.6 (174.1 to 388.7) | 14.9* (1.1 to 30.3) | 5.3 (-7.4 to 19.4) |
| Executions and police conflict | 369.8 (242.9 to 547.4) | 120.0 (79.3 to 176.3) | 13.9 (9.8 to 20.1) | -26.4* (-34.1 to -5.2) | -32.6* (-39.6 to -13.2) |
| **Nature of injury aggregates** | | | | | |
| Amputations | 32063.4 (29148.7 to 35714.4) | 664.8 (527.5 to 820.7) | 549.9 (370.3 to 815.0) | 10.7* (7.0 to 14.1) | 1.4 (-2.0 to 4.5) |
| Burns | 19145.3 (17239.5 to 21251.7) | 866.5 (672.7 to 1083.4) | 648.2 (462.4 to 874.1) | 11.9* (8.7 to 15.3) | 2.5 (-0.5 to 5.6) |
| Fractures | 25942.8 (24029.1 to 28315.5) | 8607.0 (6940.1 to 10596.9) | 1443.4 (1000.3 to 2043.3) | 14.0* (12.6 to 15.4) | 4.4* (3.1 to 5.7) |
| Head Injuries | 4075.9 (3858.3 to 4297.6) | 1458.9 (1162.2 to 1798.0) | 611.9 (431.8 to 831.4) | 15.6* (13.9 to 17.4) | 5.9* (4.4 to 7.6) |
| Spinal Injuries | 2141.9 (1935.4 to 2468.7) | 50.4 (38.3 to 64.9) | 649.7 (457.9 to 857.0) | 17.1* (13.9 to 21.0) | 7.3* (4.3 to 10.8) |
| Minor Injuries | 16189.1 (15075.6 to 17615.1) | 12201.4 (10000.1 to 14658.1) | 144.8 (68.8 to 277.0) | 15.4* (14.2 to 16.6) | 5.7* (4.6 to 6.8) |
| Other Injuries | 5723.2 (5358.2 to 6133.6) | 10081.3 (8605.7 to 11679.9) | 553.6 (392.6 to 748.6) | 14.2* (12.1 to 16.5) | 4.6* (2.7 to 6.7) |
| **Impairments** | | | | | |
| Anaemia | 112599.5 (106170.8 to 119708.8) | -- | 2990.4 (2023.3 to 4316.2) | -2.6 (-8.3 to 3.4) | -10.8* (-16.0 to -5.3) |
| Epilepsy | 3630.2 (2968.0 to 4349.1) | -- | 1452.8 (1092.7 to 1898.5) | 13.2* (7.5 to 18.9) | 3.7 (-1.6 to 8.9) |
| Guillain-Barré syndrome | 6.3 (3.6 to 10.5) | -- | 1.9 (0.9 to 3.4) | 17.6* (14.6 to 20.5) | 7.7* (4.9 to 10.4) |
| Hearing loss | 88300.3 (79855.2 to 97280.4) | -- | 1953.9 (1262.2 to 2909.8) | 8.4* (6.0 to 10.6) | -0.7 (-2.9 to 1.3) |
| Heart failure | 1015.7 (811.2 to 1250.6) | -- | 98.6 (63.7 to 140.2) | 6.6* (4.2 to 9.1) | -2.3* (-4.6 to -0.0) |
| Infertility | 25558.9 (16875.3 to 36230.4) | -- | 190.9 (95.5 to 351.6) | 11.2* (6.2 to 15.7) | 1.9 (-2.7 to 6.0) |
| Developmental intellectual disability | 10678.4 (8313.4 to 12948.8) | -- | 1489.0 (1155.7 to 1859.6) | 12.9* (9.9 to 16.3) | 3.5* (0.7 to 6.5) |
| Pelvic inflammatory disease | 161.2 (119.3 to 218.9) | -- | 21.2 (13.0 to 31.7) | 12.0* (6.6 to 17.9) | 2.6 (-2.3 to 8.0) |
| Vision loss | 100362.2 (92501.7 to 109046.9) | -- | 2025.9 (1340.2 to 3033.7) | 11.5* (10.4 to 12.6) | 2.1* (1.1 to 3.1) |

We did not calculate incidence for the nine impairments and certain NTDs. Blank cells mean that no estimate is available or that the estimate has a magnitude less than 50.
Table presents prevalence and incidence counts in thousands.
* Percentage changes that are statistically significant
‡ Incidence estimates for stroke represent first-ever stroke only.
^ Incidence of HIV/AIDS represents new infections of HIV only and does not include new infections of Tuberculosis in HIV positive cases.

**SR Table 10m: Global prevalence, incidence and YLDs for 2017, percentage change of YLD counts and percentage change of YLD rates between 2007 and 2017 by cause, age 45 to 49 years**

| Cause | Prevalence in thousands (95% UI) | Incidence in thousands (95% UI) | YLDs in thousands (95% UI) | | |
|---|---|---|---|---|---|
| | 2017 counts | 2017 counts | 2017 counts | Percentage change in counts between 2007 and 2017 | Percentage change in rates between 2007 and 2017 |
| **All causes** | **467076.4<br>(466196.2 to 467917.8)** | **2052447.8<br>(1750779.7 to 2458177.0)** | **65143.6<br>(48950.2 to 83976.3)** | **23.9*<br>(23.1 to 24.7)** | **-1.3*<br>(-1.9 to -0.6)** |
| **Communicable, maternal, neonatal, and nutritional disorders** | **311619.5<br>(287669.2 to 341501.4)** | **1408546.0<br>(1114041.0 to 1820893.0)** | **5832.1<br>(4372.7 to 7533.7)** | **18.1*<br>(13.2 to 22.7)** | **-5.9*<br>(-9.8 to -2.2)** |
| **HIV/AIDS and sexually transmitted infections** | **123584.4<br>(110060.6 to 137793.6)** | **51441.0<br>(40777.8 to 65478.7)** | **515.9<br>(340.5 to 748.4)** | **-1.8<br>(-20.9 to 21.6)** | **-21.7*<br>(-36.9 to -3.1)** |
| HIV/AIDS | 3689.4<br>(3315.9 to 4167.5) | 67.7^<br>(47.3 to 94.5) | 406.0<br>(261.4 to 597.4) | -7.3<br>(-25.6 to 19.7) | -26.1*<br>(-40.7 to -4.6) |
| HIV/AIDS - Drug-susceptible Tuberculosis | 91.5<br>(82.6 to 100.8) | 117.0<br>(104.1 to 131.7) | 34.6<br>(23.4 to 47.0) | 5.3<br>(-0.9 to 11.8) | -16.1*<br>(-21.0 to -10.9) |
| HIV/AIDS - Multidrug-resistant Tuberculosis without extensive drug resistance | 3.5<br>(2.3 to 5.2) | 4.7<br>(3.3 to 6.4) | 1.4<br>(0.8 to 2.3) | 3.8<br>(-31.0 to 55.8) | -17.2<br>(-45.0 to 24.2) |
| HIV/AIDS - Extensively drug-resistant Tuberculosis | 0.1<br>(0.1 to 0.2) | 0.1<br>(0.1 to 0.2) | 0.1<br>(0.0 to 0.1) | 88.1*<br>(23.1 to 210.9) | 50.0<br>(-1.9 to 147.8) |
| HIV/AIDS resulting in other diseases | 3594.2<br>(3222.8 to 4076.9) | 67.7<br>(47.3 to 94.5) | 369.9<br>(230.5 to 552.6) | -8.4<br>(-27.4 to 21.2) | -27.0*<br>(-42.1 to -3.4) |
| HIV/AIDS not on antiretroviral treatment without Tuberculosis | 909.6<br>(741.5 to 1143.3) | 67.7<br>(47.3 to 94.5) | 163.9<br>(84.9 to 292.0) | -54.9*<br>(-59.5 to -49.9) | -64.1*<br>(-67.8 to -60.1) |
| HIV/AIDS on antiretroviral treatment without Tuberculosis | 2684.7<br>(2409.8 to 2991.7) | -- | 206.0<br>(137.0 to 291.4) | 412.6*<br>(334.9 to 528.0) | 308.6*<br>(246.6 to 400.6) |
| Sexually transmitted infections excluding HIV | 121531.2<br>(107976.1 to 135783.5) | 51373.3<br>(40711.0 to 65419.5) | 109.9<br>(58.4 to 197.4) | 26.2*<br>(24.0 to 28.6) | 0.6<br>(-1.1 to 2.5) |
| Syphilis | 2339.6<br>(1640.6 to 3245.1) | 550.7<br>(344.1 to 781.2) | 7.8<br>(5.4 to 10.6) | 22.4*<br>(13.8 to 31.9) | -2.4<br>(-9.3 to 5.1) |
| Early syphilis | 2298.2<br>(1601.3 to 3203.0) | 550.7<br>(344.1 to 781.2) | 0.5<br>(0.1 to 1.5) | 26.6*<br>(15.7 to 37.4) | 1.0<br>(-7.8 to 9.5) |
| Tertiary syphilis | 41.3<br>(34.7 to 48.1) | -- | 7.3<br>(5.0 to 10.1) | 22.1*<br>(13.0 to 32.0) | -2.7<br>(-9.9 to 5.2) |
| Chlamydial infection | 8355.8<br>(5805.4 to 11471.3) | 12541.3<br>(8442.9 to 17434.6) | 22.6<br>(12.2 to 42.7) | 27.5*<br>(22.7 to 33.0) | 1.6<br>(-2.2 to 6.1) |
| Chlamydia episode | 7946.9<br>(5437.8 to 11061.5) | 12541.3<br>(8442.9 to 17434.6) | 13.1<br>(4.8 to 30.0) | 24.9*<br>(22.6 to 27.1) | -0.5<br>(-2.3 to 1.3) |
| Chlamydial infection complications | 408.9<br>(263.6 to 606.0) | -- | 9.6<br>(6.1 to 13.9) | 31.2*<br>(20.6 to 42.4) | 4.6<br>(-3.9 to 13.5) |
| Gonococcal infection | 1254.0<br>(741.9 to 2091.4) | 4950.4<br>(2736.6 to 8129.4) | 6.6<br>(3.5 to 12.4) | 26.1*<br>(17.8 to 35.9) | 0.5<br>(-6.1 to 8.3) |
| Gonococcal infection complications | 119.4<br>(79.6 to 170.7) | -- | 3.4<br>(2.0 to 5.4) | 32.2*<br>(18.1 to 47.4) | 5.4<br>(-5.9 to 17.5) |
| Gonorrhoea episode | 1134.6<br>(624.3 to 1968.5) | 4950.4<br>(2736.6 to 8129.4) | 3.1<br>(0.9 to 7.7) | 20.2*<br>(11.6 to 25.5) | -4.2<br>(-11.0 to 0.1) |
| Trichomoniasis | 13327.2<br>(8740.0 to 19704.2) | 29190.4<br>(20625.5 to 41333.6) | 22.0<br>(7.6 to 48.9) | 24.4*<br>(21.6 to 26.9) | -0.8<br>(-3.1 to 1.2) |
| Genital herpes | 103511.9<br>(90175.7 to 118253.6) | 4140.6<br>(2828.5 to 5528.5) | 25.9<br>(8.2 to 62.1) | 25.2*<br>(23.7 to 26.9) | -0.2<br>(-1.4 to 1.1) |
| Other sexually transmitted infections | 1093.0<br>(676.6 to 1637.6) | -- | 25.0<br>(15.5 to 41.4) | 29.2*<br>(23.9 to 34.7) | 3.0<br>(-1.3 to 7.3) |
| Other sexually transmitted diseases residual | -- | -- | 9.5<br>(4.8 to 18.8) | 26.3*<br>(21.4 to 32.5) | 0.7<br>(-3.3 to 5.6) |
| Other sexually transmitted diseases | 1093.0<br>(676.6 to 1637.6) | -- | 25.0<br>(15.5 to 41.4) | 29.2*<br>(23.9 to 34.7) | 3.0<br>(-1.3 to 7.3) |
| **Respiratory infections and tuberculosis** | **163856.8<br>(115519.8 to 227342.5)** | **966926.7<br>(675368.8 to 1383704.8)** | **667.0<br>(444.8 to 979.5)** | **20.9*<br>(18.7 to 22.8)** | **-3.7*<br>(-5.4 to -2.1)** |
| Tuberculosis | 152385.7<br>(102071.8 to 219382.6) | 544.2<br>(385.9 to 730.0) | 239.4<br>(148.3 to 364.7) | 18.0*<br>(13.5 to 22.4) | -6.0*<br>(-9.5 to -2.4) |
| Latent tuberculosis infection | 151601.2<br>(101359.3 to 218559.7) | -- | -- | -- | -- |
| Drug-susceptible tuberculosis | 746.2<br>(538.1 to 994.9) | 513.5<br>(363.6 to 690.0) | 227.5<br>(141.2 to 347.0) | 18.0*<br>(11.2 to 23.5) | -6.0*<br>(-11.3 to -1.5) |
| Multidrug-resistant tuberculosis without extensive drug resistance | 36.4<br>(16.1 to 74.7) | 28.8<br>(13.9 to 53.6) | 11.3<br>(4.7 to 23.4) | 16.5<br>(-44.5 to 107.2) | -7.1<br>(-55.7 to 65.1) |
| Extensively drug-resistant tuberculosis | 1.9<br>(1.0 to 3.6) | 1.9<br>(1.2 to 3.0) | 0.6<br>(0.3 to 1.3) | 55.1<br>(-6.8 to 173.1) | 23.7<br>(-25.7 to 117.7) |
| Lower respiratory infections | 350.3<br>(307.4 to 400.2) | 15857.3<br>(13789.8 to 18116.9) | 21.1<br>(14.0 to 30.0) | 29.6*<br>(20.2 to 38.8) | 3.3<br>(-4.2 to 10.7) |
| Guillain-Barré syndrome due to lower respiratory infections | 0.7<br>(0.3 to 1.3) | -- | 0.2<br>(0.1 to 0.4) | 26.4*<br>(23.3 to 29.3) | 0.8<br>(-1.7 to 3.1) |
| Lower respiratory infection episode | 349.6<br>(306.8 to 399.3) | 15857.3<br>(13789.8 to 18116.9) | 20.9<br>(13.9 to 29.8) | 29.7*<br>(20.1 to 39.0) | 3.4<br>(-4.2 to 10.8) |
| Upper respiratory infections | 13084.5<br>(8984.5 to 19013.3) | 946760.2<br>(653783.5 to 1363314.9) | 320.5<br>(176.4 to 542.4) | 24.0*<br>(22.4 to 25.6) | -1.2<br>(-2.4 to 0.1) |
| Guillain-Barré syndrome due to upper respiratory infections | 1.9<br>(1.1 to 3.0) | -- | 0.6<br>(0.3 to 1.0) | 26.4*<br>(23.4 to 29.4) | 0.8<br>(-1.6 to 3.1) |
| Upper respiratory infection episode | 13082.7<br>(8982.8 to 19011.9) | 946760.2<br>(653783.5 to 1363314.9) | 320.0<br>(176.0 to 541.6) | 24.0*<br>(22.4 to 25.6) | -1.2<br>(-2.4 to 0.1) |
| Otitis media | 4141.5<br>(3168.9 to 5268.6) | 3765.0<br>(2607.7 to 5072.6) | 85.9<br>(50.1 to 141.4) | 15.9*<br>(10.7 to 21.8) | -7.6*<br>(-11.8 to -2.9) |
| Acute otitis media | 207.5<br>(148.7 to 283.2) | 3764.4<br>(2606.8 to 5072.3) | 2.7<br>(1.3 to 5.2) | 22.7*<br>(15.8 to 29.9) | -2.2<br>(-7.7 to 3.6) |
| Chronic otitis media | 3934.0<br>(2977.5 to 5048.8) | 0.6<br>(0.0 to 2.5) | 83.3<br>(48.7 to 137.1) | 15.7*<br>(10.4 to 21.8) | -7.7*<br>(-12.0 to -2.9) |
| **Enteric infections** | **4443.6<br>(3924.1 to 4986.8)** | **303211.8<br>(259593.2 to 348252.1)** | **494.7<br>(343.5 to 696.3)** | **45.4*<br>(39.5 to 51.7)** | **15.9*<br>(11.2 to 20.9)** |
| Diarrhoeal diseases | 4464.7<br>(3945.1 to 5012.8) | 302928.6<br>(259289.4 to 347968.7) | 492.5<br>(341.8 to 693.4) | 45.7*<br>(39.9 to 52.0) | 16.2*<br>(11.5 to 21.2) |
| Guillain-Barré syndrome due to diarrheal diseases | 0.6<br>(0.3 to 1.0) | -- | 0.2<br>(0.1 to 0.3) | 26.4*<br>(23.3 to 29.4) | 0.8<br>(-1.7 to 3.1) |
| Diarrheal disease episode | 4464.1<br>(3944.6 to 5012.1) | 302928.6<br>(259289.4 to 347968.7) | 492.3<br>(341.7 to 693.2) | 45.7*<br>(39.9 to 52.0) | 16.2*<br>(11.5 to 21.2) |
| Typhoid and paratyphoid | 1.4<br>(0.5 to 2.6) | 269.0<br>(219.9 to 319.0) | 2.2<br>(1.3 to 3.2) | -5.0<br>(-21.7 to 14.9) | -24.3*<br>(-37.6 to -8.4) |
| Typhoid fever | 12.7<br>(9.6 to 16.2) | 201.0<br>(159.9 to 242.2) | 2.0<br>(1.2 to 2.9) | -4.0<br>(-22.7 to 19.2) | -23.5*<br>(-38.4 to -5.0) |
| Typhoid fever complications | 2.7<br>(1.9 to 3.7) | 34.6<br>(26.2 to 43.4) | 0.9<br>(0.5 to 1.3) | -4.1<br>(-29.8 to 29.1) | -23.5<br>(-44.0 to 2.9) |
| Typhoid fever episode | 10.0<br>(7.6 to 13.1) | 166.4<br>(132.4 to 200.6) | 1.1<br>(0.6 to 1.7) | -3.9<br>(-27.7 to 27.5) | -23.4<br>(-42.4 to 1.6) |
| Paratyphoid fever | 3.0<br>(2.1 to 4.1) | 68.0<br>(46.6 to 93.7) | 0.2<br>(0.1 to 0.3) | -14.8*<br>(-27.0 to -0.4) | -32.1*<br>(-41.8 to -20.6) |
| Intestinal perforation due to paratyphoid | 0.1<br>(0.1 to 0.2) | 3.5<br>(2.3 to 5.0) | -- | -14.5<br>(-33.3 to 7.6) | -31.9*<br>(-46.8 to -14.2) |
| Paratyphoid fever episode | 2.9<br>(2.0 to 3.9) | 64.5<br>(43.9 to 88.8) | 0.2<br>(0.1 to 0.3) | -14.9<br>(-27.5 to 1.2) | -32.1*<br>(-42.2 to -19.3) |
| iNTS | 0.5<br>(0.3 to 0.9) | 14.1<br>(8.4 to 23.0) | 0.1<br>(0.0 to 0.1) | -20.5*<br>(-25.8 to -15.0) | -36.6*<br>(-40.8 to -32.2) |
| Other intestinal infectious diseases | -- | -- | -- | -8.7<br>(-24.7 to 10.5) | -27.2*<br>(-40.0 to -11.9) |
| **Neglected tropical diseases and malaria** | **63662.9<br>(60493.4 to 67164.9)** | **11754.2<br>(9680.4 to 14410.6)** | **869.7<br>(607.6 to 1194.1)** | **-1.1<br>(-8.9 to 6.4)** | **-21.2*<br>(-27.4 to -15.2)** |
| Malaria | 3479.0<br>(3236.3 to 3718.6) | 3059.0<br>(2551.7 to 3600.1) | 33.0<br>(24.4 to 44.2) | -10.1<br>(-20.5 to 3.5) | -28.3*<br>(-36.6 to -17.5) |
| Malaria complications | 28.1<br>(25.5 to 31.0) | -- | 11.7<br>(9.0 to 14.5) | 36.6*<br>(22.2 to 53.3) | 8.9<br>(-2.6 to 22.2) |
| Malaria episode | 178.6<br>(116.6 to 248.9) | 3059.0<br>(2551.7 to 3600.1) | 6.1<br>(3.3 to 10.2) | -16.0*<br>(-24.9 to -6.5) | -33.1*<br>(-40.1 to -25.5) |

| | | | | | |
|---|---|---|---|---|---|
| Malaria parasitemia | 3272.3 (3029.1 to 3535.2) | -- | 15.2 (9.6 to 23.1) | -27.1* (-40.9 to -9.9) | -41.9* (-52.9 to -28.2) |
| Chagas disease | 544.6 (456.1 to 642.1) | 9.2 (6.2 to 12.8) | 4.6 (2.9 to 6.6) | -4.8 (-10.7 to 1.4) | -24.1* (-28.8 to -19.2) |
| Acute Chagas disease | 0.1 (0.0 to 0.1) | 9.2 (6.2 to 12.8) | -- | 0.7 (-4.0 to 5.0) | -19.7* (-23.5 to -16.3) |
| Asymptomatic Chagas disease | 464.8 (387.9 to 547.3) | -- | -- | -- | -- |
| Symptomatic chronic Chagas infection | 79.7 (61.8 to 99.9) | -- | 4.6 (2.9 to 6.6) | -4.8 (-10.7 to 1.3) | -24.1* (-28.8 to -19.2) |
| Leishmaniasis | 350.4 (255.0 to 484.4) | 29.0 (20.2 to 39.9) | 21.9 (13.0 to 34.8) | 116.1* (85.8 to 146.4) | 72.2* (48.1 to 96.4) |
| Visceral leishmaniasis | 0.2 (0.2 to 0.3) | 0.8 (0.6 to 1.1) | -- | -70.9* (-77.2 to -59.2) | -76.8* (-81.9 to -67.5) |
| Cutaneous and mucocutaneous leishmaniasis | 351.9 (258.5 to 485.7) | 28.3 (19.3 to 39.1) | 21.9 (13.0 to 34.8) | 117.0* (86.6 to 147.7) | 73.0* (48.8 to 97.4) |
| African trypanosomiasis | 0.3 (0.0 to 1.1) | 0.2 (0.0 to 0.6) | 0.1 (0.0 to 0.3) | -78.1 (-96.9 to 41.4) | -82.5 (-97.5 to 12.7) |
| Trypanosomiasis Gambiense | 0.2 (0.0 to 1.1) | 0.2 (0.0 to 0.6) | 0.1 (0.0 to 0.3) | -77.3 (-97.1 to 53.6) | -81.9 (-97.7 to 22.4) |
| Trypanosomiasis Rhodesiense | -- | -- | -- | -91.0* (-99.1 to -38.3) | -92.9* (-99.3 to -50.8) |
| Schistosomiasis | 10454.3 (9405.2 to 11511.8) | -- | 77.9 (38.4 to 146.7) | -15.9* (-19.0 to -11.7) | -33.0* (-35.4 to -29.6) |
| Mild schistosomiasis | 8837.8 (7897.4 to 9825.3) | -- | 49.4 (20.1 to 104.8) | -18.3* (-20.4 to -16.3) | -34.9* (-36.6 to -33.3) |
| Anemia due to schistosomiasis | 549.0 (464.6 to 653.7) | -- | 12.7 (8.0 to 19.8) | -22.1* (-35.8 to -4.8) | -37.9* (-48.8 to -24.1) |
| Schistosomiasis complications | 1069.9 (839.5 to 1351.6) | -- | 15.9 (9.2 to 26.8) | -0.3 (-7.7 to 8.3) | -20.5* (-26.4 to -13.7) |
| Cysticercosis | 440.0 (314.5 to 581.6) | -- | 128.5 (74.8 to 196.4) | 8.5* (0.8 to 15.8) | -13.5* (-19.6 to -7.7) |
| Cystic echinococcosis | 44.3 (22.6 to 90.2) | 10.0 (5.6 to 18.4) | 3.6 (1.7 to 7.7) | 14.2* (2.4 to 24.0) | -9.0* (-18.4 to -1.2) |
| Lymphatic filariasis | 4426.4 (3953.3 to 5003.2) | -- | 132.4 (73.5 to 208.0) | -35.3* (-47.1 to -24.3) | -48.5* (-57.9 to -39.6) |
| Prevalence of detectable microfiliaria due to lymphatic filariasis | 3212.6 (2938.2 to 3531.6) | -- | -- | -- | -- |
| Lymphatic filariasis complications | 1213.8 (825.0 to 1695.9) | -- | 132.4 (73.5 to 208.0) | -35.3* (-47.1 to -24.3) | -48.5* (-57.9 to -39.6) |
| Onchocerciasis | 1091.5 (681.9 to 1931.9) | -- | 78.5 (39.3 to 141.8) | -0.7 (-20.1 to 17.2) | -20.8* (-36.3 to -6.6) |
| Asymptomatic onchocerciasis | 246.1 (0.0 to 1025.0) | -- | -- | -- | -- |
| Skin disease due to onchocerciasis | 723.8 (514.6 to 971.7) | -- | 67.7 (29.3 to 131.8) | -1.4 (-25.5 to 17.8) | -21.4* (-40.6 to -6.1) |
| Vision loss due to onchocerciasis | 121.5 (85.1 to 184.8) | -- | 10.8 (6.7 to 16.2) | 4.2 (-11.5 to 22.2) | -16.9* (-29.4 to -2.6) |
| Trachoma | 80.0 (44.2 to 164.9) | -- | 10.6 (6.1 to 17.7) | -19.6 (-37.8 to 1.4) | -36.0* (-50.4 to -19.2) |
| Dengue | 288.8 (157.5 to 488.9) | 4827.4 (2938.3 to 7318.7) | 46.1 (20.5 to 85.9) | 79.9* (55.5 to 182.5) | 43.4* (24.0 to 125.2) |
| Post-dengue chronic fatigue syndrome | 203.6 (95.2 to 372.3) | -- | 41.1 (17.2 to 78.6) | 79.9* (54.3 to 182.8) | 43.4* (23.0 to 125.4) |
| Dengue episode | 85.2 (51.5 to 127.8) | 4827.4 (2938.3 to 7318.7) | 5.0 (2.6 to 8.7) | 79.7* (54.9 to 171.3) | 43.3* (23.5 to 116.3) |
| Yellow fever | 0.1 (0.0 to 0.2) | 3.0 (0.8 to 7.9) | -- | -15.3 (-39.3 to 19.0) | -32.5* (-51.6 to -5.2) |
| Asymptomatic yellow fever | 0.0 (0.0 to 0.1) | 1.7 (0.4 to 4.6) | -- | -- | -- |
| Yellow fever episode | 0.0 (0.0 to 0.1) | 1.3 (0.4 to 3.4) | -- | -15.3 (-39.3 to 19.0) | -32.5* (-51.6 to -5.2) |
| Rabies | 0.0 (0.0 to 0.1) | 1.0 (0.8 to 1.3) | -- | -22.1* (-41.4 to -1.0) | -37.9* (-53.3 to -21.1) |
| Intestinal nematode infections | 39072.6 (36157.1 to 42529.9) | -- | 70.4 (41.0 to 115.7) | -18.6* (-23.8 to -13.2) | -35.2* (-39.3 to -30.8) |
| Ascariasis | 17644.3 (15382.1 to 20274.0) | -- | 21.7 (11.6 to 37.3) | -25.3* (-33.4 to -16.5) | -40.5* (-46.9 to -33.5) |
| Asymptomatic ascariasis | 16455.2 (14327.1 to 18950.6) | -- | -- | -- | -- |
| Ascariasis complications | 1189.1 (1050.7 to 1348.8) | -- | 21.7 (11.6 to 37.3) | -25.3* (-33.4 to -16.5) | -40.5* (-46.9 to -33.5) |
| Trichuriasis | 12422.0 (10567.1 to 14730.9) | -- | 7.9 (4.4 to 13.2) | -7.1 (-16.1 to 2.8) | -26.0* (-33.1 to -18.1) |
| Asymptomatic trichuriasis | 12014.0 (10194.5 to 14296.7) | -- | -- | -- | -- |
| Trichuriasis complications | 408.1 (369.1 to 446.7) | -- | 7.9 (4.4 to 13.2) | -7.1 (-16.1 to 2.8) | -26.0* (-33.1 to -18.1) |
| Hookworm disease | 11177.4 (10165.3 to 12287.8) | -- | 40.8 (24.2 to 65.1) | -16.7* (-24.2 to -8.3) | -33.6* (-39.6 to -26.9) |
| Asymptomatic hookworm disease | 9214.8 (8365.3 to 10144.2) | -- | -- | -- | -- |
| Anemia due to hookworm disease | 520.9 (451.0 to 619.0) | -- | 11.1 (7.1 to 16.5) | -20.9* (-32.6 to -6.9) | -36.9* (-46.2 to -25.8) |
| Hookworm disease complications | 1441.8 (1320.1 to 1580.2) | -- | 29.7 (16.5 to 49.5) | -15.0* (-22.1 to -7.1) | -32.3* (-37.9 to -25.9) |
| Food-borne trematodiases | 8265.1 (7258.8 to 9505.6) | 3655.7 (2799.9 to 4551.1) | 212.6 (113.2 to 376.6) | 34.7* (25.7 to 43.2) | 7.4* (0.2 to 14.2) |
| Asymptomatic food-borne tremodiases | 6357.7 (5174.6 to 7542.0) | 2616.3 (1287.7 to 3755.2) | -- | -- | -- |
| Food-borne tremodiases complications | 1907.4 (1195.8 to 3032.6) | 1039.4 (227.2 to 2342.1) | 212.6 (113.2 to 376.6) | 34.7* (25.7 to 43.2) | 7.4* (0.2 to 14.2) |
| Leprosy | 48.8 (45.3 to 53.0) | 4.2 (3.9 to 4.6) | 3.0 (2.0 to 4.3) | 1.9 (-7.2 to 11.6) | -18.7* (-26.0 to -11.1) |
| Ebola virus disease | -- | -- | -- | -96.1* (-96.6 to -95.3) | -96.9* (-97.3 to -96.3) |
| Ebola cases | -- | -- | -- | -98.2* (-98.4 to -97.9) | -98.6* (-98.7 to -98.4) |
| Post-Ebola chronic fatigue syndrome | -- | -- | -- | -95.8* (-96.2 to -95.0) | -96.7* (-97.0 to -96.0) |
| Zika virus disease | 2.6 (1.5 to 4.8) | 155.4 (90.2 to 293.4) | 0.1 (0.0 to 0.1) | -- | -- |
| Zika virus complications | -- | -- | -- | -- | -- |
| Zika virus episode | 2.6 (1.5 to 4.8) | 155.4 (90.2 to 293.4) | 0.0 (0.0 to 0.1) | -- | -- |
| Guinea worm disease | -- | -- | -- | -99.5* (-99.7 to -99.3) | -99.6* (-99.7 to -99.4) |
| Other neglected tropical diseases | 2264.7 (2101.6 to 2444.1) | -- | 46.4 (30.1 to 68.2) | 6.8 (-2.9 to 17.3) | -14.8* (-22.6 to -6.5) |
| Acute infection due to other neglected tropical diseases | -- | -- | 0.5 (0.3 to 0.9) | 112.9* (83.5 to 218.0) | 69.7* (46.3 to 153.5) |
| Anemia due to other neglected tropical diseases | 2264.7 (2101.6 to 2444.1) | -- | 45.9 (29.9 to 67.6) | 6.2 (-3.6 to 16.9) | -15.3* (-23.2 to -6.8) |
| **Other infectious diseases** | **5120.4 (4600.8 to 5833.5)** | **15458.1 (11783.1 to 20769.7)** | **209.9 (146.8 to 287.4)** | **15.0* (6.9 to 24.2)** | **-8.3* (-14.8 to -1.0)** |
| Meningitis | 569.9 (480.3 to 671.4) | 141.3 (97.1 to 192.0) | 45.0 (31.5 to 60.2) | 2.7 (-1.8 to 7.6) | -18.1* (-21.7 to -14.2) |
| Pneumococcal meningitis | 204.7 (168.5 to 247.8) | 8.9 (5.6 to 13.8) | 16.8 (11.6 to 22.6) | -18.4* (-23.8 to -12.3) | -34.9* (-39.3 to -30.1) |

| | | | | | |
|---|---|---|---|---|---|
| Acute pneumococcal meningitis | 0.4 (0.2 to 0.6) | 8.9 (5.6 to 13.8) | 0.1 (0.0 to 0.1) | -14.9* (-25.2 to -6.0) | -32.2* (-40.4 to -25.1) |
| Pneumococcal meningitis complications | 204.3 (168.1 to 247.5) | -- | 16.8 (11.6 to 22.5) | -18.4* (-23.9 to -12.3) | -35.0* (-39.3 to -30.1) |
| H influenzae type B meningitis | 44.3 (31.6 to 59.8) | 2.9 (1.6 to 4.8) | 3.3 (2.3 to 4.5) | -48.3* (-51.4 to -45.3) | -58.8* (-61.3 to -56.4) |
| Acute H influenzae type B meningitis | 0.1 (0.1 to 0.2) | 2.9 (1.6 to 4.8) | -- | -50.4* (-56.8 to -44.7) | -60.5* (-65.6 to -56.0) |
| H Influenza type B meningitis complications | 44.2 (31.5 to 59.7) | -- | 3.3 (2.3 to 4.5) | -48.3* (-51.4 to -45.3) | -58.8* (-61.3 to -56.4) |
| Meningococcal infection | 59.4 (42.2 to 78.4) | 8.0 (4.6 to 12.6) | 5.1 (3.5 to 6.9) | 5.1* (1.4 to 9.0) | -16.2* (-19.1 to -13.1) |
| Acute meningococcal meningitis | 0.4 (0.2 to 0.5) | 8.0 (4.6 to 12.6) | 0.0 (0.0 to 0.1) | 18.1* (5.0 to 29.1) | -5.8 (-16.3 to 2.9) |
| Meningococcal meningitis complications | 59.0 (41.9 to 78.1) | -- | 5.1 (3.4 to 6.8) | 5.0* (1.3 to 9.1) | -16.3* (-19.2 to -13.1) |
| Other meningitis | 261.4 (193.5 to 331.7) | 121.4 (83.6 to 165.5) | 19.7 (13.7 to 26.8) | 66.0* (56.1 to 77.3) | 32.3* (24.4 to 41.3) |
| Other acute bacterial meningitis | 1.0 (0.7 to 1.5) | 23.6 (15.9 to 32.8) | 0.1 (0.1 to 0.2) | 65.6* (50.2 to 79.8) | 32.0* (19.7 to 43.4) |
| Acute viral meningitis | 4.3 (3.0 to 6.1) | 97.8 (67.3 to 133.0) | 0.6 (0.3 to 0.9) | 16.7* (6.0 to 25.5) | -7.0 (-15.5 to 0.0) |
| Other bacterial meningitis complications | 256.1 (186.8 to 326.1) | -- | 19.0 (13.1 to 25.7) | 68.1* (57.7 to 80.3) | 34.0* (25.7 to 43.8) |
| Encephalitis | 524.3 (290.6 to 841.8) | 101.1 (96.8 to 105.4) | 39.1 (27.2 to 51.6) | 22.4* (15.6 to 29.9) | -2.5 (-7.8 to 3.5) |
| Acute encephalitis | 5.4 (5.2 to 5.6) | 101.1 (96.8 to 105.4) | 0.7 (0.5 to 1.0) | 27.8* (27.3 to 28.4) | 1.9* (1.5 to 2.3) |
| Encephalitis complications | 518.9 (285.2 to 836.5) | -- | 38.4 (26.6 to 50.7) | 22.3* (15.4 to 29.9) | -2.5 (-8.0 to 3.6) |
| Diphtheria | -- | 0.4 (0.3 to 0.5) | -- | -24.3* (-42.4 to -0.1) | -39.6* (-54.1 to -20.4) |
| Whooping cough | 2.0 (1.6 to 2.5) | 14.7 (11.4 to 18.6) | 0.1 (0.1 to 0.2) | 6.1* (5.4 to 7.0) | -15.4* (-16.0 to -14.7) |
| Tetanus | 3.0 (2.8 to 3.1) | 3.6 (1.8 to 5.3) | 0.1 (0.0 to 0.1) | -9.8 (-22.5 to 5.5) | -28.1* (-38.2 to -15.9) |
| Severe tetanus | 0.2 (0.1 to 0.3) | 3.6 (1.8 to 5.3) | -- | -41.1* (-52.8 to -19.4) | -53.1* (-62.4 to -35.8) |
| Neonatal tetanus complications | 2.8 (2.7 to 2.9) | -- | 0.1 (0.0 to 0.1) | 23.1* (21.0 to 24.8) | -1.9* (-3.5 to -0.5) |
| Measles | 0.4 (0.1 to 0.8) | 13.3 (4.8 to 29.8) | 0.0 (0.0 to 0.1) | -17.6* (-25.6 to -9.3) | -34.3* (-40.7 to -27.7) |
| Varicella and herpes zoster | 406.5 (312.8 to 511.9) | 2518.6 (1995.0 to 3056.3) | 22.1 (12.5 to 35.9) | 28.3* (22.5 to 34.0) | 2.2 (-2.4 to 6.8) |
| Chickenpox | 4.7 (2.1 to 8.6) | 243.9 (107.9 to 451.3) | 0.0 (0.0 to 0.1) | 35.7* (31.9 to 39.6) | 8.1* (5.2 to 11.3) |
| Herpes zoster | 401.8 (309.2 to 506.8) | 2274.6 (1765.9 to 2834.2) | 22.1 (12.5 to 35.9) | 28.3* (22.5 to 33.9) | 2.2 (-2.4 to 6.8) |
| Acute hepatitis | 1371.9 (946.5 to 1985.8) | 12665.1 (8964.5 to 17997.7) | 34.5 (20.2 to 55.4) | 23.8 (-17.7 to 81.3) | -1.3 (-34.4 to 44.5) |
| Acute hepatitis A | 150.6 (124.1 to 173.8) | 1957.8 (1612.9 to 2259.4) | 6.3 (3.9 to 9.4) | 28.5* (17.0 to 40.6) | 2.5 (-6.7 to 12.0) |
| Acute hepatitis B | 1172.0 (756.3 to 1778.8) | 10157.4 (6554.9 to 15416.4) | 27.0 (14.7 to 46.1) | 22.9 (-28.2 to 100.8) | -2.1 (-42.7 to 60.1) |
| Acute hepatitis C | 21.0 (7.3 to 41.9) | 182.2 (63.6 to 363.2) | 0.3 (0.1 to 0.7) | 9.1 (-0.1 to 13.5) | -13.0* (-20.4 to -9.5) |
| Acute hepatitis E | 28.3 (19.7 to 39.7) | 367.6 (256.1 to 516.3) | 0.9 (0.5 to 1.5) | 27.1* (6.1 to 48.7) | 1.3 (-15.4 to 18.6) |
| Other unspecified infectious diseases | 2836.9 (2655.0 to 3026.2) | -- | 69.0 (45.3 to 102.2) | 12.2* (4.5 to 20.3) | -10.6* (-16.7 to -4.1) |
| Guillain-Barré syndrome due to other infectious diseases | 0.4 (0.2 to 0.7) | -- | 0.1 (0.1 to 0.2) | 26.4* (23.4 to 29.3) | 0.8 (-1.7 to 3.0) |
| Other infectious diseases | -- | -- | 12.1 (7.0 to 19.6) | 29.2* (24.6 to 34.3) | 3.0 (-0.7 to 7.0) |
| Anemia due to other infectious diseases | 2836.5 (2654.6 to 3026.0) | -- | 56.7 (37.2 to 83.7) | 9.1* (0.1 to 18.6) | -13.0* (-20.2 to -5.5) |
| **Maternal and neonatal disorders** | **8404.2 (7307.6 to 9623.3)** | **1334.0 (1119.8 to 1559.8)** | **1600.8 (1189.2 to 2065.2)** | **44.6* (36.4 to 54.3)** | **15.2* (8.7 to 23.0)** |
| Maternal disorders | 413.0 (373.1 to 456.5) | 1334.0 (1119.8 to 1559.8) | 30.3 (21.4 to 40.3) | -3.3 (-11.1 to 5.4) | -22.9* (-29.2 to -16.0) |
| Maternal haemorrhage | 15.1 (13.4 to 17.0) | 73.6 (45.5 to 105.7) | 0.8 (0.5 to 1.1) | -9.4 (-24.0 to 7.7) | -27.8* (-39.4 to -14.1) |
| Maternal hemorrhage complications | 13.5 (12.1 to 15.1) | -- | 0.5 (0.3 to 0.7) | -17.6* (-30.4 to -3.3) | -34.3* (-44.5 to -22.9) |
| Maternal hemorrhage episode | 1.6 (0.9 to 2.7) | 73.6 (45.5 to 105.7) | 0.3 (0.1 to 0.5) | 10.6 (-23.4 to 59.3) | -11.8 (-39.0 to 27.0) |
| Maternal sepsis and other pregnancy related infections | 325.0 (289.8 to 364.6) | 188.3 (134.5 to 245.7) | 2.3 (1.1 to 4.3) | 21.6 (-1.8 to 47.4) | -3.1 (-21.7 to 17.5) |
| Infertility due to puerperal sepsis | 310.8 (275.4 to 349.3) | 2.1 (1.3 to 3.1) | 1.5 (0.6 to 3.3) | 25.1* (20.2 to 30.3) | -0.3 (-4.2 to 3.9) |
| Maternal sepsis and other maternal infection episode | 14.2 (7.6 to 24.1) | 186.3 (132.2 to 243.2) | 0.7 (0.3 to 1.3) | 14.6 (-37.9 to 96.4) | -8.7 (-50.5 to 56.5) |
| Maternal hypertensive disorders | 27.3 (15.0 to 43.5) | 178.2 (136.8 to 219.8) | 1.4 (0.7 to 2.6) | 21.7 (-17.0 to 73.1) | -3.0 (-33.9 to 38.0) |
| Maternal hypertensive disorder complications | 1.9 (1.7 to 2.2) | 24.0 (21.6 to 26.7) | 0.1 (0.1 to 0.2) | 33.7* (30.9 to 37.0) | 6.6* (4.3 to 9.2) |
| Maternal hypertensive disorder episode | 25.4 (13.0 to 41.5) | 154.2 (112.9 to 195.6) | 1.3 (0.6 to 2.4) | 20.6 (-21.9 to 76.4) | -3.9 (-37.7 to 40.6) |
| Maternal obstructed labour and uterine rupture | 64.5 (54.2 to 76.4) | 43.9 (29.4 to 63.3) | 20.4 (13.6 to 28.7) | -8.5 (-17.2 to 1.2) | -27.1* (-34.0 to -19.4) |
| Obstructed labor, acute event | 0.6 (0.3 to 0.9) | 42.5 (28.1 to 62.1) | 0.2 (0.1 to 0.3) | 11.2 (-33.6 to 76.4) | -11.4 (-47.1 to 40.6) |
| Maternal obstructed labor complications | 63.9 (53.5 to 75.8) | 1.4 (0.9 to 1.9) | 20.2 (13.4 to 28.4) | -8.7 (-17.5 to 1.2) | -27.2* (-34.3 to -19.3) |
| Maternal abortion and miscarriage | 2.8 (1.7 to 4.5) | 346.8 (236.0 to 469.8) | 0.3 (0.2 to 0.6) | 11.2 (-29.3 to 67.1) | -11.3 (-43.6 to 33.2) |
| Ectopic pregnancy | 4.1 (2.4 to 6.1) | 503.2 (343.1 to 671.9) | 0.4 (0.2 to 0.7) | 26.0 (-29.8 to 121.4) | 0.4 (-44.0 to 76.4) |
| Other maternal disorders | -- | -- | 4.6 (3.3 to 6.2) | 3.8 (-4.8 to 13.2) | -17.3* (-24.1 to -9.8) |
| Neonatal disorders | 7997.5 (6897.0 to 9215.4) | -- | 1570.5 (1164.8 to 2025.3) | 46.0* (37.6 to 56.4) | 16.3* (9.7 to 24.6) |
| Neonatal preterm birth | 4507.2 (3892.0 to 5315.8) | -- | 683.1 (478.5 to 928.8) | 51.4* (30.8 to 72.5) | 20.6* (4.3 to 37.5) |
| Preterm birth complications | 3841.0 (3223.5 to 4592.8) | -- | 644.4 (443.2 to 886.1) | 53.9* (31.6 to 76.2) | 22.7* (4.9 to 40.5) |
| Retinopathy of prematurity | 666.2 (538.0 to 799.0) | -- | 38.7 (25.7 to 53.9) | 18.3* (12.4 to 25.4) | -5.7* (-10.4 to -0.0) |
| Uncomplicated preterm birth | -- | -- | -- | -- | -- |
| Neonatal encephalopathy due to birth asphyxia and trauma | 3379.9 (2503.5 to 4510.0) | -- | 596.9 (362.4 to 918.8) | 48.7* (42.5 to 55.9) | 18.6* (13.6 to 24.2) |
| Neonatal sepsis and other neonatal infections | 829.6 (512.9 to 1257.4) | -- | 246.5 (133.0 to 409.5) | 28.4* (23.1 to 33.7) | 2.3 (-1.9 to 6.6) |
| Neonatal sepsis and other neonatal infection complications | 829.6 (512.9 to 1257.4) | -- | 246.5 (133.0 to 409.5) | 28.4* (23.1 to 33.7) | 2.3 (-1.9 to 6.6) |
| Neonatal sepsis and other neonatal infection episode | -- | -- | -- | -- | -- |
| Hemolytic disease and other neonatal jaundice | 117.7 (101.6 to 134.3) | -- | 44.0 (33.9 to 55.6) | 40.3* (35.3 to 45.2) | 11.9* (7.9 to 15.8) |

| | | | | | |
|---|---|---|---|---|---|
| Other neonatal disorders | -- | -- | -- | -- | -- |
| **Nutritional deficiencies** | **94872.6 (90556.3 to 99125.0)** | **58298.4 (52749.7 to 64103.4)** | **1474.2 (981.7 to 2164.0)** | **9.1* (2.0 to 16.8)** | **-13.1* (-18.7 to -6.9)** |
| Protein-energy malnutrition | 3667.8 (3448.6 to 3891.7) | 3871.3 (3602.4 to 4132.4) | 23.9 (15.5 to 33.6) | 36.8* (25.5 to 48.0) | 9.0* (0.0 to 18.0) |
| Iodine deficiency | 7869.6 (7122.8 to 8738.7) | 48.3 (40.4 to 57.7) | 123.8 (73.7 to 201.2) | 9.4* (4.9 to 13.2) | -12.8* (-16.4 to -9.8) |
| Visible goiter without symptoms | 7737.8 (6988.2 to 8605.9) | 48.3 (40.4 to 57.7) | 82.1 (39.9 to 154.3) | 11.8* (10.2 to 13.4) | -10.9* (-12.2 to -9.6) |
| Visible goiter with complications | 131.8 (66.6 to 193.3) | -- | 41.7 (18.9 to 66.2) | 5.0 (-11.8 to 14.5) | -16.3* (-29.7 to -8.7) |
| Vitamin A deficiency | 33860.5 (30831.0 to 37038.5) | 54378.9 (48806.5 to 60178.4) | 10.3 (6.3 to 15.7) | 33.9* (23.7 to 44.9) | 6.7 (-1.4 to 15.5) |
| Asymptomatic vitamin A deficiency | 33706.4 (30663.8 to 36871.3) | 54378.9 (48806.5 to 60178.4) | -- | -- | -- |
| Vitamin A deficiency complications | 154.0 (108.5 to 216.8) | -- | 10.3 (6.3 to 15.7) | 33.9* (23.7 to 44.9) | 6.7 (-1.4 to 15.5) |
| Vitamin A deficiency with anemia | -- | -- | -- | -- | -- |
| Dietary iron deficiency | 61458.2 (57524.1 to 65567.5) | -- | 1305.7 (857.8 to 1912.3) | 8.3* (0.6 to 16.9) | -13.7* (-19.8 to -6.8) |
| Other nutritional deficiencies | -- | -- | 10.3 (6.7 to 14.5) | 34.8* (23.7 to 45.9) | 7.5 (-1.4 to 16.3) |
| **Non-communicable diseases** | **461702.6 (459708.7 to 463431.5)** | **610904.6 (562280.9 to 659746.1)** | **54269.8 (40598.3 to 69948.9)** | **24.3* (23.5 to 25.1)** | **-0.9* (-1.5 to -0.2)** |
| **Neoplasms** | **7191.2 (6981.6 to 7415.7)** | **1294.6 (1137.4 to 1638.3)** | **515.3 (369.3 to 684.0)** | **35.7* (31.9 to 39.6)** | **8.2* (5.1 to 11.2)** |
| Lip and oral cavity cancer | 126.3 (119.4 to 133.7) | 28.3 (26.6 to 30.0) | 12.4 (8.9 to 16.2) | 25.8* (16.3 to 35.8) | 0.3 (-7.3 to 8.2) |
| Diagnosis and primary therapy phase of mouth cancer | 7.1 (6.7 to 7.5) | 28.3 (26.6 to 30.0) | 2.0 (1.4 to 2.8) | 25.6* (16.2 to 35.4) | 0.1 (-7.4 to 7.9) |
| Controlled phase of mouth cancer | 107.6 (101.8 to 113.8) | -- | 5.0 (3.2 to 7.4) | 27.0* (16.1 to 39.3) | 1.2 (-7.5 to 11.1) |
| Metastatic phase of mouth cancer | 10.0 (9.4 to 10.7) | -- | 4.5 (3.0 to 6.0) | 24.6* (9.3 to 41.8) | -0.7 (-12.9 to 13.0) |
| Terminal phase of mouth cancer | 1.5 (1.4 to 1.6) | -- | 0.8 (0.6 to 1.1) | 25.8* (17.4 to 34.0) | 0.3 (-6.4 to 6.8) |
| Nasopharynx cancer | 74.5 (64.5 to 84.7) | 14.2 (12.4 to 16.1) | 7.2 (5.1 to 9.8) | 47.2* (26.4 to 70.9) | 17.3* (0.7 to 36.2) |
| Diagnosis and primary therapy phase of nasopharynx cancer | 4.3 (3.7 to 4.9) | 14.2 (12.4 to 16.1) | 1.2 (0.8 to 1.8) | 51.3* (29.5 to 75.7) | 20.6* (3.2 to 40.0) |
| Controlled phase of nasopharynx cancer | 63.8 (55.3 to 72.6) | -- | 3.0 (1.9 to 4.5) | 51.8* (28.1 to 80.8) | 21.0* (2.1 to 44.1) |
| Metastatic phase of nasopharynx cancer | 5.7 (5.0 to 6.5) | -- | 2.6 (1.7 to 3.5) | 41.7* (15.9 to 72.8) | 13.0 (-7.6 to 37.8) |
| Terminal phase of nasopharynx cancer | 0.7 (0.6 to 0.7) | -- | 0.4 (0.2 to 0.5) | 36.9* (21.5 to 54.4) | 9.1 (-3.2 to 23.1) |
| Other pharynx cancer | 37.2 (33.5 to 40.0) | 14.3 (12.7 to 15.5) | 4.9 (3.4 to 6.5) | 24.1* (13.2 to 33.7) | -1.1 (-9.8 to 6.5) |
| Diagnosis and primary therapy phase of other pharynx cancer | 2.0 (1.8 to 2.2) | 14.3 (12.7 to 15.5) | 0.6 (0.4 to 0.8) | 22.7* (13.6 to 30.2) | -2.2 (-9.4 to 3.8) |
| Controlled phase of other pharynx cancer | 28.9 (26.0 to 31.0) | -- | 1.4 (0.9 to 2.0) | 22.9* (7.9 to 39.0) | -2.0 (-14.0 to 10.8) |
| Metastatic phase of other pharynx cancer | 5.4 (4.8 to 5.8) | -- | 2.4 (1.6 to 3.2) | 24.8* (13.0 to 34.8) | -0.5 (-10.0 to 7.4) |
| Terminal phase of other pharynx cancer | 1.0 (0.9 to 1.1) | -- | 0.5 (0.4 to 0.7) | 25.4* (14.7 to 34.4) | -0.0 (-8.6 to 7.2) |
| Oesophageal cancer | 49.9 (47.8 to 52.1) | 21.4 (20.5 to 22.2) | 7.0 (5.0 to 9.2) | 16.5* (10.4 to 23.2) | -7.2* (-12.0 to -1.8) |
| Diagnosis and primary therapy phase of esophageal cancer | 4.1 (3.9 to 4.3) | 21.4 (20.5 to 22.2) | 1.2 (0.8 to 1.6) | 21.9* (15.3 to 29.3) | -2.8 (-8.1 to 3.1) |
| Controlled phase of esophageal cancer | 37.1 (35.5 to 38.8) | -- | 1.8 (1.1 to 2.6) | 25.4* (10.0 to 42.6) | -0.1 (-12.3 to 13.7) |
| Metastatic phase of esophageal cancer | 7.2 (6.9 to 7.4) | -- | 3.2 (2.2 to 4.3) | 11.6* (6.5 to 16.7) | -11.1* (-15.1 to -7.0) |
| Terminal phase of esophageal cancer | 1.6 (1.5 to 1.6) | -- | 0.8 (0.6 to 1.1) | 11.6* (6.9 to 16.3) | -11.1* (-14.8 to -7.3) |
| Stomach cancer | 160.8 (152.9 to 168.9) | 57.1 (54.8 to 59.6) | 18.9 (13.6 to 25.1) | 39.2* (30.4 to 49.4) | 10.9* (4.0 to 19.1) |
| Diagnosis and primary therapy phase of stomach cancer | 14.0 (13.3 to 14.6) | 57.1 (54.8 to 59.6) | 4.0 (2.6 to 5.6) | 47.9* (32.0 to 65.2) | 17.9* (5.2 to 31.7) |
| Controlled phase of stomach cancer | 127.8 (121.3 to 134.5) | -- | 6.0 (3.8 to 8.8) | 65.9* (50.9 to 82.4) | 32.3* (20.3 to 45.4) |
| Metastatic phase of stomach cancer | 15.1 (14.5 to 15.8) | -- | 6.8 (4.6 to 9.1) | 22.6* (14.7 to 31.4) | -2.3 (-8.6 to 4.8) |
| Terminal phase of stomach cancer | 3.9 (3.7 to 4.1) | -- | 2.1 (1.5 to 2.7) | 22.6* (17.3 to 28.0) | -2.3 (-6.5 to 2.0) |
| Colon and rectum cancer | 469.0 (453.3 to 484.5) | 77.9 (75.4 to 80.5) | 41.9 (29.9 to 55.6) | 37.3* (31.7 to 43.2) | 9.4* (5.0 to 14.1) |
| Diagnosis and primary therapy phase of colon and rectum cancers | 19.4 (18.7 to 20.1) | 77.9 (75.4 to 80.5) | 5.5 (3.7 to 7.7) | 43.7* (36.1 to 51.8) | 14.5* (8.5 to 21.0) |
| Controlled phase of colon and rectum cancers | 418.4 (404.2 to 432.5) | -- | 24.1 (15.7 to 34.4) | 39.0* (31.7 to 46.5) | 10.8* (5.0 to 16.8) |
| Metastatic phase of colon and rectum cancers | 22.3 (21.6 to 23.0) | -- | 10.0 (6.8 to 13.3) | 33.0* (26.7 to 40.1) | 6.0* (1.0 to 11.7) |
| Terminal phase of colon and rectum cancers | 3.1 (3.0 to 3.2) | -- | 1.7 (1.2 to 2.1) | 29.1* (24.4 to 33.5) | 2.9 (-0.8 to 6.4) |
| Stoma from colon and rectum cancers, beyond 10 years | 5.8 (5.2 to 6.4) | -- | 0.6 (0.4 to 0.8) | 13.6* (12.5 to 14.7) | -9.4* (-10.3 to -8.5) |
| Liver cancer | 85.3 (72.5 to 99.5) | 74.5 (69.3 to 80.9) | 19.8 (14.1 to 26.3) | 55.3* (41.2 to 72.0) | 23.8* (12.6 to 37.1) |
| Liver cancer due to hepatitis B | 92.5 (82.4 to 104.1) | 45.8 (41.1 to 51.1) | 12.1 (8.6 to 16.2) | 62.2* (42.2 to 86.8) | 29.3* (13.3 to 48.9) |
| Diagnosis and primary therapy phase of liver cancer due to hepatitis B | 11.6 (10.4 to 12.9) | 45.8 (41.1 to 51.1) | 3.2 (2.1 to 4.8) | 58.6* (20.7 to 106.4) | 26.4 (-3.8 to 64.5) |
| Controlled phase of liver cancer due to hepatitis B | 68.9 (61.2 to 77.7) | -- | 3.3 (2.1 to 4.9) | 95.3* (66.7 to 129.0) | 55.7* (32.9 to 82.6) |
| Metastatic phase of liver cancer due to hepatitis B | 8.6 (7.8 to 9.6) | -- | 3.8 (2.5 to 5.3) | 49.1* (16.7 to 86.4) | 18.8 (-7.0 to 48.5) |
| Terminal phase of liver cancer due to hepatitis B | 3.4 (3.1 to 3.8) | -- | 1.9 (1.3 to 2.4) | 50.3* (39.2 to 63.5) | 19.8* (11.0 to 30.3) |
| Liver cancer due to hepatitis C | 19.7 (16.3 to 23.3) | 10.1 (8.3 to 12.0) | 2.7 (1.9 to 3.7) | 44.8* (33.4 to 56.5) | 15.4* (6.3 to 24.7) |
| Diagnosis and primary therapy phase of liver cancer due to hepatitis C | 2.5 (2.1 to 3.0) | 10.1 (8.3 to 12.0) | 0.7 (0.5 to 1.1) | 42.2* (27.3 to 62.6) | 13.4* (1.5 to 29.6) |
| Controlled phase of liver cancer due to hepatitis C | 14.5 (12.1 to 17.1) | -- | 0.7 (0.4 to 1.1) | 65.8* (39.8 to 94.3) | 32.2* (11.4 to 54.9) |
| Metastatic phase of liver cancer due to hepatitis C | 1.9 (1.6 to 2.3) | -- | 0.9 (0.6 to 1.2) | 36.7* (28.5 to 45.9) | 9.0* (2.4 to 16.3) |
| Terminal phase of liver cancer due to hepatitis C | 0.8 (0.6 to 0.9) | -- | 0.4 (0.3 to 0.6) | 36.7* (28.5 to 45.9) | 9.0* (2.4 to 16.3) |
| Liver cancer due to alcohol use | 16.9 (12.4 to 24.0) | 8.9 (6.5 to 12.5) | 2.4 (1.5 to 3.6) | 33.8* (22.7 to 45.9) | 6.7 (-2.2 to 16.3) |
| Diagnosis and primary therapy phase of liver cancer due to alcohol use | 2.2 (1.6 to 3.1) | 8.9 (6.5 to 12.5) | 0.6 (0.4 to 1.0) | 32.2* (22.8 to 43.3) | 5.4 (-2.1 to 14.2) |
| Controlled phase of liver cancer due to alcohol use | 12.3 (9.0 to 17.5) | -- | 0.6 (0.3 to 1.0) | 49.3* (24.2 to 77.0) | 19.0 (-1.0 to 41.1) |
| Metastatic phase of liver cancer due to alcohol use | 1.7 (1.2 to 2.4) | -- | 0.8 (0.5 to 1.2) | 27.6* (19.0 to 37.6) | 1.7 (-5.2 to 9.7) |

| | | | | | |
|---|---|---|---|---|---|
| Terminal phase of liver cancer due to alcohol use | 0.7 (0.5 to 0.9) | -- | 0.4 (0.2 to 0.6) | 27.6* (19.0 to 37.6) | 1.7 (-5.2 to 9.7) |
| Liver cancer due to NASH | 6.4 (5.0 to 8.0) | 3.3 (2.6 to 4.1) | 0.9 (0.6 to 1.2) | 57.9* (47.4 to 69.9) | 25.8* (17.5 to 35.5) |
| Diagnosis and primary therapy phase of liver cancer due to NASH | 0.8 (0.6 to 1.0) | 3.3 (2.6 to 4.1) | 0.2 (0.1 to 0.3) | 55.4* (46.0 to 66.4) | 23.9* (16.4 to 32.7) |
| Controlled phase of liver cancer due to NASH | 4.6 (3.6 to 5.8) | -- | 0.2 (0.1 to 0.4) | 82.7* (66.4 to 102.7) | 45.6* (32.7 to 61.6) |
| Metastatic phase of liver cancer due to NASH | 0.6 (0.5 to 0.8) | -- | 0.3 (0.2 to 0.4) | 48.4* (39.6 to 58.4) | 18.3* (11.3 to 26.3) |
| Terminal phase of liver cancer due to NASH | 0.3 (0.2 to 0.3) | -- | 0.1 (0.1 to 0.2) | 48.4* (39.6 to 58.4) | 18.3* (11.3 to 26.3) |
| Liver cancer due to other causes | 12.9 (10.5 to 15.8) | 6.5 (5.3 to 7.9) | 1.7 (1.2 to 2.4) | 59.3* (45.6 to 75.4) | 27.0* (16.0 to 39.8) |
| Diagnosis and primary therapy phase of liver cancer due to other causes | 1.6 (1.3 to 2.0) | 6.5 (5.3 to 7.9) | 0.5 (0.3 to 0.7) | 56.7* (46.3 to 69.1) | 24.9* (16.6 to 34.8) |
| Controlled phase of liver cancer due to other causes | 9.5 (7.7 to 11.7) | -- | 0.5 (0.3 to 0.7) | 87.0* (51.6 to 136.4) | 49.0* (20.9 to 88.4) |
| Metastatic phase of liver cancer due to other causes | 1.2 (1.0 to 1.5) | -- | 0.6 (0.4 to 0.8) | 48.5* (38.8 to 59.7) | 18.3* (10.7 to 27.3) |
| Terminal phase of liver cancer due to other causes | 0.5 (0.4 to 0.6) | -- | 0.3 (0.2 to 0.4) | 48.5* (38.8 to 59.7) | 18.3* (10.7 to 27.3) |
| Gallbladder and biliary tract cancer | 8.1 (7.1 to 8.7) | 6.3 (5.4 to 6.8) | 1.6 (1.1 to 2.1) | 15.6* (10.0 to 21.8) | -7.9* (-12.3 to -2.9) |
| Diagnosis and primary therapy phase of gallbladder and biliary tract cancer | 1.0 (0.9 to 1.1) | 6.3 (5.4 to 6.8) | 0.3 (0.2 to 0.4) | 15.5* (9.9 to 21.7) | -7.9* (-12.4 to -3.0) |
| Controlled phase of gallbladder and biliary tract cancer | 4.8 (4.3 to 5.2) | -- | 0.2 (0.1 to 0.4) | 14.9* (8.8 to 21.3) | -8.4* (-13.2 to -3.3) |
| Metastatic phase of gallbladder and biliary tract cancer | 1.7 (1.5 to 1.9) | -- | 0.8 (0.5 to 1.0) | 15.8* (10.3 to 22.1) | -7.7* (-12.1 to -2.7) |
| Terminal phase of gallbladder and biliary tract cancer | 0.5 (0.4 to 0.5) | -- | 0.3 (0.2 to 0.3) | 15.8* (10.3 to 22.1) | -7.7* (-12.1 to -2.7) |
| Pancreatic cancer | 17.3 (16.8 to 17.8) | 15.1 (14.7 to 15.6) | 3.5 (2.5 to 4.6) | 19.9* (15.4 to 24.1) | -4.5* (-8.0 to -1.1) |
| Diagnosis and primary therapy phase of pancreatic cancer | 3.5 (3.4 to 3.6) | 15.1 (14.7 to 15.6) | 1.0 (0.7 to 1.4) | 20.0* (15.6 to 24.0) | -4.4* (-7.8 to -1.1) |
| Controlled phase of pancreatic cancer | 9.4 (9.1 to 9.7) | -- | 0.5 (0.3 to 0.7) | 11.0* (2.8 to 18.6) | -11.5* (-18.0 to -5.5) |
| Metastatic phase of pancreatic cancer | 3.1 (3.0 to 3.2) | -- | 1.4 (0.9 to 1.9) | 22.0* (17.6 to 26.1) | -2.8 (-6.3 to 0.5) |
| Terminal phase of pancreatic cancer | 1.2 (1.2 to 1.3) | -- | 0.7 (0.5 to 0.8) | 22.0* (17.6 to 26.1) | -2.8 (-6.3 to 0.5) |
| Larynx cancer | 69.1 (66.4 to 71.8) | 12.7 (12.1 to 13.2) | 7.0 (5.0 to 9.3) | 13.8* (7.0 to 20.6) | -9.3* (-14.7 to -3.9) |
| Diagnosis and primary therapy phase of larynx cancer | 4.1 (3.9 to 4.3) | 12.7 (12.1 to 13.2) | 1.2 (0.8 to 1.6) | 16.3* (11.0 to 21.4) | -7.3* (-11.5 to -3.2) |
| Controlled phase of larynx cancer | 59.1 (56.8 to 61.5) | -- | 3.5 (2.2 to 5.1) | 14.8* (5.1 to 25.0) | -8.5* (-16.2 to -0.3) |
| Metastatic phase of larynx cancer | 4.3 (4.1 to 4.5) | -- | 1.9 (1.3 to 2.6) | 11.1* (5.8 to 16.4) | -11.5* (-15.6 to -7.2) |
| Terminal phase of larynx cancer | 0.6 (0.6 to 0.6) | -- | 0.3 (0.2 to 0.4) | 9.5* (4.1 to 14.9) | -12.7* (-17.0 to -8.4) |
| Laryngectomy from larynx cancer, beyond 10 years | 1.0 (0.9 to 1.0) | -- | 0.0 (0.0 to 0.1) | 20.2* (19.4 to 20.9) | -4.2* (-4.8 to -3.6) |
| Tracheal, bronchus, and lung cancer | 132.6 (128.1 to 137.3) | 75.4 (73.0 to 78.0) | 21.3 (15.4 to 27.6) | 19.8* (11.5 to 28.0) | -4.5 (-11.1 to 2.0) |
| Diagnosis and primary therapy phase of lung, bronchus, and trachea cancer | 8.3 (8.0 to 8.6) | 75.4 (73.0 to 78.0) | 2.4 (1.6 to 3.3) | 25.0* (20.3 to 29.7) | -0.4 (-4.1 to 3.4) |
| Controlled phase of lung, bronchus, and trachea cancer | 92.4 (89.1 to 95.7) | -- | 4.4 (2.8 to 6.3) | 31.3* (19.7 to 44.2) | 4.7 (-4.6 to 14.9) |
| Metastatic phase of lung, bronchus, and trachea cancer | 26.1 (25.3 to 27.1) | -- | 11.4 (7.7 to 15.4) | 16.1* (3.6 to 28.8) | -7.5 (-17.5 to 2.7) |
| Terminal phase of lung, bronchus, and trachea cancer | 5.8 (5.6 to 6.0) | -- | 3.1 (2.2 to 4.0) | 15.3* (11.1 to 19.7) | -8.1* (-11.4 to -4.6) |
| Malignant skin melanoma | 177.1 (145.1 to 230.4) | 21.3 (17.7 to 27.5) | 10.2 (6.5 to 14.6) | 13.5* (6.2 to 21.2) | -9.5* (-15.3 to -3.4) |
| Diagnosis and primary therapy phase of malignant skin melanoma | 4.7 (3.8 to 6.1) | 21.3 (17.7 to 27.5) | 1.3 (0.9 to 2.0) | 14.3* (5.4 to 23.9) | -8.9* (-16.0 to -1.3) |
| Controlled phase of malignant skin melanoma | 170.5 (139.5 to 221.8) | -- | 8.0 (4.8 to 12.2) | 14.9* (6.7 to 23.6) | -8.4* (-14.9 to -1.5) |
| Metastatic phase of malignant skin melanoma | 1.6 (1.4 to 2.1) | -- | 0.7 (0.5 to 1.1) | 1.6 (-3.6 to 7.1) | -19.0* (-23.2 to -14.6) |
| Terminal phase of malignant skin melanoma | 0.3 (0.2 to 0.3) | -- | 0.1 (0.1 to 0.2) | 0.5 (-4.6 to 5.8) | -19.9* (-23.9 to -15.6) |
| Non-melanoma skin cancer | 51.7 (23.8 to 111.8) | 293.1 (141.5 to 631.7) | 2.3 (0.8 to 5.7) | -2.2 (-17.7 to 8.7) | -22.0* (-34.4 to -13.3) |
| Non-melanoma skin cancer (squamous-cell carcinoma) | 48.1 (18.8 to 117.5) | 41.7 (15.4 to 102.2) | 2.2 (0.7 to 5.5) | -2.6 (-19.8 to 8.8) | -22.4* (-36.1 to -13.3) |
| Non-melanoma skin cancer (basal-cell carcinoma) | 24.9 (9.5 to 59.8) | 251.4 (112.9 to 596.3) | 0.1 (0.0 to 0.3) | 7.3 (-5.4 to 21.0) | -14.4* (-24.6 to -3.6) |
| Breast cancer | 1901.3 (1820.2 to 2005.2) | 222.5 (212.8 to 235.3) | 148.9 (104.4 to 202.9) | 29.1* (22.9 to 35.2) | 2.9 (-2.0 to 7.8) |
| Diagnosis and primary therapy phase of breast cancer | 50.8 (48.6 to 53.7) | 222.5 (212.8 to 235.3) | 13.4 (9.3 to 18.3) | 32.6* (17.8 to 47.1) | 5.7 (-6.1 to 17.2) |
| Controlled phase of breast cancer | 1707.0 (1631.9 to 1805.0) | -- | 106.9 (68.1 to 154.6) | 29.4* (23.0 to 35.8) | 3.2 (-2.0 to 8.2) |
| Metastatic phase of breast cancer | 58.1 (55.7 to 61.8) | -- | 23.7 (16.5 to 31.2) | 28.1* (14.0 to 41.9) | 2.1 (-9.1 to 13.1) |
| Terminal phase of breast cancer | 4.1 (3.9 to 4.4) | -- | 2.2 (1.5 to 2.8) | 25.1* (19.6 to 29.9) | -0.3 (-4.7 to 3.5) |
| Mastectomy from breast cancer, beyond 10 years | 81.3 (76.4 to 86.3) | -- | 2.8 (1.6 to 4.3) | 14.5* (4.6 to 25.2) | -8.7* (-16.6 to -0.2) |
| Cervical cancer | 490.3 (435.7 to 518.1) | 76.0 (68.0 to 80.3) | 37.4 (26.7 to 49.8) | 30.3* (19.3 to 38.9) | 3.9 (-4.9 to 10.7) |
| Diagnosis and primary therapy phase of cervical cancer | 25.0 (22.2 to 26.4) | 76.0 (68.0 to 80.3) | 6.8 (4.5 to 9.4) | 31.9* (15.7 to 48.6) | 5.2 (-7.8 to 18.4) |
| Controlled phase of cervical cancer | 442.3 (392.8 to 467.4) | -- | 20.6 (13.1 to 29.8) | 33.7* (22.5 to 42.3) | 6.6 (-2.3 to 13.4) |
| Metastatic phase of cervical cancer | 20.2 (18.2 to 21.4) | -- | 8.6 (5.8 to 11.6) | 23.7* (9.2 to 38.7) | -1.4 (-13.0 to 10.6) |
| Terminal phase of cervical cancer | 2.7 (2.4 to 2.9) | -- | 1.5 (1.0 to 1.9) | 19.1* (10.0 to 25.5) | -5.1 (-12.3 to 0.0) |
| Uterine cancer | 259.2 (246.4 to 273.1) | 31.3 (29.8 to 33.0) | 17.1 (11.9 to 23.0) | 33.0* (23.8 to 43.1) | 6.0 (-1.3 to 14.0) |
| Diagnosis and primary therapy phase of uterine cancer | 11.6 (11.0 to 12.2) | 31.3 (29.8 to 33.0) | 3.2 (2.2 to 4.5) | 33.9* (19.8 to 49.9) | 6.8 (-4.5 to 19.5) |
| Controlled phase of uterine cancer | 242.0 (230.1 to 255.1) | -- | 11.3 (7.2 to 16.6) | 34.2* (23.4 to 45.6) | 7.0 (-1.6 to 16.1) |
| Metastatic phase of uterine cancer | 5.1 (4.8 to 5.4) | -- | 2.3 (1.6 to 3.0) | 26.9* (19.8 to 34.9) | 1.2 (-4.5 to 7.5) |
| Terminal phase of uterine cancer | 0.5 (0.5 to 0.5) | -- | 0.3 (0.2 to 0.3) | 23.0* (16.1 to 30.4) | -1.9 (-7.5 to 3.9) |
| Ovarian cancer | 161.0 (153.9 to 168.6) | 27.2 (25.9 to 28.5) | 19.8 (14.2 to 25.6) | 28.6* (18.5 to 39.8) | 2.5 (-5.6 to 11.4) |
| Diagnosis and primary therapy phase of ovarian cancer | 5.8 (5.5 to 6.0) | 27.2 (25.9 to 28.5) | 1.7 (1.1 to 2.3) | 29.0* (23.4 to 34.7) | 2.8 (-1.6 to 7.4) |
| Controlled phase of ovarian cancer | 126.4 (120.8 to 132.3) | -- | 5.9 (3.8 to 8.6) | 29.4* (19.3 to 40.4) | 3.1 (-4.9 to 11.9) |
| Metastatic phase of ovarian cancer | 27.5 (26.3 to 28.8) | -- | 11.6 (7.9 to 15.2) | 28.4* (13.8 to 44.6) | 2.4 (-9.3 to 15.2) |

| | | | | | |
|---|---|---|---|---|---|
| Terminal phase of ovarian cancer | 1.3 (1.3 to 1.4) | -- | 0.7 (0.5 to 0.9) | 25.5* (20.0 to 31.3) | 0.0 (-4.4 to 4.7) |
| Prostate cancer | 137.0 (118.0 to 168.1) | 15.2 (13.1 to 18.7) | 9.8 (6.6 to 14.2) | 20.1* (12.0 to 31.9) | -4.2 (-10.7 to 5.2) |
| Diagnosis and primary therapy phase of prostate cancer | 4.7 (4.1 to 5.8) | 15.2 (13.1 to 18.7) | 1.3 (0.9 to 1.9) | 25.4* (11.0 to 41.6) | -0.0 (-11.5 to 12.9) |
| Controlled phase of prostate cancer | 128.3 (110.2 to 157.9) | -- | 7.6 (4.8 to 11.5) | 20.9* (11.8 to 33.9) | -3.6 (-10.9 to 6.7) |
| Metastatic phase of prostate cancer | 1.6 (1.4 to 2.0) | -- | 0.7 (0.5 to 1.0) | 6.7* (0.6 to 15.9) | -15.0* (-19.8 to -7.7) |
| Terminal phase of prostate cancer | 0.1 (0.1 to 0.1) | -- | 0.1 (0.0 to 0.1) | 9.5* (3.9 to 17.8) | -12.7* (-17.2 to -6.1) |
| Impotence and Incontinence after 10-year survival from prostate cancer | 2.3 (2.1 to 2.4) | -- | 0.1 (0.1 to 0.2) | 10.4* (9.8 to 11.1) | -12.0* (-12.5 to -11.4) |
| Testicular cancer | 33.8 (31.1 to 37.4) | 4.1 (3.8 to 4.5) | 2.1 (1.4 to 2.9) | 19.8* (6.7 to 35.3) | -4.5 (-15.0 to 7.9) |
| Diagnosis and primary therapy phase of testicular cancer | 1.1 (1.0 to 1.2) | 4.1 (3.8 to 4.5) | 0.3 (0.2 to 0.4) | 21.4* (9.8 to 33.8) | -3.2 (-12.5 to 6.7) |
| Controlled phase of testicular cancer | 32.2 (29.5 to 35.6) | -- | 1.5 (1.0 to 2.2) | 22.3* (5.8 to 42.2) | -2.5 (-15.7 to 13.3) |
| Metastatic phase of testicular cancer | 0.5 (0.5 to 0.5) | -- | 0.2 (0.2 to 0.3) | 5.8 (-1.8 to 14.0) | -15.7* (-21.7 to -9.2) |
| Terminal phase of testicular cancer | 0.1 (0.1 to 0.1) | -- | -- | 3.5 (-3.7 to 11.4) | -17.5* (-23.2 to -11.2) |
| Kidney cancer | 179.9 (169.7 to 187.7) | 26.5 (24.9 to 27.6) | 10.6 (7.3 to 14.6) | 15.8* (8.5 to 23.7) | -7.7* (-13.5 to -1.4) |
| Diagnosis and primary therapy phase of kidney cancer | -- | 26.5 (24.9 to 27.6) | -- | -- | -- |
| Controlled phase of kidney cancer | 174.7 (165.0 to 182.4) | -- | 8.2 (5.3 to 11.9) | 16.7* (7.9 to 25.3) | -7.0* (-14.0 to -0.1) |
| Metastatic phase of kidney cancer | 4.3 (4.1 to 4.5) | -- | 2.0 (1.3 to 2.6) | 13.0* (7.9 to 19.5) | -9.9* (-14.0 to -4.7) |
| Terminal phase of kidney cancer | 0.8 (0.8 to 0.9) | -- | 0.4 (0.3 to 0.6) | 13.0* (7.9 to 19.4) | -9.9* (-14.0 to -4.8) |
| Bladder cancer | 109.9 (104.3 to 115.9) | 14.8 (14.0 to 15.7) | 9.7 (6.8 to 13.0) | 19.8* (13.0 to 26.8) | -4.5 (-9.9 to 1.1) |
| Diagnosis and primary therapy phase of bladder cancer | 5.7 (5.4 to 6.0) | 14.8 (14.0 to 15.7) | 1.6 (1.1 to 2.3) | 21.0* (13.1 to 30.1) | -3.6 (-9.9 to 3.7) |
| Controlled phase of bladder cancer | 99.8 (94.6 to 105.2) | -- | 6.5 (4.3 to 9.3) | 20.8* (12.4 to 29.6) | -3.7 (-10.4 to 3.3) |
| Metastatic phase of bladder cancer | 2.3 (2.2 to 2.5) | -- | 1.1 (0.7 to 1.4) | 13.5* (8.6 to 18.8) | -9.5* (-13.4 to -5.3) |
| Terminal phase of bladder cancer | 0.4 (0.4 to 0.4) | -- | 0.2 (0.2 to 0.3) | 13.0* (8.1 to 18.2) | -9.9* (-13.8 to -5.8) |
| Incontinence from bladder cancer, beyond 10 years | 1.7 (1.5 to 1.8) | -- | 0.2 (0.2 to 0.3) | 23.9* (22.4 to 25.5) | -1.2 (-2.5 to 0.0) |
| Brain and nervous system cancer | 127.7 (111.0 to 145.0) | 28.8 (25.5 to 32.0) | 12.5 (8.8 to 17.0) | 74.6* (59.0 to 92.5) | 39.2* (26.7 to 53.5) |
| Diagnosis and primary therapy phase of brain and nervous system cancers | 7.9 (6.8 to 9.0) | 28.8 (25.5 to 32.0) | 2.3 (1.5 to 3.2) | 97.9* (78.5 to 119.8) | 57.8* (42.3 to 75.2) |
| Controlled phase of brain and nervous system cancers | 108.9 (94.4 to 123.9) | -- | 5.1 (3.2 to 7.5) | 101.3* (77.4 to 128.4) | 60.5* (41.4 to 82.1) |
| Metastatic phase of brain and nervous system cancers | 9.4 (8.3 to 10.4) | -- | 4.2 (2.8 to 5.8) | 48.2* (37.3 to 60.2) | 18.2* (9.4 to 27.7) |
| Terminal phase of brain and nervous system cancers | 1.6 (1.4 to 1.7) | -- | 0.9 (0.6 to 1.1) | 42.2* (31.9 to 54.2) | 13.4* (5.1 to 22.9) |
| Thyroid cancer | 233.2 (220.1 to 257.4) | 26.2 (24.7 to 28.8) | 13.4 (9.1 to 18.8) | 34.2* (24.9 to 44.2) | 7.0 (-0.4 to 14.9) |
| Diagnosis and primary therapy phase of thyroid cancer | 6.2 (5.9 to 6.8) | 26.2 (24.7 to 28.8) | 1.8 (1.2 to 2.5) | 35.3* (23.3 to 46.7) | 7.9 (-1.7 to 16.9) |
| Controlled phase of thyroid cancer | 224.6 (211.9 to 248.0) | -- | 10.5 (6.7 to 15.4) | 34.9* (24.6 to 45.6) | 7.5 (-0.7 to 16.1) |
| Metastatic phase of thyroid cancer | 2.3 (2.2 to 2.4) | -- | 1.0 (0.7 to 1.4) | 26.3* (18.9 to 34.0) | 0.7 (-5.2 to 6.8) |
| Terminal phase of thyroid cancer | 0.2 (0.2 to 0.2) | -- | 0.1 (0.1 to 0.1) | 25.4* (18.3 to 32.6) | -0.1 (-5.7 to 5.7) |
| Mesothelioma | 3.9 (3.7 to 4.1) | 1.6 (1.5 to 1.7) | 0.7 (0.5 to 1.0) | 18.7* (9.6 to 26.2) | -5.4 (-12.6 to 0.6) |
| Diagnosis and primary therapy phase of mesothelioma | 0.4 (0.4 to 0.5) | 1.6 (1.5 to 1.7) | 0.1 (0.1 to 0.2) | 19.0* (9.6 to 27.0) | -5.2 (-12.7 to 1.2) |
| Controlled phase of mesothelioma | 2.4 (2.3 to 2.6) | -- | 0.1 (0.1 to 0.2) | 18.1* (9.1 to 25.4) | -5.9* (-13.0 to -0.0) |
| Metastatic phase of mesothelioma | 0.9 (0.9 to 1.0) | -- | 0.4 (0.3 to 0.6) | 18.7* (9.9 to 26.2) | -5.3 (-12.4 to 0.6) |
| Terminal phase of mesothelioma | 0.1 (0.1 to 0.1) | -- | 0.1 (0.0 to 0.1) | 18.5* (9.9 to 25.7) | -5.6 (-12.4 to 0.2) |
| Hodgkin lymphoma | 39.9 (33.8 to 48.5) | 5.9 (4.9 to 7.1) | 3.0 (2.0 to 4.1) | 25.0* (13.4 to 37.4) | -0.4 (-9.6 to 9.6) |
| Diagnosis and primary therapy phase of Hodgkin disease | 1.3 (1.1 to 1.6) | 5.9 (4.9 to 7.1) | 0.4 (0.2 to 0.5) | 30.1* (18.1 to 41.2) | 3.7 (-5.9 to 12.6) |
| Controlled phase of Hodgkin disease | 36.7 (31.2 to 44.6) | -- | 1.7 (1.1 to 2.7) | 31.3* (16.3 to 49.2) | 4.6 (-7.3 to 18.9) |
| Metastatic phase of Hodgkin disease | 1.7 (1.4 to 2.0) | -- | 0.7 (0.5 to 1.0) | 12.8* (4.8 to 20.6) | -10.1* (-16.5 to -3.9) |
| Terminal phase of Hodgkin disease | 0.2 (0.1 to 0.2) | -- | 0.1 (0.1 to 0.1) | 4.7 (-2.3 to 11.7) | -16.6* (-22.1 to -10.9) |
| Non-Hodgkin's lymphoma | 161.6 (155.1 to 167.9) | 28.0 (26.9 to 29.0) | 12.2 (8.6 to 16.5) | 43.1* (35.5 to 51.6) | 14.0* (8.0 to 20.8) |
| Diagnosis and primary therapy phase of non-Hodgkin lymphoma | 5.8 (5.6 to 6.1) | 28.0 (26.9 to 29.0) | 1.7 (1.1 to 2.3) | 46.0* (39.7 to 53.2) | 16.4* (11.3 to 22.1) |
| Controlled phase of non-Hodgkin lymphoma | 148.0 (142.0 to 153.8) | -- | 7.0 (4.4 to 10.2) | 46.9* (36.3 to 58.4) | 17.1* (8.6 to 26.3) |
| Metastatic phase of non-Hodgkin lymphoma | 6.6 (6.4 to 6.9) | -- | 3.0 (2.0 to 4.0) | 35.2* (30.0 to 41.8) | 7.8* (3.6 to 13.0) |
| Terminal phase of non-Hodgkin lymphoma | 1.1 (1.1 to 1.2) | -- | 0.6 (0.4 to 0.8) | 33.8* (28.6 to 40.4) | 6.7* (2.5 to 11.9) |
| Multiple myeloma | 28.7 (26.3 to 34.1) | 6.6 (6.0 to 7.6) | 5.5 (3.8 to 7.3) | 34.2* (26.0 to 42.8) | 7.0* (0.4 to 13.9) |
| Diagnosis and primary therapy phase of multiple myeloma | 1.9 (1.7 to 2.3) | 6.6 (6.0 to 7.6) | 0.5 (0.4 to 0.8) | 36.2* (27.6 to 44.6) | 8.6* (1.7 to 15.3) |
| Controlled phase of multiple myeloma | 17.8 (16.2 to 21.2) | -- | 0.9 (0.5 to 1.3) | 37.2* (21.4 to 53.5) | 9.3 (-3.3 to 22.3) |
| Metastatic phase of multiple myeloma | 8.6 (7.9 to 10.1) | -- | 3.9 (2.6 to 5.2) | 33.7* (25.6 to 41.4) | 6.6* (0.2 to 12.7) |
| Terminal phase of multiple myeloma | 0.4 (0.4 to 0.5) | -- | 0.2 (0.2 to 0.3) | 28.1* (21.2 to 34.6) | 2.1 (-3.4 to 7.3) |
| Leukaemia | 103.9 (90.4 to 113.3) | 24.4 (21.2 to 26.4) | 12.1 (8.7 to 16.0) | 39.3* (29.8 to 50.3) | 11.1* (3.5 to 19.8) |
| Acute lymphoid leukaemia | 11.8 (9.6 to 13.0) | 2.6 (2.1 to 2.8) | 1.3 (0.9 to 1.8) | 45.9* (29.7 to 58.6) | 16.3* (3.4 to 26.5) |
| Diagnosis and primary therapy phase of acute lymphoid leukemia | 1.2 (1.0 to 1.3) | 2.6 (2.1 to 2.8) | 0.3 (0.2 to 0.5) | 31.0* (19.4 to 39.7) | 4.4 (-4.9 to 11.4) |
| Controlled phase of acute lymphoid leukemia | 9.6 (7.8 to 10.6) | -- | 0.5 (0.3 to 0.7) | 50.3* (33.1 to 64.3) | 19.8* (6.1 to 31.0) |
| Metastatic phase of acute lymphoid leukemia | 0.9 (0.7 to 1.0) | -- | 0.4 (0.3 to 0.6) | 53.1* (33.9 to 68.9) | 22.1* (6.7 to 34.7) |
| Terminal phase of acute lymphoid leukemia | 0.2 (0.1 to 0.2) | -- | 0.1 (0.1 to 0.1) | 53.6* (34.1 to 69.4) | 22.5* (6.9 to 35.0) |
| Chronic lymphoid leukaemia | 32.4 (28.5 to 36.9) | 4.3 (3.8 to 4.9) | 3.8 (2.8 to 5.2) | 79.2* (58.5 to 103.2) | 42.8* (26.3 to 62.0) |

| | | | | | |
|---|---|---|---|---|---|
| Diagnosis and primary therapy phase of chronic lymphoid leukemia | 1.7 (1.5 to 2.0) | 4.3 (3.8 to 4.9) | 0.5 (0.3 to 0.7) | 83.5* (64.5 to 105.1) | 46.2* (31.1 to 63.5) |
| Controlled phase of chronic lymphoid leukemia | 26.0 (22.9 to 29.5) | -- | 1.2 (0.8 to 1.8) | 83.8* (54.5 to 118.8) | 46.5* (23.1 to 74.4) |
| Metastatic phase of chronic lymphoid leukemia | 4.5 (3.9 to 5.1) | -- | 2.0 (1.4 to 2.7) | 75.8* (56.2 to 99.0) | 40.2* (24.5 to 58.6) |
| Terminal phase of chronic lymphoid leukemia | 0.1 (0.1 to 0.2) | -- | 0.1 (0.1 to 0.1) | 68.6* (49.9 to 90.3) | 34.4* (19.5 to 51.7) |
| Acute myeloid leukaemia | 9.5 (8.6 to 10.3) | 6.6 (5.8 to 7.1) | 1.6 (1.1 to 2.1) | 22.3* (15.5 to 29.1) | -2.5 (-7.9 to 2.9) |
| Diagnosis and primary therapy phase of acute myeloid leukemia | -- | 6.6 (5.8 to 7.1) | -- | -- | -- |
| Controlled phase of acute myeloid leukemia | 6.8 (6.2 to 7.3) | -- | 0.3 (0.2 to 0.5) | 18.9* (11.0 to 26.9) | -5.3 (-11.5 to 1.1) |
| Metastatic phase of acute myeloid leukemia | 2.3 (2.0 to 2.5) | -- | 1.0 (0.7 to 1.4) | 23.1* (16.6 to 30.2) | -1.8 (-7.0 to 3.7) |
| Terminal phase of acute myeloid leukemia | 0.5 (0.4 to 0.5) | -- | 0.3 (0.2 to 0.4) | 23.1* (16.6 to 30.2) | -1.8 (-7.0 to 3.7) |
| Chronic myeloid leukaemia | 8.4 (7.6 to 9.3) | 2.3 (2.1 to 2.6) | 0.9 (0.6 to 1.2) | 5.8 (-0.9 to 13.3) | -15.7* (-21.0 to -9.7) |
| Diagnosis and primary therapy phase of chronic myeloid leukemia | 0.6 (0.5 to 0.6) | 2.3 (2.1 to 2.6) | 0.2 (0.1 to 0.2) | 7.9* (0.9 to 15.9) | -14.0* (-19.6 to -7.6) |
| Controlled phase of chronic myeloid leukemia | 7.1 (6.4 to 7.8) | -- | 0.3 (0.2 to 0.5) | 8.7* (1.7 to 16.7) | -13.3* (-18.9 to -6.9) |
| Metastatic phase of chronic myeloid leukemia | 0.6 (0.6 to 0.7) | -- | 0.3 (0.2 to 0.4) | 2.2 (-3.8 to 8.9) | -18.6* (-23.3 to -13.2) |
| Terminal phase of chronic myeloid leukemia | 0.1 (0.1 to 0.2) | -- | 0.1 (0.1 to 0.1) | 2.2 (-3.8 to 8.9) | -18.5* (-23.3 to -13.2) |
| Other leukaemia | 41.7 (34.4 to 46.6) | 8.7 (7.1 to 9.7) | 4.5 (3.1 to 5.9) | 27.8* (16.3 to 42.9) | 1.9 (-7.3 to 13.9) |
| Metastatic phase of other leukemia | 5.7 (4.6 to 6.3) | -- | 2.6 (1.7 to 3.5) | 25.7* (15.7 to 38.8) | 0.2 (-7.8 to 10.6) |
| Diagnosis and primary therapy phase of other leukemia | -- | 8.7 (7.1 to 9.7) | -- | -- | -- |
| Terminal phase of other leukemia | 0.4 (0.3 to 0.4) | -- | 0.2 (0.1 to 0.3) | 28.9* (18.3 to 43.6) | 2.7 (-5.7 to 14.5) |
| Controlled phase of other leukemia | 35.6 (29.4 to 40.0) | -- | 1.7 (1.0 to 2.6) | 31.1* (13.2 to 52.4) | 4.5 (-9.8 to 21.5) |
| Other malignant cancers | 518.6 (411.7 to 692.3) | 44.1 (40.4 to 47.2) | 39.6 (26.4 to 57.0) | 157.9* (104.3 to 236.1) | 105.5* (62.8 to 167.9) |
| Diagnosis and primary therapy phase of other malignant neoplasms | 12.0 (9.5 to 16.0) | 44.1 (40.4 to 47.2) | 3.4 (2.1 to 5.1) | 191.3* (123.4 to 288.5) | 132.2* (78.0 to 209.7) |
| Controlled phase of other malignant neoplasms | 475.9 (375.8 to 638.3) | -- | 22.4 (13.7 to 35.9) | 215.5* (141.6 to 321.1) | 151.5* (92.6 to 235.7) |
| Metastatic phase of other malignant neoplasms | 26.9 (22.4 to 34.7) | -- | 11.7 (7.5 to 16.9) | 96.1* (59.9 to 147.1) | 56.3* (27.4 to 96.9) |
| Terminal phase of other malignant neoplasms | 3.8 (3.2 to 4.9) | -- | 2.1 (1.4 to 2.9) | 87.5* (53.6 to 132.5) | 49.5* (22.4 to 85.3) |
| Other neoplasms | 1983.2 (1886.1 to 2074.0) | 811.5 (757.5 to 870.2) | 3.1 (1.9 to 4.6) | 28.5* (18.0 to 39.9) | 2.5 (-5.9 to 11.5) |
| Myelodysplastic, myeloproliferative, and other hematopoietic neoplasms | 65.1 (52.7 to 78.7) | 6.3 (3.5 to 9.3) | 3.1 (1.9 to 4.6) | 28.5* (18.0 to 39.9) | 2.5 (-5.9 to 11.5) |
| Benign and in situ intestinal neoplasms | 319.7 (305.2 to 337.0) | 74.6 (68.7 to 80.4) | -- | -- | -- |
| Benign and in situ cervical and uterine neoplasms | 140.8 (132.9 to 148.8) | 9.8 (9.0 to 10.6) | -- | -- | -- |
| Other benign and in situ neoplasms | 1491.9 (1406.1 to 1581.9) | 720.9 (666.6 to 779.5) | -- | -- | -- |
| **Cardiovascular diseases** | **32373.9 (30135.8 to 35196.1)** | **3754.6 (3401.8 to 4214.3)** | **2018.4 (1449.3 to 2643.9)** | **29.4* (26.9 to 32.3)** | **3.2* (1.2 to 5.5)** |
| Rheumatic heart disease | 3383.1 (3265.8 to 3511.5) | 8.2 (6.9 to 9.7) | 162.3 (106.3 to 236.0) | 32.7* (30.7 to 34.6) | 5.8* (4.2 to 7.3) |
| Rheumatic heart disease, without heart failure | 3277.8 (3153.3 to 3411.5) | 8.2 (6.9 to 9.7) | 152.9 (99.8 to 223.8) | 32.9* (30.9 to 34.9) | 6.0* (4.3 to 7.5) |
| Heart failure due to rheumatic heart disease | 105.3 (77.3 to 140.4) | -- | 9.3 (5.8 to 13.9) | 28.9* (19.5 to 39.7) | 2.8 (-4.7 to 11.4) |
| Ischaemic heart disease | 7577.9 (6962.3 to 8274.5) | 484.9 (343.8 to 651.4) | 362.5 (247.8 to 512.7) | 17.8* (16.0 to 19.8) | -6.1* (-7.5 to -4.5) |
| Asymptomatic ischemic heart disease following myocardial infarction | 1407.9 (1100.8 to 1770.3) | -- | -- | -- | -- |
| Angina due to ischemic heart disease | 5687.7 (5190.6 to 6240.4) | -- | 320.1 (216.1 to 453.4) | 17.3* (15.5 to 19.2) | -6.5* (-8.0 to -5.0) |
| Heart failure due to ischemic heart disease | 455.3 (340.3 to 582.1) | -- | 39.7 (24.8 to 58.9) | 22.9* (16.6 to 28.7) | -2.0 (-7.1 to 2.6) |
| Myocardial infarction due to ischemic heart disease | 27.0 (19.4 to 35.6) | 484.9 (343.8 to 651.4) | 2.7 (1.6 to 4.0) | 10.2* (3.2 to 16.3) | -12.2* (-17.7 to -7.3) |
| Stroke | 6522.3 (6044.1 to 7103.9) | 710.0‡ (588.8 to 854.4) | 1002.3 (707.4 to 1284.3) | 37.5* (32.4 to 42.4) | 9.6* (5.6 to 13.5) |
| Ischaemic stroke | 3899.7 (3480.1 to 4425.3) | 372.6 (261.4 to 507.9) | 591.2 (413.3 to 771.6) | 49.9* (42.9 to 56.9) | 19.5* (13.9 to 25.1) |
| Acute ischemic stroke | 29.2 (20.6 to 39.7) | 372.6 (261.4 to 507.9) | 7.5 (4.6 to 11.0) | 36.5* (32.7 to 40.5) | 8.8* (5.8 to 12.0) |
| Chronic ischemic stroke | 3870.5 (3451.6 to 4400.8) | -- | 583.8 (407.8 to 761.4) | 50.1* (43.0 to 57.3) | 19.6* (14.0 to 25.4) |
| Intracerebral hemorrhage | 1744.2 (1511.7 to 2017.6) | 224.9 (176.2 to 279.8) | 269.0 (188.9 to 358.8) | 22.7* (13.5 to 33.2) | -2.2 (-9.6 to 6.2) |
| Acute intracerebral hemorrhage | 12.3 (9.7 to 15.3) | 224.9 (176.2 to 279.8) | 3.0 (1.9 to 4.2) | 0.5 (-12.9 to 16.3) | -19.9* (-30.6 to -7.3) |
| Chronic intracerebral hemorrhage | 1731.8 (1499.0 to 2005.4) | -- | 266.0 (186.3 to 355.4) | 23.0* (13.6 to 33.5) | -1.9 (-9.4 to 6.4) |
| Subarachnoid hemorrhage | 993.7 (885.4 to 1121.9) | 112.5 (85.9 to 141.8) | 142.0 (99.3 to 185.3) | 23.1* (16.2 to 30.4) | -1.9 (-7.4 to 3.9) |
| Acute subarachnoid hemorrhage | 8.5 (6.6 to 10.7) | 112.5 (85.9 to 141.8) | 2.1 (1.3 to 3.0) | 21.7* (15.7 to 24.2) | -3.0* (-7.8 to -1.0) |
| Chronic subarachnoid hemorrhage | 985.2 (875.7 to 1113.7) | -- | 139.9 (97.7 to 182.7) | 23.1* (16.1 to 30.6) | -1.9 (-7.5 to 4.1) |
| Hypertensive heart disease | 727.5 (562.8 to 927.4) | -- | 63.2 (40.0 to 94.3) | 33.3* (27.9 to 38.5) | 6.2* (1.9 to 10.4) |
| Non-rheumatic valvular heart disease | 1308.5 (1236.5 to 1387.4) | 1353.2 (1292.4 to 1418.6) | 3.7 (1.5 to 7.6) | 16.1* (11.9 to 20.9) | -7.4* (-10.8 to -3.7) |
| Non-rheumatic calcific aortic valve disease | 326.7 (279.1 to 377.1) | 326.7 (279.1 to 377.1) | 0.8 (0.3 to 1.7) | 11.5* (5.6 to 17.3) | -11.1* (-15.8 to -6.5) |
| Heart failure due to calcific aortic valve disease | 5.3 (2.1 to 10.9) | -- | 0.5 (0.2 to 1.0) | 4.6 (-11.8 to 21.9) | -16.7* (-29.7 to -2.8) |
| Non-rheumatic calcific aortic valve disease | 321.4 (273.9 to 372.6) | 326.7 (279.1 to 377.1) | 0.3 (0.1 to 0.7) | 23.3* (7.4 to 41.8) | -1.7 (-14.4 to 13.0) |
| Non-rheumatic degenerative mitral valve disease | 1026.5 (990.4 to 1064.6) | 1026.5 (990.4 to 1064.6) | 2.8 (1.1 to 5.7) | 17.0* (11.4 to 22.9) | -6.7* (-11.2 to -2.1) |
| Heart failure due to degenerative mitral valve disease | 21.7 (9.3 to 43.8) | -- | 2.0 (0.8 to 4.1) | 10.8 (-0.7 to 23.2) | -11.7* (-20.9 to -1.8) |
| Non-rheumatic degenerative mitral valve disease | 1004.8 (966.1 to 1046.1) | 1026.5 (990.4 to 1064.6) | 0.8 (0.3 to 1.7) | 35.9* (13.5 to 64.4) | 8.4 (-9.5 to 31.1) |
| Other non-rheumatic valve diseases | 0.9 (0.6 to 1.3) | -- | 0.1 (0.0 to 0.1) | 36.6* (33.8 to 39.5) | 8.9* (6.7 to 11.2) |
| Mild heart failure due to other non-rheumatic valve disease | 0.2 (0.1 to 0.3) | -- | -- | 36.6* (33.8 to 39.4) | 8.9* (6.7 to 11.1) |
| Moderate heart failure due to other non-rheumatic valve disease | 0.1 (0.1 to 0.2) | -- | -- | 36.6* (33.8 to 39.5) | 8.9* (6.7 to 11.2) |
| Severe heart failure due to other non-rheumatic valve disease | 0.3 (0.2 to 0.4) | -- | 0.1 (0.0 to 0.1) | 36.6* (33.8 to 39.5) | 8.9* (6.7 to 11.2) |

| | | | | | |
|---|---|---|---|---|---|
| Controlled, medically managed heart failure due to other non-rheumatic valve disease | 0.3 (0.2 to 0.5) | -- | -- | 36.6* (33.8 to 39.5) | 8.9* (6.6 to 11.2) |
| Cardiomyopathy and myocarditis | 322.6 (253.4 to 401.5) | 181.3 (123.5 to 251.7) | 44.7 (28.7 to 65.3) | 16.8* (12.8 to 20.7) | -6.9* (-10.1 to -3.8) |
| Myocarditis | 129.5 (102.0 to 161.6) | 181.3 (123.5 to 251.7) | 9.9 (6.4 to 14.6) | 29.6* (22.4 to 36.4) | 3.3 (-2.4 to 8.7) |
| Acute myocarditis | 43.7 (30.1 to 58.9) | 181.3 (123.5 to 251.7) | 2.2 (1.2 to 3.6) | 24.1* (14.4 to 34.9) | -1.1 (-8.8 to 7.5) |
| Heart failure due to myocarditis | 85.8 (63.5 to 112.5) | -- | 7.8 (4.8 to 11.6) | 31.2* (22.2 to 40.1) | 4.6 (-2.6 to 11.7) |
| Alcoholic cardiomyopathy | 169.3 (130.3 to 213.6) | -- | 15.0 (9.5 to 21.7) | 3.7 (-2.0 to 9.1) | -17.3* (-21.9 to -13.0) |
| Other cardiomyopathy | 224.5 (169.4 to 287.2) | -- | 19.7 (12.6 to 29.2) | 22.5* (16.3 to 28.3) | -2.3 (-7.3 to 2.2) |
| Atrial fibrillation and flutter | 1015.6 (811.3 to 1246.3) | 105.3 (69.2 to 146.1) | 84.3 (53.8 to 122.7) | 21.3* (16.4 to 25.7) | -3.3 (-7.2 to 0.2) |
| Asymptomatic atrial fibrillation and flutter | 609.4 (485.8 to 749.7) | 105.3 (69.2 to 146.1) | -- | -- | -- |
| Symptomatic atrial fibrillation and flutter | 406.2 (322.8 to 496.3) | -- | 84.3 (53.8 to 122.7) | 21.3* (16.4 to 25.7) | -3.3 (-7.2 to 0.2) |
| Peripheral vascular disease | 7185.4 (5843.8 to 8880.1) | 855.5 (576.1 to 1224.3) | 0.6 (0.2 to 1.3) | 14.3* (3.1 to 24.7) | -8.9* (-17.8 to -0.6) |
| Asymptomatic peripheral arterial disease | 7143.0 (5805.7 to 8834.3) | 855.5 (576.1 to 1224.3) | -- | -- | -- |
| Symptomatic claudication due to peripheral arterial disease | 42.4 (23.2 to 71.1) | -- | 0.6 (0.2 to 1.3) | 14.3* (3.1 to 24.7) | -8.9* (-17.8 to -0.6) |
| Endocarditis | 28.3 (21.3 to 38.2) | 56.1 (38.6 to 79.6) | 2.5 (1.5 to 3.8) | 20.4* (11.1 to 30.2) | -4.0 (-11.4 to 3.8) |
| Acute endocarditis | 4.3 (2.9 to 6.0) | 56.1 (38.6 to 79.6) | 0.2 (0.1 to 0.4) | 31.1* (25.1 to 36.6) | 4.5 (-0.3 to 8.9) |
| Heart failure due to endocarditis | 24.0 (17.3 to 33.3) | -- | 2.2 (1.3 to 3.5) | 19.4* (9.5 to 29.8) | -4.8 (-12.7 to 3.5) |
| Other cardiovascular and circulatory diseases | 5536.0 (3933.6 to 7820.8) | -- | 292.4 (179.7 to 463.8) | 22.1* (19.7 to 24.6) | -2.6* (-4.6 to -0.7) |
| Heart failure due to other cardiovascular diseases | 39.7 (28.3 to 55.0) | -- | 3.7 (2.2 to 5.9) | 22.2* (20.1 to 24.2) | -2.6* (-4.2 to -1.0) |
| Other cardiovascular and circulatory disease episodes | 5496.3 (3905.8 to 7766.3) | -- | 288.7 (177.6 to 458.0) | 22.1* (19.7 to 24.7) | -2.6* (-4.6 to -0.6) |
| **Chronic respiratory diseases** | **34290.1 (30364.5 to 38542.6)** | **2809.6 (2119.3 to 3635.8)** | **3018.9 (2382.2 to 3665.3)** | **24.8* (19.5 to 30.1)** | **-0.5 (-4.7 to 3.7)** |
| Chronic obstructive pulmonary disease | 20466.1 (17345.3 to 24278.4) | 1496.0 (1027.1 to 2119.9) | 2196.7 (1732.5 to 2680.8) | 24.6* (17.8 to 31.3) | -0.7 (-6.1 to 4.7) |
| Chronic obstructive pulmonary disease with heart failure | 69.9 (46.6 to 99.3) | -- | 31.6 (19.3 to 49.5) | 35.1* (27.6 to 43.3) | 7.7* (1.8 to 14.2) |
| Chronic obstructive pulmonary disease without heart failure | 20396.2 (17269.5 to 24198.6) | 1496.0 (1027.1 to 2119.9) | 2165.1 (1706.4 to 2642.5) | 24.4* (17.6 to 31.3) | -0.8 (-6.2 to 4.6) |
| Pneumoconiosis | 27.9 (22.7 to 34.1) | 3.2 (2.2 to 4.4) | 4.4 (2.8 to 6.4) | 28.0* (23.7 to 32.9) | 2.0 (-1.4 to 6.0) |
| Silicosis | 8.8 (6.0 to 13.0) | 1.3 (0.6 to 2.2) | 1.4 (0.8 to 2.2) | 34.6* (25.8 to 46.5) | 7.3* (0.3 to 16.8) |
| Heart failure due to silicosis | -- | -- | -- | -- | -- |
| Silicosis without heart failure | 8.8 (6.0 to 13.0) | 1.3 (0.6 to 2.2) | 1.4 (0.8 to 2.2) | 34.6* (25.8 to 46.5) | 7.3* (0.3 to 16.8) |
| Asbestosis | 5.4 (3.2 to 8.7) | 0.5 (0.2 to 1.0) | 0.8 (0.4 to 1.4) | 30.1* (23.5 to 36.6) | 3.7 (-1.6 to 8.9) |
| Asbestosis without heart failure | 5.4 (3.1 to 8.6) | 0.5 (0.2 to 1.0) | 0.8 (0.4 to 1.4) | 30.2* (23.5 to 36.7) | 3.8 (-1.6 to 8.9) |
| Heart failure due to asbestosis | -- | -- | -- | 28.3* (25.2 to 31.6) | 2.3 (-0.2 to 4.9) |
| Coal workers pneumoconiosis | 6.1 (3.9 to 8.5) | 0.7 (0.3 to 1.3) | 1.0 (0.5 to 1.6) | 23.1* (12.4 to 33.2) | -1.9 (-10.4 to 6.2) |
| Coal workers pneumoconiosis without heart failure | 5.9 (3.7 to 8.3) | 0.7 (0.3 to 1.3) | 0.9 (0.5 to 1.5) | 22.4* (10.5 to 33.6) | -2.4 (-11.9 to 6.5) |
| Heart failure due to Coal workers pneumoconiosis | 0.2 (0.1 to 0.3) | -- | 0.1 (0.1 to 0.1) | 30.1* (23.0 to 37.0) | 3.7 (-2.0 to 9.2) |
| Other pneumoconiosis | 7.7 (5.6 to 11.0) | 0.7 (0.4 to 1.4) | 1.2 (0.7 to 1.9) | 23.6* (19.5 to 27.4) | -1.5 (-4.7 to 1.5) |
| Heart failure due to other pneumoconiosis | 0.1 (0.0 to 0.1) | -- | -- | 31.3* (27.5 to 35.8) | 4.7* (1.6 to 8.3) |
| Other pneumoconiosis without heart failure | 7.6 (5.5 to 10.9) | 0.7 (0.4 to 1.4) | 1.2 (0.7 to 1.9) | 23.4* (19.2 to 27.3) | -1.7 (-5.0 to 1.5) |
| Asthma | 14746.3 (12219.9 to 17597.6) | 1279.3 (819.7 to 1814.9) | 569.3 (372.9 to 824.1) | 24.5* (17.6 to 31.2) | -0.8 (-6.3 to 4.6) |
| Asymptomatic asthma | 5358.8 (4310.3 to 6569.0) | 463.9 (294.5 to 677.0) | -- | -- | -- |
| Symptomatic asthma | 9387.5 (7709.3 to 11341.8) | 815.4 (530.2 to 1161.2) | 569.3 (372.9 to 824.1) | 24.5* (17.6 to 31.2) | -0.8 (-6.3 to 4.6) |
| Interstitial lung disease and pulmonary sarcoidosis | 395.2 (326.1 to 479.9) | 31.2 (20.5 to 42.8) | 42.8 (27.1 to 63.6) | 16.2* (10.7 to 21.6) | -7.4* (-11.7 to -3.1) |
| Heart failure due to interstitial lung disease and pulmonary sarcoidosis | 7.8 (5.3 to 10.7) | -- | 3.6 (2.2 to 5.5) | 20.4* (18.8 to 22.2) | -4.1* (-5.3 to -2.6) |
| Interstitial lung disease and pulmonary sarcoidosis without heart failure | 387.4 (317.8 to 473.0) | 31.2 (20.5 to 42.8) | 39.2 (24.4 to 59.3) | 15.8* (9.9 to 21.6) | -7.7* (-12.4 to -3.1) |
| Other chronic respiratory diseases | -- | -- | 205.6 (161.7 to 251.2) | 29.9* (23.0 to 36.9) | 3.6 (-2.0 to 9.1) |
| **Digestive diseases** | **185561.4 (171099.6 to 201122.0)** | **40379.9 (31903.4 to 49685.3)** | **1823.5 (1209.3 to 2636.1)** | **27.4* (25.2 to 29.7)** | **1.5 (-0.2 to 3.4)** |
| Cirrhosis and other chronic liver diseases | 138073.7 (128135.1 to 147617.0) | 246.0 (212.7 to 286.7) | 181.4 (125.4 to 249.5) | 38.1* (33.3 to 42.5) | 10.0* (6.2 to 13.6) |
| Cirrhosis and other chronic liver diseases due to hepatitis B | 30910.7 (28594.1 to 33559.0) | 71.7 (59.5 to 87.1) | 56.6 (38.4 to 80.0) | 46.1* (38.4 to 53.7) | 16.4* (10.3 to 22.5) |
| Cirrhosis and other chronic liver diseases due to hepatitis B, decompensated | 339.3 (300.9 to 381.3) | 52.6 (44.4 to 61.2) | 56.6 (38.4 to 80.0) | 46.1* (38.4 to 53.7) | 16.4* (10.3 to 22.5) |
| Chronic hepatitis B without cirrhosis | 25928.8 (23538.0 to 28641.6) | -- | -- | -- | -- |
| Cirrhosis and other chronic liver diseases due to hepatitis B, compensated | 4642.6 (4104.3 to 5221.9) | 19.1 (9.4 to 32.1) | -- | -- | -- |
| Cirrhosis and other chronic liver diseases due to hepatitis C | 10744.5 (9337.2 to 12473.5) | 70.1 (57.2 to 86.9) | 49.1 (32.6 to 69.0) | 36.5* (29.9 to 42.7) | 8.8* (3.6 to 13.8) |
| Cirrhosis and other chronic liver diseases due to hepatitis C, decompensated | 295.9 (253.6 to 344.1) | 47.4 (39.4 to 56.8) | 49.1 (32.6 to 69.0) | 36.5* (29.9 to 42.7) | 8.8* (3.6 to 13.8) |
| Chronic hepatitis C without cirrhosis | 6787.8 (5319.7 to 8574.1) | -- | -- | -- | -- |
| Cirrhosis and other chronic liver diseases due to hepatitis C, compensated | 3660.8 (3148.8 to 4239.8) | 22.7 (13.3 to 36.0) | -- | -- | -- |
| Cirrhosis and other chronic liver diseases due to alcohol use | 3710.9 (3254.0 to 4222.1) | 64.8 (54.4 to 77.0) | 49.3 (33.2 to 69.3) | 29.5* (22.5 to 36.4) | 3.2 (-2.3 to 8.8) |
| Cirrhosis and other chronic liver diseases due to alcohol, decompensated | 296.9 (259.6 to 335.1) | 46.7 (39.5 to 54.4) | 49.3 (33.2 to 69.3) | 29.5* (22.5 to 36.4) | 3.2 (-2.3 to 8.8) |
| Cirrhosis and other chronic liver diseases due to alcohol, compensated | 3414.0 (2982.5 to 3881.7) | 18.1 (11.6 to 27.7) | -- | -- | -- |
| Cirrhosis due to NASH | 92003.2 (82790.4 to 101235.3) | 25.5 (20.0 to 31.5) | 17.4 (11.5 to 25.3) | 49.3* (38.3 to 60.2) | 19.0* (10.3 to 27.7) |
| Non-alcoholic fatty liver disease (NAFLD) / Non-alcoholic steatohepatitis (NASH) | 90587.0 (81464.7 to 99831.9) | -- | -- | -- | -- |
| Cirrhosis and other chronic liver diseases due to NASH | 1416.2 (1161.3 to 1702.4) | 25.5 (20.0 to 31.5) | 17.4 (11.5 to 25.3) | 49.3* (38.3 to 60.2) | 19.0* (10.3 to 27.7) |
| Cirrhosis and other chronic liver diseases due to other causes | 704.3 (508.3 to 935.2) | 13.8 (9.7 to 19.4) | 9.0 (5.4 to 13.6) | 29.1* (19.8 to 40.2) | 2.9 (-4.5 to 11.7) |

| | | | | | |
|---|---|---|---|---|---|
| Cirrhosis and other chronic liver diseases due to other cause, decompensated | 53.2 (38.8 to 70.3) | 8.8 (6.3 to 11.8) | 9.0 (5.4 to 13.6) | 29.1* (19.8 to 40.2) | 2.9 (-4.5 to 11.7) |
| Cirrhosis and other chronic liver diseases due to other cause, compensated | 651.1 (469.3 to 862.7) | 5.0 (2.9 to 8.4) | -- | -- | -- |
| Upper digestive system diseases | 75250.9 (57974.4 to 95496.4) | 35175.3 (26618.0 to 44083.5) | 1225.3 (730.3 to 1882.0) | 27.2* (24.7 to 29.9) | 1.4 (-0.6 to 3.6) |
| Peptic ulcer disease | 1416.6 (1042.7 to 1873.8) | 528.0 (367.6 to 733.8) | 70.1 (42.6 to 104.8) | 24.1* (18.0 to 29.6) | -1.1 (-6.0 to 3.3) |
| Acute peptic ulcer disease | 0.5 (0.3 to 0.7) | -- | 0.2 (0.1 to 0.3) | 2.4 (-0.1 to 4.6) | -18.4* (-20.4 to -16.6) |
| Chronic peptic ulcer disease | 1416.1 (1042.2 to 1873.1) | 528.0 (367.6 to 733.8) | 69.9 (42.5 to 104.7) | 24.2* (18.0 to 29.7) | -1.0 (-5.9 to 3.4) |
| Gastritis and duodenitis | 13147.9 (9196.5 to 18358.0) | 9180.1 (6107.3 to 12553.1) | 589.6 (352.6 to 926.8) | 31.7* (27.3 to 35.9) | 5.0* (1.5 to 8.3) |
| Acute gastritis and duodenitis | 3.0 (2.0 to 4.6) | -- | 1.0 (0.5 to 1.6) | 9.4* (7.1 to 11.4) | -12.8* (-14.6 to -11.2) |
| Chronic gastritis and duodenitis | 13144.9 (9193.4 to 18354.6) | 9180.1 (6107.3 to 12553.1) | 588.6 (352.2 to 925.6) | 31.7* (27.3 to 36.0) | 5.0* (1.5 to 8.4) |
| GERD | 66474.5 (46610.3 to 89657.2) | 25467.2 (17670.0 to 34177.9) | 565.6 (277.4 to 1003.9) | 23.1* (21.1 to 25.1) | -1.8* (-3.5 to -0.3) |
| Appendicitis | 24.5 (20.2 to 29.9) | 629.1 (521.2 to 767.7) | 7.9 (5.1 to 11.3) | 23.9* (15.7 to 32.4) | -1.2 (-7.8 to 5.6) |
| Paralytic ileus and intestinal obstruction | 8.3 (6.0 to 10.8) | 225.0 (164.7 to 296.6) | 2.7 (1.6 to 4.0) | 27.5* (22.2 to 32.1) | 1.6 (-2.6 to 5.3) |
| Inguinal, femoral, and abdominal hernia | 1966.4 (1377.3 to 2683.3) | 3026.3 (2118.5 to 4324.5) | 190.7 (114.0 to 295.1) | 19.6* (15.7 to 23.6) | -4.7* (-7.8 to -1.5) |
| Inflammatory bowel disease | 707.6 (611.0 to 824.4) | 358.5 (288.7 to 436.9) | 107.9 (71.9 to 154.0) | 25.7* (15.0 to 37.5) | 0.2 (-8.3 to 9.6) |
| Ulcerative colitis | 507.1 (415.1 to 622.5) | 265.7 (201.2 to 341.5) | 76.0 (50.1 to 110.3) | 36.0* (22.6 to 49.9) | 8.4 (-2.2 to 19.5) |
| Crohn's disease | 200.5 (169.7 to 236.7) | 92.8 (70.6 to 120.8) | 31.8 (21.3 to 45.7) | 6.6 (-3.6 to 18.9) | -15.0* (-23.2 to -5.2) |
| Vascular intestinal disorders | 3.9 (2.4 to 5.9) | 26.7 (17.9 to 37.7) | 1.3 (0.7 to 2.1) | 22.4* (15.8 to 27.6) | -2.4 (-7.7 to 1.7) |
| Gallbladder and biliary diseases | 3270.5 (2386.4 to 4392.7) | 540.4 (376.1 to 724.9) | 2.6 (1.5 to 4.1) | 27.3* (22.5 to 31.8) | 1.5 (-2.3 to 5.1) |
| Pancreatitis | 586.4 (497.5 to 680.1) | 152.5 (119.3 to 193.6) | 36.0 (18.2 to 60.3) | 20.8* (13.8 to 27.7) | -3.7 (-9.3 to 1.8) |
| Acute pancreatitis | 11.7 (8.6 to 15.7) | 124.9 (95.2 to 164.6) | 4.8 (3.1 to 7.3) | 16.2* (13.7 to 18.7) | -7.4* (-9.4 to -5.4) |
| Chronic pancreatitis | 574.7 (487.1 to 668.5) | 27.7 (18.4 to 38.6) | 31.2 (14.3 to 54.6) | 21.5* (13.6 to 30.0) | -3.1 (-9.5 to 3.6) |
| Other digestive diseases | -- | -- | 67.9 (45.1 to 97.9) | 35.8* (32.3 to 39.4) | 8.3* (5.5 to 11.1) |
| **Neurological disorders** | **228415.0 (193466.5 to 269649.5)** | **64056.5 (44473.1 to 84389.9)** | **5386.7 (3631.7 to 7595.8)** | **22.9* (21.3 to 24.7)** | **-2.1* (-3.3 to -0.6)** |
| Alzheimer's disease and other dementias | 203.7 (136.9 to 298.4) | 57.9 (38.8 to 83.0) | 26.5 (14.6 to 44.5) | 19.7* (16.1 to 23.2) | -4.6* (-7.5 to -1.8) |
| Parkinson's disease | 184.6 (126.6 to 252.1) | 26.0 (14.4 to 41.1) | 29.9 (17.6 to 46.1) | 31.5* (24.7 to 38.5) | 4.8 (-0.6 to 10.4) |
| Epilepsy | 1328.7 (977.0 to 1730.4) | 100.9 (63.5 to 143.9) | 396.3 (247.8 to 594.8) | 24.2* (11.6 to 39.0) | -1.0 (-11.0 to 10.8) |
| Multiple sclerosis | 203.4 (182.7 to 226.5) | 2.5 (1.8 to 3.4) | 53.0 (37.8 to 69.8) | 11.1* (5.4 to 17.3) | -11.4* (-16.0 to -6.5) |
| Motor neuron disease | 14.8 (10.7 to 19.4) | 4.0 (2.7 to 5.5) | 3.1 (2.0 to 4.6) | 30.3* (27.4 to 33.1) | 3.9* (1.5 to 6.1) |
| Headache disorders | 227510.5 (192335.2 to 268949.3) | 63865.2 (44268.2 to 84185.2) | 4765.5 (3116.8 to 6860.2) | 22.6* (21.5 to 23.8) | -2.3* (-3.2 to -1.3) |
| Migraine | 116199.3 (102469.5 to 133046.0) | 6154.0 (3650.3 to 8565.1) | 4173.8 (2664.6 to 6177.5) | 22.6* (21.2 to 23.9) | -2.3* (-3.4 to -1.3) |
| Medication overuse headache due to migraine | 8378.1 (5843.4 to 11315.8) | -- | 915.7 (499.4 to 1438.2) | 21.5* (19.9 to 23.0) | -3.2* (-4.5 to -1.9) |
| Migraine | 107821.2 (93889.3 to 124289.1) | 6154.0 (3650.3 to 8565.1) | 3258.1 (2009.5 to 4916.0) | 22.9* (21.3 to 24.5) | -2.0* (-3.3 to -0.8) |
| Tension-type headache | 161204.8 (115054.3 to 218255.6) | 57711.2 (38293.0 to 77936.8) | 591.7 (319.2 to 945.7) | 22.7* (21.0 to 24.3) | -2.2* (-3.6 to -1.0) |
| Medication overuse headache due to tension-type headache | 3115.9 (1917.8 to 4650.9) | -- | 337.9 (162.9 to 608.3) | 21.5* (19.1 to 23.9) | -3.2* (-5.1 to -1.3) |
| Tension-type headache | 158088.9 (112568.7 to 214846.1) | 57711.2 (38293.0 to 77936.8) | 253.8 (97.0 to 498.8) | 24.4* (23.1 to 25.7) | -0.8 (-1.9 to 0.2) |
| Other neurological disorders | 2.2 (1.2 to 3.7) | -- | 112.3 (73.6 to 160.9) | 33.2* (22.6 to 45.8) | 6.2 (-2.3 to 16.2) |
| Other neurological disorders | -- | -- | 111.7 (73.2 to 160.3) | 33.2* (22.6 to 45.9) | 6.2 (-2.3 to 16.3) |
| Guillain-Barré syndrome due to other neurological disorders | 2.2 (1.2 to 3.7) | -- | 0.7 (0.3 to 1.2) | 26.4* (23.4 to 29.3) | 0.8 (-1.6 to 3.1) |
| **Mental disorders** | **71525.1 (67354.5 to 75722.0)** | **25390.8 (22501.9 to 28484.1)** | **9914.9 (7427.9 to 12693.6)** | **22.1* (21.2 to 23.1)** | **-2.7* (-3.4 to -1.9)** |
| Schizophrenia | 2292.7 (2029.9 to 2614.0) | 67.8 (51.2 to 87.2) | 1463.0 (1089.6 to 1813.4) | 27.3* (24.9 to 29.6) | 1.5 (-0.4 to 3.3) |
| Depressive disorders | 23744.9 (20961.2 to 27255.6) | 22122.8 (19287.8 to 25184.3) | 3813.8 (2653.3 to 5208.2) | 20.2* (18.2 to 22.0) | -4.2* (-5.7 to -2.7) |
| Major depressive disorder | 14053.9 (12156.8 to 16425.3) | 20639.2 (17925.5 to 23820.3) | 2829.2 (1944.1 to 3848.3) | 18.3* (16.1 to 20.4) | -5.7* (-7.5 to -4.0) |
| Dysthymia | 10196.0 (8042.9 to 12615.8) | 1483.5 (1226.4 to 1788.9) | 984.6 (605.6 to 1457.3) | 25.9* (22.3 to 29.4) | 0.3 (-2.5 to 3.1) |
| Bipolar disorder | 3790.1 (3127.0 to 4603.8) | 328.7 (226.1 to 481.9) | 764.9 (471.9 to 1171.6) | 22.3* (20.1 to 24.5) | -2.6* (-4.3 to -0.8) |
| Anxiety disorders | 22680.7 (19804.7 to 25653.4) | 2861.9 (2563.9 to 3176.9) | 2141.7 (1497.4 to 2892.2) | 20.8* (18.6 to 22.9) | -3.7* (-5.4 to -2.0) |
| Eating disorders | 865.3 (627.5 to 1182.9) | 9.7 (6.8 to 13.8) | 181.0 (107.2 to 282.8) | 32.6* (28.4 to 36.5) | 5.7* (2.4 to 8.8) |
| Anorexia nervosa | 136.6 (98.2 to 181.1) | 9.7 (6.8 to 13.8) | 28.4 (16.7 to 43.7) | 27.4* (19.2 to 35.8) | 1.6 (-5.0 to 8.3) |
| Bulimia nervosa | 740.5 (513.7 to 1054.8) | -- | 152.5 (87.1 to 245.4) | 33.6* (29.1 to 37.8) | 6.5* (2.9 to 9.8) |
| Autism spectrum disorders | 1784.4 (1603.0 to 1974.5) | -- | 266.9 (182.5 to 364.0) | 22.3* (20.0 to 24.6) | -2.5* (-4.4 to -0.7) |
| Attention-deficit/hyperactivity disorder | 2855.3 (2285.7 to 3440.1) | -- | 34.0 (20.3 to 54.7) | 29.0* (25.2 to 33.6) | 2.8 (-0.2 to 6.5) |
| Conduct disorder | -- | -- | -- | -- | -- |
| Idiopathic developmental intellectual disability | 4373.1 (2369.3 to 6443.8) | -- | 172.3 (79.2 to 297.6) | 12.1* (6.6 to 14.6) | -10.6* (-15.0 to -8.7) |
| Other mental disorders | 14482.3 (12691.8 to 16470.9) | -- | 1077.3 (716.0 to 1470.8) | 25.0* (23.5 to 26.7) | -0.3 (-1.5 to 1.0) |
| **Substance use disorders** | **13661.7 (11892.3 to 15687.0)** | **4774.7 (3816.0 to 5795.3)** | **2194.3 (1557.0 to 2958.1)** | **27.6* (23.4 to 32.6)** | **1.7 (-1.7 to 5.7)** |
| Alcohol use disorders | 9753.0 (8012.1 to 11676.0) | 4465.5 (3527.8 to 5485.1) | 966.1 (628.1 to 1380.2) | 13.9* (10.8 to 16.7) | -9.2* (-11.7 to -6.9) |
| Alcohol dependence | 9735.7 (7996.0 to 11656.6) | 4465.5 (3527.8 to 5485.1) | 965.5 (627.8 to 1379.5) | 13.9* (10.8 to 16.8) | -9.2* (-11.7 to -6.9) |
| Fetal alcohol syndrome | 17.3 (13.8 to 21.4) | -- | 0.6 (0.3 to 0.9) | -1.8 (-8.2 to 3.4) | -21.7* (-26.8 to -17.5) |
| Drug use disorders | 4123.0 (3516.8 to 4842.4) | 309.2 (232.8 to 416.2) | 1228.1 (849.8 to 1690.8) | 41.0* (34.7 to 47.9) | 12.4* (7.4 to 17.9) |
| Opioid use disorders | 2594.2 (2030.2 to 3311.0) | 206.7 (141.9 to 306.5) | 1060.5 (715.0 to 1497.8) | 45.6* (38.6 to 53.2) | 16.0* (10.5 to 22.1) |
| Cocaine use disorders | 337.5 (294.9 to 380.8) | 3.9 (2.1 to 6.4) | 45.2 (30.0 to 64.9) | 11.2* (6.2 to 16.2) | -11.4* (-15.4 to -7.4) |

| | | | | | |
|---|---|---|---|---|---|
| Amphetamine use disorders | 156.7 (106.9 to 218.4) | 22.3 (13.4 to 34.1) | 20.7 (11.7 to 34.1) | 23.6* (13.6 to 33.9) | -1.5 (-9.5 to 6.7) |
| Cannabis use disorders | 819.3 (596.1 to 1077.3) | 76.3 (44.2 to 111.9) | 23.5 (13.9 to 36.2) | 18.4* (11.9 to 24.9) | -5.6* (-10.8 to -0.5) |
| Other drug use disorders | 328.0 (274.1 to 386.8) | -- | 78.3 (53.5 to 107.4) | 19.4* (15.0 to 23.7) | -4.8* (-8.3 to -1.4) |
| **Diabetes and kidney diseases** | **93878.5 (83235.1 to 104661.0)** | **3937.2 (3021.7 to 5059.7)** | **4487.8 (3080.7 to 6279.9)** | **29.6* (22.7 to 36.3)** | **3.3 (-2.2 to 8.6)** |
| Diabetes mellitus | 45549.2 (39619.4 to 52747.5) | 2518.9 (1790.4 to 3438.2) | 3921.5 (2651.1 to 5544.7) | 30.4* (22.7 to 37.9) | 3.9 (-2.2 to 9.9) |
| Diabetes mellitus type 1 | 1125.1 (1005.4 to 1264.5) | 13.0 (8.6 to 18.6) | 95.7 (64.7 to 135.4) | 17.2* (14.1 to 20.7) | -6.6* (-9.1 to -3.8) |
| Uncomplicated diabetes mellitus type 1 | 605.5 (479.5 to 721.2) | 13.0 (8.6 to 18.6) | 28.3 (16.6 to 43.3) | 15.1* (9.5 to 20.0) | -8.3* (-12.7 to -4.4) |
| Vision loss due to diabetes mellitus type 1 retinopathy | 11.6 (8.3 to 15.7) | -- | 1.0 (0.6 to 1.6) | 2.4 (-4.1 to 9.3) | -18.4* (-23.6 to -12.9) |
| Diabetic neuropathy due to diabetes mellitus type 1 | 508.1 (403.4 to 634.3) | -- | 66.4 (43.5 to 97.4) | 18.3* (12.8 to 23.5) | -5.7* (-10.1 to -1.5) |
| Diabetes mellitus type 2 | 44424.1 (38471.9 to 51623.7) | 2505.9 (1779.3 to 3425.2) | 3825.7 (2589.7 to 5423.3) | 30.7* (22.9 to 38.5) | 4.2 (-2.0 to 10.4) |
| Uncomplicated diabetes mellitus type 2 | 23351.6 (18091.4 to 29214.8) | 2505.9 (1779.3 to 3425.2) | 1092.5 (648.9 to 1659.6) | 29.6* (20.3 to 38.7) | 3.3 (-4.1 to 10.6) |
| Vision loss due to diabetes mellitus type 2 retinopathy | 437.6 (339.7 to 561.1) | -- | 38.8 (26.0 to 55.8) | 15.6* (9.2 to 21.5) | -7.8* (-13.0 to -3.1) |
| Diabetic neuropathy due to diabetes mellitus type 2 | 20634.9 (16119.2 to 26172.1) | -- | 2694.4 (1753.5 to 4018.9) | 31.5* (22.6 to 40.3) | 4.8 (-2.3 to 11.8) |
| Chronic kidney disease | 58188.5 (46876.2 to 69550.5) | 1373.5 (903.3 to 1930.8) | 566.2 (403.9 to 774.5) | 24.4* (17.0 to 32.7) | -0.9 (-6.8 to 5.8) |
| Chronic kidney disease due to diabetes mellitus type 1 | 330.4 (275.3 to 392.1) | 7.6 (5.0 to 11.0) | 43.0 (27.8 to 62.5) | 26.3* (17.1 to 36.1) | 0.7 (-6.7 to 8.5) |
| Albuminuria with preserved GFR due to type 1 diabetes mellitus | 129.7 (92.1 to 167.5) | -- | -- | -- | -- |
| End-stage chronic kidney disease due to diabetes mellitus type 1 | 77.4 (50.9 to 108.0) | -- | 30.3 (17.6 to 47.1) | 28.9* (16.6 to 42.2) | 2.8 (-7.1 to 13.4) |
| Stage III chronic kidney disease due to diabetes mellitus type 1 | 89.9 (70.5 to 115.0) | 7.6 (5.0 to 11.0) | 1.8 (1.1 to 2.7) | 21.9* (6.6 to 38.6) | -2.9 (-15.0 to 10.5) |
| Stage IV chronic kidney disease due to diabetes mellitus type 1 | 19.4 (11.6 to 29.6) | -- | 2.7 (1.4 to 4.5) | 23.4* (7.1 to 40.5) | -1.6 (-14.6 to 12.0) |
| Stage V chronic kidney disease due to diabetes mellitus type 1 | 14.0 (9.7 to 19.7) | -- | 8.1 (5.1 to 12.4) | 19.0* (8.6 to 30.0) | -5.2 (-13.5 to 3.7) |
| Chronic kidney disease due to diabetes mellitus type 2 | 10981.2 (8491.0 to 13395.3) | 140.0 (91.2 to 196.2) | 91.7 (62.5 to 126.5) | 25.0* (17.1 to 33.8) | -0.3 (-6.6 to 6.6) |
| Albuminuria with preserved GFR due to type 2 diabetes mellitus | 8974.4 (6521.3 to 11371.7) | -- | -- | -- | -- |
| End-stage chronic kidney disease due to diabetes mellitus type 2 | 63.1 (39.5 to 91.9) | -- | 25.1 (14.1 to 38.8) | 25.3* (13.4 to 37.4) | -0.1 (-9.6 to 9.5) |
| Stage III chronic kidney disease due to diabetes mellitus type 2 | 1751.1 (1430.1 to 2180.1) | 140.0 (91.2 to 196.2) | 9.1 (5.6 to 14.2) | 21.3* (6.2 to 37.6) | -3.3 (-15.3 to 9.7) |
| Stage IV chronic kidney disease due to diabetes mellitus type 2 | 114.5 (70.5 to 174.2) | -- | 13.8 (7.6 to 22.6) | 23.8* (7.7 to 41.4) | -1.3 (-14.2 to 12.7) |
| Stage V chronic kidney disease due to diabetes mellitus type 2 | 78.2 (55.1 to 109.7) | -- | 43.8 (27.5 to 67.8) | 26.1* (15.8 to 38.2) | 0.5 (-7.7 to 10.2) |
| Chronic kidney disease due to hypertension | 1555.2 (1317.5 to 1836.1) | 63.8 (41.6 to 90.6) | 95.6 (64.0 to 134.4) | 25.0* (16.6 to 34.4) | -0.3 (-7.0 to 7.2) |
| Albuminuria with preserved GFR due to hypertension | 396.5 (290.2 to 502.3) | -- | -- | -- | -- |
| End-stage chronic kidney disease due to hypertension | 51.8 (36.9 to 70.0) | -- | 20.7 (12.5 to 29.9) | 25.6* (14.9 to 36.3) | 0.1 (-8.4 to 8.7) |
| Stage III chronic kidney disease due to hypertension | 873.3 (713.1 to 1087.6) | 63.8 (41.6 to 90.6) | 5.9 (3.6 to 9.3) | 12.3 (-3.8 to 29.1) | -10.5 (-23.3 to 2.9) |
| Stage IV chronic kidney disease due to hypertension | 139.2 (85.7 to 212.6) | -- | 16.6 (9.2 to 27.3) | 24.3* (7.9 to 41.5) | -0.9 (-14.0 to 12.8) |
| Stage V chronic kidney disease due to hypertension | 94.3 (66.8 to 131.9) | -- | 52.4 (33.1 to 81.1) | 26.6* (15.8 to 38.6) | 0.9 (-7.7 to 10.5) |
| Chronic kidney disease due to glomerulonephritis | 1856.3 (1579.5 to 2180.8) | 84.3 (55.1 to 119.1) | 107.5 (72.2 to 152.2) | 24.6* (16.5 to 33.5) | -0.7 (-7.1 to 6.4) |
| Albuminuria with preserved GFR due to glomerulonephritis | 572.7 (420.5 to 726.2) | -- | -- | -- | -- |
| End-stage chronic kidney disease due to glomerulonephritis | 75.9 (48.6 to 110.7) | -- | 28.7 (16.7 to 46.8) | 21.8* (9.7 to 33.1) | -3.0 (-12.6 to 6.1) |
| Stage III chronic kidney disease due to glomerulonephritis | 963.9 (788.4 to 1191.4) | 84.3 (55.1 to 119.1) | 6.8 (4.2 to 10.5) | 19.3* (4.9 to 35.6) | -4.9 (-16.4 to 8.1) |
| Stage IV chronic kidney disease due to glomerulonephritis | 145.3 (88.6 to 222.5) | -- | 17.3 (9.5 to 28.8) | 24.8* (8.2 to 42.8) | -0.5 (-13.7 to 13.8) |
| Stage V chronic kidney disease due to glomerulonephritis | 98.5 (69.4 to 138.2) | -- | 54.6 (34.6 to 84.9) | 26.7* (15.5 to 38.9) | 1.0 (-7.9 to 10.7) |
| Chronic kidney disease due to other and unspecified causes | 43465.3 (35026.1 to 52062.3) | 1077.8 (708.9 to 1515.6) | 228.4 (164.3 to 316.0) | 23.3* (14.8 to 32.5) | -1.7 (-8.5 to 5.6) |
| Albuminuria with preserved GFR due to other and unspecified causes | 29418.8 (21454.7 to 36956.3) | -- | -- | -- | -- |
| End-stage chronic kidney disease due to other causes | 84.9 (56.5 to 119.0) | -- | 32.8 (18.9 to 51.0) | 25.0* (12.9 to 37.8) | -0.3 (-10.0 to 9.9) |
| Stage III chronic kidney disease due to other causes | 13567.0 (11081.7 to 16802.3) | 1077.8 (708.9 to 1515.6) | 82.5 (51.0 to 126.9) | 18.4* (5.9 to 31.4) | -5.7 (-15.6 to 4.8) |
| Stage IV chronic kidney disease due to other causes | 233.8 (143.4 to 357.0) | -- | 27.2 (15.1 to 44.9) | 25.7* (10.2 to 42.7) | 0.2 (-12.1 to 13.8) |
| Stage V chronic kidney disease due to other causes | 160.8 (113.5 to 225.0) | -- | 85.9 (54.0 to 132.0) | 27.1* (15.9 to 39.9) | 1.3 (-7.6 to 11.6) |
| Acute glomerulonephritis | 3.0 (2.0 to 4.6) | 44.8 (28.4 to 66.5) | 0.2 (0.1 to 0.3) | -1.4 (-5.8 to 2.0) | -21.4* (-24.9 to -18.7) |
| **Skin and subcutaneous diseases** | **105069.0 (96951.0 to 115014.3)** | **223096.2 (192962.7 to 257871.5)** | **2032.7 (1368.1 to 2943.8)** | **24.7* (23.6 to 25.8)** | **-0.6 (-1.5 to 0.2)** |
| Dermatitis | 12956.8 (10909.8 to 15594.6) | 19219.6 (13655.8 to 25990.3) | 436.3 (260.8 to 669.9) | 21.2* (19.5 to 22.9) | -3.4* (-4.8 to -2.1) |
| Atopic dermatitis | 6568.7 (5790.5 to 7471.2) | 950.9 (775.2 to 1129.2) | 281.1 (155.8 to 479.0) | 20.7* (18.5 to 22.9) | -3.8* (-5.6 to -2.0) |
| Contact dermatitis | 5824.6 (3853.1 to 8460.8) | 16531.9 (10906.0 to 23295.2) | 145.4 (80.9 to 235.4) | 21.6* (19.1 to 24.2) | -3.1* (-5.1 to -1.0) |
| Seborrhoeic dermatitis | 722.9 (572.1 to 894.8) | 1736.8 (1374.6 to 2118.0) | 9.8 (5.4 to 16.2) | 29.3* (23.8 to 35.6) | 3.1 (-1.3 to 8.1) |
| Psoriasis | 5730.3 (5262.6 to 6212.2) | 689.6 (594.5 to 784.8) | 495.3 (348.5 to 655.6) | 22.4* (19.9 to 24.7) | -2.4* (-4.5 to -0.6) |
| Bacterial skin diseases | 415.2 (378.5 to 452.8) | 11150.7 (10193.6 to 12204.5) | 10.0 (6.4 to 15.2) | 13.4* (8.7 to 18.0) | -9.6* (-13.3 to -6.0) |
| Cellulitis | 139.4 (113.7 to 170.6) | 2810.9 (2318.2 to 3354.1) | 8.0 (5.1 to 11.8) | 10.6* (5.5 to 15.9) | -11.8* (-15.9 to -7.6) |
| Pyoderma | 357.5 (326.0 to 390.3) | 8339.9 (7460.2 to 9218.8) | 2.0 (0.8 to 4.3) | 25.9* (20.8 to 31.0) | 0.4 (-3.7 to 4.4) |
| Impetigo | 38.1 (33.1 to 43.0) | 702.9 (620.5 to 795.7) | 0.2 (0.1 to 0.5) | 28.8* (21.5 to 36.5) | 2.6 (-3.1 to 8.8) |
| Abscess and other bacterial skin diseases | 319.5 (286.7 to 351.8) | 7637.0 (6772.8 to 8526.5) | 1.8 (0.7 to 3.8) | 25.6* (20.0 to 31.2) | 0.1 (-4.3 to 4.6) |
| Scabies | 8586.8 (5994.4 to 11773.6) | 25480.9 (17676.6 to 35005.6) | 219.4 (114.0 to 381.3) | 30.9* (29.1 to 32.8) | 4.4* (2.9 to 5.9) |
| Fungal skin diseases | 35518.0 (27127.4 to 45440.4) | 110035.8 (82645.6 to 142443.6) | 199.2 (76.3 to 421.4) | 25.1* (24.0 to 26.1) | -0.2 (-1.2 to 0.5) |
| Tinea capitis | 2522.4 (1755.8 to 3410.4) | 3926.4 (2768.7 to 5110.0) | 14.1 (5.2 to 30.6) | 6.2* (2.8 to 10.1) | -15.3* (-18.1 to -12.2) |
| Other fungal skin diseases | 32995.5 (24706.7 to 43087.0) | 106109.3 (78615.2 to 139065.5) | 185.2 (70.4 to 389.5) | 26.9* (26.1 to 27.6) | 1.1* (0.5 to 1.7) |

| | | | | | |
|---|---|---|---|---|---|
| Viral skin diseases | 3168.8 (2843.2 to 3517.6) | 3023.3 (2756.9 to 3295.0) | 95.7 (60.8 to 143.8) | 24.3* (21.6 to 26.9) | -0.9 (-3.1 to 1.2) |
| Viral warts | 2575.8 (2255.6 to 2914.0) | 2151.6 (1887.2 to 2400.0) | 77.8 (50.0 to 117.9) | 23.9* (21.0 to 26.8) | -1.3 (-3.5 to 1.1) |
| Molluscum contagiosum | 593.0 (534.0 to 659.1) | 871.7 (779.3 to 965.7) | 17.9 (11.4 to 26.6) | 26.2* (20.8 to 32.2) | 0.6 (-3.7 to 5.4) |
| Acne vulgaris | 2233.4 (1950.2 to 2582.6) | 925.4 (788.1 to 1082.8) | 47.3 (28.2 to 73.4) | 29.2* (25.9 to 32.4) | 3.0* (0.4 to 5.5) |
| Alopecia areata | 1187.4 (1055.7 to 1319.2) | 2057.8 (1851.5 to 2280.5) | 38.6 (24.3 to 58.4) | 21.9* (18.3 to 25.5) | -2.9 (-5.7 to 0.1) |
| Pruritus | 5279.8 (3726.8 to 7429.8) | 4053.7 (2817.7 to 5497.4) | 56.0 (24.6 to 108.6) | 27.9* (26.2 to 29.7) | 2.0* (0.6 to 3.4) |
| Urticaria | 3726.9 (2639.3 to 5103.4) | 6486.2 (4682.8 to 8870.6) | 218.8 (131.1 to 339.0) | 26.2* (23.6 to 28.6) | 0.6 (-1.5 to 2.5) |
| Decubitus ulcer | 40.5 (26.9 to 57.1) | 146.9 (96.6 to 208.3) | 7.2 (4.1 to 11.8) | 19.3* (17.9 to 20.6) | -4.9* (-6.0 to -3.9) |
| Other skin and subcutaneous diseases | 37835.8 (34393.8 to 41593.9) | 39826.3 (36327.6 to 43751.3) | 208.8 (98.7 to 389.4) | 29.9* (29.0 to 30.8) | 3.5* (2.8 to 4.2) |
| **Sense organ diseases** | **192138.7 (182561.3 to 201653.1)** | **--** | **4341.2 (2747.8 to 6653.1)** | **24.6* (23.0 to 26.1)** | **-0.7 (-2.0 to 0.5)** |
| Blindness and vision impairment | 118610.1 (108130.2 to 129438.7) | -- | 2140.2 (1342.4 to 3348.3) | 23.0* (21.3 to 24.4) | -2.0* (-3.3 to -0.8) |
| Glaucoma | 27.0 (16.9 to 39.2) | -- | 3.5 (1.8 to 5.7) | 20.1* (15.9 to 24.2) | -4.3* (-7.7 to -1.0) |
| Cataract | 3660.6 (2771.4 to 4821.8) | -- | 309.4 (204.0 to 449.7) | 18.6* (14.8 to 22.7) | -5.5* (-8.5 to -2.2) |
| Age-related macular degeneration | 25.4 (16.1 to 37.1) | -- | 3.1 (1.8 to 4.9) | 23.9* (17.3 to 30.8) | -1.2 (-6.5 to 4.2) |
| Refraction disorders | 11612.9 (8988.2 to 15014.6) | -- | 523.4 (342.1 to 769.8) | 22.4* (18.9 to 26.0) | -2.4 (-5.2 to 0.5) |
| Near vision loss | 105857.8 (94813.2 to 116242.6) | -- | 1085.2 (509.8 to 1995.5) | 24.9* (23.9 to 26.0) | -0.4 (-1.2 to 0.5) |
| Other vision loss | 2463.1 (1947.8 to 3223.2) | -- | 215.8 (142.9 to 307.2) | 21.3* (17.8 to 24.6) | -3.3* (-6.1 to -0.7) |
| Age-related and other hearing loss | 101421.5 (92677.4 to 110387.9) | -- | 2053.3 (1275.2 to 3144.7) | 26.2* (23.4 to 28.9) | 0.6 (-1.6 to 2.7) |
| Other sense organ diseases | 6042.7 (5331.7 to 6789.1) | -- | 147.7 (91.3 to 214.7) | 26.5* (24.1 to 28.9) | 0.8 (-1.0 to 2.7) |
| Chronic other sense organ diseases | 5268.4 (4596.3 to 5998.5) | -- | 137.1 (84.7 to 200.8) | 26.5* (24.0 to 29.2) | 0.8 (-1.2 to 3.0) |
| Acute other sense organ diseases | 774.3 (700.5 to 848.4) | -- | 10.5 (6.2 to 16.2) | 26.5* (20.3 to 33.2) | 0.8 (-4.1 to 6.2) |
| **Musculoskeletal disorders** | **132513.6 (117044.6 to 149416.9)** | **33454.1 (26140.4 to 41583.4)** | **14014.2 (9686.8 to 19406.9)** | **22.9* (21.3 to 24.6)** | **-2.0* (-3.3 to -0.7)** |
| Rheumatoid arthritis | 1796.7 (1532.8 to 2115.9) | 131.8 (90.0 to 174.7) | 242.0 (159.9 to 337.2) | 32.3* (27.8 to 36.8) | 5.5* (1.9 to 9.0) |
| Osteoarthritis | 24482.0 (20596.2 to 28843.7) | 2290.6 (1756.4 to 2961.8) | 800.9 (393.4 to 1586.1) | 29.9* (28.4 to 31.5) | 3.6* (2.3 to 4.8) |
| Osteoarthritis of the hip | 2831.2 (2571.3 to 3124.9) | 274.9 (225.1 to 327.2) | 92.8 (47.2 to 189.0) | 27.7* (24.5 to 31.1) | 1.8 (-0.7 to 4.5) |
| Osteoarthritis of the knee | 21650.9 (17804.5 to 25873.5) | 2015.7 (1475.3 to 2686.0) | 708.1 (347.0 to 1406.2) | 30.2* (28.6 to 32.0) | 3.8* (2.5 to 5.2) |
| Low back pain | 52747.5 (39144.8 to 69498.7) | 22044.3 (15678.7 to 29821.5) | 6080.7 (3749.6 to 9114.0) | 18.6* (17.4 to 19.8) | -5.5* (-6.4 to -4.5) |
| Low back pain with leg pain | 18750.9 (13487.9 to 25202.7) | 7819.2 (5521.5 to 10821.8) | 2732.0 (1654.6 to 4153.0) | 18.6* (17.2 to 20.0) | -5.4* (-6.6 to -4.3) |
| Low back pain without leg pain | 33996.6 (25012.8 to 45625.6) | 14225.1 (10070.4 to 19253.9) | 3348.7 (2078.7 to 5071.5) | 18.6* (17.3 to 19.9) | -5.5* (-6.5 to -4.4) |
| Neck pain | 36538.9 (26269.2 to 49749.4) | 8341.6 (5136.3 to 11528.4) | 3677.9 (2212.8 to 5671.9) | 24.7* (21.4 to 27.4) | -0.6 (-3.2 to 1.5) |
| Gout | 3497.9 (2747.5 to 4307.3) | 645.8 (449.5 to 874.8) | 113.7 (72.3 to 165.5) | 26.1* (21.1 to 31.0) | 0.5 (-3.4 to 4.5) |
| Other musculoskeletal disorders | 35953.9 (25217.7 to 48235.3) | -- | 3099.1 (1815.9 to 4738.5) | 27.3* (21.5 to 32.4) | 1.5 (-3.2 to 5.5) |
| **Other non-communicable diseases** | **333542.3 (314381.3 to 352217.6)** | **207144.8 (177864.9 to 235005.6)** | **4522.1 (3017.9 to 6586.8)** | **23.4* (21.9 to 24.9)** | **-1.6* (-2.8 to -0.5)** |
| Congenital anomalies | 5165.2 (4854.6 to 5523.2) | -- | 638.9 (448.0 to 861.6) | 23.4* (20.8 to 26.1) | -1.6 (-3.7 to 0.5) |
| Neural tube defects | 152.7 (136.1 to 170.4) | -- | 44.9 (31.2 to 60.0) | 30.7* (24.6 to 36.6) | 4.2 (-0.7 to 8.8) |
| Severe motor and cognitive impairment due to anencephaly | -- | -- | -- | -- | -- |
| Encephalocoele due to neural tube defects | 19.4 (15.9 to 23.0) | -- | 3.3 (2.2 to 4.7) | 24.8* (21.2 to 28.5) | -0.5 (-3.4 to 2.5) |
| Spina bifida due to neural tube defects | 133.3 (118.9 to 149.5) | -- | 41.5 (28.8 to 55.6) | 31.2* (24.7 to 37.3) | 4.6 (-0.6 to 9.4) |
| Congenital heart anomalies | 174.1 (159.4 to 190.5) | -- | 10.2 (4.7 to 17.3) | 24.6* (20.3 to 29.8) | -0.6 (-4.1 to 3.4) |
| Critical malformations of great vessels, congenital valvular heart disease and patent ductus arteriosus | 30.5 (26.3 to 35.4) | -- | 2.0 (0.9 to 3.3) | 27.6* (25.1 to 30.0) | 1.7 (-0.3 to 3.7) |
| Other congenital heart anomalies | 54.0 (49.3 to 59.0) | -- | 3.2 (1.3 to 5.6) | 24.5* (15.6 to 34.2) | -0.7 (-7.8 to 6.9) |
| Severe congenital heart anomalies excluding single ventricle heart defects | 74.4 (66.1 to 83.5) | -- | 4.5 (2.1 to 7.5) | 23.8* (15.7 to 32.4) | -1.3 (-7.8 to 5.5) |
| Single ventricle and single ventricle pathway congenital heart anomalies | 2.5 (2.1 to 3.0) | -- | 0.2 (0.1 to 0.3) | 26.4* (23.3 to 29.4) | 0.7 (-1.7 to 3.1) |
| Ventricular septal defect and atrial septal defect | 12.6 (10.2 to 15.0) | -- | 0.4 (0.2 to 0.8) | 20.8* (18.3 to 22.9) | -3.7* (-5.7 to -2.0) |
| Orofacial clefts | 642.7 (588.2 to 697.1) | -- | 17.8 (11.6 to 25.9) | 30.3* (20.2 to 41.2) | 3.8 (-4.2 to 12.6) |
| Down's syndrome | 236.3 (206.8 to 267.6) | -- | 21.2 (14.3 to 29.2) | 20.9* (17.5 to 24.3) | -3.6* (-6.3 to -0.9) |
| Turner syndrome | 19.2 (16.6 to 22.0) | -- | 0.4 (0.2 to 0.7) | 20.4* (16.5 to 24.3) | -4.0* (-7.1 to -1.0) |
| Klinefelter syndrome | 25.8 (21.8 to 30.4) | -- | 0.3 (0.1 to 0.5) | 23.7* (15.1 to 33.6) | -1.4 (-8.2 to 6.5) |
| Other chromosomal abnormalities | 376.5 (297.6 to 462.2) | -- | 33.4 (21.7 to 47.1) | 33.0* (28.3 to 37.7) | 6.0* (2.3 to 9.7) |
| Congenital musculoskeletal and limb anomalies | 1764.3 (1567.2 to 2013.7) | -- | 258.3 (169.2 to 355.1) | 22.8* (20.5 to 25.2) | -2.1* (-4.0 to -0.2) |
| Polydactyly and syndactyly | 5.4 (4.3 to 6.6) | -- | 0.1 (0.0 to 0.1) | 17.4* (15.2 to 19.6) | -6.4* (-8.2 to -4.7) |
| Congenital limb deficiency | 778.4 (690.5 to 889.2) | -- | 114.2 (75.2 to 157.4) | 22.8* (19.4 to 26.3) | -2.1 (-4.8 to 0.7) |
| Other congenital musculoskeletal anomalies | 980.4 (870.7 to 1119.2) | -- | 144.0 (95.0 to 197.6) | 22.8* (19.6 to 25.8) | -2.1 (-4.7 to 0.3) |
| Urogenital congenital anomalies | 131.6 (110.0 to 155.6) | -- | 4.0 (2.5 to 6.2) | 5.2* (0.8 to 9.5) | -16.1* (-19.7 to -12.7) |
| Digestive congenital anomalies | 1105.9 (993.6 to 1234.7) | -- | 49.6 (33.0 to 71.0) | 12.9* (7.0 to 19.3) | -10.0* (-14.7 to -4.9) |
| Congenital diaphragmatic hernia | 9.5 (8.4 to 10.8) | -- | 0.2 (0.1 to 0.3) | 26.8* (25.5 to 28.0) | 1.1* (0.1 to 2.0) |
| Congenital atresia and/or stenosis of the digestive tract | 0.3 (0.2 to 0.4) | -- | -- | 40.1* (33.8 to 46.2) | 11.7* (6.7 to 16.5) |
| Congenital malformations of the abdominal wall after treatment | 36.9 (32.3 to 41.8) | -- | 1.6 (1.0 to 2.3) | 26.2* (25.1 to 27.4) | 0.6 (-0.3 to 1.5) |
| Other congenital malformations of the digestive tract | 1059.1 (948.2 to 1189.7) | -- | 47.8 (31.7 to 68.5) | 12.5* (6.4 to 19.1) | -10.3* (-15.2 to -5.1) |
| Other congenital anomalies | 1510.3 (1301.2 to 1752.9) | -- | 199.0 (134.4 to 277.6) | 24.1* (16.6 to 32.2) | -1.1 (-7.0 to 5.4) |

| | | | | | |
|---|---|---|---|---|---|
| Other congenital birth defects | -- | -- | 14.3 (10.3 to 18.7) | 36.1* (30.8 to 41.5) | 8.5* (4.2 to 12.8) |
| Hearing loss due to other congenital anomalies | 1510.3 (1301.2 to 1752.9) | -- | 184.7 (123.2 to 260.8) | 23.3* (15.2 to 31.9) | -1.7 (-8.1 to 5.1) |
| Urinary diseases and male infertility | 5375.5 (4513.7 to 6199.8) | 28604.3 (23555.7 to 34222.7) | 191.8 (122.4 to 275.5) | 19.2* (16.7 to 21.6) | -5.0* (-7.0 to -3.1) |
| Urinary tract infections | 375.2 (290.9 to 471.2) | 19478.5 (14877.1 to 24740.7) | 12.3 (7.2 to 19.2) | 30.3* (22.2 to 38.0) | 3.9 (-2.6 to 10.0) |
| Urolithiasis | 305.4 (224.5 to 404.2) | 8068.9 (5969.4 to 10676.4) | 22.7 (13.8 to 35.0) | 16.4* (9.7 to 23.9) | -7.2* (-12.6 to -1.2) |
| Benign prostatic hyperplasia | 4207.7 (3393.3 to 4988.8) | 1056.8 (829.7 to 1273.6) | 142.1 (88.0 to 209.8) | 20.7* (18.0 to 23.3) | -3.8* (-6.0 to -1.7) |
| Male infertility | 572.3 (346.5 to 863.5) | -- | 3.2 (1.2 to 7.5) | 25.4* (15.6 to 34.6) | -0.1 (-7.9 to 7.3) |
| Other urinary diseases | -- | -- | 11.5 (7.5 to 16.4) | -1.1 (-5.9 to 3.3) | -21.2* (-25.0 to -17.6) |
| Gynecological diseases | 109953.8 (105451.1 to 115017.2) | 19893.2 (18670.0 to 21242.6) | 1704.0 (1168.5 to 2391.9) | 24.5* (22.6 to 26.4) | -0.8 (-2.3 to 0.8) |
| Uterine fibroids | 33565.6 (28309.4 to 39669.6) | 655.4 (433.9 to 929.5) | 315.7 (185.4 to 525.6) | 17.4* (10.3 to 25.8) | -6.4 (-12.1 to 0.3) |
| Polycystic ovarian syndrome | 5923.5 (4687.5 to 7555.9) | 228.2 (134.7 to 375.9) | 50.6 (23.0 to 96.7) | 29.8* (27.9 to 31.8) | 3.5* (2.0 to 5.1) |
| Female infertility | 113.1 (63.7 to 185.0) | -- | 0.6 (0.2 to 1.5) | 35.8* (20.9 to 50.2) | 8.3 (-3.7 to 19.7) |
| Endometriosis | 6305.8 (4982.1 to 8004.4) | 315.7 (170.4 to 505.5) | 574.1 (363.3 to 858.7) | 28.8* (26.5 to 31.1) | 2.7* (0.8 to 4.5) |
| Genital prolapse | 11725.4 (9486.2 to 14454.5) | 1338.2 (906.9 to 1877.1) | 36.9 (16.9 to 70.3) | 21.7* (19.3 to 23.9) | -3.0* (-4.9 to -1.3) |
| Premenstrual syndrome | 71560.0 (67495.9 to 75704.7) | 13814.9 (12744.4 to 14838.0) | 585.5 (378.5 to 881.2) | 24.0* (22.3 to 25.7) | -1.1 (-2.5 to 0.2) |
| Other gynecological diseases | 7656.8 (7177.1 to 8145.2) | 3540.7 (3115.1 to 4047.1) | 140.5 (91.0 to 206.2) | 24.7* (20.8 to 28.2) | -0.6 (-3.7 to 2.2) |
| Other gynecological diseases | 6918.6 (6437.6 to 7411.7) | 3540.7 (3115.1 to 4047.1) | 125.1 (80.8 to 183.6) | 25.0* (21.1 to 28.9) | -0.4 (-3.5 to 2.7) |
| Other gynecological diseases with anemia | 738.2 (674.9 to 807.3) | -- | 15.4 (9.6 to 22.7) | 21.9* (7.0 to 38.2) | -2.9 (-14.7 to 10.1) |
| Hemoglobinopathies and hemolytic anaemias | 110925.3 (108545.2 to 113408.3) | -- | 273.6 (180.8 to 396.9) | 17.8* (7.0 to 29.3) | -6.1 (-14.7 to 3.1) |
| Thalassemias | 0.6 (0.6 to 0.7) | -- | 0.0 (0.0 to 0.1) | 35.0* (12.9 to 58.6) | 7.6 (-10.0 to 26.4) |
| Thalassaemias trait | 20758.4 (19930.2 to 21663.0) | -- | 134.8 (86.4 to 201.1) | 26.0* (7.9 to 48.2) | 0.4 (-14.0 to 18.1) |
| Sickle cell disorders | 11.2 (9.0 to 13.8) | -- | 1.7 (1.0 to 2.6) | 42.0* (22.9 to 67.1) | 13.2 (-2.0 to 33.2) |
| Sickle cell trait | 22908.5 (20617.9 to 25491.3) | -- | 49.3 (32.6 to 71.7) | 13.1* (3.7 to 23.6) | -9.8* (-17.3 to -1.5) |
| G6PD deficiency | 19808.9 (19033.3 to 20589.5) | -- | 1.3 (0.9 to 1.9) | 10.1 (-1.9 to 24.3) | -12.2* (-21.8 to -1.0) |
| G6PD trait | 54587.0 (53706.4 to 55455.8) | -- | -- | 13.5 (-8.4 to 34.7) | -9.5 (-27.0 to 7.4) |
| Other hemoglobinopathies and hemolytic anaemias | 4501.9 (4177.5 to 4854.3) | -- | 86.3 (56.8 to 125.7) | 9.0 (-0.2 to 19.4) | -13.1* (-20.5 to -4.8) |
| Endocrine, metabolic, blood, and immune disorders | 6658.8 (6127.1 to 7346.9) | -- | 179.9 (121.8 to 253.8) | 14.6* (8.4 to 20.6) | -8.6* (-13.6 to -3.8) |
| Anemia due to endocrine, metabolic, blood, and immune disorders | 4806.1 (4536.8 to 5084.9) | -- | 112.3 (74.8 to 163.1) | 7.6 (-0.5 to 16.4) | -14.2* (-20.7 to -7.2) |
| Endocrine, metabolic, blood, and immune disorders | 1845.0 (1389.8 to 2409.7) | -- | 66.9 (41.7 to 101.0) | 28.7* (21.9 to 35.3) | 2.6 (-2.9 to 7.8) |
| Heart failure due to endocrine, metabolic, blood, and immune disorders | 7.7 (5.5 to 10.6) | -- | 0.7 (0.4 to 1.1) | 17.6* (15.6 to 19.5) | -6.3* (-7.9 to -4.8) |
| Oral disorders | 239405.0 (206684.3 to 272372.2) | 158647.3 (130222.4 to 185210.9) | 1533.9 (870.9 to 2639.5) | 24.9* (23.8 to 25.8) | -0.4 (-1.3 to 0.3) |
| Caries of deciduous teeth | -- | -- | -- | -- | -- |
| Caries of permanent teeth | 163076.0 (127955.4 to 203952.6) | 149286.2 (120523.9 to 174979.6) | 116.7 (50.8 to 221.3) | 15.9* (14.0 to 17.8) | -7.6* (-9.2 to -6.1) |
| Periodontal disease | 101671.5 (76589.6 to 128753.1) | 8059.4 (4937.9 to 11312.8) | 670.3 (258.6 to 1426.7) | 28.3* (27.4 to 29.2) | 2.3* (1.6 to 3.0) |
| Edentulism and severe tooth loss | 14476.6 (11869.3 to 17600.9) | 1301.7 (855.1 to 1981.5) | 410.4 (260.3 to 613.0) | 21.4* (20.0 to 22.7) | -3.3* (-4.4 to -2.2) |
| Other oral disorders | 11702.0 (10531.4 to 13012.7) | -- | 336.5 (209.0 to 494.9) | 26.1* (24.9 to 27.4) | 0.5 (-0.4 to 1.5) |
| **Injuries** | **133156.1 (123865.8 to 144750.4)** | **32997.2 (28328.7 to 38768.2)** | **5041.7 (3712.6 to 6647.0)** | **26.0* (24.7 to 27.3)** | **0.4 (-0.6 to 1.5)** |
| **Transport injuries** | **21782.8 (20020.8 to 23717.5)** | **4636.8 (3222.5 to 6174.1)** | **1317.9 (949.9 to 1762.2)** | **26.0* (24.6 to 27.4)** | **0.4 (-0.7 to 1.6)** |
| Road injuries | 17390.1 (15898.5 to 18986.2) | 4175.3 (2790.8 to 5716.4) | 1047.2 (752.4 to 1399.6) | 24.7* (23.2 to 26.2) | -0.6 (-1.8 to 0.6) |
| Pedestrian road injuries | 4502.7 (3783.5 to 5430.1) | 866.3 (496.2 to 1355.6) | 273.9 (191.7 to 377.9) | 24.2* (22.3 to 26.2) | -1.0 (-2.5 to 0.6) |
| Cyclist road injuries | 2922.6 (2365.7 to 3521.2) | 957.7 (574.5 to 1481.9) | 168.7 (115.7 to 233.5) | 33.6* (31.1 to 36.4) | 6.5* (4.5 to 8.8) |
| Motorcyclist road injuries | 4666.8 (3921.3 to 5526.6) | 748.7 (440.5 to 1160.6) | 279.5 (191.1 to 384.9) | 26.3* (24.3 to 28.3) | 0.7 (-0.9 to 2.3) |
| Motor vehicle road injuries | 4126.1 (3488.1 to 4998.2) | 1218.4 (715.0 to 1838.4) | 261.8 (186.5 to 356.6) | 12.8* (11.1 to 14.6) | -10.1* (-11.4 to -8.6) |
| Other road injuries | 1172.0 (933.0 to 1481.0) | 384.2 (200.3 to 682.9) | 63.4 (44.2 to 89.2) | 58.1* (54.8 to 60.9) | 26.0* (23.4 to 28.3) |
| Other transport injuries | 4392.6 (3538.9 to 5262.7) | 461.5 (286.1 to 668.7) | 270.7 (190.3 to 369.5) | 31.4* (29.1 to 33.7) | 4.7* (2.9 to 6.6) |
| **Unintentional injuries** | **80052.7 (74220.9 to 87877.1)** | **26064.2 (21621.4 to 31427.3)** | **3023.0 (2155.6 to 4098.4)** | **25.5* (24.3 to 26.5)** | **0.0 (-0.9 to 0.9)** |
| Falls | 32436.4 (28021.8 to 38059.8) | 10194.5 (7019.0 to 14383.0) | 1514.8 (1070.1 to 2068.6) | 23.5* (22.3 to 24.6) | -1.6* (-2.5 to -0.7) |
| Drowning | 186.3 (161.3 to 216.9) | 17.5 (12.5 to 23.9) | 11.1 (7.9 to 15.3) | 27.2* (23.2 to 31.0) | 1.4 (-1.8 to 4.4) |
| Fire, heat, and hot substances | 9402.9 (7979.3 to 10963.8) | 494.5 (306.1 to 766.9) | 289.8 (201.4 to 405.1) | 22.2* (18.0 to 26.1) | -2.6 (-6.0 to 0.5) |
| Poisonings | 358.4 (303.9 to 419.2) | 163.9 (97.3 to 261.4) | 34.5 (22.9 to 48.4) | 30.6* (27.4 to 34.0) | 4.1* (1.5 to 6.8) |
| Poisoning by carbon monoxide | 72.7 (61.5 to 87.7) | 57.2 (27.2 to 104.2) | 5.9 (4.0 to 8.5) | 37.2* (33.3 to 41.4) | 9.4* (6.3 to 12.7) |
| Poisoning by other means | 285.7 (231.8 to 345.6) | 106.7 (58.8 to 179.1) | 28.6 (18.6 to 41.1) | 29.3* (25.7 to 33.0) | 3.0* (0.2 to 6.0) |
| Exposure to mechanical forces | 18155.0 (15434.8 to 21257.8) | 4736.5 (2938.9 to 7111.1) | 461.2 (318.5 to 664.1) | 27.6* (26.2 to 28.9) | 1.7* (0.6 to 2.7) |
| Unintentional firearm injuries | 804.7 (644.5 to 953.4) | 95.3 (41.2 to 184.7) | 29.6 (20.4 to 40.8) | 32.0* (30.5 to 33.7) | 5.3* (4.0 to 6.6) |
| Other exposure to mechanical forces | 17350.2 (14656.0 to 20488.8) | 4641.2 (2871.4 to 6955.9) | 431.6 (295.2 to 625.3) | 27.3* (25.9 to 28.6) | 1.5* (0.3 to 2.5) |
| Adverse effects of medical treatment | 223.8 (140.2 to 334.3) | 2927.6 (1930.0 to 4258.2) | 29.8 (16.1 to 51.3) | 53.4* (44.1 to 62.9) | 22.3* (14.8 to 29.9) |
| Animal contact | 3307.4 (2820.5 to 3784.4) | 2704.6 (1707.6 to 3993.6) | 82.8 (56.3 to 116.1) | 22.1* (19.1 to 25.0) | -2.6* (-5.0 to -0.4) |
| Venomous animal contact | 1385.2 (1145.9 to 1646.4) | 1326.8 (809.9 to 2043.2) | 53.2 (35.7 to 74.6) | 22.4* (18.6 to 26.2) | -2.4 (-5.4 to 0.6) |
| Non-venomous animal contact | 1922.2 (1577.6 to 2374.8) | 1377.7 (808.4 to 2095.2) | 29.5 (18.1 to 47.8) | 21.6* (18.4 to 24.1) | -3.1* (-5.6 to -1.1) |
| Foreign body | 1623.7 (1391.6 to 1861.7) | 1325.8 (822.2 to 2054.4) | 78.5 (55.8 to 107.9) | 29.5* (27.6 to 31.3) | 3.2* (1.7 to 4.6) |

| | | | | | |
|---|---|---|---|---|---|
| Pulmonary aspiration and foreign body in airway | 289.1 (231.8 to 363.0) | 50.1 (25.1 to 86.1) | 13.5 (9.3 to 18.9) | 41.2* (37.1 to 45.2) | 12.5* (9.3 to 15.7) |
| Foreign body in eyes | 209.9 (111.2 to 368.4) | 1072.1 (581.7 to 1784.7) | 14.0 (7.1 to 25.3) | 21.8* (19.4 to 23.8) | -2.9* (-4.8 to -1.3) |
| Foreign body in other body part | 1124.7 (932.1 to 1298.2) | 203.6 (110.1 to 334.5) | 51.0 (36.4 to 66.9) | 28.9* (27.1 to 30.7) | 2.8* (1.3 to 4.1) |
| Environmental heat and cold exposure | 2939.0 (2448.9 to 3492.4) | 463.0 (280.7 to 704.3) | 123.6 (86.4 to 168.7) | 26.3* (24.0 to 28.4) | 0.7 (-1.2 to 2.3) |
| Exposure to forces of nature | 1313.2 (972.6 to 1755.7) | 31.0 (20.9 to 42.5) | 57.4 (42.0 to 77.3) | 68.2* (52.0 to 89.5) | 34.1* (21.2 to 51.0) |
| Other unintentional injuries | 10106.6 (8263.6 to 12348.3) | 3005.3 (1796.1 to 4573.0) | 339.5 (231.9 to 475.4) | 26.3* (24.7 to 27.8) | 0.6 (-0.6 to 1.9) |
| **Self-harm and interpersonal violence** | **31385.3 (24667.4 to 40301.1)** | **2296.3 (1701.8 to 2999.4)** | **700.8 (535.2 to 873.8)** | **28.3* (22.4 to 35.3)** | **2.3 (-2.4 to 7.9)** |
| Self-harm | 837.4 (677.1 to 1000.1) | 301.1 (207.0 to 437.3) | 44.2 (30.9 to 59.3) | 3.8* (2.3 to 5.1) | -17.3* (-18.4 to -16.2) |
| Self-harm by firearm | 7.7 (6.5 to 9.4) | 2.1 (0.5 to 5.9) | 0.3 (0.2 to 0.5) | 1.7 (-1.8 to 4.9) | -18.9* (-21.8 to -16.4) |
| Self-harm by other specified means | 829.7 (669.1 to 992.3) | 299.0 (206.1 to 435.2) | 43.9 (30.7 to 58.8) | 3.8* (2.3 to 5.1) | -17.3* (-18.4 to -16.2) |
| Interpersonal violence | 25146.9 (18839.1 to 33956.3) | 1570.1 (1027.9 to 2245.0) | 407.8 (302.7 to 538.7) | 24.6* (21.2 to 28.5) | -0.6 (-3.4 to 2.5) |
| Assault by firearm | 265.5 (213.4 to 333.8) | 29.9 (14.5 to 54.7) | 12.1 (8.3 to 16.8) | 16.6* (14.5 to 18.7) | -7.1* (-8.7 to -5.4) |
| Assault by sharp object | 1535.5 (1222.3 to 2150.0) | 218.5 (106.3 to 378.1) | 48.5 (33.7 to 68.0) | 16.7* (14.8 to 18.6) | -6.9* (-8.5 to -5.4) |
| Sexual violence | 19021.6 (12990.7 to 28015.4) | -- | 158.5 (88.1 to 257.6) | 40.8* (38.1 to 43.3) | 12.2* (10.1 to 14.2) |
| Assault by other means | 4324.3 (3501.7 to 5346.1) | 1321.7 (862.3 to 1902.6) | 188.7 (133.6 to 262.2) | 16.0* (14.6 to 17.5) | -7.5* (-8.7 to -6.3) |
| Conflict and terrorism | 4917.7 (3329.8 to 7081.8) | 343.1 (254.1 to 457.3) | 231.8 (156.6 to 337.1) | 42.6* (24.9 to 63.9) | 13.7 (-0.4 to 30.7) |
| Executions and police conflict | 484.2 (316.0 to 718.8) | 81.9 (53.4 to 118.7) | 16.9 (11.8 to 24.6) | 23.9* (10.1 to 43.3) | -1.3 (-12.3 to 14.2) |
| **Nature of injury aggregates** | | | | | |
| Amputations | 34363.5 (31451.3 to 38200.3) | 629.9 (503.7 to 793.7) | 580.8 (388.7 to 866.3) | 21.4* (17.2 to 25.0) | -3.2* (-6.6 to -0.4) |
| Burns | 19432.8 (17612.7 to 21422.7) | 734.1 (568.6 to 945.0) | 605.4 (426.7 to 821.0) | 21.1* (17.3 to 24.8) | -3.4* (-6.5 to -0.6) |
| Fractures | 29732.3 (27762.8 to 32938.9) | 8577.9 (7004.5 to 10717.2) | 1640.5 (1121.6 to 2284.9) | 24.6* (23.3 to 25.9) | -0.7 (-1.7 to 0.4) |
| Head Injuries | 4498.3 (4276.4 to 4742.4) | 1395.3 (1124.5 to 1747.7) | 669.6 (473.4 to 902.4) | 28.1* (26.3 to 29.9) | 2.1* (0.7 to 3.6) |
| Spinal Injuries | 2229.5 (2039.8 to 2502.6) | 49.3 (38.0 to 65.2) | 659.0 (466.8 to 862.4) | 32.7* (29.3 to 36.4) | 5.8* (3.1 to 8.7) |
| Minor Injuries | 17802.9 (16645.9 to 19296.0) | 11534.4 (9522.4 to 14052.2) | 159.4 (76.2 to 304.8) | 27.3* (26.3 to 28.5) | 1.5* (0.7 to 2.4) |
| Other Injuries | 6141.7 (5791.3 to 6533.7) | 10076.3 (8556.5 to 11807.6) | 568.6 (406.3 to 763.5) | 26.6* (24.3 to 29.2) | 0.9 (-0.9 to 3.0) |
| **Impairments** | | | | | |
| Anaemia | 103460.7 (97028.6 to 109657.3) | -- | 2736.8 (1856.6 to 3880.5) | 10.6* (3.3 to 17.6) | -11.9* (-17.6 to -6.3) |
| Epilepsy | 3409.1 (2810.3 to 4068.8) | -- | 1334.2 (1019.3 to 1736.3) | 27.1* (20.4 to 34.2) | 1.3 (-4.0 to 7.0) |
| Guillain-Barré syndrome | 5.8 (3.5 to 9.2) | -- | 1.7 (0.8 to 3.0) | 26.9* (24.0 to 29.7) | 1.1 (-1.2 to 3.4) |
| Hearing loss | 106954.6 (98425.4 to 116020.6) | -- | 2324.0 (1489.1 to 3436.1) | 25.5* (22.9 to 27.9) | 0.1 (-2.0 to 1.9) |
| Heart failure | 1992.9 (1701.0 to 2356.5) | -- | 202.9 (138.2 to 286.2) | 26.0* (23.3 to 28.7) | 0.5 (-1.7 to 2.6) |
| Infertility | 3026.7 (1926.7 to 4397.5) | -- | 28.3 (14.0 to 57.7) | 33.5* (24.8 to 41.7) | 6.4 (-0.5 to 12.9) |
| Developmental intellectual disability | 9318.0 (7247.7 to 11279.9) | -- | 1339.4 (1043.3 to 1677.3) | 27.4* (23.9 to 31.6) | 1.5 (-1.3 to 4.9) |
| Pelvic inflammatory disease | 110.7 (85.8 to 139.0) | -- | 14.5 (9.0 to 20.6) | 27.8* (20.7 to 35.4) | 1.9 (-3.8 to 7.9) |
| Vision loss | 125495.7 (114421.0 to 137079.7) | -- | 2478.1 (1630.8 to 3737.4) | 24.1* (22.8 to 25.5) | -1.1 (-2.1 to 0.1) |

We did not calculate incidence for the nine impairments and certain NTDs. Blank cells mean that no estimate is available or that the estimate has a magnitude less than 50.
Table presents prevalence and incidence counts in thousands.
* Percentage changes that are statistically significant
‡ Incidence estimates for stroke represent first-ever stroke only.
^ Incidence of HIV/AIDS represents new infections of HIV only and does not include new infections of Tuberculosis in HIV positive cases.

| SR Table 10n: Global prevalence, incidence and YLDs for 2017, percentage change of YLD counts and percentage change of YLD rates between 2007 and 2017 by cause, age 50 to 54 years | | | | | |
|---|---|---|---|---|---|
| **Cause** | **Prevalence in thousands (95% UI)** | **Incidence in thousands (95% UI)** | **YLDs in thousands (95% UI)** | | |
| | **2017 counts** | **2017 counts** | **2017 counts** | **Percentage change in counts between 2007 and 2017** | **Percentage change in rates between 2007 and 2017** |
| **All causes** | **421672.0 (421039.8 to 422297.0)** | **1801242.0 (1535172.7 to 2098115.1)** | **63995.3 (47843.5 to 82658.5)** | **26.7* (25.9 to 27.7)** | **-0.4 (-1.1 to 0.3)** |
| **Communicable, maternal, neonatal, and nutritional disorders** | **276455.7 (254325.8 to 301736.3)** | **1214520.8 (954151.9 to 1504581.4)** | **4953.5 (3710.6 to 6353.2)** | **22.5* (18.0 to 27.0)** | **-3.8* (-7.3 to -0.2)** |
| **HIV/AIDS and sexually transmitted infections** | **108208.1 (97173.2 to 120934.4)** | **31927.3 (25245.1 to 39863.0)** | **344.5 (229.2 to 500.6)** | **12.2 (-13.0 to 41.7)** | **-11.8 (-31.7 to 11.3)** |
| HIV/AIDS | 2572.1 (2277.6 to 2953.9) | 53.7^ (37.4 to 71.5) | 270.4 (175.0 to 399.2) | 8.8 (-17.8 to 48.1) | -14.6 (-35.4 to 16.3) |
| HIV/AIDS - Drug-susceptible Tuberculosis | 60.4 (54.9 to 65.9) | 78.7 (70.4 to 87.9) | 22.8 (15.4 to 30.6) | 20.8* (12.8 to 29.3) | -5.1 (-11.4 to 1.6) |
| HIV/AIDS - Multidrug-resistant Tuberculosis without extensive drug resistance | 2.4 (1.6 to 3.7) | 3.2 (2.3 to 4.4) | 1.0 (0.6 to 1.6) | 15.9 (-24.4 to 75.8) | -8.9 (-40.6 to 38.1) |
| HIV/AIDS - Extensively drug-resistant Tuberculosis | 0.1 (0.1 to 0.2) | 0.1 (0.1 to 0.1) | 0.0 (0.0 to 0.1) | 103.1* (25.7 to 262.9) | 59.5 (-1.3 to 185.1) |
| HIV/AIDS resulting in other diseases | 2509.2 (2212.8 to 2889.0) | 53.7 (37.4 to 71.5) | 246.6 (156.1 to 368.6) | 7.7 (-19.8 to 51.5) | -15.4 (-37.0 to 19.0) |
| HIV/AIDS not on antiretroviral treatment without Tuberculosis | 507.4 (404.3 to 661.7) | 53.7 (37.4 to 71.5) | 94.6 (48.0 to 179.7) | -53.3* (-59.0 to -46.4) | -63.3* (-67.8 to -57.9) |
| HIV/AIDS on antiretroviral treatment without Tuberculosis | 2001.8 (1775.5 to 2287.9) | -- | 152.0 (100.8 to 215.6) | 477.0* (386.6 to 618.6) | 353.3* (282.2 to 464.5) |
| Sexually transmitted infections excluding HIV | 106702.7 (95511.7 to 119499.4) | 31873.6 (25196.5 to 39805.3) | 74.1 (38.5 to 134.9) | 26.9* (24.6 to 28.9) | -0.3 (-2.1 to 1.2) |
| Syphilis | 1712.5 (1225.2 to 2297.1) | 354.5 (242.0 to 513.7) | 7.9 (5.3 to 10.8) | 19.7* (10.5 to 29.6) | -6.0 (-13.2 to 1.8) |
| Early syphilis | 1669.6 (1178.0 to 2255.5) | 354.5 (242.0 to 513.7) | 0.4 (0.1 to 1.0) | 31.4* (20.5 to 43.6) | 3.2 (-5.3 to 12.8) |
| Tertiary syphilis | 42.9 (36.0 to 51.4) | -- | 7.5 (5.0 to 10.5) | 19.2* (9.9 to 29.5) | -6.4 (-13.7 to 1.8) |
| Chlamydial infection | 4329.6 (2844.4 to 6061.8) | 5661.2 (3704.5 to 7995.4) | 10.8 (5.8 to 19.6) | 27.9* (23.6 to 33.1) | 0.5 (-2.9 to 4.6) |
| Chlamydia episode | 4299.7 (2813.1 to 6027.5) | 5661.2 (3704.5 to 7995.4) | 6.9 (2.5 to 15.2) | 26.4* (23.5 to 29.3) | -0.7 (-3.0 to 1.6) |
| Chlamydial infection complications | 30.0 (22.9 to 38.8) | -- | 3.9 (2.4 to 5.9) | 30.5* (20.1 to 41.3) | 2.5 (-5.7 to 11.0) |
| Gonococcal infection | 871.5 (500.6 to 1456.3) | 3304.2 (1958.1 to 5111.5) | 4.1 (2.2 to 7.3) | 25.8* (18.0 to 34.3) | -1.2 (-7.3 to 5.5) |
| Gonococcal infection complications | 13.3 (9.0 to 18.2) | -- | 1.7 (1.0 to 2.8) | 30.6* (14.2 to 45.5) | 2.6 (-10.3 to 14.3) |
| Gonorrhoea episode | 858.2 (488.3 to 1443.5) | 3304.2 (1958.1 to 5111.5) | 2.4 (0.7 to 5.4) | 22.6* (14.8 to 28.3) | -3.7 (-9.8 to 0.8) |
| Trichomoniasis | 9997.0 (6334.0 to 15466.6) | 19784.9 (13852.0 to 27061.8) | 16.6 (5.8 to 38.6) | 27.3* (24.5 to 29.7) | 0.0 (-2.2 to 1.9) |
| Genital herpes | 95349.8 (83892.2 to 108683.3) | 2768.8 (1609.0 to 4073.0) | 23.4 (7.4 to 57.1) | 28.1* (26.4 to 29.7) | 0.6 (-0.7 to 1.9) |
| Other sexually transmitted infections | 51.1 (36.4 to 69.7) | -- | 11.3 (7.2 to 17.1) | 28.9* (22.9 to 36.4) | 1.3 (-3.4 to 7.1) |
| Other sexually transmitted diseases residual | -- | -- | 4.6 (2.3 to 8.7) | 25.4* (21.2 to 30.3) | -1.5 (-4.8 to 2.3) |
| Other sexually transmitted diseases | 51.1 (36.4 to 69.7) | -- | 11.3 (7.2 to 17.1) | 28.9* (22.9 to 36.4) | 1.3 (-3.4 to 7.1) |
| **Respiratory infections and tuberculosis** | **147966.2 (104279.4 to 199737.7)** | **841576.9 (595520.5 to 1125821.8)** | **619.7 (412.2 to 904.6)** | **22.6* (20.3 to 24.7)** | **-3.7* (-5.5 to -2.1)** |
| Tuberculosis | 137615.8 (91961.4 to 191330.1) | 551.5 (405.2 to 738.2) | 235.3 (150.7 to 350.5) | 19.8* (15.6 to 24.2) | -5.9* (-9.2 to -2.4) |
| Latent tuberculosis infection | 136835.8 (91255.8 to 190409.6) | -- | -- | -- | -- |
| Drug-susceptible tuberculosis | 742.7 (550.9 to 981.5) | 521.4 (379.3 to 701.4) | 223.8 (143.4 to 335.3) | 20.0* (13.2 to 25.5) | -5.7* (-11.0 to -1.4) |
| Multidrug-resistant tuberculosis without extensive drug resistance | 35.5 (15.5 to 73.3) | 28.3 (14.6 to 52.0) | 10.9 (4.6 to 22.7) | 14.3 (-43.5 to 103.7) | -10.3 (-55.6 to 60.1) |
| Extensively drug-resistant tuberculosis | 1.9 (1.0 to 3.7) | 1.9 (1.2 to 2.9) | 0.6 (0.3 to 1.3) | 53.4 (-7.6 to 172.0) | 20.5 (-27.4 to 113.7) |
| Lower respiratory infections | 311.6 (275.4 to 350.7) | 14139.5 (12168.2 to 16182.9) | 18.6 (12.2 to 26.5) | 32.0* (21.5 to 42.2) | 3.7 (-4.6 to 11.7) |
| Guillain-Barré syndrome due to lower respiratory infections | 0.6 (0.3 to 1.2) | -- | 0.2 (0.1 to 0.4) | 30.1* (26.7 to 33.0) | 2.2 (-0.5 to 4.5) |
| Lower respiratory infection episode | 310.9 (274.3 to 350.3) | 14139.5 (12168.2 to 16182.9) | 18.5 (12.1 to 26.2) | 32.0* (21.3 to 42.4) | 3.7 (-4.7 to 11.8) |
| Upper respiratory infections | 11288.6 (7873.2 to 15483.8) | 823663.7 (576725.7 to 1108374.0) | 275.7 (152.5 to 476.6) | 26.2* (24.6 to 27.6) | -0.9 (-2.1 to 0.3) |
| Guillain-Barré syndrome due to upper respiratory infections | 1.7 (0.9 to 2.8) | -- | 0.5 (0.2 to 1.0) | 30.1* (26.7 to 33.1) | 2.2 (-0.5 to 4.5) |
| Upper respiratory infection episode | 11286.9 (7871.7 to 15481.4) | 823663.7 (576725.7 to 1108374.0) | 275.2 (151.9 to 476.1) | 26.1* (24.6 to 27.6) | -0.9 (-2.1 to 0.3) |
| Otitis media | 4352.0 (3060.5 to 5939.7) | 3222.1 (2188.6 to 4403.6) | 90.0 (50.3 to 152.5) | 17.7* (12.4 to 23.7) | -7.5* (-11.7 to -2.9) |
| Acute otitis media | 176.6 (120.8 to 251.8) | 3221.5 (2188.3 to 4402.6) | 2.3 (1.0 to 4.4) | 26.3* (18.9 to 34.0) | -0.8 (-6.6 to 5.3) |
| Chronic otitis media | 4175.4 (2873.3 to 5753.9) | 0.6 (0.0 to 2.4) | 87.8 (48.8 to 148.9) | 17.5* (11.9 to 23.6) | -7.7* (-12.1 to -2.9) |
| **Enteric infections** | **3944.5 (3420.0 to 4498.5)** | **267557.3 (222492.1 to 312112.5)** | **435.4 (300.6 to 613.7)** | **49.7* (42.7 to 56.4)** | **17.6* (12.1 to 22.9)** |
| Diarrhoeal diseases | 3964.1 (3423.2 to 4518.3) | 267332.2 (222263.7 to 311893.1) | 433.6 (299.4 to 611.5) | 50.0* (42.9 to 56.8) | 17.8* (12.3 to 23.2) |
| Guillain-Barré syndrome due to diarrheal diseases | 0.6 (0.3 to 1.0) | -- | 0.2 (0.1 to 0.3) | 30.1* (26.7 to 33.1) | 2.2 (-0.4 to 4.6) |
| Diarrheal disease episode | 3963.5 (3422.6 to 4517.8) | 267332.2 (222263.7 to 311893.1) | 433.4 (299.3 to 611.3) | 50.0* (42.9 to 56.8) | 17.8* (12.3 to 23.2) |
| Typhoid and paratyphoid | 1.1 (0.4 to 2.1) | 214.8 (174.3 to 255.5) | 1.7 (1.1 to 2.6) | -0.9 (-18.8 to 20.5) | -22.2* (-36.2 to -5.4) |
| Typhoid fever | 10.2 (7.7 to 13.1) | 161.7 (126.7 to 197.7) | 1.6 (1.0 to 2.4) | 0.7 (-19.4 to 24.2) | -20.9* (-36.7 to -2.4) |
| Typhoid fever complications | 2.2 (1.5 to 3.0) | 27.9 (21.6 to 34.9) | 0.7 (0.4 to 1.1) | 0.6 (-26.2 to 40.8) | -21.0 (-42.0 to 10.6) |
| Typhoid fever episode | 8.1 (6.0 to 10.5) | 133.8 (104.2 to 162.8) | 0.9 (0.5 to 1.4) | 0.7 (-23.7 to 35.5) | -20.9 (-40.0 to 6.4) |
| Paratyphoid fever | 2.3 (1.6 to 3.3) | 53.1 (36.0 to 74.1) | 0.1 (0.1 to 0.2) | -15.4* (-27.8 to -1.9) | -33.6* (-43.3 to -22.9) |
| Intestinal perforation due to paratyphoid | 0.1 (0.1 to 0.2) | 2.7 (1.8 to 3.9) | -- | -15.8 (-33.0 to 5.8) | -33.9* (-47.4 to -16.9) |
| Paratyphoid fever episode | 2.2 (1.5 to 3.1) | 50.4 (34.1 to 70.1) | 0.1 (0.1 to 0.2) | -15.4* (-28.9 to -0.7) | -33.5* (-44.2 to -22.0) |
| iNTS | 0.4 (0.2 to 0.7) | 10.3 (5.7 to 17.9) | 0.1 (0.0 to 0.1) | -16.9* (-22.9 to -10.1) | -34.7* (-39.4 to -29.4) |
| Other intestinal infectious diseases | -- | -- | -- | -2.3 (-19.9 to 18.9) | -23.2* (-37.1 to -6.6) |
| **Neglected tropical diseases and malaria** | **52638.5 (50104.0 to 55437.0)** | **10403.1 (8496.3 to 12684.8)** | **747.9 (522.9 to 1017.7)** | **1.8 (-5.5 to 9.3)** | **-20.0* (-25.8 to -14.2)** |
| Malaria | 2719.7 (2531.1 to 2900.8) | 2127.8 (1808.2 to 2481.8) | 24.2 (17.5 to 32.9) | -14.0 (-26.8 to 2.0) | -32.5* (-42.5 to -19.9) |
| Malaria complications | 19.6 (17.7 to 21.6) | -- | 8.2 (6.4 to 10.4) | 42.0* (27.2 to 59.4) | 11.6 (-0.1 to 25.2) |
| Malaria episode | 124.4 (81.4 to 173.1) | 2127.8 (1808.2 to 2481.8) | 4.3 (2.2 to 7.2) | -18.9* (-29.8 to -6.3) | -36.3* (-44.8 to -26.4) |

| | | | | | |
|---|---|---|---|---|---|
| Malaria parasitemia | 2575.7 (2385.4 to 2777.7) | -- | 11.8 (7.3 to 17.9) | -31.4* (-47.3 to -9.4) | -46.1* (-58.6 to -28.8) |
| Chagas disease | 530.5 (449.1 to 624.0) | 7.7 (5.2 to 11.0) | 5.0 (3.2 to 7.3) | 2.6 (-4.0 to 9.7) | -19.4* (-24.6 to -13.8) |
| Acute Chagas disease | 0.0 (0.0 to 0.1) | 7.7 (5.2 to 11.0) | -- | 12.7* (7.3 to 17.9) | -11.5* (-15.7 to -7.4) |
| Asymptomatic Chagas disease | 446.3 (373.3 to 527.1) | -- | -- | -- | -- |
| Symptomatic chronic Chagas infection | 84.2 (65.4 to 104.9) | -- | 5.0 (3.2 to 7.3) | 2.6 (-4.0 to 9.7) | -19.4* (-24.6 to -13.8) |
| Leishmaniasis | 251.2 (185.1 to 336.5) | 25.5 (18.3 to 35.2) | 15.6 (9.5 to 24.7) | 73.8* (55.6 to 93.6) | 36.5* (22.2 to 52.1) |
| Visceral leishmaniasis | 0.2 (0.1 to 0.2) | 0.6 (0.5 to 0.9) | -- | -70.3* (-76.3 to -57.5) | -76.6* (-81.4 to -66.6) |
| Cutaneous and mucocutaneous leishmaniasis | 252.6 (185.9 to 338.2) | 24.9 (17.4 to 34.6) | 15.6 (9.5 to 24.7) | 74.4* (56.2 to 94.2) | 37.0* (22.7 to 52.5) |
| African trypanosomiasis | 0.2 (0.0 to 1.0) | 0.1 (0.0 to 0.5) | 0.1 (0.0 to 0.3) | -77.0 (-97.3 to 59.5) | -82.0 (-97.9 to 25.3) |
| Trypanosomiasis Gambiense | 0.2 (0.0 to 1.0) | 0.1 (0.0 to 0.5) | 0.1 (0.0 to 0.3) | -76.4 (-97.5 to 67.7) | -81.5 (-98.0 to 31.7) |
| Trypanosomiasis Rhodesiense | -- | -- | -- | -90.0* (-99.0 to -35.4) | -92.1* (-99.2 to -49.2) |
| Schistosomiasis | 8410.8 (7637.3 to 9225.7) | -- | 62.5 (31.3 to 115.8) | -15.4* (-19.9 to -9.0) | -33.5* (-37.1 to -28.5) |
| Mild schistosomiasis | 7172.5 (6486.4 to 7960.0) | -- | 39.8 (16.1 to 83.4) | -17.3* (-19.0 to -15.3) | -35.0* (-36.4 to -33.5) |
| Anemia due to schistosomiasis | 459.8 (370.1 to 575.4) | -- | 11.0 (6.3 to 18.0) | -22.8 (-41.8 to 7.1) | -39.3* (-54.3 to -15.9) |
| Schistosomiasis complications | 780.8 (627.8 to 968.2) | -- | 11.6 (6.7 to 19.2) | 2.1 (-5.3 to 10.9) | -19.8* (-25.6 to -12.9) |
| Cysticercosis | 449.2 (330.1 to 589.5) | -- | 128.9 (78.3 to 191.0) | 12.3* (4.9 to 19.2) | -11.8* (-17.6 to -6.4) |
| Cystic echinococcosis | 43.4 (24.1 to 77.6) | 10.0 (5.7 to 17.2) | 3.5 (1.8 to 6.7) | 29.5* (15.6 to 41.3) | 1.7 (-9.2 to 11.0) |
| Lymphatic filariasis | 3629.9 (3254.6 to 4064.3) | -- | 106.6 (59.4 to 167.4) | -32.8* (-44.9 to -21.6) | -47.2* (-56.7 to -38.4) |
| Prevalence of detectable microfiliaria due to lymphatic filariasis | 2644.8 (2445.2 to 2875.8) | -- | -- | -- | -- |
| Lymphatic filariasis complications | 985.1 (667.7 to 1380.0) | -- | 106.6 (59.4 to 167.4) | -32.8* (-44.9 to -21.6) | -47.2* (-56.7 to -38.4) |
| Onchocerciasis | 1003.1 (648.5 to 1687.7) | -- | 76.8 (39.7 to 138.1) | 9.5 (-10.5 to 28.5) | -14.0 (-29.7 to 0.9) |
| Asymptomatic onchocerciasis | 200.1 (0.0 to 847.9) | -- | -- | -- | -- |
| Skin disease due to onchocerciasis | 658.1 (472.0 to 881.0) | -- | 63.9 (28.5 to 124.5) | 8.7 (-17.4 to 30.7) | -14.6 (-35.1 to 2.6) |
| Vision loss due to onchocerciasis | 144.9 (100.0 to 222.9) | -- | 12.9 (7.9 to 19.9) | 13.5 (-2.3 to 30.3) | -10.9 (-23.2 to 2.3) |
| Trachoma | 152.7 (64.8 to 313.8) | -- | 15.7 (8.3 to 26.9) | -8.3 (-28.7 to 12.0) | -27.9* (-44.0 to -12.0) |
| Dengue | 256.3 (139.9 to 433.4) | 4285.3 (2611.6 to 6491.1) | 40.5 (18.0 to 75.0) | 90.7* (65.2 to 195.2) | 49.8* (29.8 to 131.9) |
| Post-dengue chronic fatigue syndrome | 180.7 (84.5 to 330.3) | -- | 36.1 (14.9 to 67.5) | 90.7* (64.4 to 196.6) | 49.8* (29.2 to 133.0) |
| Dengue episode | 75.6 (45.8 to 113.4) | 4285.3 (2611.6 to 6491.1) | 4.4 (2.3 to 7.7) | 90.5* (64.6 to 185.8) | 49.6* (29.3 to 124.5) |
| Yellow fever | 0.1 (0.0 to 0.2) | 2.3 (0.7 to 6.1) | -- | -11.4 (-37.1 to 32.5) | -30.4 (-50.6 to 4.0) |
| Asymptomatic yellow fever | 0.0 (0.0 to 0.1) | 1.3 (0.3 to 3.7) | -- | -- | -- |
| Yellow fever episode | 0.0 (0.0 to 0.1) | 1.0 (0.3 to 2.7) | -- | -11.4 (-37.1 to 32.5) | -30.4 (-50.6 to 4.0) |
| Rabies | -- | 0.8 (0.6 to 1.0) | -- | -27.9* (-44.6 to -8.1) | -43.4* (-56.5 to -27.8) |
| Intestinal nematode infections | 31540.6 (29159.0 to 34363.6) | -- | 56.2 (32.6 to 91.5) | -17.3* (-23.1 to -11.6) | -35.1* (-39.6 to -30.6) |
| Ascariasis | 13939.0 (12187.2 to 15976.9) | -- | 16.9 (9.1 to 29.0) | -20.4* (-29.0 to -11.1) | -37.5* (-44.2 to -30.2) |
| Asymptomatic ascariasis | 13004.2 (11354.1 to 14946.0) | -- | -- | -- | -- |
| Ascariasis complications | 934.8 (824.6 to 1058.8) | -- | 16.9 (9.1 to 29.0) | -20.4* (-29.0 to -11.1) | -37.5* (-44.2 to -30.2) |
| Trichuriasis | 9684.4 (8181.4 to 11584.4) | -- | 6.0 (3.4 to 9.9) | -1.2 (-10.3 to 9.7) | -22.4* (-29.5 to -13.8) |
| Asymptomatic trichuriasis | 9374.9 (7889.6 to 11235.8) | -- | -- | -- | -- |
| Trichuriasis complications | 309.5 (280.0 to 339.7) | -- | 6.0 (3.4 to 9.9) | -1.2 (-10.3 to 9.7) | -22.4* (-29.5 to -13.8) |
| Hookworm disease | 9544.4 (8632.5 to 10614.0) | -- | 33.3 (19.6 to 53.4) | -18.1* (-26.3 to -9.8) | -35.7* (-42.1 to -29.1) |
| Asymptomatic hookworm disease | 7930.5 (7186.7 to 8818.7) | -- | -- | -- | -- |
| Anemia due to hookworm disease | 387.3 (336.5 to 450.7) | -- | 8.2 (5.3 to 12.3) | -29.1* (-42.2 to -15.1) | -44.3* (-54.6 to -33.3) |
| Hookworm disease complications | 1226.5 (1115.0 to 1355.8) | -- | 25.1 (14.2 to 42.2) | -13.7* (-21.5 to -6.2) | -32.2* (-38.3 to -26.3) |
| Food-borne trematodiases | 7282.8 (6428.1 to 8354.9) | 3803.5 (3037.2 to 4566.7) | 168.1 (86.2 to 291.0) | 33.2* (25.6 to 40.9) | 4.6 (-1.3 to 10.7) |
| Asymptomatic food-borne tremodiases | 5768.3 (4751.5 to 6834.7) | 2734.6 (1282.6 to 3951.3) | -- | -- | -- |
| Food-borne tremodiases complications | 1514.5 (891.3 to 2362.3) | 1069.0 (167.4 to 2600.2) | 168.1 (86.2 to 291.0) | 33.2* (25.6 to 40.9) | 4.6 (-1.3 to 10.7) |
| Leprosy | 51.4 (47.9 to 55.3) | 4.0 (3.7 to 4.4) | 3.2 (2.1 to 4.5) | 5.6 (-2.2 to 14.0) | -17.0* (-23.2 to -10.4) |
| Ebola virus disease | -- | -- | -- | -98.0* (-98.2 to -97.6) | -98.4* (-98.6 to -98.1) |
| Ebola cases | -- | -- | -- | -98.2* (-98.3 to -98.1) | -98.6* (-98.6 to -98.5) |
| Post-Ebola chronic fatigue syndrome | -- | -- | -- | -97.9* (-98.2 to -97.6) | -98.4* (-98.6 to -98.1) |
| Zika virus disease | 2.3 (1.3 to 4.1) | 136.0 (78.2 to 250.6) | 0.0 (0.0 to 0.1) | -- | -- |
| Zika virus complications | -- | -- | -- | -- | -- |
| Zika virus episode | 2.2 (1.3 to 4.1) | 136.0 (78.2 to 250.6) | 0.0 (0.0 to 0.1) | -- | -- |
| Guinea worm disease | -- | -- | -- | -99.5* (-99.7 to -99.3) | -99.6* (-99.7 to -99.4) |
| Other neglected tropical diseases | 2073.7 (1914.4 to 2270.2) | -- | 41.0 (27.0 to 60.3) | 12.5* (1.7 to 23.4) | -11.6* (-20.1 to -3.0) |
| Acute infection due to other neglected tropical diseases | -- | -- | 0.4 (0.2 to 0.8) | 93.5* (67.6 to 186.9) | 52.0* (31.6 to 125.3) |
| Anemia due to other neglected tropical diseases | 2073.7 (1914.4 to 2270.2) | -- | 40.5 (26.5 to 59.5) | 12.0* (1.2 to 23.1) | -12.0* (-20.5 to -3.3) |
| **Other infectious diseases** | **4648.9 (4134.5 to 5238.8)** | **13639.6 (10408.1 to 18240.0)** | **186.1 (132.3 to 255.1)** | **18.2* (10.1 to 27.8)** | **-7.2 (-13.5 to 0.4)** |
| Meningitis | 485.0 (406.3 to 575.1) | 125.1 (88.2 to 169.8) | 36.7 (25.6 to 49.1) | 6.6* (2.0 to 11.3) | -16.2* (-19.9 to -12.5) |
| Pneumococcal meningitis | 174.7 (143.6 to 211.8) | 8.3 (5.3 to 12.5) | 13.7 (9.4 to 18.8) | -14.0* (-19.5 to -7.9) | -32.4* (-36.8 to -27.6) |

| | | | | | |
|---|---|---|---|---|---|
| Acute pneumococcal meningitis | 0.4 (0.2 to 0.6) | 8.3 (5.3 to 12.5) | 0.0 (0.0 to 0.1) | -9.6* (-20.0 to -0.2) | -29.0* (-37.2 to -21.6) |
| Pneumococcal meningitis complications | 174.3 (143.2 to 211.4) | -- | 13.7 (9.4 to 18.7) | -14.0* (-19.6 to -7.9) | -32.4* (-36.8 to -27.6) |
| H influenzae type B meningitis | 36.6 (26.0 to 49.2) | 2.7 (1.5 to 4.3) | 2.6 (1.8 to 3.5) | -46.6* (-49.6 to -43.8) | -58.1* (-60.4 to -55.9) |
| Acute H influenzae type B meningitis | 0.1 (0.1 to 0.2) | 2.7 (1.5 to 4.3) | -- | -48.3* (-55.1 to -42.4) | -59.4* (-64.7 to -54.8) |
| H Influenza type B meningitis complications | 36.5 (25.9 to 49.0) | -- | 2.6 (1.8 to 3.5) | -46.6* (-49.6 to -43.8) | -58.0* (-60.4 to -55.8) |
| Meningococcal infection | 51.3 (36.4 to 67.9) | 7.7 (4.7 to 12.0) | 4.2 (2.9 to 5.7) | 9.7* (6.0 to 13.5) | -13.8* (-16.8 to -10.9) |
| Acute meningococcal meningitis | 0.3 (0.2 to 0.5) | 7.7 (4.7 to 12.0) | 0.0 (0.0 to 0.1) | 22.5* (9.8 to 34.3) | -3.7 (-13.7 to 5.5) |
| Meningococcal meningitis complications | 51.0 (36.1 to 67.5) | -- | 4.2 (2.9 to 5.7) | 9.6* (5.7 to 13.4) | -13.9* (-16.9 to -10.9) |
| Other meningitis | 222.5 (163.5 to 284.0) | 106.4 (75.2 to 144.4) | 16.1 (11.2 to 21.7) | 65.7* (56.2 to 76.7) | 30.2* (22.7 to 38.8) |
| Other acute bacterial meningitis | 1.0 (0.7 to 1.4) | 22.0 (15.4 to 30.1) | 0.1 (0.1 to 0.2) | 69.4* (54.7 to 83.5) | 33.1* (21.5 to 44.1) |
| Acute viral meningitis | 3.7 (2.6 to 5.2) | 84.4 (59.5 to 114.6) | 0.5 (0.3 to 0.8) | 21.3* (10.9 to 30.5) | -4.7 (-12.9 to 2.5) |
| Other bacterial meningitis complications | 217.8 (158.3 to 278.2) | -- | 15.5 (10.7 to 20.9) | 67.7* (57.5 to 79.1) | 31.7* (23.7 to 40.7) |
| Encephalitis | 489.0 (265.9 to 790.4) | 100.7 (96.8 to 104.3) | 34.7 (24.1 to 46.2) | 23.6* (16.5 to 31.7) | -2.9 (-8.5 to 3.5) |
| Acute encephalitis | 5.3 (5.1 to 5.5) | 100.7 (96.8 to 104.3) | 0.7 (0.5 to 1.0) | 31.1* (30.6 to 31.7) | 3.0* (2.6 to 3.4) |
| Encephalitis complications | 483.7 (260.6 to 785.3) | -- | 34.0 (23.7 to 45.2) | 23.4* (16.2 to 31.7) | -3.1 (-8.7 to 3.5) |
| Diphtheria | -- | 0.3 (0.2 to 0.4) | -- | -22.7 (-41.4 to 3.9) | -39.3* (-54.0 to -18.4) |
| Whooping cough | 1.8 (1.4 to 2.3) | 13.3 (10.3 to 16.9) | 0.1 (0.1 to 0.1) | 8.4* (7.7 to 9.1) | -14.9* (-15.4 to -14.3) |
| Tetanus | 2.5 (2.4 to 2.6) | 3.7 (2.0 to 5.5) | 0.1 (0.0 to 0.1) | -9.4 (-23.7 to 7.6) | -28.8* (-40.1 to -15.4) |
| Severe tetanus | 0.2 (0.1 to 0.3) | 3.7 (2.0 to 5.5) | -- | -37.8* (-49.9 to -12.7) | -51.1* (-60.7 to -31.4) |
| Neonatal tetanus complications | 2.3 (2.2 to 2.4) | -- | 0.0 (0.0 to 0.1) | 26.5* (24.6 to 28.2) | -0.6 (-2.1 to 0.7) |
| Measles | 0.3 (0.1 to 0.6) | 10.3 (3.7 to 22.8) | 0.0 (0.0 to 0.1) | -14.2* (-22.4 to -5.8) | -32.6* (-39.1 to -26.0) |
| Varicella and herpes zoster | 505.6 (414.5 to 612.3) | 2977.5 (2487.6 to 3583.7) | 27.5 (16.2 to 42.9) | 31.0* (25.5 to 36.7) | 2.9 (-1.4 to 7.4) |
| Chickenpox | 2.6 (1.0 to 5.1) | 135.7 (53.9 to 264.9) | -- | 40.8* (36.3 to 44.7) | 10.6* (7.1 to 13.7) |
| Herpes zoster | 503.0 (412.3 to 610.0) | 2841.7 (2344.5 to 3446.2) | 27.5 (16.2 to 42.9) | 31.0* (25.5 to 36.7) | 2.9 (-1.4 to 7.4) |
| Acute hepatitis | 1138.9 (773.0 to 1664.7) | 10408.7 (7257.8 to 14939.4) | 28.1 (16.2 to 45.3) | 22.1 (-21.9 to 91.9) | -4.1 (-38.6 to 50.7) |
| Acute hepatitis A | 102.5 (81.4 to 123.9) | 1332.6 (1058.6 to 1610.1) | 4.2 (2.6 to 6.4) | 30.1* (17.0 to 44.2) | 2.2 (-8.1 to 13.3) |
| Acute hepatitis B | 994.0 (624.6 to 1507.5) | 8615.1 (5413.3 to 13065.3) | 22.8 (12.2 to 38.8) | 20.7 (-29.5 to 111.3) | -5.2 (-44.6 to 66.0) |
| Acute hepatitis C | 20.8 (8.4 to 38.2) | 180.1 (72.8 to 331.3) | 0.3 (0.1 to 0.7) | 7.5 (-3.3 to 13.7) | -15.6* (-24.0 to -10.7) |
| Acute hepatitis E | 21.6 (14.4 to 31.3) | 281.0 (187.3 to 406.8) | 0.7 (0.4 to 1.2) | 28.6* (7.6 to 51.1) | 1.0 (-15.5 to 18.7) |
| Other unspecified infectious diseases | 2538.7 (2358.9 to 2738.7) | -- | 58.9 (38.8 to 87.2) | 16.0* (7.2 to 24.7) | -8.9* (-15.8 to -2.0) |
| Guillain-Barré syndrome due to other infectious diseases | 0.4 (0.2 to 0.7) | -- | 0.1 (0.0 to 0.2) | 30.1* (26.7 to 33.1) | 2.2 (-0.5 to 4.5) |
| Other infectious diseases | -- | -- | 10.1 (6.0 to 16.6) | 33.0* (27.6 to 38.7) | 4.5* (0.3 to 9.0) |
| Anemia due to other infectious diseases | 2538.3 (2358.4 to 2738.4) | -- | 48.7 (32.0 to 72.1) | 12.9* (2.9 to 22.9) | -11.3* (-19.2 to -3.5) |
| **Maternal and neonatal disorders** | **7178.9 (6222.9 to 8207.6)** | **26.6 (23.1 to 30.1)** | **1342.2 (991.5 to 1755.2)** | **45.9* (38.0 to 55.0)** | **14.6* (8.4 to 21.7)** |
| Maternal disorders | 296.2 (264.9 to 330.0) | 26.6 (23.1 to 30.1) | 15.3 (10.7 to 20.6) | -5.6 (-15.6 to 5.1) | -25.8* (-33.7 to -17.4) |
| Maternal haemorrhage | 0.3 (0.3 to 0.4) | 1.7 (1.2 to 2.3) | -- | 6.1 (-10.0 to 24.1) | -16.7* (-29.3 to -2.5) |
| Maternal hemorrhage complications | 0.3 (0.2 to 0.3) | -- | -- | 3.6 (-12.2 to 24.3) | -18.6* (-31.0 to -2.4) |
| Maternal hemorrhage episode | 0.0 (0.0 to 0.1) | 1.7 (1.2 to 2.3) | -- | 14.5 (-20.2 to 64.4) | -10.1 (-37.3 to 29.1) |
| Maternal sepsis and other pregnancy related infections | 264.0 (234.0 to 296.7) | 3.9 (3.0 to 4.7) | 1.3 (0.5 to 2.8) | 28.9* (23.5 to 34.5) | 1.3 (-3.0 to 5.7) |
| Infertility due to puerperal sepsis | 263.7 (233.8 to 296.4) | 0.1 (0.0 to 0.1) | 1.3 (0.5 to 2.8) | 29.1* (23.7 to 34.6) | 1.4 (-2.8 to 5.7) |
| Maternal sepsis and other maternal infection episode | 0.3 (0.2 to 0.5) | 3.8 (2.9 to 4.7) | -- | 16.3 (-36.8 to 99.3) | -8.6 (-50.4 to 56.5) |
| Maternal hypertensive disorders | 0.5 (0.3 to 0.7) | 3.9 (3.3 to 4.5) | -- | 28.2 (-1.1 to 62.3) | 0.7 (-22.3 to 27.5) |
| Maternal hypertensive disorder complications | 0.1 (0.1 to 0.2) | 1.2 (1.0 to 1.4) | -- | 43.0* (40.6 to 45.6) | 12.3* (10.5 to 14.4) |
| Maternal hypertensive disorder episode | 0.4 (0.2 to 0.6) | 2.8 (2.1 to 3.3) | -- | 23.0 (-14.2 to 71.2) | -3.4 (-32.6 to 34.5) |
| Maternal obstructed labour and uterine rupture | 34.8 (28.8 to 42.0) | 0.8 (0.6 to 1.1) | 10.9 (7.1 to 15.4) | -13.5* (-23.3 to -3.2) | -32.0* (-39.7 to -23.9) |
| Obstructed labor, acute event | -- | 0.8 (0.6 to 1.1) | -- | 10.8 (-33.5 to 76.2) | -13.0 (-47.7 to 38.4) |
| Maternal obstructed labor complications | 34.8 (28.7 to 42.0) | -- | 10.9 (7.1 to 15.4) | -13.5* (-23.3 to -3.2) | -32.0* (-39.7 to -23.9) |
| Maternal abortion and miscarriage | 0.1 (0.0 to 0.1) | 6.3 (4.5 to 8.3) | -- | 10.0 (-29.5 to 60.2) | -13.6 (-44.6 to 25.8) |
| Ectopic pregnancy | 0.1 (0.1 to 0.1) | 9.9 (7.3 to 12.5) | -- | 27.0 (-18.3 to 91.1) | -0.2 (-35.8 to 50.1) |
| Other maternal disorders | -- | -- | 3.0 (2.1 to 4.1) | 18.9* (6.3 to 32.5) | -6.6 (-16.5 to 4.1) |
| Neonatal disorders | 6887.1 (5930.2 to 7919.9) | -- | 1326.9 (977.5 to 1738.8) | 46.8* (38.7 to 56.2) | 15.3* (9.0 to 22.7) |
| Neonatal preterm birth | 3867.7 (3343.0 to 4538.1) | -- | 558.5 (389.6 to 760.7) | 53.4* (33.8 to 72.3) | 20.5* (5.1 to 35.4) |
| Preterm birth complications | 3278.4 (2765.7 to 3911.6) | -- | 524.1 (359.1 to 721.3) | 56.0* (34.8 to 76.3) | 22.6* (5.9 to 38.5) |
| Retinopathy of prematurity | 589.4 (477.4 to 705.2) | -- | 34.4 (23.4 to 47.8) | 22.1* (15.5 to 29.1) | -4.1 (-9.3 to 1.4) |
| Uncomplicated preterm birth | -- | -- | -- | -- | -- |
| Neonatal encephalopathy due to birth asphyxia and trauma | 2967.1 (2196.8 to 3962.3) | -- | 517.2 (313.6 to 795.4) | 47.2* (41.4 to 54.3) | 15.6* (11.0 to 21.2) |
| Neonatal sepsis and other neonatal infections | 712.7 (441.3 to 1086.9) | -- | 214.7 (113.7 to 359.3) | 31.7* (26.7 to 37.8) | 3.4 (-0.5 to 8.3) |
| Neonatal sepsis and other neonatal infection complications | 712.7 (441.3 to 1086.9) | -- | 214.7 (113.7 to 359.3) | 31.7* (26.7 to 37.8) | 3.4 (-0.5 to 8.3) |
| Neonatal sepsis and other neonatal infection episode | -- | -- | -- | -- | -- |
| Hemolytic disease and other neonatal jaundice | 95.3 (82.3 to 109.1) | -- | 36.4 (27.9 to 46.0) | 44.2* (39.6 to 48.4) | 13.3* (9.7 to 16.6) |

| | | | | | |
|---|---|---|---|---|---|
| Other neonatal disorders | -- | -- | -- | -- | -- |
| **Nutritional deficiencies** | **83234.0 (79673.9 to 87257.9)** | **49308.1 (44516.2 to 54218.7)** | **1277.7 (854.6 to 1880.8)** | **13.3* (5.5 to 21.3)** | **-11.0* (-17.1 to -4.7)** |
| Protein-energy malnutrition | 3637.6 (3421.1 to 3848.2) | 3764.8 (3501.5 to 4013.9) | 21.1 (13.7 to 29.2) | 42.0* (28.5 to 53.9) | 11.5* (1.0 to 20.9) |
| Iodine deficiency | 6998.1 (6333.8 to 7765.3) | 40.9 (34.3 to 48.7) | 106.5 (63.2 to 173.7) | 12.5* (7.5 to 16.2) | -11.6* (-15.6 to -8.7) |
| Visible goiter without symptoms | 6890.1 (6244.3 to 7650.5) | 40.9 (34.3 to 48.7) | 72.8 (35.1 to 136.9) | 14.7* (13.0 to 16.2) | -9.9* (-11.2 to -8.7) |
| Visible goiter with complications | 107.9 (55.8 to 158.3) | -- | 33.8 (15.6 to 52.9) | 8.1 (-8.7 to 18.3) | -15.1* (-28.3 to -7.1) |
| Vitamin A deficiency | 27979.1 (25370.0 to 30566.7) | 45502.5 (40770.6 to 50463.7) | 9.0 (5.6 to 13.6) | 37.8* (28.3 to 47.8) | 8.3* (0.8 to 16.1) |
| Asymptomatic vitamin A deficiency | 27843.6 (25229.2 to 30399.7) | 45502.5 (40770.6 to 50463.7) | -- | -- | -- |
| Vitamin A deficiency complications | 135.5 (93.3 to 192.2) | -- | 9.0 (5.6 to 13.6) | 37.8* (28.3 to 47.8) | 8.3* (0.8 to 16.1) |
| Vitamin A deficiency with anemia | -- | -- | -- | -- | -- |
| Dietary iron deficiency | 54942.0 (51144.8 to 59066.6) | -- | 1130.2 (754.2 to 1650.9) | 12.4* (3.6 to 21.3) | -11.7* (-18.6 to -4.7) |
| Other nutritional deficiencies | -- | -- | 10.9 (7.1 to 15.2) | 64.1* (48.6 to 77.9) | 28.9* (16.7 to 39.8) |
| **Non-communicable diseases** | **418204.5 (416766.0 to 419400.2)** | **555765.6 (516306.6 to 600759.7)** | **54048.3 (40246.9 to 69878.1)** | **26.9* (26.0 to 27.8)** | **-0.3 (-1.0 to 0.4)** |
| **Neoplasms** | **8980.4 (8763.1 to 9227.3)** | **1871.7 (1644.1 to 2150.2)** | **673.1 (486.2 to 880.5)** | **36.0* (32.9 to 39.3)** | **6.9* (4.4 to 9.4)** |
| Lip and oral cavity cancer | 179.9 (171.0 to 189.4) | 40.4 (38.3 to 42.7) | 17.6 (12.8 to 22.9) | 33.3* (23.7 to 43.5) | 4.7 (-2.9 to 12.7) |
| Diagnosis and primary therapy phase of mouth cancer | 10.2 (9.7 to 10.7) | 40.4 (38.3 to 42.7) | 2.9 (1.9 to 4.1) | 33.7* (18.5 to 51.0) | 5.0 (-6.9 to 18.6) |
| Controlled phase of mouth cancer | 153.0 (145.5 to 160.9) | -- | 7.1 (4.5 to 10.3) | 34.7* (24.6 to 46.5) | 5.8 (-2.1 to 15.1) |
| Metastatic phase of mouth cancer | 14.5 (13.7 to 15.4) | -- | 6.4 (4.3 to 8.6) | 31.9* (16.4 to 48.3) | 3.6 (-8.5 to 16.5) |
| Terminal phase of mouth cancer | 2.2 (2.1 to 2.4) | -- | 1.2 (0.8 to 1.5) | 32.1* (23.6 to 40.1) | 3.7 (-2.9 to 10.0) |
| Nasopharynx cancer | 66.9 (58.8 to 76.3) | 14.8 (13.1 to 16.6) | 6.9 (4.8 to 9.3) | 33.2* (14.6 to 54.1) | 4.6 (-9.9 to 21.1) |
| Diagnosis and primary therapy phase of nasopharynx cancer | 3.8 (3.3 to 4.3) | 14.8 (13.1 to 16.6) | 1.1 (0.7 to 1.6) | 34.7* (4.6 to 71.3) | 5.8 (-17.9 to 34.6) |
| Controlled phase of nasopharynx cancer | 56.3 (49.4 to 64.1) | -- | 2.7 (1.7 to 4.0) | 35.3* (14.3 to 59.7) | 6.3 (-10.2 to 25.5) |
| Metastatic phase of nasopharynx cancer | 6.1 (5.4 to 6.9) | -- | 2.7 (1.8 to 3.8) | 31.4* (7.3 to 61.1) | 3.2 (-15.7 to 26.6) |
| Terminal phase of nasopharynx cancer | 0.8 (0.7 to 0.9) | -- | 0.4 (0.3 to 0.5) | 28.5* (14.5 to 43.3) | 1.0 (-10.0 to 12.6) |
| Other pharynx cancer | 64.5 (58.8 to 69.1) | 23.6 (21.2 to 25.5) | 8.2 (5.7 to 10.9) | 35.4* (23.8 to 45.7) | 6.3 (-2.8 to 14.4) |
| Diagnosis and primary therapy phase of other pharynx cancer | 3.5 (3.2 to 3.8) | 23.6 (21.2 to 25.5) | 1.0 (0.7 to 1.4) | 36.7* (27.5 to 44.3) | 7.4* (0.2 to 13.4) |
| Controlled phase of other pharynx cancer | 50.5 (46.2 to 54.0) | -- | 2.4 (1.5 to 3.5) | 37.0* (22.5 to 52.6) | 7.6 (-3.8 to 19.8) |
| Metastatic phase of other pharynx cancer | 8.8 (7.9 to 9.6) | -- | 3.9 (2.6 to 5.2) | 34.4* (20.3 to 48.2) | 5.6 (-5.5 to 16.4) |
| Terminal phase of other pharynx cancer | 1.6 (1.4 to 1.7) | -- | 0.9 (0.6 to 1.1) | 33.9* (23.4 to 42.8) | 5.2 (-3.1 to 12.2) |
| Oesophageal cancer | 80.8 (77.8 to 83.8) | 37.8 (36.5 to 39.2) | 11.9 (8.6 to 15.5) | 3.6 (-3.4 to 11.3) | -18.6* (-24.1 to -12.6) |
| Diagnosis and primary therapy phase of esophageal cancer | 6.6 (6.3 to 6.8) | 37.8 (36.5 to 39.2) | 1.9 (1.3 to 2.6) | 5.6 (-0.3 to 11.3) | -17.0* (-21.7 to -12.5) |
| Controlled phase of esophageal cancer | 58.5 (56.3 to 60.7) | -- | 2.8 (1.8 to 4.1) | 7.2 (-4.3 to 19.8) | -15.8* (-24.8 to -5.9) |
| Metastatic phase of esophageal cancer | 12.9 (12.4 to 13.4) | -- | 5.8 (3.9 to 7.6) | 2.1 (-9.1 to 14.9) | -19.8* (-28.6 to -9.8) |
| Terminal phase of esophageal cancer | 2.8 (2.7 to 2.9) | -- | 1.5 (1.1 to 1.9) | 0.6 (-3.3 to 4.8) | -21.0* (-24.1 to -17.7) |
| Stomach cancer | 254.7 (242.1 to 269.4) | 91.1 (87.1 to 95.4) | 29.6 (21.2 to 38.9) | 31.0* (20.5 to 41.3) | 2.9 (-5.3 to 11.0) |
| Diagnosis and primary therapy phase of stomach cancer | 22.3 (21.2 to 23.5) | 91.1 (87.1 to 95.4) | 6.2 (4.2 to 8.7) | 39.8* (21.2 to 61.8) | 9.8 (-4.8 to 27.1) |
| Controlled phase of stomach cancer | 201.8 (191.6 to 213.9) | -- | 9.5 (6.1 to 13.7) | 56.7* (43.6 to 71.4) | 23.1* (12.8 to 34.6) |
| Metastatic phase of stomach cancer | 24.3 (23.2 to 25.4) | -- | 10.6 (7.1 to 14.2) | 15.0* (0.8 to 30.0) | -9.6 (-20.8 to 2.1) |
| Terminal phase of stomach cancer | 6.3 (6.0 to 6.5) | -- | 3.4 (2.4 to 4.3) | 15.0* (9.8 to 20.7) | -9.6* (-13.7 to -5.2) |
| Colon and rectum cancer | 778.0 (753.4 to 802.5) | 128.5 (124.5 to 132.4) | 68.0 (48.7 to 90.5) | 38.9* (32.4 to 45.6) | 9.1* (4.0 to 14.3) |
| Diagnosis and primary therapy phase of colon and rectum cancers | 32.4 (31.4 to 33.4) | 128.5 (124.5 to 132.4) | 8.8 (6.0 to 12.0) | 42.1* (28.5 to 56.7) | 11.6* (0.9 to 23.1) |
| Controlled phase of colon and rectum cancers | 693.5 (671.4 to 716.1) | -- | 39.6 (26.0 to 56.8) | 42.2* (35.6 to 49.2) | 11.7* (6.5 to 17.2) |
| Metastatic phase of colon and rectum cancers | 37.3 (36.2 to 38.5) | -- | 15.9 (10.8 to 21.3) | 32.8* (20.5 to 45.1) | 4.3 (-5.3 to 14.0) |
| Terminal phase of colon and rectum cancers | 5.1 (5.0 to 5.3) | -- | 2.8 (1.9 to 3.5) | 29.3* (24.2 to 34.5) | 1.6 (-2.4 to 5.6) |
| Stoma from colon and rectum cancers, beyond 10 years | 9.6 (8.7 to 10.7) | -- | 0.9 (0.6 to 1.3) | 17.4* (16.3 to 18.6) | -7.8* (-8.6 to -6.8) |
| Liver cancer | 122.9 (107.5 to 139.6) | 106.9 (99.5 to 114.8) | 27.8 (19.9 to 36.9) | 45.5* (33.5 to 59.4) | 14.3* (4.9 to 25.2) |
| Liver cancer due to hepatitis B | 114.7 (101.6 to 128.2) | 59.7 (53.3 to 66.6) | 15.3 (10.7 to 20.5) | 48.8* (30.4 to 70.4) | 16.9* (2.4 to 33.8) |
| Diagnosis and primary therapy phase of liver cancer due to hepatitis B | 14.9 (13.3 to 16.6) | 59.7 (53.3 to 66.6) | 4.1 (2.7 to 5.8) | 44.7* (16.0 to 78.8) | 13.6 (-8.9 to 40.5) |
| Controlled phase of liver cancer due to hepatitis B | 83.9 (74.0 to 93.8) | -- | 3.9 (2.5 to 5.9) | 77.4* (53.3 to 103.9) | 39.3* (20.4 to 60.2) |
| Metastatic phase of liver cancer due to hepatitis B | 11.4 (10.2 to 12.7) | -- | 4.9 (3.2 to 6.7) | 39.1* (6.9 to 77.4) | 9.2 (-16.0 to 39.4) |
| Terminal phase of liver cancer due to hepatitis B | 4.5 (4.0 to 5.0) | -- | 2.4 (1.7 to 3.2) | 38.8* (28.7 to 51.1) | 9.0* (1.1 to 18.7) |
| Liver cancer due to hepatitis C | 36.5 (31.1 to 41.9) | 19.3 (16.4 to 22.1) | 5.1 (3.5 to 6.8) | 38.8* (29.2 to 50.3) | 9.1* (1.5 to 18.1) |
| Diagnosis and primary therapy phase of liver cancer due to hepatitis C | 4.8 (4.1 to 5.5) | 19.3 (16.4 to 22.1) | 1.4 (0.9 to 2.0) | 37.3* (26.0 to 51.3) | 7.9 (-1.0 to 18.8) |
| Controlled phase of liver cancer due to hepatitis C | 26.6 (22.7 to 30.5) | -- | 1.3 (0.8 to 1.9) | 56.4* (35.5 to 82.6) | 22.9* (6.4 to 43.4) |
| Metastatic phase of liver cancer due to hepatitis C | 3.7 (3.1 to 4.2) | -- | 1.6 (1.1 to 2.3) | 31.6* (22.2 to 43.6) | 3.4 (-4.0 to 12.8) |
| Terminal phase of liver cancer due to hepatitis C | 1.5 (1.2 to 1.7) | -- | 0.8 (0.5 to 1.1) | 32.5* (25.7 to 39.8) | 4.1 (-1.3 to 9.8) |
| Liver cancer due to alcohol use | 24.6 (17.9 to 34.5) | 13.3 (9.7 to 18.7) | 3.5 (2.2 to 5.3) | 37.3* (27.4 to 49.3) | 7.9* (0.1 to 17.3) |
| Diagnosis and primary therapy phase of liver cancer due to alcohol use | 3.3 (2.4 to 4.6) | 13.3 (9.7 to 18.7) | 0.9 (0.6 to 1.5) | 36.1* (27.1 to 46.4) | 6.9 (-0.2 to 15.0) |
| Controlled phase of liver cancer due to alcohol use | 17.7 (12.9 to 24.9) | -- | 0.8 (0.5 to 1.4) | 52.0* (27.4 to 81.9) | 19.4* (0.1 to 42.9) |
| Metastatic phase of liver cancer due to alcohol use | 2.6 (1.9 to 3.6) | -- | 1.2 (0.7 to 1.8) | 31.7* (23.3 to 41.4) | 3.5 (-3.1 to 11.0) |

| | | | | | |
|---|---|---|---|---|---|
| Terminal phase of liver cancer due to alcohol use | 1.0 (0.7 to 1.4) | -- | 0.5 (0.3 to 0.8) | 31.8* (23.5 to 41.2) | 3.5 (-3.0 to 10.9) |
| Liver cancer due to NASH | 10.2 (8.1 to 12.6) | 5.5 (4.4 to 6.8) | 1.5 (1.0 to 2.0) | 51.9* (42.3 to 63.4) | 19.3* (11.8 to 28.3) |
| Diagnosis and primary therapy phase of liver cancer due to NASH | 1.4 (1.1 to 1.7) | 5.5 (4.4 to 6.8) | 0.4 (0.2 to 0.6) | 50.2* (42.2 to 59.8) | 18.0* (11.7 to 25.6) |
| Controlled phase of liver cancer due to NASH | 7.3 (5.8 to 9.0) | -- | 0.4 (0.2 to 0.6) | 71.4* (49.4 to 99.0) | 34.6* (17.4 to 56.3) |
| Metastatic phase of liver cancer due to NASH | 1.1 (0.8 to 1.3) | -- | 0.5 (0.3 to 0.7) | 44.6* (37.2 to 53.7) | 13.6* (7.8 to 20.7) |
| Terminal phase of liver cancer due to NASH | 0.4 (0.3 to 0.5) | -- | 0.2 (0.2 to 0.3) | 44.6* (37.2 to 53.7) | 13.6* (7.8 to 20.7) |
| Liver cancer due to other causes | 17.1 (13.7 to 21.1) | 9.0 (7.3 to 11.1) | 2.4 (1.6 to 3.3) | 48.0* (37.5 to 61.1) | 16.3* (8.0 to 26.6) |
| Diagnosis and primary therapy phase of liver cancer due to other causes | 2.2 (1.8 to 2.8) | 9.0 (7.3 to 11.1) | 0.6 (0.4 to 0.9) | 46.0* (37.3 to 56.8) | 14.7* (7.9 to 23.2) |
| Controlled phase of liver cancer due to other causes | 12.4 (10.0 to 15.4) | -- | 0.6 (0.4 to 0.9) | 70.8* (43.0 to 102.7) | 34.1* (12.4 to 59.3) |
| Metastatic phase of liver cancer due to other causes | 1.7 (1.4 to 2.1) | -- | 0.8 (0.5 to 1.1) | 39.4* (31.4 to 49.1) | 9.5* (3.2 to 17.2) |
| Terminal phase of liver cancer due to other causes | 0.7 (0.6 to 0.8) | -- | 0.4 (0.2 to 0.5) | 39.4* (31.4 to 49.1) | 9.5* (3.2 to 17.2) |
| Gallbladder and biliary tract cancer | 11.9 (10.3 to 12.9) | 10.9 (9.2 to 11.8) | 2.6 (1.8 to 3.5) | 21.7* (15.2 to 27.9) | -4.4 (-9.5 to 0.5) |
| Diagnosis and primary therapy phase of gallbladder and biliary tract cancer | 1.7 (1.4 to 1.8) | 10.9 (9.2 to 11.8) | 0.5 (0.3 to 0.7) | 21.2* (14.9 to 27.5) | -4.8 (-9.8 to 0.2) |
| Controlled phase of gallbladder and biliary tract cancer | 6.3 (5.6 to 6.8) | -- | 0.3 (0.2 to 0.5) | 16.9* (10.2 to 23.5) | -8.1* (-13.4 to -3.0) |
| Metastatic phase of gallbladder and biliary tract cancer | 3.0 (2.5 to 3.3) | -- | 1.4 (0.9 to 1.8) | 22.6* (16.3 to 29.3) | -3.7 (-8.7 to 1.5) |
| Terminal phase of gallbladder and biliary tract cancer | 0.9 (0.7 to 0.9) | -- | 0.5 (0.3 to 0.6) | 22.6* (16.3 to 29.3) | -3.7 (-8.7 to 1.5) |
| Pancreatic cancer | 30.0 (29.1 to 30.9) | 27.0 (26.2 to 27.8) | 6.3 (4.4 to 8.2) | 22.7* (17.9 to 26.9) | -3.6* (-7.4 to -0.3) |
| Diagnosis and primary therapy phase of pancreatic cancer | 6.3 (6.1 to 6.5) | 27.0 (26.2 to 27.8) | 1.8 (1.2 to 2.5) | 22.8* (18.5 to 26.9) | -3.5* (-6.9 to -0.3) |
| Controlled phase of pancreatic cancer | 16.0 (15.6 to 16.5) | -- | 0.8 (0.5 to 1.1) | 13.6* (3.7 to 23.1) | -10.8* (-18.5 to -3.3) |
| Metastatic phase of pancreatic cancer | 5.5 (5.4 to 5.7) | -- | 2.5 (1.7 to 3.3) | 24.7* (20.4 to 28.8) | -2.0 (-5.4 to 1.2) |
| Terminal phase of pancreatic cancer | 2.2 (2.1 to 2.2) | -- | 1.2 (0.8 to 1.5) | 24.7* (20.3 to 28.7) | -2.0 (-5.5 to 1.1) |
| Larynx cancer | 124.0 (119.6 to 128.6) | 22.6 (21.8 to 23.4) | 12.3 (8.8 to 16.4) | 21.4* (15.5 to 27.7) | -4.6 (-9.3 to 0.3) |
| Diagnosis and primary therapy phase of larynx cancer | 7.4 (7.1 to 7.6) | 22.6 (21.8 to 23.4) | 2.1 (1.4 to 2.9) | 24.0* (15.7 to 32.4) | -2.6 (-9.1 to 4.0) |
| Controlled phase of larynx cancer | 105.1 (101.4 to 109.2) | -- | 6.0 (3.9 to 8.7) | 23.5* (15.5 to 33.1) | -3.0 (-9.3 to 4.6) |
| Metastatic phase of larynx cancer | 7.8 (7.5 to 8.1) | -- | 3.5 (2.4 to 4.7) | 17.7* (10.5 to 24.9) | -7.5* (-13.2 to -1.9) |
| Terminal phase of larynx cancer | 1.1 (1.1 to 1.1) | -- | 0.6 (0.4 to 0.8) | 15.1* (10.6 to 20.2) | -9.6* (-13.1 to -5.6) |
| Laryngectomy from larynx cancer, beyond 10 years | 2.6 (2.5 to 2.8) | -- | 0.1 (0.1 to 0.2) | 17.7* (16.9 to 18.5) | -7.5* (-8.2 to -6.9) |
| Tracheal, bronchus, and lung cancer | 259.3 (250.8 to 269.0) | 147.4 (142.6 to 152.6) | 40.6 (29.0 to 52.3) | 23.4* (14.9 to 32.3) | -3.1 (-9.7 to 3.9) |
| Diagnosis and primary therapy phase of lung, bronchus, and trachea cancer | 16.3 (15.7 to 16.9) | 147.4 (142.6 to 152.6) | 4.6 (3.1 to 6.4) | 31.7* (20.3 to 44.6) | 3.4 (-5.5 to 13.6) |
| Controlled phase of lung, bronchus, and trachea cancer | 180.5 (174.4 to 187.7) | -- | 8.4 (5.5 to 12.3) | 41.4* (32.0 to 52.4) | 11.1* (3.7 to 19.7) |
| Metastatic phase of lung, bronchus, and trachea cancer | 51.2 (49.5 to 53.0) | -- | 21.4 (14.6 to 28.4) | 17.5* (4.6 to 31.0) | -7.7 (-17.8 to 2.9) |
| Terminal phase of lung, bronchus, and trachea cancer | 11.3 (11.0 to 11.7) | -- | 6.1 (4.2 to 7.8) | 17.7* (11.4 to 23.6) | -7.5* (-12.5 to -2.9) |
| Malignant skin melanoma | 214.4 (170.8 to 275.3) | 26.2 (20.9 to 33.4) | 12.5 (8.0 to 18.5) | 24.2* (15.7 to 32.3) | -2.5 (-9.1 to 3.9) |
| Diagnosis and primary therapy phase of malignant skin melanoma | 5.7 (4.5 to 7.3) | 26.2 (20.9 to 33.4) | 1.6 (1.0 to 2.4) | 25.8* (16.2 to 37.0) | -1.2 (-8.7 to 7.6) |
| Controlled phase of malignant skin melanoma | 206.0 (164.1 to 264.8) | -- | 9.6 (5.8 to 14.8) | 26.1* (17.1 to 35.6) | -1.0 (-8.0 to 6.5) |
| Metastatic phase of malignant skin melanoma | 2.3 (1.9 to 2.9) | -- | 1.0 (0.7 to 1.5) | 9.8* (4.8 to 15.2) | -13.7* (-17.7 to -9.5) |
| Terminal phase of malignant skin melanoma | 0.4 (0.3 to 0.5) | -- | 0.2 (0.1 to 0.3) | 8.3* (3.7 to 13.6) | -14.9* (-18.6 to -10.7) |
| Non-melanoma skin cancer | 103.6 (54.9 to 192.3) | 475.0 (255.1 to 753.1) | 4.1 (1.6 to 8.5) | 7.7 (-10.5 to 22.6) | -15.4* (-29.7 to -3.7) |
| Non-melanoma skin cancer (squamous-cell carcinoma) | 90.0 (41.1 to 186.4) | 78.0 (38.9 to 149.4) | 3.9 (1.5 to 8.4) | 7.4 (-11.8 to 22.8) | -15.6* (-30.7 to -3.5) |
| Non-melanoma skin cancer (basal-cell carcinoma) | 38.6 (13.5 to 77.2) | 397.0 (166.6 to 668.4) | 0.2 (0.0 to 0.4) | 15.9* (4.4 to 27.3) | -8.9* (-18.0 to -0.0) |
| Breast cancer | 2073.3 (1989.0 to 2154.3) | 242.3 (231.2 to 252.6) | 160.9 (112.0 to 219.6) | 34.0* (27.8 to 40.4) | 5.3* (0.4 to 10.3) |
| Diagnosis and primary therapy phase of breast cancer | 53.8 (51.5 to 56.1) | 242.3 (231.2 to 252.6) | 14.1 (9.6 to 19.3) | 36.6* (22.1 to 53.3) | 7.3 (-4.1 to 20.4) |
| Controlled phase of breast cancer | 1799.9 (1720.6 to 1876.0) | -- | 112.1 (70.9 to 163.9) | 34.7* (28.6 to 41.0) | 5.8* (1.0 to 10.8) |
| Metastatic phase of breast cancer | 67.2 (64.1 to 70.2) | -- | 27.0 (18.7 to 35.2) | 33.2* (21.5 to 46.6) | 4.6 (-4.6 to 15.2) |
| Terminal phase of breast cancer | 5.0 (4.8 to 5.3) | -- | 2.7 (1.9 to 3.5) | 30.6* (24.2 to 36.4) | 2.6 (-2.5 to 7.1) |
| Mastectomy from breast cancer, beyond 10 years | 147.3 (138.8 to 156.5) | -- | 5.0 (2.9 to 8.0) | 18.9* (11.7 to 26.2) | -6.6* (-12.3 to -0.9) |
| Cervical cancer | 418.7 (365.0 to 442.0) | 74.9 (66.2 to 79.0) | 33.8 (24.0 to 44.7) | 36.6* (26.3 to 45.8) | 7.3 (-0.8 to 14.6) |
| Diagnosis and primary therapy phase of cervical cancer | 21.2 (18.5 to 22.4) | 74.9 (66.2 to 79.0) | 5.7 (3.8 to 8.0) | 38.7* (19.1 to 56.7) | 9.0 (-6.5 to 23.1) |
| Controlled phase of cervical cancer | 372.4 (324.1 to 393.1) | -- | 17.2 (10.8 to 25.1) | 40.0* (29.2 to 49.5) | 10.0* (1.5 to 17.4) |
| Metastatic phase of cervical cancer | 21.9 (19.5 to 23.1) | -- | 9.1 (6.1 to 12.1) | 30.8* (16.9 to 48.9) | 2.7 (-8.2 to 17.0) |
| Terminal phase of cervical cancer | 3.2 (2.9 to 3.4) | -- | 1.7 (1.2 to 2.2) | 29.4* (21.9 to 36.0) | 1.6 (-4.3 to 6.8) |
| Uterine cancer | 435.8 (415.5 to 456.8) | 52.9 (50.4 to 55.4) | 28.6 (20.3 to 38.1) | 33.1* (25.5 to 41.9) | 4.6 (-1.4 to 11.4) |
| Diagnosis and primary therapy phase of uterine cancer | 19.5 (18.6 to 20.4) | 52.9 (50.4 to 55.4) | 5.3 (3.5 to 7.3) | 33.9* (17.2 to 53.7) | 5.2 (-7.9 to 20.8) |
| Controlled phase of uterine cancer | 406.4 (387.5 to 426.1) | -- | 18.8 (12.3 to 27.7) | 34.4* (26.6 to 43.9) | 5.6 (-0.6 to 13.0) |
| Metastatic phase of uterine cancer | 9.0 (8.6 to 9.4) | -- | 4.0 (2.7 to 5.3) | 27.2* (18.4 to 37.7) | -0.1 (-7.0 to 8.1) |
| Terminal phase of uterine cancer | 0.9 (0.8 to 0.9) | -- | 0.5 (0.3 to 0.6) | 24.9* (19.1 to 32.0) | -1.9 (-6.4 to 3.7) |
| Ovarian cancer | 169.7 (162.3 to 177.6) | 32.9 (31.4 to 34.5) | 21.4 (15.5 to 27.6) | 34.2* (24.4 to 45.1) | 5.4 (-2.3 to 14.0) |
| Diagnosis and primary therapy phase of ovarian cancer | 6.0 (5.7 to 6.3) | 32.9 (31.4 to 34.5) | 1.7 (1.1 to 2.4) | 35.3* (30.1 to 40.7) | 6.3* (2.2 to 10.5) |
| Controlled phase of ovarian cancer | 131.0 (125.3 to 137.0) | -- | 6.1 (3.8 to 8.9) | 35.6* (25.9 to 47.8) | 6.5 (-1.1 to 16.1) |
| Metastatic phase of ovarian cancer | 31.0 (29.6 to 32.5) | -- | 12.7 (8.5 to 16.8) | 33.5* (19.8 to 49.4) | 4.9 (-5.9 to 17.4) |

| | | | | | |
|---|---|---|---|---|---|
| Terminal phase of ovarian cancer | 1.8 (1.7 to 1.8) | -- | 0.9 (0.7 to 1.2) | 32.9* (26.9 to 38.5) | 4.4 (-0.3 to 8.8) |
| Prostate cancer | 423.8 (356.5 to 533.1) | 47.8 (40.2 to 59.9) | 31.0 (21.1 to 44.3) | 27.3* (19.6 to 39.0) | -0.0 (-6.1 to 9.2) |
| Diagnosis and primary therapy phase of prostate cancer | 14.9 (12.5 to 18.7) | 47.8 (40.2 to 59.9) | 4.0 (2.6 to 5.7) | 31.3* (13.9 to 52.4) | 3.1 (-10.5 to 19.7) |
| Controlled phase of prostate cancer | 399.2 (335.3 to 503.1) | -- | 23.7 (15.2 to 35.3) | 28.6* (21.0 to 40.7) | 1.0 (-5.0 to 10.5) |
| Metastatic phase of prostate cancer | 6.6 (5.5 to 8.1) | -- | 2.9 (1.9 to 3.9) | 14.1* (2.5 to 28.0) | -10.4 (-19.5 to 0.5) |
| Terminal phase of prostate cancer | 0.4 (0.3 to 0.5) | -- | 0.2 (0.2 to 0.3) | 14.6* (8.5 to 23.9) | -9.9* (-14.8 to -2.7) |
| Impotence and Incontinence after 10-year survival from prostate cancer | 2.7 (2.5 to 2.9) | -- | 0.1 (0.1 to 0.2) | 15.9* (15.1 to 16.7) | -9.0* (-9.6 to -8.4) |
| Testicular cancer | 22.3 (20.4 to 24.8) | 2.8 (2.6 to 3.1) | 1.4 (1.0 to 2.0) | 30.8* (14.4 to 51.0) | 2.7 (-10.1 to 18.6) |
| Diagnosis and primary therapy phase of testicular cancer | 0.7 (0.7 to 0.8) | 2.8 (2.6 to 3.1) | 0.2 (0.1 to 0.3) | 34.1* (21.6 to 48.2) | 5.3 (-4.5 to 16.4) |
| Controlled phase of testicular cancer | 21.1 (19.3 to 23.5) | -- | 1.0 (0.6 to 1.5) | 35.0* (14.5 to 61.1) | 6.1 (-10.0 to 26.6) |
| Metastatic phase of testicular cancer | 0.4 (0.4 to 0.4) | -- | 0.2 (0.1 to 0.2) | 11.9* (4.2 to 20.4) | -12.1* (-18.2 to -5.4) |
| Terminal phase of testicular cancer | -- | -- | -- | 7.6* (0.7 to 15.4) | -15.5* (-20.9 to -9.4) |
| Kidney cancer | 235.3 (223.4 to 245.8) | 36.3 (34.4 to 38.0) | 14.1 (9.9 to 19.3) | 19.8* (13.3 to 27.1) | -5.9* (-11.0 to -0.2) |
| Diagnosis and primary therapy phase of kidney cancer | -- | 36.3 (34.4 to 38.0) | -- | -- | -- |
| Controlled phase of kidney cancer | 227.6 (216.1 to 237.7) | -- | 10.5 (6.8 to 15.6) | 21.0* (13.4 to 29.4) | -5.0 (-10.9 to 1.7) |
| Metastatic phase of kidney cancer | 6.5 (6.1 to 6.8) | -- | 2.9 (2.0 to 3.9) | 16.8* (11.8 to 22.7) | -8.3* (-12.2 to -3.6) |
| Terminal phase of kidney cancer | 1.2 (1.2 to 1.3) | -- | 0.7 (0.5 to 0.9) | 16.4* (11.5 to 22.1) | -8.5* (-12.4 to -4.1) |
| Bladder cancer | 201.3 (193.0 to 210.9) | 27.4 (26.3 to 28.7) | 17.6 (12.4 to 23.4) | 24.5* (18.6 to 30.7) | -2.2 (-6.9 to 2.7) |
| Diagnosis and primary therapy phase of bladder cancer | 10.5 (10.0 to 11.0) | 27.4 (26.3 to 28.7) | 3.0 (2.0 to 4.2) | 25.6* (12.9 to 38.3) | -1.3 (-11.3 to 8.6) |
| Controlled phase of bladder cancer | 182.2 (174.7 to 190.9) | -- | 11.7 (7.8 to 16.4) | 26.0* (19.0 to 33.8) | -1.0 (-6.5 to 5.1) |
| Metastatic phase of bladder cancer | 4.5 (4.3 to 4.7) | -- | 2.0 (1.4 to 2.7) | 16.6* (12.5 to 20.9) | -8.4* (-11.6 to -5.0) |
| Terminal phase of bladder cancer | 0.8 (0.8 to 0.9) | -- | 0.4 (0.3 to 0.6) | 16.0* (12.0 to 20.4) | -8.9* (-12.0 to -5.4) |
| Incontinence from bladder cancer, beyond 10 years | 3.3 (3.1 to 3.5) | -- | 0.5 (0.3 to 0.7) | 24.8* (23.6 to 25.8) | -2.0* (-2.9 to -1.2) |
| Brain and nervous system cancer | 124.1 (108.2 to 138.0) | 32.1 (28.3 to 35.6) | 13.0 (9.1 to 17.5) | 61.5* (49.7 to 74.1) | 26.9* (17.6 to 36.8) |
| Diagnosis and primary therapy phase of brain and nervous system cancers | 7.6 (6.7 to 8.5) | 32.1 (28.3 to 35.6) | 2.2 (1.4 to 3.2) | 82.6* (67.0 to 98.9) | 43.5* (31.2 to 56.3) |
| Controlled phase of brain and nervous system cancers | 103.7 (90.2 to 115.3) | -- | 4.9 (3.0 to 7.1) | 84.4* (65.0 to 105.7) | 44.9* (29.6 to 61.6) |
| Metastatic phase of brain and nervous system cancers | 10.9 (9.6 to 12.1) | -- | 4.9 (3.3 to 6.7) | 42.0* (33.3 to 50.5) | 11.6* (4.7 to 18.2) |
| Terminal phase of brain and nervous system cancers | 1.9 (1.7 to 2.1) | -- | 1.0 (0.7 to 1.3) | 37.7* (29.6 to 45.5) | 8.1* (1.8 to 14.3) |
| Thyroid cancer | 262.1 (249.7 to 282.6) | 29.8 (28.5 to 32.2) | 15.3 (10.4 to 21.2) | 44.0* (35.3 to 53.9) | 13.2* (6.3 to 20.9) |
| Diagnosis and primary therapy phase of thyroid cancer | 7.0 (6.7 to 7.5) | 29.8 (28.5 to 32.2) | 2.0 (1.4 to 2.8) | 44.9* (37.8 to 52.8) | 13.8* (8.2 to 20.0) |
| Controlled phase of thyroid cancer | 251.6 (239.7 to 271.3) | -- | 11.7 (7.5 to 17.3) | 45.1* (35.2 to 56.2) | 14.0* (6.2 to 22.7) |
| Metastatic phase of thyroid cancer | 3.3 (3.1 to 3.5) | -- | 1.5 (1.0 to 2.0) | 35.9* (29.1 to 43.2) | 6.8* (1.4 to 12.5) |
| Terminal phase of thyroid cancer | 0.3 (0.3 to 0.3) | -- | 0.1 (0.1 to 0.2) | 34.2* (27.7 to 41.2) | 5.4* (0.3 to 11.0) |
| Mesothelioma | 6.1 (5.8 to 6.4) | 2.5 (2.4 to 2.7) | 1.1 (0.8 to 1.5) | 19.6* (10.7 to 28.2) | -6.1 (-13.0 to 0.7) |
| Diagnosis and primary therapy phase of mesothelioma | 0.7 (0.7 to 0.7) | 2.5 (2.4 to 2.7) | 0.2 (0.1 to 0.3) | 19.4* (10.3 to 28.7) | -6.2 (-13.4 to 1.1) |
| Controlled phase of mesothelioma | 3.7 (3.6 to 4.0) | -- | 0.2 (0.1 to 0.3) | 19.3* (10.5 to 28.0) | -6.3 (-13.2 to 0.6) |
| Metastatic phase of mesothelioma | 1.5 (1.4 to 1.5) | -- | 0.7 (0.4 to 0.9) | 19.7* (10.9 to 28.4) | -6.0 (-12.9 to 0.9) |
| Terminal phase of mesothelioma | 0.2 (0.2 to 0.2) | -- | 0.1 (0.1 to 0.1) | 19.8* (11.1 to 28.1) | -5.9 (-12.7 to 0.6) |
| Hodgkin lymphoma | 41.0 (33.5 to 49.4) | 6.2 (5.1 to 7.4) | 3.1 (2.1 to 4.3) | 34.8* (22.5 to 48.1) | 5.9 (-3.7 to 16.3) |
| Diagnosis and primary therapy phase of Hodgkin disease | 1.4 (1.1 to 1.6) | 6.2 (5.1 to 7.4) | 0.4 (0.3 to 0.6) | 42.8* (30.5 to 54.8) | 12.2* (2.5 to 21.6) |
| Controlled phase of Hodgkin disease | 37.5 (30.7 to 45.3) | -- | 1.8 (1.1 to 2.7) | 42.9* (25.2 to 62.7) | 12.3 (-1.7 to 27.8) |
| Metastatic phase of Hodgkin disease | 1.9 (1.6 to 2.2) | -- | 0.8 (0.6 to 1.1) | 20.7* (11.8 to 28.8) | -5.2 (-12.2 to 1.2) |
| Terminal phase of Hodgkin disease | 0.2 (0.2 to 0.2) | -- | 0.1 (0.1 to 0.1) | 9.6* (2.3 to 16.3) | -13.9* (-19.6 to -8.6) |
| Non-Hodgkin's lymphoma | 216.7 (208.8 to 225.1) | 38.3 (36.9 to 39.6) | 16.5 (11.8 to 21.9) | 48.4* (40.6 to 56.1) | 16.6* (10.5 to 22.6) |
| Diagnosis and primary therapy phase of non-Hodgkin lymphoma | 7.8 (7.6 to 8.2) | 38.3 (36.9 to 39.6) | 2.3 (1.5 to 3.2) | 52.4* (46.3 to 58.8) | 19.7* (15.0 to 24.8) |
| Controlled phase of non-Hodgkin lymphoma | 197.9 (190.7 to 205.7) | -- | 9.2 (5.8 to 13.5) | 52.9* (42.0 to 63.6) | 20.1* (11.5 to 28.5) |
| Metastatic phase of non-Hodgkin lymphoma | 9.3 (9.0 to 9.6) | -- | 4.2 (2.8 to 5.6) | 39.7* (34.3 to 44.6) | 9.7* (5.5 to 13.6) |
| Terminal phase of non-Hodgkin lymphoma | 1.6 (1.5 to 1.6) | -- | 0.9 (0.6 to 1.1) | 38.0* (32.7 to 43.0) | 8.4* (4.2 to 12.3) |
| Multiple myeloma | 51.6 (47.7 to 62.5) | 11.7 (10.9 to 13.8) | 9.7 (6.8 to 13.0) | 39.3* (28.0 to 52.1) | 9.4* (0.5 to 19.5) |
| Diagnosis and primary therapy phase of multiple myeloma | 3.4 (3.2 to 4.2) | 11.7 (10.9 to 13.8) | 1.0 (0.7 to 1.4) | 40.9* (32.9 to 49.5) | 10.7* (4.4 to 17.4) |
| Controlled phase of multiple myeloma | 31.9 (29.5 to 38.8) | -- | 1.5 (1.0 to 2.3) | 41.5* (26.8 to 58.6) | 11.1 (-0.4 to 24.6) |
| Metastatic phase of multiple myeloma | 15.5 (14.4 to 18.7) | -- | 6.8 (4.6 to 9.2) | 39.1* (25.2 to 55.6) | 9.3 (-1.6 to 22.2) |
| Terminal phase of multiple myeloma | 0.7 (0.7 to 0.9) | -- | 0.4 (0.3 to 0.5) | 31.1* (24.3 to 38.3) | 3.0 (-2.3 to 8.7) |
| Leukaemia | 135.4 (117.8 to 147.2) | 30.8 (26.8 to 33.1) | 15.9 (11.4 to 21.2) | 39.8* (30.8 to 49.9) | 9.8* (2.7 to 17.7) |
| Acute lymphoid leukaemia | 13.1 (10.9 to 14.3) | 2.9 (2.4 to 3.1) | 1.4 (1.0 to 2.0) | 46.8* (31.5 to 59.6) | 15.3* (3.3 to 25.4) |
| Diagnosis and primary therapy phase of acute lymphoid leukemia | 1.3 (1.1 to 1.4) | 2.9 (2.4 to 3.1) | 0.4 (0.3 to 0.5) | 32.5* (21.5 to 41.3) | 4.1 (-4.6 to 11.0) |
| Controlled phase of acute lymphoid leukemia | 10.6 (8.8 to 11.6) | -- | 0.5 (0.3 to 0.8) | 52.4* (35.8 to 67.2) | 19.7* (6.7 to 31.3) |
| Metastatic phase of acute lymphoid leukemia | 1.0 (0.8 to 1.1) | -- | 0.5 (0.3 to 0.6) | 53.0* (34.5 to 68.8) | 20.2* (5.7 to 32.6) |
| Terminal phase of acute lymphoid leukemia | 0.2 (0.1 to 0.2) | -- | 0.1 (0.1 to 0.1) | 53.3* (34.6 to 69.2) | 20.4* (5.8 to 32.9) |
| Chronic lymphoid leukaemia | 48.9 (43.4 to 54.8) | 6.5 (5.8 to 7.3) | 5.8 (4.1 to 7.7) | 60.4* (44.4 to 78.6) | 26.0* (13.4 to 40.3) |

| | | | | | |
|---|---|---|---|---|---|
| Diagnosis and primary therapy phase of chronic lymphoid leukemia | 2.6 (2.3 to 3.0) | 6.5 (5.8 to 7.3) | 0.8 (0.5 to 1.1) | 64.1* (48.9 to 80.6) | 28.9* (17.0 to 41.9) |
| Controlled phase of chronic lymphoid leukemia | 39.2 (34.7 to 43.8) | -- | 1.8 (1.2 to 2.8) | 64.0* (42.5 to 89.4) | 28.8* (11.9 to 48.8) |
| Metastatic phase of chronic lymphoid leukemia | 6.9 (6.1 to 7.8) | -- | 3.1 (2.1 to 4.2) | 57.7* (42.9 to 76.0) | 23.9* (12.3 to 38.2) |
| Terminal phase of chronic lymphoid leukemia | 0.2 (0.2 to 0.2) | -- | 0.1 (0.1 to 0.2) | 51.0* (37.8 to 66.5) | 18.6* (8.3 to 30.8) |
| Acute myeloid leukaemia | 10.6 (9.6 to 11.5) | 7.5 (6.7 to 8.2) | 1.9 (1.3 to 2.5) | 30.9* (23.1 to 38.9) | 2.8 (-3.3 to 9.1) |
| Diagnosis and primary therapy phase of acute myeloid leukemia | -- | 7.5 (6.7 to 8.2) | -- | -- | -- |
| Controlled phase of acute myeloid leukemia | 7.4 (6.7 to 8.0) | -- | 0.4 (0.2 to 0.5) | 28.9* (18.4 to 39.2) | 1.3 (-7.0 to 9.4) |
| Metastatic phase of acute myeloid leukemia | 2.6 (2.3 to 2.9) | -- | 1.2 (0.8 to 1.6) | 31.3* (23.7 to 38.9) | 3.2 (-2.8 to 9.1) |
| Terminal phase of acute myeloid leukemia | 0.6 (0.5 to 0.6) | -- | 0.3 (0.2 to 0.4) | 31.3* (23.7 to 38.9) | 3.2 (-2.8 to 9.1) |
| Chronic myeloid leukaemia | 10.1 (9.0 to 11.3) | 2.9 (2.5 to 3.2) | 1.1 (0.8 to 1.4) | 13.6* (6.4 to 21.5) | -10.8* (-16.4 to -4.6) |
| Diagnosis and primary therapy phase of chronic myeloid leukemia | 0.7 (0.6 to 0.8) | 2.9 (2.5 to 3.2) | 0.2 (0.1 to 0.3) | 15.1* (7.3 to 23.5) | -9.5* (-15.7 to -3.0) |
| Controlled phase of chronic myeloid leukemia | 8.4 (7.5 to 9.4) | -- | 0.4 (0.3 to 0.6) | 16.2* (8.5 to 24.8) | -8.8* (-14.8 to -2.0) |
| Metastatic phase of chronic myeloid leukemia | 0.8 (0.7 to 0.9) | -- | 0.4 (0.2 to 0.5) | 10.7* (3.6 to 17.9) | -13.1* (-18.6 to -7.3) |
| Terminal phase of chronic myeloid leukemia | 0.2 (0.2 to 0.2) | -- | 0.1 (0.1 to 0.1) | 10.7* (3.6 to 17.9) | -13.1* (-18.6 to -7.3) |
| Other leukaemia | 52.6 (43.4 to 58.9) | 11.0 (9.1 to 12.3) | 5.7 (4.0 to 7.6) | 29.8* (18.4 to 43.7) | 2.0 (-7.0 to 12.9) |
| Metastatic phase of other leukemia | 7.4 (6.0 to 8.3) | -- | 3.3 (2.2 to 4.5) | 27.7* (17.1 to 39.4) | 0.3 (-8.0 to 9.5) |
| Diagnosis and primary therapy phase of other leukemia | -- | 11.0 (9.1 to 12.3) | -- | -- | -- |
| Terminal phase of other leukemia | 0.5 (0.4 to 0.6) | -- | 0.3 (0.2 to 0.4) | 29.3* (18.2 to 41.5) | 1.6 (-7.1 to 11.2) |
| Controlled phase of other leukemia | 44.7 (36.9 to 50.1) | -- | 2.1 (1.3 to 3.2) | 33.3* (15.5 to 54.3) | 4.7 (-9.3 to 21.2) |
| Other malignant cancers | 463.8 (375.7 to 601.6) | 51.0 (46.0 to 54.3) | 37.5 (25.3 to 52.1) | 131.4* (89.6 to 186.9) | 81.8* (49.0 to 125.3) |
| Diagnosis and primary therapy phase of other malignant neoplasms | 10.9 (8.9 to 14.1) | 51.0 (46.0 to 54.3) | 3.1 (1.9 to 4.4) | 169.8* (112.8 to 250.8) | 111.9* (67.1 to 175.6) |
| Controlled phase of other malignant neoplasms | 419.6 (337.7 to 547.3) | -- | 19.6 (12.1 to 30.3) | 195.3* (128.7 to 282.4) | 131.9* (79.7 to 200.4) |
| Metastatic phase of other malignant neoplasms | 29.0 (25.0 to 34.8) | -- | 12.6 (8.2 to 17.3) | 77.2* (52.4 to 111.4) | 39.2* (19.7 to 66.1) |
| Terminal phase of other malignant neoplasms | 4.3 (3.7 to 5.0) | -- | 2.3 (1.5 to 3.0) | 70.4* (49.9 to 100.2) | 33.8* (17.7 to 57.3) |
| Other neoplasms | 2240.3 (2112.8 to 2376.0) | 900.1 (848.8 to 953.5) | 3.9 (2.4 to 5.8) | 31.8* (22.2 to 42.3) | 3.5 (-4.0 to 11.8) |
| Myelodysplastic, myeloproliferative, and other hematopoietic neoplasms | 82.5 (65.8 to 100.5) | 8.4 (6.0 to 11.1) | 3.9 (2.4 to 5.8) | 31.8* (22.2 to 42.3) | 3.5 (-4.0 to 11.8) |
| Benign and in situ intestinal neoplasms | 425.7 (401.1 to 453.5) | 91.5 (85.6 to 97.2) | -- | -- | -- |
| Benign and in situ cervical and uterine neoplasms | 100.7 (94.4 to 107.2) | 3.4 (2.8 to 4.1) | -- | -- | -- |
| Other benign and in situ neoplasms | 1663.0 (1545.0 to 1799.1) | 796.7 (742.5 to 850.3) | -- | -- | -- |
| **Cardiovascular diseases** | **40819.7 (37854.5 to 44360.2)** | **5108.1 (4734.4 to 5536.0)** | **2667.0 (1901.1 to 3440.3)** | **34.3* (30.9 to 38.1)** | **5.5* (2.9 to 8.5)** |
| Rheumatic heart disease | 2733.2 (2634.1 to 2839.4) | 4.3 (3.2 to 5.4) | 132.2 (86.7 to 190.1) | 32.0* (29.7 to 34.4) | 3.7* (1.9 to 5.5) |
| Rheumatic heart disease, without heart failure | 2602.4 (2497.9 to 2716.0) | 4.3 (3.2 to 5.4) | 120.7 (78.8 to 174.7) | 32.9* (30.6 to 35.3) | 4.4* (2.6 to 6.3) |
| Heart failure due to rheumatic heart disease | 130.9 (93.0 to 174.5) | -- | 11.5 (6.9 to 17.0) | 23.3* (14.2 to 32.3) | -3.1 (-10.3 to 4.0) |
| Ischaemic heart disease | 10200.0 (9237.2 to 11303.0) | 711.9 (562.1 to 871.5) | 458.7 (312.7 to 644.7) | 19.9* (18.3 to 21.5) | -5.8* (-7.1 to -4.6) |
| Asymptomatic ischemic heart disease following myocardial infarction | 2432.6 (1853.4 to 3083.5) | -- | -- | -- | -- |
| Angina due to ischemic heart disease | 6967.9 (6254.9 to 7769.4) | -- | 389.2 (259.8 to 558.3) | 19.3* (17.6 to 21.2) | -6.2* (-7.7 to -4.8) |
| Heart failure due to ischemic heart disease | 759.9 (566.4 to 984.7) | -- | 65.7 (40.9 to 97.1) | 23.6* (19.3 to 28.3) | -2.9 (-6.3 to 0.8) |
| Myocardial infarction due to ischemic heart disease | 39.6 (31.0 to 48.8) | 711.9 (562.1 to 871.5) | 3.9 (2.5 to 5.7) | 12.2* (5.6 to 18.5) | -11.9* (-17.0 to -6.9) |
| Stroke | 9404.4 (8605.8 to 10266.3) | 1004.8‡ (874.8 to 1156.2) | 1485.7 (1045.1 to 1913.6) | 43.5* (37.6 to 49.5) | 12.7* (8.1 to 17.4) |
| Ischaemic stroke | 6008.8 (5311.9 to 6834.6) | 573.2 (460.9 to 713.7) | 939.2 (650.3 to 1221.6) | 57.5* (49.2 to 65.6) | 23.7* (17.2 to 30.1) |
| Acute ischemic stroke | 44.7 (35.4 to 55.8) | 573.2 (460.9 to 713.7) | 11.4 (7.3 to 15.9) | 42.3* (37.3 to 47.6) | 11.8* (7.8 to 16.0) |
| Chronic ischemic stroke | 5964.0 (5273.0 to 6793.6) | -- | 927.8 (642.4 to 1206.2) | 57.7* (49.3 to 66.0) | 23.9* (17.3 to 30.4) |
| Intracerebral hemorrhage | 2273.1 (1921.1 to 2631.1) | 306.6 (260.4 to 356.4) | 357.9 (250.6 to 473.3) | 23.8* (13.4 to 34.6) | -2.7 (-10.9 to 5.7) |
| Acute intracerebral hemorrhage | 16.4 (13.6 to 19.7) | 306.6 (260.4 to 356.4) | 4.0 (2.7 to 5.6) | -0.9 (-12.1 to 11.0) | -22.1* (-30.9 to -12.8) |
| Chronic intracerebral hemorrhage | 2256.7 (1904.4 to 2614.1) | -- | 353.9 (247.8 to 468.3) | 24.2* (13.6 to 35.2) | -2.5 (-10.7 to 6.2) |
| Subarachnoid hemorrhage | 1305.6 (1155.2 to 1506.7) | 125.0 (99.2 to 150.9) | 188.5 (132.9 to 247.2) | 25.6* (18.8 to 32.5) | -1.3 (-6.7 to 4.1) |
| Acute subarachnoid hemorrhage | 9.9 (7.8 to 12.0) | 125.0 (99.2 to 150.9) | 2.4 (1.6 to 3.4) | 24.2* (19.4 to 26.6) | -2.4* (-6.2 to -0.5) |
| Chronic subarachnoid hemorrhage | 1295.7 (1144.6 to 1496.1) | -- | 186.1 (131.1 to 244.0) | 25.7* (18.7 to 32.6) | -1.3 (-6.8 to 4.1) |
| Hypertensive heart disease | 1081.2 (823.6 to 1375.7) | -- | 93.5 (60.2 to 142.4) | 33.9* (29.7 to 38.2) | 5.2* (1.9 to 8.6) |
| Non-rheumatic valvular heart disease | 1740.2 (1620.7 to 1867.9) | 1812.0 (1706.2 to 1930.5) | 6.8 (3.0 to 13.1) | 19.0* (14.6 to 23.2) | -6.5* (-10.0 to -3.2) |
| Non-rheumatic calcific aortic valve disease | 494.8 (407.6 to 598.3) | 494.8 (407.6 to 598.3) | 1.7 (0.7 to 3.4) | 14.9* (6.6 to 23.2) | -9.7* (-16.3 to -3.2) |
| Heart failure due to calcific aortic valve disease | 11.5 (5.1 to 22.4) | -- | 1.1 (0.4 to 2.1) | 6.9 (-8.5 to 23.1) | -16.1* (-28.1 to -3.3) |
| Non-rheumatic calcific aortic valve disease | 483.2 (397.4 to 586.0) | 494.8 (407.6 to 598.3) | 0.7 (0.3 to 1.4) | 30.6* (8.1 to 59.3) | 2.6 (-15.1 to 25.1) |
| Non-rheumatic degenerative mitral valve disease | 1317.2 (1256.4 to 1379.4) | 1317.2 (1256.4 to 1379.4) | 5.0 (2.1 to 9.6) | 20.1* (14.7 to 25.4) | -5.7* (-9.9 to -1.5) |
| Heart failure due to degenerative mitral valve disease | 38.9 (18.3 to 74.0) | -- | 3.6 (1.5 to 7.0) | 13.5* (2.3 to 25.2) | -10.9* (-19.6 to -1.7) |
| Non-rheumatic degenerative mitral valve disease | 1278.4 (1208.7 to 1344.5) | 1317.2 (1256.4 to 1379.4) | 1.4 (0.6 to 2.7) | 41.5* (18.4 to 65.9) | 11.1 (-7.0 to 30.3) |
| Other non-rheumatic valve diseases | 1.4 (0.9 to 2.0) | -- | 0.1 (0.1 to 0.2) | 35.1* (32.4 to 37.8) | 6.2* (4.0 to 8.2) |
| Mild heart failure due to other non-rheumatic valve disease | 0.3 (0.1 to 0.4) | -- | -- | 35.2* (32.4 to 37.8) | 6.2* (4.0 to 8.2) |
| Moderate heart failure due to other non-rheumatic valve disease | 0.2 (0.1 to 0.3) | -- | -- | 35.1* (32.4 to 37.8) | 6.2* (4.0 to 8.2) |
| Severe heart failure due to other non-rheumatic valve disease | 0.4 (0.3 to 0.7) | -- | 0.1 (0.0 to 0.1) | 35.1* (32.4 to 37.8) | 6.2* (4.0 to 8.2) |

| | | | | | |
|---|---|---|---|---|---|
| Controlled, medically managed heart failure due to other non-rheumatic valve disease | 0.5 (0.3 to 0.7) | -- | -- | 35.2* (32.4 to 37.8) | 6.2* (4.0 to 8.2) |
| Cardiomyopathy and myocarditis | 441.7 (330.0 to 567.7) | 207.6 (155.2 to 268.8) | 49.6 (30.1 to 74.2) | 18.9* (14.7 to 22.6) | -6.6* (-9.9 to -3.7) |
| Myocarditis | 117.0 (90.0 to 146.8) | 207.6 (155.2 to 268.8) | 8.5 (5.4 to 12.8) | 27.5* (21.3 to 33.8) | 0.2 (-4.7 to 5.1) |
| Acute myocarditis | 49.3 (35.4 to 65.7) | 207.6 (155.2 to 268.8) | 2.4 (1.3 to 3.8) | 24.9* (11.6 to 39.0) | -1.9 (-12.3 to 9.2) |
| Heart failure due to myocarditis | 67.7 (46.1 to 92.8) | -- | 6.2 (3.6 to 9.5) | 28.6* (21.7 to 35.6) | 1.0 (-4.4 to 6.5) |
| Alcoholic cardiomyopathy | 200.1 (144.9 to 262.2) | -- | 17.5 (10.8 to 26.1) | 7.6* (2.0 to 13.0) | -15.5* (-19.9 to -11.2) |
| Other cardiomyopathy | 271.4 (189.7 to 363.4) | -- | 23.6 (14.0 to 35.8) | 25.7* (19.0 to 31.9) | -1.3 (-6.5 to 3.6) |
| Atrial fibrillation and flutter | 1610.6 (1299.4 to 1972.7) | 196.0 (147.3 to 248.8) | 132.5 (85.5 to 193.9) | 24.3* (20.7 to 27.8) | -2.4 (-5.2 to 0.4) |
| Asymptomatic atrial fibrillation and flutter | 966.5 (777.0 to 1184.5) | 196.0 (147.3 to 248.8) | -- | -- | -- |
| Symptomatic atrial fibrillation and flutter | 644.2 (517.5 to 787.3) | -- | 132.5 (85.5 to 193.9) | 24.3* (20.7 to 27.8) | -2.4 (-5.2 to 0.4) |
| Peripheral vascular disease | 10010.6 (7911.1 to 12737.8) | 1107.8 (843.5 to 1442.5) | 11.3 (5.0 to 21.4) | 20.3* (16.7 to 23.9) | -5.5* (-8.4 to -2.7) |
| Asymptomatic peripheral arterial disease | 9182.4 (7268.8 to 11713.0) | 1107.8 (843.5 to 1442.5) | -- | -- | -- |
| Symptomatic claudication due to peripheral arterial disease | 828.2 (574.5 to 1174.7) | -- | 11.3 (5.0 to 21.4) | 20.3* (16.7 to 23.9) | -5.5* (-8.4 to -2.7) |
| Endocarditis | 29.7 (21.9 to 40.1) | 63.7 (46.4 to 85.3) | 2.6 (1.6 to 4.0) | 25.6* (16.8 to 34.8) | -1.4 (-8.2 to 5.9) |
| Acute endocarditis | 4.8 (3.4 to 6.5) | 63.7 (46.4 to 85.3) | 0.3 (0.2 to 0.4) | 33.7* (28.5 to 38.9) | 5.1* (0.9 to 9.1) |
| Heart failure due to endocarditis | 24.9 (17.2 to 34.9) | -- | 2.3 (1.4 to 3.6) | 24.7* (14.8 to 34.9) | -2.1 (-9.8 to 6.0) |
| Other cardiovascular and circulatory diseases | 5595.1 (3820.8 to 7807.8) | -- | 294.1 (174.7 to 469.3) | 26.7* (23.9 to 29.2) | -0.5 (-2.7 to 1.5) |
| Heart failure due to other cardiovascular diseases | 56.3 (38.2 to 79.8) | -- | 5.2 (3.1 to 8.2) | 26.5* (20.2 to 32.5) | -0.6 (-5.6 to 4.1) |
| Other cardiovascular and circulatory disease episodes | 5538.9 (3783.3 to 7727.7) | -- | 289.0 (171.5 to 461.2) | 26.7* (23.9 to 29.3) | -0.5 (-2.7 to 1.5) |
| **Chronic respiratory diseases** | **39553.6 (35050.5 to 44731.1)** | **3214.3 (2468.0 to 3996.8)** | **3558.9 (2860.1 to 4333.0)** | **26.3* (20.3 to 31.9)** | **-0.8 (-5.5 to 3.6)** |
| Chronic obstructive pulmonary disease | 26070.9 (22138.8 to 30768.9) | 1855.7 (1252.3 to 2535.7) | 2756.8 (2177.6 to 3403.0) | 25.2* (17.9 to 31.7) | -1.7 (-7.4 to 3.5) |
| Chronic obstructive pulmonary disease with heart failure | 193.7 (138.2 to 263.8) | -- | 82.7 (50.8 to 122.9) | 29.3* (21.6 to 37.2) | 1.5 (-4.4 to 7.7) |
| Chronic obstructive pulmonary disease without heart failure | 25877.2 (21967.2 to 30578.7) | 1855.7 (1252.3 to 2535.7) | 2674.1 (2104.1 to 3323.8) | 25.1* (17.4 to 31.8) | -1.8 (-7.7 to 3.5) |
| Pneumoconiosis | 37.5 (30.0 to 46.5) | 4.5 (3.4 to 5.8) | 5.9 (3.7 to 8.7) | 22.7* (17.9 to 28.3) | -3.6 (-7.4 to 0.8) |
| Silicosis | 11.0 (7.2 to 16.4) | 1.8 (1.1 to 2.6) | 1.7 (1.0 to 3.0) | 28.6* (18.2 to 41.7) | 1.0 (-7.2 to 11.3) |
| Heart failure due to silicosis | 0.3 (0.2 to 0.4) | -- | 0.1 (0.1 to 0.2) | 21.5* (18.1 to 25.4) | -4.6* (-7.2 to -1.5) |
| Silicosis without heart failure | 10.8 (6.9 to 16.2) | 1.8 (1.1 to 2.6) | 1.6 (0.9 to 2.8) | 29.2* (18.0 to 43.2) | 1.5 (-7.3 to 12.5) |
| Asbestosis | 6.9 (4.1 to 10.6) | 0.7 (0.4 to 1.1) | 1.1 (0.6 to 1.9) | 31.0* (23.8 to 39.6) | 2.9 (-2.8 to 9.7) |
| Asbestosis without heart failure | 6.8 (4.0 to 10.6) | 0.7 (0.4 to 1.1) | 1.0 (0.5 to 1.8) | 31.5* (24.1 to 40.4) | 3.3 (-2.5 to 10.3) |
| Heart failure due to asbestosis | 0.1 (0.1 to 0.1) | -- | 0.0 (0.0 to 0.1) | 18.3* (15.0 to 21.3) | -7.1* (-9.6 to -4.7) |
| Coal workers pneumoconiosis | 9.1 (5.8 to 13.6) | 1.0 (0.5 to 1.6) | 1.4 (0.8 to 2.4) | 15.2* (2.6 to 28.8) | -9.5 (-19.4 to 1.2) |
| Coal workers pneumoconiosis without heart failure | 8.7 (5.5 to 13.1) | 1.0 (0.5 to 1.6) | 1.3 (0.6 to 2.2) | 14.8* (0.2 to 30.2) | -9.8 (-21.3 to 2.3) |
| Heart failure due to Coal workers pneumoconiosis | 0.4 (0.3 to 0.5) | -- | 0.2 (0.1 to 0.3) | 17.7* (11.6 to 23.6) | -7.5* (-12.4 to -2.9) |
| Other pneumoconiosis | 10.5 (7.4 to 14.9) | 1.0 (0.6 to 1.5) | 1.7 (1.0 to 2.7) | 18.7* (14.6 to 22.5) | -6.7* (-10.0 to -3.8) |
| Heart failure due to other pneumoconiosis | 0.1 (0.1 to 0.1) | -- | 0.0 (0.0 to 0.1) | 23.0* (17.6 to 27.9) | -3.4 (-7.6 to 0.5) |
| Other pneumoconiosis without heart failure | 10.4 (7.3 to 14.8) | 1.0 (0.6 to 1.5) | 1.6 (0.9 to 2.7) | 18.6* (14.4 to 22.4) | -6.8* (-10.1 to -3.8) |
| Asthma | 14835.4 (12061.9 to 18137.1) | 1316.5 (928.4 to 1746.3) | 568.7 (364.7 to 832.1) | 30.3* (23.1 to 38.0) | 2.4 (-3.3 to 8.4) |
| Asymptomatic asthma | 5391.0 (4273.5 to 6665.9) | 477.3 (329.4 to 654.8) | -- | -- | -- |
| Symptomatic asthma | 9444.4 (7658.5 to 11626.3) | 839.1 (577.3 to 1128.1) | 568.7 (364.7 to 832.1) | 30.3* (23.1 to 38.0) | 2.4 (-3.3 to 8.4) |
| Interstitial lung disease and pulmonary sarcoidosis | 502.8 (418.1 to 599.2) | 37.6 (25.4 to 51.2) | 53.3 (34.0 to 79.2) | 19.3* (13.4 to 25.5) | -6.3* (-11.0 to -1.4) |
| Heart failure due to interstitial lung disease and pulmonary sarcoidosis | 12.8 (9.3 to 17.0) | -- | 5.9 (3.6 to 8.7) | 21.7* (20.2 to 23.4) | -4.4* (-5.6 to -3.1) |
| Interstitial lung disease and pulmonary sarcoidosis without heart failure | 490.1 (403.4 to 587.2) | 37.6 (25.4 to 51.2) | 47.4 (29.4 to 71.1) | 19.0* (12.3 to 26.2) | -6.6* (-11.8 to -0.9) |
| Other chronic respiratory diseases | -- | -- | 174.1 (138.0 to 214.4) | 35.3* (27.6 to 42.3) | 6.3* (0.2 to 11.8) |
| **Digestive diseases** | **175677.2 (162430.4 to 191157.9)** | **39633.2 (31014.9 to 48371.2)** | **1832.0 (1235.5 to 2605.9)** | **28.5* (26.3 to 30.8)** | **1.0 (-0.8 to 2.8)** |
| Cirrhosis and other chronic liver diseases | 130984.4 (121253.1 to 142207.3) | 201.5 (181.0 to 220.7) | 194.3 (135.1 to 267.6) | 40.2* (35.8 to 44.3) | 10.1* (6.7 to 13.4) |
| Cirrhosis and other chronic liver diseases due to hepatitis B | 27892.6 (25704.3 to 30179.1) | 54.8 (46.5 to 63.7) | 56.5 (38.8 to 80.9) | 42.4* (35.3 to 49.2) | 11.9* (6.3 to 17.2) |
| Cirrhosis and other chronic liver diseases due to hepatitis B, decompensated | 341.9 (297.3 to 385.3) | 52.2 (44.0 to 61.2) | 56.5 (38.8 to 80.9) | 42.4* (35.3 to 49.2) | 11.9* (6.3 to 17.2) |
| Chronic hepatitis B without cirrhosis | 23541.7 (21338.5 to 25932.5) | -- | -- | -- | -- |
| Cirrhosis and other chronic liver diseases due to hepatitis B, compensated | 4009.0 (3526.2 to 4539.2) | 2.6 (0.5 to 6.4) | -- | -- | -- |
| Cirrhosis and other chronic liver diseases due to hepatitis C | 10037.3 (8658.9 to 11636.4) | 56.4 (46.9 to 66.9) | 51.9 (33.9 to 72.9) | 38.8* (32.1 to 45.8) | 9.0* (3.8 to 14.5) |
| Cirrhosis and other chronic liver diseases due to hepatitis C, decompensated | 315.1 (265.4 to 367.1) | 50.6 (41.7 to 60.0) | 51.9 (33.9 to 72.9) | 38.8* (32.1 to 45.8) | 9.0* (3.8 to 14.5) |
| Chronic hepatitis C without cirrhosis | 6469.4 (5033.1 to 8145.1) | -- | -- | -- | -- |
| Cirrhosis and other chronic liver diseases due to hepatitis C, compensated | 3252.7 (2740.3 to 3806.2) | 5.8 (2.5 to 10.8) | -- | -- | -- |
| Cirrhosis and other chronic liver diseases due to alcohol use | 3561.5 (3127.6 to 4034.2) | 56.1 (47.7 to 65.1) | 55.2 (37.9 to 77.2) | 35.6* (28.7 to 42.2) | 6.5* (1.1 to 11.7) |
| Cirrhosis and other chronic liver diseases due to alcohol, decompensated | 334.7 (292.1 to 381.3) | 51.5 (43.6 to 60.2) | 55.2 (37.9 to 77.2) | 35.6* (28.7 to 42.2) | 6.5* (1.1 to 11.7) |
| Cirrhosis and other chronic liver diseases due to alcohol, compensated | 3226.8 (2829.3 to 3657.2) | 4.6 (2.2 to 8.0) | -- | -- | -- |
| Cirrhosis due to NASH | 88754.6 (78964.2 to 99264.4) | 20.9 (16.8 to 25.8) | 19.5 (13.0 to 28.0) | 54.9* (44.0 to 66.5) | 21.7* (13.1 to 30.8) |
| Non-alcoholic fatty liver disease (NAFLD) / Non-alcoholic steatohepatitis (NASH) | 87392.5 (77655.3 to 97976.9) | -- | -- | -- | -- |
| Cirrhosis and other chronic liver diseases due to NASH | 1362.1 (1111.3 to 1629.1) | 20.9 (16.8 to 25.8) | 19.5 (13.0 to 28.0) | 54.9* (44.0 to 66.5) | 21.7* (13.1 to 30.8) |
| Cirrhosis and other chronic liver diseases due to other causes | 738.4 (552.5 to 971.5) | 13.3 (9.9 to 17.4) | 11.2 (7.0 to 17.0) | 36.2* (26.2 to 46.7) | 7.0 (-0.8 to 15.2) |

| | | | | | |
|---|---|---|---|---|---|
| Cirrhosis and other chronic liver diseases due to other cause, decompensated | 67.5<br>(50.1 to 87.6) | 11.2<br>(8.3 to 14.6) | 11.2<br>(7.0 to 17.0) | 36.2*<br>(26.2 to 46.7) | 7.0<br>(-0.8 to 15.2) |
| Cirrhosis and other chronic liver diseases due to other cause, compensated | 670.9<br>(499.6 to 883.1) | 2.1<br>(1.0 to 3.5) | -- | -- | -- |
| Upper digestive system diseases | 72177.0<br>(55849.6 to 91403.4) | 33752.9<br>(25393.6 to 42576.7) | 1200.7<br>(737.0 to 1859.8) | 27.8*<br>(24.9 to 30.6) | 0.4<br>(-1.9 to 2.6) |
| Peptic ulcer disease | 1426.8<br>(1052.1 to 1871.0) | 546.4<br>(385.2 to 746.1) | 69.5<br>(42.4 to 104.2) | 24.3*<br>(17.6 to 30.6) | -2.3<br>(-7.6 to 2.6) |
| Acute peptic ulcer disease | 0.6<br>(0.4 to 0.8) | -- | 0.2<br>(0.1 to 0.3) | 4.8*<br>(2.8 to 6.4) | -17.7*<br>(-19.2 to -16.4) |
| Chronic peptic ulcer disease | 1426.2<br>(1051.6 to 1870.6) | 546.4<br>(385.2 to 746.1) | 69.4<br>(42.3 to 104.0) | 24.4*<br>(17.6 to 30.7) | -2.3<br>(-7.6 to 2.7) |
| Gastritis and duodenitis | 13660.8<br>(9673.1 to 19342.4) | 9250.6<br>(6331.0 to 12711.7) | 597.5<br>(359.7 to 934.5) | 29.2*<br>(24.0 to 34.1) | 1.5<br>(-2.6 to 5.4) |
| Acute gastritis and duodenitis | 3.3<br>(2.1 to 4.9) | -- | 1.1<br>(0.6 to 1.8) | 17.9*<br>(16.0 to 19.6) | -7.4*<br>(-8.9 to -6.0) |
| Chronic gastritis and duodenitis | 13657.6<br>(9669.9 to 19339.6) | 9250.6<br>(6331.0 to 12711.7) | 596.4<br>(358.8 to 932.9) | 29.2*<br>(24.0 to 34.2) | 1.5<br>(-2.6 to 5.4) |
| GERD | 63039.2<br>(44796.0 to 85191.9) | 23955.9<br>(16422.5 to 32209.3) | 533.7<br>(264.3 to 960.3) | 26.6*<br>(24.6 to 28.8) | -0.5<br>(-2.1 to 1.2) |
| Appendicitis | 19.7<br>(15.8 to 24.1) | 517.9<br>(415.1 to 626.8) | 6.4<br>(4.0 to 9.1) | 27.3*<br>(23.1 to 31.3) | 0.0<br>(-3.3 to 3.1) |
| Paralytic ileus and intestinal obstruction | 8.9<br>(6.6 to 11.7) | 242.6<br>(180.4 to 317.9) | 2.9<br>(1.8 to 4.4) | 33.2*<br>(27.4 to 38.2) | 4.6*<br>(0.0 to 8.6) |
| Inguinal, femoral, and abdominal hernia | 2011.2<br>(1417.7 to 2705.7) | 3829.6<br>(2701.4 to 5164.6) | 193.5<br>(117.3 to 292.2) | 21.8*<br>(17.6 to 26.6) | -4.3*<br>(-7.7 to -0.6) |
| Inflammatory bowel disease | 802.7<br>(685.6 to 928.6) | 336.2<br>(267.2 to 408.9) | 118.7<br>(79.5 to 163.1) | 28.0*<br>(18.5 to 36.9) | 0.5<br>(-6.9 to 7.5) |
| Ulcerative colitis | 603.1<br>(492.1 to 732.1) | 253.6<br>(189.1 to 325.2) | 87.9<br>(57.7 to 122.8) | 36.7*<br>(24.6 to 48.0) | 7.4<br>(-2.1 to 16.2) |
| Crohn's disease | 199.6<br>(167.4 to 238.3) | 82.6<br>(64.0 to 104.1) | 30.9<br>(20.1 to 43.2) | 8.2<br>(-1.0 to 17.8) | -15.0*<br>(-22.2 to -7.5) |
| Vascular intestinal disorders | 4.7<br>(2.9 to 7.1) | 33.2<br>(21.5 to 46.0) | 1.5<br>(0.8 to 2.5) | 24.2*<br>(15.8 to 31.6) | -2.4<br>(-9.0 to 3.3) |
| Gallbladder and biliary diseases | 3317.4<br>(2414.3 to 4604.0) | 575.9<br>(420.4 to 777.6) | 2.7<br>(1.6 to 4.2) | 32.8*<br>(27.1 to 39.3) | 4.3<br>(-0.1 to 9.4) |
| Pancreatitis | 616.3<br>(530.2 to 705.3) | 143.4<br>(111.9 to 177.8) | 37.0<br>(18.7 to 63.4) | 25.1*<br>(17.7 to 32.1) | -1.7<br>(-7.5 to 3.8) |
| Acute pancreatitis | 11.3<br>(8.3 to 15.0) | 117.9<br>(87.6 to 151.6) | 4.6<br>(2.9 to 7.0) | 17.9*<br>(12.3 to 23.3) | -7.3*<br>(-11.8 to -3.2) |
| Chronic pancreatitis | 605.0<br>(517.2 to 694.7) | 25.5<br>(17.0 to 37.2) | 32.4<br>(14.8 to 57.6) | 26.2*<br>(17.9 to 34.5) | -0.9<br>(-7.4 to 5.6) |
| Other digestive diseases | -- | -- | 74.2<br>(49.6 to 105.5) | 34.3*<br>(30.6 to 38.1) | 5.5*<br>(2.6 to 8.5) |
| **Neurological disorders** | **205678.2<br>(171925.4 to 240821.8)** | **58741.1<br>(41708.7 to 79790.5)** | **4701.5<br>(3145.8 to 6541.0)** | **26.3*<br>(24.7 to 28.2)** | **-0.8<br>(-2.0 to 0.7)** |
| Alzheimer's disease and other dementias | 553.5<br>(401.7 to 738.9) | 105.4<br>(71.9 to 147.4) | 69.1<br>(41.5 to 106.1) | 23.3*<br>(18.4 to 28.3) | -3.1<br>(-7.0 to 0.8) |
| Parkinson's disease | 323.6<br>(228.0 to 441.2) | 46.3<br>(32.6 to 65.4) | 50.5<br>(29.4 to 77.4) | 30.5*<br>(22.3 to 38.9) | 2.5<br>(-3.9 to 9.1) |
| Epilepsy | 1238.3<br>(892.1 to 1594.9) | 96.3<br>(64.8 to 135.5) | 361.5<br>(221.7 to 540.5) | 27.4*<br>(14.4 to 43.7) | 0.0<br>(-10.1 to 12.9) |
| Multiple sclerosis | 195.8<br>(178.0 to 215.8) | 1.7<br>(1.0 to 2.4) | 50.6<br>(36.0 to 66.6) | 20.2*<br>(14.2 to 26.7) | -5.6*<br>(-10.3 to -0.4) |
| Motor neuron disease | 17.5<br>(12.8 to 23.5) | 5.5<br>(3.7 to 7.5) | 3.7<br>(2.3 to 5.7) | 35.7*<br>(31.1 to 39.0) | 6.6*<br>(3.0 to 9.2) |
| Headache disorders | 204542.8<br>(170524.9 to 239732.1) | 58485.9<br>(41497.7 to 79531.7) | 4071.5<br>(2665.5 to 5809.8) | 26.2*<br>(25.0 to 27.4) | -0.9<br>(-1.8 to 0.1) |
| Migraine | 97176.6<br>(85340.6 to 110480.4) | 4885.7<br>(3268.5 to 6624.3) | 3518.4<br>(2225.4 to 5134.0) | 26.4*<br>(25.0 to 27.7) | -0.7<br>(-1.8 to 0.3) |
| Medication overuse headache due to migraine | 7937.9<br>(5518.9 to 10716.8) | -- | 861.3<br>(479.7 to 1364.6) | 23.8*<br>(22.4 to 25.3) | -2.7*<br>(-3.9 to -1.6) |
| Migraine | 89238.7<br>(78077.3 to 102959.5) | 4885.7<br>(3268.5 to 6624.3) | 2657.0<br>(1608.0 to 3998.4) | 27.2*<br>(25.6 to 28.9) | -0.1<br>(-1.4 to 1.2) |
| Tension-type headache | 150388.9<br>(104735.0 to 197686.7) | 53600.2<br>(35830.4 to 74068.5) | 553.1<br>(305.8 to 889.9) | 25.1*<br>(23.5 to 26.8) | -1.7*<br>(-3.0 to -0.4) |
| Medication overuse headache due to tension-type headache | 2951.6<br>(1841.8 to 4403.6) | -- | 318.2<br>(151.0 to 586.4) | 23.8*<br>(21.3 to 26.5) | -2.7*<br>(-4.7 to -0.7) |
| Tension-type headache | 147437.3<br>(101756.1 to 194762.9) | 53600.2<br>(35830.4 to 74068.5) | 235.0<br>(96.5 to 467.7) | 26.9*<br>(25.6 to 28.2) | -0.3<br>(-1.3 to 0.7) |
| Other neurological disorders | 2.1<br>(1.0 to 3.6) | -- | 94.6<br>(62.6 to 132.0) | 32.3*<br>(22.8 to 42.9) | 3.9<br>(-3.5 to 12.3) |
| Other neurological disorders | -- | -- | 94.0<br>(62.2 to 130.9) | 32.3*<br>(22.8 to 43.0) | 3.9<br>(-3.5 to 12.3) |
| Guillain-Barré syndrome due to other neurological disorders | 2.1<br>(1.0 to 3.6) | -- | 0.6<br>(0.3 to 1.2) | 30.1*<br>(26.7 to 33.1) | 2.2<br>(-0.5 to 4.5) |
| **Mental disorders** | **64256.0<br>(60575.7 to 67848.3)** | **23883.2<br>(21083.3 to 27053.9)** | **8843.2<br>(6590.4 to 11325.8)** | **24.8*<br>(23.8 to 25.9)** | **-1.9*<br>(-2.7 to -1.1)** |
| Schizophrenia | 1971.1<br>(1737.9 to 2228.6) | 51.3<br>(41.5 to 61.6) | 1244.9<br>(929.0 to 1540.1) | 28.9*<br>(26.3 to 31.4) | 1.2<br>(-0.8 to 3.2) |
| Depressive disorders | 23417.8<br>(20984.5 to 26045.7) | 21212.5<br>(18403.4 to 24347.7) | 3695.3<br>(2586.2 to 5079.3) | 23.6*<br>(21.7 to 25.5) | -2.9*<br>(-4.4 to -1.4) |
| Major depressive disorder | 13448.0<br>(11769.9 to 15511.7) | 19724.3<br>(16945.8 to 22838.6) | 2687.5<br>(1848.0 to 3713.2) | 21.9*<br>(19.6 to 24.3) | -4.2*<br>(-6.0 to -2.4) |
| Dysthymia | 10510.7<br>(8706.9 to 12603.0) | 1488.2<br>(1215.9 to 1821.0) | 1007.8<br>(654.9 to 1465.7) | 28.3*<br>(25.0 to 31.6) | 0.8<br>(-1.8 to 3.4) |
| Bipolar disorder | 3326.0<br>(2678.5 to 4220.8) | 291.7<br>(202.6 to 431.1) | 665.0<br>(410.8 to 1004.7) | 26.1*<br>(23.9 to 28.4) | -0.9<br>(-2.7 to 0.9) |
| Anxiety disorders | 20274.1<br>(18029.9 to 22822.8) | 2327.7<br>(2117.9 to 2557.0) | 1899.4<br>(1308.7 to 2552.7) | 24.1*<br>(22.0 to 26.3) | -2.5*<br>(-4.1 to -0.8) |
| Eating disorders | -- | -- | -- | -- | -- |
| Anorexia nervosa | -- | -- | -- | -- | -- |
| Bulimia nervosa | -- | -- | -- | -- | -- |
| Autism spectrum disorders | 1577.8<br>(1418.7 to 1747.8) | -- | 234.2<br>(162.2 to 321.7) | 24.8*<br>(22.3 to 27.4) | -2.0<br>(-3.9 to 0.0) |
| Attention-deficit/hyperactivity disorder | 2083.4<br>(1642.3 to 2533.2) | -- | 24.6<br>(14.6 to 40.3) | 28.5*<br>(23.7 to 33.5) | 0.9<br>(-2.8 to 4.9) |
| Conduct disorder | -- | -- | -- | -- | -- |
| Idiopathic developmental intellectual disability | 3424.5<br>(1774.7 to 5115.4) | -- | 134.8<br>(61.1 to 234.7) | 14.7*<br>(9.4 to 17.3) | -9.9*<br>(-14.1 to -7.9) |
| Other mental disorders | 12810.6<br>(11246.1 to 14570.8) | -- | 944.9<br>(623.7 to 1300.8) | 26.9*<br>(25.3 to 28.5) | -0.3<br>(-1.6 to 0.9) |
| **Substance use disorders** | **11051.9<br>(9389.8 to 12947.3)** | **3766.7<br>(2961.4 to 4693.5)** | **1732.6<br>(1228.4 to 2317.2)** | **30.3*<br>(26.4 to 34.6)** | **2.3<br>(-0.7 to 5.7)** |
| Alcohol use disorders | 8111.0<br>(6462.6 to 10029.5) | 3530.3<br>(2733.3 to 4456.4) | 795.5<br>(515.6 to 1160.8) | 17.3*<br>(13.5 to 20.7) | -7.8*<br>(-10.8 to -5.2) |
| Alcohol dependence | 8095.1<br>(6450.6 to 10013.5) | 3530.3<br>(2733.3 to 4456.4) | 795.0<br>(515.3 to 1160.1) | 17.3*<br>(13.5 to 20.7) | -7.8*<br>(-10.8 to -5.2) |
| Fetal alcohol syndrome | 16.0<br>(12.7 to 19.6) | -- | 0.5<br>(0.3 to 0.8) | 3.3<br>(-3.3 to 9.6) | -18.8*<br>(-24.0 to -13.9) |
| Drug use disorders | 3102.4<br>(2647.0 to 3683.4) | 236.4<br>(172.7 to 325.9) | 937.1<br>(647.6 to 1290.0) | 43.8*<br>(37.8 to 50.4) | 12.9*<br>(8.2 to 18.2) |
| Opioid use disorders | 1996.6<br>(1554.9 to 2544.4) | 165.7<br>(101.2 to 252.6) | 806.6<br>(548.9 to 1134.2) | 47.7*<br>(40.8 to 55.2) | 16.0*<br>(10.6 to 21.9) |
| Cocaine use disorders | 282.1<br>(247.6 to 319.8) | 3.3<br>(1.9 to 5.6) | 37.5<br>(24.4 to 54.7) | 20.2*<br>(14.9 to 26.4) | -5.6*<br>(-9.7 to -0.7) |

| | | | | | |
|---|---|---|---|---|---|
| Amphetamine use disorders | 103.0 (68.5 to 152.9) | 15.5 (7.7 to 26.4) | 13.7 (7.4 to 23.5) | 25.7* (17.7 to 33.1) | -1.2 (-7.5 to 4.6) |
| Cannabis use disorders | 540.5 (415.2 to 702.8) | 52.0 (31.7 to 74.4) | 15.6 (9.2 to 23.5) | 22.6* (17.3 to 27.9) | -3.7 (-7.9 to 0.5) |
| Other drug use disorders | 282.2 (221.9 to 359.3) | -- | 63.6 (42.8 to 87.8) | 25.6* (20.9 to 30.5) | -1.4 (-5.0 to 2.5) |
| **Diabetes and kidney diseases** | **98469.8 (89185.4 to 109219.1)** | **4173.3 (3283.1 to 5205.9)** | **5020.2 (3414.9 to 7039.4)** | **30.1* (22.3 to 38.0)** | **2.2 (-4.0 to 8.4)** |
| Diabetes mellitus | 51022.1 (44890.1 to 58305.5) | 2510.7 (1879.5 to 3295.7) | 4417.1 (2941.0 to 6279.1) | 30.9* (22.2 to 39.6) | 2.8 (-4.0 to 9.7) |
| Diabetes mellitus type 1 | 1004.3 (895.7 to 1118.9) | 10.8 (7.0 to 15.4) | 85.7 (57.9 to 120.6) | 21.7* (18.5 to 25.0) | -4.4* (-6.9 to -1.8) |
| Uncomplicated diabetes mellitus type 1 | 529.7 (424.3 to 628.8) | 10.8 (7.0 to 15.4) | 24.6 (14.8 to 37.2) | 19.6* (13.4 to 24.8) | -6.1* (-10.9 to -2.0) |
| Vision loss due to diabetes mellitus type 1 retinopathy | 10.4 (7.4 to 14.2) | -- | 0.9 (0.6 to 1.4) | 8.0* (0.7 to 15.6) | -15.2* (-20.9 to -9.2) |
| Diabetic neuropathy due to diabetes mellitus type 1 | 464.2 (376.4 to 574.1) | -- | 60.2 (39.7 to 87.3) | 22.8* (17.3 to 27.9) | -3.5 (-7.9 to 0.4) |
| Diabetes mellitus type 2 | 50017.8 (43871.7 to 57245.2) | 2500.0 (1867.9 to 3282.2) | 4331.5 (2887.5 to 6165.9) | 31.1* (22.3 to 40.0) | 3.0 (-3.9 to 10.0) |
| Uncomplicated diabetes mellitus type 2 | 25626.5 (19655.2 to 31228.5) | 2500.0 (1867.9 to 3282.2) | 1192.4 (703.6 to 1798.1) | 29.5* (18.6 to 39.5) | 1.7 (-6.8 to 9.6) |
| Vision loss due to diabetes mellitus type 2 retinopathy | 522.8 (401.6 to 679.3) | -- | 44.8 (29.4 to 64.0) | 21.7* (16.2 to 27.0) | -4.4* (-8.8 to -0.2) |
| Diabetic neuropathy due to diabetes mellitus type 2 | 23868.5 (18995.6 to 30080.1) | -- | 3094.3 (1976.2 to 4488.0) | 31.9* (22.4 to 41.5) | 3.6 (-3.9 to 11.2) |
| Chronic kidney disease | 59750.4 (49437.9 to 71134.7) | 1617.5 (1030.7 to 2243.3) | 602.9 (430.1 to 804.7) | 24.4* (16.6 to 32.6) | -2.3 (-8.4 to 4.2) |
| Chronic kidney disease due to diabetes mellitus type 1 | 285.2 (236.4 to 341.2) | 6.9 (4.4 to 9.7) | 39.3 (24.0 to 57.8) | 26.6* (16.1 to 37.2) | -0.5 (-8.8 to 7.7) |
| Albuminuria with preserved GFR due to type 1 diabetes mellitus | 99.1 (72.4 to 130.2) | -- | -- | -- | -- |
| End-stage chronic kidney disease due to diabetes mellitus type 1 | 68.8 (40.7 to 102.5) | -- | 27.4 (14.3 to 43.8) | 28.5* (14.3 to 43.3) | 1.0 (-10.2 to 12.6) |
| Stage III chronic kidney disease due to diabetes mellitus type 1 | 84.6 (67.3 to 107.3) | 6.9 (4.4 to 9.7) | 1.6 (1.0 to 2.4) | 19.5* (2.6 to 36.8) | -6.1 (-19.4 to 7.5) |
| Stage IV chronic kidney disease due to diabetes mellitus type 1 | 19.8 (12.1 to 30.3) | -- | 2.7 (1.5 to 4.5) | 27.5* (11.1 to 47.0) | 0.2 (-12.7 to 15.5) |
| Stage V chronic kidney disease due to diabetes mellitus type 1 | 12.9 (8.8 to 17.9) | -- | 7.5 (4.5 to 11.1) | 21.3* (9.3 to 33.5) | -4.7 (-14.2 to 4.9) |
| Chronic kidney disease due to diabetes mellitus type 2 | 11943.8 (9609.1 to 14540.1) | 203.1 (129.9 to 281.9) | 132.1 (91.0 to 181.5) | 26.5* (17.8 to 35.5) | -0.6 (-7.4 to 6.5) |
| Albuminuria with preserved GFR due to type 2 diabetes mellitus | 8862.6 (6580.5 to 11351.9) | -- | -- | -- | -- |
| End-stage chronic kidney disease due to diabetes mellitus type 2 | 108.6 (75.2 to 148.9) | -- | 42.4 (25.7 to 62.1) | 25.8* (13.9 to 38.3) | -1.2 (-10.5 to 8.6) |
| Stage III chronic kidney disease due to diabetes mellitus type 2 | 2694.0 (2207.2 to 3329.8) | 203.1 (129.9 to 281.9) | 12.9 (8.0 to 20.0) | 8.5 (-5.7 to 23.8) | -14.8* (-26.0 to -2.7) |
| Stage IV chronic kidney disease due to diabetes mellitus type 2 | 173.5 (110.3 to 258.1) | -- | 20.0 (10.9 to 32.9) | 30.3* (14.7 to 49.6) | 2.4 (-9.9 to 17.5) |
| Stage V chronic kidney disease due to diabetes mellitus type 2 | 105.1 (74.5 to 144.7) | -- | 56.8 (36.2 to 84.3) | 30.7* (18.8 to 43.0) | 2.7 (-6.7 to 12.3) |
| Chronic kidney disease due to hypertension | 1605.4 (1370.3 to 1894.6) | 51.6 (33.2 to 71.7) | 105.4 (72.0 to 145.7) | 27.0* (17.7 to 36.8) | -0.2 (-7.6 to 7.4) |
| Albuminuria with preserved GFR due to hypertension | 422.7 (311.3 to 542.4) | -- | -- | -- | -- |
| End-stage chronic kidney disease due to hypertension | 73.9 (53.1 to 101.2) | -- | 28.8 (17.4 to 42.7) | 27.4* (14.4 to 42.5) | 0.0 (-10.1 to 11.9) |
| Stage III chronic kidney disease due to hypertension | 860.2 (704.9 to 1067.6) | 51.6 (33.2 to 71.7) | 7.5 (4.5 to 11.8) | 3.4 (-11.6 to 18.9) | -18.8* (-30.6 to -6.6) |
| Stage IV chronic kidney disease due to hypertension | 155.1 (99.0 to 230.3) | -- | 18.0 (9.8 to 29.1) | 30.4* (13.5 to 48.5) | 2.4 (-10.8 to 16.6) |
| Stage V chronic kidney disease due to hypertension | 93.4 (66.4 to 128.7) | -- | 51.0 (31.7 to 75.9) | 30.0* (18.8 to 42.8) | 2.2 (-6.7 to 12.2) |
| Chronic kidney disease due to glomerulonephritis | 2003.3 (1713.1 to 2374.8) | 108.4 (69.5 to 150.3) | 94.9 (63.9 to 134.1) | 26.2* (17.9 to 35.0) | -0.8 (-7.4 to 6.0) |
| Albuminuria with preserved GFR due to glomerulonephritis | 468.9 (347.3 to 600.3) | -- | -- | -- | -- |
| End-stage chronic kidney disease due to glomerulonephritis | 81.7 (51.4 to 118.3) | -- | 31.1 (17.7 to 49.5) | 23.9* (11.3 to 37.5) | -2.7 (-12.6 to 8.0) |
| Stage III chronic kidney disease due to glomerulonephritis | 1245.3 (1022.3 to 1553.5) | 108.4 (69.5 to 150.3) | 5.6 (3.4 to 8.7) | 8.7 (-6.4 to 26.1) | -14.6* (-26.5 to -1.0) |
| Stage IV chronic kidney disease due to glomerulonephritis | 129.4 (82.7 to 192.7) | -- | 15.2 (8.4 to 24.8) | 29.6* (12.7 to 49.1) | 1.8 (-11.5 to 17.1) |
| Stage V chronic kidney disease due to glomerulonephritis | 77.9 (55.4 to 107.3) | -- | 43.0 (27.1 to 63.9) | 29.5* (18.7 to 41.1) | 1.8 (-6.7 to 10.8) |
| Chronic kidney disease due to other and unspecified causes | 43912.6 (36424.9 to 52422.4) | 1247.5 (793.9 to 1727.3) | 231.2 (164.6 to 316.0) | 20.9* (11.9 to 30.6) | -5.0 (-12.1 to 2.6) |
| Albuminuria with preserved GFR due to other and unspecified causes | 26752.9 (19861.4 to 34343.5) | -- | -- | -- | -- |
| End-stage chronic kidney disease due to other causes | 96.9 (64.4 to 140.4) | -- | 37.0 (20.7 to 58.6) | 25.3* (12.9 to 37.8) | -1.6 (-11.3 to 8.2) |
| Stage III chronic kidney disease due to other causes | 16672.4 (13696.6 to 20634.8) | 1247.5 (793.9 to 1727.3) | 88.3 (54.7 to 133.5) | 9.2 (-2.6 to 22.4) | -14.2* (-23.5 to -3.8) |
| Stage IV chronic kidney disease due to other causes | 242.3 (153.8 to 360.3) | -- | 27.5 (15.1 to 45.2) | 31.3* (14.7 to 50.6) | 3.1 (-9.9 to 18.3) |
| Stage V chronic kidney disease due to other causes | 148.1 (105.3 to 203.8) | -- | 78.5 (49.7 to 116.7) | 30.8* (19.0 to 42.2) | 2.8 (-6.5 to 11.7) |
| Acute glomerulonephritis | 3.0 (2.0 to 4.3) | 45.0 (29.5 to 63.6) | 0.2 (0.1 to 0.3) | -3.3 (-8.1 to 1.0) | -24.0* (-27.8 to -20.7) |
| **Skin and subcutaneous diseases** | **104947.5 (96918.7 to 114003.4)** | **222511.2 (195384.2 to 252348.2)** | **1937.9 (1315.5 to 2839.2)** | **27.5* (26.4 to 28.4)** | **0.2 (-0.7 to 0.9)** |
| Dermatitis | 12272.7 (10259.9 to 14794.6) | 18231.4 (13368.4 to 24909.6) | 408.8 (243.9 to 632.5) | 23.8* (22.0 to 25.7) | -2.7* (-4.2 to -1.2) |
| Atopic dermatitis | 6133.9 (5403.5 to 7016.4) | 917.1 (753.1 to 1104.6) | 260.9 (139.1 to 435.5) | 23.6* (21.0 to 26.1) | -2.9* (-5.0 to -0.9) |
| Contact dermatitis | 5569.9 (3706.2 to 7995.7) | 15711.5 (10857.1 to 22433.0) | 138.0 (75.4 to 228.3) | 24.0* (21.4 to 26.8) | -2.6* (-4.7 to -0.4) |
| Seborrhoeic dermatitis | 729.4 (578.4 to 912.6) | 1602.8 (1263.9 to 2018.1) | 9.8 (5.4 to 16.4) | 25.5* (19.6 to 31.2) | -1.4 (-6.1 to 3.0) |
| Psoriasis | 5873.1 (5411.5 to 6343.0) | 693.7 (603.9 to 787.3) | 503.4 (355.0 to 665.8) | 27.5* (25.1 to 29.9) | 0.1 (-1.7 to 2.0) |
| Bacterial skin diseases | 420.8 (380.8 to 461.2) | 11002.2 (9979.0 to 12018.1) | 9.7 (6.1 to 14.4) | 18.1* (13.4 to 22.6) | -7.3* (-10.9 to -3.7) |
| Cellulitis | 135.2 (110.4 to 164.6) | 2768.1 (2261.4 to 3285.1) | 7.7 (4.9 to 11.2) | 15.5* (10.5 to 20.7) | -9.3* (-13.2 to -5.2) |
| Pyoderma | 352.5 (319.1 to 389.3) | 8234.2 (7367.3 to 9182.6) | 2.0 (0.8 to 4.1) | 29.4* (24.9 to 34.0) | 1.6 (-1.9 to 5.3) |
| Impetigo | 32.3 (28.4 to 36.8) | 597.6 (530.8 to 676.7) | 0.2 (0.1 to 0.4) | 31.3* (24.7 to 38.8) | 3.2 (-2.1 to 9.0) |
| Abscess and other bacterial skin diseases | 320.2 (286.3 to 356.8) | 7636.6 (6781.2 to 8597.6) | 1.8 (0.7 to 3.8) | 29.2* (24.1 to 34.1) | 1.5 (-2.5 to 5.4) |
| Scabies | 7182.3 (4826.3 to 9823.7) | 21501.6 (15075.5 to 29662.6) | 182.5 (87.1 to 321.7) | 29.7* (28.0 to 31.5) | 1.9* (0.6 to 3.3) |
| Fungal skin diseases | 35096.4 (27148.5 to 44670.3) | 111525.9 (86435.4 to 138434.4) | 195.9 (76.1 to 405.8) | 27.6* (26.7 to 28.3) | 0.2 (-0.4 to 0.8) |
| Tinea capitis | 1906.6 (1324.7 to 2641.9) | 3090.7 (2107.1 to 4107.4) | 10.6 (4.0 to 24.3) | 7.7* (4.5 to 11.5) | -15.4* (-17.9 to -12.5) |
| Other fungal skin diseases | 33189.9 (25319.9 to 42662.8) | 108435.2 (83793.6 to 135614.7) | 185.3 (70.7 to 385.5) | 28.9* (28.3 to 29.5) | 1.3* (0.8 to 1.7) |

| | | | | | |
|---|---|---|---|---|---|
| Viral skin diseases | 2885.7<br>(2590.3 to 3232.9) | 2458.5<br>(2230.9 to 2713.1) | 86.7<br>(55.2 to 130.5) | 25.2*<br>(22.5 to 27.7) | -1.7<br>(-3.8 to 0.3) |
| Viral warts | 2458.0<br>(2173.7 to 2826.5) | 1928.8<br>(1714.3 to 2166.3) | 73.9<br>(46.7 to 112.5) | 24.8*<br>(22.0 to 27.6) | -2.0<br>(-4.2 to 0.2) |
| Molluscum contagiosum | 427.7<br>(375.4 to 481.8) | 529.7<br>(473.4 to 591.6) | 12.8<br>(8.1 to 19.2) | 27.5*<br>(20.7 to 34.5) | 0.2<br>(-5.2 to 5.6) |
| Acne vulgaris | 1472.3<br>(1285.4 to 1708.2) | 630.3<br>(531.0 to 740.1) | 31.2<br>(17.9 to 49.8) | 33.1*<br>(29.5 to 36.6) | 4.6*<br>(1.8 to 7.3) |
| Alopecia areata | 983.1<br>(878.6 to 1101.0) | 1675.6<br>(1509.3 to 1856.7) | 31.8<br>(20.5 to 46.9) | 24.4*<br>(20.6 to 28.5) | -2.2<br>(-5.2 to 0.9) |
| Pruritus | 5208.3<br>(3652.5 to 7349.3) | 4066.4<br>(2799.8 to 5722.8) | 55.0<br>(23.8 to 105.9) | 29.7*<br>(27.9 to 31.4) | 1.9*<br>(0.5 to 3.2) |
| Urticaria | 3207.4<br>(2192.3 to 4383.6) | 5543.6<br>(3808.8 to 7492.1) | 187.1<br>(108.6 to 290.5) | 27.9*<br>(25.1 to 30.6) | 0.5<br>(-1.7 to 2.6) |
| Decubitus ulcer | 49.7<br>(34.6 to 70.2) | 180.0<br>(123.1 to 257.9) | 8.8<br>(5.2 to 14.5) | 22.6*<br>(21.1 to 24.0) | -3.7*<br>(-4.9 to -2.6) |
| Other skin and subcutaneous diseases | 43190.1<br>(39288.5 to 47215.8) | 45002.0<br>(41111.9 to 49274.1) | 237.2<br>(112.2 to 446.4) | 32.9*<br>(32.0 to 33.8) | 4.4*<br>(3.7 to 5.1) |
| **Sense organ diseases** | **205293.0<br>(196538.6 to 213878.1)** | **--** | **5093.6<br>(3256.9 to 7736.1)** | **26.5*<br>(24.9 to 28.0)** | **-0.6<br>(-1.9 to 0.5)** |
| Blindness and vision impairment | 127989.4<br>(118561.5 to 137688.7) | -- | 2462.9<br>(1576.5 to 3770.8) | 25.7*<br>(23.9 to 27.3) | -1.3*<br>(-2.7 to -0.0) |
| Glaucoma | 220.4<br>(155.6 to 299.7) | -- | 26.8<br>(15.8 to 41.5) | 22.8*<br>(15.8 to 30.3) | -3.5<br>(-9.0 to 2.4) |
| Cataract | 5416.0<br>(3967.8 to 7138.1) | -- | 426.1<br>(277.0 to 620.0) | 25.1*<br>(21.3 to 29.3) | -1.7<br>(-4.7 to 1.6) |
| Age-related macular degeneration | 212.0<br>(150.5 to 294.4) | -- | 20.7<br>(13.0 to 31.7) | 27.8*<br>(19.8 to 35.5) | 0.4<br>(-5.9 to 6.4) |
| Refraction disorders | 12496.8<br>(9313.0 to 16629.1) | -- | 567.4<br>(356.2 to 831.4) | 23.4*<br>(19.0 to 27.2) | -3.1*<br>(-6.5 to -0.1) |
| Near vision loss | 114061.8<br>(104486.0 to 123360.8) | -- | 1163.7<br>(554.4 to 2121.4) | 27.6*<br>(26.8 to 28.5) | 0.3<br>(-0.4 to 1.0) |
| Other vision loss | 2959.7<br>(2303.9 to 3786.1) | -- | 258.1<br>(173.0 to 365.1) | 23.4*<br>(20.3 to 26.5) | -3.1*<br>(-5.5 to -0.7) |
| Age-related and other hearing loss | 117317.2<br>(107558.5 to 126624.3) | -- | 2455.3<br>(1547.3 to 3734.6) | 27.2*<br>(24.4 to 29.9) | -0.1<br>(-2.3 to 2.0) |
| Other sense organ diseases | 7132.8<br>(6424.5 to 7932.1) | -- | 175.4<br>(106.3 to 255.5) | 28.8*<br>(26.7 to 31.0) | 1.2<br>(-0.5 to 2.9) |
| Chronic other sense organ diseases | 6392.3<br>(5697.6 to 7194.0) | -- | 165.4<br>(100.2 to 241.2) | 28.8*<br>(26.6 to 31.1) | 1.2<br>(-0.6 to 3.0) |
| Acute other sense organ diseases | 740.5<br>(674.0 to 814.4) | -- | 10.0<br>(6.0 to 15.4) | 28.5*<br>(22.4 to 35.1) | 1.0<br>(-3.9 to 6.1) |
| **Musculoskeletal disorders** | **136444.2<br>(119764.3 to 152163.9)** | **32001.6<br>(25179.2 to 39655.6)** | **14213.0<br>(9775.2 to 19756.8)** | **25.9*<br>(24.3 to 27.8)** | **-1.1<br>(-2.4 to 0.4)** |
| Rheumatoid arthritis | 2137.7<br>(1812.5 to 2486.5) | 139.8<br>(100.9 to 182.9) | 285.2<br>(189.7 to 396.5) | 36.5*<br>(31.4 to 41.5) | 7.2*<br>(3.3 to 11.2) |
| Osteoarthritis | 33273.3<br>(28205.9 to 39129.5) | 2356.6<br>(1869.4 to 2911.7) | 1081.1<br>(538.2 to 2148.5) | 29.6*<br>(28.3 to 30.8) | 1.8*<br>(0.8 to 2.8) |
| Osteoarthritis of the hip | 3977.2<br>(3586.4 to 4409.7) | 307.5<br>(265.7 to 356.1) | 129.4<br>(66.8 to 269.4) | 30.8*<br>(28.3 to 33.4) | 2.8*<br>(0.8 to 4.8) |
| Osteoarthritis of the knee | 29296.0<br>(24242.2 to 35056.9) | 2049.1<br>(1564.2 to 2615.5) | 951.7<br>(468.9 to 1912.7) | 29.4*<br>(28.0 to 30.8) | 1.7*<br>(0.5 to 2.7) |
| Low back pain | 52335.1<br>(39720.8 to 69360.4) | 21645.6<br>(15719.8 to 29057.4) | 6012.4<br>(3839.4 to 9268.3) | 22.5*<br>(21.3 to 23.6) | -3.8*<br>(-4.7 to -2.9) |
| Low back pain with leg pain | 19084.5<br>(13958.9 to 25564.0) | 7873.3<br>(5543.6 to 10556.4) | 2762.3<br>(1739.9 to 4417.5) | 22.5*<br>(21.2 to 23.8) | -3.7*<br>(-4.8 to -2.7) |
| Low back pain without leg pain | 33250.7<br>(24779.3 to 44096.1) | 13772.4<br>(9796.2 to 18620.4) | 3250.1<br>(1997.3 to 4873.6) | 22.5*<br>(21.2 to 23.8) | -3.8*<br>(-4.8 to -2.8) |
| Neck pain | 37016.9<br>(26528.8 to 48812.2) | 7064.5<br>(4649.4 to 9995.3) | 3696.5<br>(2236.7 to 5537.0) | 26.5*<br>(23.5 to 29.5) | -0.6<br>(-3.0 to 1.8) |
| Gout | 4127.6<br>(3190.9 to 5234.2) | 795.1<br>(604.2 to 1012.8) | 132.9<br>(84.5 to 197.1) | 29.3*<br>(24.1 to 34.2) | 1.6<br>(-2.5 to 5.4) |
| Other musculoskeletal disorders | 35181.8<br>(23023.1 to 48667.7) | -- | 3004.9<br>(1713.9 to 4689.9) | 29.9*<br>(22.9 to 35.6) | 2.1<br>(-3.5 to 6.5) |
| **Other non-communicable diseases** | **295527.6<br>(277582.9 to 313168.6)** | **159961.2<br>(138231.8 to 184968.8)** | **3775.3<br>(2506.8 to 5444.0)** | **24.1*<br>(22.4 to 25.7)** | **-2.5*<br>(-3.8 to -1.3)** |
| Congenital anomalies | 4451.5<br>(4184.1 to 4773.7) | -- | 568.0<br>(401.9 to 763.7) | 25.7*<br>(23.2 to 28.4) | -1.3<br>(-3.3 to 0.8) |
| Neural tube defects | 133.9<br>(119.4 to 149.4) | -- | 39.3<br>(27.2 to 52.7) | 32.4*<br>(26.6 to 38.3) | 4.0<br>(-0.5 to 8.6) |
| Severe motor and cognitive impairment due to anencephaly | -- | -- | -- | -- | -- |
| Encephalocoele due to neural tube defects | 17.8<br>(14.6 to 21.1) | -- | 3.1<br>(2.1 to 4.3) | 26.4*<br>(22.6 to 30.3) | -0.7<br>(-3.7 to 2.4) |
| Spina bifida due to neural tube defects | 116.2<br>(103.5 to 130.4) | -- | 36.2<br>(25.2 to 48.8) | 32.9*<br>(26.9 to 39.3) | 4.4<br>(-0.3 to 9.4) |
| Congenital heart anomalies | 147.8<br>(135.3 to 161.9) | -- | 8.7<br>(3.9 to 14.9) | 28.0*<br>(22.7 to 33.3) | 0.6<br>(-3.6 to 4.7) |
| Critical malformations of great vessels, congenital valvular heart disease and patent ductus arteriosus | 25.5<br>(22.0 to 29.4) | -- | 1.6<br>(0.7 to 2.7) | 28.9*<br>(26.4 to 31.6) | 1.3<br>(-0.7 to 3.4) |
| Other congenital heart anomalies | 50.3<br>(45.9 to 55.2) | -- | 2.9<br>(1.2 to 5.2) | 27.8*<br>(17.5 to 38.3) | 0.4<br>(-7.7 to 8.7) |
| Severe congenital heart anomalies excluding single ventricle heart defects | 62.1<br>(55.2 to 69.8) | -- | 3.8<br>(1.7 to 6.3) | 28.2*<br>(19.0 to 38.3) | 0.7<br>(-6.5 to 8.6) |
| Single ventricle and single ventricle pathway congenital heart anomalies | 1.8<br>(1.5 to 2.2) | -- | 0.1<br>(0.1 to 0.2) | 28.4*<br>(24.2 to 32.0) | 0.9<br>(-2.4 to 3.7) |
| Ventricular septal defect and atrial septal defect | 8.0<br>(6.5 to 9.6) | -- | 0.3<br>(0.1 to 0.5) | 23.5*<br>(20.9 to 26.0) | -3.0*<br>(-5.0 to -1.1) |
| Orofacial clefts | 578.3<br>(528.9 to 627.2) | -- | 15.6<br>(10.4 to 22.6) | 31.5*<br>(21.6 to 41.6) | 3.3<br>(-4.5 to 11.2) |
| Down's syndrome | 161.8<br>(140.9 to 183.8) | -- | 19.4<br>(13.2 to 26.5) | 19.9*<br>(16.8 to 23.4) | -5.8*<br>(-8.2 to -3.0) |
| Turner syndrome | 14.6<br>(12.6 to 16.9) | -- | 0.2<br>(0.1 to 0.4) | 24.0*<br>(21.3 to 26.7) | -2.6*<br>(-4.7 to -0.5) |
| Klinefelter syndrome | 15.5<br>(13.0 to 18.5) | -- | 0.0<br>(0.0 to 0.1) | 27.6*<br>(25.3 to 30.1) | 0.3<br>(-1.5 to 2.2) |
| Other chromosomal abnormalities | 266.2<br>(213.0 to 323.5) | -- | 32.2<br>(21.3 to 44.6) | 28.6*<br>(24.7 to 32.3) | 1.0<br>(-2.0 to 4.0) |
| Congenital musculoskeletal and limb anomalies | 1588.6<br>(1413.3 to 1814.5) | -- | 230.7<br>(153.4 to 315.6) | 25.0*<br>(22.7 to 27.5) | -1.8<br>(-3.6 to 0.1) |
| Polydactyly and syndactyly | 4.6<br>(3.7 to 5.6) | -- | 0.1<br>(0.0 to 0.1) | 17.5*<br>(15.3 to 19.6) | -7.7*<br>(-9.5 to -6.1) |
| Congenital limb deficiency | 678.6<br>(602.9 to 775.7) | -- | 98.9<br>(65.4 to 134.8) | 25.1*<br>(21.1 to 28.7) | -1.7<br>(-4.8 to 1.1) |
| Other congenital musculoskeletal anomalies | 905.4<br>(805.4 to 1034.1) | -- | 131.8<br>(88.0 to 180.3) | 24.9*<br>(21.7 to 28.1) | -1.9<br>(-4.4 to 0.6) |
| Urogenital congenital anomalies | 78.3<br>(68.0 to 90.7) | -- | 3.0<br>(1.8 to 4.6) | 10.3*<br>(5.4 to 15.5) | -13.3*<br>(-17.2 to -9.3) |
| Digestive congenital anomalies | 1026.6<br>(922.9 to 1145.7) | -- | 45.8<br>(30.5 to 65.2) | 16.4*<br>(10.4 to 22.7) | -8.6*<br>(-13.2 to -3.6) |
| Congenital diaphragmatic hernia | 7.7<br>(6.7 to 8.7) | -- | 0.2<br>(0.1 to 0.2) | 30.0*<br>(28.6 to 31.3) | 2.2*<br>(1.1 to 3.1) |
| Congenital atresia and/or stenosis of the digestive tract | 0.3<br>(0.2 to 0.4) | -- | -- | 46.2*<br>(39.5 to 52.2) | 14.8*<br>(9.6 to 19.6) |
| Congenital malformations of the abdominal wall after treatment | 29.8<br>(26.0 to 33.8) | -- | 1.3<br>(0.8 to 1.9) | 28.9*<br>(27.6 to 30.1) | 1.3*<br>(0.2 to 2.2) |
| Other congenital malformations of the digestive tract | 988.8<br>(886.0 to 1108.8) | -- | 44.3<br>(29.5 to 63.1) | 16.0*<br>(9.9 to 22.5) | -8.9*<br>(-13.7 to -3.7) |
| Other congenital anomalies | 1327.7<br>(1140.3 to 1543.1) | -- | 173.2<br>(117.4 to 242.5) | 27.8*<br>(20.2 to 35.6) | 0.4<br>(-5.6 to 6.5) |

| | | | | | |
|---|---|---|---|---|---|
| Other congenital birth defects | -- | -- | 13.3 (9.6 to 17.6) | 44.2* (38.7 to 49.6) | 13.2* (9.0 to 17.5) |
| Hearing loss due to other congenital anomalies | 1327.7 (1140.3 to 1543.1) | -- | 159.8 (107.2 to 225.8) | 26.6* (18.6 to 34.7) | -0.6 (-6.9 to 5.8) |
| Urinary diseases and male infertility | 9413.0 (7867.2 to 10928.2) | 26864.7 (21895.6 to 32163.3) | 342.9 (217.7 to 490.4) | 23.9* (21.8 to 25.9) | -2.6* (-4.3 to -1.1) |
| Urinary tract infections | 331.2 (247.7 to 429.7) | 17152.0 (12960.4 to 22045.5) | 10.8 (6.0 to 17.0) | 35.7* (27.7 to 43.9) | 6.6* (0.3 to 13.0) |
| Urolithiasis | 318.3 (229.3 to 419.8) | 8376.5 (6150.8 to 11146.4) | 23.3 (14.4 to 35.2) | 17.8* (10.8 to 25.0) | -7.5* (-12.9 to -1.8) |
| Benign prostatic hyperplasia | 8942.4 (7355.6 to 10516.3) | 1336.2 (1111.6 to 1574.3) | 299.7 (183.9 to 438.7) | 25.2* (23.0 to 27.2) | -1.7* (-3.4 to -0.1) |
| Male infertility | -- | -- | -- | -- | -- |
| Other urinary diseases | -- | -- | 9.1 (5.9 to 13.1) | -4.1 (-8.9 to 0.7) | -24.7* (-28.4 to -20.9) |
| Gynecological diseases | 62314.2 (58884.5 to 66300.0) | 3380.3 (2846.7 to 3997.4) | 801.8 (545.4 to 1138.3) | 24.9* (22.6 to 27.1) | -1.9* (-3.7 to -0.1) |
| Uterine fibroids | 21882.7 (18590.0 to 25804.5) | 49.4 (24.3 to 79.9) | 189.0 (109.9 to 315.1) | 17.3* (9.0 to 25.2) | -7.8* (-14.3 to -1.7) |
| Polycystic ovarian syndrome | 3271.4 (2594.8 to 4154.8) | 169.6 (78.9 to 299.4) | 27.7 (12.9 to 53.1) | 33.3* (31.0 to 35.5) | 4.7* (2.9 to 6.4) |
| Female infertility | -- | -- | -- | -- | -- |
| Endometriosis | 2572.6 (1945.6 to 3430.3) | 6.8 (0.3 to 25.7) | 232.3 (143.7 to 354.0) | 28.8* (25.7 to 32.2) | 1.2 (-1.2 to 3.8) |
| Genital prolapse | 12315.5 (9804.8 to 15667.3) | 1300.5 (939.5 to 1701.6) | 38.6 (17.5 to 73.0) | 25.7* (23.5 to 28.0) | -1.3 (-3.0 to 0.5) |
| Premenstrual syndrome | 27494.7 (25855.7 to 29296.4) | -- | 223.5 (144.9 to 333.0) | 26.4* (24.0 to 28.9) | -0.7 (-2.6 to 1.3) |
| Other gynecological diseases | 4084.1 (3639.0 to 4639.3) | 1854.0 (1453.9 to 2327.9) | 90.8 (58.8 to 130.6) | 25.4* (20.9 to 29.9) | -1.5 (-5.0 to 2.1) |
| Other gynecological diseases | 3862.6 (3417.6 to 4402.5) | 1854.0 (1453.9 to 2327.9) | 87.1 (56.4 to 124.8) | 26.4* (21.9 to 31.1) | -0.7 (-4.2 to 3.0) |
| Other gynecological diseases with anemia | 221.6 (200.5 to 243.6) | -- | 3.7 (2.3 to 5.7) | 6.5 (-9.5 to 23.8) | -16.3* (-28.9 to -2.7) |
| Hemoglobinopathies and hemolytic anaemias | 97969.9 (95879.3 to 100079.8) | -- | 216.8 (141.5 to 312.7) | 8.3 (-2.5 to 21.3) | -14.9* (-23.4 to -4.8) |
| Thalassemias | 0.3 (0.3 to 0.4) | -- | -- | 25.1* (4.5 to 45.0) | -1.8 (-17.9 to 13.9) |
| Thalassaemias trait | 18608.7 (17879.1 to 19373.2) | -- | 101.1 (63.5 to 150.1) | 4.2 (-12.2 to 26.4) | -18.2* (-31.0 to -0.7) |
| Sickle cell disorders | 3.9 (3.0 to 5.0) | -- | 0.6 (0.4 to 0.9) | 44.4* (23.5 to 66.3) | 13.4 (-3.0 to 30.7) |
| Sickle cell trait | 19276.5 (17342.8 to 21462.6) | -- | 38.0 (25.2 to 55.6) | 11.7* (0.6 to 23.7) | -12.2* (-20.9 to -2.8) |
| G6PD deficiency | 17134.3 (16462.5 to 17801.6) | -- | 1.2 (0.8 to 1.7) | 11.7 (-0.0 to 24.2) | -12.2* (-21.5 to -2.4) |
| G6PD trait | 48835.3 (48048.1 to 49612.8) | -- | -- | 14.4 (-2.7 to 36.7) | -10.2 (-23.6 to 7.4) |
| Other hemoglobinopathies and hemolytic anaemias | 4089.8 (3787.2 to 4442.4) | -- | 75.8 (50.7 to 112.0) | 12.1* (2.2 to 22.0) | -11.9* (-19.7 to -4.2) |
| Endocrine, metabolic, blood, and immune disorders | 6406.0 (5800.0 to 7139.3) | -- | 177.0 (120.1 to 249.4) | 18.9* (12.2 to 25.1) | -6.6* (-11.9 to -1.8) |
| Anemia due to endocrine, metabolic, blood, and immune disorders | 4207.9 (3936.1 to 4504.6) | -- | 97.2 (66.0 to 139.0) | 9.9* (0.3 to 19.7) | -13.7* (-21.2 to -6.0) |
| Endocrine, metabolic, blood, and immune disorders | 2185.9 (1617.6 to 2863.4) | -- | 78.6 (48.7 to 118.7) | 32.4* (25.6 to 39.8) | 4.0 (-1.3 to 9.8) |
| Heart failure due to endocrine, metabolic, blood, and immune disorders | 12.2 (8.5 to 17.1) | -- | 1.1 (0.7 to 1.8) | 22.3* (20.4 to 24.3) | -3.9* (-5.4 to -2.4) |
| Oral disorders | 224486.7 (195980.8 to 252113.2) | 129716.2 (108940.8 to 154875.1) | 1668.9 (973.4 to 2761.5) | 26.1* (24.8 to 27.2) | -0.9* (-1.9 to -0.1) |
| Caries of deciduous teeth | -- | -- | -- | -- | -- |
| Caries of permanent teeth | 143384.2 (109491.4 to 179492.5) | 120309.5 (99126.9 to 145772.7) | 103.6 (43.6 to 204.0) | 19.6* (17.8 to 21.3) | -6.1* (-7.5 to -4.7) |
| Periodontal disease | 103576.6 (78101.4 to 129998.9) | 7374.3 (4166.7 to 10310.3) | 679.4 (260.2 to 1440.4) | 30.0* (29.0 to 30.9) | 2.1* (1.4 to 2.8) |
| Edentulism and severe tooth loss | 20524.0 (16569.5 to 25818.1) | 2032.3 (1253.5 to 2845.3) | 578.5 (365.7 to 858.1) | 22.2* (20.9 to 23.5) | -4.0* (-5.0 to -3.0) |
| Other oral disorders | 10754.4 (9629.7 to 11933.7) | -- | 307.5 (190.7 to 451.2) | 27.9* (26.6 to 29.3) | 0.5 (-0.6 to 1.5) |
| **Injuries** | **130396.6 (122355.0 to 140293.9)** | **30955.6 (26182.4 to 35974.4)** | **4993.5 (3654.8 to 6636.9)** | **29.6* (28.4 to 30.8)** | **1.8* (0.9 to 2.8)** |
| **Transport injuries** | **22684.3 (20874.0 to 24779.8)** | **4076.3 (2927.7 to 5517.1)** | **1333.6 (950.6 to 1774.5)** | **29.4* (28.0 to 30.7)** | **1.6* (0.6 to 2.6)** |
| Road injuries | 18282.4 (16682.9 to 20085.1) | 3658.8 (2489.7 to 5119.7) | 1070.9 (770.4 to 1430.5) | 28.5* (27.1 to 29.9) | 1.0 (-0.2 to 2.1) |
| Pedestrian road injuries | 4740.2 (3967.6 to 5757.5) | 766.5 (418.7 to 1257.8) | 279.8 (195.8 to 386.4) | 26.4* (24.5 to 28.4) | -0.7 (-2.2 to 0.8) |
| Cyclist road injuries | 3130.9 (2540.4 to 3763.7) | 908.2 (540.6 to 1407.7) | 176.4 (120.2 to 242.2) | 36.8* (34.7 to 39.1) | 7.5* (5.8 to 9.3) |
| Motorcyclist road injuries | 4816.0 (4064.4 to 5703.8) | 618.9 (336.4 to 996.7) | 280.1 (189.2 to 384.8) | 31.1* (28.8 to 33.2) | 3.0* (1.2 to 4.6) |
| Motor vehicle road injuries | 4366.4 (3722.2 to 5232.2) | 1032.7 (616.7 to 1578.1) | 269.6 (192.8 to 362.9) | 17.7* (16.0 to 19.4) | -7.5* (-8.8 to -6.2) |
| Other road injuries | 1229.0 (958.3 to 1575.4) | 332.5 (163.0 to 570.0) | 65.0 (45.4 to 90.5) | 61.2* (58.1 to 64.1) | 26.6* (24.2 to 28.9) |
| Other transport injuries | 4401.9 (3528.5 to 5227.7) | 417.5 (266.3 to 611.2) | 262.7 (187.1 to 351.4) | 32.9* (30.9 to 35.2) | 4.4* (2.8 to 6.2) |
| **Unintentional injuries** | **81995.2 (76101.1 to 90295.3)** | **25161.3 (20682.9 to 30209.5)** | **3058.2 (2191.7 to 4163.4)** | **28.5* (27.4 to 29.5)** | **1.0* (0.1 to 1.7)** |
| Falls | 34282.1 (29847.2 to 39743.7) | 10485.5 (7049.2 to 14572.8) | 1593.1 (1131.6 to 2185.1) | 26.8* (25.4 to 28.0) | -0.4 (-1.5 to 0.6) |
| Drowning | 193.5 (167.6 to 224.4) | 16.6 (11.7 to 22.8) | 11.2 (8.0 to 15.1) | 31.6* (28.3 to 35.1) | 3.4* (0.8 to 6.2) |
| Fire, heat, and hot substances | 9368.1 (7961.8 to 10954.8) | 359.6 (220.4 to 555.7) | 265.3 (180.8 to 375.4) | 25.2* (21.1 to 28.8) | -1.7 (-4.9 to 1.2) |
| Poisonings | 346.0 (293.7 to 401.1) | 126.0 (72.0 to 198.1) | 32.1 (21.3 to 44.5) | 33.7* (30.2 to 37.3) | 5.0* (2.3 to 7.8) |
| Poisoning by carbon monoxide | 71.6 (60.8 to 86.5) | 43.1 (19.6 to 84.4) | 5.5 (3.8 to 7.8) | 39.6* (35.6 to 43.8) | 9.7* (6.5 to 13.0) |
| Poisoning by other means | 274.3 (222.6 to 328.3) | 82.9 (43.4 to 137.3) | 26.5 (17.2 to 38.0) | 32.5* (28.8 to 36.2) | 4.1* (1.1 to 7.0) |
| Exposure to mechanical forces | 18380.8 (15667.7 to 21441.8) | 4119.1 (2713.2 to 5900.0) | 460.3 (312.4 to 656.1) | 29.8* (28.5 to 31.0) | 2.0* (1.0 to 2.9) |
| Unintentional firearm injuries | 796.5 (642.6 to 946.6) | 73.5 (32.3 to 140.8) | 28.0 (19.3 to 39.1) | 34.9* (33.5 to 36.2) | 6.0* (4.9 to 7.0) |
| Other exposure to mechanical forces | 17584.3 (14942.0 to 20679.6) | 4045.6 (2668.1 to 5835.0) | 432.3 (292.0 to 618.9) | 29.5* (28.1 to 30.7) | 1.7* (0.6 to 2.7) |
| Adverse effects of medical treatment | 267.0 (173.5 to 388.0) | 3492.7 (2358.9 to 4899.4) | 35.6 (19.3 to 58.9) | 68.1* (58.5 to 76.8) | 32.1* (24.5 to 38.8) |
| Animal contact | 3200.7 (2761.6 to 3670.7) | 2275.9 (1394.7 to 3340.2) | 76.3 (51.2 to 107.6) | 25.0* (21.9 to 27.9) | -1.8 (-4.3 to 0.5) |
| Venomous animal contact | 1297.8 (1055.0 to 1549.9) | 1044.6 (629.8 to 1574.3) | 47.0 (31.8 to 65.5) | 26.4* (22.3 to 30.5) | -0.7 (-3.9 to 2.5) |
| Non-venomous animal contact | 1902.9 (1585.9 to 2315.8) | 1231.3 (724.0 to 1884.6) | 29.3 (18.3 to 46.3) | 22.9* (19.8 to 25.1) | -3.5* (-5.9 to -1.7) |
| Foreign body | 1609.3 (1398.9 to 1814.9) | 1133.7 (715.7 to 1728.2) | 75.0 (52.2 to 101.6) | 29.0* (27.4 to 30.6) | 1.3* (0.1 to 2.6) |

| | | | | | |
|---|---|---|---|---|---|
| Pulmonary aspiration and foreign body in airway | 281.1 (230.2 to 348.6) | 32.7 (16.5 to 54.8) | 12.6 (8.7 to 17.6) | 38.7* (35.4 to 42.2) | 9.0* (6.4 to 11.7) |
| Foreign body in eyes | 196.7 (111.6 to 323.4) | 915.0 (513.0 to 1509.1) | 13.3 (7.1 to 23.5) | 25.9* (23.7 to 27.9) | -1.1 (-2.8 to 0.5) |
| Foreign body in other body part | 1131.5 (947.9 to 1301.1) | 186.0 (102.6 to 333.1) | 49.0 (34.9 to 64.0) | 27.6* (25.9 to 29.2) | 0.2 (-1.1 to 1.5) |
| Environmental heat and cold exposure | 2948.4 (2515.0 to 3486.1) | 439.4 (274.9 to 658.4) | 121.3 (83.0 to 164.4) | 29.2* (27.1 to 31.2) | 1.5 (-0.2 to 3.1) |
| Exposure to forces of nature | 1029.3 (757.6 to 1383.4) | 29.2 (20.0 to 40.8) | 44.0 (32.4 to 59.5) | 62.4* (49.1 to 78.3) | 27.6* (17.1 to 40.0) |
| Other unintentional injuries | 10374.1 (8513.6 to 12318.7) | 2683.6 (1511.6 to 4007.1) | 344.1 (231.2 to 482.6) | 31.2* (29.6 to 32.6) | 3.0* (1.8 to 4.2) |
| **Self-harm and interpersonal violence** | **25962.5 (21076.0 to 32428.7)** | **1718.0 (1225.0 to 2282.2)** | **601.6 (460.9 to 749.2)** | **35.8* (29.6 to 43.6)** | **6.7* (1.8 to 12.8)** |
| Self-harm | 862.1 (702.2 to 1027.8) | 220.5 (149.3 to 315.6) | 43.2 (30.3 to 57.6) | 9.6* (8.2 to 10.9) | -13.9* (-15.0 to -12.9) |
| Self-harm by firearm | 8.7 (7.3 to 10.5) | 2.9 (0.6 to 8.0) | 0.4 (0.3 to 0.5) | 2.3 (-1.6 to 6.0) | -19.7* (-22.7 to -16.7) |
| Self-harm by other specified means | 853.4 (693.0 to 1020.4) | 217.6 (147.4 to 312.5) | 42.8 (30.0 to 57.1) | 9.7* (8.2 to 10.9) | -13.9* (-15.0 to -12.9) |
| Interpersonal violence | 20414.7 (15823.6 to 26807.6) | 1208.5 (727.8 to 1738.8) | 355.9 (264.3 to 455.4) | 22.6* (20.6 to 24.8) | -3.7* (-5.2 to -2.0) |
| Assault by firearm | 270.1 (221.9 to 336.4) | 23.9 (11.1 to 44.2) | 11.8 (8.2 to 16.2) | 23.0* (21.2 to 24.8) | -3.4* (-4.8 to -1.9) |
| Assault by sharp object | 1498.5 (1215.6 to 2073.7) | 145.9 (67.6 to 255.5) | 45.2 (31.3 to 63.1) | 19.4* (17.6 to 21.1) | -6.2* (-7.6 to -4.9) |
| Sexual violence | 14231.0 (9726.4 to 20447.6) | -- | 113.4 (65.6 to 177.6) | 33.2* (31.0 to 35.3) | 4.6* (2.9 to 6.3) |
| Assault by other means | 4415.2 (3652.4 to 5359.6) | 1038.8 (617.9 to 1526.7) | 185.5 (132.0 to 256.4) | 17.7* (16.3 to 18.9) | -7.6* (-8.7 to -6.6) |
| Conflict and terrorism | 4166.7 (2833.6 to 5826.7) | 233.8 (169.9 to 304.7) | 185.1 (128.5 to 265.5) | 80.5* (56.2 to 109.0) | 41.8* (22.7 to 64.2) |
| Executions and police conflict | 525.0 (336.1 to 771.1) | 55.1 (36.0 to 78.0) | 17.4 (11.6 to 25.2) | 62.4* (40.1 to 91.9) | 27.6* (10.1 to 50.8) |
| **Nature of injury aggregates** | | | | | |
| Amputations | 34200.5 (31519.3 to 37855.9) | 570.5 (451.0 to 710.2) | 574.3 (381.4 to 857.7) | 24.8* (20.7 to 28.7) | -2.0 (-5.2 to 1.1) |
| Burns | 19195.2 (17597.1 to 21068.9) | 576.1 (444.4 to 733.5) | 542.4 (374.9 to 755.8) | 24.8* (21.1 to 28.1) | -2.0 (-4.8 to 0.6) |
| Fractures | 31910.1 (29837.8 to 35081.6) | 8320.9 (6595.6 to 10322.4) | 1749.1 (1214.5 to 2450.9) | 28.6* (27.3 to 30.0) | 1.0 (-0.0 to 2.1) |
| Head Injuries | 4629.4 (4411.4 to 4877.7) | 1266.4 (1015.9 to 1583.1) | 682.4 (482.3 to 922.1) | 31.8* (30.2 to 33.4) | 3.6* (2.3 to 4.8) |
| Spinal Injuries | 2137.5 (1970.0 to 2367.3) | 46.1 (34.5 to 60.4) | 615.3 (440.1 to 797.9) | 36.7* (33.4 to 42.1) | 7.4* (4.8 to 11.6) |
| Minor Injuries | 18140.0 (17042.0 to 19610.7) | 10371.5 (8435.2 to 12340.4) | 162.3 (77.3 to 310.3) | 30.2* (29.2 to 31.8) | 2.3* (1.5 to 3.5) |
| Other Injuries | 6227.4 (5895.6 to 6589.8) | 9804.0 (8335.7 to 11553.9) | 554.3 (394.5 to 740.6) | 31.8* (29.4 to 34.6) | 3.5* (1.7 to 5.7) |
| **Impairments** | | | | | |
| Anaemia | 89622.1 (83504.9 to 96195.0) | -- | 2290.5 (1579.2 to 3221.2) | 9.6* (1.3 to 17.4) | -13.9* (-20.4 to -7.7) |
| Epilepsy | 3107.3 (2594.3 to 3680.0) | -- | 1183.4 (892.4 to 1544.4) | 29.7* (23.3 to 36.5) | 1.8 (-3.1 to 7.2) |
| Guillain-Barré syndrome | 5.4 (2.9 to 8.8) | -- | 1.6 (0.7 to 2.9) | 30.5* (27.0 to 33.6) | 2.5 (-0.2 to 4.9) |
| Hearing loss | 122899.2 (113226.0 to 132243.4) | -- | 2705.4 (1736.6 to 4004.0) | 26.8* (24.3 to 29.2) | -0.4 (-2.4 to 1.5) |
| Heart failure | 2918.8 (2441.2 to 3486.5) | -- | 324.2 (220.0 to 457.1) | 26.8* (24.0 to 29.4) | -0.4 (-2.6 to 1.7) |
| Infertility | 264.6 (234.4 to 297.2) | -- | 1.3 (0.5 to 2.8) | 28.6* (23.2 to 33.8) | 1.0 (-3.2 to 5.1) |
| Developmental intellectual disability | 7622.3 (5932.6 to 9221.2) | -- | 1140.9 (897.7 to 1419.7) | 30.1* (26.6 to 34.3) | 2.2 (-0.6 to 5.5) |
| Pelvic inflammatory disease | 70.0 (49.5 to 95.6) | -- | 9.2 (5.6 to 14.1) | 31.1* (23.4 to 39.7) | 3.0 (-3.1 to 9.8) |
| Vision loss | 137278.0 (126599.0 to 148741.3) | -- | 2786.1 (1851.6 to 4187.5) | 26.6* (25.1 to 28.1) | -0.6 (-1.7 to 0.6) |

We did not calculate incidence for the nine impairments and certain NTDs. Blank cells mean that no estimate is available or that the estimate has a magnitude less than 50.
Table presents prevalence and incidence counts in thousands.
* Percentage changes that are statistically significant
‡ Incidence estimates for stroke represent first-ever stroke only.
^ Incidence of HIV/AIDS represents new infections of HIV only and does not include new infections of Tuberculosis in HIV positive cases.

| SR Table 10o: Global prevalence, incidence and YLDs for 2017, percentage change of YLD counts and percentage change of YLD rates between 2007 and 2017 by cause, age 55 to 59 years | | | | | |
|---|---|---|---|---|---|
| Cause | Prevalence in thousands (95% UI) | Incidence in thousands (95% UI) | YLDs in thousands (95% UI) | | |
| | 2017 counts | 2017 counts | 2017 counts | Percentage change in counts between 2007 and 2017 | Percentage change in rates between 2007 and 2017 |
| **All causes** | **345858.2 (345467.0 to 346209.0)** | **1466617.3 (1248476.8 to 1718773.3)** | **58168.0 (44138.6 to 74646.5)** | **27.8* (27.0 to 28.7)** | **0.6 (-0.1 to 1.3)** |
| **Communicable, maternal, neonatal, and nutritional disorders** | **222166.2 (204175.4 to 244601.9)** | **965821.8 (751952.7 to 1214279.9)** | **3940.5 (2962.9 to 5117.3)** | **22.0* (18.3 to 26.2)** | **-3.9* (-6.9 to -0.7)** |
| **HIV/AIDS and sexually transmitted infections** | **85092.4 (76550.6 to 95380.2)** | **14977.1 (11735.2 to 19248.2)** | **226.0 (153.0 to 323.8)** | **26.8 (-2.2 to 58.3)** | **-0.2 (-23.0 to 24.6)** |
| HIV/AIDS | 1772.7 (1534.1 to 2036.7) | 49.5^ (31.8 to 68.5) | 178.5 (120.8 to 258.5) | 26.5 (-6.1 to 74.2) | -0.5 (-26.1 to 37.1) |
| HIV/AIDS - Drug-susceptible Tuberculosis | 38.7 (35.3 to 42.1) | 51.2 (46.5 to 56.6) | 14.5 (10.0 to 19.5) | 27.9* (19.0 to 37.0) | 0.7 (-6.4 to 7.8) |
| HIV/AIDS - Multidrug-resistant Tuberculosis without extensive drug resistance | 1.6 (1.1 to 2.5) | 2.2 (1.5 to 2.9) | 0.7 (0.4 to 1.1) | 25.9 (-19.1 to 91.2) | -0.9 (-36.3 to 50.5) |
| HIV/AIDS - Extensively drug-resistant Tuberculosis | 0.1 (0.0 to 0.1) | 0.1 (0.0 to 0.1) | -- | 101.3* (29.9 to 237.8) | 58.4* (2.2 to 165.9) |
| HIV/AIDS resulting in other diseases | 1732.3 (1495.0 to 1996.1) | 49.5 (31.8 to 68.5) | 163.3 (108.9 to 240.4) | 26.3 (-8.0 to 79.3) | -0.6 (-27.6 to 41.2) |
| HIV/AIDS not on antiretroviral treatment without Tuberculosis | 327.7 (266.5 to 411.1) | 49.5 (31.8 to 68.5) | 58.0 (31.5 to 105.5) | -48.6* (-55.8 to -39.2) | -59.6* (-65.2 to -52.1) |
| HIV/AIDS on antiretroviral treatment without Tuberculosis | 1404.7 (1213.4 to 1618.2) | -- | 105.3 (69.4 to 150.0) | 542.4* (442.2 to 687.3) | 405.6* (326.8 to 519.7) |
| Sexually transmitted infections excluding HIV | 84006.7 (75350.3 to 94377.8) | 14927.6 (11679.8 to 19198.6) | 47.4 (25.1 to 86.3) | 28.0* (25.2 to 30.0) | 0.7 (-1.4 to 2.3) |
| Syphilis | 1069.0 (823.4 to 1388.6) | 158.6 (105.2 to 221.5) | 7.0 (4.8 to 9.7) | 19.7* (12.3 to 28.4) | -5.8 (-11.6 to 1.0) |
| Early syphilis | 1030.1 (786.6 to 1351.6) | 158.6 (105.2 to 221.5) | 0.2 (0.1 to 0.6) | 34.6* (23.3 to 46.8) | 5.9 (-3.0 to 15.5) |
| Tertiary syphilis | 38.9 (32.6 to 46.6) | -- | 6.8 (4.5 to 9.5) | 19.3* (11.6 to 28.2) | -6.1 (-12.1 to 0.9) |
| Chlamydial infection | 1545.5 (1096.4 to 2085.1) | 1512.0 (1010.6 to 2102.1) | 3.8 (2.1 to 6.8) | 28.6* (25.1 to 33.1) | 1.3 (-1.5 to 4.7) |
| Chlamydia episode | 1534.8 (1084.8 to 2072.3) | 1512.0 (1010.6 to 2102.1) | 2.3 (0.9 to 5.3) | 27.1* (22.6 to 31.6) | 0.0 (-3.5 to 3.6) |
| Chlamydial infection complications | 10.8 (8.3 to 13.4) | -- | 1.4 (0.9 to 2.1) | 31.3* (24.7 to 38.1) | 3.3 (-1.9 to 8.7) |
| Gonococcal infection | 389.8 (236.8 to 619.9) | 1240.0 (779.8 to 1904.1) | 1.7 (0.9 to 3.2) | 26.4* (18.8 to 36.5) | -0.5 (-6.5 to 7.4) |
| Gonococcal infection complications | 5.4 (3.8 to 7.5) | -- | 0.7 (0.4 to 1.1) | 32.1* (17.8 to 48.9) | 4.0 (-7.3 to 17.2) |
| Gonorrhoea episode | 384.4 (231.7 to 615.3) | 1240.0 (779.8 to 1904.1) | 1.0 (0.4 to 2.5) | 22.7* (14.8 to 29.6) | -3.4 (-9.6 to 2.0) |
| Trichomoniasis | 6136.3 (3763.9 to 9087.3) | 10640.1 (7641.8 to 14670.2) | 10.3 (3.8 to 23.4) | 30.5* (27.8 to 33.2) | 2.7* (0.6 to 4.9) |
| Genital herpes | 78120.1 (69137.7 to 88298.1) | 1376.8 (996.7 to 1813.3) | 18.8 (5.9 to 45.7) | 29.6* (27.9 to 31.1) | 2.0* (0.7 to 3.2) |
| Other sexually transmitted infections | 31.2 (23.0 to 40.9) | -- | 5.8 (3.7 to 8.1) | 29.2* (21.2 to 37.8) | 1.7 (-4.6 to 8.4) |
| Other sexually transmitted diseases residual | -- | -- | 1.7 (0.8 to 3.3) | 26.1* (21.6 to 31.6) | -0.7 (-4.3 to 3.6) |
| Other sexually transmitted diseases | 31.2 (23.0 to 40.9) | -- | 5.8 (3.7 to 8.1) | 29.2* (21.2 to 37.8) | 1.7 (-4.6 to 8.4) |
| **Respiratory infections and tuberculosis** | **117492.6 (81446.7 to 161981.9)** | **663538.1 (463168.5 to 915038.7)** | **516.6 (352.6 to 731.4)** | **20.2* (17.8 to 22.2)** | **-5.4* (-7.3 to -3.8)** |
| Tuberculosis | 109324.4 (72285.5 to 155594.7) | 520.6 (384.5 to 677.5) | 217.2 (133.5 to 321.7) | 15.3* (11.5 to 18.9) | -9.3* (-12.2 to -6.4) |
| Latent tuberculosis infection | 108595.4 (71516.1 to 154856.6) | -- | -- | -- | -- |
| Drug-susceptible tuberculosis | 694.0 (531.4 to 896.0) | 492.1 (365.2 to 645.0) | 206.5 (126.8 to 304.8) | 15.5* (9.5 to 20.4) | -9.1* (-13.8 to -5.3) |
| Multidrug-resistant tuberculosis without extensive drug resistance | 33.3 (15.1 to 66.2) | 26.7 (14.2 to 48.6) | 10.1 (4.3 to 20.1) | 9.3 (-46.1 to 90.0) | -14.0 (-57.6 to 49.6) |
| Extensively drug-resistant tuberculosis | 1.7 (1.0 to 3.2) | 1.7 (1.1 to 2.6) | 0.6 (0.3 to 1.1) | 47.9 (-8.2 to 158.3) | 16.4 (-27.7 to 103.3) |
| Lower respiratory infections | 274.4 (232.3 to 316.3) | 12470.7 (10358.3 to 14913.5) | 16.3 (10.6 to 23.0) | 37.8* (25.5 to 50.5) | 8.5 (-1.2 to 18.4) |
| Guillain-Barré syndrome due to lower respiratory infections | 0.6 (0.3 to 1.1) | -- | 0.2 (0.1 to 0.3) | 31.0* (27.9 to 33.8) | 3.1* (0.7 to 5.3) |
| Lower respiratory infection episode | 273.8 (232.0 to 315.8) | 12470.7 (10358.3 to 14913.5) | 16.1 (10.5 to 22.8) | 37.9* (25.4 to 50.7) | 8.5 (-1.3 to 18.6) |
| Upper respiratory infections | 8957.2 (6191.3 to 12428.9) | 647962.3 (447620.9 to 900631.0) | 216.8 (119.5 to 359.5) | 25.5* (24.0 to 27.1) | -1.2 (-2.4 to 0.0) |
| Guillain-Barré syndrome due to upper respiratory infections | 1.6 (0.9 to 2.7) | -- | 0.5 (0.2 to 0.9) | 31.0* (27.9 to 33.8) | 3.1* (0.7 to 5.3) |
| Upper respiratory infection episode | 8955.6 (6189.5 to 12427.7) | 647962.3 (447620.9 to 900631.0) | 216.3 (118.9 to 358.7) | 25.5* (24.0 to 27.1) | -1.2 (-2.4 to 0.0) |
| Otitis media | 3230.7 (2398.5 to 4151.7) | 2584.5 (1843.5 to 3424.1) | 66.4 (37.4 to 108.1) | 16.6* (11.5 to 21.9) | -8.2* (-12.2 to -4.1) |
| Acute otitis media | 139.9 (100.7 to 181.0) | 2584.0 (1842.8 to 3423.8) | 1.8 (0.8 to 3.3) | 27.0* (19.3 to 35.0) | -0.0 (-6.1 to 6.2) |
| Chronic otitis media | 3090.8 (2251.5 to 4011.2) | 0.5 (0.0 to 1.8) | 64.6 (36.2 to 105.4) | 16.4* (11.2 to 21.7) | -8.4* (-12.5 to -4.2) |
| **Enteric infections** | **3388.5 (2868.7 to 3933.6)** | **228318.5 (184035.3 to 270528.4)** | **370.4 (252.4 to 528.6)** | **55.0* (46.8 to 63.0)** | **22.0* (15.6 to 28.3)** |
| Diarrhoeal diseases | 3405.7 (2887.2 to 3948.8) | 228140.4 (183867.9 to 270344.3) | 369.0 (251.3 to 526.8) | 55.4* (47.2 to 63.4) | 22.3* (15.8 to 28.6) |
| Guillain-Barré syndrome due to diarrheal diseases | 0.6 (0.3 to 0.9) | -- | 0.2 (0.1 to 0.3) | 31.0* (28.0 to 33.8) | 3.1* (0.7 to 5.3) |
| Diarrheal disease episode | 3405.1 (2886.5 to 3948.4) | 228140.4 (183867.9 to 270344.3) | 368.8 (251.2 to 526.7) | 55.4* (47.2 to 63.4) | 22.3* (15.8 to 28.6) |
| Typhoid and paratyphoid | 0.9 (0.3 to 1.6) | 170.6 (141.5 to 204.9) | 1.4 (0.9 to 2.0) | -1.2 (-16.7 to 17.7) | -22.3* (-34.4 to -7.4) |
| Typhoid fever | 8.3 (6.4 to 10.5) | 130.6 (105.0 to 160.8) | 1.3 (0.8 to 1.9) | 0.7 (-16.1 to 21.6) | -20.7* (-33.9 to -4.3) |
| Typhoid fever complications | 1.7 (1.2 to 2.4) | 22.4 (17.4 to 28.2) | 0.6 (0.3 to 0.8) | -0.5 (-25.6 to 33.7) | -21.7 (-41.4 to 5.2) |
| Typhoid fever episode | 6.6 (5.0 to 8.5) | 108.1 (86.4 to 133.7) | 0.7 (0.4 to 1.1) | 1.7 (-22.5 to 33.9) | -20.0 (-39.0 to 5.4) |
| Paratyphoid fever | 1.8 (1.2 to 2.3) | 40.1 (28.6 to 53.5) | 0.1 (0.1 to 0.2) | -19.4* (-30.6 to -6.8) | -36.6* (-45.4 to -26.7) |
| Intestinal perforation due to paratyphoid | 0.1 (0.1 to 0.1) | 2.1 (1.4 to 2.8) | -- | -18.9 (-34.9 to 2.6) | -36.2* (-48.8 to -19.2) |
| Paratyphoid fever episode | 1.7 (1.2 to 2.2) | 38.0 (27.0 to 50.9) | 0.1 (0.1 to 0.2) | -19.5* (-30.7 to -6.2) | -36.6* (-45.5 to -26.2) |
| iNTS | 0.3 (0.2 to 0.4) | 7.4 (4.2 to 11.5) | 0.0 (0.0 to 0.1) | -12.7* (-19.5 to -5.6) | -31.3* (-36.6 to -25.7) |
| Other intestinal infectious diseases | -- | -- | -- | -4.1 (-19.1 to 14.2) | -24.5* (-36.3 to -10.1) |
| **Neglected tropical diseases and malaria** | **41268.2 (39372.7 to 43489.3)** | **9315.5 (7634.9 to 11217.6)** | **611.5 (433.1 to 835.9)** | **5.1 (-1.6 to 11.6)** | **-17.3* (-22.6 to -12.2)** |
| Malaria | 2098.9 (1954.9 to 2235.7) | 1479.4 (1285.4 to 1708.7) | 17.9 (12.9 to 24.4) | -0.9 (-13.5 to 15.6) | -22.0* (-31.9 to -9.0) |
| Malaria complications | 12.9 (11.6 to 14.3) | -- | 5.5 (4.2 to 6.9) | 47.8* (31.9 to 64.8) | 16.3* (3.8 to 29.7) |
| Malaria episode | 86.7 (57.5 to 119.5) | 1479.4 (1285.4 to 1708.7) | 3.1 (1.6 to 5.1) | -12.6 (-23.5 to 0.4) | -31.2* (-39.8 to -20.9) |

| | | | | | |
|---|---|---|---|---|---|
| Malaria parasitemia | 1999.3<br>(1851.8 to 2150.8) | -- | 9.4<br>(5.8 to 14.7) | -13.7<br>(-31.5 to 9.6) | -32.1*<br>(-46.1 to -13.7) |
| Chagas disease | 489.3<br>(416.5 to 572.5) | 6.2<br>(3.8 to 9.1) | 5.3<br>(3.5 to 7.7) | 9.9*<br>(2.4 to 17.4) | -13.5*<br>(-19.4 to -7.6) |
| Acute Chagas disease | 0.0<br>(0.0 to 0.1) | 6.2<br>(3.8 to 9.1) | -- | 24.4*<br>(19.2 to 29.9) | -2.1<br>(-6.1 to 2.2) |
| Asymptomatic Chagas disease | 404.2<br>(340.1 to 473.6) | -- | -- | -- | -- |
| Symptomatic chronic Chagas infection | 85.1<br>(67.1 to 106.0) | -- | 5.3<br>(3.5 to 7.7) | 9.9*<br>(2.4 to 17.4) | -13.5*<br>(-19.4 to -7.6) |
| Leishmaniasis | 167.4<br>(127.5 to 213.2) | 22.1<br>(15.6 to 30.8) | 10.4<br>(6.5 to 15.9) | 13.1*<br>(2.7 to 27.5) | -11.0<br>(-19.1 to 0.3) |
| Visceral leishmaniasis | 0.1<br>(0.1 to 0.2) | 0.5<br>(0.4 to 0.7) | -- | -71.4*<br>(-77.2 to -59.5) | -77.5*<br>(-82.1 to -68.2) |
| Cutaneous and mucocutaneous leishmaniasis | 168.6<br>(129.0 to 215.4) | 21.6<br>(15.1 to 30.4) | 10.4<br>(6.5 to 15.9) | 13.4*<br>(3.0 to 27.9) | -10.7<br>(-18.9 to 0.7) |
| African trypanosomiasis | 0.2<br>(0.0 to 0.8) | 0.1<br>(0.0 to 0.5) | 0.0<br>(0.0 to 0.2) | -73.8<br>(-97.3 to 84.6) | -79.4<br>(-97.9 to 45.3) |
| Trypanosomiasis Gambiense | 0.2<br>(0.0 to 0.8) | 0.1<br>(0.0 to 0.5) | 0.0<br>(0.0 to 0.2) | -73.0<br>(-97.4 to 95.2) | -78.7<br>(-98.0 to 53.7) |
| Trypanosomiasis Rhodesiense | -- | -- | -- | -90.3*<br>(-99.1 to -29.0) | -92.4*<br>(-99.3 to -44.1) |
| Schistosomiasis | 6517.0<br>(5768.4 to 7259.6) | -- | 46.7<br>(22.3 to 89.5) | -13.7*<br>(-17.5 to -8.9) | -32.1*<br>(-35.1 to -28.3) |
| Mild schistosomiasis | 5637.1<br>(4911.4 to 6318.7) | -- | 31.1<br>(12.4 to 66.4) | -16.3*<br>(-18.6 to -14.1) | -34.1*<br>(-35.9 to -32.4) |
| Anemia due to schistosomiasis | 326.6<br>(263.1 to 408.3) | -- | 7.3<br>(4.3 to 11.9) | -19.2<br>(-36.3 to 0.9) | -36.4*<br>(-49.9 to -20.6) |
| Schistosomiasis complications | 554.9<br>(451.2 to 680.3) | -- | 8.3<br>(4.9 to 13.9) | 4.3<br>(-2.9 to 13.5) | -17.9*<br>(-23.6 to -10.6) |
| Cysticercosis | 434.4<br>(326.2 to 559.5) | -- | 122.6<br>(74.8 to 180.5) | 18.6*<br>(10.9 to 25.7) | -6.6*<br>(-12.7 to -1.1) |
| Cystic echinococcosis | 41.6<br>(23.4 to 69.6) | 9.7<br>(5.1 to 16.8) | 3.3<br>(1.6 to 6.0) | 52.1*<br>(32.5 to 68.0) | 19.7*<br>(4.3 to 32.3) |
| Lymphatic filariasis | 2867.6<br>(2585.1 to 3190.4) | -- | 77.3<br>(43.3 to 121.1) | -30.6*<br>(-43.0 to -18.6) | -45.4*<br>(-55.1 to -36.0) |
| Prevalence of detectable microfiliaria due to lymphatic filariasis | 2145.7<br>(2000.7 to 2312.6) | -- | -- | -- | -- |
| Lymphatic filariasis complications | 721.9<br>(491.0 to 1023.3) | -- | 77.3<br>(43.3 to 121.1) | -30.6*<br>(-43.0 to -18.6) | -45.4*<br>(-55.1 to -36.0) |
| Onchocerciasis | 857.5<br>(575.2 to 1390.3) | -- | 68.6<br>(36.8 to 122.1) | 20.3*<br>(0.7 to 40.1) | -5.3<br>(-20.8 to 10.3) |
| Asymptomatic onchocerciasis | 149.6<br>(0.0 to 650.6) | -- | -- | -- | -- |
| Skin disease due to onchocerciasis | 540.7<br>(390.3 to 733.5) | -- | 54.4<br>(25.2 to 106.2) | 19.7<br>(-6.7 to 43.5) | -5.8<br>(-26.6 to 13.0) |
| Vision loss due to onchocerciasis | 167.2<br>(116.8 to 257.0) | -- | 14.1<br>(8.8 to 20.8) | 23.0*<br>(8.0 to 39.4) | -3.2<br>(-15.0 to 9.7) |
| Trachoma | 309.9<br>(153.5 to 544.3) | -- | 24.9<br>(14.1 to 39.9) | 1.3<br>(-16.9 to 18.4) | -20.3*<br>(-34.6 to -6.8) |
| Dengue | 216.3<br>(118.2 to 365.5) | 3617.3<br>(2205.9 to 5476.4) | 33.7<br>(14.9 to 61.9) | 96.4*<br>(69.8 to 202.2) | 54.6*<br>(33.7 to 137.9) |
| Post-dengue chronic fatigue syndrome | 152.4<br>(71.4 to 278.6) | -- | 30.1<br>(12.6 to 55.7) | 96.4*<br>(68.7 to 205.4) | 54.6*<br>(32.8 to 140.4) |
| Dengue episode | 63.8<br>(38.7 to 95.7) | 3617.3<br>(2205.9 to 5476.4) | 3.7<br>(1.9 to 6.4) | 96.4*<br>(69.6 to 191.9) | 54.6*<br>(33.5 to 129.8) |
| Yellow fever | 0.0<br>(0.0 to 0.1) | 1.7<br>(0.5 to 4.6) | -- | -10.1<br>(-35.9 to 29.5) | -29.2<br>(-49.6 to 1.9) |
| Asymptomatic yellow fever | 0.0<br>(0.0 to 0.1) | 1.0<br>(0.2 to 2.6) | -- | -- | -- |
| Yellow fever episode | 0.0<br>(0.0 to 0.1) | 0.8<br>(0.2 to 2.1) | -- | -10.1<br>(-35.9 to 29.5) | -29.2<br>(-49.6 to 1.9) |
| Rabies | -- | 0.7<br>(0.5 to 0.9) | -- | -29.7*<br>(-45.0 to -12.7) | -44.6*<br>(-56.7 to -31.3) |
| Intestinal nematode infections | 24962.3<br>(23164.8 to 27071.5) | -- | 45.5<br>(26.7 to 74.0) | -15.0*<br>(-20.7 to -9.4) | -33.1*<br>(-37.6 to -28.7) |
| Ascariasis | 11440.5<br>(9974.4 to 13113.5) | -- | 13.9<br>(7.6 to 23.8) | -14.4*<br>(-23.4 to -4.3) | -32.6*<br>(-39.7 to -24.7) |
| Asymptomatic ascariasis | 10665.7<br>(9287.9 to 12234.1) | -- | -- | -- | -- |
| Ascariasis complications | 774.7<br>(683.4 to 880.6) | -- | 13.9<br>(7.6 to 23.8) | -14.4*<br>(-23.4 to -4.3) | -32.6*<br>(-39.7 to -24.7) |
| Trichuriasis | 6945.6<br>(5945.1 to 8188.7) | -- | 4.6<br>(2.5 to 7.6) | 5.9<br>(-4.3 to 17.1) | -16.7*<br>(-24.6 to -7.8) |
| Asymptomatic trichuriasis | 6708.5<br>(5721.4 to 7922.7) | -- | -- | -- | -- |
| Trichuriasis complications | 237.1<br>(214.5 to 260.0) | -- | 4.6<br>(2.5 to 7.6) | 5.9<br>(-4.3 to 17.1) | -16.7*<br>(-24.6 to -7.8) |
| Hookworm disease | 7913.0<br>(7202.5 to 8709.9) | -- | 27.0<br>(16.0 to 43.2) | -18.0*<br>(-25.8 to -9.6) | -35.5*<br>(-41.6 to -28.9) |
| Asymptomatic hookworm disease | 6584.8<br>(5995.1 to 7255.5) | -- | -- | -- | -- |
| Anemia due to hookworm disease | 314.8<br>(273.2 to 368.6) | -- | 6.5<br>(4.1 to 9.9) | -23.8*<br>(-36.8 to -8.1) | -40.0*<br>(-50.3 to -27.6) |
| Hookworm disease complications | 1013.3<br>(927.9 to 1108.9) | -- | 20.5<br>(11.6 to 34.2) | -16.1*<br>(-23.3 to -8.5) | -33.9*<br>(-39.7 to -28.0) |
| Food-borne trematodiases | 5100.2<br>(4516.3 to 5807.7) | 4068.2<br>(3371.0 to 4772.4) | 116.6<br>(61.2 to 194.1) | 28.1*<br>(22.7 to 34.3) | 0.8<br>(-3.4 to 5.7) |
| Asymptomatic food-borne tremodiases | 4036.4<br>(3346.2 to 4739.9) | 2916.0<br>(1240.7 to 4165.2) | -- | -- | -- |
| Food-borne tremodiases complications | 1063.7<br>(651.6 to 1626.0) | 1152.2<br>(160.9 to 2751.5) | 116.6<br>(61.2 to 194.1) | 28.1*<br>(22.7 to 34.3) | 0.8<br>(-3.4 to 5.7) |
| Leprosy | 52.2<br>(48.8 to 55.9) | 3.7<br>(3.3 to 4.2) | 3.2<br>(2.2 to 4.5) | 8.6*<br>(0.6 to 17.2) | -14.5*<br>(-20.8 to -7.8) |
| Ebola virus disease | -- | -- | -- | -96.6*<br>(-97.2 to -96.0) | -97.4*<br>(-97.8 to -96.8) |
| Ebola cases | -- | -- | -- | -98.7*<br>(-98.9 to -98.5) | -99.0*<br>(-99.1 to -98.8) |
| Post-Ebola chronic fatigue syndrome | -- | -- | -- | -96.4*<br>(-96.8 to -95.6) | -97.1*<br>(-97.4 to -96.6) |
| Zika virus disease | 1.8<br>(1.0 to 3.4) | 106.5<br>(60.7 to 202.0) | 0.0<br>(0.0 to 0.1) | -- | -- |
| Zika virus complications | -- | -- | -- | -- | -- |
| Zika virus episode | 1.7<br>(1.0 to 3.3) | 106.5<br>(60.7 to 202.0) | 0.0<br>(0.0 to 0.1) | -- | -- |
| Guinea worm disease | -- | -- | -- | -99.5*<br>(-99.7 to -99.3) | -99.6*<br>(-99.7 to -99.4) |
| Other neglected tropical diseases | 1689.8<br>(1559.0 to 1838.8) | -- | 35.3<br>(23.4 to 52.7) | 10.3<br>(-0.5 to 22.7) | -13.2*<br>(-21.7 to -3.4) |
| Acute infection due to other neglected tropical diseases | -- | -- | 0.4<br>(0.2 to 0.7) | 103.3*<br>(75.6 to 199.8) | 60.0*<br>(38.2 to 136.0) |
| Anemia due to other neglected tropical diseases | 1689.8<br>(1559.0 to 1838.8) | -- | 34.9<br>(23.0 to 52.2) | 9.7<br>(-1.1 to 22.2) | -13.6*<br>(-22.2 to -3.8) |
| **Other infectious diseases** | **3847.8<br>(3440.5 to 4351.3)** | **10438.7<br>(8043.0 to 13985.6)** | **154.0<br>(107.1 to 211.4)** | **17.2*<br>(9.8 to 25.3)** | **-7.8*<br>(-13.5 to -1.4)** |
| Meningitis | 407.7<br>(340.9 to 484.3) | 110.1<br>(77.7 to 153.9) | 28.8<br>(20.1 to 38.6) | 10.4*<br>(5.9 to 15.1) | -13.1*<br>(-16.7 to -9.4) |
| Pneumococcal meningitis | 147.9<br>(120.4 to 180.3) | 8.0<br>(4.7 to 11.7) | 10.8<br>(7.5 to 14.8) | -9.7*<br>(-14.9 to -4.1) | -29.0*<br>(-33.0 to -24.6) |

| | | | | | |
|---|---|---|---|---|---|
| Acute pneumococcal meningitis | 0.4 (0.2 to 0.5) | 8.0 (4.7 to 11.7) | 0.0 (0.0 to 0.1) | -4.2 (-14.3 to 5.4) | -24.6* (-32.6 to -17.1) |
| Pneumococcal meningitis complications | 147.5 (120.1 to 180.0) | -- | 10.8 (7.5 to 14.7) | -9.8* (-15.0 to -4.1) | -29.0* (-33.1 to -24.6) |
| H influenzae type B meningitis | 29.9 (21.1 to 40.4) | 2.5 (1.4 to 4.1) | 2.0 (1.3 to 2.7) | -45.9* (-48.5 to -43.4) | -57.4* (-59.5 to -55.4) |
| Acute H influenzae type B meningitis | 0.1 (0.1 to 0.2) | 2.5 (1.4 to 4.1) | -- | -46.9* (-54.0 to -40.4) | -58.2* (-63.8 to -53.1) |
| H Influenza type B meningitis complications | 29.8 (21.0 to 40.3) | -- | 1.9 (1.3 to 2.6) | -45.9* (-48.5 to -43.4) | -57.4* (-59.5 to -55.4) |
| Meningococcal infection | 44.2 (31.5 to 58.6) | 7.7 (4.6 to 12.3) | 3.4 (2.3 to 4.7) | 14.7* (10.0 to 18.8) | -9.7* (-13.4 to -6.5) |
| Acute meningococcal meningitis | 0.3 (0.2 to 0.5) | 7.7 (4.6 to 12.3) | 0.0 (0.0 to 0.1) | 26.8* (14.0 to 38.3) | -0.2 (-10.3 to 8.8) |
| Meningococcal meningitis complications | 43.8 (31.1 to 58.3) | -- | 3.4 (2.3 to 4.6) | 14.5* (9.9 to 18.6) | -9.9* (-13.5 to -6.6) |
| Other meningitis | 185.8 (136.1 to 237.2) | 92.0 (64.9 to 129.0) | 12.6 (8.8 to 17.1) | 68.5* (59.1 to 78.6) | 32.6* (25.2 to 40.6) |
| Other acute bacterial meningitis | 0.9 (0.6 to 1.3) | 20.5 (14.1 to 29.4) | 0.1 (0.1 to 0.2) | 75.2* (60.8 to 89.6) | 37.9* (26.6 to 49.2) |
| Acute viral meningitis | 3.2 (2.2 to 4.5) | 71.5 (50.4 to 100.0) | 0.4 (0.2 to 0.7) | 25.7* (14.8 to 35.7) | -1.1 (-9.7 to 6.8) |
| Other bacterial meningitis complications | 181.6 (132.0 to 233.3) | -- | 12.0 (8.4 to 16.5) | 70.5* (60.6 to 81.1) | 34.2* (26.4 to 42.5) |
| Encephalitis | 410.2 (218.5 to 669.6) | 96.5 (93.0 to 99.7) | 27.6 (19.1 to 36.6) | 21.4* (15.1 to 28.8) | -4.4 (-9.4 to 1.4) |
| Acute encephalitis | 5.0 (4.9 to 5.2) | 96.5 (93.0 to 99.7) | 0.7 (0.4 to 1.0) | 32.4* (31.8 to 33.0) | 4.2* (3.7 to 4.7) |
| Encephalitis complications | 405.1 (213.6 to 664.6) | -- | 26.9 (18.6 to 35.8) | 21.2* (14.8 to 28.7) | -4.6 (-9.7 to 1.3) |
| Diphtheria | -- | 0.2 (0.1 to 0.2) | -- | -19.1 (-38.8 to 8.2) | -36.3* (-51.9 to -14.9) |
| Whooping cough | 2.2 (1.7 to 2.8) | 15.9 (12.3 to 20.2) | 0.1 (0.1 to 0.2) | 11.4* (10.9 to 11.9) | -12.3* (-12.7 to -11.9) |
| Tetanus | 2.0 (1.9 to 2.2) | 3.4 (1.9 to 5.3) | 0.1 (0.0 to 0.1) | -9.9 (-23.8 to 6.6) | -29.1* (-40.0 to -16.1) |
| Severe tetanus | 0.2 (0.1 to 0.3) | 3.4 (1.9 to 5.3) | -- | -35.6* (-47.8 to -11.9) | -49.3* (-58.9 to -30.6) |
| Neonatal tetanus complications | 1.8 (1.8 to 1.9) | -- | 0.0 (0.0 to 0.1) | 26.5* (24.6 to 28.0) | -0.5 (-1.9 to 0.7) |
| Measles | 0.2 (0.1 to 0.6) | 9.1 (3.3 to 20.3) | 0.0 (0.0 to 0.1) | -18.1* (-25.6 to -10.5) | -35.5* (-41.4 to -29.5) |
| Varicella and herpes zoster | 515.0 (413.0 to 624.3) | 2934.7 (2338.1 to 3624.5) | 27.9 (16.7 to 43.4) | 29.4* (24.4 to 34.5) | 1.8 (-2.1 to 5.9) |
| Chickenpox | 0.9 (0.3 to 1.9) | 47.7 (16.9 to 100.1) | -- | 43.6* (37.2 to 48.2) | 13.1* (8.0 to 16.7) |
| Herpes zoster | 514.1 (411.0 to 623.7) | 2886.9 (2291.6 to 3578.6) | 27.9 (16.7 to 43.4) | 29.4* (24.4 to 34.5) | 1.8 (-2.1 to 5.9) |
| Acute hepatitis | 801.8 (541.8 to 1194.9) | 7268.8 (5067.5 to 10675.3) | 19.4 (11.0 to 32.5) | 16.9 (-23.2 to 81.9) | -8.0 (-39.6 to 43.2) |
| Acute hepatitis A | 58.1 (44.9 to 73.0) | 755.9 (584.2 to 948.9) | 2.4 (1.4 to 3.8) | 24.7* (11.2 to 38.7) | -1.8 (-12.5 to 9.2) |
| Acute hepatitis B | 709.3 (456.5 to 1099.6) | 6147.5 (3956.0 to 9530.0) | 16.2 (8.7 to 28.2) | 15.8 (-29.2 to 94.9) | -8.8 (-44.2 to 53.4) |
| Acute hepatitis C | 18.8 (8.0 to 32.9) | 162.6 (69.2 to 285.2) | 0.3 (0.1 to 0.6) | 1.4 (-12.4 to 11.8) | -20.2* (-31.0 to -12.0) |
| Acute hepatitis E | 15.6 (10.3 to 22.6) | 202.8 (134.0 to 293.5) | 0.5 (0.3 to 0.9) | 27.7* (8.3 to 49.9) | 0.5 (-14.8 to 18.0) |
| Other unspecified infectious diseases | 2138.1 (1995.3 to 2299.4) | -- | 50.2 (32.9 to 74.1) | 13.2* (4.4 to 21.9) | -10.9* (-17.8 to -4.1) |
| Guillain-Barré syndrome due to other infectious diseases | 0.4 (0.2 to 0.6) | -- | 0.1 (0.0 to 0.2) | 31.0* (27.9 to 33.8) | 3.1* (0.7 to 5.3) |
| Other infectious diseases | -- | -- | 7.3 (4.2 to 11.6) | 29.9* (24.9 to 35.0) | 2.2 (-1.7 to 6.3) |
| Anemia due to other infectious diseases | 2137.8 (1995.1 to 2299.0) | -- | 42.8 (28.1 to 63.4) | 10.7* (0.8 to 20.8) | -12.8* (-20.6 to -4.9) |
| **Maternal and neonatal disorders** | **5201.8 (4501.3 to 5951.2)** | **--** | **972.3 (708.4 to 1260.2)** | **43.9* (37.0 to 51.8)** | **13.3* (7.8 to 19.5)** |
| Maternal disorders | 19.3 (15.5 to 23.7) | -- | 6.4 (4.1 to 9.0) | -16.0* (-25.8 to -4.7) | -33.9* (-41.6 to -25.0) |
| Maternal haemorrhage | -- | -- | -- | -- | -- |
| Maternal hemorrhage complications | -- | -- | -- | -- | -- |
| Maternal hemorrhage episode | -- | -- | -- | -- | -- |
| Maternal sepsis and other pregnancy related infections | -- | -- | -- | -- | -- |
| Infertility due to puerperal sepsis | -- | -- | -- | -- | -- |
| Maternal sepsis and other maternal infection episode | -- | -- | -- | -- | -- |
| Maternal hypertensive disorders | -- | -- | -- | -- | -- |
| Maternal hypertensive disorder complications | -- | -- | -- | -- | -- |
| Maternal hypertensive disorder episode | -- | -- | -- | -- | -- |
| Maternal obstructed labour and uterine rupture | 20.4 (16.7 to 24.7) | -- | 6.4 (4.1 to 9.0) | -16.0* (-25.8 to -4.7) | -33.9* (-41.6 to -25.0) |
| Obstructed labor, acute event | -- | -- | -- | -- | -- |
| Maternal obstructed labor complications | 20.4 (16.7 to 24.7) | -- | 6.4 (4.1 to 9.0) | -16.0* (-25.8 to -4.7) | -33.9* (-41.6 to -25.0) |
| Maternal abortion and miscarriage | -- | -- | -- | -- | -- |
| Ectopic pregnancy | -- | -- | -- | -- | -- |
| Other maternal disorders | -- | -- | -- | -- | -- |
| Neonatal disorders | 5182.8 (4482.6 to 5929.6) | -- | 966.0 (702.8 to 1254.2) | 44.6* (37.6 to 52.8) | 13.8* (8.3 to 20.3) |
| Neonatal preterm birth | 2924.1 (2571.1 to 3367.0) | -- | 383.1 (270.7 to 519.0) | 51.5* (33.0 to 70.2) | 19.2* (4.7 to 34.0) |
| Preterm birth complications | 2437.7 (2105.6 to 2845.1) | -- | 355.0 (245.8 to 488.2) | 54.3* (33.9 to 74.0) | 21.4* (5.4 to 37.0) |
| Retinopathy of prematurity | 486.4 (394.7 to 581.1) | -- | 28.1 (18.8 to 39.1) | 23.1* (16.8 to 30.6) | -3.1 (-8.1 to 2.8) |
| Uncomplicated preterm birth | -- | -- | -- | -- | -- |
| Neonatal encephalopathy due to birth asphyxia and trauma | 2230.2 (1661.4 to 2939.0) | -- | 383.1 (231.2 to 587.7) | 41.9* (36.8 to 47.8) | 11.7* (7.7 to 16.3) |
| Neonatal sepsis and other neonatal infections | 563.6 (349.8 to 866.7) | -- | 172.5 (92.1 to 292.9) | 36.2* (30.7 to 42.0) | 7.2* (2.9 to 11.7) |
| Neonatal sepsis and other neonatal infection complications | 563.6 (349.8 to 866.7) | -- | 172.5 (92.1 to 292.9) | 36.2* (30.7 to 42.0) | 7.2* (2.9 to 11.7) |
| Neonatal sepsis and other neonatal infection episode | -- | -- | -- | -- | -- |
| Hemolytic disease and other neonatal jaundice | 68.6 (59.0 to 78.6) | -- | 27.2 (20.9 to 34.0) | 47.3* (43.2 to 51.2) | 15.9* (12.7 to 19.0) |

| | | | | | |
|---|---|---|---|---|---|
| Other neonatal disorders | -- | -- | -- | -- | -- |
| **Nutritional deficiencies** | **68971.4 (66050.8 to 72322.5)** | **39180.4 (35268.8 to 43213.8)** | **1089.6 (726.3 to 1602.2)** | **9.7* (1.9 to 18.2)** | **-13.6* (-19.8 to -6.9)** |
| Protein-energy malnutrition | 3090.8 (2905.5 to 3264.1) | 3135.1 (2913.1 to 3345.5) | 18.0 (11.9 to 25.2) | 49.2* (32.6 to 61.5) | 17.4* (4.4 to 27.1) |
| Iodine deficiency | 5791.3 (5247.0 to 6425.7) | 29.3 (24.8 to 34.8) | 87.4 (52.1 to 141.7) | 12.1* (7.2 to 16.0) | -11.8* (-15.6 to -8.7) |
| Visible goiter without symptoms | 5701.8 (5168.1 to 6325.2) | 29.3 (24.8 to 34.8) | 59.8 (29.2 to 112.7) | 13.9* (12.2 to 15.5) | -10.3* (-11.7 to -9.1) |
| Visible goiter with complications | 89.5 (46.2 to 132.1) | -- | 27.6 (13.2 to 43.9) | 8.3 (-9.3 to 19.0) | -14.7* (-28.6 to -6.3) |
| Vitamin A deficiency | 22048.0 (20003.1 to 24094.5) | 36016.0 (32153.1 to 40043.3) | 7.3 (4.7 to 10.9) | 39.9* (30.2 to 50.8) | 10.1* (2.4 to 18.7) |
| Asymptomatic vitamin A deficiency | 21933.2 (19861.4 to 23995.3) | 36016.0 (32153.1 to 40043.3) | -- | -- | -- |
| Vitamin A deficiency complications | 114.8 (81.0 to 157.1) | -- | 7.3 (4.7 to 10.9) | 39.9* (30.2 to 50.8) | 10.1* (2.4 to 18.7) |
| Vitamin A deficiency with anemia | -- | -- | -- | -- | -- |
| Dietary iron deficiency | 46672.3 (43575.9 to 50104.5) | -- | 968.3 (639.7 to 1430.7) | 8.5 (-0.2 to 18.1) | -14.6* (-21.5 to -7.0) |
| Other nutritional deficiencies | -- | -- | 8.5 (5.6 to 11.9) | 62.6* (44.5 to 76.0) | 28.0* (13.7 to 38.5) |
| **Non-communicable diseases** | **343951.5 (343162.9 to 344706.1)** | **473878.8 (438934.2 to 510906.4)** | **49630.4 (37844.0 to 63959.0)** | **27.8* (26.9 to 28.8)** | **0.6 (-0.1 to 1.4)** |
| **Neoplasms** | **9938.9 (9706.1 to 10242.8)** | **2315.5 (1940.5 to 2838.2)** | **750.7 (543.0 to 980.1)** | **28.9* (26.4 to 31.6)** | **1.4 (-0.5 to 3.6)** |
| Lip and oral cavity cancer | 206.4 (197.4 to 215.0) | 47.3 (45.1 to 49.4) | 20.2 (14.8 to 26.3) | 38.9* (29.4 to 48.5) | 9.3* (1.8 to 16.9) |
| Diagnosis and primary therapy phase of mouth cancer | 11.8 (11.3 to 12.3) | 47.3 (45.1 to 49.4) | 3.3 (2.2 to 4.7) | 38.9* (24.2 to 54.6) | 9.3 (-2.2 to 21.7) |
| Controlled phase of mouth cancer | 174.5 (166.9 to 181.7) | -- | 8.0 (5.2 to 11.7) | 40.2* (30.4 to 50.5) | 10.4* (2.7 to 18.5) |
| Metastatic phase of mouth cancer | 17.4 (16.6 to 18.2) | -- | 7.4 (4.9 to 10.0) | 37.4* (22.1 to 54.4) | 8.2 (-3.9 to 21.5) |
| Terminal phase of mouth cancer | 2.7 (2.5 to 2.8) | -- | 1.4 (1.0 to 1.8) | 38.8* (31.6 to 46.2) | 9.2* (3.6 to 15.1) |
| Nasopharynx cancer | 43.5 (40.5 to 47.2) | 11.1 (10.3 to 11.9) | 4.8 (3.5 to 6.4) | 11.1 (-0.6 to 25.4) | -12.6* (-21.7 to -1.3) |
| Diagnosis and primary therapy phase of nasopharynx cancer | 2.4 (2.3 to 2.6) | 11.1 (10.3 to 11.9) | 0.7 (0.5 to 1.0) | 11.3 (-6.6 to 28.7) | -12.4 (-26.5 to 1.3) |
| Controlled phase of nasopharynx cancer | 35.8 (33.3 to 38.8) | -- | 1.7 (1.1 to 2.5) | 10.3 (-3.3 to 26.2) | -13.2* (-23.9 to -0.7) |
| Metastatic phase of nasopharynx cancer | 4.6 (4.3 to 5.0) | -- | 2.1 (1.4 to 2.8) | 11.4 (-5.8 to 31.8) | -12.3 (-25.9 to 3.7) |
| Terminal phase of nasopharynx cancer | 0.6 (0.6 to 0.7) | -- | 0.3 (0.2 to 0.4) | 12.3* (3.3 to 22.9) | -11.6* (-18.7 to -3.3) |
| Other pharynx cancer | 78.0 (71.4 to 82.3) | 29.3 (26.4 to 31.2) | 9.9 (7.1 to 13.0) | 50.2* (37.3 to 62.7) | 18.3* (8.1 to 28.0) |
| Diagnosis and primary therapy phase of other pharynx cancer | 4.3 (4.0 to 4.5) | 29.3 (26.4 to 31.2) | 1.2 (0.8 to 1.7) | 53.4* (43.7 to 61.4) | 20.8* (13.1 to 27.1) |
| Controlled phase of other pharynx cancer | 60.6 (55.5 to 63.9) | -- | 2.8 (1.8 to 4.1) | 53.3* (39.0 to 70.1) | 20.6* (9.4 to 33.9) |
| Metastatic phase of other pharynx cancer | 11.1 (10.0 to 11.8) | -- | 4.8 (3.2 to 6.4) | 47.9* (29.4 to 67.5) | 16.4* (1.9 to 31.8) |
| Terminal phase of other pharynx cancer | 2.0 (1.8 to 2.1) | -- | 1.1 (0.7 to 1.4) | 49.4* (38.8 to 58.4) | 17.6* (9.3 to 24.6) |
| Oesophageal cancer | 94.2 (91.2 to 97.2) | 46.7 (45.3 to 48.2) | 14.2 (10.2 to 18.4) | -4.9 (-11.7 to 2.1) | -25.2* (-30.5 to -19.7) |
| Diagnosis and primary therapy phase of esophageal cancer | 7.7 (7.5 to 8.0) | 46.7 (45.3 to 48.2) | 2.2 (1.5 to 3.1) | -3.3 (-13.4 to 8.7) | -23.9* (-31.8 to -14.5) |
| Controlled phase of esophageal cancer | 66.9 (64.7 to 69.1) | -- | 3.1 (2.0 to 4.5) | -3.5 (-11.7 to 5.3) | -24.1* (-30.5 to -17.1) |
| Metastatic phase of esophageal cancer | 16.1 (15.6 to 16.6) | -- | 7.0 (4.7 to 9.3) | -5.4 (-16.5 to 7.8) | -25.5* (-34.3 to -15.2) |
| Terminal phase of esophageal cancer | 3.5 (3.4 to 3.6) | -- | 1.9 (1.3 to 2.4) | -7.4* (-10.5 to -4.0) | -27.1* (-29.5 to -24.5) |
| Stomach cancer | 276.8 (264.9 to 289.1) | 103.1 (99.5 to 106.9) | 32.6 (23.8 to 42.6) | 10.4* (3.3 to 17.9) | -13.1* (-18.7 to -7.2) |
| Diagnosis and primary therapy phase of stomach cancer | 24.7 (23.6 to 25.7) | 103.1 (99.5 to 106.9) | 6.8 (4.5 to 9.4) | 16.5* (3.3 to 30.9) | -8.3 (-18.7 to 3.1) |
| Controlled phase of stomach cancer | 217.1 (207.5 to 227.2) | -- | 10.1 (6.6 to 14.7) | 27.0* (18.1 to 36.7) | -0.0 (-7.0 to 7.6) |
| Metastatic phase of stomach cancer | 27.9 (26.9 to 28.8) | -- | 11.9 (8.2 to 15.7) | 0.0 (-10.0 to 12.1) | -21.3* (-29.2 to -11.7) |
| Terminal phase of stomach cancer | 7.2 (6.9 to 7.4) | -- | 3.9 (2.7 to 5.0) | -1.0 (-5.5 to 3.5) | -22.1* (-25.6 to -18.5) |
| Colon and rectum cancer | 957.4 (932.8 to 984.1) | 161.4 (157.2 to 165.9) | 84.0 (61.0 to 109.9) | 27.7* (22.3 to 32.9) | 0.5 (-3.7 to 4.6) |
| Diagnosis and primary therapy phase of colon and rectum cancers | 40.0 (39.0 to 41.2) | 161.4 (157.2 to 165.9) | 10.6 (7.3 to 14.5) | 29.5* (16.9 to 42.8) | 1.9 (-8.0 to 12.4) |
| Controlled phase of colon and rectum cancers | 848.1 (826.0 to 872.2) | -- | 48.4 (32.0 to 68.9) | 30.0* (24.9 to 35.1) | 2.3 (-1.7 to 6.3) |
| Metastatic phase of colon and rectum cancers | 48.5 (47.2 to 49.9) | -- | 20.0 (13.6 to 26.3) | 23.6* (13.2 to 34.1) | -2.7 (-10.9 to 5.5) |
| Terminal phase of colon and rectum cancers | 6.7 (6.5 to 6.9) | -- | 3.6 (2.5 to 4.6) | 20.8* (16.4 to 25.1) | -4.9* (-8.4 to -1.6) |
| Stoma from colon and rectum cancers, beyond 10 years | 14.1 (12.8 to 15.5) | -- | 1.3 (0.9 to 1.8) | 17.2* (6.5 to 28.9) | -7.7 (-16.2 to 1.4) |
| Liver cancer | 102.4 (92.3 to 113.6) | 100.8 (95.8 to 106.9) | 25.8 (18.4 to 34.0) | 19.5* (11.9 to 28.8) | -5.9 (-11.9 to 1.4) |
| Liver cancer due to hepatitis B | 82.3 (73.5 to 93.0) | 45.1 (40.2 to 51.3) | 11.4 (8.1 to 15.2) | 12.8* (1.0 to 26.9) | -11.2* (-20.5 to -0.1) |
| Diagnosis and primary therapy phase of liver cancer due to hepatitis B | 11.1 (9.9 to 12.6) | 45.1 (40.2 to 51.3) | 3.0 (2.0 to 4.3) | 10.6 (-9.3 to 36.5) | -13.0 (-28.6 to 7.5) |
| Controlled phase of liver cancer due to hepatitis B | 59.1 (52.9 to 66.8) | -- | 2.8 (1.7 to 4.1) | 34.4* (18.1 to 53.7) | 5.8 (-7.1 to 21.0) |
| Metastatic phase of liver cancer due to hepatitis B | 8.7 (7.7 to 9.8) | -- | 3.7 (2.5 to 5.2) | 5.8 (-15.0 to 30.4) | -16.7 (-33.1 to 2.7) |
| Terminal phase of liver cancer due to hepatitis B | 3.4 (3.1 to 3.9) | -- | 1.9 (1.3 to 2.5) | 5.0 (-1.5 to 14.8) | -17.3* (-22.5 to -9.7) |
| Liver cancer due to hepatitis C | 47.3 (41.1 to 53.4) | 26.0 (22.5 to 29.6) | 6.7 (4.7 to 9.2) | 21.1* (13.9 to 29.1) | -4.7 (-10.3 to 1.6) |
| Diagnosis and primary therapy phase of liver cancer due to hepatitis C | 6.4 (5.6 to 7.3) | 26.0 (22.5 to 29.6) | 1.8 (1.2 to 2.6) | 20.2* (10.1 to 31.1) | -5.4 (-13.4 to 3.2) |
| Controlled phase of liver cancer due to hepatitis C | 33.9 (29.6 to 38.2) | -- | 1.6 (1.0 to 2.4) | 33.5* (18.0 to 50.0) | 5.1 (-7.1 to 18.1) |
| Metastatic phase of liver cancer due to hepatitis C | 5.0 (4.3 to 5.7) | -- | 2.2 (1.5 to 3.1) | 16.4* (6.7 to 27.7) | -8.4 (-16.0 to 0.5) |
| Terminal phase of liver cancer due to hepatitis C | 2.0 (1.7 to 2.3) | -- | 1.1 (0.7 to 1.4) | 16.4* (11.5 to 22.4) | -8.4* (-12.2 to -3.7) |
| Liver cancer due to alcohol use | 25.5 (19.1 to 33.2) | 14.7 (11.1 to 19.1) | 3.8 (2.5 to 5.4) | 32.4* (24.8 to 42.3) | 4.2 (-1.8 to 12.0) |
| Diagnosis and primary therapy phase of liver cancer due to alcohol use | 3.6 (2.7 to 4.7) | 14.7 (11.1 to 19.1) | 1.0 (0.6 to 1.5) | 31.4* (25.1 to 39.4) | 3.4 (-1.5 to 9.7) |
| Controlled phase of liver cancer due to alcohol use | 17.9 (13.5 to 23.4) | -- | 0.8 (0.5 to 1.3) | 44.3* (24.0 to 67.6) | 13.5 (-2.4 to 31.9) |
| Metastatic phase of liver cancer due to alcohol use | 2.8 (2.1 to 3.7) | -- | 1.3 (0.8 to 1.9) | 28.3* (22.0 to 35.9) | 1.0 (-4.0 to 7.0) |

| | | | | | |
|---|---|---|---|---|---|
| Terminal phase of liver cancer due to alcohol use | 1.1 (0.9 to 1.5) | -- | 0.6 (0.4 to 0.9) | 28.3* (22.1 to 36.0) | 1.0 (-3.9 to 7.0) |
| Liver cancer due to NASH | 11.7 (9.2 to 14.4) | 6.7 (5.3 to 8.3) | 1.8 (1.2 to 2.5) | 35.1* (28.7 to 43.3) | 6.4* (1.3 to 12.8) |
| Diagnosis and primary therapy phase of liver cancer due to NASH | 1.6 (1.3 to 2.0) | 6.7 (5.3 to 8.3) | 0.5 (0.3 to 0.7) | 34.0* (28.2 to 42.1) | 5.4* (0.9 to 11.9) |
| Controlled phase of liver cancer due to NASH | 8.2 (6.5 to 10.2) | -- | 0.4 (0.2 to 0.6) | 49.8* (36.6 to 65.8) | 17.9* (7.5 to 30.5) |
| Metastatic phase of liver cancer due to NASH | 1.3 (1.0 to 1.6) | -- | 0.6 (0.4 to 0.8) | 30.0* (24.3 to 37.8) | 2.3 (-2.1 to 8.5) |
| Terminal phase of liver cancer due to NASH | 0.5 (0.4 to 0.6) | -- | 0.3 (0.2 to 0.4) | 30.0* (24.3 to 37.8) | 2.3 (-2.1 to 8.5) |
| Liver cancer due to other causes | 14.9 (12.0 to 18.0) | 8.2 (6.6 to 9.9) | 2.1 (1.5 to 3.0) | 20.9* (13.4 to 30.8) | -4.8 (-10.7 to 2.9) |
| Diagnosis and primary therapy phase of liver cancer due to other causes | 2.0 (1.6 to 2.4) | 8.2 (6.6 to 9.9) | 0.6 (0.4 to 0.8) | 19.1* (12.8 to 27.5) | -6.2 (-11.2 to 0.4) |
| Controlled phase of liver cancer due to other causes | 10.7 (8.6 to 12.9) | -- | 0.5 (0.3 to 0.8) | 40.9* (20.3 to 62.7) | 10.9 (-5.3 to 28.0) |
| Metastatic phase of liver cancer due to other causes | 1.6 (1.3 to 1.9) | -- | 0.7 (0.5 to 1.0) | 13.9* (7.8 to 21.8) | -10.4* (-15.1 to -4.1) |
| Terminal phase of liver cancer due to other causes | 0.6 (0.5 to 0.8) | -- | 0.3 (0.2 to 0.5) | 13.9* (7.8 to 21.8) | -10.4* (-15.1 to -4.1) |
| Gallbladder and biliary tract cancer | 20.6 (18.4 to 22.4) | 16.1 (14.0 to 17.3) | 4.0 (2.8 to 5.4) | 23.8* (17.5 to 31.0) | -2.5 (-7.5 to 3.1) |
| Diagnosis and primary therapy phase of gallbladder and biliary tract cancer | 2.7 (2.3 to 2.9) | 16.1 (14.0 to 17.3) | 0.8 (0.5 to 1.1) | 24.3* (17.8 to 31.8) | -2.2 (-7.2 to 3.8) |
| Controlled phase of gallbladder and biliary tract cancer | 12.2 (11.0 to 13.3) | -- | 0.6 (0.4 to 0.9) | 29.7* (15.5 to 45.8) | 2.1 (-9.1 to 14.8) |
| Metastatic phase of gallbladder and biliary tract cancer | 4.4 (3.8 to 4.8) | -- | 2.0 (1.3 to 2.7) | 22.5* (16.4 to 29.4) | -3.6 (-8.4 to 1.8) |
| Terminal phase of gallbladder and biliary tract cancer | 1.3 (1.1 to 1.4) | -- | 0.7 (0.5 to 0.9) | 22.3* (16.6 to 29.0) | -3.7 (-8.2 to 1.5) |
| Pancreatic cancer | 38.7 (37.8 to 39.6) | 38.3 (37.5 to 39.2) | 8.7 (6.0 to 11.4) | 23.1* (19.3 to 26.6) | -3.1* (-6.1 to -0.4) |
| Diagnosis and primary therapy phase of pancreatic cancer | 8.7 (8.5 to 8.9) | 38.3 (37.5 to 39.2) | 2.5 (1.7 to 3.5) | 23.2* (19.7 to 26.3) | -3.0* (-5.8 to -0.6) |
| Controlled phase of pancreatic cancer | 18.9 (18.5 to 19.4) | -- | 0.9 (0.6 to 1.3) | 14.2* (4.7 to 25.3) | -10.1* (-17.6 to -1.3) |
| Metastatic phase of pancreatic cancer | 7.9 (7.7 to 8.1) | -- | 3.6 (2.4 to 4.8) | 24.8* (21.3 to 27.9) | -1.8 (-4.5 to 0.7) |
| Terminal phase of pancreatic cancer | 3.1 (3.0 to 3.2) | -- | 1.7 (1.2 to 2.1) | 24.8* (21.3 to 27.9) | -1.8 (-4.5 to 0.7) |
| Larynx cancer | 171.8 (166.5 to 177.4) | 31.1 (30.1 to 32.1) | 16.7 (12.1 to 22.1) | 28.4* (21.1 to 35.5) | 1.1 (-4.7 to 6.6) |
| Diagnosis and primary therapy phase of larynx cancer | 10.3 (9.9 to 10.6) | 31.1 (30.1 to 32.1) | 2.9 (1.9 to 4.0) | 31.5* (18.8 to 45.6) | 3.5 (-6.5 to 14.6) |
| Controlled phase of larynx cancer | 144.6 (139.9 to 149.5) | -- | 8.1 (5.3 to 11.6) | 30.2* (21.6 to 38.9) | 2.5 (-4.3 to 9.3) |
| Metastatic phase of larynx cancer | 10.9 (10.6 to 11.3) | -- | 4.8 (3.3 to 6.3) | 25.0* (12.8 to 38.5) | -1.6 (-11.2 to 9.1) |
| Terminal phase of larynx cancer | 1.5 (1.5 to 1.6) | -- | 0.8 (0.6 to 1.0) | 23.1* (18.3 to 27.9) | -3.1 (-6.9 to 0.7) |
| Laryngectomy from larynx cancer, beyond 10 years | 4.5 (4.2 to 4.8) | -- | 0.2 (0.1 to 0.4) | 21.2* (20.3 to 22.1) | -4.6* (-5.3 to -3.9) |
| Tracheal, bronchus, and lung cancer | 348.9 (338.0 to 358.8) | 200.3 (194.5 to 205.8) | 53.7 (38.9 to 68.9) | 19.2* (12.6 to 26.0) | -6.2* (-11.4 to -0.8) |
| Diagnosis and primary therapy phase of lung, bronchus, and trachea cancer | 22.0 (21.3 to 22.7) | 200.3 (194.5 to 205.8) | 6.0 (4.1 to 8.2) | 27.9* (15.0 to 42.6) | 0.7 (-9.5 to 12.3) |
| Controlled phase of lung, bronchus, and trachea cancer | 241.8 (233.8 to 248.9) | -- | 11.2 (7.2 to 16.3) | 38.3* (30.8 to 46.0) | 8.9* (2.9 to 14.9) |
| Metastatic phase of lung, bronchus, and trachea cancer | 69.7 (67.6 to 71.6) | -- | 28.4 (19.7 to 37.4) | 13.0* (3.9 to 22.3) | -11.0* (-18.2 to -3.7) |
| Terminal phase of lung, bronchus, and trachea cancer | 15.4 (15.0 to 15.9) | -- | 8.1 (5.6 to 10.3) | 13.3* (3.4 to 24.5) | -10.8* (-18.6 to -2.0) |
| Malignant skin melanoma | 235.3 (186.8 to 304.9) | 29.4 (23.3 to 37.9) | 13.8 (8.9 to 20.5) | 30.7* (21.5 to 41.9) | 2.9 (-4.4 to 11.7) |
| Diagnosis and primary therapy phase of malignant skin melanoma | 6.3 (5.0 to 8.2) | 29.4 (23.3 to 37.9) | 1.8 (1.1 to 2.6) | 32.6* (20.1 to 48.8) | 4.3 (-5.5 to 17.1) |
| Controlled phase of malignant skin melanoma | 225.4 (179.1 to 292.7) | -- | 10.4 (6.2 to 16.1) | 32.7* (22.7 to 45.2) | 4.4 (-3.4 to 14.3) |
| Metastatic phase of malignant skin melanoma | 3.0 (2.4 to 3.7) | -- | 1.4 (0.9 to 2.0) | 18.1* (12.2 to 26.0) | -7.1* (-11.7 to -0.8) |
| Terminal phase of malignant skin melanoma | 0.5 (0.4 to 0.6) | -- | 0.3 (0.2 to 0.4) | 16.4* (10.8 to 24.0) | -8.4* (-12.8 to -2.4) |
| Non-melanoma skin cancer | 192.6 (99.6 to 349.9) | 721.9 (352.2 to 1235.4) | 6.9 (2.9 to 15.1) | 24.4* (9.3 to 36.6) | -2.1 (-13.9 to 7.5) |
| Non-melanoma skin cancer (squamous-cell carcinoma) | 155.6 (67.8 to 317.5) | 127.4 (52.7 to 261.2) | 6.6 (2.6 to 14.9) | 24.3* (8.1 to 36.9) | -2.2 (-14.9 to 7.8) |
| Non-melanoma skin cancer (basal-cell carcinoma) | 60.3 (22.2 to 121.9) | 594.5 (211.8 to 1114.8) | 0.3 (0.1 to 0.6) | 29.1* (15.6 to 41.3) | 1.6 (-9.0 to 11.2) |
| Breast cancer | 2015.3 (1923.3 to 2085.2) | 229.4 (218.6 to 238.8) | 154.2 (108.2 to 210.3) | 30.5* (23.9 to 35.9) | 2.7 (-2.4 to 6.9) |
| Diagnosis and primary therapy phase of breast cancer | 50.2 (47.7 to 52.1) | 229.4 (218.6 to 238.8) | 13.0 (8.9 to 17.8) | 32.5* (20.1 to 46.3) | 4.3 (-5.4 to 15.2) |
| Controlled phase of breast cancer | 1667.7 (1583.5 to 1732.3) | -- | 104.0 (65.2 to 151.7) | 31.3* (24.5 to 37.0) | 3.4 (-2.0 to 7.8) |
| Metastatic phase of breast cancer | 67.3 (64.3 to 70.0) | -- | 26.9 (18.9 to 34.9) | 29.7* (18.4 to 42.4) | 2.1 (-6.8 to 12.1) |
| Terminal phase of breast cancer | 5.2 (4.9 to 5.4) | -- | 2.8 (1.9 to 3.6) | 26.7* (19.8 to 32.2) | -0.3 (-5.7 to 4.0) |
| Mastectomy from breast cancer, beyond 10 years | 224.9 (213.0 to 235.7) | -- | 7.5 (4.3 to 11.8) | 20.2* (15.1 to 25.7) | -5.4* (-9.4 to -1.1) |
| Cervical cancer | 311.2 (292.4 to 326.2) | 60.1 (56.5 to 63.0) | 26.0 (18.8 to 34.5) | 24.8* (17.7 to 32.7) | -1.8 (-7.3 to 4.4) |
| Diagnosis and primary therapy phase of cervical cancer | 15.8 (14.8 to 16.5) | 60.1 (56.5 to 63.0) | 4.3 (2.8 to 6.0) | 27.3* (14.2 to 42.1) | 0.2 (-10.1 to 11.9) |
| Controlled phase of cervical cancer | 274.2 (257.5 to 287.4) | -- | 12.6 (8.1 to 18.6) | 27.2* (19.9 to 35.5) | 0.2 (-5.7 to 6.6) |
| Metastatic phase of cervical cancer | 18.5 (17.4 to 19.4) | -- | 7.7 (5.2 to 10.1) | 20.7* (8.0 to 35.8) | -5.0 (-15.0 to 6.9) |
| Terminal phase of cervical cancer | 2.8 (2.6 to 2.9) | -- | 1.5 (1.0 to 1.9) | 19.4* (14.5 to 25.3) | -6.0* (-9.9 to -1.4) |
| Uterine cancer | 488.2 (468.3 to 507.2) | 60.1 (57.7 to 62.5) | 32.0 (22.6 to 42.5) | 31.1* (24.5 to 38.9) | 3.2 (-2.0 to 9.3) |
| Diagnosis and primary therapy phase of uterine cancer | 22.0 (21.1 to 22.8) | 60.1 (57.7 to 62.5) | 5.8 (3.9 to 8.0) | 32.4* (18.1 to 48.2) | 4.2 (-7.0 to 16.6) |
| Controlled phase of uterine cancer | 454.1 (435.6 to 471.8) | -- | 20.9 (13.5 to 30.3) | 32.3* (25.5 to 39.6) | 4.2 (-1.2 to 9.9) |
| Metastatic phase of uterine cancer | 11.0 (10.6 to 11.4) | -- | 4.7 (3.2 to 6.3) | 25.5* (11.5 to 41.5) | -1.2 (-12.2 to 11.4) |
| Terminal phase of uterine cancer | 1.1 (1.0 to 1.1) | -- | 0.6 (0.4 to 0.7) | 22.3* (17.1 to 28.5) | -3.7 (-7.8 to 1.1) |
| Ovarian cancer | 151.5 (145.8 to 157.7) | 32.3 (31.1 to 33.7) | 19.5 (14.0 to 25.0) | 29.6* (20.0 to 40.6) | 2.0 (-5.5 to 10.7) |
| Diagnosis and primary therapy phase of ovarian cancer | 5.3 (5.1 to 5.5) | 32.3 (31.1 to 33.7) | 1.5 (1.0 to 2.1) | 31.2* (26.5 to 36.0) | 3.3 (-0.4 to 7.1) |
| Controlled phase of ovarian cancer | 115.3 (110.9 to 119.9) | -- | 5.3 (3.4 to 7.7) | 31.2* (22.5 to 40.5) | 3.3 (-3.6 to 10.6) |
| Metastatic phase of ovarian cancer | 29.1 (28.0 to 30.3) | -- | 11.7 (8.0 to 15.4) | 28.8* (14.5 to 45.8) | 1.4 (-9.9 to 14.7) |

| | | | | | |
|---|---|---|---|---|---|
| Terminal phase of ovarian cancer | 1.8<br>(1.8 to 1.9) | -- | 1.0<br>(0.7 to 1.3) | 29.5*<br>(24.3 to 34.5) | 1.9<br>(-2.1 to 5.9) |
| Prostate cancer | 897.3<br>(761.0 to 1136.6) | 102.9<br>(87.0 to 130.4) | 67.5<br>(46.1 to 93.1) | 33.2*<br>(25.2 to 45.0) | 4.8<br>(-1.5 to 14.1) |
| Diagnosis and primary therapy phase of prostate cancer | 31.6<br>(26.7 to 40.1) | 102.9<br>(87.0 to 130.4) | 8.3<br>(5.5 to 11.9) | 36.3*<br>(22.1 to 55.0) | 7.3<br>(-3.9 to 22.0) |
| Controlled phase of prostate cancer | 837.7<br>(710.2 to 1062.9) | -- | 50.1<br>(31.9 to 73.3) | 34.6*<br>(26.4 to 46.7) | 6.0<br>(-0.5 to 15.5) |
| Metastatic phase of prostate cancer | 19.8<br>(16.4 to 24.0) | -- | 8.1<br>(5.4 to 10.9) | 23.1*<br>(9.6 to 39.1) | -3.1<br>(-13.7 to 9.5) |
| Terminal phase of prostate cancer | 1.1<br>(0.9 to 1.4) | -- | 0.6<br>(0.4 to 0.8) | 22.7*<br>(15.9 to 31.8) | -3.4<br>(-8.7 to 3.7) |
| Impotence and Incontinence after 10-year survival from prostate cancer | 7.0<br>(6.8 to 7.3) | -- | 0.3<br>(0.2 to 0.5) | 24.8*<br>(20.6 to 28.9) | -1.8<br>(-5.1 to 1.5) |
| Testicular cancer | 14.1<br>(13.0 to 15.5) | 1.9<br>(1.7 to 2.0) | 0.9<br>(0.6 to 1.3) | 40.0*<br>(22.3 to 61.5) | 10.2<br>(-3.7 to 27.1) |
| Diagnosis and primary therapy phase of testicular cancer | 0.5<br>(0.4 to 0.5) | 1.9<br>(1.7 to 2.0) | 0.1<br>(0.1 to 0.2) | 42.6*<br>(29.9 to 57.4) | 12.2*<br>(2.3 to 23.9) |
| Controlled phase of testicular cancer | 13.3<br>(12.2 to 14.6) | -- | 0.6<br>(0.4 to 0.9) | 44.3*<br>(20.3 to 73.8) | 13.6<br>(-5.3 to 36.8) |
| Metastatic phase of testicular cancer | 0.4<br>(0.3 to 0.4) | -- | 0.2<br>(0.1 to 0.2) | 25.8*<br>(16.7 to 36.1) | -1.0<br>(-8.2 to 7.2) |
| Terminal phase of testicular cancer | -- | -- | -- | 24.1*<br>(15.4 to 33.4) | -2.4<br>(-9.2 to 5.0) |
| Kidney cancer | 258.8<br>(245.3 to 270.4) | 42.7<br>(40.5 to 44.6) | 16.1<br>(11.3 to 21.8) | 23.5*<br>(17.3 to 31.1) | -2.8<br>(-7.7 to 3.2) |
| Diagnosis and primary therapy phase of kidney cancer | -- | 42.7<br>(40.5 to 44.6) | -- | -- | -- |
| Controlled phase of kidney cancer | 248.7<br>(235.8 to 259.8) | -- | 11.4<br>(7.4 to 16.7) | 24.6*<br>(17.5 to 33.1) | -1.9<br>(-7.5 to 4.8) |
| Metastatic phase of kidney cancer | 8.5<br>(8.0 to 8.9) | -- | 3.8<br>(2.6 to 5.1) | 21.1*<br>(13.0 to 29.9) | -4.7<br>(-11.0 to 2.2) |
| Terminal phase of kidney cancer | 1.6<br>(1.5 to 1.7) | -- | 0.9<br>(0.6 to 1.1) | 19.9*<br>(15.0 to 25.8) | -5.6*<br>(-9.5 to -0.9) |
| Bladder cancer | 275.5<br>(265.6 to 286.4) | 39.0<br>(37.5 to 40.6) | 23.9<br>(17.1 to 31.8) | 24.7*<br>(19.3 to 30.3) | -1.9<br>(-6.1 to 2.6) |
| Diagnosis and primary therapy phase of bladder cancer | 14.4<br>(13.9 to 15.0) | 39.0<br>(37.5 to 40.6) | 3.9<br>(2.7 to 5.4) | 25.8*<br>(12.2 to 41.0) | -1.0<br>(-11.7 to 11.0) |
| Controlled phase of bladder cancer | 248.1<br>(238.8 to 257.8) | -- | 15.5<br>(10.4 to 21.7) | 26.0*<br>(19.6 to 32.3) | -0.8<br>(-5.9 to 4.2) |
| Metastatic phase of bladder cancer | 7.0<br>(6.7 to 7.3) | -- | 3.1<br>(2.1 to 4.2) | 18.6*<br>(9.7 to 28.4) | -6.6<br>(-13.7 to 1.1) |
| Terminal phase of bladder cancer | 1.3<br>(1.2 to 1.3) | -- | 0.7<br>(0.5 to 0.9) | 17.1*<br>(13.7 to 21.1) | -7.8*<br>(-10.5 to -4.7) |
| Incontinence from bladder cancer, beyond 10 years | 4.8<br>(4.4 to 5.2) | -- | 0.7<br>(0.4 to 1.0) | 25.9*<br>(24.7 to 27.0) | -0.9*<br>(-1.9 to -0.0) |
| Brain and nervous system cancer | 101.8<br>(89.4 to 114.2) | 31.0<br>(27.5 to 34.5) | 11.7<br>(8.3 to 15.5) | 38.8*<br>(29.9 to 52.0) | 9.3*<br>(2.2 to 19.6) |
| Diagnosis and primary therapy phase of brain and nervous system cancers | 6.2<br>(5.4 to 7.0) | 31.0<br>(27.5 to 34.5) | 1.8<br>(1.2 to 2.6) | 48.4*<br>(37.6 to 64.8) | 16.8*<br>(8.3 to 29.7) |
| Controlled phase of brain and nervous system cancers | 82.5<br>(72.0 to 92.8) | -- | 3.8<br>(2.4 to 5.8) | 49.2*<br>(32.8 to 69.2) | 17.4*<br>(4.5 to 33.2) |
| Metastatic phase of brain and nervous system cancers | 11.1<br>(9.9 to 12.3) | -- | 5.0<br>(3.4 to 6.7) | 31.0*<br>(23.9 to 41.1) | 3.1<br>(-2.5 to 11.0) |
| Terminal phase of brain and nervous system cancers | 2.0<br>(1.8 to 2.2) | -- | 1.1<br>(0.7 to 1.4) | 29.1*<br>(22.3 to 38.6) | 1.6<br>(-3.7 to 9.1) |
| Thyroid cancer | 240.8<br>(230.0 to 257.1) | 28.0<br>(26.7 to 29.9) | 14.4<br>(10.0 to 20.0) | 39.5*<br>(31.7 to 47.4) | 9.8*<br>(3.6 to 16.1) |
| Diagnosis and primary therapy phase of thyroid cancer | 6.5<br>(6.2 to 6.9) | 28.0<br>(26.7 to 29.9) | 1.9<br>(1.2 to 2.6) | 40.4*<br>(33.7 to 47.5) | 10.5*<br>(5.2 to 16.1) |
| Controlled phase of thyroid cancer | 230.1<br>(219.8 to 245.7) | -- | 10.6<br>(6.9 to 15.6) | 40.6*<br>(31.8 to 50.0) | 10.7*<br>(3.8 to 18.0) |
| Metastatic phase of thyroid cancer | 3.9<br>(3.7 to 4.1) | -- | 1.8<br>(1.2 to 2.4) | 32.8*<br>(26.4 to 39.7) | 4.6<br>(-0.5 to 9.9) |
| Terminal phase of thyroid cancer | 0.3<br>(0.3 to 0.3) | -- | 0.2<br>(0.1 to 0.2) | 31.1*<br>(24.8 to 37.8) | 3.2<br>(-1.8 to 8.5) |
| Mesothelioma | 8.6<br>(8.2 to 9.0) | 3.5<br>(3.4 to 3.7) | 1.6<br>(1.1 to 2.1) | 16.0*<br>(8.5 to 22.8) | -8.7*<br>(-14.6 to -3.3) |
| Diagnosis and primary therapy phase of mesothelioma | 1.0<br>(0.9 to 1.0) | 3.5<br>(3.4 to 3.7) | 0.3<br>(0.2 to 0.4) | 16.0*<br>(8.8 to 22.9) | -8.7*<br>(-14.4 to -3.3) |
| Controlled phase of mesothelioma | 5.3<br>(5.0 to 5.5) | -- | 0.3<br>(0.2 to 0.4) | 16.2*<br>(5.6 to 27.3) | -8.5<br>(-16.9 to 0.2) |
| Metastatic phase of mesothelioma | 2.1<br>(2.0 to 2.2) | -- | 0.9<br>(0.6 to 1.2) | 15.9*<br>(8.9 to 22.6) | -8.8*<br>(-14.3 to -3.5) |
| Terminal phase of mesothelioma | 0.3<br>(0.3 to 0.3) | -- | 0.2<br>(0.1 to 0.2) | 15.8*<br>(8.8 to 22.5) | -8.8*<br>(-14.3 to -3.6) |
| Hodgkin lymphoma | 35.1<br>(29.4 to 42.9) | 5.8<br>(4.8 to 7.0) | 2.8<br>(1.9 to 3.9) | 29.3*<br>(19.3 to 39.5) | 1.7<br>(-6.1 to 9.8) |
| Diagnosis and primary therapy phase of Hodgkin disease | 1.2<br>(1.0 to 1.4) | 5.8<br>(4.8 to 7.0) | 0.3<br>(0.2 to 0.5) | 36.2*<br>(28.0 to 45.0) | 7.2*<br>(0.8 to 14.1) |
| Controlled phase of Hodgkin disease | 31.8<br>(26.6 to 38.9) | -- | 1.5<br>(0.9 to 2.3) | 36.8*<br>(22.0 to 53.8) | 7.7<br>(-4.0 to 21.0) |
| Metastatic phase of Hodgkin disease | 1.9<br>(1.6 to 2.4) | -- | 0.9<br>(0.6 to 1.2) | 18.3*<br>(12.1 to 24.8) | -6.9*<br>(-11.8 to -1.8) |
| Terminal phase of Hodgkin disease | 0.2<br>(0.2 to 0.3) | -- | 0.1<br>(0.1 to 0.1) | 10.5*<br>(4.7 to 16.2) | -13.0*<br>(-17.6 to -8.5) |
| Non-Hodgkin's lymphoma | 232.5<br>(224.7 to 241.0) | 42.5<br>(41.2 to 43.9) | 18.0<br>(13.0 to 24.1) | 37.3*<br>(31.3 to 43.7) | 8.1*<br>(3.3 to 13.1) |
| Diagnosis and primary therapy phase of non-Hodgkin lymphoma | 8.5<br>(8.2 to 8.8) | 42.5<br>(41.2 to 43.9) | 2.4<br>(1.6 to 3.4) | 40.7*<br>(33.3 to 48.2) | 10.8*<br>(4.9 to 16.6) |
| Controlled phase of non-Hodgkin lymphoma | 211.4<br>(204.3 to 219.2) | -- | 9.8<br>(6.3 to 14.2) | 40.4*<br>(32.2 to 48.8) | 10.5*<br>(4.1 to 17.2) |
| Metastatic phase of non-Hodgkin lymphoma | 10.8<br>(10.5 to 11.1) | -- | 4.9<br>(3.3 to 6.5) | 31.5*<br>(26.3 to 36.6) | 3.5<br>(-0.6 to 7.6) |
| Terminal phase of non-Hodgkin lymphoma | 1.8<br>(1.8 to 1.9) | -- | 1.0<br>(0.7 to 1.3) | 30.0*<br>(25.5 to 34.4) | 2.4<br>(-1.2 to 5.8) |
| Multiple myeloma | 63.2<br>(58.4 to 77.6) | 15.7<br>(14.6 to 18.8) | 11.9<br>(8.3 to 16.2) | 39.2*<br>(28.2 to 51.6) | 9.5*<br>(0.9 to 19.3) |
| Diagnosis and primary therapy phase of multiple myeloma | 4.2<br>(3.8 to 5.1) | 15.7<br>(14.6 to 18.8) | 1.2<br>(0.8 to 1.7) | 41.4*<br>(34.1 to 48.7) | 11.3*<br>(5.6 to 17.0) |
| Controlled phase of multiple myeloma | 38.3<br>(35.4 to 47.4) | -- | 1.8<br>(1.1 to 2.7) | 41.4*<br>(27.6 to 56.6) | 11.3*<br>(0.4 to 23.3) |
| Metastatic phase of multiple myeloma | 19.6<br>(18.2 to 24.2) | -- | 8.4<br>(5.6 to 11.5) | 38.9*<br>(25.4 to 55.4) | 9.4<br>(-1.3 to 22.4) |
| Terminal phase of multiple myeloma | 1.0<br>(1.0 to 1.2) | -- | 0.6<br>(0.4 to 0.7) | 31.4*<br>(25.1 to 37.1) | 3.4<br>(-1.5 to 7.9) |
| Leukaemia | 138.0<br>(125.7 to 148.5) | 31.6<br>(28.6 to 33.7) | 16.7<br>(12.0 to 22.0) | 23.0*<br>(16.5 to 30.8) | -3.2<br>(-8.3 to 2.9) |
| Acute lymphoid leukaemia | 11.7<br>(10.0 to 12.8) | 3.1<br>(2.6 to 3.3) | 1.4<br>(1.0 to 1.8) | 34.4*<br>(22.8 to 42.9) | 5.8<br>(-3.4 to 12.5) |
| Diagnosis and primary therapy phase of acute lymphoid leukemia | 1.4<br>(1.2 to 1.5) | 3.1<br>(2.6 to 3.3) | 0.4<br>(0.3 to 0.6) | 30.6*<br>(22.1 to 37.3) | 2.8<br>(-3.9 to 8.1) |
| Controlled phase of acute lymphoid leukemia | 9.2<br>(7.9 to 10.1) | -- | 0.5<br>(0.3 to 0.7) | 37.1*<br>(24.8 to 46.6) | 7.9<br>(-1.8 to 15.4) |
| Metastatic phase of acute lymphoid leukemia | 0.9<br>(0.8 to 1.0) | -- | 0.4<br>(0.3 to 0.6) | 35.1*<br>(22.4 to 45.1) | 6.3<br>(-3.7 to 14.2) |
| Terminal phase of acute lymphoid leukemia | 0.2<br>(0.1 to 0.2) | -- | 0.1<br>(0.1 to 0.1) | 35.2*<br>(22.4 to 45.3) | 6.4<br>(-3.6 to 14.4) |
| Chronic lymphoid leukaemia | 58.3<br>(54.0 to 63.0) | 7.9<br>(7.3 to 8.5) | 7.0<br>(5.1 to 9.2) | 32.2*<br>(20.8 to 44.2) | 4.0<br>(-4.9 to 13.5) |

| | | | | | |
|---|---|---|---|---|---|
| Diagnosis and primary therapy phase of chronic lymphoid leukemia | 3.1 (2.9 to 3.4) | 7.9 (7.3 to 8.5) | 0.9 (0.6 to 1.3) | 33.9* (24.9 to 42.7) | 5.4 (-1.7 to 12.3) |
| Controlled phase of chronic lymphoid leukemia | 46.3 (42.9 to 50.1) | -- | 2.1 (1.4 to 3.1) | 34.3* (20.8 to 49.2) | 5.7 (-5.0 to 17.5) |
| Metastatic phase of chronic lymphoid leukemia | 8.6 (8.0 to 9.3) | -- | 3.8 (2.6 to 5.1) | 30.8* (16.8 to 46.3) | 3.0 (-8.0 to 15.2) |
| Terminal phase of chronic lymphoid leukemia | 0.3 (0.2 to 0.3) | -- | 0.1 (0.1 to 0.2) | 26.0* (18.3 to 34.0) | -0.8 (-6.9 to 5.4) |
| Acute myeloid leukaemia | 9.0 (8.1 to 9.8) | 7.5 (6.6 to 8.1) | 1.8 (1.3 to 2.4) | 27.7* (20.5 to 34.4) | 0.5 (-5.1 to 5.8) |
| Diagnosis and primary therapy phase of acute myeloid leukemia | -- | 7.5 (6.6 to 8.1) | -- | -- | -- |
| Controlled phase of acute myeloid leukemia | 5.8 (5.2 to 6.2) | -- | 0.3 (0.2 to 0.4) | 23.4* (14.2 to 33.1) | -2.9 (-10.1 to 4.7) |
| Metastatic phase of acute myeloid leukemia | 2.7 (2.4 to 2.9) | -- | 1.2 (0.8 to 1.6) | 28.5* (21.6 to 35.1) | 1.1 (-4.3 to 6.3) |
| Terminal phase of acute myeloid leukemia | 0.6 (0.5 to 0.6) | -- | 0.3 (0.2 to 0.4) | 28.5* (21.6 to 35.1) | 1.1 (-4.3 to 6.3) |
| Chronic myeloid leukaemia | 8.3 (7.5 to 9.0) | 2.7 (2.4 to 2.9) | 0.9 (0.7 to 1.3) | 10.0* (3.6 to 16.5) | -13.4* (-18.4 to -8.3) |
| Diagnosis and primary therapy phase of chronic myeloid leukemia | 0.6 (0.5 to 0.6) | 2.7 (2.4 to 2.9) | 0.2 (0.1 to 0.2) | 10.7* (4.1 to 17.4) | -12.9* (-18.1 to -7.6) |
| Controlled phase of chronic myeloid leukemia | 6.7 (6.1 to 7.3) | -- | 0.3 (0.2 to 0.5) | 11.4* (3.7 to 19.2) | -12.4* (-18.4 to -6.2) |
| Metastatic phase of chronic myeloid leukemia | 0.8 (0.7 to 0.9) | -- | 0.4 (0.2 to 0.5) | 8.8* (3.0 to 14.7) | -14.3* (-18.9 to -9.7) |
| Terminal phase of chronic myeloid leukemia | 0.2 (0.2 to 0.2) | -- | 0.1 (0.1 to 0.1) | 8.8* (3.0 to 14.7) | -14.3* (-18.9 to -9.7) |
| Other leukaemia | 50.6 (43.7 to 56.9) | 10.5 (9.0 to 11.8) | 5.6 (4.0 to 7.6) | 12.0* (2.9 to 23.6) | -11.9* (-19.0 to -2.7) |
| Metastatic phase of other leukemia | 7.4 (6.4 to 8.3) | -- | 3.4 (2.2 to 4.6) | 9.9* (1.9 to 20.1) | -13.5* (-19.8 to -5.4) |
| Diagnosis and primary therapy phase of other leukemia | -- | 10.5 (9.0 to 11.8) | -- | -- | -- |
| Terminal phase of other leukemia | 0.5 (0.4 to 0.6) | -- | 0.3 (0.2 to 0.3) | 8.8* (1.4 to 19.3) | -14.3* (-20.2 to -6.1) |
| Controlled phase of other leukemia | 42.7 (36.6 to 48.1) | -- | 2.0 (1.2 to 2.9) | 16.1* (2.8 to 32.7) | -8.6 (-19.1 to 4.5) |
| Other malignant cancers | 395.3 (332.3 to 489.9) | 52.4 (48.6 to 55.5) | 33.6 (23.8 to 46.0) | 87.5* (55.3 to 124.8) | 47.6* (22.2 to 77.0) |
| Diagnosis and primary therapy phase of other malignant neoplasms | 9.4 (7.9 to 11.6) | 52.4 (48.6 to 55.5) | 2.7 (1.7 to 3.8) | 116.5* (71.1 to 165.0) | 70.4* (34.7 to 108.6) |
| Controlled phase of other malignant neoplasms | 353.0 (294.2 to 441.4) | -- | 16.4 (10.1 to 24.9) | 135.1* (81.3 to 192.4) | 85.1* (42.7 to 130.2) |
| Metastatic phase of other malignant neoplasms | 28.7 (25.7 to 32.6) | -- | 12.2 (8.4 to 16.5) | 50.5* (28.9 to 73.5) | 18.5* (1.5 to 36.5) |
| Terminal phase of other malignant neoplasms | 4.3 (3.9 to 4.9) | -- | 2.3 (1.6 to 3.1) | 46.1* (30.1 to 63.1) | 15.0* (2.4 to 28.4) |
| Other neoplasms | 2390.4 (2279.7 to 2508.1) | 955.5 (894.8 to 1025.0) | 4.5 (2.9 to 6.8) | 38.0* (29.6 to 46.4) | 8.6* (2.0 to 15.3) |
| Myelodysplastic, myeloproliferative, and other hematopoietic neoplasms | 98.0 (81.6 to 115.3) | 10.4 (6.8 to 14.6) | 4.5 (2.9 to 6.8) | 38.0* (29.6 to 46.4) | 8.6* (2.0 to 15.3) |
| Benign and in situ intestinal neoplasms | 501.0 (477.4 to 528.7) | 106.3 (98.2 to 115.9) | -- | -- | -- |
| Benign and in situ cervical and uterine neoplasms | 63.9 (60.1 to 67.9) | 3.1 (2.7 to 3.5) | -- | -- | -- |
| Other benign and in situ neoplasms | 1761.8 (1662.1 to 1871.6) | 835.7 (776.5 to 902.0) | -- | -- | -- |
| **Cardiovascular diseases** | **47655.7 (44327.7 to 51320.4)** | **6322.7 (5734.2 to 6974.3)** | **3221.7 (2347.2 to 4139.8)** | **35.4* (32.4 to 38.7)** | **6.6* (4.2 to 9.2)** |
| Rheumatic heart disease | 1831.2 (1765.2 to 1900.7) | 5.4 (3.9 to 7.0) | 89.1 (59.0 to 127.9) | 28.0* (25.8 to 30.2) | 0.7 (-1.0 to 2.5) |
| Rheumatic heart disease, without heart failure | 1712.6 (1636.5 to 1792.4) | 5.4 (3.9 to 7.0) | 78.8 (51.8 to 114.4) | 29.6* (27.5 to 31.8) | 2.0* (0.4 to 3.8) |
| Heart failure due to rheumatic heart disease | 118.6 (83.3 to 158.7) | -- | 10.3 (6.3 to 15.5) | 17.0* (8.2 to 26.0) | -7.9* (-14.9 to -0.8) |
| Ischaemic heart disease | 12447.4 (11345.5 to 13696.7) | 920.7 (665.4 to 1213.5) | 516.1 (346.8 to 726.1) | 21.9* (20.5 to 23.3) | -4.1* (-5.1 to -2.9) |
| Asymptomatic ischemic heart disease following myocardial infarction | 3680.6 (2961.3 to 4468.0) | -- | -- | -- | -- |
| Angina due to ischemic heart disease | 7753.1 (6991.9 to 8534.3) | -- | 428.8 (288.3 to 614.6) | 21.6* (20.2 to 23.2) | -4.3* (-5.4 to -3.0) |
| Heart failure due to ischemic heart disease | 962.0 (702.1 to 1269.5) | -- | 82.3 (51.1 to 126.1) | 23.7* (19.7 to 27.5) | -2.6 (-5.7 to 0.4) |
| Myocardial infarction due to ischemic heart disease | 51.8 (37.8 to 67.1) | 920.7 (665.4 to 1213.5) | 5.0 (3.1 to 7.7) | 13.6* (5.9 to 20.4) | -10.6* (-16.7 to -5.3) |
| Stroke | 11388.6 (10582.7 to 12307.9) | 1153.3‡ (966.0 to 1393.5) | 1857.3 (1321.2 to 2369.9) | 43.9* (38.8 to 49.1) | 13.2* (9.2 to 17.4) |
| Ischaemic stroke | 7798.0 (7041.9 to 8659.0) | 695.4 (519.6 to 927.0) | 1252.7 (881.4 to 1623.1) | 57.0* (50.2 to 64.3) | 23.6* (18.2 to 29.3) |
| Acute ischemic stroke | 55.0 (41.3 to 73.3) | 695.4 (519.6 to 927.0) | 14.1 (9.1 to 21.0) | 43.8* (34.2 to 52.8) | 13.2* (5.7 to 20.3) |
| Chronic ischemic stroke | 7743.0 (6992.1 to 8606.5) | -- | 1238.5 (872.2 to 1606.8) | 57.2* (50.2 to 64.5) | 23.7* (18.2 to 29.5) |
| Intracerebral hemorrhage | 2447.5 (2124.7 to 2785.6) | 338.5 (269.2 to 408.4) | 394.2 (279.6 to 511.9) | 21.4* (11.5 to 31.3) | -4.5 (-12.2 to 3.4) |
| Acute intracerebral hemorrhage | 18.3 (14.4 to 22.3) | 338.5 (269.2 to 408.4) | 4.6 (3.1 to 6.3) | -5.1 (-18.1 to 7.0) | -25.3* (-35.5 to -15.8) |
| Chronic intracerebral hemorrhage | 2429.2 (2106.1 to 2769.6) | -- | 389.6 (276.6 to 506.3) | 21.8* (11.7 to 31.9) | -4.2 (-12.1 to 3.8) |
| Subarachnoid hemorrhage | 1415.3 (1253.1 to 1620.8) | 119.4 (91.3 to 154.1) | 210.4 (148.4 to 274.2) | 24.9* (18.8 to 30.6) | -1.7 (-6.5 to 2.8) |
| Acute subarachnoid hemorrhage | 9.5 (7.4 to 12.1) | 119.4 (91.3 to 154.1) | 2.4 (1.6 to 3.5) | 22.6* (18.9 to 24.8) | -3.5* (-6.4 to -1.7) |
| Chronic subarachnoid hemorrhage | 1405.8 (1242.5 to 1612.7) | -- | 208.0 (146.6 to 271.9) | 24.9* (18.8 to 30.6) | -1.7 (-6.5 to 2.8) |
| Hypertensive heart disease | 1176.4 (864.8 to 1526.1) | -- | 100.7 (64.3 to 151.4) | 30.0* (26.5 to 33.4) | 2.3 (-0.4 to 5.0) |
| Non-rheumatic valvular heart disease | 2244.9 (2103.8 to 2394.7) | 2346.2 (2210.2 to 2488.4) | 12.0 (5.7 to 21.4) | 22.3* (18.2 to 26.5) | -3.8* (-6.9 to -0.4) |
| Non-rheumatic calcific aortic valve disease | 737.3 (623.9 to 858.2) | 737.3 (623.9 to 858.2) | 3.5 (1.6 to 6.4) | 19.8* (12.0 to 27.7) | -5.7 (-11.9 to 0.5) |
| Heart failure due to calcific aortic valve disease | 24.5 (12.6 to 43.2) | -- | 2.3 (1.0 to 4.1) | 13.0 (-1.9 to 28.3) | -11.0 (-22.8 to 1.0) |
| Non-rheumatic calcific aortic valve disease | 712.8 (604.2 to 832.1) | 737.3 (623.9 to 858.2) | 1.3 (0.5 to 2.5) | 33.9* (11.8 to 58.8) | 5.4 (-12.0 to 25.0) |
| Non-rheumatic degenerative mitral valve disease | 1608.9 (1541.1 to 1681.2) | 1608.9 (1541.1 to 1681.2) | 8.3 (3.8 to 15.0) | 23.2* (18.2 to 28.4) | -3.0 (-7.0 to 1.1) |
| Heart failure due to degenerative mitral valve disease | 66.4 (34.1 to 117.9) | -- | 6.1 (2.8 to 11.1) | 17.9* (7.3 to 29.4) | -7.2 (-15.6 to 1.9) |
| Non-rheumatic degenerative mitral valve disease | 1542.6 (1461.9 to 1622.0) | 1608.9 (1541.1 to 1681.2) | 2.2 (1.0 to 4.2) | 40.7* (18.5 to 65.9) | 10.7 (-6.7 to 30.6) |
| Other non-rheumatic valve diseases | 1.5 (1.0 to 2.2) | -- | 0.1 (0.1 to 0.2) | 30.1* (27.5 to 32.9) | 2.4* (0.3 to 4.6) |
| Mild heart failure due to other non-rheumatic valve disease | 0.3 (0.2 to 0.5) | -- | -- | 30.1* (27.5 to 32.9) | 2.4* (0.3 to 4.6) |
| Moderate heart failure due to other non-rheumatic valve disease | 0.2 (0.1 to 0.3) | -- | -- | 30.1* (27.5 to 32.9) | 2.4* (0.3 to 4.6) |
| Severe heart failure due to other non-rheumatic valve disease | 0.5 (0.3 to 0.7) | -- | 0.1 (0.1 to 0.1) | 30.1* (27.5 to 32.9) | 2.4* (0.4 to 4.6) |

| | | | | | |
|---|---|---|---|---|---|
| Controlled, medically managed heart failure due to other non-rheumatic valve disease | 0.6<br>(0.4 to 0.8) | -- | -- | 30.1*<br>(27.5 to 32.9) | 2.4*<br>(0.3 to 4.6) |
| Cardiomyopathy and myocarditis | 509.7<br>(384.7 to 659.0) | 218.0<br>(158.6 to 298.3) | 51.7<br>(32.8 to 77.6) | 23.1*<br>(19.5 to 26.6) | -3.1*<br>(-6.0 to -0.3) |
| Myocarditis | 110.6<br>(87.4 to 137.9) | 218.0<br>(158.6 to 298.3) | 7.8<br>(5.0 to 11.5) | 21.5*<br>(16.0 to 26.7) | -4.4*<br>(-8.7 to -0.3) |
| Acute myocarditis | 52.0<br>(37.5 to 70.2) | 218.0<br>(158.6 to 298.3) | 2.5<br>(1.4 to 4.0) | 24.0*<br>(12.9 to 36.8) | -2.4<br>(-11.1 to 7.7) |
| Heart failure due to myocarditis | 58.6<br>(41.2 to 80.8) | -- | 5.3<br>(3.2 to 8.2) | 20.3*<br>(14.4 to 26.3) | -5.3*<br>(-10.0 to -0.6) |
| Alcoholic cardiomyopathy | 216.7<br>(156.9 to 287.6) | -- | 18.7<br>(11.7 to 28.6) | 19.1*<br>(13.6 to 24.8) | -6.2*<br>(-10.6 to -1.8) |
| Other cardiomyopathy | 293.6<br>(209.9 to 392.9) | -- | 25.2<br>(15.6 to 38.2) | 26.7*<br>(21.3 to 32.2) | -0.3<br>(-4.5 to 4.0) |
| Atrial fibrillation and flutter | 2484.6<br>(1997.2 to 3021.6) | 293.1<br>(192.3 to 422.2) | 202.0<br>(131.6 to 290.5) | 26.4*<br>(23.6 to 29.2) | -0.5<br>(-2.7 to 1.7) |
| Asymptomatic atrial fibrillation and flutter | 1490.9<br>(1201.9 to 1811.5) | 293.1<br>(192.3 to 422.2) | -- | -- | -- |
| Symptomatic atrial fibrillation and flutter | 993.7<br>(793.2 to 1208.3) | -- | 202.0<br>(131.6 to 290.5) | 26.4*<br>(23.6 to 29.2) | -0.5<br>(-2.7 to 1.7) |
| Peripheral vascular disease | 12212.8<br>(9967.6 to 14991.3) | 1318.2<br>(879.7 to 1813.2) | 38.5<br>(17.5 to 72.0) | 21.3*<br>(19.3 to 23.4) | -4.5*<br>(-6.1 to -2.8) |
| Asymptomatic peripheral arterial disease | 9365.2<br>(7536.2 to 11685.1) | 1318.2<br>(879.7 to 1813.2) | -- | -- | -- |
| Symptomatic claudication due to peripheral arterial disease | 2847.6<br>(1998.1 to 4017.2) | -- | 38.5<br>(17.5 to 72.0) | 21.3*<br>(19.3 to 23.4) | -4.5*<br>(-6.1 to -2.8) |
| Endocarditis | 35.6<br>(26.1 to 48.3) | 67.8<br>(47.9 to 89.8) | 3.1<br>(1.9 to 4.7) | 27.7*<br>(20.6 to 35.6) | 0.5<br>(-5.1 to 6.7) |
| Acute endocarditis | 5.1<br>(3.7 to 6.8) | 67.8<br>(47.9 to 89.8) | 0.3<br>(0.2 to 0.5) | 35.1*<br>(28.8 to 40.6) | 6.3*<br>(1.4 to 10.6) |
| Heart failure due to endocarditis | 30.5<br>(21.2 to 42.6) | -- | 2.8<br>(1.7 to 4.3) | 26.9*<br>(18.8 to 35.3) | -0.1<br>(-6.5 to 6.5) |
| Other cardiovascular and circulatory diseases | 6734.9<br>(4609.9 to 9542.6) | -- | 351.3<br>(207.9 to 548.5) | 29.4*<br>(26.8 to 31.9) | 1.8<br>(-0.2 to 3.9) |
| Heart failure due to other cardiovascular diseases | 77.9<br>(53.5 to 109.4) | -- | 7.0<br>(4.1 to 10.9) | 29.1*<br>(21.6 to 36.9) | 1.6<br>(-4.3 to 7.8) |
| Other cardiovascular and circulatory disease episodes | 6657.1<br>(4557.0 to 9433.2) | -- | 344.3<br>(203.9 to 537.5) | 29.4*<br>(26.8 to 31.9) | 1.8<br>(-0.2 to 3.8) |
| **Chronic respiratory diseases** | **42199.9<br>(37336.5 to 47178.2)** | **3304.7<br>(2492.6 to 4337.4)** | **3898.3<br>(3152.9 to 4665.0)** | **26.4*<br>(19.5 to 32.4)** | **-0.5<br>(-5.9 to 4.2)** |
| Chronic obstructive pulmonary disease | 29984.1<br>(25317.9 to 35038.8) | 1985.1<br>(1327.6 to 2721.0) | 3122.7<br>(2508.7 to 3817.5) | 24.7*<br>(16.9 to 31.6) | -1.8<br>(-8.0 to 3.6) |
| Chronic obstructive pulmonary disease with heart failure | 374.3<br>(248.9 to 532.1) | -- | 154.7<br>(93.7 to 235.0) | 22.6*<br>(17.7 to 27.8) | -3.5<br>(-7.4 to 0.6) |
| Chronic obstructive pulmonary disease without heart failure | 29609.7<br>(24925.3 to 34597.2) | 1985.1<br>(1327.6 to 2721.0) | 2968.0<br>(2374.7 to 3632.0) | 24.8*<br>(16.8 to 32.0) | -1.8<br>(-8.1 to 3.9) |
| Pneumoconiosis | 43.4<br>(35.9 to 52.2) | 5.0<br>(3.6 to 6.8) | 6.8<br>(4.4 to 10.0) | 18.1*<br>(11.6 to 24.3) | -7.1*<br>(-12.1 to -2.1) |
| Silicosis | 12.5<br>(8.5 to 17.3) | 2.0<br>(1.1 to 3.3) | 2.0<br>(1.1 to 3.3) | 20.7*<br>(10.1 to 31.9) | -5.0<br>(-13.4 to 3.8) |
| Heart failure due to silicosis | 0.7<br>(0.4 to 0.9) | -- | 0.3<br>(0.2 to 0.5) | 9.7*<br>(5.8 to 13.8) | -13.6*<br>(-16.7 to -10.5) |
| Silicosis without heart failure | 11.8<br>(7.9 to 16.7) | 2.0<br>(1.1 to 3.3) | 1.7<br>(0.9 to 2.9) | 22.9*<br>(10.1 to 36.1) | -3.2<br>(-13.3 to 7.1) |
| Asbestosis | 8.2<br>(5.4 to 12.0) | 0.8<br>(0.4 to 1.5) | 1.3<br>(0.7 to 2.1) | 29.8*<br>(22.4 to 38.4) | 2.1<br>(-3.6 to 8.9) |
| Asbestosis without heart failure | 8.0<br>(5.2 to 11.9) | 0.8<br>(0.4 to 1.5) | 1.2<br>(0.7 to 2.0) | 31.0*<br>(23.5 to 39.8) | 3.1<br>(-2.8 to 10.0) |
| Heart failure due to asbestosis | 0.1<br>(0.1 to 0.2) | -- | 0.1<br>(0.0 to 0.1) | 8.5*<br>(4.8 to 12.3) | -14.6*<br>(-17.5 to -11.6) |
| Coal workers pneumoconiosis | 10.8<br>(7.2 to 15.5) | 1.1<br>(0.5 to 2.0) | 1.7<br>(0.9 to 2.7) | 9.9<br>(-6.8 to 27.7) | -13.5<br>(-26.6 to 0.5) |
| Coal workers pneumoconiosis without heart failure | 10.4<br>(6.8 to 15.2) | 1.1<br>(0.5 to 2.0) | 1.5<br>(0.8 to 2.5) | 9.7<br>(-8.7 to 29.7) | -13.6<br>(-28.1 to 2.1) |
| Heart failure due to Coal workers pneumoconiosis | 0.3<br>(0.2 to 0.4) | -- | 0.2<br>(0.1 to 0.2) | 11.1*<br>(5.3 to 16.6) | -12.6*<br>(-17.1 to -8.2) |
| Other pneumoconiosis | 12.0<br>(8.7 to 16.2) | 1.1<br>(0.5 to 1.9) | 1.9<br>(1.1 to 2.9) | 16.0*<br>(8.8 to 23.9) | -8.7*<br>(-14.4 to -2.5) |
| Heart failure due to other pneumoconiosis | 0.2<br>(0.1 to 0.2) | -- | 0.1<br>(0.0 to 0.1) | 12.2*<br>(3.5 to 23.1) | -11.7*<br>(-18.5 to -3.1) |
| Other pneumoconiosis without heart failure | 11.8<br>(8.6 to 16.0) | 1.1<br>(0.5 to 1.9) | 1.8<br>(1.1 to 2.8) | 16.1*<br>(8.8 to 24.4) | -8.6*<br>(-14.4 to -2.0) |
| Asthma | 14147.6<br>(11720.7 to 16982.5) | 1272.2<br>(827.9 to 1840.8) | 537.5<br>(348.1 to 775.3) | 33.1*<br>(26.0 to 40.9) | 4.8<br>(-0.8 to 10.9) |
| Asymptomatic asthma | 5140.8<br>(4147.9 to 6300.0) | 461.1<br>(302.4 to 668.6) | -- | -- | -- |
| Symptomatic asthma | 9006.8<br>(7401.5 to 10899.7) | 811.1<br>(521.1 to 1195.0) | 537.5<br>(348.1 to 775.3) | 33.1*<br>(26.0 to 40.9) | 4.8<br>(-0.8 to 10.9) |
| Interstitial lung disease and pulmonary sarcoidosis | 590.6<br>(496.2 to 694.5) | 42.3<br>(28.4 to 60.3) | 62.1<br>(39.4 to 90.8) | 22.2*<br>(17.0 to 27.4) | -3.8<br>(-7.9 to 0.3) |
| Heart failure due to interstitial lung disease and pulmonary sarcoidosis | 21.8<br>(14.8 to 30.3) | -- | 10.0<br>(6.0 to 15.0) | 18.5*<br>(14.9 to 22.3) | -6.7*<br>(-9.5 to -3.8) |
| Interstitial lung disease and pulmonary sarcoidosis without heart failure | 568.8<br>(472.2 to 674.5) | 42.3<br>(28.4 to 60.3) | 52.2<br>(32.0 to 79.4) | 22.9*<br>(16.7 to 28.8) | -3.2<br>(-8.1 to 1.4) |
| Other chronic respiratory diseases | -- | -- | 169.1<br>(136.2 to 206.0) | 42.4*<br>(33.9 to 50.1) | 12.1*<br>(5.4 to 18.1) |
| **Digestive diseases** | **146651.9<br>(135260.8 to 159024.3)** | **35393.7<br>(28092.8 to 43965.1)** | **1571.2<br>(1090.5 to 2201.2)** | **28.8*<br>(26.7 to 31.2)** | **1.4<br>(-0.3 to 3.3)** |
| Cirrhosis and other chronic liver diseases | 107273.2<br>(99062.7 to 115669.2) | 209.4<br>(191.5 to 228.3) | 180.4<br>(125.4 to 249.3) | 39.7*<br>(35.6 to 43.9) | 10.0*<br>(6.7 to 13.3) |
| Cirrhosis and other chronic liver diseases due to hepatitis B | 21448.2<br>(19873.4 to 23109.7) | 51.4<br>(43.7 to 60.0) | 46.6<br>(31.9 to 64.0) | 37.6*<br>(30.8 to 44.4) | 8.3*<br>(2.9 to 13.6) |
| Cirrhosis and other chronic liver diseases due to hepatitis B, decompensated | 284.8<br>(250.4 to 323.0) | 50.9<br>(43.3 to 59.6) | 46.6<br>(31.9 to 64.0) | 37.6*<br>(30.8 to 44.4) | 8.3*<br>(2.9 to 13.6) |
| Chronic hepatitis B without cirrhosis | 18336.3<br>(16713.3 to 20083.6) | -- | -- | -- | -- |
| Cirrhosis and other chronic liver diseases due to hepatitis B, compensated | 2827.1<br>(2488.1 to 3176.8) | 0.5<br>(0.2 to 1.0) | -- | -- | -- |
| Cirrhosis and other chronic liver diseases due to hepatitis C | 8362.7<br>(7203.0 to 9742.0) | 58.4<br>(50.3 to 66.4) | 48.6<br>(32.5 to 68.0) | 35.4*<br>(29.3 to 41.4) | 6.6*<br>(1.8 to 11.3) |
| Cirrhosis and other chronic liver diseases due to hepatitis C, decompensated | 298.5<br>(262.7 to 333.4) | 55.1<br>(47.2 to 62.8) | 48.6<br>(32.5 to 68.0) | 35.4*<br>(29.3 to 41.4) | 6.6*<br>(1.8 to 11.3) |
| Chronic hepatitis C without cirrhosis | 5545.7<br>(4315.1 to 6956.0) | -- | -- | -- | -- |
| Cirrhosis and other chronic liver diseases due to hepatitis C, compensated | 2518.6<br>(2216.5 to 2829.6) | 3.3<br>(1.8 to 5.3) | -- | -- | -- |
| Cirrhosis and other chronic liver diseases due to alcohol use | 2938.9<br>(2585.2 to 3275.6) | 61.7<br>(53.7 to 70.0) | 53.8<br>(36.5 to 74.4) | 39.8*<br>(33.2 to 46.5) | 10.0*<br>(4.8 to 15.3) |
| Cirrhosis and other chronic liver diseases due to alcohol, decompensated | 330.5<br>(290.7 to 370.4) | 58.8<br>(51.0 to 67.2) | 53.8<br>(36.5 to 74.4) | 39.8*<br>(33.2 to 46.5) | 10.0*<br>(4.8 to 15.3) |
| Cirrhosis and other chronic liver diseases due to alcohol, compensated | 2608.4<br>(2289.4 to 2915.9) | 2.9<br>(1.6 to 4.3) | -- | -- | -- |
| Cirrhosis due to NASH | 73812.6<br>(65928.7 to 82184.6) | 21.7<br>(18.6 to 25.0) | 18.5<br>(12.5 to 26.1) | 58.6*<br>(47.8 to 69.5) | 24.8*<br>(16.3 to 33.4) |
| Non-alcoholic fatty liver disease (NAFLD) / Non-alcoholic steatohepatitis (NASH) | 72712.2<br>(64781.8 to 80971.7) | -- | -- | -- | -- |
| Cirrhosis and other chronic liver diseases due to NASH | 1100.4<br>(960.6 to 1244.3) | 21.7<br>(18.6 to 25.0) | 18.5<br>(12.5 to 26.1) | 58.6*<br>(47.8 to 69.5) | 24.8*<br>(16.3 to 33.4) |
| Cirrhosis and other chronic liver diseases due to other causes | 710.8<br>(522.9 to 931.9) | 16.2<br>(12.0 to 21.4) | 12.9<br>(8.2 to 19.2) | 40.8*<br>(31.1 to 51.0) | 10.8*<br>(3.2 to 18.8) |

| | | | | | |
|---|---|---|---|---|---|
| Cirrhosis and other chronic liver diseases due to other cause, decompensated | 78.6<br>(58.2 to 101.3) | 14.6<br>(10.7 to 19.3) | 12.9<br>(8.2 to 19.2) | 40.8*<br>(31.1 to 51.0) | 10.8*<br>(3.2 to 18.8) |
| Cirrhosis and other chronic liver diseases due to other cause, compensated | 632.2<br>(465.1 to 831.1) | 1.7<br>(0.9 to 2.6) | -- | -- | -- |
| Upper digestive system diseases | 62206.7<br>(48694.2 to 78410.4) | 29010.1<br>(22225.6 to 37178.4) | 987.6<br>(611.1 to 1496.9) | 27.3*<br>(24.3 to 30.3) | 0.2<br>(-2.1 to 2.5) |
| Peptic ulcer disease | 1288.6<br>(959.8 to 1669.3) | 503.3<br>(369.2 to 651.6) | 61.8<br>(38.6 to 89.3) | 26.3*<br>(19.9 to 32.5) | -0.6<br>(-5.6 to 4.3) |
| Acute peptic ulcer disease | 0.7<br>(0.4 to 0.9) | -- | 0.2<br>(0.1 to 0.3) | 7.6*<br>(5.7 to 9.4) | -15.3*<br>(-16.8 to -13.9) |
| Chronic peptic ulcer disease | 1288.0<br>(959.0 to 1668.7) | 503.3<br>(369.2 to 651.6) | 61.6<br>(38.4 to 89.1) | 26.4*<br>(19.9 to 32.6) | -0.5<br>(-5.6 to 4.4) |
| Gastritis and duodenitis | 10948.7<br>(7389.5 to 15292.7) | 7503.9<br>(5167.8 to 10338.3) | 461.5<br>(273.1 to 727.3) | 24.7*<br>(19.2 to 30.3) | -1.9<br>(-6.2 to 2.6) |
| Acute gastritis and duodenitis | 3.1<br>(2.1 to 4.5) | -- | 1.0<br>(0.6 to 1.7) | 20.1*<br>(18.6 to 21.6) | -5.5*<br>(-6.6 to -4.3) |
| Chronic gastritis and duodenitis | 10945.6<br>(7386.3 to 15288.6) | 7503.9<br>(5167.8 to 10338.3) | 460.5<br>(272.0 to 726.3) | 24.7*<br>(19.2 to 30.3) | -1.9<br>(-6.2 to 2.6) |
| GERD | 55365.6<br>(40405.9 to 74605.0) | 21002.9<br>(14359.2 to 28430.0) | 464.2<br>(228.9 to 813.7) | 30.2*<br>(28.1 to 32.1) | 2.5*<br>(0.8 to 4.0) |
| Appendicitis | 17.6<br>(14.3 to 21.4) | 468.7<br>(379.6 to 571.8) | 5.6<br>(3.5 to 8.0) | 30.5*<br>(20.8 to 41.0) | 2.7<br>(-4.9 to 11.0) |
| Paralytic ileus and intestinal obstruction | 9.3<br>(6.9 to 12.5) | 257.2<br>(191.3 to 348.3) | 3.0<br>(1.8 to 4.7) | 37.5*<br>(26.8 to 47.3) | 8.2<br>(-0.2 to 15.9) |
| Inguinal, femoral, and abdominal hernia | 1923.5<br>(1392.1 to 2550.7) | 4469.3<br>(3200.2 to 5993.7) | 183.2<br>(111.9 to 280.2) | 23.4*<br>(18.2 to 28.6) | -2.9<br>(-6.9 to 1.2) |
| Inflammatory bowel disease | 749.6<br>(642.9 to 888.3) | 253.8<br>(203.9 to 311.4) | 109.5<br>(73.3 to 155.9) | 31.3*<br>(23.6 to 40.6) | 3.4<br>(-2.8 to 10.7) |
| Ulcerative colitis | 563.2<br>(464.7 to 692.6) | 189.4<br>(141.3 to 245.8) | 81.1<br>(53.3 to 117.4) | 37.4*<br>(28.0 to 48.7) | 8.2*<br>(0.8 to 17.0) |
| Crohn's disease | 186.4<br>(159.4 to 220.3) | 64.4<br>(48.7 to 83.1) | 28.4<br>(18.9 to 39.9) | 16.6*<br>(7.8 to 27.2) | -8.2<br>(-15.1 to 0.1) |
| Vascular intestinal disorders | 5.8<br>(3.8 to 8.4) | 41.0<br>(28.3 to 54.0) | 1.9<br>(1.1 to 3.0) | 26.0*<br>(17.0 to 34.4) | -0.8<br>(-7.9 to 5.8) |
| Gallbladder and biliary diseases | 2962.3<br>(2137.2 to 3971.2) | 554.6<br>(409.6 to 727.8) | 2.5<br>(1.5 to 4.0) | 36.2*<br>(28.4 to 45.1) | 7.2*<br>(1.0 to 14.2) |
| Pancreatitis | 604.5<br>(523.6 to 695.8) | 129.5<br>(104.8 to 160.0) | 35.3<br>(18.2 to 60.9) | 29.1*<br>(21.9 to 35.4) | 1.6<br>(-4.0 to 6.6) |
| Acute pancreatitis | 10.1<br>(7.8 to 13.1) | 104.7<br>(80.9 to 134.9) | 4.1<br>(2.6 to 6.0) | 19.7*<br>(12.4 to 26.7) | -5.8*<br>(-11.5 to -0.3) |
| Chronic pancreatitis | 594.4<br>(512.9 to 685.9) | 24.8<br>(16.8 to 35.4) | 31.2<br>(14.4 to 55.5) | 30.4*<br>(22.2 to 38.1) | 2.7<br>(-3.8 to 8.7) |
| Other digestive diseases | -- | -- | 62.4<br>(41.6 to 87.4) | 35.8*<br>(32.3 to 39.9) | 6.9*<br>(4.1 to 10.1) |
| **Neurological disorders** | **161128.6<br>(132644.6 to 197388.6)** | **43194.2<br>(30595.3 to 59546.9)** | **3818.9<br>(2546.2 to 5296.7)** | **28.8*<br>(27.1 to 30.6)** | **1.4*<br>(0.0 to 2.8)** |
| Alzheimer's disease and other dementias | 1037.0<br>(805.6 to 1298.9) | 192.8<br>(131.8 to 267.8) | 125.9<br>(78.5 to 186.0) | 21.6*<br>(18.0 to 25.2) | -4.3*<br>(-7.1 to -1.4) |
| Parkinson's disease | 491.1<br>(359.4 to 657.0) | 63.7<br>(36.8 to 98.0) | 73.9<br>(46.6 to 109.4) | 27.5*<br>(21.2 to 33.7) | 0.3<br>(-4.6 to 5.3) |
| Epilepsy | 1128.4<br>(835.0 to 1454.3) | 84.8<br>(54.5 to 125.4) | 323.0<br>(200.5 to 478.7) | 31.5*<br>(18.3 to 46.5) | 3.5<br>(-6.9 to 15.3) |
| Multiple sclerosis | 176.3<br>(161.1 to 193.9) | 1.0<br>(0.7 to 1.3) | 45.2<br>(32.3 to 58.4) | 27.0*<br>(20.4 to 33.7) | -0.1<br>(-5.2 to 5.2) |
| Motor neuron disease | 18.1<br>(13.6 to 24.3) | 6.5<br>(4.7 to 8.9) | 3.8<br>(2.4 to 5.8) | 35.2*<br>(30.6 to 39.5) | 6.4*<br>(2.8 to 9.8) |
| Headache disorders | 159616.0<br>(130819.1 to 196151.7) | 42845.3<br>(30238.6 to 59225.1) | 3165.8<br>(2074.6 to 4553.7) | 28.7*<br>(27.5 to 29.9) | 1.3*<br>(0.4 to 2.3) |
| Migraine | 74317.9<br>(65386.2 to 83694.3) | 3016.4<br>(1949.2 to 4391.8) | 2713.6<br>(1731.3 to 4001.6) | 29.0*<br>(27.7 to 30.4) | 1.5*<br>(0.5 to 2.6) |
| Medication overuse headache due to migraine | 6842.9<br>(4762.4 to 9189.2) | -- | 735.8<br>(416.4 to 1181.2) | 26.7*<br>(25.4 to 28.1) | -0.3<br>(-1.3 to 0.8) |
| Migraine | 67475.0<br>(58493.7 to 76644.5) | 3016.4<br>(1949.2 to 4391.8) | 1977.8<br>(1183.2 to 3041.1) | 29.9*<br>(28.2 to 31.7) | 2.2*<br>(0.9 to 3.7) |
| Tension-type headache | 116699.5<br>(80285.3 to 164620.0) | 39828.9<br>(27230.1 to 56224.9) | 452.1<br>(241.6 to 737.0) | 27.0*<br>(25.3 to 28.4) | -0.1<br>(-1.4 to 1.1) |
| Medication overuse headache due to tension-type headache | 2543.9<br>(1519.6 to 3750.0) | -- | 271.7<br>(129.9 to 506.3) | 26.7*<br>(24.3 to 29.0) | -0.3<br>(-2.2 to 1.6) |
| Tension-type headache | 114155.7<br>(78288.1 to 161819.6) | 39828.9<br>(27230.1 to 56224.9) | 180.4<br>(72.4 to 351.2) | 27.3*<br>(26.0 to 28.6) | 0.2<br>(-0.8 to 1.2) |
| Other neurological disorders | 1.9<br>(1.1 to 3.3) | -- | 81.3<br>(55.8 to 111.9) | 36.2*<br>(28.4 to 44.9) | 7.2*<br>(1.1 to 14.1) |
| Other neurological disorders | -- | -- | 80.7<br>(55.4 to 111.3) | 36.2*<br>(28.4 to 45.0) | 7.2*<br>(1.1 to 14.1) |
| Guillain-Barré syndrome due to other neurological disorders | 1.9<br>(1.1 to 3.3) | -- | 0.6<br>(0.3 to 1.0) | 31.0*<br>(28.0 to 33.8) | 3.1*<br>(0.7 to 5.3) |
| **Mental disorders** | **52826.7<br>(49712.0 to 55896.2)** | **20250.0<br>(17102.6 to 23656.3)** | **7230.0<br>(5367.4 to 9294.8)** | **24.6*<br>(23.6 to 25.7)** | **-1.9*<br>(-2.7 to -1.0)** |
| Schizophrenia | 1473.8<br>(1311.0 to 1658.1) | 32.9<br>(26.6 to 39.9) | 917.1<br>(693.0 to 1129.0) | 27.0*<br>(24.9 to 29.5) | -0.0<br>(-1.7 to 1.9) |
| Depressive disorders | 20574.0<br>(18091.3 to 23119.3) | 18314.9<br>(15205.4 to 21706.7) | 3196.1<br>(2205.8 to 4395.1) | 23.3*<br>(21.2 to 25.5) | -3.0*<br>(-4.6 to -1.2) |
| Major depressive disorder | 11616.6<br>(9714.5 to 13685.5) | 16999.2<br>(13806.3 to 20308.7) | 2296.4<br>(1539.3 to 3248.0) | 21.6*<br>(18.9 to 24.3) | -4.3*<br>(-6.4 to -2.1) |
| Dysthymia | 9474.2<br>(7808.9 to 11258.7) | 1315.6<br>(1002.7 to 1690.0) | 899.7<br>(591.6 to 1298.0) | 27.8*<br>(24.4 to 31.0) | 0.6<br>(-2.1 to 3.1) |
| Bipolar disorder | 2733.4<br>(2218.9 to 3464.8) | 208.0<br>(163.3 to 259.8) | 540.4<br>(330.5 to 817.8) | 27.9*<br>(25.7 to 30.2) | 0.7<br>(-1.1 to 2.5) |
| Anxiety disorders | 16410.3<br>(14762.0 to 18282.1) | 1694.4<br>(1499.8 to 1935.4) | 1520.9<br>(1066.7 to 2017.8) | 24.6*<br>(22.5 to 26.7) | -1.9*<br>(-3.6 to -0.3) |
| Eating disorders | -- | -- | -- | -- | -- |
| Anorexia nervosa | -- | -- | -- | -- | -- |
| Bulimia nervosa | -- | -- | -- | -- | -- |
| Autism spectrum disorders | 1284.4<br>(1154.0 to 1420.9) | -- | 188.6<br>(130.4 to 258.0) | 24.3*<br>(22.1 to 26.7) | -2.1*<br>(-3.9 to -0.2) |
| Attention-deficit/hyperactivity disorder | 1199.1<br>(932.4 to 1484.4) | -- | 14.1<br>(8.2 to 23.2) | 26.6*<br>(21.3 to 31.6) | -0.4<br>(-4.5 to 3.6) |
| Conduct disorder | -- | -- | -- | -- | -- |
| Idiopathic developmental intellectual disability | 2569.3<br>(1299.5 to 3870.9) | -- | 100.7<br>(44.4 to 175.8) | 14.5*<br>(8.8 to 17.6) | -9.8*<br>(-14.4 to -7.4) |
| Other mental disorders | 10314.8<br>(9067.5 to 11681.4) | -- | 752.2<br>(501.3 to 1034.0) | 26.7*<br>(25.1 to 28.3) | -0.2<br>(-1.6 to 1.0) |
| **Substance use disorders** | **8246.9<br>(7155.8 to 9487.6)** | **2740.8<br>(2255.6 to 3296.7)** | **1253.2<br>(903.1 to 1680.6)** | **32.8*<br>(29.4 to 37.2)** | **4.5*<br>(1.9 to 8.0)** |
| Alcohol use disorders | 6291.1<br>(5209.7 to 7483.1) | 2586.6<br>(2097.3 to 3139.8) | 609.7<br>(411.5 to 879.2) | 22.6*<br>(19.6 to 25.6) | -3.5*<br>(-5.9 to -1.1) |
| Alcohol dependence | 6277.4<br>(5196.0 to 7468.7) | 2586.6<br>(2097.3 to 3139.8) | 609.2<br>(411.1 to 878.4) | 22.6*<br>(19.6 to 25.7) | -3.5*<br>(-5.9 to -1.1) |
| Fetal alcohol syndrome | 13.7<br>(11.0 to 16.9) | -- | 0.5<br>(0.3 to 0.7) | 8.8*<br>(2.0 to 14.2) | -14.4*<br>(-19.7 to -10.1) |
| Drug use disorders | 2076.3<br>(1753.7 to 2462.3) | 154.2<br>(112.7 to 211.0) | 643.5<br>(451.9 to 882.8) | 44.3*<br>(38.3 to 50.6) | 13.6*<br>(8.9 to 18.6) |
| Opioid use disorders | 1398.1<br>(1103.0 to 1774.9) | 111.1<br>(71.7 to 165.0) | 556.8<br>(381.6 to 778.6) | 47.1*<br>(40.3 to 54.6) | 15.8*<br>(10.4 to 21.7) |
| Cocaine use disorders | 229.6<br>(202.4 to 258.8) | 3.1<br>(1.9 to 4.4) | 30.2<br>(19.5 to 43.6) | 28.2*<br>(21.4 to 35.4) | 0.9<br>(-4.5 to 6.6) |

| | | | | | |
|---|---|---|---|---|---|
| Amphetamine use disorders | 64.3 (43.2 to 94.6) | 8.9 (5.0 to 13.5) | 8.5 (4.5 to 14.4) | 29.1* (19.9 to 37.9) | 1.6 (-5.7 to 8.5) |
| Cannabis use disorders | 335.1 (259.6 to 428.2) | 31.2 (24.2 to 39.2) | 9.7 (5.8 to 15.0) | 25.0* (19.7 to 30.4) | -1.6 (-5.8 to 2.7) |
| Other drug use disorders | 123.5 (99.8 to 150.9) | -- | 38.3 (25.7 to 52.1) | 28.9* (23.7 to 34.2) | 1.5 (-2.6 to 5.6) |
| **Diabetes and kidney diseases** | **95955.1 (87508.2 to 105026.9)** | **4207.8 (3307.4 to 5176.8)** | **4969.4 (3433.5 to 6892.9)** | **32.4* (25.4 to 40.2)** | **4.2 (-1.3 to 10.4)** |
| Diabetes mellitus | 51351.1 (45722.8 to 58171.8) | 2256.3 (1690.3 to 2934.5) | 4375.8 (2939.1 to 6201.4) | 33.1* (25.2 to 41.9) | 4.8 (-1.4 to 11.7) |
| Diabetes mellitus type 1 | 835.8 (745.1 to 925.5) | 8.4 (5.7 to 11.6) | 69.8 (47.4 to 96.8) | 24.6* (21.5 to 27.8) | -2.0 (-4.3 to 0.6) |
| Uncomplicated diabetes mellitus type 1 | 453.1 (379.6 to 525.1) | 8.4 (5.7 to 11.6) | 20.9 (12.7 to 30.5) | 20.5* (15.6 to 25.1) | -5.2* (-9.0 to -1.5) |
| Vision loss due to diabetes mellitus type 1 retinopathy | 8.9 (6.5 to 11.9) | -- | 0.7 (0.5 to 1.1) | 9.8* (2.5 to 16.4) | -13.6* (-19.3 to -8.4) |
| Diabetic neuropathy due to diabetes mellitus type 1 | 373.8 (313.2 to 448.5) | -- | 48.2 (32.2 to 69.2) | 26.7* (21.5 to 31.4) | -0.3 (-4.4 to 3.4) |
| Diabetes mellitus type 2 | 50515.3 (44841.5 to 57375.5) | 2248.0 (1684.0 to 2925.9) | 4306.0 (2888.4 to 6106.1) | 33.3* (25.2 to 42.3) | 4.9 (-1.4 to 12.0) |
| Uncomplicated diabetes mellitus type 2 | 26367.6 (21290.1 to 31220.6) | 2248.0 (1684.0 to 2925.9) | 1217.7 (736.0 to 1839.8) | 30.6* (21.1 to 39.7) | 2.8 (-4.7 to 9.9) |
| Vision loss due to diabetes mellitus type 2 retinopathy | 572.8 (446.1 to 733.6) | -- | 46.4 (31.2 to 65.3) | 26.7* (22.2 to 31.7) | -0.2 (-3.8 to 3.6) |
| Diabetic neuropathy due to diabetes mellitus type 2 | 23574.9 (19615.6 to 28540.8) | -- | 3041.9 (2016.6 to 4335.6) | 34.5* (25.4 to 43.9) | 5.9 (-1.3 to 13.3) |
| Chronic kidney disease | 59313.2 (50420.1 to 69904.3) | 1907.0 (1228.6 to 2724.2) | 593.4 (430.1 to 799.6) | 27.0* (19.8 to 34.4) | -0.1 (-5.7 to 5.8) |
| Chronic kidney disease due to diabetes mellitus type 1 | 232.6 (192.8 to 279.9) | 6.2 (4.0 to 9.0) | 29.7 (18.0 to 45.4) | 23.5* (14.7 to 33.0) | -2.8 (-9.7 to 4.7) |
| Albuminuria with preserved GFR due to type 1 diabetes mellitus | 78.4 (57.7 to 103.1) | -- | -- | -- | -- |
| End-stage chronic kidney disease due to diabetes mellitus type 1 | 48.3 (26.7 to 77.6) | -- | 19.9 (9.9 to 34.3) | 23.1* (11.0 to 36.4) | -3.1 (-12.6 to 7.3) |
| Stage III chronic kidney disease due to diabetes mellitus type 1 | 76.7 (61.8 to 95.6) | 6.2 (4.0 to 9.0) | 1.5 (1.0 to 2.2) | 36.2* (19.1 to 57.2) | 7.2 (-6.2 to 23.7) |
| Stage IV chronic kidney disease due to diabetes mellitus type 1 | 19.6 (12.3 to 29.1) | -- | 2.6 (1.4 to 4.3) | 25.5* (9.5 to 44.2) | -1.2 (-13.8 to 13.5) |
| Stage V chronic kidney disease due to diabetes mellitus type 1 | 9.7 (6.7 to 13.8) | -- | 5.6 (3.5 to 8.6) | 21.1* (10.7 to 32.4) | -4.6 (-12.9 to 4.2) |
| Chronic kidney disease due to diabetes mellitus type 2 | 12308.3 (10115.3 to 14851.8) | 277.2 (178.5 to 396.8) | 159.8 (113.5 to 219.9) | 27.9* (20.3 to 36.3) | 0.7 (-5.3 to 7.3) |
| Albuminuria with preserved GFR due to type 2 diabetes mellitus | 8089.6 (6069.0 to 10519.3) | -- | -- | -- | -- |
| End-stage chronic kidney disease due to diabetes mellitus type 2 | 141.2 (98.7 to 185.2) | -- | 55.6 (34.6 to 81.5) | 21.4* (12.2 to 31.4) | -4.4 (-11.7 to 3.4) |
| Stage III chronic kidney disease due to diabetes mellitus type 2 | 3708.9 (3070.1 to 4502.7) | 277.2 (178.5 to 396.8) | 15.2 (9.2 to 24.4) | 25.7* (8.4 to 42.7) | -1.1 (-14.7 to 12.4) |
| Stage IV chronic kidney disease due to diabetes mellitus type 2 | 253.2 (161.6 to 371.8) | -- | 28.3 (15.6 to 46.5) | 33.6* (18.6 to 51.3) | 5.2 (-6.7 to 19.1) |
| Stage V chronic kidney disease due to diabetes mellitus type 2 | 115.4 (81.4 to 161.9) | -- | 60.7 (37.8 to 91.2) | 32.3* (20.6 to 45.1) | 4.1 (-5.0 to 14.2) |
| Chronic kidney disease due to hypertension | 1608.9 (1381.1 to 1875.9) | 41.6 (26.8 to 59.4) | 110.3 (77.0 to 149.6) | 29.5* (21.8 to 38.2) | 2.0 (-4.2 to 8.8) |
| Albuminuria with preserved GFR due to hypertension | 419.6 (312.1 to 544.0) | -- | -- | -- | -- |
| End-stage chronic kidney disease due to hypertension | 90.4 (67.0 to 120.1) | -- | 35.6 (22.0 to 51.2) | 25.8* (15.1 to 37.6) | -1.0 (-9.4 to 8.3) |
| Stage III chronic kidney disease due to hypertension | 834.6 (689.6 to 1016.9) | 41.6 (26.8 to 59.4) | 9.4 (5.8 to 14.6) | 26.0* (9.3 to 44.4) | -0.9 (-14.0 to 13.7) |
| Stage IV chronic kidney disease due to hypertension | 181.7 (115.6 to 268.8) | -- | 20.7 (11.5 to 33.9) | 33.6* (18.6 to 51.6) | 5.2 (-6.7 to 19.3) |
| Stage V chronic kidney disease due to hypertension | 82.5 (58.0 to 115.9) | -- | 44.7 (27.8 to 66.8) | 31.6* (20.8 to 43.9) | 3.6 (-4.9 to 13.3) |
| Chronic kidney disease due to glomerulonephritis | 2157.6 (1838.9 to 2513.2) | 138.9 (89.4 to 198.4) | 76.4 (51.5 to 107.8) | 29.5* (21.1 to 38.2) | 2.0 (-4.6 to 8.8) |
| Albuminuria with preserved GFR due to glomerulonephritis | 353.4 (263.3 to 461.2) | -- | -- | -- | -- |
| End-stage chronic kidney disease due to glomerulonephritis | 73.2 (47.1 to 109.4) | -- | 28.5 (16.3 to 45.7) | 23.7* (11.7 to 35.4) | -2.6 (-12.1 to 6.6) |
| Stage III chronic kidney disease due to glomerulonephritis | 1563.5 (1295.0 to 1894.7) | 138.9 (89.4 to 198.4) | 5.2 (3.3 to 7.9) | 45.4* (24.6 to 66.8) | 14.4 (-1.9 to 31.3) |
| Stage IV chronic kidney disease due to glomerulonephritis | 115.2 (73.4 to 170.3) | -- | 13.4 (7.4 to 21.9) | 32.3* (16.3 to 50.1) | 4.1 (-8.4 to 18.1) |
| Stage V chronic kidney disease due to glomerulonephritis | 52.3 (36.8 to 73.8) | -- | 29.3 (18.2 to 44.2) | 31.8* (20.4 to 42.8) | 3.7 (-5.3 to 12.4) |
| Chronic kidney disease due to other and unspecified causes | 43005.8 (36553.2 to 50647.5) | 1443.0 (929.4 to 2060.6) | 217.2 (155.6 to 294.9) | 24.7* (15.8 to 33.8) | -1.9 (-8.8 to 5.4) |
| Albuminuria with preserved GFR due to other and unspecified causes | 23016.6 (17313.1 to 29978.1) | -- | -- | -- | -- |
| End-stage chronic kidney disease due to other causes | 101.6 (66.8 to 143.3) | -- | 39.4 (22.4 to 60.9) | 21.0* (11.0 to 32.2) | -4.8 (-12.6 to 4.1) |
| Stage III chronic kidney disease due to other causes | 19493.7 (16189.8 to 23701.2) | 1443.0 (929.4 to 2060.6) | 82.8 (50.9 to 130.4) | 17.9* (4.4 to 30.9) | -7.2 (-17.8 to 3.1) |
| Stage IV chronic kidney disease due to other causes | 270.1 (172.1 to 397.9) | -- | 30.0 (16.7 to 49.0) | 33.5* (18.4 to 51.6) | 5.1 (-6.8 to 19.3) |
| Stage V chronic kidney disease due to other causes | 123.9 (87.3 to 174.7) | -- | 65.0 (40.3 to 96.3) | 32.8* (21.3 to 45.3) | 4.5 (-4.5 to 14.4) |
| Acute glomerulonephritis | 3.0 (1.9 to 4.2) | 44.4 (28.8 to 63.1) | 0.2 (0.1 to 0.3) | -0.5 (-6.9 to 4.0) | -21.7* (-26.7 to -18.2) |
| **Skin and subcutaneous diseases** | **97529.3 (91088.5 to 104727.7)** | **208766.7 (185738.3 to 236112.9)** | **1725.7 (1178.4 to 2535.0)** | **27.9* (27.0 to 28.7)** | **0.7 (-0.0 to 1.3)** |
| Dermatitis | 11019.6 (9315.4 to 13153.8) | 16247.0 (11844.1 to 21271.4) | 363.8 (224.5 to 567.5) | 24.3* (22.6 to 25.9) | -2.2* (-3.5 to -0.9) |
| Atopic dermatitis | 5466.8 (4782.0 to 6217.7) | 816.9 (658.3 to 979.0) | 230.4 (129.1 to 382.5) | 24.0* (21.7 to 26.1) | -2.4* (-4.2 to -0.7) |
| Contact dermatitis | 5084.7 (3457.4 to 7235.9) | 14157.5 (9655.5 to 19146.0) | 125.0 (69.7 to 204.1) | 25.2* (22.9 to 27.7) | -1.4 (-3.2 to 0.5) |
| Seborrhoeic dermatitis | 622.6 (503.9 to 770.2) | 1272.6 (1009.8 to 1553.5) | 8.3 (4.5 to 13.8) | 18.3* (13.5 to 23.4) | -6.9* (-10.7 to -2.9) |
| Psoriasis | 5557.7 (5130.2 to 5999.3) | 607.8 (529.6 to 689.1) | 471.1 (330.7 to 620.1) | 30.0* (27.8 to 32.3) | 2.3* (0.6 to 4.1) |
| Bacterial skin diseases | 419.1 (381.2 to 455.8) | 10810.0 (9750.0 to 11780.8) | 9.2 (5.8 to 13.7) | 24.4* (19.5 to 29.0) | -2.1 (-5.9 to 1.6) |
| Cellulitis | 130.3 (106.2 to 159.1) | 2659.3 (2222.6 to 3201.3) | 7.3 (4.7 to 10.7) | 22.4* (17.0 to 28.1) | -3.7 (-7.9 to 0.8) |
| Pyoderma | 340.7 (310.8 to 371.9) | 8150.7 (7234.1 to 9039.9) | 1.9 (0.7 to 4.0) | 32.8* (28.3 to 37.5) | 4.5* (1.0 to 8.2) |
| Impetigo | 26.2 (23.3 to 29.4) | 488.2 (437.0 to 544.1) | 0.1 (0.1 to 0.3) | 32.7* (25.3 to 40.3) | 4.5 (-1.4 to 10.4) |
| Abscess and other bacterial skin diseases | 314.5 (284.5 to 345.8) | 7662.5 (6730.7 to 8552.1) | 1.7 (0.7 to 3.7) | 32.8* (28.1 to 37.9) | 4.5* (0.8 to 8.6) |
| Scabies | 5240.0 (3516.0 to 7304.3) | 15672.5 (10556.6 to 21372.2) | 132.2 (63.5 to 236.1) | 26.0* (24.2 to 27.8) | -0.8 (-2.2 to 0.6) |
| Fungal skin diseases | 33868.3 (27640.6 to 41126.9) | 107765.7 (85145.3 to 133696.6) | 187.7 (74.2 to 380.4) | 27.6* (26.9 to 28.2) | 0.4 (-0.1 to 0.9) |
| Tinea capitis | 1544.6 (1040.3 to 2112.3) | 2576.5 (1837.7 to 3443.8) | 8.5 (3.3 to 18.9) | 9.0* (5.7 to 13.0) | -14.2* (-16.8 to -11.0) |
| Other fungal skin diseases | 32323.7 (25942.4 to 39699.5) | 105189.2 (82554.2 to 131780.0) | 179.2 (70.4 to 366.7) | 28.7* (28.1 to 29.2) | 1.3* (0.8 to 1.7) |

| | | | | | |
|---|---|---|---|---|---|
| Viral skin diseases | 2362.5 (2103.2 to 2644.9) | 1664.2 (1488.5 to 1830.5) | 70.4 (44.7 to 106.0) | 21.8* (19.3 to 24.4) | -4.1* (-6.1 to -2.1) |
| Viral warts | 2087.7 (1828.5 to 2389.3) | 1327.1 (1158.8 to 1485.3) | 62.2 (39.5 to 94.3) | 21.1* (18.5 to 23.9) | -4.7* (-6.8 to -2.5) |
| Molluscum contagiosum | 274.8 (242.7 to 313.9) | 337.1 (298.8 to 378.4) | 8.2 (5.2 to 12.2) | 27.6* (20.6 to 35.5) | 0.4 (-5.1 to 6.6) |
| Acne vulgaris | 953.5 (833.7 to 1078.2) | 425.6 (368.5 to 483.3) | 20.1 (11.8 to 32.2) | 34.8* (30.7 to 39.0) | 6.1* (2.9 to 9.4) |
| Alopecia areata | 797.2 (718.5 to 881.9) | 1430.7 (1302.3 to 1565.0) | 25.5 (16.2 to 38.2) | 24.0* (20.4 to 27.8) | -2.4 (-5.2 to 0.6) |
| Pruritus | 4734.1 (3313.5 to 6824.2) | 3671.5 (2575.3 to 5027.4) | 49.6 (22.0 to 93.2) | 29.1* (27.5 to 30.7) | 1.7* (0.4 to 2.9) |
| Urticaria | 2561.4 (1835.0 to 3491.6) | 4498.9 (3282.8 to 6218.5) | 148.0 (88.6 to 232.4) | 27.9* (25.5 to 30.4) | 0.7 (-1.2 to 2.6) |
| Decubitus ulcer | 57.3 (40.0 to 80.1) | 207.9 (143.0 to 285.1) | 10.1 (6.0 to 16.1) | 26.8* (25.0 to 28.6) | -0.2 (-1.6 to 1.2) |
| Other skin and subcutaneous diseases | 43649.3 (39785.5 to 47141.3) | 45764.8 (41589.4 to 49739.0) | 238.0 (116.5 to 437.8) | 32.7* (31.8 to 33.5) | 4.4* (3.8 to 5.1) |
| **Sense organ diseases** | **195443.4 (188950.8 to 202317.6)** | **--** | **5648.2 (3737.9 to 8315.7)** | **27.1* (25.6 to 28.5)** | **0.1 (-1.2 to 1.2)** |
| Blindness and vision impairment | 121418.2 (113038.9 to 129805.4) | -- | 2649.4 (1737.0 to 3999.2) | 27.3* (25.6 to 29.0) | 0.2 (-1.1 to 1.5) |
| Glaucoma | 581.0 (458.4 to 732.7) | -- | 68.8 (45.1 to 99.4) | 24.6* (20.6 to 28.9) | -1.9 (-5.1 to 1.5) |
| Cataract | 8181.4 (6136.1 to 10634.0) | -- | 593.1 (399.0 to 857.6) | 32.9* (29.0 to 36.9) | 4.6* (1.5 to 7.7) |
| Age-related macular degeneration | 574.2 (428.3 to 745.7) | -- | 46.3 (30.1 to 66.3) | 25.7* (20.3 to 30.8) | -1.1 (-5.3 to 2.9) |
| Refraction disorders | 13210.9 (9903.0 to 16744.3) | -- | 592.5 (378.1 to 890.2) | 21.9* (17.5 to 26.0) | -4.0* (-7.5 to -0.8) |
| Near vision loss | 106054.2 (97109.4 to 114522.7) | -- | 1074.6 (518.9 to 1944.5) | 28.4* (27.5 to 29.3) | 1.0* (0.3 to 1.8) |
| Other vision loss | 3203.7 (2523.3 to 4031.1) | -- | 274.1 (182.9 to 379.5) | 24.8* (22.0 to 27.5) | -1.8 (-4.0 to 0.4) |
| Age-related and other hearing loss | 124416.8 (117194.5 to 131145.6) | -- | 2780.6 (1805.9 to 4156.6) | 26.9* (24.3 to 29.5) | -0.1 (-2.2 to 1.9) |
| Other sense organ diseases | 8833.3 (7915.5 to 9803.3) | -- | 218.1 (130.5 to 325.2) | 28.3* (26.3 to 30.2) | 1.0 (-0.6 to 2.5) |
| Chronic other sense organ diseases | 8145.1 (7224.9 to 9091.2) | -- | 208.9 (124.5 to 308.9) | 28.3* (26.2 to 30.3) | 1.0 (-0.7 to 2.6) |
| Acute other sense organ diseases | 688.2 (619.5 to 761.2) | -- | 9.3 (5.5 to 14.3) | 28.2* (22.5 to 33.8) | 0.9 (-3.6 to 5.3) |
| **Musculoskeletal disorders** | **122951.7 (110088.6 to 137037.3)** | **27887.1 (21199.7 to 36363.7)** | **12506.6 (8862.0 to 17536.8)** | **26.7* (25.3 to 28.3)** | **-0.3 (-1.4 to 1.0)** |
| Rheumatoid arthritis | 2235.4 (1890.6 to 2585.8) | 128.8 (86.8 to 178.8) | 294.9 (196.1 to 402.4) | 36.6* (31.5 to 41.3) | 7.5* (3.5 to 11.2) |
| Osteoarthritis | 37443.9 (32266.9 to 43247.4) | 2036.3 (1529.5 to 2593.4) | 1206.9 (598.4 to 2403.0) | 28.4* (27.4 to 29.5) | 1.1* (0.3 to 2.0) |
| Osteoarthritis of the hip | 4775.3 (4343.3 to 5272.2) | 289.8 (234.9 to 346.2) | 154.0 (78.3 to 315.2) | 33.4* (31.5 to 35.6) | 5.0* (3.5 to 6.7) |
| Osteoarthritis of the knee | 32668.6 (27625.4 to 38327.4) | 1746.5 (1249.1 to 2290.9) | 1052.9 (515.8 to 2110.2) | 27.8* (26.7 to 28.9) | 0.6 (-0.3 to 1.4) |
| Low back pain | 48281.6 (35930.9 to 63937.3) | 20096.1 (13912.1 to 28304.9) | 5486.5 (3573.4 to 8311.4) | 25.2* (24.0 to 26.3) | -1.4* (-2.4 to -0.6) |
| Low back pain with leg pain | 17886.5 (13033.7 to 24435.2) | 7429.2 (4908.7 to 10698.8) | 2545.5 (1614.8 to 3941.4) | 25.2* (23.8 to 26.5) | -1.5* (-2.5 to -0.5) |
| Low back pain without leg pain | 30395.1 (22389.3 to 40913.5) | 12666.9 (8620.0 to 17726.0) | 2941.0 (1858.9 to 4481.2) | 25.2* (23.9 to 26.4) | -1.4* (-2.5 to -0.5) |
| Neck pain | 28140.1 (19969.4 to 39140.3) | 4735.1 (2952.6 to 7168.4) | 2777.1 (1746.4 to 4290.4) | 26.1* (23.4 to 28.8) | -0.8 (-2.9 to 1.4) |
| Gout | 4553.2 (3680.8 to 5528.8) | 890.8 (632.2 to 1168.1) | 144.6 (94.4 to 205.7) | 29.8* (25.6 to 34.0) | 2.2 (-1.1 to 5.5) |
| Other musculoskeletal disorders | 30753.6 (21944.3 to 41069.7) | -- | 2596.6 (1563.5 to 3936.0) | 28.7* (22.1 to 34.7) | 1.3 (-3.9 to 6.0) |
| **Other non-communicable diseases** | **240509.3 (226053.4 to 255940.8)** | **118540.2 (102417.1 to 136689.7)** | **3036.6 (1971.7 to 4428.8)** | **24.4* (23.1 to 25.7)** | **-2.1* (-3.1 to -1.1)** |
| Congenital anomalies | 3807.2 (3571.7 to 4081.6) | -- | 483.3 (339.1 to 647.2) | 27.0* (24.5 to 29.7) | -0.1 (-2.0 to 2.1) |
| Neural tube defects | 106.8 (95.2 to 119.1) | -- | 31.2 (21.8 to 41.7) | 31.4* (25.4 to 37.4) | 3.4 (-1.3 to 8.2) |
| Severe motor and cognitive impairment due to anencephaly | -- | -- | -- | -- | -- |
| Encephalocoele due to neural tube defects | 14.9 (12.3 to 17.7) | -- | 2.6 (1.7 to 3.6) | 26.2* (22.5 to 30.0) | -0.7 (-3.6 to 2.3) |
| Spina bifida due to neural tube defects | 91.9 (82.0 to 103.2) | -- | 28.6 (19.8 to 38.4) | 31.9* (25.6 to 38.3) | 3.8 (-1.2 to 8.8) |
| Congenital heart anomalies | 117.1 (107.0 to 128.4) | -- | 6.9 (3.1 to 11.7) | 27.9* (22.3 to 32.9) | 0.7 (-3.8 to 4.6) |
| Critical malformations of great vessels, congenital valvular heart disease and patent ductus arteriosus | 19.2 (16.6 to 22.4) | -- | 1.2 (0.5 to 2.1) | 27.0* (24.2 to 29.7) | -0.1 (-2.3 to 2.1) |
| Other congenital heart anomalies | 41.7 (38.1 to 45.8) | -- | 2.4 (1.0 to 4.3) | 28.3* (18.1 to 37.9) | 1.0 (-7.0 to 8.6) |
| Severe congenital heart anomalies excluding single ventricle heart defects | 50.0 (44.4 to 56.0) | -- | 3.0 (1.4 to 5.0) | 28.4* (20.3 to 36.6) | 1.1 (-5.3 to 7.5) |
| Single ventricle and single ventricle pathway congenital heart anomalies | 1.3 (1.1 to 1.6) | -- | 0.1 (0.1 to 0.1) | 25.5* (19.6 to 30.3) | -1.2 (-5.9 to 2.6) |
| Ventricular septal defect and atrial septal defect | 4.8 (3.9 to 5.8) | -- | 0.2 (0.1 to 0.3) | 21.5* (19.0 to 23.8) | -4.4* (-6.3 to -2.6) |
| Orofacial clefts | 469.4 (429.4 to 508.8) | -- | 12.4 (8.3 to 17.9) | 29.7* (20.6 to 40.0) | 2.1 (-5.1 to 10.2) |
| Down's syndrome | 109.6 (95.7 to 124.9) | -- | 16.2 (11.1 to 22.0) | 19.7* (16.2 to 23.2) | -5.8* (-8.5 to -3.0) |
| Turner syndrome | 10.2 (8.8 to 11.8) | -- | 0.1 (0.1 to 0.3) | 23.7* (20.4 to 26.5) | -2.7* (-5.2 to -0.5) |
| Klinefelter syndrome | 8.3 (7.0 to 9.9) | -- | -- | 31.6* (29.6 to 33.6) | 3.6* (2.0 to 5.2) |
| Other chromosomal abnormalities | 187.5 (150.1 to 228.9) | -- | 28.0 (18.5 to 38.9) | 24.1* (19.7 to 27.9) | -2.3 (-5.8 to 0.7) |
| Congenital musculoskeletal and limb anomalies | 1440.0 (1279.8 to 1649.8) | -- | 207.1 (138.9 to 283.0) | 27.4* (25.1 to 29.8) | 0.3 (-1.5 to 2.1) |
| Polydactyly and syndactyly | 3.7 (2.9 to 4.4) | -- | 0.0 (0.0 to 0.1) | 15.6* (13.4 to 17.7) | -9.0* (-10.7 to -7.4) |
| Congenital limb deficiency | 555.6 (493.4 to 636.8) | -- | 80.1 (54.4 to 109.2) | 27.6* (24.0 to 31.4) | 0.4 (-2.4 to 3.4) |
| Other congenital musculoskeletal anomalies | 880.8 (782.8 to 1009.1) | -- | 126.9 (84.9 to 173.5) | 27.3* (24.5 to 30.2) | 0.2 (-2.0 to 2.5) |
| Urogenital congenital anomalies | 55.4 (49.4 to 61.9) | -- | 2.5 (1.5 to 3.7) | 13.9* (8.3 to 19.8) | -10.3* (-14.8 to -5.7) |
| Digestive congenital anomalies | 900.2 (810.1 to 1004.3) | -- | 39.8 (26.5 to 56.7) | 18.1* (12.2 to 24.0) | -7.1* (-11.7 to -2.4) |
| Congenital diaphragmatic hernia | 5.7 (5.0 to 6.4) | -- | 0.1 (0.1 to 0.2) | 29.1* (27.6 to 30.5) | 1.6* (0.5 to 2.7) |
| Congenital atresia and/or stenosis of the digestive tract | 0.2 (0.2 to 0.3) | -- | -- | 40.6* (34.7 to 46.0) | 10.6* (6.0 to 14.9) |
| Congenital malformations of the abdominal wall after treatment | 22.2 (19.3 to 25.2) | -- | 1.0 (0.6 to 1.4) | 28.0* (26.6 to 29.3) | 0.8 (-0.3 to 1.7) |
| Other congenital malformations of the digestive tract | 872.2 (782.5 to 976.2) | -- | 38.7 (25.7 to 55.1) | 17.8* (11.8 to 23.9) | -7.3* (-12.0 to -2.5) |
| Other congenital anomalies | 1064.7 (916.3 to 1236.9) | -- | 139.1 (93.9 to 194.2) | 29.6* (22.1 to 37.7) | 2.0 (-3.9 to 8.4) |

| | | | | | |
|---|---|---|---|---|---|
| Other congenital birth defects | -- | -- | 10.7<br>(7.6 to 14.1) | 47.6*<br>(41.9 to 53.1) | 16.1*<br>(11.7 to 20.5) |
| Hearing loss due to other congenital anomalies | 1064.7<br>(916.3 to 1236.9) | -- | 128.4<br>(85.7 to 182.2) | 28.3*<br>(20.2 to 37.0) | 1.0<br>(-5.4 to 7.8) |
| Urinary diseases and male infertility | 12013.9<br>(10233.8 to 13875.1) | 22748.1<br>(18999.3 to 27132.9) | 428.9<br>(273.4 to 616.3) | 28.1*<br>(26.1 to 29.8) | 0.8<br>(-0.7 to 2.2) |
| Urinary tract infections | 261.3<br>(203.0 to 331.5) | 13536.4<br>(10403.6 to 17448.8) | 8.5<br>(4.8 to 13.1) | 38.2*<br>(29.7 to 46.6) | 8.8*<br>(2.1 to 15.4) |
| Urolithiasis | 298.9<br>(220.5 to 399.8) | 7867.3<br>(5832.6 to 10414.4) | 21.6<br>(13.4 to 32.0) | 20.4*<br>(13.7 to 28.1) | -5.3<br>(-10.5 to 0.8) |
| Benign prostatic hyperplasia | 11766.1<br>(9888.6 to 13708.6) | 1344.4<br>(1103.4 to 1616.6) | 390.9<br>(245.2 to 566.1) | 28.9*<br>(27.0 to 30.7) | 1.5<br>(-0.0 to 2.9) |
| Male infertility | -- | -- | -- | -- | -- |
| Other urinary diseases | -- | -- | 8.0<br>(5.3 to 11.3) | 3.7<br>(-1.0 to 9.0) | -18.3*<br>(-22.1 to -14.2) |
| Gynecological diseases | 22659.2<br>(20005.0 to 25894.8) | 1208.2<br>(813.3 to 1681.9) | 125.6<br>(70.1 to 224.9) | 18.7*<br>(13.5 to 22.6) | -6.6*<br>(-10.7 to -3.5) |
| Uterine fibroids | 12212.7<br>(10425.6 to 14427.6) | 30.3<br>(19.1 to 46.7) | 89.2<br>(50.2 to 154.1) | 16.0*<br>(9.2 to 21.3) | -8.7*<br>(-14.0 to -4.5) |
| Polycystic ovarian syndrome | -- | -- | -- | -- | -- |
| Female infertility | -- | -- | -- | -- | -- |
| Endometriosis | -- | -- | -- | -- | -- |
| Genital prolapse | 11681.7<br>(9544.1 to 14381.2) | 1177.9<br>(789.9 to 1649.6) | 36.4<br>(17.4 to 68.3) | 25.8*<br>(23.7 to 27.9) | -1.0<br>(-2.6 to 0.6) |
| Premenstrual syndrome | -- | -- | -- | -- | -- |
| Other gynecological diseases | -- | -- | -- | -- | -- |
| Other gynecological diseases | -- | -- | -- | -- | -- |
| Other gynecological diseases with anemia | -- | -- | -- | -- | -- |
| Hemoglobinopathies and hemolytic anaemias | 78807.4<br>(77086.7 to 80598.1) | -- | 156.1<br>(102.9 to 232.8) | 5.7<br>(-3.6 to 16.1) | -16.8*<br>(-24.1 to -8.6) |
| Thalassemias | 0.1<br>(0.1 to 0.1) | -- | -- | 24.0*<br>(5.6 to 45.2) | -2.4<br>(-16.9 to 14.3) |
| Thalassaemias trait | 13673.7<br>(13133.8 to 14243.5) | -- | 60.3<br>(39.3 to 92.0) | -0.9<br>(-14.1 to 16.0) | -22.0*<br>(-32.4 to -8.7) |
| Sickle cell disorders | 1.1<br>(0.9 to 1.4) | -- | 0.2<br>(0.1 to 0.3) | 65.7*<br>(53.0 to 79.4) | 30.4*<br>(20.4 to 41.2) |
| Sickle cell trait | 15827.3<br>(14246.4 to 17627.4) | -- | 28.8<br>(18.5 to 42.9) | 16.5*<br>(3.7 to 29.4) | -8.3<br>(-18.4 to 1.8) |
| G6PD deficiency | 13710.2<br>(13172.4 to 14247.8) | -- | 1.0<br>(0.7 to 1.4) | 6.9<br>(-6.7 to 20.2) | -15.8*<br>(-26.5 to -5.4) |
| G6PD trait | 40102.8<br>(39455.4 to 40753.9) | -- | -- | -0.9<br>(-30.2 to 18.6) | -22.0*<br>(-45.1 to -6.7) |
| Other hemoglobinopathies and hemolytic anaemias | 3401.2<br>(3150.6 to 3667.6) | -- | 65.7<br>(43.4 to 98.2) | 7.9<br>(-1.3 to 18.0) | -15.1*<br>(-22.3 to -7.1) |
| Endocrine, metabolic, blood, and immune disorders | 5900.4<br>(5304.4 to 6612.3) | -- | 166.6<br>(117.0 to 237.1) | 20.4*<br>(14.1 to 26.9) | -5.3*<br>(-10.2 to -0.1) |
| Anemia due to endocrine, metabolic, blood, and immune disorders | 3650.9<br>(3425.7 to 3912.8) | -- | 85.5<br>(57.1 to 123.4) | 10.7*<br>(1.2 to 20.6) | -12.9*<br>(-20.4 to -5.0) |
| Endocrine, metabolic, blood, and immune disorders | 2231.8<br>(1711.7 to 2879.3) | -- | 79.5<br>(50.9 to 117.6) | 32.7*<br>(26.4 to 39.8) | 4.5<br>(-0.5 to 10.0) |
| Heart failure due to endocrine, metabolic, blood, and immune disorders | 17.7<br>(12.4 to 24.5) | -- | 1.6<br>(1.0 to 2.5) | 23.6*<br>(19.1 to 27.9) | -2.7<br>(-6.3 to 0.7) |
| Oral disorders | 192390.6<br>(171701.0 to 215188.1) | 94583.9<br>(79055.7 to 112633.9) | 1676.2<br>(995.4 to 2691.2) | 25.7*<br>(24.7 to 26.5) | -1.1*<br>(-1.8 to -0.4) |
| Caries of deciduous teeth | -- | -- | -- | -- | -- |
| Caries of permanent teeth | 119063.9<br>(95037.1 to 151422.0) | 85836.8<br>(70963.6 to 103576.9) | 86.9<br>(38.3 to 169.8) | 22.3*<br>(20.7 to 23.8) | -3.8*<br>(-5.0 to -2.6) |
| Periodontal disease | 89425.6<br>(69274.9 to 108047.5) | 6198.6<br>(3771.8 to 8487.8) | 582.4<br>(235.6 to 1223.0) | 28.7*<br>(27.9 to 29.6) | 1.3*<br>(0.7 to 2.0) |
| Edentulism and severe tooth loss | 27069.1<br>(21756.3 to 32914.0) | 2548.4<br>(1628.6 to 3581.8) | 757.7<br>(473.8 to 1110.4) | 23.4*<br>(22.2 to 24.5) | -2.9*<br>(-3.8 to -2.0) |
| Other oral disorders | 8779.9<br>(7773.2 to 9824.6) | -- | 249.2<br>(153.0 to 379.3) | 27.2*<br>(25.8 to 28.5) | 0.1<br>(-0.9 to 1.2) |
| **Injuries** | **118602.1<br>(112253.1 to 126504.1)** | **26916.8<br>(22395.0 to 31568.8)** | **4597.2<br>(3335.1 to 6145.9)** | **32.8*<br>(31.7 to 33.9)** | **4.5*<br>(3.7 to 5.4)** |
| **Transport injuries** | **20603.0<br>(19036.7 to 22324.0)** | **3027.6<br>(2145.6 to 4085.7)** | **1176.5<br>(842.4 to 1565.1)** | **30.5*<br>(29.3 to 31.6)** | **2.7*<br>(1.8 to 3.6)** |
| Road injuries | 16751.6<br>(15431.4 to 18308.8) | 2691.8<br>(1844.6 to 3739.7) | 954.2<br>(684.0 to 1282.3) | 30.2*<br>(29.1 to 31.5) | 2.5*<br>(1.6 to 3.5) |
| Pedestrian road injuries | 4314.0<br>(3656.5 to 5250.1) | 546.9<br>(319.1 to 899.3) | 247.2<br>(173.6 to 344.9) | 26.1*<br>(24.4 to 27.7) | -0.8<br>(-2.1 to 0.5) |
| Cyclist road injuries | 2943.9<br>(2435.0 to 3479.5) | 702.4<br>(398.9 to 1122.4) | 161.5<br>(111.8 to 223.5) | 35.9*<br>(34.0 to 37.9) | 7.0*<br>(5.5 to 8.5) |
| Motorcyclist road injuries | 4176.6<br>(3556.2 to 4951.3) | 419.7<br>(233.6 to 659.9) | 236.1<br>(162.0 to 323.0) | 32.3*<br>(30.1 to 34.2) | 4.1*<br>(2.4 to 5.6) |
| Motor vehicle road injuries | 4226.4<br>(3622.7 to 4974.8) | 777.8<br>(476.6 to 1212.1) | 253.0<br>(180.2 to 344.1) | 24.2*<br>(22.6 to 25.6) | -2.3*<br>(-3.5 to -1.1) |
| Other road injuries | 1090.7<br>(871.8 to 1358.1) | 244.9<br>(125.3 to 419.4) | 56.3<br>(39.4 to 78.0) | 58.2*<br>(55.8 to 60.7) | 24.5*<br>(22.6 to 26.5) |
| Other transport injuries | 3851.4<br>(3208.7 to 4476.2) | 335.8<br>(194.5 to 495.5) | 222.3<br>(157.0 to 294.4) | 31.6*<br>(29.7 to 33.5) | 3.5*<br>(2.1 to 5.1) |
| **Unintentional injuries** | **80348.9<br>(74682.4 to 87916.8)** | **22740.3<br>(18390.6 to 27490.5)** | **2969.6<br>(2116.9 to 4063.2)** | **32.8*<br>(31.7 to 33.9)** | **4.5*<br>(3.7 to 5.4)** |
| Falls | 35493.4<br>(30940.1 to 40898.7) | 9913.2<br>(6248.6 to 14507.9) | 1632.7<br>(1165.4 to 2266.6) | 32.2*<br>(30.5 to 33.7) | 4.0*<br>(2.7 to 5.2) |
| Drowning | 175.2<br>(151.4 to 204.9) | 14.7<br>(10.2 to 20.3) | 9.8<br>(7.0 to 13.2) | 32.9*<br>(29.6 to 36.1) | 4.6*<br>(2.0 to 7.1) |
| Fire, heat, and hot substances | 8403.5<br>(7166.9 to 9868.2) | 263.7<br>(159.9 to 401.9) | 220.6<br>(148.5 to 317.2) | 27.9*<br>(24.2 to 30.9) | 0.7<br>(-2.2 to 3.0) |
| Poisonings | 315.8<br>(265.5 to 366.1) | 90.2<br>(52.3 to 149.8) | 28.9<br>(19.3 to 40.4) | 38.2*<br>(34.8 to 41.6) | 8.8*<br>(6.1 to 11.5) |
| Poisoning by carbon monoxide | 64.3<br>(54.7 to 77.1) | 31.3<br>(14.5 to 57.3) | 4.9<br>(3.3 to 6.9) | 41.9*<br>(38.0 to 46.1) | 11.7*<br>(8.6 to 15.0) |
| Poisoning by other means | 251.5<br>(202.3 to 300.7) | 58.9<br>(31.2 to 104.2) | 24.1<br>(15.7 to 34.7) | 37.5*<br>(33.9 to 41.1) | 8.3*<br>(5.4 to 11.0) |
| Exposure to mechanical forces | 17624.9<br>(15216.5 to 20628.4) | 3192.9<br>(2143.4 to 4476.3) | 431.8<br>(294.0 to 617.6) | 33.9*<br>(32.7 to 35.0) | 5.4*<br>(4.5 to 6.3) |
| Unintentional firearm injuries | 721.2<br>(594.7 to 845.5) | 54.4<br>(24.4 to 109.2) | 24.4<br>(16.9 to 33.9) | 38.4*<br>(37.1 to 39.8) | 9.0*<br>(7.9 to 10.1) |
| Other exposure to mechanical forces | 16903.6<br>(14516.9 to 19966.3) | 3138.5<br>(2109.6 to 4387.8) | 407.4<br>(275.7 to 584.6) | 33.6*<br>(32.4 to 34.8) | 5.1*<br>(4.2 to 6.1) |
| Adverse effects of medical treatment | 282.4<br>(179.5 to 420.1) | 3691.8<br>(2431.5 to 5273.4) | 37.7<br>(19.9 to 62.8) | 76.2*<br>(64.7 to 87.7) | 38.7*<br>(29.6 to 47.8) |
| Animal contact | 3019.6<br>(2654.1 to 3446.6) | 1862.1<br>(1136.0 to 2666.0) | 69.1<br>(46.9 to 98.1) | 26.3*<br>(23.7 to 28.7) | -0.6<br>(-2.6 to 1.3) |
| Venomous animal contact | 1217.2<br>(991.1 to 1444.3) | 831.7<br>(492.9 to 1214.1) | 41.4<br>(28.5 to 58.3) | 28.6*<br>(25.1 to 32.2) | 1.2<br>(-1.5 to 4.1) |
| Non-venomous animal contact | 1802.3<br>(1505.6 to 2180.3) | 1030.4<br>(602.6 to 1531.3) | 27.7<br>(17.3 to 43.8) | 22.9*<br>(20.2 to 24.7) | -3.2*<br>(-5.4 to -1.8) |
| Foreign body | 1420.9<br>(1251.2 to 1585.1) | 956.0<br>(563.1 to 1414.6) | 64.4<br>(45.6 to 86.8) | 27.5*<br>(26.0 to 28.9) | 0.4<br>(-0.8 to 1.4) |

| | | | | | |
|---|---|---|---|---|---|
| Pulmonary aspiration and foreign body in airway | 228.2 (189.3 to 276.6) | 26.0 (14.7 to 42.2) | 10.0 (6.9 to 13.6) | 36.0* (33.2 to 38.7) | 7.1* (4.9 to 9.2) |
| Foreign body in eyes | 181.9 (108.0 to 278.0) | 767.3 (394.9 to 1224.8) | 12.4 (6.8 to 20.4) | 29.5* (27.6 to 31.1) | 1.9* (0.4 to 3.2) |
| Foreign body in other body part | 1010.8 (855.8 to 1147.8) | 162.7 (94.5 to 281.1) | 42.1 (29.9 to 54.5) | 25.1* (23.6 to 26.5) | -1.5* (-2.7 to -0.5) |
| Environmental heat and cold exposure | 2730.1 (2375.7 to 3225.0) | 410.3 (248.5 to 646.5) | 110.3 (77.0 to 151.0) | 32.9* (31.0 to 34.8) | 4.6* (3.1 to 6.1) |
| Exposure to forces of nature | 841.8 (606.2 to 1125.2) | 22.6 (15.6 to 31.4) | 34.3 (25.3 to 46.4) | 70.5* (55.1 to 89.8) | 34.2* (22.0 to 49.4) |
| Other unintentional injuries | 10090.7 (8393.0 to 11950.7) | 2322.7 (1463.5 to 3508.5) | 330.0 (220.9 to 464.5) | 33.1* (31.6 to 34.4) | 4.7* (3.6 to 5.8) |
| **Self-harm and interpersonal violence** | **18147.2 (15170.9 to 21453.7)** | **1148.9 (848.1 to 1492.8)** | **451.0 (348.7 to 565.5)** | **39.5* (33.4 to 48.1)** | **9.8* (5.0 to 16.6)** |
| Self-harm | 845.7 (690.9 to 995.1) | 150.7 (99.9 to 215.7) | 39.9 (28.0 to 53.3) | 13.8* (12.3 to 15.2) | -10.4* (-11.6 to -9.3) |
| Self-harm by firearm | 9.7 (8.1 to 11.6) | 3.5 (0.8 to 9.5) | 0.4 (0.3 to 0.6) | 5.7* (2.3 to 9.9) | -16.8* (-19.5 to -13.5) |
| Self-harm by other specified means | 836.0 (681.3 to 986.5) | 147.3 (97.6 to 210.8) | 39.5 (27.7 to 52.7) | 13.9* (12.4 to 15.3) | -10.3* (-11.6 to -9.3) |
| Interpersonal violence | 13805.1 (11200.8 to 16989.7) | 790.7 (519.5 to 1122.2) | 272.0 (203.5 to 354.5) | 23.9* (22.9 to 24.9) | -2.5* (-3.3 to -1.7) |
| Assault by firearm | 252.9 (209.8 to 313.6) | 16.8 (8.1 to 30.9) | 10.4 (7.2 to 14.3) | 29.8* (27.9 to 31.6) | 2.2* (0.6 to 3.6) |
| Assault by sharp object | 1300.5 (1058.7 to 1781.2) | 88.3 (40.1 to 154.5) | 37.4 (25.8 to 52.1) | 24.2* (22.4 to 26.0) | -2.3* (-3.7 to -0.8) |
| Sexual violence | 8239.7 (5880.9 to 11251.9) | -- | 62.0 (35.8 to 101.3) | 25.1* (23.0 to 27.2) | -1.5 (-3.2 to 0.1) |
| Assault by other means | 4012.1 (3328.0 to 4790.4) | 685.6 (436.5 to 992.0) | 162.2 (113.3 to 223.4) | 23.0* (21.8 to 24.1) | -3.2* (-4.2 to -2.3) |
| Conflict and terrorism | 3125.3 (2126.6 to 4364.4) | 168.1 (121.4 to 226.7) | 126.9 (90.3 to 180.1) | 104.4* (74.9 to 144.4) | 60.9* (37.7 to 92.3) |
| Executions and police conflict | 377.2 (243.2 to 560.9) | 39.3 (25.5 to 56.9) | 12.2 (8.1 to 17.7) | 85.3* (56.0 to 127.4) | 45.8* (22.8 to 79.0) |
| **Nature of injury aggregates** | | | | | |
| Amputations | 31998.7 (29676.8 to 35231.5) | 473.7 (375.2 to 594.5) | 532.8 (349.9 to 785.1) | 27.1* (22.8 to 30.9) | 0.1 (-3.4 to 3.0) |
| Burns | 17419.1 (16025.5 to 19099.9) | 435.2 (346.2 to 559.6) | 447.4 (300.3 to 636.8) | 28.7* (25.2 to 31.6) | 1.3 (-1.4 to 3.5) |
| Fractures | 32295.4 (30129.8 to 35355.7) | 7484.0 (5806.3 to 9543.5) | 1756.2 (1221.3 to 2476.9) | 32.5* (31.3 to 33.8) | 4.3* (3.3 to 5.3) |
| Head Injuries | 4339.0 (4119.0 to 4543.8) | 1041.3 (813.3 to 1320.8) | 632.0 (448.2 to 852.2) | 35.9* (34.3 to 37.5) | 7.0* (5.7 to 8.2) |
| Spinal Injuries | 1831.9 (1703.2 to 1990.3) | 38.9 (28.2 to 51.7) | 511.3 (365.9 to 662.9) | 38.3* (34.9 to 43.1) | 8.8* (6.2 to 12.6) |
| Minor Injuries | 17257.2 (16200.0 to 18692.3) | 8487.8 (6842.6 to 10283.1) | 154.1 (73.2 to 291.4) | 32.7* (31.6 to 34.3) | 4.4* (3.6 to 5.7) |
| Other Injuries | 5801.9 (5488.3 to 6117.2) | 8955.8 (7455.5 to 10689.7) | 501.4 (357.6 to 671.5) | 35.8* (33.5 to 38.4) | 6.9* (5.1 to 8.9) |
| **Impairments** | | | | | |
| Anaemia | 74184.5 (69299.2 to 79654.9) | -- | 1839.5 (1272.6 to 2659.3) | 10.4* (2.4 to 19.2) | -13.1* (-19.4 to -6.2) |
| Epilepsy | 2689.4 (2250.1 to 3172.1) | -- | 987.3 (741.0 to 1292.2) | 32.9* (26.5 to 39.4) | 4.6 (-0.4 to 9.7) |
| Guillain-Barré syndrome | 5.1 (3.0 to 8.2) | -- | 1.5 (0.8 to 2.7) | 31.3* (28.5 to 34.1) | 3.4* (1.2 to 5.5) |
| Hearing loss | 128644.6 (121427.3 to 135474.4) | -- | 2976.0 (1945.6 to 4414.0) | 26.7* (24.1 to 29.2) | -0.3 (-2.3 to 1.7) |
| Heart failure | 3506.5 (2872.8 to 4265.4) | -- | 432.5 (289.6 to 607.7) | 24.2* (21.7 to 26.5) | -2.3* (-4.2 to -0.4) |
| Infertility | 0.1 (0.0 to 0.3) | -- | -- | -16.7* (-25.3 to -11.1) | -34.4* (-41.2 to -30.0) |
| Developmental intellectual disability | 5869.5 (4559.5 to 7107.1) | -- | 889.4 (697.5 to 1110.6) | 31.2* (27.2 to 35.5) | 3.3* (0.1 to 6.6) |
| Pelvic inflammatory disease | 39.4 (28.9 to 51.7) | -- | 5.2 (3.1 to 7.6) | 30.9* (22.2 to 40.4) | 3.0 (-3.8 to 10.5) |
| Vision loss | 133745.0 (123512.9 to 144285.8) | -- | 2927.9 (1960.7 to 4290.0) | 27.8* (26.2 to 29.5) | 0.6 (-0.6 to 1.9) |

We did not calculate incidence for the nine impairments and certain NTDs. Blank cells mean that no estimate is available or that the estimate has a magnitude less than 50.
Table presents prevalence and incidence counts in thousands.
* Percentage changes that are statistically significant
‡ Incidence estimates for stroke represent first-ever stroke only.
^ Incidence of HIV/AIDS represents new infections of HIV only and does not include new infections of Tuberculosis in HIV positive cases.

**SR Table 10p: Global prevalence, incidence and YLDs for 2017, percentage change of YLD counts and percentage change of YLD rates between 2007 and 2017 by cause, age 60 to 64 years**

| Cause | Prevalence in thousands (95% UI) | Incidence in thousands (95% UI) | YLDs in thousands (95% UI) | | |
|---|---|---|---|---|---|
| | 2017 counts | 2017 counts | 2017 counts | Percentage change in counts between 2007 and 2017 | Percentage change in rates between 2007 and 2017 |
| **All causes** | **305220.0 (304980.3 to 305430.6)** | **1292577.7 (1108085.8 to 1501693.9)** | **56935.3 (42829.5 to 72944.0)** | **47.0* (45.9 to 48.1)** | **0.1 (-0.6 to 0.9)** |
| **Communicable, maternal, neonatal, and nutritional disorders** | **188735.6 (172514.3 to 208629.1)** | **824211.7 (640484.0 to 1023974.0)** | **3210.7 (2400.3 to 4143.3)** | **35.7* (31.1 to 40.7)** | **-7.5* (-10.7 to -4.1)** |
| **HIV/AIDS and sexually transmitted infections** | **69727.8 (62467.9 to 78202.5)** | **7840.6 (6878.9 to 9110.0)** | **149.7 (103.0 to 210.9)** | **38.6* (6.2 to 73.6)** | **-5.6 (-27.6 to 18.3)** |
| HIV/AIDS | 1189.3 (1025.0 to 1373.2) | 39.6^ (28.4 to 53.0) | 118.7 (80.9 to 169.4) | 36.0* (0.9 to 84.8) | -7.3 (-31.3 to 25.9) |
| HIV/AIDS - Drug-susceptible Tuberculosis | 27.0 (24.7 to 29.4) | 32.8 (29.9 to 36.0) | 10.1 (6.8 to 13.7) | 36.3* (26.2 to 47.5) | -7.1 (-14.0 to 0.5) |
| HIV/AIDS - Multidrug-resistant Tuberculosis without extensive drug resistance | 1.2 (0.7 to 2.0) | 1.4 (1.0 to 1.9) | 0.5 (0.3 to 0.8) | 41.5 (-10.8 to 130.5) | -3.6 (-39.2 to 57.1) |
| HIV/AIDS - Extensively drug-resistant Tuberculosis | 0.1 (0.0 to 0.1) | 0.0 (0.0 to 0.1) | -- | 153.6* (45.5 to 398.0) | 72.8 (-0.8 to 239.3) |
| HIV/AIDS resulting in other diseases | 1161.1 (996.3 to 1345.8) | 39.6 (28.4 to 53.0) | 108.0 (72.8 to 156.7) | 36.0 (-1.3 to 90.5) | -7.4 (-32.8 to 29.8) |
| HIV/AIDS not on antiretroviral treatment without Tuberculosis | 228.2 (185.1 to 282.9) | 39.6 (28.4 to 53.0) | 38.9 (21.4 to 71.6) | -43.8* (-52.4 to -32.8) | -61.7* (-67.6 to -54.2) |
| HIV/AIDS on antiretroviral treatment without Tuberculosis | 932.9 (799.2 to 1079.1) | -- | 69.2 (45.3 to 98.5) | 568.7* (492.4 to 681.8) | 355.7* (303.6 to 432.7) |
| Sexually transmitted infections excluding HIV | 68976.7 (61760.3 to 77542.5) | 7801.0 (6842.2 to 9071.4) | 31.0 (16.0 to 59.3) | 49.4* (46.2 to 52.7) | 1.8 (-0.4 to 4.0) |
| Syphilis | 607.0 (480.4 to 785.8) | 39.4 (21.8 to 60.9) | 7.1 (4.7 to 9.9) | 48.8* (40.0 to 60.3) | 1.4 (-4.6 to 9.2) |
| Early syphilis | 566.8 (440.2 to 745.3) | 39.4 (21.8 to 60.9) | 0.1 (0.0 to 0.3) | 48.5* (36.2 to 60.5) | 1.2 (-7.2 to 9.4) |
| Tertiary syphilis | 40.2 (33.8 to 47.4) | -- | 6.9 (4.5 to 9.7) | 48.8* (39.9 to 60.3) | 1.4 (-4.7 to 9.2) |
| Chlamydial infection | 435.7 (315.0 to 579.5) | 353.2 (233.2 to 508.0) | 0.7 (0.3 to 1.5) | 48.7* (39.7 to 58.2) | 1.3 (-4.8 to 7.8) |
| Chlamydia episode | 434.9 (314.3 to 578.6) | 353.2 (233.2 to 508.0) | 0.6 (0.2 to 1.4) | 47.8* (38.3 to 57.4) | 0.7 (-5.7 to 7.3) |
| Chlamydial infection complications | 0.8 (0.5 to 1.1) | -- | 0.1 (0.1 to 0.2) | 54.6* (28.4 to 82.0) | 5.4 (-12.5 to 24.0) |
| Gonococcal infection | 114.2 (64.8 to 187.2) | 310.6 (187.6 to 475.2) | 0.4 (0.2 to 0.8) | 46.0* (34.1 to 59.3) | -0.5 (-8.6 to 8.5) |
| Gonococcal infection complications | 1.0 (0.7 to 1.5) | -- | 0.1 (0.1 to 0.2) | 51.4* (25.9 to 77.0) | 3.2 (-14.2 to 20.6) |
| Gonorrhoea episode | 113.2 (63.7 to 186.4) | 310.6 (187.6 to 475.2) | 0.3 (0.1 to 0.7) | 43.7* (28.9 to 56.3) | -2.1 (-12.2 to 6.5) |
| Trichomoniasis | 3949.6 (3091.1 to 5081.6) | 6449.7 (5545.0 to 7692.4) | 6.7 (2.8 to 14.9) | 52.0* (47.5 to 56.2) | 3.6* (0.5 to 6.4) |
| Genital herpes | 65837.7 (58494.9 to 74751.7) | 648.2 (411.6 to 934.8) | 15.6 (4.8 to 37.9) | 48.7* (46.6 to 50.6) | 1.3 (-0.1 to 2.6) |
| Other sexually transmitted infections | -- | -- | 0.5 (0.2 to 1.0) | 47.5* (40.2 to 54.7) | 0.5 (-4.4 to 5.4) |
| Other sexually transmitted diseases residual | -- | -- | 0.5 (0.2 to 1.0) | 47.5* (40.2 to 54.7) | 0.5 (-4.4 to 5.4) |
| Other sexually transmitted diseases | -- | -- | 0.5 (0.2 to 1.0) | 47.5* (40.2 to 54.7) | 0.5 (-4.4 to 5.4) |
| **Respiratory infections and tuberculosis** | **100049.7 (70195.5 to 139612.2)** | **543133.5 (365924.0 to 748080.0)** | **455.3 (309.4 to 636.4)** | **34.0* (30.6 to 37.1)** | **-8.7* (-11.0 to -6.6)** |
| Tuberculosis | 93531.2 (61830.5 to 134048.3) | 463.3 (357.4 to 590.3) | 217.6 (137.2 to 319.7) | 26.1* (21.8 to 30.2) | -14.1* (-17.0 to -11.3) |
| Latent tuberculosis infection | 92790.5 (61037.3 to 133175.7) | -- | -- | -- | -- |
| Drug-susceptible tuberculosis | 705.8 (539.1 to 925.1) | 439.0 (338.4 to 557.9) | 207.1 (130.9 to 304.5) | 26.3* (19.8 to 31.9) | -13.9* (-18.4 to -10.1) |
| Multidrug-resistant tuberculosis without extensive drug resistance | 33.1 (14.5 to 63.9) | 23.0 (12.2 to 41.8) | 9.9 (4.0 to 20.5) | 19.1 (-42.4 to 113.2) | -18.9 (-60.7 to 45.3) |
| Extensively drug-resistant tuberculosis | 1.8 (0.9 to 3.5) | 1.4 (0.9 to 2.1) | 0.6 (0.3 to 1.2) | 75.7* (0.6 to 229.3) | 19.7 (-31.5 to 124.4) |
| Lower respiratory infections | 250.9 (214.6 to 288.4) | 11607.6 (9893.3 to 13620.1) | 14.7 (9.8 to 21.0) | 52.2* (39.8 to 64.3) | 3.7 (-4.7 to 12.0) |
| Guillain-Barré syndrome due to lower respiratory infections | 0.6 (0.3 to 1.2) | -- | 0.2 (0.1 to 0.4) | 46.0* (42.2 to 49.7) | -0.5 (-3.1 to 2.0) |
| Lower respiratory infection episode | 250.3 (214.1 to 287.9) | 11607.6 (9893.3 to 13620.1) | 14.6 (9.7 to 20.8) | 52.3* (39.7 to 64.5) | 3.8 (-4.8 to 12.1) |
| Upper respiratory infections | 7290.2 (4858.2 to 9907.6) | 528958.3 (352169.8 to 733765.6) | 175.0 (92.1 to 295.7) | 44.3* (42.3 to 46.2) | -1.7* (-3.0 to -0.4) |
| Guillain-Barré syndrome due to upper respiratory infections | 1.6 (0.9 to 2.9) | -- | 0.5 (0.2 to 0.9) | 46.0* (42.2 to 49.7) | -0.5 (-3.1 to 2.0) |
| Upper respiratory infection episode | 7288.5 (4856.5 to 9906.6) | 528958.3 (352169.8 to 733765.6) | 174.6 (91.3 to 295.2) | 44.3* (42.3 to 46.2) | -1.7* (-3.0 to -0.4) |
| Otitis media | 2352.1 (1626.8 to 3140.9) | 2104.2 (1440.3 to 2795.1) | 48.0 (25.3 to 79.6) | 32.3* (26.2 to 39.0) | -9.9* (-14.0 to -5.3) |
| Acute otitis media | 114.2 (76.3 to 160.0) | 2103.9 (1439.7 to 2794.3) | 1.4 (0.7 to 2.8) | 43.1* (34.8 to 52.9) | -2.5 (-8.2 to 4.2) |
| Chronic otitis media | 2237.9 (1506.4 to 3047.2) | 0.4 (0.0 to 1.4) | 46.5 (24.2 to 76.9) | 32.0* (25.7 to 38.9) | -10.1* (-14.4 to -5.4) |
| **Enteric infections** | **3254.6 (2828.0 to 3708.2)** | **223182.8 (188962.7 to 259090.5)** | **351.7 (241.3 to 494.7)** | **65.9* (58.2 to 73.9)** | **13.1* (7.8 to 18.5)** |
| Diarrhoeal diseases | 3272.9 (2843.9 to 3731.7) | 223030.2 (188788.9 to 258925.9) | 350.5 (240.4 to 492.9) | 66.3* (58.5 to 74.5) | 13.3* (8.0 to 18.9) |
| Guillain-Barré syndrome due to diarrheal diseases | 0.6 (0.3 to 1.0) | -- | 0.2 (0.1 to 0.3) | 46.0* (42.2 to 49.7) | -0.5 (-3.1 to 2.0) |
| Diarrheal disease episode | 3272.4 (2843.3 to 3731.4) | 223030.2 (188788.9 to 258925.9) | 350.3 (240.2 to 492.7) | 66.3* (58.6 to 74.5) | 13.3* (8.0 to 18.9) |
| Typhoid and paratyphoid | 0.8 (0.2 to 1.5) | 147.3 (122.8 to 177.5) | 1.2 (0.8 to 1.8) | 2.0 (-14.6 to 23.1) | -30.5* (-41.8 to -16.2) |
| Typhoid fever | 7.2 (5.4 to 9.3) | 113.6 (92.3 to 139.4) | 1.1 (0.7 to 1.6) | 4.1 (-14.3 to 27.9) | -29.1* (-41.6 to -12.9) |
| Typhoid fever complications | 1.5 (1.1 to 2.0) | 19.5 (15.2 to 24.9) | 0.5 (0.3 to 0.8) | 3.1 (-22.5 to 38.6) | -29.7* (-47.2 to -5.5) |
| Typhoid fever episode | 5.7 (4.2 to 7.5) | 94.1 (76.2 to 114.8) | 0.6 (0.4 to 1.0) | 4.9 (-23.4 to 38.2) | -28.5* (-47.8 to -5.8) |
| Paratyphoid fever | 1.5 (1.0 to 2.0) | 33.7 (24.2 to 44.8) | 0.1 (0.1 to 0.1) | -18.0* (-29.8 to -4.9) | -44.1* (-52.2 to -35.2) |
| Intestinal perforation due to paratyphoid | 0.1 (0.0 to 0.1) | 1.7 (1.2 to 2.4) | -- | -18.2 (-34.4 to 1.6) | -44.3* (-55.3 to -30.8) |
| Paratyphoid fever episode | 1.4 (1.0 to 1.9) | 31.9 (23.0 to 42.4) | 0.1 (0.0 to 0.1) | -18.0* (-30.3 to -4.0) | -44.1* (-52.5 to -34.6) |
| iNTS | 0.2 (0.1 to 0.3) | 5.3 (3.0 to 8.4) | -- | -7.8 (-16.9 to 1.4) | -37.2* (-43.4 to -30.9) |
| Other intestinal infectious diseases | -- | -- | -- | 3.4 (-13.4 to 24.8) | -29.5* (-41.0 to -14.9) |
| **Neglected tropical diseases and malaria** | **31999.6 (30440.6 to 33791.7)** | **7977.3 (6375.7 to 9795.0)** | **504.4 (358.4 to 684.3)** | **13.9* (5.8 to 22.5)** | **-22.4* (-27.9 to -16.5)** |
| Malaria | 1553.9 (1450.2 to 1646.6) | 1027.1 (916.9 to 1166.4) | 12.6 (8.8 to 17.9) | -14.7 (-29.8 to 2.6) | -41.9* (-52.1 to -30.1) |
| Malaria complications | 7.6 (6.8 to 8.5) | -- | 3.3 (2.5 to 4.1) | 47.8* (36.2 to 60.7) | 0.7 (-7.2 to 9.5) |
| Malaria episode | 60.1 (40.3 to 82.6) | 1027.1 (916.9 to 1166.4) | 2.1 (1.1 to 3.5) | -18.7* (-28.3 to -7.4) | -44.6* (-51.1 to -36.9) |

| | | | | | |
|---|---|---|---|---|---|
| Malaria parasitemia | 1486.1 (1380.4 to 1590.8) | -- | 7.2 (4.3 to 11.5) | -27.7* (-45.1 to -4.5) | -50.8* (-62.6 to -34.9) |
| Chagas disease | 422.9 (362.8 to 491.3) | 4.6 (2.9 to 7.0) | 5.3 (3.5 to 7.7) | 16.5* (9.1 to 24.0) | -20.6* (-25.7 to -15.5) |
| Acute Chagas disease | 0.0 (0.0 to 0.1) | 4.6 (2.9 to 7.0) | -- | 34.3* (28.7 to 40.0) | -8.5* (-12.3 to -4.6) |
| Asymptomatic Chagas disease | 341.6 (290.9 to 397.5) | -- | -- | -- | -- |
| Symptomatic chronic Chagas infection | 81.3 (64.4 to 101.3) | -- | 5.3 (3.5 to 7.7) | 16.5* (9.1 to 24.0) | -20.6* (-25.7 to -15.5) |
| Leishmaniasis | 141.9 (108.7 to 182.4) | 18.9 (13.3 to 26.3) | 8.7 (5.5 to 13.4) | -1.1 (-13.5 to 15.9) | -32.6* (-41.1 to -21.1) |
| Visceral leishmaniasis | 0.1 (0.1 to 0.1) | 0.4 (0.3 to 0.6) | -- | -72.0* (-77.3 to -61.3) | -80.9* (-84.6 to -73.6) |
| Cutaneous and mucocutaneous leishmaniasis | 142.9 (108.8 to 183.0) | 18.5 (12.9 to 25.9) | 8.7 (5.5 to 13.4) | -0.9 (-13.4 to 16.2) | -32.5* (-41.0 to -20.8) |
| African trypanosomiasis | 0.1 (0.0 to 0.6) | 0.1 (0.0 to 0.3) | 0.0 (0.0 to 0.2) | -74.8 (-97.4 to 63.5) | -82.8 (-98.3 to 11.4) |
| Trypanosomiasis Gambiense | 0.1 (0.0 to 0.6) | 0.1 (0.0 to 0.3) | 0.0 (0.0 to 0.2) | -73.8 (-97.7 to 69.0) | -82.2 (-98.4 to 15.2) |
| Trypanosomiasis Rhodesiense | -- | -- | -- | -90.5* (-99.2 to -33.7) | -93.5* (-99.5 to -54.9) |
| Schistosomiasis | 5057.4 (4491.8 to 5629.5) | -- | 35.6 (17.6 to 68.5) | -12.9* (-17.4 to -7.3) | -40.6* (-43.7 to -36.8) |
| Mild schistosomiasis | 4427.5 (3879.2 to 4971.9) | -- | 24.2 (9.8 to 51.7) | -13.9* (-16.2 to -11.8) | -41.3* (-42.9 to -39.9) |
| Anemia due to schistosomiasis | 251.6 (199.4 to 314.5) | -- | 5.8 (3.4 to 9.7) | -22.3 (-42.2 to 3.4) | -47.1* (-60.6 to -29.5) |
| Schistosomiasis complications | 379.6 (307.1 to 459.9) | -- | 5.6 (3.3 to 9.4) | 5.8 (-1.6 to 15.5) | -27.9* (-32.9 to -21.3) |
| Cysticercosis | 426.9 (320.9 to 559.6) | -- | 118.1 (74.8 to 177.6) | 31.7* (23.4 to 38.8) | -10.3* (-15.9 to -5.4) |
| Cystic echinococcosis | 34.0 (18.3 to 58.3) | 7.3 (3.9 to 12.7) | 2.7 (1.3 to 5.0) | 80.4* (64.4 to 96.8) | 22.9* (12.0 to 34.1) |
| Lymphatic filariasis | 2130.4 (1941.2 to 2343.4) | -- | 49.1 (27.8 to 76.8) | -31.6* (-44.3 to -19.7) | -53.4* (-62.1 to -45.3) |
| Prevalence of detectable microfiliaria due to lymphatic filariasis | 1665.9 (1559.9 to 1789.3) | -- | -- | -- | -- |
| Lymphatic filariasis complications | 464.5 (314.0 to 657.3) | -- | 49.1 (27.8 to 76.8) | -31.6* (-44.3 to -19.7) | -53.4* (-62.1 to -45.3) |
| Onchocerciasis | 718.8 (488.2 to 1128.9) | -- | 59.0 (32.6 to 103.5) | 20.1* (1.9 to 38.8) | -18.2* (-30.6 to -5.4) |
| Asymptomatic onchocerciasis | 115.7 (0.0 to 497.4) | -- | -- | -- | -- |
| Skin disease due to onchocerciasis | 435.6 (312.4 to 586.2) | -- | 45.3 (21.3 to 87.7) | 19.1 (-6.6 to 41.8) | -18.8* (-36.3 to -3.4) |
| Vision loss due to onchocerciasis | 167.6 (114.3 to 253.3) | -- | 13.8 (8.6 to 20.5) | 23.4* (8.6 to 40.0) | -15.9* (-26.0 to -4.6) |
| Trachoma | 510.2 (280.1 to 845.7) | -- | 38.5 (23.1 to 60.3) | 2.4 (-13.6 to 18.5) | -30.2* (-41.2 to -19.3) |
| Dengue | 189.7 (103.5 to 321.0) | 3173.7 (1933.1 to 4809.3) | 29.2 (13.0 to 54.0) | 99.9* (71.9 to 208.1) | 36.2* (17.1 to 110.0) |
| Post-dengue chronic fatigue syndrome | 133.7 (62.5 to 244.4) | -- | 26.0 (11.0 to 49.1) | 100.0* (71.4 to 207.8) | 36.3* (16.8 to 109.7) |
| Dengue episode | 56.0 (33.9 to 84.0) | 3173.7 (1933.1 to 4809.3) | 3.2 (1.6 to 5.6) | 99.6* (70.6 to 199.5) | 36.0* (16.3 to 104.1) |
| Yellow fever | 0.0 (0.0 to 0.1) | 1.2 (0.3 to 3.2) | -- | -12.1 (-38.4 to 27.4) | -40.1* (-58.1 to -13.2) |
| Asymptomatic yellow fever | 0.0 (0.0 to 0.1) | 0.7 (0.2 to 2.0) | -- | -- | -- |
| Yellow fever episode | -- | 0.5 (0.1 to 1.4) | -- | -12.1 (-38.4 to 27.4) | -40.1* (-58.1 to -13.2) |
| Rabies | -- | 0.6 (0.4 to 0.7) | -- | -21.2* (-39.1 to -0.1) | -46.3* (-58.5 to -32.0) |
| Intestinal nematode infections | 19013.0 (17544.3 to 20768.3) | -- | 34.6 (20.1 to 56.6) | -12.3* (-19.0 to -5.0) | -40.2* (-44.8 to -35.3) |
| Ascariasis | 8319.5 (7210.1 to 9622.5) | -- | 9.8 (5.3 to 16.8) | -13.1* (-22.9 to -2.3) | -40.8* (-47.5 to -33.5) |
| Asymptomatic ascariasis | 7768.5 (6722.9 to 9021.6) | -- | -- | -- | -- |
| Ascariasis complications | 551.0 (484.0 to 628.1) | -- | 9.8 (5.3 to 16.8) | -13.1* (-22.9 to -2.3) | -40.8* (-47.5 to -33.5) |
| Trichuriasis | 5034.4 (4211.7 to 6055.1) | -- | 3.0 (1.7 to 5.0) | 13.2* (1.7 to 25.3) | -22.9* (-30.7 to -14.6) |
| Asymptomatic trichuriasis | 4876.1 (4069.0 to 5876.4) | -- | -- | -- | -- |
| Trichuriasis complications | 158.3 (142.8 to 173.6) | -- | 3.0 (1.7 to 5.0) | 13.2* (1.7 to 25.3) | -22.9* (-30.7 to -14.6) |
| Hookworm disease | 6561.8 (5906.3 to 7336.5) | -- | 21.8 (12.9 to 35.7) | -14.6* (-23.9 to -4.2) | -41.8* (-48.1 to -34.8) |
| Asymptomatic hookworm disease | 5471.5 (4926.3 to 6114.5) | -- | -- | -- | -- |
| Anemia due to hookworm disease | 252.1 (213.6 to 301.4) | -- | 4.9 (3.2 to 7.5) | -29.1* (-42.8 to -9.9) | -51.7* (-61.0 to -38.6) |
| Hookworm disease complications | 838.2 (757.7 to 933.1) | -- | 16.8 (9.6 to 28.1) | -9.1* (-17.5 to -0.2) | -38.1* (-43.8 to -32.0) |
| Food-borne trematodiases | 4267.1 (3772.8 to 4864.2) | 3659.0 (2927.8 to 4472.7) | 93.6 (49.7 to 167.3) | 71.7* (63.6 to 80.3) | 17.0* (11.5 to 22.9) |
| Asymptomatic food-borne tremodiases | 3408.5 (2776.1 to 4011.7) | 2761.0 (1422.2 to 3872.6) | -- | -- | -- |
| Food-borne tremodiases complications | 858.7 (514.1 to 1382.0) | 898.0 (109.6 to 2204.9) | 93.6 (49.7 to 167.3) | 71.7* (63.6 to 80.3) | 17.0* (11.5 to 22.9) |
| Leprosy | 53.0 (49.7 to 56.6) | 4.0 (3.5 to 4.4) | 3.2 (2.2 to 4.5) | 12.2* (4.8 to 20.0) | -23.5* (-28.6 to -18.2) |
| Ebola virus disease | -- | -- | -- | -96.9* (-98.8 to -92.0) | -97.9* (-99.2 to -94.6) |
| Ebola cases | -- | -- | -- | -98.7* (-98.8 to -98.7) | -99.1* (-99.2 to -99.1) |
| Post-Ebola chronic fatigue syndrome | -- | -- | -- | -96.8* (-98.8 to -91.7) | -97.8* (-99.2 to -94.4) |
| Zika virus disease | 1.3 (0.7 to 2.7) | 80.7 (44.5 to 161.7) | 0.0 (0.0 to 0.1) | -- | -- |
| Zika virus complications | -- | -- | -- | -- | -- |
| Zika virus episode | 1.3 (0.7 to 2.7) | 80.7 (44.5 to 161.7) | -- | -- | -- |
| Guinea worm disease | -- | -- | -- | -99.5* (-99.7 to -99.3) | -99.7* (-99.8 to -99.5) |
| Other neglected tropical diseases | 736.2 (674.3 to 804.8) | -- | 14.1 (9.2 to 21.0) | 12.6* (0.4 to 25.4) | -23.3* (-31.6 to -14.6) |
| Acute infection due to other neglected tropical diseases | -- | -- | 0.4 (0.2 to 0.6) | 119.8* (87.8 to 226.8) | 49.7* (28.0 to 122.7) |
| Anemia due to other neglected tropical diseases | 736.2 (674.3 to 804.8) | -- | 13.8 (9.0 to 20.5) | 11.2 (-1.2 to 24.0) | -24.2* (-32.7 to -15.5) |
| **Other infectious diseases** | **2507.7 (2180.0 to 2913.1)** | **9231.3 (7166.2 to 12146.3)** | **114.5 (79.6 to 158.5)** | **32.0* (23.5 to 42.2)** | **-10.1* (-15.9 to -3.1)** |
| Meningitis | 331.3 (276.2 to 394.5) | 95.2 (69.7 to 129.5) | 21.6 (15.1 to 28.8) | 15.6* (10.8 to 20.5) | -21.2* (-24.5 to -17.9) |
| Pneumococcal meningitis | 119.9 (97.2 to 147.7) | 7.6 (4.9 to 11.1) | 8.1 (5.6 to 10.9) | -5.5 (-11.0 to 0.6) | -35.6* (-39.4 to -31.4) |

| | | | | | |
|---|---|---|---|---|---|
| Acute pneumococcal meningitis | 0.3 (0.2 to 0.5) | 7.6 (4.9 to 11.1) | 0.0 (0.0 to 0.1) | 0.3 (-10.5 to 11.2) | -31.6* (-39.0 to -24.2) |
| Pneumococcal meningitis complications | 119.6 (96.8 to 147.2) | -- | 8.1 (5.6 to 10.8) | -5.6 (-11.1 to 0.6) | -35.7* (-39.4 to -31.4) |
| H influenzae type B meningitis | 23.9 (16.6 to 32.4) | 2.4 (1.4 to 3.9) | 1.4 (1.0 to 1.9) | -44.3* (-46.6 to -42.2) | -62.0* (-63.6 to -60.6) |
| Acute H influenzae type B meningitis | 0.1 (0.1 to 0.2) | 2.4 (1.4 to 3.9) | -- | -44.5* (-52.2 to -37.7) | -62.2* (-67.5 to -57.5) |
| H Influenza type B meningitis complications | 23.8 (16.4 to 32.3) | -- | 1.4 (1.0 to 1.9) | -44.3* (-46.6 to -42.2) | -62.0* (-63.6 to -60.6) |
| Meningococcal infection | 36.2 (25.5 to 48.3) | 7.2 (4.4 to 11.3) | 2.6 (1.8 to 3.5) | 18.7* (14.0 to 23.4) | -19.1* (-22.3 to -15.9) |
| Acute meningococcal meningitis | 0.3 (0.2 to 0.5) | 7.2 (4.4 to 11.3) | 0.0 (0.0 to 0.1) | 32.4* (18.0 to 45.8) | -9.8* (-19.6 to -0.7) |
| Meningococcal meningitis complications | 35.8 (25.2 to 48.0) | -- | 2.5 (1.7 to 3.5) | 18.5* (13.8 to 23.2) | -19.2* (-22.5 to -16.1) |
| Other meningitis | 151.4 (109.1 to 194.4) | 78.0 (57.1 to 105.6) | 9.5 (6.5 to 12.9) | 76.6* (67.4 to 86.4) | 20.4* (14.1 to 27.0) |
| Other acute bacterial meningitis | 0.8 (0.6 to 1.2) | 19.0 (13.5 to 25.9) | 0.1 (0.1 to 0.2) | 86.4* (69.7 to 102.2) | 27.0* (15.6 to 37.8) |
| Acute viral meningitis | 2.6 (1.8 to 3.7) | 59.0 (43.2 to 80.2) | 0.4 (0.2 to 0.6) | 31.7* (20.8 to 42.0) | -10.3* (-17.7 to -3.2) |
| Other bacterial meningitis complications | 147.9 (105.8 to 190.9) | -- | 9.0 (6.2 to 12.3) | 78.9* (69.1 to 89.1) | 21.9* (15.2 to 28.9) |
| Encephalitis | 384.3 (196.4 to 635.6) | 95.5 (92.4 to 98.5) | 23.7 (16.5 to 32.1) | 34.3* (27.5 to 42.1) | -8.5* (-13.2 to -3.2) |
| Acute encephalitis | 4.9 (4.7 to 5.1) | 95.5 (92.4 to 98.5) | 0.7 (0.4 to 0.9) | 45.3* (44.7 to 45.9) | -1.0* (-1.4 to -0.6) |
| Encephalitis complications | 379.4 (191.4 to 630.6) | -- | 23.0 (16.0 to 31.2) | 34.0* (27.1 to 42.0) | -8.7* (-13.4 to -3.2) |
| Diphtheria | -- | -- | -- | -- | -- |
| Whooping cough | -- | -- | -- | -- | -- |
| Tetanus | 1.7 (1.6 to 1.8) | 2.6 (1.5 to 3.8) | 0.0 (0.0 to 0.1) | -7.0 (-22.1 to 9.4) | -36.7* (-46.9 to -25.5) |
| Severe tetanus | 0.1 (0.1 to 0.2) | 2.6 (1.5 to 3.8) | -- | -35.2* (-49.5 to -9.0) | -55.8* (-65.6 to -38.0) |
| Neonatal tetanus complications | 1.6 (1.5 to 1.6) | -- | -- | 29.8* (27.8 to 31.7) | -11.5* (-12.9 to -10.3) |
| Measles | -- | -- | -- | -- | -- |
| Varicella and herpes zoster | 558.9 (429.8 to 707.2) | 3136.9 (2381.5 to 4004.1) | 30.0 (17.3 to 47.1) | 51.9* (46.0 to 58.0) | 3.5 (-0.5 to 7.7) |
| Chickenpox | 0.2 (0.1 to 0.5) | 11.7 (3.5 to 26.9) | -- | 63.2* (52.9 to 70.9) | 11.2* (4.2 to 16.5) |
| Herpes zoster | 558.6 (429.6 to 707.2) | 3125.2 (2373.4 to 3998.0) | 30.0 (17.3 to 47.1) | 51.9* (46.0 to 58.0) | 3.5 (-0.5 to 7.7) |
| Acute hepatitis | 658.3 (446.6 to 971.3) | 5901.2 (4051.4 to 8600.3) | 15.5 (8.7 to 25.6) | 39.2 (-10.0 to 122.9) | -5.1 (-38.7 to 51.9) |
| Acute hepatitis A | 32.0 (22.4 to 43.4) | 415.9 (291.5 to 563.8) | 1.3 (0.7 to 2.2) | 41.0* (24.4 to 56.1) | -3.9 (-15.2 to 6.4) |
| Acute hepatitis B | 595.8 (383.6 to 913.2) | 5163.8 (3324.4 to 7914.3) | 13.5 (7.3 to 23.3) | 39.4 (-15.7 to 134.9) | -5.0 (-42.5 to 60.1) |
| Acute hepatitis C | 17.2 (8.7 to 28.1) | 149.1 (75.3 to 243.4) | 0.2 (0.1 to 0.6) | 12.7 (-2.8 to 23.7) | -23.2* (-33.8 to -15.7) |
| Acute hepatitis E | 13.3 (8.8 to 19.5) | 172.4 (114.3 to 253.4) | 0.4 (0.2 to 0.7) | 47.8* (24.0 to 75.9) | 0.7 (-15.5 to 19.8) |
| Other unspecified infectious diseases | 927.5 (857.3 to 1005.7) | -- | 23.8 (15.6 to 34.6) | 21.3* (11.4 to 31.3) | -17.4* (-24.1 to -10.5) |
| Guillain-Barré syndrome due to other infectious diseases | 0.4 (0.2 to 0.7) | -- | 0.1 (0.0 to 0.2) | 46.0* (42.2 to 49.7) | -0.5 (-3.1 to 2.0) |
| Other infectious diseases | -- | -- | 5.9 (3.4 to 9.5) | 45.5* (40.4 to 51.2) | -0.9 (-4.3 to 3.0) |
| Anemia due to other infectious diseases | 927.2 (856.9 to 1005.4) | -- | 17.8 (11.6 to 26.3) | 14.9* (3.5 to 27.3) | -21.7* (-29.5 to -13.3) |
| **Maternal and neonatal disorders** | **4271.8 (3677.1 to 4875.9)** | **--** | **765.0 (552.5 to 994.0)** | **77.1* (67.7 to 88.5)** | **20.7* (14.2 to 28.4)** |
| Maternal disorders | 11.2 (9.0 to 13.9) | -- | 3.9 (2.6 to 5.4) | -19.6* (-29.5 to -8.0) | -45.2* (-52.0 to -37.3) |
| Maternal haemorrhage | -- | -- | -- | -- | -- |
| Maternal hemorrhage complications | -- | -- | -- | -- | -- |
| Maternal hemorrhage episode | -- | -- | -- | -- | -- |
| Maternal sepsis and other pregnancy related infections | -- | -- | -- | -- | -- |
| Infertility due to puerperal sepsis | -- | -- | -- | -- | -- |
| Maternal sepsis and other maternal infection episode | -- | -- | -- | -- | -- |
| Maternal hypertensive disorders | -- | -- | -- | -- | -- |
| Maternal hypertensive disorder complications | -- | -- | -- | -- | -- |
| Maternal hypertensive disorder episode | -- | -- | -- | -- | -- |
| Maternal obstructed labour and uterine rupture | 12.5 (10.4 to 15.0) | -- | 3.9 (2.6 to 5.4) | -19.6* (-29.5 to -8.0) | -45.2* (-52.0 to -37.3) |
| Obstructed labor, acute event | -- | -- | -- | -- | -- |
| Maternal obstructed labor complications | 12.5 (10.4 to 15.0) | -- | 3.9 (2.6 to 5.4) | -19.6* (-29.5 to -8.0) | -45.2* (-52.0 to -37.3) |
| Maternal abortion and miscarriage | -- | -- | -- | -- | -- |
| Ectopic pregnancy | -- | -- | -- | -- | -- |
| Other maternal disorders | -- | -- | -- | -- | -- |
| Neonatal disorders | 4260.7 (3666.5 to 4865.6) | -- | 761.2 (549.7 to 989.8) | 78.2* (68.7 to 90.1) | 21.4* (14.9 to 29.5) |
| Neonatal preterm birth | 2391.3 (2107.2 to 2736.9) | -- | 284.3 (199.3 to 384.8) | 85.4* (60.7 to 111.0) | 26.3* (9.5 to 43.8) |
| Preterm birth complications | 1970.1 (1694.5 to 2294.7) | -- | 260.1 (179.8 to 358.5) | 91.7* (63.9 to 119.8) | 30.6* (11.7 to 49.8) |
| Retinopathy of prematurity | 421.2 (343.5 to 503.0) | -- | 24.3 (16.4 to 33.8) | 37.3* (30.3 to 45.0) | -6.4* (-11.2 to -1.2) |
| Uncomplicated preterm birth | -- | -- | -- | -- | -- |
| Neonatal encephalopathy due to birth asphyxia and trauma | 1907.1 (1415.5 to 2525.9) | -- | 319.2 (194.1 to 492.1) | 79.8* (71.9 to 89.7) | 22.5* (17.2 to 29.3) |
| Neonatal sepsis and other neonatal infections | 442.1 (275.7 to 674.3) | -- | 137.0 (71.1 to 235.3) | 62.8* (56.9 to 70.1) | 11.0* (6.9 to 15.9) |
| Neonatal sepsis and other neonatal infection complications | 442.1 (275.7 to 674.3) | -- | 137.0 (71.1 to 235.3) | 62.8* (56.9 to 70.1) | 11.0* (6.9 to 15.9) |
| Neonatal sepsis and other neonatal infection episode | -- | -- | -- | -- | -- |
| Hemolytic disease and other neonatal jaundice | 50.7 (43.5 to 57.8) | -- | 20.6 (15.7 to 25.8) | 68.7* (63.6 to 73.2) | 14.9* (11.5 to 18.0) |

| | | | | | |
|---|---|---|---|---|---|
| Other neonatal disorders | -- | -- | -- | -- | -- |
| **Nutritional deficiencies** | **57634.0 (54865.9 to 60521.2)** | **32812.1 (29442.6 to 36249.8)** | **870.1 (582.6 to 1273.7)** | **16.9* (7.4 to 26.5)** | **-20.4* (-26.8 to -13.8)** |
| Protein-energy malnutrition | 3006.5 (2825.0 to 3182.5) | 2988.7 (2772.6 to 3196.1) | 15.1 (9.9 to 21.2) | 51.9* (37.8 to 65.0) | 3.5 (-6.1 to 12.4) |
| Iodine deficiency | 5154.6 (4676.8 to 5704.6) | 24.4 (20.6 to 29.3) | 76.0 (45.1 to 124.1) | 21.2* (15.4 to 25.3) | -17.4* (-21.4 to -14.6) |
| Visible goiter without symptoms | 5078.1 (4610.4 to 5629.0) | 24.4 (20.6 to 29.3) | 52.8 (25.7 to 98.8) | 26.3* (24.3 to 28.4) | -13.9* (-15.3 to -12.5) |
| Visible goiter with complications | 76.4 (39.9 to 113.3) | -- | 23.2 (11.3 to 36.2) | 10.9 (-7.0 to 23.1) | -24.5* (-36.6 to -16.1) |
| Vitamin A deficiency | 18098.3 (16405.5 to 19902.3) | 29799.0 (26479.8 to 33294.5) | 5.9 (3.7 to 8.8) | 46.3* (36.2 to 57.3) | -0.3 (-7.2 to 7.2) |
| Asymptomatic vitamin A deficiency | 18005.4 (16290.8 to 19794.9) | 29799.0 (26479.8 to 33294.5) | -- | -- | -- |
| Vitamin A deficiency complications | 92.9 (62.6 to 129.5) | -- | 5.9 (3.7 to 8.8) | 46.3* (36.2 to 57.3) | -0.3 (-7.2 to 7.2) |
| Vitamin A deficiency with anemia | -- | -- | -- | -- | -- |
| Dietary iron deficiency | 38319.5 (35527.3 to 41447.9) | -- | 766.1 (505.2 to 1122.5) | 15.5* (4.9 to 26.3) | -21.3* (-28.5 to -13.9) |
| Other nutritional deficiencies | -- | -- | 7.1 (4.7 to 10.0) | 52.1* (37.9 to 65.1) | 3.6 (-6.0 to 12.5) |
| **Non-communicable diseases** | **304190.6 (303769.5 to 304568.4)** | **443914.7 (403589.8 to 485604.3)** | **49349.3 (37197.3 to 63021.7)** | **46.9* (45.8 to 48.1)** | **0.1 (-0.7 to 0.9)** |
| **Neoplasms** | **12138.7 (11790.9 to 12569.0)** | **3062.4 (2617.1 to 3633.2)** | **963.9 (709.2 to 1244.1)** | **60.6* (57.5 to 64.0)** | **9.4* (7.3 to 11.7)** |
| Lip and oral cavity cancer | 216.5 (206.5 to 227.5) | 51.5 (48.9 to 54.3) | 21.4 (15.7 to 27.9) | 54.9* (45.1 to 65.7) | 5.5 (-1.1 to 12.9) |
| Diagnosis and primary therapy phase of mouth cancer | 12.5 (12.0 to 13.2) | 51.5 (48.9 to 54.3) | 3.5 (2.3 to 4.8) | 54.6* (38.4 to 72.8) | 5.3 (-5.7 to 17.8) |
| Controlled phase of mouth cancer | 181.6 (173.3 to 190.6) | -- | 8.3 (5.4 to 12.0) | 56.3* (46.3 to 67.2) | 6.5 (-0.3 to 14.0) |
| Metastatic phase of mouth cancer | 19.5 (18.5 to 20.5) | -- | 8.1 (5.5 to 10.8) | 53.7* (36.8 to 71.7) | 4.8 (-6.8 to 17.0) |
| Terminal phase of mouth cancer | 3.0 (2.8 to 3.1) | -- | 1.6 (1.1 to 2.1) | 53.9* (45.3 to 62.7) | 4.9 (-1.0 to 10.8) |
| Nasopharynx cancer | 44.5 (41.4 to 48.8) | 11.6 (10.8 to 12.7) | 5.0 (3.7 to 6.7) | 54.4* (38.8 to 74.2) | 5.2 (-5.4 to 18.7) |
| Diagnosis and primary therapy phase of nasopharynx cancer | 2.5 (2.3 to 2.8) | 11.6 (10.8 to 12.7) | 0.7 (0.5 to 1.0) | 53.2* (33.7 to 81.7) | 4.4 (-8.9 to 23.8) |
| Controlled phase of nasopharynx cancer | 36.3 (33.7 to 39.7) | -- | 1.7 (1.1 to 2.5) | 55.1* (35.6 to 77.3) | 5.7 (-7.6 to 20.8) |
| Metastatic phase of nasopharynx cancer | 5.0 (4.7 to 5.5) | -- | 2.2 (1.5 to 3.1) | 54.2* (32.9 to 82.7) | 5.0 (-9.4 to 24.5) |
| Terminal phase of nasopharynx cancer | 0.7 (0.6 to 0.7) | -- | 0.4 (0.3 to 0.5) | 55.2* (44.4 to 71.3) | 5.8 (-1.6 to 16.7) |
| Other pharynx cancer | 79.7 (73.0 to 84.5) | 31.2 (28.2 to 33.3) | 10.3 (7.4 to 13.5) | 62.0* (47.0 to 75.7) | 10.4* (0.2 to 19.7) |
| Diagnosis and primary therapy phase of other pharynx cancer | 4.4 (4.1 to 4.7) | 31.2 (28.2 to 33.3) | 1.3 (0.8 to 1.8) | 66.1* (55.2 to 75.6) | 13.2* (5.7 to 19.7) |
| Controlled phase of other pharynx cancer | 61.1 (56.2 to 64.6) | -- | 2.8 (1.8 to 4.1) | 67.0* (52.1 to 82.8) | 13.8* (3.7 to 24.5) |
| Metastatic phase of other pharynx cancer | 12.0 (10.8 to 12.8) | -- | 5.1 (3.4 to 6.9) | 58.8* (37.3 to 80.7) | 8.2 (-6.4 to 23.2) |
| Terminal phase of other pharynx cancer | 2.2 (1.9 to 2.3) | -- | 1.2 (0.8 to 1.5) | 60.7* (48.5 to 70.5) | 9.5* (1.2 to 16.2) |
| Oesophageal cancer | 136.2 (131.2 to 141.4) | 70.8 (68.2 to 73.4) | 20.6 (14.9 to 26.6) | 33.7* (23.1 to 44.6) | -8.9* (-16.1 to -1.5) |
| Diagnosis and primary therapy phase of esophageal cancer | 11.2 (10.8 to 11.6) | 70.8 (68.2 to 73.4) | 3.1 (2.0 to 4.3) | 38.9* (19.6 to 63.3) | -5.3 (-18.5 to 11.3) |
| Controlled phase of esophageal cancer | 95.0 (91.4 to 98.8) | -- | 4.4 (2.8 to 6.4) | 40.8* (30.1 to 53.5) | -4.0 (-11.4 to 4.6) |
| Metastatic phase of esophageal cancer | 24.6 (23.7 to 25.5) | -- | 10.3 (7.0 to 13.6) | 30.3* (14.5 to 48.4) | -11.2 (-22.0 to 1.1) |
| Terminal phase of esophageal cancer | 5.4 (5.2 to 5.5) | -- | 2.9 (2.0 to 3.7) | 30.6* (16.8 to 47.4) | -11.0 (-20.4 to 0.5) |
| Stomach cancer | 434.6 (412.6 to 457.9) | 164.8 (157.6 to 172.5) | 50.4 (37.3 to 65.6) | 63.3* (52.3 to 75.5) | 11.3* (3.8 to 19.6) |
| Diagnosis and primary therapy phase of stomach cancer | 39.2 (37.3 to 41.3) | 164.8 (157.6 to 172.5) | 10.5 (7.1 to 14.3) | 73.9* (55.7 to 96.1) | 18.5* (6.1 to 33.6) |
| Controlled phase of stomach cancer | 338.9 (321.3 to 357.7) | -- | 15.6 (10.1 to 23.2) | 95.4* (82.3 to 109.5) | 33.1* (24.2 to 42.8) |
| Metastatic phase of stomach cancer | 44.9 (43.0 to 46.9) | -- | 18.4 (12.7 to 24.5) | 44.6* (30.8 to 60.2) | -1.4 (-10.9 to 9.1) |
| Terminal phase of stomach cancer | 11.6 (11.1 to 12.1) | -- | 6.0 (4.1 to 7.8) | 43.5* (25.7 to 62.8) | -2.2 (-14.3 to 11.0) |
| Colon and rectum cancer | 1330.5 (1294.4 to 1368.4) | 229.9 (223.6 to 236.6) | 116.9 (85.8 to 152.5) | 62.1* (55.7 to 68.7) | 10.4* (6.1 to 14.9) |
| Diagnosis and primary therapy phase of colon and rectum cancers | 56.1 (54.6 to 57.8) | 229.9 (223.6 to 236.6) | 14.6 (9.9 to 19.9) | 66.7* (52.9 to 81.1) | 13.6* (4.2 to 23.4) |
| Controlled phase of colon and rectum cancers | 1171.8 (1139.7 to 1206.0) | -- | 66.4 (44.4 to 93.8) | 65.3* (59.4 to 71.2) | 12.6* (8.6 to 16.7) |
| Metastatic phase of colon and rectum cancers | 72.2 (70.2 to 74.3) | -- | 28.8 (20.3 to 37.5) | 56.1* (44.1 to 68.8) | 6.4 (-1.8 to 15.0) |
| Terminal phase of colon and rectum cancers | 9.9 (9.6 to 10.2) | -- | 5.3 (3.7 to 6.8) | 53.7* (48.0 to 59.0) | 4.7* (0.8 to 8.3) |
| Stoma from colon and rectum cancers, beyond 10 years | 20.4 (18.7 to 22.2) | -- | 1.9 (1.2 to 2.6) | 39.7* (27.1 to 53.8) | -4.8 (-13.4 to 4.8) |
| Liver cancer | 138.6 (126.2 to 152.5) | 139.2 (131.6 to 147.9) | 34.2 (24.4 to 45.1) | 71.2* (60.1 to 83.3) | 16.6* (9.1 to 24.9) |
| Liver cancer due to hepatitis B | 96.2 (85.6 to 107.9) | 58.8 (52.3 to 66.0) | 14.1 (10.0 to 19.1) | 73.9* (56.6 to 95.0) | 18.5* (6.7 to 32.9) |
| Diagnosis and primary therapy phase of liver cancer due to hepatitis B | 14.2 (12.6 to 15.9) | 58.8 (52.3 to 66.0) | 3.8 (2.5 to 5.4) | 70.9* (40.7 to 108.0) | 16.5 (-4.1 to 41.8) |
| Controlled phase of liver cancer due to hepatitis B | 66.0 (58.8 to 74.1) | -- | 3.1 (1.9 to 4.6) | 110.1* (85.8 to 136.6) | 43.2* (26.6 to 61.2) |
| Metastatic phase of liver cancer due to hepatitis B | 11.4 (10.2 to 12.9) | -- | 4.8 (3.1 to 6.8) | 63.9* (33.5 to 100.3) | 11.6 (-9.0 to 36.5) |
| Terminal phase of liver cancer due to hepatitis B | 4.6 (4.1 to 5.1) | -- | 2.4 (1.7 to 3.2) | 62.7* (46.0 to 82.2) | 10.9 (-0.5 to 24.1) |
| Liver cancer due to hepatitis C | 63.6 (56.2 to 71.8) | 39.1 (34.2 to 44.4) | 9.7 (6.7 to 13.2) | 62.9* (52.9 to 73.6) | 11.0* (4.2 to 18.3) |
| Diagnosis and primary therapy phase of liver cancer due to hepatitis C | 9.4 (8.3 to 10.7) | 39.1 (34.2 to 44.4) | 2.7 (1.7 to 3.8) | 62.0* (47.8 to 78.3) | 10.4* (0.7 to 21.5) |
| Controlled phase of liver cancer due to hepatitis C | 43.5 (38.5 to 49.0) | -- | 2.0 (1.3 to 3.0) | 74.1* (56.3 to 94.5) | 18.7* (6.5 to 32.5) |
| Metastatic phase of liver cancer due to hepatitis C | 7.6 (6.7 to 8.7) | -- | 3.4 (2.2 to 4.6) | 59.2* (45.1 to 74.3) | 8.4 (-1.1 to 18.8) |
| Terminal phase of liver cancer due to hepatitis C | 3.0 (2.7 to 3.5) | -- | 1.6 (1.1 to 2.2) | 59.4* (52.4 to 66.8) | 8.6* (3.9 to 13.7) |
| Liver cancer due to alcohol use | 31.9 (24.5 to 41.3) | 20.3 (15.7 to 26.4) | 5.0 (3.3 to 7.3) | 69.1* (57.2 to 82.3) | 15.3* (7.1 to 24.2) |
| Diagnosis and primary therapy phase of liver cancer due to alcohol use | 4.9 (3.7 to 6.3) | 20.3 (15.7 to 26.4) | 1.4 (0.9 to 2.1) | 68.4* (51.2 to 88.1) | 14.8* (3.0 to 28.2) |
| Controlled phase of liver cancer due to alcohol use | 21.4 (16.5 to 27.7) | -- | 1.0 (0.6 to 1.6) | 82.8* (57.1 to 113.1) | 24.5* (7.1 to 45.2) |
| Metastatic phase of liver cancer due to alcohol use | 4.0 (3.1 to 5.2) | -- | 1.8 (1.1 to 2.6) | 64.8* (49.5 to 82.5) | 12.3* (1.8 to 24.4) |

| | | | | | |
|---|---|---|---|---|---|
| Terminal phase of liver cancer due to alcohol use | 1.6 (1.2 to 2.1) | -- | 0.9 (0.6 to 1.2) | 64.9* (55.8 to 75.0) | 12.4* (6.1 to 19.2) |
| Liver cancer due to NASH | 15.8 (12.7 to 19.4) | 10.1 (8.1 to 12.3) | 2.5 (1.8 to 3.6) | 83.9* (73.6 to 95.1) | 25.3* (18.3 to 32.9) |
| Diagnosis and primary therapy phase of liver cancer due to NASH | 2.4 (1.9 to 2.9) | 10.1 (8.1 to 12.3) | 0.7 (0.5 to 1.0) | 82.7* (73.9 to 92.2) | 24.5* (18.5 to 31.0) |
| Controlled phase of liver cancer due to NASH | 10.7 (8.6 to 13.0) | -- | 0.5 (0.3 to 0.8) | 103.1* (80.0 to 131.6) | 38.4* (22.6 to 57.8) |
| Metastatic phase of liver cancer due to NASH | 2.0 (1.6 to 2.4) | -- | 0.9 (0.6 to 1.3) | 78.0* (69.6 to 87.3) | 21.3* (15.6 to 27.6) |
| Terminal phase of liver cancer due to NASH | 0.8 (0.6 to 1.0) | -- | 0.4 (0.3 to 0.6) | 78.0* (69.6 to 87.3) | 21.3* (15.6 to 27.6) |
| Liver cancer due to other causes | 17.7 (14.3 to 21.4) | 11.0 (8.9 to 13.3) | 2.8 (1.9 to 3.8) | 81.2* (69.7 to 93.7) | 23.5* (15.6 to 32.0) |
| Diagnosis and primary therapy phase of liver cancer due to other causes | 2.6 (2.1 to 3.2) | 11.0 (8.9 to 13.3) | 0.8 (0.5 to 1.1) | 79.6* (69.6 to 90.7) | 22.4* (15.6 to 29.9) |
| Controlled phase of liver cancer due to other causes | 12.1 (9.8 to 14.6) | -- | 0.6 (0.3 to 0.9) | 106.3* (75.2 to 140.7) | 40.6* (19.4 to 64.0) |
| Metastatic phase of liver cancer due to other causes | 2.1 (1.7 to 2.6) | -- | 1.0 (0.6 to 1.4) | 73.5* (64.1 to 84.0) | 18.2* (11.8 to 25.4) |
| Terminal phase of liver cancer due to other causes | 0.9 (0.7 to 1.0) | -- | 0.5 (0.3 to 0.6) | 73.5* (64.1 to 84.0) | 18.2* (11.8 to 25.4) |
| Gallbladder and biliary tract cancer | 31.7 (27.2 to 35.7) | 24.9 (21.1 to 27.4) | 6.2 (4.3 to 8.2) | 48.8* (37.4 to 62.5) | 1.4 (-6.4 to 10.7) |
| Diagnosis and primary therapy phase of gallbladder and biliary tract cancer | 4.1 (3.6 to 4.6) | 24.9 (21.1 to 27.4) | 1.2 (0.8 to 1.7) | 50.5* (39.7 to 64.6) | 2.6 (-4.8 to 12.2) |
| Controlled phase of gallbladder and biliary tract cancer | 18.7 (16.1 to 21.7) | -- | 0.9 (0.5 to 1.3) | 68.8* (42.9 to 102.8) | 15.1 (-2.6 to 38.2) |
| Metastatic phase of gallbladder and biliary tract cancer | 6.8 (5.8 to 7.5) | -- | 3.1 (2.0 to 4.2) | 44.8* (34.1 to 57.4) | -1.3 (-8.6 to 7.2) |
| Terminal phase of gallbladder and biliary tract cancer | 2.0 (1.7 to 2.2) | -- | 1.1 (0.7 to 1.4) | 44.4* (35.2 to 54.7) | -1.6 (-7.9 to 5.4) |
| Pancreatic cancer | 48.0 (46.7 to 49.3) | 56.3 (54.9 to 57.7) | 12.2 (8.5 to 16.0) | 52.4* (47.0 to 57.5) | 3.9* (0.2 to 7.3) |
| Diagnosis and primary therapy phase of pancreatic cancer | 12.4 (12.1 to 12.8) | 56.3 (54.9 to 57.7) | 3.5 (2.4 to 4.9) | 53.0* (44.0 to 62.2) | 4.3 (-1.9 to 10.5) |
| Controlled phase of pancreatic cancer | 19.2 (18.7 to 19.8) | -- | 0.9 (0.6 to 1.3) | 28.9* (16.8 to 42.3) | -12.2* (-20.4 to -3.1) |
| Metastatic phase of pancreatic cancer | 11.7 (11.4 to 12.0) | -- | 5.3 (3.6 to 7.1) | 55.5* (50.1 to 59.9) | 5.9* (2.3 to 9.0) |
| Terminal phase of pancreatic cancer | 4.6 (4.5 to 4.7) | -- | 2.5 (1.7 to 3.2) | 55.5* (50.6 to 59.6) | 6.0* (2.6 to 8.7) |
| Larynx cancer | 206.8 (201.1 to 213.2) | 38.0 (36.9 to 39.2) | 20.1 (14.6 to 26.2) | 58.7* (50.4 to 67.3) | 8.1* (2.5 to 14.0) |
| Diagnosis and primary therapy phase of larynx cancer | 12.4 (12.1 to 12.8) | 38.0 (36.9 to 39.2) | 3.4 (2.3 to 4.7) | 64.1* (47.3 to 82.6) | 11.8* (0.4 to 24.4) |
| Controlled phase of larynx cancer | 172.2 (167.2 to 177.7) | -- | 9.4 (6.2 to 13.6) | 62.7* (52.9 to 72.7) | 10.9* (4.2 to 17.7) |
| Metastatic phase of larynx cancer | 13.8 (13.4 to 14.2) | -- | 5.9 (4.0 to 7.8) | 53.0* (38.0 to 68.1) | 4.3 (-6.0 to 14.5) |
| Terminal phase of larynx cancer | 1.9 (1.9 to 2.0) | -- | 1.0 (0.7 to 1.3) | 47.4* (41.7 to 53.1) | 0.4 (-3.5 to 4.3) |
| Laryngectomy from larynx cancer, beyond 10 years | 6.5 (6.1 to 6.9) | -- | 0.3 (0.2 to 0.5) | 40.1* (31.0 to 49.7) | -4.5 (-10.8 to 2.0) |
| Tracheal, bronchus, and lung cancer | 569.6 (551.1 to 588.0) | 325.8 (316.0 to 335.6) | 85.4 (62.9 to 108.4) | 69.8* (60.0 to 79.4) | 15.7* (9.0 to 22.3) |
| Diagnosis and primary therapy phase of lung, bronchus, and trachea cancer | 36.3 (35.1 to 37.4) | 325.8 (316.0 to 335.6) | 9.5 (6.5 to 13.0) | 83.4* (63.2 to 104.3) | 25.0* (11.2 to 39.2) |
| Controlled phase of lung, bronchus, and trachea cancer | 394.7 (381.1 to 408.5) | -- | 18.1 (11.7 to 26.2) | 102.0* (91.8 to 113.0) | 37.6* (30.7 to 45.2) |
| Metastatic phase of lung, bronchus, and trachea cancer | 113.5 (110.2 to 116.9) | -- | 45.2 (31.1 to 59.0) | 59.8* (48.9 to 71.6) | 8.9* (1.4 to 16.9) |
| Terminal phase of lung, bronchus, and trachea cancer | 25.2 (24.4 to 25.9) | -- | 12.5 (8.8 to 16.1) | 60.0* (42.7 to 79.7) | 9.0 (-2.7 to 22.4) |
| Malignant skin melanoma | 245.5 (183.8 to 294.8) | 31.3 (23.6 to 37.3) | 14.5 (9.1 to 21.1) | 48.7* (39.1 to 61.8) | 1.3 (-5.2 to 10.3) |
| Diagnosis and primary therapy phase of malignant skin melanoma | 6.6 (5.0 to 8.0) | 31.3 (23.6 to 37.3) | 1.8 (1.1 to 2.6) | 49.0* (32.5 to 71.3) | 1.5 (-9.7 to 16.7) |
| Controlled phase of malignant skin melanoma | 234.5 (175.5 to 281.9) | -- | 10.7 (6.3 to 16.4) | 49.8* (39.5 to 64.1) | 2.1 (-5.0 to 11.8) |
| Metastatic phase of malignant skin melanoma | 3.7 (2.8 to 4.4) | -- | 1.7 (1.0 to 2.3) | 42.8* (33.2 to 54.5) | -2.7 (-9.2 to 5.3) |
| Terminal phase of malignant skin melanoma | 0.6 (0.5 to 0.7) | -- | 0.3 (0.2 to 0.5) | 42.1* (35.0 to 51.5) | -3.1 (-8.0 to 3.3) |
| Non-melanoma skin cancer | 289.3 (157.6 to 512.1) | 949.1 (507.5 to 1518.3) | 10.2 (4.6 to 21.1) | 41.7* (26.9 to 54.8) | -3.4 (-13.5 to 5.5) |
| Non-melanoma skin cancer (squamous-cell carcinoma) | 233.6 (113.1 to 458.8) | 197.5 (108.2 to 342.2) | 9.9 (4.3 to 20.7) | 41.6* (26.3 to 55.0) | -3.5 (-13.9 to 5.6) |
| Non-melanoma skin cancer (basal-cell carcinoma) | 78.5 (31.9 to 158.6) | 751.6 (325.2 to 1306.0) | 0.3 (0.1 to 0.8) | 45.7* (31.4 to 57.7) | -0.7 (-10.5 to 7.5) |
| Breast cancer | 2130.9 (2046.9 to 2201.1) | 236.8 (226.7 to 246.0) | 162.4 (115.9 to 219.8) | 53.6* (47.4 to 59.8) | 4.7* (0.4 to 8.9) |
| Diagnosis and primary therapy phase of breast cancer | 51.7 (49.5 to 53.7) | 236.8 (226.7 to 246.0) | 13.2 (9.1 to 18.2) | 57.2* (43.3 to 72.5) | 7.1 (-2.3 to 17.5) |
| Controlled phase of breast cancer | 1702.9 (1628.7 to 1768.1) | -- | 106.9 (68.6 to 154.6) | 55.2* (48.4 to 61.8) | 5.7* (1.1 to 10.3) |
| Metastatic phase of breast cancer | 74.6 (71.6 to 77.7) | -- | 29.5 (20.3 to 38.4) | 52.3* (40.2 to 65.5) | 3.8 (-4.5 to 12.8) |
| Terminal phase of breast cancer | 5.6 (5.4 to 5.9) | -- | 3.0 (2.1 to 3.9) | 47.1* (40.0 to 53.5) | 0.2 (-4.6 to 4.6) |
| Mastectomy from breast cancer, beyond 10 years | 296.1 (277.6 to 313.8) | -- | 9.9 (5.7 to 15.4) | 40.0* (34.7 to 45.4) | -4.6* (-8.2 to -0.9) |
| Cervical cancer | 246.7 (224.7 to 258.4) | 51.5 (46.9 to 54.0) | 21.5 (15.6 to 28.2) | 46.2* (34.8 to 56.0) | -0.4 (-8.2 to 6.3) |
| Diagnosis and primary therapy phase of cervical cancer | 12.5 (11.4 to 13.1) | 51.5 (46.9 to 54.0) | 3.4 (2.2 to 4.7) | 47.7* (32.8 to 62.1) | 0.6 (-9.5 to 10.5) |
| Controlled phase of cervical cancer | 214.9 (195.7 to 225.1) | -- | 9.8 (6.3 to 14.4) | 48.1* (35.5 to 58.3) | 0.9 (-7.7 to 7.8) |
| Metastatic phase of cervical cancer | 16.7 (15.3 to 17.5) | -- | 6.9 (4.6 to 9.2) | 43.6* (27.6 to 61.7) | -2.1 (-13.1 to 10.2) |
| Terminal phase of cervical cancer | 2.6 (2.4 to 2.7) | -- | 1.4 (1.0 to 1.8) | 42.1* (31.6 to 49.4) | -3.2 (-10.3 to 1.8) |
| Uterine cancer | 518.0 (497.1 to 537.7) | 65.9 (63.2 to 68.3) | 34.3 (24.4 to 46.1) | 65.2* (56.1 to 73.7) | 12.6* (6.4 to 18.3) |
| Diagnosis and primary therapy phase of uterine cancer | 23.5 (22.5 to 24.3) | 65.9 (63.2 to 68.3) | 6.1 (4.2 to 8.4) | 66.5* (48.9 to 84.7) | 13.5* (1.5 to 25.9) |
| Controlled phase of uterine cancer | 479.7 (460.3 to 498.0) | -- | 21.9 (14.2 to 31.5) | 67.5* (59.1 to 76.4) | 14.2* (8.4 to 20.2) |
| Metastatic phase of uterine cancer | 13.4 (12.9 to 14.0) | -- | 5.6 (3.7 to 7.3) | 57.1* (39.4 to 77.0) | 7.0 (-5.0 to 20.6) |
| Terminal phase of uterine cancer | 1.4 (1.3 to 1.4) | -- | 0.7 (0.5 to 1.0) | 52.6* (46.6 to 58.6) | 4.0 (-0.1 to 8.1) |
| Ovarian cancer | 131.8 (127.5 to 136.4) | 32.8 (31.7 to 34.0) | 17.7 (12.9 to 22.7) | 48.8* (38.1 to 61.0) | 1.4 (-5.9 to 9.7) |
| Diagnosis and primary therapy phase of ovarian cancer | 4.6 (4.4 to 4.7) | 32.8 (31.7 to 34.0) | 1.3 (0.9 to 1.8) | 49.8* (44.5 to 55.7) | 2.1 (-1.5 to 6.1) |
| Controlled phase of ovarian cancer | 97.8 (94.6 to 101.2) | -- | 4.5 (2.8 to 6.5) | 50.4* (40.3 to 62.0) | 2.5 (-4.4 to 10.4) |
| Metastatic phase of ovarian cancer | 27.5 (26.5 to 28.5) | -- | 10.9 (7.4 to 14.3) | 47.8* (32.0 to 65.6) | 0.7 (-10.1 to 12.9) |

| | | | | | |
|---|---|---|---|---|---|
| Terminal phase of ovarian cancer | 2.0 (1.9 to 2.1) | -- | 1.1 (0.8 to 1.4) | 50.3* (44.3 to 56.4) | 2.4 (-1.7 to 6.6) |
| Prostate cancer | 1582.6 (1361.9 to 2011.0) | 185.1 (158.2 to 234.5) | 122.4 (86.6 to 168.4) | 51.4* (42.6 to 64.9) | 3.2 (-2.8 to 12.3) |
| Diagnosis and primary therapy phase of prostate cancer | 55.6 (47.7 to 70.8) | 185.1 (158.2 to 234.5) | 14.3 (9.7 to 20.3) | 54.1* (40.0 to 73.2) | 5.0 (-4.6 to 18.0) |
| Controlled phase of prostate cancer | 1451.3 (1246.1 to 1852.4) | -- | 86.0 (55.5 to 126.4) | 52.0* (43.1 to 66.3) | 3.6 (-2.5 to 13.3) |
| Metastatic phase of prostate cancer | 49.4 (41.5 to 60.4) | -- | 19.6 (13.4 to 26.6) | 47.9* (34.9 to 63.0) | 0.7 (-8.1 to 11.1) |
| Terminal phase of prostate cancer | 2.7 (2.3 to 3.3) | -- | 1.5 (1.0 to 1.9) | 43.8* (36.8 to 53.1) | -2.0 (-6.8 to 4.3) |
| Impotence and Incontinence after 10-year survival from prostate cancer | 23.7 (22.7 to 24.8) | -- | 1.1 (0.8 to 1.6) | 42.3* (26.0 to 60.6) | -3.0 (-14.2 to 9.4) |
| Testicular cancer | 10.9 (10.1 to 11.9) | 1.6 (1.5 to 1.7) | 0.8 (0.5 to 1.1) | 75.5* (54.1 to 99.1) | 19.6* (5.0 to 35.7) |
| Diagnosis and primary therapy phase of testicular cancer | 0.4 (0.3 to 0.4) | 1.6 (1.5 to 1.7) | 0.1 (0.1 to 0.1) | 80.5* (65.8 to 98.3) | 23.0* (13.0 to 35.2) |
| Controlled phase of testicular cancer | 10.1 (9.4 to 11.0) | -- | 0.5 (0.3 to 0.7) | 80.1* (50.1 to 116.2) | 22.7* (2.3 to 47.3) |
| Metastatic phase of testicular cancer | 0.4 (0.4 to 0.4) | -- | 0.2 (0.1 to 0.2) | 63.5* (53.0 to 77.0) | 11.4* (4.2 to 20.6) |
| Terminal phase of testicular cancer | -- | -- | -- | 61.5* (51.1 to 73.3) | 10.0* (3.0 to 18.1) |
| Kidney cancer | 291.3 (278.7 to 304.5) | 50.7 (48.5 to 52.9) | 18.5 (13.2 to 25.0) | 49.0* (41.2 to 57.9) | 1.5 (-3.8 to 7.6) |
| Diagnosis and primary therapy phase of kidney cancer | -- | 50.7 (48.5 to 52.9) | -- | -- | -- |
| Controlled phase of kidney cancer | 278.3 (266.3 to 291.0) | -- | 12.7 (8.2 to 18.4) | 49.6* (41.1 to 59.0) | 2.0 (-3.8 to 8.3) |
| Metastatic phase of kidney cancer | 10.9 (10.4 to 11.4) | -- | 4.7 (3.2 to 6.3) | 47.7* (34.4 to 61.4) | 0.6 (-8.4 to 10.0) |
| Terminal phase of kidney cancer | 2.1 (2.0 to 2.2) | -- | 1.1 (0.8 to 1.5) | 47.7* (41.4 to 55.1) | 0.6 (-3.6 to 5.7) |
| Bladder cancer | 386.9 (374.4 to 407.3) | 57.9 (55.9 to 61.1) | 34.0 (24.3 to 45.2) | 52.0* (45.5 to 58.8) | 3.5 (-0.8 to 8.2) |
| Diagnosis and primary therapy phase of bladder cancer | 20.4 (19.8 to 21.5) | 57.9 (55.9 to 61.1) | 5.5 (3.7 to 7.5) | 54.9* (40.2 to 71.1) | 5.6 (-4.5 to 16.6) |
| Controlled phase of bladder cancer | 346.1 (334.9 to 364.6) | -- | 21.4 (14.4 to 30.4) | 53.4* (46.2 to 61.0) | 4.5 (-0.4 to 9.7) |
| Metastatic phase of bladder cancer | 11.5 (11.1 to 12.3) | -- | 5.0 (3.4 to 6.5) | 45.4* (30.8 to 62.0) | -0.9 (-10.9 to 10.4) |
| Terminal phase of bladder cancer | 2.1 (2.0 to 2.2) | -- | 1.1 (0.8 to 1.5) | 43.2* (38.4 to 47.9) | -2.4 (-5.7 to 0.8) |
| Incontinence from bladder cancer, beyond 10 years | 6.7 (6.2 to 7.2) | -- | 0.9 (0.6 to 1.4) | 49.0* (40.6 to 57.5) | 1.5 (-4.2 to 7.3) |
| Brain and nervous system cancer | 118.6 (101.1 to 129.7) | 36.7 (32.0 to 39.9) | 13.7 (9.7 to 18.1) | 76.8* (66.4 to 88.2) | 20.5* (13.4 to 28.3) |
| Diagnosis and primary therapy phase of brain and nervous system cancers | 7.3 (6.2 to 8.0) | 36.7 (32.0 to 39.9) | 2.1 (1.4 to 2.9) | 88.7* (74.2 to 104.3) | 28.6* (18.7 to 39.2) |
| Controlled phase of brain and nervous system cancers | 95.7 (81.2 to 105.0) | -- | 4.4 (2.8 to 6.4) | 90.7* (72.9 to 109.9) | 30.0* (17.8 to 43.0) |
| Metastatic phase of brain and nervous system cancers | 13.3 (11.6 to 14.4) | -- | 6.0 (4.0 to 8.1) | 66.7* (57.4 to 76.9) | 13.6* (7.2 to 20.5) |
| Terminal phase of brain and nervous system cancers | 2.4 (2.1 to 2.6) | -- | 1.3 (0.9 to 1.7) | 64.8* (55.9 to 73.5) | 12.3* (6.2 to 18.2) |
| Thyroid cancer | 215.3 (205.8 to 230.2) | 25.8 (24.6 to 27.5) | 13.2 (9.2 to 18.2) | 65.3* (57.3 to 74.5) | 12.7* (7.2 to 18.9) |
| Diagnosis and primary therapy phase of thyroid cancer | 5.8 (5.5 to 6.2) | 25.8 (24.6 to 27.5) | 1.7 (1.1 to 2.3) | 67.2* (57.4 to 77.1) | 13.9* (7.3 to 20.7) |
| Controlled phase of thyroid cancer | 204.6 (195.6 to 218.8) | -- | 9.3 (6.1 to 13.5) | 67.0* (57.3 to 78.0) | 13.8* (7.2 to 21.3) |
| Metastatic phase of thyroid cancer | 4.5 (4.3 to 4.8) | -- | 2.0 (1.4 to 2.7) | 57.8* (51.5 to 65.0) | 7.5* (3.2 to 12.4) |
| Terminal phase of thyroid cancer | 0.4 (0.4 to 0.4) | -- | 0.2 (0.1 to 0.3) | 55.7* (49.2 to 62.9) | 6.1* (1.7 to 11.0) |
| Mesothelioma | 8.7 (8.4 to 9.2) | 3.7 (3.6 to 3.9) | 1.7 (1.2 to 2.2) | 23.8* (15.3 to 32.1) | -15.7* (-21.4 to -10.0) |
| Diagnosis and primary therapy phase of mesothelioma | 1.0 (1.0 to 1.1) | 3.7 (3.6 to 3.9) | 0.3 (0.2 to 0.4) | 22.5* (14.7 to 30.9) | -16.5* (-21.8 to -10.8) |
| Controlled phase of mesothelioma | 5.3 (5.1 to 5.6) | -- | 0.3 (0.2 to 0.4) | 24.0* (6.7 to 45.1) | -15.5* (-27.3 to -1.1) |
| Metastatic phase of mesothelioma | 2.1 (2.1 to 2.3) | -- | 1.0 (0.6 to 1.3) | 24.0* (15.8 to 32.2) | -15.5* (-21.1 to -9.9) |
| Terminal phase of mesothelioma | 0.3 (0.3 to 0.3) | -- | 0.2 (0.1 to 0.2) | 24.1* (16.3 to 31.6) | -15.4* (-20.8 to -10.3) |
| Hodgkin lymphoma | 40.4 (32.3 to 49.3) | 6.5 (5.3 to 7.9) | 3.3 (2.2 to 4.5) | 58.1* (38.1 to 75.6) | 7.7 (-5.9 to 19.6) |
| Diagnosis and primary therapy phase of Hodgkin disease | 1.3 (1.1 to 1.6) | 6.5 (5.3 to 7.9) | 0.4 (0.2 to 0.6) | 67.6* (48.5 to 84.3) | 14.2* (1.2 to 25.6) |
| Controlled phase of Hodgkin disease | 36.4 (29.0 to 44.5) | -- | 1.7 (1.0 to 2.6) | 68.2* (41.3 to 94.9) | 14.6 (-3.7 to 32.8) |
| Metastatic phase of Hodgkin disease | 2.4 (2.0 to 3.0) | -- | 1.1 (0.7 to 1.5) | 45.2* (30.7 to 57.1) | -1.1 (-10.9 to 7.0) |
| Terminal phase of Hodgkin disease | 0.2 (0.2 to 0.3) | -- | 0.1 (0.1 to 0.2) | 30.6* (20.0 to 39.7) | -11.0* (-18.2 to -4.8) |
| Non-Hodgkin's lymphoma | 285.2 (275.9 to 295.4) | 54.2 (52.6 to 56.0) | 22.4 (16.2 to 29.5) | 63.9* (57.0 to 71.6) | 11.7* (7.0 to 16.9) |
| Diagnosis and primary therapy phase of non-Hodgkin lymphoma | 10.5 (10.1 to 10.8) | 54.2 (52.6 to 56.0) | 2.9 (2.0 to 4.0) | 68.2* (53.1 to 84.5) | 14.6* (4.3 to 25.7) |
| Controlled phase of non-Hodgkin lymphoma | 257.8 (249.4 to 267.1) | -- | 11.8 (7.6 to 17.1) | 67.8* (59.2 to 76.6) | 14.3* (8.4 to 20.4) |
| Metastatic phase of non-Hodgkin lymphoma | 14.5 (14.0 to 14.9) | -- | 6.4 (4.3 to 8.5) | 57.3* (47.6 to 67.4) | 7.2* (0.6 to 14.1) |
| Terminal phase of non-Hodgkin lymphoma | 2.5 (2.4 to 2.5) | -- | 1.3 (0.9 to 1.7) | 55.3* (50.2 to 61.5) | 5.8* (2.3 to 10.0) |
| Multiple myeloma | 70.5 (65.3 to 85.3) | 20.1 (18.7 to 23.5) | 13.6 (9.5 to 18.1) | 61.0* (46.9 to 74.4) | 9.7* (0.1 to 18.9) |
| Diagnosis and primary therapy phase of multiple myeloma | 4.5 (4.2 to 5.5) | 20.1 (18.7 to 23.5) | 1.3 (0.8 to 1.9) | 64.4* (56.6 to 73.6) | 12.0* (6.7 to 18.3) |
| Controlled phase of multiple myeloma | 41.5 (38.3 to 50.6) | -- | 1.9 (1.2 to 2.9) | 64.8* (49.1 to 82.7) | 12.3* (1.6 to 24.5) |
| Metastatic phase of multiple myeloma | 23.1 (21.4 to 27.6) | -- | 9.7 (6.5 to 13.0) | 60.7* (43.3 to 77.8) | 9.5 (-2.4 to 21.2) |
| Terminal phase of multiple myeloma | 1.4 (1.3 to 1.6) | -- | 0.7 (0.5 to 1.0) | 50.4* (44.0 to 57.1) | 2.5 (-1.9 to 7.0) |
| Leukaemia | 176.5 (156.2 to 189.6) | 40.5 (35.8 to 43.1) | 21.7 (15.7 to 28.6) | 53.3* (43.6 to 62.4) | 4.4 (-2.1 to 10.6) |
| Acute lymphoid leukaemia | 12.6 (10.3 to 13.8) | 3.4 (2.9 to 3.7) | 1.5 (1.0 to 2.1) | 67.6* (49.6 to 81.9) | 14.2* (1.9 to 23.9) |
| Diagnosis and primary therapy phase of acute lymphoid leukemia | 1.5 (1.3 to 1.6) | 3.4 (2.9 to 3.7) | 0.4 (0.3 to 0.6) | 61.3* (47.3 to 71.3) | 9.9* (0.4 to 16.7) |
| Controlled phase of acute lymphoid leukemia | 9.8 (8.0 to 10.8) | -- | 0.5 (0.3 to 0.7) | 72.0* (51.7 to 89.2) | 17.2* (3.3 to 28.9) |
| Metastatic phase of acute lymphoid leukemia | 1.1 (0.8 to 1.2) | -- | 0.5 (0.3 to 0.7) | 69.0* (48.3 to 85.9) | 15.2* (1.0 to 26.6) |
| Terminal phase of acute lymphoid leukemia | 0.2 (0.2 to 0.2) | -- | 0.1 (0.1 to 0.1) | 69.3* (48.5 to 86.2) | 15.3* (1.2 to 26.8) |
| Chronic lymphoid leukaemia | 71.8 (65.8 to 78.0) | 10.1 (9.3 to 11.0) | 8.8 (6.3 to 11.6) | 55.0* (40.0 to 71.2) | 5.6 (-4.6 to 16.6) |

| | | | | | |
|---|---|---|---|---|---|
| Diagnosis and primary therapy phase of chronic lymphoid leukemia | 3.9 (3.5 to 4.2) | 10.1 (9.3 to 11.0) | 1.1 (0.7 to 1.6) | 56.8* (46.0 to 69.3) | 6.8 (-0.5 to 15.3) |
| Controlled phase of chronic lymphoid leukemia | 56.1 (51.5 to 61.0) | -- | 2.6 (1.7 to 3.8) | 56.3* (41.2 to 73.3) | 6.5 (-3.8 to 18.1) |
| Metastatic phase of chronic lymphoid leukemia | 11.4 (10.5 to 12.4) | -- | 4.9 (3.2 to 6.6) | 54.3* (34.8 to 75.9) | 5.1 (-8.1 to 19.9) |
| Terminal phase of chronic lymphoid leukemia | 0.4 (0.3 to 0.4) | -- | 0.2 (0.1 to 0.3) | 48.5* (38.9 to 59.8) | 1.2 (-5.4 to 8.9) |
| Acute myeloid leukaemia | 10.0 (9.0 to 10.7) | 8.8 (7.8 to 9.4) | 2.1 (1.5 to 2.8) | 41.5* (34.3 to 49.2) | -3.5 (-8.5 to 1.7) |
| Diagnosis and primary therapy phase of acute myeloid leukemia | -- | 8.8 (7.8 to 9.4) | -- | -- | -- |
| Controlled phase of acute myeloid leukemia | 6.2 (5.6 to 6.6) | -- | 0.3 (0.2 to 0.5) | 39.9* (29.3 to 51.3) | -4.7 (-11.9 to 3.1) |
| Metastatic phase of acute myeloid leukemia | 3.2 (2.8 to 3.4) | -- | 1.4 (0.9 to 1.9) | 41.8* (34.7 to 49.6) | -3.4 (-8.2 to 1.9) |
| Terminal phase of acute myeloid leukemia | 0.7 (0.6 to 0.7) | -- | 0.4 (0.3 to 0.5) | 41.8* (34.7 to 49.6) | -3.4 (-8.2 to 1.9) |
| Chronic myeloid leukaemia | 7.6 (6.9 to 8.4) | 2.7 (2.4 to 3.0) | 0.9 (0.7 to 1.2) | 22.6* (14.4 to 30.5) | -16.5* (-22.0 to -11.1) |
| Diagnosis and primary therapy phase of chronic myeloid leukemia | 0.5 (0.5 to 0.6) | 2.7 (2.4 to 3.0) | 0.2 (0.1 to 0.2) | 25.2* (16.9 to 33.7) | -14.7* (-20.3 to -8.9) |
| Controlled phase of chronic myeloid leukemia | 6.0 (5.5 to 6.7) | -- | 0.3 (0.2 to 0.4) | 26.2* (16.6 to 35.1) | -14.0* (-20.6 to -7.9) |
| Metastatic phase of chronic myeloid leukemia | 0.8 (0.7 to 0.9) | -- | 0.4 (0.2 to 0.5) | 19.7* (12.2 to 27.1) | -18.5* (-23.5 to -13.4) |
| Terminal phase of chronic myeloid leukemia | 0.2 (0.2 to 0.2) | -- | 0.1 (0.1 to 0.1) | 19.6* (12.2 to 27.1) | -18.5* (-23.5 to -13.4) |
| Other leukaemia | 74.4 (61.7 to 82.1) | 15.4 (12.8 to 17.0) | 8.4 (5.9 to 11.2) | 56.6* (43.9 to 69.8) | 6.7 (-2.0 to 15.7) |
| Metastatic phase of other leukemia | 11.5 (9.6 to 12.6) | -- | 5.1 (3.4 to 7.1) | 52.2* (39.2 to 65.6) | 3.7 (-5.1 to 12.9) |
| Diagnosis and primary therapy phase of other leukemia | -- | 15.4 (12.8 to 17.0) | -- | -- | -- |
| Terminal phase of other leukemia | 0.7 (0.6 to 0.8) | -- | 0.4 (0.3 to 0.5) | 49.5* (38.3 to 61.7) | 1.9 (-5.8 to 10.2) |
| Controlled phase of other leukemia | 62.2 (51.6 to 68.7) | -- | 2.9 (1.8 to 4.3) | 66.2* (46.8 to 86.0) | 13.2* (0.0 to 26.7) |
| Other malignant cancers | 613.0 (493.5 to 793.3) | 68.6 (61.0 to 72.8) | 49.4 (34.3 to 68.0) | 171.0* (115.0 to 239.2) | 84.7* (46.5 to 131.1) |
| Diagnosis and primary therapy phase of other malignant neoplasms | 14.6 (11.8 to 18.9) | 68.6 (61.0 to 72.8) | 4.0 (2.6 to 5.8) | 212.2* (133.7 to 301.9) | 112.8* (59.3 to 173.8) |
| Controlled phase of other malignant neoplasms | 551.3 (440.7 to 719.6) | -- | 25.4 (15.7 to 38.8) | 253.1* (160.4 to 364.6) | 140.6* (77.4 to 216.6) |
| Metastatic phase of other malignant neoplasms | 41.0 (35.3 to 48.7) | -- | 16.8 (11.4 to 23.1) | 106.6* (71.1 to 146.5) | 40.8* (16.6 to 68.0) |
| Terminal phase of other malignant neoplasms | 6.1 (5.3 to 7.1) | -- | 3.3 (2.3 to 4.4) | 99.6* (73.1 to 132.7) | 36.0* (18.0 to 58.6) |
| Other neoplasms | 2500.8 (2368.7 to 2638.5) | 1059.5 (1008.3 to 1121.5) | 5.6 (3.4 to 8.4) | 69.4* (59.9 to 79.1) | 15.5* (9.0 to 22.0) |
| Myelodysplastic, myeloproliferative, and other hematopoietic neoplasms | 122.1 (98.7 to 145.3) | 14.3 (10.7 to 18.4) | 5.6 (3.4 to 8.4) | 69.4* (59.9 to 79.1) | 15.5* (9.0 to 22.0) |
| Benign and in situ intestinal neoplasms | 516.0 (486.7 to 550.5) | 133.8 (125.9 to 143.1) | -- | -- | -- |
| Benign and in situ cervical and uterine neoplasms | 42.1 (39.6 to 45.0) | 3.1 (2.6 to 3.5) | -- | -- | -- |
| Other benign and in situ neoplasms | 1855.3 (1723.4 to 1989.4) | 908.2 (857.4 to 969.0) | -- | -- | -- |
| **Cardiovascular diseases** | **58414.1 (54627.3 to 62791.4)** | **8136.7 (7588.4 to 8790.6)** | **4199.7 (3025.5 to 5396.3)** | **63.0* (58.5 to 67.9)** | **11.1* (8.0 to 14.4)** |
| Rheumatic heart disease | 1370.9 (1316.2 to 1427.2) | 6.5 (5.2 to 7.8) | 68.5 (45.7 to 97.5) | 49.9* (46.9 to 52.9) | 2.2* (0.1 to 4.2) |
| Rheumatic heart disease, without heart failure | 1223.6 (1145.6 to 1298.7) | 6.5 (5.2 to 7.8) | 55.8 (36.5 to 81.6) | 50.8* (47.9 to 54.1) | 2.8* (0.7 to 5.0) |
| Heart failure due to rheumatic heart disease | 147.2 (102.6 to 199.7) | -- | 12.6 (7.6 to 19.5) | 46.2* (36.1 to 56.6) | -0.4 (-7.2 to 6.7) |
| Ischaemic heart disease | 15403.2 (13954.7 to 16973.1) | 1188.2 (947.6 to 1436.8) | 623.1 (424.1 to 874.4) | 40.9* (39.6 to 42.3) | -4.0* (-4.9 to -3.0) |
| Asymptomatic ischemic heart disease following myocardial infarction | 4859.3 (3802.7 to 6023.4) | -- | -- | -- | -- |
| Angina due to ischemic heart disease | 9011.9 (8154.6 to 9963.5) | -- | 492.9 (332.9 to 699.1) | 40.8* (39.3 to 42.2) | -4.1* (-5.1 to -3.1) |
| Heart failure due to ischemic heart disease | 1466.4 (1097.9 to 1908.9) | -- | 124.0 (78.0 to 189.7) | 42.3* (38.5 to 45.6) | -3.0* (-5.6 to -0.8) |
| Myocardial infarction due to ischemic heart disease | 65.6 (51.9 to 80.7) | 1188.2 (947.6 to 1436.8) | 6.2 (4.1 to 9.2) | 28.6* (21.1 to 36.3) | -12.4* (-17.5 to -7.1) |
| Stroke | 14225.8 (13076.5 to 15517.4) | 1452.2‡ (1268.5 to 1674.9) | 2447.8 (1767.8 to 3143.8) | 79.7* (72.4 to 87.2) | 22.4* (17.4 to 27.5) |
| Ischaemic stroke | 10644.4 (9539.6 to 11912.9) | 947.8 (775.7 to 1168.2) | 1789.6 (1275.7 to 2335.9) | 96.8* (86.8 to 106.6) | 34.1* (27.3 to 40.8) |
| Acute ischemic stroke | 73.9 (59.3 to 92.8) | 947.8 (775.7 to 1168.2) | 19.0 (12.5 to 27.2) | 76.9* (66.9 to 87.5) | 20.6* (13.7 to 27.8) |
| Chronic ischemic stroke | 10570.5 (9469.7 to 11826.8) | -- | 1770.6 (1262.1 to 2309.1) | 97.0* (86.9 to 107.0) | 34.2* (27.3 to 41.1) |
| Intracerebral hemorrhage | 2639.6 (2293.2 to 3041.8) | 384.6 (323.0 to 449.4) | 443.4 (314.2 to 579.0) | 45.4* (34.2 to 58.4) | -0.9 (-8.5 to 7.9) |
| Acute intracerebral hemorrhage | 21.2 (17.2 to 25.5) | 384.6 (323.0 to 449.4) | 5.5 (3.6 to 7.5) | 14.9* (0.8 to 27.0) | -21.7* (-31.3 to -13.5) |
| Chronic intracerebral hemorrhage | 2618.4 (2273.4 to 3020.8) | -- | 438.0 (310.4 to 572.2) | 45.9* (34.5 to 58.9) | -0.6 (-8.4 to 8.3) |
| Subarachnoid hemorrhage | 1379.1 (1198.4 to 1581.1) | 119.7 (94.2 to 148.1) | 214.7 (151.5 to 280.2) | 44.9* (37.7 to 53.1) | -1.2 (-6.2 to 4.3) |
| Acute subarachnoid hemorrhage | 9.6 (7.4 to 12.1) | 119.7 (94.2 to 148.1) | 2.5 (1.6 to 3.6) | 41.2* (37.9 to 43.2) | -3.8* (-6.0 to -2.4) |
| Chronic subarachnoid hemorrhage | 1369.5 (1189.2 to 1571.9) | -- | 212.2 (149.6 to 277.0) | 45.0* (37.6 to 53.2) | -1.2 (-6.2 to 4.4) |
| Hypertensive heart disease | 1672.4 (1236.7 to 2176.6) | -- | 141.5 (92.6 to 212.6) | 54.3* (50.6 to 57.9) | 5.1* (2.6 to 7.6) |
| Non-rheumatic valvular heart disease | 2973.5 (2775.5 to 3201.8) | 3078.4 (2880.8 to 3305.9) | 20.6 (10.5 to 34.9) | 40.9* (34.9 to 46.6) | -4.0* (-8.1 to -0.1) |
| Non-rheumatic calcific aortic valve disease | 1065.7 (885.9 to 1284.6) | 1065.7 (885.9 to 1284.6) | 6.9 (3.3 to 12.1) | 39.6* (30.8 to 47.9) | -4.9 (-10.9 to 0.8) |
| Heart failure due to calcific aortic valve disease | 49.8 (27.2 to 83.7) | -- | 4.5 (2.2 to 7.7) | 32.5* (13.6 to 49.6) | -9.7 (-22.6 to 1.9) |
| Non-rheumatic calcific aortic valve disease | 1015.9 (843.4 to 1224.9) | 1065.7 (885.9 to 1284.6) | 2.4 (1.1 to 4.4) | 55.1* (31.2 to 82.6) | 5.7 (-10.6 to 24.4) |
| Non-rheumatic degenerative mitral valve disease | 2012.7 (1922.6 to 2107.7) | 2012.7 (1922.6 to 2107.7) | 13.5 (6.7 to 23.4) | 41.3* (33.9 to 49.1) | -3.7 (-8.8 to 1.6) |
| Heart failure due to degenerative mitral valve disease | 114.8 (65.0 to 189.3) | -- | 9.9 (4.7 to 17.0) | 34.1* (20.6 to 47.7) | -8.6 (-17.8 to 0.7) |
| Non-rheumatic degenerative mitral valve disease | 1898.0 (1790.9 to 1997.9) | 2012.7 (1922.6 to 2107.7) | 3.6 (1.7 to 6.5) | 66.1* (41.3 to 91.3) | 13.2 (-3.7 to 30.4) |
| Other non-rheumatic valve diseases | 1.9 (1.3 to 2.7) | -- | 0.2 (0.1 to 0.3) | 60.3* (50.5 to 69.5) | 9.2* (2.6 to 15.5) |
| Mild heart failure due to other non-rheumatic valve disease | 0.4 (0.2 to 0.5) | -- | -- | 60.3* (57.1 to 63.4) | 9.3* (7.0 to 11.4) |
| Moderate heart failure due to other non-rheumatic valve disease | 0.2 (0.1 to 0.4) | -- | -- | 60.3* (57.1 to 63.5) | 9.2* (7.0 to 11.4) |
| Severe heart failure due to other non-rheumatic valve disease | 0.6 (0.4 to 0.9) | -- | 0.1 (0.1 to 0.2) | 60.3* (45.6 to 74.4) | 9.2 (-0.8 to 18.8) |

| | | | | | |
|---|---|---|---|---|---|
| Controlled, medically managed heart failure due to other non-rheumatic valve disease | 0.7 (0.5 to 1.0) | -- | 0.0 (0.0 to 0.1) | 60.3* (45.2 to 75.1) | 9.2 (-1.0 to 19.3) |
| Cardiomyopathy and myocarditis | 619.5 (458.3 to 811.7) | 252.5 (192.8 to 335.6) | 58.4 (36.8 to 89.8) | 43.3* (39.2 to 47.3) | -2.4 (-5.2 to 0.4) |
| Myocarditis | 114.0 (90.6 to 139.8) | 252.5 (192.8 to 335.6) | 7.7 (4.9 to 11.5) | 47.6* (40.3 to 54.9) | 0.6 (-4.4 to 5.6) |
| Acute myocarditis | 59.9 (44.4 to 79.4) | 252.5 (192.8 to 335.6) | 2.8 (1.7 to 4.5) | 46.7* (34.8 to 60.7) | -0.0 (-8.2 to 9.5) |
| Heart failure due to myocarditis | 54.1 (36.1 to 75.2) | -- | 4.9 (2.9 to 7.8) | 48.1* (39.6 to 56.6) | 0.9 (-4.9 to 6.7) |
| Alcoholic cardiomyopathy | 217.1 (155.4 to 289.2) | -- | 18.5 (11.7 to 28.2) | 44.5* (37.9 to 51.5) | -1.5 (-6.0 to 3.2) |
| Other cardiomyopathy | 380.4 (265.8 to 510.7) | -- | 32.2 (19.6 to 49.9) | 41.6* (36.4 to 46.8) | -3.5 (-7.1 to 0.0) |
| Atrial fibrillation and flutter | 3844.6 (3005.4 to 4803.4) | 432.5 (330.5 to 540.7) | 308.5 (202.0 to 451.9) | 47.4* (44.7 to 49.9) | 0.4 (-1.4 to 2.2) |
| Asymptomatic atrial fibrillation and flutter | 2307.0 (1792.8 to 2883.8) | 432.5 (330.5 to 540.7) | -- | -- | -- |
| Symptomatic atrial fibrillation and flutter | 1537.5 (1201.7 to 1911.8) | -- | 308.5 (202.0 to 451.9) | 47.4* (44.7 to 49.9) | 0.4 (-1.4 to 2.2) |
| Peripheral vascular disease | 15454.8 (12491.5 to 18874.5) | 1642.9 (1231.6 to 2102.0) | 71.7 (33.4 to 131.4) | 39.2* (37.4 to 41.0) | -5.2* (-6.4 to -3.9) |
| Asymptomatic peripheral arterial disease | 10099.1 (7747.0 to 12736.2) | 1642.9 (1231.6 to 2102.0) | -- | -- | -- |
| Symptomatic claudication due to peripheral arterial disease | 5355.7 (3924.3 to 7178.5) | -- | 71.7 (33.4 to 131.4) | 39.2* (37.4 to 41.0) | -5.2* (-6.4 to -3.9) |
| Endocarditis | 54.7 (41.1 to 72.4) | 83.5 (63.2 to 105.3) | 4.7 (2.9 to 7.1) | 42.3* (32.9 to 52.7) | -3.0 (-9.5 to 4.1) |
| Acute endocarditis | 6.2 (4.6 to 8.0) | 83.5 (63.2 to 105.3) | 0.4 (0.2 to 0.6) | 55.6* (48.1 to 61.8) | 6.1* (0.9 to 10.2) |
| Heart failure due to endocarditis | 48.5 (34.8 to 66.0) | -- | 4.3 (2.7 to 6.6) | 41.3* (30.9 to 52.3) | -3.7 (-10.8 to 3.8) |
| Other cardiovascular and circulatory diseases | 8807.0 (6312.7 to 12132.2) | -- | 455.1 (274.0 to 694.5) | 44.4* (41.9 to 46.9) | -1.6 (-3.3 to 0.1) |
| Heart failure due to other cardiovascular diseases | 127.2 (90.3 to 176.7) | -- | 11.0 (6.6 to 16.9) | 44.2* (35.7 to 53.1) | -1.8 (-7.6 to 4.3) |
| Other cardiovascular and circulatory disease episodes | 8679.8 (6220.5 to 11968.2) | -- | 444.0 (267.2 to 677.4) | 44.4* (41.9 to 46.9) | -1.6 (-3.3 to 0.1) |
| **Chronic respiratory diseases** | **46599.3 (41375.9 to 52564.3)** | **3655.5 (2767.7 to 4651.2)** | **4324.0 (3491.2 to 5167.5)** | **39.8* (31.7 to 46.7)** | **-4.7* (-10.3 to -0.0)** |
| Chronic obstructive pulmonary disease | 35593.8 (29984.2 to 41728.0) | 2254.9 (1516.0 to 3088.5) | 3675.9 (2961.2 to 4454.9) | 38.4* (29.1 to 46.6) | -5.7* (-12.0 to -0.1) |
| Chronic obstructive pulmonary disease with heart failure | 938.3 (688.8 to 1228.0) | -- | 379.7 (246.5 to 535.9) | 46.5* (42.2 to 50.6) | -0.2 (-3.1 to 2.6) |
| Chronic obstructive pulmonary disease without heart failure | 34655.5 (28951.0 to 40797.0) | 2254.9 (1516.0 to 3088.5) | 3296.2 (2632.3 to 4026.7) | 37.5* (27.3 to 46.5) | -6.3* (-13.3 to -0.2) |
| Pneumoconiosis | 61.6 (50.9 to 74.3) | 8.0 (6.4 to 10.0) | 9.6 (6.3 to 14.0) | 47.8* (37.4 to 58.4) | 0.7 (-6.4 to 7.9) |
| Silicosis | 18.5 (12.5 to 27.0) | 3.2 (2.1 to 4.7) | 2.9 (1.7 to 4.9) | 52.5* (31.4 to 76.0) | 3.9 (-10.5 to 19.9) |
| Heart failure due to silicosis | 0.7 (0.5 to 0.9) | -- | 0.3 (0.2 to 0.5) | 42.3* (36.1 to 48.5) | -3.0 (-7.3 to 1.2) |
| Silicosis without heart failure | 17.8 (11.9 to 26.3) | 3.2 (2.1 to 4.7) | 2.6 (1.4 to 4.5) | 54.0* (29.5 to 81.4) | 4.9 (-11.8 to 23.6) |
| Asbestosis | 10.5 (6.9 to 15.6) | 1.1 (0.7 to 1.8) | 1.7 (0.9 to 2.8) | 49.3* (38.9 to 60.8) | 1.7 (-5.4 to 9.6) |
| Asbestosis without heart failure | 10.2 (6.6 to 15.3) | 1.1 (0.7 to 1.8) | 1.5 (0.8 to 2.6) | 51.8* (40.5 to 64.1) | 3.4 (-4.3 to 11.8) |
| Heart failure due to asbestosis | 0.3 (0.2 to 0.4) | -- | 0.1 (0.1 to 0.2) | 25.7* (19.0 to 32.1) | -14.4* (-18.9 to -10.0) |
| Coal workers pneumoconiosis | 16.8 (11.2 to 24.8) | 2.1 (1.3 to 3.2) | 2.6 (1.5 to 4.3) | 44.7* (21.4 to 69.4) | -1.4 (-17.3 to 15.4) |
| Coal workers pneumoconiosis without heart failure | 16.1 (10.5 to 24.0) | 2.1 (1.3 to 3.2) | 2.3 (1.2 to 3.9) | 44.9* (17.4 to 74.3) | -1.3 (-20.0 to 18.8) |
| Heart failure due to Coal workers pneumoconiosis | 0.7 (0.5 to 0.9) | -- | 0.3 (0.2 to 0.5) | 42.9* (36.8 to 49.0) | -2.6 (-6.8 to 1.5) |
| Other pneumoconiosis | 15.8 (11.3 to 21.5) | 1.5 (1.0 to 2.2) | 2.5 (1.5 to 3.8) | 44.9* (32.4 to 59.6) | -1.2 (-9.8 to 8.7) |
| Heart failure due to other pneumoconiosis | 0.3 (0.2 to 0.4) | -- | 0.1 (0.1 to 0.2) | 30.2* (19.2 to 39.8) | -11.3* (-18.7 to -4.7) |
| Other pneumoconiosis without heart failure | 15.5 (11.0 to 21.2) | 1.5 (1.0 to 2.2) | 2.3 (1.3 to 3.6) | 46.0* (32.8 to 61.7) | -0.5 (-9.5 to 10.2) |
| Asthma | 13774.8 (11311.9 to 17051.6) | 1337.7 (931.6 to 1851.8) | 517.7 (340.8 to 760.6) | 48.6* (39.8 to 58.5) | 1.3 (-4.8 to 8.0) |
| Asymptomatic asthma | 5004.9 (3997.8 to 6322.2) | 484.9 (339.0 to 681.4) | -- | -- | -- |
| Symptomatic asthma | 8769.8 (7039.2 to 11022.9) | 852.8 (591.5 to 1198.0) | 517.7 (340.8 to 760.6) | 48.6* (39.8 to 58.5) | 1.3 (-4.8 to 8.0) |
| Interstitial lung disease and pulmonary sarcoidosis | 705.6 (602.8 to 826.8) | 54.9 (36.9 to 75.9) | 74.1 (49.7 to 108.7) | 45.6* (40.2 to 51.7) | -0.8 (-4.5 to 3.4) |
| Heart failure due to interstitial lung disease and pulmonary sarcoidosis | 36.0 (27.0 to 45.3) | -- | 16.1 (10.5 to 23.0) | 30.6* (26.2 to 35.1) | -11.0* (-14.0 to -7.9) |
| Interstitial lung disease and pulmonary sarcoidosis without heart failure | 669.6 (567.2 to 788.3) | 54.9 (36.9 to 75.9) | 57.9 (36.9 to 88.2) | 50.4* (43.3 to 58.5) | 2.5 (-2.4 to 8.0) |
| Other chronic respiratory diseases | -- | -- | 46.7 (37.7 to 56.5) | 53.1* (43.0 to 61.9) | 4.3 (-2.5 to 10.3) |
| **Digestive diseases** | **132358.7 (121560.1 to 143551.6)** | **33991.1 (26935.8 to 41068.3)** | **1523.4 (1042.0 to 2126.5)** | **54.7* (51.7 to 58.0)** | **5.4* (3.4 to 7.7)** |
| Cirrhosis and other chronic liver diseases | 96336.9 (88679.5 to 103625.1) | 209.1 (190.0 to 229.7) | 185.0 (130.1 to 254.0) | 71.0* (66.2 to 76.0) | 16.5* (13.2 to 19.9) |
| Cirrhosis and other chronic liver diseases due to hepatitis B | 19443.8 (18023.6 to 20911.0) | 56.4 (47.5 to 65.7) | 49.9 (34.1 to 69.4) | 78.2* (70.0 to 86.6) | 21.4* (15.9 to 27.1) |
| Cirrhosis and other chronic liver diseases due to hepatitis B, decompensated | 308.8 (270.0 to 350.7) | 56.2 (47.3 to 65.5) | 49.9 (34.1 to 69.4) | 78.2* (70.0 to 86.6) | 21.4* (15.9 to 27.1) |
| Chronic hepatitis B without cirrhosis | 16574.8 (15132.2 to 18119.3) | -- | -- | -- | -- |
| Cirrhosis and other chronic liver diseases due to hepatitis B, compensated | 2560.2 (2250.4 to 2879.3) | 0.2 (0.1 to 0.5) | -- | -- | -- |
| Cirrhosis and other chronic liver diseases due to hepatitis C | 7531.2 (6447.0 to 8713.3) | 55.7 (48.0 to 64.6) | 48.4 (32.5 to 67.7) | 63.1* (56.1 to 70.1) | 11.1* (6.4 to 15.9) |
| Cirrhosis and other chronic liver diseases due to hepatitis C, decompensated | 300.8 (262.4 to 337.9) | 54.3 (46.6 to 63.2) | 48.4 (32.5 to 67.7) | 63.1* (56.1 to 70.1) | 11.1* (6.4 to 15.9) |
| Chronic hepatitis C without cirrhosis | 5186.7 (4058.9 to 6437.2) | -- | -- | -- | -- |
| Cirrhosis and other chronic liver diseases due to hepatitis C, compensated | 2043.7 (1765.5 to 2326.1) | 1.5 (0.4 to 3.0) | -- | -- | -- |
| Cirrhosis and other chronic liver diseases due to alcohol use | 2416.3 (2109.2 to 2707.8) | 57.3 (49.5 to 65.8) | 53.0 (35.9 to 73.9) | 69.0* (61.7 to 76.8) | 15.1* (10.2 to 20.5) |
| Cirrhosis and other chronic liver diseases due to alcohol, decompensated | 329.9 (289.5 to 369.9) | 56.1 (48.1 to 64.8) | 53.0 (35.9 to 73.9) | 69.0* (61.7 to 76.8) | 15.1* (10.2 to 20.5) |
| Cirrhosis and other chronic liver diseases due to alcohol, compensated | 2086.4 (1816.1 to 2346.8) | 1.2 (0.4 to 2.4) | -- | -- | -- |
| Cirrhosis due to NASH | 66243.3 (59252.1 to 73700.5) | 21.6 (18.2 to 25.3) | 18.5 (12.6 to 25.8) | 88.9* (77.7 to 101.7) | 28.7* (21.1 to 37.5) |
| Non-alcoholic fatty liver disease (NAFLD) / Non-alcoholic steatohepatitis (NASH) | 65313.1 (58242.8 to 72812.0) | -- | -- | -- | -- |
| Cirrhosis and other chronic liver diseases due to NASH | 930.2 (794.7 to 1067.1) | 21.6 (18.2 to 25.3) | 18.5 (12.6 to 25.8) | 88.9* (77.7 to 101.7) | 28.7* (21.1 to 37.5) |
| Cirrhosis and other chronic liver diseases due to other causes | 702.4 (533.6 to 892.3) | 18.0 (13.4 to 23.1) | 15.2 (9.9 to 22.5) | 62.9* (52.8 to 74.3) | 11.0* (4.1 to 18.7) |

| | | | | | |
|---|---|---|---|---|---|
| Cirrhosis and other chronic liver diseases due to other cause, decompensated | 93.7 (71.7 to 119.8) | 17.0 (12.6 to 22.1) | 15.2 (9.9 to 22.5) | 62.9* (52.8 to 74.3) | 11.0* (4.1 to 18.7) |
| Cirrhosis and other chronic liver diseases due to other cause, compensated | 608.6 (463.6 to 771.9) | 1.0 (0.4 to 1.7) | -- | -- | -- |
| Upper digestive system diseases | 56989.9 (43739.6 to 71400.1) | 27136.7 (20284.7 to 34376.0) | 913.2 (566.8 to 1399.4) | 51.1* (47.4 to 55.6) | 3.0* (0.4 to 6.0) |
| Peptic ulcer disease | 1276.5 (981.8 to 1618.8) | 518.8 (376.1 to 675.6) | 60.1 (39.2 to 89.6) | 47.6* (40.7 to 54.9) | 0.6 (-4.1 to 5.5) |
| Acute peptic ulcer disease | 0.8 (0.5 to 1.0) | -- | 0.3 (0.1 to 0.4) | 30.8* (28.3 to 33.2) | -10.9* (-12.5 to -9.2) |
| Chronic peptic ulcer disease | 1275.8 (981.0 to 1617.9) | 518.8 (376.1 to 675.6) | 59.9 (39.0 to 89.3) | 47.7* (40.7 to 55.0) | 0.7 (-4.1 to 5.6) |
| Gastritis and duodenitis | 10244.4 (7109.9 to 13958.2) | 7216.7 (4974.3 to 9962.7) | 431.4 (258.0 to 663.8) | 53.6* (46.3 to 61.5) | 4.7 (-0.3 to 10.1) |
| Acute gastritis and duodenitis | 2.9 (2.0 to 4.4) | -- | 1.0 (0.5 to 1.6) | 30.1* (28.2 to 32.0) | -11.3* (-12.6 to -10.1) |
| Chronic gastritis and duodenitis | 10241.5 (7105.6 to 13955.4) | 7216.7 (4974.3 to 9962.7) | 430.4 (257.3 to 662.8) | 53.7* (46.4 to 61.6) | 4.7 (-0.3 to 10.1) |
| GERD | 50620.0 (35368.2 to 67799.2) | 19401.2 (13113.4 to 26180.2) | 421.8 (211.7 to 729.3) | 49.2* (46.7 to 51.7) | 1.6 (-0.1 to 3.3) |
| Appendicitis | 17.1 (14.0 to 20.6) | 452.8 (375.2 to 542.7) | 5.2 (3.4 to 7.5) | 43.3* (29.0 to 57.9) | -2.4 (-12.1 to 7.6) |
| Paralytic ileus and intestinal obstruction | 9.9 (7.4 to 12.9) | 276.8 (207.1 to 355.5) | 3.1 (2.0 to 4.8) | 54.3* (40.5 to 67.6) | 5.2 (-4.3 to 14.2) |
| Inguinal, femoral, and abdominal hernia | 2030.5 (1526.8 to 2697.6) | 4975.0 (3592.4 to 6601.8) | 191.0 (120.7 to 288.2) | 47.1* (40.3 to 54.2) | 0.3 (-4.4 to 5.1) |
| Inflammatory bowel disease | 779.6 (658.7 to 910.8) | 219.0 (170.8 to 269.0) | 111.3 (73.6 to 154.4) | 63.3* (54.1 to 74.3) | 11.3* (5.0 to 18.7) |
| Ulcerative colitis | 599.5 (486.0 to 727.5) | 165.6 (120.4 to 216.2) | 84.4 (54.7 to 118.8) | 73.4* (62.6 to 85.3) | 18.2* (10.8 to 26.3) |
| Crohn's disease | 180.1 (151.0 to 213.4) | 53.4 (40.5 to 68.6) | 26.9 (17.9 to 37.4) | 38.0* (27.4 to 49.2) | -6.0 (-13.2 to 1.7) |
| Vascular intestinal disorders | 7.4 (5.2 to 10.3) | 52.1 (37.8 to 68.5) | 2.3 (1.4 to 3.7) | 46.7* (31.4 to 63.1) | -0.1 (-10.4 to 11.1) |
| Gallbladder and biliary diseases | 2734.6 (2026.4 to 3637.6) | 536.4 (398.9 to 695.7) | 2.4 (1.5 to 3.8) | 61.9* (51.0 to 73.1) | 10.3* (2.9 to 17.9) |
| Pancreatitis | 605.3 (527.5 to 698.8) | 133.2 (103.2 to 170.9) | 34.9 (17.6 to 59.4) | 56.7* (48.6 to 65.2) | 6.8* (1.2 to 12.6) |
| Acute pancreatitis | 10.3 (7.6 to 13.7) | 106.2 (76.4 to 143.5) | 4.1 (2.6 to 6.0) | 47.1* (38.3 to 55.5) | 0.2 (-5.7 to 6.0) |
| Chronic pancreatitis | 595.0 (517.6 to 686.8) | 27.0 (18.7 to 36.7) | 30.8 (14.2 to 54.3) | 58.1* (48.9 to 67.9) | 7.7* (1.4 to 14.4) |
| Other digestive diseases | -- | -- | 74.8 (50.3 to 103.8) | 71.7* (66.8 to 77.2) | 17.0* (13.6 to 20.7) |
| **Neurological disorders** | **132352.9 (111933.5 to 158745.8)** | **37063.2 (25303.3 to 49950.2)** | **3195.7 (2182.0 to 4462.9)** | **47.0* (44.9 to 49.3)** | **0.2 (-1.2 to 1.7)** |
| Alzheimer's disease and other dementias | 2184.4 (1745.7 to 2773.8) | 393.6 (271.2 to 539.0) | 262.0 (165.2 to 380.7) | 47.2* (44.1 to 50.3) | 0.3 (-1.8 to 2.4) |
| Parkinson's disease | 816.4 (597.5 to 1078.7) | 113.2 (79.8 to 150.7) | 121.2 (79.0 to 182.1) | 52.3* (46.9 to 58.2) | 3.8* (0.1 to 7.8) |
| Epilepsy | 1104.6 (810.9 to 1447.3) | 80.4 (50.3 to 120.4) | 308.7 (188.2 to 466.1) | 47.6* (31.0 to 65.1) | 0.5 (-10.7 to 12.5) |
| Multiple sclerosis | 147.0 (134.8 to 161.5) | 0.7 (0.4 to 0.9) | 37.3 (26.8 to 48.4) | 41.6* (33.7 to 49.9) | -3.5 (-8.9 to 2.1) |
| Motor neuron disease | 20.9 (15.4 to 27.3) | 8.4 (6.2 to 11.0) | 4.4 (2.8 to 6.4) | 55.7* (47.3 to 62.8) | 6.1* (0.4 to 10.9) |
| Headache disorders | 129897.7 (109237.4 to 156714.7) | 36467.0 (24644.3 to 49289.1) | 2405.1 (1578.3 to 3501.2) | 46.6* (45.2 to 47.9) | -0.1 (-1.1 to 0.8) |
| Migraine | 54513.4 (48455.8 to 61079.8) | 1519.4 (974.9 to 2334.4) | 2021.2 (1289.9 to 3042.6) | 46.5* (44.9 to 48.0) | -0.2 (-1.3 to 0.9) |
| Medication overuse headache due to migraine | 5995.9 (4316.3 to 8234.4) | -- | 636.0 (364.2 to 1010.9) | 48.2* (46.4 to 49.8) | 1.0 (-0.3 to 2.1) |
| Migraine | 48517.4 (42747.4 to 54589.5) | 1519.4 (974.9 to 2334.4) | 1385.1 (834.8 to 2138.0) | 45.7* (43.5 to 47.9) | -0.7 (-2.2 to 0.8) |
| Tension-type headache | 97492.7 (71227.8 to 129985.9) | 34947.5 (23268.8 to 47952.8) | 383.9 (216.5 to 626.1) | 47.1* (45.3 to 48.9) | 0.3 (-1.0 to 1.5) |
| Medication overuse headache due to tension-type headache | 2228.9 (1389.9 to 3346.4) | -- | 234.6 (116.0 to 432.2) | 48.1* (45.2 to 50.9) | 0.9 (-1.1 to 2.8) |
| Tension-type headache | 95263.8 (69133.2 to 127711.0) | 34947.5 (23268.8 to 47952.8) | 149.3 (60.3 to 301.5) | 45.6* (44.1 to 47.2) | -0.8 (-1.8 to 0.3) |
| Other neurological disorders | 1.9 (1.0 to 3.5) | -- | 57.0 (39.0 to 77.3) | 56.0* (48.5 to 64.2) | 6.3* (1.2 to 11.9) |
| Other neurological disorders | -- | -- | 56.4 (38.6 to 76.6) | 56.2* (48.6 to 64.3) | 6.4* (1.2 to 12.0) |
| Guillain-Barré syndrome due to other neurological disorders | 1.9 (1.0 to 3.5) | -- | 0.6 (0.3 to 1.0) | 46.0* (42.2 to 49.7) | -0.5 (-3.1 to 2.0) |
| **Mental disorders** | **46406.4 (43341.9 to 49347.4)** | **18323.1 (15488.7 to 21221.6)** | **6299.2 (4669.1 to 8110.6)** | **44.2* (42.9 to 45.6)** | **-1.7* (-2.7 to -0.8)** |
| Schizophrenia | 1177.3 (1057.4 to 1314.3) | 22.5 (16.7 to 29.4) | 720.9 (542.4 to 885.2) | 49.0* (46.0 to 52.1) | 1.6 (-0.5 to 3.7) |
| Depressive disorders | 19502.5 (16924.7 to 22107.9) | 16818.5 (14009.4 to 19702.6) | 2969.7 (2033.8 to 4072.4) | 44.0* (41.4 to 46.6) | -1.9* (-3.6 to -0.1) |
| Major depressive disorder | 10736.2 (8861.1 to 12683.3) | 15664.3 (12767.0 to 18498.2) | 2095.9 (1411.4 to 2922.9) | 40.8* (37.5 to 44.0) | -4.1* (-6.3 to -1.9) |
| Dysthymia | 9302.5 (7463.8 to 11339.0) | 1154.2 (883.6 to 1485.4) | 873.8 (566.0 to 1272.9) | 52.4* (47.4 to 57.3) | 3.9* (0.5 to 7.2) |
| Bipolar disorder | 2282.0 (1923.9 to 2719.9) | 159.4 (110.9 to 212.2) | 445.4 (281.6 to 663.3) | 44.5* (41.9 to 47.2) | -1.6 (-3.3 to 0.3) |
| Anxiety disorders | 13941.6 (12368.7 to 15746.7) | 1322.7 (1142.6 to 1538.9) | 1275.9 (890.6 to 1711.6) | 42.8* (40.3 to 45.5) | -2.7* (-4.4 to -0.9) |
| Eating disorders | -- | -- | -- | -- | -- |
| Anorexia nervosa | -- | -- | -- | -- | -- |
| Bulimia nervosa | -- | -- | -- | -- | -- |
| Autism spectrum disorders | 1098.0 (985.9 to 1216.2) | -- | 159.6 (110.7 to 216.6) | 42.5* (39.9 to 45.0) | -2.9* (-4.7 to -1.2) |
| Attention-deficit/hyperactivity disorder | 684.7 (528.1 to 852.8) | -- | 8.0 (4.5 to 13.2) | 51.0* (43.4 to 59.0) | 2.9 (-2.3 to 8.3) |
| Conduct disorder | -- | -- | -- | -- | -- |
| Idiopathic developmental intellectual disability | 1977.5 (975.2 to 3010.5) | -- | 77.6 (33.4 to 135.7) | 22.0* (14.0 to 25.9) | -16.9* (-22.3 to -14.2) |
| Other mental disorders | 8917.7 (7880.5 to 10071.4) | -- | 642.2 (431.0 to 876.0) | 46.2* (44.4 to 48.2) | -0.4 (-1.6 to 1.0) |
| **Substance use disorders** | **6237.0 (5202.6 to 7399.9)** | **1954.0 (1549.9 to 2445.1)** | **941.0 (669.0 to 1275.1)** | **57.9* (53.7 to 62.8)** | **7.6* (4.7 to 10.9)** |
| Alcohol use disorders | 4873.2 (3911.1 to 6028.3) | 1844.2 (1452.2 to 2321.3) | 466.4 (311.5 to 682.4) | 49.4* (44.9 to 53.5) | 1.8 (-1.3 to 4.6) |
| Alcohol dependence | 4861.9 (3900.0 to 6016.2) | 1844.2 (1452.2 to 2321.3) | 466.0 (311.2 to 681.9) | 49.4* (44.9 to 53.5) | 1.8 (-1.3 to 4.6) |
| Fetal alcohol syndrome | 11.3 (9.0 to 13.9) | -- | 0.4 (0.2 to 0.6) | 24.9* (16.2 to 31.6) | -14.9* (-20.8 to -10.3) |
| Drug use disorders | 1450.4 (1183.4 to 1778.8) | 109.8 (74.3 to 155.8) | 474.6 (321.6 to 655.5) | 67.3* (60.1 to 75.2) | 14.0* (9.1 to 19.4) |
| Opioid use disorders | 1069.7 (803.9 to 1397.5) | 85.1 (50.8 to 133.4) | 420.2 (280.1 to 590.8) | 71.1* (63.0 to 79.9) | 16.6* (11.1 to 22.6) |
| Cocaine use disorders | 173.5 (152.3 to 195.8) | 2.0 (1.0 to 3.4) | 22.6 (14.6 to 32.3) | 40.5* (32.5 to 48.9) | -4.3 (-9.7 to 1.5) |

| | | | | | |
|---|---|---|---|---|---|
| Amphetamine use disorders | 38.2 (24.8 to 56.2) | 5.7 (2.4 to 9.9) | 5.1 (2.7 to 9.1) | 49.5* (38.5 to 60.1) | 1.9 (-5.6 to 9.1) |
| Cannabis use disorders | 223.1 (177.2 to 279.3) | 16.9 (10.9 to 23.4) | 6.4 (3.9 to 9.8) | 43.9* (37.6 to 50.9) | -1.9 (-6.3 to 2.8) |
| Other drug use disorders | 20.7 (15.5 to 27.7) | -- | 20.3 (13.3 to 28.1) | 43.2* (36.0 to 50.6) | -2.4 (-7.3 to 2.6) |
| **Diabetes and kidney diseases** | **97702.4 (89691.5 to 106451.0)** | **4068.5 (3192.9 to 5131.2)** | **4994.2 (3467.2 to 6807.8)** | **48.4* (41.4 to 56.2)** | **1.1 (-3.7 to 6.4)** |
| Diabetes mellitus | 52291.2 (46689.3 to 58554.7) | 1857.3 (1400.4 to 2403.1) | 4348.6 (2935.6 to 6070.1) | 49.7* (41.6 to 58.6) | 2.0 (-3.5 to 8.1) |
| Diabetes mellitus type 1 | 666.0 (586.2 to 745.2) | 6.8 (4.4 to 9.4) | 54.2 (37.3 to 75.7) | 38.4* (34.7 to 41.9) | -5.7* (-8.2 to -3.3) |
| Uncomplicated diabetes mellitus type 1 | 374.9 (321.8 to 433.3) | 6.8 (4.4 to 9.4) | 17.1 (10.4 to 25.0) | 34.8* (29.6 to 39.9) | -8.2* (-11.7 to -4.7) |
| Vision loss due to diabetes mellitus type 1 retinopathy | 6.9 (5.0 to 9.4) | -- | 0.6 (0.4 to 0.9) | 19.4* (12.0 to 26.6) | -18.6* (-23.7 to -13.7) |
| Diabetic neuropathy due to diabetes mellitus type 1 | 284.2 (234.0 to 336.0) | -- | 36.5 (24.2 to 52.8) | 40.5* (35.0 to 45.9) | -4.2* (-8.0 to -0.6) |
| Diabetes mellitus type 2 | 51625.2 (45995.7 to 57805.4) | 1850.5 (1391.2 to 2396.9) | 4294.3 (2893.5 to 5994.0) | 49.8* (41.6 to 58.9) | 2.1 (-3.5 to 8.2) |
| Uncomplicated diabetes mellitus type 2 | 27981.3 (23296.4 to 32828.8) | 1850.5 (1391.2 to 2396.9) | 1280.7 (792.1 to 1913.8) | 49.8* (40.5 to 59.0) | 2.1 (-4.3 to 8.3) |
| Vision loss due to diabetes mellitus type 2 retinopathy | 588.5 (445.8 to 763.0) | -- | 46.2 (31.4 to 64.6) | 36.4* (30.9 to 41.8) | -7.1* (-10.8 to -3.4) |
| Diabetic neuropathy due to diabetes mellitus type 2 | 23055.4 (19327.9 to 27533.1) | -- | 2967.4 (2020.9 to 4124.5) | 50.1* (41.4 to 59.3) | 2.3 (-3.7 to 8.5) |
| Chronic kidney disease | 63347.2 (53767.2 to 74138.0) | 2166.7 (1386.2 to 3034.3) | 645.5 (469.9 to 856.6) | 40.6* (32.0 to 49.9) | -4.2 (-10.0 to 2.1) |
| Chronic kidney disease due to diabetes mellitus type 1 | 186.1 (152.5 to 222.1) | 5.1 (3.3 to 7.3) | 21.4 (12.8 to 33.4) | 40.6* (31.4 to 50.4) | -4.2 (-10.5 to 2.5) |
| Albuminuria with preserved GFR due to type 1 diabetes mellitus | 65.5 (48.6 to 85.5) | -- | -- | -- | -- |
| End-stage chronic kidney disease due to diabetes mellitus type 1 | 31.2 (15.5 to 52.2) | -- | 13.7 (6.3 to 25.2) | 44.7* (31.4 to 58.0) | -1.4 (-10.5 to 7.6) |
| Stage III chronic kidney disease due to diabetes mellitus type 1 | 64.7 (52.7 to 80.7) | 5.1 (3.3 to 7.3) | 1.1 (0.7 to 1.6) | 31.8* (13.7 to 50.8) | -10.2 (-22.5 to 2.8) |
| Stage IV chronic kidney disease due to diabetes mellitus type 1 | 17.3 (10.9 to 26.4) | -- | 2.3 (1.2 to 3.7) | 33.2* (15.6 to 52.6) | -9.2 (-21.2 to 4.0) |
| Stage V chronic kidney disease due to diabetes mellitus type 1 | 7.3 (5.1 to 10.1) | -- | 4.3 (2.6 to 6.4) | 34.3* (22.0 to 48.1) | -8.5 (-16.9 to 0.9) |
| Chronic kidney disease due to diabetes mellitus type 2 | 13552.0 (11315.9 to 16010.1) | 343.9 (221.2 to 480.5) | 197.9 (139.3 to 268.5) | 42.3* (33.0 to 51.4) | -3.0 (-9.4 to 3.1) |
| Albuminuria with preserved GFR due to type 2 diabetes mellitus | 8102.2 (5991.4 to 10380.2) | -- | -- | -- | -- |
| End-stage chronic kidney disease due to diabetes mellitus type 2 | 177.4 (126.3 to 232.8) | -- | 71.8 (44.4 to 103.6) | 43.3* (34.0 to 52.5) | -2.4 (-8.7 to 3.9) |
| Stage III chronic kidney disease due to diabetes mellitus type 2 | 4776.9 (3968.7 to 5794.3) | 343.9 (221.2 to 480.5) | 17.5 (10.8 to 27.7) | 25.1* (8.5 to 42.2) | -14.7* (-26.1 to -3.1) |
| Stage IV chronic kidney disease due to diabetes mellitus type 2 | 356.2 (225.0 to 540.7) | -- | 37.7 (21.0 to 61.2) | 41.7* (24.3 to 60.8) | -3.5 (-15.3 to 9.6) |
| Stage V chronic kidney disease due to diabetes mellitus type 2 | 139.2 (101.2 to 189.9) | -- | 70.9 (45.9 to 105.6) | 46.7* (33.0 to 61.9) | -0.0 (-9.4 to 10.3) |
| Chronic kidney disease due to hypertension | 1711.9 (1468.0 to 2002.8) | 32.2 (20.8 to 44.8) | 126.3 (90.9 to 170.0) | 43.7* (34.7 to 53.3) | -2.1 (-8.2 to 4.4) |
| Albuminuria with preserved GFR due to hypertension | 457.4 (340.0 to 589.2) | -- | -- | -- | -- |
| End-stage chronic kidney disease due to hypertension | 112.7 (83.3 to 149.5) | -- | 45.5 (27.8 to 66.7) | 50.3* (38.7 to 63.5) | 2.4 (-5.5 to 11.4) |
| Stage III chronic kidney disease due to hypertension | 830.2 (684.2 to 1013.1) | 32.2 (20.8 to 44.8) | 10.4 (6.3 to 16.3) | 19.2* (4.2 to 35.6) | -18.8* (-29.0 to -7.6) |
| Stage IV chronic kidney disease due to hypertension | 223.8 (141.6 to 341.2) | -- | 24.3 (13.5 to 38.6) | 40.9* (24.0 to 60.4) | -4.0 (-15.5 to 9.3) |
| Stage V chronic kidney disease due to hypertension | 87.7 (63.9 to 120.9) | -- | 46.2 (29.2 to 68.8) | 45.6* (32.3 to 60.1) | -0.8 (-9.8 to 9.1) |
| Chronic kidney disease due to glomerulonephritis | 2482.4 (2112.2 to 2935.1) | 171.4 (110.3 to 239.8) | 63.9 (42.5 to 87.4) | 43.2* (33.9 to 52.7) | -2.4 (-8.8 to 4.0) |
| Albuminuria with preserved GFR due to glomerulonephritis | 290.5 (214.1 to 377.7) | -- | -- | -- | -- |
| End-stage chronic kidney disease due to glomerulonephritis | 65.7 (40.8 to 99.0) | -- | 26.5 (15.0 to 41.5) | 46.4* (33.1 to 60.6) | -0.3 (-9.3 to 9.5) |
| Stage III chronic kidney disease due to glomerulonephritis | 1983.3 (1641.0 to 2403.0) | 171.4 (110.3 to 239.8) | 3.3 (2.1 to 5.0) | 21.8* (5.3 to 41.8) | -17.0* (-28.2 to -3.4) |
| Stage IV chronic kidney disease due to glomerulonephritis | 102.6 (64.2 to 155.1) | -- | 11.7 (6.4 to 18.7) | 39.3* (21.4 to 57.7) | -5.1 (-17.3 to 7.5) |
| Stage V chronic kidney disease due to glomerulonephritis | 40.2 (28.9 to 55.1) | -- | 22.5 (14.2 to 33.6) | 45.4* (33.9 to 59.2) | -0.9 (-8.7 to 8.5) |
| Chronic kidney disease due to other and unspecified causes | 45414.8 (38502.1 to 53229.6) | 1614.0 (1030.7 to 2259.8) | 236.0 (167.6 to 318.7) | 36.9* (26.4 to 48.0) | -6.7 (-13.8 to 0.9) |
| Albuminuria with preserved GFR due to other and unspecified causes | 22209.3 (16455.3 to 28499.3) | -- | -- | -- | -- |
| End-stage chronic kidney disease due to other causes | 109.3 (68.7 to 161.5) | -- | 43.8 (24.2 to 69.5) | 43.2* (31.5 to 55.7) | -2.4 (-10.4 to 6.1) |
| Stage III chronic kidney disease due to other causes | 22644.5 (18756.2 to 27344.0) | 1614.0 (1030.7 to 2259.8) | 92.6 (58.0 to 144.4) | 26.5* (12.9 to 41.5) | -13.8* (-23.1 to -3.6) |
| Stage IV chronic kidney disease due to other causes | 324.7 (205.3 to 492.7) | -- | 34.6 (19.0 to 56.0) | 42.0* (24.9 to 60.8) | -3.3 (-14.9 to 9.6) |
| Stage V chronic kidney disease due to other causes | 126.9 (92.0 to 173.6) | -- | 65.0 (41.6 to 96.4) | 46.9* (33.1 to 62.6) | 0.1 (-9.3 to 10.8) |
| Acute glomerulonephritis | 3.0 (2.0 to 4.3) | 44.5 (30.6 to 62.3) | 0.2 (0.1 to 0.3) | 13.1* (5.1 to 18.1) | -22.9* (-28.4 to -19.6) |
| **Skin and subcutaneous diseases** | **96542.5 (89387.3 to 105621.9)** | **207378.0 (174336.5 to 245130.9)** | **1619.9 (1082.5 to 2420.1)** | **47.4* (46.2 to 48.3)** | **0.4 (-0.4 to 1.1)** |
| Dermatitis | 10312.5 (8537.8 to 12534.6) | 15450.6 (11112.9 to 20790.9) | 334.3 (203.7 to 520.7) | 43.1* (41.2 to 45.0) | -2.5* (-3.8 to -1.2) |
| Atopic dermatitis | 5005.9 (4368.6 to 5689.7) | 762.1 (635.1 to 895.4) | 208.8 (117.3 to 343.7) | 41.9* (39.3 to 44.5) | -3.3* (-5.1 to -1.6) |
| Contact dermatitis | 4794.3 (3210.2 to 6948.0) | 13424.0 (9075.0 to 18825.5) | 116.6 (67.5 to 198.1) | 44.8* (42.4 to 47.5) | -1.3 (-3.0 to 0.5) |
| Seborrhoeic dermatitis | 668.6 (529.4 to 817.6) | 1264.5 (1029.4 to 1510.8) | 8.9 (4.9 to 14.3) | 50.2* (44.6 to 56.0) | 2.3 (-1.4 to 6.3) |
| Psoriasis | 5118.8 (4743.2 to 5506.1) | 510.5 (449.6 to 582.9) | 428.4 (303.7 to 569.5) | 47.2* (44.8 to 49.6) | 0.3 (-1.3 to 2.0) |
| Bacterial skin diseases | 413.0 (375.4 to 451.9) | 10548.9 (9595.3 to 11533.1) | 8.3 (5.3 to 12.4) | 43.2* (37.4 to 48.6) | -2.5 (-6.3 to 1.2) |
| Cellulitis | 116.7 (94.1 to 141.3) | 2428.6 (1992.6 to 2881.8) | 6.5 (4.2 to 9.5) | 40.7* (34.6 to 47.4) | -4.1 (-8.3 to 0.5) |
| Pyoderma | 337.9 (307.4 to 372.0) | 8120.2 (7265.4 to 9077.4) | 1.9 (0.8 to 3.9) | 52.3* (47.5 to 57.4) | 3.7* (0.5 to 7.3) |
| Impetigo | 22.5 (19.7 to 25.4) | 421.3 (374.6 to 467.3) | 0.1 (0.1 to 0.3) | 46.9* (36.9 to 57.3) | 0.1 (-6.7 to 7.2) |
| Abscess and other bacterial skin diseases | 315.4 (284.8 to 349.8) | 7698.9 (6863.2 to 8654.4) | 1.7 (0.7 to 3.6) | 52.7* (47.4 to 58.1) | 4.0* (0.4 to 7.7) |
| Scabies | 4464.2 (3060.3 to 6228.0) | 13373.5 (9298.1 to 18344.8) | 111.8 (55.9 to 197.1) | 52.6* (50.3 to 55.0) | 4.0* (2.4 to 5.6) |
| Fungal skin diseases | 34188.6 (26073.5 to 44630.0) | 108201.0 (77784.3 to 144653.4) | 188.0 (71.7 to 395.4) | 46.0* (44.8 to 46.9) | -0.5 (-1.3 to 0.1) |
| Tinea capitis | 1322.1 (894.3 to 1865.1) | 2260.1 (1576.4 to 2969.2) | 7.2 (2.7 to 16.0) | 15.0* (11.4 to 19.0) | -21.7* (-24.1 to -18.9) |
| Other fungal skin diseases | 32866.4 (24658.7 to 43355.4) | 105940.9 (75564.2 to 142433.8) | 180.8 (68.5 to 379.5) | 47.6* (46.8 to 48.3) | 0.6* (0.0 to 1.0) |

| | | | | | |
|---|---|---|---|---|---|
| Viral skin diseases | 2228.4 (1998.9 to 2473.7) | 1295.7 (1173.8 to 1416.5) | 65.8 (42.8 to 97.0) | 46.3* (43.2 to 49.2) | -0.3 (-2.4 to 1.7) |
| Viral warts | 2016.5 (1790.0 to 2261.3) | 1028.2 (911.4 to 1140.5) | 59.6 (38.6 to 87.8) | 46.2* (42.9 to 49.3) | -0.4 (-2.6 to 1.7) |
| Molluscum contagiosum | 211.8 (188.4 to 237.8) | 267.4 (237.3 to 300.0) | 6.2 (4.0 to 9.4) | 47.6* (38.5 to 57.9) | 0.6 (-5.6 to 7.6) |
| Acne vulgaris | 691.0 (664.4 to 717.5) | 322.8 (311.5 to 334.5) | 14.5 (8.7 to 22.6) | 56.2* (51.8 to 61.0) | 6.4* (3.5 to 9.7) |
| Alopecia areata | 731.4 (657.3 to 813.8) | 1243.1 (1121.7 to 1373.7) | 23.2 (14.9 to 34.3) | 42.2* (37.9 to 46.7) | -3.1* (-6.1 to -0.0) |
| Pruritus | 4545.3 (3127.9 to 6647.6) | 3444.6 (2385.6 to 4822.7) | 47.3 (20.1 to 92.8) | 49.9* (48.1 to 51.8) | 2.1* (0.9 to 3.4) |
| Urticaria | 2310.5 (1593.4 to 3216.2) | 4061.2 (2848.1 to 5576.6) | 132.3 (78.9 to 210.9) | 47.8* (44.9 to 50.8) | 0.7 (-1.3 to 2.7) |
| Decubitus ulcer | 70.3 (48.2 to 94.7) | 259.0 (171.4 to 351.9) | 12.1 (7.3 to 18.2) | 43.7* (38.9 to 48.5) | -2.1 (-5.4 to 1.2) |
| Other skin and subcutaneous diseases | 46890.1 (42842.2 to 50884.9) | 48667.1 (44392.4 to 53495.0) | 253.8 (122.3 to 472.7) | 52.5* (51.5 to 53.3) | 3.9* (3.3 to 4.5) |
| **Sense organ diseases** | **194416.3 (188889.8 to 200108.0)** | **--** | **6390.4 (4278.1 to 9310.4)** | **44.8* (42.8 to 46.5)** | **-1.3* (-2.7 to -0.2)** |
| Blindness and vision impairment | 120902.3 (113381.3 to 129111.5) | -- | 3037.9 (2032.2 to 4452.3) | 42.5* (40.1 to 44.7) | -2.9* (-4.5 to -1.4) |
| Glaucoma | 1020.1 (830.1 to 1255.8) | -- | 118.5 (78.7 to 167.3) | 39.9* (36.4 to 43.6) | -4.7* (-7.1 to -2.1) |
| Cataract | 12336.7 (9559.4 to 15648.4) | -- | 868.2 (591.0 to 1216.9) | 44.5* (40.6 to 48.7) | -1.5 (-4.2 to 1.3) |
| Age-related macular degeneration | 1095.2 (816.5 to 1448.2) | -- | 78.9 (52.7 to 112.4) | 42.6* (37.7 to 47.3) | -2.9 (-6.2 to 0.4) |
| Refraction disorders | 13816.3 (10365.5 to 18058.9) | -- | 630.0 (403.1 to 944.3) | 34.0* (29.4 to 38.3) | -8.7* (-11.8 to -5.8) |
| Near vision loss | 103422.7 (95581.0 to 111209.9) | -- | 1040.0 (500.7 to 1900.3) | 46.6* (45.5 to 47.8) | -0.1 (-0.9 to 0.7) |
| Other vision loss | 3591.2 (2827.8 to 4546.3) | -- | 302.3 (200.8 to 417.0) | 42.7* (39.9 to 45.5) | -2.8* (-4.6 to -0.9) |
| Age-related and other hearing loss | 136985.6 (129595.3 to 143371.7) | -- | 3092.0 (2015.9 to 4608.7) | 46.8* (43.6 to 50.0) | 0.0 (-2.2 to 2.2) |
| Other sense organ diseases | 10581.1 (9435.4 to 11825.2) | -- | 260.5 (161.7 to 385.5) | 48.6* (46.7 to 50.6) | 1.3 (-0.1 to 2.6) |
| Chronic other sense organ diseases | 9896.8 (8750.1 to 11124.9) | -- | 251.3 (155.7 to 372.6) | 48.6* (46.6 to 50.7) | 1.3 (-0.1 to 2.7) |
| Acute other sense organ diseases | 684.3 (613.9 to 752.0) | -- | 9.1 (5.5 to 14.1) | 48.2* (41.9 to 54.8) | 1.0 (-3.3 to 5.5) |
| **Musculoskeletal disorders** | **118262.4 (105878.2 to 131434.6)** | **27054.7 (21224.9 to 34256.5)** | **11834.9 (8125.3 to 16154.3)** | **45.1* (43.1 to 47.3)** | **-1.2 (-2.5 to 0.4)** |
| Rheumatoid arthritis | 2398.7 (2026.5 to 2800.4) | 127.1 (87.7 to 166.8) | 312.5 (210.3 to 427.4) | 56.1* (50.6 to 61.5) | 6.4* (2.6 to 10.1) |
| Osteoarthritis | 41866.2 (36411.7 to 47936.5) | 1736.0 (1288.7 to 2189.9) | 1337.4 (658.9 to 2643.0) | 46.4* (45.4 to 47.6) | -0.2 (-0.9 to 0.6) |
| Osteoarthritis of the hip | 5483.1 (5010.7 to 6013.4) | 269.8 (222.7 to 316.7) | 175.1 (87.7 to 358.0) | 51.6* (49.5 to 53.7) | 3.3* (1.9 to 4.7) |
| Osteoarthritis of the knee | 36383.1 (30832.7 to 42433.1) | 1466.1 (1028.0 to 1913.2) | 1162.4 (565.4 to 2314.1) | 45.6* (44.5 to 46.9) | -0.8 (-1.5 to 0.1) |
| Low back pain | 46265.5 (34198.9 to 63118.1) | 19275.7 (13876.3 to 26748.1) | 5200.2 (3234.0 to 7681.0) | 42.3* (41.1 to 43.6) | -3.0* (-3.9 to -2.2) |
| Low back pain with leg pain | 17272.2 (12385.9 to 23727.6) | 7212.3 (4995.3 to 10139.6) | 2432.6 (1483.2 to 3819.8) | 42.3* (41.0 to 43.8) | -3.0* (-3.9 to -2.0) |
| Low back pain without leg pain | 28993.3 (21150.6 to 39864.7) | 12063.4 (8530.1 to 16943.7) | 2767.7 (1692.6 to 4212.9) | 42.3* (41.0 to 43.6) | -3.0* (-3.9 to -2.1) |
| Neck pain | 24993.7 (18101.6 to 32988.6) | 4969.0 (2980.7 to 7068.6) | 2440.4 (1515.8 to 3688.2) | 50.0* (46.8 to 53.2) | 2.2* (0.0 to 4.4) |
| Gout | 5113.8 (4024.2 to 6422.1) | 946.9 (723.5 to 1201.9) | 159.2 (100.7 to 228.7) | 48.9* (44.7 to 53.0) | 1.4 (-1.4 to 4.3) |
| Other musculoskeletal disorders | 28637.8 (19477.0 to 39840.3) | -- | 2385.2 (1351.6 to 3767.1) | 43.9* (34.3 to 52.9) | -2.0 (-8.5 to 4.2) |
| **Other non-communicable diseases** | **216257.9 (203599.8 to 229437.9)** | **98168.0 (84499.4 to 113086.2)** | **3063.2 (2017.2 to 4444.4)** | **41.5* (39.8 to 43.0)** | **-3.6* (-4.7 to -2.5)** |
| Congenital anomalies | 3434.5 (3224.3 to 3680.0) | -- | 431.5 (302.7 to 576.1) | 44.0* (41.2 to 46.7) | -1.9* (-3.8 to -0.1) |
| Neural tube defects | 91.9 (81.9 to 102.7) | -- | 26.8 (18.6 to 35.9) | 50.8* (44.5 to 57.1) | 2.8 (-1.6 to 7.0) |
| Severe motor and cognitive impairment due to anencephaly | -- | -- | -- | -- | -- |
| Encephalocoele due to neural tube defects | 13.3 (11.0 to 15.8) | -- | 2.3 (1.5 to 3.2) | 42.2* (37.9 to 46.6) | -3.1* (-6.0 to -0.1) |
| Spina bifida due to neural tube defects | 78.6 (70.0 to 88.4) | -- | 24.5 (17.0 to 32.8) | 51.7* (45.1 to 58.6) | 3.4 (-1.1 to 8.1) |
| Congenital heart anomalies | 99.9 (91.3 to 109.6) | -- | 5.9 (2.8 to 9.9) | 45.7* (39.8 to 51.5) | -0.7 (-4.7 to 3.2) |
| Critical malformations of great vessels, congenital valvular heart disease and patent ductus arteriosus | 16.5 (14.3 to 19.2) | -- | 1.0 (0.5 to 1.8) | 49.7* (46.8 to 52.6) | 2.0* (0.0 to 4.0) |
| Other congenital heart anomalies | 38.0 (34.7 to 41.9) | -- | 2.2 (0.9 to 3.8) | 46.5* (35.8 to 57.4) | -0.2 (-7.5 to 7.2) |
| Severe congenital heart anomalies excluding single ventricle heart defects | 41.3 (36.6 to 46.1) | -- | 2.5 (1.3 to 4.1) | 44.0* (34.9 to 53.4) | -1.9 (-8.1 to 4.5) |
| Single ventricle and single ventricle pathway congenital heart anomalies | 1.0 (0.9 to 1.3) | -- | 0.1 (0.1 to 0.1) | 41.4* (34.2 to 48.4) | -3.7 (-8.6 to 1.1) |
| Ventricular septal defect and atrial septal defect | 3.0 (2.4 to 3.7) | -- | 0.1 (0.0 to 0.2) | 40.5* (37.2 to 43.3) | -4.3* (-6.5 to -2.4) |
| Orofacial clefts | 416.8 (381.3 to 452.0) | -- | 10.9 (7.2 to 15.7) | 47.8* (36.5 to 60.5) | 0.7 (-7.0 to 9.4) |
| Down's syndrome | 92.6 (80.5 to 105.7) | -- | 13.6 (9.4 to 18.7) | 36.9* (32.7 to 41.2) | -6.7* (-9.6 to -3.8) |
| Turner syndrome | 7.2 (6.1 to 8.3) | -- | 0.1 (0.0 to 0.2) | 43.7* (40.7 to 46.4) | -2.1* (-4.1 to -0.2) |
| Klinefelter syndrome | 4.5 (3.8 to 5.5) | -- | -- | 52.3* (50.2 to 54.5) | 3.8* (2.3 to 5.3) |
| Other chromosomal abnormalities | 162.6 (131.2 to 196.5) | -- | 24.1 (16.1 to 33.5) | 36.0* (31.5 to 39.9) | -7.3* (-10.4 to -4.7) |
| Congenital musculoskeletal and limb anomalies | 1375.0 (1222.3 to 1574.8) | -- | 195.5 (131.4 to 267.1) | 46.4* (43.7 to 49.0) | -0.3 (-2.1 to 1.5) |
| Polydactyly and syndactyly | 3.1 (2.5 to 3.7) | -- | 0.0 (0.0 to 0.1) | 32.5* (29.7 to 35.0) | -9.7* (-11.6 to -8.0) |
| Congenital limb deficiency | 472.6 (419.7 to 542.5) | -- | 67.4 (45.0 to 92.1) | 46.4* (42.3 to 50.8) | -0.2 (-3.1 to 2.7) |
| Other congenital musculoskeletal anomalies | 899.3 (799.4 to 1030.0) | -- | 128.0 (86.3 to 174.8) | 46.4* (43.3 to 49.3) | -0.3 (-2.4 to 1.7) |
| Urogenital congenital anomalies | 46.4 (41.3 to 51.8) | -- | 2.1 (1.3 to 3.2) | 27.2* (20.6 to 34.1) | -13.3* (-17.8 to -8.6) |
| Digestive congenital anomalies | 797.0 (717.2 to 889.5) | -- | 34.9 (23.2 to 49.8) | 37.5* (30.6 to 44.7) | -6.3* (-11.0 to -1.4) |
| Congenital diaphragmatic hernia | 4.6 (4.0 to 5.1) | -- | 0.1 (0.1 to 0.1) | 51.5* (49.6 to 53.2) | 3.3* (2.0 to 4.4) |
| Congenital atresia and/or stenosis of the digestive tract | 0.2 (0.1 to 0.3) | -- | -- | 61.9* (54.2 to 68.6) | 10.3* (5.1 to 14.9) |
| Congenital malformations of the abdominal wall after treatment | 17.9 (15.6 to 20.3) | -- | 0.8 (0.5 to 1.1) | 49.3* (47.6 to 50.9) | 1.8* (0.6 to 2.8) |
| Other congenital malformations of the digestive tract | 774.3 (694.8 to 866.0) | -- | 34.0 (22.6 to 48.5) | 37.2* (30.1 to 44.6) | -6.5* (-11.3 to -1.5) |
| Other congenital anomalies | 921.1 (791.2 to 1071.9) | -- | 117.5 (79.9 to 161.8) | 43.0* (34.2 to 51.9) | -2.5 (-8.5 to 3.5) |

| | | | | | |
|---|---|---|---|---|---|
| Other congenital birth defects | -- | -- | 9.2<br>(6.6 to 12.1) | 58.0*<br>(52.8 to 63.7) | 7.7*<br>(4.1 to 11.6) |
| Hearing loss due to other congenital anomalies | 921.1<br>(791.2 to 1071.9) | -- | 108.4<br>(72.8 to 150.7) | 41.9*<br>(32.3 to 51.6) | -3.3<br>(-9.9 to 3.3) |
| Urinary diseases and male infertility | 12847.6<br>(10979.6 to 14911.2) | 20902.6<br>(17394.1 to 24710.7) | 454.0<br>(291.1 to 662.1) | 44.7*<br>(42.8 to 46.6) | -1.4*<br>(-2.7 to -0.1) |
| Urinary tract infections | 228.1<br>(177.0 to 295.1) | 11894.3<br>(9295.4 to 15252.4) | 7.3<br>(4.2 to 11.4) | 66.8*<br>(55.6 to 77.6) | 13.6*<br>(6.0 to 21.0) |
| Urolithiasis | 292.3<br>(209.3 to 386.5) | 7669.6<br>(5519.2 to 9965.2) | 20.8<br>(12.0 to 31.2) | 46.5*<br>(37.8 to 55.6) | -0.2<br>(-6.1 to 6.0) |
| Benign prostatic hyperplasia | 12715.5<br>(10718.6 to 14967.6) | 1338.6<br>(1135.4 to 1616.6) | 418.4<br>(264.6 to 614.5) | 44.9*<br>(43.0 to 46.9) | -1.3<br>(-2.5 to 0.1) |
| Male infertility | -- | -- | -- | -- | -- |
| Other urinary diseases | -- | -- | 7.6<br>(4.9 to 11.0) | 16.0*<br>(10.3 to 22.0) | -21.0*<br>(-24.8 to -16.9) |
| Gynecological diseases | 17841.8<br>(15398.2 to 20813.7) | 1207.9<br>(879.7 to 1611.4) | 88.9<br>(49.2 to 157.8) | 39.7*<br>(34.2 to 44.4) | -4.8*<br>(-8.6 to -1.6) |
| Uterine fibroids | 7182.2<br>(6186.1 to 8384.6) | 31.5<br>(19.9 to 46.7) | 53.5<br>(29.8 to 90.9) | 39.7*<br>(31.1 to 47.3) | -4.8<br>(-10.7 to 0.4) |
| Polycystic ovarian syndrome | -- | -- | -- | -- | -- |
| Female infertility | -- | -- | -- | -- | -- |
| Endometriosis | -- | -- | -- | -- | -- |
| Genital prolapse | 11460.4<br>(9102.1 to 14278.3) | 1176.4<br>(847.4 to 1578.0) | 35.4<br>(16.4 to 67.7) | 39.7*<br>(37.3 to 41.8) | -4.8*<br>(-6.4 to -3.4) |
| Premenstrual syndrome | -- | -- | -- | -- | -- |
| Other gynecological diseases | -- | -- | -- | -- | -- |
| Other gynecological diseases | -- | -- | -- | -- | -- |
| Other gynecological diseases with anemia | -- | -- | -- | -- | -- |
| Hemoglobinopathies and hemolytic anaemias | 69961.1<br>(68448.8 to 71474.9) | -- | 177.8<br>(115.4 to 260.6) | 15.6*<br>(5.0 to 26.0) | -21.2*<br>(-28.5 to -14.1) |
| Thalassemias | 0.1<br>(0.0 to 0.1) | -- | -- | 29.3*<br>(3.8 to 64.7) | -11.9<br>(-29.3 to 12.2) |
| Thalassaemias trait | 12626.9<br>(12162.5 to 13138.8) | -- | 58.2<br>(36.3 to 89.3) | 17.8<br>(-0.2 to 38.1) | -19.7*<br>(-32.0 to -5.9) |
| Sickle cell disorders | 0.3<br>(0.2 to 0.4) | -- | 0.0<br>(0.0 to 0.1) | 53.9*<br>(44.0 to 63.5) | 4.8<br>(-1.9 to 11.4) |
| Sickle cell trait | 13250.1<br>(11897.1 to 14771.2) | -- | 23.1<br>(15.1 to 33.8) | 10.1<br>(-0.5 to 23.0) | -25.0*<br>(-32.2 to -16.2) |
| G6PD deficiency | 11660.0<br>(11204.5 to 12113.7) | -- | 0.8<br>(0.5 to 1.1) | 31.0*<br>(14.4 to 51.0) | -10.8<br>(-22.0 to 2.9) |
| G6PD trait | 35085.0<br>(34514.9 to 35643.8) | -- | -- | 32.9*<br>(13.9 to 56.0) | -9.4<br>(-22.4 to 6.3) |
| Other hemoglobinopathies and hemolytic anaemias | 4681.9<br>(4334.7 to 5065.1) | -- | 95.6<br>(63.8 to 140.2) | 15.6*<br>(5.5 to 26.2) | -21.3*<br>(-28.1 to -14.0) |
| Endocrine, metabolic, blood, and immune disorders | 5222.9<br>(4625.0 to 5937.0) | -- | 142.6<br>(99.3 to 202.5) | 35.9*<br>(27.5 to 43.6) | -7.4*<br>(-13.1 to -2.1) |
| Anemia due to endocrine, metabolic, blood, and immune disorders | 2791.0<br>(2584.7 to 3011.6) | -- | 55.3<br>(37.0 to 80.3) | 12.8*<br>(2.6 to 24.1) | -23.1*<br>(-30.1 to -15.4) |
| Endocrine, metabolic, blood, and immune disorders | 2403.6<br>(1863.7 to 3103.6) | -- | 84.8<br>(55.5 to 124.9) | 56.7*<br>(47.3 to 66.5) | 6.8*<br>(0.3 to 13.5) |
| Heart failure due to endocrine, metabolic, blood, and immune disorders | 28.2<br>(20.3 to 38.5) | -- | 2.6<br>(1.5 to 4.0) | 38.9*<br>(30.6 to 47.8) | -5.4<br>(-11.0 to 0.7) |
| Oral disorders | 175132.8<br>(156811.0 to 194401.7) | 76057.5<br>(63307.4 to 90092.1) | 1768.4<br>(1074.7 to 2736.0) | 44.0*<br>(42.6 to 45.2) | -1.9*<br>(-2.9 to -1.0) |
| Caries of deciduous teeth | -- | -- | -- | -- | -- |
| Caries of permanent teeth | 103763.6<br>(80015.6 to 132560.9) | 67613.4<br>(54697.6 to 81470.7) | 74.8<br>(31.6 to 147.8) | 38.9*<br>(37.1 to 40.8) | -5.3*<br>(-6.6 to -4.1) |
| Periodontal disease | 81375.5<br>(64271.7 to 96766.8) | 5482.9<br>(3390.0 to 7476.0) | 525.5<br>(206.9 to 1094.9) | 49.9*<br>(48.9 to 51.1) | 2.1*<br>(1.5 to 2.9) |
| Edentulism and severe tooth loss | 34396.7<br>(27771.6 to 41982.6) | 2961.2<br>(2042.6 to 4005.9) | 954.8<br>(601.0 to 1388.8) | 40.6*<br>(39.3 to 41.9) | -4.2*<br>(-5.1 to -3.3) |
| Other oral disorders | 7580.6<br>(6732.0 to 8454.3) | -- | 213.3<br>(131.2 to 326.8) | 47.0*<br>(45.3 to 48.4) | 0.2<br>(-1.0 to 1.1) |
| **Injuries** | **113718.3<br>(107858.7 to 120929.5)** | **24451.3<br>(20321.4 to 28583.4)** | **4375.3<br>(3163.0 to 5893.4)** | **57.4*<br>(56.1 to 58.8)** | **7.3*<br>(6.4 to 8.2)** |
| **Transport injuries** | **20373.4<br>(18816.3 to 22005.9)** | **2567.6<br>(1786.9 to 3490.2)** | **1124.5<br>(812.9 to 1502.1)** | **55.9*<br>(54.4 to 57.3)** | **6.2*<br>(5.2 to 7.2)** |
| Road injuries | 16630.7<br>(15344.6 to 18060.6) | 2257.4<br>(1483.1 to 3193.3) | 917.0<br>(664.2 to 1238.3) | 57.2*<br>(55.8 to 58.6) | 7.1*<br>(6.2 to 8.1) |
| Pedestrian road injuries | 4384.9<br>(3747.7 to 5242.8) | 499.5<br>(277.5 to 829.5) | 244.0<br>(170.6 to 335.4) | 55.7*<br>(53.4 to 57.9) | 6.1*<br>(4.5 to 7.6) |
| Cyclist road injuries | 3049.4<br>(2560.1 to 3541.4) | 585.7<br>(327.9 to 961.7) | 162.8<br>(114.3 to 224.0) | 64.1*<br>(61.6 to 66.6) | 11.8*<br>(10.1 to 13.5) |
| Motorcyclist road injuries | 4042.0<br>(3480.6 to 4796.8) | 348.1<br>(183.1 to 599.3) | 220.8<br>(152.0 to 303.2) | 57.3*<br>(54.7 to 59.8) | 7.2*<br>(5.4 to 8.9) |
| Motor vehicle road injuries | 4057.9<br>(3456.6 to 4729.9) | 606.3<br>(357.0 to 958.9) | 234.4<br>(166.5 to 318.2) | 48.2*<br>(46.4 to 50.0) | 0.9<br>(-0.3 to 2.2) |
| Other road injuries | 1096.5<br>(888.4 to 1358.3) | 217.8<br>(115.2 to 367.0) | 55.0<br>(37.8 to 76.4) | 91.5*<br>(88.1 to 94.4) | 30.5*<br>(28.2 to 32.4) |
| Other transport injuries | 3742.7<br>(3162.3 to 4290.1) | 310.2<br>(195.7 to 462.7) | 207.5<br>(148.7 to 276.8) | 50.1*<br>(47.7 to 52.7) | 2.3*<br>(0.6 to 4.0) |
| **Unintentional injuries** | **78878.7<br>(73432.8 to 85830.0)** | **21099.0<br>(17168.1 to 25296.2)** | **2892.7<br>(2038.5 to 3977.8)** | **57.4*<br>(55.7 to 59.0)** | **7.3*<br>(6.1 to 8.3)** |
| Falls | 36141.9<br>(31691.2 to 41636.0) | 9410.9<br>(6199.2 to 13413.5) | 1650.2<br>(1153.4 to 2254.4) | 59.4*<br>(57.4 to 61.4) | 8.6*<br>(7.2 to 10.0) |
| Drowning | 180.9<br>(156.9 to 213.0) | 15.1<br>(10.9 to 21.1) | 9.8<br>(6.9 to 13.1) | 60.8*<br>(56.1 to 65.9) | 9.6*<br>(6.4 to 13.0) |
| Fire, heat, and hot substances | 7766.7<br>(6612.1 to 9126.3) | 200.3<br>(121.6 to 301.2) | 185.3<br>(121.6 to 273.3) | 49.6*<br>(43.9 to 54.3) | 1.9<br>(-2.0 to 5.2) |
| Poisonings | 290.3<br>(245.9 to 335.5) | 76.1<br>(44.9 to 115.7) | 25.6<br>(17.1 to 35.7) | 65.4*<br>(61.4 to 69.3) | 12.7*<br>(10.0 to 15.4) |
| Poisoning by carbon monoxide | 60.6<br>(51.6 to 72.2) | 27.4<br>(12.3 to 51.1) | 4.3<br>(3.0 to 6.1) | 71.2*<br>(66.4 to 75.6) | 16.6*<br>(13.4 to 19.7) |
| Poisoning by other means | 229.7<br>(186.5 to 274.1) | 48.7<br>(26.1 to 77.7) | 21.2<br>(13.9 to 30.6) | 64.3*<br>(60.1 to 68.4) | 11.9*<br>(9.1 to 14.8) |
| Exposure to mechanical forces | 16925.7<br>(14628.3 to 19791.0) | 2446.9<br>(1569.7 to 3590.9) | 404.1<br>(272.5 to 574.5) | 59.9*<br>(58.2 to 61.5) | 8.9*<br>(7.8 to 10.1) |
| Unintentional firearm injuries | 675.1<br>(555.7 to 789.0) | 43.2<br>(19.2 to 83.5) | 21.5<br>(14.9 to 29.7) | 59.0*<br>(57.2 to 60.8) | 8.4*<br>(7.1 to 9.6) |
| Other exposure to mechanical forces | 16250.6<br>(13948.0 to 19170.1) | 2403.7<br>(1546.8 to 3506.0) | 382.6<br>(257.0 to 546.6) | 59.9*<br>(58.2 to 61.6) | 9.0*<br>(7.8 to 10.1) |
| Adverse effects of medical treatment | 313.2<br>(200.7 to 456.9) | 4099.4<br>(2740.5 to 5876.3) | 41.8<br>(22.4 to 71.5) | 113.1*<br>(96.1 to 130.2) | 45.2*<br>(33.7 to 56.8) |
| Animal contact | 2922.9<br>(2561.4 to 3406.6) | 1606.4<br>(1033.6 to 2345.7) | 64.0<br>(43.6 to 91.7) | 36.6*<br>(33.3 to 39.5) | -6.9*<br>(-9.2 to -4.9) |
| Venomous animal contact | 1133.5<br>(929.8 to 1339.2) | 706.2<br>(439.5 to 1048.9) | 36.8<br>(25.1 to 50.8) | 36.2*<br>(32.5 to 39.8) | -7.2*<br>(-9.7 to -4.8) |
| Non-venomous animal contact | 1789.5<br>(1467.3 to 2200.7) | 900.2<br>(542.3 to 1356.3) | 27.2<br>(16.7 to 42.8) | 37.1*<br>(33.5 to 39.8) | -6.6*<br>(-9.0 to -4.7) |
| Foreign body | 1381.7<br>(1228.4 to 1545.9) | 791.3<br>(505.3 to 1179.3) | 60.2<br>(43.2 to 81.0) | 48.9*<br>(47.1 to 50.6) | 1.5*<br>(0.3 to 2.6) |

| | | | | | |
|---|---|---|---|---|---|
| Pulmonary aspiration and foreign body in airway | 222.3 (184.6 to 270.2) | 29.5 (16.3 to 45.8) | 9.3 (6.4 to 12.7) | 68.9* (65.5 to 72.3) | 15.1* (12.8 to 17.4) |
| Foreign body in eyes | 168.3 (110.6 to 250.4) | 622.0 (339.8 to 1012.9) | 11.5 (6.9 to 18.3) | 41.9* (40.2 to 43.5) | -3.3* (-4.5 to -2.2) |
| Foreign body in other body part | 991.1 (843.5 to 1130.0) | 139.9 (79.1 to 217.6) | 39.4 (28.2 to 51.8) | 47.0* (45.0 to 48.8) | 0.2 (-1.2 to 1.4) |
| Environmental heat and cold exposure | 2747.6 (2392.9 to 3261.2) | 391.4 (230.4 to 617.4) | 108.8 (76.3 to 150.5) | 59.3* (56.2 to 62.2) | 8.6* (6.4 to 10.5) |
| Exposure to forces of nature | 695.3 (504.4 to 944.2) | 19.1 (12.7 to 27.0) | 27.2 (20.1 to 36.1) | 87.2* (68.6 to 109.2) | 27.6* (14.9 to 42.6) |
| Other unintentional injuries | 9737.5 (8277.0 to 11397.7) | 2042.3 (1256.5 to 3152.3) | 315.7 (214.4 to 445.6) | 47.3* (45.7 to 48.7) | 0.3 (-0.7 to 1.3) |
| **Self-harm and interpersonal violence** | **15307.5 (13534.8 to 17369.8)** | **784.7 (571.2 to 1045.2)** | **358.2 (277.5 to 452.6)** | **62.6* (57.0 to 69.9)** | **10.8* (7.0 to 15.7)** |
| Self-harm | 784.0 (643.0 to 923.2) | 121.0 (75.8 to 185.7) | 35.4 (25.0 to 47.0) | 29.8* (28.1 to 31.4) | -11.6* (-12.7 to -10.5) |
| Self-harm by firearm | 11.2 (9.2 to 13.7) | 3.9 (1.0 to 10.4) | 0.5 (0.3 to 0.6) | 22.1* (18.5 to 26.8) | -16.8* (-19.2 to -13.6) |
| Self-harm by other specified means | 772.8 (631.5 to 912.1) | 117.1 (73.6 to 181.9) | 34.9 (24.7 to 46.5) | 29.9* (28.2 to 31.6) | -11.5* (-12.7 to -10.4) |
| Interpersonal violence | 12097.6 (10459.9 to 13994.7) | 535.0 (330.9 to 786.0) | 236.0 (178.7 to 310.0) | 53.2* (51.6 to 54.9) | 4.4* (3.3 to 5.5) |
| Assault by firearm | 231.5 (192.9 to 283.5) | 12.7 (6.2 to 23.2) | 9.0 (6.3 to 12.3) | 46.8* (44.5 to 48.7) | 0.0 (-1.5 to 1.4) |
| Assault by sharp object | 1176.8 (961.4 to 1598.4) | 58.7 (28.0 to 104.2) | 32.0 (22.1 to 44.7) | 47.7* (45.4 to 49.9) | 0.6 (-0.9 to 2.2) |
| Sexual violence | 6924.9 (5519.8 to 8762.6) | -- | 50.1 (31.6 to 74.3) | 65.5* (62.2 to 68.8) | 12.7* (10.5 to 15.0) |
| Assault by other means | 3764.4 (3170.8 to 4465.1) | 463.6 (280.8 to 685.5) | 144.9 (101.5 to 197.1) | 51.0* (49.4 to 52.5) | 2.9* (1.8 to 3.9) |
| Conflict and terrorism | 2131.9 (1398.1 to 3007.0) | 107.9 (75.9 to 144.7) | 77.7 (55.8 to 107.9) | 119.9* (87.9 to 160.3) | 49.9* (28.1 to 77.4) |
| Executions and police conflict | 296.6 (184.9 to 445.8) | 20.7 (13.3 to 30.1) | 9.1 (5.9 to 13.4) | 151.3* (105.4 to 211.9) | 71.2* (40.0 to 112.6) |
| **Nature of injury aggregates** | | | | | |
| Amputations | 30352.7 (28143.1 to 33312.3) | 403.4 (314.4 to 505.9) | 504.5 (335.1 to 741.9) | 46.9* (40.9 to 52.5) | 0.1 (-4.0 to 3.9) |
| Burns | 16049.6 (14779.9 to 17551.8) | 340.1 (261.6 to 432.7) | 369.5 (240.9 to 542.7) | 49.3* (43.5 to 53.7) | 1.7 (-2.2 to 4.7) |
| Fractures | 33203.0 (30838.0 to 36152.0) | 6980.3 (5394.4 to 8895.2) | 1790.8 (1229.0 to 2499.7) | 57.7* (55.9 to 59.3) | 7.4* (6.2 to 8.6) |
| Head Injuries | 4153.4 (3947.1 to 4340.5) | 904.0 (698.5 to 1150.1) | 596.6 (422.1 to 806.3) | 63.4* (61.6 to 65.2) | 11.3* (10.1 to 12.6) |
| Spinal Injuries | 1651.7 (1545.5 to 1776.9) | 34.6 (25.5 to 46.5) | 446.1 (319.8 to 581.1) | 64.7* (61.2 to 68.6) | 12.2* (9.8 to 14.9) |
| Minor Injuries | 16836.8 (15803.3 to 18131.9) | 7168.6 (5808.3 to 8623.8) | 149.7 (70.6 to 283.2) | 58.0* (56.6 to 59.6) | 7.6* (6.7 to 8.8) |
| Other Injuries | 5623.0 (5314.5 to 5941.3) | 8620.2 (7077.8 to 10394.7) | 468.0 (333.3 to 626.7) | 60.6* (57.5 to 63.7) | 9.4* (7.3 to 11.6) |
| **Impairments** | | | | | |
| Anaemia | 62842.2 (58310.6 to 67940.0) | -- | 1547.8 (1061.9 to 2185.4) | 18.6* (8.6 to 27.9) | -19.2* (-26.0 to -12.8) |
| Epilepsy | 2462.7 (2052.9 to 2943.7) | -- | 867.6 (650.3 to 1141.0) | 53.8* (45.7 to 62.3) | 4.8 (-0.7 to 10.6) |
| Guillain-Barré syndrome | 5.1 (2.8 to 8.6) | -- | 1.5 (0.7 to 2.6) | 46.3* (42.4 to 50.0) | -0.3 (-2.9 to 2.2) |
| Hearing loss | 140206.8 (132874.0 to 146567.3) | -- | 3248.9 (2138.3 to 4803.0) | 46.3* (43.2 to 49.4) | -0.3 (-2.4 to 1.8) |
| Heart failure | 5362.2 (4490.2 to 6318.3) | -- | 768.8 (526.0 to 1043.8) | 46.0* (43.6 to 48.4) | -0.5 (-2.1 to 1.1) |
| Infertility | -- | -- | -- | -17.6* (-29.5 to -9.2) | -43.8* (-52.0 to -38.1) |
| Developmental intellectual disability | 4726.3 (3676.7 to 5702.7) | -- | 719.8 (557.7 to 899.9) | 52.8* (47.4 to 58.8) | 4.1* (0.4 to 8.2) |
| Pelvic inflammatory disease | -- | -- | -- | -- | -- |
| Vision loss | 137308.8 (127119.8 to 149169.6) | -- | 3295.2 (2249.5 to 4736.7) | 42.7* (40.6 to 44.9) | -2.8* (-4.2 to -1.3) |

We did not calculate incidence for the nine impairments and certain NTDs. Blank cells mean that no estimate is available or that the estimate has a magnitude less than 50.
Table presents prevalence and incidence counts in thousands.
* Percentage changes that are statistically significant
‡ Incidence estimates for stroke represent first-ever stroke only.
^ Incidence of HIV/AIDS represents new infections of HIV only and does not include new infections of Tuberculosis in HIV positive cases.

| SR Table 10q: Global prevalence, incidence and YLDs for 2017, percentage change of YLD counts and percentage change of YLD rates between 2007 and 2017 by cause, age 65 to 69 years | | | | | |
|---|---|---|---|---|---|
| Cause | Prevalence in thousands (95% UI) | Incidence in thousands (95% UI) | YLDs in thousands (95% UI) | | |
| | 2017 counts | 2017 counts | 2017 counts | Percentage change in counts between 2007 and 2017 | Percentage change in rates between 2007 and 2017 |
| **All causes** | **240505.5 (240414.8 to 240590.0)** | **1100502.1 (972661.5 to 1268447.3)** | **50592.6 (38170.8 to 64499.5)** | **38.6* (37.6 to 39.7)** | **0.1 (-0.7 to 0.8)** |
| **Communicable, maternal, neonatal, and nutritional disorders** | **144678.5 (131060.9 to 160478.3)** | **674864.0 (547386.2 to 833932.4)** | **2402.1 (1805.2 to 3089.5)** | **28.1* (23.7 to 32.6)** | **-7.6* (-10.7 to -4.3)** |
| **HIV/AIDS and sexually transmitted infections** | **51419.2 (45881.2 to 57742.0)** | **4790.0 (4174.5 to 5621.6)** | **100.2 (69.4 to 140.5)** | **37.3* (5.8 to 71.6)** | **-0.9 (-23.6 to 23.8)** |
| HIV/AIDS | 776.1 (670.1 to 890.4) | 23.9^ (17.7 to 31.0) | 77.8 (53.5 to 109.4) | 36.6* (0.9 to 88.0) | -1.4 (-27.2 to 35.7) |
| HIV/AIDS - Drug-susceptible Tuberculosis | 20.0 (18.3 to 21.8) | 21.7 (19.8 to 23.8) | 7.5 (5.1 to 10.1) | 50.9* (38.9 to 63.3) | 8.9* (0.3 to 17.9) |
| HIV/AIDS - Multidrug-resistant Tuberculosis without extensive drug resistance | 0.8 (0.5 to 1.5) | 0.9 (0.6 to 1.2) | 0.3 (0.2 to 0.6) | 44.2 (-11.7 to 151.2) | 4.1 (-36.2 to 81.3) |
| HIV/AIDS - Extensively drug-resistant Tuberculosis | 0.0 (0.0 to 0.1) | 0.0 (0.0 to 0.1) | -- | 182.8* (44.7 to 522.8) | 104.2* (4.5 to 349.6) |
| HIV/AIDS resulting in other diseases | 755.2 (649.3 to 868.3) | 23.9 (17.7 to 31.0) | 69.9 (47.2 to 100.5) | 35.2 (-2.7 to 93.4) | -2.4 (-29.8 to 39.6) |
| HIV/AIDS not on antiretroviral treatment without Tuberculosis | 149.5 (119.4 to 188.5) | 23.9 (17.7 to 31.0) | 25.3 (13.8 to 45.4) | -43.8* (-53.9 to -32.4) | -59.4* (-66.8 to -51.2) |
| HIV/AIDS on antiretroviral treatment without Tuberculosis | 605.7 (523.5 to 690.9) | -- | 44.6 (29.8 to 63.2) | 575.2* (514.9 to 666.3) | 387.4* (343.9 to 453.2) |
| Sexually transmitted infections excluding HIV | 50916.3 (45339.9 to 57228.0) | 4766.1 (4152.6 to 5595.7) | 22.5 (11.9 to 42.0) | 39.6* (36.7 to 42.4) | 0.7 (-1.3 to 2.8) |
| Syphilis | 290.8 (231.2 to 366.2) | -- | 6.1 (4.1 to 8.8) | 38.7* (31.8 to 46.6) | 0.1 (-4.8 to 5.8) |
| Early syphilis | 255.2 (195.8 to 330.5) | -- | 0.1 (0.0 to 0.2) | 44.4* (32.7 to 56.1) | 4.2 (-4.2 to 12.7) |
| Tertiary syphilis | 35.6 (30.1 to 41.9) | -- | 6.1 (4.1 to 8.7) | 38.7* (31.7 to 46.6) | 0.1 (-4.9 to 5.8) |
| Chlamydial infection | 128.1 (87.1 to 181.7) | 92.0 (60.7 to 132.4) | 0.2 (0.1 to 0.4) | 38.6* (26.1 to 52.4) | 0.1 (-8.9 to 10.0) |
| Chlamydia episode | 127.9 (86.9 to 181.5) | 92.0 (60.7 to 132.4) | 0.2 (0.1 to 0.4) | 37.6* (23.8 to 52.7) | -0.7 (-10.6 to 10.2) |
| Chlamydial infection complications | 0.2 (0.1 to 0.3) | -- | -- | 45.7* (20.0 to 72.7) | 5.2 (-13.4 to 24.7) |
| Gonococcal infection | 35.8 (19.9 to 61.4) | 81.1 (49.0 to 124.1) | 0.1 (0.1 to 0.3) | 36.2* (25.9 to 47.8) | -1.7 (-9.1 to 6.7) |
| Gonococcal infection complications | 0.3 (0.2 to 0.4) | -- | 0.0 (0.0 to 0.1) | 42.7* (17.3 to 67.6) | 3.0 (-15.3 to 21.0) |
| Gonorrhoea episode | 35.6 (19.6 to 61.1) | 81.1 (49.0 to 124.1) | 0.1 (0.0 to 0.2) | 33.9* (23.0 to 42.8) | -3.3 (-11.2 to 3.1) |
| Trichomoniasis | 2678.6 (2127.9 to 3387.8) | 4366.2 (3752.9 to 5204.2) | 4.5 (1.8 to 9.8) | 41.7* (37.5 to 45.8) | 2.3 (-0.8 to 5.2) |
| Genital herpes | 49031.6 (43273.2 to 55737.5) | 226.9 (161.9 to 312.3) | 11.4 (3.4 to 28.0) | 39.3* (37.3 to 41.1) | 0.5 (-0.9 to 1.8) |
| Other sexually transmitted infections | -- | -- | 0.1 (0.0 to 0.2) | 36.6* (28.4 to 45.1) | -1.4 (-7.3 to 4.8) |
| Other sexually transmitted diseases residual | -- | -- | 0.1 (0.0 to 0.2) | 36.6* (28.4 to 45.1) | -1.4 (-7.3 to 4.8) |
| Other sexually transmitted diseases | -- | -- | 0.1 (0.0 to 0.2) | 36.6* (28.4 to 45.1) | -1.4 (-7.3 to 4.8) |
| **Respiratory infections and tuberculosis** | **77770.8 (54202.3 to 108102.0)** | **416054.5 (297975.3 to 574039.4)** | **377.7 (261.4 to 531.6)** | **27.0* (23.5 to 30.1)** | **-8.3* (-10.9 to -6.0)** |
| Tuberculosis | 73020.2 (49226.5 to 104559.0) | 395.6 (306.8 to 508.2) | 202.7 (129.0 to 299.6) | 20.9* (16.7 to 25.2) | -12.7* (-15.7 to -9.6) |
| Latent tuberculosis infection | 72317.3 (48521.9 to 103851.4) | -- | -- | -- | -- |
| Drug-susceptible tuberculosis | 670.3 (511.0 to 873.8) | 375.8 (290.0 to 484.8) | 193.2 (122.9 to 283.1) | 21.4* (14.8 to 27.1) | -12.4* (-17.1 to -8.3) |
| Multidrug-resistant tuberculosis without extensive drug resistance | 31.0 (13.3 to 61.1) | 18.8 (9.4 to 35.2) | 9.0 (3.6 to 19.5) | 9.4 (-48.3 to 98.5) | -21.0 (-62.7 to 43.3) |
| Extensively drug-resistant tuberculosis | 1.6 (0.8 to 3.5) | 1.0 (0.6 to 1.7) | 0.5 (0.2 to 1.2) | 51.4 (-19.2 to 198.7) | 9.3 (-41.7 to 115.6) |
| Lower respiratory infections | 229.0 (205.9 to 254.4) | 10648.9 (9570.5 to 11986.1) | 13.3 (9.0 to 18.7) | 40.2* (30.8 to 50.4) | 1.2 (-5.6 to 8.5) |
| Guillain-Barré syndrome due to lower respiratory infections | 0.6 (0.2 to 1.0) | -- | 0.2 (0.1 to 0.3) | 41.8* (37.8 to 45.5) | 2.3 (-0.5 to 5.0) |
| Lower respiratory infection episode | 228.4 (205.6 to 253.9) | 10648.9 (9570.5 to 11986.1) | 13.1 (8.9 to 18.6) | 40.2* (30.7 to 50.4) | 1.2 (-5.6 to 8.6) |
| Upper respiratory infections | 5545.0 (3847.0 to 7792.0) | 403495.2 (285898.5 to 561504.7) | 131.8 (73.3 to 226.1) | 36.9* (35.0 to 38.7) | -1.2 (-2.5 to 0.1) |
| Guillain-Barré syndrome due to upper respiratory infections | 1.5 (0.9 to 2.5) | -- | 0.4 (0.2 to 0.8) | 41.8* (37.9 to 45.6) | 2.3 (-0.5 to 5.1) |
| Upper respiratory infection episode | 5543.5 (3845.5 to 7791.0) | 403495.2 (285898.5 to 561504.7) | 131.4 (73.0 to 225.7) | 36.8* (35.0 to 38.7) | -1.2 (-2.5 to 0.1) |
| Otitis media | 1481.0 (1120.6 to 1919.2) | 1514.7 (1109.7 to 2039.5) | 29.9 (17.1 to 49.7) | 25.4* (19.8 to 31.3) | -9.5* (-13.5 to -5.2) |
| Acute otitis media | 83.1 (59.6 to 111.4) | 1514.5 (1109.6 to 2039.2) | 1.0 (0.5 to 2.0) | 36.4* (27.9 to 45.3) | -1.5 (-7.7 to 4.9) |
| Chronic otitis media | 1397.9 (1038.6 to 1831.8) | 0.3 (0.0 to 1.0) | 28.8 (16.5 to 47.9) | 25.0* (19.2 to 31.1) | -9.7* (-14.0 to -5.4) |
| **Enteric infections** | **3156.5 (2813.1 to 3585.3)** | **215880.7 (187895.7 to 250225.0)** | **336.2 (231.1 to 462.2)** | **60.0* (52.7 to 67.1)** | **15.5* (10.2 to 20.6)** |
| Diarrhoeal diseases | 3176.4 (2832.7 to 3597.9) | 215755.8 (187756.9 to 250102.9) | 335.2 (230.5 to 460.7) | 60.3* (53.0 to 67.4) | 15.7* (10.5 to 20.8) |
| Guillain-Barré syndrome due to diarrheal diseases | 0.5 (0.3 to 0.9) | -- | 0.2 (0.1 to 0.3) | 41.8* (37.8 to 45.5) | 2.3 (-0.5 to 5.0) |
| Diarrheal disease episode | 3175.9 (2832.3 to 3597.1) | 215755.8 (187756.9 to 250102.9) | 335.0 (230.4 to 460.6) | 60.3* (53.0 to 67.4) | 15.7* (10.5 to 20.8) |
| Typhoid and paratyphoid | 0.5 (0.1 to 0.9) | 121.2 (101.0 to 144.1) | 1.0 (0.6 to 1.4) | 1.4 (-16.4 to 21.5) | -26.8* (-39.7 to -12.3) |
| Typhoid fever | 5.9 (4.6 to 7.5) | 94.3 (75.5 to 114.9) | 0.9 (0.6 to 1.3) | 3.4 (-16.0 to 25.8) | -25.4* (-39.4 to -9.2) |
| Typhoid fever complications | 1.2 (0.9 to 1.7) | 16.2 (12.7 to 20.3) | 0.4 (0.2 to 0.6) | 2.1 (-23.5 to 36.0) | -26.3* (-44.8 to -1.9) |
| Typhoid fever episode | 4.7 (3.6 to 6.0) | 78.1 (62.5 to 95.0) | 0.5 (0.3 to 0.8) | 4.3 (-20.9 to 34.2) | -24.7* (-42.9 to -3.1) |
| Paratyphoid fever | 1.2 (0.8 to 1.6) | 26.9 (18.6 to 37.0) | 0.1 (0.0 to 0.1) | -18.2* (-30.3 to -3.4) | -40.9* (-49.7 to -30.3) |
| Intestinal perforation due to paratyphoid | 0.1 (0.0 to 0.1) | 1.4 (0.9 to 1.9) | -- | -18.5 (-36.7 to 2.3) | -41.2* (-54.3 to -26.2) |
| Paratyphoid fever episode | 1.1 (0.8 to 1.5) | 25.5 (17.7 to 34.8) | 0.1 (0.0 to 0.1) | -18.2* (-30.8 to -3.0) | -40.9* (-50.1 to -30.0) |
| iNTS | 0.1 (0.1 to 0.2) | 3.7 (2.1 to 5.9) | -- | -9.3 (-18.8 to 0.8) | -34.5* (-41.4 to -27.2) |
| Other intestinal infectious diseases | -- | -- | -- | -0.0 (-17.5 to 19.8) | -27.8* (-40.5 to -13.5) |
| **Neglected tropical diseases and malaria** | **23096.6 (21978.8 to 24382.8)** | **6288.5 (4885.2 to 7773.8)** | **400.9 (290.0 to 539.9)** | **8.5* (1.7 to 15.9)** | **-21.7* (-26.6 to -16.3)** |
| Malaria | 1091.2 (1018.7 to 1153.5) | 683.6 (623.5 to 770.3) | 9.2 (6.3 to 12.9) | -16.0 (-28.5 to 3.6) | -39.3* (-48.4 to -25.2) |
| Malaria complications | 3.8 (3.3 to 4.3) | -- | 1.7 (1.3 to 2.1) | 39.4* (36.3 to 42.0) | 0.6 (-1.6 to 2.5) |
| Malaria episode | 40.3 (27.2 to 54.9) | 683.6 (623.5 to 770.3) | 1.6 (0.9 to 2.5) | -17.6* (-27.9 to -3.6) | -40.5* (-48.0 to -30.4) |

| | | | | | |
|---|---|---|---|---|---|
| Malaria parasitemia | 1047.1 (972.8 to 1116.5) | -- | 6.0 (3.7 to 8.9) | -24.1* (-40.0 to -0.5) | -45.2* (-56.7 to -28.1) |
| Chagas disease | 342.0 (292.9 to 396.1) | 3.3 (2.2 to 5.0) | 5.0 (3.3 to 7.3) | 17.2* (10.6 to 23.7) | -15.4* (-20.1 to -10.7) |
| Acute Chagas disease | -- | 3.3 (2.2 to 5.0) | -- | 34.2* (28.1 to 39.5) | -3.1 (-7.5 to 0.7) |
| Asymptomatic Chagas disease | 268.7 (228.3 to 311.8) | -- | -- | -- | -- |
| Symptomatic chronic Chagas infection | 73.3 (58.2 to 90.4) | -- | 5.0 (3.3 to 7.3) | 17.2* (10.6 to 23.7) | -15.4* (-20.2 to -10.7) |
| Leishmaniasis | 132.6 (97.5 to 175.4) | 15.6 (11.2 to 21.3) | 8.0 (4.9 to 12.3) | 10.3* (1.2 to 23.2) | -20.4* (-26.9 to -11.0) |
| Visceral leishmaniasis | 0.1 (0.1 to 0.1) | 0.4 (0.3 to 0.5) | -- | -72.5* (-77.3 to -63.7) | -80.1* (-83.6 to -73.8) |
| Cutaneous and mucocutaneous leishmaniasis | 133.3 (98.7 to 175.6) | 15.3 (10.9 to 21.0) | 8.0 (4.9 to 12.3) | 10.5* (1.4 to 23.7) | -20.2* (-26.8 to -10.7) |
| African trypanosomiasis | 0.1 (0.0 to 0.4) | 0.1 (0.0 to 0.2) | 0.0 (0.0 to 0.1) | -76.5 (-97.2 to 56.3) | -83.1 (-98.0 to 12.9) |
| Trypanosomiasis Gambiense | 0.1 (0.0 to 0.4) | 0.1 (0.0 to 0.2) | 0.0 (0.0 to 0.1) | -75.5 (-97.3 to 69.8) | -82.3 (-98.0 to 22.6) |
| Trypanosomiasis Rhodesiense | -- | -- | -- | -91.8* (-99.2 to -32.3) | -94.1* (-99.4 to -51.1) |
| Schistosomiasis | 3723.7 (3384.8 to 4082.0) | -- | 25.8 (12.7 to 49.5) | -18.3* (-22.9 to -13.1) | -41.0* (-44.3 to -37.2) |
| Mild schistosomiasis | 3270.1 (2958.9 to 3608.2) | -- | 17.7 (7.2 to 37.3) | -18.7* (-20.5 to -16.9) | -41.3* (-42.6 to -40.0) |
| Anemia due to schistosomiasis | 215.5 (172.4 to 268.7) | -- | 4.6 (2.7 to 7.3) | -26.9* (-44.0 to -5.5) | -47.2* (-59.5 to -31.8) |
| Schistosomiasis complications | 239.0 (187.6 to 298.0) | -- | 3.5 (2.0 to 5.9) | -0.4 (-7.9 to 8.5) | -28.1* (-33.5 to -21.7) |
| Cysticercosis | 392.5 (292.7 to 507.6) | -- | 105.9 (65.7 to 163.2) | 28.2* (21.8 to 34.5) | -7.5* (-12.1 to -2.9) |
| Cystic echinococcosis | 22.5 (13.2 to 37.2) | 4.4 (2.7 to 7.4) | 1.8 (0.9 to 3.2) | 15.7* (4.9 to 26.4) | -16.5* (-24.3 to -8.8) |
| Lymphatic filariasis | 1498.1 (1374.2 to 1635.6) | -- | 28.6 (16.3 to 44.5) | -36.2* (-48.2 to -24.8) | -53.9* (-62.6 to -45.7) |
| Prevalence of detectable microfiliaria due to lymphatic filariasis | 1222.2 (1142.4 to 1317.8) | -- | -- | -- | -- |
| Lymphatic filariasis complications | 275.9 (189.6 to 392.4) | -- | 28.6 (16.3 to 44.5) | -36.2* (-48.2 to -24.8) | -53.9* (-62.6 to -45.7) |
| Onchocerciasis | 503.4 (346.3 to 770.4) | -- | 42.1 (24.0 to 72.8) | 11.5 (-3.2 to 28.3) | -19.5* (-30.2 to -7.4) |
| Asymptomatic onchocerciasis | 72.8 (0.0 to 312.2) | -- | -- | -- | -- |
| Skin disease due to onchocerciasis | 292.3 (206.3 to 403.8) | -- | 31.2 (15.0 to 61.7) | 9.8 (-11.9 to 30.4) | -20.7* (-36.4 to -5.9) |
| Vision loss due to onchocerciasis | 138.4 (94.3 to 208.7) | -- | 11.0 (6.6 to 16.8) | 16.6* (2.7 to 32.8) | -15.8* (-25.9 to -4.1) |
| Trachoma | 682.2 (424.6 to 1091.9) | -- | 50.7 (31.7 to 76.6) | -2.4 (-16.0 to 12.8) | -29.5* (-39.4 to -18.5) |
| Dengue | 150.3 (81.9 to 254.4) | 2515.0 (1530.9 to 3812.8) | 22.8 (10.1 to 42.0) | 93.0* (66.6 to 199.7) | 39.3* (20.3 to 116.4) |
| Post-dengue chronic fatigue syndrome | 105.9 (49.5 to 193.6) | -- | 20.3 (8.6 to 38.6) | 93.0* (65.9 to 201.6) | 39.3* (19.8 to 117.7) |
| Dengue episode | 44.4 (26.8 to 66.6) | 2515.0 (1530.9 to 3812.8) | 2.5 (1.3 to 4.3) | 93.1* (65.3 to 189.0) | 39.4* (19.4 to 108.7) |
| Yellow fever | 0.0 (0.0 to 0.1) | 0.8 (0.2 to 2.3) | -- | -21.3 (-43.9 to 13.4) | -43.2* (-59.5 to -18.1) |
| Asymptomatic yellow fever | -- | 0.5 (0.1 to 1.3) | -- | -- | -- |
| Yellow fever episode | -- | 0.4 (0.1 to 1.0) | -- | -21.3 (-43.9 to 13.4) | -43.2* (-59.5 to -18.1) |
| Rabies | -- | 0.5 (0.3 to 0.6) | -- | -25.0* (-42.5 to -6.9) | -45.8* (-58.5 to -32.8) |
| Intestinal nematode infections | 13471.0 (12421.9 to 14666.3) | -- | 25.7 (15.3 to 41.7) | -16.1* (-23.3 to -8.9) | -39.4* (-44.6 to -34.3) |
| Ascariasis | 5871.4 (5046.9 to 6848.3) | -- | 6.7 (3.6 to 11.4) | -19.8* (-29.0 to -9.6) | -42.1* (-48.7 to -34.8) |
| Asymptomatic ascariasis | 5488.4 (4711.4 to 6406.3) | -- | -- | -- | -- |
| Ascariasis complications | 383.0 (334.0 to 437.3) | -- | 6.7 (3.6 to 11.4) | -19.8* (-29.0 to -9.6) | -42.1* (-48.7 to -34.8) |
| Trichuriasis | 3238.1 (2700.9 to 3903.5) | -- | 1.9 (1.0 to 3.1) | 5.4 (-5.4 to 17.1) | -23.9* (-31.7 to -15.5) |
| Asymptomatic trichuriasis | 3139.0 (2611.5 to 3791.6) | -- | -- | -- | -- |
| Trichuriasis complications | 99.0 (89.3 to 108.7) | -- | 1.9 (1.0 to 3.1) | 5.4 (-5.4 to 17.1) | -23.9* (-31.7 to -15.5) |
| Hookworm disease | 4975.4 (4455.9 to 5582.1) | -- | 17.1 (10.2 to 27.7) | -16.4* (-26.2 to -6.3) | -39.7* (-46.7 to -32.4) |
| Asymptomatic hookworm disease | 4097.3 (3670.0 to 4596.4) | -- | -- | -- | -- |
| Anemia due to hookworm disease | 245.2 (207.3 to 291.2) | -- | 4.6 (2.9 to 6.8) | -23.4* (-39.1 to -5.7) | -44.7* (-56.0 to -31.9) |
| Hookworm disease complications | 632.9 (569.9 to 707.0) | -- | 12.5 (7.2 to 20.9) | -13.6* (-21.7 to -5.0) | -37.6* (-43.5 to -31.4) |
| Food-borne trematodiases | 2821.3 (2485.9 to 3206.6) | 3005.1 (2239.4 to 3843.2) | 61.9 (32.8 to 109.7) | 47.8* (36.9 to 58.7) | 6.7 (-1.1 to 14.6) |
| Asymptomatic food-borne tremodiases | 2247.4 (1815.7 to 2641.6) | 2265.4 (1142.2 to 3265.8) | -- | -- | -- |
| Food-borne tremodiases complications | 573.9 (337.2 to 926.6) | 739.6 (86.4 to 1841.0) | 61.9 (32.8 to 109.7) | 47.8* (36.9 to 58.7) | 6.7 (-1.1 to 14.6) |
| Leprosy | 49.6 (46.7 to 52.9) | 3.3 (3.1 to 3.6) | 3.0 (2.0 to 4.2) | 8.7* (2.0 to 16.2) | -21.5* (-26.4 to -16.1) |
| Ebola virus disease | -- | -- | -- | -98.7* (-98.9 to -98.5) | -99.1* (-99.2 to -98.9) |
| Ebola cases | -- | -- | -- | -99.0* (-99.0 to -98.9) | -99.3* (-99.3 to -99.2) |
| Post-Ebola chronic fatigue syndrome | -- | -- | -- | -98.7* (-98.9 to -98.5) | -99.1* (-99.2 to -98.9) |
| Zika virus disease | 0.9 (0.5 to 1.8) | 56.7 (32.1 to 106.8) | -- | -- | -- |
| Zika virus complications | -- | -- | -- | -- | -- |
| Zika virus episode | 0.9 (0.5 to 1.8) | 56.7 (32.1 to 106.8) | -- | -- | -- |
| Guinea worm disease | -- | -- | -- | -99.6* (-99.7 to -99.4) | -99.7* (-99.8 to -99.5) |
| Other neglected tropical diseases | 524.6 (478.2 to 576.5) | -- | 10.4 (6.8 to 15.0) | 7.5 (-4.7 to 19.2) | -22.4* (-31.2 to -14.0) |
| Acute infection due to other neglected tropical diseases | -- | -- | 0.3 (0.2 to 0.5) | 117.4* (86.3 to 221.0) | 56.9* (34.5 to 131.7) |
| Anemia due to other neglected tropical diseases | 524.6 (478.2 to 576.5) | -- | 10.1 (6.6 to 14.5) | 5.8 (-6.5 to 17.0) | -23.6* (-32.5 to -15.5) |
| **Other infectious diseases** | **1944.4 (1663.6 to 2277.2)** | **6988.6 (5468.9 to 9019.0)** | **87.8 (60.4 to 122.2)** | **25.6* (18.1 to 34.5)** | **-9.3* (-14.8 to -2.9)** |
| Meningitis | 257.3 (212.8 to 307.9) | 78.5 (54.9 to 107.8) | 15.3 (10.8 to 20.6) | 8.4* (3.8 to 13.1) | -21.7* (-25.1 to -18.4) |
| Pneumococcal meningitis | 93.6 (75.9 to 115.4) | 7.1 (4.4 to 10.6) | 5.8 (4.0 to 7.8) | -10.5* (-15.9 to -4.8) | -35.4* (-39.3 to -31.3) |

| | | | | | |
|---|---|---|---|---|---|
| Acute pneumococcal meningitis | 0.3 (0.2 to 0.5) | 7.1 (4.4 to 10.6) | 0.0 (0.0 to 0.1) | -4.8 (-15.6 to 5.4) | -31.2* (-39.0 to -23.9) |
| Pneumococcal meningitis complications | 93.3 (75.6 to 115.1) | -- | 5.8 (4.0 to 7.7) | -10.6* (-16.0 to -4.9) | -35.4* (-39.4 to -31.3) |
| H influenzae type B meningitis | 18.5 (12.8 to 25.1) | 2.2 (1.2 to 3.7) | 1.0 (0.7 to 1.4) | -47.5* (-49.8 to -45.4) | -62.1* (-63.7 to -60.6) |
| Acute H influenzae type B meningitis | 0.1 (0.1 to 0.2) | 2.2 (1.2 to 3.7) | -- | -46.9* (-53.7 to -40.2) | -61.6* (-66.6 to -56.9) |
| H Influenza type B meningitis complications | 18.4 (12.7 to 25.0) | -- | 1.0 (0.7 to 1.3) | -47.5* (-49.8 to -45.4) | -62.1* (-63.7 to -60.6) |
| Meningococcal infection | 28.2 (19.6 to 37.9) | 6.2 (3.5 to 9.9) | 1.8 (1.3 to 2.5) | 7.9* (3.6 to 12.3) | -22.1* (-25.2 to -18.9) |
| Acute meningococcal meningitis | 0.3 (0.2 to 0.4) | 6.2 (3.5 to 9.9) | 0.0 (0.0 to 0.1) | 20.9* (7.8 to 32.4) | -12.7* (-22.2 to -4.4) |
| Meningococcal meningitis complications | 28.0 (19.3 to 37.5) | -- | 1.8 (1.2 to 2.5) | 7.7* (3.3 to 12.2) | -22.3* (-25.4 to -19.0) |
| Other meningitis | 116.9 (82.3 to 151.2) | 63.0 (43.8 to 87.8) | 6.7 (4.6 to 9.2) | 65.5* (57.4 to 75.7) | 19.5* (13.6 to 26.8) |
| Other acute bacterial meningitis | 0.7 (0.5 to 1.0) | 16.9 (11.4 to 24.0) | 0.1 (0.1 to 0.2) | 78.2* (61.0 to 94.2) | 28.6* (16.2 to 40.2) |
| Acute viral meningitis | 2.0 (1.4 to 2.8) | 46.1 (32.2 to 63.3) | 0.3 (0.2 to 0.4) | 23.5* (12.3 to 32.7) | -10.8* (-18.9 to -4.2) |
| Other bacterial meningitis complications | 114.2 (80.2 to 148.4) | -- | 6.3 (4.3 to 8.7) | 67.7* (59.1 to 78.5) | 21.1* (14.8 to 28.8) |
| Encephalitis | 316.2 (157.2 to 532.9) | 84.4 (80.7 to 87.6) | 17.9 (12.5 to 24.5) | 30.5* (24.9 to 36.9) | -5.8* (-9.9 to -1.2) |
| Acute encephalitis | 4.2 (4.0 to 4.3) | 84.4 (80.7 to 87.6) | 0.6 (0.4 to 0.8) | 41.8* (41.2 to 42.5) | 2.4* (1.9 to 2.9) |
| Encephalitis complications | 312.0 (153.1 to 528.6) | -- | 17.4 (12.1 to 23.7) | 30.2* (24.3 to 36.7) | -6.0* (-10.3 to -1.3) |
| Diphtheria | -- | -- | -- | -- | -- |
| Whooping cough | -- | -- | -- | -- | -- |
| Tetanus | 1.3 (1.3 to 1.5) | 2.6 (1.6 to 4.4) | 0.0 (0.0 to 0.1) | -13.2 (-29.4 to 4.8) | -37.4* (-49.0 to -24.4) |
| Severe tetanus | 0.1 (0.1 to 0.2) | 2.6 (1.6 to 4.4) | -- | -36.7* (-49.8 to -12.7) | -54.3* (-63.8 to -37.0) |
| Neonatal tetanus complications | 1.2 (1.1 to 1.3) | -- | -- | 27.5* (25.7 to 29.2) | -7.9* (-9.3 to -6.8) |
| Measles | -- | -- | -- | -- | -- |
| Varicella and herpes zoster | 491.1 (384.4 to 620.5) | 2705.7 (2140.7 to 3370.4) | 26.0 (15.6 to 39.8) | 43.1* (37.6 to 48.5) | 3.3 (-0.7 to 7.2) |
| Chickenpox | 0.0 (0.0 to 0.1) | 2.2 (0.5 to 5.8) | -- | 66.6* (56.8 to 76.5) | 20.3* (13.2 to 27.4) |
| Herpes zoster | 491.0 (384.3 to 620.4) | 2703.6 (2139.4 to 3367.7) | 26.0 (15.6 to 39.8) | 43.1* (37.6 to 48.5) | 3.3 (-0.7 to 7.2) |
| Acute hepatitis | 462.3 (299.6 to 692.0) | 4117.3 (2697.5 to 6073.4) | 10.6 (5.7 to 18.1) | 28.8 (-17.6 to 109.0) | -7.0 (-40.5 to 50.9) |
| Acute hepatitis A | 14.6 (9.0 to 21.0) | 189.9 (117.5 to 272.6) | 0.6 (0.3 to 1.0) | 35.4* (15.5 to 57.0) | -2.3 (-16.6 to 13.3) |
| Acute hepatitis B | 422.5 (265.0 to 650.0) | 3661.8 (2296.4 to 5633.0) | 9.5 (4.9 to 16.4) | 28.6 (-22.2 to 120.3) | -7.2 (-43.8 to 59.0) |
| Acute hepatitis C | 14.2 (6.5 to 27.9) | 122.9 (56.4 to 241.5) | 0.2 (0.1 to 0.5) | 6.6 (-5.9 to 16.0) | -23.0* (-32.1 to -16.2) |
| Acute hepatitis E | 11.0 (7.1 to 16.4) | 142.7 (92.2 to 213.3) | 0.4 (0.2 to 0.6) | 38.5* (14.5 to 62.9) | -0.0 (-17.3 to 17.6) |
| Other unspecified infectious diseases | 691.4 (637.6 to 750.9) | -- | 17.9 (11.7 to 26.1) | 14.8* (4.7 to 24.8) | -17.2* (-24.4 to -9.9) |
| Guillain-Barré syndrome due to other infectious diseases | 0.3 (0.2 to 0.6) | -- | 0.1 (0.0 to 0.2) | 41.8* (37.8 to 45.5) | 2.3 (-0.5 to 5.0) |
| Other infectious diseases | -- | -- | 4.2 (2.5 to 6.9) | 37.5* (32.4 to 42.7) | -0.7 (-4.4 to 3.0) |
| Anemia due to other infectious diseases | 691.1 (637.2 to 750.6) | -- | 13.6 (8.9 to 19.5) | 9.0 (-2.8 to 21.6) | -21.3* (-29.9 to -12.2) |
| **Maternal and neonatal disorders** | **2881.4 (2498.2 to 3276.9)** | **--** | **447.8 (324.1 to 581.5)** | **72.3* (62.5 to 83.5)** | **24.4* (17.3 to 32.4)** |
| Maternal disorders | 6.4 (5.1 to 8.0) | -- | 2.3 (1.5 to 3.1) | -27.0* (-36.9 to -15.9) | -47.3* (-54.4 to -39.3) |
| Maternal haemorrhage | -- | -- | -- | -- | -- |
| Maternal hemorrhage complications | -- | -- | -- | -- | -- |
| Maternal hemorrhage episode | -- | -- | -- | -- | -- |
| Maternal sepsis and other pregnancy related infections | -- | -- | -- | -- | -- |
| Infertility due to puerperal sepsis | -- | -- | -- | -- | -- |
| Maternal sepsis and other maternal infection episode | -- | -- | -- | -- | -- |
| Maternal hypertensive disorders | -- | -- | -- | -- | -- |
| Maternal hypertensive disorder complications | -- | -- | -- | -- | -- |
| Maternal hypertensive disorder episode | -- | -- | -- | -- | -- |
| Maternal obstructed labour and uterine rupture | 7.4 (6.1 to 8.9) | -- | 2.3 (1.5 to 3.1) | -27.0* (-36.9 to -15.9) | -47.3* (-54.4 to -39.3) |
| Obstructed labor, acute event | -- | -- | -- | -- | -- |
| Maternal obstructed labor complications | 7.4 (6.1 to 8.9) | -- | 2.3 (1.5 to 3.1) | -27.0* (-36.9 to -15.9) | -47.3* (-54.4 to -39.3) |
| Maternal abortion and miscarriage | -- | -- | -- | -- | -- |
| Ectopic pregnancy | -- | -- | -- | -- | -- |
| Other maternal disorders | -- | -- | -- | -- | -- |
| Neonatal disorders | 2875.1 (2492.7 to 3270.7) | -- | 445.6 (322.3 to 578.8) | 73.5* (63.9 to 84.9) | 25.3* (18.3 to 33.5) |
| Neonatal preterm birth | 1690.1 (1515.4 to 1888.9) | -- | 165.6 (116.8 to 226.0) | 80.6* (56.9 to 106.7) | 30.4* (13.3 to 49.2) |
| Preterm birth complications | 1359.6 (1198.8 to 1556.9) | -- | 146.9 (100.3 to 205.4) | 89.9* (62.0 to 118.9) | 37.1* (17.0 to 58.0) |
| Retinopathy of prematurity | 330.5 (269.6 to 395.0) | -- | 18.7 (12.7 to 25.9) | 30.3* (23.3 to 37.8) | -5.9* (-11.0 to -0.5) |
| Uncomplicated preterm birth | -- | -- | -- | -- | -- |
| Neonatal encephalopathy due to birth asphyxia and trauma | 1245.6 (913.3 to 1626.8) | -- | 174.9 (104.0 to 271.6) | 77.8* (68.9 to 88.1) | 28.4* (22.0 to 35.8) |
| Neonatal sepsis and other neonatal infections | 300.4 (188.7 to 450.4) | -- | 92.3 (48.2 to 158.6) | 57.3* (50.8 to 65.5) | 13.5* (8.8 to 19.5) |
| Neonatal sepsis and other neonatal infection complications | 300.4 (188.7 to 450.4) | -- | 92.3 (48.2 to 158.6) | 57.3* (50.8 to 65.5) | 13.5* (8.8 to 19.5) |
| Neonatal sepsis and other neonatal infection episode | -- | -- | -- | -- | -- |
| Hemolytic disease and other neonatal jaundice | 30.5 (26.0 to 35.1) | -- | 12.7 (9.7 to 16.0) | 59.3* (54.4 to 63.8) | 15.0* (11.5 to 18.3) |

| | | | | | |
|---|---|---|---|---|---|
| Other neonatal disorders | -- | -- | -- | -- | -- |
| **Nutritional deficiencies** | **43655.7 (41730.7 to 46017.2)** | **24839.1 (22266.4 to 27564.4)** | **651.5 (435.1 to 935.5)** | **9.3* (1.1 to 18.1)** | **-21.1* (-27.0 to -14.7)** |
| Protein-energy malnutrition | 2523.3 (2368.6 to 2674.0) | 2456.2 (2271.2 to 2633.7) | 11.5 (7.6 to 16.0) | 42.1* (27.6 to 55.3) | 2.6 (-7.9 to 12.1) |
| Iodine deficiency | 4102.8 (3728.1 to 4542.8) | 17.0 (14.3 to 20.6) | 60.7 (36.7 to 99.4) | 18.6* (14.0 to 22.7) | -14.4* (-17.7 to -11.4) |
| Visible goiter without symptoms | 4037.6 (3671.0 to 4476.8) | 17.0 (14.3 to 20.6) | 41.5 (20.2 to 77.8) | 20.4* (18.5 to 22.3) | -13.1* (-14.5 to -11.7) |
| Visible goiter with complications | 65.2 (36.8 to 93.6) | -- | 19.2 (10.1 to 29.9) | 14.9* (0.8 to 25.7) | -17.1* (-27.2 to -9.2) |
| Vitamin A deficiency | 13552.6 (12237.1 to 14969.7) | 22366.0 (19781.8 to 25107.2) | 4.2 (2.6 to 6.2) | 37.5* (27.1 to 47.6) | -0.7 (-8.3 to 6.5) |
| Asymptomatic vitamin A deficiency | 13485.8 (12175.4 to 14900.5) | 22366.0 (19781.8 to 25107.2) | -- | -- | -- |
| Vitamin A deficiency complications | 66.9 (45.6 to 92.8) | -- | 4.2 (2.6 to 6.2) | 37.5* (27.1 to 47.6) | -0.7 (-8.3 to 6.5) |
| Vitamin A deficiency with anemia | -- | -- | -- | -- | -- |
| Dietary iron deficiency | 28550.1 (26413.3 to 30793.7) | -- | 569.6 (375.5 to 813.4) | 7.5 (-1.9 to 17.3) | -22.4* (-29.2 to -15.3) |
| Other nutritional deficiencies | -- | -- | 5.4 (3.6 to 7.6) | 47.5* (32.4 to 61.1) | 6.5 (-4.4 to 16.3) |
| **Non-communicable diseases** | **240091.7 (239928.0 to 240246.0)** | **404790.4 (373479.2 to 435908.2)** | **44413.9 (33581.3 to 56635.1)** | **38.5* (37.4 to 39.7)** | **0.0 (-0.8 to 0.8)** |
| **Neoplasms** | **12520.3 (12169.0 to 13054.1)** | **3391.2 (2915.8 to 3981.2)** | **1012.2 (753.0 to 1302.7)** | **56.7* (53.5 to 60.2)** | **13.1* (10.8 to 15.6)** |
| Lip and oral cavity cancer | 199.1 (190.8 to 206.9) | 47.7 (45.7 to 49.8) | 19.8 (14.5 to 25.5) | 52.2* (43.5 to 61.9) | 9.9* (3.6 to 16.9) |
| Diagnosis and primary therapy phase of mouth cancer | 11.7 (11.2 to 12.2) | 47.7 (45.7 to 49.8) | 3.2 (2.2 to 4.4) | 53.5* (40.6 to 68.1) | 10.8* (1.5 to 21.4) |
| Controlled phase of mouth cancer | 166.0 (159.3 to 172.5) | -- | 7.5 (4.9 to 10.9) | 55.2* (45.7 to 64.8) | 12.0* (5.2 to 19.0) |
| Metastatic phase of mouth cancer | 18.5 (17.7 to 19.4) | -- | 7.6 (5.2 to 10.0) | 49.5* (33.3 to 67.8) | 7.9 (-3.8 to 21.1) |
| Terminal phase of mouth cancer | 2.8 (2.7 to 2.9) | -- | 1.5 (1.1 to 1.9) | 49.2* (42.2 to 56.1) | 7.7* (2.7 to 12.7) |
| Nasopharynx cancer | 37.2 (34.5 to 40.7) | 9.9 (9.2 to 10.9) | 4.3 (3.1 to 5.7) | 51.6* (38.1 to 68.1) | 9.4 (-0.3 to 21.4) |
| Diagnosis and primary therapy phase of nasopharynx cancer | 2.1 (2.0 to 2.4) | 9.9 (9.2 to 10.9) | 0.6 (0.4 to 0.9) | 51.1* (34.8 to 72.7) | 9.1 (-2.7 to 24.7) |
| Controlled phase of nasopharynx cancer | 29.9 (27.8 to 32.8) | -- | 1.4 (0.9 to 2.0) | 53.0* (34.5 to 73.7) | 10.5 (-2.9 to 25.4) |
| Metastatic phase of nasopharynx cancer | 4.5 (4.2 to 4.9) | -- | 2.0 (1.3 to 2.7) | 50.6* (32.0 to 72.2) | 8.7 (-4.7 to 24.3) |
| Terminal phase of nasopharynx cancer | 0.6 (0.6 to 0.6) | -- | 0.3 (0.2 to 0.4) | 52.1* (42.2 to 65.4) | 9.8* (2.6 to 19.4) |
| Other pharynx cancer | 60.0 (55.1 to 63.5) | 25.2 (22.9 to 26.8) | 8.2 (5.8 to 10.6) | 57.8* (44.6 to 70.9) | 13.9* (4.4 to 23.4) |
| Diagnosis and primary therapy phase of other pharynx cancer | 3.4 (3.1 to 3.5) | 25.2 (22.9 to 26.8) | 1.0 (0.6 to 1.4) | 63.6* (51.4 to 74.7) | 18.1* (9.3 to 26.1) |
| Controlled phase of other pharynx cancer | 45.0 (41.4 to 47.5) | -- | 2.0 (1.3 to 3.0) | 65.2* (49.3 to 82.2) | 19.3* (7.8 to 31.5) |
| Metastatic phase of other pharynx cancer | 9.9 (9.0 to 10.5) | -- | 4.2 (2.8 to 5.6) | 53.8* (35.3 to 74.0) | 11.0 (-2.3 to 25.6) |
| Terminal phase of other pharynx cancer | 1.8 (1.6 to 1.9) | -- | 1.0 (0.7 to 1.2) | 55.0* (43.1 to 64.8) | 11.9* (3.3 to 19.0) |
| Oesophageal cancer | 141.5 (135.2 to 148.3) | 79.2 (75.9 to 82.8) | 22.0 (16.1 to 28.0) | 42.3* (31.2 to 54.3) | 2.7 (-5.3 to 11.4) |
| Diagnosis and primary therapy phase of esophageal cancer | 11.7 (11.2 to 12.3) | 79.2 (75.9 to 82.8) | 3.2 (2.1 to 4.5) | 48.2* (27.6 to 74.7) | 7.0 (-7.9 to 26.1) |
| Controlled phase of esophageal cancer | 95.8 (91.2 to 100.6) | -- | 4.4 (2.9 to 6.3) | 53.1* (40.6 to 67.1) | 10.5* (1.5 to 20.6) |
| Metastatic phase of esophageal cancer | 27.9 (26.7 to 29.1) | -- | 11.3 (7.8 to 14.8) | 38.0* (22.3 to 55.2) | -0.3 (-11.7 to 12.0) |
| Terminal phase of esophageal cancer | 6.1 (5.8 to 6.3) | -- | 3.2 (2.2 to 4.2) | 38.8* (20.5 to 59.8) | 0.2 (-13.0 to 15.3) |
| Stomach cancer | 477.2 (454.2 to 504.5) | 191.7 (183.3 to 201.1) | 56.3 (41.6 to 72.2) | 62.5* (50.7 to 74.4) | 17.3* (8.8 to 25.9) |
| Diagnosis and primary therapy phase of stomach cancer | 44.3 (42.2 to 46.9) | 191.7 (183.3 to 201.1) | 11.5 (7.9 to 15.7) | 73.9* (56.4 to 92.8) | 25.5* (12.9 to 39.2) |
| Controlled phase of stomach cancer | 366.0 (348.0 to 387.5) | -- | 16.6 (10.7 to 24.1) | 95.1* (83.3 to 108.1) | 40.8* (32.3 to 50.2) |
| Metastatic phase of stomach cancer | 53.2 (50.9 to 55.8) | -- | 21.2 (14.7 to 28.0) | 44.9* (31.7 to 59.1) | 4.6 (-4.9 to 14.8) |
| Terminal phase of stomach cancer | 13.7 (13.1 to 14.4) | -- | 6.9 (4.8 to 9.1) | 42.8* (24.6 to 62.8) | 3.1 (-10.1 to 17.5) |
| Colon and rectum cancer | 1508.7 (1464.8 to 1557.0) | 267.7 (259.8 to 276.0) | 134.0 (98.4 to 175.0) | 56.1* (50.2 to 62.6) | 12.7* (8.4 to 17.4) |
| Diagnosis and primary therapy phase of colon and rectum cancers | 64.3 (62.4 to 66.4) | 267.7 (259.8 to 276.0) | 16.4 (11.2 to 22.1) | 61.6* (48.7 to 74.7) | 16.7* (7.4 to 26.1) |
| Controlled phase of colon and rectum cancers | 1313.7 (1274.4 to 1355.7) | -- | 74.0 (49.1 to 104.0) | 59.8* (53.7 to 65.6) | 15.4* (10.9 to 19.6) |
| Metastatic phase of colon and rectum cancers | 89.0 (86.4 to 91.7) | -- | 34.7 (24.1 to 44.8) | 49.5* (38.9 to 60.5) | 7.9* (0.3 to 15.9) |
| Terminal phase of colon and rectum cancers | 12.2 (11.8 to 12.6) | -- | 6.4 (4.4 to 8.2) | 47.6* (36.2 to 60.2) | 6.6 (-1.7 to 15.7) |
| Stoma from colon and rectum cancers, beyond 10 years | 29.4 (27.3 to 31.8) | -- | 2.6 (1.8 to 3.7) | 37.8* (27.1 to 48.2) | -0.5 (-8.2 to 7.0) |
| Liver cancer | 125.1 (113.8 to 137.2) | 136.7 (129.5 to 144.1) | 32.5 (23.2 to 42.8) | 65.6* (55.6 to 76.7) | 19.5* (12.4 to 27.5) |
| Liver cancer due to hepatitis B | 81.0 (72.7 to 90.1) | 53.7 (48.2 to 59.7) | 12.5 (8.8 to 16.7) | 76.3* (57.7 to 97.5) | 27.3* (13.8 to 42.6) |
| Diagnosis and primary therapy phase of liver cancer due to hepatitis B | 12.8 (11.5 to 14.2) | 53.7 (48.2 to 59.7) | 3.4 (2.2 to 4.8) | 74.1* (44.1 to 109.6) | 25.7* (4.0 to 51.3) |
| Controlled phase of liver cancer due to hepatitis B | 53.4 (47.8 to 59.6) | -- | 2.5 (1.5 to 3.7) | 126.4* (98.2 to 159.9) | 63.5* (43.1 to 87.6) |
| Metastatic phase of liver cancer due to hepatitis B | 10.6 (9.5 to 11.8) | -- | 4.4 (2.9 to 6.1) | 65.2* (36.9 to 100.3) | 19.2 (-1.2 to 44.6) |
| Terminal phase of liver cancer due to hepatitis B | 4.2 (3.8 to 4.7) | -- | 2.2 (1.5 to 3.0) | 61.6* (38.2 to 88.0) | 16.6 (-0.2 to 35.7) |
| Liver cancer due to hepatitis C | 58.3 (53.4 to 63.7) | 39.1 (35.5 to 42.8) | 9.4 (6.6 to 12.5) | 53.2* (44.8 to 63.0) | 10.6* (4.5 to 17.7) |
| Diagnosis and primary therapy phase of liver cancer due to hepatitis C | 9.3 (8.4 to 10.1) | 39.1 (35.5 to 42.8) | 2.6 (1.7 to 3.6) | 51.1* (38.4 to 66.4) | 9.1 (-0.1 to 20.1) |
| Controlled phase of liver cancer due to hepatitis C | 38.2 (35.1 to 41.7) | -- | 1.8 (1.1 to 2.6) | 65.5* (48.3 to 84.5) | 19.5* (7.1 to 33.2) |
| Metastatic phase of liver cancer due to hepatitis C | 7.7 (7.0 to 8.4) | -- | 3.4 (2.3 to 4.6) | 50.4* (38.9 to 63.3) | 8.6* (0.2 to 17.9) |
| Terminal phase of liver cancer due to hepatitis C | 3.1 (2.8 to 3.4) | -- | 1.7 (1.1 to 2.2) | 50.2* (44.2 to 57.3) | 8.4* (4.1 to 13.5) |
| Liver cancer due to alcohol use | 34.4 (28.6 to 41.2) | 23.8 (19.8 to 28.4) | 5.7 (4.0 to 8.0) | 57.1* (46.9 to 68.0) | 13.4* (6.1 to 21.3) |
| Diagnosis and primary therapy phase of liver cancer due to alcohol use | 5.6 (4.7 to 6.7) | 23.8 (19.8 to 28.4) | 1.6 (1.1 to 2.3) | 56.1* (43.5 to 69.6) | 12.7* (3.6 to 22.5) |
| Controlled phase of liver cancer due to alcohol use | 22.3 (18.5 to 26.6) | -- | 1.0 (0.6 to 1.5) | 74.7* (51.8 to 99.2) | 26.1* (9.6 to 43.8) |
| Metastatic phase of liver cancer due to alcohol use | 4.7 (3.9 to 5.6) | -- | 2.1 (1.4 to 3.0) | 52.6* (38.9 to 65.6) | 10.1* (0.3 to 19.5) |

| | | | | | |
|---|---|---|---|---|---|
| Terminal phase of liver cancer due to alcohol use | 1.9 (1.6 to 2.2) | -- | 1.0 (0.7 to 1.4) | 52.4* (45.0 to 60.8) | 10.0* (4.7 to 16.1) |
| Liver cancer due to NASH | 15.0 (12.1 to 18.4) | 10.5 (8.5 to 12.9) | 2.6 (1.8 to 3.6) | 75.8* (67.4 to 85.3) | 26.9* (20.8 to 33.8) |
| Diagnosis and primary therapy phase of liver cancer due to NASH | 2.5 (2.0 to 3.0) | 10.5 (8.5 to 12.9) | 0.7 (0.5 to 1.0) | 74.7* (67.1 to 83.2) | 26.1* (20.6 to 32.2) |
| Controlled phase of liver cancer due to NASH | 9.6 (7.7 to 11.8) | -- | 0.5 (0.3 to 0.7) | 98.5* (76.0 to 122.0) | 43.3* (27.0 to 60.3) |
| Metastatic phase of liver cancer due to NASH | 2.1 (1.7 to 2.6) | -- | 0.9 (0.6 to 1.3) | 70.1* (62.8 to 78.1) | 22.8* (17.5 to 28.6) |
| Terminal phase of liver cancer due to NASH | 0.8 (0.7 to 1.0) | -- | 0.5 (0.3 to 0.6) | 70.1* (62.8 to 78.1) | 22.8* (17.5 to 28.6) |
| Liver cancer due to other causes | 13.9 (11.5 to 16.9) | 9.6 (7.9 to 11.5) | 2.3 (1.6 to 3.3) | 77.6* (67.2 to 90.1) | 28.2* (20.7 to 37.2) |
| Diagnosis and primary therapy phase of liver cancer due to other causes | 2.3 (1.9 to 2.7) | 9.6 (7.9 to 11.5) | 0.7 (0.4 to 1.0) | 76.0* (67.0 to 86.7) | 27.1* (20.5 to 34.8) |
| Controlled phase of liver cancer due to other causes | 9.0 (7.4 to 10.9) | -- | 0.4 (0.3 to 0.7) | 107.1* (76.3 to 139.6) | 49.5* (27.2 to 72.9) |
| Metastatic phase of liver cancer due to other causes | 1.9 (1.6 to 2.3) | -- | 0.9 (0.6 to 1.2) | 70.2* (61.8 to 80.4) | 22.8* (16.8 to 30.3) |
| Terminal phase of liver cancer due to other causes | 0.8 (0.6 to 0.9) | -- | 0.4 (0.3 to 0.6) | 70.2* (61.8 to 80.4) | 22.8* (16.8 to 30.3) |
| Gallbladder and biliary tract cancer | 35.3 (30.6 to 40.8) | 29.1 (25.3 to 32.3) | 7.1 (4.9 to 9.5) | 43.9* (31.0 to 59.8) | 3.9 (-5.4 to 15.3) |
| Diagnosis and primary therapy phase of gallbladder and biliary tract cancer | 4.8 (4.1 to 5.4) | 29.1 (25.3 to 32.3) | 1.4 (0.9 to 1.9) | 45.5* (33.0 to 62.0) | 5.0 (-4.0 to 16.9) |
| Controlled phase of gallbladder and biliary tract cancer | 20.2 (17.3 to 24.0) | -- | 0.9 (0.6 to 1.4) | 75.9* (44.5 to 116.0) | 27.0* (4.3 to 56.0) |
| Metastatic phase of gallbladder and biliary tract cancer | 8.0 (7.0 to 8.9) | -- | 3.6 (2.4 to 4.8) | 38.5* (26.7 to 51.8) | -0.0 (-8.5 to 9.6) |
| Terminal phase of gallbladder and biliary tract cancer | 2.3 (2.0 to 2.6) | -- | 1.2 (0.9 to 1.6) | 38.8* (28.6 to 51.1) | 0.2 (-7.1 to 9.1) |
| Pancreatic cancer | 52.0 (50.6 to 53.4) | 66.4 (64.8 to 68.0) | 13.8 (9.5 to 18.1) | 49.8* (42.8 to 57.4) | 8.2* (3.1 to 13.7) |
| Diagnosis and primary therapy phase of pancreatic cancer | 14.5 (14.1 to 14.8) | 66.4 (64.8 to 68.0) | 4.0 (2.7 to 5.5) | 50.2* (38.4 to 64.4) | 8.4 (-0.1 to 18.7) |
| Controlled phase of pancreatic cancer | 18.2 (17.6 to 18.9) | -- | 0.8 (0.5 to 1.2) | 31.5* (18.1 to 46.3) | -5.1 (-14.7 to 5.6) |
| Metastatic phase of pancreatic cancer | 13.9 (13.5 to 14.2) | -- | 6.0 (4.1 to 8.1) | 52.1* (40.9 to 64.2) | 9.8* (1.8 to 18.5) |
| Terminal phase of pancreatic cancer | 5.5 (5.3 to 5.6) | -- | 2.9 (2.0 to 3.8) | 50.9* (46.6 to 54.9) | 8.9* (5.9 to 11.8) |
| Larynx cancer | 181.7 (176.3 to 187.4) | 34.7 (33.7 to 35.9) | 17.8 (13.1 to 23.2) | 51.2* (42.8 to 60.1) | 9.1* (3.1 to 15.5) |
| Diagnosis and primary therapy phase of larynx cancer | 10.9 (10.6 to 11.3) | 34.7 (33.7 to 35.9) | 3.0 (2.0 to 4.1) | 58.3* (40.2 to 77.8) | 14.2* (1.2 to 28.3) |
| Controlled phase of larynx cancer | 147.4 (142.8 to 152.3) | -- | 7.9 (5.2 to 11.3) | 55.8* (46.9 to 65.1) | 12.5* (6.0 to 19.2) |
| Metastatic phase of larynx cancer | 13.2 (12.8 to 13.6) | -- | 5.5 (3.7 to 7.2) | 45.1* (30.1 to 62.3) | 4.7 (-6.1 to 17.2) |
| Terminal phase of larynx cancer | 1.9 (1.8 to 1.9) | -- | 1.0 (0.7 to 1.3) | 41.2* (36.1 to 46.5) | 1.9 (-1.8 to 5.8) |
| Laryngectomy from larynx cancer, beyond 10 years | 8.3 (7.9 to 8.7) | -- | 0.4 (0.3 to 0.6) | 30.3* (16.5 to 45.8) | -5.9 (-15.9 to 5.2) |
| Tracheal, bronchus, and lung cancer | 645.1 (623.2 to 666.6) | 380.5 (368.2 to 392.1) | 97.0 (71.1 to 123.9) | 68.7* (59.7 to 77.6) | 21.8* (15.3 to 28.2) |
| Diagnosis and primary therapy phase of lung, bronchus, and trachea cancer | 41.6 (40.2 to 43.0) | 380.5 (368.2 to 392.1) | 10.7 (7.3 to 14.6) | 83.9* (66.3 to 102.9) | 32.8* (20.1 to 46.5) |
| Controlled phase of lung, bronchus, and trachea cancer | 440.6 (425.2 to 456.4) | -- | 20.0 (13.0 to 29.1) | 105.5* (96.0 to 116.3) | 48.4* (41.5 to 56.2) |
| Metastatic phase of lung, bronchus, and trachea cancer | 133.3 (129.0 to 137.5) | -- | 52.1 (36.3 to 67.6) | 58.4* (48.2 to 68.3) | 14.4* (7.0 to 21.5) |
| Terminal phase of lung, bronchus, and trachea cancer | 29.6 (28.6 to 30.5) | -- | 14.2 (10.1 to 18.0) | 57.0* (41.7 to 74.4) | 13.4* (2.3 to 25.9) |
| Malignant skin melanoma | 245.5 (172.0 to 284.8) | 32.2 (22.9 to 37.3) | 14.7 (8.8 to 21.0) | 54.3* (44.7 to 64.9) | 11.4* (4.4 to 19.0) |
| Diagnosis and primary therapy phase of malignant skin melanoma | 6.7 (4.7 to 7.8) | 32.2 (22.9 to 37.3) | 1.8 (1.1 to 2.6) | 55.6* (35.5 to 78.0) | 12.3 (-2.2 to 28.5) |
| Controlled phase of malignant skin melanoma | 233.6 (163.6 to 270.9) | -- | 10.6 (6.2 to 16.0) | 57.8* (47.6 to 69.4) | 13.9* (6.6 to 22.3) |
| Metastatic phase of malignant skin melanoma | 4.4 (3.2 to 5.2) | -- | 1.9 (1.2 to 2.7) | 40.1* (29.4 to 51.7) | 1.1 (-6.6 to 9.5) |
| Terminal phase of malignant skin melanoma | 0.7 (0.5 to 0.8) | -- | 0.4 (0.2 to 0.5) | 38.1* (31.0 to 44.8) | -0.3 (-5.4 to 4.5) |
| Non-melanoma skin cancer | 391.1 (232.9 to 616.3) | 1117.2 (648.7 to 1689.4) | 13.8 (6.7 to 25.1) | 57.2* (43.9 to 69.1) | 13.5* (3.9 to 22.0) |
| Non-melanoma skin cancer (squamous-cell carcinoma) | 324.4 (166.2 to 549.7) | 268.7 (144.8 to 423.9) | 13.5 (6.2 to 24.9) | 57.4* (43.4 to 69.4) | 13.6* (3.5 to 22.3) |
| Non-melanoma skin cancer (basal-cell carcinoma) | 86.1 (37.1 to 153.2) | 848.5 (393.4 to 1401.4) | 0.4 (0.1 to 0.8) | 52.1* (37.8 to 63.0) | 9.8 (-0.5 to 17.7) |
| Breast cancer | 1901.1 (1835.5 to 1965.3) | 211.2 (203.0 to 219.1) | 144.8 (104.5 to 194.4) | 50.1* (44.2 to 56.0) | 8.3* (4.1 to 12.6) |
| Diagnosis and primary therapy phase of breast cancer | 45.5 (43.7 to 47.2) | 211.2 (203.0 to 219.1) | 11.5 (7.9 to 15.5) | 54.1* (39.6 to 69.5) | 11.3* (0.8 to 22.4) |
| Controlled phase of breast cancer | 1473.7 (1415.4 to 1529.6) | -- | 91.8 (59.6 to 133.0) | 52.1* (45.9 to 58.4) | 9.8* (5.3 to 14.4) |
| Metastatic phase of breast cancer | 73.8 (71.0 to 76.8) | -- | 28.6 (19.6 to 37.2) | 48.0* (37.1 to 61.3) | 6.8 (-1.0 to 16.4) |
| Terminal phase of breast cancer | 5.5 (5.3 to 5.8) | -- | 3.0 (2.0 to 3.8) | 41.9* (35.9 to 47.7) | 2.4 (-1.9 to 6.6) |
| Mastectomy from breast cancer, beyond 10 years | 302.6 (287.9 to 317.1) | -- | 10.0 (5.8 to 15.4) | 37.1* (32.4 to 41.9) | -1.0 (-4.4 to 2.4) |
| Cervical cancer | 182.3 (169.7 to 190.7) | 39.5 (36.8 to 41.3) | 16.3 (11.7 to 21.5) | 35.2* (24.8 to 44.1) | -2.4 (-9.9 to 4.0) |
| Diagnosis and primary therapy phase of cervical cancer | 9.4 (8.7 to 9.8) | 39.5 (36.8 to 41.3) | 2.6 (1.7 to 3.6) | 37.0* (22.5 to 51.2) | -1.1 (-11.6 to 9.2) |
| Controlled phase of cervical cancer | 157.5 (146.5 to 164.7) | -- | 7.1 (4.5 to 10.4) | 36.7* (27.1 to 45.7) | -1.3 (-8.3 to 5.2) |
| Metastatic phase of cervical cancer | 13.4 (12.5 to 14.0) | -- | 5.5 (3.8 to 7.3) | 33.2* (17.6 to 48.9) | -3.9 (-15.1 to 7.5) |
| Terminal phase of cervical cancer | 2.1 (1.9 to 2.2) | -- | 1.1 (0.8 to 1.4) | 32.1* (23.1 to 38.6) | -4.6 (-11.1 to 0.1) |
| Uterine cancer | 426.3 (408.5 to 446.5) | 56.4 (54.2 to 59.0) | 28.8 (20.5 to 37.8) | 56.8* (48.0 to 66.5) | 13.2* (6.8 to 20.2) |
| Diagnosis and primary therapy phase of uterine cancer | 19.5 (18.7 to 20.4) | 56.4 (54.2 to 59.0) | 5.0 (3.4 to 7.0) | 58.6* (40.7 to 79.7) | 14.5* (1.6 to 29.7) |
| Controlled phase of uterine cancer | 392.3 (375.8 to 411.0) | -- | 17.7 (11.5 to 25.6) | 60.0* (51.8 to 69.4) | 15.5* (9.6 to 22.3) |
| Metastatic phase of uterine cancer | 13.1 (12.6 to 13.7) | -- | 5.3 (3.7 to 7.0) | 47.3* (30.2 to 67.7) | 6.4 (-6.0 to 21.1) |
| Terminal phase of uterine cancer | 1.4 (1.3 to 1.5) | -- | 0.8 (0.5 to 1.0) | 43.0* (37.0 to 49.2) | 3.2 (-1.1 to 7.7) |
| Ovarian cancer | 110.1 (106.4 to 114.0) | 30.3 (29.3 to 31.4) | 15.3 (11.1 to 19.4) | 39.1* (27.8 to 50.5) | 0.4 (-7.8 to 8.6) |
| Diagnosis and primary therapy phase of ovarian cancer | 3.8 (3.7 to 3.9) | 30.3 (29.3 to 31.4) | 1.1 (0.7 to 1.5) | 40.9* (35.9 to 46.2) | 1.7 (-1.9 to 5.6) |
| Controlled phase of ovarian cancer | 79.7 (77.0 to 82.5) | -- | 3.6 (2.3 to 5.2) | 41.3* (31.8 to 51.3) | 2.0 (-4.8 to 9.2) |
| Metastatic phase of ovarian cancer | 24.7 (23.8 to 25.5) | -- | 9.6 (6.5 to 12.5) | 37.9* (21.9 to 55.0) | -0.4 (-12.0 to 11.9) |

| | | | | | |
|---|---|---|---|---|---|
| Terminal phase of ovarian cancer | 1.9 (1.9 to 2.0) | -- | 1.1 (0.7 to 1.3) | 40.6* (35.7 to 46.3) | 1.5 (-2.0 to 5.6) |
| Prostate cancer | 2070.3 (1821.8 to 2733.7) | 250.3 (219.6 to 327.0) | 164.2 (116.5 to 228.8) | 48.7* (39.8 to 62.2) | 7.4* (0.9 to 17.1) |
| Diagnosis and primary therapy phase of prostate cancer | 72.9 (64.0 to 96.7) | 250.3 (219.6 to 327.0) | 18.4 (12.2 to 26.3) | 52.1* (38.2 to 68.7) | 9.8 (-0.2 to 21.8) |
| Controlled phase of prostate cancer | 1861.0 (1633.3 to 2478.1) | -- | 106.4 (69.3 to 157.7) | 51.2* (42.1 to 65.8) | 9.2* (2.6 to 19.7) |
| Metastatic phase of prostate cancer | 90.3 (77.7 to 114.9) | -- | 35.0 (23.8 to 47.5) | 41.1* (30.0 to 55.3) | 1.9 (-6.2 to 12.1) |
| Terminal phase of prostate cancer | 4.8 (4.1 to 5.9) | -- | 2.5 (1.7 to 3.3) | 37.3* (25.4 to 51.8) | -0.9 (-9.5 to 9.6) |
| Impotence and Incontinence after 10-year survival from prostate cancer | 41.3 (39.6 to 43.2) | -- | 1.9 (1.3 to 2.7) | 44.0* (30.4 to 58.8) | 3.9 (-5.9 to 14.6) |
| Testicular cancer | 6.6 (6.2 to 7.1) | 1.2 (1.1 to 1.3) | 0.6 (0.4 to 0.7) | 74.0* (56.8 to 93.6) | 25.6* (13.2 to 39.8) |
| Diagnosis and primary therapy phase of testicular cancer | 0.2 (0.2 to 0.2) | 1.2 (1.1 to 1.3) | 0.1 (0.0 to 0.1) | 78.5* (65.4 to 92.8) | 28.8* (19.4 to 39.2) |
| Controlled phase of testicular cancer | 5.9 (5.5 to 6.4) | -- | 0.3 (0.2 to 0.4) | 80.0* (52.2 to 115.4) | 29.9* (9.9 to 55.5) |
| Metastatic phase of testicular cancer | 0.4 (0.4 to 0.4) | -- | 0.2 (0.1 to 0.2) | 64.8* (53.1 to 77.7) | 19.0* (10.5 to 28.3) |
| Terminal phase of testicular cancer | 0.0 (0.0 to 0.1) | -- | -- | 66.9* (54.9 to 79.7) | 20.5* (11.8 to 29.7) |
| Kidney cancer | 275.7 (263.2 to 286.9) | 51.6 (49.1 to 53.6) | 18.2 (13.0 to 24.4) | 42.0* (34.5 to 50.6) | 2.5 (-2.9 to 8.7) |
| Diagnosis and primary therapy phase of kidney cancer | -- | 51.6 (49.1 to 53.6) | -- | -- | -- |
| Controlled phase of kidney cancer | 261.2 (249.5 to 271.9) | -- | 11.8 (7.6 to 17.2) | 43.5* (35.8 to 52.2) | 3.6 (-2.0 to 9.9) |
| Metastatic phase of kidney cancer | 12.2 (11.5 to 12.6) | -- | 5.1 (3.4 to 6.9) | 39.5* (26.7 to 53.5) | 0.7 (-8.6 to 10.8) |
| Terminal phase of kidney cancer | 2.3 (2.2 to 2.4) | -- | 1.3 (0.9 to 1.6) | 38.7* (32.9 to 45.6) | 0.1 (-4.0 to 5.1) |
| Bladder cancer | 428.8 (415.0 to 448.8) | 68.4 (66.1 to 72.0) | 38.4 (27.5 to 50.5) | 44.8* (38.4 to 51.2) | 4.5 (-0.1 to 9.1) |
| Diagnosis and primary therapy phase of bladder cancer | 22.9 (22.1 to 24.0) | 68.4 (66.1 to 72.0) | 6.0 (4.0 to 8.3) | 47.4* (32.3 to 61.9) | 6.4 (-4.5 to 16.9) |
| Controlled phase of bladder cancer | 378.3 (365.5 to 395.5) | -- | 23.2 (15.7 to 32.5) | 48.0* (41.4 to 54.7) | 6.8* (2.0 to 11.7) |
| Metastatic phase of bladder cancer | 15.3 (14.8 to 16.2) | -- | 6.4 (4.3 to 8.5) | 35.5* (22.5 to 49.7) | -2.2 (-11.5 to 8.1) |
| Terminal phase of bladder cancer | 2.8 (2.7 to 3.0) | -- | 1.5 (1.1 to 1.9) | 34.0* (29.7 to 39.0) | -3.2 (-6.3 to 0.4) |
| Incontinence from bladder cancer, beyond 10 years | 9.6 (8.7 to 10.5) | -- | 1.3 (0.8 to 1.8) | 39.2* (24.4 to 54.8) | 0.5 (-10.2 to 11.7) |
| Brain and nervous system cancer | 106.6 (86.8 to 116.1) | 34.7 (29.4 to 37.4) | 12.6 (8.8 to 16.9) | 73.7* (61.4 to 85.4) | 25.4* (16.5 to 33.9) |
| Diagnosis and primary therapy phase of brain and nervous system cancers | 6.6 (5.4 to 7.2) | 34.7 (29.4 to 37.4) | 1.9 (1.2 to 2.7) | 84.8* (69.4 to 100.7) | 33.4* (22.3 to 44.9) |
| Controlled phase of brain and nervous system cancers | 84.8 (68.5 to 92.7) | -- | 3.9 (2.4 to 5.7) | 88.3* (70.5 to 106.7) | 35.9* (23.1 to 49.2) |
| Metastatic phase of brain and nervous system cancers | 12.8 (11.0 to 13.8) | -- | 5.7 (3.7 to 7.8) | 64.0* (51.1 to 77.5) | 18.4* (9.1 to 28.1) |
| Terminal phase of brain and nervous system cancers | 2.3 (2.0 to 2.5) | -- | 1.2 (0.8 to 1.6) | 63.1* (52.8 to 72.1) | 17.8* (10.3 to 24.2) |
| Thyroid cancer | 179.5 (171.3 to 190.8) | 22.3 (21.3 to 23.7) | 11.5 (8.2 to 15.7) | 57.0* (48.8 to 66.1) | 13.3* (7.4 to 19.9) |
| Diagnosis and primary therapy phase of thyroid cancer | 4.9 (4.7 to 5.2) | 22.3 (21.3 to 23.7) | 1.4 (0.9 to 2.0) | 60.1* (50.2 to 70.1) | 15.6* (8.4 to 22.8) |
| Controlled phase of thyroid cancer | 169.1 (161.3 to 179.8) | -- | 7.6 (4.9 to 11.1) | 59.7* (49.6 to 71.1) | 15.3* (8.0 to 23.5) |
| Metastatic phase of thyroid cancer | 5.0 (4.8 to 5.4) | -- | 2.3 (1.5 to 3.1) | 47.9* (41.6 to 54.4) | 6.8* (2.2 to 11.4) |
| Terminal phase of thyroid cancer | 0.4 (0.4 to 0.4) | -- | 0.2 (0.2 to 0.3) | 46.4* (40.4 to 52.7) | 5.7* (1.3 to 10.2) |
| Mesothelioma | 10.8 (10.2 to 11.3) | 4.6 (4.4 to 4.8) | 2.1 (1.4 to 2.7) | 28.1* (18.2 to 37.0) | -7.6* (-14.6 to -1.1) |
| Diagnosis and primary therapy phase of mesothelioma | 1.3 (1.2 to 1.3) | 4.6 (4.4 to 4.8) | 0.4 (0.2 to 0.5) | 27.6* (19.3 to 35.8) | -7.9* (-13.9 to -2.0) |
| Controlled phase of mesothelioma | 6.5 (6.1 to 6.8) | -- | 0.3 (0.2 to 0.5) | 28.3* (9.2 to 52.4) | -7.4 (-21.1 to 10.0) |
| Metastatic phase of mesothelioma | 2.7 (2.6 to 2.8) | -- | 1.2 (0.8 to 1.6) | 28.2* (17.1 to 38.5) | -7.5 (-15.4 to 0.0) |
| Terminal phase of mesothelioma | 0.4 (0.4 to 0.4) | -- | 0.2 (0.1 to 0.3) | 27.8* (20.1 to 35.0) | -7.8* (-13.3 to -2.5) |
| Hodgkin lymphoma | 31.1 (24.2 to 36.4) | 5.6 (4.4 to 6.6) | 2.8 (1.8 to 3.8) | 53.3* (33.4 to 71.6) | 10.7 (-3.7 to 23.8) |
| Diagnosis and primary therapy phase of Hodgkin disease | 1.0 (0.8 to 1.2) | 5.6 (4.4 to 6.6) | 0.3 (0.2 to 0.4) | 67.4* (45.7 to 85.2) | 20.9* (5.2 to 33.7) |
| Controlled phase of Hodgkin disease | 27.4 (21.3 to 32.0) | -- | 1.3 (0.8 to 1.9) | 67.1* (40.3 to 95.6) | 20.6* (1.3 to 41.2) |
| Metastatic phase of Hodgkin disease | 2.4 (1.9 to 2.9) | -- | 1.1 (0.7 to 1.5) | 40.8* (23.5 to 54.8) | 1.6 (-10.9 to 11.7) |
| Terminal phase of Hodgkin disease | 0.2 (0.2 to 0.3) | -- | 0.1 (0.1 to 0.2) | 22.8* (11.0 to 33.1) | -11.3* (-19.8 to -3.9) |
| Non-Hodgkin's lymphoma | 286.7 (276.8 to 296.3) | 57.4 (55.7 to 59.1) | 22.9 (16.6 to 30.3) | 60.4* (52.8 to 68.0) | 15.8* (10.3 to 21.2) |
| Diagnosis and primary therapy phase of non-Hodgkin lymphoma | 10.6 (10.3 to 11.0) | 57.4 (55.7 to 59.1) | 2.9 (2.0 to 4.0) | 64.5* (47.8 to 83.1) | 18.8* (6.7 to 32.2) |
| Controlled phase of non-Hodgkin lymphoma | 257.0 (248.0 to 265.7) | -- | 11.6 (7.5 to 16.7) | 65.3* (56.8 to 73.6) | 19.3* (13.2 to 25.4) |
| Metastatic phase of non-Hodgkin lymphoma | 16.3 (15.8 to 16.8) | -- | 7.0 (4.7 to 9.2) | 53.2* (42.0 to 65.7) | 10.6* (2.5 to 19.6) |
| Terminal phase of non-Hodgkin lymphoma | 2.8 (2.7 to 2.9) | -- | 1.5 (1.0 to 1.9) | 51.0* (46.1 to 56.3) | 9.0* (5.4 to 12.9) |
| Multiple myeloma | 67.6 (62.0 to 81.8) | 22.1 (20.5 to 25.8) | 13.4 (9.4 to 17.7) | 55.7* (42.0 to 69.7) | 12.4* (2.5 to 22.5) |
| Diagnosis and primary therapy phase of multiple myeloma | 4.3 (3.9 to 5.2) | 22.1 (20.5 to 25.8) | 1.2 (0.8 to 1.7) | 61.8* (53.4 to 71.7) | 16.8* (10.8 to 23.9) |
| Controlled phase of multiple myeloma | 38.4 (34.9 to 46.8) | -- | 1.8 (1.1 to 2.6) | 62.2* (44.9 to 80.2) | 17.1* (4.6 to 30.1) |
| Metastatic phase of multiple myeloma | 23.4 (21.6 to 28.1) | -- | 9.5 (6.5 to 12.7) | 54.8* (38.1 to 72.9) | 11.8 (-0.3 to 24.8) |
| Terminal phase of multiple myeloma | 1.6 (1.5 to 1.8) | -- | 0.8 (0.6 to 1.1) | 45.1* (39.2 to 51.4) | 4.7* (0.5 to 9.3) |
| Leukaemia | 194.5 (172.1 to 209.4) | 46.8 (41.7 to 50.0) | 24.9 (17.8 to 32.8) | 50.7* (37.9 to 61.8) | 8.8 (-0.4 to 16.8) |
| Acute lymphoid leukaemia | 13.5 (11.0 to 14.8) | 3.3 (2.7 to 3.5) | 1.6 (1.1 to 2.2) | 65.0* (43.9 to 79.6) | 19.1* (3.9 to 29.7) |
| Diagnosis and primary therapy phase of acute lymphoid leukemia | 1.5 (1.2 to 1.6) | 3.3 (2.7 to 3.5) | 0.4 (0.3 to 0.6) | 51.2* (36.1 to 61.2) | 9.2 (-1.7 to 16.3) |
| Controlled phase of acute lymphoid leukemia | 10.6 (8.6 to 11.7) | -- | 0.5 (0.3 to 0.8) | 73.3* (49.4 to 92.3) | 25.1* (7.9 to 38.8) |
| Metastatic phase of acute lymphoid leukemia | 1.2 (1.0 to 1.3) | -- | 0.5 (0.4 to 0.7) | 68.5* (44.1 to 86.1) | 21.6* (4.1 to 34.4) |
| Terminal phase of acute lymphoid leukemia | 0.2 (0.2 to 0.2) | -- | 0.1 (0.1 to 0.2) | 68.7* (44.2 to 86.4) | 21.8* (4.1 to 34.6) |
| Chronic lymphoid leukaemia | 79.4 (72.5 to 86.1) | 11.8 (10.8 to 12.7) | 10.0 (7.1 to 13.1) | 47.5* (32.3 to 64.0) | 6.4 (-4.5 to 18.4) |

| | | | | | |
|---|---|---|---|---|---|
| Diagnosis and primary therapy phase of chronic lymphoid leukemia | 4.3 (3.9 to 4.6) | 11.8 (10.8 to 12.7) | 1.2 (0.8 to 1.7) | 49.5* (36.9 to 62.0) | 7.9 (-1.2 to 17.0) |
| Controlled phase of chronic lymphoid leukemia | 60.9 (55.7 to 66.1) | -- | 2.8 (1.8 to 4.0) | 49.9* (34.2 to 66.4) | 8.2 (-3.1 to 20.1) |
| Metastatic phase of chronic lymphoid leukemia | 13.8 (12.6 to 15.0) | -- | 5.7 (3.8 to 7.8) | 46.3* (26.8 to 66.8) | 5.6 (-8.5 to 20.4) |
| Terminal phase of chronic lymphoid leukemia | 0.5 (0.4 to 0.5) | -- | 0.3 (0.2 to 0.3) | 40.1* (28.6 to 51.3) | 1.1 (-7.1 to 9.2) |
| Acute myeloid leukaemia | 11.5 (10.3 to 12.3) | 10.6 (9.4 to 11.4) | 2.5 (1.8 to 3.3) | 42.8* (34.1 to 51.8) | 3.1 (-3.2 to 9.6) |
| Diagnosis and primary therapy phase of acute myeloid leukemia | -- | 10.6 (9.4 to 11.4) | -- | -- | -- |
| Controlled phase of acute myeloid leukemia | 6.8 (6.1 to 7.3) | -- | 0.3 (0.2 to 0.5) | 42.8* (32.2 to 53.7) | 3.1 (-4.6 to 10.9) |
| Metastatic phase of acute myeloid leukemia | 3.9 (3.4 to 4.2) | -- | 1.7 (1.2 to 2.3) | 42.8* (34.2 to 51.9) | 3.1 (-3.2 to 9.6) |
| Terminal phase of acute myeloid leukemia | 0.8 (0.7 to 0.9) | -- | 0.5 (0.3 to 0.6) | 42.8* (34.2 to 51.7) | 3.1 (-3.1 to 9.5) |
| Chronic myeloid leukaemia | 7.2 (6.5 to 7.9) | 3.0 (2.7 to 3.3) | 1.0 (0.7 to 1.3) | 15.0* (6.7 to 23.4) | -17.0* (-23.0 to -10.9) |
| Diagnosis and primary therapy phase of chronic myeloid leukemia | 0.5 (0.5 to 0.6) | 3.0 (2.7 to 3.3) | 0.2 (0.1 to 0.2) | 16.8* (8.2 to 25.2) | -15.7* (-21.9 to -9.6) |
| Controlled phase of chronic myeloid leukemia | 5.5 (5.0 to 6.0) | -- | 0.3 (0.2 to 0.4) | 18.1* (8.5 to 27.0) | -14.7* (-21.7 to -8.3) |
| Metastatic phase of chronic myeloid leukemia | 1.0 (0.9 to 1.1) | -- | 0.4 (0.3 to 0.6) | 13.1* (4.9 to 21.1) | -18.3* (-24.2 to -12.6) |
| Terminal phase of chronic myeloid leukemia | 0.2 (0.2 to 0.2) | -- | 0.1 (0.1 to 0.1) | 13.1* (4.9 to 21.1) | -18.3* (-24.2 to -12.6) |
| Other leukaemia | 83.0 (68.6 to 92.2) | 18.1 (15.0 to 20.2) | 9.8 (6.9 to 13.1) | 59.0* (40.5 to 76.9) | 14.8* (1.5 to 27.7) |
| Metastatic phase of other leukemia | 14.6 (12.1 to 16.2) | -- | 6.3 (4.2 to 8.6) | 54.5* (35.6 to 74.7) | 11.6 (-2.1 to 26.1) |
| Diagnosis and primary therapy phase of other leukemia | -- | 18.1 (15.0 to 20.2) | -- | -- | -- |
| Terminal phase of other leukemia | 0.9 (0.8 to 1.0) | -- | 0.5 (0.3 to 0.6) | 53.8* (37.6 to 67.9) | 11.0 (-0.7 to 21.2) |
| Controlled phase of other leukemia | 67.5 (55.8 to 74.9) | -- | 3.1 (1.9 to 4.5) | 70.1* (49.1 to 92.0) | 22.8* (7.6 to 38.6) |
| Other malignant cancers | 577.5 (454.6 to 745.0) | 70.6 (62.6 to 75.0) | 48.1 (33.0 to 68.3) | 156.9* (104.3 to 222.3) | 85.5* (47.5 to 132.6) |
| Diagnosis and primary therapy phase of other malignant neoplasms | 14.1 (11.2 to 18.0) | 70.6 (62.6 to 75.0) | 3.8 (2.4 to 5.6) | 196.6* (125.8 to 276.1) | 114.2* (63.0 to 171.5) |
| Controlled phase of other malignant neoplasms | 513.2 (400.7 to 665.1) | -- | 23.3 (14.3 to 36.8) | 247.0* (155.1 to 353.4) | 150.5* (84.2 to 227.3) |
| Metastatic phase of other malignant neoplasms | 43.7 (37.0 to 52.0) | -- | 17.5 (11.6 to 24.3) | 97.0* (66.6 to 132.7) | 42.2* (20.2 to 68.0) |
| Terminal phase of other malignant neoplasms | 6.5 (5.5 to 7.7) | -- | 3.5 (2.4 to 4.8) | 89.5* (61.1 to 119.8) | 36.8* (16.3 to 58.7) |
| Other neoplasms | 2583.2 (2473.7 to 2691.0) | 1111.2 (1046.7 to 1182.4) | 6.4 (4.0 to 9.5) | 71.1* (62.9 to 79.8) | 23.6* (17.6 to 29.8) |
| Myelodysplastic, myeloproliferative, and other hematopoietic neoplasms | 141.7 (117.9 to 165.3) | 17.7 (12.3 to 24.0) | 6.4 (4.0 to 9.5) | 71.1* (62.9 to 79.8) | 23.6* (17.6 to 29.8) |
| Benign and in situ intestinal neoplasms | 566.8 (539.5 to 597.6) | 166.5 (155.8 to 179.6) | -- | -- | -- |
| Benign and in situ cervical and uterine neoplasms | 25.7 (24.2 to 27.4) | 2.7 (2.4 to 3.0) | -- | -- | -- |
| Other benign and in situ neoplasms | 1887.6 (1785.1 to 1992.1) | 924.4 (860.0 to 994.3) | -- | -- | -- |
| **Cardiovascular diseases** | **61838.5 (57983.3 to 65946.1)** | **9382.9 (8639.5 to 10207.9)** | **4471.4 (3240.3 to 5778.7)** | **49.2* (45.2 to 53.3)** | **7.7* (4.9 to 10.7)** |
| Rheumatic heart disease | 851.2 (815.6 to 886.9) | 7.1 (5.3 to 9.0) | 44.6 (30.4 to 62.4) | 40.3* (36.9 to 43.8) | 1.3 (-1.2 to 3.8) |
| Rheumatic heart disease, without heart failure | 692.8 (629.2 to 749.4) | 7.1 (5.3 to 9.0) | 31.2 (20.7 to 45.3) | 41.2* (37.8 to 44.7) | 1.9 (-0.5 to 4.5) |
| Heart failure due to rheumatic heart disease | 158.4 (116.5 to 209.3) | -- | 13.4 (8.2 to 19.8) | 38.3* (31.1 to 46.1) | -0.1 (-5.3 to 5.5) |
| Ischaemic heart disease | 17894.5 (16305.7 to 19689.8) | 1349.7 (1042.7 to 1766.5) | 704.9 (489.1 to 972.6) | 29.4* (28.2 to 30.6) | -6.6* (-7.5 to -5.7) |
| Asymptomatic ischemic heart disease following myocardial infarction | 6106.0 (4884.1 to 7542.2) | -- | -- | -- | -- |
| Angina due to ischemic heart disease | 9439.1 (8573.7 to 10289.3) | -- | 508.6 (347.0 to 712.3) | 29.0* (27.8 to 30.2) | -6.9* (-7.8 to -6.0) |
| Heart failure due to ischemic heart disease | 2275.1 (1744.9 to 2968.8) | -- | 189.3 (122.6 to 286.6) | 30.8* (27.6 to 33.7) | -5.6* (-7.9 to -3.5) |
| Myocardial infarction due to ischemic heart disease | 74.4 (56.1 to 95.6) | 1349.7 (1042.7 to 1766.5) | 7.0 (4.5 to 10.6) | 19.3* (10.9 to 26.9) | -13.9* (-19.9 to -8.4) |
| Stroke | 14023.7 (12841.1 to 15199.8) | 1511.8‡ (1267.8 to 1804.3) | 2465.4 (1776.3 to 3164.8) | 63.2* (56.4 to 70.2) | 17.8* (12.9 to 22.8) |
| Ischaemic stroke | 11571.2 (10355.4 to 12787.8) | 1043.2 (805.4 to 1334.5) | 1971.9 (1403.5 to 2556.3) | 74.0* (65.8 to 82.6) | 25.6* (19.7 to 31.8) |
| Acute ischemic stroke | 81.5 (63.3 to 102.8) | 1043.2 (805.4 to 1334.5) | 20.3 (13.2 to 28.8) | 55.7* (39.0 to 72.5) | 12.4* (0.4 to 24.5) |
| Chronic ischemic stroke | 11489.7 (10277.2 to 12705.3) | -- | 1951.6 (1388.3 to 2532.9) | 74.2* (65.7 to 83.0) | 25.8* (19.6 to 32.1) |
| Intracerebral hemorrhage | 1898.2 (1627.0 to 2205.7) | 353.6 (278.2 to 436.9) | 324.1 (229.8 to 427.7) | 26.8* (16.7 to 38.0) | -8.5* (-15.7 to -0.3) |
| Acute intracerebral hemorrhage | 19.9 (15.5 to 24.7) | 353.6 (278.2 to 436.9) | 5.2 (3.2 to 7.2) | 10.6 (-2.6 to 23.1) | -20.2* (-29.7 to -11.1) |
| Chronic intracerebral hemorrhage | 1878.4 (1606.6 to 2188.6) | -- | 318.9 (225.0 to 421.8) | 27.1* (16.9 to 38.6) | -8.2 (-15.6 to 0.1) |
| Subarachnoid hemorrhage | 1058.9 (917.2 to 1225.9) | 115.0 (88.4 to 142.9) | 169.3 (119.2 to 223.0) | 39.2* (32.0 to 46.2) | 0.5 (-4.7 to 5.5) |
| Acute subarachnoid hemorrhage | 9.2 (7.2 to 11.4) | 115.0 (88.4 to 142.9) | 2.4 (1.5 to 3.4) | 39.2* (35.9 to 41.5) | 0.5 (-1.9 to 2.2) |
| Chronic subarachnoid hemorrhage | 1049.8 (908.1 to 1217.5) | -- | 166.9 (117.1 to 219.9) | 39.2* (31.9 to 46.3) | 0.5 (-4.8 to 5.6) |
| Hypertensive heart disease | 2302.3 (1757.0 to 2942.5) | -- | 191.8 (123.7 to 288.2) | 43.6* (40.6 to 46.4) | 3.6* (1.5 to 5.7) |
| Non-rheumatic valvular heart disease | 3797.4 (3584.8 to 4046.6) | 3882.4 (3663.1 to 4140.7) | 33.9 (18.1 to 55.4) | 35.9* (30.8 to 41.1) | -1.9 (-5.5 to 1.9) |
| Non-rheumatic calcific aortic valve disease | 1497.3 (1295.2 to 1746.0) | 1497.3 (1295.2 to 1746.0) | 12.7 (6.4 to 21.1) | 35.1* (26.8 to 43.2) | -2.4 (-8.5 to 3.4) |
| Heart failure due to calcific aortic valve disease | 94.3 (53.5 to 149.7) | -- | 8.1 (4.2 to 13.7) | 25.3* (8.6 to 41.6) | -9.5 (-21.6 to 2.2) |
| Non-rheumatic calcific aortic valve disease | 1403.0 (1210.9 to 1632.8) | 1497.3 (1295.2 to 1746.0) | 4.5 (2.2 to 7.9) | 57.1* (35.3 to 81.7) | 13.4 (-2.3 to 31.1) |
| Non-rheumatic degenerative mitral valve disease | 2385.2 (2299.2 to 2473.0) | 2385.2 (2299.2 to 2473.0) | 21.0 (11.2 to 34.4) | 36.3* (29.8 to 43.0) | -1.6 (-6.3 to 3.2) |
| Heart failure due to degenerative mitral valve disease | 182.9 (106.4 to 278.6) | -- | 15.2 (8.3 to 25.0) | 27.6* (15.6 to 40.3) | -7.9 (-16.5 to 1.2) |
| Non-rheumatic degenerative mitral valve disease | 2202.2 (2086.3 to 2312.9) | 2385.2 (2299.2 to 2473.0) | 5.7 (2.8 to 10.0) | 66.6* (44.4 to 92.2) | 20.3* (4.2 to 38.7) |
| Other non-rheumatic valve diseases | 2.4 (1.7 to 3.5) | -- | 0.2 (0.1 to 0.4) | 48.2* (40.6 to 55.8) | 7.0* (1.5 to 12.5) |
| Mild heart failure due to other non-rheumatic valve disease | 0.5 (0.3 to 0.7) | -- | -- | 48.7* (35.7 to 62.8) | 7.4 (-2.1 to 17.5) |
| Moderate heart failure due to other non-rheumatic valve disease | 0.3 (0.2 to 0.5) | -- | -- | 48.1* (32.3 to 64.7) | 6.9 (-4.5 to 18.9) |
| Severe heart failure due to other non-rheumatic valve disease | 0.8 (0.5 to 1.1) | -- | 0.1 (0.1 to 0.2) | 48.2* (37.8 to 58.4) | 7.0 (-0.5 to 14.3) |

| | | | | | |
|---|---|---|---|---|---|
| Controlled, medically managed heart failure due to other non-rheumatic valve disease | 0.9<br>(0.6 to 1.3) | -- | 0.0<br>(0.0 to 0.1) | 48.2*<br>(38.2 to 57.5) | 7.0<br>(-0.2 to 13.7) |
| Cardiomyopathy and myocarditis | 761.3<br>(582.6 to 967.6) | 254.5<br>(178.7 to 344.3) | 67.3<br>(43.5 to 100.0) | 33.7*<br>(30.2 to 37.3) | -3.5*<br>(-6.0 to -0.9) |
| Myocarditis | 127.3<br>(101.5 to 155.6) | 254.5<br>(178.7 to 344.3) | 8.7<br>(5.6 to 12.8) | 39.1*<br>(32.2 to 46.1) | 0.4<br>(-4.6 to 5.5) |
| Acute myocarditis | 60.8<br>(45.0 to 80.3) | 254.5<br>(178.7 to 344.3) | 2.8<br>(1.6 to 4.6) | 35.5*<br>(25.3 to 46.3) | -2.2<br>(-9.5 to 5.6) |
| Heart failure due to myocarditis | 66.5<br>(48.1 to 88.1) | -- | 5.8<br>(3.6 to 8.9) | 41.0*<br>(32.6 to 49.6) | 1.8<br>(-4.3 to 8.0) |
| Alcoholic cardiomyopathy | 231.6<br>(173.9 to 296.9) | -- | 19.4<br>(12.5 to 29.0) | 22.5*<br>(16.8 to 28.5) | -11.6*<br>(-15.7 to -7.2) |
| Other cardiomyopathy | 470.8<br>(350.9 to 609.3) | -- | 39.3<br>(25.2 to 59.1) | 38.8*<br>(34.5 to 43.6) | 0.2<br>(-2.9 to 3.6) |
| Atrial fibrillation and flutter | 5183.2<br>(4244.1 to 6211.5) | 526.4<br>(340.0 to 710.1) | 409.4<br>(266.7 to 580.2) | 39.0*<br>(36.9 to 41.0) | 0.3<br>(-1.1 to 1.8) |
| Asymptomatic atrial fibrillation and flutter | 3110.2<br>(2526.3 to 3719.5) | 526.4<br>(340.0 to 710.1) | -- | -- | -- |
| Symptomatic atrial fibrillation and flutter | 2073.0<br>(1692.6 to 2488.9) | -- | 409.4<br>(266.7 to 580.2) | 39.0*<br>(36.9 to 41.0) | 0.3<br>(-1.1 to 1.8) |
| Peripheral vascular disease | 17221.0<br>(14282.4 to 20644.1) | 1758.3<br>(1213.9 to 2414.6) | 96.7<br>(44.5 to 183.5) | 31.4*<br>(29.4 to 33.7) | -5.1*<br>(-6.6 to -3.5) |
| Asymptomatic peripheral arterial disease | 9919.1<br>(7037.5 to 13039.3) | 1758.3<br>(1213.9 to 2414.6) | -- | -- | -- |
| Symptomatic claudication due to peripheral arterial disease | 7301.9<br>(4986.4 to 10088.7) | -- | 96.7<br>(44.5 to 183.5) | 31.4*<br>(29.4 to 33.7) | -5.1*<br>(-6.6 to -3.5) |
| Endocarditis | 58.2<br>(44.7 to 77.5) | 92.6<br>(66.9 to 121.0) | 4.9<br>(3.0 to 7.5) | 38.5*<br>(28.6 to 48.9) | -0.0<br>(-7.2 to 7.5) |
| Acute endocarditis | 6.9<br>(5.1 to 9.0) | 92.6<br>(66.9 to 121.0) | 0.4<br>(0.2 to 0.6) | 47.2*<br>(36.1 to 56.0) | 6.2<br>(-1.7 to 12.6) |
| Heart failure due to endocarditis | 51.2<br>(37.5 to 70.2) | -- | 4.5<br>(2.7 to 7.1) | 37.8*<br>(27.0 to 48.9) | -0.5<br>(-8.3 to 7.5) |
| Other cardiovascular and circulatory diseases | 8827.8<br>(6418.6 to 12007.0) | -- | 452.5<br>(276.9 to 700.5) | 37.4*<br>(35.1 to 39.7) | -0.8<br>(-2.5 to 0.8) |
| Heart failure due to other cardiovascular diseases | 200.3<br>(145.6 to 276.2) | -- | 16.8<br>(10.3 to 26.6) | 37.4*<br>(31.0 to 44.4) | -0.8<br>(-5.4 to 4.2) |
| Other cardiovascular and circulatory disease episodes | 8627.4<br>(6266.8 to 11733.8) | -- | 435.7<br>(266.2 to 674.1) | 37.4*<br>(35.0 to 39.8) | -0.8<br>(-2.5 to 0.9) |
| **Chronic respiratory diseases** | **46158.5<br>(40884.1 to 51741.8)** | **3556.8<br>(2770.4 to 4363.6)** | **4373.2<br>(3569.6 to 5147.8)** | **32.7*<br>(24.8 to 39.3)** | **-4.2<br>(-9.9 to 0.5)** |
| Chronic obstructive pulmonary disease | 36959.6<br>(30947.2 to 42957.0) | 2174.7<br>(1533.3 to 2884.1) | 3769.4<br>(3079.9 to 4468.0) | 31.3*<br>(22.5 to 38.7) | -5.2<br>(-11.5 to 0.2) |
| Chronic obstructive pulmonary disease with heart failure | 2051.6<br>(1523.6 to 2644.9) | -- | 813.4<br>(526.2 to 1122.7) | 41.5*<br>(38.7 to 44.4) | 2.2*<br>(0.1 to 4.2) |
| Chronic obstructive pulmonary disease without heart failure | 34908.1<br>(29093.0 to 41034.6) | 2174.7<br>(1533.3 to 2884.1) | 2956.0<br>(2324.4 to 3612.3) | 28.8*<br>(17.8 to 38.2) | -7.0*<br>(-14.9 to -0.2) |
| Pneumoconiosis | 73.4<br>(61.9 to 87.4) | 9.6<br>(7.1 to 12.7) | 11.3<br>(7.6 to 16.0) | 33.4*<br>(23.1 to 43.7) | -3.7<br>(-11.2 to 3.8) |
| Silicosis | 22.6<br>(15.6 to 31.4) | 3.8<br>(1.9 to 5.9) | 3.6<br>(2.1 to 5.6) | 33.9*<br>(15.8 to 54.2) | -3.3<br>(-16.4 to 11.3) |
| Heart failure due to silicosis | 2.8<br>(2.1 to 3.4) | -- | 1.3<br>(0.8 to 1.8) | 36.0*<br>(31.0 to 40.7) | -1.8<br>(-5.4 to 1.6) |
| Silicosis without heart failure | 19.9<br>(13.0 to 28.3) | 3.8<br>(1.9 to 5.9) | 2.3<br>(1.1 to 4.0) | 32.8*<br>(5.5 to 66.0) | -4.1<br>(-23.8 to 19.8) |
| Asbestosis | 12.1<br>(8.7 to 16.9) | 1.4<br>(0.8 to 2.3) | 1.9<br>(1.1 to 3.0) | 49.1*<br>(38.1 to 60.9) | 7.7<br>(-0.3 to 16.2) |
| Asbestosis without heart failure | 11.5<br>(8.1 to 16.3) | 1.4<br>(0.8 to 2.3) | 1.6<br>(0.9 to 2.6) | 52.5*<br>(39.1 to 66.5) | 10.1*<br>(0.4 to 20.2) |
| Heart failure due to asbestosis | 0.6<br>(0.5 to 0.8) | -- | 0.3<br>(0.2 to 0.4) | 32.2*<br>(26.2 to 37.7) | -4.5*<br>(-8.9 to -0.6) |
| Coal workers pneumoconiosis | 21.4<br>(15.1 to 30.5) | 2.7<br>(1.4 to 4.5) | 3.2<br>(1.9 to 5.0) | 27.8*<br>(6.7 to 52.9) | -7.7<br>(-23.0 to 10.4) |
| Coal workers pneumoconiosis without heart failure | 20.5<br>(14.2 to 29.6) | 2.7<br>(1.4 to 4.5) | 2.8<br>(1.6 to 4.5) | 26.9*<br>(3.3 to 56.5) | -8.4<br>(-25.4 to 13.0) |
| Heart failure due to Coal workers pneumoconiosis | 0.9<br>(0.7 to 1.1) | -- | 0.4<br>(0.3 to 0.6) | 34.7*<br>(28.8 to 40.7) | -2.8<br>(-7.0 to 1.6) |
| Other pneumoconiosis | 17.2<br>(13.1 to 22.1) | 1.7<br>(1.0 to 2.8) | 2.7<br>(1.6 to 4.0) | 29.7*<br>(15.8 to 44.7) | -6.4<br>(-16.4 to 4.5) |
| Heart failure due to other pneumoconiosis | 0.3<br>(0.3 to 0.4) | -- | 0.2<br>(0.1 to 0.2) | 32.1*<br>(23.2 to 39.4) | -4.7<br>(-11.0 to 0.7) |
| Other pneumoconiosis without heart failure | 16.8<br>(12.8 to 21.8) | 1.7<br>(1.0 to 2.8) | 2.5<br>(1.5 to 3.8) | 29.6*<br>(14.9 to 45.7) | -6.5<br>(-17.1 to 5.2) |
| Asthma | 12778.3<br>(10492.8 to 15641.2) | 1305.0<br>(887.9 to 1769.9) | 473.9<br>(310.9 to 687.5) | 42.2*<br>(34.1 to 51.0) | 2.6<br>(-3.2 to 9.0) |
| Asymptomatic asthma | 4642.5<br>(3735.4 to 5785.2) | 473.1<br>(314.6 to 646.5) | -- | -- | -- |
| Symptomatic asthma | 8135.8<br>(6632.6 to 10010.2) | 831.8<br>(561.9 to 1143.7) | 473.9<br>(310.9 to 687.5) | 42.2*<br>(34.1 to 51.0) | 2.6<br>(-3.2 to 9.0) |
| Interstitial lung disease and pulmonary sarcoidosis | 788.7<br>(670.4 to 917.1) | 67.5<br>(45.2 to 92.9) | 82.3<br>(55.0 to 117.7) | 39.5*<br>(34.6 to 44.7) | 0.7<br>(-2.8 to 4.4) |
| Heart failure due to interstitial lung disease and pulmonary sarcoidosis | 63.4<br>(47.6 to 79.6) | -- | 26.7<br>(17.5 to 37.9) | 35.4*<br>(29.7 to 41.9) | -2.2<br>(-6.4 to 2.5) |
| Interstitial lung disease and pulmonary sarcoidosis without heart failure | 725.3<br>(608.9 to 853.9) | 67.5<br>(45.2 to 92.9) | 55.6<br>(35.8 to 83.3) | 41.5*<br>(35.0 to 48.4) | 2.2<br>(-2.6 to 7.2) |
| Other chronic respiratory diseases | -- | -- | 36.2<br>(29.6 to 42.9) | 44.8*<br>(35.3 to 52.8) | 4.5<br>(-2.3 to 10.3) |
| **Digestive diseases** | **109057.6<br>(100839.3 to 118020.3)** | **28913.4<br>(23667.0 to 34448.3)** | **1287.4<br>(892.4 to 1793.1)** | **45.3*<br>(42.1 to 48.5)** | **4.9*<br>(2.6 to 7.2)** |
| Cirrhosis and other chronic liver diseases | 79685.7<br>(73759.1 to 86181.2) | 171.4<br>(153.5 to 189.0) | 152.8<br>(106.7 to 208.2) | 59.4*<br>(54.8 to 64.3) | 15.0*<br>(11.7 to 18.6) |
| Cirrhosis and other chronic liver diseases due to hepatitis B | 14690.3<br>(13593.6 to 15782.7) | 51.5<br>(43.9 to 59.4) | 42.7<br>(29.2 to 59.2) | 63.2*<br>(56.6 to 70.2) | 17.8*<br>(13.1 to 22.9) |
| Cirrhosis and other chronic liver diseases due to hepatitis B, decompensated | 267.7<br>(234.5 to 301.7) | 51.1<br>(43.3 to 59.1) | 42.7<br>(29.2 to 59.2) | 63.2*<br>(56.6 to 70.2) | 17.8*<br>(13.1 to 22.9) |
| Chronic hepatitis B without cirrhosis | 12477.3<br>(11369.5 to 13589.9) | -- | -- | -- | -- |
| Cirrhosis and other chronic liver diseases due to hepatitis B, compensated | 1945.4<br>(1734.1 to 2163.2) | 0.4<br>(0.1 to 1.1) | -- | -- | -- |
| Cirrhosis and other chronic liver diseases due to hepatitis C | 5971.4<br>(5137.1 to 6916.8) | 44.5<br>(38.2 to 50.8) | 40.6<br>(27.5 to 56.0) | 55.7*<br>(49.1 to 62.2) | 12.4*<br>(7.6 to 17.1) |
| Cirrhosis and other chronic liver diseases due to hepatitis C, decompensated | 255.4<br>(227.6 to 282.8) | 44.0<br>(37.5 to 50.4) | 40.6<br>(27.5 to 56.0) | 55.7*<br>(49.1 to 62.2) | 12.4*<br>(7.6 to 17.1) |
| Chronic hepatitis C without cirrhosis | 4231.1<br>(3384.6 to 5162.8) | -- | -- | -- | -- |
| Cirrhosis and other chronic liver diseases due to hepatitis C, compensated | 1485.0<br>(1324.8 to 1649.9) | 0.5<br>(0.2 to 1.4) | -- | -- | -- |
| Cirrhosis and other chronic liver diseases due to alcohol use | 1631.2<br>(1430.3 to 1820.5) | 40.5<br>(34.4 to 46.3) | 39.3<br>(27.4 to 54.4) | 55.3*<br>(48.4 to 62.4) | 12.1*<br>(7.2 to 17.3) |
| Cirrhosis and other chronic liver diseases due to alcohol, decompensated | 248.2<br>(220.1 to 277.7) | 39.9<br>(33.8 to 45.9) | 39.3<br>(27.4 to 54.4) | 55.3*<br>(48.4 to 62.4) | 12.1*<br>(7.2 to 17.3) |
| Cirrhosis and other chronic liver diseases due to alcohol, compensated | 1383.1<br>(1207.4 to 1551.4) | 0.6<br>(0.3 to 1.1) | -- | -- | -- |
| Cirrhosis due to NASH | 56754.9<br>(50908.7 to 63215.8) | 17.7<br>(15.1 to 20.5) | 14.9<br>(10.2 to 20.9) | 74.0*<br>(63.0 to 84.5) | 25.6*<br>(17.7 to 33.2) |
| Non-alcoholic fatty liver disease (NAFLD) / Non-alcoholic steatohepatitis (NASH) | 56083.7<br>(50256.9 to 62473.1) | -- | -- | -- | -- |
| Cirrhosis and other chronic liver diseases due to NASH | 671.2<br>(596.6 to 754.1) | 17.7<br>(15.1 to 20.5) | 14.9<br>(10.2 to 20.9) | 74.0*<br>(63.0 to 84.5) | 25.6*<br>(17.7 to 33.2) |
| Cirrhosis and other chronic liver diseases due to other causes | 637.8<br>(489.1 to 815.1) | 17.2<br>(12.7 to 22.9) | 15.3<br>(9.8 to 23.0) | 56.5*<br>(46.9 to 66.8) | 13.0*<br>(6.1 to 20.4) |

| | | | | | |
|---|---|---|---|---|---|
| Cirrhosis and other chronic liver diseases due to other cause, decompensated | 96.5 (72.7 to 125.2) | 17.1 (12.6 to 22.8) | 15.3 (9.8 to 23.0) | 56.5* (46.9 to 66.8) | 13.0* (6.1 to 20.4) |
| Cirrhosis and other chronic liver diseases due to other cause, compensated | 541.4 (414.7 to 692.5) | 0.1 (0.0 to 0.4) | -- | -- | -- |
| Upper digestive system diseases | 47474.9 (37142.4 to 58954.4) | 22637.6 (17617.2 to 28112.4) | 766.4 (463.9 to 1141.2) | 42.7* (38.8 to 46.9) | 3.0* (0.2 to 6.0) |
| Peptic ulcer disease | 1157.5 (878.0 to 1469.9) | 491.4 (350.2 to 629.3) | 55.2 (35.0 to 81.2) | 34.8* (27.8 to 41.3) | -2.7 (-7.7 to 2.0) |
| Acute peptic ulcer disease | 0.8 (0.6 to 1.1) | -- | 0.3 (0.2 to 0.4) | 38.9* (36.3 to 42.3) | 0.3 (-1.6 to 2.7) |
| Chronic peptic ulcer disease | 1156.7 (877.2 to 1469.1) | 491.4 (350.2 to 629.3) | 54.9 (34.8 to 80.9) | 34.8* (27.8 to 41.3) | -2.7 (-7.7 to 2.0) |
| Gastritis and duodenitis | 8466.9 (6026.5 to 11611.4) | 5956.0 (4170.8 to 8156.1) | 361.7 (220.4 to 546.4) | 46.4* (38.5 to 53.9) | 5.7 (-0.0 to 11.1) |
| Acute gastritis and duodenitis | 2.9 (1.9 to 4.2) | -- | 0.9 (0.5 to 1.5) | 30.3* (28.3 to 32.1) | -5.9* (-7.4 to -4.6) |
| Chronic gastritis and duodenitis | 8464.0 (6022.9 to 11609.4) | 5956.0 (4170.8 to 8156.1) | 360.8 (219.3 to 545.0) | 46.4* (38.5 to 53.9) | 5.7 (-0.0 to 11.1) |
| GERD | 42397.1 (30647.0 to 55792.4) | 16190.3 (11540.9 to 21225.9) | 349.5 (174.5 to 607.5) | 40.4* (38.0 to 43.0) | 1.4 (-0.4 to 3.2) |
| Appendicitis | 15.7 (12.9 to 18.8) | 422.7 (346.4 to 501.7) | 4.7 (3.0 to 6.6) | 32.5* (19.7 to 45.8) | -4.3 (-13.6 to 5.3) |
| Paralytic ileus and intestinal obstruction | 9.9 (7.7 to 12.4) | 278.8 (216.0 to 355.7) | 3.1 (1.9 to 4.4) | 52.0* (38.7 to 67.1) | 9.8* (0.1 to 20.7) |
| Inguinal, femoral, and abdominal hernia | 1886.6 (1396.6 to 2530.1) | 4582.2 (3259.1 to 6085.1) | 174.6 (109.8 to 261.0) | 35.4* (27.8 to 44.0) | -2.3 (-7.8 to 3.9) |
| Inflammatory bowel disease | 646.9 (551.5 to 748.0) | 164.6 (129.5 to 205.1) | 91.5 (61.5 to 128.6) | 57.6* (48.1 to 67.7) | 13.7* (6.9 to 21.0) |
| Ulcerative colitis | 493.7 (404.1 to 590.3) | 126.1 (93.3 to 165.6) | 68.9 (45.0 to 97.5) | 63.8* (52.6 to 75.1) | 18.3* (10.1 to 26.4) |
| Crohn's disease | 153.2 (132.1 to 178.6) | 38.5 (29.0 to 49.6) | 22.6 (15.0 to 31.8) | 41.2* (30.6 to 51.8) | 1.9 (-5.7 to 9.6) |
| Vascular intestinal disorders | 9.2 (7.0 to 12.0) | 65.9 (48.9 to 85.3) | 2.8 (1.7 to 4.2) | 45.2* (27.6 to 62.2) | 4.8 (-7.9 to 17.1) |
| Gallbladder and biliary diseases | 2334.9 (1772.9 to 3064.0) | 465.6 (349.0 to 607.9) | 2.1 (1.3 to 3.2) | 49.9* (36.8 to 62.4) | 8.2 (-1.3 to 17.2) |
| Pancreatitis | 559.3 (488.4 to 629.2) | 124.6 (99.0 to 151.0) | 31.8 (16.2 to 54.9) | 47.1* (38.9 to 55.2) | 6.2* (0.2 to 12.1) |
| Acute pancreatitis | 9.4 (7.0 to 12.3) | 97.2 (73.1 to 124.1) | 3.7 (2.4 to 5.5) | 39.3* (32.8 to 46.6) | 0.6 (-4.2 to 5.8) |
| Chronic pancreatitis | 549.9 (479.0 to 620.2) | 27.4 (18.3 to 37.4) | 28.0 (12.9 to 50.3) | 48.2* (39.0 to 57.7) | 7.0* (0.3 to 13.8) |
| Other digestive diseases | -- | -- | 57.7 (38.3 to 80.2) | 61.0* (55.3 to 66.4) | 16.2* (12.1 to 20.1) |
| **Neurological disorders** | **106367.4 (88833.4 to 126367.7)** | **34006.5 (24009.2 to 45098.1)** | **2527.9 (1785.2 to 3395.4)** | **37.5* (35.5 to 39.7)** | **-0.7 (-2.2 to 0.8)** |
| Alzheimer's disease and other dementias | 3657.1 (2951.1 to 4483.6) | 623.4 (439.3 to 812.5) | 432.0 (284.1 to 646.6) | 38.1* (36.0 to 40.2) | -0.3 (-1.8 to 1.2) |
| Parkinson's disease | 1185.4 (889.3 to 1508.5) | 156.3 (91.8 to 237.2) | 172.9 (113.1 to 250.2) | 41.4* (37.4 to 45.4) | 2.1 (-0.8 to 4.9) |
| Epilepsy | 1009.2 (740.8 to 1324.0) | 71.6 (47.6 to 101.1) | 273.5 (168.1 to 412.0) | 39.3* (24.0 to 55.1) | 0.6 (-10.5 to 12.0) |
| Multiple sclerosis | 116.1 (106.3 to 127.6) | 0.5 (0.3 to 0.6) | 29.0 (20.6 to 37.7) | 41.0* (33.2 to 49.3) | 1.8 (-3.8 to 7.8) |
| Motor neuron disease | 22.5 (16.9 to 29.7) | 9.2 (6.9 to 11.7) | 4.8 (3.1 to 7.0) | 55.9* (47.9 to 62.8) | 12.6* (6.7 to 17.5) |
| Headache disorders | 102948.0 (84867.5 to 123479.5) | 33145.5 (23197.2 to 44171.8) | 1575.9 (1051.4 to 2244.0) | 36.3* (34.9 to 37.6) | -1.6* (-2.6 to -0.7) |
| Migraine | 34274.5 (30531.3 to 38604.7) | 718.4 (429.8 to 1085.1) | 1281.7 (820.1 to 1881.0) | 36.3* (34.7 to 37.8) | -1.6* (-2.8 to -0.5) |
| Medication overuse headache due to migraine | 4337.7 (2995.0 to 5857.0) | -- | 452.9 (254.1 to 719.2) | 34.4* (32.7 to 36.1) | -3.0* (-4.2 to -1.7) |
| Migraine | 29936.8 (26274.6 to 33886.4) | 718.4 (429.8 to 1085.1) | 828.8 (497.2 to 1300.2) | 37.3* (35.0 to 39.6) | -0.9 (-2.6 to 0.8) |
| Tension-type headache | 83798.6 (62466.1 to 108660.4) | 32427.1 (22602.0 to 43488.8) | 294.2 (164.2 to 476.6) | 36.3* (34.3 to 38.1) | -1.6* (-3.1 to -0.3) |
| Medication overuse headache due to tension-type headache | 1610.8 (1003.1 to 2380.4) | -- | 166.9 (80.5 to 296.4) | 34.4* (31.6 to 36.9) | -3.0* (-5.0 to -1.1) |
| Tension-type headache | 82187.8 (61105.1 to 107290.8) | 32427.1 (22602.0 to 43488.8) | 127.3 (54.4 to 245.2) | 38.9* (37.3 to 40.5) | 0.3 (-0.9 to 1.4) |
| Other neurological disorders | 1.8 (0.9 to 3.0) | -- | 39.9 (27.5 to 54.6) | 50.4* (45.3 to 56.1) | 8.6* (4.9 to 12.7) |
| Other neurological disorders | -- | -- | 39.3 (27.2 to 53.8) | 50.5* (45.4 to 56.3) | 8.6* (4.9 to 12.8) |
| Guillain-Barré syndrome due to other neurological disorders | 1.8 (0.9 to 3.0) | -- | 0.5 (0.2 to 1.0) | 41.8* (37.8 to 45.6) | 2.3 (-0.5 to 5.1) |
| **Mental disorders** | **35774.9 (33431.5 to 38016.9)** | **14484.1 (12787.2 to 16296.1)** | **4769.6 (3556.2 to 6114.8)** | **36.2* (35.1 to 37.4)** | **-1.7* (-2.5 to -0.8)** |
| Schizophrenia | 797.3 (712.4 to 890.0) | 12.4 (9.2 to 16.2) | 478.5 (362.7 to 584.5) | 42.9* (39.7 to 46.0) | 3.1* (0.8 to 5.4) |
| Depressive disorders | 15578.6 (13827.4 to 17457.2) | 13446.4 (11794.6 to 15259.8) | 2343.0 (1636.9 to 3160.8) | 34.7* (32.7 to 36.7) | -2.7* (-4.2 to -1.3) |
| Major depressive disorder | 8671.7 (7530.2 to 9815.2) | 12644.6 (10966.9 to 14454.9) | 1663.0 (1151.0 to 2262.5) | 32.0* (29.8 to 34.2) | -4.7* (-6.3 to -3.1) |
| Dysthymia | 7345.9 (5904.9 to 8981.6) | 801.8 (648.4 to 980.8) | 680.0 (445.0 to 990.1) | 42.0* (37.3 to 46.6) | 2.5 (-0.9 to 5.9) |
| Bipolar disorder | 1663.3 (1373.1 to 1970.2) | 113.6 (88.8 to 142.5) | 318.9 (198.7 to 478.9) | 37.0* (34.6 to 39.8) | -1.1 (-2.8 to 0.9) |
| Anxiety disorders | 10655.0 (9320.2 to 12132.0) | 911.7 (806.9 to 1025.1) | 958.9 (672.9 to 1287.4) | 36.2* (33.5 to 38.7) | -1.7 (-3.6 to 0.1) |
| Eating disorders | -- | -- | -- | -- | -- |
| Anorexia nervosa | -- | -- | -- | -- | -- |
| Bulimia nervosa | -- | -- | -- | -- | -- |
| Autism spectrum disorders | 858.6 (770.8 to 951.6) | -- | 123.0 (85.4 to 165.8) | 37.8* (35.2 to 40.8) | -0.5 (-2.4 to 1.6) |
| Attention-deficit/hyperactivity disorder | 298.3 (221.4 to 385.5) | -- | 3.4 (1.9 to 5.8) | 41.2* (31.7 to 51.4) | 1.9 (-5.0 to 9.3) |
| Conduct disorder | -- | -- | -- | -- | -- |
| Idiopathic developmental intellectual disability | 1368.7 (667.9 to 2101.6) | -- | 54.0 (23.1 to 93.4) | 20.2* (13.4 to 23.6) | -13.3* (-18.1 to -10.8) |
| Other mental disorders | 6914.4 (6130.9 to 7805.8) | -- | 490.0 (330.3 to 667.6) | 38.5* (36.6 to 40.5) | 0.0 (-1.4 to 1.4) |
| **Substance use disorders** | **4021.8 (3483.1 to 4624.7)** | **1132.2 (911.3 to 1393.4)** | **601.0 (426.1 to 801.5)** | **44.8* (40.4 to 49.2)** | **4.5* (1.4 to 7.7)** |
| Alcohol use disorders | 3110.9 (2589.5 to 3711.4) | 1063.5 (842.7 to 1326.0) | 292.9 (198.4 to 417.3) | 32.2* (28.5 to 35.7) | -4.5* (-7.2 to -2.0) |
| Alcohol dependence | 3102.6 (2582.8 to 3700.9) | 1063.5 (842.7 to 1326.0) | 292.6 (198.1 to 416.9) | 32.3* (28.5 to 35.7) | -4.5* (-7.2 to -2.0) |
| Fetal alcohol syndrome | 8.3 (6.5 to 10.3) | -- | 0.3 (0.2 to 0.4) | 19.3* (10.1 to 26.7) | -13.9* (-20.5 to -8.5) |
| Drug use disorders | 959.6 (787.1 to 1160.7) | 68.7 (45.8 to 99.7) | 308.1 (209.1 to 419.8) | 59.1* (52.0 to 67.1) | 14.9* (9.7 to 20.6) |
| Opioid use disorders | 703.2 (535.3 to 905.6) | 59.2 (36.2 to 90.4) | 270.9 (179.0 to 376.9) | 61.8* (53.4 to 70.8) | 16.8* (10.7 to 23.3) |
| Cocaine use disorders | 126.2 (110.4 to 142.8) | 0.6 (0.3 to 0.9) | 16.2 (10.5 to 23.2) | 42.4* (33.7 to 51.6) | 2.8 (-3.5 to 9.4) |

| | | | | | |
|---|---|---|---|---|---|
| Amphetamine use disorders | 23.4 (14.6 to 35.4) | 4.4 (2.4 to 6.8) | 3.2 (1.6 to 5.6) | 42.4* (35.9 to 48.6) | 2.8 (-1.9 to 7.3) |
| Cannabis use disorders | 151.1 (118.1 to 191.9) | 4.5 (2.9 to 6.2) | 4.3 (2.6 to 6.6) | 40.6* (33.7 to 46.9) | 1.5 (-3.5 to 6.0) |
| Other drug use disorders | 7.7 (6.2 to 9.2) | -- | 13.5 (8.8 to 18.7) | 42.6* (35.3 to 50.2) | 3.0 (-2.3 to 8.4) |
| **Diabetes and kidney diseases** | **87795.1 (81023.2 to 95642.3)** | **3591.5 (2742.4 to 4530.9)** | **4411.3 (3070.0 to 6015.0)** | **40.4* (33.5 to 47.3)** | **1.3 (-3.6 to 6.3)** |
| Diabetes mellitus | 45901.3 (40922.5 to 51273.5) | 1282.2 (899.0 to 1680.5) | 3718.0 (2531.9 to 5143.5) | 41.0* (33.0 to 48.9) | 1.8 (-4.0 to 7.5) |
| Diabetes mellitus type 1 | 493.7 (434.4 to 552.5) | 5.6 (3.5 to 8.2) | 39.0 (26.9 to 54.9) | 36.6* (32.9 to 40.3) | -1.4 (-4.1 to 1.3) |
| Uncomplicated diabetes mellitus type 1 | 289.5 (241.9 to 340.6) | 5.6 (3.5 to 8.2) | 13.1 (8.0 to 19.2) | 35.0* (29.7 to 40.2) | -2.5 (-6.4 to 1.2) |
| Vision loss due to diabetes mellitus type 1 retinopathy | 5.3 (3.9 to 7.1) | -- | 0.4 (0.3 to 0.6) | 12.9* (5.9 to 20.3) | -18.5* (-23.6 to -13.1) |
| Diabetic neuropathy due to diabetes mellitus type 1 | 198.8 (159.5 to 244.4) | -- | 25.5 (16.7 to 37.1) | 37.9* (31.9 to 43.6) | -0.5 (-4.8 to 3.6) |
| Diabetes mellitus type 2 | 45407.6 (40428.0 to 50781.8) | 1276.6 (894.2 to 1674.3) | 3679.1 (2503.2 to 5086.9) | 41.1* (32.9 to 49.1) | 1.8 (-4.0 to 7.6) |
| Uncomplicated diabetes mellitus type 2 | 25412.2 (20983.4 to 30015.1) | 1276.6 (894.2 to 1674.3) | 1149.0 (706.9 to 1734.9) | 42.8* (33.6 to 51.8) | 3.1 (-3.5 to 9.6) |
| Vision loss due to diabetes mellitus type 2 retinopathy | 545.0 (424.2 to 692.9) | -- | 41.7 (28.8 to 58.3) | 25.4* (20.8 to 29.8) | -9.4* (-12.8 to -6.3) |
| Diabetic neuropathy due to diabetes mellitus type 2 | 19450.5 (15650.5 to 24044.1) | -- | 2488.4 (1649.5 to 3548.7) | 40.6* (32.0 to 49.2) | 1.5 (-4.7 to 7.7) |
| Chronic kidney disease | 61158.3 (52158.5 to 71421.9) | 2267.4 (1534.4 to 3110.6) | 693.1 (499.1 to 916.3) | 37.0* (28.5 to 45.8) | -1.1 (-7.2 to 5.3) |
| Chronic kidney disease due to diabetes mellitus type 1 | 138.5 (113.9 to 167.3) | 3.9 (2.6 to 5.5) | 15.0 (8.9 to 23.3) | 40.0* (31.9 to 47.9) | 1.1 (-4.8 to 6.8) |
| Albuminuria with preserved GFR due to type 1 diabetes mellitus | 51.4 (38.1 to 68.6) | -- | -- | -- | -- |
| End-stage chronic kidney disease due to diabetes mellitus type 1 | 20.0 (9.8 to 35.3) | -- | 9.4 (4.2 to 17.2) | 42.9* (33.8 to 52.0) | 3.1 (-3.4 to 9.7) |
| Stage III chronic kidney disease due to diabetes mellitus type 1 | 49.1 (40.5 to 60.7) | 3.9 (2.6 to 5.5) | 0.9 (0.6 to 1.4) | 33.1* (15.7 to 53.5) | -3.9 (-16.4 to 10.8) |
| Stage IV chronic kidney disease due to diabetes mellitus type 1 | 12.9 (8.1 to 19.0) | -- | 1.7 (0.9 to 2.7) | 33.5* (17.5 to 51.3) | -3.6 (-15.2 to 9.2) |
| Stage V chronic kidney disease due to diabetes mellitus type 1 | 5.0 (3.4 to 7.1) | -- | 2.9 (1.8 to 4.4) | 37.4* (24.6 to 51.6) | -0.8 (-10.0 to 9.4) |
| Chronic kidney disease due to diabetes mellitus type 2 | 13040.1 (11008.9 to 15459.7) | 370.3 (250.3 to 508.3) | 219.2 (157.6 to 292.3) | 38.6* (30.3 to 47.2) | 0.1 (-6.0 to 6.2) |
| Albuminuria with preserved GFR due to type 2 diabetes mellitus | 7012.5 (5320.9 to 9175.4) | -- | -- | -- | -- |
| End-stage chronic kidney disease due to diabetes mellitus type 2 | 176.7 (132.6 to 226.0) | -- | 73.7 (46.0 to 103.3) | 43.6* (34.3 to 52.3) | 3.6 (-3.0 to 9.9) |
| Stage III chronic kidney disease due to diabetes mellitus type 2 | 5283.1 (4424.0 to 6252.5) | 370.3 (250.3 to 508.3) | 24.1 (15.1 to 36.5) | 26.2* (11.0 to 43.3) | -8.9 (-19.9 to 3.4) |
| Stage IV chronic kidney disease due to diabetes mellitus type 2 | 410.5 (261.9 to 608.3) | -- | 43.4 (23.3 to 69.5) | 35.1* (19.8 to 51.7) | -2.5 (-13.5 to 9.5) |
| Stage V chronic kidney disease due to diabetes mellitus type 2 | 157.3 (108.8 to 217.8) | -- | 77.9 (48.6 to 114.9) | 40.4* (26.5 to 53.8) | 1.4 (-8.7 to 11.0) |
| Chronic kidney disease due to hypertension | 1848.6 (1584.3 to 2152.2) | 22.9 (15.5 to 31.6) | 139.0 (98.3 to 185.6) | 40.1* (31.1 to 49.3) | 1.1 (-5.3 to 7.8) |
| Albuminuria with preserved GFR due to hypertension | 433.1 (327.9 to 571.2) | -- | -- | -- | -- |
| End-stage chronic kidney disease due to hypertension | 117.5 (88.3 to 153.0) | -- | 48.7 (31.2 to 70.1) | 47.4* (37.8 to 57.1) | 6.4 (-0.6 to 13.4) |
| Stage III chronic kidney disease due to hypertension | 954.2 (793.2 to 1140.7) | 22.9 (15.5 to 31.6) | 15.0 (9.5 to 22.8) | 26.4* (9.6 to 43.6) | -8.8 (-20.9 to 3.6) |
| Stage IV chronic kidney disease due to hypertension | 248.4 (160.2 to 370.0) | -- | 26.8 (14.6 to 43.1) | 35.5* (20.0 to 52.2) | -2.2 (-13.4 to 9.9) |
| Stage V chronic kidney disease due to hypertension | 95.5 (66.3 to 131.6) | -- | 48.5 (30.0 to 72.2) | 40.4* (26.6 to 54.8) | 1.3 (-8.6 to 11.8) |
| Chronic kidney disease due to glomerulonephritis | 2514.1 (2139.7 to 2936.0) | 195.0 (131.9 to 268.0) | 52.8 (35.3 to 73.6) | 38.8* (30.7 to 47.6) | 0.2 (-5.6 to 6.5) |
| Albuminuria with preserved GFR due to glomerulonephritis | 206.3 (155.1 to 270.7) | -- | -- | -- | -- |
| End-stage chronic kidney disease due to glomerulonephritis | 56.7 (36.1 to 86.5) | -- | 23.7 (13.1 to 37.4) | 40.9* (27.9 to 54.1) | 1.7 (-7.6 to 11.2) |
| Stage III chronic kidney disease due to glomerulonephritis | 2142.7 (1791.4 to 2546.0) | 195.0 (131.9 to 268.0) | 3.1 (1.9 to 4.7) | 37.5* (17.5 to 62.6) | -0.8 (-15.2 to 17.4) |
| Stage IV chronic kidney disease due to glomerulonephritis | 78.2 (50.3 to 116.4) | -- | 9.1 (5.0 to 14.7) | 33.6* (18.0 to 51.0) | -3.5 (-14.8 to 9.0) |
| Stage V chronic kidney disease due to glomerulonephritis | 30.1 (20.9 to 41.8) | -- | 16.9 (10.4 to 25.1) | 39.1* (27.7 to 51.7) | 0.4 (-7.8 to 9.5) |
| Chronic kidney disease due to other and unspecified causes | 43617.1 (37264.0 to 50934.0) | 1675.2 (1133.8 to 2298.2) | 267.2 (189.8 to 359.4) | 33.6* (23.5 to 44.9) | -3.6 (-10.9 to 4.6) |
| Albuminuria with preserved GFR due to other and unspecified causes | 18942.6 (14360.9 to 24903.2) | -- | -- | -- | -- |
| End-stage chronic kidney disease due to other causes | 93.3 (59.3 to 134.1) | -- | 38.8 (21.3 to 60.3) | 40.4* (28.7 to 51.2) | 1.4 (-7.1 to 9.1) |
| Stage III chronic kidney disease due to other causes | 24084.9 (20172.7 to 28550.8) | 1675.2 (1133.8 to 2298.2) | 121.9 (78.1 to 183.5) | 27.6* (13.5 to 42.7) | -7.9 (-18.1 to 3.0) |
| Stage IV chronic kidney disease due to other causes | 359.1 (231.7 to 532.4) | -- | 38.2 (20.8 to 61.9) | 35.2* (20.1 to 52.0) | -2.4 (-13.3 to 9.7) |
| Stage V chronic kidney disease due to other causes | 137.3 (95.3 to 189.9) | -- | 68.3 (42.7 to 101.8) | 40.5* (27.3 to 54.8) | 1.4 (-8.1 to 11.7) |
| Acute glomerulonephritis | 2.8 (1.9 to 3.8) | 41.9 (27.7 to 58.4) | 0.1 (0.1 to 0.2) | 7.2* (2.4 to 11.6) | -22.6* (-26.0 to -19.4) |
| **Skin and subcutaneous diseases** | **89556.2 (83359.5 to 96752.8)** | **207923.8 (181092.2 to 233941.9)** | **1388.6 (926.4 to 2090.3)** | **41.2* (40.4 to 42.3)** | **1.9* (1.3 to 2.7)** |
| Dermatitis | 8736.2 (7399.1 to 10480.3) | 13064.7 (9357.1 to 17005.7) | 280.0 (170.5 to 426.2) | 38.8* (36.8 to 40.7) | 0.2 (-1.3 to 1.6) |
| Atopic dermatitis | 4280.0 (3793.0 to 4815.6) | 666.6 (551.0 to 799.0) | 176.2 (99.3 to 289.0) | 37.6* (35.1 to 40.2) | -0.7 (-2.5 to 1.2) |
| Contact dermatitis | 4007.4 (2732.5 to 5712.9) | 11320.6 (7607.7 to 15277.0) | 96.1 (54.5 to 155.6) | 40.2* (37.6 to 43.0) | 1.2 (-0.6 to 3.2) |
| Seborrhoeic dermatitis | 591.6 (482.5 to 725.6) | 1077.5 (888.2 to 1257.4) | 7.8 (4.2 to 12.7) | 49.4* (44.4 to 54.8) | 7.9* (4.2 to 11.7) |
| Psoriasis | 4198.7 (3925.5 to 4498.5) | 366.9 (320.6 to 419.1) | 345.6 (245.9 to 455.3) | 43.7* (41.0 to 46.3) | 3.7* (1.8 to 5.6) |
| Bacterial skin diseases | 377.6 (345.4 to 414.2) | 9649.0 (8768.3 to 10615.3) | 7.3 (4.7 to 10.8) | 39.9* (34.8 to 44.8) | 1.0 (-2.7 to 4.6) |
| Cellulitis | 103.0 (84.4 to 126.7) | 2209.5 (1816.1 to 2605.7) | 5.6 (3.6 to 8.4) | 40.0* (33.7 to 46.4) | 1.0 (-3.5 to 5.7) |
| Pyoderma | 305.0 (278.3 to 335.9) | 7439.5 (6680.5 to 8275.2) | 1.7 (0.7 to 3.4) | 39.6* (35.0 to 44.0) | 0.8 (-2.6 to 4.0) |
| Impetigo | 17.3 (15.2 to 19.4) | 323.5 (287.0 to 362.1) | 0.1 (0.0 to 0.2) | 40.5* (31.8 to 49.6) | 1.5 (-4.9 to 8.0) |
| Abscess and other bacterial skin diseases | 287.7 (260.3 to 319.3) | 7116.0 (6357.3 to 7943.3) | 1.6 (0.6 to 3.2) | 39.5* (34.6 to 44.1) | 0.7 (-2.8 to 4.0) |
| Scabies | 3236.3 (2279.2 to 4388.4) | 9709.9 (7001.7 to 12983.7) | 79.9 (42.4 to 136.5) | 41.9* (39.8 to 44.0) | 2.4* (0.9 to 4.0) |
| Fungal skin diseases | 36371.0 (28960.0 to 46093.0) | 119464.3 (93395.4 to 145030.5) | 197.9 (77.8 to 421.0) | 36.4* (35.7 to 37.0) | -1.6* (-2.0 to -1.1) |
| Tinea capitis | 952.2 (664.6 to 1345.3) | 1637.9 (1151.4 to 2143.6) | 5.1 (2.0 to 11.3) | 8.9* (5.5 to 12.6) | -21.4* (-23.8 to -18.7) |
| Other fungal skin diseases | 35418.8 (28019.3 to 45279.2) | 117826.4 (91744.6 to 143393.1) | 192.8 (75.2 to 409.4) | 37.3* (36.8 to 37.9) | -0.9* (-1.3 to -0.4) |

| | | | | | |
|---|---|---|---|---|---|
| Viral skin diseases | 1871.0 (1676.0 to 2074.7) | 872.8 (783.4 to 970.2) | 54.6 (35.5 to 81.8) | 43.3* (40.4 to 46.1) | 3.5* (1.3 to 5.5) |
| Viral warts | 1715.6 (1519.0 to 1923.1) | 685.4 (597.0 to 785.3) | 50.1 (32.4 to 75.2) | 43.8* (40.6 to 47.0) | 3.8* (1.5 to 6.1) |
| Molluscum contagiosum | 155.5 (137.7 to 176.1) | 187.4 (166.3 to 210.1) | 4.5 (2.8 to 6.9) | 38.6* (30.1 to 48.2) | 0.0 (-6.1 to 7.0) |
| Acne vulgaris | 498.0 (483.5 to 512.6) | 231.3 (223.3 to 239.7) | 10.4 (6.3 to 16.3) | 54.8* (50.2 to 59.3) | 11.8* (8.5 to 15.0) |
| Alopecia areata | 502.3 (451.3 to 560.6) | 873.1 (788.8 to 962.2) | 15.8 (10.0 to 23.5) | 37.6* (33.2 to 42.2) | -0.7 (-3.9 to 2.6) |
| Pruritus | 3929.3 (2694.5 to 5607.3) | 3077.5 (2167.5 to 4405.2) | 40.6 (17.3 to 78.7) | 40.8* (39.1 to 42.5) | 1.6* (0.4 to 2.9) |
| Urticaria | 1788.7 (1272.6 to 2434.2) | 3120.1 (2239.2 to 4159.8) | 100.7 (61.0 to 156.4) | 38.2* (35.2 to 41.3) | -0.3 (-2.4 to 2.0) |
| Decubitus ulcer | 89.9 (62.7 to 120.9) | 333.8 (232.7 to 450.2) | 14.8 (8.8 to 22.0) | 39.4* (32.3 to 45.9) | 0.6 (-4.5 to 5.3) |
| Other skin and subcutaneous diseases | 44982.4 (41418.6 to 48621.2) | 47160.3 (42853.9 to 51265.1) | 240.9 (115.9 to 446.2) | 45.3* (44.6 to 46.2) | 4.9* (4.4 to 5.5) |
| **Sense organ diseases** | **168870.7 (165485.0 to 172625.8)** | **--** | **6629.7 (4498.1 to 9468.5)** | **34.9* (33.1 to 36.5)** | **-2.6* (-3.9 to -1.4)** |
| Blindness and vision impairment | 104550.8 (98757.4 to 110904.2) | -- | 3146.8 (2180.5 to 4485.1) | 31.6* (29.5 to 33.5) | -5.0* (-6.5 to -3.7) |
| Glaucoma | 1187.3 (966.2 to 1446.1) | -- | 135.7 (88.5 to 193.0) | 28.9* (25.9 to 31.9) | -6.9* (-9.1 to -4.8) |
| Cataract | 16510.1 (13369.4 to 20306.7) | -- | 1164.8 (794.1 to 1594.5) | 33.2* (30.0 to 36.2) | -3.8* (-6.1 to -1.7) |
| Age-related macular degeneration | 1301.4 (987.5 to 1675.5) | -- | 91.6 (62.0 to 129.4) | 33.4* (29.2 to 37.6) | -3.7* (-6.7 to -0.7) |
| Refraction disorders | 12893.4 (9943.2 to 16392.3) | -- | 595.3 (383.9 to 861.9) | 23.5* (19.8 to 27.0) | -10.9* (-13.5 to -8.3) |
| Near vision loss | 86526.4 (80377.4 to 92980.8) | -- | 860.7 (411.4 to 1583.4) | 36.7* (35.7 to 37.6) | -1.3* (-2.0 to -0.7) |
| Other vision loss | 3547.8 (2835.5 to 4427.1) | -- | 298.7 (198.2 to 413.5) | 29.7* (27.1 to 32.2) | -6.4* (-8.2 to -4.5) |
| Age-related and other hearing loss | 133542.3 (129837.7 to 137494.7) | -- | 3211.0 (2123.8 to 4665.5) | 37.9* (34.8 to 40.9) | -0.5 (-2.7 to 1.7) |
| Other sense organ diseases | 11165.4 (10082.7 to 12260.7) | -- | 271.9 (168.1 to 396.7) | 40.0* (38.3 to 41.6) | 1.1 (-0.2 to 2.3) |
| Chronic other sense organ diseases | 10502.8 (9415.3 to 11584.1) | -- | 263.1 (162.6 to 384.8) | 40.0* (38.3 to 41.8) | 1.1 (-0.1 to 2.3) |
| Acute other sense organ diseases | 662.6 (597.8 to 732.0) | -- | 8.7 (5.3 to 13.4) | 39.9* (34.1 to 46.2) | 1.0 (-3.2 to 5.5) |
| **Musculoskeletal disorders** | **102401.8 (93684.5 to 111448.9)** | **23012.4 (17924.9 to 28881.4)** | **10177.7 (7137.6 to 13870.8)** | **38.4* (36.4 to 40.6)** | **-0.1 (-1.5 to 1.5)** |
| Rheumatoid arthritis | 2249.9 (1939.7 to 2576.6) | 107.6 (75.7 to 143.7) | 288.2 (196.3 to 389.4) | 49.9* (45.1 to 54.5) | 8.2* (4.7 to 11.5) |
| Osteoarthritis | 40287.7 (35425.2 to 45412.2) | 1319.2 (1009.2 to 1727.6) | 1270.7 (625.6 to 2514.0) | 40.0* (39.1 to 41.1) | 1.1* (0.4 to 1.9) |
| Osteoarthritis of the hip | 5508.1 (5076.7 to 5998.5) | 225.6 (184.0 to 270.1) | 173.7 (88.6 to 351.1) | 45.5* (43.5 to 47.5) | 5.0* (3.6 to 6.5) |
| Osteoarthritis of the knee | 34779.6 (29899.3 to 39806.5) | 1093.7 (790.9 to 1511.4) | 1097.0 (535.3 to 2195.9) | 39.2* (38.3 to 40.4) | 0.5 (-0.2 to 1.4) |
| Low back pain | 39952.8 (30138.7 to 51601.9) | 15919.3 (11365.4 to 21066.2) | 4398.8 (2816.6 to 6458.7) | 34.3* (33.1 to 35.4) | -3.1* (-3.9 to -2.2) |
| Low back pain with leg pain | 14823.4 (10668.1 to 19463.7) | 5930.2 (3998.5 to 8045.7) | 2046.0 (1262.7 to 3113.9) | 34.3* (32.9 to 35.6) | -3.1* (-4.0 to -2.1) |
| Low back pain without leg pain | 25129.4 (18840.8 to 32915.6) | 9989.0 (7212.3 to 13261.1) | 2352.9 (1515.4 to 3505.7) | 34.2* (32.9 to 35.6) | -3.1* (-4.0 to -2.1) |
| Neck pain | 22836.3 (16553.8 to 30311.6) | 4817.2 (3138.4 to 6892.5) | 2200.3 (1373.3 to 3339.4) | 42.4* (39.6 to 45.2) | 2.8* (0.7 to 4.8) |
| Gout | 5036.4 (4076.5 to 6084.7) | 849.1 (612.9 to 1154.4) | 153.8 (101.6 to 215.4) | 44.2* (40.5 to 48.3) | 4.1* (1.4 to 7.0) |
| Other musculoskeletal disorders | 22827.0 (16182.5 to 29656.1) | -- | 1865.8 (1121.3 to 2769.9) | 40.7* (30.2 to 51.2) | 1.5 (-6.0 to 9.2) |
| **Other non-communicable diseases** | **172742.5 (164428.6 to 180379.3)** | **74284.4 (66064.9 to 83542.9)** | **2763.9 (1863.9 to 3950.4)** | **32.4* (31.0 to 33.9)** | **-4.4* (-5.4 to -3.4)** |
| Congenital anomalies | 2860.6 (2686.3 to 3066.9) | -- | 354.0 (252.0 to 468.7) | 35.8* (33.5 to 38.4) | -1.9* (-3.6 to -0.1) |
| Neural tube defects | 70.6 (62.9 to 78.9) | -- | 20.5 (14.5 to 27.5) | 40.5* (34.5 to 46.3) | 1.4 (-2.9 to 5.6) |
| Severe motor and cognitive impairment due to anencephaly | -- | -- | -- | -- | -- |
| Encephalocoele due to neural tube defects | 10.6 (8.7 to 12.6) | -- | 1.8 (1.2 to 2.6) | 32.8* (28.8 to 36.8) | -4.2* (-7.0 to -1.3) |
| Spina bifida due to neural tube defects | 60.1 (53.5 to 67.5) | -- | 18.7 (13.1 to 25.2) | 41.3* (34.8 to 47.5) | 2.0 (-2.7 to 6.5) |
| Congenital heart anomalies | 83.5 (76.3 to 91.6) | -- | 5.0 (2.4 to 8.3) | 40.8* (34.7 to 46.9) | 1.6 (-2.7 to 6.1) |
| Critical malformations of great vessels, congenital valvular heart disease and patent ductus arteriosus | 12.7 (11.0 to 14.6) | -- | 0.8 (0.3 to 1.4) | 42.5* (39.8 to 45.4) | 2.9* (0.9 to 5.0) |
| Other congenital heart anomalies | 35.5 (32.4 to 39.1) | -- | 2.0 (0.8 to 3.5) | 40.6* (28.8 to 52.7) | 1.5 (-7.0 to 10.3) |
| Severe congenital heart anomalies excluding single ventricle heart defects | 32.7 (29.0 to 36.4) | -- | 2.1 (1.2 to 3.3) | 40.5* (31.8 to 49.9) | 1.4 (-4.8 to 8.2) |
| Single ventricle and single ventricle pathway congenital heart anomalies | 0.8 (0.7 to 1.0) | -- | 0.1 (0.0 to 0.1) | 43.8* (39.6 to 48.2) | 3.8* (0.8 to 7.0) |
| Ventricular septal defect and atrial septal defect | 1.8 (1.5 to 2.2) | -- | 0.1 (0.0 to 0.1) | 35.4* (32.1 to 38.3) | -2.3* (-4.6 to -0.2) |
| Orofacial clefts | 328.1 (300.0 to 355.8) | -- | 8.4 (5.6 to 12.1) | 40.5* (29.2 to 53.0) | 1.4 (-6.7 to 10.5) |
| Down's syndrome | 72.1 (62.6 to 82.3) | -- | 10.5 (7.2 to 14.4) | 29.7* (25.3 to 34.1) | -6.3* (-9.6 to -3.2) |
| Turner syndrome | 4.7 (4.0 to 5.5) | -- | 0.1 (0.0 to 0.1) | 41.2* (38.3 to 43.8) | 1.9 (-0.1 to 3.8) |
| Klinefelter syndrome | 2.2 (1.8 to 2.7) | -- | -- | 47.5* (45.3 to 49.6) | 6.5* (4.9 to 8.0) |
| Other chromosomal abnormalities | 132.4 (107.9 to 159.2) | -- | 19.5 (13.0 to 27.0) | 31.0* (27.0 to 34.4) | -5.4* (-8.3 to -3.0) |
| Congenital musculoskeletal and limb anomalies | 1220.5 (1084.7 to 1396.5) | -- | 171.0 (115.4 to 231.7) | 37.0* (34.6 to 39.6) | -1.1 (-2.8 to 0.8) |
| Polydactyly and syndactyly | 2.4 (1.9 to 2.8) | -- | 0.0 (0.0 to 0.1) | 28.5* (26.0 to 30.9) | -7.2* (-9.0 to -5.5) |
| Congenital limb deficiency | 364.3 (323.7 to 417.3) | -- | 51.2 (34.6 to 69.3) | 37.1* (32.6 to 41.1) | -1.1 (-4.2 to 1.9) |
| Other congenital musculoskeletal anomalies | 853.9 (758.9 to 977.0) | -- | 119.7 (80.8 to 162.2) | 36.9* (34.1 to 39.9) | -1.1 (-3.2 to 1.0) |
| Urogenital congenital anomalies | 41.2 (36.7 to 46.0) | -- | 1.9 (1.2 to 2.8) | 31.6* (24.9 to 38.7) | -5.0 (-9.8 to 0.1) |
| Digestive congenital anomalies | 662.1 (597.0 to 738.3) | -- | 28.6 (19.3 to 40.6) | 28.7* (22.1 to 35.9) | -7.1* (-11.9 to -1.9) |
| Congenital diaphragmatic hernia | 3.4 (2.9 to 3.8) | -- | 0.1 (0.0 to 0.1) | 44.4* (42.6 to 46.1) | 4.2* (2.9 to 5.5) |
| Congenital atresia and/or stenosis of the digestive tract | 0.2 (0.1 to 0.2) | -- | -- | 61.3* (54.8 to 67.6) | 16.4* (11.8 to 21.0) |
| Congenital malformations of the abdominal wall after treatment | 13.3 (11.5 to 15.1) | -- | 0.6 (0.4 to 0.8) | 43.1* (41.4 to 44.6) | 3.3* (2.1 to 4.4) |
| Other congenital malformations of the digestive tract | 645.3 (580.0 to 720.6) | -- | 28.0 (18.8 to 39.6) | 28.4* (21.6 to 35.7) | -7.3* (-12.2 to -2.0) |
| Other congenital anomalies | 703.3 (604.7 to 819.2) | -- | 88.5 (60.0 to 122.7) | 36.3* (28.7 to 45.0) | -1.6 (-7.1 to 4.7) |

| | | | | | |
|---|---|---|---|---|---|
| Other congenital birth defects | -- | -- | 6.6 (4.7 to 8.7) | 46.8* (41.5 to 51.8) | 6.0* (2.2 to 9.6) |
| Hearing loss due to other congenital anomalies | 703.3 (604.7 to 819.2) | -- | 81.9 (55.2 to 114.9) | 35.5* (27.3 to 44.7) | -2.2 (-8.1 to 4.5) |
| Urinary diseases and male infertility | 11834.3 (10228.3 to 13629.7) | 17267.3 (14550.4 to 19956.2) | 412.8 (268.4 to 595.4) | 35.7* (34.0 to 37.4) | -2.1* (-3.3 to -0.8) |
| Urinary tract infections | 183.6 (148.1 to 226.4) | 9582.5 (7723.1 to 11809.5) | 5.8 (3.4 to 8.8) | 63.0* (53.1 to 73.7) | 17.7* (10.6 to 25.4) |
| Urolithiasis | 249.2 (191.1 to 315.2) | 6522.7 (4977.4 to 8120.7) | 17.4 (11.3 to 25.1) | 37.8* (29.3 to 47.5) | -0.5 (-6.6 to 6.5) |
| Benign prostatic hyperplasia | 11810.2 (10119.7 to 13735.1) | 1162.1 (963.7 to 1410.3) | 383.8 (243.5 to 559.8) | 35.6* (33.9 to 37.4) | -2.1* (-3.4 to -0.8) |
| Male infertility | -- | -- | -- | -- | -- |
| Other urinary diseases | -- | -- | 5.8 (3.9 to 8.1) | 13.3* (7.5 to 19.7) | -18.2* (-22.4 to -13.6) |
| Gynecological diseases | 13792.4 (11976.4 to 15858.4) | 1115.2 (834.7 to 1425.0) | 62.6 (33.6 to 111.7) | 30.4* (25.8 to 34.4) | -5.9* (-9.2 to -3.0) |
| Uterine fibroids | 3879.2 (3381.7 to 4463.4) | 19.0 (12.4 to 27.5) | 30.7 (17.6 to 52.2) | 29.2* (20.7 to 36.8) | -6.8* (-12.9 to -1.3) |
| Polycystic ovarian syndrome | -- | -- | -- | -- | -- |
| Female infertility | -- | -- | -- | -- | -- |
| Endometriosis | -- | -- | -- | -- | -- |
| Genital prolapse | 10447.1 (8605.3 to 12647.9) | 1096.2 (815.5 to 1412.0) | 31.9 (15.0 to 61.6) | 31.6* (29.6 to 33.6) | -5.0* (-6.4 to -3.6) |
| Premenstrual syndrome | -- | -- | -- | -- | -- |
| Other gynecological diseases | -- | -- | -- | -- | -- |
| Other gynecological diseases | -- | -- | -- | -- | -- |
| Other gynecological diseases with anemia | -- | -- | -- | -- | -- |
| Hemoglobinopathies and hemolytic anaemias | 54253.2 (53073.7 to 55466.8) | -- | 159.1 (103.8 to 228.6) | 17.7* (7.3 to 30.1) | -15.0* (-22.6 to -6.1) |
| Thalassemias | -- | -- | -- | 34.8* (11.4 to 61.6) | -2.7 (-19.5 to 16.6) |
| Thalassaemias trait | 9609.4 (9254.1 to 9993.4) | -- | 60.0 (38.0 to 90.1) | 26.8* (6.7 to 51.6) | -8.5 (-23.0 to 9.5) |
| Sickle cell disorders | 0.1 (0.1 to 0.1) | -- | -- | 59.7* (50.2 to 70.2) | 15.3* (8.5 to 22.9) |
| Sickle cell trait | 10368.1 (9303.0 to 11560.5) | -- | 23.4 (15.0 to 34.6) | 27.1* (12.3 to 46.8) | -8.3 (-18.9 to 5.9) |
| G6PD deficiency | 8844.2 (8499.9 to 9185.6) | -- | 0.9 (0.6 to 1.3) | 27.8* (11.8 to 46.4) | -7.7 (-19.3 to 5.7) |
| G6PD trait | 27557.5 (27107.3 to 27998.8) | -- | -- | 37.1* (16.4 to 68.0) | -1.0 (-15.9 to 21.3) |
| Other hemoglobinopathies and hemolytic anaemias | 3511.3 (3249.9 to 3795.0) | -- | 74.9 (50.3 to 105.9) | 8.8* (0.1 to 18.2) | -21.4* (-27.8 to -14.6) |
| Endocrine, metabolic, blood, and immune disorders | 4476.3 (3943.3 to 5049.9) | -- | 127.9 (87.9 to 177.7) | 29.6* (21.8 to 37.4) | -6.5* (-12.1 to -0.8) |
| Anemia due to endocrine, metabolic, blood, and immune disorders | 2083.5 (1925.1 to 2250.1) | -- | 42.1 (28.4 to 59.8) | 4.5 (-5.8 to 15.8) | -24.6* (-32.0 to -16.4) |
| Endocrine, metabolic, blood, and immune disorders | 2342.7 (1830.5 to 2901.0) | -- | 81.4 (53.2 to 115.1) | 47.3* (38.0 to 56.9) | 6.4 (-0.4 to 13.3) |
| Heart failure due to endocrine, metabolic, blood, and immune disorders | 50.1 (36.8 to 67.7) | -- | 4.4 (2.7 to 7.0) | 38.3* (28.9 to 48.5) | -0.1 (-6.9 to 7.2) |
| Oral disorders | 140909.9 (128763.8 to 151833.1) | 55901.9 (48328.7 to 65086.9) | 1647.4 (1041.4 to 2491.8) | 32.8* (31.3 to 34.4) | -4.1* (-5.2 to -3.0) |
| Caries of deciduous teeth | -- | -- | -- | -- | -- |
| Caries of permanent teeth | 81202.4 (66799.8 to 96714.3) | 48964.2 (41507.1 to 57937.2) | 58.7 (25.9 to 110.4) | 30.9* (29.2 to 32.5) | -5.5* (-6.7 to -4.4) |
| Periodontal disease | 61607.1 (48368.7 to 73293.5) | 4188.8 (2549.1 to 5906.3) | 393.0 (152.7 to 800.0) | 41.3* (40.4 to 42.4) | 2.0* (1.3 to 2.8) |
| Edentulism and severe tooth loss | 37761.4 (31489.0 to 45272.6) | 2749.0 (1865.2 to 3826.8) | 1036.3 (681.7 to 1500.4) | 29.2* (27.8 to 30.6) | -6.8* (-7.7 to -5.8) |
| Other oral disorders | 5730.8 (5117.3 to 6403.7) | -- | 159.3 (99.4 to 234.5) | 38.8* (37.4 to 40.4) | 0.2 (-0.8 to 1.4) |
| **Injuries** | **98461.9 (93331.6 to 104610.3)** | **20847.7 (17621.4 to 24705.5)** | **3776.7 (2731.6 to 5086.6)** | **47.4* (46.3 to 48.6)** | **6.4* (5.6 to 7.2)** |
| **Transport injuries** | **17205.3 (15846.8 to 18635.7)** | **1876.5 (1318.4 to 2485.5)** | **915.2 (670.7 to 1217.3)** | **47.6* (46.3 to 48.9)** | **6.6* (5.6 to 7.5)** |
| Road injuries | 14007.1 (12904.0 to 15283.0) | 1607.5 (1060.6 to 2191.7) | 744.6 (537.9 to 999.5) | 48.2* (46.9 to 49.5) | 7.0* (6.1 to 7.9) |
| Pedestrian road injuries | 3724.2 (3185.5 to 4405.2) | 393.9 (215.3 to 608.6) | 200.9 (140.2 to 278.1) | 45.3* (43.1 to 47.3) | 4.9* (3.3 to 6.4) |
| Cyclist road injuries | 2593.3 (2170.1 to 3059.4) | 402.2 (231.2 to 619.0) | 133.5 (92.2 to 181.2) | 56.1* (54.0 to 58.1) | 12.7* (11.2 to 14.1) |
| Motorcyclist road injuries | 3289.0 (2838.7 to 3866.0) | 227.3 (125.6 to 377.1) | 172.2 (121.0 to 235.4) | 53.5* (50.9 to 56.1) | 10.8* (9.0 to 12.7) |
| Motor vehicle road injuries | 3484.5 (2985.7 to 4066.8) | 423.2 (258.5 to 644.7) | 193.3 (138.6 to 261.7) | 37.1* (35.5 to 38.7) | -1.0 (-2.2 to 0.1) |
| Other road injuries | 916.2 (747.6 to 1127.2) | 160.8 (82.7 to 268.8) | 44.7 (31.4 to 62.2) | 76.1* (73.3 to 78.6) | 27.1* (25.1 to 29.0) |
| Other transport injuries | 3198.2 (2733.5 to 3648.4) | 269.0 (163.8 to 400.9) | 170.6 (120.9 to 227.4) | 45.1* (42.9 to 47.4) | 4.8* (3.1 to 6.4) |
| **Unintentional injuries** | **70714.1 (65671.9 to 76855.7)** | **18458.9 (15294.4 to 22084.6)** | **2603.8 (1842.4 to 3529.5)** | **47.2* (45.8 to 48.5)** | **6.3* (5.3 to 7.2)** |
| Falls | 34382.4 (30005.1 to 39936.5) | 8620.0 (5851.3 to 11601.7) | 1563.6 (1088.9 to 2127.0) | 47.9* (46.0 to 49.7) | 6.8* (5.4 to 8.1) |
| Drowning | 160.8 (139.4 to 188.5) | 13.8 (10.1 to 18.8) | 8.4 (6.0 to 11.3) | 49.4* (45.2 to 53.4) | 7.8* (4.8 to 10.7) |
| Fire, heat, and hot substances | 6288.3 (5371.6 to 7380.7) | 140.7 (86.8 to 207.4) | 137.5 (87.2 to 208.3) | 40.6* (35.9 to 44.2) | 1.5 (-1.9 to 4.1) |
| Poisonings | 246.2 (208.2 to 284.1) | 61.2 (35.9 to 94.5) | 21.3 (14.2 to 30.1) | 51.7* (48.6 to 54.9) | 9.5* (7.3 to 11.8) |
| Poisoning by carbon monoxide | 51.2 (43.7 to 60.5) | 21.0 (9.6 to 40.3) | 3.6 (2.5 to 5.0) | 55.7* (52.0 to 59.4) | 12.4* (9.7 to 15.1) |
| Poisoning by other means | 194.9 (157.8 to 231.4) | 40.2 (22.0 to 63.5) | 17.7 (11.6 to 25.7) | 51.0* (47.7 to 54.4) | 9.0* (6.6 to 11.5) |
| Exposure to mechanical forces | 14504.1 (12517.3 to 16818.4) | 1731.0 (1181.0 to 2520.4) | 337.5 (230.0 to 486.1) | 50.0* (48.6 to 51.5) | 8.3* (7.3 to 9.4) |
| Unintentional firearm injuries | 558.9 (461.9 to 656.6) | 31.3 (13.7 to 58.4) | 17.0 (11.8 to 23.6) | 48.8* (46.8 to 50.7) | 7.4* (6.0 to 8.8) |
| Other exposure to mechanical forces | 13945.3 (11980.7 to 16313.2) | 1699.7 (1158.6 to 2477.6) | 320.5 (216.9 to 462.5) | 50.1* (48.7 to 51.6) | 8.3* (7.3 to 9.4) |
| Adverse effects of medical treatment | 290.5 (179.8 to 441.5) | 3805.1 (2488.5 to 5600.8) | 38.8 (20.3 to 64.8) | 109.6* (90.6 to 126.4) | 51.3* (37.6 to 63.4) |
| Animal contact | 2594.7 (2296.9 to 3006.4) | 1290.2 (840.8 to 1858.7) | 54.9 (37.6 to 77.7) | 31.5* (28.3 to 34.5) | -5.1* (-7.4 to -2.9) |
| Venomous animal contact | 988.4 (817.2 to 1142.3) | 574.3 (364.2 to 873.3) | 30.7 (21.2 to 41.8) | 30.1* (26.6 to 33.5) | -6.1* (-8.6 to -3.7) |
| Non-venomous animal contact | 1606.3 (1318.0 to 1973.5) | 715.9 (435.8 to 1059.0) | 24.3 (14.9 to 38.8) | 33.3* (29.6 to 36.1) | -3.8* (-6.5 to -1.7) |
| Foreign body | 1180.5 (1050.9 to 1315.0) | 653.9 (422.1 to 963.6) | 49.8 (35.8 to 66.9) | 41.2* (39.6 to 42.8) | 1.9* (0.8 to 3.1) |

| | | | | | |
|---|---|---|---|---|---|
| Pulmonary aspiration and foreign body in airway | 182.8 (154.6 to 218.8) | 33.6 (19.6 to 54.6) | 7.4 (5.1 to 10.1) | 56.5* (53.1 to 59.8) | 13.0* (10.5 to 15.4) |
| Foreign body in eyes | 151.4 (103.5 to 215.2) | 509.8 (287.2 to 820.3) | 10.3 (6.2 to 16.0) | 38.6* (37.0 to 40.1) | 0.0 (-1.1 to 1.1) |
| Foreign body in other body part | 846.2 (726.2 to 963.4) | 110.4 (63.8 to 172.4) | 32.2 (23.3 to 42.0) | 38.9* (37.2 to 40.6) | 0.3 (-1.0 to 1.5) |
| Environmental heat and cold exposure | 2351.9 (2021.6 to 2765.7) | 347.4 (209.9 to 532.5) | 92.3 (64.5 to 128.5) | 41.3* (38.7 to 43.7) | 2.0* (0.1 to 3.7) |
| Exposure to forces of nature | 492.8 (360.3 to 669.9) | 13.0 (8.6 to 18.1) | 18.8 (13.8 to 25.0) | 71.8* (54.5 to 89.1) | 24.1* (11.5 to 36.5) |
| Other unintentional injuries | 8689.9 (7364.4 to 10121.9) | 1782.7 (1018.0 to 2819.3) | 280.9 (191.1 to 391.4) | 42.3* (41.1 to 43.5) | 2.7* (1.8 to 3.6) |
| **Self-harm and interpersonal violence** | **11556.1 (10280.9 to 13043.1)** | **512.3 (378.6 to 672.0)** | **257.7 (198.6 to 326.0)** | **49.2* (44.6 to 55.1)** | **7.7* (4.4 to 12.0)** |
| Self-harm | 694.2 (573.5 to 815.6) | 105.6 (65.9 to 154.7) | 29.8 (21.0 to 39.7) | 25.1* (23.6 to 26.4) | -9.7* (-10.8 to -8.7) |
| Self-harm by firearm | 11.7 (9.6 to 14.2) | 4.2 (1.1 to 10.4) | 0.5 (0.3 to 0.6) | 15.6* (12.5 to 20.0) | -16.5* (-18.8 to -13.4) |
| Self-harm by other specified means | 682.5 (561.3 to 804.8) | 101.4 (62.9 to 149.2) | 29.4 (20.7 to 39.2) | 25.3* (23.8 to 26.6) | -9.6* (-10.6 to -8.6) |
| Interpersonal violence | 9392.9 (8214.7 to 10813.4) | 331.2 (216.1 to 485.0) | 178.0 (133.1 to 230.6) | 41.6* (39.9 to 43.5) | 2.2* (1.0 to 3.6) |
| Assault by firearm | 191.3 (160.4 to 233.1) | 9.2 (4.1 to 17.6) | 7.0 (5.0 to 9.6) | 45.9* (43.6 to 47.9) | 5.3* (3.6 to 6.8) |
| Assault by sharp object | 923.2 (756.5 to 1245.4) | 39.6 (19.4 to 73.1) | 23.9 (16.5 to 33.7) | 37.0* (35.0 to 38.9) | -1.1 (-2.5 to 0.3) |
| Sexual violence | 5267.8 (4196.0 to 6628.4) | -- | 36.8 (23.4 to 54.8) | 55.2* (52.3 to 58.1) | 12.0* (9.9 to 14.1) |
| Assault by other means | 3010.6 (2542.9 to 3555.9) | 282.4 (183.4 to 408.4) | 110.3 (77.4 to 150.1) | 38.3* (36.9 to 39.7) | -0.1 (-1.1 to 0.9) |
| Conflict and terrorism | 1295.4 (840.8 to 1866.3) | 65.2 (44.0 to 88.1) | 44.6 (31.7 to 61.9) | 111.1* (79.5 to 151.0) | 52.4* (29.6 to 81.2) |
| Executions and police conflict | 174.0 (108.1 to 258.6) | 10.2 (5.9 to 15.3) | 5.2 (3.3 to 7.5) | 157.8* (105.9 to 231.5) | 86.1* (48.7 to 139.3) |
| **Nature of injury aggregates** | | | | | |
| Amputations | 26144.8 (24218.4 to 28655.6) | 324.7 (255.1 to 408.4) | 435.4 (288.4 to 640.7) | 38.2* (33.0 to 42.8) | -0.2 (-4.0 to 3.1) |
| Burns | 12884.3 (11873.3 to 14070.8) | 251.6 (192.8 to 324.3) | 268.4 (167.3 to 405.7) | 40.4* (36.2 to 43.5) | 1.3 (-1.6 to 3.6) |
| Fractures | 31079.1 (28653.8 to 33921.7) | 6256.9 (4870.5 to 7841.9) | 1663.4 (1138.6 to 2321.7) | 46.0* (44.5 to 47.6) | 5.4* (4.3 to 6.6) |
| Head Injuries | 3464.7 (3307.4 to 3621.7) | 734.0 (563.1 to 951.5) | 488.6 (346.6 to 654.7) | 50.7* (49.1 to 52.3) | 8.8* (7.6 to 9.9) |
| Spinal Injuries | 1361.3 (1271.0 to 1468.5) | 29.0 (21.4 to 40.3) | 358.0 (257.7 to 462.7) | 61.1* (57.9 to 64.4) | 16.3* (14.0 to 18.7) |
| Minor Injuries | 14875.9 (13929.0 to 15963.5) | 5715.7 (4594.0 to 6985.1) | 131.2 (61.7 to 246.0) | 49.9* (48.6 to 51.3) | 8.2* (7.3 to 9.2) |
| Other Injuries | 4866.6 (4607.6 to 5147.7) | 7535.9 (6056.3 to 9381.2) | 395.0 (277.0 to 526.8) | 52.9* (49.9 to 56.4) | 10.4* (8.2 to 12.9) |
| **Impairments** | | | | | |
| Anaemia | 52971.4 (49140.3 to 57101.1) | -- | 1365.7 (948.9 to 1907.4) | 16.6* (8.3 to 25.2) | -15.8* (-21.8 to -9.6) |
| Epilepsy | 1989.4 (1654.7 to 2375.0) | -- | 652.4 (483.4 to 870.8) | 47.2* (38.4 to 56.7) | 6.2 (-0.1 to 13.1) |
| Guillain-Barré syndrome | 4.7 (2.8 to 7.5) | -- | 1.4 (0.7 to 2.5) | 42.0* (38.1 to 45.7) | 2.5 (-0.3 to 5.2) |
| Hearing loss | 135696.0 (131895.3 to 139565.8) | -- | 3323.6 (2219.5 to 4796.4) | 37.7* (34.6 to 40.9) | -0.6 (-2.8 to 1.7) |
| Heart failure | 8294.9 (7083.2 to 9726.6) | -- | 1357.4 (929.7 to 1811.9) | 39.3* (37.5 to 41.3) | 0.5 (-0.8 to 2.0) |
| Infertility | -- | -- | -- | -29.7* (-40.3 to -23.0) | -49.3* (-56.9 to -44.4) |
| Developmental intellectual disability | 3338.8 (2583.9 to 4048.1) | -- | 478.7 (367.4 to 600.9) | 48.8* (43.1 to 55.2) | 7.4* (3.3 to 12.0) |
| Pelvic inflammatory disease | -- | -- | -- | -- | -- |
| Vision loss | 123882.6 (115415.8 to 134102.7) | -- | 3350.6 (2342.8 to 4716.7) | 31.5* (29.4 to 33.4) | -5.0* (-6.6 to -3.7) |

We did not calculate incidence for the nine impairments and certain NTDs. Blank cells mean that no estimate is available or that the estimate has a magnitude less than 50.
Table presents prevalence and incidence counts in thousands.
* Percentage changes that are statistically significant
‡ Incidence estimates for stroke represent first-ever stroke only.
^ Incidence of HIV/AIDS represents new infections of HIV only and does not include new infections of Tuberculosis in HIV positive cases.

**SR Table 10r: Global prevalence, incidence and YLDs for 2017, percentage change of YLD counts and percentage change of YLD rates between 2007 and 2017 by cause, age 70 to 74 years**

| Cause | Prevalence in thousands (95% UI) | Incidence in thousands (95% UI) | YLDs in thousands (95% UI) | | |
|---|---|---|---|---|---|
| | 2017 counts | 2017 counts | 2017 counts | Percentage change in counts between 2007 and 2017 | Percentage change in rates between 2007 and 2017 |
| **All causes** | **167911.0 (167876.1 to 167943.6)** | **826156.5 (737654.1 to 938493.5)** | **39586.9 (30035.4 to 50233.9)** | **22.5* (21.5 to 23.4)** | **0.2 (-0.6 to 1.0)** |
| **Communicable, maternal, neonatal, and nutritional disorders** | **97132.8 (89502.8 to 106910.6)** | **484745.8 (402928.3 to 589511.3)** | **1658.1 (1227.0 to 2147.6)** | **15.9* (12.6 to 19.5)** | **-5.2* (-7.9 to -2.3)** |
| **HIV/AIDS and sexually transmitted infections** | **33629.8 (30004.1 to 37669.5)** | **2623.7 (2278.8 to 3116.5)** | **57.5 (39.7 to 79.9)** | **21.9 (-5.5 to 51.1)** | **-0.3 (-22.7 to 23.6)** |
| HIV/AIDS | 432.1 (375.3 to 494.3) | 9.4^ (7.1 to 12.2) | 42.8 (29.5 to 60.3) | 21.8 (-10.1 to 68.9) | -0.3 (-26.4 to 38.2) |
| HIV/AIDS - Drug-susceptible Tuberculosis | 11.6 (10.6 to 12.6) | 12.5 (11.5 to 13.6) | 4.3 (3.0 to 5.8) | 27.0* (16.5 to 37.9) | 3.9 (-4.7 to 12.8) |
| HIV/AIDS - Multidrug-resistant Tuberculosis without extensive drug resistance | 0.5 (0.3 to 0.9) | 0.5 (0.3 to 0.7) | 0.2 (0.1 to 0.4) | 7.5 (-34.8 to 94.6) | -12.0 (-46.7 to 59.2) |
| HIV/AIDS - Extensively drug-resistant Tuberculosis | 0.0 (0.0 to 0.1) | -- | -- | 86.8 (-12.4 to 336.9) | 52.8 (-28.3 to 257.4) |
| HIV/AIDS resulting in other diseases | 420.0 (363.1 to 482.2) | 9.4 (7.1 to 12.2) | 38.4 (25.7 to 55.2) | 21.3 (-13.0 to 75.8) | -0.7 (-28.8 to 43.8) |
| HIV/AIDS not on antiretroviral treatment without Tuberculosis | 74.4 (57.2 to 97.9) | 9.4 (7.1 to 12.2) | 13.2 (6.9 to 24.8) | -52.1* (-60.1 to -42.6) | -60.8* (-67.4 to -53.1) |
| HIV/AIDS on antiretroviral treatment without Tuberculosis | 345.6 (298.1 to 392.8) | -- | 25.1 (16.9 to 35.8) | 529.9* (464.0 to 614.5) | 415.3* (361.4 to 484.5) |
| Sexually transmitted infections excluding HIV | 33345.1 (29708.4 to 37386.2) | 2614.3 (2268.0 to 3106.1) | 14.7 (8.1 to 26.9) | 22.2* (19.3 to 24.6) | -0.0 (-2.4 to 1.9) |
| Syphilis | 151.6 (122.0 to 188.4) | -- | 4.5 (3.1 to 6.4) | 19.5* (13.5 to 26.3) | -2.2 (-7.1 to 3.4) |
| Early syphilis | 125.0 (95.0 to 161.4) | -- | 0.0 (0.0 to 0.1) | 34.7* (25.7 to 44.0) | 10.2* (2.8 to 17.8) |
| Tertiary syphilis | 26.6 (22.5 to 31.7) | -- | 4.5 (3.0 to 6.4) | 19.5* (13.4 to 26.3) | -2.3 (-7.2 to 3.3) |
| Chlamydial infection | 9.8 (6.3 to 13.8) | -- | -- | 20.1* (18.5 to 22.2) | -1.7* (-3.1 to -0.0) |
| Chlamydia episode | 9.8 (6.3 to 13.8) | -- | -- | 19.9* (18.3 to 21.6) | -1.9* (-3.2 to -0.5) |
| Chlamydial infection complications | -- | -- | -- | 29.0* (6.2 to 52.9) | 5.5 (-13.1 to 25.1) |
| Gonococcal infection | 0.9 (0.3 to 2.7) | -- | -- | 23.5* (10.2 to 33.0) | 1.0 (-9.9 to 8.8) |
| Gonococcal infection complications | -- | -- | -- | 27.7* (5.0 to 49.9) | 4.5 (-14.1 to 22.6) |
| Gonorrhoea episode | 0.9 (0.3 to 2.7) | -- | -- | 22.8* (4.4 to 33.4) | 0.4 (-14.6 to 9.1) |
| Trichomoniasis | 1619.6 (1328.0 to 1969.7) | 2570.8 (2209.9 to 3061.0) | 2.7 (1.1 to 5.7) | 24.5* (20.9 to 27.9) | 1.8 (-1.1 to 4.6) |
| Genital herpes | 32270.4 (28507.8 to 36560.3) | 43.4 (26.2 to 71.0) | 7.4 (2.2 to 17.9) | 23.0* (21.3 to 24.7) | 0.7 (-0.8 to 2.0) |
| Other sexually transmitted infections | -- | -- | -- | 19.9* (17.6 to 23.0) | -1.9 (-3.8 to 0.7) |
| Other sexually transmitted diseases residual | -- | -- | -- | 19.9* (17.6 to 23.0) | -1.9 (-3.8 to 0.7) |
| Other sexually transmitted diseases | -- | -- | -- | 19.9* (17.6 to 23.0) | -1.9 (-3.8 to 0.7) |
| **Respiratory infections and tuberculosis** | **49555.1 (35731.6 to 66433.2)** | **275329.9 (197884.9 to 382311.3)** | **297.4 (206.5 to 402.5)** | **12.8* (9.8 to 15.5)** | **-7.7* (-10.2 to -5.5)** |
| Tuberculosis | 46382.1 (32379.8 to 63897.8) | 259.8 (205.6 to 319.5) | 183.5 (116.8 to 270.5) | 8.7* (5.1 to 12.5) | -11.1* (-14.0 to -8.0) |
| Latent tuberculosis infection | 45732.1 (31802.7 to 63306.6) | -- | -- | -- | -- |
| Drug-susceptible tuberculosis | 620.5 (464.4 to 814.8) | 247.2 (196.1 to 306.0) | 175.1 (111.6 to 256.6) | 9.4* (3.7 to 14.2) | -10.5* (-15.1 to -6.6) |
| Multidrug-resistant tuberculosis without extensive drug resistance | 28.1 (12.0 to 56.4) | 12.1 (5.8 to 23.2) | 8.0 (3.3 to 16.7) | -5.2 (-56.4 to 75.2) | -22.4 (-64.3 to 43.3) |
| Extensively drug-resistant tuberculosis | 1.5 (0.7 to 3.3) | 0.6 (0.3 to 1.0) | 0.5 (0.2 to 1.1) | 27.5 (-35.7 to 156.5) | 4.3 (-47.4 to 109.8) |
| Lower respiratory infections | 180.1 (159.5 to 203.1) | 8409.3 (7424.7 to 9540.7) | 10.3 (7.0 to 14.5) | 26.7* (17.0 to 36.5) | 3.7 (-4.3 to 11.7) |
| Guillain-Barré syndrome due to lower respiratory infections | 0.4 (0.2 to 0.8) | -- | 0.1 (0.1 to 0.3) | 28.7* (25.2 to 32.2) | 5.3* (2.4 to 8.1) |
| Lower respiratory infection episode | 179.7 (159.1 to 202.5) | 8409.3 (7424.7 to 9540.7) | 10.2 (6.8 to 14.3) | 26.7* (16.8 to 36.5) | 3.7 (-4.5 to 11.7) |
| Upper respiratory infections | 3677.0 (2569.8 to 5192.2) | 265663.1 (187734.6 to 372708.3) | 86.2 (44.6 to 143.8) | 20.9* (19.3 to 22.5) | -1.1 (-2.4 to 0.2) |
| Guillain-Barré syndrome due to upper respiratory infections | 1.2 (0.7 to 2.0) | -- | 0.4 (0.2 to 0.6) | 28.7* (25.2 to 32.1) | 5.3* (2.4 to 8.1) |
| Upper respiratory infection episode | 3675.8 (2568.9 to 5191.5) | 265663.1 (187734.6 to 372708.3) | 85.8 (44.2 to 143.3) | 20.9* (19.2 to 22.5) | -1.1 (-2.5 to 0.2) |
| Otitis media | 873.6 (618.3 to 1185.3) | 997.7 (704.8 to 1352.0) | 17.4 (9.5 to 29.6) | 12.2* (7.1 to 17.7) | -8.2* (-12.4 to -3.7) |
| Acute otitis media | 54.8 (36.6 to 75.2) | 997.5 (704.8 to 1351.9) | 0.7 (0.3 to 1.3) | 21.6* (12.9 to 30.7) | -0.5 (-7.6 to 6.9) |
| Chronic otitis media | 818.9 (563.8 to 1132.8) | 0.2 (0.0 to 0.6) | 16.7 (9.1 to 28.4) | 11.8* (6.4 to 17.6) | -8.5* (-13.0 to -3.8) |
| **Enteric infections** | **2654.9 (2312.2 to 3087.9)** | **180863.4 (154476.9 to 216181.5)** | **278.5 (190.3 to 383.6)** | **47.3* (39.8 to 55.2)** | **20.5* (14.4 to 27.0)** |
| Diarrhoeal diseases | 2674.1 (2323.7 to 3110.4) | 180778.3 (154402.8 to 216090.6) | 277.8 (189.7 to 382.7) | 47.4* (40.0 to 55.4) | 20.6* (14.5 to 27.1) |
| Guillain-Barré syndrome due to diarrheal diseases | 0.4 (0.2 to 0.7) | -- | 0.1 (0.1 to 0.2) | 28.7* (25.2 to 32.2) | 5.3* (2.4 to 8.2) |
| Diarrheal disease episode | 2673.7 (2323.4 to 3109.9) | 180778.3 (154402.8 to 216090.6) | 277.7 (189.6 to 382.5) | 47.5* (40.0 to 55.4) | 20.6* (14.5 to 27.1) |
| Typhoid and paratyphoid | 0.3 (0.1 to 0.7) | 82.6 (68.4 to 97.2) | 0.7 (0.4 to 1.0) | -1.9 (-17.6 to 16.4) | -19.8* (-32.6 to -4.8) |
| Typhoid fever | 4.1 (3.2 to 5.2) | 64.8 (51.9 to 78.6) | 0.6 (0.4 to 0.9) | 0.1 (-17.5 to 20.9) | -18.1* (-32.5 to -1.1) |
| Typhoid fever complications | 0.9 (0.6 to 1.2) | 11.1 (8.6 to 13.9) | 0.3 (0.2 to 0.4) | -1.8 (-25.6 to 32.9) | -19.7 (-39.1 to 8.7) |
| Typhoid fever episode | 3.3 (2.5 to 4.2) | 53.7 (42.9 to 65.1) | 0.4 (0.2 to 0.5) | 1.6 (-23.1 to 33.8) | -16.9 (-37.1 to 9.4) |
| Paratyphoid fever | 0.8 (0.5 to 1.1) | 17.7 (12.3 to 24.6) | 0.0 (0.0 to 0.1) | -22.1* (-34.5 to -8.4) | -36.3* (-46.5 to -25.1) |
| Intestinal perforation due to paratyphoid | 0.0 (0.0 to 0.1) | 0.9 (0.6 to 1.3) | -- | -22.0* (-39.1 to -1.4) | -36.2* (-50.2 to -19.3) |
| Paratyphoid fever episode | 0.7 (0.5 to 1.0) | 16.8 (11.6 to 23.3) | 0.0 (0.0 to 0.1) | -22.2* (-35.1 to -8.0) | -36.3* (-46.9 to -24.7) |
| iNTS | 0.1 (0.1 to 0.2) | 2.5 (1.5 to 4.0) | -- | -12.1* (-20.2 to -3.9) | -28.1* (-34.8 to -21.4) |
| Other intestinal infectious diseases | -- | -- | -- | -8.6 (-23.2 to 8.5) | -25.2* (-37.1 to -11.2) |
| **Neglected tropical diseases and malaria** | **16088.2 (15303.7 to 16982.6)** | **4244.3 (3320.2 to 5252.4)** | **289.3 (209.8 to 388.6)** | **-0.5 (-6.2 to 5.4)** | **-18.6* (-23.3 to -13.8)** |
| Malaria | 748.8 (697.8 to 791.5) | 420.9 (389.5 to 477.0) | 5.6 (3.8 to 8.1) | -26.4* (-39.5 to -5.6) | -39.8* (-50.5 to -22.8) |
| Malaria complications | 1.6 (1.3 to 1.8) | -- | 0.7 (0.5 to 0.9) | 36.5* (33.2 to 39.7) | 11.7* (9.0 to 14.2) |
| Malaria episode | 24.8 (16.7 to 33.6) | 420.9 (389.5 to 477.0) | 0.9 (0.5 to 1.6) | -28.3* (-37.4 to -16.8) | -41.3* (-48.8 to -31.9) |

| | | | | | |
|---|---|---|---|---|---|
| Malaria parasitemia | 722.4 (670.7 to 768.8) | -- | 3.9 (2.5 to 6.2) | -31.6* (-47.2 to -4.8) | -44.0* (-56.8 to -22.1) |
| Chagas disease | 258.8 (221.4 to 299.0) | 2.3 (1.5 to 3.4) | 4.5 (2.9 to 6.5) | 12.7* (6.6 to 19.0) | -7.8* (-12.8 to -2.7) |
| Acute Chagas disease | -- | 2.3 (1.5 to 3.4) | -- | 30.1* (25.0 to 35.1) | 6.4* (2.3 to 10.5) |
| Asymptomatic Chagas disease | 196.5 (166.0 to 228.0) | -- | -- | -- | -- |
| Symptomatic chronic Chagas infection | 62.3 (49.4 to 76.4) | -- | 4.5 (2.9 to 6.5) | 12.7* (6.6 to 19.0) | -7.8* (-12.8 to -2.7) |
| Leishmaniasis | 111.9 (81.2 to 149.3) | 11.9 (8.4 to 16.6) | 6.7 (4.1 to 10.2) | 20.8* (14.3 to 30.6) | -1.2 (-6.5 to 6.8) |
| Visceral leishmaniasis | 0.1 (0.1 to 0.1) | 0.2 (0.2 to 0.3) | -- | -72.2* (-76.9 to -63.9) | -77.3* (-81.1 to -70.4) |
| Cutaneous and mucocutaneous leishmaniasis | 112.4 (81.4 to 150.0) | 11.6 (8.1 to 16.4) | 6.6 (4.1 to 10.2) | 21.1* (14.5 to 31.0) | -0.9 (-6.3 to 7.1) |
| African trypanosomiasis | 0.1 (0.0 to 0.2) | 0.0 (0.0 to 0.1) | 0.0 (0.0 to 0.1) | -79.1 (-98.0 to 20.4) | -82.9* (-98.3 to -1.5) |
| Trypanosomiasis Gambiense | 0.1 (0.0 to 0.2) | 0.0 (0.0 to 0.1) | 0.0 (0.0 to 0.1) | -78.4 (-98.2 to 29.8) | -82.4 (-98.5 to 6.2) |
| Trypanosomiasis Rhodesiense | -- | -- | -- | -89.5* (-99.1 to -24.1) | -91.4* (-99.3 to -37.9) |
| Schistosomiasis | 2618.2 (2370.5 to 2880.0) | -- | 17.7 (8.7 to 33.4) | -20.3* (-24.9 to -15.2) | -34.8* (-38.5 to -30.6) |
| Mild schistosomiasis | 2315.5 (2090.1 to 2559.8) | -- | 12.3 (5.0 to 25.8) | -20.3* (-22.2 to -18.3) | -34.8* (-36.3 to -33.2) |
| Anemia due to schistosomiasis | 156.9 (122.7 to 198.4) | -- | 3.1 (1.9 to 4.9) | -29.2* (-45.5 to -8.2) | -42.1* (-55.4 to -24.9) |
| Schistosomiasis complications | 146.4 (111.7 to 185.6) | -- | 2.2 (1.2 to 3.6) | -2.3 (-9.6 to 6.7) | -20.1* (-26.1 to -12.8) |
| Cysticercosis | 308.9 (220.9 to 404.2) | -- | 81.4 (49.5 to 124.1) | 17.9* (12.0 to 23.7) | -3.6 (-8.4 to 1.2) |
| Cystic echinococcosis | 11.6 (7.1 to 19.5) | 2.2 (1.3 to 3.9) | 0.9 (0.5 to 1.6) | -9.1 (-19.1 to 1.1) | -25.7* (-33.8 to -17.3) |
| Lymphatic filariasis | 996.9 (917.1 to 1087.6) | -- | 16.3 (9.3 to 25.0) | -38.9* (-50.2 to -28.1) | -50.0* (-59.3 to -41.2) |
| Prevalence of detectable microfiliaria due to lymphatic filariasis | 836.6 (780.6 to 905.9) | -- | -- | -- | -- |
| Lymphatic filariasis complications | 160.3 (110.8 to 225.1) | -- | 16.3 (9.3 to 25.0) | -38.9* (-50.2 to -28.1) | -50.0* (-59.3 to -41.2) |
| Onchocerciasis | 322.2 (218.3 to 496.0) | -- | 27.4 (15.7 to 47.5) | 0.4 (-13.6 to 16.0) | -17.9* (-29.3 to -5.1) |
| Asymptomatic onchocerciasis | 45.2 (0.0 to 191.9) | -- | -- | -- | -- |
| Skin disease due to onchocerciasis | 183.9 (127.4 to 260.9) | -- | 20.2 (9.9 to 39.4) | -2.3 (-22.8 to 16.6) | -20.1* (-36.8 to -4.7) |
| Vision loss due to onchocerciasis | 93.1 (60.9 to 143.9) | -- | 7.2 (4.3 to 11.3) | 9.0 (-5.0 to 26.2) | -10.8 (-22.2 to 3.3) |
| Trachoma | 678.5 (411.9 to 1067.9) | -- | 49.4 (30.5 to 76.0) | -9.8 (-24.2 to 5.3) | -26.2* (-38.0 to -13.9) |
| Dengue | 107.5 (58.7 to 181.9) | 1799.6 (1096.0 to 2727.4) | 16.0 (7.1 to 29.5) | 81.5* (57.6 to 180.4) | 48.5* (28.9 to 129.4) |
| Post-dengue chronic fatigue syndrome | 75.7 (35.4 to 138.5) | -- | 14.2 (6.1 to 26.7) | 81.5* (56.4 to 181.5) | 48.5* (28.0 to 130.3) |
| Dengue episode | 31.8 (19.2 to 47.6) | 1799.6 (1096.0 to 2727.4) | 1.8 (0.9 to 3.1) | 81.3* (55.0 to 172.4) | 48.3* (26.8 to 122.9) |
| Yellow fever | -- | 0.5 (0.2 to 1.5) | -- | -24.7 (-47.0 to 7.4) | -38.4* (-56.6 to -12.2) |
| Asymptomatic yellow fever | -- | 0.3 (0.1 to 0.9) | -- | -- | -- |
| Yellow fever episode | -- | 0.2 (0.1 to 0.7) | -- | -24.7 (-47.0 to 7.4) | -38.4* (-56.6 to -12.2) |
| Rabies | -- | 0.4 (0.3 to 0.5) | -- | -30.9* (-47.4 to -13.8) | -43.5* (-57.0 to -29.5) |
| Intestinal nematode infections | 9537.7 (8796.4 to 10387.1) | -- | 18.0 (10.5 to 29.1) | -24.3* (-30.5 to -18.3) | -38.1* (-43.1 to -33.2) |
| Ascariasis | 4246.3 (3655.5 to 4939.4) | -- | 4.8 (2.6 to 8.1) | -26.5* (-34.9 to -17.3) | -39.8* (-46.7 to -32.3) |
| Asymptomatic ascariasis | 3968.3 (3414.1 to 4618.4) | -- | -- | -- | -- |
| Ascariasis complications | 278.0 (243.2 to 316.9) | -- | 4.8 (2.6 to 8.1) | -26.5* (-34.9 to -17.3) | -39.8* (-46.7 to -32.3) |
| Trichuriasis | 2076.9 (1737.1 to 2496.0) | -- | 1.2 (0.7 to 1.9) | -4.8 (-14.2 to 5.5) | -22.1* (-29.8 to -13.7) |
| Asymptomatic trichuriasis | 2013.8 (1680.3 to 2427.8) | -- | -- | -- | -- |
| Trichuriasis complications | 63.0 (57.0 to 69.1) | -- | 1.2 (0.7 to 1.9) | -4.8 (-14.2 to 5.5) | -22.1* (-29.8 to -13.7) |
| Hookworm disease | 3664.0 (3288.7 to 4099.5) | -- | 12.0 (7.2 to 19.2) | -24.9* (-33.4 to -16.4) | -38.6* (-45.5 to -31.6) |
| Asymptomatic hookworm disease | 3032.7 (2714.8 to 3394.3) | -- | -- | -- | -- |
| Anemia due to hookworm disease | 166.0 (142.2 to 194.7) | -- | 2.9 (1.9 to 4.6) | -38.2* (-50.1 to -24.4) | -49.4* (-59.2 to -38.2) |
| Hookworm disease complications | 465.3 (418.5 to 519.0) | -- | 9.1 (5.2 to 15.1) | -19.3* (-26.7 to -11.2) | -34.0* (-40.1 to -27.4) |
| Food-borne trematodiases | 1598.0 (1408.0 to 1816.9) | 1966.7 (1518.5 to 2465.0) | 35.1 (18.5 to 61.4) | 15.3* (6.9 to 23.4) | -5.6 (-12.5 to 0.9) |
| Asymptomatic food-borne tremodiases | 1269.0 (1029.7 to 1497.9) | 1481.7 (754.4 to 2104.0) | -- | -- | -- |
| Food-borne tremodiases complications | 329.0 (189.9 to 532.5) | 485.1 (53.9 to 1193.6) | 35.1 (18.5 to 61.4) | 15.3* (6.9 to 23.4) | -5.6 (-12.5 to 0.9) |
| Leprosy | 42.4 (40.0 to 44.9) | 2.6 (2.4 to 2.8) | 2.5 (1.7 to 3.5) | 4.2 (-2.0 to 10.8) | -14.8* (-19.8 to -9.3) |
| Ebola virus disease | -- | -- | -- | -91.7* (-97.8 to -74.7) | -93.2* (-98.2 to -79.3) |
| Ebola cases | -- | -- | -- | -97.9* (-98.0 to -97.8) | -98.3* (-98.4 to -98.2) |
| Post-Ebola chronic fatigue syndrome | -- | -- | -- | -91.3* (-97.8 to -73.6) | -92.9* (-98.2 to -78.4) |
| Zika virus disease | 0.6 (0.4 to 1.1) | 37.2 (21.2 to 68.4) | -- | -- | -- |
| Zika virus complications | -- | -- | -- | -- | -- |
| Zika virus episode | 0.6 (0.3 to 1.1) | 37.2 (21.2 to 68.4) | -- | -- | -- |
| Guinea worm disease | -- | -- | -- | -99.6* (-99.7 to -99.4) | -99.7* (-99.8 to -99.5) |
| Other neglected tropical diseases | 383.0 (351.1 to 420.2) | -- | 7.9 (5.2 to 11.4) | 7.8 (-4.2 to 20.7) | -11.8* (-21.6 to -1.2) |
| Acute infection due to other neglected tropical diseases | -- | -- | 0.2 (0.1 to 0.3) | 58.5* (35.5 to 135.7) | 29.7* (10.8 to 92.8) |
| Anemia due to other neglected tropical diseases | 383.0 (351.1 to 420.2) | -- | 7.7 (5.1 to 11.1) | 7.0 (-5.0 to 20.1) | -12.5* (-22.3 to -1.7) |
| **Other infectious diseases** | **1373.3 (1191.7 to 1580.6)** | **4717.7 (3705.4 to 6056.7)** | **60.1 (42.3 to 82.2)** | **13.4* (6.8 to 20.5)** | **-7.3* (-12.6 to -1.4)** |
| Meningitis | 187.0 (153.7 to 224.8) | 60.7 (44.2 to 80.9) | 10.2 (7.1 to 13.8) | -0.5 (-4.7 to 4.0) | -18.6* (-22.0 to -14.9) |
| Pneumococcal meningitis | 68.4 (54.8 to 84.8) | 6.2 (4.0 to 9.0) | 3.9 (2.7 to 5.2) | -17.4* (-22.5 to -11.4) | -32.5* (-36.6 to -27.5) |

| | | | | | |
|---|---|---|---|---|---|
| Acute pneumococcal meningitis | 0.3<br>(0.2 to 0.4) | 6.2<br>(4.0 to 9.0) | 0.0<br>(0.0 to 0.1) | -11.0*<br>(-20.6 to -1.6) | -27.2*<br>(-35.1 to -19.5) |
| Pneumococcal meningitis complications | 68.1<br>(54.5 to 84.5) | -- | 3.9<br>(2.7 to 5.2) | -17.5*<br>(-22.6 to -11.4) | -32.5*<br>(-36.7 to -27.5) |
| H influenzae type B meningitis | 13.4<br>(9.0 to 18.1) | 1.9<br>(1.1 to 3.0) | 0.7<br>(0.4 to 0.9) | -52.0*<br>(-54.2 to -49.7) | -60.7*<br>(-62.5 to -58.9) |
| Acute H influenzae type B meningitis | 0.1<br>(0.0 to 0.1) | 1.9<br>(1.1 to 3.0) | -- | -49.9*<br>(-56.3 to -44.4) | -59.0*<br>(-64.3 to -54.5) |
| H Influenza type B meningitis complications | 13.3<br>(9.0 to 18.0) | -- | 0.6<br>(0.4 to 0.9) | -52.0*<br>(-54.3 to -49.7) | -60.7*<br>(-62.6 to -58.9) |
| Meningococcal infection | 20.7<br>(14.1 to 28.0) | 5.1<br>(3.0 to 8.0) | 1.2<br>(0.9 to 1.7) | -1.2<br>(-5.4 to 3.1) | -19.2*<br>(-22.6 to -15.7) |
| Acute meningococcal meningitis | 0.2<br>(0.1 to 0.4) | 5.1<br>(3.0 to 8.0) | 0.0<br>(0.0 to 0.1) | 12.8*<br>(1.4 to 22.9) | -7.7<br>(-17.0 to 0.6) |
| Meningococcal meningitis complications | 20.5<br>(13.9 to 27.7) | -- | 1.2<br>(0.8 to 1.7) | -1.5<br>(-5.8 to 2.8) | -19.4*<br>(-22.9 to -15.9) |
| Other meningitis | 84.5<br>(59.0 to 109.4) | 47.5<br>(35.0 to 63.7) | 4.4<br>(3.0 to 6.2) | 51.2*<br>(43.2 to 60.1) | 23.7*<br>(17.2 to 31.0) |
| Other acute bacterial meningitis | 0.6<br>(0.4 to 0.9) | 13.9<br>(10.0 to 19.3) | 0.1<br>(0.0 to 0.1) | 66.8*<br>(52.2 to 82.1) | 36.5*<br>(24.5 to 49.0) |
| Acute viral meningitis | 1.4<br>(1.0 to 2.0) | 33.6<br>(24.5 to 44.8) | 0.2<br>(0.1 to 0.3) | 15.0*<br>(5.3 to 23.6) | -5.9<br>(-13.9 to 1.1) |
| Other bacterial meningitis complications | 82.5<br>(57.1 to 107.3) | -- | 4.2<br>(2.8 to 5.8) | 53.1*<br>(44.7 to 62.8) | 25.2*<br>(18.4 to 33.2) |
| Encephalitis | 232.6<br>(111.7 to 397.2) | 71.2<br>(68.8 to 73.8) | 12.1<br>(8.3 to 16.6) | 19.6*<br>(14.5 to 25.3) | -2.2<br>(-6.4 to 2.5) |
| Acute encephalitis | 3.3<br>(3.2 to 3.5) | 71.2<br>(68.8 to 73.8) | 0.4<br>(0.3 to 0.6) | 30.9*<br>(30.3 to 31.5) | 7.1*<br>(6.6 to 7.6) |
| Encephalitis complications | 229.3<br>(108.4 to 393.9) | -- | 11.7<br>(7.9 to 16.1) | 19.2*<br>(13.9 to 25.1) | -2.5<br>(-6.8 to 2.3) |
| Diphtheria | -- | -- | -- | -- | -- |
| Whooping cough | -- | -- | -- | -- | -- |
| Tetanus | 0.9<br>(0.9 to 1.0) | 2.0<br>(1.2 to 3.0) | -- | -20.3*<br>(-36.7 to -3.1) | -34.8*<br>(-48.2 to -20.7) |
| Severe tetanus | 0.1<br>(0.1 to 0.2) | 2.0<br>(1.2 to 3.0) | -- | -41.3*<br>(-55.0 to -19.1) | -52.0*<br>(-63.2 to -33.8) |
| Neonatal tetanus complications | 0.8<br>(0.8 to 0.9) | -- | -- | 21.6*<br>(19.8 to 23.1) | -0.5<br>(-2.0 to 0.7) |
| Measles | -- | -- | -- | -- | -- |
| Varicella and herpes zoster | 357.5<br>(298.3 to 428.0) | 1967.3<br>(1626.0 to 2328.9) | 18.7<br>(11.4 to 28.3) | 24.6*<br>(20.5 to 29.1) | 1.9<br>(-1.4 to 5.6) |
| Chickenpox | -- | 0.3<br>(0.0 to 0.9) | -- | 50.9*<br>(39.7 to 65.1) | 23.5*<br>(14.3 to 35.1) |
| Herpes zoster | 357.5<br>(298.3 to 428.0) | 1967.0<br>(1626.0 to 2328.8) | 18.7<br>(11.4 to 28.3) | 24.6*<br>(20.5 to 29.1) | 1.9<br>(-1.4 to 5.6) |
| Acute hepatitis | 295.0<br>(193.7 to 449.2) | 2616.6<br>(1734.6 to 3955.1) | 6.7<br>(3.6 to 11.1) | 11.6<br>(-31.1 to 79.1) | -8.7<br>(-43.6 to 46.5) |
| Acute hepatitis A | 4.9<br>(2.5 to 8.2) | 63.9<br>(32.4 to 106.8) | 0.2<br>(0.1 to 0.4) | 20.3<br>(-0.4 to 42.6) | -1.6<br>(-18.5 to 16.7) |
| Acute hepatitis B | 271.0<br>(170.8 to 425.6) | 2348.5<br>(1480.1 to 3688.8) | 6.0<br>(3.2 to 10.3) | 11.4<br>(-35.3 to 85.9) | -8.9<br>(-47.1 to 52.1) |
| Acute hepatitis C | 10.0<br>(4.3 to 19.2) | 87.1<br>(37.6 to 166.1) | 0.1<br>(0.0 to 0.4) | -5.9<br>(-16.5 to 1.2) | -23.0*<br>(-31.7 to -17.2) |
| Acute hepatitis E | 9.0<br>(6.3 to 12.5) | 117.1<br>(81.3 to 162.8) | 0.3<br>(0.2 to 0.5) | 22.9*<br>(2.4 to 45.7) | 0.5<br>(-16.2 to 19.2) |
| Other unspecified infectious diseases | 495.7<br>(460.5 to 534.9) | -- | 12.4<br>(8.2 to 18.1) | 6.8<br>(-2.3 to 15.9) | -12.7*<br>(-20.1 to -5.2) |
| Guillain-Barré syndrome due to other infectious diseases | 0.3<br>(0.1 to 0.5) | -- | 0.1<br>(0.0 to 0.2) | 28.7*<br>(25.2 to 32.1) | 5.3*<br>(2.4 to 8.1) |
| Other infectious diseases | -- | -- | 2.4<br>(1.4 to 3.8) | 21.3*<br>(17.2 to 26.0) | -0.7<br>(-4.1 to 3.1) |
| Anemia due to other infectious diseases | 495.4<br>(460.4 to 534.7) | -- | 9.9<br>(6.5 to 14.4) | 3.6<br>(-7.2 to 14.6) | -15.2*<br>(-24.1 to -6.3) |
| **Maternal and neonatal disorders** | **1698.4<br>(1470.9 to 1935.4)** | **--** | **196.2<br>(143.0 to 257.9)** | **53.1*<br>(44.4 to 63.2)** | **25.2*<br>(18.1 to 33.5)** |
| Maternal disorders | 3.6<br>(2.8 to 4.4) | -- | 1.3<br>(0.9 to 1.8) | -30.2*<br>(-40.1 to -18.6) | -42.9*<br>(-51.0 to -33.4) |
| Maternal haemorrhage | -- | -- | -- | -- | -- |
| Maternal hemorrhage complications | -- | -- | -- | -- | -- |
| Maternal hemorrhage episode | -- | -- | -- | -- | -- |
| Maternal sepsis and other pregnancy related infections | -- | -- | -- | -- | -- |
| Infertility due to puerperal sepsis | -- | -- | -- | -- | -- |
| Maternal sepsis and other maternal infection episode | -- | -- | -- | -- | -- |
| Maternal hypertensive disorders | -- | -- | -- | -- | -- |
| Maternal hypertensive disorder complications | -- | -- | -- | -- | -- |
| Maternal hypertensive disorder episode | -- | -- | -- | -- | -- |
| Maternal obstructed labour and uterine rupture | 4.2<br>(3.5 to 5.1) | -- | 1.3<br>(0.9 to 1.8) | -30.2*<br>(-40.1 to -18.6) | -42.9*<br>(-51.0 to -33.4) |
| Obstructed labor, acute event | -- | -- | -- | -- | -- |
| Maternal obstructed labor complications | 4.2<br>(3.5 to 5.1) | -- | 1.3<br>(0.9 to 1.8) | -30.2*<br>(-40.1 to -18.6) | -42.9*<br>(-51.0 to -33.4) |
| Maternal abortion and miscarriage | -- | -- | -- | -- | -- |
| Ectopic pregnancy | -- | -- | -- | -- | -- |
| Other maternal disorders | -- | -- | -- | -- | -- |
| Neonatal disorders | 1694.8<br>(1467.4 to 1932.2) | -- | 194.9<br>(141.7 to 256.8) | 54.3*<br>(45.2 to 65.1) | 26.2*<br>(18.8 to 35.0) |
| Neonatal preterm birth | 1057.3<br>(967.0 to 1156.9) | -- | 76.4<br>(53.7 to 103.5) | 58.5*<br>(38.4 to 82.0) | 29.7*<br>(13.2 to 48.9) |
| Preterm birth complications | 826.9<br>(746.5 to 919.4) | -- | 63.5<br>(42.2 to 89.4) | 70.5*<br>(45.0 to 99.3) | 39.5*<br>(18.7 to 63.1) |
| Retinopathy of prematurity | 230.4<br>(187.6 to 275.1) | -- | 12.8<br>(8.7 to 17.7) | 17.5*<br>(11.2 to 24.3) | -3.9<br>(-9.0 to 1.7) |
| Uncomplicated preterm birth | -- | -- | -- | -- | -- |
| Neonatal encephalopathy due to birth asphyxia and trauma | 682.4<br>(488.1 to 902.4) | -- | 63.0<br>(37.0 to 98.0) | 62.1*<br>(52.9 to 72.6) | 32.6*<br>(25.1 to 41.2) |
| Neonatal sepsis and other neonatal infections | 168.1<br>(108.9 to 249.3) | -- | 49.4<br>(26.1 to 87.0) | 41.1*<br>(34.7 to 49.1) | 15.5*<br>(10.2 to 22.0) |
| Neonatal sepsis and other neonatal infection complications | 168.1<br>(108.9 to 249.3) | -- | 49.4<br>(26.1 to 87.0) | 41.1*<br>(34.7 to 49.1) | 15.5*<br>(10.2 to 22.0) |
| Neonatal sepsis and other neonatal infection episode | -- | -- | -- | -- | -- |
| Hemolytic disease and other neonatal jaundice | 14.5<br>(12.1 to 17.0) | -- | 6.1<br>(4.6 to 7.8) | 44.1*<br>(38.6 to 49.2) | 17.8*<br>(13.4 to 22.1) |

| | | | | | |
|---|---|---|---|---|---|
| Other neonatal disorders | -- | -- | -- | -- | -- |
| **Nutritional deficiencies** | **30620.5 (29119.6 to 32266.1)** | **16953.7 (15073.5 to 18923.9)** | **479.1 (326.7 to 686.6)** | **4.4 (-2.7 to 11.9)** | **-14.6* (-20.4 to -8.4)** |
| Protein-energy malnutrition | 1908.6 (1788.9 to 2026.8) | 1818.1 (1681.3 to 1955.0) | 8.2 (5.3 to 11.3) | 33.7* (19.0 to 45.7) | 9.4 (-2.7 to 19.2) |
| Iodine deficiency | 2913.9 (2646.5 to 3224.1) | 10.5 (8.8 to 12.9) | 42.4 (25.4 to 68.6) | 12.2* (8.7 to 16.1) | -8.2* (-11.1 to -5.0) |
| Visible goiter without symptoms | 2867.3 (2604.7 to 3178.3) | 10.5 (8.8 to 12.9) | 29.1 (14.3 to 54.1) | 10.3* (8.7 to 11.8) | -9.8* (-11.1 to -8.5) |
| Visible goiter with complications | 46.6 (28.5 to 64.8) | -- | 13.3 (7.2 to 20.7) | 16.5* (4.7 to 25.5) | -4.7 (-14.4 to 2.7) |
| Vitamin A deficiency | 9118.1 (8181.8 to 10142.5) | 15125.1 (13246.1 to 17118.8) | 2.7 (1.7 to 4.0) | 26.0* (15.7 to 36.5) | 3.0 (-5.4 to 11.7) |
| Asymptomatic vitamin A deficiency | 9075.8 (8138.5 to 10084.5) | 15125.1 (13246.1 to 17118.8) | -- | -- | -- |
| Vitamin A deficiency complications | 42.4 (29.3 to 57.8) | -- | 2.7 (1.7 to 4.0) | 26.0* (15.7 to 36.5) | 3.0 (-5.4 to 11.7) |
| Vitamin A deficiency with anemia | -- | -- | -- | -- | -- |
| Dietary iron deficiency | 20449.2 (18969.4 to 21996.3) | -- | 422.4 (286.7 to 611.1) | 2.9 (-4.8 to 11.4) | -15.8* (-22.1 to -8.9) |
| Other nutritional deficiencies | -- | -- | 3.4 (2.2 to 4.7) | 41.9* (26.3 to 54.6) | 16.1* (3.3 to 26.5) |
| **Non-communicable diseases** | **167776.3 (167708.5 to 167835.2)** | **325189.0 (296051.0 to 353156.6)** | **35113.5 (26822.1 to 44613.3)** | **22.4* (21.3 to 23.4)** | **0.1 (-0.8 to 1.0)** |
| **Neoplasms** | **10197.6 (9866.2 to 10699.5)** | **2993.6 (2508.9 to 3621.5)** | **842.4 (628.7 to 1083.9)** | **33.4* (30.4 to 36.7)** | **9.1* (6.7 to 11.8)** |
| Lip and oral cavity cancer | 151.2 (144.6 to 157.5) | 38.0 (36.2 to 39.7) | 15.4 (11.4 to 19.7) | 36.0* (28.0 to 44.7) | 11.3* (4.7 to 18.4) |
| Diagnosis and primary therapy phase of mouth cancer | 9.1 (8.7 to 9.5) | 38.0 (36.2 to 39.7) | 2.5 (1.7 to 3.4) | 36.6* (25.0 to 49.3) | 11.8* (2.3 to 22.1) |
| Controlled phase of mouth cancer | 124.5 (119.1 to 129.6) | -- | 5.5 (3.6 to 7.9) | 39.1* (31.3 to 47.6) | 13.8* (7.4 to 20.7) |
| Metastatic phase of mouth cancer | 15.3 (14.6 to 16.0) | -- | 6.1 (4.3 to 8.1) | 33.4* (20.1 to 49.3) | 9.1 (-1.8 to 22.1) |
| Terminal phase of mouth cancer | 2.3 (2.2 to 2.4) | -- | 1.2 (0.9 to 1.6) | 34.7* (28.4 to 41.0) | 10.2* (5.0 to 15.3) |
| Nasopharynx cancer | 22.1 (21.0 to 23.2) | 6.3 (6.0 to 6.6) | 2.7 (1.9 to 3.5) | 16.3* (8.1 to 25.2) | -4.9 (-11.6 to 2.4) |
| Diagnosis and primary therapy phase of nasopharynx cancer | 1.3 (1.2 to 1.4) | 6.3 (6.0 to 6.6) | 0.4 (0.2 to 0.5) | 16.2* (10.7 to 22.1) | -5.0* (-9.5 to -0.1) |
| Controlled phase of nasopharynx cancer | 17.4 (16.6 to 18.3) | -- | 0.8 (0.5 to 1.2) | 17.5* (4.2 to 32.4) | -3.9 (-14.8 to 8.3) |
| Metastatic phase of nasopharynx cancer | 3.0 (2.8 to 3.1) | -- | 1.3 (0.9 to 1.8) | 15.6* (4.2 to 28.7) | -5.4 (-14.7 to 5.3) |
| Terminal phase of nasopharynx cancer | 0.4 (0.4 to 0.4) | -- | 0.2 (0.1 to 0.3) | 15.9* (10.6 to 21.7) | -5.2* (-9.6 to -0.5) |
| Other pharynx cancer | 39.7 (36.0 to 42.0) | 18.1 (16.2 to 19.2) | 5.7 (4.1 to 7.4) | 43.3* (30.0 to 55.7) | 17.2* (6.4 to 27.4) |
| Diagnosis and primary therapy phase of other pharynx cancer | 2.3 (2.0 to 2.4) | 18.1 (16.2 to 19.2) | 0.6 (0.4 to 0.9) | 47.7* (36.7 to 58.1) | 20.8* (11.8 to 29.4) |
| Controlled phase of other pharynx cancer | 28.8 (26.2 to 30.4) | -- | 1.3 (0.8 to 1.9) | 48.6* (33.9 to 63.2) | 21.5* (9.5 to 33.5) |
| Metastatic phase of other pharynx cancer | 7.3 (6.5 to 7.8) | -- | 3.0 (2.0 to 4.0) | 40.2* (24.0 to 58.3) | 14.7* (1.5 to 29.5) |
| Terminal phase of other pharynx cancer | 1.3 (1.2 to 1.4) | -- | 0.7 (0.5 to 0.9) | 43.6* (31.7 to 54.3) | 17.4* (7.7 to 26.2) |
| Oesophageal cancer | 109.8 (104.3 to 116.3) | 68.0 (65.2 to 71.5) | 18.0 (12.8 to 23.3) | 13.6* (4.0 to 24.6) | -7.1 (-14.9 to 1.9) |
| Diagnosis and primary therapy phase of esophageal cancer | 9.2 (8.7 to 9.8) | 68.0 (65.2 to 71.5) | 2.5 (1.6 to 3.5) | 22.5* (3.7 to 46.8) | 0.2 (-15.2 to 20.1) |
| Controlled phase of esophageal cancer | 71.0 (67.0 to 75.6) | -- | 3.2 (2.1 to 4.7) | 27.4* (14.3 to 40.8) | 4.3 (-6.5 to 15.2) |
| Metastatic phase of esophageal cancer | 24.3 (23.4 to 25.6) | -- | 9.6 (6.6 to 12.8) | 8.9 (-4.3 to 22.7) | -10.9 (-21.7 to 0.4) |
| Terminal phase of esophageal cancer | 5.3 (5.1 to 5.6) | -- | 2.7 (1.9 to 3.6) | 9.3 (-7.8 to 29.6) | -10.6 (-24.6 to 6.1) |
| Stomach cancer | 380.0 (362.7 to 399.4) | 166.1 (159.2 to 173.6) | 46.4 (34.0 to 59.0) | 23.1* (14.8 to 31.6) | 0.7 (-6.1 to 7.7) |
| Diagnosis and primary therapy phase of stomach cancer | 36.5 (34.9 to 38.3) | 166.1 (159.2 to 173.6) | 9.3 (6.2 to 12.6) | 30.8* (18.9 to 44.3) | 7.0 (-2.7 to 18.0) |
| Controlled phase of stomach cancer | 284.2 (270.7 to 299.3) | -- | 12.8 (8.3 to 18.5) | 46.6* (38.2 to 56.3) | 20.0* (13.1 to 27.8) |
| Metastatic phase of stomach cancer | 47.2 (45.2 to 49.3) | -- | 18.3 (12.8 to 23.9) | 11.4* (1.4 to 21.6) | -8.9* (-17.1 to -0.5) |
| Terminal phase of stomach cancer | 12.2 (11.7 to 12.7) | -- | 6.0 (4.2 to 7.8) | 10.9 (-2.5 to 26.4) | -9.3 (-20.2 to 3.4) |
| Colon and rectum cancer | 1275.5 (1241.3 to 1313.2) | 241.2 (234.7 to 248.3) | 116.2 (86.2 to 149.0) | 28.5* (23.6 to 33.3) | 5.1* (1.1 to 9.1) |
| Diagnosis and primary therapy phase of colon and rectum cancers | 54.9 (53.4 to 56.6) | 241.2 (234.7 to 248.3) | 13.7 (9.5 to 18.4) | 32.7* (23.4 to 42.6) | 8.5* (0.9 to 16.7) |
| Controlled phase of colon and rectum cancers | 1089.6 (1059.4 to 1122.4) | -- | 60.8 (41.3 to 84.8) | 32.5* (27.8 to 37.9) | 8.4* (4.5 to 12.8) |
| Metastatic phase of colon and rectum cancers | 85.9 (83.6 to 88.4) | -- | 32.8 (22.5 to 42.6) | 22.5* (15.0 to 30.7) | 0.2 (-6.0 to 6.9) |
| Terminal phase of colon and rectum cancers | 12.0 (11.6 to 12.3) | -- | 6.0 (4.3 to 7.7) | 19.7* (9.9 to 30.1) | -2.1 (-10.1 to 6.4) |
| Stoma from colon and rectum cancers, beyond 10 years | 33.2 (30.9 to 35.8) | -- | 2.9 (1.9 to 3.9) | 19.3* (10.3 to 29.2) | -2.4 (-9.7 to 5.7) |
| Liver cancer | 76.2 (69.7 to 83.1) | 104.3 (99.7 to 109.8) | 23.9 (17.1 to 31.3) | 25.2* (18.6 to 32.8) | 2.4 (-3.0 to 8.7) |
| Liver cancer due to hepatitis B | 45.7 (41.0 to 51.7) | 35.2 (31.4 to 39.6) | 7.8 (5.4 to 10.4) | 27.1* (14.3 to 42.2) | 4.0 (-6.5 to 16.4) |
| Diagnosis and primary therapy phase of liver cancer due to hepatitis B | 8.2 (7.3 to 9.2) | 35.2 (31.4 to 39.6) | 2.2 (1.4 to 3.1) | 24.4* (4.9 to 49.7) | 1.8 (-14.2 to 22.5) |
| Controlled phase of liver cancer due to hepatitis B | 27.7 (24.7 to 31.2) | -- | 1.3 (0.8 to 1.9) | 70.4* (47.5 to 97.5) | 39.4* (20.6 to 61.6) |
| Metastatic phase of liver cancer due to hepatitis B | 7.0 (6.3 to 7.9) | -- | 2.9 (1.9 to 4.0) | 19.6* (0.6 to 41.4) | -2.2 (-17.7 to 15.7) |
| Terminal phase of liver cancer due to hepatitis B | 2.8 (2.5 to 3.2) | -- | 1.5 (1.0 to 2.0) | 19.5* (4.3 to 37.3) | -2.2 (-14.7 to 12.3) |
| Liver cancer due to hepatitis C | 43.5 (39.8 to 47.5) | 32.8 (29.9 to 36.1) | 7.6 (5.3 to 10.1) | 19.3* (13.0 to 26.8) | -2.4 (-7.5 to 3.8) |
| Diagnosis and primary therapy phase of liver cancer due to hepatitis C | 7.6 (7.0 to 8.4) | 32.8 (29.9 to 36.1) | 2.1 (1.4 to 3.0) | 18.3* (7.8 to 29.8) | -3.2 (-11.8 to 6.2) |
| Controlled phase of liver cancer due to hepatitis C | 26.7 (24.4 to 29.2) | -- | 1.2 (0.8 to 1.8) | 32.8* (20.1 to 47.7) | 8.6 (-1.7 to 20.8) |
| Metastatic phase of liver cancer due to hepatitis C | 6.5 (6.0 to 7.2) | -- | 2.8 (1.9 to 3.8) | 16.3* (6.7 to 27.1) | -4.9 (-12.7 to 4.0) |
| Terminal phase of liver cancer due to hepatitis C | 2.6 (2.4 to 2.9) | -- | 1.4 (1.0 to 1.8) | 16.6* (11.2 to 22.0) | -4.6* (-9.0 to -0.2) |
| Liver cancer due to alcohol use | 25.3 (21.2 to 30.0) | 20.0 (16.8 to 23.8) | 4.7 (3.2 to 6.4) | 25.4* (17.8 to 33.0) | 2.6 (-3.6 to 8.8) |
| Diagnosis and primary therapy phase of liver cancer due to alcohol use | 4.6 (3.9 to 5.5) | 20.0 (16.8 to 23.8) | 1.3 (0.9 to 1.9) | 24.9* (12.8 to 37.7) | 2.2 (-7.7 to 12.6) |
| Controlled phase of liver cancer due to alcohol use | 15.0 (12.7 to 17.8) | -- | 0.7 (0.4 to 1.0) | 41.7* (24.9 to 61.2) | 15.9* (2.2 to 31.9) |
| Metastatic phase of liver cancer due to alcohol use | 4.0 (3.4 to 4.8) | -- | 1.8 (1.2 to 2.5) | 22.1* (13.6 to 31.6) | -0.1 (-7.1 to 7.7) |

| | | | | | |
|---|---|---|---|---|---|
| Terminal phase of liver cancer due to alcohol use | 1.6 (1.3 to 1.9) | -- | 0.9 (0.6 to 1.2) | 21.7* (16.4 to 27.5) | -0.4 (-4.8 to 4.3) |
| Liver cancer due to NASH | 11.4 (9.3 to 14.1) | 9.3 (7.6 to 11.5) | 2.2 (1.5 to 3.0) | 36.7* (30.6 to 43.9) | 11.8* (6.8 to 17.7) |
| Diagnosis and primary therapy phase of liver cancer due to NASH | 2.1 (1.7 to 2.6) | 9.3 (7.6 to 11.5) | 0.6 (0.4 to 0.9) | 35.9* (30.3 to 42.6) | 11.1* (6.6 to 16.6) |
| Controlled phase of liver cancer due to NASH | 6.7 (5.5 to 8.2) | -- | 0.3 (0.2 to 0.5) | 57.5* (39.1 to 77.8) | 28.8* (13.8 to 45.4) |
| Metastatic phase of liver cancer due to NASH | 1.9 (1.5 to 2.3) | -- | 0.8 (0.6 to 1.2) | 32.7* (27.4 to 39.0) | 8.5* (4.2 to 13.7) |
| Terminal phase of liver cancer due to NASH | 0.7 (0.6 to 0.9) | -- | 0.4 (0.3 to 0.6) | 32.7* (27.4 to 39.1) | 8.5* (4.2 to 13.8) |
| Liver cancer due to other causes | 8.7 (7.0 to 10.5) | 6.9 (5.6 to 8.4) | 1.6 (1.1 to 2.3) | 30.5* (23.4 to 39.6) | 6.7* (1.0 to 14.2) |
| Diagnosis and primary therapy phase of liver cancer due to other causes | 1.6 (1.3 to 1.9) | 6.9 (5.6 to 8.4) | 0.5 (0.3 to 0.7) | 29.4* (23.0 to 37.2) | 5.8* (0.7 to 12.2) |
| Controlled phase of liver cancer due to other causes | 5.1 (4.1 to 6.2) | -- | 0.2 (0.1 to 0.4) | 57.3* (35.0 to 83.5) | 28.7* (10.4 to 50.1) |
| Metastatic phase of liver cancer due to other causes | 1.4 (1.1 to 1.7) | -- | 0.6 (0.4 to 0.9) | 25.4* (19.2 to 32.8) | 2.6 (-2.5 to 8.6) |
| Terminal phase of liver cancer due to other causes | 0.6 (0.4 to 0.7) | -- | 0.3 (0.2 to 0.4) | 25.4* (19.2 to 32.8) | 2.6 (-2.5 to 8.6) |
| Gallbladder and biliary tract cancer | 30.9 (27.0 to 35.4) | 28.2 (24.5 to 31.1) | 6.6 (4.6 to 8.9) | 21.4* (12.0 to 33.3) | -0.7 (-8.4 to 9.1) |
| Diagnosis and primary therapy phase of gallbladder and biliary tract cancer | 4.5 (3.9 to 5.0) | 28.2 (24.5 to 31.1) | 1.3 (0.8 to 1.8) | 22.4* (11.3 to 37.4) | 0.1 (-9.0 to 12.4) |
| Controlled phase of gallbladder and biliary tract cancer | 16.4 (14.1 to 19.5) | -- | 0.7 (0.5 to 1.1) | 40.7* (17.6 to 71.9) | 15.1 (-3.8 to 40.6) |
| Metastatic phase of gallbladder and biliary tract cancer | 7.8 (6.8 to 8.6) | -- | 3.4 (2.3 to 4.6) | 18.7* (8.3 to 30.7) | -2.9 (-11.4 to 6.9) |
| Terminal phase of gallbladder and biliary tract cancer | 2.3 (2.0 to 2.5) | -- | 1.2 (0.8 to 1.6) | 18.1* (10.3 to 28.9) | -3.4 (-9.7 to 5.5) |
| Pancreatic cancer | 49.0 (47.4 to 50.5) | 63.4 (61.7 to 65.1) | 12.7 (8.9 to 16.5) | 31.4* (24.3 to 39.3) | 7.5* (1.7 to 13.9) |
| Diagnosis and primary therapy phase of pancreatic cancer | 13.8 (13.4 to 14.2) | 63.4 (61.7 to 65.1) | 3.6 (2.5 to 5.0) | 31.2* (18.8 to 45.1) | 7.3 (-2.8 to 18.7) |
| Controlled phase of pancreatic cancer | 16.7 (16.0 to 17.5) | -- | 0.8 (0.5 to 1.1) | 33.7* (20.2 to 48.3) | 9.4 (-1.6 to 21.3) |
| Metastatic phase of pancreatic cancer | 13.2 (12.9 to 13.6) | -- | 5.5 (3.7 to 7.3) | 31.1* (19.1 to 44.6) | 7.2 (-2.6 to 18.3) |
| Terminal phase of pancreatic cancer | 5.2 (5.1 to 5.4) | -- | 2.8 (2.0 to 3.6) | 31.8* (27.7 to 35.6) | 7.8* (4.4 to 11.0) |
| Larynx cancer | 126.8 (123.3 to 130.9) | 25.3 (24.5 to 26.1) | 12.6 (9.3 to 16.3) | 29.4* (22.4 to 37.0) | 5.9* (0.2 to 12.1) |
| Diagnosis and primary therapy phase of larynx cancer | 7.6 (7.4 to 7.9) | 25.3 (24.5 to 26.1) | 2.1 (1.4 to 2.9) | 33.6* (19.3 to 49.1) | 9.3 (-2.4 to 22.0) |
| Controlled phase of larynx cancer | 99.3 (96.2 to 102.8) | -- | 5.2 (3.4 to 7.5) | 33.6* (25.8 to 41.6) | 9.3* (2.9 to 15.8) |
| Metastatic phase of larynx cancer | 10.1 (9.8 to 10.4) | -- | 4.1 (2.8 to 5.5) | 25.4* (12.3 to 40.4) | 2.6 (-8.1 to 14.8) |
| Terminal phase of larynx cancer | 1.4 (1.4 to 1.5) | -- | 0.8 (0.5 to 1.0) | 22.1* (17.9 to 26.0) | -0.2 (-3.6 to 3.1) |
| Laryngectomy from larynx cancer, beyond 10 years | 8.4 (8.0 to 8.8) | -- | 0.4 (0.3 to 0.6) | 14.7* (2.1 to 29.4) | -6.2 (-16.5 to 5.8) |
| Tracheal, bronchus, and lung cancer | 501.2 (486.3 to 517.7) | 326.7 (317.8 to 336.9) | 79.3 (58.1 to 100.6) | 34.0* (27.7 to 40.8) | 9.6* (4.5 to 15.1) |
| Diagnosis and primary therapy phase of lung, bronchus, and trachea cancer | 33.1 (32.1 to 34.2) | 326.7 (317.8 to 336.9) | 8.4 (5.9 to 11.3) | 46.0* (33.8 to 60.5) | 19.5* (9.4 to 31.3) |
| Controlled phase of lung, bronchus, and trachea cancer | 326.6 (316.3 to 337.9) | -- | 14.6 (9.5 to 21.0) | 63.8* (56.8 to 71.4) | 34.0* (28.2 to 40.2) |
| Metastatic phase of lung, bronchus, and trachea cancer | 115.9 (112.7 to 119.5) | -- | 44.4 (31.0 to 57.8) | 26.6* (19.8 to 33.9) | 3.6 (-2.0 to 9.5) |
| Terminal phase of lung, bronchus, and trachea cancer | 25.7 (25.0 to 26.5) | -- | 12.0 (8.4 to 15.2) | 25.8* (13.4 to 40.3) | 2.9 (-7.2 to 14.8) |
| Malignant skin melanoma | 221.5 (148.2 to 247.7) | 29.8 (20.3 to 33.2) | 13.4 (7.9 to 19.1) | 46.8* (35.5 to 56.8) | 20.1* (10.8 to 28.3) |
| Diagnosis and primary therapy phase of malignant skin melanoma | 6.2 (4.1 to 7.0) | 29.8 (20.3 to 33.2) | 1.6 (0.9 to 2.3) | 47.9* (27.4 to 70.5) | 21.0* (4.2 to 39.5) |
| Controlled phase of malignant skin melanoma | 209.6 (140.0 to 234.6) | -- | 9.3 (5.2 to 14.1) | 52.0* (40.4 to 62.8) | 24.4* (14.8 to 33.2) |
| Metastatic phase of malignant skin melanoma | 4.8 (3.3 to 5.4) | -- | 2.0 (1.2 to 2.8) | 29.2* (16.0 to 43.9) | 5.7 (-5.1 to 17.7) |
| Terminal phase of malignant skin melanoma | 0.8 (0.5 to 0.9) | -- | 0.4 (0.3 to 0.6) | 29.5* (21.5 to 36.8) | 5.9 (-0.6 to 11.9) |
| Non-melanoma skin cancer | 396.3 (221.2 to 631.9) | 1057.2 (573.4 to 1672.1) | 13.9 (6.4 to 25.7) | 46.3* (33.3 to 57.1) | 19.7* (9.0 to 28.5) |
| Non-melanoma skin cancer (squamous-cell carcinoma) | 333.1 (160.5 to 568.7) | 272.4 (135.0 to 440.1) | 13.5 (6.1 to 25.3) | 46.4* (33.1 to 57.7) | 19.8* (8.9 to 29.0) |
| Non-melanoma skin cancer (basal-cell carcinoma) | 78.3 (31.9 to 146.0) | 784.9 (337.1 to 1378.7) | 0.3 (0.1 to 0.8) | 39.4* (24.1 to 49.0) | 14.0* (1.5 to 21.9) |
| Breast cancer | 1350.0 (1301.0 to 1392.9) | 153.6 (147.4 to 159.6) | 104.1 (74.9 to 140.0) | 33.3* (27.6 to 38.3) | 9.1* (4.4 to 13.2) |
| Diagnosis and primary therapy phase of breast cancer | 31.6 (30.3 to 32.8) | 153.6 (147.4 to 159.6) | 7.9 (5.4 to 10.6) | 37.3* (24.7 to 50.9) | 12.3* (2.0 to 23.5) |
| Controlled phase of breast cancer | 1000.5 (958.7 to 1038.7) | -- | 62.0 (40.0 to 89.3) | 35.3* (29.5 to 40.9) | 10.7* (5.9 to 15.2) |
| Metastatic phase of breast cancer | 61.6 (59.0 to 64.2) | -- | 23.5 (16.3 to 30.5) | 31.6* (22.1 to 40.9) | 7.7 (-0.1 to 15.3) |
| Terminal phase of breast cancer | 4.8 (4.6 to 5.0) | -- | 2.5 (1.8 to 3.2) | 28.6* (20.4 to 37.2) | 5.2 (-1.5 to 12.2) |
| Mastectomy from breast cancer, beyond 10 years | 251.5 (237.1 to 266.8) | -- | 8.2 (4.8 to 12.6) | 22.2* (17.6 to 26.5) | -0.0 (-3.8 to 3.5) |
| Cervical cancer | 111.4 (104.8 to 116.4) | 25.4 (23.8 to 26.6) | 10.3 (7.5 to 13.5) | 15.2* (7.6 to 22.6) | -5.8 (-12.0 to 0.3) |
| Diagnosis and primary therapy phase of cervical cancer | 5.9 (5.5 to 6.2) | 25.4 (23.8 to 26.6) | 1.6 (1.1 to 2.2) | 16.0* (6.4 to 25.9) | -5.1 (-13.0 to 3.0) |
| Controlled phase of cervical cancer | 95.0 (89.3 to 99.2) | -- | 4.2 (2.7 to 6.1) | 16.3* (8.8 to 23.9) | -4.9 (-11.0 to 1.3) |
| Metastatic phase of cervical cancer | 9.2 (8.6 to 9.6) | -- | 3.7 (2.6 to 4.9) | 14.1* (2.3 to 27.1) | -6.7 (-16.4 to 4.0) |
| Terminal phase of cervical cancer | 1.4 (1.3 to 1.5) | -- | 0.8 (0.5 to 1.0) | 12.9* (7.6 to 17.8) | -7.6* (-12.0 to -3.6) |
| Uterine cancer | 286.1 (274.2 to 298.9) | 39.6 (38.1 to 41.3) | 19.9 (14.5 to 26.6) | 33.2* (25.6 to 41.1) | 9.0* (2.7 to 15.5) |
| Diagnosis and primary therapy phase of uterine cancer | 13.3 (12.8 to 13.9) | 39.6 (38.1 to 41.3) | 3.4 (2.3 to 4.6) | 34.4* (18.0 to 54.0) | 9.9 (-3.5 to 26.0) |
| Controlled phase of uterine cancer | 260.9 (250.0 to 272.7) | -- | 11.6 (7.6 to 16.7) | 36.2* (28.8 to 44.1) | 11.4* (5.4 to 17.9) |
| Metastatic phase of uterine cancer | 10.7 (10.3 to 11.1) | -- | 4.3 (3.0 to 5.7) | 26.2* (10.5 to 44.7) | 3.2 (-9.6 to 18.3) |
| Terminal phase of uterine cancer | 1.2 (1.1 to 1.2) | -- | 0.6 (0.4 to 0.8) | 23.9* (19.3 to 28.8) | 1.3 (-2.4 to 5.4) |
| Ovarian cancer | 76.4 (74.2 to 79.2) | 23.0 (22.3 to 23.8) | 11.0 (8.2 to 14.0) | 21.0* (12.0 to 30.8) | -1.1 (-8.3 to 7.0) |
| Diagnosis and primary therapy phase of ovarian cancer | 2.6 (2.6 to 2.7) | 23.0 (22.3 to 23.8) | 0.8 (0.5 to 1.0) | 22.5* (18.5 to 27.0) | 0.2 (-3.0 to 3.9) |
| Controlled phase of ovarian cancer | 53.7 (52.1 to 55.6) | -- | 2.4 (1.5 to 3.5) | 23.0* (14.3 to 32.6) | 0.6 (-6.5 to 8.5) |
| Metastatic phase of ovarian cancer | 18.5 (18.0 to 19.3) | -- | 7.0 (4.9 to 9.1) | 19.9* (7.9 to 33.8) | -1.9 (-11.7 to 9.5) |

| | | | | | |
|---|---|---|---|---|---|
| Terminal phase of ovarian cancer | 1.5 (1.5 to 1.6) | -- | 0.8 (0.6 to 1.1) | 23.2* (19.1 to 27.7) | 0.8 (-2.6 to 4.5) |
| Prostate cancer | 1942.7 (1752.5 to 2585.1) | 248.5 (221.8 to 327.8) | 160.8 (114.8 to 222.7) | 34.6* (26.7 to 47.3) | 10.1* (3.6 to 20.5) |
| Diagnosis and primary therapy phase of prostate cancer | 68.5 (61.6 to 91.9) | 248.5 (221.8 to 327.8) | 16.9 (11.2 to 24.2) | 38.0* (26.1 to 54.2) | 12.9* (3.2 to 26.1) |
| Controlled phase of prostate cancer | 1693.0 (1524.2 to 2276.9) | -- | 92.9 (60.2 to 138.3) | 38.5* (30.2 to 51.9) | 13.3* (6.5 to 24.3) |
| Metastatic phase of prostate cancer | 119.4 (106.2 to 153.5) | -- | 45.3 (30.8 to 62.3) | 26.8* (18.8 to 38.8) | 3.7 (-2.8 to 13.6) |
| Terminal phase of prostate cancer | 6.4 (5.6 to 8.0) | -- | 3.1 (2.1 to 4.2) | 25.7* (8.3 to 43.7) | 2.8 (-11.4 to 17.6) |
| Impotence and Incontinence after 10-year survival from prostate cancer | 55.4 (53.4 to 57.5) | -- | 2.5 (1.7 to 3.5) | 30.4* (20.1 to 42.1) | 6.7 (-1.7 to 16.3) |
| Testicular cancer | 4.0 (3.8 to 4.3) | 0.8 (0.8 to 0.9) | 0.4 (0.3 to 0.5) | 36.8* (27.3 to 47.6) | 11.9* (4.1 to 20.7) |
| Diagnosis and primary therapy phase of testicular cancer | 0.1 (0.1 to 0.1) | 0.8 (0.8 to 0.9) | 0.0 (0.0 to 0.1) | 39.4* (30.0 to 49.2) | 14.1* (6.4 to 22.1) |
| Controlled phase of testicular cancer | 3.5 (3.3 to 3.8) | -- | 0.2 (0.1 to 0.3) | 41.2* (30.1 to 53.4) | 15.5* (6.4 to 25.5) |
| Metastatic phase of testicular cancer | 0.3 (0.3 to 0.3) | -- | 0.1 (0.1 to 0.2) | 31.7* (22.8 to 40.9) | 7.7* (0.4 to 15.3) |
| Terminal phase of testicular cancer | -- | -- | -- | 35.0* (25.9 to 44.3) | 10.4* (3.0 to 18.1) |
| Kidney cancer | 209.4 (197.0 to 217.8) | 43.0 (40.4 to 44.7) | 14.5 (10.5 to 19.0) | 22.7* (16.3 to 30.3) | 0.4 (-4.9 to 6.6) |
| Diagnosis and primary therapy phase of kidney cancer | -- | 43.0 (40.4 to 44.7) | -- | -- | -- |
| Controlled phase of kidney cancer | 196.1 (184.6 to 203.9) | -- | 8.7 (5.7 to 12.5) | 24.9* (18.2 to 32.6) | 2.2 (-3.3 to 8.5) |
| Metastatic phase of kidney cancer | 11.2 (10.5 to 11.6) | -- | 4.6 (3.2 to 6.1) | 19.8* (8.6 to 33.4) | -2.0 (-11.2 to 9.1) |
| Terminal phase of kidney cancer | 2.2 (2.0 to 2.2) | -- | 1.2 (0.8 to 1.5) | 18.7* (13.8 to 24.5) | -2.9 (-6.9 to 1.8) |
| Bladder cancer | 370.0 (356.0 to 385.0) | 65.3 (63.0 to 68.6) | 34.2 (24.6 to 44.3) | 23.6* (18.4 to 29.0) | 1.1 (-3.2 to 5.5) |
| Diagnosis and primary therapy phase of bladder cancer | 19.8 (19.1 to 20.7) | 65.3 (63.0 to 68.6) | 5.1 (3.5 to 6.9) | 26.0* (13.8 to 40.2) | 3.1 (-6.9 to 14.7) |
| Controlled phase of bladder cancer | 318.8 (306.8 to 332.0) | -- | 19.2 (13.2 to 26.8) | 27.1* (21.3 to 33.2) | 4.0 (-0.8 to 9.0) |
| Metastatic phase of bladder cancer | 16.6 (16.1 to 17.7) | -- | 6.6 (4.6 to 8.7) | 15.6* (5.1 to 27.5) | -5.4 (-14.0 to 4.3) |
| Terminal phase of bladder cancer | 3.1 (3.0 to 3.3) | -- | 1.7 (1.2 to 2.1) | 15.3* (11.9 to 19.2) | -5.7* (-8.4 to -2.5) |
| Incontinence from bladder cancer, beyond 10 years | 11.7 (11.0 to 12.4) | -- | 1.5 (1.0 to 2.2) | 20.1* (7.9 to 34.0) | -1.7 (-11.7 to 9.6) |
| Brain and nervous system cancer | 82.0 (63.2 to 90.4) | 26.9 (21.5 to 29.4) | 9.7 (6.6 to 13.1) | 48.3* (37.3 to 59.6) | 21.3* (12.3 to 30.6) |
| Diagnosis and primary therapy phase of brain and nervous system cancers | 5.2 (4.0 to 5.7) | 26.9 (21.5 to 29.4) | 1.4 (0.9 to 2.1) | 58.3* (42.9 to 72.5) | 29.5* (16.9 to 41.1) |
| Controlled phase of brain and nervous system cancers | 65.0 (49.6 to 71.7) | -- | 2.9 (1.8 to 4.3) | 63.0* (46.2 to 79.3) | 33.4* (19.6 to 46.7) |
| Metastatic phase of brain and nervous system cancers | 10.1 (8.1 to 11.0) | -- | 4.4 (2.8 to 6.0) | 39.2* (28.9 to 50.8) | 13.9* (5.4 to 23.3) |
| Terminal phase of brain and nervous system cancers | 1.8 (1.4 to 2.0) | -- | 1.0 (0.6 to 1.3) | 38.5* (31.1 to 45.5) | 13.3* (7.3 to 19.0) |
| Thyroid cancer | 114.9 (109.8 to 121.5) | 15.2 (14.5 to 16.0) | 8.0 (5.7 to 10.7) | 33.7* (27.1 to 40.3) | 9.3* (4.0 to 14.8) |
| Diagnosis and primary therapy phase of thyroid cancer | 3.2 (3.0 to 3.4) | 15.2 (14.5 to 16.0) | 0.9 (0.6 to 1.3) | 35.9* (30.5 to 41.6) | 11.2* (6.7 to 15.8) |
| Controlled phase of thyroid cancer | 106.7 (101.9 to 112.8) | -- | 4.8 (3.1 to 6.9) | 36.8* (28.3 to 45.9) | 11.9* (5.0 to 19.4) |
| Metastatic phase of thyroid cancer | 4.7 (4.5 to 4.9) | -- | 2.1 (1.4 to 2.8) | 26.8* (21.6 to 32.0) | 3.8 (-0.5 to 8.0) |
| Terminal phase of thyroid cancer | 0.4 (0.4 to 0.4) | -- | 0.2 (0.1 to 0.3) | 25.6* (20.3 to 30.7) | 2.7 (-1.6 to 6.9) |
| Mesothelioma | 11.0 (10.5 to 11.6) | 4.8 (4.6 to 5.0) | 2.1 (1.4 to 2.7) | 21.7* (8.2 to 36.7) | -0.4 (-11.5 to 11.8) |
| Diagnosis and primary therapy phase of mesothelioma | 1.3 (1.2 to 1.4) | 4.8 (4.6 to 5.0) | 0.4 (0.2 to 0.5) | 23.4* (10.9 to 37.5) | 1.0 (-9.3 to 12.5) |
| Controlled phase of mesothelioma | 6.6 (6.2 to 6.9) | -- | 0.3 (0.2 to 0.4) | 23.5* (3.9 to 46.7) | 1.1 (-15.0 to 20.0) |
| Metastatic phase of mesothelioma | 2.8 (2.7 to 2.9) | -- | 1.2 (0.8 to 1.6) | 20.7* (0.8 to 43.2) | -1.2 (-17.5 to 17.2) |
| Terminal phase of mesothelioma | 0.4 (0.4 to 0.4) | -- | 0.2 (0.1 to 0.3) | 22.1* (14.7 to 29.5) | -0.2 (-6.2 to 5.9) |
| Hodgkin lymphoma | 21.0 (17.2 to 24.0) | 4.3 (3.5 to 5.0) | 2.1 (1.4 to 2.8) | 24.8* (14.9 to 34.3) | 2.1 (-6.0 to 9.9) |
| Diagnosis and primary therapy phase of Hodgkin disease | 0.7 (0.6 to 0.8) | 4.3 (3.5 to 5.0) | 0.2 (0.1 to 0.3) | 36.6* (27.8 to 44.7) | 11.7* (4.5 to 18.4) |
| Controlled phase of Hodgkin disease | 18.0 (14.7 to 20.6) | -- | 0.8 (0.5 to 1.2) | 37.3* (20.2 to 54.4) | 12.3 (-1.7 to 26.3) |
| Metastatic phase of Hodgkin disease | 2.1 (1.7 to 2.5) | -- | 1.0 (0.6 to 1.3) | 16.1* (8.5 to 23.0) | -5.0 (-11.2 to 0.6) |
| Terminal phase of Hodgkin disease | 0.2 (0.2 to 0.2) | -- | 0.1 (0.1 to 0.1) | 4.9 (-1.5 to 10.6) | -14.2* (-19.4 to -9.5) |
| Non-Hodgkin's lymphoma | 249.3 (240.1 to 257.7) | 52.6 (50.9 to 54.2) | 20.3 (14.8 to 26.5) | 40.8* (33.5 to 48.2) | 15.2* (9.2 to 21.2) |
| Diagnosis and primary therapy phase of non-Hodgkin lymphoma | 9.4 (9.0 to 9.7) | 52.6 (50.9 to 54.2) | 2.5 (1.7 to 3.4) | 45.0* (26.9 to 65.5) | 18.6* (3.8 to 35.4) |
| Controlled phase of non-Hodgkin lymphoma | 221.3 (212.9 to 228.9) | -- | 9.9 (6.5 to 14.2) | 47.1* (39.7 to 55.1) | 20.4* (14.3 to 26.9) |
| Metastatic phase of non-Hodgkin lymphoma | 15.9 (15.4 to 16.4) | -- | 6.5 (4.5 to 8.5) | 32.8* (20.6 to 46.9) | 8.6 (-1.4 to 20.1) |
| Terminal phase of non-Hodgkin lymphoma | 2.7 (2.6 to 2.8) | -- | 1.5 (1.0 to 1.9) | 32.0* (27.6 to 36.2) | 7.9* (4.4 to 11.5) |
| Multiple myeloma | 54.4 (48.1 to 63.7) | 20.5 (18.5 to 23.6) | 11.1 (7.9 to 14.4) | 39.1* (27.6 to 50.2) | 13.8* (4.4 to 22.9) |
| Diagnosis and primary therapy phase of multiple myeloma | 3.3 (3.0 to 3.9) | 20.5 (18.5 to 23.6) | 0.9 (0.6 to 1.3) | 46.3* (35.9 to 56.5) | 19.7* (11.2 to 28.1) |
| Controlled phase of multiple myeloma | 29.3 (26.0 to 34.6) | -- | 1.3 (0.8 to 2.0) | 47.8* (33.9 to 64.7) | 20.9* (9.5 to 34.8) |
| Metastatic phase of multiple myeloma | 20.2 (17.9 to 23.5) | -- | 8.0 (5.5 to 10.7) | 38.1* (24.0 to 52.7) | 13.0* (1.4 to 24.9) |
| Terminal phase of multiple myeloma | 1.5 (1.4 to 1.7) | -- | 0.8 (0.6 to 1.1) | 28.3* (22.9 to 33.9) | 5.0* (0.5 to 9.5) |
| Leukaemia | 152.8 (139.0 to 163.3) | 39.9 (36.2 to 42.4) | 20.7 (14.9 to 26.8) | 22.8* (15.5 to 30.6) | 0.5 (-5.5 to 6.8) |
| Acute lymphoid leukaemia | 9.2 (7.8 to 10.0) | 2.6 (2.2 to 2.7) | 1.2 (0.8 to 1.5) | 33.7* (22.4 to 44.2) | 9.4* (0.2 to 17.9) |
| Diagnosis and primary therapy phase of acute lymphoid leukemia | 1.1 (1.0 to 1.2) | 2.6 (2.2 to 2.7) | 0.3 (0.2 to 0.5) | 28.1* (19.0 to 35.7) | 4.8 (-2.6 to 11.0) |
| Controlled phase of acute lymphoid leukemia | 7.0 (6.0 to 7.6) | -- | 0.3 (0.2 to 0.5) | 38.3* (24.7 to 51.3) | 13.1* (2.0 to 23.8) |
| Metastatic phase of acute lymphoid leukemia | 0.9 (0.8 to 1.0) | -- | 0.4 (0.3 to 0.5) | 34.5* (22.2 to 46.8) | 10.0 (-0.1 to 20.1) |
| Terminal phase of acute lymphoid leukemia | 0.2 (0.1 to 0.2) | -- | 0.1 (0.1 to 0.1) | 34.8* (22.4 to 47.2) | 10.3* (0.1 to 20.4) |
| Chronic lymphoid leukaemia | 62.0 (57.3 to 67.4) | 9.9 (9.1 to 10.7) | 8.1 (5.9 to 10.5) | 20.7* (9.5 to 33.2) | -1.3 (-10.4 to 9.0) |

| | | | | | |
|---|---|---|---|---|---|
| Diagnosis and primary therapy phase of chronic lymphoid leukemia | 3.3 (3.1 to 3.6) | 9.9 (9.1 to 10.7) | 0.9 (0.6 to 1.3) | 22.4* (13.6 to 32.7) | 0.1 (-7.1 to 8.6) |
| Controlled phase of chronic lymphoid leukemia | 46.3 (42.7 to 50.3) | -- | 2.1 (1.3 to 3.0) | 22.4* (10.7 to 36.1) | 0.1 (-9.4 to 11.3) |
| Metastatic phase of chronic lymphoid leukemia | 12.0 (11.1 to 13.0) | -- | 4.8 (3.3 to 6.4) | 19.8* (5.4 to 36.9) | -2.0 (-13.8 to 12.0) |
| Terminal phase of chronic lymphoid leukemia | 0.4 (0.4 to 0.5) | -- | 0.2 (0.2 to 0.3) | 16.1* (8.3 to 24.5) | -5.0 (-11.4 to 1.9) |
| Acute myeloid leukaemia | 10.3 (9.1 to 11.1) | 9.9 (8.8 to 10.7) | 2.3 (1.6 to 3.1) | 28.5* (21.3 to 36.3) | 5.1 (-0.7 to 11.5) |
| Diagnosis and primary therapy phase of acute myeloid leukemia | -- | 9.9 (8.8 to 10.7) | -- | -- | -- |
| Controlled phase of acute myeloid leukemia | 5.9 (5.2 to 6.3) | -- | 0.3 (0.2 to 0.4) | 27.5* (18.9 to 36.3) | 4.3 (-2.7 to 11.5) |
| Metastatic phase of acute myeloid leukemia | 3.6 (3.2 to 3.9) | -- | 1.6 (1.1 to 2.2) | 28.7* (21.4 to 36.6) | 5.3 (-0.7 to 11.8) |
| Terminal phase of acute myeloid leukemia | 0.8 (0.7 to 0.8) | -- | 0.4 (0.3 to 0.6) | 28.4* (21.4 to 36.5) | 5.0 (-0.7 to 11.7) |
| Chronic myeloid leukaemia | 6.0 (5.4 to 6.5) | 2.6 (2.4 to 2.9) | 0.8 (0.6 to 1.1) | 0.8 (-5.5 to 7.9) | -17.5* (-22.7 to -11.7) |
| Diagnosis and primary therapy phase of chronic myeloid leukemia | 0.4 (0.4 to 0.5) | 2.6 (2.4 to 2.9) | 0.1 (0.1 to 0.2) | 1.9 (-4.4 to 9.0) | -16.6* (-21.8 to -10.8) |
| Controlled phase of chronic myeloid leukemia | 4.5 (4.1 to 4.9) | -- | 0.2 (0.1 to 0.3) | 3.6 (-4.8 to 13.1) | -15.3* (-22.1 to -7.5) |
| Metastatic phase of chronic myeloid leukemia | 0.9 (0.8 to 0.9) | -- | 0.4 (0.3 to 0.5) | -0.6 (-6.7 to 6.2) | -18.7* (-23.7 to -13.2) |
| Terminal phase of chronic myeloid leukemia | 0.2 (0.2 to 0.2) | -- | 0.1 (0.1 to 0.1) | -0.6 (-6.7 to 6.2) | -18.7* (-23.7 to -13.2) |
| Other leukaemia | 65.4 (56.4 to 70.9) | 14.9 (12.8 to 16.2) | 8.3 (5.9 to 10.6) | 24.9* (14.0 to 36.5) | 2.1 (-6.7 to 11.7) |
| Metastatic phase of other leukemia | 13.2 (11.4 to 14.3) | -- | 5.5 (3.7 to 7.3) | 22.0* (9.1 to 35.0) | -0.2 (-10.7 to 10.4) |
| Diagnosis and primary therapy phase of other leukemia | -- | 14.9 (12.8 to 16.2) | -- | -- | -- |
| Terminal phase of other leukemia | 0.8 (0.7 to 0.9) | -- | 0.4 (0.3 to 0.6) | 19.1* (12.1 to 26.1) | -2.6 (-8.3 to 3.2) |
| Controlled phase of other leukemia | 51.4 (44.3 to 55.7) | -- | 2.3 (1.5 to 3.4) | 33.5* (21.5 to 47.1) | 9.2 (-0.6 to 20.3) |
| Other malignant cancers | 479.0 (378.9 to 656.0) | 57.6 (52.0 to 60.6) | 40.6 (27.9 to 57.7) | 113.9* (70.7 to 169.2) | 75.0* (39.7 to 120.2) |
| Diagnosis and primary therapy phase of other malignant neoplasms | 12.0 (9.6 to 16.2) | 57.6 (52.0 to 60.6) | 3.1 (2.0 to 4.6) | 143.4* (83.7 to 223.1) | 99.1* (50.3 to 164.3) |
| Controlled phase of other malignant neoplasms | 421.7 (329.9 to 586.2) | -- | 18.9 (11.2 to 31.2) | 184.4* (110.2 to 283.6) | 132.6* (72.0 to 213.8) |
| Metastatic phase of other malignant neoplasms | 39.6 (33.8 to 48.5) | -- | 15.3 (10.2 to 21.4) | 69.3* (41.3 to 104.2) | 38.5* (15.6 to 67.1) |
| Terminal phase of other malignant neoplasms | 5.9 (5.1 to 7.2) | -- | 3.2 (2.1 to 4.3) | 60.9* (37.6 to 89.8) | 31.6* (12.6 to 55.2) |
| Other neoplasms | 2194.5 (2080.4 to 2308.0) | 885.8 (839.8 to 935.6) | 6.1 (3.8 to 9.3) | 50.0* (43.4 to 56.3) | 22.7* (17.3 to 27.9) |
| Myelodysplastic, myeloproliferative, and other hematopoietic neoplasms | 137.9 (113.6 to 165.5) | 16.4 (12.5 to 20.4) | 6.1 (3.8 to 9.3) | 50.0* (43.4 to 56.3) | 22.7* (17.3 to 27.9) |
| Benign and in situ intestinal neoplasms | 481.5 (450.5 to 513.4) | 125.4 (117.5 to 134.7) | -- | -- | -- |
| Benign and in situ cervical and uterine neoplasms | 14.3 (13.3 to 15.3) | 1.7 (1.5 to 2.0) | -- | -- | -- |
| Other benign and in situ neoplasms | 1595.8 (1488.0 to 1707.0) | 742.3 (697.9 to 790.6) | -- | -- | -- |
| **Cardiovascular diseases** | **54834.8 (51400.3 to 58586.9)** | **8995.5 (8417.4 to 9630.2)** | **4061.2 (2973.6 to 5236.7)** | **28.1* (24.7 to 31.6)** | **4.8* (2.0 to 7.6)** |
| Rheumatic heart disease | 474.0 (451.0 to 495.8) | 7.1 (5.8 to 8.4) | 26.0 (17.6 to 35.3) | 25.6* (22.4 to 29.0) | 2.8* (0.2 to 5.5) |
| Rheumatic heart disease, without heart failure | 346.3 (288.8 to 389.2) | 7.1 (5.8 to 8.4) | 15.4 (10.0 to 22.5) | 26.4* (23.0 to 29.6) | 3.4* (0.6 to 6.0) |
| Heart failure due to rheumatic heart disease | 127.7 (88.6 to 177.7) | -- | 10.6 (6.3 to 16.2) | 24.6* (17.8 to 31.2) | 2.0 (-3.6 to 7.3) |
| Ischaemic heart disease | 16730.1 (14876.8 to 18650.6) | 1325.4 (1095.4 to 1612.1) | 658.4 (457.4 to 906.9) | 13.4* (12.2 to 14.6) | -7.3* (-8.2 to -6.3) |
| Asymptomatic ischemic heart disease following myocardial infarction | 5726.8 (4332.7 to 7164.5) | -- | -- | -- | -- |
| Angina due to ischemic heart disease | 8444.6 (7632.9 to 9283.9) | -- | 448.1 (303.6 to 628.9) | 13.6* (12.6 to 14.6) | -7.1* (-7.9 to -6.2) |
| Heart failure due to ischemic heart disease | 2487.4 (1877.9 to 3259.5) | -- | 203.8 (130.8 to 309.8) | 13.2* (10.2 to 15.9) | -7.4* (-9.8 to -5.2) |
| Myocardial infarction due to ischemic heart disease | 71.2 (57.6 to 88.3) | 1325.4 (1095.4 to 1612.1) | 6.6 (4.3 to 9.5) | 4.5 (-2.0 to 10.7) | -14.5* (-19.8 to -9.4) |
| Stroke | 11606.3 (10468.1 to 12644.5) | 1370.8‡ (1189.2 to 1582.6) | 2133.4 (1539.5 to 2754.6) | 37.7* (31.3 to 43.9) | 12.6* (7.4 to 17.7) |
| Ischaemic stroke | 10378.9 (9195.3 to 11481.3) | 973.2 (801.7 to 1177.2) | 1836.1 (1309.8 to 2379.7) | 43.6* (36.0 to 50.5) | 17.5* (11.2 to 23.2) |
| Acute ischemic stroke | 74.8 (60.4 to 91.1) | 973.2 (801.7 to 1177.2) | 17.8 (11.6 to 24.7) | 32.8* (21.1 to 45.7) | 8.6 (-0.9 to 19.2) |
| Chronic ischemic stroke | 10304.1 (9120.5 to 11417.2) | -- | 1818.3 (1296.0 to 2358.3) | 43.7* (36.0 to 50.7) | 17.6* (11.2 to 23.3) |
| Intracerebral hemorrhage | 1083.3 (895.4 to 1300.3) | 308.3 (257.3 to 370.9) | 192.6 (137.5 to 259.8) | 4.2 (-5.8 to 15.2) | -14.8* (-22.9 to -5.8) |
| Acute intracerebral hemorrhage | 16.8 (13.5 to 21.0) | 308.3 (257.3 to 370.9) | 4.4 (2.8 to 6.1) | -4.6 (-14.2 to 6.3) | -21.9* (-29.8 to -13.0) |
| Chronic intracerebral hemorrhage | 1066.5 (877.4 to 1282.9) | -- | 188.3 (134.3 to 255.5) | 4.4 (-5.8 to 15.6) | -14.6* (-22.9 to -5.4) |
| Subarachnoid hemorrhage | 618.6 (508.7 to 751.0) | 89.4 (72.1 to 108.7) | 104.7 (72.6 to 143.0) | 21.4* (15.3 to 27.6) | -0.7 (-5.6 to 4.4) |
| Acute subarachnoid hemorrhage | 7.1 (5.6 to 8.9) | 89.4 (72.1 to 108.7) | 1.9 (1.2 to 2.6) | 24.1* (20.6 to 26.2) | 1.5 (-1.3 to 3.2) |
| Chronic subarachnoid hemorrhage | 611.5 (501.1 to 745.0) | -- | 102.8 (71.4 to 140.6) | 21.4* (15.1 to 27.6) | -0.7 (-5.8 to 4.4) |
| Hypertensive heart disease | 2366.8 (1728.8 to 3116.1) | -- | 194.2 (123.7 to 292.8) | 23.6* (21.5 to 25.8) | 1.1 (-0.6 to 2.9) |
| Non-rheumatic valvular heart disease | 4046.4 (3794.8 to 4326.5) | 4118.6 (3862.2 to 4415.2) | 46.9 (25.5 to 75.1) | 22.5* (18.1 to 27.1) | 0.2 (-3.4 to 4.0) |
| Non-rheumatic calcific aortic valve disease | 1733.8 (1489.9 to 2025.6) | 1733.8 (1489.9 to 2025.6) | 19.0 (10.1 to 31.4) | 22.2* (14.5 to 30.2) | -0.0 (-6.3 to 6.5) |
| Heart failure due to calcific aortic valve disease | 147.1 (86.8 to 227.7) | -- | 12.2 (6.5 to 20.2) | 12.8 (-2.5 to 28.4) | -7.7 (-20.3 to 5.1) |
| Non-rheumatic calcific aortic valve disease | 1586.7 (1354.2 to 1843.6) | 1733.8 (1489.9 to 2025.6) | 6.9 (3.4 to 11.8) | 43.4* (22.4 to 65.7) | 17.3* (0.2 to 35.5) |
| Non-rheumatic degenerative mitral valve disease | 2384.8 (2307.9 to 2471.8) | 2384.8 (2307.9 to 2471.8) | 27.6 (14.9 to 44.1) | 22.7* (17.5 to 27.6) | 0.4 (-3.9 to 4.4) |
| Heart failure due to degenerative mitral valve disease | 246.9 (150.9 to 370.7) | -- | 20.2 (11.3 to 32.4) | 15.4* (5.2 to 26.6) | -5.6 (-13.9 to 3.6) |
| Non-rheumatic degenerative mitral valve disease | 2138.0 (2012.6 to 2266.3) | 2384.8 (2307.9 to 2471.8) | 7.5 (3.7 to 12.7) | 48.1* (27.4 to 69.2) | 21.1* (4.2 to 38.4) |
| Other non-rheumatic valve diseases | 2.7 (1.8 to 3.9) | -- | 0.2 (0.1 to 0.4) | 27.1* (22.6 to 32.0) | 4.0* (0.3 to 8.0) |
| Mild heart failure due to other non-rheumatic valve disease | 0.5 (0.3 to 0.8) | -- | -- | 27.2* (19.4 to 35.4) | 4.1 (-2.3 to 10.7) |
| Moderate heart failure due to other non-rheumatic valve disease | 0.3 (0.2 to 0.5) | -- | -- | 26.9* (17.3 to 37.2) | 3.8 (-4.1 to 12.3) |
| Severe heart failure due to other non-rheumatic valve disease | 0.9 (0.6 to 1.3) | -- | 0.2 (0.1 to 0.3) | 27.2* (21.2 to 33.5) | 4.0 (-0.8 to 9.2) |

| | | | | | |
|---|---|---|---|---|---|
| Controlled, medically managed heart failure due to other non-rheumatic valve disease | 1.0 (0.7 to 1.4) | -- | 0.0 (0.0 to 0.1) | 27.1* (21.3 to 33.5) | 4.0 (-0.8 to 9.2) |
| Cardiomyopathy and myocarditis | 630.3 (455.1 to 856.2) | 226.5 (172.9 to 288.4) | 54.1 (32.9 to 82.0) | 19.5* (16.0 to 23.1) | -2.2 (-5.1 to 0.7) |
| Myocarditis | 95.3 (75.8 to 117.5) | 226.5 (172.9 to 288.4) | 6.1 (3.9 to 9.1) | 21.0* (15.6 to 26.1) | -1.0 (-5.5 to 3.2) |
| Acute myocarditis | 54.2 (41.0 to 70.2) | 226.5 (172.9 to 288.4) | 2.5 (1.5 to 3.9) | 18.6* (11.0 to 26.6) | -3.0 (-9.2 to 3.6) |
| Heart failure due to myocarditis | 41.1 (28.0 to 58.5) | -- | 3.6 (2.1 to 5.7) | 22.7* (15.7 to 30.2) | 0.4 (-5.4 to 6.5) |
| Alcoholic cardiomyopathy | 137.0 (95.4 to 189.3) | -- | 11.3 (6.9 to 17.6) | 9.9* (3.8 to 17.0) | -10.1* (-15.1 to -4.3) |
| Other cardiomyopathy | 445.6 (311.8 to 622.7) | -- | 36.6 (22.3 to 55.6) | 22.6* (18.6 to 26.4) | 0.3 (-3.0 to 3.4) |
| Atrial fibrillation and flutter | 5710.9 (4575.6 to 6856.2) | 487.6 (367.5 to 622.4) | 443.9 (287.0 to 628.9) | 23.1* (21.5 to 24.6) | 0.7 (-0.6 to 1.9) |
| Asymptomatic atrial fibrillation and flutter | 3426.7 (2732.3 to 4095.8) | 487.6 (367.5 to 622.4) | -- | -- | -- |
| Symptomatic atrial fibrillation and flutter | 2284.2 (1821.5 to 2757.0) | -- | 443.9 (287.0 to 628.9) | 23.1* (21.5 to 24.6) | 0.7 (-0.6 to 1.9) |
| Peripheral vascular disease | 16257.1 (13192.3 to 19899.0) | 1368.5 (999.1 to 1796.8) | 97.6 (43.0 to 182.4) | 16.7* (15.0 to 18.5) | -4.5* (-5.9 to -3.0) |
| Asymptomatic peripheral arterial disease | 8808.0 (6073.1 to 11727.8) | 1368.5 (999.1 to 1796.8) | -- | -- | -- |
| Symptomatic claudication due to peripheral arterial disease | 7449.2 (5206.7 to 10371.6) | -- | 97.6 (43.0 to 182.4) | 16.7* (15.0 to 18.5) | -4.5* (-5.9 to -3.0) |
| Endocarditis | 61.0 (45.3 to 80.9) | 91.1 (71.8 to 111.4) | 5.0 (3.1 to 7.8) | 22.0* (14.3 to 30.5) | -0.2 (-6.5 to 6.8) |
| Acute endocarditis | 6.7 (5.3 to 8.4) | 91.1 (71.8 to 111.4) | 0.4 (0.2 to 0.6) | 29.6* (21.2 to 37.8) | 6.0 (-0.9 to 12.7) |
| Heart failure due to endocarditis | 54.3 (38.4 to 74.6) | -- | 4.7 (2.9 to 7.3) | 21.5* (12.9 to 30.6) | -0.6 (-7.6 to 6.8) |
| Other cardiovascular and circulatory diseases | 7920.5 (5629.8 to 10832.2) | -- | 401.7 (245.8 to 628.4) | 21.3* (19.0 to 23.3) | -0.8 (-2.7 to 0.9) |
| Heart failure due to other cardiovascular diseases | 237.7 (166.9 to 329.3) | -- | 19.6 (11.9 to 30.6) | 20.8* (15.6 to 26.0) | -1.2 (-5.4 to 3.0) |
| Other cardiovascular and circulatory disease episodes | 7682.8 (5469.4 to 10498.8) | -- | 382.1 (233.6 to 598.0) | 21.3* (19.0 to 23.4) | -0.8 (-2.7 to 0.9) |
| **Chronic respiratory diseases** | **39050.5 (35048.8 to 43624.3)** | **2656.3 (2011.2 to 3418.0)** | **3762.0 (3141.0 to 4386.7)** | **19.4* (13.0 to 25.5)** | **-2.3 (-7.6 to 2.7)** |
| Chronic obstructive pulmonary disease | 32538.0 (27737.7 to 37722.9) | 1666.0 (1090.5 to 2366.7) | 3271.2 (2704.1 to 3837.2) | 18.3* (10.9 to 25.6) | -3.2 (-9.3 to 2.8) |
| Chronic obstructive pulmonary disease with heart failure | 2828.4 (2174.5 to 3544.9) | -- | 1096.5 (723.1 to 1461.2) | 25.7* (21.6 to 28.7) | 2.8 (-0.6 to 5.3) |
| Chronic obstructive pulmonary disease without heart failure | 29709.6 (25004.6 to 34975.1) | 1666.0 (1090.5 to 2366.7) | 2174.7 (1670.6 to 2745.2) | 14.9* (4.2 to 25.4) | -6.0 (-14.7 to 2.6) |
| Pneumoconiosis | 74.1 (60.3 to 89.7) | 8.5 (6.7 to 10.4) | 11.2 (7.3 to 15.7) | 14.6* (6.5 to 23.2) | -6.2 (-12.9 to 0.8) |
| Silicosis | 23.2 (14.5 to 34.0) | 3.3 (2.1 to 4.8) | 3.5 (2.0 to 5.5) | 12.3 (-2.8 to 28.4) | -8.1 (-20.5 to 5.1) |
| Heart failure due to silicosis | 2.1 (1.6 to 2.6) | -- | 1.0 (0.6 to 1.4) | 14.1* (7.1 to 21.3) | -6.7* (-12.4 to -0.7) |
| Silicosis without heart failure | 21.1 (12.3 to 31.6) | 3.3 (2.1 to 4.8) | 2.5 (1.2 to 4.3) | 11.6 (-8.9 to 33.7) | -8.7 (-25.5 to 9.4) |
| Asbestosis | 12.1 (8.8 to 17.0) | 1.3 (0.8 to 1.9) | 1.9 (1.1 to 2.9) | 37.9* (26.5 to 50.4) | 12.8* (3.5 to 23.1) |
| Asbestosis without heart failure | 11.1 (7.9 to 16.1) | 1.3 (0.8 to 1.9) | 1.4 (0.8 to 2.4) | 41.8* (26.8 to 58.2) | 16.0* (3.7 to 29.5) |
| Heart failure due to asbestosis | 1.0 (0.8 to 1.2) | -- | 0.4 (0.3 to 0.6) | 26.7* (22.0 to 30.9) | 3.6 (-0.2 to 7.1) |
| Coal workers pneumoconiosis | 22.9 (15.5 to 33.1) | 2.4 (1.6 to 3.7) | 3.4 (2.0 to 5.3) | 8.3 (-8.2 to 25.7) | -11.4 (-24.9 to 2.8) |
| Coal workers pneumoconiosis without heart failure | 21.4 (14.1 to 31.5) | 2.4 (1.6 to 3.7) | 2.7 (1.4 to 4.5) | 6.6 (-14.3 to 27.5) | -12.8 (-29.9 to 4.3) |
| Heart failure due to Coal workers pneumoconiosis | 1.5 (1.2 to 1.9) | -- | 0.7 (0.4 to 1.0) | 15.6* (7.1 to 25.5) | -5.4 (-12.4 to 2.7) |
| Other pneumoconiosis | 15.9 (11.9 to 20.9) | 1.5 (1.0 to 2.2) | 2.4 (1.5 to 3.7) | 12.6* (2.5 to 24.1) | -7.9 (-16.2 to 1.6) |
| Heart failure due to other pneumoconiosis | 0.7 (0.6 to 0.9) | -- | 0.3 (0.2 to 0.5) | 11.6* (7.5 to 16.1) | -8.7* (-12.1 to -5.0) |
| Other pneumoconiosis without heart failure | 15.2 (11.2 to 20.1) | 1.5 (1.0 to 2.2) | 2.1 (1.2 to 3.2) | 12.8* (1.2 to 26.0) | -7.7 (-17.2 to 3.1) |
| Asthma | 10257.7 (8240.4 to 12699.9) | 915.3 (637.4 to 1209.4) | 375.1 (242.4 to 548.6) | 27.6* (19.7 to 35.7) | 4.4 (-2.0 to 11.0) |
| Asymptomatic asthma | 3726.5 (2942.8 to 4674.1) | 331.9 (224.0 to 441.9) | -- | -- | -- |
| Symptomatic asthma | 6531.2 (5202.1 to 8165.5) | 583.4 (405.2 to 778.5) | 375.1 (242.4 to 548.6) | 27.6* (19.7 to 35.7) | 4.4 (-2.0 to 11.0) |
| Interstitial lung disease and pulmonary sarcoidosis | 759.5 (644.7 to 901.1) | 66.5 (46.6 to 90.0) | 78.1 (51.5 to 111.8) | 24.8* (20.3 to 29.4) | 2.1 (-1.6 to 5.9) |
| Heart failure due to interstitial lung disease and pulmonary sarcoidosis | 73.4 (53.1 to 92.2) | -- | 29.7 (19.2 to 42.5) | 24.0* (18.6 to 30.2) | 1.5 (-3.0 to 6.5) |
| Interstitial lung disease and pulmonary sarcoidosis without heart failure | 686.1 (577.1 to 816.3) | 66.5 (46.6 to 90.0) | 48.4 (31.2 to 72.2) | 25.2* (19.1 to 31.3) | 2.4 (-2.6 to 7.4) |
| Other chronic respiratory diseases | -- | -- | 26.4 (21.8 to 30.8) | 37.0* (28.7 to 45.3) | 12.1* (5.2 to 18.8) |
| **Digestive diseases** | **81216.9 (75075.5 to 87258.5)** | **21500.4 (17436.3 to 25554.2)** | **926.6 (634.3 to 1286.8)** | **27.1* (24.3 to 30.5)** | **3.9* (1.7 to 6.7)** |
| Cirrhosis and other chronic liver diseases | 61190.6 (56604.3 to 66189.8) | 135.4 (121.3 to 148.9) | 105.7 (75.2 to 144.4) | 40.0* (36.0 to 44.2) | 14.5* (11.3 to 18.0) |
| Cirrhosis and other chronic liver diseases due to hepatitis B | 9936.7 (9182.0 to 10688.2) | 42.1 (35.8 to 49.0) | 30.7 (21.1 to 42.7) | 38.9* (33.1 to 45.0) | 13.6* (8.9 to 18.7) |
| Cirrhosis and other chronic liver diseases due to hepatitis B, decompensated | 195.6 (172.7 to 220.1) | 41.7 (35.3 to 48.8) | 30.7 (21.1 to 42.7) | 38.9* (33.1 to 45.0) | 13.6* (8.9 to 18.7) |
| Chronic hepatitis B without cirrhosis | 8456.0 (7677.3 to 9224.5) | -- | -- | -- | -- |
| Cirrhosis and other chronic liver diseases due to hepatitis B, compensated | 1285.1 (1143.7 to 1437.7) | 0.4 (0.0 to 1.3) | -- | -- | -- |
| Cirrhosis and other chronic liver diseases due to hepatitis C | 4147.2 (3535.7 to 4820.2) | 33.8 (29.3 to 38.9) | 27.6 (19.2 to 38.2) | 37.4* (31.2 to 43.2) | 12.4* (7.3 to 17.1) |
| Cirrhosis and other chronic liver diseases due to hepatitis C, decompensated | 176.2 (157.0 to 196.3) | 33.3 (28.7 to 38.6) | 27.6 (19.2 to 38.2) | 37.4* (31.2 to 43.2) | 12.4* (7.3 to 17.1) |
| Chronic hepatitis C without cirrhosis | 3056.4 (2452.5 to 3744.4) | -- | -- | -- | -- |
| Cirrhosis and other chronic liver diseases due to hepatitis C, compensated | 914.6 (819.5 to 1014.8) | 0.5 (0.0 to 1.4) | -- | -- | -- |
| Cirrhosis and other chronic liver diseases due to alcohol use | 984.4 (856.1 to 1119.5) | 29.2 (24.7 to 34.4) | 24.5 (17.0 to 33.7) | 37.4* (30.8 to 44.6) | 12.4* (7.0 to 18.3) |
| Cirrhosis and other chronic liver diseases due to alcohol, decompensated | 157.2 (137.0 to 178.7) | 29.0 (24.4 to 34.2) | 24.5 (17.0 to 33.7) | 37.4* (30.8 to 44.6) | 12.4* (7.0 to 18.3) |
| Cirrhosis and other chronic liver diseases due to alcohol, compensated | 827.2 (715.3 to 942.8) | 0.2 (0.0 to 0.5) | -- | -- | -- |
| Cirrhosis due to NASH | 45632.8 (41059.7 to 50544.2) | 14.1 (12.2 to 16.3) | 10.2 (7.0 to 14.3) | 55.4* (46.0 to 64.9) | 27.2* (19.4 to 34.9) |
| Non-alcoholic fatty liver disease (NAFLD) / Non-alcoholic steatohepatitis (NASH) | 45206.5 (40635.4 to 50098.4) | -- | -- | -- | -- |
| Cirrhosis and other chronic liver diseases due to NASH | 426.3 (378.6 to 478.2) | 14.1 (12.2 to 16.3) | 10.2 (7.0 to 14.3) | 55.4* (46.0 to 64.9) | 27.2* (19.4 to 34.9) |
| Cirrhosis and other chronic liver diseases due to other causes | 491.0 (389.5 to 630.0) | 16.1 (12.5 to 21.0) | 12.7 (8.2 to 18.8) | 42.3* (33.6 to 51.7) | 16.4* (9.3 to 24.1) |

| | | | | | |
|---|---|---|---|---|---|
| Cirrhosis and other chronic liver diseases due to other cause, decompensated | 81.6 (63.6 to 105.4) | 16.0 (12.4 to 20.9) | 12.7 (8.2 to 18.8) | 42.3* (33.6 to 51.7) | 16.4* (9.3 to 24.1) |
| Cirrhosis and other chronic liver diseases due to other cause, compensated | 409.4 (324.0 to 528.0) | 0.1 (0.0 to 0.3) | -- | -- | -- |
| Upper digestive system diseases | 34708.4 (27726.6 to 41632.2) | 16684.8 (12792.4 to 20817.3) | 551.5 (341.0 to 839.6) | 24.9* (21.3 to 29.0) | 2.1 (-0.8 to 5.5) |
| Peptic ulcer disease | 938.8 (731.6 to 1149.0) | 416.3 (313.6 to 531.5) | 44.3 (28.0 to 63.5) | 14.4* (8.8 to 20.4) | -6.4* (-11.0 to -1.5) |
| Acute peptic ulcer disease | 0.8 (0.5 to 1.1) | -- | 0.3 (0.1 to 0.4) | 22.3* (20.0 to 25.3) | 0.0 (-1.9 to 2.5) |
| Chronic peptic ulcer disease | 938.0 (730.7 to 1148.3) | 416.3 (313.6 to 531.5) | 44.0 (27.9 to 63.3) | 14.4* (8.7 to 20.4) | -6.4* (-11.1 to -1.5) |
| Gastritis and duodenitis | 6021.4 (4368.2 to 7950.7) | 4285.4 (3099.8 to 5709.4) | 252.8 (152.7 to 384.5) | 27.1* (19.9 to 35.5) | 4.0 (-1.9 to 10.8) |
| Acute gastritis and duodenitis | 2.8 (2.0 to 3.8) | -- | 0.9 (0.5 to 1.4) | 21.0* (19.1 to 22.9) | -1.0 (-2.6 to 0.5) |
| Chronic gastritis and duodenitis | 6018.6 (4365.7 to 7948.1) | 4285.4 (3099.8 to 5709.4) | 251.9 (152.1 to 383.2) | 27.1* (19.9 to 35.5) | 4.0 (-1.9 to 10.9) |
| GERD | 31218.0 (22899.5 to 39723.3) | 11983.2 (8438.9 to 15684.7) | 254.4 (122.4 to 457.8) | 24.7* (22.5 to 26.8) | 2.0* (0.2 to 3.7) |
| Appendicitis | 12.1 (10.1 to 14.5) | 317.3 (265.3 to 376.9) | 3.5 (2.3 to 5.0) | 20.0* (8.9 to 31.8) | -1.8 (-10.9 to 7.8) |
| Paralytic ileus and intestinal obstruction | 9.2 (7.1 to 11.6) | 262.3 (201.4 to 330.3) | 2.8 (1.8 to 4.1) | 36.0* (23.2 to 49.0) | 11.2* (0.8 to 21.9) |
| Inguinal, femoral, and abdominal hernia | 1403.7 (1040.4 to 1892.6) | 3450.3 (2350.2 to 4731.7) | 127.7 (81.6 to 189.2) | 20.9* (13.4 to 28.8) | -1.1 (-7.3 to 5.3) |
| Inflammatory bowel disease | 428.0 (369.3 to 497.2) | 103.5 (80.9 to 129.6) | 59.6 (40.6 to 82.4) | 31.5* (22.9 to 40.0) | 7.5* (0.6 to 14.5) |
| Ulcerative colitis | 324.1 (269.3 to 391.6) | 78.0 (55.7 to 103.4) | 44.4 (29.9 to 63.0) | 34.3* (24.7 to 43.7) | 9.8* (2.1 to 17.5) |
| Crohn's disease | 103.9 (89.0 to 121.7) | 25.5 (18.9 to 33.7) | 15.1 (10.4 to 21.3) | 23.9* (13.6 to 34.2) | 1.3 (-7.1 to 9.8) |
| Vascular intestinal disorders | 9.4 (7.3 to 12.1) | 68.6 (52.5 to 88.4) | 2.8 (1.7 to 4.1) | 24.8* (6.8 to 43.8) | 2.1 (-12.7 to 17.6) |
| Gallbladder and biliary diseases | 1665.5 (1259.6 to 2152.6) | 374.2 (290.7 to 482.1) | 1.6 (1.0 to 2.4) | 28.0* (16.9 to 39.7) | 4.7 (-4.4 to 14.3) |
| Pancreatitis | 448.9 (388.5 to 506.2) | 103.8 (83.4 to 127.7) | 25.2 (12.9 to 42.6) | 28.4* (22.2 to 34.7) | 5.0 (-0.0 to 10.2) |
| Acute pancreatitis | 7.9 (6.0 to 10.2) | 81.2 (62.2 to 103.7) | 3.1 (2.0 to 4.5) | 22.7* (16.1 to 29.2) | 0.4 (-5.0 to 5.7) |
| Chronic pancreatitis | 441.1 (380.2 to 498.5) | 22.6 (15.0 to 31.3) | 22.1 (10.1 to 39.4) | 29.2* (22.4 to 36.8) | 5.7* (0.1 to 11.9) |
| Other digestive diseases | -- | -- | 46.2 (31.2 to 63.4) | 39.7* (35.0 to 45.0) | 14.3* (10.4 to 18.7) |
| **Neurological disorders** | **74670.2 (61028.2 to 89882.2)** | **21786.5 (15683.0 to 29172.0)** | **2058.2 (1462.7 to 2767.9)** | **21.2* (19.6 to 23.1)** | **-0.8 (-2.2 to 0.7)** |
| Alzheimer's disease and other dementias | 5289.2 (4304.6 to 6406.2) | 926.8 (644.3 to 1288.8) | 723.5 (474.5 to 1031.8) | 19.9* (18.6 to 21.2) | -1.9* (-3.0 to -0.8) |
| Parkinson's disease | 1387.7 (1016.7 to 1823.9) | 172.1 (123.5 to 225.8) | 198.5 (126.4 to 298.1) | 22.6* (19.7 to 25.3) | 0.3 (-2.1 to 2.5) |
| Epilepsy | 820.0 (613.3 to 1072.6) | 56.2 (37.9 to 78.2) | 216.0 (132.8 to 322.0) | 25.7* (11.6 to 40.2) | 2.8 (-8.7 to 14.7) |
| Multiple sclerosis | 79.0 (72.1 to 87.2) | 0.3 (0.2 to 0.4) | 19.6 (14.2 to 25.2) | 28.6* (20.4 to 37.4) | 5.2 (-1.5 to 12.4) |
| Motor neuron disease | 19.7 (15.3 to 25.8) | 8.0 (6.2 to 10.2) | 4.2 (2.8 to 6.1) | 41.4* (33.5 to 48.5) | 15.7* (9.2 to 21.5) |
| Headache disorders | 70313.0 (55960.0 to 86247.4) | 20623.0 (14454.2 to 27984.2) | 869.6 (578.2 to 1262.1) | 20.2* (18.9 to 21.5) | -1.6* (-2.7 to -0.6) |
| Migraine | 18664.7 (16514.0 to 21013.2) | 451.6 (289.0 to 660.5) | 685.7 (436.5 to 1033.9) | 20.2* (18.6 to 21.8) | -1.6* (-3.0 to -0.4) |
| Medication overuse headache due to migraine | 2453.6 (1760.9 to 3365.2) | -- | 252.1 (141.5 to 396.7) | 17.6* (15.7 to 19.4) | -3.8* (-5.4 to -2.3) |
| Migraine | 16211.1 (14167.0 to 18388.4) | 451.6 (289.0 to 660.5) | 433.5 (257.3 to 695.9) | 21.8* (19.7 to 23.9) | -0.3 (-2.1 to 1.3) |
| Tension-type headache | 60209.0 (43483.4 to 78850.5) | 20171.4 (13945.6 to 27376.6) | 183.9 (103.5 to 304.6) | 20.2* (18.1 to 22.1) | -1.6* (-3.4 to -0.1) |
| Medication overuse headache due to tension-type headache | 912.2 (564.7 to 1338.5) | -- | 93.0 (43.8 to 170.3) | 17.7* (15.0 to 20.5) | -3.7* (-5.9 to -1.5) |
| Tension-type headache | 59296.8 (42704.2 to 77906.6) | 20171.4 (13945.6 to 27376.6) | 91.0 (35.2 to 184.9) | 23.0* (21.4 to 24.3) | 0.6 (-0.6 to 1.6) |
| Other neurological disorders | 1.4 (0.7 to 2.4) | -- | 26.8 (18.9 to 36.0) | 37.3* (34.4 to 40.8) | 12.3* (9.9 to 15.1) |
| Other neurological disorders | -- | -- | 26.4 (18.6 to 35.5) | 37.5* (34.5 to 40.9) | 12.5* (10.0 to 15.3) |
| Guillain-Barré syndrome due to other neurological disorders | 1.4 (0.7 to 2.4) | -- | 0.4 (0.2 to 0.8) | 28.7* (25.2 to 32.3) | 5.3* (2.5 to 8.2) |
| **Mental disorders** | **24183.9 (22612.3 to 25622.7)** | **10098.9 (8822.4 to 11625.7)** | **3153.1 (2335.7 to 4033.4)** | **19.8* (18.7 to 20.8)** | **-2.0* (-2.9 to -1.2)** |
| Schizophrenia | 456.1 (410.4 to 509.0) | 5.1 (3.8 to 6.7) | 267.5 (203.2 to 324.7) | 25.8* (22.8 to 29.1) | 2.9* (0.5 to 5.6) |
| Depressive disorders | 10757.5 (9536.5 to 12053.0) | 9475.0 (8198.5 to 10989.8) | 1606.9 (1123.3 to 2166.0) | 17.8* (16.1 to 19.4) | -3.6* (-5.0 to -2.3) |
| Major depressive disorder | 6168.2 (5339.0 to 7089.3) | 8986.1 (7701.8 to 10504.0) | 1161.1 (795.5 to 1603.8) | 16.1* (14.2 to 17.8) | -5.1* (-6.6 to -3.6) |
| Dysthymia | 4891.6 (4059.9 to 5949.6) | 488.9 (403.1 to 583.7) | 445.8 (293.6 to 649.7) | 22.8* (19.1 to 26.6) | 0.4 (-2.6 to 3.5) |
| Bipolar disorder | 1021.4 (846.4 to 1204.5) | 71.1 (58.3 to 85.3) | 192.3 (120.5 to 289.3) | 21.5* (18.9 to 24.5) | -0.6 (-2.8 to 1.8) |
| Anxiety disorders | 7217.9 (6376.4 to 8124.8) | 547.6 (497.3 to 598.7) | 638.0 (450.9 to 850.8) | 20.2* (17.9 to 22.5) | -1.7 (-3.5 to 0.2) |
| Eating disorders | -- | -- | -- | -- | -- |
| Anorexia nervosa | -- | -- | -- | -- | -- |
| Bulimia nervosa | -- | -- | -- | -- | -- |
| Autism spectrum disorders | 589.3 (528.6 to 653.8) | -- | 83.1 (57.9 to 111.6) | 23.0* (20.8 to 25.5) | 0.7 (-1.2 to 2.6) |
| Attention-deficit/hyperactivity disorder | 103.9 (67.1 to 147.8) | -- | 1.2 (0.6 to 2.0) | 22.8* (12.2 to 34.6) | 0.5 (-8.2 to 10.1) |
| Conduct disorder | -- | -- | -- | -- | -- |
| Idiopathic developmental intellectual disability | 797.5 (377.9 to 1253.6) | -- | 31.7 (13.3 to 54.9) | 12.0* (7.4 to 14.9) | -8.4* (-12.1 to -6.0) |
| Other mental disorders | 4771.9 (4234.7 to 5369.2) | -- | 332.3 (224.4 to 451.7) | 22.7* (20.9 to 24.4) | 0.4 (-1.1 to 1.7) |
| **Substance use disorders** | **2166.1 (1813.4 to 2541.6)** | **568.3 (439.3 to 744.8)** | **328.9 (231.4 to 438.2)** | **28.4* (24.2 to 33.1)** | **5.1* (1.6 to 8.9)** |
| Alcohol use disorders | 1615.1 (1264.5 to 1995.0) | 525.4 (401.2 to 697.4) | 149.6 (100.1 to 217.5) | 16.5* (12.9 to 19.8) | -4.7* (-7.7 to -2.0) |
| Alcohol dependence | 1610.0 (1260.5 to 1990.4) | 525.4 (401.2 to 697.4) | 149.4 (100.0 to 217.2) | 16.5* (12.9 to 19.8) | -4.7* (-7.6 to -2.0) |
| Fetal alcohol syndrome | 5.1 (4.0 to 6.4) | -- | 0.2 (0.1 to 0.3) | 3.2 (-6.0 to 10.6) | -15.6* (-23.1 to -9.5) |
| Drug use disorders | 573.7 (468.8 to 690.5) | 42.9 (27.3 to 65.7) | 179.3 (123.3 to 245.6) | 40.4* (33.6 to 47.9) | 14.9* (9.3 to 21.0) |
| Opioid use disorders | 414.3 (310.6 to 523.6) | 39.9 (24.2 to 62.7) | 156.2 (104.8 to 216.9) | 42.4* (34.7 to 50.5) | 16.5* (10.2 to 23.1) |
| Cocaine use disorders | 80.3 (70.0 to 91.4) | -- | 10.2 (6.6 to 14.6) | 29.3* (20.2 to 39.2) | 5.8 (-1.7 to 13.9) |

| | | | | | |
|---|---|---|---|---|---|
| Amphetamine use disorders | 14.0 (8.7 to 20.1) | 3.0 (1.6 to 4.5) | 1.9 (1.0 to 3.2) | 26.5* (22.7 to 30.0) | 3.5* (0.4 to 6.4) |
| Cannabis use disorders | 94.5 (73.3 to 120.1) | -- | 2.7 (1.6 to 4.2) | 24.9* (19.0 to 30.8) | 2.2 (-2.7 to 7.0) |
| Other drug use disorders | 4.1 (3.5 to 4.8) | -- | 8.3 (5.4 to 11.6) | 28.7* (21.0 to 36.5) | 5.3 (-1.1 to 11.7) |
| **Diabetes and kidney diseases** | **69555.9 (64537.7 to 75165.8)** | **3041.4 (2340.1 to 3890.5)** | **3404.7 (2410.2 to 4660.6)** | **25.1* (19.4 to 30.9)** | **2.4 (-2.3 to 7.1)** |
| Diabetes mellitus | 34796.8 (31057.9 to 38787.7) | 875.3 (622.6 to 1153.1) | 2793.1 (1926.4 to 3888.5) | 26.0* (19.3 to 33.0) | 3.1 (-2.4 to 8.8) |
| Diabetes mellitus type 1 | 310.2 (269.9 to 355.1) | 4.2 (2.8 to 6.1) | 24.2 (16.5 to 33.8) | 23.6* (20.1 to 27.2) | 1.1 (-1.7 to 4.0) |
| Uncomplicated diabetes mellitus type 1 | 183.1 (152.1 to 216.0) | 4.2 (2.8 to 6.1) | 8.2 (5.0 to 12.1) | 22.7* (17.3 to 28.4) | 0.4 (-4.0 to 5.0) |
| Vision loss due to diabetes mellitus type 1 retinopathy | 3.4 (2.5 to 4.6) | -- | 0.3 (0.2 to 0.4) | 0.2 (-5.7 to 6.5) | -18.0* (-22.9 to -12.9) |
| Diabetic neuropathy due to diabetes mellitus type 1 | 123.7 (99.6 to 153.3) | -- | 15.8 (10.3 to 22.9) | 24.6* (18.9 to 29.8) | 1.9 (-2.7 to 6.2) |
| Diabetes mellitus type 2 | 34486.6 (30751.9 to 38468.9) | 871.2 (619.3 to 1149.1) | 2768.9 (1907.3 to 3856.0) | 26.1* (19.3 to 33.0) | 3.1 (-2.4 to 8.8) |
| Uncomplicated diabetes mellitus type 2 | 19267.4 (15788.1 to 22731.9) | 871.2 (619.3 to 1149.1) | 859.5 (533.2 to 1301.6) | 27.6* (19.0 to 35.5) | 4.4 (-2.6 to 10.9) |
| Vision loss due to diabetes mellitus type 2 retinopathy | 416.8 (322.7 to 534.8) | -- | 31.5 (21.9 to 44.8) | 11.3* (7.5 to 14.7) | -8.9* (-12.0 to -6.1) |
| Diabetic neuropathy due to diabetes mellitus type 2 | 14802.4 (11837.3 to 18337.6) | -- | 1877.9 (1247.1 to 2690.8) | 25.6* (18.1 to 33.2) | 2.8 (-3.3 to 9.0) |
| Chronic kidney disease | 52949.2 (45770.1 to 61437.3) | 2132.4 (1504.3 to 3014.5) | 611.5 (441.7 to 820.3) | 21.1* (13.5 to 28.8) | -1.0 (-7.2 to 5.4) |
| Chronic kidney disease due to diabetes mellitus type 1 | 86.6 (72.6 to 103.6) | 2.5 (1.7 to 3.5) | 9.0 (5.1 to 14.5) | 24.9* (18.2 to 31.5) | 2.2 (-3.3 to 7.6) |
| Albuminuria with preserved GFR due to type 1 diabetes mellitus | 33.3 (24.5 to 44.1) | -- | -- | -- | -- |
| End-stage chronic kidney disease due to diabetes mellitus type 1 | 12.1 (5.8 to 21.9) | -- | 5.9 (2.6 to 11.1) | 24.7* (16.4 to 32.6) | 2.0 (-4.8 to 8.5) |
| Stage III chronic kidney disease due to diabetes mellitus type 1 | 30.6 (25.1 to 37.2) | 2.5 (1.7 to 3.5) | 0.6 (0.4 to 0.9) | 16.1* (1.0 to 32.3) | -5.0 (-17.4 to 8.2) |
| Stage IV chronic kidney disease due to diabetes mellitus type 1 | 8.2 (5.3 to 12.0) | -- | 1.1 (0.6 to 1.8) | 26.8* (10.1 to 42.8) | 3.7 (-9.9 to 16.8) |
| Stage V chronic kidney disease due to diabetes mellitus type 1 | 2.5 (1.7 to 3.4) | -- | 1.4 (0.9 to 2.2) | 28.5* (17.2 to 41.4) | 5.1 (-4.2 to 15.7) |
| Chronic kidney disease due to diabetes mellitus type 2 | 10881.8 (9285.2 to 12722.1) | 338.3 (238.7 to 475.8) | 192.7 (136.7 to 260.0) | 23.4* (16.1 to 31.3) | 1.0 (-5.0 to 7.4) |
| Albuminuria with preserved GFR due to type 2 diabetes mellitus | 5307.0 (3976.2 to 6827.3) | -- | -- | -- | -- |
| End-stage chronic kidney disease due to diabetes mellitus type 2 | 136.2 (101.1 to 174.6) | -- | 58.3 (36.9 to 82.7) | 26.7* (19.0 to 33.6) | 3.7 (-2.7 to 9.3) |
| Stage III chronic kidney disease due to diabetes mellitus type 2 | 4858.1 (4123.6 to 5741.8) | 338.3 (238.7 to 475.8) | 20.5 (13.0 to 30.8) | 7.4 (-4.5 to 20.5) | -12.2* (-21.9 to -1.4) |
| Stage IV chronic kidney disease due to diabetes mellitus type 2 | 439.9 (287.6 to 635.1) | -- | 45.7 (26.0 to 74.5) | 23.8* (10.3 to 37.6) | 1.2 (-9.7 to 12.6) |
| Stage V chronic kidney disease due to diabetes mellitus type 2 | 140.6 (100.5 to 194.2) | -- | 68.2 (42.3 to 102.4) | 26.0* (15.8 to 37.0) | 3.1 (-5.3 to 12.0) |
| Chronic kidney disease due to hypertension | 1689.1 (1455.5 to 1954.4) | 14.6 (10.4 to 20.6) | 131.2 (92.2 to 175.9) | 24.1* (16.5 to 32.0) | 1.6 (-4.7 to 7.9) |
| Albuminuria with preserved GFR due to hypertension | 360.2 (270.8 to 464.4) | -- | -- | -- | -- |
| End-stage chronic kidney disease due to hypertension | 102.2 (76.8 to 136.0) | -- | 43.4 (28.9 to 62.4) | 28.9* (20.8 to 37.3) | 5.5 (-1.1 to 12.3) |
| Stage III chronic kidney disease due to hypertension | 855.0 (718.1 to 1021.9) | 14.6 (10.4 to 20.6) | 14.1 (8.9 to 21.1) | 6.6 (-5.5 to 21.1) | -12.8* (-22.7 to -1.0) |
| Stage IV chronic kidney disease due to hypertension | 281.6 (183.7 to 409.1) | -- | 29.5 (16.6 to 48.4) | 24.2* (10.7 to 37.7) | 1.6 (-9.4 to 12.6) |
| Stage V chronic kidney disease due to hypertension | 90.0 (64.5 to 123.8) | -- | 44.1 (27.4 to 66.0) | 26.1* (14.9 to 38.0) | 3.2 (-6.0 to 12.9) |
| Chronic kidney disease due to glomerulonephritis | 2467.8 (2117.6 to 2878.0) | 200.0 (141.6 to 282.9) | 37.8 (25.8 to 52.1) | 22.4* (14.7 to 30.1) | 0.1 (-6.2 to 6.4) |
| Albuminuria with preserved GFR due to glomerulonephritis | 128.7 (95.9 to 166.2) | -- | -- | -- | -- |
| End-stage chronic kidney disease due to glomerulonephritis | 43.1 (27.0 to 63.8) | -- | 18.5 (10.3 to 29.2) | 23.0* (11.4 to 34.0) | 0.6 (-8.9 to 9.6) |
| Stage III chronic kidney disease due to glomerulonephritis | 2219.4 (1885.4 to 2622.2) | 200.0 (141.6 to 282.9) | 2.0 (1.3 to 3.0) | 7.5 (-7.3 to 22.5) | -12.0 (-24.2 to 0.2) |
| Stage IV chronic kidney disease due to glomerulonephritis | 58.1 (37.8 to 84.4) | -- | 6.7 (3.8 to 10.9) | 22.9* (7.7 to 37.6) | 0.5 (-11.9 to 12.5) |
| Stage V chronic kidney disease due to glomerulonephritis | 18.6 (13.3 to 25.5) | -- | 10.6 (6.4 to 15.9) | 24.4* (14.5 to 34.2) | 1.7 (-6.3 to 9.8) |
| Chronic kidney disease due to other and unspecified causes | 37823.9 (32736.6 to 43856.2) | 1576.9 (1112.2 to 2228.7) | 240.8 (172.1 to 322.5) | 17.4* (8.1 to 26.3) | -4.0 (-11.6 to 3.3) |
| Albuminuria with preserved GFR due to other and unspecified causes | 14464.2 (10810.7 to 18561.2) | -- | -- | -- | -- |
| End-stage chronic kidney disease due to other causes | 65.8 (42.6 to 93.5) | -- | 28.1 (16.1 to 42.7) | 23.2* (12.0 to 33.3) | 0.8 (-8.4 to 9.0) |
| Stage III chronic kidney disease due to other causes | 22744.2 (19303.3 to 26829.2) | 1576.9 (1112.2 to 2228.7) | 104.8 (67.3 to 157.0) | 9.2 (-1.6 to 20.8) | -10.7* (-19.5 to -1.2) |
| Stage IV chronic kidney disease due to other causes | 416.7 (272.7 to 600.9) | -- | 43.3 (24.3 to 71.1) | 23.4* (10.3 to 36.9) | 0.9 (-9.7 to 12.0) |
| Stage V chronic kidney disease due to other causes | 133.0 (95.0 to 183.1) | -- | 64.6 (40.0 to 97.2) | 26.0* (14.8 to 37.2) | 3.1 (-6.1 to 12.2) |
| Acute glomerulonephritis | 2.3 (1.5 to 3.1) | 33.7 (23.5 to 45.2) | 0.1 (0.1 to 0.2) | -2.2 (-6.4 to 1.9) | -20.0* (-23.4 to -16.7) |
| **Skin and subcutaneous diseases** | **75569.9 (69975.0 to 82045.8)** | **187197.3 (160500.7 to 214144.3)** | **1067.3 (695.7 to 1643.2)** | **24.8* (24.0 to 25.7)** | **2.1* (1.4 to 2.8)** |
| Dermatitis | 6569.9 (5563.3 to 7849.8) | 9600.5 (6975.3 to 12611.9) | 208.3 (127.9 to 319.0) | 23.1* (21.5 to 24.7) | 0.7 (-0.6 to 2.0) |
| Atopic dermatitis | 3266.0 (2905.9 to 3700.5) | 502.8 (414.0 to 604.9) | 132.5 (71.8 to 219.9) | 22.1* (20.1 to 24.3) | -0.1 (-1.7 to 1.7) |
| Contact dermatitis | 2953.7 (2047.3 to 4192.8) | 8290.0 (5667.9 to 11340.9) | 69.8 (39.7 to 114.4) | 24.3* (22.0 to 26.9) | 1.7 (-0.2 to 3.8) |
| Seborrhoeic dermatitis | 464.9 (386.1 to 567.2) | 807.8 (669.3 to 929.9) | 6.0 (3.4 to 9.8) | 30.2* (25.6 to 34.7) | 6.5* (2.8 to 10.2) |
| Psoriasis | 2867.0 (2683.5 to 3068.3) | 207.0 (176.9 to 239.6) | 231.7 (165.2 to 302.6) | 28.6* (26.3 to 31.2) | 5.2* (3.3 to 7.3) |
| Bacterial skin diseases | 311.4 (286.1 to 339.0) | 8043.8 (7339.5 to 8715.4) | 5.7 (3.6 to 8.5) | 27.8* (23.3 to 32.9) | 4.6* (0.9 to 8.7) |
| Cellulitis | 80.6 (66.5 to 96.3) | 1796.6 (1510.9 to 2109.2) | 4.4 (2.8 to 6.4) | 29.3* (23.5 to 35.7) | 5.8* (1.0 to 11.0) |
| Pyoderma | 251.5 (229.0 to 276.3) | 6247.3 (5592.7 to 6889.2) | 1.3 (0.6 to 2.8) | 23.2* (19.1 to 27.2) | 0.8 (-2.6 to 4.1) |
| Impetigo | 13.3 (11.9 to 14.9) | 259.5 (235.8 to 285.6) | 0.1 (0.0 to 0.2) | 29.6* (21.6 to 38.4) | 6.0 (-0.5 to 13.2) |
| Abscess and other bacterial skin diseases | 238.2 (215.7 to 263.0) | 5987.8 (5319.6 to 6641.2) | 1.3 (0.5 to 2.7) | 22.9* (18.6 to 27.0) | 0.5 (-2.9 to 3.9) |
| Scabies | 2281.1 (1597.3 to 3076.7) | 6928.0 (4905.9 to 9354.5) | 55.6 (29.0 to 96.4) | 22.6* (20.8 to 24.5) | 0.3 (-1.2 to 1.9) |
| Fungal skin diseases | 36988.5 (29754.3 to 45740.5) | 117480.2 (91070.9 to 144278.8) | 199.0 (78.5 to 415.3) | 20.5* (20.1 to 20.9) | -1.4* (-1.7 to -1.1) |
| Tinea capitis | 663.7 (465.4 to 964.5) | 1134.8 (791.0 to 1517.2) | 3.5 (1.3 to 8.1) | 1.8 (-1.3 to 5.0) | -16.7* (-19.2 to -14.1) |
| Other fungal skin diseases | 36324.8 (29180.6 to 45008.2) | 116345.4 (89751.4 to 142937.8) | 195.5 (77.0 to 404.2) | 20.9* (20.5 to 21.3) | -1.1* (-1.4 to -0.7) |

| | | | | | |
|---|---|---|---|---|---|
| Viral skin diseases | 1289.1 (1158.5 to 1422.2) | 499.0 (450.4 to 554.6) | 37.1 (23.7 to 54.5) | 25.2* (22.9 to 27.8) | 2.4* (0.5 to 4.5) |
| Viral warts | 1193.0 (1061.5 to 1327.2) | 386.2 (338.9 to 440.5) | 34.4 (21.9 to 50.4) | 25.5* (23.0 to 28.1) | 2.7* (0.7 to 4.8) |
| Molluscum contagiosum | 96.1 (84.6 to 108.8) | 112.8 (100.8 to 125.7) | 2.8 (1.8 to 4.2) | 21.9* (13.6 to 30.5) | -0.3 (-7.1 to 6.7) |
| Acne vulgaris | 314.8 (305.8 to 323.7) | 141.8 (136.8 to 147.0) | 6.6 (4.0 to 10.3) | 37.3* (33.8 to 41.2) | 12.4* (9.5 to 15.5) |
| Alopecia areata | 330.7 (299.3 to 363.4) | 561.9 (512.9 to 615.9) | 10.3 (6.6 to 15.2) | 22.1* (18.3 to 26.4) | -0.1 (-3.2 to 3.4) |
| Pruritus | 3181.9 (2207.3 to 4464.7) | 2470.4 (1653.9 to 3372.4) | 32.5 (14.3 to 62.7) | 23.6* (22.2 to 25.0) | 1.1 (-0.0 to 2.3) |
| Urticaria | 1168.0 (805.5 to 1601.2) | 2024.6 (1396.4 to 2783.1) | 64.8 (38.8 to 99.1) | 21.4* (18.8 to 24.1) | -0.7 (-2.8 to 1.5) |
| Decubitus ulcer | 105.6 (76.6 to 142.8) | 392.1 (280.3 to 532.6) | 16.4 (10.2 to 24.4) | 26.0* (18.9 to 33.3) | 3.1 (-2.8 to 9.0) |
| Other skin and subcutaneous diseases | 37702.1 (34502.4 to 41082.3) | 38847.8 (35437.1 to 42156.3) | 199.3 (96.2 to 373.0) | 28.2* (27.4 to 28.9) | 4.8* (4.2 to 5.4) |
| **Sense organ diseases** | **126606.2 (124509.3 to 128842.3)** | **--** | **6015.3 (4175.2 to 8393.6)** | **19.3* (17.4 to 20.9)** | **-2.4* (-4.0 to -1.1)** |
| Blindness and vision impairment | 79100.9 (74772.5 to 83610.3) | -- | 2752.9 (1913.7 to 3828.6) | 16.6* (14.7 to 18.3) | -4.7* (-6.1 to -3.2) |
| Glaucoma | 1006.4 (818.8 to 1226.1) | -- | 113.7 (76.2 to 160.7) | 14.3* (12.0 to 16.5) | -6.5* (-8.4 to -4.7) |
| Cataract | 16914.4 (13641.8 to 20908.6) | -- | 1213.1 (834.1 to 1644.7) | 18.6* (16.0 to 21.4) | -2.9* (-5.1 to -0.7) |
| Age-related macular degeneration | 1126.9 (851.2 to 1452.3) | -- | 80.9 (53.3 to 113.7) | 17.7* (14.5 to 20.7) | -3.7* (-6.3 to -1.2) |
| Refraction disorders | 9968.4 (7519.2 to 12914.6) | -- | 467.2 (301.6 to 678.8) | 8.9* (5.7 to 12.2) | -10.9* (-13.5 to -8.2) |
| Near vision loss | 63907.8 (60005.6 to 68186.7) | -- | 627.8 (296.6 to 1156.0) | 20.6* (19.9 to 21.4) | -1.3* (-2.0 to -0.7) |
| Other vision loss | 2978.8 (2391.6 to 3694.6) | -- | 250.1 (168.9 to 348.0) | 12.7* (10.6 to 14.8) | -7.8* (-9.5 to -6.1) |
| Age-related and other hearing loss | 110474.6 (107880.4 to 112783.9) | -- | 3049.4 (2072.8 to 4238.0) | 21.5* (18.3 to 24.7) | -0.6 (-3.2 to 2.0) |
| Other sense organ diseases | 8886.4 (7950.0 to 9901.1) | -- | 212.9 (131.3 to 304.3) | 23.3* (21.9 to 24.8) | 0.9 (-0.3 to 2.1) |
| Chronic other sense organ diseases | 8327.7 (7419.6 to 9350.3) | -- | 205.6 (126.4 to 294.8) | 23.3* (21.8 to 24.7) | 0.9 (-0.3 to 2.0) |
| Acute other sense organ diseases | 558.8 (509.8 to 612.9) | -- | 7.3 (4.4 to 11.0) | 23.5* (19.0 to 28.3) | 1.0 (-2.7 to 4.9) |
| **Musculoskeletal disorders** | **75581.7 (69497.5 to 82250.5)** | **16249.6 (12748.0 to 19946.4)** | **7369.1 (5249.1 to 10146.6)** | **23.1* (21.2 to 25.4)** | **0.7 (-0.9 to 2.6)** |
| Rheumatoid arthritis | 1722.4 (1517.5 to 1951.1) | 66.9 (45.2 to 92.9) | 216.7 (149.2 to 291.2) | 33.9* (29.6 to 38.0) | 9.5* (6.0 to 12.9) |
| Osteoarthritis | 32970.5 (29205.4 to 36970.7) | 815.1 (583.9 to 1109.1) | 1025.3 (513.5 to 2049.4) | 24.4* (23.6 to 25.3) | 1.8* (1.1 to 2.5) |
| Osteoarthritis of the hip | 4675.3 (4346.5 to 5048.9) | 142.5 (111.9 to 176.4) | 145.4 (73.8 to 293.4) | 29.5* (27.9 to 31.1) | 5.9* (4.6 to 7.2) |
| Osteoarthritis of the knee | 28295.1 (24513.8 to 32360.1) | 672.6 (447.3 to 969.6) | 880.0 (438.8 to 1752.4) | 23.6* (22.8 to 24.5) | 1.1* (0.5 to 1.9) |
| Low back pain | 29126.6 (21638.3 to 37880.1) | 11623.5 (8288.1 to 14843.3) | 3160.7 (2006.9 to 4651.7) | 19.4* (18.3 to 20.5) | -2.3* (-3.2 to -1.4) |
| Low back pain with leg pain | 10695.9 (7691.4 to 14547.7) | 4249.8 (3008.3 to 5755.6) | 1460.6 (889.0 to 2225.3) | 19.4* (18.2 to 20.6) | -2.3* (-3.3 to -1.4) |
| Low back pain without leg pain | 18430.7 (13734.0 to 24430.2) | 7373.7 (5227.5 to 9505.4) | 1700.1 (1080.6 to 2475.3) | 19.4* (18.1 to 20.6) | -2.3* (-3.4 to -1.4) |
| Neck pain | 17076.8 (12260.7 to 22937.5) | 3077.1 (1878.8 to 4681.1) | 1618.3 (1043.1 to 2412.8) | 24.1* (21.6 to 26.9) | 1.6 (-0.6 to 3.8) |
| Gout | 4235.3 (3337.1 to 5312.3) | 666.9 (500.9 to 869.9) | 126.9 (83.8 to 179.6) | 29.3* (26.1 to 32.8) | 5.7* (3.2 to 8.6) |
| Other musculoskeletal disorders | 15247.4 (10107.7 to 20922.2) | -- | 1221.2 (707.3 to 1854.2) | 28.2* (16.3 to 39.2) | 4.9 (-4.8 to 13.9) |
| **Other non-communicable diseases** | **122333.2 (116184.4 to 128346.8)** | **49215.5 (42718.9 to 55329.6)** | **2124.9 (1438.2 to 2984.3)** | **16.8* (15.6 to 17.9)** | **-4.5* (-5.4 to -3.5)** |
| Congenital anomalies | 2107.9 (1973.6 to 2262.2) | -- | 232.9 (166.0 to 308.5) | 20.4* (18.1 to 23.0) | -1.5 (-3.4 to 0.6) |
| Neural tube defects | 48.6 (43.3 to 54.2) | -- | 14.1 (9.8 to 18.8) | 23.7* (18.2 to 28.7) | 1.2 (-3.3 to 5.3) |
| Severe motor and cognitive impairment due to anencephaly | -- | -- | -- | -- | -- |
| Encephalocoele due to neural tube defects | 7.5 (6.2 to 8.9) | -- | 1.3 (0.9 to 1.8) | 17.5* (13.9 to 21.1) | -3.9* (-6.9 to -0.9) |
| Spina bifida due to neural tube defects | 41.1 (36.6 to 46.2) | -- | 12.8 (8.8 to 17.2) | 24.4* (18.5 to 29.7) | 1.8 (-3.1 to 6.1) |
| Congenital heart anomalies | 58.4 (53.2 to 64.2) | -- | 3.3 (1.4 to 5.8) | 26.2* (20.3 to 32.2) | 3.2 (-1.6 to 8.1) |
| Critical malformations of great vessels, congenital valvular heart disease and patent ductus arteriosus | 8.8 (7.6 to 10.2) | -- | 0.5 (0.2 to 0.9) | 25.0* (22.2 to 27.3) | 2.2 (-0.0 to 4.1) |
| Other congenital heart anomalies | 27.5 (25.0 to 30.2) | -- | 1.5 (0.6 to 2.7) | 26.0* (15.8 to 37.4) | 3.1 (-5.3 to 12.4) |
| Severe congenital heart anomalies excluding single ventricle heart defects | 20.8 (18.4 to 23.3) | -- | 1.2 (0.5 to 2.1) | 27.1* (16.5 to 38.1) | 4.0 (-4.7 to 13.0) |
| Single ventricle and single ventricle pathway congenital heart anomalies | 0.4 (0.3 to 0.4) | -- | -- | 29.5* (26.1 to 32.8) | 5.9* (3.1 to 8.6) |
| Ventricular septal defect and atrial septal defect | 1.0 (0.8 to 1.2) | -- | 0.0 (0.0 to 0.1) | 18.2* (15.3 to 21.1) | -3.3* (-5.7 to -0.9) |
| Orofacial clefts | 229.6 (210.0 to 249.2) | -- | 5.8 (3.8 to 8.2) | 25.8* (15.7 to 35.8) | 2.9 (-5.3 to 11.1) |
| Down's syndrome | -- | -- | -- | -- | -- |
| Turner syndrome | 2.6 (2.2 to 3.1) | -- | 0.0 (0.0 to 0.1) | 23.3* (20.2 to 26.3) | 0.8 (-1.6 to 3.3) |
| Klinefelter syndrome | 0.9 (0.8 to 1.1) | -- | -- | 31.1* (29.3 to 33.0) | 7.3* (5.8 to 8.8) |
| Other chromosomal abnormalities | -- | -- | -- | -- | -- |
| Congenital musculoskeletal and limb anomalies | 962.9 (856.9 to 1100.4) | -- | 132.7 (89.8 to 178.8) | 20.0* (18.3 to 22.0) | -1.8* (-3.3 to -0.2) |
| Polydactyly and syndactyly | 1.6 (1.3 to 1.9) | -- | -- | 13.9* (12.0 to 16.0) | -6.8* (-8.3 to -5.1) |
| Congenital limb deficiency | 247.3 (219.9 to 282.9) | -- | 34.2 (23.1 to 46.4) | 20.1* (16.4 to 24.0) | -1.7 (-4.8 to 1.4) |
| Other congenital musculoskeletal anomalies | 714.0 (635.4 to 815.9) | -- | 98.5 (66.5 to 132.5) | 20.0* (17.8 to 22.3) | -1.8 (-3.6 to 0.1) |
| Urogenital congenital anomalies | 32.1 (28.6 to 35.8) | -- | 1.5 (0.9 to 2.2) | 18.5* (11.9 to 24.9) | -3.0 (-8.5 to 2.2) |
| Digestive congenital anomalies | 472.1 (425.7 to 525.9) | -- | 20.2 (13.5 to 28.2) | 12.9* (6.8 to 19.6) | -7.6* (-12.7 to -2.2) |
| Congenital diaphragmatic hernia | 2.2 (1.9 to 2.5) | -- | 0.0 (0.0 to 0.1) | 26.9* (25.1 to 28.6) | 3.8* (2.4 to 5.2) |
| Congenital atresia and/or stenosis of the digestive tract | 0.1 (0.1 to 0.2) | -- | -- | 42.2* (36.9 to 47.8) | 16.3* (12.0 to 20.9) |
| Congenital malformations of the abdominal wall after treatment | 8.8 (7.6 to 10.0) | -- | 0.4 (0.2 to 0.6) | 26.5* (25.0 to 27.8) | 3.5* (2.2 to 4.5) |
| Other congenital malformations of the digestive tract | 461.0 (414.5 to 514.3) | -- | 19.7 (13.3 to 27.6) | 12.6* (6.4 to 19.5) | -7.9* (-13.0 to -2.2) |
| Other congenital anomalies | 480.9 (413.5 to 560.2) | -- | 55.4 (37.3 to 77.1) | 22.7* (14.7 to 31.8) | 0.4 (-6.2 to 7.8) |

| | | | | | |
|---|---|---|---|---|---|
| Other congenital birth defects | -- | -- | -- | -- | -- |
| Hearing loss due to other congenital anomalies | 480.9 (413.5 to 560.2) | -- | 55.4 (37.3 to 77.1) | 22.7* (14.7 to 31.8) | 0.4 (-6.2 to 7.8) |
| Urinary diseases and male infertility | 9255.4 (7935.3 to 10657.0) | 12488.1 (10611.9 to 14369.8) | 319.1 (205.8 to 460.9) | 22.1* (20.6 to 23.7) | -0.1 (-1.3 to 1.2) |
| Urinary tract infections | 133.4 (104.3 to 163.3) | 7001.7 (5556.2 to 8433.1) | 4.2 (2.5 to 6.3) | 47.3* (37.8 to 57.9) | 20.5* (12.7 to 29.2) |
| Urolithiasis | 177.1 (128.9 to 228.0) | 4615.4 (3395.1 to 5928.9) | 12.2 (7.7 to 18.0) | 21.6* (13.4 to 30.1) | -0.6 (-7.2 to 6.4) |
| Benign prostatic hyperplasia | 9301.1 (7903.1 to 10821.0) | 871.0 (730.2 to 1052.3) | 298.1 (189.8 to 432.5) | 22.2* (20.7 to 23.7) | -0.0 (-1.3 to 1.2) |
| Male infertility | -- | -- | -- | -- | -- |
| Other urinary diseases | -- | -- | 4.6 (3.1 to 6.5) | 3.4 (-2.1 to 9.6) | -15.4* (-19.9 to -10.3) |
| Gynecological diseases | 9763.0 (8332.1 to 11364.4) | 676.5 (480.2 to 900.0) | 38.6 (20.1 to 70.7) | 16.5* (13.7 to 19.2) | -4.7* (-7.0 to -2.5) |
| Uterine fibroids | 1875.0 (1641.0 to 2156.4) | 9.2 (6.3 to 13.0) | 13.8 (7.7 to 23.3) | 14.4* (8.4 to 21.6) | -6.4* (-11.3 to -0.5) |
| Polycystic ovarian syndrome | -- | -- | -- | -- | -- |
| Female infertility | -- | -- | -- | -- | -- |
| Endometriosis | -- | -- | -- | -- | -- |
| Genital prolapse | 8222.6 (6739.5 to 9886.7) | 667.3 (472.2 to 889.3) | 24.8 (11.8 to 47.8) | 17.7* (16.0 to 19.5) | -3.7* (-5.1 to -2.2) |
| Premenstrual syndrome | -- | -- | -- | -- | -- |
| Other gynecological diseases | -- | -- | -- | -- | -- |
| Other gynecological diseases | -- | -- | -- | -- | -- |
| Other gynecological diseases with anemia | -- | -- | -- | -- | -- |
| Hemoglobinopathies and hemolytic anaemias | 37978.2 (37146.4 to 38820.5) | -- | 112.6 (76.0 to 162.6) | 5.3 (-3.2 to 15.1) | -13.8* (-20.8 to -5.8) |
| Thalassemias | -- | -- | -- | 14.8* (4.3 to 28.0) | -6.1 (-14.7 to 4.7) |
| Thalassaemias trait | 6603.3 (6336.5 to 6887.1) | -- | 40.0 (25.4 to 61.2) | 10.4 (-6.2 to 31.1) | -9.7 (-23.3 to 7.2) |
| Sickle cell disorders | -- | -- | -- | 45.8* (35.2 to 58.4) | 19.3* (10.6 to 29.6) |
| Sickle cell trait | 7244.2 (6499.3 to 8074.4) | -- | 14.8 (10.0 to 21.5) | 5.8 (-5.4 to 18.2) | -13.4* (-22.6 to -3.3) |
| G6PD deficiency | 6089.0 (5852.5 to 6324.7) | -- | 0.6 (0.4 to 0.8) | 10.8 (-4.3 to 27.4) | -9.4 (-21.7 to 4.2) |
| G6PD trait | 19469.8 (19150.8 to 19786.7) | -- | -- | 18.6* (3.4 to 39.4) | -3.0 (-15.5 to 14.1) |
| Other hemoglobinopathies and hemolytic anaemias | 2530.0 (2354.8 to 2727.3) | -- | 57.2 (38.4 to 82.0) | 1.9 (-5.3 to 10.0) | -16.6* (-22.5 to -10.0) |
| Endocrine, metabolic, blood, and immune disorders | 3595.3 (3164.3 to 4078.8) | -- | 106.5 (73.6 to 149.1) | 19.3* (12.7 to 26.8) | -2.4 (-7.8 to 3.7) |
| Anemia due to endocrine, metabolic, blood, and immune disorders | 1494.0 (1387.9 to 1616.0) | -- | 31.5 (21.2 to 44.6) | 0.0 (-9.7 to 9.9) | -18.2* (-26.2 to -10.1) |
| Endocrine, metabolic, blood, and immune disorders | 2041.4 (1631.5 to 2514.1) | -- | 69.9 (46.0 to 101.3) | 30.2* (21.3 to 40.0) | 6.5 (-0.8 to 14.5) |
| Heart failure due to endocrine, metabolic, blood, and immune disorders | 59.9 (42.7 to 82.1) | -- | 5.1 (3.1 to 8.1) | 23.7* (15.1 to 32.4) | 1.2 (-5.8 to 8.3) |
| Oral disorders | 99999.2 (90955.2 to 109007.1) | 36051.0 (30358.8 to 41767.8) | 1315.1 (841.9 to 1955.9) | 15.8* (14.4 to 17.2) | -5.3* (-6.4 to -4.1) |
| Caries of deciduous teeth | -- | -- | -- | -- | -- |
| Caries of permanent teeth | 56119.6 (43514.2 to 69639.8) | 31119.5 (25735.7 to 36952.0) | 40.4 (17.4 to 77.6) | 15.9* (14.2 to 17.5) | -5.2* (-6.6 to -3.9) |
| Periodontal disease | 40777.1 (32209.1 to 48972.2) | 2979.3 (1882.2 to 4097.4) | 257.0 (96.5 to 525.8) | 25.7* (24.7 to 26.7) | 2.8* (2.0 to 3.6) |
| Edentulism and severe tooth loss | 33674.0 (28145.8 to 39925.1) | 1952.2 (1323.8 to 2742.0) | 912.9 (598.2 to 1314.4) | 12.6* (11.5 to 13.9) | -7.9* (-8.8 to -6.8) |
| Other oral disorders | 3818.4 (3404.4 to 4337.1) | -- | 104.8 (65.9 to 152.9) | 22.3* (20.9 to 23.7) | 0.1 (-1.1 to 1.2) |
| **Injuries** | **73791.3 (69764.0 to 79143.8)** | **16221.6 (13464.1 to 19409.6)** | **2815.4 (2024.6 to 3805.3)** | **28.9* (27.9 to 29.8)** | **5.4* (4.7 to 6.2)** |
| **Transport injuries** | **12763.8 (11701.2 to 13901.3)** | **1239.7 (917.0 to 1605.1)** | **652.8 (475.9 to 873.3)** | **29.2* (28.3 to 30.1)** | **5.7* (4.9 to 6.4)** |
| Road injuries | 10327.5 (9516.2 to 11340.1) | 1000.8 (683.8 to 1352.8) | 526.7 (379.3 to 710.9) | 29.0* (28.0 to 29.9) | 5.5* (4.7 to 6.3) |
| Pedestrian road injuries | 2780.5 (2411.4 to 3312.3) | 287.3 (172.1 to 431.0) | 144.4 (101.4 to 199.2) | 26.4* (24.9 to 28.0) | 3.4* (2.2 to 4.7) |
| Cyclist road injuries | 1962.6 (1659.0 to 2323.6) | 239.6 (138.8 to 369.0) | 96.8 (67.1 to 131.4) | 34.1* (32.4 to 35.7) | 9.7* (8.3 to 11.0) |
| Motorcyclist road injuries | 2363.8 (2033.1 to 2747.2) | 114.0 (61.9 to 185.7) | 118.1 (83.7 to 161.5) | 36.5* (34.5 to 38.5) | 11.7* (10.1 to 13.3) |
| Motor vehicle road injuries | 2533.0 (2168.0 to 2975.3) | 265.5 (169.6 to 396.9) | 135.1 (97.4 to 181.7) | 18.3* (17.1 to 19.6) | -3.2* (-4.2 to -2.1) |
| Other road injuries | 687.6 (566.4 to 819.4) | 94.3 (51.1 to 159.9) | 32.2 (22.9 to 45.2) | 51.7* (49.5 to 53.9) | 24.1* (22.3 to 25.9) |
| Other transport injuries | 2436.2 (2081.6 to 2795.2) | 238.9 (159.2 to 348.4) | 126.1 (90.6 to 168.1) | 30.1* (28.5 to 31.7) | 6.4* (5.1 to 7.8) |
| **Unintentional injuries** | **54027.0 (49948.5 to 59347.9)** | **14632.2 (11963.5 to 17796.1)** | **1998.7 (1410.2 to 2719.0)** | **28.9* (27.7 to 30.0)** | **5.4* (4.4 to 6.3)** |
| Falls | 27195.2 (23803.5 to 32262.7) | 7330.5 (5078.3 to 10045.9) | 1237.5 (861.7 to 1691.0) | 28.3* (26.5 to 29.9) | 4.9* (3.5 to 6.3) |
| Drowning | 125.8 (109.0 to 146.3) | 12.0 (8.7 to 16.7) | 6.5 (4.6 to 8.7) | 31.3* (28.1 to 34.3) | 7.4* (4.8 to 9.8) |
| Fire, heat, and hot substances | 4385.0 (3754.9 to 5135.7) | 85.3 (53.9 to 127.3) | 88.2 (55.0 to 136.4) | 25.5* (22.9 to 27.7) | 2.6* (0.5 to 4.4) |
| Poisonings | 175.4 (149.5 to 202.2) | 47.2 (26.8 to 73.7) | 14.7 (9.8 to 20.6) | 29.9* (26.4 to 33.3) | 6.2* (3.4 to 9.0) |
| Poisoning by carbon monoxide | 36.7 (31.5 to 43.0) | 15.1 (6.8 to 27.2) | 2.5 (1.7 to 3.4) | 33.5* (30.2 to 37.2) | 9.2* (6.5 to 12.2) |
| Poisoning by other means | 138.7 (113.4 to 164.4) | 32.1 (17.5 to 53.6) | 12.2 (8.0 to 17.6) | 29.1* (25.3 to 32.7) | 5.6* (2.5 to 8.6) |
| Exposure to mechanical forces | 10596.4 (9169.3 to 12327.5) | 1157.7 (744.1 to 1808.4) | 241.3 (164.7 to 344.9) | 31.2* (30.2 to 32.3) | 7.3* (6.5 to 8.3) |
| Unintentional firearm injuries | 409.3 (342.9 to 478.6) | 21.4 (9.2 to 41.4) | 11.8 (8.3 to 16.5) | 32.3* (30.8 to 33.9) | 8.2* (7.0 to 9.6) |
| Other exposure to mechanical forces | 10187.0 (8747.4 to 11920.6) | 1136.2 (727.9 to 1765.3) | 229.5 (155.5 to 328.5) | 31.2* (30.1 to 32.3) | 7.3* (6.4 to 8.2) |
| Adverse effects of medical treatment | 218.0 (145.1 to 315.3) | 2857.1 (2013.8 to 4067.6) | 29.1 (16.5 to 48.3) | 80.1* (64.2 to 96.9) | 47.3* (34.3 to 61.0) |
| Animal contact | 1977.6 (1741.3 to 2286.8) | 902.6 (548.1 to 1325.4) | 40.5 (27.5 to 57.7) | 20.9* (18.1 to 23.3) | -1.1 (-3.4 to 0.9) |
| Venomous animal contact | 738.0 (602.5 to 845.9) | 389.7 (227.7 to 617.8) | 21.9 (15.2 to 29.7) | 20.7* (17.5 to 23.8) | -1.3 (-3.9 to 1.3) |
| Non-venomous animal contact | 1239.7 (1023.9 to 1506.8) | 512.9 (302.3 to 778.4) | 18.7 (11.6 to 29.9) | 21.2* (18.4 to 23.4) | -0.8 (-3.2 to 0.9) |
| Foreign body | 902.7 (809.3 to 1011.4) | 531.4 (353.9 to 819.8) | 37.3 (26.7 to 49.8) | 24.2* (23.0 to 25.4) | 1.6* (0.7 to 2.6) |

| | | | | | |
|---|---|---|---|---|---|
| Pulmonary aspiration and foreign body in airway | 136.7 (117.1 to 163.9) | 32.4 (19.2 to 49.3) | 5.3 (3.7 to 7.3) | 33.7* (31.2 to 36.0) | 9.3* (7.3 to 11.3) |
| Foreign body in eyes | 129.4 (92.1 to 181.8) | 412.1 (235.8 to 697.3) | 8.6 (5.2 to 13.7) | 26.5* (25.0 to 28.0) | 3.5* (2.3 to 4.7) |
| Foreign body in other body part | 636.6 (549.7 to 730.0) | 87.0 (47.5 to 145.2) | 23.3 (16.7 to 30.7) | 21.4* (20.2 to 22.7) | -0.6 (-1.7 to 0.4) |
| Environmental heat and cold exposure | 1774.5 (1540.4 to 2081.4) | 268.3 (165.2 to 410.7) | 69.3 (48.3 to 96.8) | 26.1* (24.1 to 28.0) | 3.1* (1.5 to 4.7) |
| Exposure to forces of nature | 322.7 (236.8 to 439.5) | 9.2 (6.3 to 12.6) | 11.9 (8.7 to 15.7) | 56.3* (42.5 to 73.6) | 27.8* (16.6 to 42.0) |
| Other unintentional injuries | 6882.5 (5927.4 to 7874.5) | 1431.0 (806.2 to 2220.6) | 222.3 (150.9 to 308.6) | 28.3* (27.5 to 29.3) | 5.0* (4.3 to 5.8) |
| **Self-harm and interpersonal violence** | **7725.3 (6899.1 to 8672.4)** | **349.7 (265.9 to 468.6)** | **163.9 (126.7 to 207.0)** | **27.7* (24.5 to 31.6)** | **4.4* (1.9 to 7.7)** |
| Self-harm | 523.4 (434.4 to 611.7) | 86.9 (54.0 to 129.1) | 21.6 (15.4 to 28.8) | 12.3* (11.1 to 13.4) | -8.1* (-9.1 to -7.2) |
| Self-harm by firearm | 10.8 (8.7 to 13.8) | 3.6 (0.9 to 8.6) | 0.4 (0.3 to 0.6) | 8.9* (6.0 to 12.6) | -10.9* (-13.3 to -7.9) |
| Self-harm by other specified means | 512.6 (423.5 to 600.6) | 83.3 (51.5 to 125.0) | 21.2 (15.0 to 28.2) | 12.4* (11.2 to 13.5) | -8.1* (-9.0 to -7.2) |
| Interpersonal violence | 6417.5 (5634.8 to 7320.1) | 215.8 (140.3 to 324.0) | 116.5 (87.6 to 151.0) | 22.2* (21.0 to 23.5) | -0.1 (-1.0 to 1.0) |
| Assault by firearm | 141.3 (119.8 to 170.3) | 7.4 (3.4 to 13.7) | 4.9 (3.4 to 6.7) | 32.9* (31.0 to 34.7) | 8.7* (7.2 to 10.2) |
| Assault by sharp object | 636.6 (526.2 to 859.8) | 28.6 (14.5 to 50.3) | 15.7 (10.8 to 22.3) | 20.4* (18.8 to 22.1) | -1.5* (-2.8 to -0.1) |
| Sexual violence | 3580.9 (2912.2 to 4437.0) | -- | 24.1 (15.6 to 35.1) | 30.3* (28.0 to 32.6) | 6.6* (4.7 to 8.4) |
| Assault by other means | 2058.6 (1756.3 to 2430.2) | 179.9 (115.1 to 274.1) | 71.8 (50.2 to 97.6) | 19.4* (18.3 to 20.6) | -2.3* (-3.2 to -1.3) |
| Conflict and terrorism | 697.2 (447.6 to 1031.7) | 42.0 (28.3 to 56.7) | 23.3 (16.3 to 32.7) | 82.2* (56.4 to 113.1) | 49.1* (28.0 to 74.3) |
| Executions and police conflict | 87.3 (54.0 to 133.1) | 5.0 (3.1 to 7.0) | 2.6 (1.7 to 3.8) | 144.2* (97.3 to 208.9) | 99.7* (61.4 to 152.7) |
| **Nature of injury aggregates** | | | | | |
| Amputations | 19355.1 (17906.9 to 21212.3) | 244.7 (190.4 to 309.4) | 325.6 (218.6 to 474.9) | 22.5* (18.8 to 25.6) | 0.2 (-2.8 to 2.8) |
| Burns | 8961.8 (8292.0 to 9771.3) | 170.2 (131.5 to 219.9) | 169.8 (103.1 to 265.3) | 24.9* (22.9 to 26.5) | 2.2* (0.6 to 3.5) |
| Fractures | 24866.9 (22768.8 to 27858.0) | 5282.8 (3995.6 to 6857.9) | 1328.1 (913.4 to 1844.9) | 27.2* (25.9 to 28.6) | 4.0* (3.0 to 5.2) |
| Head Injuries | 2453.5 (2350.1 to 2563.0) | 554.0 (428.4 to 736.7) | 339.4 (240.4 to 452.8) | 30.9* (29.4 to 32.4) | 7.1* (5.9 to 8.3) |
| Spinal Injuries | 934.5 (863.2 to 1009.6) | 22.8 (16.4 to 31.9) | 239.7 (173.0 to 308.6) | 41.3* (38.8 to 43.9) | 15.6* (13.6 to 17.7) |
| Minor Injuries | 11232.8 (10539.2 to 12151.1) | 4212.1 (3344.6 to 5231.4) | 98.1 (46.5 to 183.1) | 30.9* (29.8 to 32.1) | 7.1* (6.2 to 8.1) |
| Other Injuries | 3660.5 (3463.6 to 3873.3) | 5734.9 (4710.6 to 7007.7) | 290.6 (204.4 to 385.8) | 34.5* (31.9 to 37.2) | 10.0* (7.9 to 12.3) |
| **Impairments** | | | | | |
| Anaemia | 38982.8 (36130.7 to 41952.2) | -- | 1032.0 (724.8 to 1426.8) | 5.4 (-1.8 to 12.6) | -13.8* (-19.7 to -7.9) |
| Epilepsy | 1390.9 (1141.9 to 1660.7) | -- | 417.5 (301.8 to 561.6) | 30.3* (21.8 to 39.0) | 6.6 (-0.3 to 13.7) |
| Guillain-Barré syndrome | 3.7 (2.2 to 6.0) | -- | 1.1 (0.6 to 2.0) | 28.9* (25.4 to 32.4) | 5.4* (2.6 to 8.3) |
| Hearing loss | 111817.2 (109213.1 to 114110.8) | -- | 3123.2 (2127.6 to 4327.1) | 21.5* (18.3 to 24.5) | -0.6 (-3.2 to 1.9) |
| Heart failure | 9339.0 (7775.9 to 10992.7) | -- | 1656.3 (1162.7 to 2192.5) | 23.2* (20.5 to 25.3) | 0.8 (-1.4 to 2.5) |
| Infertility | -- | -- | -- | -37.9* (-50.0 to -31.9) | -49.2* (-59.1 to -44.3) |
| Developmental intellectual disability | 1860.8 (1408.8 to 2302.7) | -- | 234.4 (177.6 to 297.1) | 34.4* (29.3 to 40.5) | 9.9* (5.8 to 14.9) |
| Pelvic inflammatory disease | -- | -- | -- | -- | -- |
| Vision loss | 97436.0 (90062.1 to 105520.4) | -- | 2891.1 (2029.0 to 3986.9) | 16.2* (14.5 to 18.0) | -4.9* (-6.4 to -3.5) |

We did not calculate incidence for the nine impairments and certain NTDs. Blank cells mean that no estimate is available or that the estimate has a magnitude less than 50.
Table presents prevalence and incidence counts in thousands.
* Percentage changes that are statistically significant
‡ Incidence estimates for stroke represent first-ever stroke only.
^ Incidence of HIV/AIDS represents new infections of HIV only and does not include new infections of Tuberculosis in HIV positive cases.

**SR Table 10s: Global prevalence, incidence and YLDs for 2017, percentage change of YLD counts and percentage change of YLD rates between 2007 and 2017 by cause, age 75 to 79 years**

| Cause | Prevalence in thousands (95% UI) | Incidence in thousands (95% UI) | YLDs in thousands (95% UI) | | |
|---|---|---|---|---|---|
| | 2017 counts | 2017 counts | 2017 counts | Percentage change in counts between 2007 and 2017 | Percentage change in rates between 2007 and 2017 |
| **All causes** | **123644.8 (123628.6 to 123658.9)** | **635989.5 (564815.8 to 723831.2)** | **32512.2 (24766.7 to 41137.0)** | **26.1* (25.2 to 27.1)** | **0.4 (-0.3 to 1.2)** |
| **Communicable, maternal, neonatal, and nutritional disorders** | **66816.7 (61776.0 to 72194.5)** | **355363.8 (293423.0 to 436339.1)** | **1127.5 (825.9 to 1471.7)** | **20.0* (16.1 to 23.9)** | **-4.5* (-7.6 to -1.4)** |
| **HIV/AIDS and sexually transmitted infections** | **23235.4 (20574.9 to 26094.5)** | **1544.2 (1331.2 to 1836.5)** | **33.3 (22.9 to 47.1)** | **16.3 (-5.5 to 39.8)** | **-7.4 (-24.8 to 11.3)** |
| HIV/AIDS | 236.0 (206.5 to 272.0) | 6.1^ (4.4 to 8.2) | 23.1 (15.6 to 32.8) | 13.1 (-13.1 to 51.5) | -10.0 (-30.8 to 20.6) |
| HIV/AIDS - Drug-susceptible Tuberculosis | 6.4 (5.8 to 6.9) | 7.0 (6.4 to 7.6) | 2.3 (1.6 to 3.1) | 5.0 (-4.8 to 14.8) | -16.4* (-24.2 to -8.6) |
| HIV/AIDS - Multidrug-resistant Tuberculosis without extensive drug resistance | 0.3 (0.2 to 0.5) | 0.3 (0.2 to 0.4) | 0.1 (0.1 to 0.2) | -11.7 (-47.6 to 66.7) | -29.7 (-58.3 to 32.7) |
| HIV/AIDS - Extensively drug-resistant Tuberculosis | -- | -- | -- | 33.0 (-42.4 to 219.5) | 5.9 (-54.1 to 154.4) |
| HIV/AIDS resulting in other diseases | 229.3 (199.8 to 265.7) | 6.1 (4.4 to 8.2) | 20.7 (13.7 to 29.9) | 14.2 (-14.2 to 60.6) | -9.0 (-31.7 to 27.8) |
| HIV/AIDS not on antiretroviral treatment without Tuberculosis | 40.9 (31.0 to 55.7) | 6.1 (4.4 to 8.2) | 7.1 (3.5 to 13.3) | -54.8* (-61.8 to -48.4) | -64.0* (-69.6 to -58.9) |
| HIV/AIDS on antiretroviral treatment without Tuberculosis | 188.4 (163.6 to 213.5) | -- | 13.6 (9.2 to 19.3) | 459.3* (393.9 to 547.0) | 345.3* (293.2 to 415.2) |
| Sexually transmitted infections excluding HIV | 23077.6 (20411.1 to 25952.8) | 1538.1 (1325.7 to 1830.0) | 10.2 (5.8 to 18.5) | 24.4* (20.9 to 26.6) | -1.0 (-3.7 to 0.8) |
| Syphilis | 89.0 (72.2 to 110.9) | -- | 3.5 (2.3 to 4.8) | 21.1* (15.6 to 27.1) | -3.6 (-7.9 to 1.2) |
| Early syphilis | 68.4 (51.7 to 89.2) | -- | -- | 38.4* (29.9 to 47.6) | 10.2* (3.4 to 17.5) |
| Tertiary syphilis | 20.6 (17.3 to 24.3) | -- | 3.4 (2.3 to 4.8) | 21.1* (15.5 to 27.1) | -3.6 (-8.0 to 1.2) |
| Chlamydial infection | 0.3 (0.2 to 0.4) | -- | -- | 24.3* (20.6 to 28.8) | -1.0 (-4.0 to 2.6) |
| Chlamydia episode | 0.3 (0.2 to 0.4) | -- | -- | 24.3* (20.6 to 28.8) | -1.0 (-4.0 to 2.6) |
| Chlamydial infection complications | -- | -- | -- | 33.4* (10.2 to 58.0) | 6.2 (-12.3 to 25.8) |
| Gonococcal infection | -- | -- | -- | 91.5 (-61.8 to 788.9) | 52.5 (-69.6 to 607.8) |
| Gonococcal infection complications | -- | -- | -- | 31.6* (8.1 to 54.5) | 4.8 (-13.9 to 23.0) |
| Gonorrhoea episode | -- | -- | -- | 91.5 (-61.8 to 788.9) | 52.5 (-69.6 to 607.8) |
| Trichomoniasis | 1020.0 (853.7 to 1222.0) | 1527.3 (1313.8 to 1818.8) | 1.7 (0.7 to 3.6) | 27.2* (23.4 to 30.8) | 1.3 (-1.7 to 4.1) |
| Genital herpes | 22401.4 (19713.5 to 25348.4) | 10.8 (6.5 to 17.7) | 5.1 (1.5 to 12.5) | 25.7* (23.9 to 27.7) | 0.1 (-1.3 to 1.7) |
| Other sexually transmitted infections | -- | -- | -- | 23.2* (19.1 to 28.9) | -1.9 (-5.1 to 2.6) |
| Other sexually transmitted diseases residual | -- | -- | -- | 23.2* (19.1 to 28.9) | -1.9 (-5.1 to 2.6) |
| Other sexually transmitted diseases | -- | -- | -- | 23.2* (19.1 to 28.9) | -1.9 (-5.1 to 2.6) |
| **Respiratory infections and tuberculosis** | **31296.9 (22953.2 to 41568.7)** | **188729.1 (130186.4 to 265503.2)** | **212.1 (149.6 to 291.4)** | **21.5* (19.0 to 23.9)** | **-3.3* (-5.3 to -1.4)** |
| Tuberculosis | 28962.3 (20249.0 to 39125.0) | 178.0 (141.6 to 217.5) | 133.7 (87.7 to 188.8) | 20.6* (17.0 to 24.4) | -4.0* (-6.8 to -0.9) |
| Latent tuberculosis infection | 28480.1 (19793.0 to 38662.6) | -- | -- | -- | -- |
| Drug-susceptible tuberculosis | 459.9 (366.3 to 568.6) | 169.0 (134.3 to 205.9) | 127.4 (83.2 to 179.6) | 21.4* (15.3 to 26.4) | -3.4 (-8.2 to 0.7) |
| Multidrug-resistant tuberculosis without extensive drug resistance | 21.2 (8.9 to 42.2) | 8.5 (4.1 to 16.2) | 5.9 (2.4 to 12.5) | 4.5 (-51.3 to 93.2) | -16.8 (-61.2 to 53.8) |
| Extensively drug-resistant tuberculosis | 1.2 (0.5 to 2.6) | 0.5 (0.3 to 0.8) | 0.4 (0.2 to 0.9) | 45.3 (-27.6 to 192.8) | 15.7 (-42.3 to 133.1) |
| Lower respiratory infections | 147.0 (126.9 to 168.4) | 6888.6 (5846.2 to 7955.6) | 8.3 (5.6 to 11.7) | 30.6* (18.2 to 43.1) | 4.0 (-5.8 to 14.0) |
| Guillain-Barré syndrome due to lower respiratory infections | 0.3 (0.2 to 0.6) | -- | 0.1 (0.0 to 0.2) | 31.3* (28.2 to 34.6) | 4.5* (2.1 to 7.2) |
| Lower respiratory infection episode | 146.7 (126.5 to 168.2) | 6888.6 (5846.2 to 7955.6) | 8.2 (5.5 to 11.5) | 30.6* (18.1 to 43.4) | 4.0 (-5.9 to 14.2) |
| Upper respiratory infections | 2497.2 (1686.9 to 3571.1) | 180946.3 (122279.1 to 257104.8) | 57.7 (30.5 to 97.2) | 23.3* (21.6 to 25.1) | -1.8* (-3.2 to -0.4) |
| Guillain-Barré syndrome due to upper respiratory infections | 0.9 (0.5 to 1.5) | -- | 0.3 (0.1 to 0.5) | 31.3* (28.1 to 34.5) | 4.6* (2.0 to 7.1) |
| Upper respiratory infection episode | 2496.2 (1685.0 to 3570.1) | 180946.3 (122279.1 to 257104.8) | 57.5 (30.3 to 96.9) | 23.3* (21.6 to 25.1) | -1.8* (-3.2 to -0.4) |
| Otitis media | 629.4 (478.8 to 807.4) | 716.2 (522.1 to 951.3) | 12.4 (7.2 to 20.3) | 17.0* (11.8 to 22.1) | -6.8* (-10.9 to -2.8) |
| Acute otitis media | 39.3 (28.5 to 52.8) | 716.1 (522.1 to 951.2) | 0.5 (0.2 to 0.9) | 25.3* (17.2 to 33.8) | -0.3 (-6.6 to 6.5) |
| Chronic otitis media | 590.1 (437.8 to 767.5) | 0.1 (0.0 to 0.5) | 11.9 (7.0 to 19.4) | 16.7* (11.2 to 22.0) | -7.1* (-11.5 to -2.9) |
| **Enteric infections** | **2160.7 (1816.7 to 2584.4)** | **146472.7 (120372.6 to 180651.3)** | **223.1 (152.9 to 312.0)** | **50.7* (41.8 to 60.2)** | **20.0* (12.9 to 27.6)** |
| Diarrhoeal diseases | 2177.8 (1827.6 to 2608.4) | 146420.9 (120324.4 to 180599.9) | 222.7 (152.6 to 311.6) | 50.8* (41.9 to 60.4) | 20.1* (13.0 to 27.7) |
| Guillain-Barré syndrome due to diarrheal diseases | 0.3 (0.2 to 0.5) | -- | 0.1 (0.0 to 0.2) | 31.3* (28.2 to 34.5) | 4.6* (2.1 to 7.1) |
| Diarrheal disease episode | 2177.5 (1827.1 to 2608.1) | 146420.9 (120324.4 to 180599.9) | 222.6 (152.5 to 311.5) | 50.8* (41.9 to 60.4) | 20.1* (13.0 to 27.7) |
| Typhoid and paratyphoid | 0.1 (0.0 to 0.3) | 50.0 (43.3 to 57.1) | 0.4 (0.3 to 0.6) | 3.3 (-14.2 to 24.6) | -17.7* (-31.7 to -0.8) |
| Typhoid fever | 2.5 (2.0 to 3.0) | 39.4 (33.2 to 46.1) | 0.4 (0.2 to 0.6) | 5.4 (-14.5 to 29.7) | -16.1 (-31.9 to 3.3) |
| Typhoid fever complications | 0.5 (0.4 to 0.7) | 6.8 (5.5 to 8.3) | 0.2 (0.1 to 0.3) | 3.4 (-25.1 to 41.0) | -17.6 (-40.4 to 12.3) |
| Typhoid fever episode | 2.0 (1.5 to 2.4) | 32.6 (27.5 to 38.1) | 0.2 (0.1 to 0.3) | 6.9 (-17.3 to 42.4) | -14.8 (-34.2 to 13.4) |
| Paratyphoid fever | 0.5 (0.3 to 0.6) | 10.6 (7.9 to 13.9) | -- | -17.5* (-29.6 to -3.4) | -34.3* (-44.0 to -23.1) |
| Intestinal perforation due to paratyphoid | -- | 0.5 (0.4 to 0.7) | -- | -17.3 (-35.6 to 6.3) | -34.1* (-48.7 to -15.4) |
| Paratyphoid fever episode | 0.4 (0.3 to 0.6) | 10.1 (7.5 to 13.2) | -- | -17.5* (-30.6 to -2.5) | -34.3* (-44.8 to -22.4) |
| iNTS | 0.1 (0.0 to 0.1) | 1.8 (1.1 to 3.0) | -- | -7.7 (-16.0 to 0.0) | -26.5* (-33.1 to -20.4) |
| Other intestinal infectious diseases | -- | -- | -- | -5.2 (-21.3 to 14.3) | -24.5* (-37.4 to -9.0) |
| **Neglected tropical diseases and malaria** | **10740.1 (10223.9 to 11291.5)** | **3685.5 (3011.7 to 4468.4)** | **208.9 (150.5 to 276.6)** | **5.1 (-1.2 to 11.5)** | **-16.3* (-21.4 to -11.2)** |
| Malaria | 486.3 (452.2 to 518.7) | 246.5 (230.4 to 280.8) | 3.2 (2.2 to 4.7) | -29.3* (-42.5 to -13.2) | -43.7* (-54.2 to -30.9) |
| Malaria complications | 0.5 (0.4 to 0.6) | -- | 0.2 (0.2 to 0.3) | 50.1* (45.7 to 54.4) | 19.5* (16.0 to 22.9) |
| Malaria episode | 14.4 (9.6 to 19.4) | 246.5 (230.4 to 280.8) | 0.5 (0.3 to 0.9) | -28.8* (-37.2 to -19.5) | -43.3* (-50.0 to -35.9) |

| | | | | | |
|---|---|---|---|---|---|
| Malaria parasitemia | 471.4 (437.4 to 503.7) | -- | 2.5 (1.6 to 3.6) | -32.4* (-47.3 to -13.2) | -46.2* (-58.1 to -30.9) |
| Chagas disease | 185.4 (158.8 to 214.9) | 1.5 (1.0 to 2.4) | 3.8 (2.4 to 5.6) | 9.8* (4.4 to 14.9) | -12.6* (-16.9 to -8.5) |
| Acute Chagas disease | -- | 1.5 (1.0 to 2.4) | -- | 29.9* (24.9 to 34.7) | 3.4 (-0.5 to 7.2) |
| Asymptomatic Chagas disease | 135.2 (113.8 to 156.9) | -- | -- | -- | -- |
| Symptomatic chronic Chagas infection | 50.2 (39.4 to 62.0) | -- | 3.8 (2.4 to 5.6) | 9.8* (4.4 to 14.9) | -12.6* (-16.9 to -8.5) |
| Leishmaniasis | 76.1 (55.1 to 101.2) | 8.4 (5.8 to 12.1) | 4.5 (2.8 to 6.8) | 23.3* (17.7 to 32.5) | -1.8 (-6.3 to 5.5) |
| Visceral leishmaniasis | 0.0 (0.0 to 0.1) | 0.2 (0.1 to 0.2) | -- | -70.9* (-75.8 to -62.8) | -76.8* (-80.7 to -70.4) |
| Cutaneous and mucocutaneous leishmaniasis | 76.4 (55.3 to 101.4) | 8.3 (5.6 to 11.9) | 4.5 (2.8 to 6.8) | 23.6* (17.9 to 32.9) | -1.6 (-6.1 to 5.8) |
| African trypanosomiasis | 0.0 (0.0 to 0.1) | 0.0 (0.0 to 0.1) | -- | -79.0 (-97.5 to 35.7) | -83.3 (-98.0 to 8.1) |
| Trypanosomiasis Gambiense | 0.0 (0.0 to 0.1) | 0.0 (0.0 to 0.1) | -- | -78.4 (-97.8 to 57.5) | -82.8 (-98.3 to 25.4) |
| Trypanosomiasis Rhodesiense | -- | -- | -- | -88.4* (-98.9 to -17.6) | -90.8* (-99.2 to -34.4) |
| Schistosomiasis | 1775.9 (1574.4 to 1990.7) | -- | 11.8 (5.7 to 22.2) | -15.1* (-20.2 to -9.8) | -32.4* (-36.5 to -28.2) |
| Mild schistosomiasis | 1591.6 (1398.1 to 1789.0) | -- | 8.4 (3.5 to 17.3) | -14.3* (-16.7 to -11.8) | -31.8* (-33.7 to -29.8) |
| Anemia due to schistosomiasis | 95.4 (76.6 to 123.6) | -- | 2.2 (1.3 to 3.4) | -25.6* (-41.7 to -4.5) | -40.8* (-53.6 to -23.9) |
| Schistosomiasis complications | 89.4 (69.3 to 112.7) | -- | 1.3 (0.7 to 2.2) | 3.0 (-4.9 to 12.6) | -18.0* (-24.3 to -10.3) |
| Cysticercosis | 243.3 (177.9 to 321.3) | -- | 62.3 (38.2 to 91.4) | 23.0* (16.5 to 29.1) | -2.0 (-7.3 to 2.8) |
| Cystic echinococcosis | 9.1 (5.7 to 14.8) | 1.6 (0.9 to 3.1) | 0.7 (0.4 to 1.2) | 15.7* (1.7 to 30.7) | -7.9 (-19.0 to 4.1) |
| Lymphatic filariasis | 649.2 (595.8 to 710.0) | -- | 9.4 (5.5 to 14.5) | -37.6* (-49.4 to -26.3) | -50.3* (-59.7 to -41.3) |
| Prevalence of detectable microfiliaria due to lymphatic filariasis | 554.6 (511.6 to 605.8) | -- | -- | -- | -- |
| Lymphatic filariasis complications | 94.6 (65.3 to 132.5) | -- | 9.4 (5.5 to 14.5) | -37.6* (-49.4 to -26.3) | -50.3* (-59.7 to -41.3) |
| Onchocerciasis | 187.6 (124.9 to 291.4) | -- | 16.4 (9.4 to 28.5) | 4.4 (-10.5 to 20.7) | -16.9* (-28.7 to -3.9) |
| Asymptomatic onchocerciasis | 27.3 (0.0 to 114.4) | -- | -- | -- | -- |
| Skin disease due to onchocerciasis | 109.4 (74.0 to 161.7) | -- | 12.4 (6.2 to 24.2) | 2.0 (-17.1 to 21.8) | -18.8* (-34.0 to -3.0) |
| Vision loss due to onchocerciasis | 50.9 (33.1 to 78.3) | -- | 4.0 (2.3 to 6.2) | 12.8 (-3.1 to 32.1) | -10.2 (-22.8 to 5.2) |
| Trachoma | 573.1 (379.9 to 820.6) | -- | 42.3 (26.3 to 63.2) | -5.1 (-21.5 to 13.7) | -24.4* (-37.5 to -9.4) |
| Dengue | 80.7 (44.1 to 136.4) | 1351.2 (824.5 to 2044.8) | 11.8 (5.3 to 21.6) | 93.7* (68.2 to 199.9) | 54.3* (34.0 to 138.8) |
| Post-dengue chronic fatigue syndrome | 56.9 (26.6 to 103.9) | -- | 10.5 (4.5 to 19.6) | 93.7* (67.7 to 200.7) | 54.2* (33.5 to 139.5) |
| Dengue episode | 23.8 (14.5 to 35.7) | 1351.2 (824.5 to 2044.8) | 1.3 (0.7 to 2.3) | 94.1* (66.0 to 193.5) | 54.5* (32.2 to 133.7) |
| Yellow fever | -- | 0.3 (0.1 to 1.0) | -- | -23.3 (-46.1 to 5.0) | -39.0* (-57.1 to -16.4) |
| Asymptomatic yellow fever | -- | 0.2 (0.0 to 0.5) | -- | -- | -- |
| Yellow fever episode | -- | 0.1 (0.0 to 0.4) | -- | -23.3 (-46.1 to 5.0) | -39.0* (-57.1 to -16.4) |
| Rabies | -- | 0.3 (0.2 to 0.4) | -- | -31.3* (-49.8 to -13.7) | -45.3* (-60.0 to -31.3) |
| Intestinal nematode infections | 6130.4 (5664.7 to 6674.6) | -- | 11.6 (6.8 to 18.9) | -20.7* (-27.3 to -14.0) | -36.9* (-42.1 to -31.5) |
| Ascariasis | 2752.7 (2379.3 to 3178.2) | -- | 3.1 (1.7 to 5.2) | -23.9* (-32.2 to -14.5) | -39.4* (-46.0 to -32.0) |
| Asymptomatic ascariasis | 2570.7 (2219.2 to 2981.9) | -- | -- | -- | -- |
| Ascariasis complications | 181.9 (159.7 to 207.0) | -- | 3.1 (1.7 to 5.2) | -23.9* (-32.2 to -14.5) | -39.4* (-46.0 to -32.0) |
| Trichuriasis | 1232.6 (1029.8 to 1482.3) | -- | 0.7 (0.4 to 1.1) | -1.3 (-11.6 to 10.2) | -21.4* (-29.6 to -12.3) |
| Asymptomatic trichuriasis | 1195.5 (995.8 to 1439.7) | -- | -- | -- | -- |
| Trichuriasis complications | 37.1 (33.4 to 40.6) | -- | 0.7 (0.4 to 1.1) | -1.3 (-11.6 to 10.2) | -21.4* (-29.6 to -12.3) |
| Hookworm disease | 2417.3 (2164.2 to 2708.7) | -- | 7.8 (4.7 to 12.5) | -20.8* (-29.4 to -11.8) | -36.9* (-43.8 to -29.7) |
| Asymptomatic hookworm disease | 2007.3 (1798.7 to 2250.8) | -- | -- | -- | -- |
| Anemia due to hookworm disease | 104.0 (89.6 to 120.8) | -- | 1.9 (1.2 to 2.8) | -34.7* (-47.7 to -19.6) | -48.0* (-58.4 to -36.0) |
| Hookworm disease complications | 305.9 (274.6 to 341.9) | -- | 5.9 (3.4 to 9.8) | -15.0* (-23.0 to -6.8) | -32.3* (-38.7 to -25.8) |
| Food-borne trematodiases | 1107.3 (980.0 to 1247.8) | 2049.6 (1693.0 to 2440.8) | 23.9 (12.3 to 40.6) | 25.8* (19.1 to 32.2) | 0.2 (-5.2 to 5.3) |
| Asymptomatic food-borne tremodiases | 877.8 (713.1 to 1035.8) | 1542.8 (787.9 to 2145.8) | -- | -- | -- |
| Food-borne tremodiases complications | 229.5 (132.9 to 364.3) | 506.7 (53.6 to 1286.6) | 23.9 (12.3 to 40.6) | 25.8* (19.1 to 32.2) | 0.2 (-5.2 to 5.3) |
| Leprosy | 34.1 (32.4 to 36.0) | 1.8 (1.6 to 1.9) | 2.0 (1.4 to 2.8) | 8.2* (2.5 to 14.4) | -13.8* (-18.4 to -8.9) |
| Ebola virus disease | -- | -- | -- | -98.7* (-98.9 to -98.4) | -99.0* (-99.1 to -98.7) |
| Ebola cases | -- | -- | -- | -98.5* (-98.6 to -98.3) | -98.8* (-98.9 to -98.7) |
| Post-Ebola chronic fatigue syndrome | -- | -- | -- | -98.7* (-98.9 to -98.4) | -99.0* (-99.1 to -98.7) |
| Zika virus disease | 0.4 (0.2 to 0.7) | 24.3 (13.4 to 43.6) | -- | -- | -- |
| Zika virus complications | -- | -- | -- | -- | -- |
| Zika virus episode | 0.4 (0.2 to 0.7) | 24.3 (13.4 to 43.6) | -- | -- | -- |
| Guinea worm disease | -- | -- | -- | -99.6* (-99.7 to -99.4) | -99.7* (-99.8 to -99.5) |
| Other neglected tropical diseases | 264.3 (243.7 to 287.1) | -- | 5.2 (3.5 to 7.9) | 5.0 (-7.5 to 17.2) | -16.4* (-26.3 to -6.7) |
| Acute infection due to other neglected tropical diseases | -- | -- | 0.2 (0.1 to 0.3) | 71.2* (46.7 to 156.6) | 36.3* (16.8 to 104.3) |
| Anemia due to other neglected tropical diseases | 264.3 (243.7 to 287.1) | -- | 5.1 (3.4 to 7.6) | 3.7 (-8.4 to 16.2) | -17.4* (-27.1 to -7.5) |
| **Other infectious diseases** | **998.9 (852.4 to 1166.7)** | **3390.1 (2676.6 to 4418.3)** | **42.3 (29.3 to 57.8)** | **17.4* (10.8 to 25.0)** | **-6.5* (-11.8 to -0.4)** |
| Meningitis | 138.7 (112.8 to 166.9) | 46.6 (33.6 to 63.6) | 7.1 (4.8 to 9.7) | 1.8 (-2.1 to 6.3) | -19.0* (-22.0 to -15.4) |
| Pneumococcal meningitis | 51.1 (40.2 to 63.8) | 5.2 (3.2 to 7.8) | 2.7 (1.8 to 3.7) | -15.7* (-20.2 to -10.3) | -32.9* (-36.4 to -28.5) |

| | | | | | |
|---|---|---|---|---|---|
| Acute pneumococcal meningitis | 0.2 (0.1 to 0.3) | 5.2 (3.2 to 7.8) | -- | -8.0 (-17.9 to 2.5) | -26.7* (-34.6 to -18.4) |
| Pneumococcal meningitis complications | 50.9 (39.9 to 63.6) | -- | 2.7 (1.8 to 3.7) | -15.8* (-20.3 to -10.2) | -32.9* (-36.5 to -28.5) |
| H influenzae type B meningitis | 9.8 (6.5 to 13.3) | 1.5 (0.9 to 2.5) | 0.4 (0.3 to 0.6) | -50.6* (-53.0 to -48.1) | -60.7* (-62.6 to -58.7) |
| Acute H influenzae type B meningitis | 0.1 (0.0 to 0.1) | 1.5 (0.9 to 2.5) | -- | -47.4* (-53.6 to -41.8) | -58.1* (-63.0 to -53.7) |
| H Influenza type B meningitis complications | 9.7 (6.5 to 13.3) | -- | 0.4 (0.3 to 0.6) | -50.6* (-53.1 to -48.2) | -60.7* (-62.7 to -58.7) |
| Meningococcal infection | 15.7 (10.6 to 21.3) | 4.3 (2.6 to 7.0) | 0.9 (0.6 to 1.3) | 0.5 (-3.8 to 4.8) | -19.9* (-23.4 to -16.5) |
| Acute meningococcal meningitis | 0.2 (0.1 to 0.3) | 4.3 (2.6 to 7.0) | -- | 16.7* (4.4 to 28.1) | -7.1 (-16.9 to 2.0) |
| Meningococcal meningitis complications | 15.5 (10.4 to 21.0) | -- | 0.9 (0.6 to 1.2) | 0.1 (-4.3 to 4.5) | -20.3* (-23.8 to -16.8) |
| Other meningitis | 62.1 (43.6 to 80.6) | 35.5 (25.2 to 47.7) | 3.1 (2.1 to 4.3) | 55.4* (48.3 to 63.8) | 23.7* (18.1 to 30.4) |
| Other acute bacterial meningitis | 0.5 (0.3 to 0.7) | 11.3 (7.9 to 15.8) | 0.1 (0.0 to 0.1) | 73.7* (57.1 to 89.3) | 38.3* (25.1 to 50.7) |
| Acute viral meningitis | 1.0 (0.8 to 1.4) | 24.3 (17.5 to 33.1) | 0.1 (0.1 to 0.2) | 18.9* (9.1 to 28.4) | -5.3 (-13.2 to 2.3) |
| Other bacterial meningitis complications | 60.5 (42.1 to 79.0) | -- | 2.8 (1.9 to 4.0) | 57.4* (49.9 to 66.5) | 25.3* (19.3 to 32.5) |
| Encephalitis | 176.4 (81.9 to 302.6) | 61.7 (59.3 to 63.9) | 8.4 (5.7 to 11.4) | 25.7* (20.1 to 31.9) | 0.1 (-4.3 to 5.0) |
| Acute encephalitis | 2.8 (2.7 to 2.9) | 61.7 (59.3 to 63.9) | 0.4 (0.2 to 0.5) | 37.5* (22.6 to 54.6) | 9.5 (-2.4 to 23.1) |
| Encephalitis complications | 173.6 (79.2 to 299.8) | -- | 8.0 (5.5 to 11.0) | 25.2* (19.8 to 31.6) | -0.3 (-4.6 to 4.8) |
| Diphtheria | -- | -- | -- | -- | -- |
| Whooping cough | -- | -- | -- | -- | -- |
| Tetanus | 0.6 (0.6 to 0.7) | 1.4 (0.8 to 2.2) | -- | -17.4 (-33.7 to 2.1) | -34.2* (-47.2 to -18.7) |
| Severe tetanus | 0.1 (0.0 to 0.1) | 1.4 (0.8 to 2.2) | -- | -38.3* (-51.5 to -13.7) | -50.9* (-61.4 to -31.3) |
| Neonatal tetanus complications | 0.5 (0.5 to 0.6) | -- | -- | 30.1* (27.9 to 31.8) | 3.6* (1.9 to 4.9) |
| Measles | -- | -- | -- | -- | -- |
| Varicella and herpes zoster | 273.0 (221.1 to 331.8) | 1496.7 (1194.3 to 1806.0) | 14.1 (8.4 to 21.3) | 27.7* (24.0 to 31.6) | 1.7 (-1.3 to 4.8) |
| Chickenpox | -- | 0.0 (0.0 to 0.1) | -- | 55.4* (41.4 to 75.6) | 23.7* (12.6 to 39.8) |
| Herpes zoster | 273.0 (221.1 to 331.8) | 1496.7 (1194.3 to 1805.9) | 14.1 (8.4 to 21.3) | 27.7* (24.0 to 31.6) | 1.7 (-1.3 to 4.8) |
| Acute hepatitis | 200.9 (124.5 to 314.1) | 1783.6 (1120.2 to 2757.9) | 4.5 (2.3 to 7.8) | 18.3 (-28.5 to 88.6) | -5.8 (-43.1 to 50.1) |
| Acute hepatitis A | 1.4 (0.6 to 2.9) | 18.5 (7.2 to 37.5) | 0.1 (0.0 to 0.1) | 19.5* (10.8 to 28.3) | -4.8 (-11.8 to 2.1) |
| Acute hepatitis B | 183.6 (108.1 to 295.2) | 1591.1 (937.2 to 2558.0) | 4.0 (2.0 to 7.2) | 18.4 (-32.2 to 96.1) | -5.7 (-46.0 to 56.2) |
| Acute hepatitis C | 7.6 (3.2 to 13.6) | 65.7 (27.5 to 117.4) | 0.1 (0.0 to 0.2) | -0.2 (-10.3 to 5.8) | -20.5* (-28.5 to -15.8) |
| Acute hepatitis E | 8.3 (5.2 to 12.6) | 108.4 (67.0 to 163.4) | 0.3 (0.1 to 0.5) | 26.2* (6.7 to 48.5) | 0.5 (-15.0 to 18.2) |
| Other unspecified infectious diseases | 350.0 (324.0 to 376.9) | -- | 8.2 (5.4 to 12.1) | 9.2 (-1.8 to 19.3) | -13.0* (-21.8 to -5.0) |
| Guillain-Barré syndrome due to other infectious diseases | 0.2 (0.1 to 0.4) | -- | 0.1 (0.0 to 0.1) | 31.3* (28.1 to 34.6) | 4.5* (2.0 to 7.2) |
| Other infectious diseases | -- | -- | 1.4 (0.8 to 2.3) | 22.3* (17.7 to 27.0) | -2.6 (-6.3 to 1.2) |
| Anemia due to other infectious diseases | 349.8 (323.8 to 376.6) | -- | 6.7 (4.4 to 9.9) | 6.6 (-6.3 to 18.8) | -15.1* (-25.4 to -5.4) |
| **Maternal and neonatal disorders** | **1134.1 (977.3 to 1306.2)** | **--** | **83.4 (61.2 to 110.9)** | **56.0* (47.3 to 66.2)** | **24.2* (17.3 to 32.4)** |
| Maternal disorders | 1.9 (1.5 to 2.5) | -- | 0.7 (0.5 to 1.0) | -25.5* (-37.1 to -12.6) | -40.6* (-49.9 to -30.4) |
| Maternal haemorrhage | -- | -- | -- | -- | -- |
| Maternal hemorrhage complications | -- | -- | -- | -- | -- |
| Maternal hemorrhage episode | -- | -- | -- | -- | -- |
| Maternal sepsis and other pregnancy related infections | -- | -- | -- | -- | -- |
| Infertility due to puerperal sepsis | -- | -- | -- | -- | -- |
| Maternal sepsis and other maternal infection episode | -- | -- | -- | -- | -- |
| Maternal hypertensive disorders | -- | -- | -- | -- | -- |
| Maternal hypertensive disorder complications | -- | -- | -- | -- | -- |
| Maternal hypertensive disorder episode | -- | -- | -- | -- | -- |
| Maternal obstructed labour and uterine rupture | 2.4 (1.9 to 2.9) | -- | 0.7 (0.5 to 1.0) | -25.5* (-37.1 to -12.6) | -40.6* (-49.9 to -30.4) |
| Obstructed labor, acute event | -- | -- | -- | -- | -- |
| Maternal obstructed labor complications | 2.4 (1.9 to 2.9) | -- | 0.7 (0.5 to 1.0) | -25.5* (-37.1 to -12.6) | -40.6* (-49.9 to -30.4) |
| Maternal abortion and miscarriage | -- | -- | -- | -- | -- |
| Ectopic pregnancy | -- | -- | -- | -- | -- |
| Other maternal disorders | -- | -- | -- | -- | -- |
| Neonatal disorders | 1132.1 (975.3 to 1304.2) | -- | 82.7 (60.5 to 110.1) | 57.4* (48.6 to 68.1) | 25.4* (18.3 to 33.9) |
| Neonatal preterm birth | 705.7 (655.5 to 759.8) | -- | 33.3 (23.9 to 44.4) | 56.0* (37.9 to 77.8) | 24.2* (9.8 to 41.6) |
| Preterm birth complications | 540.9 (497.2 to 586.2) | -- | 24.2 (16.3 to 33.8) | 73.6* (47.6 to 104.5) | 38.2* (17.5 to 62.9) |
| Retinopathy of prematurity | 164.7 (133.9 to 196.7) | -- | 9.1 (6.2 to 12.4) | 22.8* (16.5 to 29.9) | -2.2 (-7.3 to 3.4) |
| Uncomplicated preterm birth | -- | -- | -- | -- | -- |
| Neonatal encephalopathy due to birth asphyxia and trauma | 437.7 (299.7 to 594.4) | -- | 21.1 (12.3 to 32.9) | 71.8* (58.3 to 84.7) | 36.8* (26.1 to 47.1) |
| Neonatal sepsis and other neonatal infections | 95.5 (63.9 to 140.1) | -- | 25.6 (13.1 to 45.2) | 49.2* (40.9 to 59.0) | 18.8* (12.2 to 26.6) |
| Neonatal sepsis and other neonatal infection complications | 95.5 (63.9 to 140.1) | -- | 25.6 (13.1 to 45.2) | 49.2* (40.9 to 59.0) | 18.8* (12.2 to 26.6) |
| Neonatal sepsis and other neonatal infection episode | -- | -- | -- | -- | -- |
| Hemolytic disease and other neonatal jaundice | 6.3 (4.9 to 7.7) | -- | 2.6 (1.9 to 3.5) | 54.3* (46.5 to 62.6) | 22.8* (16.6 to 29.5) |

| | | | | | |
|---|---|---|---|---|---|
| Other neonatal disorders | -- | -- | -- | -- | -- |
| **Nutritional deficiencies** | **21557.1 (20471.2 to 22669.6)** | **11534.9 (10149.6 to 12992.1)** | **324.4 (217.3 to 469.7)** | **8.0 (-0.1 to 16.2)** | **-14.0* (-20.4 to -7.5)** |
| Protein-energy malnutrition | 1478.9 (1383.3 to 1572.8) | 1379.7 (1273.6 to 1490.0) | 5.6 (3.7 to 7.8) | 40.0* (23.1 to 53.5) | 11.5 (-1.9 to 22.2) |
| Iodine deficiency | 2130.6 (1934.6 to 2363.4) | 6.8 (5.5 to 8.5) | 29.3 (17.3 to 47.5) | 20.3* (17.4 to 24.6) | -4.2* (-6.5 to -0.8) |
| Visible goiter without symptoms | 2101.3 (1906.7 to 2332.4) | 6.8 (5.5 to 8.5) | 21.0 (10.4 to 39.0) | 17.4* (15.8 to 18.9) | -6.5* (-7.8 to -5.3) |
| Visible goiter with complications | 29.3 (18.3 to 41.2) | -- | 8.2 (4.5 to 12.8) | 28.6* (18.7 to 37.6) | 2.4 (-5.5 to 9.6) |
| Vitamin A deficiency | 6047.0 (5356.2 to 6808.9) | 10148.4 (8782.2 to 11596.9) | 1.7 (1.1 to 2.5) | 33.0* (24.1 to 43.3) | 5.9 (-1.1 to 14.1) |
| Asymptomatic vitamin A deficiency | 6021.9 (5326.7 to 6782.4) | 10148.4 (8782.2 to 11596.9) | -- | -- | -- |
| Vitamin A deficiency complications | 25.1 (18.4 to 33.1) | -- | 1.7 (1.1 to 2.5) | 33.0* (24.1 to 43.3) | 5.9 (-1.1 to 14.1) |
| Vitamin A deficiency with anemia | -- | -- | -- | -- | -- |
| Dietary iron deficiency | 14440.7 (13383.9 to 15500.6) | -- | 285.7 (190.0 to 415.2) | 6.1 (-3.1 to 15.2) | -15.5* (-22.8 to -8.3) |
| Other nutritional deficiencies | -- | -- | 2.2 (1.4 to 3.0) | 41.6* (24.6 to 55.3) | 12.8 (-0.8 to 23.6) |
| **Non-communicable diseases** | **123591.5 (123563.3 to 123617.2)** | **266762.8 (237981.9 to 296706.0)** | **29046.6 (22196.5 to 36628.8)** | **26.0* (24.9 to 27.1)** | **0.3 (-0.6 to 1.2)** |
| **Neoplasms** | **7938.1 (7658.2 to 8296.0)** | **2576.3 (2124.1 to 3185.0)** | **701.9 (528.3 to 892.4)** | **29.9* (27.1 to 33.1)** | **3.4* (1.2 to 6.0)** |
| Lip and oral cavity cancer | 111.9 (107.3 to 116.3) | 29.9 (28.6 to 31.1) | 11.8 (8.7 to 15.0) | 35.5* (27.8 to 43.4) | 7.9* (1.8 to 14.2) |
| Diagnosis and primary therapy phase of mouth cancer | 6.9 (6.6 to 7.2) | 29.9 (28.6 to 31.1) | 1.9 (1.3 to 2.6) | 36.6* (26.5 to 48.0) | 8.8* (0.7 to 17.8) |
| Controlled phase of mouth cancer | 90.4 (86.8 to 94.0) | -- | 4.0 (2.6 to 5.7) | 38.8* (31.1 to 46.5) | 10.6* (4.4 to 16.7) |
| Metastatic phase of mouth cancer | 12.6 (12.1 to 13.2) | -- | 5.0 (3.4 to 6.5) | 32.9* (20.5 to 47.1) | 5.8 (-4.1 to 17.1) |
| Terminal phase of mouth cancer | 1.9 (1.8 to 2.0) | -- | 1.0 (0.7 to 1.3) | 34.1* (28.3 to 40.0) | 6.8* (2.1 to 11.5) |
| Nasopharynx cancer | 14.1 (13.4 to 14.9) | 4.2 (4.0 to 4.4) | 1.8 (1.3 to 2.4) | 19.7* (11.4 to 28.4) | -4.7 (-11.3 to 2.3) |
| Diagnosis and primary therapy phase of nasopharynx cancer | 0.9 (0.8 to 0.9) | 4.2 (4.0 to 4.4) | 0.2 (0.2 to 0.3) | 20.2* (14.8 to 26.2) | -4.3 (-8.6 to 0.5) |
| Controlled phase of nasopharynx cancer | 10.9 (10.4 to 11.5) | -- | 0.5 (0.3 to 0.7) | 23.2* (8.8 to 39.4) | -1.9 (-13.4 to 11.0) |
| Metastatic phase of nasopharynx cancer | 2.1 (2.0 to 2.2) | -- | 0.9 (0.6 to 1.3) | 17.9* (6.9 to 29.9) | -6.1 (-14.9 to 3.5) |
| Terminal phase of nasopharynx cancer | 0.3 (0.3 to 0.3) | -- | 0.1 (0.1 to 0.2) | 18.9* (13.6 to 24.7) | -5.3* (-9.5 to -0.7) |
| Other pharynx cancer | 19.2 (16.9 to 20.4) | 9.8 (8.6 to 10.4) | 3.0 (2.1 to 4.0) | 39.5* (27.2 to 50.3) | 11.1* (1.3 to 19.6) |
| Diagnosis and primary therapy phase of other pharynx cancer | 1.1 (1.0 to 1.2) | 9.8 (8.6 to 10.4) | 0.3 (0.2 to 0.4) | 40.6* (29.4 to 49.4) | 12.0* (3.0 to 18.9) |
| Controlled phase of other pharynx cancer | 13.3 (11.8 to 14.1) | -- | 0.6 (0.4 to 0.9) | 42.9* (27.8 to 60.4) | 13.8* (1.8 to 27.7) |
| Metastatic phase of other pharynx cancer | 4.1 (3.5 to 4.3) | -- | 1.7 (1.2 to 2.3) | 38.2* (23.7 to 52.3) | 10.0 (-1.5 to 21.3) |
| Terminal phase of other pharynx cancer | 0.7 (0.6 to 0.8) | -- | 0.4 (0.3 to 0.5) | 39.6* (28.7 to 48.2) | 11.2* (2.5 to 18.0) |
| Oesophageal cancer | 75.2 (71.4 to 79.1) | 56.3 (54.1 to 58.6) | 13.8 (10.1 to 17.9) | 17.1* (8.2 to 27.3) | -6.8 (-13.8 to 1.4) |
| Diagnosis and primary therapy phase of esophageal cancer | 6.3 (6.0 to 6.7) | 56.3 (54.1 to 58.6) | 1.7 (1.1 to 2.3) | 29.3* (10.4 to 50.9) | 3.0 (-12.1 to 20.1) |
| Controlled phase of esophageal cancer | 43.8 (41.2 to 46.4) | -- | 1.9 (1.3 to 2.8) | 42.0* (29.4 to 55.9) | 13.1* (3.0 to 24.1) |
| Metastatic phase of esophageal cancer | 20.6 (19.8 to 21.4) | -- | 8.0 (5.5 to 10.4) | 11.3* (0.6 to 23.9) | -11.4* (-19.9 to -1.4) |
| Terminal phase of esophageal cancer | 4.5 (4.3 to 4.7) | -- | 2.3 (1.6 to 2.9) | 12.8 (-2.6 to 30.0) | -10.2 (-22.5 to 3.5) |
| Stomach cancer | 282.3 (271.2 to 292.9) | 145.4 (140.1 to 150.4) | 37.5 (27.6 to 47.5) | 18.9* (13.1 to 25.3) | -5.3* (-10.0 to -0.2) |
| Diagnosis and primary therapy phase of stomach cancer | 28.8 (27.7 to 29.9) | 145.4 (140.1 to 150.4) | 7.2 (4.9 to 9.7) | 22.5* (12.8 to 33.2) | -2.5 (-10.2 to 6.0) |
| Controlled phase of stomach cancer | 199.8 (191.6 to 207.5) | -- | 8.8 (5.9 to 12.8) | 32.3* (25.8 to 39.1) | 5.3* (0.2 to 10.8) |
| Metastatic phase of stomach cancer | 42.6 (41.1 to 44.1) | -- | 16.1 (11.3 to 21.0) | 12.8* (5.2 to 21.0) | -10.2* (-16.2 to -3.7) |
| Terminal phase of stomach cancer | 11.0 (10.6 to 11.4) | -- | 5.3 (3.8 to 6.8) | 13.8* (2.8 to 26.0) | -9.4 (-18.2 to 0.3) |
| Colon and rectum cancer | 1106.7 (1075.3 to 1139.0) | 232.2 (225.8 to 238.9) | 106.8 (79.9 to 136.8) | 28.3* (23.6 to 33.3) | 2.2 (-1.6 to 6.1) |
| Diagnosis and primary therapy phase of colon and rectum cancers | 48.4 (47.0 to 49.9) | 232.2 (225.8 to 238.9) | 11.9 (8.1 to 15.9) | 32.0* (22.9 to 42.2) | 5.1 (-2.2 to 13.2) |
| Controlled phase of colon and rectum cancers | 916.0 (889.4 to 943.5) | -- | 51.8 (35.0 to 72.1) | 33.2* (28.3 to 38.6) | 6.1* (2.2 to 10.3) |
| Metastatic phase of colon and rectum cancers | 90.3 (87.8 to 92.8) | -- | 33.7 (23.2 to 43.4) | 22.3* (15.9 to 29.4) | -2.7 (-7.7 to 3.1) |
| Terminal phase of colon and rectum cancers | 12.8 (12.5 to 13.2) | -- | 6.1 (4.3 to 7.8) | 21.6* (10.7 to 34.0) | -3.2 (-11.9 to 6.7) |
| Stoma from colon and rectum cancers, beyond 10 years | 39.2 (36.4 to 42.0) | -- | 3.3 (2.3 to 4.6) | 19.4* (10.9 to 28.8) | -5.0 (-11.7 to 2.6) |
| Liver cancer | 45.2 (41.1 to 49.8) | 85.4 (82.1 to 89.7) | 18.5 (13.1 to 24.1) | 25.5* (19.3 to 33.1) | -0.1 (-5.0 to 6.0) |
| Liver cancer due to hepatitis B | 23.6 (21.2 to 26.3) | 23.3 (21.0 to 25.9) | 4.9 (3.3 to 6.5) | 26.1* (12.5 to 41.8) | 0.4 (-10.4 to 12.9) |
| Diagnosis and primary therapy phase of liver cancer due to hepatitis B | 5.2 (4.7 to 5.8) | 23.3 (21.0 to 25.9) | 1.4 (0.9 to 1.9) | 24.0* (4.6 to 47.7) | -1.3 (-16.7 to 17.6) |
| Controlled phase of liver cancer due to hepatitis B | 11.7 (10.5 to 13.1) | -- | 0.5 (0.3 to 0.8) | 92.8* (64.8 to 130.8) | 53.5* (31.2 to 83.8) |
| Metastatic phase of liver cancer due to hepatitis B | 4.7 (4.3 to 5.3) | -- | 1.9 (1.3 to 2.6) | 19.0 (-0.3 to 41.7) | -5.2 (-20.6 to 12.8) |
| Terminal phase of liver cancer due to hepatitis B | 1.9 (1.7 to 2.1) | -- | 1.0 (0.7 to 1.3) | 20.7* (5.7 to 38.4) | -3.9 (-15.8 to 10.2) |
| Liver cancer due to hepatitis C | 33.6 (30.5 to 36.9) | 31.2 (28.3 to 34.2) | 6.7 (4.7 to 8.9) | 21.0* (14.2 to 28.8) | -3.6 (-9.1 to 2.6) |
| Diagnosis and primary therapy phase of liver cancer due to hepatitis C | 7.0 (6.4 to 7.7) | 31.2 (28.3 to 34.2) | 1.9 (1.3 to 2.7) | 20.4* (8.8 to 33.8) | -4.1 (-13.3 to 6.5) |
| Controlled phase of liver cancer due to hepatitis C | 17.8 (16.0 to 19.5) | -- | 0.8 (0.5 to 1.2) | 35.6* (20.6 to 52.0) | 8.0 (-3.9 to 21.0) |
| Metastatic phase of liver cancer due to hepatitis C | 6.3 (5.7 to 6.9) | -- | 2.7 (1.8 to 3.6) | 18.9* (8.3 to 30.2) | -5.3 (-13.8 to 3.6) |
| Terminal phase of liver cancer due to hepatitis C | 2.5 (2.3 to 2.8) | -- | 1.3 (0.9 to 1.8) | 18.5* (12.9 to 24.8) | -5.6* (-10.1 to -0.6) |
| Liver cancer due to alcohol use | 16.1 (14.2 to 18.3) | 15.8 (13.9 to 18.1) | 3.5 (2.4 to 4.7) | 26.5* (19.3 to 35.0) | 0.7 (-5.0 to 7.5) |
| Diagnosis and primary therapy phase of liver cancer due to alcohol use | 3.5 (3.1 to 4.1) | 15.8 (13.9 to 18.1) | 1.0 (0.6 to 1.4) | 25.8* (12.7 to 40.2) | 0.2 (-10.3 to 11.6) |
| Controlled phase of liver cancer due to alcohol use | 8.1 (7.1 to 9.2) | -- | 0.4 (0.2 to 0.5) | 47.8* (25.0 to 74.4) | 17.7 (-0.5 to 38.8) |
| Metastatic phase of liver cancer due to alcohol use | 3.2 (2.8 to 3.7) | -- | 1.4 (1.0 to 1.9) | 23.7* (14.8 to 34.4) | -1.5 (-8.6 to 7.0) |

| | | | | | |
|---|---|---|---|---|---|
| Terminal phase of liver cancer due to alcohol use | 1.3 (1.1 to 1.5) | -- | 0.7 (0.5 to 0.9) | 23.5* (18.4 to 29.6) | -1.7 (-5.7 to 3.2) |
| Liver cancer due to NASH | 8.8 (7.3 to 10.7) | 9.2 (7.5 to 11.1) | 2.0 (1.4 to 2.8) | 35.6* (30.1 to 43.1) | 8.0* (3.6 to 13.9) |
| Diagnosis and primary therapy phase of liver cancer due to NASH | 2.0 (1.7 to 2.5) | 9.2 (7.5 to 11.1) | 0.6 (0.4 to 0.8) | 35.1* (30.0 to 41.9) | 7.6* (3.5 to 13.0) |
| Controlled phase of liver cancer due to NASH | 4.1 (3.4 to 5.1) | -- | 0.2 (0.1 to 0.3) | 63.4* (42.5 to 87.2) | 30.1* (13.5 to 49.0) |
| Metastatic phase of liver cancer due to NASH | 1.9 (1.5 to 2.3) | -- | 0.8 (0.6 to 1.2) | 32.4* (27.5 to 38.9) | 5.4* (1.5 to 10.6) |
| Terminal phase of liver cancer due to NASH | 0.7 (0.6 to 0.9) | -- | 0.4 (0.3 to 0.6) | 32.4* (27.4 to 39.0) | 5.4* (1.4 to 10.6) |
| Liver cancer due to other causes | 5.8 (4.9 to 7.0) | 5.9 (5.0 to 7.1) | 1.3 (0.9 to 1.8) | 29.5* (23.3 to 37.7) | 3.1 (-1.8 to 9.7) |
| Diagnosis and primary therapy phase of liver cancer due to other causes | 1.3 (1.1 to 1.6) | 5.9 (5.0 to 7.1) | 0.4 (0.2 to 0.6) | 28.3* (22.4 to 35.9) | 2.2 (-2.5 to 8.2) |
| Controlled phase of liver cancer due to other causes | 2.8 (2.3 to 3.4) | -- | 0.1 (0.1 to 0.2) | 73.4* (52.1 to 100.1) | 38.1* (21.1 to 59.3) |
| Metastatic phase of liver cancer due to other causes | 1.2 (1.0 to 1.4) | -- | 0.5 (0.4 to 0.8) | 24.8* (19.1 to 32.3) | -0.6 (-5.1 to 5.3) |
| Terminal phase of liver cancer due to other causes | 0.5 (0.4 to 0.6) | -- | 0.3 (0.2 to 0.4) | 24.8* (19.1 to 32.3) | -0.6 (-5.1 to 5.3) |
| Gallbladder and biliary tract cancer | 29.5 (25.9 to 34.2) | 28.4 (25.1 to 31.8) | 6.5 (4.5 to 8.5) | 21.4* (9.9 to 36.6) | -3.3 (-12.5 to 8.8) |
| Diagnosis and primary therapy phase of gallbladder and biliary tract cancer | 4.4 (3.9 to 5.0) | 28.4 (25.1 to 31.8) | 1.2 (0.8 to 1.7) | 21.0* (7.9 to 39.1) | -3.6 (-14.1 to 10.8) |
| Controlled phase of gallbladder and biliary tract cancer | 14.9 (12.7 to 17.9) | -- | 0.7 (0.4 to 1.0) | 39.9* (13.7 to 73.9) | 11.4 (-9.4 to 38.5) |
| Metastatic phase of gallbladder and biliary tract cancer | 7.9 (7.0 to 8.9) | -- | 3.4 (2.2 to 4.5) | 19.4* (6.9 to 34.5) | -5.0 (-14.9 to 7.1) |
| Terminal phase of gallbladder and biliary tract cancer | 2.3 (2.0 to 2.6) | -- | 1.2 (0.8 to 1.6) | 18.8* (9.7 to 31.3) | -5.4 (-12.6 to 4.6) |
| Pancreatic cancer | 45.4 (43.8 to 46.9) | 59.7 (58.0 to 61.4) | 11.6 (8.1 to 15.3) | 33.7* (25.4 to 42.5) | 6.5 (-0.1 to 13.5) |
| Diagnosis and primary therapy phase of pancreatic cancer | 13.0 (12.6 to 13.3) | 59.7 (58.0 to 61.4) | 3.3 (2.3 to 4.6) | 33.5* (18.7 to 49.9) | 6.3 (-5.5 to 19.4) |
| Controlled phase of pancreatic cancer | 15.1 (14.3 to 15.8) | -- | 0.7 (0.4 to 1.0) | 70.0* (52.4 to 89.4) | 35.4* (21.4 to 50.8) |
| Metastatic phase of pancreatic cancer | 12.5 (12.1 to 12.8) | -- | 5.0 (3.4 to 6.5) | 31.4* (17.3 to 47.5) | 4.6 (-6.6 to 17.4) |
| Terminal phase of pancreatic cancer | 4.9 (4.8 to 5.1) | -- | 2.6 (1.8 to 3.4) | 31.0* (26.8 to 35.3) | 4.3* (1.0 to 7.7) |
| Larynx cancer | 84.7 (82.4 to 87.3) | 17.7 (17.2 to 18.3) | 8.7 (6.3 to 11.1) | 29.8* (22.5 to 36.8) | 3.3 (-2.5 to 8.9) |
| Diagnosis and primary therapy phase of larynx cancer | 5.1 (4.9 to 5.3) | 17.7 (17.2 to 18.3) | 1.4 (0.9 to 1.9) | 34.8* (21.9 to 49.0) | 7.4 (-2.9 to 18.6) |
| Controlled phase of larynx cancer | 62.7 (60.8 to 64.8) | -- | 3.3 (2.1 to 4.6) | 34.4* (26.4 to 43.3) | 7.0* (0.7 to 14.1) |
| Metastatic phase of larynx cancer | 7.5 (7.3 to 7.8) | -- | 3.0 (2.1 to 4.0) | 25.9* (12.0 to 40.9) | 0.3 (-10.8 to 12.2) |
| Terminal phase of larynx cancer | 1.1 (1.0 to 1.1) | -- | 0.6 (0.4 to 0.7) | 22.6* (19.0 to 26.6) | -2.4 (-5.3 to 0.8) |
| Laryngectomy from larynx cancer, beyond 10 years | 8.3 (7.8 to 8.8) | -- | 0.4 (0.2 to 0.6) | 18.1* (4.9 to 32.3) | -5.9 (-16.4 to 5.3) |
| Tracheal, bronchus, and lung cancer | 379.5 (368.8 to 390.2) | 279.9 (272.0 to 288.1) | 64.5 (47.3 to 81.1) | 30.8* (25.2 to 37.2) | 4.1 (-0.3 to 9.3) |
| Diagnosis and primary therapy phase of lung, bronchus, and trachea cancer | 25.9 (25.1 to 26.6) | 279.9 (272.0 to 288.1) | 6.4 (4.5 to 8.7) | 39.8* (28.6 to 52.4) | 11.3* (2.4 to 21.3) |
| Controlled phase of lung, bronchus, and trachea cancer | 230.7 (223.9 to 237.5) | -- | 10.2 (6.6 to 14.5) | 55.5* (49.1 to 62.1) | 23.8* (18.8 to 29.1) |
| Metastatic phase of lung, bronchus, and trachea cancer | 100.6 (97.8 to 103.6) | -- | 37.7 (26.5 to 48.4) | 25.6* (19.1 to 33.0) | -0.0 (-5.2 to 5.9) |
| Terminal phase of lung, bronchus, and trachea cancer | 22.3 (21.7 to 23.0) | -- | 10.2 (7.2 to 12.8) | 25.0* (14.7 to 36.5) | -0.5 (-8.7 to 8.7) |
| Malignant skin melanoma | 168.5 (107.7 to 189.6) | 24.9 (16.2 to 27.8) | 10.8 (6.3 to 15.0) | 34.7* (23.2 to 44.5) | 7.2 (-1.9 to 15.1) |
| Diagnosis and primary therapy phase of malignant skin melanoma | 4.9 (3.1 to 5.5) | 24.9 (16.2 to 27.8) | 1.2 (0.7 to 1.8) | 34.9* (13.9 to 56.6) | 7.4 (-9.3 to 24.7) |
| Controlled phase of malignant skin melanoma | 157.5 (100.4 to 177.3) | -- | 6.9 (3.8 to 10.3) | 39.4* (27.2 to 50.0) | 11.0* (1.3 to 19.4) |
| Metastatic phase of malignant skin melanoma | 5.3 (3.5 to 5.9) | -- | 2.2 (1.2 to 3.0) | 23.2* (9.3 to 38.1) | -1.9 (-13.0 to 10.0) |
| Terminal phase of malignant skin melanoma | 0.9 (0.6 to 1.0) | -- | 0.5 (0.3 to 0.6) | 23.9* (14.7 to 31.1) | -1.3 (-8.7 to 4.4) |
| Non-melanoma skin cancer | 346.9 (183.7 to 574.2) | 905.8 (458.1 to 1515.1) | 12.0 (5.3 to 22.2) | 22.9* (11.8 to 32.5) | -2.1 (-11.0 to 5.5) |
| Non-melanoma skin cancer (squamous-cell carcinoma) | 292.5 (135.0 to 519.0) | 238.0 (112.0 to 403.9) | 11.7 (5.1 to 21.9) | 22.9* (11.6 to 32.9) | -2.1 (-11.2 to 5.8) |
| Non-melanoma skin cancer (basal-cell carcinoma) | 67.3 (25.2 to 138.1) | 667.8 (253.2 to 1258.4) | 0.3 (0.1 to 0.7) | 22.7* (8.2 to 32.4) | -2.3 (-13.9 to 5.4) |
| Breast cancer | 1050.5 (1015.6 to 1086.5) | 121.9 (117.0 to 126.9) | 82.8 (60.6 to 109.4) | 28.0* (22.7 to 33.4) | 1.9 (-2.3 to 6.2) |
| Diagnosis and primary therapy phase of breast cancer | 23.9 (23.0 to 24.9) | 121.9 (117.0 to 126.9) | 5.9 (4.1 to 7.9) | 32.1* (19.2 to 46.4) | 5.2 (-5.1 to 16.6) |
| Controlled phase of breast cancer | 729.6 (700.0 to 761.1) | -- | 45.7 (29.6 to 66.3) | 30.6* (25.0 to 36.5) | 4.0 (-0.5 to 8.7) |
| Metastatic phase of breast cancer | 57.4 (55.1 to 59.7) | -- | 21.4 (15.0 to 27.6) | 25.2* (16.6 to 35.6) | -0.3 (-7.2 to 8.0) |
| Terminal phase of breast cancer | 4.5 (4.3 to 4.7) | -- | 2.3 (1.6 to 2.9) | 24.9* (10.4 to 41.5) | -0.5 (-12.1 to 12.6) |
| Mastectomy from breast cancer, beyond 10 years | 235.0 (223.4 to 247.4) | -- | 7.5 (4.4 to 11.8) | 19.0* (15.2 to 22.9) | -5.3* (-8.3 to -2.1) |
| Cervical cancer | 79.4 (74.8 to 82.9) | 18.9 (17.8 to 19.8) | 7.7 (5.6 to 9.9) | 16.5* (9.3 to 23.6) | -7.2* (-13.0 to -1.6) |
| Diagnosis and primary therapy phase of cervical cancer | 4.3 (4.1 to 4.5) | 18.9 (17.8 to 19.8) | 1.2 (0.8 to 1.6) | 17.2* (8.2 to 27.3) | -6.7 (-13.9 to 1.3) |
| Controlled phase of cervical cancer | 66.7 (62.8 to 69.7) | -- | 2.9 (1.9 to 4.2) | 18.7* (10.9 to 26.4) | -5.5 (-11.7 to 0.6) |
| Metastatic phase of cervical cancer | 7.3 (6.8 to 7.6) | -- | 3.0 (2.0 to 3.9) | 14.8* (3.8 to 26.2) | -8.6 (-17.3 to 0.5) |
| Terminal phase of cervical cancer | 1.1 (1.0 to 1.2) | -- | 0.6 (0.4 to 0.8) | 13.7* (8.4 to 18.5) | -9.4* (-13.7 to -5.7) |
| Uterine cancer | 197.6 (188.3 to 207.6) | 29.3 (28.0 to 30.7) | 14.4 (10.7 to 18.6) | 24.8* (16.4 to 33.7) | -0.6 (-7.3 to 6.5) |
| Diagnosis and primary therapy phase of uterine cancer | 9.5 (9.1 to 10.0) | 29.3 (28.0 to 30.7) | 2.4 (1.6 to 3.3) | 25.9* (7.1 to 46.6) | 0.3 (-14.7 to 16.7) |
| Controlled phase of uterine cancer | 177.6 (169.1 to 186.6) | -- | 7.8 (5.1 to 11.3) | 28.1* (19.8 to 37.0) | 2.0 (-4.6 to 9.1) |
| Metastatic phase of uterine cancer | 9.5 (9.1 to 9.9) | -- | 3.7 (2.5 to 4.8) | 18.8* (3.5 to 36.4) | -5.4 (-17.6 to 8.6) |
| Terminal phase of uterine cancer | 1.0 (1.0 to 1.1) | -- | 0.6 (0.4 to 0.7) | 18.4* (13.1 to 23.7) | -5.7* (-10.0 to -1.5) |
| Ovarian cancer | 54.6 (52.8 to 56.6) | 17.3 (16.7 to 17.9) | 8.2 (5.9 to 10.4) | 15.2* (5.9 to 25.8) | -8.2 (-15.6 to 0.2) |
| Diagnosis and primary therapy phase of ovarian cancer | 1.9 (1.8 to 2.0) | 17.3 (16.7 to 17.9) | 0.5 (0.4 to 0.8) | 16.8* (12.5 to 21.1) | -7.0* (-10.4 to -3.6) |
| Controlled phase of ovarian cancer | 37.2 (36.0 to 38.6) | -- | 1.6 (1.1 to 2.3) | 17.4* (8.0 to 27.2) | -6.5 (-14.0 to 1.3) |
| Metastatic phase of ovarian cancer | 14.3 (13.9 to 14.8) | -- | 5.3 (3.8 to 7.0) | 14.3* (1.8 to 28.8) | -9.0 (-18.9 to 2.6) |

| | | | | | |
|---|---|---|---|---|---|
| Terminal phase of ovarian cancer | 1.2 (1.1 to 1.2) | -- | 0.6 (0.4 to 0.8) | 16.3* (12.2 to 20.5) | -7.4* (-10.7 to -4.0) |
| Prostate cancer | 1508.6 (1348.9 to 2006.7) | 211.5 (187.3 to 279.2) | 135.7 (98.3 to 185.2) | 29.6* (22.8 to 40.3) | 3.2 (-2.3 to 11.7) |
| Diagnosis and primary therapy phase of prostate cancer | 52.6 (46.7 to 71.0) | 211.5 (187.3 to 279.2) | 12.8 (8.6 to 18.1) | 34.3* (23.7 to 48.8) | 7.0 (-1.5 to 18.5) |
| Controlled phase of prostate cancer | 1230.7 (1089.2 to 1665.0) | -- | 65.1 (42.1 to 98.3) | 36.0* (27.9 to 48.4) | 8.3* (1.9 to 18.2) |
| Metastatic phase of prostate cancer | 136.6 (120.5 to 178.2) | -- | 50.9 (34.6 to 69.0) | 22.1* (15.3 to 31.6) | -2.8 (-8.2 to 4.8) |
| Terminal phase of prostate cancer | 7.6 (6.7 to 9.6) | -- | 3.5 (2.4 to 4.7) | 21.6* (7.8 to 37.6) | -3.2 (-14.1 to 9.6) |
| Impotence and Incontinence after 10-year survival from prostate cancer | 81.1 (78.9 to 83.4) | -- | 3.5 (2.4 to 4.8) | 22.8* (15.3 to 30.7) | -2.2 (-8.2 to 4.1) |
| Testicular cancer | 3.1 (2.9 to 3.3) | 0.7 (0.6 to 0.7) | 0.3 (0.2 to 0.4) | 37.9* (27.9 to 49.3) | 9.8* (1.8 to 18.9) |
| Diagnosis and primary therapy phase of testicular cancer | 0.1 (0.1 to 0.1) | 0.7 (0.6 to 0.7) | -- | 41.7* (32.2 to 52.2) | 12.8* (5.2 to 21.2) |
| Controlled phase of testicular cancer | 2.6 (2.4 to 2.8) | -- | 0.1 (0.1 to 0.2) | 45.2* (31.9 to 61.0) | 15.6* (5.0 to 28.2) |
| Metastatic phase of testicular cancer | 0.3 (0.3 to 0.3) | -- | 0.1 (0.1 to 0.2) | 31.8* (22.9 to 41.4) | 4.9 (-2.2 to 12.6) |
| Terminal phase of testicular cancer | -- | -- | -- | 35.3* (26.3 to 45.0) | 7.7* (0.5 to 15.5) |
| Kidney cancer | 143.1 (131.5 to 149.3) | 36.7 (33.7 to 38.2) | 11.3 (8.3 to 14.6) | 21.4* (14.2 to 28.8) | -3.4 (-9.1 to 2.6) |
| Diagnosis and primary therapy phase of kidney cancer | -- | 36.7 (33.7 to 38.2) | -- | -- | -- |
| Controlled phase of kidney cancer | 129.8 (119.1 to 135.4) | -- | 5.7 (3.7 to 8.2) | 22.1* (15.5 to 29.5) | -2.8 (-8.0 to 3.1) |
| Metastatic phase of kidney cancer | 11.1 (10.2 to 11.6) | -- | 4.4 (3.1 to 5.8) | 20.8* (8.2 to 35.2) | -3.8 (-13.9 to 7.6) |
| Terminal phase of kidney cancer | 2.2 (2.0 to 2.3) | -- | 1.2 (0.8 to 1.5) | 20.1* (15.3 to 24.7) | -4.4* (-8.2 to -0.7) |
| Bladder cancer | 316.6 (304.7 to 329.2) | 64.6 (62.2 to 67.9) | 31.0 (23.0 to 40.2) | 20.4* (15.3 to 25.9) | -4.1 (-8.2 to 0.3) |
| Diagnosis and primary therapy phase of bladder cancer | 17.2 (16.5 to 18.0) | 64.6 (62.2 to 67.9) | 4.3 (2.9 to 5.8) | 20.9* (9.2 to 34.2) | -3.7 (-13.0 to 6.9) |
| Controlled phase of bladder cancer | 263.6 (253.1 to 274.7) | -- | 15.8 (11.0 to 21.9) | 22.8* (17.0 to 28.9) | -2.2 (-6.9 to 2.6) |
| Metastatic phase of bladder cancer | 18.9 (18.2 to 20.0) | -- | 7.3 (5.1 to 9.7) | 15.7* (6.0 to 26.1) | -7.9 (-15.6 to 0.4) |
| Terminal phase of bladder cancer | 3.5 (3.4 to 3.8) | -- | 1.9 (1.3 to 2.4) | 16.5* (5.7 to 29.1) | -7.2 (-15.8 to 2.8) |
| Incontinence from bladder cancer, beyond 10 years | 13.3 (12.6 to 14.1) | -- | 1.7 (1.1 to 2.4) | 22.2* (9.8 to 36.5) | -2.7 (-12.6 to 8.7) |
| Brain and nervous system cancer | 58.3 (41.8 to 65.3) | 21.4 (16.0 to 23.7) | 7.4 (4.8 to 10.0) | 46.2* (34.2 to 57.6) | 16.4* (6.9 to 25.5) |
| Diagnosis and primary therapy phase of brain and nervous system cancers | 3.7 (2.7 to 4.2) | 21.4 (16.0 to 23.7) | 1.1 (0.6 to 1.5) | 53.8* (38.1 to 69.0) | 22.4* (10.0 to 34.6) |
| Controlled phase of brain and nervous system cancers | 44.8 (31.8 to 50.3) | -- | 2.0 (1.1 to 2.9) | 59.1* (41.4 to 76.5) | 26.7* (12.6 to 40.5) |
| Metastatic phase of brain and nervous system cancers | 8.3 (6.3 to 9.2) | -- | 3.5 (2.2 to 4.8) | 39.2* (25.8 to 52.6) | 10.9* (0.2 to 21.5) |
| Terminal phase of brain and nervous system cancers | 1.5 (1.1 to 1.6) | -- | 0.8 (0.5 to 1.1) | 39.8* (31.8 to 47.9) | 11.3* (4.9 to 17.7) |
| Thyroid cancer | 78.8 (75.4 to 83.0) | 11.5 (11.0 to 12.1) | 6.2 (4.5 to 8.1) | 31.0* (24.7 to 38.2) | 4.3 (-0.7 to 10.0) |
| Diagnosis and primary therapy phase of thyroid cancer | 2.2 (2.1 to 2.4) | 11.5 (11.0 to 12.1) | 0.6 (0.4 to 0.9) | 33.7* (27.9 to 39.8) | 6.5* (1.8 to 11.3) |
| Controlled phase of thyroid cancer | 71.3 (68.2 to 75.1) | -- | 3.1 (2.1 to 4.5) | 35.1* (26.6 to 45.1) | 7.6* (0.8 to 15.5) |
| Metastatic phase of thyroid cancer | 4.9 (4.7 to 5.2) | -- | 2.2 (1.5 to 2.9) | 25.3* (19.8 to 32.1) | -0.2 (-4.6 to 5.2) |
| Terminal phase of thyroid cancer | 0.4 (0.4 to 0.4) | -- | 0.2 (0.1 to 0.3) | 26.9* (21.9 to 32.5) | 1.1 (-2.9 to 5.5) |
| Mesothelioma | 10.3 (9.8 to 10.8) | 4.6 (4.4 to 4.8) | 1.9 (1.4 to 2.6) | 26.5* (12.1 to 43.5) | 0.7 (-10.7 to 14.2) |
| Diagnosis and primary therapy phase of mesothelioma | 1.2 (1.2 to 1.3) | 4.6 (4.4 to 4.8) | 0.3 (0.2 to 0.5) | 27.7* (16.5 to 41.1) | 1.7 (-7.2 to 12.3) |
| Controlled phase of mesothelioma | 6.0 (5.7 to 6.3) | -- | 0.3 (0.2 to 0.4) | 27.8* (9.7 to 52.6) | 1.8 (-12.7 to 21.5) |
| Metastatic phase of mesothelioma | 2.7 (2.6 to 2.8) | -- | 1.1 (0.8 to 1.5) | 26.0* (4.6 to 54.0) | 0.4 (-16.7 to 22.6) |
| Terminal phase of mesothelioma | 0.4 (0.4 to 0.4) | -- | 0.2 (0.1 to 0.3) | 25.1* (17.7 to 32.6) | -0.4 (-6.3 to 5.6) |
| Hodgkin lymphoma | 11.4 (8.8 to 12.5) | 2.7 (2.1 to 3.0) | 1.3 (0.9 to 1.7) | 25.1* (16.3 to 33.9) | -0.4 (-7.4 to 6.6) |
| Diagnosis and primary therapy phase of Hodgkin disease | 0.4 (0.3 to 0.4) | 2.7 (2.1 to 3.0) | 0.1 (0.1 to 0.2) | 40.1* (31.5 to 48.9) | 11.5* (4.7 to 18.5) |
| Controlled phase of Hodgkin disease | 9.4 (7.2 to 10.3) | -- | 0.4 (0.3 to 0.6) | 40.3* (24.9 to 58.4) | 11.7 (-0.6 to 26.2) |
| Metastatic phase of Hodgkin disease | 1.5 (1.2 to 1.7) | -- | 0.7 (0.4 to 0.9) | 17.4* (9.9 to 24.1) | -6.5* (-12.5 to -1.2) |
| Terminal phase of Hodgkin disease | 0.1 (0.1 to 0.2) | -- | 0.1 (0.1 to 0.1) | 6.1* (0.1 to 11.6) | -15.5* (-20.3 to -11.2) |
| Non-Hodgkin's lymphoma | 190.8 (183.1 to 197.9) | 44.7 (43.1 to 46.2) | 16.4 (12.2 to 21.2) | 33.5* (26.0 to 40.7) | 6.3* (0.3 to 12.0) |
| Diagnosis and primary therapy phase of non-Hodgkin lymphoma | 7.4 (7.1 to 7.6) | 44.7 (43.1 to 46.2) | 1.9 (1.3 to 2.6) | 38.6* (20.7 to 57.3) | 10.4 (-3.9 to 25.2) |
| Controlled phase of non-Hodgkin lymphoma | 166.0 (159.3 to 172.4) | -- | 7.3 (4.7 to 10.5) | 39.6* (32.1 to 47.9) | 11.1* (5.2 to 17.8) |
| Metastatic phase of non-Hodgkin lymphoma | 14.8 (14.3 to 15.3) | -- | 5.9 (4.0 to 7.7) | 26.9* (15.0 to 40.1) | 1.0 (-8.4 to 11.6) |
| Terminal phase of non-Hodgkin lymphoma | 2.5 (2.5 to 2.6) | -- | 1.4 (0.9 to 1.8) | 25.5* (21.4 to 29.6) | -0.1 (-3.3 to 3.2) |
| Multiple myeloma | 41.3 (35.9 to 46.8) | 18.9 (16.4 to 21.0) | 8.9 (6.2 to 11.7) | 34.4* (22.4 to 47.8) | 7.0 (-2.5 to 17.7) |
| Diagnosis and primary therapy phase of multiple myeloma | 2.4 (2.1 to 2.8) | 18.9 (16.4 to 21.0) | 0.7 (0.4 to 1.0) | 42.4* (32.1 to 53.1) | 13.4* (5.2 to 21.9) |
| Controlled phase of multiple myeloma | 20.6 (17.6 to 23.5) | -- | 0.9 (0.6 to 1.3) | 44.6* (28.9 to 61.9) | 15.2* (2.6 to 28.9) |
| Metastatic phase of multiple myeloma | 16.9 (14.7 to 19.0) | -- | 6.6 (4.5 to 8.7) | 33.5* (19.0 to 49.7) | 6.3 (-5.2 to 19.2) |
| Terminal phase of multiple myeloma | 1.4 (1.3 to 1.6) | -- | 0.8 (0.5 to 1.0) | 25.4* (19.8 to 30.8) | -0.1 (-4.6 to 4.2) |
| Leukaemia | 135.8 (125.9 to 145.3) | 39.5 (36.3 to 42.2) | 19.8 (14.4 to 25.4) | 22.0* (13.6 to 31.2) | -2.9 (-9.6 to 4.5) |
| Acute lymphoid leukaemia | 6.8 (5.9 to 7.4) | 1.7 (1.5 to 1.9) | 0.9 (0.6 to 1.2) | 44.3* (30.4 to 55.7) | 14.9* (3.8 to 24.0) |
| Diagnosis and primary therapy phase of acute lymphoid leukemia | 0.8 (0.7 to 0.8) | 1.7 (1.5 to 1.9) | 0.2 (0.1 to 0.3) | 34.3* (24.1 to 41.8) | 7.0 (-1.2 to 12.9) |
| Controlled phase of acute lymphoid leukemia | 5.2 (4.5 to 5.7) | -- | 0.3 (0.2 to 0.4) | 51.7* (34.3 to 67.0) | 20.8* (6.9 to 32.9) |
| Metastatic phase of acute lymphoid leukemia | 0.7 (0.6 to 0.8) | -- | 0.3 (0.2 to 0.4) | 45.7* (30.0 to 59.5) | 16.0* (3.5 to 27.0) |
| Terminal phase of acute lymphoid leukemia | 0.1 (0.1 to 0.1) | -- | 0.1 (0.0 to 0.1) | 46.1* (30.2 to 60.0) | 16.3* (3.7 to 27.4) |
| Chronic lymphoid leukaemia | 59.2 (54.5 to 64.4) | 10.5 (9.7 to 11.4) | 8.2 (6.0 to 10.5) | 20.5* (8.0 to 34.8) | -4.1 (-14.0 to 7.4) |

| | | | | | |
|---|---|---|---|---|---|
| Diagnosis and primary therapy phase of chronic lymphoid leukemia | 3.2 (2.9 to 3.4) | 10.5 (9.7 to 11.4) | 0.9 (0.6 to 1.2) | 23.2* (6.0 to 42.4) | -1.9 (-15.6 to 13.4) |
| Controlled phase of chronic lymphoid leukemia | 42.2 (38.9 to 46.0) | -- | 1.9 (1.2 to 2.8) | 23.6* (9.8 to 38.2) | -1.6 (-12.6 to 10.1) |
| Metastatic phase of chronic lymphoid leukemia | 13.2 (12.2 to 14.4) | -- | 5.1 (3.5 to 6.9) | 19.2* (3.8 to 37.7) | -5.1 (-17.4 to 9.6) |
| Terminal phase of chronic lymphoid leukemia | 0.5 (0.5 to 0.6) | -- | 0.3 (0.2 to 0.4) | 16.3* (7.3 to 26.7) | -7.4 (-14.6 to 0.9) |
| Acute myeloid leukaemia | 10.1 (9.0 to 11.0) | 10.0 (9.0 to 10.9) | 2.3 (1.6 to 3.1) | 29.3* (19.7 to 40.2) | 3.0 (-4.7 to 11.6) |
| Diagnosis and primary therapy phase of acute myeloid leukemia | -- | 10.0 (9.0 to 10.9) | -- | -- | -- |
| Controlled phase of acute myeloid leukemia | 5.6 (5.0 to 6.1) | -- | 0.3 (0.2 to 0.4) | 30.0* (11.4 to 49.2) | 3.5 (-11.3 to 18.8) |
| Metastatic phase of acute myeloid leukemia | 3.7 (3.3 to 4.0) | -- | 1.6 (1.1 to 2.2) | 29.3* (19.5 to 40.0) | 2.9 (-4.9 to 11.5) |
| Terminal phase of acute myeloid leukemia | 0.8 (0.7 to 0.9) | -- | 0.4 (0.3 to 0.6) | 29.3* (20.4 to 39.3) | 2.9 (-4.1 to 10.9) |
| Chronic myeloid leukaemia | 6.4 (5.9 to 7.1) | 3.1 (2.8 to 3.4) | 1.0 (0.7 to 1.3) | 1.9 (-6.7 to 11.1) | -18.9* (-25.7 to -11.5) |
| Diagnosis and primary therapy phase of chronic myeloid leukemia | 0.5 (0.4 to 0.5) | 3.1 (2.8 to 3.4) | 0.1 (0.1 to 0.2) | 2.0 (-5.9 to 10.9) | -18.8* (-25.1 to -11.7) |
| Controlled phase of chronic myeloid leukemia | 4.7 (4.3 to 5.2) | -- | 0.2 (0.1 to 0.3) | 3.8 (-11.6 to 21.5) | -17.4* (-29.6 to -3.2) |
| Metastatic phase of chronic myeloid leukemia | 1.0 (0.9 to 1.1) | -- | 0.5 (0.3 to 0.6) | 1.2 (-6.4 to 9.9) | -19.4* (-25.5 to -12.5) |
| Terminal phase of chronic myeloid leukemia | 0.2 (0.2 to 0.2) | -- | 0.1 (0.1 to 0.2) | 1.2 (-6.4 to 9.9) | -19.4* (-25.5 to -12.5) |
| Other leukaemia | 53.2 (47.7 to 57.7) | 14.2 (12.7 to 15.4) | 7.5 (5.5 to 9.6) | 22.3* (12.2 to 33.6) | -2.6 (-10.6 to 6.4) |
| Metastatic phase of other leukemia | 13.4 (11.9 to 14.5) | -- | 5.3 (3.7 to 7.1) | 20.2* (8.0 to 33.8) | -4.3 (-14.0 to 6.6) |
| Diagnosis and primary therapy phase of other leukemia | -- | 14.2 (12.7 to 15.4) | -- | -- | -- |
| Terminal phase of other leukemia | 0.8 (0.8 to 0.9) | -- | 0.5 (0.3 to 0.6) | 19.5* (12.5 to 27.0) | -4.8 (-10.4 to 1.1) |
| Controlled phase of other leukemia | 39.0 (35.1 to 42.3) | -- | 1.7 (1.1 to 2.5) | 30.2* (19.1 to 42.7) | 3.7 (-5.2 to 13.6) |
| Other malignant cancers | 401.3 (310.8 to 575.3) | 52.7 (46.3 to 55.7) | 35.6 (24.4 to 51.5) | 106.3* (61.9 to 170.1) | 64.3* (28.9 to 115.1) |
| Diagnosis and primary therapy phase of other malignant neoplasms | 10.4 (8.1 to 14.6) | 52.7 (46.3 to 55.7) | 2.7 (1.7 to 4.1) | 131.3* (72.6 to 217.5) | 84.2* (37.4 to 152.8) |
| Controlled phase of other malignant neoplasms | 346.8 (265.5 to 506.3) | -- | 15.3 (9.1 to 24.9) | 169.4* (91.9 to 286.9) | 114.5* (52.8 to 208.1) |
| Metastatic phase of other malignant neoplasms | 38.6 (32.0 to 48.4) | -- | 14.5 (9.5 to 20.4) | 71.0* (39.0 to 113.3) | 36.2* (10.7 to 69.8) |
| Terminal phase of other malignant neoplasms | 5.8 (4.8 to 7.3) | -- | 3.0 (2.0 to 4.1) | 59.6* (31.8 to 92.2) | 27.1* (5.0 to 53.0) |
| Other neoplasms | 1663.8 (1597.8 to 1734.3) | 658.7 (621.1 to 698.2) | 5.6 (3.6 to 8.3) | 37.7* (32.3 to 43.6) | 9.7* (5.3 to 14.4) |
| Myelodysplastic, myeloproliferative, and other hematopoietic neoplasms | 128.6 (107.8 to 151.2) | 14.4 (10.0 to 20.1) | 5.6 (3.6 to 8.3) | 37.7* (32.3 to 43.6) | 9.7* (5.3 to 14.4) |
| Benign and in situ intestinal neoplasms | 344.8 (326.6 to 364.6) | 72.4 (67.9 to 77.1) | -- | -- | -- |
| Benign and in situ cervical and uterine neoplasms | 7.6 (7.1 to 8.2) | 0.9 (0.8 to 1.0) | -- | -- | -- |
| Other benign and in situ neoplasms | 1207.8 (1145.6 to 1271.5) | 570.9 (533.0 to 609.8) | -- | -- | -- |
| **Cardiovascular diseases** | **48800.2 (45813.6 to 51834.4)** | **8654.0 (8044.5 to 9333.5)** | **3867.2 (2859.7 to 4971.8)** | **32.4* (28.9 to 36.5)** | **5.4* (2.6 to 8.7)** |
| Rheumatic heart disease | 270.6 (256.9 to 283.9) | 7.6 (6.1 to 9.4) | 15.9 (11.1 to 21.4) | 33.9* (29.6 to 37.9) | 6.6* (3.2 to 9.8) |
| Rheumatic heart disease, without heart failure | 165.4 (127.9 to 199.0) | 7.6 (6.1 to 9.4) | 7.3 (4.5 to 10.9) | 32.9* (27.7 to 37.6) | 5.8* (1.7 to 9.6) |
| Heart failure due to rheumatic heart disease | 105.2 (76.8 to 139.2) | -- | 8.6 (5.2 to 12.7) | 34.7* (27.9 to 41.5) | 7.3* (1.8 to 12.7) |
| Ischaemic heart disease | 15912.0 (14181.6 to 17615.9) | 1375.6 (1050.3 to 1792.0) | 615.9 (424.0 to 846.5) | 19.1* (17.9 to 20.2) | -5.2* (-6.1 to -4.3) |
| Asymptomatic ischemic heart disease following myocardial infarction | 5619.5 (4176.5 to 7025.2) | -- | -- | -- | -- |
| Angina due to ischemic heart disease | 7553.9 (6829.7 to 8307.1) | -- | 394.3 (268.2 to 541.5) | 20.9* (19.8 to 22.0) | -3.7* (-4.6 to -2.9) |
| Heart failure due to ischemic heart disease | 2666.3 (1997.7 to 3497.3) | -- | 215.0 (140.5 to 320.2) | 16.2* (13.4 to 18.9) | -7.4* (-9.7 to -5.3) |
| Myocardial infarction due to ischemic heart disease | 72.3 (54.6 to 94.0) | 1375.6 (1050.3 to 1792.0) | 6.6 (4.2 to 9.6) | 5.9 (-1.8 to 13.6) | -15.7* (-21.8 to -9.6) |
| Stroke | 10228.2 (9316.2 to 11126.4) | 1321.8‡ (1126.7 to 1524.2) | 2026.0 (1463.7 to 2578.6) | 43.3* (36.5 to 50.0) | 14.1* (8.7 to 19.4) |
| Ischaemic stroke | 9682.3 (8674.3 to 10666.3) | 961.0 (768.3 to 1170.3) | 1831.8 (1321.4 to 2335.2) | 47.8* (40.3 to 55.2) | 17.7* (11.7 to 23.6) |
| Acute ischemic stroke | 73.2 (59.7 to 88.7) | 961.0 (768.3 to 1170.3) | 17.0 (11.5 to 23.5) | 38.6* (23.9 to 53.9) | 10.4 (-1.4 to 22.6) |
| Chronic ischemic stroke | 9609.1 (8605.4 to 10588.9) | -- | 1814.8 (1307.9 to 2315.2) | 47.9* (40.3 to 55.3) | 17.8* (11.7 to 23.7) |
| Intracerebral hemorrhage | 671.9 (557.0 to 812.4) | 291.4 (240.5 to 350.8) | 128.4 (90.5 to 172.3) | 6.2 (-3.2 to 16.7) | -15.5* (-22.9 to -7.1) |
| Acute intracerebral hemorrhage | 14.9 (12.3 to 17.9) | 291.4 (240.5 to 350.8) | 4.0 (2.7 to 5.5) | -5.3 (-14.8 to 4.6) | -24.6* (-32.2 to -16.7) |
| Chronic intracerebral hemorrhage | 657.0 (543.1 to 797.7) | -- | 124.4 (87.1 to 167.6) | 6.6 (-3.2 to 17.6) | -15.1* (-22.9 to -6.3) |
| Subarachnoid hemorrhage | 357.7 (295.9 to 434.2) | 69.4 (55.8 to 86.0) | 65.9 (46.2 to 88.2) | 22.4* (16.3 to 28.6) | -2.6 (-7.4 to 2.4) |
| Acute subarachnoid hemorrhage | 5.4 (4.4 to 6.6) | 69.4 (55.8 to 86.0) | 1.5 (1.0 to 2.1) | 26.1* (20.9 to 29.3) | 0.4 (-3.8 to 3.0) |
| Chronic subarachnoid hemorrhage | 352.3 (290.5 to 428.5) | -- | 64.4 (45.0 to 86.5) | 22.3* (16.1 to 28.7) | -2.6 (-7.5 to 2.5) |
| Hypertensive heart disease | 2353.2 (1727.1 to 3139.9) | -- | 189.9 (119.5 to 280.9) | 27.8* (25.3 to 29.9) | 1.7 (-0.3 to 3.5) |
| Non-rheumatic valvular heart disease | 4150.3 (3892.4 to 4422.8) | 4233.8 (3966.7 to 4511.9) | 62.5 (35.1 to 98.2) | 25.2* (20.5 to 29.7) | -0.3 (-4.1 to 3.3) |
| Non-rheumatic calcific aortic valve disease | 2028.7 (1778.7 to 2300.0) | 2028.7 (1778.7 to 2300.0) | 28.9 (15.7 to 46.0) | 26.1* (18.0 to 33.5) | 0.4 (-6.0 to 6.3) |
| Heart failure due to calcific aortic valve disease | 232.9 (144.3 to 352.7) | -- | 18.7 (10.4 to 30.2) | 17.4* (1.2 to 33.3) | -6.5 (-19.4 to 6.2) |
| Non-rheumatic calcific aortic valve disease | 1795.8 (1556.9 to 2054.2) | 2028.7 (1778.7 to 2300.0) | 10.1 (5.1 to 16.9) | 46.2* (25.6 to 69.1) | 16.4* (0.0 to 34.7) |
| Non-rheumatic degenerative mitral valve disease | 2205.2 (2135.7 to 2283.4) | 2205.2 (2135.7 to 2283.4) | 33.3 (18.9 to 51.4) | 24.3* (19.4 to 29.0) | -1.0 (-4.9 to 2.7) |
| Heart failure due to degenerative mitral valve disease | 305.8 (189.8 to 448.1) | -- | 24.6 (13.9 to 37.9) | 18.4* (8.3 to 29.6) | -5.7 (-13.8 to 3.2) |
| Non-rheumatic degenerative mitral valve disease | 1899.4 (1750.0 to 2034.3) | 2205.2 (2135.7 to 2283.4) | 8.7 (4.5 to 14.4) | 44.5* (23.3 to 67.2) | 15.0 (-1.8 to 33.2) |
| Other non-rheumatic valve diseases | 3.9 (2.6 to 5.5) | -- | 0.4 (0.2 to 0.6) | 36.6* (32.5 to 40.5) | 8.8* (5.5 to 11.9) |
| Mild heart failure due to other non-rheumatic valve disease | 0.7 (0.4 to 1.1) | -- | 0.0 (0.0 to 0.1) | 36.6* (31.8 to 41.4) | 8.7* (4.9 to 12.6) |
| Moderate heart failure due to other non-rheumatic valve disease | 0.5 (0.3 to 0.7) | -- | 0.0 (0.0 to 0.1) | 36.7* (31.4 to 42.0) | 8.8* (4.6 to 13.1) |
| Severe heart failure due to other non-rheumatic valve disease | 1.3 (0.8 to 1.9) | -- | 0.2 (0.1 to 0.4) | 36.6* (31.7 to 41.5) | 8.8* (4.9 to 12.6) |

| | | | | | |
|---|---|---|---|---|---|
| Controlled, medically managed heart failure due to other non-rheumatic valve disease | 1.4 (1.0 to 2.1) | -- | 0.1 (0.0 to 0.1) | 36.5* (31.5 to 41.3) | 8.7* (4.7 to 12.5) |
| Cardiomyopathy and myocarditis | 610.6 (448.3 to 816.1) | 212.7 (156.9 to 283.1) | 50.5 (31.2 to 75.1) | 19.5* (16.0 to 23.4) | -4.9* (-7.7 to -1.7) |
| Myocarditis | 85.8 (68.4 to 104.5) | 212.7 (156.9 to 283.1) | 5.3 (3.4 to 7.8) | 24.1* (18.3 to 30.0) | -1.2 (-5.8 to 3.5) |
| Acute myocarditis | 51.3 (38.1 to 66.4) | 212.7 (156.9 to 283.1) | 2.3 (1.4 to 3.6) | 23.4* (16.3 to 30.8) | -1.7 (-7.4 to 4.2) |
| Heart failure due to myocarditis | 34.5 (24.0 to 48.6) | -- | 3.0 (1.8 to 4.6) | 24.6* (15.8 to 33.5) | -0.8 (-7.8 to 6.3) |
| Alcoholic cardiomyopathy | 107.8 (76.4 to 146.7) | -- | 8.8 (5.4 to 13.3) | 16.9* (9.2 to 23.8) | -6.9* (-13.1 to -1.4) |
| Other cardiomyopathy | 451.3 (320.1 to 618.0) | -- | 36.4 (22.2 to 54.3) | 19.5* (15.4 to 23.8) | -4.9* (-8.1 to -1.4) |
| Atrial fibrillation and flutter | 6078.8 (5038.8 to 7175.8) | 430.3 (290.6 to 580.3) | 464.3 (306.4 to 653.7) | 24.3* (22.9 to 25.6) | -1.0* (-2.1 to -0.0) |
| Asymptomatic atrial fibrillation and flutter | 3647.4 (3012.3 to 4299.7) | 430.3 (290.6 to 580.3) | -- | -- | -- |
| Symptomatic atrial fibrillation and flutter | 2431.5 (2010.5 to 2893.5) | -- | 464.3 (306.4 to 653.7) | 24.3* (22.9 to 25.6) | -1.0* (-2.1 to -0.0) |
| Peripheral vascular disease | 14715.2 (12024.5 to 17755.9) | 981.6 (692.7 to 1348.2) | 85.2 (37.9 to 161.3) | 20.1* (18.3 to 22.8) | -4.4* (-5.8 to -2.3) |
| Asymptomatic peripheral arterial disease | 8124.6 (5451.4 to 10946.8) | 981.6 (692.7 to 1348.2) | -- | -- | -- |
| Symptomatic claudication due to peripheral arterial disease | 6590.6 (4556.6 to 9454.6) | -- | 85.2 (37.9 to 161.3) | 20.1* (18.3 to 22.8) | -4.4* (-5.8 to -2.3) |
| Endocarditis | 80.5 (57.7 to 107.4) | 90.5 (68.4 to 112.7) | 6.5 (4.0 to 10.0) | 20.2* (12.9 to 27.4) | -4.3 (-10.1 to 1.5) |
| Acute endocarditis | 6.7 (5.2 to 8.3) | 90.5 (68.4 to 112.7) | 0.4 (0.2 to 0.6) | 29.6* (18.1 to 39.5) | 3.2 (-5.9 to 11.1) |
| Heart failure due to endocarditis | 73.8 (51.4 to 101.0) | -- | 6.1 (3.8 to 9.4) | 19.7* (12.0 to 27.5) | -4.7 (-10.8 to 1.5) |
| Other cardiovascular and circulatory diseases | 7003.6 (4901.8 to 9582.3) | -- | 350.6 (216.3 to 540.3) | 22.2* (19.5 to 24.6) | -2.7* (-4.8 to -0.8) |
| Heart failure due to other cardiovascular diseases | 252.7 (174.3 to 348.8) | -- | 20.4 (12.5 to 31.5) | 21.6* (16.6 to 26.2) | -3.2 (-7.2 to 0.5) |
| Other cardiovascular and circulatory disease episodes | 6750.9 (4721.3 to 9241.0) | -- | 330.2 (202.9 to 508.3) | 22.2* (19.6 to 24.6) | -2.7* (-4.8 to -0.8) |
| **Chronic respiratory diseases** | **32521.6 (29210.6 to 36572.8)** | **1820.4 (1387.4 to 2337.7)** | **3099.4 (2548.4 to 3647.5)** | **23.6* (16.2 to 30.2)** | **-1.6 (-7.5 to 3.7)** |
| Chronic obstructive pulmonary disease | 28238.9 (24249.8 to 33134.4) | 1215.1 (805.0 to 1733.3) | 2719.2 (2245.2 to 3208.9) | 22.7* (14.4 to 30.1) | -2.3 (-8.9 to 3.6) |
| Chronic obstructive pulmonary disease with heart failure | 3131.5 (2364.5 to 3984.0) | -- | 1186.1 (789.7 to 1567.7) | 27.3* (22.6 to 30.8) | 1.4 (-2.4 to 4.2) |
| Chronic obstructive pulmonary disease without heart failure | 25107.4 (21125.1 to 29928.6) | 1215.1 (805.0 to 1733.3) | 1533.1 (1093.4 to 2036.1) | 19.3* (6.4 to 31.4) | -5.0 (-15.3 to 4.7) |
| Pneumoconiosis | 70.1 (58.5 to 83.4) | 7.0 (5.3 to 9.1) | 10.3 (6.9 to 14.3) | 22.4* (14.6 to 30.3) | -2.5 (-8.8 to 3.7) |
| Silicosis | 22.0 (15.0 to 30.8) | 2.8 (1.6 to 4.3) | 3.2 (1.9 to 4.8) | 23.5* (6.9 to 41.4) | -1.7 (-14.9 to 12.6) |
| Heart failure due to silicosis | 1.4 (1.0 to 1.8) | -- | 0.6 (0.4 to 0.9) | 17.8* (9.5 to 26.1) | -6.2 (-12.8 to 0.4) |
| Silicosis without heart failure | 20.6 (13.7 to 29.2) | 2.8 (1.6 to 4.3) | 2.5 (1.4 to 4.0) | 25.0* (3.8 to 47.6) | -0.5 (-17.3 to 17.5) |
| Asbestosis | 10.5 (7.9 to 14.0) | 1.0 (0.6 to 1.7) | 1.6 (1.0 to 2.5) | 31.6* (21.1 to 42.4) | 4.8 (-3.5 to 13.4) |
| Asbestosis without heart failure | 9.3 (6.9 to 12.8) | 1.0 (0.6 to 1.7) | 1.1 (0.6 to 1.8) | 38.1* (22.5 to 54.6) | 9.9 (-2.5 to 23.1) |
| Heart failure due to asbestosis | 1.2 (0.9 to 1.5) | -- | 0.5 (0.3 to 0.8) | 20.2* (16.3 to 24.0) | -4.3* (-7.4 to -1.3) |
| Coal workers pneumoconiosis | 22.9 (16.3 to 33.3) | 2.0 (1.1 to 3.3) | 3.3 (2.0 to 5.1) | 18.7* (2.0 to 36.8) | -5.4 (-18.8 to 9.0) |
| Coal workers pneumoconiosis without heart failure | 21.6 (15.2 to 31.9) | 2.0 (1.1 to 3.3) | 2.7 (1.5 to 4.3) | 17.8 (-2.4 to 39.8) | -6.2 (-22.3 to 11.3) |
| Heart failure due to Coal workers pneumoconiosis | 1.3 (1.0 to 1.6) | -- | 0.6 (0.4 to 0.8) | 23.3* (14.5 to 33.1) | -1.8 (-8.8 to 6.0) |
| Other pneumoconiosis | 14.7 (11.4 to 18.8) | 1.2 (0.7 to 1.9) | 2.2 (1.4 to 3.2) | 20.1* (9.9 to 30.4) | -4.4 (-12.5 to 3.9) |
| Heart failure due to other pneumoconiosis | 0.7 (0.6 to 0.9) | -- | 0.3 (0.2 to 0.5) | 21.0* (16.8 to 24.6) | -3.6* (-7.0 to -0.7) |
| Other pneumoconiosis without heart failure | 13.9 (10.8 to 18.0) | 1.2 (0.7 to 1.9) | 1.9 (1.1 to 2.8) | 19.9* (8.0 to 32.5) | -4.5 (-14.0 to 5.5) |
| Asthma | 7722.0 (6344.9 to 9498.1) | 546.2 (412.0 to 715.6) | 278.1 (181.0 to 409.1) | 30.3* (23.4 to 38.6) | 3.8 (-1.8 to 10.4) |
| Asymptomatic asthma | 2805.3 (2244.3 to 3486.3) | 198.1 (147.5 to 264.7) | -- | -- | -- |
| Symptomatic asthma | 4916.7 (4014.3 to 6068.1) | 348.1 (264.1 to 454.8) | 278.1 (181.0 to 409.1) | 30.3* (23.4 to 38.6) | 3.8 (-1.8 to 10.4) |
| Interstitial lung disease and pulmonary sarcoidosis | 732.1 (611.1 to 865.8) | 52.1 (38.1 to 68.0) | 73.9 (49.0 to 106.0) | 30.7* (25.9 to 35.2) | 4.1* (0.2 to 7.6) |
| Heart failure due to interstitial lung disease and pulmonary sarcoidosis | 76.2 (53.3 to 99.0) | -- | 30.0 (19.1 to 43.4) | 27.1* (21.4 to 32.5) | 1.2 (-3.3 to 5.5) |
| Interstitial lung disease and pulmonary sarcoidosis without heart failure | 655.9 (543.5 to 782.7) | 52.1 (38.1 to 68.0) | 44.0 (28.9 to 64.1) | 33.3* (27.2 to 39.2) | 6.1* (1.3 to 10.9) |
| Other chronic respiratory diseases | -- | -- | 17.9 (14.8 to 21.1) | 41.6* (32.3 to 49.9) | 12.7* (5.3 to 19.4) |
| **Digestive diseases** | **61781.2 (57422.2 to 66270.9)** | **16301.7 (13520.1 to 19318.4)** | **703.0 (484.7 to 983.9)** | **29.6* (26.6 to 33.0)** | **3.2* (0.8 to 5.9)** |
| Cirrhosis and other chronic liver diseases | 46561.1 (42943.2 to 50357.3) | 113.2 (102.4 to 124.6) | 75.6 (53.2 to 102.4) | 45.7* (41.7 to 50.3) | 16.0* (12.8 to 19.7) |
| Cirrhosis and other chronic liver diseases due to hepatitis B | 7037.8 (6493.1 to 7599.8) | 36.0 (31.3 to 41.1) | 21.8 (14.8 to 30.0) | 45.6* (39.7 to 51.8) | 15.9* (11.2 to 20.9) |
| Cirrhosis and other chronic liver diseases due to hepatitis B, decompensated | 141.1 (125.3 to 157.9) | 35.6 (30.8 to 40.8) | 21.8 (14.8 to 30.0) | 45.6* (39.7 to 51.8) | 15.9* (11.2 to 20.9) |
| Chronic hepatitis B without cirrhosis | 6025.5 (5481.3 to 6597.5) | -- | -- | -- | -- |
| Cirrhosis and other chronic liver diseases due to hepatitis B, compensated | 871.1 (789.5 to 962.5) | 0.4 (0.0 to 1.3) | -- | -- | -- |
| Cirrhosis and other chronic liver diseases due to hepatitis C | 3103.1 (2635.9 to 3649.9) | 26.6 (22.7 to 30.7) | 19.3 (13.3 to 26.6) | 42.9* (36.9 to 49.1) | 13.7* (9.0 to 18.7) |
| Cirrhosis and other chronic liver diseases due to hepatitis C, decompensated | 125.4 (110.3 to 141.2) | 26.2 (22.3 to 30.4) | 19.3 (13.3 to 26.6) | 42.9* (36.9 to 49.1) | 13.7* (9.0 to 18.7) |
| Chronic hepatitis C without cirrhosis | 2373.0 (1898.0 to 2918.8) | -- | -- | -- | -- |
| Cirrhosis and other chronic liver diseases due to hepatitis C, compensated | 604.7 (530.5 to 680.9) | 0.4 (0.0 to 1.2) | -- | -- | -- |
| Cirrhosis and other chronic liver diseases due to alcohol use | 680.9 (601.1 to 771.0) | 23.9 (20.4 to 27.5) | 17.1 (11.9 to 23.5) | 44.4* (37.0 to 51.8) | 14.9* (9.1 to 20.9) |
| Cirrhosis and other chronic liver diseases due to alcohol, decompensated | 111.4 (98.1 to 125.1) | 23.8 (20.3 to 27.4) | 17.1 (11.9 to 23.5) | 44.4* (37.0 to 51.8) | 14.9* (9.1 to 20.9) |
| Cirrhosis and other chronic liver diseases due to alcohol, compensated | 569.5 (498.5 to 648.3) | 0.2 (0.0 to 0.6) | -- | -- | -- |
| Cirrhosis due to NASH | 35359.4 (31900.6 to 39058.0) | 11.7 (9.9 to 13.8) | 7.1 (4.9 to 10.0) | 60.5* (49.8 to 71.9) | 27.8* (19.3 to 36.9) |
| Non-alcoholic fatty liver disease (NAFLD) / Non-alcoholic steatohepatitis (NASH) | 35076.0 (31621.2 to 38783.2) | -- | -- | -- | -- |
| Cirrhosis and other chronic liver diseases due to NASH | 283.4 (243.8 to 327.4) | 11.7 (9.9 to 13.8) | 7.1 (4.9 to 10.0) | 60.5* (49.8 to 71.9) | 27.8* (19.3 to 36.9) |
| Cirrhosis and other chronic liver diseases due to other causes | 383.7 (302.2 to 480.2) | 14.9 (11.5 to 19.0) | 10.4 (6.8 to 15.1) | 44.2* (35.8 to 53.6) | 14.8* (8.1 to 22.3) |

| | | | | | |
|---|---|---|---|---|---|
| Cirrhosis and other chronic liver diseases due to other cause, decompensated | 67.8 (53.6 to 84.2) | 14.8 (11.5 to 18.9) | 10.4 (6.8 to 15.1) | 44.2* (35.8 to 53.6) | 14.8* (8.1 to 22.3) |
| Cirrhosis and other chronic liver diseases due to other cause, compensated | 315.9 (248.1 to 395.4) | 0.1 (0.0 to 0.3) | -- | -- | -- |
| Upper digestive system diseases | 26743.8 (21263.1 to 32902.2) | 12300.1 (9712.6 to 15214.9) | 421.7 (255.3 to 640.9) | 28.0* (24.1 to 32.5) | 1.9 (-1.2 to 5.5) |
| Peptic ulcer disease | 877.6 (678.0 to 1109.2) | 390.1 (290.2 to 497.1) | 40.6 (26.0 to 59.6) | 16.8* (11.0 to 22.5) | -7.0* (-11.6 to -2.4) |
| Acute peptic ulcer disease | 0.7 (0.5 to 1.0) | -- | 0.2 (0.1 to 0.4) | 10.9* (8.7 to 12.8) | -11.7* (-13.4 to -10.2) |
| Chronic peptic ulcer disease | 876.9 (677.1 to 1108.5) | 390.1 (290.2 to 497.1) | 40.4 (25.8 to 59.4) | 16.8* (11.0 to 22.6) | -7.0* (-11.6 to -2.4) |
| Gastritis and duodenitis | 4457.7 (3260.8 to 5981.7) | 3091.0 (2156.2 to 4272.4) | 186.4 (114.0 to 280.7) | 32.5* (24.1 to 41.2) | 5.5 (-1.2 to 12.4) |
| Acute gastritis and duodenitis | 2.8 (2.1 to 3.7) | -- | 0.9 (0.6 to 1.4) | 18.4* (15.7 to 20.9) | -5.7* (-7.9 to -3.7) |
| Chronic gastritis and duodenitis | 4454.9 (3257.7 to 5979.1) | 3091.0 (2156.2 to 4272.4) | 185.4 (113.1 to 279.7) | 32.6* (24.2 to 41.3) | 5.5 (-1.1 to 12.5) |
| GERD | 24213.3 (17983.9 to 31694.1) | 8819.0 (6423.3 to 11819.4) | 194.7 (94.1 to 360.4) | 26.4* (24.5 to 28.4) | 0.6 (-0.9 to 2.2) |
| Appendicitis | 12.3 (10.4 to 14.2) | 347.2 (300.2 to 398.4) | 3.4 (2.3 to 4.7) | 22.9* (13.7 to 33.4) | -2.1 (-9.5 to 6.2) |
| Paralytic ileus and intestinal obstruction | 8.8 (6.9 to 11.1) | 255.2 (200.3 to 321.1) | 2.7 (1.7 to 3.9) | 30.1* (18.1 to 43.1) | 3.6 (-6.0 to 13.9) |
| Inguinal, femoral, and abdominal hernia | 1130.4 (860.5 to 1472.0) | 2718.7 (1917.9 to 3662.4) | 101.0 (64.3 to 149.8) | 26.4* (18.3 to 35.6) | 0.6 (-5.8 to 8.0) |
| Inflammatory bowel disease | 273.4 (235.1 to 316.2) | 69.4 (55.4 to 86.5) | 37.5 (25.1 to 51.9) | 23.1* (15.8 to 30.3) | -2.0 (-7.8 to 3.8) |
| Ulcerative colitis | 203.9 (168.2 to 241.8) | 49.1 (35.7 to 65.1) | 27.5 (18.2 to 38.8) | 26.2* (18.2 to 34.4) | 0.5 (-5.9 to 7.0) |
| Crohn's disease | 69.5 (58.8 to 81.2) | 20.3 (15.4 to 25.6) | 10.0 (6.6 to 14.0) | 15.3* (6.6 to 25.1) | -8.2* (-15.1 to -0.4) |
| Vascular intestinal disorders | 10.2 (7.8 to 13.1) | 76.8 (58.2 to 98.6) | 2.9 (1.9 to 4.2) | 20.4* (2.0 to 40.1) | -4.2 (-18.8 to 11.5) |
| Gallbladder and biliary diseases | 1374.6 (1057.9 to 1728.2) | 329.7 (252.7 to 424.9) | 1.3 (0.9 to 2.1) | 29.4* (15.6 to 43.3) | 3.1 (-8.0 to 14.1) |
| Pancreatitis | 385.2 (330.9 to 443.8) | 91.4 (71.8 to 112.7) | 21.4 (11.1 to 36.1) | 32.3* (25.4 to 39.5) | 5.4 (-0.1 to 11.1) |
| Acute pancreatitis | 7.4 (5.6 to 9.4) | 75.2 (56.3 to 95.5) | 2.9 (1.8 to 4.1) | 23.5* (15.6 to 31.3) | -1.6 (-8.0 to 4.6) |
| Chronic pancreatitis | 377.7 (323.0 to 435.9) | 16.2 (11.1 to 22.2) | 18.6 (8.7 to 32.9) | 33.8* (25.9 to 42.6) | 6.5* (0.2 to 13.5) |
| Other digestive diseases | -- | -- | 35.4 (24.2 to 48.0) | 34.8* (30.0 to 40.1) | 7.3* (3.5 to 11.6) |
| **Neurological disorders** | **51577.6 (42714.3 to 63386.4)** | **15522.4 (10730.9 to 21323.9)** | **2020.9 (1438.7 to 2695.8)** | **24.4* (22.9 to 26.1)** | **-1.0 (-2.1 to 0.4)** |
| Alzheimer's disease and other dementias | 8011.7 (6412.5 to 9885.1) | 1398.1 (996.8 to 1906.9) | 1076.8 (721.0 to 1538.8) | 23.2* (22.0 to 24.3) | -1.9* (-2.8 to -1.0) |
| Parkinson's disease | 1558.5 (1198.0 to 1969.7) | 185.8 (115.9 to 270.7) | 218.2 (141.5 to 308.9) | 26.0* (23.5 to 28.5) | 0.4 (-1.7 to 2.4) |
| Epilepsy | 689.8 (522.8 to 903.3) | 45.6 (29.9 to 65.0) | 175.2 (107.0 to 262.0) | 29.5* (15.2 to 45.5) | 3.1 (-8.3 to 15.9) |
| Multiple sclerosis | 54.5 (49.6 to 60.3) | 0.3 (0.2 to 0.3) | 13.3 (9.5 to 17.4) | 23.4* (14.6 to 33.9) | -1.7 (-8.8 to 6.6) |
| Motor neuron disease | 15.2 (11.6 to 19.7) | 6.4 (4.9 to 8.2) | 3.2 (2.1 to 4.7) | 37.6* (29.0 to 46.1) | 9.6* (2.7 to 16.3) |
| Headache disorders | 45191.8 (35826.8 to 57867.4) | 13886.3 (9169.7 to 19534.9) | 516.5 (346.1 to 743.8) | 24.0* (22.6 to 25.6) | -1.2 (-2.4 to 0.0) |
| Migraine | 11259.0 (10002.7 to 12669.7) | 391.0 (250.2 to 564.4) | 403.1 (253.5 to 601.1) | 24.0* (22.2 to 25.9) | -1.2 (-2.7 to 0.3) |
| Medication overuse headache due to migraine | 1507.0 (1067.2 to 2041.4) | -- | 152.0 (85.2 to 236.2) | 22.5* (20.5 to 24.3) | -2.5* (-4.1 to -1.0) |
| Migraine | 9752.0 (8499.4 to 10983.7) | 391.0 (250.2 to 564.4) | 251.1 (147.3 to 400.9) | 25.0* (22.3 to 27.7) | -0.5 (-2.6 to 1.6) |
| Tension-type headache | 38434.4 (27909.5 to 52626.2) | 13495.2 (8794.4 to 19197.1) | 113.4 (62.6 to 188.2) | 24.1* (21.8 to 25.9) | -1.2 (-3.0 to 0.2) |
| Medication overuse headache due to tension-type headache | 560.7 (342.7 to 850.1) | -- | 56.1 (26.4 to 101.8) | 22.5* (19.1 to 25.6) | -2.5 (-5.2 to 0.0) |
| Tension-type headache | 37873.7 (27378.8 to 52177.6) | 13495.2 (8794.4 to 19197.1) | 57.2 (22.2 to 114.2) | 25.6* (24.1 to 27.2) | 0.0 (-1.2 to 1.3) |
| Other neurological disorders | 1.1 (0.6 to 1.8) | -- | 17.6 (12.1 to 23.8) | 37.1* (35.1 to 39.5) | 9.2* (7.6 to 11.0) |
| Other neurological disorders | -- | -- | 17.2 (12.0 to 23.4) | 37.2* (35.2 to 39.6) | 9.3* (7.6 to 11.2) |
| Guillain-Barré syndrome due to other neurological disorders | 1.1 (0.6 to 1.8) | -- | 0.3 (0.2 to 0.6) | 31.3* (28.2 to 34.5) | 4.6* (2.0 to 7.1) |
| **Mental disorders** | **17162.0 (15977.1 to 18376.2)** | **7504.2 (6210.3 to 9018.8)** | **2187.9 (1610.4 to 2824.8)** | **23.7* (22.5 to 24.8)** | **-1.5* (-2.5 to -0.6)** |
| Schizophrenia | 259.6 (231.7 to 289.7) | 1.2 (0.9 to 1.6) | 148.7 (112.8 to 178.9) | 29.1* (25.5 to 32.7) | 2.8 (-0.1 to 5.7) |
| Depressive disorders | 7814.5 (6824.0 to 8939.9) | 7119.8 (5809.5 to 8639.2) | 1163.6 (801.6 to 1602.4) | 22.9* (20.9 to 24.9) | -2.1* (-3.7 to -0.6) |
| Major depressive disorder | 4675.5 (3821.9 to 5653.8) | 6809.1 (5546.9 to 8338.7) | 862.5 (581.9 to 1212.8) | 21.8* (19.3 to 24.2) | -3.0* (-5.0 to -1.1) |
| Dysthymia | 3358.3 (2776.7 to 4101.5) | 310.7 (237.0 to 393.2) | 301.1 (199.1 to 439.0) | 26.1* (22.8 to 29.5) | 0.4 (-2.3 to 3.1) |
| Bipolar disorder | 637.1 (539.4 to 745.8) | 46.7 (35.7 to 58.7) | 117.5 (74.5 to 177.0) | 24.3* (21.4 to 27.3) | -1.0 (-3.3 to 1.4) |
| Anxiety disorders | 5118.7 (4606.4 to 5664.4) | 336.5 (305.5 to 371.5) | 443.9 (314.1 to 591.0) | 22.6* (20.6 to 24.7) | -2.3* (-4.0 to -0.7) |
| Eating disorders | -- | -- | -- | -- | -- |
| Anorexia nervosa | -- | -- | -- | -- | -- |
| Bulimia nervosa | -- | -- | -- | -- | -- |
| Autism spectrum disorders | 419.4 (375.8 to 465.7) | -- | 58.2 (40.9 to 77.9) | 25.7* (23.0 to 28.3) | 0.1 (-2.1 to 2.2) |
| Attention-deficit/hyperactivity disorder | 30.8 (16.5 to 48.8) | -- | 0.4 (0.2 to 0.7) | 24.8* (11.2 to 37.5) | -0.7 (-11.4 to 9.5) |
| Conduct disorder | -- | -- | -- | -- | -- |
| Idiopathic developmental intellectual disability | 434.5 (188.5 to 707.5) | -- | 17.4 (7.1 to 31.0) | 15.9* (10.8 to 19.3) | -7.7* (-11.8 to -5.0) |
| Other mental disorders | 3482.7 (3104.6 to 3916.9) | -- | 238.1 (162.1 to 322.2) | 25.8* (23.9 to 27.5) | 0.2 (-1.4 to 1.5) |
| **Substance use disorders** | **1338.9 (1137.5 to 1563.5)** | **333.5 (267.0 to 411.7)** | **204.5 (145.4 to 270.5)** | **34.0* (30.0 to 38.2)** | **6.7* (3.5 to 10.1)** |
| Alcohol use disorders | 982.5 (801.8 to 1201.4) | 300.1 (233.1 to 374.8) | 89.4 (60.3 to 126.6) | 26.4* (22.9 to 30.2) | 0.7 (-2.1 to 3.6) |
| Alcohol dependence | 979.2 (799.0 to 1198.1) | 300.1 (233.1 to 374.8) | 89.3 (60.2 to 126.5) | 26.5* (23.0 to 30.2) | 0.7 (-2.1 to 3.7) |
| Fetal alcohol syndrome | 3.2 (2.5 to 4.1) | -- | 0.1 (0.1 to 0.2) | -3.3 (-10.9 to 3.0) | -23.0* (-29.0 to -18.0) |
| Drug use disorders | 371.6 (307.9 to 446.2) | 33.4 (21.5 to 48.2) | 115.1 (80.5 to 155.6) | 40.6* (33.3 to 47.3) | 11.9* (6.2 to 17.3) |
| Opioid use disorders | 271.9 (206.7 to 348.9) | 31.3 (19.4 to 46.2) | 100.3 (68.7 to 138.9) | 43.9* (35.7 to 51.5) | 14.6* (8.1 to 20.7) |
| Cocaine use disorders | 51.9 (45.2 to 59.4) | -- | 6.5 (4.2 to 9.3) | 20.2* (11.2 to 29.6) | -4.3 (-11.5 to 3.2) |

| | | | | | |
|---|---|---|---|---|---|
| Amphetamine use disorders | 9.4 (5.2 to 14.6) | 2.1 (0.9 to 3.8) | 1.3 (0.6 to 2.3) | 26.9* (23.2 to 29.6) | 1.0 (-1.9 to 3.2) |
| Cannabis use disorders | 60.5 (46.7 to 76.6) | -- | 1.7 (1.0 to 2.7) | 22.2* (16.3 to 28.5) | -2.7 (-7.4 to 2.3) |
| Other drug use disorders | 2.5 (2.2 to 3.0) | -- | 5.4 (3.5 to 7.4) | 21.5* (14.2 to 29.5) | -3.3 (-9.1 to 3.1) |
| **Diabetes and kidney diseases** | **58284.8 (54332.5 to 62527.1)** | **2583.2 (1936.6 to 3289.5)** | **2772.8 (1973.6 to 3718.2)** | **29.1* (23.4 to 34.7)** | **2.8 (-1.8 to 7.2)** |
| Diabetes mellitus | 27395.7 (24458.5 to 30334.3) | 699.2 (532.4 to 900.1) | 2201.3 (1515.8 to 3042.7) | 29.7* (23.0 to 36.8) | 3.3 (-2.1 to 8.9) |
| Diabetes mellitus type 1 | 199.1 (171.6 to 230.1) | 3.3 (2.0 to 4.9) | 15.6 (10.6 to 21.6) | 21.8* (18.0 to 25.8) | -3.0 (-6.0 to 0.1) |
| Uncomplicated diabetes mellitus type 1 | 114.9 (96.3 to 135.5) | 3.3 (2.0 to 4.9) | 5.0 (3.1 to 7.4) | 21.9* (16.3 to 27.6) | -2.9 (-7.4 to 1.6) |
| Vision loss due to diabetes mellitus type 1 retinopathy | 2.3 (1.7 to 3.1) | -- | 0.2 (0.1 to 0.3) | 3.2 (-3.1 to 9.5) | -17.9* (-22.8 to -12.8) |
| Diabetic neuropathy due to diabetes mellitus type 1 | 81.8 (67.2 to 98.7) | -- | 10.3 (6.9 to 14.6) | 22.1* (16.8 to 27.7) | -2.8 (-7.0 to 1.7) |
| Diabetes mellitus type 2 | 27196.6 (24253.4 to 30162.2) | 695.8 (529.0 to 897.0) | 2185.8 (1505.3 to 3022.6) | 29.7* (23.0 to 36.9) | 3.3 (-2.0 to 9.0) |
| Uncomplicated diabetes mellitus type 2 | 14813.9 (12598.9 to 17304.9) | 695.8 (529.0 to 897.0) | 651.3 (417.3 to 975.2) | 31.6* (23.3 to 39.7) | 4.8 (-1.8 to 11.2) |
| Vision loss due to diabetes mellitus type 2 retinopathy | 339.1 (269.0 to 423.5) | -- | 25.2 (17.4 to 34.7) | 16.6* (13.1 to 19.9) | -7.2* (-10.0 to -4.5) |
| Diabetic neuropathy due to diabetes mellitus type 2 | 12043.6 (10159.1 to 14210.0) | -- | 1509.2 (1014.3 to 2120.4) | 29.2* (22.2 to 36.7) | 2.9 (-2.7 to 8.8) |
| Chronic kidney disease | 49280.4 (43297.0 to 56486.2) | 1857.7 (1239.8 to 2564.9) | 571.4 (410.5 to 749.2) | 26.7* (18.7 to 34.8) | 0.9 (-5.5 to 7.4) |
| Chronic kidney disease due to diabetes mellitus type 1 | 55.1 (45.7 to 65.4) | 1.3 (0.9 to 1.8) | 5.5 (3.0 to 9.1) | 28.2* (20.8 to 36.5) | 2.1 (-3.8 to 8.7) |
| Albuminuria with preserved GFR due to type 1 diabetes mellitus | 23.0 (17.0 to 30.5) | -- | -- | -- | -- |
| End-stage chronic kidney disease due to diabetes mellitus type 1 | 7.7 (3.6 to 14.0) | -- | 3.9 (1.7 to 7.4) | 27.4* (18.2 to 37.5) | 1.5 (-5.9 to 9.5) |
| Stage III chronic kidney disease due to diabetes mellitus type 1 | 18.9 (15.4 to 22.9) | 1.3 (0.9 to 1.8) | 0.4 (0.3 to 0.6) | 29.3* (11.4 to 49.8) | 3.0 (-11.3 to 19.2) |
| Stage IV chronic kidney disease due to diabetes mellitus type 1 | 4.4 (3.0 to 6.5) | -- | 0.6 (0.3 to 0.9) | 30.2* (14.7 to 46.6) | 3.7 (-8.7 to 16.8) |
| Stage V chronic kidney disease due to diabetes mellitus type 1 | 1.1 (0.7 to 1.4) | -- | 0.6 (0.4 to 0.9) | 30.8* (19.6 to 42.2) | 4.1 (-4.7 to 13.2) |
| Chronic kidney disease due to diabetes mellitus type 2 | 9324.5 (8139.2 to 10808.3) | 269.3 (179.5 to 371.4) | 161.9 (112.6 to 218.6) | 27.5* (20.0 to 35.6) | 1.5 (-4.4 to 8.0) |
| Albuminuria with preserved GFR due to type 2 diabetes mellitus | 4227.8 (3170.8 to 5411.1) | -- | -- | -- | -- |
| End-stage chronic kidney disease due to diabetes mellitus type 2 | 97.2 (68.3 to 126.7) | -- | 42.9 (27.1 to 61.4) | 28.9* (21.1 to 36.1) | 2.6 (-3.6 to 8.4) |
| Stage III chronic kidney disease due to diabetes mellitus type 2 | 4448.8 (3786.9 to 5224.4) | 269.3 (179.5 to 371.4) | 19.7 (12.3 to 30.4) | 20.3* (7.1 to 34.9) | -4.2 (-14.7 to 7.4) |
| Stage IV chronic kidney disease due to diabetes mellitus type 2 | 435.2 (298.0 to 615.5) | -- | 44.4 (26.5 to 69.6) | 27.8* (14.6 to 41.6) | 1.8 (-8.8 to 12.8) |
| Stage V chronic kidney disease due to diabetes mellitus type 2 | 115.5 (83.3 to 153.9) | -- | 54.8 (34.3 to 78.1) | 28.8* (18.3 to 40.1) | 2.6 (-5.8 to 11.6) |
| Chronic kidney disease due to hypertension | 1701.8 (1488.6 to 1987.3) | 8.6 (5.7 to 11.8) | 126.6 (90.3 to 167.1) | 27.1* (19.8 to 34.8) | 1.2 (-4.6 to 7.3) |
| Albuminuria with preserved GFR due to hypertension | 317.4 (238.4 to 408.7) | -- | -- | -- | -- |
| End-stage chronic kidney disease due to hypertension | 83.8 (64.5 to 107.8) | -- | 36.7 (23.9 to 50.6) | 27.9* (20.2 to 35.5) | 1.8 (-4.3 to 7.9) |
| Stage III chronic kidney disease due to hypertension | 892.1 (752.8 to 1066.9) | 8.6 (5.7 to 11.8) | 15.6 (9.9 to 23.9) | 19.7* (6.2 to 33.9) | -4.7 (-15.4 to 6.6) |
| Stage IV chronic kidney disease due to hypertension | 322.6 (220.4 to 455.7) | -- | 33.2 (19.8 to 51.9) | 28.0* (14.0 to 42.7) | 2.0 (-9.3 to 13.6) |
| Stage V chronic kidney disease due to hypertension | 85.9 (62.2 to 114.9) | -- | 41.0 (25.8 to 58.3) | 28.8* (16.9 to 40.0) | 2.5 (-6.9 to 11.5) |
| Chronic kidney disease due to glomerulonephritis | 2552.8 (2186.2 to 2977.6) | 190.0 (127.1 to 262.0) | 26.3 (17.5 to 35.9) | 26.1* (18.5 to 33.6) | 0.4 (-5.6 to 6.4) |
| Albuminuria with preserved GFR due to glomerulonephritis | 85.3 (63.6 to 109.6) | -- | -- | -- | -- |
| End-stage chronic kidney disease due to glomerulonephritis | 31.3 (19.7 to 46.6) | -- | 13.9 (7.5 to 21.1) | 26.5* (15.0 to 38.2) | 0.8 (-8.4 to 10.1) |
| Stage III chronic kidney disease due to glomerulonephritis | 2383.5 (2024.0 to 2803.2) | 190.0 (127.1 to 262.0) | 1.2 (0.8 to 1.8) | 8.1 (-8.1 to 25.8) | -13.9 (-26.8 to 0.2) |
| Stage IV chronic kidney disease due to glomerulonephritis | 41.5 (28.4 to 58.2) | -- | 4.8 (2.8 to 7.6) | 27.1* (11.4 to 43.1) | 1.2 (-11.3 to 14.0) |
| Stage V chronic kidney disease due to glomerulonephritis | 11.1 (8.0 to 14.8) | -- | 6.4 (3.9 to 9.2) | 28.6* (19.8 to 37.8) | 2.4 (-4.6 to 9.7) |
| Chronic kidney disease due to other and unspecified causes | 35646.3 (31216.2 to 40921.7) | 1388.4 (926.4 to 1917.9) | 251.0 (177.6 to 334.3) | 26.1* (16.9 to 36.3) | 0.4 (-6.9 to 8.6) |
| Albuminuria with preserved GFR due to other and unspecified causes | 11940.5 (8995.0 to 15320.9) | -- | -- | -- | -- |
| End-stage chronic kidney disease due to other causes | 46.9 (30.4 to 70.0) | -- | 20.6 (11.8 to 31.9) | 25.2* (15.3 to 36.3) | -0.3 (-8.2 to 8.5) |
| Stage III chronic kidney disease due to other causes | 23017.6 (19582.9 to 26988.2) | 1388.4 (926.4 to 1917.9) | 115.0 (72.9 to 176.0) | 24.0* (11.2 to 37.8) | -1.2 (-11.4 to 9.7) |
| Stage IV chronic kidney disease due to other causes | 507.2 (346.9 to 714.7) | -- | 51.8 (30.7 to 80.5) | 27.6* (13.2 to 40.9) | 1.6 (-9.9 to 12.2) |
| Stage V chronic kidney disease due to other causes | 134.0 (96.9 to 178.6) | -- | 63.6 (40.3 to 90.0) | 29.1* (19.1 to 40.3) | 2.8 (-5.2 to 11.7) |
| Acute glomerulonephritis | 1.8 (1.2 to 2.5) | 26.3 (18.9 to 36.1) | 0.1 (0.1 to 0.2) | 6.2* (1.6 to 10.2) | -15.4* (-19.1 to -12.3) |
| **Skin and subcutaneous diseases** | **62774.2 (57197.1 to 69404.7)** | **162860.1 (135033.2 to 191408.6)** | **824.6 (532.8 to 1282.3)** | **26.2* (25.3 to 26.9)** | **0.5 (-0.2 to 1.0)** |
| Dermatitis | 5014.4 (4231.8 to 5951.8) | 7111.3 (5070.2 to 9499.3) | 157.9 (93.6 to 242.0) | 23.1* (21.4 to 24.9) | -2.0* (-3.3 to -0.6) |
| Atopic dermatitis | 2570.1 (2266.1 to 2905.3) | 375.2 (315.4 to 449.1) | 102.6 (56.4 to 167.4) | 22.8* (20.6 to 25.1) | -2.2* (-4.0 to -0.4) |
| Contact dermatitis | 2185.2 (1458.5 to 3131.2) | 6135.8 (4102.0 to 8544.9) | 50.9 (28.6 to 83.0) | 23.8* (21.6 to 26.3) | -1.4 (-3.1 to 0.5) |
| Seborrhoeic dermatitis | 349.6 (285.1 to 419.4) | 600.4 (499.9 to 692.1) | 4.5 (2.6 to 7.3) | 23.8* (20.0 to 27.8) | -1.4 (-4.4 to 1.7) |
| Psoriasis | 1891.8 (1762.7 to 2024.3) | 110.8 (92.9 to 129.7) | 150.0 (107.8 to 195.9) | 26.3* (23.8 to 28.9) | 0.5 (-1.4 to 2.6) |
| Bacterial skin diseases | 279.1 (255.7 to 302.4) | 7204.4 (6554.5 to 7863.8) | 4.8 (3.1 to 7.0) | 25.3* (20.0 to 30.5) | -0.2 (-4.4 to 3.9) |
| Cellulitis | 67.6 (56.5 to 82.5) | 1538.8 (1280.8 to 1818.0) | 3.6 (2.4 to 5.1) | 24.0* (17.3 to 30.5) | -1.3 (-6.6 to 3.9) |
| Pyoderma | 226.1 (205.9 to 246.7) | 5665.6 (5054.4 to 6299.2) | 1.2 (0.5 to 2.5) | 29.5* (25.7 to 33.2) | 3.1* (0.1 to 6.0) |
| Impetigo | 9.8 (8.7 to 11.0) | 184.6 (167.2 to 202.1) | 0.1 (0.0 to 0.1) | 31.6* (24.3 to 39.1) | 4.8 (-1.0 to 10.8) |
| Abscess and other bacterial skin diseases | 216.2 (195.9 to 236.9) | 5481.0 (4867.2 to 6117.0) | 1.1 (0.5 to 2.4) | 29.4* (25.6 to 33.2) | 3.0 (-0.0 to 6.1) |
| Scabies | 1815.6 (1268.0 to 2408.5) | 5526.9 (3842.1 to 7352.9) | 43.7 (22.0 to 73.8) | 27.7* (26.0 to 29.5) | 1.7* (0.3 to 3.1) |
| Fungal skin diseases | 34938.6 (27202.1 to 44400.3) | 107358.7 (79212.1 to 135913.6) | 185.5 (72.2 to 400.4) | 25.5* (25.1 to 25.9) | -0.1 (-0.4 to 0.2) |
| Tinea capitis | 440.3 (311.5 to 615.1) | 825.4 (595.0 to 1086.9) | 2.3 (0.9 to 5.0) | 7.1* (4.1 to 10.2) | -14.7* (-17.1 to -12.2) |
| Other fungal skin diseases | 34498.3 (26751.4 to 43998.5) | 106533.3 (78335.4 to 135078.2) | 183.2 (71.0 to 396.4) | 25.8* (25.4 to 26.2) | 0.1 (-0.2 to 0.5) |

| | | | | | |
|---|---|---|---|---|---|
| Viral skin diseases | 855.2 (764.4 to 950.5) | 322.8 (290.4 to 355.3) | 24.3 (15.8 to 35.2) | 24.4* (21.8 to 26.9) | -1.0 (-3.0 to 1.1) |
| Viral warts | 799.3 (707.9 to 896.8) | 249.7 (219.5 to 280.7) | 22.7 (14.7 to 33.3) | 24.3* (21.7 to 27.0) | -1.0 (-3.1 to 1.2) |
| Molluscum contagiosum | 55.9 (49.2 to 63.9) | 73.2 (64.4 to 82.8) | 1.6 (1.0 to 2.3) | 25.7* (16.1 to 36.0) | 0.1 (-7.6 to 8.3) |
| Acne vulgaris | 201.9 (196.3 to 207.4) | 87.6 (84.6 to 90.8) | 4.2 (2.5 to 6.6) | 36.0* (32.5 to 39.7) | 8.3* (5.5 to 11.2) |
| Alopecia areata | 192.6 (172.6 to 216.2) | 322.6 (292.0 to 355.8) | 5.9 (3.8 to 8.9) | 21.3* (16.5 to 25.8) | -3.4 (-7.2 to 0.2) |
| Pruritus | 2508.1 (1801.8 to 3374.1) | 1906.1 (1349.8 to 2608.0) | 25.2 (11.4 to 48.9) | 27.6* (26.0 to 29.1) | 1.6* (0.4 to 2.8) |
| Urticaria | 813.3 (558.6 to 1145.9) | 1423.6 (981.3 to 1993.4) | 44.5 (26.1 to 69.8) | 25.4* (22.7 to 28.3) | -0.1 (-2.3 to 2.2) |
| Decubitus ulcer | 130.2 (94.5 to 179.4) | 484.2 (347.3 to 685.0) | 19.2 (12.0 to 28.1) | 25.9* (17.1 to 34.4) | 0.3 (-6.8 to 7.0) |
| Other skin and subcutaneous diseases | 30586.1 (28019.9 to 33125.7) | 31000.9 (28510.2 to 33591.2) | 159.5 (77.8 to 291.2) | 29.9* (29.1 to 30.6) | 3.4* (2.8 to 4.0) |
| **Sense organ diseases** | **97696.4 (96333.9 to 99030.2)** | **--** | **5600.9 (3933.7 to 7629.9)** | **23.9* (21.9 to 25.8)** | **-1.4 (-3.0 to 0.2)** |
| Blindness and vision impairment | 62264.7 (59143.8 to 65615.3) | -- | 2421.0 (1687.1 to 3300.3) | 22.5* (21.1 to 24.0) | -2.4* (-3.6 to -1.3) |
| Glaucoma | 853.8 (698.8 to 1034.2) | -- | 96.4 (63.9 to 135.3) | 20.8* (18.5 to 23.0) | -3.8* (-5.7 to -2.1) |
| Cataract | 16191.2 (13699.9 to 19061.0) | -- | 1185.5 (826.9 to 1605.3) | 24.0* (21.8 to 26.2) | -1.3 (-3.0 to 0.5) |
| Age-related macular degeneration | 962.4 (762.5 to 1214.0) | -- | 73.9 (48.8 to 103.4) | 22.4* (19.3 to 25.4) | -2.5* (-5.0 to -0.2) |
| Refraction disorders | 7810.1 (6168.4 to 9945.3) | -- | 364.2 (241.9 to 518.0) | 16.1* (13.4 to 19.0) | -7.5* (-9.7 to -5.3) |
| Near vision loss | 49205.8 (45968.7 to 52501.0) | -- | 476.7 (223.9 to 866.8) | 26.5* (25.7 to 27.3) | 0.7* (0.1 to 1.4) |
| Other vision loss | 2651.8 (2161.0 to 3200.8) | -- | 224.3 (153.5 to 313.7) | 18.7* (16.6 to 20.7) | -5.5* (-7.1 to -3.9) |
| Age-related and other hearing loss | 90996.2 (89574.2 to 92372.4) | -- | 2998.8 (2113.6 to 4084.2) | 24.9* (21.2 to 28.2) | -0.6 (-3.5 to 2.1) |
| Other sense organ diseases | 7673.1 (6891.6 to 8550.5) | -- | 181.0 (112.8 to 258.6) | 26.3* (24.9 to 27.6) | 0.6 (-0.5 to 1.6) |
| Chronic other sense organ diseases | 7198.5 (6418.4 to 8053.9) | -- | 175.0 (109.2 to 249.4) | 26.3* (24.9 to 27.7) | 0.6 (-0.6 to 1.7) |
| Acute other sense organ diseases | 474.6 (430.6 to 519.8) | -- | 6.1 (3.7 to 9.3) | 26.7* (22.2 to 31.4) | 0.9 (-2.7 to 4.7) |
| **Musculoskeletal disorders** | **56866.2 (52352.2 to 61480.6)** | **12922.2 (10179.9 to 15816.0)** | **5369.3 (3782.1 to 7343.1)** | **25.7* (24.1 to 27.6)** | **0.1 (-1.2 to 1.6)** |
| Rheumatoid arthritis | 1261.4 (1100.6 to 1439.4) | 35.0 (27.4 to 42.6) | 155.8 (109.0 to 209.1) | 34.7* (30.5 to 39.2) | 7.2* (3.9 to 10.8) |
| Osteoarthritis | 27212.6 (24295.5 to 30493.0) | 473.0 (337.1 to 621.0) | 833.7 (421.1 to 1672.6) | 26.0* (25.2 to 26.7) | 0.3 (-0.3 to 0.9) |
| Osteoarthritis of the hip | 3917.3 (3635.3 to 4219.5) | 75.0 (59.8 to 91.8) | 120.0 (61.7 to 243.2) | 29.6* (28.0 to 31.1) | 3.2* (2.0 to 4.4) |
| Osteoarthritis of the knee | 23295.3 (20328.7 to 26639.8) | 398.0 (265.3 to 550.4) | 713.7 (360.2 to 1424.6) | 25.4* (24.6 to 26.1) | -0.2 (-0.8 to 0.4) |
| Low back pain | 24028.3 (18386.8 to 29855.5) | 9612.7 (7010.1 to 12403.0) | 2532.5 (1652.2 to 3626.5) | 23.3* (21.9 to 24.5) | -1.8* (-2.9 to -0.9) |
| Low back pain with leg pain | 8410.3 (6329.1 to 10937.7) | 3367.7 (2330.3 to 4507.8) | 1122.6 (702.9 to 1636.9) | 23.3* (21.8 to 24.6) | -1.8* (-3.0 to -0.8) |
| Low back pain without leg pain | 15618.0 (11856.8 to 19646.9) | 6245.1 (4555.3 to 8214.8) | 1409.8 (907.6 to 2052.3) | 23.3* (21.9 to 24.5) | -1.8* (-2.9 to -0.9) |
| Neck pain | 11793.8 (8593.8 to 16098.6) | 2258.9 (1341.7 to 3328.0) | 1099.6 (697.1 to 1677.0) | 26.2* (23.9 to 29.0) | 0.5 (-1.3 to 2.7) |
| Gout | 3493.5 (2848.4 to 4295.9) | 542.5 (385.4 to 707.0) | 102.6 (67.6 to 145.3) | 30.7* (27.5 to 33.8) | 4.1* (1.5 to 6.6) |
| Other musculoskeletal disorders | 8218.4 (5680.4 to 11105.2) | -- | 645.1 (383.3 to 958.1) | 31.5* (18.3 to 43.7) | 4.7 (-5.8 to 14.4) |
| **Other non-communicable diseases** | **90260.0 (86482.5 to 93874.1)** | **35026.1 (30755.6 to 39714.7)** | **1694.4 (1152.7 to 2371.9)** | **20.8* (19.6 to 21.9)** | **-3.8* (-4.7 to -2.9)** |
| Congenital anomalies | 1595.2 (1495.5 to 1712.7) | -- | 172.8 (123.9 to 228.3) | 23.7* (21.4 to 26.1) | -1.5 (-3.4 to 0.4) |
| Neural tube defects | 35.2 (31.4 to 39.4) | -- | 10.2 (7.1 to 13.6) | 28.6* (23.0 to 34.4) | 2.4 (-2.1 to 7.0) |
| Severe motor and cognitive impairment due to anencephaly | -- | -- | -- | -- | -- |
| Encephalocoele due to neural tube defects | 5.5 (4.6 to 6.6) | -- | 1.0 (0.6 to 1.4) | 22.1* (18.2 to 25.9) | -2.8 (-5.9 to 0.3) |
| Spina bifida due to neural tube defects | 29.7 (26.4 to 33.4) | -- | 9.2 (6.4 to 12.4) | 29.4* (23.2 to 35.4) | 3.0 (-1.9 to 7.8) |
| Congenital heart anomalies | 42.3 (38.5 to 46.6) | -- | 2.4 (1.0 to 4.1) | 27.7* (20.5 to 35.5) | 1.7 (-4.1 to 7.9) |
| Critical malformations of great vessels, congenital valvular heart disease and patent ductus arteriosus | 6.4 (5.6 to 7.4) | -- | 0.4 (0.2 to 0.7) | 27.6* (24.9 to 30.3) | 1.6 (-0.5 to 3.8) |
| Other congenital heart anomalies | 20.1 (18.3 to 22.1) | -- | 1.1 (0.5 to 1.9) | 27.7* (15.9 to 39.8) | 1.7 (-7.7 to 11.4) |
| Severe congenital heart anomalies excluding single ventricle heart defects | 15.0 (13.2 to 16.8) | -- | 0.8 (0.3 to 1.5) | 27.9* (15.6 to 41.1) | 1.9 (-7.9 to 12.3) |
| Single ventricle and single ventricle pathway congenital heart anomalies | 0.2 (0.2 to 0.3) | -- | -- | 30.3* (26.6 to 34.0) | 3.8* (0.8 to 6.7) |
| Ventricular septal defect and atrial septal defect | 0.6 (0.4 to 0.7) | -- | -- | 18.5* (15.0 to 21.9) | -5.7* (-8.4 to -3.0) |
| Orofacial clefts | 168.8 (154.3 to 183.2) | -- | 4.1 (2.7 to 5.8) | 28.9* (19.2 to 39.7) | 2.6 (-5.1 to 11.2) |
| Down's syndrome | -- | -- | -- | -- | -- |
| Turner syndrome | 1.6 (1.3 to 1.8) | -- | -- | 23.5* (22.2 to 24.9) | -1.7* (-2.7 to -0.6) |
| Klinefelter syndrome | 0.4 (0.3 to 0.5) | -- | -- | 33.4* (31.4 to 35.3) | 6.2* (4.7 to 7.7) |
| Other chromosomal abnormalities | -- | -- | -- | -- | -- |
| Congenital musculoskeletal and limb anomalies | 747.1 (664.5 to 853.2) | -- | 101.3 (68.8 to 135.9) | 23.1* (21.1 to 25.3) | -2.0* (-3.6 to -0.2) |
| Polydactyly and syndactyly | 1.1 (0.9 to 1.3) | -- | -- | 16.7* (14.8 to 18.6) | -7.1* (-8.6 to -5.5) |
| Congenital limb deficiency | 180.1 (160.1 to 205.8) | -- | 24.5 (16.6 to 33.1) | 23.3* (19.4 to 27.4) | -1.8 (-4.9 to 1.4) |
| Other congenital musculoskeletal anomalies | 565.9 (503.4 to 646.3) | -- | 76.7 (52.3 to 103.0) | 23.1* (20.7 to 25.4) | -2.0* (-3.9 to -0.1) |
| Urogenital congenital anomalies | 26.1 (23.3 to 29.1) | -- | 1.2 (0.7 to 1.8) | 12.6* (6.4 to 19.2) | -10.3* (-15.3 to -5.1) |
| Digestive congenital anomalies | 372.4 (336.8 to 414.4) | -- | 15.7 (10.7 to 22.0) | 16.9* (10.6 to 23.3) | -6.9* (-11.9 to -1.8) |
| Congenital diaphragmatic hernia | 1.6 (1.4 to 1.8) | -- | 0.0 (0.0 to 0.1) | 29.9* (28.0 to 31.8) | 3.5* (1.9 to 4.9) |
| Congenital atresia and/or stenosis of the digestive tract | 0.1 (0.1 to 0.1) | -- | -- | 38.7* (33.3 to 44.3) | 10.5* (6.1 to 14.9) |
| Congenital malformations of the abdominal wall after treatment | 6.3 (5.4 to 7.1) | -- | 0.3 (0.2 to 0.4) | 29.7* (28.0 to 31.1) | 3.3* (2.0 to 4.4) |
| Other congenital malformations of the digestive tract | 364.4 (328.6 to 406.1) | -- | 15.4 (10.4 to 21.5) | 16.7* (10.3 to 23.2) | -7.1* (-12.2 to -1.9) |
| Other congenital anomalies | 334.6 (286.7 to 390.1) | -- | 38.0 (25.7 to 52.2) | 26.7* (17.7 to 35.3) | 0.9 (-6.3 to 7.8) |

| | | | | | |
|---|---|---|---|---|---|
| Other congenital birth defects | -- | -- | -- | -- | -- |
| Hearing loss due to other congenital anomalies | 334.6 (286.7 to 390.1) | -- | 38.0 (25.7 to 52.2) | 26.7* (17.7 to 35.3) | 0.9 (-6.3 to 7.8) |
| Urinary diseases and male infertility | 7159.4 (6204.4 to 8233.9) | 9542.7 (8208.6 to 11127.9) | 243.1 (158.5 to 349.3) | 26.1* (24.6 to 27.6) | 0.4 (-0.8 to 1.6) |
| Urinary tract infections | 103.9 (84.4 to 125.6) | 5462.8 (4424.6 to 6648.8) | 3.2 (1.9 to 4.9) | 45.8* (37.4 to 54.9) | 16.1* (9.4 to 23.3) |
| Urolithiasis | 132.2 (98.3 to 171.9) | 3423.8 (2541.5 to 4480.1) | 9.0 (5.5 to 13.2) | 29.2* (20.7 to 38.5) | 2.9 (-3.9 to 10.3) |
| Benign prostatic hyperplasia | 7228.1 (6189.6 to 8393.3) | 656.1 (546.8 to 801.3) | 228.2 (147.1 to 331.2) | 26.0* (24.4 to 27.6) | 0.3 (-1.0 to 1.6) |
| Male infertility | -- | -- | -- | -- | -- |
| Other urinary diseases | -- | -- | 2.7 (1.8 to 3.8) | 10.6* (4.9 to 16.6) | -11.9* (-16.5 to -7.1) |
| Gynecological diseases | 6703.7 (5701.8 to 7875.4) | 265.7 (191.5 to 363.2) | 25.2 (12.9 to 46.4) | 22.4* (19.8 to 25.1) | -2.5* (-4.6 to -0.4) |
| Uterine fibroids | 1027.7 (898.5 to 1184.9) | 3.9 (2.4 to 6.0) | 7.7 (4.3 to 12.9) | 23.4* (16.2 to 30.6) | -1.8 (-7.5 to 4.0) |
| Polycystic ovarian syndrome | -- | -- | -- | -- | -- |
| Female infertility | -- | -- | -- | -- | -- |
| Endometriosis | -- | -- | -- | -- | -- |
| Genital prolapse | 5866.7 (4833.6 to 7118.2) | 261.8 (187.9 to 359.3) | 17.5 (8.3 to 33.7) | 22.0* (20.0 to 24.0) | -2.9* (-4.5 to -1.3) |
| Premenstrual syndrome | -- | -- | -- | -- | -- |
| Other gynecological diseases | -- | -- | -- | -- | -- |
| Other gynecological diseases | -- | -- | -- | -- | -- |
| Other gynecological diseases with anemia | -- | -- | -- | -- | -- |
| Hemoglobinopathies and hemolytic anaemias | 27538.9 (26984.3 to 28103.9) | -- | 82.7 (55.5 to 118.1) | 11.8* (2.3 to 23.5) | -11.0* (-18.5 to -1.7) |
| Thalassemias | -- | -- | -- | 8.7 (-2.0 to 23.4) | -13.4* (-21.9 to -1.7) |
| Thalassaemias trait | 4739.7 (4561.4 to 4944.8) | -- | 30.3 (19.0 to 45.0) | 20.5* (2.4 to 45.5) | -4.0 (-18.5 to 15.8) |
| Sickle cell disorders | -- | -- | -- | 37.6* (27.1 to 50.5) | 9.6* (1.2 to 19.8) |
| Sickle cell trait | 5000.9 (4492.5 to 5574.3) | -- | 10.4 (6.8 to 15.0) | 18.7* (7.6 to 30.0) | -5.5 (-14.3 to 3.5) |
| G6PD deficiency | 4185.6 (4022.4 to 4348.6) | -- | 0.5 (0.3 to 0.7) | 20.6* (5.9 to 39.0) | -4.0 (-15.6 to 10.6) |
| G6PD trait | 14602.7 (14362.2 to 14847.5) | -- | -- | 34.5* (18.1 to 55.5) | 7.1 (-5.9 to 23.8) |
| Other hemoglobinopathies and hemolytic anaemias | 1790.3 (1662.5 to 1919.2) | -- | 41.5 (28.2 to 59.4) | 4.6 (-3.4 to 13.0) | -16.7* (-23.1 to -10.0) |
| Endocrine, metabolic, blood, and immune disorders | 2964.5 (2588.1 to 3450.0) | -- | 88.5 (60.0 to 123.4) | 22.1* (14.3 to 30.1) | -2.8 (-9.0 to 3.6) |
| Anemia due to endocrine, metabolic, blood, and immune disorders | 1054.6 (981.1 to 1135.5) | -- | 21.2 (14.2 to 30.8) | 2.9 (-7.7 to 14.3) | -18.1* (-26.5 to -9.0) |
| Endocrine, metabolic, blood, and immune disorders | 1850.7 (1480.4 to 2331.0) | -- | 62.4 (41.1 to 90.5) | 30.6* (20.5 to 40.6) | 4.0 (-4.0 to 11.9) |
| Heart failure due to endocrine, metabolic, blood, and immune disorders | 59.1 (41.1 to 80.4) | -- | 4.9 (3.0 to 7.6) | 19.4* (11.4 to 27.4) | -4.9 (-11.3 to 1.5) |
| Oral disorders | 73806.4 (68208.8 to 79102.2) | 25217.8 (20876.0 to 29543.6) | 1082.2 (702.2 to 1575.6) | 19.8* (18.4 to 21.0) | -4.6* (-5.7 to -3.6) |
| Caries of deciduous teeth | -- | -- | -- | -- | -- |
| Caries of permanent teeth | 39237.4 (31644.0 to 47668.9) | 21699.1 (17481.7 to 25882.9) | 28.6 (12.6 to 51.7) | 18.5* (16.8 to 20.0) | -5.6* (-7.0 to -4.5) |
| Periodontal disease | 28096.9 (22315.5 to 33810.6) | 2215.8 (1400.0 to 3051.6) | 174.8 (65.5 to 360.7) | 30.4* (29.4 to 31.5) | 3.9* (3.1 to 4.7) |
| Edentulism and severe tooth loss | 30177.8 (25700.4 to 35464.1) | 1302.9 (851.8 to 1858.5) | 806.4 (530.6 to 1128.1) | 17.3* (16.1 to 18.5) | -6.6* (-7.6 to -5.7) |
| Other oral disorders | 2678.7 (2423.4 to 2982.4) | -- | 72.5 (45.1 to 105.6) | 25.5* (24.1 to 27.0) | -0.1 (-1.2 to 1.1) |
| **Injuries** | **61412.0 (57938.1 to 65560.7)** | **13862.9 (11619.1 to 16566.8)** | **2338.1 (1682.5 to 3150.9)** | **31.1* (30.1 to 32.1)** | **4.4* (3.6 to 5.2)** |
| **Transport injuries** | **10019.9 (9247.2 to 10891.7)** | **969.4 (729.6 to 1246.4)** | **487.0 (357.2 to 651.2)** | **30.5* (29.6 to 31.4)** | **3.9* (3.2 to 4.6)** |
| Road injuries | 8066.8 (7481.2 to 8848.9) | 746.3 (528.7 to 1017.4) | 390.3 (281.4 to 526.5) | 29.8* (28.8 to 30.8) | 3.4* (2.5 to 4.2) |
| Pedestrian road injuries | 2252.0 (1939.0 to 2677.1) | 242.2 (155.5 to 352.9) | 112.0 (76.9 to 155.9) | 29.9* (28.6 to 31.3) | 3.4* (2.4 to 4.5) |
| Cyclist road injuries | 1549.9 (1324.1 to 1835.0) | 169.8 (99.6 to 261.1) | 72.5 (50.2 to 98.0) | 34.5* (32.9 to 36.3) | 7.1* (5.8 to 8.5) |
| Motorcyclist road injuries | 1693.0 (1455.9 to 1959.9) | 63.4 (34.3 to 109.7) | 79.3 (56.3 to 108.2) | 38.9* (37.0 to 40.7) | 10.6* (9.1 to 12.1) |
| Motor vehicle road injuries | 2034.4 (1737.1 to 2397.9) | 208.9 (136.5 to 309.9) | 102.5 (73.1 to 138.9) | 16.9* (15.5 to 18.2) | -7.0* (-8.0 to -5.8) |
| Other road injuries | 537.5 (442.1 to 642.3) | 61.8 (31.4 to 103.6) | 24.0 (16.8 to 33.3) | 52.5* (50.4 to 54.6) | 21.5* (19.7 to 23.1) |
| Other transport injuries | 1953.1 (1679.0 to 2225.9) | 223.1 (141.9 to 336.8) | 96.7 (68.1 to 128.7) | 33.2* (31.8 to 34.8) | 6.1* (4.9 to 7.3) |
| **Unintentional injuries** | **46627.6 (43185.4 to 50861.6)** | **12624.6 (10389.6 to 15343.2)** | **1738.4 (1226.6 to 2370.6)** | **31.5* (30.2 to 32.8)** | **4.7* (3.7 to 5.7)** |
| Falls | 25144.6 (22072.6 to 29341.3) | 7322.3 (5392.5 to 9737.4) | 1137.6 (801.6 to 1539.0) | 30.5* (28.5 to 32.3) | 3.9* (2.4 to 5.4) |
| Drowning | 110.7 (95.4 to 131.0) | 11.5 (8.3 to 15.7) | 5.5 (3.9 to 7.4) | 36.4* (33.5 to 38.9) | 8.6* (6.3 to 10.6) |
| Fire, heat, and hot substances | 3468.8 (2986.6 to 4039.5) | 58.1 (36.9 to 82.8) | 64.1 (38.5 to 100.3) | 31.3* (29.3 to 32.9) | 4.5* (3.0 to 5.8) |
| Poisonings | 146.0 (124.2 to 168.1) | 43.6 (25.3 to 70.8) | 11.9 (8.0 to 16.6) | 36.6* (33.3 to 40.2) | 8.8* (6.1 to 11.7) |
| Poisoning by carbon monoxide | 30.5 (26.0 to 35.6) | 12.9 (5.9 to 24.0) | 2.0 (1.4 to 2.7) | 40.1* (36.9 to 43.3) | 11.5* (9.0 to 14.1) |
| Poisoning by other means | 115.5 (94.6 to 137.1) | 30.6 (17.0 to 50.6) | 9.9 (6.5 to 14.2) | 36.0* (32.4 to 39.7) | 8.3* (5.5 to 11.3) |
| Exposure to mechanical forces | 8440.9 (7339.1 to 9836.8) | 838.7 (545.6 to 1189.2) | 187.3 (126.8 to 268.8) | 34.7* (33.8 to 35.7) | 7.3* (6.5 to 8.0) |
| Unintentional firearm injuries | 311.7 (261.8 to 362.3) | 14.6 (6.6 to 28.9) | 8.6 (5.9 to 11.9) | 37.6* (36.2 to 39.0) | 9.6* (8.5 to 10.7) |
| Other exposure to mechanical forces | 8129.1 (7039.5 to 9507.0) | 824.0 (534.8 to 1171.6) | 178.7 (121.1 to 256.3) | 34.6* (33.6 to 35.6) | 7.2* (6.4 to 7.9) |
| Adverse effects of medical treatment | 140.3 (94.5 to 193.5) | 1839.0 (1305.9 to 2509.6) | 18.7 (10.8 to 30.7) | 46.8* (34.3 to 59.2) | 16.9* (6.9 to 26.7) |
| Animal contact | 1533.9 (1341.4 to 1781.1) | 610.7 (390.0 to 948.3) | 30.5 (20.6 to 43.4) | 26.3* (23.8 to 28.5) | 0.6 (-1.4 to 2.3) |
| Venomous animal contact | 553.8 (453.1 to 634.2) | 245.6 (146.8 to 388.4) | 15.8 (11.0 to 21.6) | 28.1* (24.9 to 31.5) | 2.0 (-0.5 to 4.7) |
| Non-venomous animal contact | 980.1 (811.6 to 1189.9) | 365.0 (227.8 to 570.2) | 14.7 (9.2 to 23.4) | 24.4* (21.9 to 26.3) | -1.0 (-2.9 to 0.6) |
| Foreign body | 742.9 (666.1 to 832.3) | 433.8 (295.0 to 646.2) | 29.3 (20.9 to 39.5) | 27.0* (26.0 to 28.0) | 1.1* (0.3 to 1.9) |

| | | | | | |
|---|---|---|---|---|---|
| Pulmonary aspiration and foreign body in airway | 112.5 (96.1 to 133.1) | 29.6 (18.5 to 44.9) | 4.2 (2.9 to 5.8) | 35.1* (33.2 to 36.9) | 7.6* (6.1 to 9.0) |
| Foreign body in eyes | 107.8 (77.0 to 150.6) | 322.6 (192.5 to 520.3) | 7.0 (4.3 to 10.6) | 24.7* (23.1 to 26.9) | -0.7 (-2.0 to 1.0) |
| Foreign body in other body part | 522.6 (454.4 to 602.3) | 81.6 (45.1 to 137.0) | 18.1 (12.8 to 24.0) | 26.2* (25.1 to 27.2) | 0.5 (-0.4 to 1.3) |
| Environmental heat and cold exposure | 1506.6 (1306.5 to 1730.6) | 216.9 (139.5 to 315.8) | 57.3 (39.7 to 79.9) | 34.3* (32.6 to 36.1) | 7.0* (5.6 to 8.3) |
| Exposure to forces of nature | 221.5 (162.3 to 302.9) | 6.6 (4.8 to 8.9) | 7.7 (5.7 to 10.0) | 57.1* (42.5 to 73.7) | 25.1* (13.4 to 38.3) |
| Other unintentional injuries | 5885.7 (5045.6 to 6641.1) | 1243.5 (735.6 to 1844.8) | 188.6 (127.3 to 263.6) | 32.7* (31.6 to 33.8) | 5.6* (4.8 to 6.5) |
| **Self-harm and interpersonal violence** | **5564.9 (5035.1 to 6169.5)** | **268.9 (200.7 to 351.2)** | **112.6 (86.2 to 143.5)** | **28.2* (25.6 to 31.2)** | **2.1 (-0.0 to 4.5)** |
| Self-harm | 438.7 (367.2 to 507.4) | 77.7 (50.2 to 112.1) | 17.0 (12.1 to 22.9) | 15.0* (13.8 to 16.1) | -8.4* (-9.4 to -7.6) |
| Self-harm by firearm | 9.9 (8.3 to 12.0) | 3.2 (0.9 to 7.8) | 0.4 (0.2 to 0.5) | 16.6* (13.7 to 19.9) | -7.2* (-9.5 to -4.5) |
| Self-harm by other specified means | 428.7 (357.8 to 498.4) | 74.6 (48.1 to 108.5) | 16.7 (11.8 to 22.4) | 15.0* (13.8 to 16.0) | -8.5* (-9.4 to -7.6) |
| Interpersonal violence | 4693.6 (4172.1 to 5291.0) | 161.5 (103.4 to 239.8) | 82.1 (61.8 to 106.2) | 25.3* (24.1 to 26.7) | -0.2 (-1.2 to 0.9) |
| Assault by firearm | 103.0 (87.7 to 123.1) | 6.3 (2.7 to 11.3) | 3.4 (2.3 to 4.7) | 29.7* (27.9 to 31.4) | 3.3* (1.9 to 4.6) |
| Assault by sharp object | 465.8 (387.4 to 622.5) | 22.7 (10.7 to 38.0) | 10.9 (7.5 to 15.6) | 23.0* (21.3 to 24.6) | -2.1* (-3.4 to -0.8) |
| Sexual violence | 2566.1 (2119.6 to 3076.5) | -- | 16.7 (10.8 to 24.5) | 33.6* (31.4 to 35.7) | 6.3* (4.6 to 8.1) |
| Assault by other means | 1558.7 (1337.0 to 1827.8) | 132.5 (83.1 to 202.0) | 51.2 (35.9 to 70.1) | 23.1* (21.9 to 24.3) | -2.0* (-2.9 to -1.0) |
| Conflict and terrorism | 388.7 (247.0 to 579.1) | 27.1 (19.0 to 36.3) | 12.2 (8.5 to 17.3) | 74.2* (49.5 to 106.3) | 38.7* (19.0 to 64.3) |
| Executions and police conflict | 44.0 (26.9 to 65.8) | 2.6 (1.7 to 3.7) | 1.2 (0.8 to 1.8) | 149.4* (95.2 to 215.8) | 98.6* (55.4 to 151.4) |
| **Nature of injury aggregates** | | | | | |
| Amputations | 15735.1 (14580.3 to 17214.7) | 207.1 (165.4 to 260.8) | 261.8 (174.6 to 380.1) | 25.5* (22.2 to 28.6) | -0.0 (-2.7 to 2.4) |
| Burns | 7327.0 (6793.9 to 7953.5) | 129.3 (101.5 to 170.0) | 125.4 (72.5 to 203.2) | 31.1* (29.8 to 32.2) | 4.4* (3.3 to 5.3) |
| Fractures | 22571.6 (20593.7 to 25134.9) | 5264.9 (4084.5 to 6653.1) | 1206.4 (837.8 to 1659.3) | 29.6* (28.1 to 31.0) | 3.2* (2.0 to 4.3) |
| Head Injuries | 1895.1 (1801.8 to 2001.2) | 495.5 (383.6 to 662.5) | 256.4 (184.4 to 339.7) | 35.6* (34.2 to 37.1) | 8.0* (6.8 to 9.2) |
| Spinal Injuries | 651.4 (590.6 to 719.0) | 20.0 (14.7 to 27.8) | 161.5 (116.0 to 207.6) | 42.4* (39.9 to 45.0) | 13.4* (11.4 to 15.5) |
| Minor Injuries | 9247.8 (8702.5 to 9934.2) | 3454.9 (2781.9 to 4274.0) | 79.7 (37.9 to 146.9) | 32.3* (31.4 to 33.4) | 5.4* (4.6 to 6.2) |
| Other Injuries | 2951.2 (2783.0 to 3148.7) | 4291.2 (3618.2 to 5041.6) | 230.2 (162.3 to 306.1) | 33.6* (31.4 to 35.6) | 6.4* (4.6 to 8.0) |
| **Impairments** | | | | | |
| Anaemia | 30446.2 (28047.8 to 33019.3) | -- | 819.9 (577.2 to 1139.6) | 12.8* (4.9 to 21.4) | -10.2* (-16.5 to -3.3) |
| Epilepsy | 1043.9 (860.3 to 1261.2) | -- | 286.6 (199.1 to 390.0) | 32.1* (22.8 to 43.0) | 5.2 (-2.2 to 13.9) |
| Guillain-Barré syndrome | 2.8 (1.7 to 4.5) | -- | 0.8 (0.4 to 1.5) | 31.4* (28.3 to 34.7) | 4.7* (2.2 to 7.3) |
| Hearing loss | 91956.8 (90596.3 to 93330.3) | -- | 3050.3 (2155.3 to 4141.2) | 24.8* (21.2 to 28.1) | -0.6 (-3.5 to 2.0) |
| Heart failure | 9922.4 (8027.4 to 11955.2) | -- | 1759.5 (1220.4 to 2290.8) | 25.3* (22.0 to 27.8) | -0.2 (-2.8 to 1.8) |
| Infertility | -- | -- | -- | -40.9* (-57.2 to -33.3) | -52.9* (-65.9 to -46.9) |
| Developmental intellectual disability | 1089.1 (823.7 to 1361.5) | -- | 121.6 (91.5 to 155.9) | 36.9* (31.7 to 43.2) | 9.0* (4.9 to 14.0) |
| Pelvic inflammatory disease | -- | -- | -- | -- | -- |
| Vision loss | 78862.6 (73347.3 to 84966.8) | -- | 2517.8 (1743.6 to 3435.8) | 22.0* (20.5 to 23.5) | -2.8* (-4.0 to -1.6) |

We did not calculate incidence for the nine impairments and certain NTDs. Blank cells mean that no estimate is available or that the estimate has a magnitude less than 50.
Table presents prevalence and incidence counts in thousands.
* Percentage changes that are statistically significant
‡ Incidence estimates for stroke represent first-ever stroke only.
^ Incidence of HIV/AIDS represents new infections of HIV only and does not include new infections of Tuberculosis in HIV positive cases.

| SR Table 10t: Global prevalence, incidence and YLDs for 2017, percentage change of YLD counts and percentage change of YLD rates between 2007 and 2017 by cause, age 80 to 84 | | | | | |
|---|---|---|---|---|---|
| **Cause** | **Prevalence in thousands (95% UI)** | **Incidence in thousands (95% UI)** | **YLDs in thousands (95% UI)** | | |
| | **2017 counts** | **2017 counts** | **2017 counts** | **Percentage change in counts between 2007 and 2017** | **Percentage change in rates between 2007 and 2017** |
| **All causes** | **80225.5 (80218.9 to 80231.3)** | **451063.5 (409741.7 to 499381.7)** | **23641.8 (18164.9 to 29654.6)** | **36.5* (35.5 to 37.6)** | **0.3 (-0.4 to 1.2)** |
| **Communicable, maternal, neonatal, and nutritional disorders** | **40916.0 (38300.5 to 43610.6)** | **239751.3 (202686.1 to 285690.0)** | **677.5 (494.3 to 884.5)** | **33.8* (29.8 to 38.5)** | **-1.6 (-4.6 to 1.8)** |
| **HIV/AIDS and sexually transmitted infections** | **14039.8 (12441.9 to 15867.6)** | **757.0 (652.1 to 903.0)** | **11.9 (7.9 to 17.6)** | **55.6* (35.2 to 73.2)** | **14.4 (-0.6 to 27.3)** |
| HIV/AIDS | 52.0 (42.3 to 65.0) | -- | 5.7 (3.5 to 8.7) | 87.2* (36.3 to 154.8) | 37.6* (0.2 to 87.3) |
| HIV/AIDS - Drug-susceptible Tuberculosis | 2.0 (1.8 to 2.2) | 1.7 (1.5 to 1.8) | 0.8 (0.5 to 1.0) | 191.4* (157.1 to 223.5) | 114.2* (89.0 to 137.8) |
| HIV/AIDS - Multidrug-resistant Tuberculosis without extensive drug resistance | 0.1 (0.0 to 0.2) | 0.1 (0.0 to 0.1) | 0.0 (0.0 to 0.1) | 169.5* (25.4 to 548.5) | 98.1 (-7.8 to 376.7) |
| HIV/AIDS - Extensively drug-resistant Tuberculosis | -- | -- | -- | 555.0* (125.8 to 1585.0) | 381.5* (66.0 to 1138.6) |
| HIV/AIDS resulting in other diseases | 49.9 (40.2 to 62.9) | -- | 4.9 (2.9 to 7.8) | 76.9* (26.8 to 149.3) | 30.0 (-6.8 to 83.2) |
| HIV/AIDS not on antiretroviral treatment without Tuberculosis | 9.0 (5.3 to 14.1) | -- | 1.9 (0.8 to 4.0) | -17.7 (-37.2 to 3.3) | -39.5* (-53.8 to -24.0) |
| HIV/AIDS on antiretroviral treatment without Tuberculosis | 40.9 (34.6 to 49.0) | -- | 3.0 (2.0 to 4.3) | 572.9* (490.9 to 677.8) | 394.6* (334.4 to 471.7) |
| Sexually transmitted infections excluding HIV | 14006.5 (12410.2 to 15831.0) | 757.0 (652.1 to 903.0) | 6.2 (3.6 to 11.0) | 34.8* (32.0 to 38.3) | -0.9 (-2.9 to 1.7) |
| Syphilis | 47.4 (39.0 to 57.5) | -- | 2.3 (1.5 to 3.2) | 36.7* (29.4 to 43.7) | 0.5 (-4.9 to 5.6) |
| Early syphilis | 33.4 (24.9 to 43.5) | -- | -- | 51.8* (42.1 to 61.0) | 11.6* (4.5 to 18.3) |
| Tertiary syphilis | 14.0 (11.8 to 16.6) | -- | 2.3 (1.5 to 3.2) | 36.6* (29.3 to 43.7) | 0.4 (-4.9 to 5.6) |
| Chlamydial infection | -- | -- | -- | 33.2* (26.6 to 41.6) | -2.1 (-7.0 to 4.1) |
| Chlamydia episode | -- | -- | -- | 33.2* (26.6 to 41.6) | -2.1 (-7.0 to 4.1) |
| Chlamydial infection complications | -- | -- | -- | 52.7* (26.5 to 80.8) | 12.2 (-7.0 to 32.9) |
| Gonococcal infection | -- | -- | -- | 161.7 (-93.0 to 3277.4) | 92.4 (-94.9 to 2382.7) |
| Gonococcal infection complications | -- | -- | -- | 50.5* (23.7 to 76.4) | 10.7 (-9.0 to 29.7) |
| Gonorrhoea episode | -- | -- | -- | 161.7 (-93.0 to 3277.4) | 92.4 (-94.9 to 2382.7) |
| Trichomoniasis | 546.1 (460.2 to 649.1) | 757.0 (652.1 to 903.0) | 0.9 (0.4 to 1.9) | 33.0* (29.2 to 37.0) | -2.2 (-5.1 to 0.7) |
| Genital herpes | 13648.2 (11977.5 to 15522.9) | -- | 3.0 (0.9 to 7.5) | 34.0* (31.9 to 36.2) | -1.5 (-3.0 to 0.2) |
| Other sexually transmitted infections | -- | -- | -- | 34.3* (27.1 to 45.6) | -1.3 (-6.6 to 7.0) |
| Other sexually transmitted diseases residual | -- | -- | -- | 34.3* (27.1 to 45.6) | -1.3 (-6.6 to 7.0) |
| Other sexually transmitted diseases | -- | -- | -- | 34.3* (27.1 to 45.6) | -1.3 (-6.6 to 7.0) |
| **Respiratory infections and tuberculosis** | **18739.6 (14807.0 to 23212.9)** | **120073.8 (87361.8 to 162690.8)** | **133.2 (96.2 to 177.7)** | **38.0* (35.3 to 41.0)** | **1.5 (-0.5 to 3.6)** |
| Tuberculosis | 17192.2 (13215.0 to 21852.2) | 131.1 (114.0 to 148.3) | 82.3 (55.1 to 112.8) | 41.1* (37.1 to 44.9) | 3.7* (0.8 to 6.5) |
| Latent tuberculosis infection | 16889.2 (12927.9 to 21550.8) | -- | -- | -- | -- |
| Drug-susceptible tuberculosis | 289.0 (240.5 to 342.8) | 124.7 (108.2 to 141.8) | 78.4 (52.7 to 108.7) | 42.0* (34.0 to 48.0) | 4.4 (-1.5 to 8.8) |
| Multidrug-resistant tuberculosis without extensive drug resistance | 13.3 (5.8 to 26.6) | 6.1 (3.1 to 11.0) | 3.6 (1.5 to 7.7) | 22.2 (-42.4 to 122.2) | -10.2 (-57.7 to 63.3) |
| Extensively drug-resistant tuberculosis | 0.8 (0.3 to 1.6) | 0.3 (0.2 to 0.5) | 0.2 (0.1 to 0.6) | 74.5 (-13.0 to 258.2) | 28.3 (-36.0 to 163.3) |
| Lower respiratory infections | 133.5 (120.1 to 148.6) | 6623.8 (5957.0 to 7368.3) | 7.4 (5.0 to 10.3) | 42.9* (33.2 to 52.8) | 5.0 (-2.1 to 12.3) |
| Guillain-Barré syndrome due to lower respiratory infections | 0.2 (0.1 to 0.4) | -- | 0.1 (0.0 to 0.1) | 39.6* (35.7 to 43.4) | 2.6 (-0.2 to 5.4) |
| Lower respiratory infection episode | 133.2 (119.8 to 148.4) | 6623.8 (5957.0 to 7368.3) | 7.3 (4.9 to 10.2) | 42.9* (33.1 to 52.9) | 5.0 (-2.1 to 12.4) |
| Upper respiratory infections | 1553.5 (1101.7 to 2146.7) | 112896.8 (80382.3 to 155290.7) | 35.4 (19.8 to 59.5) | 32.7* (30.2 to 34.9) | -2.5* (-4.3 to -0.8) |
| Guillain-Barré syndrome due to upper respiratory infections | 0.6 (0.3 to 1.0) | -- | 0.2 (0.1 to 0.3) | 39.6* (35.7 to 43.4) | 2.6 (-0.3 to 5.4) |
| Upper respiratory infection episode | 1552.9 (1101.3 to 2146.3) | 112896.8 (80382.3 to 155290.7) | 35.2 (19.5 to 59.3) | 32.6* (30.2 to 34.9) | -2.5* (-4.3 to -0.8) |
| Otitis media | 420.5 (301.4 to 556.9) | 422.0 (294.9 to 566.3) | 8.2 (4.4 to 13.6) | 28.5* (22.9 to 34.5) | -5.5* (-9.7 to -1.1) |
| Acute otitis media | 23.6 (16.0 to 33.4) | 422.0 (294.9 to 566.0) | 0.3 (0.1 to 0.5) | 34.8* (22.6 to 47.5) | -0.9 (-9.9 to 8.4) |
| Chronic otitis media | 396.9 (275.2 to 533.2) | 0.1 (0.0 to 0.3) | 7.9 (4.2 to 13.2) | 28.3* (22.4 to 34.6) | -5.7* (-10.0 to -1.1) |
| **Enteric infections** | **1581.9 (1362.6 to 1827.5)** | **107634.6 (91727.1 to 127658.2)** | **160.6 (111.6 to 220.3)** | **61.3* (52.3 to 71.2)** | **18.5* (12.0 to 25.9)** |
| Diarrhoeal diseases | 1595.4 (1374.1 to 1843.7) | 107605.8 (91701.6 to 127626.6) | 160.4 (111.4 to 219.8) | 61.4* (52.4 to 71.3) | 18.6* (12.0 to 26.0) |
| Guillain-Barré syndrome due to diarrheal diseases | 0.2 (0.1 to 0.4) | -- | 0.1 (0.0 to 0.1) | 39.6* (35.8 to 43.3) | 2.6 (-0.2 to 5.4) |
| Diarrheal disease episode | 1595.2 (1373.8 to 1843.5) | 107605.8 (91701.6 to 127626.6) | 160.3 (111.4 to 219.8) | 61.4* (52.4 to 71.4) | 18.6* (12.0 to 26.0) |
| Typhoid and paratyphoid | 0.0 (0.0 to 0.1) | 27.6 (23.6 to 32.0) | 0.2 (0.1 to 0.3) | 18.8 (-0.6 to 43.0) | -12.7 (-26.9 to 5.1) |
| Typhoid fever | 1.4 (1.1 to 1.7) | 21.8 (18.2 to 25.5) | 0.2 (0.1 to 0.3) | 21.2 (-0.2 to 48.7) | -10.9 (-26.7 to 9.3) |
| Typhoid fever complications | 0.3 (0.2 to 0.4) | 3.7 (3.0 to 4.7) | 0.1 (0.1 to 0.1) | 19.2 (-13.6 to 64.2) | -12.4 (-36.5 to 20.7) |
| Typhoid fever episode | 1.1 (0.9 to 1.4) | 18.0 (15.0 to 21.3) | 0.1 (0.1 to 0.2) | 22.8 (-5.5 to 59.5) | -9.7 (-30.5 to 17.2) |
| Paratyphoid fever | 0.3 (0.2 to 0.3) | 5.8 (4.3 to 7.6) | -- | -6.2 (-20.7 to 11.0) | -31.1* (-41.7 to -18.4) |
| Intestinal perforation due to paratyphoid | -- | 0.3 (0.2 to 0.4) | -- | -6.3 (-28.4 to 21.9) | -31.1* (-47.3 to -10.4) |
| Paratyphoid fever episode | 0.2 (0.2 to 0.3) | 5.5 (4.0 to 7.1) | -- | -6.2 (-21.6 to 13.5) | -31.0* (-42.4 to -16.6) |
| iNTS | 0.0 (0.0 to 0.1) | 1.2 (0.7 to 2.0) | -- | -1.2 (-10.5 to 7.1) | -27.4* (-34.2 to -21.3) |
| Other intestinal infectious diseases | -- | -- | -- | 10.3 (-7.7 to 32.7) | -18.9* (-32.2 to -2.4) |
| **Neglected tropical diseases and malaria** | **6092.7 (5804.6 to 6428.8)** | **2350.6 (1955.6 to 2861.3)** | **129.6 (94.7 to 171.1)** | **15.8* (8.0 to 24.0)** | **-14.9* (-20.6 to -8.9)** |
| Malaria | 263.9 (245.3 to 283.5) | 124.3 (117.0 to 142.2) | 1.7 (1.1 to 2.4) | -23.7* (-36.5 to -5.5) | -43.9* (-53.3 to -30.5) |
| Malaria complications | 0.1 (0.1 to 0.1) | -- | 0.0 (0.0 to 0.1) | 69.5* (62.2 to 77.5) | 24.6* (19.3 to 30.5) |
| Malaria episode | 7.3 (4.9 to 9.8) | 124.3 (117.0 to 142.2) | 0.3 (0.1 to 0.4) | -16.0* (-25.6 to -4.2) | -38.3* (-45.3 to -29.6) |

| | | | | | |
|---|---|---|---|---|---|
| Malaria parasitemia | 256.5 (237.6 to 276.2) | -- | 1.3 (0.9 to 2.0) | -26.3* (-40.6 to -5.2) | -45.8* (-56.4 to -30.3) |
| Chagas disease | 120.4 (102.9 to 139.2) | 0.9 (0.6 to 1.4) | 2.9 (1.8 to 4.3) | 13.6* (7.7 to 19.6) | -16.5* (-20.8 to -12.1) |
| Acute Chagas disease | -- | 0.9 (0.6 to 1.4) | -- | 36.1* (30.9 to 41.2) | 0.0 (-3.8 to 3.8) |
| Asymptomatic Chagas disease | 83.6 (70.6 to 98.0) | -- | -- | -- | -- |
| Symptomatic chronic Chagas infection | 36.8 (28.4 to 45.7) | -- | 2.9 (1.8 to 4.3) | 13.6* (7.7 to 19.6) | -16.5* (-20.8 to -12.1) |
| Leishmaniasis | 43.7 (31.9 to 57.5) | 5.6 (3.8 to 8.1) | 2.5 (1.6 to 3.8) | 30.2* (24.3 to 39.2) | -4.3 (-8.6 to 2.4) |
| Visceral leishmaniasis | -- | 0.1 (0.1 to 0.1) | -- | -67.1* (-72.6 to -58.0) | -75.8* (-79.8 to -69.1) |
| Cutaneous and mucocutaneous leishmaniasis | 43.9 (32.0 to 57.9) | 5.5 (3.7 to 8.0) | 2.5 (1.6 to 3.8) | 30.5* (24.5 to 39.6) | -4.1 (-8.5 to 2.6) |
| African trypanosomiasis | 0.0 (0.0 to 0.1) | -- | -- | -73.9 (-97.3 to 57.9) | -80.8 (-98.0 to 16.1) |
| Trypanosomiasis Gambiense | 0.0 (0.0 to 0.1) | -- | -- | -73.1 (-97.6 to 80.3) | -80.2 (-98.2 to 32.6) |
| Trypanosomiasis Rhodesiense | -- | -- | -- | -86.5 (-98.8 to 0.9) | -90.1* (-99.1 to -25.9) |
| Schistosomiasis | 1026.5 (907.0 to 1144.3) | -- | 6.6 (3.2 to 12.6) | -9.3* (-14.8 to -4.3) | -33.3* (-37.3 to -29.6) |
| Mild schistosomiasis | 924.3 (812.4 to 1035.4) | -- | 4.8 (2.0 to 10.0) | -6.8* (-9.3 to -3.9) | -31.5* (-33.3 to -29.4) |
| Anemia due to schistosomiasis | 54.8 (44.3 to 67.6) | -- | 1.2 (0.7 to 1.9) | -25.2* (-40.9 to -5.4) | -45.0* (-56.6 to -30.5) |
| Schistosomiasis complications | 47.6 (37.5 to 59.6) | -- | 0.7 (0.4 to 1.1) | 10.7* (2.5 to 21.1) | -18.6* (-24.6 to -11.0) |
| Cysticercosis | 166.7 (125.1 to 216.8) | -- | 41.1 (25.6 to 59.6) | 34.0* (27.0 to 40.5) | -1.5 (-6.6 to 3.2) |
| Cystic echinococcosis | 4.5 (2.6 to 8.2) | 0.9 (0.4 to 1.8) | 0.3 (0.2 to 0.7) | 27.6* (6.2 to 47.3) | -6.2 (-21.9 to 8.3) |
| Lymphatic filariasis | 362.0 (327.5 to 401.1) | -- | 5.2 (3.0 to 8.0) | -33.5* (-46.5 to -21.3) | -51.1* (-60.6 to -42.1) |
| Prevalence of detectable microfiliaria due to lymphatic filariasis | 309.0 (282.7 to 341.2) | -- | -- | -- | -- |
| Lymphatic filariasis complications | 52.9 (35.7 to 74.9) | -- | 5.2 (3.0 to 8.0) | -33.5* (-46.5 to -21.3) | -51.1* (-60.6 to -42.1) |
| Onchocerciasis | 80.9 (52.2 to 131.4) | -- | 7.1 (4.0 to 12.7) | 5.6 (-9.5 to 24.2) | -22.3* (-33.5 to -8.7) |
| Asymptomatic onchocerciasis | 14.3 (0.0 to 57.8) | -- | -- | -- | -- |
| Skin disease due to onchocerciasis | 49.1 (32.6 to 75.2) | -- | 5.8 (2.9 to 11.0) | 3.7 (-13.5 to 25.0) | -23.8* (-36.4 to -8.1) |
| Vision loss due to onchocerciasis | 17.4 (11.3 to 29.3) | -- | 1.3 (0.8 to 2.1) | 15.3 (-3.5 to 37.7) | -15.2 (-29.1 to 1.2) |
| Trachoma | 412.4 (271.8 to 622.4) | -- | 30.7 (19.1 to 46.4) | 4.0 (-14.8 to 25.5) | -23.6* (-37.4 to -7.8) |
| Dengue | 46.6 (25.6 to 78.6) | 780.4 (477.8 to 1178.2) | 6.7 (3.0 to 12.3) | 119.9* (90.8 to 238.7) | 61.7* (40.2 to 149.0) |
| Post-dengue chronic fatigue syndrome | 32.8 (15.4 to 60.0) | -- | 5.9 (2.5 to 11.0) | 120.0* (89.2 to 241.8) | 61.7* (39.1 to 151.2) |
| Dengue episode | 13.8 (8.4 to 20.6) | 780.4 (477.8 to 1178.2) | 0.7 (0.4 to 1.3) | 119.4* (89.5 to 230.8) | 61.3* (39.3 to 143.2) |
| Yellow fever | -- | 0.2 (0.1 to 0.5) | -- | -25.4 (-47.3 to 2.4) | -45.2* (-61.3 to -24.7) |
| Asymptomatic yellow fever | -- | 0.1 (0.0 to 0.3) | -- | -- | -- |
| Yellow fever episode | -- | 0.1 (0.0 to 0.2) | -- | -25.4 (-47.3 to 2.4) | -45.2* (-61.3 to -24.7) |
| Rabies | -- | 0.2 (0.1 to 0.2) | -- | 7.3 (-20.0 to 39.6) | -21.1 (-41.2 to 2.6) |
| Intestinal nematode infections | 3297.3 (3045.1 to 3583.8) | -- | 6.3 (3.8 to 10.1) | -9.2* (-17.1 to -1.8) | -33.3* (-39.1 to -27.8) |
| Ascariasis | 1488.7 (1293.3 to 1706.6) | -- | 1.7 (0.9 to 2.8) | -12.0* (-21.6 to -1.2) | -35.3* (-42.4 to -27.4) |
| Asymptomatic ascariasis | 1388.9 (1207.3 to 1598.3) | -- | -- | -- | -- |
| Ascariasis complications | 99.8 (87.7 to 113.1) | -- | 1.7 (0.9 to 2.8) | -12.0* (-21.6 to -1.2) | -35.3* (-42.4 to -27.4) |
| Trichuriasis | 619.7 (515.4 to 748.1) | -- | 0.3 (0.2 to 0.6) | 11.2 (-0.5 to 24.3) | -18.2* (-26.9 to -8.7) |
| Asymptomatic trichuriasis | 601.1 (498.4 to 726.7) | -- | -- | -- | -- |
| Trichuriasis complications | 18.6 (16.7 to 20.4) | -- | 0.3 (0.2 to 0.6) | 11.2 (-0.5 to 24.3) | -18.2* (-26.9 to -8.7) |
| Hookworm disease | 1320.8 (1181.6 to 1491.3) | -- | 4.3 (2.6 to 6.9) | -9.4 (-19.8 to 0.9) | -33.4* (-41.0 to -25.8) |
| Asymptomatic hookworm disease | 1094.8 (977.5 to 1236.2) | -- | -- | -- | -- |
| Anemia due to hookworm disease | 59.4 (50.9 to 69.9) | -- | 1.1 (0.7 to 1.7) | -19.5* (-35.1 to -0.7) | -40.8* (-52.3 to -27.0) |
| Hookworm disease complications | 166.6 (149.0 to 187.7) | -- | 3.2 (1.8 to 5.3) | -5.1 (-14.4 to 4.7) | -30.3* (-37.1 to -23.1) |
| Food-borne trematodiases | 665.0 (583.0 to 751.2) | 1422.8 (1191.1 to 1709.8) | 14.3 (7.0 to 24.5) | 52.2* (43.6 to 61.5) | 11.9* (5.5 to 18.7) |
| Asymptomatic food-borne tremodiases | 525.3 (425.4 to 621.4) | 1070.7 (536.0 to 1501.7) | -- | -- | -- |
| Food-borne tremodiases complications | 139.6 (76.6 to 224.3) | 352.1 (37.4 to 882.8) | 14.3 (7.0 to 24.5) | 52.2* (43.6 to 61.5) | 11.9* (5.5 to 18.7) |
| Leprosy | 23.1 (22.0 to 24.2) | 1.0 (0.9 to 1.1) | 1.3 (0.9 to 1.8) | 22.1* (16.1 to 28.4) | -10.3* (-14.7 to -5.6) |
| Ebola virus disease | -- | -- | -- | -92.1* (-92.7 to -90.4) | -94.2* (-94.7 to -92.9) |
| Ebola cases | -- | -- | -- | -91.7* (-91.9 to -91.3) | -93.9* (-94.1 to -93.6) |
| Post-Ebola chronic fatigue syndrome | -- | -- | -- | -92.1* (-92.8 to -90.4) | -94.2* (-94.7 to -92.9) |
| Zika virus disease | 0.2 (0.1 to 0.5) | 14.5 (8.0 to 27.8) | -- | -- | -- |
| Zika virus complications | -- | -- | -- | -- | -- |
| Zika virus episode | 0.2 (0.1 to 0.5) | 14.5 (8.0 to 27.8) | -- | -- | -- |
| Guinea worm disease | -- | -- | -- | -99.6* (-99.8 to -99.4) | -99.7* (-99.8 to -99.6) |
| Other neglected tropical diseases | 156.0 (142.7 to 170.6) | -- | 2.9 (1.9 to 4.2) | 15.9* (2.8 to 29.3) | -14.8* (-24.4 to -4.9) |
| Acute infection due to other neglected tropical diseases | -- | -- | 0.1 (0.0 to 0.1) | 49.2* (28.7 to 122.5) | 9.7 (-5.4 to 63.6) |
| Anemia due to other neglected tropical diseases | 156.0 (142.7 to 170.6) | -- | 2.8 (1.8 to 4.1) | 15.4* (2.2 to 28.8) | -15.2* (-24.9 to -5.3) |
| **Other infectious diseases** | **637.2 (541.7 to 748.8)** | **2153.3 (1650.3 to 2758.5)** | **26.3 (18.2 to 36.7)** | **29.8* (23.3 to 36.4)** | **-4.6 (-9.4 to 0.3)** |
| Meningitis | 89.6 (71.6 to 108.1) | 33.8 (25.7 to 43.4) | 4.5 (3.0 to 6.2) | 10.5* (6.6 to 14.9) | -18.8* (-21.6 to -15.5) |
| Pneumococcal meningitis | 33.6 (26.2 to 41.4) | 4.2 (2.6 to 6.2) | 1.8 (1.1 to 2.4) | -7.9* (-12.4 to -2.8) | -32.3* (-35.6 to -28.5) |

| | | | | | |
|---|---|---|---|---|---|
| Acute pneumococcal meningitis | 0.2 (0.1 to 0.3) | 4.2 (2.6 to 6.2) | -- | 3.8 (-6.6 to 15.2) | -23.7* (-31.4 to -15.3) |
| Pneumococcal meningitis complications | 33.4 (26.1 to 41.2) | -- | 1.7 (1.1 to 2.4) | -8.0* (-12.6 to -2.9) | -32.4* (-35.7 to -28.6) |
| H influenzae type B meningitis | 6.2 (4.2 to 8.5) | 1.2 (0.7 to 1.9) | 0.3 (0.2 to 0.4) | -45.2* (-48.0 to -42.3) | -59.7* (-61.8 to -57.6) |
| Acute H influenzae type B meningitis | 0.1 (0.0 to 0.1) | 1.2 (0.7 to 1.9) | -- | -40.3* (-47.4 to -34.2) | -56.1* (-61.3 to -51.6) |
| H Influenza type B meningitis complications | 6.2 (4.1 to 8.4) | -- | 0.3 (0.2 to 0.4) | -45.3* (-48.1 to -42.3) | -59.8* (-61.9 to -57.6) |
| Meningococcal infection | 10.2 (6.9 to 13.8) | 3.3 (2.1 to 5.2) | 0.6 (0.4 to 0.8) | 7.3* (2.7 to 11.6) | -21.1* (-24.5 to -18.0) |
| Acute meningococcal meningitis | 0.1 (0.1 to 0.2) | 3.3 (2.1 to 5.2) | -- | 27.3* (14.3 to 39.9) | -6.4 (-16.0 to 2.8) |
| Meningococcal meningitis complications | 10.1 (6.8 to 13.7) | -- | 0.6 (0.4 to 0.8) | 6.8* (2.2 to 11.2) | -21.5* (-24.9 to -18.3) |
| Other meningitis | 39.6 (27.9 to 51.5) | 25.1 (18.9 to 32.5) | 1.9 (1.2 to 2.7) | 67.3* (59.6 to 75.7) | 23.0* (17.3 to 29.2) |
| Other acute bacterial meningitis | 0.4 (0.3 to 0.5) | 8.6 (6.2 to 11.3) | 0.0 (0.0 to 0.1) | 87.9* (71.2 to 104.0) | 38.1* (25.8 to 49.9) |
| Acute viral meningitis | 0.7 (0.5 to 0.9) | 16.4 (12.5 to 21.1) | 0.1 (0.1 to 0.1) | 31.2* (20.2 to 41.3) | -3.5 (-11.6 to 3.9) |
| Other bacterial meningitis complications | 38.5 (27.0 to 50.3) | -- | 1.8 (1.1 to 2.5) | 69.3* (61.0 to 78.8) | 24.4* (18.3 to 31.4) |
| Encephalitis | 115.7 (51.9 to 201.1) | 41.4 (40.1 to 42.7) | 5.0 (3.4 to 7.0) | 38.6* (33.3 to 44.7) | 1.9 (-2.0 to 6.3) |
| Acute encephalitis | 1.8 (1.7 to 1.9) | 41.4 (40.1 to 42.7) | 0.2 (0.2 to 0.3) | 53.6* (33.3 to 77.6) | 12.9 (-2.0 to 30.6) |
| Encephalitis complications | 113.9 (50.1 to 199.3) | -- | 4.8 (3.2 to 6.7) | 37.9* (32.3 to 44.4) | 1.4 (-2.8 to 6.2) |
| Diphtheria | -- | -- | -- | -- | -- |
| Whooping cough | -- | -- | -- | -- | -- |
| Tetanus | 0.4 (0.3 to 0.4) | 0.9 (0.5 to 1.4) | -- | -3.4 (-21.9 to 19.5) | -29.0* (-42.6 to -12.2) |
| Severe tetanus | 0.0 (0.0 to 0.1) | 0.9 (0.5 to 1.4) | -- | -28.1 (-44.5 to 1.9) | -47.1* (-59.2 to -25.1) |
| Neonatal tetanus complications | 0.3 (0.3 to 0.3) | -- | -- | 52.2* (50.6 to 53.6) | 11.8* (10.7 to 12.9) |
| Measles | -- | -- | -- | -- | -- |
| Varicella and herpes zoster | 185.7 (137.8 to 236.3) | 1013.2 (743.8 to 1299.5) | 9.4 (5.4 to 14.7) | 38.8* (33.3 to 44.0) | 2.1 (-2.0 to 5.8) |
| Chickenpox | -- | -- | -- | 65.9* (49.1 to 92.0) | 21.9* (9.6 to 41.1) |
| Herpes zoster | 185.7 (137.8 to 236.3) | 1013.2 (743.8 to 1299.5) | 9.4 (5.4 to 14.7) | 38.8* (33.3 to 44.0) | 2.1 (-2.0 to 5.8) |
| Acute hepatitis | 119.2 (70.9 to 184.5) | 1064.0 (641.4 to 1631.3) | 2.6 (1.4 to 4.6) | 34.1 (-13.2 to 108.1) | -1.4 (-36.2 to 53.0) |
| Acute hepatitis A | 0.3 (0.1 to 0.8) | 4.0 (1.0 to 9.8) | -- | 21.8* (11.1 to 31.9) | -10.5* (-18.3 to -3.0) |
| Acute hepatitis B | 106.6 (57.9 to 170.2) | 923.7 (501.8 to 1475.1) | 2.3 (1.1 to 4.2) | 34.9 (-19.5 to 121.3) | -0.9 (-40.8 to 62.7) |
| Acute hepatitis C | 5.4 (2.1 to 9.8) | 46.7 (18.1 to 84.6) | 0.1 (0.0 to 0.2) | 11.0* (1.3 to 16.8) | -18.4* (-25.6 to -14.1) |
| Acute hepatitis E | 6.9 (4.5 to 9.7) | 89.7 (59.1 to 125.7) | 0.2 (0.1 to 0.4) | 36.8* (14.0 to 62.7) | 0.5 (-16.2 to 19.6) |
| Other unspecified infectious diseases | 217.0 (200.4 to 234.4) | -- | 4.7 (3.1 to 6.7) | 24.0* (12.9 to 35.7) | -8.9* (-17.0 to -0.2) |
| Guillain-Barré syndrome due to other infectious diseases | 0.1 (0.1 to 0.2) | -- | 0.0 (0.0 to 0.1) | 39.6* (35.7 to 43.4) | 2.6 (-0.2 to 5.4) |
| Other infectious diseases | -- | -- | 0.8 (0.5 to 1.3) | 38.7* (34.7 to 43.9) | 1.9 (-1.0 to 5.8) |
| Anemia due to other infectious diseases | 216.9 (200.2 to 234.3) | -- | 3.9 (2.6 to 5.6) | 21.2* (8.5 to 35.7) | -10.9* (-20.2 to -0.3) |
| **Maternal and neonatal disorders** | **114.1 (94.4 to 133.8)** | **--** | **23.5 (16.5 to 33.0)** | **68.7* (57.6 to 80.6)** | **24.0* (15.8 to 32.8)** |
| Maternal disorders | 0.9 (0.7 to 1.1) | -- | 0.3 (0.2 to 0.5) | -23.6* (-35.4 to -9.6) | -43.9* (-52.5 to -33.6) |
| Maternal haemorrhage | -- | -- | -- | -- | -- |
| Maternal hemorrhage complications | -- | -- | -- | -- | -- |
| Maternal hemorrhage episode | -- | -- | -- | -- | -- |
| Maternal sepsis and other pregnancy related infections | -- | -- | -- | -- | -- |
| Infertility due to puerperal sepsis | -- | -- | -- | -- | -- |
| Maternal sepsis and other maternal infection episode | -- | -- | -- | -- | -- |
| Maternal hypertensive disorders | -- | -- | -- | -- | -- |
| Maternal hypertensive disorder complications | -- | -- | -- | -- | -- |
| Maternal hypertensive disorder episode | -- | -- | -- | -- | -- |
| Maternal obstructed labour and uterine rupture | 1.1 (0.9 to 1.4) | -- | 0.3 (0.2 to 0.5) | -23.6* (-35.4 to -9.6) | -43.9* (-52.5 to -33.6) |
| Obstructed labor, acute event | -- | -- | -- | -- | -- |
| Maternal obstructed labor complications | 1.1 (0.9 to 1.4) | -- | 0.3 (0.2 to 0.5) | -23.6* (-35.4 to -9.6) | -43.9* (-52.5 to -33.6) |
| Maternal abortion and miscarriage | -- | -- | -- | -- | -- |
| Ectopic pregnancy | -- | -- | -- | -- | -- |
| Other maternal disorders | -- | -- | -- | -- | -- |
| Neonatal disorders | 113.2 (93.3 to 132.9) | -- | 23.2 (16.1 to 32.7) | 71.6* (60.3 to 83.7) | 26.2* (17.9 to 35.1) |
| Neonatal preterm birth | 111.5 (92.0 to 131.8) | -- | 8.9 (6.3 to 11.9) | 56.0* (39.0 to 79.4) | 14.7* (2.2 to 31.9) |
| Preterm birth complications | 8.6 (4.9 to 13.7) | -- | 3.4 (1.8 to 5.7) | 111.3* (59.3 to 170.5) | 55.3* (17.1 to 98.8) |
| Retinopathy of prematurity | 102.9 (84.0 to 123.0) | -- | 5.5 (3.8 to 7.6) | 34.3* (26.3 to 42.1) | -1.3 (-7.2 to 4.4) |
| Uncomplicated preterm birth | -- | -- | -- | -- | -- |
| Neonatal encephalopathy due to birth asphyxia and trauma | 9.7 (4.6 to 17.1) | -- | 3.8 (1.5 to 7.6) | 132.3* (115.5 to 154.3) | 70.8* (58.4 to 86.9) |
| Neonatal sepsis and other neonatal infections | 26.1 (14.2 to 42.7) | -- | 9.8 (4.9 to 17.4) | 70.4* (58.4 to 84.7) | 25.3* (16.4 to 35.8) |
| Neonatal sepsis and other neonatal infection complications | 26.1 (14.2 to 42.7) | -- | 9.8 (4.9 to 17.4) | 70.4* (58.4 to 84.7) | 25.3* (16.4 to 35.8) |
| Neonatal sepsis and other neonatal infection episode | -- | -- | -- | -- | -- |
| Hemolytic disease and other neonatal jaundice | 1.8 (1.4 to 2.4) | -- | 0.8 (0.5 to 1.1) | 67.0* (55.5 to 78.1) | 22.8* (14.3 to 30.9) |

| | | | | | |
|---|---|---|---|---|---|
| Other neonatal disorders | -- | -- | -- | -- | -- |
| **Nutritional deficiencies** | **13339.1 (12647.0 to 14056.3)** | **6780.3 (5905.4 to 7727.9)** | **192.3 (128.8 to 278.1)** | **22.9* (14.3 to 32.7)** | **-9.6* (-16.0 to -2.5)** |
| Protein-energy malnutrition | 1041.7 (972.8 to 1110.9) | 950.6 (874.0 to 1030.1) | 3.3 (2.1 to 4.5) | 53.3* (36.0 to 68.1) | 12.7 (-0.0 to 23.6) |
| Iodine deficiency | 1349.3 (1222.8 to 1503.0) | 3.8 (3.0 to 5.1) | 17.0 (9.9 to 28.3) | 33.9* (30.4 to 38.6) | -1.6 (-4.1 to 1.9) |
| Visible goiter without symptoms | 1335.3 (1211.0 to 1484.7) | 3.8 (3.0 to 5.1) | 13.2 (6.5 to 24.4) | 30.1* (28.2 to 31.8) | -4.4* (-5.7 to -3.1) |
| Visible goiter with complications | 14.0 (8.6 to 20.2) | -- | 3.9 (2.1 to 6.0) | 48.4* (35.1 to 59.2) | 9.1 (-0.7 to 17.0) |
| Vitamin A deficiency | 3422.4 (2972.8 to 3922.8) | 5825.8 (4947.4 to 6778.2) | 0.9 (0.5 to 1.3) | 48.8* (37.8 to 60.2) | 9.4* (1.3 to 17.7) |
| Asymptomatic vitamin A deficiency | 3409.8 (2963.2 to 3906.2) | 5825.8 (4947.4 to 6778.2) | -- | -- | -- |
| Vitamin A deficiency complications | 12.6 (9.0 to 17.0) | -- | 0.9 (0.5 to 1.3) | 48.8* (37.8 to 60.2) | 9.4* (1.3 to 17.7) |
| Vitamin A deficiency with anemia | -- | -- | -- | -- | -- |
| Dietary iron deficiency | 8926.4 (8255.1 to 9599.5) | -- | 170.0 (113.8 to 245.0) | 21.2* (11.5 to 32.2) | -10.9* (-18.0 to -2.8) |
| Other nutritional deficiencies | -- | -- | 1.1 (0.7 to 1.5) | 64.9* (46.2 to 80.8) | 21.2* (7.5 to 32.9) |
| **Non-communicable diseases** | **80206.0 (80194.4 to 80216.0)** | **199691.4 (180878.8 to 218559.4)** | **21243.1 (16450.8 to 26722.6)** | **36.5* (35.3 to 37.7)** | **0.3 (-0.6 to 1.2)** |
| **Neoplasms** | **4605.7 (4445.3 to 4822.3)** | **1887.6 (1580.7 to 2286.0)** | **436.1 (328.4 to 548.7)** | **36.5* (33.6 to 39.9)** | **0.3 (-1.8 to 2.8)** |
| Lip and oral cavity cancer | 67.8 (65.2 to 70.5) | 20.8 (20.0 to 21.6) | 7.8 (5.8 to 9.9) | 49.7* (41.0 to 59.2) | 10.1* (3.7 to 17.0) |
| Diagnosis and primary therapy phase of mouth cancer | 4.4 (4.2 to 4.6) | 20.8 (20.0 to 21.6) | 1.2 (0.8 to 1.6) | 49.0* (37.3 to 60.6) | 9.5* (0.9 to 18.1) |
| Controlled phase of mouth cancer | 52.6 (50.6 to 54.6) | -- | 2.3 (1.5 to 3.2) | 53.7* (44.1 to 63.1) | 13.0* (5.9 to 19.9) |
| Metastatic phase of mouth cancer | 9.4 (9.0 to 9.7) | -- | 3.6 (2.5 to 4.7) | 47.7* (34.7 to 62.5) | 8.6 (-1.0 to 19.4) |
| Terminal phase of mouth cancer | 1.4 (1.4 to 1.5) | -- | 0.8 (0.5 to 1.0) | 49.4* (42.5 to 57.1) | 9.8* (4.7 to 15.5) |
| Nasopharynx cancer | 8.6 (8.2 to 9.0) | 2.8 (2.7 to 3.0) | 1.2 (0.9 to 1.6) | 37.9* (28.6 to 47.3) | 1.4 (-5.5 to 8.3) |
| Diagnosis and primary therapy phase of nasopharynx cancer | 0.6 (0.5 to 0.6) | 2.8 (2.7 to 3.0) | 0.2 (0.1 to 0.2) | 39.3* (33.3 to 45.7) | 2.4 (-2.0 to 7.1) |
| Controlled phase of nasopharynx cancer | 6.3 (6.0 to 6.6) | -- | 0.3 (0.2 to 0.4) | 43.6* (26.1 to 62.8) | 5.5 (-7.3 to 19.7) |
| Metastatic phase of nasopharynx cancer | 1.5 (1.5 to 1.6) | -- | 0.7 (0.5 to 0.9) | 35.5* (23.8 to 49.0) | -0.4 (-9.0 to 9.5) |
| Terminal phase of nasopharynx cancer | 0.2 (0.2 to 0.2) | -- | 0.1 (0.1 to 0.1) | 36.7* (31.0 to 43.0) | 0.5 (-3.7 to 5.1) |
| Other pharynx cancer | 10.3 (8.8 to 11.0) | 5.8 (4.9 to 6.2) | 1.7 (1.2 to 2.3) | 58.1* (43.8 to 72.1) | 16.2* (5.7 to 26.5) |
| Diagnosis and primary therapy phase of other pharynx cancer | 0.6 (0.5 to 0.7) | 5.8 (4.9 to 6.2) | 0.2 (0.1 to 0.3) | 60.3* (46.1 to 73.4) | 17.9* (7.4 to 27.5) |
| Controlled phase of other pharynx cancer | 6.7 (5.8 to 7.1) | -- | 0.3 (0.2 to 0.4) | 61.7* (43.5 to 80.5) | 18.8* (5.5 to 32.7) |
| Metastatic phase of other pharynx cancer | 2.5 (2.1 to 2.7) | -- | 1.0 (0.7 to 1.3) | 56.4* (40.2 to 75.0) | 15.0* (3.1 to 28.7) |
| Terminal phase of other pharynx cancer | 0.4 (0.4 to 0.5) | -- | 0.2 (0.2 to 0.3) | 59.4* (45.4 to 71.5) | 17.2* (6.9 to 26.1) |
| Oesophageal cancer | 43.1 (41.1 to 45.1) | 40.4 (38.8 to 42.0) | 9.2 (6.7 to 11.7) | 33.0* (23.0 to 44.5) | -2.2 (-9.6 to 6.3) |
| Diagnosis and primary therapy phase of esophageal cancer | 3.7 (3.5 to 3.9) | 40.4 (38.8 to 42.0) | 1.0 (0.7 to 1.3) | 49.8* (29.7 to 75.3) | 10.1 (-4.6 to 28.9) |
| Controlled phase of esophageal cancer | 21.1 (19.9 to 22.3) | -- | 0.9 (0.6 to 1.3) | 73.4* (56.3 to 91.3) | 27.5* (14.9 to 40.6) |
| Metastatic phase of esophageal cancer | 15.1 (14.5 to 15.6) | -- | 5.7 (4.0 to 7.4) | 26.8* (15.3 to 39.3) | -6.8 (-15.3 to 2.4) |
| Terminal phase of esophageal cancer | 3.3 (3.2 to 3.4) | -- | 1.6 (1.2 to 2.1) | 29.4* (11.5 to 50.1) | -4.9 (-18.0 to 10.3) |
| Stomach cancer | 177.6 (171.9 to 183.7) | 109.6 (106.2 to 113.3) | 26.0 (19.2 to 32.8) | 29.4* (23.0 to 36.1) | -4.9 (-9.6 to 0.0) |
| Diagnosis and primary therapy phase of stomach cancer | 19.7 (19.1 to 20.4) | 109.6 (106.2 to 113.3) | 4.8 (3.3 to 6.4) | 31.7* (21.6 to 44.1) | -3.2 (-10.6 to 5.9) |
| Controlled phase of stomach cancer | 116.2 (112.3 to 120.2) | -- | 5.1 (3.3 to 7.1) | 38.6* (32.2 to 45.5) | 1.9 (-2.8 to 6.9) |
| Metastatic phase of stomach cancer | 33.2 (32.1 to 34.3) | -- | 12.2 (8.6 to 15.6) | 26.2* (17.0 to 35.5) | -7.2* (-14.0 to -0.4) |
| Terminal phase of stomach cancer | 8.6 (8.3 to 8.8) | -- | 4.0 (2.8 to 5.1) | 26.0* (14.0 to 39.3) | -7.4 (-16.2 to 2.4) |
| Colon and rectum cancer | 608.8 (591.9 to 625.7) | 166.9 (162.5 to 171.5) | 67.4 (50.9 to 86.0) | 31.7* (27.1 to 36.8) | -3.2 (-6.6 to 0.5) |
| Diagnosis and primary therapy phase of colon and rectum cancers | 26.6 (25.8 to 27.3) | 166.9 (162.5 to 171.5) | 6.4 (4.4 to 8.4) | 34.4* (23.7 to 45.1) | -1.2 (-9.1 to 6.7) |
| Controlled phase of colon and rectum cancers | 460.6 (447.6 to 474.6) | -- | 26.7 (18.3 to 37.2) | 36.0* (31.4 to 41.7) | -0.0 (-3.4 to 4.1) |
| Metastatic phase of colon and rectum cancers | 71.7 (69.8 to 73.7) | -- | 26.1 (18.4 to 33.6) | 28.3* (21.4 to 35.6) | -5.7* (-10.8 to -0.3) |
| Terminal phase of colon and rectum cancers | 10.9 (10.6 to 11.2) | -- | 5.0 (3.5 to 6.3) | 28.8* (15.5 to 43.1) | -5.3 (-15.1 to 5.2) |
| Stoma from colon and rectum cancers, beyond 10 years | 38.9 (36.3 to 41.8) | -- | 3.2 (2.2 to 4.3) | 24.7* (17.3 to 32.5) | -8.3* (-13.8 to -2.6) |
| Liver cancer | 29.9 (26.4 to 33.1) | 63.0 (60.4 to 65.6) | 13.1 (9.3 to 17.1) | 51.8* (44.2 to 62.1) | 11.6* (6.0 to 19.2) |
| Liver cancer due to hepatitis B | 12.6 (11.2 to 14.2) | 14.2 (12.5 to 16.0) | 2.9 (2.0 to 3.8) | 56.9* (40.8 to 76.7) | 15.4* (3.5 to 29.9) |
| Diagnosis and primary therapy phase of liver cancer due to hepatitis B | 3.1 (2.8 to 3.5) | 14.2 (12.5 to 16.0) | 0.8 (0.5 to 1.2) | 51.9* (28.8 to 81.1) | 11.6 (-5.3 to 33.1) |
| Controlled phase of liver cancer due to hepatitis B | 5.4 (4.7 to 6.1) | -- | 0.2 (0.2 to 0.4) | 275.8* (218.0 to 343.9) | 176.2* (133.8 to 226.3) |
| Metastatic phase of liver cancer due to hepatitis B | 2.9 (2.6 to 3.3) | -- | 1.2 (0.8 to 1.6) | 46.6* (25.5 to 74.7) | 7.8 (-7.7 to 28.4) |
| Terminal phase of liver cancer due to hepatitis B | 1.2 (1.0 to 1.3) | -- | 0.6 (0.4 to 0.8) | 49.8* (34.6 to 67.5) | 10.1 (-1.0 to 23.1) |
| Liver cancer due to hepatitis C | 25.5 (22.8 to 28.0) | 26.5 (24.0 to 28.8) | 5.4 (3.8 to 7.2) | 47.0* (36.6 to 58.0) | 8.1* (0.4 to 16.1) |
| Diagnosis and primary therapy phase of liver cancer due to hepatitis C | 5.9 (5.3 to 6.4) | 26.5 (24.0 to 28.8) | 1.5 (1.0 to 2.1) | 44.0* (29.8 to 59.7) | 5.9 (-4.6 to 17.4) |
| Controlled phase of liver cancer due to hepatitis C | 12.0 (10.5 to 13.5) | -- | 0.5 (0.3 to 0.8) | 139.4* (106.5 to 171.0) | 76.0* (51.8 to 99.2) |
| Metastatic phase of liver cancer due to hepatitis C | 5.4 (4.9 to 5.9) | -- | 2.2 (1.5 to 3.0) | 39.4* (25.7 to 53.5) | 2.5 (-7.6 to 12.8) |
| Terminal phase of liver cancer due to hepatitis C | 2.2 (2.0 to 2.3) | -- | 1.1 (0.8 to 1.5) | 40.6* (32.6 to 49.6) | 3.4 (-2.5 to 10.0) |
| Liver cancer due to alcohol use | 9.3 (8.0 to 10.7) | 10.4 (9.0 to 12.0) | 2.2 (1.5 to 3.0) | 49.2* (40.5 to 58.3) | 9.7* (3.3 to 16.3) |
| Diagnosis and primary therapy phase of liver cancer due to alcohol use | 2.3 (2.0 to 2.7) | 10.4 (9.0 to 12.0) | 0.6 (0.4 to 0.9) | 47.3* (37.3 to 59.1) | 8.3* (0.9 to 16.9) |
| Controlled phase of liver cancer due to alcohol use | 4.0 (3.4 to 4.7) | -- | 0.2 (0.1 to 0.3) | 135.6* (98.3 to 174.1) | 73.2* (45.8 to 101.5) |
| Metastatic phase of liver cancer due to alcohol use | 2.1 (1.8 to 2.5) | -- | 0.9 (0.6 to 1.3) | 43.4* (33.1 to 53.7) | 5.4 (-2.2 to 13.0) |

| | | | | | |
|---|---|---|---|---|---|
| Terminal phase of liver cancer due to alcohol use | 0.9 (0.7 to 1.0) | -- | 0.5 (0.3 to 0.6) | 43.2* (37.0 to 50.2) | 5.3* (0.7 to 10.4) |
| Liver cancer due to NASH | 6.3 (5.1 to 7.6) | 7.4 (6.1 to 9.0) | 1.6 (1.1 to 2.2) | 61.2* (54.4 to 70.7) | 18.5* (13.5 to 25.5) |
| Diagnosis and primary therapy phase of liver cancer due to NASH | 1.6 (1.3 to 2.0) | 7.4 (6.1 to 9.0) | 0.5 (0.3 to 0.7) | 59.7* (53.0 to 68.1) | 17.4* (12.5 to 23.5) |
| Controlled phase of liver cancer due to NASH | 2.5 (2.0 to 3.1) | -- | 0.1 (0.1 to 0.2) | 174.5* (138.1 to 219.7) | 101.8* (75.0 to 135.0) |
| Metastatic phase of liver cancer due to NASH | 1.5 (1.3 to 1.8) | -- | 0.7 (0.4 to 1.0) | 54.8* (48.6 to 63.2) | 13.8* (9.3 to 20.0) |
| Terminal phase of liver cancer due to NASH | 0.6 (0.5 to 0.7) | -- | 0.3 (0.2 to 0.5) | 54.7* (48.6 to 63.0) | 13.7* (9.2 to 19.8) |
| Liver cancer due to other causes | 3.8 (3.1 to 4.6) | 4.5 (3.6 to 5.4) | 1.0 (0.6 to 1.4) | 56.7* (48.2 to 67.1) | 15.2* (9.0 to 22.8) |
| Diagnosis and primary therapy phase of liver cancer due to other causes | 1.0 (0.8 to 1.2) | 4.5 (3.6 to 5.4) | 0.3 (0.2 to 0.4) | 54.5* (46.5 to 64.5) | 13.6* (7.7 to 20.9) |
| Controlled phase of liver cancer due to other causes | 1.5 (1.2 to 1.9) | -- | 0.1 (0.0 to 0.1) | 209.8* (169.2 to 261.7) | 127.8* (97.9 to 165.9) |
| Metastatic phase of liver cancer due to other causes | 0.9 (0.7 to 1.1) | -- | 0.4 (0.3 to 0.6) | 48.8* (41.2 to 58.4) | 9.4* (3.8 to 16.4) |
| Terminal phase of liver cancer due to other causes | 0.4 (0.3 to 0.4) | -- | 0.2 (0.1 to 0.3) | 48.8* (41.2 to 58.4) | 9.4* (3.8 to 16.4) |
| Gallbladder and biliary tract cancer | 25.4 (22.0 to 29.1) | 26.1 (23.0 to 29.2) | 5.7 (3.9 to 7.6) | 40.1* (25.9 to 58.1) | 3.0 (-7.4 to 16.2) |
| Diagnosis and primary therapy phase of gallbladder and biliary tract cancer | 4.0 (3.5 to 4.5) | 26.1 (23.0 to 29.2) | 1.1 (0.7 to 1.5) | 41.7* (25.6 to 60.2) | 4.2 (-7.7 to 17.7) |
| Controlled phase of gallbladder and biliary tract cancer | 11.9 (10.0 to 14.1) | -- | 0.5 (0.3 to 0.8) | 94.9* (57.6 to 136.4) | 43.3* (15.8 to 73.8) |
| Metastatic phase of gallbladder and biliary tract cancer | 7.3 (6.5 to 8.2) | -- | 3.0 (1.9 to 4.0) | 34.9* (18.3 to 57.3) | -0.8 (-13.0 to 15.6) |
| Terminal phase of gallbladder and biliary tract cancer | 2.1 (1.9 to 2.4) | -- | 1.1 (0.7 to 1.5) | 34.4* (22.2 to 48.8) | -1.2 (-10.2 to 9.4) |
| Pancreatic cancer | 39.9 (38.4 to 41.4) | 50.7 (49.1 to 52.5) | 9.7 (6.8 to 12.5) | 45.8* (36.2 to 55.9) | 7.2* (0.2 to 14.6) |
| Diagnosis and primary therapy phase of pancreatic cancer | 11.1 (10.7 to 11.5) | 50.7 (49.1 to 52.5) | 2.8 (1.9 to 3.7) | 45.8* (30.8 to 62.3) | 7.2 (-3.9 to 19.3) |
| Controlled phase of pancreatic cancer | 13.9 (13.2 to 14.7) | -- | 0.6 (0.4 to 0.9) | 117.0* (96.8 to 139.9) | 59.5* (44.6 to 76.3) |
| Metastatic phase of pancreatic cancer | 10.6 (10.3 to 11.0) | -- | 4.1 (2.8 to 5.3) | 42.0* (26.9 to 57.3) | 4.4 (-6.7 to 15.7) |
| Terminal phase of pancreatic cancer | 4.2 (4.0 to 4.3) | -- | 2.2 (1.5 to 2.8) | 39.7* (31.9 to 49.8) | 2.7 (-3.0 to 10.1) |
| Larynx cancer | 38.9 (37.9 to 40.0) | 9.6 (9.3 to 9.9) | 4.4 (3.2 to 5.6) | 37.2* (30.0 to 45.4) | 0.8 (-4.5 to 6.9) |
| Diagnosis and primary therapy phase of larynx cancer | 2.2 (2.2 to 2.3) | 9.6 (9.3 to 9.9) | 0.6 (0.4 to 0.9) | 39.1* (29.0 to 48.6) | 2.2 (-5.2 to 9.2) |
| Controlled phase of larynx cancer | 24.8 (24.0 to 25.7) | -- | 1.3 (0.8 to 1.8) | 40.1* (31.2 to 49.7) | 3.0 (-3.6 to 10.0) |
| Metastatic phase of larynx cancer | 4.4 (4.3 to 4.5) | -- | 1.8 (1.2 to 2.3) | 36.2* (21.3 to 52.7) | 0.1 (-10.8 to 12.3) |
| Terminal phase of larynx cancer | 0.7 (0.6 to 0.7) | -- | 0.4 (0.2 to 0.5) | 34.6* (30.3 to 38.9) | -1.1 (-4.3 to 2.1) |
| Laryngectomy from larynx cancer, beyond 10 years | 6.8 (6.5 to 7.1) | -- | 0.3 (0.2 to 0.5) | 30.0* (17.2 to 45.0) | -4.4 (-13.8 to 6.6) |
| Tracheal, bronchus, and lung cancer | 219.5 (213.5 to 225.2) | 197.4 (191.6 to 202.9) | 42.4 (30.7 to 52.8) | 43.9* (37.2 to 50.2) | 5.8* (0.9 to 10.4) |
| Diagnosis and primary therapy phase of lung, bronchus, and trachea cancer | 15.8 (15.3 to 16.2) | 197.4 (191.6 to 202.9) | 3.8 (2.6 to 5.2) | 48.7* (37.0 to 62.4) | 9.3* (0.7 to 19.4) |
| Controlled phase of lung, bronchus, and trachea cancer | 115.6 (112.5 to 118.5) | -- | 5.0 (3.2 to 7.2) | 61.6* (54.0 to 69.4) | 18.8* (13.2 to 24.5) |
| Metastatic phase of lung, bronchus, and trachea cancer | 72.2 (70.0 to 74.2) | -- | 26.4 (18.5 to 33.6) | 41.1* (33.8 to 48.2) | 3.8 (-1.6 to 8.9) |
| Terminal phase of lung, bronchus, and trachea cancer | 16.0 (15.5 to 16.5) | -- | 7.1 (5.1 to 8.9) | 40.6* (28.7 to 54.0) | 3.3 (-5.4 to 13.2) |
| Malignant skin melanoma | 104.8 (65.4 to 116.7) | 18.2 (11.7 to 20.1) | 7.4 (4.2 to 10.3) | 37.8* (24.2 to 48.5) | 1.3 (-8.7 to 9.2) |
| Diagnosis and primary therapy phase of malignant skin melanoma | 3.2 (2.0 to 3.6) | 18.2 (11.7 to 20.1) | 0.8 (0.4 to 1.2) | 40.7* (22.8 to 60.1) | 3.4 (-9.7 to 17.7) |
| Controlled phase of malignant skin melanoma | 95.7 (59.6 to 106.6) | -- | 4.1 (2.3 to 6.2) | 44.9* (30.6 to 56.6) | 6.5 (-4.0 to 15.1) |
| Metastatic phase of malignant skin melanoma | 5.1 (3.4 to 5.7) | -- | 2.0 (1.1 to 2.8) | 27.0* (9.8 to 45.5) | -6.7 (-19.3 to 7.0) |
| Terminal phase of malignant skin melanoma | 0.8 (0.6 to 0.9) | -- | 0.4 (0.3 to 0.6) | 24.6* (13.4 to 34.6) | -8.4* (-16.6 to -1.1) |
| Non-melanoma skin cancer | 297.2 (169.1 to 473.5) | 727.4 (422.7 to 1126.8) | 10.1 (4.7 to 18.6) | 15.7* (6.3 to 25.5) | -14.9* (-21.8 to -7.8) |
| Non-melanoma skin cancer (squamous-cell carcinoma) | 253.9 (131.1 to 429.9) | 212.0 (116.0 to 352.1) | 9.9 (4.5 to 18.3) | 15.7* (6.1 to 25.6) | -15.0* (-22.0 to -7.7) |
| Non-melanoma skin cancer (basal-cell carcinoma) | 53.1 (22.3 to 110.5) | 515.4 (242.3 to 910.2) | 0.2 (0.1 to 0.5) | 18.1* (5.5 to 27.8) | -13.2* (-22.4 to -6.1) |
| Breast cancer | 619.0 (599.6 to 640.6) | 77.0 (74.0 to 80.2) | 49.7 (37.1 to 64.6) | 22.4* (17.6 to 27.3) | -10.0* (-13.6 to -6.4) |
| Diagnosis and primary therapy phase of breast cancer | 12.8 (12.3 to 13.3) | 77.0 (74.0 to 80.2) | 3.1 (2.1 to 4.2) | 26.1* (11.3 to 42.1) | -7.3 (-18.2 to 4.5) |
| Controlled phase of breast cancer | 363.1 (348.9 to 378.6) | -- | 22.8 (14.9 to 32.7) | 24.5* (18.9 to 30.4) | -8.5* (-12.6 to -4.2) |
| Metastatic phase of breast cancer | 43.6 (41.9 to 45.3) | -- | 15.9 (11.3 to 20.4) | 20.6* (12.8 to 29.2) | -11.4* (-17.1 to -5.0) |
| Terminal phase of breast cancer | 3.8 (3.7 to 3.9) | -- | 1.8 (1.3 to 2.4) | 24.6* (6.5 to 46.8) | -8.4 (-21.7 to 7.9) |
| Mastectomy from breast cancer, beyond 10 years | 195.7 (184.3 to 208.6) | -- | 6.2 (3.7 to 9.6) | 17.2* (13.0 to 21.3) | -13.8* (-16.9 to -10.9) |
| Cervical cancer | 45.2 (42.9 to 47.0) | 11.8 (11.2 to 12.2) | 4.7 (3.4 to 6.1) | 21.8* (15.3 to 28.5) | -10.5* (-15.3 to -5.5) |
| Diagnosis and primary therapy phase of cervical cancer | 2.6 (2.5 to 2.7) | 11.8 (11.2 to 12.2) | 0.7 (0.5 to 1.0) | 23.2* (15.4 to 31.3) | -9.4* (-15.2 to -3.5) |
| Controlled phase of cervical cancer | 36.8 (35.0 to 38.2) | -- | 1.6 (1.0 to 2.3) | 24.0* (16.1 to 32.3) | -8.8* (-14.6 to -2.8) |
| Metastatic phase of cervical cancer | 5.0 (4.7 to 5.2) | -- | 2.0 (1.4 to 2.6) | 19.9* (8.9 to 31.9) | -11.8* (-19.9 to -3.1) |
| Terminal phase of cervical cancer | 0.7 (0.7 to 0.8) | -- | 0.4 (0.3 to 0.5) | 19.9* (15.6 to 24.0) | -11.8* (-15.0 to -8.8) |
| Uterine cancer | 97.7 (93.5 to 102.7) | 16.6 (15.9 to 17.4) | 7.9 (5.8 to 10.2) | 20.7* (11.5 to 29.8) | -11.3* (-18.0 to -4.6) |
| Diagnosis and primary therapy phase of uterine cancer | 5.0 (4.7 to 5.2) | 16.6 (15.9 to 17.4) | 1.2 (0.8 to 1.7) | 22.2* (1.1 to 46.6) | -10.2 (-25.7 to 7.8) |
| Controlled phase of uterine cancer | 85.2 (81.5 to 89.6) | -- | 3.7 (2.4 to 5.2) | 24.2* (16.4 to 33.7) | -8.7* (-14.4 to -1.8) |
| Metastatic phase of uterine cancer | 6.8 (6.5 to 7.1) | -- | 2.6 (1.8 to 3.4) | 16.2* (0.2 to 33.0) | -14.6* (-26.3 to -2.2) |
| Terminal phase of uterine cancer | 0.8 (0.7 to 0.8) | -- | 0.4 (0.3 to 0.5) | 15.4* (10.7 to 20.9) | -15.2* (-18.7 to -11.1) |
| Ovarian cancer | 37.9 (36.5 to 39.3) | 12.7 (12.3 to 13.2) | 6.0 (4.4 to 7.6) | 16.9* (6.2 to 28.5) | -14.1* (-22.0 to -5.5) |
| Diagnosis and primary therapy phase of ovarian cancer | 1.4 (1.3 to 1.4) | 12.7 (12.3 to 13.2) | 0.4 (0.3 to 0.5) | 18.1* (13.7 to 22.8) | -13.2* (-16.5 to -9.7) |
| Controlled phase of ovarian cancer | 24.6 (23.7 to 25.5) | -- | 1.1 (0.7 to 1.5) | 19.3* (8.2 to 32.8) | -12.3* (-20.5 to -2.4) |
| Metastatic phase of ovarian cancer | 11.1 (10.7 to 11.5) | -- | 4.1 (2.8 to 5.3) | 16.2* (1.4 to 32.1) | -14.6* (-25.4 to -2.9) |

| | | | | | |
|---|---|---|---|---|---|
| Terminal phase of ovarian cancer | 0.9 (0.9 to 0.9) | -- | 0.5 (0.3 to 0.6) | 16.6* (12.4 to 21.2) | -14.3* (-17.3 to -10.9) |
| Prostate cancer | 605.8 (535.6 to 752.1) | 116.2 (99.2 to 144.3) | 63.4 (47.2 to 82.8) | 36.9* (30.6 to 48.2) | 0.7 (-4.0 to 8.9) |
| Diagnosis and primary therapy phase of prostate cancer | 19.0 (16.4 to 24.4) | 116.2 (99.2 to 144.3) | 4.5 (3.0 to 6.3) | 44.5* (32.0 to 59.9) | 6.2 (-3.0 to 17.6) |
| Controlled phase of prostate cancer | 403.2 (348.9 to 524.0) | -- | 20.6 (13.3 to 29.6) | 47.5* (39.6 to 60.9) | 8.4* (2.6 to 18.3) |
| Metastatic phase of prostate cancer | 87.2 (74.2 to 108.0) | -- | 31.7 (21.8 to 42.0) | 31.6* (24.3 to 42.8) | -3.2 (-8.6 to 5.0) |
| Terminal phase of prostate cancer | 6.6 (5.6 to 8.0) | -- | 2.9 (2.0 to 3.7) | 29.7* (14.8 to 49.5) | -4.7 (-15.6 to 9.9) |
| Impotence and Incontinence after 10-year survival from prostate cancer | 89.8 (87.0 to 92.4) | -- | 3.7 (2.6 to 5.2) | 27.8* (21.3 to 34.7) | -6.0* (-10.8 to -1.0) |
| Testicular cancer | 2.5 (2.3 to 2.6) | 0.6 (0.5 to 0.6) | 0.3 (0.2 to 0.4) | 67.3* (53.8 to 81.8) | 23.0* (13.0 to 33.6) |
| Diagnosis and primary therapy phase of testicular cancer | 0.1 (0.1 to 0.1) | 0.6 (0.5 to 0.6) | -- | 72.9* (60.3 to 86.7) | 27.1* (17.8 to 37.3) |
| Controlled phase of testicular cancer | 2.0 (1.9 to 2.1) | -- | 0.1 (0.1 to 0.1) | 75.7* (55.0 to 98.7) | 29.2* (14.0 to 46.1) |
| Metastatic phase of testicular cancer | 0.3 (0.3 to 0.4) | -- | 0.2 (0.1 to 0.2) | 62.0* (49.9 to 74.8) | 19.1* (10.2 to 28.5) |
| Terminal phase of testicular cancer | -- | -- | -- | 64.6* (52.4 to 77.2) | 21.0* (12.1 to 30.2) |
| Kidney cancer | 55.9 (49.3 to 58.5) | 19.7 (17.5 to 20.5) | 5.5 (4.0 to 7.0) | 25.0* (15.9 to 34.6) | -8.1* (-14.8 to -1.1) |
| Diagnosis and primary therapy phase of kidney cancer | -- | 19.7 (17.5 to 20.5) | -- | -- | -- |
| Controlled phase of kidney cancer | 47.7 (42.0 to 49.9) | -- | 2.1 (1.3 to 3.0) | 24.0* (16.2 to 32.3) | -8.9* (-14.6 to -2.8) |
| Metastatic phase of kidney cancer | 6.9 (6.1 to 7.2) | -- | 2.7 (1.8 to 3.5) | 25.9* (10.4 to 43.8) | -7.5 (-18.8 to 5.7) |
| Terminal phase of kidney cancer | 1.3 (1.2 to 1.4) | -- | 0.7 (0.5 to 0.9) | 24.4* (19.6 to 29.6) | -8.5* (-12.1 to -4.7) |
| Bladder cancer | 179.4 (173.6 to 186.5) | 48.0 (46.2 to 50.2) | 19.9 (14.9 to 25.4) | 30.1* (24.4 to 36.6) | -4.4 (-8.6 to 0.4) |
| Diagnosis and primary therapy phase of bladder cancer | 9.9 (9.5 to 10.3) | 48.0 (46.2 to 50.2) | 2.5 (1.7 to 3.4) | 30.3* (17.0 to 46.0) | -4.2 (-14.0 to 7.3) |
| Controlled phase of bladder cancer | 138.1 (133.2 to 143.8) | -- | 8.2 (5.6 to 11.2) | 33.4* (26.6 to 40.7) | -2.0 (-6.9 to 3.4) |
| Metastatic phase of bladder cancer | 16.5 (15.9 to 17.4) | -- | 6.2 (4.3 to 8.0) | 26.3* (14.6 to 39.7) | -7.1 (-15.7 to 2.7) |
| Terminal phase of bladder cancer | 3.1 (3.0 to 3.3) | -- | 1.6 (1.1 to 2.0) | 27.0* (10.9 to 44.8) | -6.7 (-18.5 to 6.5) |
| Incontinence from bladder cancer, beyond 10 years | 11.8 (11.2 to 12.4) | -- | 1.4 (1.0 to 2.0) | 32.0* (18.3 to 46.8) | -3.0 (-13.1 to 7.9) |
| Brain and nervous system cancer | 38.8 (25.0 to 44.9) | 14.7 (10.1 to 16.8) | 5.0 (3.0 to 6.9) | 61.4* (44.0 to 78.2) | 18.6* (5.8 to 31.0) |
| Diagnosis and primary therapy phase of brain and nervous system cancers | 2.6 (1.7 to 3.0) | 14.7 (10.1 to 16.8) | 0.7 (0.4 to 1.1) | 68.2* (45.4 to 87.6) | 23.7* (6.9 to 37.9) |
| Controlled phase of brain and nervous system cancers | 29.2 (18.5 to 34.0) | -- | 1.3 (0.7 to 2.0) | 76.4* (49.2 to 100.9) | 29.7* (9.7 to 47.7) |
| Metastatic phase of brain and nervous system cancers | 5.9 (4.1 to 6.7) | -- | 2.5 (1.5 to 3.5) | 53.9* (36.5 to 72.3) | 13.1* (0.4 to 26.7) |
| Terminal phase of brain and nervous system cancers | 1.1 (0.7 to 1.2) | -- | 0.6 (0.4 to 0.8) | 56.6* (43.3 to 68.9) | 15.1* (5.3 to 24.2) |
| Thyroid cancer | 37.2 (35.8 to 39.0) | 6.2 (5.9 to 6.5) | 3.5 (2.5 to 4.5) | 38.9* (32.8 to 45.2) | 2.1 (-2.4 to 6.8) |
| Diagnosis and primary therapy phase of thyroid cancer | 1.1 (1.1 to 1.2) | 6.2 (5.9 to 6.5) | 0.3 (0.2 to 0.4) | 41.6* (36.4 to 47.0) | 4.1* (0.3 to 8.1) |
| Controlled phase of thyroid cancer | 32.2 (31.0 to 33.8) | -- | 1.4 (0.9 to 2.0) | 43.7* (34.0 to 55.2) | 5.6 (-1.5 to 14.1) |
| Metastatic phase of thyroid cancer | 3.6 (3.5 to 3.8) | -- | 1.6 (1.1 to 2.1) | 34.8* (28.6 to 41.4) | -0.9 (-5.5 to 3.9) |
| Terminal phase of thyroid cancer | 0.3 (0.3 to 0.3) | -- | 0.1 (0.1 to 0.2) | 36.2* (31.4 to 41.3) | 0.1 (-3.4 to 3.8) |
| Mesothelioma | 6.8 (6.5 to 7.2) | 3.2 (3.0 to 3.4) | 1.3 (0.9 to 1.7) | 34.8* (20.6 to 50.6) | -0.9 (-11.3 to 10.7) |
| Diagnosis and primary therapy phase of mesothelioma | 0.8 (0.8 to 0.9) | 3.2 (3.0 to 3.4) | 0.2 (0.2 to 0.3) | 37.4* (23.8 to 51.6) | 1.0 (-9.0 to 11.4) |
| Controlled phase of mesothelioma | 3.9 (3.7 to 4.1) | -- | 0.2 (0.1 to 0.3) | 38.2* (17.6 to 61.5) | 1.6 (-13.5 to 18.7) |
| Metastatic phase of mesothelioma | 1.8 (1.8 to 2.0) | -- | 0.8 (0.5 to 1.0) | 33.6* (12.7 to 57.9) | -1.8 (-17.1 to 16.1) |
| Terminal phase of mesothelioma | 0.3 (0.2 to 0.3) | -- | 0.1 (0.1 to 0.2) | 33.6* (26.2 to 41.4) | -1.8 (-7.3 to 4.0) |
| Hodgkin lymphoma | 4.5 (3.5 to 4.9) | 1.6 (1.2 to 1.7) | 0.7 (0.5 to 0.9) | 27.6* (18.6 to 35.7) | -6.2* (-12.8 to -0.2) |
| Diagnosis and primary therapy phase of Hodgkin disease | 0.2 (0.1 to 0.2) | 1.6 (1.2 to 1.7) | 0.0 (0.0 to 0.1) | 37.1* (26.1 to 45.7) | 0.8 (-7.3 to 7.1) |
| Controlled phase of Hodgkin disease | 3.3 (2.6 to 3.6) | -- | 0.2 (0.1 to 0.2) | 40.2* (27.0 to 53.0) | 3.1 (-6.7 to 12.5) |
| Metastatic phase of Hodgkin disease | 0.9 (0.7 to 1.0) | -- | 0.4 (0.3 to 0.6) | 23.7* (15.3 to 31.1) | -9.0* (-15.2 to -3.6) |
| Terminal phase of Hodgkin disease | 0.1 (0.1 to 0.1) | -- | 0.1 (0.0 to 0.1) | 19.3* (12.7 to 26.1) | -12.3* (-17.2 to -7.3) |
| Non-Hodgkin's lymphoma | 111.8 (108.1 to 115.8) | 33.6 (32.5 to 34.7) | 11.1 (8.3 to 14.1) | 37.6* (29.3 to 45.6) | 1.2 (-4.9 to 7.0) |
| Diagnosis and primary therapy phase of non-Hodgkin lymphoma | 4.5 (4.3 to 4.6) | 33.6 (32.5 to 34.7) | 1.1 (0.8 to 1.6) | 40.6* (25.8 to 57.4) | 3.4 (-7.5 to 15.7) |
| Controlled phase of non-Hodgkin lymphoma | 92.6 (89.4 to 95.9) | -- | 4.0 (2.6 to 5.7) | 46.0* (37.8 to 54.8) | 7.3* (1.3 to 13.8) |
| Metastatic phase of non-Hodgkin lymphoma | 12.6 (12.2 to 13.0) | -- | 4.8 (3.3 to 6.2) | 32.2* (19.7 to 45.1) | -2.9 (-12.0 to 6.7) |
| Terminal phase of non-Hodgkin lymphoma | 2.2 (2.1 to 2.3) | -- | 1.2 (0.8 to 1.5) | 31.5* (26.5 to 36.8) | -3.3 (-7.0 to 0.5) |
| Multiple myeloma | 19.2 (16.0 to 21.0) | 14.1 (12.0 to 15.3) | 4.9 (3.4 to 6.4) | 39.9* (27.8 to 53.8) | 2.8 (-6.0 to 13.1) |
| Diagnosis and primary therapy phase of multiple myeloma | 0.9 (0.8 to 1.0) | 14.1 (12.0 to 15.3) | 0.3 (0.2 to 0.4) | 53.7* (44.1 to 63.5) | 13.0* (5.9 to 20.2) |
| Controlled phase of multiple myeloma | 7.4 (6.0 to 8.1) | -- | 0.3 (0.2 to 0.5) | 58.3* (39.8 to 80.2) | 16.4* (2.8 to 32.4) |
| Metastatic phase of multiple myeloma | 9.8 (8.2 to 10.7) | -- | 3.7 (2.5 to 4.9) | 39.2* (24.6 to 57.0) | 2.3 (-8.4 to 15.4) |
| Terminal phase of multiple myeloma | 1.1 (1.0 to 1.2) | -- | 0.6 (0.4 to 0.8) | 30.7* (24.7 to 36.6) | -3.9 (-8.3 to 0.4) |
| Leukaemia | 92.8 (86.5 to 98.9) | 31.8 (29.5 to 33.6) | 15.2 (11.2 to 19.4) | 28.1* (18.8 to 37.3) | -5.8 (-12.7 to 1.0) |
| Acute lymphoid leukaemia | 4.7 (4.1 to 5.1) | 1.2 (1.0 to 1.3) | 0.6 (0.5 to 0.9) | 61.1* (46.3 to 73.8) | 18.4* (7.5 to 27.7) |
| Diagnosis and primary therapy phase of acute lymphoid leukemia | 0.5 (0.4 to 0.5) | 1.2 (1.0 to 1.3) | 0.1 (0.1 to 0.2) | 48.2* (37.5 to 56.7) | 9.0* (1.1 to 15.2) |
| Controlled phase of acute lymphoid leukemia | 3.5 (3.1 to 3.8) | -- | 0.2 (0.1 to 0.3) | 70.9* (52.2 to 88.2) | 25.6* (11.9 to 38.3) |
| Metastatic phase of acute lymphoid leukemia | 0.6 (0.5 to 0.6) | -- | 0.3 (0.2 to 0.4) | 62.6* (46.6 to 77.1) | 19.6* (7.7 to 30.2) |
| Terminal phase of acute lymphoid leukemia | 0.1 (0.1 to 0.1) | -- | 0.1 (0.0 to 0.1) | 62.7* (46.5 to 77.2) | 19.6* (7.7 to 30.3) |
| Chronic lymphoid leukaemia | 41.5 (38.0 to 45.1) | 8.4 (7.7 to 9.1) | 6.3 (4.7 to 8.1) | 23.8* (10.0 to 39.5) | -9.0 (-19.1 to 2.6) |

| | | | | | |
|---|---|---|---|---|---|
| Diagnosis and primary therapy phase of chronic lymphoid leukemia | 2.2 (2.0 to 2.4) | 8.4 (7.7 to 9.1) | 0.6 (0.4 to 0.9) | 27.1* (10.1 to 47.2) | -6.6 (-19.1 to 8.2) |
| Controlled phase of chronic lymphoid leukemia | 27.5 (25.1 to 30.2) | -- | 1.2 (0.8 to 1.7) | 28.0* (13.6 to 45.0) | -5.9 (-16.5 to 6.6) |
| Metastatic phase of chronic lymphoid leukemia | 11.2 (10.3 to 12.2) | -- | 4.3 (3.0 to 5.6) | 22.3* (5.9 to 41.5) | -10.1 (-22.1 to 4.1) |
| Terminal phase of chronic lymphoid leukemia | 0.5 (0.4 to 0.5) | -- | 0.3 (0.2 to 0.3) | 21.8* (12.5 to 31.4) | -10.5* (-17.3 to -3.4) |
| Acute myeloid leukaemia | 7.6 (6.8 to 8.3) | 7.9 (7.1 to 8.6) | 1.8 (1.2 to 2.4) | 38.4* (25.3 to 54.0) | 1.7 (-7.9 to 13.2) |
| Diagnosis and primary therapy phase of acute myeloid leukemia | -- | 7.9 (7.1 to 8.6) | -- | -- | -- |
| Controlled phase of acute myeloid leukemia | 4.1 (3.6 to 4.4) | -- | 0.2 (0.1 to 0.3) | 39.4* (19.1 to 62.0) | 2.5 (-12.5 to 19.1) |
| Metastatic phase of acute myeloid leukemia | 2.9 (2.6 to 3.2) | -- | 1.3 (0.9 to 1.7) | 38.1* (22.8 to 58.0) | 1.5 (-9.8 to 16.1) |
| Terminal phase of acute myeloid leukemia | 0.6 (0.6 to 0.7) | -- | 0.3 (0.2 to 0.4) | 38.9* (29.6 to 49.2) | 2.1 (-4.7 to 9.7) |
| Chronic myeloid leukaemia | 5.1 (4.7 to 5.6) | 2.6 (2.4 to 2.8) | 0.8 (0.6 to 1.0) | 10.9* (1.4 to 22.9) | -18.5* (-25.4 to -9.6) |
| Diagnosis and primary therapy phase of chronic myeloid leukemia | 0.4 (0.4 to 0.4) | 2.6 (2.4 to 2.8) | 0.1 (0.1 to 0.2) | 10.6* (1.9 to 20.5) | -18.7* (-25.1 to -11.4) |
| Controlled phase of chronic myeloid leukemia | 3.6 (3.3 to 4.0) | -- | 0.2 (0.1 to 0.3) | 14.3 (-5.1 to 38.6) | -16.0 (-30.2 to 1.9) |
| Metastatic phase of chronic myeloid leukemia | 0.9 (0.8 to 1.0) | -- | 0.4 (0.3 to 0.5) | 10.0* (1.6 to 19.6) | -19.2* (-25.3 to -12.1) |
| Terminal phase of chronic myeloid leukemia | 0.2 (0.2 to 0.2) | -- | 0.1 (0.1 to 0.1) | 10.0* (1.6 to 19.6) | -19.2* (-25.3 to -12.1) |
| Other leukaemia | 33.9 (31.2 to 36.4) | 11.6 (10.7 to 12.6) | 5.6 (4.1 to 7.2) | 29.9* (17.5 to 42.8) | -4.5 (-13.7 to 5.0) |
| Metastatic phase of other leukemia | 11.2 (10.3 to 12.1) | -- | 4.3 (3.0 to 5.5) | 28.3* (13.6 to 43.5) | -5.7 (-16.5 to 5.5) |
| Diagnosis and primary therapy phase of other leukemia | -- | 11.6 (10.7 to 12.6) | -- | -- | -- |
| Terminal phase of other leukemia | 0.8 (0.7 to 0.9) | -- | 0.4 (0.3 to 0.5) | 30.2* (22.9 to 37.9) | -4.3 (-9.7 to 1.4) |
| Controlled phase of other leukemia | 21.9 (20.2 to 23.5) | -- | 1.0 (0.6 to 1.4) | 37.3* (24.0 to 52.1) | 0.9 (-8.9 to 11.8) |
| Other malignant cancers | 272.2 (202.3 to 413.8) | 41.3 (35.5 to 44.1) | 26.4 (18.0 to 39.0) | 122.9* (66.0 to 191.0) | 63.8* (22.0 to 113.9) |
| Diagnosis and primary therapy phase of other malignant neoplasms | 7.5 (5.6 to 11.2) | 41.3 (35.5 to 44.1) | 1.9 (1.2 to 3.0) | 145.7* (77.7 to 237.8) | 80.6* (30.6 to 148.3) |
| Controlled phase of other malignant neoplasms | 227.2 (166.9 to 351.4) | -- | 9.9 (5.7 to 16.4) | 189.8* (95.5 to 299.9) | 113.1* (43.7 to 194.0) |
| Metastatic phase of other malignant neoplasms | 33.0 (26.2 to 44.6) | -- | 12.1 (7.8 to 17.5) | 93.5* (50.2 to 144.7) | 42.3* (10.4 to 79.9) |
| Terminal phase of other malignant neoplasms | 5.0 (4.0 to 6.8) | -- | 2.5 (1.7 to 3.5) | 78.1* (42.7 to 119.1) | 30.9* (4.9 to 61.0) |
| Other neoplasms | 1108.4 (1055.8 to 1170.1) | 411.0 (386.3 to 437.5) | 4.4 (2.8 to 6.6) | 35.6* (30.3 to 41.2) | -0.4 (-4.2 to 3.8) |
| Myelodysplastic, myeloproliferative, and other hematopoietic neoplasms | 102.9 (84.5 to 125.7) | 11.0 (7.9 to 14.8) | 4.4 (2.8 to 6.6) | 35.6* (30.3 to 41.2) | -0.4 (-4.2 to 3.8) |
| Benign and in situ intestinal neoplasms | 201.3 (188.7 to 213.6) | 34.8 (32.1 to 37.8) | -- | -- | -- |
| Benign and in situ cervical and uterine neoplasms | 3.6 (3.3 to 3.9) | 0.5 (0.4 to 0.6) | -- | -- | -- |
| Other benign and in situ neoplasms | 815.8 (766.1 to 874.4) | 364.8 (340.4 to 390.5) | -- | -- | -- |
| **Cardiovascular diseases** | **35554.5 (33480.7 to 37758.1)** | **7098.0 (6645.2 to 7612.5)** | **3031.1 (2237.1 to 3867.1)** | **43.8* (39.6 to 48.4)** | **5.7* (2.6 to 9.1)** |
| Rheumatic heart disease | 134.5 (127.2 to 142.1) | 9.6 (8.2 to 11.1) | 8.5 (5.9 to 11.4) | 49.5* (44.4 to 54.7) | 9.9* (6.1 to 13.7) |
| Rheumatic heart disease, without heart failure | 62.2 (44.6 to 80.5) | 9.6 (8.2 to 11.1) | 2.7 (1.6 to 4.2) | 49.0* (42.4 to 56.2) | 9.6* (4.7 to 14.9) |
| Heart failure due to rheumatic heart disease | 72.3 (55.5 to 89.5) | -- | 5.8 (3.8 to 8.2) | 49.6* (42.5 to 57.0) | 10.0* (4.7 to 15.4) |
| Ischaemic heart disease | 11785.0 (10332.3 to 13286.1) | 1225.5 (1015.1 to 1478.7) | 464.0 (321.6 to 631.4) | 29.6* (28.0 to 31.2) | -4.7* (-5.9 to -3.5) |
| Asymptomatic ischemic heart disease following myocardial infarction | 4137.2 (2900.5 to 5448.3) | -- | -- | -- | -- |
| Angina due to ischemic heart disease | 5098.7 (4549.8 to 5704.4) | -- | 261.4 (179.9 to 363.5) | 35.4* (34.2 to 36.8) | -0.5 (-1.4 to 0.5) |
| Heart failure due to ischemic heart disease | 2487.0 (1879.7 to 3221.0) | -- | 197.0 (126.3 to 291.1) | 23.2* (19.4 to 26.5) | -9.4* (-12.2 to -7.0) |
| Myocardial infarction due to ischemic heart disease | 62.2 (49.9 to 77.2) | 1225.5 (1015.1 to 1478.7) | 5.6 (3.8 to 7.9) | 10.7* (3.5 to 18.5) | -18.6* (-23.9 to -12.9) |
| Stroke | 7683.0 (6976.8 to 8439.0) | 1122.4‡ (985.9 to 1276.4) | 1607.6 (1156.3 to 2031.0) | 57.2* (48.7 to 65.4) | 15.5* (9.3 to 21.6) |
| Ischaemic stroke | 7481.8 (6710.0 to 8269.2) | 808.6 (671.4 to 959.1) | 1485.5 (1067.3 to 1885.1) | 61.2* (52.0 to 70.0) | 18.5* (11.7 to 25.0) |
| Acute ischemic stroke | 58.8 (48.6 to 70.3) | 808.6 (671.4 to 959.1) | 13.6 (9.2 to 18.4) | 47.6* (30.4 to 64.1) | 8.5 (-4.1 to 20.6) |
| Chronic ischemic stroke | 7423.0 (6649.4 to 8210.7) | -- | 1471.9 (1058.8 to 1865.7) | 61.4* (52.1 to 70.3) | 18.6* (11.8 to 25.2) |
| Intracerebral hemorrhage | 416.7 (335.1 to 510.9) | 264.4 (227.8 to 308.7) | 84.1 (59.2 to 113.0) | 16.7* (3.6 to 30.0) | -14.2* (-23.9 to -4.4) |
| Acute intracerebral hemorrhage | 12.8 (10.7 to 15.2) | 264.4 (227.8 to 308.7) | 3.6 (2.4 to 4.9) | 1.1 (-8.3 to 10.7) | -25.7* (-32.6 to -18.6) |
| Chronic intracerebral hemorrhage | 403.9 (321.4 to 497.3) | -- | 80.5 (56.1 to 108.6) | 17.5* (3.5 to 31.2) | -13.6* (-23.9 to -3.6) |
| Subarachnoid hemorrhage | 195.3 (155.6 to 240.2) | 49.4 (40.2 to 62.5) | 38.0 (26.9 to 51.0) | 29.2* (21.7 to 37.0) | -5.0 (-10.6 to 0.7) |
| Acute subarachnoid hemorrhage | 3.7 (2.9 to 4.7) | 49.4 (40.2 to 62.5) | 1.0 (0.7 to 1.5) | 35.0* (28.6 to 39.3) | -0.7 (-5.4 to 2.4) |
| Chronic subarachnoid hemorrhage | 191.6 (152.2 to 236.2) | -- | 37.0 (26.1 to 49.6) | 29.1* (21.3 to 37.0) | -5.1 (-10.8 to 0.7) |
| Hypertensive heart disease | 2091.1 (1541.1 to 2764.4) | -- | 165.9 (103.0 to 246.1) | 38.4* (35.5 to 41.0) | 1.7 (-0.4 to 3.7) |
| Non-rheumatic valvular heart disease | 3514.9 (3250.4 to 3800.0) | 3607.8 (3333.4 to 3911.0) | 67.5 (39.0 to 105.3) | 32.1* (26.7 to 36.9) | -2.9 (-6.9 to 0.6) |
| Non-rheumatic calcific aortic valve disease | 1935.9 (1667.0 to 2229.1) | 1935.9 (1667.0 to 2229.1) | 35.1 (20.1 to 55.5) | 32.5* (23.8 to 40.7) | -2.6 (-9.0 to 3.4) |
| Heart failure due to calcific aortic valve disease | 293.1 (182.0 to 434.7) | -- | 23.2 (13.2 to 37.0) | 24.6* (7.5 to 42.6) | -8.4 (-21.0 to 4.8) |
| Non-rheumatic calcific aortic valve disease | 1642.8 (1382.2 to 1927.7) | 1935.9 (1667.0 to 2229.1) | 11.9 (6.3 to 19.8) | 51.1* (28.0 to 77.3) | 11.1 (-5.9 to 30.4) |
| Non-rheumatic degenerative mitral valve disease | 1671.8 (1619.6 to 1727.7) | 1671.8 (1619.6 to 1727.7) | 32.0 (18.5 to 48.7) | 31.4* (26.2 to 36.6) | -3.4 (-7.2 to 0.4) |
| Heart failure due to degenerative mitral valve disease | 301.5 (195.6 to 435.9) | -- | 23.8 (13.7 to 35.8) | 26.4* (14.5 to 39.2) | -7.1 (-15.9 to 2.3) |
| Non-rheumatic degenerative mitral valve disease | 1370.3 (1226.7 to 1491.3) | 1671.8 (1619.6 to 1727.7) | 8.3 (4.5 to 13.5) | 48.1* (25.9 to 73.6) | 8.9 (-7.5 to 27.6) |
| Other non-rheumatic valve diseases | 4.0 (2.7 to 5.8) | -- | 0.4 (0.2 to 0.6) | 54.4* (49.3 to 59.4) | 13.5* (9.8 to 17.1) |
| Mild heart failure due to other non-rheumatic valve disease | 0.7 (0.4 to 1.2) | -- | 0.0 (0.0 to 0.1) | 54.7* (48.5 to 60.1) | 13.7* (9.2 to 17.7) |
| Moderate heart failure due to other non-rheumatic valve disease | 0.5 (0.3 to 0.8) | -- | 0.0 (0.0 to 0.1) | 54.6* (48.7 to 60.5) | 13.7* (9.3 to 18.0) |
| Severe heart failure due to other non-rheumatic valve disease | 1.3 (0.8 to 1.9) | -- | 0.2 (0.1 to 0.4) | 54.5* (48.4 to 60.7) | 13.6* (9.1 to 18.2) |

| | | | | | |
|---|---|---|---|---|---|
| Controlled, medically managed heart failure due to other non-rheumatic valve disease | 1.5 (1.0 to 2.2) | -- | 0.1 (0.0 to 0.1) | 53.9* (45.6 to 62.3) | 13.1* (7.1 to 19.3) |
| Cardiomyopathy and myocarditis | 555.8 (397.4 to 736.6) | 163.2 (127.3 to 208.9) | 44.5 (27.2 to 66.7) | 23.9* (19.7 to 27.8) | -8.9* (-12.0 to -6.1) |
| Myocarditis | 81.9 (65.8 to 101.4) | 163.2 (127.3 to 208.9) | 5.2 (3.3 to 7.6) | 34.9* (28.3 to 41.5) | -0.8 (-5.7 to 4.0) |
| Acute myocarditis | 39.6 (29.9 to 51.7) | 163.2 (127.3 to 208.9) | 1.8 (1.1 to 2.7) | 35.5* (28.0 to 43.6) | -0.4 (-5.9 to 5.6) |
| Heart failure due to myocarditis | 42.3 (28.4 to 57.5) | -- | 3.5 (2.0 to 5.3) | 34.6* (25.3 to 44.3) | -1.1 (-7.9 to 6.1) |
| Alcoholic cardiomyopathy | 62.4 (42.5 to 85.0) | -- | 5.0 (3.0 to 7.5) | 17.5* (9.4 to 25.7) | -13.6* (-19.5 to -7.6) |
| Other cardiomyopathy | 432.9 (297.8 to 583.6) | -- | 34.3 (20.9 to 51.9) | 23.3* (18.7 to 27.6) | -9.3* (-12.7 to -6.2) |
| Atrial fibrillation and flutter | 5227.0 (4311.3 to 6231.5) | 283.4 (197.7 to 375.7) | 391.6 (262.0 to 549.1) | 31.5* (30.0 to 32.9) | -3.3* (-4.4 to -2.3) |
| Asymptomatic atrial fibrillation and flutter | 3136.2 (2582.4 to 3735.9) | 283.4 (197.7 to 375.7) | -- | -- | -- |
| Symptomatic atrial fibrillation and flutter | 2090.7 (1714.8 to 2518.5) | -- | 391.6 (262.0 to 549.1) | 31.5* (30.0 to 32.9) | -3.3* (-4.4 to -2.3) |
| Peripheral vascular disease | 10997.2 (8934.1 to 13414.7) | 611.2 (426.9 to 840.1) | 61.3 (26.7 to 116.3) | 30.4* (28.4 to 33.0) | -4.1* (-5.6 to -2.2) |
| Asymptomatic peripheral arterial disease | 6176.2 (4024.3 to 8256.1) | 611.2 (426.9 to 840.1) | -- | -- | -- |
| Symptomatic claudication due to peripheral arterial disease | 4821.0 (3225.0 to 7031.4) | -- | 61.3 (26.7 to 116.3) | 30.4* (28.4 to 33.0) | -4.1* (-5.6 to -2.2) |
| Endocarditis | 71.1 (51.2 to 97.1) | 74.9 (60.9 to 89.3) | 5.6 (3.5 to 8.5) | 25.9* (19.0 to 33.1) | -7.5* (-12.6 to -2.1) |
| Acute endocarditis | 5.4 (4.2 to 6.5) | 74.9 (60.9 to 89.3) | 0.3 (0.2 to 0.5) | 40.6* (30.2 to 50.4) | 3.4 (-4.3 to 10.5) |
| Heart failure due to endocarditis | 65.7 (45.9 to 91.5) | -- | 5.3 (3.3 to 8.2) | 25.1* (18.0 to 32.7) | -8.0* (-13.3 to -2.5) |
| Other cardiovascular and circulatory diseases | 4314.4 (3057.0 to 5955.1) | -- | 214.5 (134.0 to 326.4) | 30.3* (26.9 to 33.2) | -4.2* (-6.7 to -2.1) |
| Heart failure due to other cardiovascular diseases | 230.6 (159.9 to 322.9) | -- | 18.3 (11.3 to 28.3) | 29.3* (24.1 to 34.3) | -4.9* (-8.8 to -1.3) |
| Other cardiovascular and circulatory disease episodes | 4083.7 (2894.5 to 5642.2) | -- | 196.2 (122.7 to 298.9) | 30.4* (26.9 to 33.4) | -4.2* (-6.7 to -2.0) |
| **Chronic respiratory diseases** | **22651.5 (20387.7 to 25556.6)** | **1172.3 (870.6 to 1567.4)** | **2124.4 (1788.2 to 2486.9)** | **36.2* (27.6 to 43.6)** | **0.1 (-6.2 to 5.5)** |
| Chronic obstructive pulmonary disease | 20347.8 (17607.3 to 23843.9) | 829.5 (543.0 to 1201.7) | 1880.1 (1572.3 to 2215.4) | 35.3* (26.2 to 43.5) | -0.5 (-7.3 to 5.5) |
| Chronic obstructive pulmonary disease with heart failure | 2787.0 (2131.6 to 3490.1) | -- | 1028.1 (749.1 to 1276.5) | 39.3* (33.7 to 43.9) | 2.4 (-1.7 to 5.8) |
| Chronic obstructive pulmonary disease without heart failure | 17560.8 (14916.7 to 20905.2) | 829.5 (543.0 to 1201.7) | 852.0 (578.3 to 1203.6) | 30.9* (14.4 to 45.0) | -3.8 (-15.9 to 6.6) |
| Pneumoconiosis | 51.9 (43.0 to 61.8) | 4.8 (3.6 to 6.2) | 7.6 (5.1 to 10.7) | 43.9* (35.5 to 52.8) | 5.7 (-0.4 to 12.4) |
| Silicosis | 15.7 (10.8 to 21.8) | 1.9 (1.1 to 2.9) | 2.3 (1.4 to 3.6) | 52.9* (35.5 to 71.8) | 12.4 (-0.4 to 26.3) |
| Heart failure due to silicosis | 1.9 (1.4 to 2.4) | -- | 0.8 (0.5 to 1.2) | 46.3* (33.6 to 61.2) | 7.6 (-1.8 to 18.5) |
| Silicosis without heart failure | 13.9 (9.1 to 20.0) | 1.9 (1.1 to 2.9) | 1.5 (0.7 to 2.6) | 57.0* (28.9 to 84.7) | 15.4 (-5.3 to 35.8) |
| Asbestosis | 7.7 (5.7 to 10.6) | 0.7 (0.5 to 1.2) | 1.2 (0.8 to 1.8) | 35.4* (24.3 to 47.2) | -0.5 (-8.6 to 8.2) |
| Asbestosis without heart failure | 6.6 (4.7 to 9.3) | 0.7 (0.5 to 1.2) | 0.7 (0.4 to 1.2) | 48.0* (28.5 to 68.5) | 8.8 (-5.6 to 23.8) |
| Heart failure due to asbestosis | 1.1 (0.9 to 1.4) | -- | 0.5 (0.3 to 0.7) | 21.1* (15.6 to 26.7) | -11.0* (-15.1 to -6.9) |
| Coal workers pneumoconiosis | 17.8 (12.8 to 25.0) | 1.3 (0.6 to 2.1) | 2.5 (1.5 to 3.8) | 45.6* (25.9 to 66.9) | 7.0 (-7.4 to 22.7) |
| Coal workers pneumoconiosis without heart failure | 16.8 (11.9 to 24.0) | 1.3 (0.6 to 2.1) | 2.0 (1.2 to 3.3) | 46.4* (22.7 to 73.0) | 7.6 (-9.8 to 27.2) |
| Heart failure due to Coal workers pneumoconiosis | 1.0 (0.7 to 1.2) | -- | 0.4 (0.3 to 0.6) | 41.8* (27.5 to 58.2) | 4.3 (-6.3 to 16.3) |
| Other pneumoconiosis | 10.7 (8.1 to 13.7) | 0.9 (0.5 to 1.4) | 1.6 (1.0 to 2.5) | 36.1* (24.8 to 46.5) | 0.0 (-8.3 to 7.7) |
| Heart failure due to other pneumoconiosis | 0.8 (0.7 to 1.1) | -- | 0.4 (0.2 to 0.6) | 31.3* (24.0 to 38.9) | -3.5 (-8.9 to 2.1) |
| Other pneumoconiosis without heart failure | 9.9 (7.5 to 12.7) | 0.9 (0.5 to 1.4) | 1.2 (0.7 to 1.9) | 37.7* (23.1 to 51.7) | 1.2 (-9.5 to 11.5) |
| Asthma | 4783.8 (4026.3 to 5725.7) | 304.0 (209.8 to 418.5) | 169.3 (110.6 to 247.2) | 41.5* (34.2 to 50.2) | 4.0 (-1.4 to 10.4) |
| Asymptomatic asthma | 1738.0 (1430.4 to 2128.0) | 110.2 (74.4 to 152.8) | -- | -- | -- |
| Symptomatic asthma | 3045.9 (2534.9 to 3692.1) | 193.8 (131.4 to 269.1) | 169.3 (110.6 to 247.2) | 41.5* (34.2 to 50.2) | 4.0 (-1.4 to 10.4) |
| Interstitial lung disease and pulmonary sarcoidosis | 557.0 (470.9 to 642.6) | 34.0 (25.0 to 44.1) | 55.0 (36.7 to 77.8) | 44.3* (39.2 to 49.4) | 6.1* (2.3 to 9.8) |
| Heart failure due to interstitial lung disease and pulmonary sarcoidosis | 61.8 (42.0 to 81.6) | -- | 23.6 (14.7 to 34.8) | 37.5* (31.2 to 43.9) | 1.1 (-3.6 to 5.8) |
| Interstitial lung disease and pulmonary sarcoidosis without heart failure | 495.2 (412.7 to 578.1) | 34.0 (25.0 to 44.1) | 31.5 (21.6 to 44.1) | 49.8* (43.8 to 56.4) | 10.1* (5.7 to 15.0) |
| Other chronic respiratory diseases | -- | -- | 12.3 (10.3 to 14.5) | 55.8* (45.5 to 64.8) | 14.5* (6.9 to 21.2) |
| **Digestive diseases** | **37559.2 (34553.5 to 40591.4)** | **10071.3 (8416.2 to 11963.9)** | **431.1 (300.0 to 596.9)** | **38.1* (34.8 to 42.1)** | **1.5 (-0.9 to 4.4)** |
| Cirrhosis and other chronic liver diseases | 27213.4 (25045.7 to 29414.7) | 85.4 (77.9 to 93.2) | 45.1 (31.7 to 61.9) | 59.9* (54.9 to 64.8) | 17.5* (13.9 to 21.2) |
| Cirrhosis and other chronic liver diseases due to hepatitis B | 4352.5 (3994.9 to 4729.6) | 26.1 (22.6 to 29.9) | 12.7 (8.8 to 17.6) | 66.7* (59.6 to 74.0) | 22.6* (17.3 to 27.9) |
| Cirrhosis and other chronic liver diseases due to hepatitis B, decompensated | 83.9 (73.7 to 94.7) | 25.7 (22.0 to 29.4) | 12.7 (8.8 to 17.6) | 66.7* (59.6 to 74.0) | 22.6* (17.3 to 27.9) |
| Chronic hepatitis B without cirrhosis | 3769.0 (3406.0 to 4152.4) | -- | -- | -- | -- |
| Cirrhosis and other chronic liver diseases due to hepatitis B, compensated | 499.6 (448.3 to 552.0) | 0.5 (0.0 to 1.4) | -- | -- | -- |
| Cirrhosis and other chronic liver diseases due to hepatitis C | 2005.6 (1681.2 to 2363.3) | 19.9 (17.1 to 22.9) | 11.6 (8.1 to 16.2) | 56.4* (49.5 to 63.9) | 15.0* (9.9 to 20.5) |
| Cirrhosis and other chronic liver diseases due to hepatitis C, decompensated | 76.6 (67.4 to 86.0) | 19.4 (16.6 to 22.5) | 11.6 (8.1 to 16.2) | 56.4* (49.5 to 63.9) | 15.0* (9.9 to 20.5) |
| Chronic hepatitis C without cirrhosis | 1569.8 (1249.6 to 1930.6) | -- | -- | -- | -- |
| Cirrhosis and other chronic liver diseases due to hepatitis C, compensated | 359.2 (314.1 to 406.7) | 0.5 (0.1 to 1.2) | -- | -- | -- |
| Cirrhosis and other chronic liver diseases due to alcohol use | 384.6 (332.8 to 440.5) | 17.2 (14.6 to 20.2) | 9.5 (6.6 to 13.3) | 57.2* (49.2 to 65.8) | 15.5* (9.7 to 21.9) |
| Cirrhosis and other chronic liver diseases due to alcohol, decompensated | 63.3 (54.8 to 72.9) | 17.0 (14.5 to 20.0) | 9.5 (6.6 to 13.3) | 57.2* (49.2 to 65.8) | 15.5* (9.7 to 21.9) |
| Cirrhosis and other chronic liver diseases due to alcohol, compensated | 321.3 (275.3 to 372.1) | 0.2 (0.0 to 0.8) | -- | -- | -- |
| Cirrhosis due to NASH | 20216.9 (18157.6 to 22477.6) | 9.4 (7.9 to 10.9) | 4.3 (2.9 to 6.0) | 74.2* (62.6 to 87.9) | 28.1* (19.5 to 38.1) |
| Non-alcoholic fatty liver disease (NAFLD) / Non-alcoholic steatohepatitis (NASH) | 20047.4 (17983.4 to 22305.2) | -- | -- | -- | -- |
| Cirrhosis and other chronic liver diseases due to NASH | 169.5 (145.8 to 194.5) | 9.4 (7.9 to 10.9) | 4.3 (2.9 to 6.0) | 74.2* (62.6 to 87.9) | 28.1* (19.5 to 38.1) |
| Cirrhosis and other chronic liver diseases due to other causes | 254.0 (202.0 to 317.0) | 12.8 (10.0 to 16.0) | 6.9 (4.6 to 10.2) | 50.0* (40.1 to 60.4) | 10.3* (3.0 to 17.9) |

| | | | | | |
|---|---|---|---|---|---|
| Cirrhosis and other chronic liver diseases due to other cause, decompensated | 46.1 (37.1 to 56.6) | 12.6 (9.9 to 15.8) | 6.9 (4.6 to 10.2) | 50.0* (40.1 to 60.4) | 10.3* (3.0 to 17.9) |
| Cirrhosis and other chronic liver diseases due to other cause, compensated | 207.9 (164.6 to 258.4) | 0.2 (0.0 to 0.5) | -- | -- | -- |
| Upper digestive system diseases | 16608.5 (13001.4 to 20596.6) | 7367.7 (5789.3 to 9178.8) | 260.1 (165.4 to 400.1) | 36.7* (32.4 to 41.8) | 0.5 (-2.7 to 4.3) |
| Peptic ulcer disease | 706.3 (557.2 to 893.9) | 331.5 (249.5 to 418.4) | 32.1 (21.4 to 46.9) | 23.1* (17.5 to 29.0) | -9.5* (-13.6 to -5.2) |
| Acute peptic ulcer disease | 0.6 (0.5 to 0.8) | -- | 0.2 (0.1 to 0.3) | 9.2* (6.5 to 11.6) | -19.7* (-21.7 to -17.9) |
| Chronic peptic ulcer disease | 705.6 (556.6 to 893.2) | 331.5 (249.5 to 418.4) | 31.9 (21.3 to 46.7) | 23.2* (17.5 to 29.2) | -9.4* (-13.6 to -5.0) |
| Gastritis and duodenitis | 2653.4 (1882.1 to 3708.6) | 1767.3 (1219.8 to 2416.9) | 110.0 (65.9 to 167.5) | 44.6* (34.6 to 54.5) | 6.3 (-1.1 to 13.6) |
| Acute gastritis and duodenitis | 2.4 (1.7 to 3.2) | -- | 0.8 (0.5 to 1.2) | 19.8* (16.5 to 23.2) | -11.9* (-14.4 to -9.4) |
| Chronic gastritis and duodenitis | 2651.0 (1879.7 to 3706.1) | 1767.3 (1219.8 to 2416.9) | 109.2 (65.2 to 166.5) | 44.8* (34.7 to 54.8) | 6.5 (-1.0 to 13.8) |
| GERD | 14921.6 (10644.2 to 19769.4) | 5268.8 (3765.5 to 7105.2) | 118.0 (57.4 to 215.8) | 34.0* (31.9 to 35.9) | -1.5* (-3.0 to -0.1) |
| Appendicitis | 13.3 (12.3 to 14.4) | 365.0 (337.5 to 394.3) | 3.6 (2.5 to 4.8) | 35.4* (27.3 to 44.0) | -0.5 (-6.4 to 5.8) |
| Paralytic ileus and intestinal obstruction | 7.5 (6.0 to 9.3) | 222.1 (176.4 to 273.5) | 2.2 (1.4 to 3.2) | 33.8* (18.5 to 50.1) | -1.6 (-12.9 to 10.3) |
| Inguinal, femoral, and abdominal hernia | 667.7 (501.7 to 852.1) | 1605.1 (1183.7 to 2103.3) | 58.4 (38.0 to 84.2) | 36.7* (25.8 to 47.8) | 0.5 (-7.5 to 8.7) |
| Inflammatory bowel disease | 140.0 (119.8 to 161.9) | 38.9 (31.2 to 47.3) | 18.8 (12.8 to 25.6) | 21.4* (15.1 to 27.5) | -10.8* (-15.4 to -6.3) |
| Ulcerative colitis | 100.5 (83.3 to 120.6) | 24.7 (18.2 to 32.1) | 13.2 (9.0 to 18.1) | 25.9* (17.8 to 34.0) | -7.4* (-13.4 to -1.5) |
| Crohn's disease | 39.5 (33.4 to 46.4) | 14.2 (10.7 to 18.1) | 5.6 (3.6 to 7.8) | 11.9* (3.6 to 19.7) | -17.8* (-23.8 to -12.0) |
| Vascular intestinal disorders | 9.3 (7.1 to 11.9) | 69.8 (54.0 to 88.9) | 2.6 (1.6 to 3.8) | 22.6* (2.8 to 44.5) | -9.8 (-24.5 to 6.2) |
| Gallbladder and biliary diseases | 952.4 (744.6 to 1234.4) | 247.8 (195.1 to 311.5) | 1.0 (0.6 to 1.5) | 38.0* (23.8 to 52.5) | 1.5 (-9.0 to 12.1) |
| Pancreatitis | 274.7 (235.2 to 315.5) | 69.5 (56.0 to 86.1) | 15.2 (7.8 to 25.3) | 42.0* (34.4 to 48.8) | 4.4 (-1.2 to 9.4) |
| Acute pancreatitis | 5.8 (4.5 to 7.5) | 57.2 (44.1 to 73.1) | 2.2 (1.4 to 3.2) | 29.1* (18.7 to 38.9) | -5.1 (-12.7 to 2.1) |
| Chronic pancreatitis | 268.9 (229.6 to 310.2) | 12.2 (8.7 to 16.3) | 12.9 (5.9 to 22.5) | 44.4* (35.8 to 53.0) | 6.2 (-0.2 to 12.5) |
| Other digestive diseases | -- | -- | 24.3 (16.5 to 32.7) | 36.9* (31.6 to 42.8) | 0.6 (-3.3 to 5.0) |
| **Neurological disorders** | **36478.5 (30584.9 to 43223.0)** | **10910.7 (7800.9 to 14157.4)** | **2218.8 (1557.8 to 2978.3)** | **33.7* (32.3 to 35.2)** | **-1.7* (-2.8 to -0.6)** |
| Alzheimer's disease and other dementias | 9803.8 (7846.5 to 12154.1) | 1551.0 (1110.5 to 2060.7) | 1598.9 (1058.9 to 2213.6) | 33.2* (31.7 to 34.5) | -2.1* (-3.2 to -1.1) |
| Parkinson's disease | 1328.5 (967.3 to 1750.3) | 136.9 (92.4 to 186.1) | 181.7 (112.5 to 261.2) | 36.7* (33.4 to 39.7) | 0.5 (-1.9 to 2.7) |
| Epilepsy | 498.3 (367.5 to 647.3) | 29.9 (19.4 to 42.2) | 121.3 (73.8 to 183.2) | 38.2* (21.9 to 55.8) | 1.6 (-10.4 to 14.6) |
| Multiple sclerosis | 34.3 (31.1 to 38.0) | 0.2 (0.2 to 0.2) | 8.2 (5.9 to 10.7) | 23.7* (13.4 to 34.9) | -9.1* (-16.6 to -0.8) |
| Motor neuron disease | 9.3 (7.0 to 11.8) | 4.2 (3.3 to 5.2) | 2.0 (1.3 to 2.9) | 43.3* (33.0 to 52.5) | 5.3 (-2.2 to 12.1) |
| Headache disorders | 29444.5 (23043.5 to 36835.7) | 9188.6 (6159.5 to 12450.8) | 294.4 (191.6 to 422.6) | 32.9* (31.1 to 34.8) | -2.3* (-3.7 to -0.9) |
| Migraine | 6398.6 (5607.3 to 7277.1) | 273.2 (178.8 to 387.3) | 224.4 (139.5 to 337.2) | 32.9* (30.5 to 35.1) | -2.3* (-4.1 to -0.7) |
| Medication overuse headache due to migraine | 884.0 (621.7 to 1187.6) | -- | 87.3 (49.9 to 138.2) | 31.0* (28.3 to 33.4) | -3.7* (-5.7 to -1.9) |
| Migraine | 5514.6 (4776.6 to 6358.7) | 273.2 (178.8 to 387.3) | 137.1 (75.8 to 224.3) | 34.1* (30.5 to 37.1) | -1.4 (-4.1 to 0.8) |
| Tension-type headache | 25698.2 (18432.5 to 34122.1) | 8915.4 (5917.5 to 12180.2) | 70.1 (37.8 to 111.5) | 33.2* (30.7 to 35.3) | -2.1* (-3.9 to -0.5) |
| Medication overuse headache due to tension-type headache | 328.4 (206.1 to 479.3) | -- | 32.2 (15.8 to 57.4) | 31.0* (27.2 to 34.8) | -3.7* (-6.5 to -0.9) |
| Tension-type headache | 25369.7 (18124.2 to 33757.0) | 8915.4 (5917.5 to 12180.2) | 37.9 (15.4 to 75.5) | 35.1* (33.2 to 36.7) | -0.7 (-2.1 to 0.5) |
| Other neurological disorders | 0.7 (0.4 to 1.3) | -- | 12.3 (8.4 to 16.6) | 42.1* (40.3 to 43.7) | 4.4* (3.2 to 5.6) |
| Other neurological disorders | -- | -- | 12.1 (8.2 to 16.4) | 42.1* (40.3 to 43.8) | 4.5* (3.2 to 5.7) |
| Guillain-Barré syndrome due to other neurological disorders | 0.7 (0.4 to 1.3) | -- | 0.2 (0.1 to 0.4) | 39.7* (35.7 to 43.4) | 2.7 (-0.2 to 5.4) |
| **Mental disorders** | **10739.5 (9933.1 to 11622.0)** | **4797.9 (3977.2 to 5759.6)** | **1323.7 (970.2 to 1715.4)** | **34.6* (33.2 to 36.0)** | **-1.0* (-2.1 to -0.0)** |
| Schizophrenia | 124.7 (109.3 to 141.3) | -- | 69.6 (51.6 to 84.9) | 42.0* (37.1 to 47.1) | 4.4* (0.8 to 8.2) |
| Depressive disorders | 4897.5 (4208.4 to 5723.0) | 4599.7 (3786.4 to 5556.5) | 724.0 (496.9 to 992.7) | 34.7* (32.2 to 37.1) | -1.0 (-2.8 to 0.8) |
| Major depressive disorder | 3040.3 (2476.3 to 3695.3) | 4419.6 (3609.5 to 5370.3) | 548.9 (365.6 to 768.1) | 34.0* (30.9 to 36.8) | -1.5 (-3.7 to 0.6) |
| Dysthymia | 1989.6 (1552.4 to 2488.1) | 180.2 (134.6 to 231.8) | 175.1 (113.5 to 262.3) | 37.0* (33.1 to 41.2) | 0.7 (-2.2 to 3.8) |
| Bipolar disorder | 343.2 (287.7 to 409.4) | 25.8 (18.6 to 33.8) | 61.9 (39.2 to 95.9) | 34.0* (30.2 to 37.7) | -1.5 (-4.3 to 1.2) |
| Anxiety disorders | 3210.0 (2876.8 to 3536.8) | 172.3 (152.2 to 196.1) | 272.3 (194.8 to 361.9) | 31.7* (29.3 to 34.2) | -3.2* (-4.9 to -1.3) |
| Eating disorders | -- | -- | -- | -- | -- |
| Anorexia nervosa | -- | -- | -- | -- | -- |
| Bulimia nervosa | -- | -- | -- | -- | -- |
| Autism spectrum disorders | 262.5 (234.7 to 291.7) | -- | 35.8 (25.2 to 47.6) | 37.1* (34.3 to 39.9) | 0.8 (-1.3 to 2.8) |
| Attention-deficit/hyperactivity disorder | 4.3 (1.9 to 7.7) | -- | 0.1 (0.0 to 0.1) | 42.1* (30.0 to 55.0) | 4.4 (-4.5 to 13.9) |
| Conduct disorder | -- | -- | -- | -- | -- |
| Idiopathic developmental intellectual disability | 250.7 (130.9 to 388.9) | -- | 8.4 (3.4 to 15.0) | 29.1* (24.1 to 32.9) | -5.1* (-8.8 to -2.3) |
| Other mental disorders | 2265.8 (2019.0 to 2540.4) | -- | 151.7 (104.0 to 202.7) | 36.7* (34.8 to 38.8) | 0.5 (-0.9 to 2.0) |
| **Substance use disorders** | **734.9 (610.2 to 873.1)** | **187.7 (149.2 to 233.1)** | **117.1 (82.7 to 155.3)** | **48.0* (42.6 to 53.4)** | **8.8* (4.8 to 12.8)** |
| Alcohol use disorders | 515.8 (394.9 to 649.2) | 162.8 (125.5 to 208.1) | 46.1 (30.2 to 64.7) | 42.4* (37.7 to 47.2) | 4.7* (1.2 to 8.2) |
| Alcohol dependence | 512.9 (391.7 to 646.3) | 162.8 (125.5 to 208.1) | 46.0 (30.1 to 64.5) | 42.5* (37.8 to 47.3) | 4.8* (1.3 to 8.3) |
| Fetal alcohol syndrome | 2.8 (2.2 to 3.6) | -- | 0.1 (0.1 to 0.2) | 6.1 (-2.1 to 14.8) | -22.0* (-28.0 to -15.6) |
| Drug use disorders | 228.5 (185.7 to 284.6) | 24.9 (17.5 to 34.8) | 71.0 (48.5 to 98.7) | 51.8* (43.1 to 60.0) | 11.6* (5.2 to 17.6) |
| Opioid use disorders | 171.6 (130.0 to 226.6) | 22.6 (15.4 to 32.3) | 61.7 (40.7 to 87.9) | 57.1* (47.6 to 66.3) | 15.5* (8.5 to 22.2) |
| Cocaine use disorders | 31.8 (27.6 to 36.6) | -- | 3.9 (2.6 to 5.6) | 21.3* (10.8 to 31.5) | -10.9* (-18.6 to -3.4) |

| | | | | | |
|---|---|---|---|---|---|
| Amphetamine use disorders | 7.2 (4.0 to 12.1) | 2.4 (1.5 to 3.5) | 1.0 (0.5 to 1.9) | 33.4* (29.4 to 36.7) | -1.9 (-4.9 to 0.5) |
| Cannabis use disorders | 34.4 (26.5 to 43.9) | -- | 1.0 (0.6 to 1.5) | 27.7* (21.1 to 34.5) | -6.1* (-11.0 to -1.1) |
| Other drug use disorders | 1.7 (1.5 to 2.0) | -- | 3.4 (2.2 to 4.8) | 24.2* (15.0 to 32.7) | -8.7* (-15.5 to -2.4) |
| **Diabetes and kidney diseases** | **41695.3 (38846.5 to 44596.3)** | **1554.1 (1121.0 to 2053.4)** | **1954.2 (1403.6 to 2596.3)** | **39.2* (33.4 to 45.2)** | **2.3 (-2.0 to 6.7)** |
| Diabetes mellitus | 18729.5 (16901.5 to 20781.0) | 364.5 (256.7 to 487.3) | 1496.6 (1031.5 to 2055.6) | 40.7* (33.4 to 48.7) | 3.5 (-2.0 to 9.3) |
| Diabetes mellitus type 1 | 112.4 (95.5 to 131.8) | 2.5 (1.5 to 3.6) | 8.8 (5.8 to 12.2) | 26.0* (21.3 to 30.6) | -7.4* (-10.8 to -4.0) |
| Uncomplicated diabetes mellitus type 1 | 63.6 (52.1 to 76.0) | 2.5 (1.5 to 3.6) | 2.7 (1.7 to 4.0) | 26.4* (19.8 to 33.1) | -7.1* (-11.9 to -2.1) |
| Vision loss due to diabetes mellitus type 1 retinopathy | 1.4 (1.0 to 1.9) | -- | 0.1 (0.1 to 0.2) | 7.8* (1.2 to 14.7) | -20.7* (-25.6 to -15.6) |
| Diabetic neuropathy due to diabetes mellitus type 1 | 47.4 (38.6 to 58.7) | -- | 5.9 (3.9 to 8.4) | 26.2* (19.9 to 32.4) | -7.3* (-11.9 to -2.7) |
| Diabetes mellitus type 2 | 18617.2 (16792.9 to 20669.9) | 362.0 (254.9 to 484.5) | 1487.8 (1025.1 to 2044.4) | 40.8* (33.4 to 48.8) | 3.5 (-1.9 to 9.4) |
| Uncomplicated diabetes mellitus type 2 | 9967.5 (8459.8 to 11565.0) | 362.0 (254.9 to 484.5) | 431.4 (276.4 to 650.5) | 42.5* (33.7 to 51.4) | 4.7 (-1.7 to 11.3) |
| Vision loss due to diabetes mellitus type 2 retinopathy | 237.9 (186.5 to 298.4) | -- | 17.5 (12.0 to 24.6) | 26.4* (22.7 to 30.1) | -7.1* (-9.8 to -4.3) |
| Diabetic neuropathy due to diabetes mellitus type 2 | 8411.8 (7095.8 to 9755.0) | -- | 1038.9 (700.5 to 1426.7) | 40.4* (33.1 to 48.7) | 3.2 (-2.2 to 9.3) |
| Chronic kidney disease | 38095.4 (33411.6 to 43339.2) | 1170.8 (763.9 to 1659.6) | 457.5 (326.6 to 601.5) | 34.2* (26.0 to 42.7) | -1.3 (-7.4 to 4.9) |
| Chronic kidney disease due to diabetes mellitus type 1 | 29.8 (24.6 to 36.4) | 0.5 (0.3 to 0.7) | 3.0 (1.6 to 4.8) | 35.2* (24.6 to 45.3) | -0.6 (-8.4 to 6.8) |
| Albuminuria with preserved GFR due to type 1 diabetes mellitus | 13.7 (10.2 to 18.0) | -- | -- | -- | -- |
| End-stage chronic kidney disease due to diabetes mellitus type 1 | 4.5 (2.3 to 8.1) | -- | 2.3 (1.0 to 4.1) | 35.5* (22.5 to 47.9) | -0.4 (-10.0 to 8.8) |
| Stage III chronic kidney disease due to diabetes mellitus type 1 | 9.3 (7.6 to 11.3) | 0.5 (0.3 to 0.7) | 0.2 (0.1 to 0.3) | 33.3* (13.5 to 55.8) | -2.0 (-16.6 to 14.6) |
| Stage IV chronic kidney disease due to diabetes mellitus type 1 | 1.9 (1.3 to 2.7) | -- | 0.3 (0.1 to 0.4) | 31.3* (16.1 to 47.0) | -3.5 (-14.6 to 8.0) |
| Stage V chronic kidney disease due to diabetes mellitus type 1 | 0.4 (0.3 to 0.5) | -- | 0.2 (0.1 to 0.3) | 38.3* (26.4 to 51.4) | 1.6 (-7.1 to 11.3) |
| Chronic kidney disease due to diabetes mellitus type 2 | 6352.3 (5501.4 to 7284.2) | 146.0 (95.2 to 207.3) | 111.5 (79.4 to 147.1) | 36.3* (28.2 to 45.0) | 0.2 (-5.8 to 6.6) |
| Albuminuria with preserved GFR due to type 2 diabetes mellitus | 2738.5 (2120.4 to 3455.9) | -- | -- | -- | -- |
| End-stage chronic kidney disease due to diabetes mellitus type 2 | 59.7 (40.5 to 79.7) | -- | 26.5 (16.4 to 38.6) | 41.2* (31.4 to 50.4) | 3.8 (-3.4 to 10.5) |
| Stage III chronic kidney disease due to diabetes mellitus type 2 | 3124.3 (2687.1 to 3673.5) | 146.0 (95.2 to 207.3) | 13.1 (8.3 to 19.5) | 27.8* (14.8 to 41.0) | -6.0 (-15.6 to 3.6) |
| Stage IV chronic kidney disease due to diabetes mellitus type 2 | 349.5 (239.5 to 488.5) | -- | 34.8 (20.9 to 53.9) | 32.6* (19.4 to 47.2) | -2.5 (-12.2 to 8.2) |
| Stage V chronic kidney disease due to diabetes mellitus type 2 | 80.2 (59.0 to 105.0) | -- | 37.1 (23.6 to 51.6) | 39.7* (28.5 to 50.4) | 2.7 (-5.5 to 10.6) |
| Chronic kidney disease due to hypertension | 1403.7 (1200.3 to 1639.3) | 3.7 (2.4 to 5.2) | 108.6 (76.7 to 143.1) | 35.8* (28.0 to 44.3) | -0.2 (-5.9 to 6.1) |
| Albuminuria with preserved GFR due to hypertension | 230.6 (177.5 to 292.1) | -- | -- | -- | -- |
| End-stage chronic kidney disease due to hypertension | 61.0 (45.9 to 79.8) | -- | 27.0 (17.0 to 38.1) | 37.5* (28.3 to 45.9) | 1.1 (-5.7 to 7.3) |
| Stage III chronic kidney disease due to hypertension | 704.7 (599.2 to 841.7) | 3.7 (2.4 to 5.2) | 12.9 (8.3 to 19.2) | 28.1* (15.1 to 41.3) | -5.9 (-15.4 to 3.9) |
| Stage IV chronic kidney disease due to hypertension | 330.9 (225.3 to 463.1) | -- | 33.2 (19.8 to 51.8) | 33.8* (20.2 to 48.4) | -1.7 (-11.7 to 9.1) |
| Stage V chronic kidney disease due to hypertension | 76.4 (56.0 to 100.3) | -- | 35.4 (22.6 to 49.5) | 39.6* (27.8 to 52.0) | 2.6 (-6.0 to 11.7) |
| Chronic kidney disease due to glomerulonephritis | 2269.6 (1949.2 to 2665.8) | 130.9 (85.3 to 185.6) | 15.5 (10.1 to 21.4) | 36.2* (27.4 to 45.5) | 0.1 (-6.4 to 7.0) |
| Albuminuria with preserved GFR due to glomerulonephritis | 47.0 (35.9 to 59.3) | -- | -- | -- | -- |
| End-stage chronic kidney disease due to glomerulonephritis | 19.0 (11.7 to 29.5) | -- | 8.5 (4.6 to 13.3) | 36.6* (23.0 to 49.9) | 0.4 (-9.6 to 10.2) |
| Stage III chronic kidney disease due to glomerulonephritis | 2172.4 (1857.6 to 2564.2) | 130.9 (85.3 to 185.6) | 0.7 (0.4 to 1.0) | 30.3* (11.9 to 51.7) | -4.2 (-17.7 to 11.5) |
| Stage IV chronic kidney disease due to glomerulonephritis | 25.4 (17.2 to 35.6) | -- | 2.9 (1.7 to 4.5) | 32.6* (18.0 to 48.2) | -2.6 (-13.3 to 9.0) |
| Stage V chronic kidney disease due to glomerulonephritis | 5.9 (4.3 to 7.7) | -- | 3.4 (2.1 to 4.9) | 39.8* (30.3 to 50.1) | 2.8 (-4.2 to 10.4) |
| Chronic kidney disease due to other and unspecified causes | 28040.0 (24632.0 to 31850.9) | 889.8 (580.6 to 1260.2) | 219.0 (153.2 to 291.1) | 32.3* (22.7 to 42.2) | -2.8 (-9.8 to 4.5) |
| Albuminuria with preserved GFR due to other and unspecified causes | 8217.2 (6374.1 to 10393.3) | -- | -- | -- | -- |
| End-stage chronic kidney disease due to other causes | 28.5 (18.6 to 41.7) | -- | 12.6 (7.6 to 19.6) | 36.8* (25.3 to 50.4) | 0.5 (-7.9 to 10.5) |
| Stage III chronic kidney disease due to other causes | 19096.7 (16432.0 to 22465.1) | 889.8 (580.6 to 1260.2) | 90.3 (57.2 to 133.2) | 27.3* (14.1 to 40.5) | -6.5 (-16.1 to 3.3) |
| Stage IV chronic kidney disease due to other causes | 568.1 (388.3 to 794.7) | -- | 56.5 (33.9 to 88.0) | 32.1* (19.0 to 46.9) | -2.9 (-12.5 to 8.0) |
| Stage V chronic kidney disease due to other causes | 129.6 (94.9 to 170.0) | -- | 59.7 (38.3 to 83.2) | 39.8* (29.8 to 50.3) | 2.8 (-4.6 to 10.5) |
| Acute glomerulonephritis | 1.2 (0.9 to 1.7) | 18.9 (13.8 to 25.8) | 0.1 (0.0 to 0.1) | 22.2* (16.5 to 26.9) | -10.2* (-14.3 to -6.7) |
| **Skin and subcutaneous diseases** | **46562.7 (42900.2 to 50956.5)** | **131411.2 (113322.6 to 148606.6)** | **572.7 (362.5 to 915.9)** | **35.4* (34.0 to 36.5)** | **-0.4 (-1.5 to 0.3)** |
| Dermatitis | 3433.7 (2916.0 to 4057.3) | 4842.7 (3386.9 to 6312.7) | 106.6 (65.0 to 162.6) | 30.4* (28.7 to 32.0) | -4.2* (-5.4 to -2.9) |
| Atopic dermatitis | 1779.0 (1566.6 to 2020.4) | 254.5 (215.9 to 300.6) | 69.7 (38.9 to 114.7) | 30.2* (27.8 to 32.6) | -4.3* (-6.1 to -2.5) |
| Contact dermatitis | 1487.9 (1026.9 to 2070.1) | 4192.1 (2714.1 to 5646.2) | 34.0 (19.6 to 54.2) | 30.8* (28.3 to 33.2) | -3.9* (-5.7 to -2.1) |
| Seborrhoeic dermatitis | 230.1 (189.1 to 279.0) | 396.1 (330.7 to 459.5) | 2.9 (1.6 to 4.8) | 29.5* (25.3 to 33.8) | -4.8* (-7.9 to -1.6) |
| Psoriasis | 1058.2 (987.4 to 1130.4) | 51.6 (42.7 to 61.7) | 82.1 (59.4 to 106.1) | 31.8* (28.6 to 34.9) | -3.1* (-5.5 to -0.8) |
| Bacterial skin diseases | 211.0 (194.8 to 228.7) | 5512.7 (5083.2 to 5976.2) | 3.6 (2.4 to 5.4) | 26.5* (20.4 to 32.3) | -7.0* (-11.5 to -2.8) |
| Cellulitis | 52.9 (43.5 to 63.1) | 1227.2 (1031.3 to 1426.5) | 2.7 (1.8 to 3.9) | 23.1* (16.3 to 30.5) | -9.5* (-14.5 to -4.1) |
| Pyoderma | 167.8 (153.2 to 181.8) | 4285.5 (3899.1 to 4702.8) | 0.9 (0.4 to 1.8) | 38.7* (34.6 to 42.7) | 2.0 (-1.0 to 4.9) |
| Impetigo | 5.7 (5.2 to 6.3) | 114.0 (103.1 to 124.8) | 0.0 (0.0 to 0.1) | 42.3* (37.0 to 48.4) | 4.6* (0.7 to 9.1) |
| Abscess and other bacterial skin diseases | 162.1 (147.6 to 176.0) | 4171.5 (3785.4 to 4584.5) | 0.8 (0.3 to 1.8) | 38.6* (34.4 to 42.8) | 1.9 (-1.2 to 4.9) |
| Scabies | 1315.3 (931.5 to 1808.7) | 4000.9 (2854.4 to 5531.0) | 31.2 (16.0 to 53.1) | 45.8* (43.8 to 47.7) | 7.2* (5.7 to 8.5) |
| Fungal skin diseases | 30386.0 (25232.7 to 36747.7) | 93173.2 (74761.8 to 110308.4) | 158.7 (62.7 to 336.9) | 37.5* (37.0 to 38.0) | 1.1* (0.7 to 1.5) |
| Tinea capitis | 263.1 (185.5 to 370.5) | 517.3 (361.6 to 682.8) | 1.4 (0.5 to 2.9) | 18.8* (15.8 to 22.2) | -12.7* (-14.8 to -10.2) |
| Other fungal skin diseases | 30123.0 (24979.1 to 36498.8) | 92655.9 (74324.6 to 109739.9) | 157.4 (62.0 to 333.7) | 37.7* (37.2 to 38.2) | 1.2* (0.8 to 1.6) |

| | | | | | |
|---|---|---|---|---|---|
| Viral skin diseases | 519.7 (464.2 to 571.2) | 208.5 (189.2 to 230.6) | 14.5 (9.2 to 21.4) | 33.2* (30.2 to 36.1) | -2.1 (-4.3 to 0.1) |
| Viral warts | 491.4 (436.4 to 542.1) | 166.3 (147.3 to 188.5) | 13.7 (8.7 to 20.3) | 33.0* (29.8 to 36.0) | -2.3* (-4.6 to -0.0) |
| Molluscum contagiosum | 28.3 (25.2 to 32.1) | 42.2 (37.9 to 47.3) | 0.8 (0.5 to 1.2) | 37.6* (25.8 to 49.8) | 1.1 (-7.5 to 10.1) |
| Acne vulgaris | 113.4 (110.3 to 116.7) | 46.6 (44.9 to 48.3) | 2.3 (1.4 to 3.7) | 42.1* (37.8 to 46.5) | 4.5* (1.3 to 7.7) |
| Alopecia areata | 102.6 (91.6 to 113.1) | 175.0 (158.3 to 192.3) | 3.1 (2.0 to 4.5) | 25.5* (19.7 to 31.8) | -7.8* (-12.0 to -3.1) |
| Pruritus | 1674.0 (1159.9 to 2346.2) | 1295.9 (901.4 to 1793.3) | 16.6 (7.6 to 32.8) | 38.8* (36.9 to 40.4) | 2.1* (0.7 to 3.2) |
| Urticaria | 502.2 (350.8 to 690.7) | 866.2 (597.0 to 1191.2) | 27.0 (16.5 to 42.2) | 36.1* (32.7 to 39.2) | 0.1 (-2.5 to 2.3) |
| Decubitus ulcer | 143.7 (101.6 to 199.9) | 534.8 (372.7 to 742.4) | 20.7 (13.0 to 31.2) | 30.8* (22.4 to 39.2) | -3.8 (-10.0 to 2.3) |
| Other skin and subcutaneous diseases | 20683.8 (18988.2 to 22401.2) | 20702.9 (19075.7 to 22442.1) | 106.2 (51.3 to 195.2) | 38.8* (38.0 to 39.6) | 2.0* (1.5 to 2.6) |
| **Sense organ diseases** | **65499.2 (64667.6 to 66408.9)** | **--** | **4417.5 (3131.7 to 5926.9)** | **35.3* (32.9 to 37.5)** | **-0.5 (-2.3 to 1.1)** |
| Blindness and vision impairment | 42426.6 (40327.5 to 44917.5) | -- | 1788.4 (1240.8 to 2423.1) | 35.1* (33.3 to 36.8) | -0.7 (-2.0 to 0.6) |
| Glaucoma | 597.2 (485.5 to 736.0) | -- | 66.9 (43.8 to 95.3) | 31.2* (28.8 to 33.7) | -3.5* (-5.4 to -1.7) |
| Cataract | 12590.4 (10554.8 to 14943.2) | -- | 936.8 (636.2 to 1255.1) | 35.7* (33.5 to 38.0) | -0.3 (-1.8 to 1.5) |
| Age-related macular degeneration | 731.0 (571.8 to 918.6) | -- | 61.8 (40.3 to 88.1) | 29.9* (26.3 to 33.6) | -4.5* (-7.2 to -1.8) |
| Refraction disorders | 5164.2 (4000.5 to 6619.4) | -- | 238.1 (156.0 to 340.8) | 31.5* (28.7 to 34.0) | -3.3* (-5.4 to -1.5) |
| Near vision loss | 32966.0 (30901.9 to 35154.9) | -- | 314.5 (148.1 to 567.9) | 41.3* (40.4 to 42.1) | 3.8* (3.2 to 4.5) |
| Other vision loss | 2034.1 (1642.1 to 2514.5) | -- | 170.2 (115.6 to 235.5) | 29.9* (27.9 to 31.9) | -4.5* (-6.0 to -3.0) |
| Age-related and other hearing loss | 64515.3 (63667.7 to 65331.7) | -- | 2501.5 (1792.3 to 3391.0) | 35.4* (31.7 to 39.3) | -0.5 (-3.2 to 2.4) |
| Other sense organ diseases | 5508.0 (4911.2 to 6137.7) | -- | 127.6 (79.6 to 182.2) | 36.7* (35.1 to 38.4) | 0.5 (-0.7 to 1.7) |
| Chronic other sense organ diseases | 5151.2 (4558.1 to 5784.0) | -- | 123.1 (76.6 to 177.1) | 36.7* (35.0 to 38.3) | 0.5 (-0.8 to 1.7) |
| Acute other sense organ diseases | 356.8 (324.5 to 393.1) | -- | 4.5 (2.7 to 6.8) | 37.3* (32.8 to 42.2) | 1.0 (-2.4 to 4.6) |
| **Musculoskeletal disorders** | **38051.0 (34995.6 to 41104.9)** | **8511.0 (6422.3 to 10560.6)** | **3497.7 (2473.1 to 4813.4)** | **34.2* (32.3 to 36.5)** | **-1.4 (-2.8 to 0.3)** |
| Rheumatoid arthritis | 763.3 (674.2 to 861.0) | 15.5 (11.8 to 19.8) | 92.4 (64.6 to 123.7) | 40.7* (35.6 to 45.9) | 3.4 (-0.3 to 7.3) |
| Osteoarthritis | 19419.4 (17428.3 to 21616.1) | 255.5 (172.3 to 356.5) | 584.9 (298.3 to 1161.7) | 33.6* (32.9 to 34.5) | -1.8* (-2.3 to -1.2) |
| Osteoarthritis of the hip | 2796.3 (2604.2 to 3008.3) | 38.2 (29.3 to 49.4) | 84.2 (43.4 to 171.2) | 36.4* (34.8 to 38.1) | 0.3 (-0.9 to 1.5) |
| Osteoarthritis of the knee | 16623.1 (14606.7 to 18869.5) | 217.3 (137.1 to 317.8) | 500.7 (255.1 to 991.8) | 33.2* (32.4 to 34.1) | -2.1* (-2.7 to -1.5) |
| Low back pain | 16862.0 (12718.6 to 21640.9) | 6370.6 (4494.6 to 8379.7) | 1726.7 (1116.5 to 2469.9) | 31.7* (30.0 to 33.3) | -3.2* (-4.4 to -2.0) |
| Low back pain with leg pain | 5442.1 (3943.4 to 7202.8) | 2051.7 (1405.3 to 2778.7) | 710.1 (432.7 to 1044.8) | 31.7* (29.8 to 33.6) | -3.2* (-4.6 to -1.8) |
| Low back pain without leg pain | 11419.9 (8641.7 to 14709.3) | 4318.9 (3001.7 to 5768.8) | 1016.7 (662.6 to 1487.2) | 31.7* (29.9 to 33.4) | -3.2* (-4.5 to -2.0) |
| Neck pain | 7713.6 (5541.2 to 10557.2) | 1513.5 (914.7 to 2234.4) | 704.8 (446.4 to 1043.0) | 35.9* (33.2 to 38.4) | -0.1 (-2.1 to 1.7) |
| Gout | 2433.9 (1889.7 to 3028.1) | 356.0 (265.4 to 448.0) | 70.0 (43.5 to 100.1) | 41.4* (38.0 to 45.0) | 3.9* (1.4 to 6.6) |
| Other musculoskeletal disorders | 4155.5 (2572.0 to 6404.0) | -- | 318.9 (172.1 to 526.1) | 42.3* (20.6 to 61.4) | 4.6 (-11.3 to 18.7) |
| **Other non-communicable diseases** | **57947.4 (55182.5 to 60623.3)** | **21678.5 (18862.2 to 24539.2)** | **1118.7 (764.1 to 1567.0)** | **31.7* (30.3 to 32.9)** | **-3.2* (-4.2 to -2.3)** |
| Congenital anomalies | 1010.8 (947.2 to 1084.8) | -- | 106.1 (76.2 to 139.6) | 34.1* (31.5 to 37.0) | -1.4 (-3.4 to 0.7) |
| Neural tube defects | 22.5 (20.0 to 25.1) | -- | 6.5 (4.5 to 8.6) | 40.6* (33.9 to 46.7) | 3.3 (-1.6 to 7.8) |
| Severe motor and cognitive impairment due to anencephaly | -- | -- | -- | -- | -- |
| Encephalocoele due to neural tube defects | 3.5 (2.9 to 4.2) | -- | 0.6 (0.4 to 0.9) | 33.8* (29.6 to 38.1) | -1.6 (-4.7 to 1.5) |
| Spina bifida due to neural tube defects | 18.9 (16.8 to 21.3) | -- | 5.9 (4.1 to 7.9) | 41.3* (34.1 to 47.9) | 3.9 (-1.4 to 8.8) |
| Congenital heart anomalies | 28.0 (25.4 to 30.8) | -- | 1.5 (0.6 to 2.7) | 36.8* (28.8 to 45.1) | 0.5 (-5.3 to 6.7) |
| Critical malformations of great vessels, congenital valvular heart disease and patent ductus arteriosus | 4.2 (3.6 to 4.9) | -- | 0.3 (0.1 to 0.5) | 39.4* (36.4 to 42.4) | 2.5* (0.3 to 4.6) |
| Other congenital heart anomalies | 13.7 (12.4 to 15.0) | -- | 0.7 (0.3 to 1.3) | 37.4* (24.7 to 51.3) | 1.0 (-8.3 to 11.2) |
| Severe congenital heart anomalies excluding single ventricle heart defects | 9.6 (8.5 to 10.8) | -- | 0.5 (0.2 to 0.9) | 34.9* (21.5 to 49.0) | -0.8 (-10.7 to 9.5) |
| Single ventricle and single ventricle pathway congenital heart anomalies | 0.1 (0.1 to 0.2) | -- | -- | 35.3* (31.0 to 39.5) | -0.5 (-3.7 to 2.6) |
| Ventricular septal defect and atrial septal defect | 0.3 (0.2 to 0.4) | -- | -- | 22.2* (18.0 to 26.1) | -10.2* (-13.3 to -7.3) |
| Orofacial clefts | 109.4 (99.9 to 118.7) | -- | 2.5 (1.7 to 3.6) | 41.2* (30.6 to 53.7) | 3.8 (-4.0 to 13.0) |
| Down's syndrome | -- | -- | -- | -- | -- |
| Turner syndrome | 0.9 (0.7 to 1.0) | -- | -- | 27.6* (26.0 to 29.3) | -6.2* (-7.4 to -4.9) |
| Klinefelter syndrome | 0.2 (0.1 to 0.2) | -- | -- | 46.7* (44.5 to 48.8) | 7.8* (6.2 to 9.4) |
| Other chromosomal abnormalities | -- | -- | -- | -- | -- |
| Congenital musculoskeletal and limb anomalies | 461.8 (410.7 to 526.9) | -- | 61.4 (41.8 to 82.2) | 33.4* (31.0 to 35.9) | -1.9* (-3.7 to -0.1) |
| Polydactyly and syndactyly | 0.7 (0.6 to 0.8) | -- | -- | 25.6* (23.4 to 27.7) | -7.6* (-9.3 to -6.1) |
| Congenital limb deficiency | 114.6 (101.9 to 130.9) | -- | 15.3 (10.5 to 20.6) | 33.7* (28.9 to 38.3) | -1.7 (-5.2 to 1.7) |
| Other congenital musculoskeletal anomalies | 346.6 (308.2 to 395.4) | -- | 46.1 (31.4 to 61.6) | 33.3* (30.6 to 36.1) | -2.0 (-4.0 to 0.1) |
| Urogenital congenital anomalies | 19.9 (17.8 to 22.2) | -- | 0.9 (0.6 to 1.4) | 10.9* (4.4 to 18.0) | -18.4* (-23.3 to -13.3) |
| Digestive congenital anomalies | 253.7 (229.6 to 282.4) | -- | 10.5 (7.2 to 14.6) | 24.2* (16.5 to 31.2) | -8.7* (-14.4 to -3.5) |
| Congenital diaphragmatic hernia | 1.0 (0.9 to 1.2) | -- | -- | 39.6* (37.2 to 41.9) | 2.6* (0.9 to 4.3) |
| Congenital atresia and/or stenosis of the digestive tract | 0.1 (0.1 to 0.1) | -- | -- | 43.0* (37.1 to 48.9) | 5.1* (0.8 to 9.5) |
| Congenital malformations of the abdominal wall after treatment | 4.0 (3.5 to 4.6) | -- | 0.2 (0.1 to 0.3) | 38.5* (36.5 to 40.2) | 1.8* (0.3 to 3.1) |
| Other congenital malformations of the digestive tract | 248.6 (224.4 to 276.9) | -- | 10.3 (7.0 to 14.3) | 24.0* (16.1 to 31.1) | -8.9* (-14.6 to -3.6) |
| Other congenital anomalies | 203.3 (173.5 to 238.6) | -- | 22.7 (15.5 to 31.4) | 39.7* (30.4 to 50.2) | 2.7 (-4.2 to 10.4) |

| | | | | | |
|---|---|---|---|---|---|
| Other congenital birth defects | -- | -- | -- | -- | -- |
| Hearing loss due to other congenital anomalies | 203.3 (173.5 to 238.6) | -- | 22.7 (15.5 to 31.4) | 39.7* (30.4 to 50.2) | 2.7 (-4.2 to 10.4) |
| Urinary diseases and male infertility | 4574.4 (3971.5 to 5299.7) | 6433.1 (5556.9 to 7398.0) | 153.1 (99.3 to 218.0) | 41.8* (39.9 to 43.7) | 4.2* (2.8 to 5.7) |
| Urinary tract infections | 75.6 (62.2 to 91.6) | 4004.1 (3334.7 to 4793.9) | 2.3 (1.4 to 3.5) | 49.7* (41.4 to 58.1) | 10.1* (3.9 to 16.2) |
| Urolithiasis | 77.5 (58.3 to 99.5) | 1998.0 (1519.0 to 2585.4) | 5.2 (3.3 to 7.6) | 43.9* (32.7 to 56.0) | 5.8 (-2.5 to 14.7) |
| Benign prostatic hyperplasia | 4630.6 (3993.1 to 5428.4) | 431.0 (367.8 to 524.1) | 143.9 (92.0 to 206.2) | 41.7* (39.7 to 43.7) | 4.2* (2.7 to 5.7) |
| Male infertility | -- | -- | -- | -- | -- |
| Other urinary diseases | -- | -- | 1.7 (1.2 to 2.4) | 31.2* (23.9 to 38.9) | -3.5 (-8.9 to 2.1) |
| Gynecological diseases | 3667.4 (3085.9 to 4353.0) | 106.5 (66.2 to 162.3) | 13.1 (6.7 to 24.1) | 26.3* (23.3 to 29.2) | -7.1* (-9.4 to -5.0) |
| Uterine fibroids | 493.5 (429.1 to 571.8) | 1.8 (0.9 to 3.2) | 3.6 (2.0 to 6.1) | 26.5* (18.8 to 33.6) | -7.0* (-12.7 to -1.8) |
| Polycystic ovarian syndrome | -- | -- | -- | -- | -- |
| Female infertility | -- | -- | -- | -- | -- |
| Endometriosis | -- | -- | -- | -- | -- |
| Genital prolapse | 3254.9 (2648.5 to 3960.2) | 104.7 (64.6 to 160.2) | 9.5 (4.5 to 18.1) | 26.3* (23.6 to 28.9) | -7.2* (-9.2 to -5.3) |
| Premenstrual syndrome | -- | -- | -- | -- | -- |
| Other gynecological diseases | -- | -- | -- | -- | -- |
| Other gynecological diseases | -- | -- | -- | -- | -- |
| Other gynecological diseases with anemia | -- | -- | -- | -- | -- |
| Hemoglobinopathies and hemolytic anaemias | 17604.6 (17249.8 to 17973.0) | -- | 56.8 (38.9 to 81.9) | 24.1* (13.9 to 34.7) | -8.8* (-16.3 to -1.0) |
| Thalassemias | -- | -- | -- | -2.4 (-9.2 to 5.4) | -28.3* (-33.3 to -22.5) |
| Thalassaemias trait | 2944.5 (2832.3 to 3053.5) | -- | 20.7 (13.1 to 31.5) | 31.1* (11.0 to 55.7) | -3.6 (-18.4 to 14.5) |
| Sickle cell disorders | -- | -- | -- | 29.6* (18.1 to 41.6) | -4.7 (-13.2 to 4.1) |
| Sickle cell trait | 2992.1 (2689.3 to 3330.5) | -- | 6.0 (4.1 to 8.5) | 29.8* (19.1 to 41.8) | -4.6 (-12.5 to 4.3) |
| G6PD deficiency | 2507.9 (2410.3 to 2606.3) | -- | 0.3 (0.2 to 0.4) | 31.1* (16.5 to 49.3) | -3.6 (-14.3 to 9.7) |
| G6PD trait | 9700.8 (9539.2 to 9866.3) | -- | -- | 29.4* (13.6 to 47.4) | -4.9 (-16.5 to 8.3) |
| Other hemoglobinopathies and hemolytic anaemias | 1141.2 (1058.5 to 1231.3) | -- | 29.9 (20.6 to 42.1) | 18.5* (10.3 to 27.0) | -12.9* (-19.0 to -6.7) |
| Endocrine, metabolic, blood, and immune disorders | 2130.3 (1844.7 to 2472.2) | -- | 63.2 (43.6 to 88.1) | 31.3* (23.1 to 39.7) | -3.5 (-9.5 to 2.7) |
| Anemia due to endocrine, metabolic, blood, and immune disorders | 652.3 (603.2 to 703.1) | -- | 11.5 (7.6 to 16.4) | 15.4* (3.8 to 28.1) | -15.2* (-23.7 to -5.8) |
| Endocrine, metabolic, blood, and immune disorders | 1419.6 (1148.9 to 1755.1) | -- | 47.0 (31.4 to 68.2) | 36.9* (25.6 to 48.0) | 0.6 (-7.7 to 8.8) |
| Heart failure due to endocrine, metabolic, blood, and immune disorders | 58.4 (40.8 to 81.7) | -- | 4.7 (2.9 to 7.3) | 23.4* (16.2 to 30.6) | -9.3* (-14.6 to -4.0) |
| Oral disorders | 47268.6 (43303.3 to 51251.2) | 15138.9 (12511.2 to 17824.6) | 726.3 (468.7 to 1056.3) | 30.1* (28.6 to 31.6) | -4.3* (-5.5 to -3.3) |
| Caries of deciduous teeth | -- | -- | -- | -- | -- |
| Caries of permanent teeth | 23923.3 (18066.1 to 30281.3) | 13101.9 (10453.1 to 15785.0) | 17.6 (7.8 to 32.7) | 26.6* (24.1 to 28.8) | -7.0* (-8.8 to -5.3) |
| Periodontal disease | 17222.0 (13674.6 to 20730.0) | 1394.7 (847.7 to 1932.4) | 105.6 (41.9 to 215.9) | 42.8* (41.6 to 44.0) | 4.9* (4.1 to 5.9) |
| Edentulism and severe tooth loss | 21401.4 (18164.4 to 25183.1) | 642.3 (405.6 to 931.3) | 562.5 (374.9 to 777.2) | 27.8* (26.3 to 29.1) | -6.1* (-7.1 to -5.1) |
| Other oral disorders | 1529.1 (1361.5 to 1709.6) | -- | 40.7 (25.2 to 59.9) | 35.4* (33.5 to 37.3) | -0.5 (-1.9 to 1.0) |
| **Injuries** | **44686.8 (42463.2 to 47674.7)** | **11620.8 (9819.5 to 13738.9)** | **1721.2 (1227.1 to 2325.2)** | **38.3* (36.8 to 39.6)** | **1.6* (0.5 to 2.6)** |
| **Transport injuries** | **7048.8 (6502.5 to 7667.0)** | **704.1 (555.0 to 883.8)** | **325.8 (235.4 to 436.0)** | **41.0* (39.7 to 42.1)** | **3.6* (2.7 to 4.5)** |
| Road injuries | 5594.8 (5173.4 to 6140.3) | 508.2 (367.6 to 682.1) | 256.3 (183.8 to 346.4) | 39.8* (38.4 to 41.1) | 2.7* (1.7 to 3.7) |
| Pedestrian road injuries | 1607.0 (1376.6 to 1917.1) | 165.4 (109.5 to 240.9) | 76.2 (52.2 to 105.7) | 43.1* (41.5 to 44.7) | 5.2* (4.0 to 6.4) |
| Cyclist road injuries | 1093.7 (940.3 to 1284.5) | 111.4 (67.7 to 170.5) | 48.4 (33.8 to 65.8) | 45.2* (43.3 to 47.1) | 6.8* (5.3 to 8.2) |
| Motorcyclist road injuries | 1090.2 (941.7 to 1267.9) | 36.3 (20.0 to 60.5) | 47.7 (33.8 to 65.4) | 55.3* (53.2 to 57.4) | 14.2* (12.6 to 15.7) |
| Motor vehicle road injuries | 1431.1 (1227.3 to 1693.7) | 153.4 (102.9 to 219.0) | 68.2 (48.1 to 92.7) | 20.5* (18.9 to 22.2) | -11.4* (-12.6 to -10.2) |
| Other road injuries | 372.9 (306.2 to 443.6) | 41.7 (22.9 to 68.6) | 15.7 (11.0 to 21.7) | 66.1* (63.5 to 68.8) | 22.1* (20.2 to 24.1) |
| Other transport injuries | 1453.9 (1264.9 to 1636.6) | 196.0 (137.6 to 273.6) | 69.5 (49.1 to 92.9) | 45.8* (43.9 to 47.4) | 7.2* (5.8 to 8.4) |
| **Unintentional injuries** | **34669.7 (32430.7 to 37742.6)** | **10717.1 (8924.8 to 12780.3)** | **1326.3 (932.6 to 1812.6)** | **37.5* (35.7 to 39.1)** | **1.1 (-0.3 to 2.3)** |
| Falls | 19735.9 (17484.2 to 22740.7) | 7139.7 (5439.7 to 9148.6) | 910.0 (636.9 to 1238.9) | 35.2* (32.5 to 37.6) | -0.6 (-2.6 to 1.2) |
| Drowning | 85.8 (73.6 to 99.8) | 10.1 (7.5 to 13.8) | 4.0 (2.7 to 5.4) | 53.6* (50.3 to 56.7) | 12.9* (10.5 to 15.2) |
| Fire, heat, and hot substances | 2359.5 (2042.7 to 2730.8) | 31.7 (19.4 to 46.4) | 40.7 (23.7 to 65.7) | 43.1* (41.2 to 44.9) | 5.2* (3.8 to 6.5) |
| Poisonings | 103.5 (88.9 to 118.8) | 37.9 (23.1 to 56.7) | 8.0 (5.5 to 11.1) | 44.9* (40.4 to 49.4) | 6.5* (3.2 to 9.8) |
| Poisoning by carbon monoxide | 21.8 (18.7 to 25.5) | 9.8 (4.4 to 17.2) | 1.3 (0.9 to 1.8) | 50.9* (46.7 to 55.3) | 10.9* (7.9 to 14.2) |
| Poisoning by other means | 81.7 (68.1 to 96.5) | 28.2 (16.9 to 43.7) | 6.7 (4.5 to 9.5) | 43.8* (39.1 to 48.5) | 5.7* (2.3 to 9.2) |
| Exposure to mechanical forces | 5831.4 (5119.2 to 6720.1) | 575.5 (398.3 to 805.1) | 125.8 (85.7 to 179.0) | 46.4* (45.4 to 47.4) | 7.6* (6.9 to 8.4) |
| Unintentional firearm injuries | 209.4 (175.4 to 241.8) | 10.8 (5.4 to 20.9) | 5.4 (3.7 to 7.7) | 51.6* (50.0 to 53.3) | 11.5* (10.3 to 12.7) |
| Other exposure to mechanical forces | 5622.0 (4906.7 to 6499.5) | 564.6 (391.5 to 788.8) | 120.4 (81.9 to 171.3) | 46.2* (45.2 to 47.2) | 7.5* (6.7 to 8.2) |
| Adverse effects of medical treatment | 81.3 (56.2 to 116.3) | 1065.6 (777.9 to 1461.6) | 10.9 (6.2 to 18.2) | 23.6* (10.9 to 34.7) | -9.1* (-18.4 to -1.0) |
| Animal contact | 1022.8 (890.5 to 1187.7) | 351.4 (226.3 to 519.9) | 19.5 (13.2 to 27.6) | 39.0* (36.5 to 40.9) | 2.1* (0.3 to 3.6) |
| Venomous animal contact | 340.6 (279.0 to 389.5) | 132.2 (80.3 to 199.7) | 9.5 (6.6 to 12.9) | 43.0* (39.3 to 46.7) | 5.1* (2.4 to 7.8) |
| Non-venomous animal contact | 682.2 (572.0 to 823.2) | 219.1 (136.8 to 324.1) | 10.0 (6.4 to 15.8) | 35.4* (33.1 to 37.1) | -0.5 (-2.1 to 0.8) |
| Foreign body | 547.6 (490.0 to 612.5) | 297.7 (196.1 to 428.5) | 20.7 (14.5 to 27.7) | 36.8* (35.7 to 37.9) | 0.6 (-0.2 to 1.4) |

| | | | | | |
|---|---|---|---|---|---|
| Pulmonary aspiration and foreign body in airway | 84.8<br>(71.8 to 102.8) | 27.0<br>(16.6 to 41.2) | 3.0<br>(2.1 to 4.1) | 47.0*<br>(44.6 to 49.5) | 8.0*<br>(6.3 to 9.9) |
| Foreign body in eyes | 79.5<br>(57.5 to 107.0) | 203.1<br>(108.1 to 335.1) | 5.1<br>(3.1 to 7.9) | 30.8*<br>(29.2 to 32.7) | -3.8*<br>(-5.0 to -2.4) |
| Foreign body in other body part | 383.2<br>(328.2 to 440.5) | 67.6<br>(44.7 to 102.1) | 12.6<br>(8.8 to 17.1) | 37.1*<br>(35.9 to 38.3) | 0.8<br>(-0.1 to 1.7) |
| Environmental heat and cold exposure | 1067.9<br>(945.7 to 1210.6) | 174.4<br>(120.6 to 237.4) | 40.0<br>(27.6 to 55.8) | 48.3*<br>(46.4 to 50.1) | 9.0*<br>(7.6 to 10.4) |
| Exposure to forces of nature | 136.4<br>(100.5 to 184.8) | 4.4<br>(3.2 to 5.9) | 4.5<br>(3.3 to 5.9) | 88.1*<br>(71.4 to 109.3) | 38.3*<br>(26.0 to 53.9) |
| Other unintentional injuries | 4434.5<br>(3799.8 to 5038.0) | 1028.6<br>(665.7 to 1495.0) | 142.3<br>(96.5 to 199.8) | 40.2*<br>(39.0 to 41.4) | 3.0*<br>(2.1 to 3.9) |
| **Self-harm and interpersonal violence** | **3615.9<br>(3243.5 to 4006.6)** | **199.6<br>(155.5 to 251.1)** | **69.1<br>(52.1 to 88.3)** | **40.3*<br>(37.8 to 43.3)** | **3.1*<br>(1.3 to 5.4)** |
| Self-harm | 318.0<br>(266.2 to 369.1) | 59.5<br>(39.6 to 84.9) | 11.7<br>(8.2 to 15.7) | 21.3*<br>(19.7 to 22.8) | -10.8*<br>(-12.0 to -9.8) |
| Self-harm by firearm | 7.9<br>(6.7 to 9.5) | 2.4<br>(0.6 to 6.1) | 0.3<br>(0.2 to 0.4) | 31.0*<br>(28.1 to 34.6) | -3.7*<br>(-5.9 to -1.0) |
| Self-harm by other specified means | 310.1<br>(257.5 to 361.2) | 57.1<br>(37.7 to 81.1) | 11.4<br>(8.0 to 15.4) | 21.1*<br>(19.5 to 22.6) | -11.0*<br>(-12.2 to -9.9) |
| Interpersonal violence | 3063.9<br>(2713.0 to 3430.0) | 103.2<br>(64.9 to 150.9) | 50.3<br>(37.5 to 65.4) | 39.3*<br>(37.9 to 40.9) | 2.4*<br>(1.4 to 3.6) |
| Assault by firearm | 69.2<br>(58.8 to 82.8) | 4.4<br>(2.0 to 8.1) | 2.1<br>(1.5 to 2.9) | 38.2*<br>(36.0 to 40.6) | 1.6<br>(-0.1 to 3.3) |
| Assault by sharp object | 298.3<br>(248.6 to 394.9) | 16.5<br>(8.3 to 28.7) | 6.6<br>(4.5 to 9.4) | 35.9*<br>(34.0 to 37.8) | -0.1<br>(-1.5 to 1.3) |
| Sexual violence | 1688.6<br>(1368.7 to 2019.8) | -- | 10.6<br>(6.8 to 15.6) | 48.4*<br>(46.3 to 50.8) | 9.1*<br>(7.5 to 10.8) |
| Assault by other means | 1007.9<br>(863.3 to 1174.9) | 82.3<br>(51.5 to 121.0) | 31.0<br>(21.4 to 42.8) | 37.3*<br>(36.0 to 38.7) | 0.9*<br>(0.0 to 1.9) |
| Conflict and terrorism | 213.5<br>(134.5 to 321.8) | 32.7<br>(22.4 to 46.9) | 6.5<br>(4.5 to 9.1) | 97.6*<br>(72.0 to 130.2) | 45.3*<br>(26.4 to 69.2) |
| Executions and police conflict | 20.6<br>(12.9 to 30.0) | 4.1<br>(2.7 to 6.0) | 0.6<br>(0.4 to 0.8) | 168.9*<br>(114.6 to 251.5) | 97.6*<br>(57.8 to 158.4) |
| **Nature of injury aggregates** | | | | | |
| Amputations | 11054.4<br>(10325.8 to 12083.4) | 169.9<br>(134.2 to 213.0) | 182.9<br>(122.0 to 266.7) | 35.7*<br>(32.3 to 38.5) | -0.3<br>(-2.7 to 1.8) |
| Burns | 5126.8<br>(4768.5 to 5566.8) | 92.1<br>(71.5 to 122.7) | 81.6<br>(45.8 to 136.0) | 41.8*<br>(40.4 to 43.4) | 4.3*<br>(3.2 to 5.4) |
| Fractures | 17798.8<br>(16393.8 to 19771.9) | 5129.4<br>(4086.8 to 6253.7) | 965.1<br>(673.6 to 1328.6) | 36.1*<br>(34.0 to 37.9) | 0.0<br>(-1.5 to 1.4) |
| Head Injuries | 1245.8<br>(1174.6 to 1326.1) | 425.8<br>(319.2 to 562.8) | 164.5<br>(117.4 to 218.8) | 44.9*<br>(42.8 to 47.0) | 6.5*<br>(5.0 to 8.1) |
| Spinal Injuries | 392.4<br>(347.9 to 448.1) | 17.4<br>(12.5 to 23.5) | 93.7<br>(67.7 to 122.3) | 50.3*<br>(46.6 to 54.1) | 10.5*<br>(7.8 to 13.3) |
| Minor Injuries | 6688.6<br>(6314.6 to 7199.0) | 2748.0<br>(2204.5 to 3400.3) | 56.8<br>(27.0 to 104.8) | 40.9*<br>(39.9 to 42.0) | 3.6*<br>(2.8 to 4.4) |
[truncated: 986,042 more chars]
